# Protein Phosphorylation in Depolarized Synaptosomes: Dissecting Primary Effects of Calcium from Synaptic Vesicle Cycling

**DOI:** 10.1016/j.mcpro.2021.100061

**Published:** 2021-02-12

**Authors:** Ivan Silbern, Kuan-Ting Pan, Maksims Fiosins, Stefan Bonn, Silvio O. Rizzoli, Eugenio F. Fornasiero, Henning Urlaub, Reinhard Jahn

**Affiliations:** 1 Institute of Clinical Chemistry, University Medical Center Goettingen, Goettingen, Germany; 2 Bioanalytical Mass Spectrometry Group, Max Planck Institute for Biophysical Chemistry, Goettingen, Germany; 3 German Center for Neurodegenerative Diseases, Tübingen, Germany; 4 Institute for Medical Systems Biology, University Medical Center Hamburg-Eppendorf, Hamburg, Germany; 5 Department of Neuro- and Sensory Physiology, University Medical Center Göttingen, Göttingen, Germany; 6 Cluster of Excellence “Multiscale Bioimaging: from Molecular Machines to Networks of Excitable Cells” (MBExC), University of Goettingen, Göttingen, Germany; 7 Laboratory of Neurobiology, Max Planck Institute for Biophysical Chemistry, Göttingen, Germany

**Keywords:** Endocytosis, exocytosis, phosphorylation, synapse, SNARE, botulinum neurotoxins, phosphomimetic studies, synaptobrevin, syntaxin, cannabinoid receptor, AAV, adeno-associated virus, ACN, acetonitrile, AGC, protein kinase A, G, C kinase group, AGC, automatic gain control, AMPAR, α-amino-3-hydroxy-5-methyl-4-isoxazolepropionic acid receptor, AP, action potential, BoNT, botulinum neurotoxin, bRP, basic reversed-phase chromatography, CAA, chloroacetamide, CaMKII, calcium-calmodulin kinase 2, CDK, cyclin-dependent kinase, CK1, casein kinase 1, CK2, casein kinase 2, CLK, SRPK1 and Clk/Sty protein kinase, CMGC, CDK, MAP, GSK, CDKL kinase group, CREB1, CAMP-responsive element binding protein 1, DAPK, death-associated protein kinase, FA, formic acid, GABA, γ-aminobutyric acid, GluDH, glutamate dehydrogenase, GO, gene ontology, GRK, G-protein-coupled receptor kinase, GSK3, glycogen synthase kinase 3, ITR, inverted terminal repeat (sequence), MAPK, mitogen-activated protein kinase, NMDAR, N-methyl-d-aspartate receptor, PAK, p21-activated kinase, PKC, protein kinase C, PNGase F, peptide-N(4)-(N-acetyl-beta-glucosaminyl)asparagine amidase, POI, protein of interest, PP1, protein phosphatase 1, RAS, rat sarcoma gene, RT, room temperature, SNAP-25, synaptosomal-associated protein, 25kDa, SNARE, N-ethylmaleimide-sensitive factor-attachment protein receptors, STE, “sterile” serine/threonine protein kinases, SV, synaptic vesicle, TCEP, tris(2-carboxyethyl)phosphine, TEAB, triethylammonium bicarbonate buffer, TFE, trifluoroethanol

## Abstract

Synaptic transmission is mediated by the regulated exocytosis of synaptic vesicles. When the presynaptic membrane is depolarized by an incoming action potential, voltage-gated calcium channels open, resulting in the influx of calcium ions that triggers the fusion of synaptic vesicles (SVs) with the plasma membrane. SVs are recycled by endocytosis. Phosphorylation of synaptic proteins plays a major role in these processes, and several studies have shown that the synaptic phosphoproteome changes rapidly in response to depolarization. However, it is unclear which of these changes are directly linked to SV cycling and which might regulate other presynaptic functions that are also controlled by calcium-dependent kinases and phosphatases. To address this question, we analyzed changes in the phosphoproteome using rat synaptosomes in which exocytosis was blocked with botulinum neurotoxins (BoNTs) while depolarization-induced calcium influx remained unchanged. BoNT-treatment significantly alters the response of the synaptic phoshoproteome to depolarization and results in reduced phosphorylation levels when compared with stimulation of synaptosomes by depolarization with KCl alone. We dissect the primary Ca^2+^-dependent phosphorylation from SV-cycling-dependent phosphorylation and confirm an effect of such SV-cycling-dependent phosphorylation events on syntaxin-1a-T21/T23, synaptobrevin-S75, and cannabinoid receptor-1-S314/T322 on exo- and endocytosis in cultured hippocampal neurons.

## Highlights

• KCl-depolarization induces phosphoproteome changes in isolated nerve terminals (synaptosomes).
• Botulinum neurotoxin affects protein phosphorylation in depolarized synaptosomes.
• BoNT treatment reveals phosphorylation sites that depend on SV-cycling activity.
• SV-cycling-dependent sites on Vamp2, Stx1a, and Cnr1 affect exo- and endocytosis.

## Graphical Abstract

**Figure:**
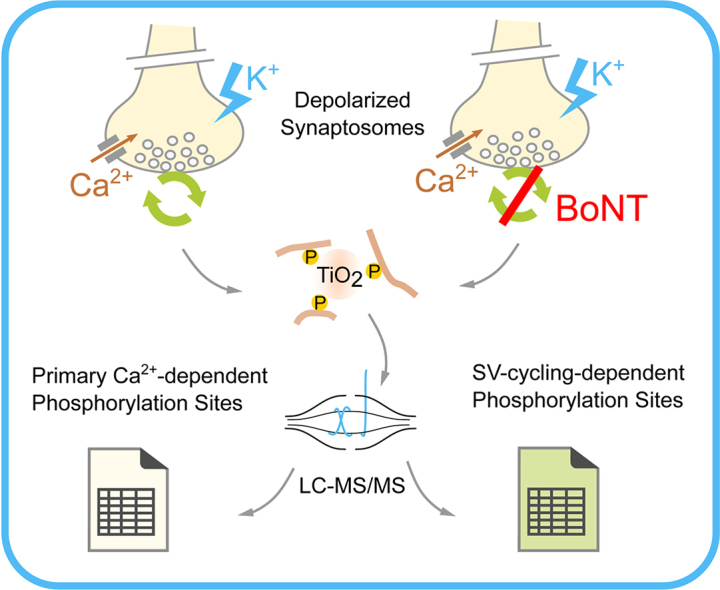

## In Brief

Analysis of protein phosphorylation in isolated nerve terminals (synaptosomes) treated with *C. botulinum* neurotoxins (BoNT) to inhibit synaptic vesicle (SV) cycling reveals phosphorylation events that are primarily dependent on depolarization-induced Ca^2+^ influx and those that also require active SV-cycling machinery. In particular, SV-cycling-dependent phosphorylation sites on synaptobrevin (*Vamp2*), syntaxin-1 (*Stx1a*), and cannabinoid receptor-1 (*Cnr1*) are capable of changing the rate of exo- and endocytosis in cultured hippocampal neurons.

In the nervous system, synapses represent specialized cellular junctions that are able to transmit signals from the presynaptic to the postsynaptic neuron. Signaling is mediated by the release of neurotransmitter molecules that are stored in the presynaptic compartment (1). When an action potential arrives in the nerve terminal, the presynaptic membrane depolarizes, which elicits a rapid influx of calcium ions through voltage-dependent calcium channels clustered around the active zone, an electron-dense protein complex connecting the presynaptic release site with the neurotransmitter receptors in the postsynaptic membrane (2). Major protein constituents of the active zone include RIM proteins, RIM-binding protein, and Munc13, as well as bassoon, piccolo, and liprins. Among other functions, these proteins are required for the clustering of calcium channels and govern the priming and docking of SVs. The calcium ions entering the presynapse are recognized by the calcium-sensor synaptotagmin 1 that triggers exocytosis by lowering the energy barrier for fusion. Exocytotic fusion itself is accomplished by soluble *N*-ethylmaleimide-sensitive factor-attachment protein receptors (SNARE) (3, 4, 5, 6). This process requires additional cytoplasmic proteins, such as Munc18 and complexins, which modulate the efficiency of SNARE zippering (7, 8).

In order to sustain continuous neurotransmitter release and to maintain the structure and composition of the plasma membrane, fused SVs are retrieved by endocytosis, followed by rapid regeneration of SV precursors that are filled with neurotransmitters and prepared for the next round of neurotransmitter release. It is still debated which mechanisms are primarily responsible for endocytosis and recycling, with several occurring in parallel (9, 10, 11, 12, 13, 14, 15, 16). The classical pathway of clathrin-mediated endocytosis requires coordinated actions of a protein machinery responsible for coat formation, invagination, and formation of a coated pit, followed by scission, removal of the vesicle from the plasma membrane, and disassembly of the clathrin coat. Key proteins involved in these steps include, among others, clathrin adaptor proteins (AP2, AP180), clathrin itself, the GTPase dynamin-1, the phosphatase synaptojanin-1, endophilins, and related proteins, as well as the clathrin uncoating ATPase Hsc70 and its cofactor auxilin (17). Finally, re-endocytosed SVs are filled with the respective neurotransmitter by specific vesicular neurotransmitter transporters that are fueled by the activity of a vacuolar proton pump (18). In this article, we refer to SV cycling as a multistep process, which includes SV fusion with the plasma membrane mediated by the exocytotic machinery (exocytosis) and SV recycling through available endocytotic mechanisms (endocytosis), followed by the steps in which SVs become ready for the next round of SV cycle (*i.e.*, refilling of the neurotransmitter, SV priming, and docking).

An increasing body of data suggests that the basic exo–endocytotic machinery is fine-tuned by regulatory control mechanisms that mainly operate by phosphorylations and dephosphorylations of proteins involved in the SV cycle. In particular, calcium plays an important role not only as a trigger of exocytosis, but also as a modulator of kinase and phosphatase activity in the presynapse (19). Calcium/calmodulin-dependent kinase 2 (CaMKII) residing in synapses is one of the major targets activated by calcium influx (20, 21, 22, 23). Its first known substrate was the SV resident protein synapsin-1, and it is now established that its phosphorylation influences the available pool of SVs for exocytosis (24, 25, 26). Other kinases such as protein kinase C (PKC) and cyclin-dependent kinase 5 (CDK5) have been shown to be important for SV endocytosis and control, together with the calcium-dependent protein phosphatase calcineurin, the phosphorylation status of many endocytosis-associated proteins such as dynamin-1, amphiphysin, and AP180 (27, 28, 29, 30, 31, 32, 33, 34, 35, 36). Mitogen-activated protein kinases (MAPK) appear to operate on parallel pathways as these can regulate synaptic plasticity either by directly modifying synaptic proteins (37, 38) or by affecting gene expression (39, 40), thereby contributing to both rapid and long-lasting modulatory effects.

Earlier phosphoproteomics studies have shown systematic changes in phosphorylation of synaptic proteins caused by depolarization-evoked calcium influx, and they have pointed to involvement of CaMKII and MAP kinases in this process (41, 42). However, it remains elusive which of the observed phosphorylation changes caused by calcium influx have a direct functional impact on SV cycling. Indeed, following the initial discovery of synapsin-1 phosphorylation, many studies have demonstrated that phosphorylation of specific synaptic proteins at specific sites does have an impact on exo- or endocytosis. For instance, phosphorylation of SNARE proteins (*Snap25*-S187, *Stx1a*-S14, S188, *Vamp2*-T35, S75), as well as of the SNARE-interacting protein Munc18 (*Stxbp1-*S313), can indeed interfere with the formation of the SNARE complex, which results in altered exocytosis (43, 44, 45, 46, 47, 48). Munc18 phosphorylation can further be a result of the MAP kinase activation due to cannabinoid receptor-1 signaling (37); the latter was also shown to bear a phosphorylation site (*Cnr1*-S317) that is controlled by PKC (49).

However, it remains to be established which of the vast amount of phosphosite changes observed in recent phosphoproteomic studies are dependent on the membrane trafficking steps of the SV cycle and which are, while being targeted by the activated network of kinases and phosphatases, not directly related to exo–endocytotic cycling. Changes in the protein conformation that accompany assembly and disassembly of multimolecular protein complexes during SV cycling may alter the accessibility of phosphorylation sites as well as the availability of docking sites for kinases and phosphatases. To address this issue, we conducted a quantitative phosphoproteome analysis using synaptosomes as a functional model of the synapse. Synaptosomes contain the complete SV-cycling machinery, can maintain the membrane potential and ATP at physiological levels, and respond to depolarization by Ca^2+^-dependent neurotransmitter release (50, 51, 52, 53). To differentiate between calcium-induced changes and phosphorylation events that are directly connected to SV cycling, we utilized botulinum neurotoxins (BoNT). These bacterial protein toxins represent a group of endoproteinases that selectively cleave individual SNARE proteins and thus block exocytosis and, consequently, SV recycling without compromising Ca^2+^ influx or synaptosomal ATP levels (54, 55). We stimulated mock- and BoNT-treated synaptosomes with potassium chloride, which is known to elicit dose-dependent Ca^2+^ influx and neurotransmitter release (56, 57). By quantitatively monitoring changes of phosphorylation sites of synaptosomal proteins, we found that BoNT treatment affects almost 1500 phosphorylation sites implying that they are directly dependent on SV cycling. Strikingly, most of the sites show reduced phosphorylation intensity in depolarized synaptosomes following BoNT treatment. We further identified SV-cycling-dependent phosphorylation sites on syntaxin-1a (*Stx1a*-T21, T23), synaptobrevin (*Vamp2*-S75), and cannabinoid receptor-1 (*Cnr1*-T314, T322). Finally, we demonstrated that phosphorylation of these sites elicits a pronounced effect on exo- and endocytosis in cultured hippocampal neurons by expressing phosphomimetic and nonphosphorylatable variants of these proteins.

## Experimental Procedures

### Ethical Statement

All experiments involving animals complied with the regulations as designated in the section 4 of the Animal Welfare Law of the Federal Republic of Germany (section 4 Tierschutzgesetz der Bundesrepublik Deutschland, TierSchG). All experiments were conducted in the animal facility at the Max-Planck-Institute for Biophysical Chemistry, Göttingen, Germany, and registered accordingly to the section 11 Abs. 1 TierSchG as documented by 39 20 00_2a Si/rö, dated Dec 11, 2013 (“Erlaubnis, Wirbeltiere zu Versuchszwecken zu züchten und zu halten”) by the Veterinär-und Verbraucherschutzamt für den Landkreis und die Stadt Göttingen and examined regularly by the supervisory veterinary authority of the Landkreis Göttingen. All procedures were supervised by the animal welfare officer and the animal welfare committee of the Max-Planck-Institute for Biophysical Chemistry, Göttingen, Germany, established in accordance with the TierSchG and the regulation of animal experiments, dated Aug 31, 2015 (Tierschutz-Versuchstier-Verordung, TierSchVersV).

### Chemicals

LC/MS-grade water, methanol, and acetonitrile (ACN) were used in this study if not otherwise stated and were purchased together with chloroform, 25% (v/v) NH_4_OH, CaCl_2_, KCl, KH_2_PO_4_, MgCl_2_, NaCl, NaHCO_3_, NaHPO_4_, sucrose, and glucose from Merck, Darmstadt, Germany. Trifluoroethanol (TFE), triethylammonium bicarbonate buffer (TEAB), formic acid (FA), EGTA, PM400-Ficoll (Ficoll), NADP, L-Glutamic dehydrogenase from bovine liver (GluDH), guanidine hydrochloride, tris(2-carboxyethyl)phosphine (TCEP), chloroacetamide (CAA), Triton-X100, and MS-grade trypsin were obtained from Sigma-Aldrich, Taufkirchen, Germany. HEPES was purchased from VWR Chemicals, Darmstadt, Germany. SDS was purchased from Serva Electrophoresis GmbH, Heidelberg, Germany. TFA was obtained from Roth, Karlsruhe, Germany. Peptide-N(4)-(N-acetyl-beta-glucosaminyl)asparagine amidase (PNGase F) was obtained from Roche, Mannheim, Germany. Rapigest was obtained from Waters, Milford, USA. Pierce 660 nm protein assay, Halt Protease and phosphatase inhibitor cocktail, and TMTsixplex isobaric labeling reagents (TMT6) were purchased from Thermo Fisher Scientific, Bleiswijk, Netherlands. *C. botulinum strain A–D* cell culture supernatants (BoNT A, B, C1, D) were obtained from Miprolab, Göttingen, Germany.

### Isolated Nerve Terminals

Isolated nerve terminals were prepared from brains of 5–6 weeks old Wistar rats as described previously (58). Briefly, brains were quickly homogenized in ice-cold homogenization buffer (320 mM sucrose, 5 mM HEPES is water) using a Teflon/glass homogenizer. The homogenate was cleared by centrifugation for 2 min at 2988×*g* in an SS-34 fixed angle rotor (Thermo Fisher Scientific, Waltham, USA). The supernatant was collected and recentrifuged for 12 min at 14,462×*g*. The synaptosomal pellet was resuspended in the homogenization buffer and loaded onto discontinuous Ficoll gradient (6%/9%/13% (wt/v) Ficoll in the homogenization buffer) and centrifuged for 35 min at 86,575×*g* in an SW-41 swinging bucket rotor (Beckman Coulter, Krefeld, Germany). The synaptosomal band at the interface between 9% and 13% Ficoll was collected and washed with the homogenization buffer. Synaptosomal protein concentration was estimated using Pierce 660 nm protein assay according to manufacturer’s instructions. The viability of synaptosomes was confirmed by glutamate release assay (59).

### BoNT Treatment and Glutamate Release Assay

In total, 1–1.5 ml of synaptosomal suspension was centrifuged at 6900×*g* for 3 min in a table-top centrifuge, and synaptosomal pellet was resuspended in the sodium-containing buffer (10 mM glucose, 5 mM KCl, 140 mM NaCl, 5 mM NaHCO_3_, 1 mM MgCl_2_, 1.2 mM NaHPO_4_, 20 mM HEPES in water) to achieve a final concentration of 1 mg/ml of synaptosomal proteins. CaCl_2_ was added to a final concentration of 1.3 mM, and synaptosomes were incubated for 5 min at 37 °C before adding BoNT. For Ca versus EGTA experiments, synaptosomes were preincubated with 1.3 mM CaCl_2_ or 0.5 mM EGTA for 15 min at 37 °C before stimulation with KCl (50 mM). To block SNARE-assisted neurotransmitter release, 20 μl of each BoNT was added per 1 mg of estimated synaptosomal protein (the final BoNT concentration of 100–200 nM was estimated on the basis of the total protein content of the semipurified BoNT, which amounted to approximately 10 μg/μl). The following combinations of *C. botulinum* cell culture supernatants were used: (i) *C. botulinum* A and D or (ii) *C. botulinum* C1 and B. Alternatively, synaptosomes were treated with the same amount of respective cell culture supernatants inactivated by heating at 95 °C for 1 h (mock control). BoNT- and mock-treated synaptosomes were incubated at 37 °C for 90 min and then directly subjected to the glutamate release assay. In brief, synaptosomal suspension was transferred into a quartz glass cuvette (Hellma, Müllheim, Germany) and kept stirred at 37 °C during the measurements. In total, 1 mM NADP and 50 U per 1 mg of synaptosomal protein of GluDH were stepwise added to the suspension. Synaptosomes were depolarized by adding KCl to a final concentration of 50 mM. NADPH-fluorescence at 440 nm was monitored at each step using Fluorolog-3 fluorometer (Horiba Jobin Yvon, Bensheim, Germany). After 2 min of stimulation, the reaction was quenched with an equal volume of lysis buffer (6 M Guanidine hydrochloride, 200 mM HEPES, 20 mM TCEP, 80 mM CAA, 1 × Halt Protease, and phosphatase inhibitor cocktail). The synaptosomal sample was incubated for 5 min at 95 °C, chilled on ice, and then sonicated for 10 min using 30 s on/30 s off cycles at the maximum output of Bioruptor ultrasonication device (Diagenode, Seraing, Belgium). The sample was repeatedly incubated for 5 min at 95 °C, and proteins were precipitated following methanol/chloroform protein precipitation method (60).

### Phosphopeptide Enrichment and TMT Labeling

Precipitated synaptosomal proteins (1–2 mg per condition) were redissolved in a digestion buffer (100 mM TEAB, 10% TFE, 0.1% Rapigest) and sonicated in the Bioruptor for 10 min at maximum intensity using 30 s on/30 s off cycles. Trypsin was added at 1:40 trypsin-to-protein ratio (wt/wt), and proteins were digested overnight at 37 °C. Next day, the sample was treated with 2.5 U of PNGase F per 1 mg of the initial protein amount for 1 h. Afterward, ACN was added to a final concentration of 45% (v/v), and the sample was incubated with a second portion of trypsin (1:100 trypsin-to-protein ratio, wt/wt) for 1 h. The sample was centrifuged for 15 min at 17,000×*g* in a table-top centrifuge, and the cleared supernatant was subjected to phosphopeptide enrichment as previously described with modifications (61). Briefly, equal input peptide amounts were assured by measuring peptide concentration in Nanodrop-1000 (Thermo Fisher Scientific, Waltham, USA) using an A280 method. KCl, KH_2_PO_4_, and TFA were added to the peptide sample to reach final concentrations of 228 mM, 3.9 mM, and 38% (v/v), respectively. Peptides were incubated with TiO_2_ beads (GL Sciences, Tokyo, Japan) at 1:10 protein-to-beads ratio for 20 min at 40 °C. Unbound peptide fraction (nonphosphorylated peptides) was collected, and TiO_2_ beads were subjected to four washing steps using washing buffer (60% (v/v) ACN, 1% (v/v) TFA in water). Bound phosphopeptides were eluted using 3.75% (v/v) NH_4_OH 40% (v/v) ACN in water, snap-frozen in liquid nitrogen, and dried in a centrifugal Savant SpeedVac vacuum concentrator (Thermo Fisher Scientific, Waltham, USA). Dried phosphopeptides and peptides in the unbound fraction were subjected to desalting using prepacked C18 spin columns (Harvard Apparatus, Holliston, USA). Desalted and dried peptides were redissolved in 50 mM TEAB 50% (v/v) ACN in water, and TMT6-labeling reaction was conducted according to manufacturer’s instructions. After the labeling, peptide samples were accordingly combined, cleaned using the prepacked C18 spin columns, and concentrated in a SpeedVac.

### Basic Reversed-Phase (bRP) Chromatography

TMT6-labeled peptides were fractionated using Agilent 1100 series HPLC system (Agilent, Santa Clara, USA) equipped with a C18-X-Bridge column (3.5 μm particles, 1.0 mm inner diameter, 150 mm length; Waters, Milford, USA). The HPLC was operated at the flow rate of 60 μl/min under basic pH (buffer A: 10 mM NH_4_OH in water, pH ∼10; buffer B: 10 mM NH_4_OH and 80% (v/v) ACN in water, pH ∼10). The column was equilibrated with the 95% buffer A and 5% buffer B mixture. Peptides were resolved using a linear gradient ranging from 10% to 35% buffer B for 35 min followed by a linear increase to 60% over 5 min and a washing step at 90% buffer B for 5 min. One-minute fractions were collected and concatenated into 24 (for Mock/BoNT phosphorylated peptides) or 12 (for Mock/BoNT nonphosphorylated peptides and Ca/EGTA phosphorylated peptides) final fractions as suggested elsewhere (62). BRP fractions were snap-frozen in liquid nitrogen and dried in the SpeedVac.

### LC-MS/MS

Dried peptides were redissolved in 2% (v/v) ACN 0.1% (v/v) TFA in water. Each concatenated BRP fraction was analyzed in triplicates (phosphorylated peptides) or as a single injection (nonphosphorylated peptides). Dissolved peptides were injected onto a C18 PepMap100-trapping column (0.3 x 5 mm, 5 μm, Thermo Fisher Scientific, Waltham, USA) connected to an in-house packed C18 analytical column (75 μm x 300 mm; Reprosil-Pur 120C18-AQ, 1.9 μm, Dr Maisch GmbH, Ammerbuch, Germany). The columns were pre-equilibrated using a mixture of 98% buffer A (0.1% (v/v) FA in water), 2% buffer B (80% (v/v) ACN, 0.1% (v/v) FA in water), or 95% buffer A, 5% buffer B (nonphosphorylated peptides). Liquid chromatography was operated on an UltiMate-3000 RSLC nanosystem (Thermo Fisher Scientific, Waltham, USA). Phosphopeptides were eluted using a 60 min-linear gradient ranging from 10% to 25% buffer B (80% (v/v) ACN, 0.1% (v/v) FA in water) followed by a linear increase to 45% buffer B over 10 min and a washing step at 90% of buffer B for 5 min. For nonphosphorylated peptides, a steeper gradient was applied ranging from 10% to 45% buffer B followed by an increase to 60% buffer B and a washing step at 90% buffer B. Eluting peptides were sprayed into a QExactive HF-X or Orbitrap Exploris 480 (Thermo Fisher Scientific, Bremen, Germany). For the analysis of phosphorylated peptides, MS1 scans (350–1600 m/z) were acquired with a resolution of 120,000 at 200 m/z, 3e6 automatic gain control (AGC) target, and 50 ms maximum injection time. Precursor ions were isolated using a 0.8 m/z isolation window, and a normalized collision energy of 33% was applied to obtain fragment spectra. Only charge states from 2+ to 6+ were considered, and the dynamic exclusion was set to 25 s. The fragment spectra were acquired with a resolution of 30,000, 1e5 AGC target, and 130 ms maximum injection time. For the analysis of nonphosphorylated peptides, following parameters were set differently: MS1 scans were acquired with a resolution of 60,000 at 200 m/z, 300% normalized AGC target. Fragment spectra were acquired with the resolution of 15,000 at 200 m/z, 50% of the normalized AGC target and 54 ms maximum injection time.

### Data Analysis

Raw files were processed using MaxQuant version 1.6.10.2 (63, 64) under default settings with some exceptions. Specifically, cysteine carbamidomethylation was selected as a fixed modification, whereas methionine oxidation, acetylation of protein N termini, and phosphorylation of serine, threonine, and tyrosine (only for phosphorylated peptides) were set as variable modifications. Up to five variable modifications were allowed per peptide. Specific trypsin digestion was selected with up to two missed cleavage sites allowed per peptide. Maximal peptide mass was increased to 6000. Quantification using reporter ions in MS2 (TMT6plex) and minimal parent ion fraction of 0.75 were selected. MS1 and MS2 mass tolerances were kept at 4.5 ppm and 20 ppm, respectively. Canonical amino acid sequences of *Rattus norvegicus* proteins were retrieved from Uniprot (65) (February 2019, 29,951 entries). Reference proteome of *C. botulinum* (strain Hall/ATCC 3502/NCTC 13319/Type A) containing canonical protein sequences (Uniprot, January 2020, 3590 entries) was supplemented with sequences of BoNT B-D (strain Okra/Type B1, *C. botulinum* C phage, *C. botulinum* D phage, respectively). Peptide-spectrum matches (PSM) and protein FDR threshold were kept at 0.01. Following steps in data analysis were conducted in R statistical programming language using custom scripts. The scripts are available at GitHub (https://github.com/IvanSilbern/2021_Silbern_etal_Synapt_BoNT_KCl). For each phosphorylation site, a leading protein was selected based on the list of potential candidate proteins for this site reported by MaxQuant. Proteins were ranked based on (listed from the most to the least significant) (i) the number of the unique phosphorylation sites assigned to the protein in the data set; (ii) the number of all phosphorylation sites per protein in the data set; (iii) “reviewed” status and Uniprot-annotation score of the protein sequence; (iv) isoform status of the protein sequence. The official gene name and Uniprot accession of the leading protein were used in the subsequent analyses. Impurity-corrected reporter ion intensities for each phosphorylation site identified at one of the three multiplicity levels were extracted from MaxQuant “Phospho(S, T, Y).txt” output table. Potential contaminants, reversed sequences, and phosphorylation sites identified with localization probability <0.75 as determined by MaxQuant (63, 64) were excluded from further analysis. Phosphorylation sites quantified at different multiplicity levels (“1” means that the phosphorylation site was quantified on singly, “2” on doubly, and “3” on multiply phosphorylated peptides) were considered as separate entities or phosphorylation events. If a phosphorylation event contained <3 nonzero intensity values for a given labeling experiment, the event was considered as not quantified in this labeling experiment. Otherwise, missing values were imputed for each TMT channel/experiment pair individually by random sampling from a Gauss distribution with a mean at the 5% quantile and a double-standard deviation of the log-transformed intensities. Only a minor proportion of phosphorylation events required imputation (<1% per channel). Log_2_-transformed reporter ion intensities were then normalized using Tukey median polishing individually for each labeling experiment and subjected to statistical testing using limma package (66).

### Experimental Design and Statistical Rationale

In total, 216 .raw files were analyzed in order to quantify changes in phosphorylation site intensities in BoNT- or mock-treated synaptosomes or synaptosomes stimulated in the presence of calcium or EGTA. Out of the 216 .raw files, 144 .raw files were obtained from two TMT6-labeling sets of mock- versus BoNT-treated synaptosomes. These files correspond to 24 concatenated BRP-fractions and three injection replicates per fraction. Remaining 72 .raw files were obtained from another two TMT6-labeling experiments of synaptosomes stimulated in the presence of Ca^2+^ or EGTA. The files correspond to 12 concatenated bRP-fractions and three injection replicates per fraction. Each set of TMT6-labeled samples contained three independent replicates for each of the two treatment conditions, resulting in six independent replicates for each treatment regime (Mock, BoNT, Ca, or EGTA). The choice for this number of replicates was dictated by the constraints of the TMT6 labeling and the previous study (41). The phosphorylation site intensities resulting from the three technical replicates were summed in order to obtain a single value reported by MaxQuant (64). We used log_2_-transformed and normalized reporter ion intensities to assess changes in the phosphorylation site intensities. Specifically, we used the limma package (66) to test the difference in phosphorylation site intensities between mock- and BoNT-treated synaptosomes and synaptosomes stimulated in the presence of calcium or EGTA. Linear models were specified to account for treatment differences (Mock, BoNT, Ca, EGTA) and batch effects between TMT6 sets. Differences in the peptide intensities between mock- and BoNT-treated synaptosomes and synaptosomes stimulated in the presence/absence of calcium were tested. Empirical-Bayes moderated *p*-values (66) were subjected to multiple testing correction using q-value approach (67). Phosphorylation events (phosphorylation site at a given multiplicity) were considered as significantly regulated when showing at least 1.2 intensity fold change (FC, corresponds to absolute log_2_ FC > 0.263) and q-value < 0.01 (corresponds to false discovery rate (FDR) < 1%).

### Defining Primary Ca^2+^-Dependent and SV-Cycling-Dependent Phosphorylation Sites

Phophorylation events were defined as “primary Ca^2+^-dependent” if they show absolute log_2_ (Ca/EGTA) > 0.263 and absolute log_2_ (Mock/BoNT) < 0.263 or were not quantified in the Mock/BoNT experiment. Conversely, events showing absolute log_2_ (Mock/BoNT) > 0.263 were termed as “SV-cycling-dependent.” Phosphorylation events that exhibited only small changes in both experiments (absolute log_2_ (Ca/EGTA) < 0.263 and absolute log_2_ (Mock/BoNT) < 0.263) were considered as being “not-affected.” In the following analyses, only phosphorylation events that surpassed an FDR-threshold of <1% were considered. Specifically, “primary Ca^2+^-dependent” phosphorylation events had to satisfy a q-value threshold of <0.01 in the Ca/EGTA experiment, whereas “SV-cycling-dependent” sites had to meet a q-value threshold of <0.01 in Mock/BoNT experiment or a q-value-threshold of <0.01 and an absolute log_2_ FC threshold of >0.263 in Ca/EGTA experiment. Phosphorylation events that did not match these criteria were considered as being “not-affected” as well. If a phosphorylation site was quantified at different multiplicities (phosphorylation events), whereby one of which was classified as being “SV-cycling-dependent,” the site was considered as being “SV-cycling-dependent.” To define regulation groups at protein level, number of significantly regulated phosphorylation sites were counted per protein (gene). Proteins were distributed in the following groups (i) “mostly primary Ca^2+^-dependent” (>60% of sites being categorized as “primary Ca^2+^-dependent”); (ii) “mostly SV-cycling-dependent” (>60% of sites being categorized as “SV-cycling-dependent”); (iii) “mixed” (none of the groups was dominating).

### Bioinformatic Analysis

Gene enrichment analyses were conducted using gene names of proteins carrying significantly regulated phosphorylation sites in Ca/EGTA or Mock/BoNT experiments. Gene ontology enrichment analysis was performed using David web interface and whole *R. norvegicus* proteome as a background (68). Direct GO term categories surpassing Benjamini–Hochberg-adjusted *p*-value threshold of 0.001 were considered for further analysis. For reactome pathway analysis, only enriched (Benjamini–Hochberg-adjusted *p*-value < 0.1) “neuronal system” categories were selected (69). Synapse-specific SynGO database was used to retrieve significantly enriched (GSEA “gene cluster” FDR corrected *p*-value < 0.001) GO-biological function terms (70). Experimentally validated kinase–substrate interactions were derived from PhosphoSitePlus database (“Kinase_Substrate_Dataset.txt”, version of 10.04.2018) (71). In order to account for possible differences in phosphorylation site annotation, sequence windows of the phosphorylation sites in the data set were aligned against sequence windows reported in the PhosphoSitePlus database using BLAST+ software (version: 2.7.1, additional command line parameters: -max_target_seqs 20) (72). Only exact alignments of the phosphorylation sites showing the minimum bitscore of 20 were considered. The best match was selected from filtered alignments ranked based on the alignment bitscore, number of amino acid positions considered identical (nident), number of open sequence gaps, kinase, and substrate species (rat > mouse > human > rabbit). Phosphorylation sites with no assignment were subjected to NetworKIN kinase–substrate prediction tool (73, 74). Specifically, rat protein sequences were aligned against the reference set of human protein sequences (STRING (75) database version 9.05) by using BLAST+ (additional command line parameters: -max_target_seqs 40 -word_size 6 -gapopen 12 -gapextend 1). The best alignment for each rat protein sequence was selected based on the rat protein sequence coverage, number of amino acid positions considered identical, alignment bitscore, number of open sequence gaps (listed with decreasing importance). Positions of phosphorylation sites in the best matching human protein sequences were subjected to NerworKIN tool. The kinase prediction with the highest NetworKIN score was selected for each phosphorylation site. Predicted kinases were organized in the kinase groups introduced by NetPhorest classification (73). The second-level and top-level kinase groups in the kinase hierarchy were used for enrichment analyses and visualizations. The overrepresentation analysis of predicted kinase–substrate interactions for phosphorylation sites significantly regulated in Ca/EGTA or Mock/BoNT experiments was conducted in comparision with NetworKIN kinase–substrate predictions for human proteome. To assess the disbalance in the number of up/downregulated phosphorylation events, the number of up/downregulated phosphorylation events controlled by each kinase group was compared with the total number of up/down phosphorylation events among significantly regulated phosphorylated sites. All tests of differential term or kinase enrichment between two groups were done using Fisher’s exact test. Benjamini–Hochberg adjusted *p*-values are reported throughout the study if not specified otherwise. The anticipated phosphatase–substrate interactions rely upon described protein phosphatase-1(PP1) and Calcineurin docking motifs (76). To demonstrate possible phosphatase–substrate connections, interaction maps were extracted from protein–protein interactions annotated for *R. norvegicus* (STRING (75) database version 10.5). Shortest paths from phosphatase to target proteins (phosphoproteins involved in endo/exocytosis and containing PP1 or Calcineurin docking motif) were computed using *shortest_path* function from *igraph* package (77) (version 1.4.6). Only proteins previously identified in synaptosomes and interactions with experimental evidence or annotated in curated databases were included in the analysis. Phosphorylation site occupancy was calculated using a 3D model presented by Hoegrebe *et al* (78). In brief, a linear model of the form *Ph = m*_*1*_*∙Prot – m*_*2*_*∙NPh*, where *Ph* is the normalized (by median polishing) intensity of the phosphorylated peptide; *Prot* is the normalized protein intensity, and *NPh* is the normalized intensity of nonphosphorylated peptide. The model was fit by using intensity data from Mock/BoNT experiments. Phosphorylation occupancy was then calculated using the following formula: *Occupancy = a/(1+a)*, where *a = m*_*2*_*∙Ph/NPh*. “Illegal” stoichiometry values, *i.e.*, values outside the interval [0;1] were excluded from the analysis. Only *m*_*2*_ values with a *p* value below 0.1 were considered.

### *C. botulinum* Cell Culture Supernatants Profiling

For protein profiling, supernatants of *C. botulinum* cell culture were digested overnight using trypsin. Digested peptides were desalted using prepacked C18 spin columns. Samples were analyzed on QExactive HF-X and liquid chromatography setup as described above.

To test for unspecific phosphatase activity, *C. botulinum* cell culture supernatants were incubated with the nuclear extract of HeLa cells. The nuclear extract was prepared as previously described (79) in Roeder D buffer (20 mM Hepes-KOH pH 7.9, 100 mM KCl, 1.5 mM MgCl_2_, 10% (v/v) glycerol, 0.5 mM DTT, 0.5 mM PMSF, 0.2 mM EDTA) and was a generous gift of Prof. Dr Lührmann laboratory. The extract was supplemented with 1.3 mM CaCl_2_ and 20 μl of each *C. botulinum* cell culture supernatant (A and D or C1 and B), heat-inactivated cell culture supernatants, or 40 μl of the sodium buffer per 1 mg of nuclear proteins. Each treatment condition was performed in triplicate, resulting in a total of nine samples. The samples were incubated for 1.5 h at 37 °C. Afterward, urea in 100 mM HEPES pH 8 was added to the final concentration of 1 M and proteins were digested overnight at 30 °C using MS-grade trypsin at a trypsin-to-protein ration (wt/wt) of 1:50. Next day, peptides were subjected to phosphopeptide enrichment as described above excluding the deglycosylation and second endoproteinase digestion steps. Enriched phosphopeptides were cleaned using the prepacked C18 spin columns, redissolved in 2% (v/v) ACN, 0.1% (v/v) TFA in water, and analyzed on UltiMate 3000 RSLC nanosystem connected to Orbitrap Exploris 480 mass spectrometer. Raw phosphorylation site intensities as reported by MaxQuant were analyzed as described before using R scripts and the limma package for data normalization, visualization, and statistical testing.

### Calcium Influx in Synaptosomes Following BoNT Treatment and KCl Stimulation

Synaptosome suspensions containing 1 mg of synaptosomal proteins were prepared as described for glutamate release assay. Suspensions were supplemented with 1.3 mM CaCl_2_ and additional 40 μl of the sodium-containing buffer or 1.3 mM CaCl_2_ and 20 μl of each *C. botulinum* cell culture supernatants in two combinations (A and D or C1 and B). Synaptosomes were then incubated for 1 h at 37 °C under mild agitation. Afterward, synaptosome suspensions were supplemented with 5 μM of Fura-2AM dye (Molecular probes, Eugene, USA) and incubated for additional 30 min at 37 °C. Following steps were conducted as previously described (80). Briefly, synaptosomes were resuspended in 1 ml of fresh sodium-containing buffer and loaded into the quartz glass cuvette. The synaptosomal suspension was stirred and kept at 37 °C during the measurements. The fluorescence was continuously measured using Fluorolog-3 at excitation wavelengths of 340 and 380 nm and emission at 505 nm, for 2 s at each wavelength. In total, 1.3 mM CaCl_2_, 50 mM KCl, 0.4% (wt/v) SDS, 1 mM EGTA were added stepwise and the signal was acquired for 3 min at each step. Fluorescence intensity at 340 nm excitation was divided by the intensity at 380 nm excitation and then by the maximum fluorescence ratio following SDS lysis. Median fluorescence ratio before stimulation with KCl was subtracted to obtain proportional changes in fluorescence intensity relatively to its maximum.

### Cell Cultures and Transfections

Primary hippocampal neuronal cultures were prepared from P2 rats as previously described (81). HEK293 T cells were obtained by a commercial supplier (Sirion Biotech GmbH; Germany). HEK293 T cells were grown in DMEM supplemented with 10% FBS, 4 mM L-glutamine, and 600 U/ml penicillin-streptomycin (Lonza). For transfection Lipofectamine 2000 transfection reagent was used following the guidelines of the manufacturer (Thermo Fisher Scientific, Invitrogen, USA). All plasmids for transfection were produced in bacteria and purified with the NucleoBond Xtra Midi endotoxin-free plasmid DNA kit (Macherey-Nagel, Germany).

### DNA Cloning of Wild-Type Proteins and Respective Phosphomimetic Mutants

All the sequences (including mutants) were designed *in silico* based on rat genes with the appropriate restriction enzymes for viral cloning and ordered at GenScript (USA). The synaptobrevin (*Vamp2*) mutants were based on rat *Vamp2* (Uniprot: P63045) fused to the optimized version of superecliptic GFP (pHluorin) (82) by mean of a short peptide linker. We used AgeI and SdaI (SbfI) restriction sites for inserting *Vamp2*-pHluorin and the respective mutants (S75D pseudo-phosphorylated and S75A non-phosphorylatable) in the viral vector (previously described, (83)) allowing neuron-specific expression under the human synapsin-1 promoter. For all the following subcloning steps, we relied on commercial chemically competent SURE bacteria from Agilent, to avoid the loss of ITR sequences. Positive clones were confirmed by sequencing, and before transfection in the packaging HEK293 T cells, the presence of ITRs and the correct length of all plasmids were checked by restriction enzyme analysis. Syntaxin-1a (*Stx1a*; P32851) and cannabinoid receptor 1 (*Cnr1*; P20272) and their mutants (respectively T21E-T23 E pseudo-phosphorylated *Stx1a*, T21A-T23 A nonphosphorylatable *Stx1a*, T314E-T322 E pseudo-phosphorylated *Cnr1* and T314A-T322 A nonphosphorylatable *Cnr1*) were inserted in the WT *Vamp2*-pHluorin viral vector by cutting with the enzymes SgsI (AscI) and SbfI (SdaI). Their expression was driven by mean of a second human synapsin-1 promoter from the same viral plasmid to allow consistent coexpression. To distinguish these proteins from the endogenous, an ALFA-tag was added (84). Also in this case, positive clones were confirmed by sequencing, and before transfection in the packaging HEK293 T cells, the presence of ITRs and the correct length of all plasmids were checked by restriction enzyme analysis. All synthetic plasmids and their respective sequences are available from the authors upon request.

### Immuno Staining with NbALFA

The expression of *Stx1a* and *Cnr1* in all viruses and mutants was confirmed by staining with the NbALFA (84). Briefly, neurons were fixed with 4% paraformaldehyde (PFA; w/v) for 30 min at room temperature (RT). Cells were quenched in 100 mM NH_4_Cl and permeabilized in PBS containing 4% bovine serum albumin (BSA; wt/v) and 0.1% (v/v) Triton-X 100 for 15 min at RT. Fluorescently labeled NbALFA (FluoTag-X2 anti-ALFA AbberiorStar635P, NanoTag Biotechnologies #N1502-Ab635P-L, Germany) was diluted 1:200 in blocking solution for 1 h at RT and subsequently washed three times for 5 min with PBS. Coverslips were mounted on glass slides using Mowiol solution, dried, and imaged. Note that superecliptic GFP (pHluorin) keeps its fluorescence in Mowiol solution since its pH is ∼7.5 and can thus be imaged with no additional staining.

### Adeno-Associated Virus Preparation

Recombinant AAV particles were prepared as previously described (83). Briefly, vectors were packaged in HEK293 T cells using the pDP6 helper plasmid (avoiding adenoviral contamination). Packaging cells were lysed in Tyrode’s solution (124 mM NaCl, 5 mM KCl, 30 mM glucose, 25 mM HEPES, 2 mM CaCl_2_, 1 mM MgCl_2_, pH 7.4), passed through 0.22 μm syringe filters, and viral titer was adjusted by serial dilution on primary hippocampal neurons monitoring pHluorin levels (always expressed under the neurospecific human synapsin-1 promoter).

### Synaptic Vesicle Exo-/Endocytosis Measurements

SV exo/endocytosis assays were performed analogously to what previously described (81, 85). Briefly, neurons were field-stimulated with platinum electrodes in custom-made chambers housing 18 mm coverslips (8 mm distance between electrodes). We used a Stimulus Isolator combined with an A310 Accupulser Stimulator (both from World Precision Instruments, Sarasota, USA) with a nominal output of 100 mA for stimulation. In the case of *Stx1a* and *Cnr1* coexpression was confirmed by imaging. Live experiments were performed in Tyrode’s solution (124 mM NaCl, 5 mM KCl, 30 mM glucose, 25 mM HEPES, 2 mM CaCl_2_, 1 mM MgCl_2_, pH 7.4) supplemented with 10 μM 6-cyano-7-nitroquinoxaline-2,3-dione (CNQX; Tocris Bioscience, Cambridge, UK) and 50 μM 2-amino-5-phosphonopentanoic acid (D-AP5; Tocris Bioscience, Cambridge, UK) to avoid spontaneous network activation. At the end of the imaging, 50 mM NH_4_Cl was applied to evaluate total SV content. Live imaging was performed with an inverted Nikon Ti epifluorescence microscope (Nikon, Tokyo, Japan) equipped with a Plan Apochromat 60x 1.4 NA oil-immersion objective, an HBO-100W Lamp, an IXON X3897 Andor camera (Northern Ireland, UK), and an OKOLab cage incubator system (OKOLab, Ottaviano, Italy) to maintain a constant temperature of 37 °C. ND2 Images were imported using the Bio-Formats (OME) plug-in. For quantifications, the fluorescence intensity was measured with custom-made Matlab (USA) code that can be provided on an individual basis if requested. Briefly, multiple regions containing synaptic boutons were manually selected, in 5–8 independent experiments. Between 140 and 345 regions were selected for each condition, over all independent experiments. The *Vamp2*-pHluorin signal was then measured in the respective regions, and the background intensity (obtained in identically sized regions from the neuron-free adjoining regions) was subtracted. The *Vamp2*-pHluorin signal curves were then normalized to the maximum intensity obtained during the addition of NH_4_Cl. To compare easily different conditions and mutant variants, all curves were furthermore normalized to their initial (prestimulus) baselines. Statistical differences were tested in Matlab, relying on Kruskal–Wallis tests, followed by Tukey–Kramer post-hoc tests.

## Results

**Fig. 1:**
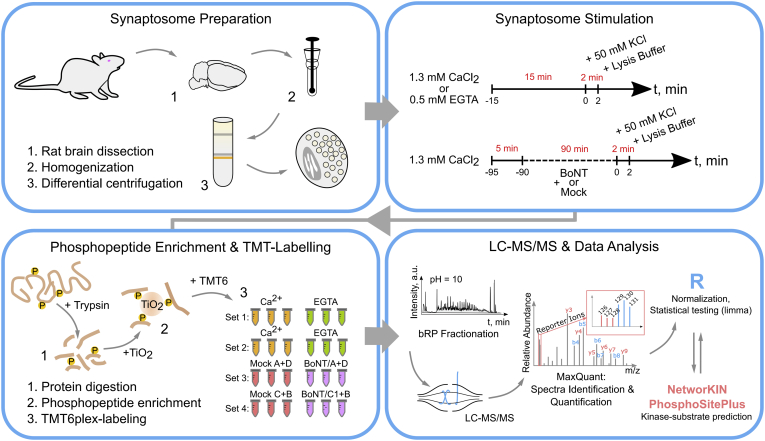
**Workflow for the phosphoproteome analyses of synaptosomes under different stimuli.** Brains of 5–6 week-old Wistar rats were homogenized and the synaptosomal fraction was enriched by differential centrifugation in discontinuous Ficoll gradient. Two sets of experiments were performed that compared a) stimulation of synaptosomes in the presence of Ca^2+^ (1.3 mM) or Ca^2+^-chelator (EGTA, 0.5 mM), or b) stimulation of synaptosomes pretreated with inactivated toxins (Mock), or a combination of two botulinum toxins (BoNT, A + D or C1+B). Synaptosomes were depolarized by 50 mM KCl for 2 min. Each experiment included three independent control stimulations (EGTA or Mock-treated) and three independent test stimulations (Ca or BoNT). Samples were processed in a conventional bottom-up proteomics workflow and phosphorylated peptides were enriched by metal-oxide chromatography (TiO_2_). Replicates within one experiment were labeled with a set of TMT6 isobaric labeling reagents. A pooled peptide sample was fractionated by reversed-phased chromatography at basic pH and measured in a high-resolution quadrupole-orbitrap mass spectrometer. Reporter ion intensities were utilized to infer relative abundances of phosphorylated peptides.

We performed a quantitative in-depth analysis of changes in protein phosphorylation events in active synaptosomes. We first quantitatively compared the phosphorylation status of proteins in chemically stimulated synaptosomes under Ca^2+^-deprived and free Ca^2+^ conditions; for the latter, depolarization of the plasma membrane of synaptosomes was induced by KCl in buffer containing EGTA or CaCl_2_ (Fig. 1). The viability of the synaptosomes was confirmed by monitoring release of the neurotransmitter (glutamate) (59) (supplemental Fig. S1*A*). We applied depolarization for 2 min; this duration was chosen in order (i) to achieve comparability with our previous studies (41) and (ii) to allow the capture of short- and medium-term changes in phosphorylation. Proteins from stimulated and nonstimulated synaptosomes were extracted, digested, and enriched for phosphopeptides in parallel; thereafter, the (phospho)peptides were labeled with unique isotopically labeled TMT6 reagents. Peptides were then pooled, prefractionated by basic reverse-phase chromatography, and finally analyzed by LC-MS/MS (Fig. 1). In the second approach, we inhibited SV cycling using BoNT and monitored quantitatively the changes in the phosphoproteome after K^+^-mediated depolarization, comparing synaptosomes that were treated with active or, respectively, with inactive BoNT (Fig. 1). Phosphopeptides were enriched and analyzed as described above. This second approach should differentiate phosphorylation events that are dependent on SV exo–endocytotic cycling.

**Fig. 2:**
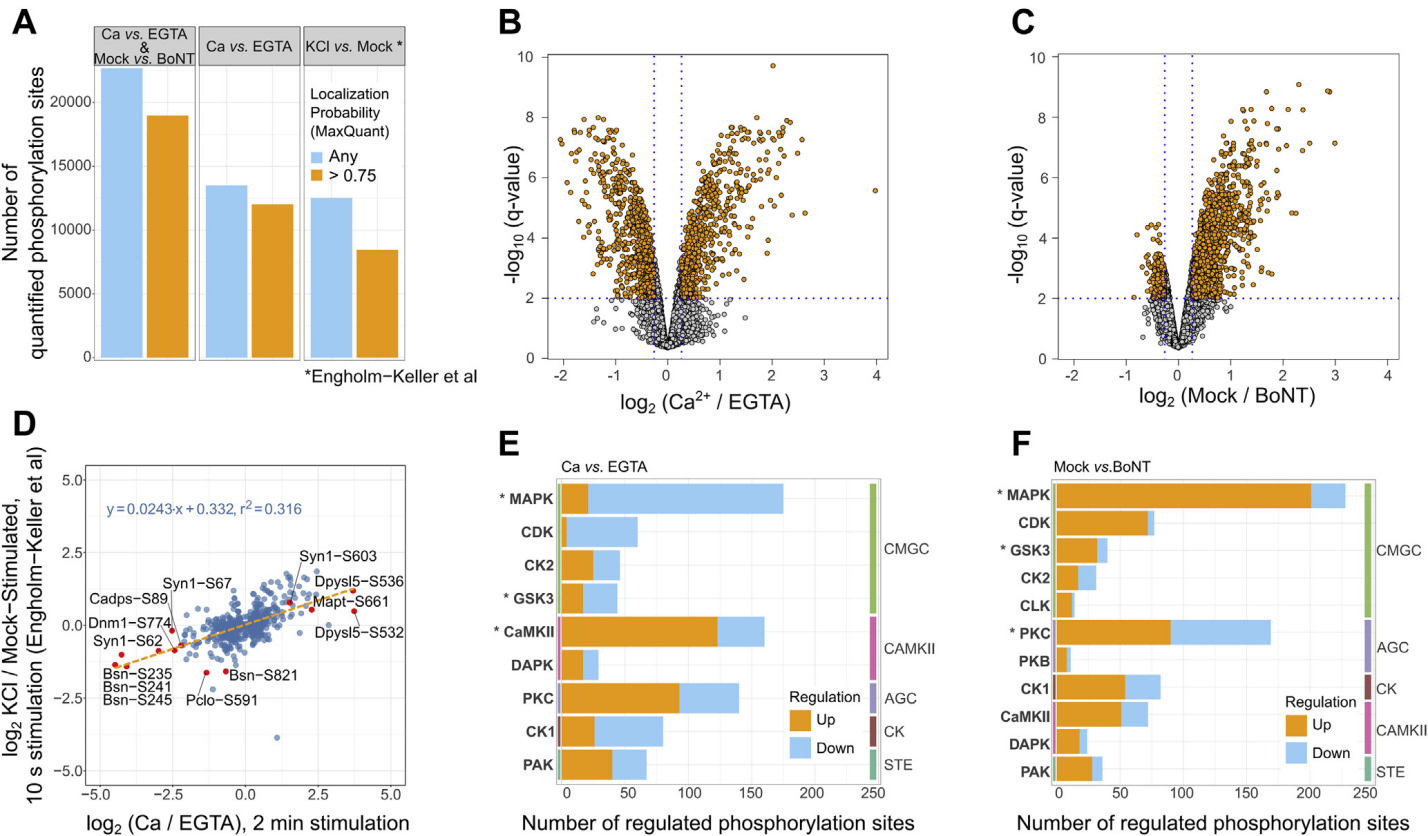
**Substantial changes in synaptic phosphoproteome following depolarization and responsible kinase groups.***A*, Number of quantified phosphorylation sites in Ca versus EGTA and Mock versus BoNT experiments (*panel 1*), Ca versus EGTA experiments only (*panel 2*) and recently published data set (Engholm-Keller (42), *panel 3*). *B* and *C*, volcano plots depicting -log_10_ (q-value) versus log_2_ (intensity fold change) of phosphorylation sites quantified within Ca versus EGTA and Mock versus BoNT experiments, respectively. *D*, Comparison of log_2_ fold changes between our and Engholm-Keller *et al* (42) data sets. Selected phosphorylation sites are marked as official gene symbol with phosphorylated amino acid and position. *E* and *F*, The stacked bar graphs show the total number of significantly regulated phosphorylation events in Ca versus EGTA (*E*) or Mock versus BoNT (*F*) experiments that can be a result of known (PhosphositePlus database (71)) or predicted (NetworKIN (73, 74)) kinase–substrate interactions. Orange bars depict the number of phosphorylation events that show significant intensity increase in Ca^2+^ or Mock-treated synaptosomes versus respective control (EGTA or BoNT). *Blue bars* correspond to downregulated events, respectively. The kinase classification (NetPhorest Groups) follows one introduced by NetPhorest (73) and uses its second-level group annotation. Colored vertical bars delineate the highest-level group in the kinase classification. Asterisks mark kinase groups that are predicted to control significantly more phosphorylation sites than expected as based on predicted kinase–substrate interactions in human proteome (*p* < 0.01). AGC, protein kinase A, G, C kinase group; CaMKII, calcium-calmodulin kinase 2; CDK, cyclin-dependent kinase; CK, casein kinase; CK1, casein kinase 1; CK2, casein kinase 2; CLK, SRPK1 and Clk/Sty protein kinase; CMGC, CDK, MAP, GSK, CDKL kinase group; DAPK, death-associated protein kinase; GSK3, glycogen synthase kinase 3; MAPK, mitogen-activated protein kinase; PAK, p21-activated kinase; PKC, protein kinase C; STE, “sterile” serine/threonine protein kinases.

Our analysis shows that phosphorylation is a prominent modification of synaptosomal proteins. Specifically, we identified and quantified phosphorylation under stimulated and nonstimulated conditions on 12,021 sites belonging to more than 2600 proteins (unique gene names) with a localization probability of >75% as determined by MaxQuant (63, 64). This coverage greatly exceeds our earlier one (41) and is comparable to a recent study from another laboratory (42). When the phosphosites quantified from BoNT-treated synaptosomes are included, the number of sites quantified in this study increases to 18,981 sites belonging to 3840 proteins (Fig. 2*A*, left panel).

### Synaptosome Depolarization by KCl in the Presence of Ca^2+^ Results in Substantial Changes in Protein Phosphorylation

When analyzing synaptosomes depolarized in the presence of calcium ions in comparison with those depolarized in the absence of calcium (EGTA control), we detected substantial changes in protein phosphorylation. Specifically, there are 1363 phosphorylated sites mapped to approximately 470 proteins that show intensity changes of at least 20% at an FDR of 1% (Fig. 2*B*). Using the PhosphositePlus database (71) to annotate known kinase–substrate relations and the NetworKIN tool (73, 74) to predict possible kinase–substrate pairs, we observed that certain kinase families were overrepresented including the CaMKII, MAPK, and GSK3 kinase groups (supplemental Table S1). Importantly, the distribution of up- or downregulated sites between kinase groups is not random: CaMKII and PKC kinase groups are responsible for phosphorylation sites that appear upregulated, while the majority of sites controlled by MAPK, CDK, and CK1 show reduced intensity in the presence of Ca^2+^ as compared with EGTA controls (Fig. 2*E* and supplemental Fig. S2). The latter observation is in line with previous studies that demonstrated upregulation of putative CaMKII substrates and downregulation of predicted MAPK substrates following potassium-triggered depolarization of synaptosomes (41, 42).

We also observed changes of phosphorylated sites in response to KCl treatment that were similar to changes reported in earlier studies (42) for bassoon (*Bsn*), piccolo (*Pclo*), synapsin-1 (*Syn1*), and dynamin-1 (*Dnm1*), as well as the microtubule-associated protein Tau (*Mapt*) and the dihydropyrimidinase-related proteins 2 and 5 (*Dpysl2* and *Dpysl5*), and others (Fig. 2*D*). Overall, the overlap of identified and quantified sites with the results of previous studies is approximately 60% as determined by the overlap in protein sequences flanking phosphorylation sites (sequence windows).

**Fig. 3:**
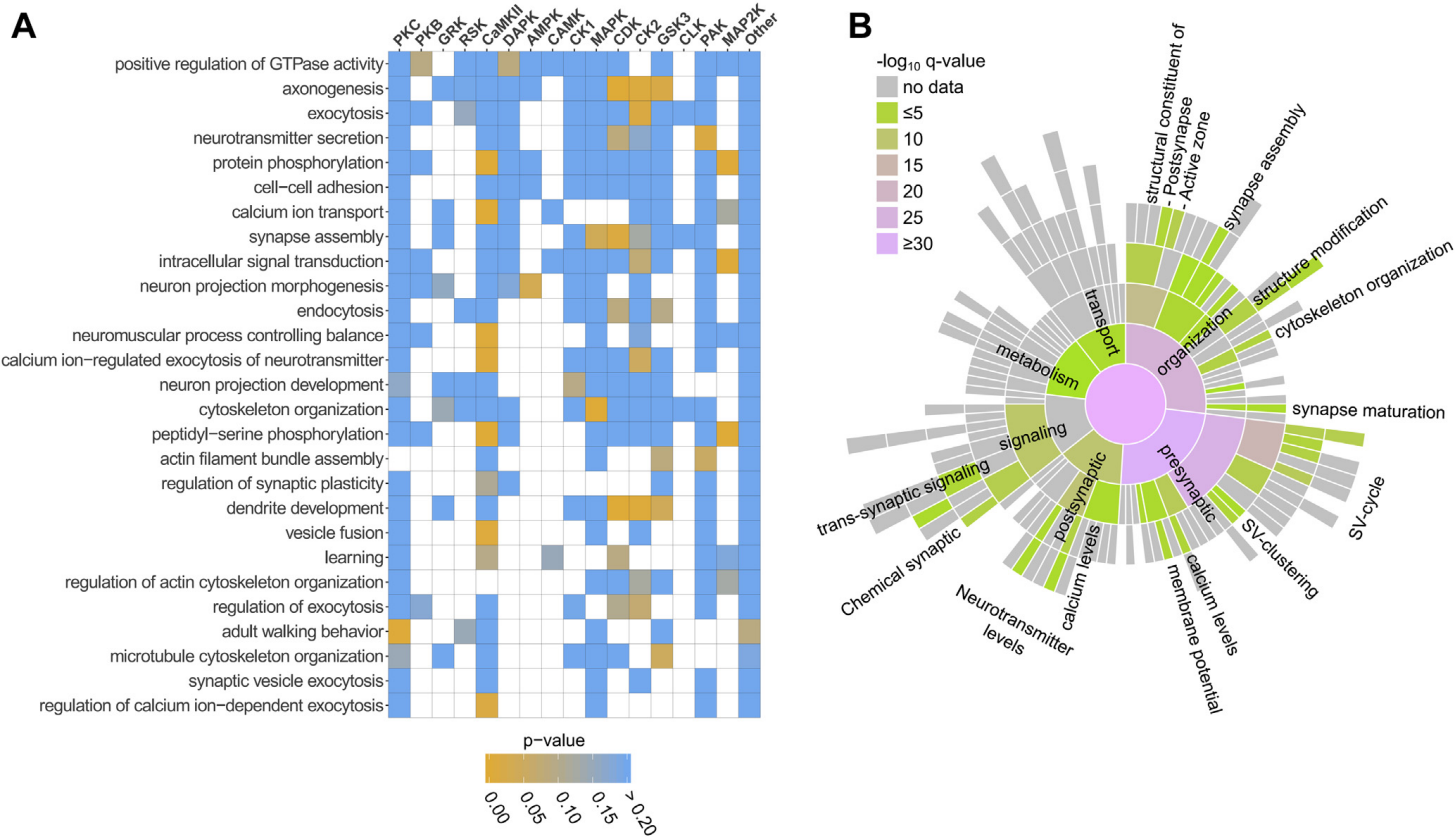
**Functional annotation of proteins carrying regulated phosphorylation sites based on Ca versus EGTA experiments.***A*, Association between significantly enriched GO terms and kinase groups. The enriched biological function-GO terms (background: *Rattus norvegicus*, *p* < 0.001) were derived using DAVID web service (68) for proteins carrying regulated phosphorylation sites. Overrepresentation of putative kinase–substrates relationships was tested for proteins annotated by each GO term. Frequencies of putative kinase–substrate relationships across all regulated sites for Ca versus EGTA experiments were taken as a background. The color encodes raw *p*-values (Fisher’s exact test). *B*, Sunburst diagram showing enriched biological function terms based on the synapse-specific SynGo database annotation (70). The color encodes the significance of the enrichment (-log_10_ (q-value)). Full list of enriched biological functions can be found in supplemental Table S2.

Enrichment analysis of the biological function of proteins carrying regulated phosphorylation sites (DAVID (68)) revealed gene ontology (GO) terms tightly connected with synaptic functioning, cytoskeleton organization, regulation of synaptic functions, and neuronal projections development (Fig. 3*A*). A similar picture (Fig. 3*B*) was obtained when using the synapse-specific SynGo database in which a large set of synaptic proteins was annotated by experts (70). It shows that the significantly enriched terms describe biological functions connected with SV cycling, regulation of neurotransmitter release, and synapse organization (for a complete list of enriched terms, see supplemental Table S2). Interestingly, regulation of some functions might be linked to the activity of certain kinase groups (Fig. 3*A*). For example, the number of sites predicted to be substrates for GSK3 and CDK is proportionally higher among proteins involved into the GO term “*axonogenesis*” as compared with other GO terms. Similarly, many phosphorylated sites on proteins that are involved in the “*calcium-dependent regulation of synaptic functions*” were also predicted as CaMKII substrates. The proportion of putative MAPK substrates is higher for proteins associated with “*synapse assembly*” and “*cytoskeleton organization*” as compared with other GO terms.

This analysis reflects, in accordance with recent studies (41, 42), the overall changes of phosphorylation in isolated nerve terminals upon stimulation—including the phosphosites that are important for exocytosis and endocytosis, *i.e.*, for SV cycling. However, the observed effects can be a result of both Ca^2+^ influx and active SV cycling.

### Block of SV Cycling with Botulinum Neurotoxins Induces Changes in Protein Phosphorylation

As indicated above, our first quantitative analysis does not allow for differentiating phosphorylation changes that are directly dependent on active membrane trafficking in the synapse rather than being caused by the activation of calcium-dependent kinases and phosphatases such as CaMKII (23), calcineurin (86), etc. Of course, these processes are intertwined since these enzymes are known to regulate the phosphorylation status of proteins closely associated with the process of SV cycling (87, 88).

We suppressed SV cycling by incubating synaptosomes with BoNT that block exocytosis by cleaving SNARE proteins. Toxin-treated synaptosomes and (as controls) synaptosomes incubated with inactivated toxins were stimulated with the high-potassium buffer in the presence of Ca^2+^ ions (Fig. 1). The toxin treatment greatly inhibited neurotransmitter release (supplemental Fig. S1*B*) but did not compromise Ca^2+^ influx (supplemental Fig. S1*C*). It led to significant changes in the phosphoproteome of the synaptosomes: 1497 sites showed significant difference with a minimum of 20% intensity changes at 1% FDR that can be mapped to approximately 700 proteins. Strikingly, BoNT treatment reduced the intensity of many phosphorylation sites (Fig. 2*C*). The phosphorylation status of these sites and their corresponding proteins is apparently dependent on the Ca^2+^-induced SV cycling activity in the presynapse.

The enrichment analyses for GO terms did not show substantial differences when compared with proteins carrying regulated sites in Ca versus EGTA experiments (not shown). The impact of BoNT treatment became apparent when we investigated possible substrate–kinase relationships. The enrichment analysis showed significant overrepresentation (*p* < 0.01) of phosphorylation sites that can be attributed to MAPK, PKC, and GSK3 kinase groups (supplemental Table S3). When comparing the quantitative Ca versus EGTA phosphorylation data, significantly fewer (*P* ≅ 1.0 × 10^-5^) regulated phosphorylation sites could be ascribed to being CaMKII substrates, whereas putative sites for MAPK (1), CLK (2), and PAK (3) were slightly over- (1, 2) or under- (3) represented, respectively (Fig. 2*F*, supplemental Table S4). Therefore, the crucial point in this analysis is the distribution of up- or downregulated phosphorylation sites for the different kinase groups. While depolarization of synaptosomes by KCl in the presence of Ca^2+^ leads to significantly reduced intensities of phosphorylation sites presumably regulated by MAPK and CDK kinases, the intensities of the putative MAPK, CDK, and GSK3 substrates appear higher when comparing mock-treated with BoNT-treated synaptosomes (compare Fig. 2, *E* and *F*). The balance of up- and downregulated phosphorylation sites was also significantly altered (*p* < 0.01) for CLK, CK1, and PAK kinases.

### Dissecting Primary Ca^2+^-Driven Phosphorylation Events from Events Linked to SV Cycling

**Fig. 4:**
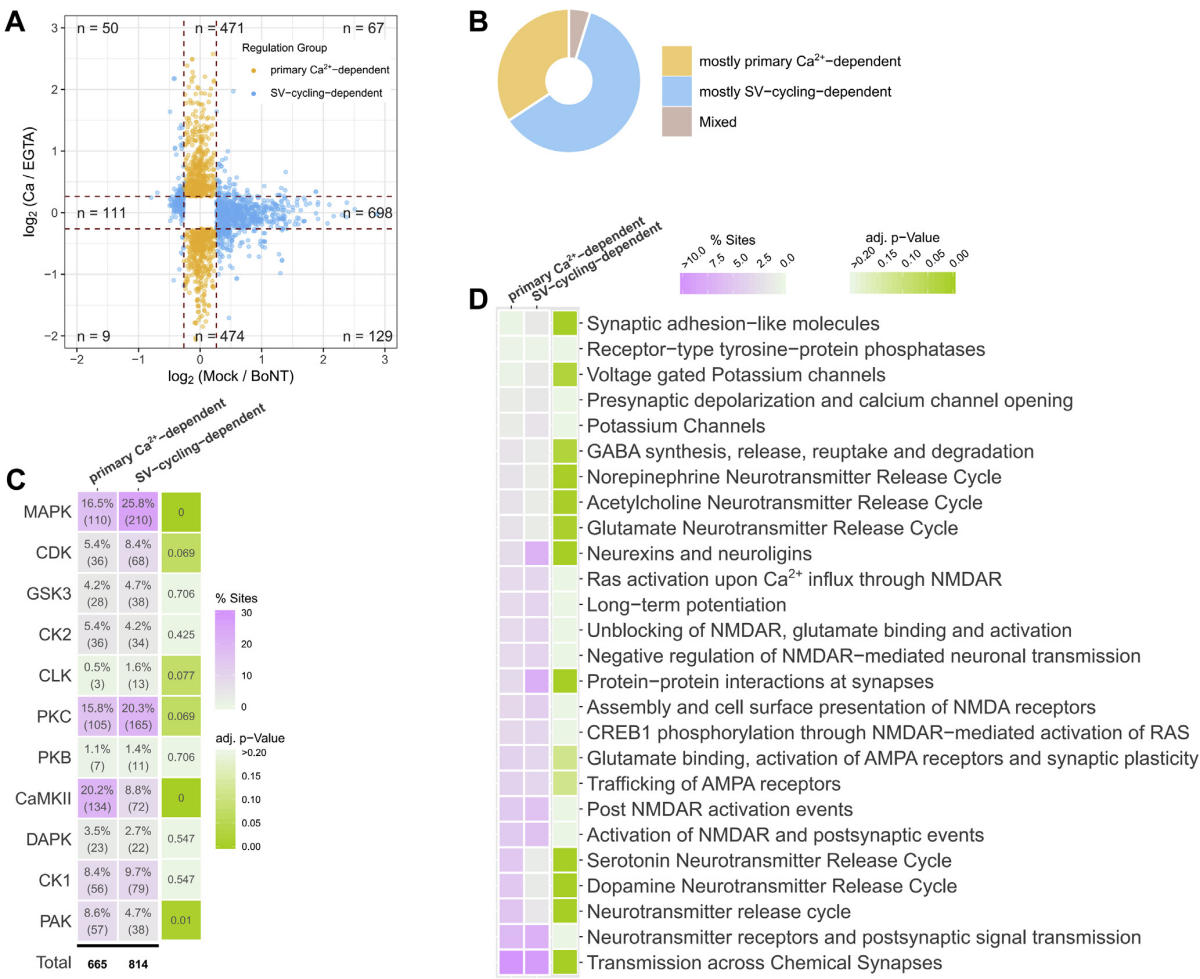
**Dependence of regulated phosphorylation sites on calcium influx and SV cycling.***A*, Categorization of phosphorylation events based on the magnitude of the log_2_ fold changes in Mock versus BoNT experiment (x-axis) and log_2_ fold changes in Ca versus EGTA experiment (y-axis). Only events quantified in both experiments and satisfying a q-value threshold of <0.01 are shown. The events are classified into two regulation groups: (i) “primary Ca^2+^-dependent” (sites with no significant or not-quantified SV cycling effect, *i.e.*, absolute log_2_ (Mock/BoNT) < 0.263 and absolute log_2_ (Ca/EGTA) > 0.263), shown as orange dots; and (ii) “SV-cycling-dependent” (absolute log_2_ (Mock/BoNT) > 0.263). *B*, Proportion of proteins that can be organized in one of the regulation groups based on the categorization of phosphorylation sites they carry. Only sites that surpassed the FDR-threshold of <1% were considered. If > 60% of the significantly regulated sites on a protein belong to one of the two regulation groups (“primary Ca^2+^-dependent” or “SV-cycling-dependent”), the protein is categorized in “mostly primary Ca^2+^-dependent” or “mostly SV-cycling-dependent,” respectively. *C*, Enrichment of predicted kinase–substrate interactions for “primary Ca^2+^-dependent” and “SV-cycling-dependent” regulation groups. The *left panel* shows the percentage and the absolute number of sites in each category (x-axis) that can be regulated by a particular kinase group (y-axis). The number under the *black line* provides the total number of sites with predicted kinase–substrate relationship in each category. The *right panel* shows Benjamini–Hochberg adjusted *p*-values after a Fisher’s exact test. An equal distribution of kinase–substrates relationships between the “primary Ca^2+^-dependent” and “SV-cycling-dependent” categories was set as a null hypothesis. *D*, Reactome pathway terms of neuronal origin (69). Reactome pathway terms of neuronal origin were selected based on their enrichment (Benjamini–Hochberg adjusted *p*-value < 0.1) for all proteins carrying significantly regulated phosphorylation sites. Proportion of “primary Ca^2+^-dependent” and “SV-cycling-dependent” sites of all “primary Ca^2+^-dependent” or “SV-cycling-dependent” sites per functional term is shown as color code (*magenta*). Fisher’s exact test was applied to test equal distribution of “primary Ca^2+^-dependent” and “SV-cycling-dependent” sites for each term. Benjamini–Hochberg adjusted *p*-values are represented as a color code (*green*). Abbreviations: AMPAR stays for α-amino-3-hydroxy-5-methyl-4-isoxazolepropionic acid receptor; CREB1 ∼ CAMP-responsive element binding protein 1; GABA ∼ γ-amminobutyric acid; NMDAR ∼ N-methyl-d-aspartate receptor; RAS ∼ rat sarcoma gene.

Assuming that the comparison of mock-treated synaptosomes with the BoNT-treated ones reveals the phosphorylation changes caused by Ca^2+^-induced SV cycling, we were able to identify phosphorylation events that are primarily dependent on Ca^2+^ influx and are not affected if SV cycling is inhibited (Figs. 4*A* and S3, orange dots). In the following, we refer to these sites as “primary Ca^2+^-dependent” sites. They are presumably directly (de)phosphorylated by Ca^2+^-dependent protein kinases and phosphatases upon Ca^2+^ influx. Conversely, a large group of phosphorylation events has shown changes when comparing mock- and BoNT-treated synaptosomes. Hence, we termed these sites “SV-cycling-dependent.” We noted with interest that the majority of “SV-cycling-dependent” sites did not show significant intensity changes in the Ca/EGTA experiment. We also noted that many proteins tend to carry regulated phosphorylation sites of one of the two groups introduced above, *e.g.*, mostly “primary Ca^2+^-dependent” or “SV-cycling-dependent” sites, while only a few contain sites of various categories (Fig. 4*B*). Interestingly, the number of proteins that carry mostly “primarily Ca^2+^-dependent” sites is lower than the number of proteins that carry mostly “SV-cycling-dependent” sites (Fig. 4*B*). It indicates the tendency that “primarily Ca^2+^-dependent” phosphorylation sites appear clustered on a few proteins, whereas “SV-cycling-dependent” sites are often found as the only significantly regulated phosphorylation site in a single protein. The site occupancy assessment (based on the model of Hoegrebe *et al*. (78)) suggests high phosphorylation levels (>60%) for a number of phosphoproteins, including *Bsn*, *Pclo*, *Dnm1*, *Camk2a*, *Mapt*, and *Dplysl5*, underlining the importance of the observed phosphorylation events (supplemental Fig. S4). It should be noted that the estimated phosphorylation levels represent only a rough estimate, as the ratio compression, which is a known issue for the TMT-based quantification, might severely affect the occupancy calculation. In terms of probable kinase regulation (Fig. 4*C*), sites predicted to be CaMKII substrates include significantly more “primary Ca^2+^-dependent” sites than sites classified as “SV-cycling dependent.” This harmonizes well with the strict Ca^2+^ dependence of CaMKII activity (89). Conversely, the MAPK group controls significantly more “SV-cycling dependent” sites than “primary Ca^2+^-dependent” sites. Similarly, predicted PAK substrates are overrepresented among “primary Ca^2+^-dependent” sites, whereas predicted PKC, CDK, and CLK-substrates appear more often among “SV-cycling-dependent” sites (Fig. 4*C*). The majority of phosphorylation sites on CAMKII, MAPK1, MAPK3, and PRKCB themselves show dependence on calcium ions. Increased phosphorylation of the regulatory sites of CaMKII, MAPK1, MAPK3, and PRKCB (*Camk2a-*T286, *Camk2b-*T287, *Camk2d-*T287, *Mapk1*-T183, Y185, *Mapk3*-T203, Y205, *Prkcb-*T640) points to the activation of those kinases following depolarization of synaptosmes by KCl (supplemental Figs. S5 and S6).

We noticed that numerous phosphorylation events on phosphatases and phosphatase regulators were significantly regulated in our study (supplemental Fig. S7*A*). Particularly, Neurabin-1 (*Ppp1r9a*), a PP1 regulator, showed increased phosphorylation in the Mock/BoNT experiment, suggesting the involvement of PP1 in regulating SV cycling. Although phosphatase–substrate interactions are more difficult to analyze due to the degenerated nature of phosphatase docking motifs, previous studies led to the proposal of docking motifs of PP1 and the Ca^2+^-calmodulin-dependent phosphatase calcineurin (76). We therefore examined possible interactions between exo- and endocytosis-related proteins containing PP1 or Calcineurin docking motifs and the respective phosphatase (supplemental Fig. S7, *B* and *C*). Available interactome data suggest a partially overlapping set of proteins that can be regulated by the phosphatases (*e.g.*, *Rims2*, *Bsn*, *Bin1*, *Stxbp1*), which does not allow confident assignment of a phosphatase to a particular phosphorylation event. When considering all potential targets, our results show a minor increase in the percentage of “SV-cycling-dependent” sites over “primary Ca^2+^-dependent” sites on proteins that can be regulated by PP1 (Fisher’s exact test, *p* = 0.075, supplemental Fig. S7*D*).

**Fig. 5:**
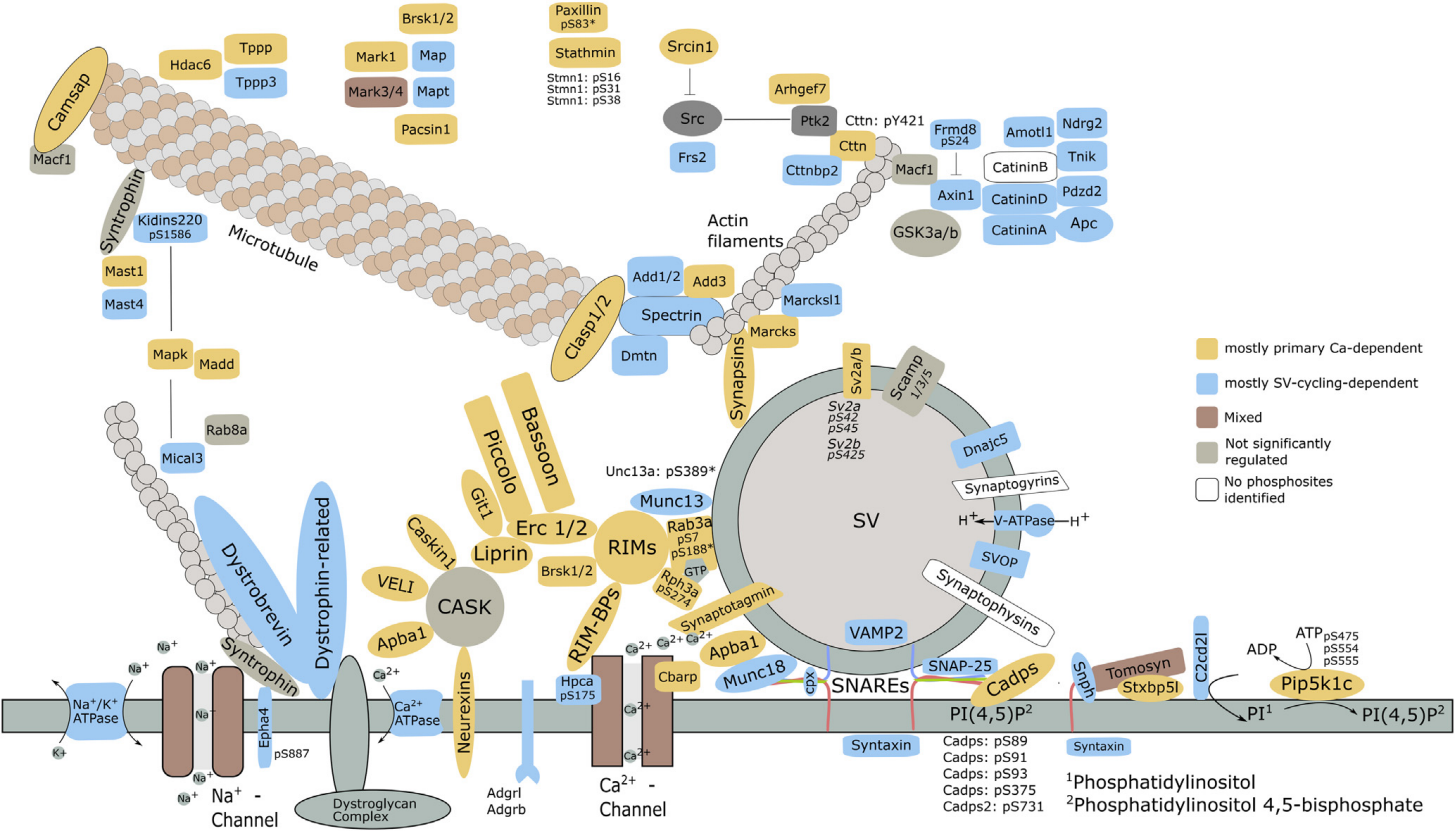
**Schematic representation of presynaptic compartment with a single docked synaptic vesicle, plasma membrane, and cytoskeleton elements, based on Chua *et al* (****97****)**. The represented proteins are categorized in “mostly primary Ca^2+^-dependent” (*yellow*), “mostly SV-cycling-dependent” (*blue*), “Mixed” (*brown*) based on the regulation type of the phosphorylation sites they carry. Proteins with no significantly regulated phosphorylation sites are represented in *gray*; proteins for which no first-class phosphorylation sites (MaxQuant localization probability <0.75) were identified are represented in *white*. In most cases, an official gene symbol was used to name proteins, except: Bassoon stays for Bsn; Ca^2+^-ATPase ∼ *Atp2b1*, *Atp2b2*, *Atp2b3*; Camsap ∼ *Camsap1*, *Camsap3*; CatininA ∼ *Cttna2*; CatininD ∼ *Ctnnd1*, *Ctnnd2*; Dystrobrevin ∼ *Dtnb*; Dystrophin-related ∼ *Drp2*; Liprin ∼ *Ppfia1*, *Ppfia2*, *Ppfia3*, *Ppfia4*; Map ∼ *Map1a*, *Map1b*, *Map1s*, *Map2*, *Map4*, *Map6*, *Map7d1*, *Map7d2*; Mapk ∼ *Mapk1*, *Mapk3*; Na^+^/K^+^ ATPase ∼ *Atp1a1*, *Atp1a2*, *Atp1a3*; Na^+^-Channel ∼ *Scn1a*, *Scn1b*, *Scn2b*, *Scn3a*; Neurexins ∼ *Nrxn1*; Piccolo ∼ *Pcl*; RIM-BPs ∼ *Rimbp1*, *Rimbp2*; RIMs ∼ *Rim1*, *Rim2*; Spectrin ∼ *Sptan1*, *Sptb*, *Sptbn1*, *Sptbn2*, *Sptbn4*; Stathmin ∼ *Stmn1*; Synaptotagmin ∼ *Syt1*, *Syt6*, *Syt9*, *Syt10*, *Syt17*, *Styl5*, *Esyt2*; Syntaxin ∼ *Stx1a*, *Stx1b*, *Stx12*; Syntrophin ∼ *Sntb2*; Tomosyn ∼ *Stxbp5*; VAMP ∼ *Vamp2*; V-ATPase ∼ *Atp6v1d*; VELI ∼ *Lin7a*, *Lin7b*, *Lin7c*. *Asterisks* mark sites that do not meet significance criteria of q-value < 0.01: Unc13a-pS389 (q-value = 0.013), Rab3a-pS188 (q-value = 0.012).

Because of the tendency of proteins to carry preferentially one of the two groups of significantly regulated phosphorylation sites, we set out to investigate the putative biological functions of these proteins in order to delineate processes that can be controlled by “primary Ca^2+^-dependent” or “SV-cycling dependent” phosphorylations. Reactome pathway analysis (69) sheds light on the putative different function of the involved phosphorylated proteins (Fig. 4*D*). One obvious difference regarding the two groups is that proteins are connected to potassium channels (“*Voltage-gated potassium channels*”), which are more enriched in the “SV-cycling-dependent” group. Another striking difference is that phosphorylated proteins termed with “*Synaptic adhesion-like molecules*” and “*Neurexins and neuroligins*” are enriched in SV-cycling-dependent group. In contrast, terms associated with “*Neurotransmitter release cycle*”, *e.g.*, glutamate, acetylcholine, etc., release, are barely enriched in phosphorylated proteins that carry “SV-cycling-dependent” sites (Fig. 4*D*, top). Reactome pathway annotation aligned well with the manual annotation of keywords describing protein function or location according to information available in the literature (supplemental Fig. S8). On the basis of our annotation, we noted that 234 out of 301 phosphorylation sites on proteins directly involved in the processes of exo- and endocytosis belong to the “primary Ca^2+^-dependent” group. For example, phosphorylation of RIM, RIM-binding protein, synapsin-1, and “dephosphins” such as dynamin-1 (*Dnm1*), amphiphysin (*Amph*), and AP180 (*Snap91*) showed a clear Ca^2+^-dependent character (supplemental Figs. S9 and S10). Importantly, phosphorylation sites on synapsin-1 followed the previously described pattern of phosphorylation changes when comparing Ca with the EGTA condition: increased phosphorylation of S9 and S603 and dephosphorylation of S62, S67, S549, and S551 (90, 91, 92, 93, 94, 95) (supplemental Fig. S11). In contrast, the “SV-cycling-dependent” phosphorylation sites tended to be present on proteins that are described as being associated with cytoskeleton elements and channels, for example, spectrin beta (*Sptb, Sptan1, Sptbn1, Sptbn2, Sptbn4*) and spectrin-binding proteins adducin alpha (*Add1*) and beta (*Add2*), as well as potassium channel subunits that are known constituents or modulators of Kv1 (*Kcna2, Kcna4, Kcnab2),* Kv2 (*Kcnb1, Kcnb2*), Kv4.3 (*Kcnd2*), Kv7.2 (*Kcnq2*), K2p10.1 *(Kcnk10*), Hcn (*Hcn1, Hcn2, Kctd3*) potassium channels (96) (supplemental Figs. S12–S15). As mentioned above, the observed SV cycling effect on several potassium channels seems to be very specific for the particular proteins and results in increased phosphorylation of the majority of those proteins except *Add1 and Kcnma2* (supplemental Fig. S16). Conversely, the Ca^2+^-activated potassium channel subunit alpha-1 (*Kcnma1*) and several other potassium channel subunits and modulators (*Kcnc3, Kcnh1, Kcnip2, Kcnq5*) have shown the “primary Ca^2+^-dependent” character of phosphorylation (supplemental Fig. S17). We summarize changes in phosphorylation site intensities for protein sequences that contain phosphorylation sites significantly regulated in one of the experiments and provide them as graphics in supplemental Data 5 and 6 (log_2_(Ca/EGTA) and log_2_(Mock/BoNT), respectively). These can be also visualized using a shiny app accessible at https://s1608-shiny.mpibpc.mpg.de. Figure 5 summarizes our results according to the schematic representation of proteins at the presynapse (97). Importantly, the proteins of the core exocytotic machinery including synaptobrevin, SNAP-25, syntaxin-1a, and Munc18 exhibit SV-cycling-dependent sites, while Munc13 reveals one SV-cycling-dependent site, which is close to the significance cutoff.

### Phosphomimetic Sites on SNARE Proteins and Cannabinoid Receptor-1 Modulate Neurotransmitter Release

Since protein phosphorylation can be an epiphenomenon of intracellular signaling, we selected a few of the changing phosphosites for determining directly whether they indeed have a modulatory effect on synaptic transmission.

**Fig. 6:**
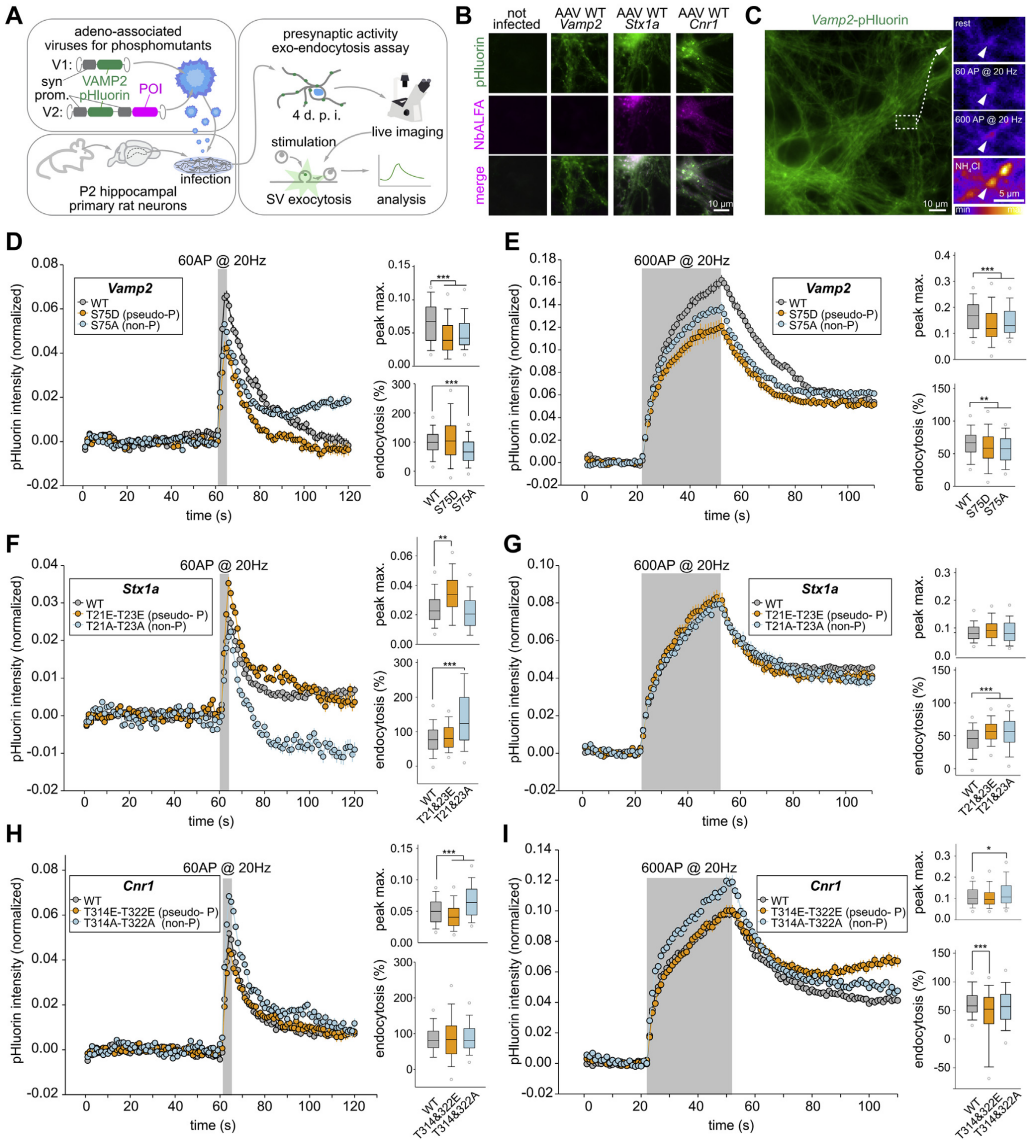
**Phosphomimetic modulation of phosphorylation sites found differentially regulated in synaptobrevin (*Vamp2*), syntaxin-1, and cannabinoid receptor-1 affects SV recycling properties.***A*, Experimental scheme. Two versions of the adeno-associated viral (AAV) constructs were prepared (V1 and V2). V1 contained *Vamp2* wild-type or the respective *Vamp2* mutants (*Vamp2*-S75D and *Vamp2*-S75A) fused to the superecliptic (pH-dependent) *green* fluorescence protein (pHluorin; (82)). The expression of recombinant proteins is driven by the neuron-specific synapsin-1 promoter. In these experiments the *Vamp2*-pHluorin is used both as the test protein and as a sensor for exo- and endocytosis. V2 contained both WT *Vamp2*-pHluorin (used as an exo-/endocytosis sensor) and the proteins of interest (POI; respectively *Stx1a*-WT, *Stx1a*-T21E-T23E; *Stx1a*-T21A-T23A; *Cnr1*-WT; *Cnr1*-T314E-T322E, and *Cnr1*-T314A-T322A); see methods for details.To distinguish these proteins from the endogenous versions, an ALFA-tag sequence has been added (84). Mutants were expressed in rat primary hippocampal neurons and exo-/endocytosis was measured 4 days post infection (d.p.i.). In order to evaluate exo-/endocytosis, neurons were field-stimulated and changes in fluorescence were measured (82). *B*, Immunofluorescence of infected neurons. The high efficiency of infection and the coexpression of the POIs (*Stx1a*; *Cnr1*) is confirmed by immunofluorescence using a nanobody directed against the ALFA tag (NbALFA; (84)). Scale bar 10 μm (*C*) The basal fluorescence of *Vamp2*-pHluorin (*left image*) allows to identify single synaptic boutons (*inset* on the *right*). Following a defined number of action potentials (AP), SVs fuse with the plasma membrane (*middle insets* on the *right*). Upon fusion, the intravesicular pH increases, resulting in higher pHluorin fluorescence intensity. Ammonium chloride (NH_4_Cl) is used at the end of each experiment to reveal the localization of SVs. *Scale bars*: 10 μm (*left image*) and 5 μm (*right insets*). *D* and *E*, Both the phosphomimetic and the phosphorylation-null mutants of Vamp2 showed decreased exocytosis, for both short and long train of stimuli, respectively 60 and 600 action potentials (APs). The maximum peak intensity was significantly lower in both stimuli (*p* < 0.001). The fraction of endocytosed *Vamp2* (measured at 30 s after the stimulation, as % of the exocytosed *Vamp2*) was also decreased, especially for the longer stimulus. *F* and *G*, The phosphomimetic mutant of *Stx1a* (T21-T23) enhanced exocytosis for short stimuli, while the phosphorylation-null mutant left exocytosis unaffected, while enhancing endocytosis. Under long stimuli the enhancement of exocytosis was no longer significant, but the increase in endocytosis was, for both phosphomimetic and phosphorylation-null mutants. *H* and *I*, The phosphomimetic mutants of *Cnr1* (T314E-T322 E) reduced exocytosis during short stimuli, while the nonphosphorylatable variant increased exocytosis. The latter effect was also observed during long stimuli. Neither of the Cnr1 variants had a substantial effect on endocytosis. In all *panels* statistical differences were tested relying on Kruskal–Wallis tests, followed by Tukey–Kramer post-hoc tests (∗ *p* < 0.05, ∗∗ *p* < 0.01 and ∗∗∗ *p* < 0.001).

We noted with interest that certain phosphorylation sites on SNARE proteins, including synaptobrevin (*Vamp2*) and syntaxin-1a (*Stx1a*), have a phenotype, which belongs to the “SV-cycling-dependent” group (supplemental Fig. S18). More intriguing, some of those sites (such as *Vamp2-*S75) were previously described as being important for the interaction with Munc18-1, a protein that can exhibit a stimulatory effect on trans-SNARE zippering (46). Similarly, we noted that two sites on the cannabinoid receptor-1 (*Cnr1*), T314 and T322, belong to the “SV-cycling-dependent” group as well. In order to test the effects of phosphorylation at these sites, we expressed the respective wild-type (WT) proteins, their phosphomimetic mutants, and phosphorylation-null mutants in cultured hippocampal neurons (Fig. 6*A*).

With this strategy, we addressed *Vamp2-*S75, *Stx1-*S21/S23, *Cnr1*-T314/T322, and their respective mutants (*Vamp2-*S75D; *Vamp2-*S75A; *Stx1-*S21E/S23E; *Stx1-*S21A/S23A; *Cnr1*-T314E/T322E, and *Cnr1*-T314A/T322A). For these proteins we created adeno-associated viruses (AAVs) that allowed us to mimic the effects of phosphorylation, and we expressed them in neurons together with *Vamp2-*pHluorin, as a sensor for exo-/endocytosis (Fig. 6, *B* and *C*).

Our stimulation experiments demonstrated that both phosphomimetic and nonphosphorylatable amino-acid substitutions, in both proteins, influence exo- and endocytosis in the cultured neurons. Specifically, we stimulated neurons with either 60 action potentials (AP) at 20 Hz (short stimulation) targeting the readily releasable pool of SVs and thus reflecting the immediate exocytotic response, or using 600 AP at 20 Hz (long stimulation) to mobilize the more inert recycling SV pools, reaching a state in which the entire vesicle cycle is fully activated (98). The expression of phosphomimetic *Vamp2-*S75D reduced exocytosis following short and long stimulation; it also reduced endocytosis in response to long stimulation. Expression of nonphosphorylatable *Vamp2*-S75A reduced exo- and endocytosis following both long and short stimulations (Fig. 6, *D* and *E*). Conversely, pseudo-phosphorylated *Stx1a*-T21E/T23E enhances exocytosis efficiency during short stimulation, while the nonphosphorylatable variant does the opposite (Fig. 6*F*). The effects on exocytosis become negligible during long stimulation, but both mutant variants result in an enhancement of endocytosis under these conditions (Fig. 6*G*).

The cannabinoid receptor-1 is known for modulation of the neurotransmitter release (49, 99, 100), and its activation can also be linked, through the activation of MAP kinases, to the phosphorylation of Munc-18 (37). Our experiments show that the pseudo-phosphorylated variant *Cnr1*-T314E/T322E reduced exocytosis (most evident during short stimulation), while the nonphosphorylatable variant *Cnr1*-T314A/T322A increased exocytosis in response to long or short stimulation. The effect on endocytosis was observed only for the pseudo-phosphorylatable *Cnr1* variant after long stimulation (Fig. 6, *H* and *I*).

These experiments tested only three of the synaptic phosphorylation sites that we found differentially phosphorylated in this work. Nevertheless, even if these experiments are based on overexpression in a background containing normal levels of WT proteins, all tested candidates resulted in significant modulations of exo- or endocytosis. Although in the case of *Vamp2* both mutants led to a substantial reduction in exocytosis rates, its pseudo-phosphorylated form (*Vamp2*-S75D) showed a greater effect on exocytosis than the nonphosphorylatable form (*Vamp2*-S75A). Presumably, the absence of a charged residue did not fully compensate the steric effect caused by the amino acid substitution at position 75 of *Vamp2*, which has apparently a stronger impact on protein function than the double mutant at positions 21/23 of *Stx1a*. The S75 phosphorylation site is located within the R-SNARE motif required for forming a coiled-coil complex with *Stx1a* and *Snap25* during exocytosis. In contrast, the T21/T23 positions of *Stx1a* are located in the regulatory N-terminal helical bundle, explaining why mutations show different effects on exocytosis. Overall, these findings support the concept that protein phosphorylation is a key regulatory mechanism in neurotransmitter release that modulates different steps in the SV cycle.

## Discussion

In this study, we have used isolated nerve terminals as a model to differentiate phosphorylation changes that are induced by calcium influx alone from those that in addition require active endo–exocytotic SV cycling. Surprisingly, we found that inhibition of exocytosis by BoNT had a profound effect on the phosphoproteome despite unchanged calcium influx. These findings reveal complex signaling cascades during which regulatory changes in protein phosphorylation are not only dependent on the activation of kinase/phosphatase networks but also on the progression of vesicular trafficking.

By using isobaric tandem-mass tags for labeling of phosphorylated peptides, we were able to substantially increase the phosphoproteome coverage and to achieve reliable quantification for thousands of phosphorylation sites. Despite differences in the synaptosome preparation (Ficoll-purified synaptosomes versus crude synaptosomal pellet) and stimulation (50 mM KCl for 2 min versus 75 mM KCl for 10 s), many of the regulated phosphorylation sites observed by us are in excellent agreement with earlier results from another laboratory (Fig. 2*D*) (42). Our data show higher magnitudes of changes, which are probably a consequence of the prolonged stimulation time used in our study. The most drastic changes were observed for pacsin (*Pacs1-*S517, log_2_ fold change of 3.98), thymosin beta (*Tmsb4x*-T23, 2.63), ADP-ribosylation factor GTPase activating protein 1 (*Arfgap1*-S247 2.57), calcium/calmodulin-dependent protein kinase type II subunit delta (*Camk2d*-S315/S319, 2.49), protein bassoon (*Bsn*-S1354, –2.06), septin 5 (*Sept5*-S17, –1.93), and RIM-binding protein 2 (*Rimbp2*-S554, –1.86). We also confirm phosphorylation changes in response to stimulation that were reported earlier for synapsin-1, dynamin-1, Tau protein, as well as the multisite phosphorylations of the giant active zone proteins bassoon and piccolo (26, 31, 36, 101).

It seems that several proteins known to function at the postsynapse respond to potassium chloride stimulation and BoNT treatment in synaptosomes (*e.g.*, *Shank1, 2, 3*; components of NMDAR, *Grin1, Grin2a, Grin2b*). It would be of interest to investigate whether these proteins are also present at measurable levels in the presynapse and whether they are affected by synaptic activity. Although synaptosomal preparations are known to contain fragments of the postsynaptic membrane (102), they usually do not retain metabolic activity, in contrast to functionally active membrane-enclosed nerve terminals that can maintain the ATP levels needed for phosphorylation (50, 51, 52, 53).

Clearly, the primary trigger for the phosphorylation changes is calcium that enters synaptosomes following potassium-induced depolarization. Calcium directly regulates the activity of kinases and phosphatases, including CaMKII, PKC, calcineurin, PP1, etc. (23, 89, 103, 104). Accordingly, we observe increased phosphorylation of specific sites on CaMKII and MAP kinases that are known to result in activation of the kinases as well as enrichment of possible substrates for CaMKII, MAPK, and GSK3 kinases among regulated phosphorylation sites.

However, the fact that selective inhibition of calcium-activated SV cycling results in major changes of the phosphoproteome documents that the activation status of the kinase/phosphatase networks is directly dependent on the progression of the exo–endocytotic cycle. In most cases, BoNT treatment inhibited phosphorylation, and these results are in line with a very early study that used BoNT-treated synaptosomes from the electric organ of *Torpedo marmorata* and radioactive phosphate for protein labeling (105). Interestingly, phosphorylation events known to be directly regulated by some of the major calcium-dependent kinases and phosphatases such as those on synapsin-1 and dynamin-1, as well as on many of the predicted CaMKII-phosphorylation sites, were almost unaffected by the BoNT treatment. Conversely, predicted sites of MAPK, PKC, and CDK were affected more strongly by the BoNT treatment. These observations support the view that the activity of MAPK is dependent on active SV cycling, resulting in the phosphorylation of delta catenin, metabotropic glutamate receptor, and other proteins in the synapse (106, 107, 108, 109). Indeed, we observe SV-cycling-dependent phosphorylation changes not only in catenin-delta (*Cttnd2*) but also in other proteins that might take part in the Wnt pathway such as *Apc, Amotl1*, and *Tnik*. Given the connection between the Wnt pathway and the regulation of cell–cell contacts and cytoskeletal elements, it is not surprising that many cytoskeleton- and cell-adhesion-related proteins contain BoNT-responsive phosphorylation sites (*e.g.*, spectrin beta, adducin alpha and beta, neurofascin, neuronal cell adhesion molecule). It is tempting to speculate that these phosphorylation changes represent the activation of pathways directed at increasing synaptic strength (most importantly LTP) by mechanisms such as size increase of the synaptic contact zone, enlargement of the presynaptic bouton, and adaptations of the presynaptic cytoskeleton to increase the efficiency of SV cycling (110, 111, 112, 113).

How can the wide-ranging neurotoxin effects be explained when considering the extraordinary specificity of the SNARE proteases, which should have no direct effect on kinases or phosphatases? Given that toxin treatment causes dephosphorylation of many substrates, the observed effect can be explained either by activation of phosphatase or by suppression of kinase activities that are not caused directly by calcium but by changes in proteins that are themselves regulated by the activity of the SV cycle and in turn control kinases or phosphatases. Moreover, it is conceivable that the availability of phosphorylation sites on substrate proteins is coupled to its “activity cycle.” Trafficking steps such as SV docking, priming, fusion, coat formation, fission, and SV movement are associated with numerous changes in protein conformation and the dynamic assembly and disassembly of multimolecular complexes. Thus, it is to be expected that phosphorylation site accessibility is dependent on these dynamic changes, many of which are likely to be arrested following inhibition of the SV cycle.

An interesting example is given by the PP1 whose postsynaptic activation appears to be dependent on synaptic activity (114). Neurabin-1 (*Ppp1r9a*) can play a crucial role in this, by recruiting PP1 to the synapse by regulating its binding to actin filaments, which impacts PP1 activity (115, 116). Our observations suggest that PP1 undergoes similar activity-dependent regulation also in the presynapse as we observe “SV-cycling-dependent” sites within the actin-binding region of Neurabin-1. It is tempting to speculate whether SV-cycling-dependent activity of PP1 or other phosphatases can be opposed by Ca^2+^-activated kinases such as CaMKII, MAPK1, MAPK3. This would explain reduced intensities of predicted MAPK substrates in Ca/EGTA experiments despite apparent activation of MAPK following K^+^-induced depolarization and Ca^2+^ influx. Furthermore, it would explain why many phosphosites appear significantly regulated when comparing mock-with BoNT-treated synaptosomes, but do not show significant changes in Ca/EGTA experiments, since comparison of Ca^2+^ with EGTA conditions reveals a composite effect of Ca^2+^ influx and SV cycling on protein phosphorylation.

Experiments involving BoNT also led us to the discovery of phosphorylation sites on the core SNARE proteins, sites that otherwise did not appear significant. We showed that these sites can affect exo- and endocytosis in hippocampal neurons under stimulation. One possible mechanism is the impaired interaction with Munc18-1, which can facilitate SNARE zippering and thereby promote SV fusion with the plasma membrane (46). The changes of endocytosis are possibly a consequence of altered exocytosis. In contrast to many other BoNT-responsive phosphorylation sites, the phosphorylation sites on *Vamp2* and *Stx1a* that we examined showed increased phosphorylation following toxin treatment, suggesting that the phosphorylation is due to adaptive changes in the synapse rather than a result of SNARE cleavage by BoNT.

In contrast to the phosphomimetic and nonphosphorylatable variants of the SNARE proteins, the corresponding variants of cannabinoid recepor-1 (*Cnr1*) had an effect only on exocytosis in cultured hippocampal neurons. The effect can be also linked through the activation of MAPK to Munc-18 phosphorylation and regulation of SNARE assembly (99). Similarly, the intensity of phosphorylation of T314/T321 sites was increased following toxin treatment. Although *Vamp*2-S75, *Stx1a*-S21/S23, *Cnr1*-T314/T321 phosphorylation sites were identified in previous studies (46, 47, 117, 118), it is the first time, to the best of our knowledge, that phosphorylation of these sites has been shown to have an impact on the exo- and endocytosis in the context of stimulation experiments.

Interestingly, we also found phosphorylation sites on Munc18 that are regulated in SV-cycling-dependent manner, namely *Stxbp1*-S313 and *Stxbp1*-S506. Both sites were significantly downregulated in the BoNT-treated samples. Although not much known about S506, S313 site was previously described as a possible target of PKC and that its phosphorylation can negatively affect interaction of Munc18 with syntaxin-1 (48, 119).

A remarkable aspect of the synapse phosphoproteome regulation has recently been addressed by F. Brüning, *et al.* (120). Their analysis shows that phosphorylation sites on many synaptic proteins undergo oscillations during the course of the day. Interestingly, a few of these sites are shown to be regulated upon depolarization by potassium chloride and treatment with botulinum neurotoxins, including phosphorylation sites on proteins previously discussed: *Cnr1, Vamp2, Stx1a, Cttnd2, Add1, Add2, Sptbn1, Sptbn4, Kcnb1, Kcnd2, Hcn2, Kcnma1, Tnik, Apc, Camk2a,* etc. This also suggests that, despite the caveat that potassium-induced depolarization is nonphysiological (42), it allows for identifying regulated phosphorylation sites that are of high functional relevance for normal synapse physiology.

Despite major progress, many aspects of synapse regulation remain to be uncovered. Our study has added another layer of complexity by showing how intricately the phosphorylation–dephosphorylation cascade in the synapse is linked to SV cycling. We believe that the results presented here provide a firm basis for future investigations about how synaptic activity and synaptic plasticity are regulated at the molecular level.

## Data Availability

The mass spectrometry data have been deposited to the ProteomeXchange Consortium *via* the PRIDE (121) partner repository with the data set identifier PXD020564.

R scripts used for data analysis are available at GitHub: https://github.com/IvanSilbern/2021_Silbern_etal_Synapt_BoNT_KCl.

We also provide a visualization tool in a shiny-app (https://cran.r-project.org/web/packages/shiny/index.html) format. The source data and code are available at GitHub: https://github.com/IvanSilbern/2021_Silbern_etal_ShinyApp. The app is deployed and accessible at: https://s1608-shiny.mpibpc.mpg.de.

## Supplemental data

This article contains supplemental data (59, 65, 66, 70, 71, 73, 74, 75, 76, 78, 122, 123).

## Conflict of interest

The authors declare that they have no conflicts of interest with the contents of this article.
