# Supplementary material for: Protein Phosphorylation in Depolarized Synaptosomes: Dissecting Primary Effects of Calcium from Synaptic Vesicle Cycling
Source: Mol Cell Proteomics. 2021 Feb 12;20:100061. doi: 10.1016/j.mcpro.2021.100061 (PMC7995663; doi:10.1016/j.mcpro.2021.100061)
Supplement: Supplemental Material 6 [file mmc6.pdf]

Uniprot Accession: A0A0G2JW01
Uncharacterized protein

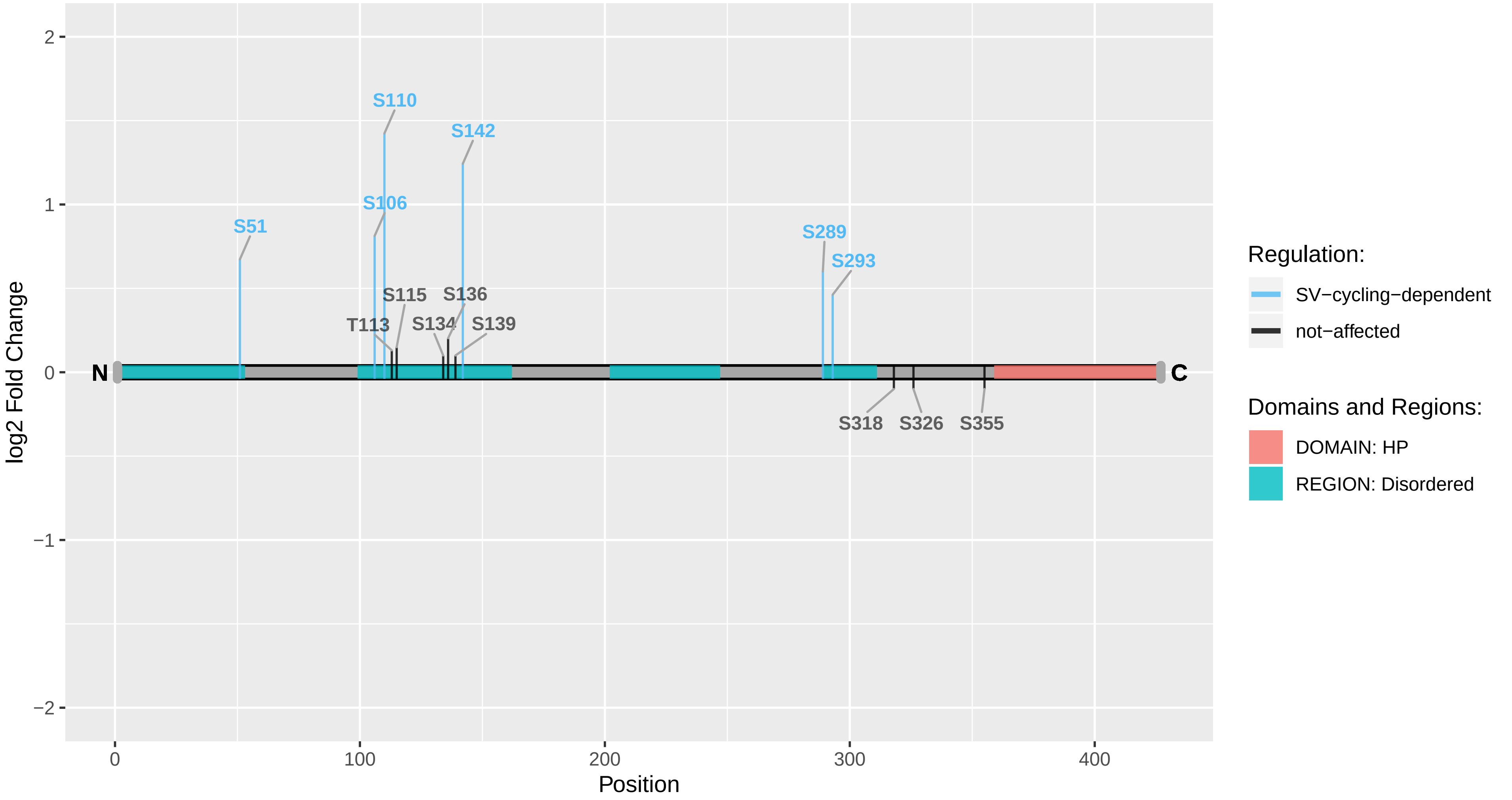

Uniprot Accession: A0A0G2JW65
Uncharacterized protein

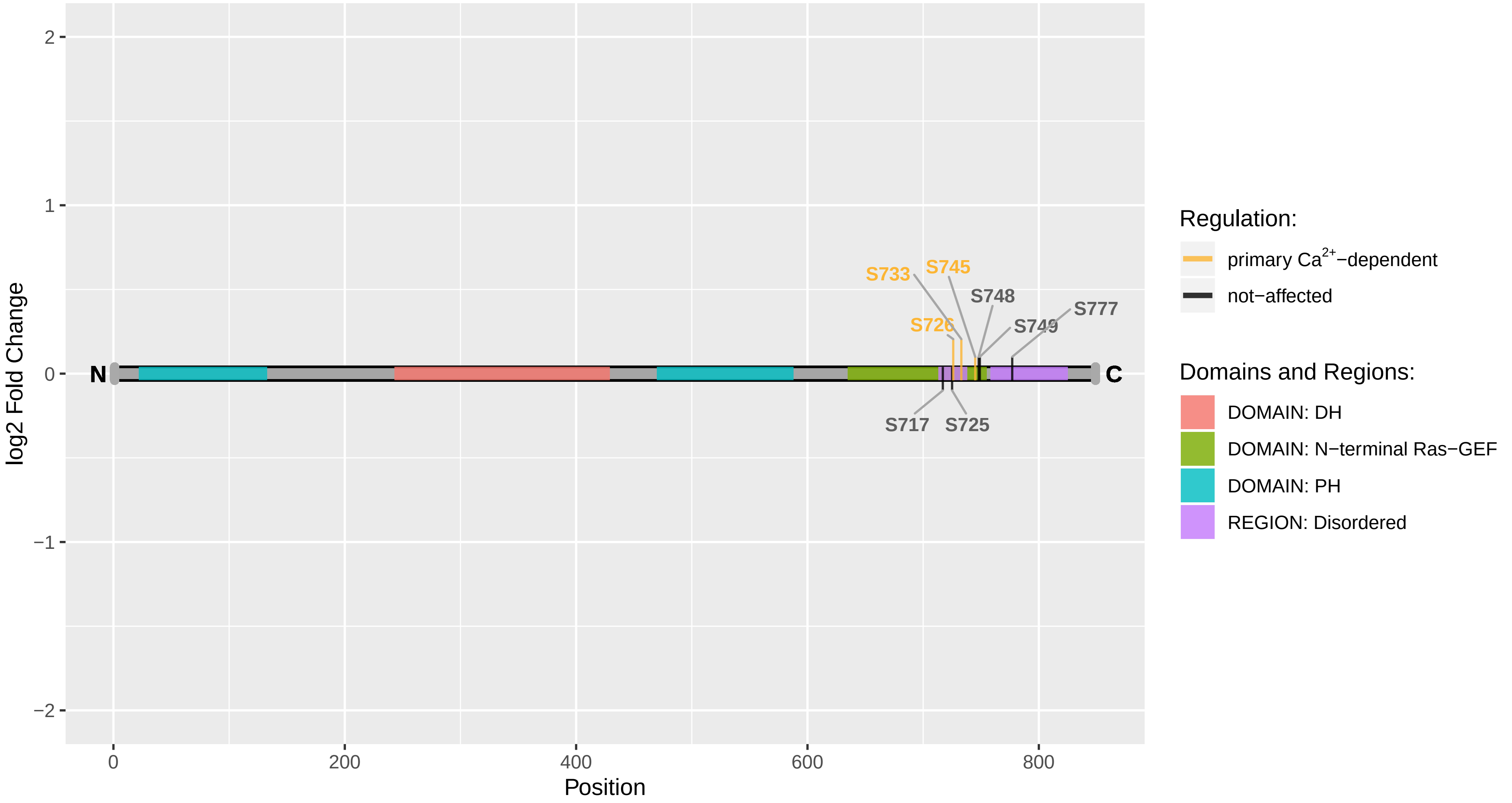

Uniprot Accession: A0A0G2JZ27
Uncharacterized protein

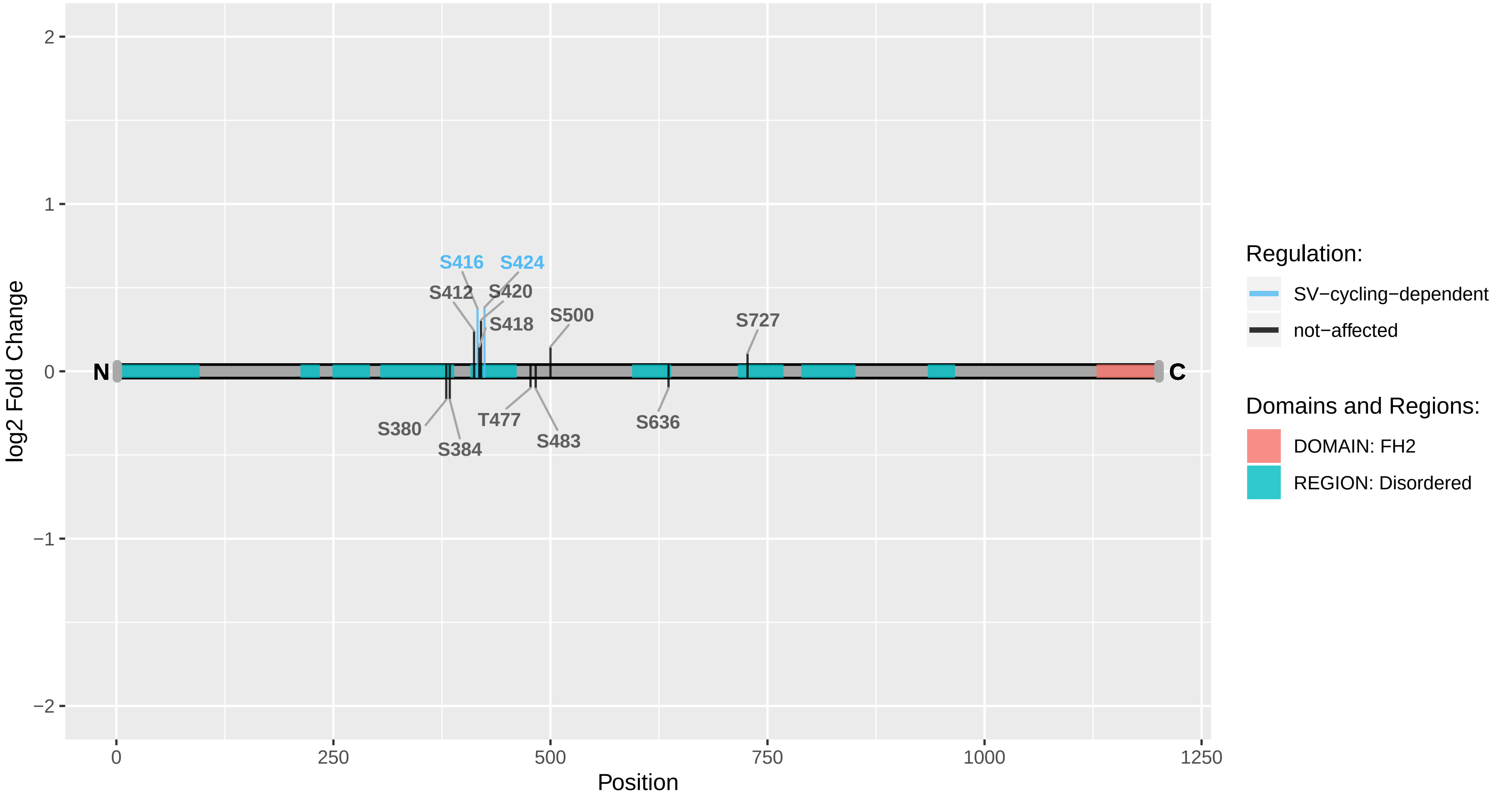

Uniprot Accession: D3ZJK8
Uncharacterized protein

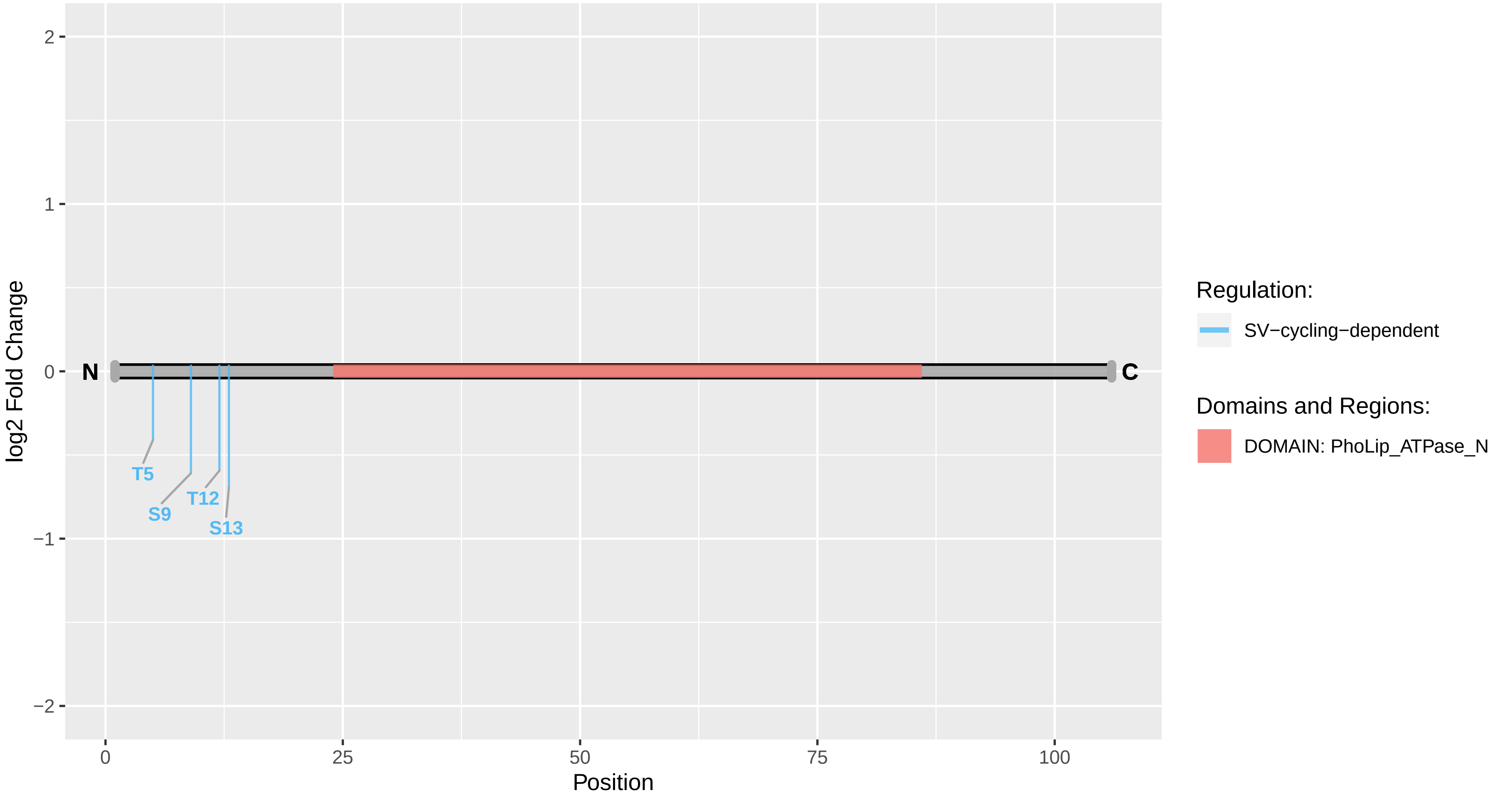

Uniprot Accession: D3ZZH5
Uncharacterized protein

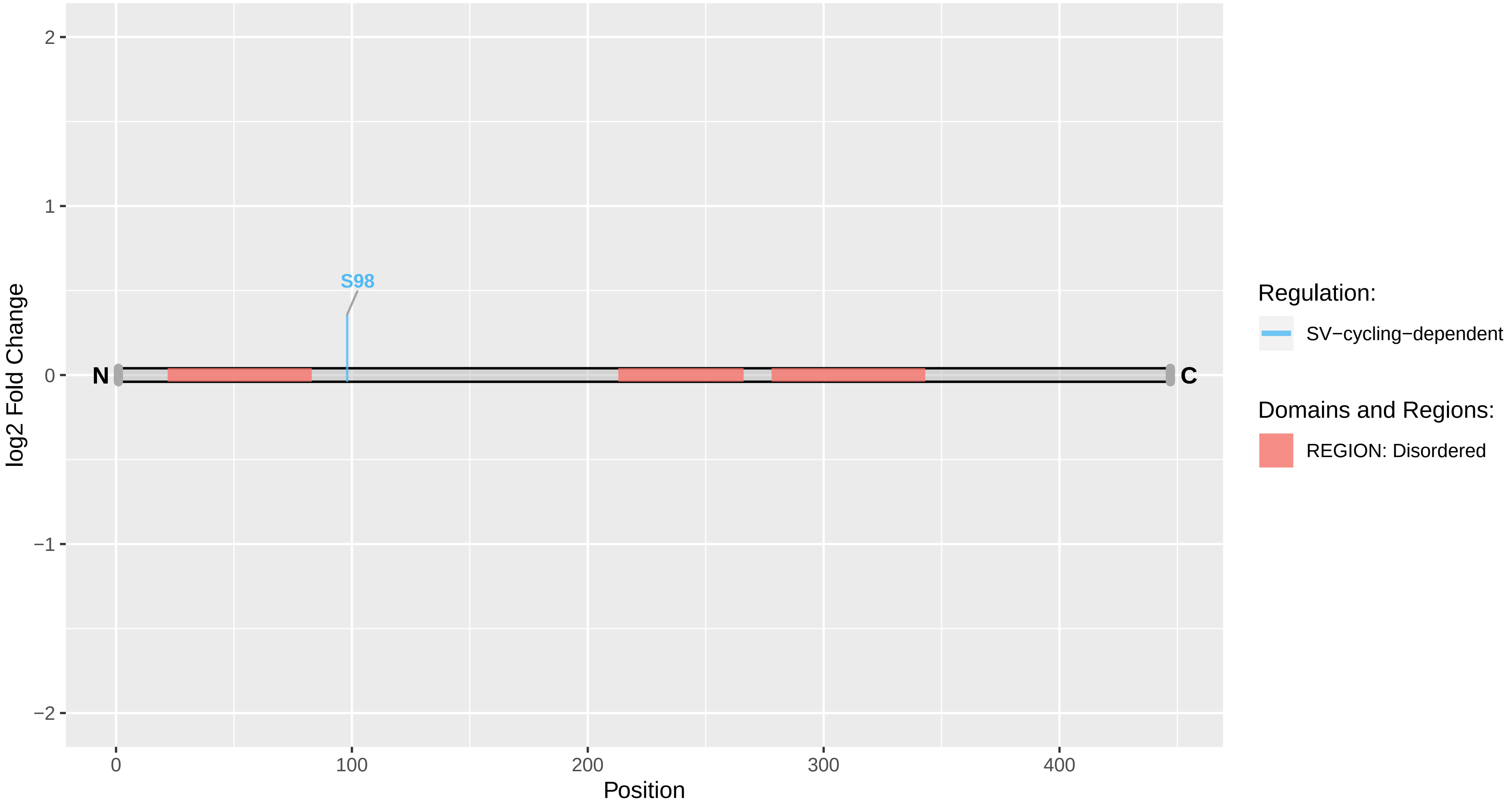

Uniprot Accession: M0R970
Uncharacterized protein

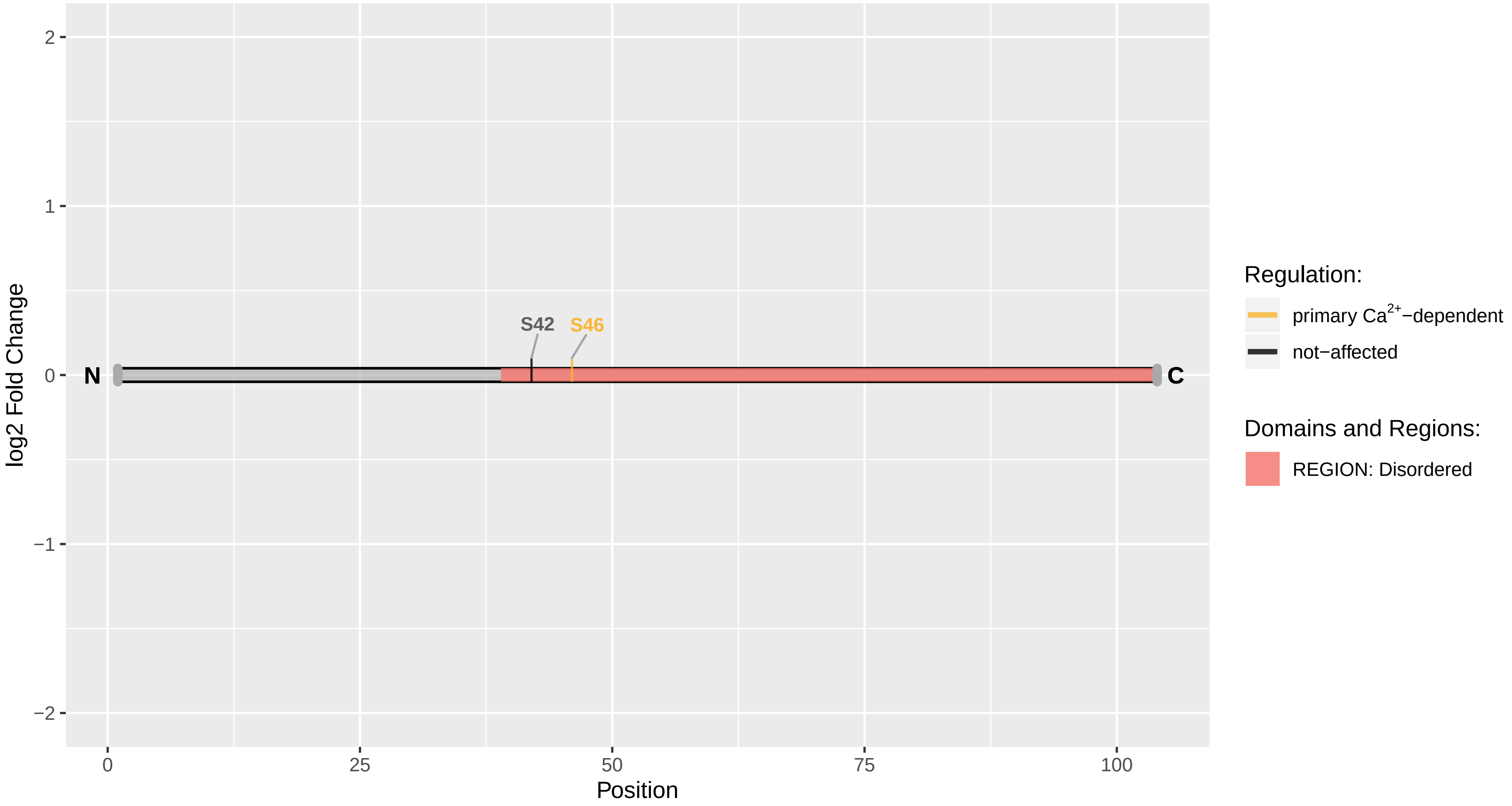

Uniprot Accession: M0RDY2
Uncharacterized protein

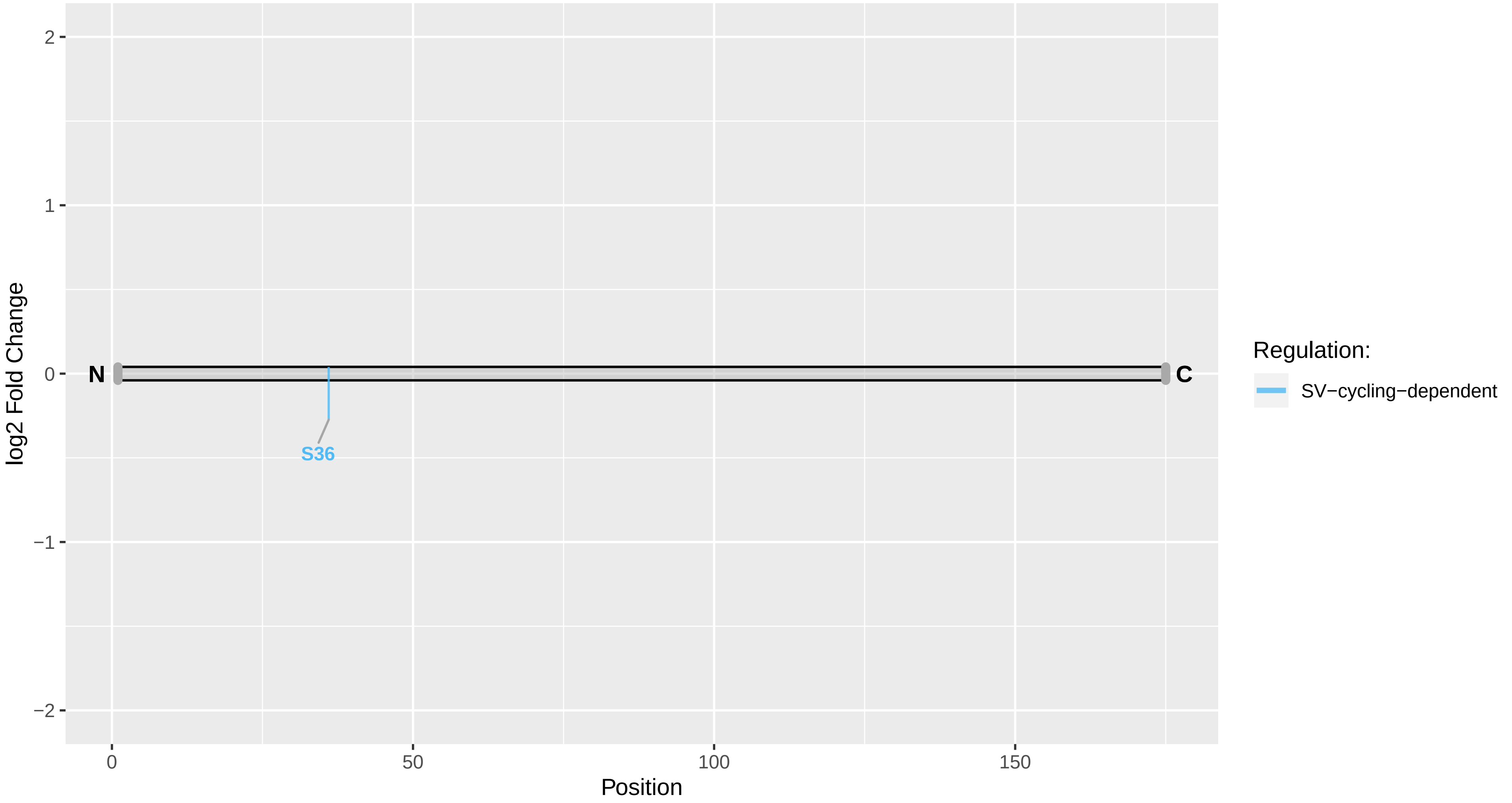

Uniprot Accession: O08654
UPF0183 protein C16orf70 homolog

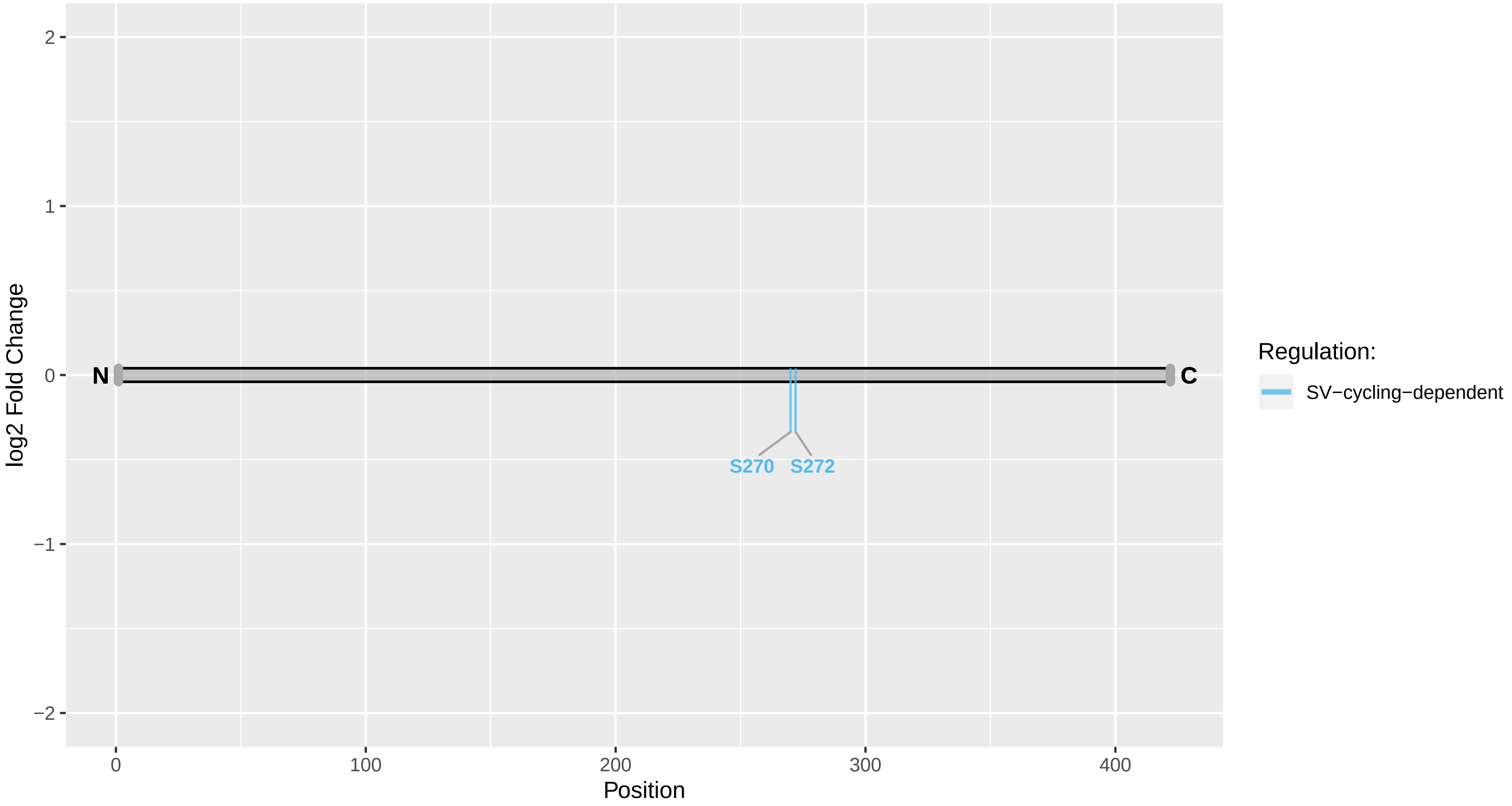

Uniprot Accession: Q63003
5E5 antigen

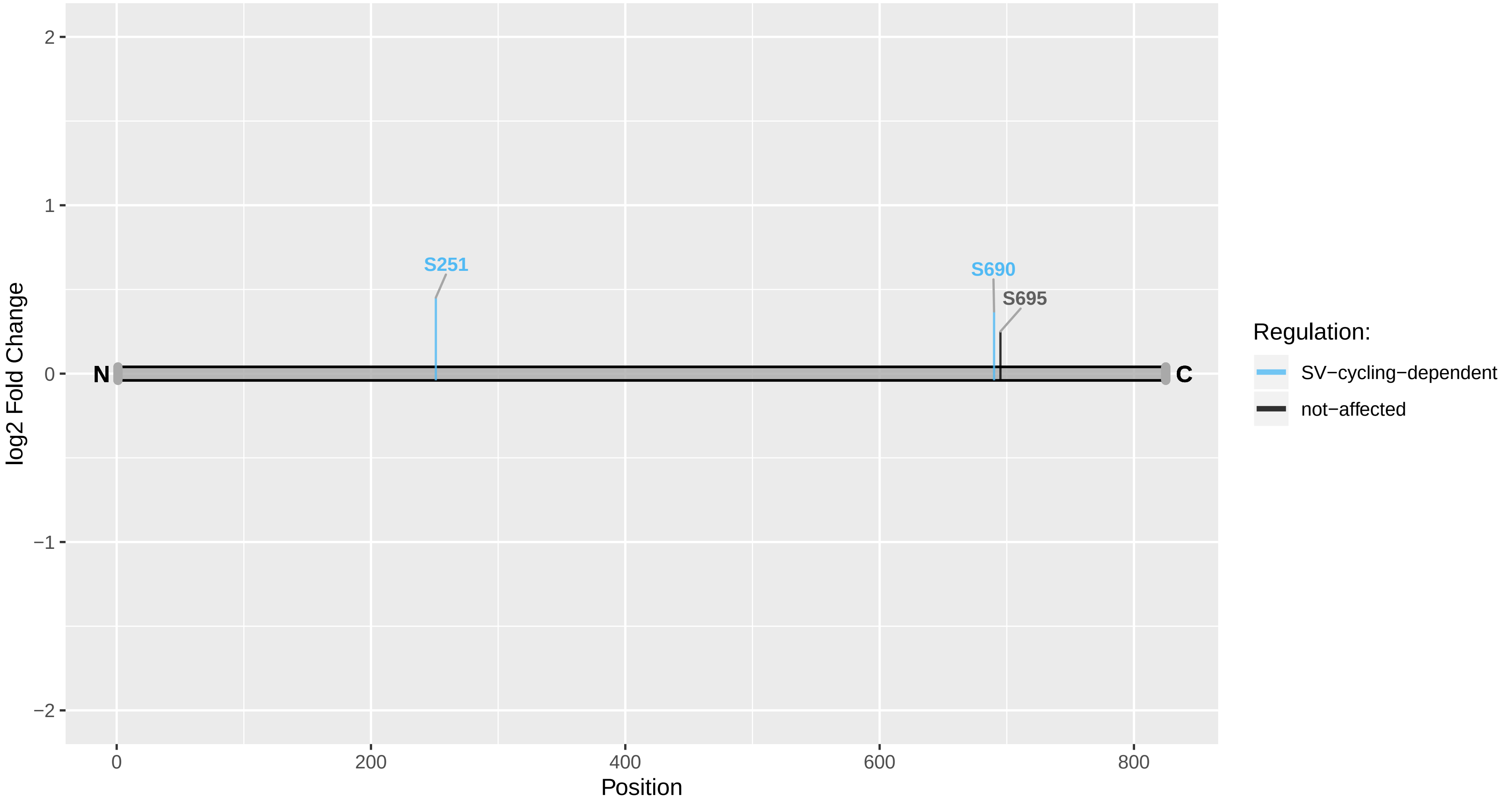

Aak1

Uniprot Accession: P0C1X8
AP2−associated protein kinase 1

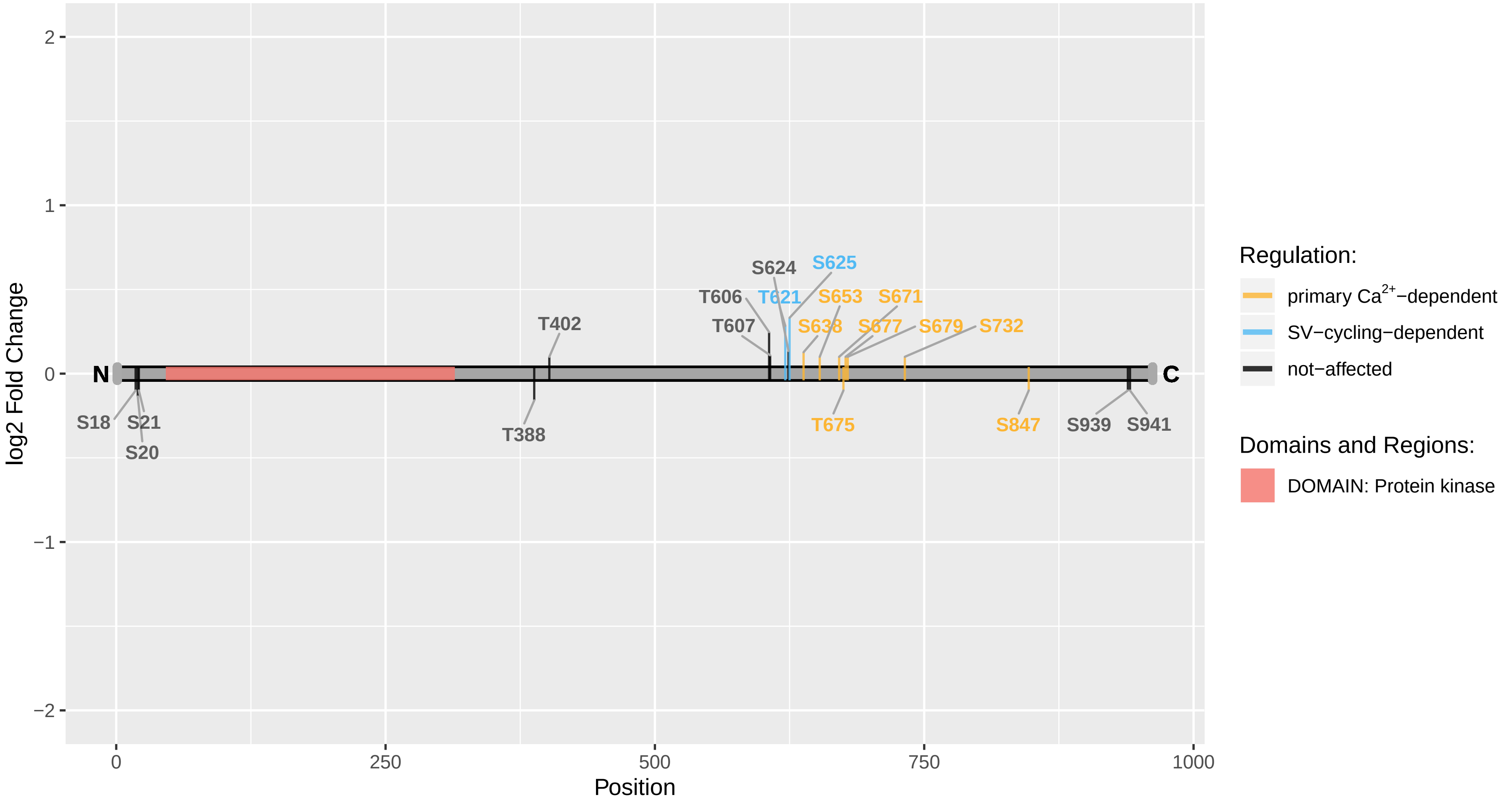

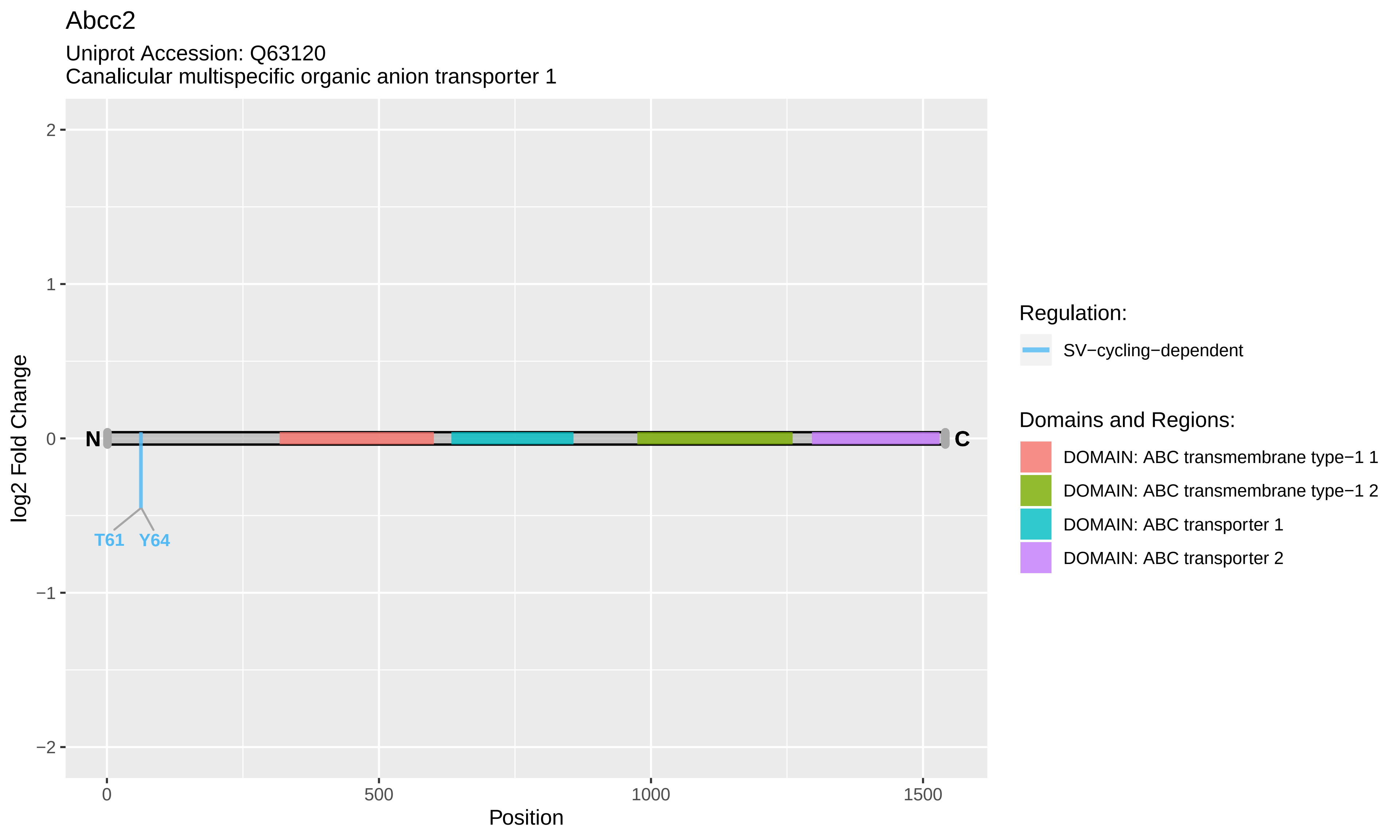

Abcc2
Uniprot Accession: Q63120
Canalicular multispecific organic anion transporter 1
2
1
0
−1
−2
log2 Fold Change
N
C
T61
Y64
0
500
1000
1500
Position
Regulation:
SV−cycling−dependent
Domains and Regions:
DOMAIN: ABC transmembrane type−1 1
DOMAIN: ABC transmembrane type−1 2
DOMAIN: ABC transporter 1
DOMAIN: ABC transporter 2

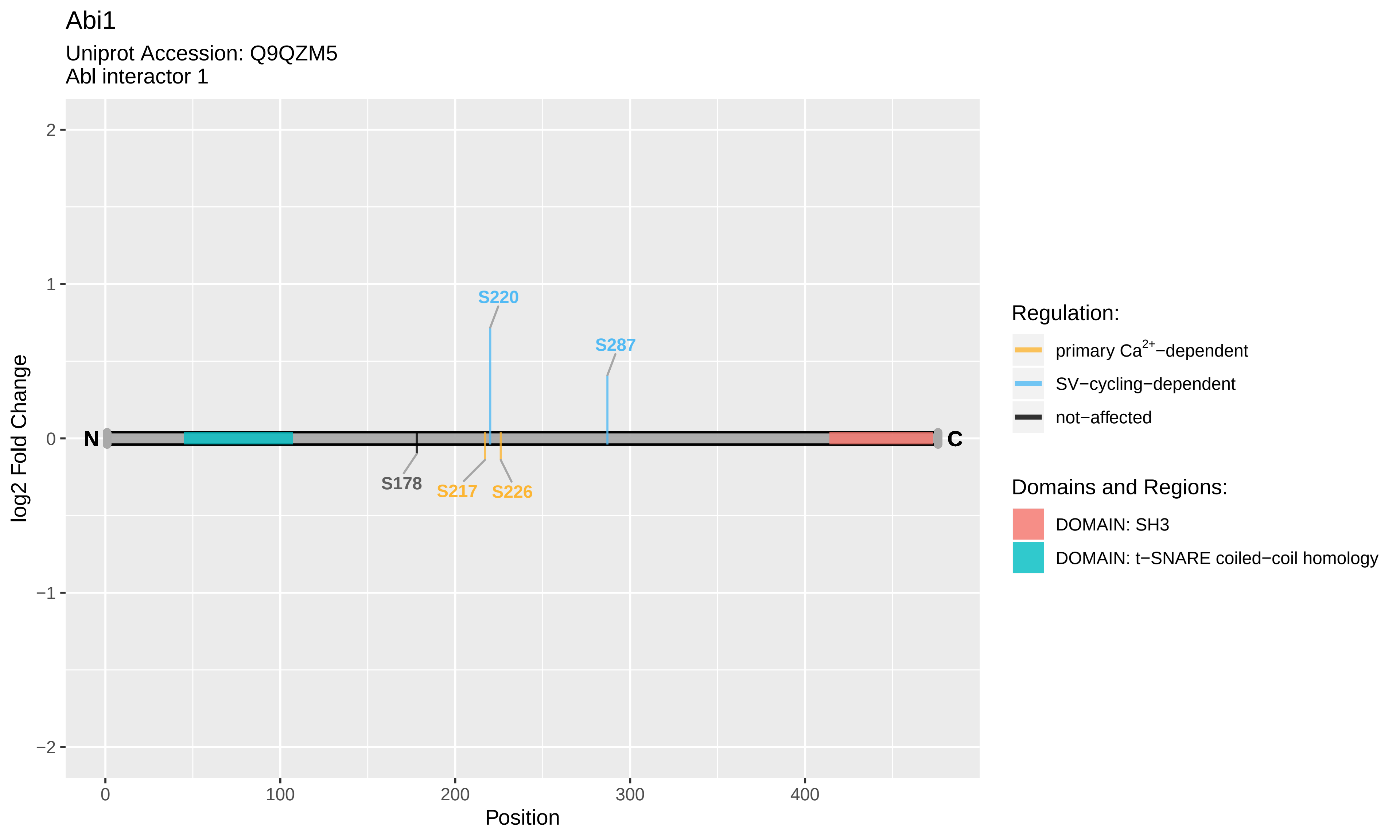

Abi1
Uniprot Accession: Q9QZM5
Abl interactor 1
log2 Fold Change
2
1
0
−1
−2
N
C
S220
S287
S178
S217
S226
0
100
200
300
400
Position
Regulation:
primary Ca2+−dependent
SV−cycling−dependent
not−affected
Domains and Regions:
DOMAIN: SH3
DOMAIN: t−SNARE coiled−coil homology

Abi2

Uniprot Accession: F1LYA6
Abl−interactor 2

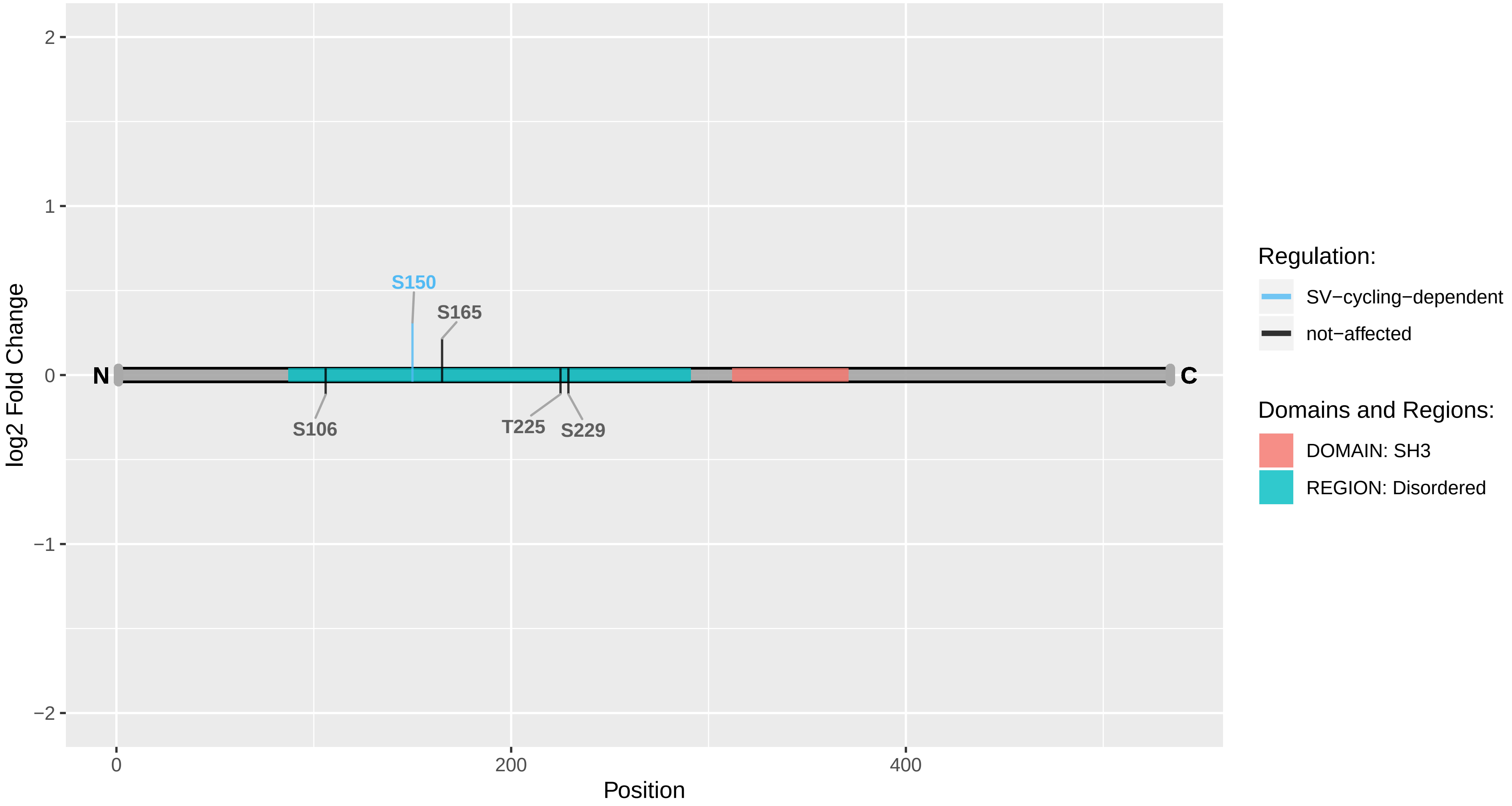

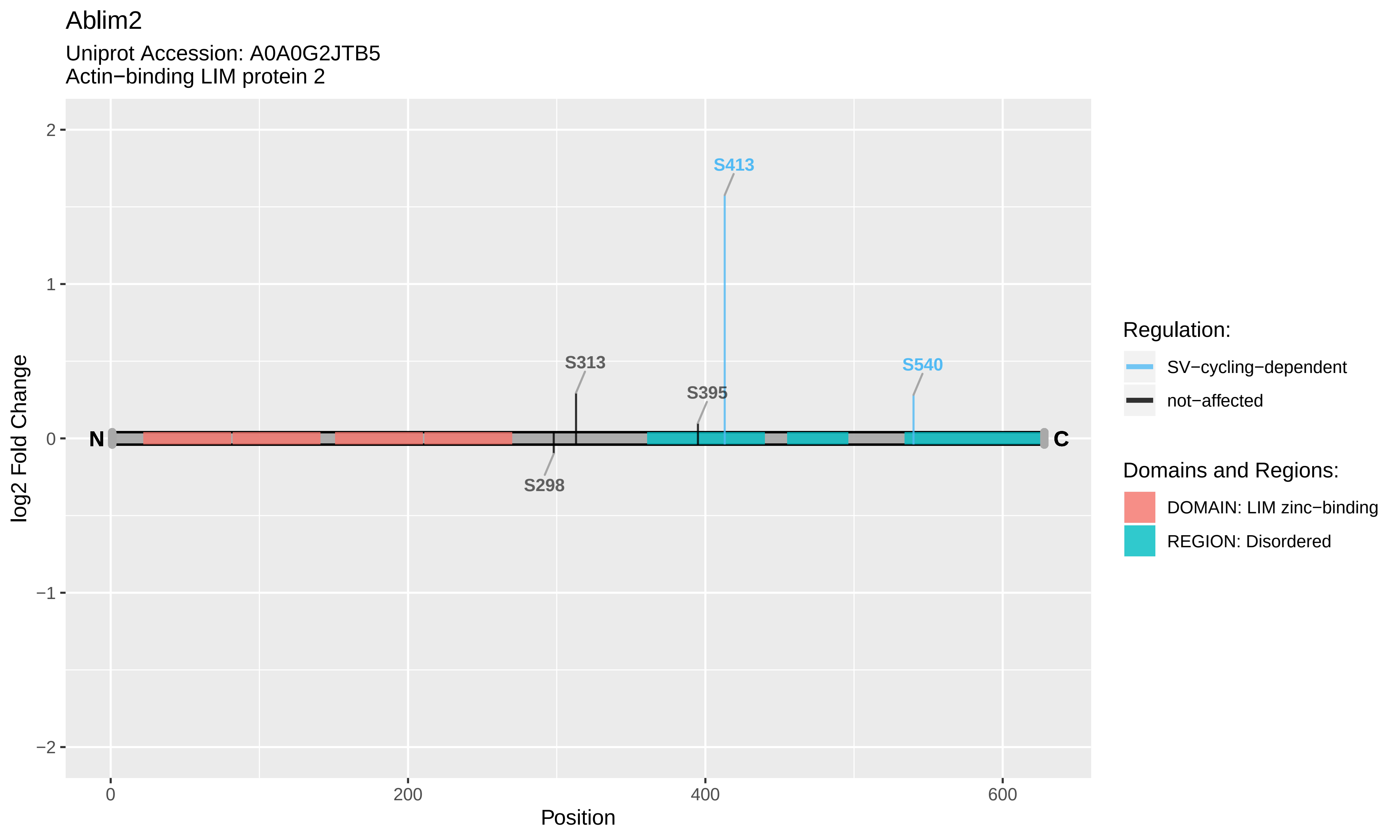

Ablim2
Uniprot Accession: A0A0G2JTB5
Actin−binding LIM protein 2
S413
S313
S540
S395
S298
N
C
2
1
0
−1
−2
log2 Fold Change
0
200
400
600
Position
Regulation:
SV−cycling−dependent
not−affected
Domains and Regions:
DOMAIN: LIM zinc−binding
REGION: Disordered

Ablim2

Uniprot Accession: Q6KC51
Actin−binding LIM protein 2

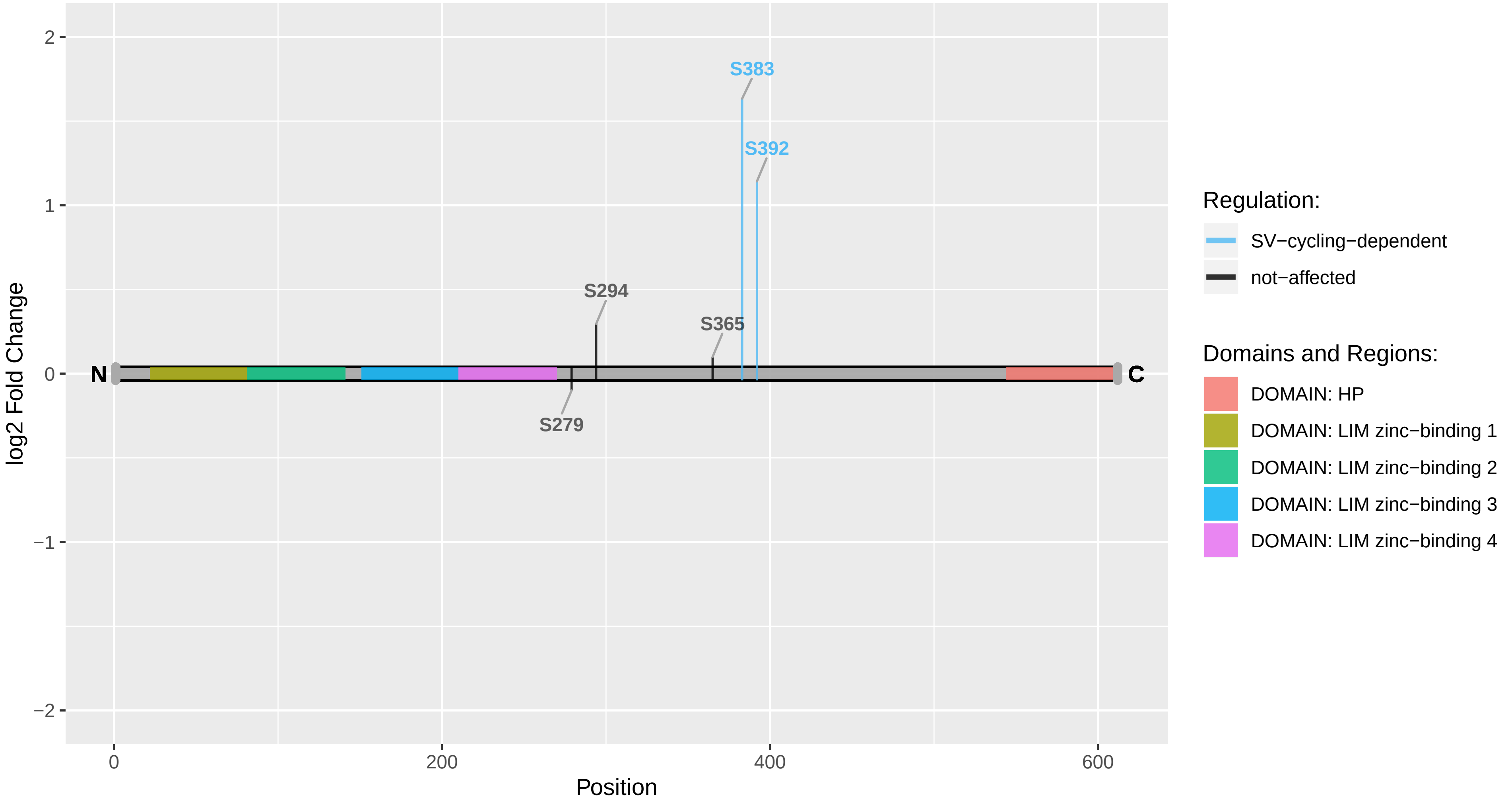

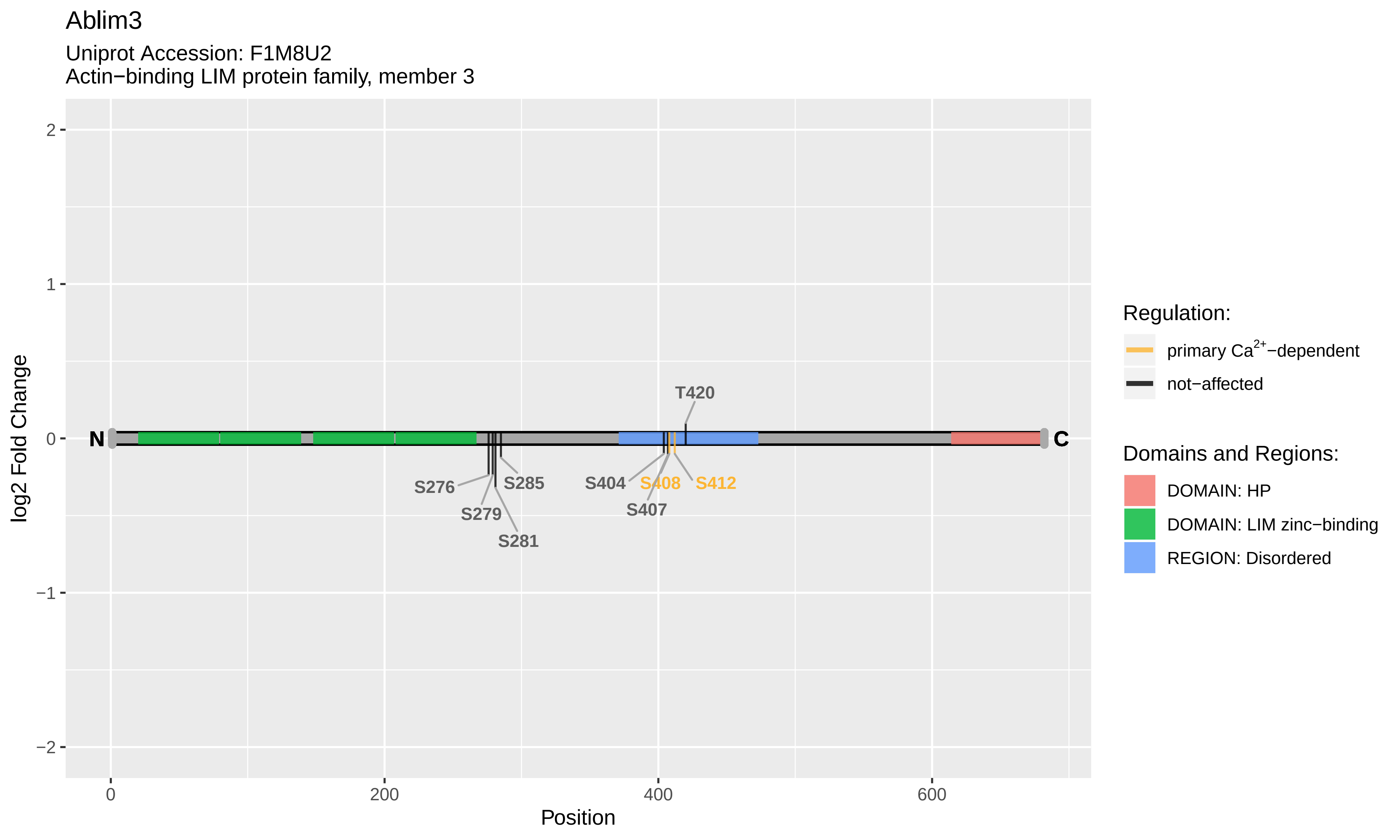

Ablim3
Uniprot Accession: F1M8U2
Actin−binding LIM protein family, member 3
log2 Fold Change
2
1
0
−1
−2
N
C
T420
S276
S279
S281
S285
S404
S408
S407
S412
0
200
400
600
Position
Regulation:
primary Ca2+−dependent
not−affected
Domains and Regions:
DOMAIN: HP
DOMAIN: LIM zinc−binding
REGION: Disordered

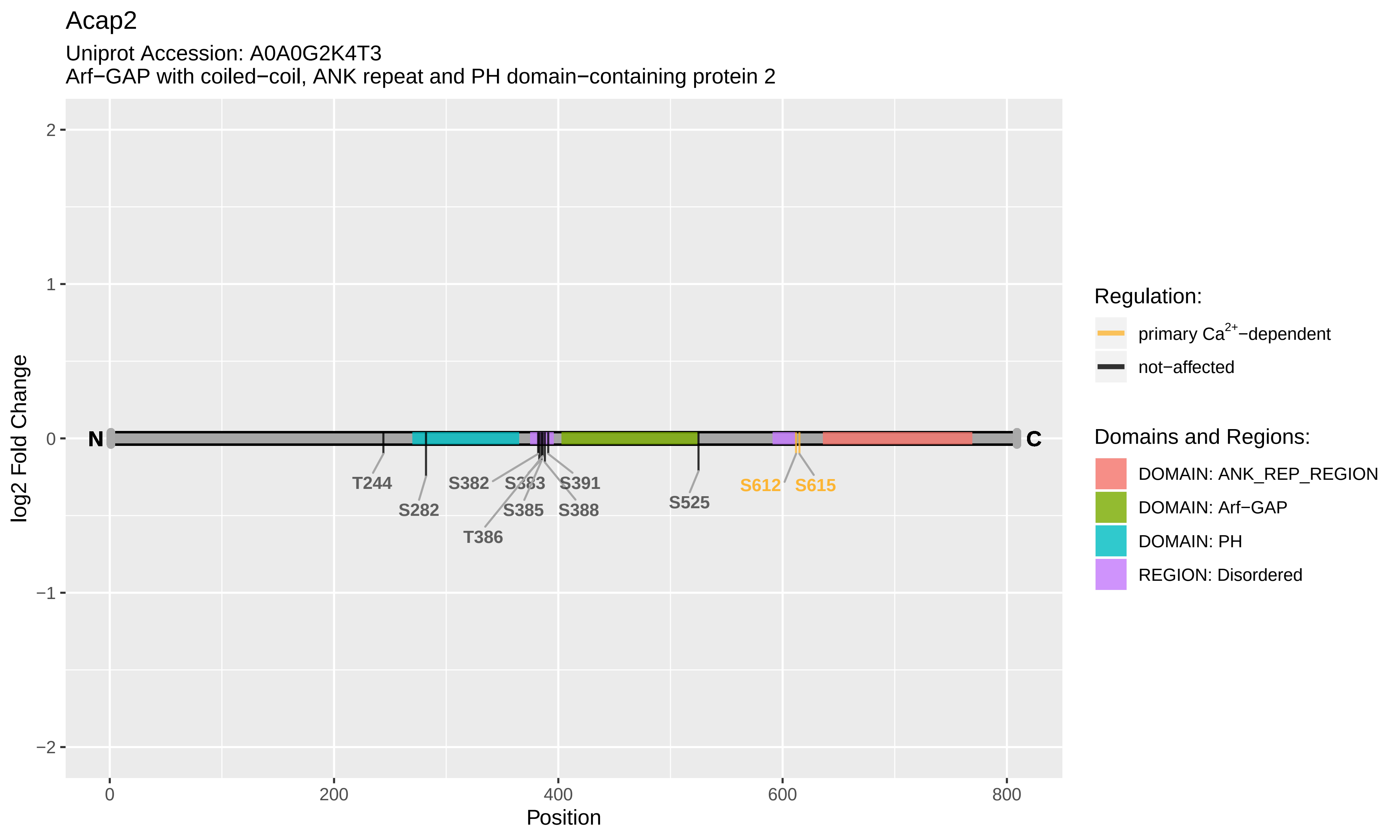

Acap2
Uniprot Accession: A0A0G2K4T3
Arf−GAP with coiled−coil, ANK repeat and PH domain−containing protein 2
log2 Fold Change
2
1
0
−1
−2
N
C
T244
S282
S382
S383
S391
T386
S385
S388
S525
S612
S615
0
200
400
600
800
Position
Regulation:
primary Ca2+−dependent
not−affected
Domains and Regions:
DOMAIN: ANK_REP_REGION
DOMAIN: Arf−GAP
DOMAIN: PH
REGION: Disordered

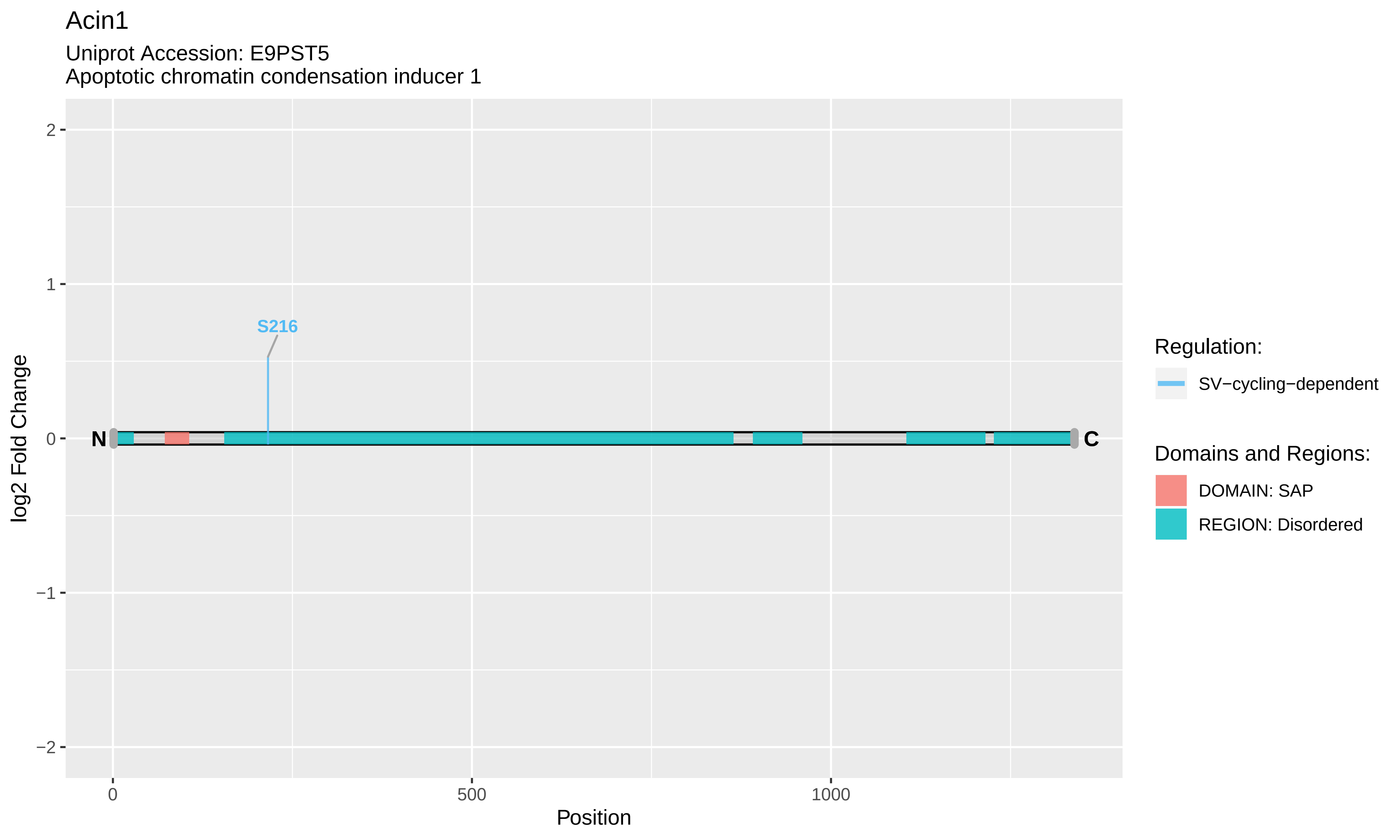
Acin1
Uniprot Accession: E9PST5
Apoptotic chromatin condensation inducer 1
log2 Fold Change
2
1
0
−1
−2
N
C
S216
0
500
1000
Position
Regulation:
SV−cycling−dependent
Domains and Regions:
DOMAIN: SAP
REGION: Disordered

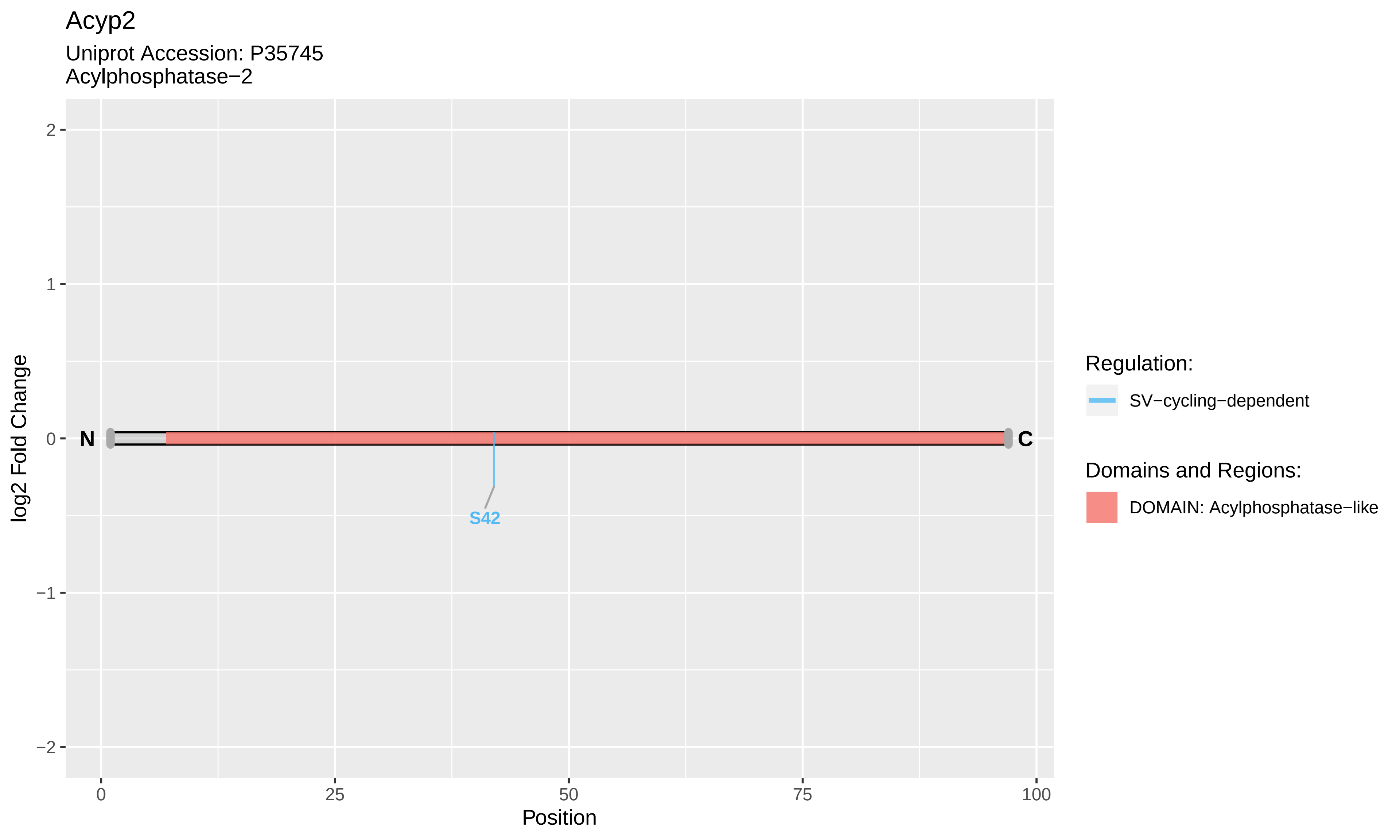
Acyp2
Uniprot Accession: P35745
Acylphosphatase−2
log2 Fold Change
2
1
0
−1
−2
N
C
S42
0
25
50
75
100
Position
Regulation:
SV−cycling−dependent
Domains and Regions:
DOMAIN: Acylphosphatase−like

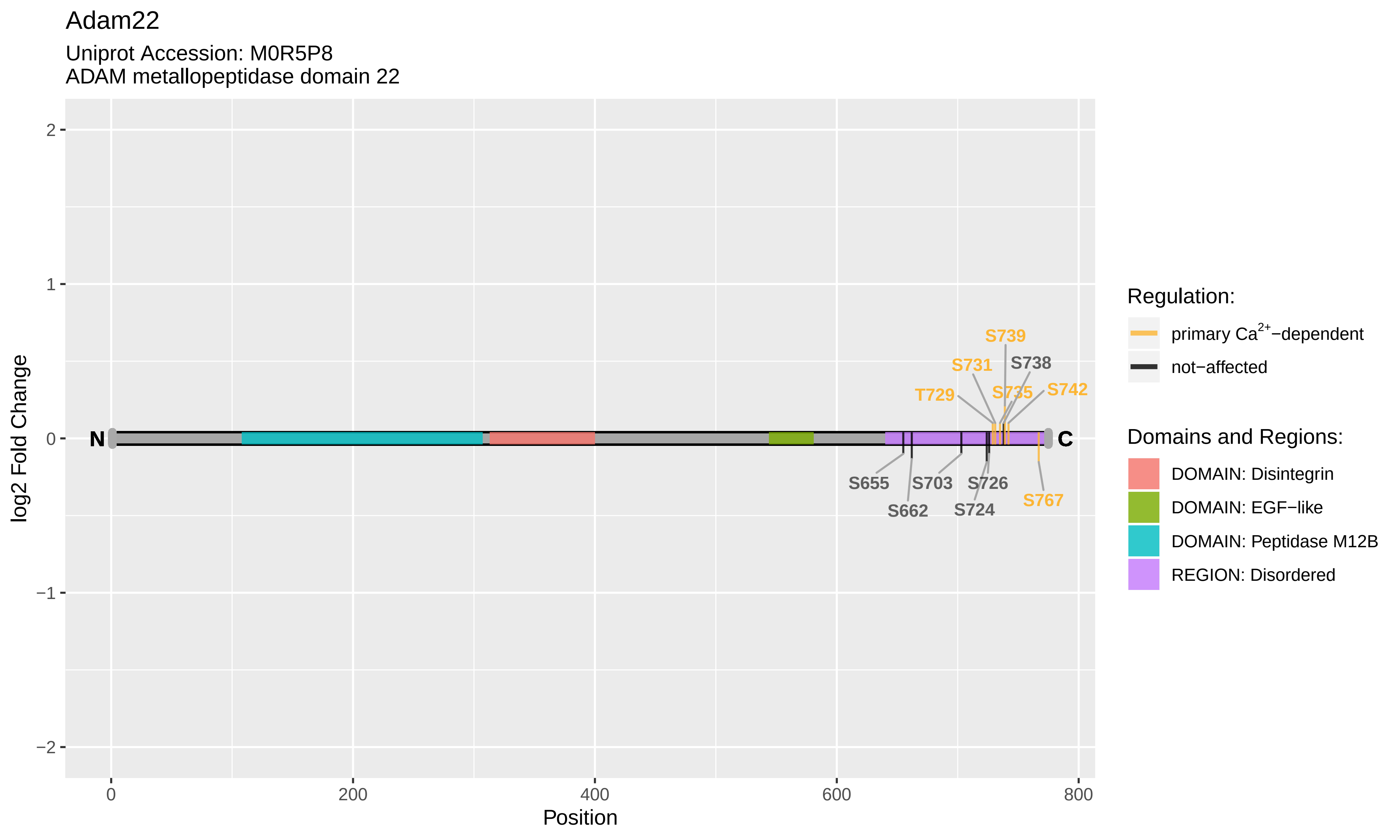
Adam22
Uniprot Accession: M0R5P8
ADAM metallopeptidase domain 22
log2 Fold Change
2
1
0
−1
−2
N
C
S739
S731
S738
T729
S735
S742
S655
S703
S726
S767
S662
S724
0
200
400
600
800
Position
Regulation:
primary Ca2+−dependent
not−affected
Domains and Regions:
DOMAIN: Disintegrin
DOMAIN: EGF−like
DOMAIN: Peptidase M12B
REGION: Disordered

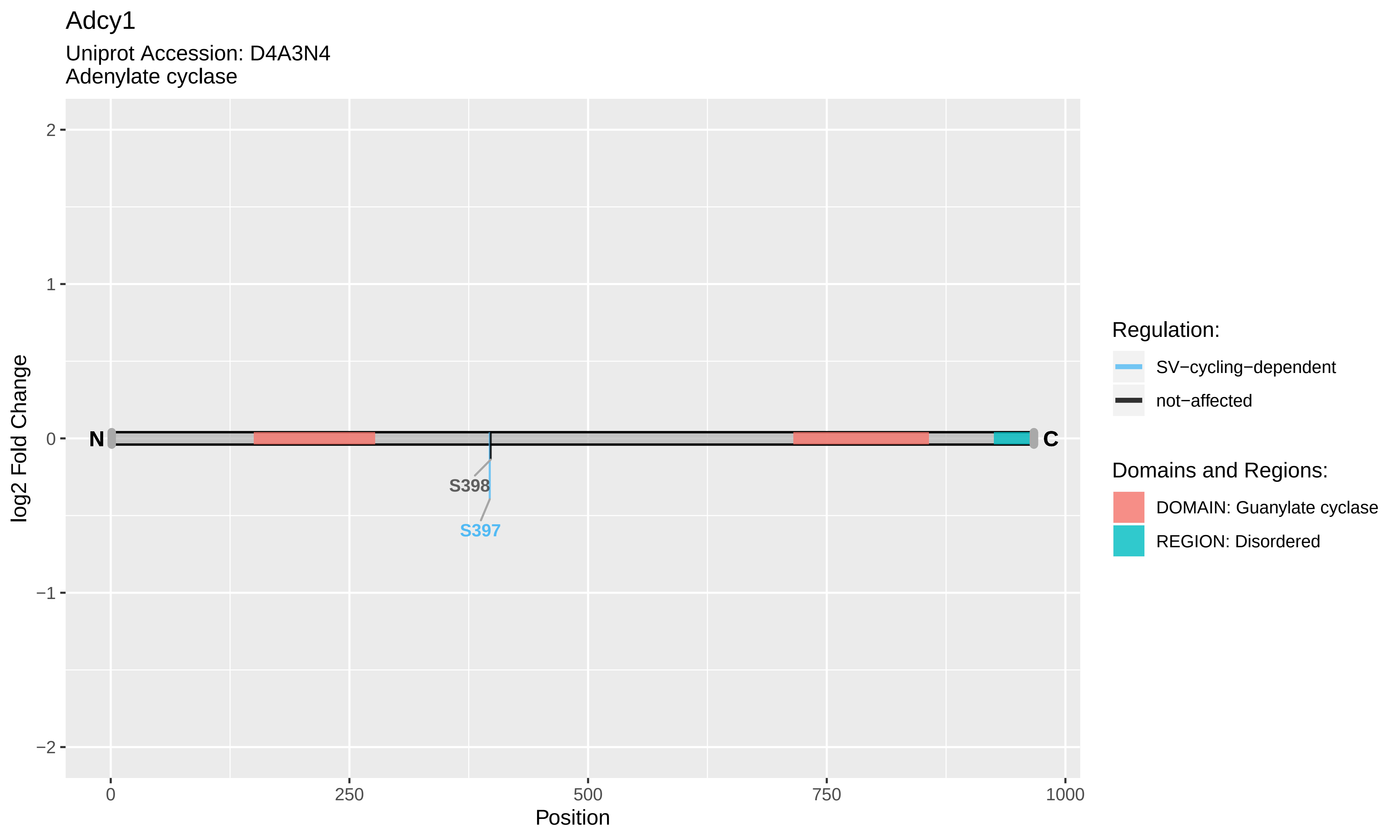

Adcy1
Uniprot Accession: D4A3N4
Adenylate cyclase
log2 Fold Change
2
1
0
−1
−2
N
C
S398
S397
0
250
500
750
1000
Position
Regulation:
SV−cycling−dependent
not−affected
Domains and Regions:
DOMAIN: Guanylate cyclase
REGION: Disordered

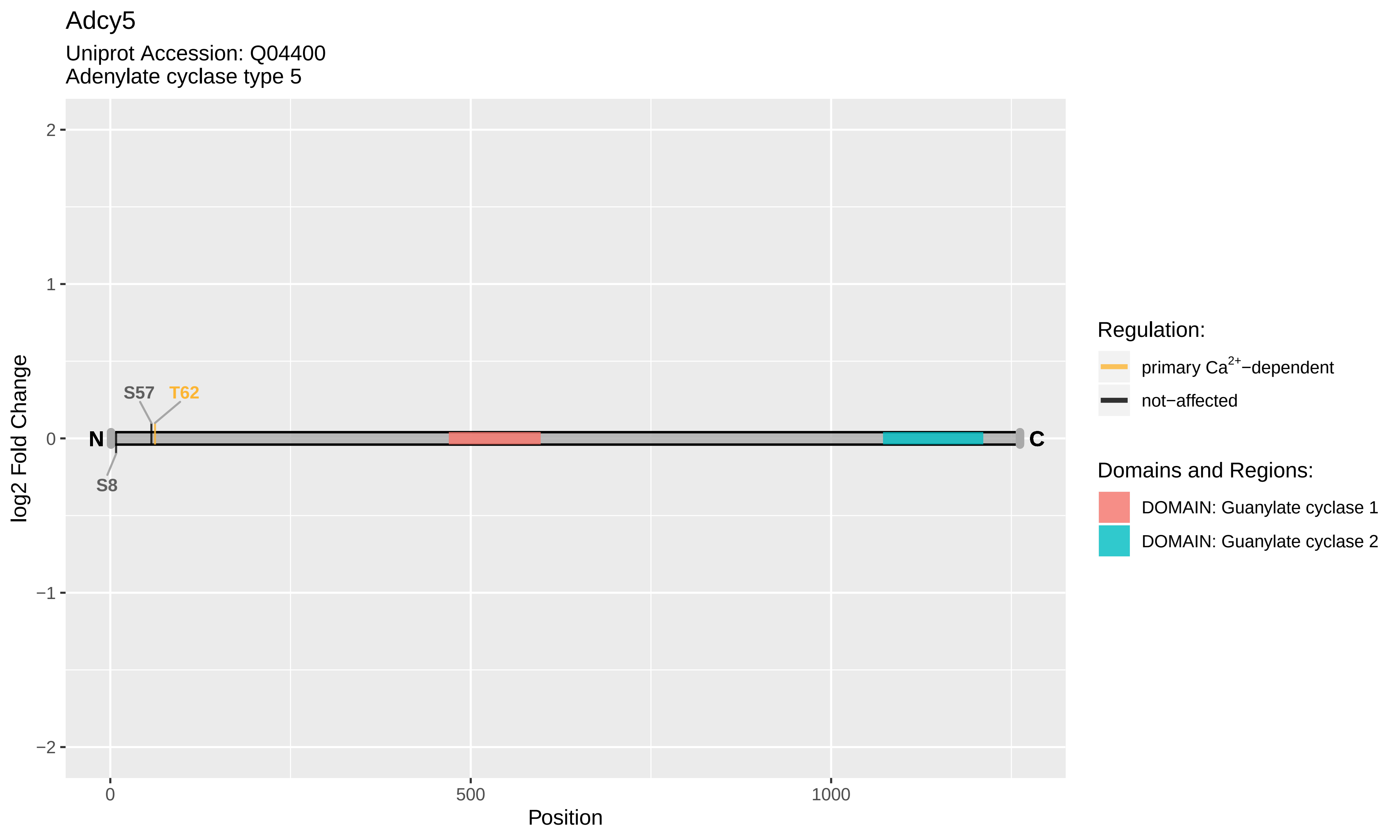

Adcy5
Uniprot Accession: Q04400
Adenylate cyclase type 5
S57
T62
N
C
S8
2
1
0
−1
−2
log2 Fold Change
0
500
1000
Position
Regulation:
primary Ca2+−dependent
not−affected
Domains and Regions:
DOMAIN: Guanylate cyclase 1
DOMAIN: Guanylate cyclase 2

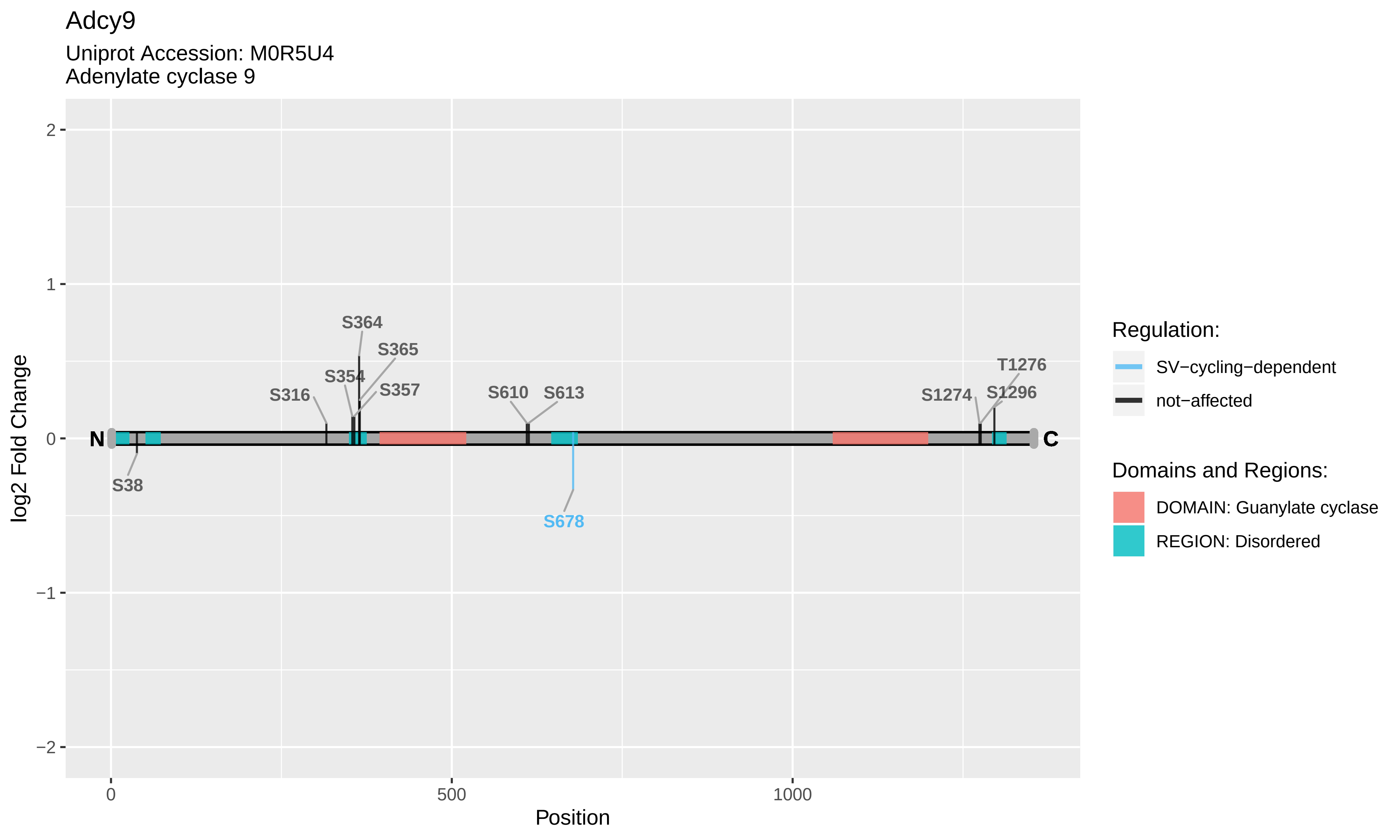

Adcy9
Uniprot Accession: M0R5U4
Adenylate cyclase 9
log2 Fold Change
2
1
0
−1
−2
N
C
S364
S365
S354
S357
S316
S610
S613
T1276
S1274
S1296
S38
S678
0
500
1000
Position
Regulation:
SV−cycling−dependent
not−affected
Domains and Regions:
DOMAIN: Guanylate cyclase
REGION: Disordered

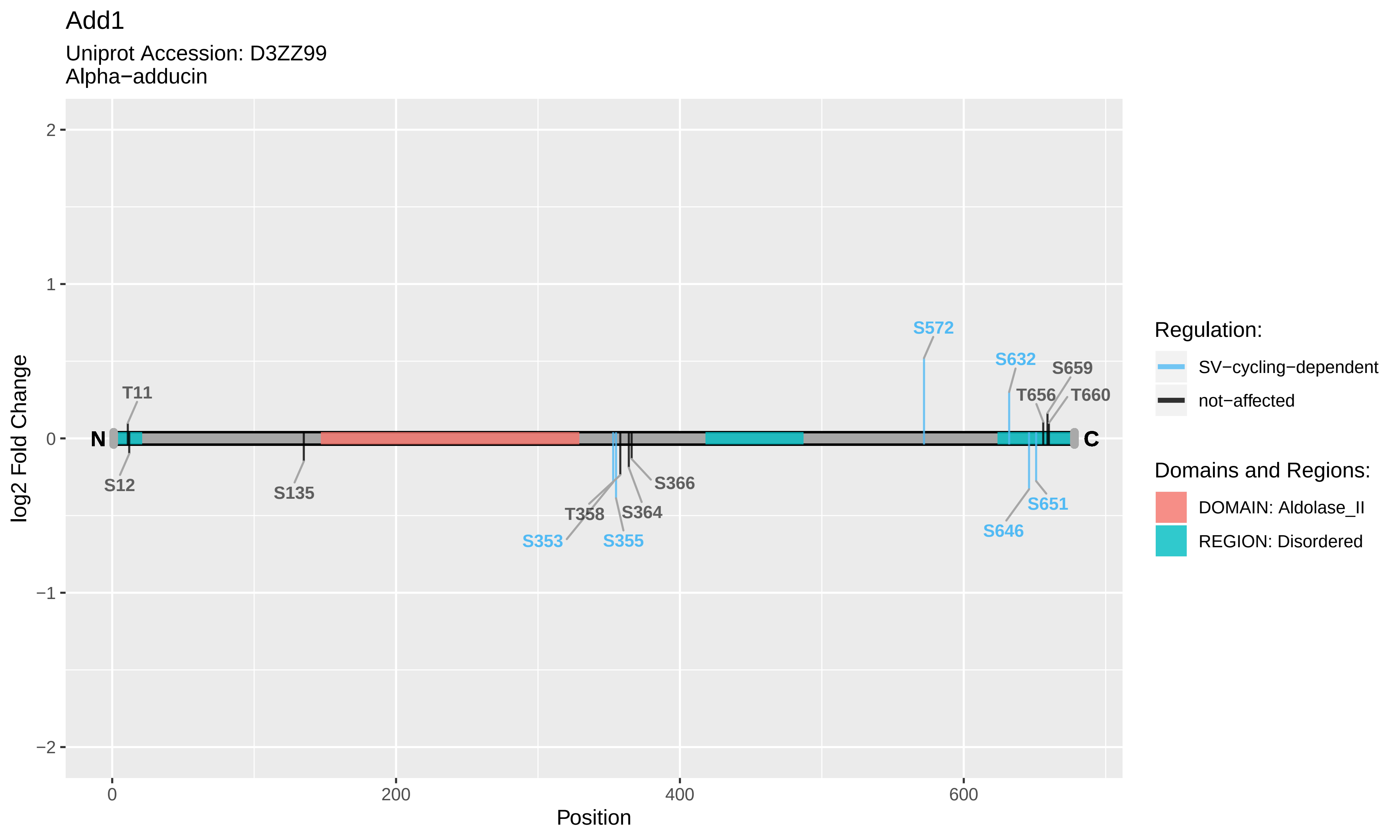
Add1
Uniprot Accession: D3ZZ99
Alpha−adducin
log2 Fold Change
2
1
0
−1
−2
0
200
400
600
Position
N
C
T11
S12
S135
T358
S353
S355
S364
S366
S572
S632
S659
T656
T660
S646
S651
Regulation:
SV−cycling−dependent
not−affected
Domains and Regions:
DOMAIN: Aldolase_II
REGION: Disordered

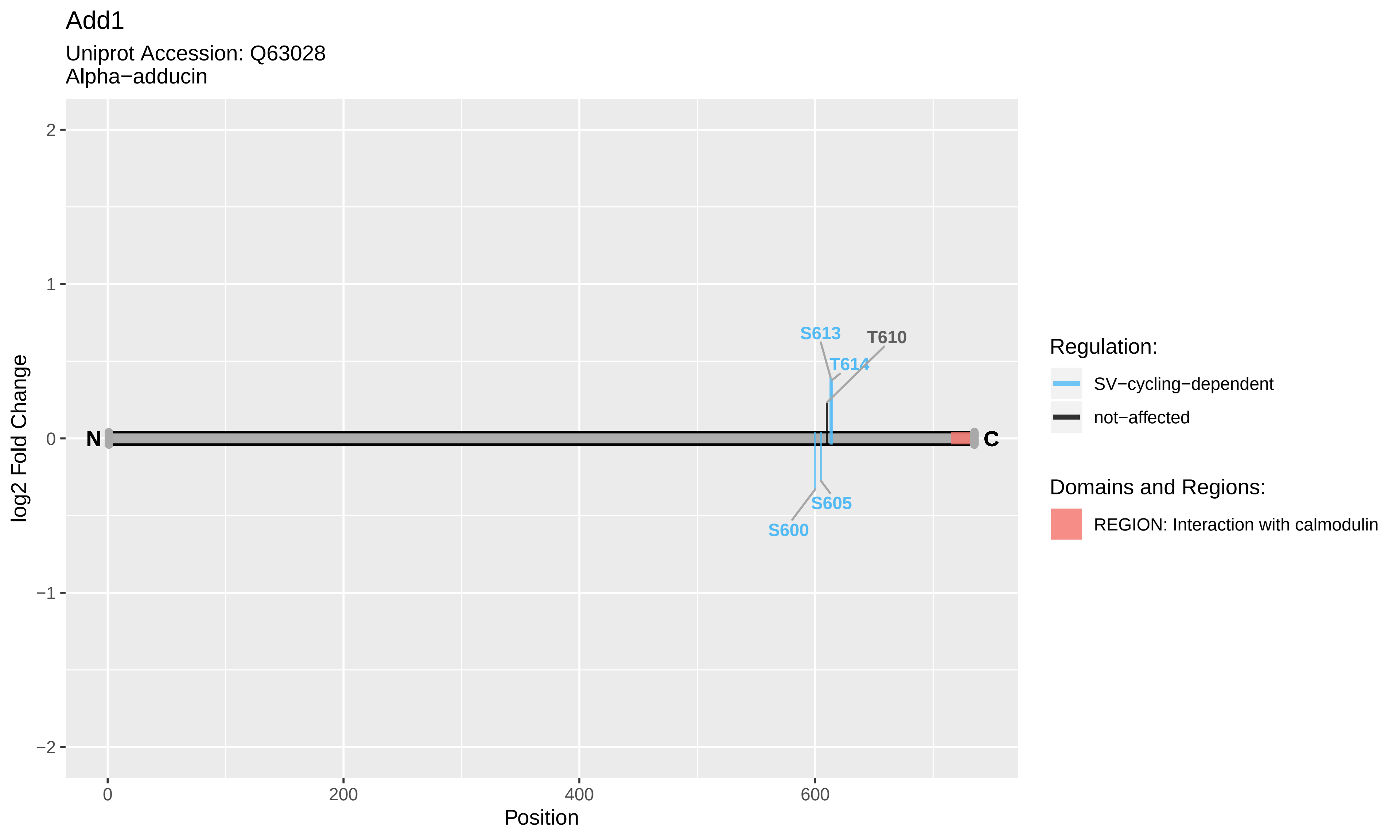
Add1
Uniprot Accession: Q63028
Alpha−adducin
log2 Fold Change
Position
N
C
S613
T610
T614
S605
S600
2
1
0
−1
−2
0
200
400
600
Regulation:
SV−cycling−dependent
not−affected
Domains and Regions:
REGION: Interaction with calmodulin

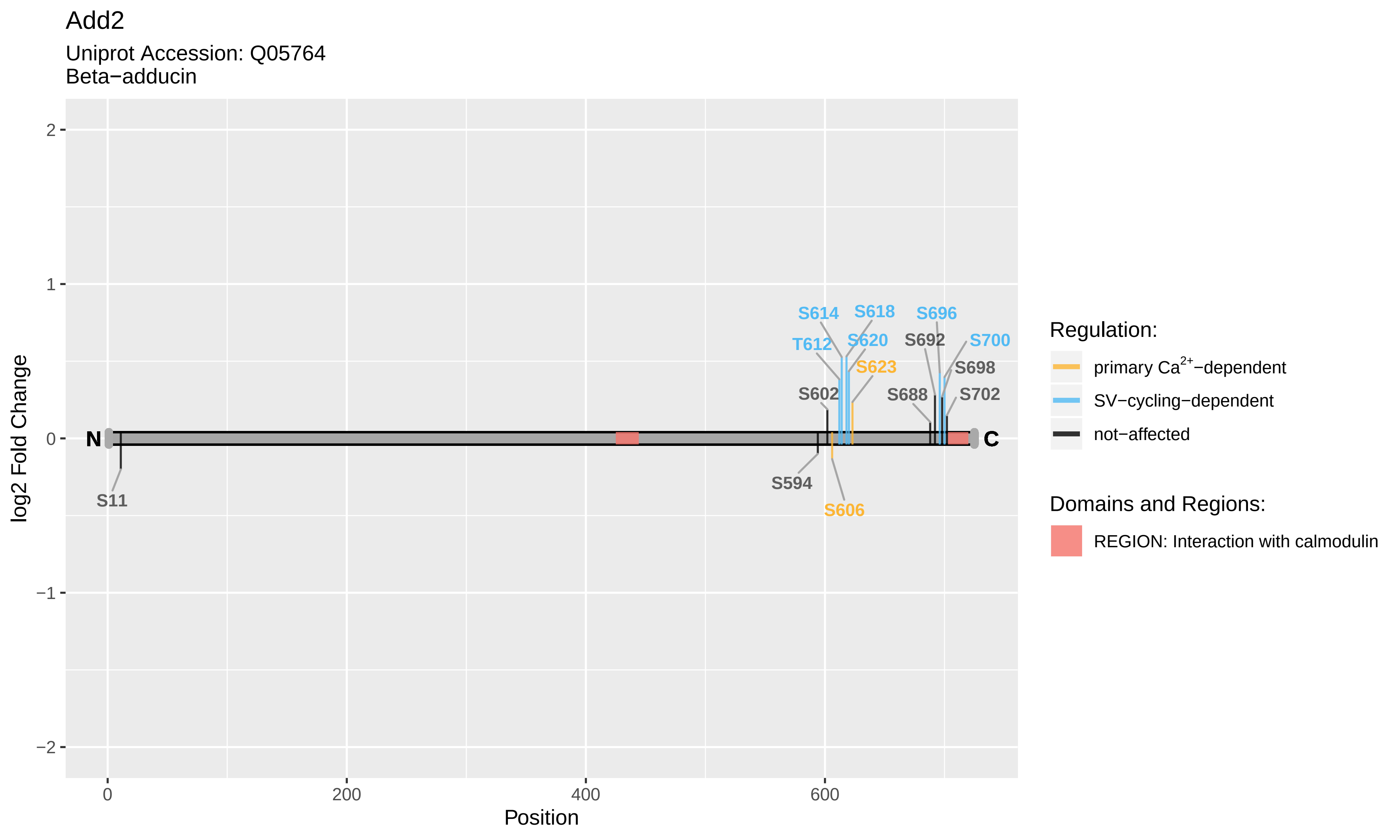

Add2
Uniprot Accession: Q05764
Beta−adducin
log2 Fold Change
2
1
0
−1
−2
Position
0
200
400
600
N
C
S11
S594
S602
S606
T612
S614
S618
S620
S623
S688
S692
S696
S698
S700
S702
Regulation:
primary Ca2+−dependent
SV−cycling−dependent
not−affected
Domains and Regions:
REGION: Interaction with calmodulin

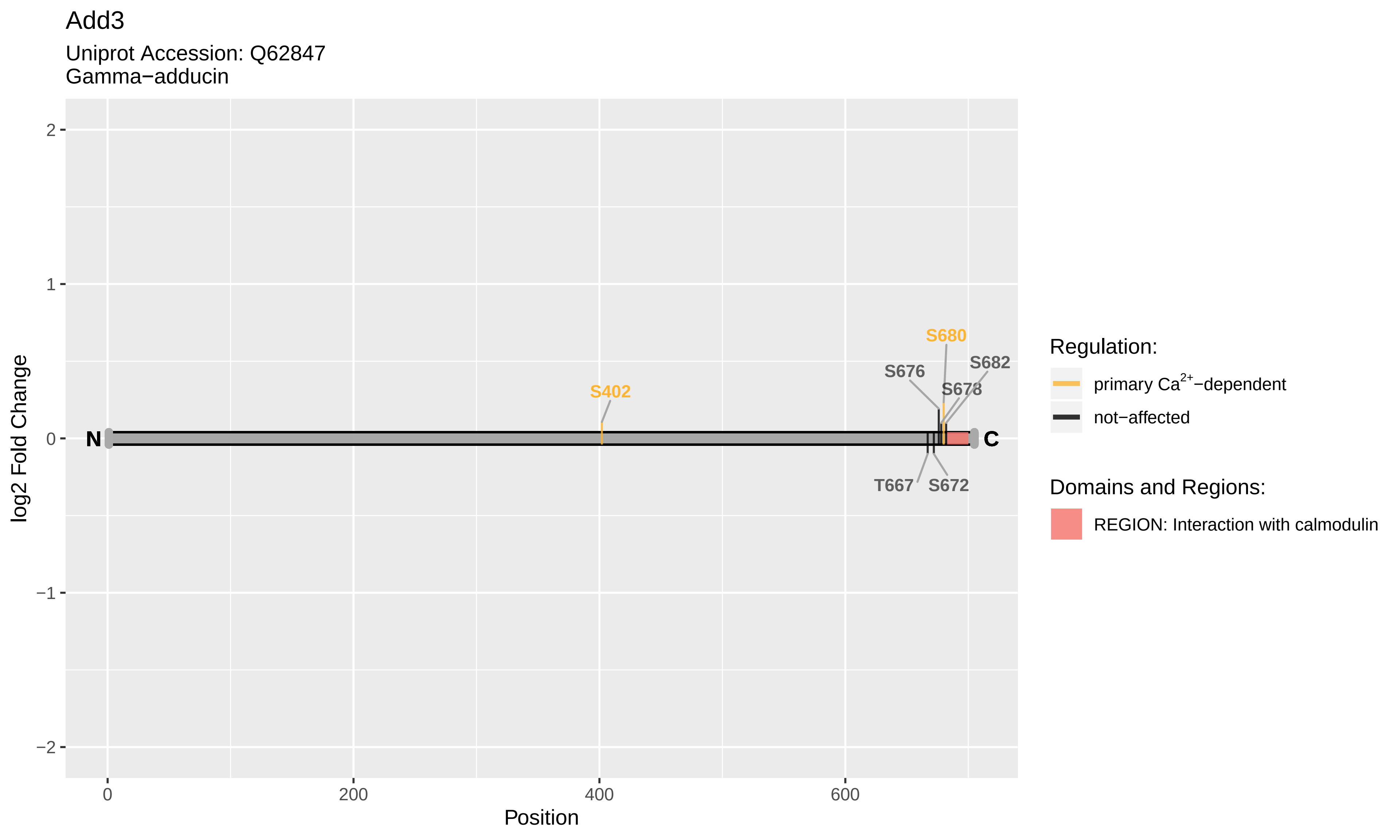
Add3
Uniprot Accession: Q62847
Gamma−adducin
2
1
0
−1
−2
log2 Fold Change
N
C
S402
S680
S676
S682
S678
T667
S672
0
200
400
600
Position
Regulation:
primary Ca2+−dependent
not−affected
Domains and Regions:
REGION: Interaction with calmodulin

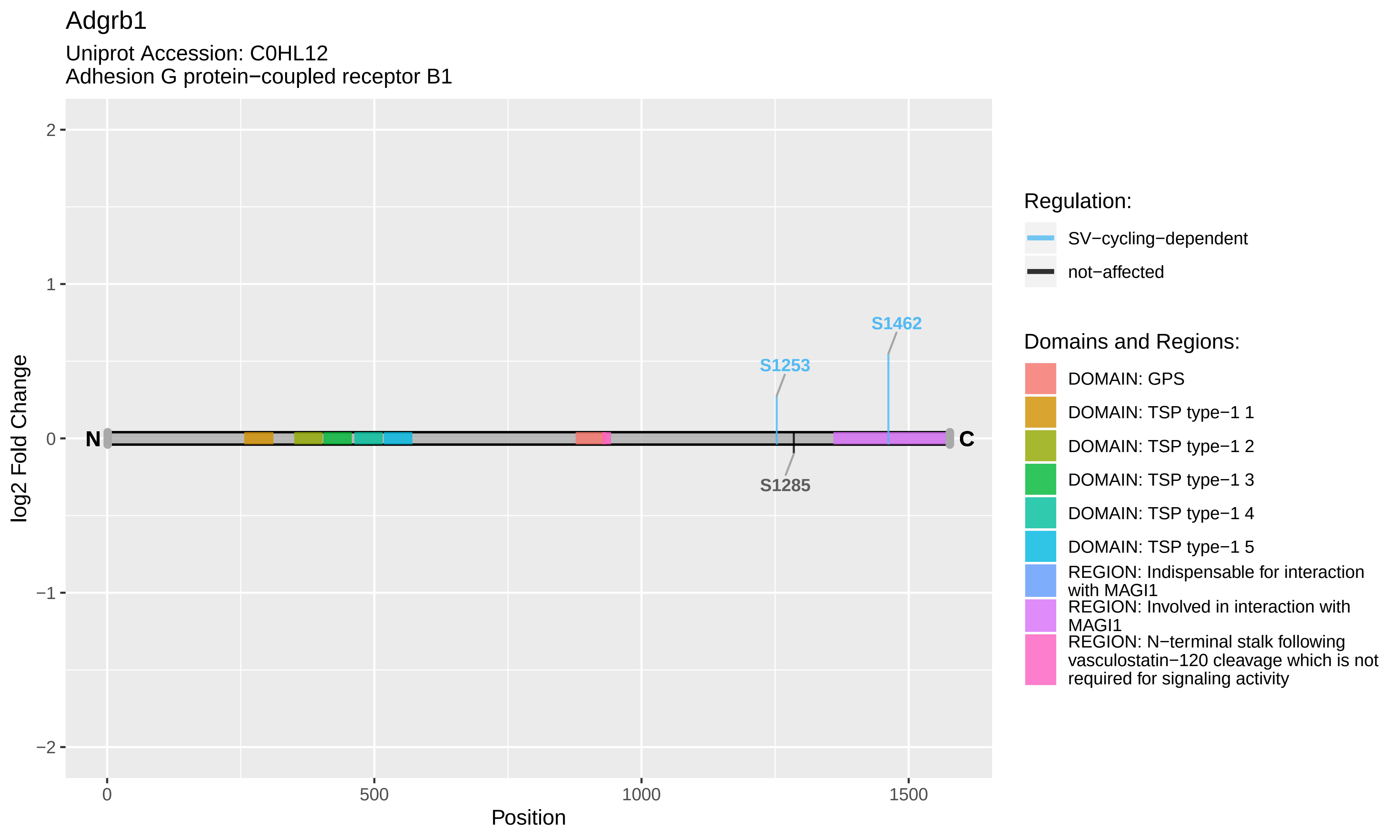

Adgrb1
Uniprot Accession: C0HL12
Adhesion G protein−coupled receptor B1
log2 Fold Change
2
1
0
−1
−2
N
C
S1253
S1462
S1285
0
500
1000
1500
Position
Regulation:
SV−cycling−dependent
not−affected
Domains and Regions:
DOMAIN: GPS
DOMAIN: TSP type−1 1
DOMAIN: TSP type−1 2
DOMAIN: TSP type−1 3
DOMAIN: TSP type−1 4
DOMAIN: TSP type−1 5
REGION: Indispensable for interaction with MAGI1
REGION: Involved in interaction with MAGI1
REGION: N−terminal stalk following vasculostatin−120 cleavage which is not required for signaling activity

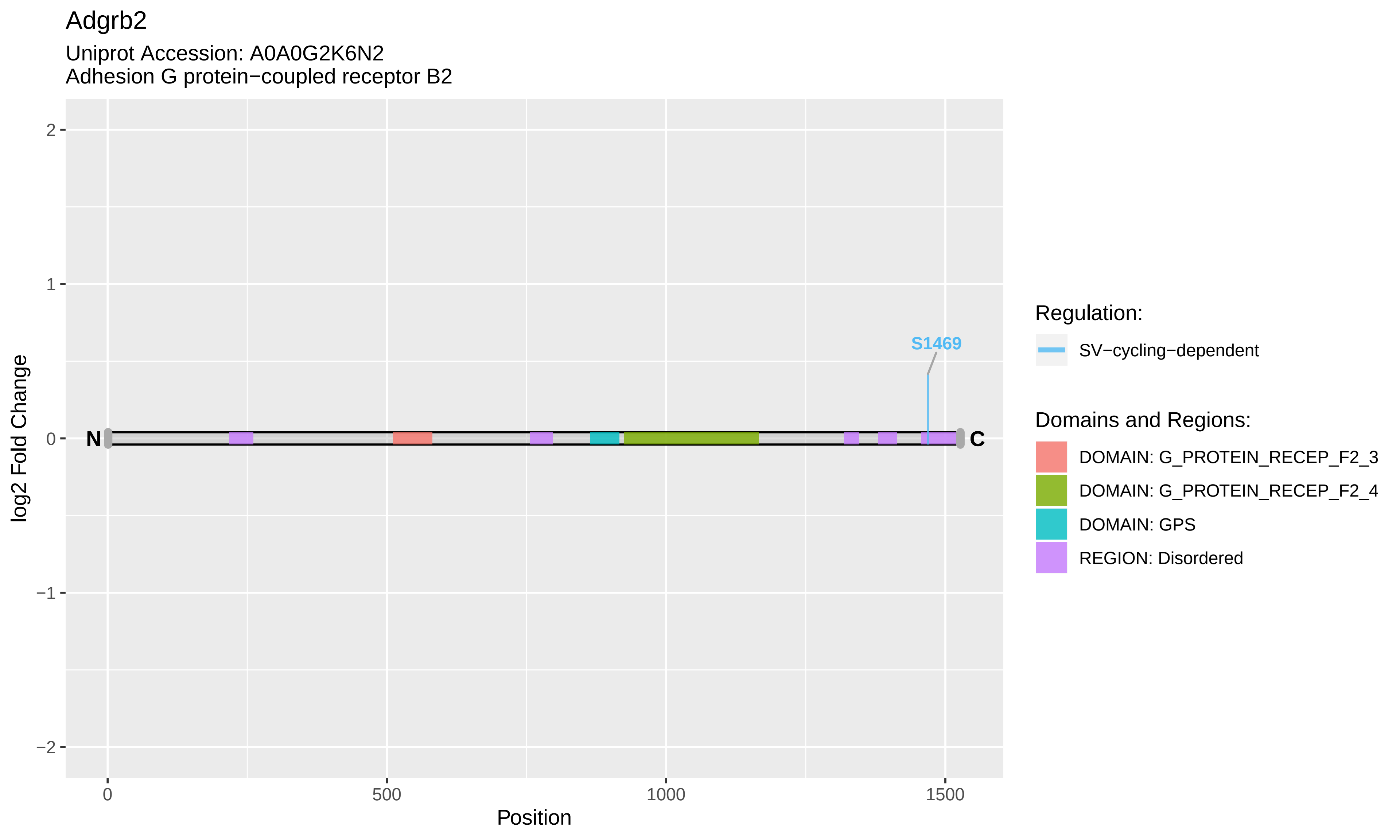

Adgrb2
Uniprot Accession: A0A0G2K6N2
Adhesion G protein−coupled receptor B2
S1469
N
C
2
1
0
−1
−2
log2 Fold Change
0
500
1000
1500
Position
Regulation:
SV−cycling−dependent
Domains and Regions:
DOMAIN: G_PROTEIN_RECEP_F2_3
DOMAIN: G_PROTEIN_RECEP_F2_4
DOMAIN: GPS
REGION: Disordered

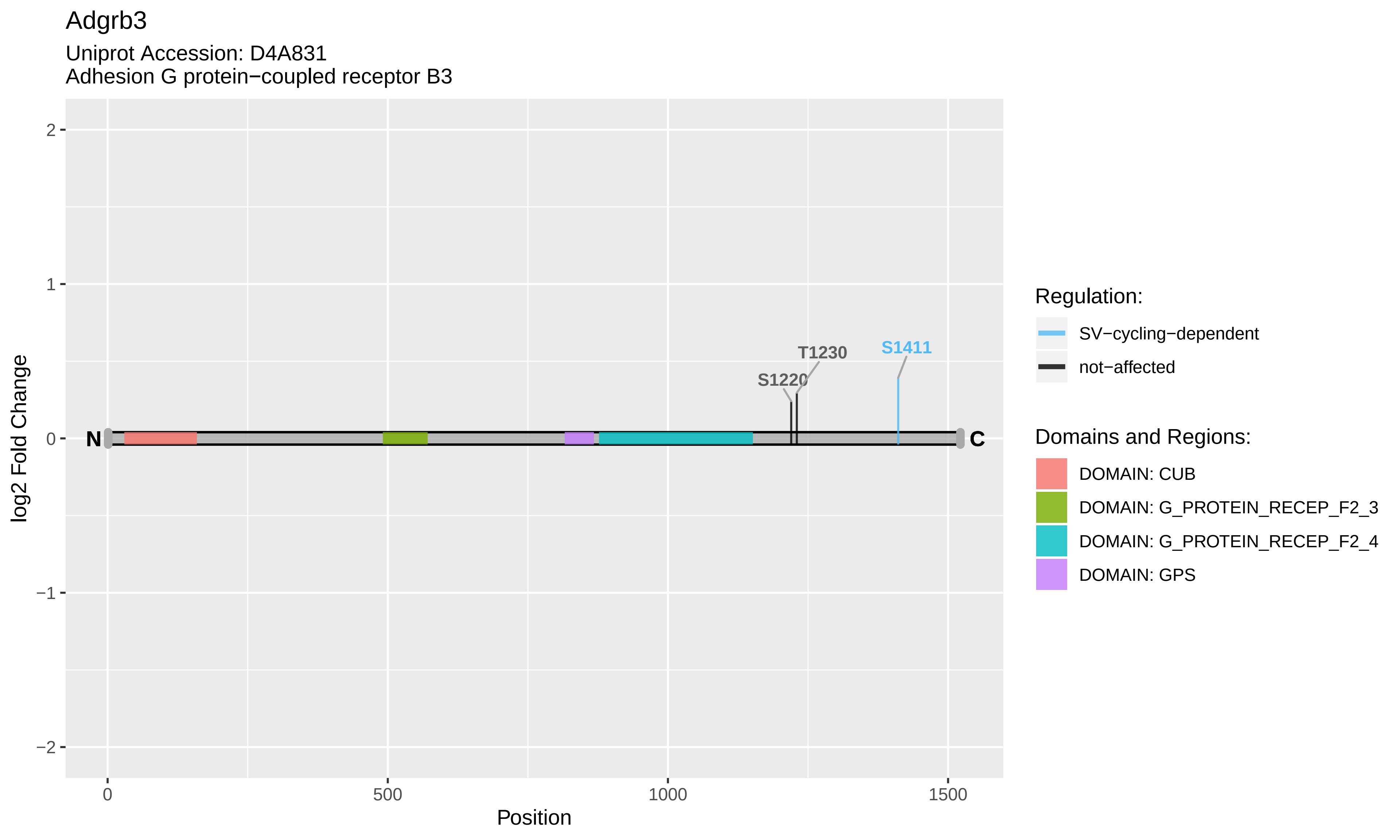
Adgrb3
Uniprot Accession: D4A831
Adhesion G protein−coupled receptor B3
log2 Fold Change
2
1
0
−1
−2
N
C
S1220
T1230
S1411
0
500
1000
1500
Position
Regulation:
SV−cycling−dependent
not−affected
Domains and Regions:
DOMAIN: CUB
DOMAIN: G_PROTEIN_RECEP_F2_3
DOMAIN: G_PROTEIN_RECEP_F2_4
DOMAIN: GPS

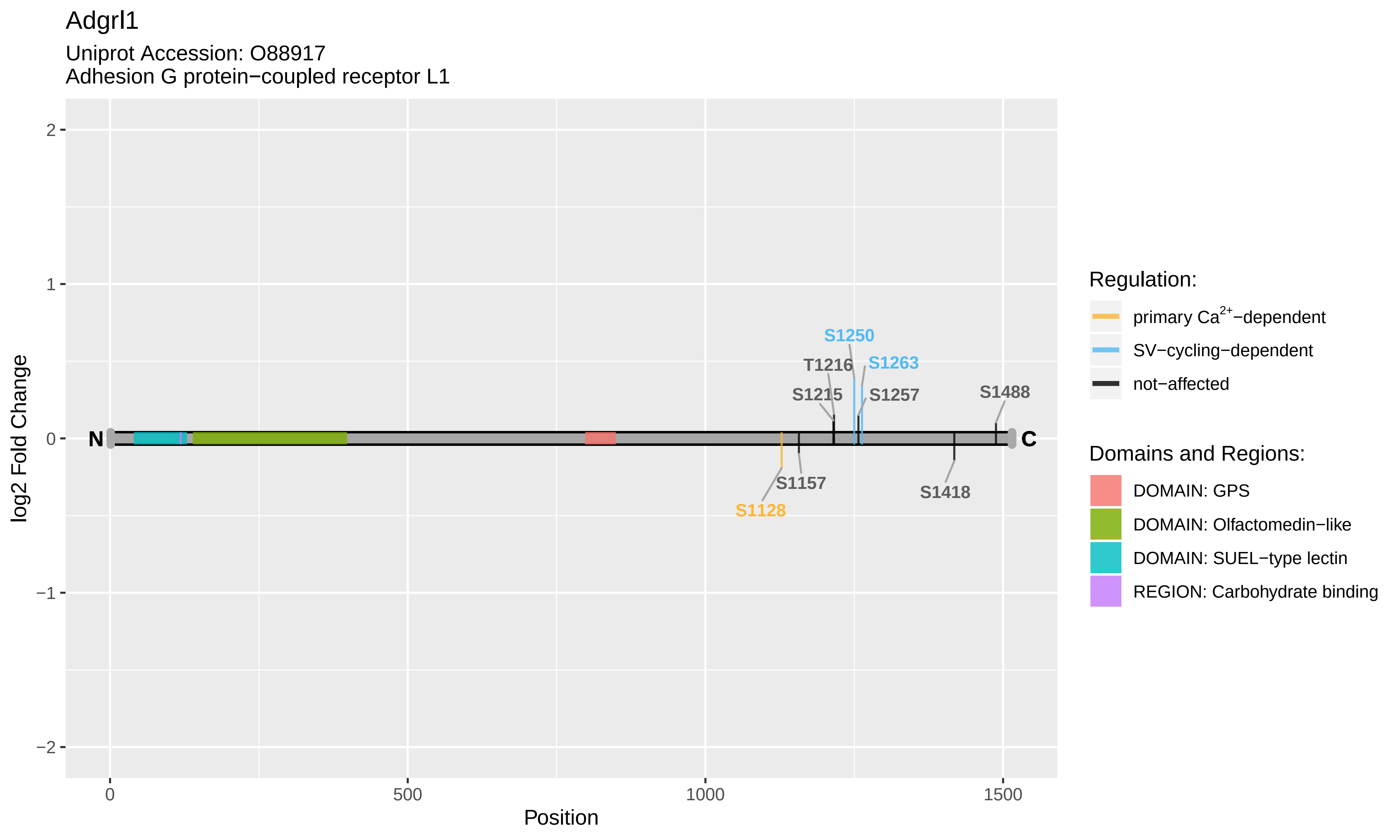
Adgrl1
Uniprot Accession: O88917
Adhesion G protein−coupled receptor L1
log2 Fold Change
2
1
0
−1
−2
N
C
S1250
T1216
S1263
S1215
S1257
S1488
S1157
S1128
S1418
0
500
1000
1500
Position
Regulation:
primary Ca2+−dependent
SV−cycling−dependent
not−affected
Domains and Regions:
DOMAIN: GPS
DOMAIN: Olfactomedin−like
DOMAIN: SUEL−type lectin
REGION: Carbohydrate binding

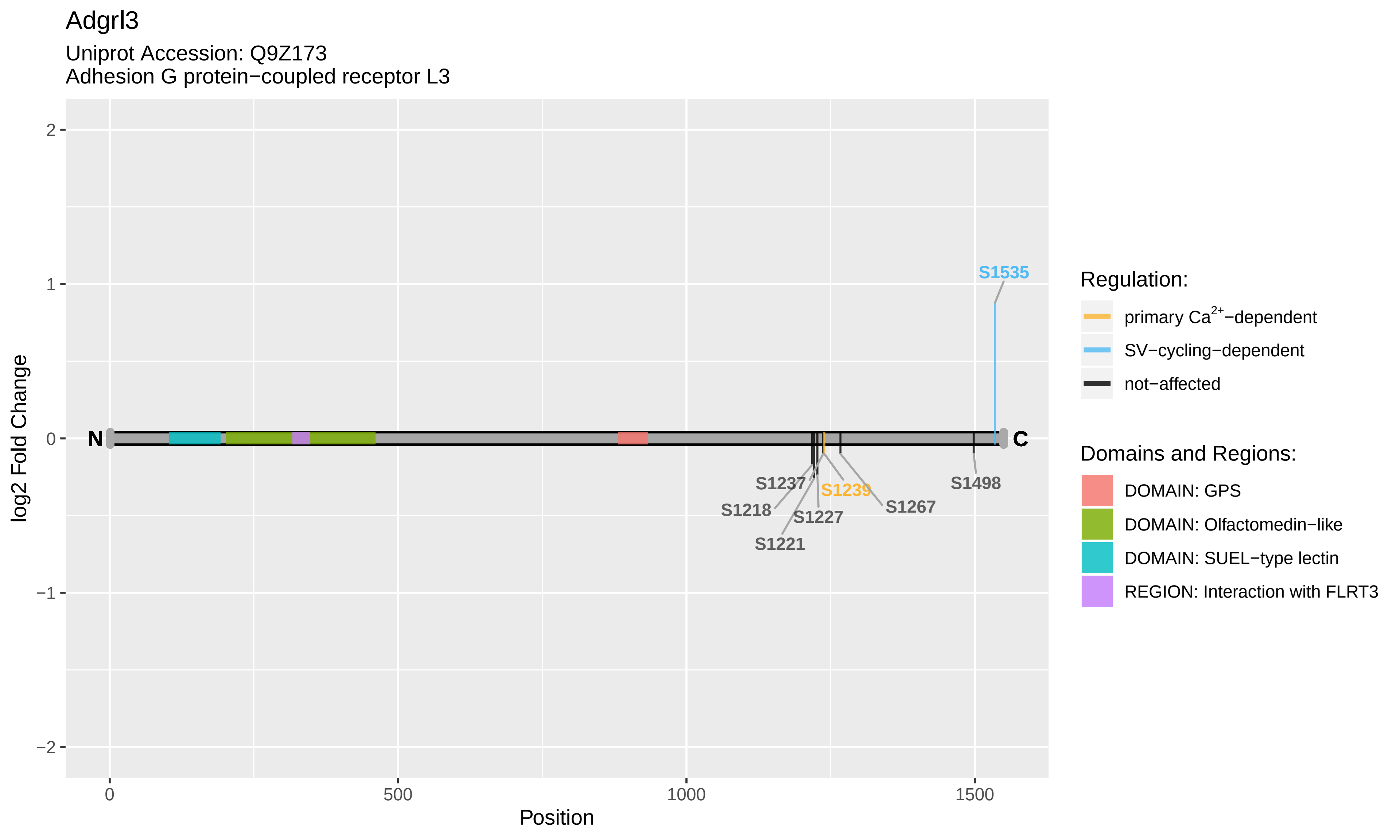

Adgrl3
Uniprot Accession: Q9Z173
Adhesion G protein−coupled receptor L3
log2 Fold Change
Position
2
1
0
−1
−2
0
500
1000
1500
N
C
S1535
S1237
S1239
S1218
S1227
S1221
S1267
S1498
Regulation:
primary Ca2+−dependent
SV−cycling−dependent
not−affected
Domains and Regions:
DOMAIN: GPS
DOMAIN: Olfactomedin−like
DOMAIN: SUEL−type lectin
REGION: Interaction with FLRT3

Adra2a

Uniprot Accession: P22909
Alpha−2A adrenergic receptor

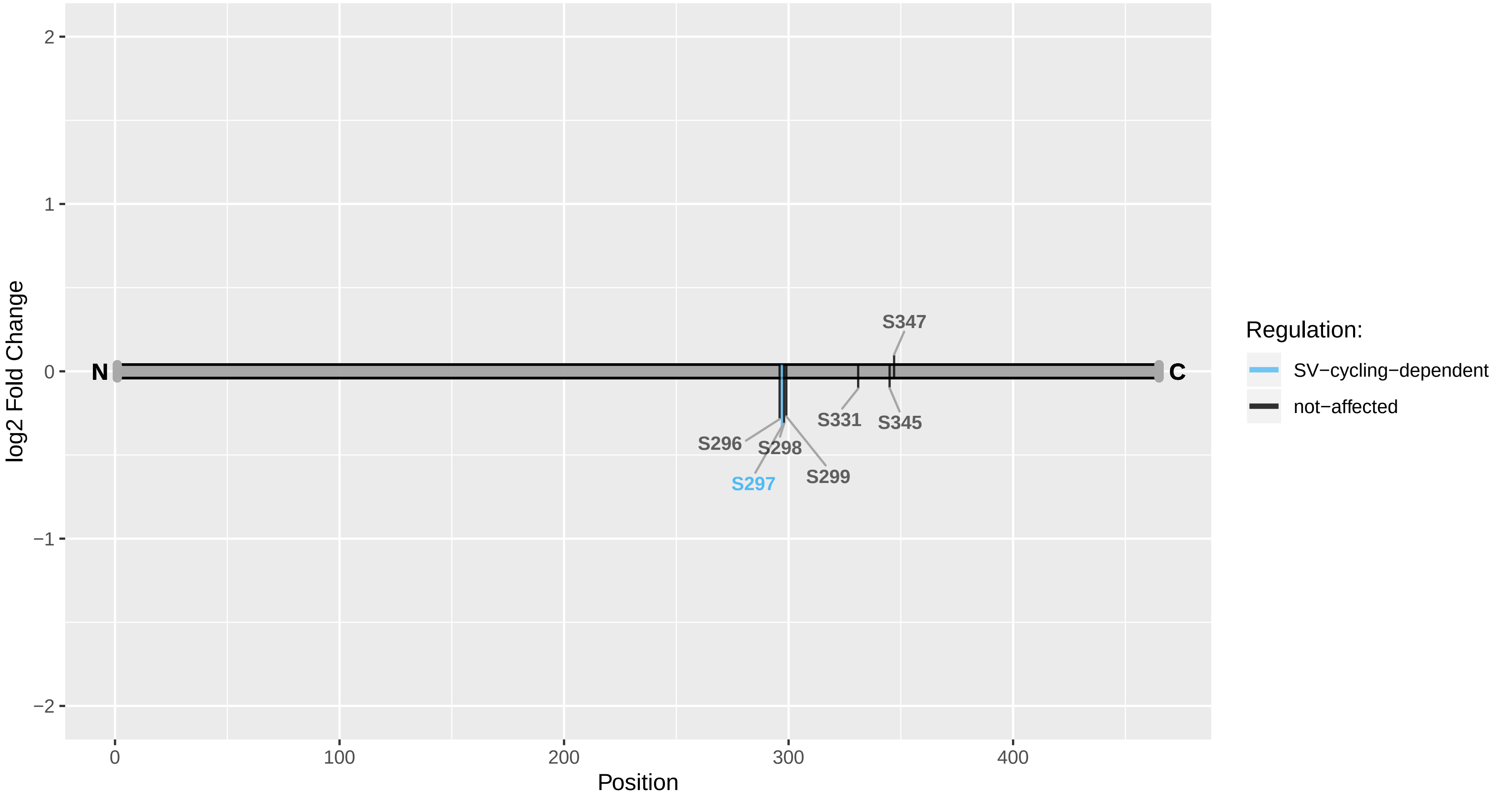

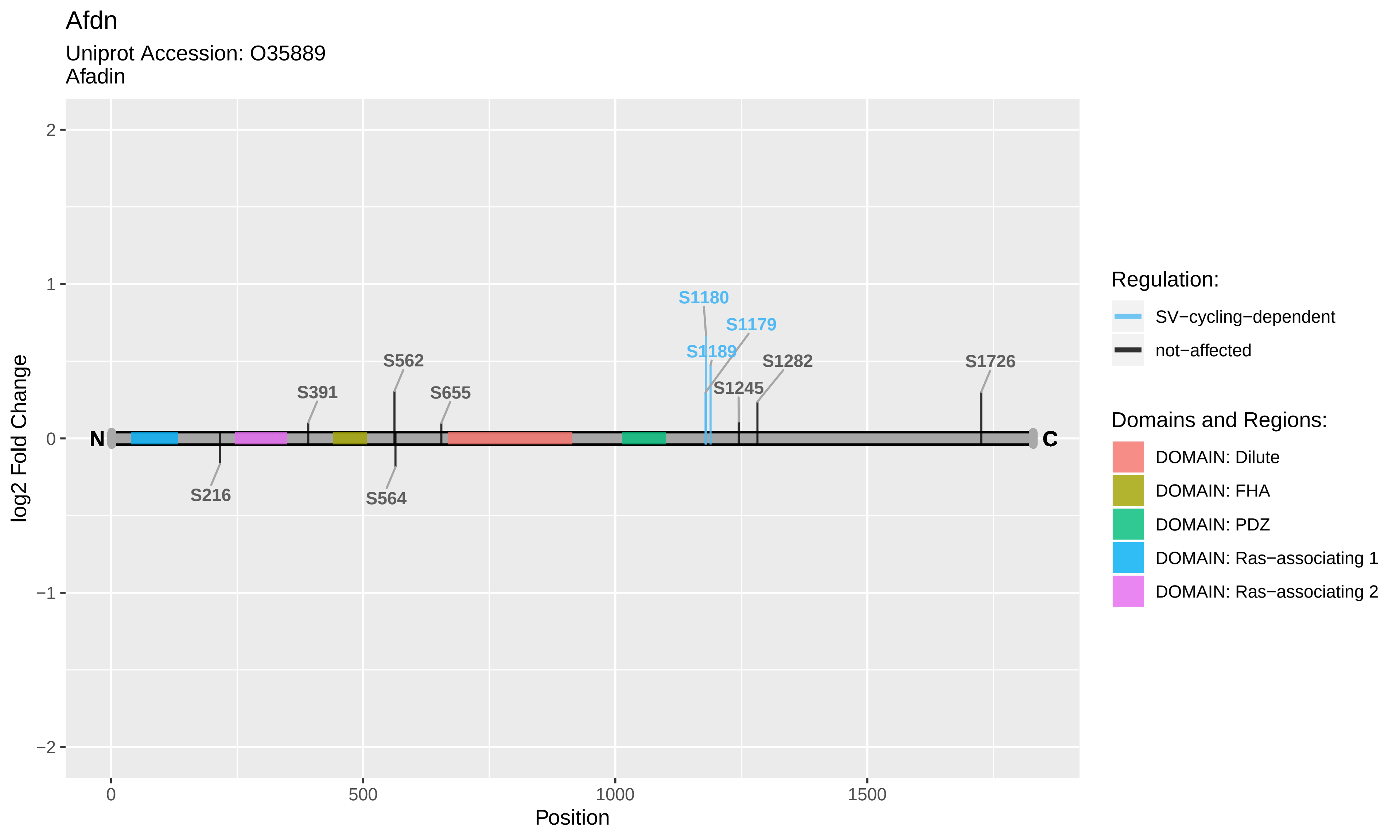
Afdn
Uniprot Accession: O35889
Afadin
log2 Fold Change
2
1
0
−1
−2
N
C
S1180
S1179
S1189
S1282
S1726
S562
S391
S655
S1245
S216
S564
0
500
1000
1500
Position
Regulation:
SV−cycling−dependent
not−affected
Domains and Regions:
DOMAIN: Dilute
DOMAIN: FHA
DOMAIN: PDZ
DOMAIN: Ras−associating 1
DOMAIN: Ras−associating 2

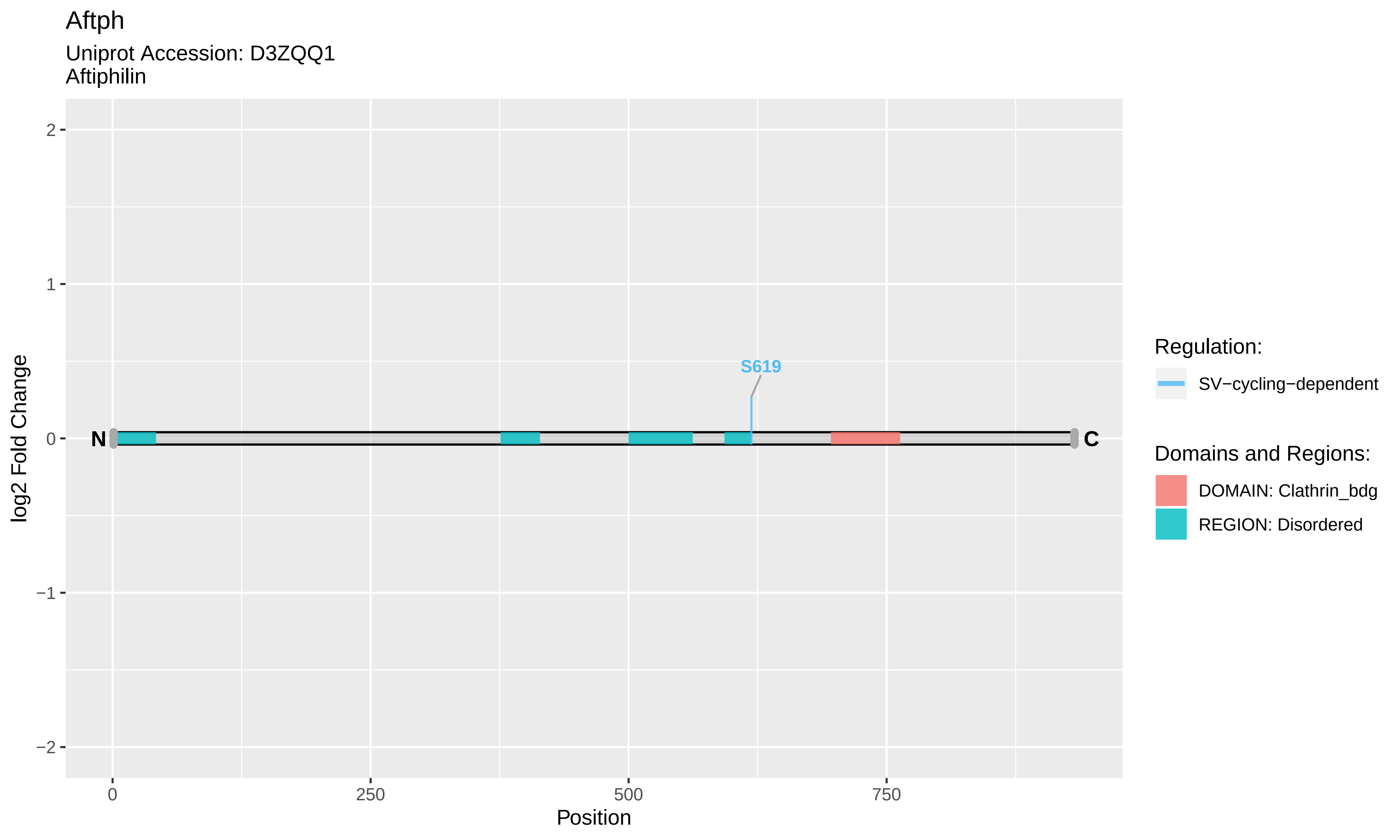

Aftph
Uniprot Accession: D3ZQQ1
Aftiphilin
log2 Fold Change
2
1
0
−1
−2
N
C
S619
0
250
500
750
Position
Regulation:
SV−cycling−dependent
Domains and Regions:
DOMAIN: Clathrin_bdg
REGION: Disordered

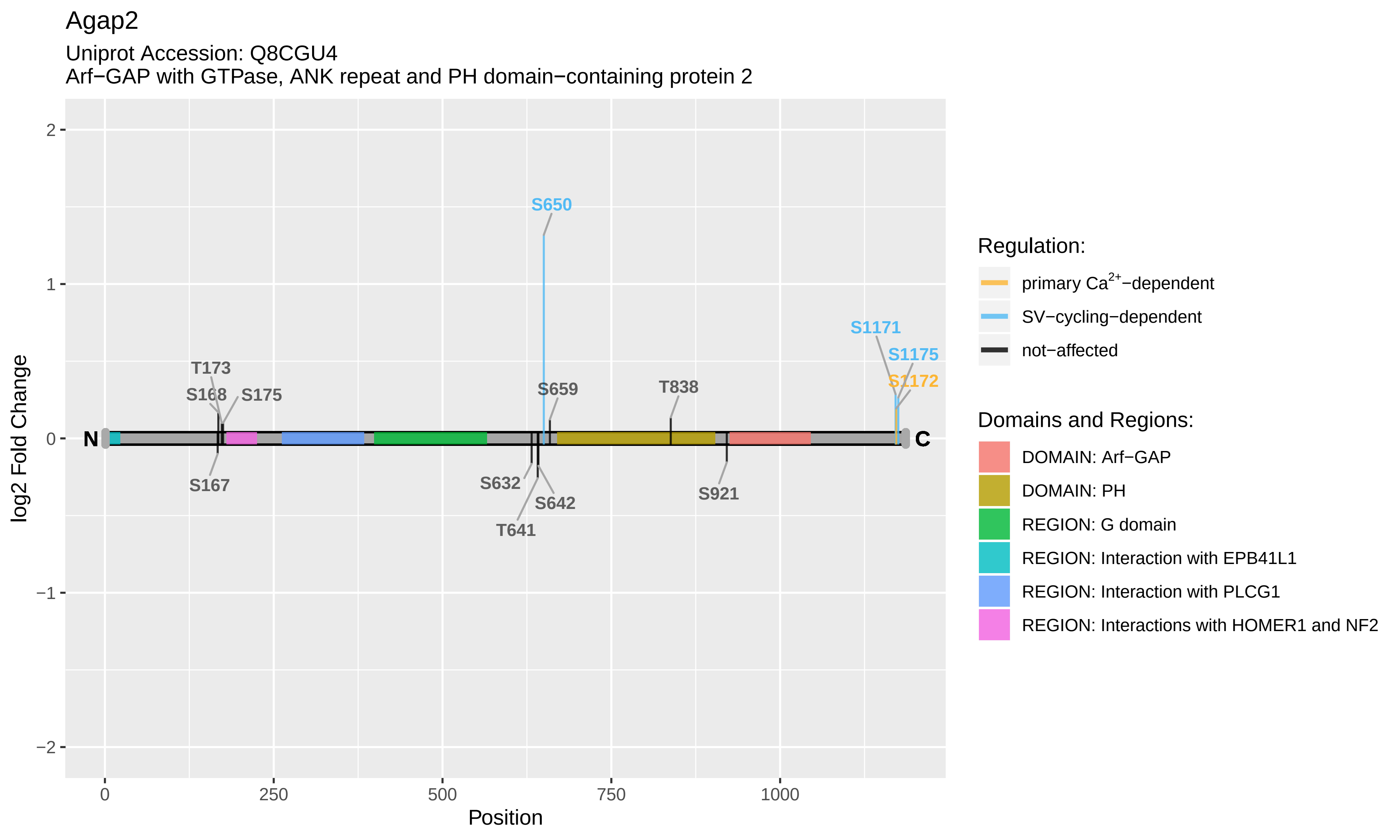
Agap2
Uniprot Accession: Q8CGU4
Arf−GAP with GTPase, ANK repeat and PH domain−containing protein 2
log2 Fold Change
Position
2
1
0
−1
−2
0
250
500
750
1000
N
C
S650
S1171
S1175
S1172
T173
S168
S175
S659
T838
S167
S632
S642
T641
S921
Regulation:
primary Ca2+−dependent
SV−cycling−dependent
not−affected
Domains and Regions:
DOMAIN: Arf−GAP
DOMAIN: PH
REGION: G domain
REGION: Interaction with EPB41L1
REGION: Interaction with PLCG1
REGION: Interactions with HOMER1 and NF2

Agap3

Uniprot Accession: A0A0G2JZ83
ArfGAP with GTPase domain, ankyrin repeat and PH domain 3

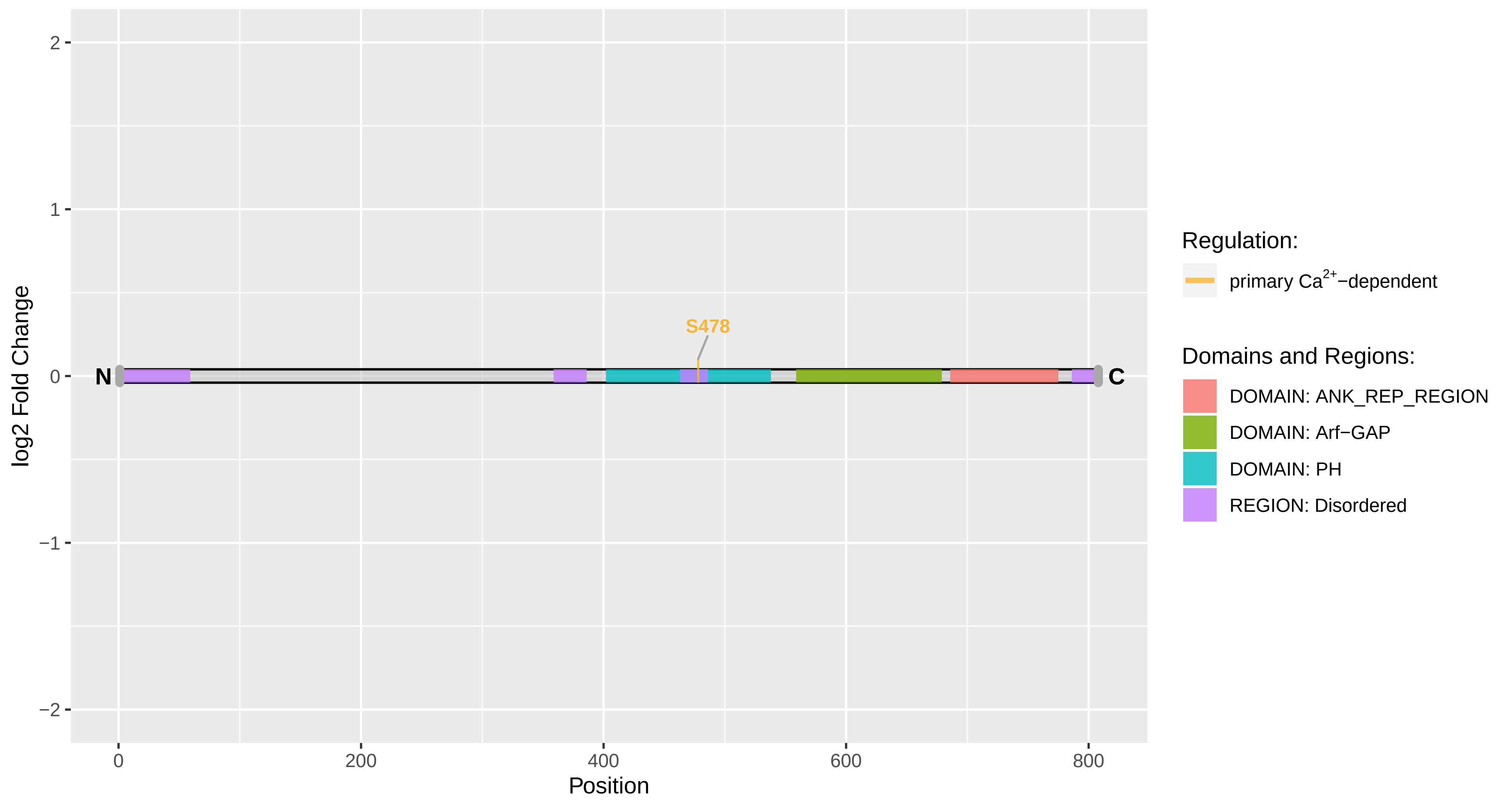

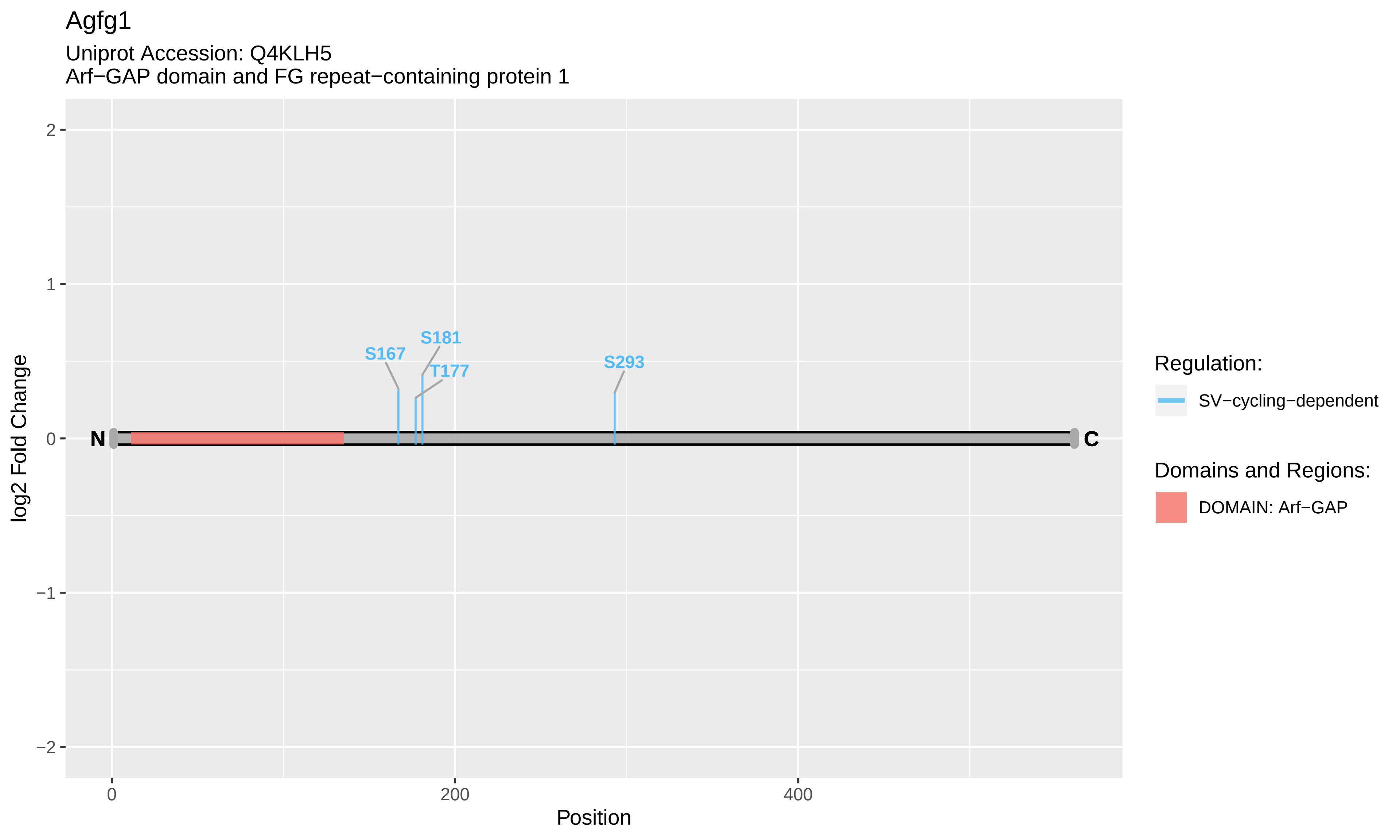

Agfg1
Uniprot Accession: Q4KLH5
Arf−GAP domain and FG repeat−containing protein 1
S181
S167
T177
S293
N
C
2
1
0
−1
−2
log2 Fold Change
0
200
400
Position
Regulation:
SV−cycling−dependent
Domains and Regions:
DOMAIN: Arf−GAP

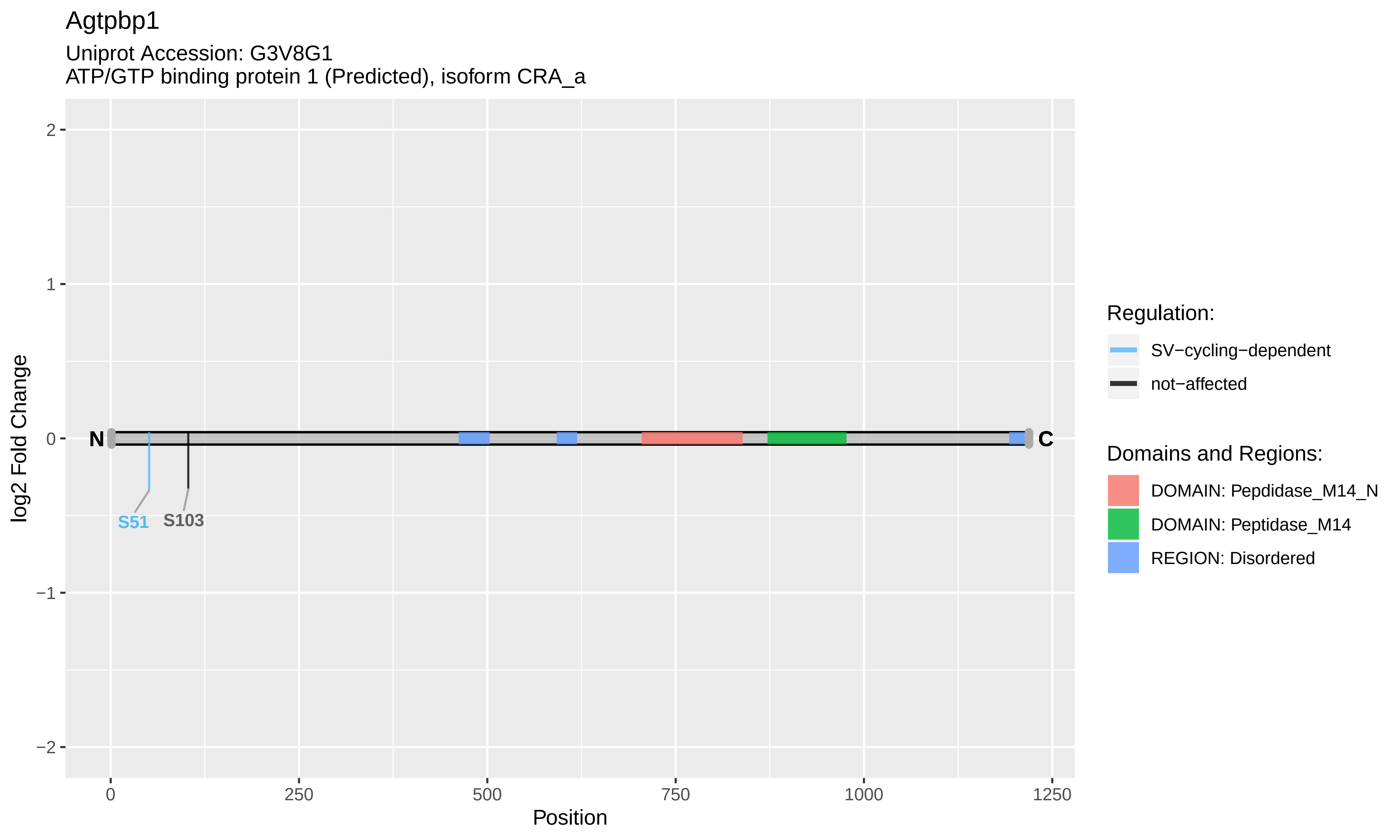

Agtpbp1
Uniprot Accession: G3V8G1
ATP/GTP binding protein 1 (Predicted), isoform CRA_a
log2 Fold Change
2
1
0
−1
−2
N
C
S51
S103
0
250
500
750
1000
1250
Position
Regulation:
SV−cycling−dependent
not−affected
Domains and Regions:
DOMAIN: Pepdidase_M14_N
DOMAIN: Peptidase_M14
REGION: Disordered

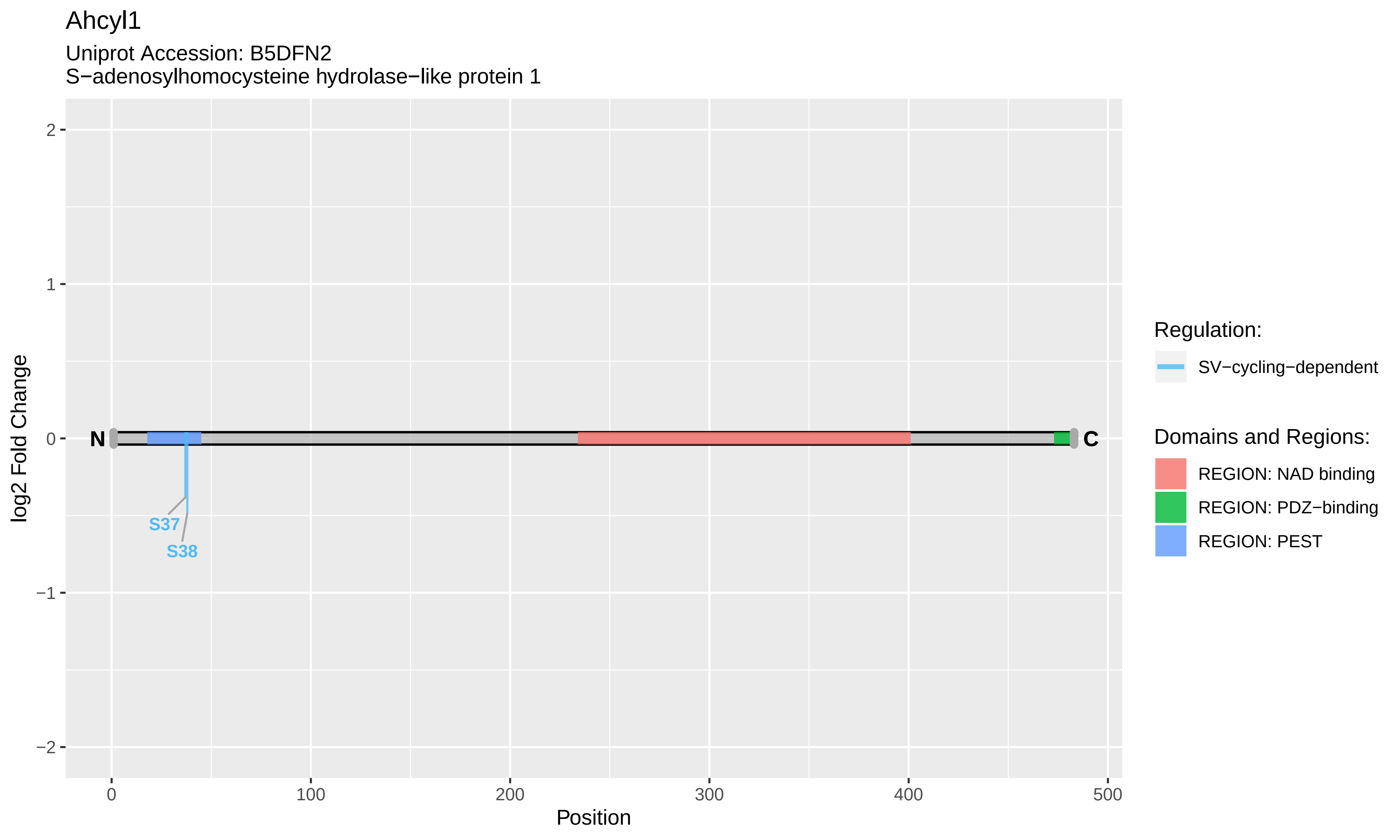

Ahcyl1
Uniprot Accession: B5DFN2
S−adenosylhomocysteine hydrolase−like protein 1
log2 Fold Change
2
1
0
−1
−2
N
C
S37
S38
0
100
200
300
400
500
Position
Regulation:
SV−cycling−dependent
Domains and Regions:
REGION: NAD binding
REGION: PDZ−binding
REGION: PEST

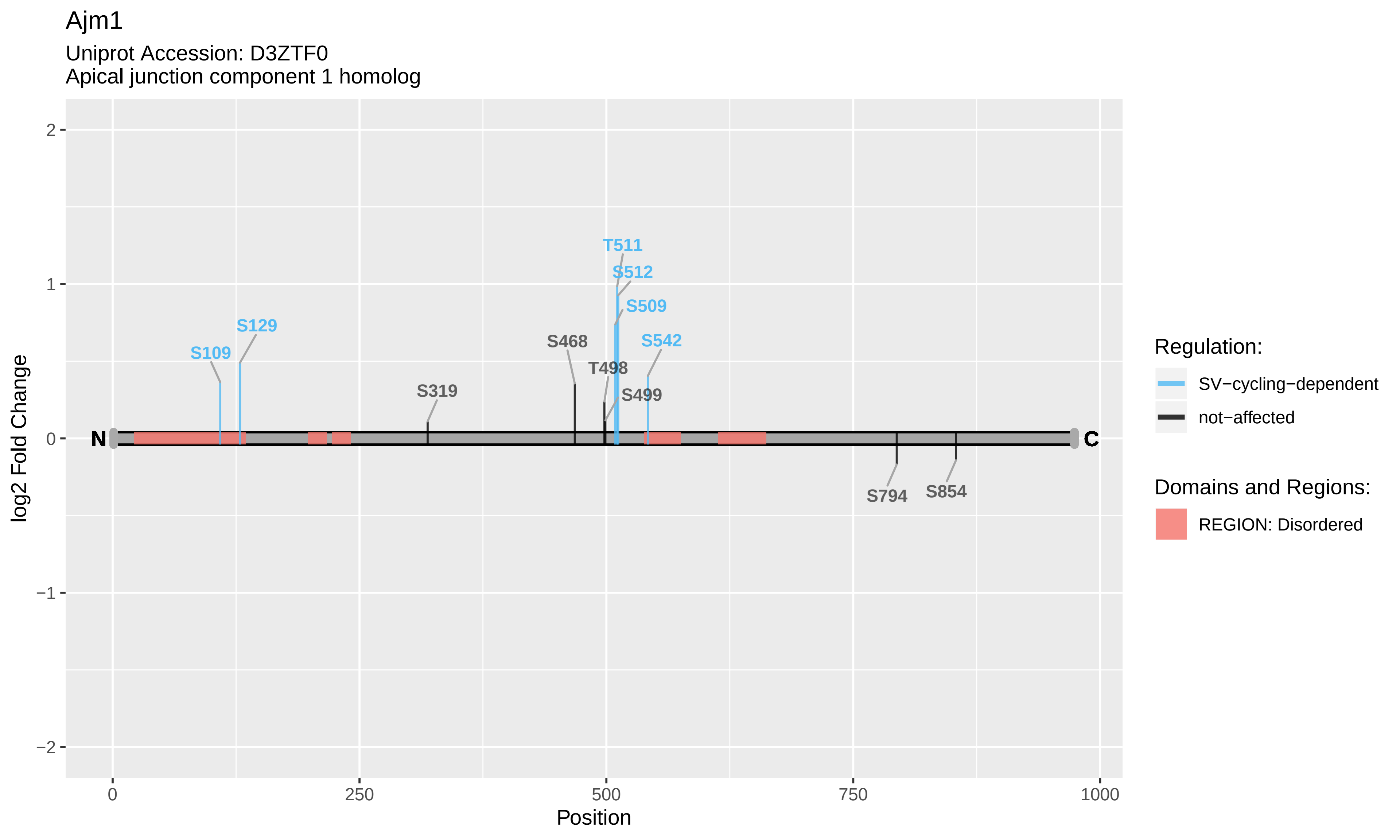

Ajm1
Uniprot Accession: D3ZTF0
Apical junction component 1 homolog
T511
S512
S509
S129
S109
S468
S542
T498
S319
S499
N
C
S794
S854
log2 Fold Change
Position
2
1
0
−1
−2
0
250
500
750
1000
Regulation:
SV−cycling−dependent
not−affected
Domains and Regions:
REGION: Disordered

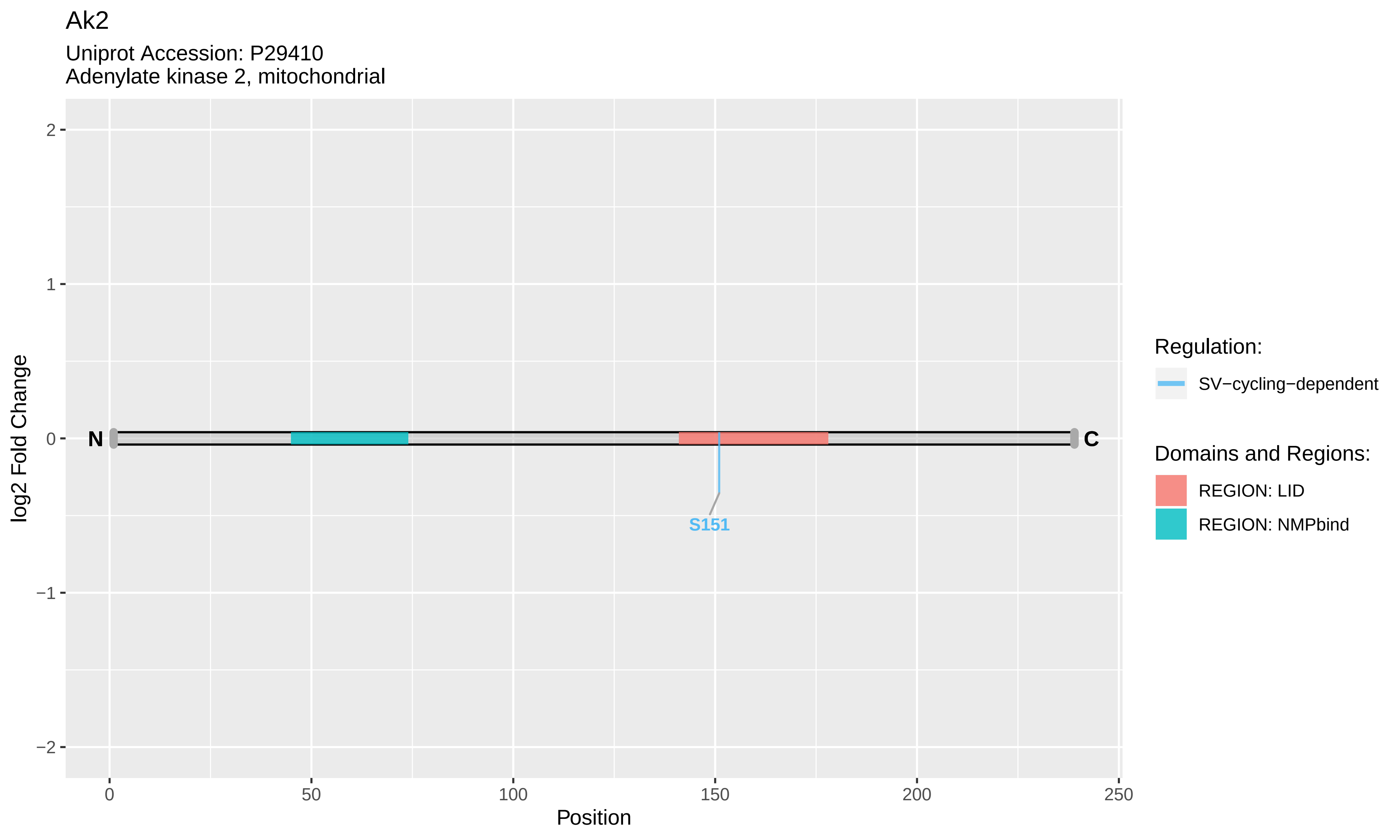

Ak2
Uniprot Accession: P29410
Adenylate kinase 2, mitochondrial
log2 Fold Change
2
1
0
−1
−2
N
C
S151
0
50
100
150
200
250
Position
Regulation:
SV−cycling−dependent
Domains and Regions:
REGION: LID
REGION: NMPbind

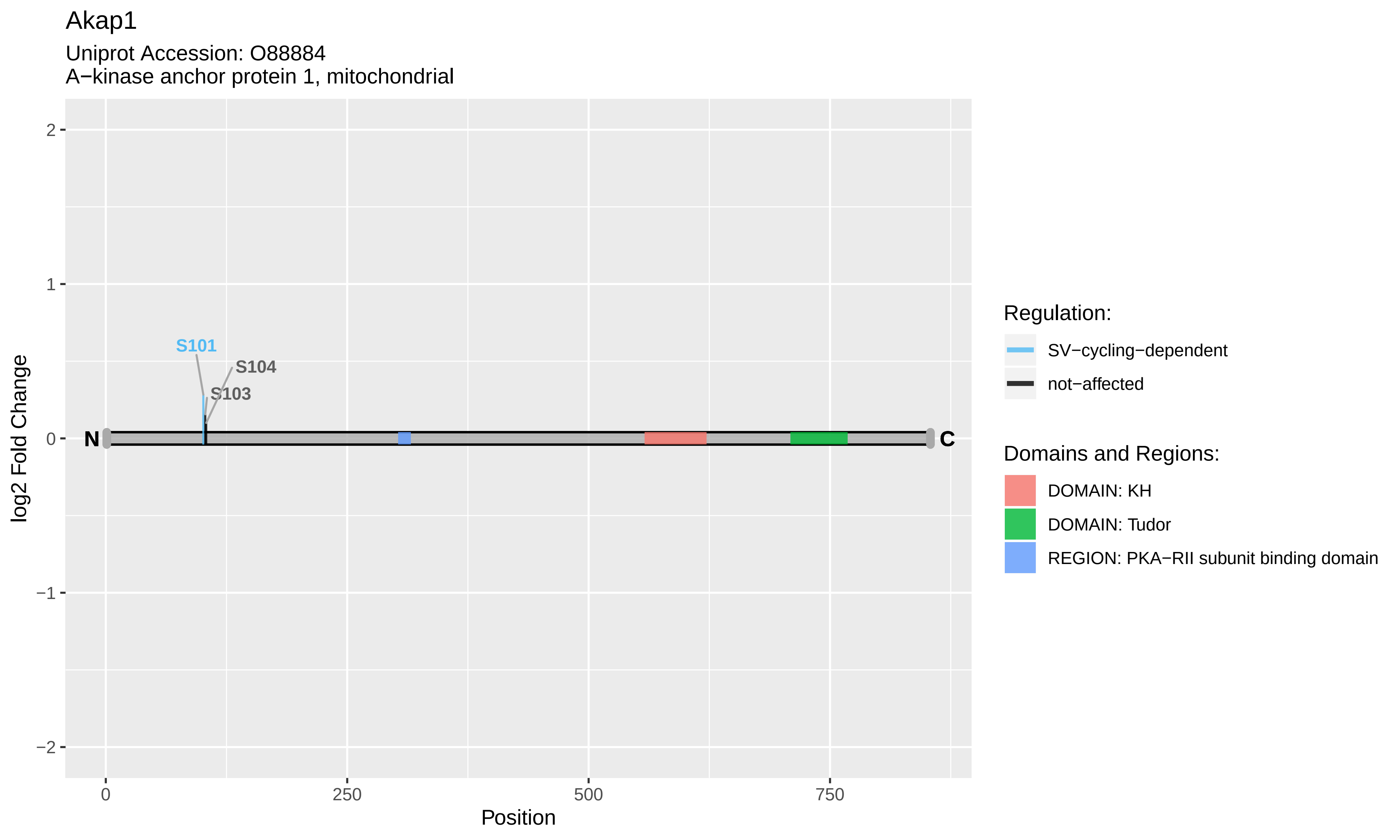

Akap1
Uniprot Accession: O88884
A−kinase anchor protein 1, mitochondrial
S101
S104
S103
N
C
2
1
0
−1
−2
log2 Fold Change
0
250
500
750
Position
Regulation:
SV−cycling−dependent
not−affected
Domains and Regions:
DOMAIN: KH
DOMAIN: Tudor
REGION: PKA−RII subunit binding domain

Akap12

Uniprot Accession: Q5QD51
A−kinase anchor protein 12

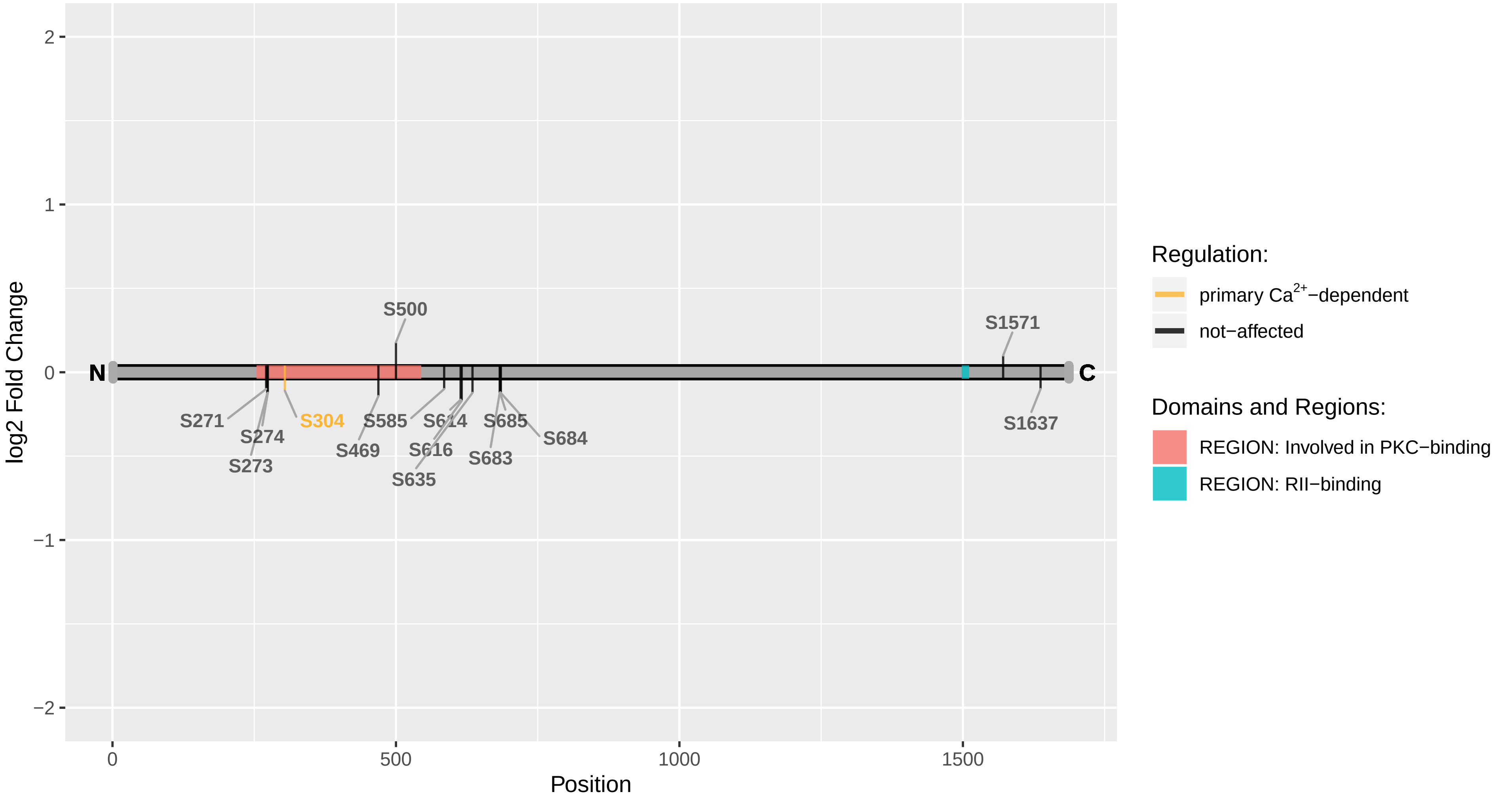

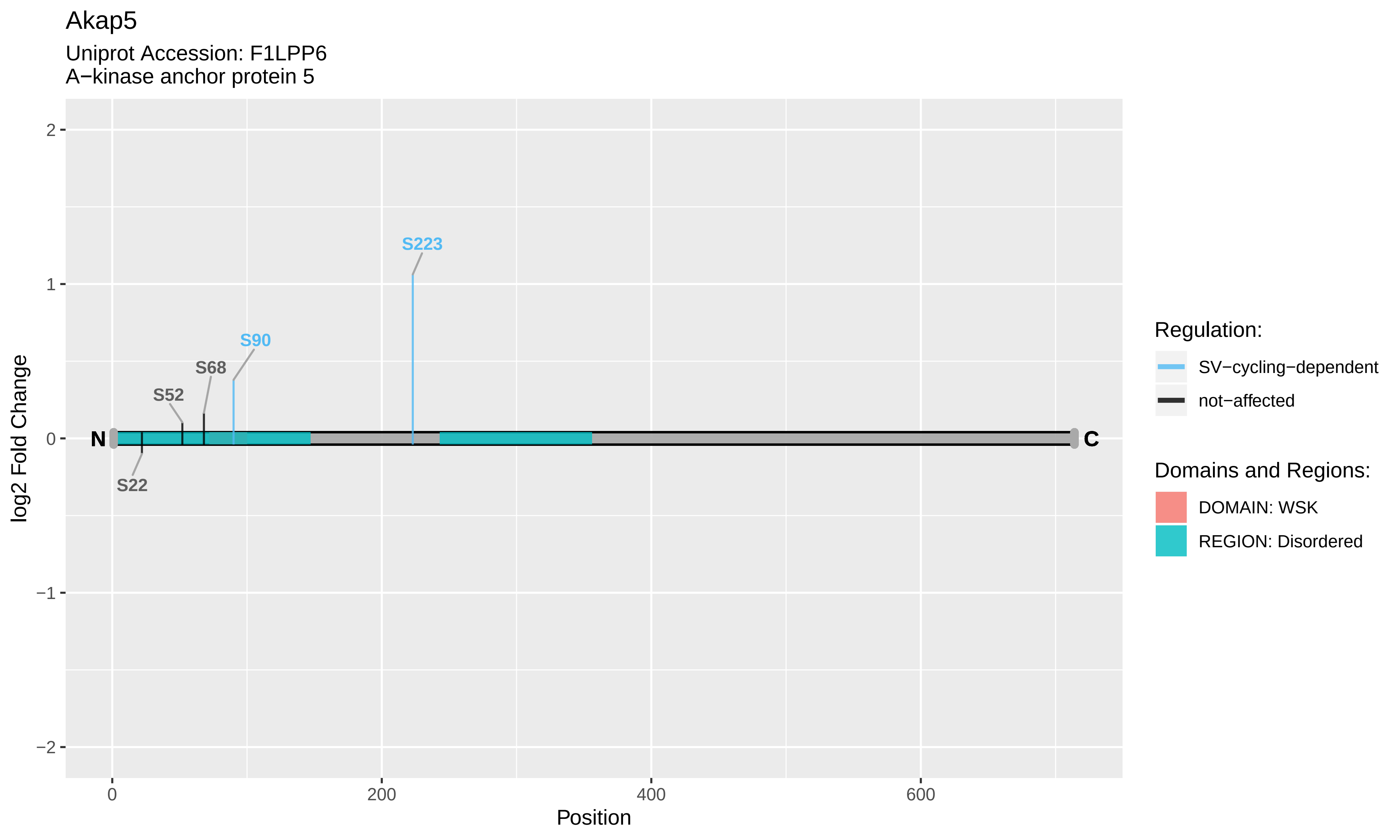

Akap5
Uniprot Accession: F1LPP6
A−kinase anchor protein 5
S223
S90
S68
S52
N
C
S22
2
1
0
−1
−2
log2 Fold Change
0
200
400
600
Position
Regulation:
SV−cycling−dependent
not−affected
Domains and Regions:
DOMAIN: WSK
REGION: Disordered

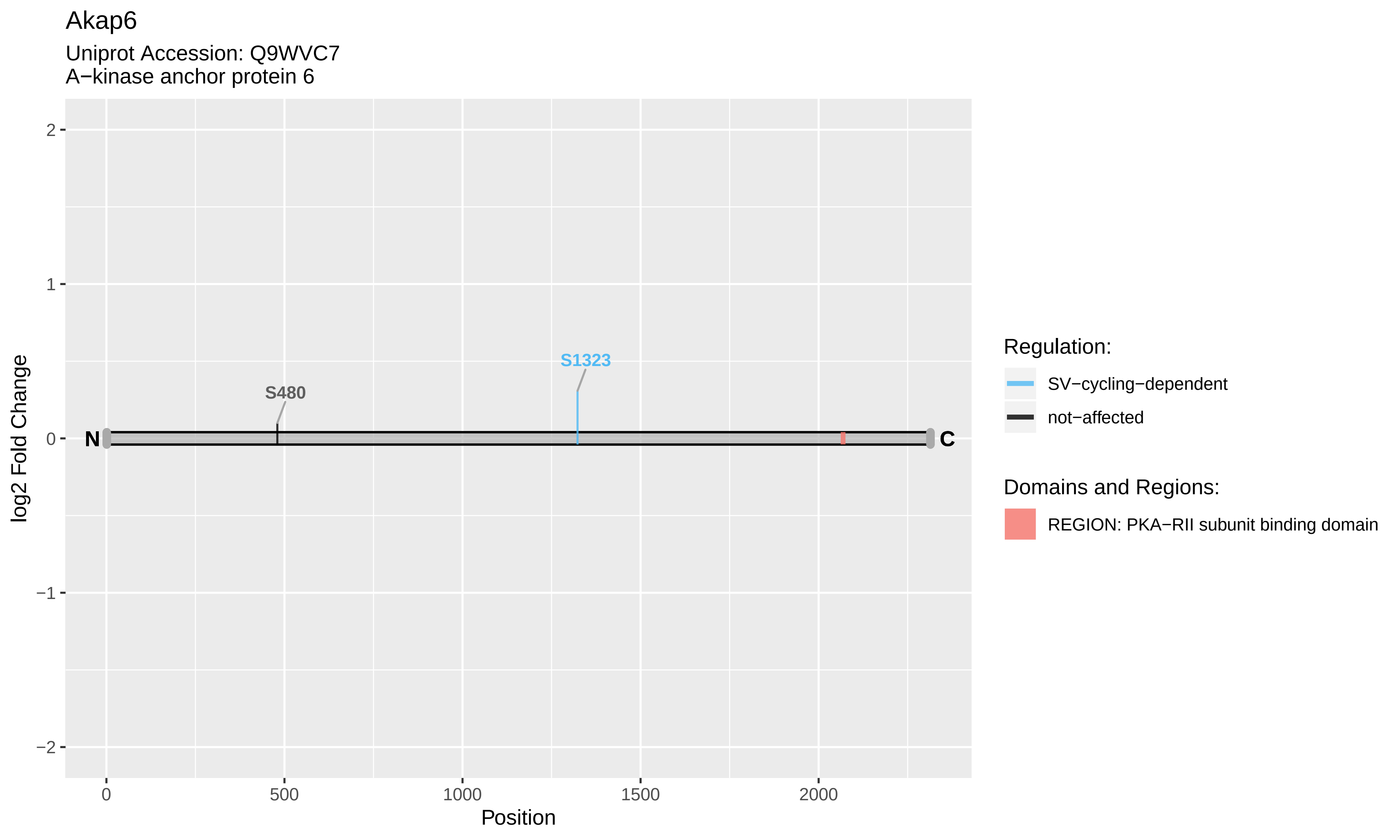

Akap6
Uniprot Accession: Q9WVC7
A−kinase anchor protein 6
S1323
S480
N
C
2
1
0
−1
−2
log2 Fold Change
0
500
1000
1500
2000
Position
Regulation:
SV−cycling−dependent
not−affected
Domains and Regions:
REGION: PKA−RII subunit binding domain

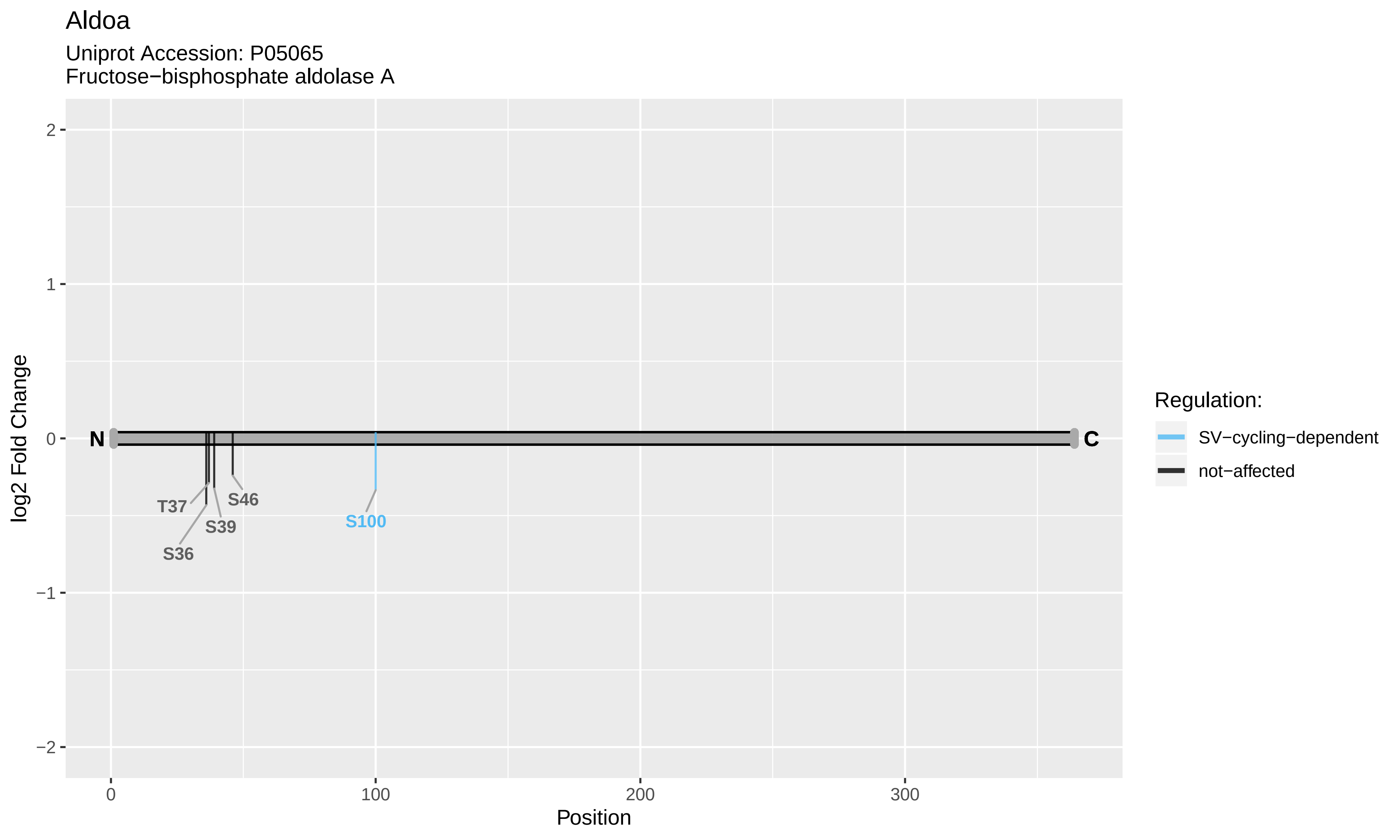

Aldoa
Uniprot Accession: P05065
Fructose−bisphosphate aldolase A
log2 Fold Change
Position
N
C
T37
S46
S39
S36
S100
Regulation:
SV−cycling−dependent
not−affected
2
1
0
−1
−2
0
100
200
300

Aldoart2

Uniprot Accession: Q6AY07
Fructose−bisphosphate aldolase

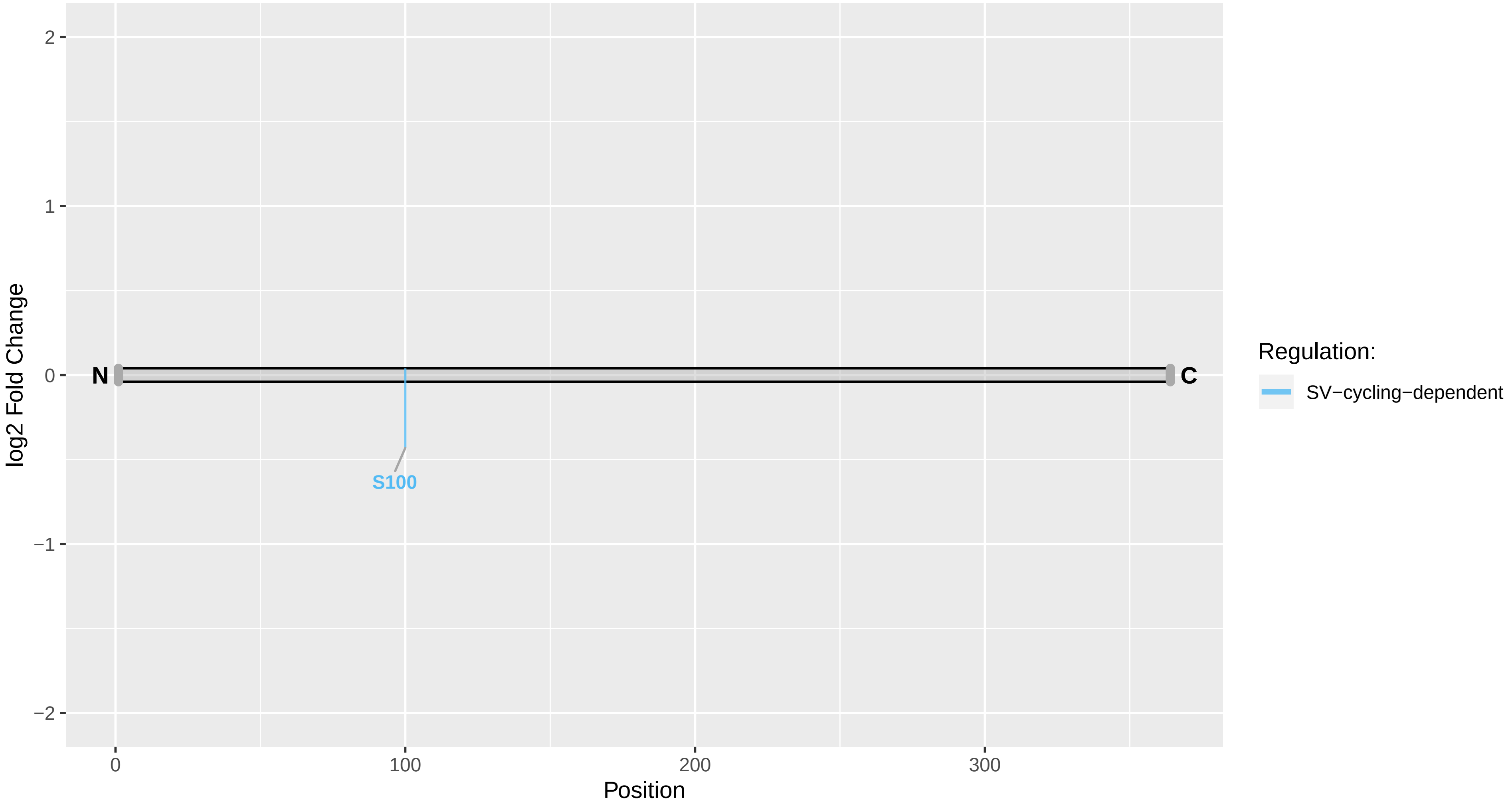

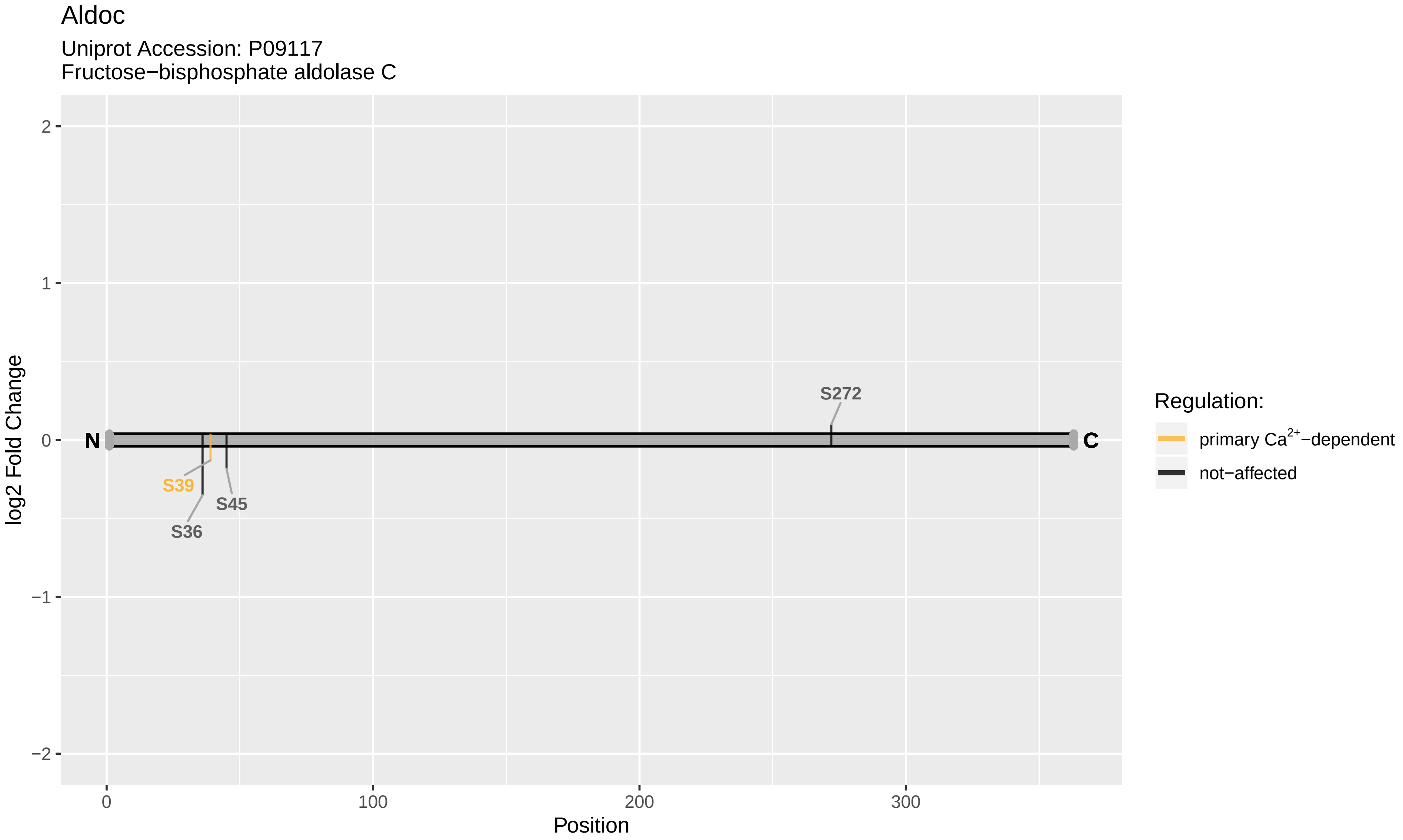

Aldoc
Uniprot Accession: P09117
Fructose−bisphosphate aldolase C
log2 Fold Change
Position
N
C
S272
S39
S45
S36
Regulation:
primary Ca2+−dependent
not−affected
2
1
0
−1
−2
0
100
200
300

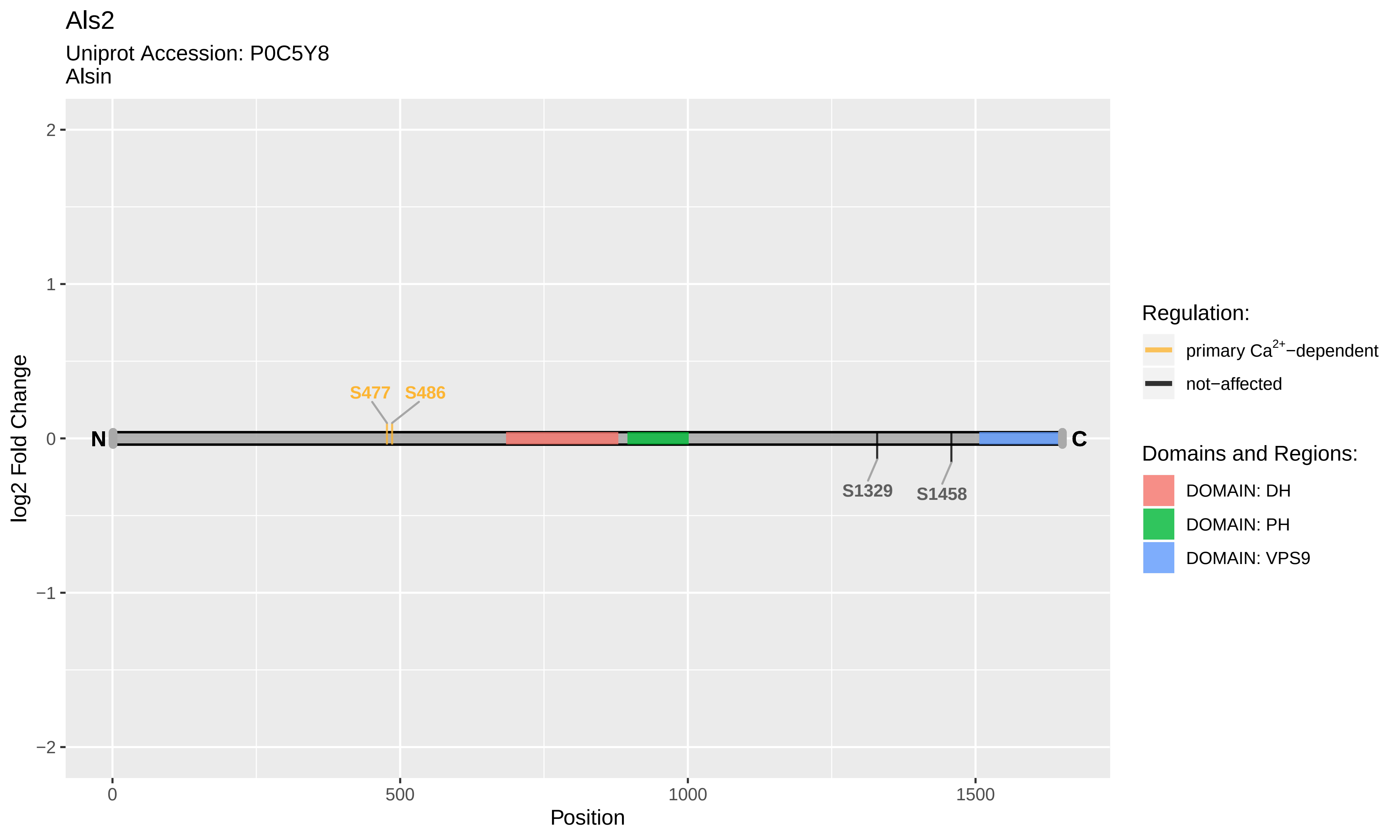

Als2
Uniprot Accession: P0C5Y8
Alsin
log2 Fold Change
2
1
0
−1
−2
N
C
S477
S486
S1329
S1458
0
500
1000
1500
Position
Regulation:
primary Ca2+−dependent
not−affected
Domains and Regions:
DOMAIN: DH
DOMAIN: PH
DOMAIN: VPS9

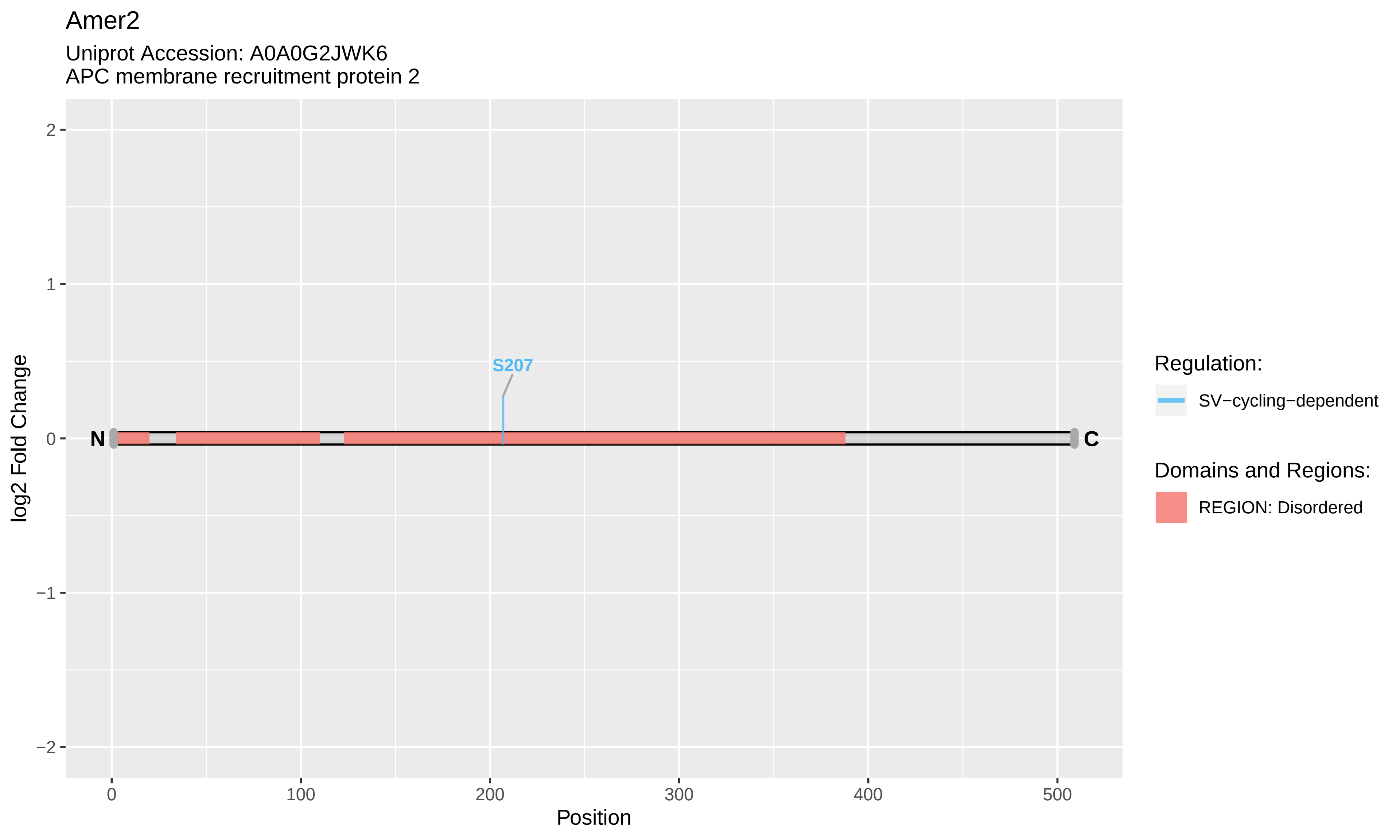
Amer2
Uniprot Accession: A0A0G2JWK6
APC membrane recruitment protein 2
S207
N
C
log2 Fold Change
2
1
0
−1
−2
0
100
200
300
400
500
Position
Regulation:
SV−cycling−dependent
Domains and Regions:
REGION: Disordered

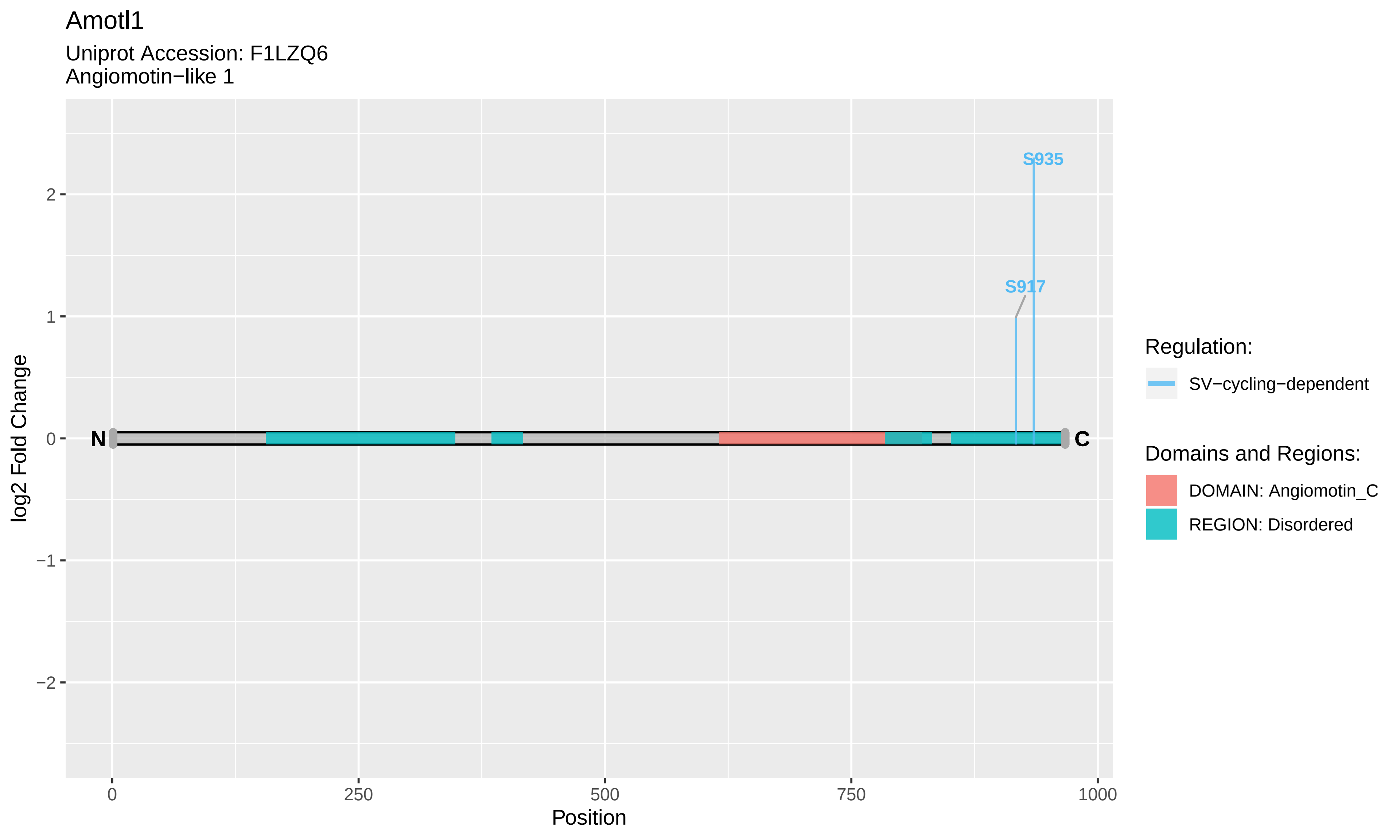
Amotl1
Uniprot Accession: F1LZQ6
Angiomotin−like 1
S935
S917
N
C
2
1
0
−1
−2
log2 Fold Change
0
250
500
750
1000
Position
Regulation:
SV−cycling−dependent
Domains and Regions:
DOMAIN: Angiomotin_C
REGION: Disordered

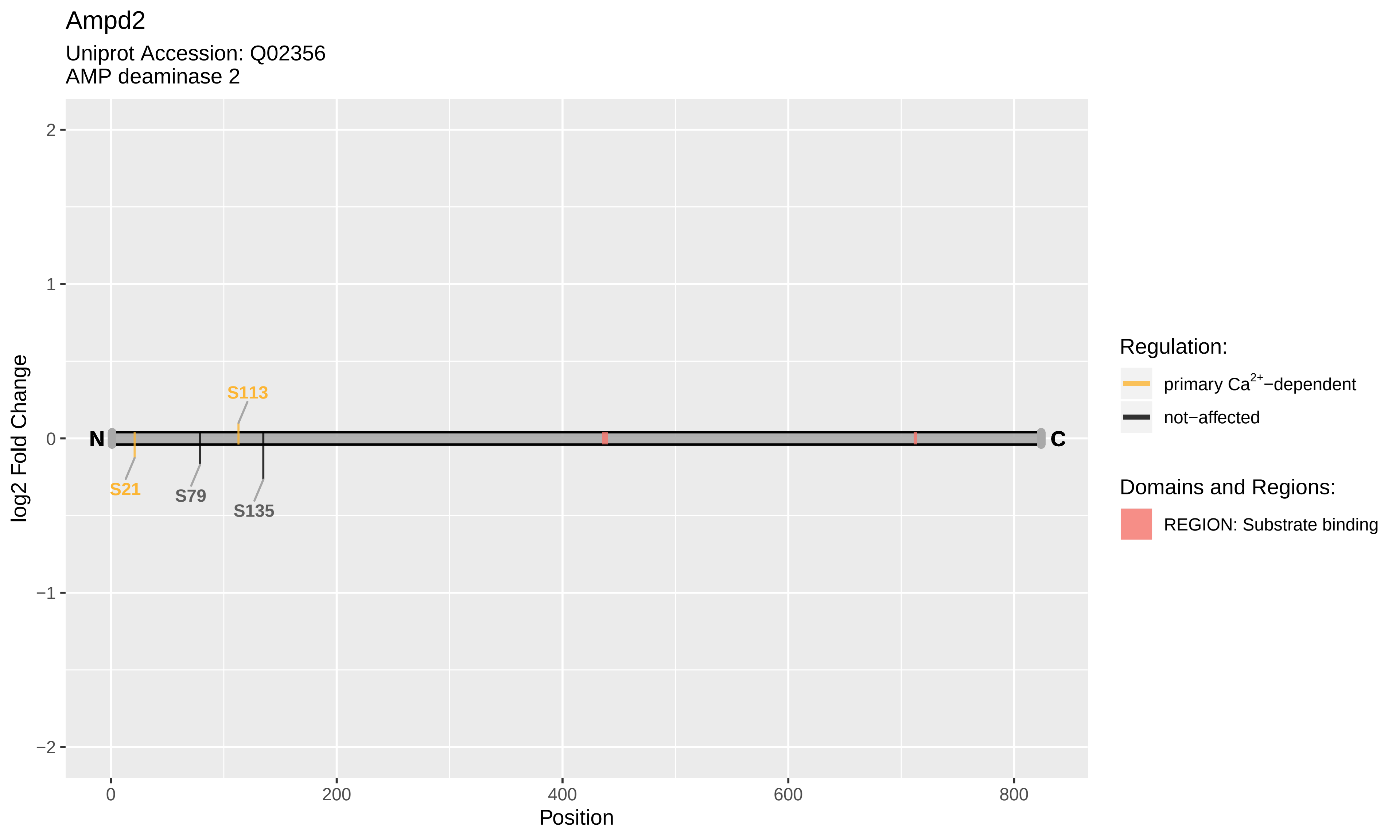

Ampd2
Uniprot Accession: Q02356
AMP deaminase 2
log2 Fold Change
2
1
0
−1
−2
N
C
S113
S21
S79
S135
0
200
400
600
800
Position
Regulation:
primary Ca2+−dependent
not−affected
Domains and Regions:
REGION: Substrate binding

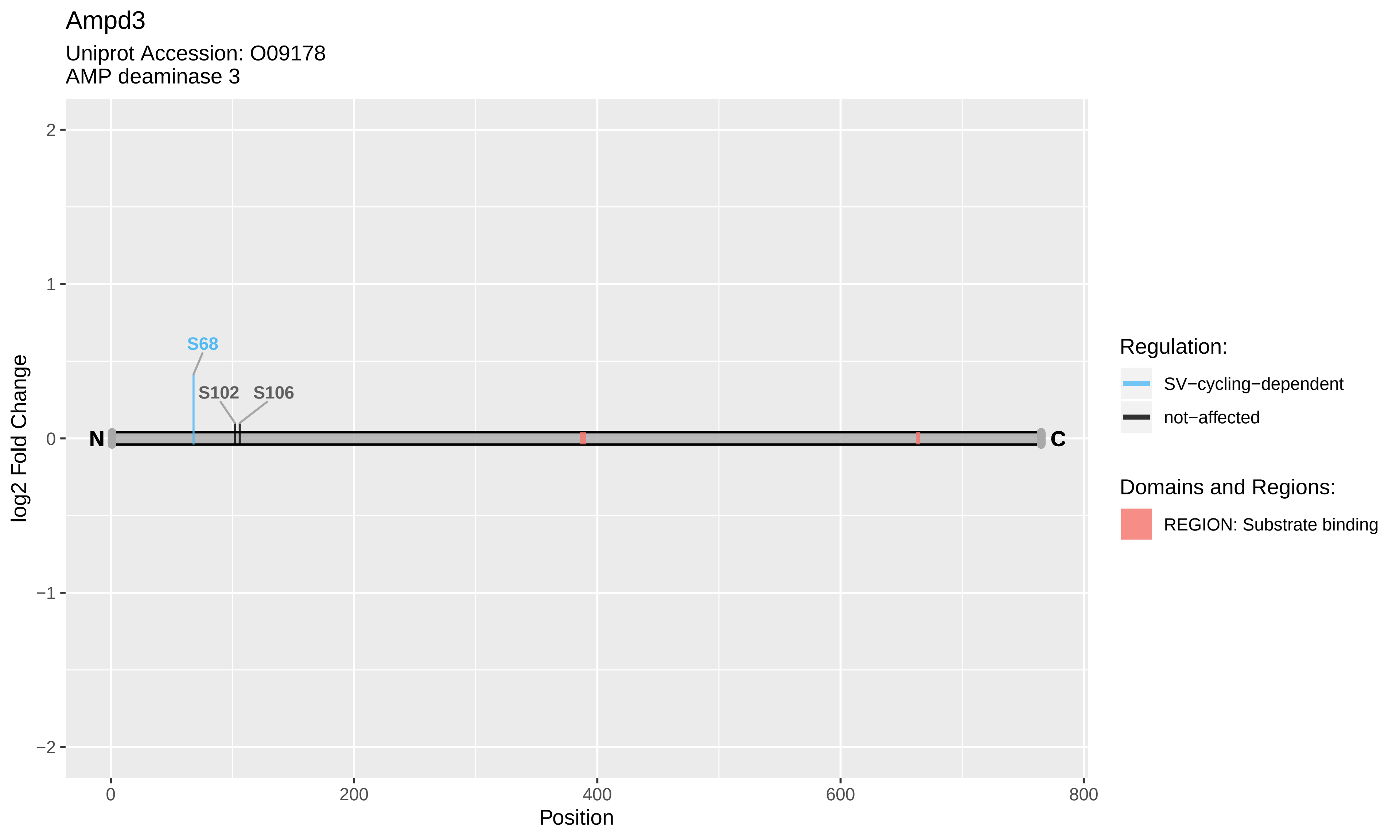
Ampd3
Uniprot Accession: O09178
AMP deaminase 3
S68
S102
S106
N
C
2
1
0
−1
−2
log2 Fold Change
0
200
400
600
800
Position
Regulation:
SV−cycling−dependent
not−affected
Domains and Regions:
REGION: Substrate binding

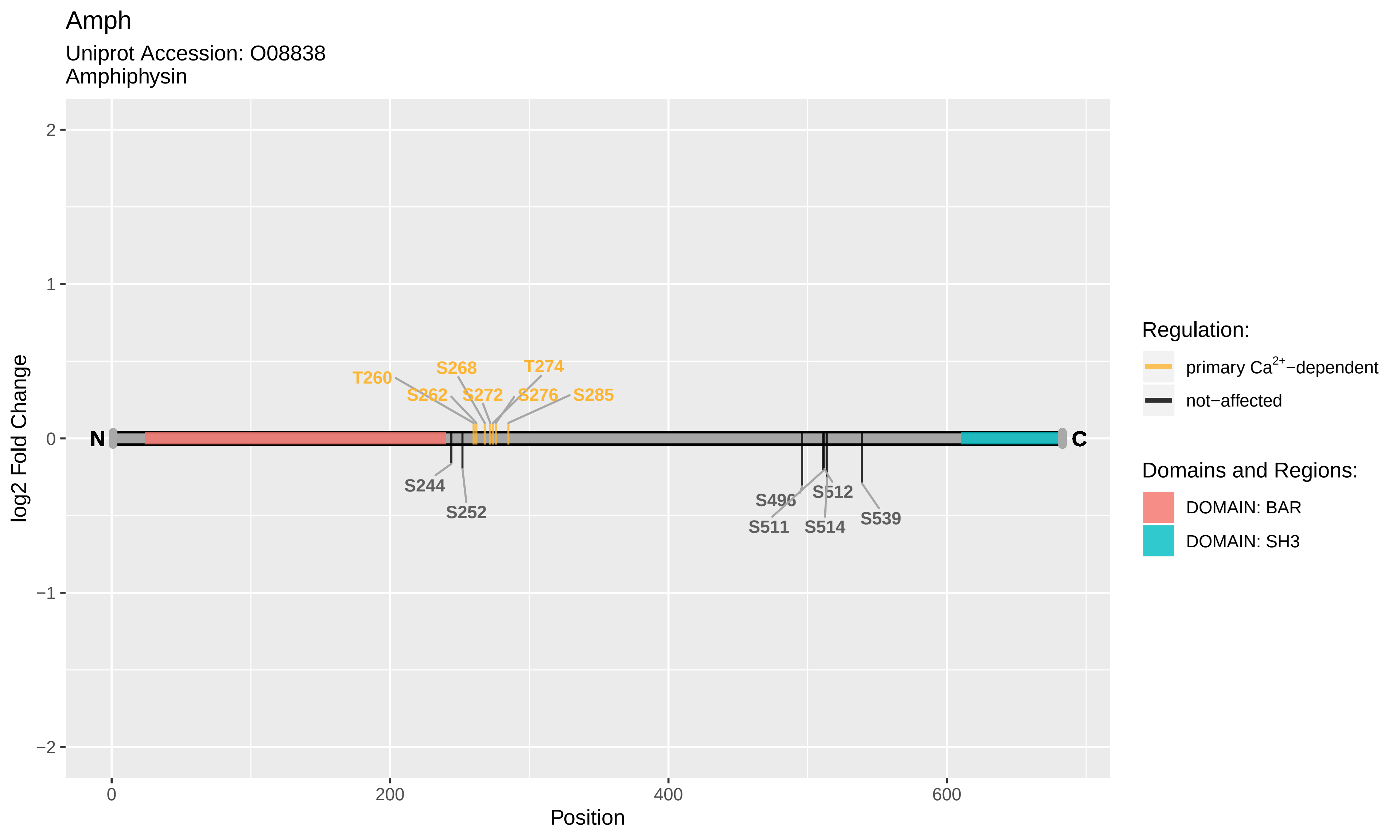

Amph
Uniprot Accession: O08838
Amphiphysin
T260
S268
T274
S262
S272
S276
S285
N
C
S244
S252
S496
S512
S511
S514
S539
log2 Fold Change
Position
2
1
0
−1
−2
0
200
400
600
Regulation:
primary Ca2+−dependent
not−affected
Domains and Regions:
DOMAIN: BAR
DOMAIN: SH3

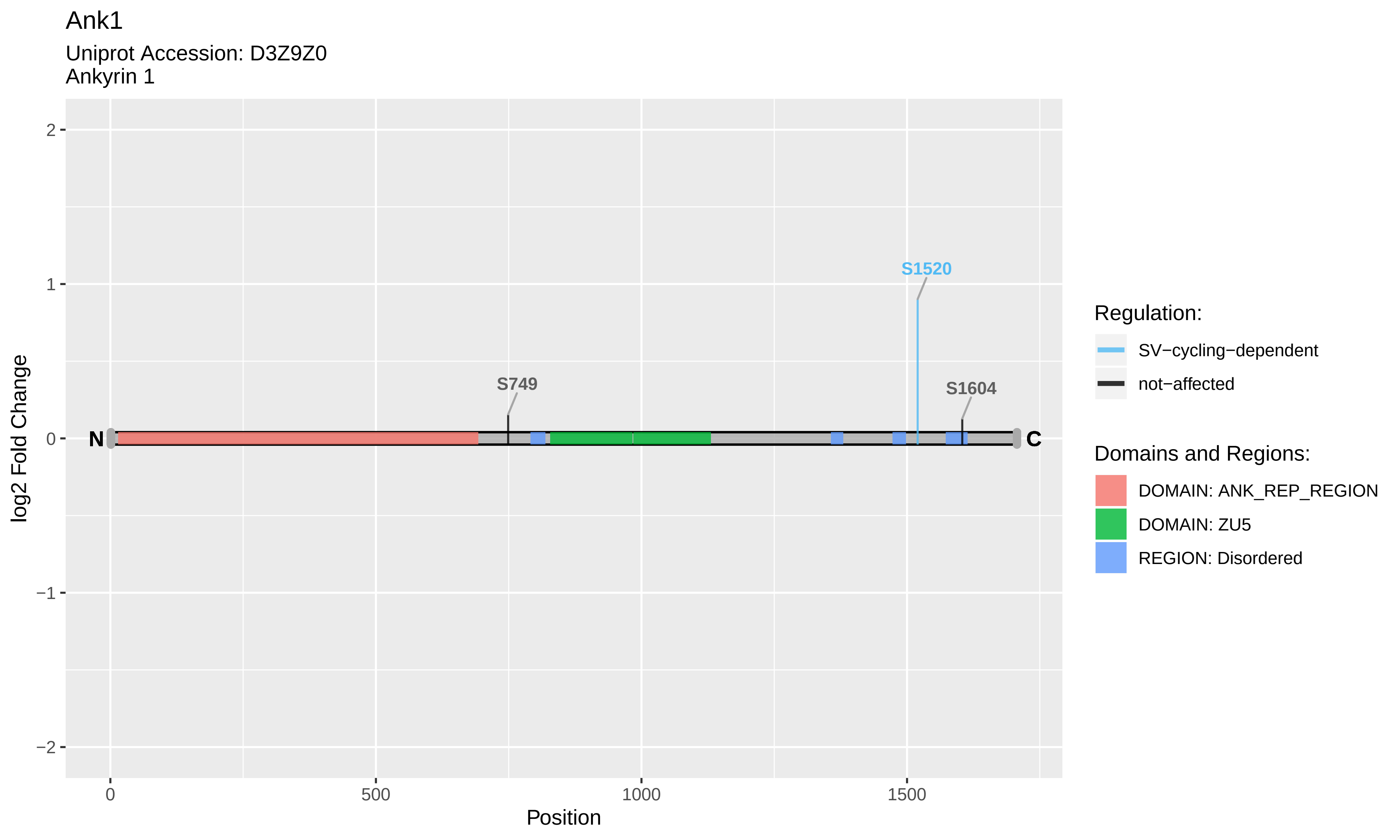

Ank1
Uniprot Accession: D3Z9Z0
Ankyrin 1
S1520
S749
S1604
N
C
log2 Fold Change
Position
Regulation:
SV−cycling−dependent
not−affected
Domains and Regions:
DOMAIN: ANK_REP_REGION
DOMAIN: ZU5
REGION: Disordered

Ank2

Uniprot Accession: A0A0G2JZ56
Ankyrin 2

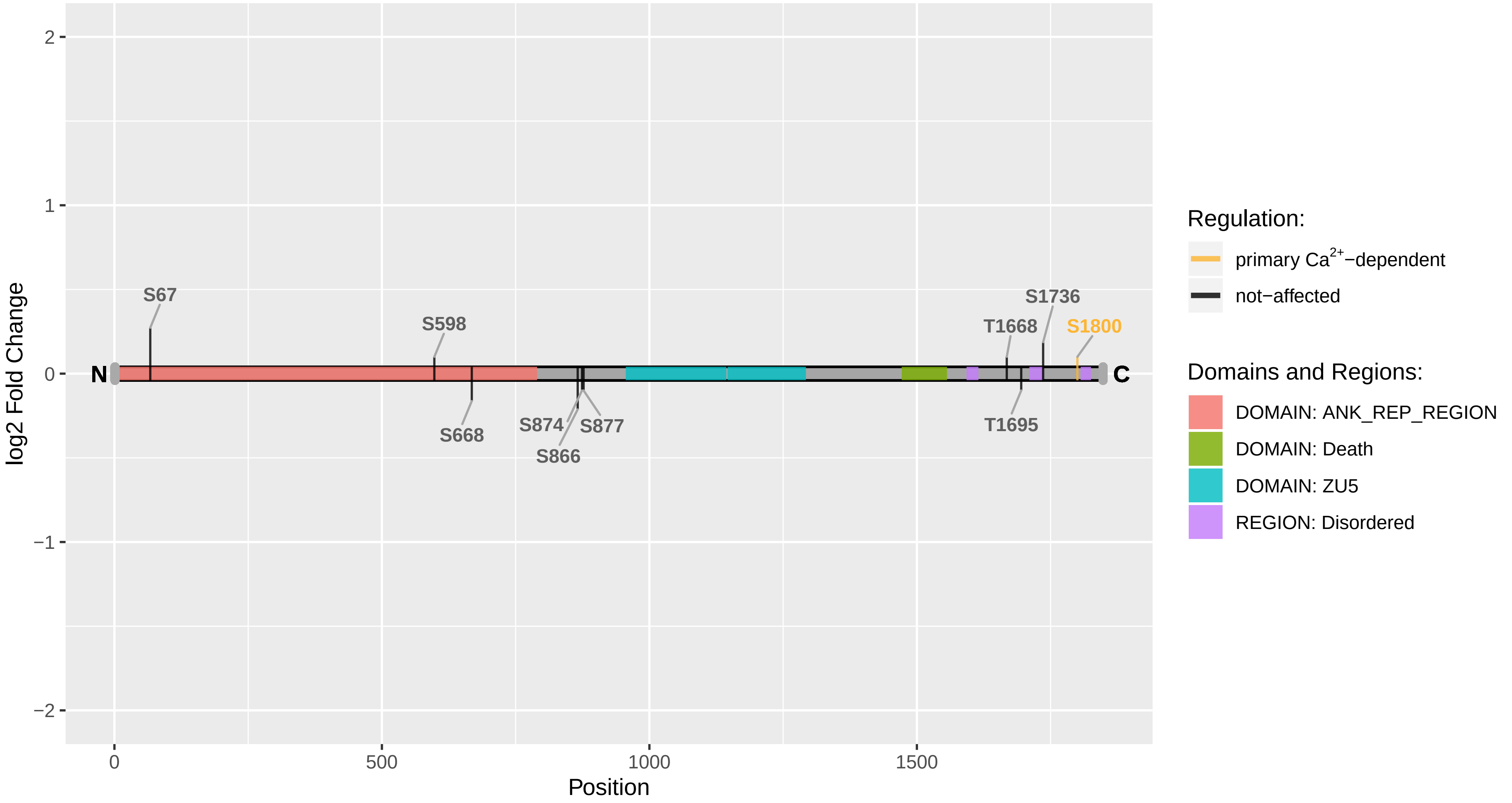

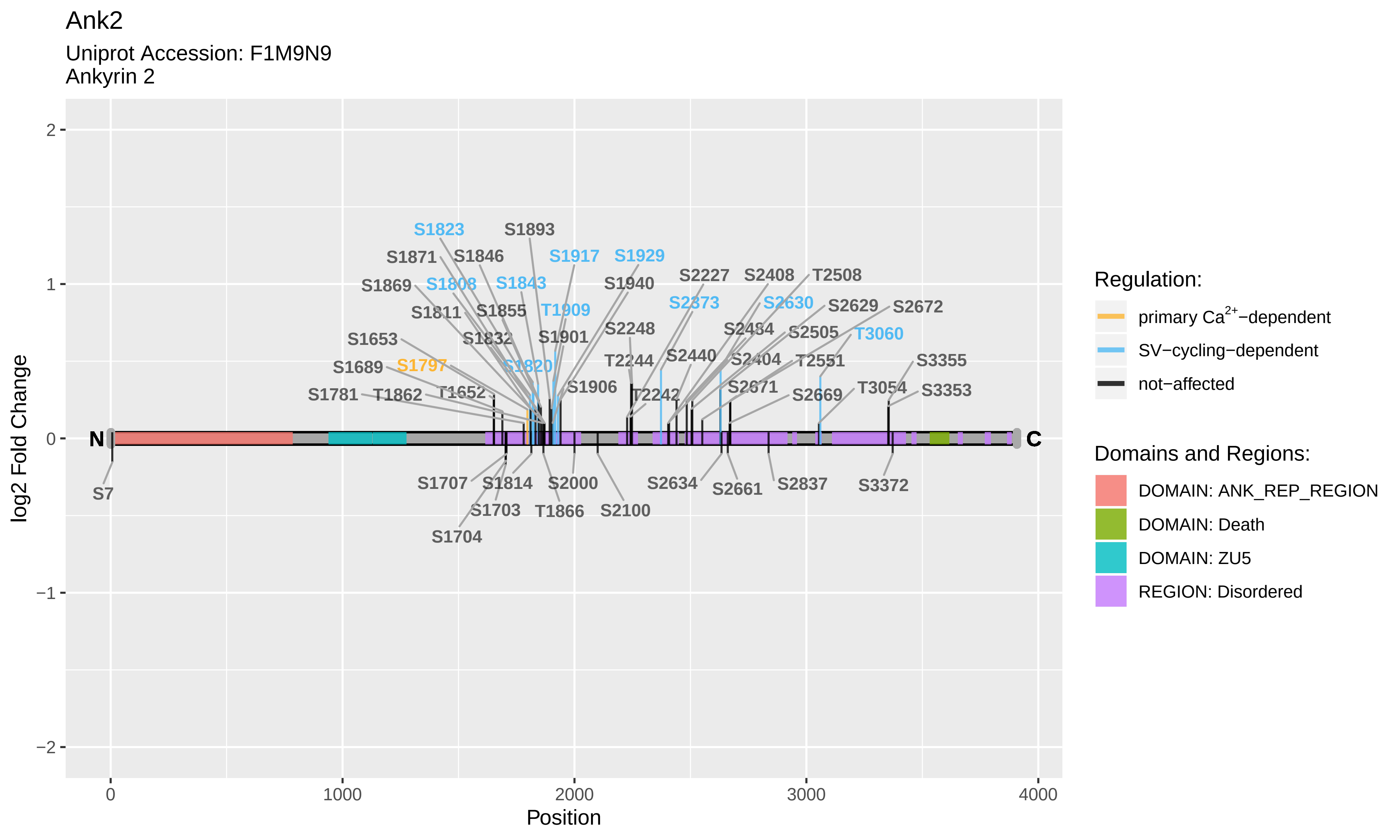

Ank2
Uniprot Accession: F1M9N9
Ankyrin 2
log2 Fold Change
Position
2
1
0
−1
−2
0
1000
2000
3000
4000
N
C
S1823
S1893
S1871
S1846
S1917
S1929
S1869
S1808
S1843
S1940
S2227
S2408
T2508
S1811
S1855
T1909
S2373
S2630
S2629
S2672
S1653
S1832
S1901
S2248
S2484
S2505
T3060
S1689
S1797
S1820
T2244
S2440
S2404
T2551
S3355
S1781
T1862
T1652
S1906
T2242
S2671
S2669
T3054
S3353
S7
S1707
S1814
S2000
S2634
S2661
S2837
S3372
S1703
T1866
S2100
S1704
Regulation:
primary Ca2+−dependent
SV−cycling−dependent
not−affected
Domains and Regions:
DOMAIN: ANK_REP_REGION
DOMAIN: Death
DOMAIN: ZU5
REGION: Disordered

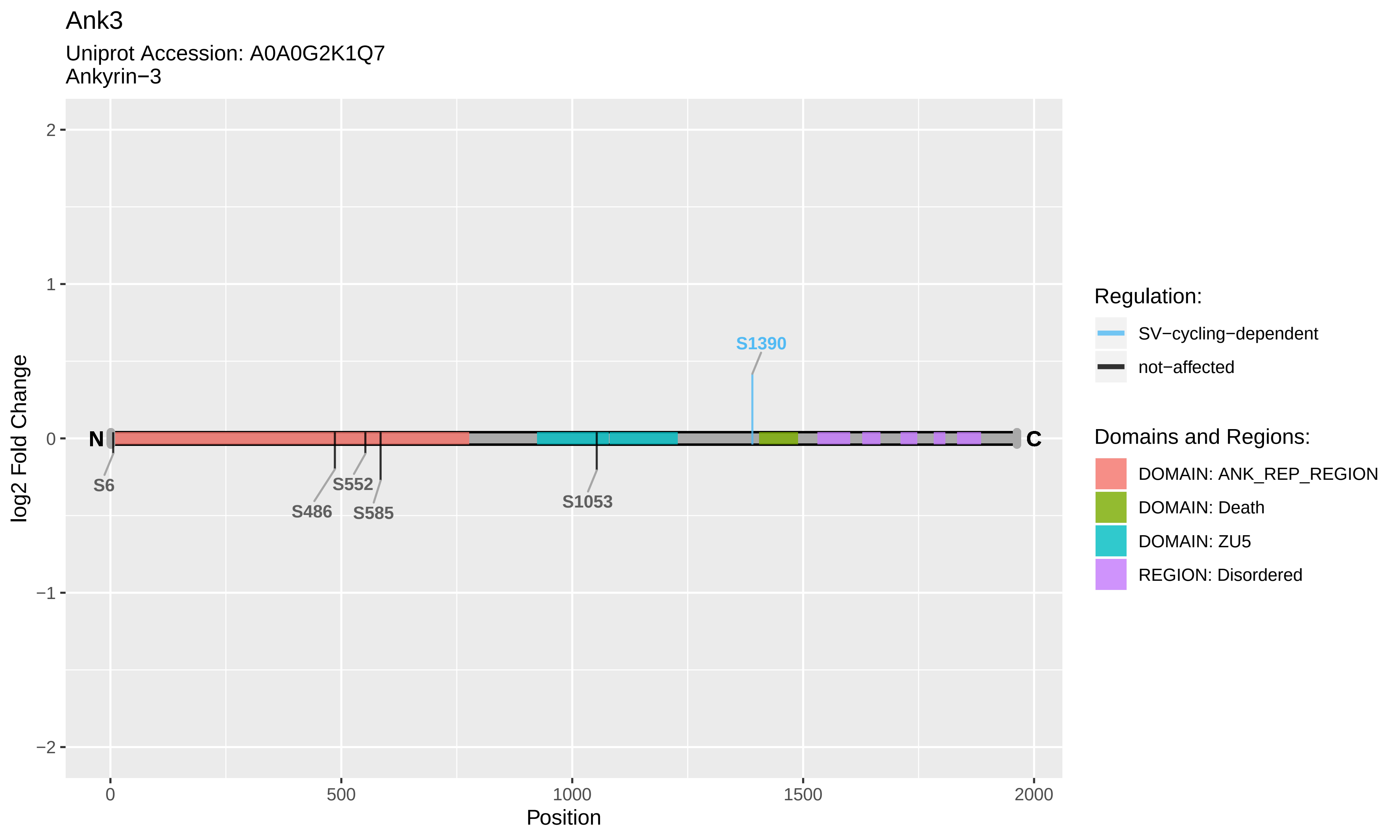
Ank3
Uniprot Accession: A0A0G2K1Q7
Ankyrin−3
log2 Fold Change
2
1
0
−1
−2
N
C
S6
S486
S552
S585
S1053
S1390
0
500
1000
1500
2000
Position
Regulation:
SV−cycling−dependent
not−affected
Domains and Regions:
DOMAIN: ANK_REP_REGION
DOMAIN: Death
DOMAIN: ZU5
REGION: Disordered

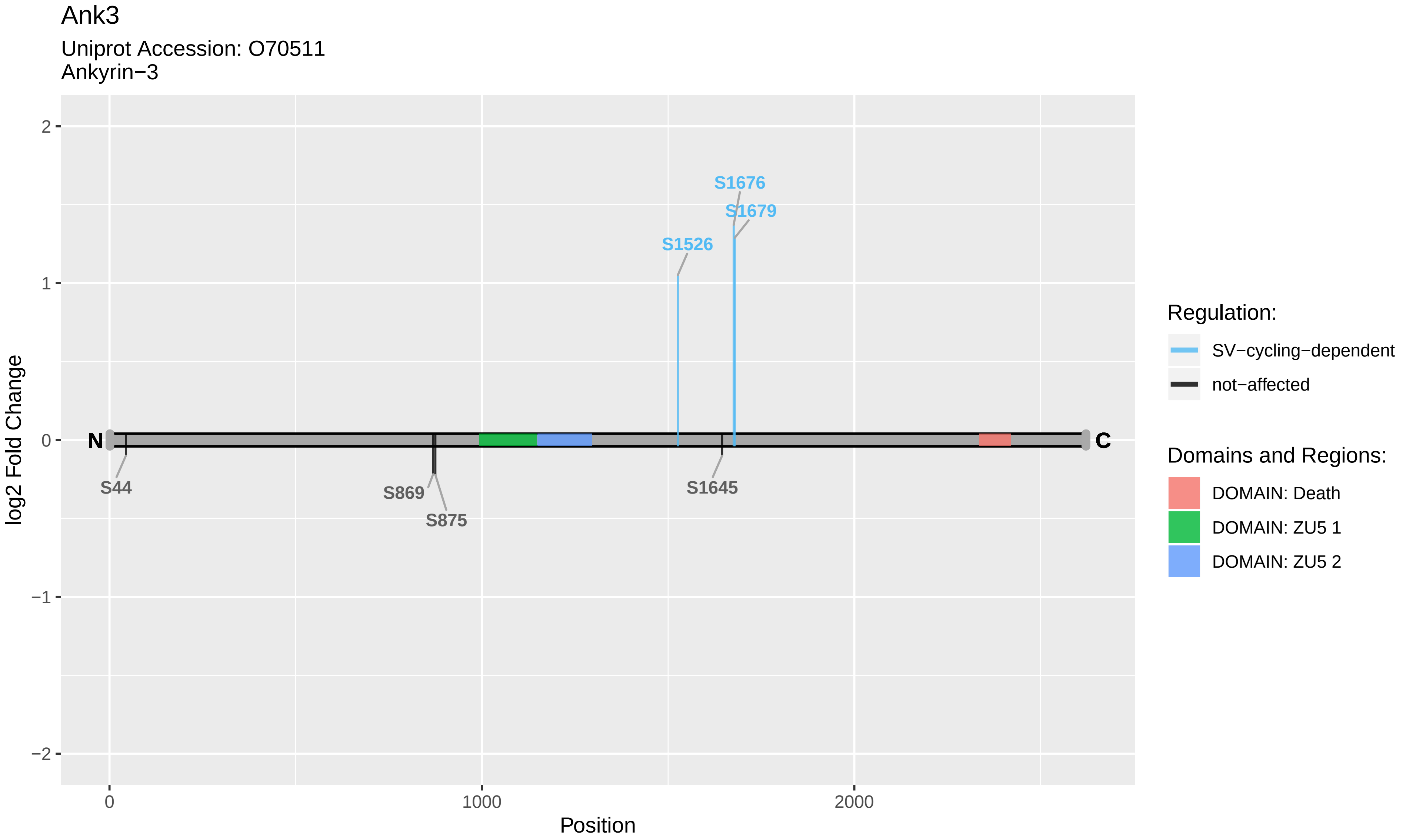

Ank3
Uniprot Accession: O70511
Ankyrin−3
S1676
S1679
S1526
S44
S869
S875
S1645
N
C
2
1
0
−1
−2
log2 Fold Change
0
1000
2000
Position
Regulation:
SV−cycling−dependent
not−affected
Domains and Regions:
DOMAIN: Death
DOMAIN: ZU5 1
DOMAIN: ZU5 2

Ankrd34a

Uniprot Accession: Q5BJT1
Ankyrin repeat domain−containing protein 34A

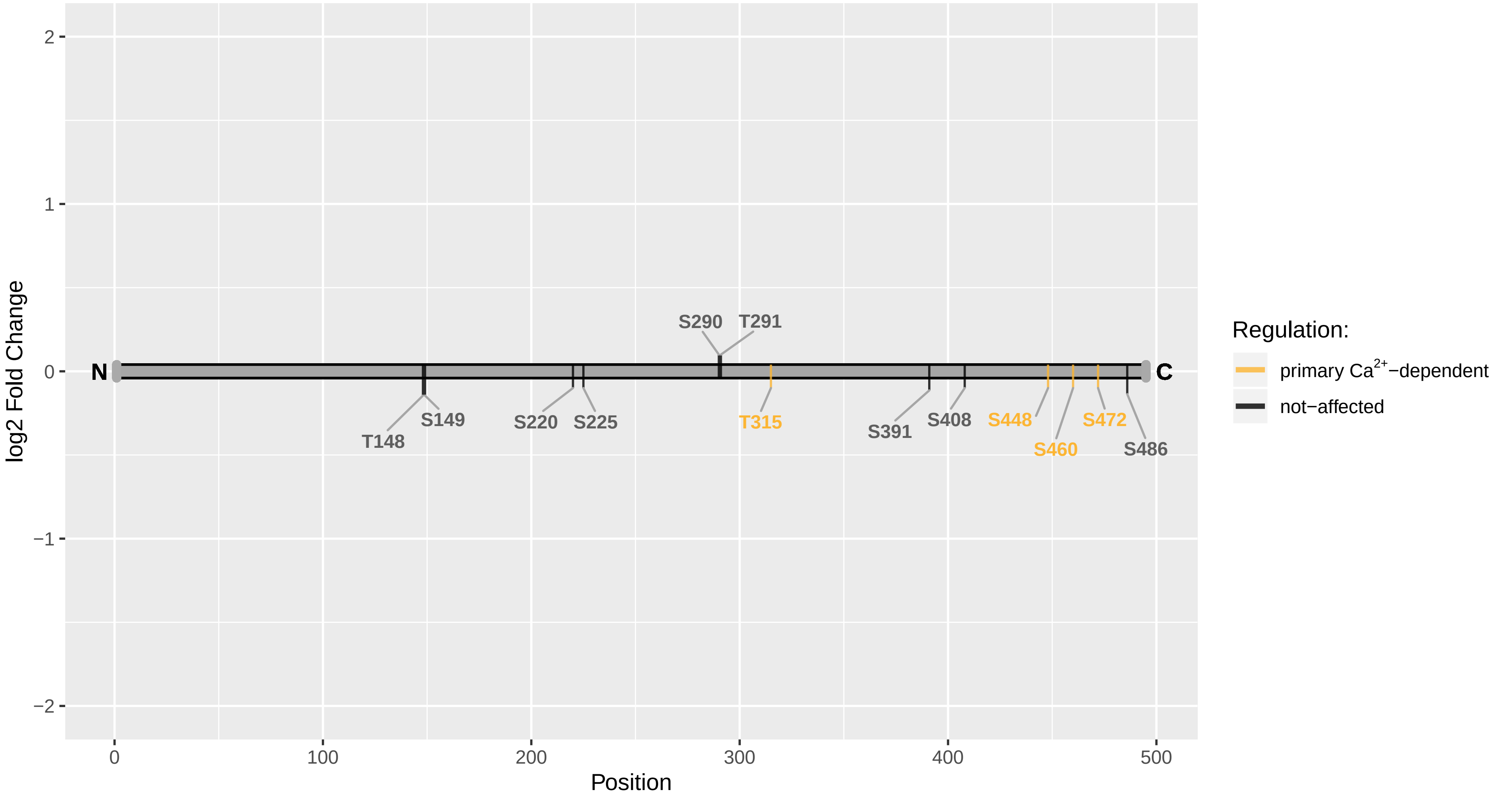

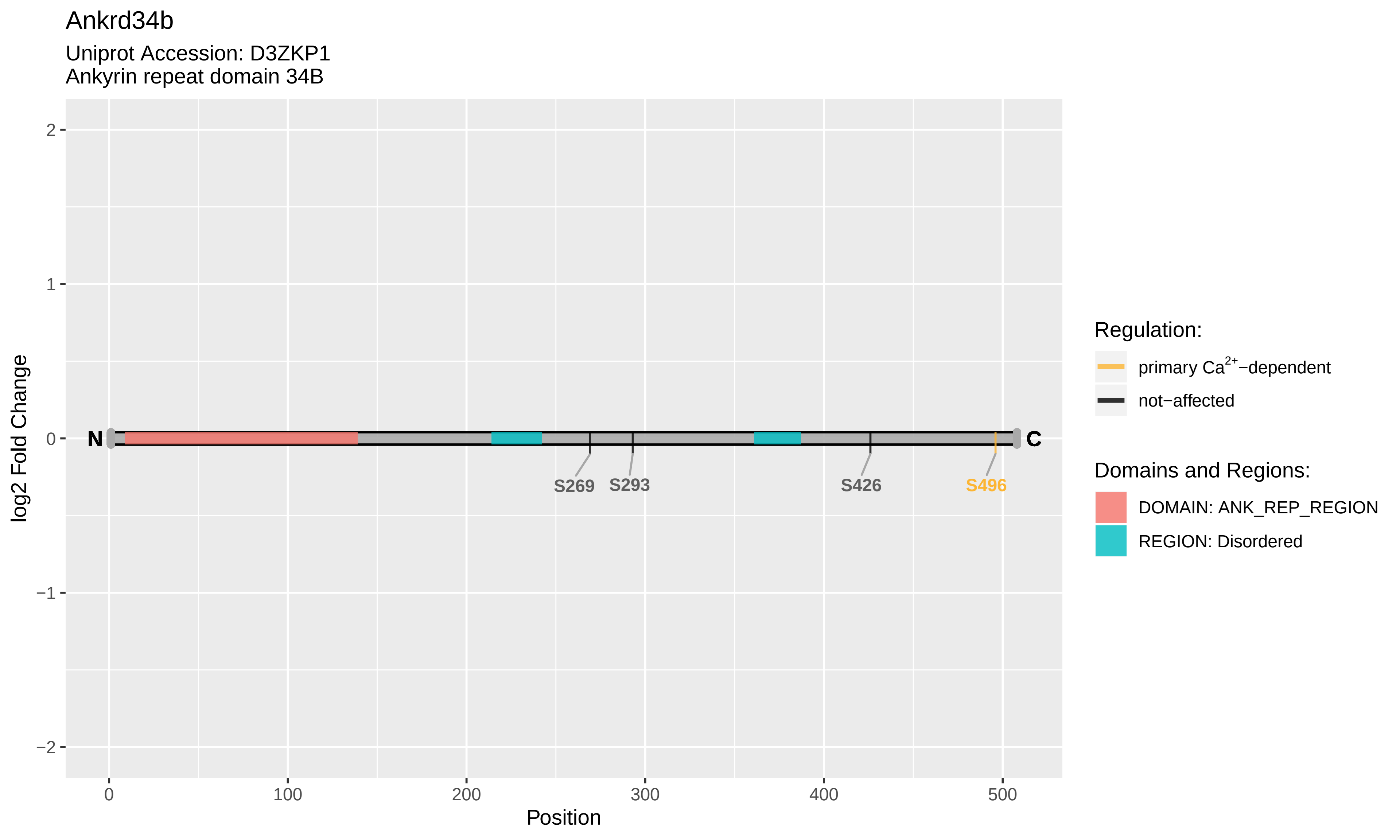
Ankrd34b
Uniprot Accession: D3ZKP1
Ankyrin repeat domain 34B
2
1
0
−1
−2
log2 Fold Change
N
C
S269
S293
S426
S496
0
100
200
300
400
500
Position
Regulation:
primary Ca2+−dependent
not−affected
Domains and Regions:
DOMAIN: ANK_REP_REGION
REGION: Disordered

Anks1a

Uniprot Accession: A0A0G2K2G7
Ankyrin repeat and sterile alpha motif domain−containing 1A

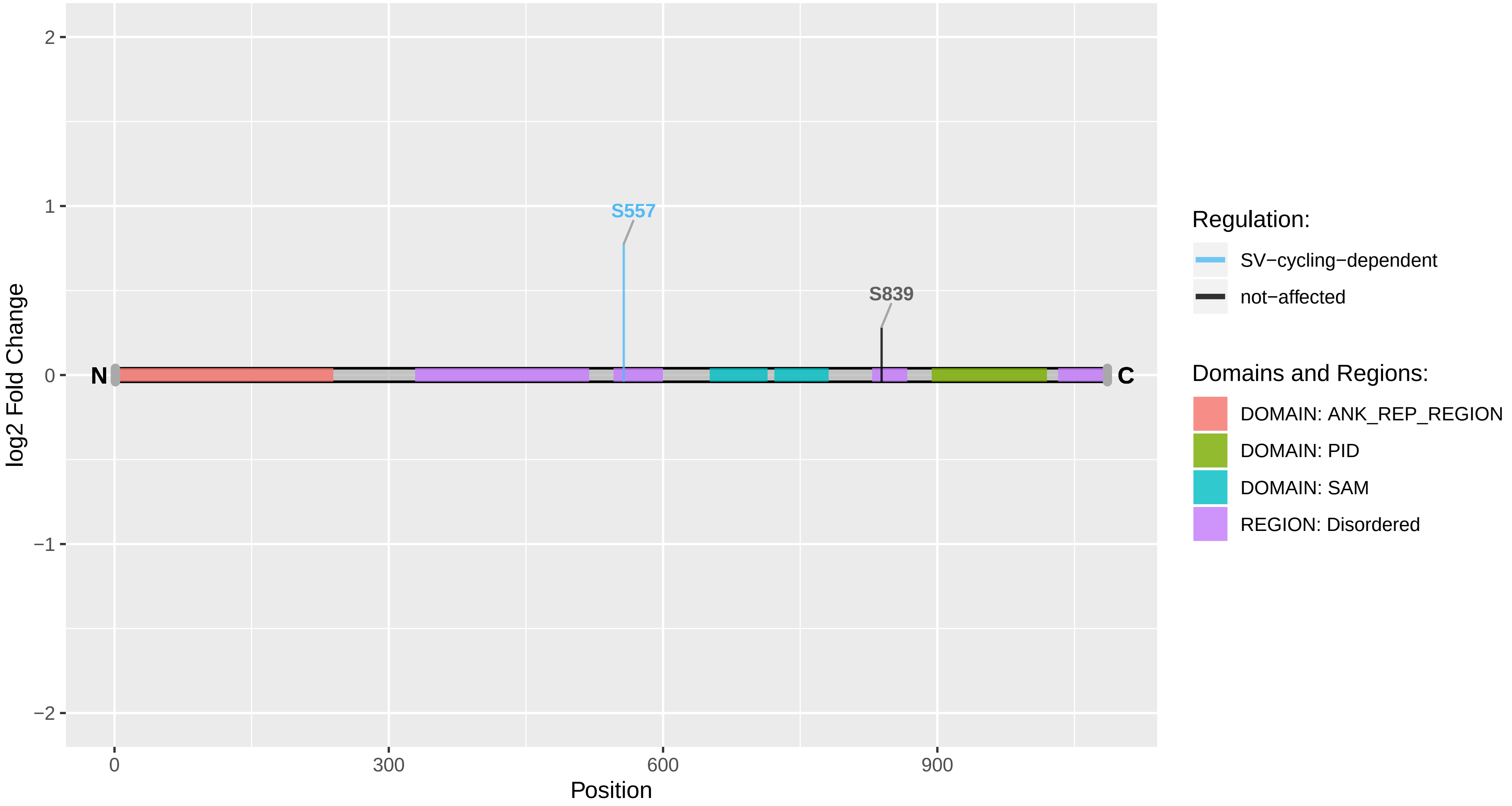

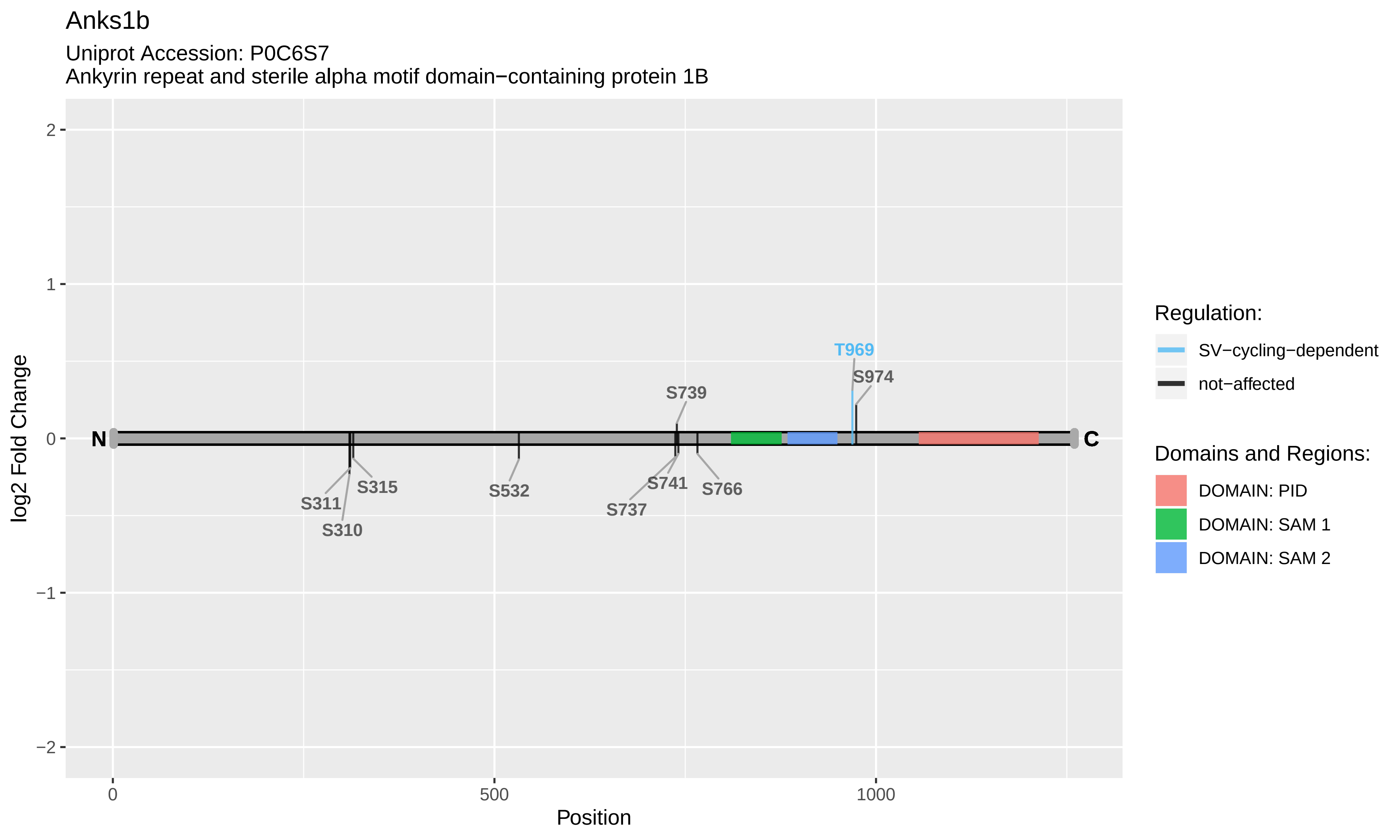
Anks1b
Uniprot Accession: P0C6S7
Ankyrin repeat and sterile alpha motif domain−containing protein 1B
log2 Fold Change
Position
N
C
T969
S974
S739
S315
S311
S310
S532
S741
S737
S766
Regulation:
SV−cycling−dependent
not−affected
Domains and Regions:
DOMAIN: PID
DOMAIN: SAM 1
DOMAIN: SAM 2
2
1
0
−1
−2
0
500
1000

Anln

Uniprot Accession: D4A0V6
Anillin, actin−binding protein

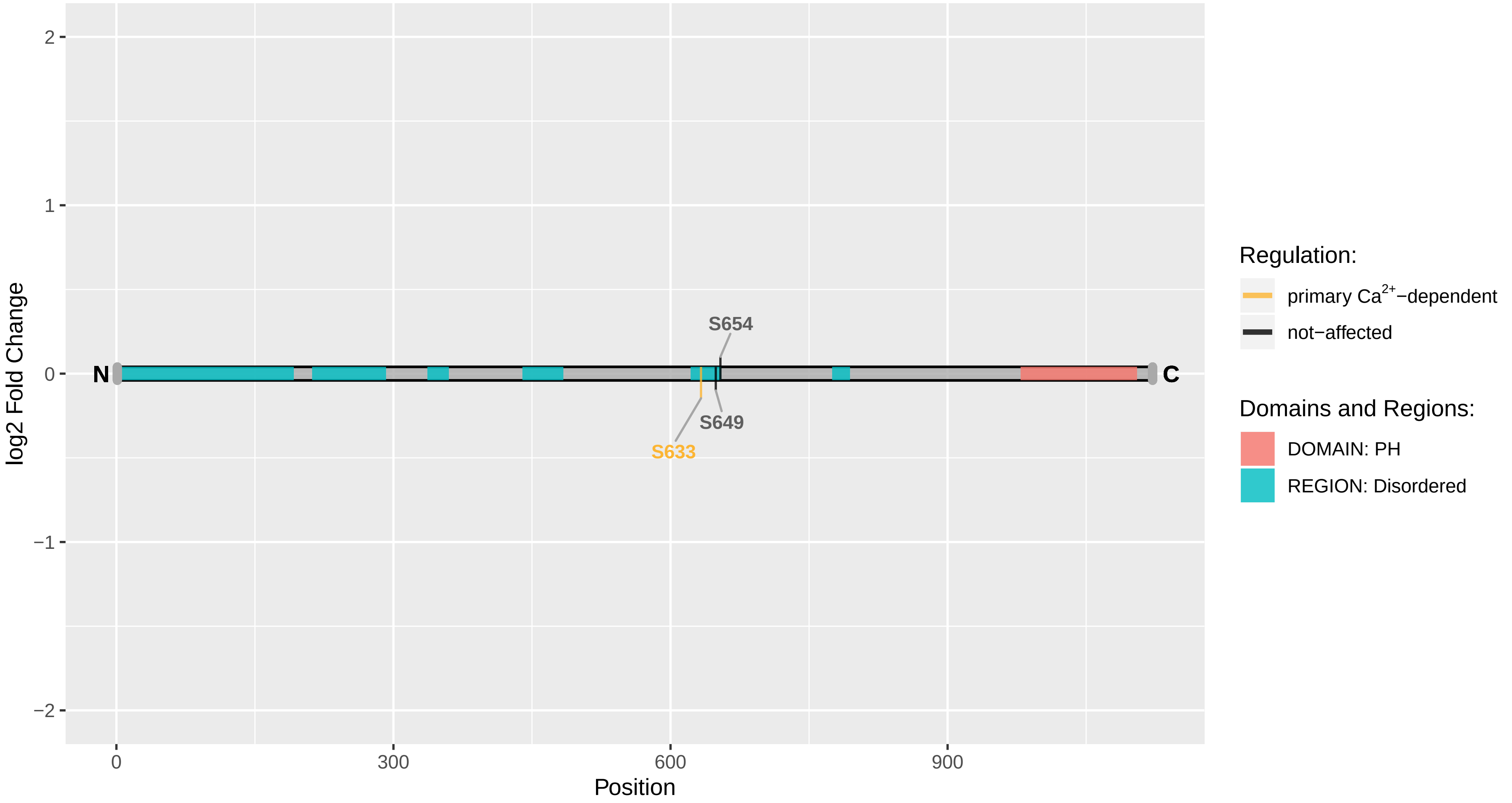

Ano8

Uniprot Accession: A0A0G2K5K1
Anoctamin

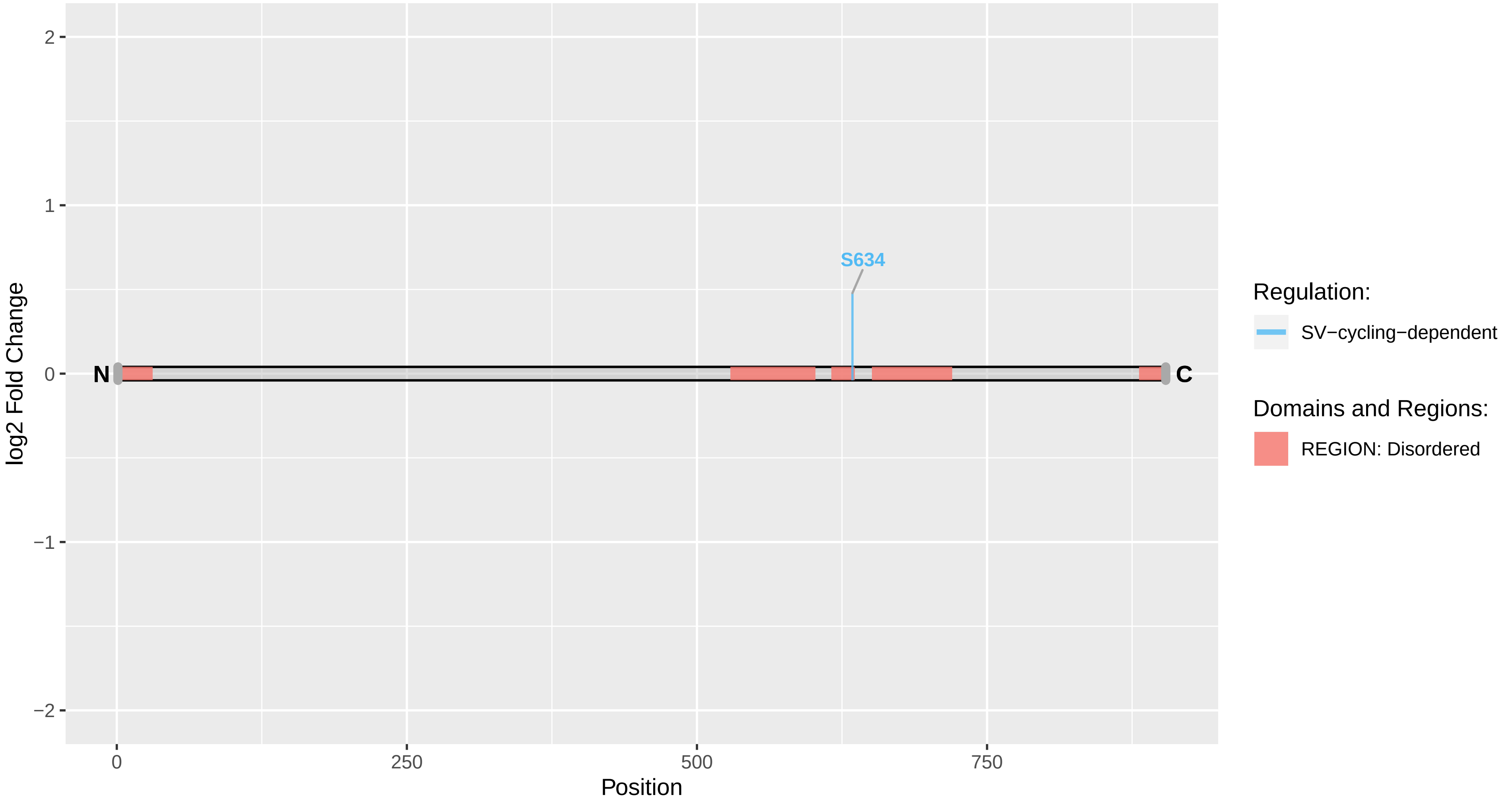

Ap2b1

Uniprot Accession: P62944
AP−2 complex subunit beta

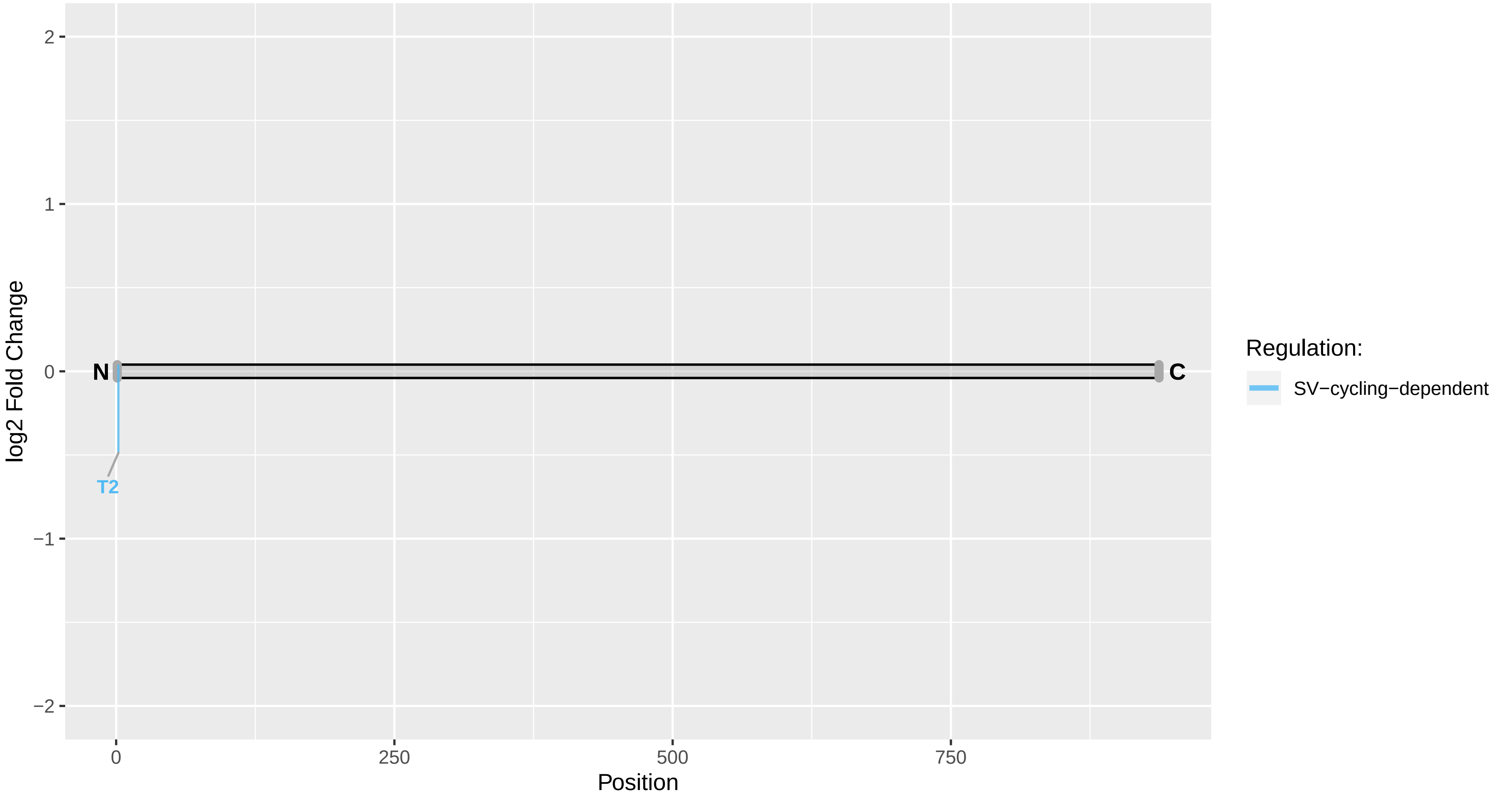

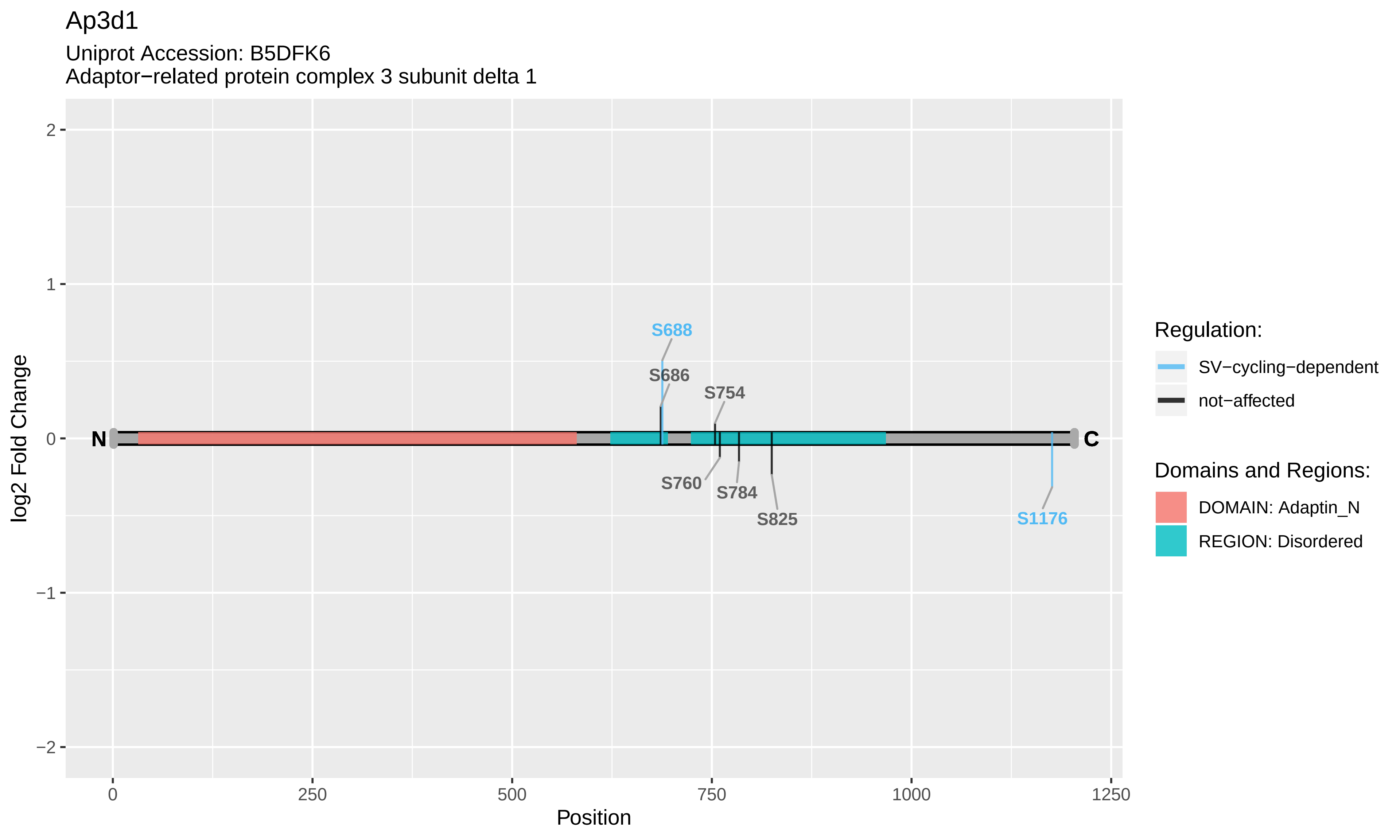

Ap3d1
Uniprot Accession: B5DFK6
Adaptor−related protein complex 3 subunit delta 1
S688
S686
S754
N
C
S760
S784
S825
S1176
2
1
0
−1
−2
log2 Fold Change
0
250
500
750
1000
1250
Position
Regulation:
SV−cycling−dependent
not−affected
Domains and Regions:
DOMAIN: Adaptin_N
REGION: Disordered

Apc

Uniprot Accession: A0A0G2K8G0
Adenomatous polyposis coli protein

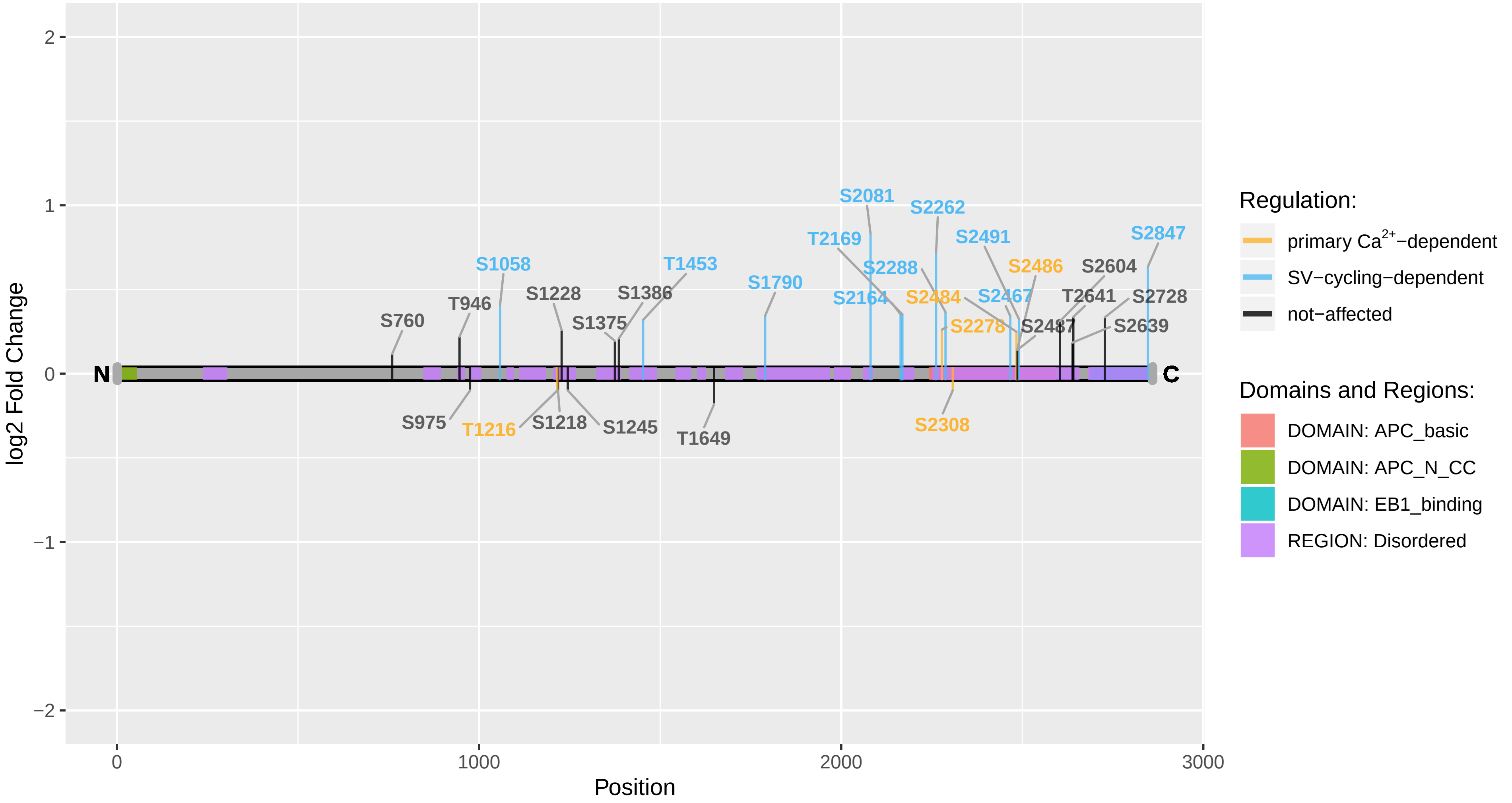

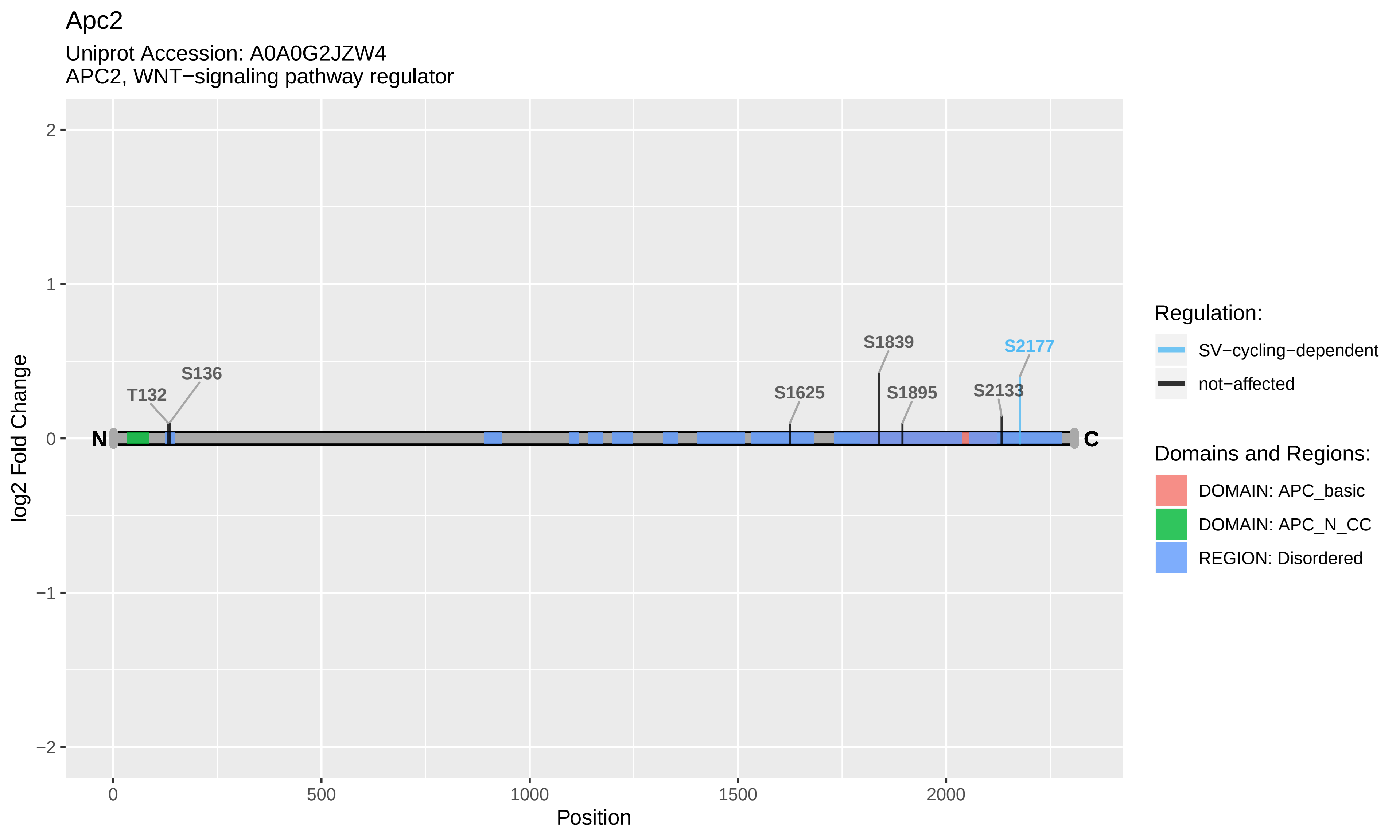

Apc2
Uniprot Accession: A0A0G2JZW4
APC2, WNT−signaling pathway regulator
log2 Fold Change
Position
2
1
0
−1
−2
0
500
1000
1500
2000
N
C
T132
S136
S1625
S1839
S1895
S2133
S2177
Regulation:
SV−cycling−dependent
not−affected
Domains and Regions:
DOMAIN: APC_basic
DOMAIN: APC_N_CC
REGION: Disordered

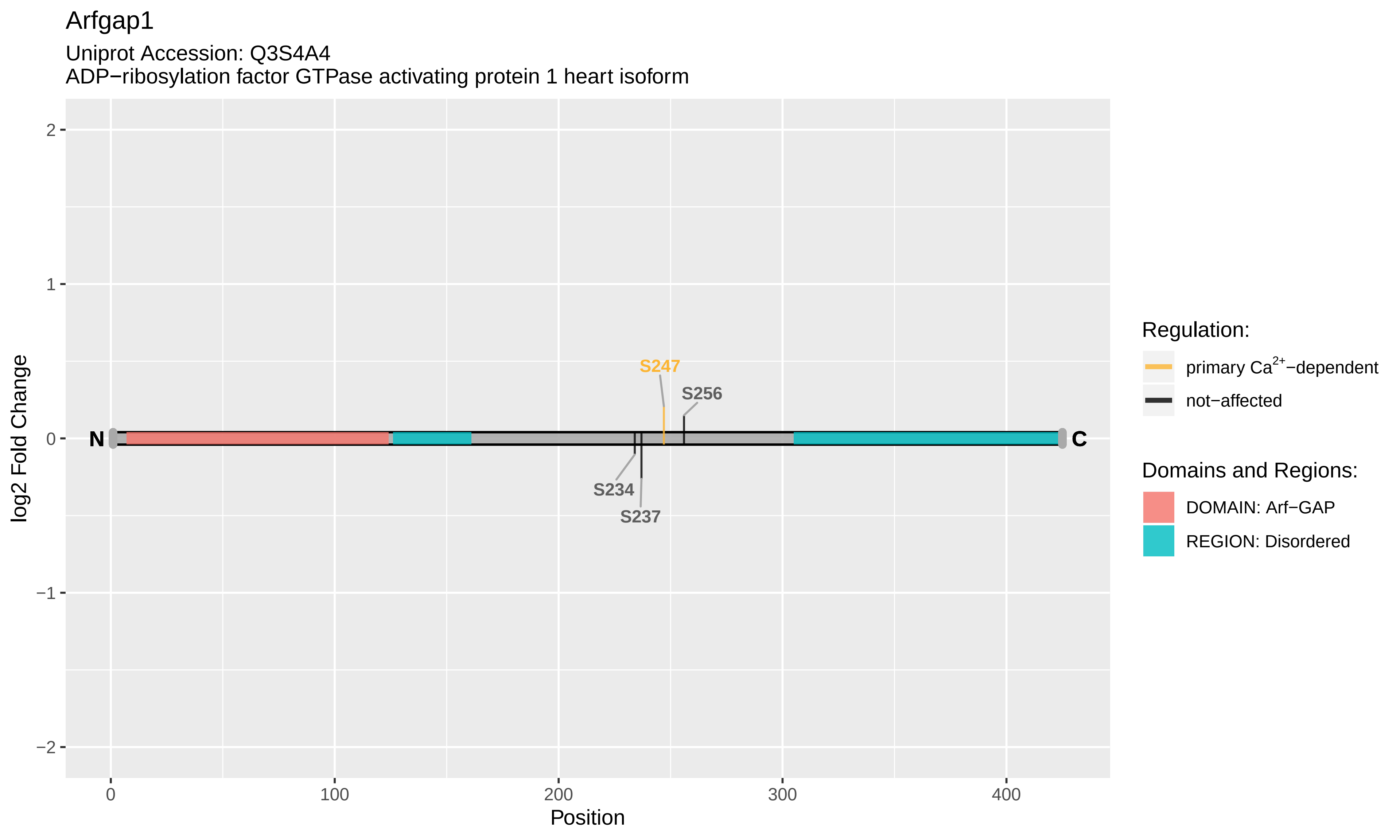

Arfgap1
Uniprot Accession: Q3S4A4
ADP−ribosylation factor GTPase activating protein 1 heart isoform
log2 Fold Change
Position
N
C
S247
S256
S234
S237
2
1
0
−1
−2
0
100
200
300
400
Regulation:
primary Ca2+−dependent
not−affected
Domains and Regions:
DOMAIN: Arf−GAP
REGION: Disordered

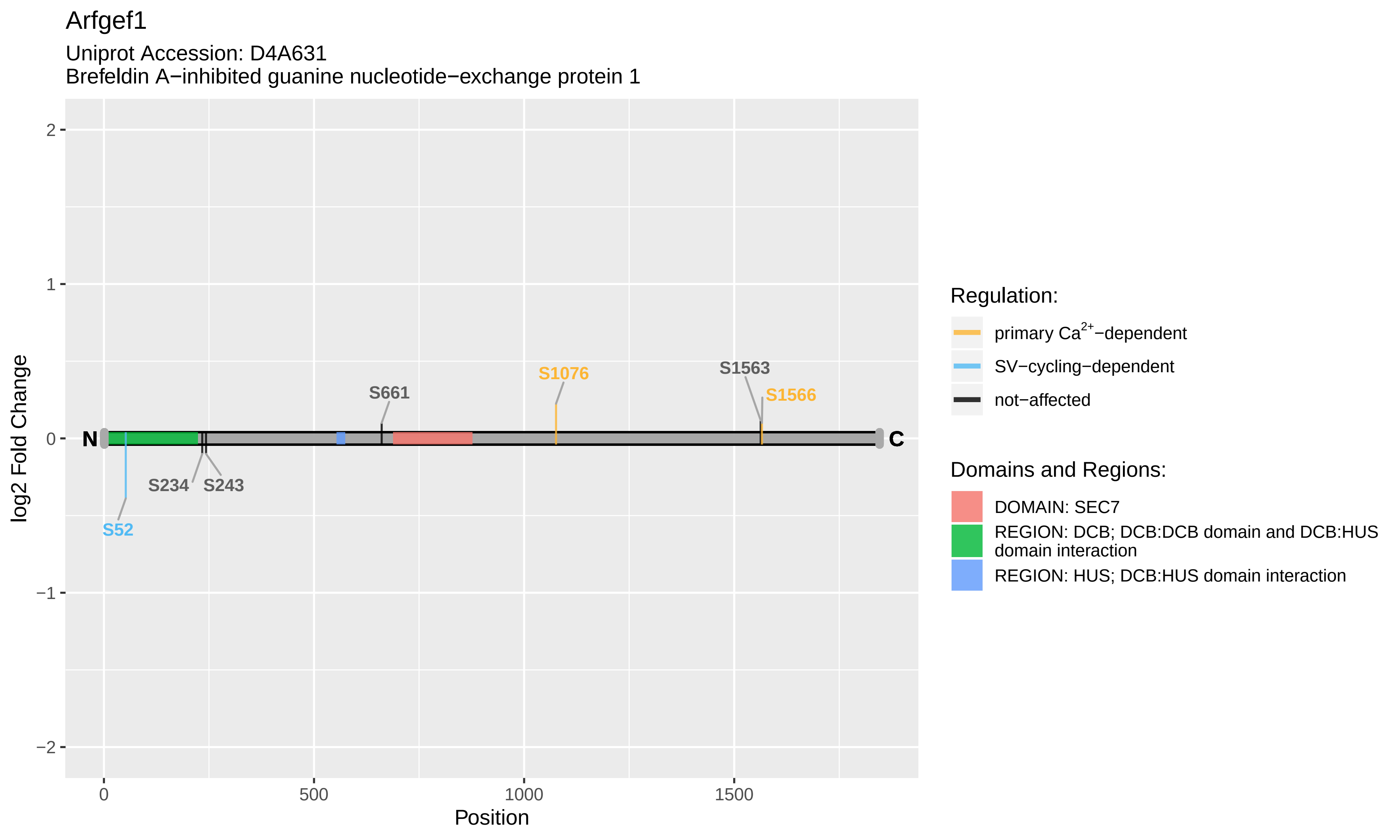

Arfgef1
Uniprot Accession: D4A631
Brefeldin A−inhibited guanine nucleotide−exchange protein 1
log2 Fold Change
2
1
0
−1
−2
N
C
S52
S234
S243
S661
S1076
S1563
S1566
0
500
1000
1500
Position
Regulation:
primary Ca2+−dependent
SV−cycling−dependent
not−affected
Domains and Regions:
DOMAIN: SEC7
REGION: DCB; DCB:DCB domain and DCB:HUS domain interaction
REGION: HUS; DCB:HUS domain interaction

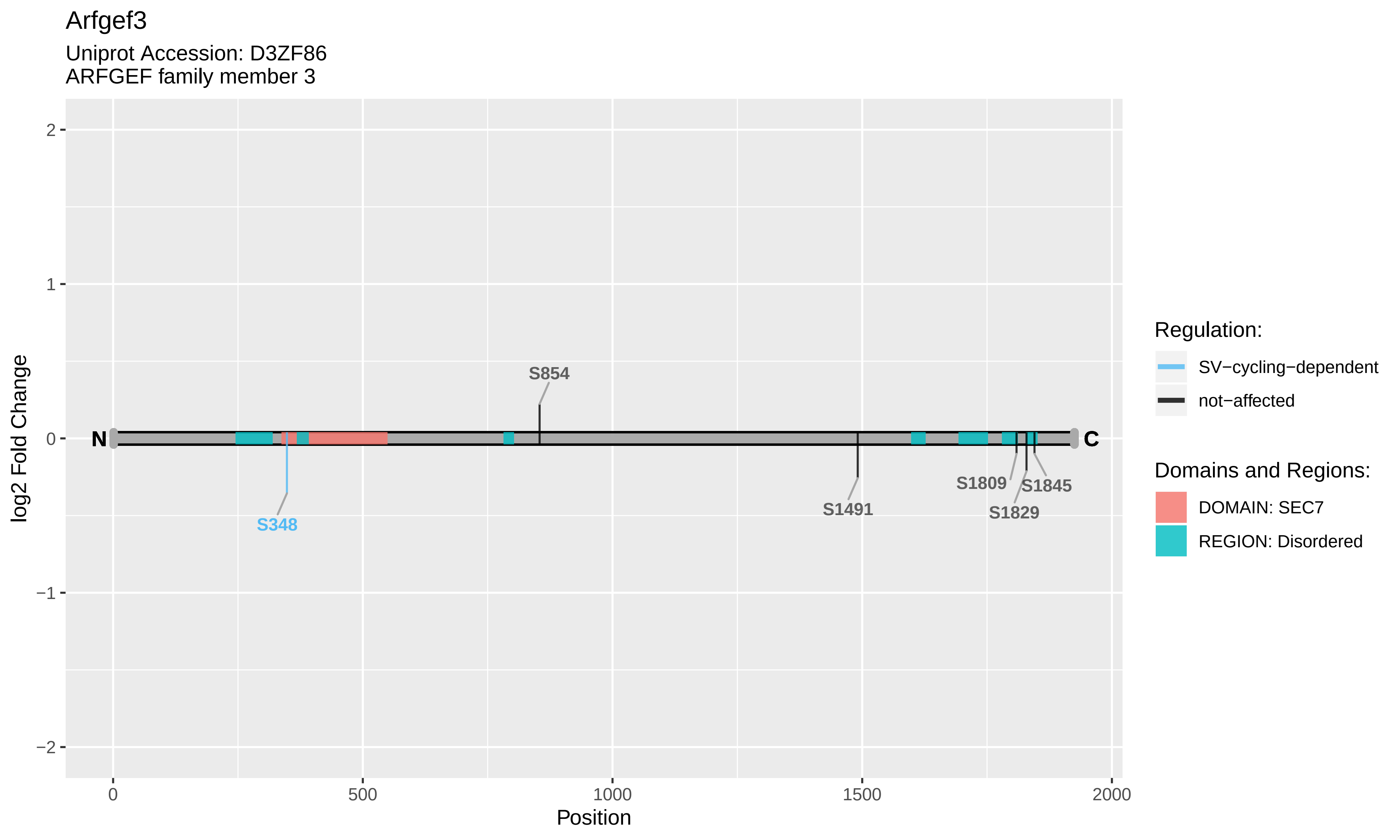
Arfgef3
Uniprot Accession: D3ZF86
ARFGEF family member 3
log2 Fold Change
Position
N
C
S854
S348
S1491
S1809
S1829
S1845
Regulation:
SV−cycling−dependent
not−affected
Domains and Regions:
DOMAIN: SEC7
REGION: Disordered
2
1
0
−1
−2
0
500
1000
1500
2000

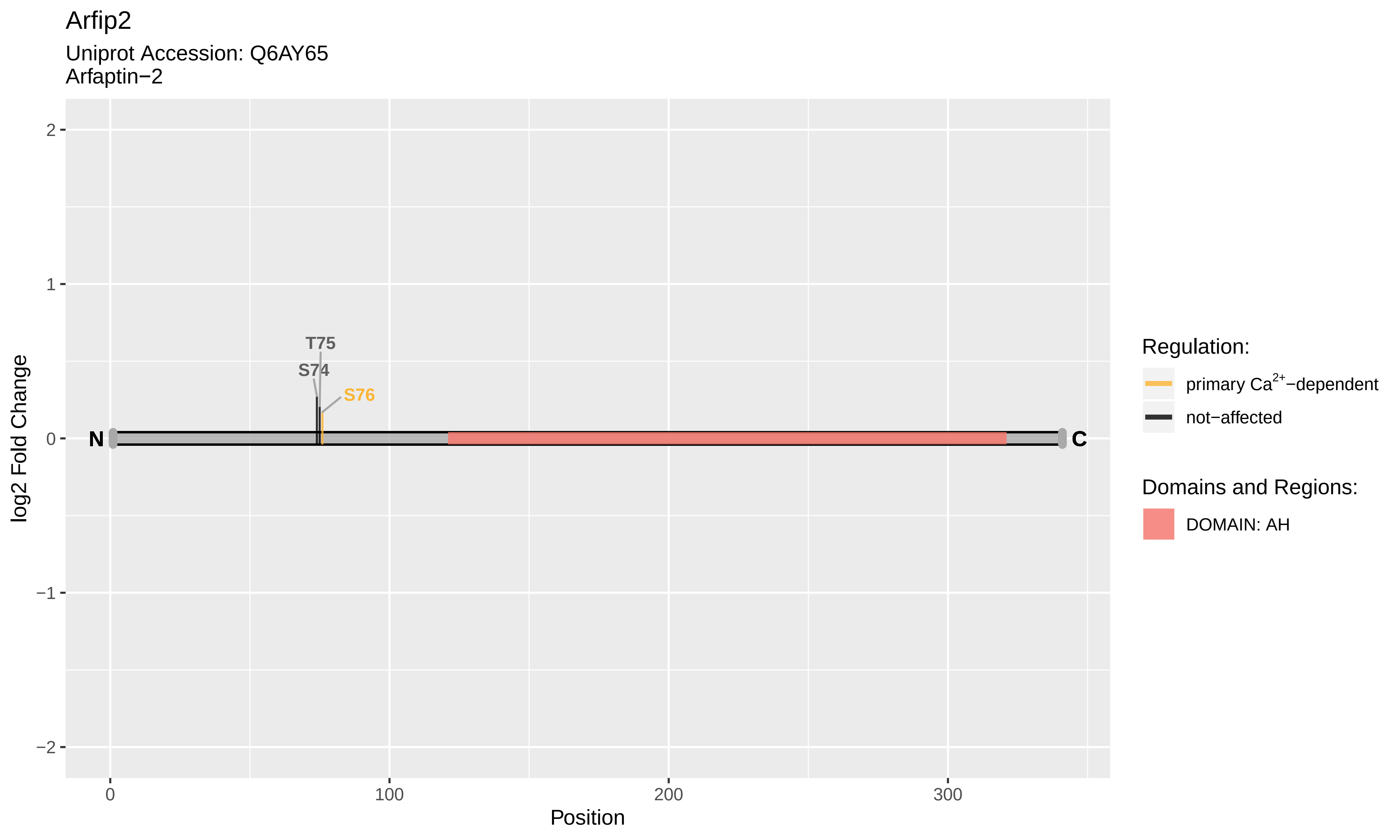
Arfip2
Uniprot Accession: Q6AY65
Arfaptin−2
T75
S74
S76
N
C
2
1
0
−1
−2
log2 Fold Change
0
100
200
300
Position
Regulation:
primary Ca2+−dependent
not−affected
Domains and Regions:
DOMAIN: AH

Arhgap1

Uniprot Accession: D4A6C5
Rho GTPase−activating protein 1

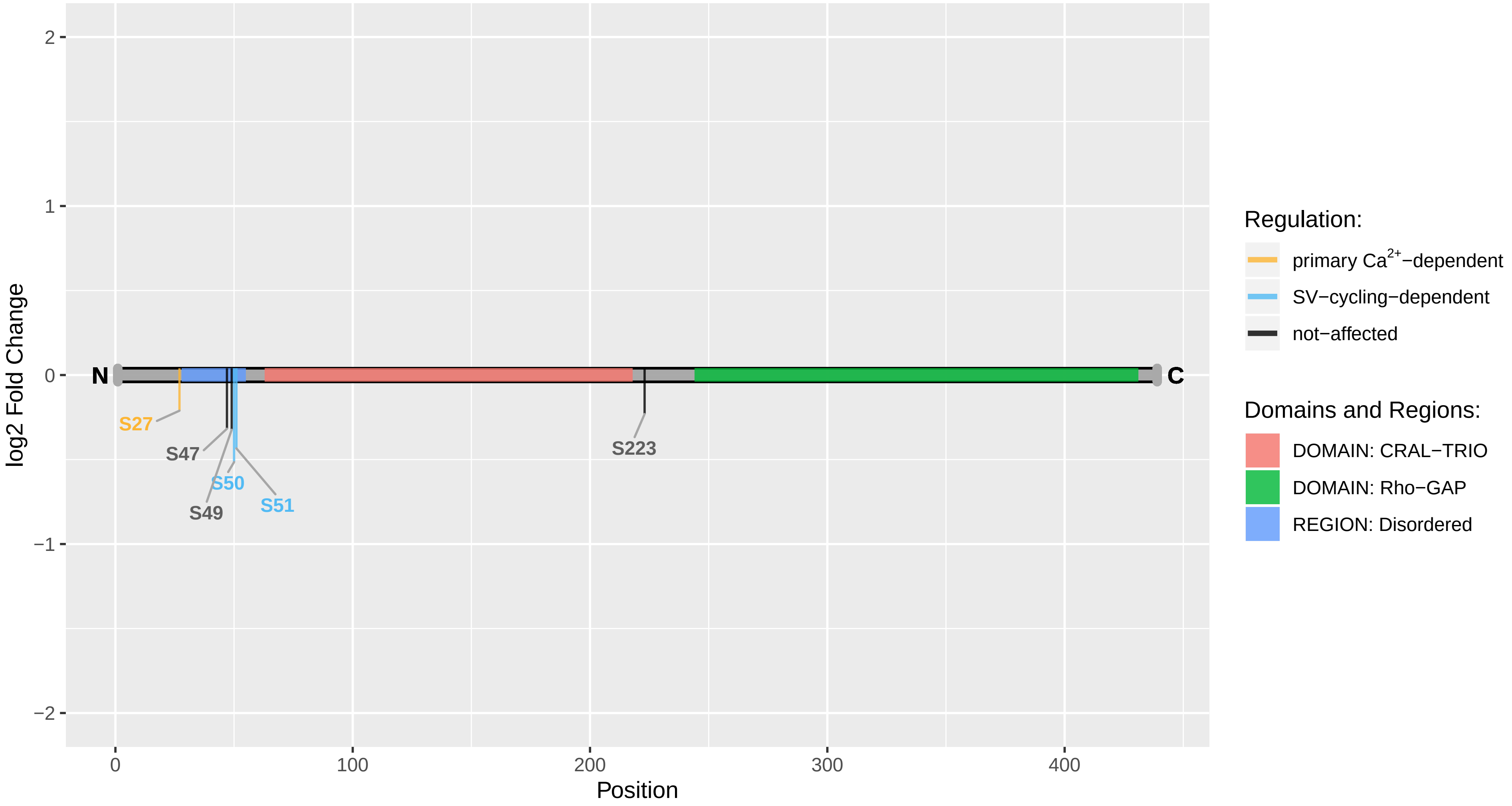

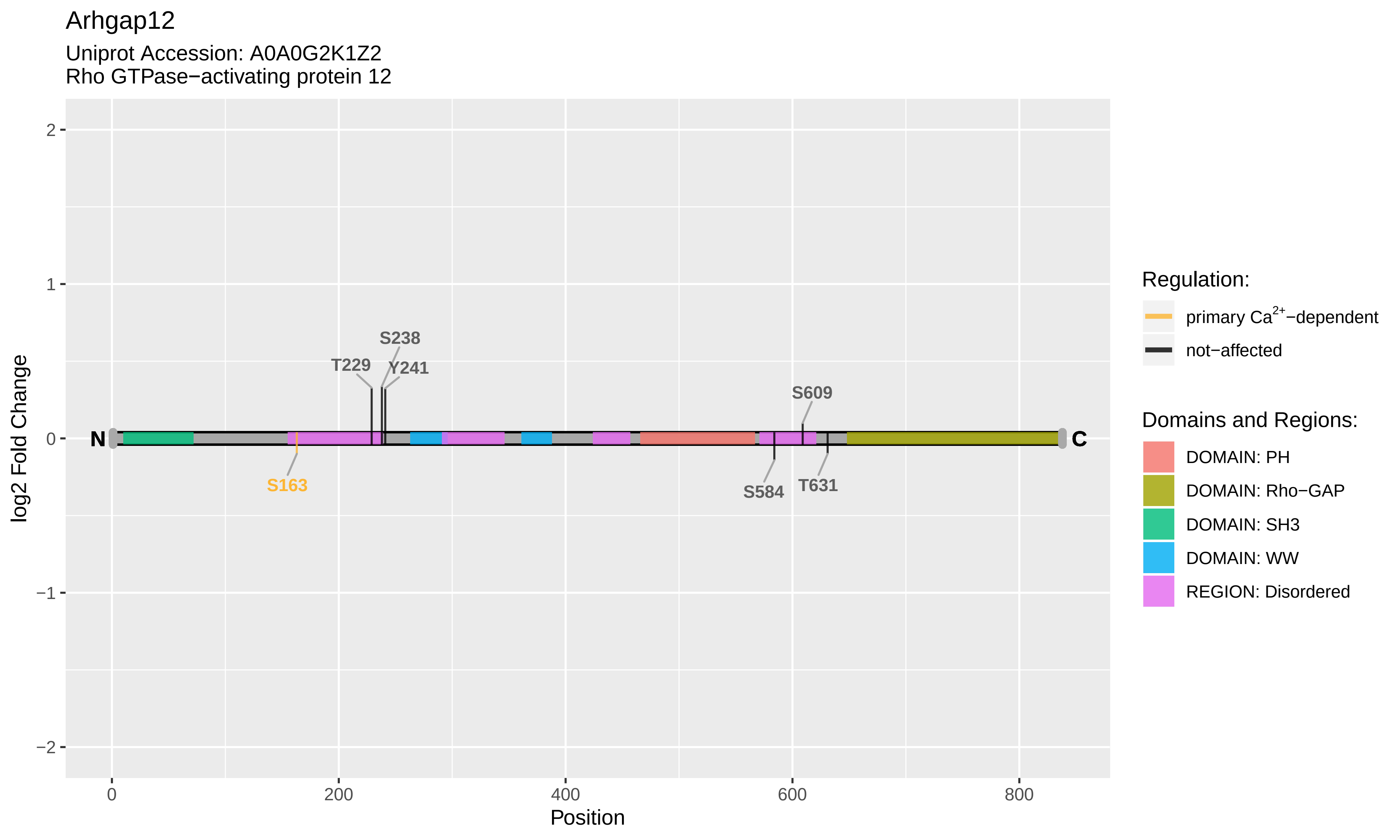

Arhgap12
Uniprot Accession: A0A0G2K1Z2
Rho GTPase−activating protein 12
log2 Fold Change
2
1
0
−1
−2
N
C
S238
T229
Y241
S609
S163
S584
T631
0
200
400
600
800
Position
Regulation:
primary Ca2+−dependent
not−affected
Domains and Regions:
DOMAIN: PH
DOMAIN: Rho−GAP
DOMAIN: SH3
DOMAIN: WW
REGION: Disordered

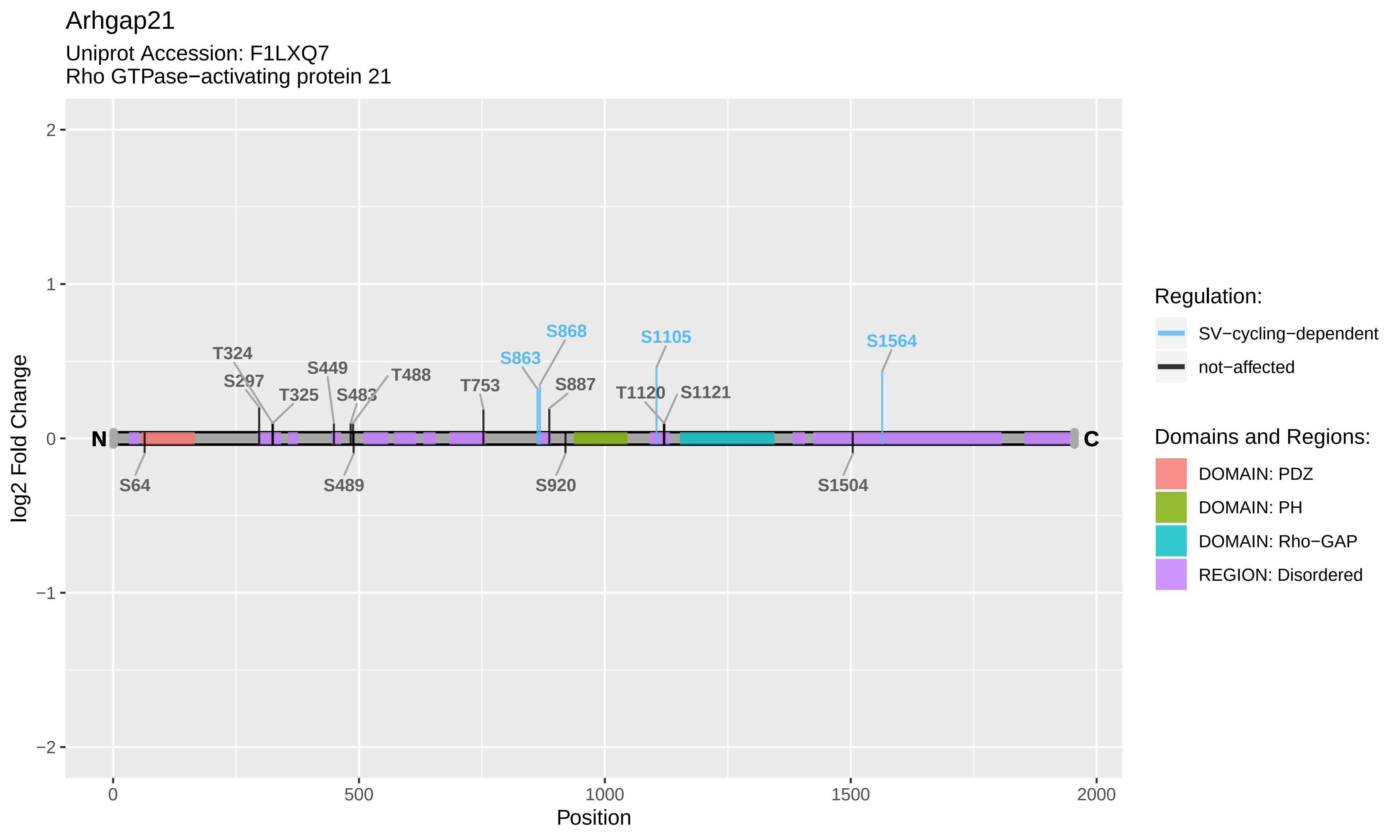
Arhgap21
Uniprot Accession: F1LXQ7
Rho GTPase−activating protein 21
log2 Fold Change
Position
N
C
S64
S297
T324
T325
S449
S483
T488
S489
T753
S863
S868
S887
S920
S1105
T1120
S1121
S1504
S1564
Regulation:
SV−cycling−dependent
not−affected
Domains and Regions:
DOMAIN: PDZ
DOMAIN: PH
DOMAIN: Rho−GAP
REGION: Disordered

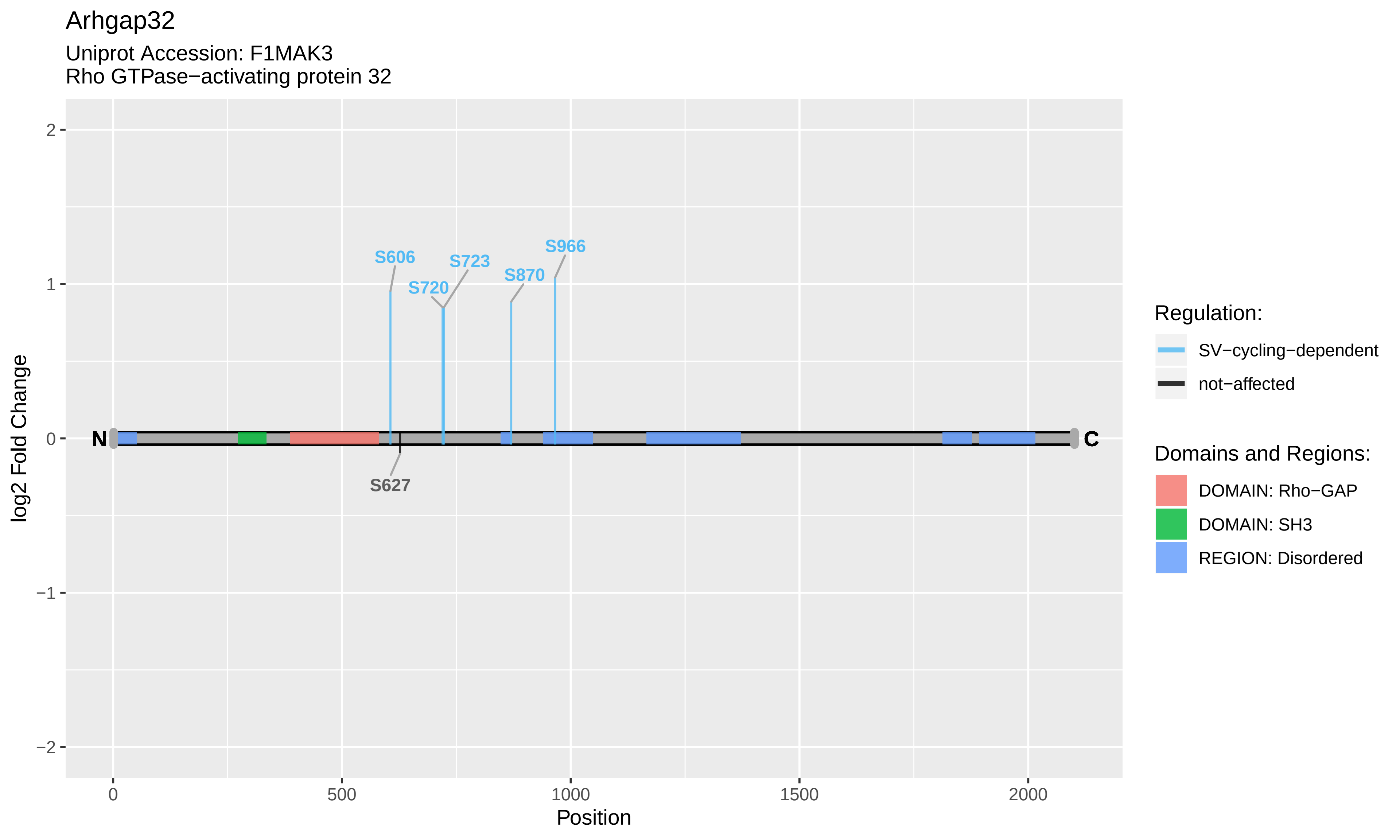

Arhgap32
Uniprot Accession: F1MAK3
Rho GTPase−activating protein 32
S606
S723
S966
S720
S870
N
C
S627
2
1
0
−1
−2
log2 Fold Change
0
500
1000
1500
2000
Position
Regulation:
SV−cycling−dependent
not−affected
Domains and Regions:
DOMAIN: Rho−GAP
DOMAIN: SH3
REGION: Disordered

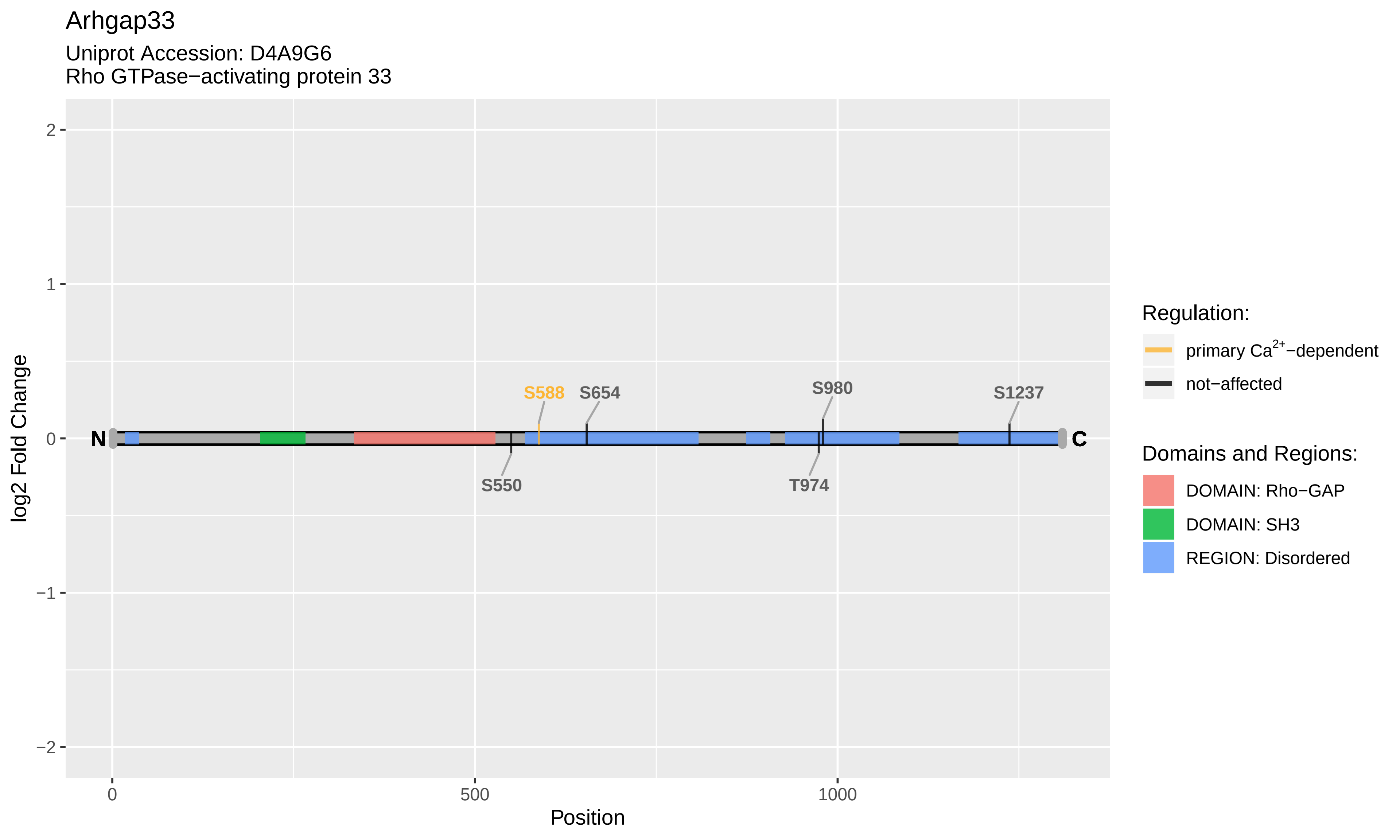

Arhgap33
Uniprot Accession: D4A9G6
Rho GTPase−activating protein 33
log2 Fold Change
Position
2
1
0
−1
−2
0
500
1000
N
C
S588
S654
S980
S1237
S550
T974
Regulation:
primary Ca2+−dependent
not−affected
Domains and Regions:
DOMAIN: Rho−GAP
DOMAIN: SH3
REGION: Disordered

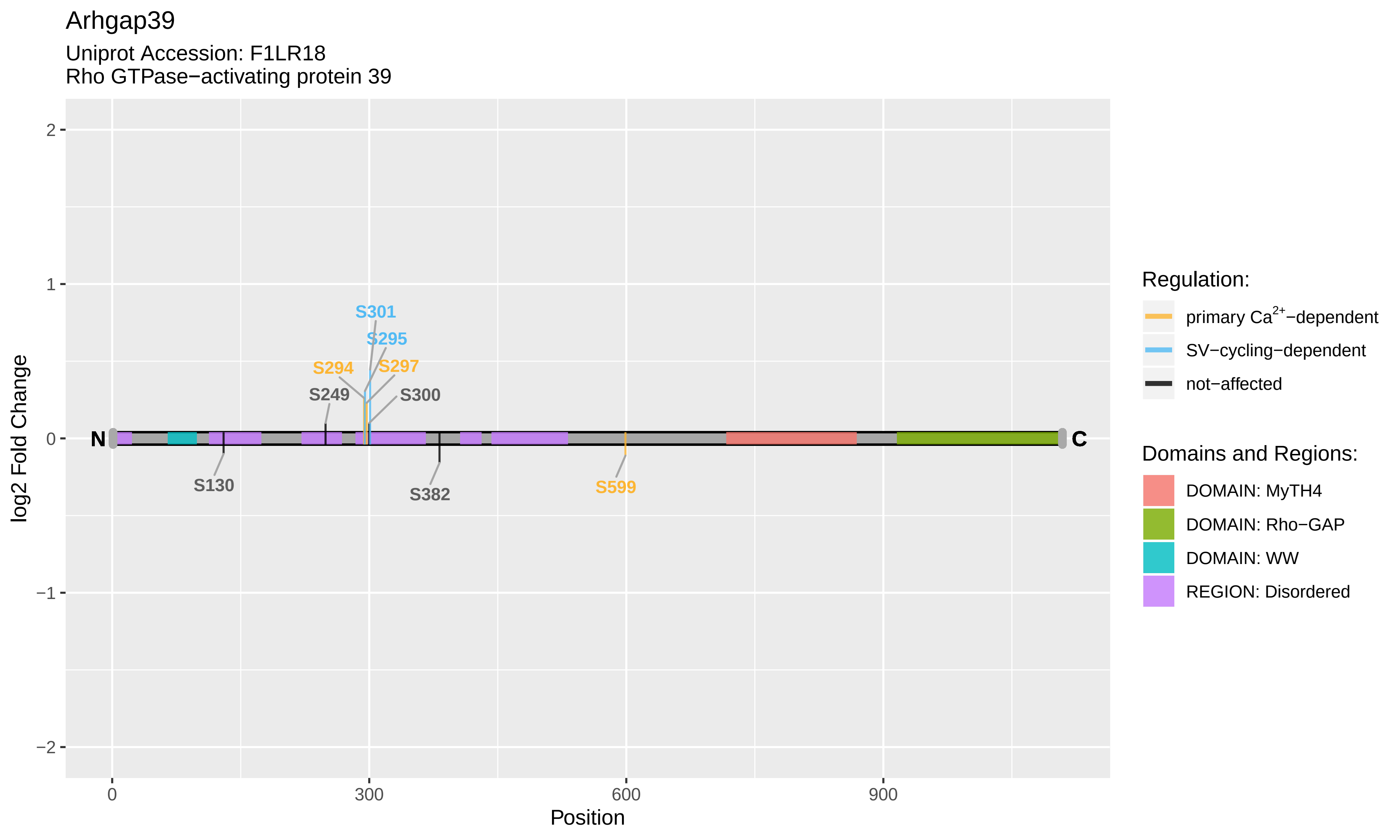

Arhgap39
Uniprot Accession: F1LR18
Rho GTPase−activating protein 39
S301
S295
S294
S297
S249
S300
N
C
S130
S382
S599
log2 Fold Change
2
1
0
−1
−2
0
300
600
900
Position
Regulation:
primary Ca2+−dependent
SV−cycling−dependent
not−affected
Domains and Regions:
DOMAIN: MyTH4
DOMAIN: Rho−GAP
DOMAIN: WW
REGION: Disordered

Arhgap44

Uniprot Accession: F1LQX4
Rho GTPase−activating protein 44

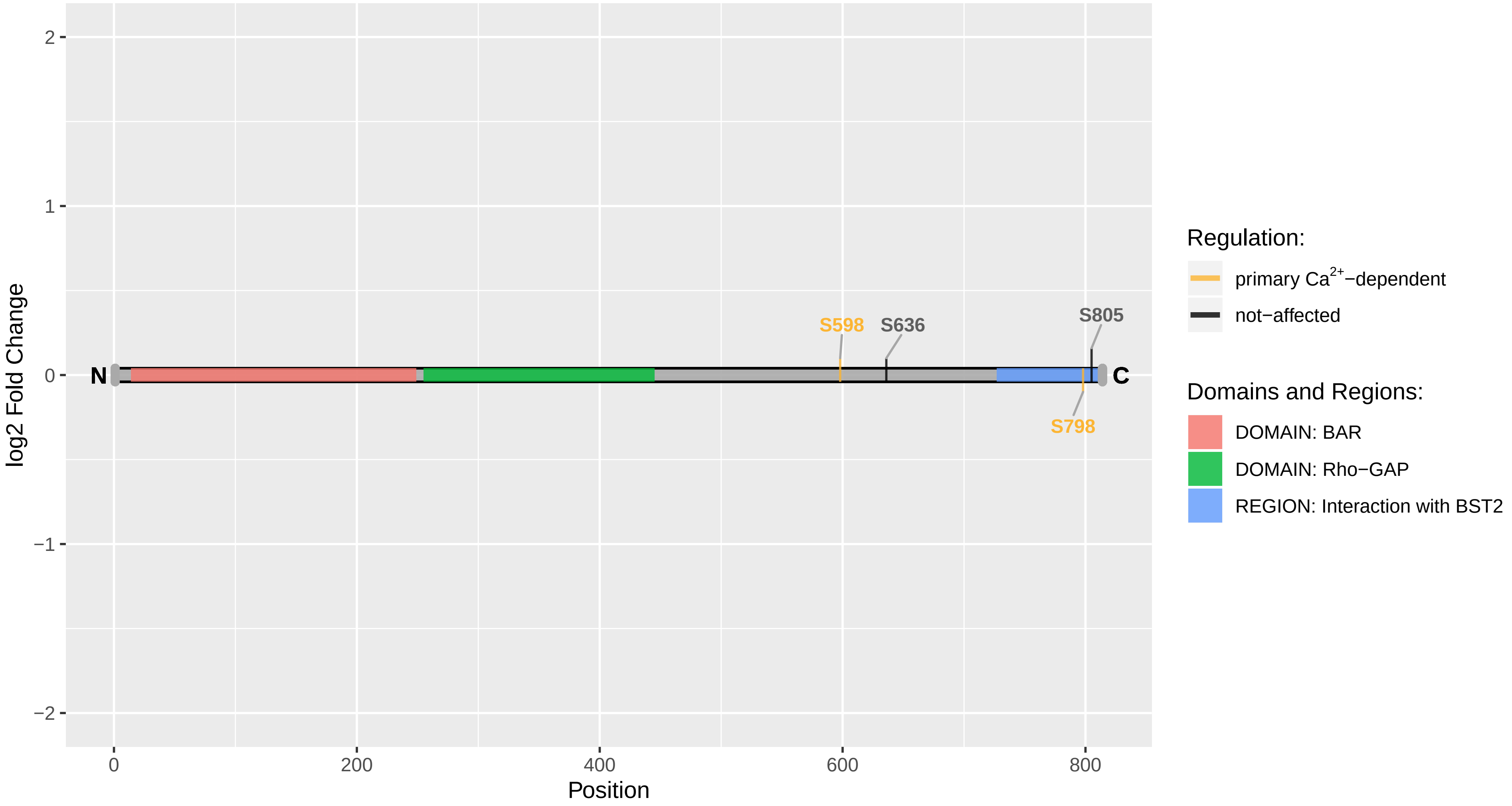

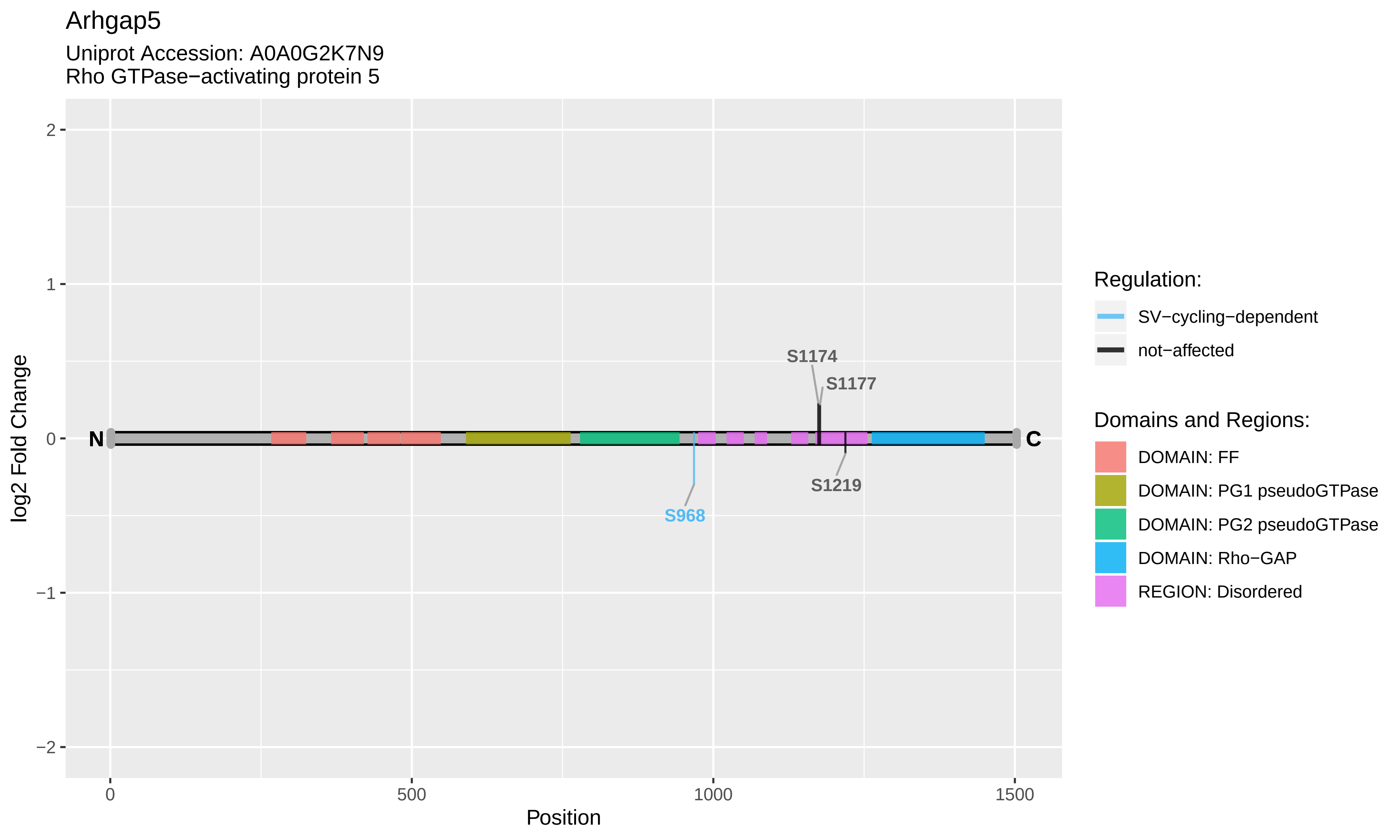

Arhgap5
Uniprot Accession: A0A0G2K7N9
Rho GTPase−activating protein 5
log2 Fold Change
Position
N
C
S1174
S1177
S1219
S968
2
1
0
−1
−2
0
500
1000
1500
Regulation:
SV−cycling−dependent
not−affected
Domains and Regions:
DOMAIN: FF
DOMAIN: PG1 pseudoGTPase
DOMAIN: PG2 pseudoGTPase
DOMAIN: Rho−GAP
REGION: Disordered

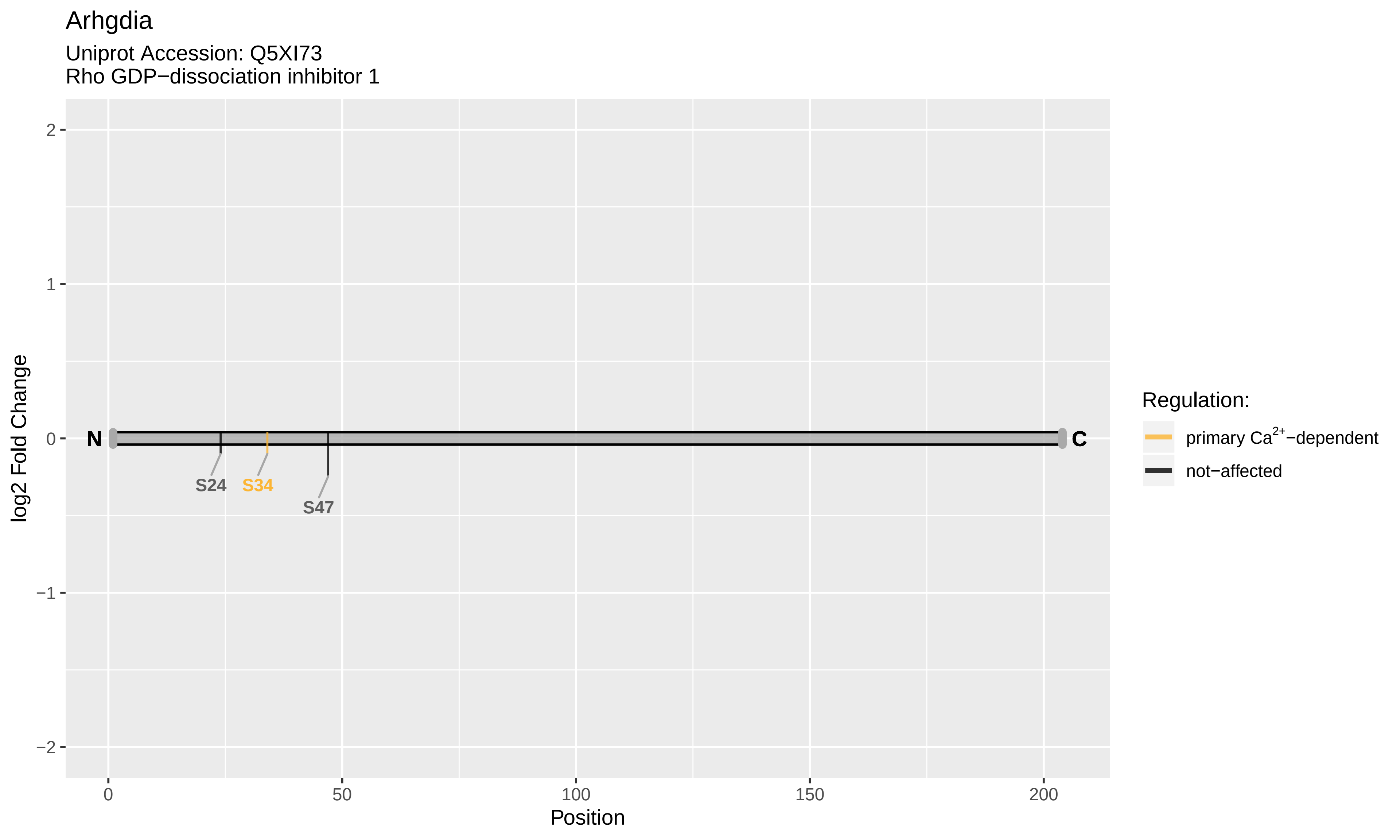

Arhgdia
Uniprot Accession: Q5XI73
Rho GDP−dissociation inhibitor 1
log2 Fold Change
2
1
0
−1
−2
N
C
S24
S34
S47
0
50
100
150
200
Position
Regulation:
primary Ca2+−dependent
not−affected

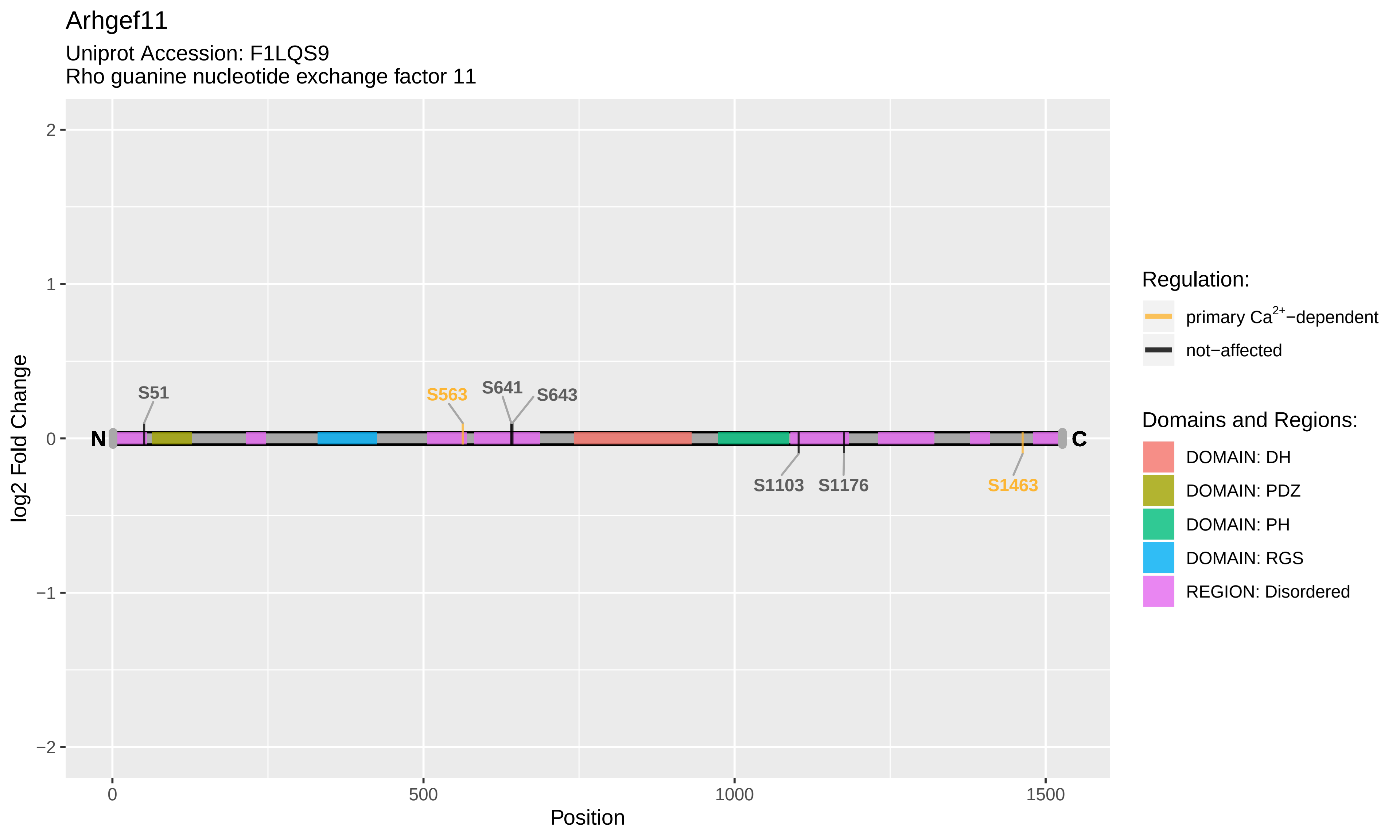
Arhgef11
Uniprot Accession: F1LQS9
Rho guanine nucleotide exchange factor 11
log2 Fold Change
Position
2
1
0
−1
−2
0
500
1000
1500
N
C
S51
S563
S641
S643
S1103
S1176
S1463
Regulation:
primary Ca2+−dependent
not−affected
Domains and Regions:
DOMAIN: DH
DOMAIN: PDZ
DOMAIN: PH
DOMAIN: RGS
REGION: Disordered

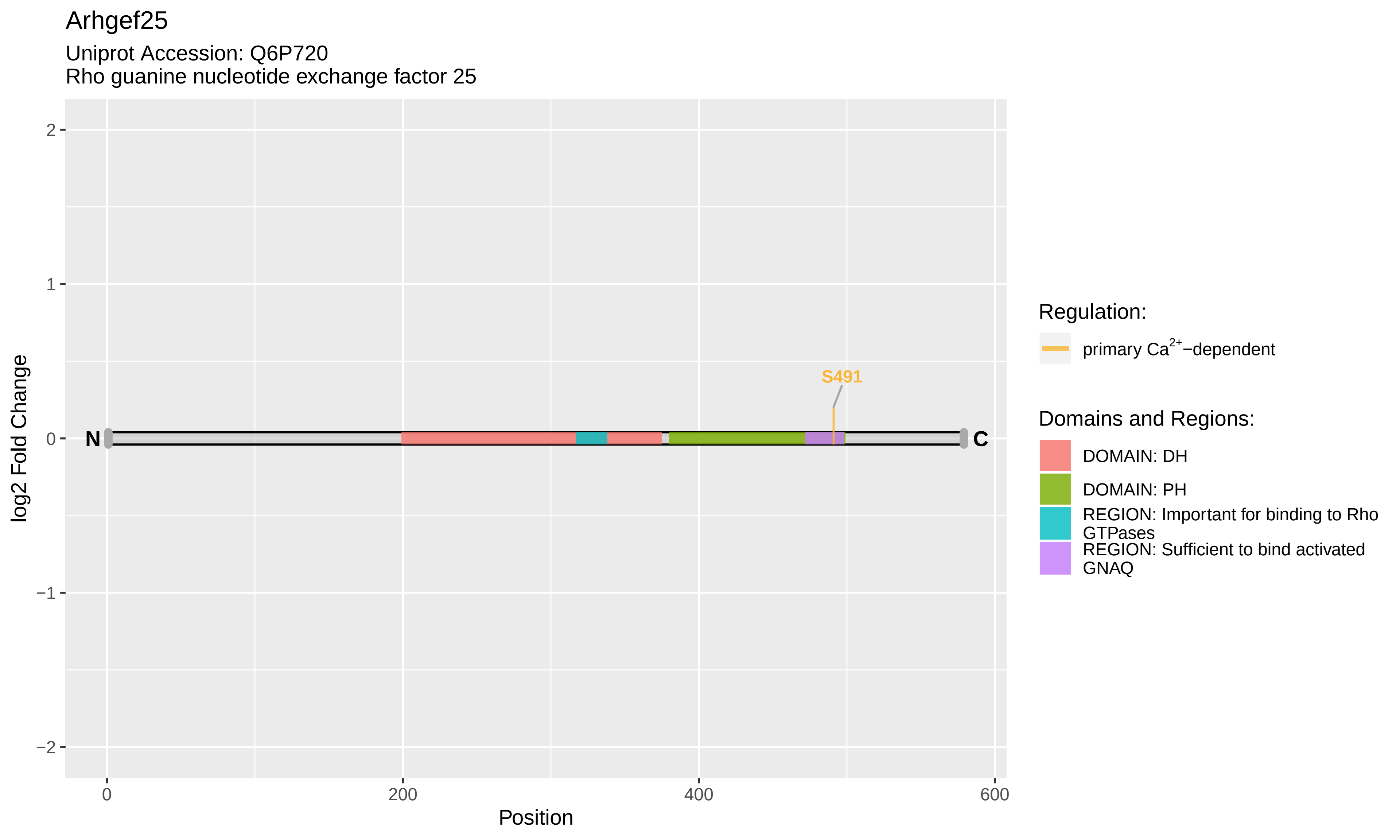

Arhgef25
Uniprot Accession: Q6P720
Rho guanine nucleotide exchange factor 25
log2 Fold Change
2
1
0
−1
−2
N
C
S491
0
200
400
600
Position
Regulation:
primary Ca2+−dependent
Domains and Regions:
DOMAIN: DH
DOMAIN: PH
REGION: Important for binding to Rho GTPases
REGION: Sufficient to bind activated GNAQ

Arhgef33

Uniprot Accession: D3Z8W6
Rho guanine nucleotide exchange factor 33

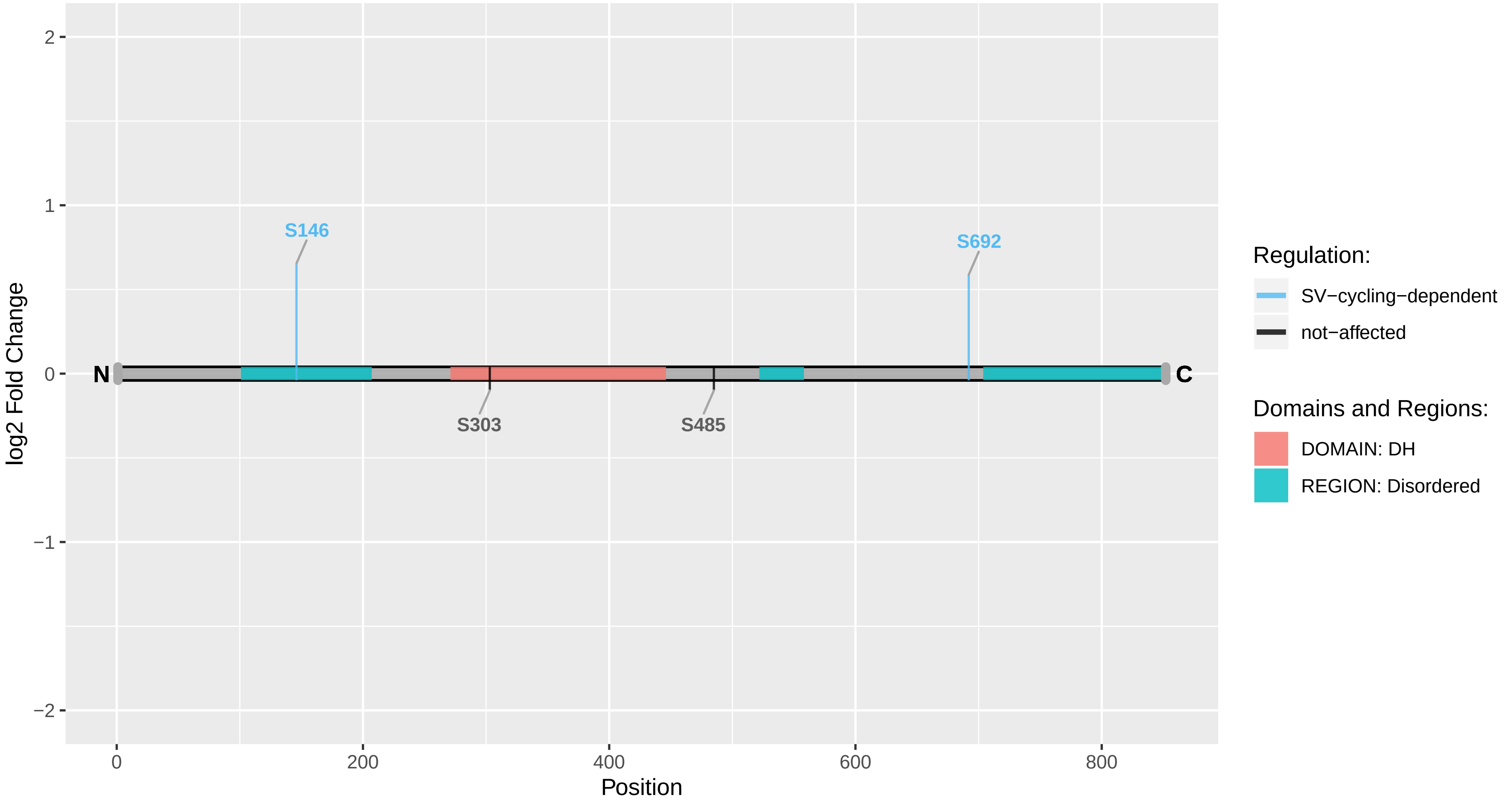

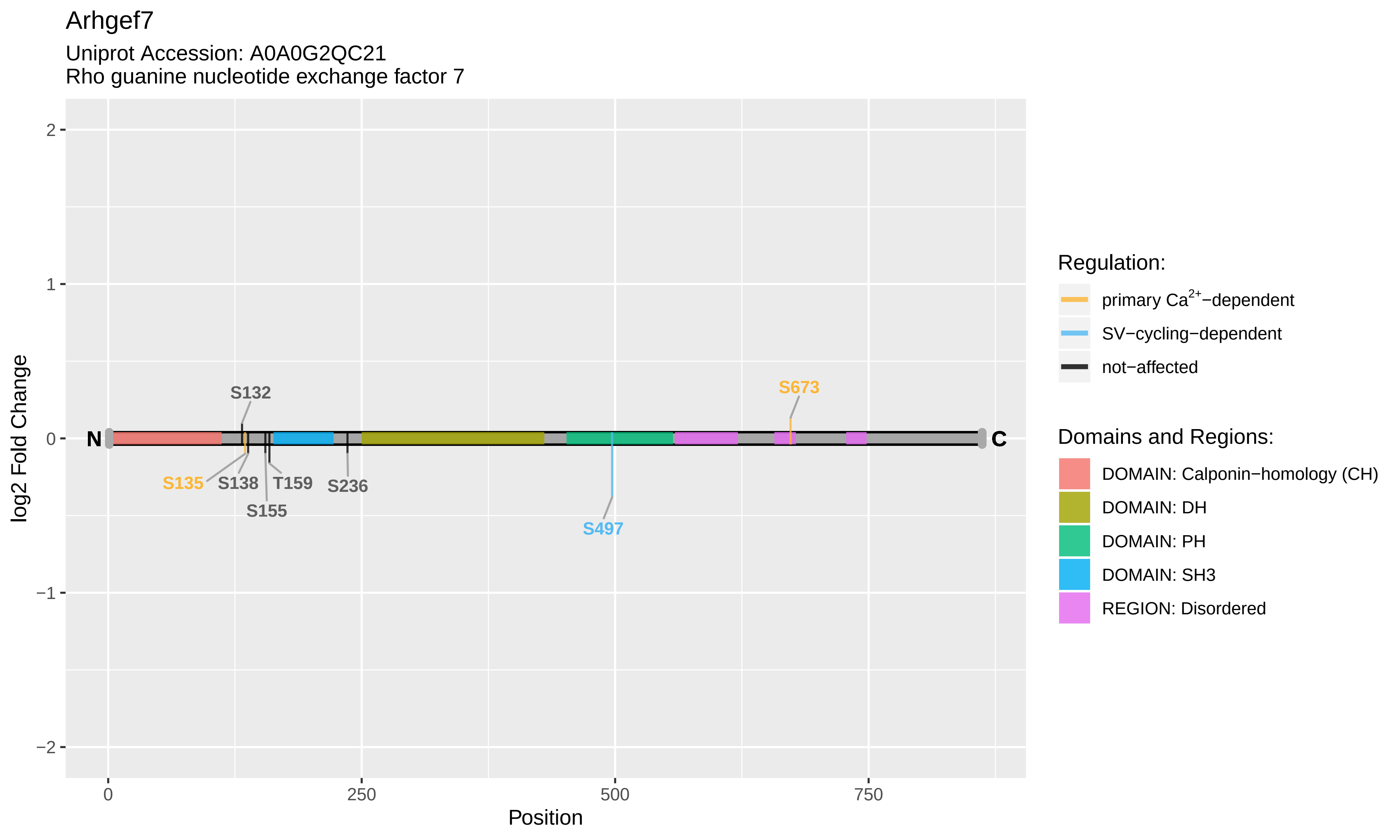
Arhgef7
Uniprot Accession: A0A0G2QC21
Rho guanine nucleotide exchange factor 7
log2 Fold Change
Position
N
C
S132
S135
S138
S155
T159
S236
S497
S673
Regulation:
primary Ca2+−dependent
SV−cycling−dependent
not−affected
Domains and Regions:
DOMAIN: Calponin−homology (CH)
DOMAIN: DH
DOMAIN: PH
DOMAIN: SH3
REGION: Disordered

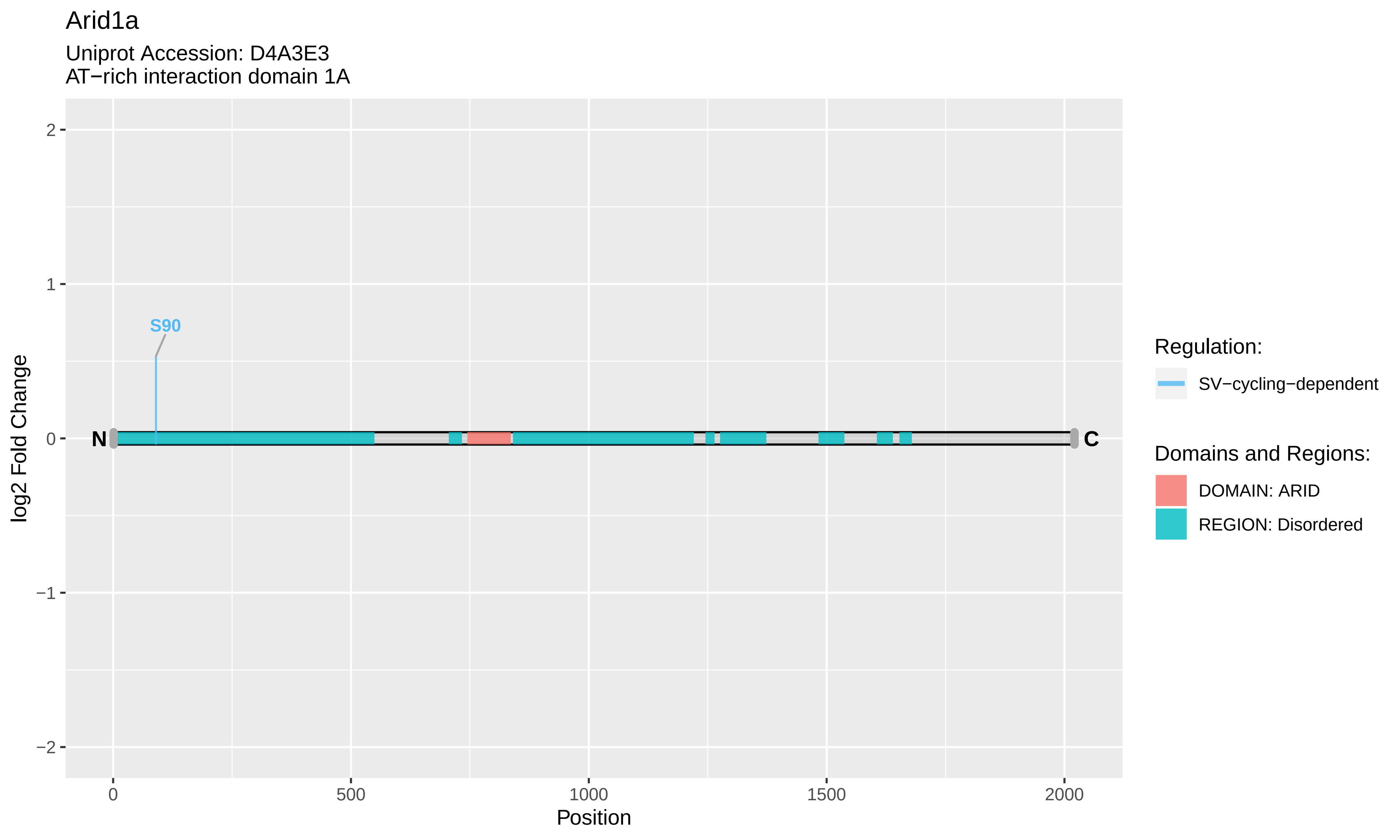
Arid1a
Uniprot Accession: D4A3E3
AT−rich interaction domain 1A
S90
N
C
2
1
0
−1
−2
log2 Fold Change
0
500
1000
1500
2000
Position
Regulation:
SV−cycling−dependent
Domains and Regions:
DOMAIN: ARID
REGION: Disordered

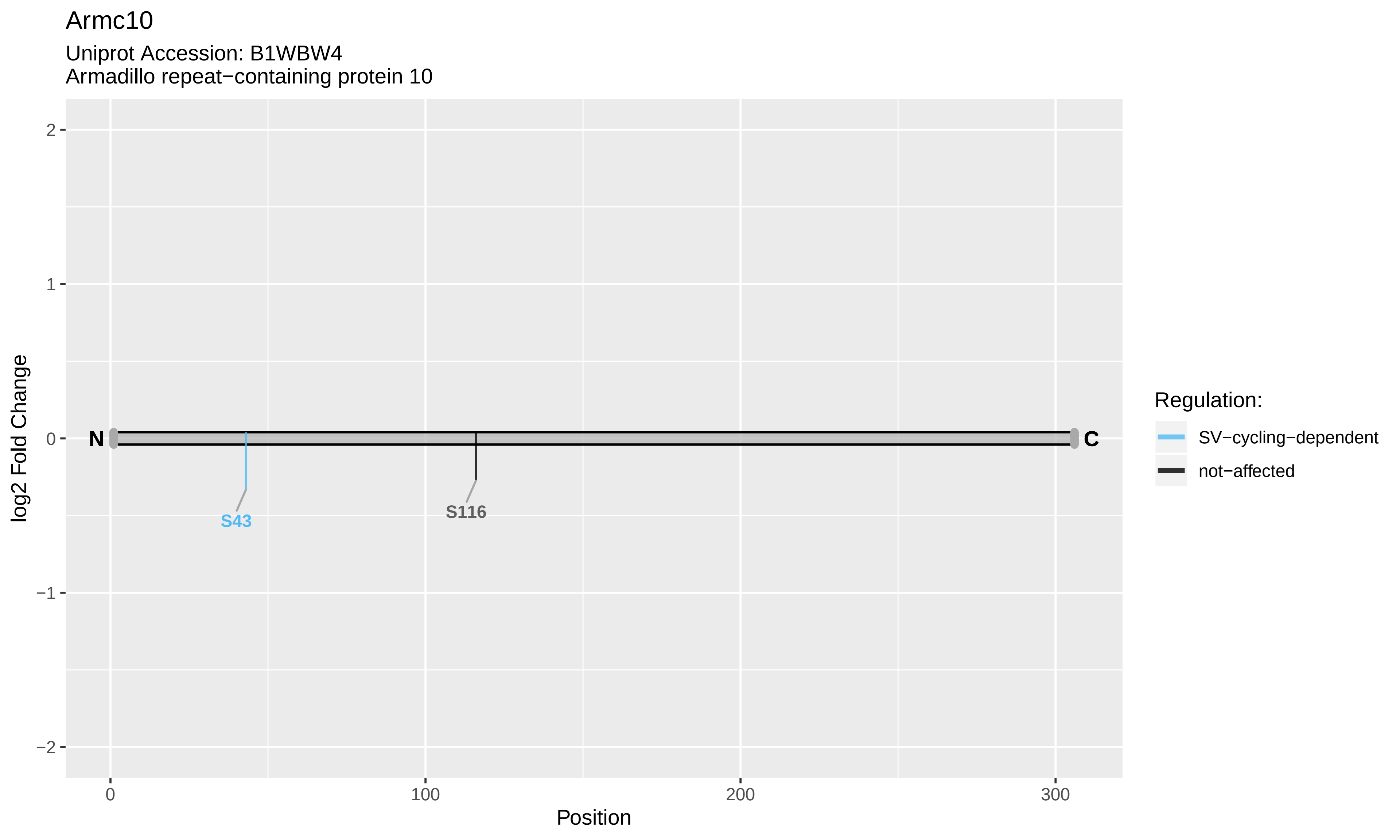

Armc10
Uniprot Accession: B1WBW4
Armadillo repeat−containing protein 10
log2 Fold Change
2
1
0
−1
−2
N
C
S43
S116
0
100
200
300
Position
Regulation:
SV−cycling−dependent
not−affected

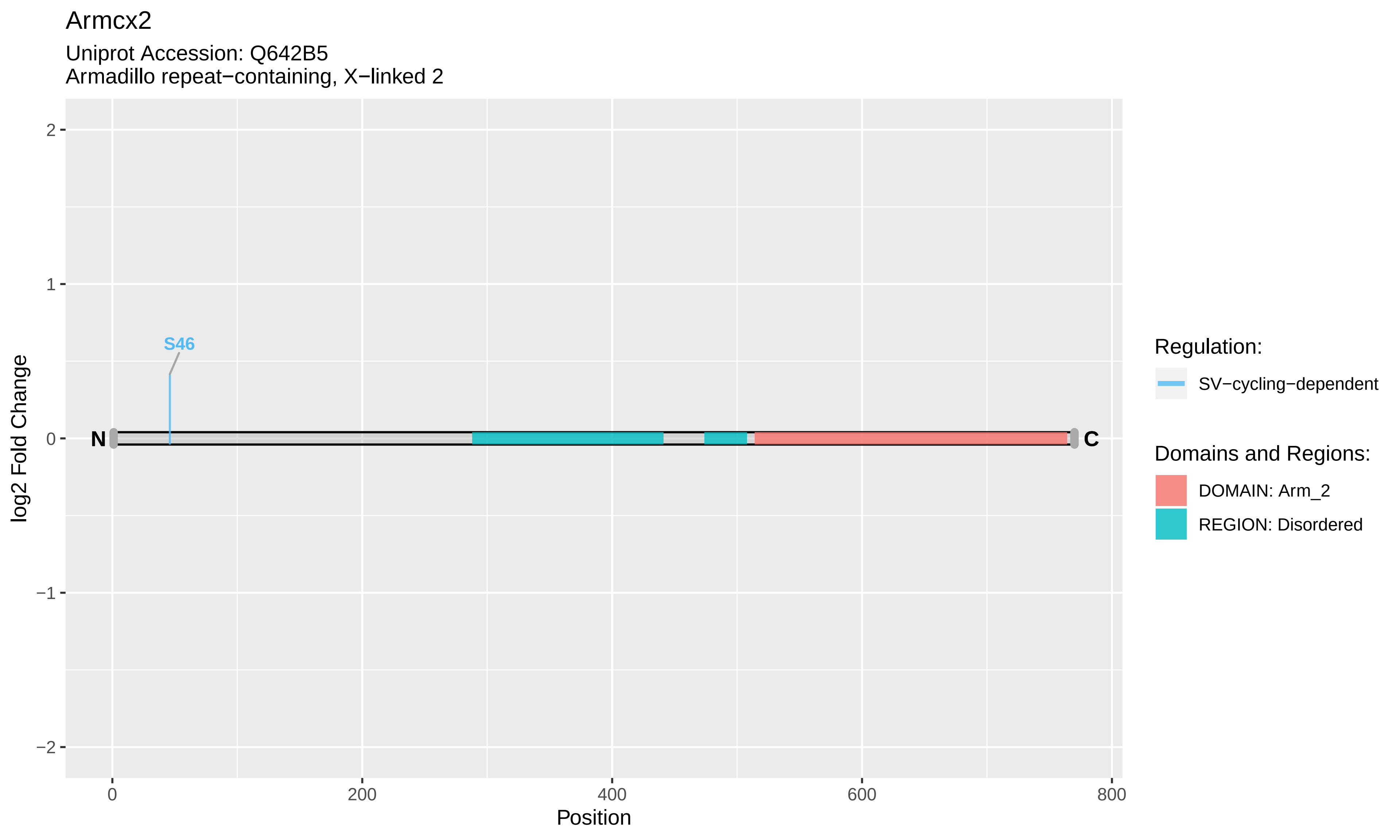
Armcx2
Uniprot Accession: Q642B5
Armadillo repeat−containing, X−linked 2
S46
N
C
log2 Fold Change
Position
Regulation:
SV−cycling−dependent
Domains and Regions:
DOMAIN: Arm_2
REGION: Disordered

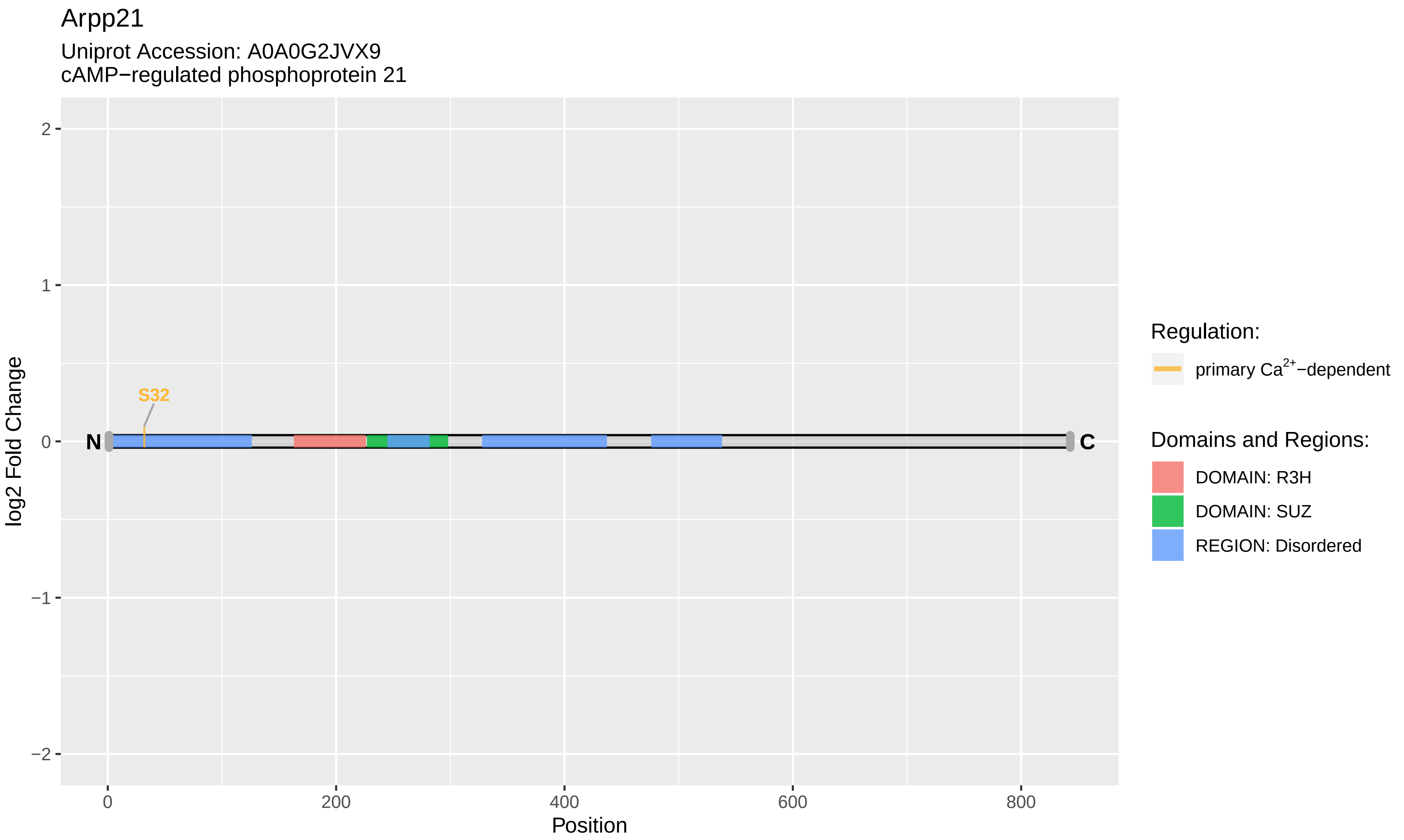

Arpp21
Uniprot Accession: A0A0G2JVX9
cAMP−regulated phosphoprotein 21
S32
N
C
log2 Fold Change
2
1
0
−1
−2
0
200
400
600
800
Position
Regulation:
primary Ca2+−dependent
Domains and Regions:
DOMAIN: R3H
DOMAIN: SUZ
REGION: Disordered

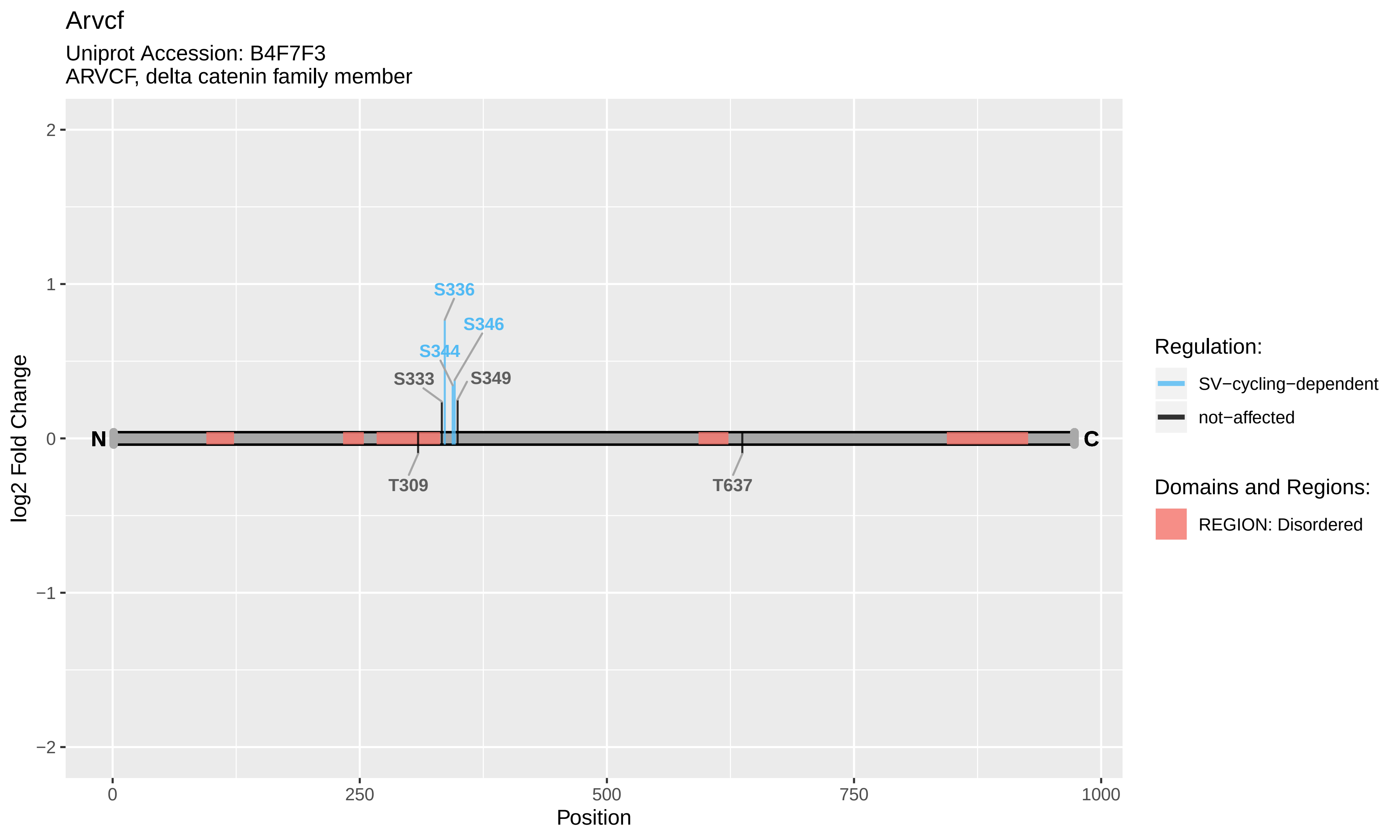
Arvcf
Uniprot Accession: B4F7F3
ARVCF, delta catenin family member
S336
S346
S344
S333
S349
N
C
T309
T637
log2 Fold Change
2
1
0
−1
−2
0
250
500
750
1000
Position
Regulation:
SV−cycling−dependent
not−affected
Domains and Regions:
REGION: Disordered

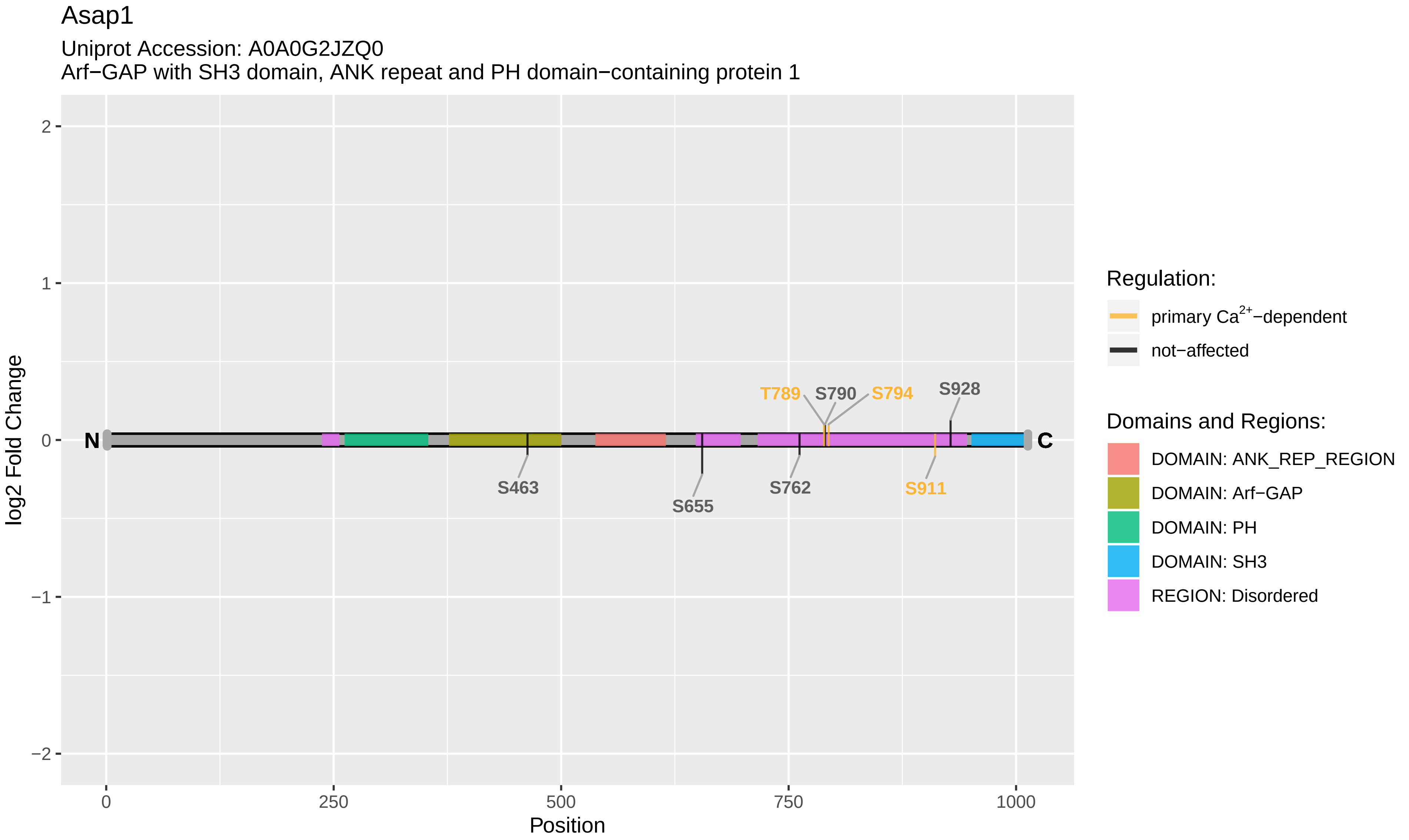
Asap1
Uniprot Accession: A0A0G2JZQ0
Arf−GAP with SH3 domain, ANK repeat and PH domain−containing protein 1
log2 Fold Change
2
1
0
−1
−2
N
C
T789
S790
S794
S928
S463
S655
S762
S911
0
250
500
750
1000
Position
Regulation:
primary Ca2+−dependent
not−affected
Domains and Regions:
DOMAIN: ANK_REP_REGION
DOMAIN: Arf−GAP
DOMAIN: PH
DOMAIN: SH3
REGION: Disordered

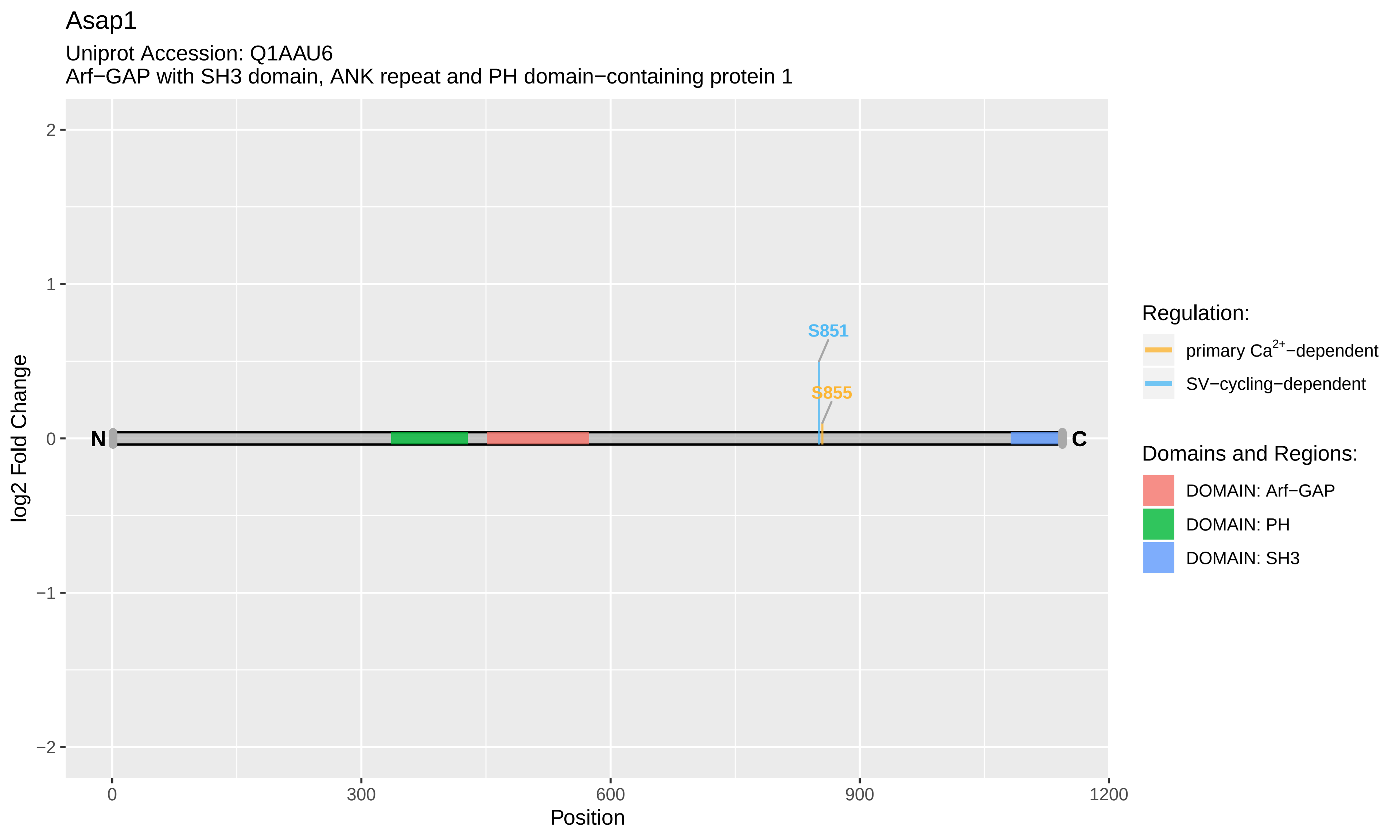

Asap1
Uniprot Accession: Q1AAU6
Arf−GAP with SH3 domain, ANK repeat and PH domain−containing protein 1
S851
S855
N
C
log2 Fold Change
2
1
0
−1
−2
0
300
600
900
1200
Position
Regulation:
primary Ca2+−dependent
SV−cycling−dependent
Domains and Regions:
DOMAIN: Arf−GAP
DOMAIN: PH
DOMAIN: SH3

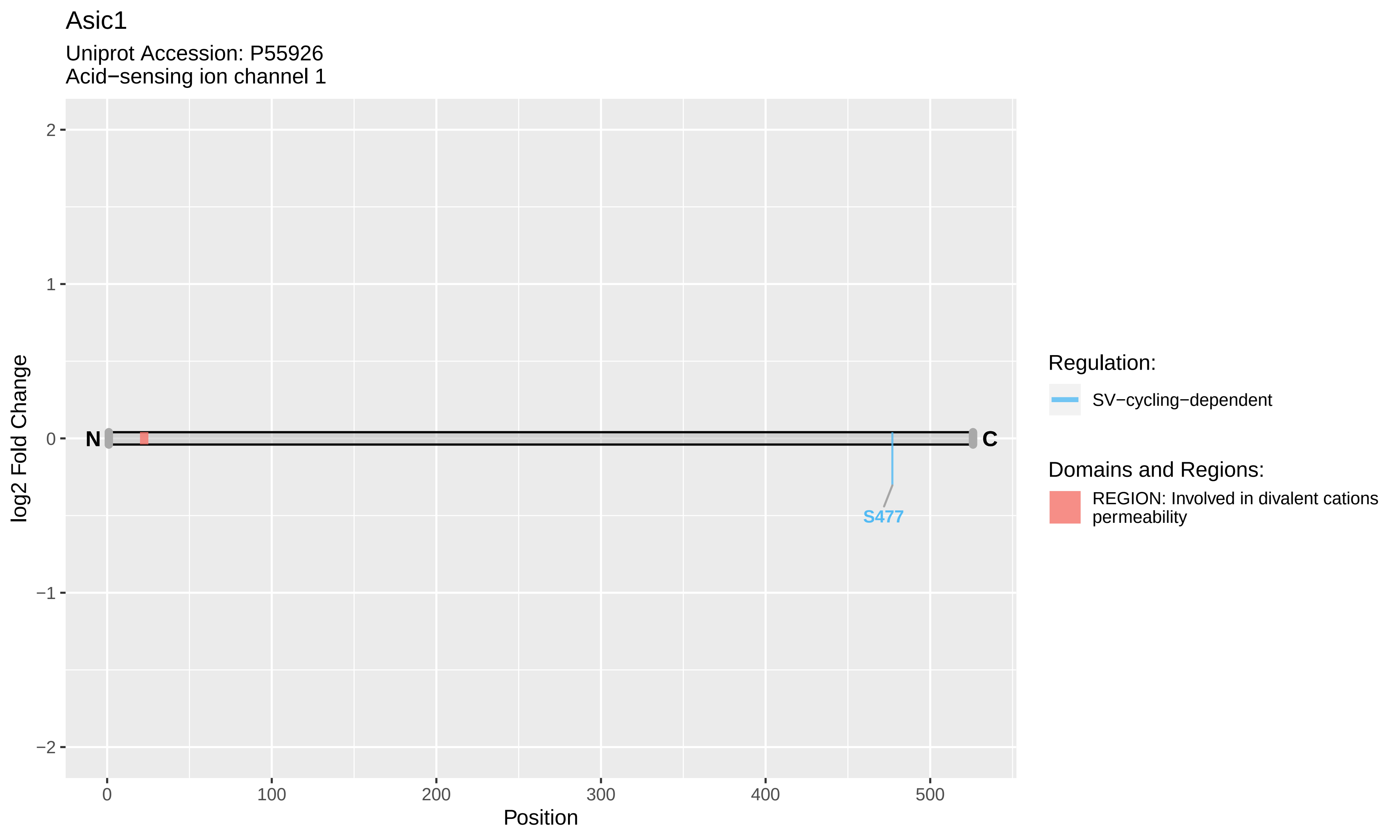
Asic1
Uniprot Accession: P55926
Acid−sensing ion channel 1
log2 Fold Change
Position
2
1
0
−1
−2
0
100
200
300
400
500
N
C
S477
Regulation:
SV−cycling−dependent
Domains and Regions:
REGION: Involved in divalent cations permeability

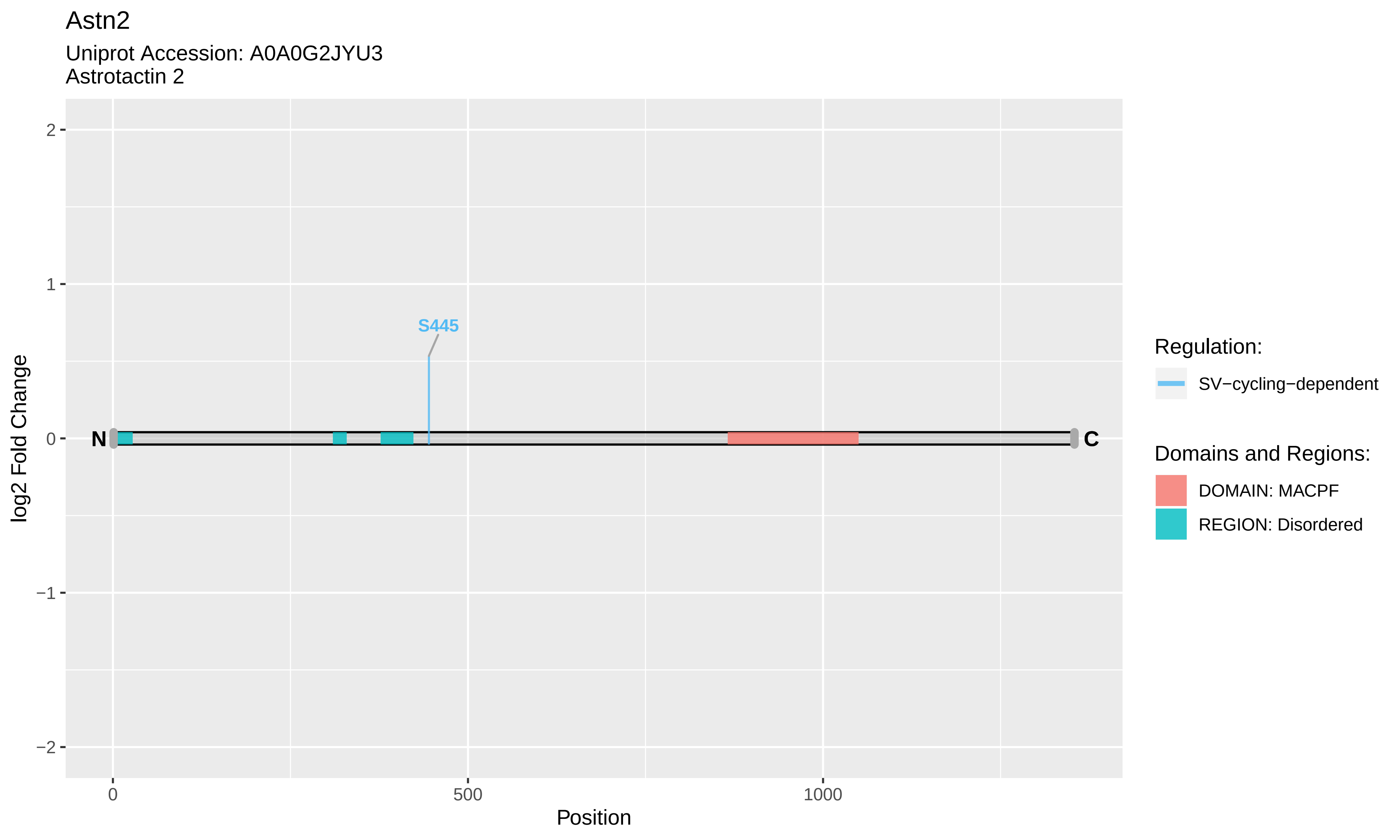
Astn2
Uniprot Accession: A0A0G2JYU3
Astrotactin 2
S445
N
C
2
1
0
−1
−2
log2 Fold Change
0
500
1000
Position
Regulation:
SV−cycling−dependent
Domains and Regions:
DOMAIN: MACPF
REGION: Disordered

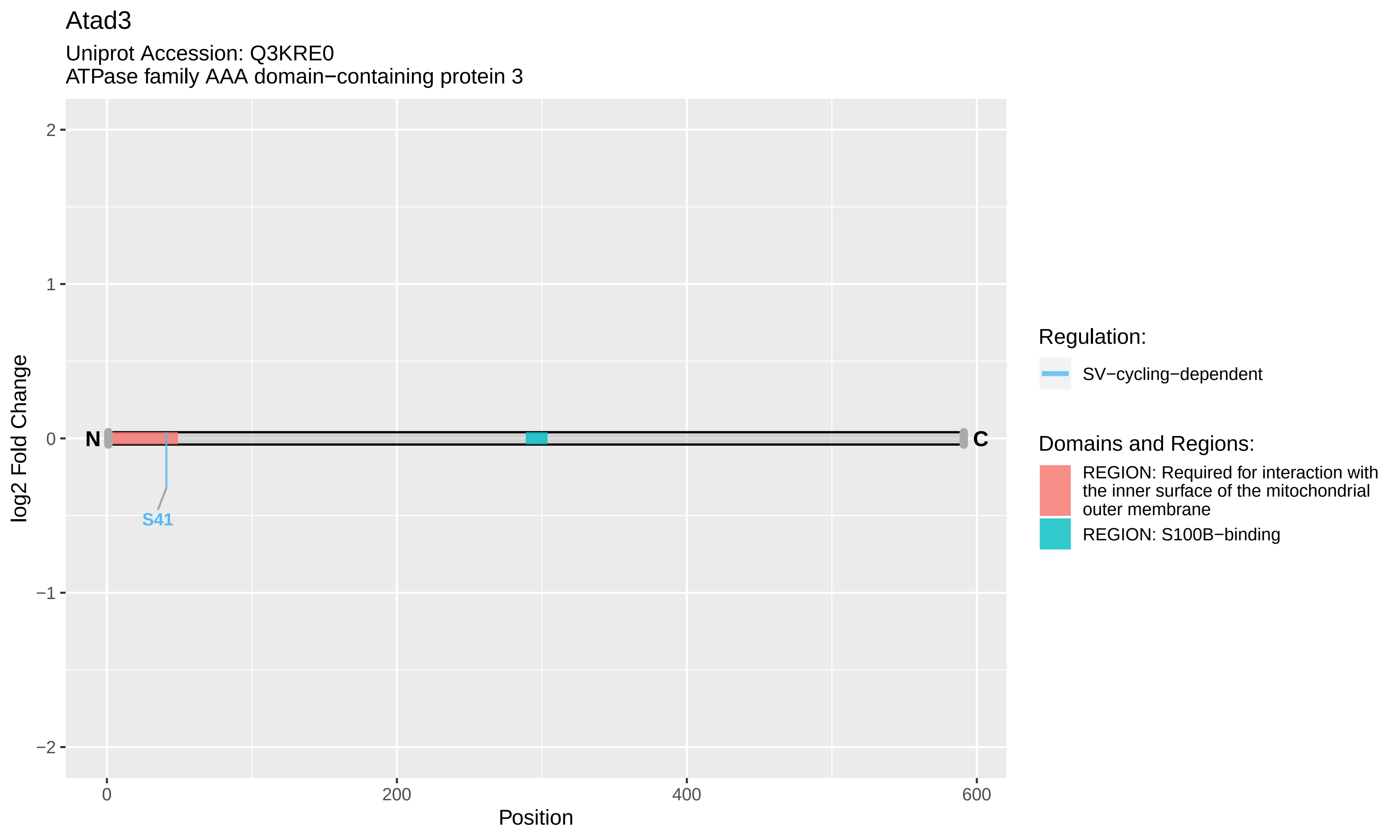

Atad3
Uniprot Accession: Q3KRE0
ATPase family AAA domain−containing protein 3
log2 Fold Change
2
1
0
−1
−2
N
C
S41
0
200
400
600
Position
Regulation:
SV−cycling−dependent
Domains and Regions:
REGION: Required for interaction with the inner surface of the mitochondrial outer membrane
REGION: S100B−binding

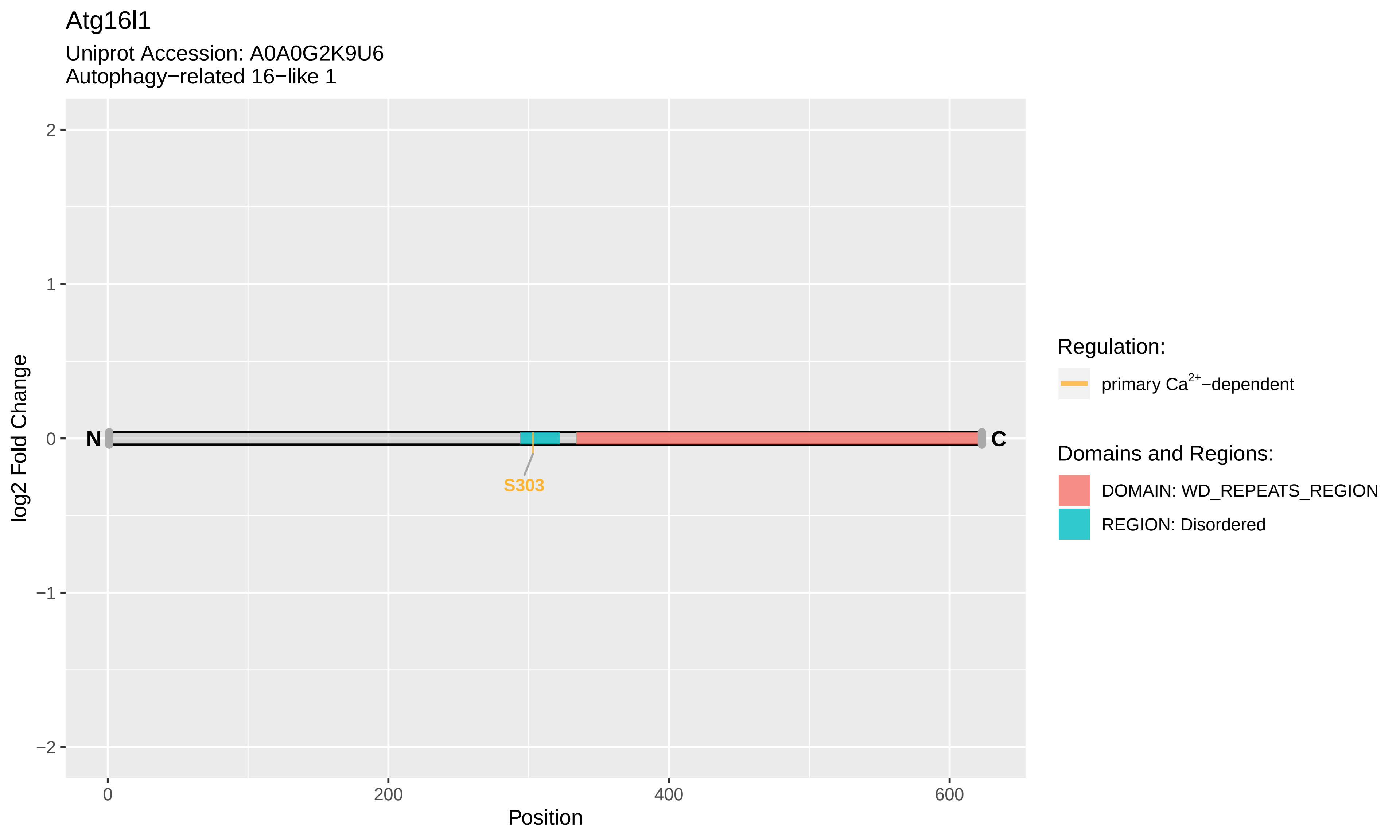

Atg16l1
Uniprot Accession: A0A0G2K9U6
Autophagy−related 16−like 1
log2 Fold Change
2
1
0
−1
−2
N
C
S303
0
200
400
600
Position
Regulation:
primary Ca2+−dependent
Domains and Regions:
DOMAIN: WD_REPEATS_REGION
REGION: Disordered

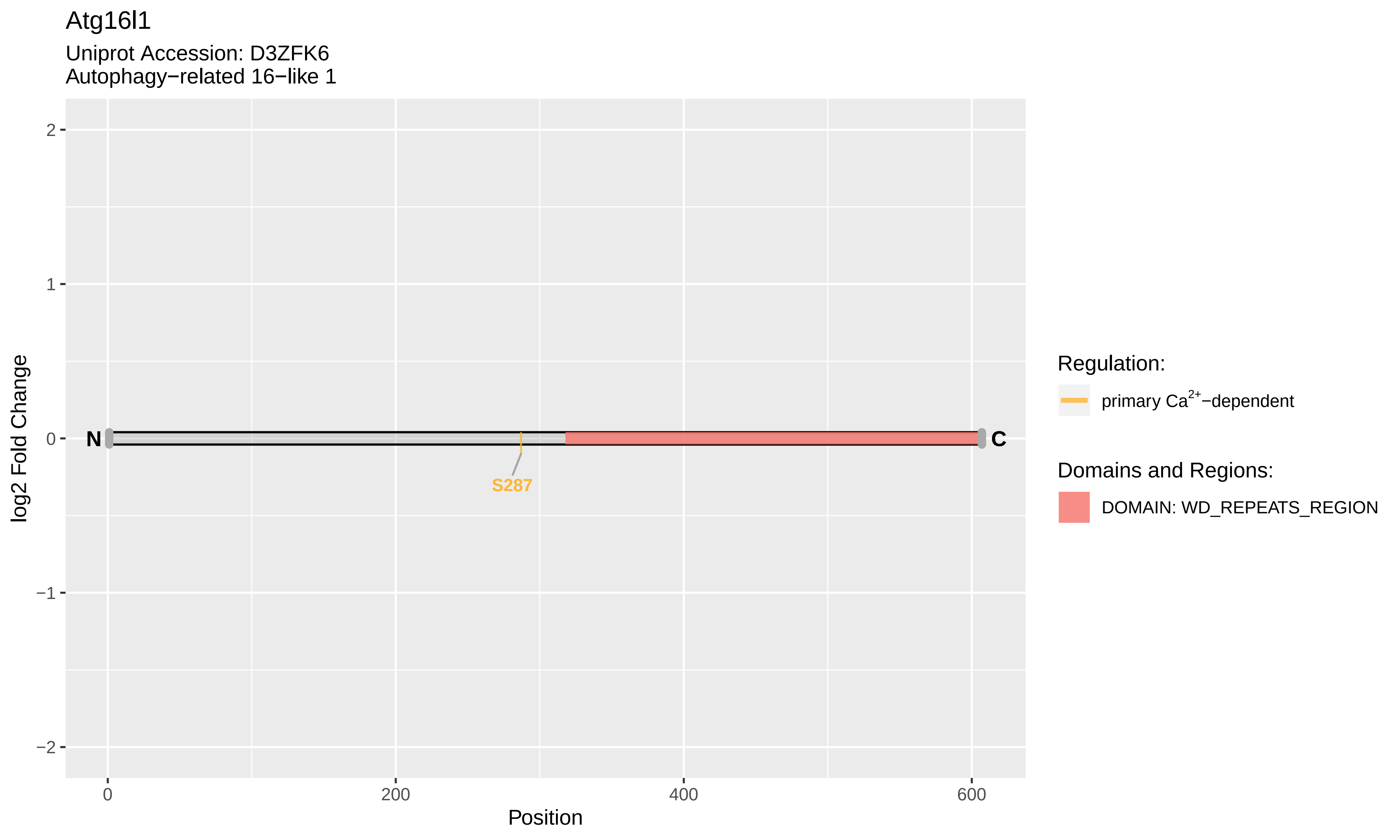

Atg16l1
Uniprot Accession: D3ZFK6
Autophagy−related 16−like 1
log2 Fold Change
Position
N
C
S287
Regulation:
primary Ca2+−dependent
Domains and Regions:
DOMAIN: WD_REPEATS_REGION

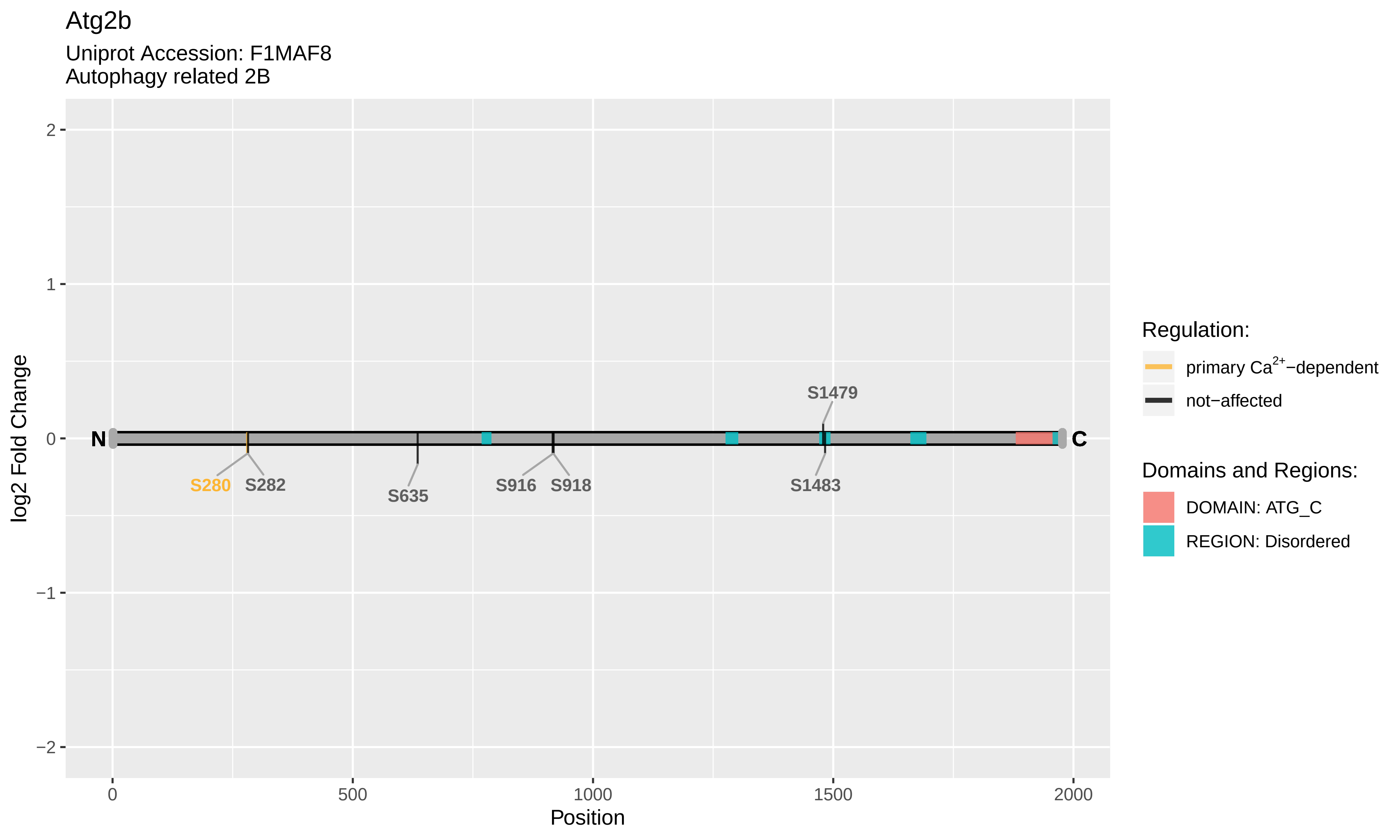

Atg2b
Uniprot Accession: F1MAF8
Autophagy related 2B
log2 Fold Change
2
1
0
−1
−2
N
C
S1479
S280
S282
S635
S916
S918
S1483
0
500
1000
1500
2000
Position
Regulation:
primary Ca2+−dependent
not−affected
Domains and Regions:
DOMAIN: ATG_C
REGION: Disordered

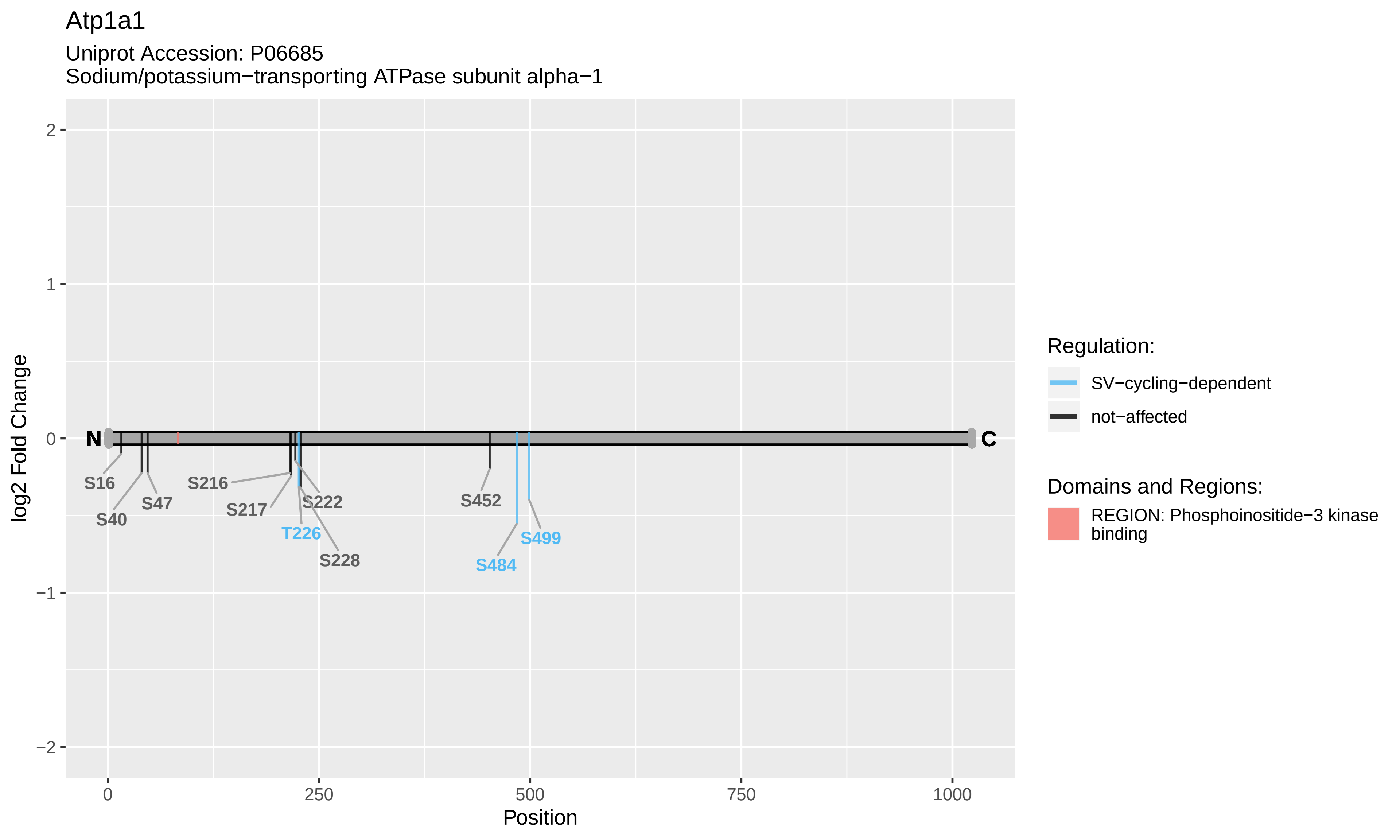
Atp1a1
Uniprot Accession: P06685
Sodium/potassium−transporting ATPase subunit alpha−1
log2 Fold Change
2
1
0
−1
−2
N
C
S16
S40
S47
S216
S217
S222
T226
S228
S452
S484
S499
0
250
500
750
1000
Position
Regulation:
SV−cycling−dependent
not−affected
Domains and Regions:
REGION: Phosphoinositide−3 kinase binding

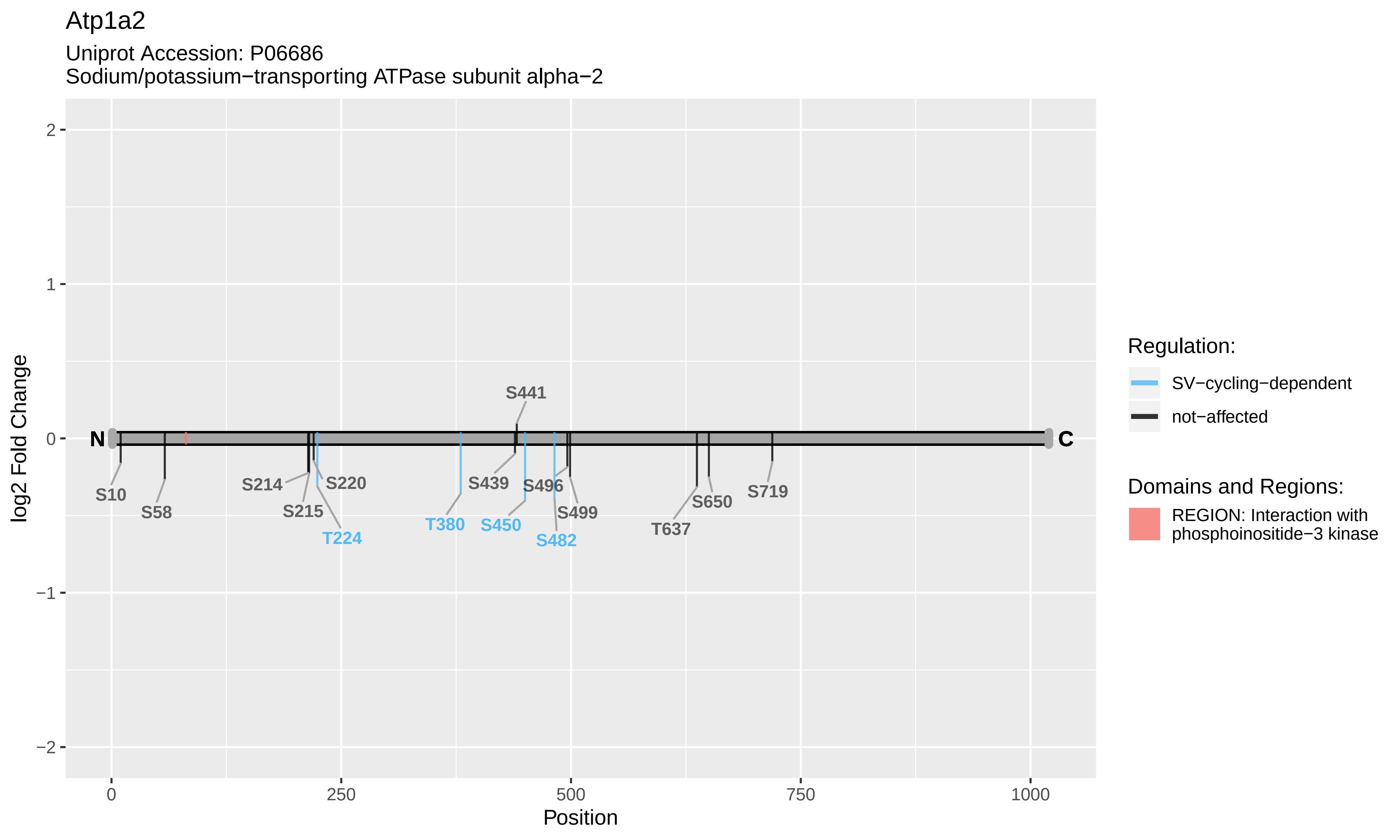

Atp1a2
Uniprot Accession: P06686
Sodium/potassium−transporting ATPase subunit alpha−2
log2 Fold Change
Position
2
1
0
−1
−2
0
250
500
750
1000
N
C
S441
S10
S58
S214
S215
S220
T224
T380
S439
S450
S496
S482
S499
T637
S650
S719
Regulation:
SV−cycling−dependent
not−affected
Domains and Regions:
REGION: Interaction with phosphoinositide−3 kinase

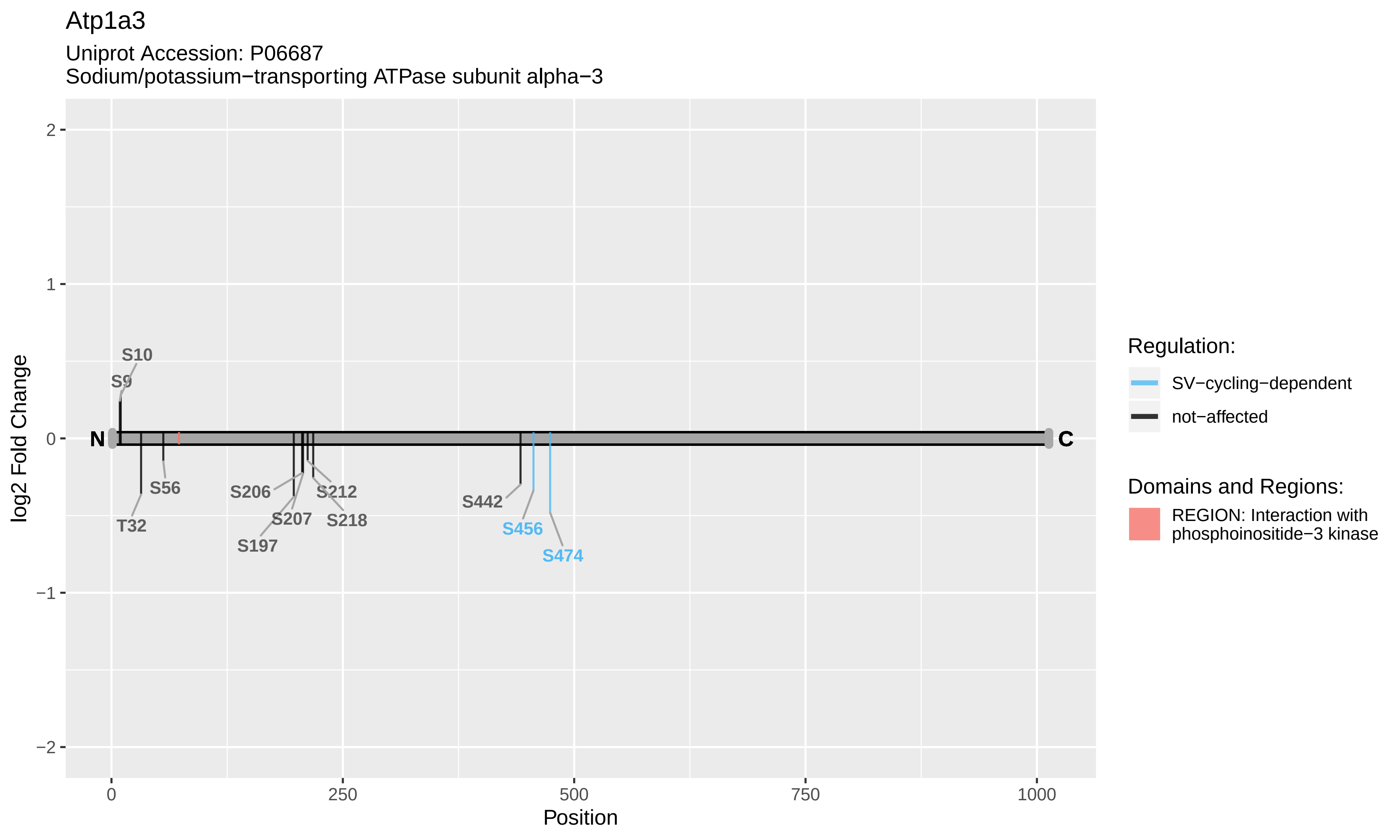

Atp1a3
Uniprot Accession: P06687
Sodium/potassium−transporting ATPase subunit alpha−3
log2 Fold Change
2
1
0
−1
−2
Position
0
250
500
750
1000
N
C
S10
S9
T32
S56
S206
S207
S197
S212
S218
S442
S456
S474
Regulation:
SV−cycling−dependent
not−affected
Domains and Regions:
REGION: Interaction with phosphoinositide−3 kinase

Atp2b1

Uniprot Accession: P11505
Plasma membrane calcium−transporting ATPase 1

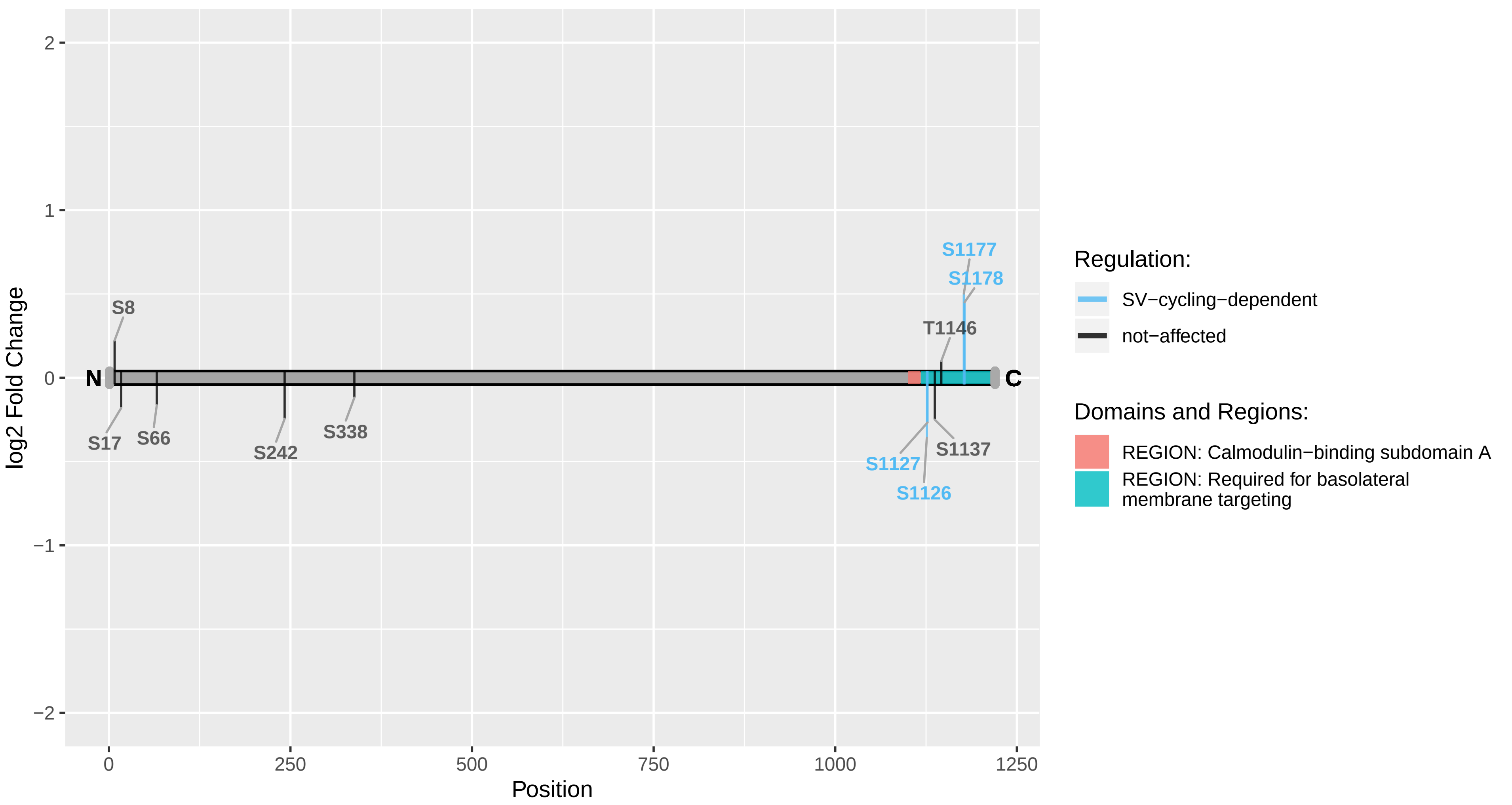

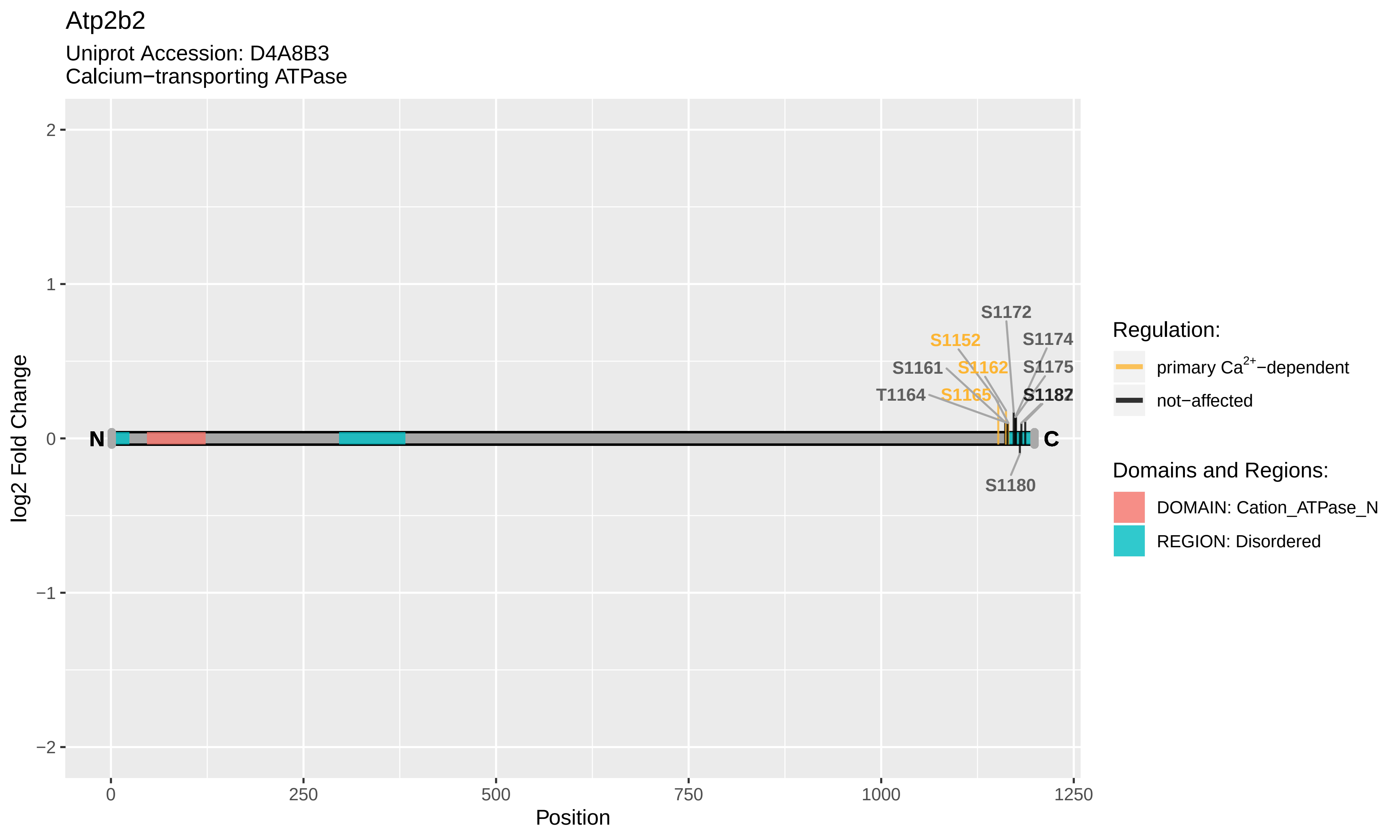

Atp2b2
Uniprot Accession: D4A8B3
Calcium−transporting ATPase
log2 Fold Change
Position
S1172
S1152
S1174
S1161
S1162
S1175
T1164
S1165
S1182
S1180
N
C
Regulation:
primary Ca2+−dependent
not−affected
Domains and Regions:
DOMAIN: Cation_ATPase_N
REGION: Disordered

Atp2b2

Uniprot Accession: P11506
Plasma membrane calcium−transporting ATPase 2

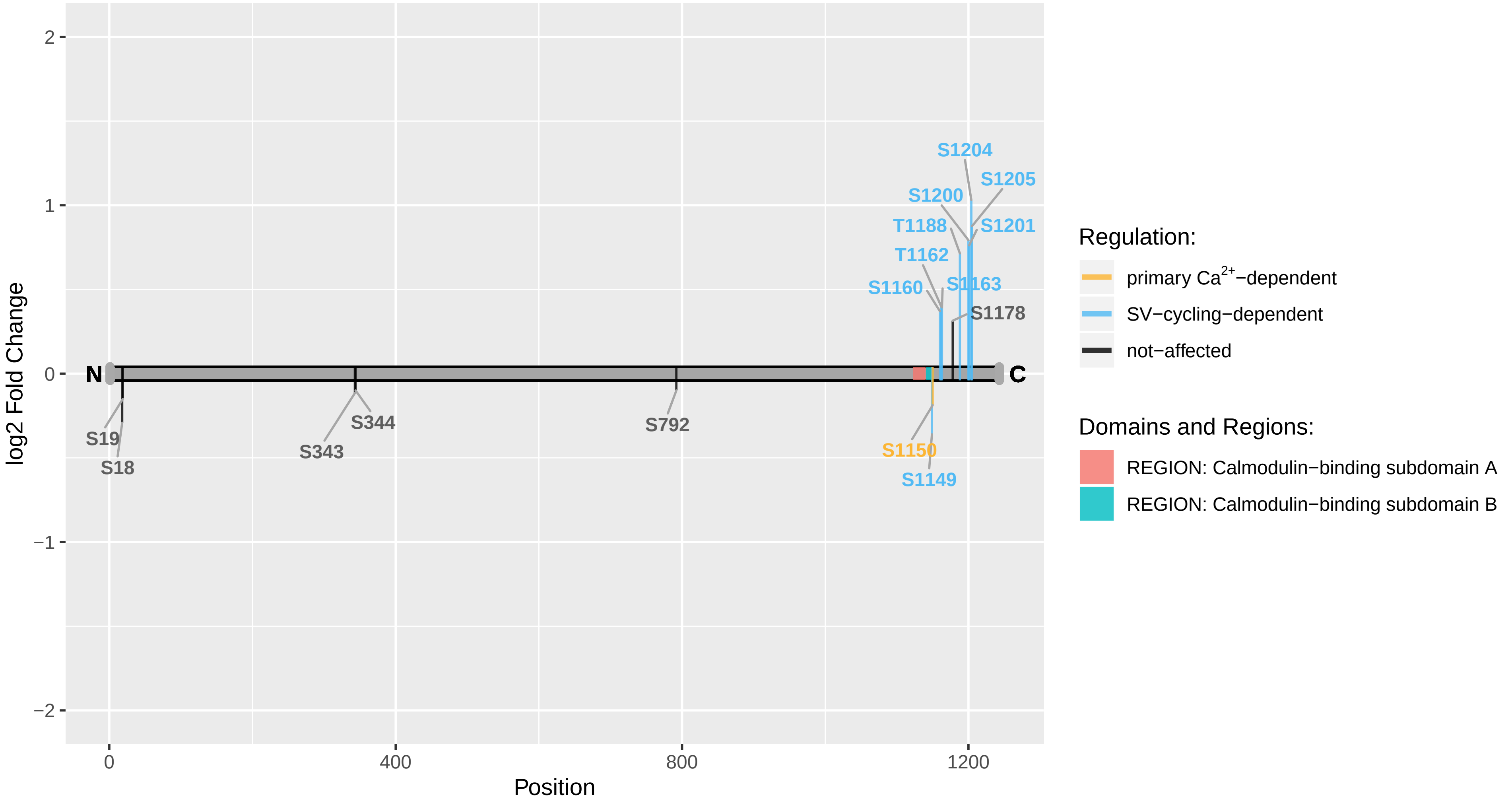

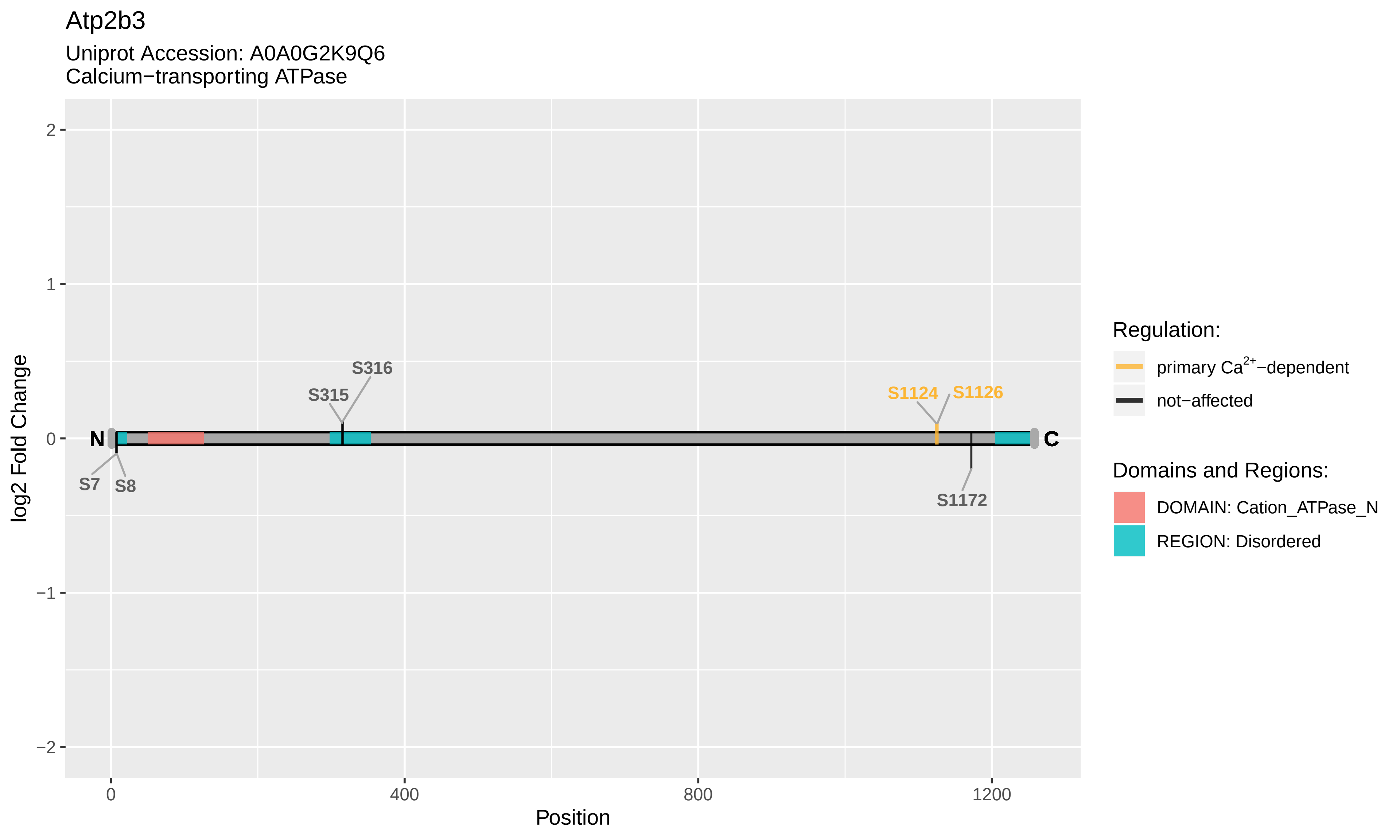

Atp2b3
Uniprot Accession: A0A0G2K9Q6
Calcium−transporting ATPase
log2 Fold Change
Position
N
C
S7
S8
S315
S316
S1124
S1126
S1172
2
1
0
−1
−2
0
400
800
1200
Regulation:
primary Ca2+−dependent
not−affected
Domains and Regions:
DOMAIN: Cation_ATPase_N
REGION: Disordered

Atp5f1a

Uniprot Accession: P15999
ATP synthase subunit alpha, mitochondrial

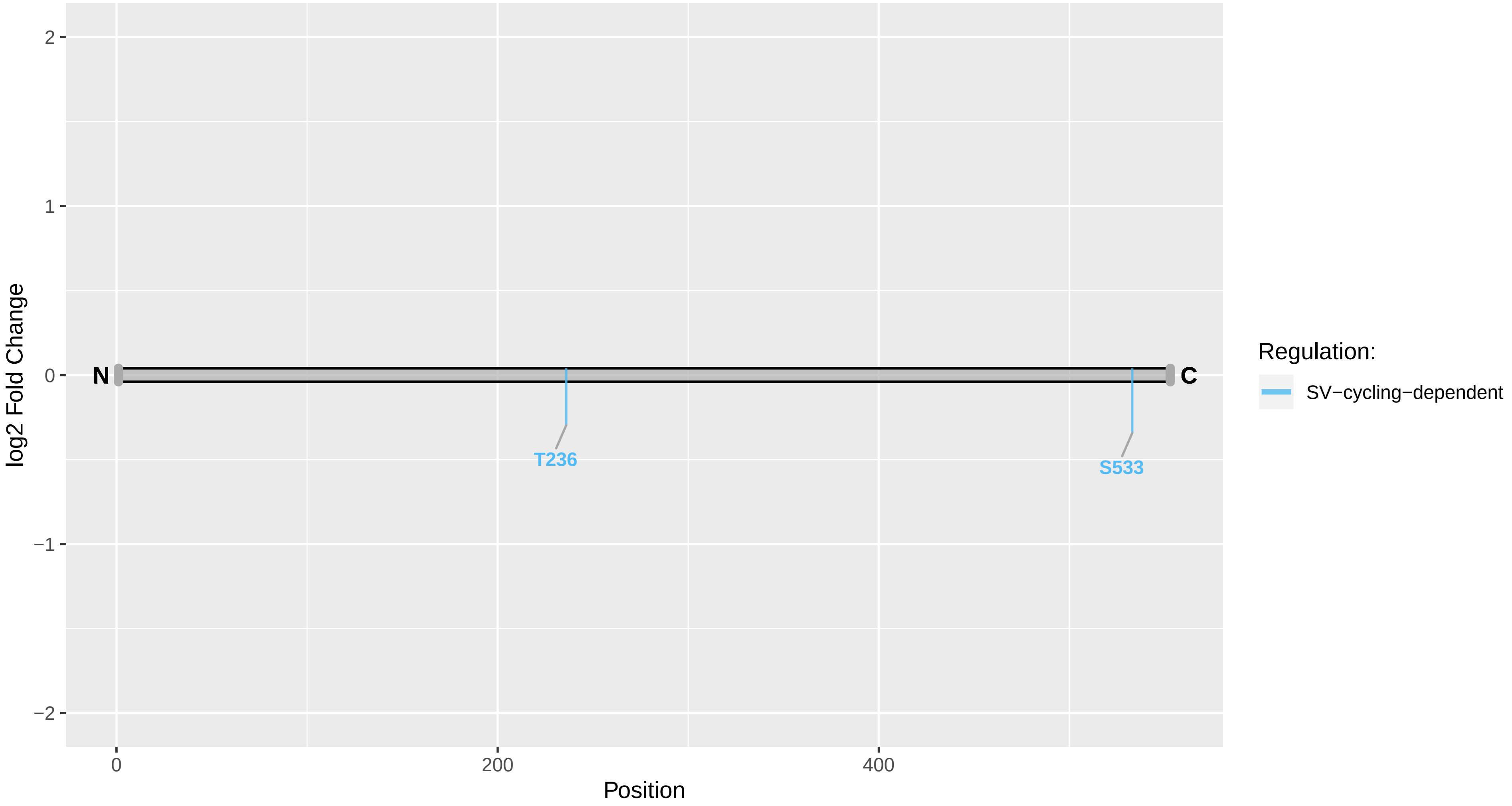

Atp6v1d

Uniprot Accession: Q6P503
ATPase H+−transporting V1 subunit D

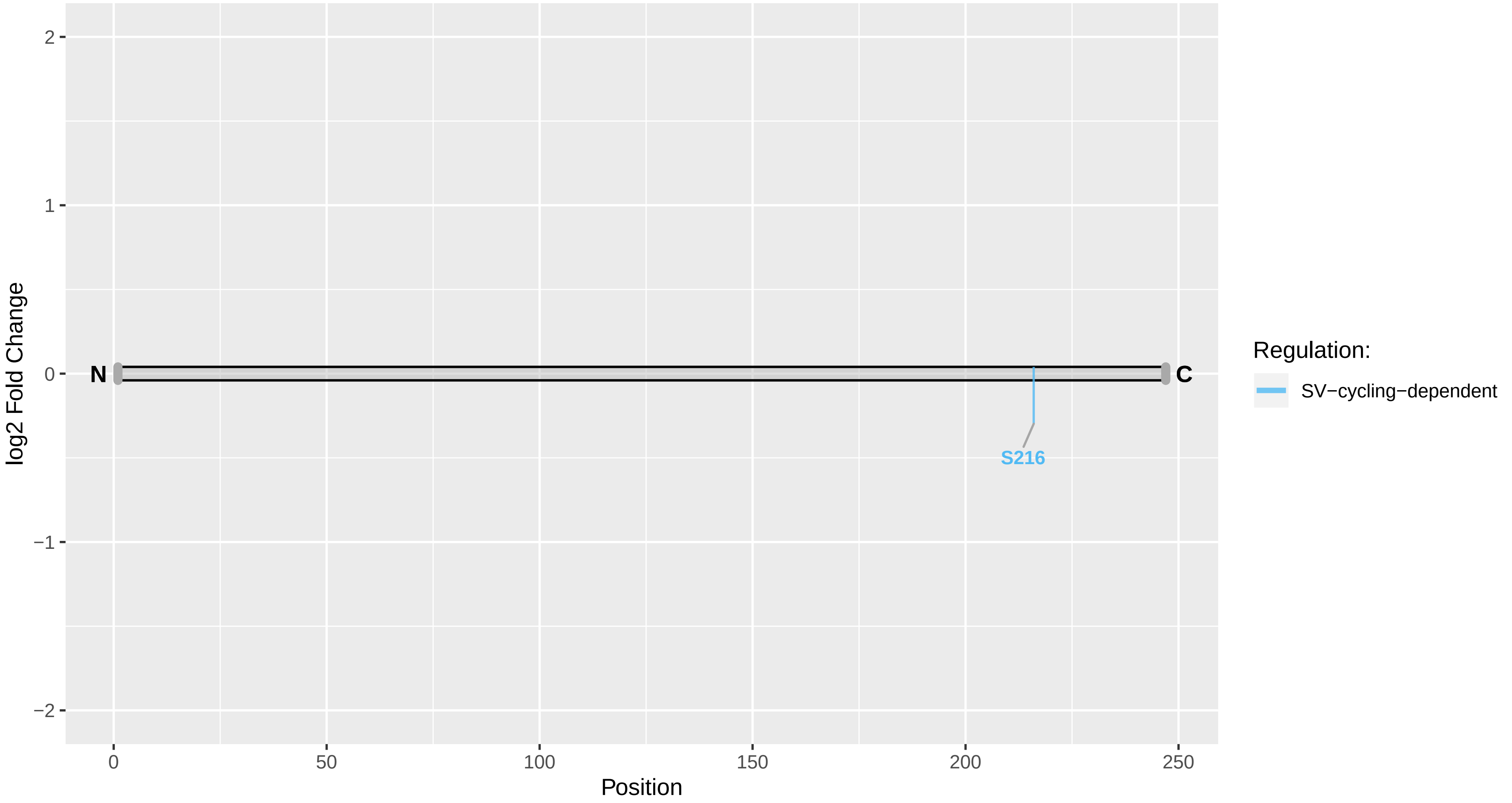

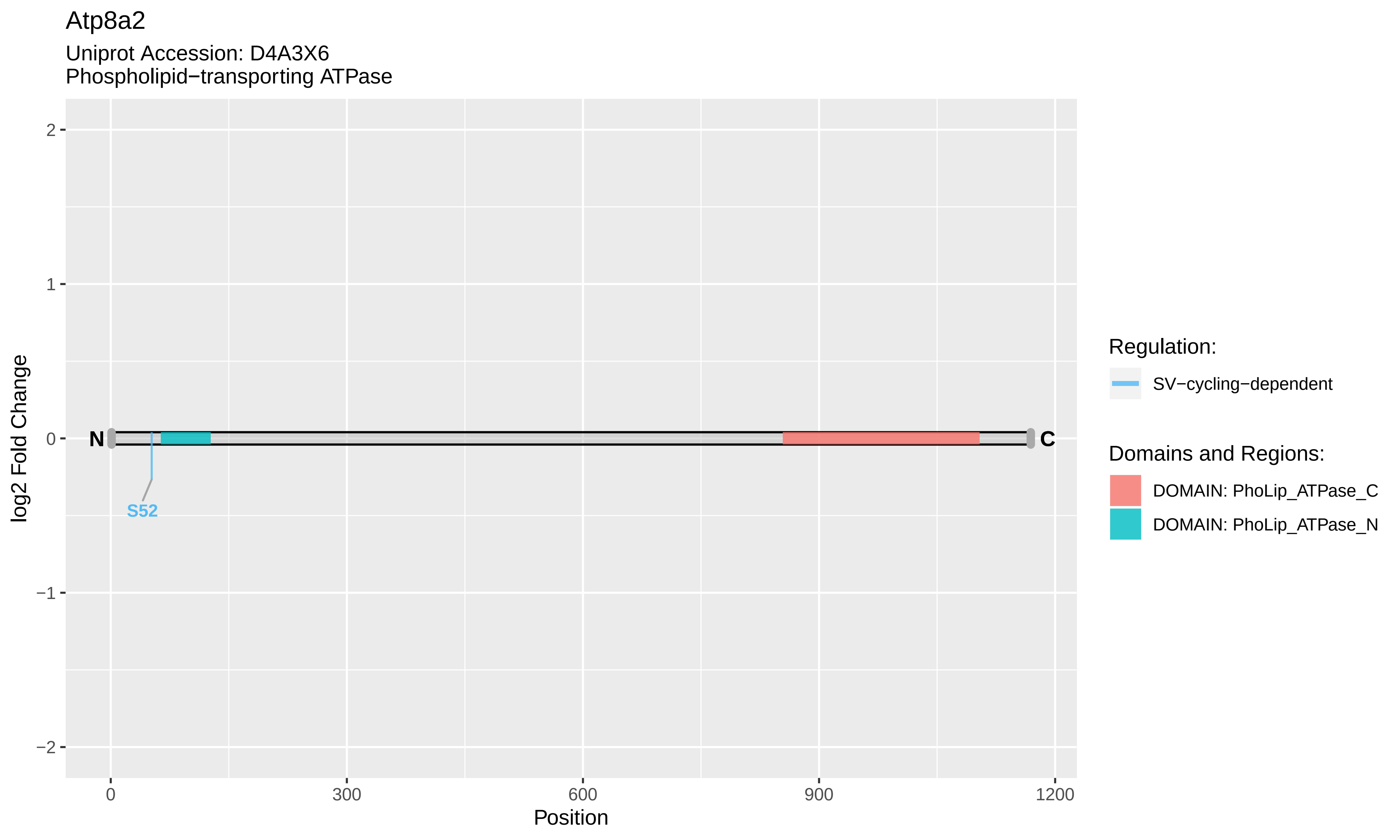

Atp8a2
Uniprot Accession: D4A3X6
Phospholipid−transporting ATPase
log2 Fold Change
2
1
0
−1
−2
N
C
S52
0
300
600
900
1200
Position
Regulation:
SV−cycling−dependent
Domains and Regions:
DOMAIN: PhoLip_ATPase_C
DOMAIN: PhoLip_ATPase_N

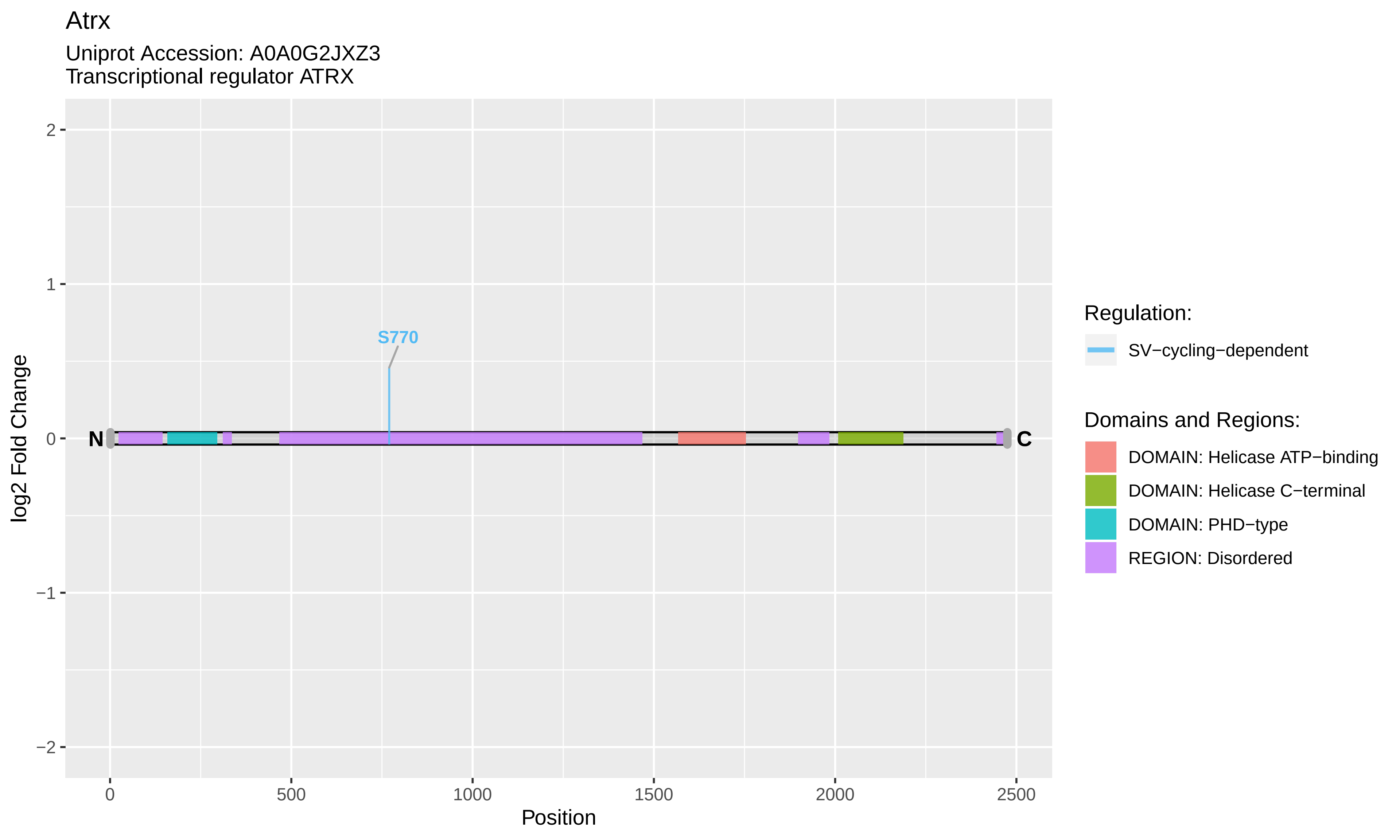
Atrx
Uniprot Accession: A0A0G2JXZ3
Transcriptional regulator ATRX
S770
N
C
log2 Fold Change
Position
2
1
0
−1
−2
0
500
1000
1500
2000
2500
Regulation:
SV−cycling−dependent
Domains and Regions:
DOMAIN: Helicase ATP−binding
DOMAIN: Helicase C−terminal
DOMAIN: PHD−type
REGION: Disordered

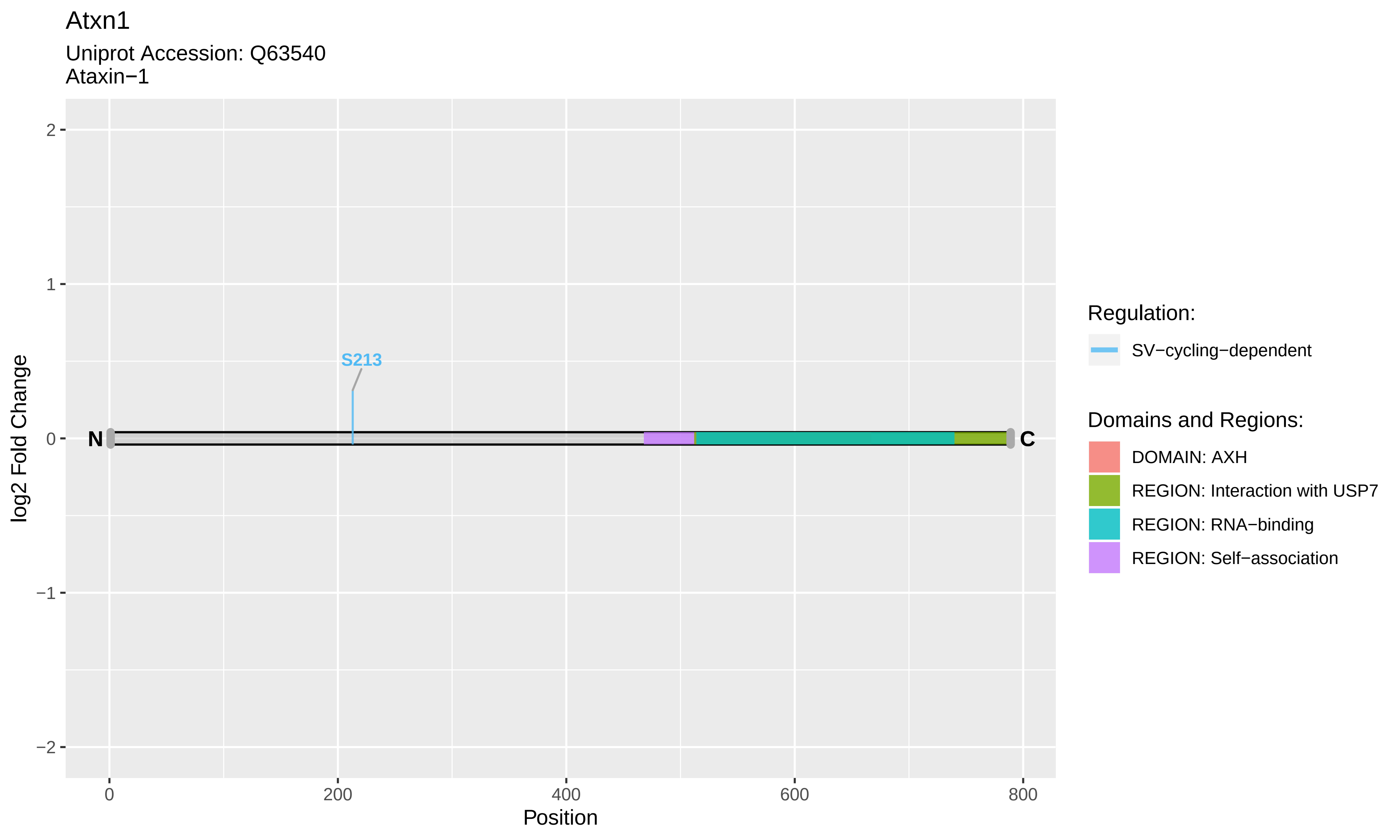

Atxn1
Uniprot Accession: Q63540
Ataxin−1
S213
N
C
log2 Fold Change
2
1
0
−1
−2
0
200
400
600
800
Position
Regulation:
SV−cycling−dependent
Domains and Regions:
DOMAIN: AXH
REGION: Interaction with USP7
REGION: RNA−binding
REGION: Self−association

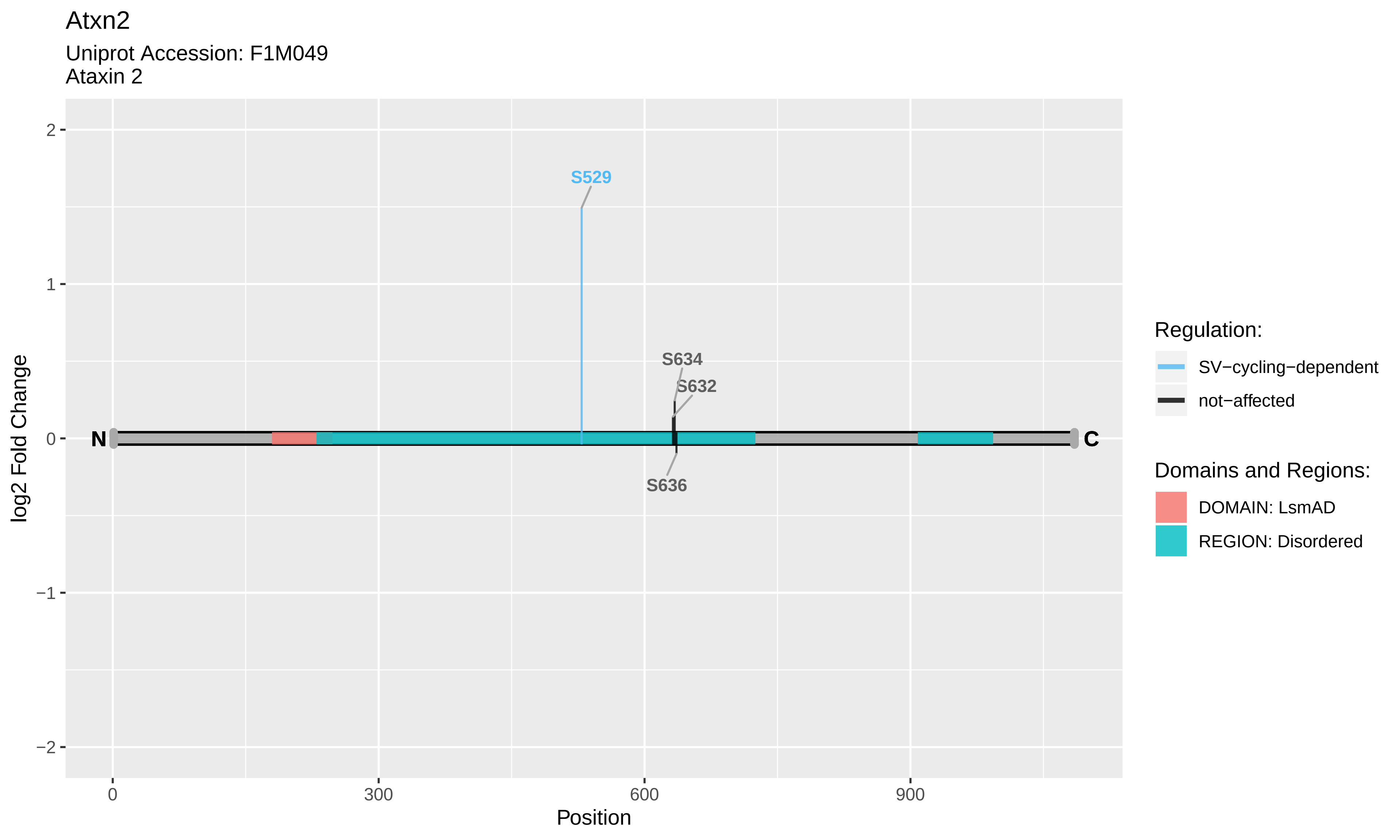

Atxn2
Uniprot Accession: F1M049
Ataxin 2
S529
S634
S632
S636
N
C
log2 Fold Change
Position
2
1
0
−1
−2
0
300
600
900
Regulation:
SV−cycling−dependent
not−affected
Domains and Regions:
DOMAIN: LsmAD
REGION: Disordered

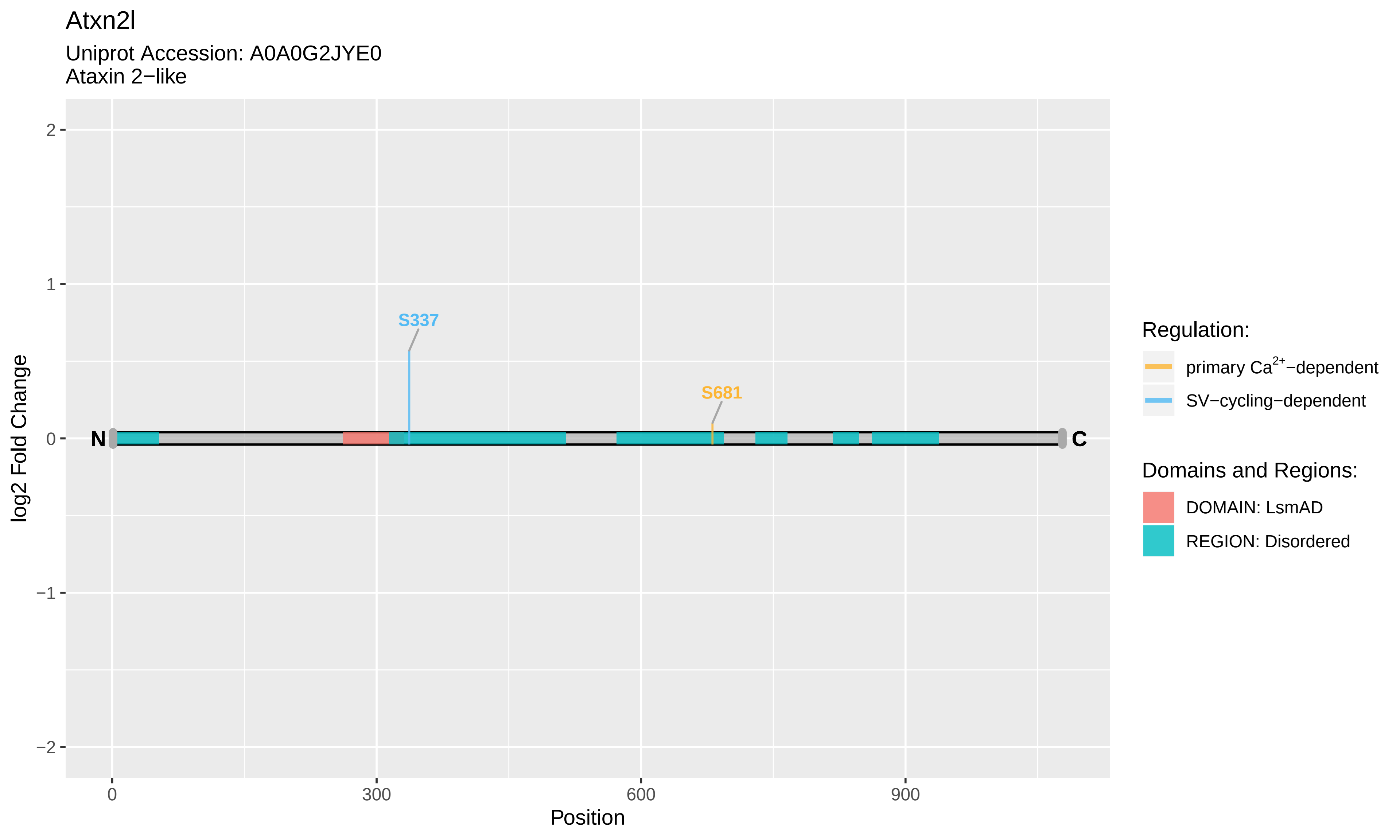

Atxn2l
Uniprot Accession: A0A0G2JYE0
Ataxin 2−like
S337
S681
N
C
2
1
0
−1
−2
log2 Fold Change
0
300
600
900
Position
Regulation:
primary Ca2+−dependent
SV−cycling−dependent
Domains and Regions:
DOMAIN: LsmAD
REGION: Disordered

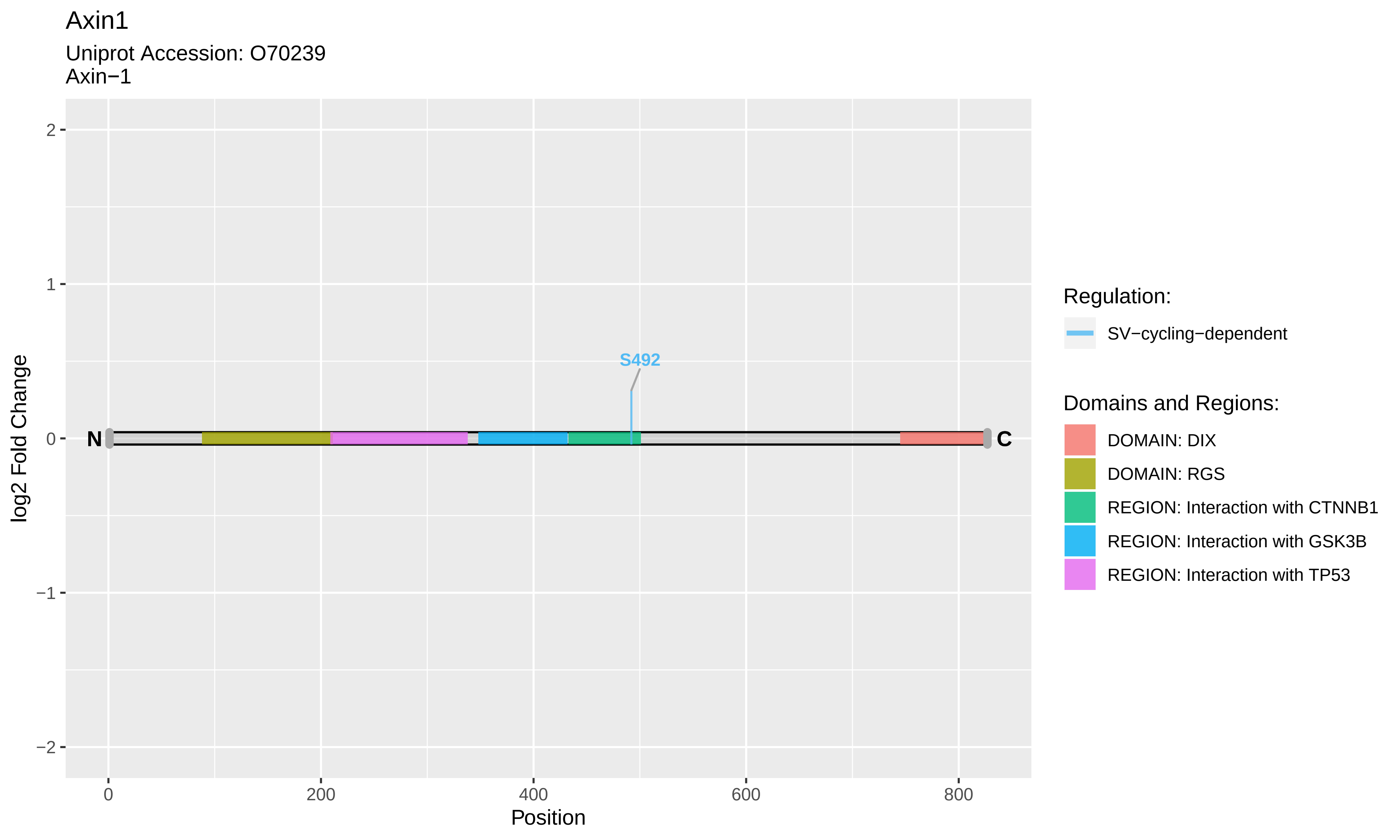

Axin1
Uniprot Accession: O70239
Axin−1
log2 Fold Change
Position
S492
N
C
Regulation:
SV−cycling−dependent
Domains and Regions:
DOMAIN: DIX
DOMAIN: RGS
REGION: Interaction with CTNNB1
REGION: Interaction with GSK3B
REGION: Interaction with TP53

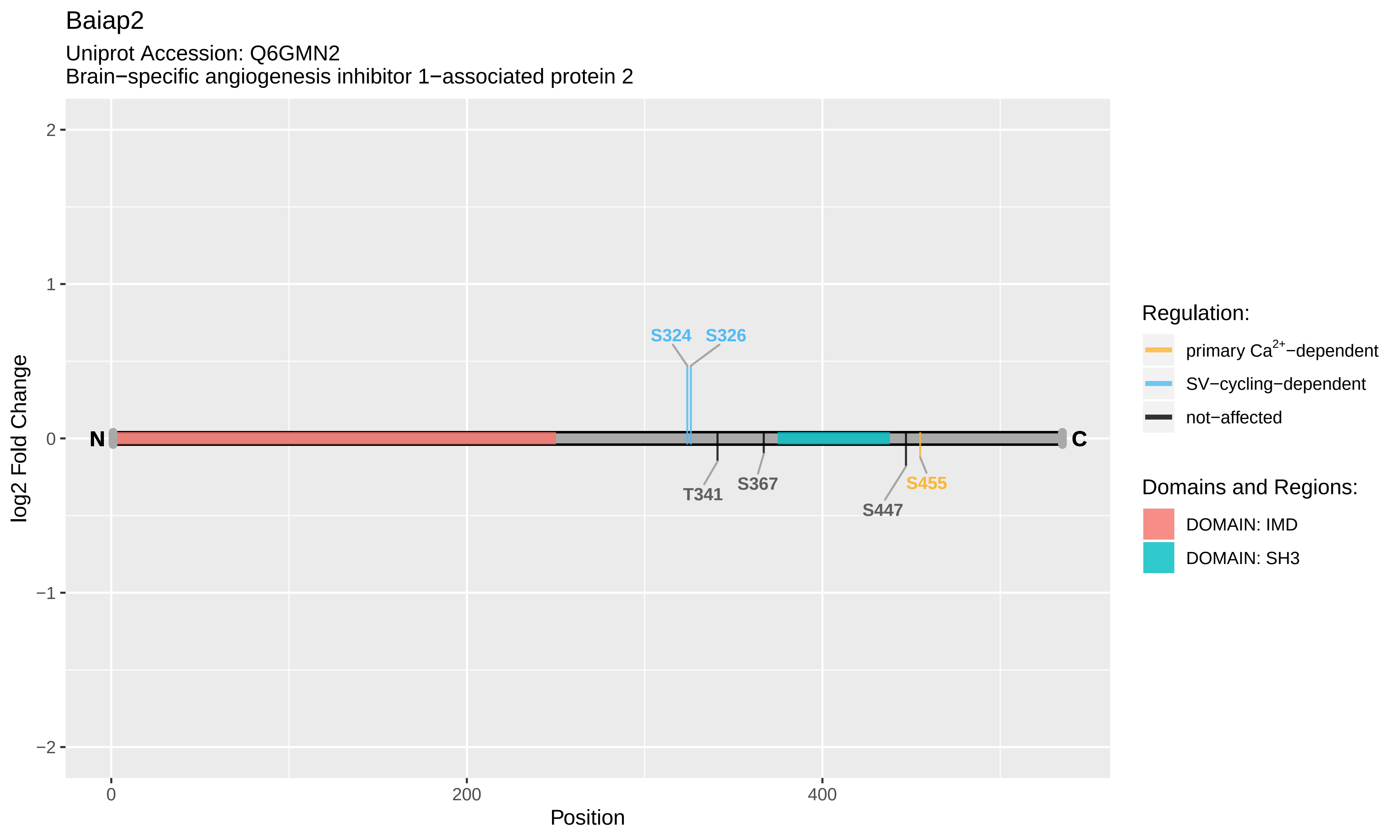

Baiap2
Uniprot Accession: Q6GMN2
Brain−specific angiogenesis inhibitor 1−associated protein 2
log2 Fold Change
2
1
0
−1
−2
N
C
S324
S326
T341
S367
S447
S455
0
200
400
Position
Regulation:
primary Ca2+−dependent
SV−cycling−dependent
not−affected
Domains and Regions:
DOMAIN: IMD
DOMAIN: SH3

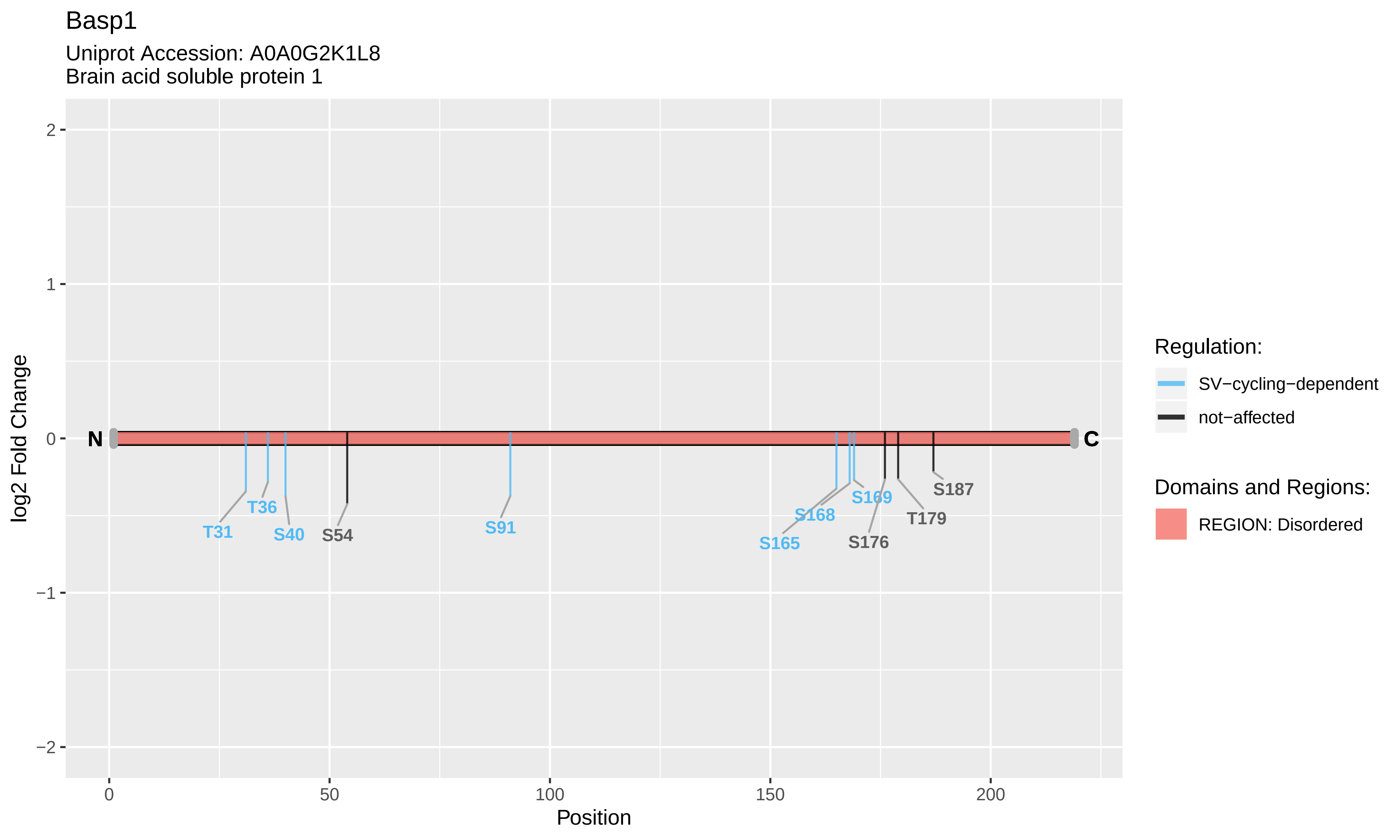

Basp1
Uniprot Accession: A0A0G2K1L8
Brain acid soluble protein 1
log2 Fold Change
2
1
0
−1
−2
N
C
T31
T36
S40
S54
S91
S165
S168
S169
S176
T179
S187
0
50
100
150
200
Position
Regulation:
SV−cycling−dependent
not−affected
Domains and Regions:
REGION: Disordered

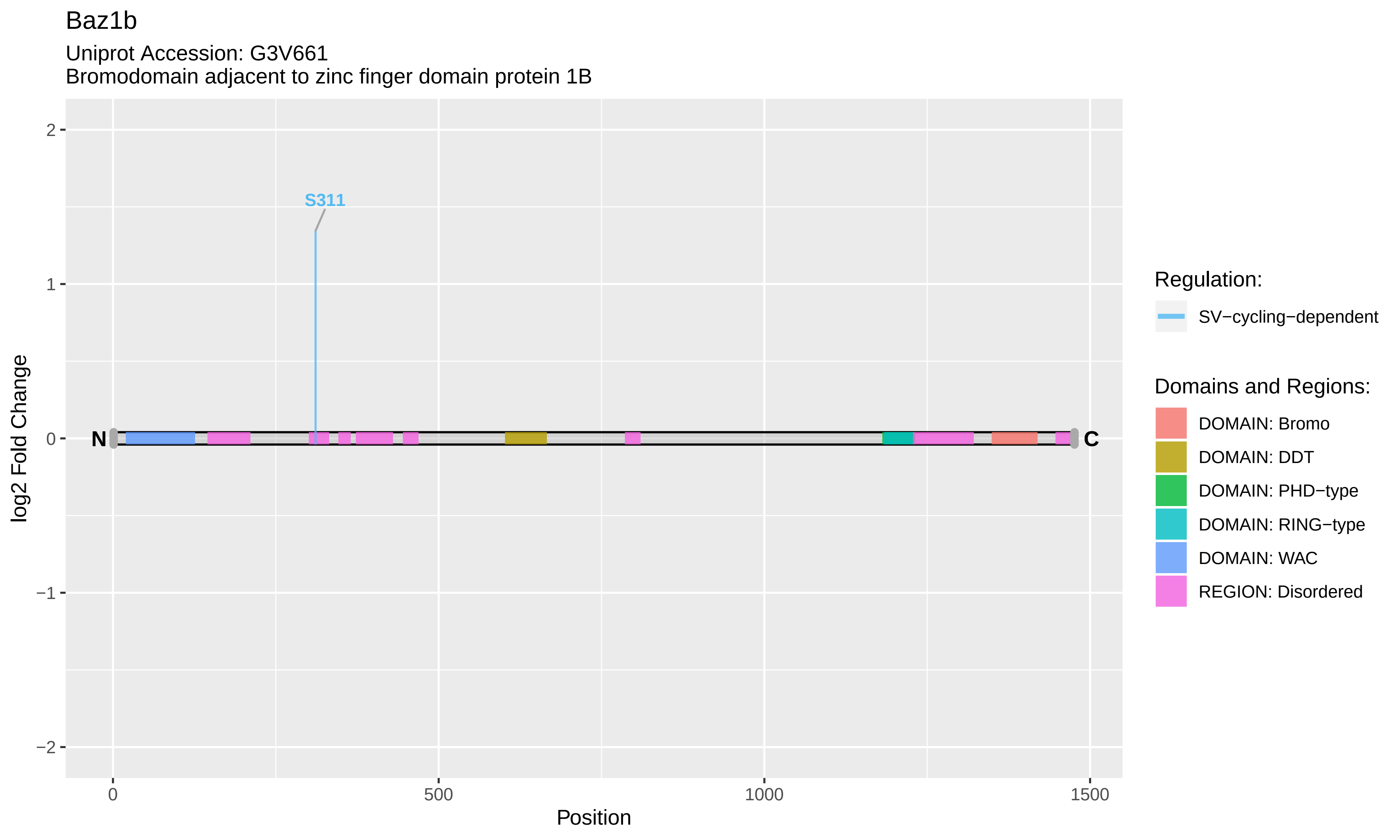

Baz1b
Uniprot Accession: G3V661
Bromodomain adjacent to zinc finger domain protein 1B
S311
N
C
2
1
0
−1
−2
log2 Fold Change
0
500
1000
1500
Position
Regulation:
SV−cycling−dependent
Domains and Regions:
DOMAIN: Bromo
DOMAIN: DDT
DOMAIN: PHD−type
DOMAIN: RING−type
DOMAIN: WAC
REGION: Disordered

Bcl10

Uniprot Accession: Q9QYN5
B−cell lymphoma/leukemia 10

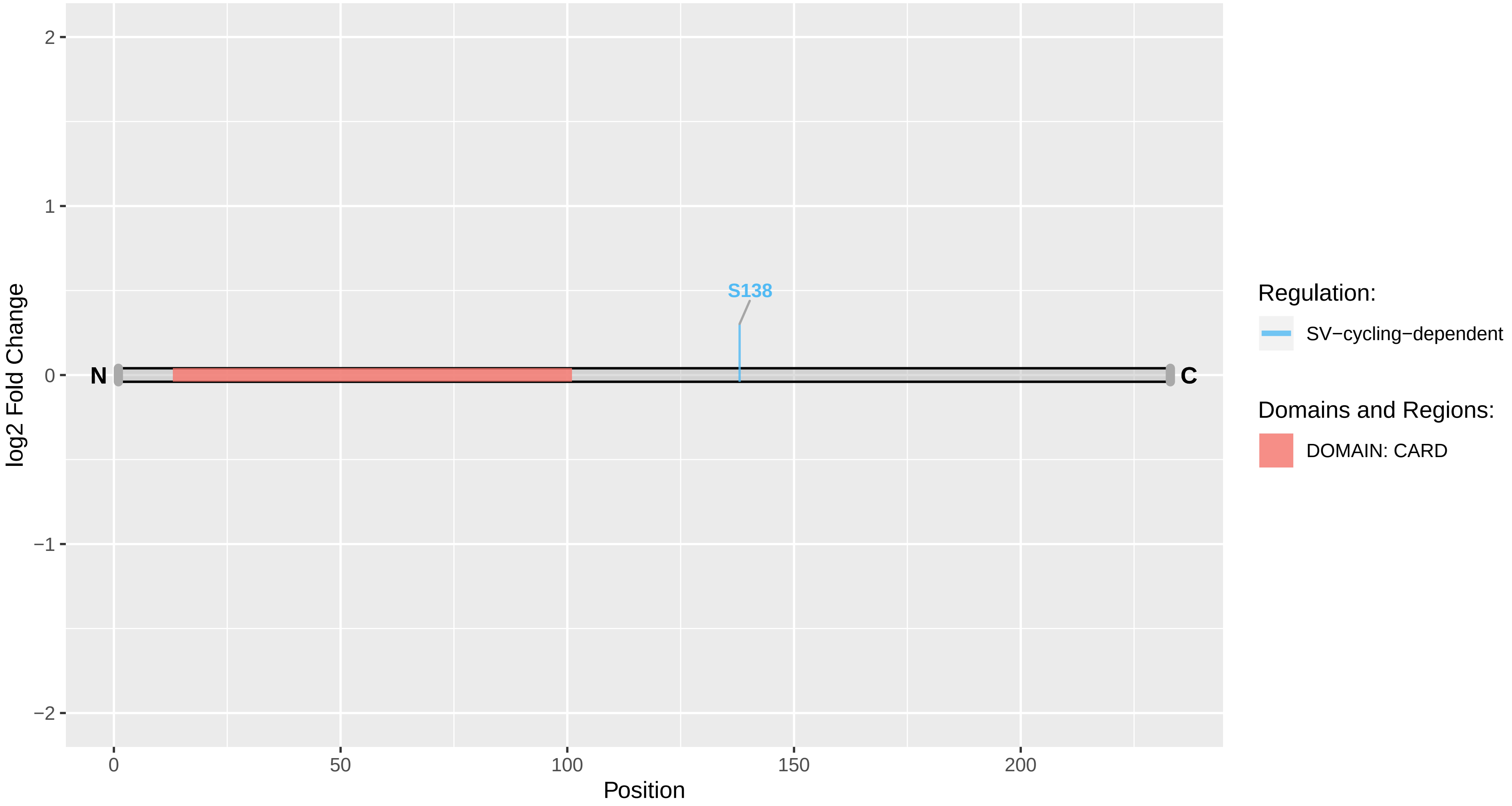

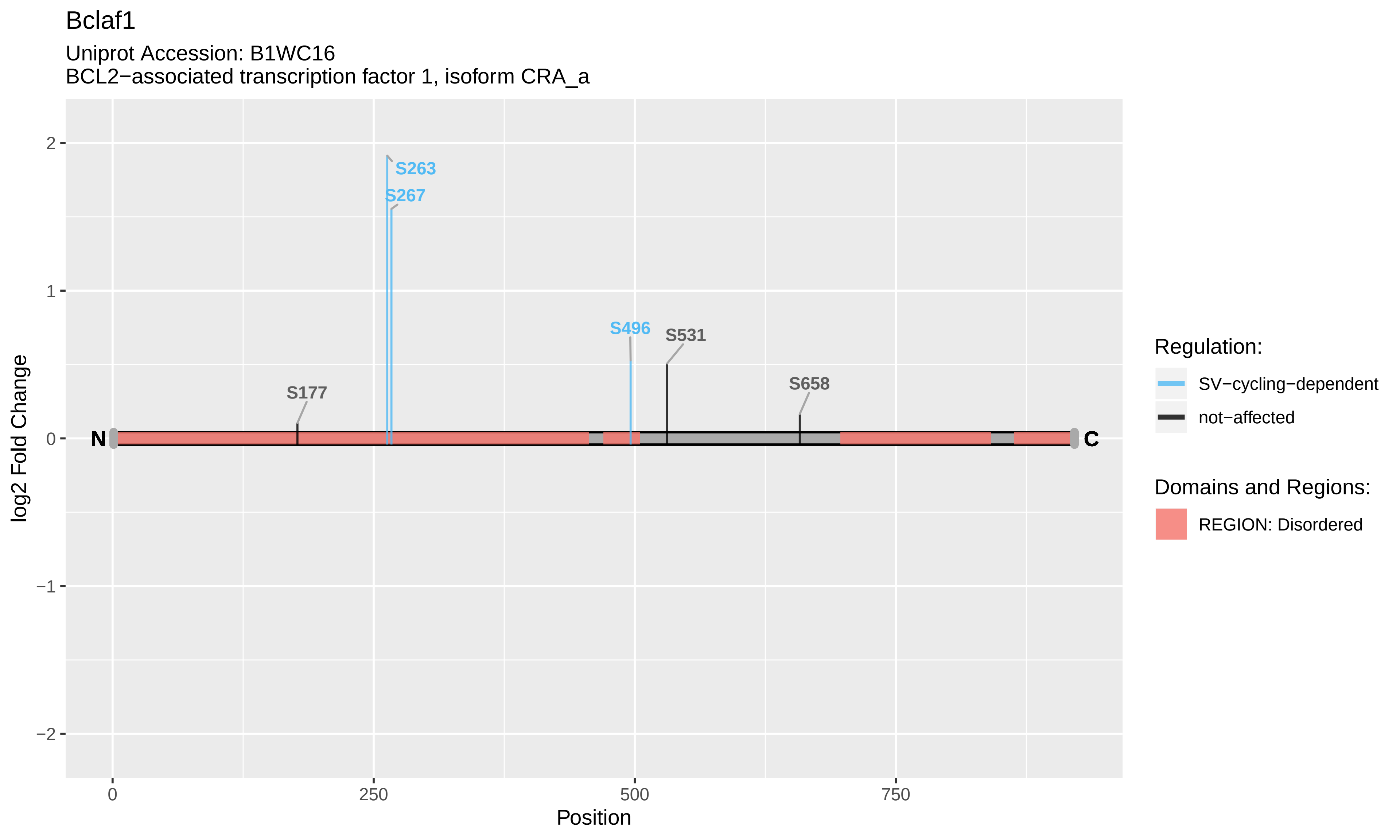
Bclaf1
Uniprot Accession: B1WC16
BCL2−associated transcription factor 1, isoform CRA_a
S263
S267
S496
S531
S658
S177
N
C
2
1
0
−1
−2
log2 Fold Change
0
250
500
750
Position
Regulation:
SV−cycling−dependent
not−affected
Domains and Regions:
REGION: Disordered

Begain

Uniprot Accession: O88881
Brain−enriched guanylate kinase−associated protein

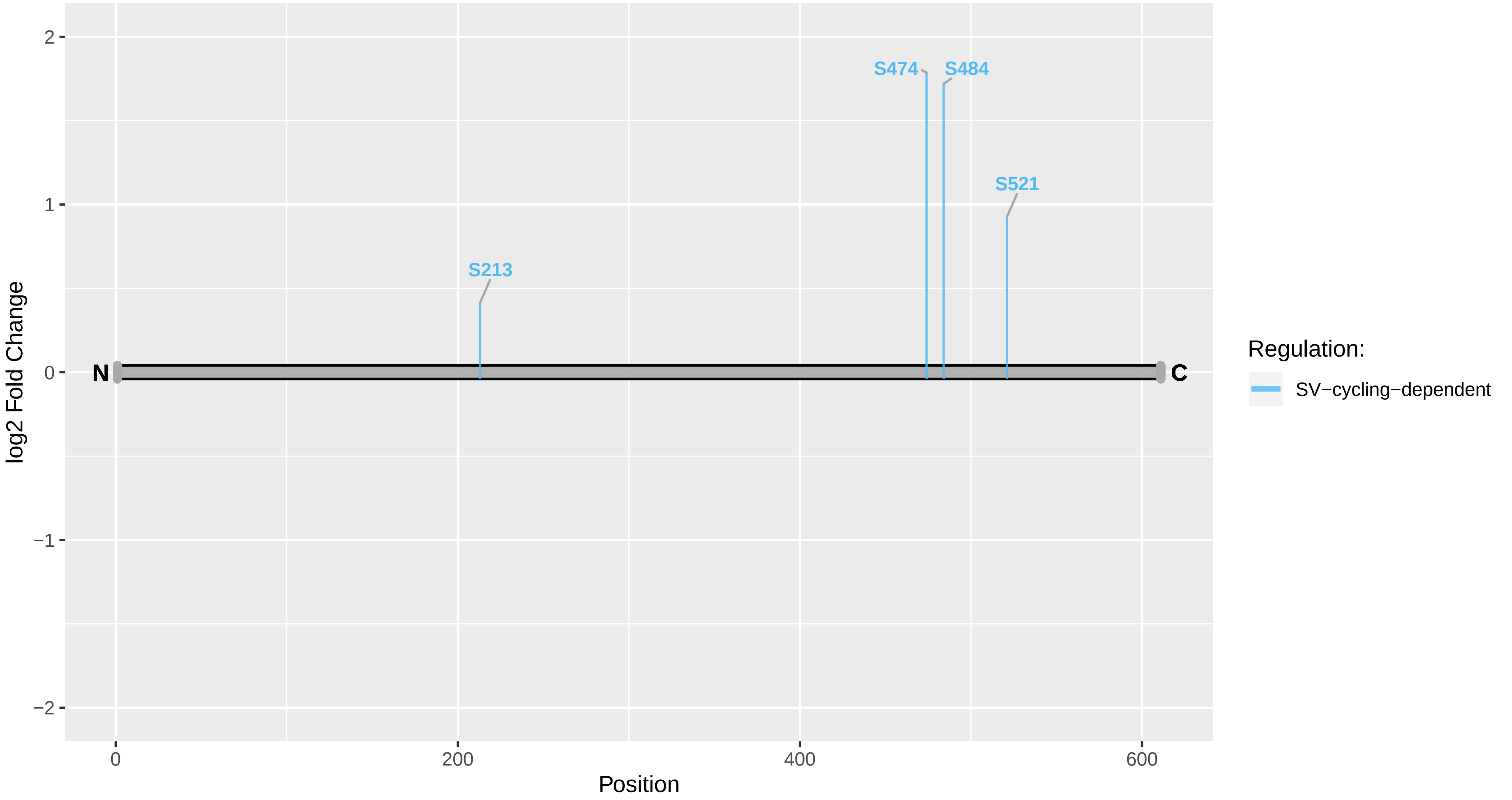

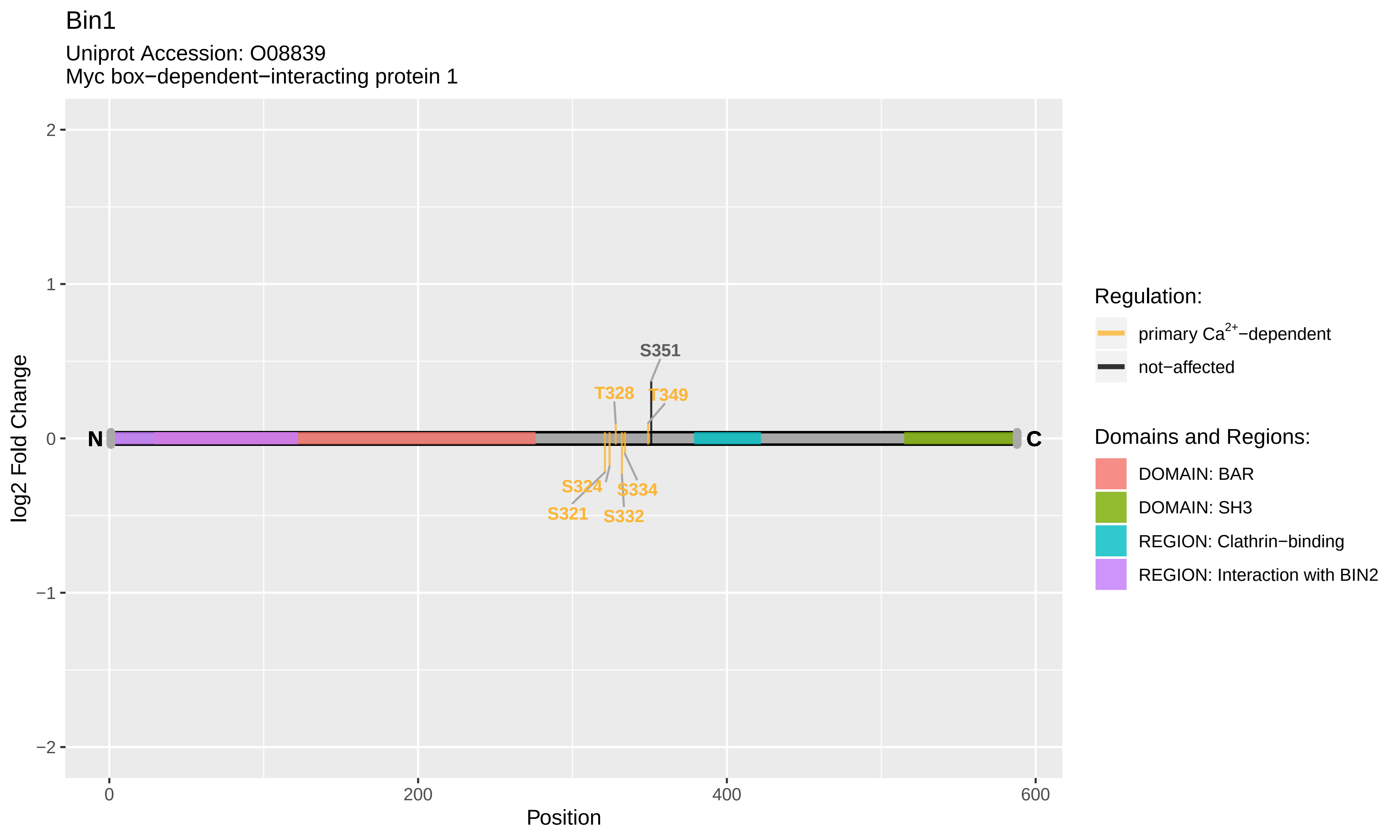

Bin1
Uniprot Accession: O08839
Myc box−dependent−interacting protein 1
log2 Fold Change
Position
2
1
0
−1
−2
0
200
400
600
N
C
S351
T328
T349
S324
S334
S321
S332
Regulation:
primary Ca2+−dependent
not−affected
Domains and Regions:
DOMAIN: BAR
DOMAIN: SH3
REGION: Clathrin−binding
REGION: Interaction with BIN2

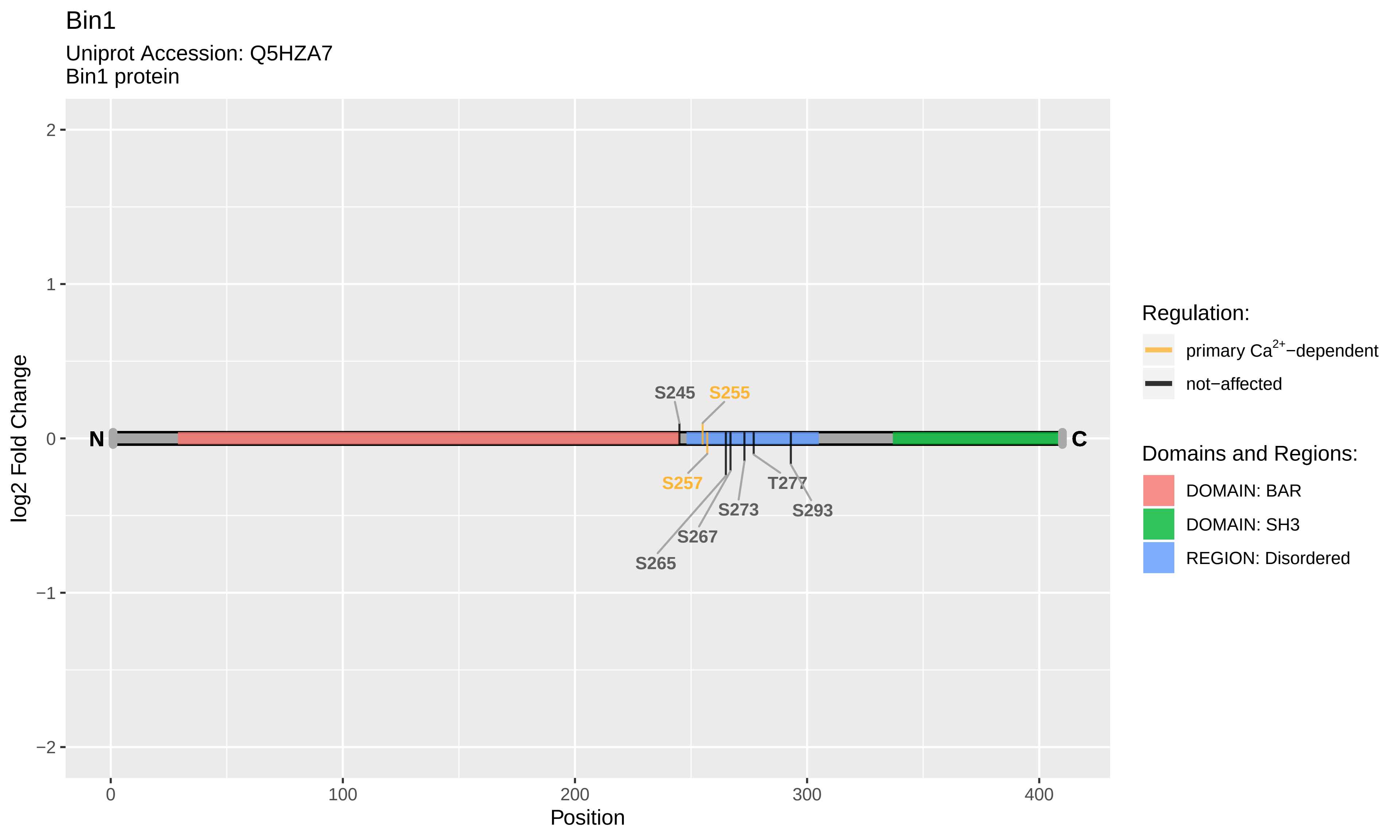

Bin1
Uniprot Accession: Q5HZA7
Bin1 protein
log2 Fold Change
2
1
0
−1
−2
N
C
S245
S255
S257
S265
S267
S273
T277
S293
0
100
200
300
400
Position
Regulation:
primary Ca2+−dependent
not−affected
Domains and Regions:
DOMAIN: BAR
DOMAIN: SH3
REGION: Disordered

Bri3bp

Uniprot Accession: Q5U3Z5
BRI3−binding protein

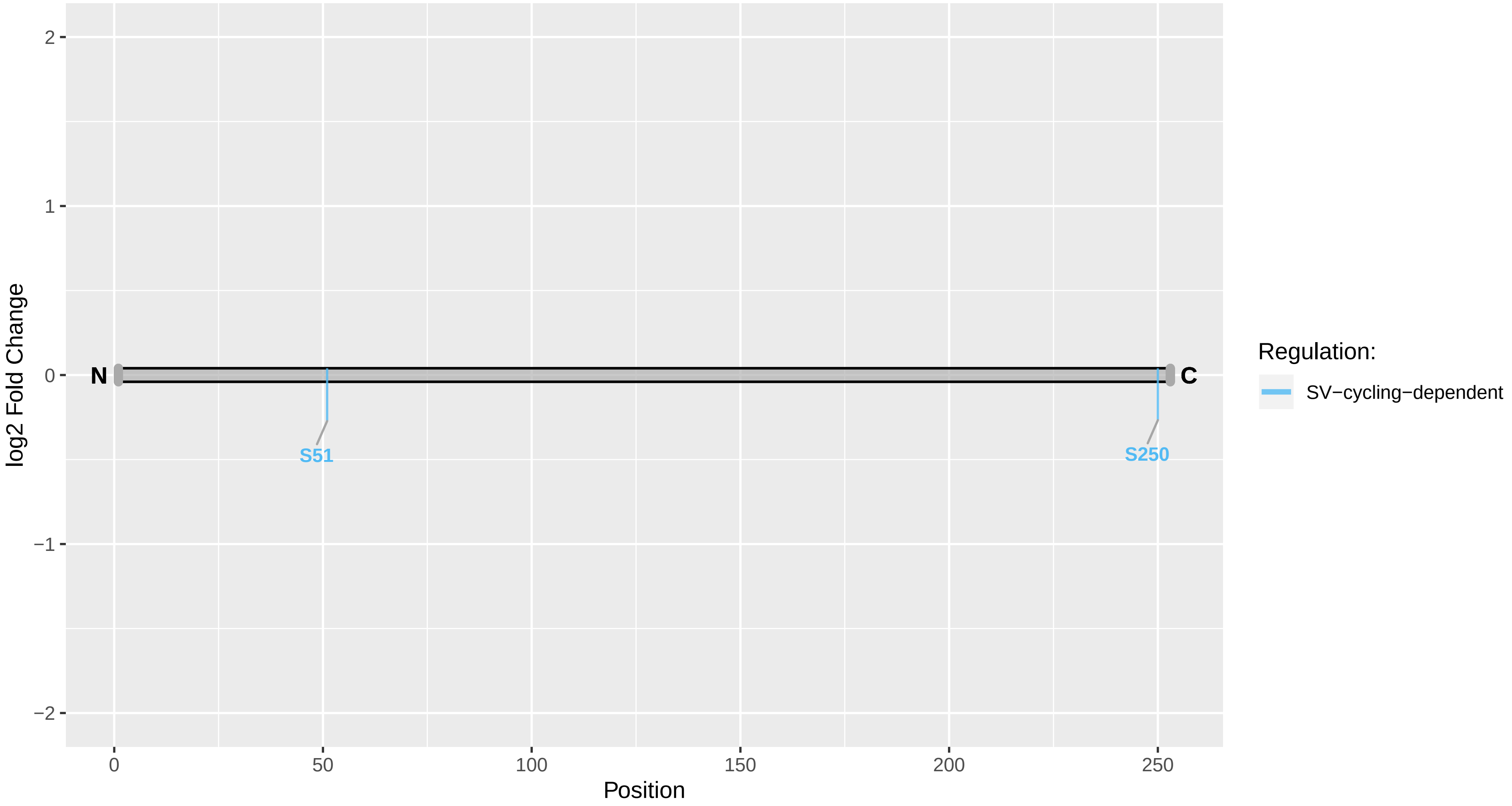

Brsk1

Uniprot Accession: B2DD29
Serine/threonine−protein kinase BRSK1

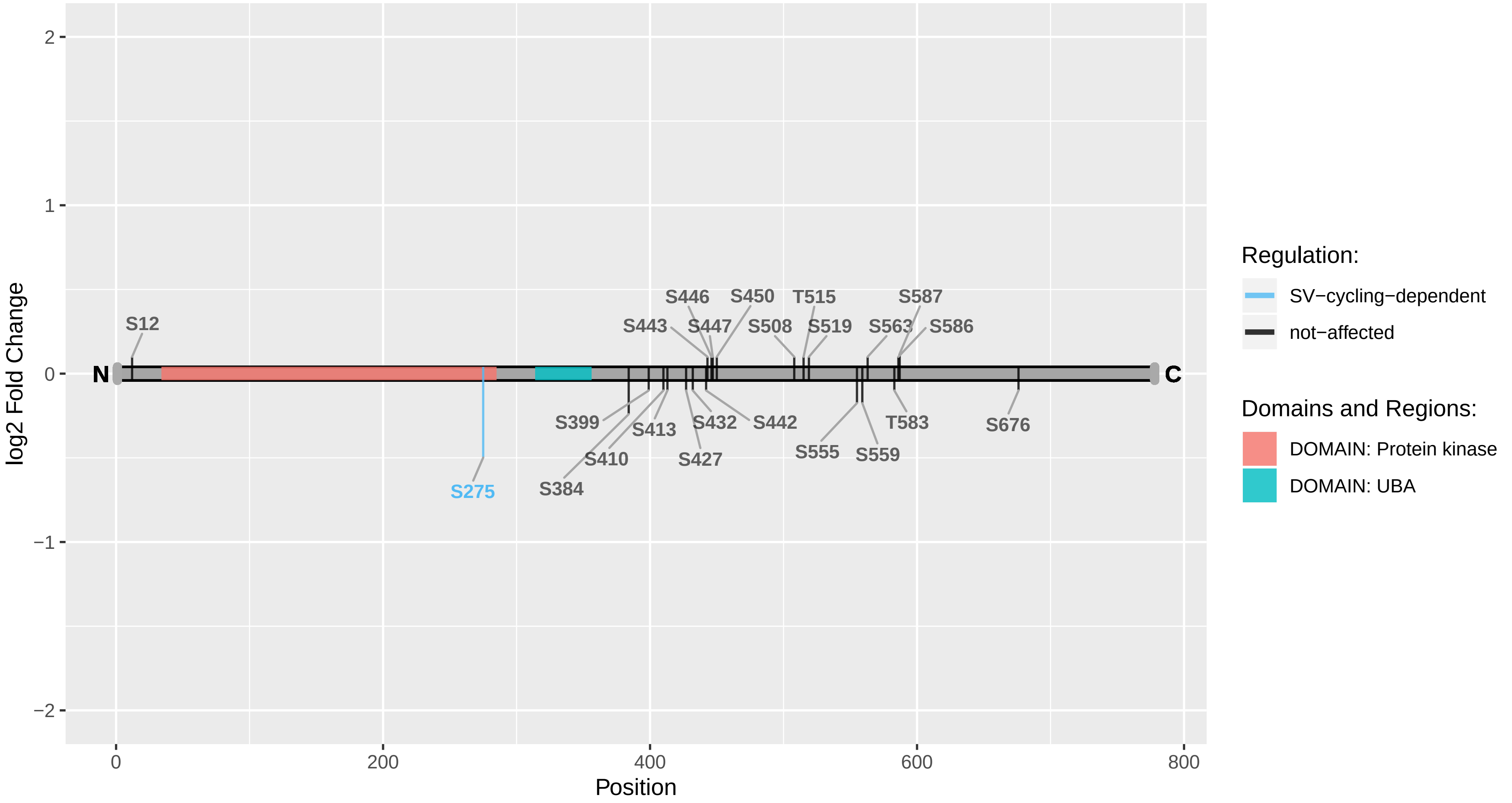

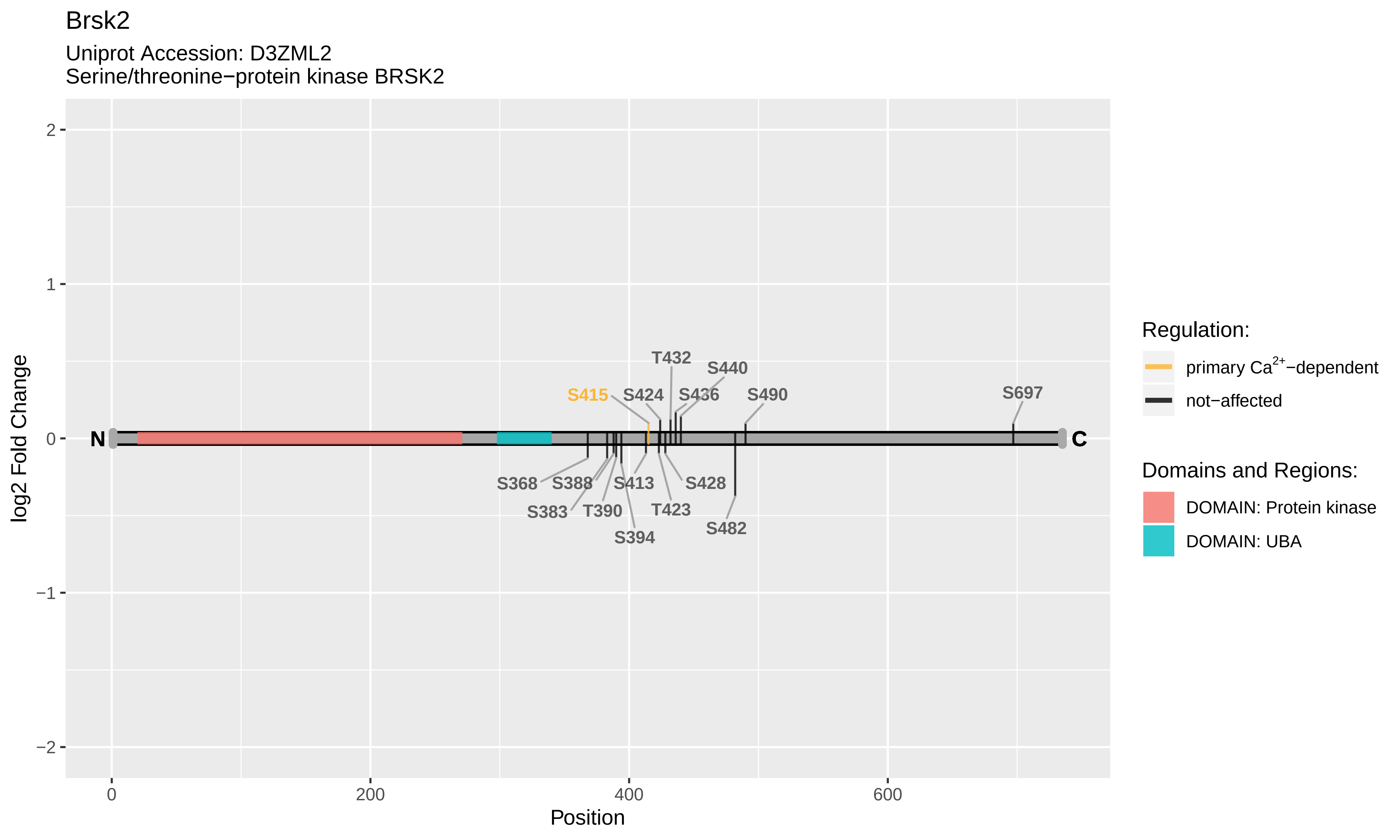
Brsk2
Uniprot Accession: D3ZML2
Serine/threonine−protein kinase BRSK2
log2 Fold Change
Position
N
C
T432
S440
S415
S424
S436
S490
S697
S368
S388
S413
S428
S383
T390
T423
S394
S482
Regulation:
primary Ca2+−dependent
not−affected
Domains and Regions:
DOMAIN: Protein kinase
DOMAIN: UBA

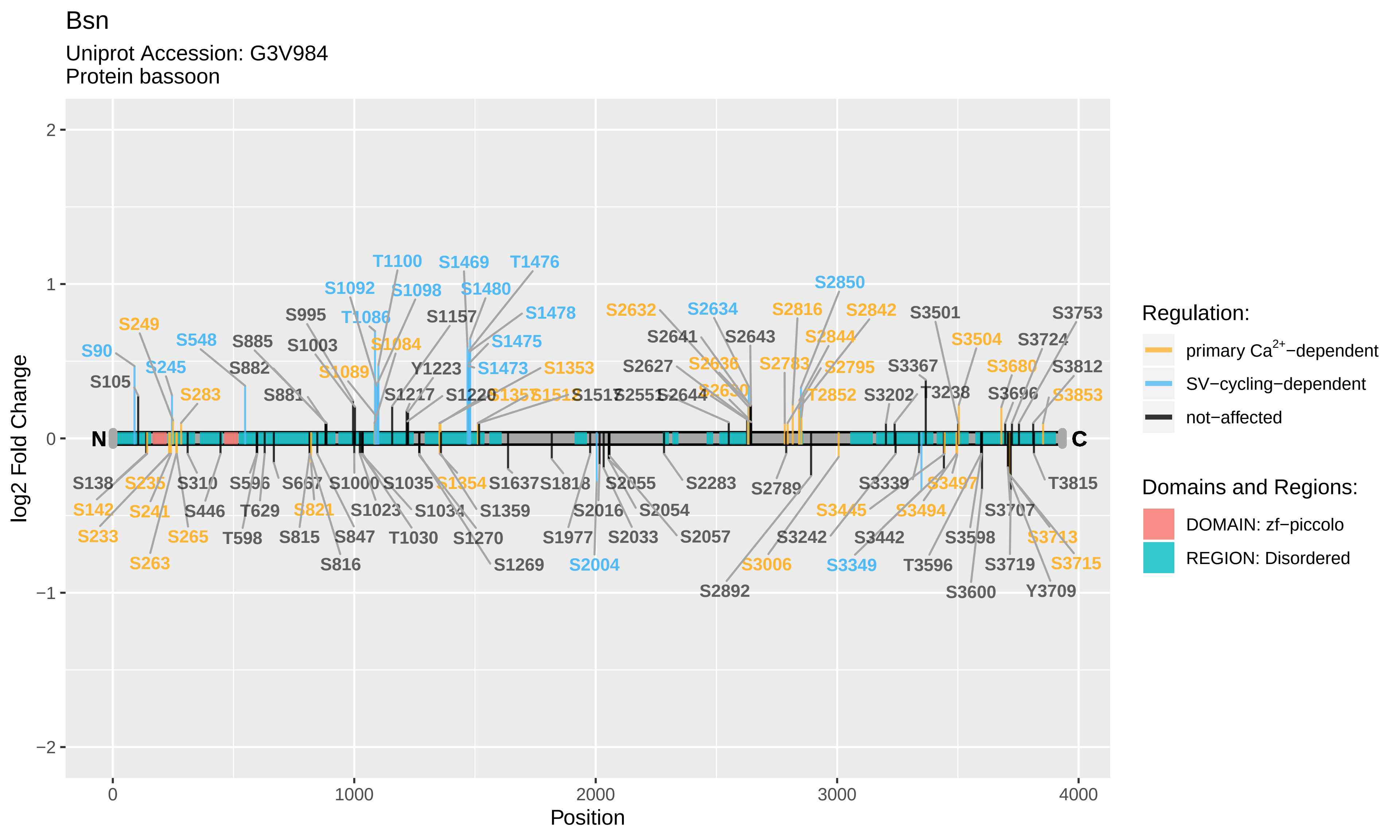

Bsn
Uniprot Accession: G3V984
Protein bassoon
log2 Fold Change
Position
2
1
0
−1
−2
0
1000
2000
3000
4000
N
C
Regulation:
primary Ca2+−dependent
SV−cycling−dependent
not−affected
Domains and Regions:
DOMAIN: zf−piccolo
REGION: Disordered
T1100
S1469
T1476
S1092
S1098
S1480
S2850
S995
T1086
S1157
S1478
S2632
S2634
S2816
S2842
S3501
S3753
S249
S885
S1003
S1084
S1475
S2641
S2643
S2844
S3504
S3724
S90
S548
S882
S1089
Y1223
S1473
S1353
S2627
S2636
S2783
S2795
S3367
S3680
S3812
S245
S105
S283
S881
S1217
S1220
S1357
S1512
S1517
S2551
S2644
S2630
T2852
S3202
T3238
S3696
S3853
S138
S235
S310
S596
S667
S1000
S1035
S1354
S1637
S1818
S2055
S2283
S2789
S3339
S3497
T3815
S142
S241
S446
T629
S821
S1023
S1034
S1359
S2016
S2054
S3445
S3494
S3707
S233
S265
T598
S815
S847
T1030
S1270
S1977
S2033
S2057
S3242
S3442
S3598
S3713
S263
S816
S1269
S2004
S3006
S3349
T3596
S3719
S3715
S2892
S3600
Y3709

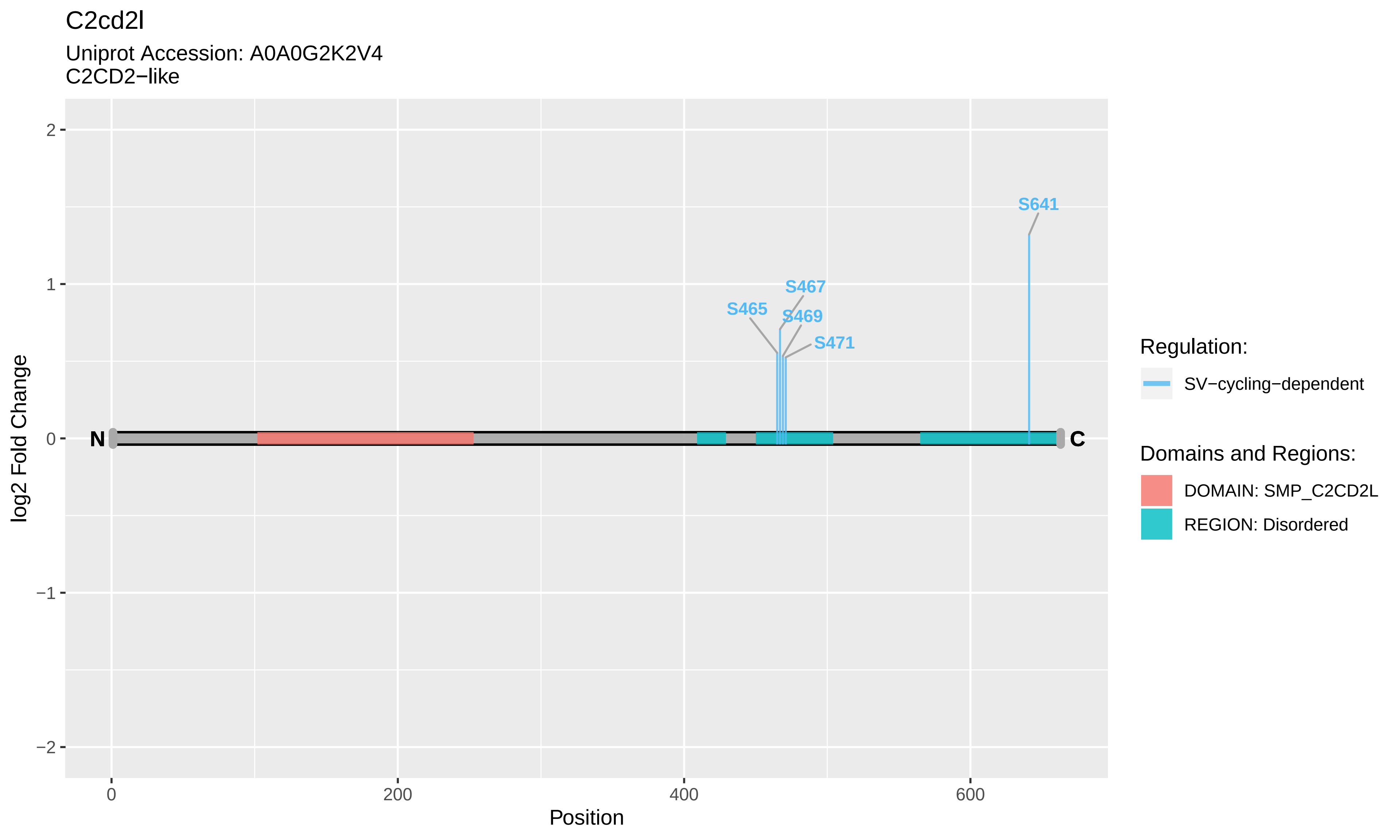
C2cd2l
Uniprot Accession: A0A0G2K2V4
C2CD2−like
S641
S467
S465
S469
S471
N
C
2
1
0
−1
−2
0
200
400
600
Position
log2 Fold Change
Regulation:
SV−cycling−dependent
Domains and Regions:
DOMAIN: SMP_C2CD2L
REGION: Disordered

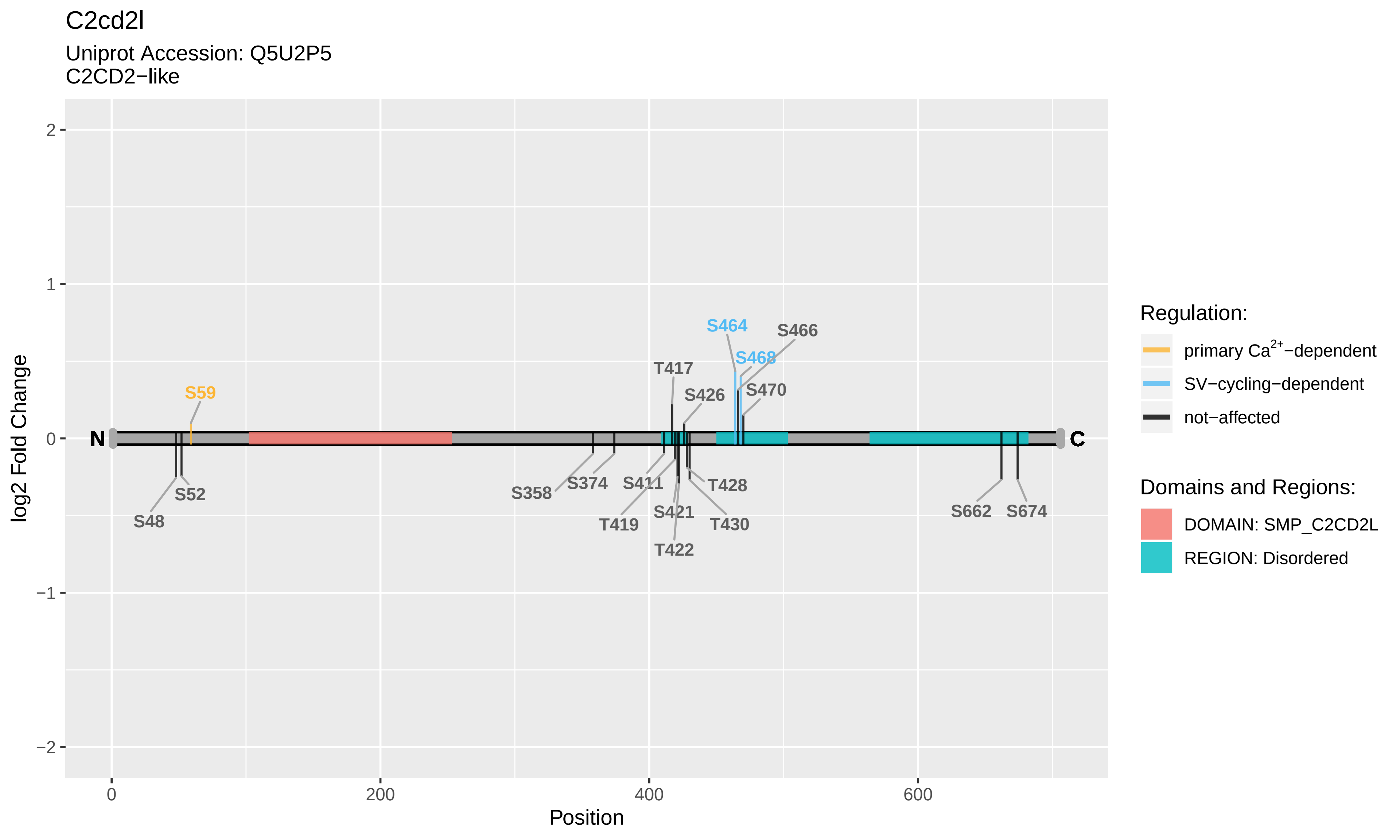
C2cd2l
Uniprot Accession: Q5U2P5
C2CD2−like
log2 Fold Change
Position
N
C
S59
S48
S52
S358
S374
S411
T417
T419
S421
T422
S426
T428
T430
S464
S466
S468
S470
S662
S674
Regulation:
primary Ca2+−dependent
SV−cycling−dependent
not−affected
Domains and Regions:
DOMAIN: SMP_C2CD2L
REGION: Disordered

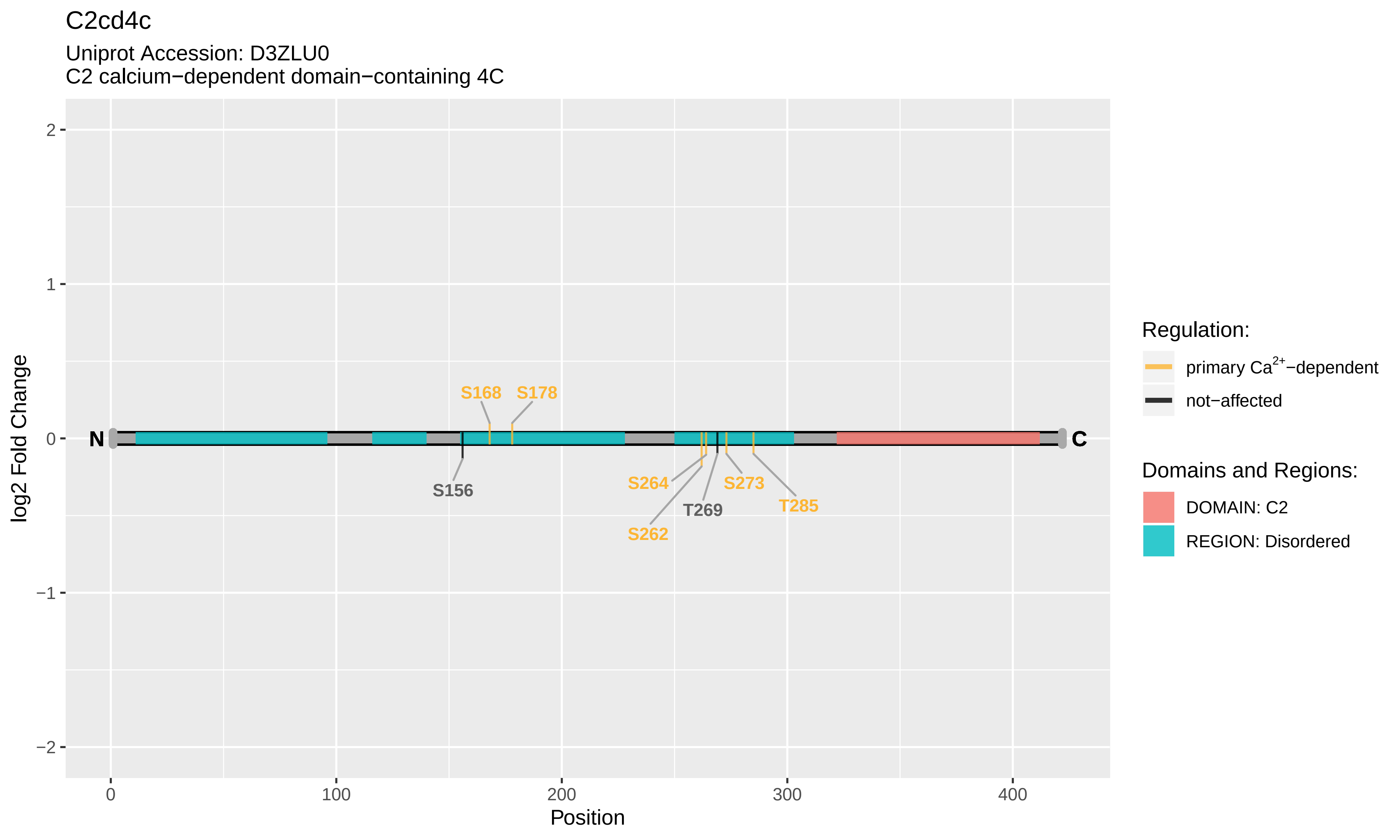

C2cd4c
Uniprot Accession: D3ZLU0
C2 calcium−dependent domain−containing 4C
log2 Fold Change
2
1
0
−1
−2
N
C
S168
S178
S156
S264
S262
T269
S273
T285
0
100
200
300
400
Position
Regulation:
primary Ca2+−dependent
not−affected
Domains and Regions:
DOMAIN: C2
REGION: Disordered

C2cd5

Uniprot Accession: A0A0G2K543
C2 calcium−dependent domain−containing 5

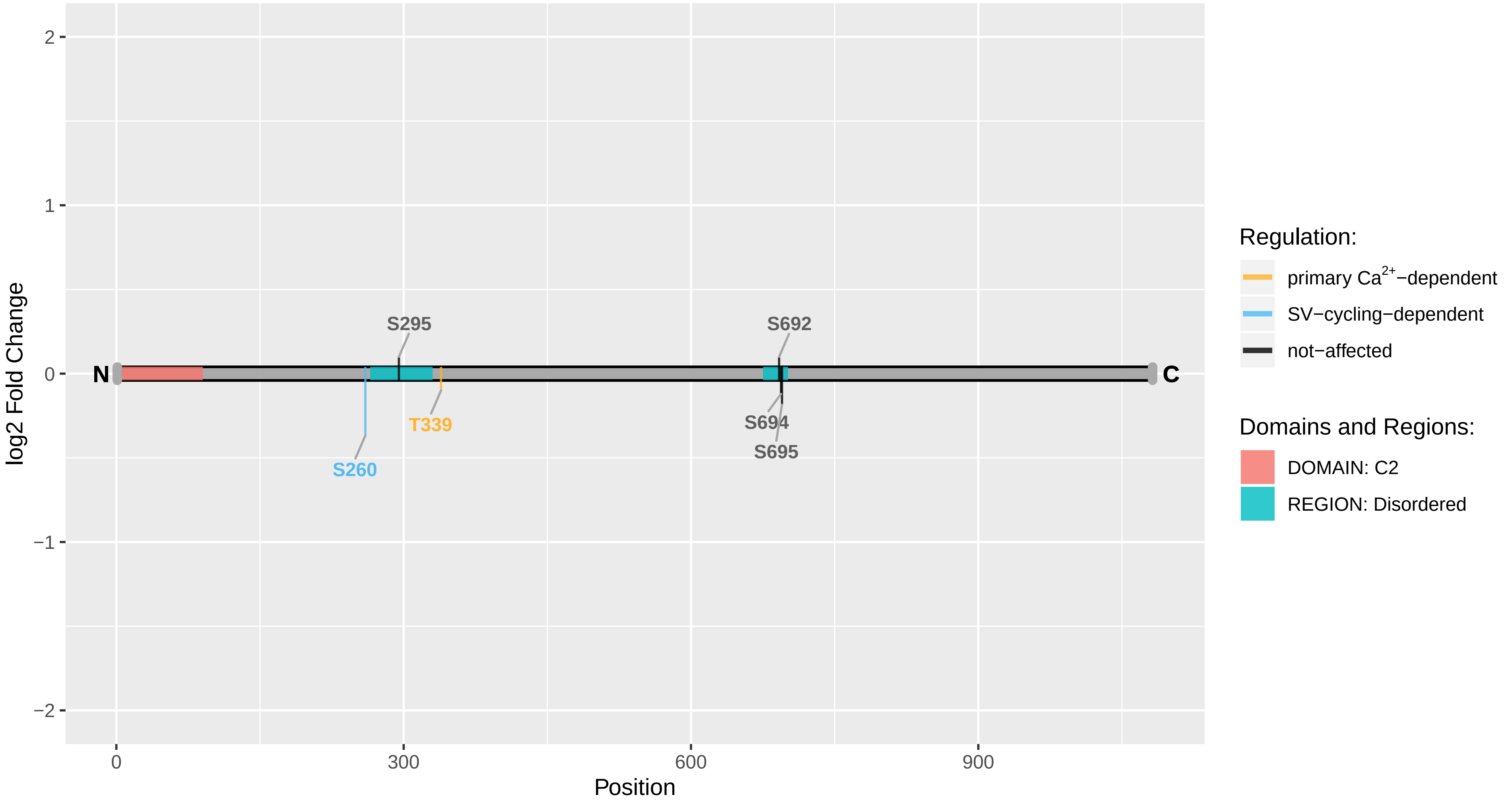

Cacna1a

Uniprot Accession: A0A0G2JXK1
Voltage−dependent P/Q−type calcium channel subunit alpha

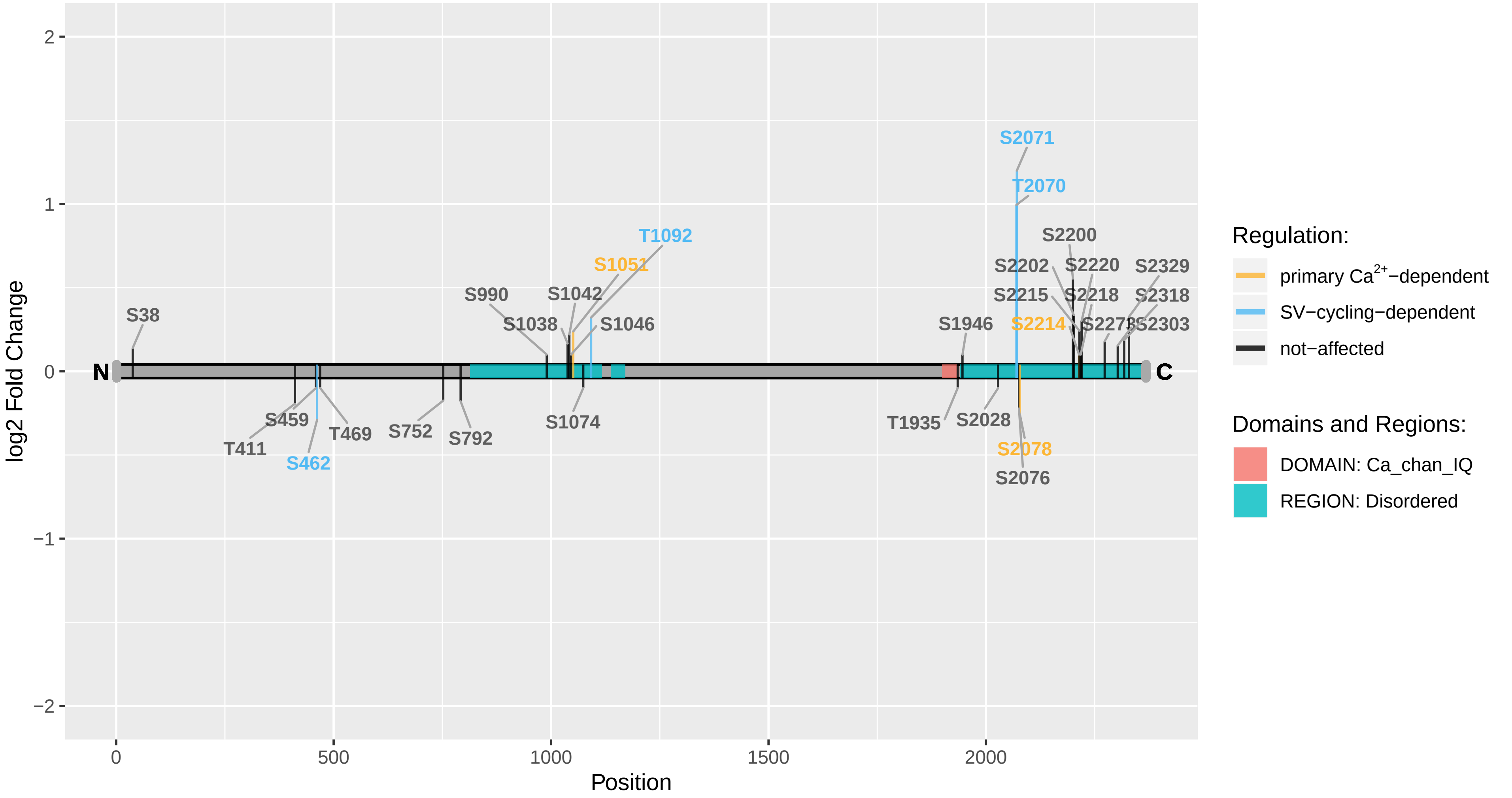

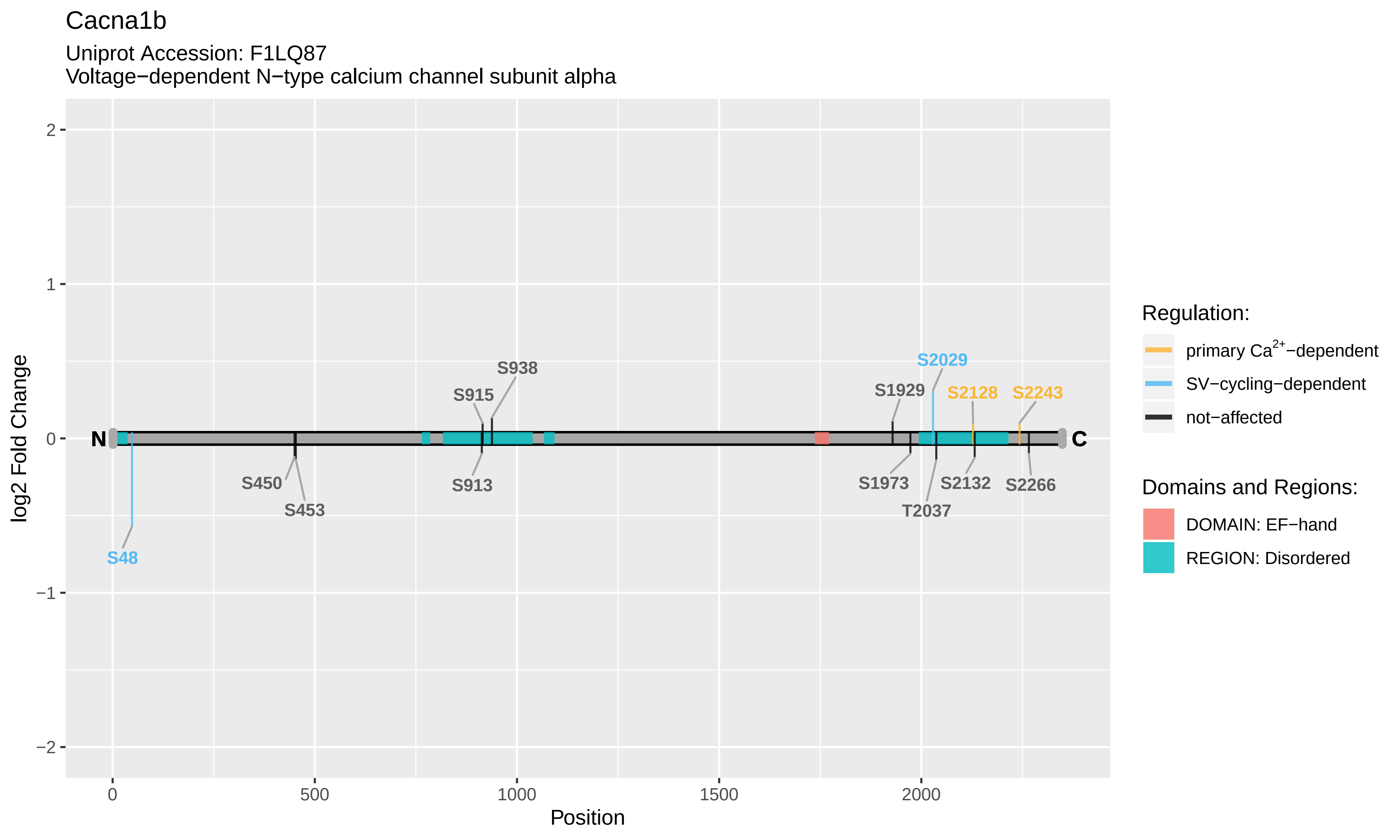
Cacna1b
Uniprot Accession: F1LQ87
Voltage−dependent N−type calcium channel subunit alpha
log2 Fold Change
Position
N
C
S48
S450
S453
S913
S915
S938
S1929
S1973
S2029
T2037
S2128
S2132
S2243
S2266
Regulation:
primary Ca2+−dependent
SV−cycling−dependent
not−affected
Domains and Regions:
DOMAIN: EF−hand
REGION: Disordered

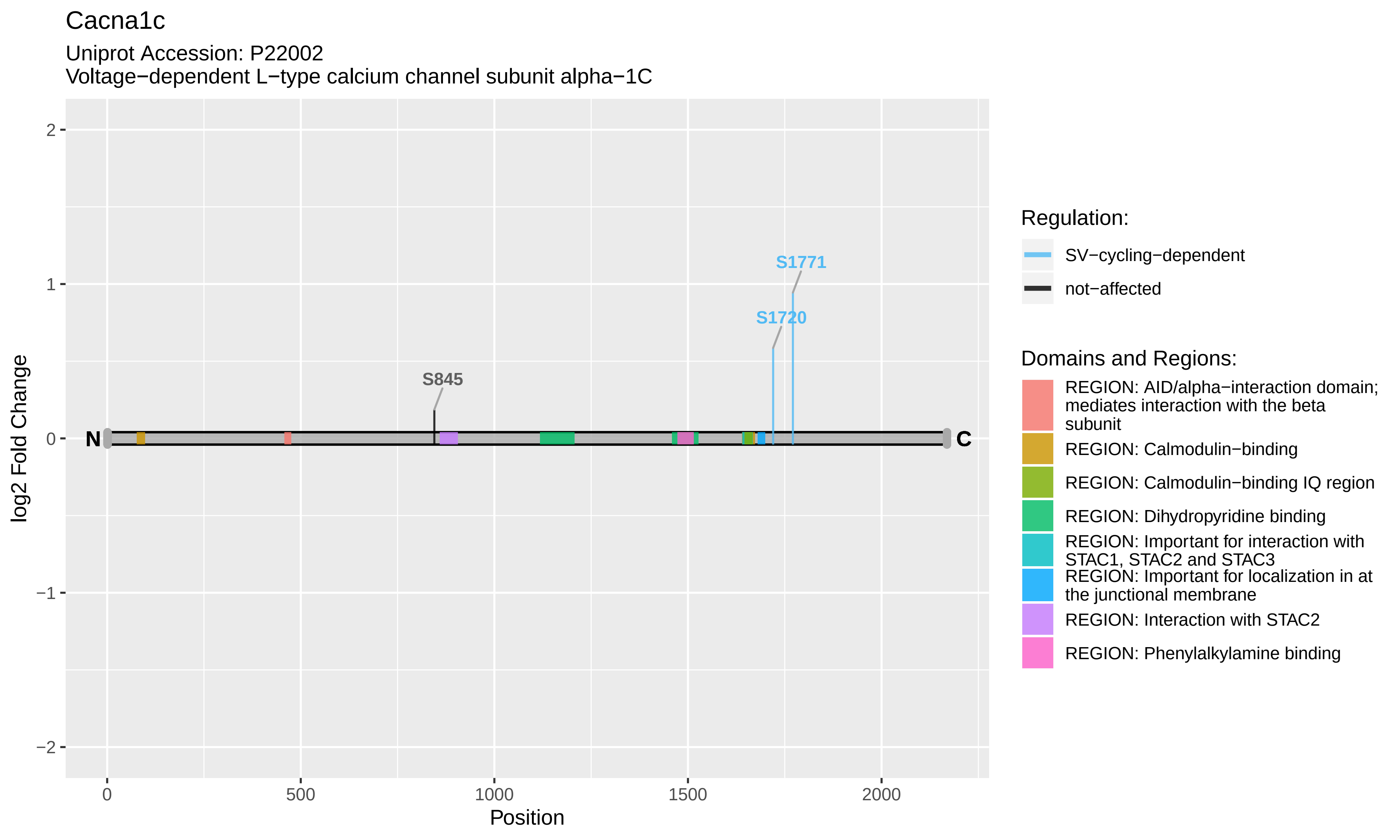
Cacna1c
Uniprot Accession: P22002
Voltage−dependent L−type calcium channel subunit alpha−1C
S1771
S1720
S845
N
C
2
1
0
−1
−2
log2 Fold Change
0
500
1000
1500
2000
Position
Regulation:
SV−cycling−dependent
not−affected
Domains and Regions:
REGION: AID/alpha−interaction domain; mediates interaction with the beta subunit
REGION: Calmodulin−binding
REGION: Calmodulin−binding IQ region
REGION: Dihydropyridine binding
REGION: Important for interaction with STAC1, STAC2 and STAC3
REGION: Important for localization in at the junctional membrane
REGION: Interaction with STAC2
REGION: Phenylalkylamine binding

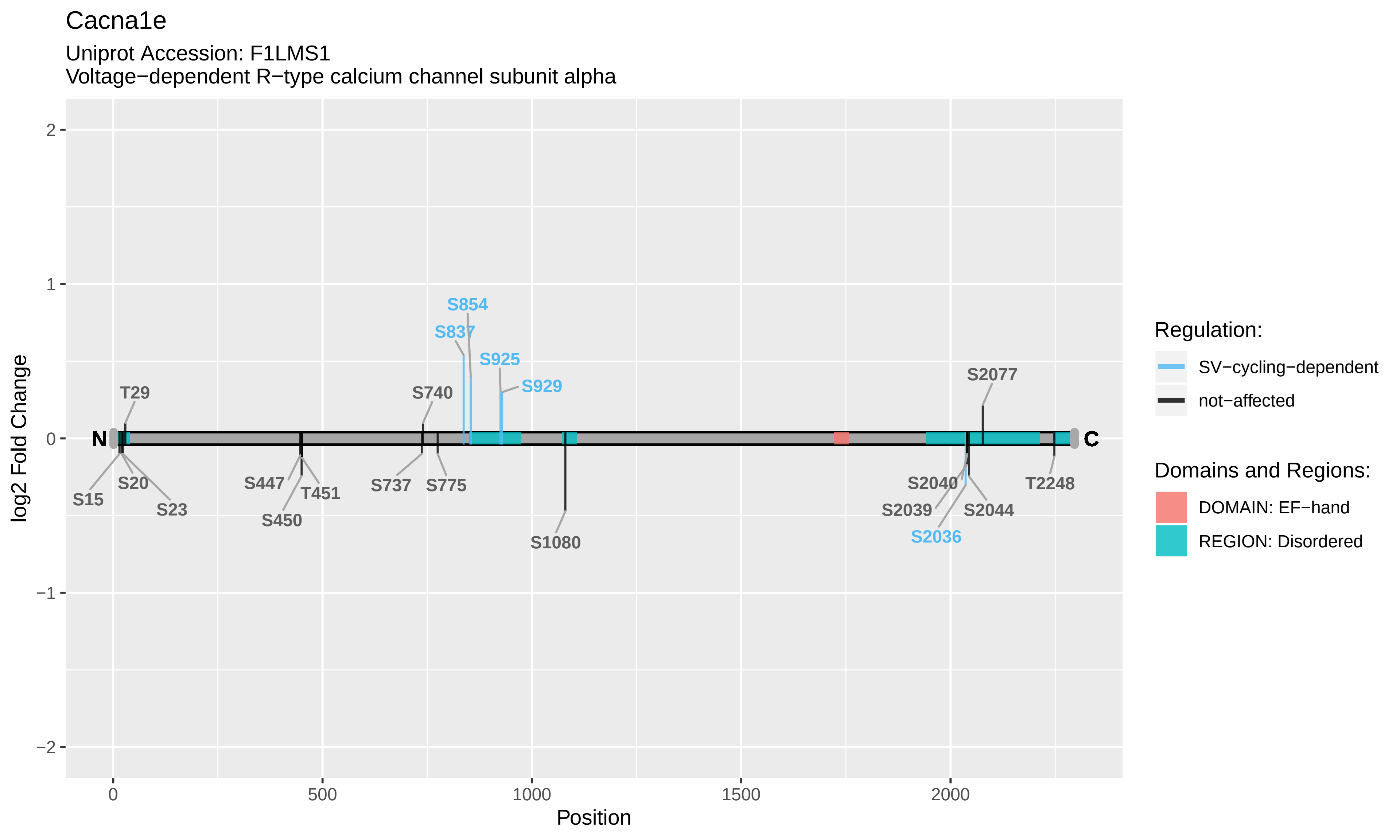
Cacna1e
Uniprot Accession: F1LMS1
Voltage−dependent R−type calcium channel subunit alpha
log2 Fold Change
Position
2
1
0
−1
−2
0
500
1000
1500
2000
N
C
S854
S837
S925
S929
T29
S740
S2077
S15
S20
S23
S447
T451
S450
S737
S775
S1080
S2040
S2039
S2044
S2036
T2248
Regulation:
SV−cycling−dependent
not−affected
Domains and Regions:
DOMAIN: EF−hand
REGION: Disordered

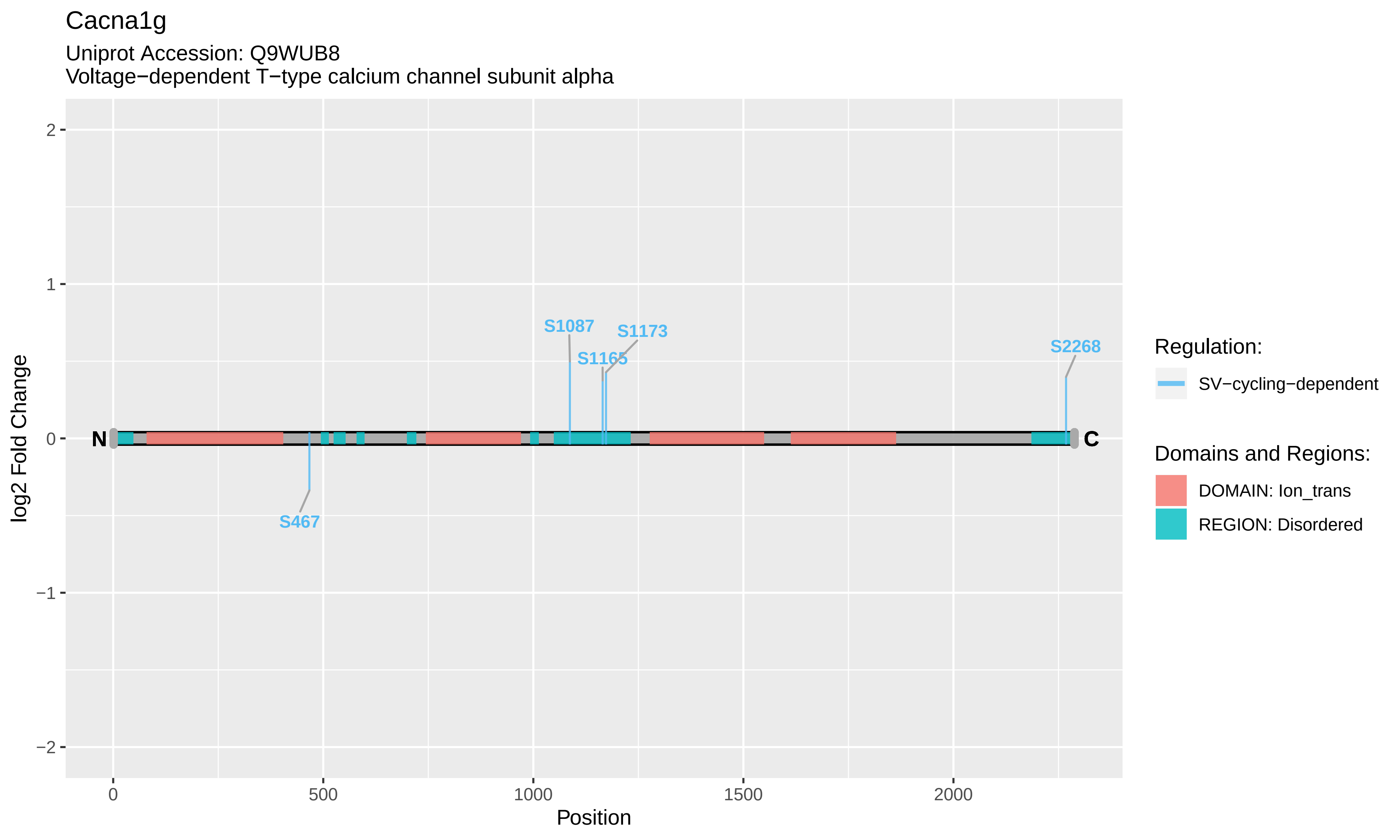

Cacna1g
Uniprot Accession: Q9WUB8
Voltage−dependent T−type calcium channel subunit alpha
log2 Fold Change
Position
N
C
S1087
S1173
S1165
S2268
S467
Regulation:
SV−cycling−dependent
Domains and Regions:
DOMAIN: Ion_trans
REGION: Disordered
2
1
0
−1
−2
0
500
1000
1500
2000

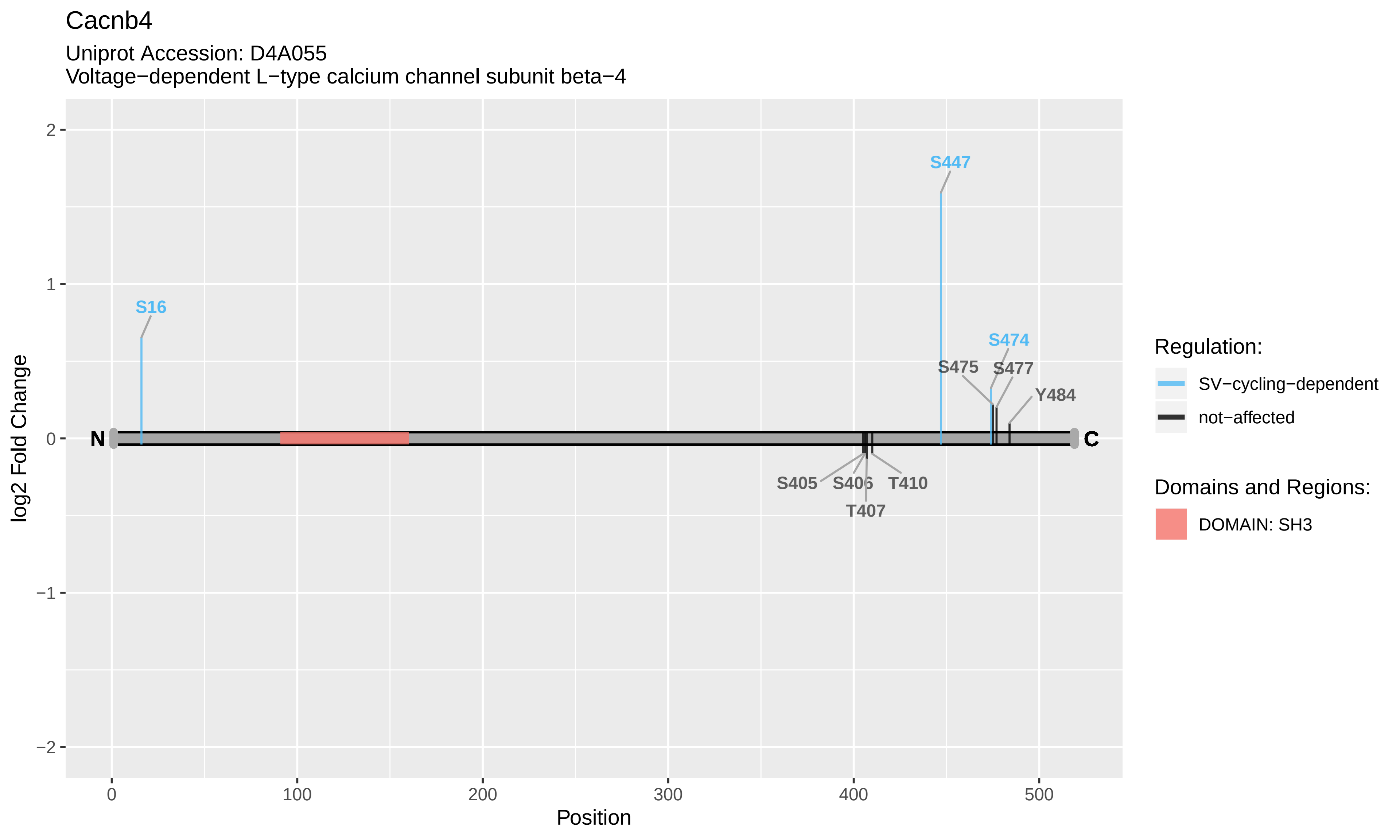

Cacnb4
Uniprot Accession: D4A055
Voltage−dependent L−type calcium channel subunit beta−4
log2 Fold Change
Position
2
1
0
−1
−2
0
100
200
300
400
500
N
C
S16
S447
S474
S475
S477
Y484
S405
S406
T407
T410
Regulation:
SV−cycling−dependent
not−affected
Domains and Regions:
DOMAIN: SH3

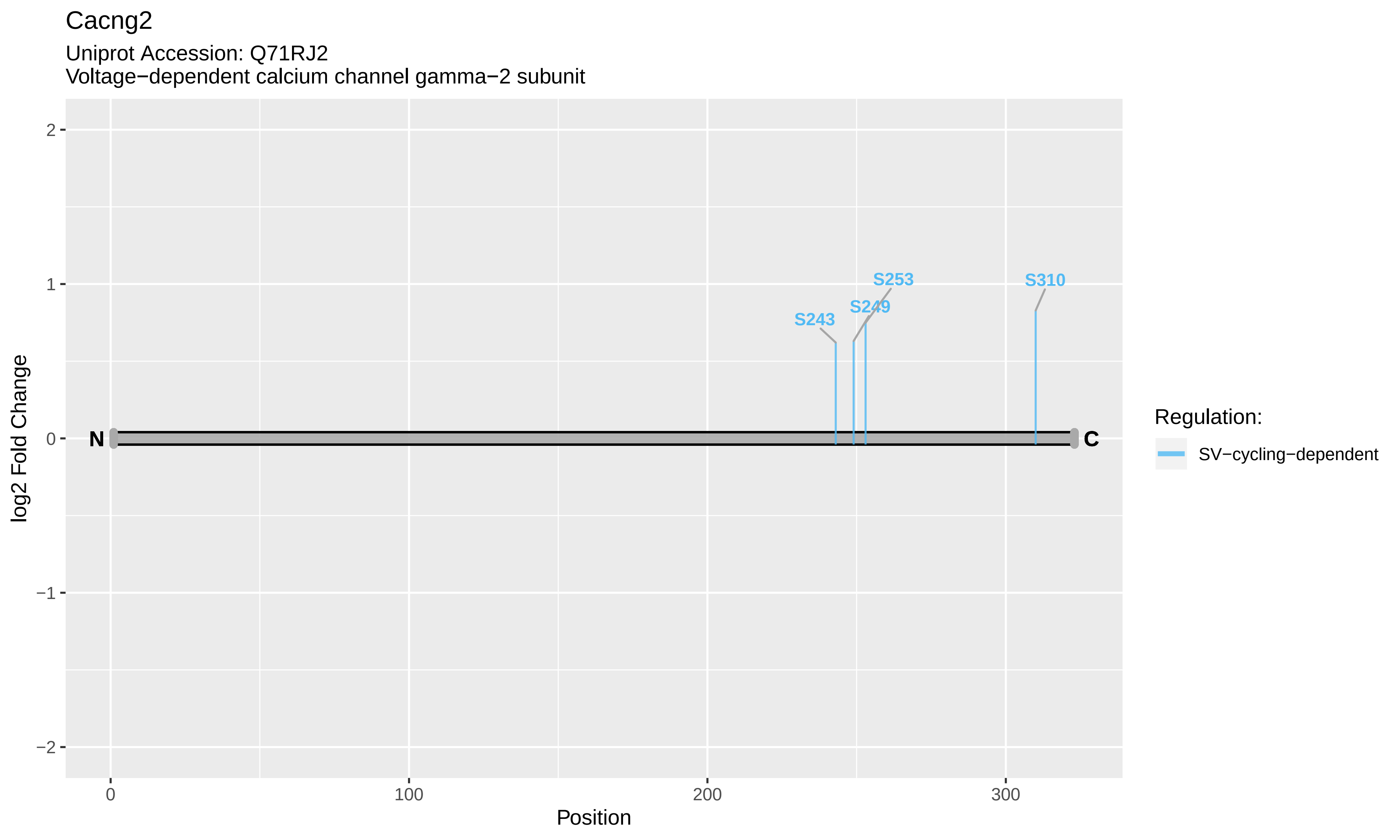
Cacng2
Uniprot Accession: Q71RJ2
Voltage−dependent calcium channel gamma−2 subunit
log2 Fold Change
Position
N
C
S243
S249
S253
S310
Regulation:
SV−cycling−dependent
2
1
0
−1
−2
0
100
200
300

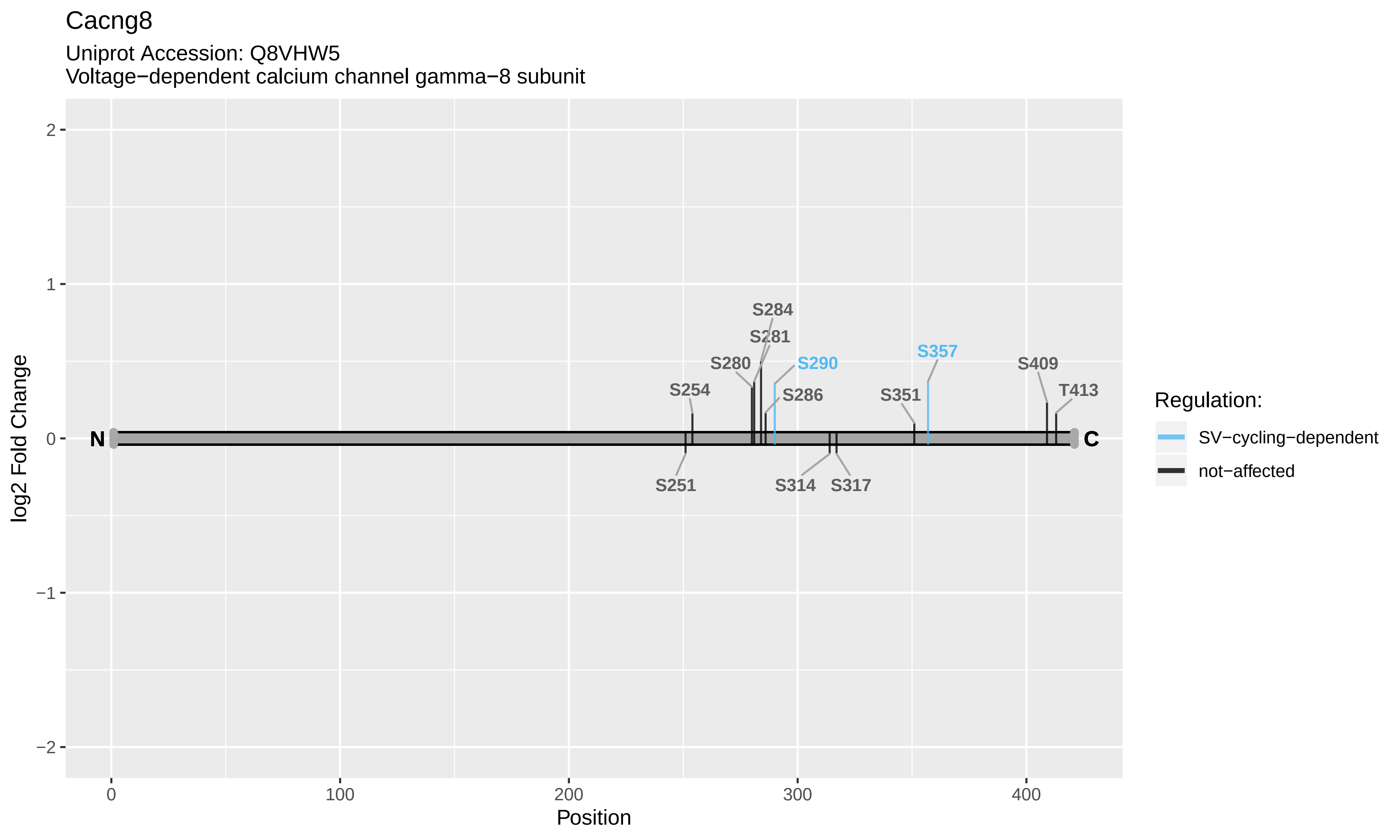
Cacng8
Uniprot Accession: Q8VHW5
Voltage−dependent calcium channel gamma−8 subunit
log2 Fold Change
Position
N
C
S251
S254
S280
S281
S284
S286
S290
S314
S317
S351
S357
S409
T413
Regulation:
SV−cycling−dependent
not−affected

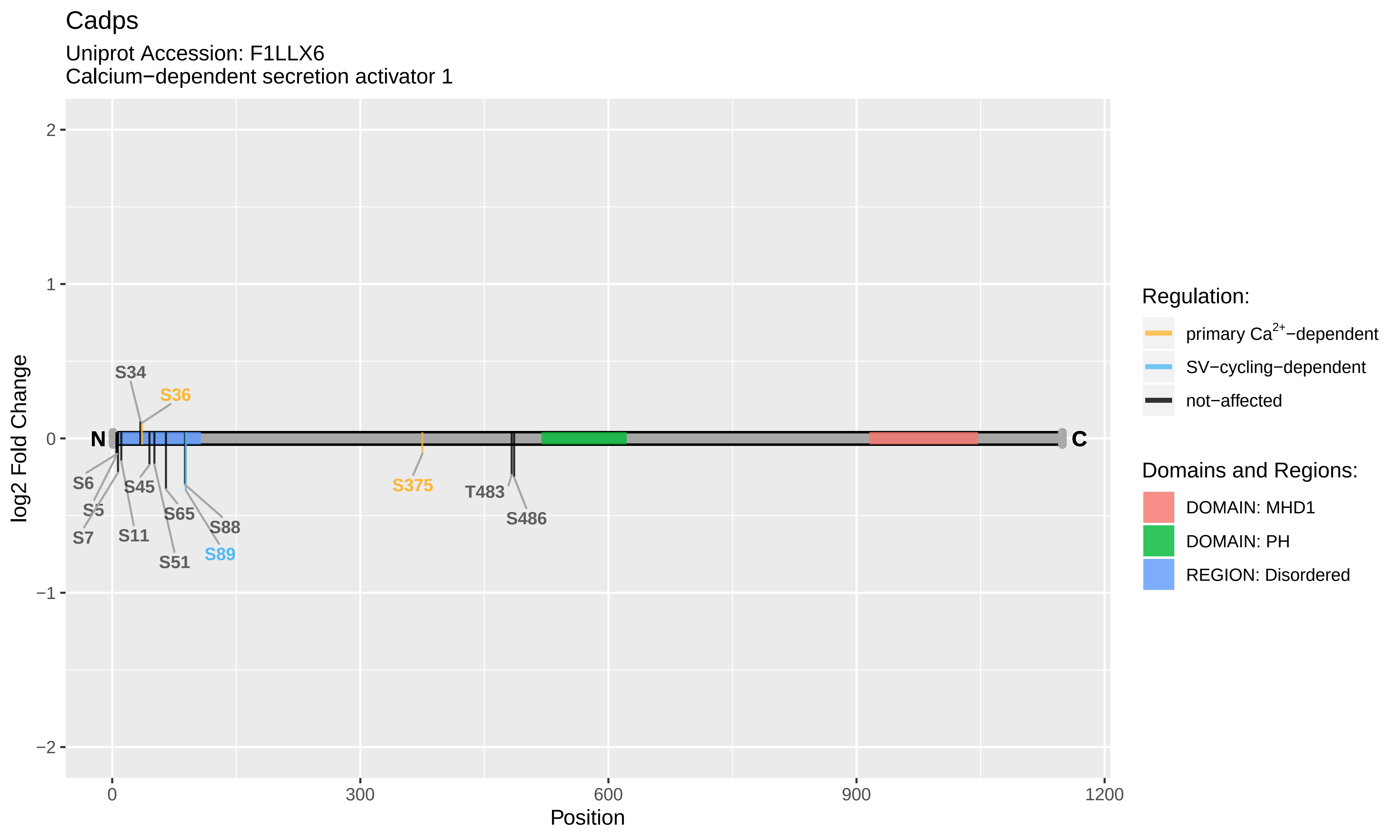
Cadps
Uniprot Accession: F1LLX6
Calcium−dependent secretion activator 1
log2 Fold Change
2
1
0
−1
−2
N
C
S34
S36
S6
S5
S7
S11
S45
S65
S51
S88
S89
S375
T483
S486
0
300
600
900
1200
Position
Regulation:
primary Ca2+−dependent
SV−cycling−dependent
not−affected
Domains and Regions:
DOMAIN: MHD1
DOMAIN: PH
REGION: Disordered

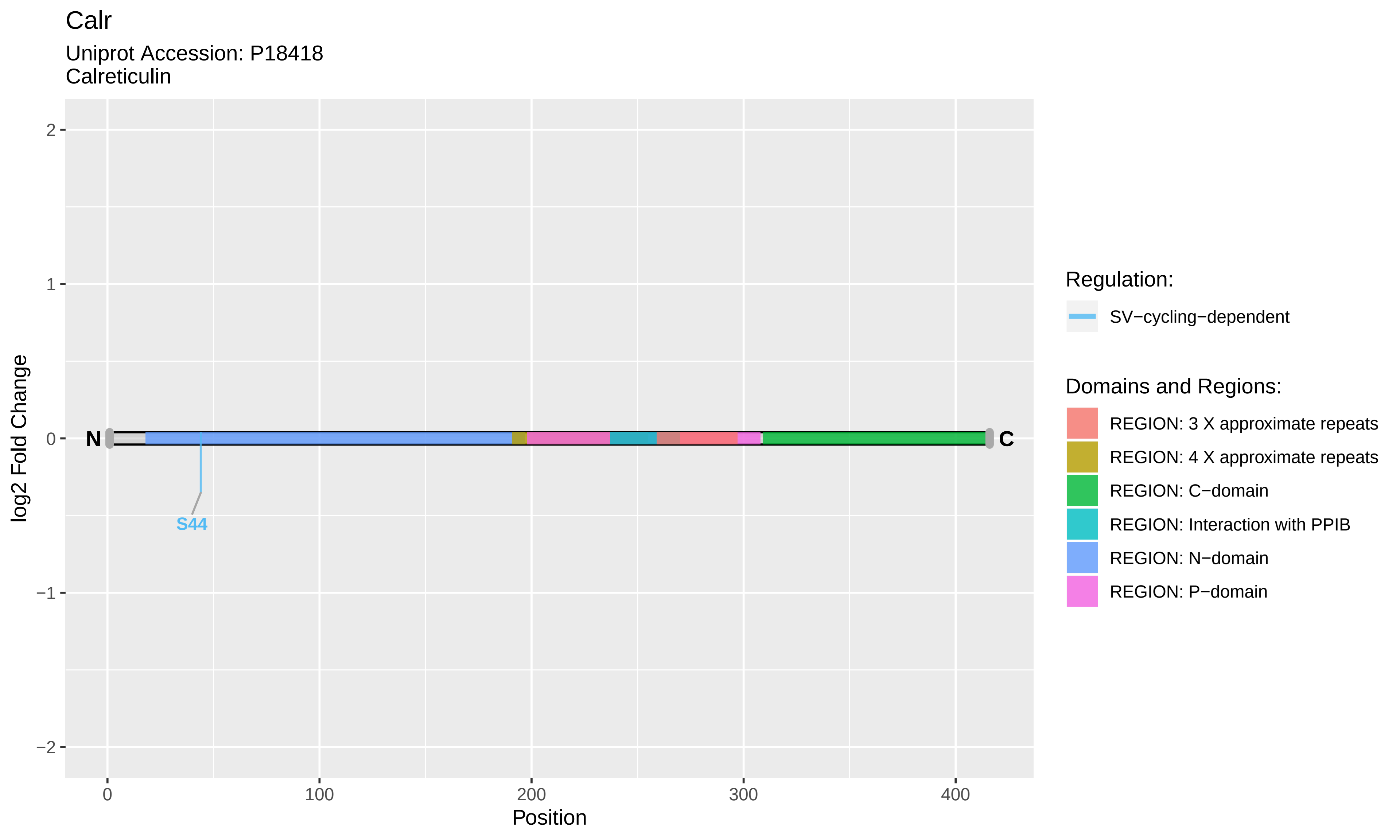

Calr
Uniprot Accession: P18418
Calreticulin
log2 Fold Change
2
1
0
−1
−2
N
C
S44
0
100
200
300
400
Position
Regulation:
SV−cycling−dependent
Domains and Regions:
REGION: 3 X approximate repeats
REGION: 4 X approximate repeats
REGION: C−domain
REGION: Interaction with PPIB
REGION: N−domain
REGION: P−domain

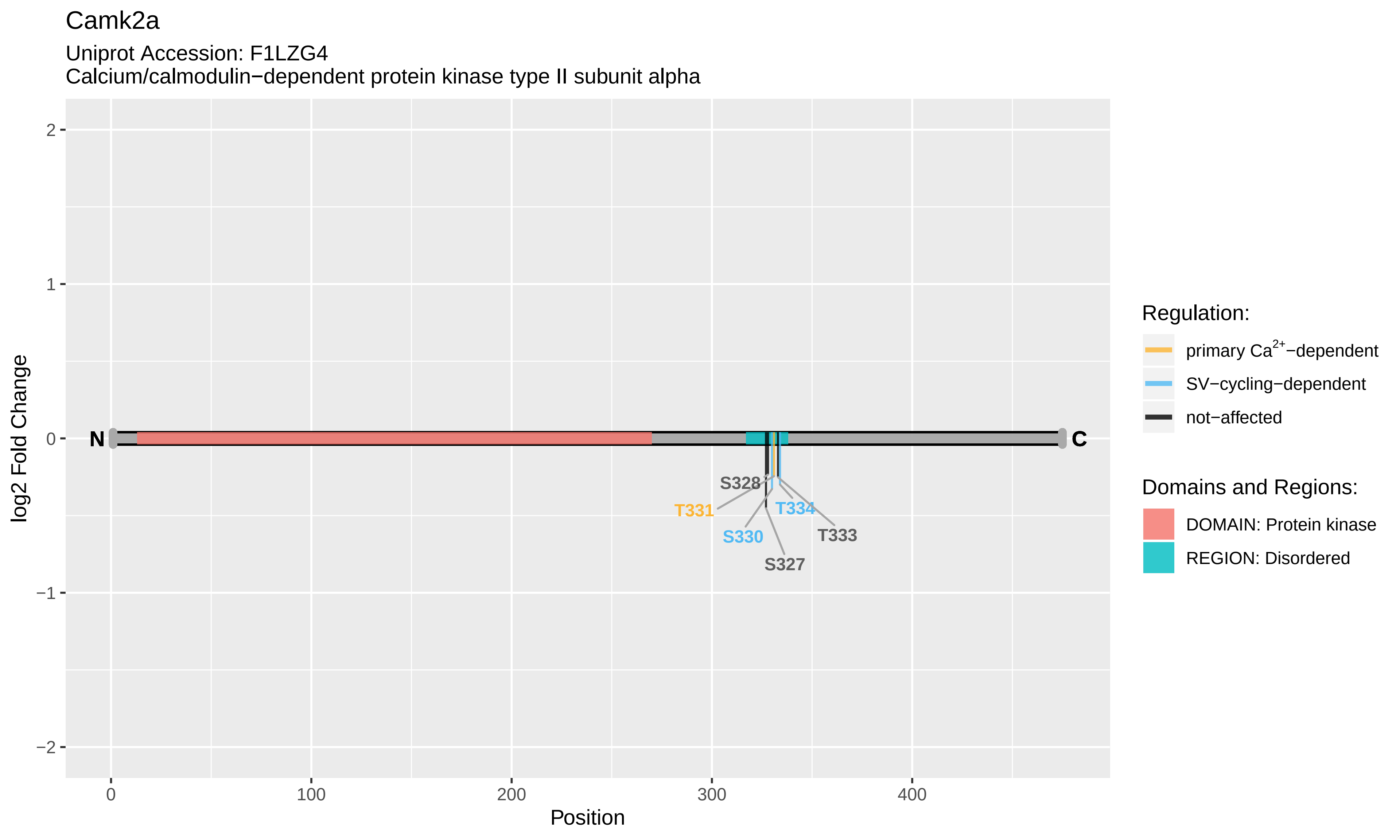

Camk2a
Uniprot Accession: F1LZG4
Calcium/calmodulin−dependent protein kinase type II subunit alpha
log2 Fold Change
2
1
0
−1
−2
N
C
S328
T331
T334
S330
T333
S327
0
100
200
300
400
Position
Regulation:
primary Ca2+−dependent
SV−cycling−dependent
not−affected
Domains and Regions:
DOMAIN: Protein kinase
REGION: Disordered

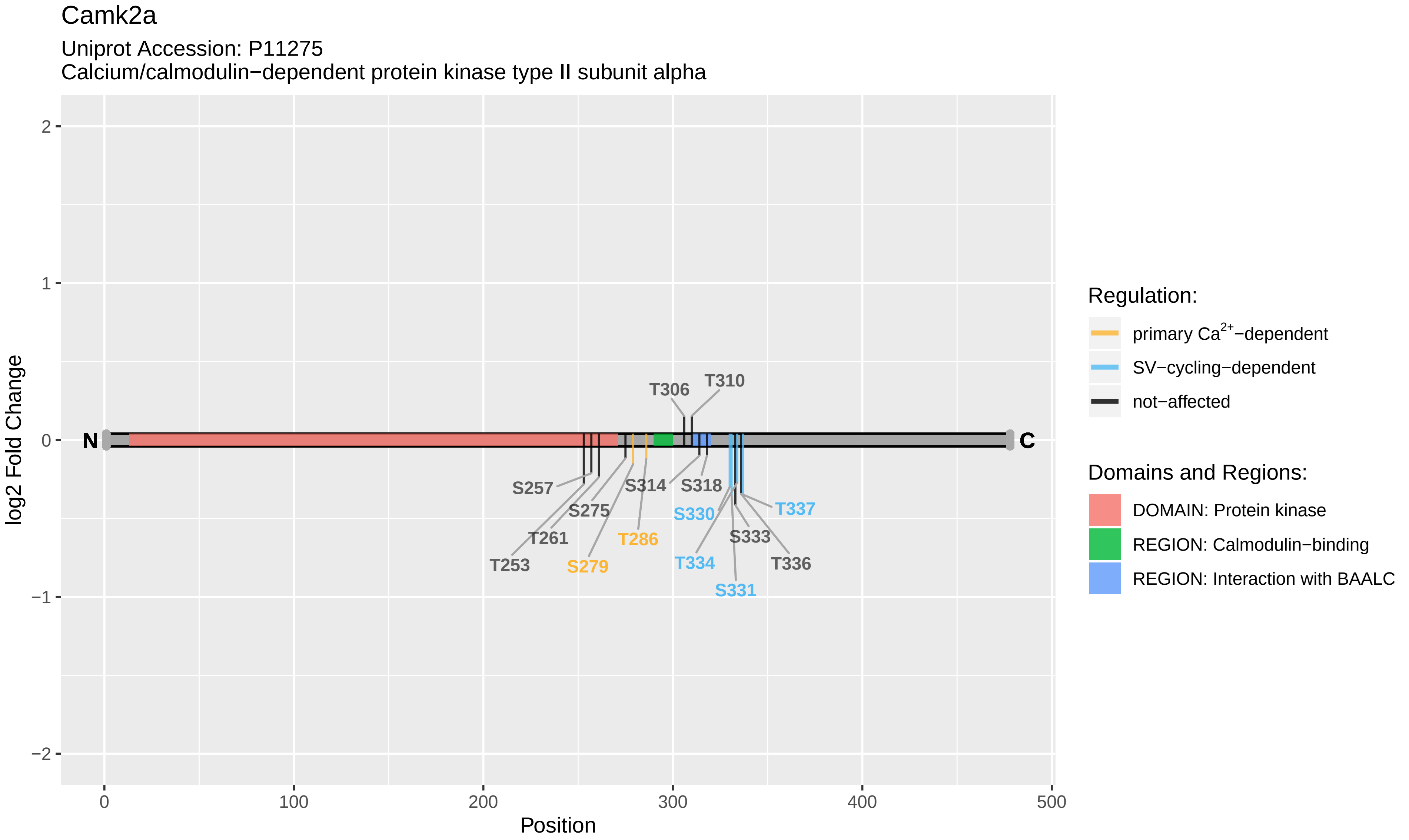

Camk2a
Uniprot Accession: P11275
Calcium/calmodulin−dependent protein kinase type II subunit alpha
log2 Fold Change
Position
N
C
T306
T310
S257
S314
S318
S275
S330
T337
T261
T286
S333
T253
S279
T334
T336
S331
Regulation:
primary Ca2+−dependent
SV−cycling−dependent
not−affected
Domains and Regions:
DOMAIN: Protein kinase
REGION: Calmodulin−binding
REGION: Interaction with BAALC

Camk2b

Uniprot Accession: F1LNI8
Calcium/calmodulin−dependent protein kinase type II subunit beta

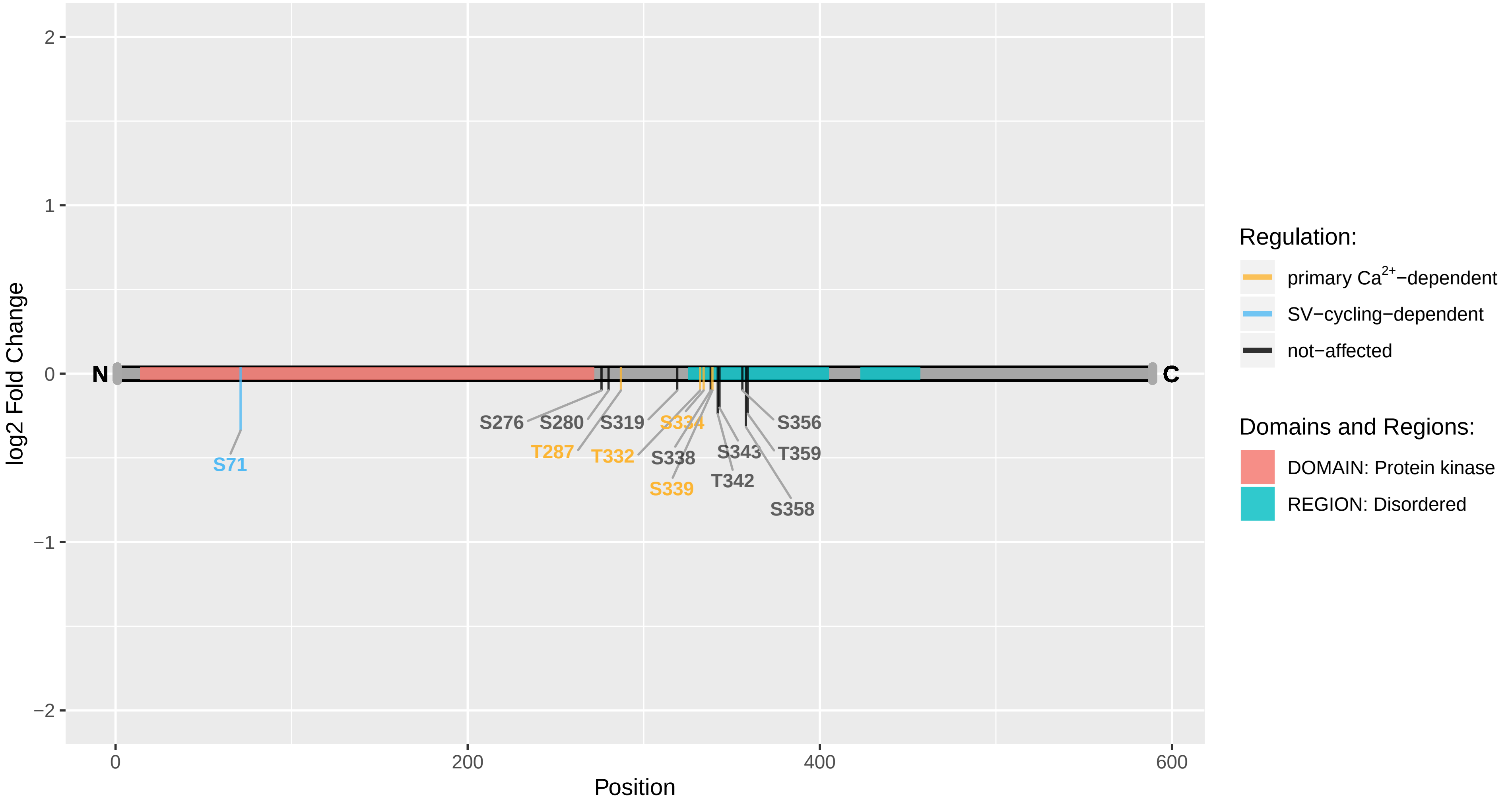

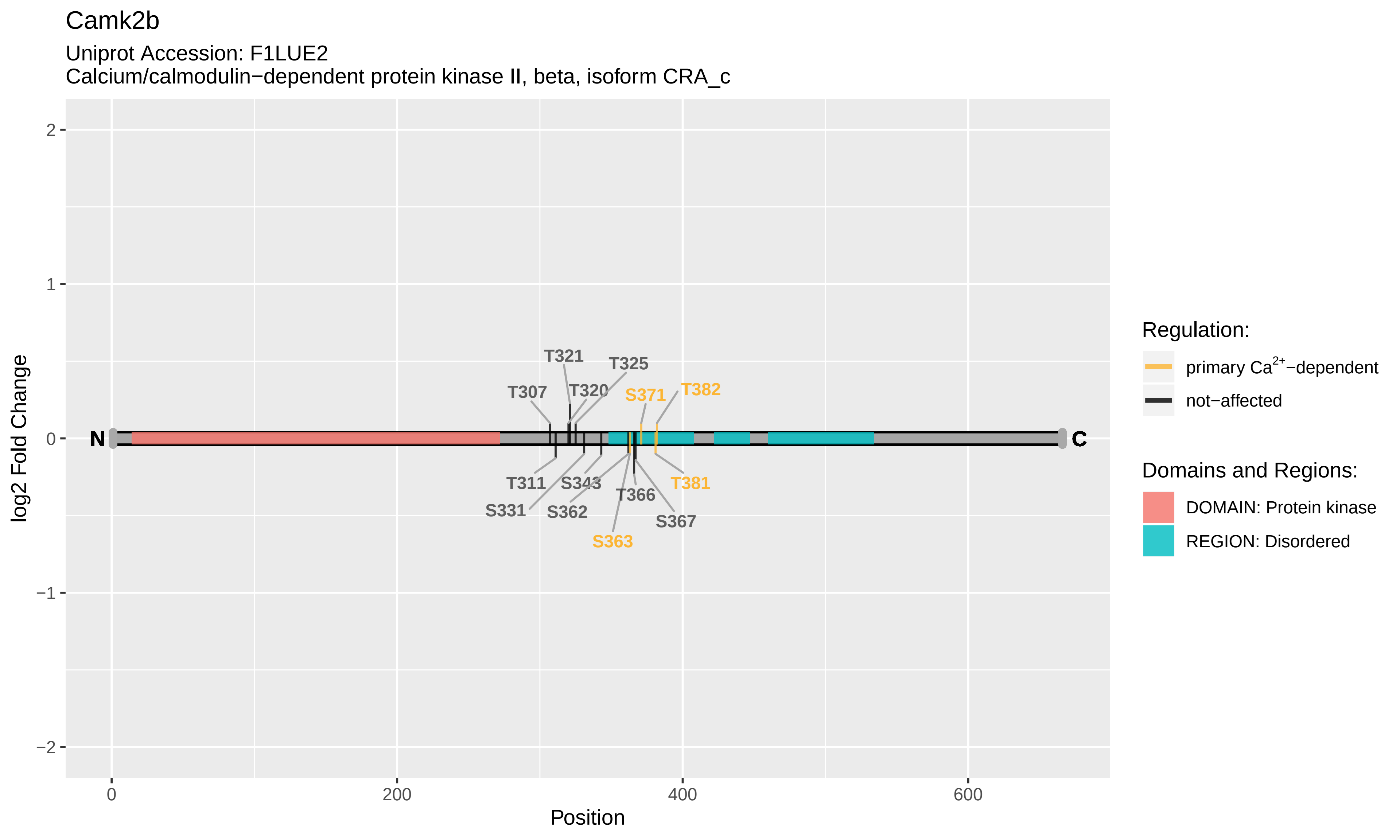
Camk2b
Uniprot Accession: F1LUE2
Calcium/calmodulin−dependent protein kinase II, beta, isoform CRA_c
log2 Fold Change
Position
N
C
T321
T325
T307
T320
S371
T382
T311
S343
T381
T366
S331
S362
S367
S363
Regulation:
primary Ca2+−dependent
not−affected
Domains and Regions:
DOMAIN: Protein kinase
REGION: Disordered

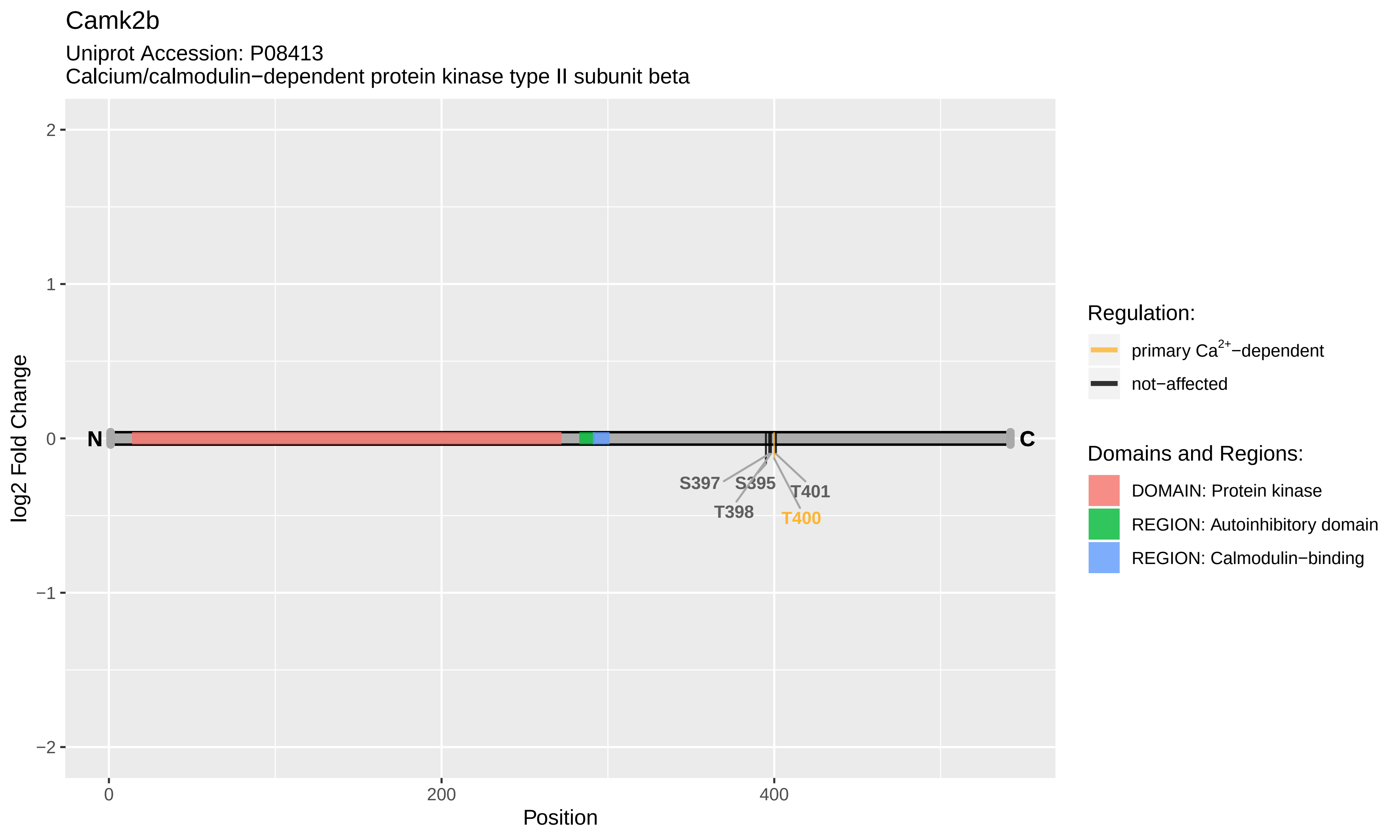

Camk2b
Uniprot Accession: P08413
Calcium/calmodulin−dependent protein kinase type II subunit beta
log2 Fold Change
2
1
0
−1
−2
N
C
S397
S395
T401
T398
T400
0
200
400
Position
Regulation:
primary Ca2+−dependent
not−affected
Domains and Regions:
DOMAIN: Protein kinase
REGION: Autoinhibitory domain
REGION: Calmodulin−binding

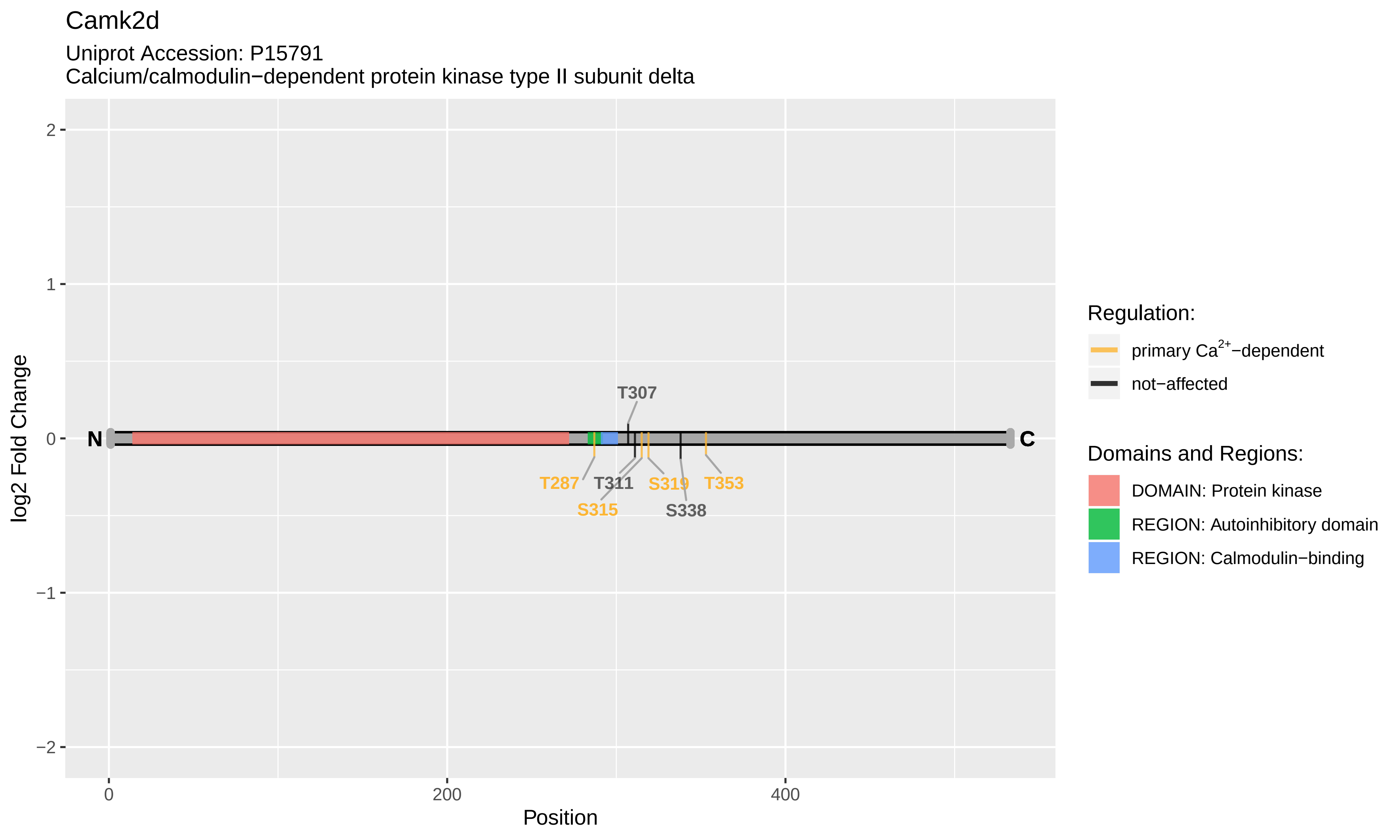
Camk2d
Uniprot Accession: P15791
Calcium/calmodulin−dependent protein kinase type II subunit delta
log2 Fold Change
2
1
0
−1
−2
N
C
T307
T287
T311
S319
T353
S315
S338
0
200
400
Position
Regulation:
primary Ca2+−dependent
not−affected
Domains and Regions:
DOMAIN: Protein kinase
REGION: Autoinhibitory domain
REGION: Calmodulin−binding

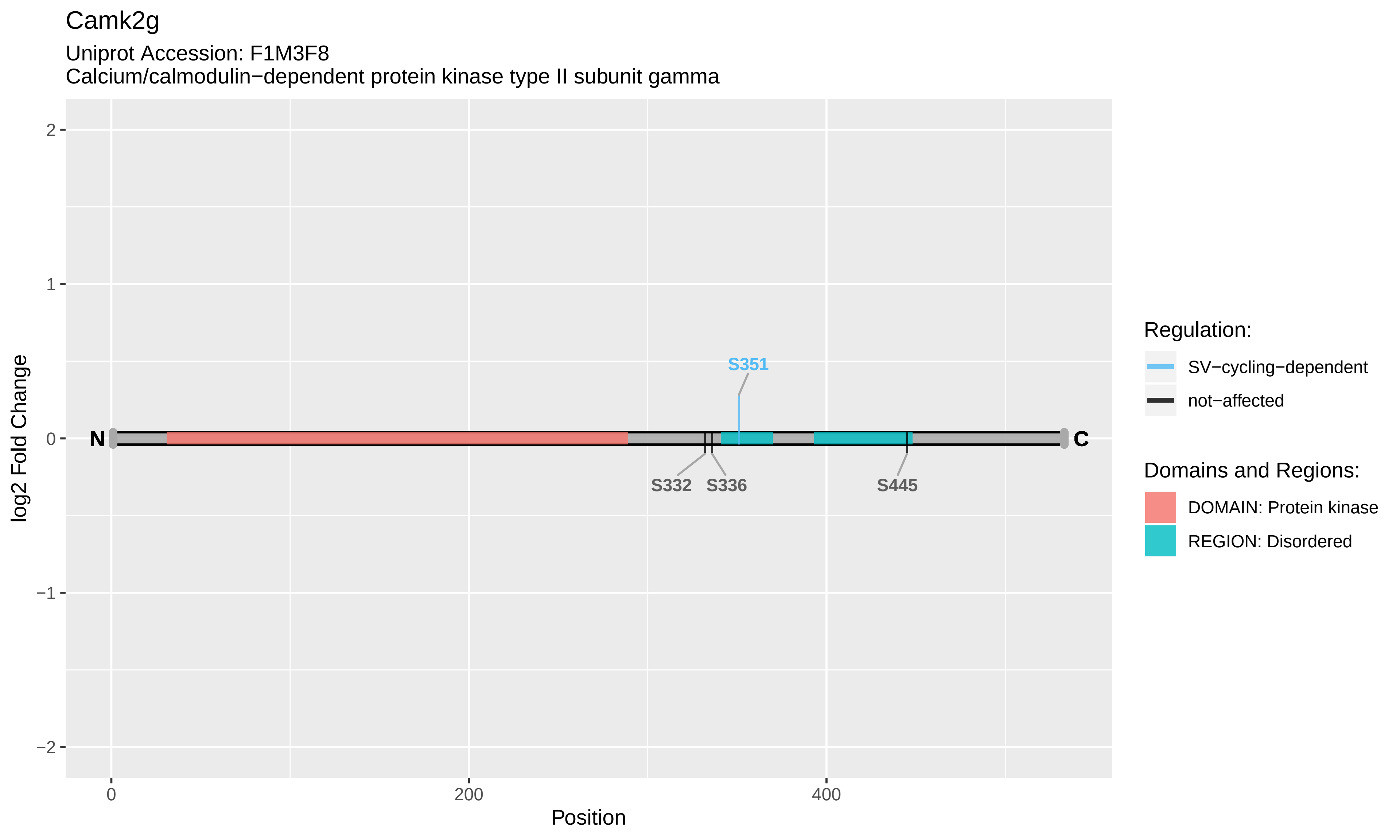
Camk2g
Uniprot Accession: F1M3F8
Calcium/calmodulin−dependent protein kinase type II subunit gamma
log2 Fold Change
2
1
0
−1
−2
N
C
S351
S332
S336
S445
0
200
400
Position
Regulation:
SV−cycling−dependent
not−affected
Domains and Regions:
DOMAIN: Protein kinase
REGION: Disordered

Camk2g

Uniprot Accession: P11730
Calcium/calmodulin−dependent protein kinase type II subunit gamma

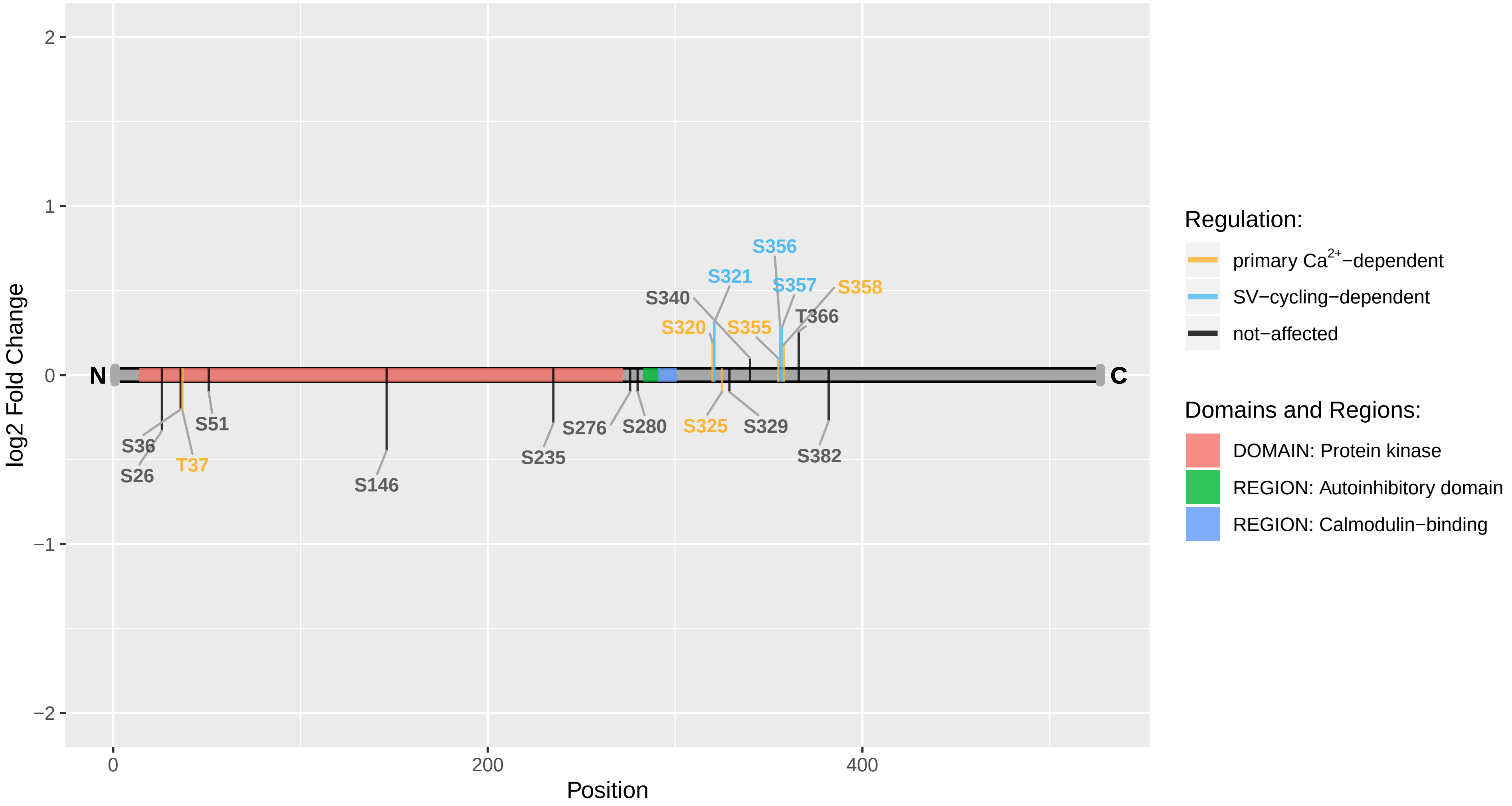

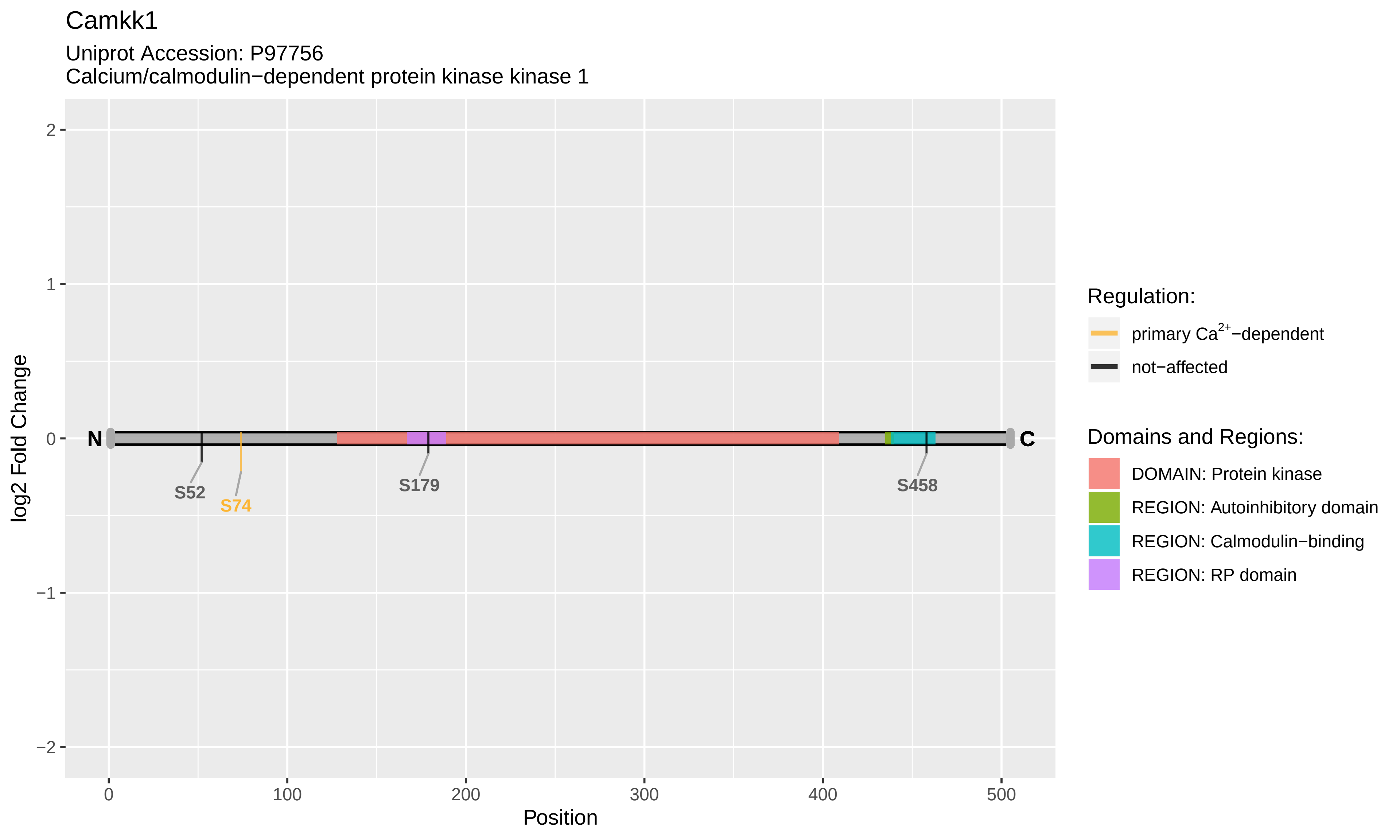
Camkk1
Uniprot Accession: P97756
Calcium/calmodulin−dependent protein kinase kinase 1
log2 Fold Change
Position
N
C
S52
S74
S179
S458
Regulation:
primary Ca2+−dependent
not−affected
Domains and Regions:
DOMAIN: Protein kinase
REGION: Autoinhibitory domain
REGION: Calmodulin−binding
REGION: RP domain

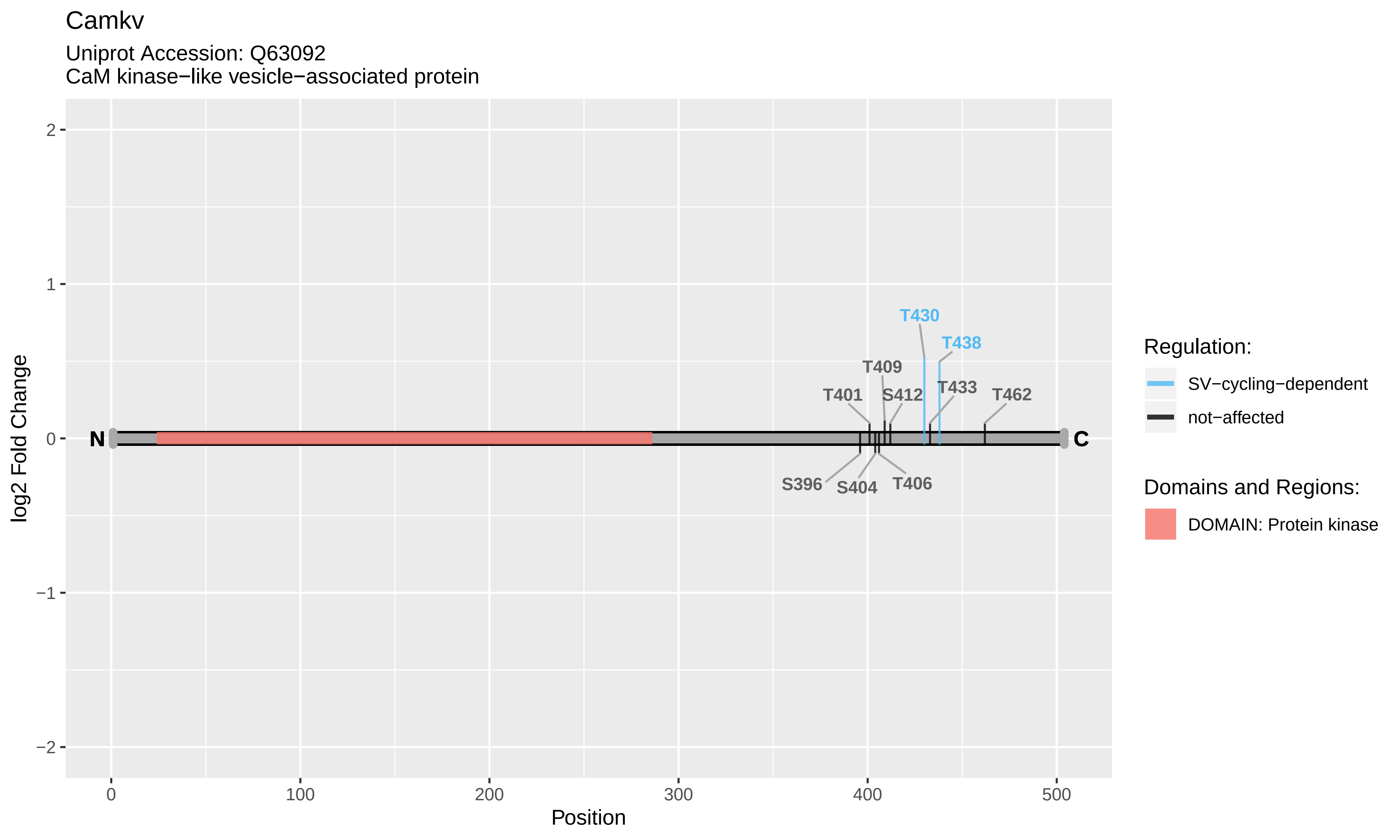
Camkv
Uniprot Accession: Q63092
CaM kinase−like vesicle−associated protein
log2 Fold Change
2
1
0
−1
−2
N
C
T430
T438
T409
T401
S412
T433
T462
S396
S404
T406
0
100
200
300
400
500
Position
Regulation:
SV−cycling−dependent
not−affected
Domains and Regions:
DOMAIN: Protein kinase

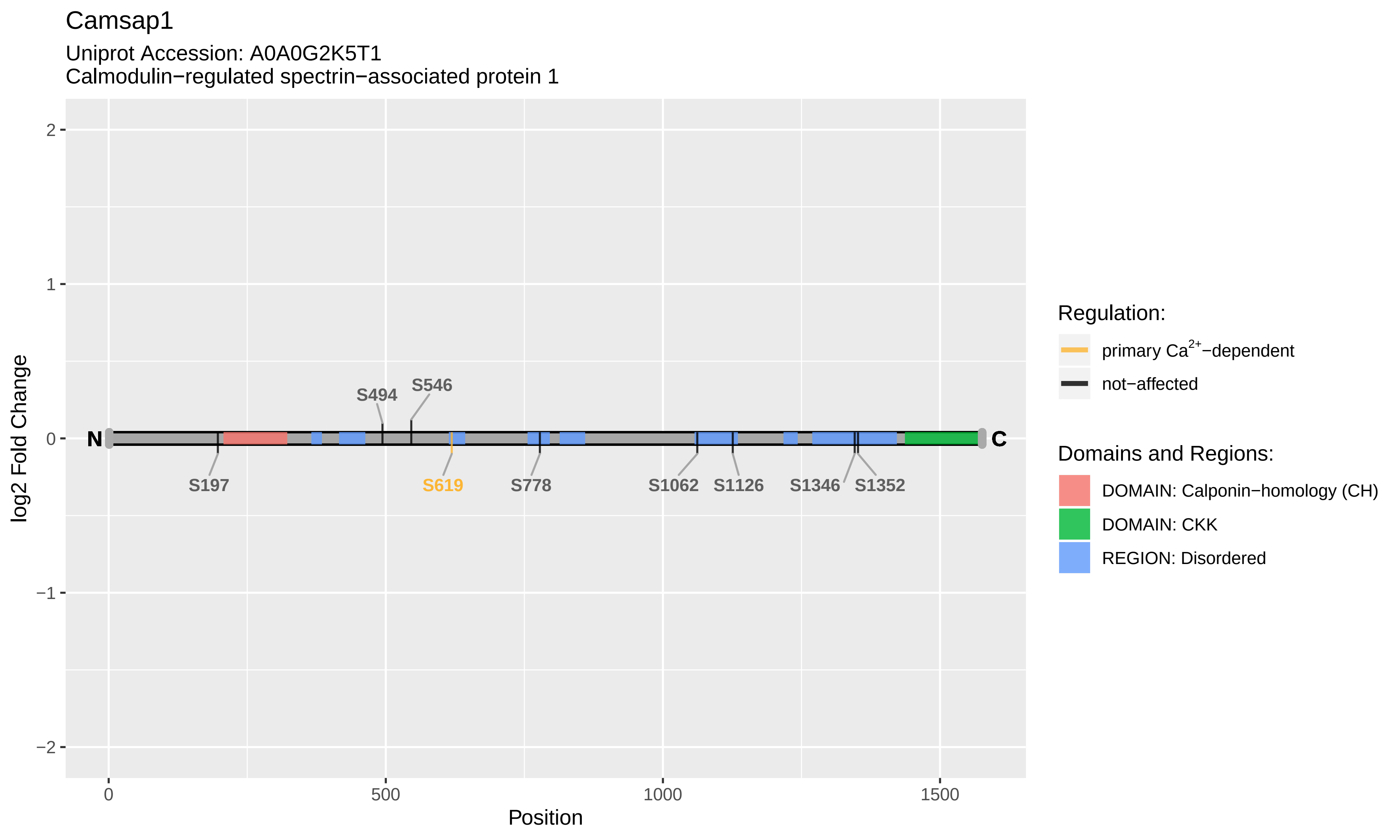
Camsap1
Uniprot Accession: A0A0G2K5T1
Calmodulin−regulated spectrin−associated protein 1
log2 Fold Change
2
1
0
−1
−2
N
C
S494
S546
S197
S619
S778
S1062
S1126
S1346
S1352
0
500
1000
1500
Position
Regulation:
primary Ca2+−dependent
not−affected
Domains and Regions:
DOMAIN: Calponin−homology (CH)
DOMAIN: CKK
REGION: Disordered

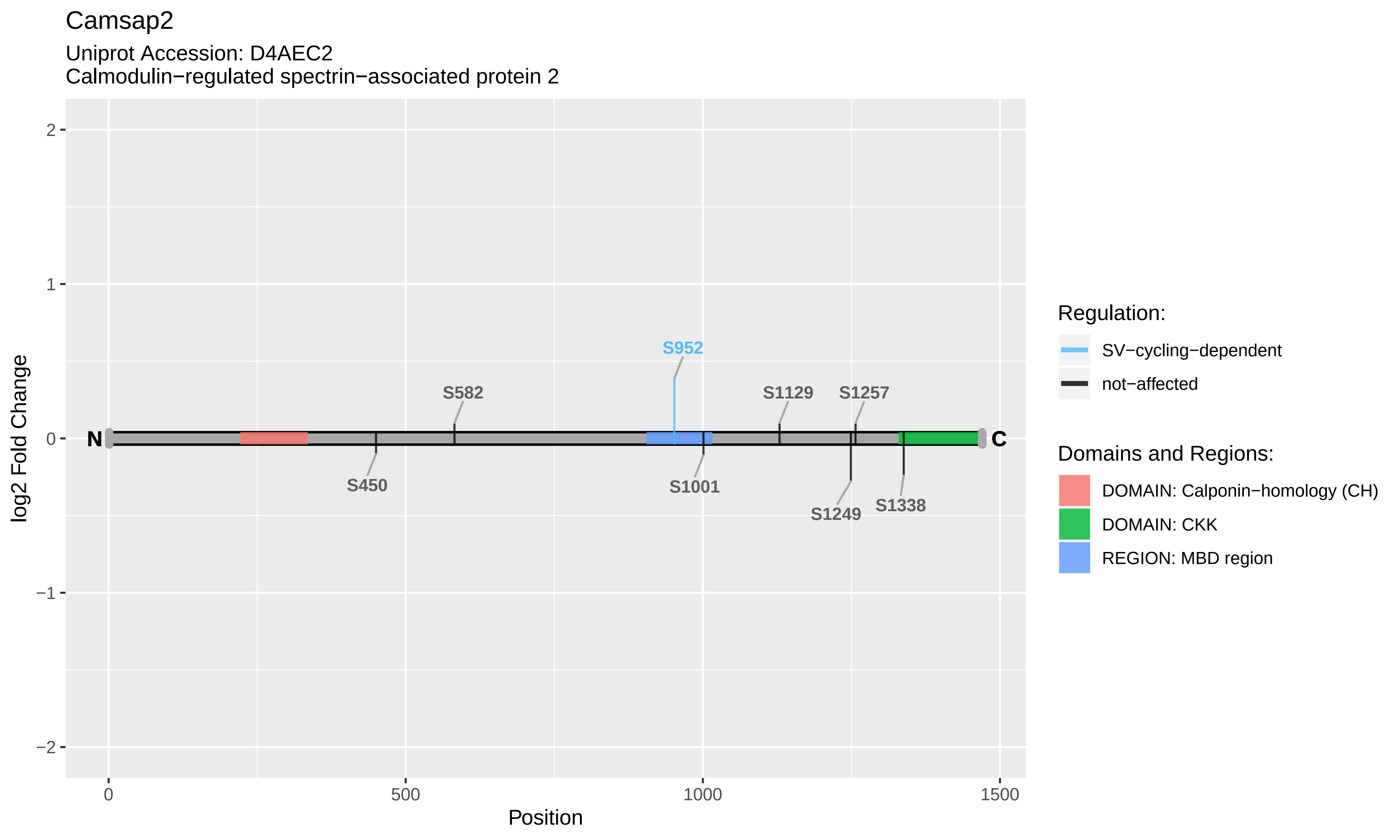

Camsap2
Uniprot Accession: D4AEC2
Calmodulin−regulated spectrin−associated protein 2
log2 Fold Change
Position
N
C
S952
S582
S1129
S1257
S450
S1001
S1249
S1338
Regulation:
SV−cycling−dependent
not−affected
Domains and Regions:
DOMAIN: Calponin−homology (CH)
DOMAIN: CKK
REGION: MBD region

Camsap3

Uniprot Accession: D3ZXQ2
Calmodulin−regulated spectrin−associated protein family, member 3

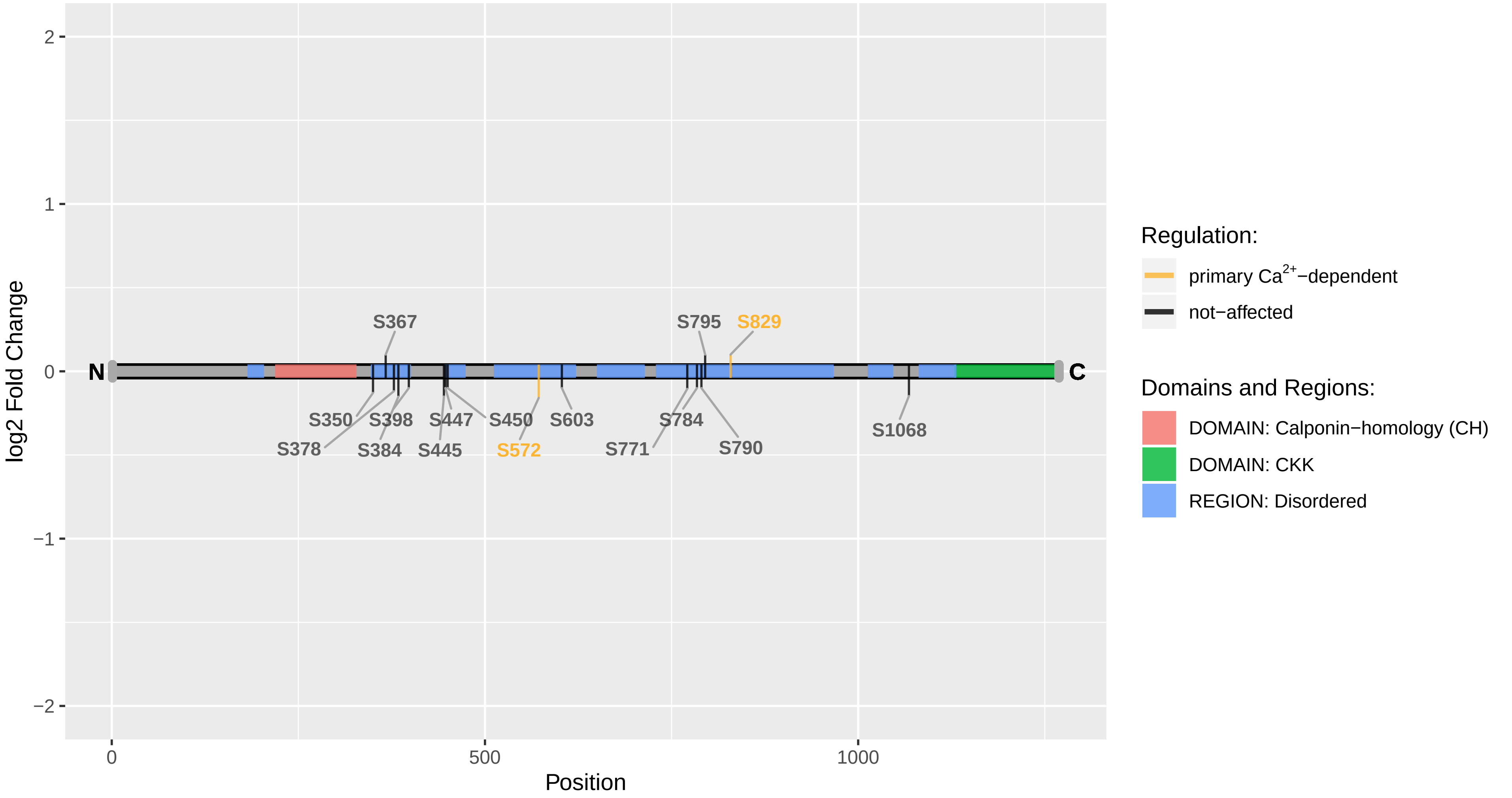

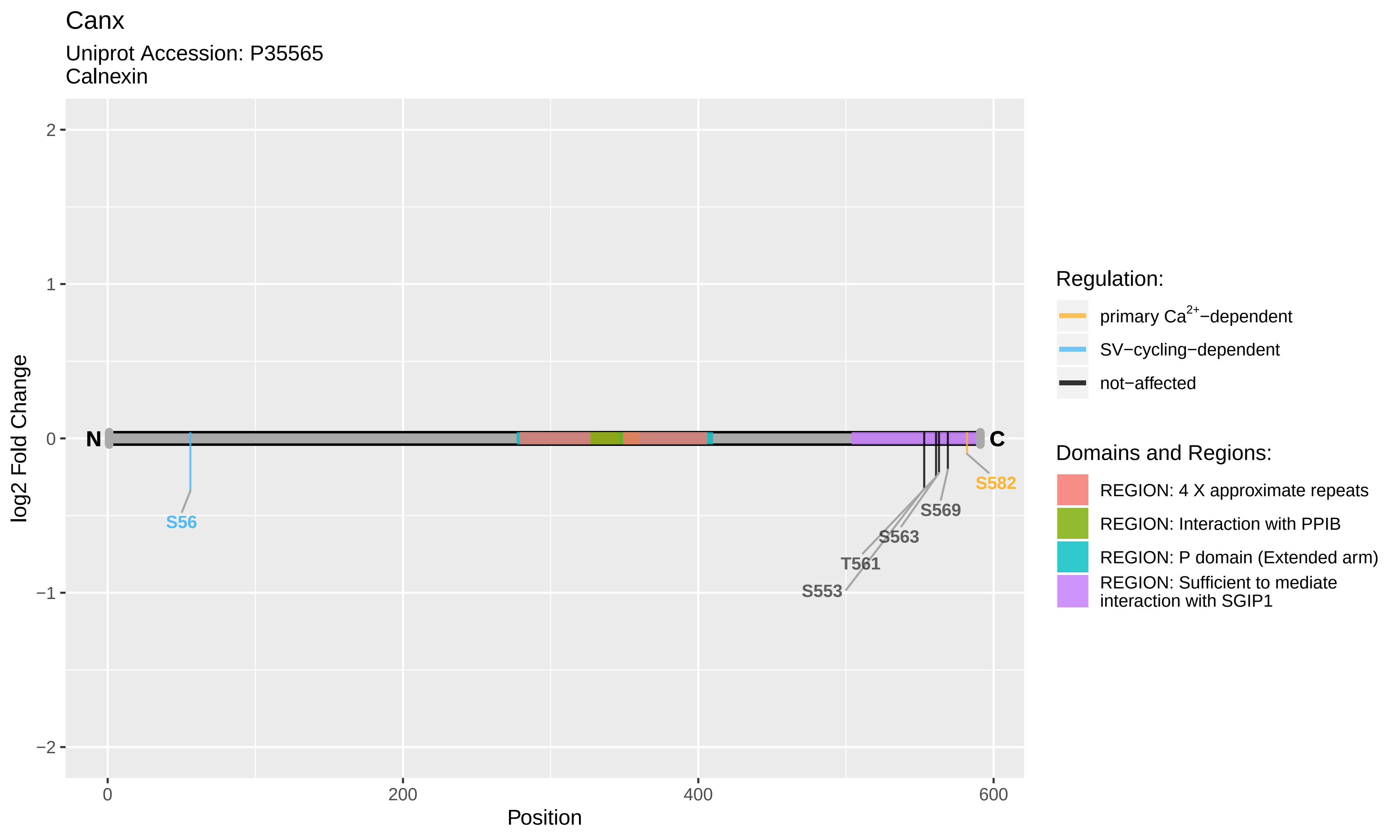

Canx
Uniprot Accession: P35565
Calnexin
log2 Fold Change
2
1
0
−1
−2
N
C
S56
S582
S569
S563
T561
S553
0
200
400
600
Position
Regulation:
primary Ca2+−dependent
SV−cycling−dependent
not−affected
Domains and Regions:
REGION: 4 X approximate repeats
REGION: Interaction with PPIB
REGION: P domain (Extended arm)
REGION: Sufficient to mediate interaction with SGIP1

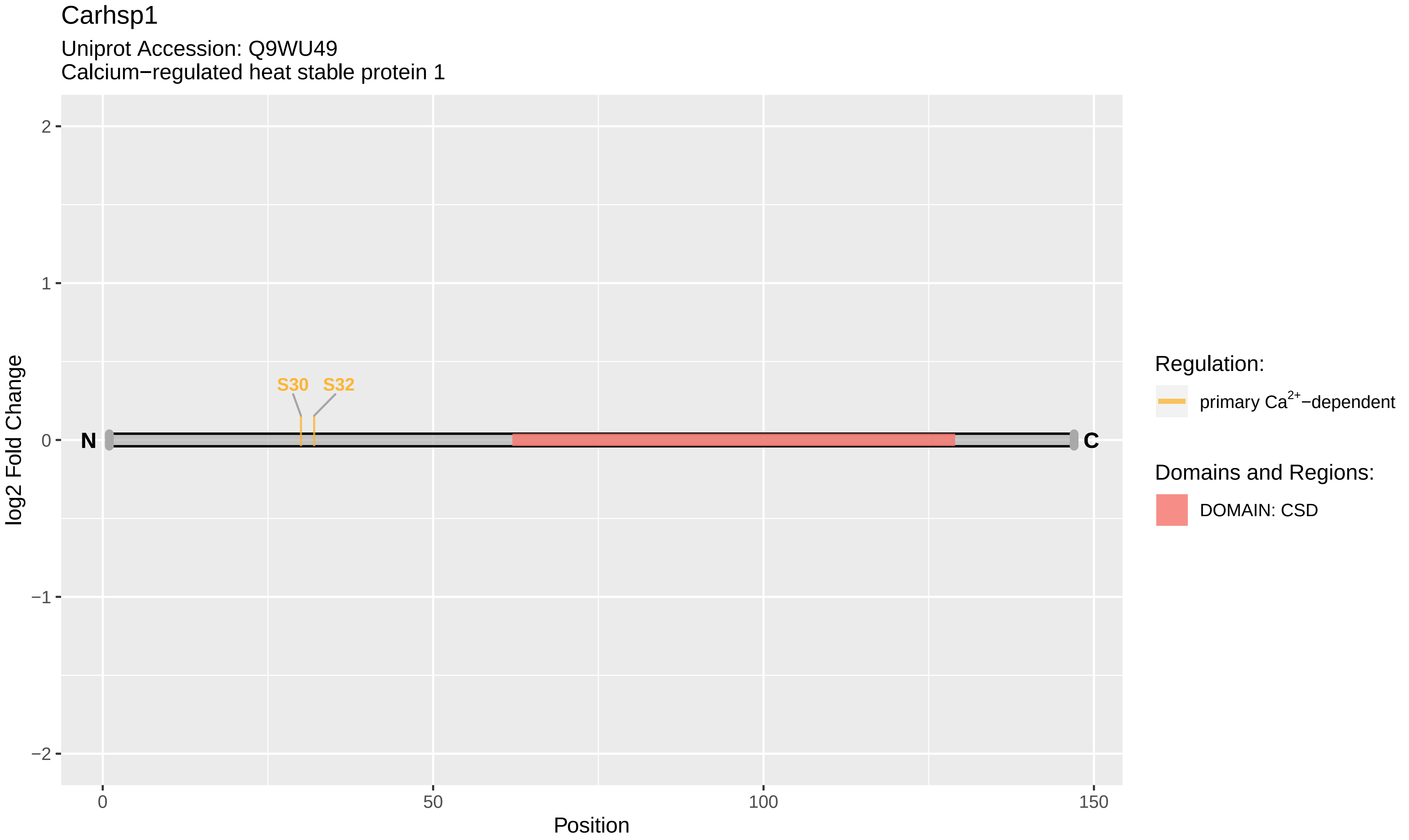

Carhsp1
Uniprot Accession: Q9WU49
Calcium−regulated heat stable protein 1
log2 Fold Change
2
1
0
−1
−2
0
50
100
150
Position
N
C
S30
S32
Regulation:
primary Ca2+−dependent
Domains and Regions:
DOMAIN: CSD

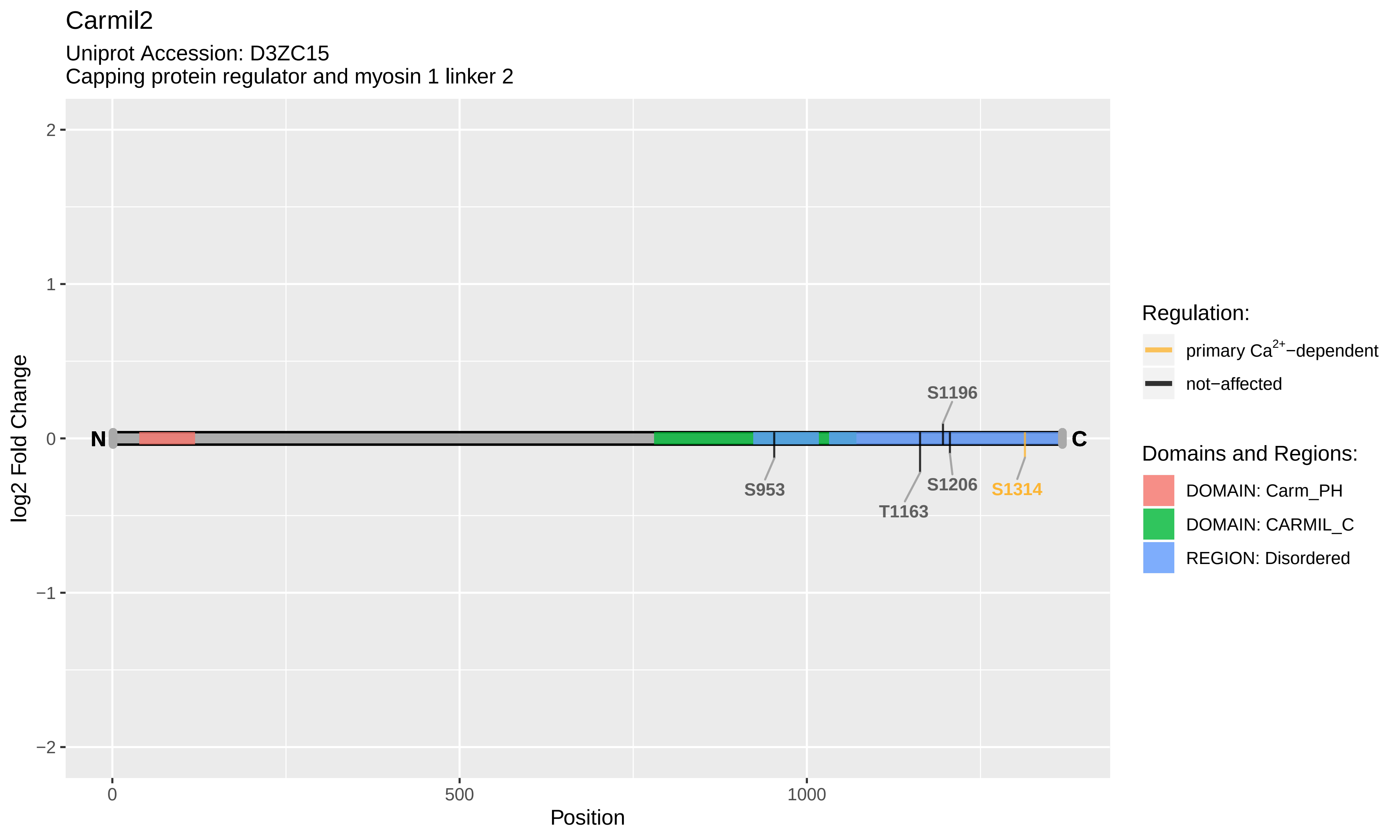
Carmil2
Uniprot Accession: D3ZC15
Capping protein regulator and myosin 1 linker 2
log2 Fold Change
2
1
0
−1
−2
N
C
S1196
S953
T1163
S1206
S1314
0
500
1000
Position
Regulation:
primary Ca2+−dependent
not−affected
Domains and Regions:
DOMAIN: Carm_PH
DOMAIN: CARMIL_C
REGION: Disordered

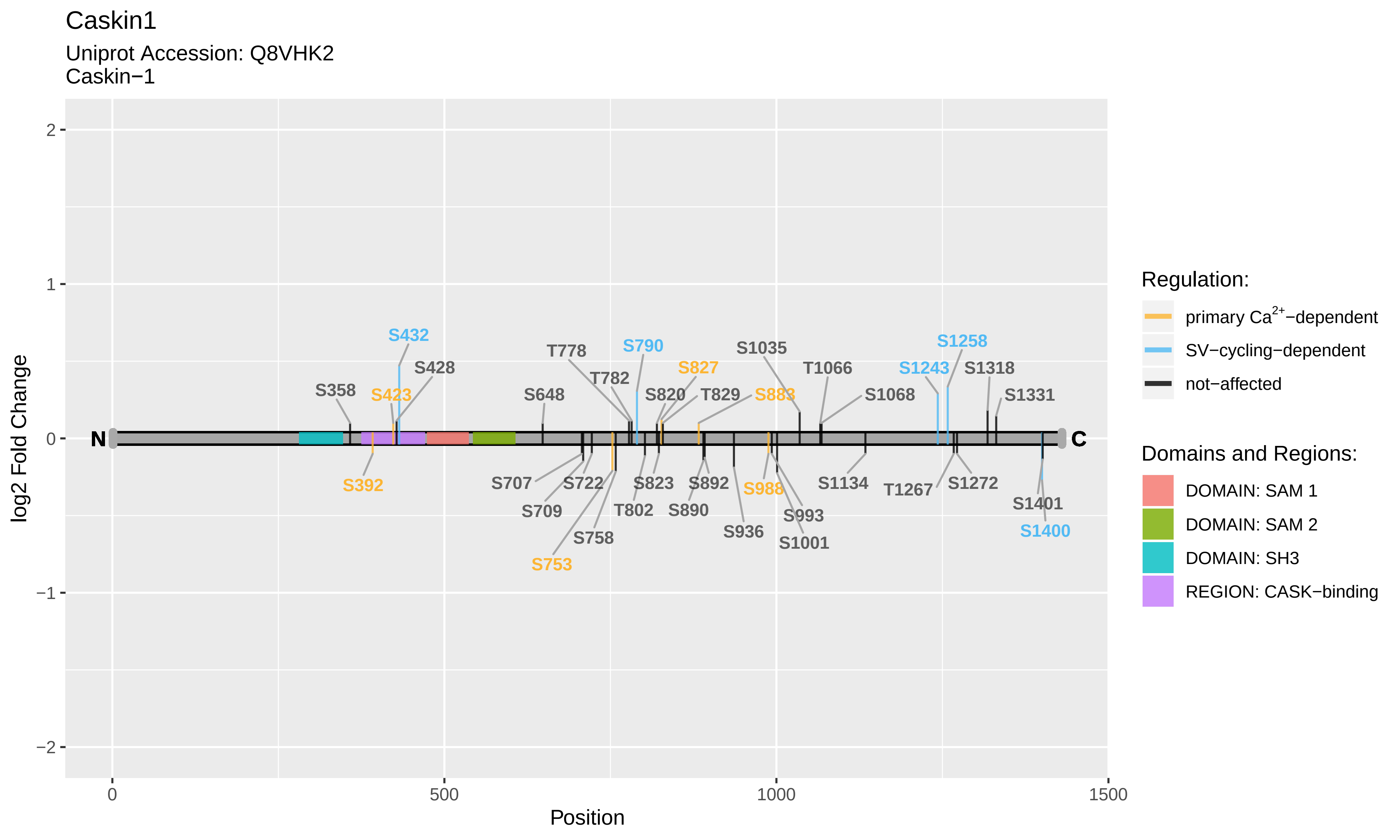
Caskin1
Uniprot Accession: Q8VHK2
Caskin−1
log2 Fold Change
Position
2
1
0
−1
−2
0
500
1000
1500
N
C
S432
S428
S358
S423
S392
S648
T778
S790
T782
S827
S820
T829
S883
S1035
T1066
S1068
S1258
S1243
S1318
S1331
S707
S709
S722
S753
S758
T802
S823
S890
S892
S936
S988
S993
S1001
S1134
T1267
S1272
S1401
S1400
Regulation:
primary Ca2+−dependent
SV−cycling−dependent
not−affected
Domains and Regions:
DOMAIN: SAM 1
DOMAIN: SAM 2
DOMAIN: SH3
REGION: CASK−binding

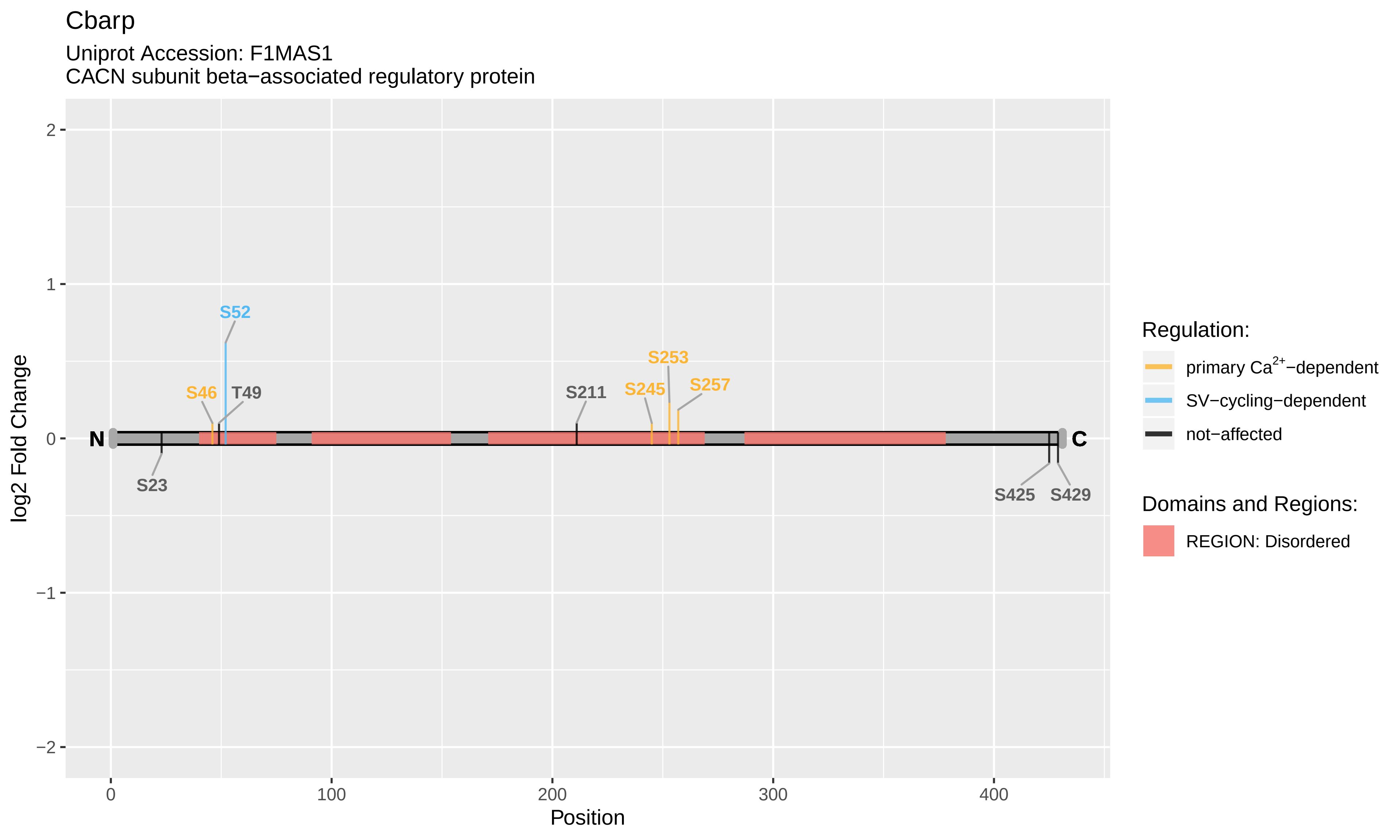

Cbarp
Uniprot Accession: F1MAS1
CACN subunit beta−associated regulatory protein
log2 Fold Change
Position
N
C
S52
S46
T49
S23
S211
S245
S253
S257
S425
S429
Regulation:
primary Ca2+−dependent
SV−cycling−dependent
not−affected
Domains and Regions:
REGION: Disordered
2
1
0
−1
−2
0
100
200
300
400

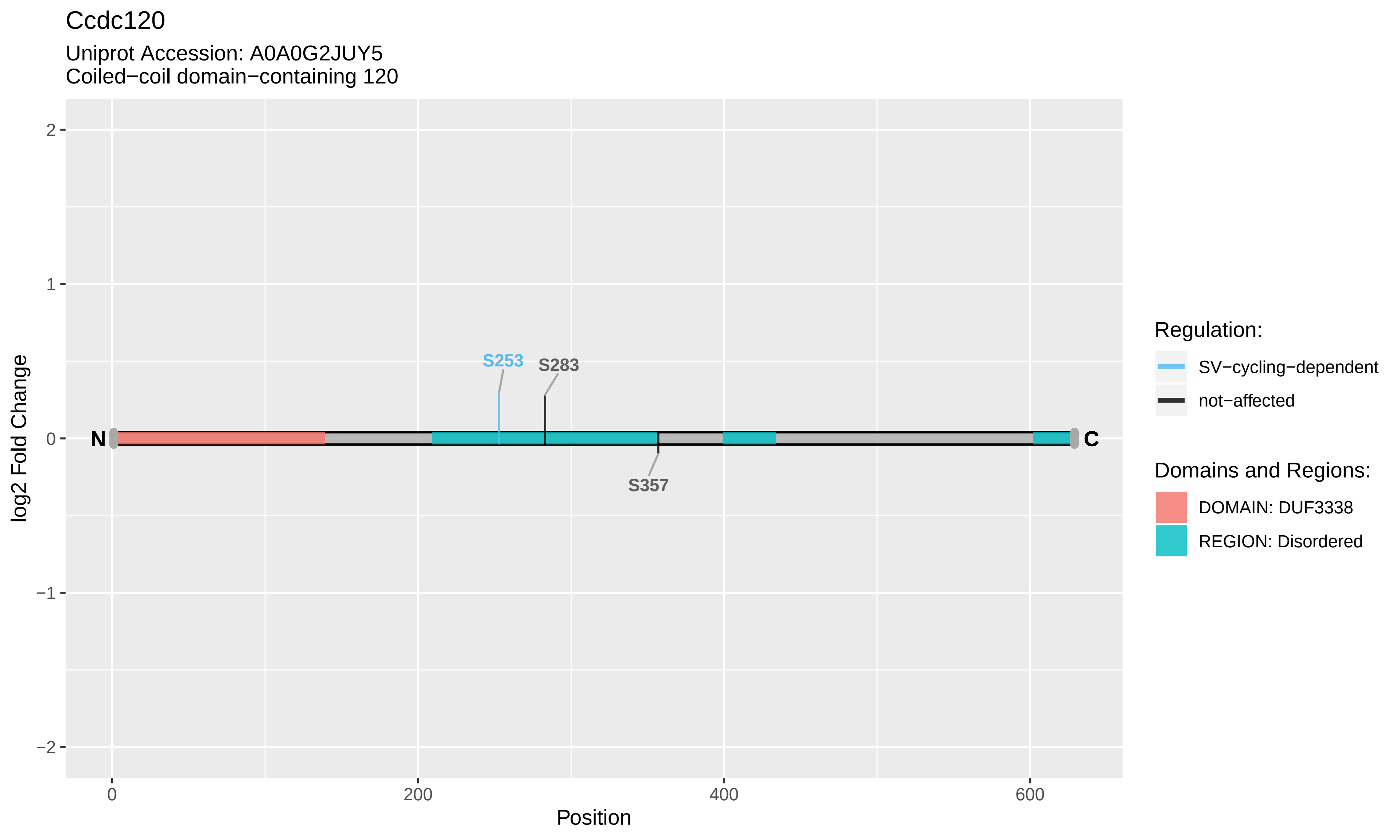

Ccdc120
Uniprot Accession: A0A0G2JUY5
Coiled−coil domain−containing 120
log2 Fold Change
2
1
0
−1
−2
N
C
S253
S283
S357
0
200
400
600
Position
Regulation:
SV−cycling−dependent
not−affected
Domains and Regions:
DOMAIN: DUF3338
REGION: Disordered

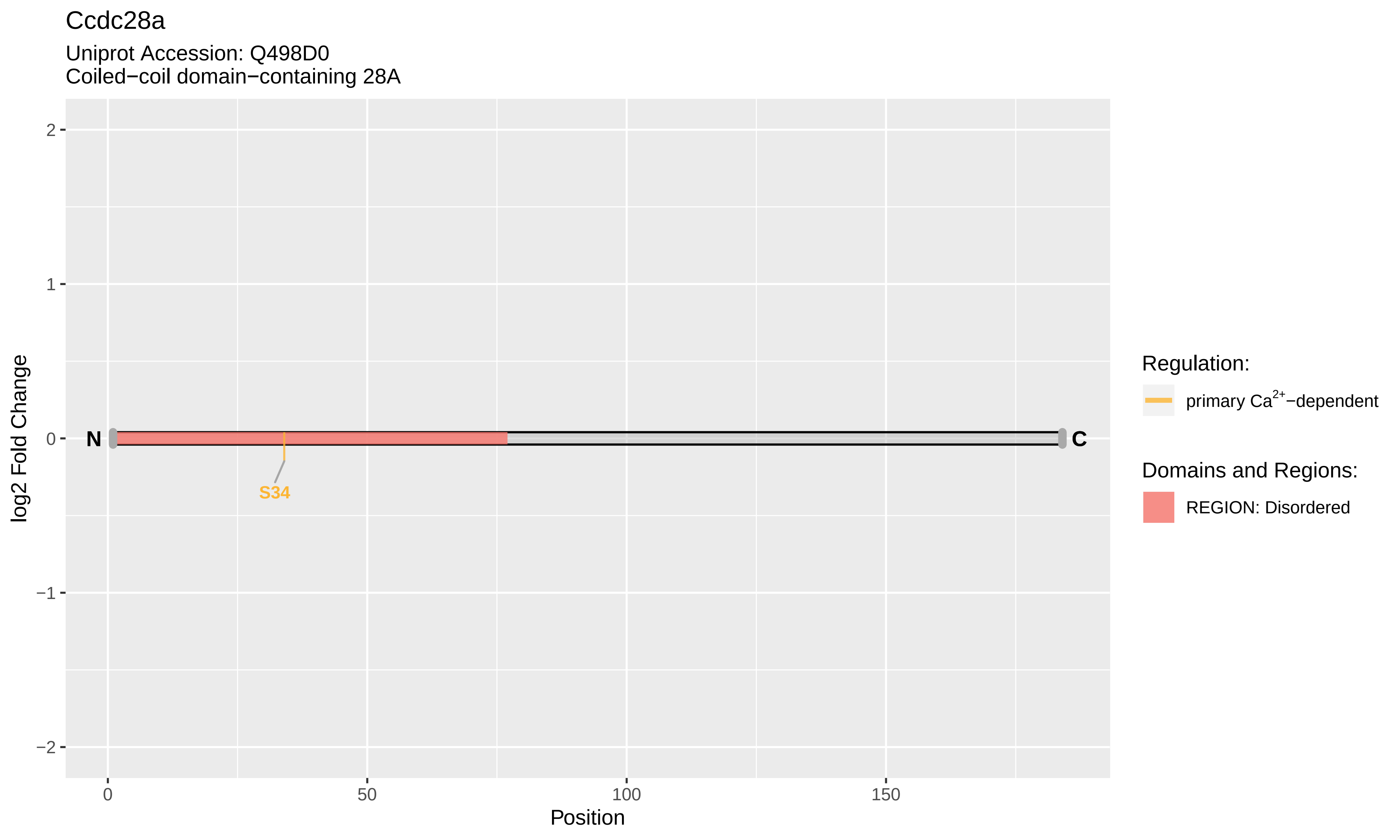
Ccdc28a
Uniprot Accession: Q498D0
Coiled−coil domain−containing 28A
log2 Fold Change
Position
N
C
S34
Regulation:
primary Ca2+−dependent
Domains and Regions:
REGION: Disordered

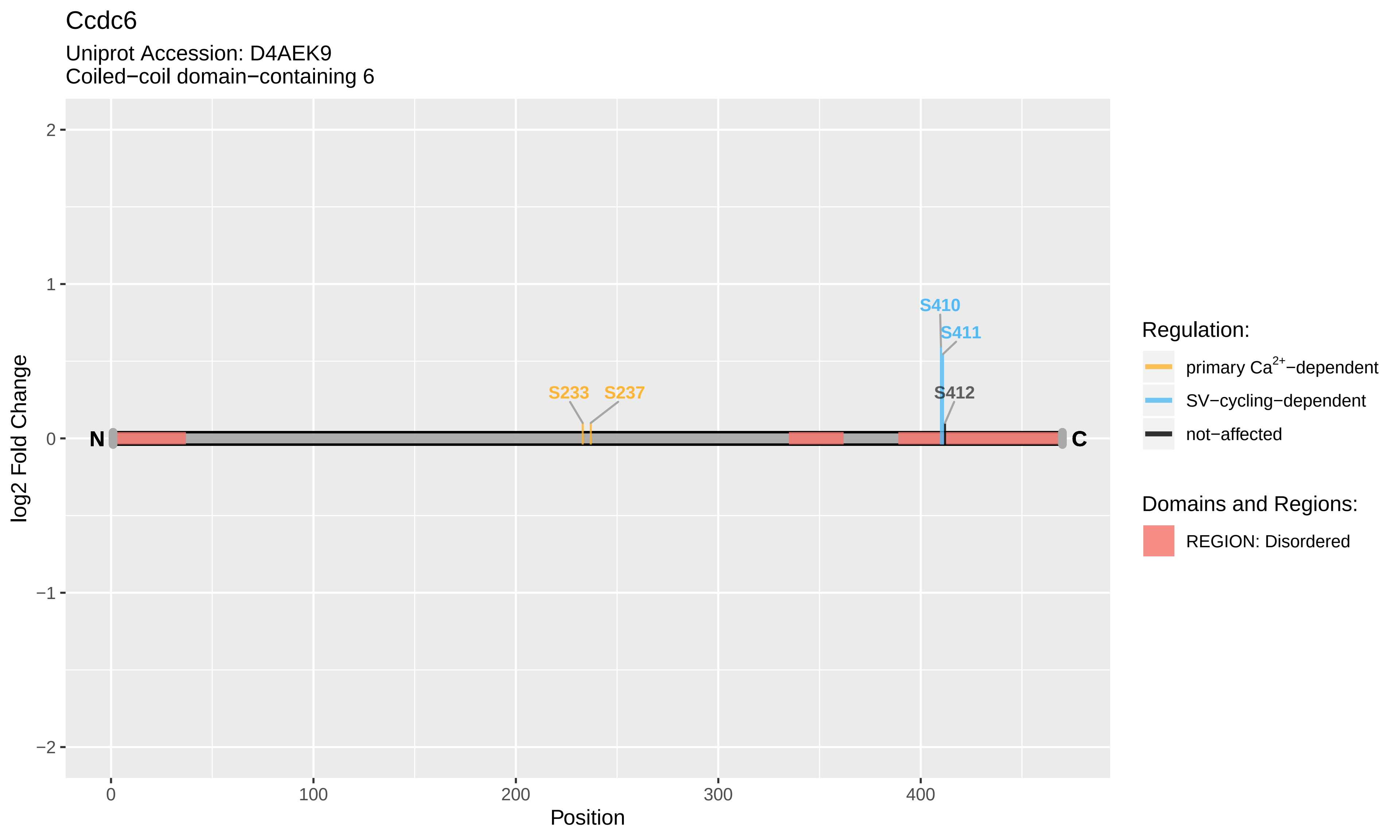

Ccdc6
Uniprot Accession: D4AEK9
Coiled−coil domain−containing 6
S410
S411
S233
S237
S412
N
C
2
1
0
−1
−2
log2 Fold Change
0
100
200
300
400
Position
Regulation:
primary Ca2+−dependent
SV−cycling−dependent
not−affected
Domains and Regions:
REGION: Disordered

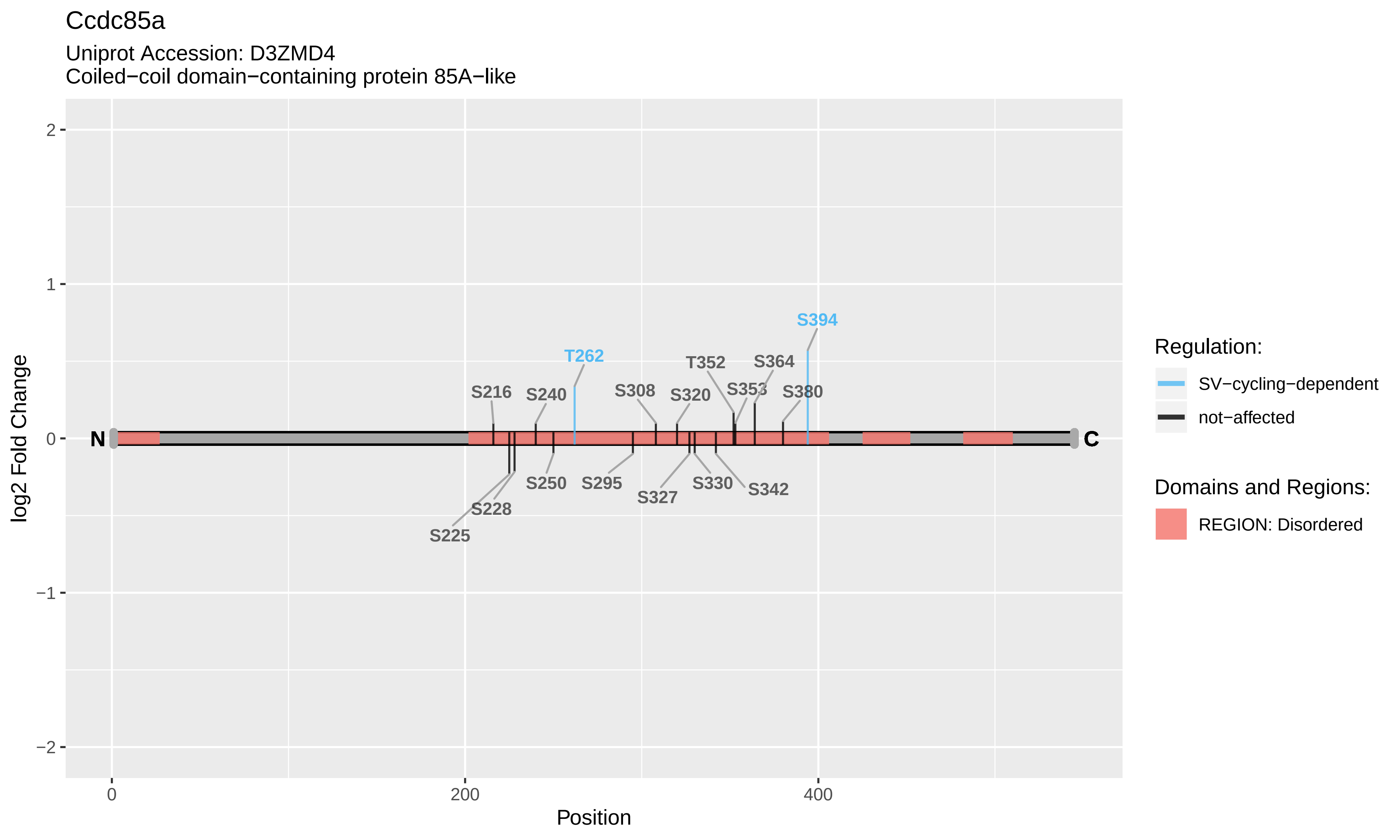

Ccdc85a
Uniprot Accession: D3ZMD4
Coiled−coil domain−containing protein 85A−like
log2 Fold Change
Position
2
1
0
−1
−2
0
200
400
N
C
T262
S394
S216
S240
S308
S320
T352
S353
S364
S380
S225
S228
S250
S295
S327
S330
S342
Regulation:
SV−cycling−dependent
not−affected
Domains and Regions:
REGION: Disordered

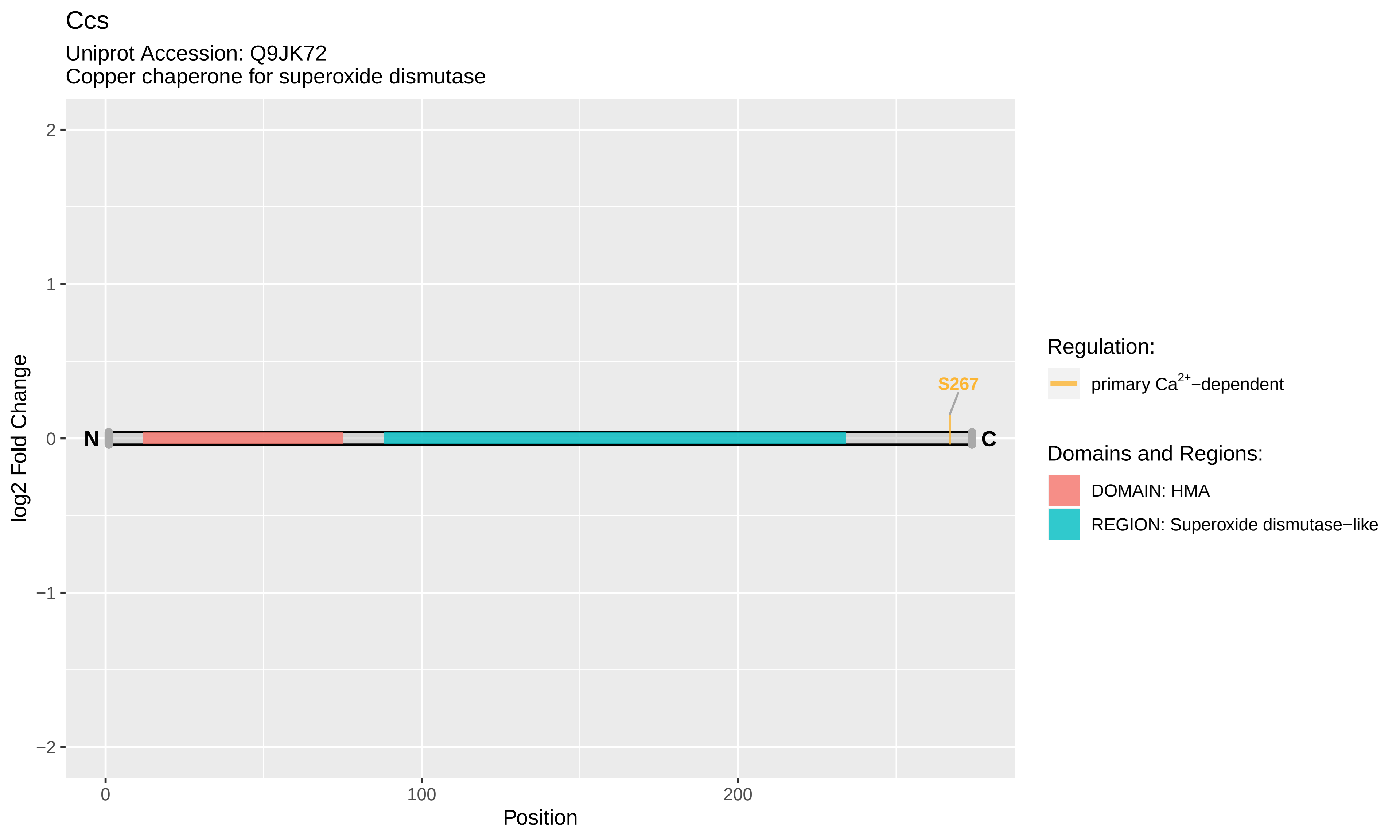
Ccs
Uniprot Accession: Q9JK72
Copper chaperone for superoxide dismutase
log2 Fold Change
2
1
0
−1
−2
0
100
200
Position
N
C
S267
Regulation:
primary Ca2+−dependent
Domains and Regions:
DOMAIN: HMA
REGION: Superoxide dismutase−like

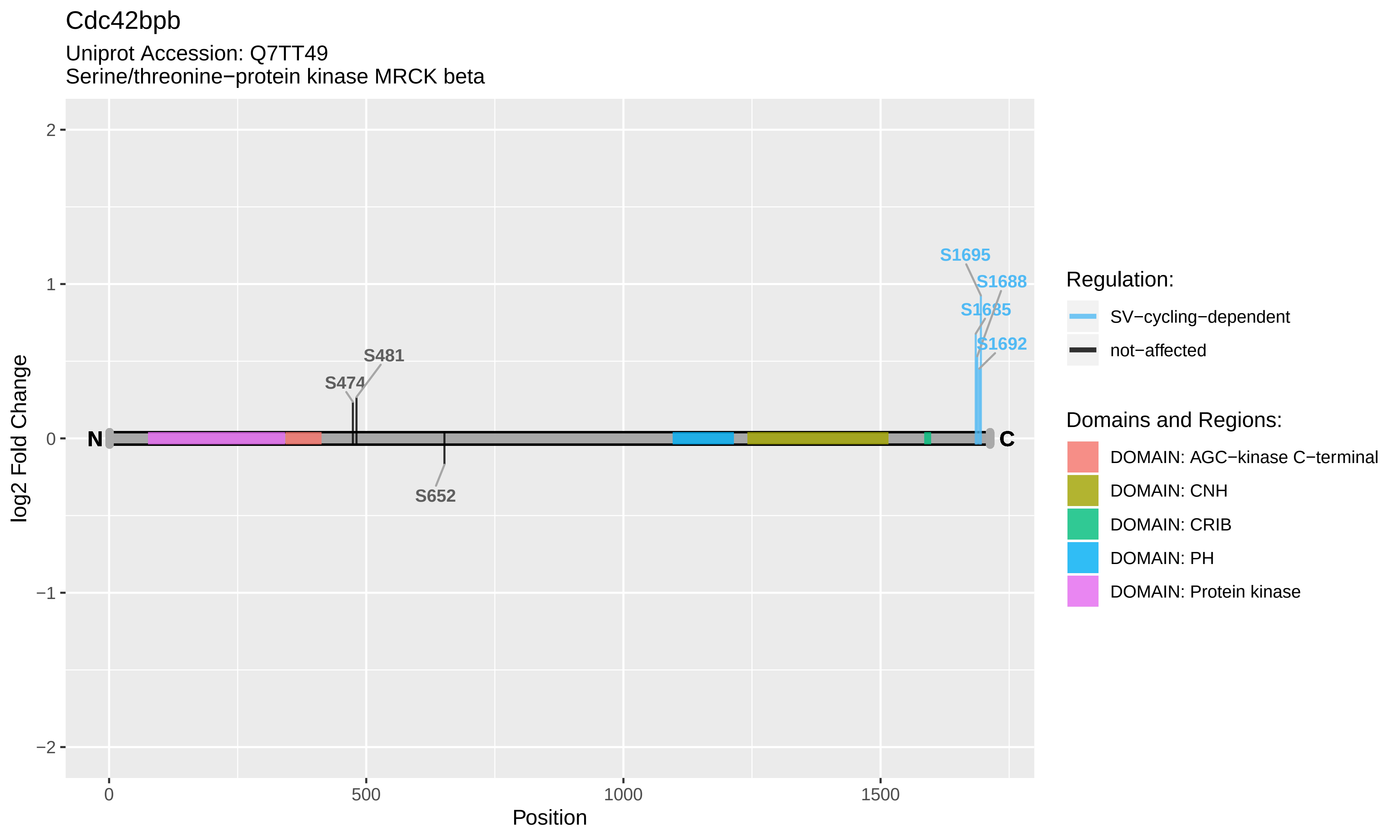

Cdc42bpb
Uniprot Accession: Q7TT49
Serine/threonine−protein kinase MRCK beta
log2 Fold Change
Position
2
1
0
−1
−2
0
500
1000
1500
N
C
S1695
S1688
S1685
S1692
S481
S474
S652
Regulation:
SV−cycling−dependent
not−affected
Domains and Regions:
DOMAIN: AGC−kinase C−terminal
DOMAIN: CNH
DOMAIN: CRIB
DOMAIN: PH
DOMAIN: Protein kinase

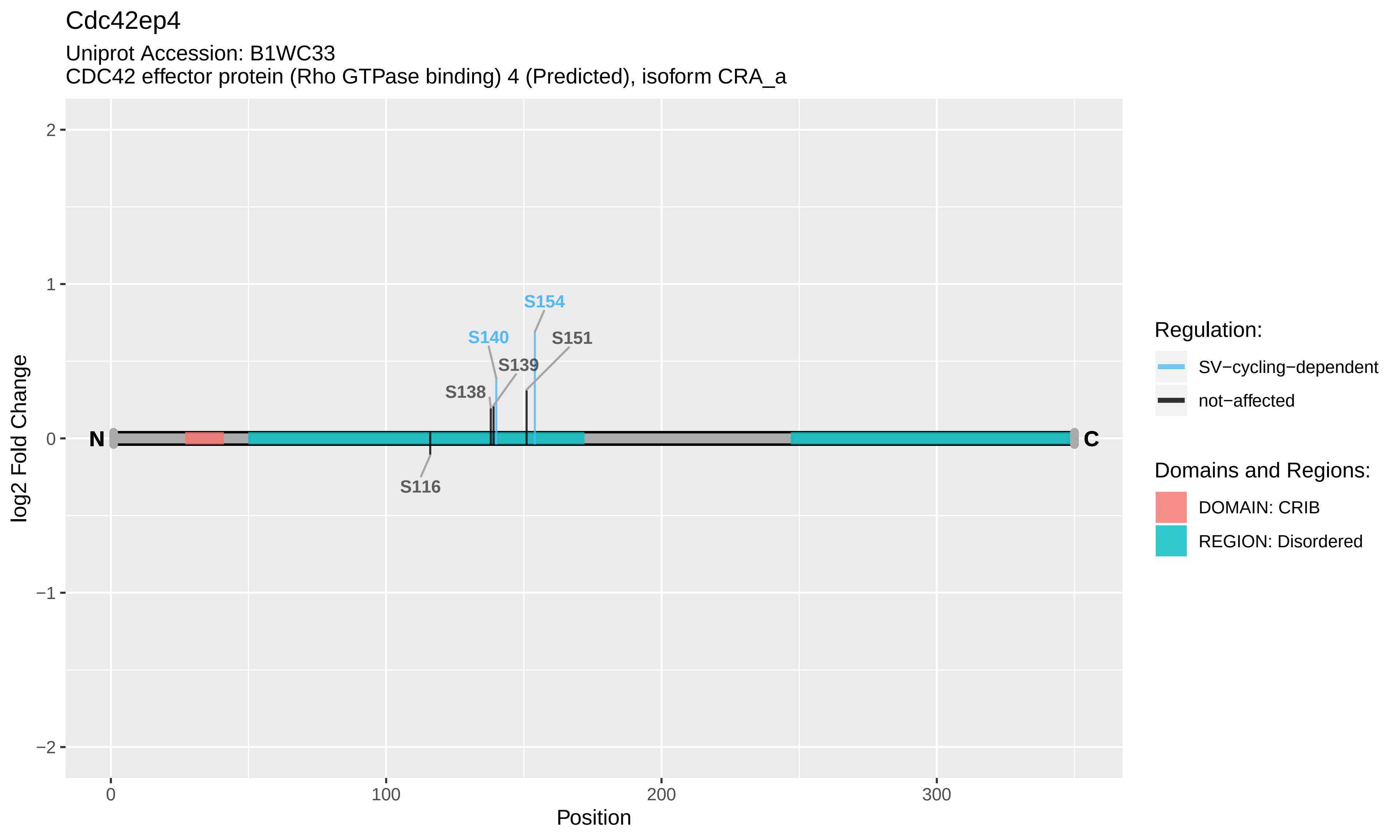

Cdc42ep4
Uniprot Accession: B1WC33
CDC42 effector protein (Rho GTPase binding) 4 (Predicted), isoform CRA_a
log2 Fold Change
Position
N
C
S154
S140
S151
S139
S138
S116
2
1
0
−1
−2
0
100
200
300
Regulation:
SV−cycling−dependent
not−affected
Domains and Regions:
DOMAIN: CRIB
REGION: Disordered

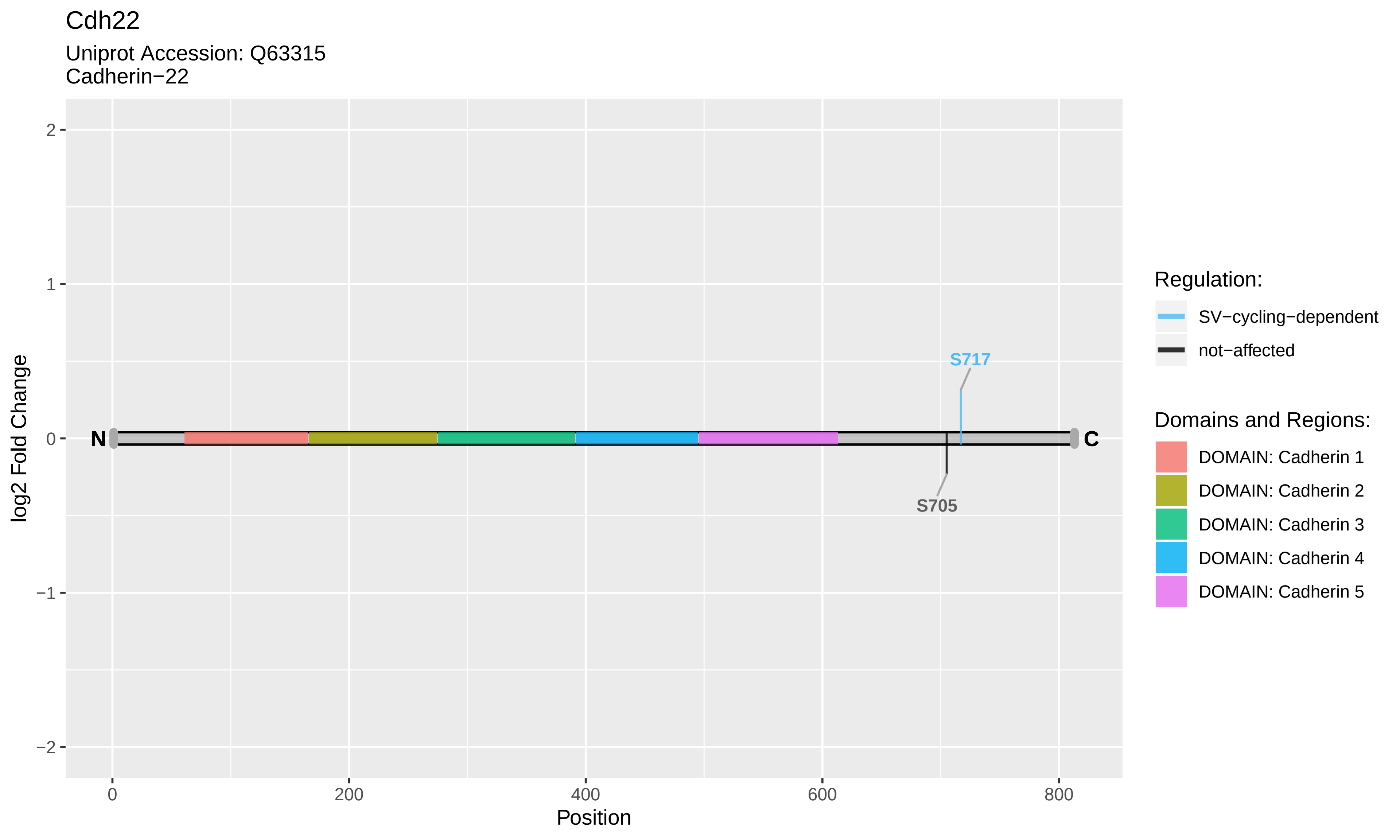

Cdh22
Uniprot Accession: Q63315
Cadherin−22
S717
S705
N
C
log2 Fold Change
Position
Regulation:
SV−cycling−dependent
not−affected
Domains and Regions:
DOMAIN: Cadherin 1
DOMAIN: Cadherin 2
DOMAIN: Cadherin 3
DOMAIN: Cadherin 4
DOMAIN: Cadherin 5

Cdk18

Uniprot Accession: O35832
Cyclin−dependent kinase 18

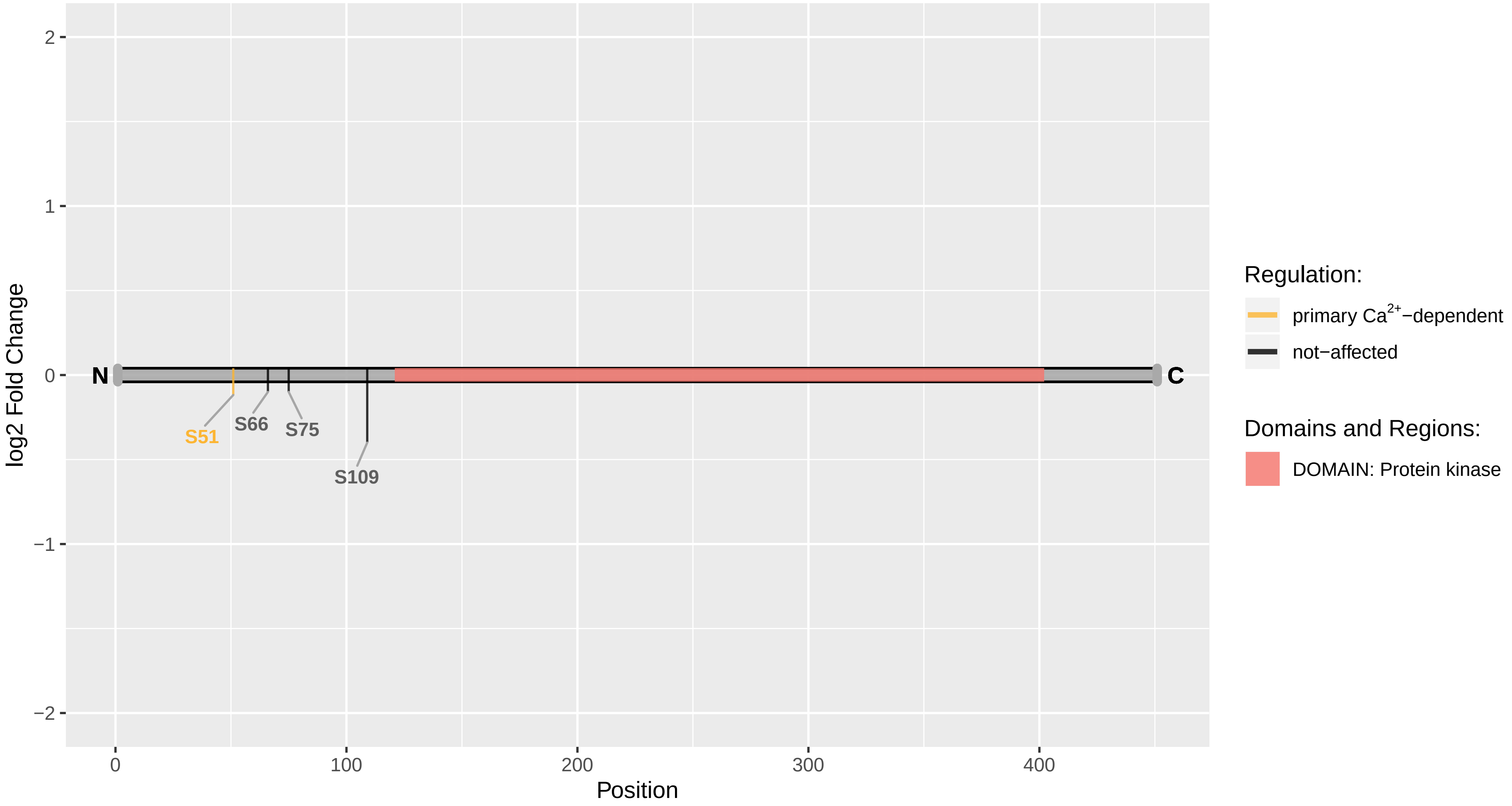

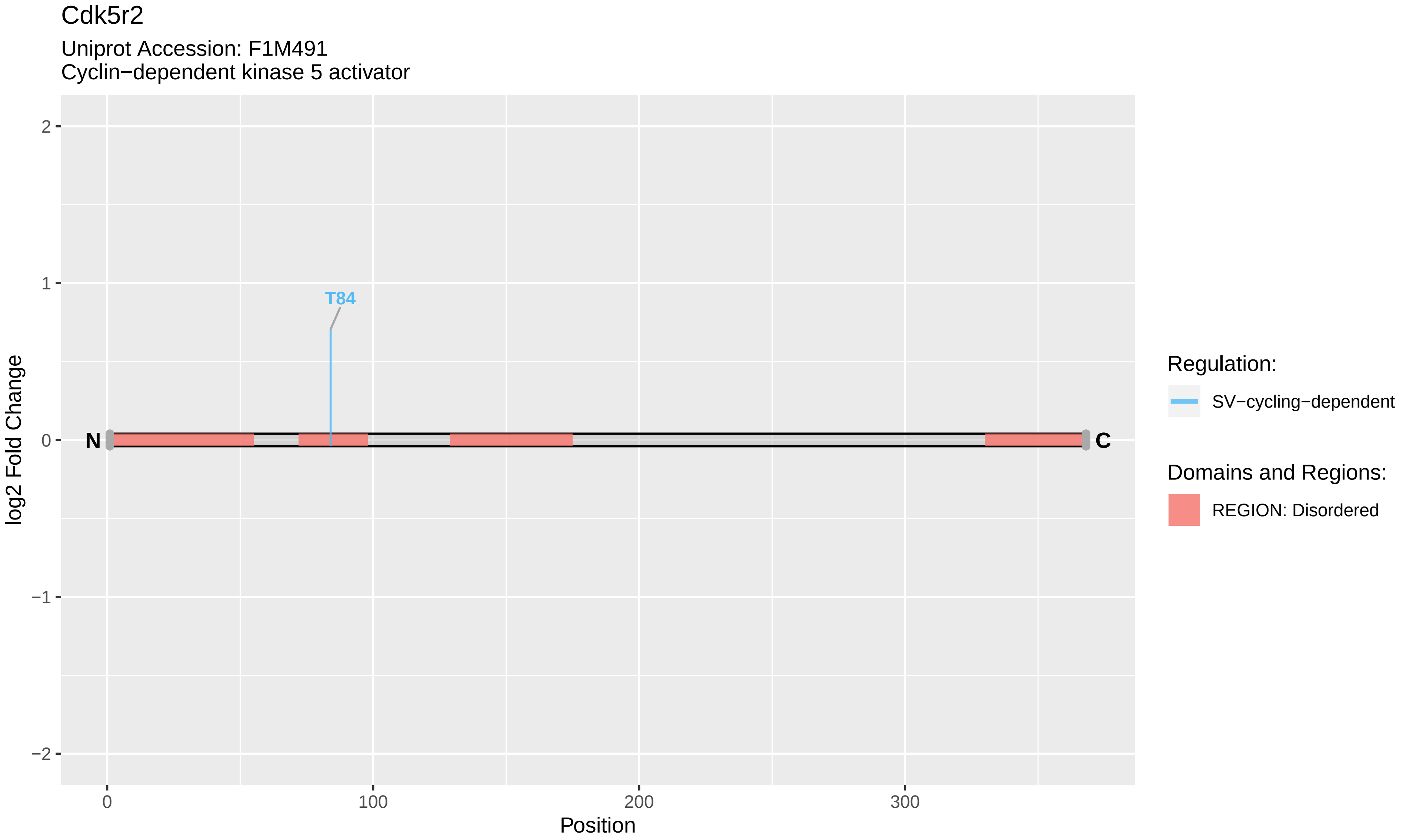
Cdk5r2
Uniprot Accession: F1M491
Cyclin−dependent kinase 5 activator
T84
N
C
2
1
0
−1
−2
log2 Fold Change
0
100
200
300
Position
Regulation:
SV−cycling−dependent
Domains and Regions:
REGION: Disordered

Cdk7

Uniprot Accession: F1LQC8
Cyclin−dependent kinase 7

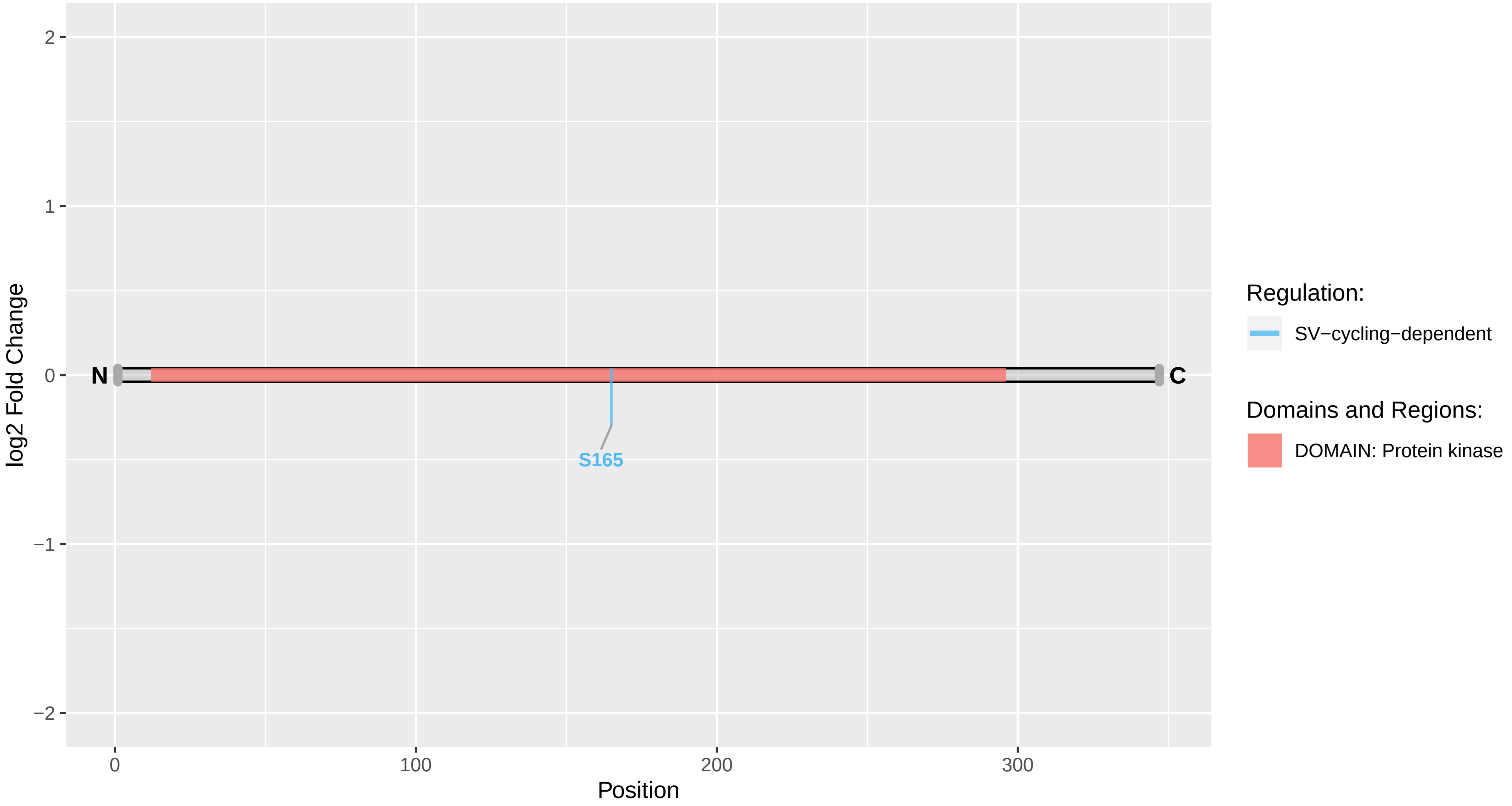

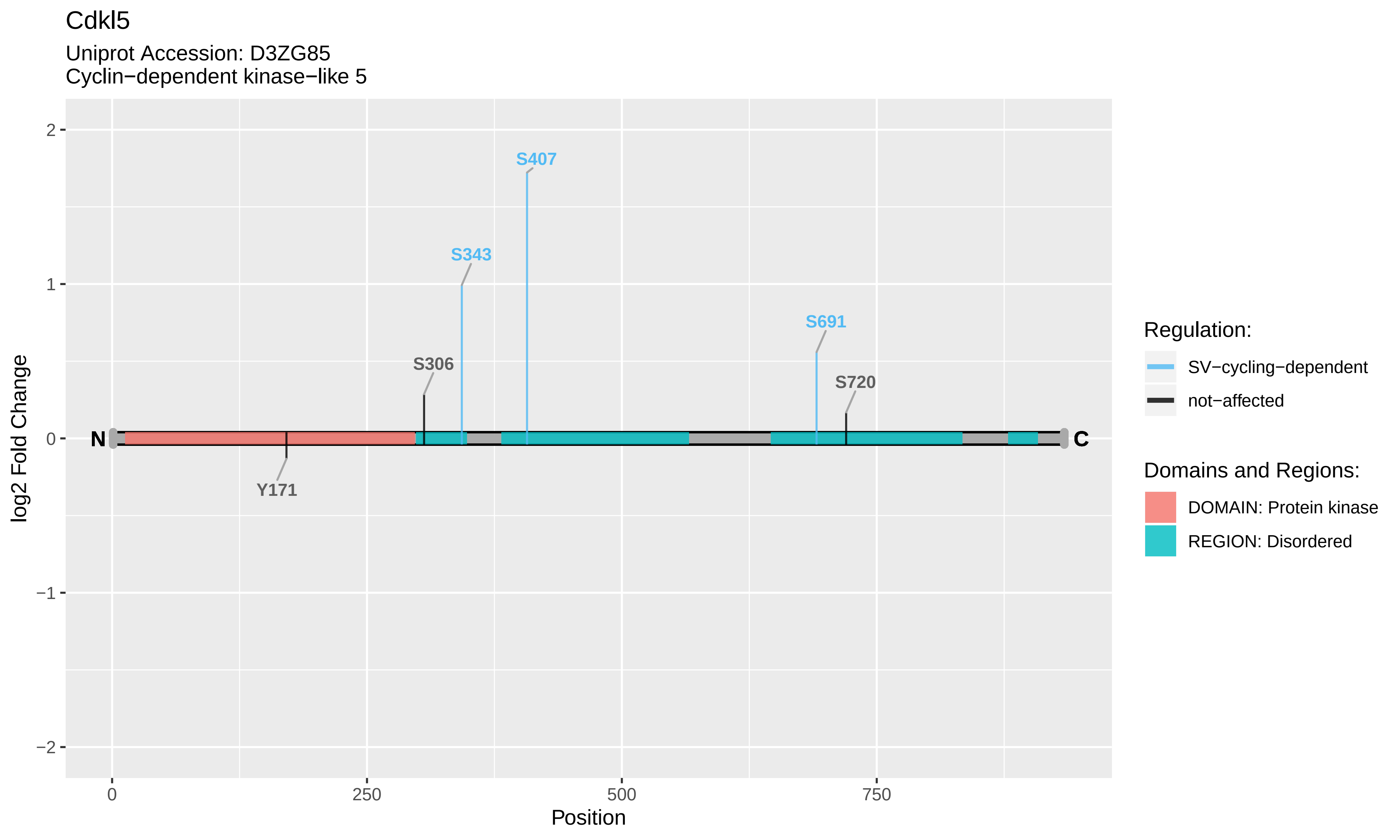

Cdkl5
Uniprot Accession: D3ZG85
Cyclin−dependent kinase−like 5
log2 Fold Change
Position
2
1
0
−1
−2
0
250
500
750
N
C
S407
S343
S306
S691
S720
Y171
Regulation:
SV−cycling−dependent
not−affected
Domains and Regions:
DOMAIN: Protein kinase
REGION: Disordered

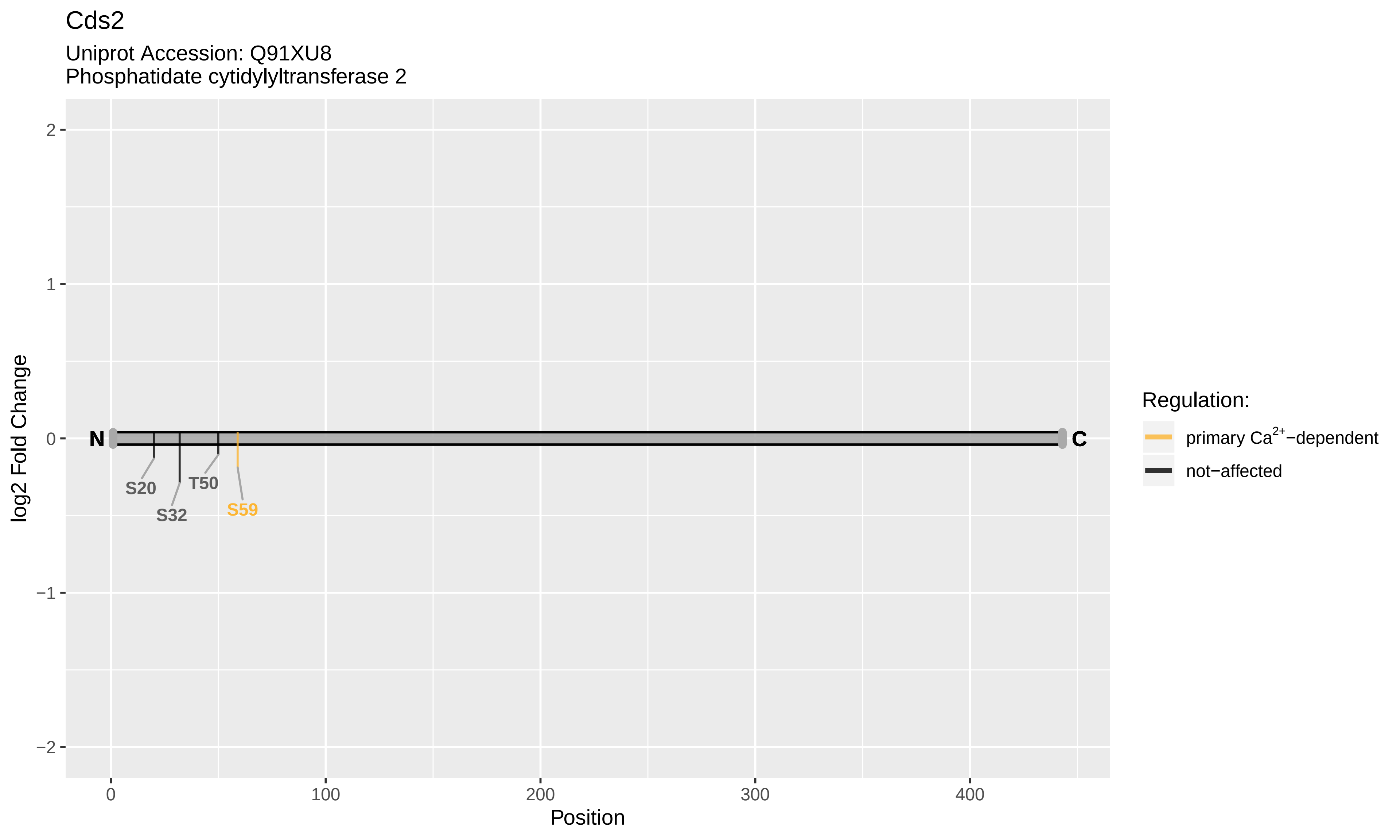

Cds2
Uniprot Accession: Q91XU8
Phosphatidate cytidylyltransferase 2
log2 Fold Change
2
1
0
−1
−2
N
C
S20
S32
T50
S59
0
100
200
300
400
Position
Regulation:
primary Ca2+−dependent
not−affected

Cdv3

Uniprot Accession: Q5XIM5
Protein CDV3 homolog

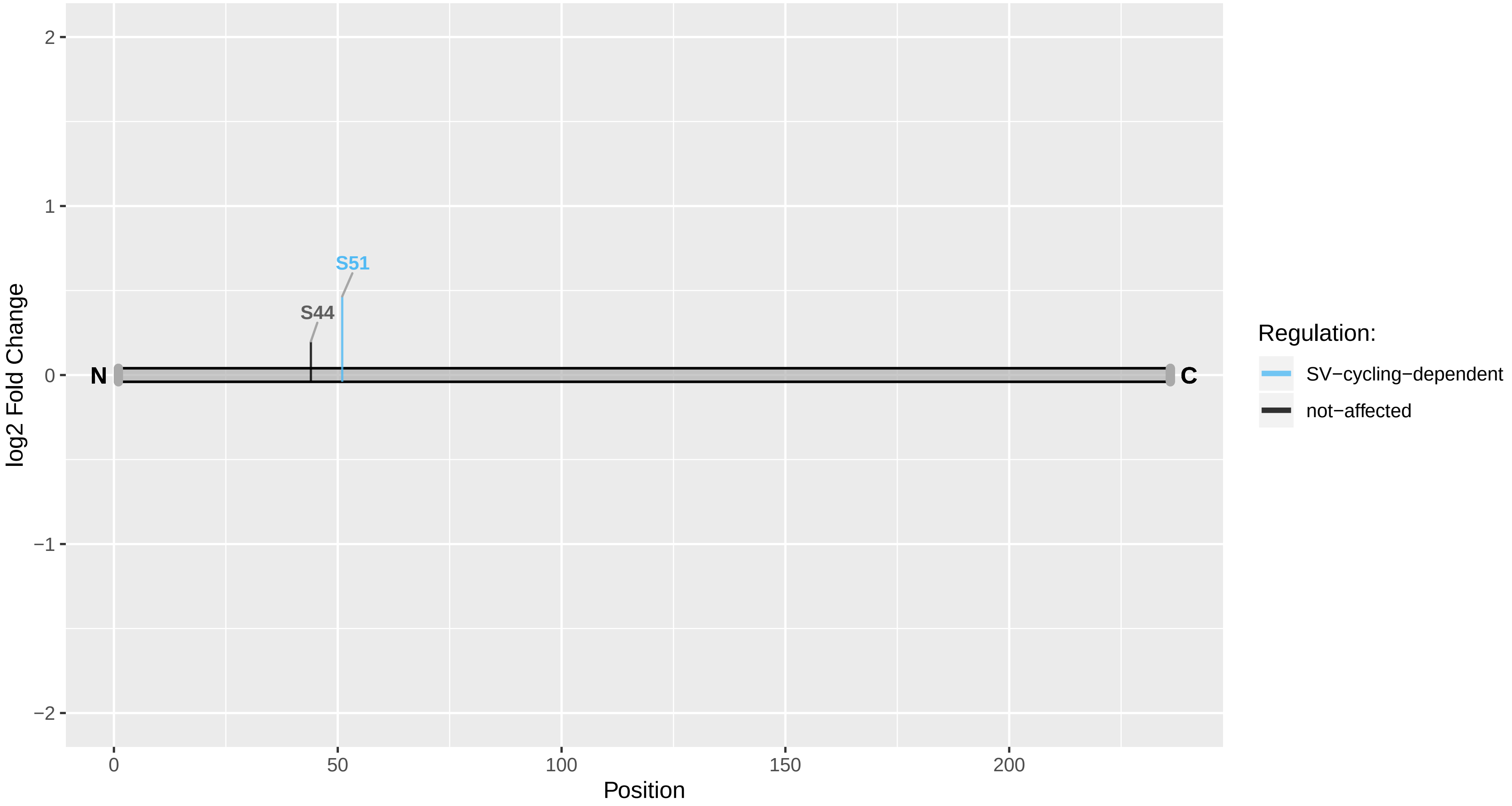

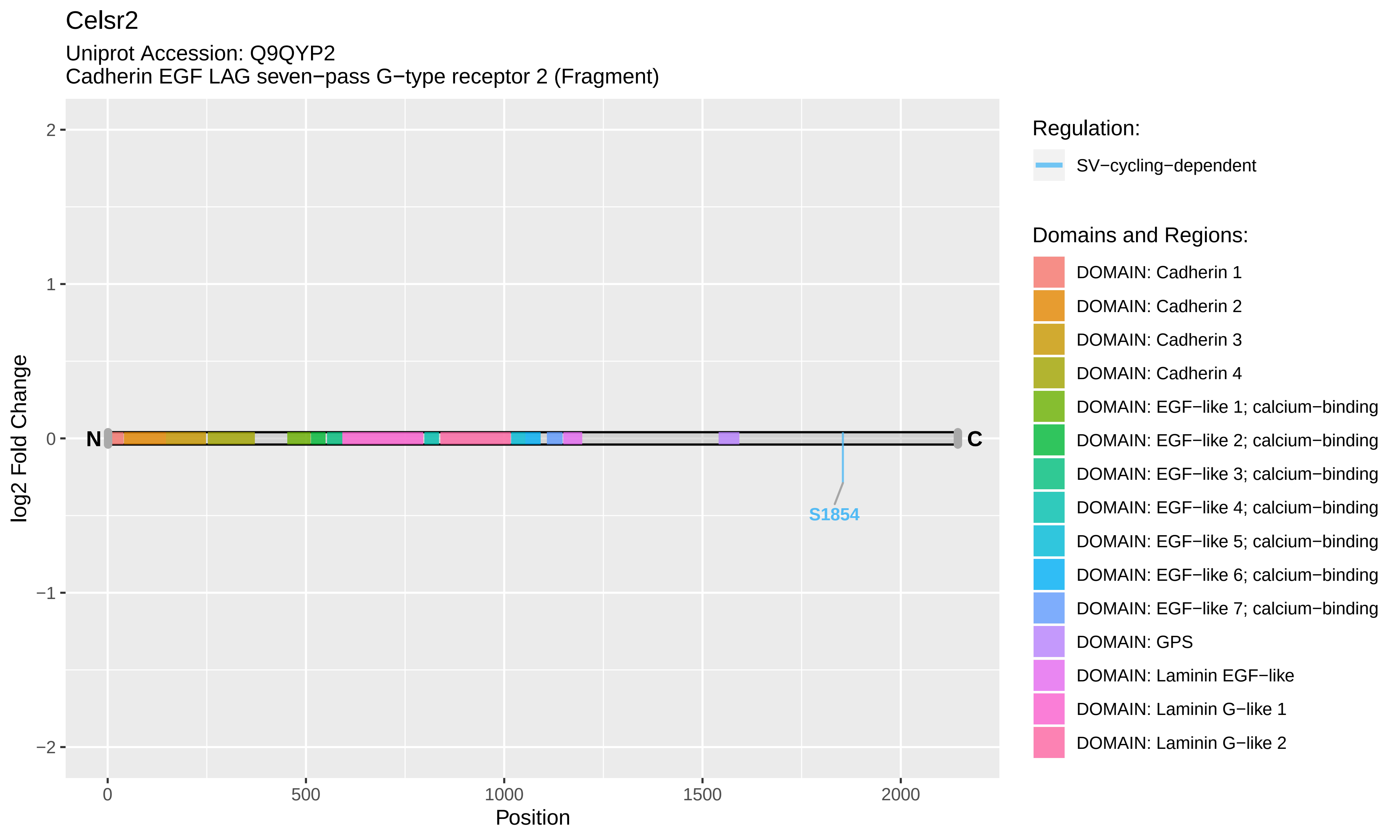
Celsr2
Uniprot Accession: Q9QYP2
Cadherin EGF LAG seven−pass G−type receptor 2 (Fragment)
log2 Fold Change
2
1
0
−1
−2
N
C
S1854
0
500
1000
1500
2000
Position
Regulation:
SV−cycling−dependent
Domains and Regions:
DOMAIN: Cadherin 1
DOMAIN: Cadherin 2
DOMAIN: Cadherin 3
DOMAIN: Cadherin 4
DOMAIN: EGF−like 1; calcium−binding
DOMAIN: EGF−like 2; calcium−binding
DOMAIN: EGF−like 3; calcium−binding
DOMAIN: EGF−like 4; calcium−binding
DOMAIN: EGF−like 5; calcium−binding
DOMAIN: EGF−like 6; calcium−binding
DOMAIN: EGF−like 7; calcium−binding
DOMAIN: GPS
DOMAIN: Laminin EGF−like
DOMAIN: Laminin G−like 1
DOMAIN: Laminin G−like 2

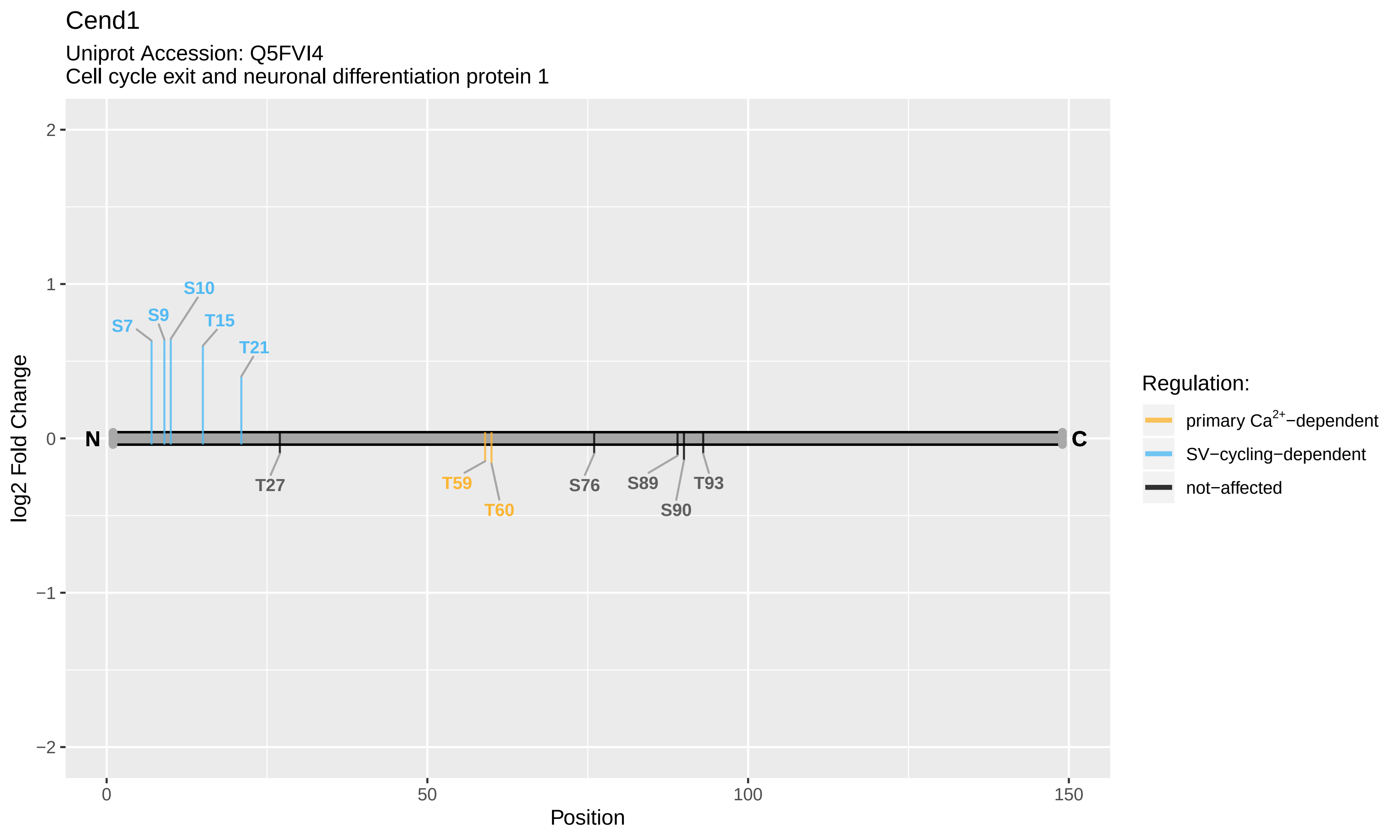
Cend1
Uniprot Accession: Q5FVI4
Cell cycle exit and neuronal differentiation protein 1
log2 Fold Change
Position
N
C
S7
S9
S10
T15
T21
T27
T59
T60
S76
S89
S90
T93
Regulation:
primary Ca2+−dependent
SV−cycling−dependent
not−affected

Cenpa

Uniprot Accession: B2RZ23
Centromere protein A

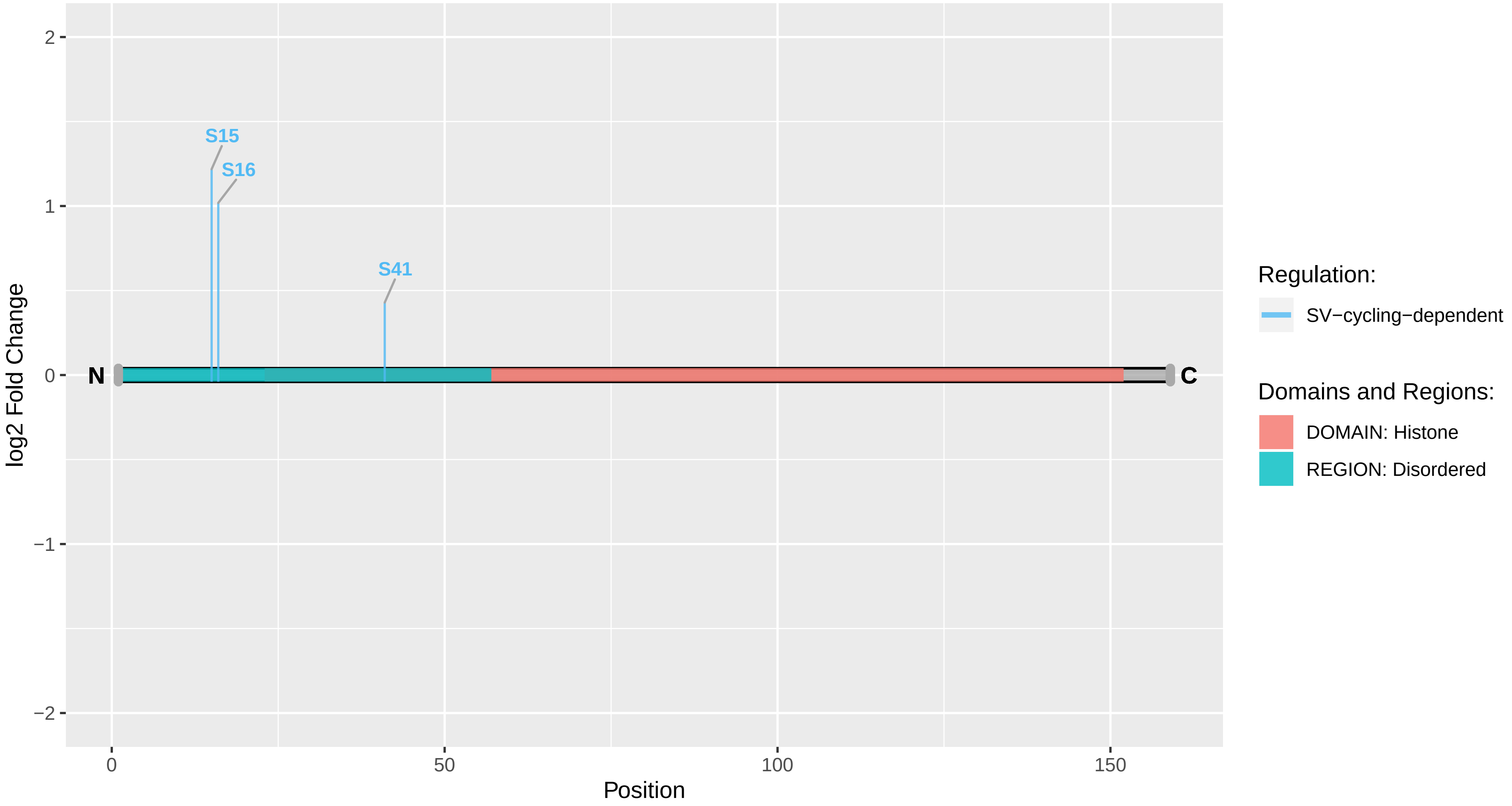

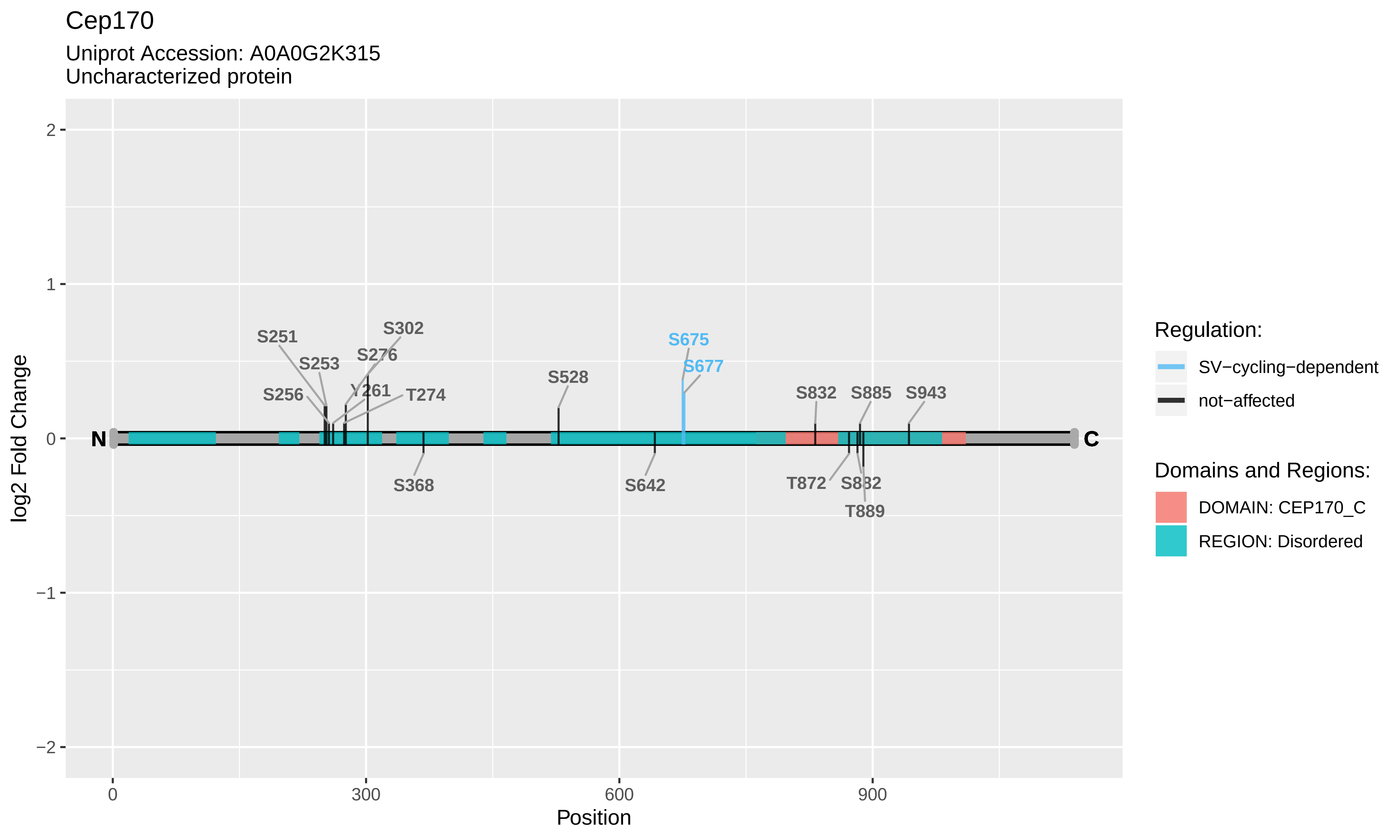
Cep170
Uniprot Accession: A0A0G2K315
Uncharacterized protein
S251
S302
S253
S276
S256
Y261
T274
S528
S675
S677
S832
S885
S943
S368
S642
T872
S882
T889
N
C
log2 Fold Change
Position
Regulation:
SV−cycling−dependent
not−affected
Domains and Regions:
DOMAIN: CEP170_C
REGION: Disordered

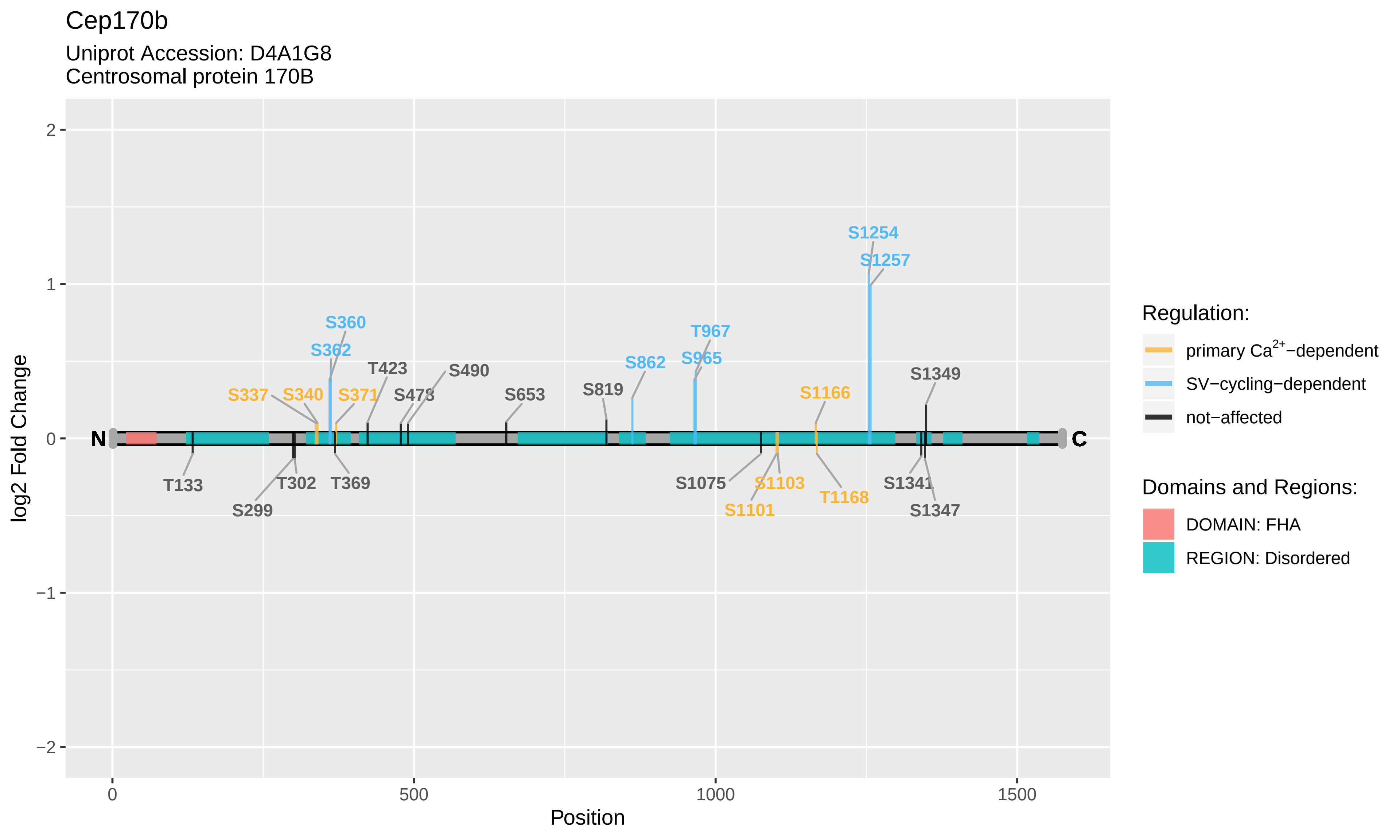
Cep170b
Uniprot Accession: D4A1G8
Centrosomal protein 170B
log2 Fold Change
Position
N
C
S1254
S1257
S360
S362
T967
S965
S862
T423
S490
S1349
S337
S340
S371
S478
S653
S819
S1166
T133
S299
T302
T369
S1075
S1103
S1101
T1168
S1341
S1347
Regulation:
primary Ca2+−dependent
SV−cycling−dependent
not−affected
Domains and Regions:
DOMAIN: FHA
REGION: Disordered

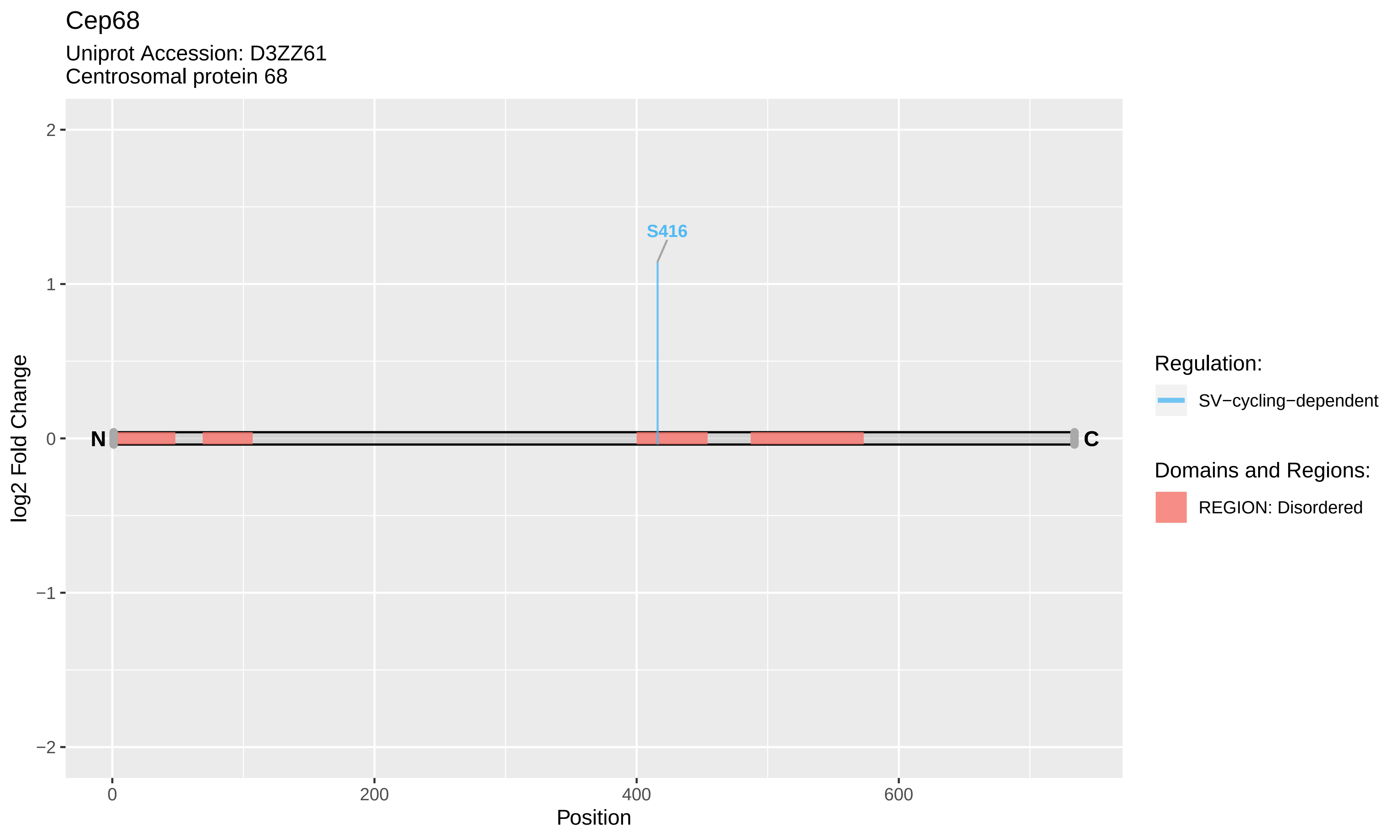

Cep68
Uniprot Accession: D3ZZ61
Centrosomal protein 68
S416
N
C
2
1
0
−1
−2
log2 Fold Change
0
200
400
600
Position
Regulation:
SV−cycling−dependent
Domains and Regions:
REGION: Disordered

Cfl2

Uniprot Accession: M0RC65
Cofilin 2

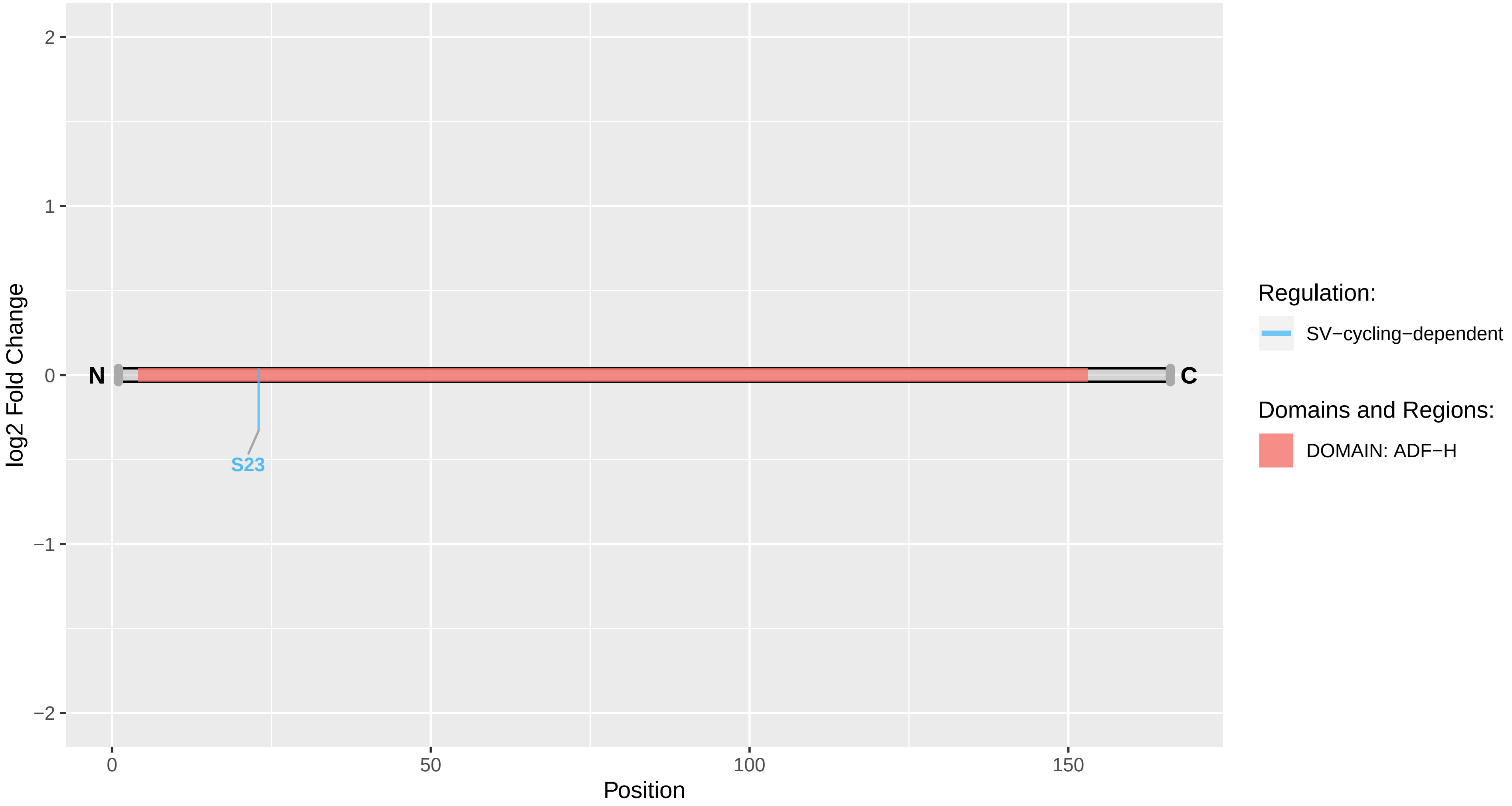

Chchd3

Uniprot Accession: D3ZUX5
MICOS complex subunit

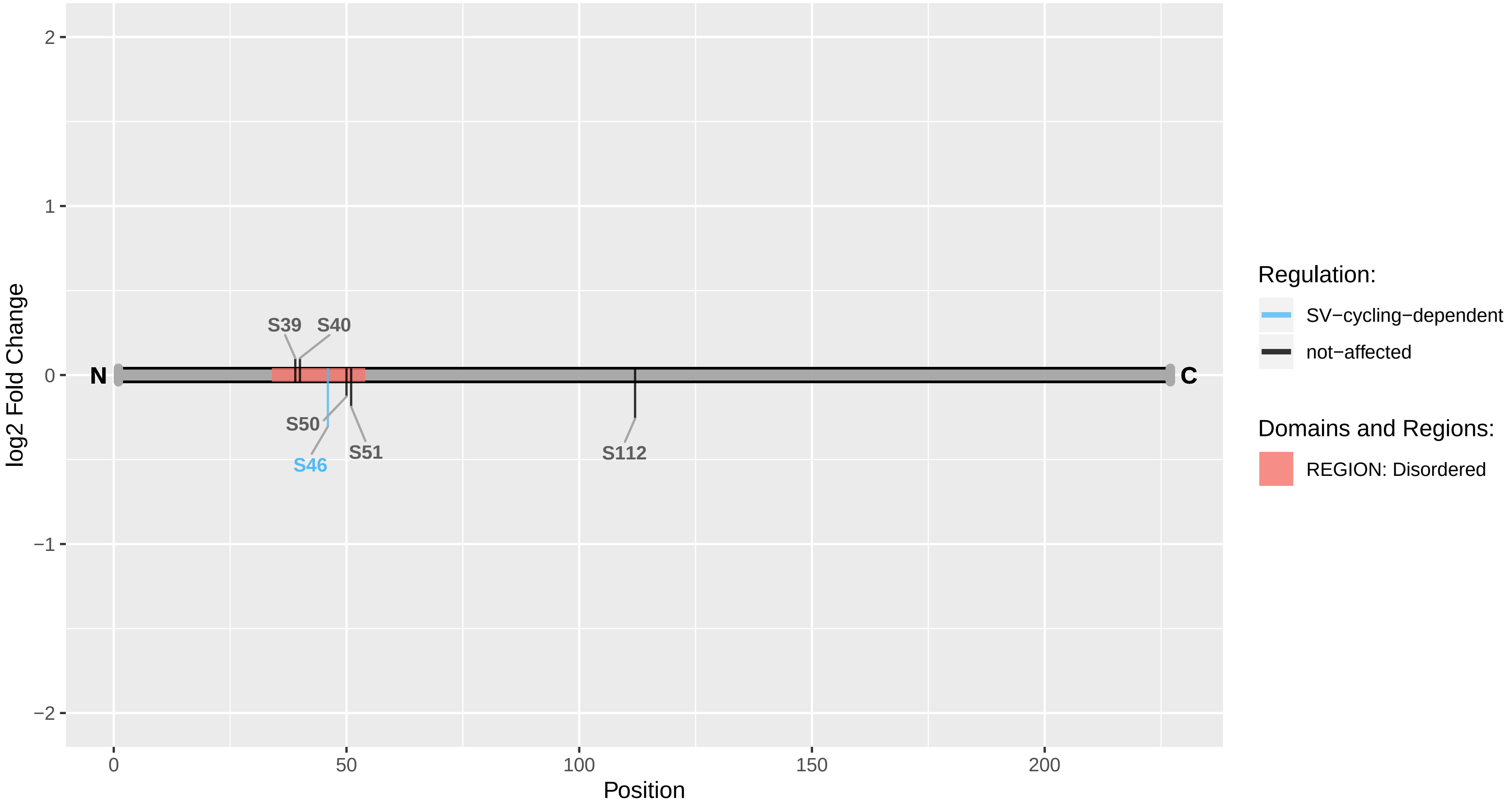

Chchd6

Uniprot Accession: D4A7N1
MICOS complex subunit Mic25

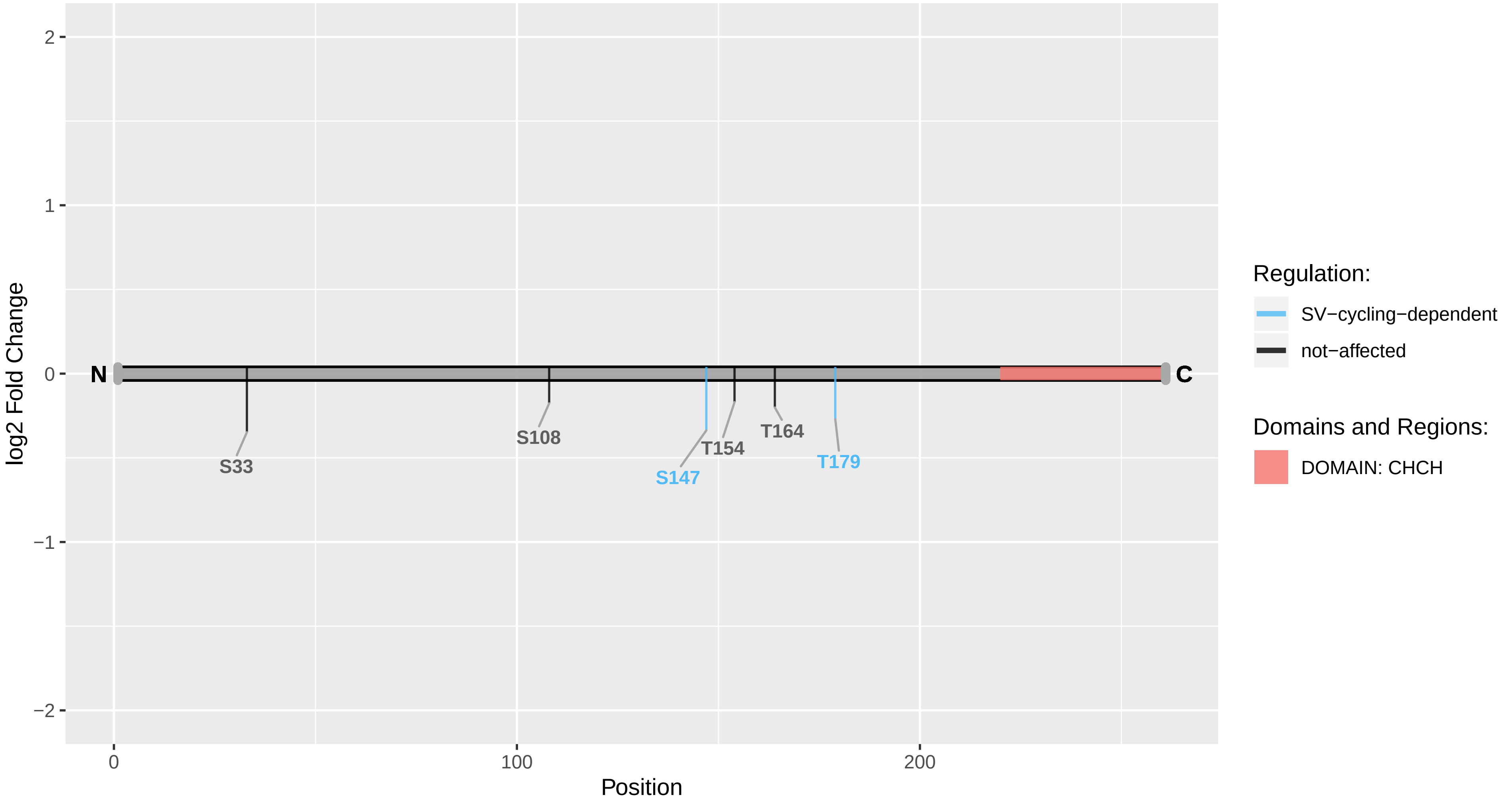

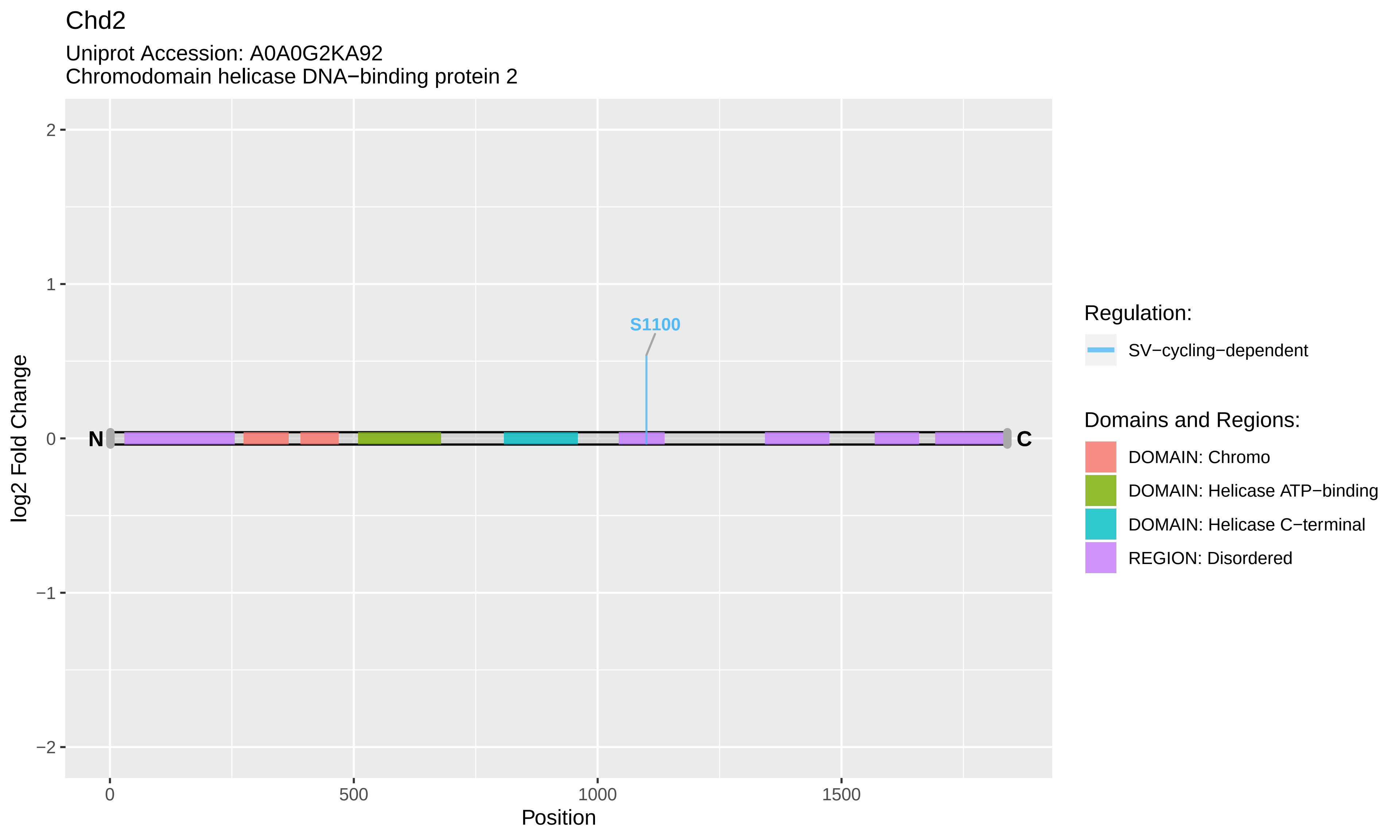

Chd2
Uniprot Accession: A0A0G2KA92
Chromodomain helicase DNA−binding protein 2
S1100
N
C
log2 Fold Change
2
1
0
−1
−2
0
500
1000
1500
Position
Regulation:
SV−cycling−dependent
Domains and Regions:
DOMAIN: Chromo
DOMAIN: Helicase ATP−binding
DOMAIN: Helicase C−terminal
REGION: Disordered

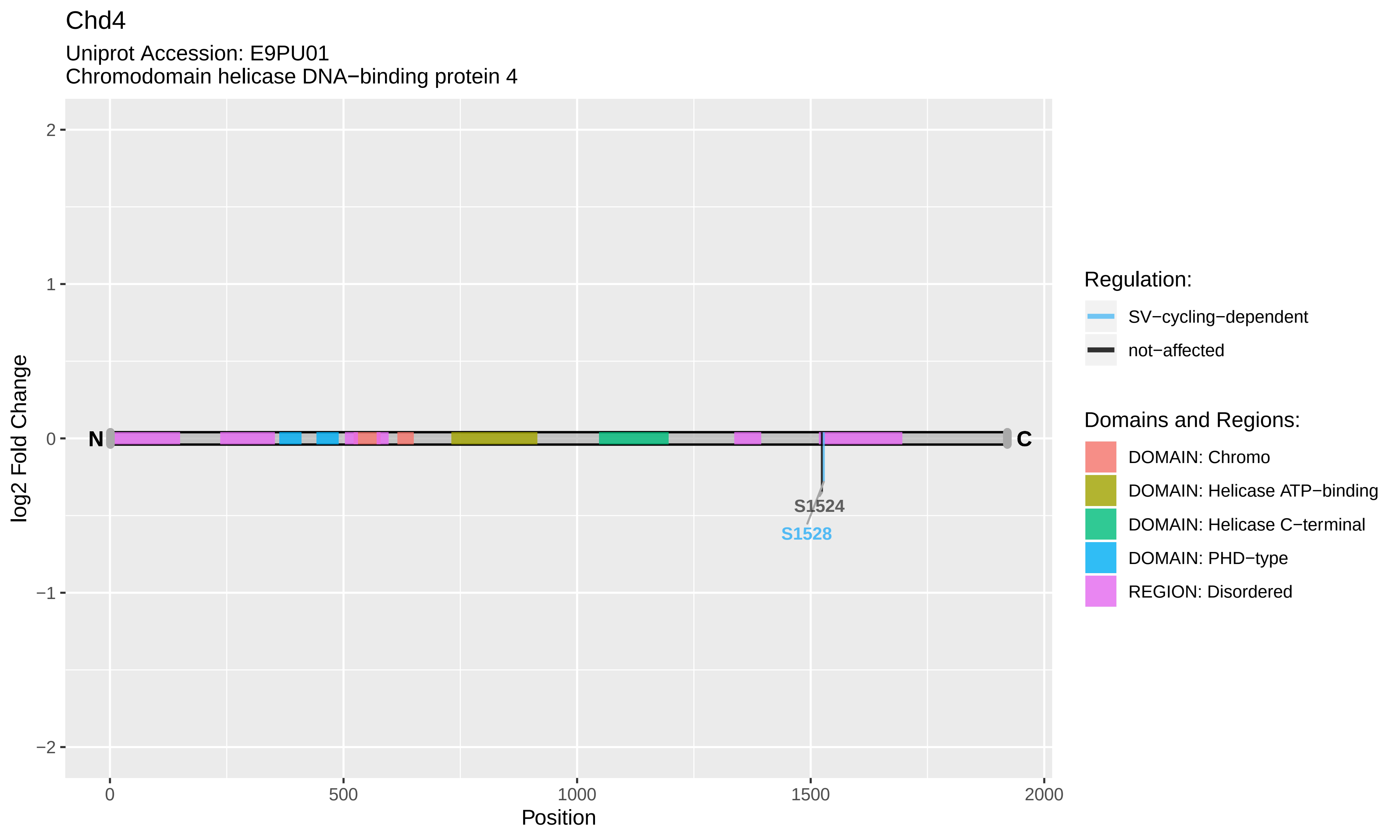
Chd4
Uniprot Accession: E9PU01
Chromodomain helicase DNA−binding protein 4
log2 Fold Change
2
1
0
−1
−2
N
C
S1524
S1528
0
500
1000
1500
2000
Position
Regulation:
SV−cycling−dependent
not−affected
Domains and Regions:
DOMAIN: Chromo
DOMAIN: Helicase ATP−binding
DOMAIN: Helicase C−terminal
DOMAIN: PHD−type
REGION: Disordered

Chgb

Uniprot Accession: O35314
Secretogranin−1

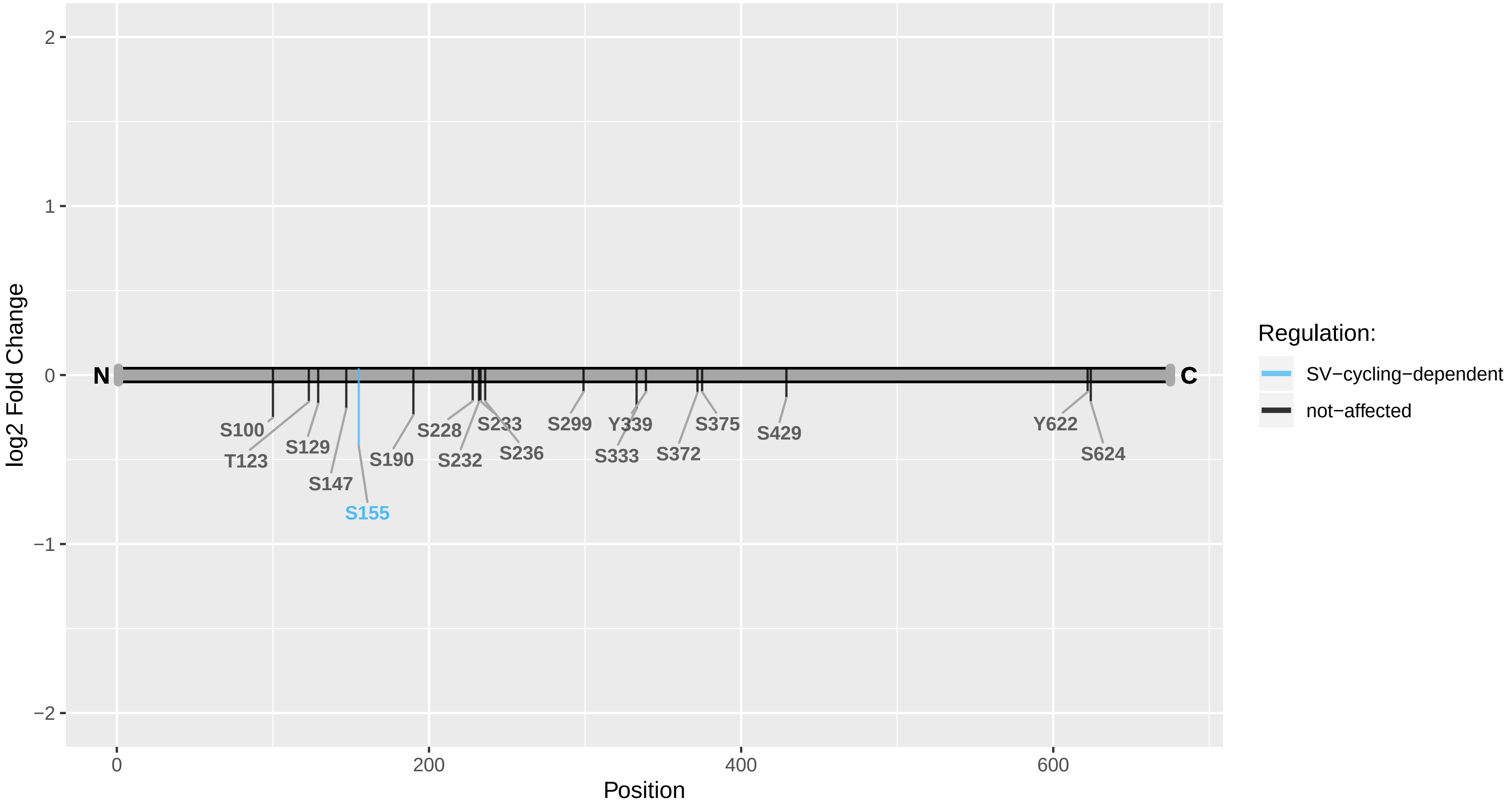

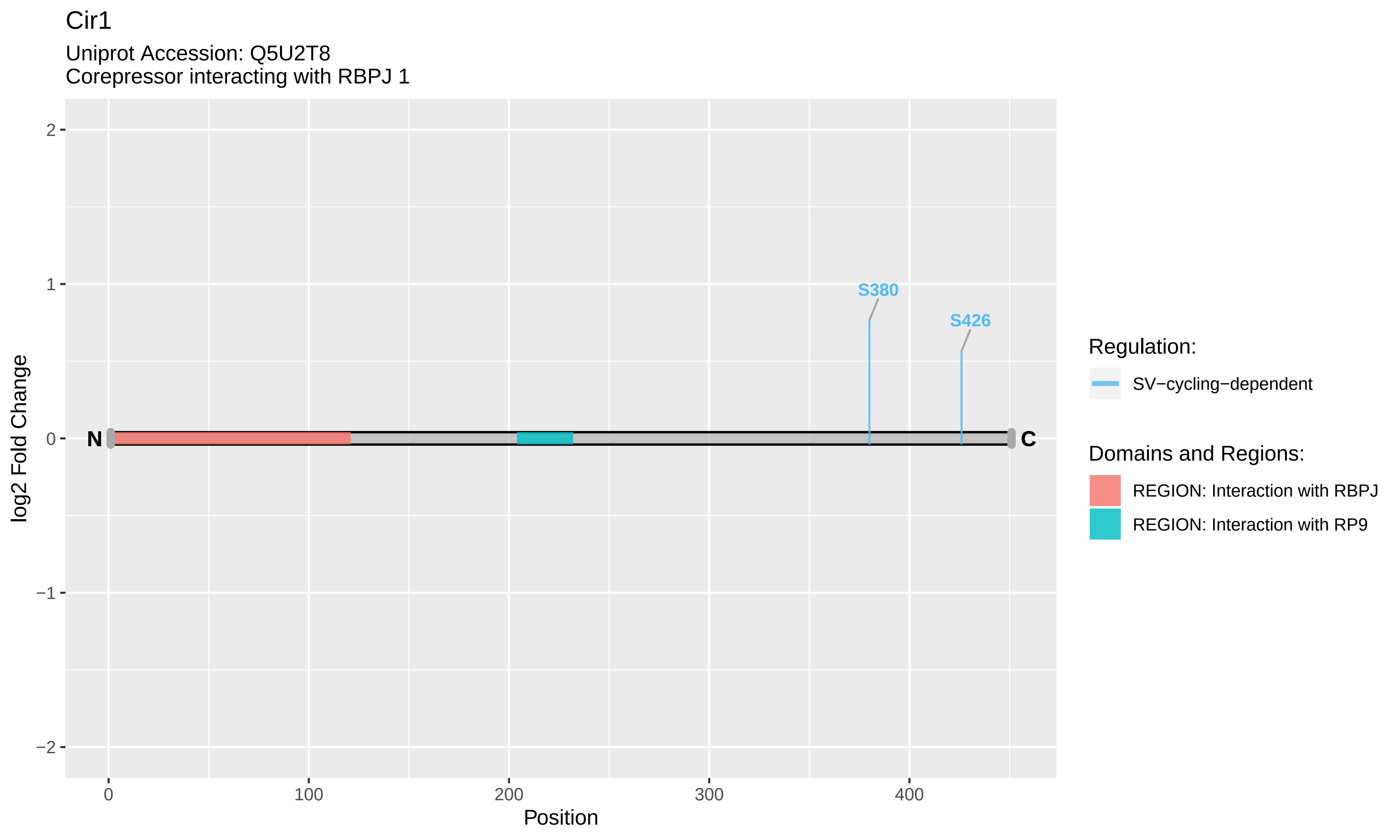
Cir1
Uniprot Accession: Q5U2T8
Corepressor interacting with RBPJ 1
S380
S426
N
C
2
1
0
−1
−2
log2 Fold Change
0
100
200
300
400
Position
Regulation:
SV−cycling−dependent
Domains and Regions:
REGION: Interaction with RBPJ
REGION: Interaction with RP9

Cit

Uniprot Accession: E9PSL7
Citron rho−interacting serine/threonine kinase

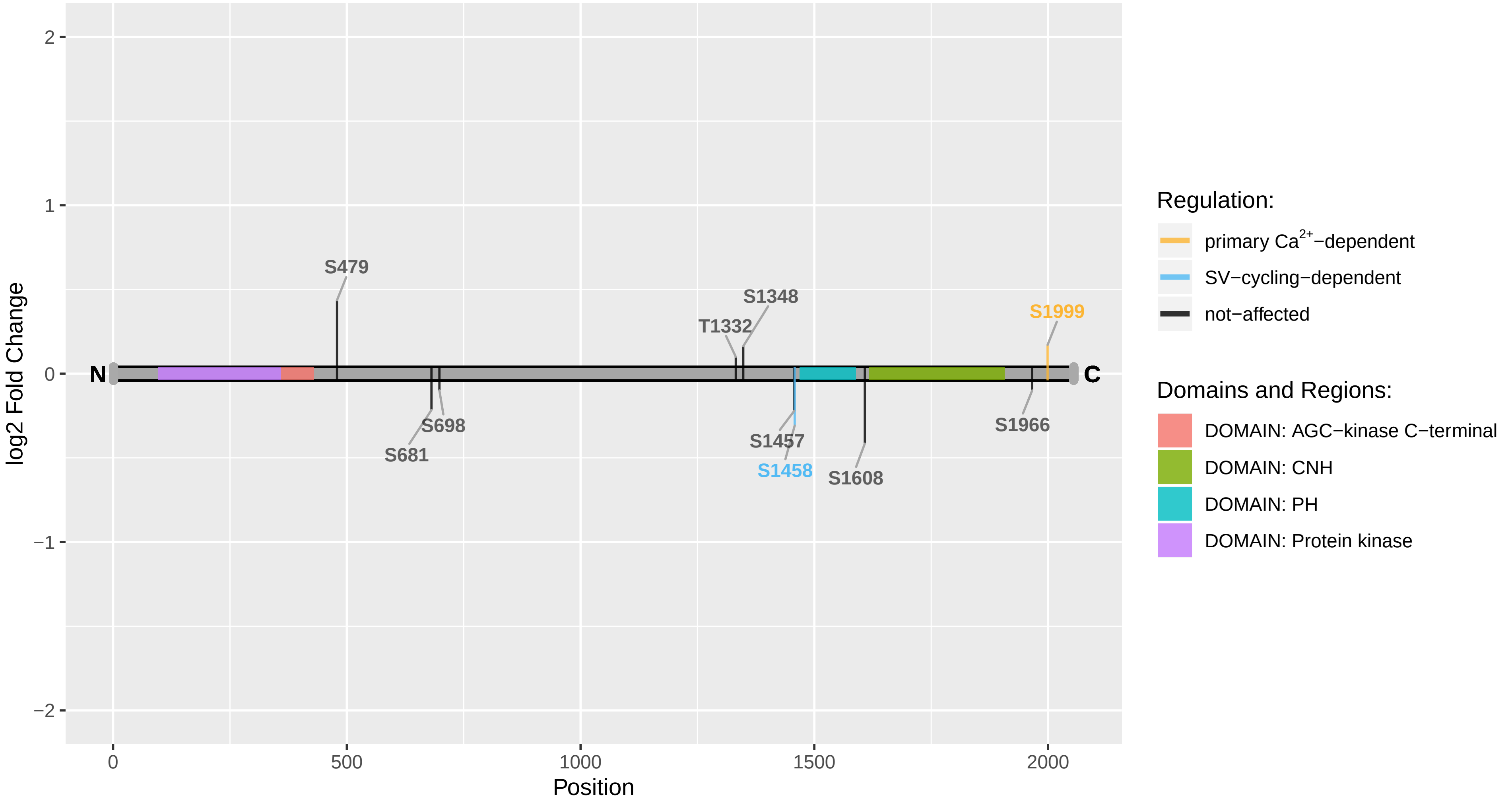

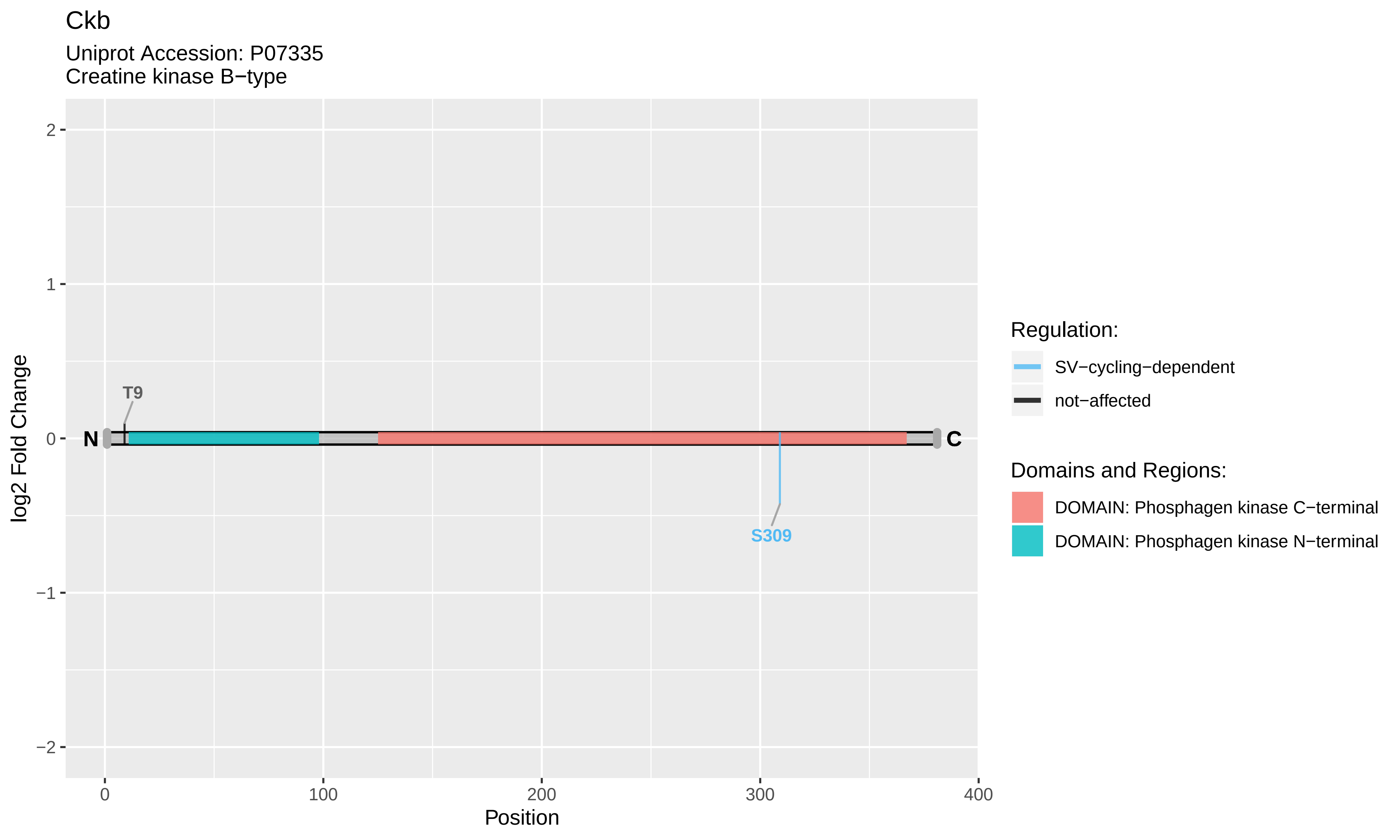
Ckb
Uniprot Accession: P07335
Creatine kinase B−type
2
1
0
−1
−2
log2 Fold Change
T9
N
C
S309
0
100
200
300
400
Position
Regulation:
SV−cycling−dependent
not−affected
Domains and Regions:
DOMAIN: Phosphagen kinase C−terminal
DOMAIN: Phosphagen kinase N−terminal

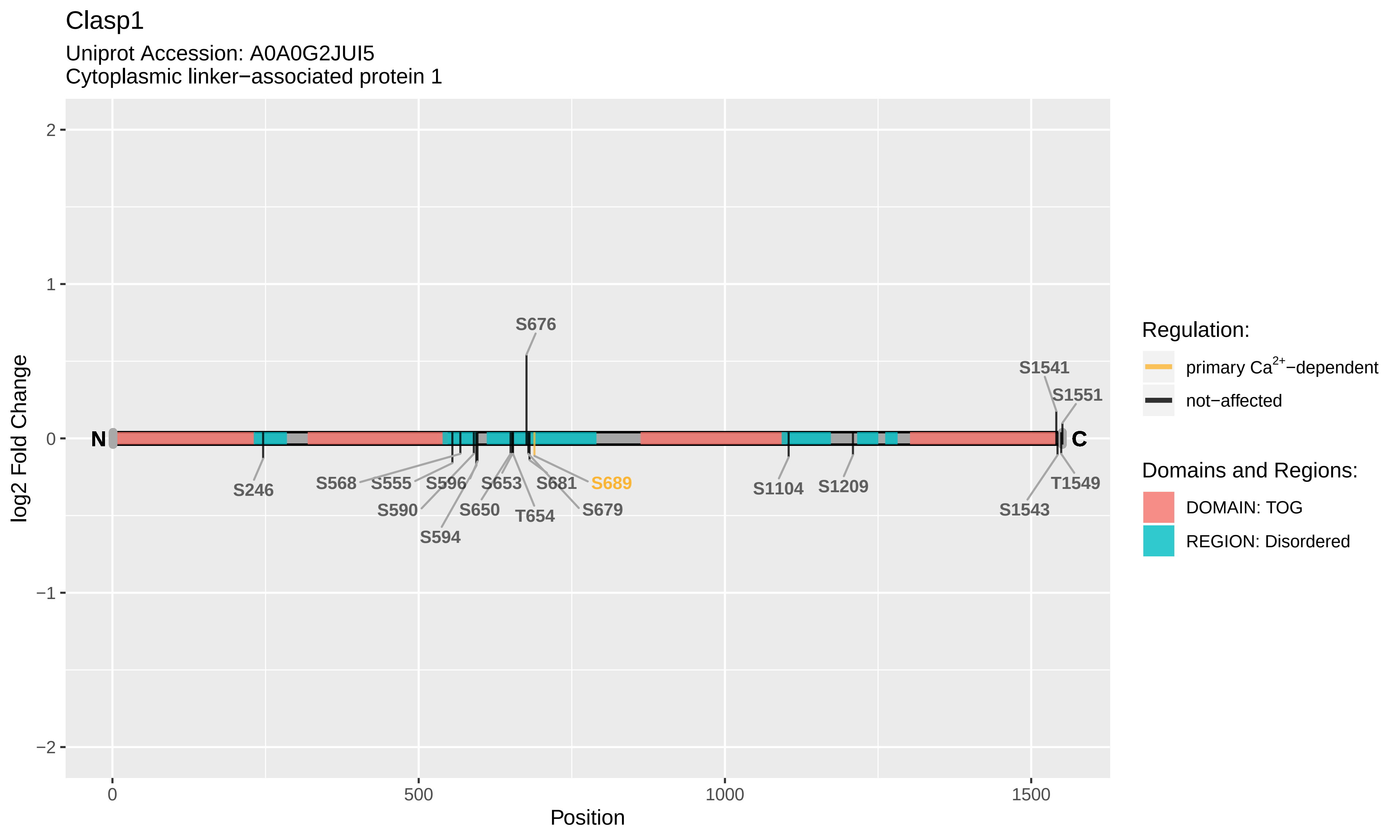

Clasp1
Uniprot Accession: A0A0G2JUI5
Cytoplasmic linker−associated protein 1
log2 Fold Change
2
1
0
−1
−2
Position
0
500
1000
1500
N
C
S676
S246
S568
S555
S596
S653
S681
S689
S590
S650
T654
S679
S594
S1104
S1209
S1541
S1551
T1549
S1543
Regulation:
primary Ca2+−dependent
not−affected
Domains and Regions:
DOMAIN: TOG
REGION: Disordered

Clasp2

Uniprot Accession: A0A0G2JZM8
CLIP−associating protein 2

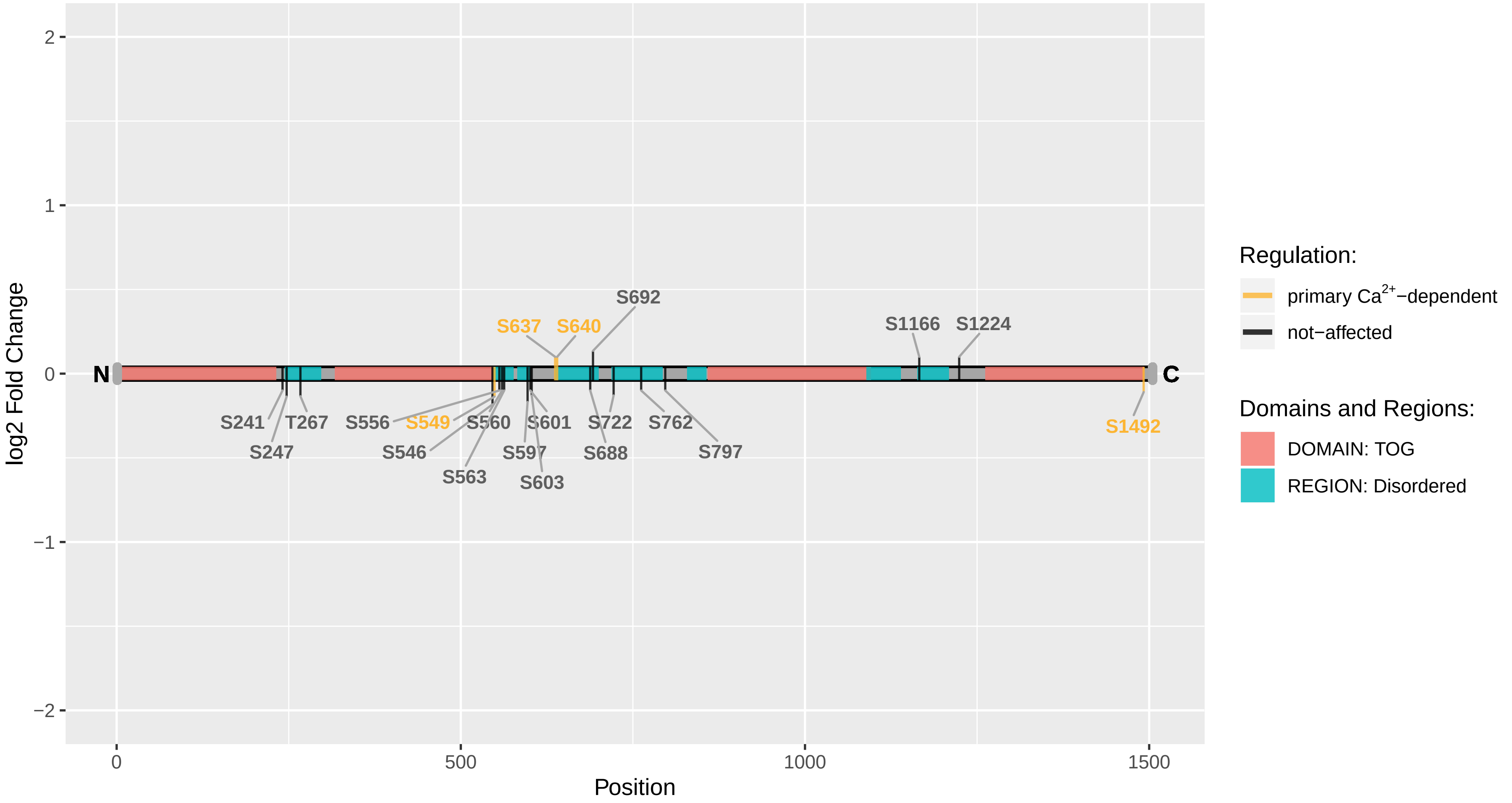

Clint1

Uniprot Accession: A0A0G2JW94
Clathrin interactor 1

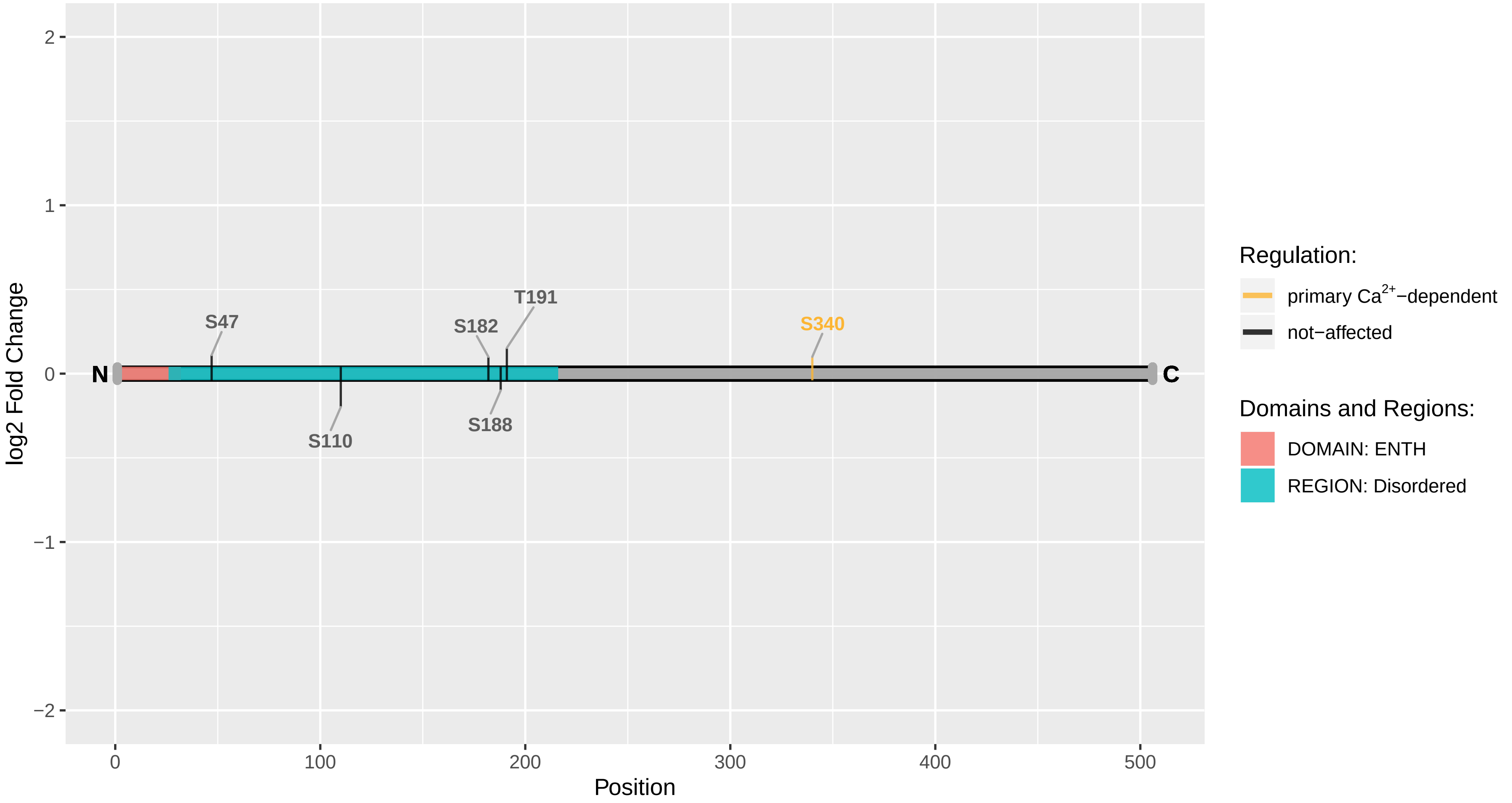

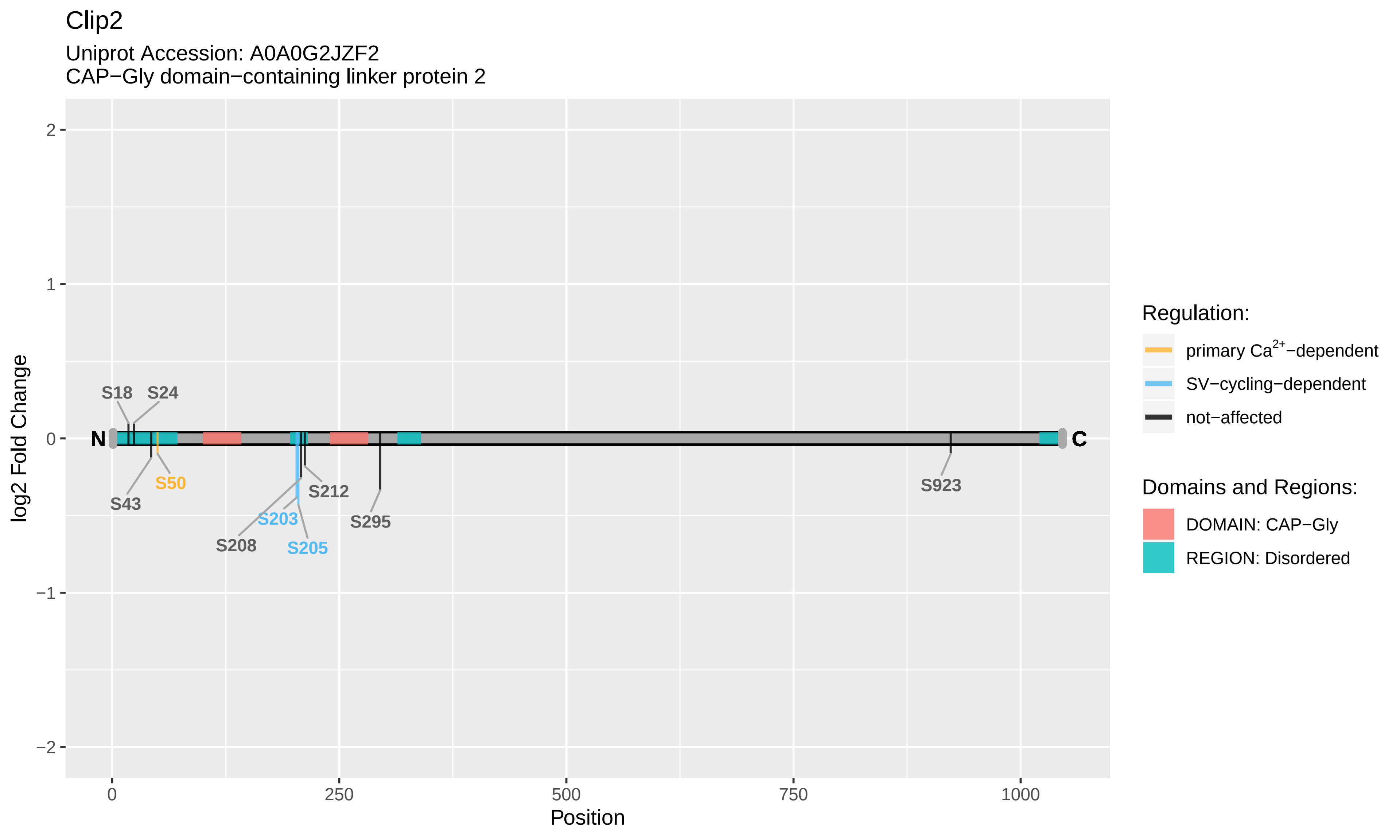

Clip2
Uniprot Accession: A0A0G2JZF2
CAP−Gly domain−containing linker protein 2
log2 Fold Change
2
1
0
−1
−2
N
C
S18
S24
S43
S50
S203
S205
S208
S212
S295
S923
0
250
500
750
1000
Position
Regulation:
primary Ca2+−dependent
SV−cycling−dependent
not−affected
Domains and Regions:
DOMAIN: CAP−Gly
REGION: Disordered

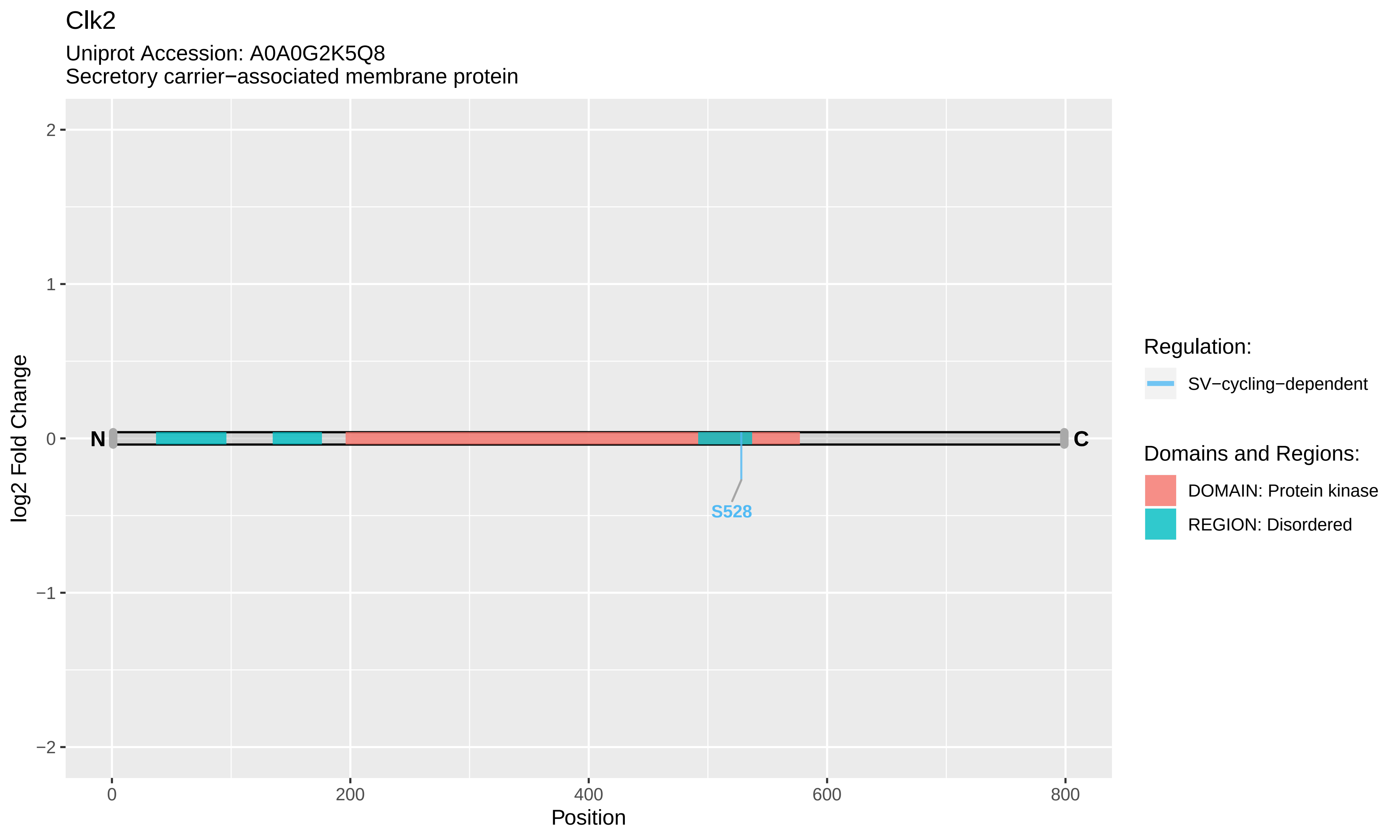

Clk2
Uniprot Accession: A0A0G2K5Q8
Secretory carrier−associated membrane protein
log2 Fold Change
2
1
0
−1
−2
N
C
S528
0
200
400
600
800
Position
Regulation:
SV−cycling−dependent
Domains and Regions:
DOMAIN: Protein kinase
REGION: Disordered

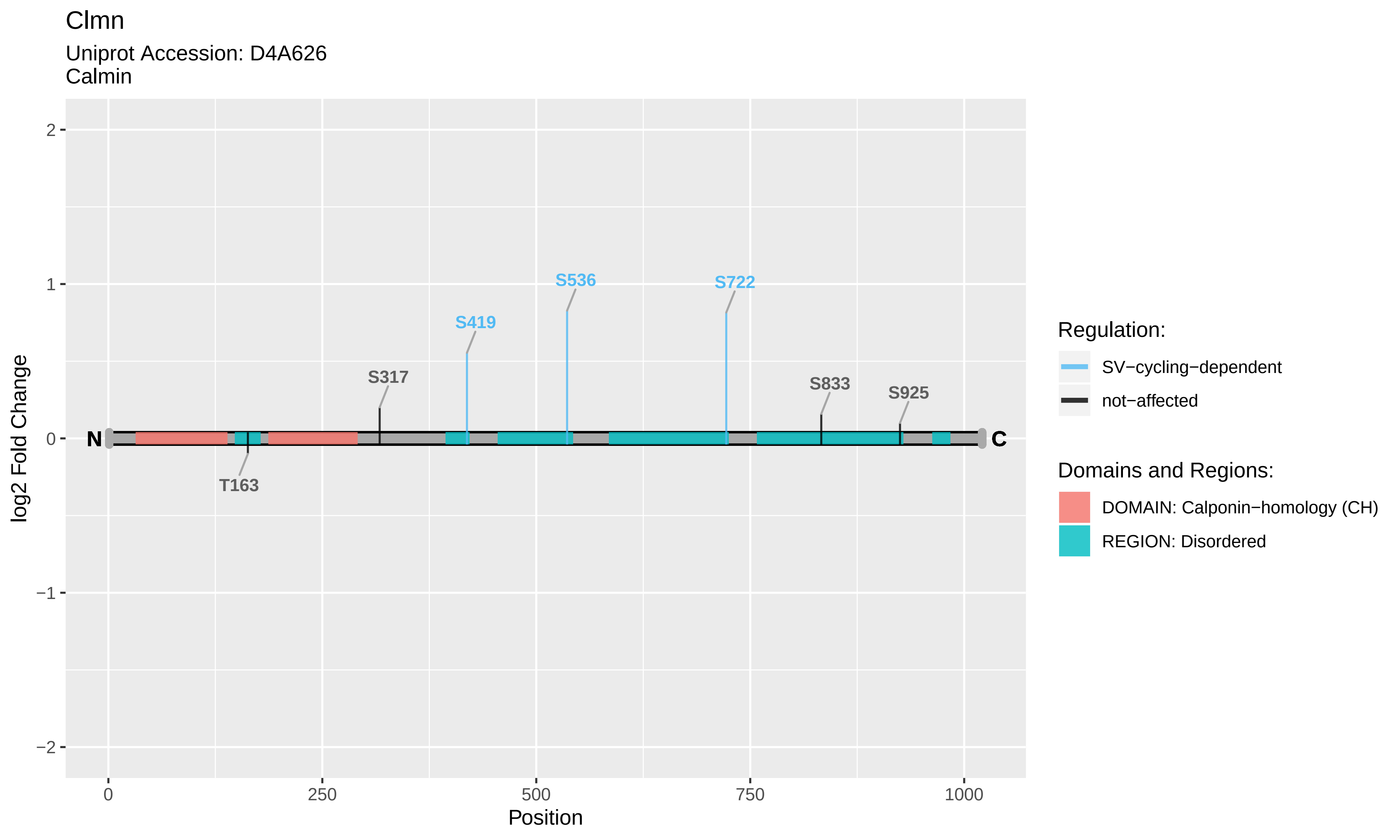
Clmn
Uniprot Accession: D4A626
Calmin
log2 Fold Change
2
1
0
−1
−2
N
C
S317
S419
S536
S722
S833
S925
T163
0
250
500
750
1000
Position
Regulation:
SV−cycling−dependent
not−affected
Domains and Regions:
DOMAIN: Calponin−homology (CH)
REGION: Disordered

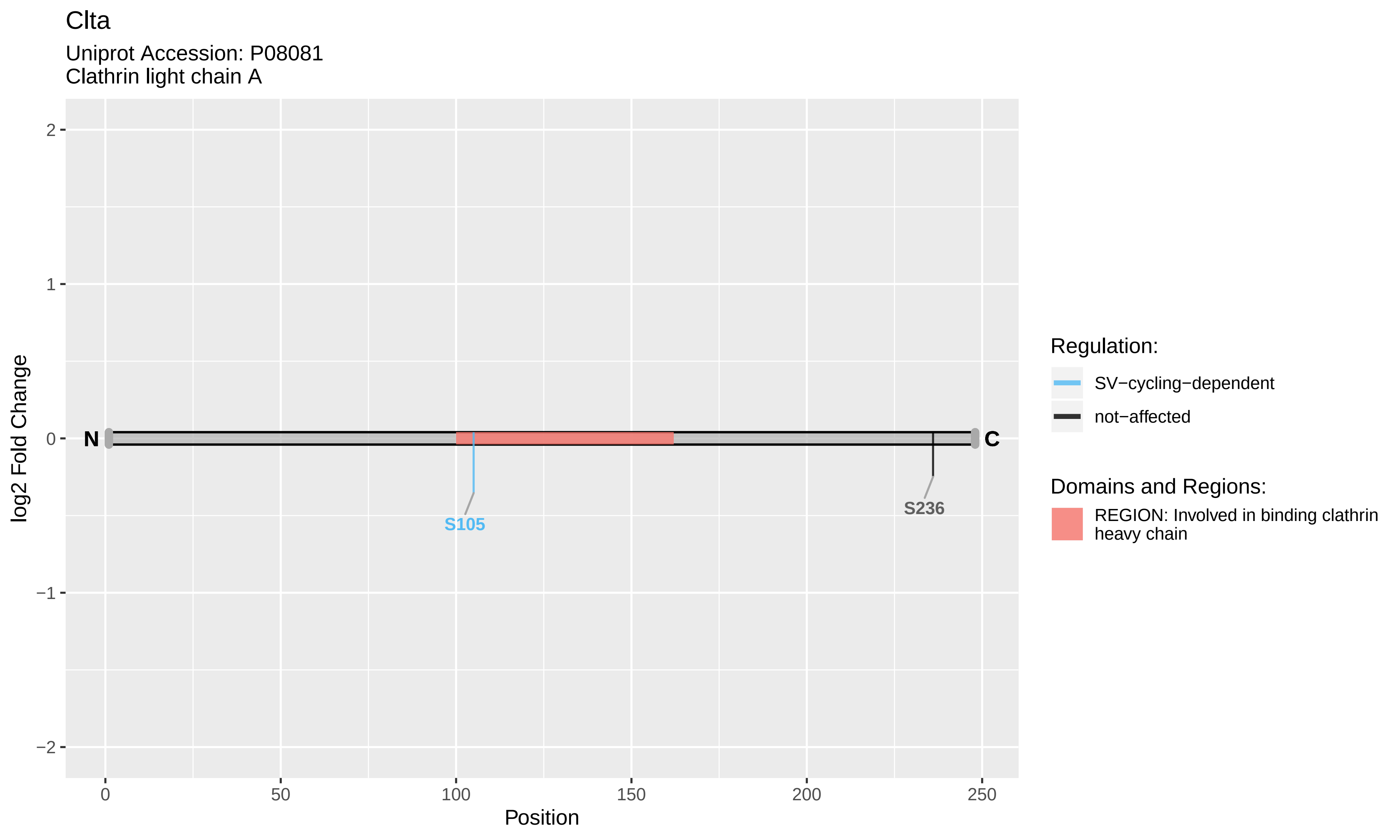

Clta
Uniprot Accession: P08081
Clathrin light chain A
log2 Fold Change
2
1
0
−1
−2
N
C
S105
S236
0
50
100
150
200
250
Position
Regulation:
SV−cycling−dependent
not−affected
Domains and Regions:
REGION: Involved in binding clathrin heavy chain

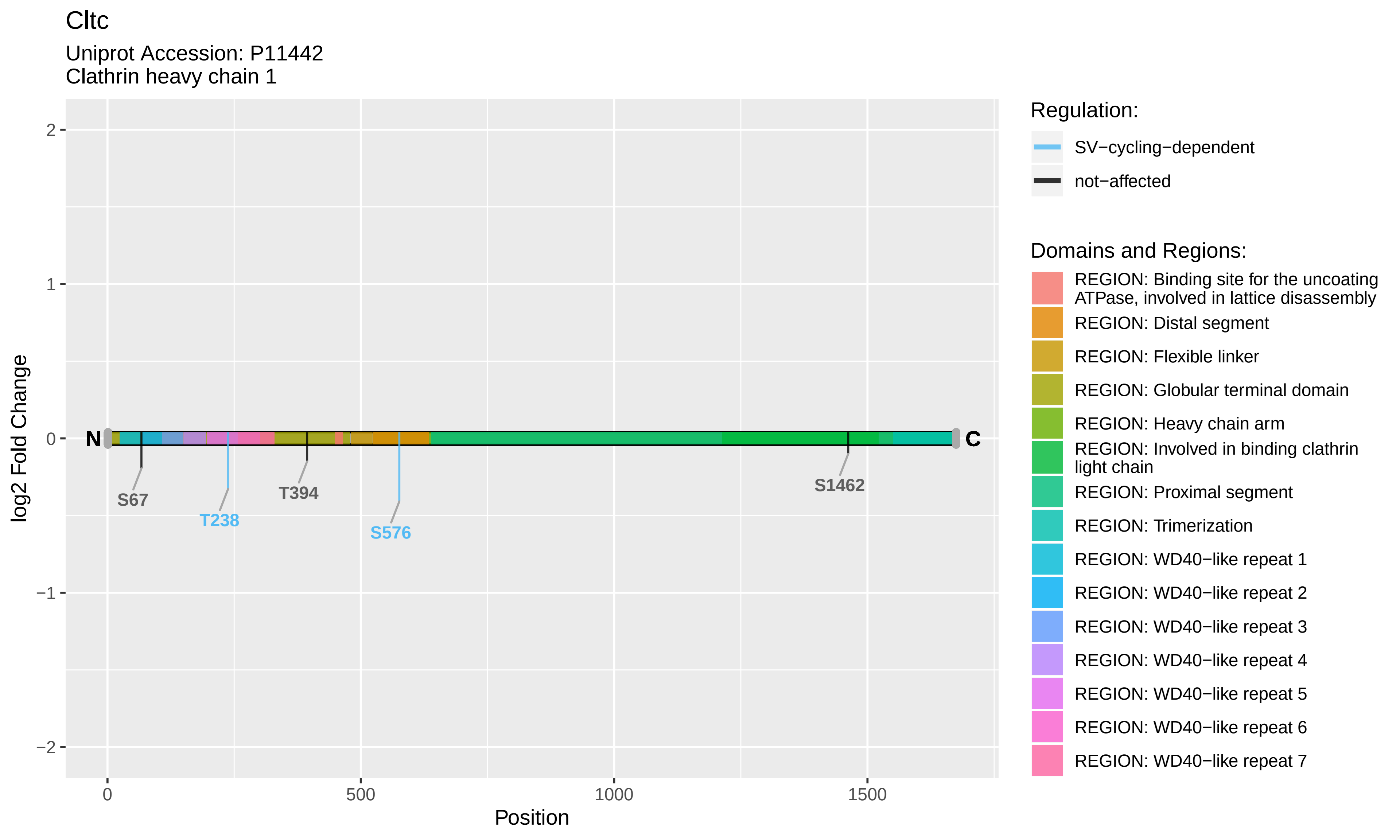

Cltc
Uniprot Accession: P11442
Clathrin heavy chain 1
log2 Fold Change
Position
N
C
S67
T238
T394
S576
S1462
Regulation:
SV−cycling−dependent
not−affected
Domains and Regions:
REGION: Binding site for the uncoating ATPase, involved in lattice disassembly
REGION: Distal segment
REGION: Flexible linker
REGION: Globular terminal domain
REGION: Heavy chain arm
REGION: Involved in binding clathrin light chain
REGION: Proximal segment
REGION: Trimerization
REGION: WD40−like repeat 1
REGION: WD40−like repeat 2
REGION: WD40−like repeat 3
REGION: WD40−like repeat 4
REGION: WD40−like repeat 5
REGION: WD40−like repeat 6
REGION: WD40−like repeat 7

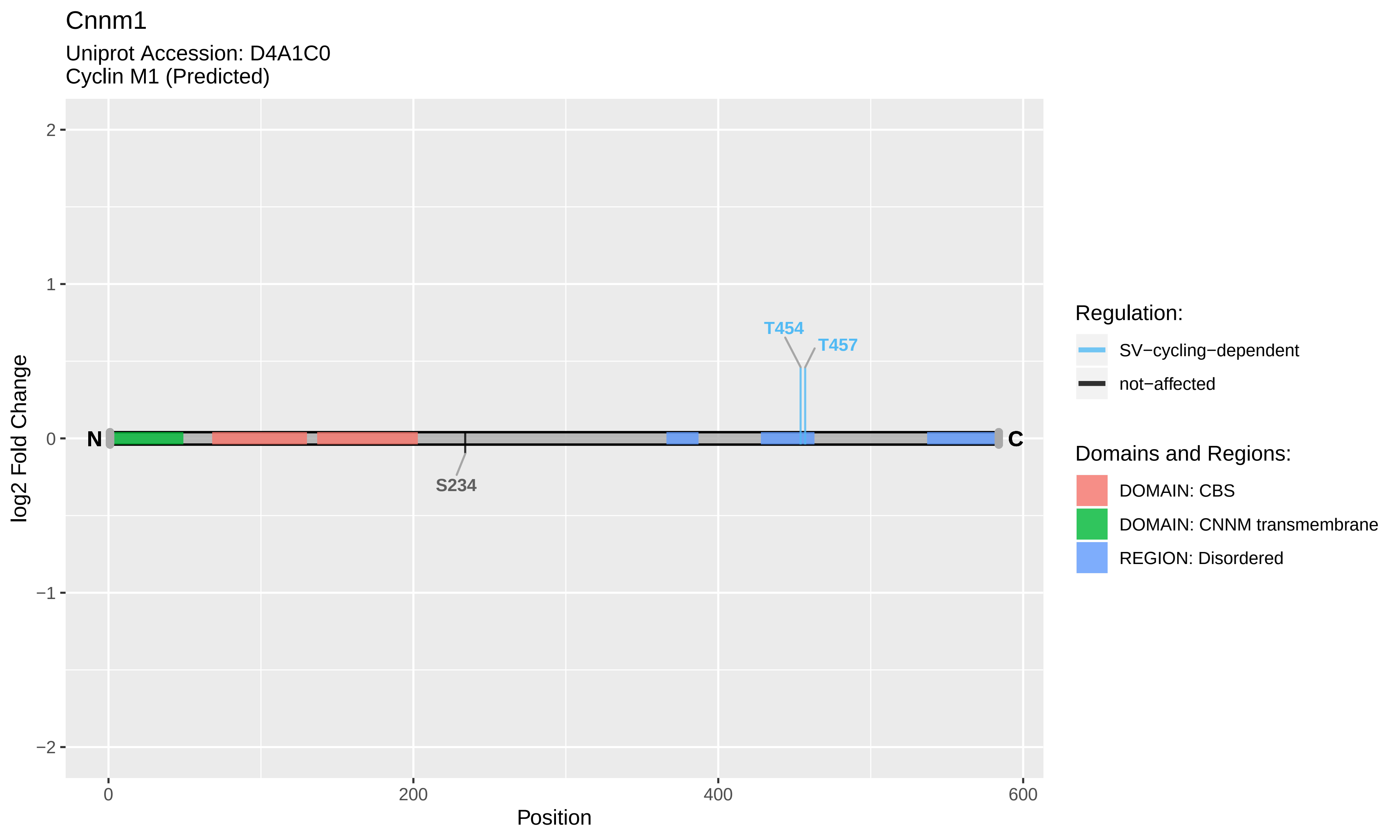

Cnnm1
Uniprot Accession: D4A1C0
Cyclin M1 (Predicted)
log2 Fold Change
2
1
0
−1
−2
N
C
T454
T457
S234
0
200
400
600
Position
Regulation:
SV−cycling−dependent
not−affected
Domains and Regions:
DOMAIN: CBS
DOMAIN: CNNM transmembrane
REGION: Disordered

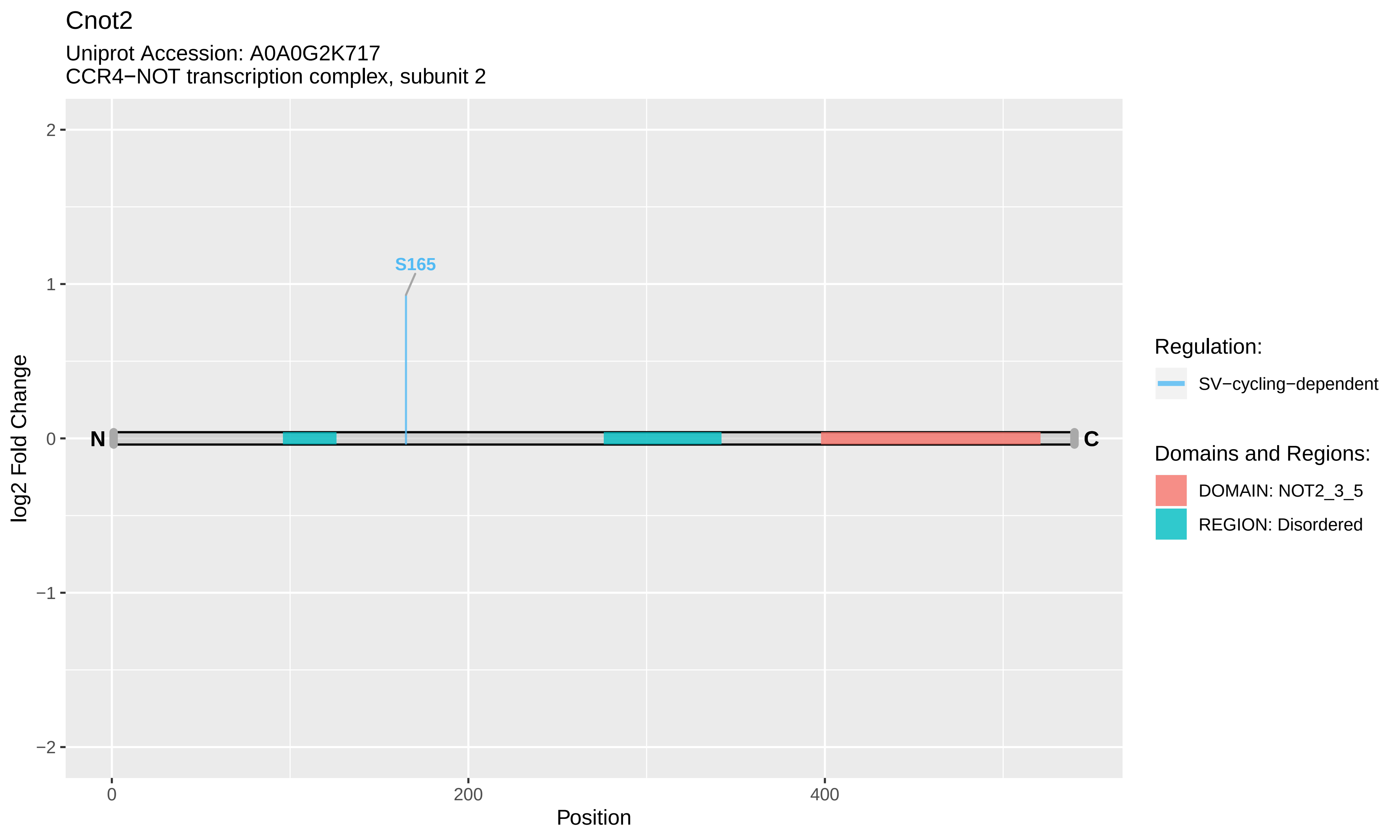

Cnot2
Uniprot Accession: A0A0G2K717
CCR4−NOT transcription complex, subunit 2
S165
N
C
2
1
0
−1
−2
0
200
400
log2 Fold Change
Position
Regulation:
SV−cycling−dependent
Domains and Regions:
DOMAIN: NOT2_3_5
REGION: Disordered

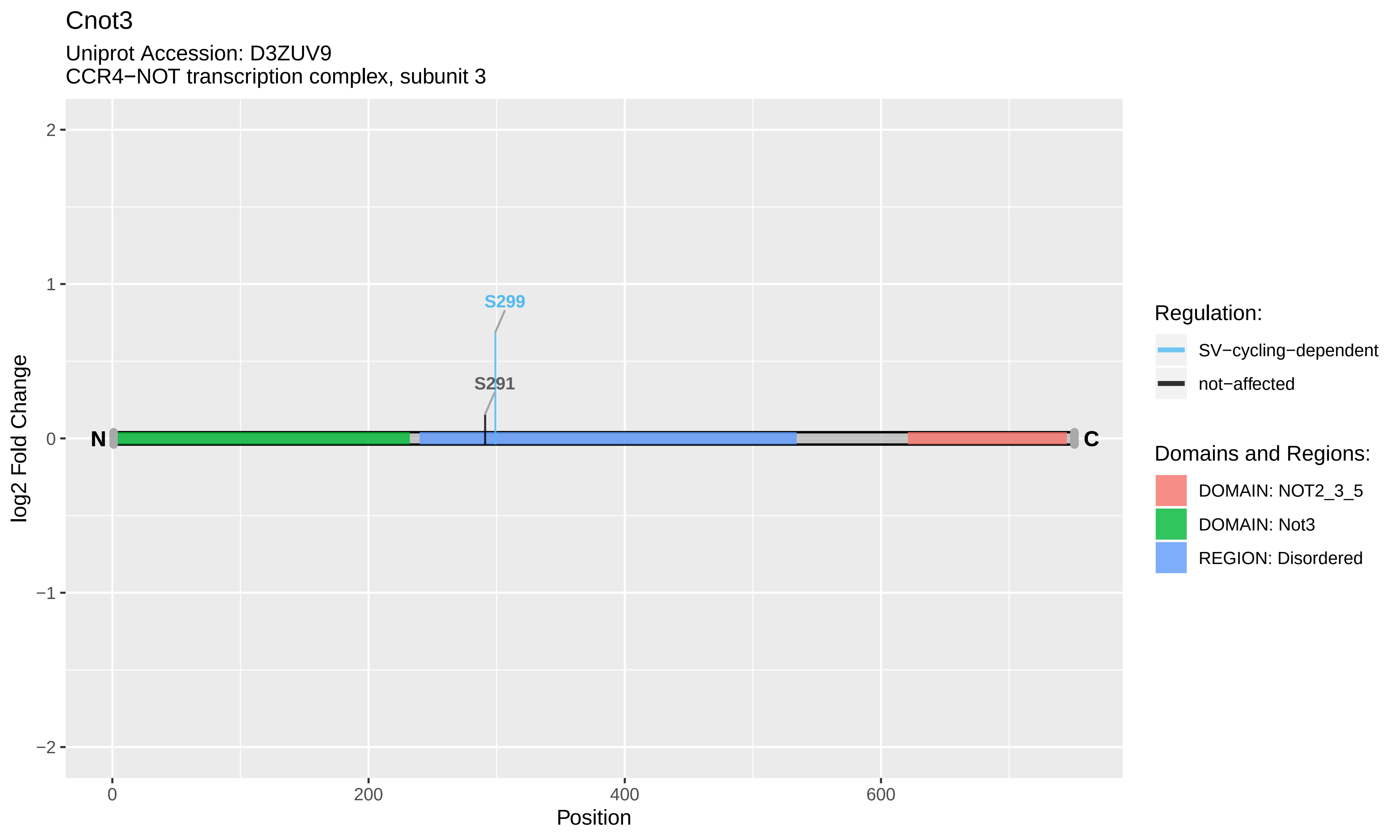

Cnot3
Uniprot Accession: D3ZUV9
CCR4−NOT transcription complex, subunit 3
log2 Fold Change
Position
S299
S291
N
C
2
1
0
−1
−2
0
200
400
600
Regulation:
SV−cycling−dependent
not−affected
Domains and Regions:
DOMAIN: NOT2_3_5
DOMAIN: Not3
REGION: Disordered

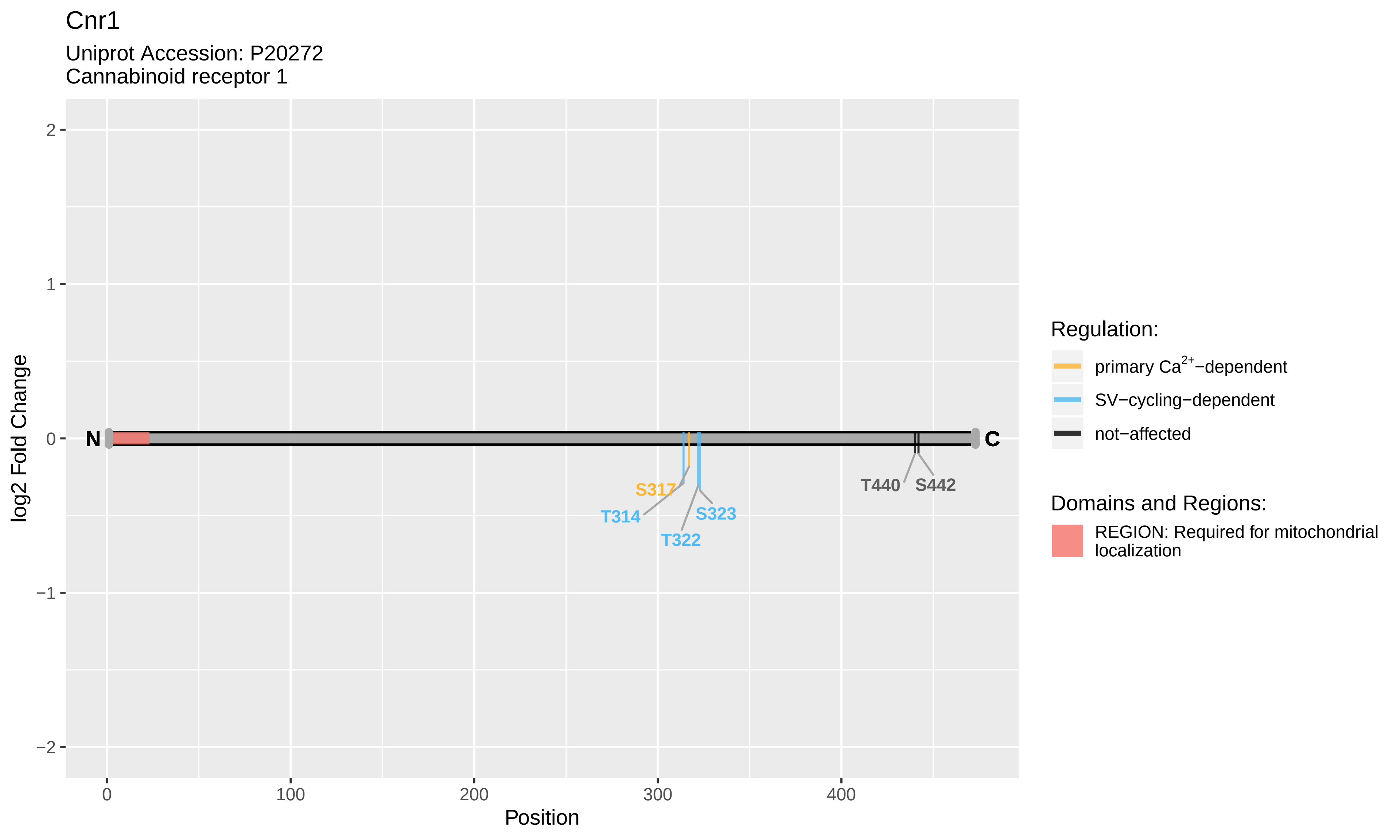
Cnr1
Uniprot Accession: P20272
Cannabinoid receptor 1
log2 Fold Change
2
1
0
−1
−2
N
C
S317
T314
S323
T322
T440
S442
0
100
200
300
400
Position
Regulation:
primary Ca2+−dependent
SV−cycling−dependent
not−affected
Domains and Regions:
REGION: Required for mitochondrial localization

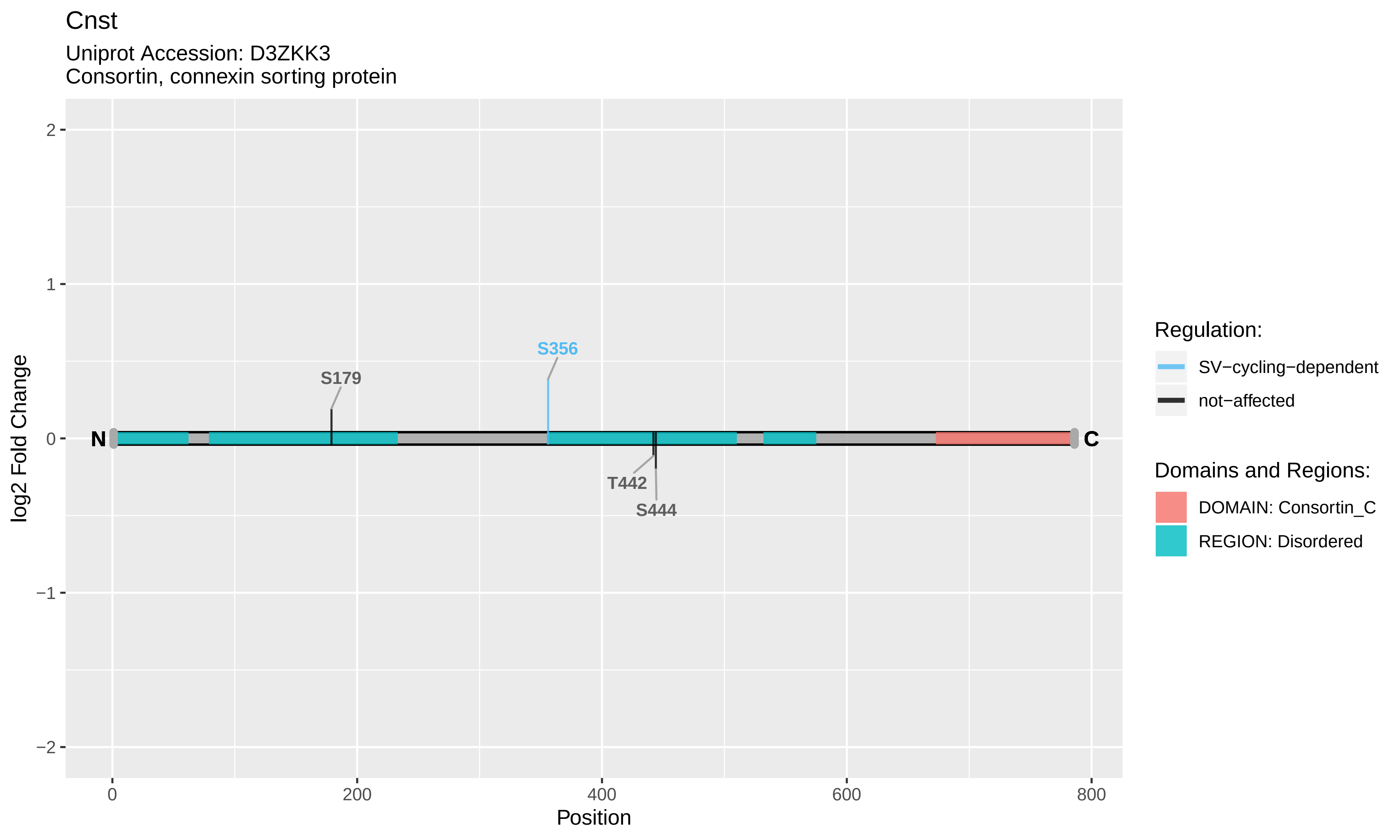

Cnst
Uniprot Accession: D3ZKK3
Consortin, connexin sorting protein
log2 Fold Change
2
1
0
−1
−2
Position
0
200
400
600
800
N
C
S179
S356
T442
S444
Regulation:
SV−cycling−dependent
not−affected
Domains and Regions:
DOMAIN: Consortin_C
REGION: Disordered

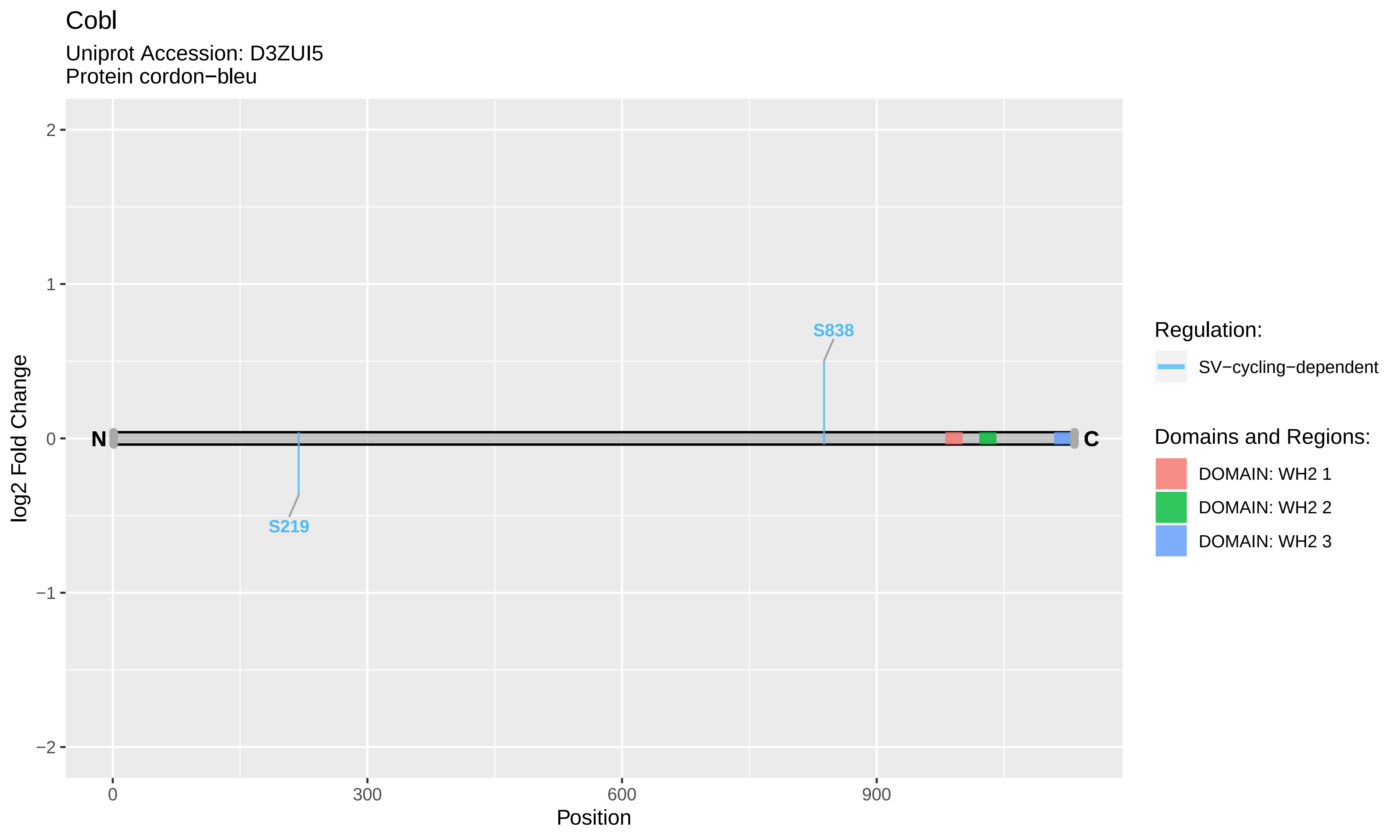

Cobl
Uniprot Accession: D3ZUI5
Protein cordon−bleu
log2 Fold Change
Position
2
1
0
−1
−2
0
300
600
900
N
C
S838
S219
Regulation:
SV−cycling−dependent
Domains and Regions:
DOMAIN: WH2 1
DOMAIN: WH2 2
DOMAIN: WH2 3

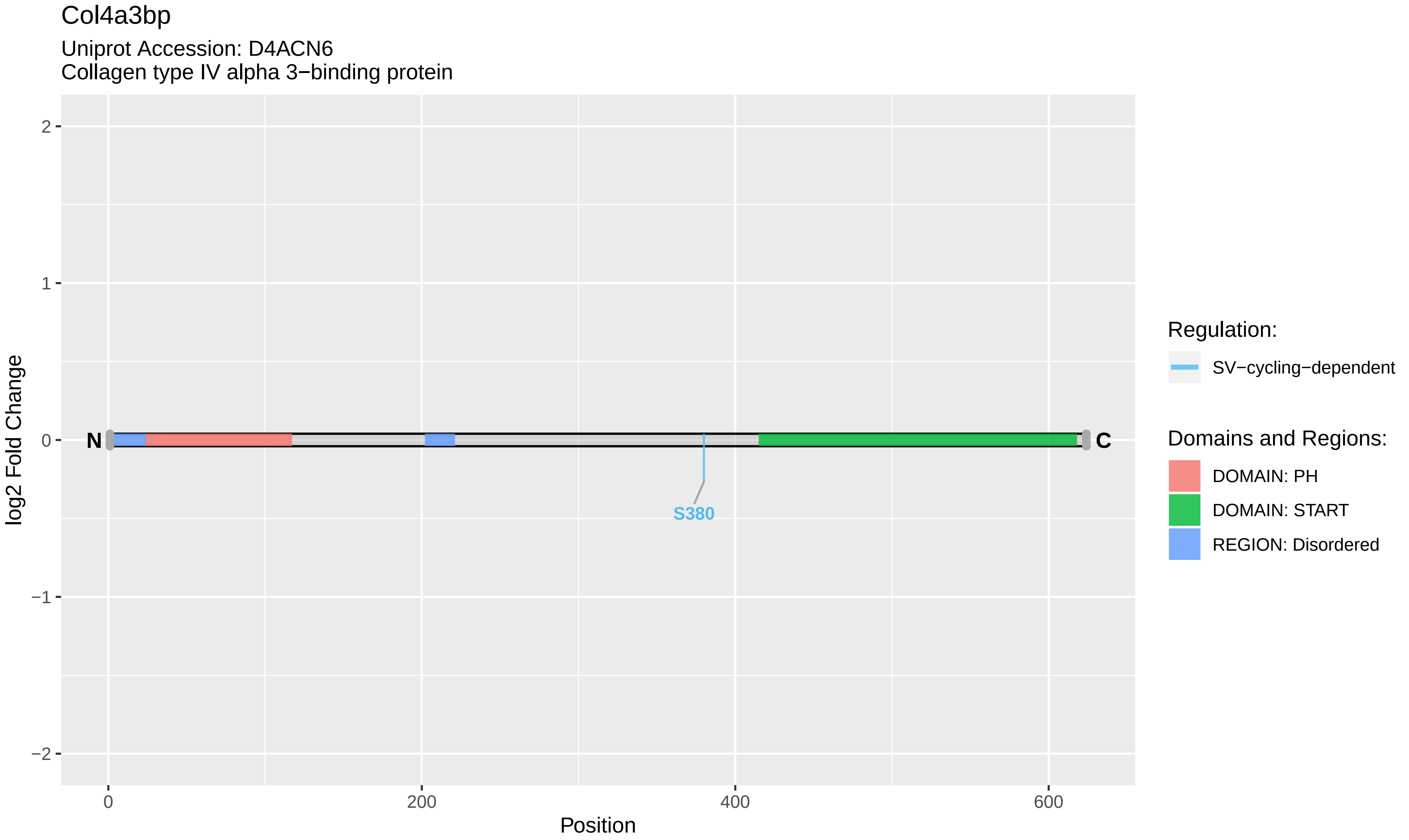
Col4a3bp
Uniprot Accession: D4ACN6
Collagen type IV alpha 3−binding protein
log2 Fold Change
2
1
0
−1
−2
N
C
S380
0
200
400
600
Position
Regulation:
SV−cycling−dependent
Domains and Regions:
DOMAIN: PH
DOMAIN: START
REGION: Disordered

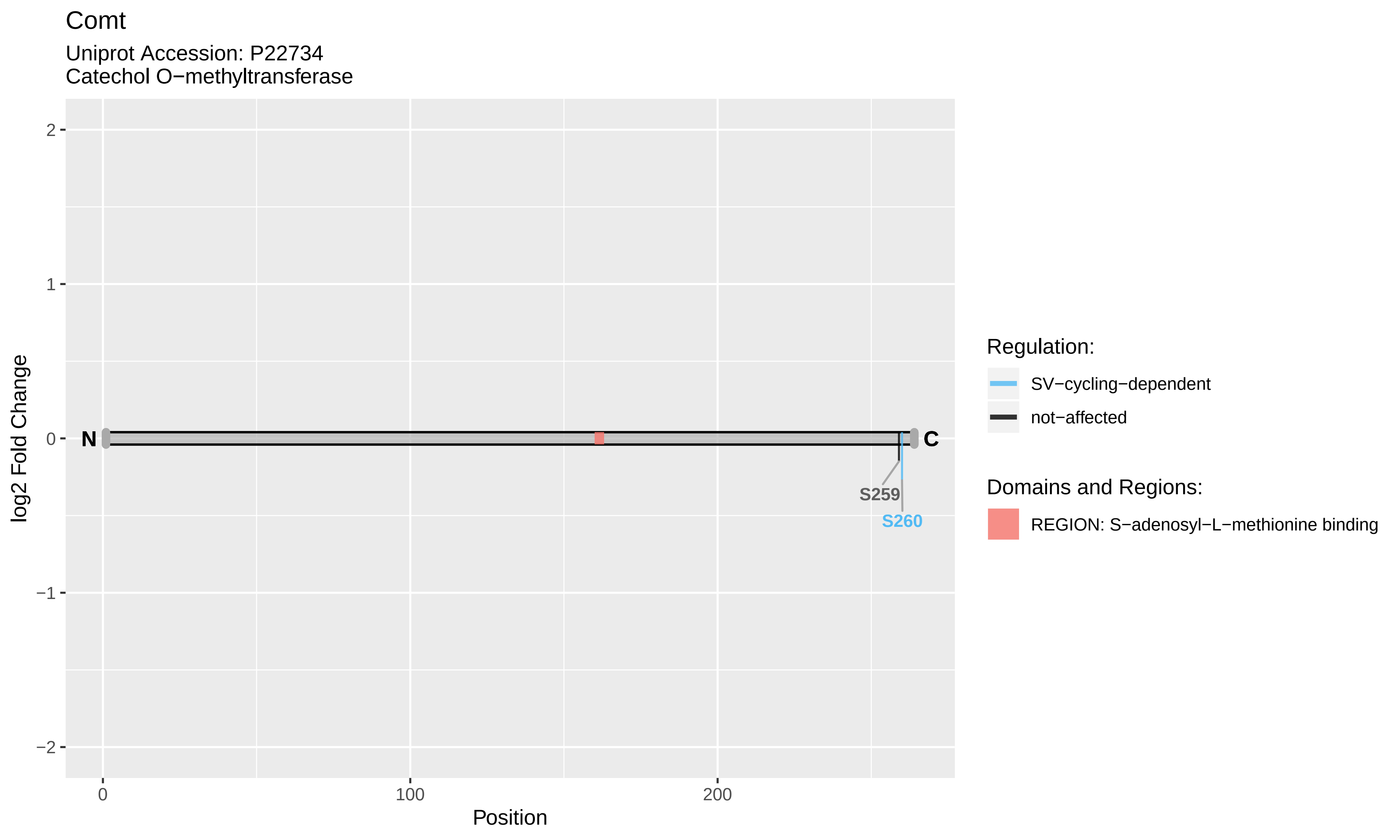

Comt
Uniprot Accession: P22734
Catechol O−methyltransferase
log2 Fold Change
2
1
0
−1
−2
N
C
S259
S260
0
100
200
Position
Regulation:
SV−cycling−dependent
not−affected
Domains and Regions:
REGION: S−adenosyl−L−methionine binding

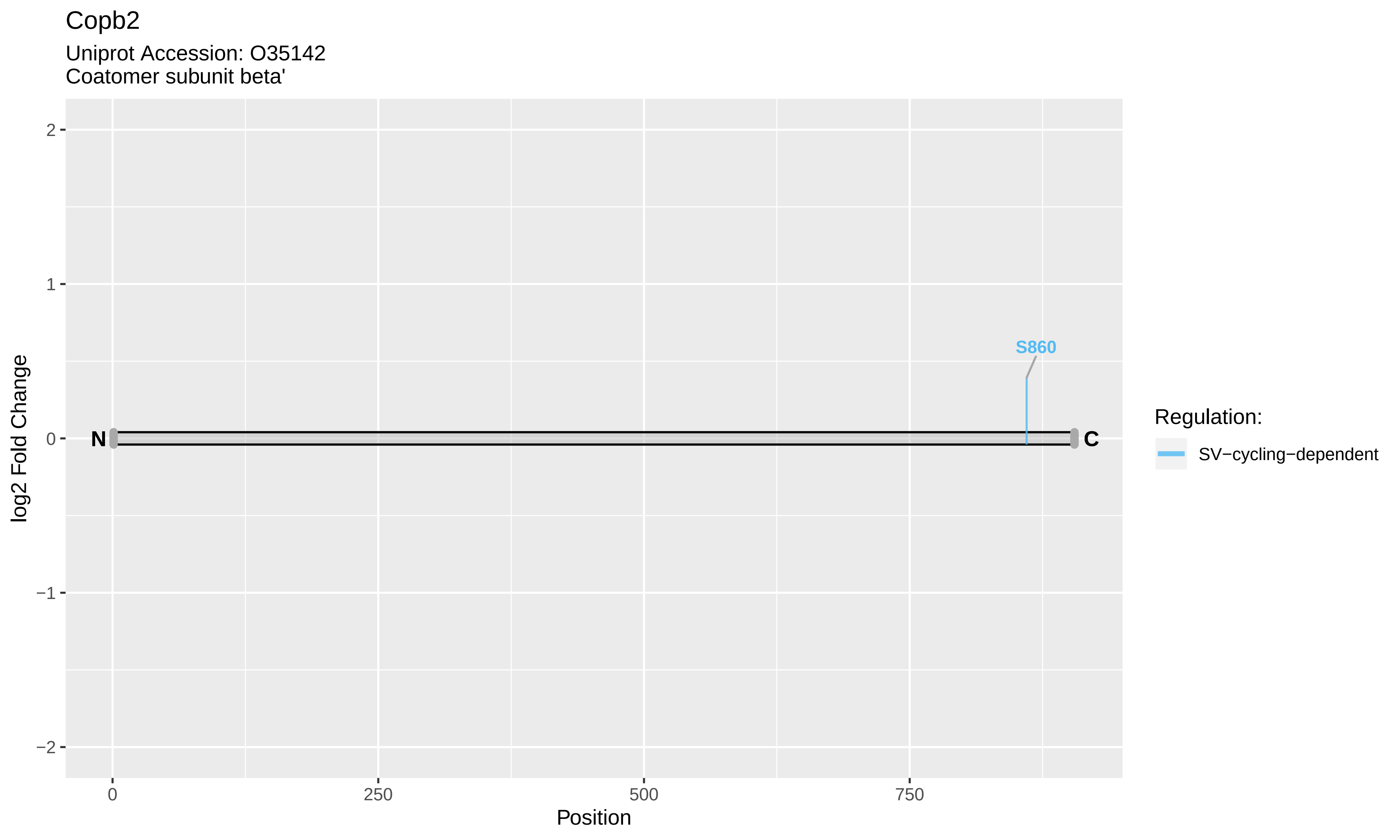

Copb2
Uniprot Accession: O35142
Coatomer subunit beta'
S860
N
C
log2 Fold Change
2
1
0
−1
−2
0
250
500
750
Position
Regulation:
SV−cycling−dependent

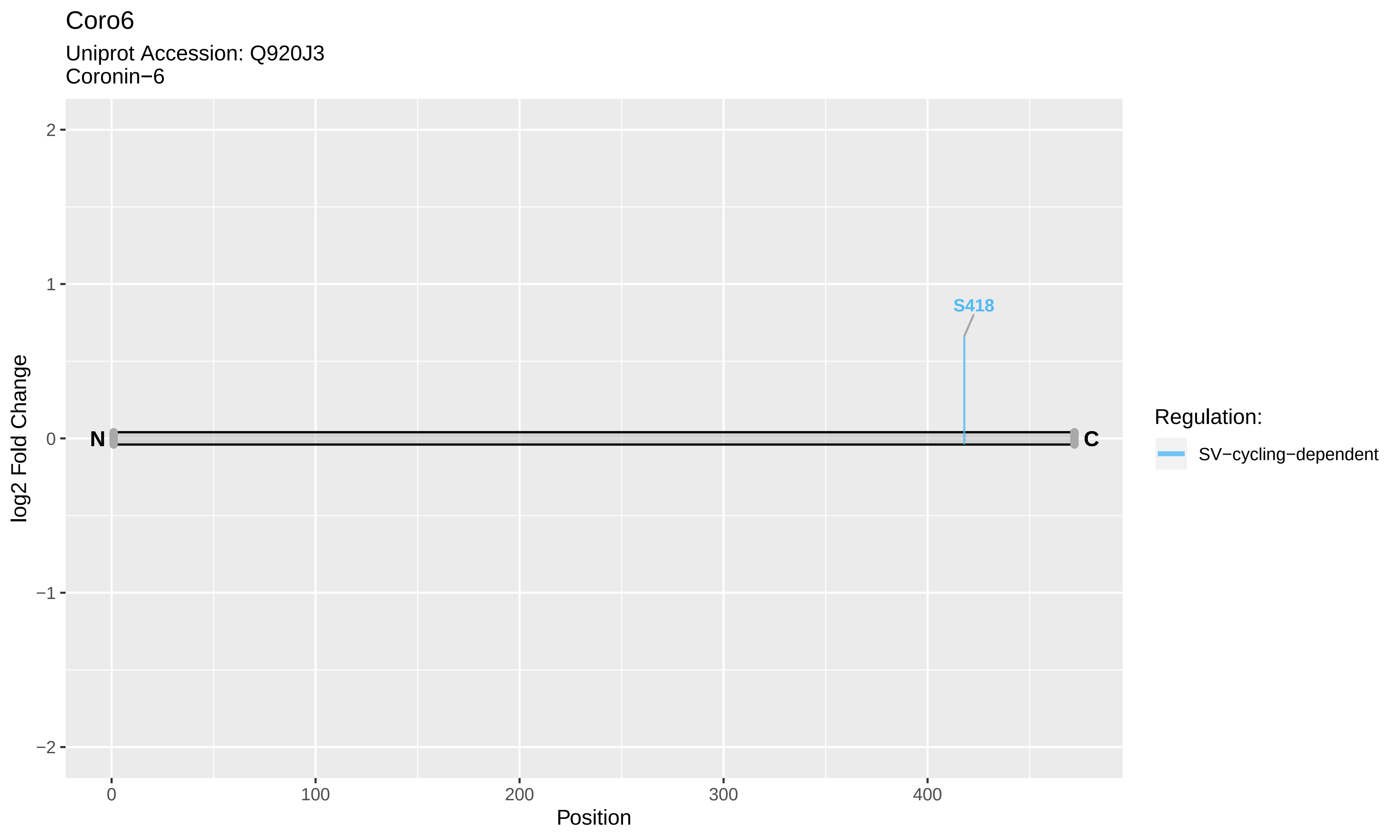

Coro6
Uniprot Accession: Q920J3
Coronin−6
S418
N
C
log2 Fold Change
2
1
0
−1
−2
0
100
200
300
400
Position
Regulation:
SV−cycling−dependent

Cox4i1

Uniprot Accession: P10888
Cytochrome c oxidase subunit 4 isoform 1, mitochondrial

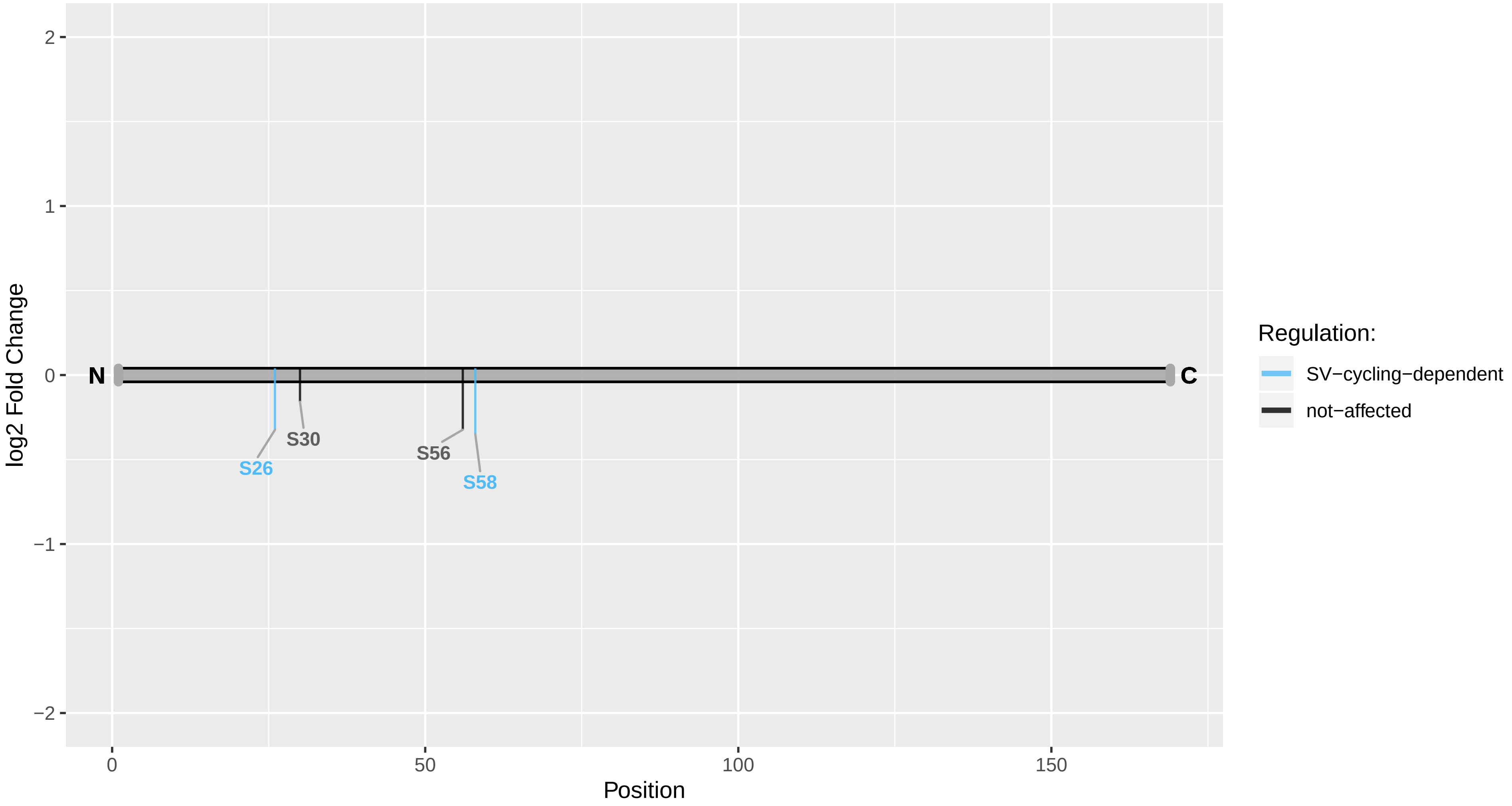

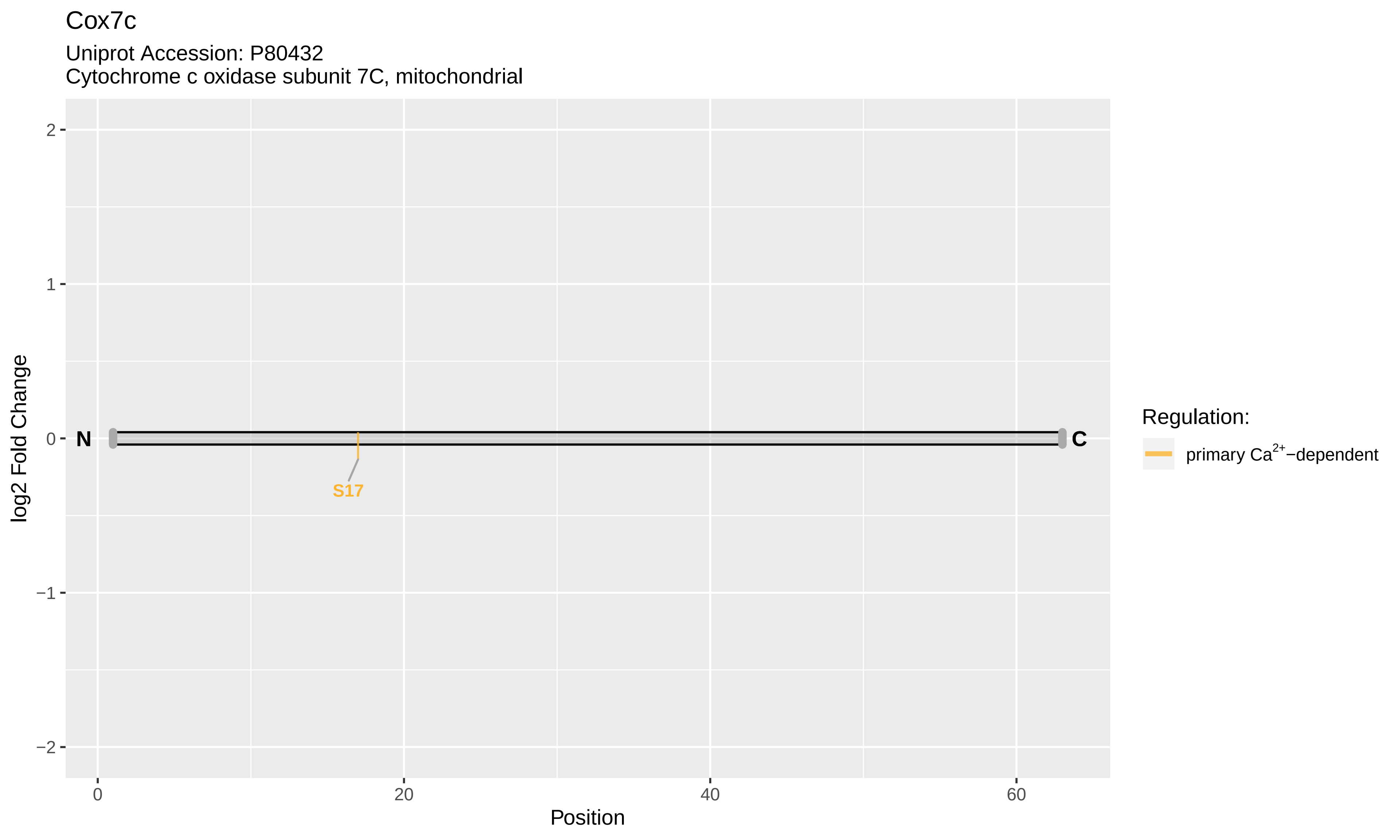

Cox7c
Uniprot Accession: P80432
Cytochrome c oxidase subunit 7C, mitochondrial
log2 Fold Change
2
1
0
−1
−2
N
C
S17
0
20
40
60
Position
Regulation:
primary Ca2+−dependent

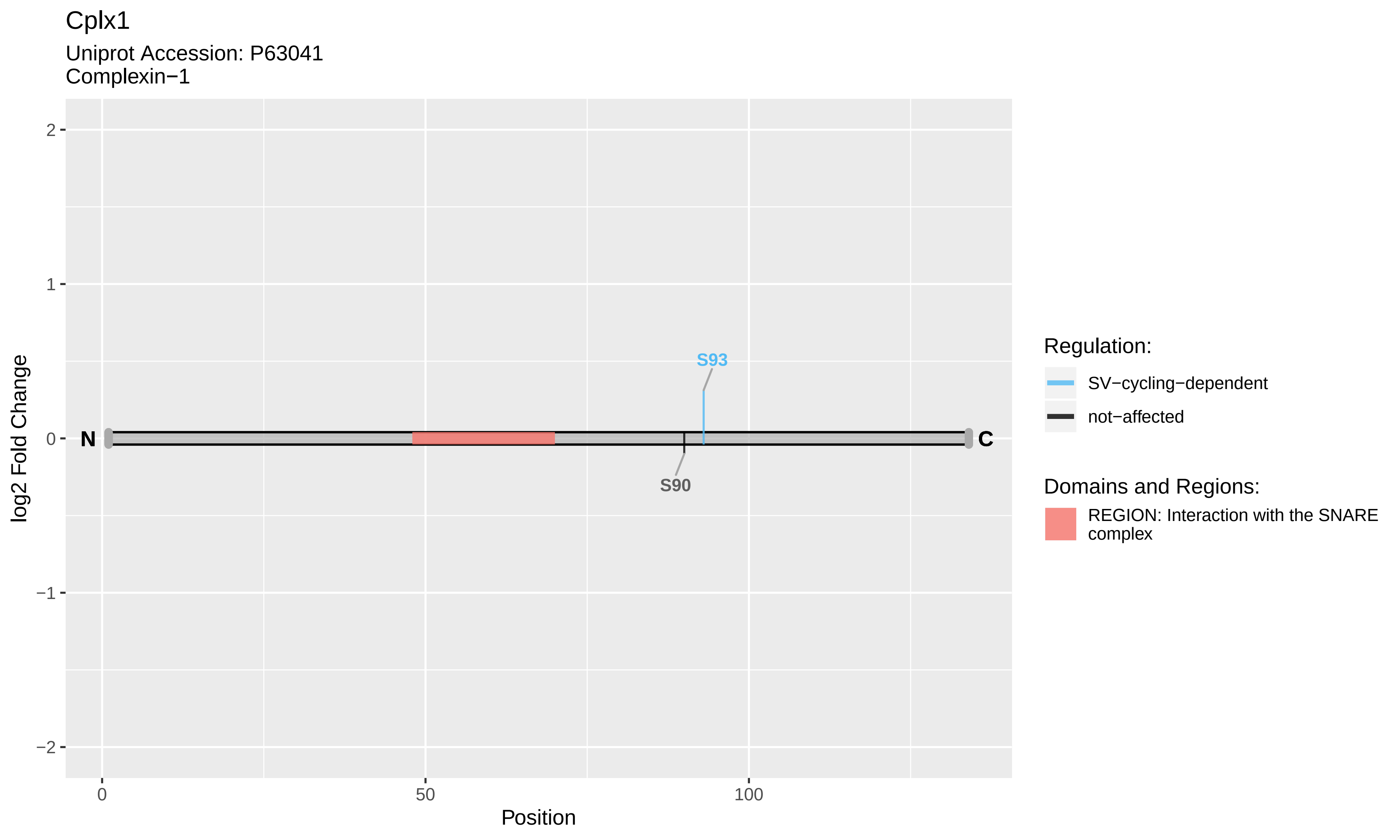

Cplx1
Uniprot Accession: P63041
Complexin−1
log2 Fold Change
2
1
0
−1
−2
N
C
S93
S90
0
50
100
Position
Regulation:
SV−cycling−dependent
not−affected
Domains and Regions:
REGION: Interaction with the SNARE complex

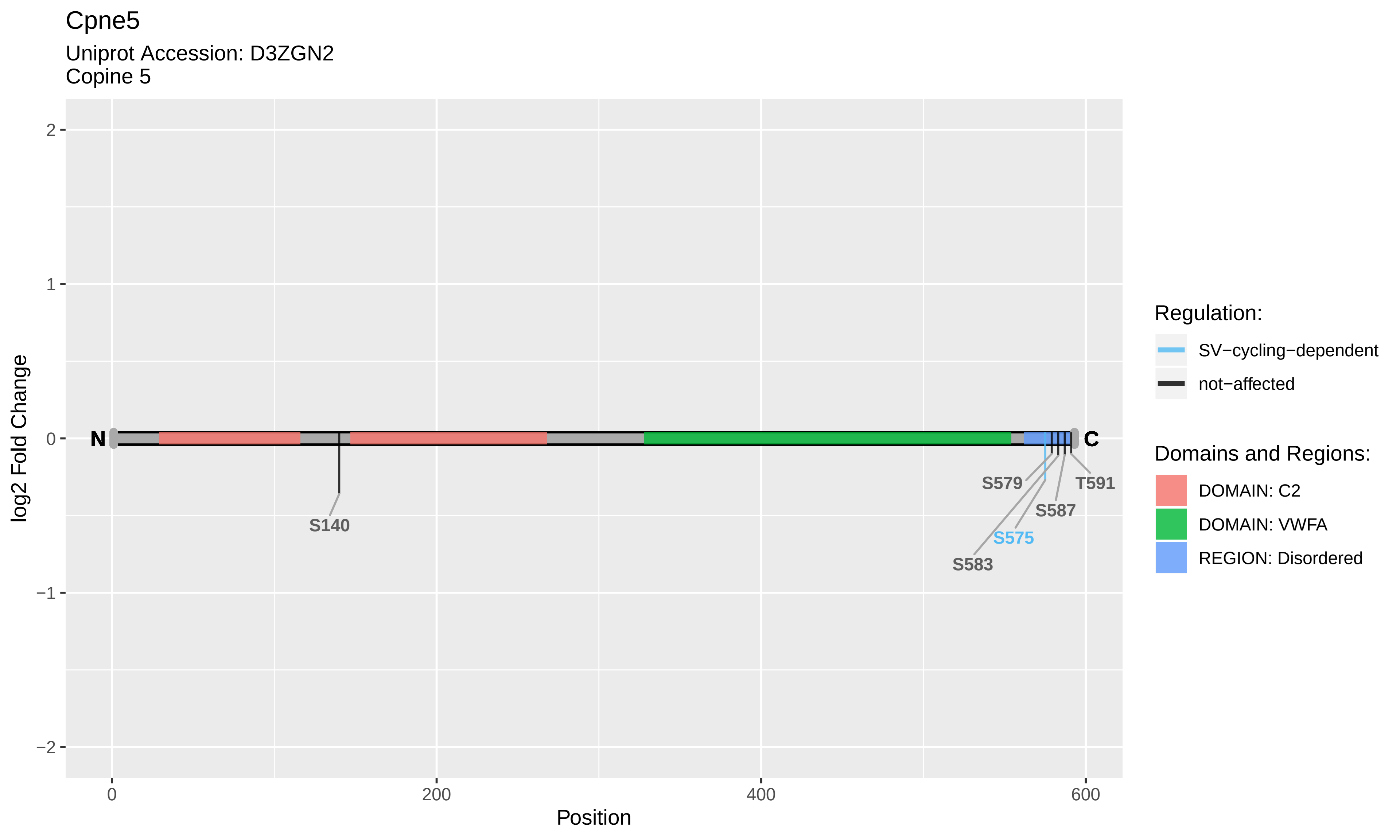

Cpne5
Uniprot Accession: D3ZGN2
Copine 5
log2 Fold Change
Position
N
C
S140
S579
S587
S575
S583
T591
Regulation:
SV−cycling−dependent
not−affected
Domains and Regions:
DOMAIN: C2
DOMAIN: VWFA
REGION: Disordered

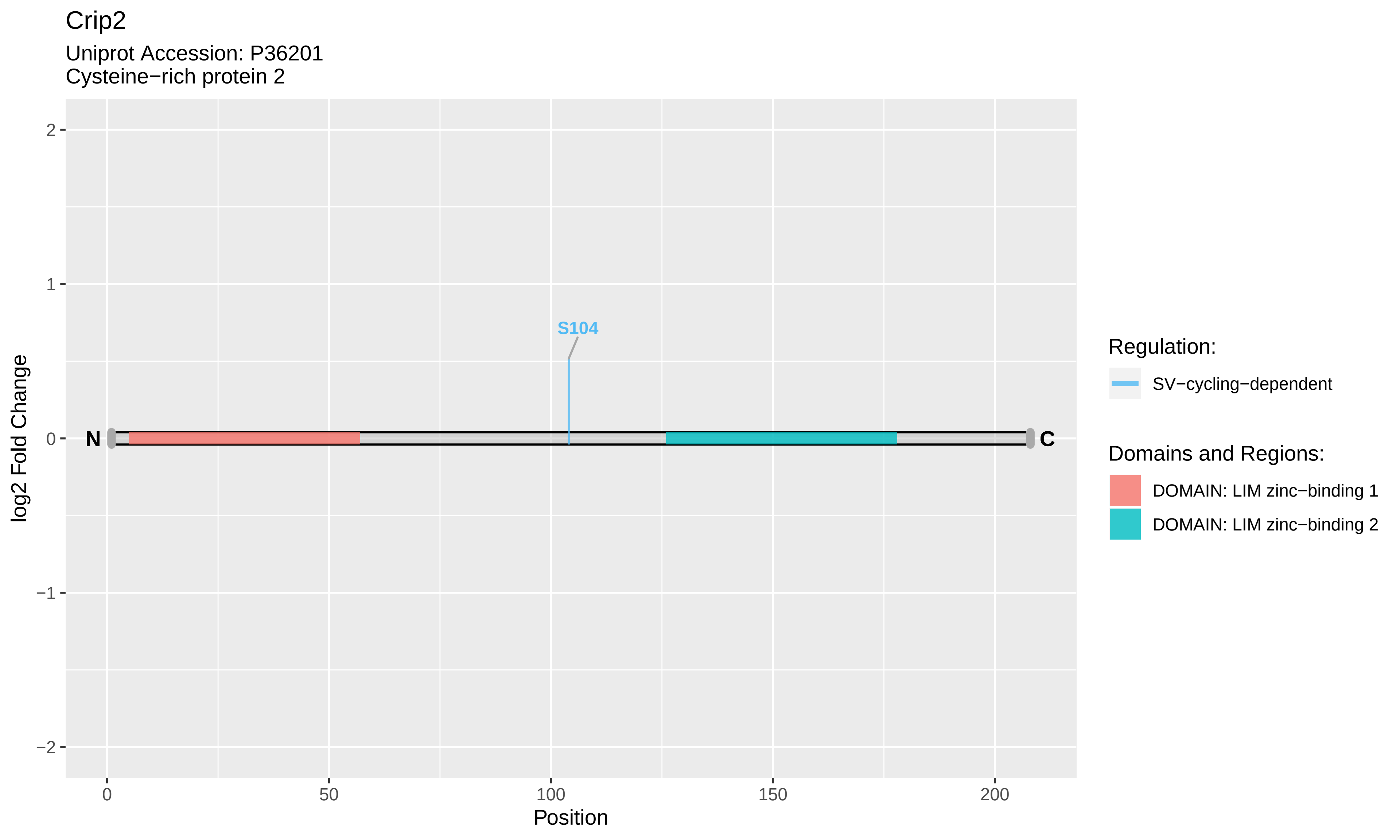

Crip2
Uniprot Accession: P36201
Cysteine−rich protein 2
S104
N
C
2
1
0
−1
−2
log2 Fold Change
0
50
100
150
200
Position
Regulation:
SV−cycling−dependent
Domains and Regions:
DOMAIN: LIM zinc−binding 1
DOMAIN: LIM zinc−binding 2

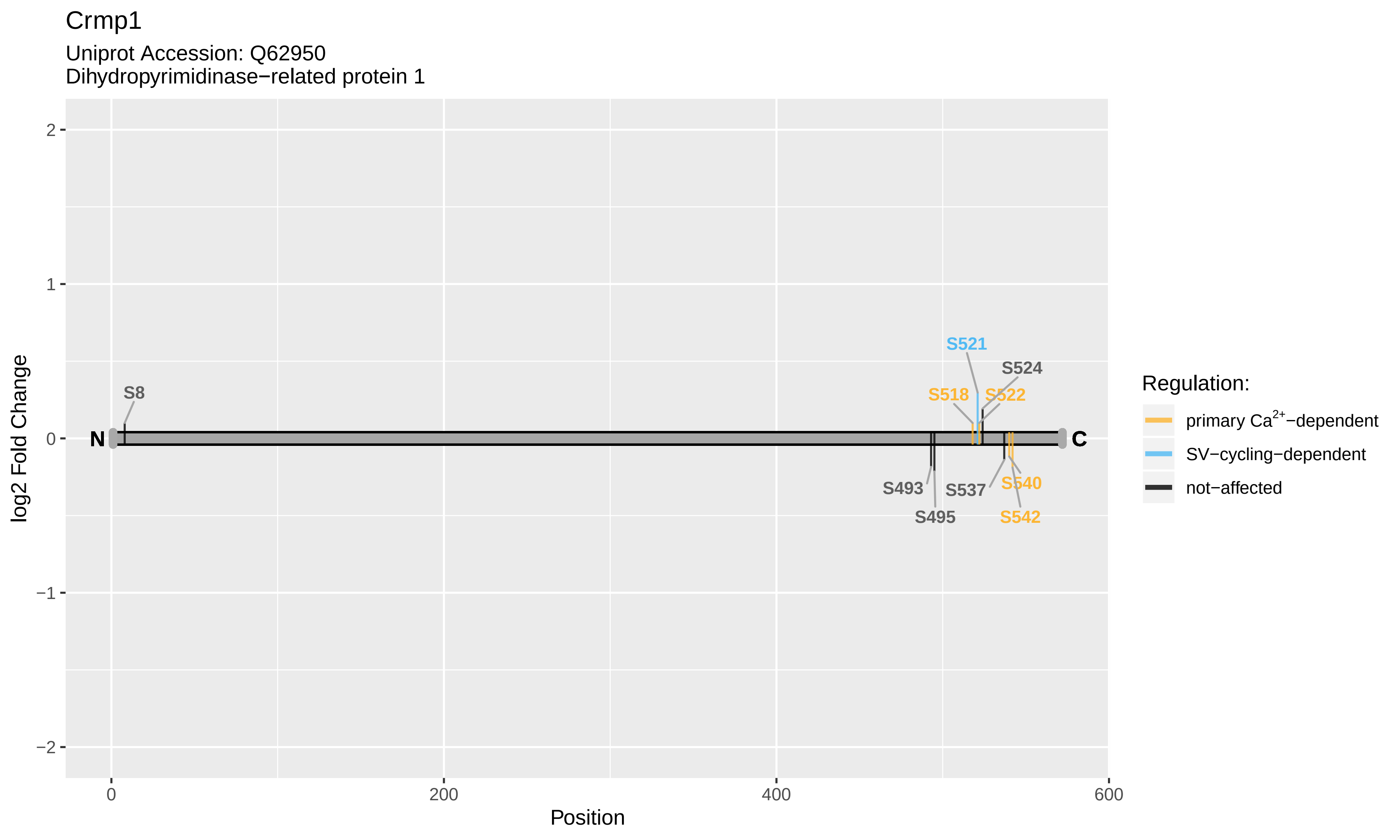
Crmp1
Uniprot Accession: Q62950
Dihydropyrimidinase−related protein 1
log2 Fold Change
Position
N
C
S8
S521
S524
S518
S522
S493
S537
S540
S495
S542
2
1
0
−1
−2
0
200
400
600
Regulation:
primary Ca2+−dependent
SV−cycling−dependent
not−affected

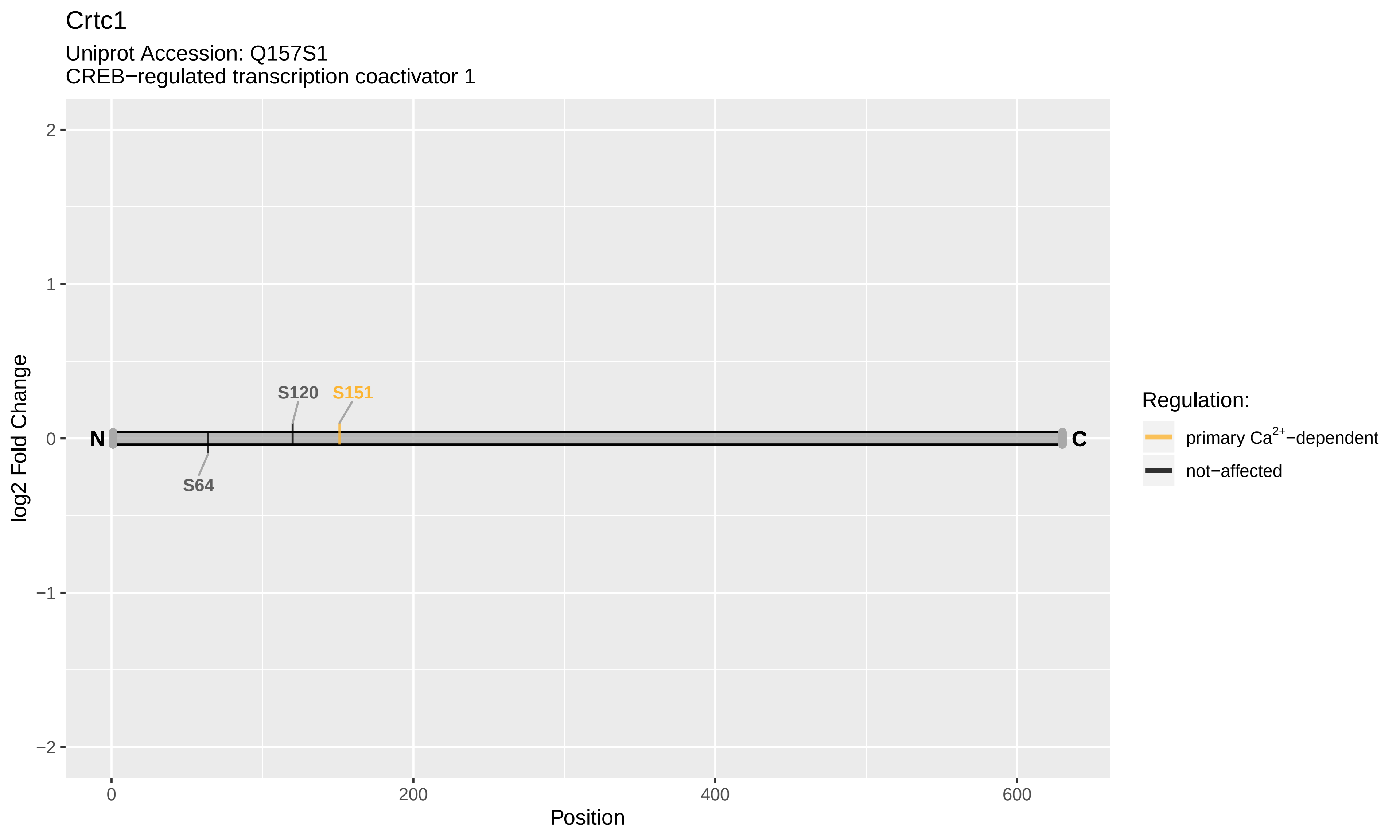
Crtc1
Uniprot Accession: Q157S1
CREB−regulated transcription coactivator 1
log2 Fold Change
2
1
0
−1
−2
N
C
S120
S151
S64
0
200
400
600
Position
Regulation:
primary Ca2+−dependent
not−affected

Cs

Uniprot Accession: Q8VHF5
Citrate synthase, mitochondrial

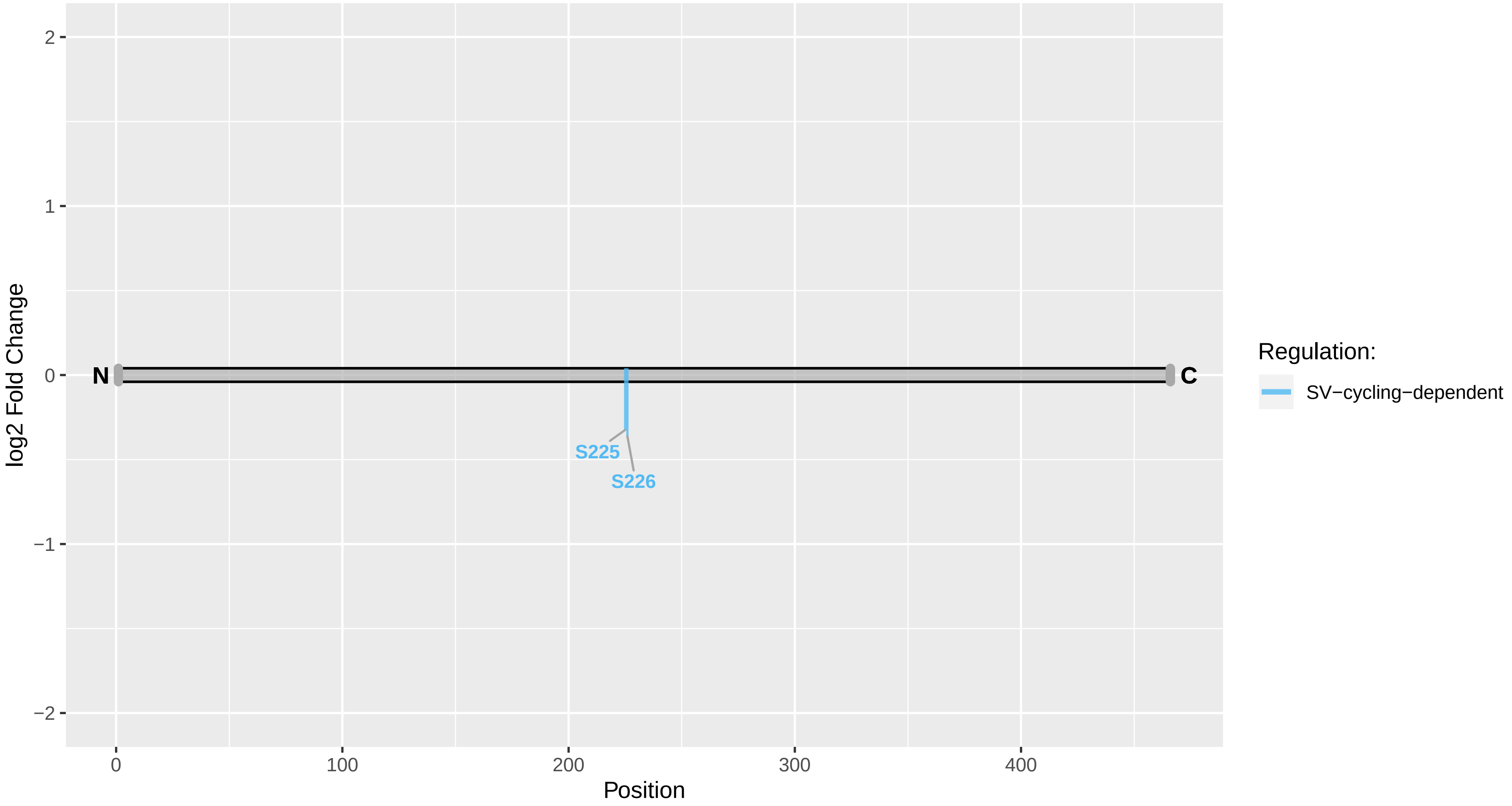

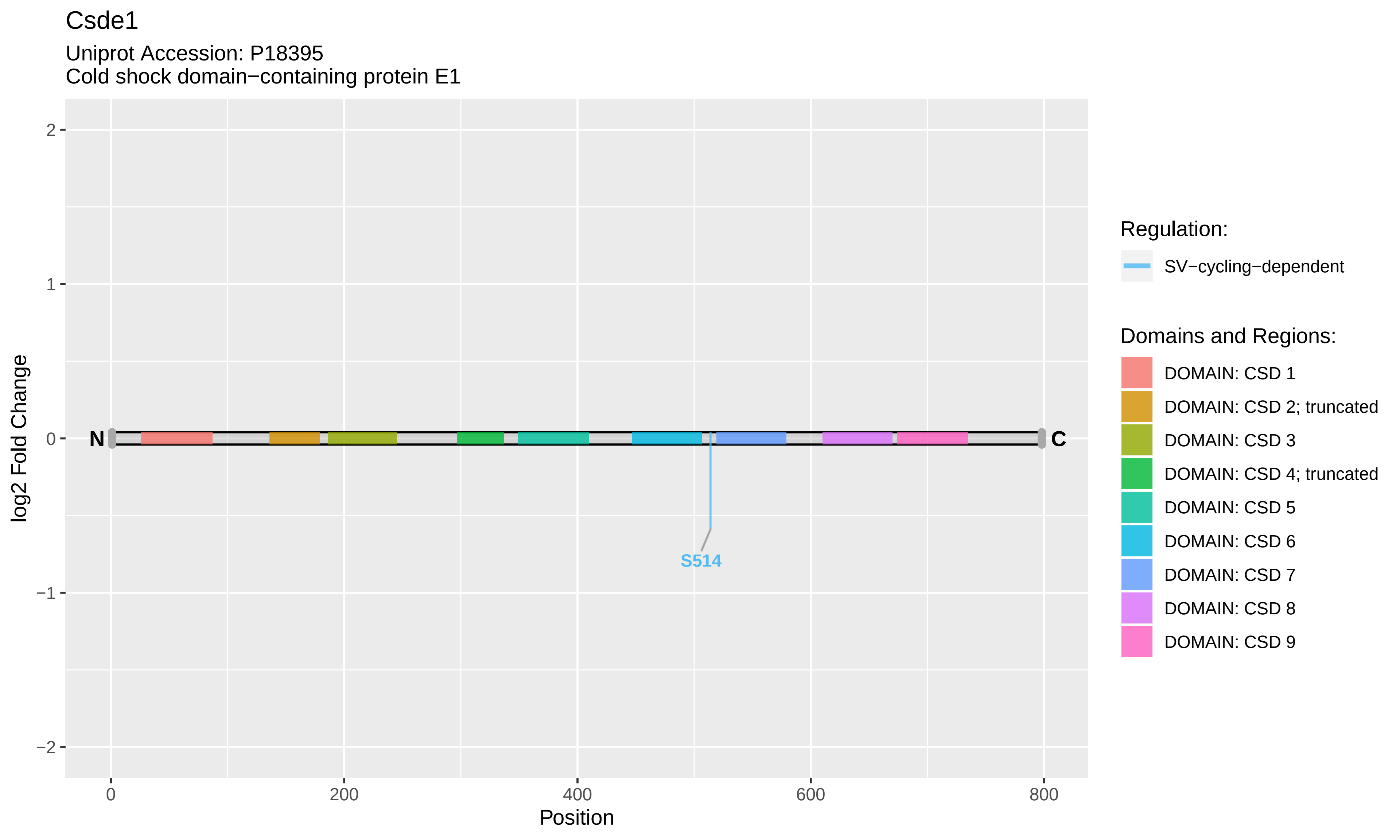

Csde1
Uniprot Accession: P18395
Cold shock domain−containing protein E1
log2 Fold Change
2
1
0
−1
−2
N
C
S514
0
200
400
600
800
Position
Regulation:
SV−cycling−dependent
Domains and Regions:
DOMAIN: CSD 1
DOMAIN: CSD 2; truncated
DOMAIN: CSD 3
DOMAIN: CSD 4; truncated
DOMAIN: CSD 5
DOMAIN: CSD 6
DOMAIN: CSD 7
DOMAIN: CSD 8
DOMAIN: CSD 9

Csnk1e

Uniprot Accession: Q9JJ76
Casein kinase 1 epsilon

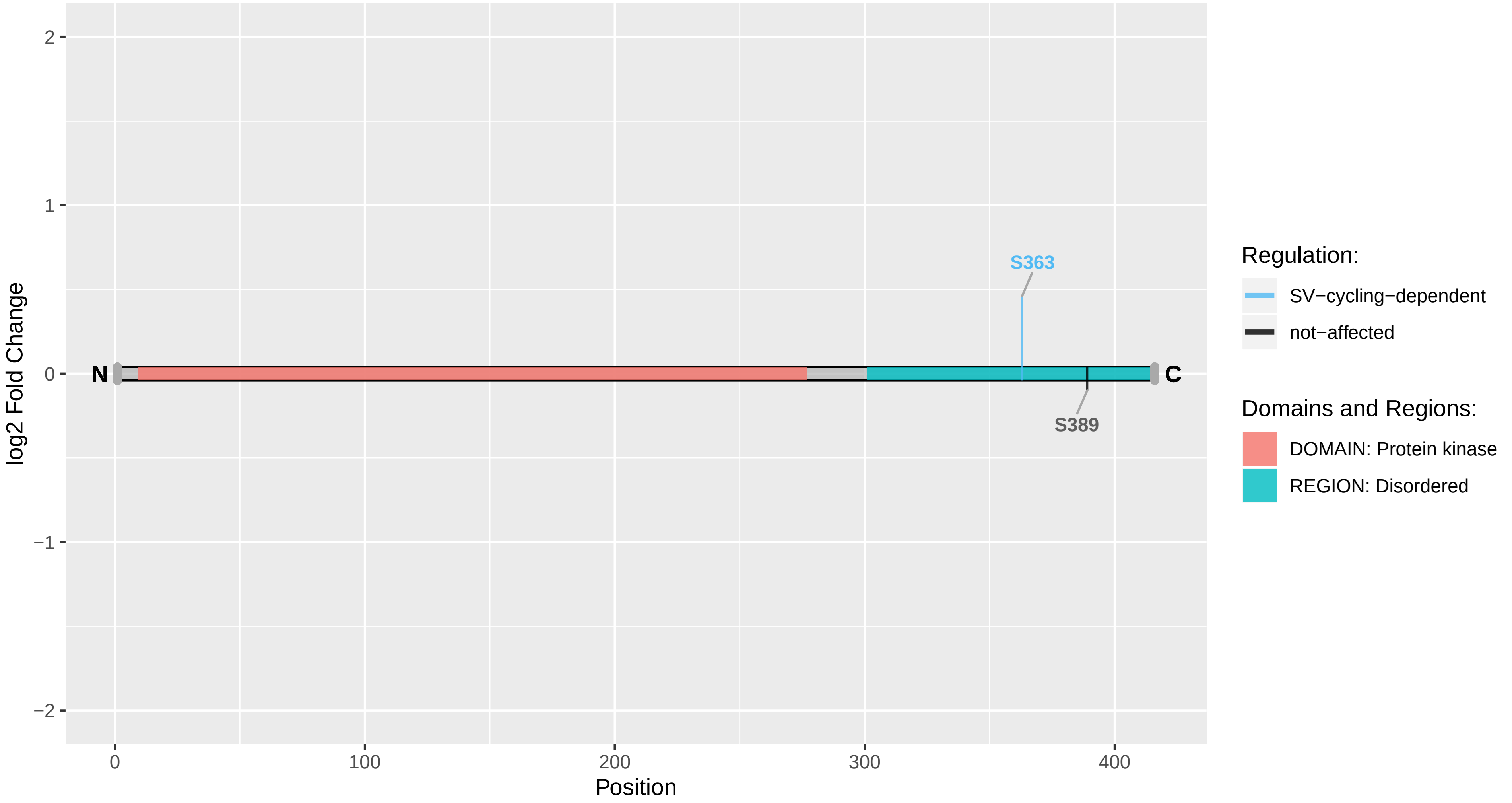

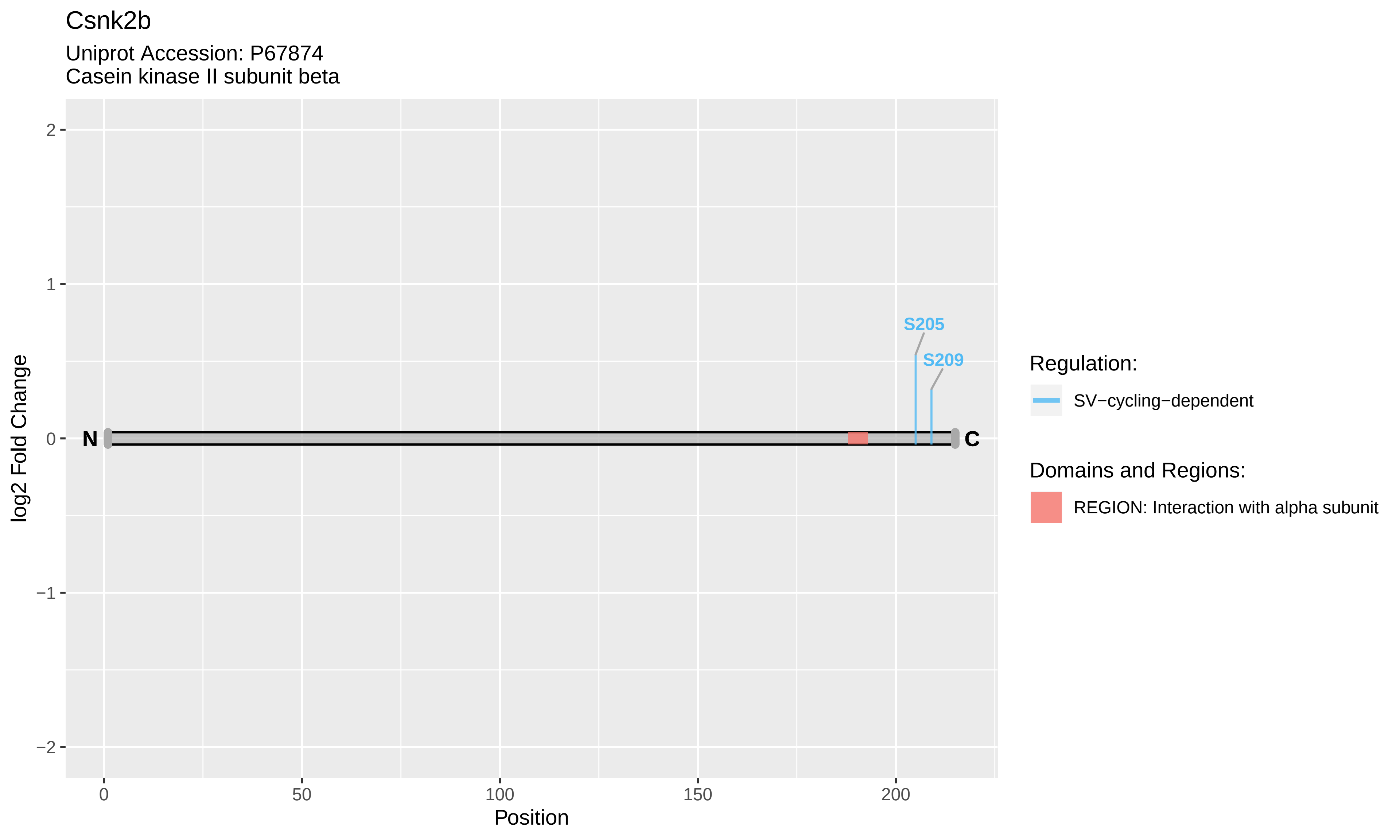
Csnk2b
Uniprot Accession: P67874
Casein kinase II subunit beta
log2 Fold Change
Position
N
C
S205
S209
2
1
0
−1
−2
0
50
100
150
200
Regulation:
SV−cycling−dependent
Domains and Regions:
REGION: Interaction with alpha subunit

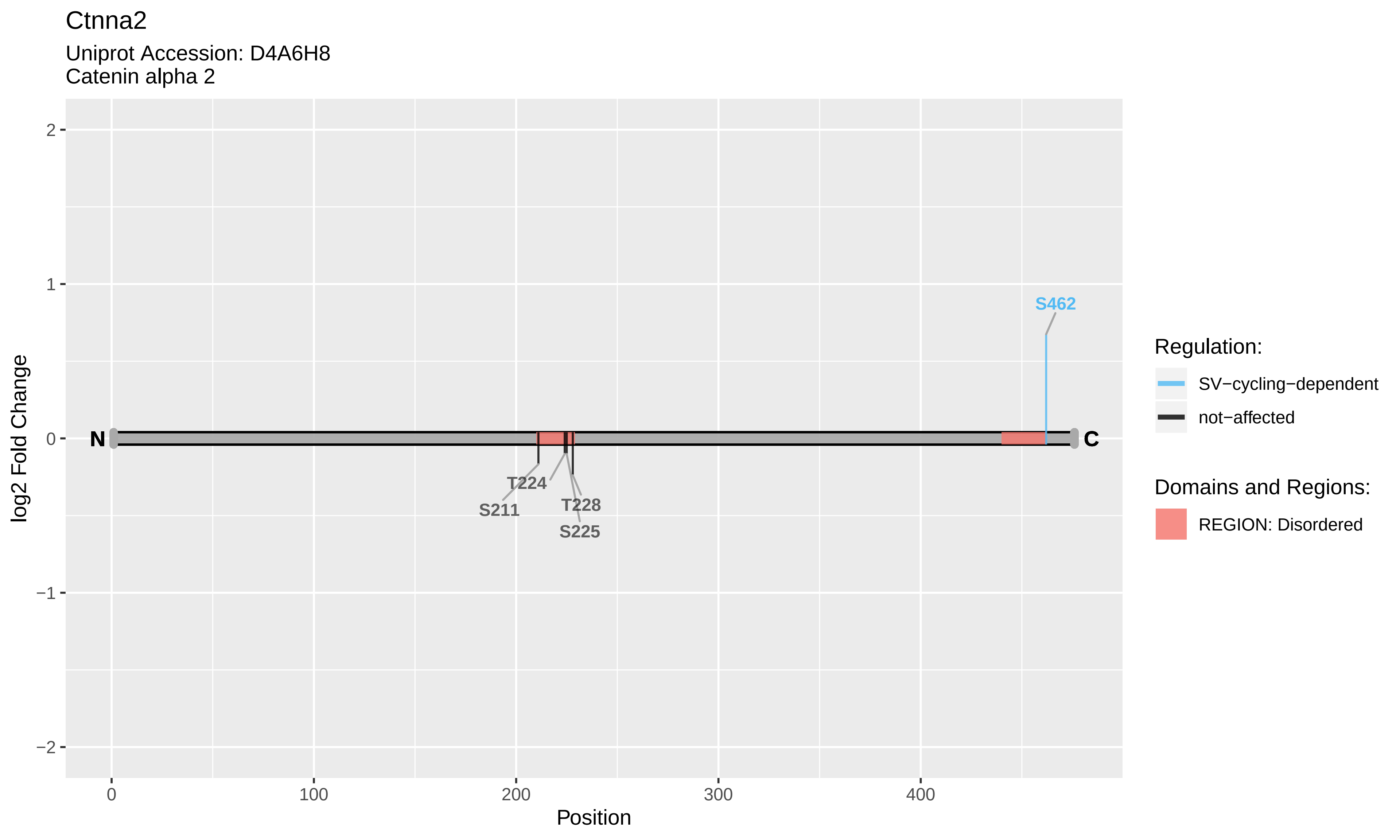
Ctnna2
Uniprot Accession: D4A6H8
Catenin alpha 2
log2 Fold Change
2
1
0
−1
−2
N
C
S462
T224
S211
T228
S225
0
100
200
300
400
Position
Regulation:
SV−cycling−dependent
not−affected
Domains and Regions:
REGION: Disordered

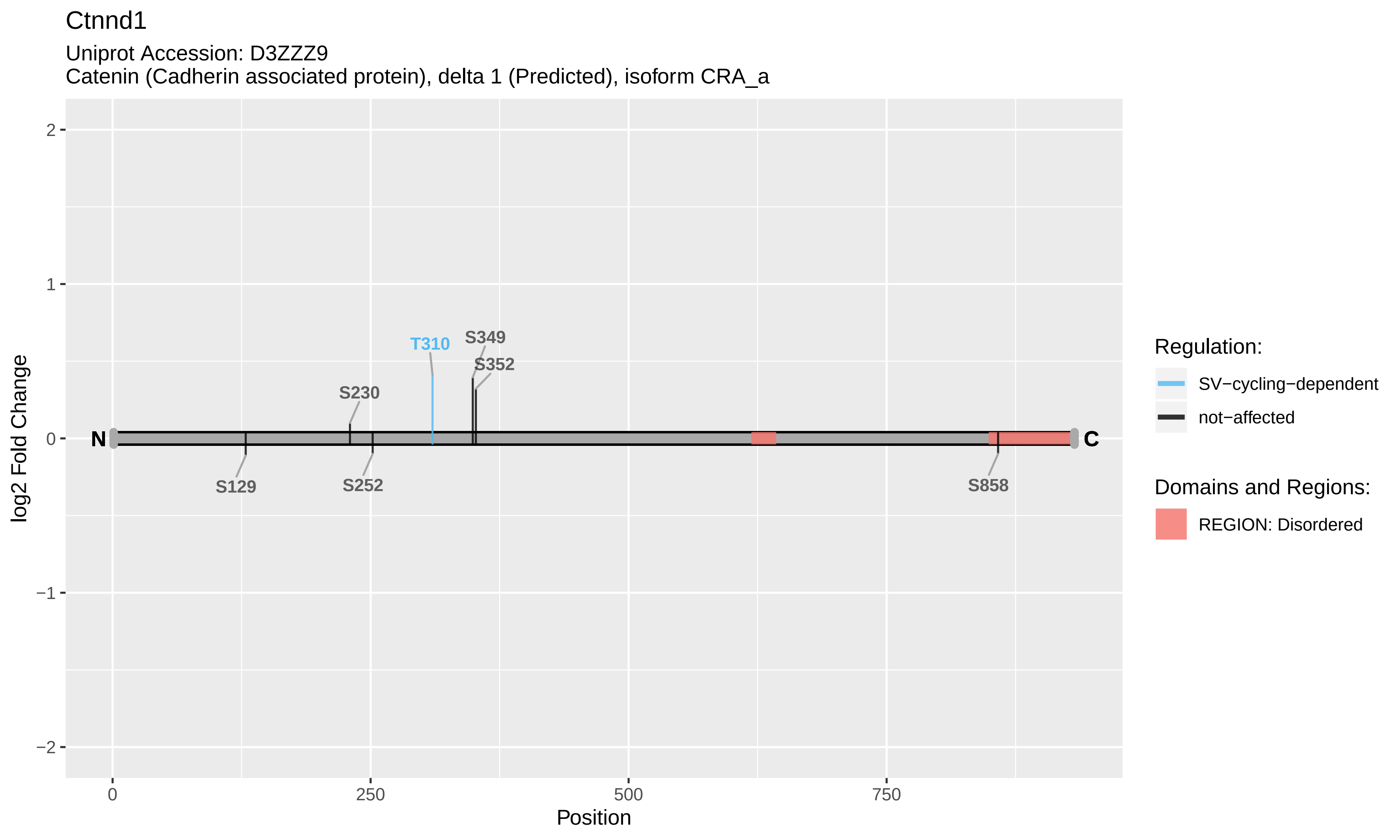

Ctnnd1
Uniprot Accession: D3ZZZ9
Catenin (Cadherin associated protein), delta 1 (Predicted), isoform CRA_a
log2 Fold Change
2
1
0
−1
−2
N
C
T310
S349
S352
S230
S129
S252
S858
0
250
500
750
Position
Regulation:
SV−cycling−dependent
not−affected
Domains and Regions:
REGION: Disordered

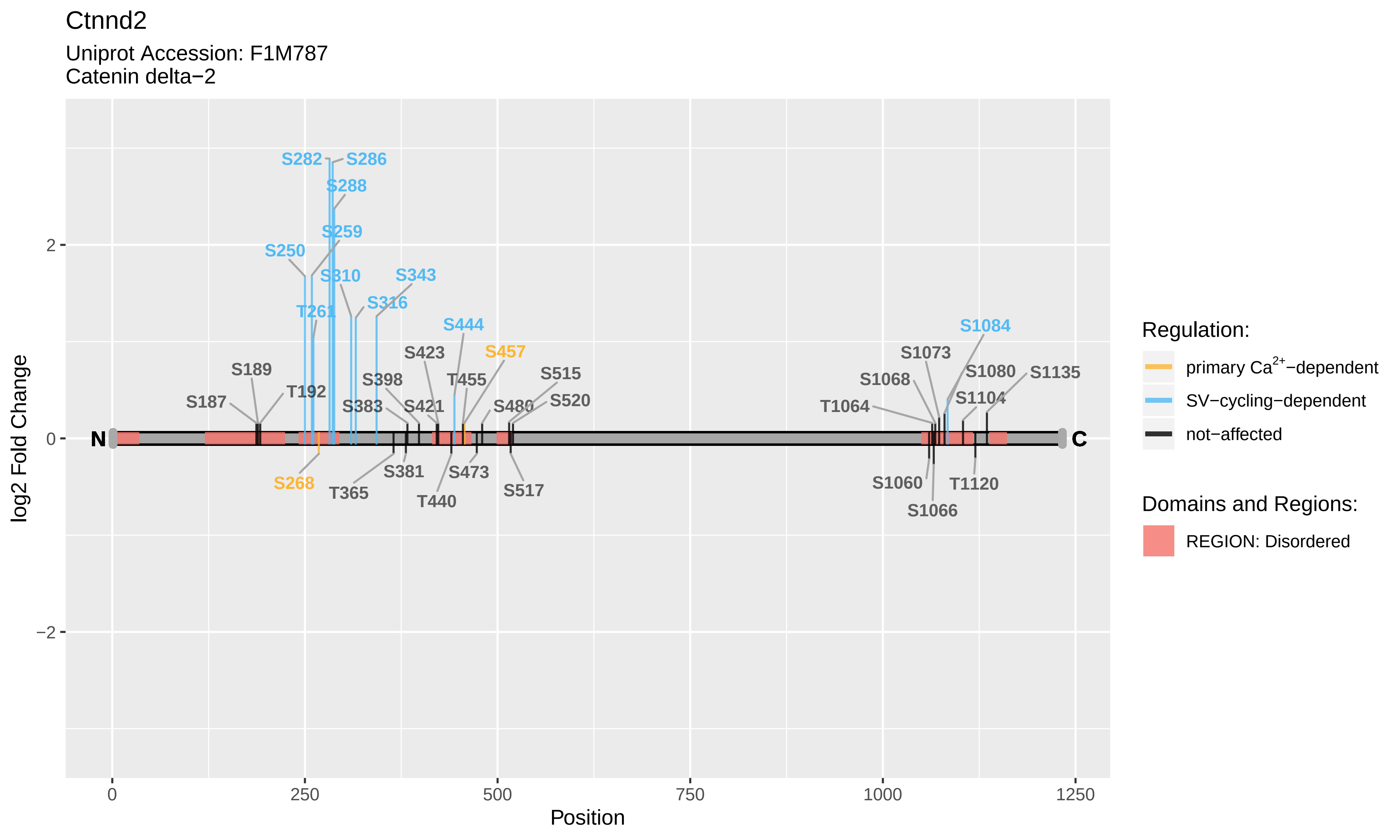

Ctnnd2
Uniprot Accession: F1M787
Catenin delta−2
log2 Fold Change
Position
N
C
S282
S286
S288
S259
S250
S310
S343
T261
S316
S444
S423
S457
S189
T192
S187
S398
T455
S515
S383
S421
S480
S520
S268
T365
S381
T440
S473
S517
S1084
S1073
S1068
S1080
S1135
T1064
S1104
S1060
S1066
T1120
2
0
−2
0
250
500
750
1000
1250
Regulation:
primary Ca2+−dependent
SV−cycling−dependent
not−affected
Domains and Regions:
REGION: Disordered

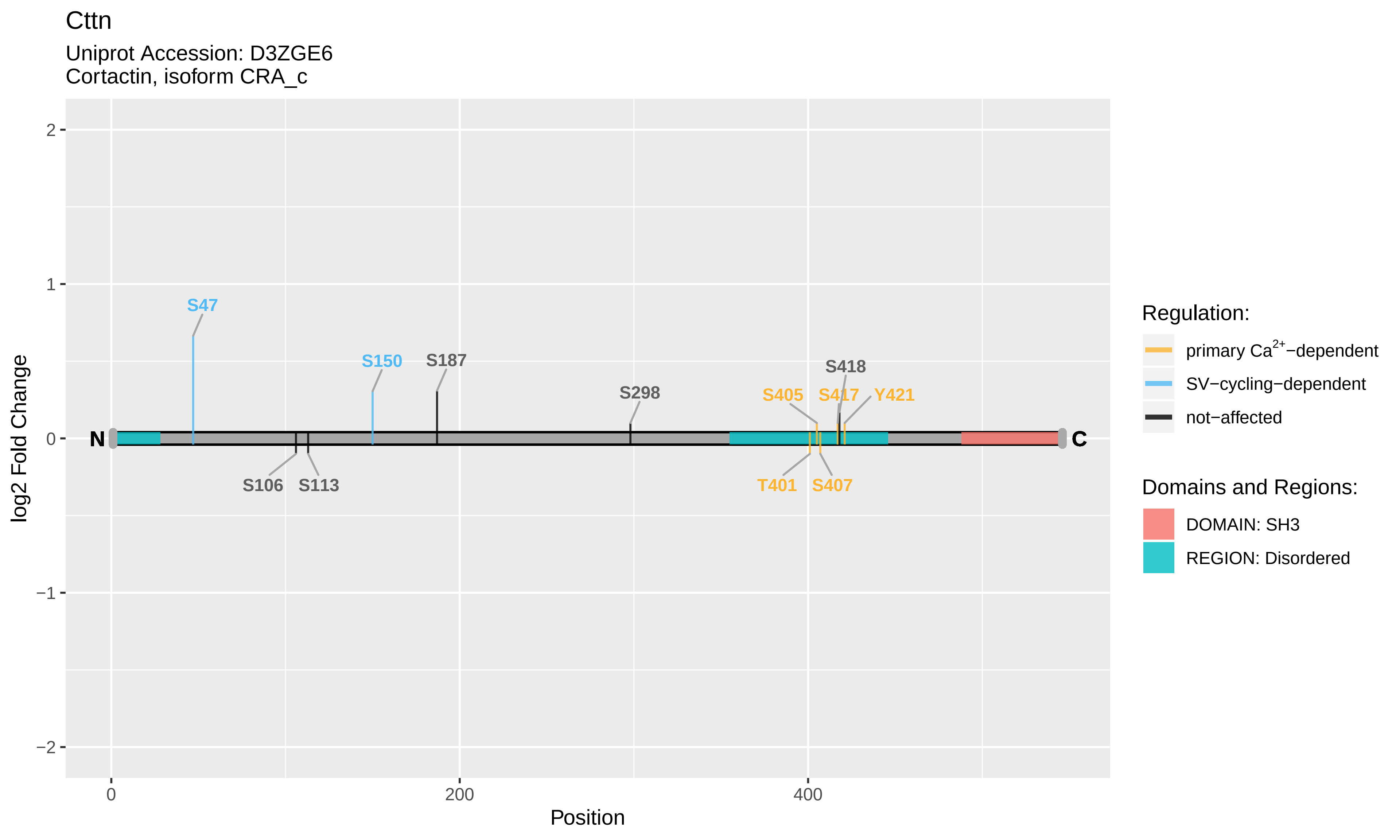

Cttn
Uniprot Accession: D3ZGE6
Cortactin, isoform CRA_c
log2 Fold Change
Position
2
1
0
−1
−2
0
200
400
N
C
S47
S150
S187
S298
S418
S405
S417
Y421
S106
S113
T401
S407
Regulation:
primary Ca2+−dependent
SV−cycling−dependent
not−affected
Domains and Regions:
DOMAIN: SH3
REGION: Disordered

Cttn

Uniprot Accession: Q66HL2
Src substrate cortactin

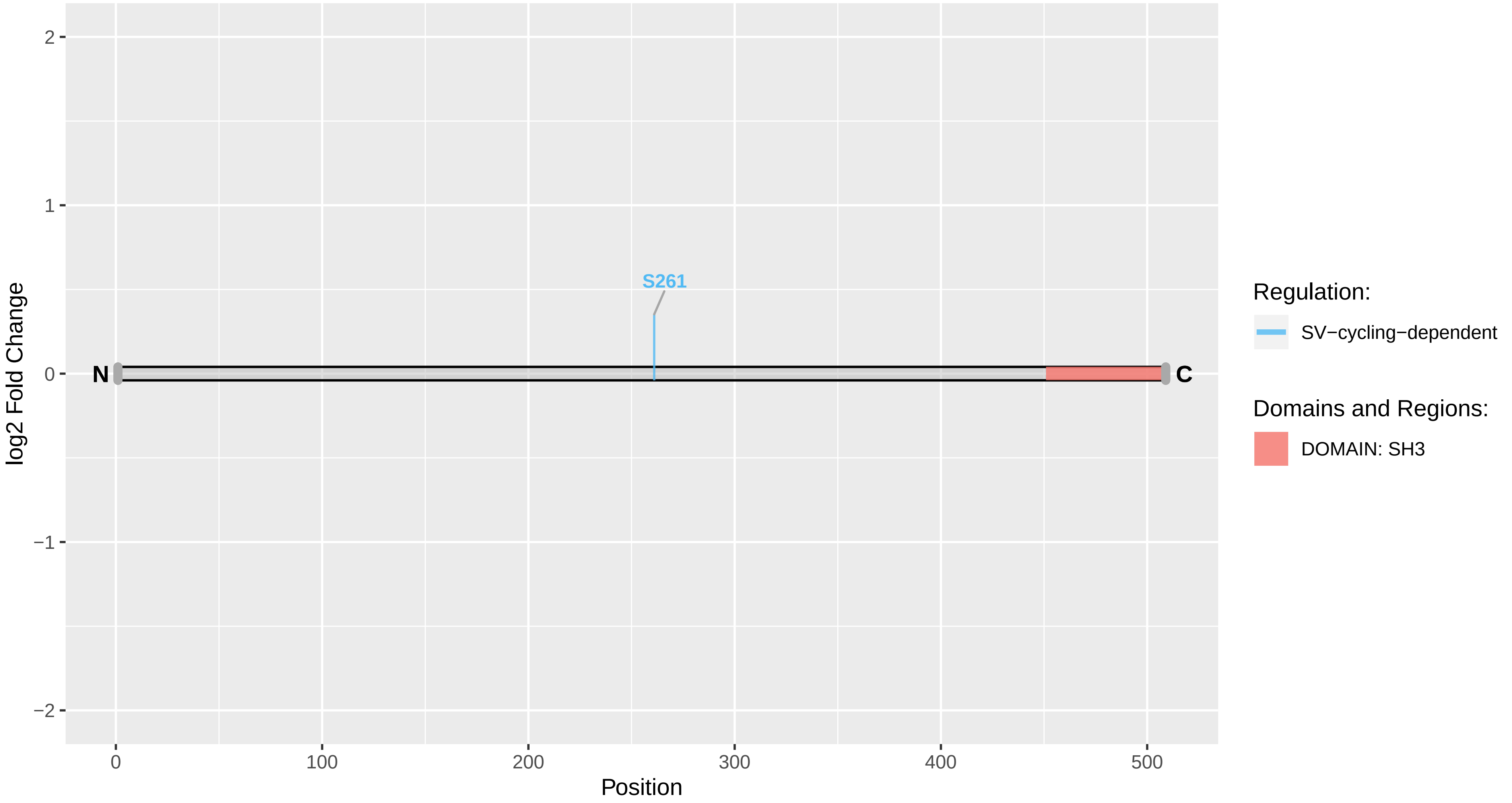

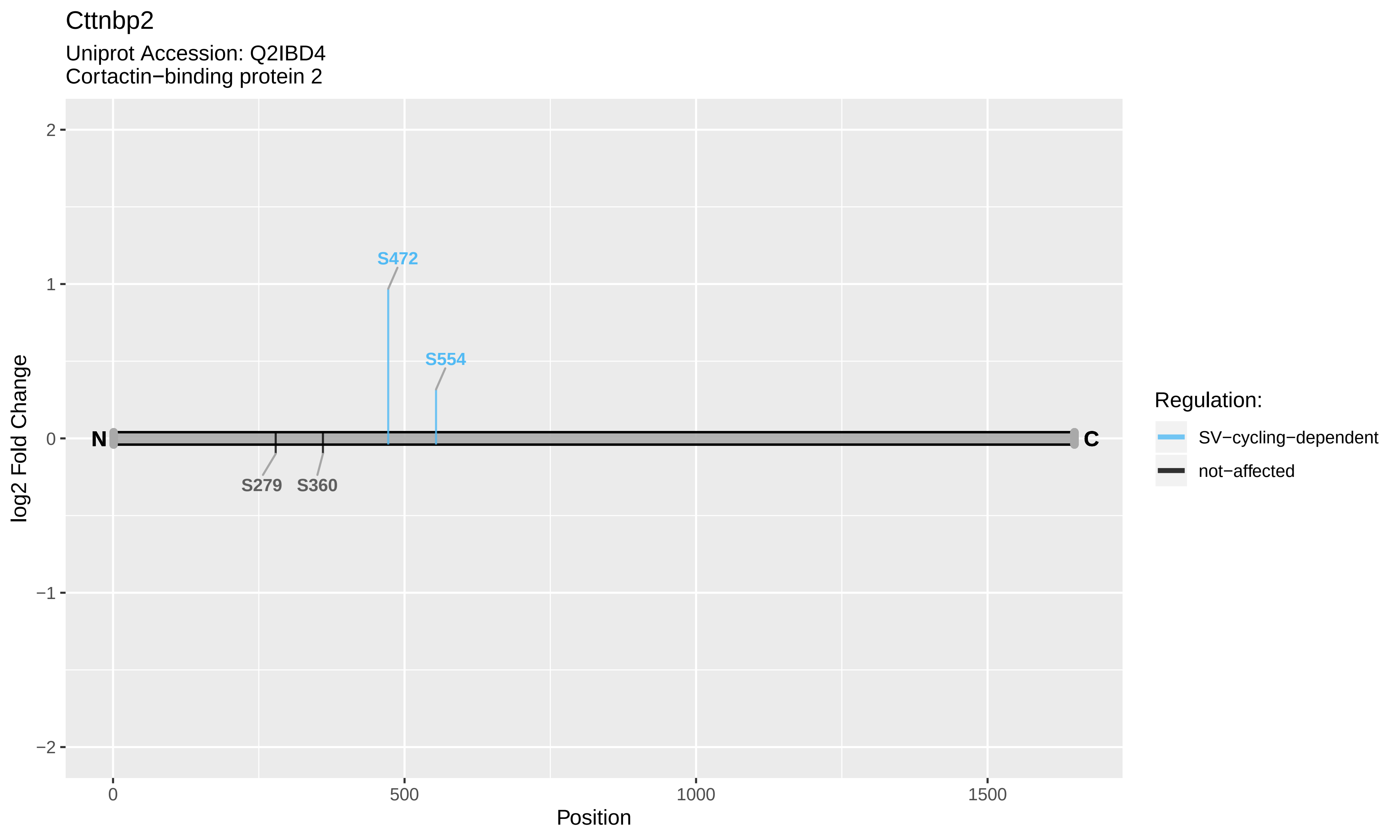

Cttnbp2
Uniprot Accession: Q2IBD4
Cortactin−binding protein 2
log2 Fold Change
2
1
0
−1
−2
N
C
S472
S554
S279
S360
0
500
1000
1500
Position
Regulation:
SV−cycling−dependent
not−affected

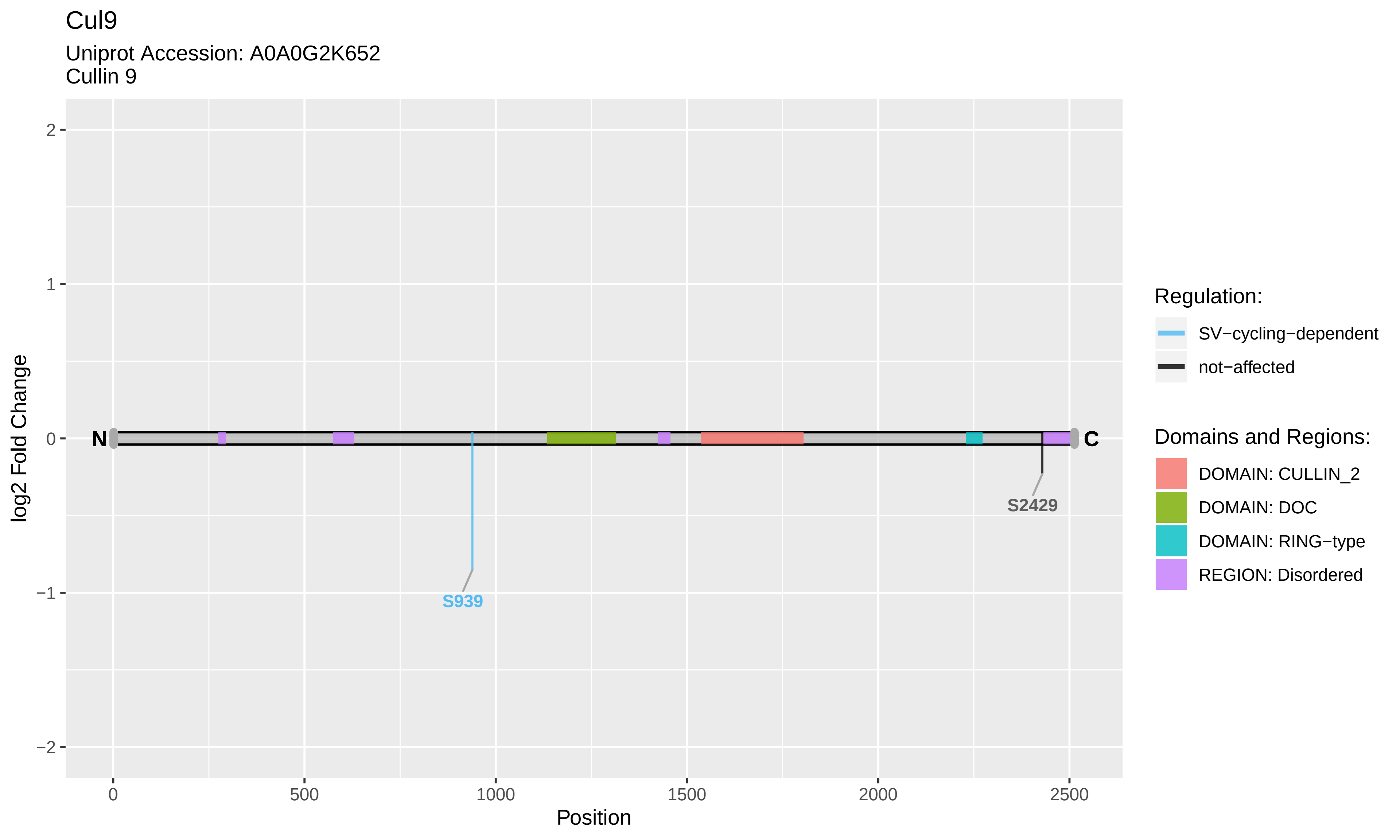
Cul9
Uniprot Accession: A0A0G2K652
Cullin 9
log2 Fold Change
2
1
0
−1
−2
N
C
S939
S2429
0
500
1000
1500
2000
2500
Position
Regulation:
SV−cycling−dependent
not−affected
Domains and Regions:
DOMAIN: CULLIN_2
DOMAIN: DOC
DOMAIN: RING−type
REGION: Disordered

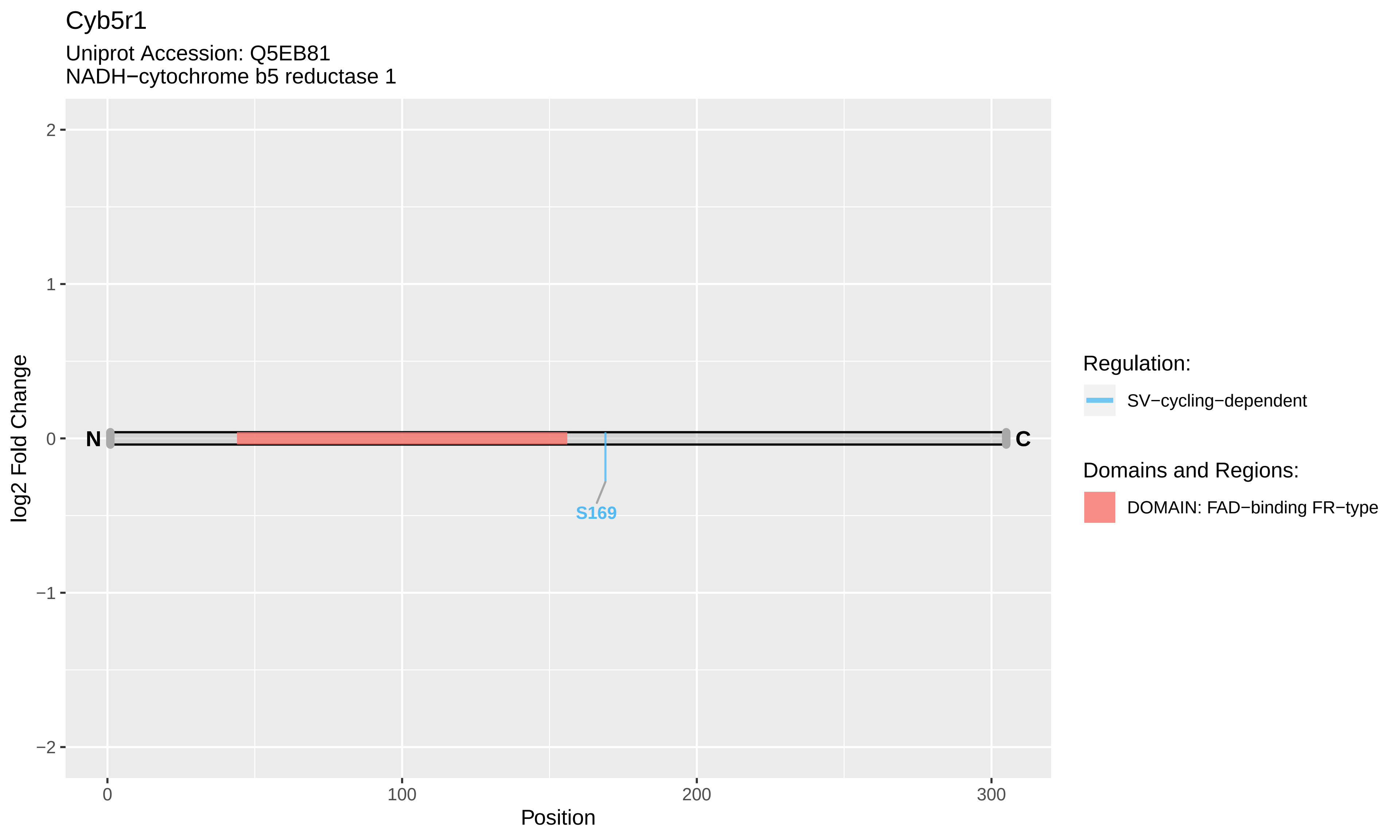
Cyb5r1
Uniprot Accession: Q5EB81
NADH−cytochrome b5 reductase 1
log2 Fold Change
2
1
0
−1
−2
N
C
S169
0
100
200
300
Position
Regulation:
SV−cycling−dependent
Domains and Regions:
DOMAIN: FAD−binding FR−type

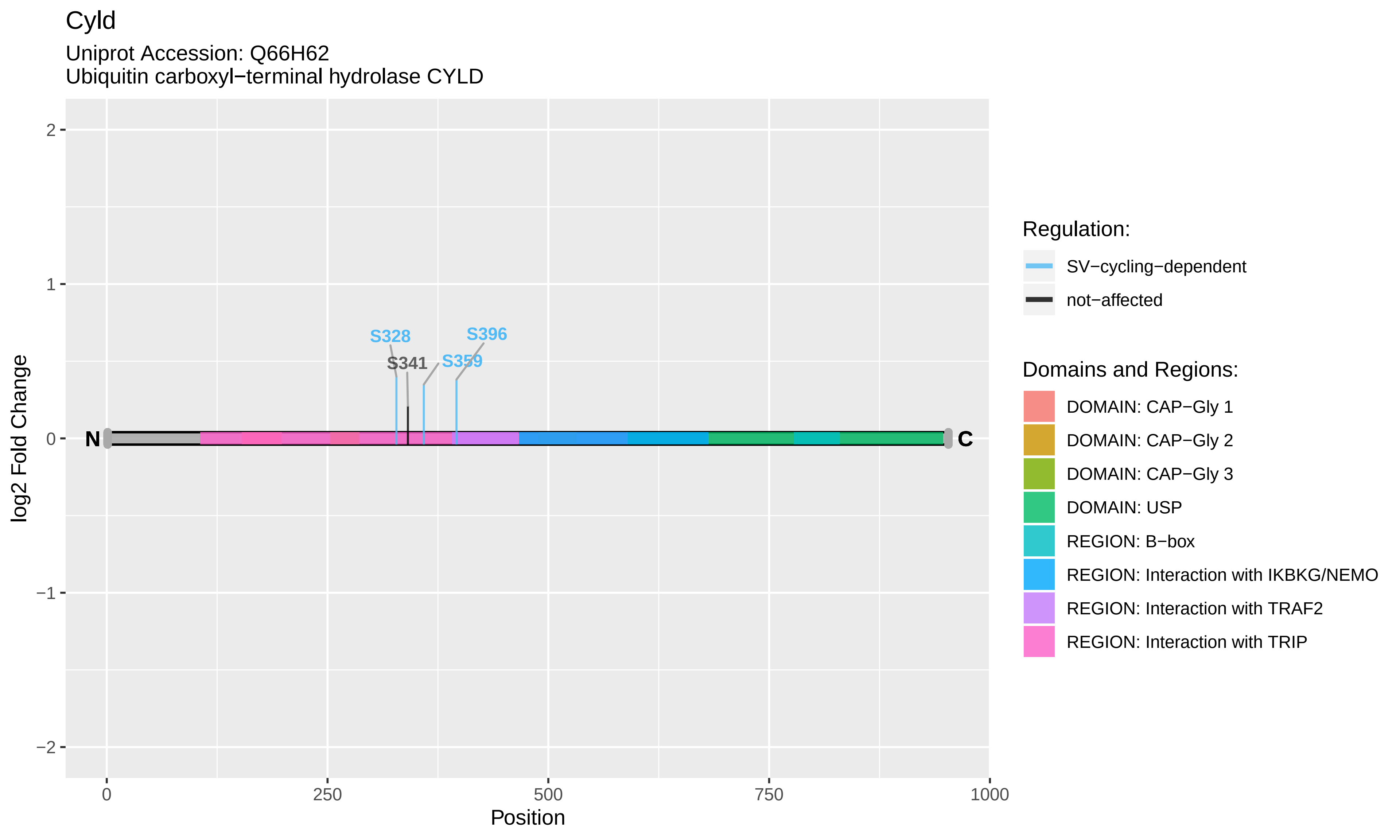

Cyld
Uniprot Accession: Q66H62
Ubiquitin carboxyl−terminal hydrolase CYLD
S328
S341
S359
S396
N
C
log2 Fold Change
Position
Regulation:
SV−cycling−dependent
not−affected
Domains and Regions:
DOMAIN: CAP−Gly 1
DOMAIN: CAP−Gly 2
DOMAIN: CAP−Gly 3
DOMAIN: USP
REGION: B−box
REGION: Interaction with IKBKG/NEMO
REGION: Interaction with TRAF2
REGION: Interaction with TRIP

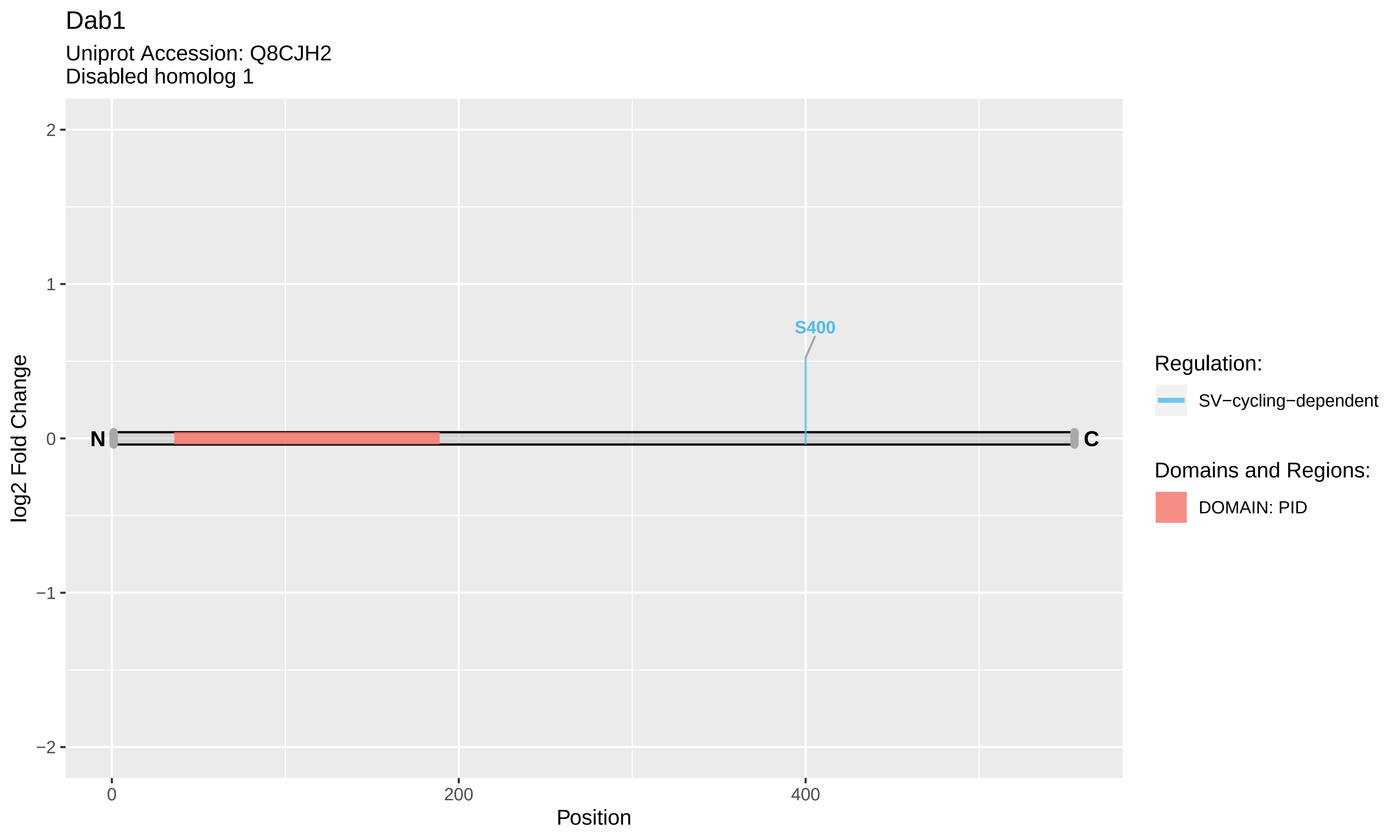

Dab1
Uniprot Accession: Q8CJH2
Disabled homolog 1
S400
N
C
2
1
0
−1
−2
log2 Fold Change
0
200
400
Position
Regulation:
SV−cycling−dependent
Domains and Regions:
DOMAIN: PID

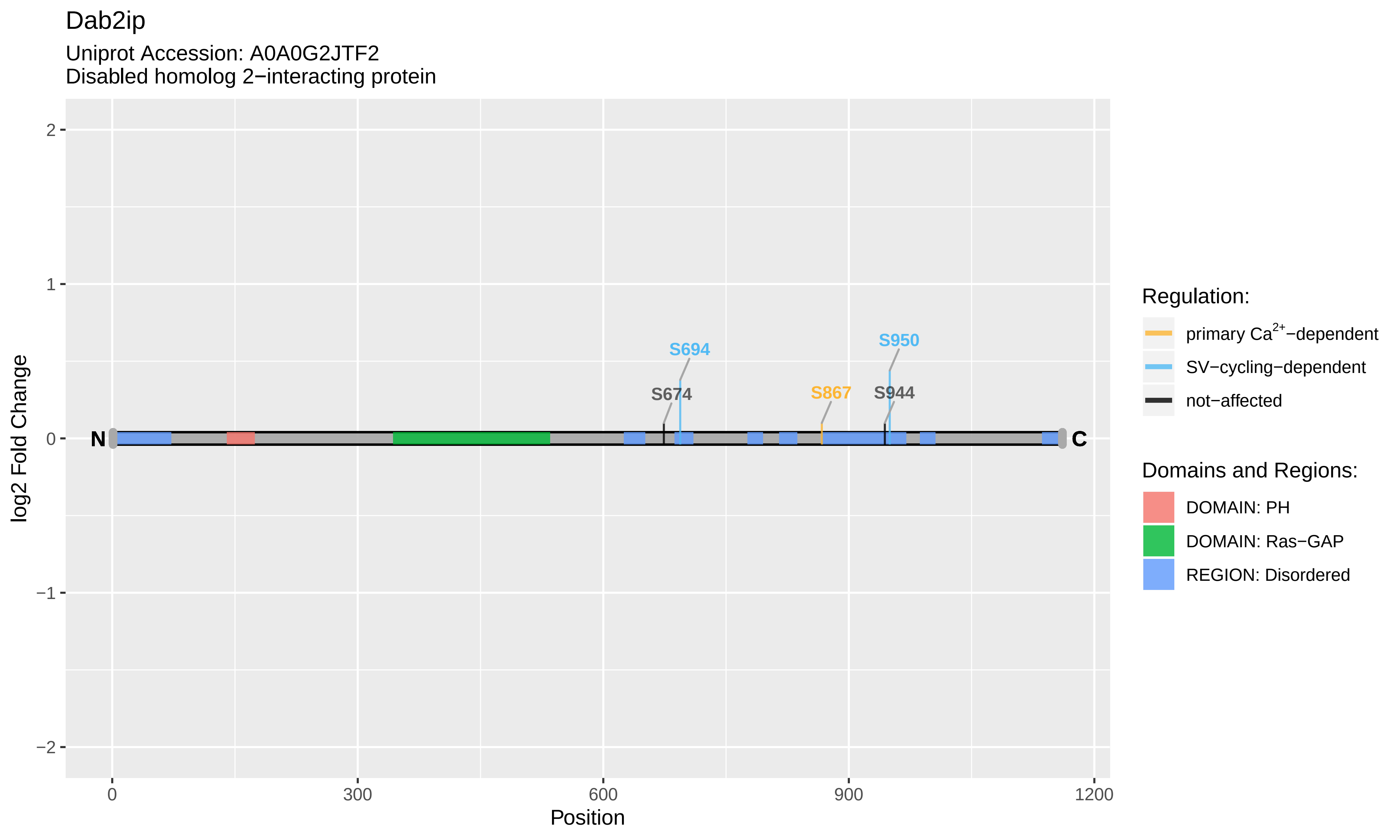
Dab2ip
Uniprot Accession: A0A0G2JTF2
Disabled homolog 2−interacting protein
log2 Fold Change
2
1
0
−1
−2
N
C
S694
S950
S674
S867
S944
0
300
600
900
1200
Position
Regulation:
primary Ca2+−dependent
SV−cycling−dependent
not−affected
Domains and Regions:
DOMAIN: PH
DOMAIN: Ras−GAP
REGION: Disordered

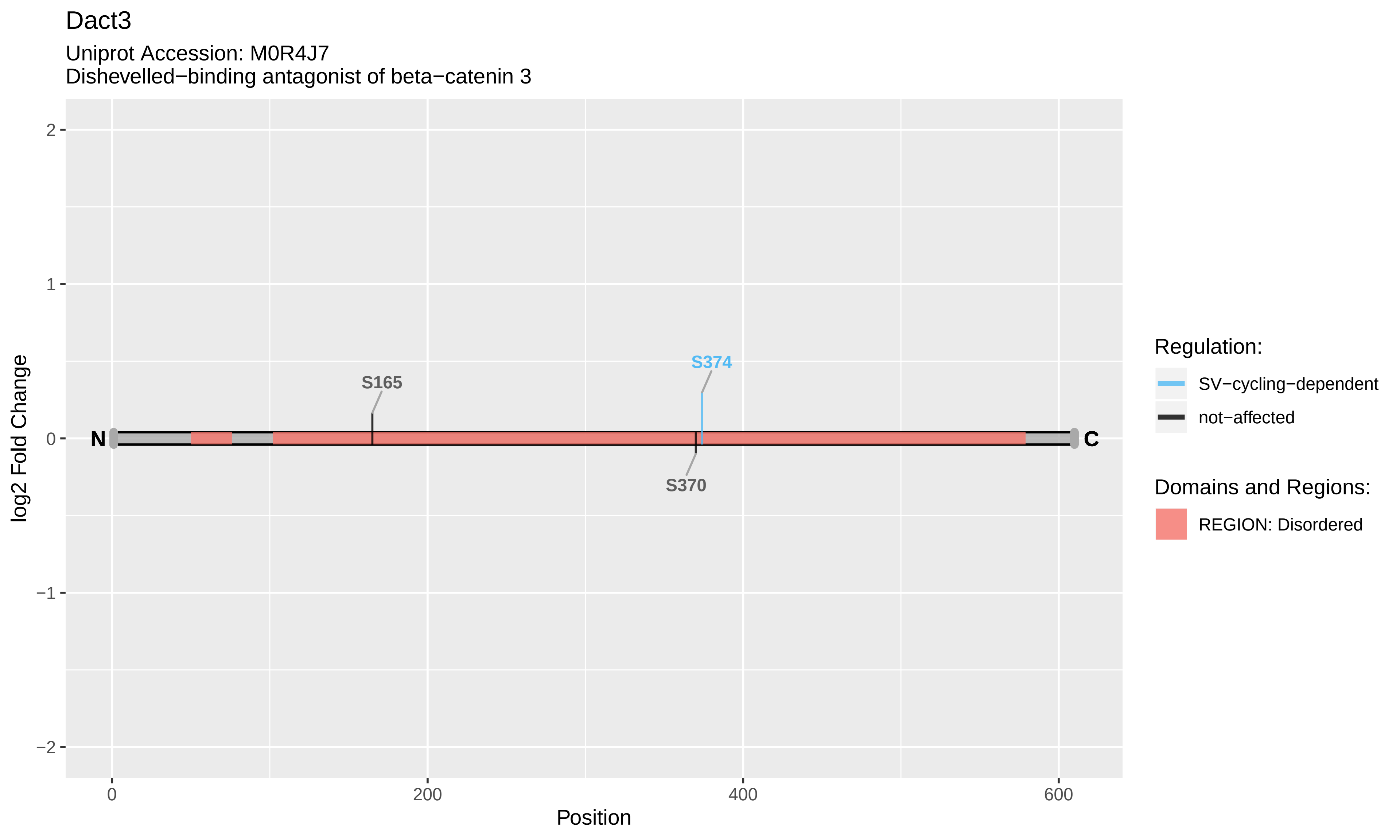

Dact3
Uniprot Accession: M0R4J7
Dishevelled−binding antagonist of beta−catenin 3
log2 Fold Change
2
1
0
−1
−2
N
C
S165
S374
S370
0
200
400
600
Position
Regulation:
SV−cycling−dependent
not−affected
Domains and Regions:
REGION: Disordered

Dagla

Uniprot Accession: Q5YLM1
Sn1−specific diacylglycerol lipase alpha

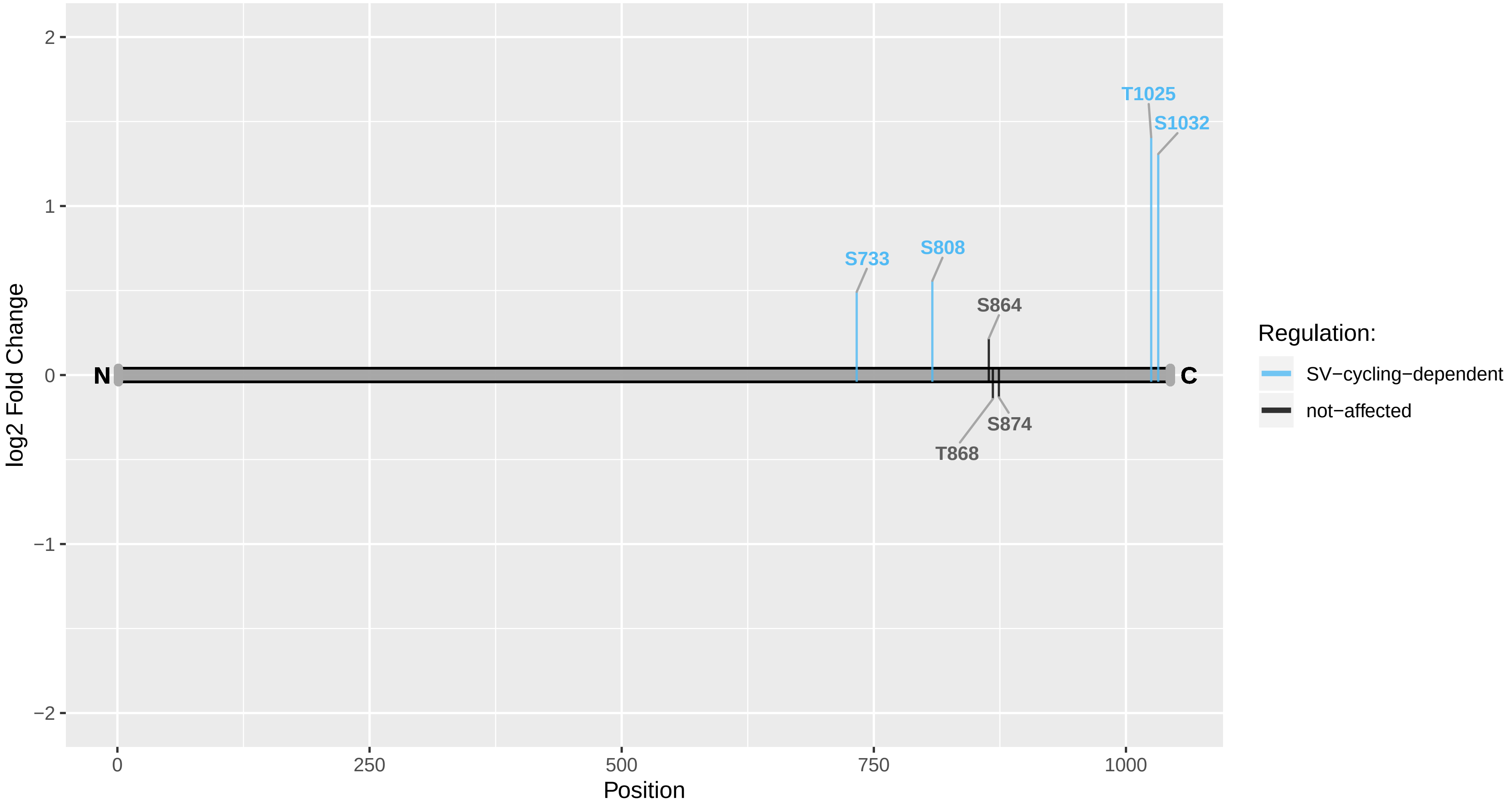

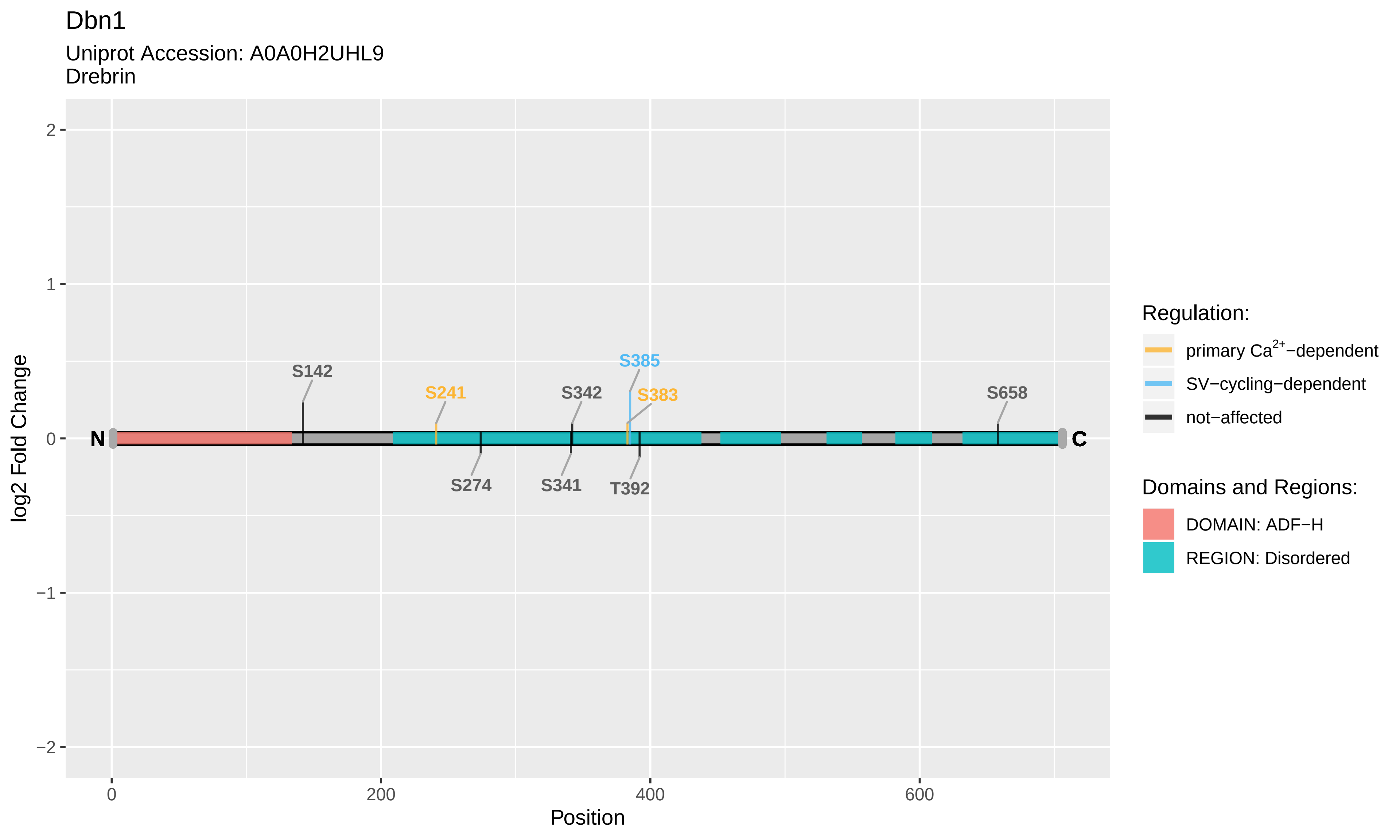

Dbn1
Uniprot Accession: A0A0H2UHL9
Drebrin
log2 Fold Change
2
1
0
−1
−2
N
C
S142
S241
S342
S385
S383
S658
S274
S341
T392
0
200
400
600
Position
Regulation:
primary Ca2+−dependent
SV−cycling−dependent
not−affected
Domains and Regions:
DOMAIN: ADF−H
REGION: Disordered

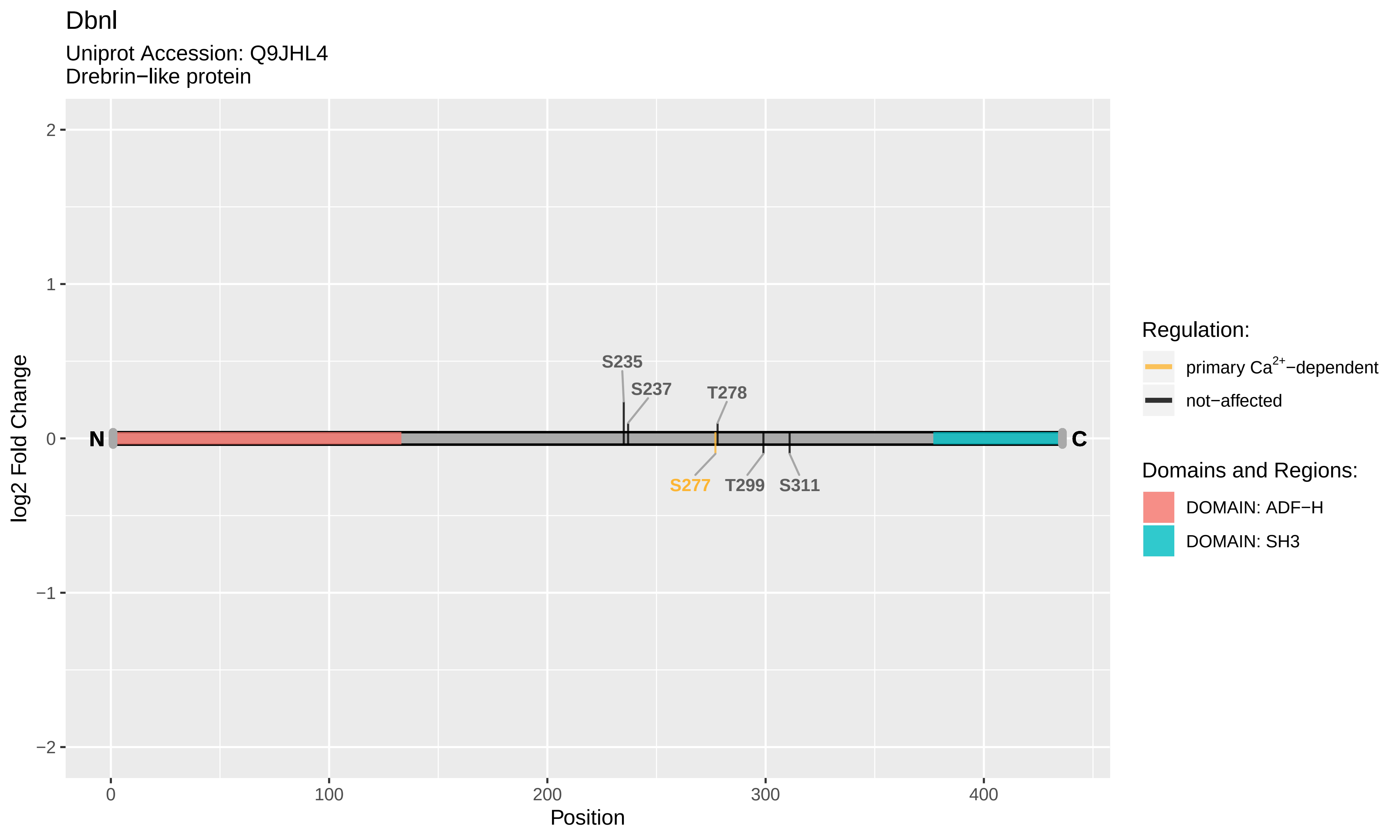
Dbnl
Uniprot Accession: Q9JHL4
Drebrin−like protein
log2 Fold Change
2
1
0
−1
−2
0
100
200
300
400
Position
N
C
S235
S237
T278
S277
T299
S311
Regulation:
primary Ca2+−dependent
not−affected
Domains and Regions:
DOMAIN: ADF−H
DOMAIN: SH3

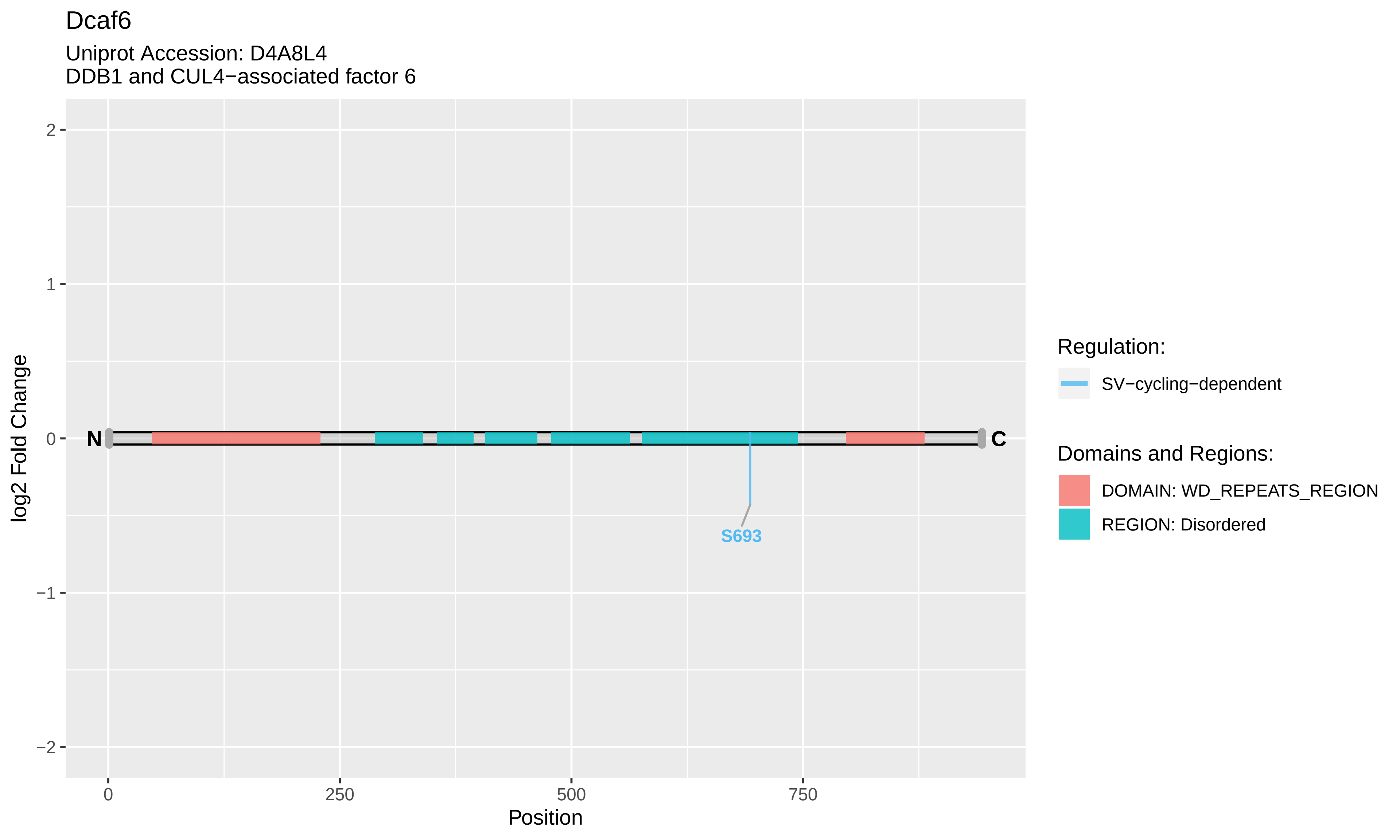

Dcaf6
Uniprot Accession: D4A8L4
DDB1 and CUL4−associated factor 6
log2 Fold Change
2
1
0
−1
−2
N
C
S693
0
250
500
750
Position
Regulation:
SV−cycling−dependent
Domains and Regions:
DOMAIN: WD_REPEATS_REGION
REGION: Disordered

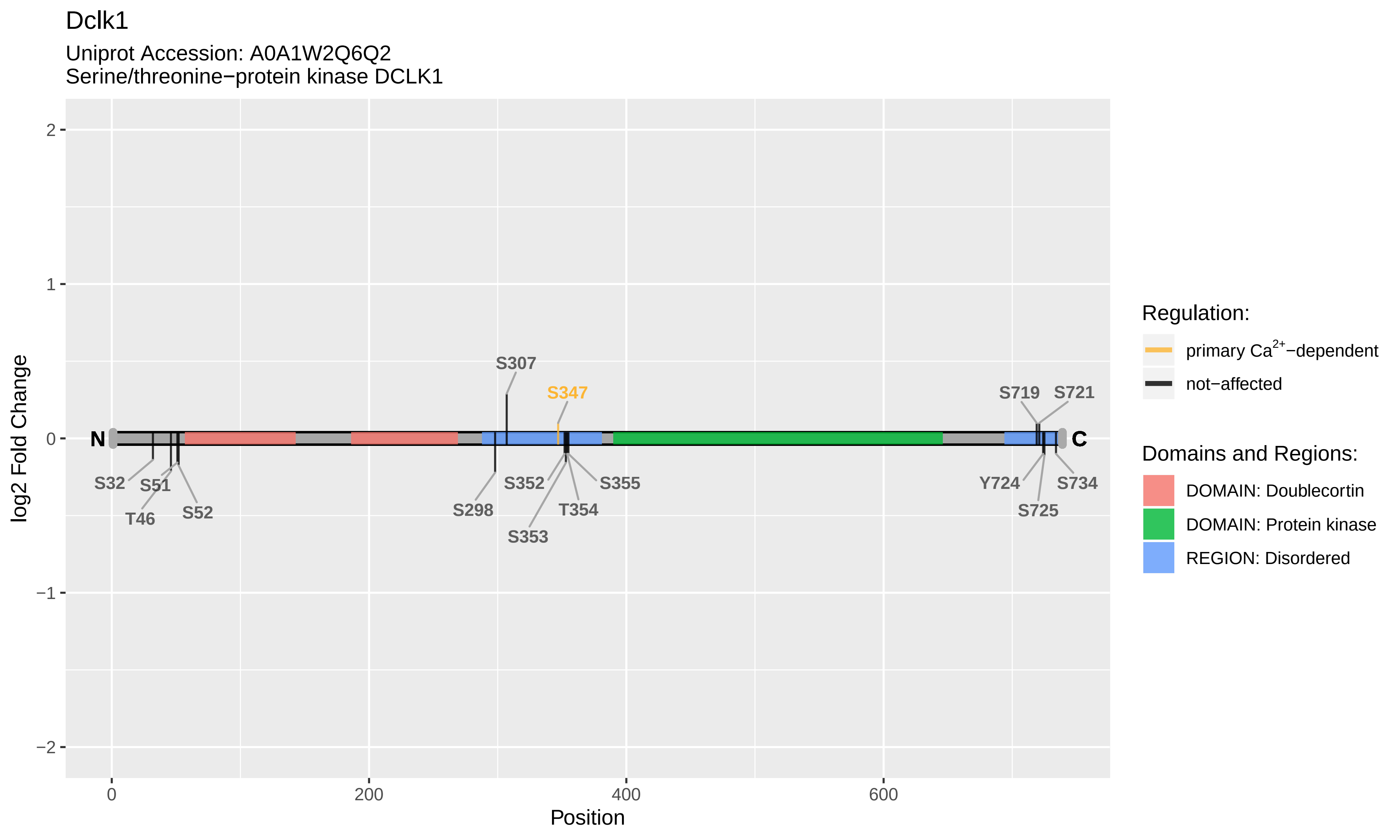
Dclk1
Uniprot Accession: A0A1W2Q6Q2
Serine/threonine−protein kinase DCLK1
log2 Fold Change
Position
N
C
S307
S347
S719
S721
S32
S51
T46
S52
S298
S352
S353
T354
S355
Y724
S725
S734
Regulation:
primary Ca2+−dependent
not−affected
Domains and Regions:
DOMAIN: Doublecortin
DOMAIN: Protein kinase
REGION: Disordered

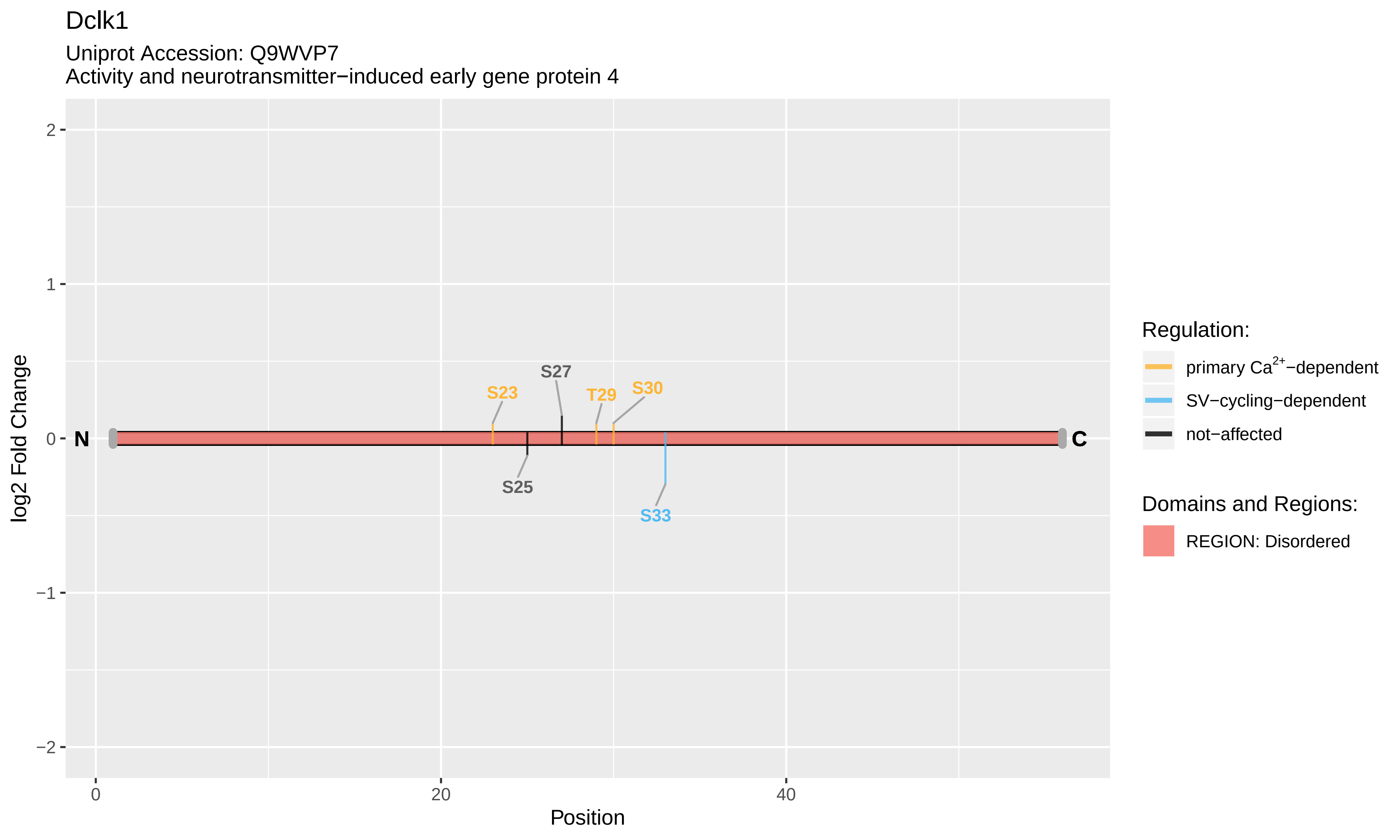

Dclk1
Uniprot Accession: Q9WVP7
Activity and neurotransmitter−induced early gene protein 4
log2 Fold Change
Position
2
1
0
−1
−2
0
20
40
N
C
S23
S25
S27
T29
S30
S33
Regulation:
primary Ca2+−dependent
SV−cycling−dependent
not−affected
Domains and Regions:
REGION: Disordered

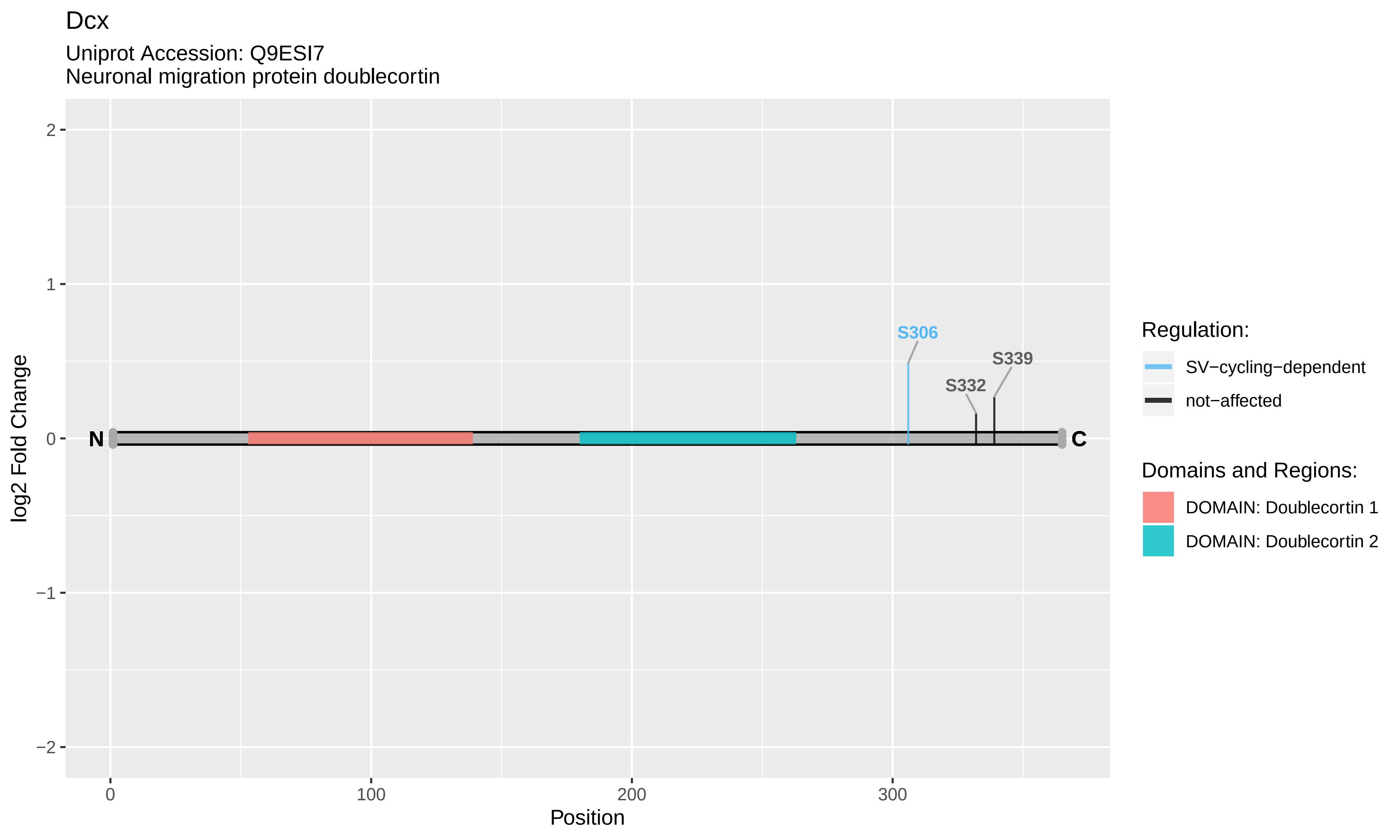
Dcx
Uniprot Accession: Q9ESI7
Neuronal migration protein doublecortin
S306
S339
S332
N
C
2
1
0
−1
−2
log2 Fold Change
0
100
200
300
Position
Regulation:
SV−cycling−dependent
not−affected
Domains and Regions:
DOMAIN: Doublecortin 1
DOMAIN: Doublecortin 2

Ddhd1

Uniprot Accession: Q3ZAU5
DDHD domain−containing 1

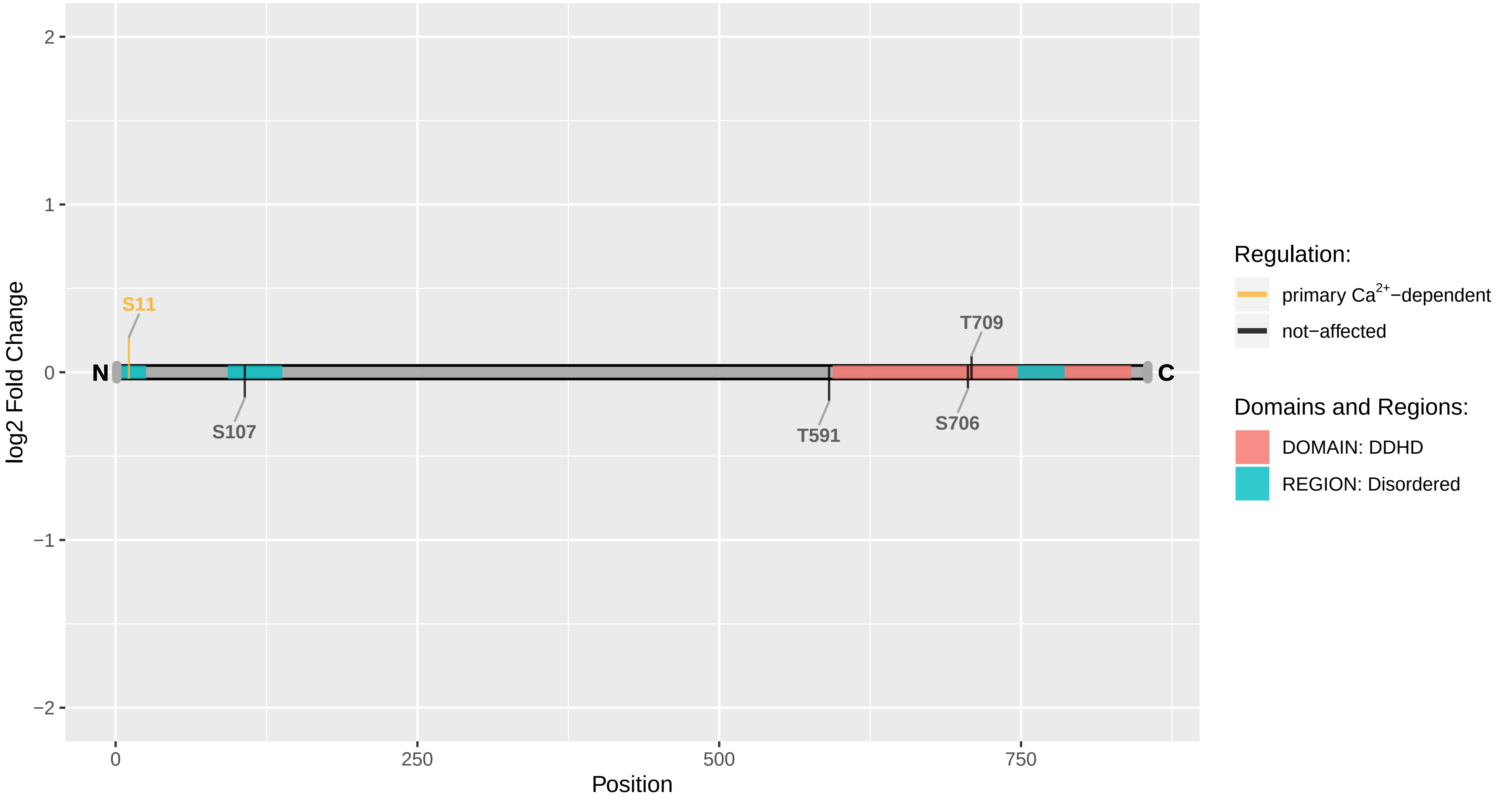

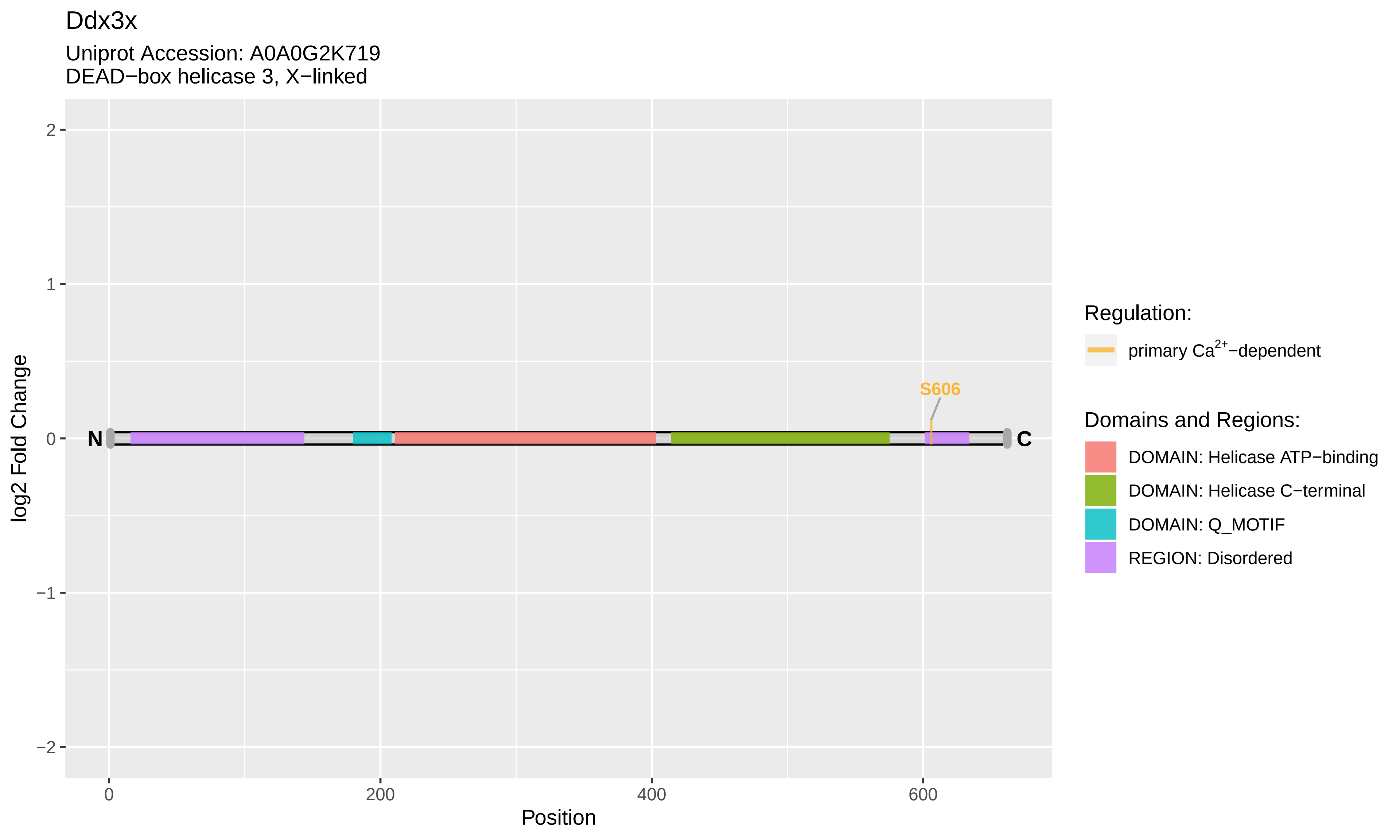
Ddx3x
Uniprot Accession: A0A0G2K719
DEAD−box helicase 3, X−linked
2
1
0
−1
−2
log2 Fold Change
0
200
400
600
Position
N
C
S606
Regulation:
primary Ca2+−dependent
Domains and Regions:
DOMAIN: Helicase ATP−binding
DOMAIN: Helicase C−terminal
DOMAIN: Q_MOTIF
REGION: Disordered

Dgkb

Uniprot Accession: P49621
Diacylglycerol kinase beta

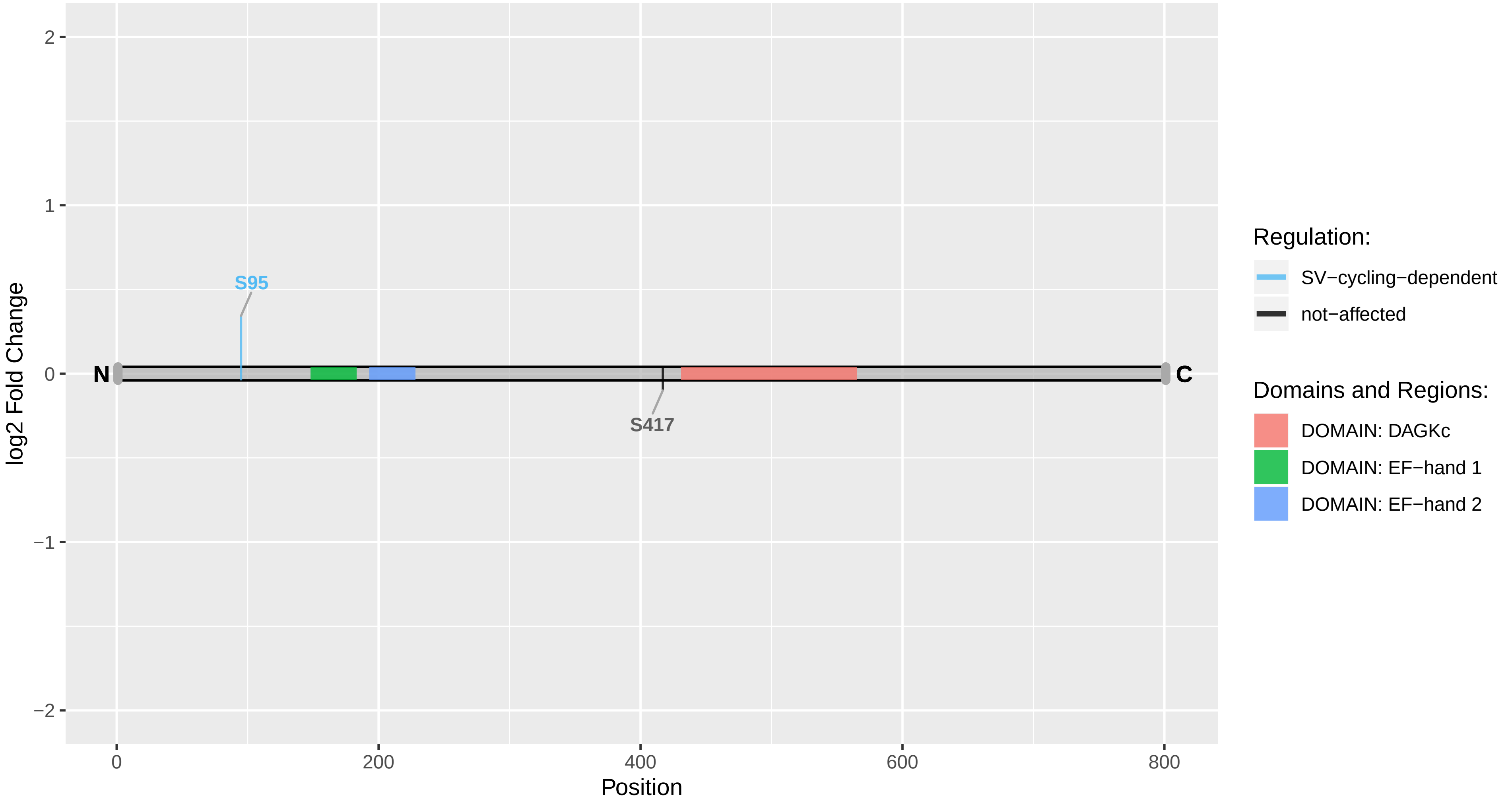

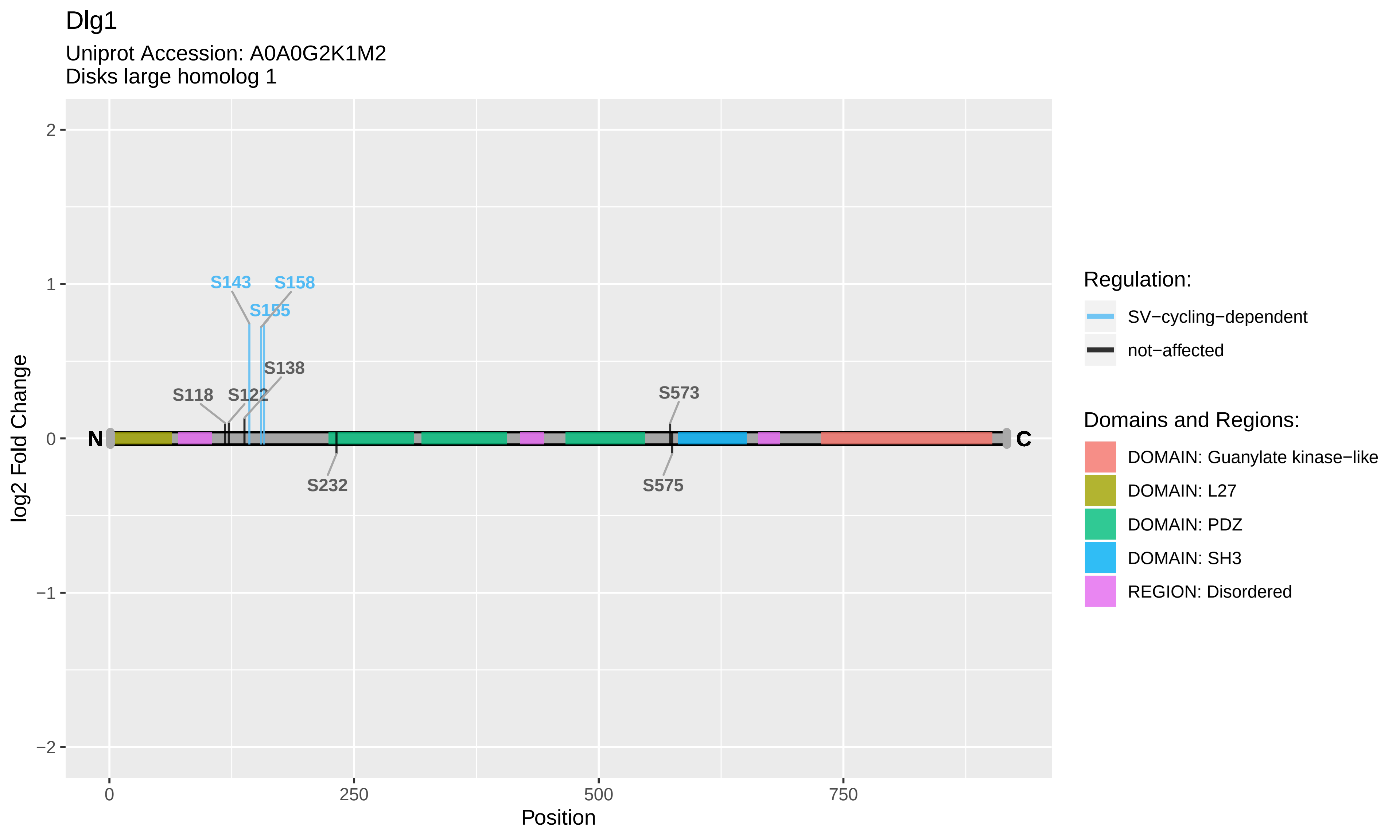
Dlg1
Uniprot Accession: A0A0G2K1M2
Disks large homolog 1
S143
S158
S155
S138
S118
S122
S573
S232
S575
N
C
2
1
0
−1
−2
log2 Fold Change
0
250
500
750
Position
Regulation:
SV−cycling−dependent
not−affected
Domains and Regions:
DOMAIN: Guanylate kinase−like
DOMAIN: L27
DOMAIN: PDZ
DOMAIN: SH3
REGION: Disordered

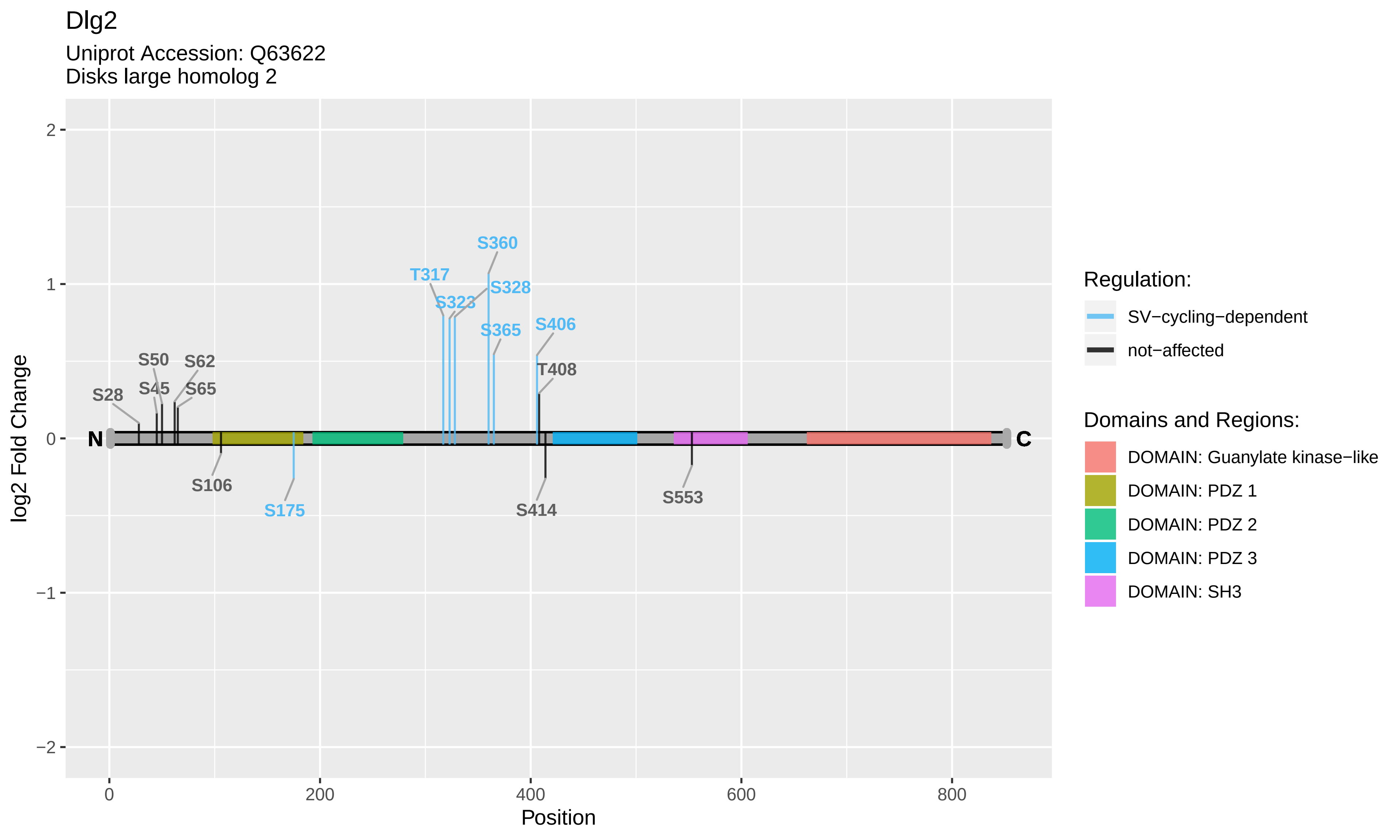
Dlg2
Uniprot Accession: Q63622
Disks large homolog 2
log2 Fold Change
Position
N
C
S28
S45
S50
S62
S65
S106
S175
T317
S323
S328
S360
S365
S406
T408
S414
S553
Regulation:
SV−cycling−dependent
not−affected
Domains and Regions:
DOMAIN: Guanylate kinase−like
DOMAIN: PDZ 1
DOMAIN: PDZ 2
DOMAIN: PDZ 3
DOMAIN: SH3

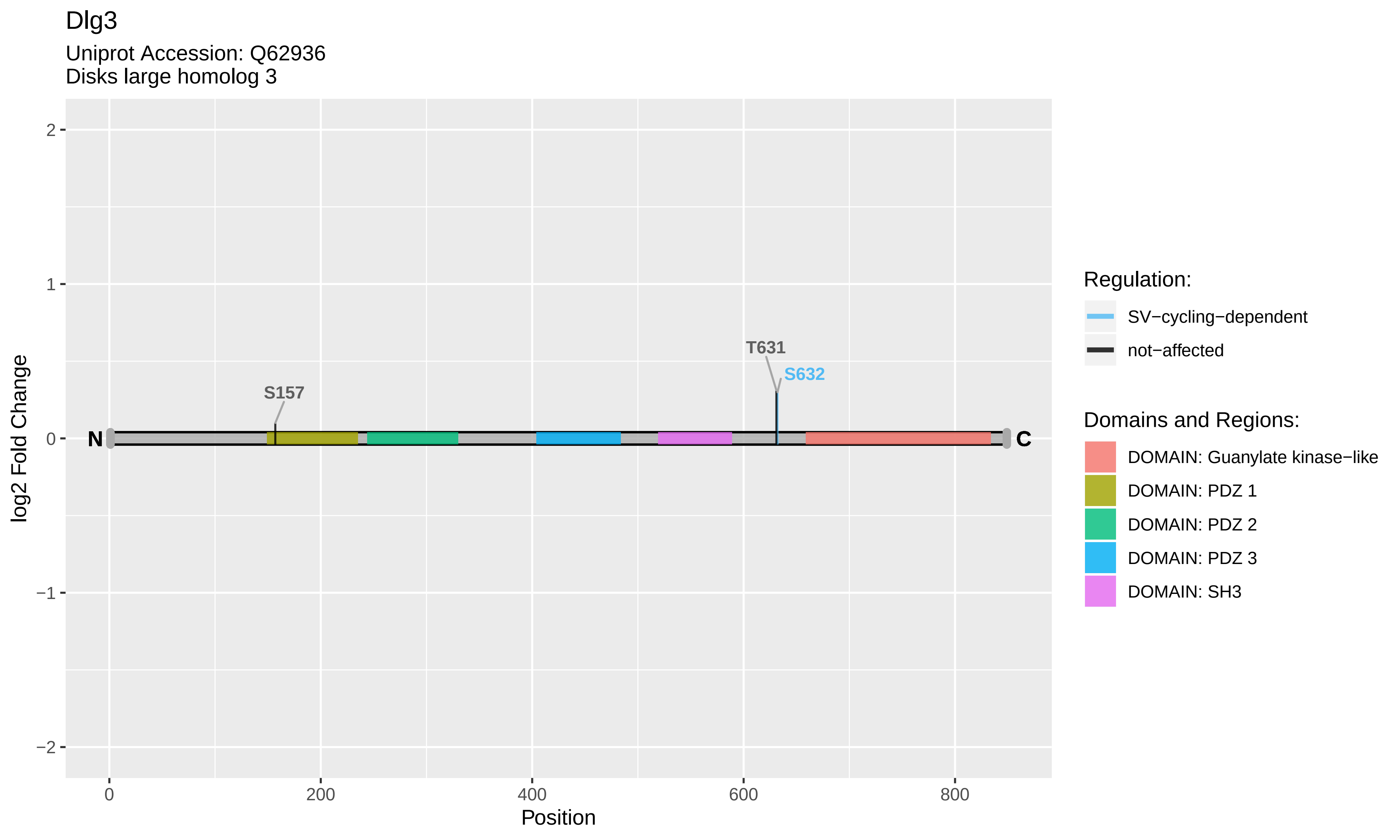
Dlg3
Uniprot Accession: Q62936
Disks large homolog 3
log2 Fold Change
2
1
0
−1
−2
N
C
S157
T631
S632
0
200
400
600
800
Position
Regulation:
SV−cycling−dependent
not−affected
Domains and Regions:
DOMAIN: Guanylate kinase−like
DOMAIN: PDZ 1
DOMAIN: PDZ 2
DOMAIN: PDZ 3
DOMAIN: SH3

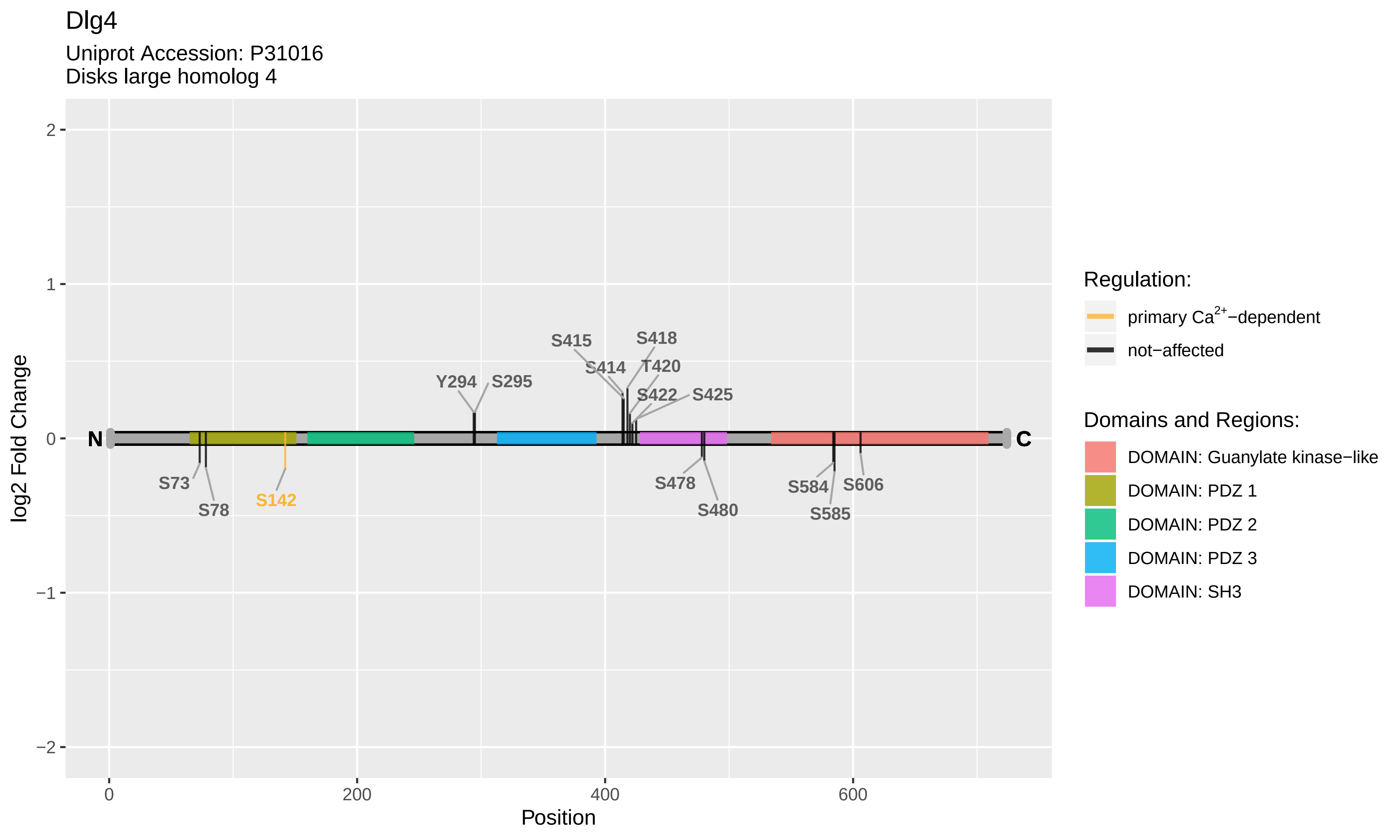
Dlg4
Uniprot Accession: P31016
Disks large homolog 4
log2 Fold Change
2
1
0
−1
−2
0
200
400
600
Position
N
C
S73
S78
S142
Y294
S295
S415
S414
S418
T420
S422
S425
S478
S480
S584
S585
S606
Regulation:
primary Ca2+−dependent
not−affected
Domains and Regions:
DOMAIN: Guanylate kinase−like
DOMAIN: PDZ 1
DOMAIN: PDZ 2
DOMAIN: PDZ 3
DOMAIN: SH3

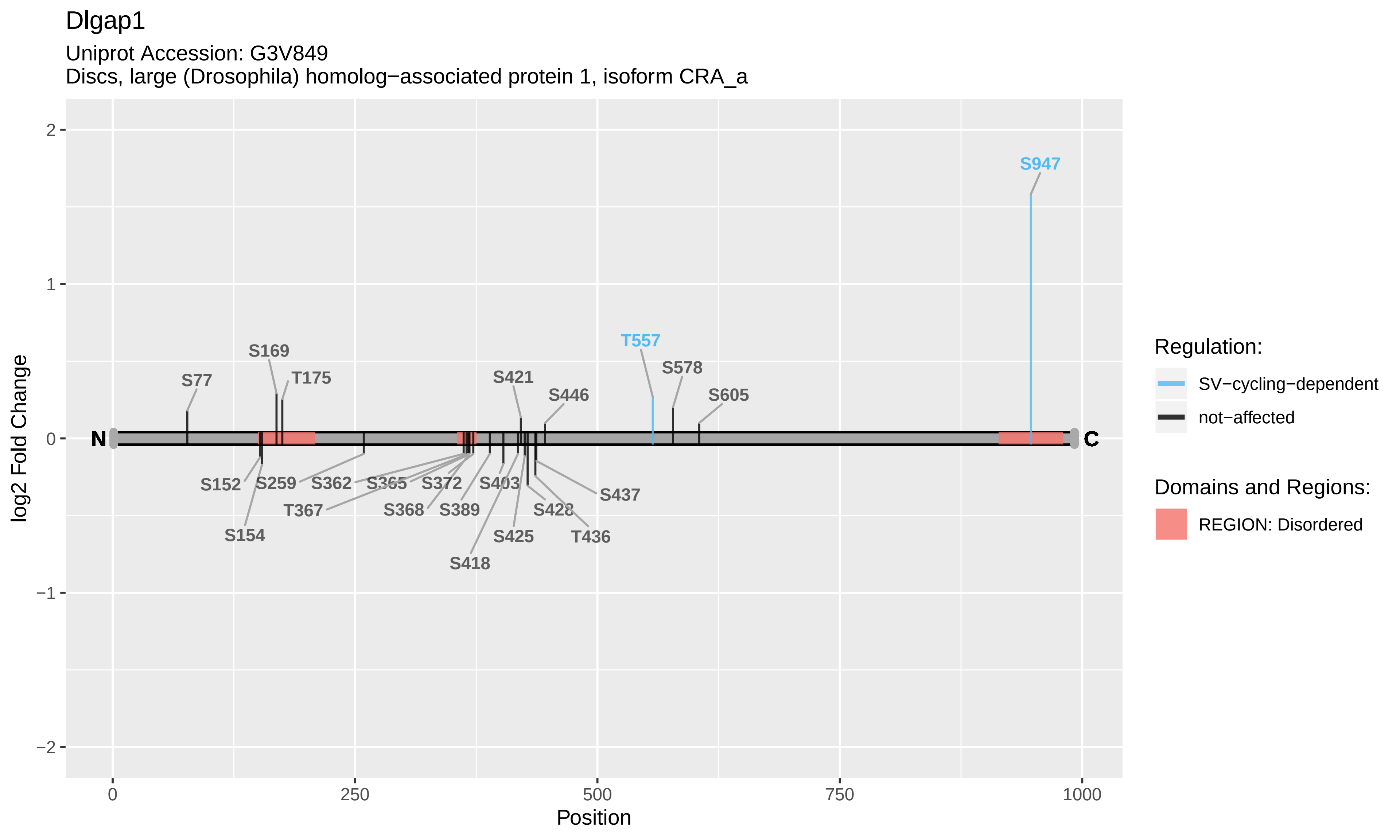
Dlgap1
Uniprot Accession: G3V849
Discs, large (Drosophila) homolog−associated protein 1, isoform CRA_a
log2 Fold Change
Position
N
C
S947
T557
S578
S605
S446
S421
S77
S169
T175
S152
S259
S362
S365
S372
S403
S437
T367
S368
S389
S428
S154
S425
T436
S418
Regulation:
SV−cycling−dependent
not−affected
Domains and Regions:
REGION: Disordered

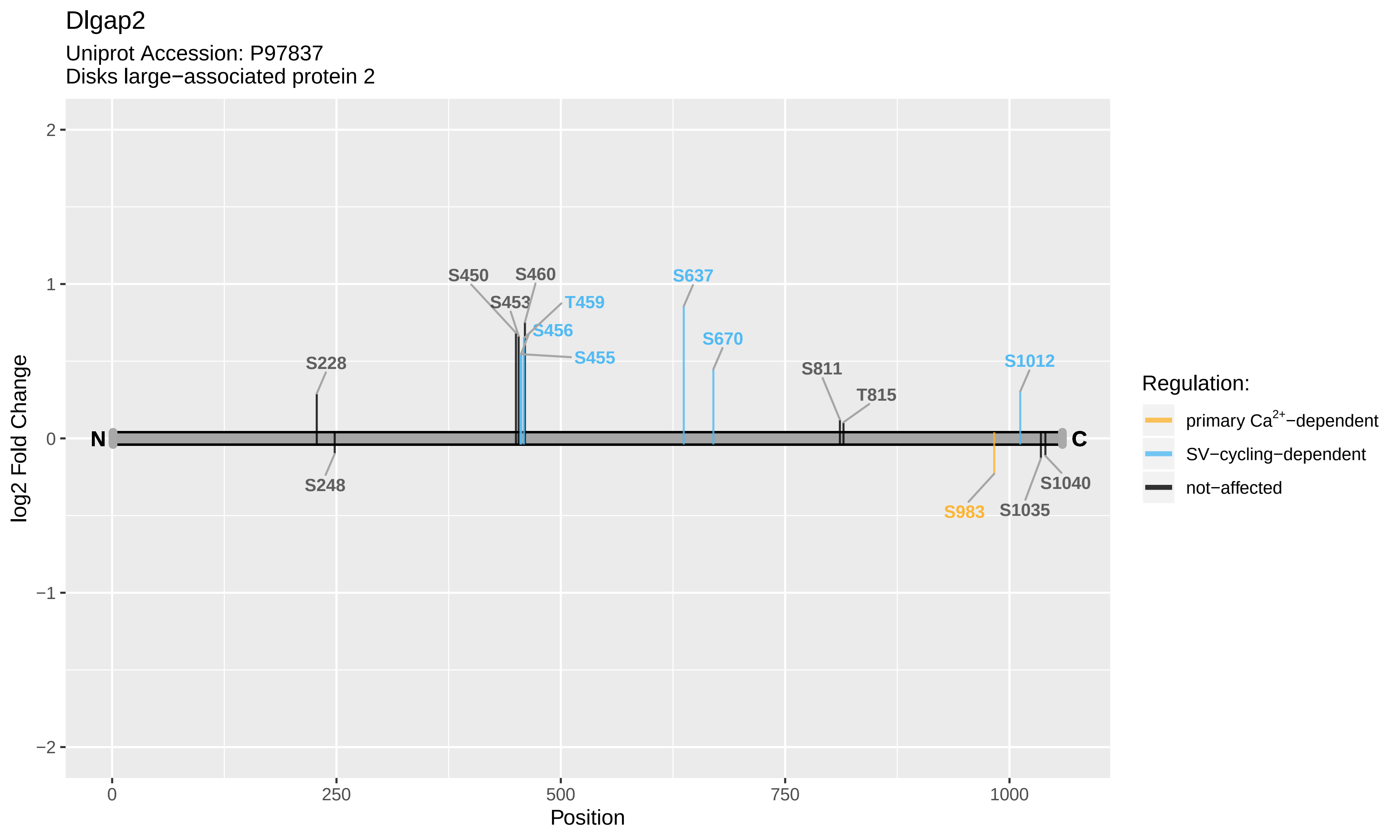

Dlgap2
Uniprot Accession: P97837
Disks large−associated protein 2
log2 Fold Change
Position
N
C
S228
S248
S450
S453
S460
T459
S456
S455
S637
S670
S811
T815
S983
S1012
S1035
S1040
Regulation:
primary Ca2+−dependent
SV−cycling−dependent
not−affected
2
1
0
−1
−2
0
250
500
750
1000

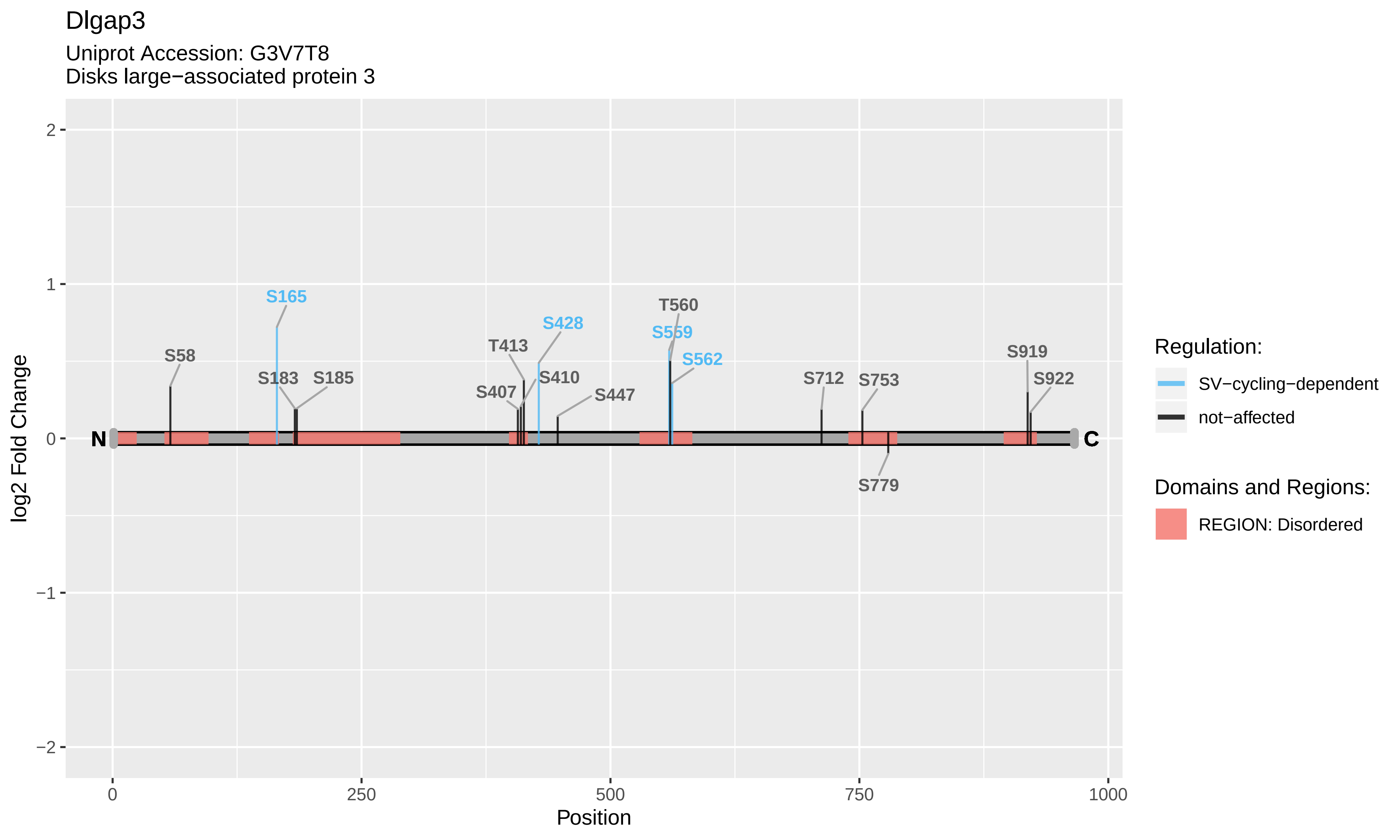
Dlgap3
Uniprot Accession: G3V7T8
Disks large−associated protein 3
log2 Fold Change
2
1
0
−1
−2
N
C
S165
T560
S428
S559
T413
S562
S919
S58
S410
S183
S185
S712
S753
S922
S407
S447
S779
0
250
500
750
1000
Position
Regulation:
SV−cycling−dependent
not−affected
Domains and Regions:
REGION: Disordered

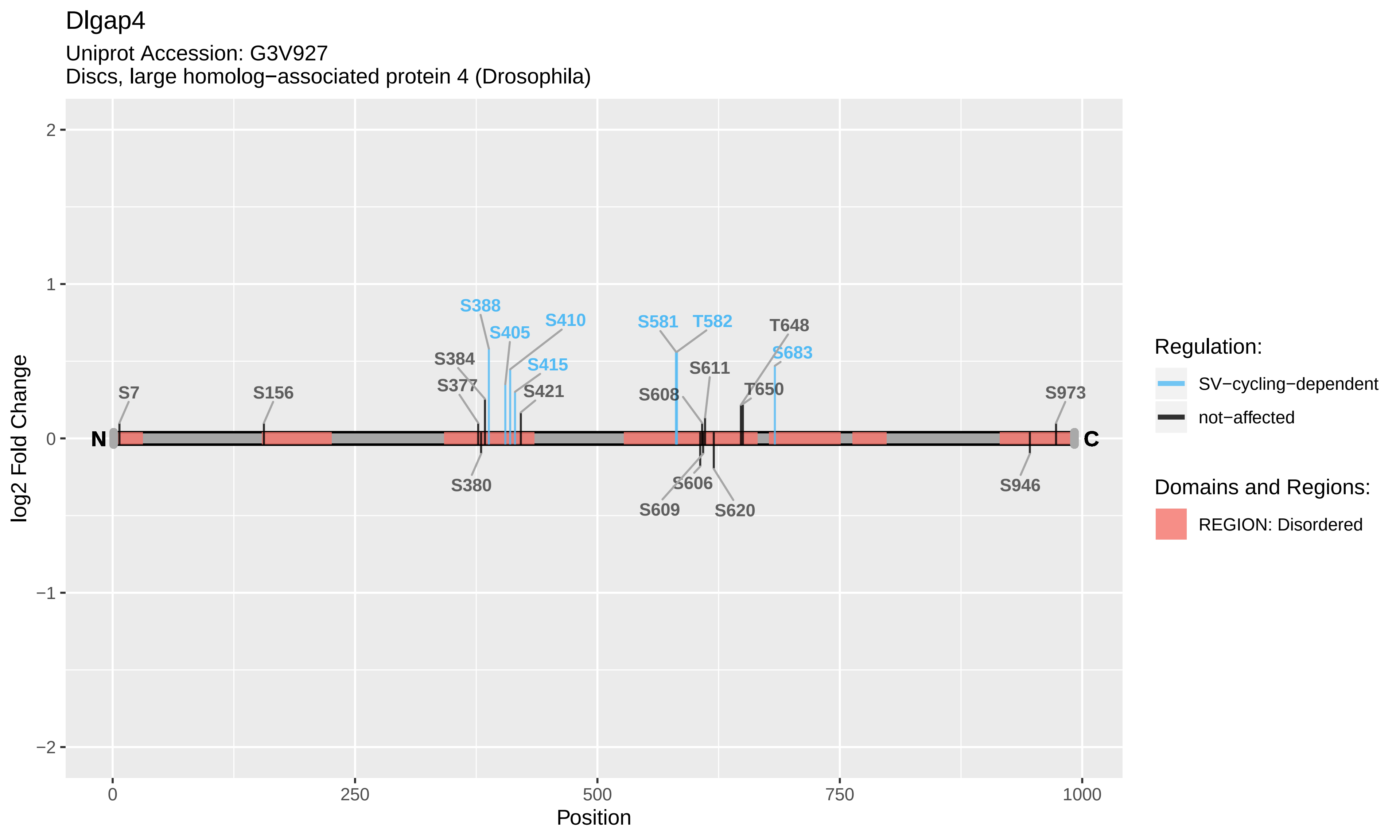

Dlgap4
Uniprot Accession: G3V927
Discs, large homolog−associated protein 4 (Drosophila)
log2 Fold Change
2
1
0
−1
−2
0
250
500
750
1000
Position
N
C
S7
S156
S377
S380
S384
S388
S405
S410
S415
S421
S581
T582
S606
S608
S609
S611
S620
T648
T650
S683
S946
S973
Regulation:
SV−cycling−dependent
not−affected
Domains and Regions:
REGION: Disordered

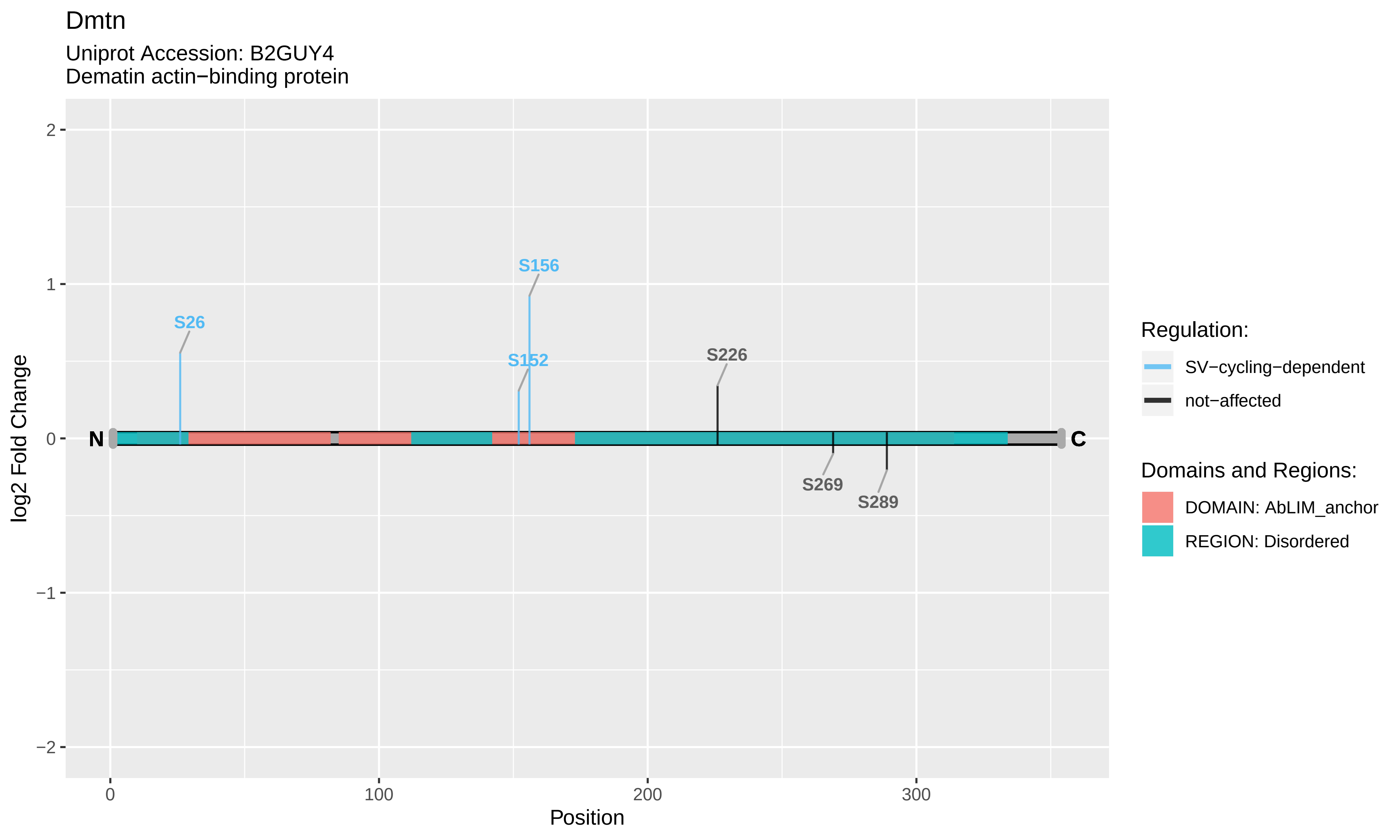

Dmtn
Uniprot Accession: B2GUY4
Dematin actin−binding protein
log2 Fold Change
2
1
0
−1
−2
0
100
200
300
Position
N
C
S26
S156
S152
S226
S269
S289
Regulation:
SV−cycling−dependent
not−affected
Domains and Regions:
DOMAIN: AbLIM_anchor
REGION: Disordered

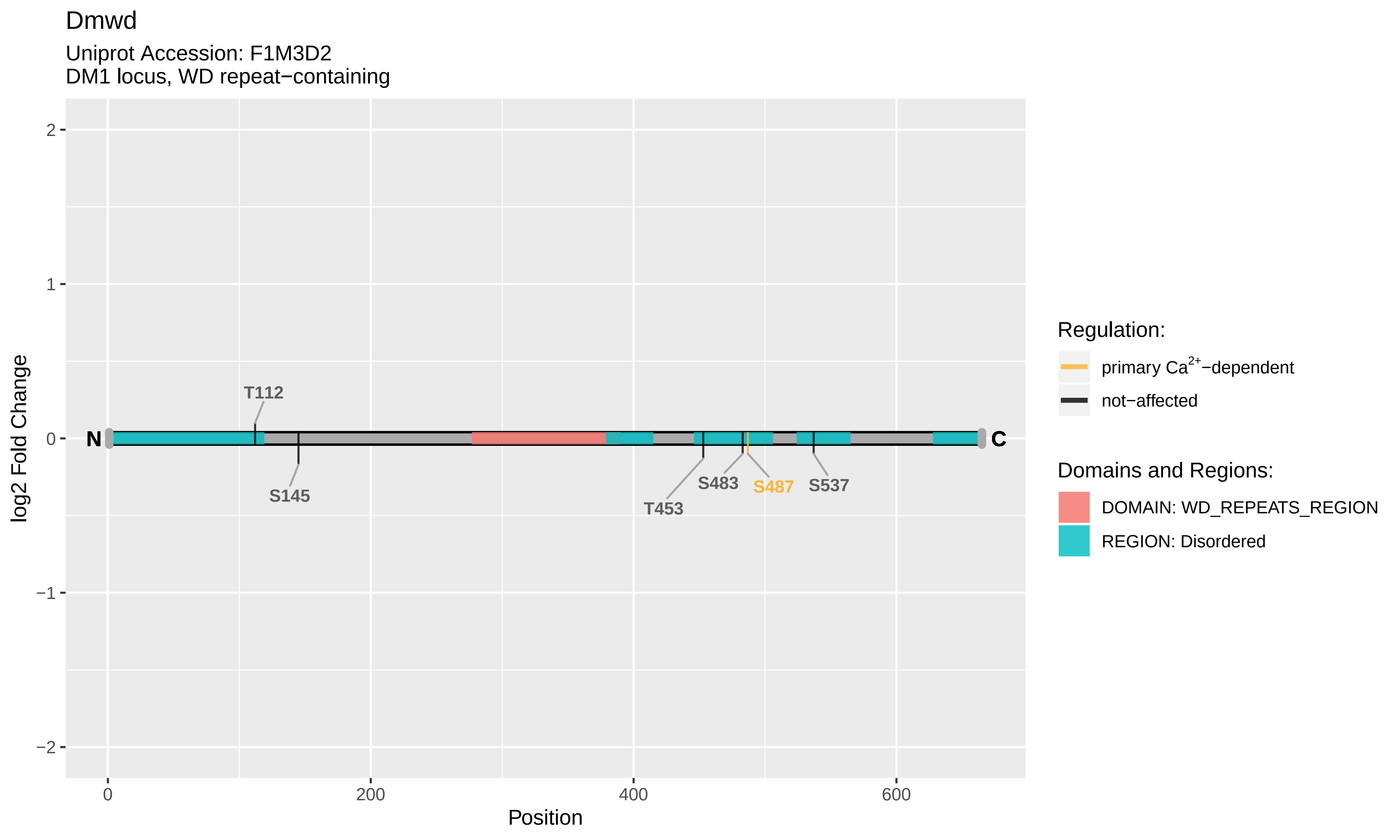
Dmwd
Uniprot Accession: F1M3D2
DM1 locus, WD repeat−containing
log2 Fold Change
2
1
0
−1
−2
N
C
T112
S145
T453
S483
S487
S537
0
200
400
600
Position
Regulation:
primary Ca2+−dependent
not−affected
Domains and Regions:
DOMAIN: WD_REPEATS_REGION
REGION: Disordered

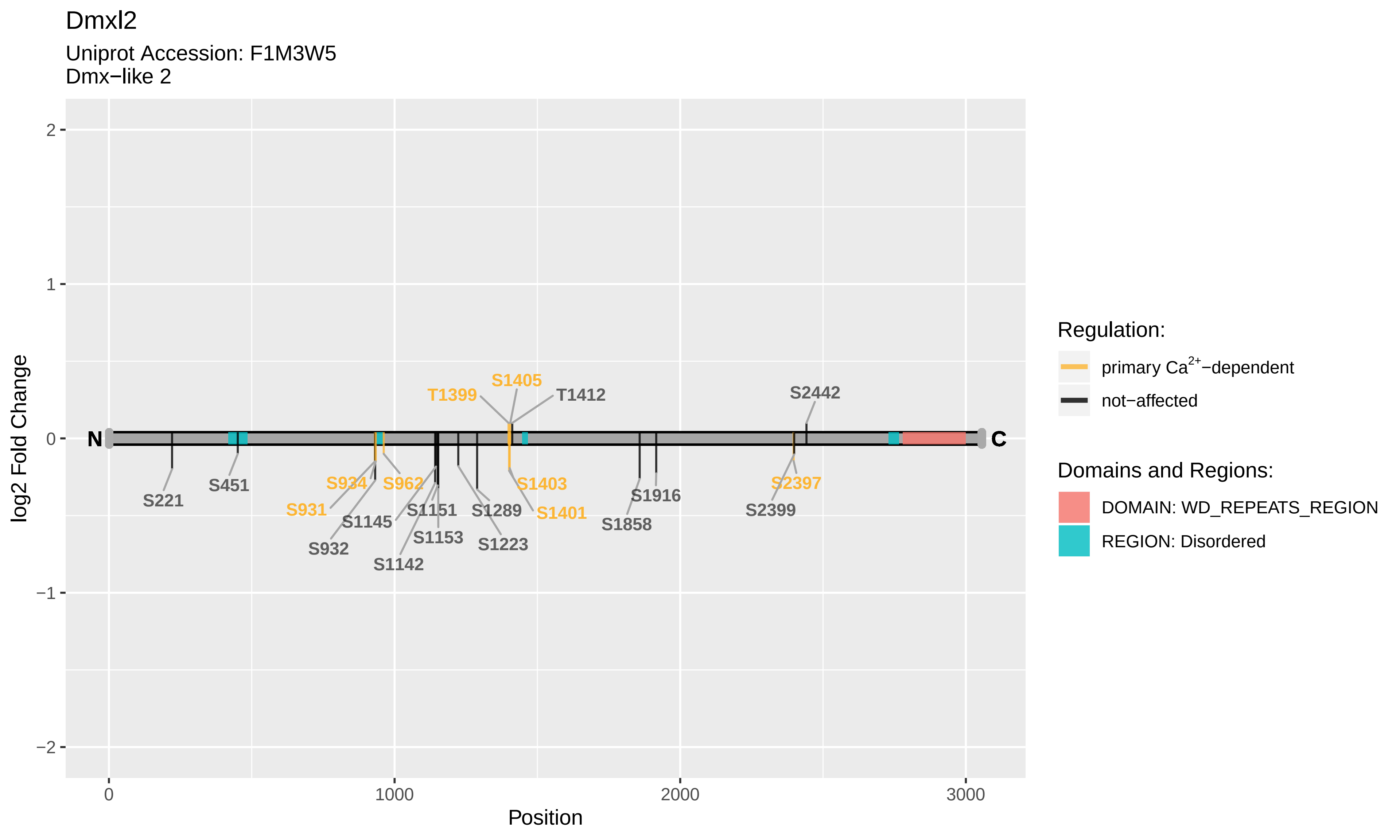
Dmxl2
Uniprot Accession: F1M3W5
Dmx−like 2
log2 Fold Change
2
1
0
−1
−2
Position
0
1000
2000
3000
N
C
S221
S451
S934
S962
S931
S932
S1145
S1151
S1153
S1142
S1289
S1223
T1399
S1405
T1412
S1403
S1401
S1916
S1858
S2397
S2399
S2442
Regulation:
primary Ca2+−dependent
not−affected
Domains and Regions:
DOMAIN: WD_REPEATS_REGION
REGION: Disordered

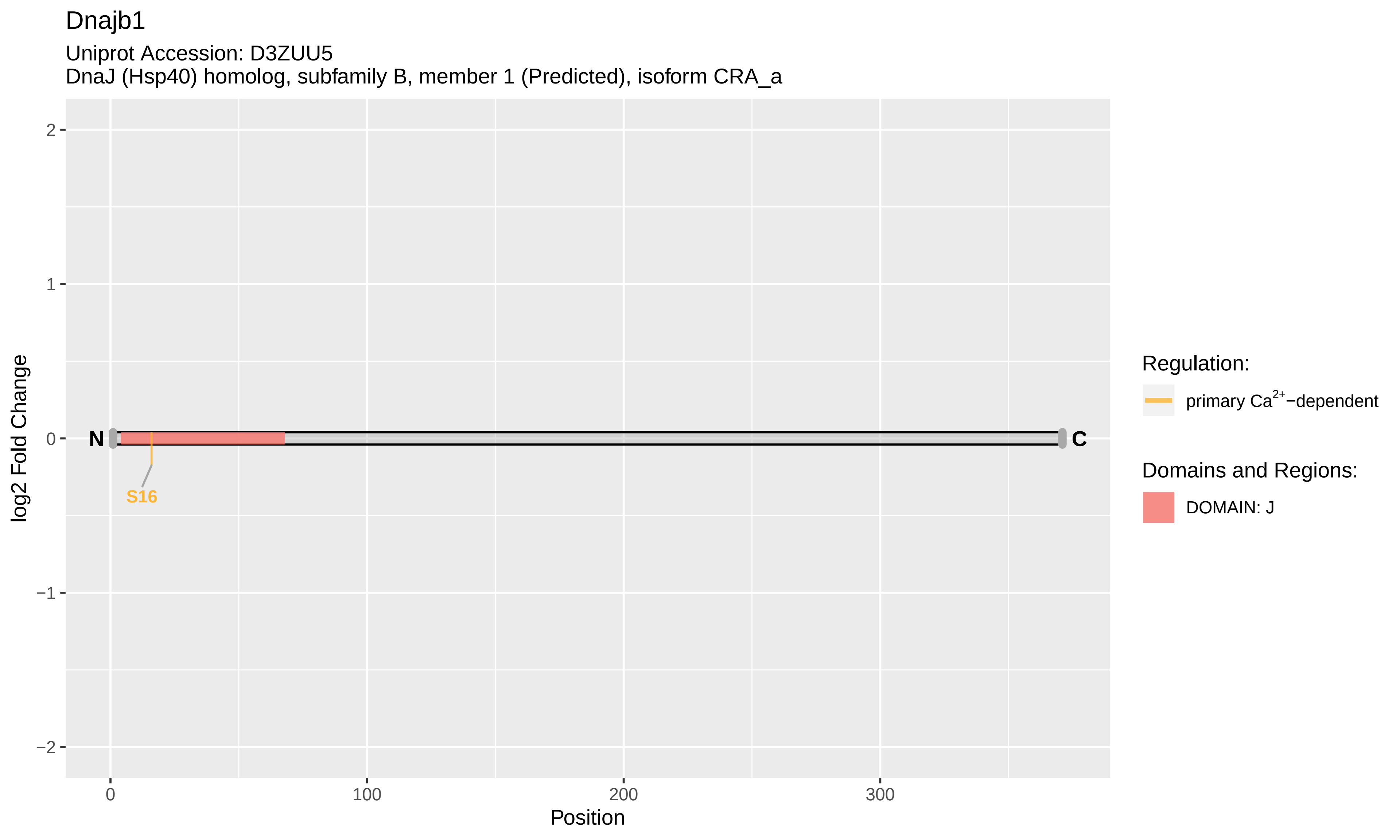

Dnajb1
Uniprot Accession: D3ZUU5
DnaJ (Hsp40) homolog, subfamily B, member 1 (Predicted), isoform CRA_a
log2 Fold Change
Position
N
C
S16
Regulation:
primary Ca2+−dependent
Domains and Regions:
DOMAIN: J

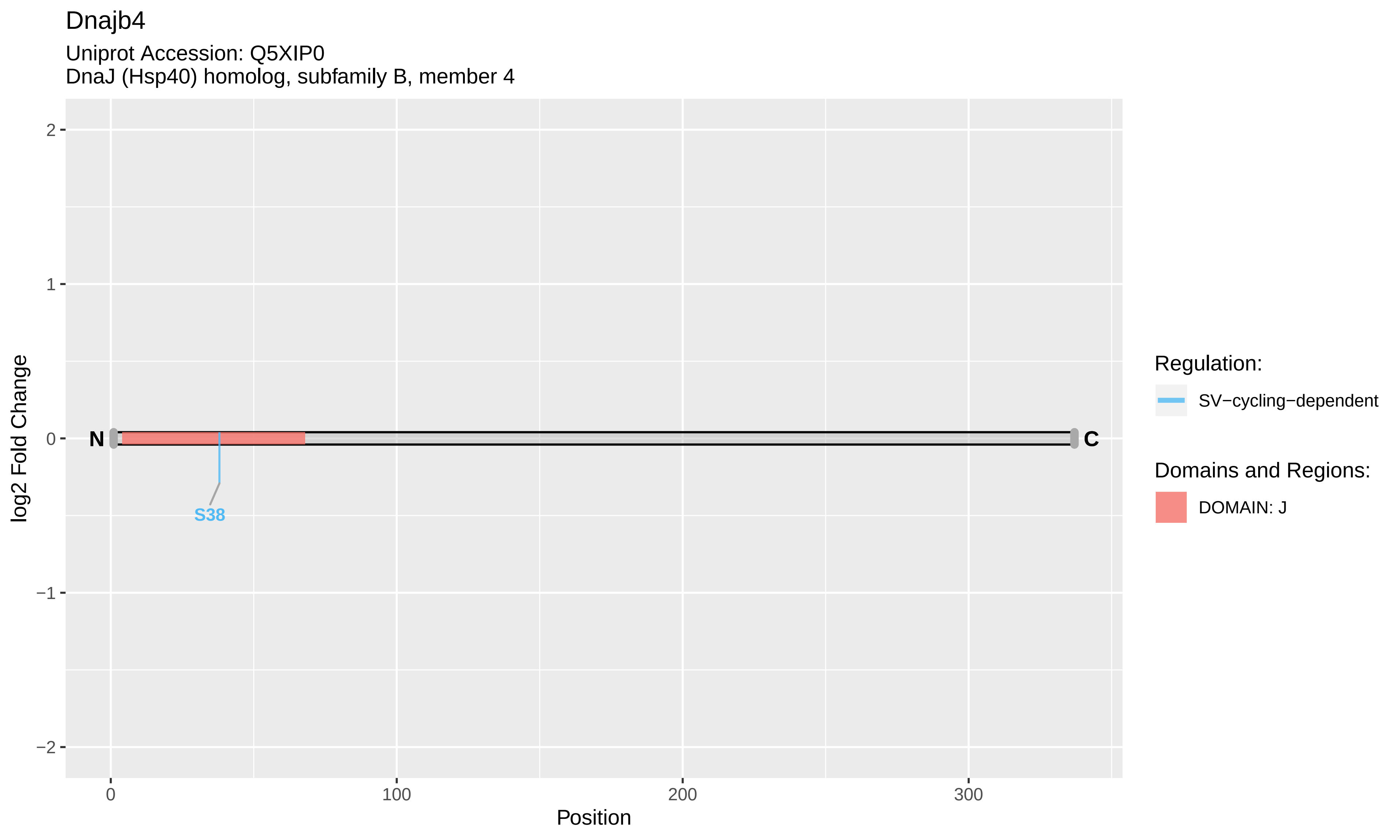

Dnajb4
Uniprot Accession: Q5XIP0
DnaJ (Hsp40) homolog, subfamily B, member 4
log2 Fold Change
2
1
0
−1
−2
N
C
S38
0
100
200
300
Position
Regulation:
SV−cycling−dependent
Domains and Regions:
DOMAIN: J

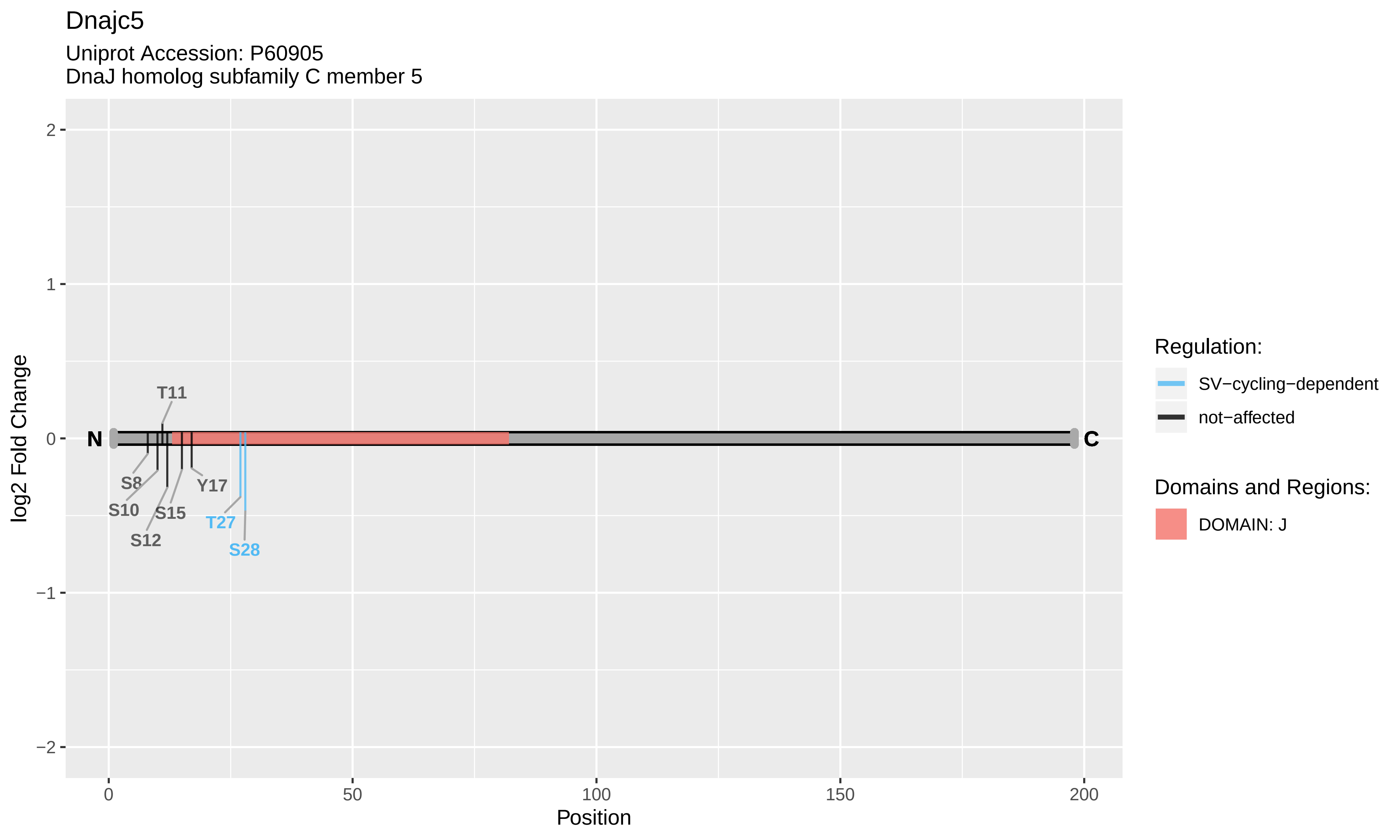

Dnajc5
Uniprot Accession: P60905
DnaJ homolog subfamily C member 5
log2 Fold Change
2
1
0
−1
−2
N
C
T11
S8
S10
S12
S15
Y17
T27
S28
0
50
100
150
200
Position
Regulation:
SV−cycling−dependent
not−affected
Domains and Regions:
DOMAIN: J

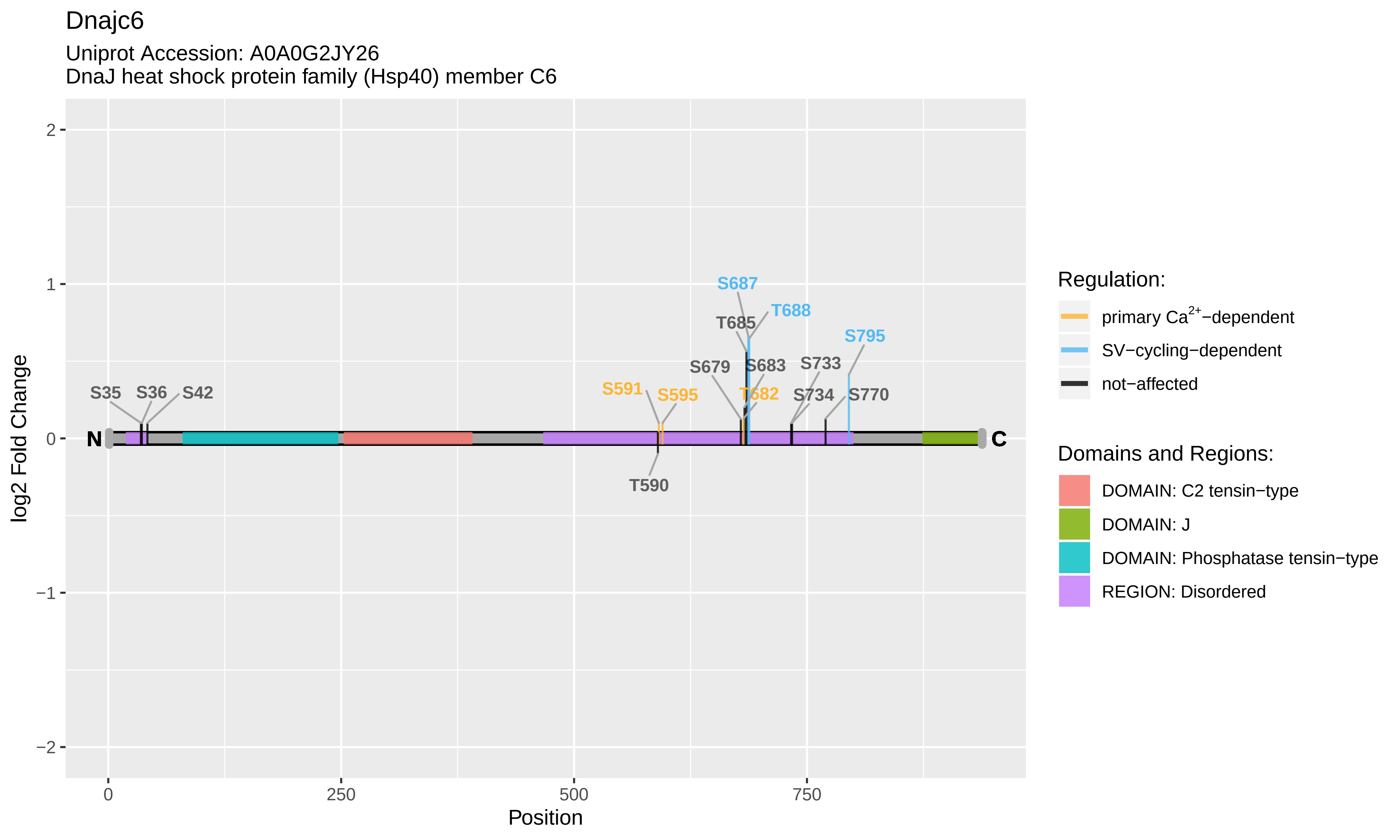

Dnajc6
Uniprot Accession: A0A0G2JY26
DnaJ heat shock protein family (Hsp40) member C6
log2 Fold Change
Position
N
C
S35
S36
S42
S591
S595
T590
S679
S683
T682
T685
S687
T688
S733
S734
S770
S795
Regulation:
primary Ca2+−dependent
SV−cycling−dependent
not−affected
Domains and Regions:
DOMAIN: C2 tensin−type
DOMAIN: J
DOMAIN: Phosphatase tensin−type
REGION: Disordered

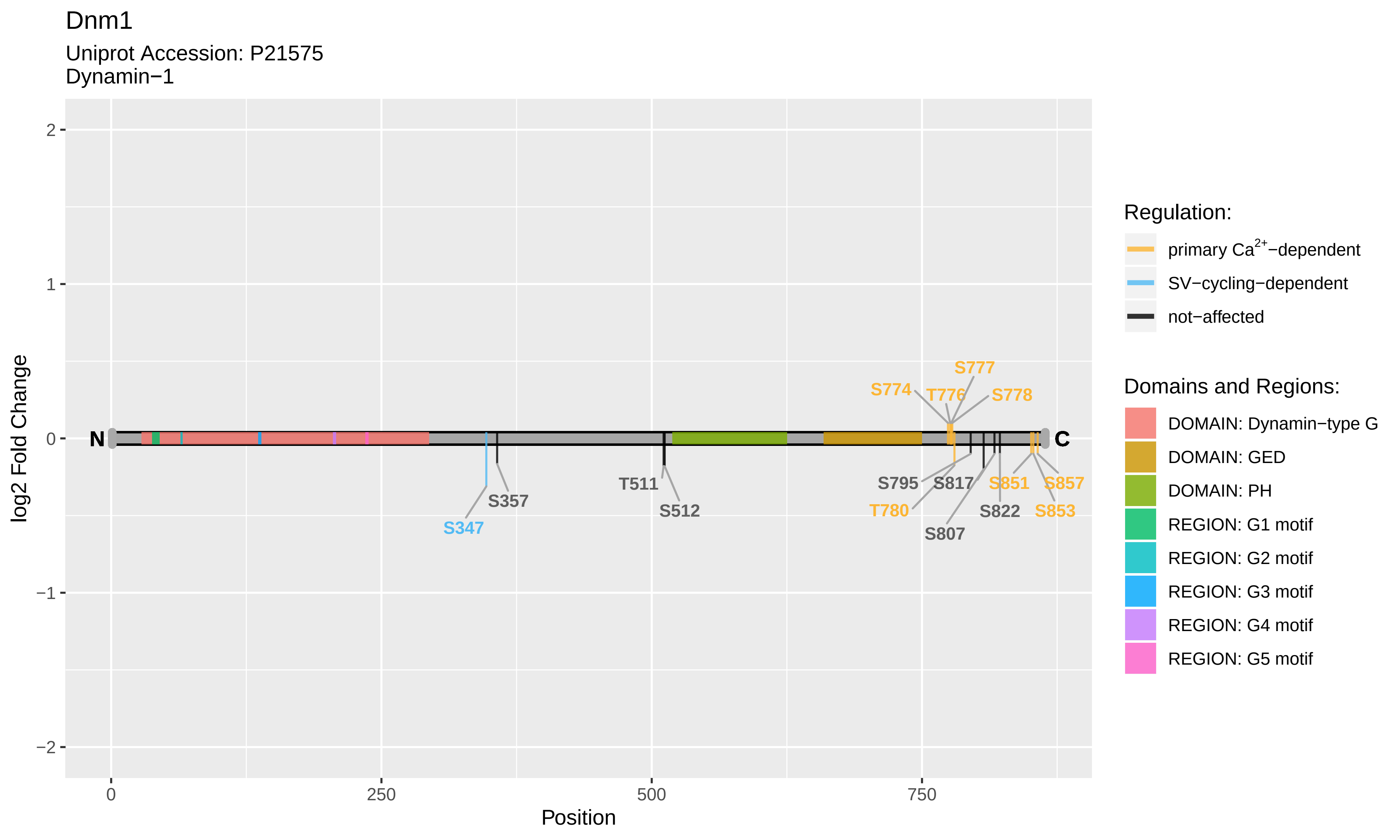
Dnm1
Uniprot Accession: P21575
Dynamin−1
log2 Fold Change
Position
2
1
0
−1
−2
0
250
500
750
N
C
S777
S774
T776
S778
S347
S357
T511
S512
S795
S817
S851
S857
T780
S822
S853
S807
Regulation:
primary Ca2+−dependent
SV−cycling−dependent
not−affected
Domains and Regions:
DOMAIN: Dynamin−type G
DOMAIN: GED
DOMAIN: PH
REGION: G1 motif
REGION: G2 motif
REGION: G3 motif
REGION: G4 motif
REGION: G5 motif

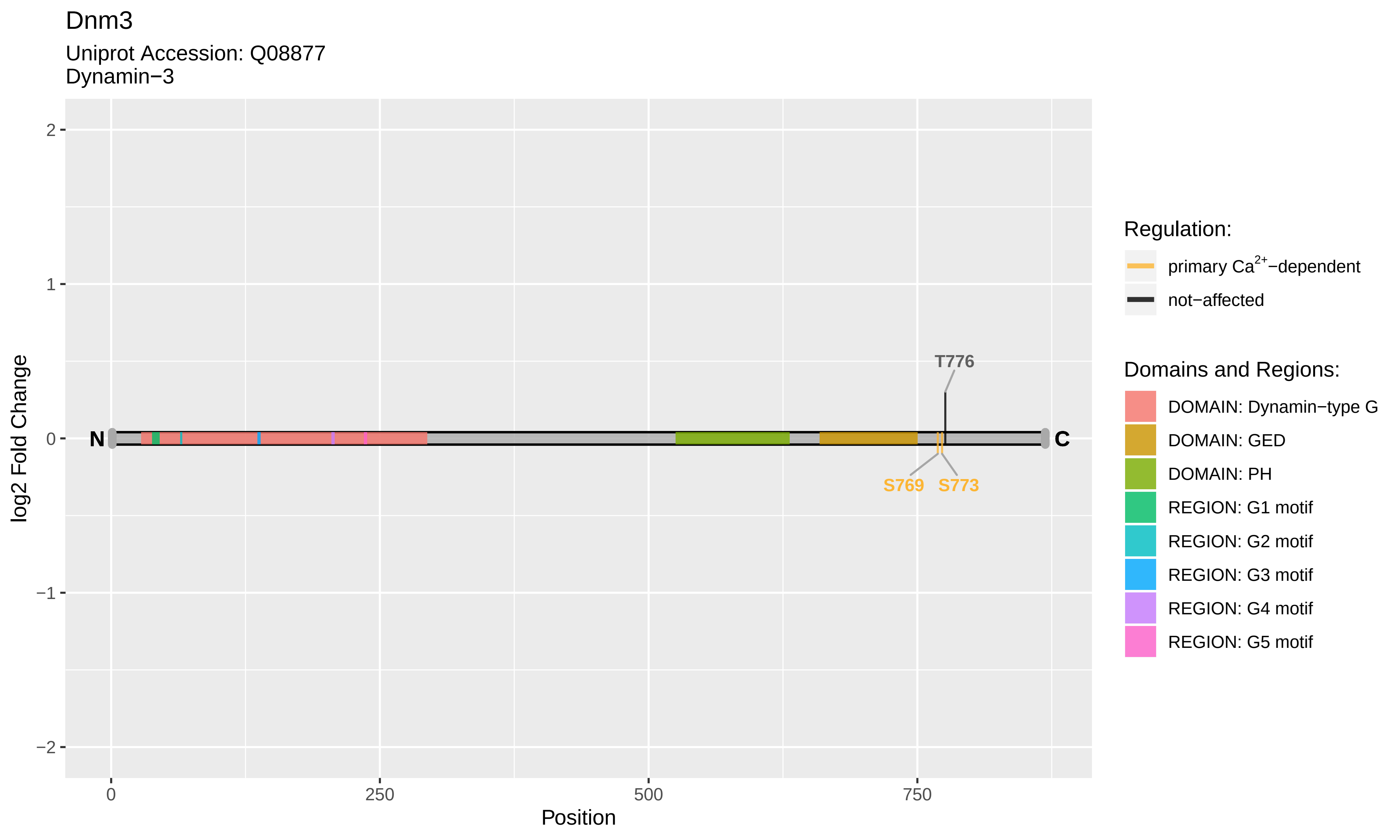
Dnm3
Uniprot Accession: Q08877
Dynamin−3
log2 Fold Change
Position
2
1
0
−1
−2
0
250
500
750
N
C
T776
S769
S773
Regulation:
primary Ca2+−dependent
not−affected
Domains and Regions:
DOMAIN: Dynamin−type G
DOMAIN: GED
DOMAIN: PH
REGION: G1 motif
REGION: G2 motif
REGION: G3 motif
REGION: G4 motif
REGION: G5 motif

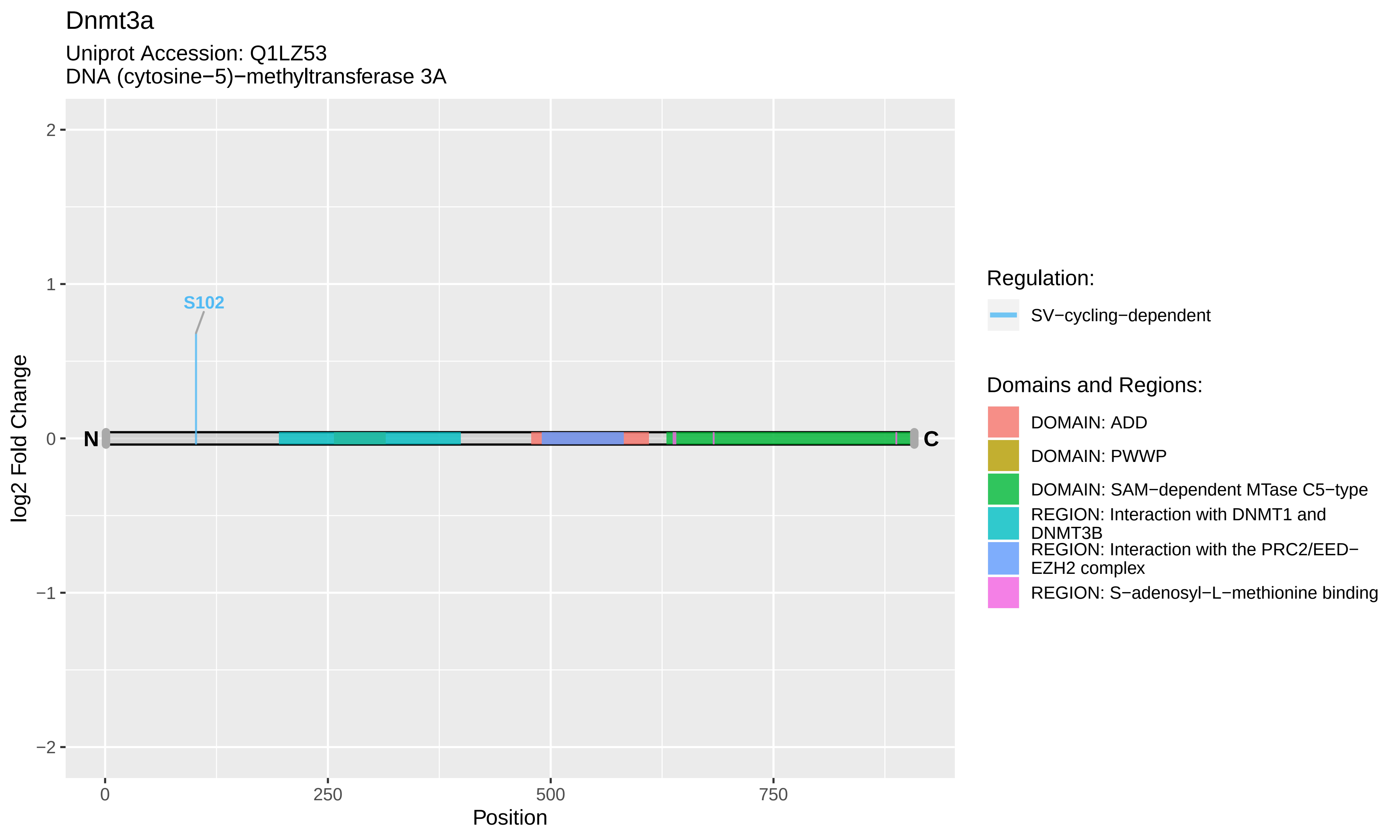
Dnmt3a
Uniprot Accession: Q1LZ53
DNA (cytosine−5)−methyltransferase 3A
S102
N
C
log2 Fold Change
2
1
0
−1
−2
0
250
500
750
Position
Regulation:
SV−cycling−dependent
Domains and Regions:
DOMAIN: ADD
DOMAIN: PWWP
DOMAIN: SAM−dependent MTase C5−type
REGION: Interaction with DNMT1 and DNMT3B
REGION: Interaction with the PRC2/EED−EZH2 complex
REGION: S−adenosyl−L−methionine binding

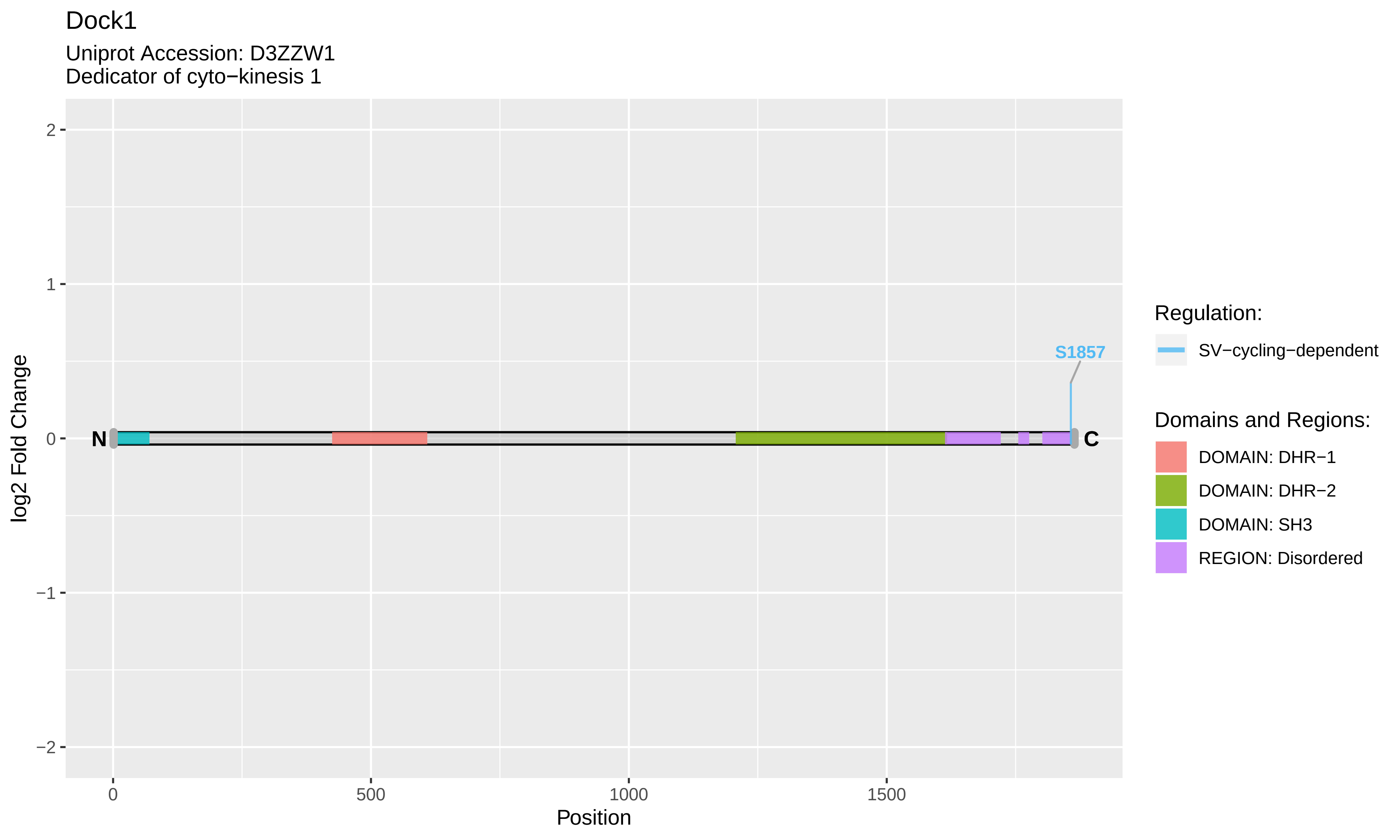
Dock1
Uniprot Accession: D3ZZW1
Dedicator of cyto−kinesis 1
log2 Fold Change
Position
N
C
S1857
Regulation:
SV−cycling−dependent
Domains and Regions:
DOMAIN: DHR−1
DOMAIN: DHR−2
DOMAIN: SH3
REGION: Disordered
2
1
0
−1
−2
0
500
1000
1500

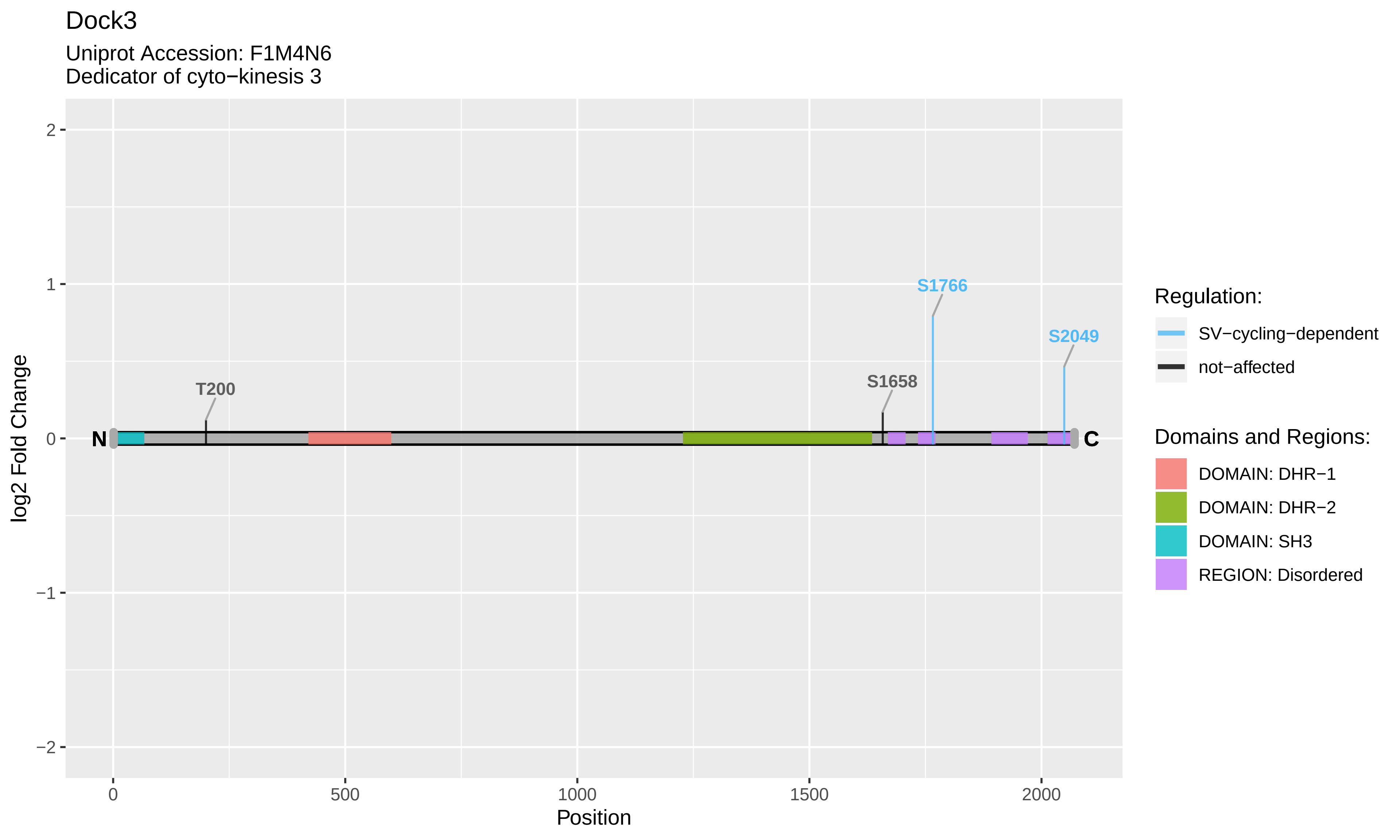
Dock3
Uniprot Accession: F1M4N6
Dedicator of cyto−kinesis 3
log2 Fold Change
Position
N
C
T200
S1658
S1766
S2049
2
1
0
−1
−2
0
500
1000
1500
2000
Regulation:
SV−cycling−dependent
not−affected
Domains and Regions:
DOMAIN: DHR−1
DOMAIN: DHR−2
DOMAIN: SH3
REGION: Disordered

Dock4

Uniprot Accession: M0R6K4
Dedicator of cytokinesis 4

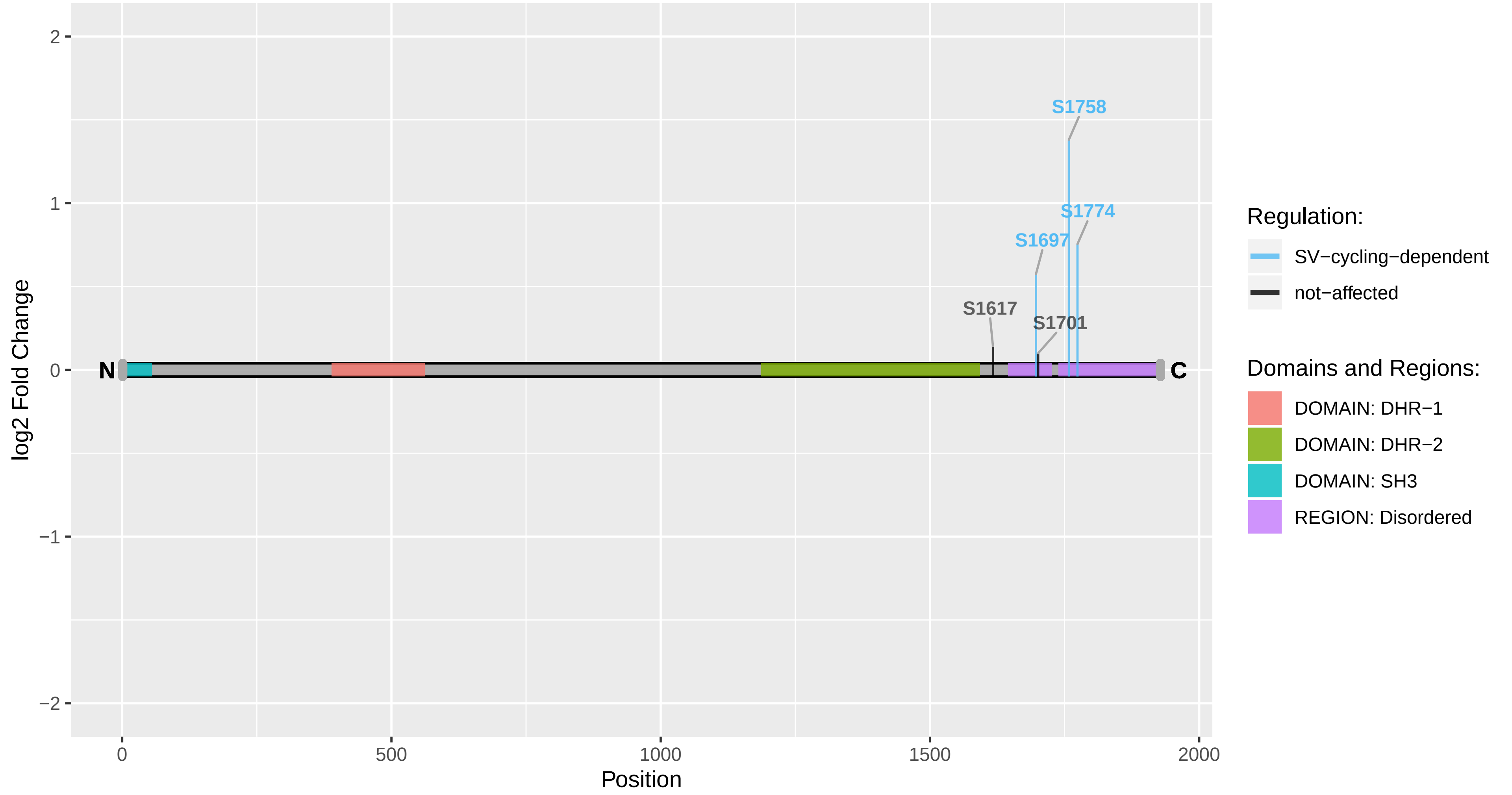

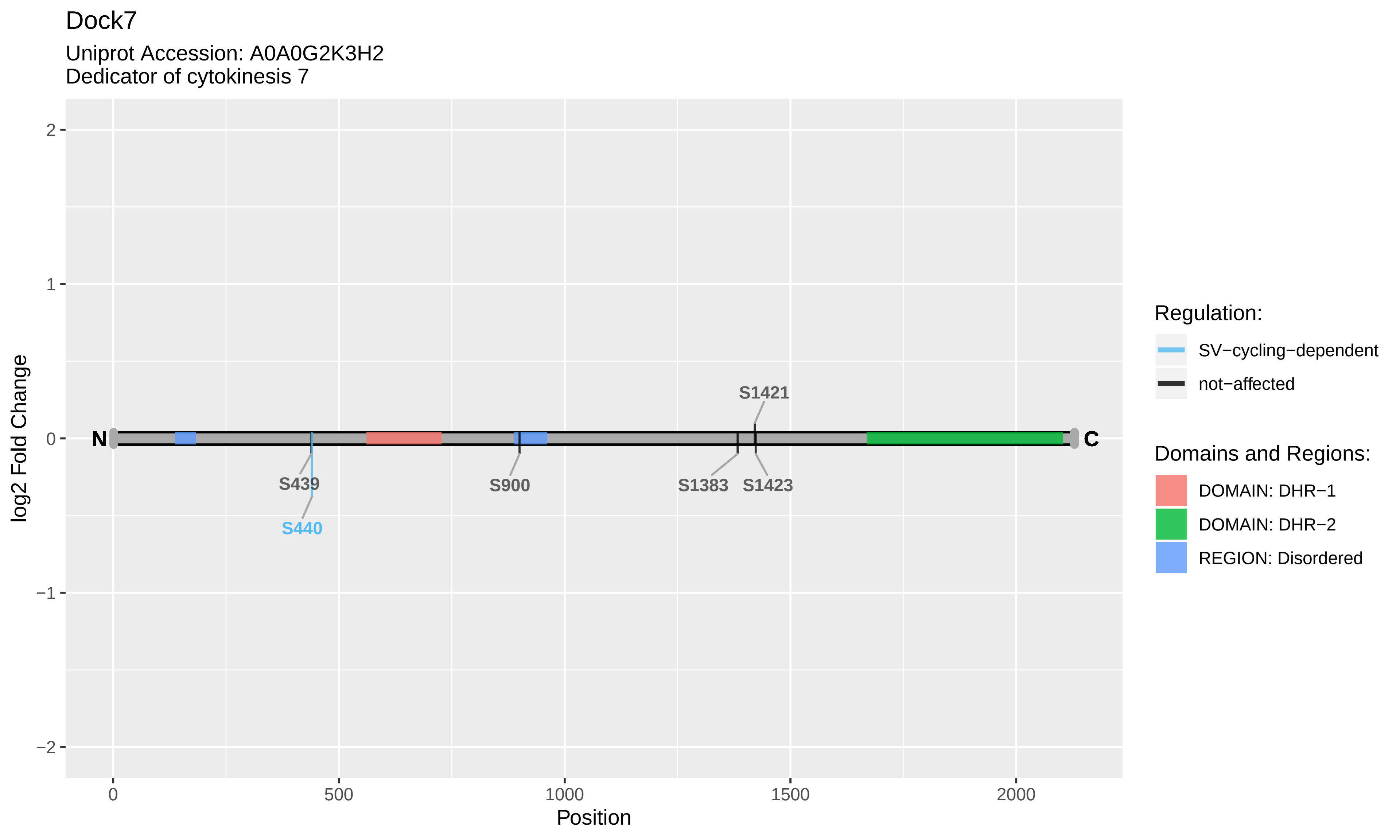
Dock7
Uniprot Accession: A0A0G2K3H2
Dedicator of cytokinesis 7
2
1
0
−1
−2
log2 Fold Change
0
500
1000
1500
2000
Position
N
C
S1421
S439
S440
S900
S1383
S1423
Regulation:
SV−cycling−dependent
not−affected
Domains and Regions:
DOMAIN: DHR−1
DOMAIN: DHR−2
REGION: Disordered

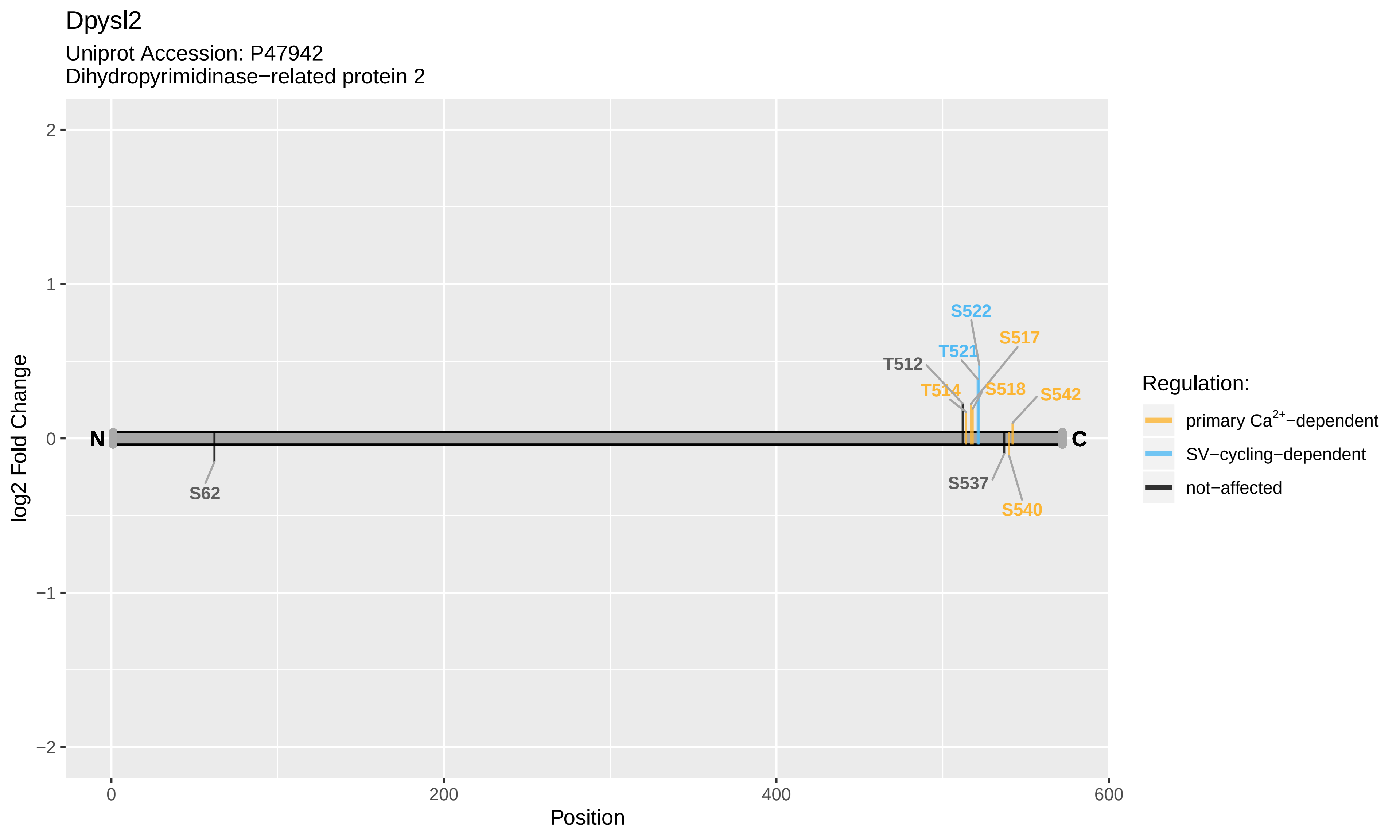
Dpysl2
Uniprot Accession: P47942
Dihydropyrimidinase−related protein 2
S522
S517
T521
T512
T514
S518
S542
N
C
S62
S537
S540
log2 Fold Change
Position
Regulation:
primary Ca2+−dependent
SV−cycling−dependent
not−affected

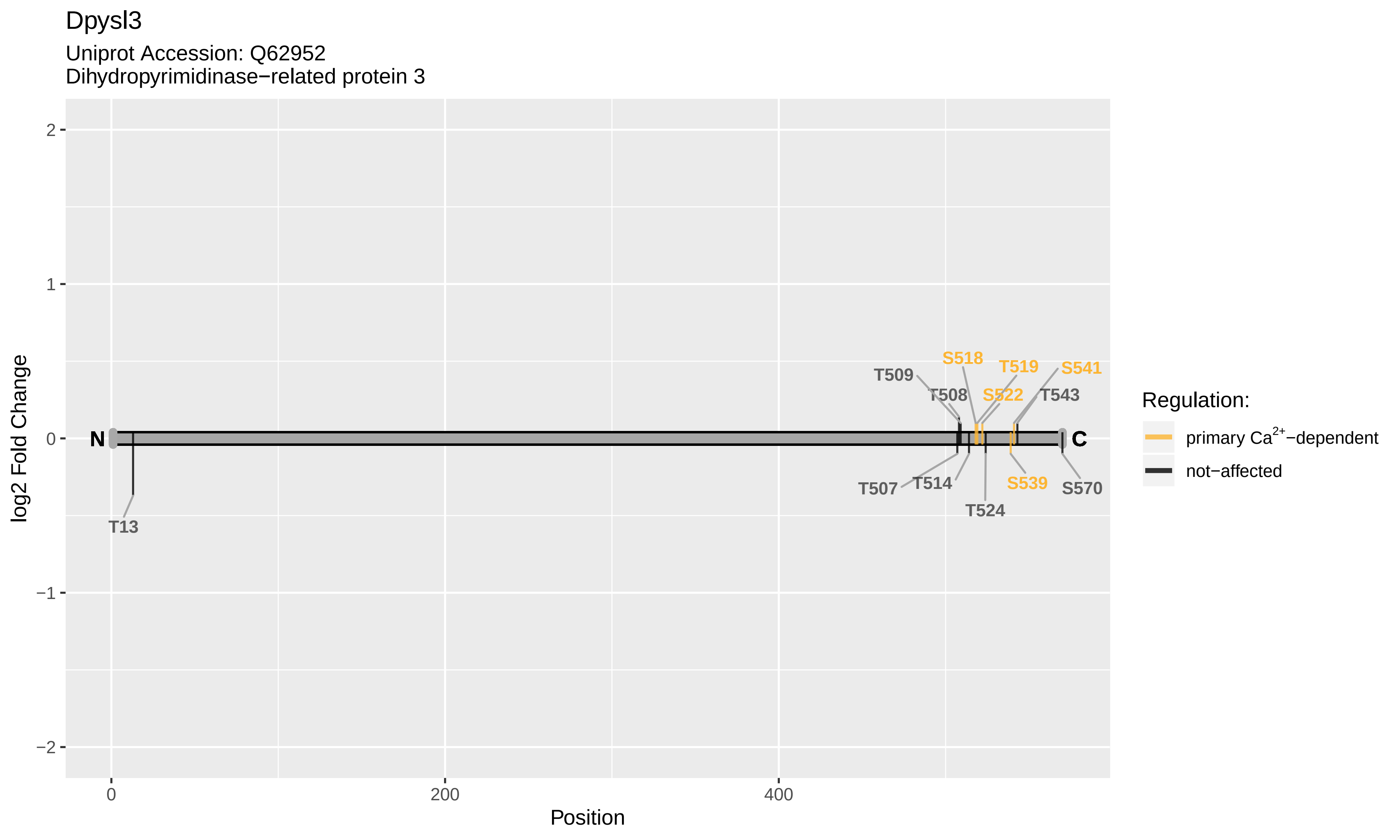

Dpysl3
Uniprot Accession: Q62952
Dihydropyrimidinase−related protein 3
log2 Fold Change
2
1
0
−1
−2
0
200
400
Position
N
C
T13
T507
T508
T509
T514
S518
T519
S522
T524
S539
S541
T543
S570
Regulation:
primary Ca2+−dependent
not−affected

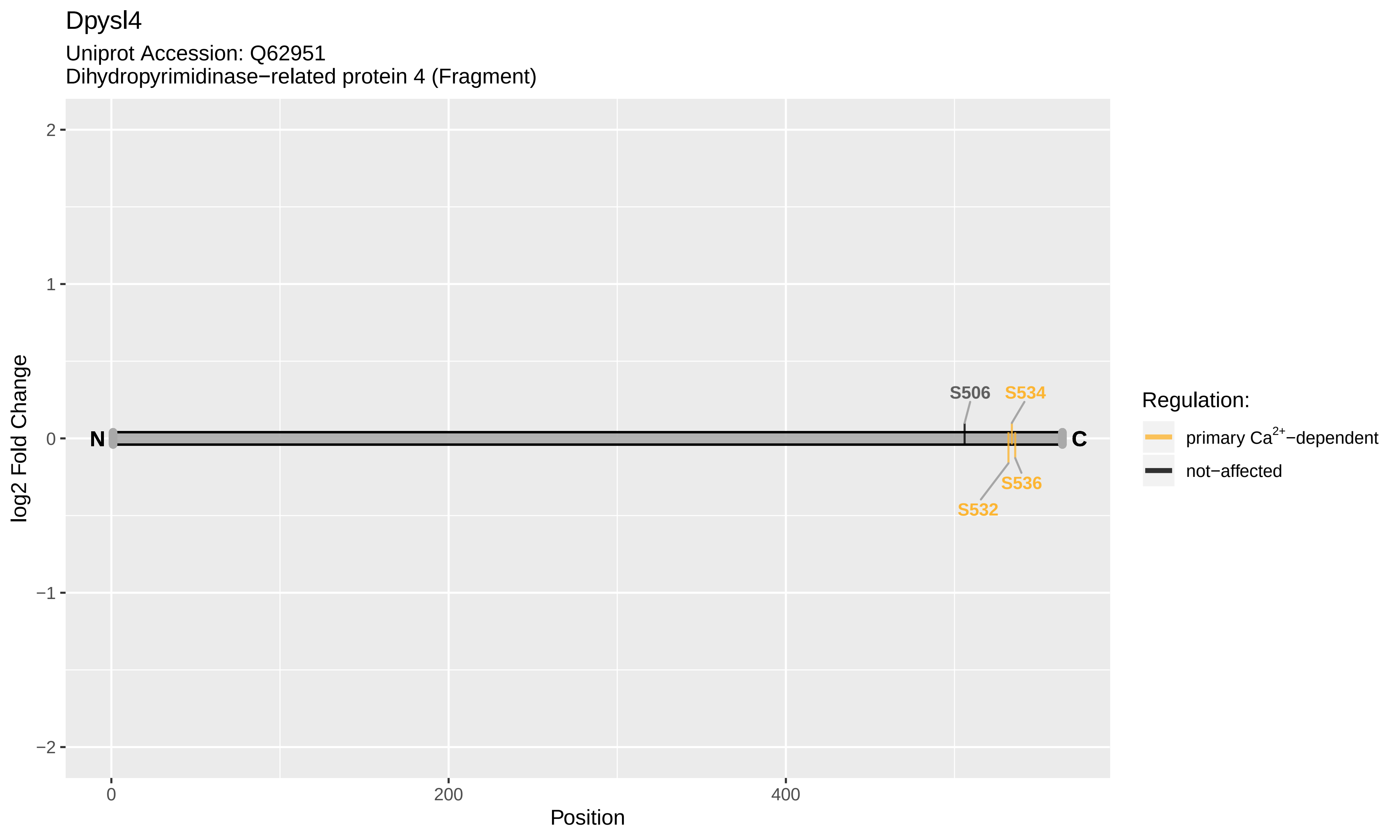

Dpysl4
Uniprot Accession: Q62951
Dihydropyrimidinase−related protein 4 (Fragment)
log2 Fold Change
2
1
0
−1
−2
N
C
S506
S534
S536
S532
0
200
400
Position
Regulation:
primary Ca2+−dependent
not−affected

Dpysl5

Uniprot Accession: Q9JHU0
Dihydropyrimidinase−related protein 5

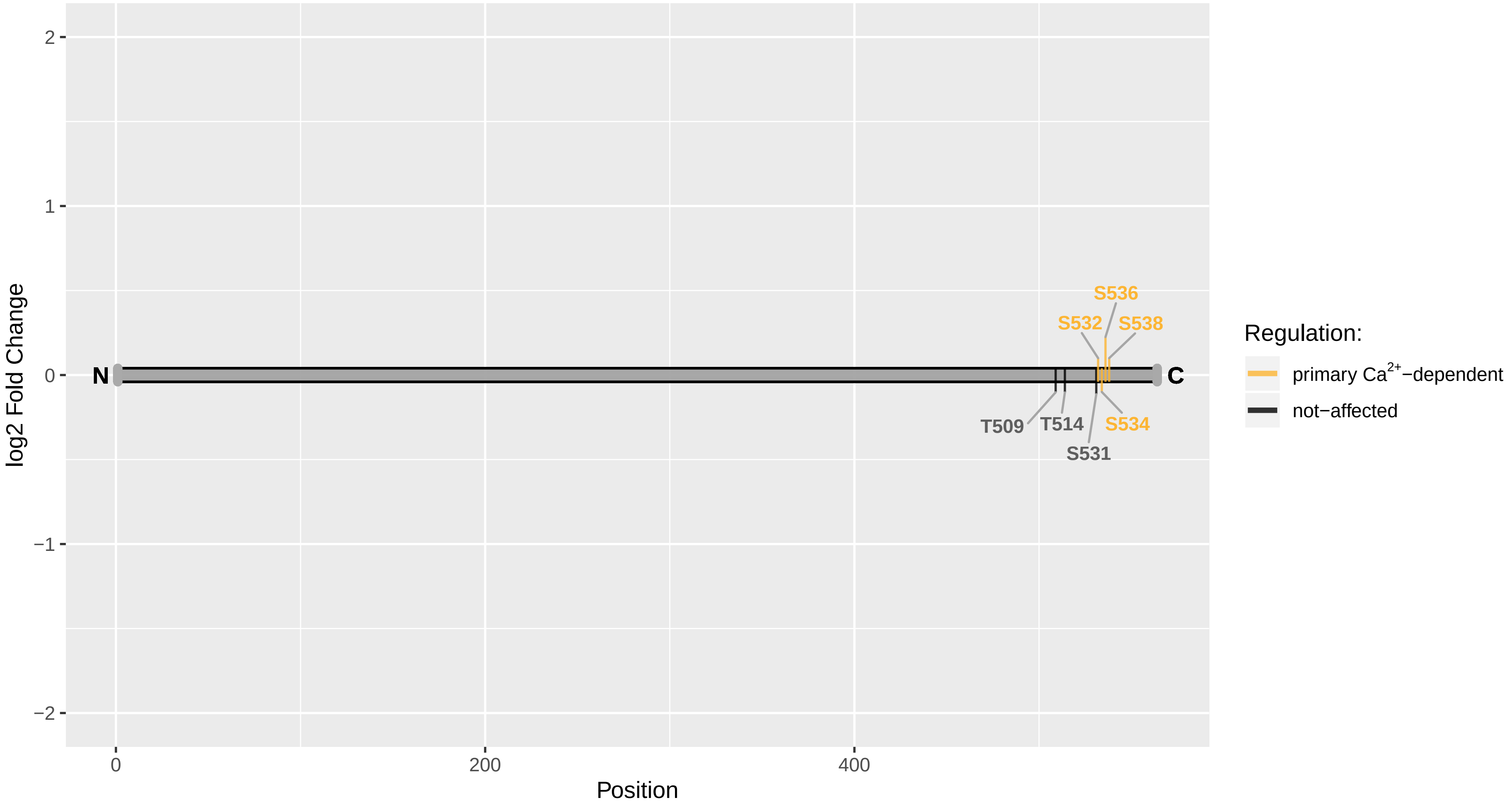

Drd1

Uniprot Accession: P18901
D(1A) dopamine receptor

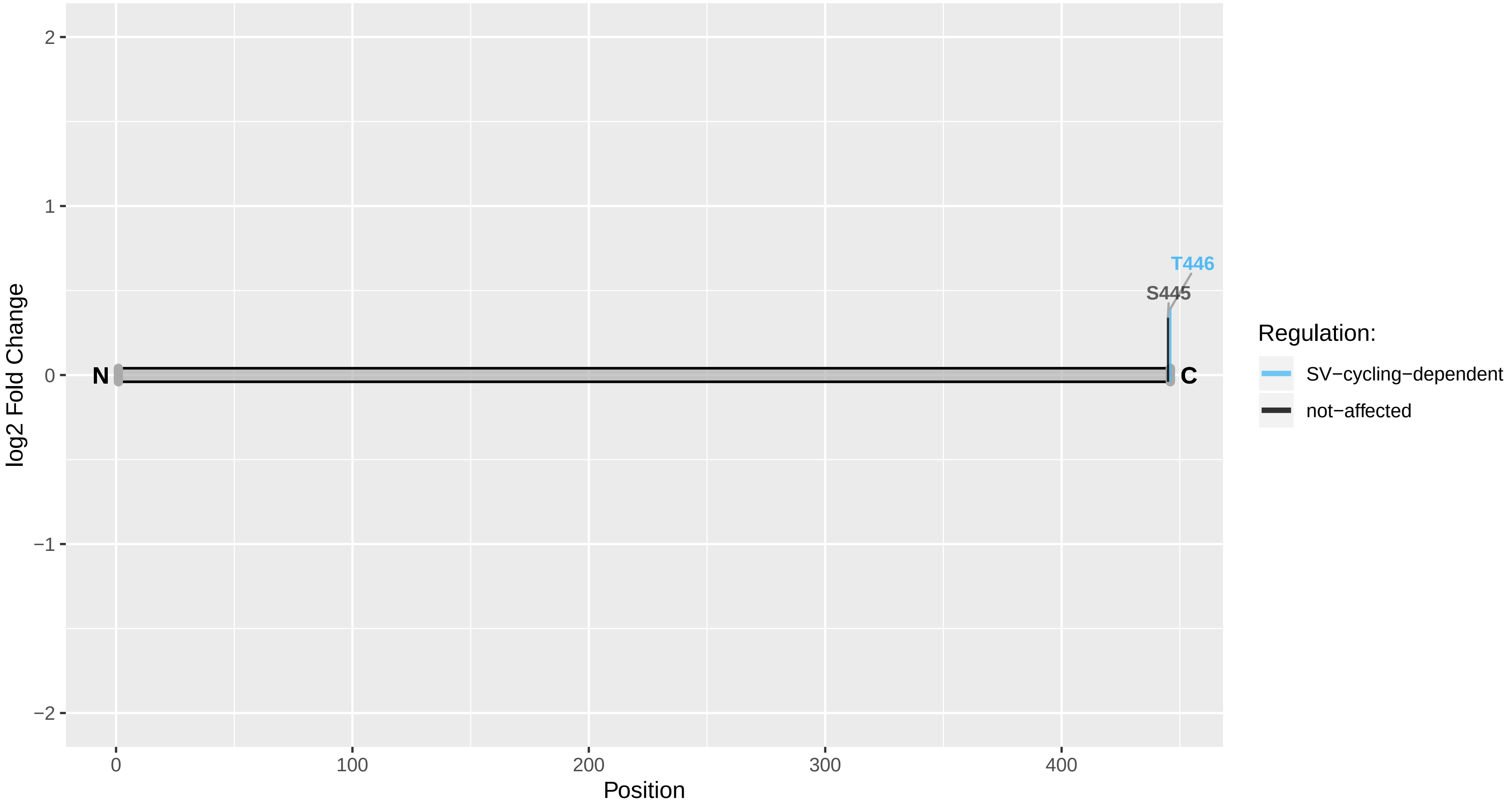

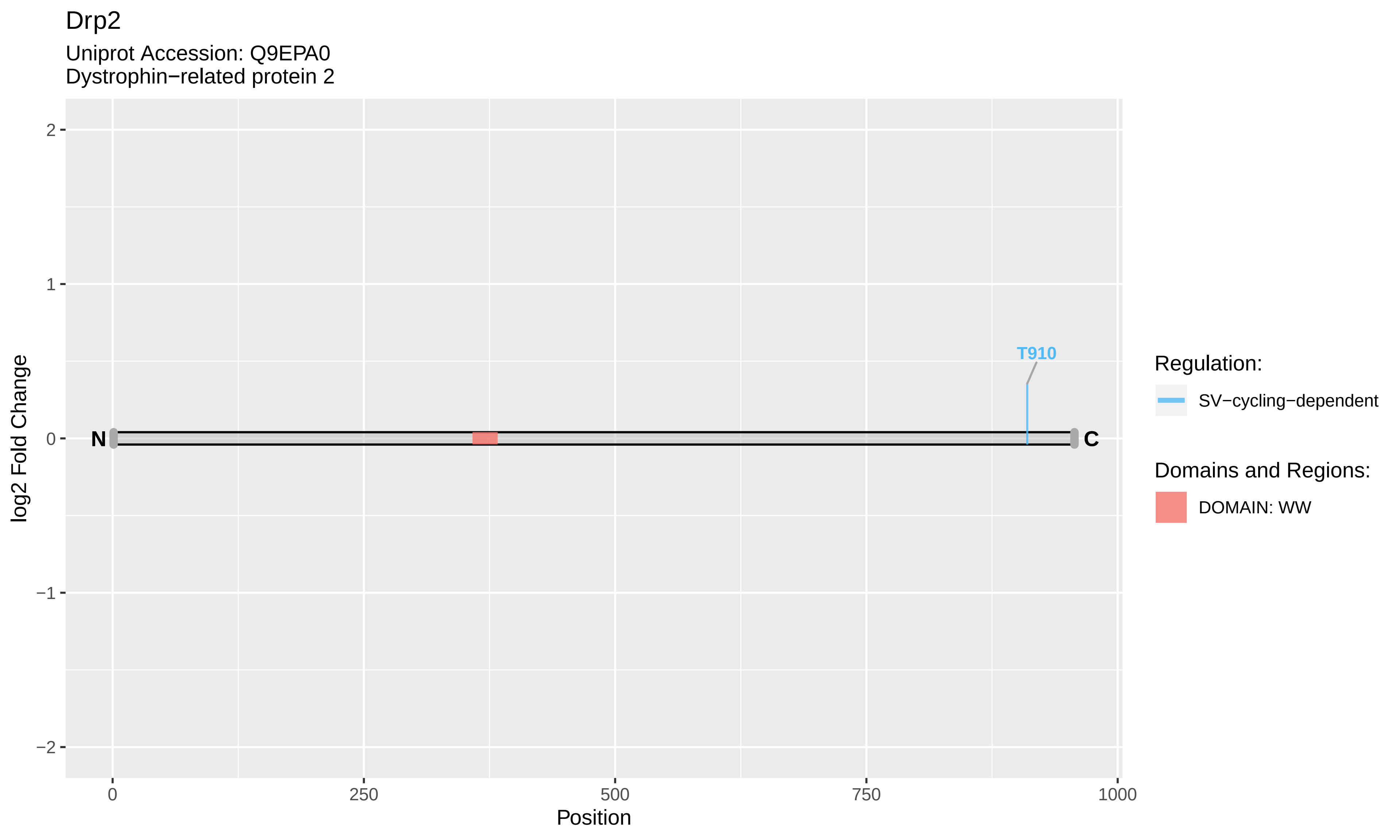

Drp2
Uniprot Accession: Q9EPA0
Dystrophin−related protein 2
T910
N
C
2
1
0
−1
−2
log2 Fold Change
0
250
500
750
1000
Position
Regulation:
SV−cycling−dependent
Domains and Regions:
DOMAIN: WW

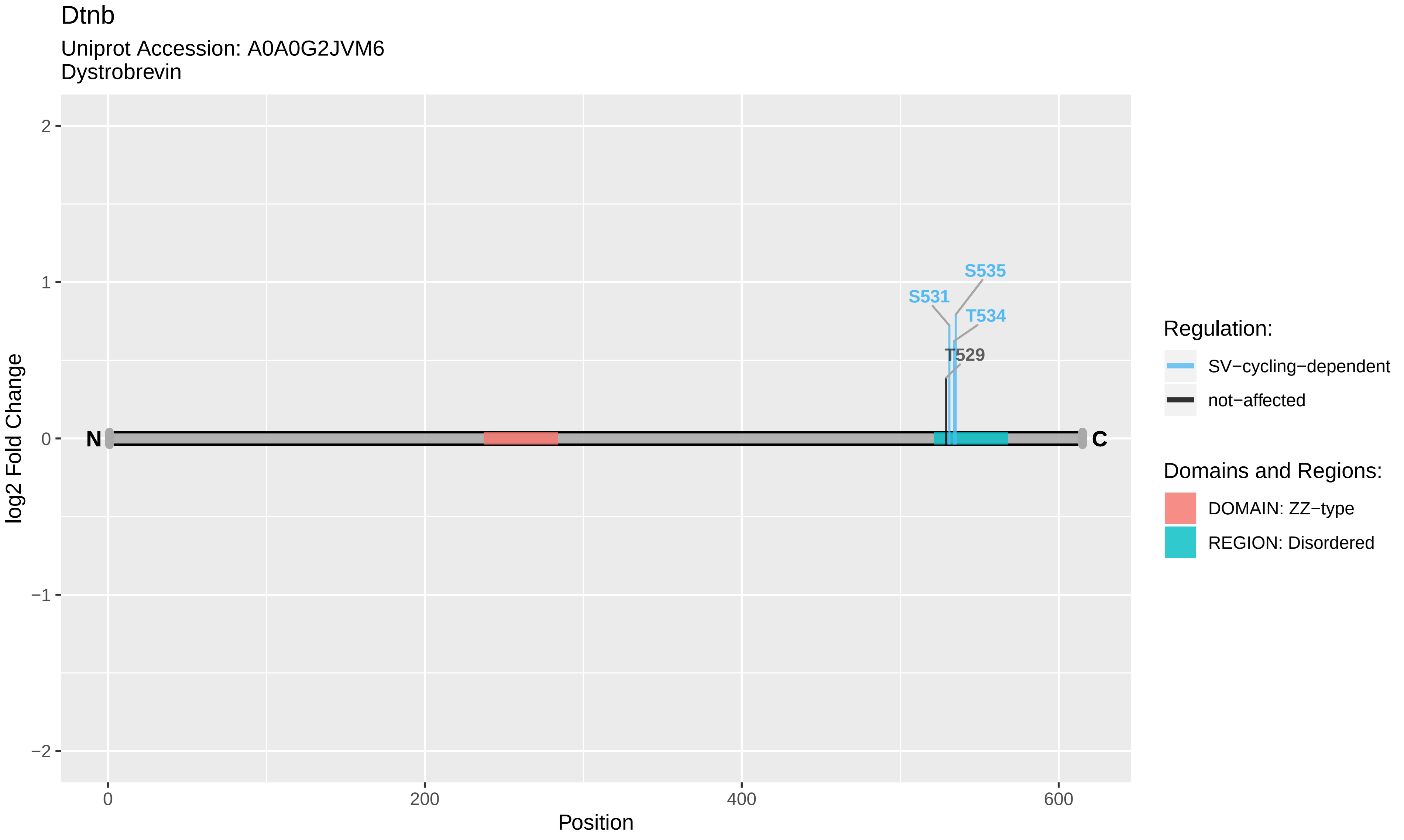

Dtnb
Uniprot Accession: A0A0G2JVM6
Dystrobrevin
log2 Fold Change
Position
N
C
S535
S531
T534
T529
Regulation:
SV−cycling−dependent
not−affected
Domains and Regions:
DOMAIN: ZZ−type
REGION: Disordered
2
1
0
−1
−2
0
200
400
600

Dync1li1

Uniprot Accession: Q9QXU8
Cytoplasmic dynein 1 light intermediate chain 1

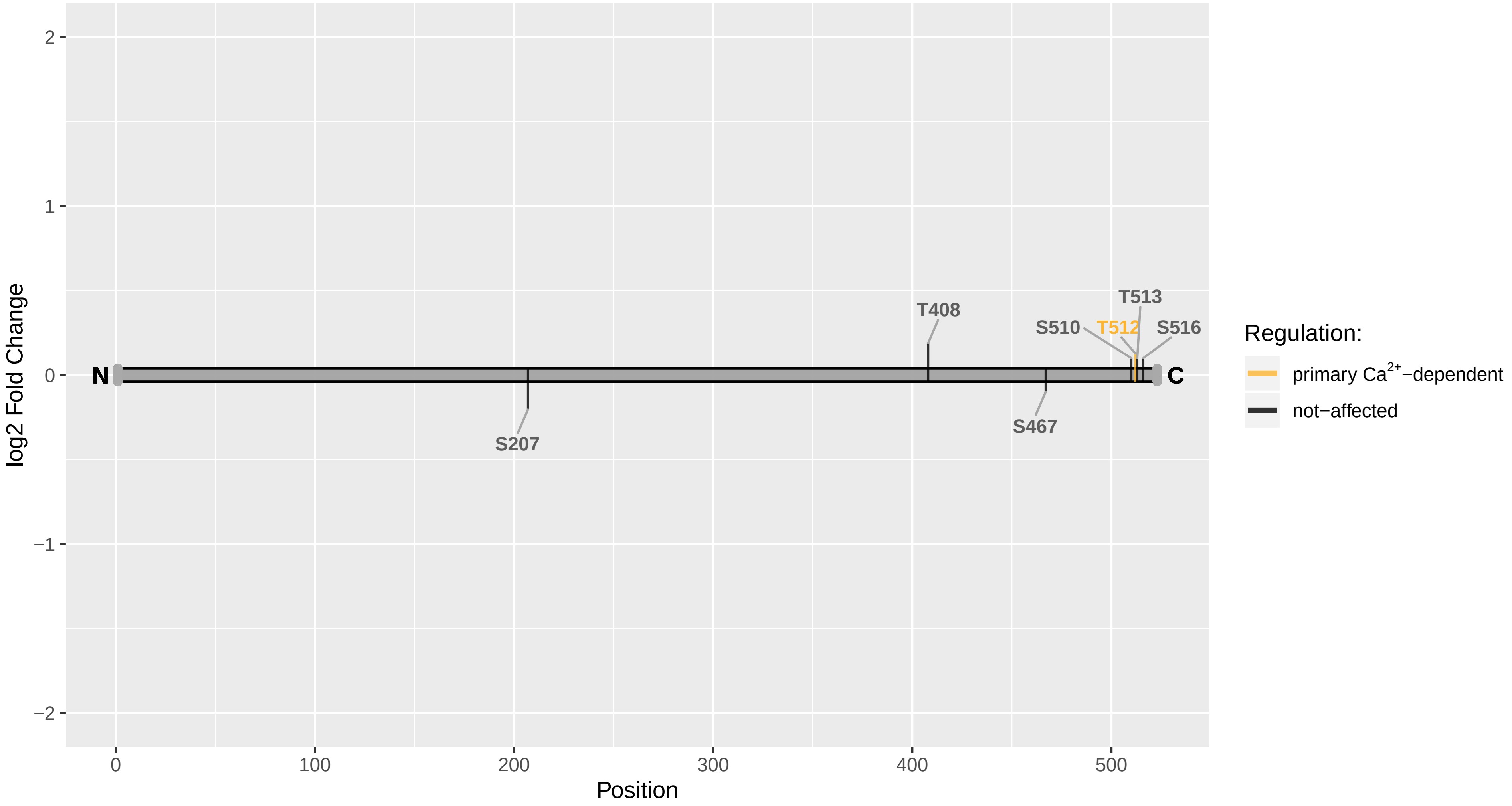

Dync1li2

Uniprot Accession: Q62698
Cytoplasmic dynein 1 light intermediate chain 2

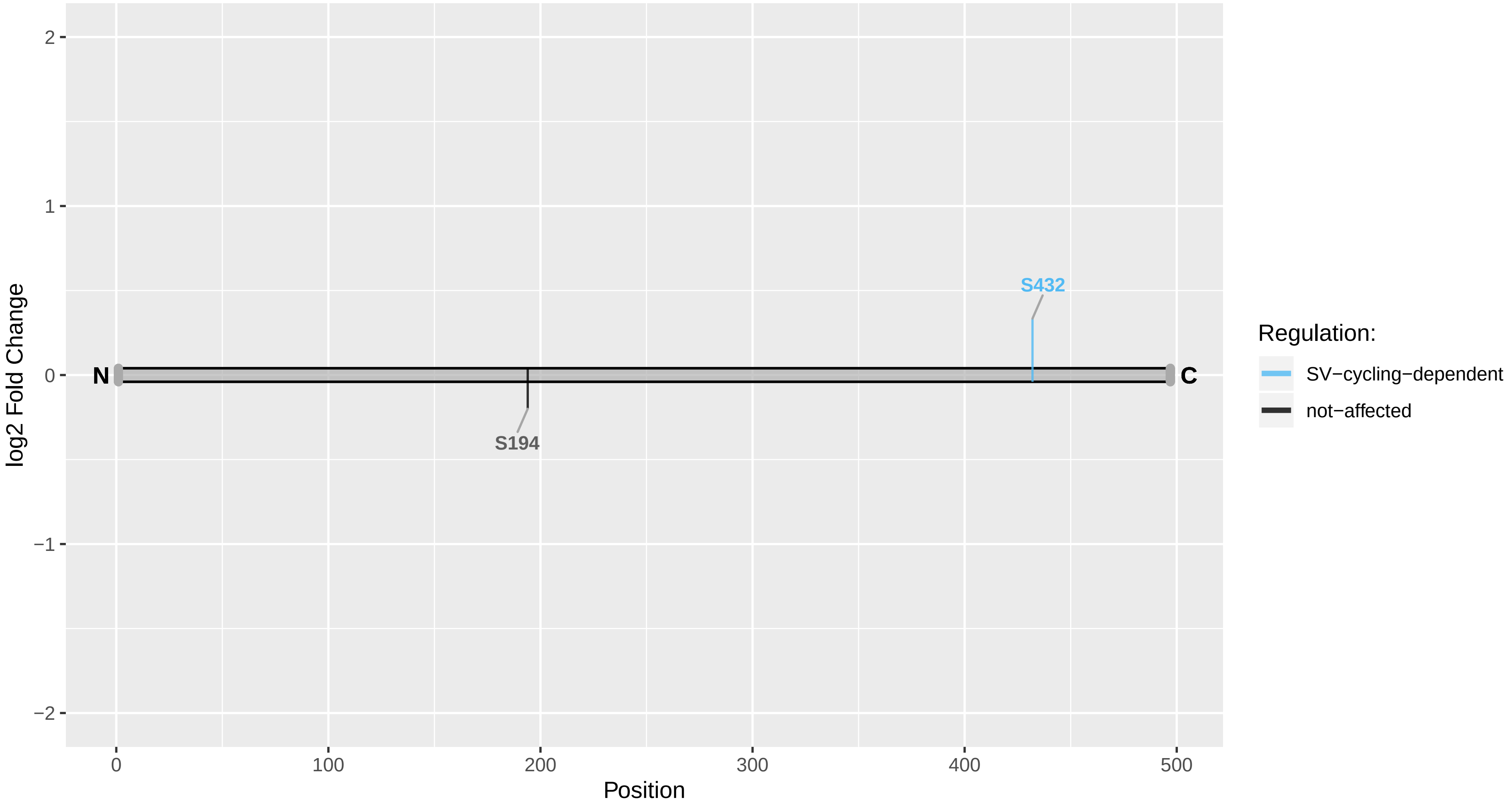

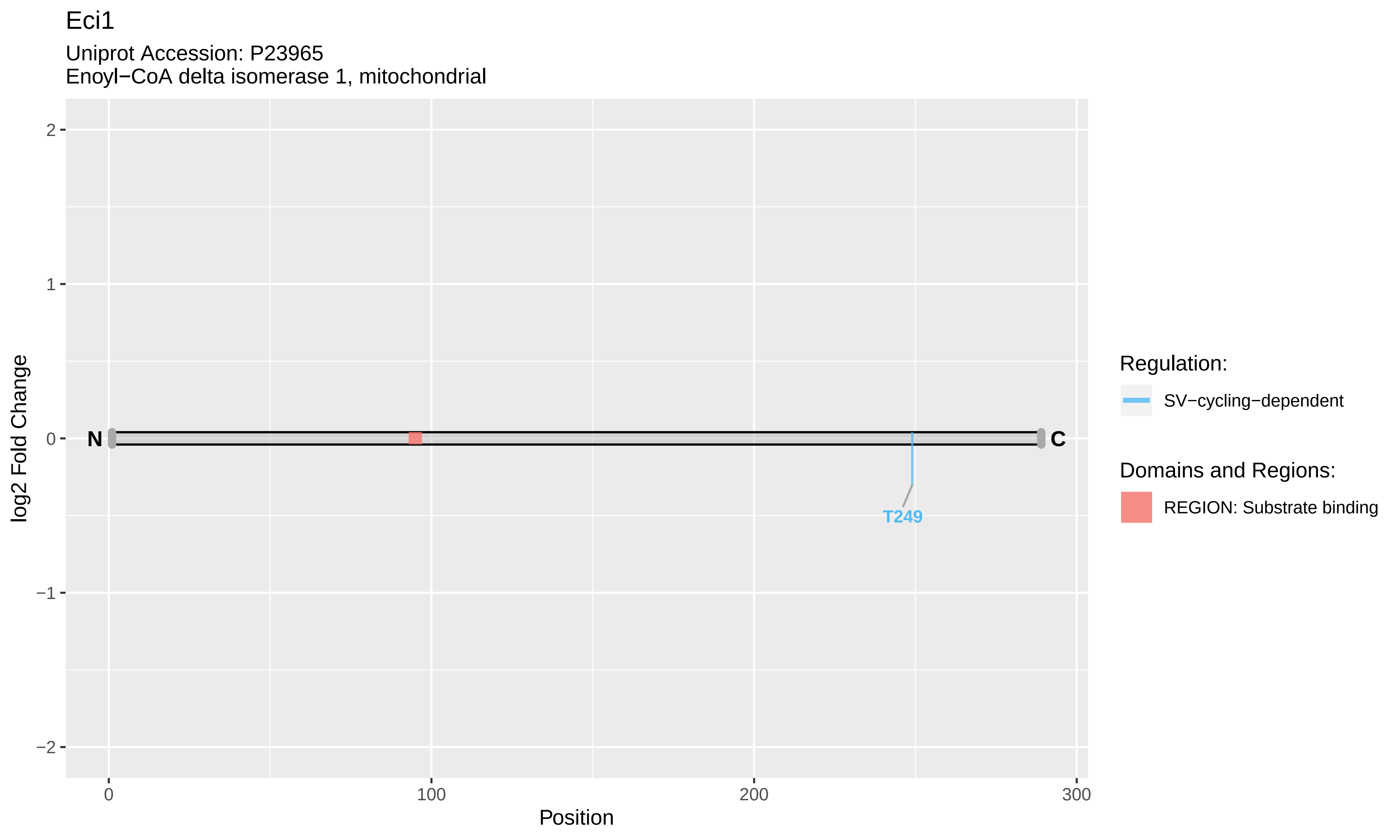
Eci1
Uniprot Accession: P23965
Enoyl−CoA delta isomerase 1, mitochondrial
log2 Fold Change
2
1
0
−1
−2
N
C
T249
0
100
200
300
Position
Regulation:
SV−cycling−dependent
Domains and Regions:
REGION: Substrate binding

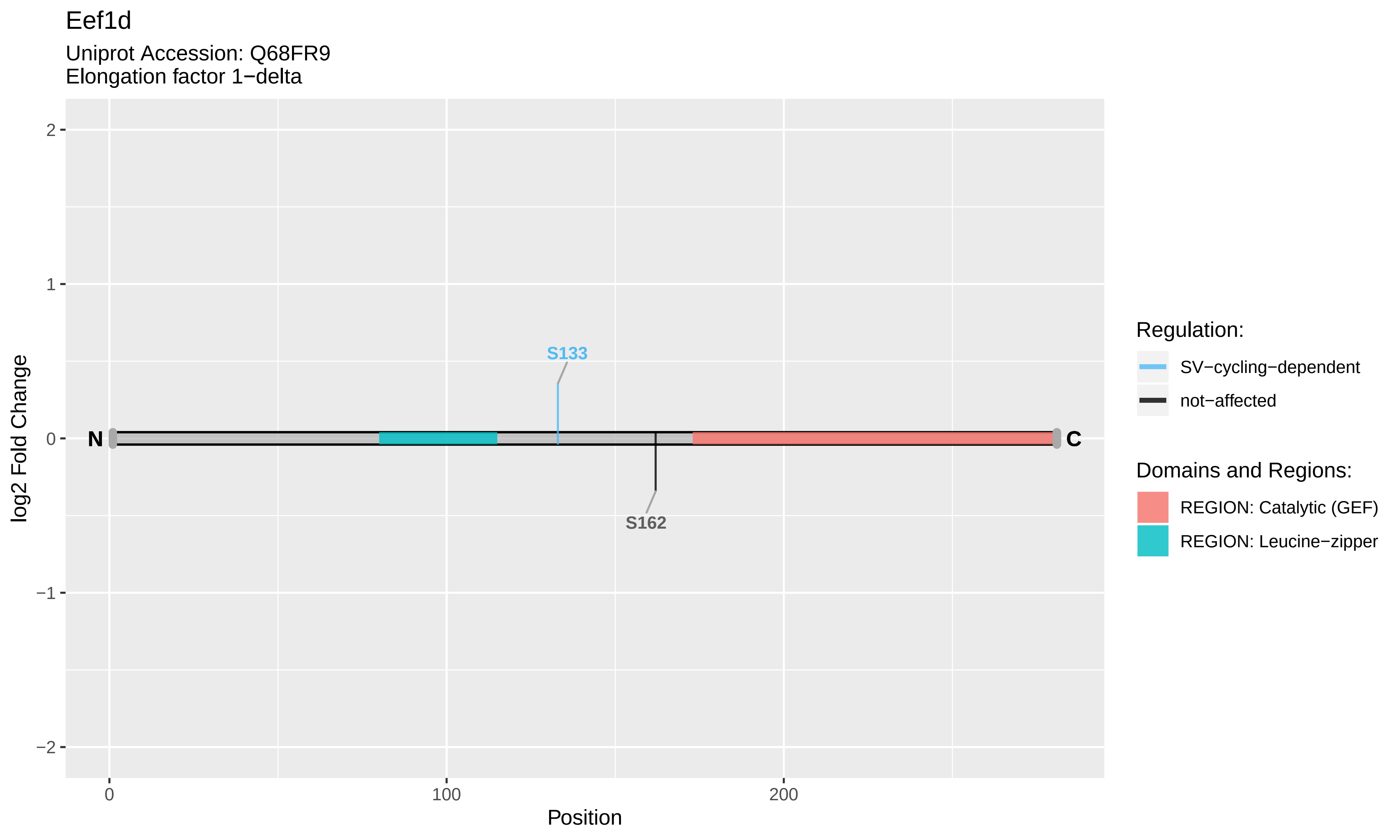

Eef1d
Uniprot Accession: Q68FR9
Elongation factor 1−delta
2
1
0
−1
−2
log2 Fold Change
N
C
S133
S162
0
100
200
Position
Regulation:
SV−cycling−dependent
not−affected
Domains and Regions:
REGION: Catalytic (GEF)
REGION: Leucine−zipper

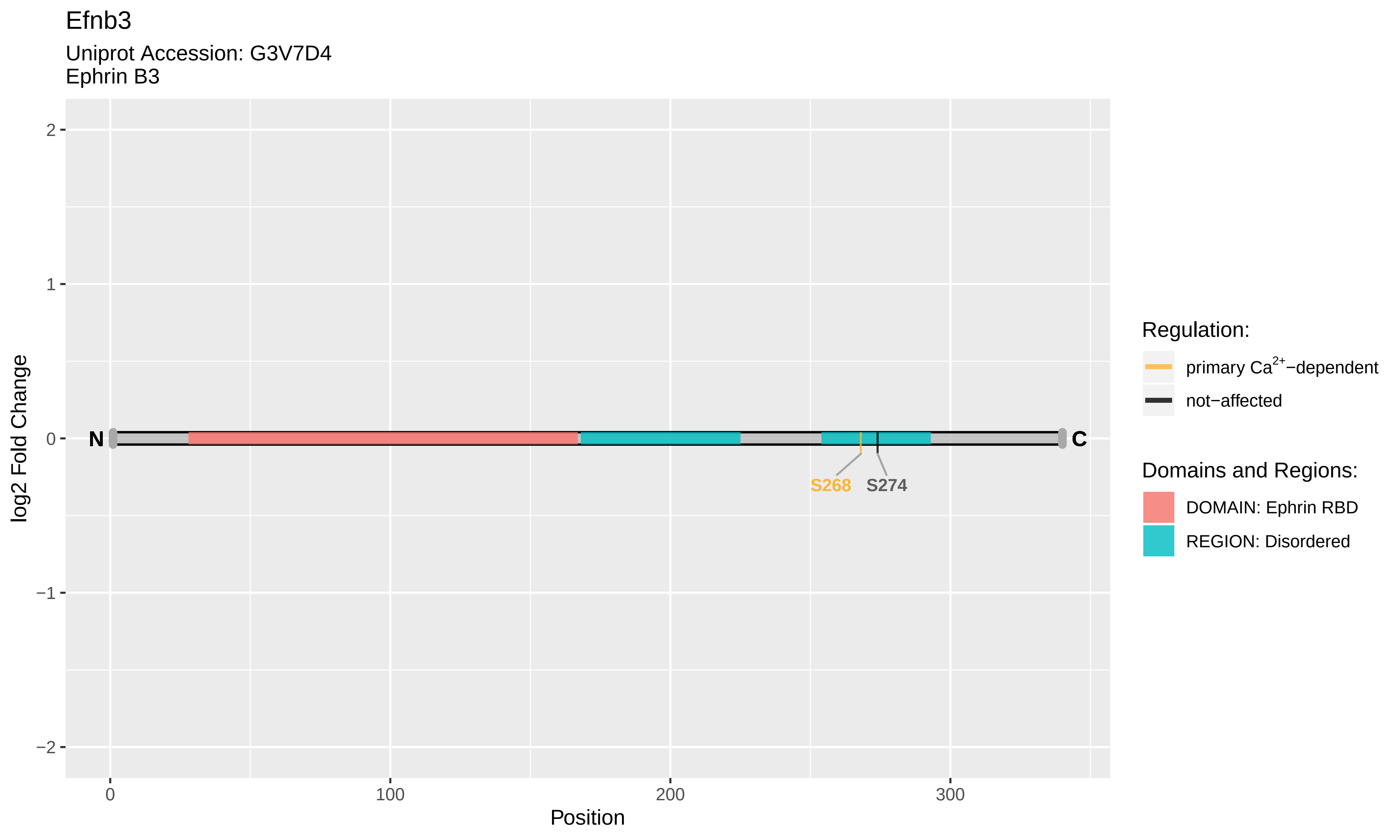

Efnb3
Uniprot Accession: G3V7D4
Ephrin B3
log2 Fold Change
2
1
0
−1
−2
N
C
S268
S274
0
100
200
300
Position
Regulation:
primary Ca2+−dependent
not−affected
Domains and Regions:
DOMAIN: Ephrin RBD
REGION: Disordered

Efr3b

Uniprot Accession: F1LTW9
EFR3 homolog B

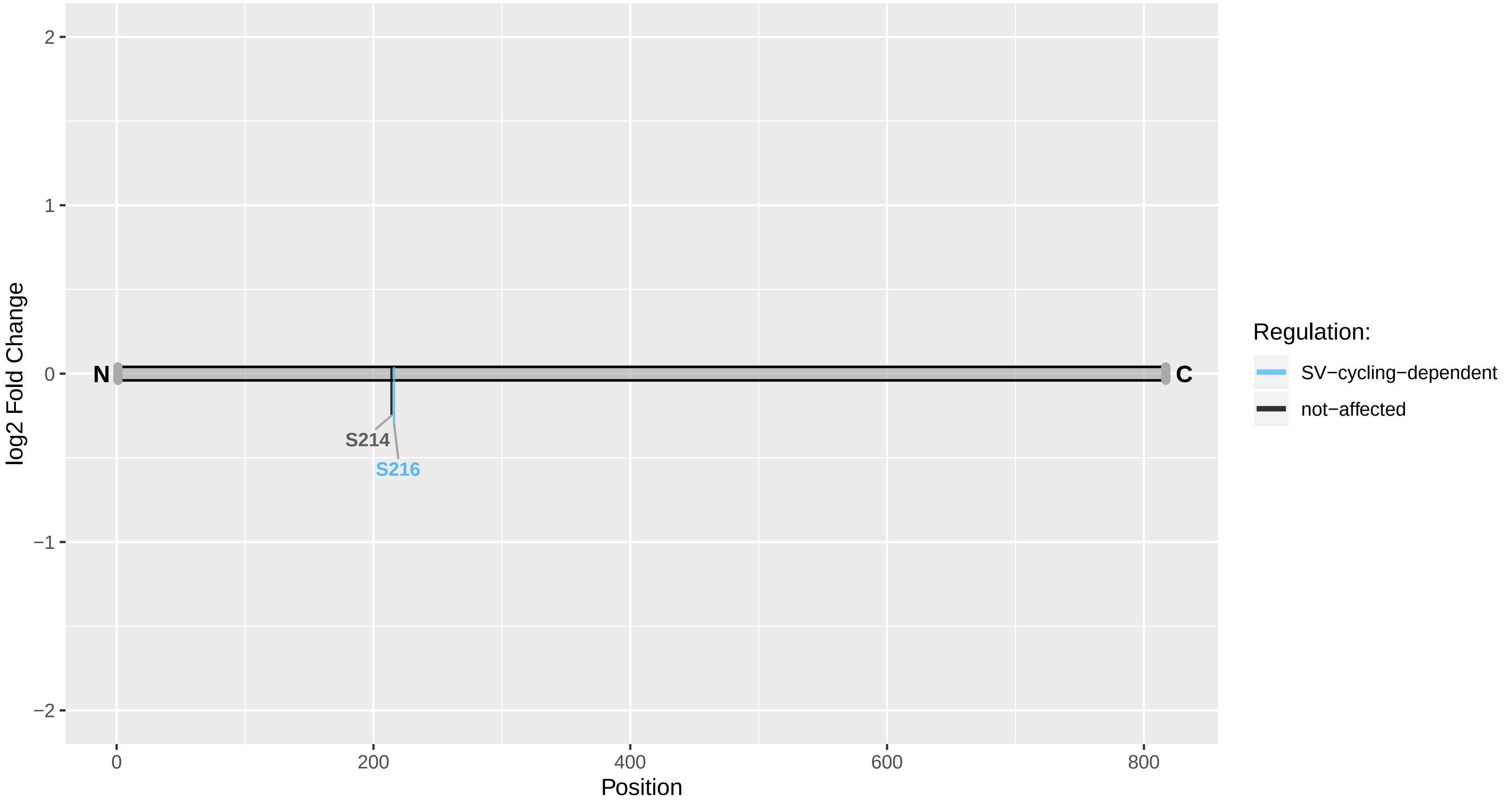

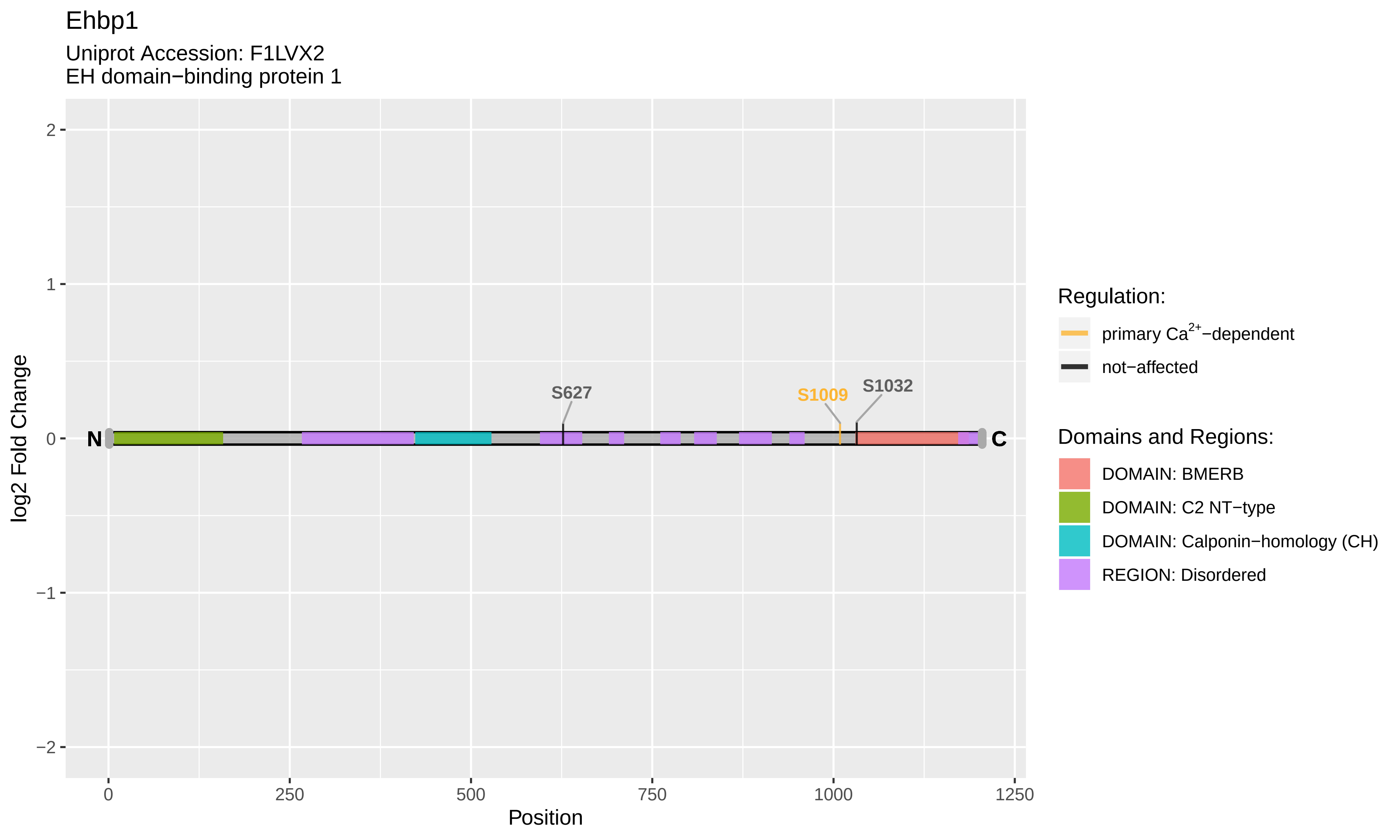

Ehbp1
Uniprot Accession: F1LVX2
EH domain−binding protein 1
log2 Fold Change
Position
N
C
S627
S1009
S1032
2
1
0
−1
−2
0
250
500
750
1000
1250
Regulation:
primary Ca2+−dependent
not−affected
Domains and Regions:
DOMAIN: BMERB
DOMAIN: C2 NT−type
DOMAIN: Calponin−homology (CH)
REGION: Disordered

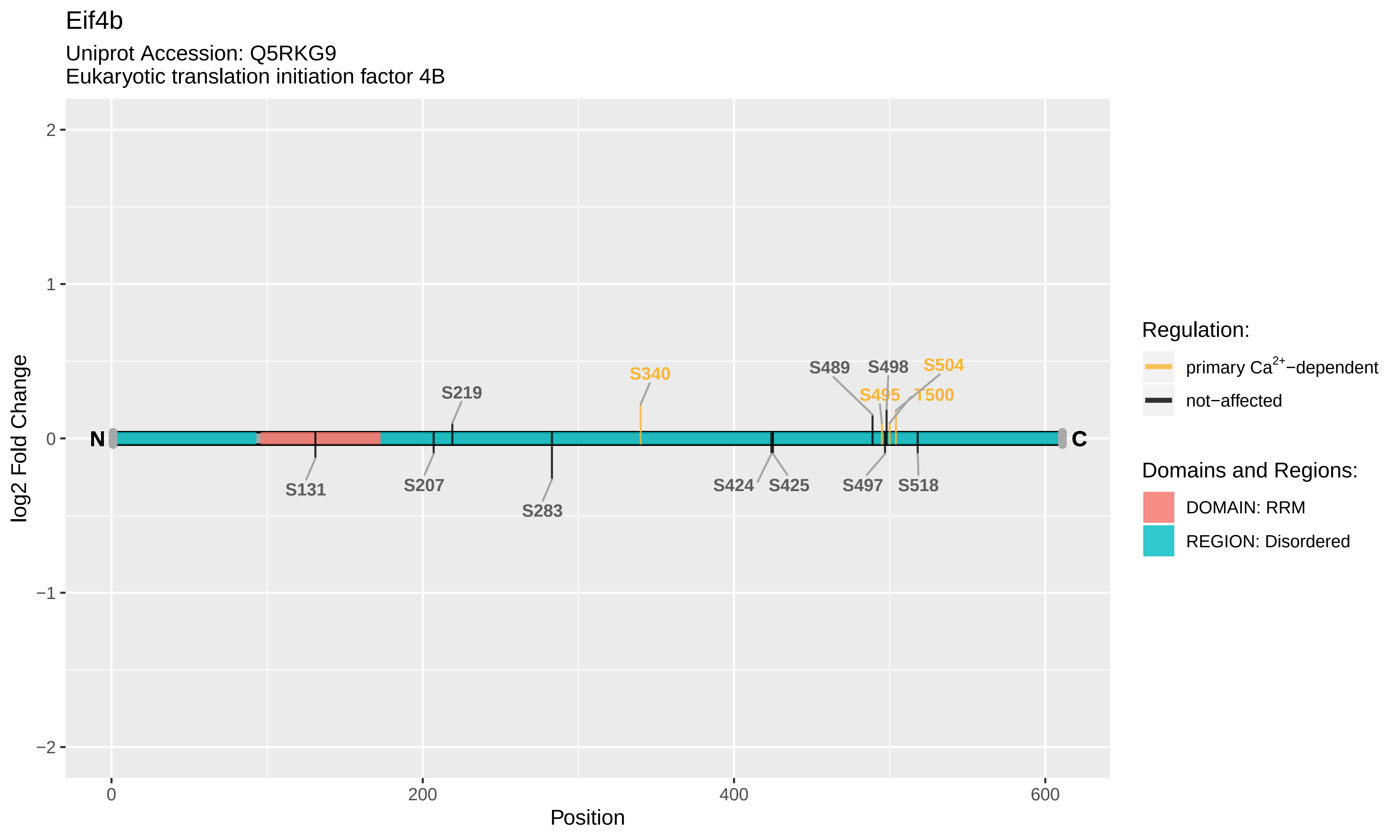

Eif4b
Uniprot Accession: Q5RKG9
Eukaryotic translation initiation factor 4B
log2 Fold Change
Position
N
C
S131
S207
S219
S283
S340
S424
S425
S489
S495
S497
S498
T500
S504
S518
Regulation:
primary Ca2+−dependent
not−affected
Domains and Regions:
DOMAIN: RRM
REGION: Disordered

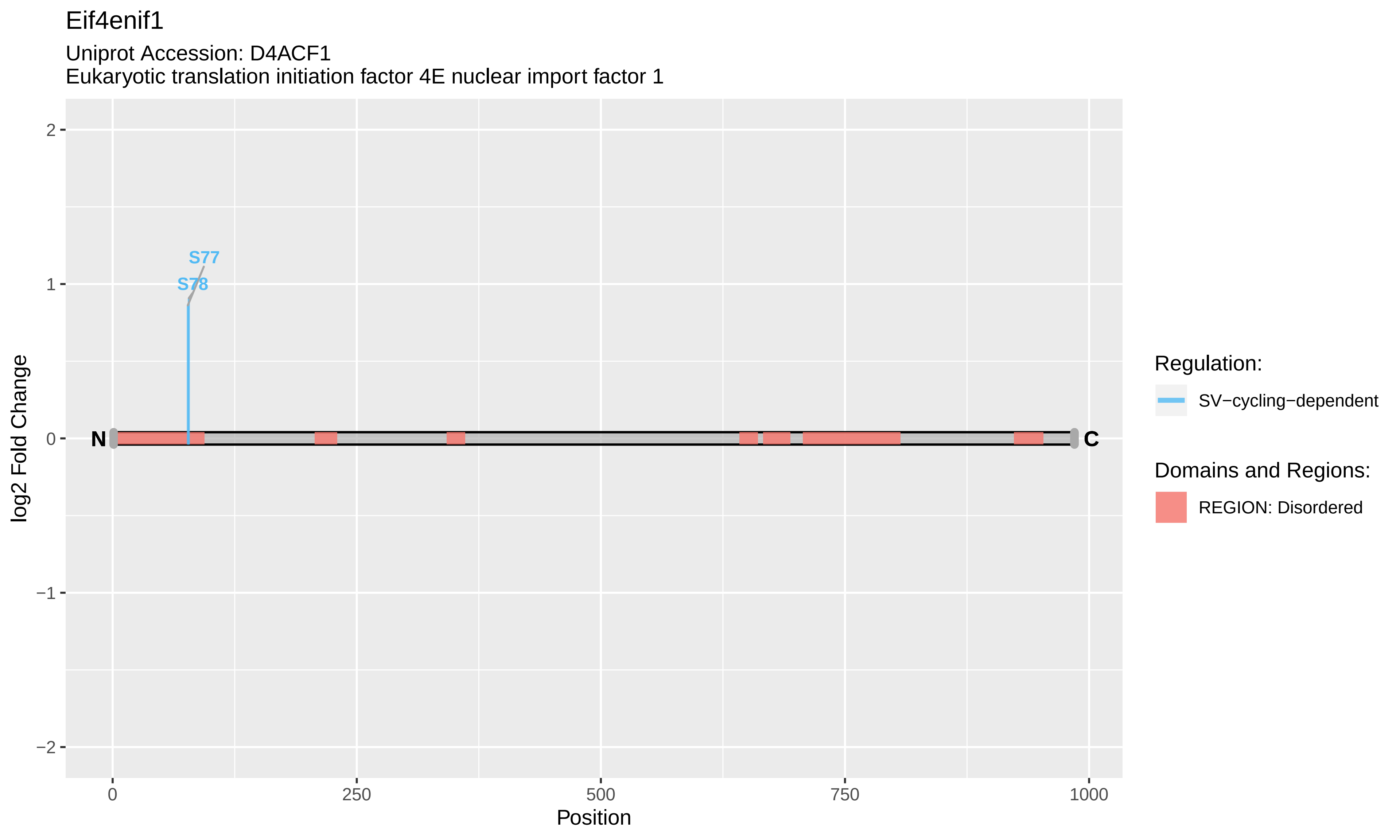

Eif4enif1
Uniprot Accession: D4ACF1
Eukaryotic translation initiation factor 4E nuclear import factor 1
S77
S78
N
C
2
1
0
−1
−2
log2 Fold Change
0
250
500
750
1000
Position
Regulation:
SV−cycling−dependent
Domains and Regions:
REGION: Disordered

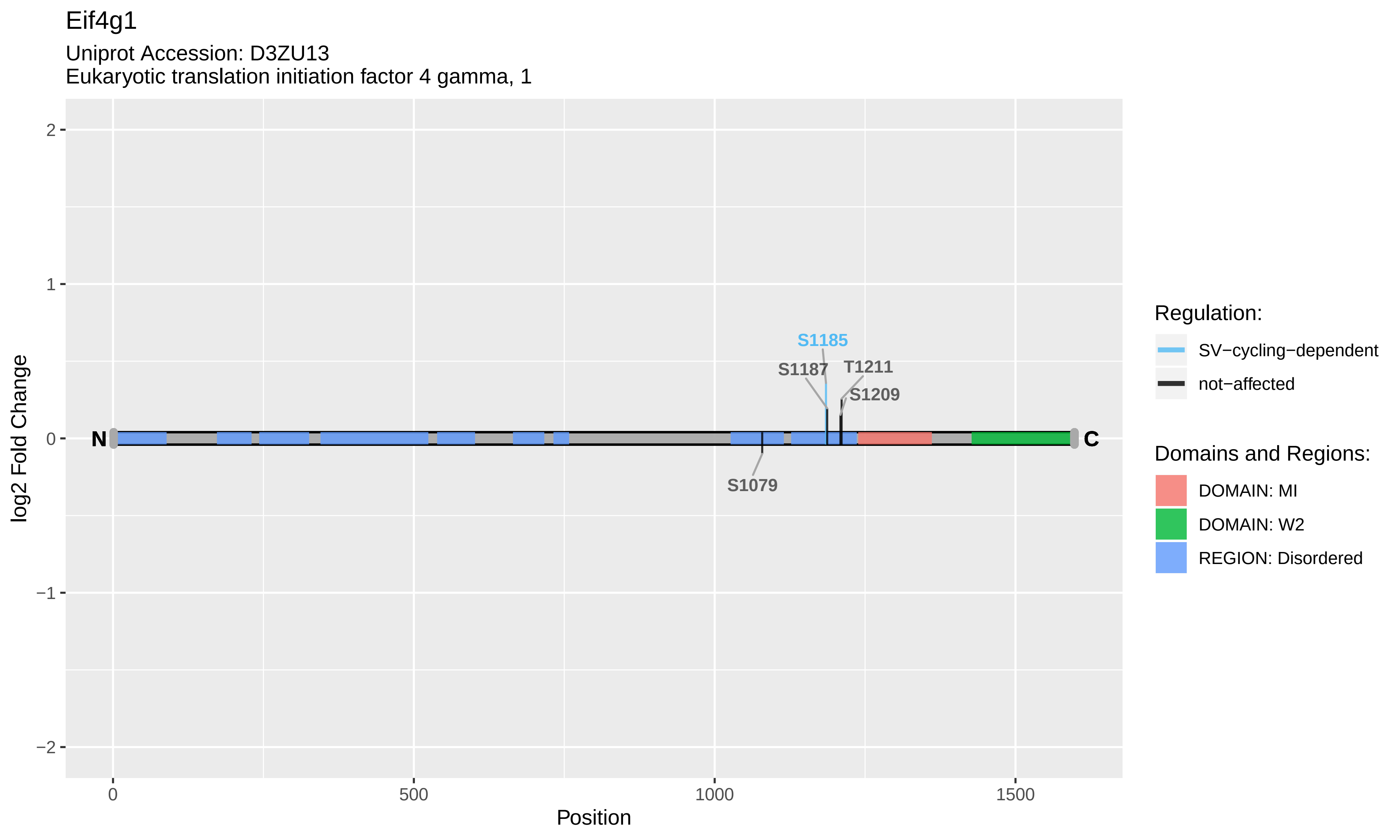

Eif4g1
Uniprot Accession: D3ZU13
Eukaryotic translation initiation factor 4 gamma, 1
log2 Fold Change
2
1
0
−1
−2
N
C
S1185
S1187
T1211
S1209
S1079
0
500
1000
1500
Position
Regulation:
SV−cycling−dependent
not−affected
Domains and Regions:
DOMAIN: MI
DOMAIN: W2
REGION: Disordered

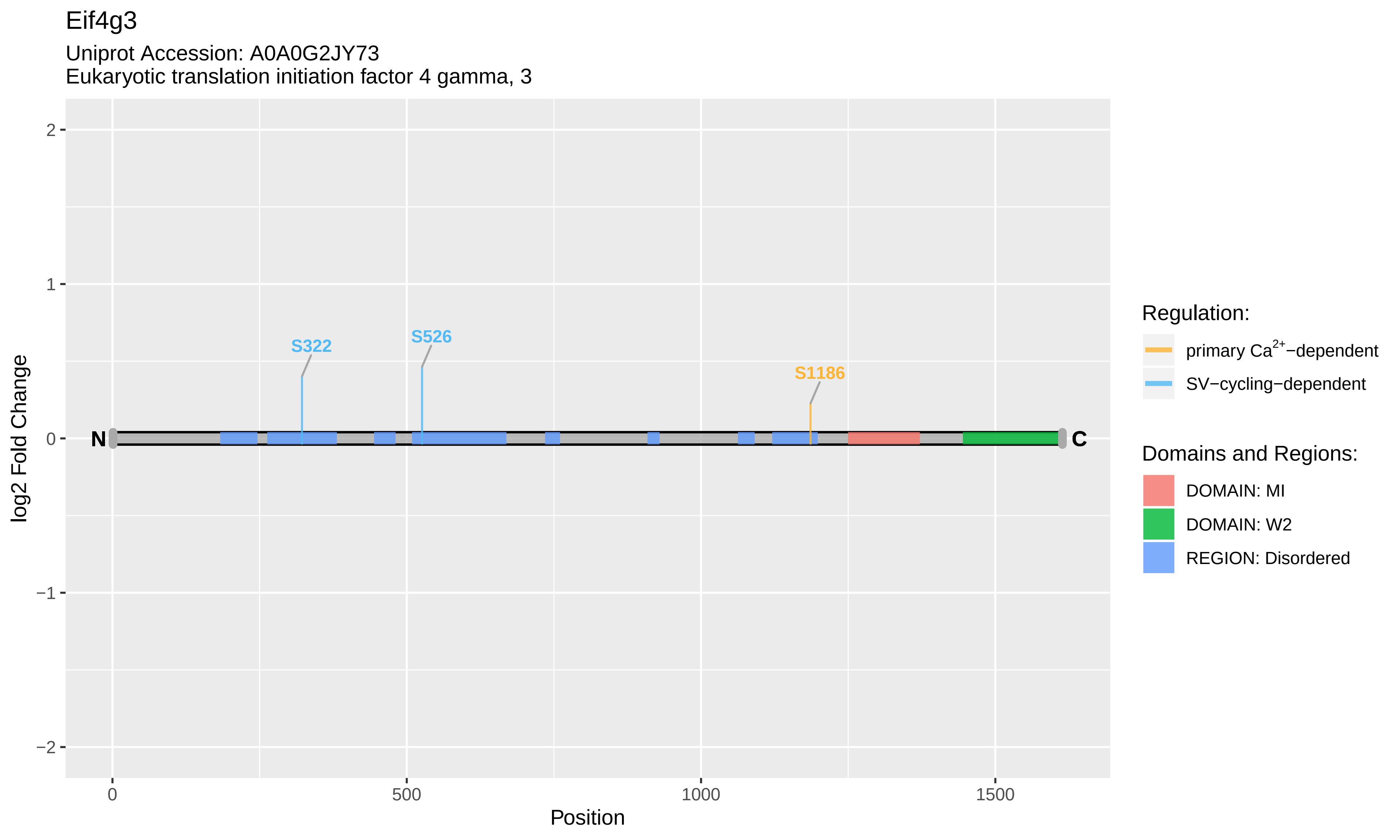

Eif4g3
Uniprot Accession: A0A0G2JY73
Eukaryotic translation initiation factor 4 gamma, 3
S322
S526
S1186
N
C
2
1
0
−1
−2
log2 Fold Change
0
500
1000
1500
Position
Regulation:
primary Ca2+−dependent
SV−cycling−dependent
Domains and Regions:
DOMAIN: MI
DOMAIN: W2
REGION: Disordered

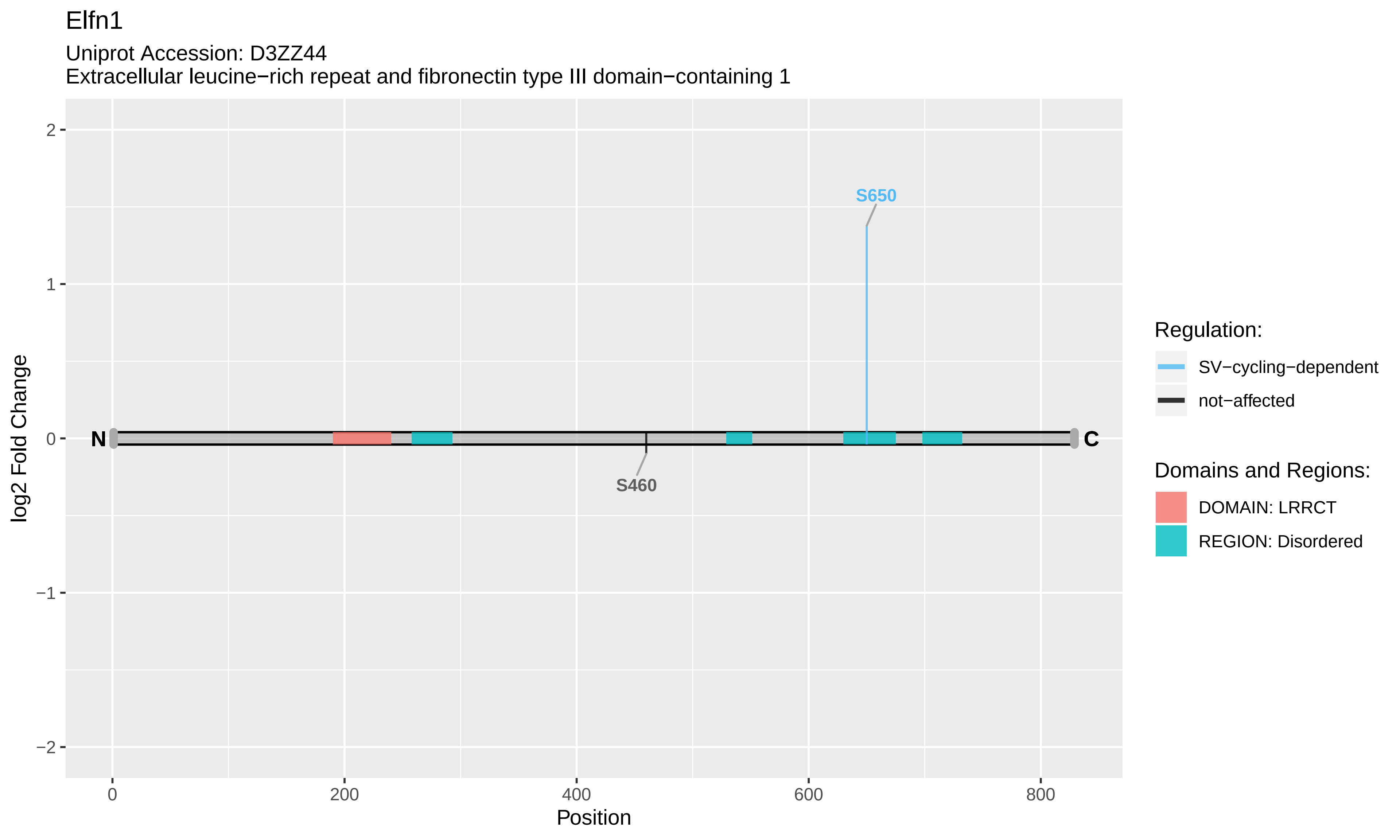

Elfn1
Uniprot Accession: D3ZZ44
Extracellular leucine−rich repeat and fibronectin type III domain−containing 1
S650
S460
N
C
log2 Fold Change
Position
2
1
0
−1
−2
0
200
400
600
800
Regulation:
SV−cycling−dependent
not−affected
Domains and Regions:
DOMAIN: LRRCT
REGION: Disordered

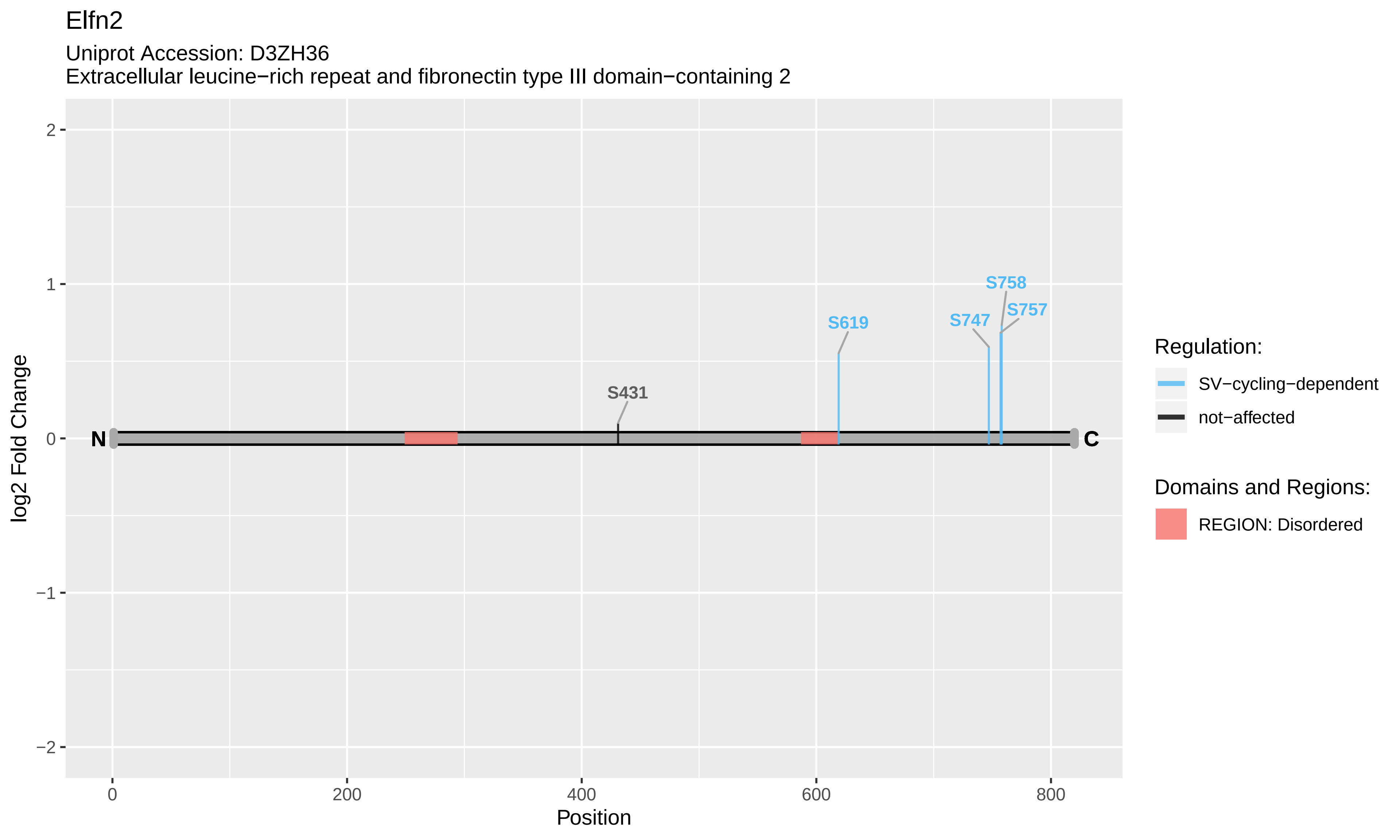

Elfn2
Uniprot Accession: D3ZH36
Extracellular leucine−rich repeat and fibronectin type III domain−containing 2
log2 Fold Change
Position
N
C
S431
S619
S747
S758
S757
2
1
0
−1
−2
0
200
400
600
800
Regulation:
SV−cycling−dependent
not−affected
Domains and Regions:
REGION: Disordered

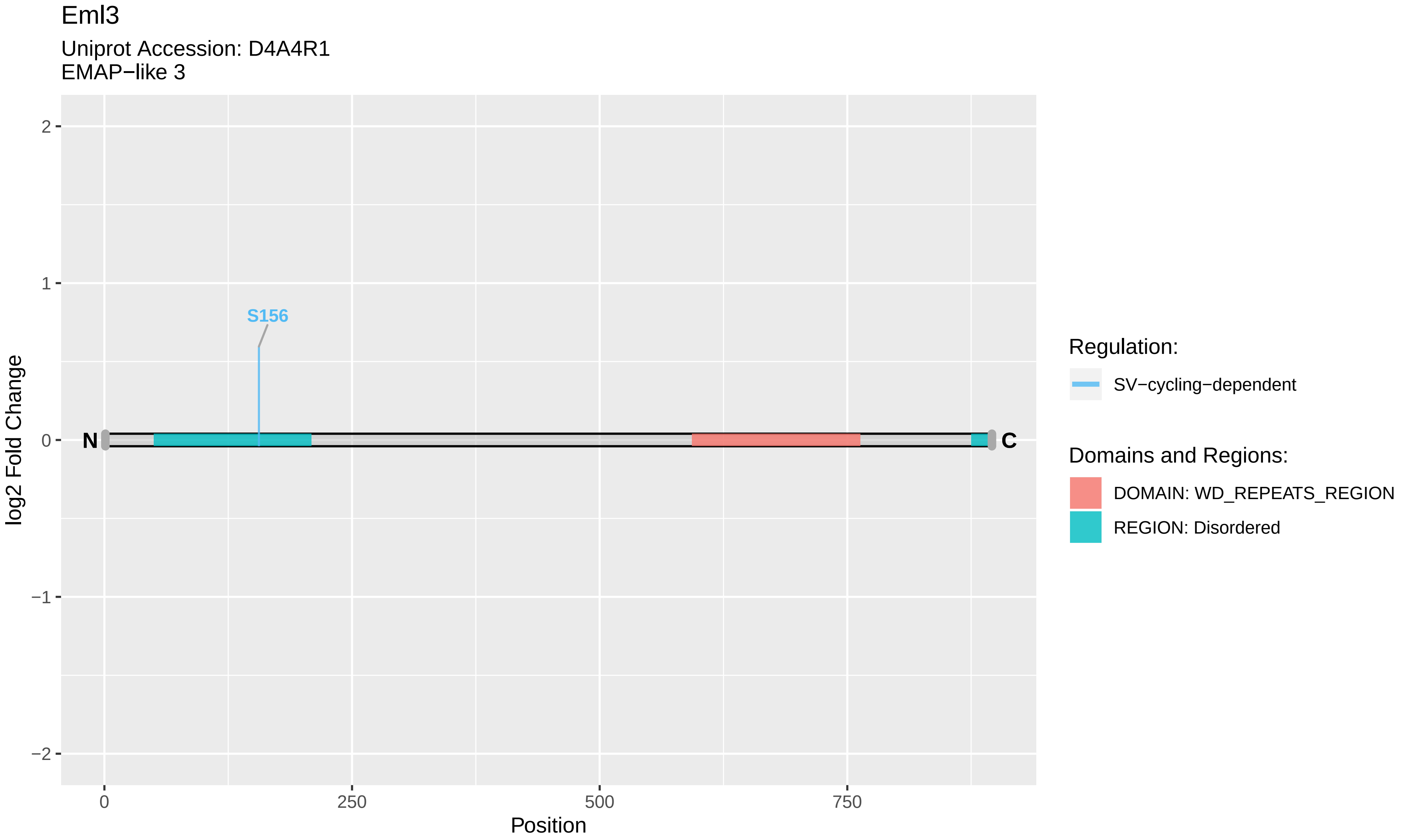

Eml3
Uniprot Accession: D4A4R1
EMAP−like 3
S156
N
C
2
1
0
−1
−2
log2 Fold Change
0
250
500
750
Position
Regulation:
SV−cycling−dependent
Domains and Regions:
DOMAIN: WD_REPEATS_REGION
REGION: Disordered

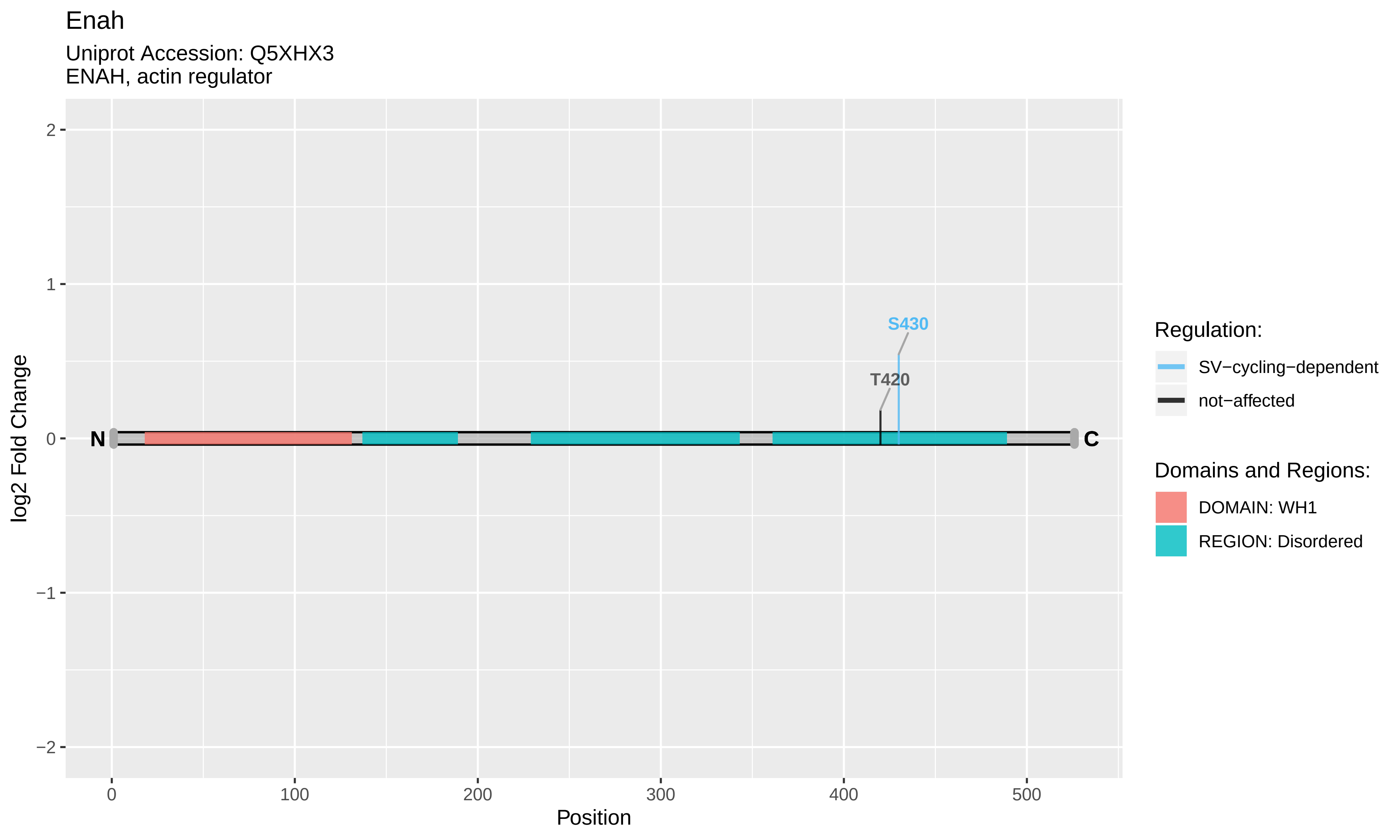

Enah
Uniprot Accession: Q5XHX3
ENAH, actin regulator
log2 Fold Change
Position
N
C
S430
T420
Regulation:
SV−cycling−dependent
not−affected
Domains and Regions:
DOMAIN: WH1
REGION: Disordered
2
1
0
−1
−2
0
100
200
300
400
500

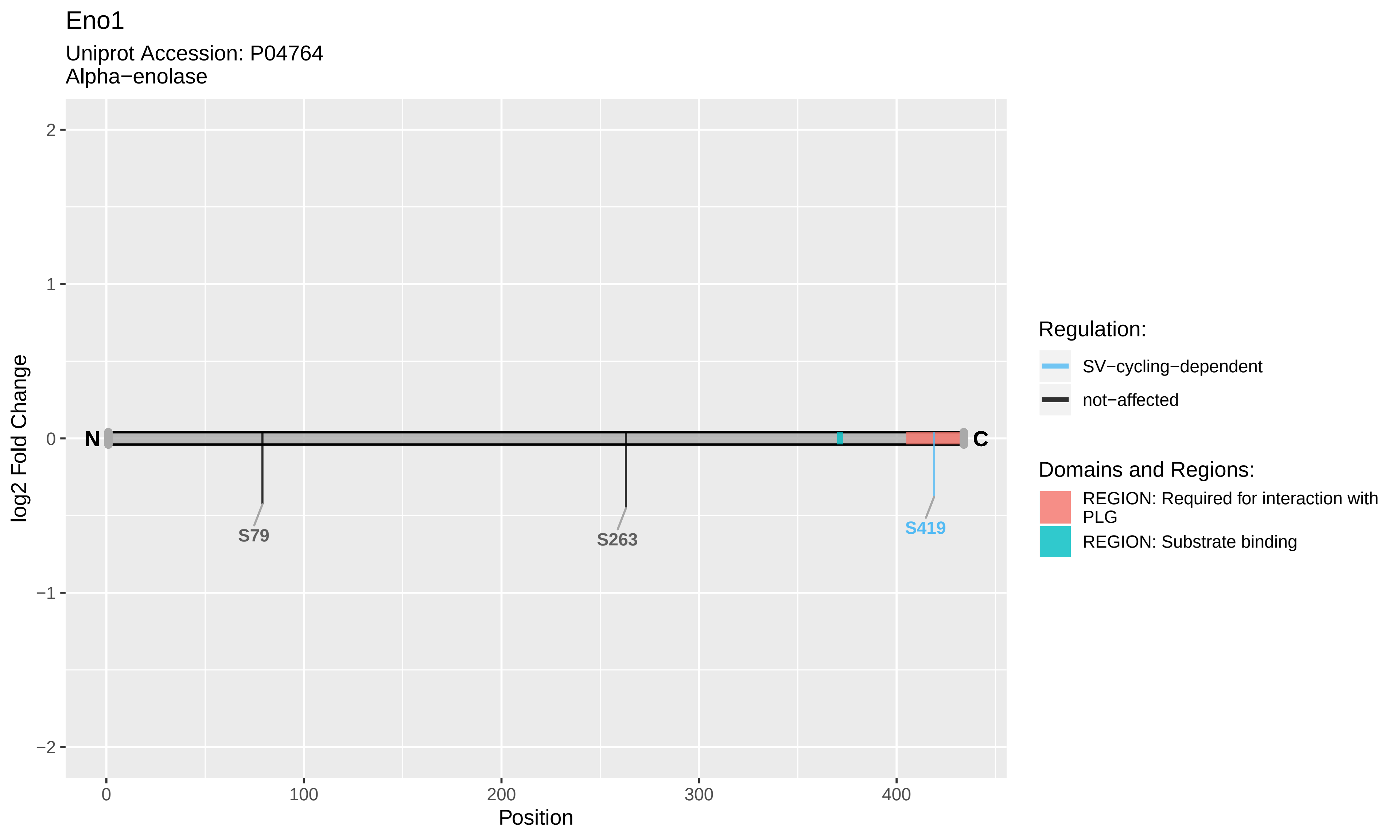
Eno1
Uniprot Accession: P04764
Alpha−enolase
log2 Fold Change
2
1
0
−1
−2
N
C
S79
S263
S419
0
100
200
300
400
Position
Regulation:
SV−cycling−dependent
not−affected
Domains and Regions:
REGION: Required for interaction with PLG
REGION: Substrate binding

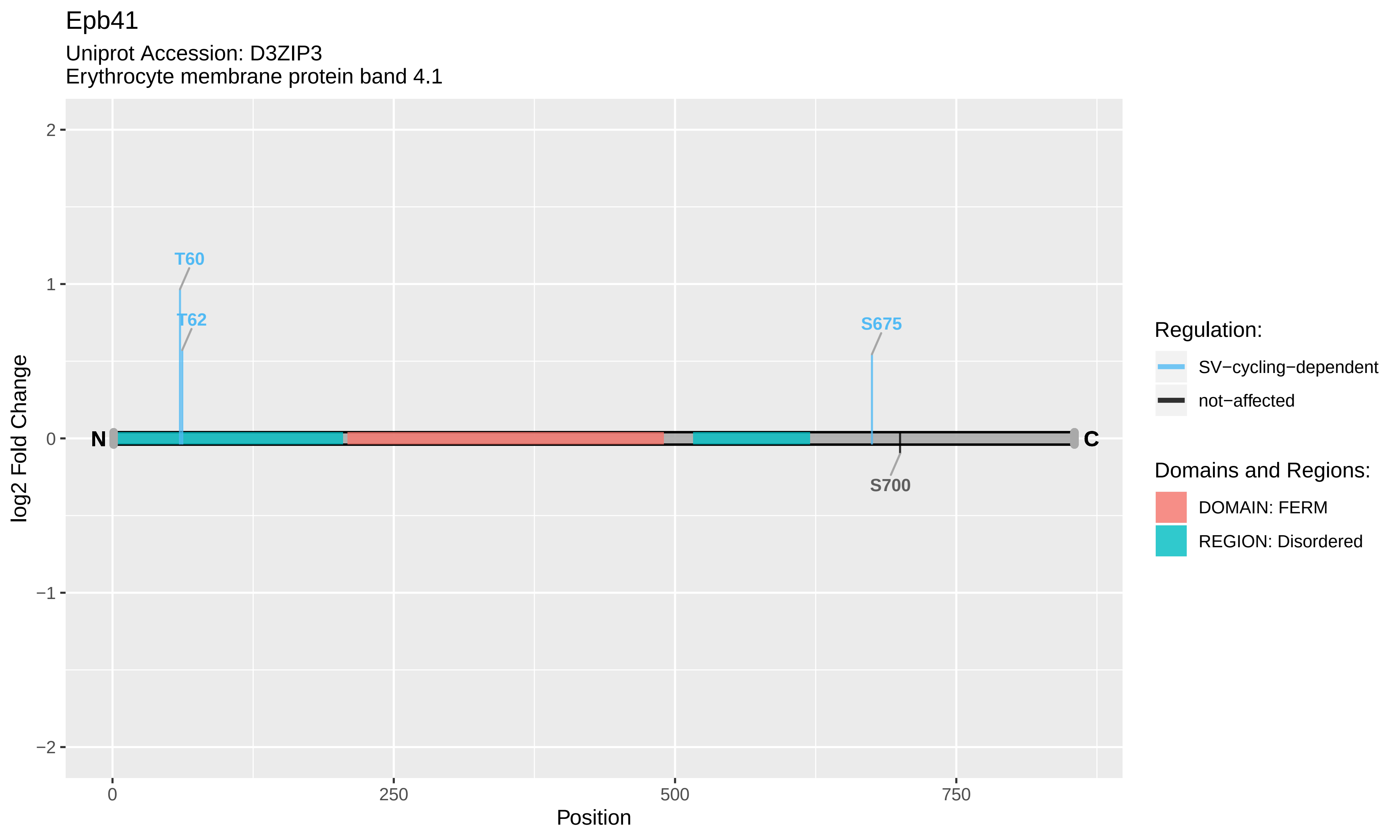

Epb41
Uniprot Accession: D3ZIP3
Erythrocyte membrane protein band 4.1
T60
T62
S675
S700
N
C
2
1
0
−1
−2
log2 Fold Change
0
250
500
750
Position
Regulation:
SV−cycling−dependent
not−affected
Domains and Regions:
DOMAIN: FERM
REGION: Disordered

Epb41l1

Uniprot Accession: A0A0G2K0F3
Band 4.1−like protein 1−like

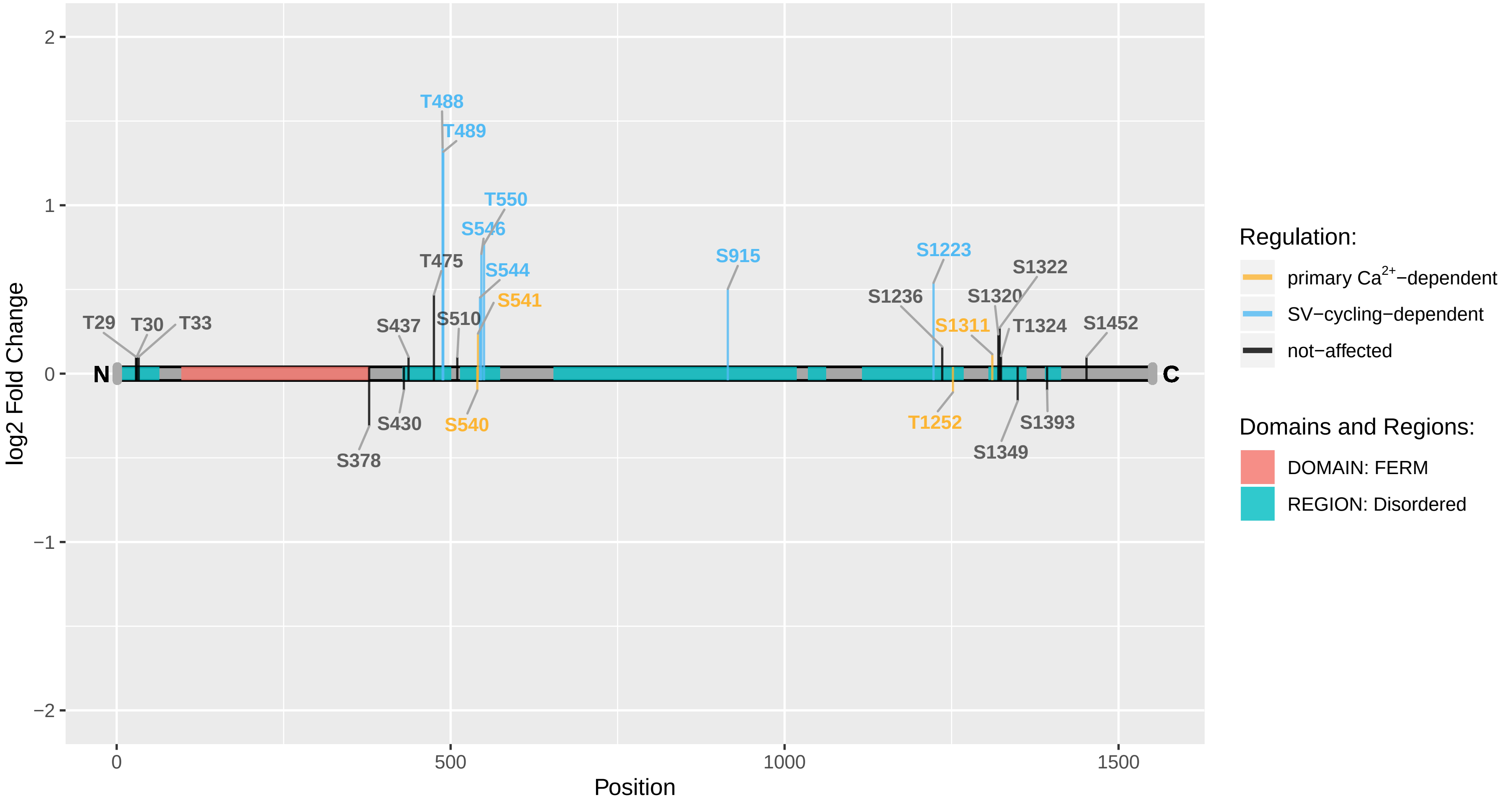

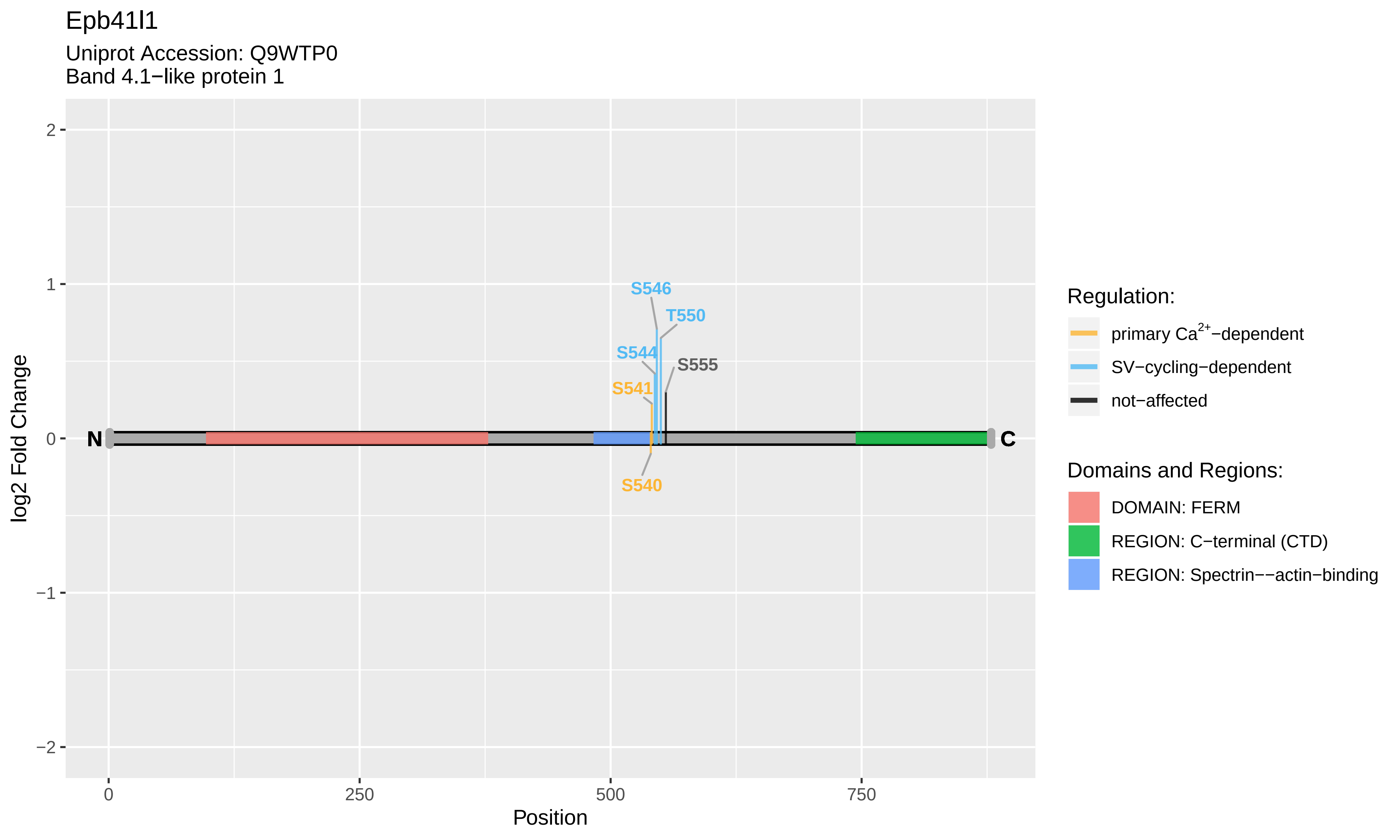

Epb41l1
Uniprot Accession: Q9WTP0
Band 4.1−like protein 1
log2 Fold Change
2
1
0
−1
−2
N
C
S546
T550
S544
S555
S541
S540
0
250
500
750
Position
Regulation:
primary Ca2+−dependent
SV−cycling−dependent
not−affected
Domains and Regions:
DOMAIN: FERM
REGION: C−terminal (CTD)
REGION: Spectrin−−actin−binding

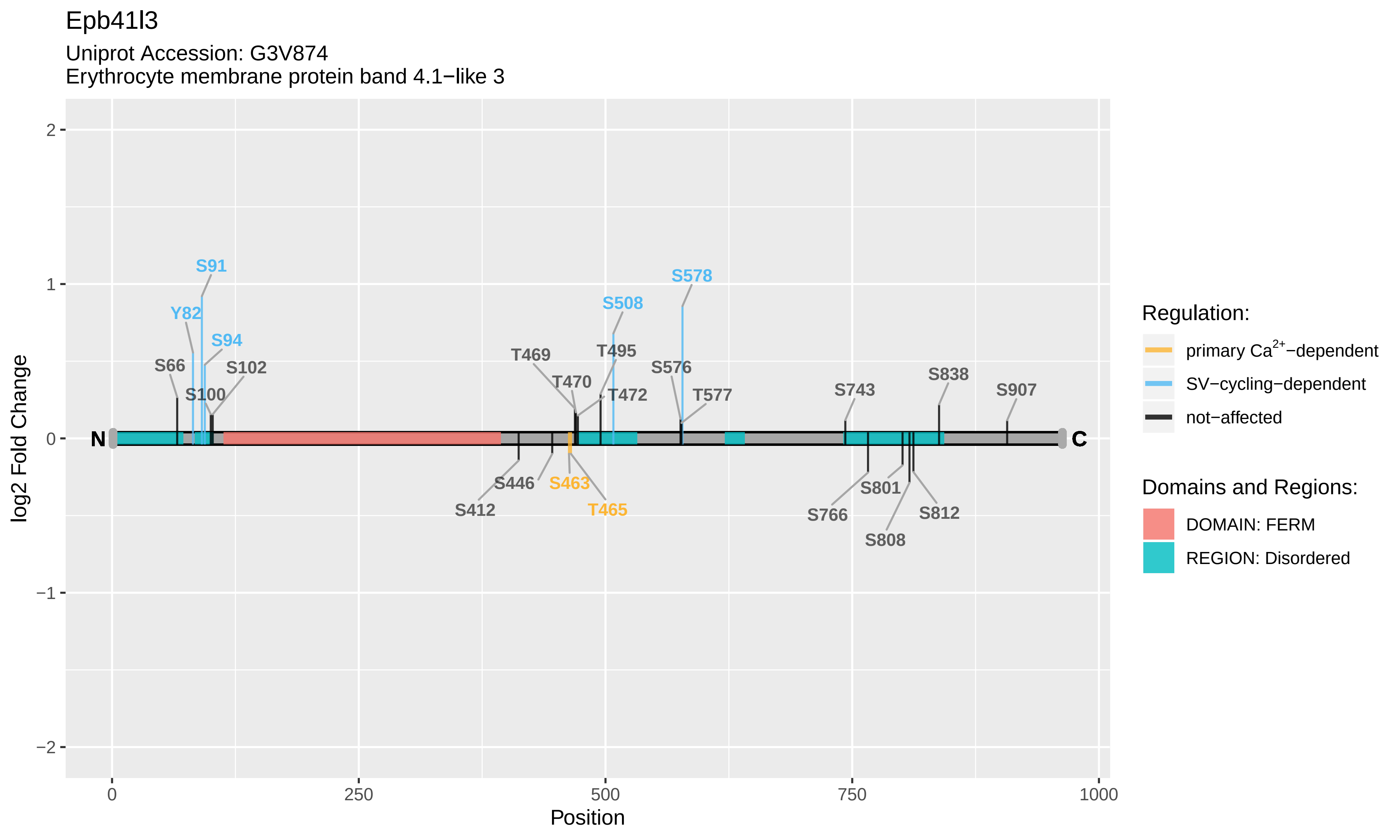

Epb41l3
Uniprot Accession: G3V874
Erythrocyte membrane protein band 4.1−like 3
log2 Fold Change
Position
2
1
0
−1
−2
0
250
500
750
1000
N
C
S91
Y82
S94
S66
S102
S100
T469
T495
T470
T472
S508
S578
S576
T577
S743
S838
S907
S446
S463
S412
T465
S801
S766
S808
S812
Regulation:
primary Ca2+−dependent
SV−cycling−dependent
not−affected
Domains and Regions:
DOMAIN: FERM
REGION: Disordered

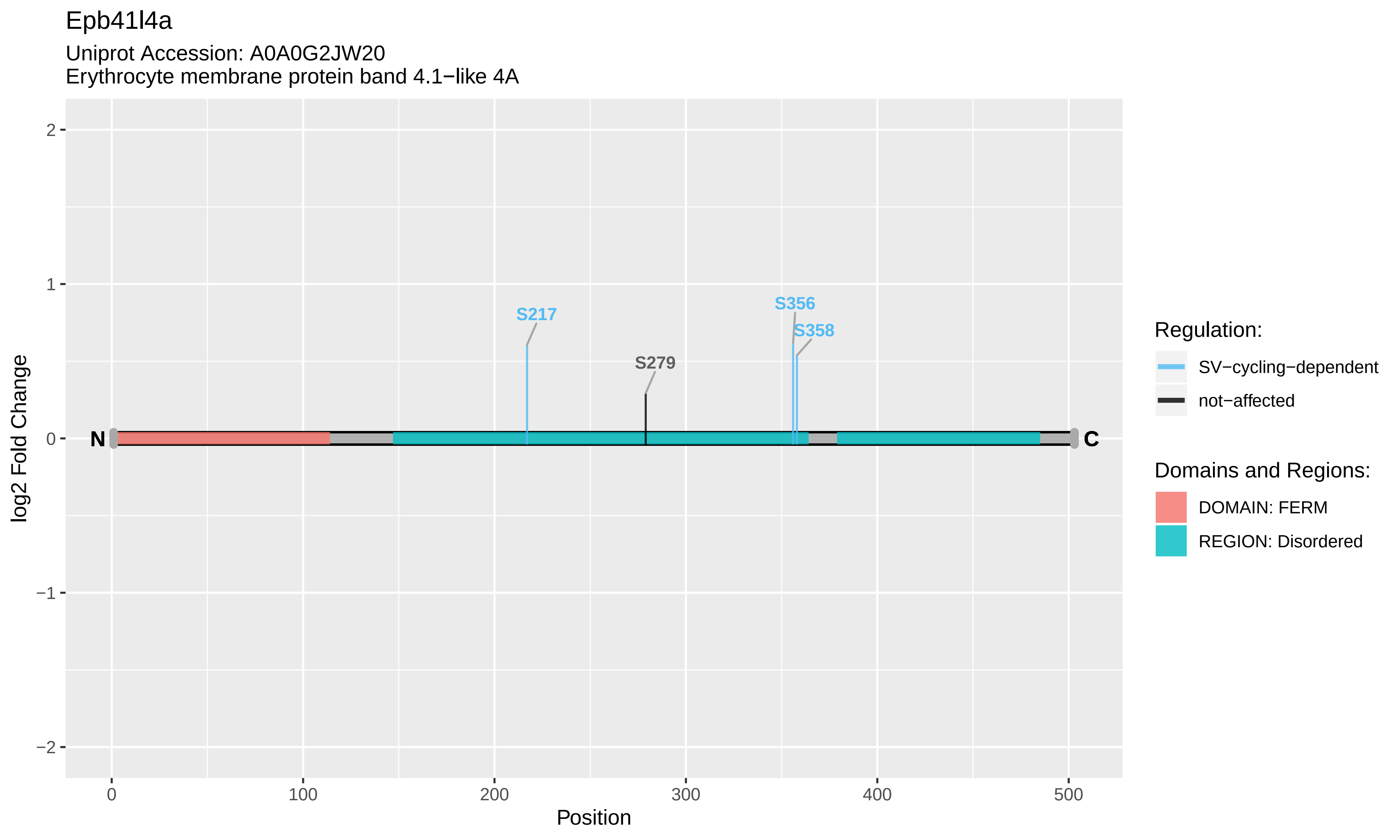

Epb41l4a
Uniprot Accession: A0A0G2JW20
Erythrocyte membrane protein band 4.1−like 4A
S217
S279
S356
S358
N
C
2
1
0
−1
−2
log2 Fold Change
0
100
200
300
400
500
Position
Regulation:
SV−cycling−dependent
not−affected
Domains and Regions:
DOMAIN: FERM
REGION: Disordered

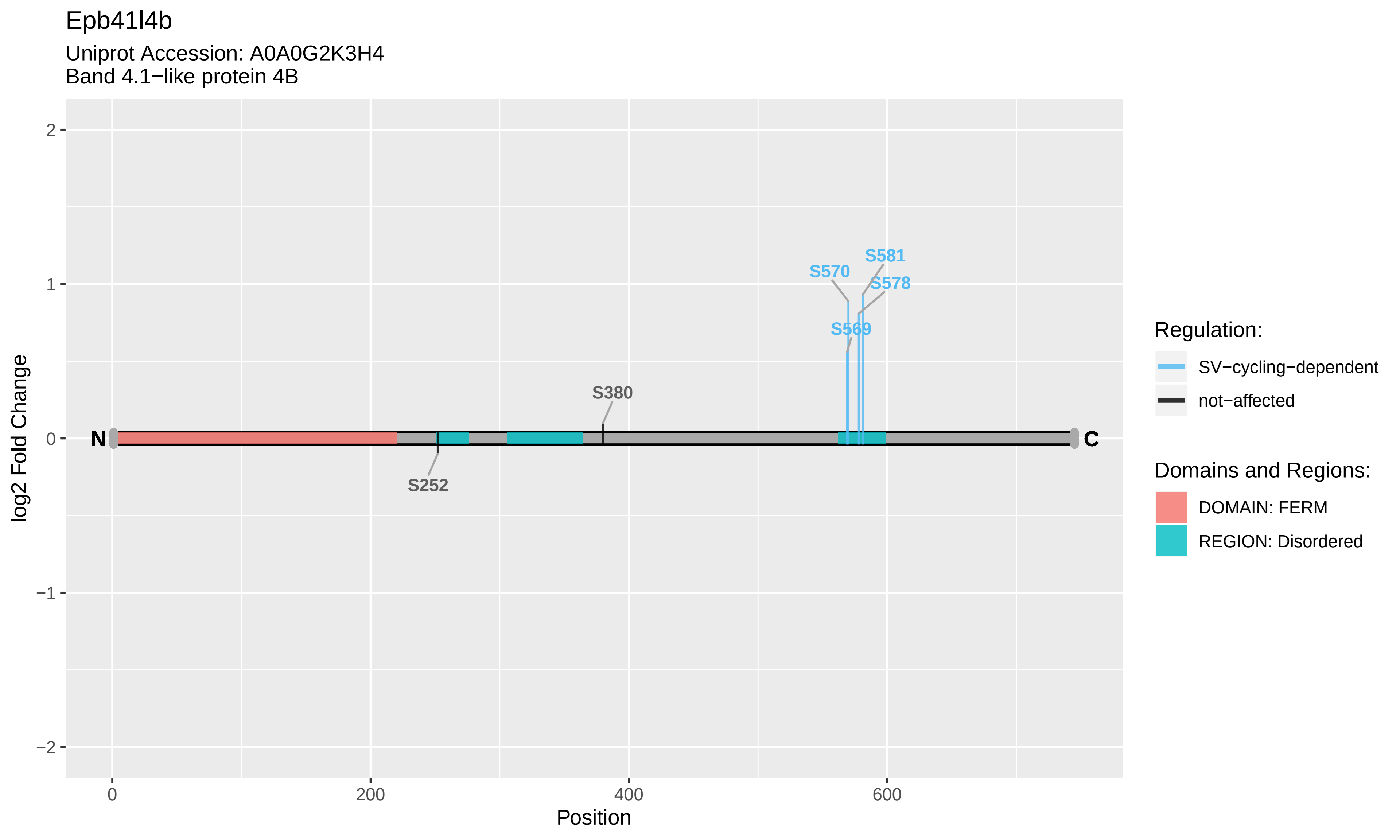

Epb41l4b
Uniprot Accession: A0A0G2K3H4
Band 4.1−like protein 4B
log2 Fold Change
2
1
0
−1
−2
N
C
S570
S581
S578
S569
S380
S252
0
200
400
600
Position
Regulation:
SV−cycling−dependent
not−affected
Domains and Regions:
DOMAIN: FERM
REGION: Disordered

Epha4

Uniprot Accession: D3ZZK3
Eph receptor A4

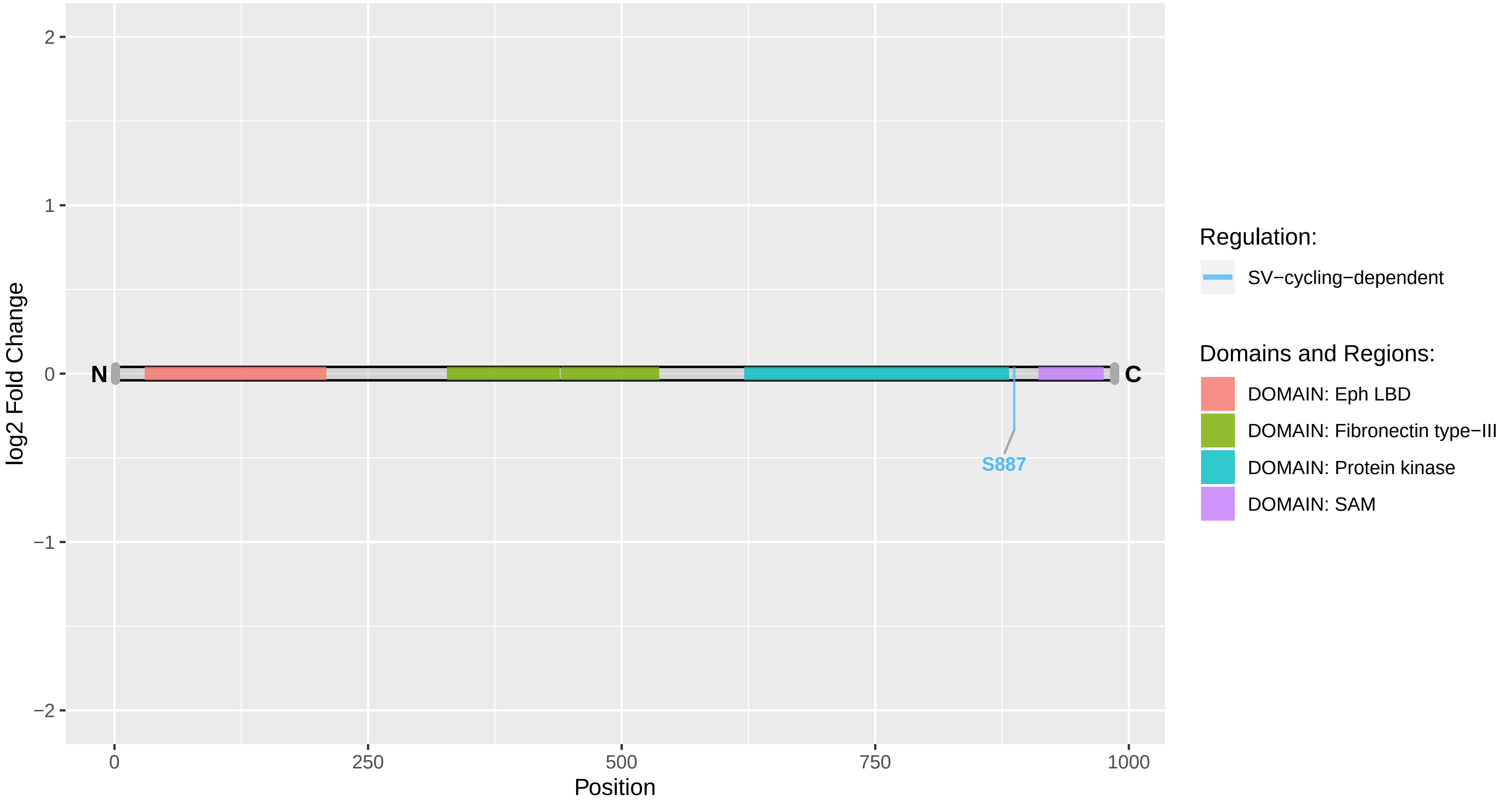

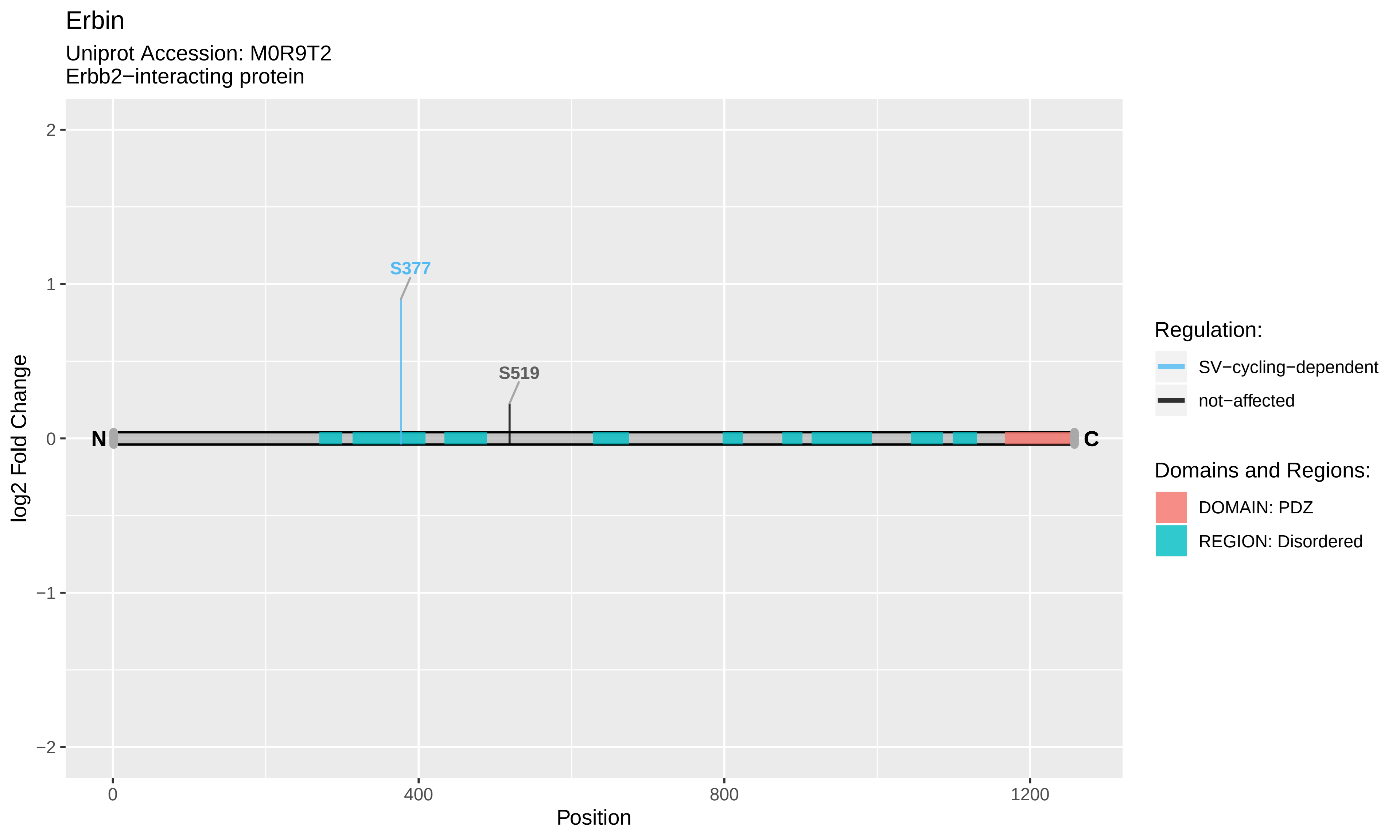

Erbin
Uniprot Accession: M0R9T2
Erbb2−interacting protein
S377
S519
N
C
2
1
0
−1
−2
log2 Fold Change
0
400
800
1200
Position
Regulation:
SV−cycling−dependent
not−affected
Domains and Regions:
DOMAIN: PDZ
REGION: Disordered

Erc1

Uniprot Accession: A0A0G2JYT1
ELKS/Rab6−interacting/CAST family member 1

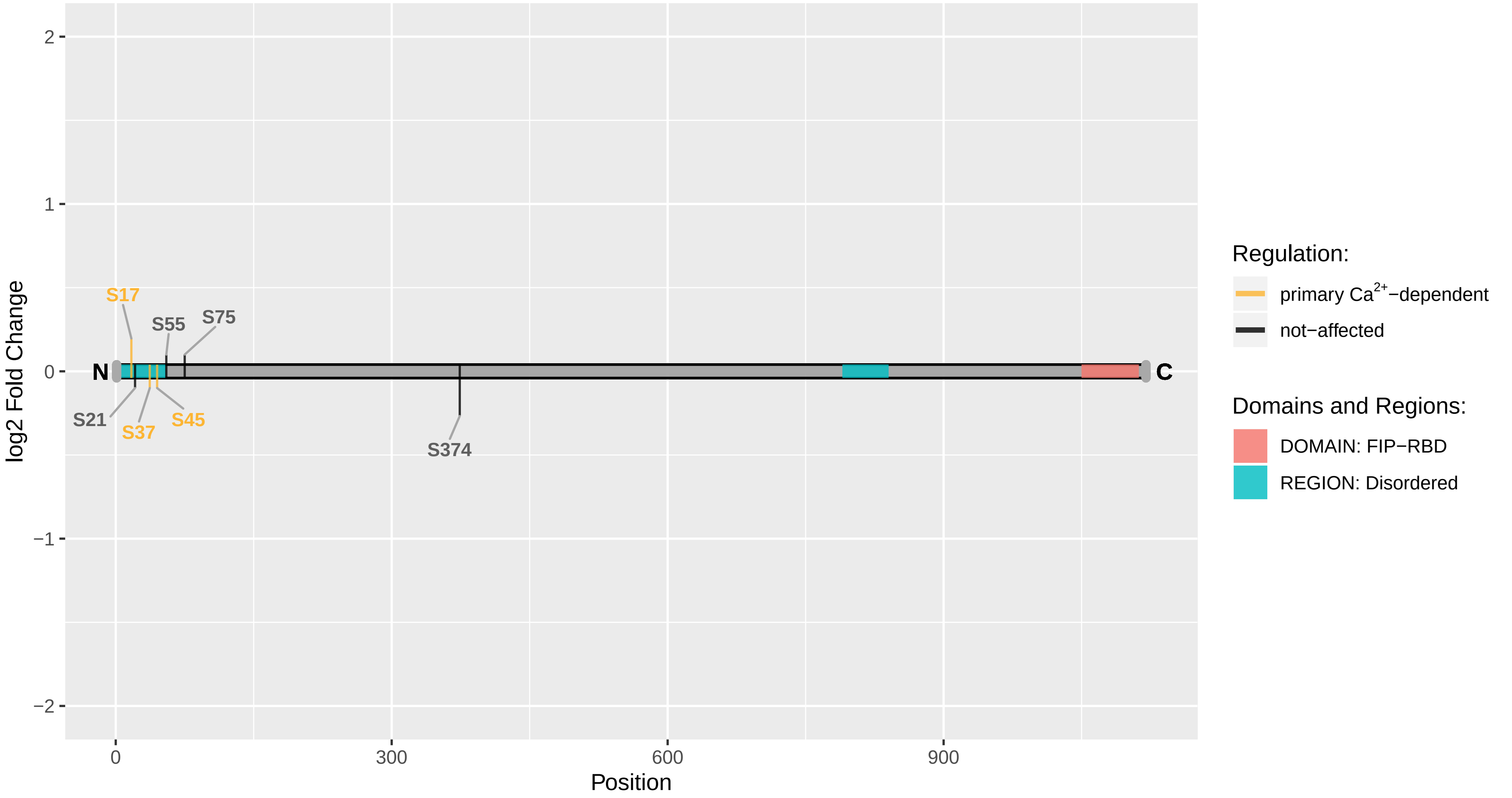

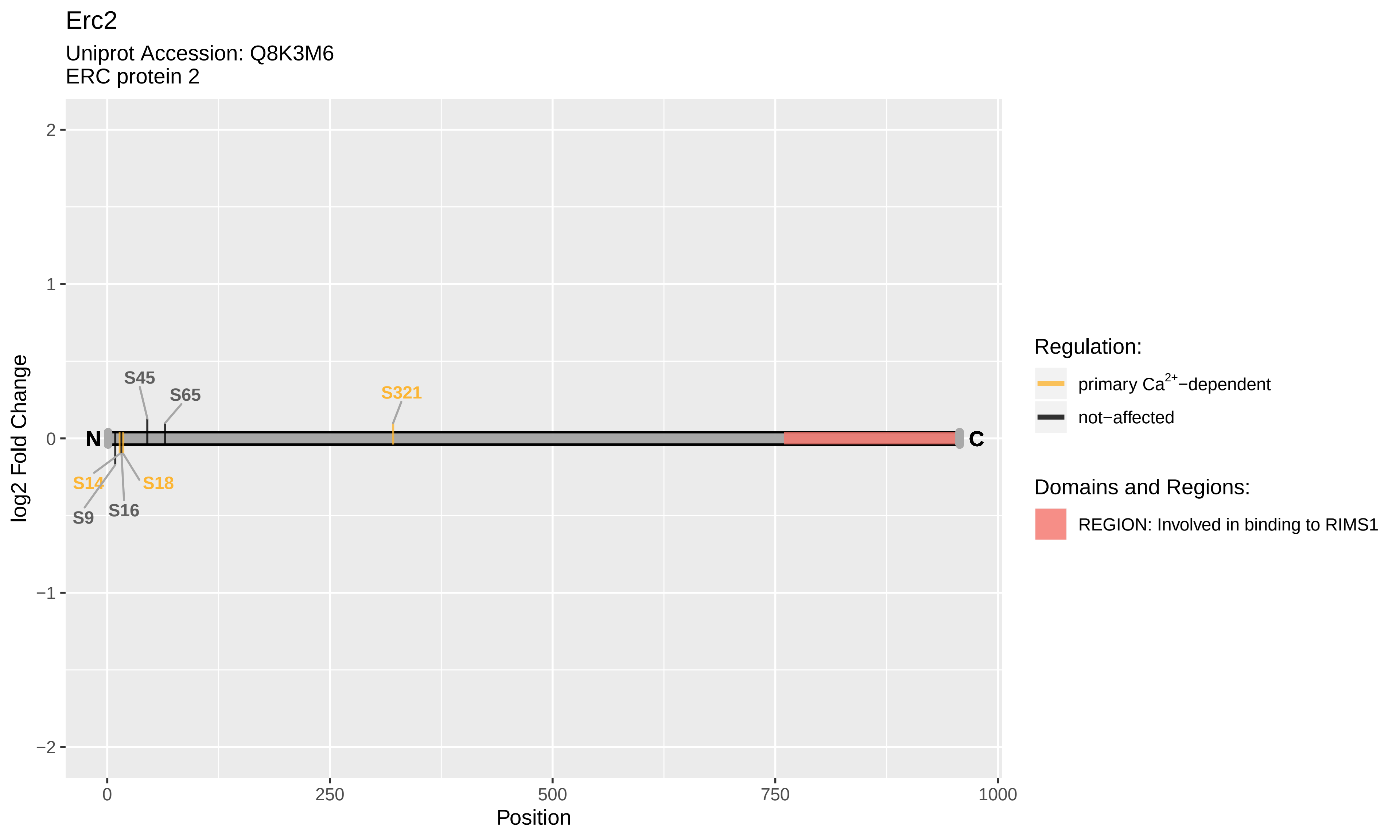

Erc2
Uniprot Accession: Q8K3M6
ERC protein 2
log2 Fold Change
Position
2
1
0
−1
−2
0
250
500
750
1000
N
C
S45
S65
S321
S14
S18
S9
S16
Regulation:
primary Ca2+−dependent
not−affected
Domains and Regions:
REGION: Involved in binding to RIMS1

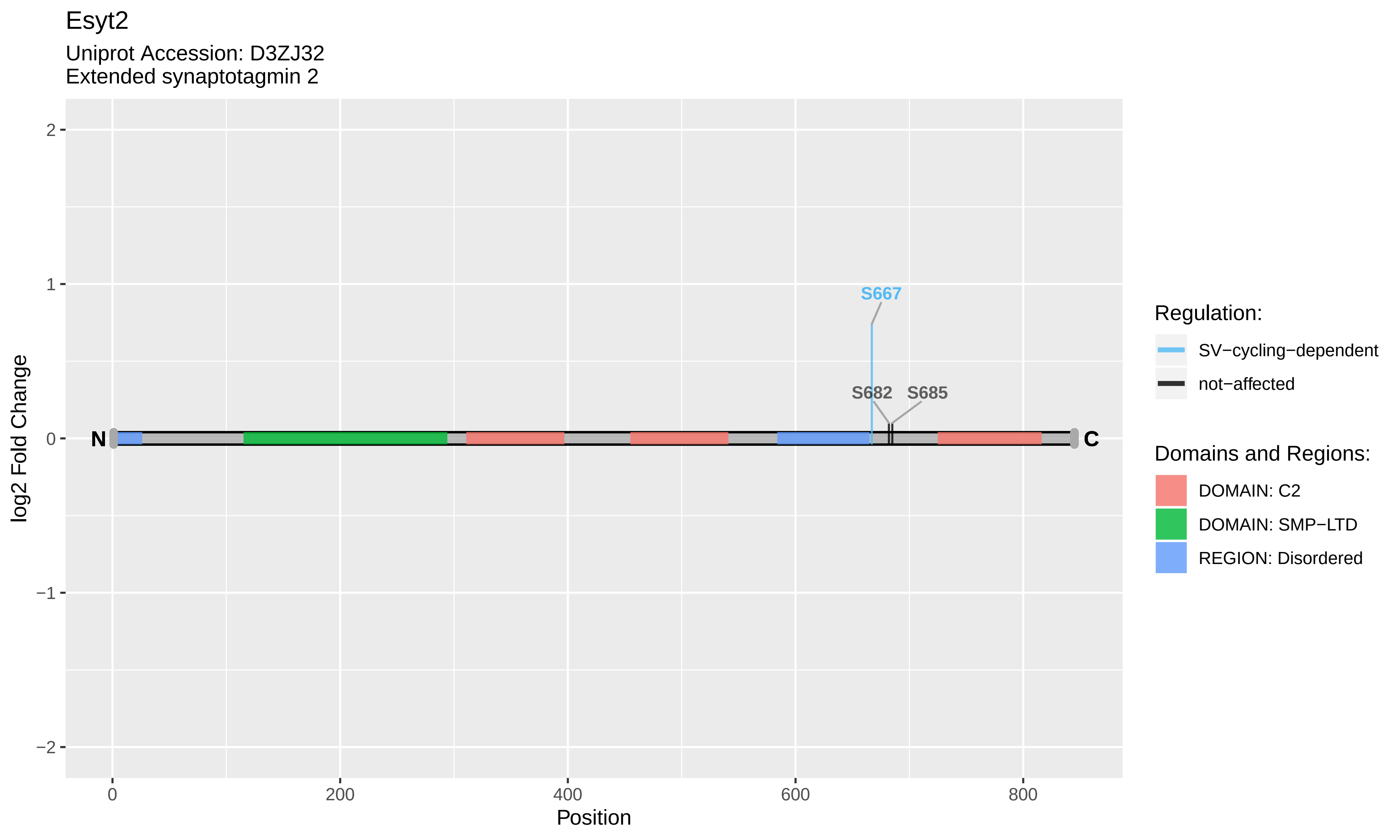

Esyt2
Uniprot Accession: D3ZJ32
Extended synaptotagmin 2
S667
S682
S685
N
C
2
1
0
−1
−2
log2 Fold Change
0
200
400
600
800
Position
Regulation:
SV−cycling−dependent
not−affected
Domains and Regions:
DOMAIN: C2
DOMAIN: SMP−LTD
REGION: Disordered

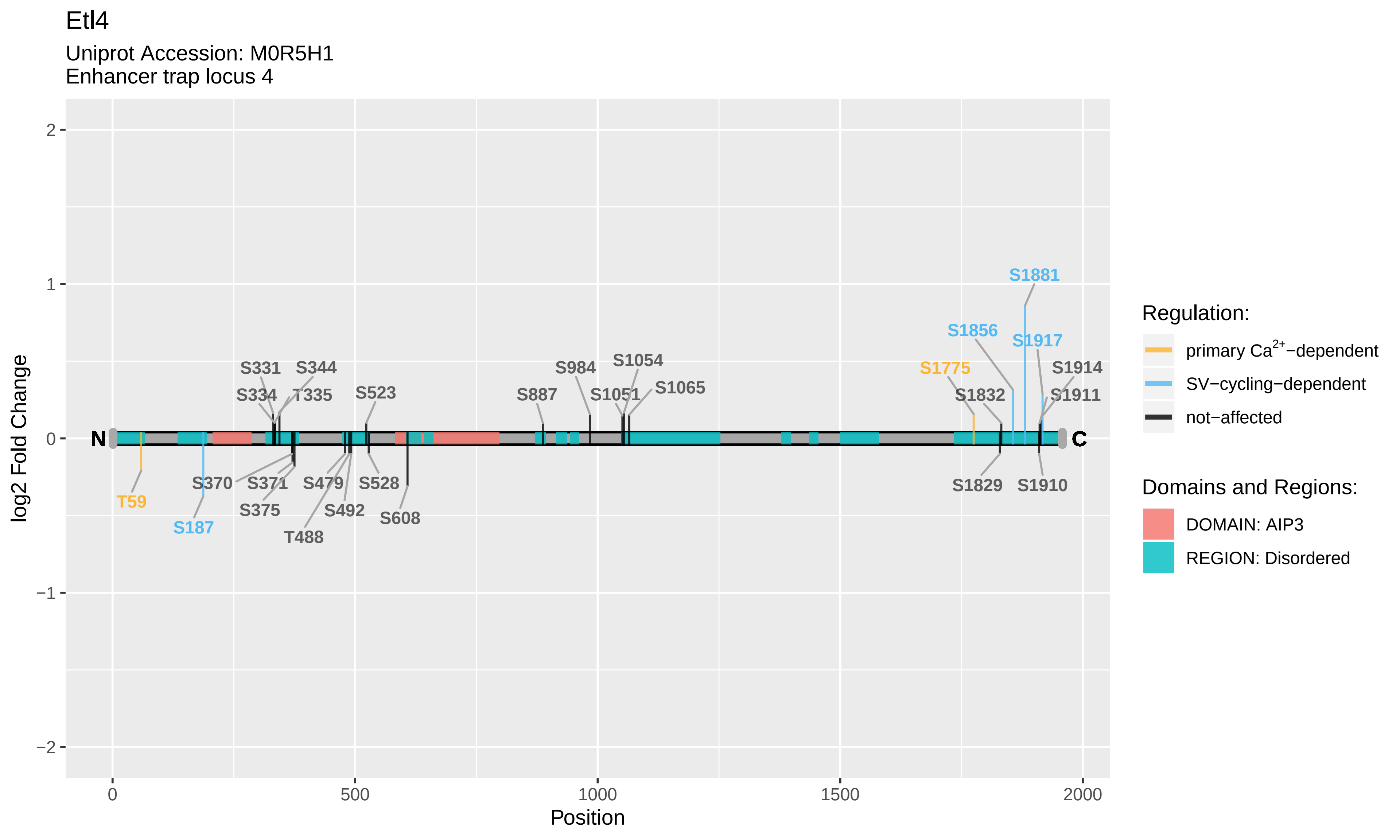

Etl4
Uniprot Accession: M0R5H1
Enhancer trap locus 4
log2 Fold Change
Position
N
C
T59
S187
S331
S334
S344
T335
S370
S371
S375
T488
S479
S492
S523
S528
S608
S887
S984
S1051
S1054
S1065
S1775
S1829
S1832
S1856
S1881
S1917
S1914
S1911
S1910
Regulation:
primary Ca^{2+}−dependent
SV−cycling−dependent
not−affected
Domains and Regions:
DOMAIN: AIP3
REGION: Disordered

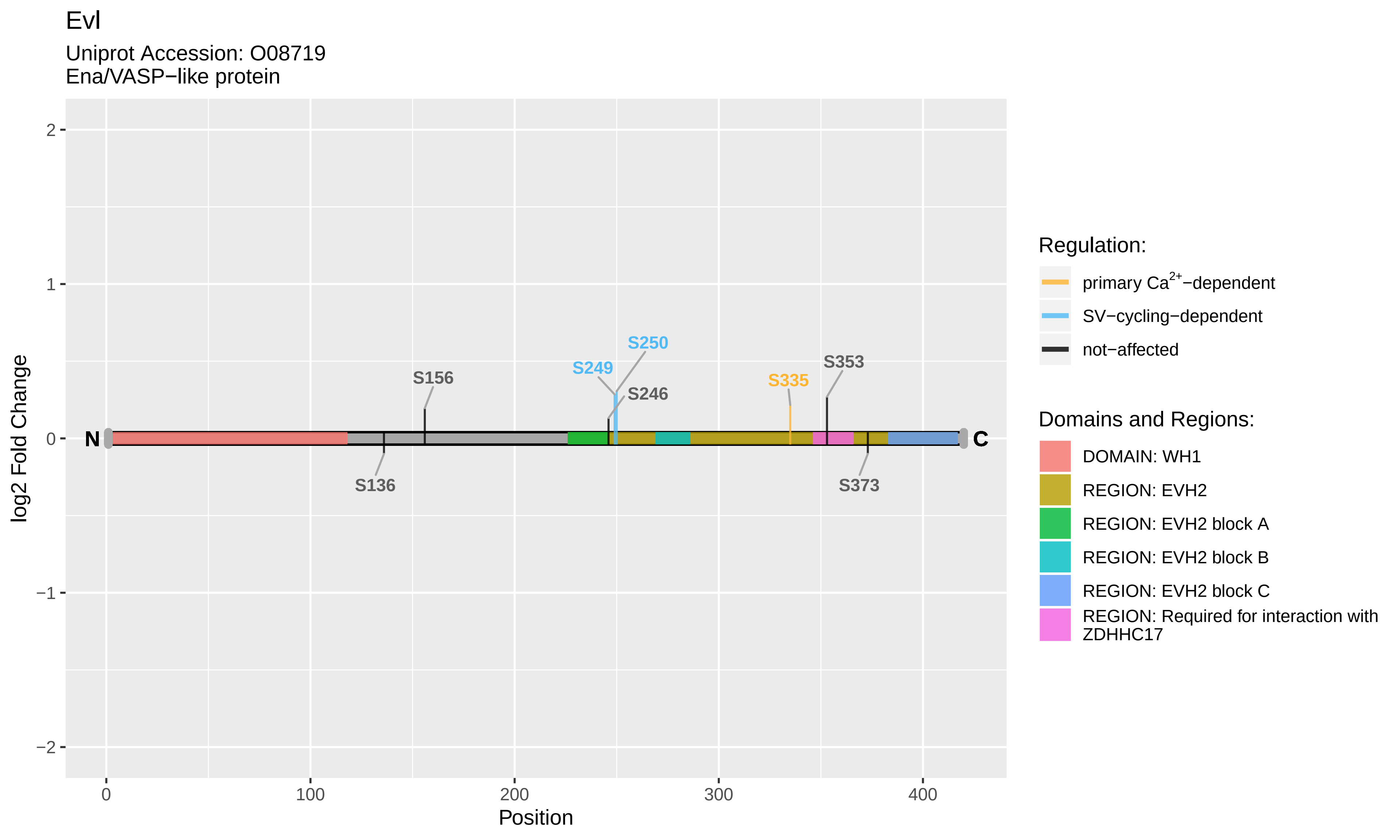
Evl
Uniprot Accession: O08719
Ena/VASP−like protein
log2 Fold Change
Position
2
1
0
−1
−2
0
100
200
300
400
N
C
S136
S156
S246
S249
S250
S335
S353
S373
Regulation:
primary Ca2+−dependent
SV−cycling−dependent
not−affected
Domains and Regions:
DOMAIN: WH1
REGION: EVH2
REGION: EVH2 block A
REGION: EVH2 block B
REGION: EVH2 block C
REGION: Required for interaction with ZDHHC17

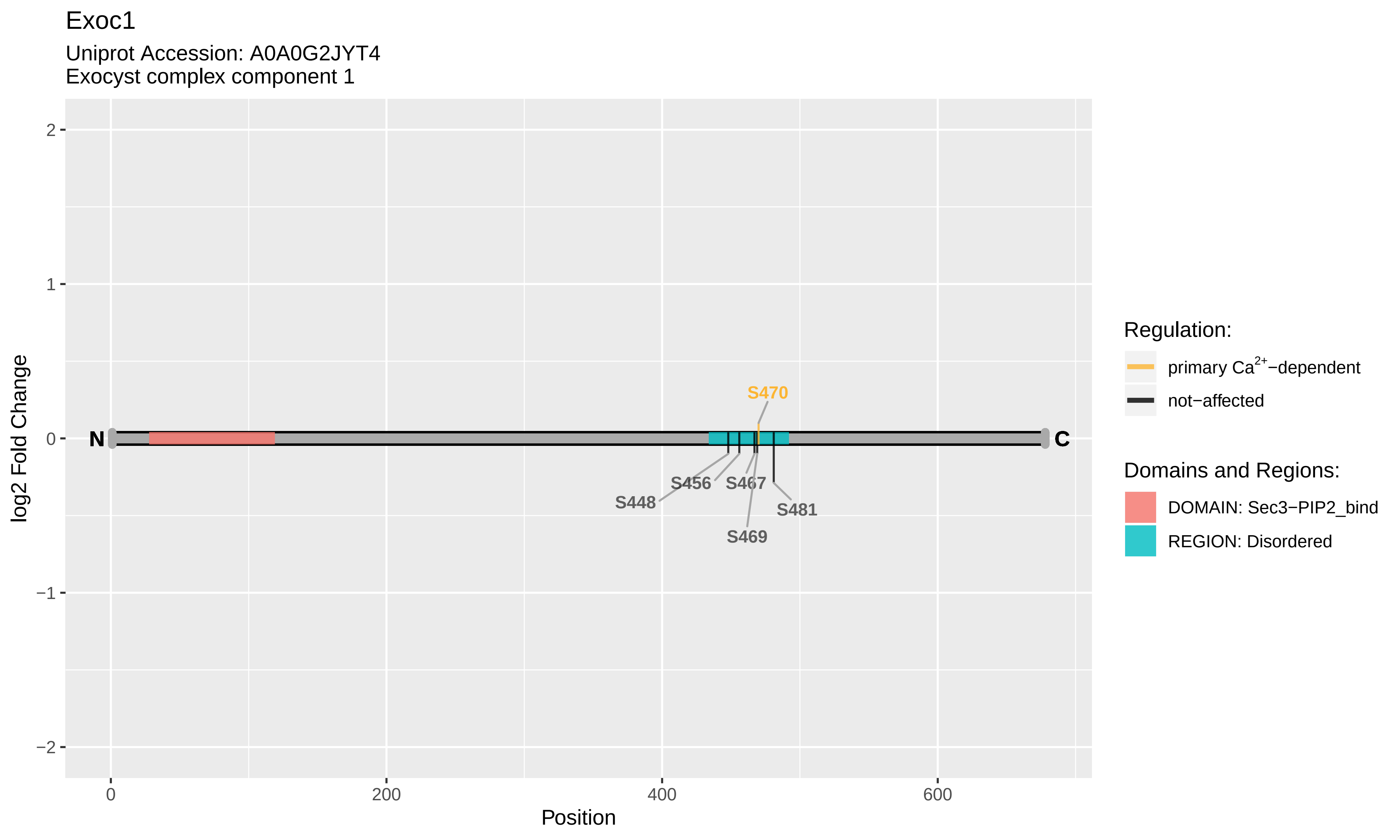

Exoc1
Uniprot Accession: A0A0G2JYT4
Exocyst complex component 1
log2 Fold Change
2
1
0
−1
−2
Position
0
200
400
600
N
C
S470
S448
S456
S467
S469
S481
Regulation:
primary Ca2+−dependent
not−affected
Domains and Regions:
DOMAIN: Sec3−PIP2_bind
REGION: Disordered

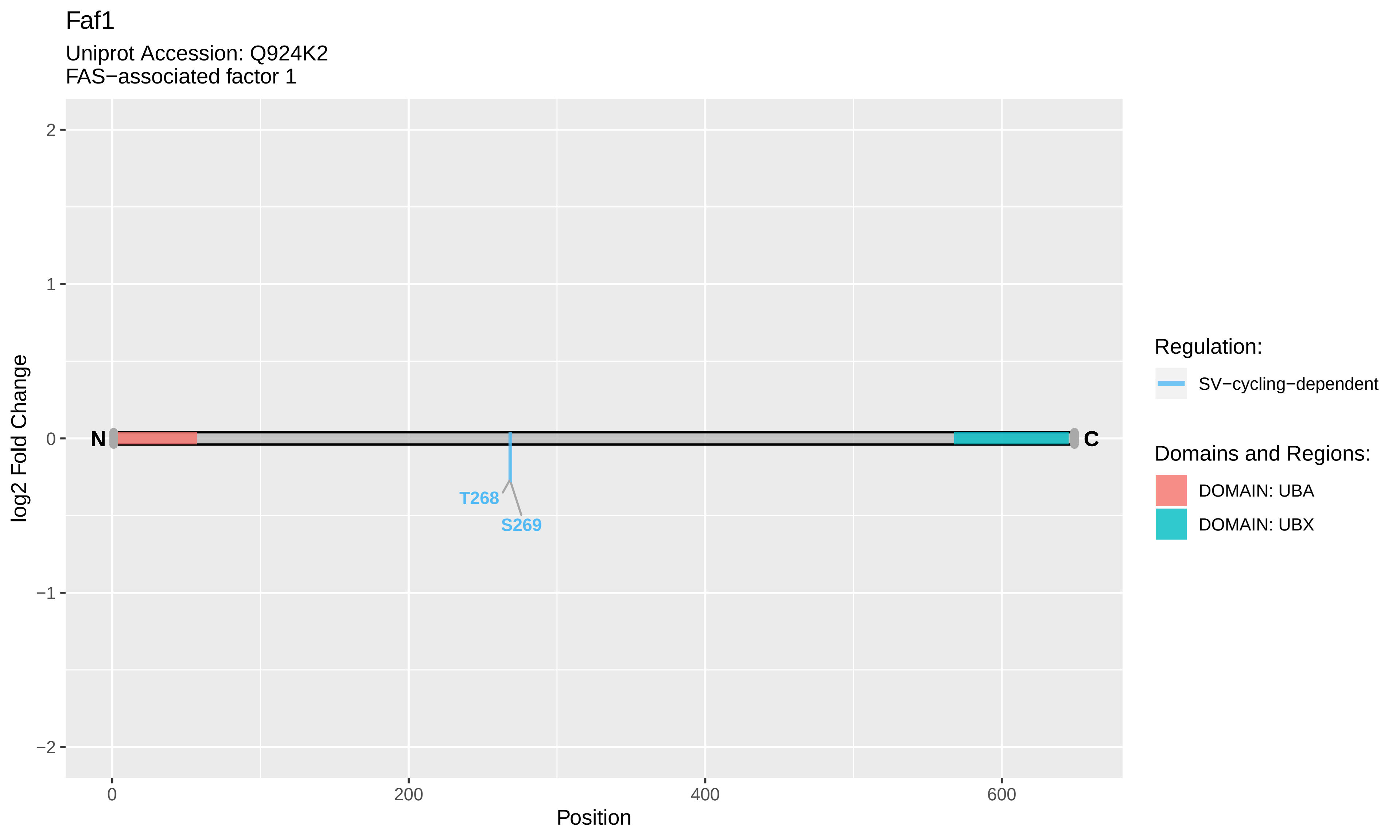
Faf1
Uniprot Accession: Q924K2
FAS−associated factor 1
log2 Fold Change
2
1
0
−1
−2
N
C
T268
S269
0
200
400
600
Position
Regulation:
SV−cycling−dependent
Domains and Regions:
DOMAIN: UBA
DOMAIN: UBX

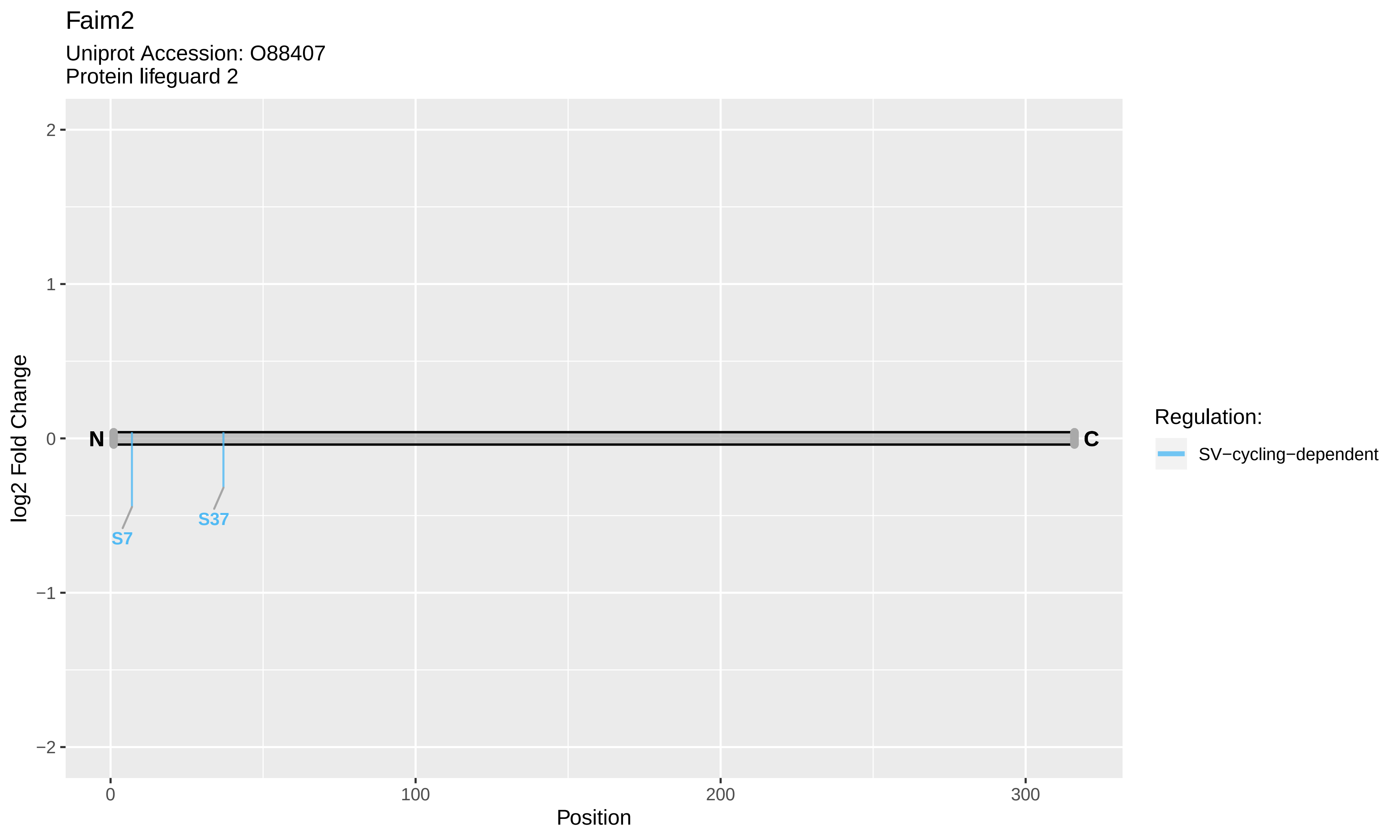
Faim2
Uniprot Accession: O88407
Protein lifeguard 2
log2 Fold Change
2
1
0
−1
−2
N
C
S7
S37
0
100
200
300
Position
Regulation:
SV−cycling−dependent

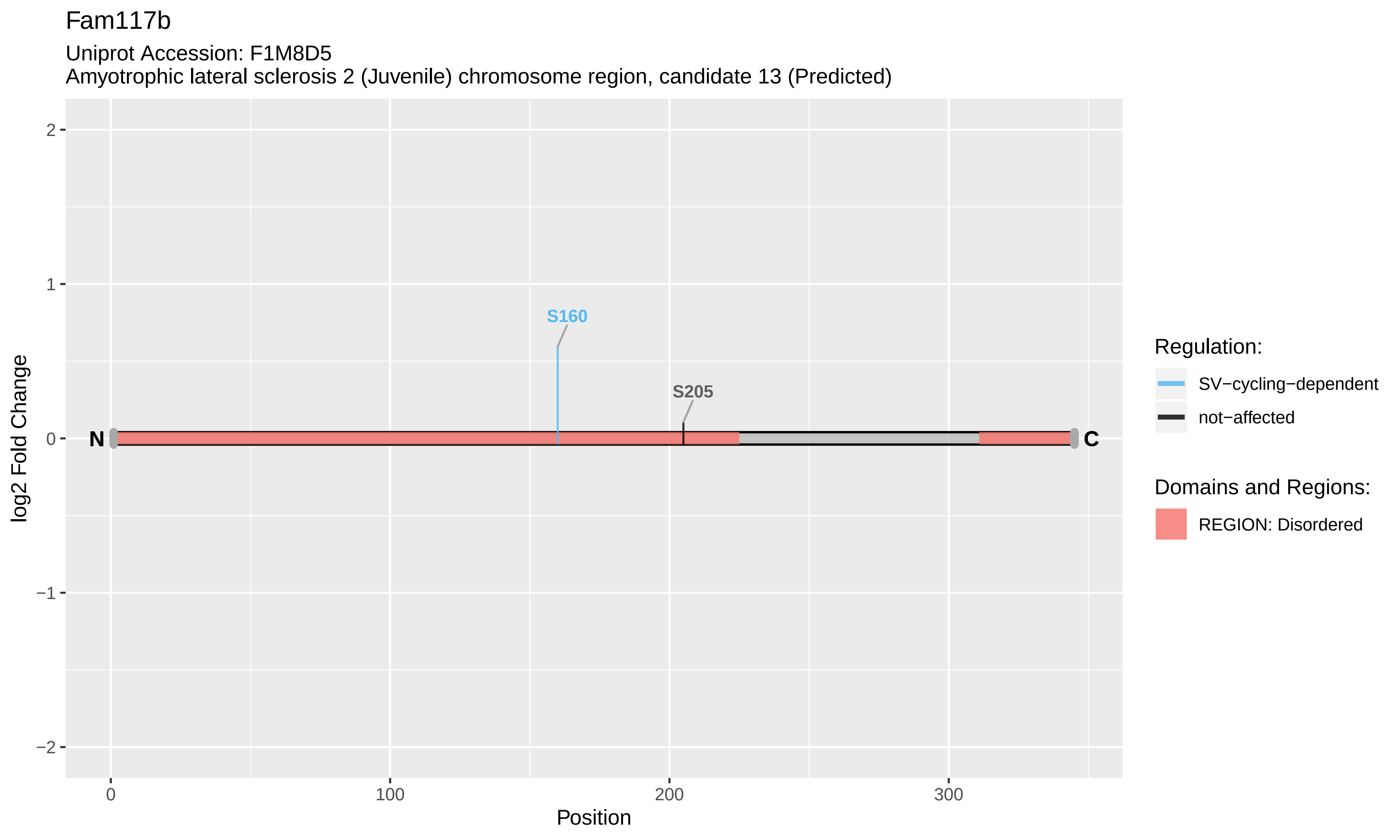

Fam117b
Uniprot Accession: F1M8D5
Amyotrophic lateral sclerosis 2 (Juvenile) chromosome region, candidate 13 (Predicted)
S160
S205
N
C
2
1
0
−1
−2
0
100
200
300
Position
log2 Fold Change
Regulation:
SV−cycling−dependent
not−affected
Domains and Regions:
REGION: Disordered

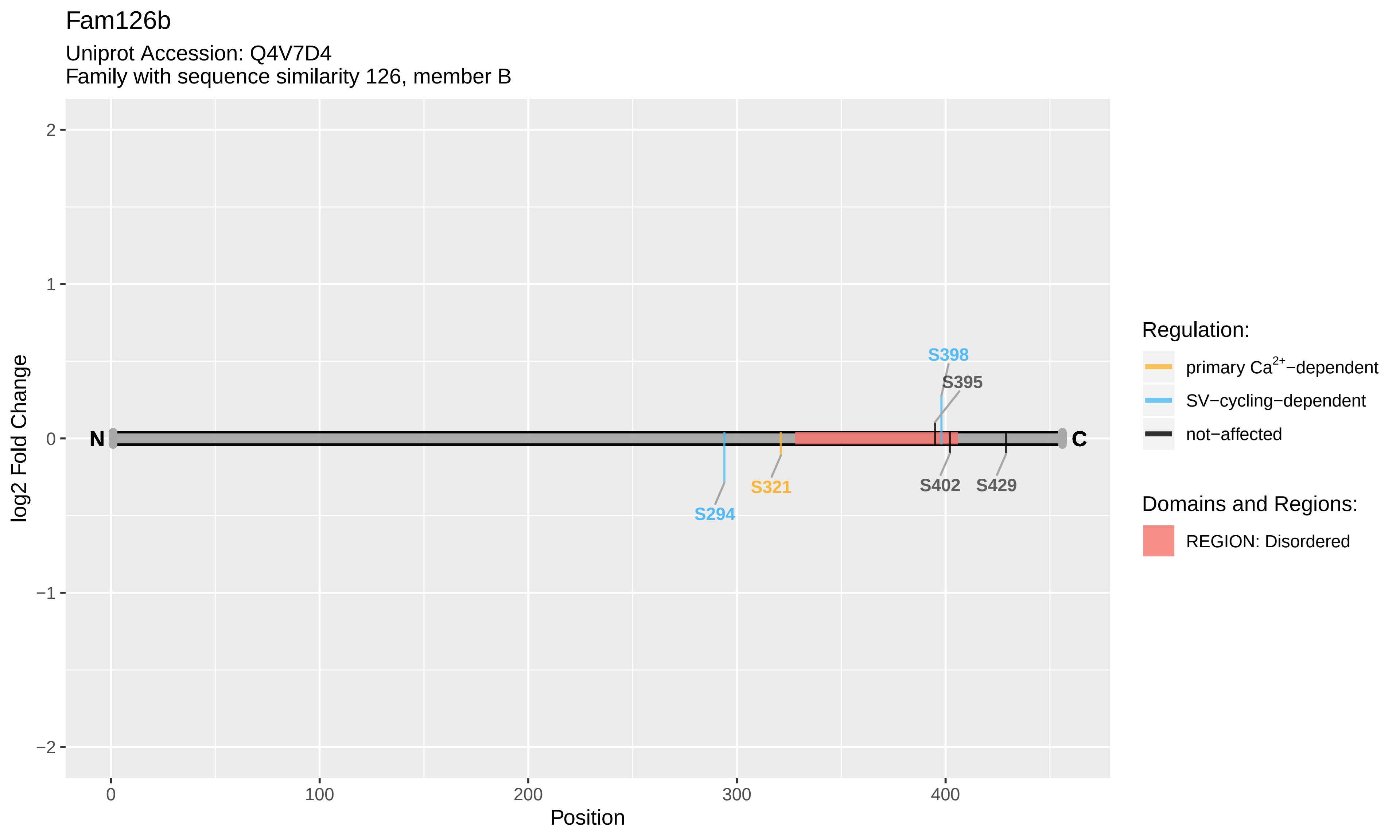

Fam126b
Uniprot Accession: Q4V7D4
Family with sequence similarity 126, member B
log2 Fold Change
2
1
0
−1
−2
N
C
S398
S395
S294
S321
S402
S429
0
100
200
300
400
Position
Regulation:
primary Ca2+−dependent
SV−cycling−dependent
not−affected
Domains and Regions:
REGION: Disordered

Fam131b

Uniprot Accession: F8WFH6
Protein FAM131B

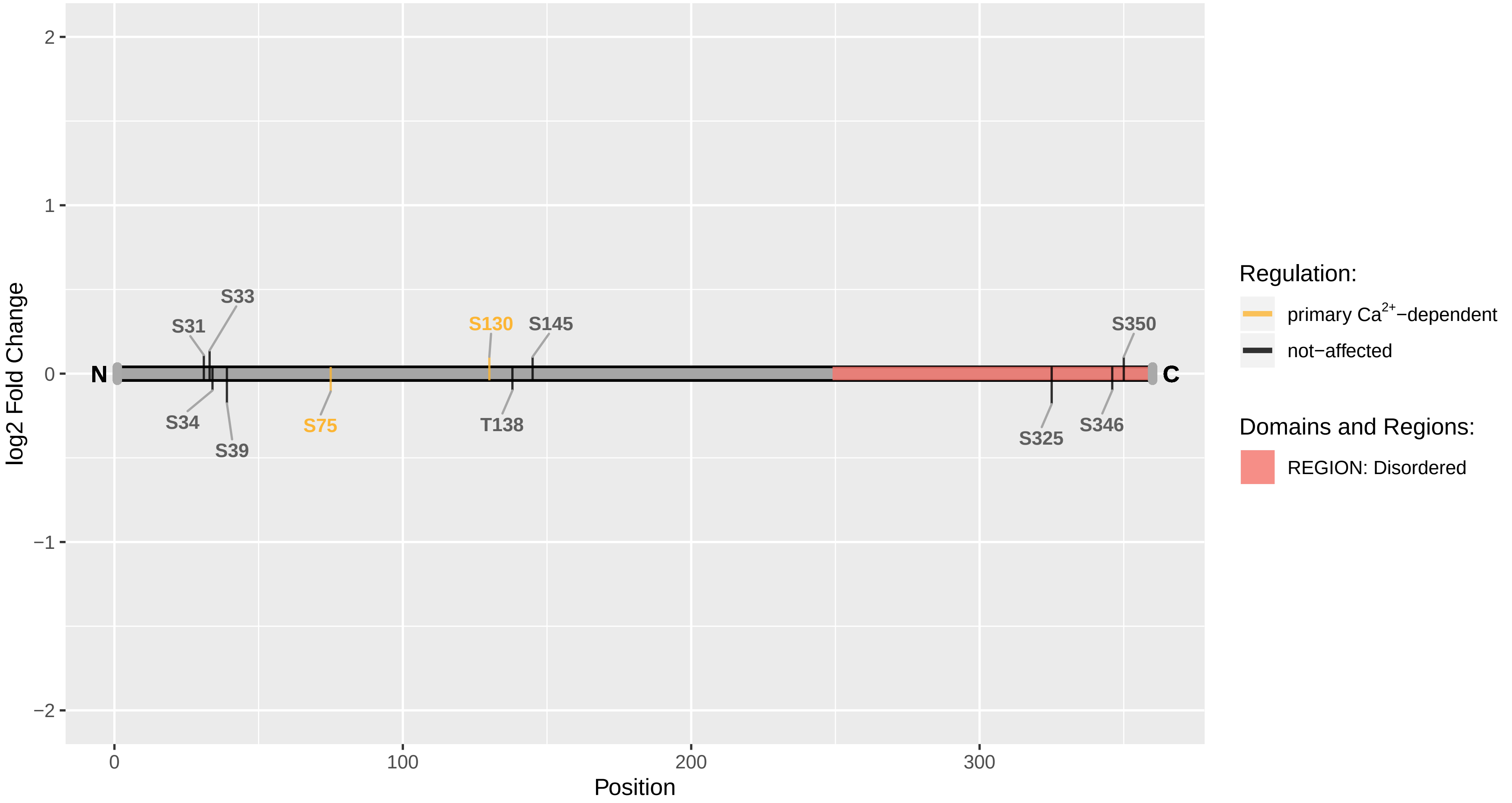

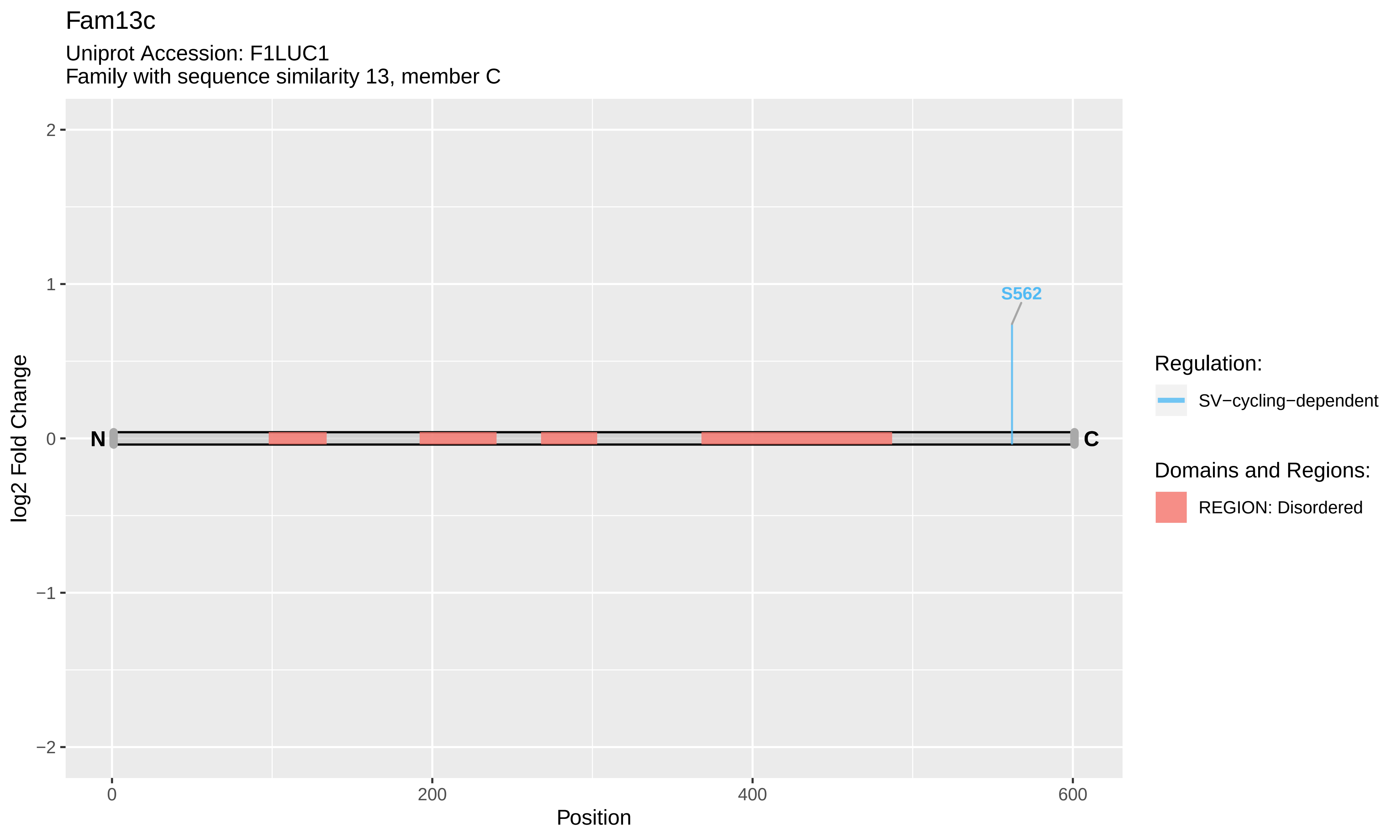
Fam13c
Uniprot Accession: F1LUC1
Family with sequence similarity 13, member C
S562
N
C
log2 Fold Change
2
1
0
−1
−2
0
200
400
600
Position
Regulation:
SV−cycling−dependent
Domains and Regions:
REGION: Disordered

Fam171a1

Uniprot Accession: D4A7Y4
Family with sequence similarity 171, member A1

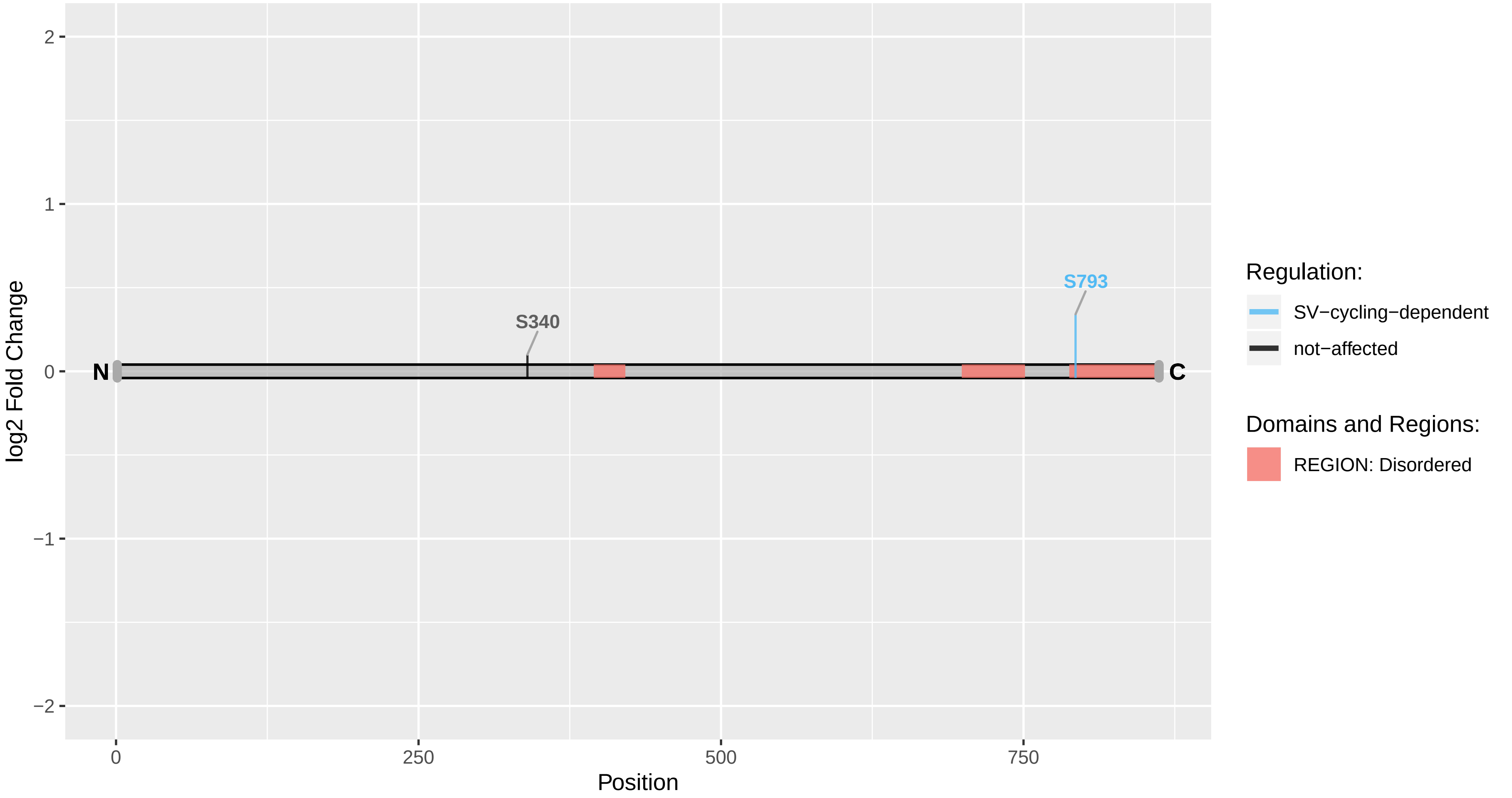

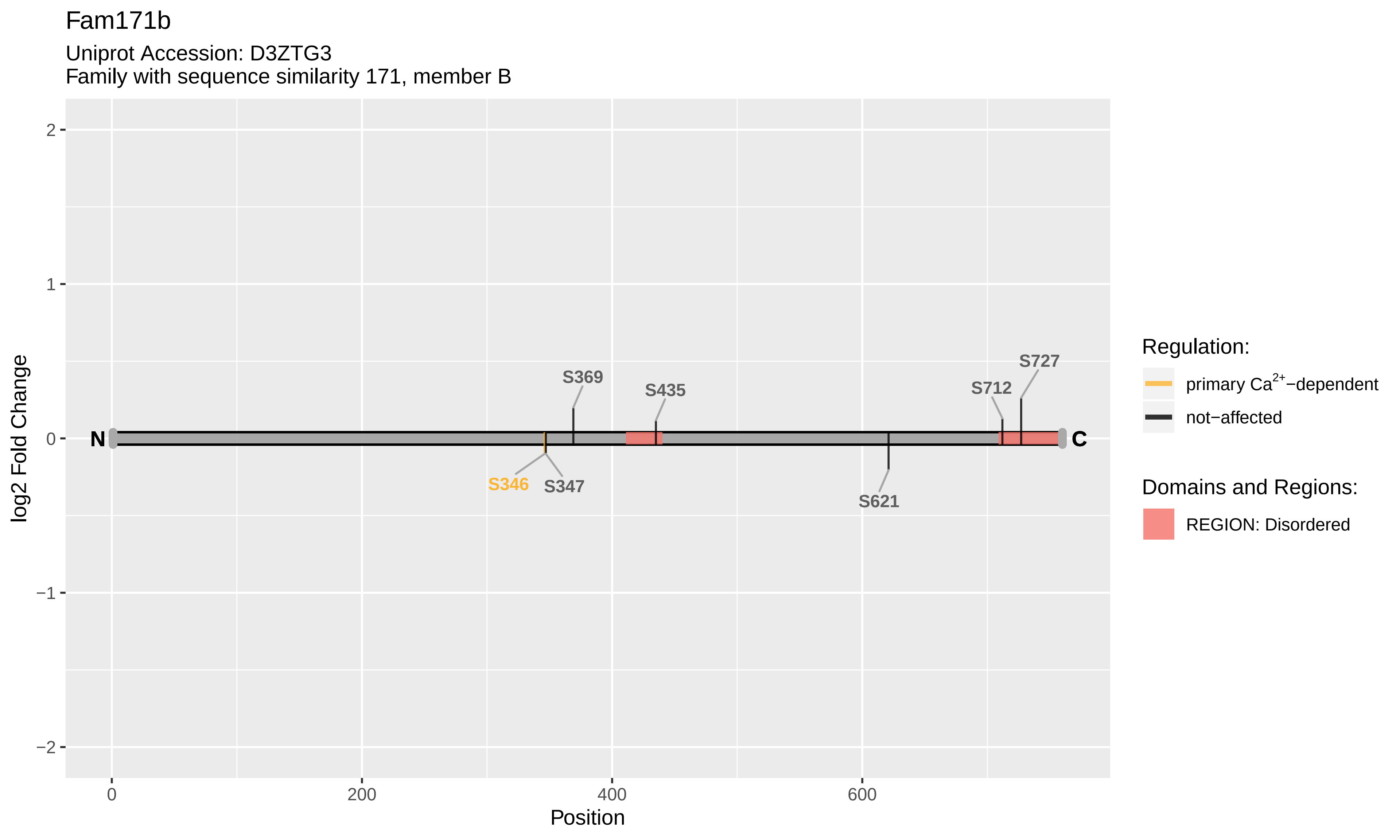

Fam171b
Uniprot Accession: D3ZTG3
Family with sequence similarity 171, member B
log2 Fold Change
Position
2
1
0
−1
−2
0
200
400
600
N
C
S369
S435
S727
S712
S346
S347
S621
Regulation:
primary Ca2+−dependent
not−affected
Domains and Regions:
REGION: Disordered

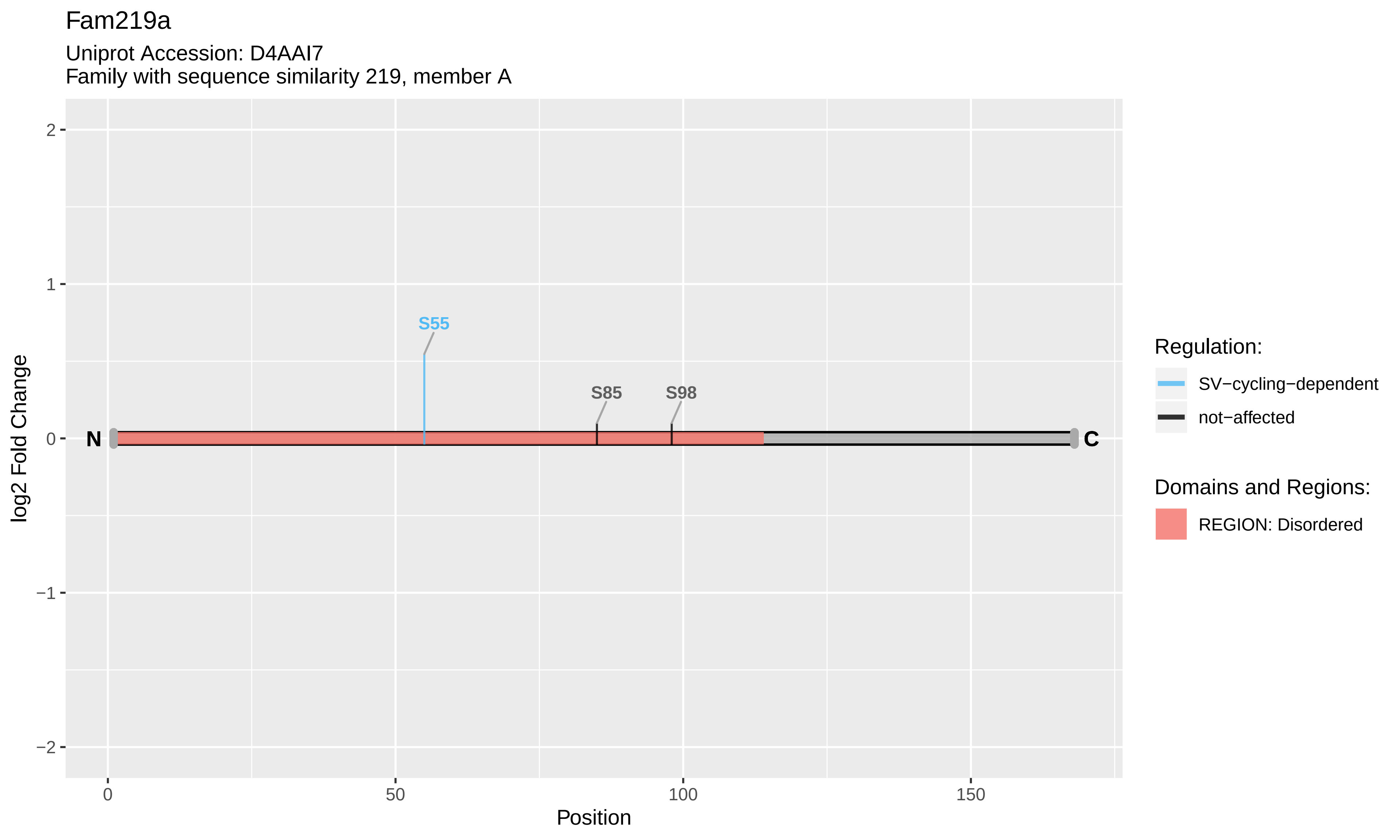
Fam219a
Uniprot Accession: D4AAI7
Family with sequence similarity 219, member A
log2 Fold Change
2
1
0
−1
−2
N
C
S55
S85
S98
0
50
100
150
Position
Regulation:
SV−cycling−dependent
not−affected
Domains and Regions:
REGION: Disordered

Farp1

Uniprot Accession: F1LYQ8
FERM, ARHGEF and pleckstrin domain−containing protein 1

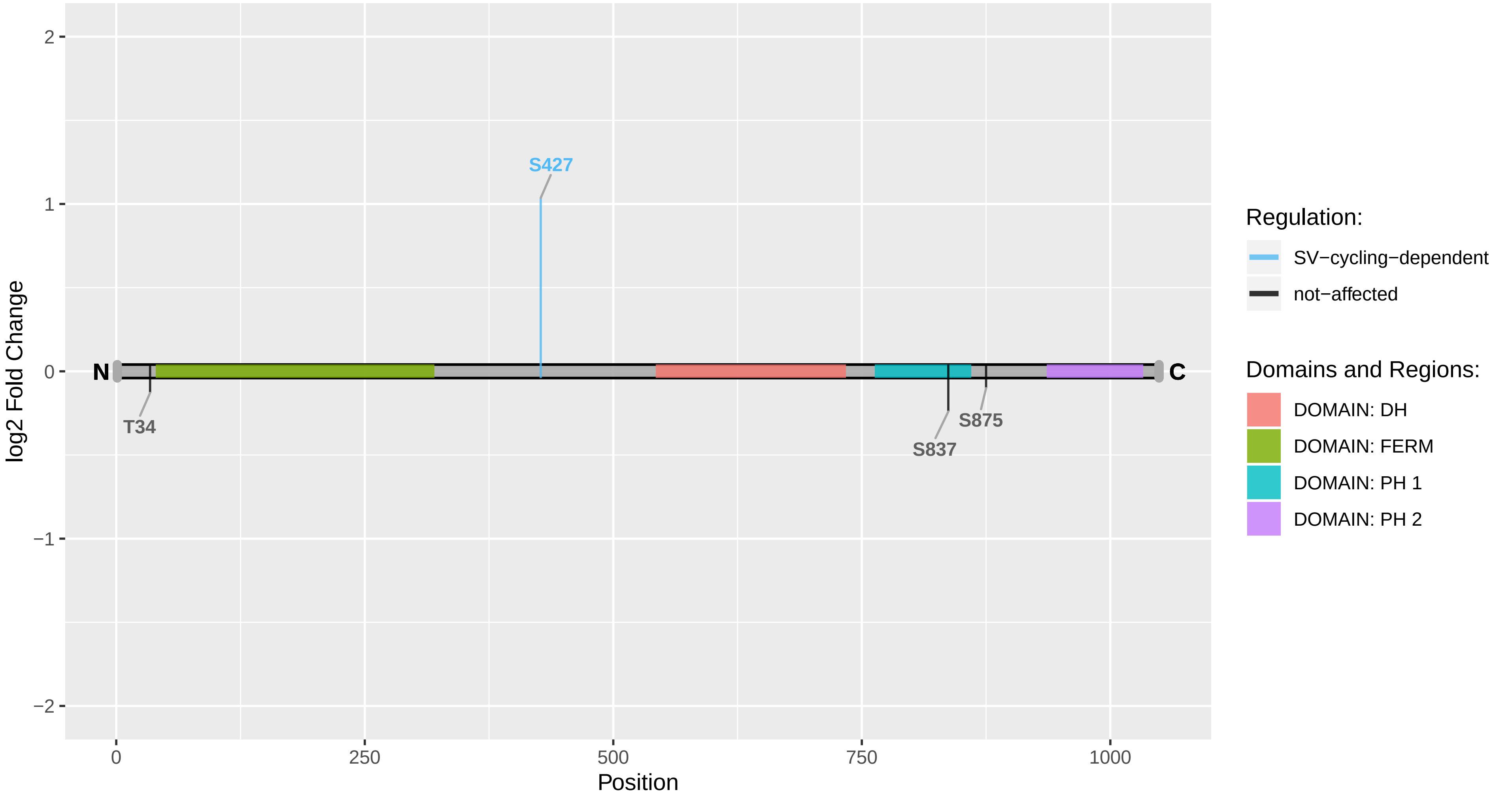

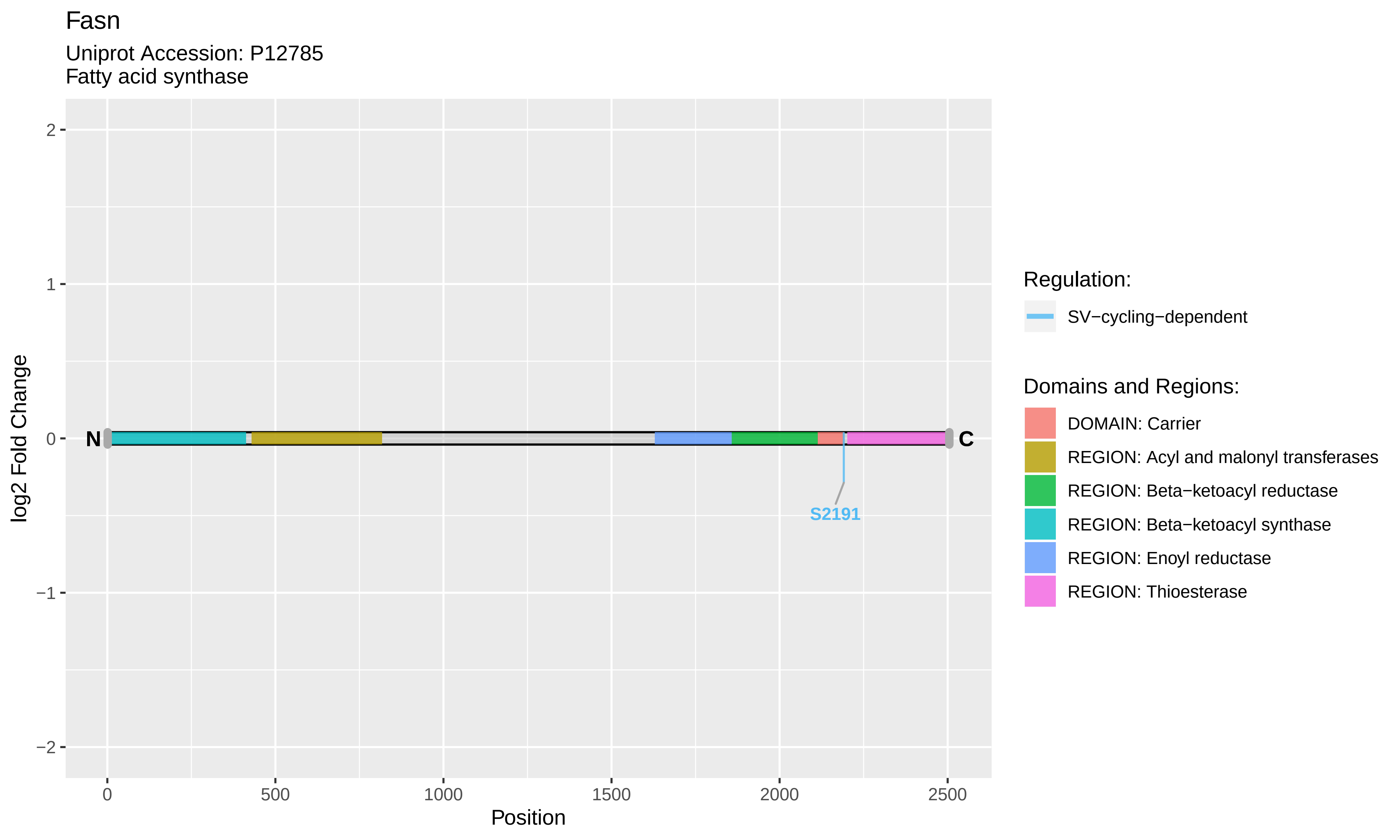

Fasn
Uniprot Accession: P12785
Fatty acid synthase
log2 Fold Change
2
1
0
−1
−2
N
C
S2191
0
500
1000
1500
2000
2500
Position
Regulation:
SV−cycling−dependent
Domains and Regions:
DOMAIN: Carrier
REGION: Acyl and malonyl transferases
REGION: Beta−ketoacyl reductase
REGION: Beta−ketoacyl synthase
REGION: Enoyl reductase
REGION: Thioesterase

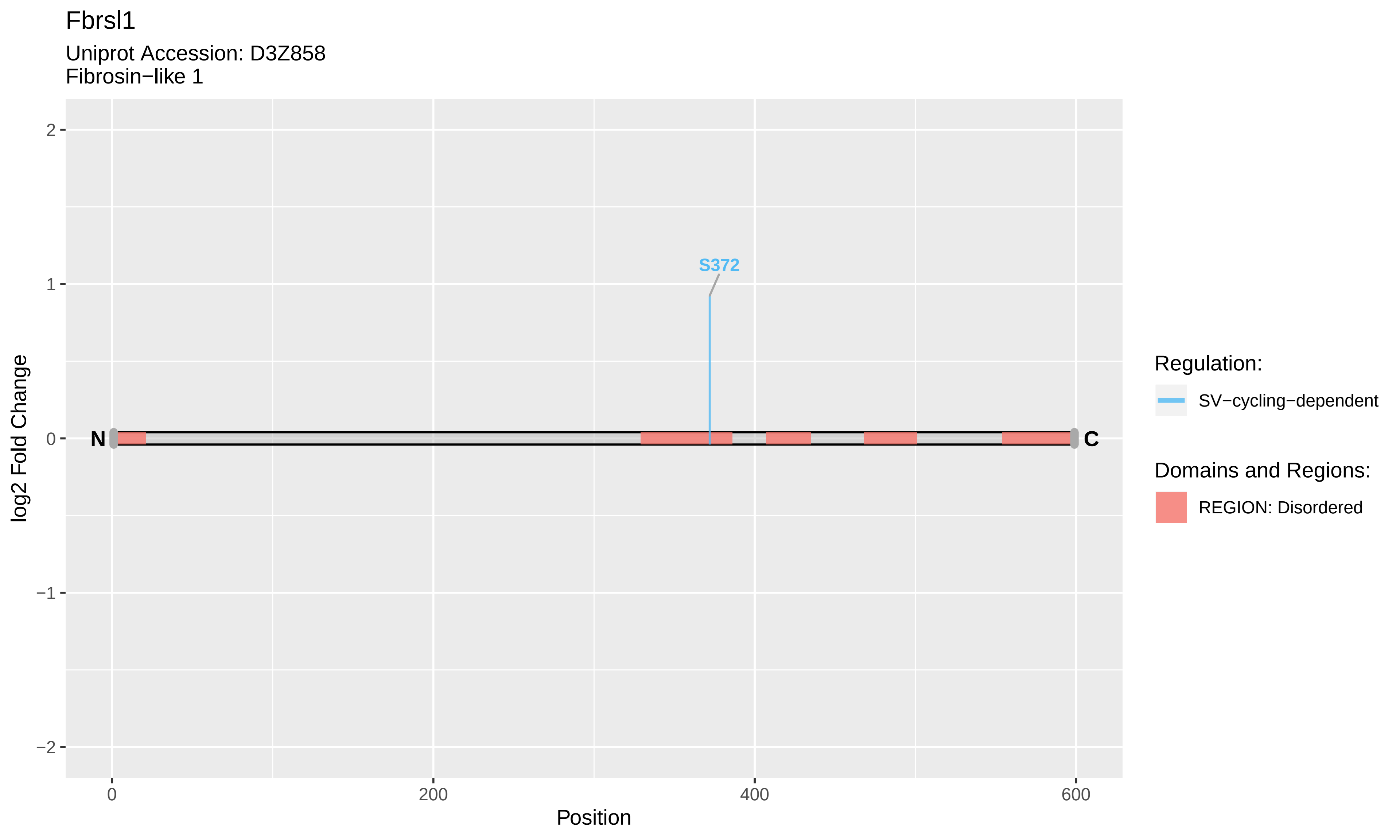

Fbrsl1
Uniprot Accession: D3Z858
Fibrosin−like 1
S372
N
C
2
1
0
−1
−2
0
200
400
600
log2 Fold Change
Position
Regulation:
SV−cycling−dependent
Domains and Regions:
REGION: Disordered

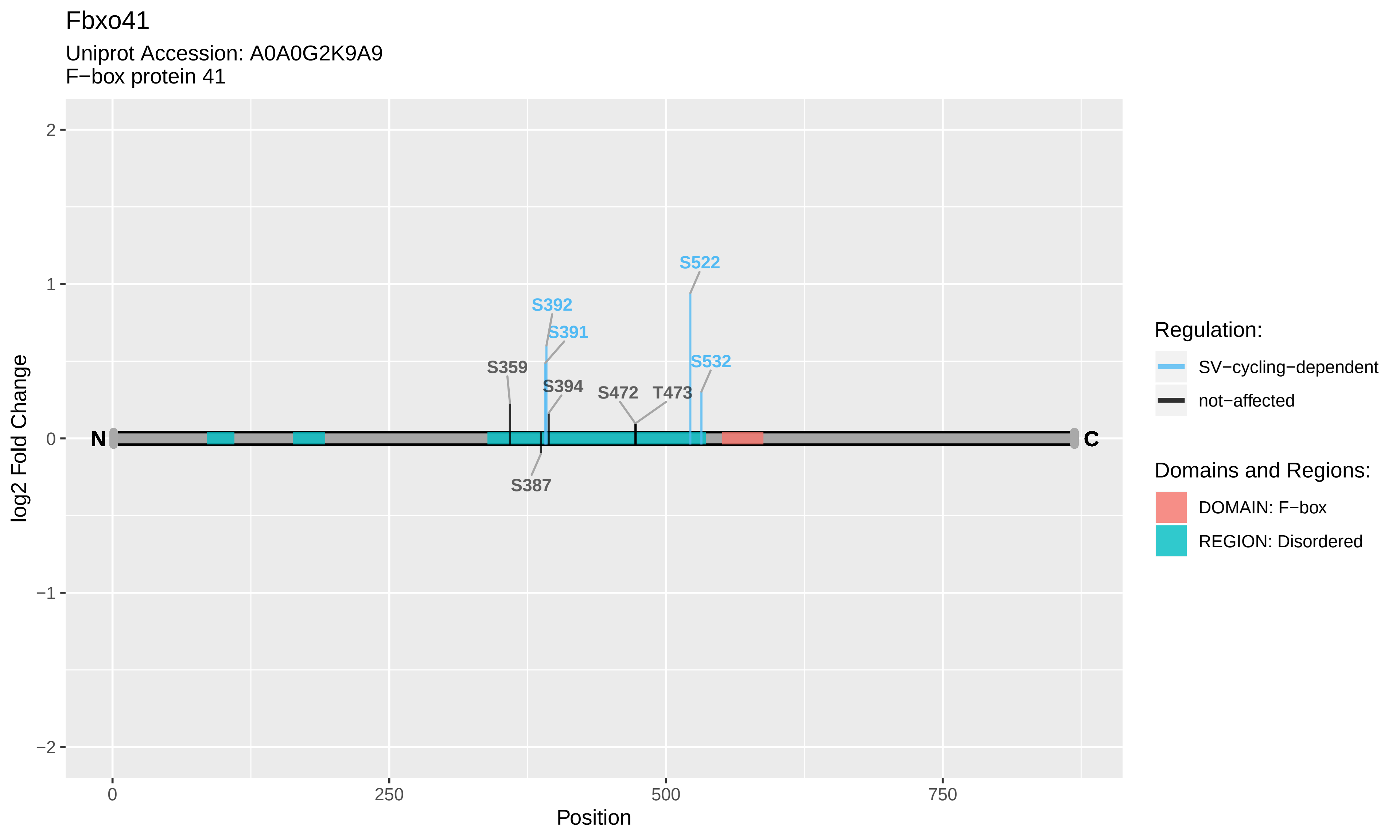
Fbxo41
Uniprot Accession: A0A0G2K9A9
F−box protein 41
log2 Fold Change
2
1
0
−1
−2
N
C
S522
S392
S391
S359
S394
S472
T473
S532
S387
0
250
500
750
Position
Regulation:
SV−cycling−dependent
not−affected
Domains and Regions:
DOMAIN: F−box
REGION: Disordered

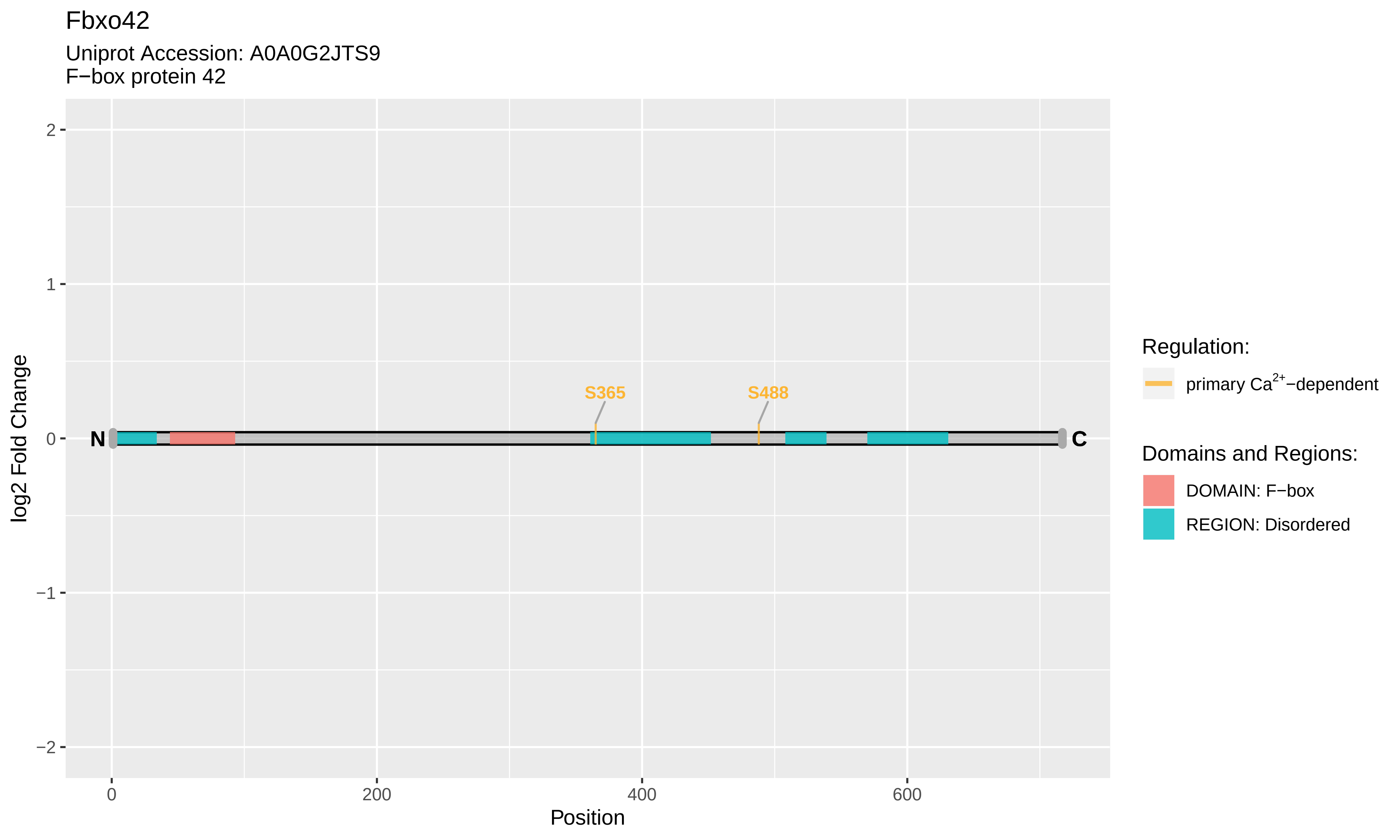
Fbxo42
Uniprot Accession: A0A0G2JTS9
F−box protein 42
S365
S488
N
C
2
1
0
−1
−2
log2 Fold Change
0
200
400
600
Position
Regulation:
primary Ca2+−dependent
Domains and Regions:
DOMAIN: F−box
REGION: Disordered

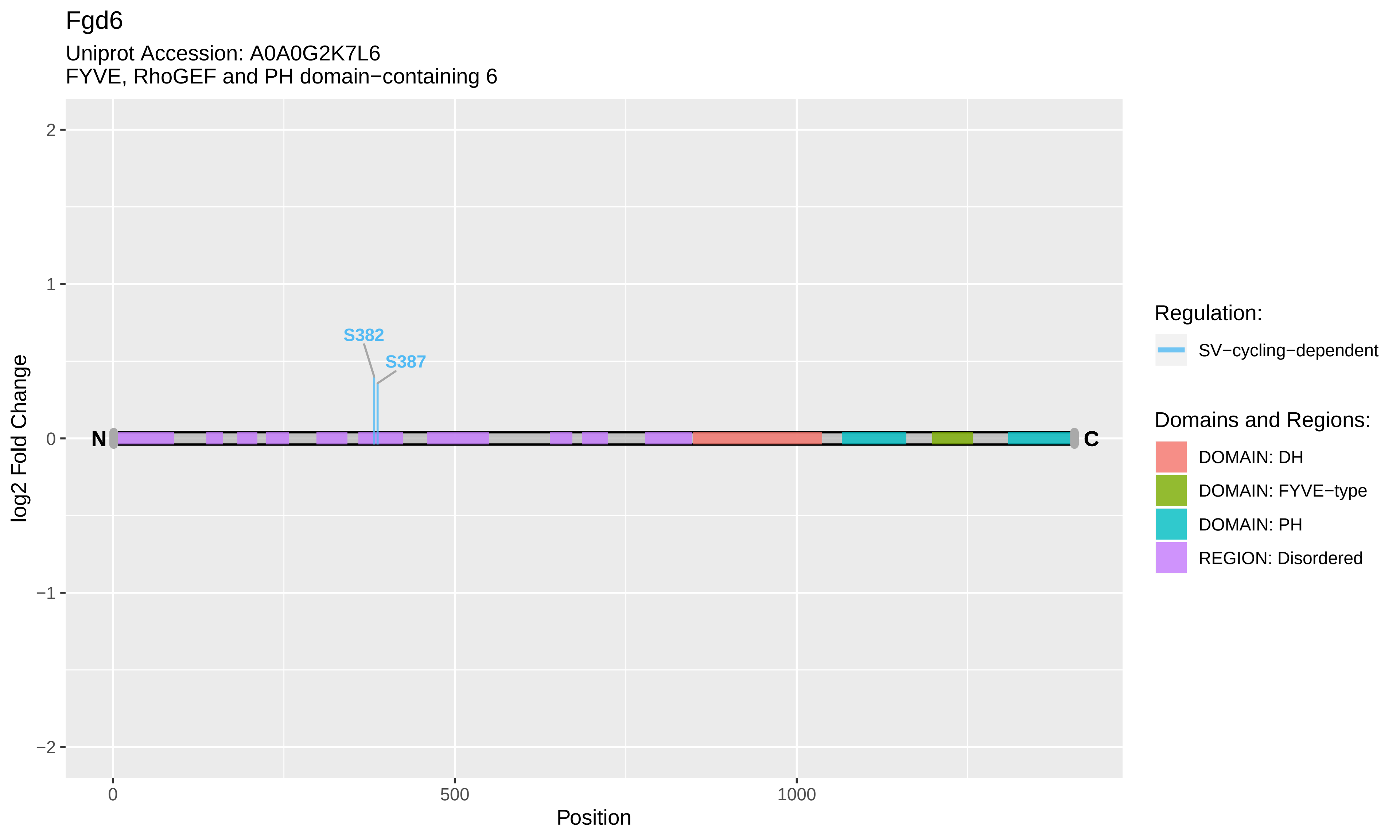
Fgd6
Uniprot Accession: A0A0G2K7L6
FYVE, RhoGEF and PH domain−containing 6
S382
S387
N
C
2
1
0
−1
−2
log2 Fold Change
0
500
1000
Position
Regulation:
SV−cycling−dependent
Domains and Regions:
DOMAIN: DH
DOMAIN: FYVE−type
DOMAIN: PH
REGION: Disordered

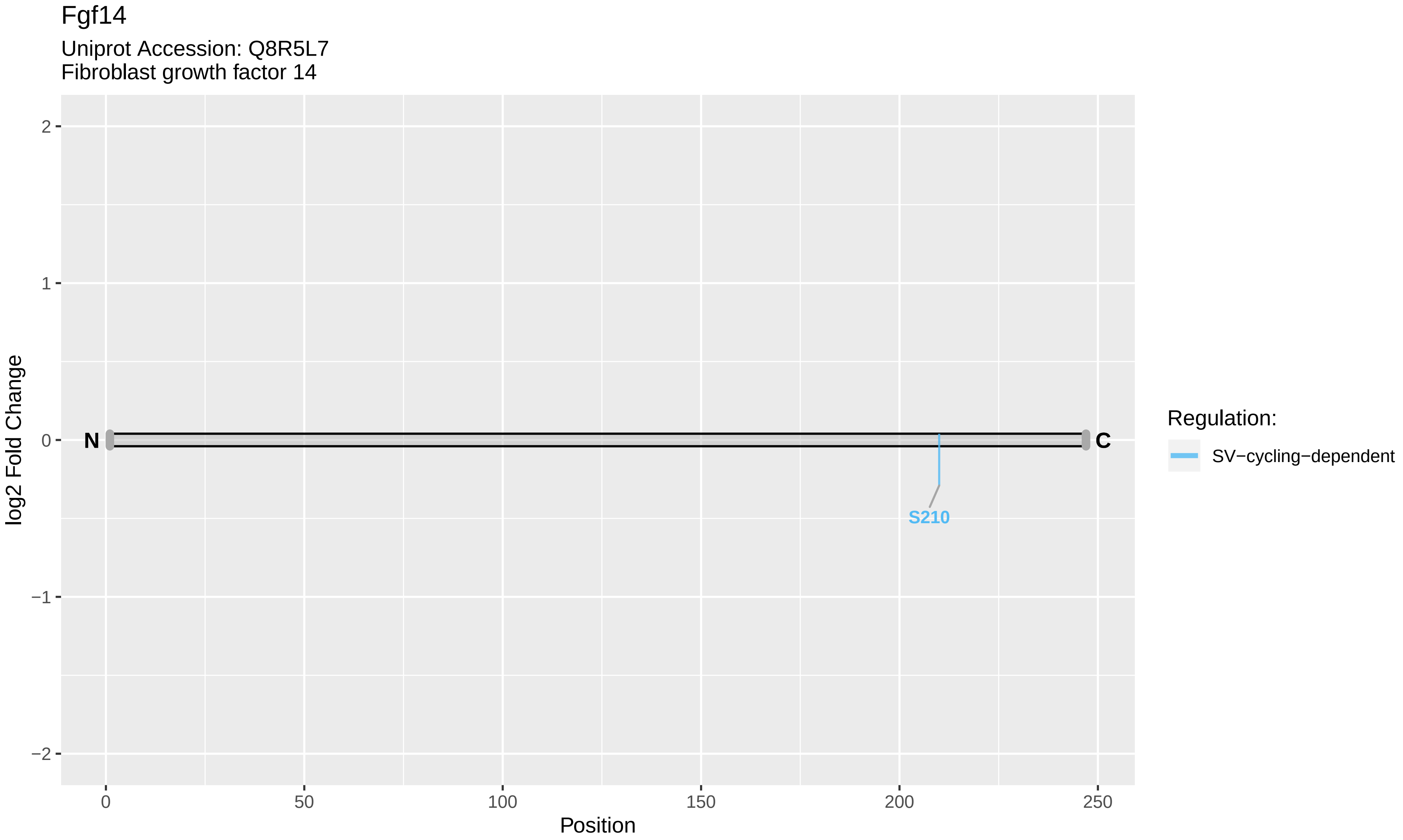

Fgf14
Uniprot Accession: Q8R5L7
Fibroblast growth factor 14
log2 Fold Change
2
1
0
−1
−2
N
C
S210
0
50
100
150
200
250
Position
Regulation:
SV−cycling−dependent

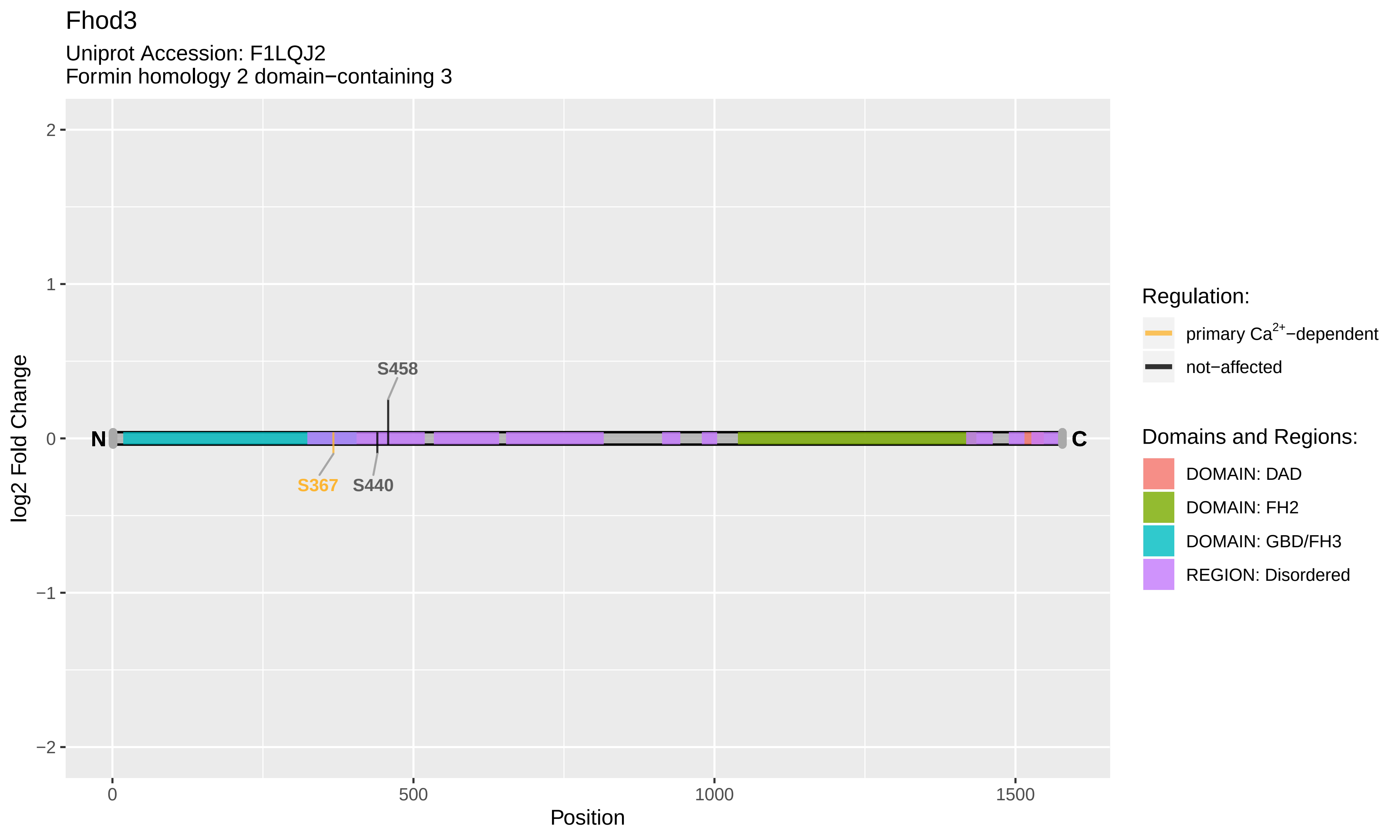

Fhod3
Uniprot Accession: F1LQJ2
Formin homology 2 domain−containing 3
S458
S367
S440
N
C
log2 Fold Change
2
1
0
−1
−2
0
500
1000
1500
Position
Regulation:
primary Ca2+−dependent
not−affected
Domains and Regions:
DOMAIN: DAD
DOMAIN: FH2
DOMAIN: GBD/FH3
REGION: Disordered

Fis1

Uniprot Accession: P84817
Mitochondrial fission 1 protein

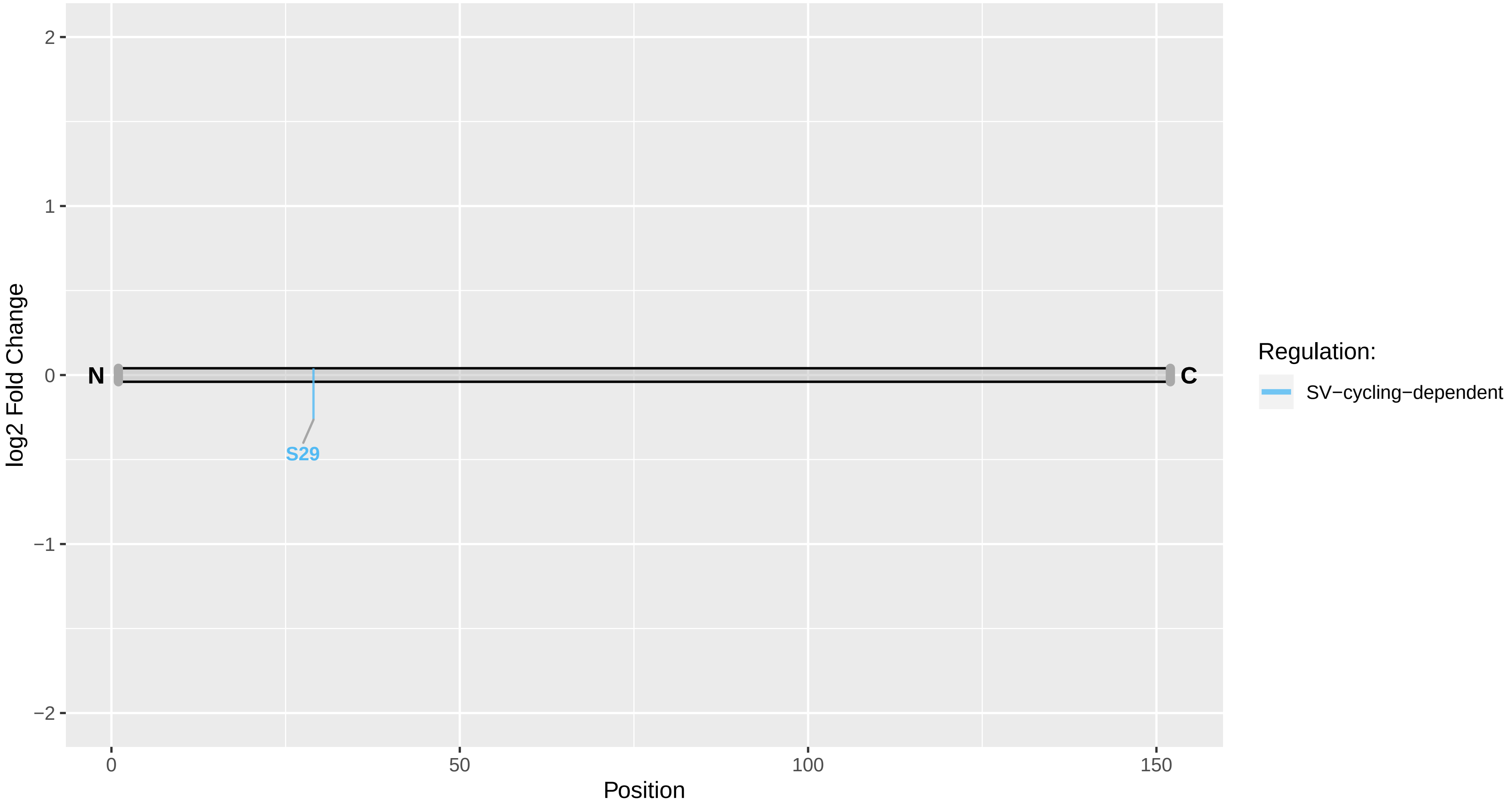

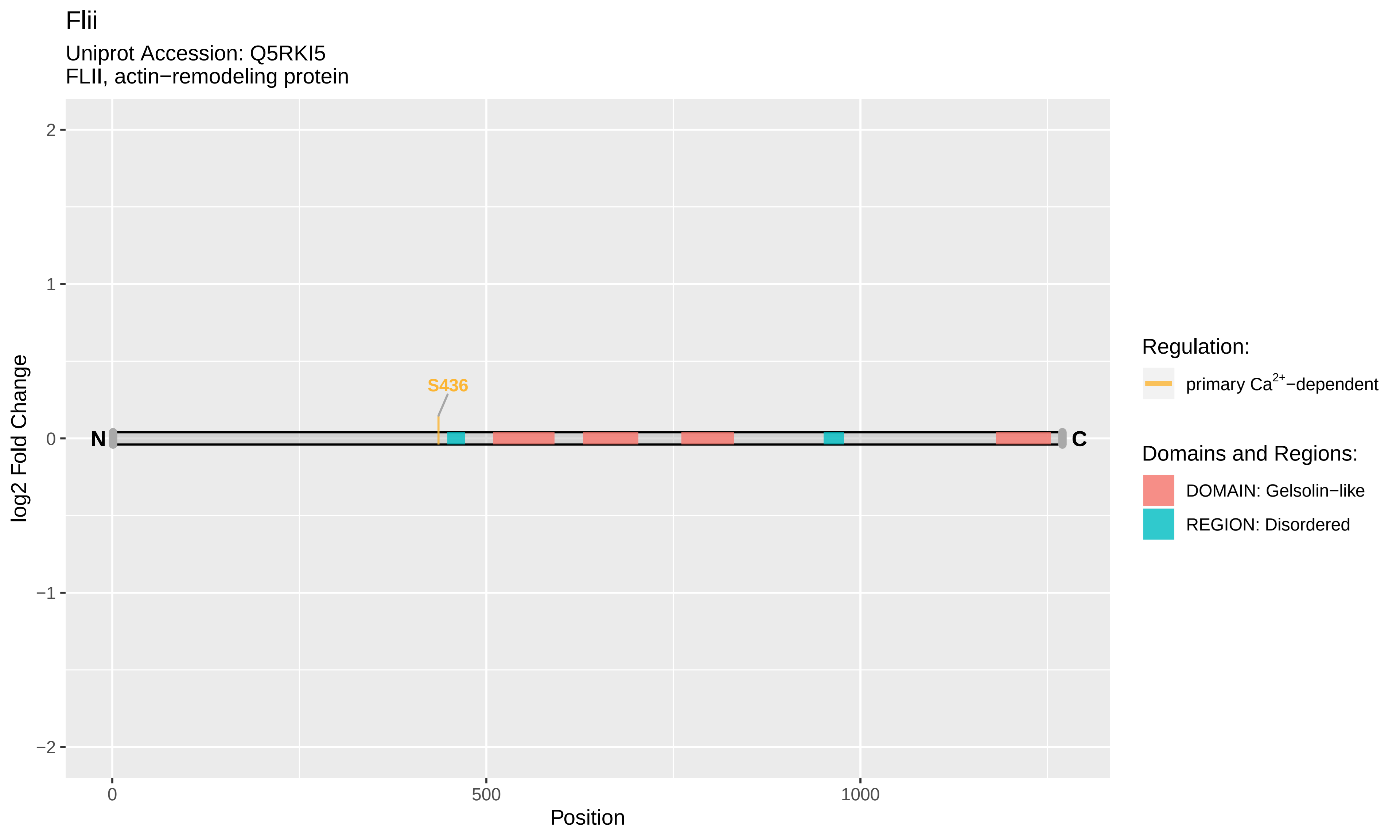
Flii
Uniprot Accession: Q5RKI5
FLII, actin−remodeling protein
log2 Fold Change
2
1
0
−1
−2
S436
N
C
0
500
1000
Position
Regulation:
primary Ca2+−dependent
Domains and Regions:
DOMAIN: Gelsolin−like
REGION: Disordered

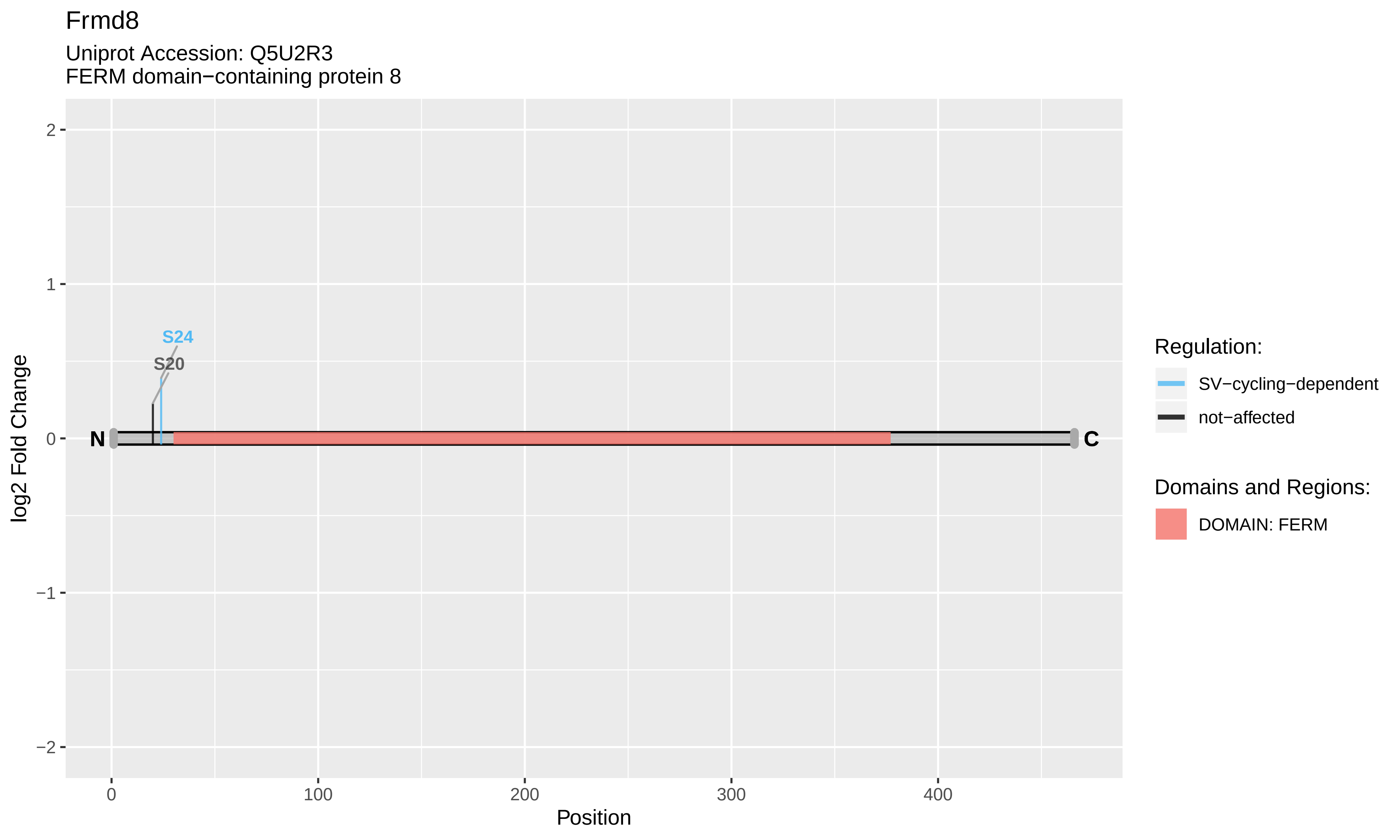
Frmd8
Uniprot Accession: Q5U2R3
FERM domain−containing protein 8
S24
S20
N
C
log2 Fold Change
2
1
0
−1
−2
0
100
200
300
400
Position
Regulation:
SV−cycling−dependent
not−affected
Domains and Regions:
DOMAIN: FERM

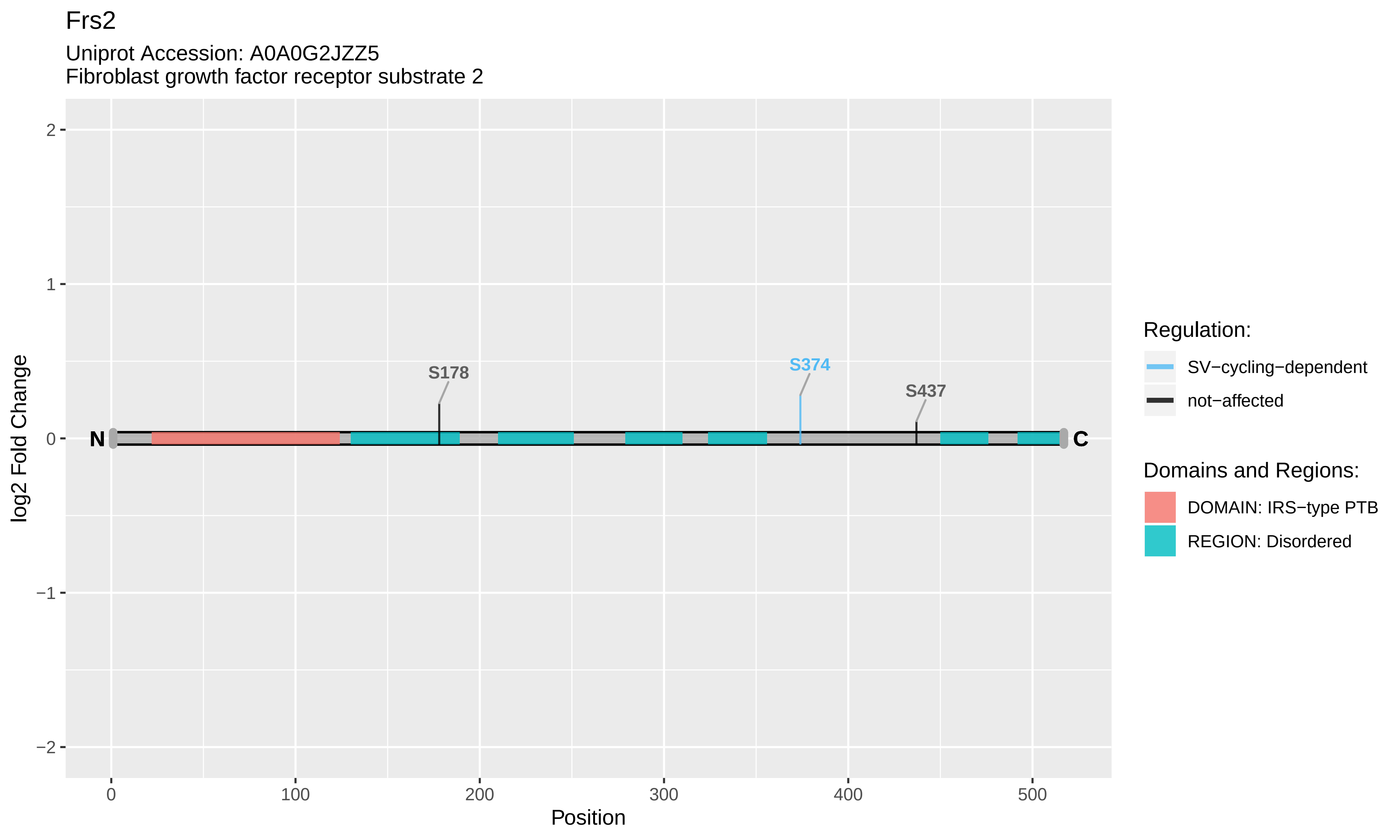
Frs2
Uniprot Accession: A0A0G2JZZ5
Fibroblast growth factor receptor substrate 2
log2 Fold Change
2
1
0
−1
−2
N
C
S178
S374
S437
0
100
200
300
400
500
Position
Regulation:
SV−cycling−dependent
not−affected
Domains and Regions:
DOMAIN: IRS−type PTB
REGION: Disordered

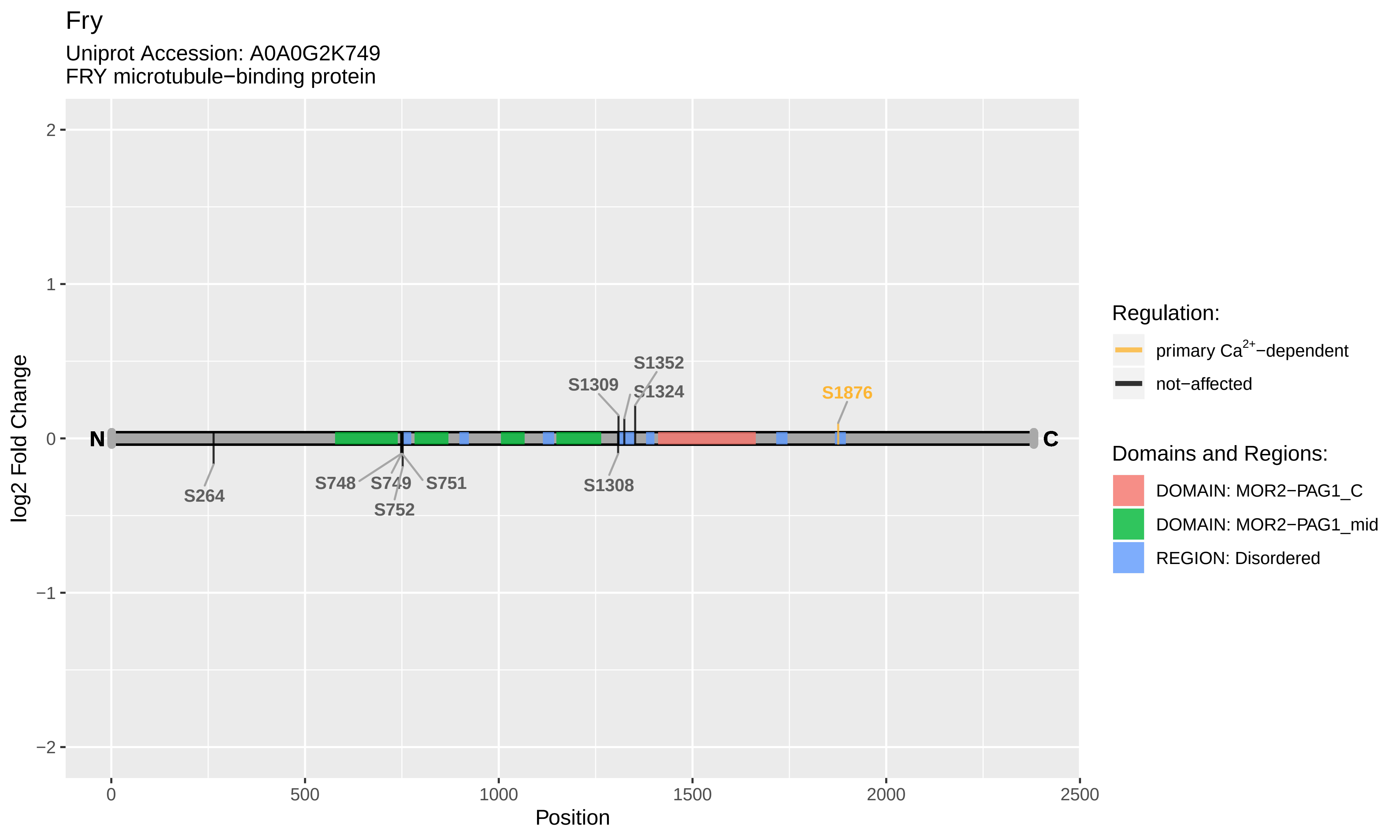
Fry
Uniprot Accession: A0A0G2K749
FRY microtubule−binding protein
log2 Fold Change
Position
N
C
S264
S748
S749
S751
S752
S1308
S1309
S1324
S1352
S1876
Regulation:
primary Ca2+−dependent
not−affected
Domains and Regions:
DOMAIN: MOR2−PAG1_C
DOMAIN: MOR2−PAG1_mid
REGION: Disordered

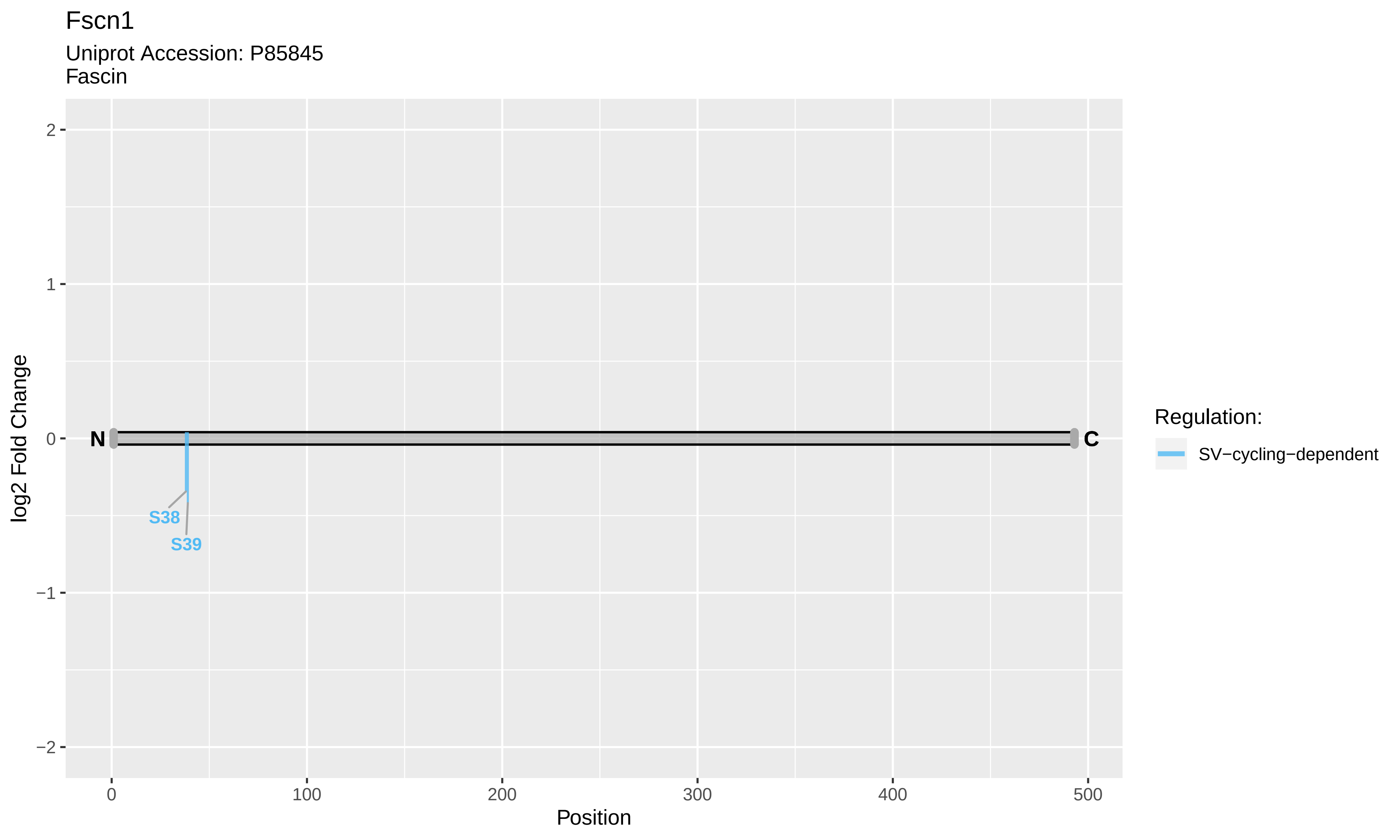

Fscn1
Uniprot Accession: P85845
Fascin
log2 Fold Change
2
1
0
−1
−2
N
C
S38
S39
0
100
200
300
400
500
Position
Regulation:
SV−cycling−dependent

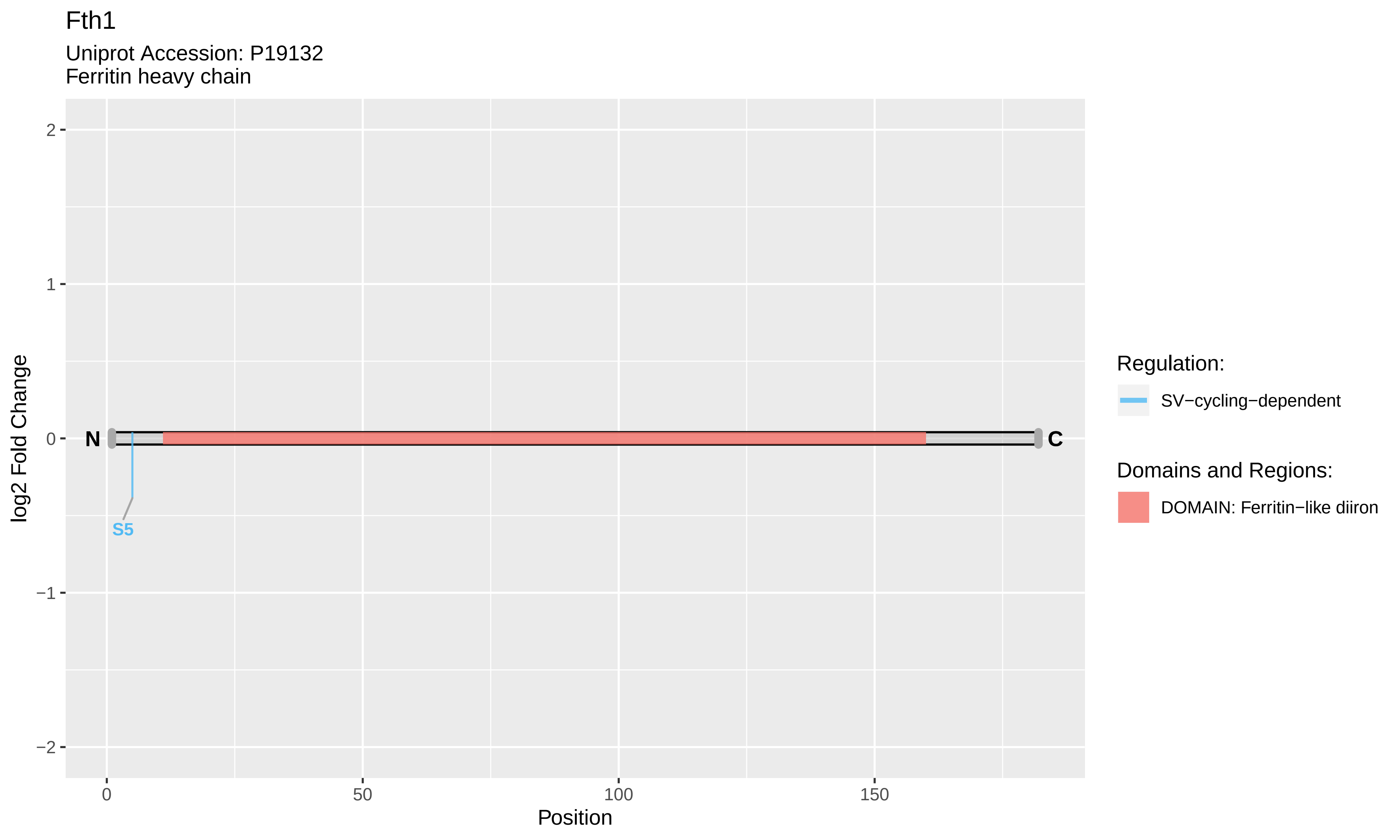

Fth1
Uniprot Accession: P19132
Ferritin heavy chain
log2 Fold Change
2
1
0
−1
−2
N
C
S5
0
50
100
150
Position
Regulation:
SV−cycling−dependent
Domains and Regions:
DOMAIN: Ferritin−like diiron

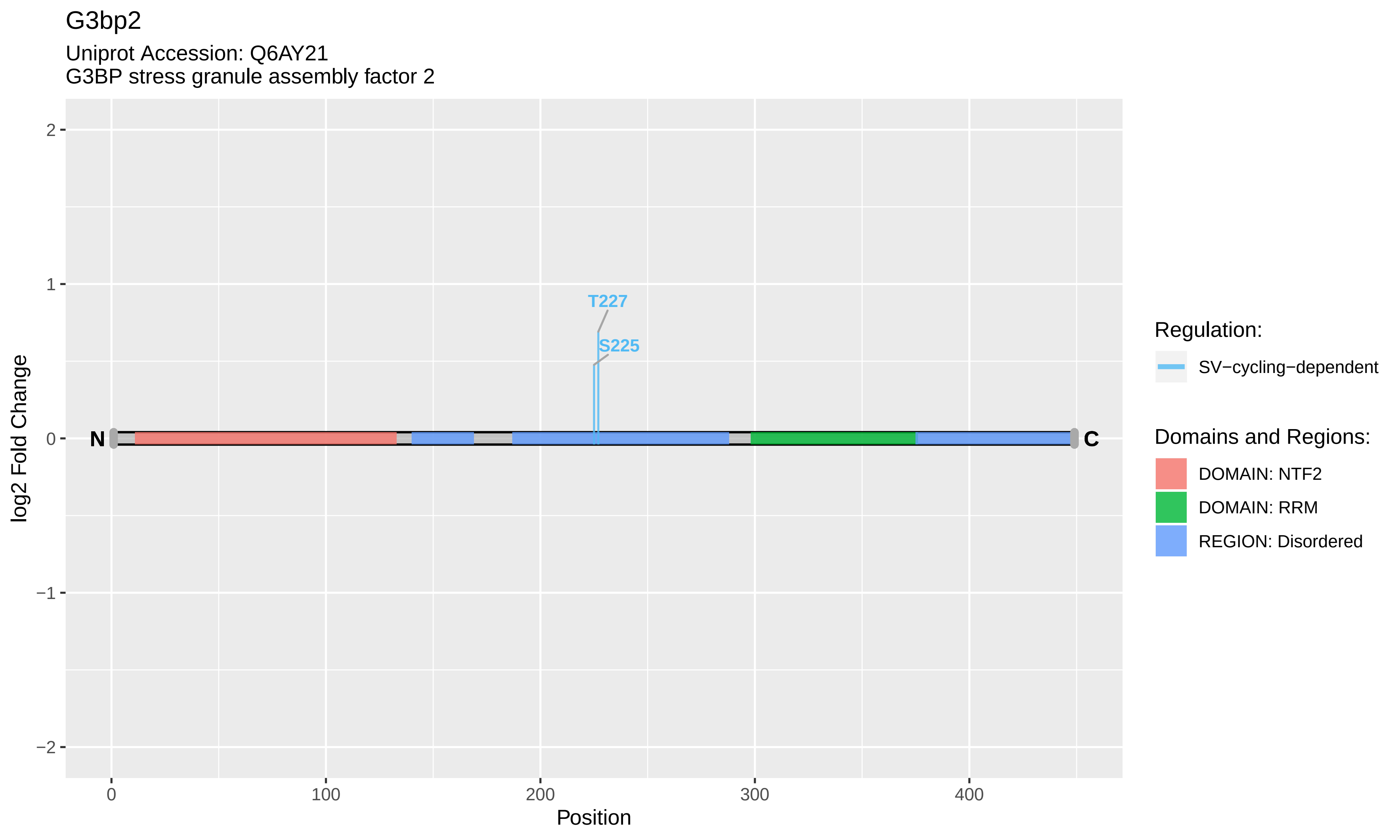
G3bp2
Uniprot Accession: Q6AY21
G3BP stress granule assembly factor 2
T227
S225
N
C
2
1
0
−1
−2
log2 Fold Change
0
100
200
300
400
Position
Regulation:
SV−cycling−dependent
Domains and Regions:
DOMAIN: NTF2
DOMAIN: RRM
REGION: Disordered

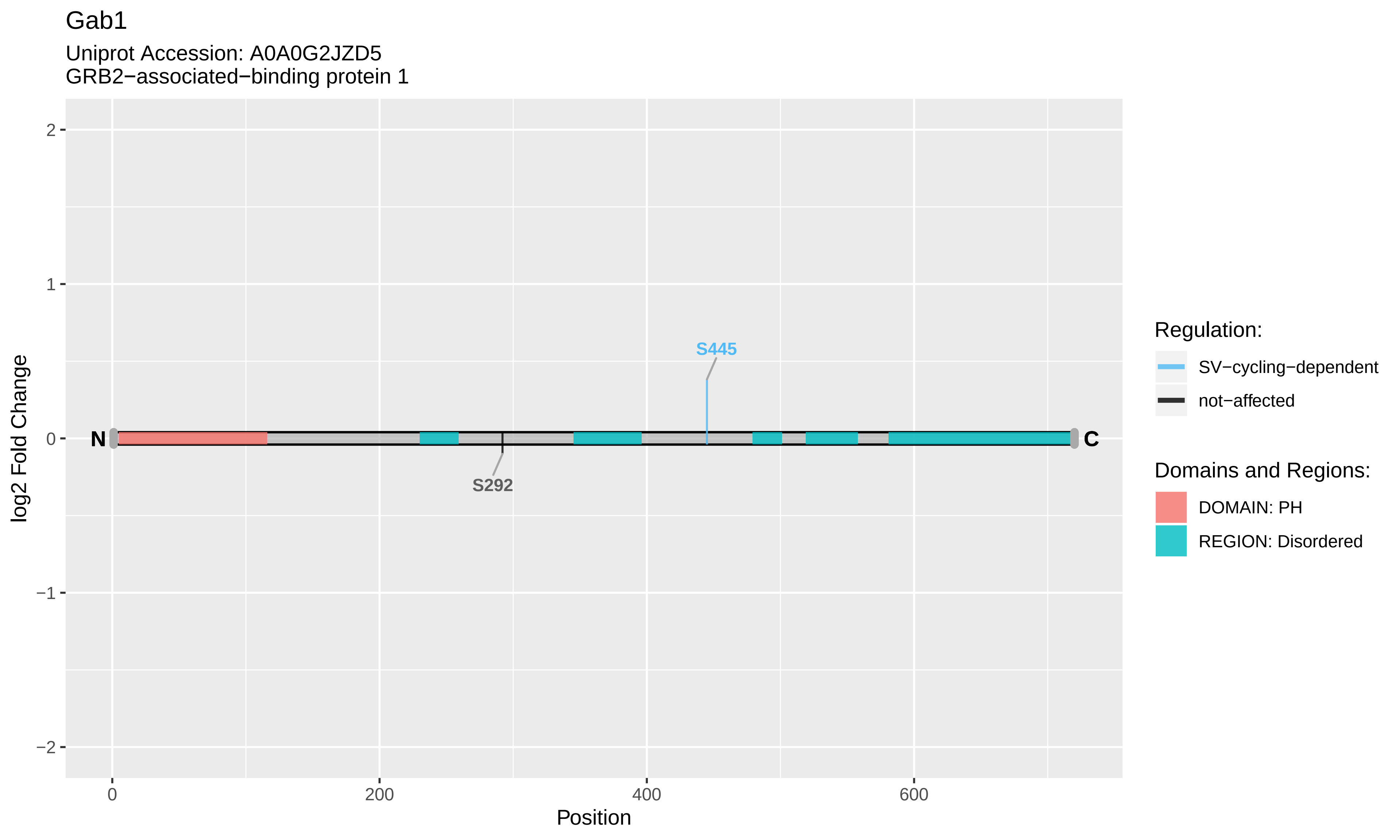
Gab1
Uniprot Accession: A0A0G2JZD5
GRB2−associated−binding protein 1
log2 Fold Change
2
1
0
−1
−2
N
C
S445
S292
0
200
400
600
Position
Regulation:
SV−cycling−dependent
not−affected
Domains and Regions:
DOMAIN: PH
REGION: Disordered

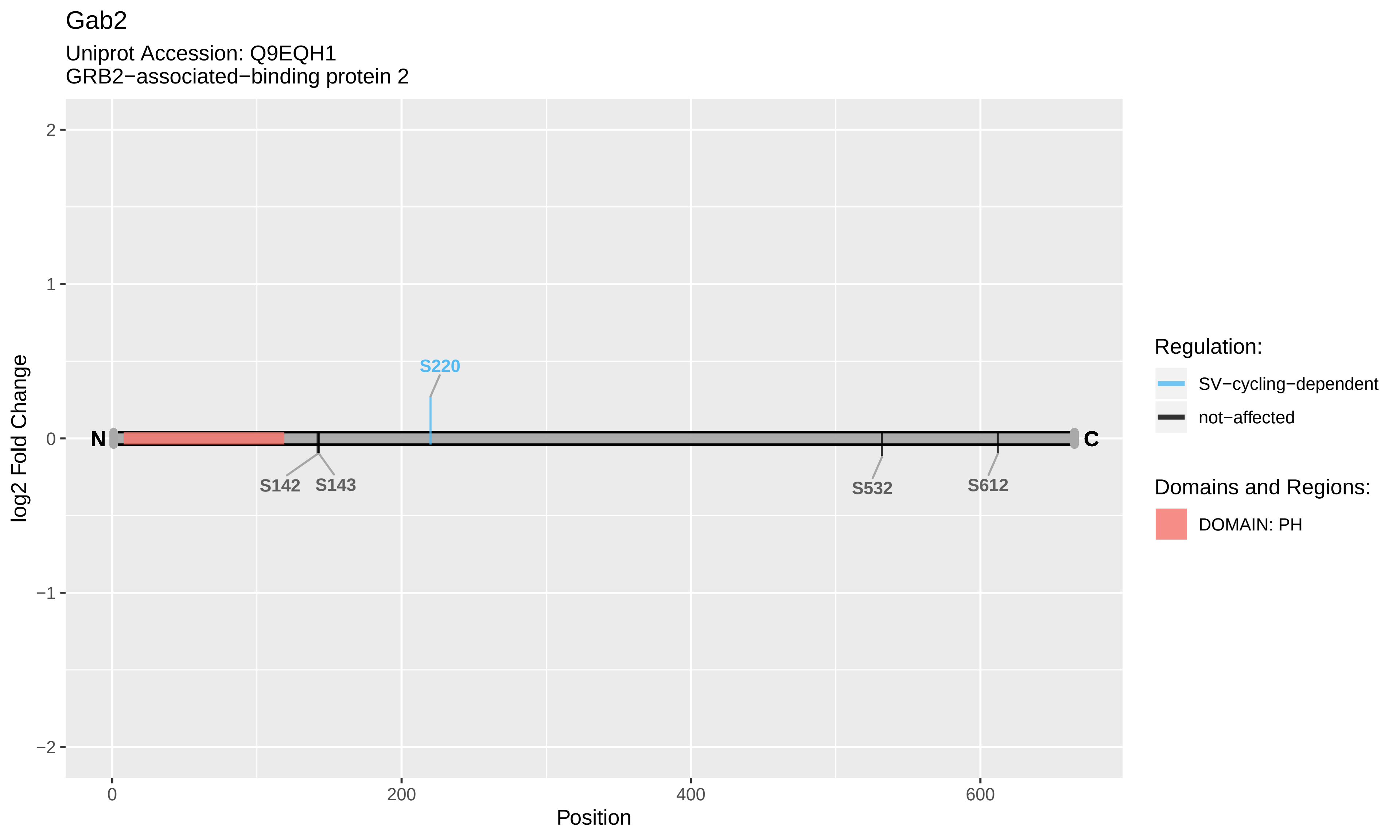
Gab2
Uniprot Accession: Q9EQH1
GRB2−associated−binding protein 2
log2 Fold Change
2
1
0
−1
−2
N
C
S220
S142
S143
S532
S612
0
200
400
600
Position
Regulation:
SV−cycling−dependent
not−affected
Domains and Regions:
DOMAIN: PH

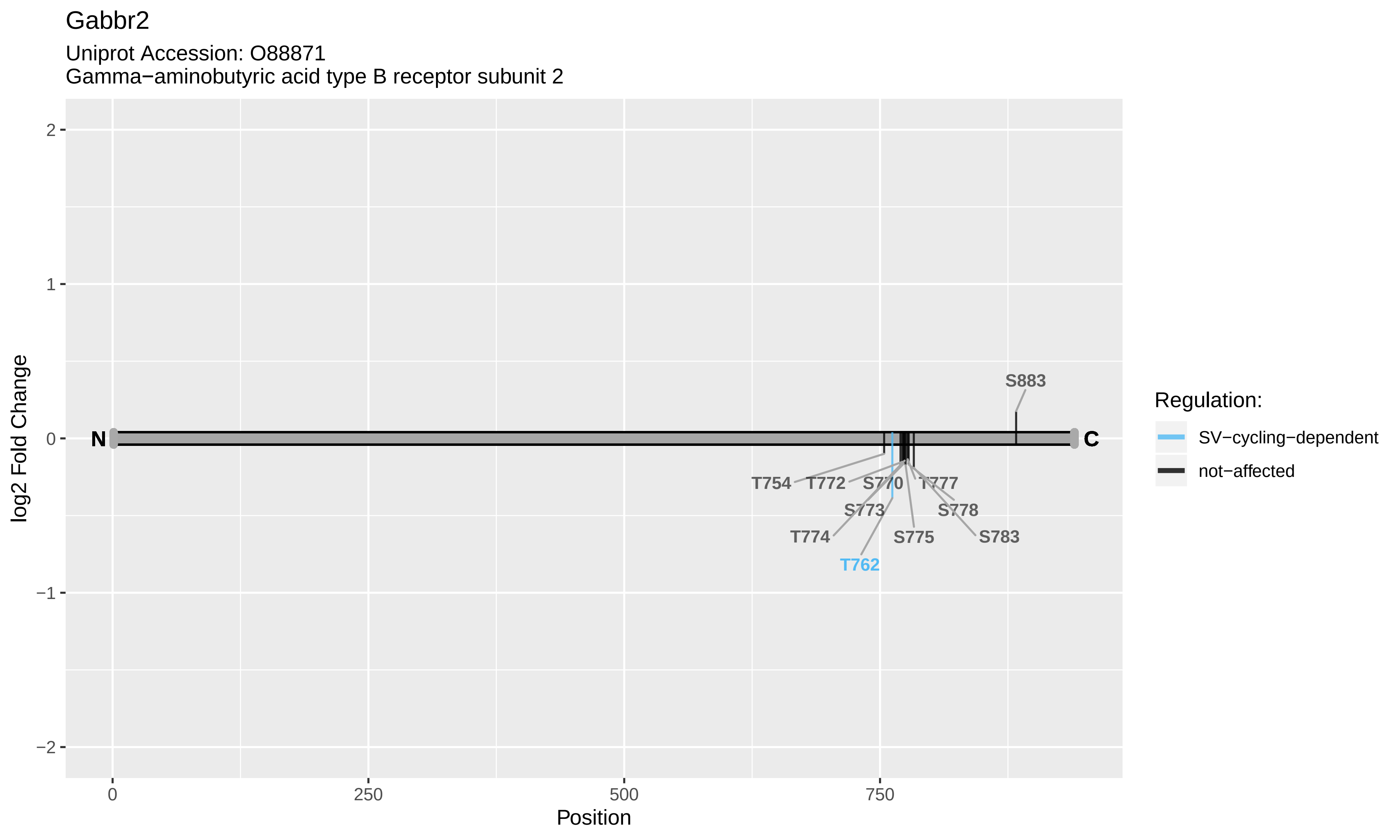
Gabbr2
Uniprot Accession: O88871
Gamma−aminobutyric acid type B receptor subunit 2
log2 Fold Change
Position
N
C
S883
T754
T772
S770
T777
S773
S778
T774
S775
S783
T762
Regulation:
SV−cycling−dependent
not−affected
2
1
0
−1
−2
0
250
500
750

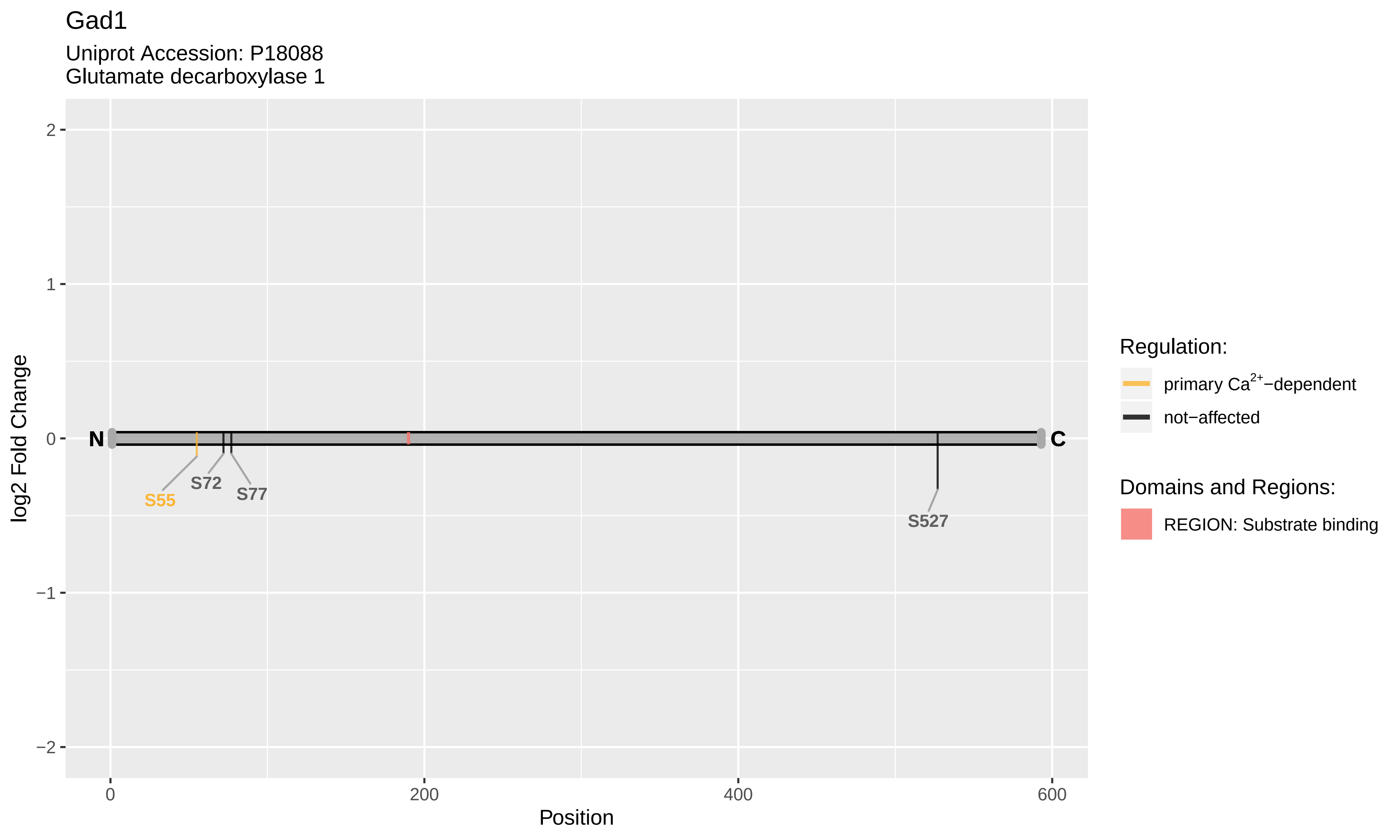

Gad1
Uniprot Accession: P18088
Glutamate decarboxylase 1
log2 Fold Change
2
1
0
−1
−2
N
C
S55
S72
S77
S527
0
200
400
600
Position
Regulation:
primary Ca2+−dependent
not−affected
Domains and Regions:
REGION: Substrate binding

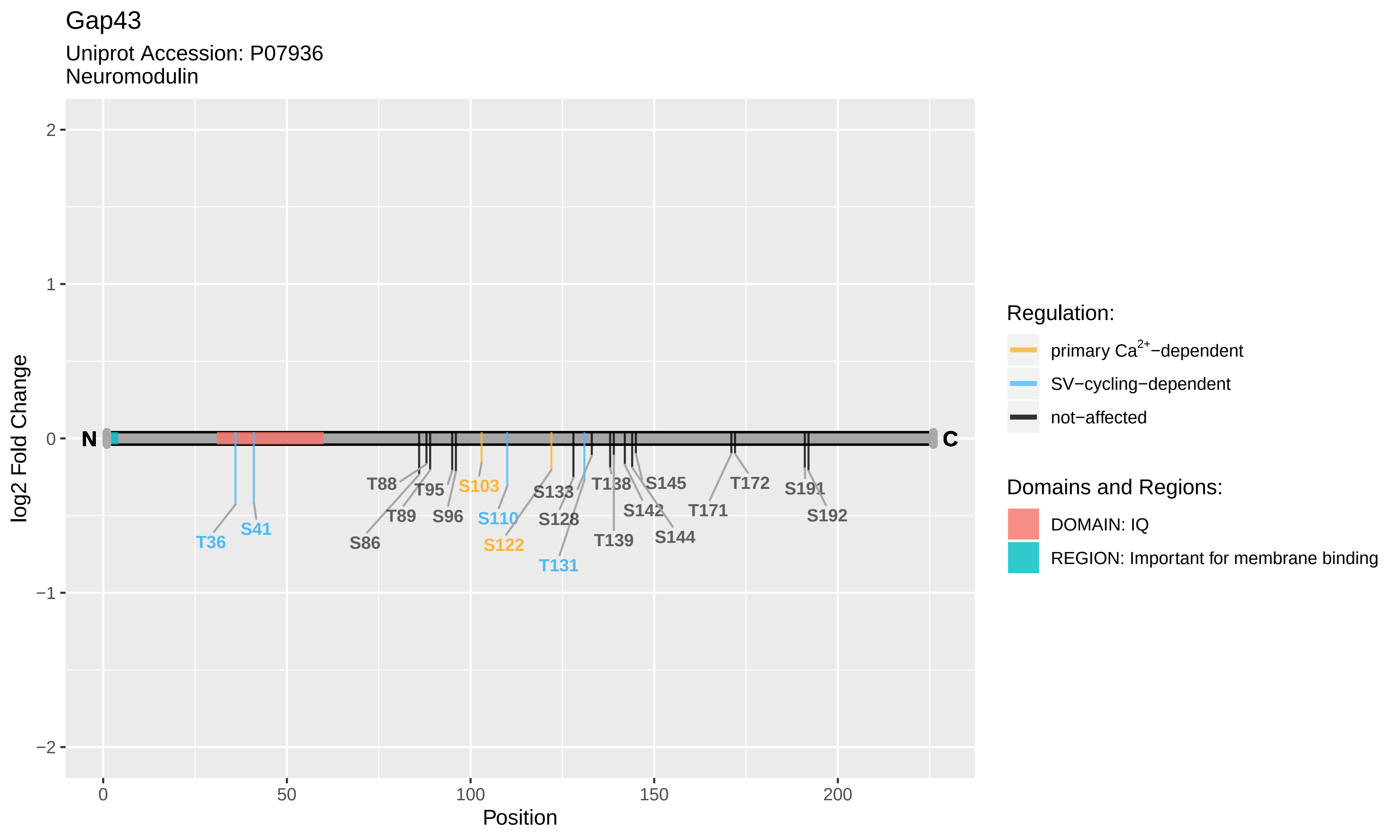

Gap43
Uniprot Accession: P07936
Neuromodulin
log2 Fold Change
Position
N
C
T36
S41
S86
T88
T89
T95
S96
S103
S110
S122
S128
T131
S133
T138
T139
S142
S144
S145
T171
T172
S191
S192
Regulation:
primary Ca2+−dependent
SV−cycling−dependent
not−affected
Domains and Regions:
DOMAIN: IQ
REGION: Important for membrane binding

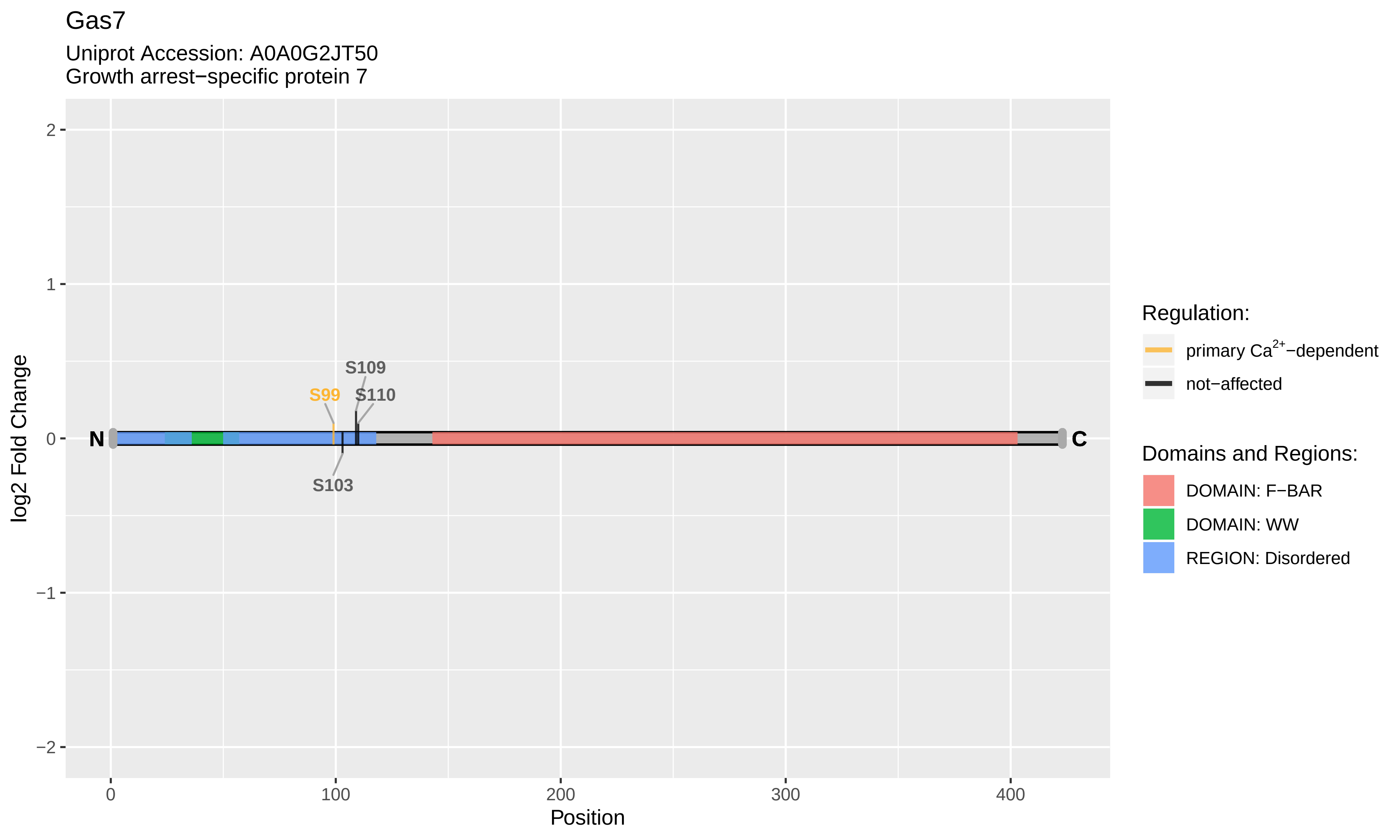

Gas7
Uniprot Accession: A0A0G2JT50
Growth arrest−specific protein 7
log2 Fold Change
Position
2
1
0
−1
−2
0
100
200
300
400
N
C
S109
S99
S110
S103
Regulation:
primary Ca2+−dependent
not−affected
Domains and Regions:
DOMAIN: F−BAR
DOMAIN: WW
REGION: Disordered

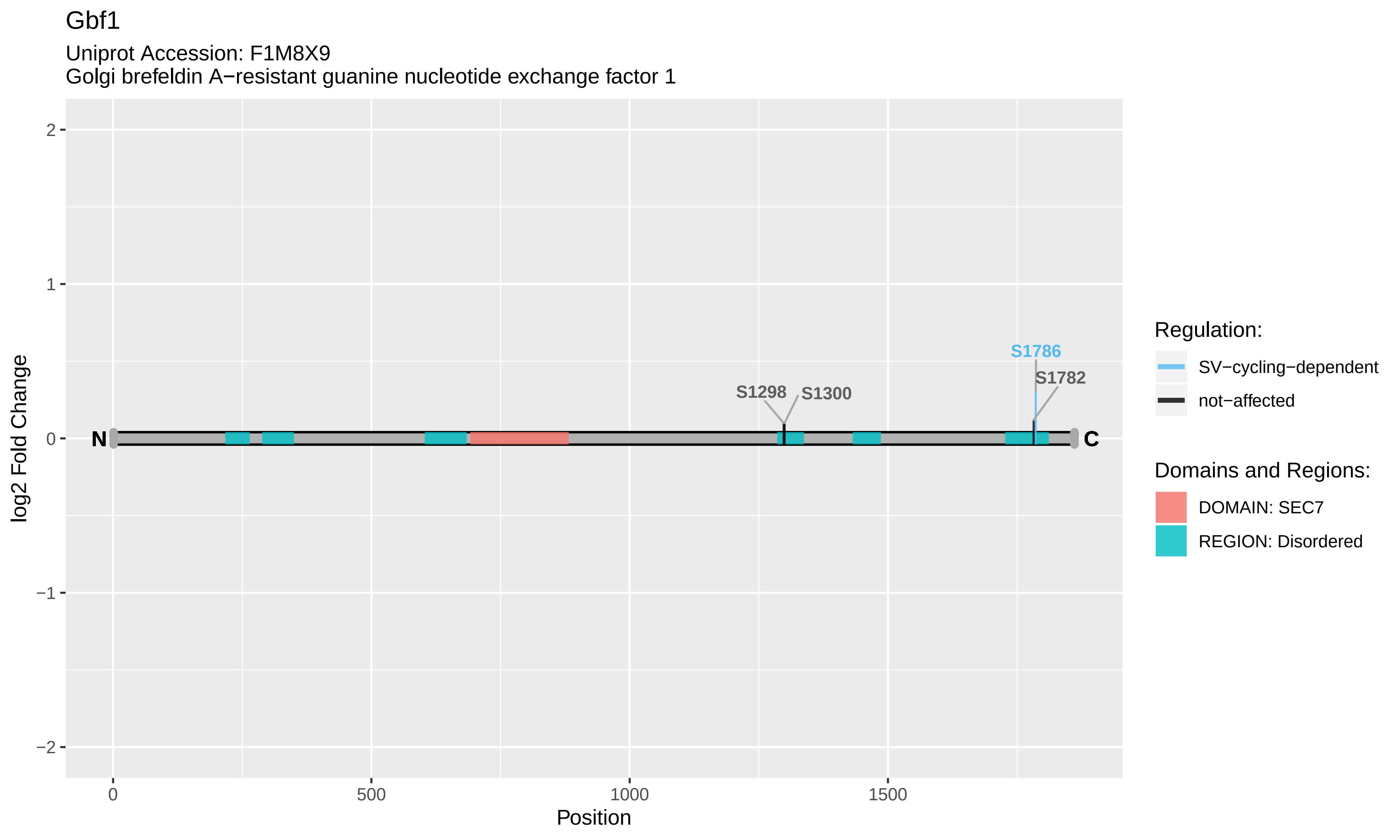
Gbf1
Uniprot Accession: F1M8X9
Golgi brefeldin A−resistant guanine nucleotide exchange factor 1
log2 Fold Change
2
1
0
−1
−2
N
C
S1298
S1300
S1786
S1782
0
500
1000
1500
Position
Regulation:
SV−cycling−dependent
not−affected
Domains and Regions:
DOMAIN: SEC7
REGION: Disordered

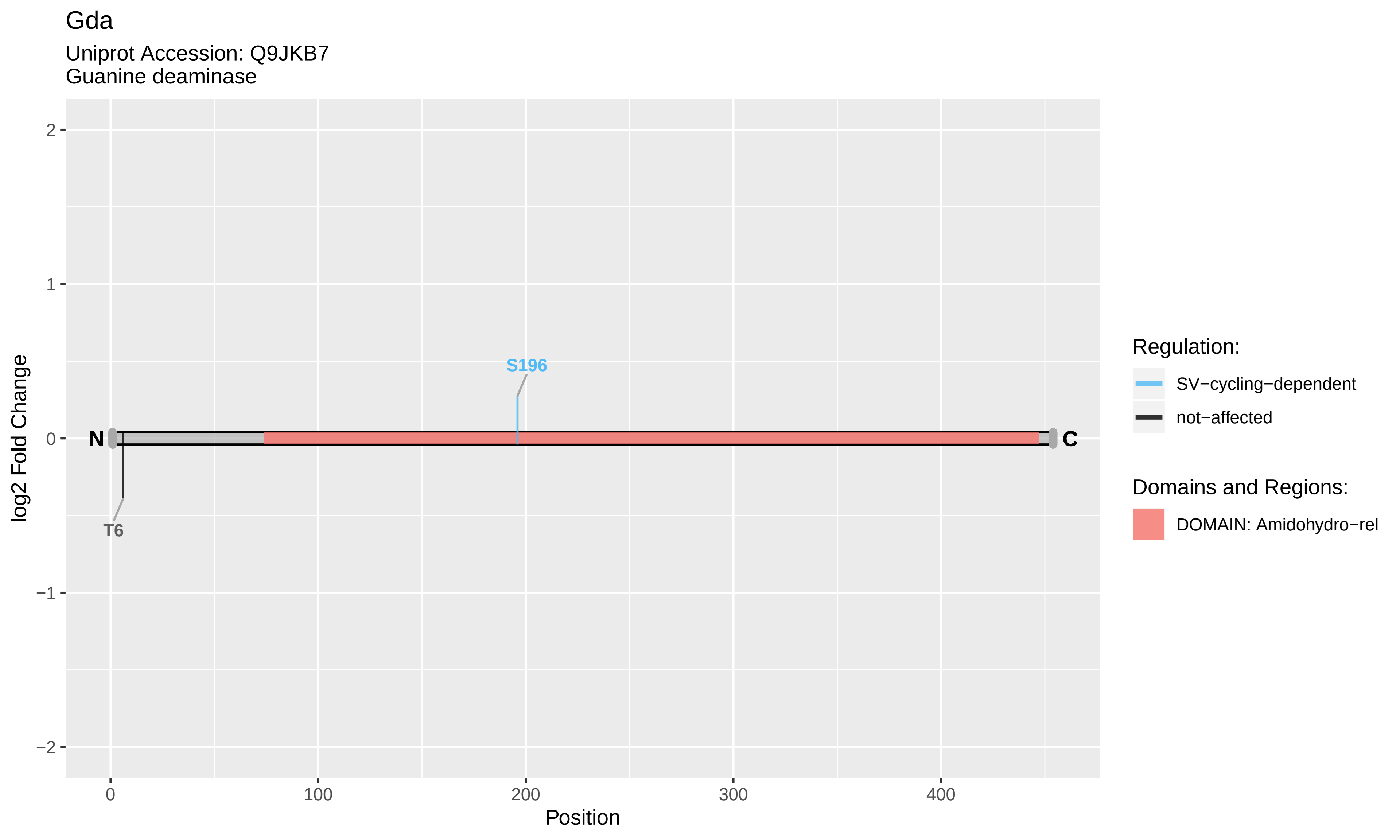

Gda
Uniprot Accession: Q9JKB7
Guanine deaminase
S196
T6
N
C
log2 Fold Change
2
1
0
−1
−2
0
100
200
300
400
Position
Regulation:
SV−cycling−dependent
not−affected
Domains and Regions:
DOMAIN: Amidohydro−rel

Gipc1

Uniprot Accession: Q9Z254
PDZ domain−containing protein GIPC1

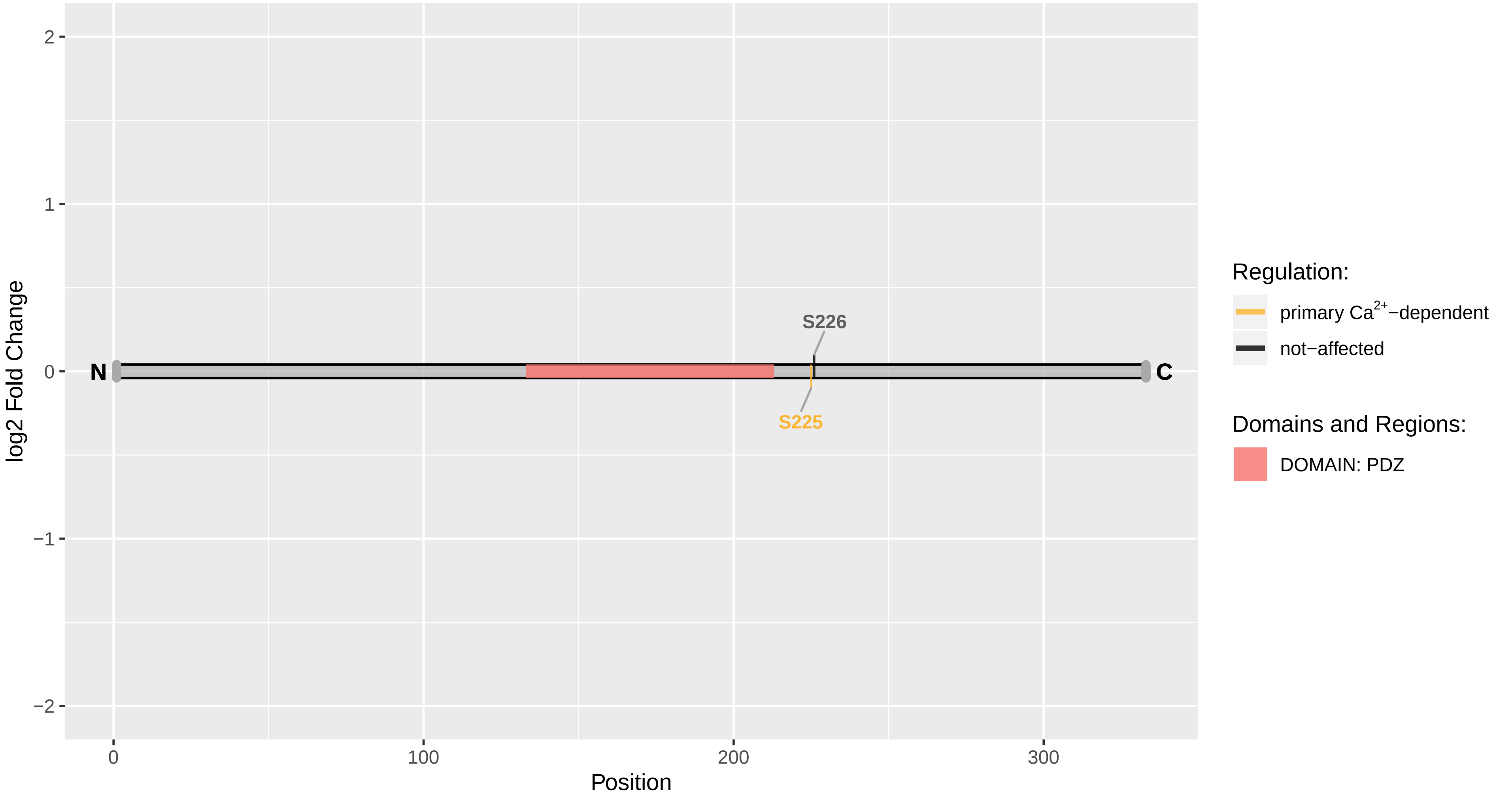

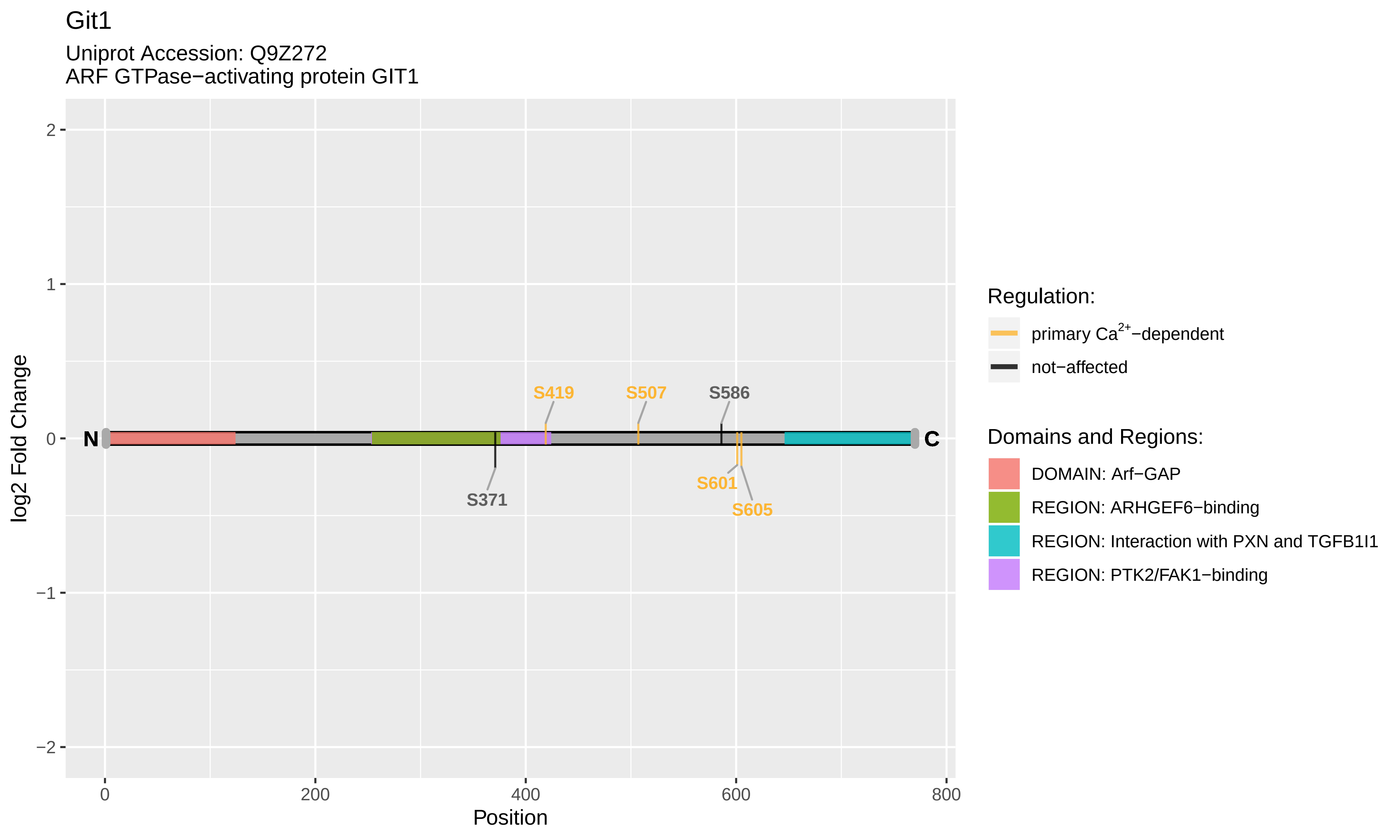

Git1
Uniprot Accession: Q9Z272
ARF GTPase−activating protein GIT1
log2 Fold Change
2
1
0
−1
−2
N
C
S419
S507
S586
S371
S601
S605
0
200
400
600
800
Position
Regulation:
primary Ca2+−dependent
not−affected
Domains and Regions:
DOMAIN: Arf−GAP
REGION: ARHGEF6−binding
REGION: Interaction with PXN and TGFB1I1
REGION: PTK2/FAK1−binding

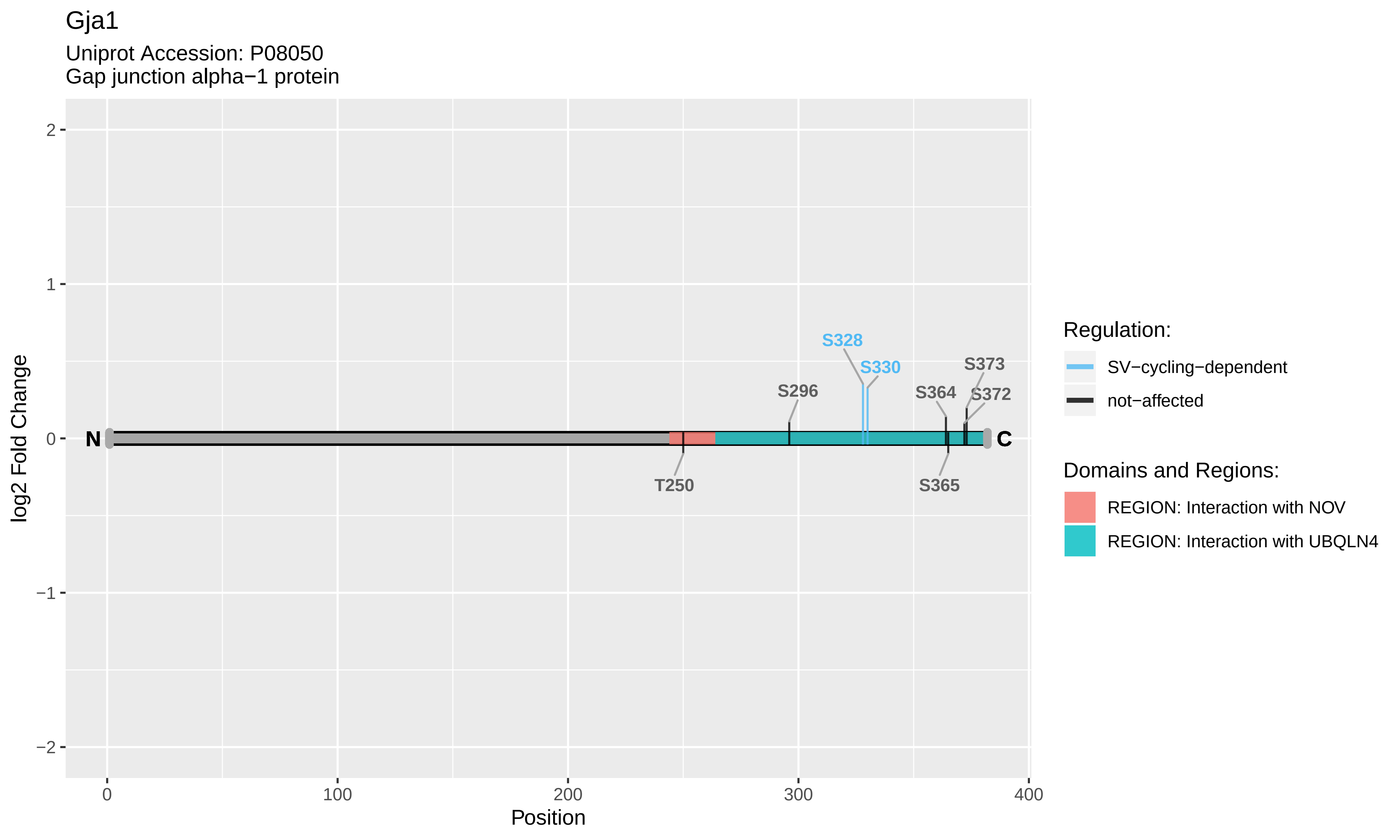

Gja1
Uniprot Accession: P08050
Gap junction alpha−1 protein
log2 Fold Change
2
1
0
−1
−2
Position
0
100
200
300
400
N
C
S328
S330
S296
S364
S373
S372
T250
S365
Regulation:
SV−cycling−dependent
not−affected
Domains and Regions:
REGION: Interaction with NOV
REGION: Interaction with UBQLN4

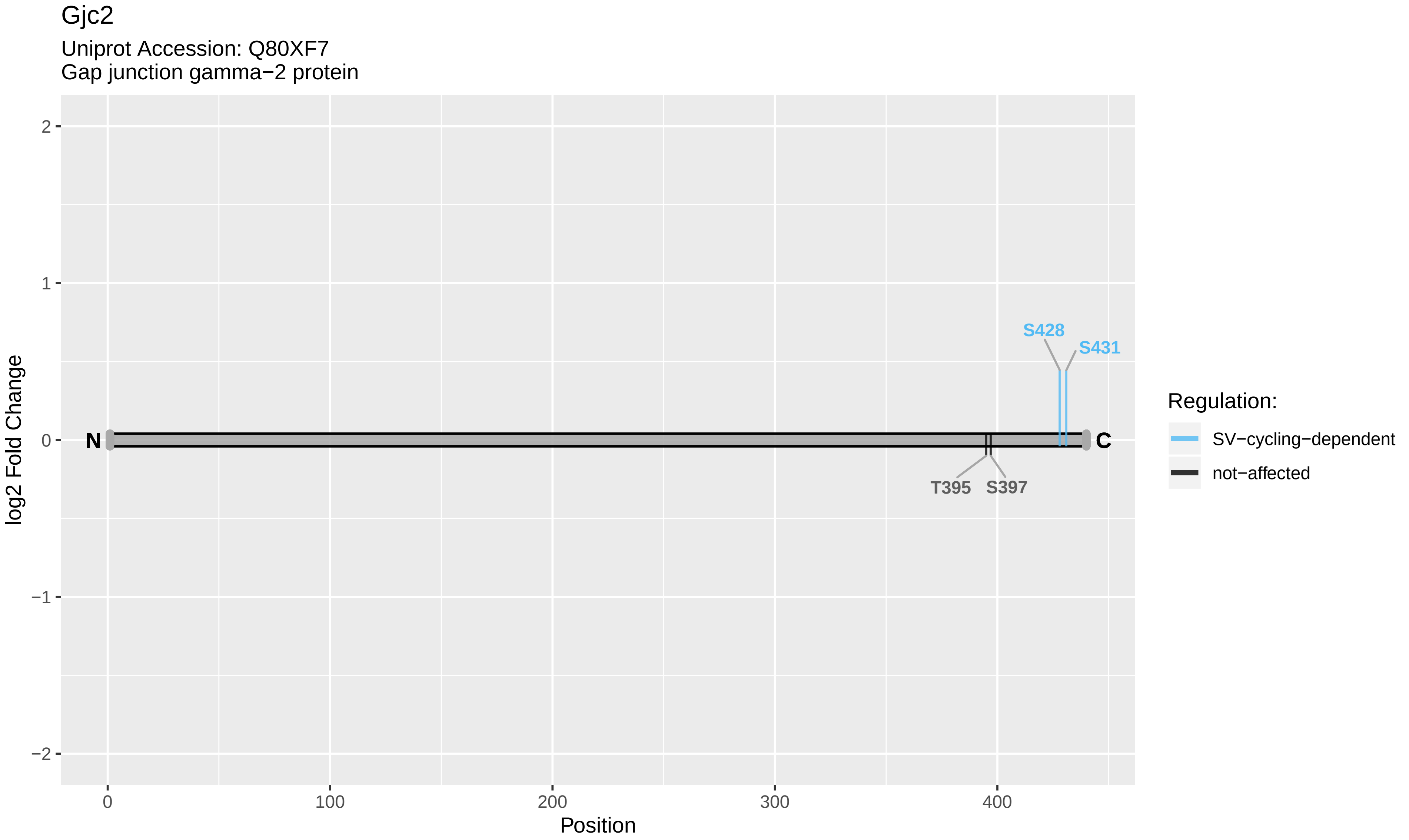

Gjc2
Uniprot Accession: Q80XF7
Gap junction gamma−2 protein
log2 Fold Change
2
1
0
−1
−2
N
C
S428
S431
T395
S397
0
100
200
300
400
Position
Regulation:
SV−cycling−dependent
not−affected

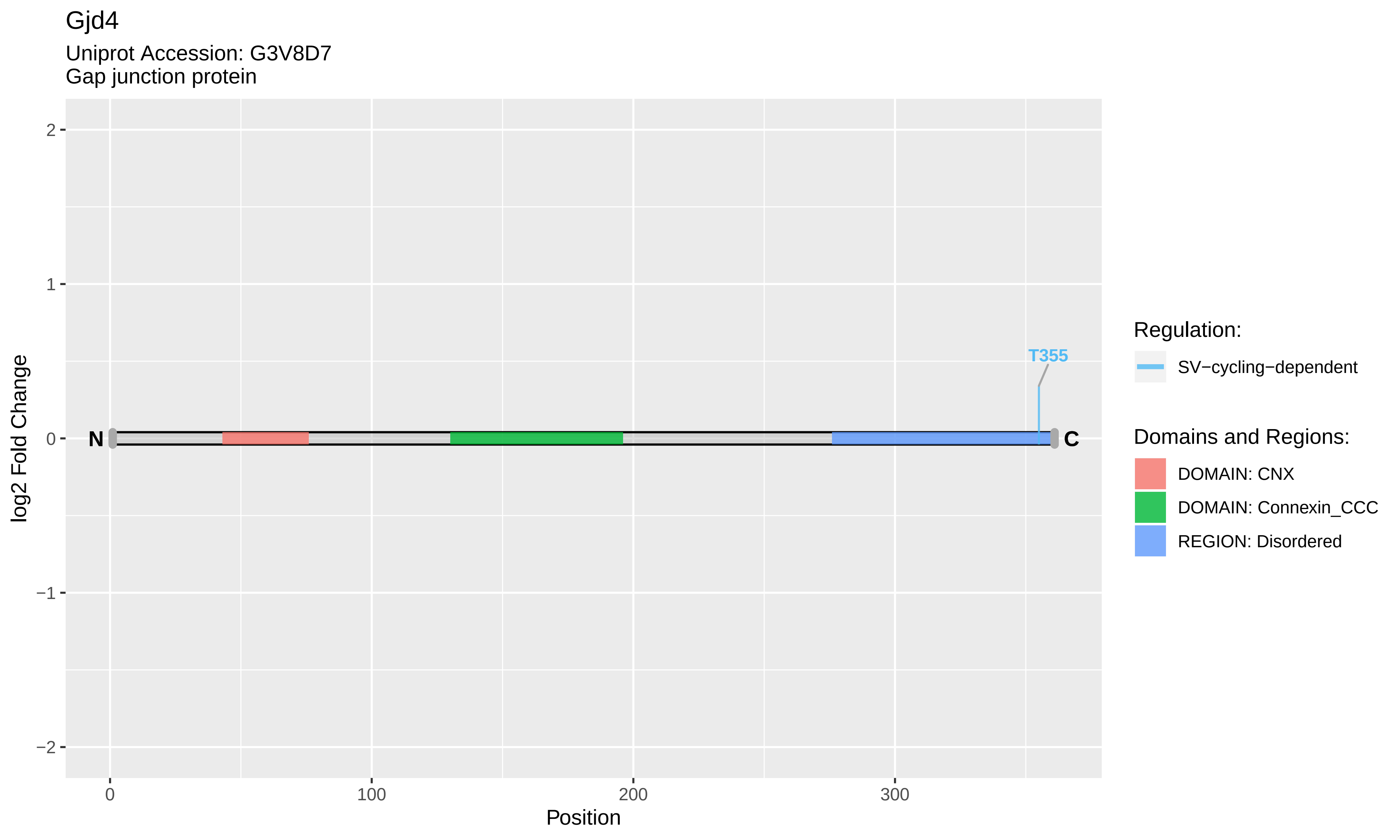
Gjd4
Uniprot Accession: G3V8D7
Gap junction protein
log2 Fold Change
2
1
0
−1
−2
N
C
T355
0
100
200
300
Position
Regulation:
SV−cycling−dependent
Domains and Regions:
DOMAIN: CNX
DOMAIN: Connexin_CCC
REGION: Disordered

Glrx5

Uniprot Accession: D4ADD7
Glutaredoxin 5

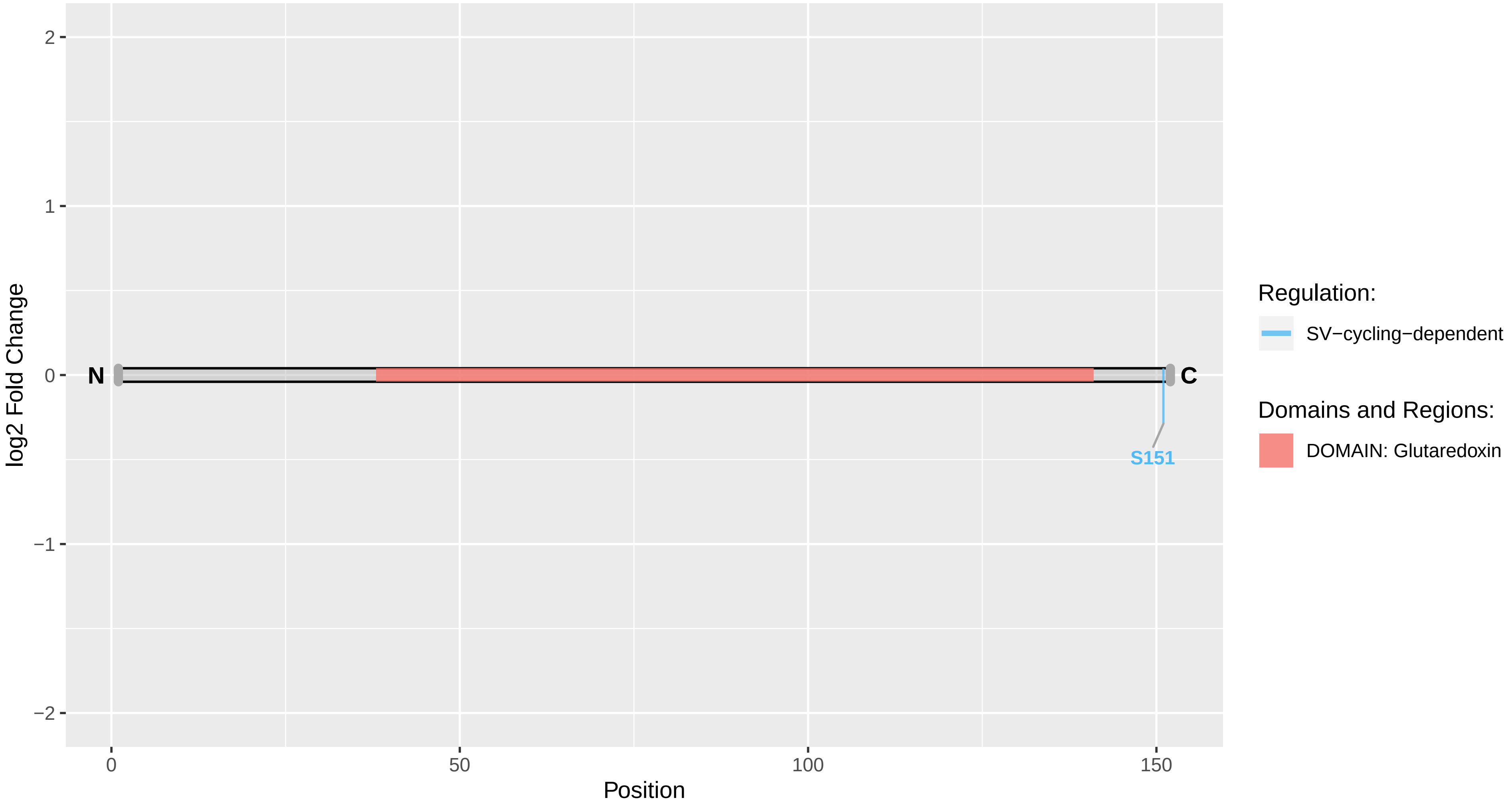

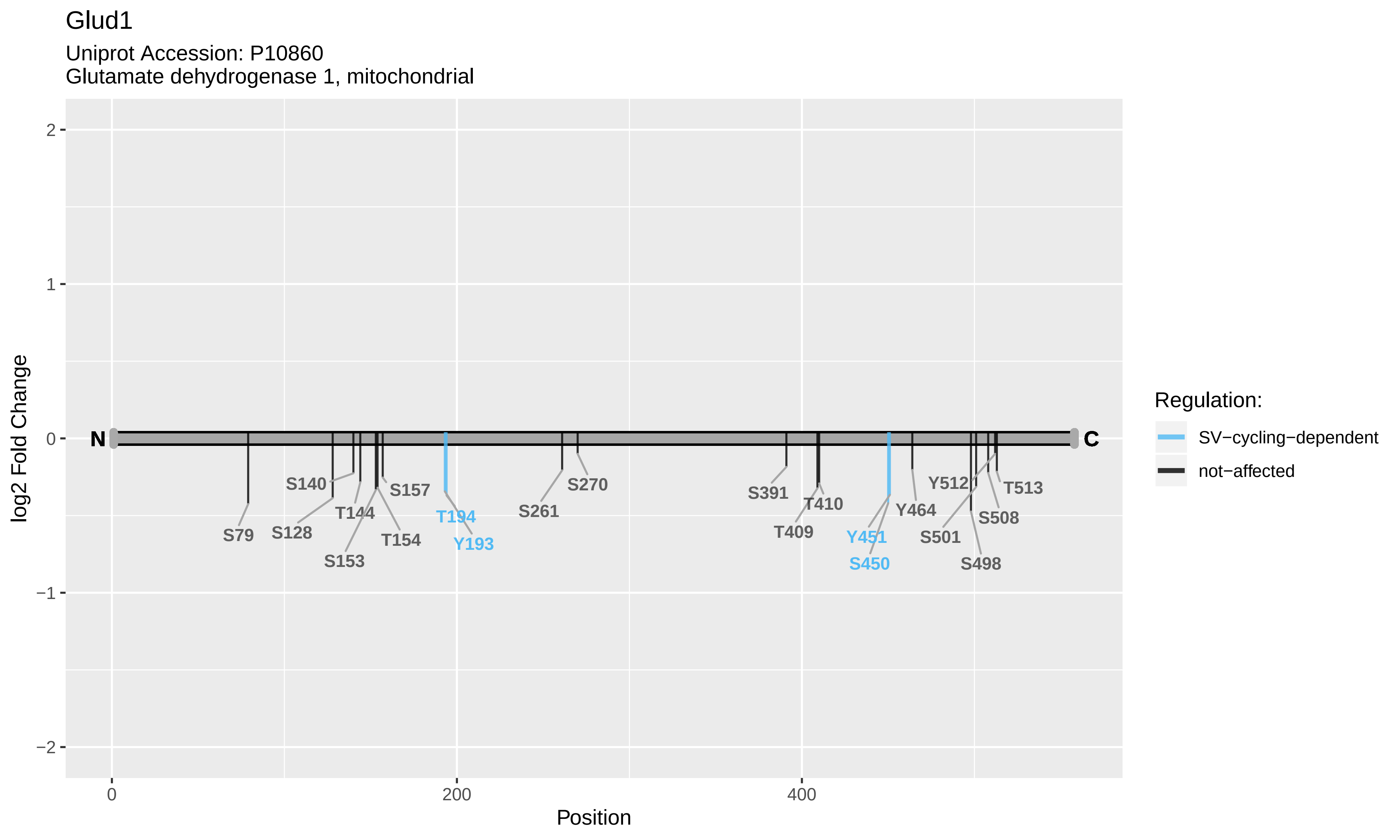
Glud1
Uniprot Accession: P10860
Glutamate dehydrogenase 1, mitochondrial
log2 Fold Change
2
1
0
−1
−2
N
C
S79
S128
S140
T144
S153
T154
S157
T194
Y193
S261
S270
S391
T409
T410
Y451
S450
Y464
S498
S501
S508
Y512
T513
0
200
400
Position
Regulation:
SV−cycling−dependent
not−affected

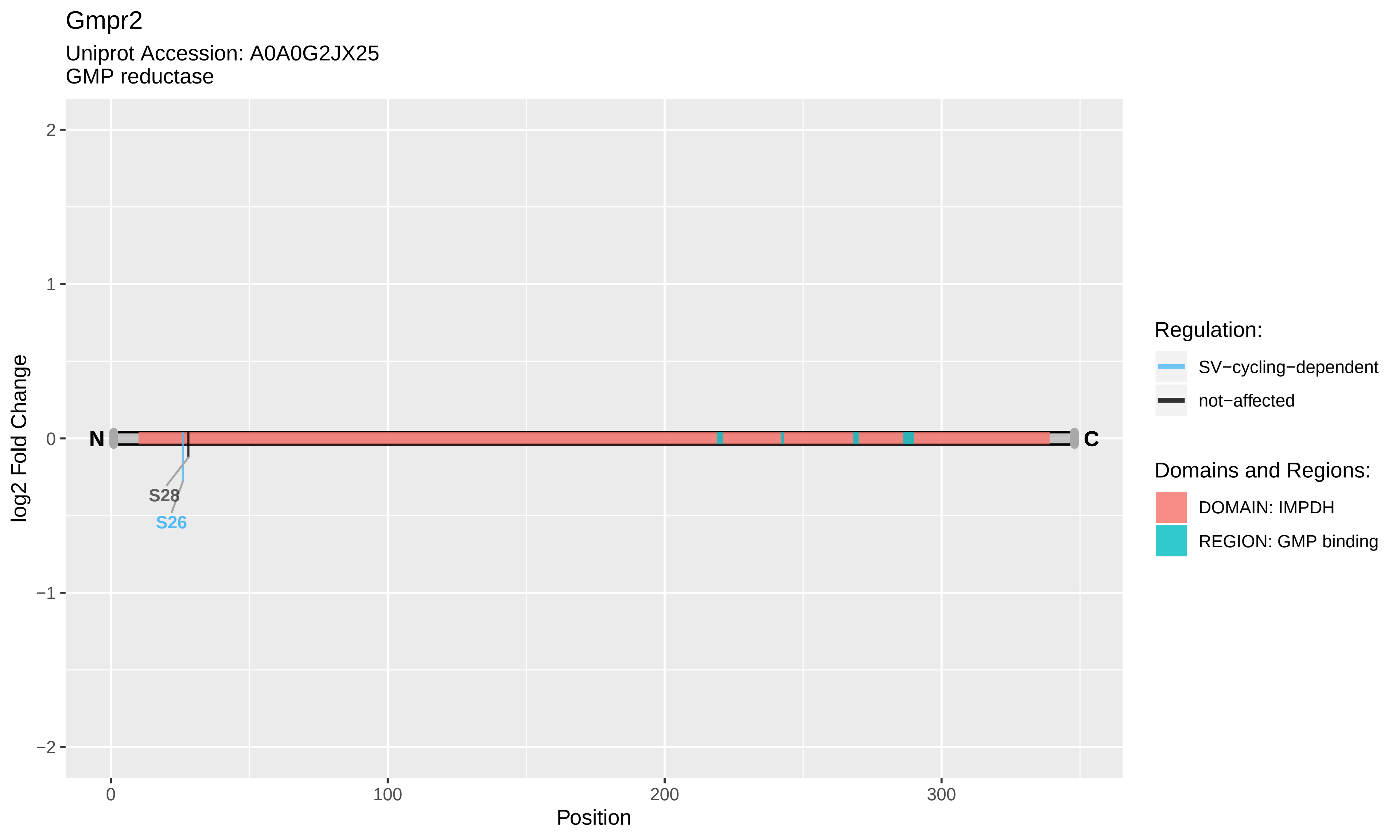

Gmpr2
Uniprot Accession: A0A0G2JX25
GMP reductase
log2 Fold Change
Position
N
C
S28
S26
Regulation:
SV−cycling−dependent
not−affected
Domains and Regions:
DOMAIN: IMPDH
REGION: GMP binding
2
1
0
−1
−2
0
100
200
300

Gnb1

Uniprot Accession: P54311
Guanine nucleotide−binding protein G(I)/G(S)/G(T) subunit beta−1

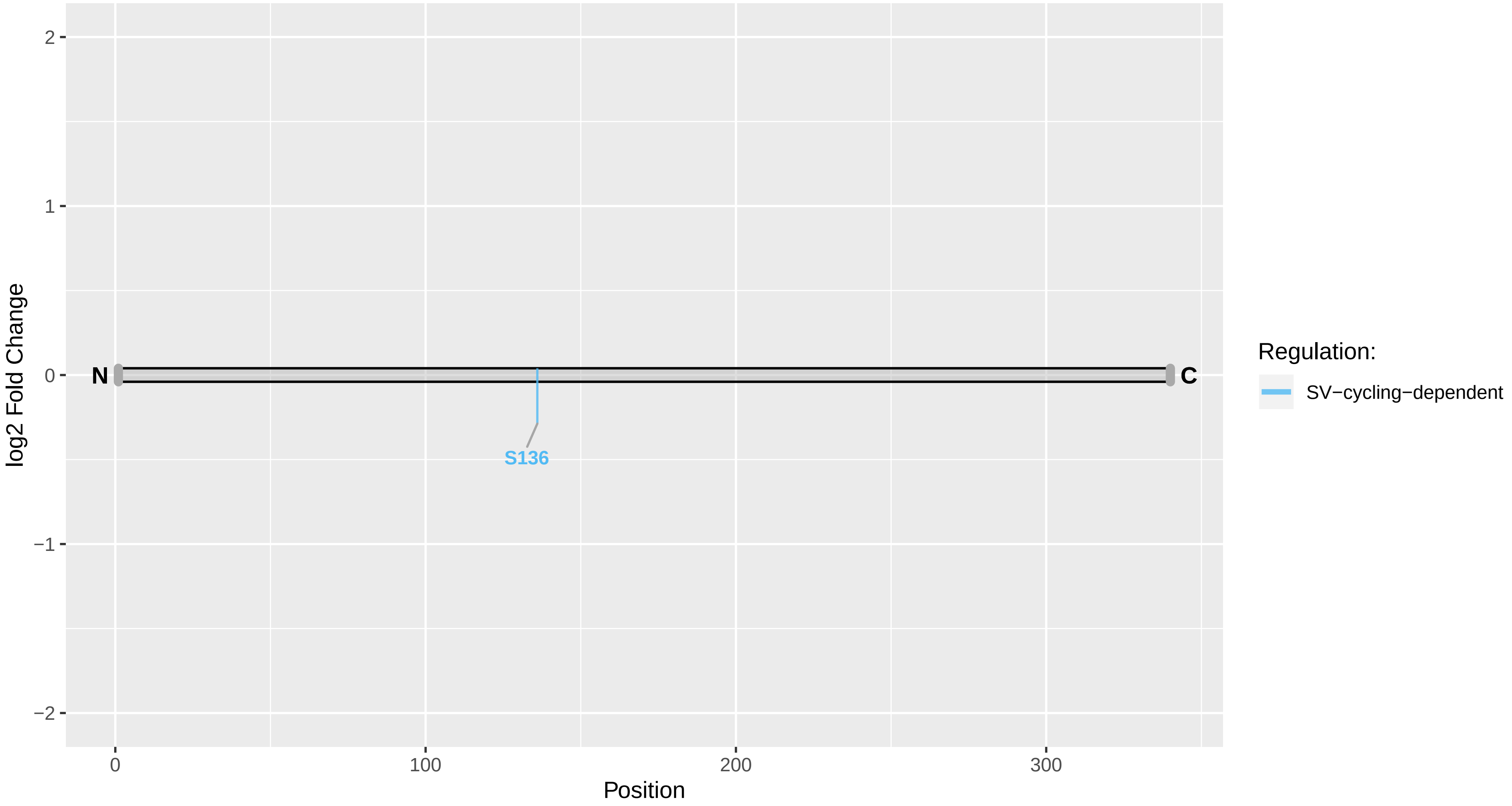

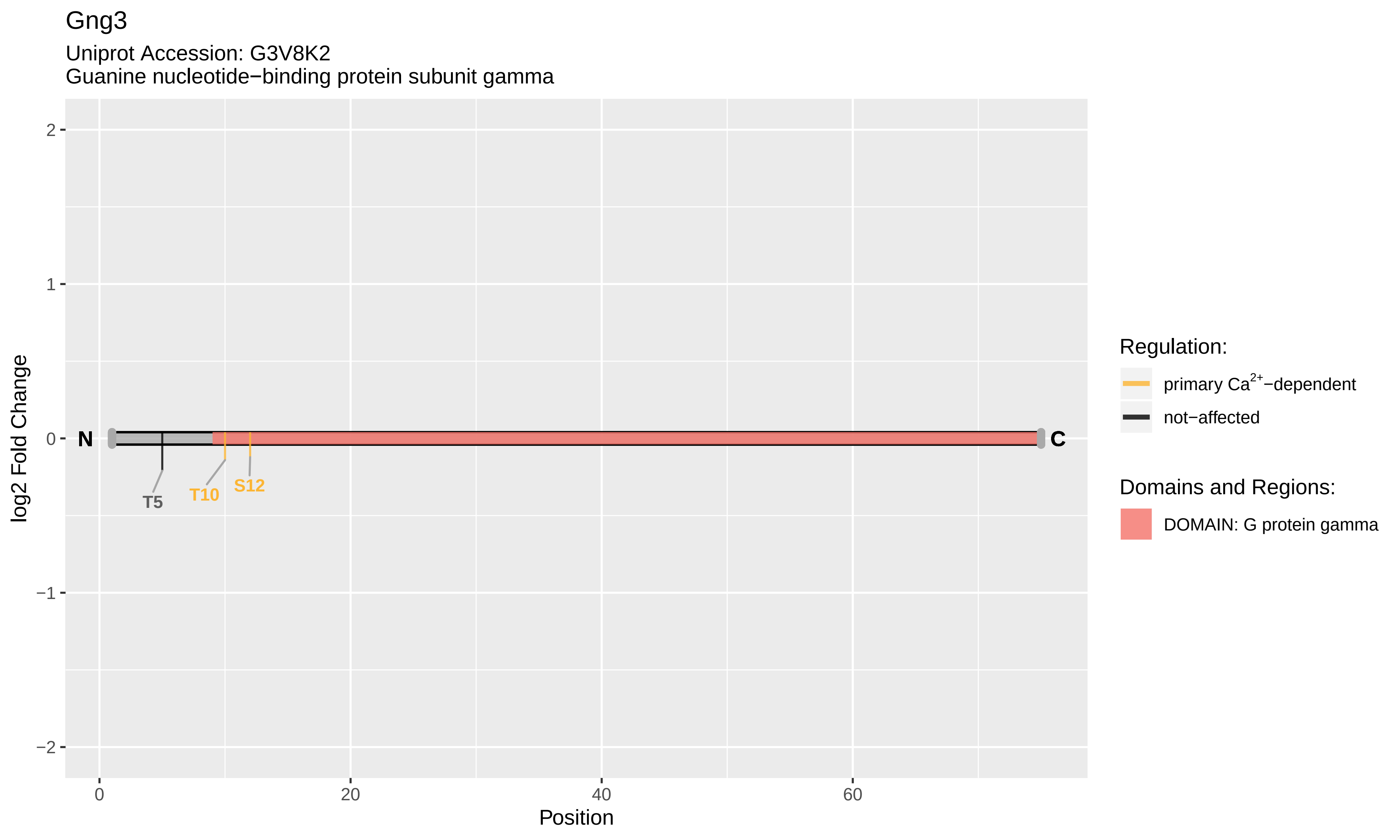
Gng3
Uniprot Accession: G3V8K2
Guanine nucleotide−binding protein subunit gamma
log2 Fold Change
2
1
0
−1
−2
N
C
T5
T10
S12
0
20
40
60
Position
Regulation:
primary Ca2+−dependent
not−affected
Domains and Regions:
DOMAIN: G protein gamma

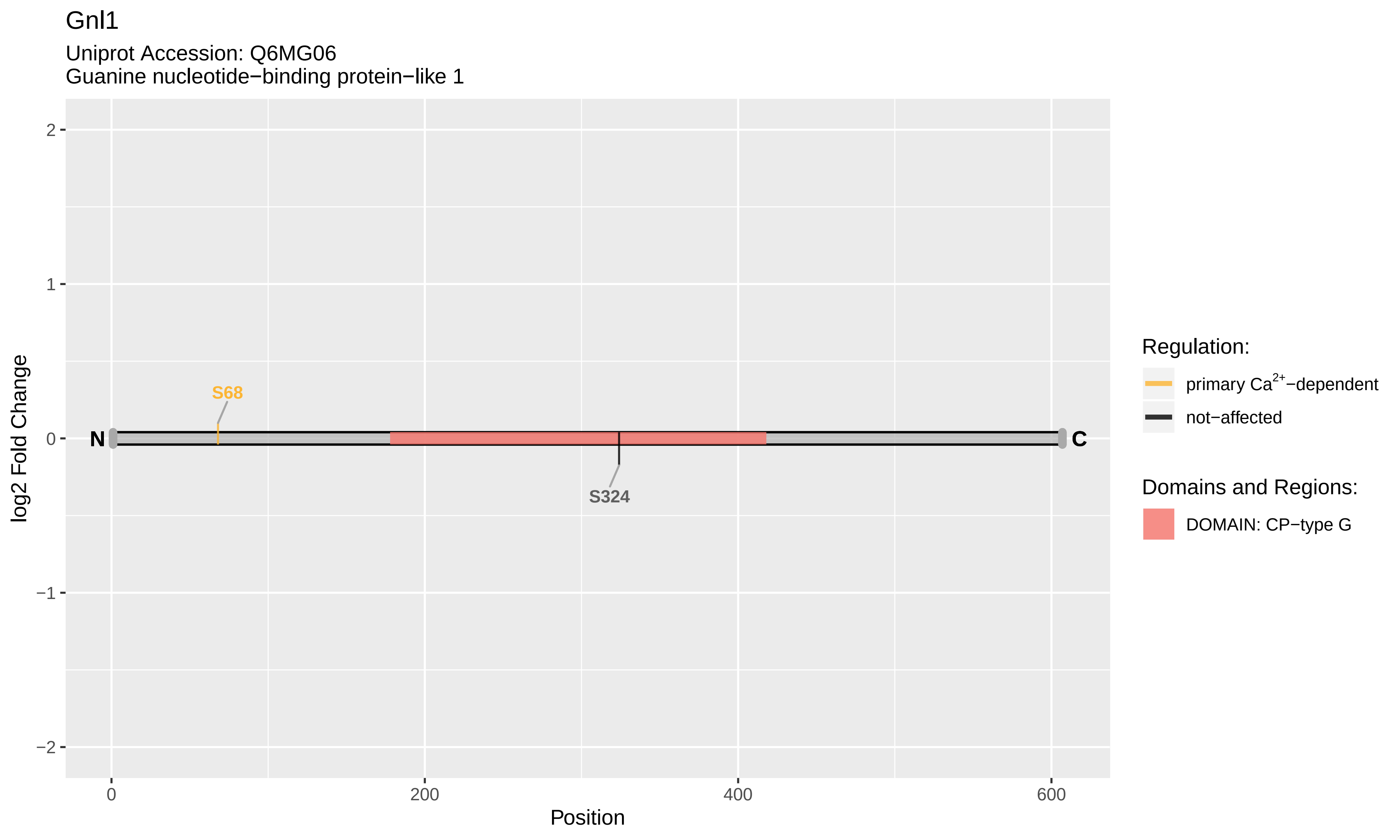

Gnl1
Uniprot Accession: Q6MG06
Guanine nucleotide−binding protein−like 1
log2 Fold Change
2
1
0
−1
−2
S68
N
C
S324
0
200
400
600
Position
Regulation:
primary Ca2+−dependent
not−affected
Domains and Regions:
DOMAIN: CP−type G

Got2

Uniprot Accession: P00507
Aspartate aminotransferase, mitochondrial

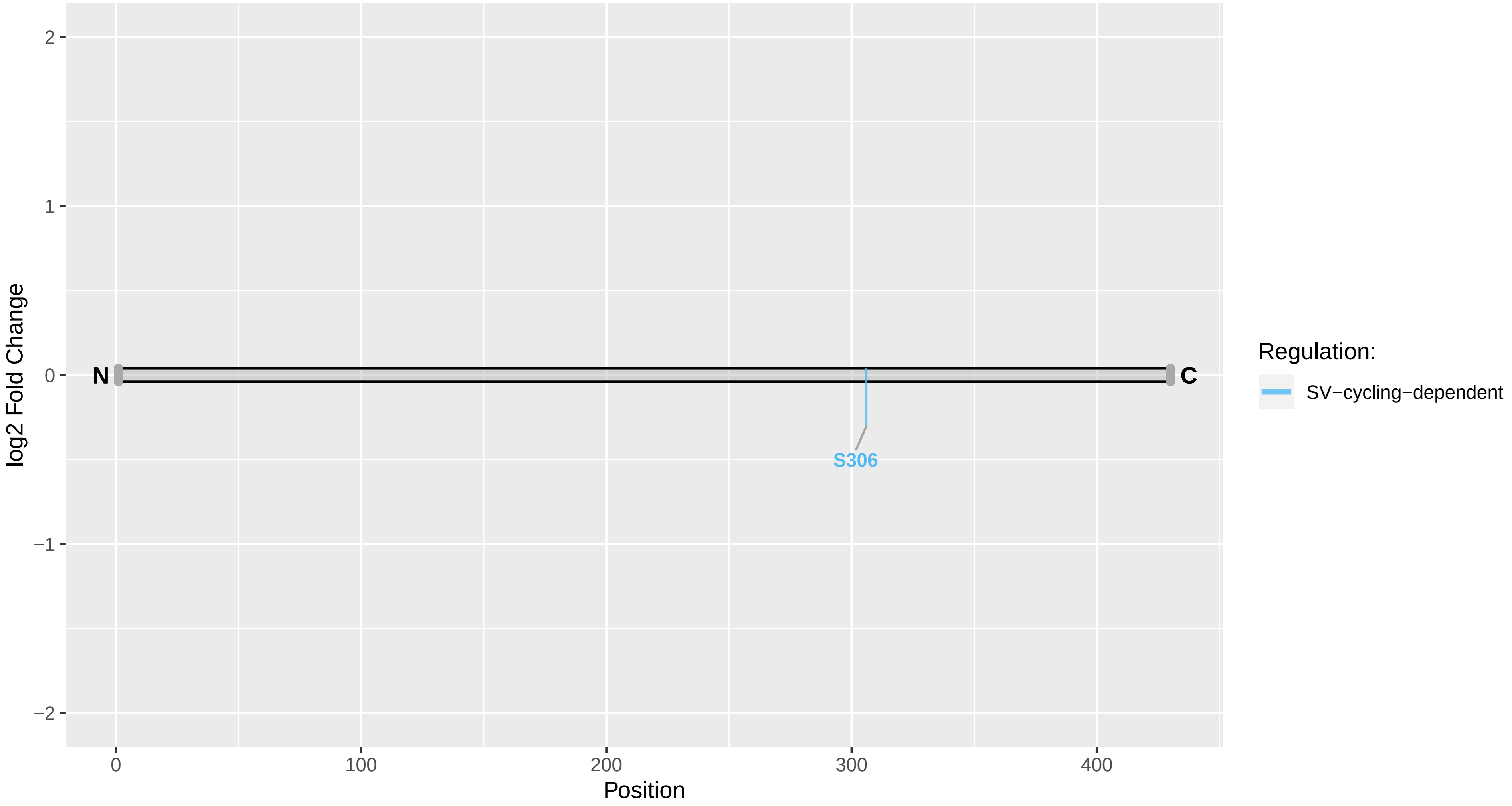

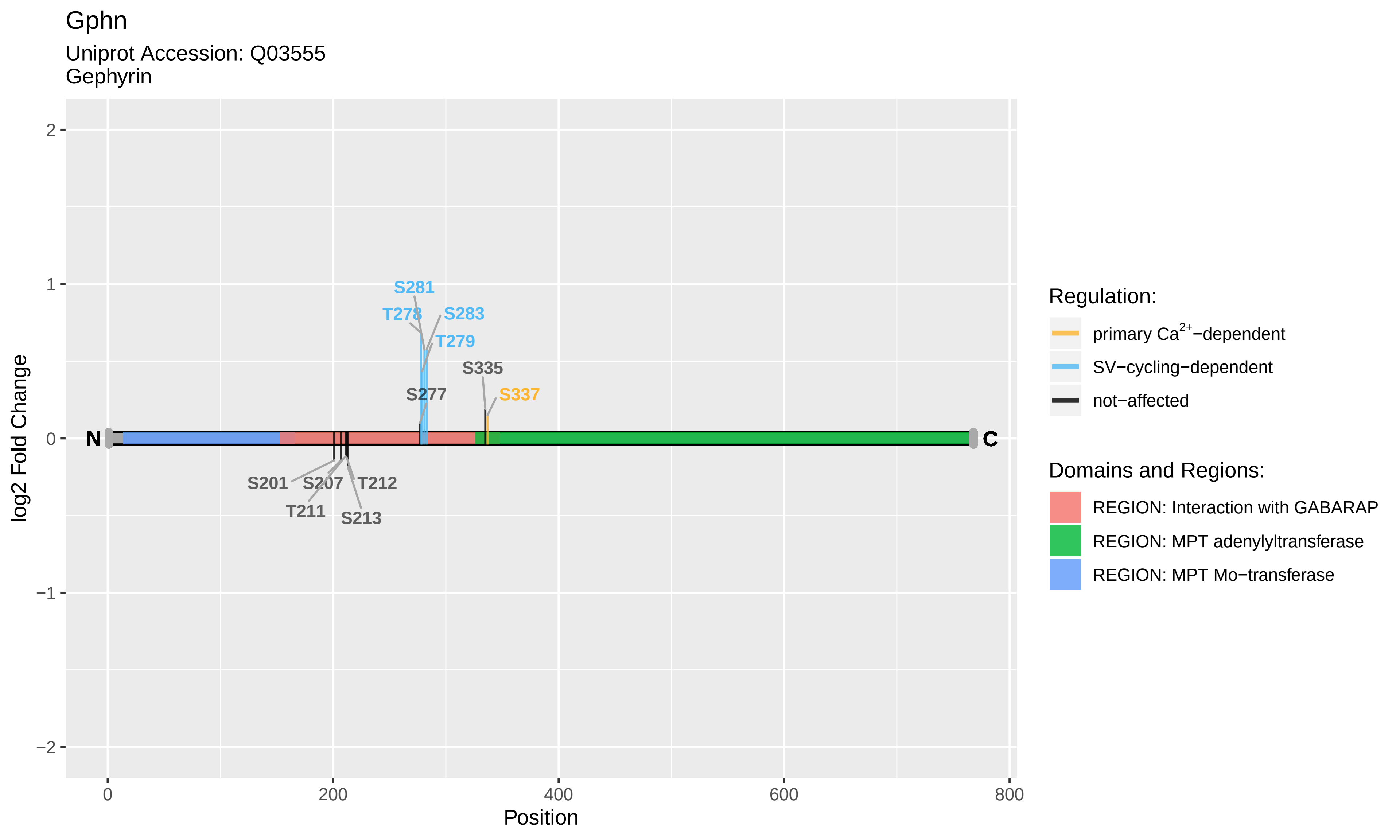

Gphn
Uniprot Accession: Q03555
Gephyrin
log2 Fold Change
Position
2
1
0
−1
−2
0
200
400
600
800
N
C
S281
T278
S283
T279
S335
S277
S337
S201
S207
T212
T211
S213
Regulation:
primary Ca2+−dependent
SV−cycling−dependent
not−affected
Domains and Regions:
REGION: Interaction with GABARAP
REGION: MPT adenylyltransferase
REGION: MPT Mo−transferase

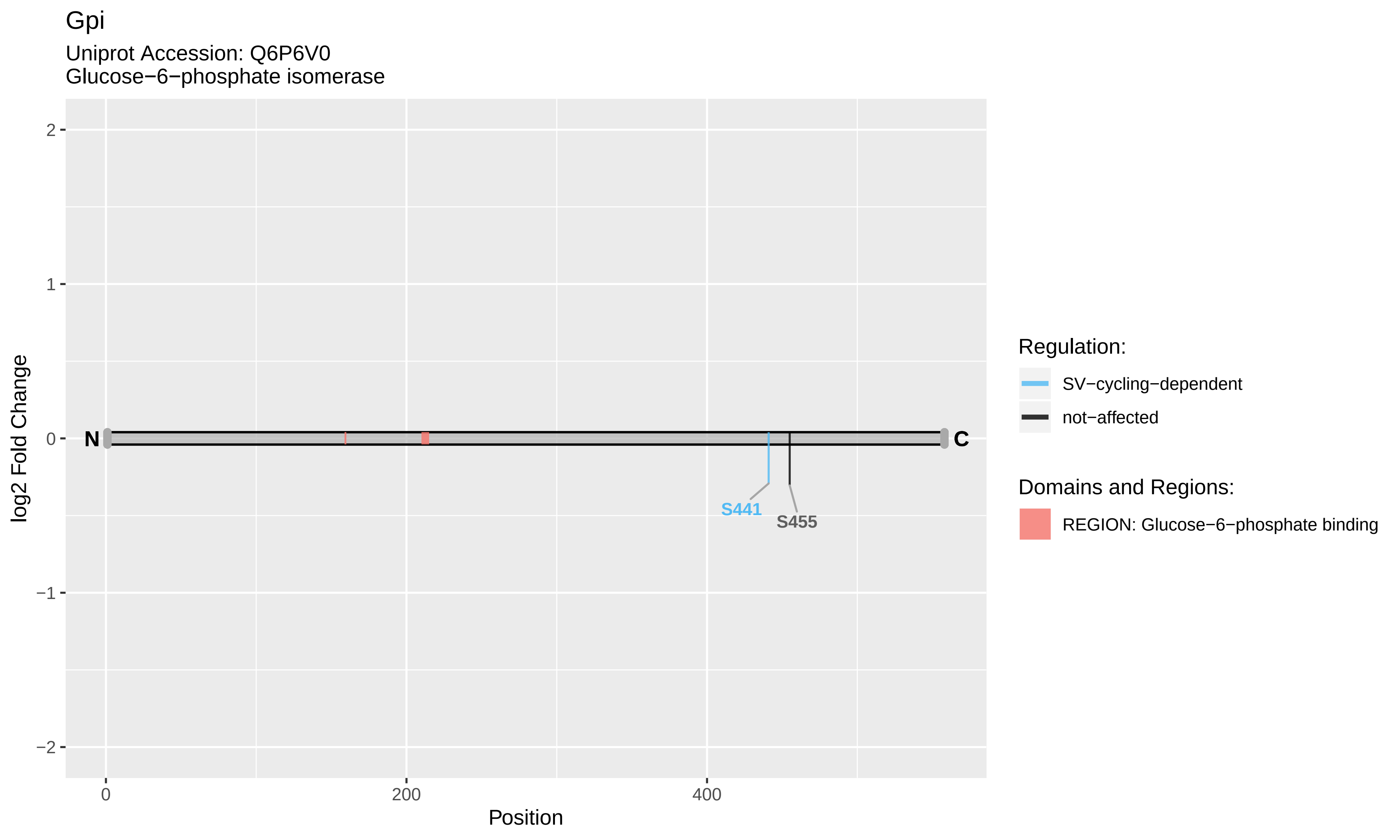

Gpi
Uniprot Accession: Q6P6V0
Glucose−6−phosphate isomerase
log2 Fold Change
2
1
0
−1
−2
N
C
S441
S455
0
200
400
Position
Regulation:
SV−cycling−dependent
not−affected
Domains and Regions:
REGION: Glucose−6−phosphate binding

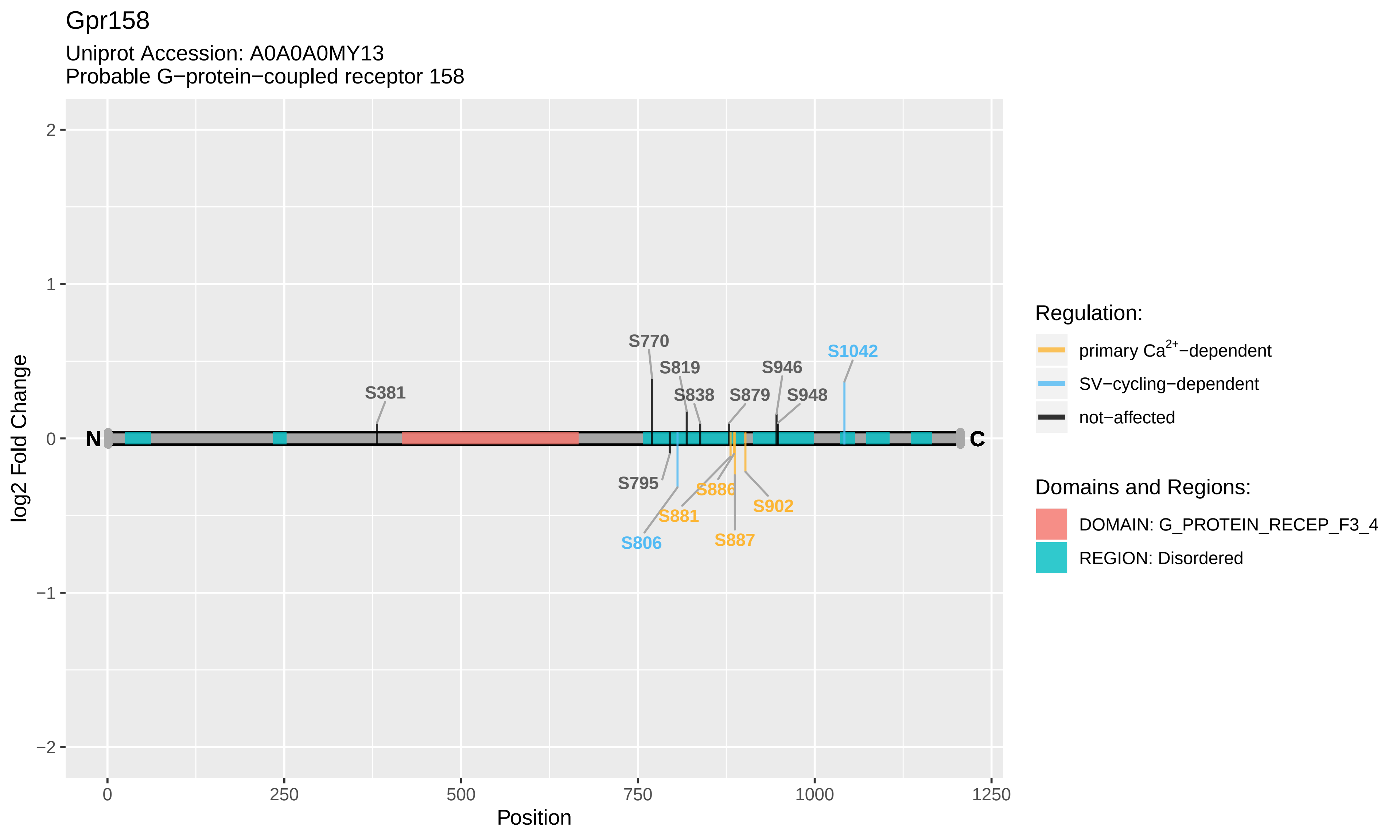
Gpr158
Uniprot Accession: A0A0A0MY13
Probable G−protein−coupled receptor 158
log2 Fold Change
Position
N
C
S381
S770
S819
S838
S879
S946
S948
S1042
S795
S886
S881
S902
S806
S887
Regulation:
primary Ca2+−dependent
SV−cycling−dependent
not−affected
Domains and Regions:
DOMAIN: G_PROTEIN_RECEP_F3_4
REGION: Disordered

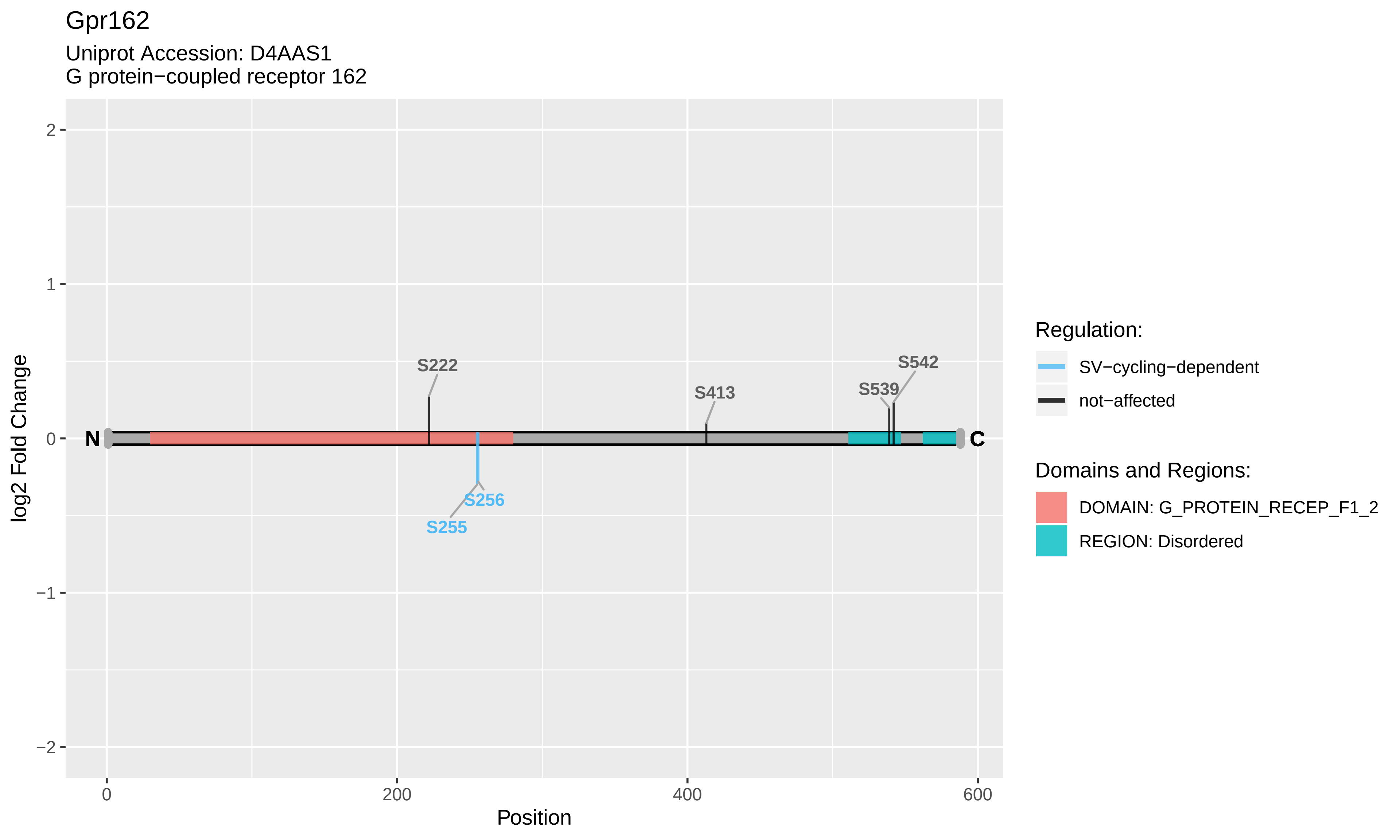

Gpr162
Uniprot Accession: D4AAS1
G protein−coupled receptor 162
log2 Fold Change
Position
2
1
0
−1
−2
0
200
400
600
N
C
S222
S413
S539
S542
S256
S255
Regulation:
SV−cycling−dependent
not−affected
Domains and Regions:
DOMAIN: G_PROTEIN_RECEP_F1_2
REGION: Disordered

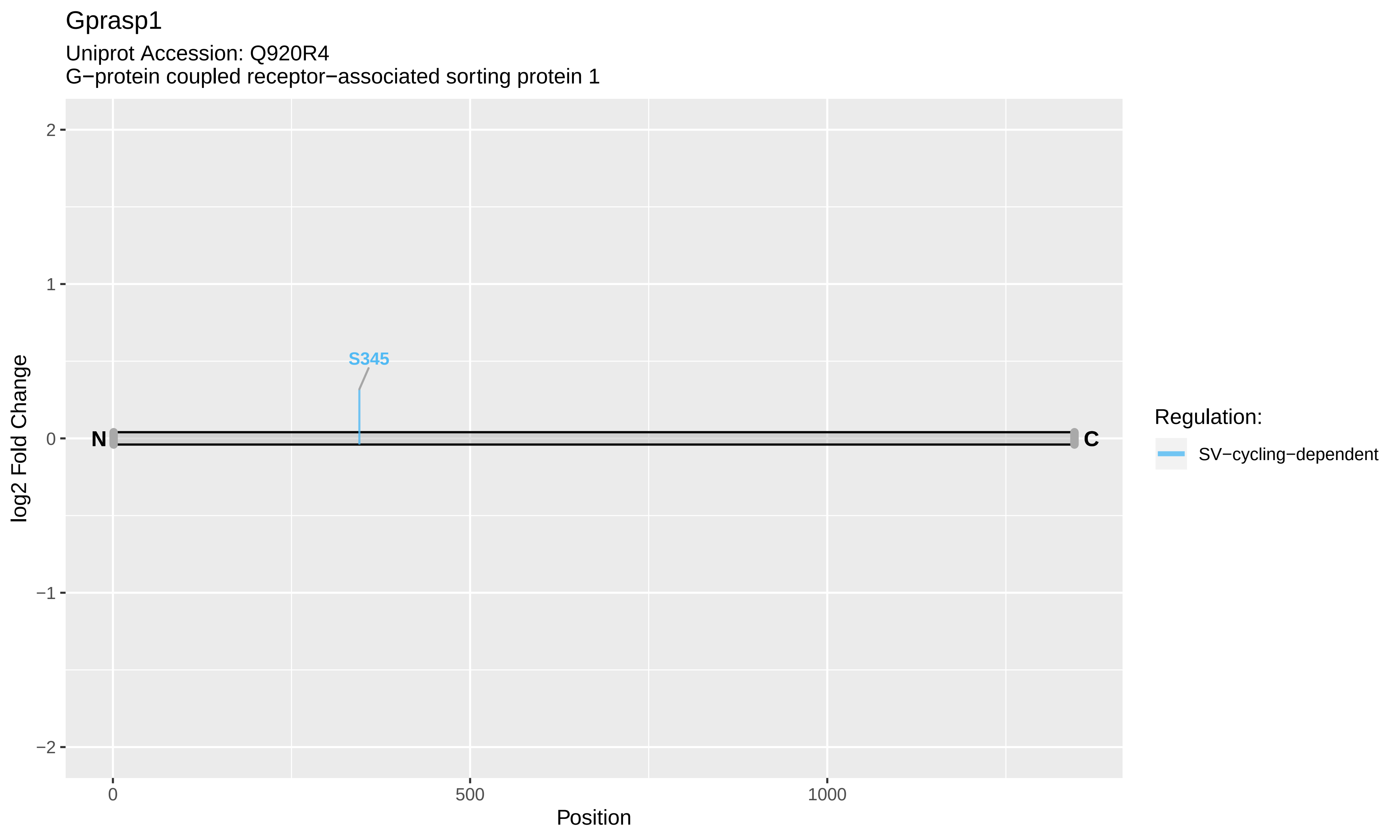
Gprasp1
Uniprot Accession: Q920R4
G−protein coupled receptor−associated sorting protein 1
log2 Fold Change
2
1
0
−1
−2
N
C
S345
0
500
1000
Position
Regulation:
SV−cycling−dependent

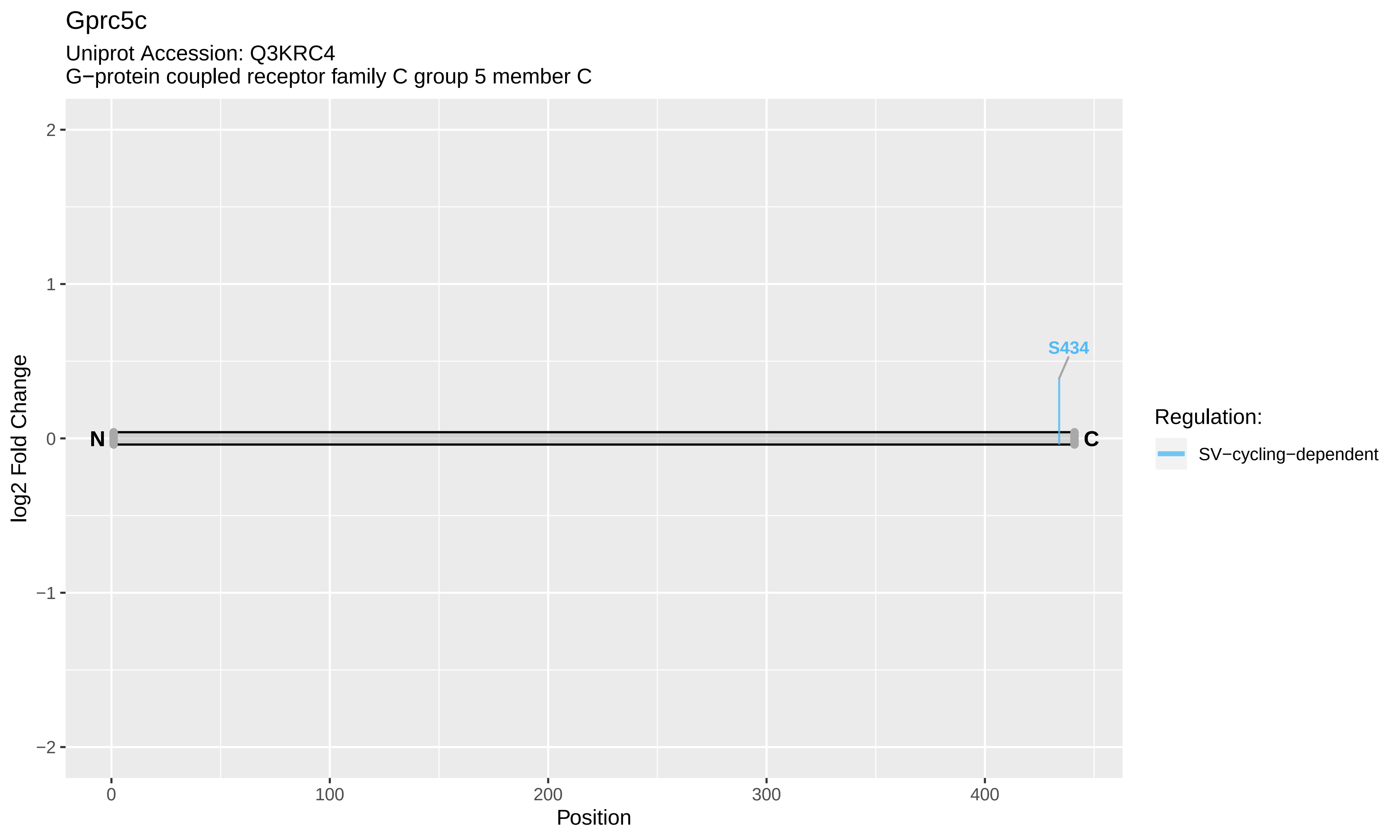
Gprc5c
Uniprot Accession: Q3KRC4
G−protein coupled receptor family C group 5 member C
log2 Fold Change
2
1
0
−1
−2
N
C
S434
0
100
200
300
400
Position
Regulation:
SV−cycling−dependent

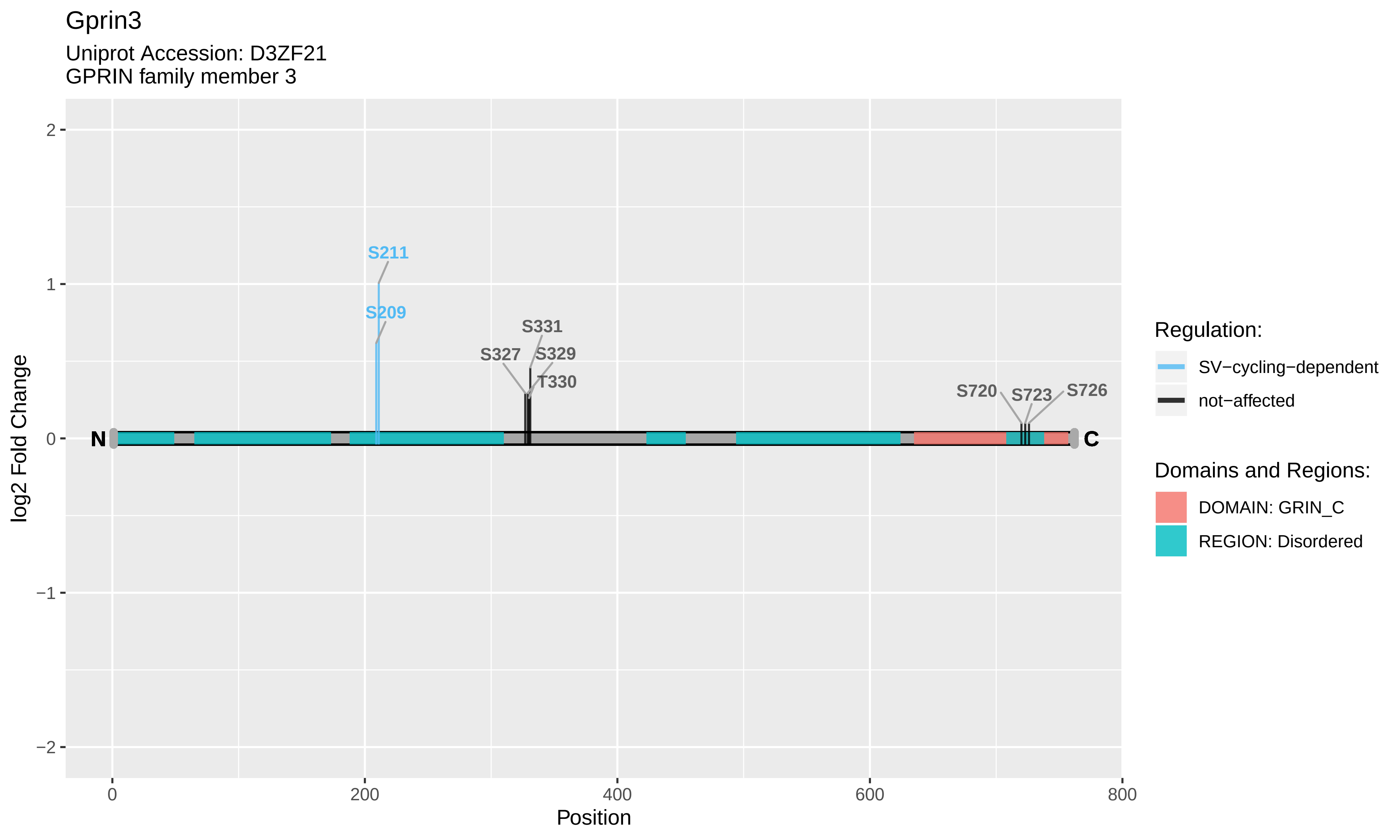

Gprin3
Uniprot Accession: D3ZF21
GPRIN family member 3
S211
S209
S331
S327
S329
T330
S720
S723
S726
N
C
2
1
0
−1
−2
log2 Fold Change
0
200
400
600
800
Position
Regulation:
SV−cycling−dependent
not−affected
Domains and Regions:
DOMAIN: GRIN_C
REGION: Disordered

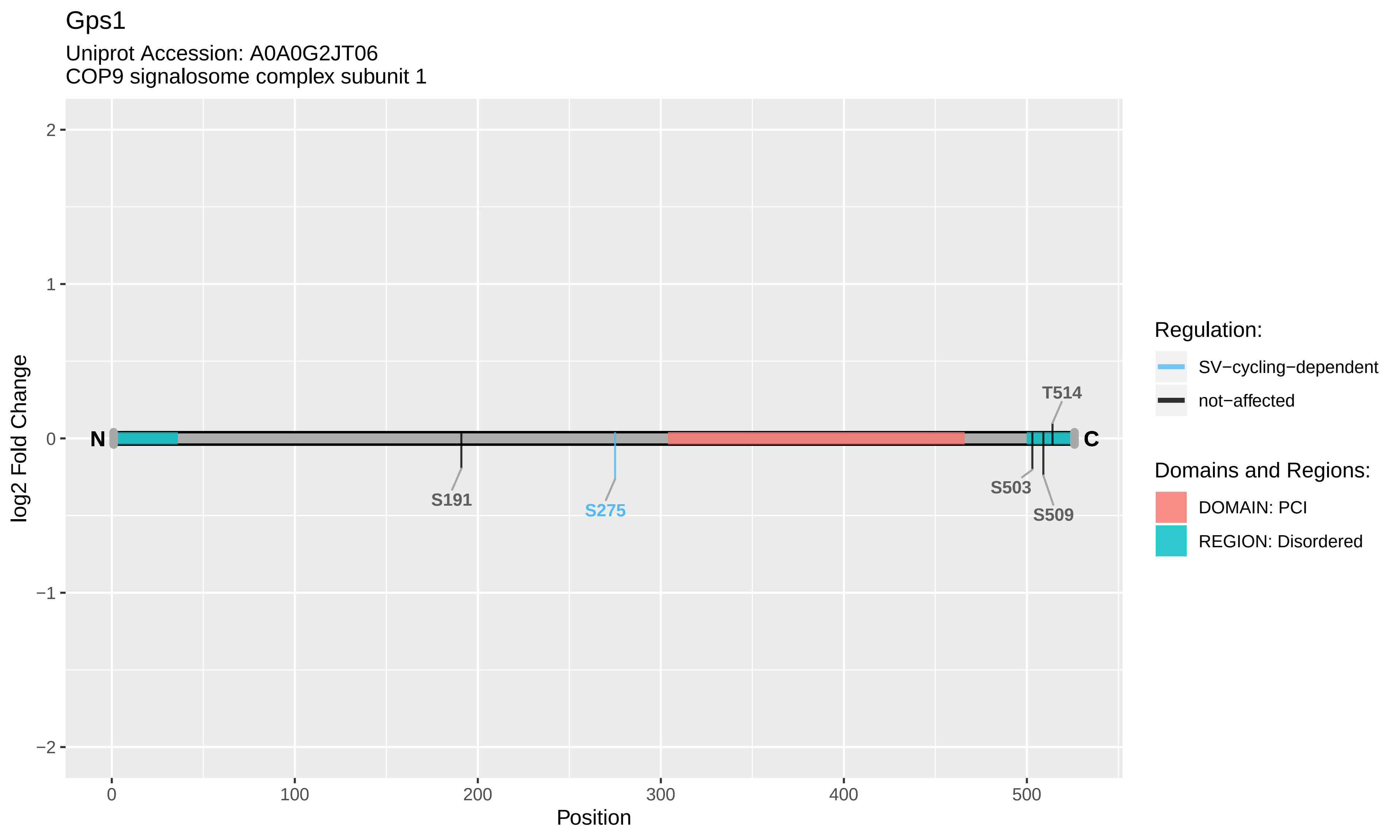

Gps1
Uniprot Accession: A0A0G2JT06
COP9 signalosome complex subunit 1
log2 Fold Change
Position
N
C
S191
S275
S503
S509
T514
Regulation:
SV−cycling−dependent
not−affected
Domains and Regions:
DOMAIN: PCI
REGION: Disordered

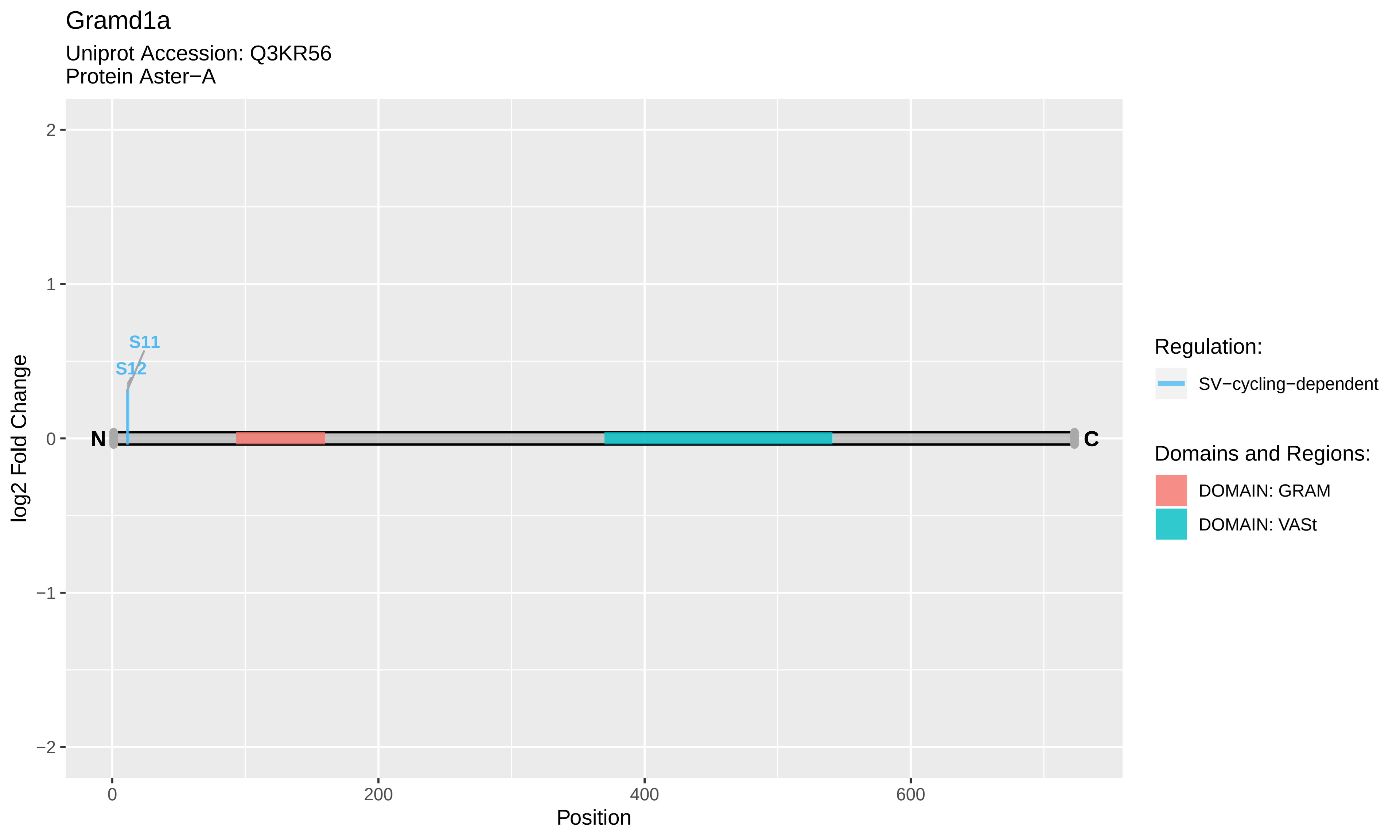

Gramd1a
Uniprot Accession: Q3KR56
Protein Aster−A
S11
S12
N
C
2
1
0
−1
−2
log2 Fold Change
0
200
400
600
Position
Regulation:
SV−cycling−dependent
Domains and Regions:
DOMAIN: GRAM
DOMAIN: VASt

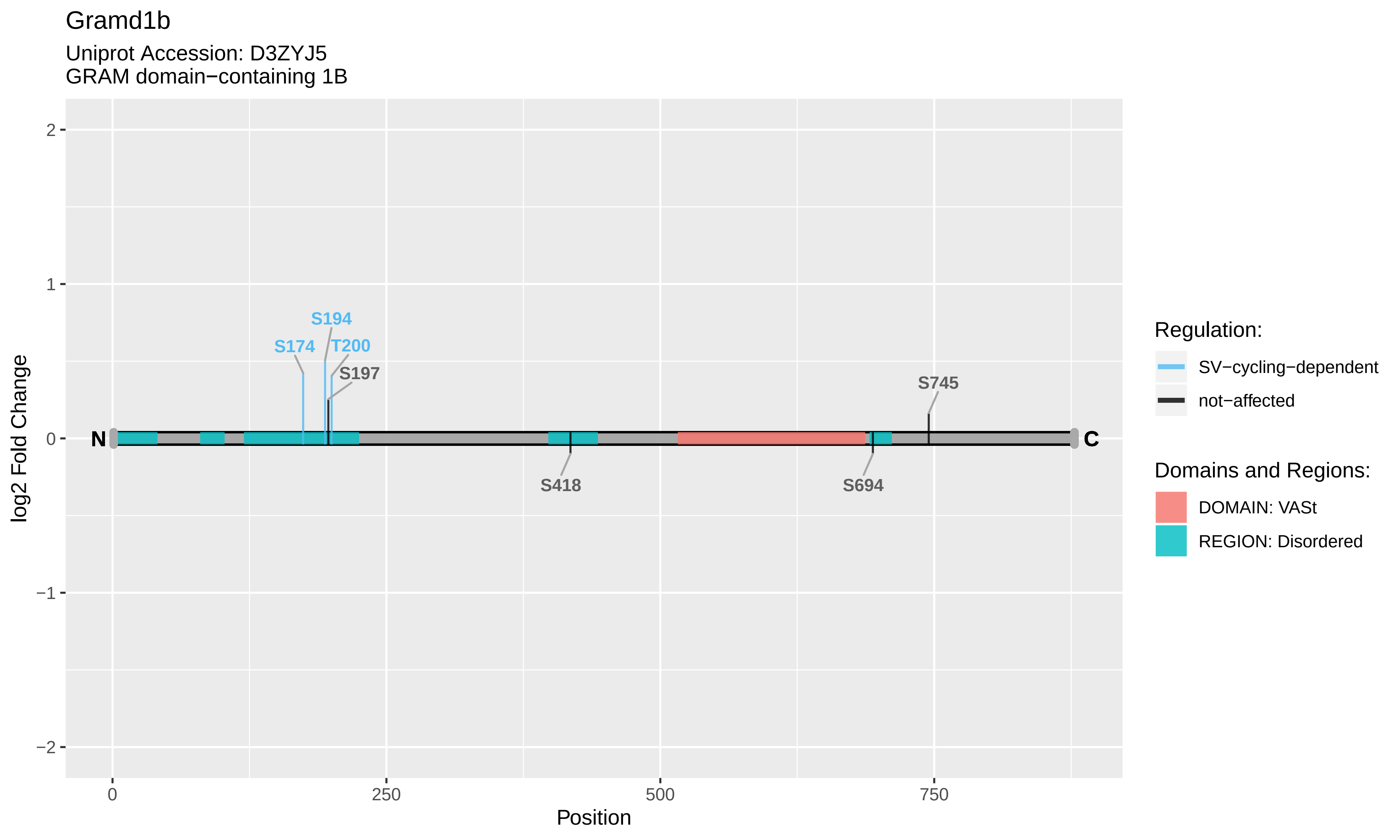

Gramd1b
Uniprot Accession: D3ZYJ5
GRAM domain−containing 1B
log2 Fold Change
Position
2
1
0
−1
−2
0
250
500
750
N
C
S194
S174
T200
S197
S745
S418
S694
Regulation:
SV−cycling−dependent
not−affected
Domains and Regions:
DOMAIN: VASt
REGION: Disordered

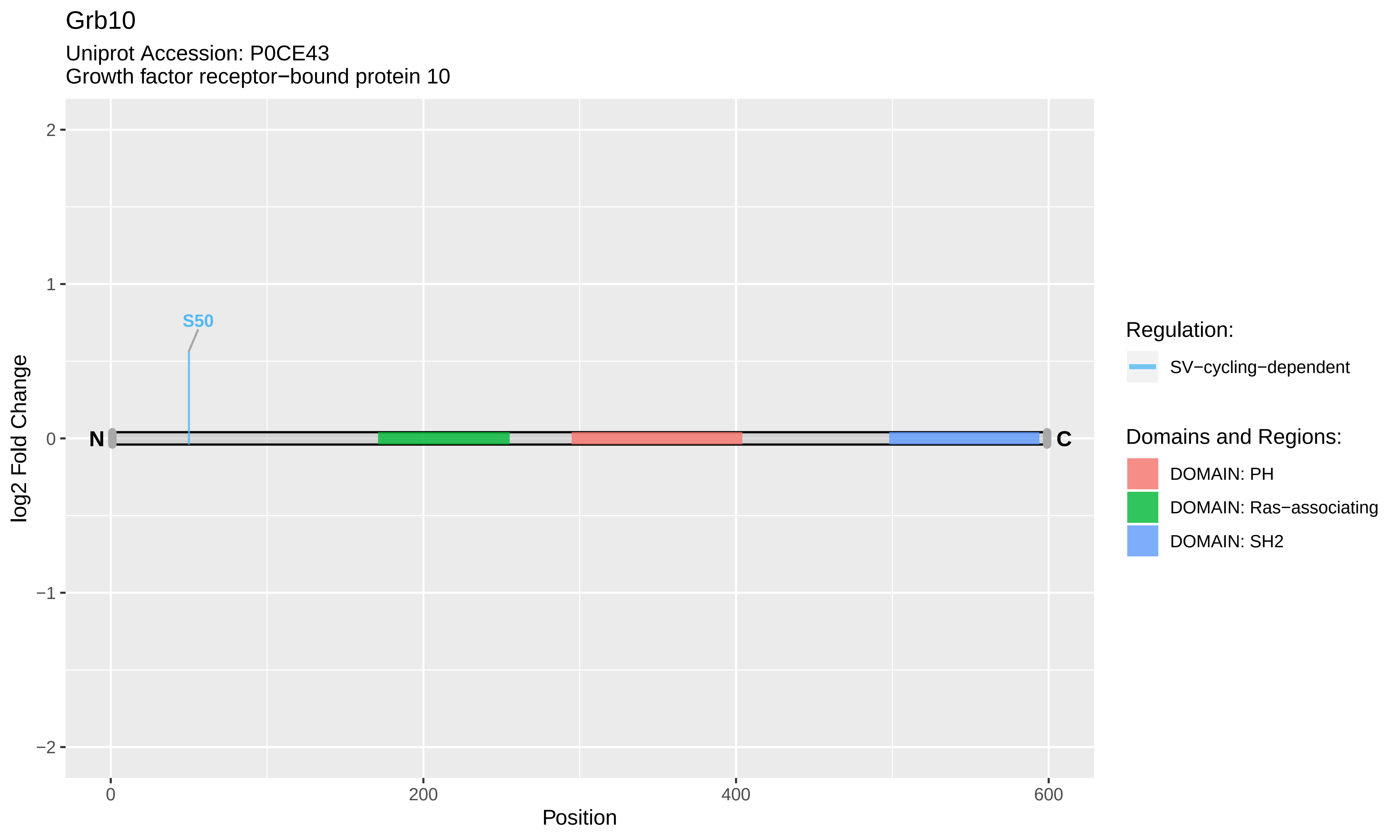

Grb10
Uniprot Accession: P0CE43
Growth factor receptor−bound protein 10
S50
N
C
log2 Fold Change
2
1
0
−1
−2
0
200
400
600
Position
Regulation:
SV−cycling−dependent
Domains and Regions:
DOMAIN: PH
DOMAIN: Ras−associating
DOMAIN: SH2

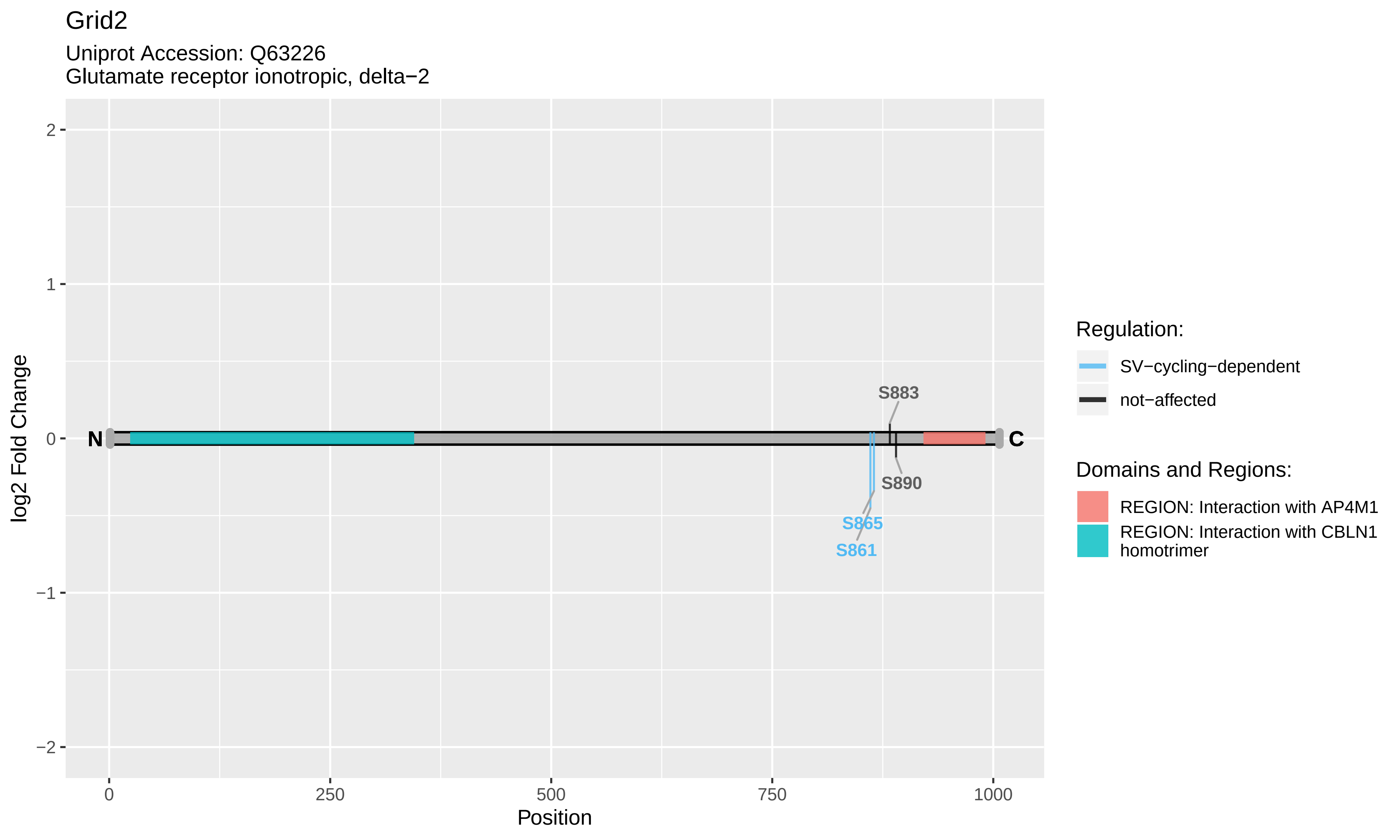

Grid2
Uniprot Accession: Q63226
Glutamate receptor ionotropic, delta−2
log2 Fold Change
2
1
0
−1
−2
N
C
S883
S890
S865
S861
0
250
500
750
1000
Position
Regulation:
SV−cycling−dependent
not−affected
Domains and Regions:
REGION: Interaction with AP4M1
REGION: Interaction with CBLN1 homotrimer

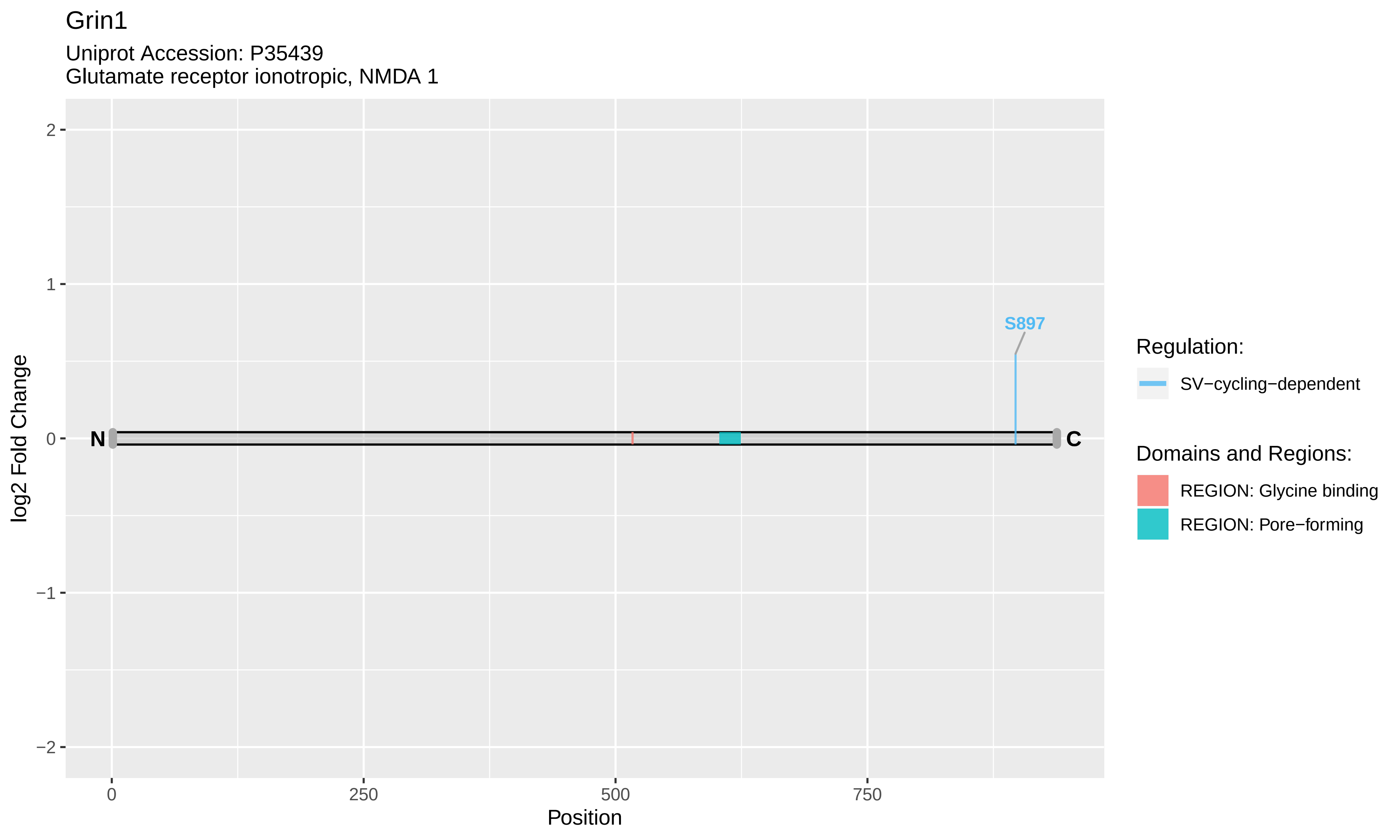
Grin1
Uniprot Accession: P35439
Glutamate receptor ionotropic, NMDA 1
S897
N
C
2
1
0
−1
−2
log2 Fold Change
0
250
500
750
Position
Regulation:
SV−cycling−dependent
Domains and Regions:
REGION: Glycine binding
REGION: Pore−forming

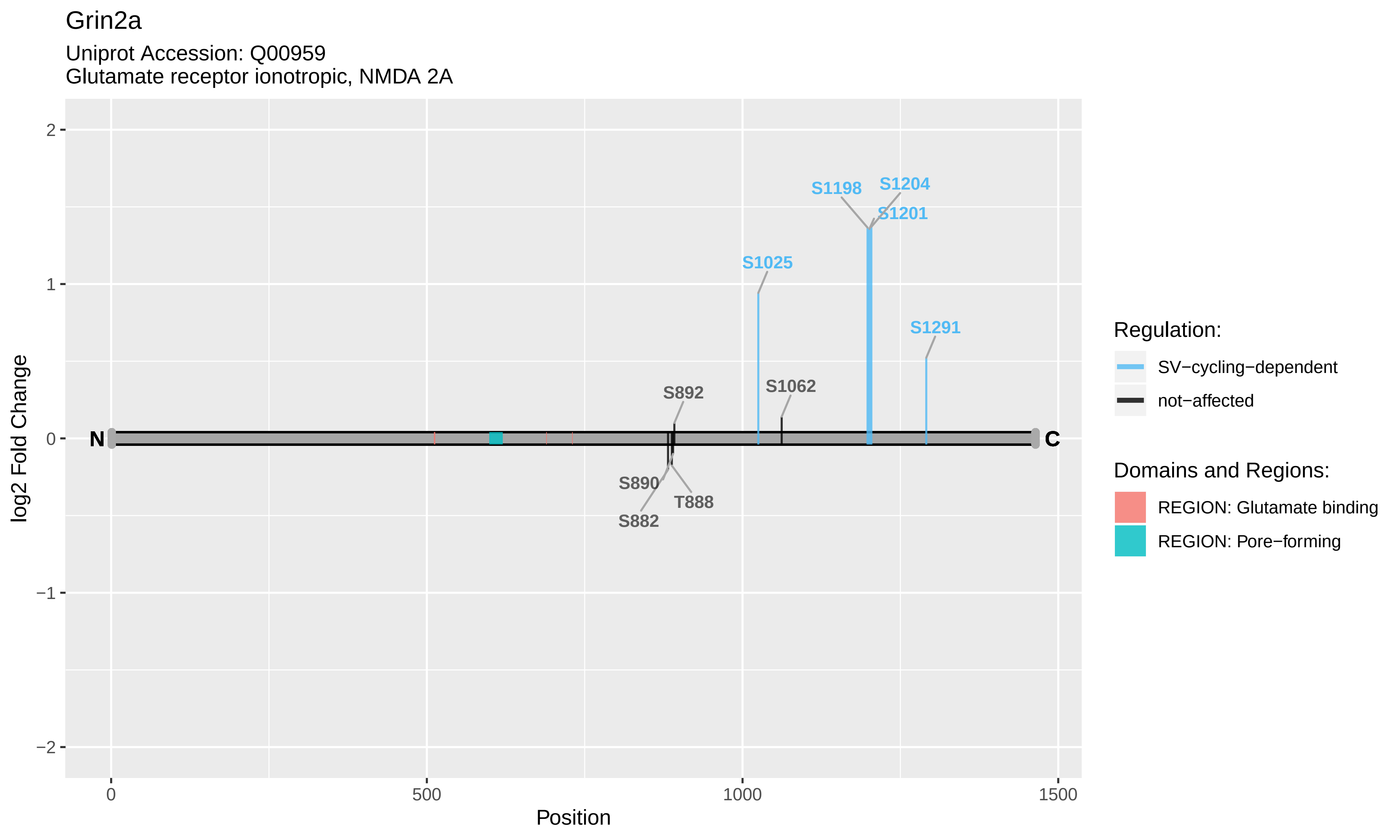
Grin2a
Uniprot Accession: Q00959
Glutamate receptor ionotropic, NMDA 2A
S1198
S1204
S1201
S1025
S1291
S892
S1062
S890
T888
S882
N
C
2
1
0
−1
−2
0
500
1000
1500
Position
log2 Fold Change
Regulation:
SV−cycling−dependent
not−affected
Domains and Regions:
REGION: Glutamate binding
REGION: Pore−forming

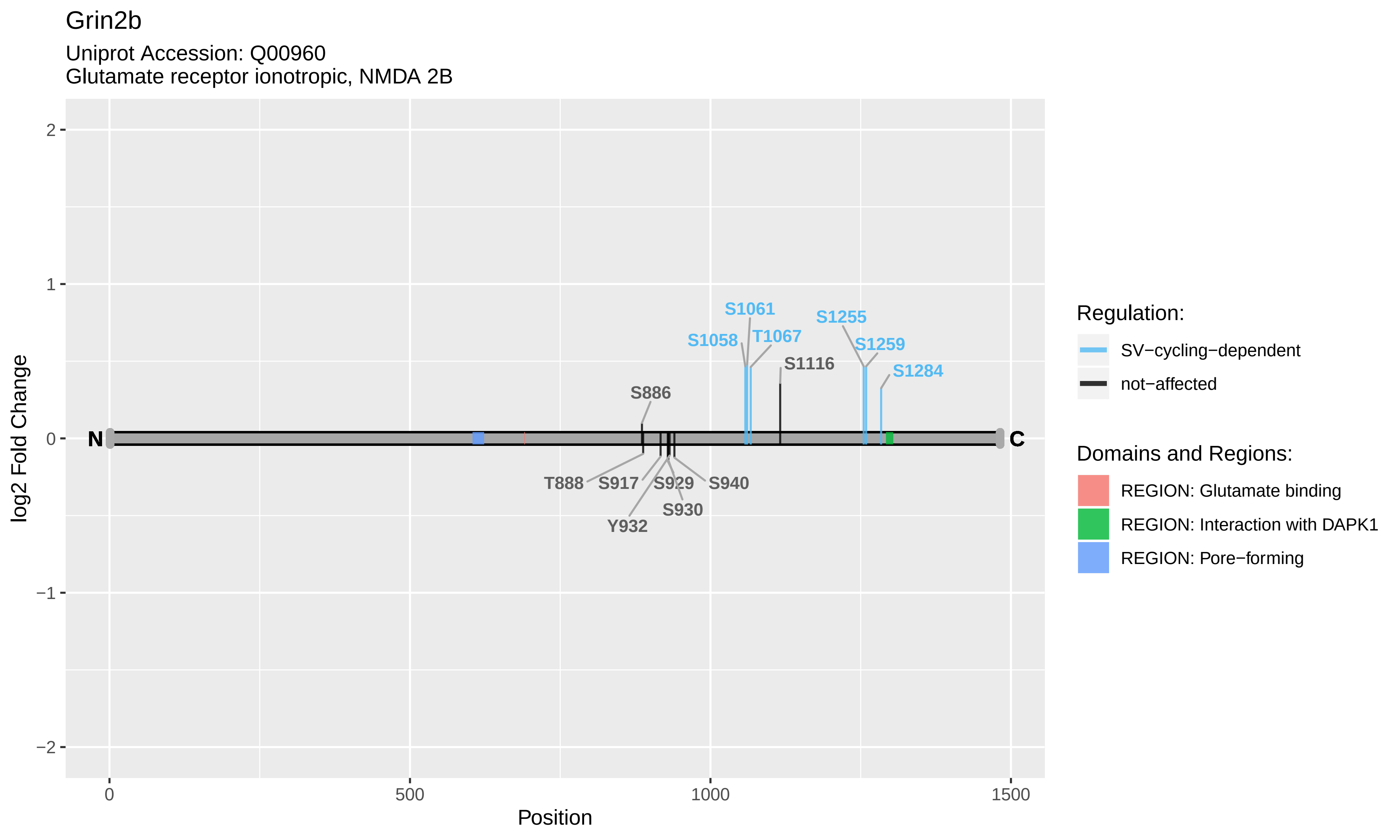

Grin2b
Uniprot Accession: Q00960
Glutamate receptor ionotropic, NMDA 2B
log2 Fold Change
Position
N
C
S886
T888
S917
S929
S930
Y932
S940
S1058
S1061
T1067
S1116
S1255
S1259
S1284
Regulation:
SV−cycling−dependent
not−affected
Domains and Regions:
REGION: Glutamate binding
REGION: Interaction with DAPK1
REGION: Pore−forming

Grip1

Uniprot Accession: P97879
Glutamate receptor−interacting protein 1

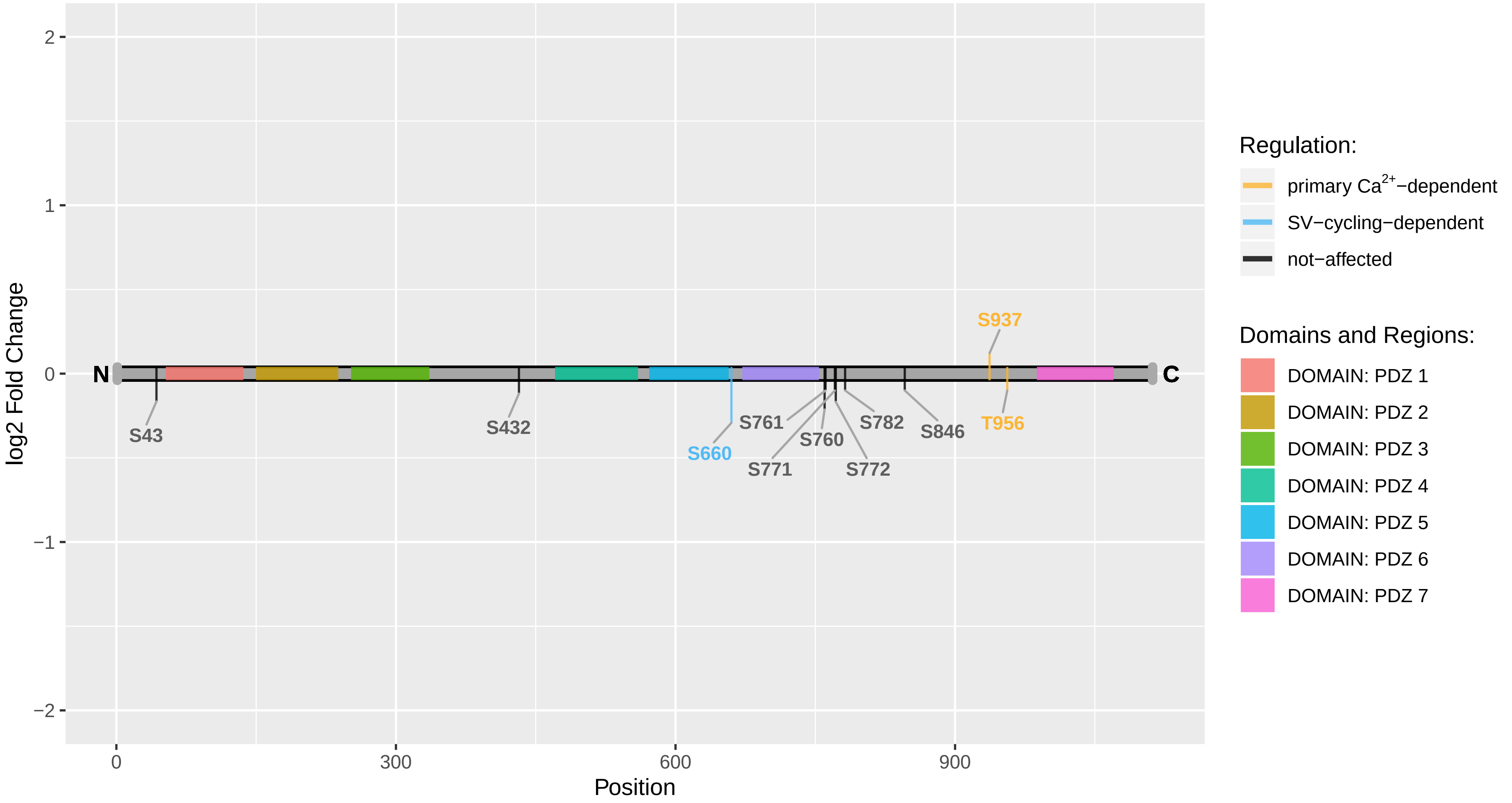

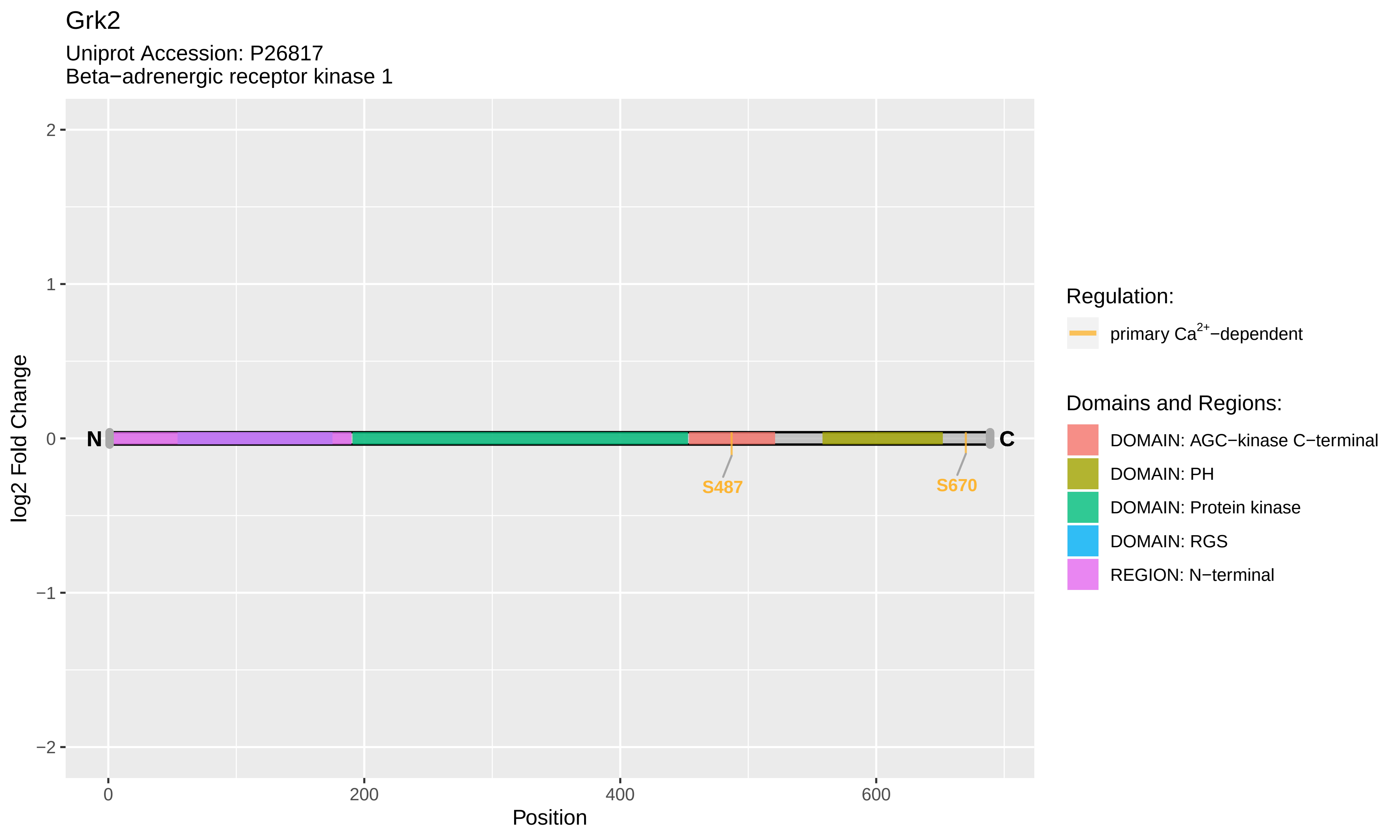
Grk2
Uniprot Accession: P26817
Beta−adrenergic receptor kinase 1
2
1
0
−1
−2
log2 Fold Change
0
200
400
600
Position
N
C
S487
S670
Regulation:
primary Ca2+−dependent
Domains and Regions:
DOMAIN: AGC−kinase C−terminal
DOMAIN: PH
DOMAIN: Protein kinase
DOMAIN: RGS
REGION: N−terminal

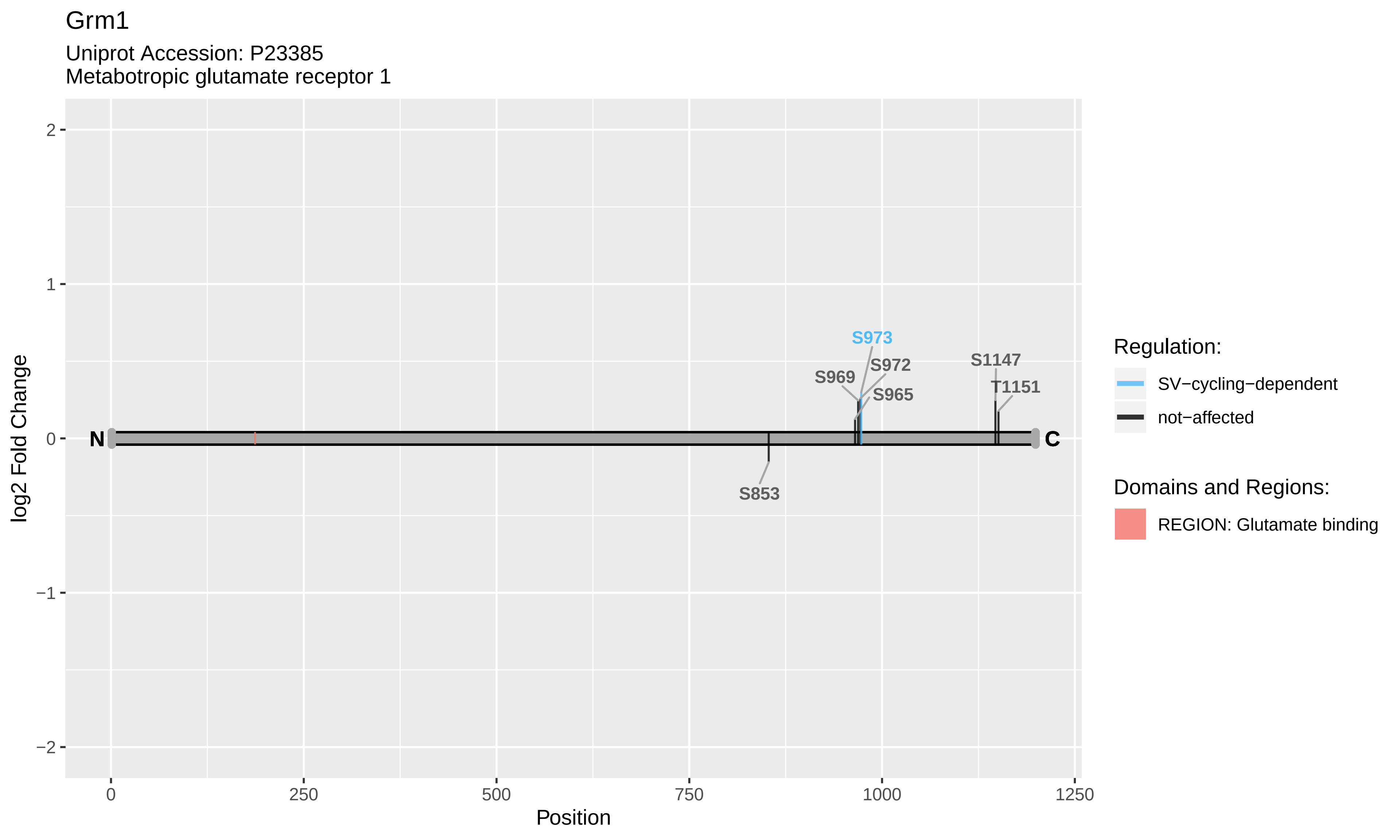

Grm1
Uniprot Accession: P23385
Metabotropic glutamate receptor 1
log2 Fold Change
Position
S973
S972
S969
S965
S1147
T1151
S853
N
C
Regulation:
SV−cycling−dependent
not−affected
Domains and Regions:
REGION: Glutamate binding

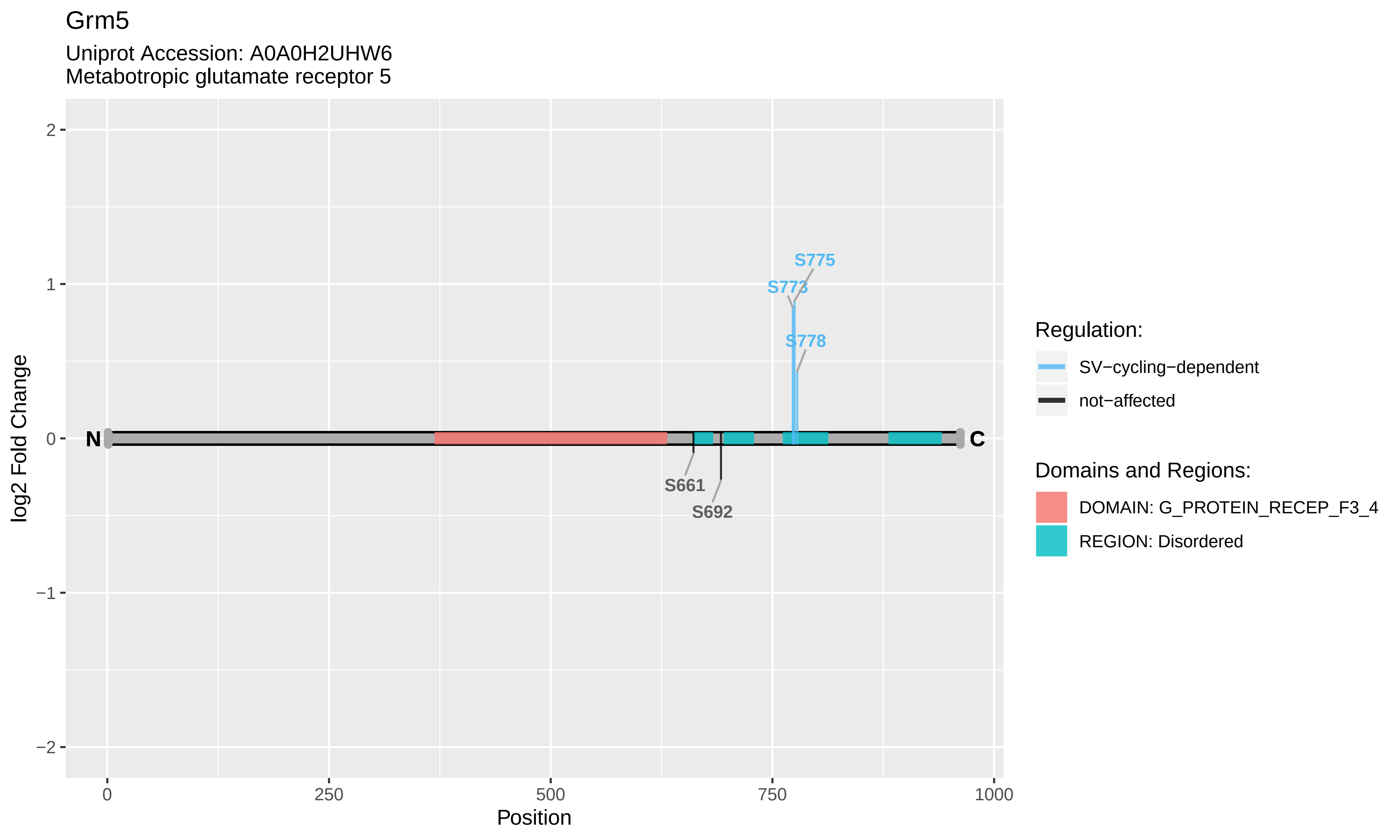

Grm5
Uniprot Accession: A0A0H2UHW6
Metabotropic glutamate receptor 5
log2 Fold Change
Position
2
1
0
−1
−2
0
250
500
750
1000
N
C
S775
S773
S778
S661
S692
Regulation:
SV−cycling−dependent
not−affected
Domains and Regions:
DOMAIN: G_PROTEIN_RECEP_F3_4
REGION: Disordered

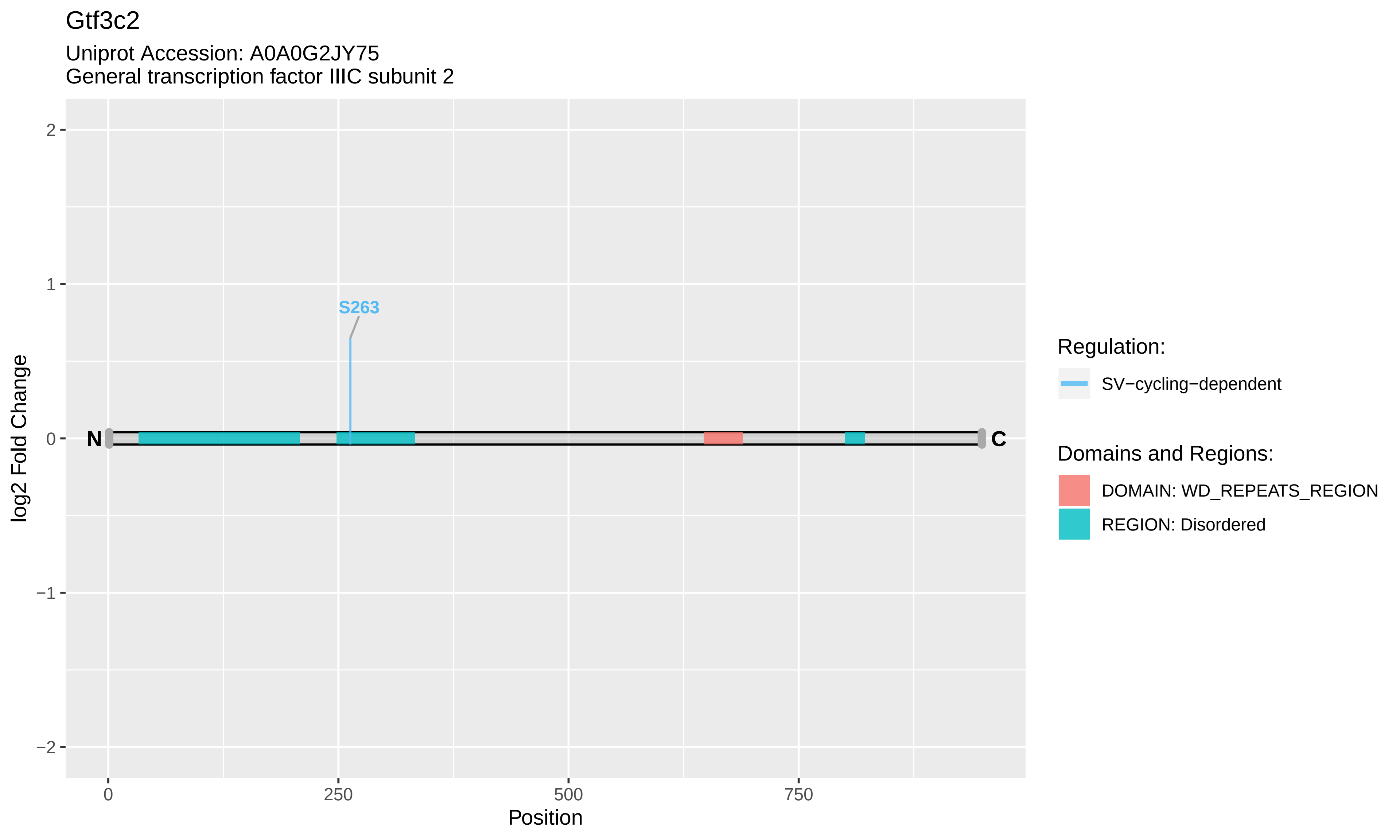

Gtf3c2
Uniprot Accession: A0A0G2JY75
General transcription factor IIIC subunit 2
S263
N
C
log2 Fold Change
2
1
0
−1
−2
0
250
500
750
Position
Regulation:
SV−cycling−dependent
Domains and Regions:
DOMAIN: WD_REPEATS_REGION
REGION: Disordered

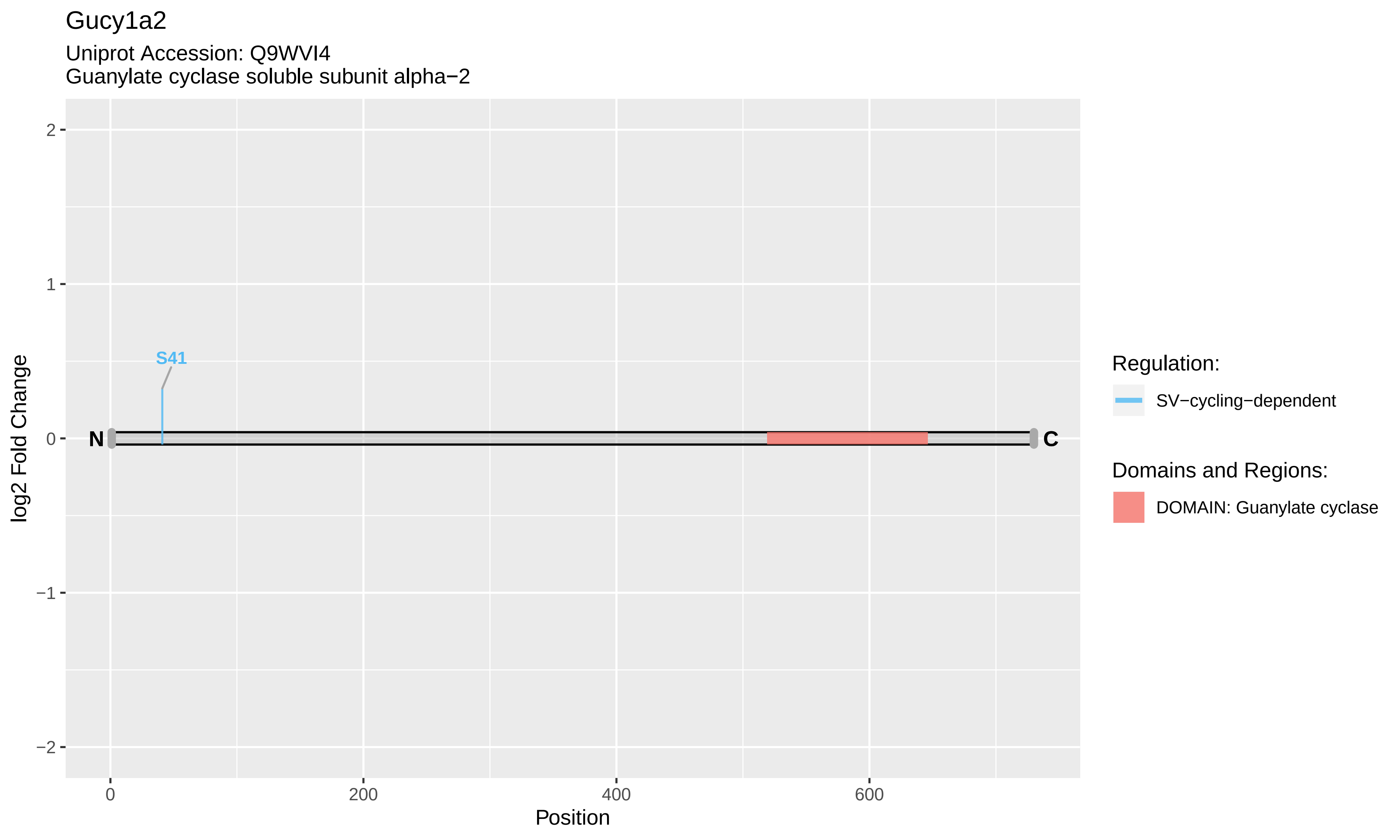
Gucy1a2
Uniprot Accession: Q9WVI4
Guanylate cyclase soluble subunit alpha−2
S41
N
C
log2 Fold Change
Position
Regulation:
SV−cycling−dependent
Domains and Regions:
DOMAIN: Guanylate cyclase
2
1
0
−1
−2
0
200
400
600

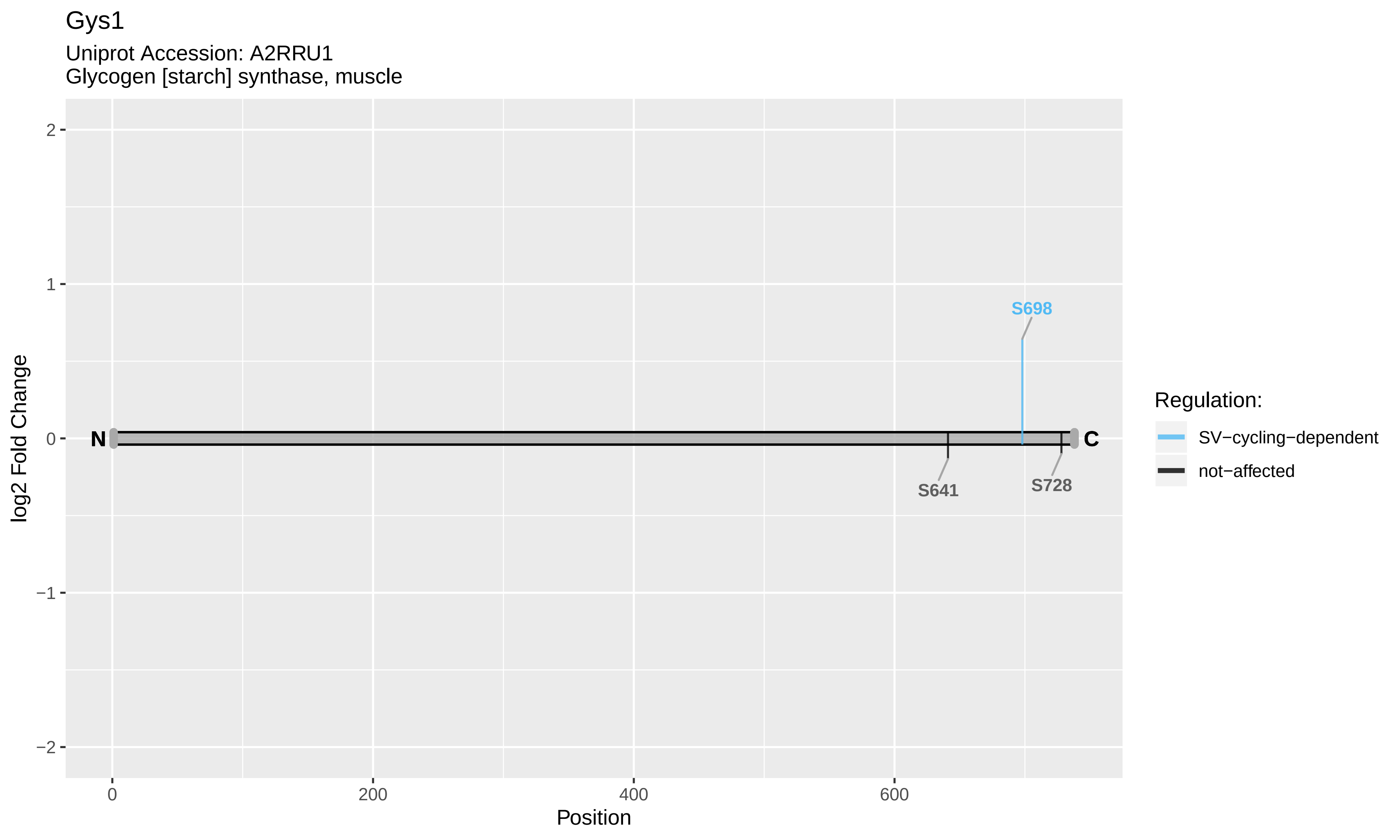
Gys1
Uniprot Accession: A2RRU1
Glycogen [starch] synthase, muscle
log2 Fold Change
2
1
0
−1
−2
N
C
S698
S641
S728
0
200
400
600
Position
Regulation:
SV−cycling−dependent
not−affected

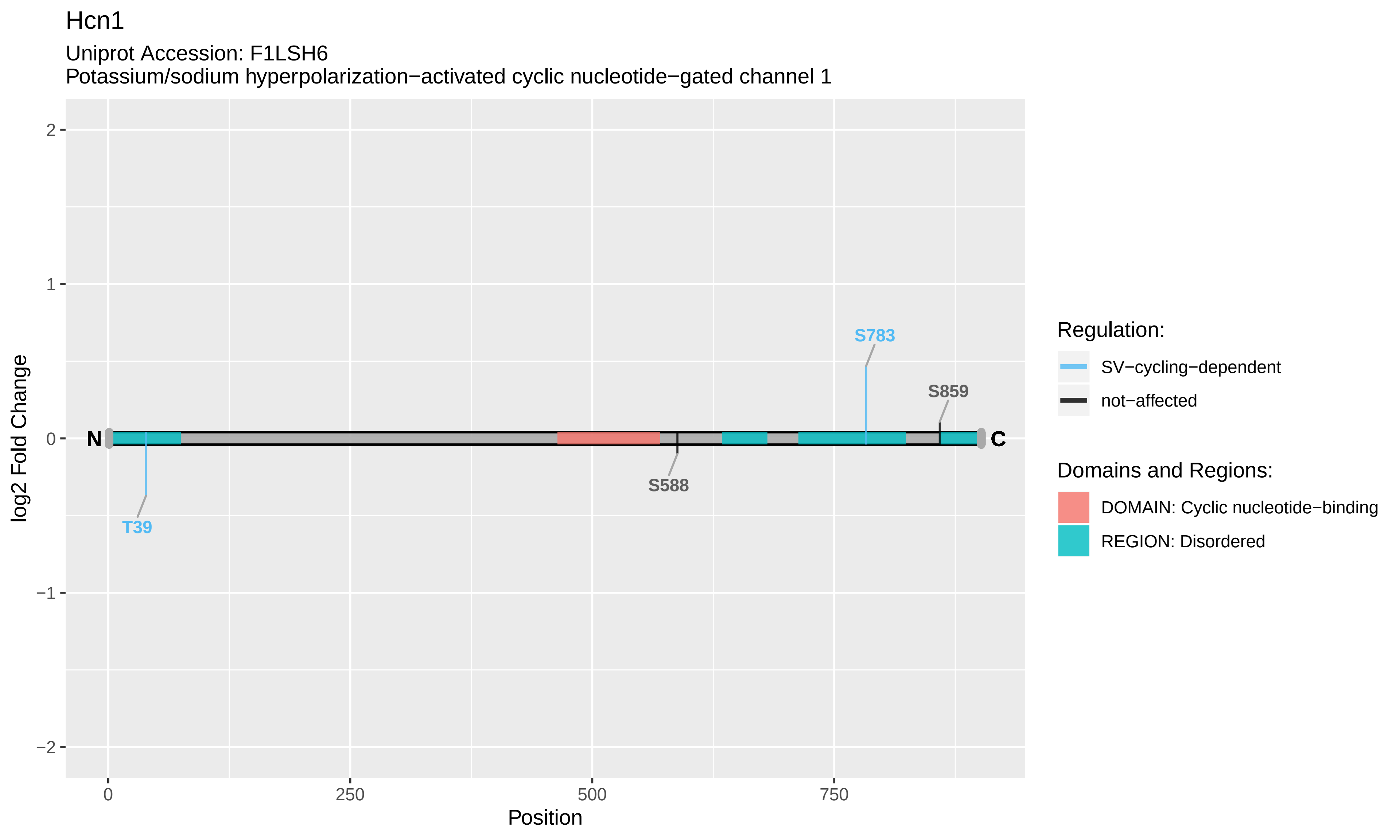
Hcn1
Uniprot Accession: F1LSH6
Potassium/sodium hyperpolarization−activated cyclic nucleotide−gated channel 1
log2 Fold Change
2
1
0
−1
−2
N
C
T39
S588
S783
S859
0
250
500
750
Position
Regulation:
SV−cycling−dependent
not−affected
Domains and Regions:
DOMAIN: Cyclic nucleotide−binding
REGION: Disordered

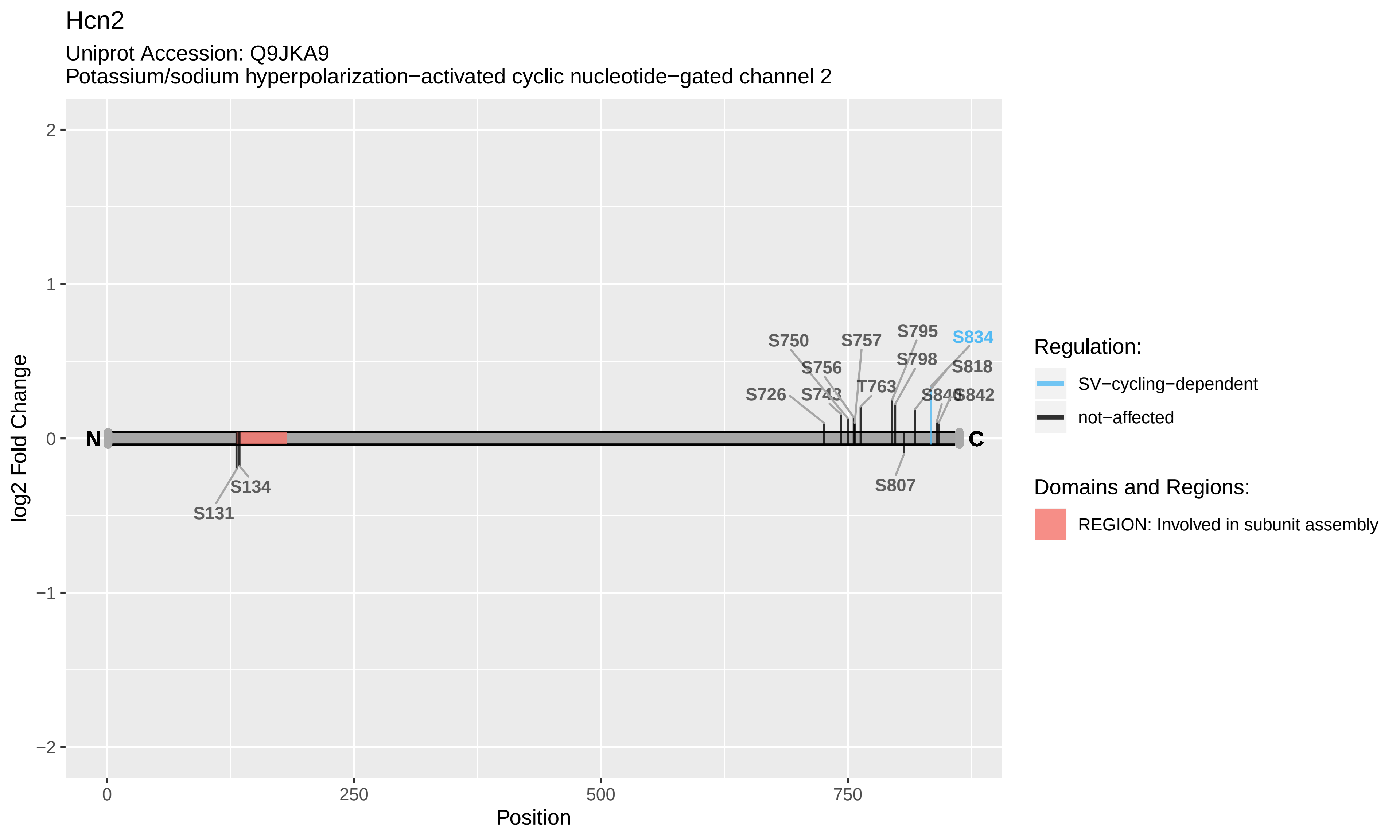

Hcn2
Uniprot Accession: Q9JKA9
Potassium/sodium hyperpolarization−activated cyclic nucleotide−gated channel 2
log2 Fold Change
Position
2
1
0
−1
−2
0
250
500
750
N
C
S131
S134
S726
S743
S750
S756
S757
T763
S795
S798
S807
S818
S834
S840
S842
Regulation:
SV−cycling−dependent
not−affected
Domains and Regions:
REGION: Involved in subunit assembly

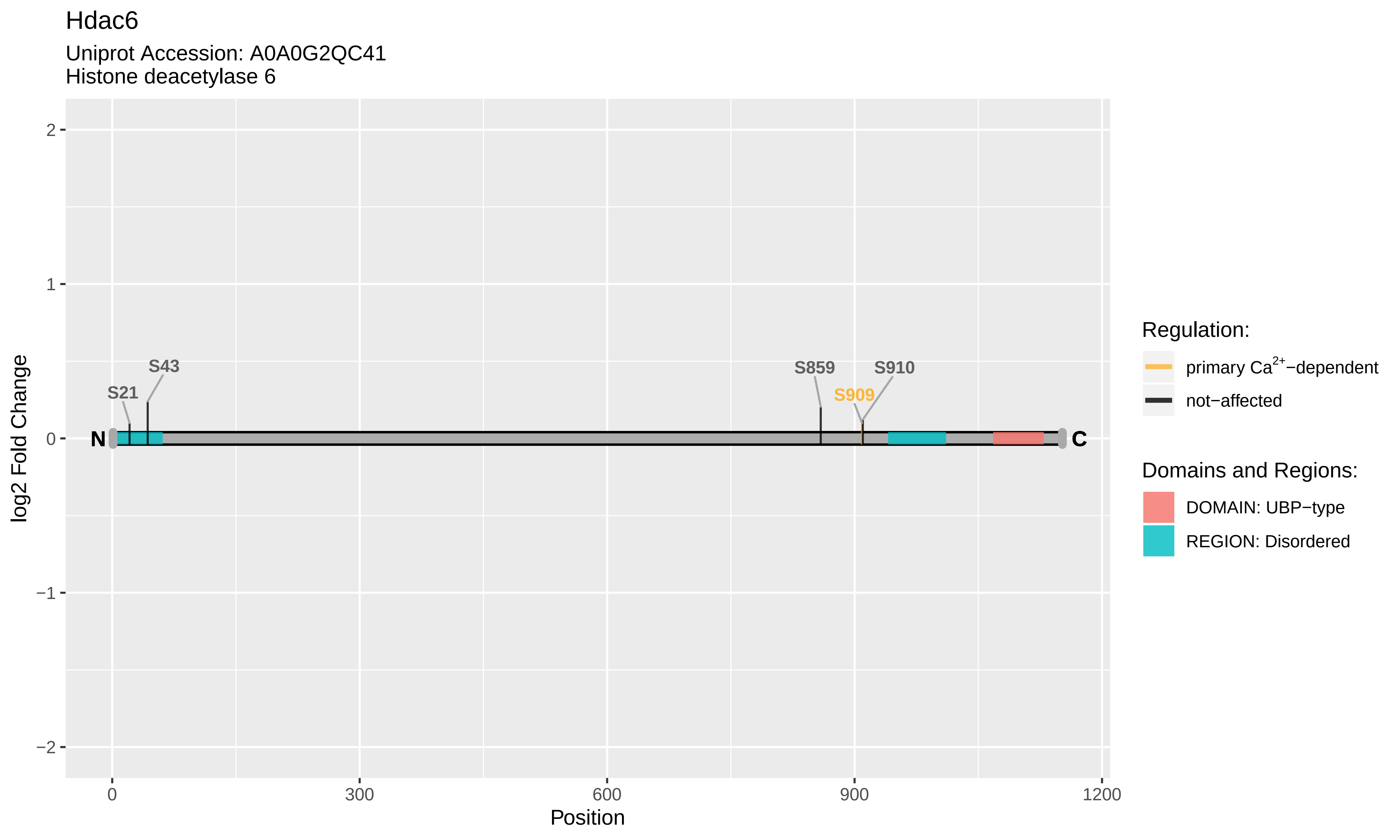

Hdac6
Uniprot Accession: A0A0G2QC41
Histone deacetylase 6
log2 Fold Change
Position
N
C
S21
S43
S859
S909
S910
Regulation:
primary Ca2+−dependent
not−affected
Domains and Regions:
DOMAIN: UBP−type
REGION: Disordered

Hectd1

Uniprot Accession: D3ZLS5
HECT domain E3 ubiquitin protein ligase 1

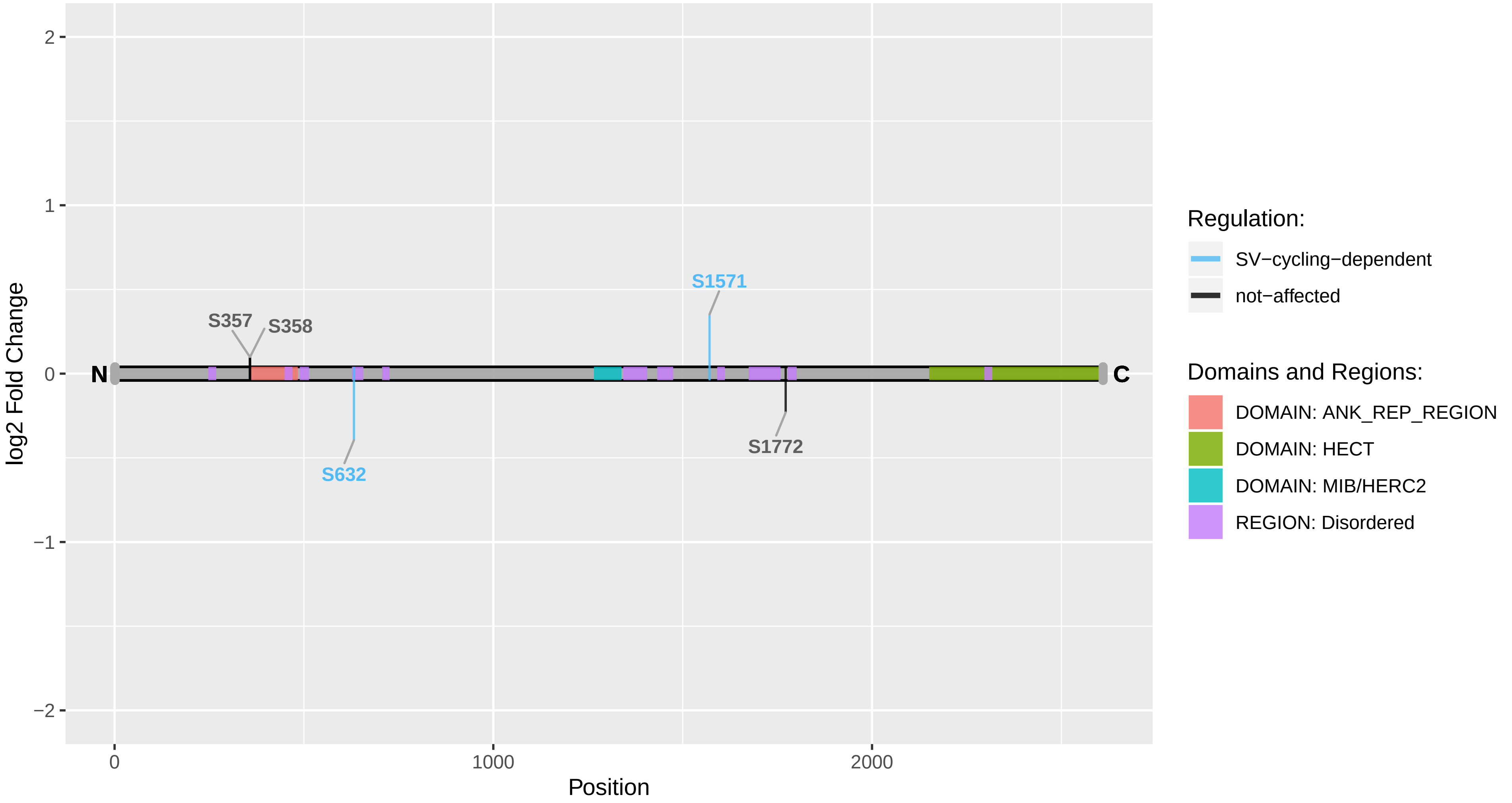

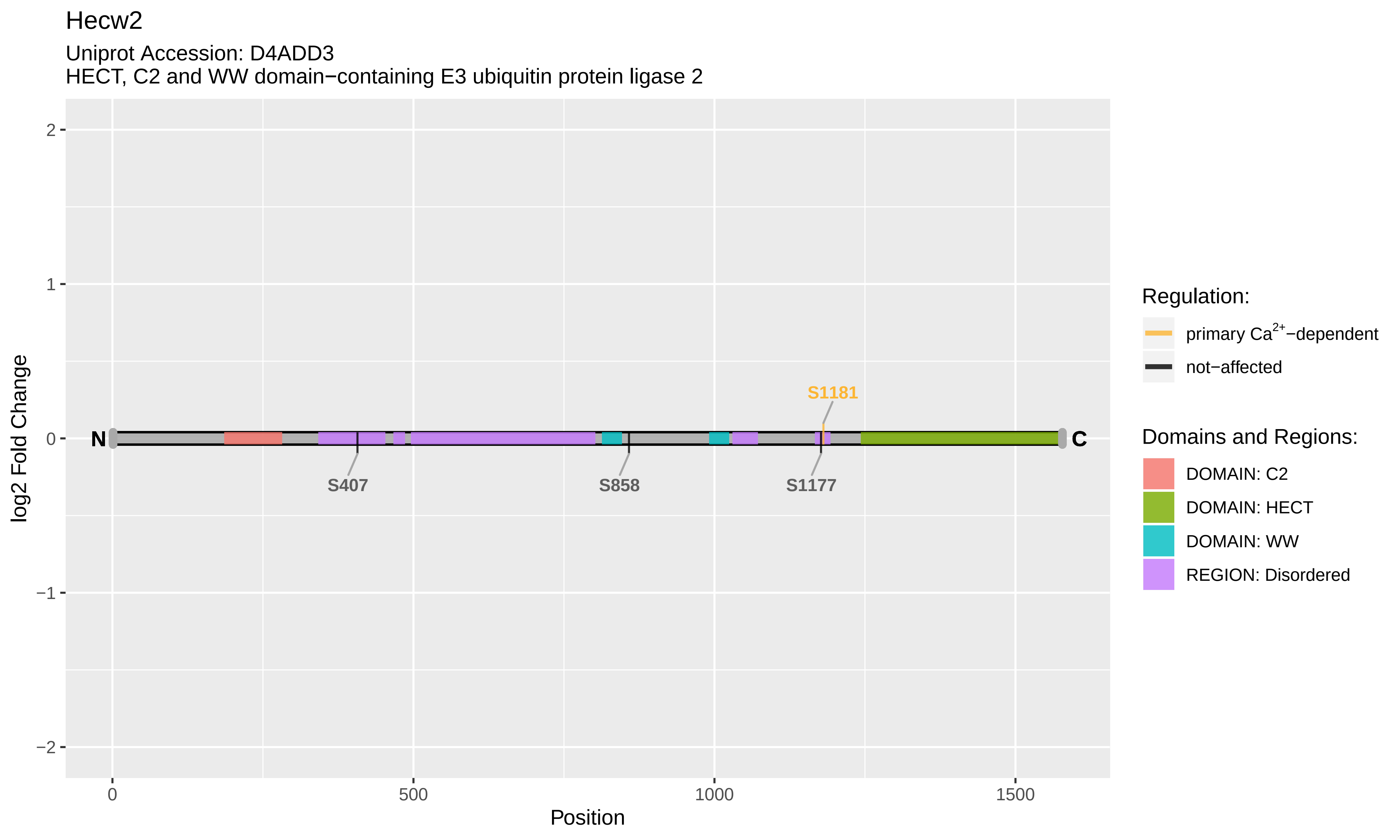
Hecw2
Uniprot Accession: D4ADD3
HECT, C2 and WW domain−containing E3 ubiquitin protein ligase 2
2
1
0
−1
−2
log2 Fold Change
0
500
1000
1500
Position
N
C
S1181
S407
S858
S1177
Regulation:
primary Ca2+−dependent
not−affected
Domains and Regions:
DOMAIN: C2
DOMAIN: HECT
DOMAIN: WW
REGION: Disordered

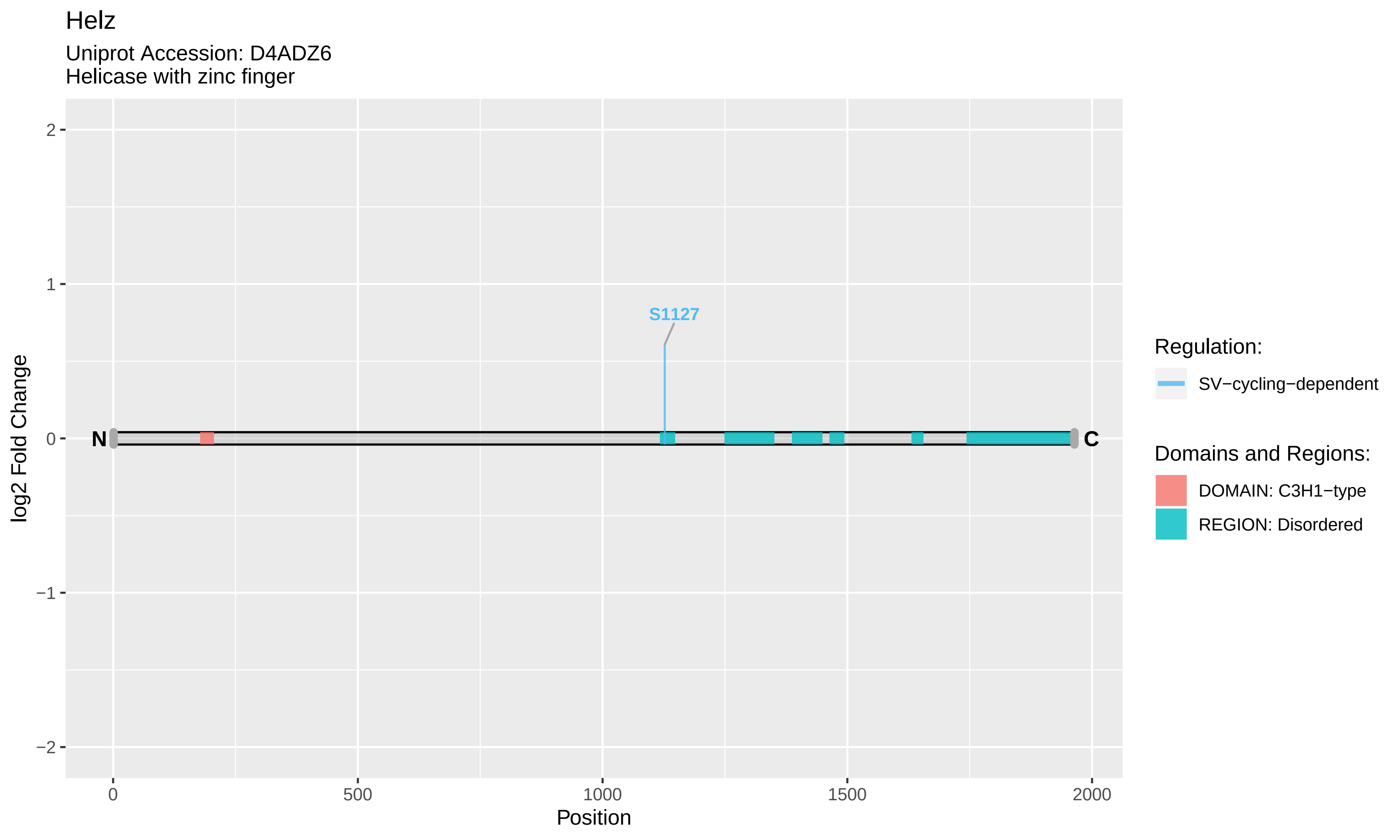

Helz
Uniprot Accession: D4ADZ6
Helicase with zinc finger
S1127
N
C
log2 Fold Change
Position
2
1
0
−1
−2
0
500
1000
1500
2000
Regulation:
SV−cycling−dependent
Domains and Regions:
DOMAIN: C3H1−type
REGION: Disordered

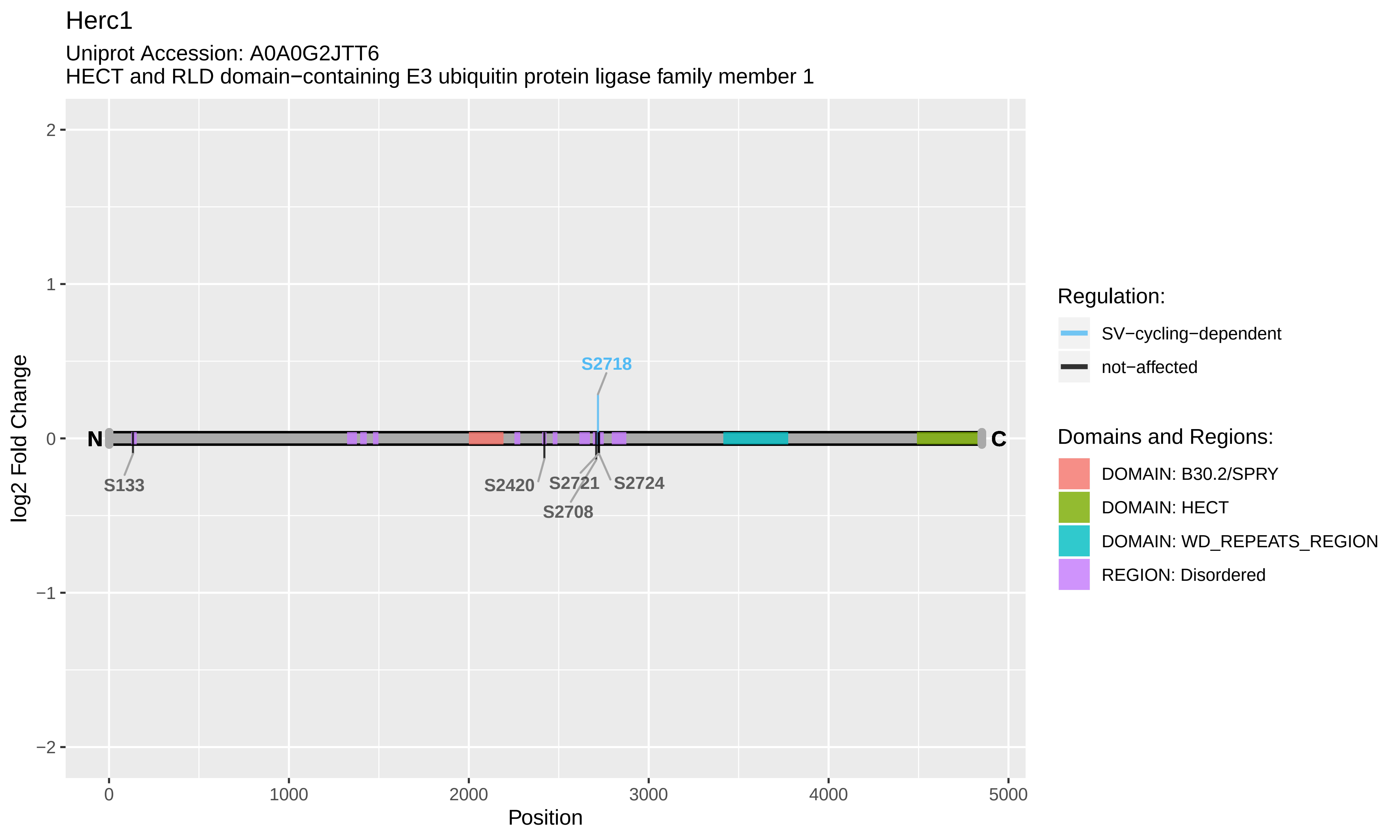

Herc1
Uniprot Accession: A0A0G2JTT6
HECT and RLD domain−containing E3 ubiquitin protein ligase family member 1
log2 Fold Change
2
1
0
−1
−2
N
C
S2718
S133
S2420
S2721
S2724
S2708
0
1000
2000
3000
4000
5000
Position
Regulation:
SV−cycling−dependent
not−affected
Domains and Regions:
DOMAIN: B30.2/SPRY
DOMAIN: HECT
DOMAIN: WD_REPEATS_REGION
REGION: Disordered

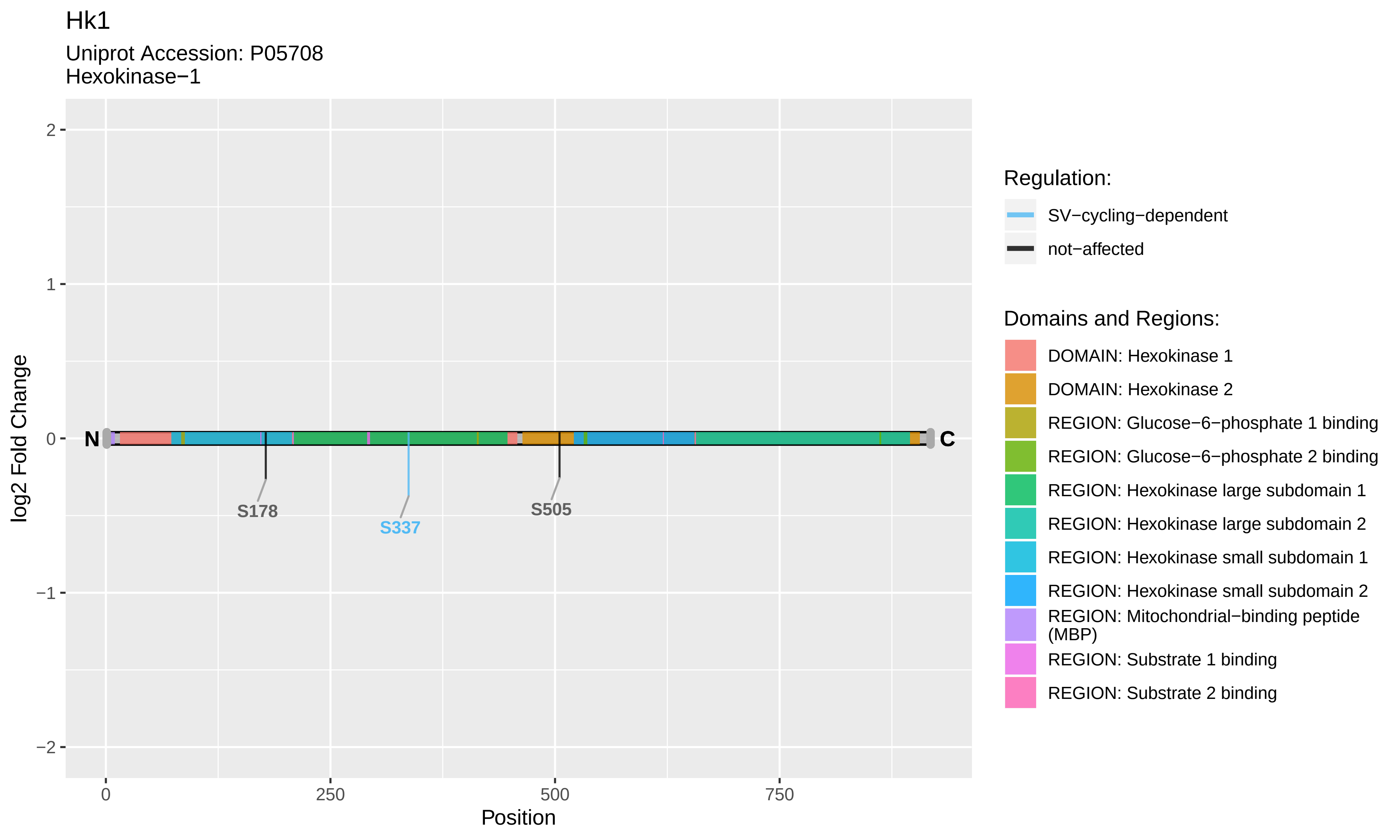
Hk1
Uniprot Accession: P05708
Hexokinase−1
log2 Fold Change
Position
N
C
S178
S337
S505
2
1
0
−1
−2
0
250
500
750
Regulation:
SV−cycling−dependent
not−affected
Domains and Regions:
DOMAIN: Hexokinase 1
DOMAIN: Hexokinase 2
REGION: Glucose−6−phosphate 1 binding
REGION: Glucose−6−phosphate 2 binding
REGION: Hexokinase large subdomain 1
REGION: Hexokinase large subdomain 2
REGION: Hexokinase small subdomain 1
REGION: Hexokinase small subdomain 2
REGION: Mitochondrial−binding peptide (MBP)
REGION: Substrate 1 binding
REGION: Substrate 2 binding

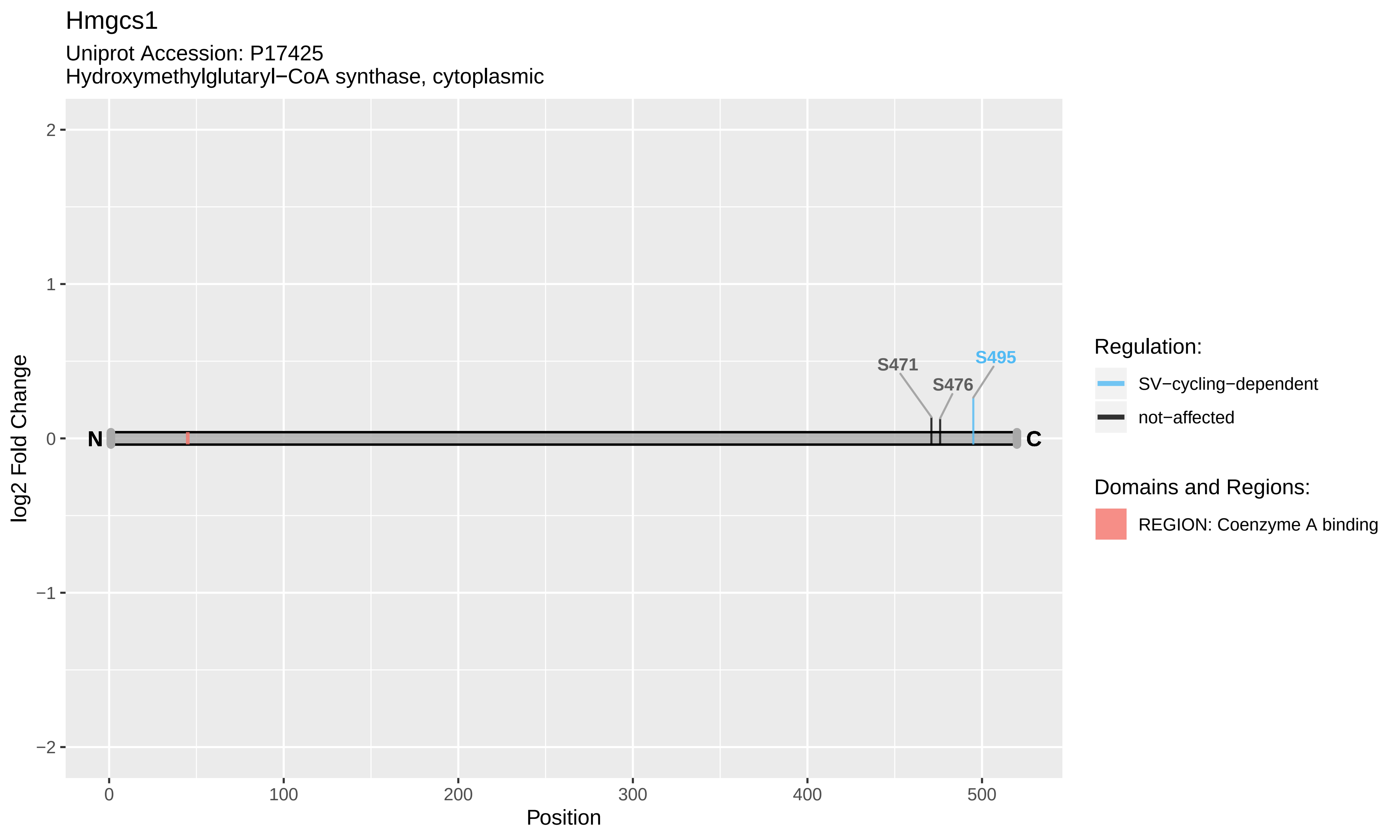

Hmgcs1
Uniprot Accession: P17425
Hydroxymethylglutaryl−CoA synthase, cytoplasmic
S471
S476
S495
N
C
log2 Fold Change
Position
Regulation:
SV−cycling−dependent
not−affected
Domains and Regions:
REGION: Coenzyme A binding

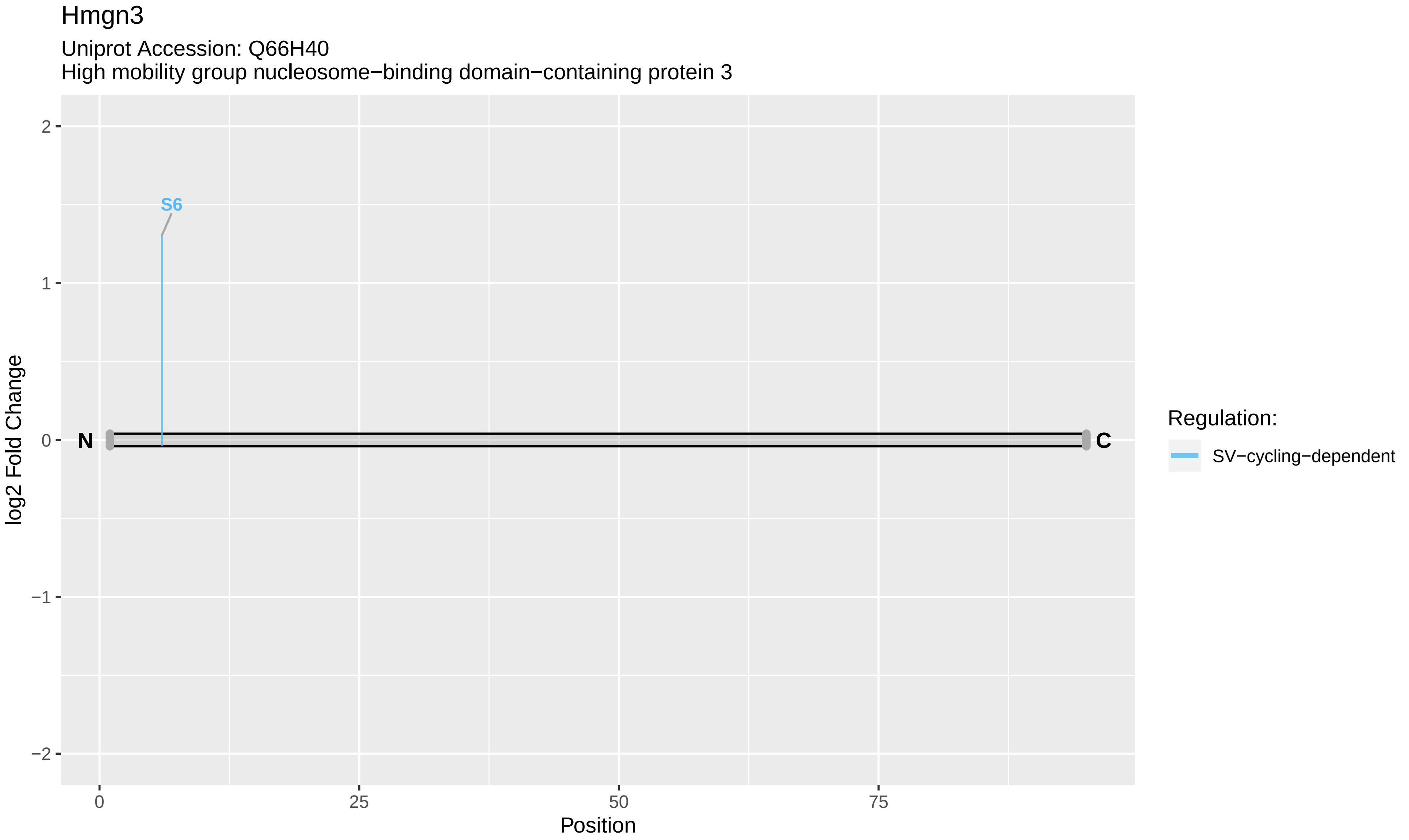

Hmgn3
Uniprot Accession: Q66H40
High mobility group nucleosome−binding domain−containing protein 3
S6
N
C
2
1
0
−1
−2
log2 Fold Change
0
25
50
75
Position
Regulation:
SV−cycling−dependent

Hnrnpa1

Uniprot Accession: P04256
Heterogeneous nuclear ribonucleoprotein A1

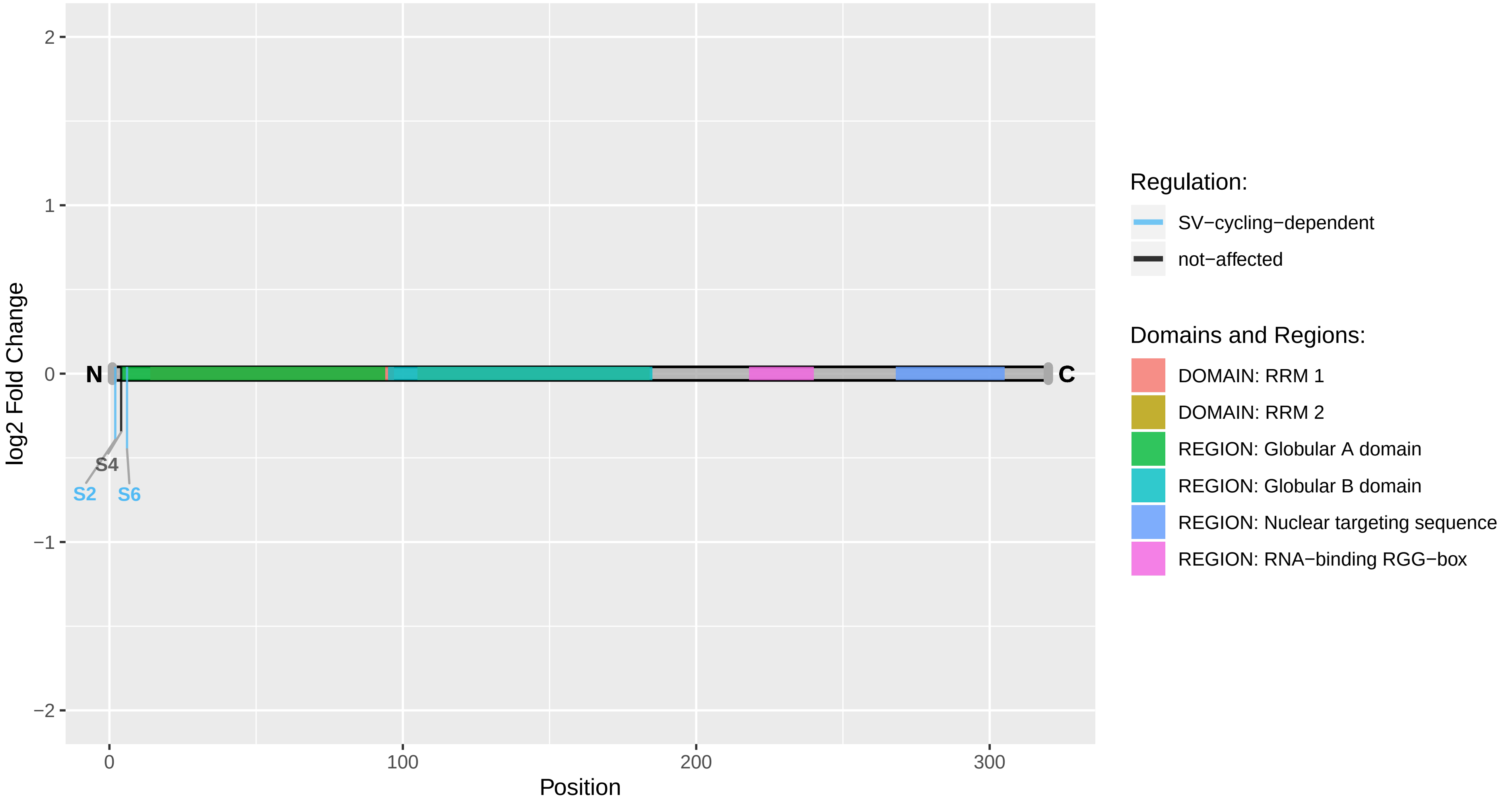

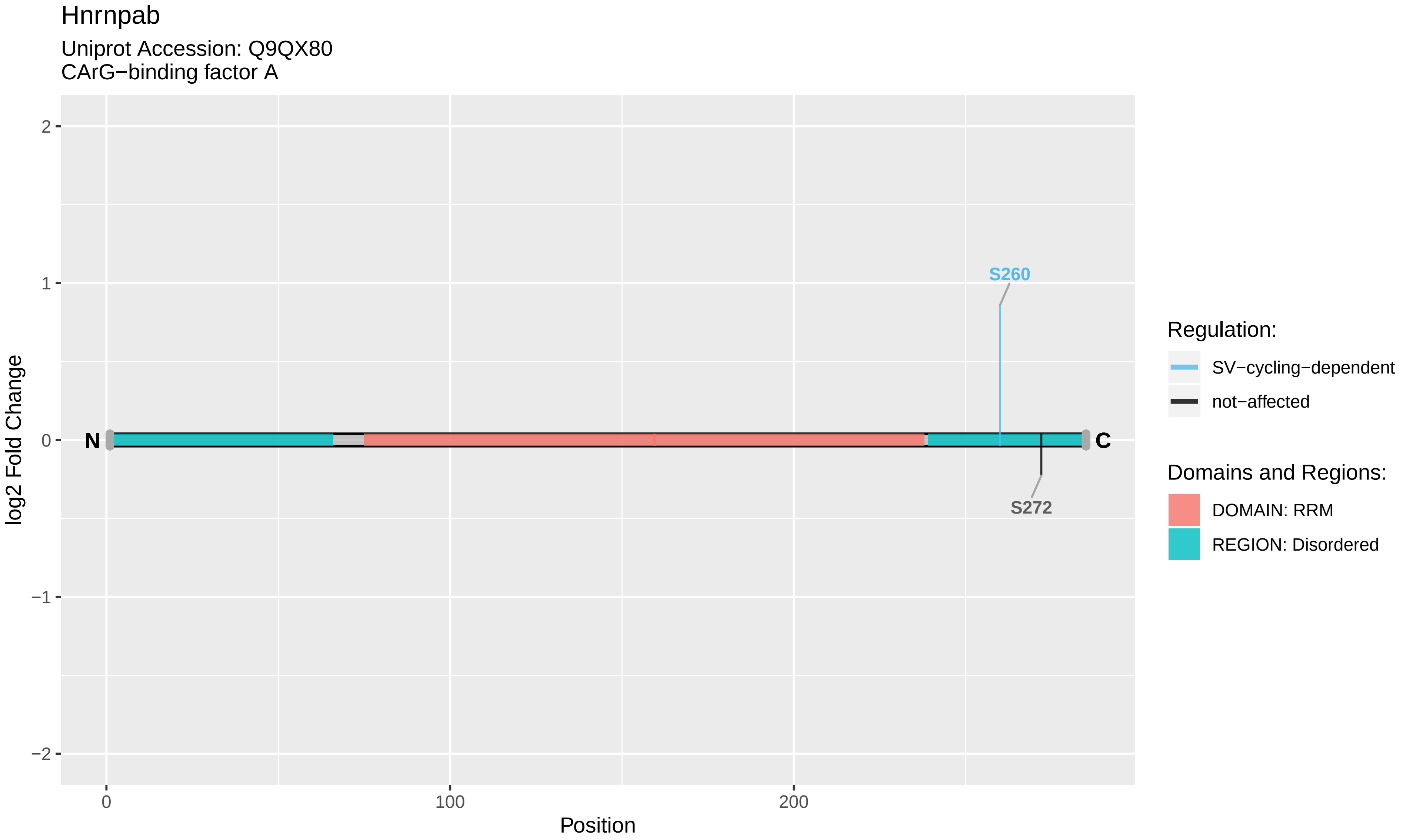
Hnrnpab
Uniprot Accession: Q9QX80
CArG−binding factor A
S260
S272
N
C
2
1
0
−1
−2
log2 Fold Change
0
100
200
Position
Regulation:
SV−cycling−dependent
not−affected
Domains and Regions:
DOMAIN: RRM
REGION: Disordered

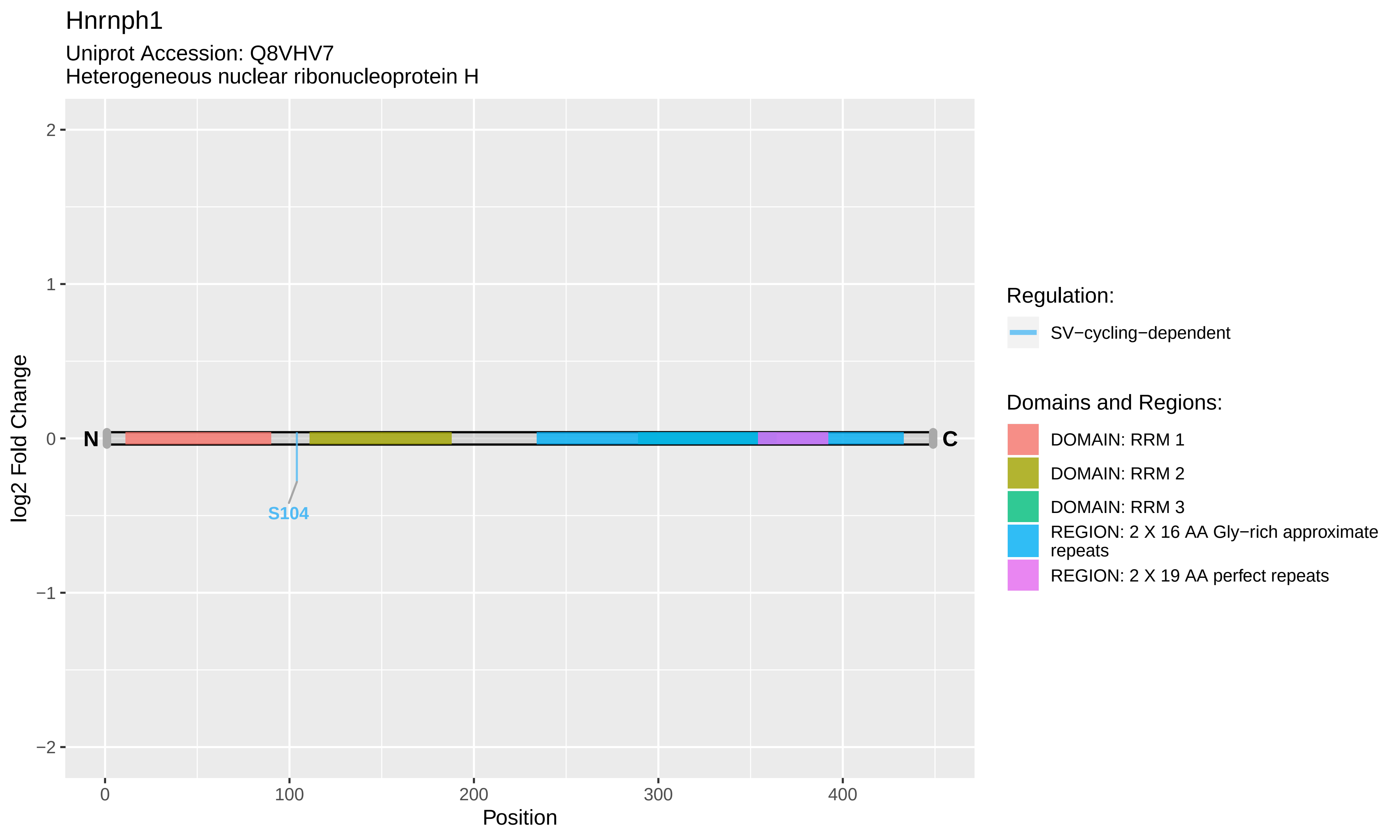
Hnrnph1
Uniprot Accession: Q8VHV7
Heterogeneous nuclear ribonucleoprotein H
log2 Fold Change
2
1
0
−1
−2
N
C
S104
0
100
200
300
400
Position
Regulation:
SV−cycling−dependent
Domains and Regions:
DOMAIN: RRM 1
DOMAIN: RRM 2
DOMAIN: RRM 3
REGION: 2 X 16 AA Gly−rich approximate repeats
REGION: 2 X 19 AA perfect repeats

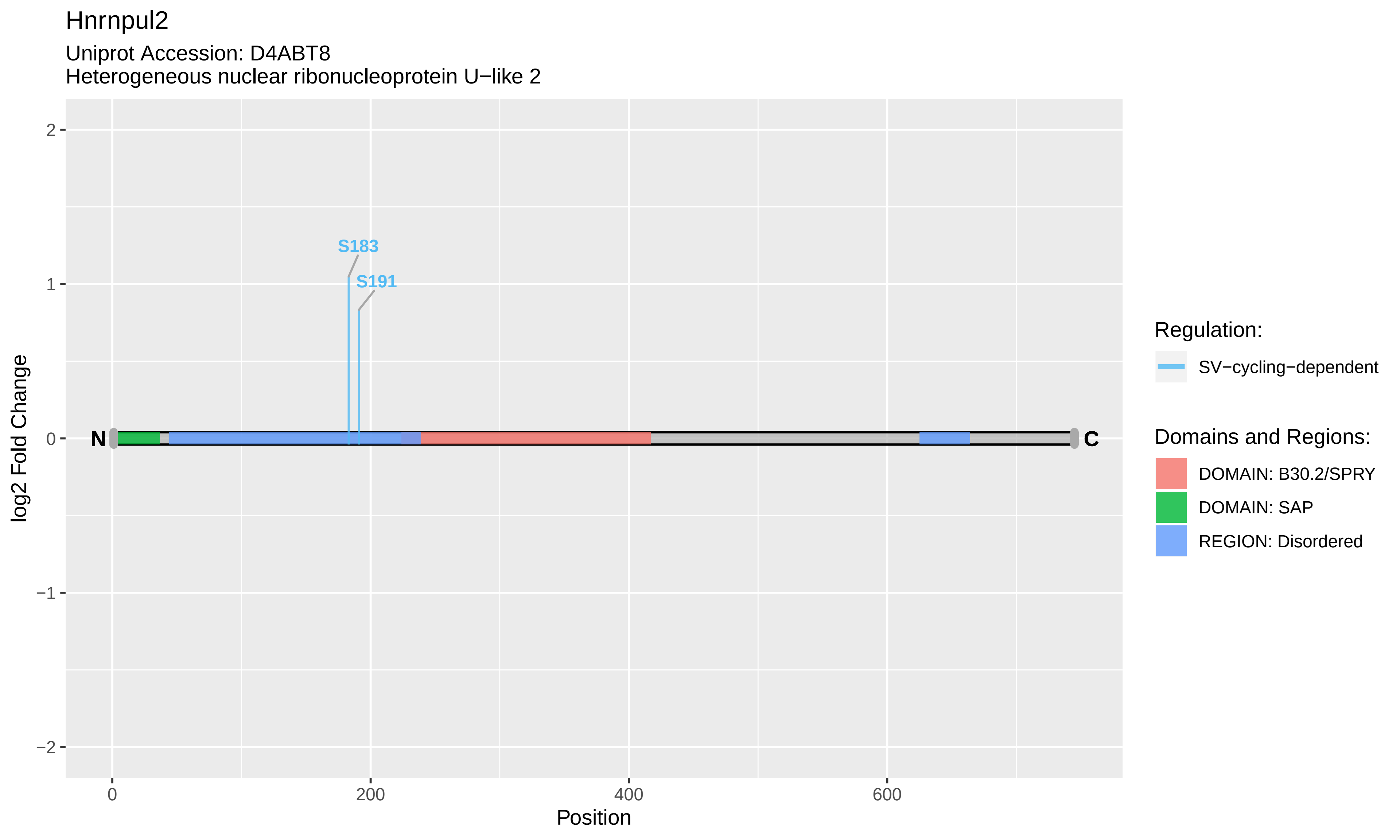

Hnrnpul2
Uniprot Accession: D4ABT8
Heterogeneous nuclear ribonucleoprotein U−like 2
S183
S191
N
C
2
1
0
−1
−2
log2 Fold Change
0
200
400
600
Position
Regulation:
SV−cycling−dependent
Domains and Regions:
DOMAIN: B30.2/SPRY
DOMAIN: SAP
REGION: Disordered

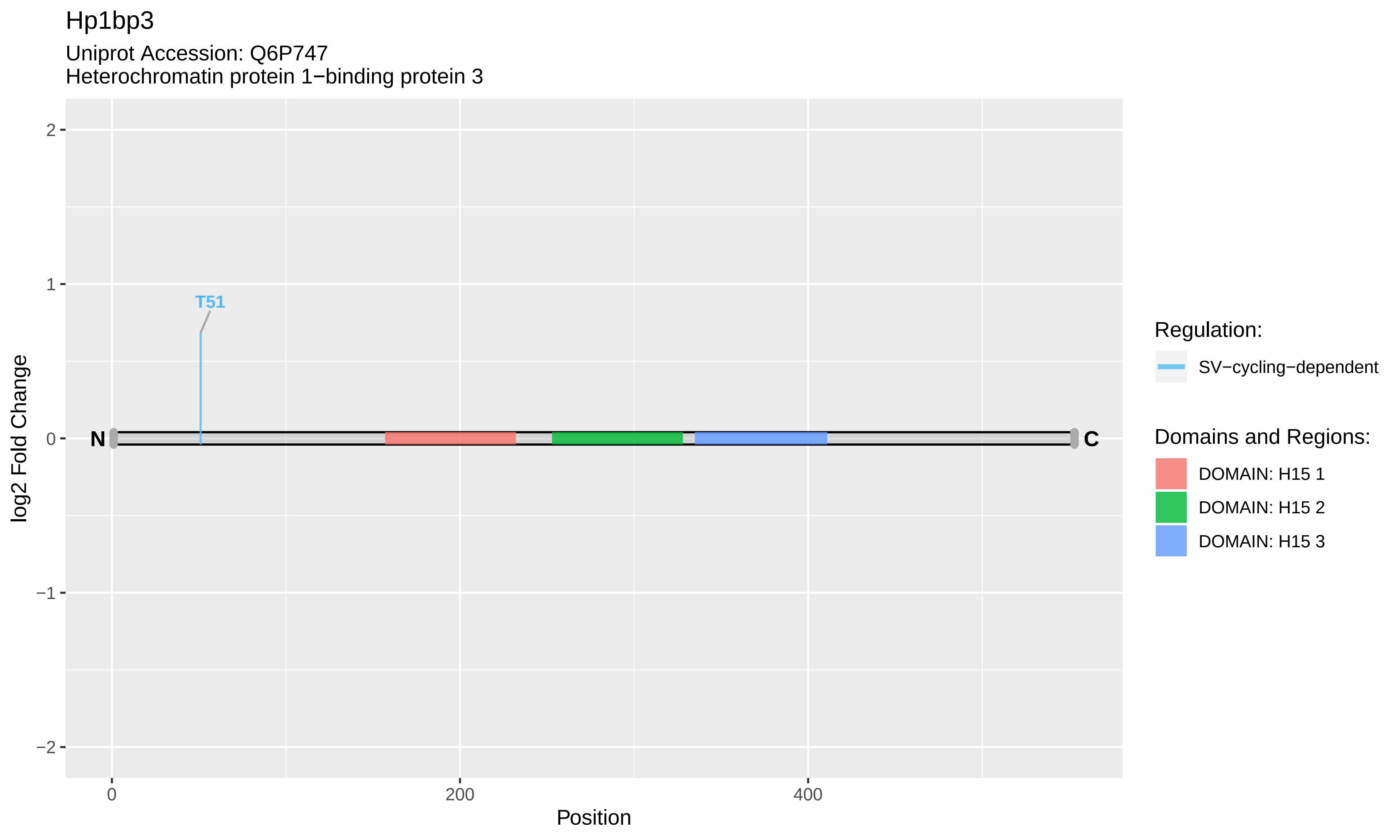

Hp1bp3
Uniprot Accession: Q6P747
Heterochromatin protein 1−binding protein 3
T51
N
C
log2 Fold Change
2
1
0
−1
−2
0
200
400
Position
Regulation:
SV−cycling−dependent
Domains and Regions:
DOMAIN: H15 1
DOMAIN: H15 2
DOMAIN: H15 3

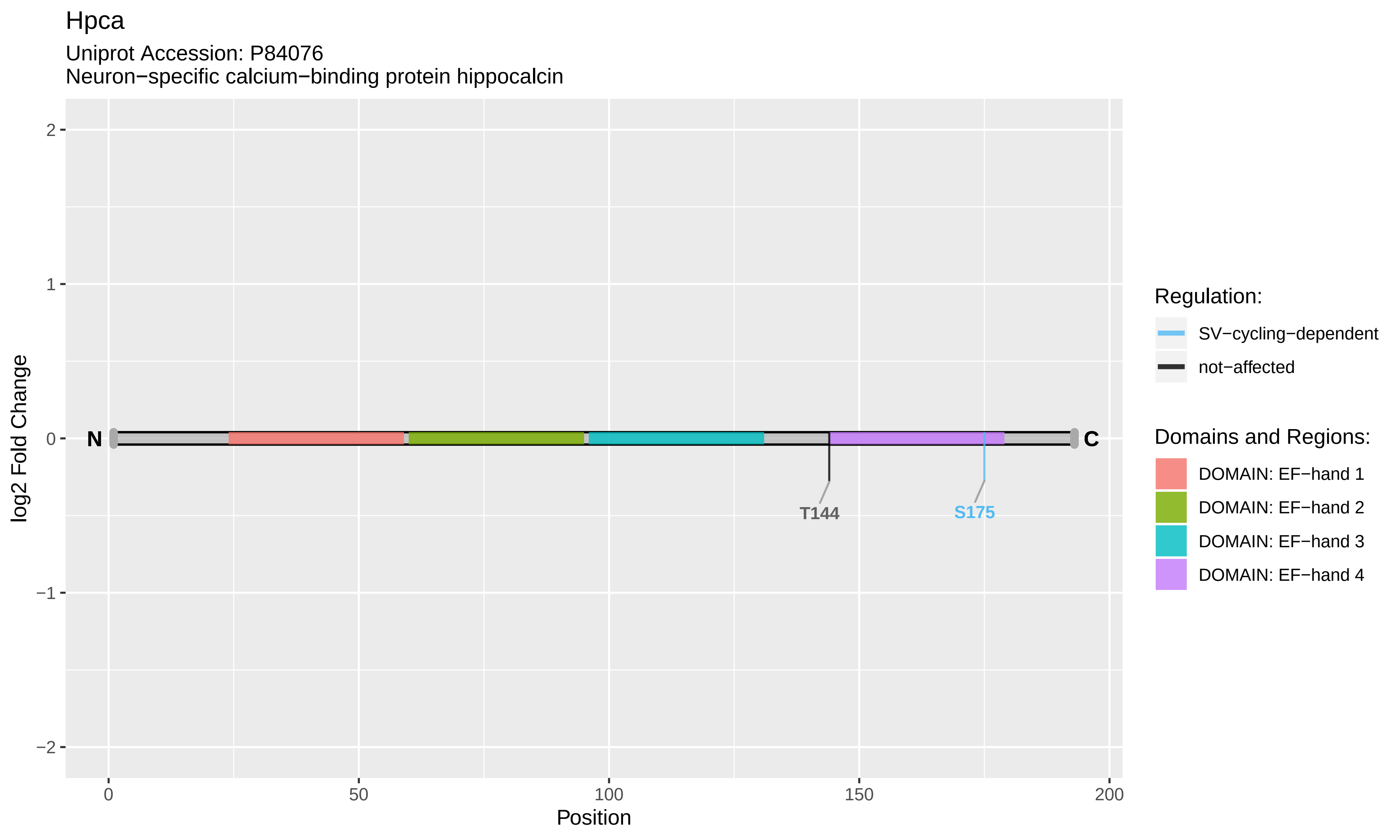
Hpca
Uniprot Accession: P84076
Neuron−specific calcium−binding protein hippocalcin
log2 Fold Change
2
1
0
−1
−2
N
C
T144
S175
0
50
100
150
200
Position
Regulation:
SV−cycling−dependent
not−affected
Domains and Regions:
DOMAIN: EF−hand 1
DOMAIN: EF−hand 2
DOMAIN: EF−hand 3
DOMAIN: EF−hand 4

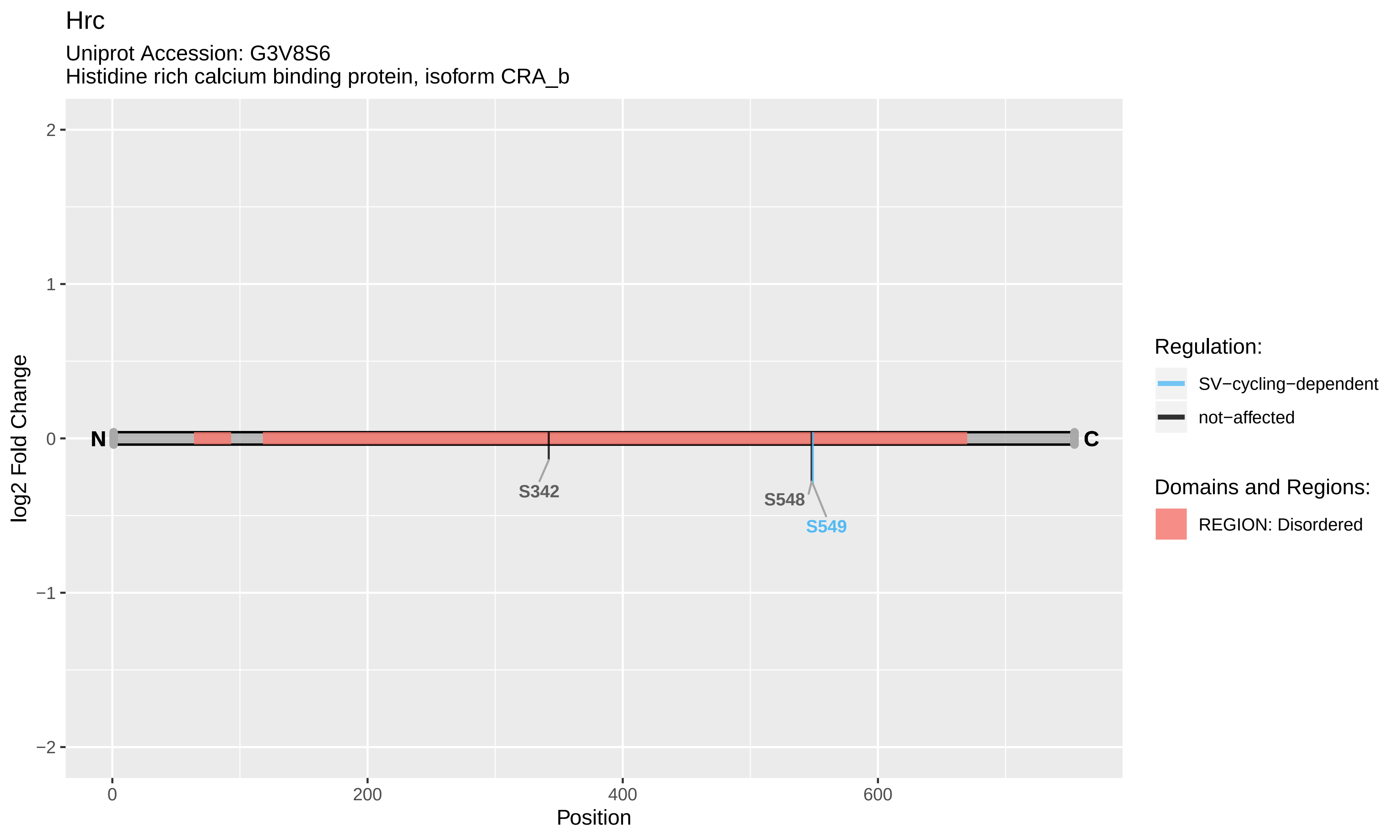
Hrc
Uniprot Accession: G3V8S6
Histidine rich calcium binding protein, isoform CRA_b
log2 Fold Change
2
1
0
−1
−2
N
C
S342
S548
S549
0
200
400
600
Position
Regulation:
SV−cycling−dependent
not−affected
Domains and Regions:
REGION: Disordered

Hs1bp3

Uniprot Accession: D4A6D9
HCLS1−binding protein 3

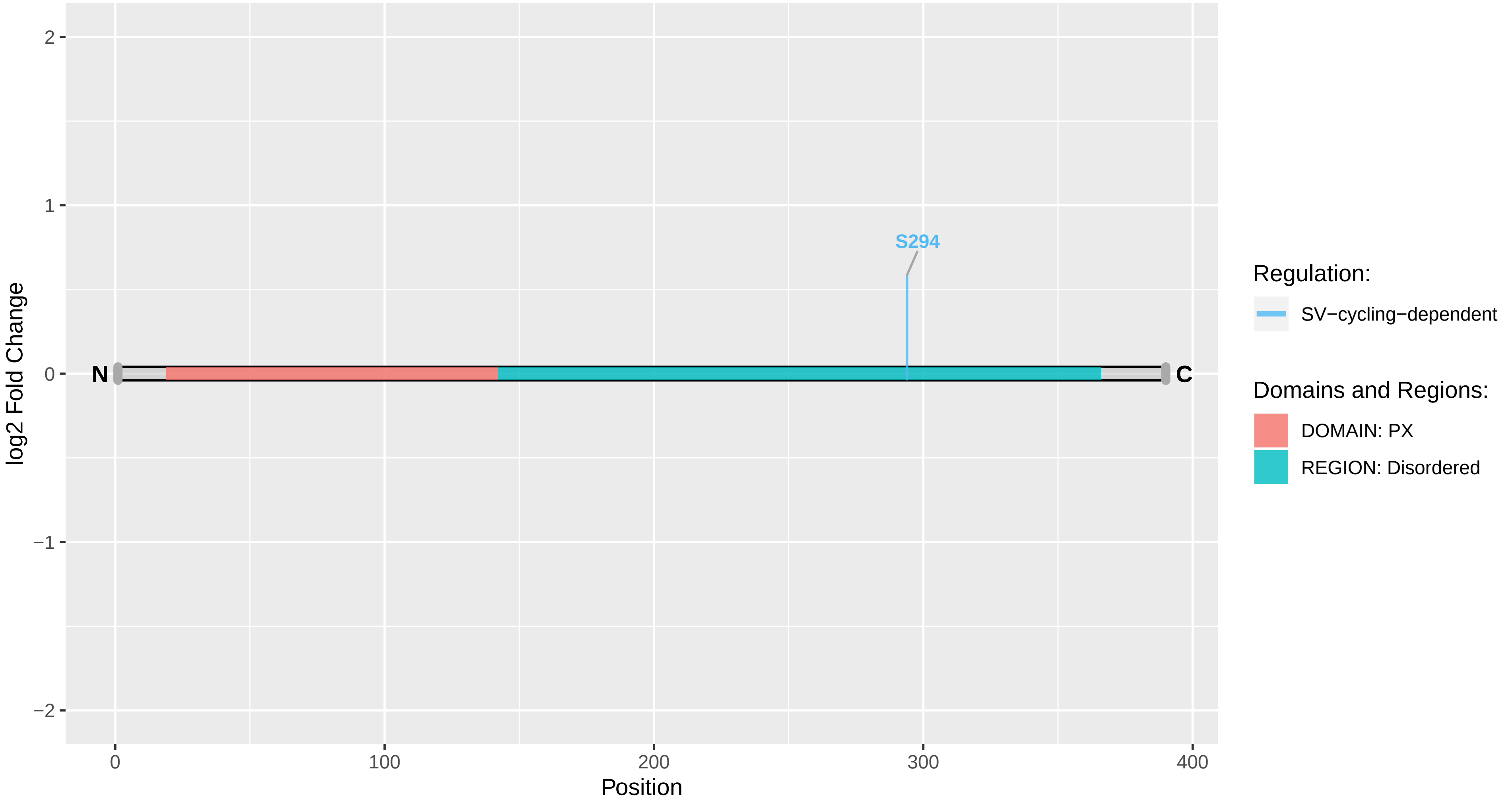

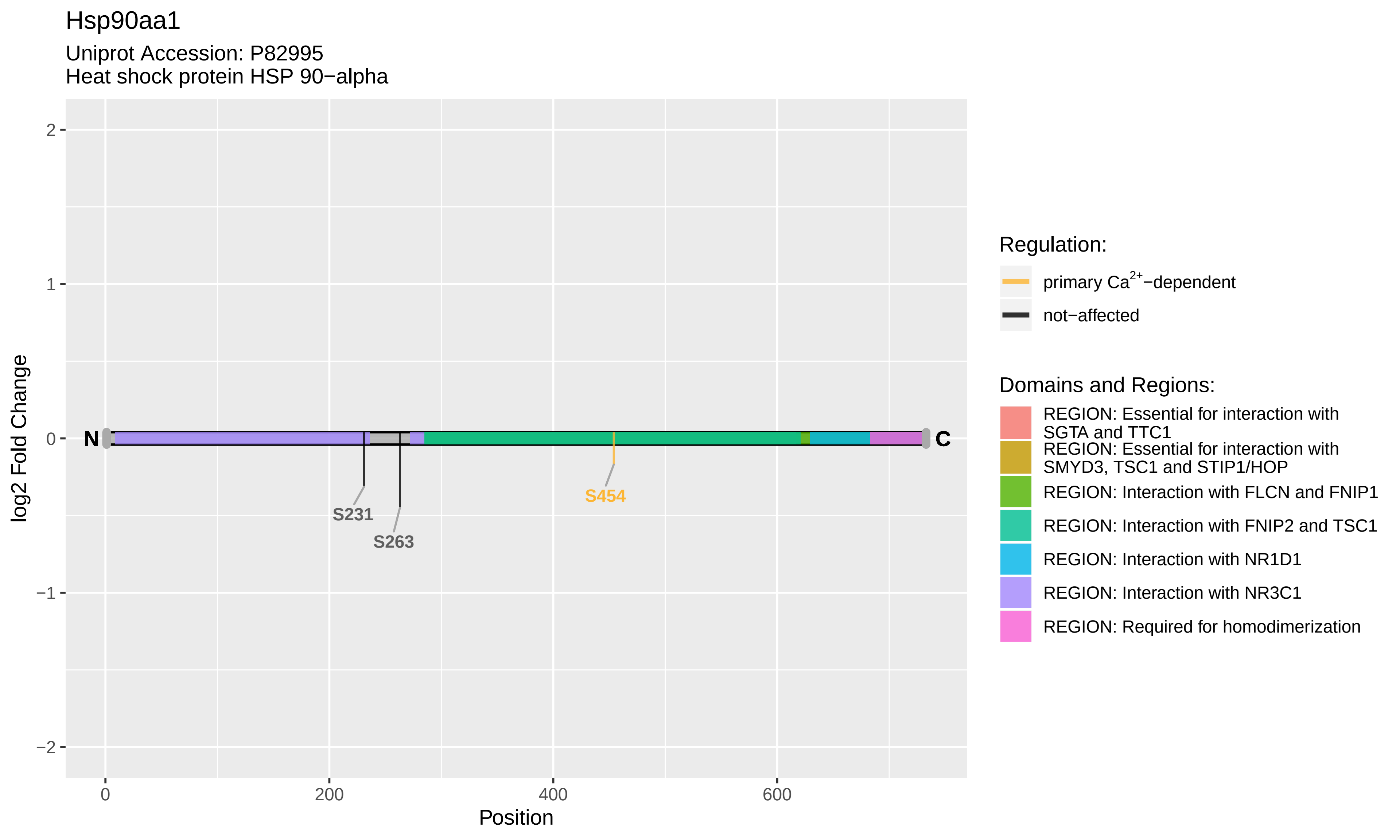

Hsp90aa1
Uniprot Accession: P82995
Heat shock protein HSP 90−alpha
log2 Fold Change
2
1
0
−1
−2
N
C
S231
S263
S454
0
200
400
600
Position
Regulation:
primary Ca2+−dependent
not−affected
Domains and Regions:
REGION: Essential for interaction with SGTA and TTC1
REGION: Essential for interaction with SMYD3, TSC1 and STIP1/HOP
REGION: Interaction with FLCN and FNIP1
REGION: Interaction with FNIP2 and TSC1
REGION: Interaction with NR1D1
REGION: Interaction with NR3C1
REGION: Required for homodimerization

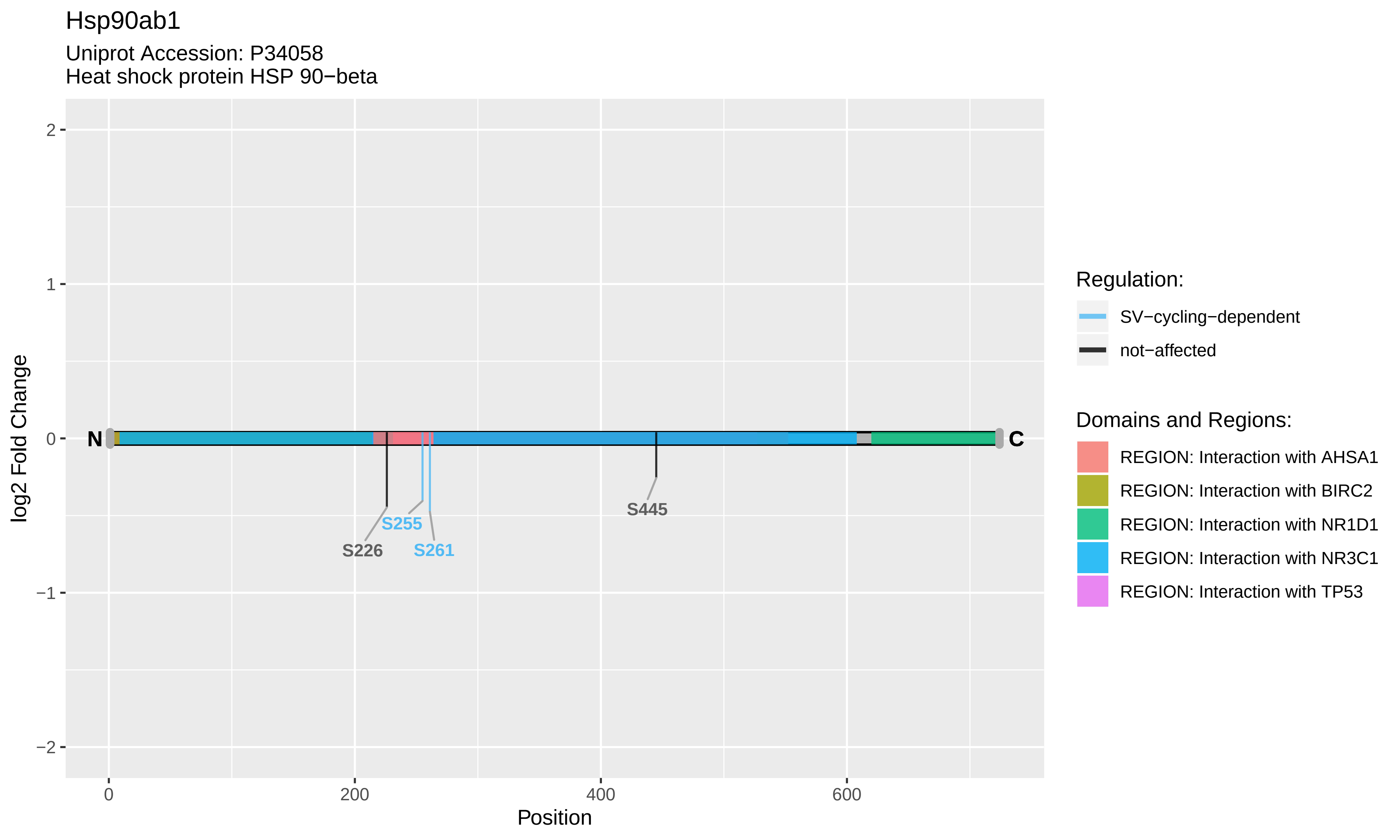

Hsp90ab1
Uniprot Accession: P34058
Heat shock protein HSP 90−beta
log2 Fold Change
2
1
0
−1
−2
N
C
S226
S255
S261
S445
0
200
400
600
Position
Regulation:
SV−cycling−dependent
not−affected
Domains and Regions:
REGION: Interaction with AHSA1
REGION: Interaction with BIRC2
REGION: Interaction with NR1D1
REGION: Interaction with NR3C1
REGION: Interaction with TP53

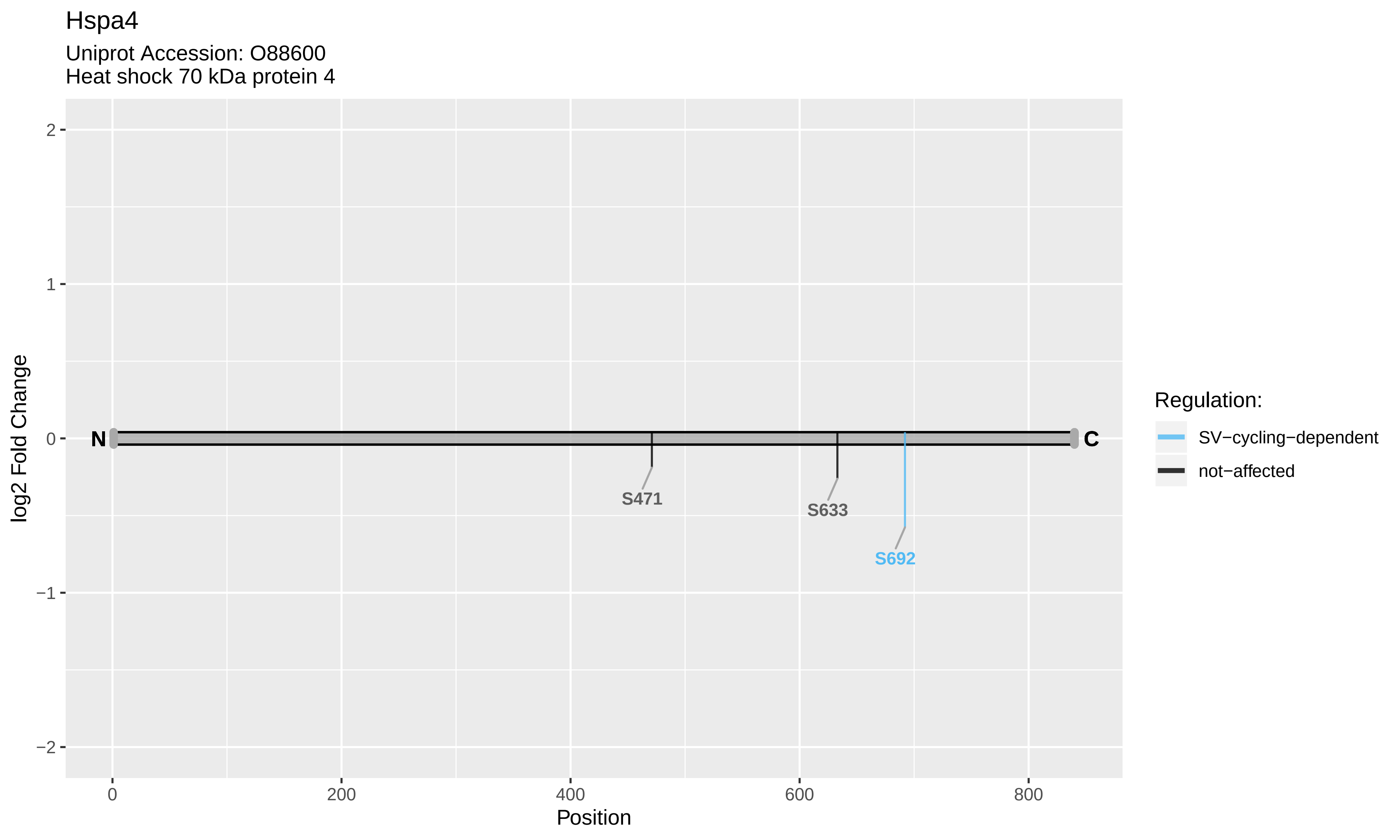

Hspa4
Uniprot Accession: O88600
Heat shock 70 kDa protein 4
log2 Fold Change
2
1
0
−1
−2
N
C
S471
S633
S692
0
200
400
600
800
Position
Regulation:
SV−cycling−dependent
not−affected

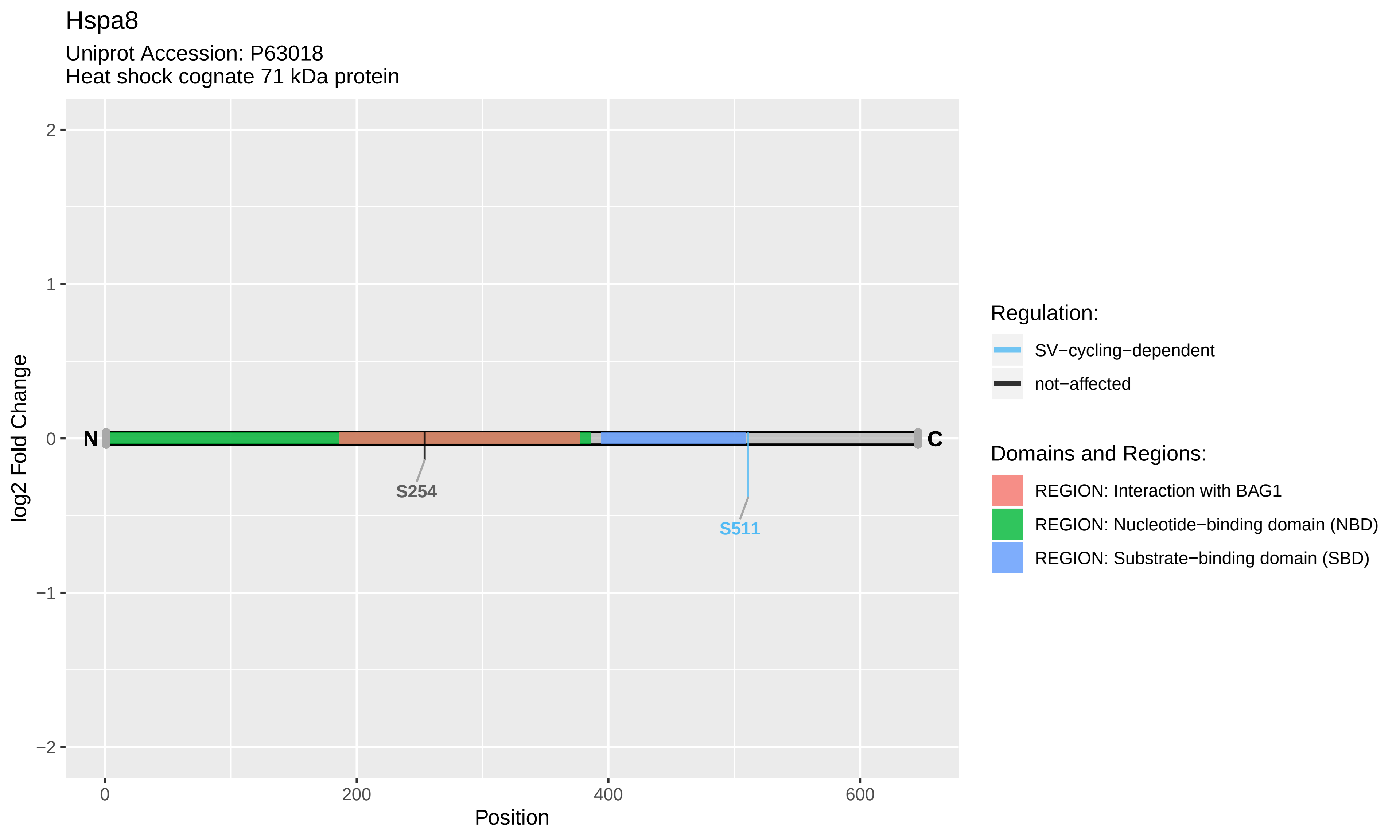
Hspa8
Uniprot Accession: P63018
Heat shock cognate 71 kDa protein
log2 Fold Change
Position
N
C
S254
S511
Regulation:
SV−cycling−dependent
not−affected
Domains and Regions:
REGION: Interaction with BAG1
REGION: Nucleotide−binding domain (NBD)
REGION: Substrate−binding domain (SBD)

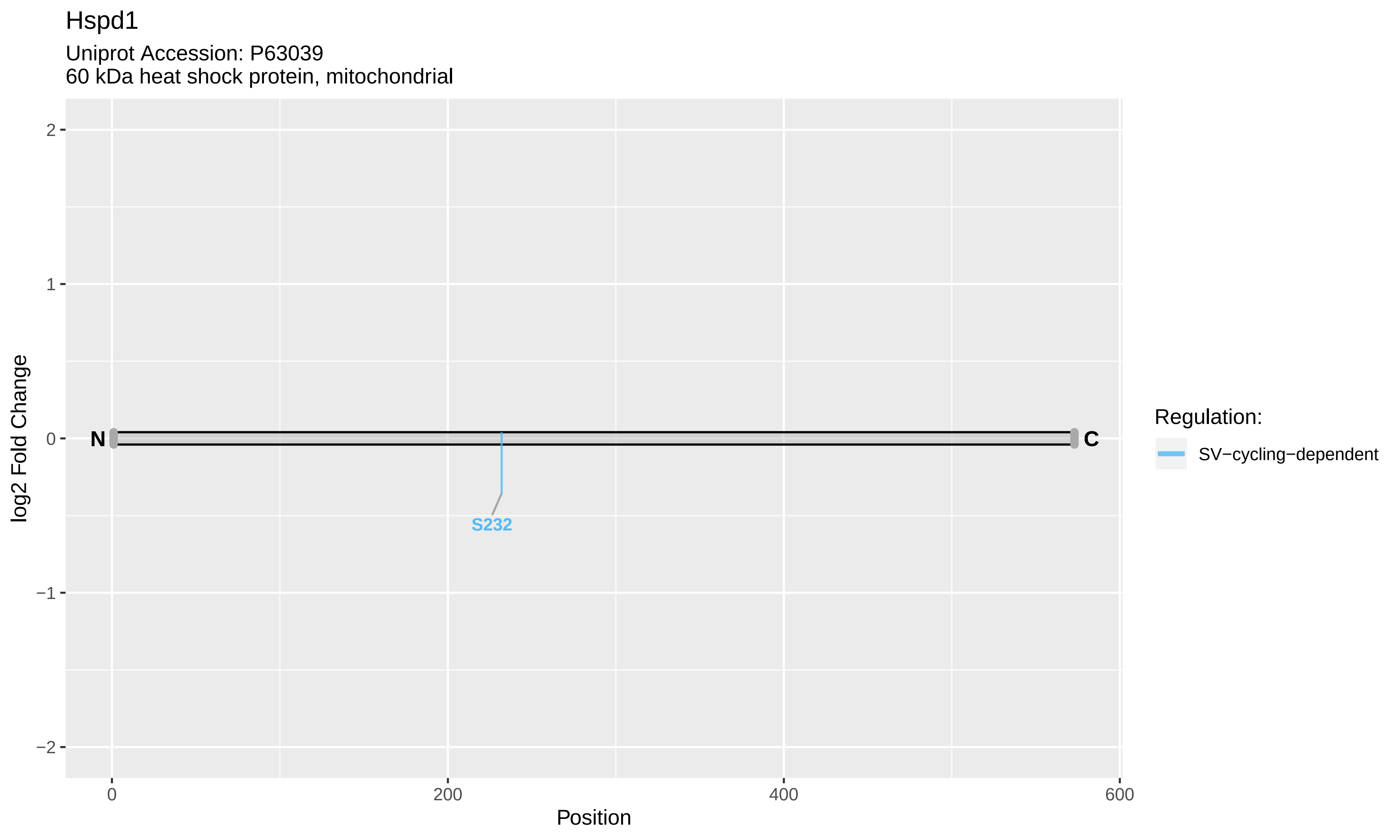

Hspd1
Uniprot Accession: P63039
60 kDa heat shock protein, mitochondrial
log2 Fold Change
2
1
0
−1
−2
N
C
S232
0
200
400
600
Position
Regulation:
SV−cycling−dependent

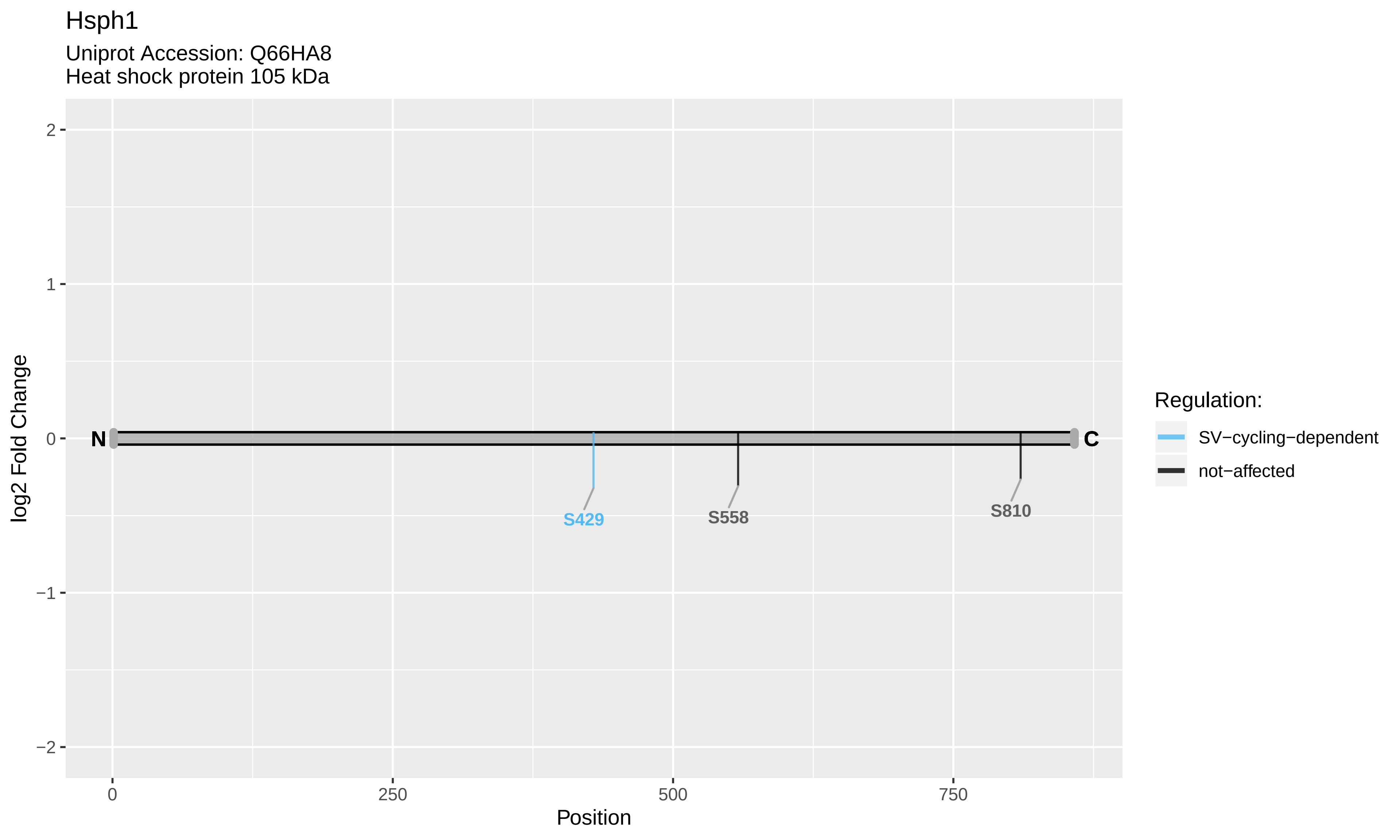

Hsph1
Uniprot Accession: Q66HA8
Heat shock protein 105 kDa
log2 Fold Change
2
1
0
−1
−2
N
C
S429
S558
S810
0
250
500
750
Position
Regulation:
SV−cycling−dependent
not−affected

Idh3a

Uniprot Accession: Q99NA5
Isocitrate dehydrogenase [NAD] subunit alpha, mitochondrial

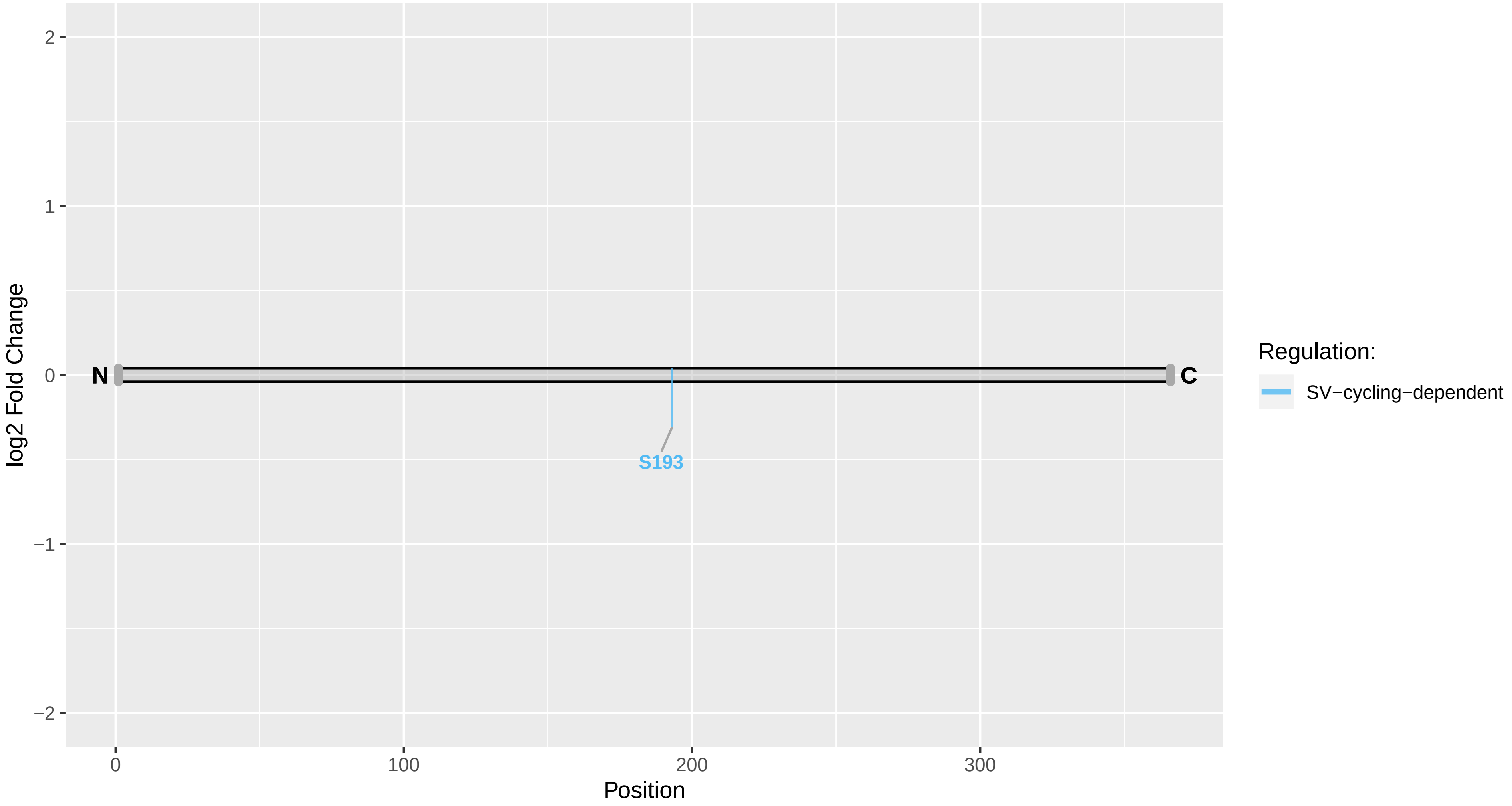

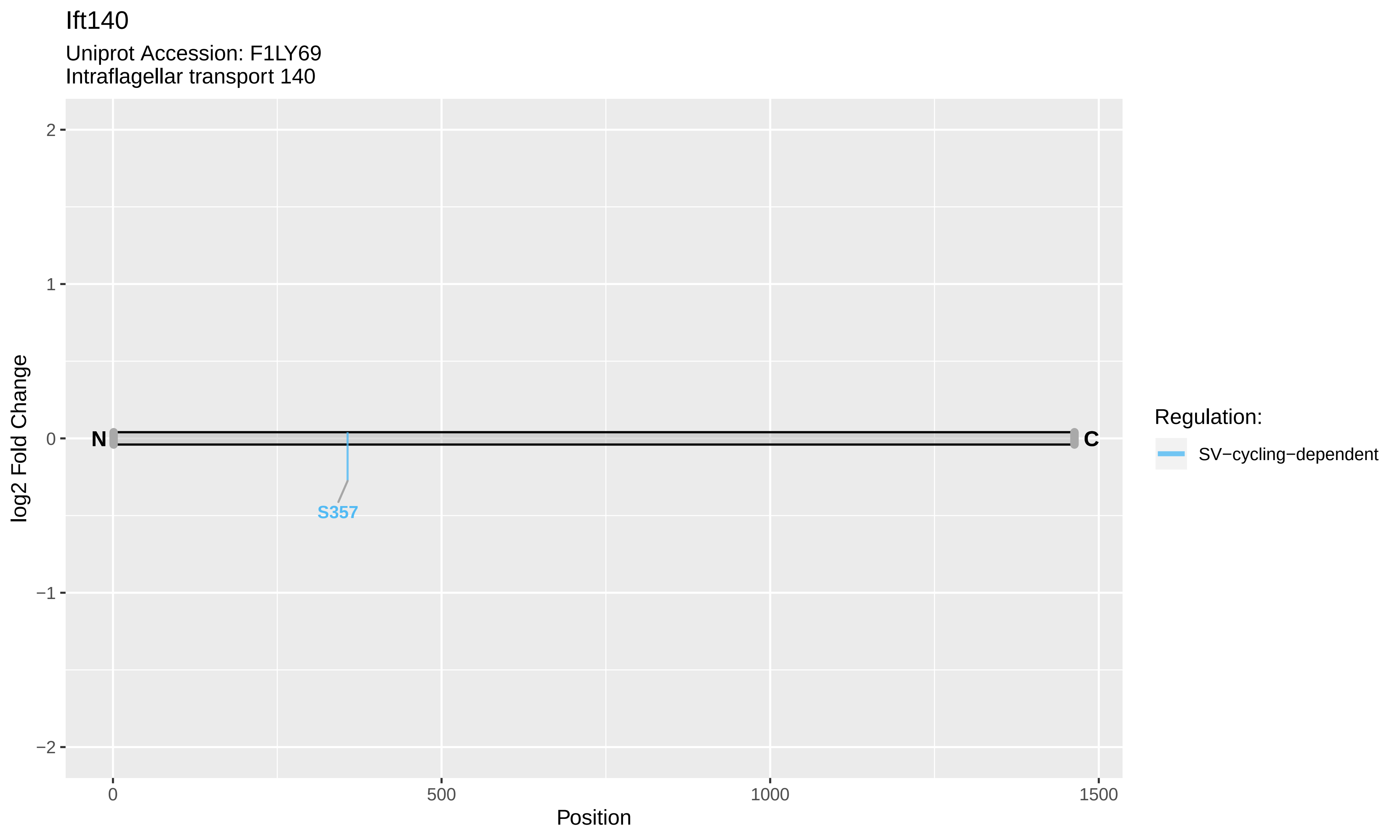
Ift140
Uniprot Accession: F1LY69
Intraflagellar transport 140
log2 Fold Change
2
1
0
−1
−2
N
C
S357
0
500
1000
1500
Position
Regulation:
SV−cycling−dependent

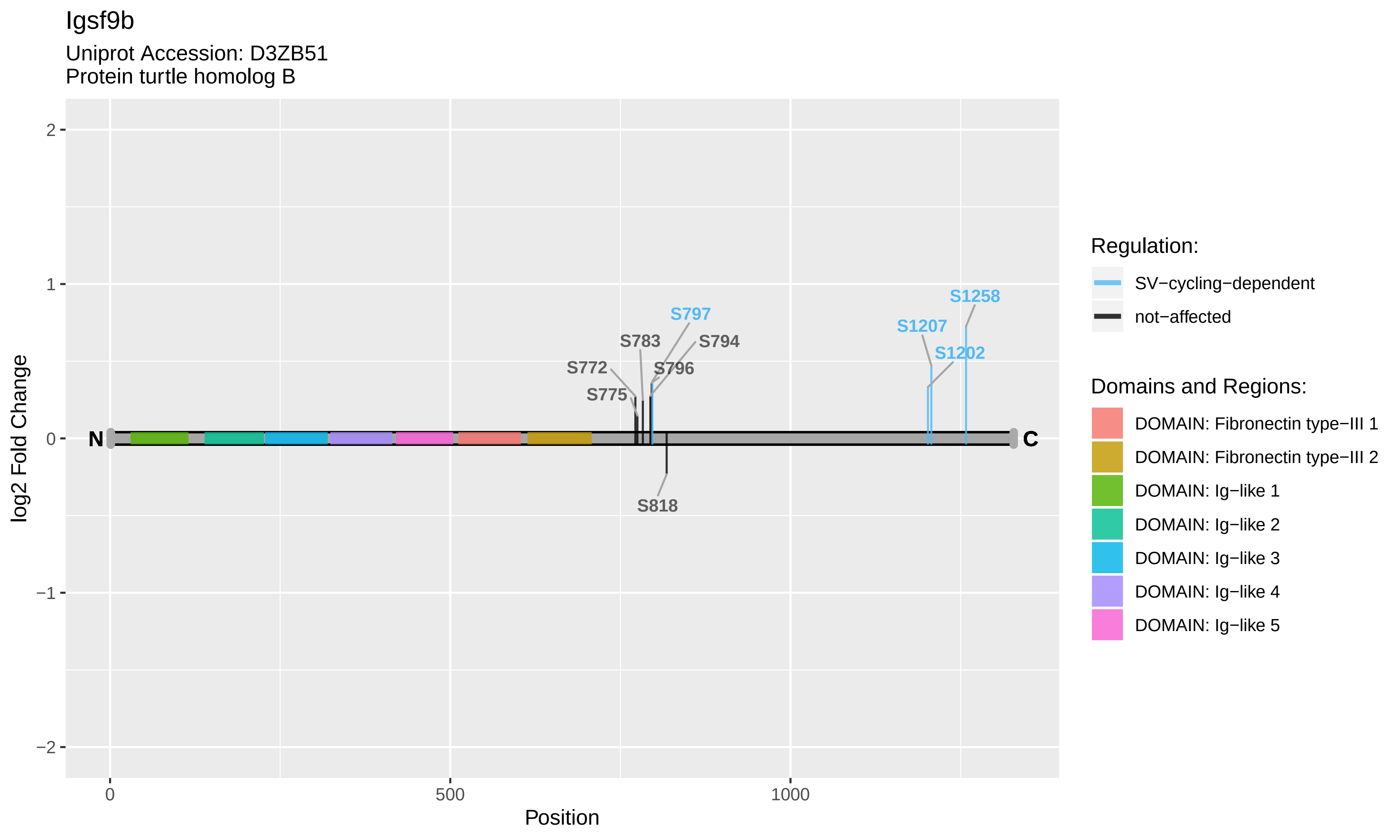
Igsf9b
Uniprot Accession: D3ZB51
Protein turtle homolog B
log2 Fold Change
Position
N
C
S772
S775
S783
S794
S796
S797
S818
S1202
S1207
S1258
Regulation:
SV−cycling−dependent
not−affected
Domains and Regions:
DOMAIN: Fibronectin type−III 1
DOMAIN: Fibronectin type−III 2
DOMAIN: Ig−like 1
DOMAIN: Ig−like 2
DOMAIN: Ig−like 3
DOMAIN: Ig−like 4
DOMAIN: Ig−like 5

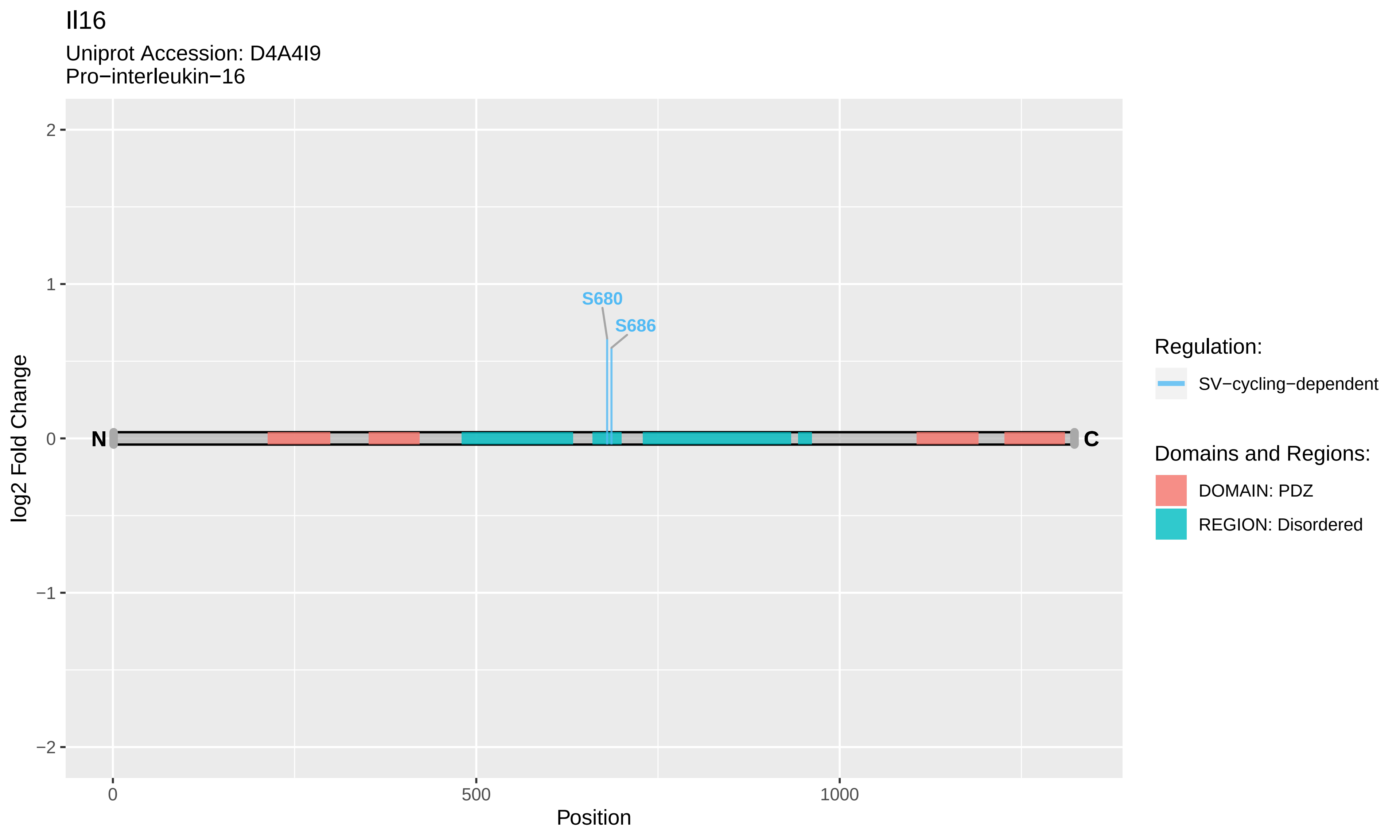
Il16
Uniprot Accession: D4A4I9
Pro−interleukin−16
S680
S686
N
C
2
1
0
−1
−2
log2 Fold Change
0
500
1000
Position
Regulation:
SV−cycling−dependent
Domains and Regions:
DOMAIN: PDZ
REGION: Disordered

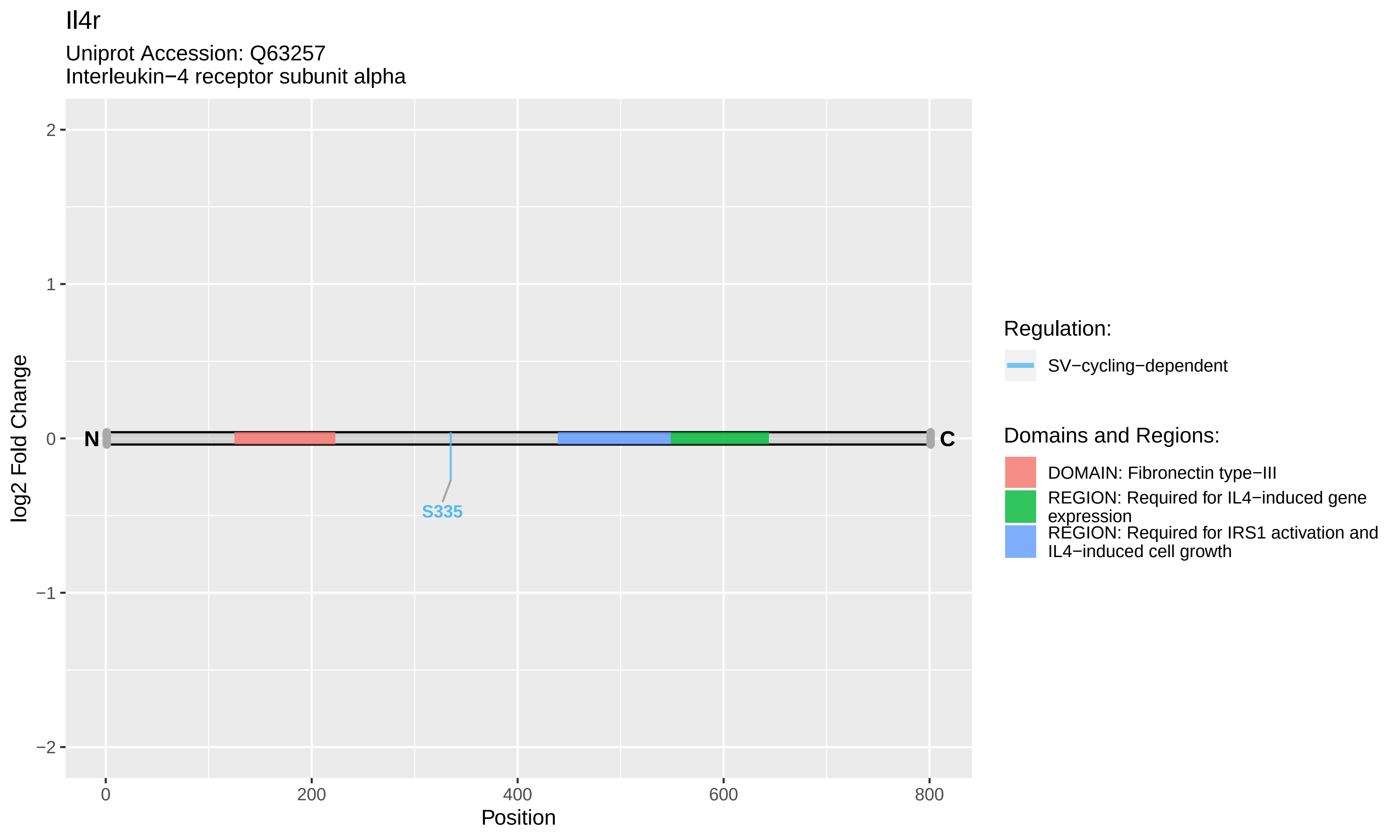
Il4r
Uniprot Accession: Q63257
Interleukin−4 receptor subunit alpha
2
1
0
−1
−2
log2 Fold Change
N
C
S335
0
200
400
600
800
Position
Regulation:
SV−cycling−dependent
Domains and Regions:
DOMAIN: Fibronectin type−III
REGION: Required for IL4−induced gene expression
REGION: Required for IRS1 activation and IL4−induced cell growth

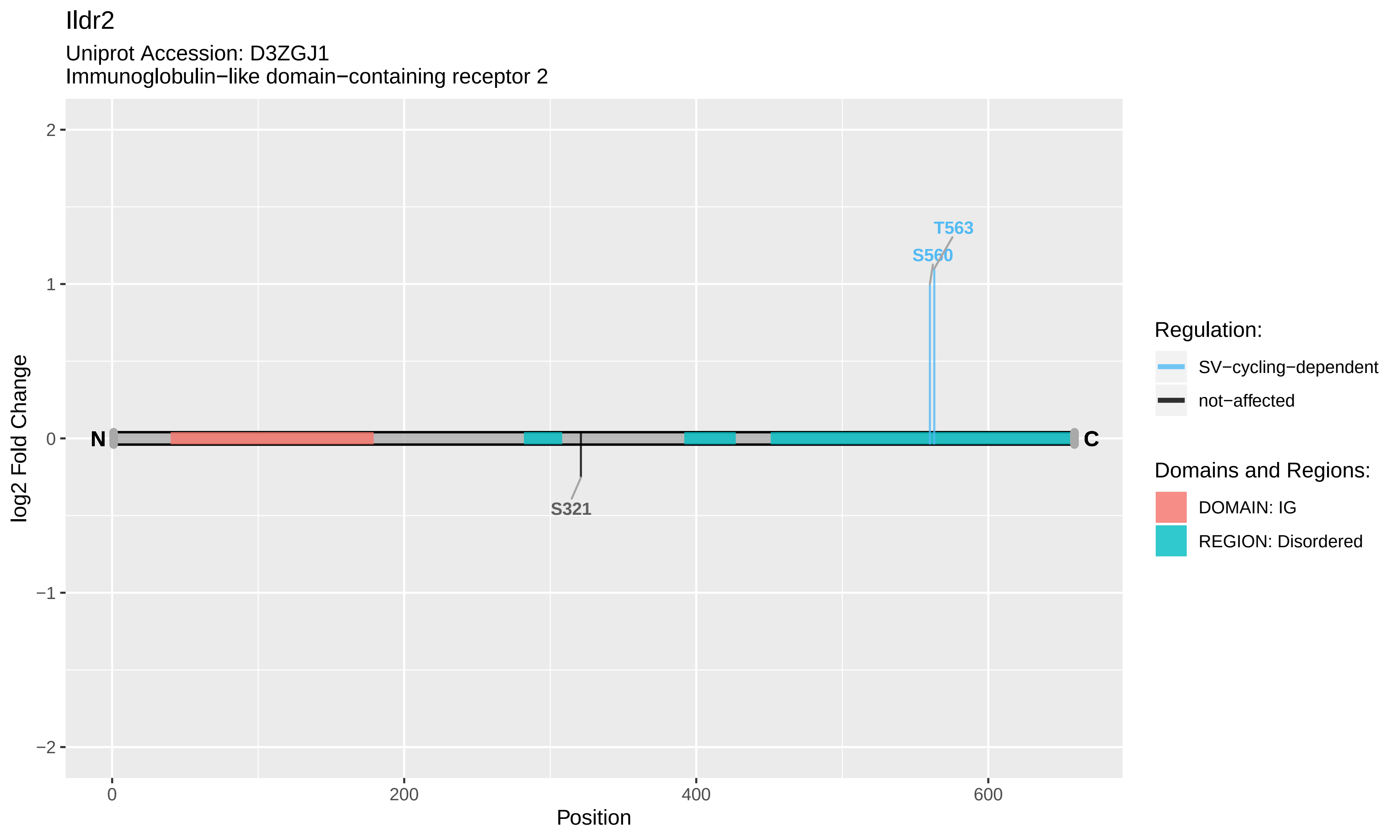

Ildr2
Uniprot Accession: D3ZGJ1
Immunoglobulin−like domain−containing receptor 2
T563
S560
S321
N
C
2
1
0
−1
−2
0
200
400
600
log2 Fold Change
Position
Regulation:
SV−cycling−dependent
not−affected
Domains and Regions:
DOMAIN: IG
REGION: Disordered

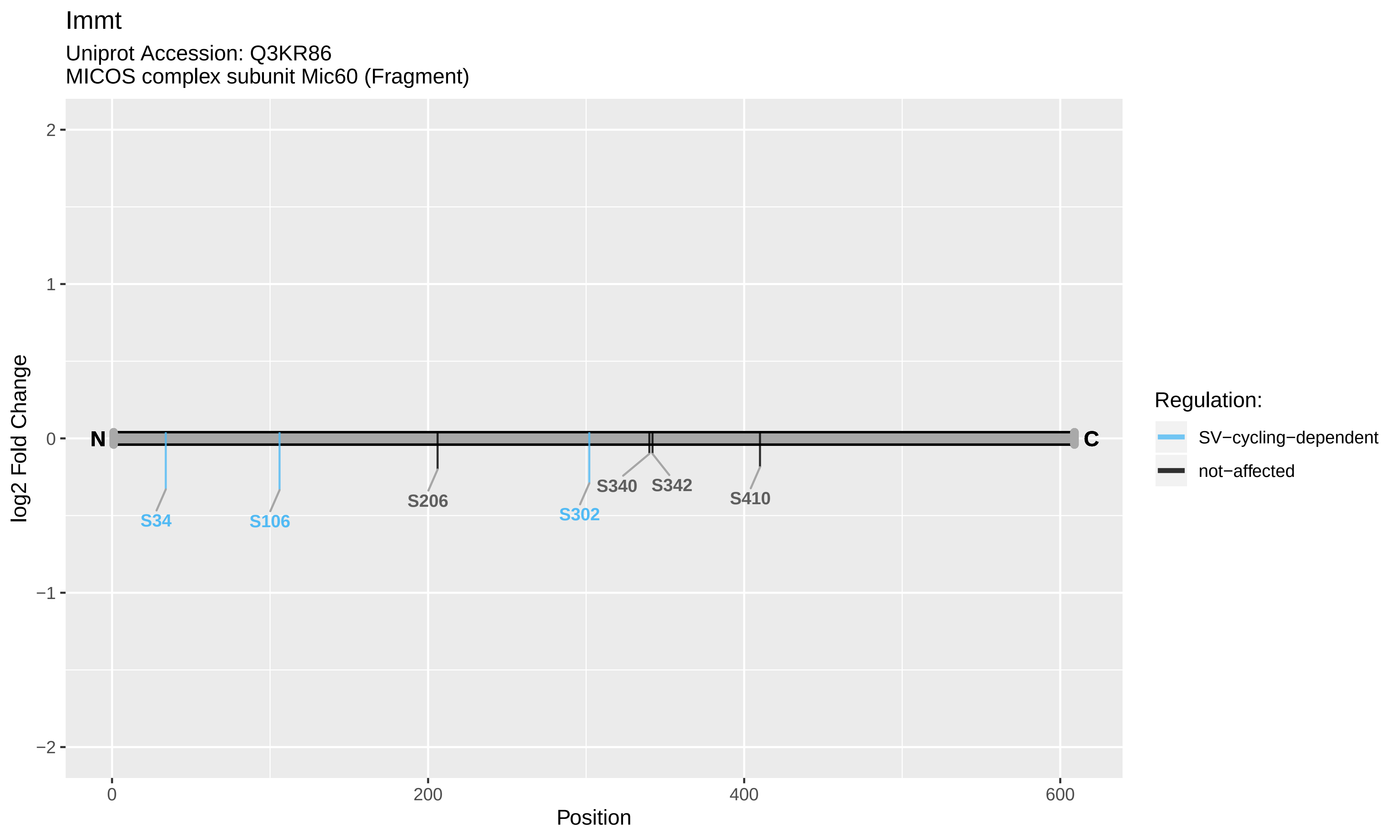

Immt
Uniprot Accession: Q3KR86
MICOS complex subunit Mic60 (Fragment)
log2 Fold Change
Position
N
C
S34
S106
S206
S302
S340
S342
S410
Regulation:
SV−cycling−dependent
not−affected

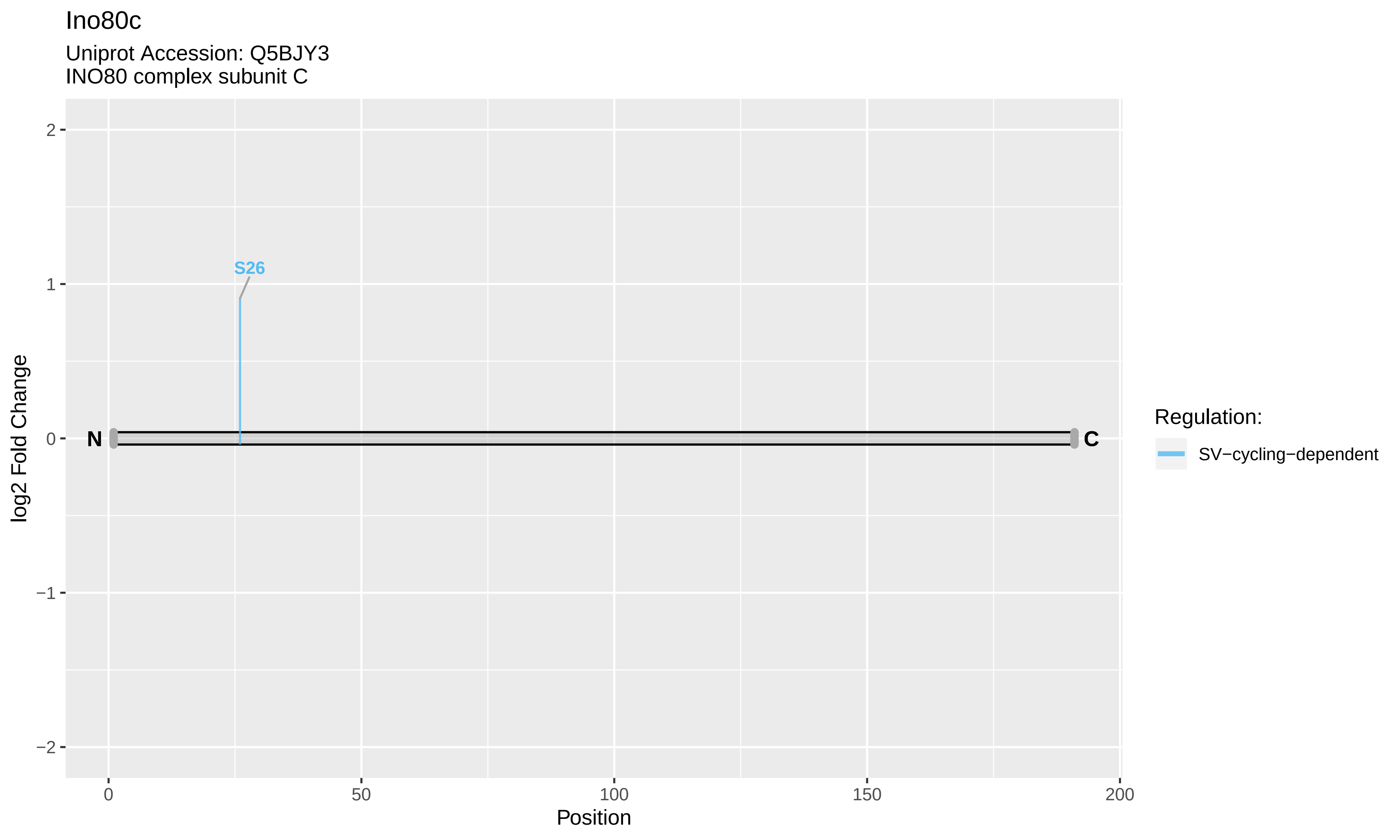

Ino80c
Uniprot Accession: Q5BJY3
INO80 complex subunit C
S26
N
C
2
1
0
−1
−2
log2 Fold Change
0
50
100
150
200
Position
Regulation:
SV−cycling−dependent

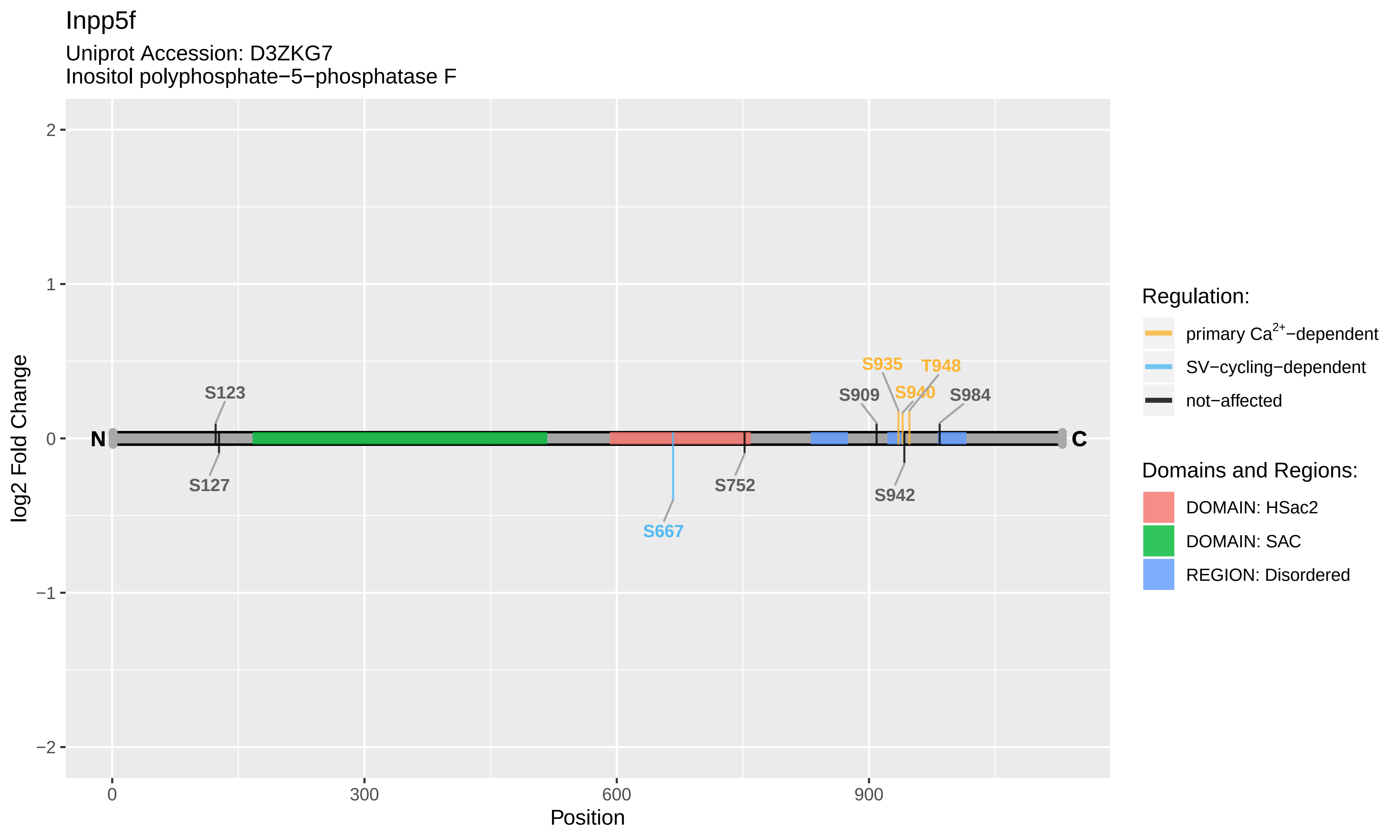
Inpp5f
Uniprot Accession: D3ZKG7
Inositol polyphosphate−5−phosphatase F
log2 Fold Change
Position
N
C
S123
S127
S667
S752
S909
S935
S940
T948
S942
S984
Regulation:
primary Ca2+−dependent
SV−cycling−dependent
not−affected
Domains and Regions:
DOMAIN: HSac2
DOMAIN: SAC
REGION: Disordered

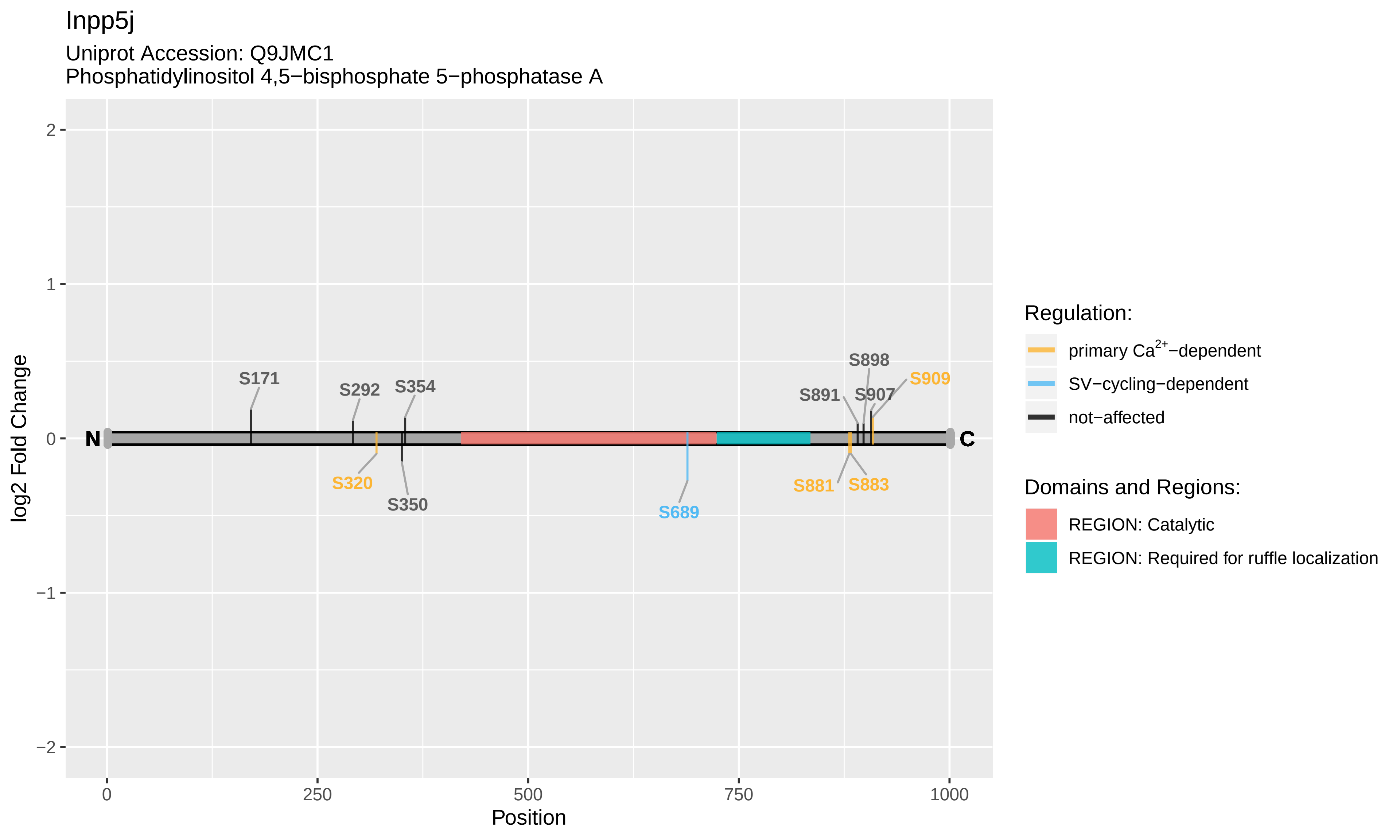

Inpp5j
Uniprot Accession: Q9JMC1
Phosphatidylinositol 4,5−bisphosphate 5−phosphatase A
S171
S292
S354
S898
S909
S891
S907
N
C
S320
S350
S689
S881
S883
2
1
0
−1
−2
log2 Fold Change
0
250
500
750
1000
Position
Regulation:
primary Ca^{2+}−dependent
SV−cycling−dependent
not−affected
Domains and Regions:
REGION: Catalytic
REGION: Required for ruffle localization

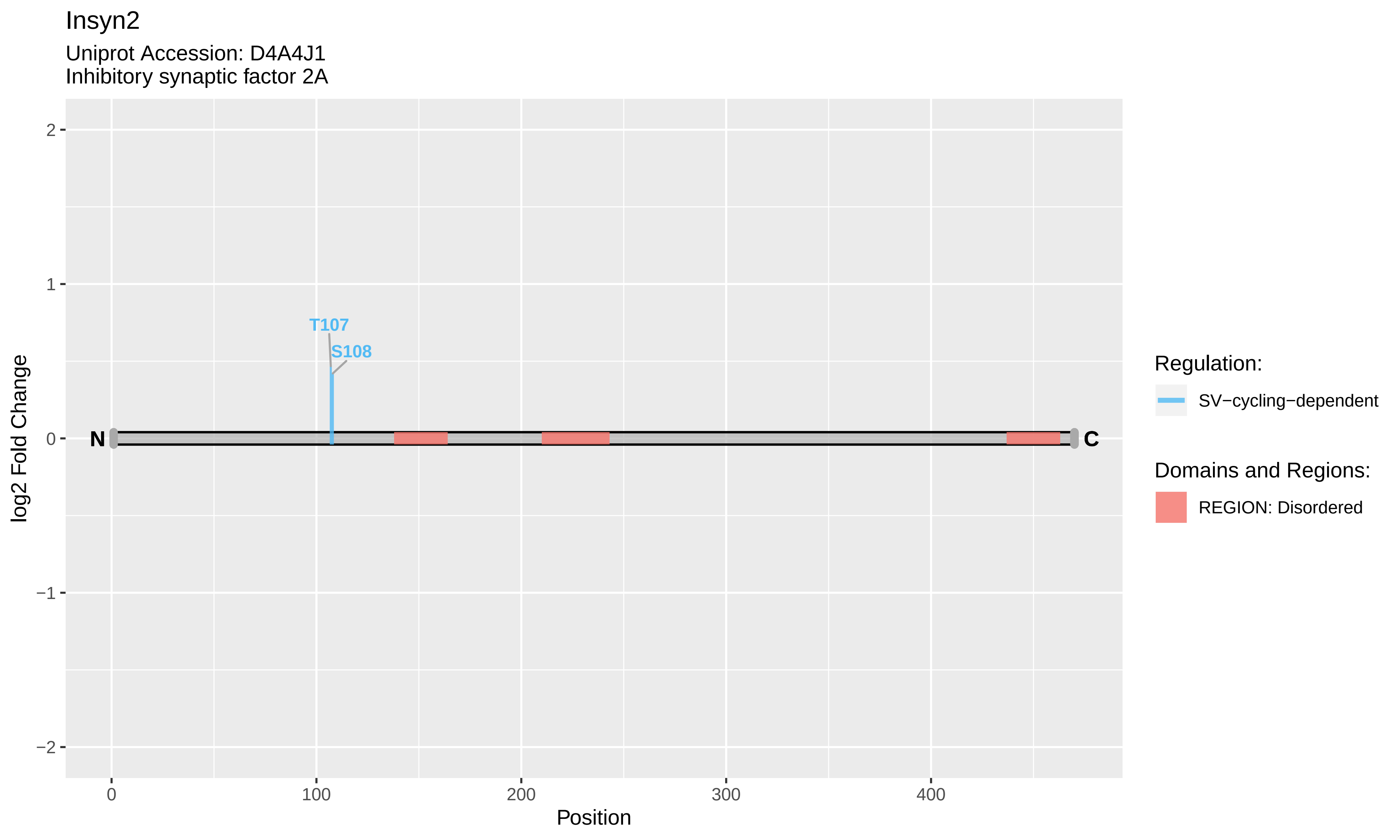

Insyn2
Uniprot Accession: D4A4J1
Inhibitory synaptic factor 2A
T107
S108
N
C
2
1
0
−1
−2
log2 Fold Change
0
100
200
300
400
Position
Regulation:
SV−cycling−dependent
Domains and Regions:
REGION: Disordered

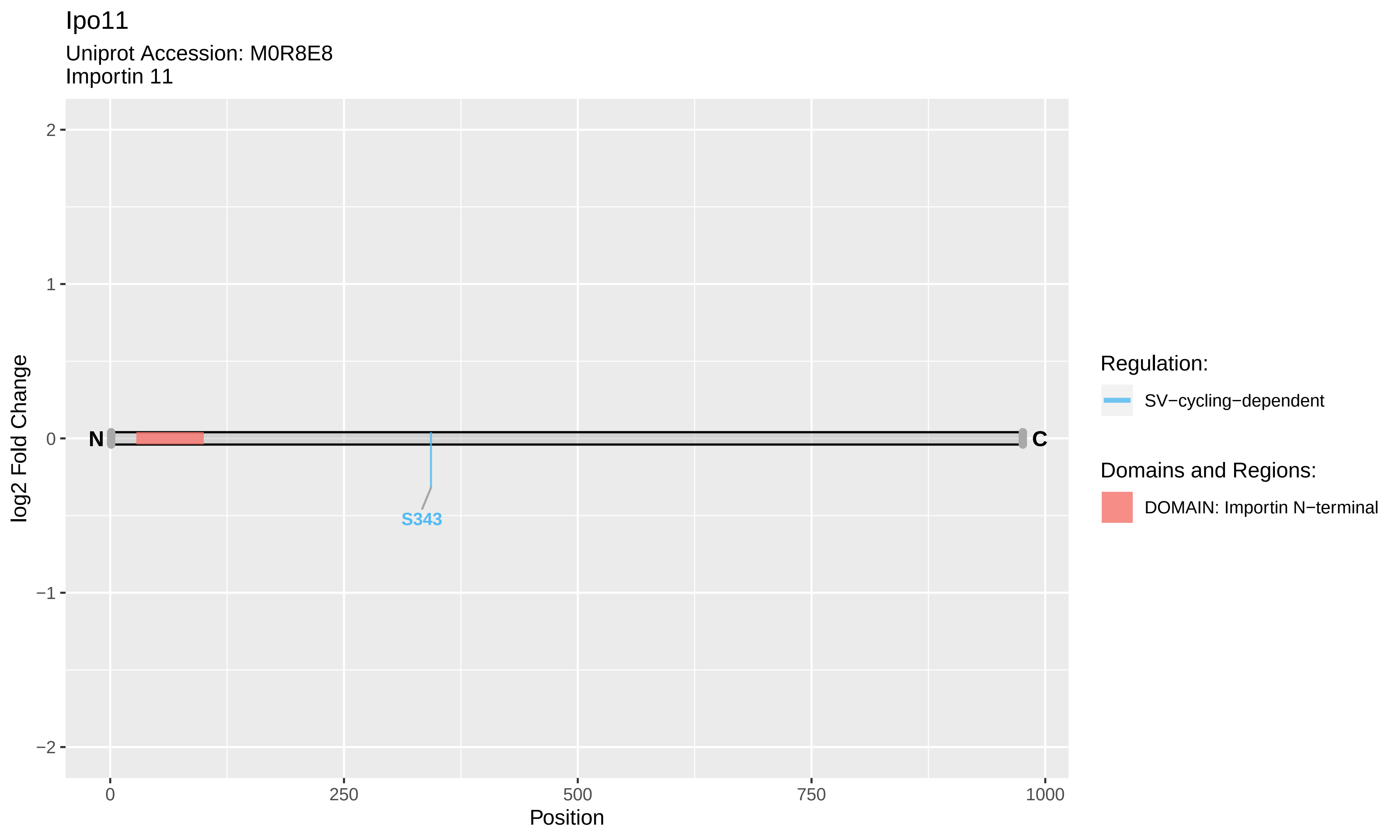

Ipo11
Uniprot Accession: M0R8E8
Importin 11
2
1
0
−1
−2
log2 Fold Change
N
C
S343
0
250
500
750
1000
Position
Regulation:
SV−cycling−dependent
Domains and Regions:
DOMAIN: Importin N−terminal

Iqsec1

Uniprot Accession: A0A0G2JUG7
IQ motif and Sec7 domain 1

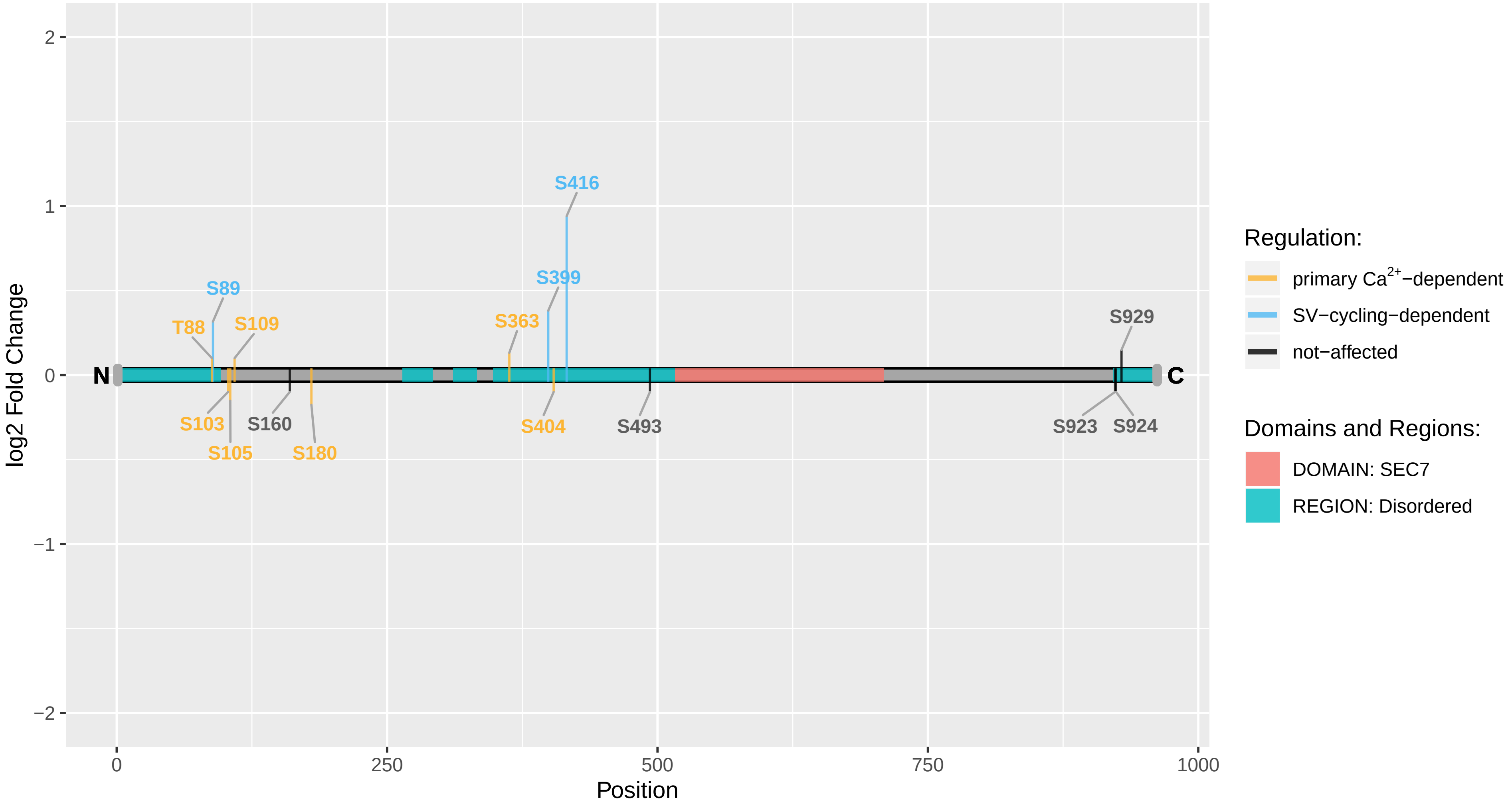

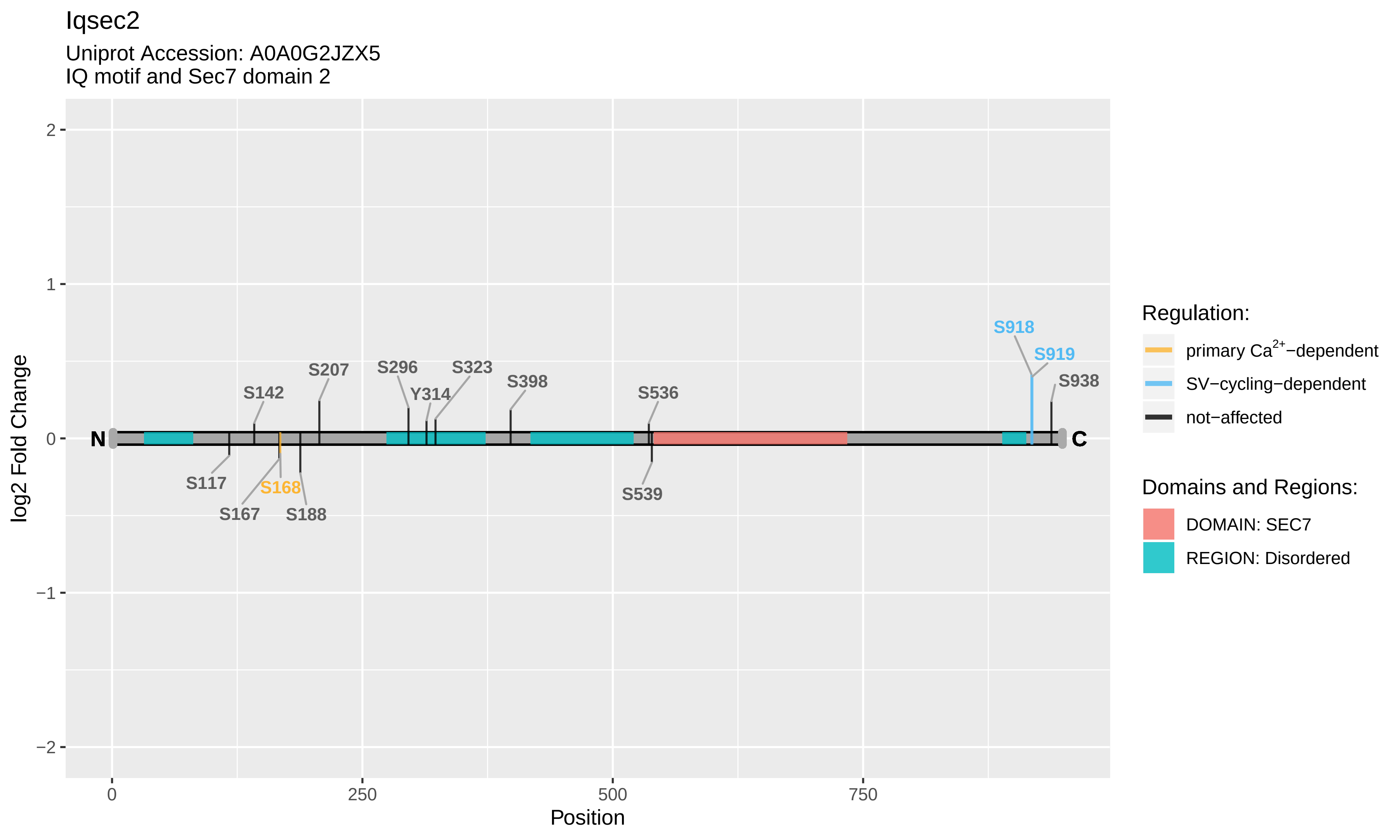

Iqsec2
Uniprot Accession: A0A0G2JZX5
IQ motif and Sec7 domain 2
log2 Fold Change
Position
N
C
S117
S142
S167
S168
S188
S207
S296
Y314
S323
S398
S536
S539
S918
S919
S938
2
1
0
−1
−2
0
250
500
750
Regulation:
primary Ca2+−dependent
SV−cycling−dependent
not−affected
Domains and Regions:
DOMAIN: SEC7
REGION: Disordered

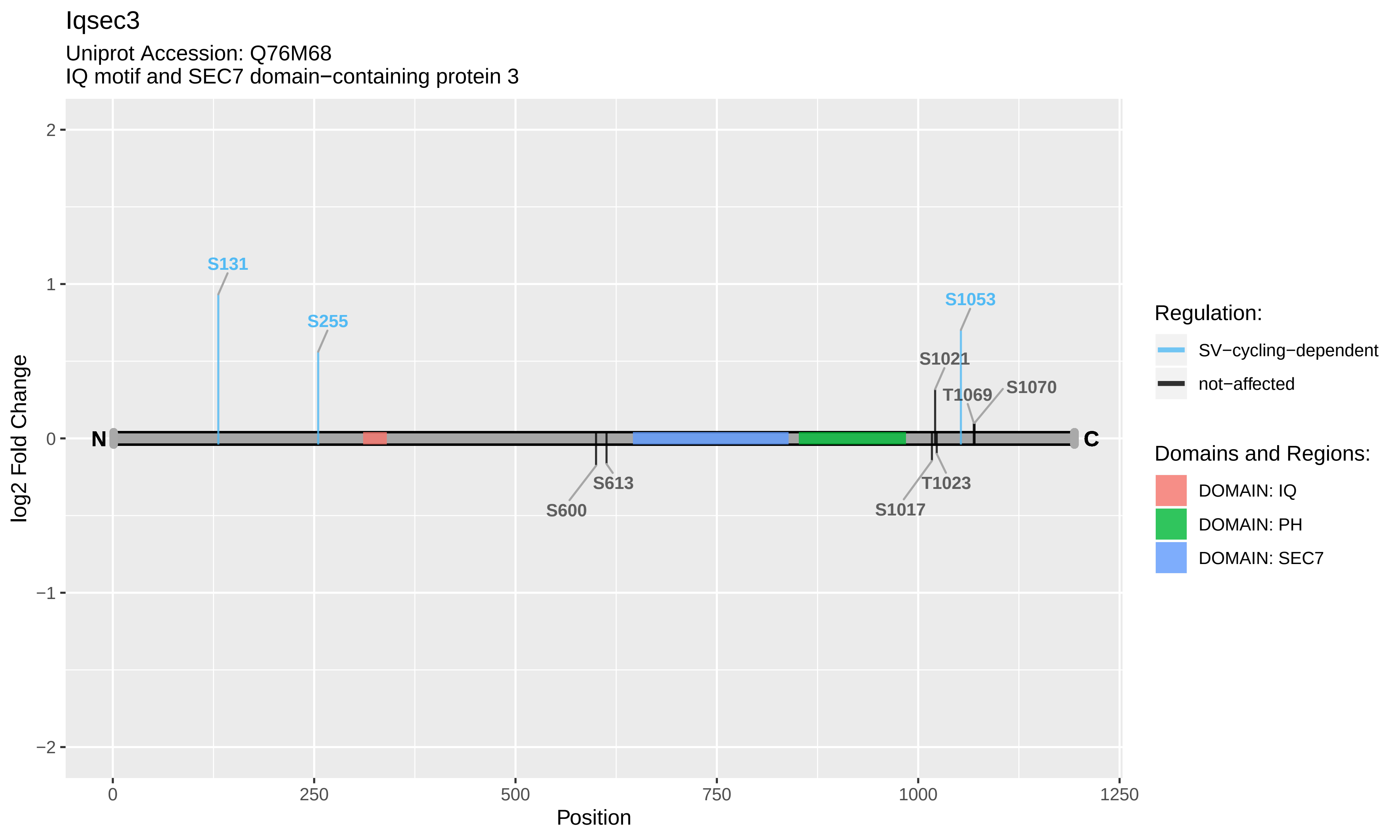
Iqsec3
Uniprot Accession: Q76M68
IQ motif and SEC7 domain−containing protein 3
log2 Fold Change
Position
S131
S255
S1053
S1021
T1069
S1070
S600
S613
S1017
T1023
N
C
Regulation:
SV−cycling−dependent
not−affected
Domains and Regions:
DOMAIN: IQ
DOMAIN: PH
DOMAIN: SEC7

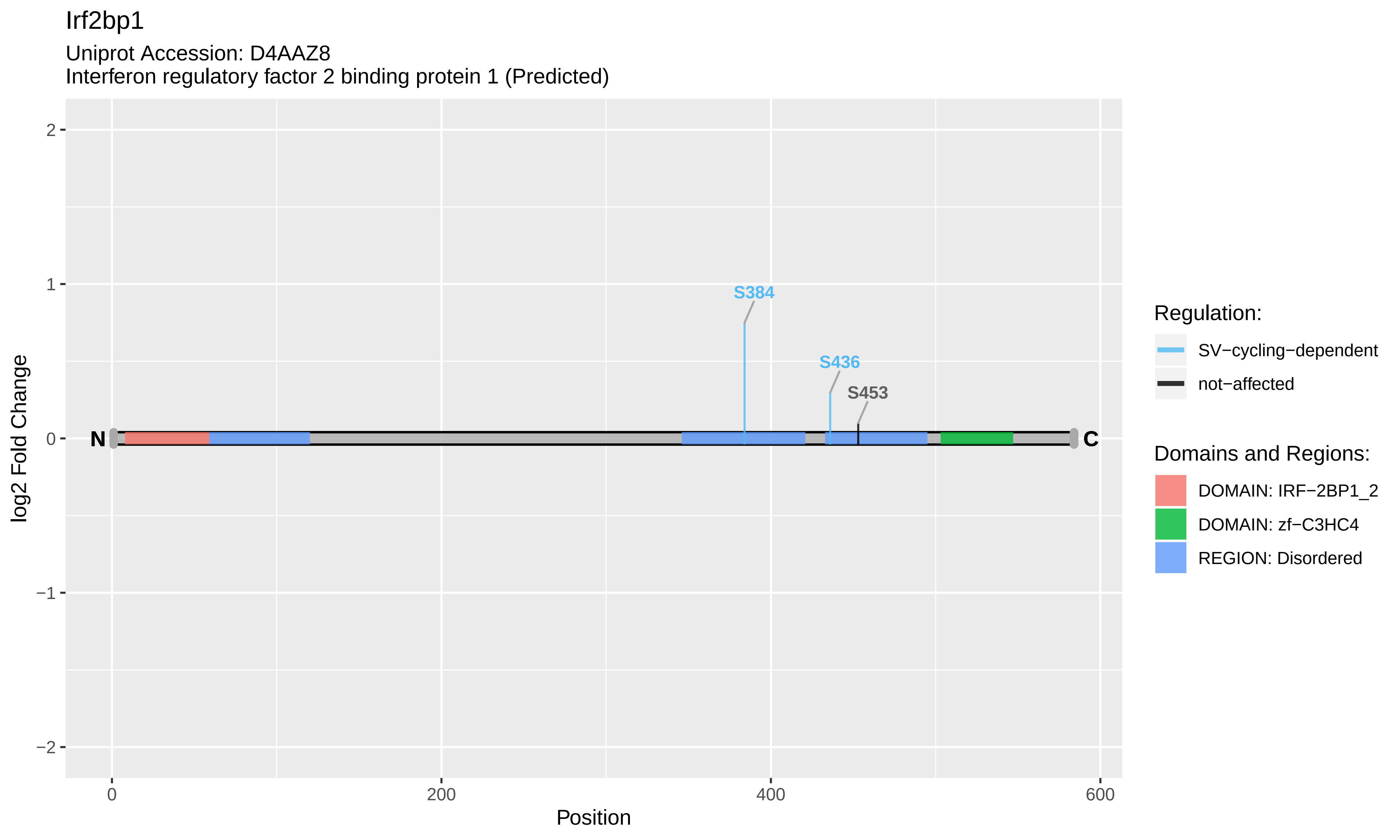

Irf2bp1
Uniprot Accession: D4AAZ8
Interferon regulatory factor 2 binding protein 1 (Predicted)
S384
S436
S453
N
C
2
1
0
−1
−2
log2 Fold Change
0
200
400
600
Position
Regulation:
SV−cycling−dependent
not−affected
Domains and Regions:
DOMAIN: IRF−2BP1_2
DOMAIN: zf−C3HC4
REGION: Disordered

Irf2bpl

Uniprot Accession: Q5EIC4
Interferon regulatory factor 2−binding protein−like

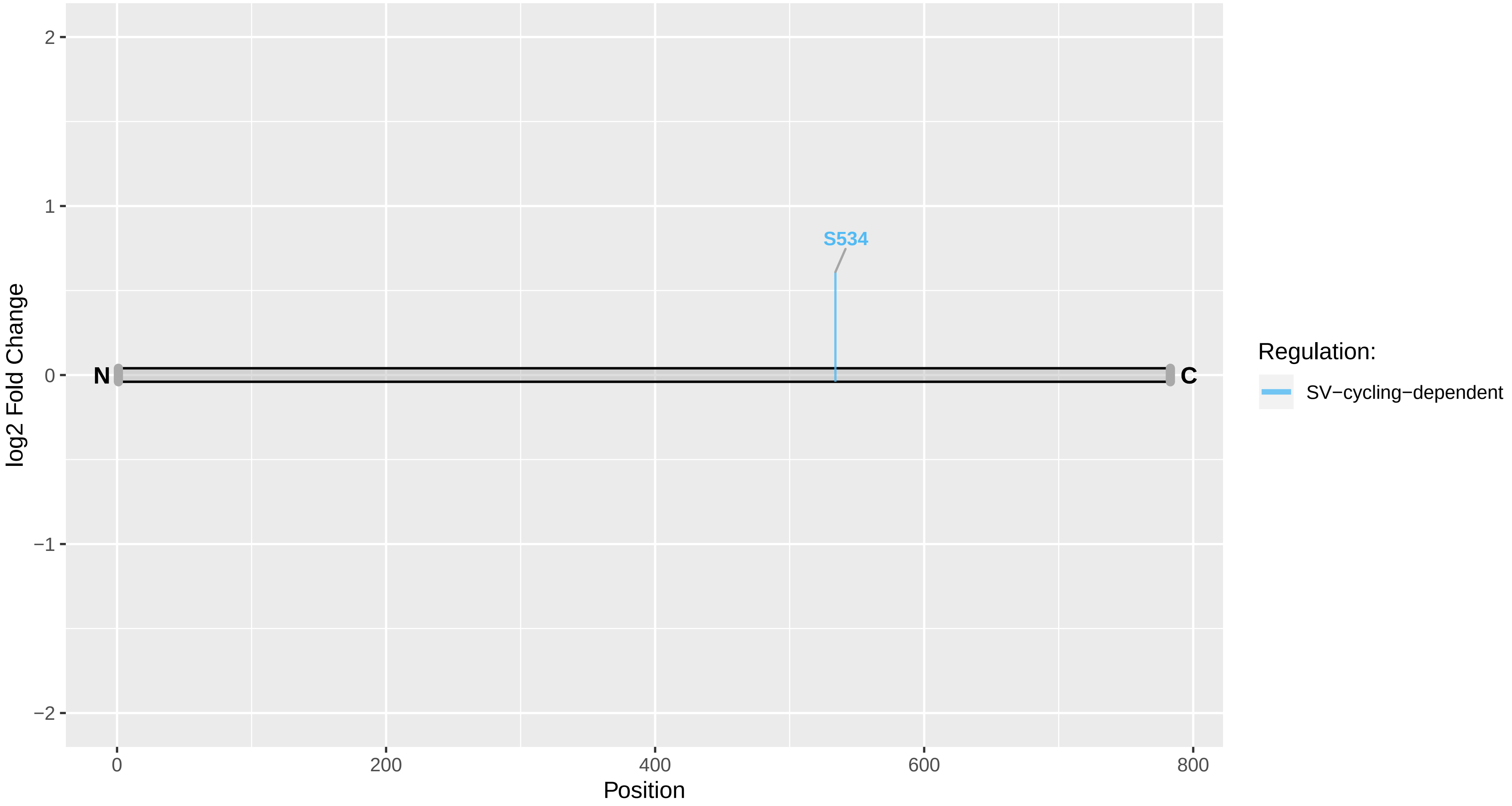

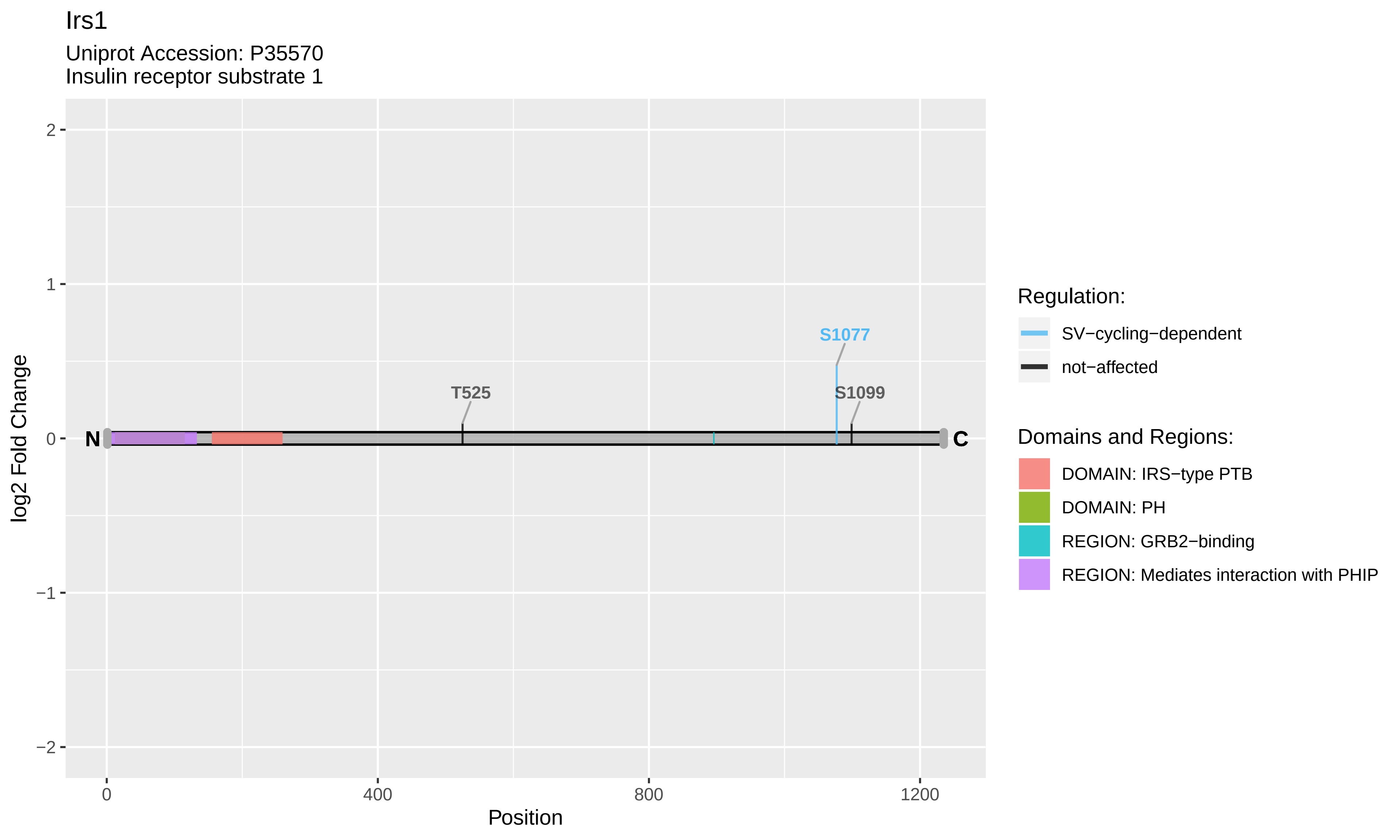

Irs1
Uniprot Accession: P35570
Insulin receptor substrate 1
log2 Fold Change
Position
2
1
0
−1
−2
0
400
800
1200
N
C
T525
S1077
S1099
Regulation:
SV−cycling−dependent
not−affected
Domains and Regions:
DOMAIN: IRS−type PTB
DOMAIN: PH
REGION: GRB2−binding
REGION: Mediates interaction with PHIP

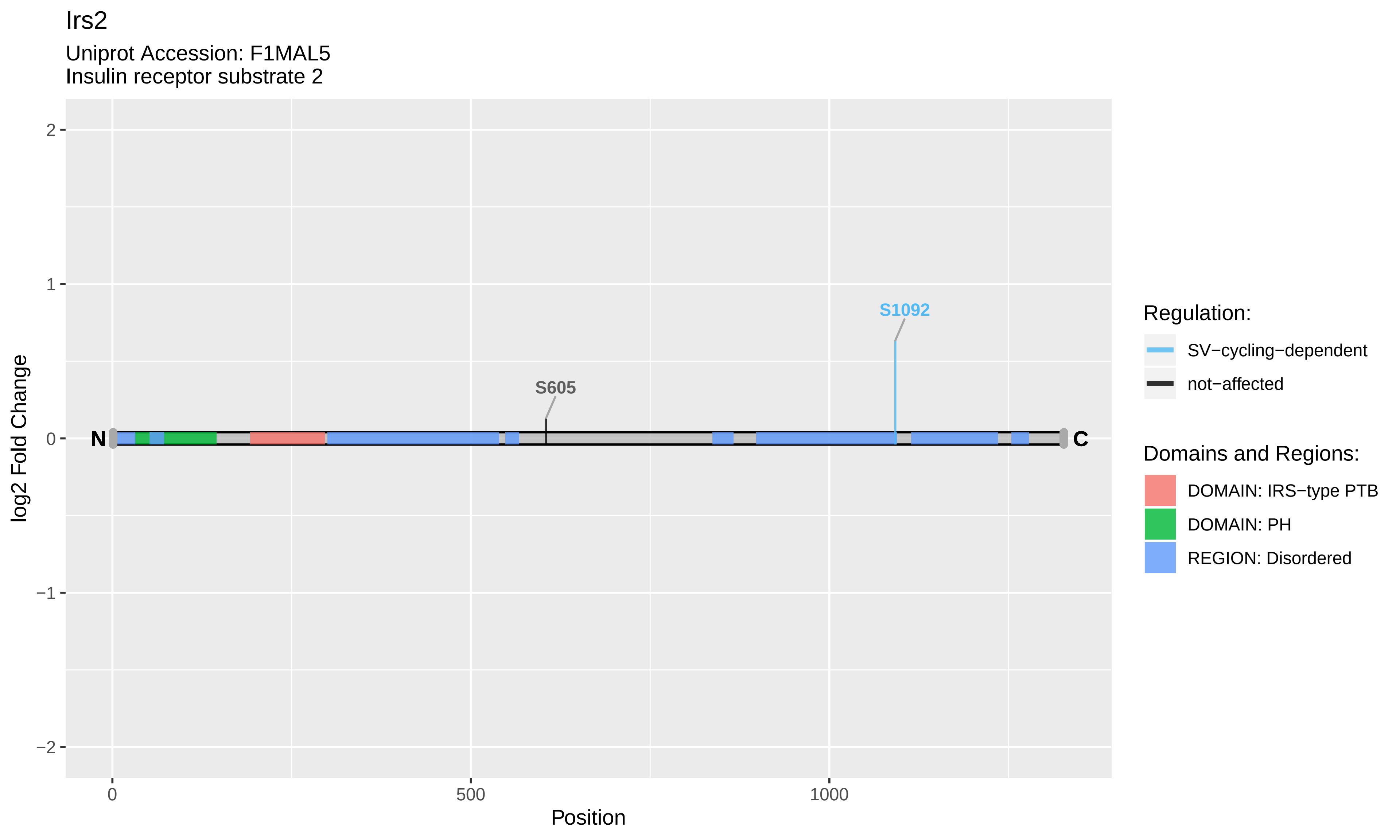

Irs2
Uniprot Accession: F1MAL5
Insulin receptor substrate 2
log2 Fold Change
2
1
0
−1
−2
N
C
S605
S1092
0
500
1000
Position
Regulation:
SV−cycling−dependent
not−affected
Domains and Regions:
DOMAIN: IRS−type PTB
DOMAIN: PH
REGION: Disordered

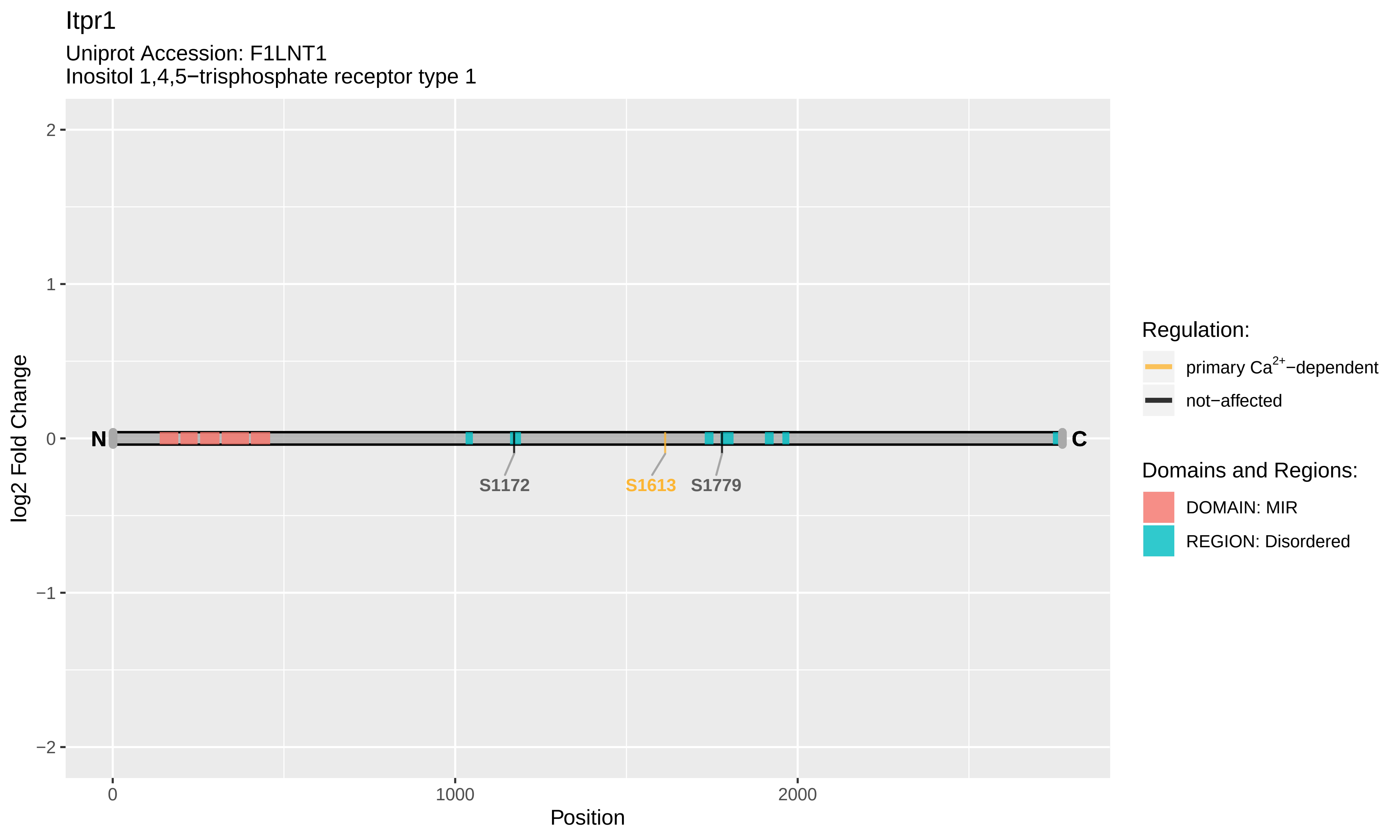

Itpr1
Uniprot Accession: F1LNT1
Inositol 1,4,5−trisphosphate receptor type 1
log2 Fold Change
2
1
0
−1
−2
N
C
S1172
S1613
S1779
0
1000
2000
Position
Regulation:
primary Ca2+−dependent
not−affected
Domains and Regions:
DOMAIN: MIR
REGION: Disordered

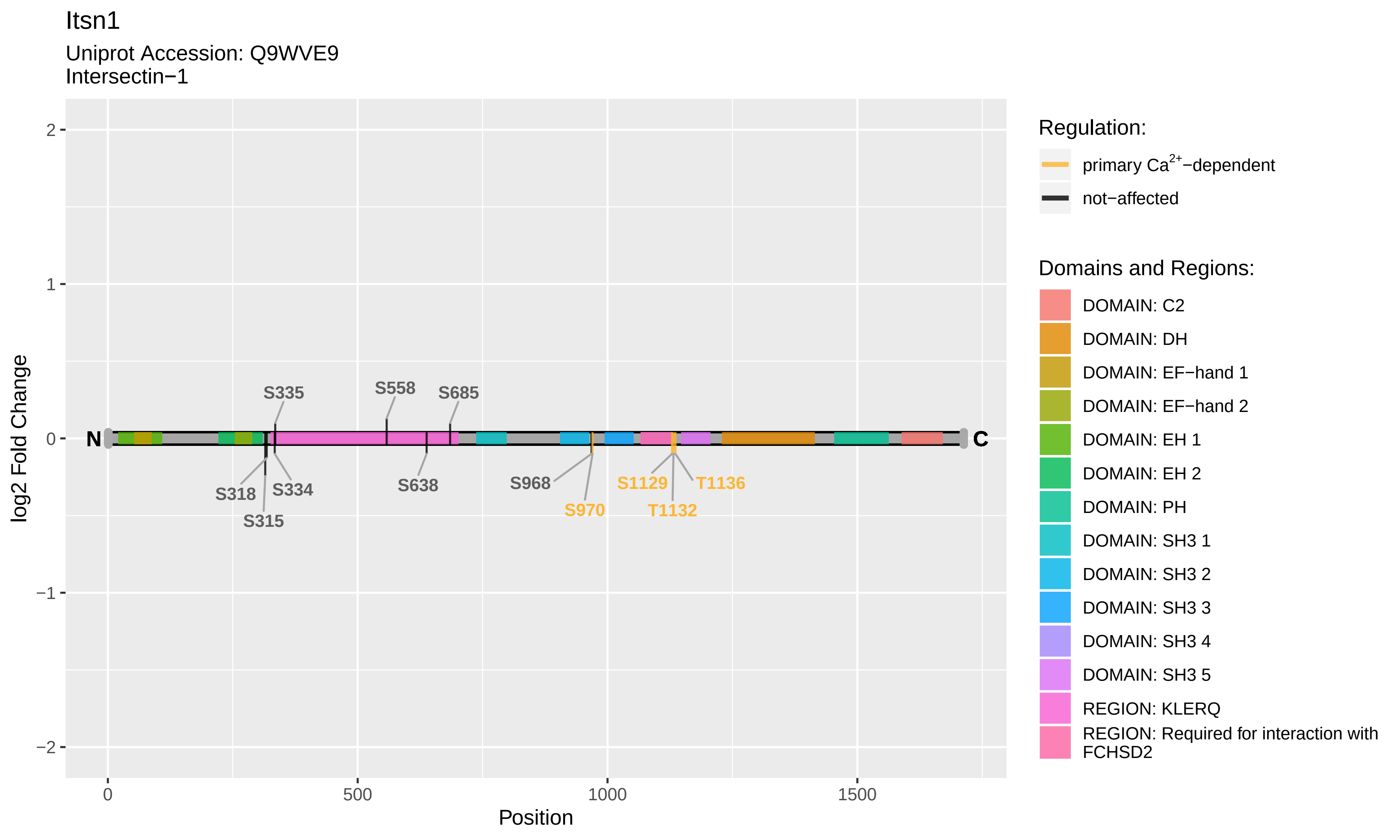
Itsn1
Uniprot Accession: Q9WVE9
Intersectin−1
log2 Fold Change
Position
N
C
S335
S558
S685
S318
S334
S315
S638
S968
S970
S1129
T1136
T1132
2
1
0
−1
−2
0
500
1000
1500
Regulation:
primary Ca2+−dependent
not−affected
Domains and Regions:
DOMAIN: C2
DOMAIN: DH
DOMAIN: EF−hand 1
DOMAIN: EF−hand 2
DOMAIN: EH 1
DOMAIN: EH 2
DOMAIN: PH
DOMAIN: SH3 1
DOMAIN: SH3 2
DOMAIN: SH3 3
DOMAIN: SH3 4
DOMAIN: SH3 5
REGION: KLERQ
REGION: Required for interaction with FCHSD2

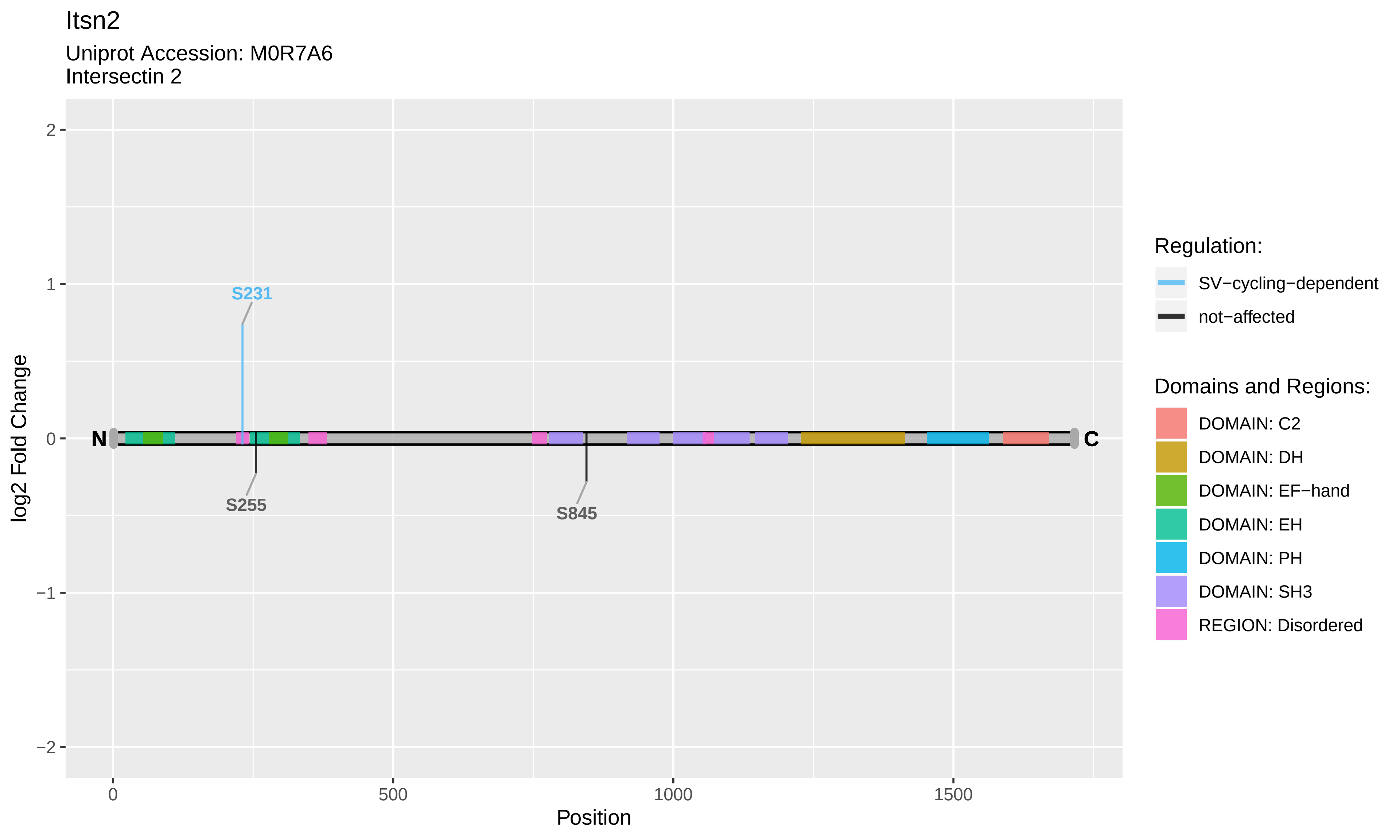
Itsn2
Uniprot Accession: M0R7A6
Intersectin 2
log2 Fold Change
2
1
0
−1
−2
N
C
S231
S255
S845
0
500
1000
1500
Position
Regulation:
SV−cycling−dependent
not−affected
Domains and Regions:
DOMAIN: C2
DOMAIN: DH
DOMAIN: EF−hand
DOMAIN: EH
DOMAIN: PH
DOMAIN: SH3
REGION: Disordered

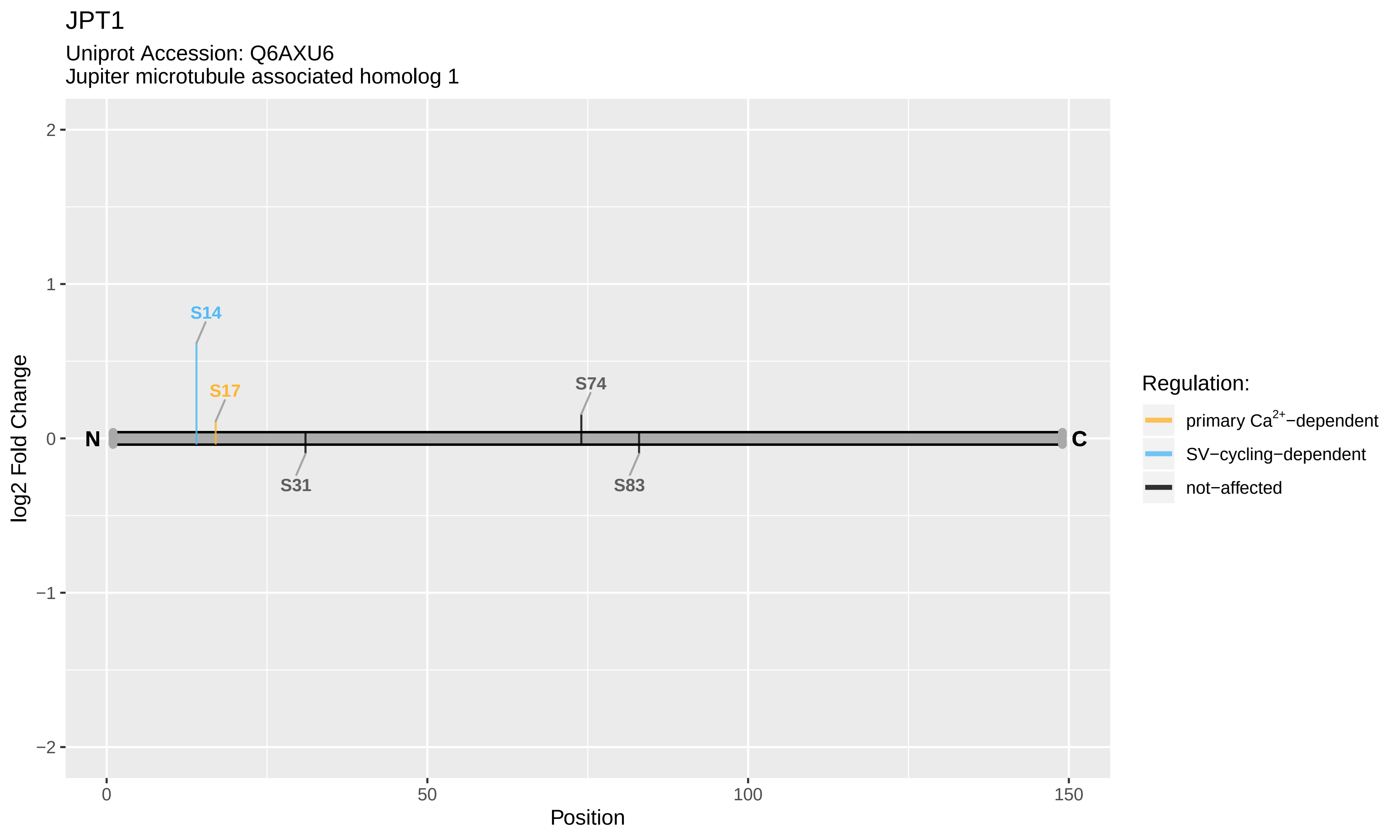
JPT1
Uniprot Accession: Q6AXU6
Jupiter microtubule associated homolog 1
log2 Fold Change
Position
S14
S17
S74
S31
S83
N
C
Regulation:
primary Ca2+−dependent
SV−cycling−dependent
not−affected
2
1
0
−1
−2
0
50
100
150

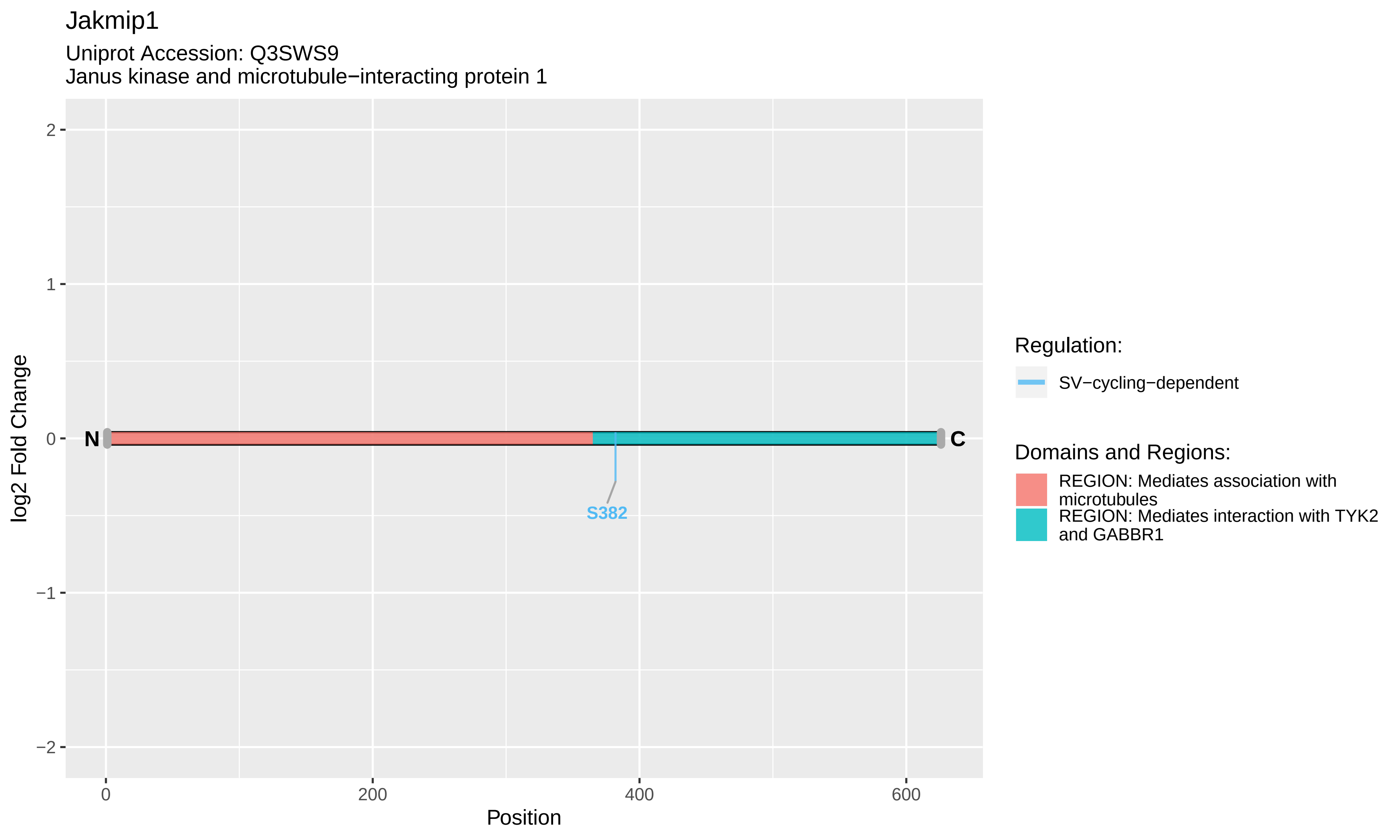
Jakmip1
Uniprot Accession: Q3SWS9
Janus kinase and microtubule−interacting protein 1
log2 Fold Change
2
1
0
−1
−2
N
C
S382
0
200
400
600
Position
Regulation:
SV−cycling−dependent
Domains and Regions:
REGION: Mediates association with microtubules
REGION: Mediates interaction with TYK2 and GABBR1

Jam2

Uniprot Accession: F1M065
Junctional adhesion molecule 2

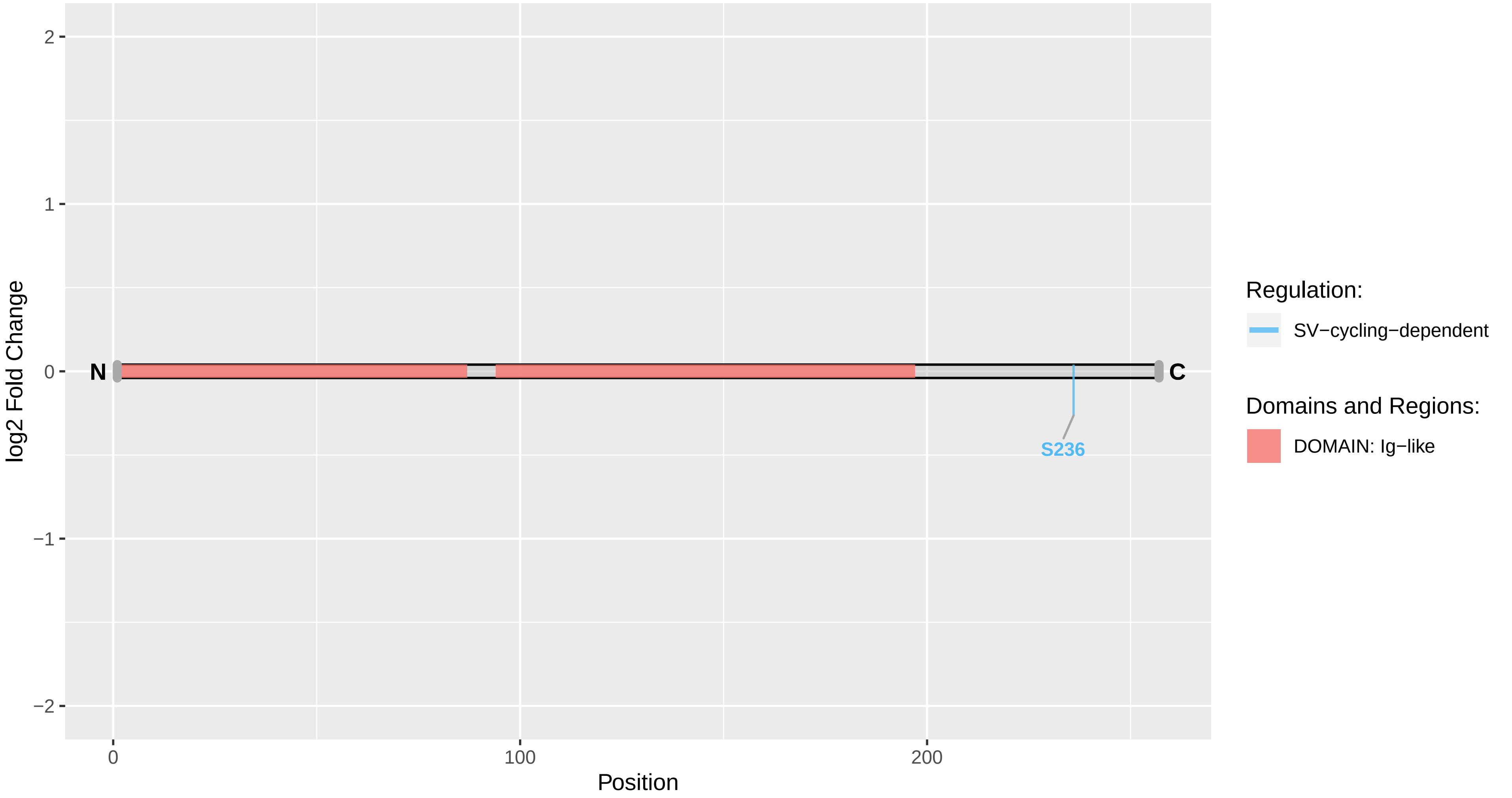

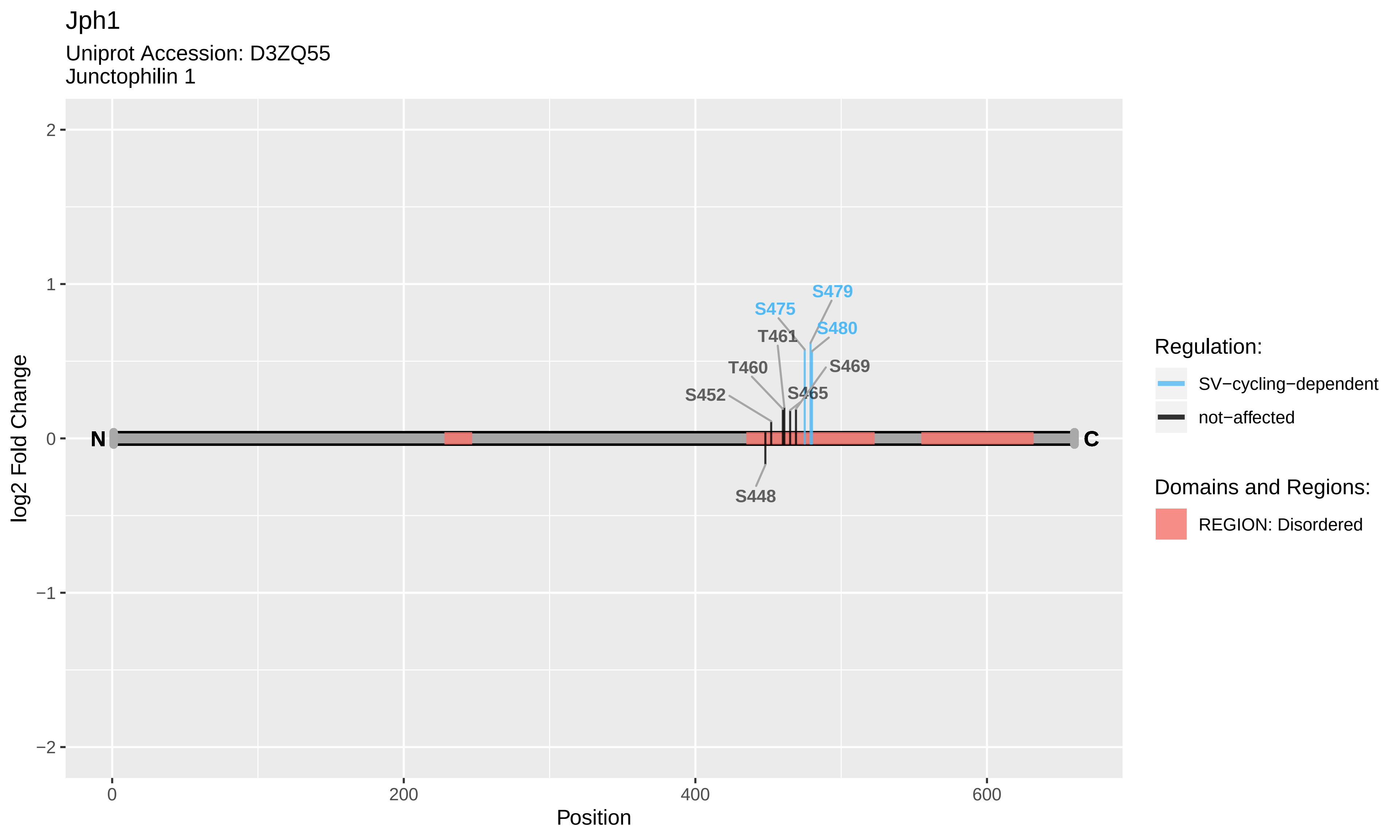

Jph1
Uniprot Accession: D3ZQ55
Junctophilin 1
log2 Fold Change
Position
2
1
0
−1
−2
0
200
400
600
N
C
S479
S475
S480
T461
T460
S469
S452
S465
S448
Regulation:
SV−cycling−dependent
not−affected
Domains and Regions:
REGION: Disordered

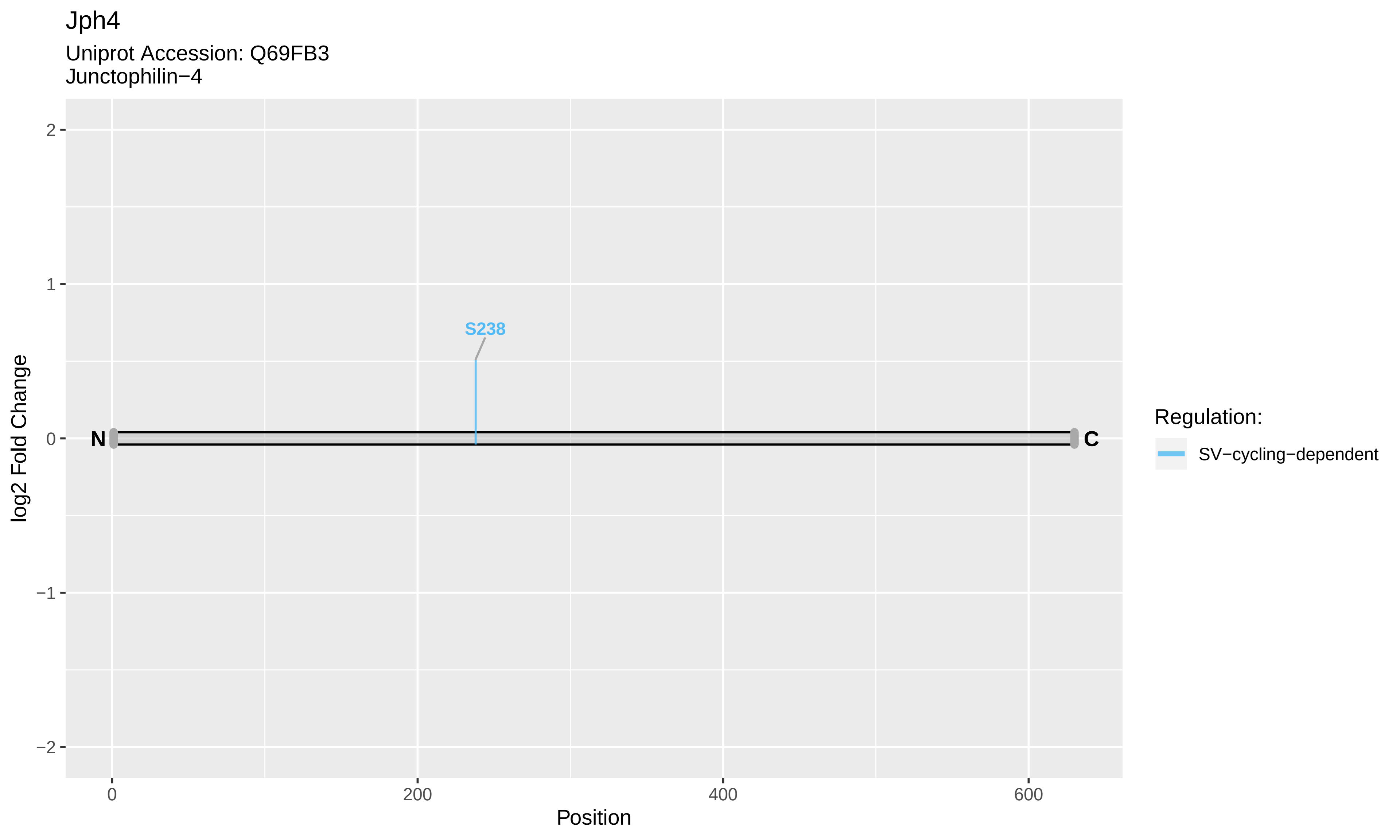

Jph4
Uniprot Accession: Q69FB3
Junctophilin−4
log2 Fold Change
2
1
0
−1
−2
0
200
400
600
Position
N
C
S238
Regulation:
SV−cycling−dependent

Kazn

Uniprot Accession: Q5FWS6
Kazrin

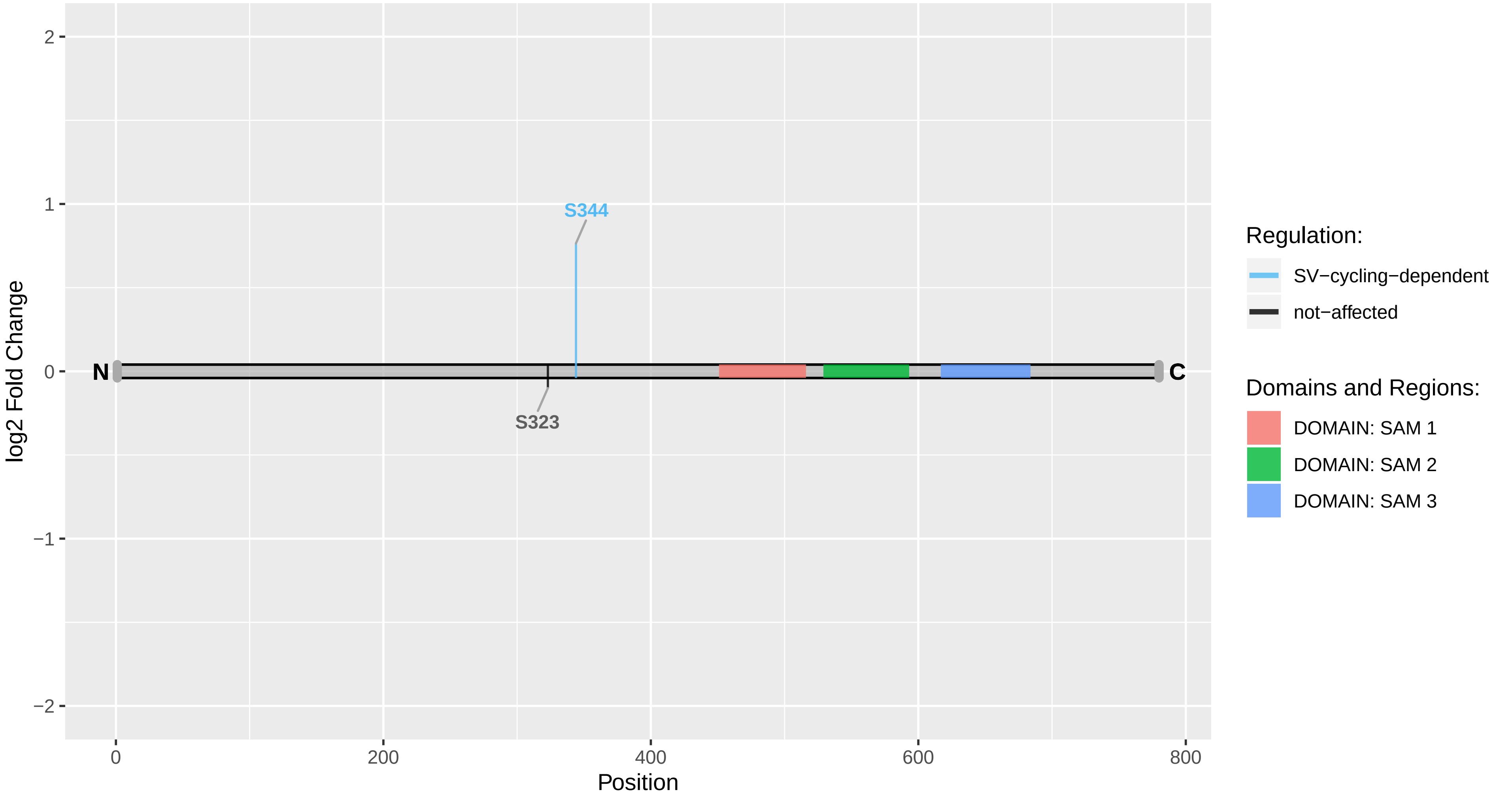

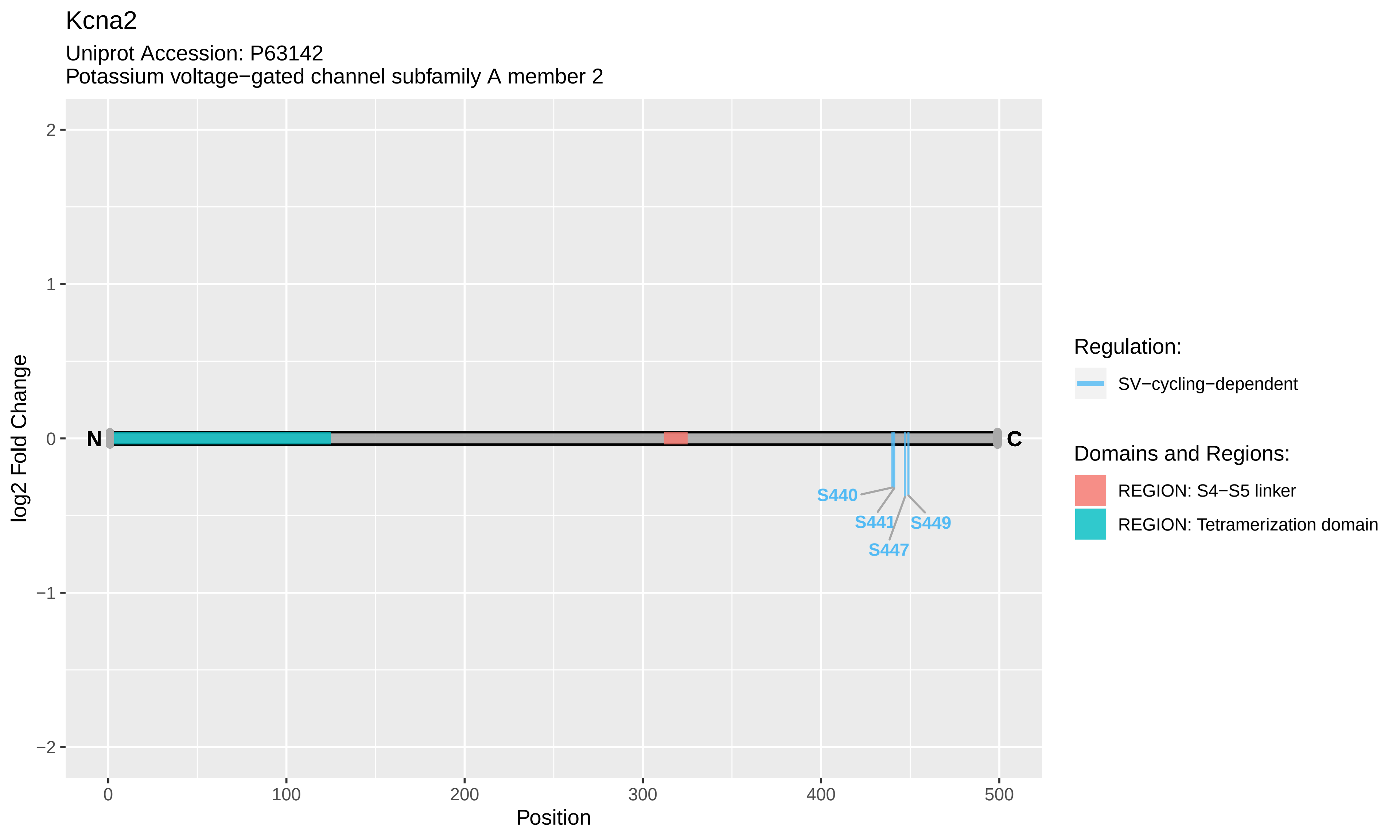
Kcna2
Uniprot Accession: P63142
Potassium voltage−gated channel subfamily A member 2
log2 Fold Change
2
1
0
−1
−2
N
C
S440
S441
S447
S449
0
100
200
300
400
500
Position
Regulation:
SV−cycling−dependent
Domains and Regions:
REGION: S4−S5 linker
REGION: Tetramerization domain

Kcna4

Uniprot Accession: P15385
Potassium voltage−gated channel subfamily A member 4

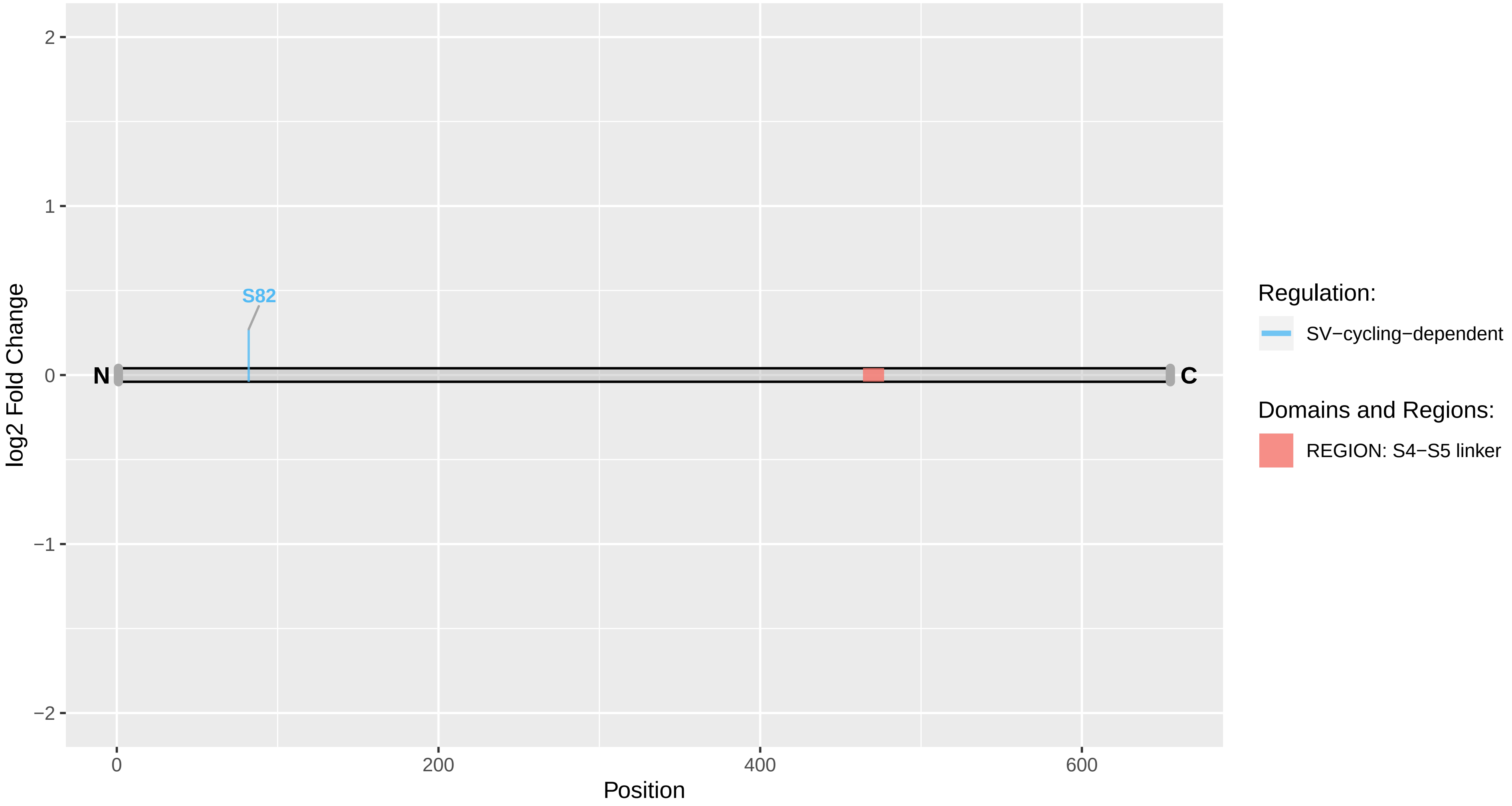

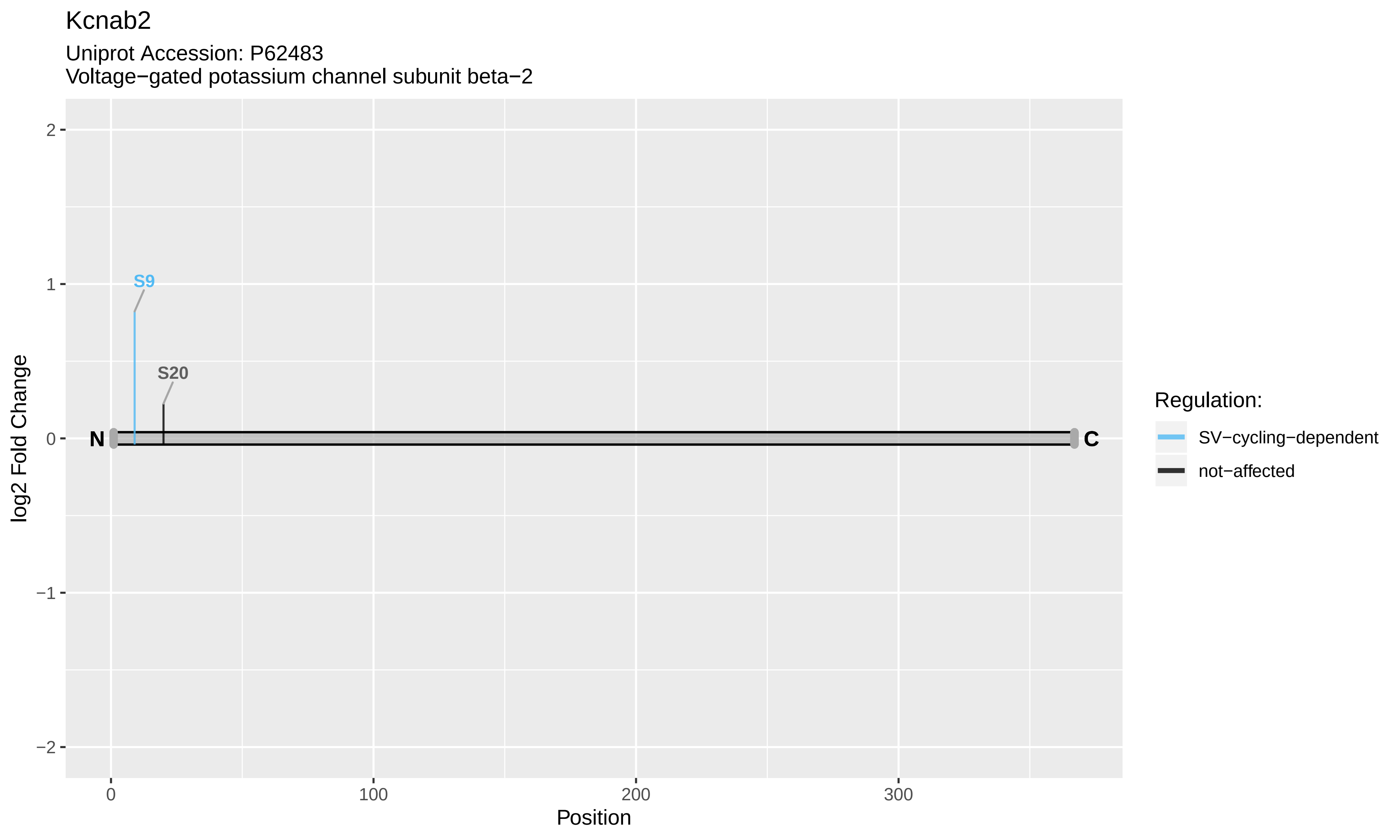

Kcnab2
Uniprot Accession: P62483
Voltage−gated potassium channel subunit beta−2
S9
S20
N
C
2
1
0
−1
−2
log2 Fold Change
0
100
200
300
Position
Regulation:
SV−cycling−dependent
not−affected

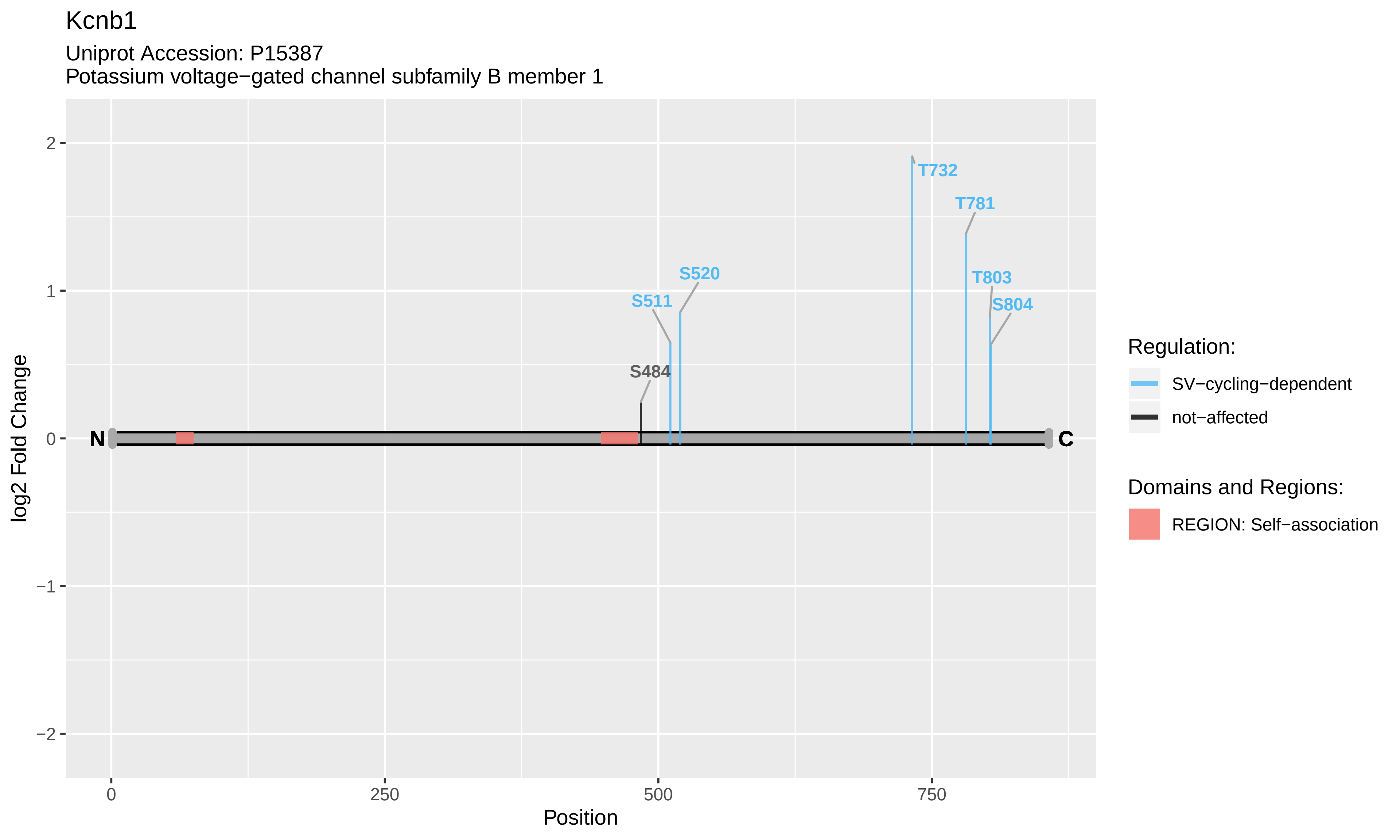
Kcnb1
Uniprot Accession: P15387
Potassium voltage−gated channel subfamily B member 1
log2 Fold Change
2
1
0
−1
−2
N
C
S484
S511
S520
T732
T781
T803
S804
0
250
500
750
Position
Regulation:
SV−cycling−dependent
not−affected
Domains and Regions:
REGION: Self−association

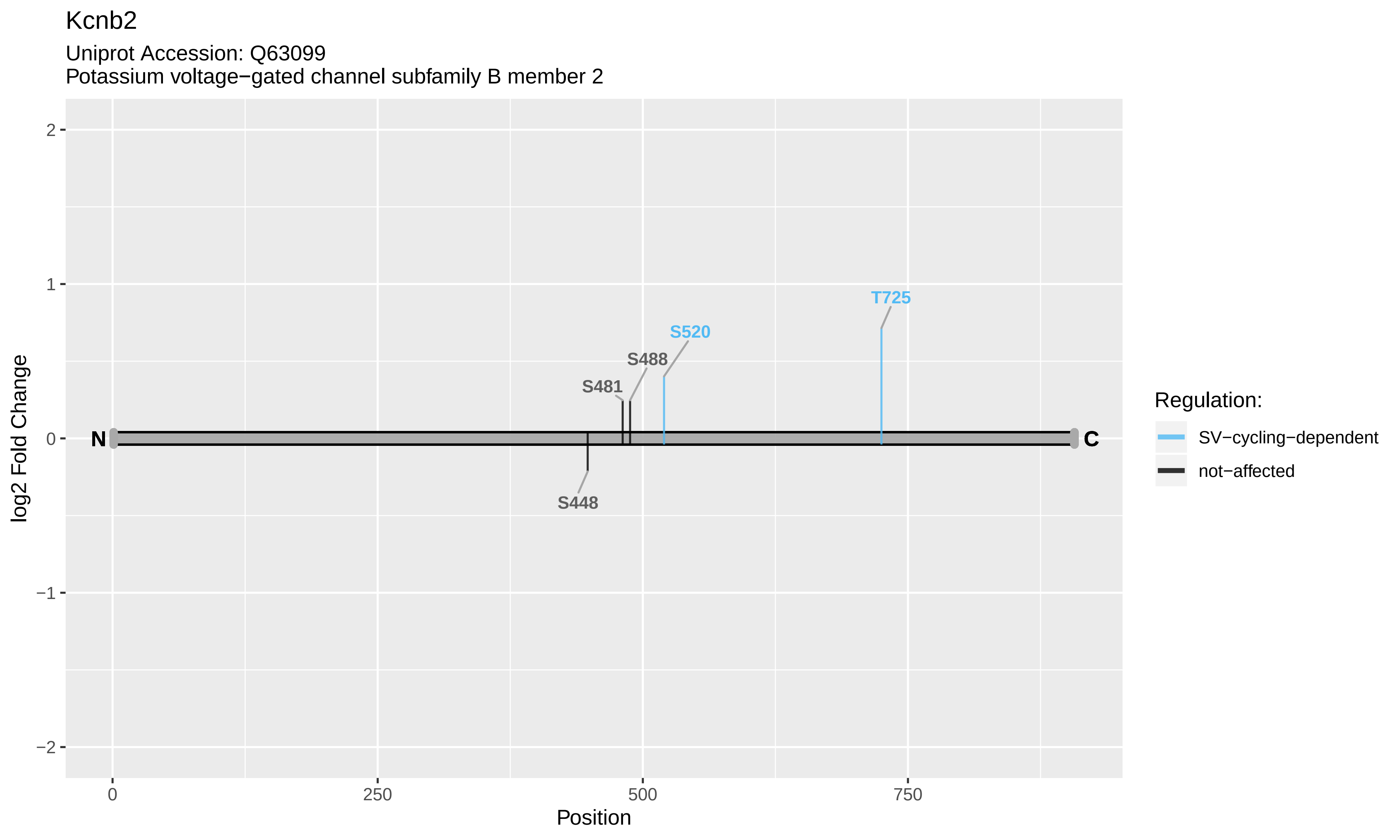

Kcnb2
Uniprot Accession: Q63099
Potassium voltage−gated channel subfamily B member 2
log2 Fold Change
2
1
0
−1
−2
N
C
S448
S481
S488
S520
T725
0
250
500
750
Position
Regulation:
SV−cycling−dependent
not−affected

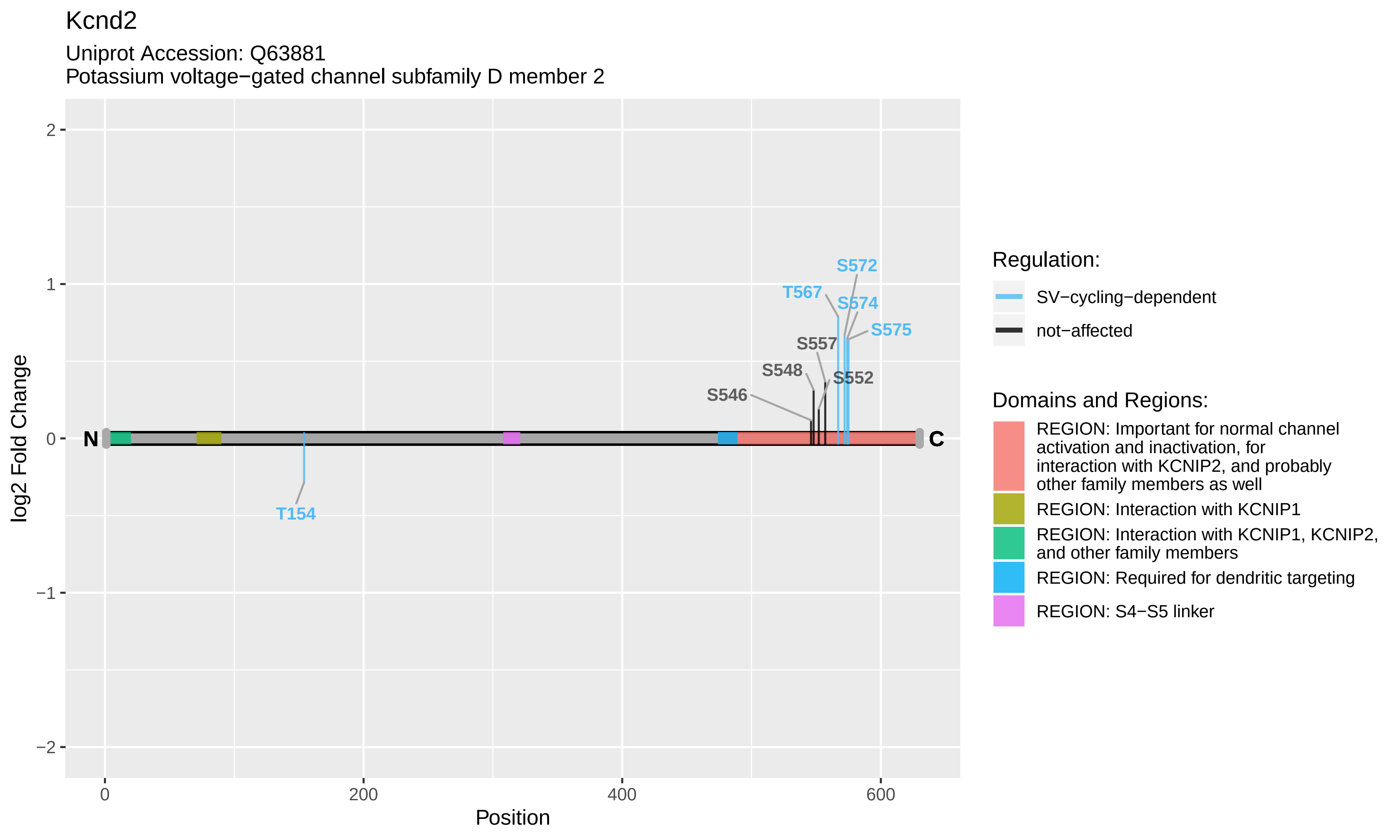

Kcnd2
Uniprot Accession: Q63881
Potassium voltage−gated channel subfamily D member 2
log2 Fold Change
Position
N
C
T154
S546
S548
S552
S557
T567
S572
S574
S575
Regulation:
SV−cycling−dependent
not−affected
Domains and Regions:
REGION: Important for normal channel activation and inactivation, for interaction with KCNIP2, and probably other family members as well
REGION: Interaction with KCNIP1
REGION: Interaction with KCNIP1, KCNIP2, and other family members
REGION: Required for dendritic targeting
REGION: S4−S5 linker

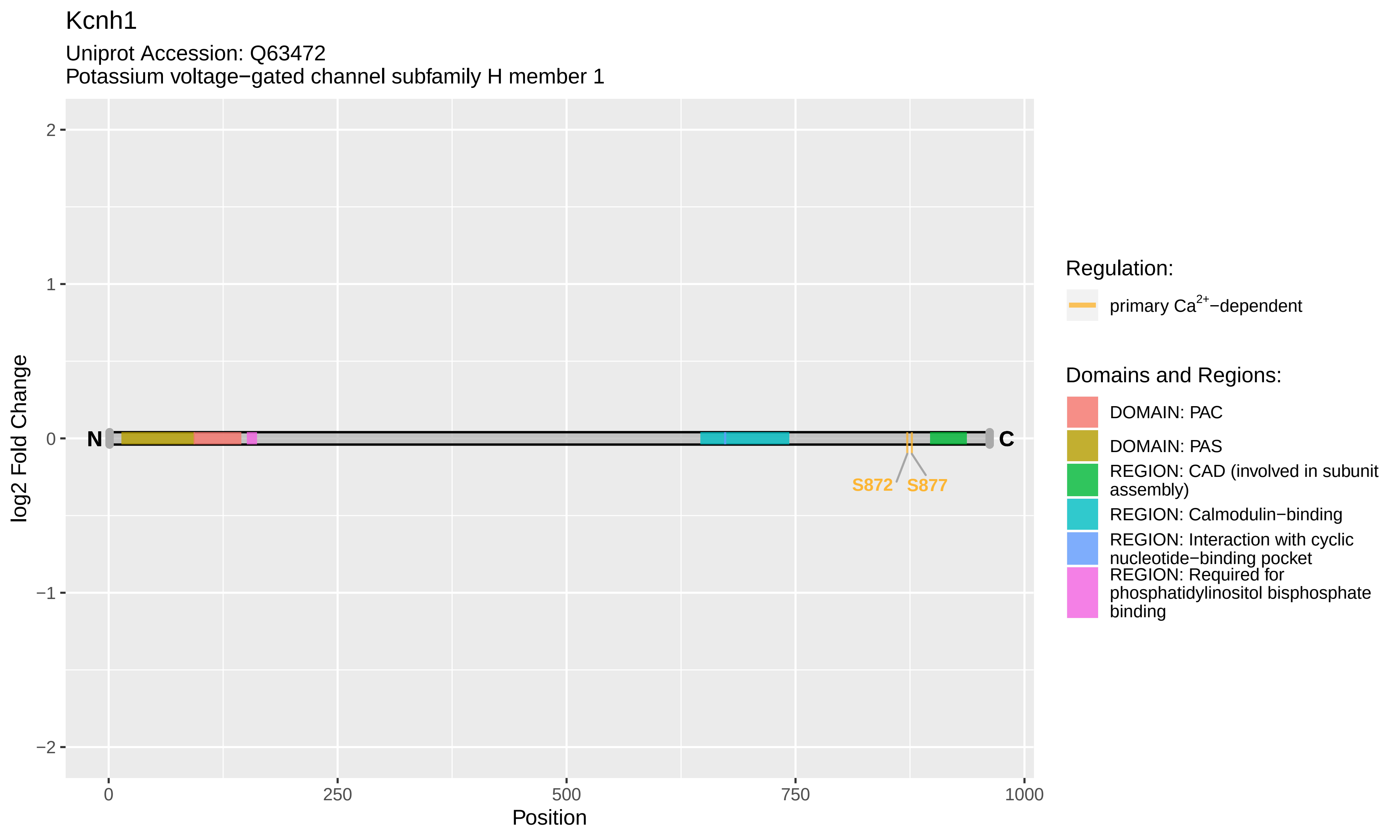
Kcnh1
Uniprot Accession: Q63472
Potassium voltage−gated channel subfamily H member 1
log2 Fold Change
2
1
0
−1
−2
N
C
S872
S877
0
250
500
750
1000
Position
Regulation:
primary Ca2+−dependent
Domains and Regions:
DOMAIN: PAC
DOMAIN: PAS
REGION: CAD (involved in subunit assembly)
REGION: Calmodulin−binding
REGION: Interaction with cyclic nucleotide−binding pocket
REGION: Required for phosphatidylinositol bisphosphate binding

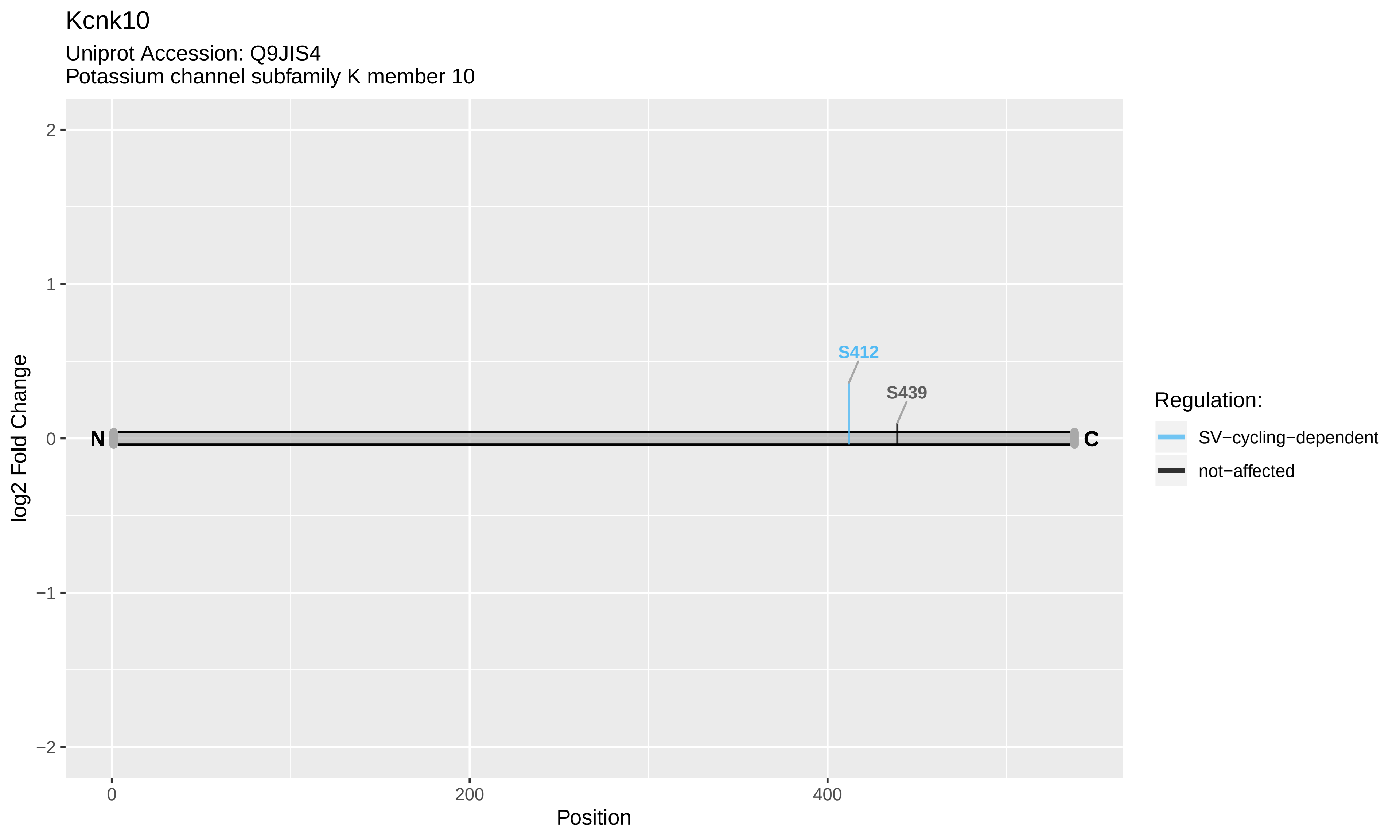
Kcnk10
Uniprot Accession: Q9JIS4
Potassium channel subfamily K member 10
log2 Fold Change
Position
S412
S439
N
C
2
1
0
−1
−2
0
200
400
Regulation:
SV−cycling−dependent
not−affected

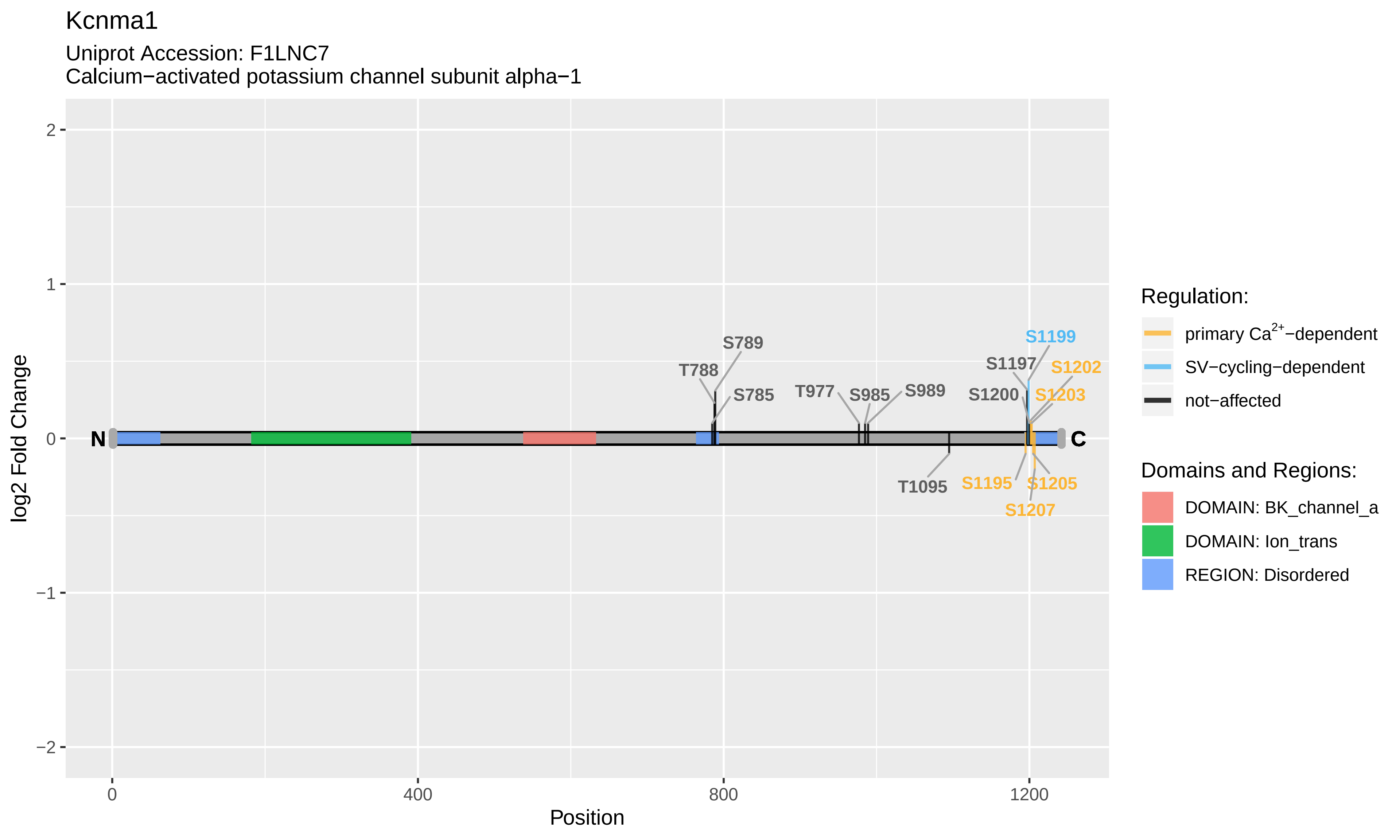

Kcnma1
Uniprot Accession: F1LNC7
Calcium−activated potassium channel subunit alpha−1
log2 Fold Change
2
1
0
−1
−2
N
C
S789
T788
S785
T977
S985
S989
T1095
S1199
S1197
S1202
S1200
S1203
S1195
S1205
S1207
0
400
800
1200
Position
Regulation:
primary Ca2+−dependent
SV−cycling−dependent
not−affected
Domains and Regions:
DOMAIN: BK_channel_a
DOMAIN: Ion_trans
REGION: Disordered

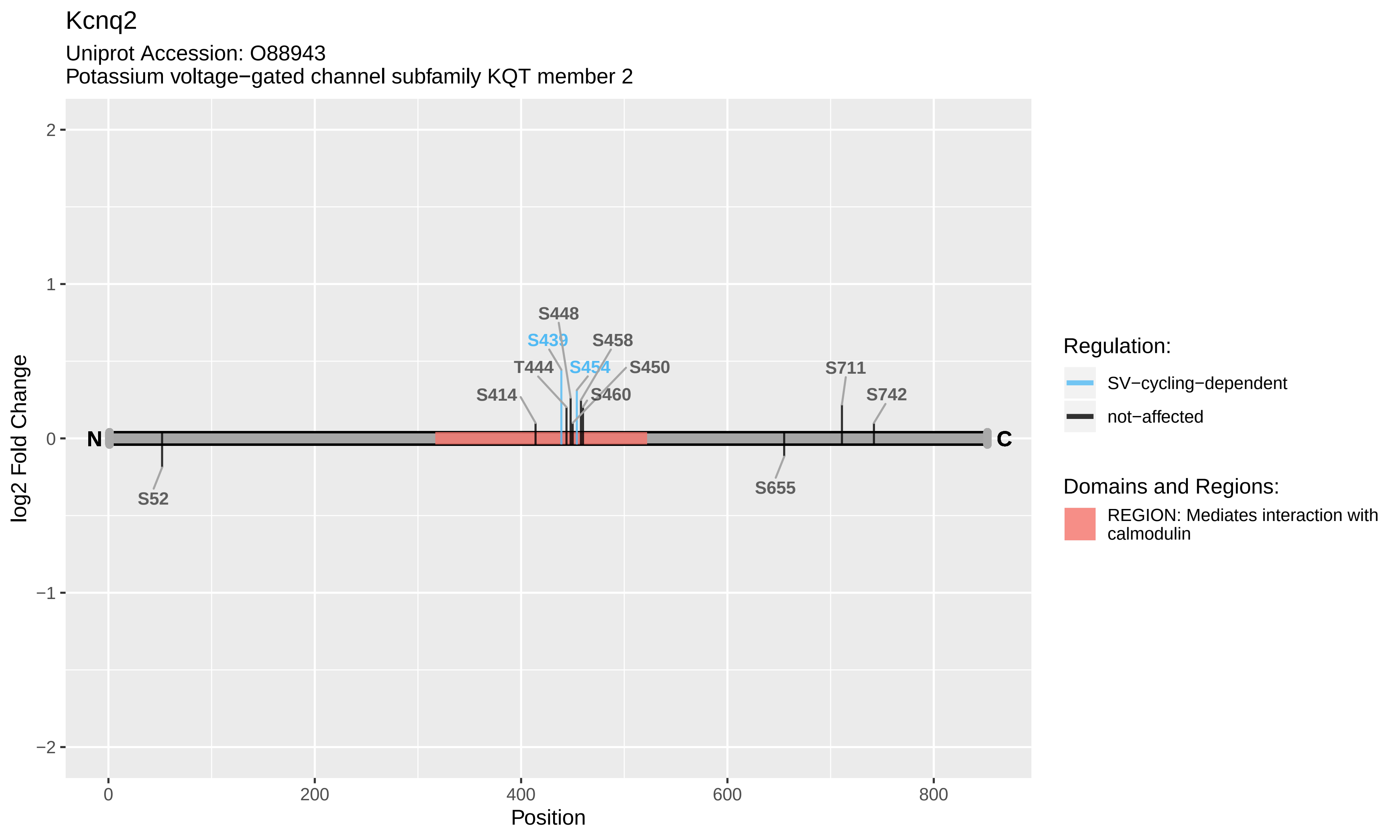
Kcnq2
Uniprot Accession: O88943
Potassium voltage−gated channel subfamily KQT member 2
log2 Fold Change
Position
S448
S439
S458
T444
S454
S450
S414
S460
S711
S742
S52
S655
N
C
2
1
0
−1
−2
0
200
400
600
800
Regulation:
SV−cycling−dependent
not−affected
Domains and Regions:
REGION: Mediates interaction with calmodulin

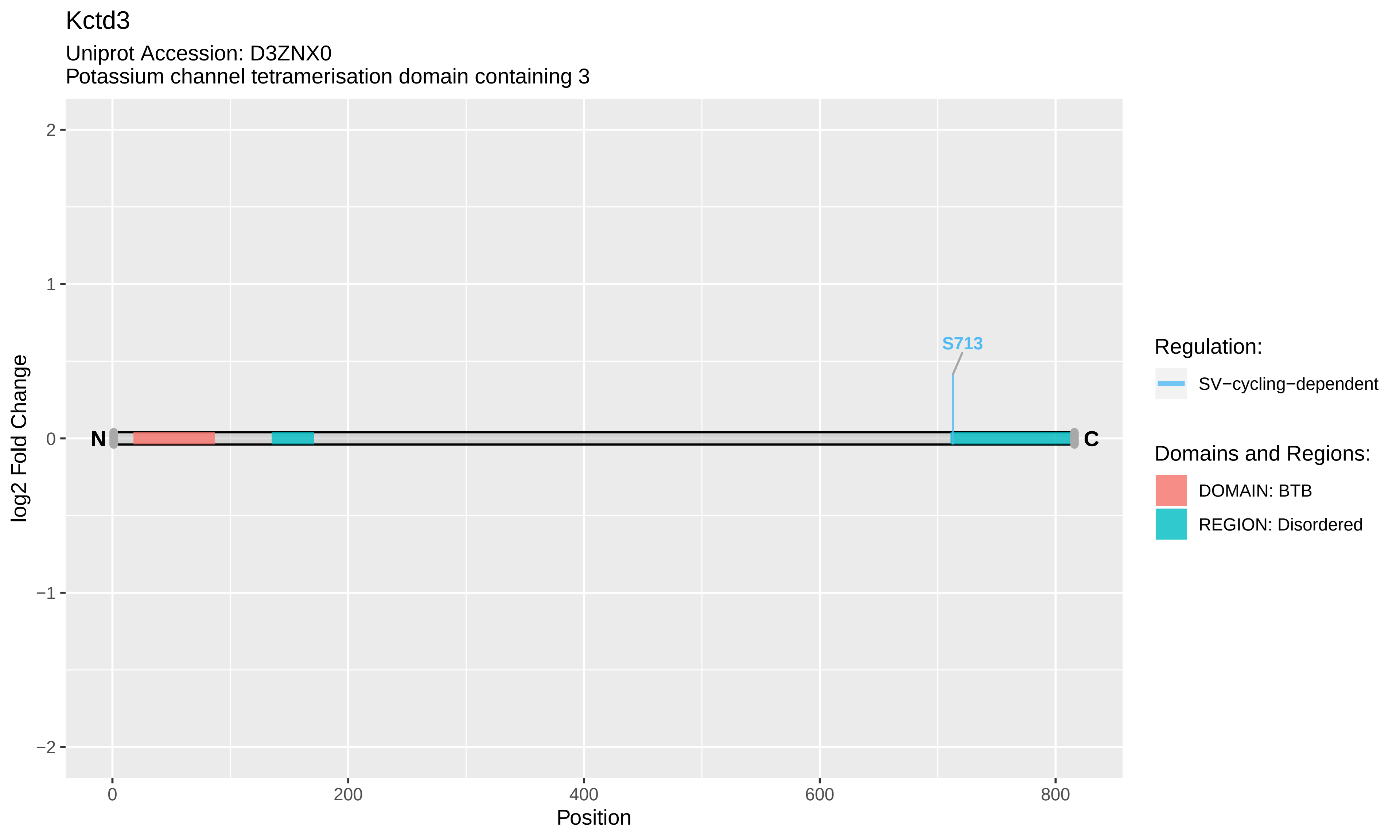

Kctd3
Uniprot Accession: D3ZNX0
Potassium channel tetramerisation domain containing 3
log2 Fold Change
2
1
0
−1
−2
N
C
S713
0
200
400
600
800
Position
Regulation:
SV−cycling−dependent
Domains and Regions:
DOMAIN: BTB
REGION: Disordered

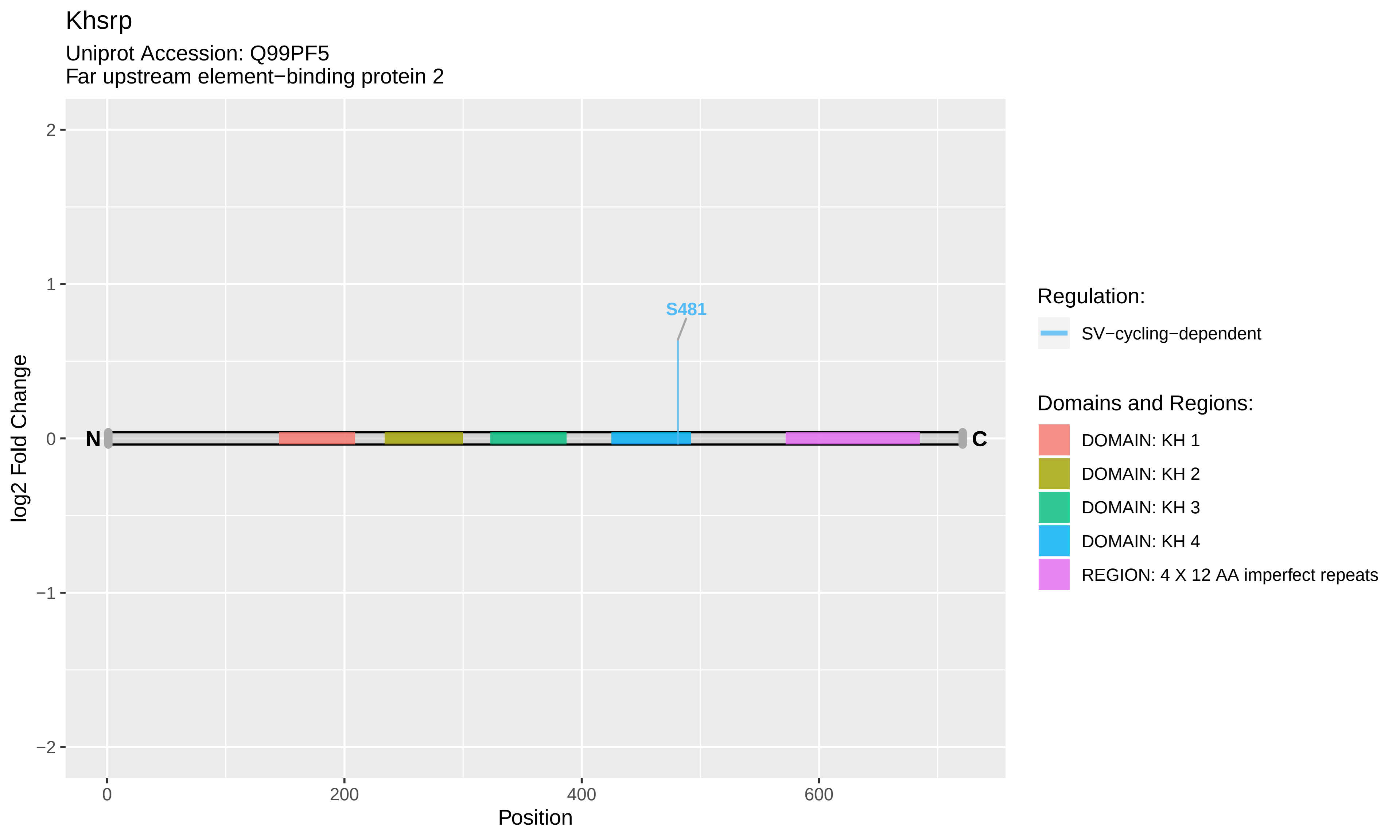

Khsrp
Uniprot Accession: Q99PF5
Far upstream element−binding protein 2
S481
N
C
2
1
0
−1
−2
log2 Fold Change
0
200
400
600
Position
Regulation:
SV−cycling−dependent
Domains and Regions:
DOMAIN: KH 1
DOMAIN: KH 2
DOMAIN: KH 3
DOMAIN: KH 4
REGION: 4 X 12 AA imperfect repeats

Kiaa1107

Uniprot Accession: D4A0X3
AP2−interacting clathrin−endocytosis protein

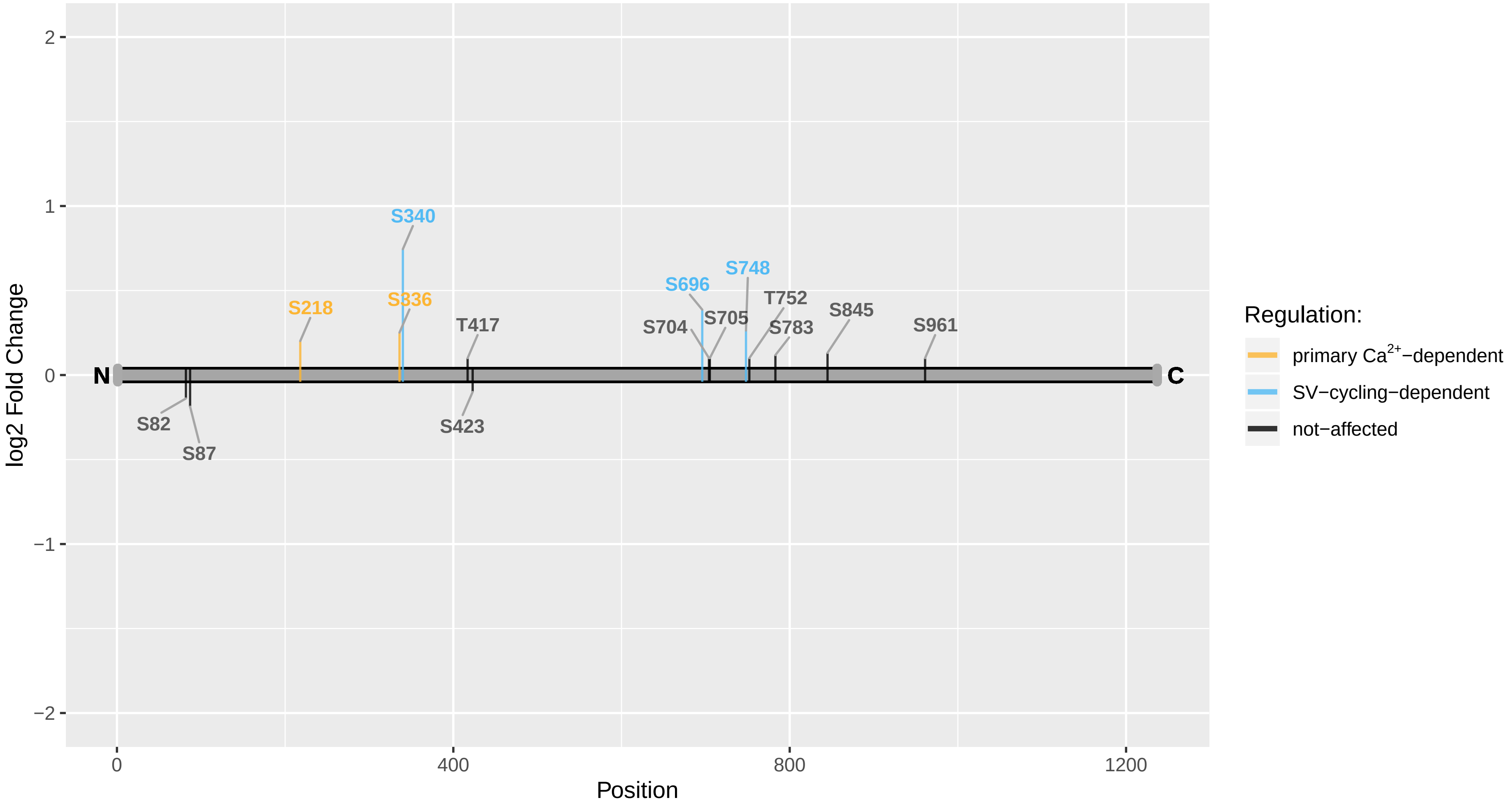

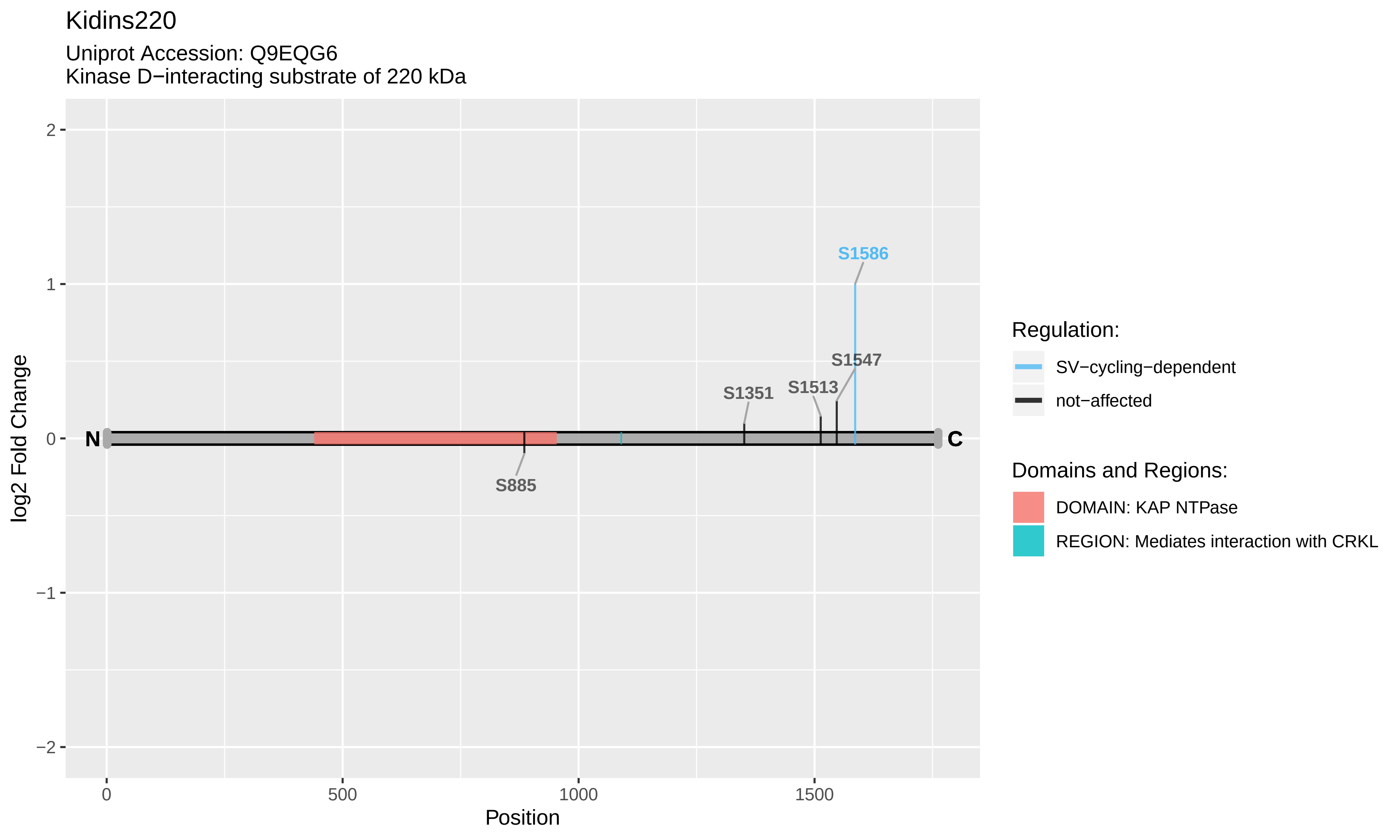

Kidins220
Uniprot Accession: Q9EQG6
Kinase D−interacting substrate of 220 kDa
log2 Fold Change
2
1
0
−1
−2
N
C
S1586
S1547
S1513
S1351
S885
0
500
1000
1500
Position
Regulation:
SV−cycling−dependent
not−affected
Domains and Regions:
DOMAIN: KAP NTPase
REGION: Mediates interaction with CRKL

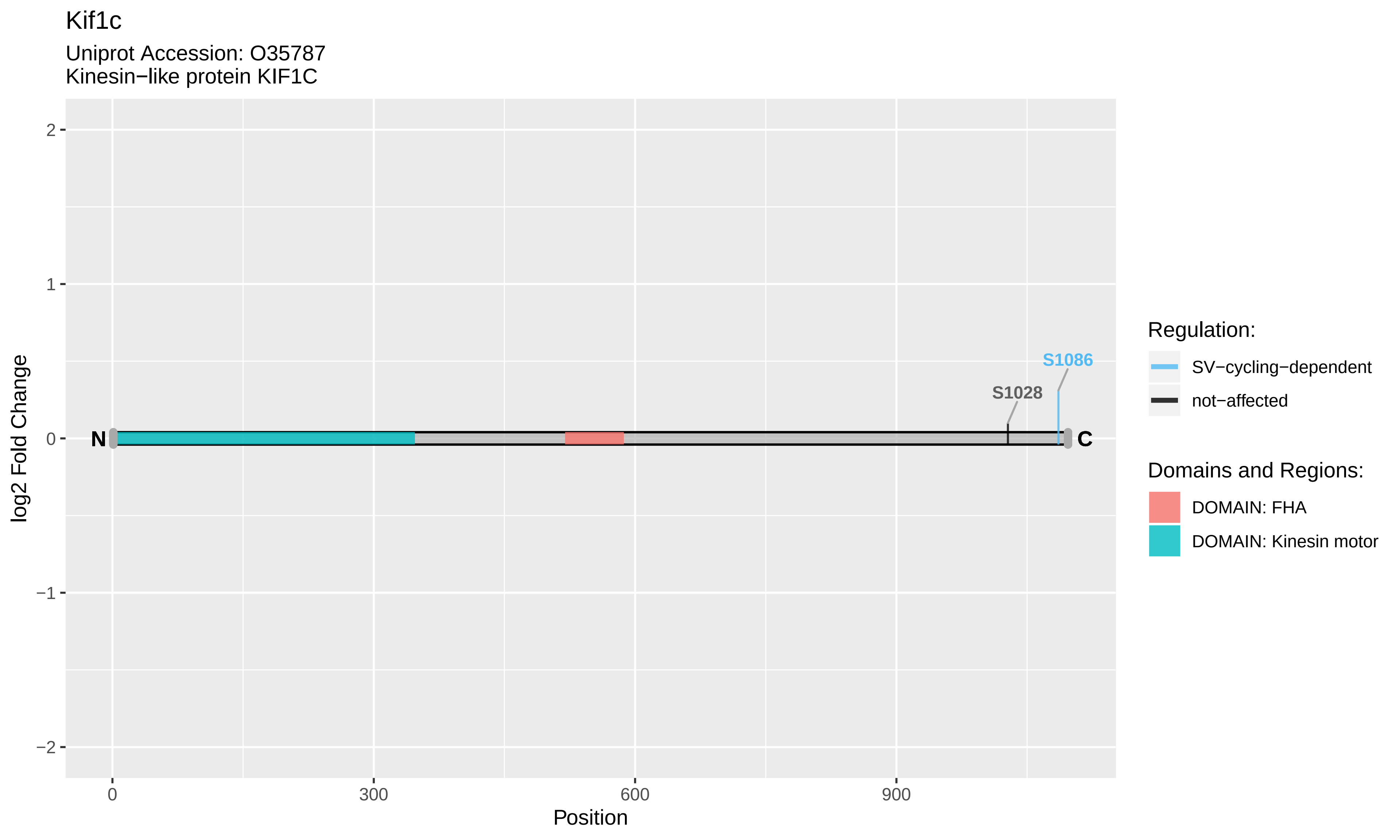
Kif1c
Uniprot Accession: O35787
Kinesin−like protein KIF1C
log2 Fold Change
Position
2
1
0
−1
−2
0
300
600
900
N
C
S1086
S1028
Regulation:
SV−cycling−dependent
not−affected
Domains and Regions:
DOMAIN: FHA
DOMAIN: Kinesin motor

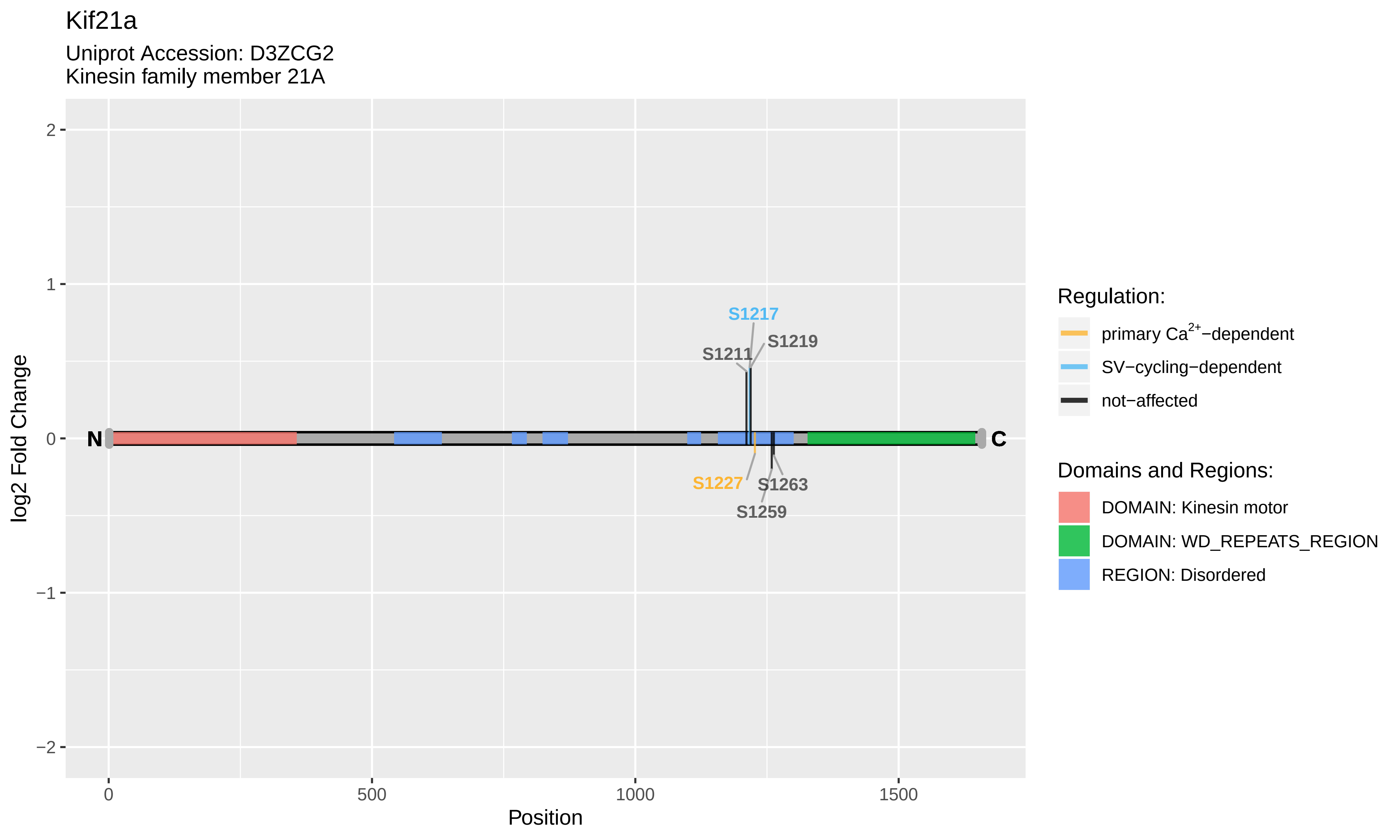
Kif21a
Uniprot Accession: D3ZCG2
Kinesin family member 21A
log2 Fold Change
Position
N
C
S1217
S1219
S1211
S1227
S1263
S1259
Regulation:
primary Ca2+−dependent
SV−cycling−dependent
not−affected
Domains and Regions:
DOMAIN: Kinesin motor
DOMAIN: WD_REPEATS_REGION
REGION: Disordered

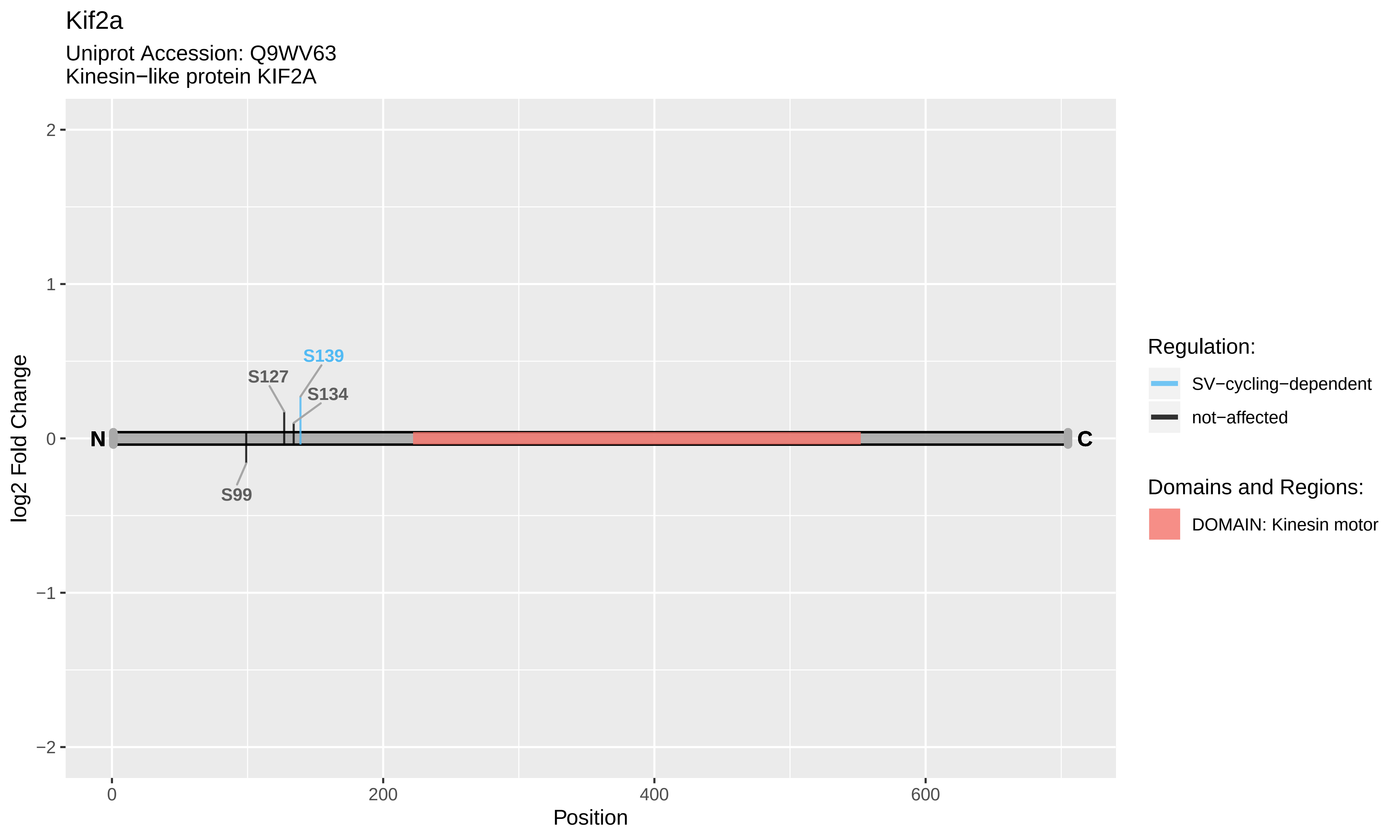
Kif2a
Uniprot Accession: Q9WV63
Kinesin−like protein KIF2A
S139
S127
S134
S99
N
C
2
1
0
−1
−2
log2 Fold Change
0
200
400
600
Position
Regulation:
SV−cycling−dependent
not−affected
Domains and Regions:
DOMAIN: Kinesin motor

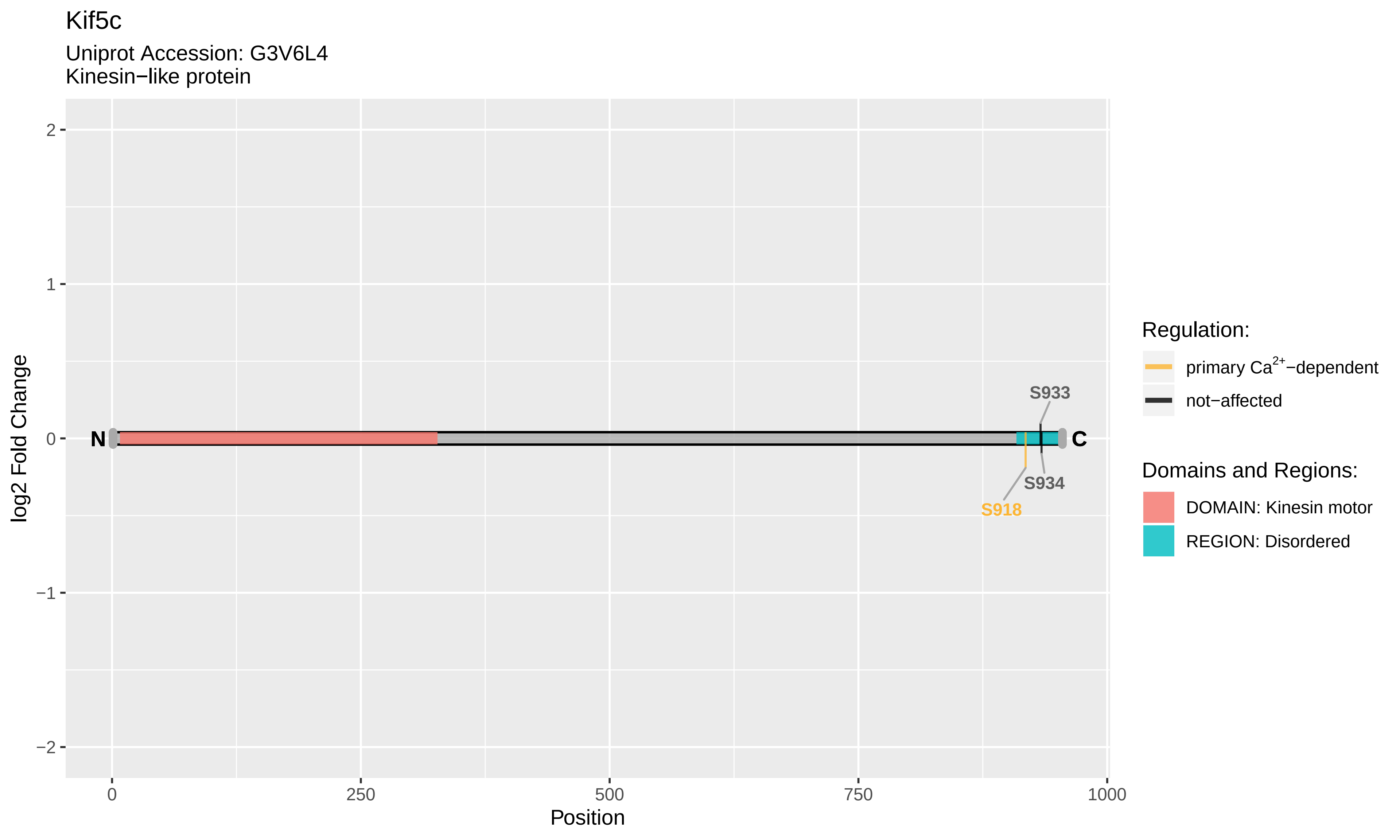

Kif5c
Uniprot Accession: G3V6L4
Kinesin−like protein
log2 Fold Change
2
1
0
−1
−2
0
250
500
750
1000
Position
N
C
S933
S934
S918
Regulation:
primary Ca2+−dependent
not−affected
Domains and Regions:
DOMAIN: Kinesin motor
REGION: Disordered

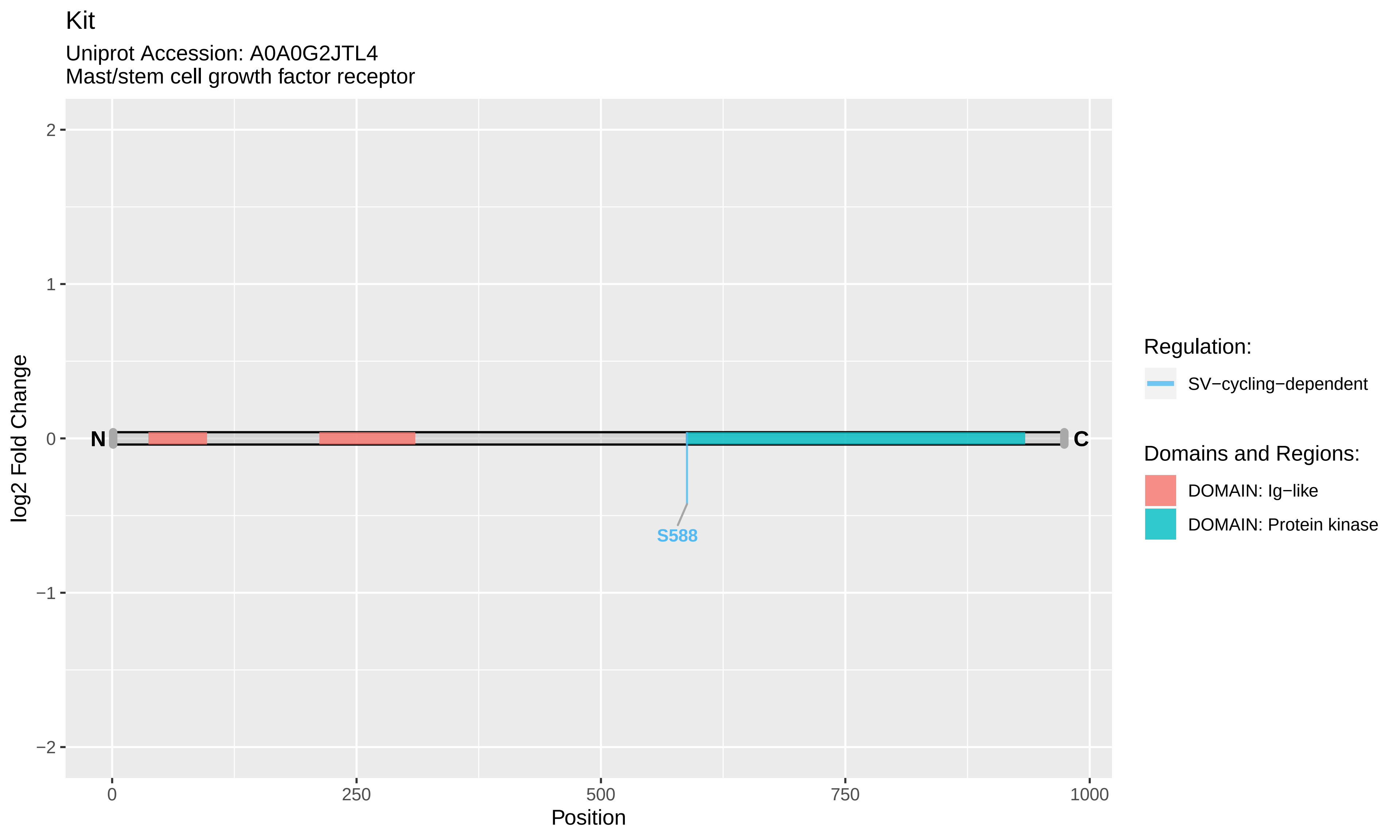

Kit
Uniprot Accession: A0A0G2JTL4
Mast/stem cell growth factor receptor
log2 Fold Change
Position
N
C
S588
Regulation:
SV−cycling−dependent
Domains and Regions:
DOMAIN: Ig−like
DOMAIN: Protein kinase
2
1
0
−1
−2
0
250
500
750
1000

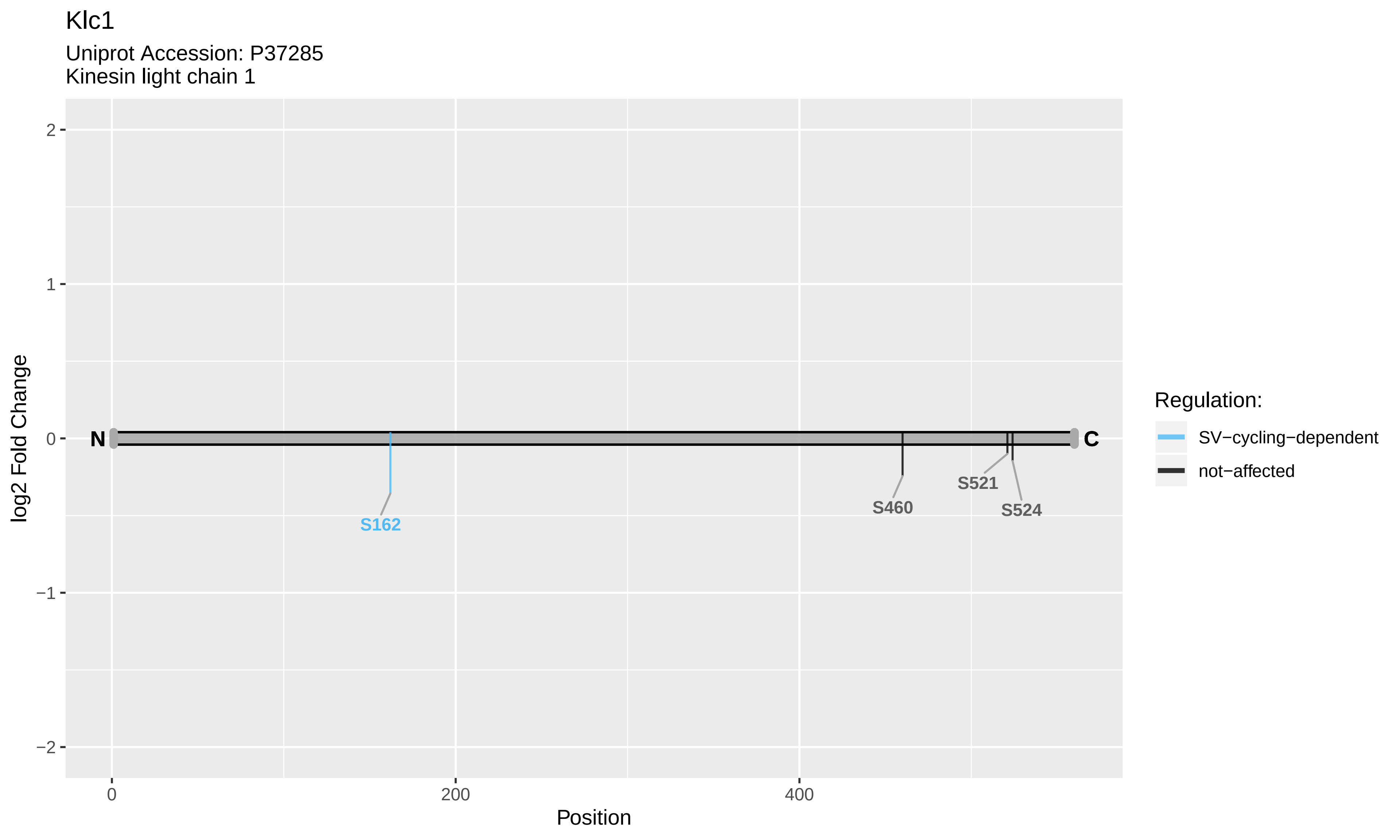

Klc1
Uniprot Accession: P37285
Kinesin light chain 1
log2 Fold Change
2
1
0
−1
−2
N
C
S162
S460
S521
S524
0
200
400
Position
Regulation:
SV−cycling−dependent
not−affected

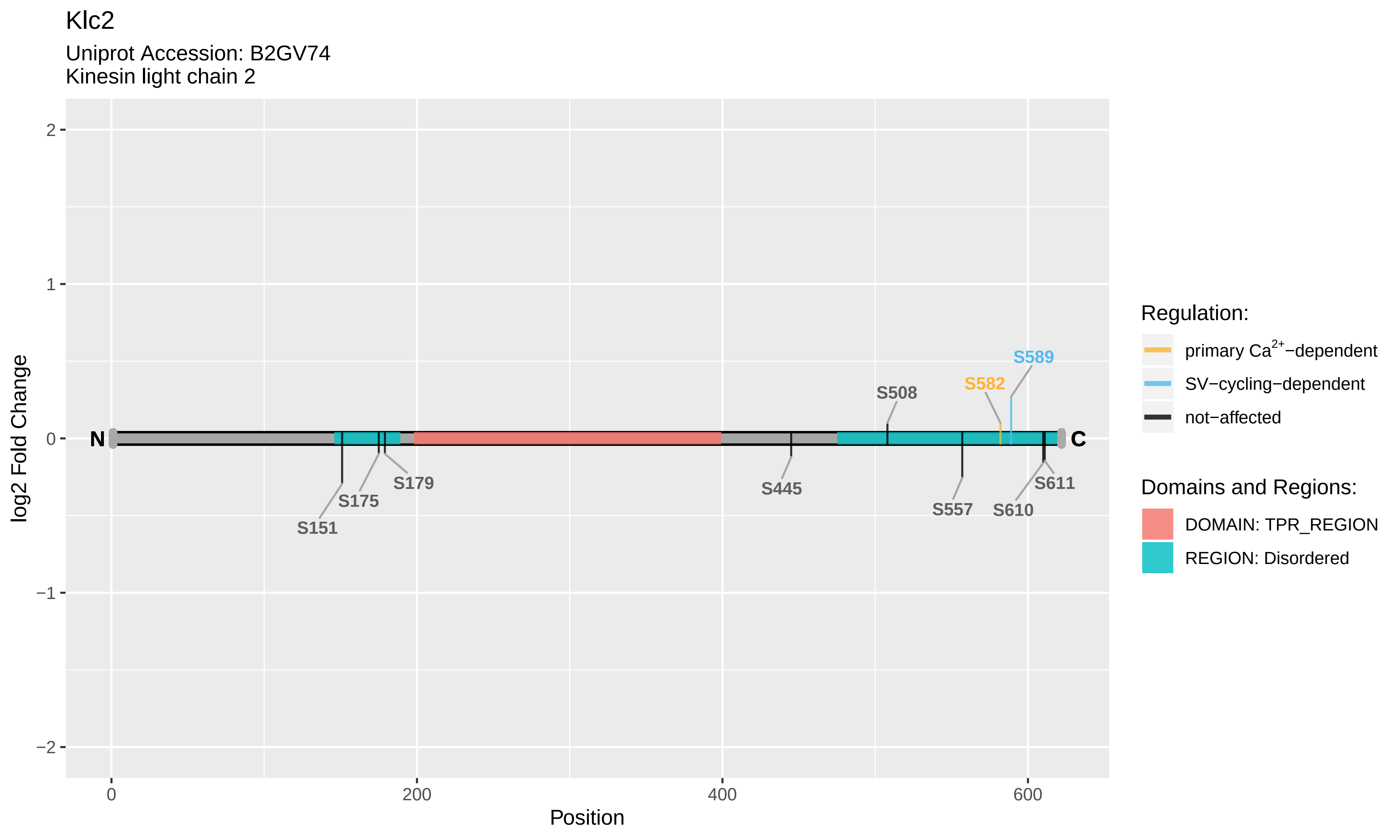
Klc2
Uniprot Accession: B2GV74
Kinesin light chain 2
log2 Fold Change
2
1
0
−1
−2
N
C
S151
S175
S179
S445
S508
S557
S582
S589
S610
S611
0
200
400
600
Position
Regulation:
primary Ca2+−dependent
SV−cycling−dependent
not−affected
Domains and Regions:
DOMAIN: TPR_REGION
REGION: Disordered

Klc4

Uniprot Accession: Q5PQM2
Kinesin light chain 4

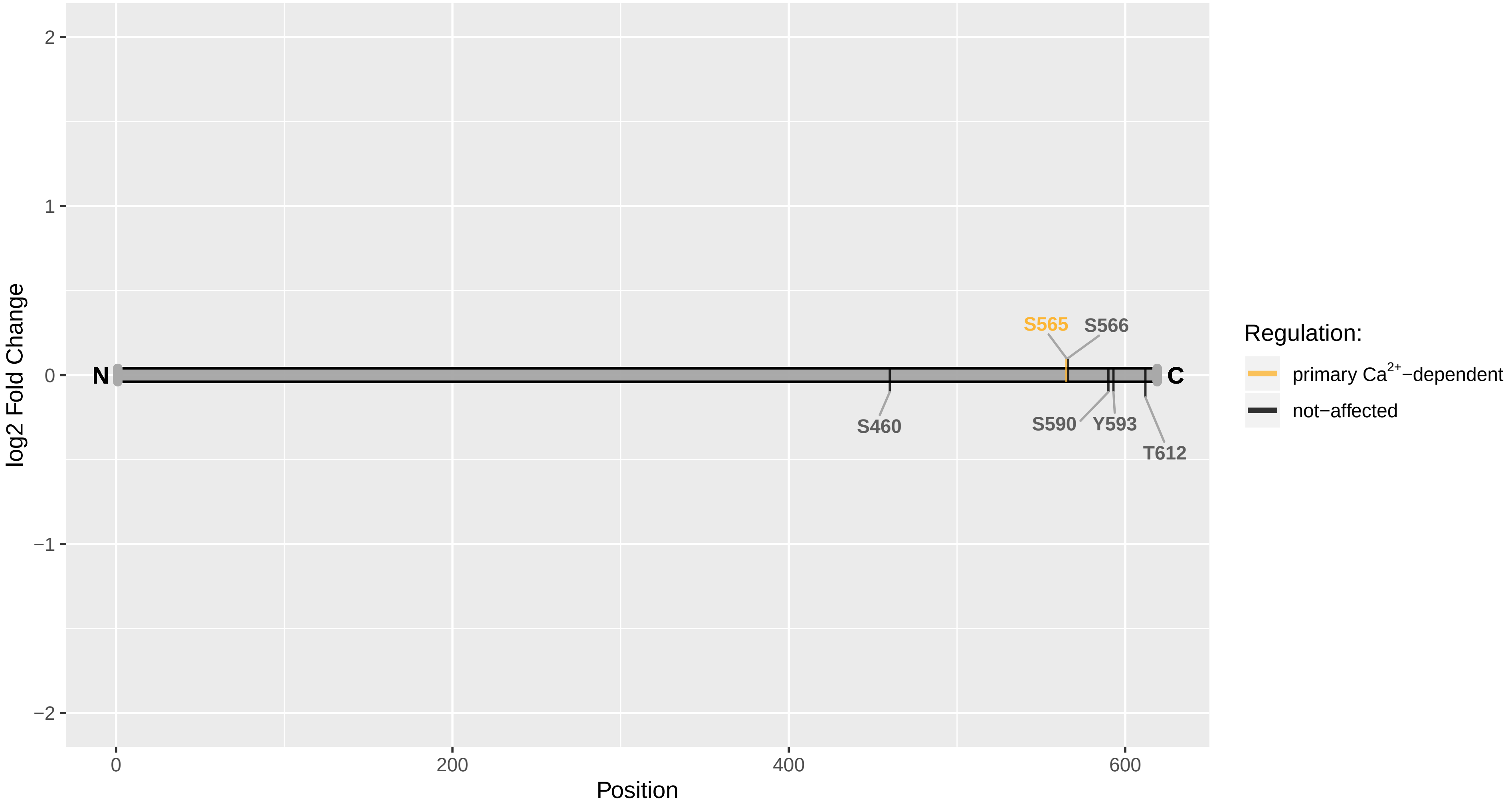

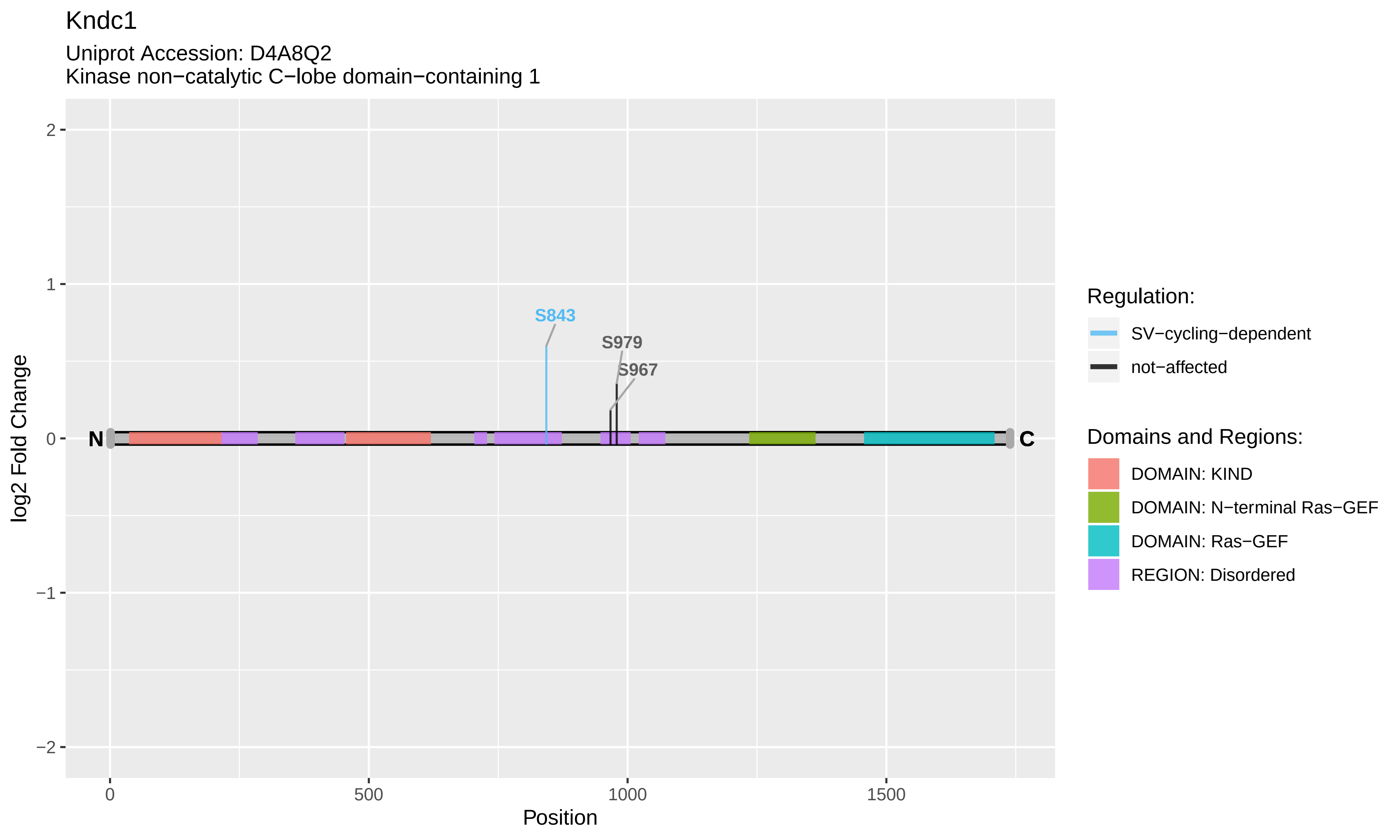
Kndc1
Uniprot Accession: D4A8Q2
Kinase non−catalytic C−lobe domain−containing 1
S843
S979
S967
N
C
2
1
0
−1
−2
log2 Fold Change
0
500
1000
1500
Position
Regulation:
SV−cycling−dependent
not−affected
Domains and Regions:
DOMAIN: KIND
DOMAIN: N−terminal Ras−GEF
DOMAIN: Ras−GEF
REGION: Disordered

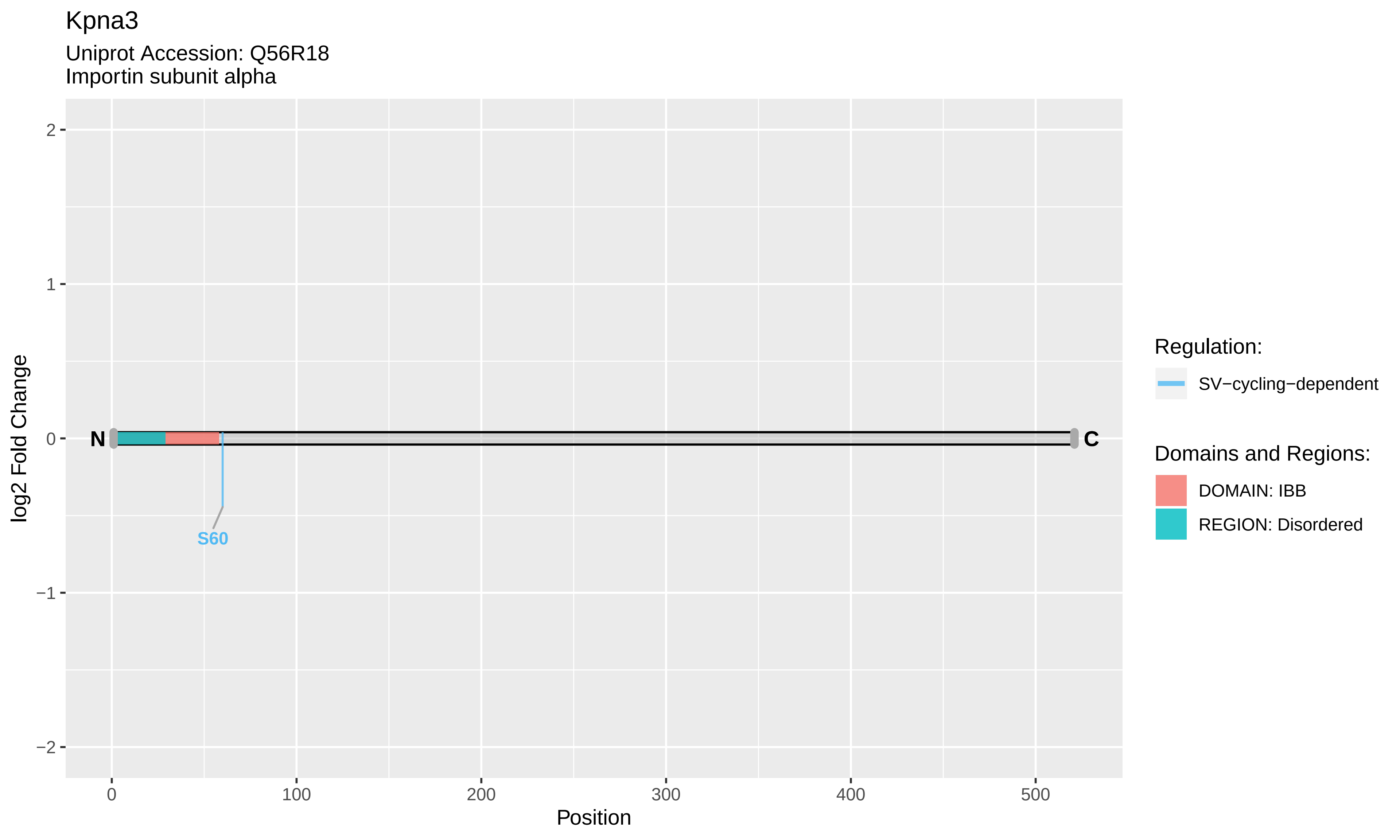

Kpna3
Uniprot Accession: Q56R18
Importin subunit alpha
log2 Fold Change
2
1
0
−1
−2
N
C
S60
0
100
200
300
400
500
Position
Regulation:
SV−cycling−dependent
Domains and Regions:
DOMAIN: IBB
REGION: Disordered

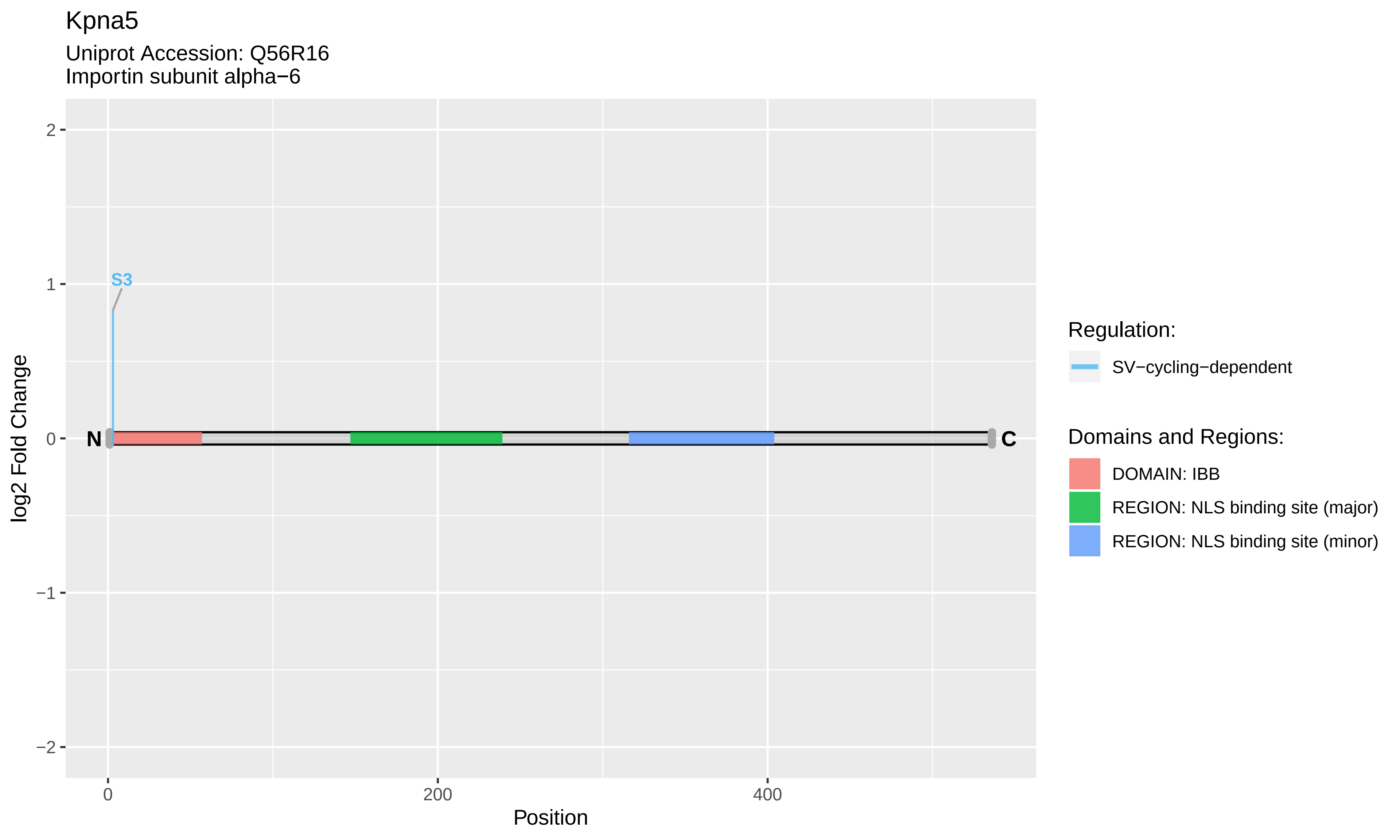
Kpna5
Uniprot Accession: Q56R16
Importin subunit alpha−6
S3
N
C
2
1
0
−1
−2
log2 Fold Change
0
200
400
Position
Regulation:
SV−cycling−dependent
Domains and Regions:
DOMAIN: IBB
REGION: NLS binding site (major)
REGION: NLS binding site (minor)

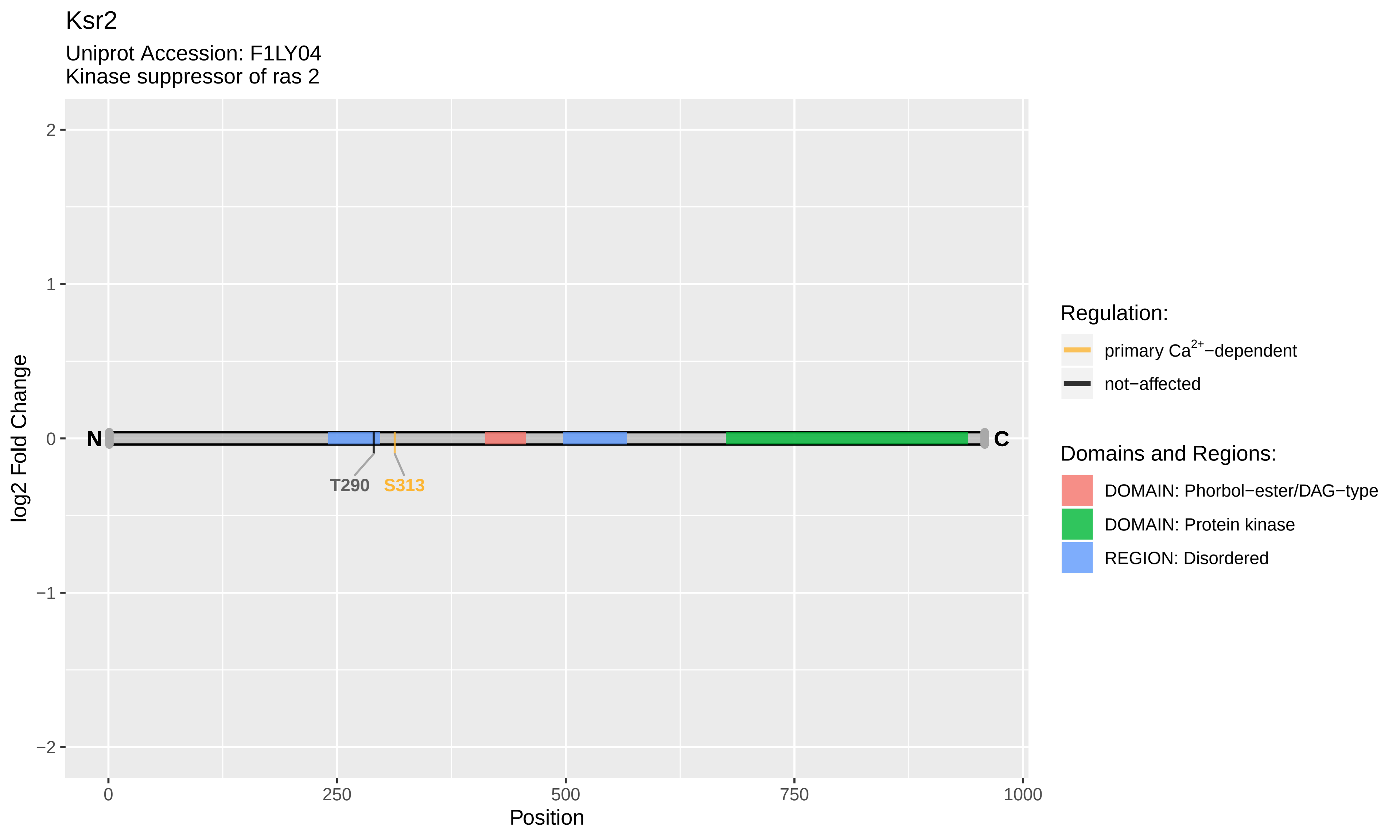

Ksr2
Uniprot Accession: F1LY04
Kinase suppressor of ras 2
log2 Fold Change
2
1
0
−1
−2
N
C
T290
S313
0
250
500
750
1000
Position
Regulation:
primary Ca2+−dependent
not−affected
Domains and Regions:
DOMAIN: Phorbol−ester/DAG−type
DOMAIN: Protein kinase
REGION: Disordered

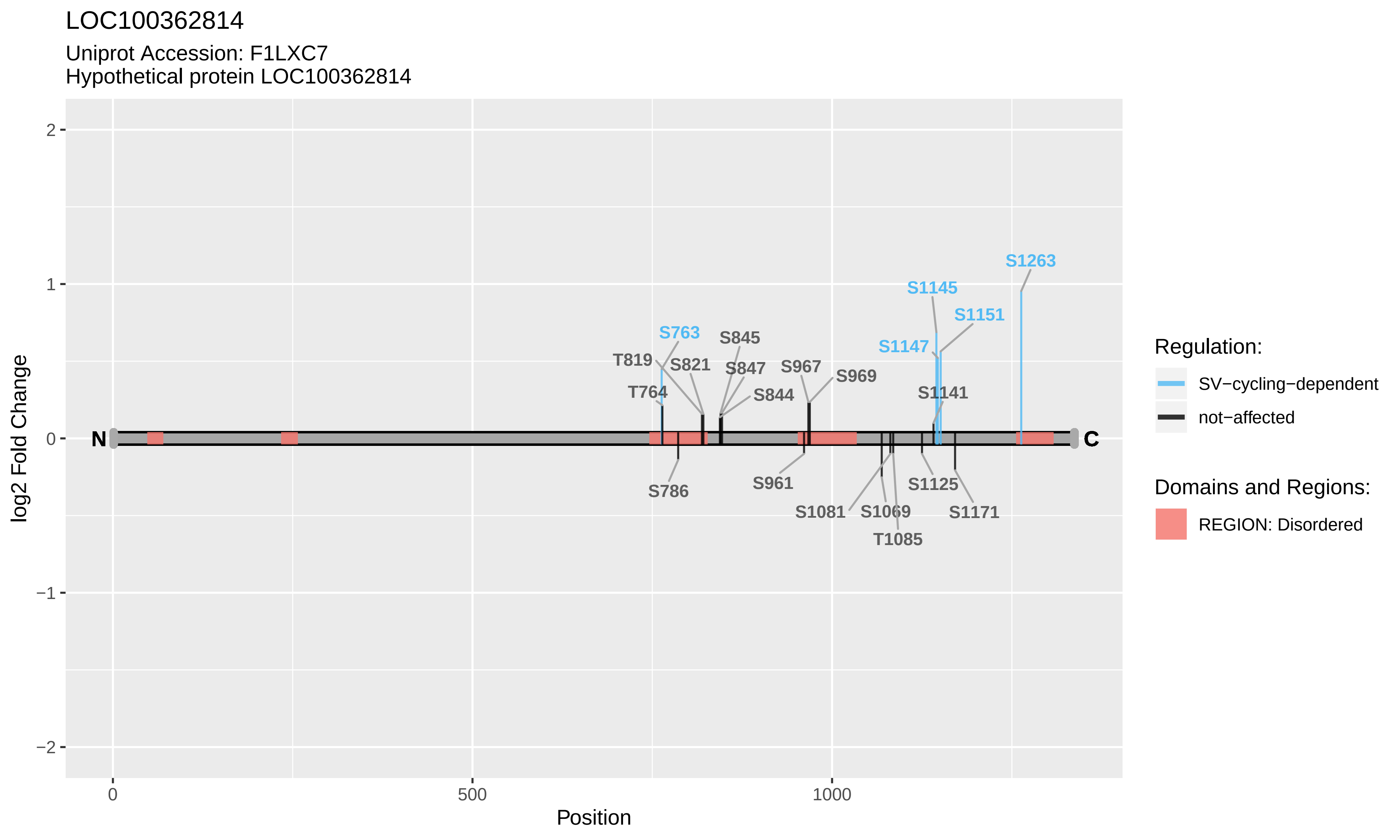

LOC100362814
Uniprot Accession: F1LXC7
Hypothetical protein LOC100362814
log2 Fold Change
2
1
0
−1
−2
Position
0
500
1000
N
C
S763
T764
T819
S821
S845
S847
S844
S967
S969
S1145
S1147
S1151
S1141
S1263
S786
S961
S1081
S1069
T1085
S1125
S1171
Regulation:
SV−cycling−dependent
not−affected
Domains and Regions:
REGION: Disordered

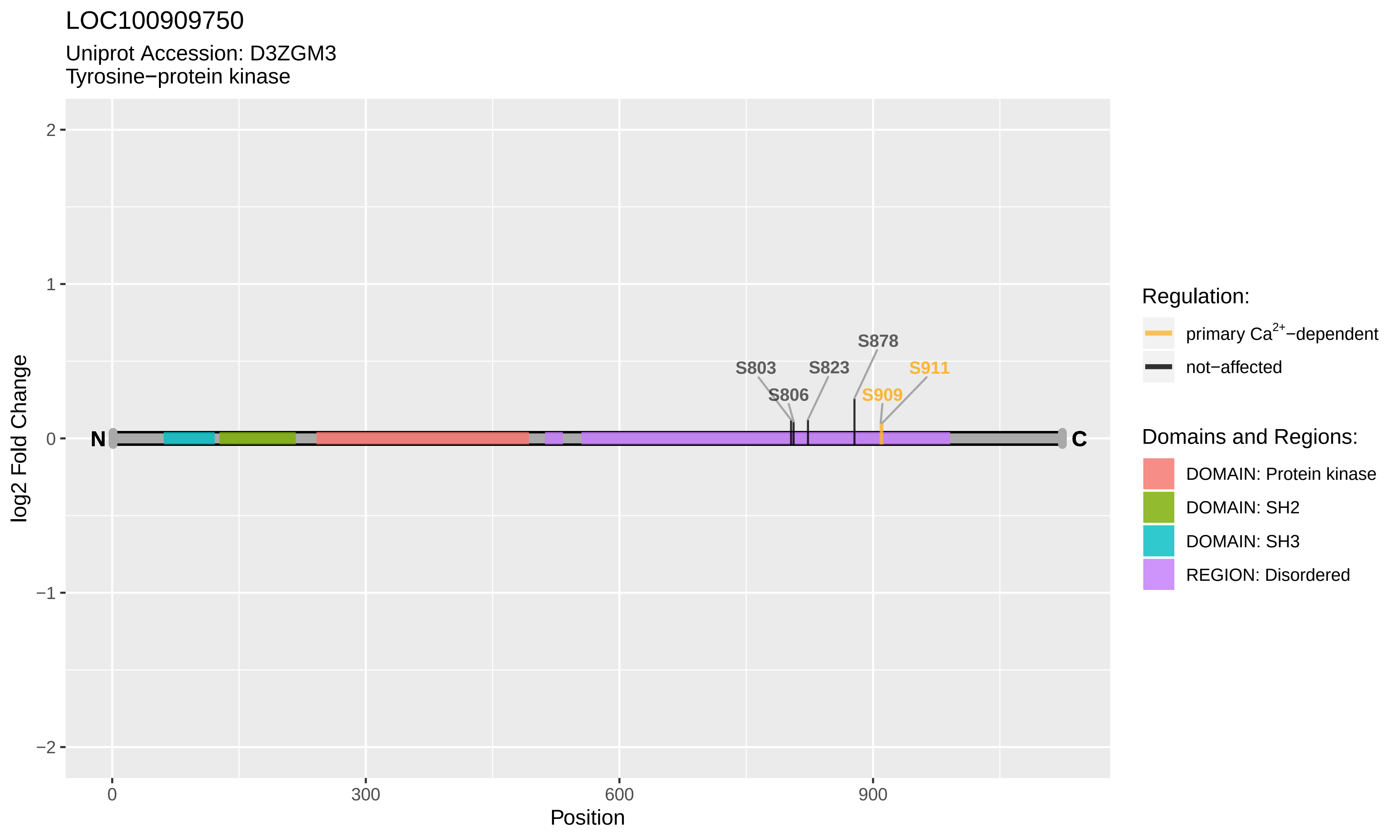

LOC100909750
Uniprot Accession: D3ZGM3
Tyrosine−protein kinase
log2 Fold Change
2
1
0
−1
−2
Position
0
300
600
900
N
C
S803
S806
S823
S878
S909
S911
Regulation:
primary Ca2+−dependent
not−affected
Domains and Regions:
DOMAIN: Protein kinase
DOMAIN: SH2
DOMAIN: SH3
REGION: Disordered

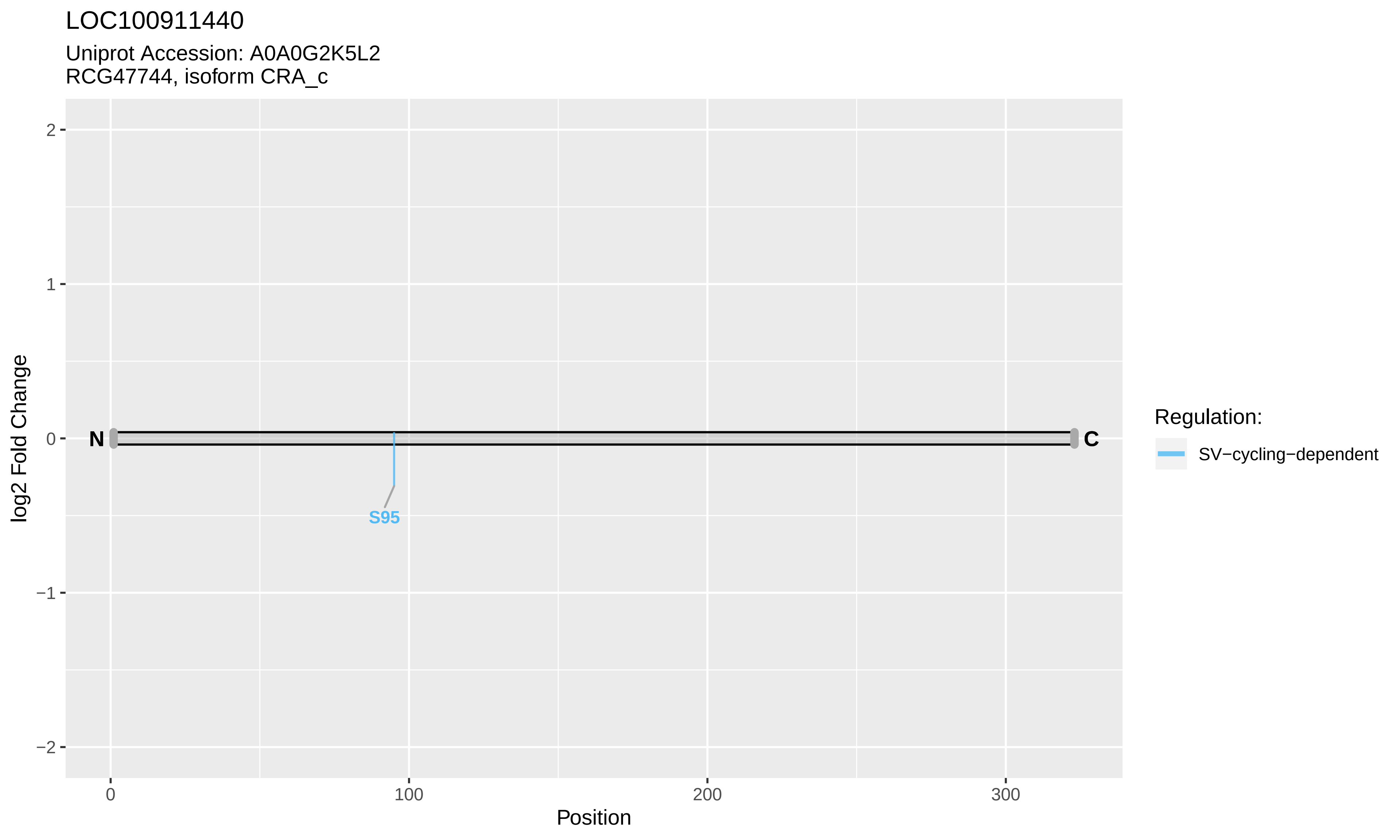

LOC100911440
Uniprot Accession: A0A0G2K5L2
RCG47744, isoform CRA_c
log2 Fold Change
Position
N
C
S95
Regulation:
SV−cycling−dependent

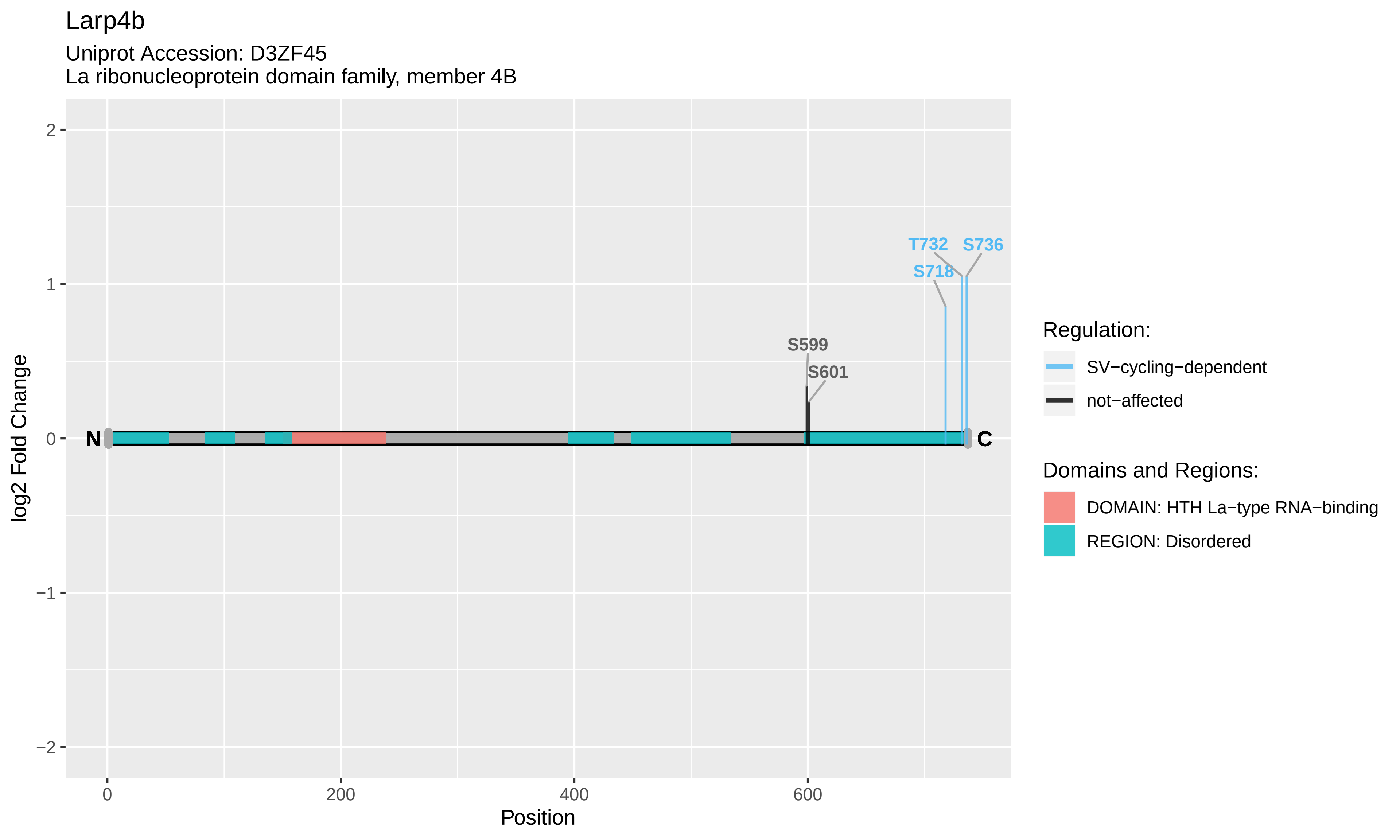

Larp4b
Uniprot Accession: D3ZF45
La ribonucleoprotein domain family, member 4B
log2 Fold Change
Position
2
1
0
−1
−2
0
200
400
600
N
C
S599
S601
T732
S736
S718
Regulation:
SV−cycling−dependent
not−affected
Domains and Regions:
DOMAIN: HTH La−type RNA−binding
REGION: Disordered

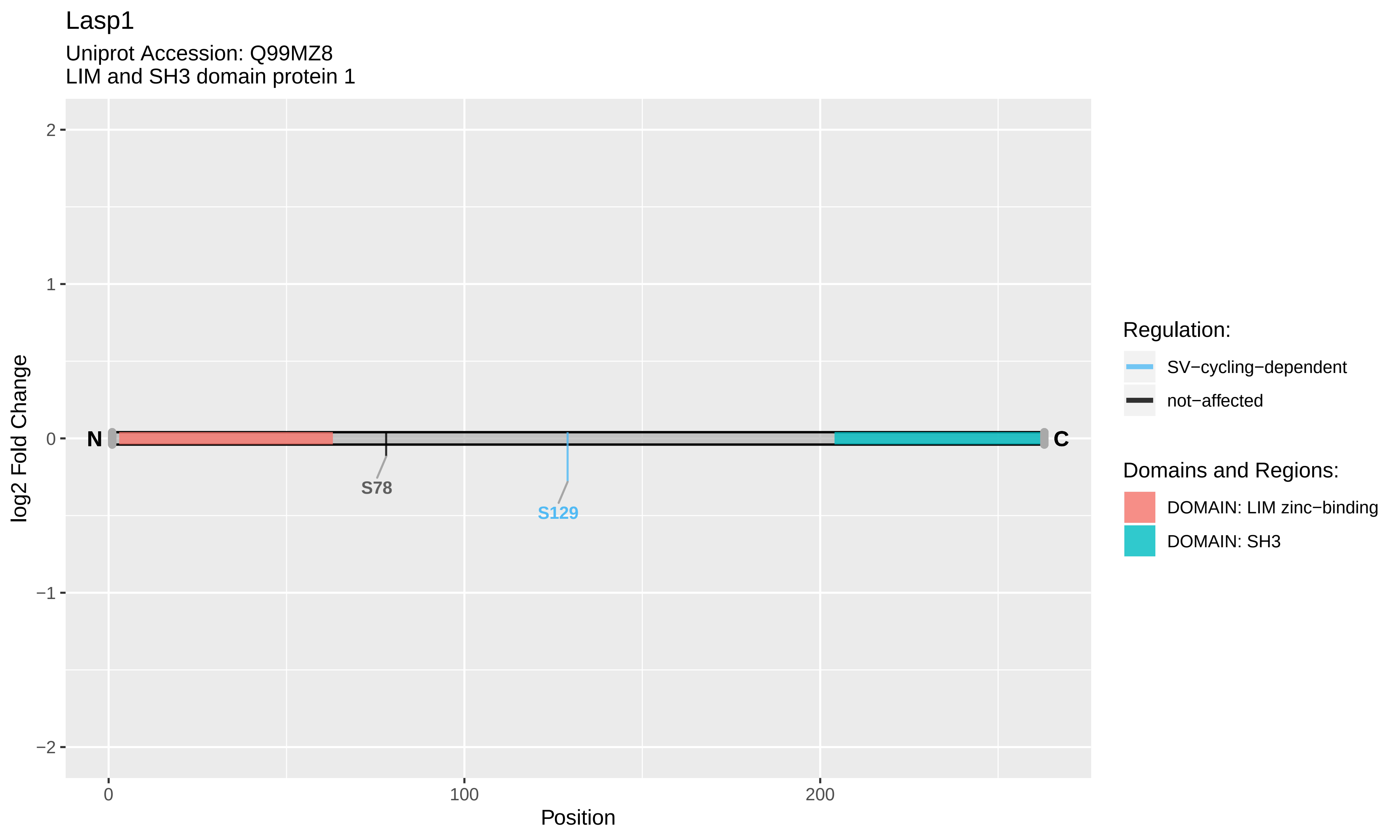

Lasp1
Uniprot Accession: Q99MZ8
LIM and SH3 domain protein 1
log2 Fold Change
2
1
0
−1
−2
N
C
S78
S129
0
100
200
Position
Regulation:
SV−cycling−dependent
not−affected
Domains and Regions:
DOMAIN: LIM zinc−binding
DOMAIN: SH3

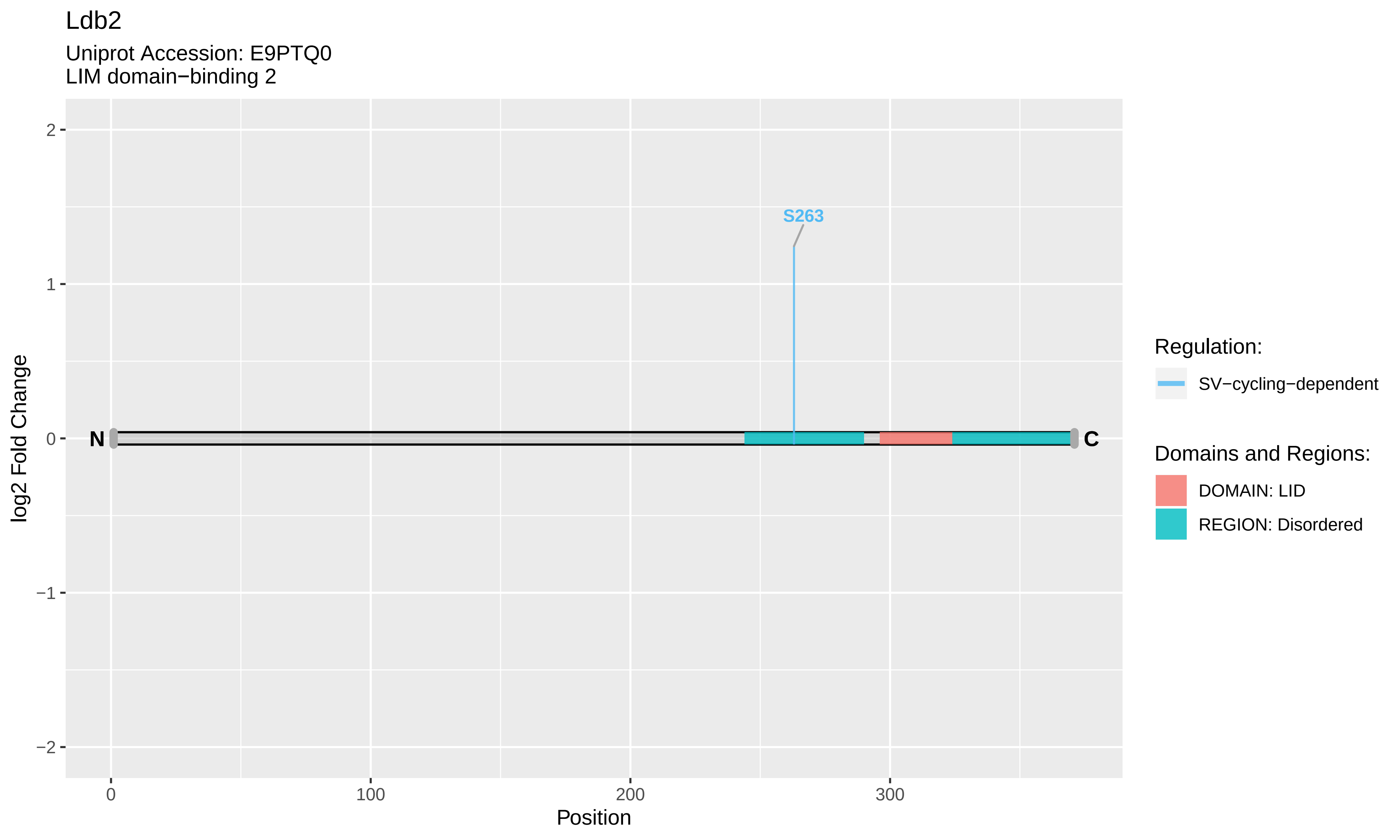
Ldb2
Uniprot Accession: E9PTQ0
LIM domain−binding 2
S263
N
C
2
1
0
−1
−2
log2 Fold Change
0
100
200
300
Position
Regulation:
SV−cycling−dependent
Domains and Regions:
DOMAIN: LID
REGION: Disordered

Leo1

Uniprot Accession: Q641X2
RNA polymerase−associated protein LEO1

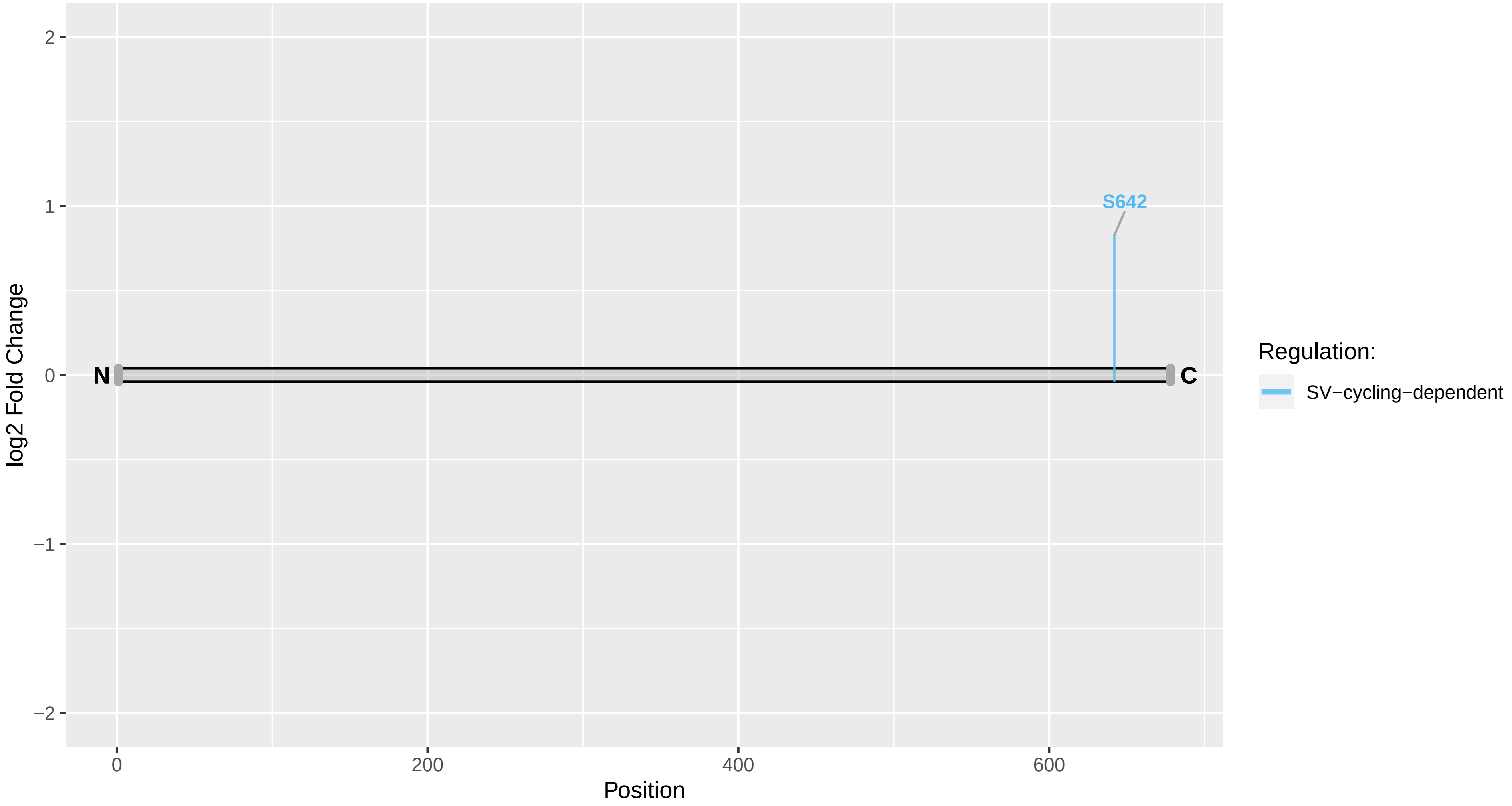

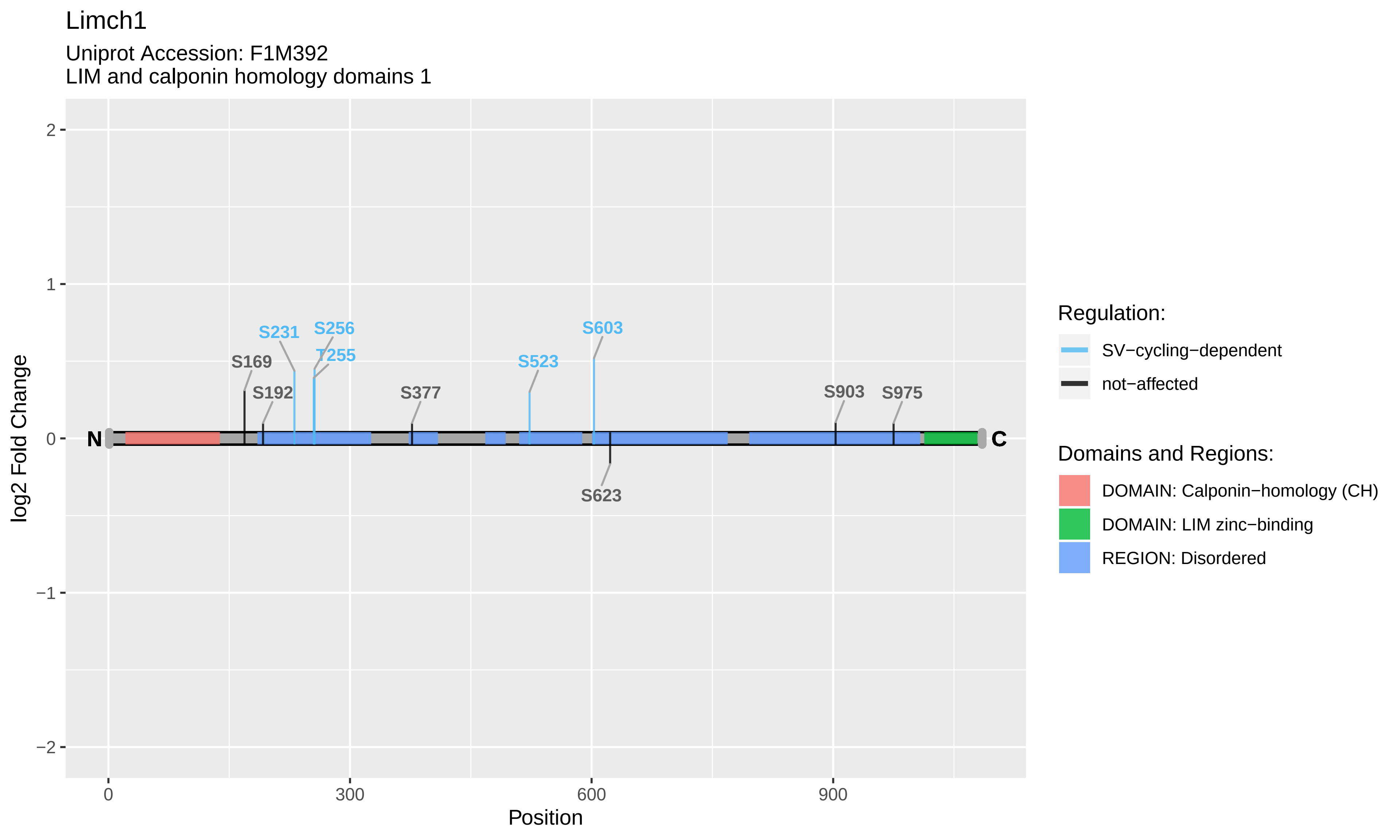
Limch1
Uniprot Accession: F1M392
LIM and calponin homology domains 1
S231
S256
T255
S169
S192
S377
S523
S603
S623
S903
S975
N
C
2
1
0
−1
−2
0
300
600
900
log2 Fold Change
Position
Regulation:
SV−cycling−dependent
not−affected
Domains and Regions:
DOMAIN: Calponin−homology (CH)
DOMAIN: LIM zinc−binding
REGION: Disordered

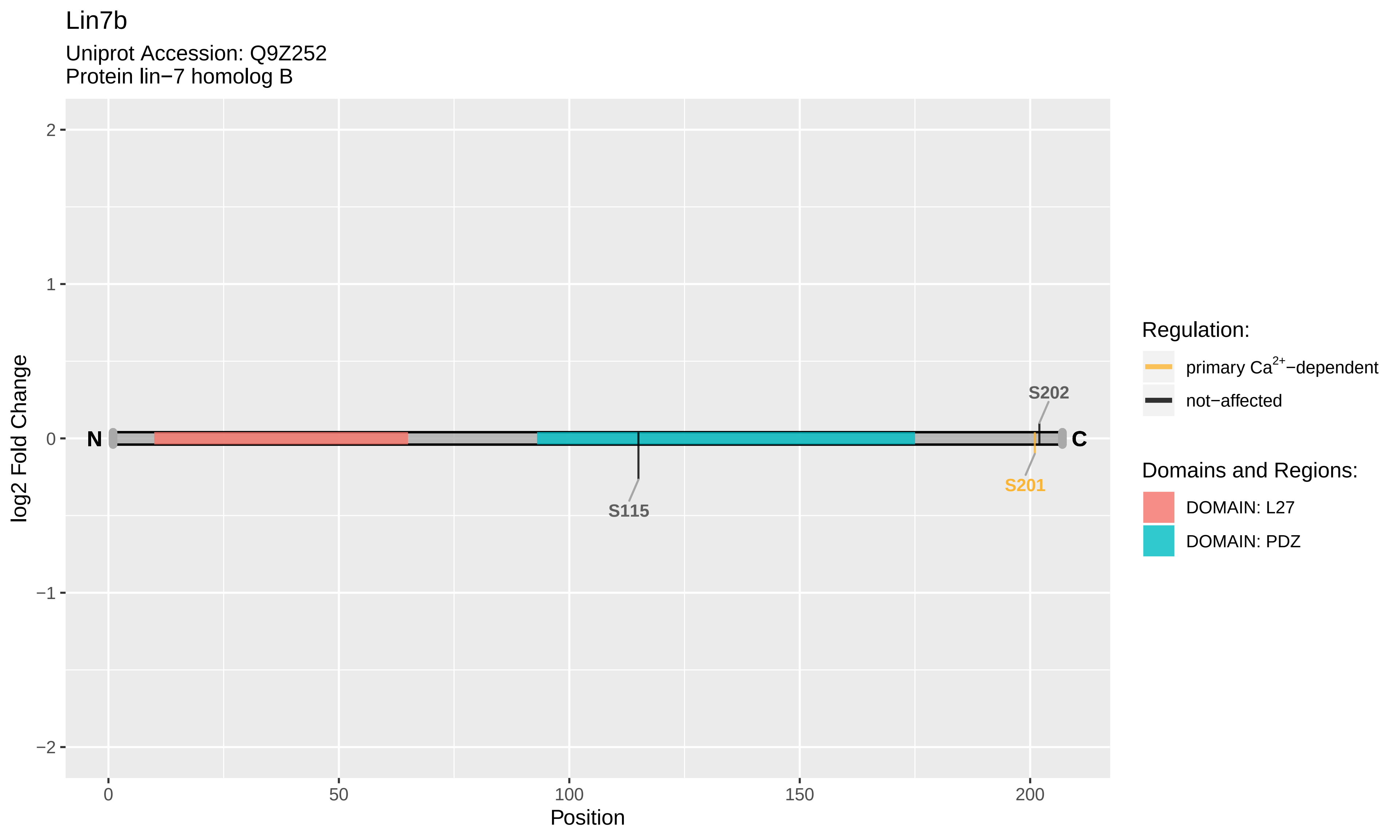

Lin7b
Uniprot Accession: Q9Z252
Protein lin−7 homolog B
log2 Fold Change
Position
N
C
S202
S201
S115
Regulation:
primary Ca2+−dependent
not−affected
Domains and Regions:
DOMAIN: L27
DOMAIN: PDZ

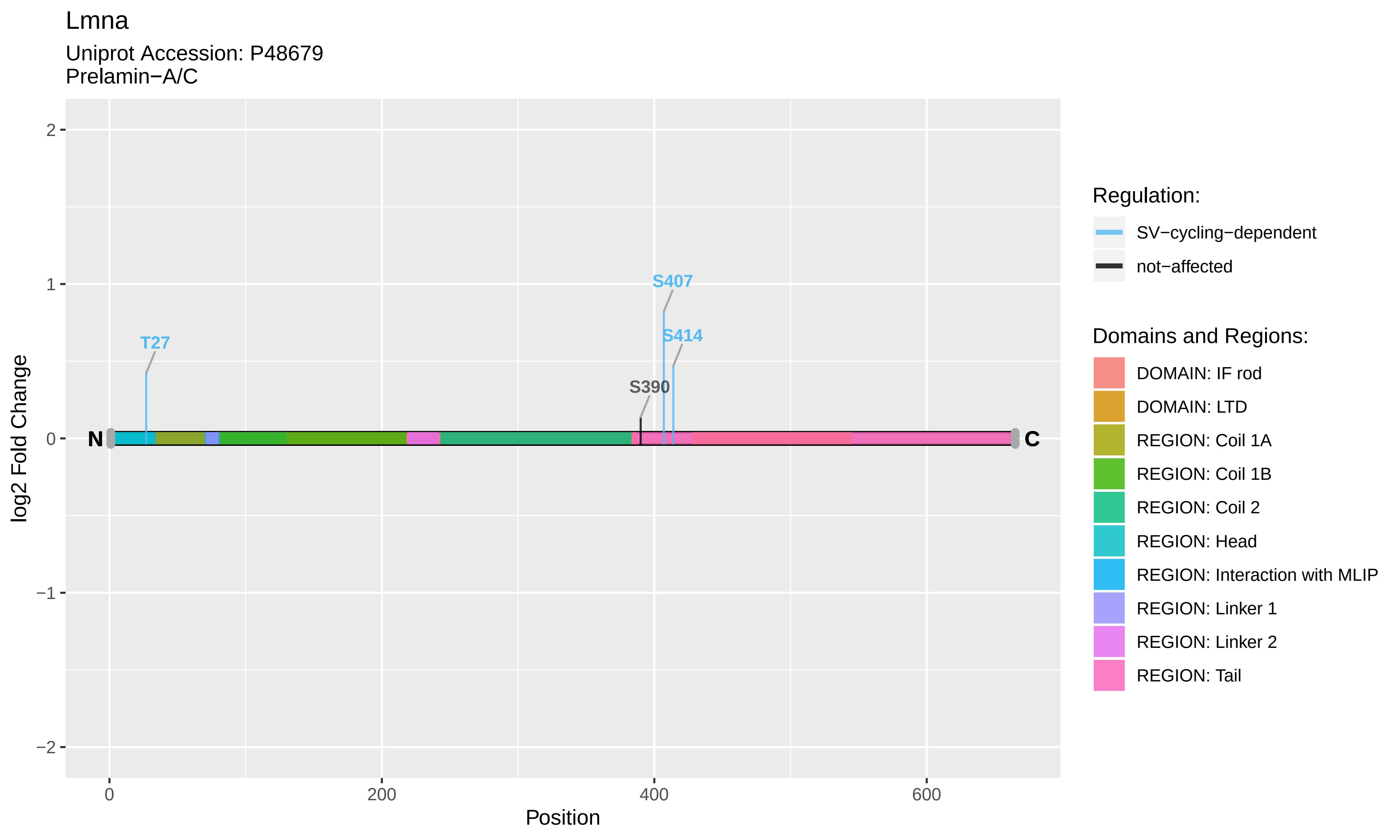

Lmna
Uniprot Accession: P48679
Prelamin−A/C
log2 Fold Change
2
1
0
−1
−2
N
C
T27
S407
S414
S390
0
200
400
600
Position
Regulation:
SV−cycling−dependent
not−affected
Domains and Regions:
DOMAIN: IF rod
DOMAIN: LTD
REGION: Coil 1A
REGION: Coil 1B
REGION: Coil 2
REGION: Head
REGION: Interaction with MLIP
REGION: Linker 1
REGION: Linker 2
REGION: Tail

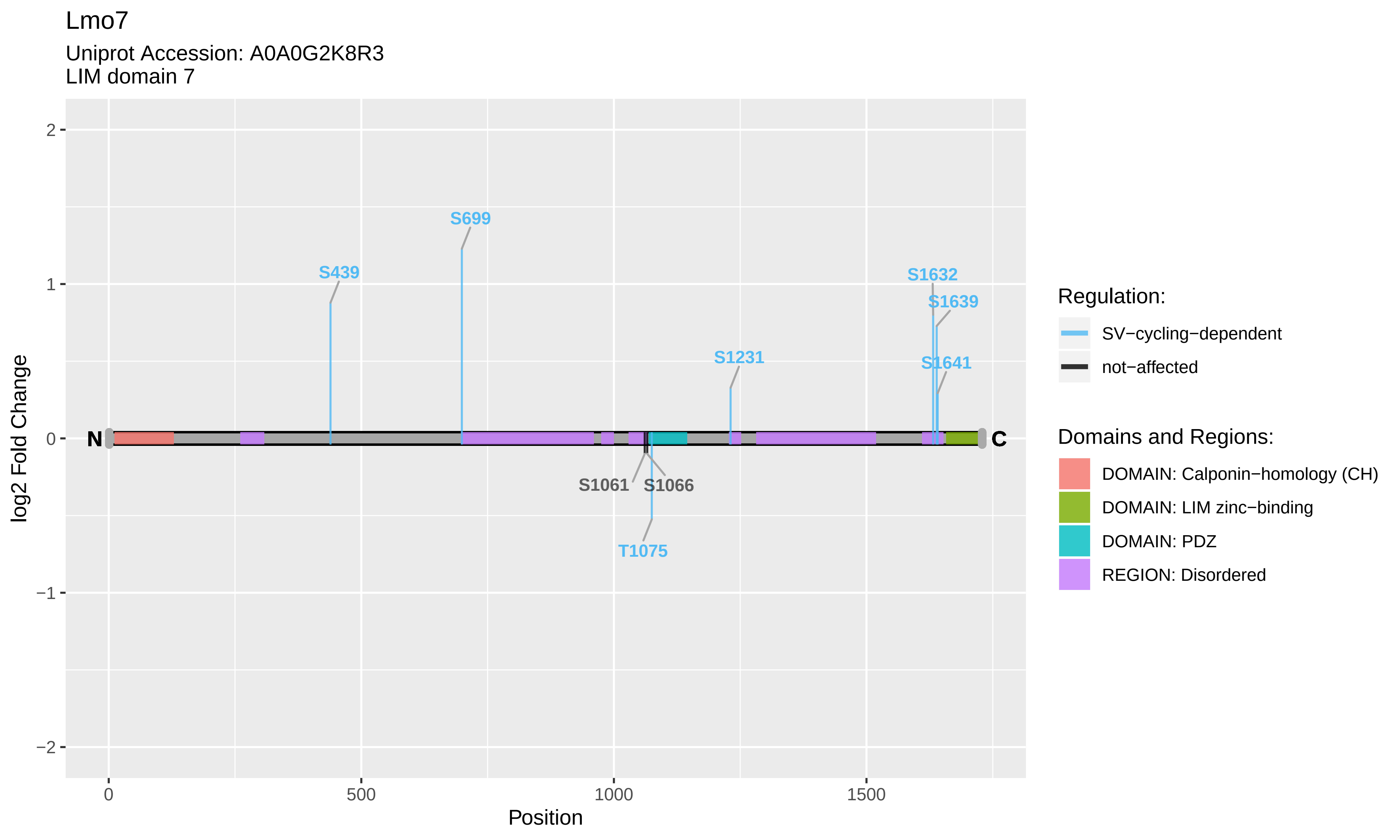

Lmo7
Uniprot Accession: A0A0G2K8R3
LIM domain 7
S699
S439
S1632
S1639
S1231
S1641
N
C
S1061
S1066
T1075
2
1
0
−1
−2
0
500
1000
1500
Position
log2 Fold Change
Regulation:
SV−cycling−dependent
not−affected
Domains and Regions:
DOMAIN: Calponin−homology (CH)
DOMAIN: LIM zinc−binding
DOMAIN: PDZ
REGION: Disordered

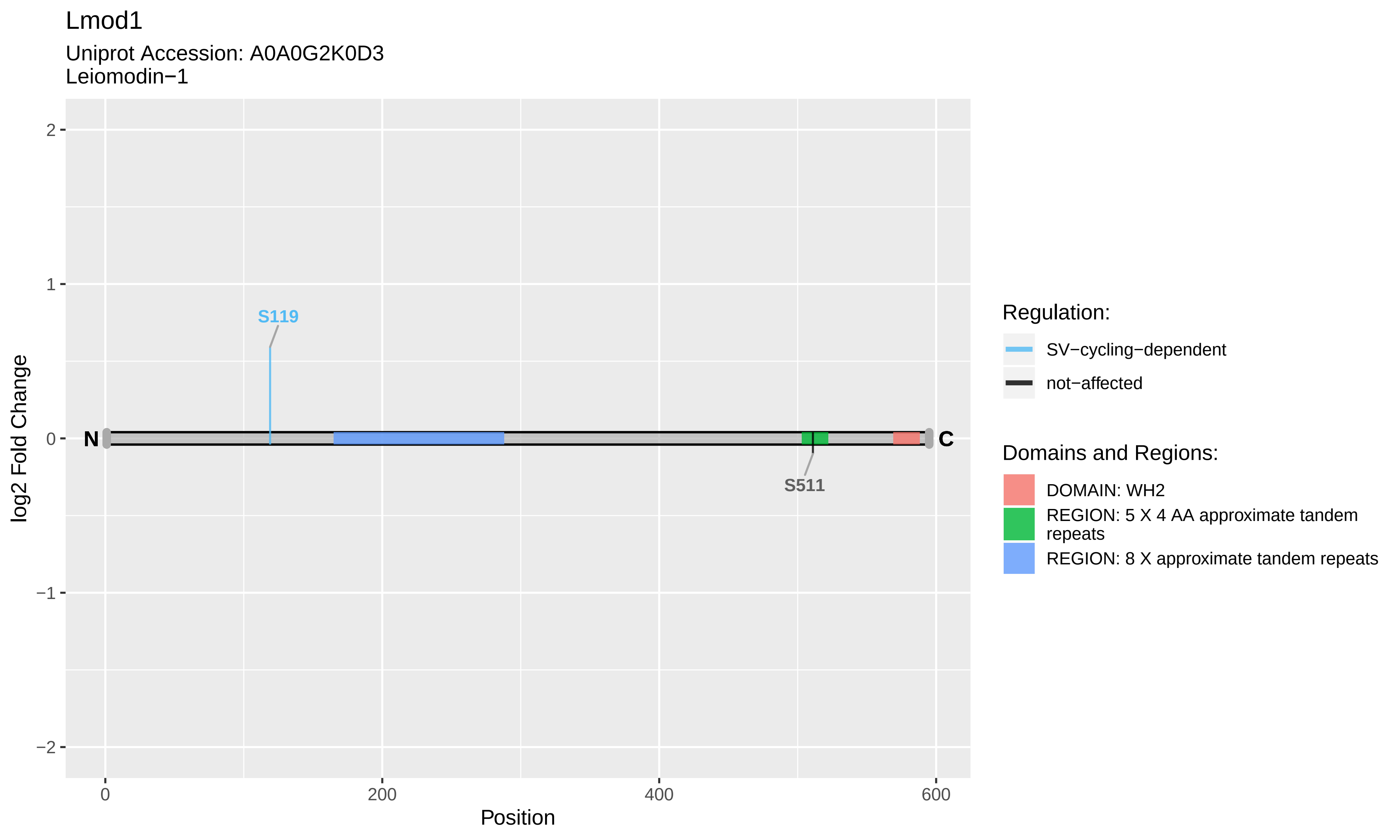
Lmod1
Uniprot Accession: A0A0G2K0D3
Leiomodin−1
S119
S511
N
C
2
1
0
−1
−2
log2 Fold Change
0
200
400
600
Position
Regulation:
SV−cycling−dependent
not−affected
Domains and Regions:
DOMAIN: WH2
REGION: 5 X 4 AA approximate tandem repeats
REGION: 8 X approximate tandem repeats

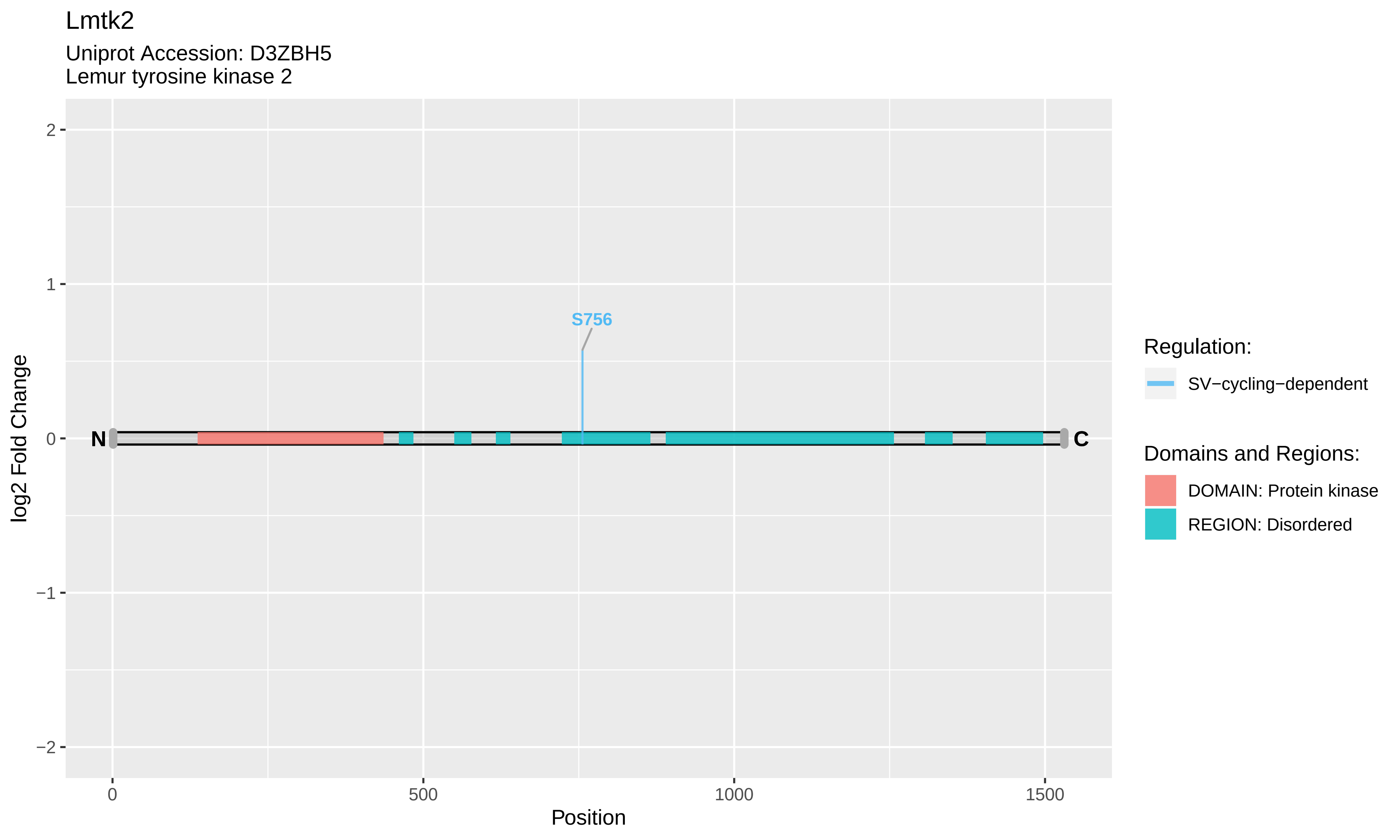

Lmtk2
Uniprot Accession: D3ZBH5
Lemur tyrosine kinase 2
S756
N
C
2
1
0
−1
−2
log2 Fold Change
0
500
1000
1500
Position
Regulation:
SV−cycling−dependent
Domains and Regions:
DOMAIN: Protein kinase
REGION: Disordered

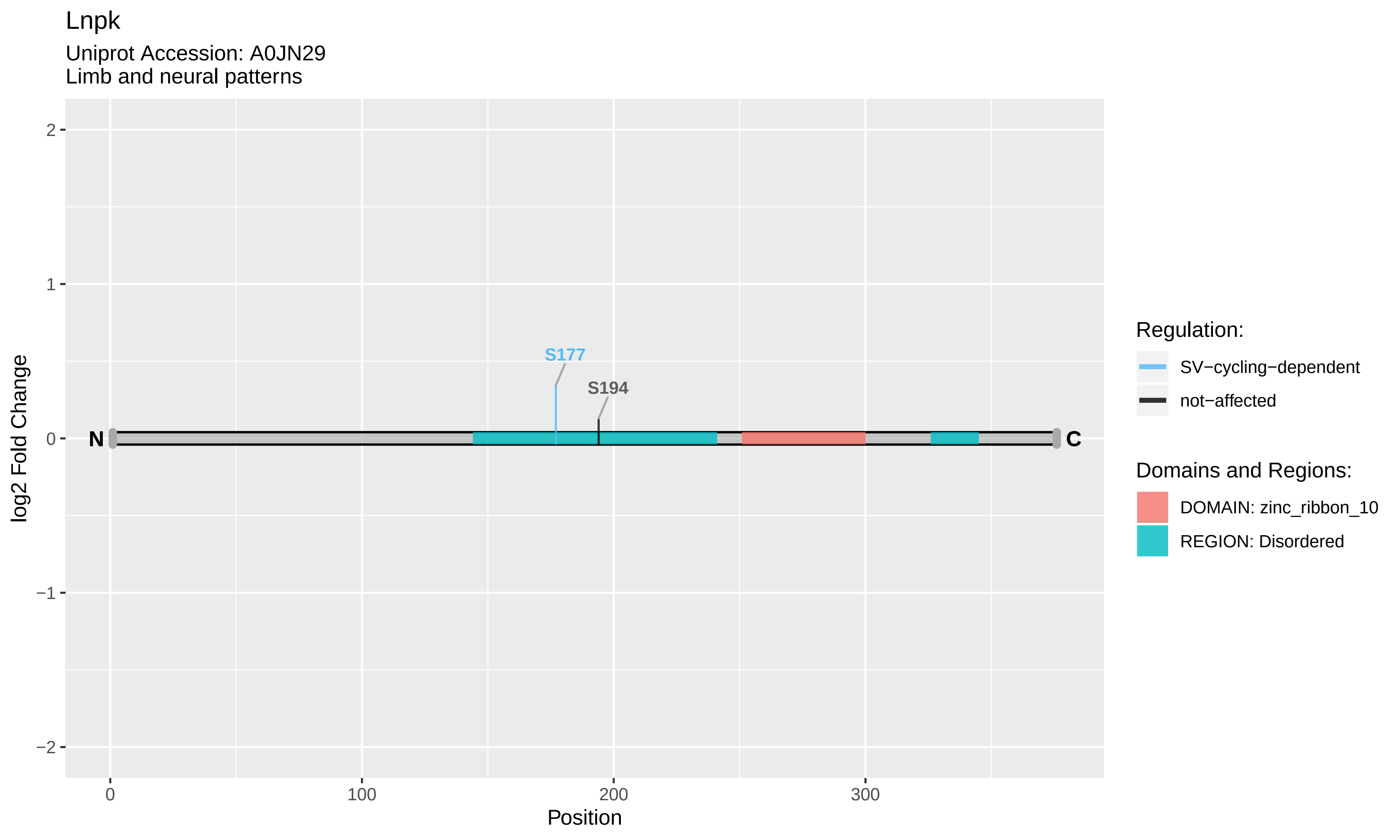

Lnpk
Uniprot Accession: A0JN29
Limb and neural patterns
S177
S194
N
C
2
1
0
−1
−2
log2 Fold Change
0
100
200
300
Position
Regulation:
SV−cycling−dependent
not−affected
Domains and Regions:
DOMAIN: zinc_ribbon_10
REGION: Disordered

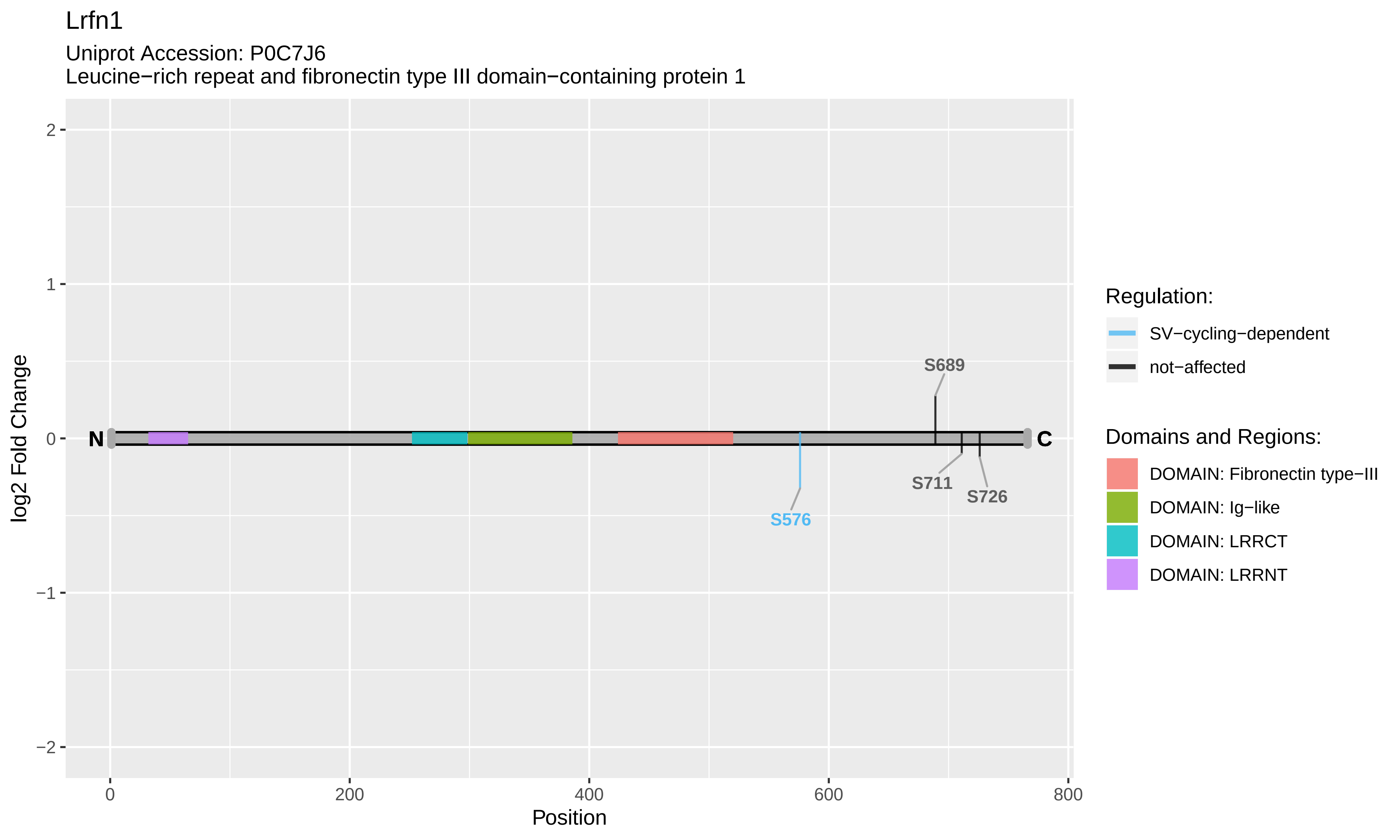
Lrfn1
Uniprot Accession: P0C7J6
Leucine−rich repeat and fibronectin type III domain−containing protein 1
log2 Fold Change
2
1
0
−1
−2
N
C
S689
S711
S726
S576
0
200
400
600
800
Position
Regulation:
SV−cycling−dependent
not−affected
Domains and Regions:
DOMAIN: Fibronectin type−III
DOMAIN: Ig−like
DOMAIN: LRRCT
DOMAIN: LRRNT

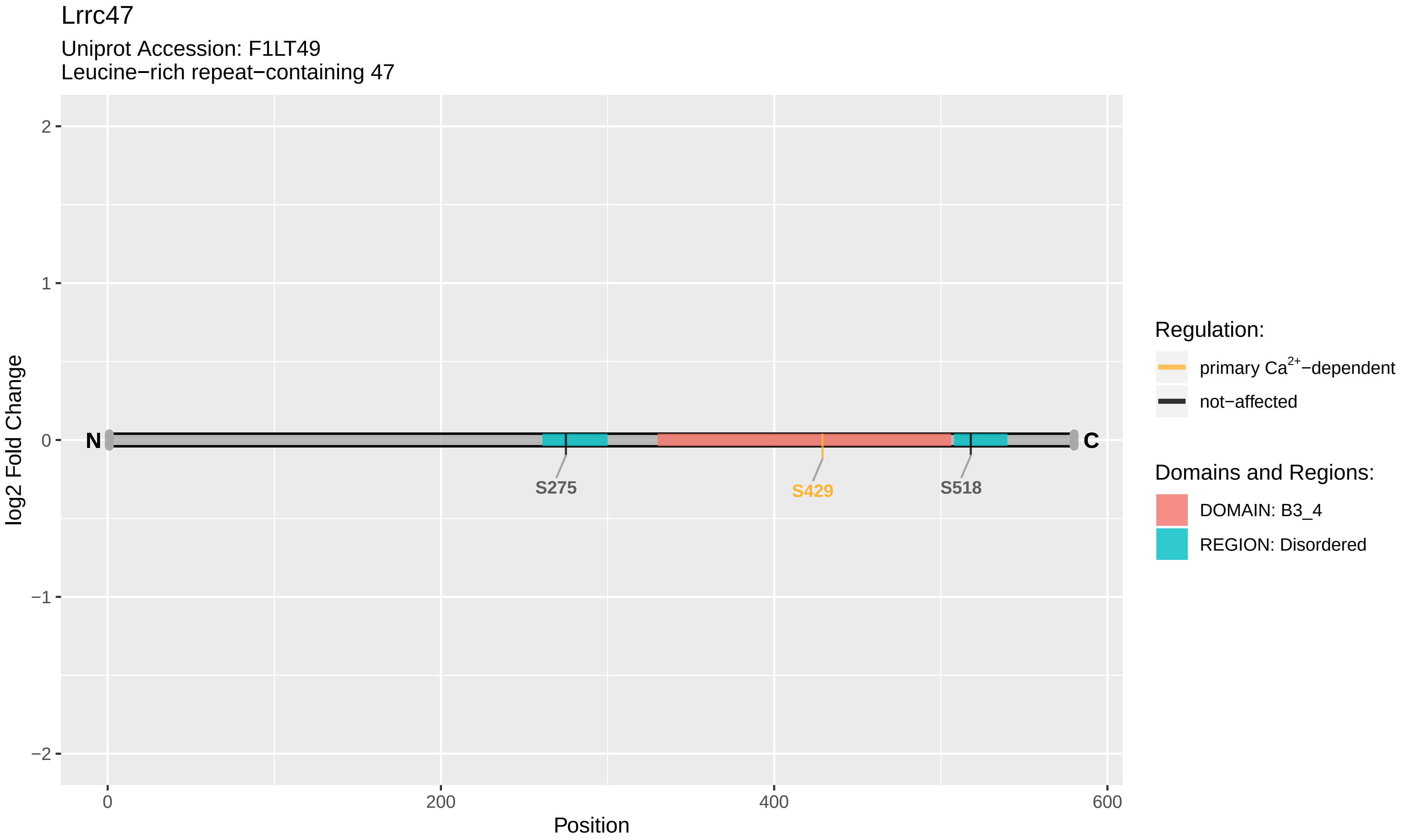
Lrrc47
Uniprot Accession: F1LT49
Leucine−rich repeat−containing 47
log2 Fold Change
2
1
0
−1
−2
0
200
400
600
Position
N
C
S275
S429
S518
Regulation:
primary Ca2+−dependent
not−affected
Domains and Regions:
DOMAIN: B3_4
REGION: Disordered

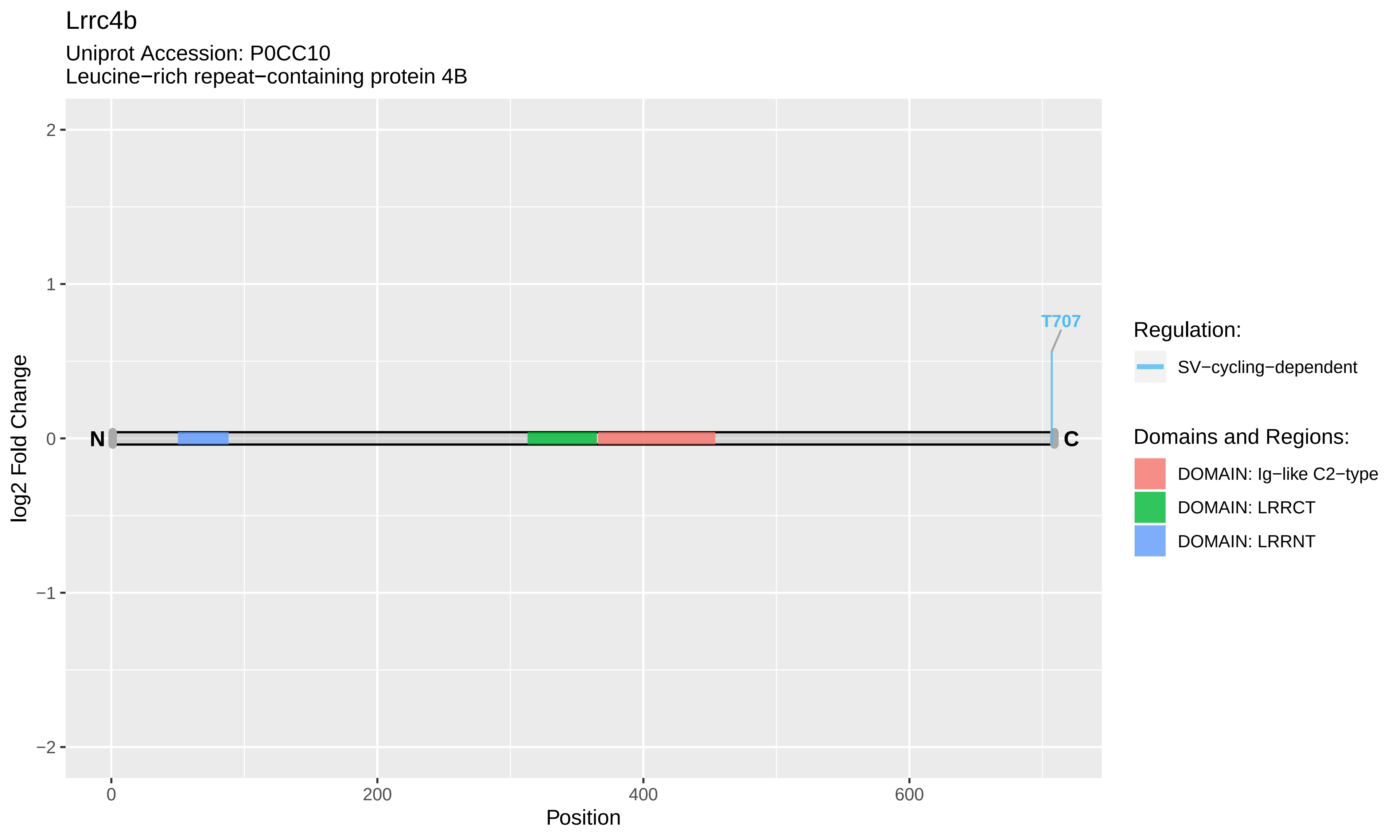
Lrrc4b
Uniprot Accession: P0CC10
Leucine−rich repeat−containing protein 4B
log2 Fold Change
Position
T707
N
C
2
1
0
−1
−2
0
200
400
600
Regulation:
SV−cycling−dependent
Domains and Regions:
DOMAIN: Ig−like C2−type
DOMAIN: LRRCT
DOMAIN: LRRNT

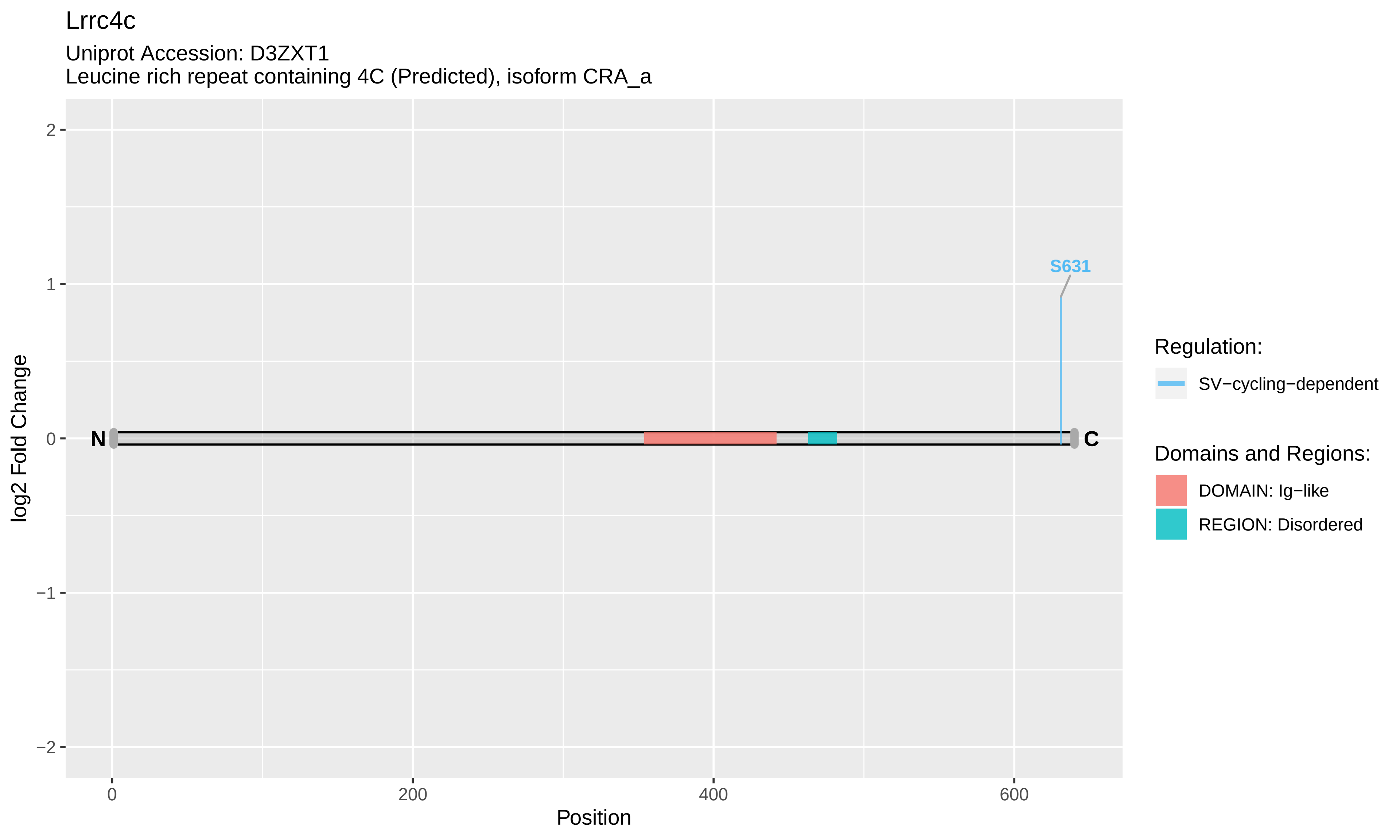

Lrrc4c
Uniprot Accession: D3ZXT1
Leucine rich repeat containing 4C (Predicted), isoform CRA_a
S631
N
C
2
1
0
−1
−2
0
200
400
600
Position
log2 Fold Change
Regulation:
SV−cycling−dependent
Domains and Regions:
DOMAIN: Ig−like
REGION: Disordered

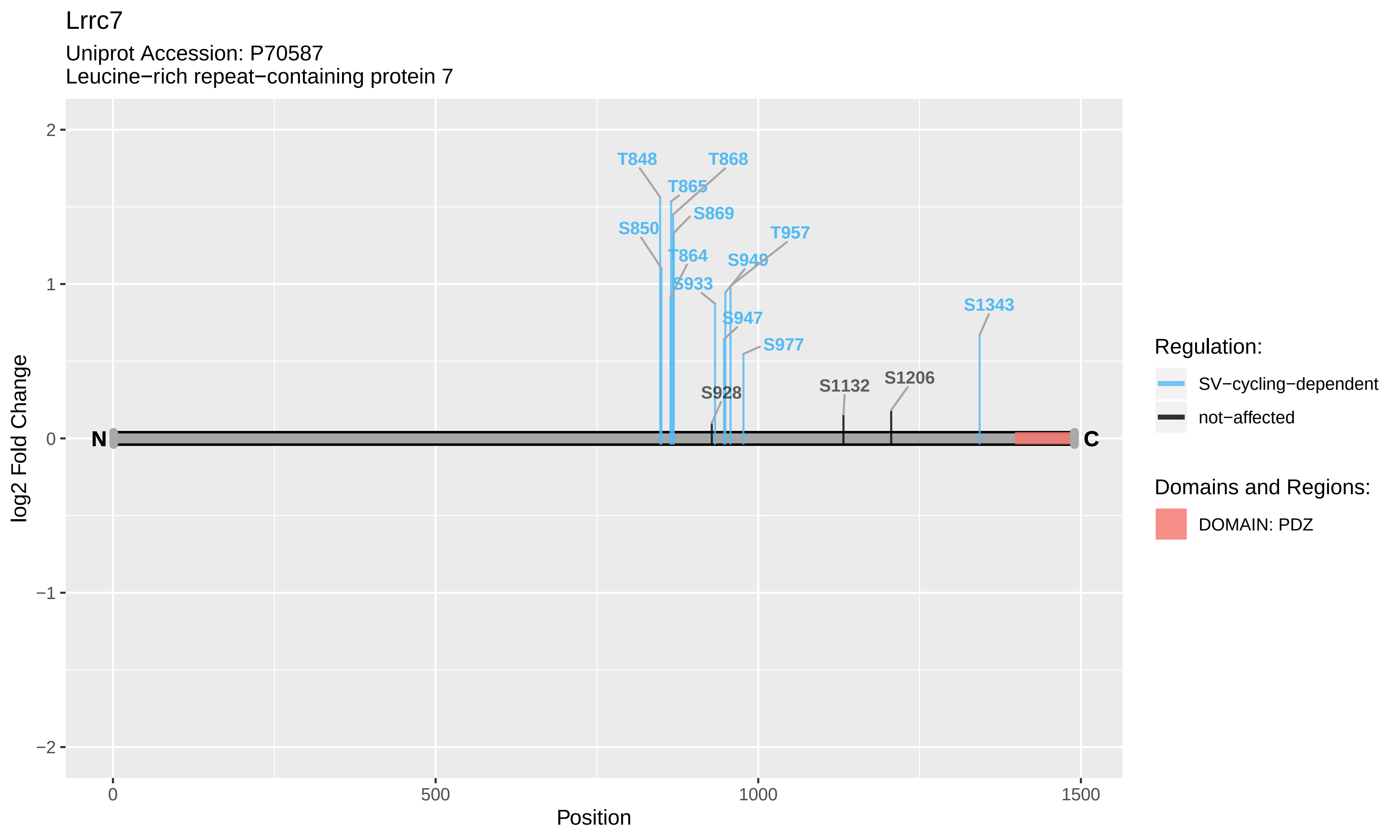

Lrrc7
Uniprot Accession: P70587
Leucine−rich repeat−containing protein 7
T848
T868
T865
S869
S850
T957
T864
S949
S933
S947
S977
S1343
S928
S1132
S1206
N
C
2
1
0
−1
−2
log2 Fold Change
0
500
1000
1500
Position
Regulation:
SV−cycling−dependent
not−affected
Domains and Regions:
DOMAIN: PDZ

Lrrfip2

Uniprot Accession: Q4V7E8
Leucine−rich repeat flightless−interacting protein 2

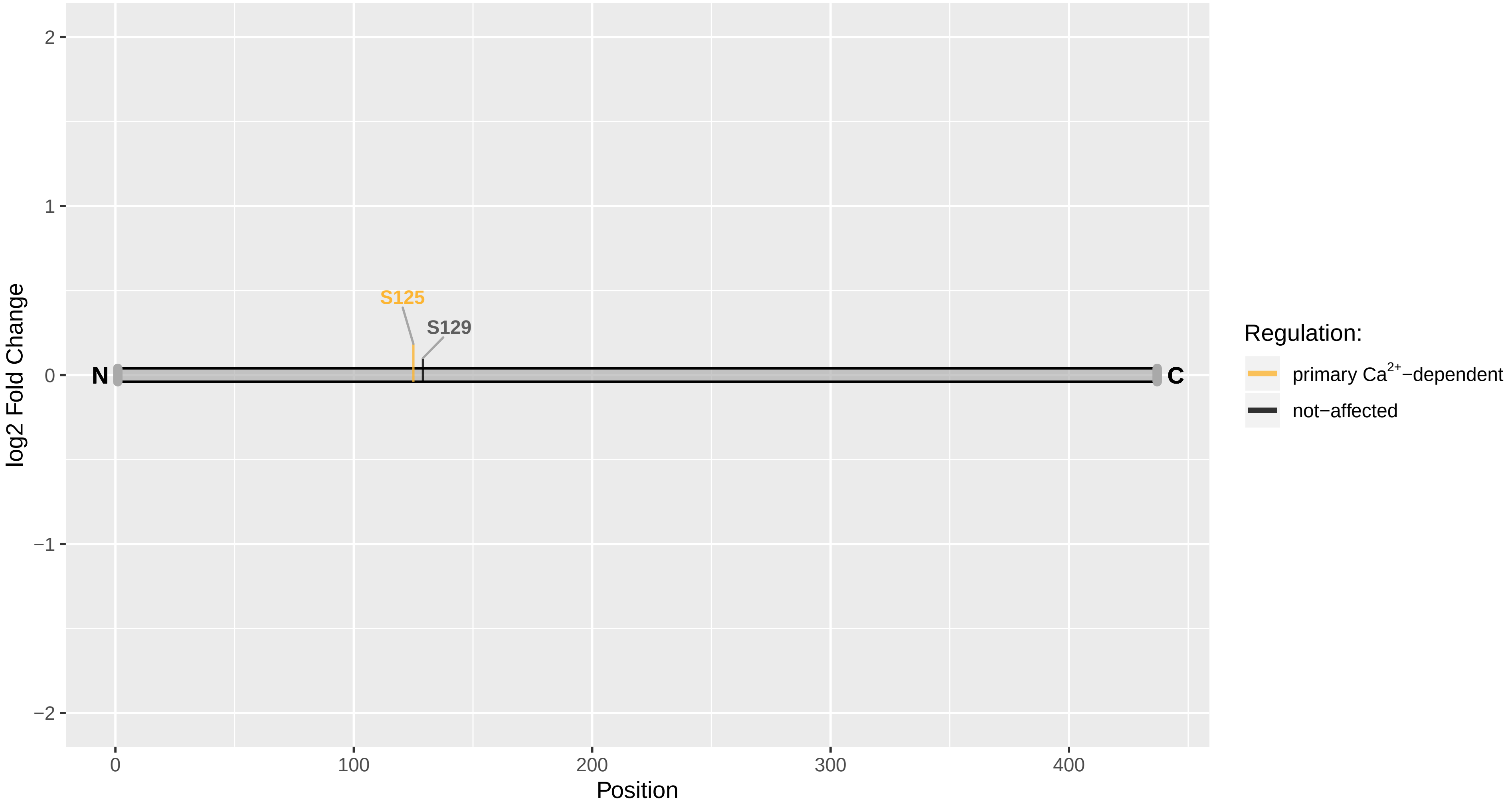

Lrrn1

Uniprot Accession: Q32Q07
Leucine−rich repeat neuronal protein 1

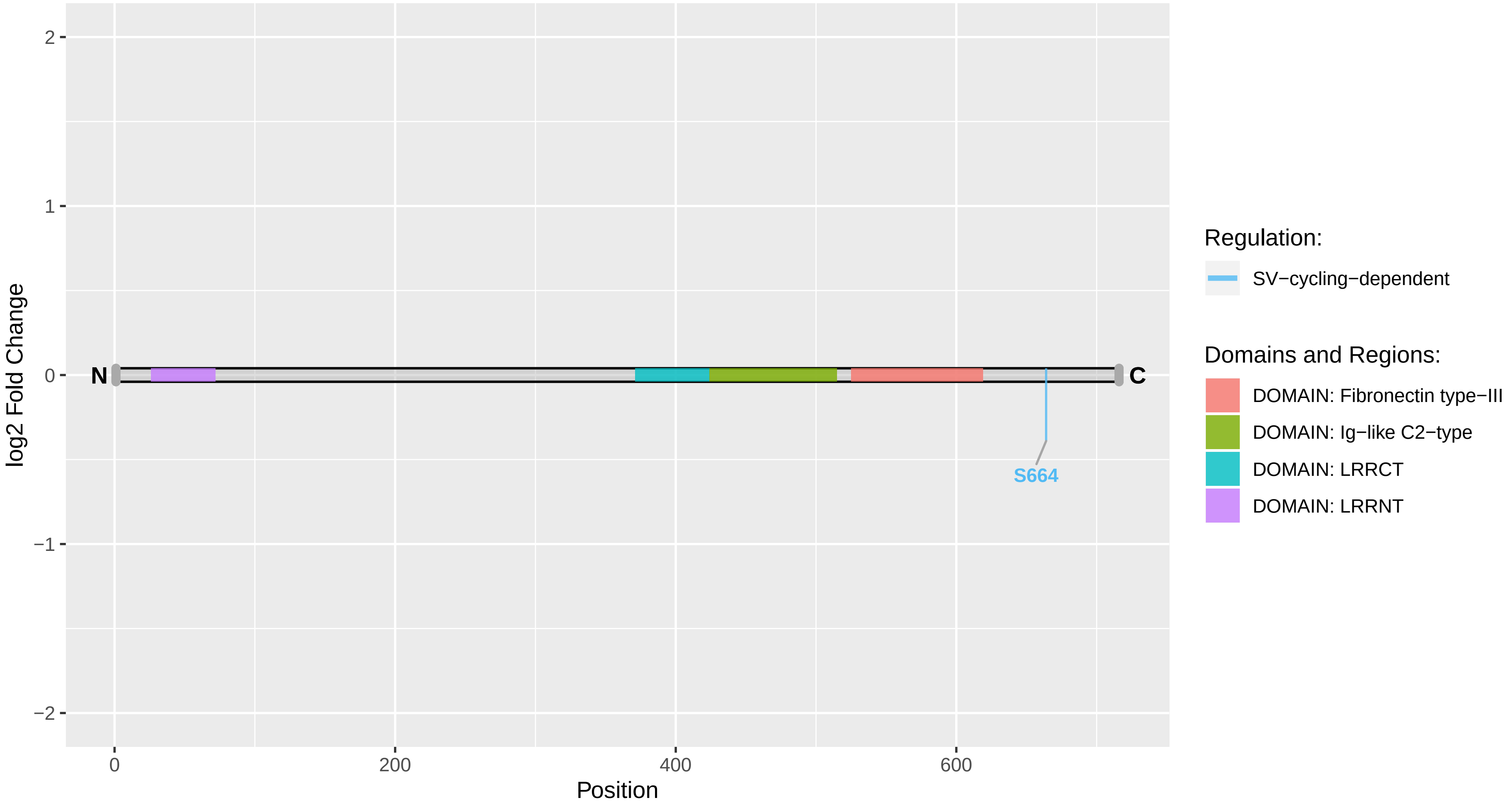

Lsm11

Uniprot Accession: B0BNI4
LSM11, U7 small nuclear RNA−associated

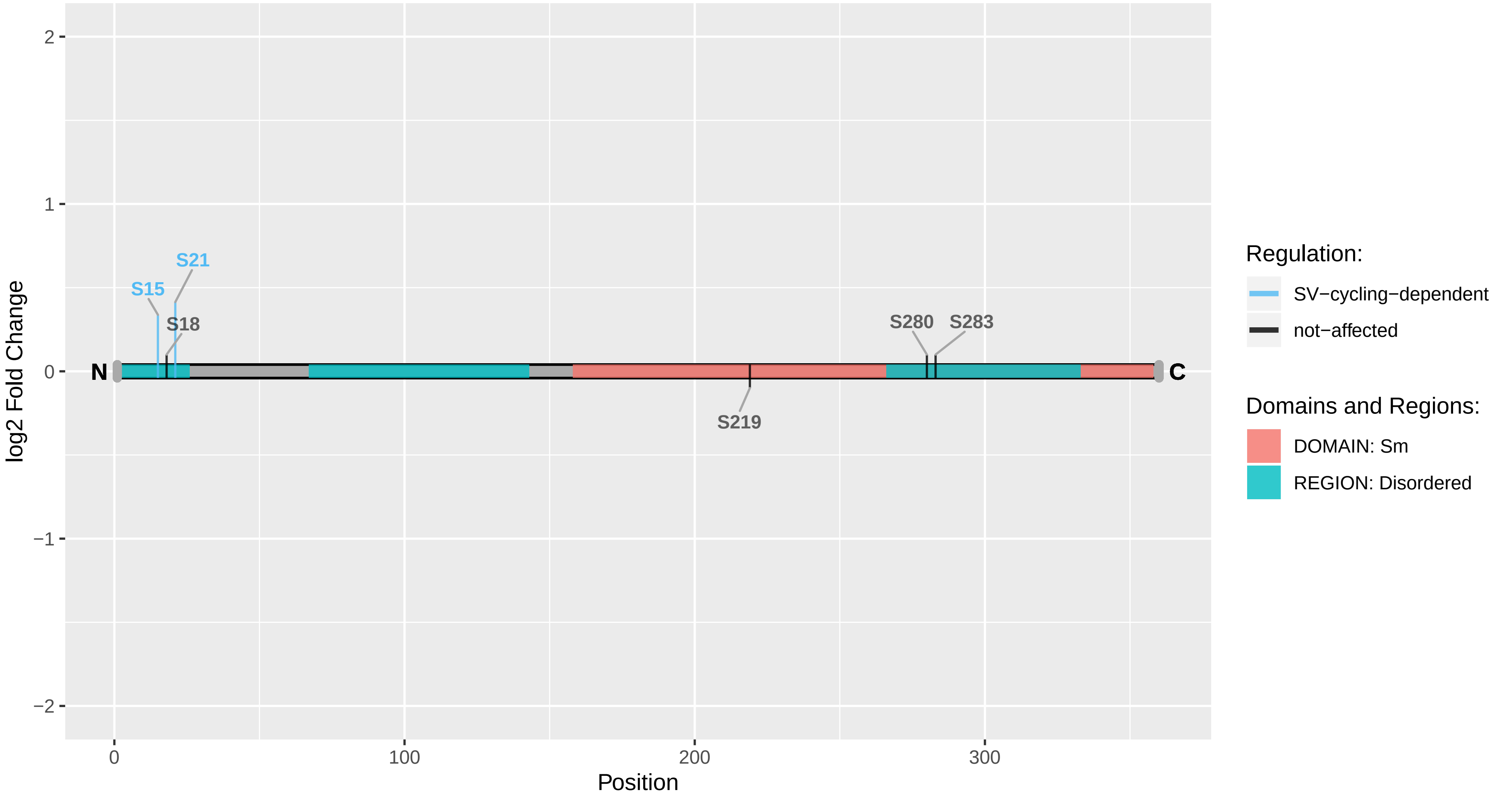

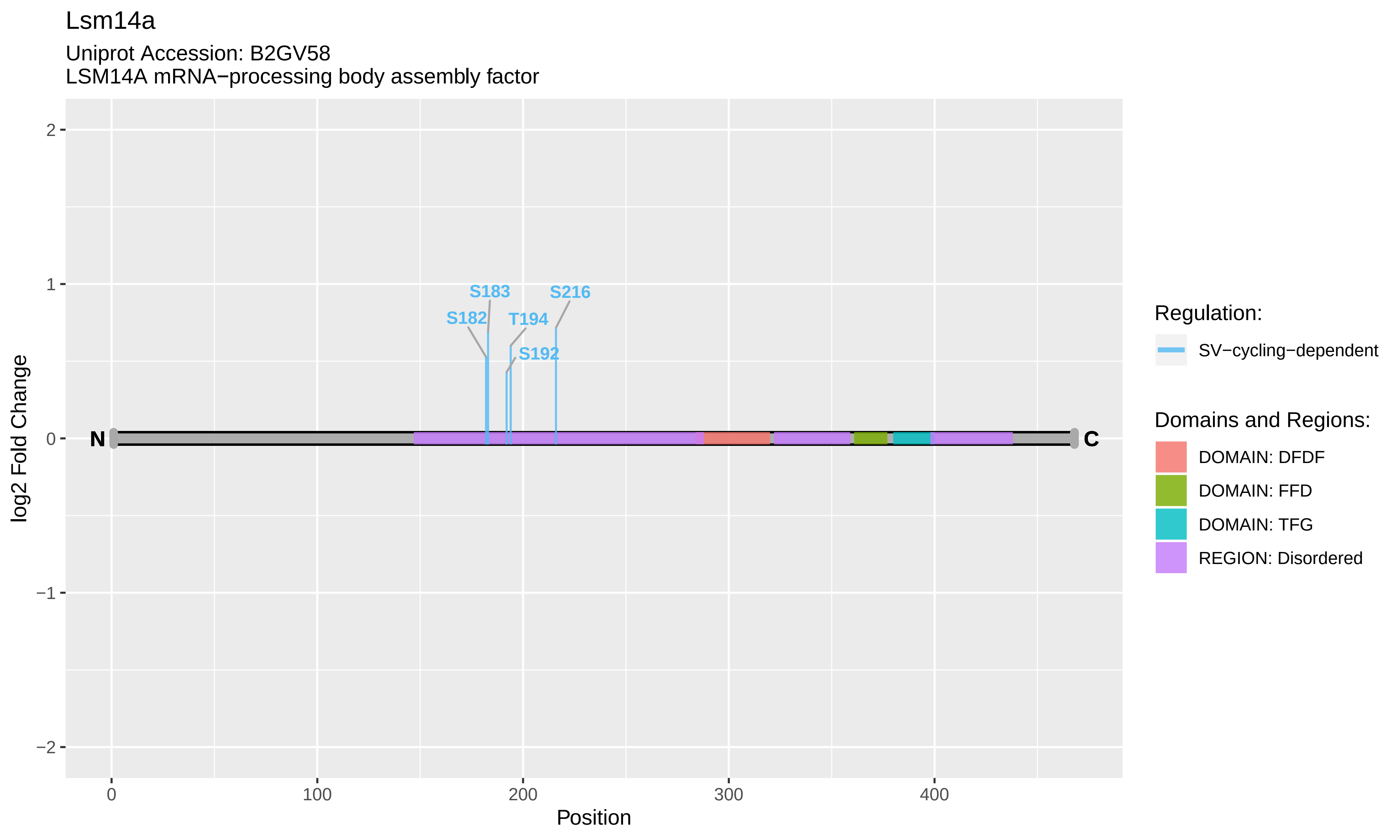

Lsm14a
Uniprot Accession: B2GV58
LSM14A mRNA−processing body assembly factor
S183
S216
S182
T194
S192
N
C
2
1
0
−1
−2
log2 Fold Change
0
100
200
300
400
Position
Regulation:
SV−cycling−dependent
Domains and Regions:
DOMAIN: DFDF
DOMAIN: FFD
DOMAIN: TFG
REGION: Disordered

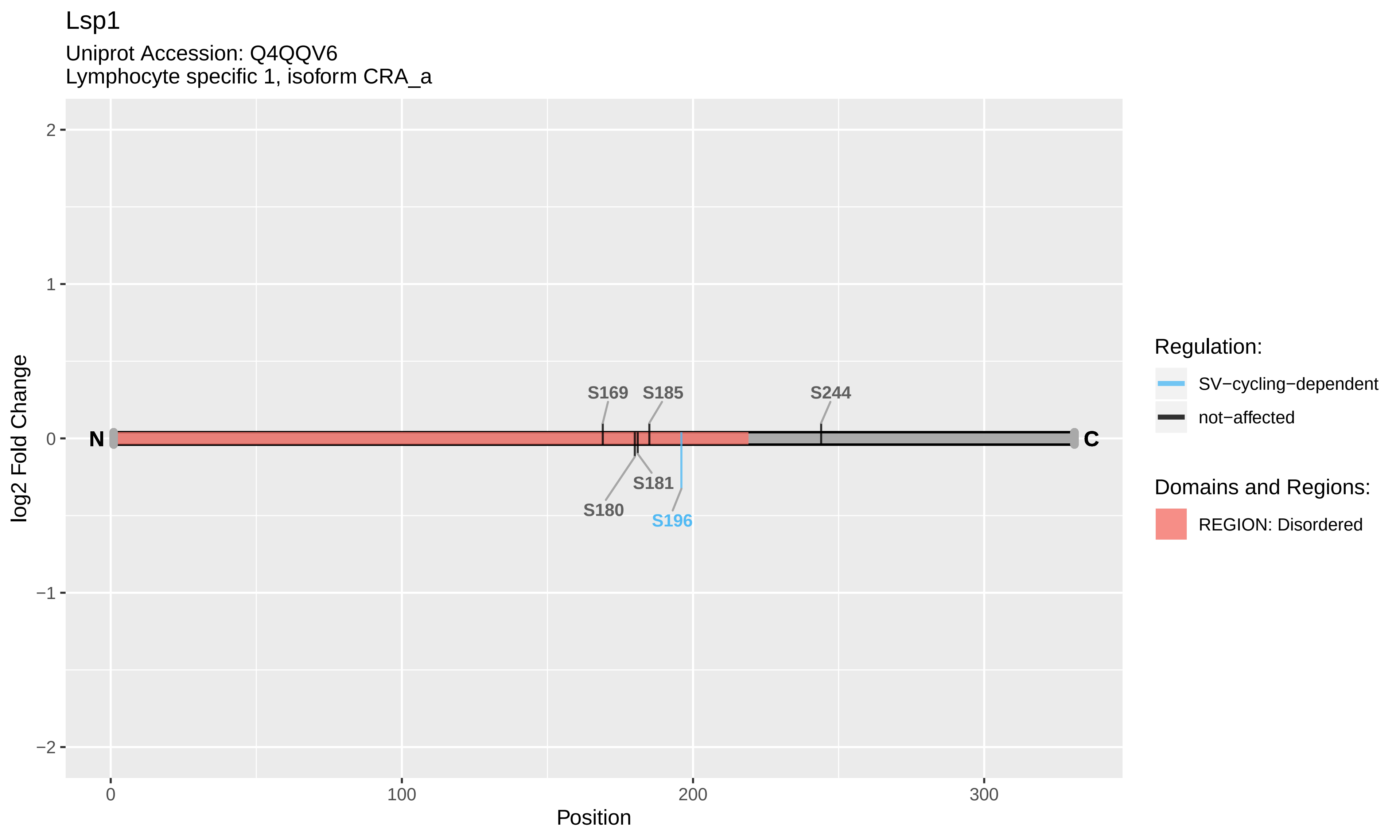
Lsp1
Uniprot Accession: Q4QQV6
Lymphocyte specific 1, isoform CRA_a
log2 Fold Change
2
1
0
−1
−2
N
C
S169
S185
S244
S181
S180
S196
0
100
200
300
Position
Regulation:
SV−cycling−dependent
not−affected
Domains and Regions:
REGION: Disordered

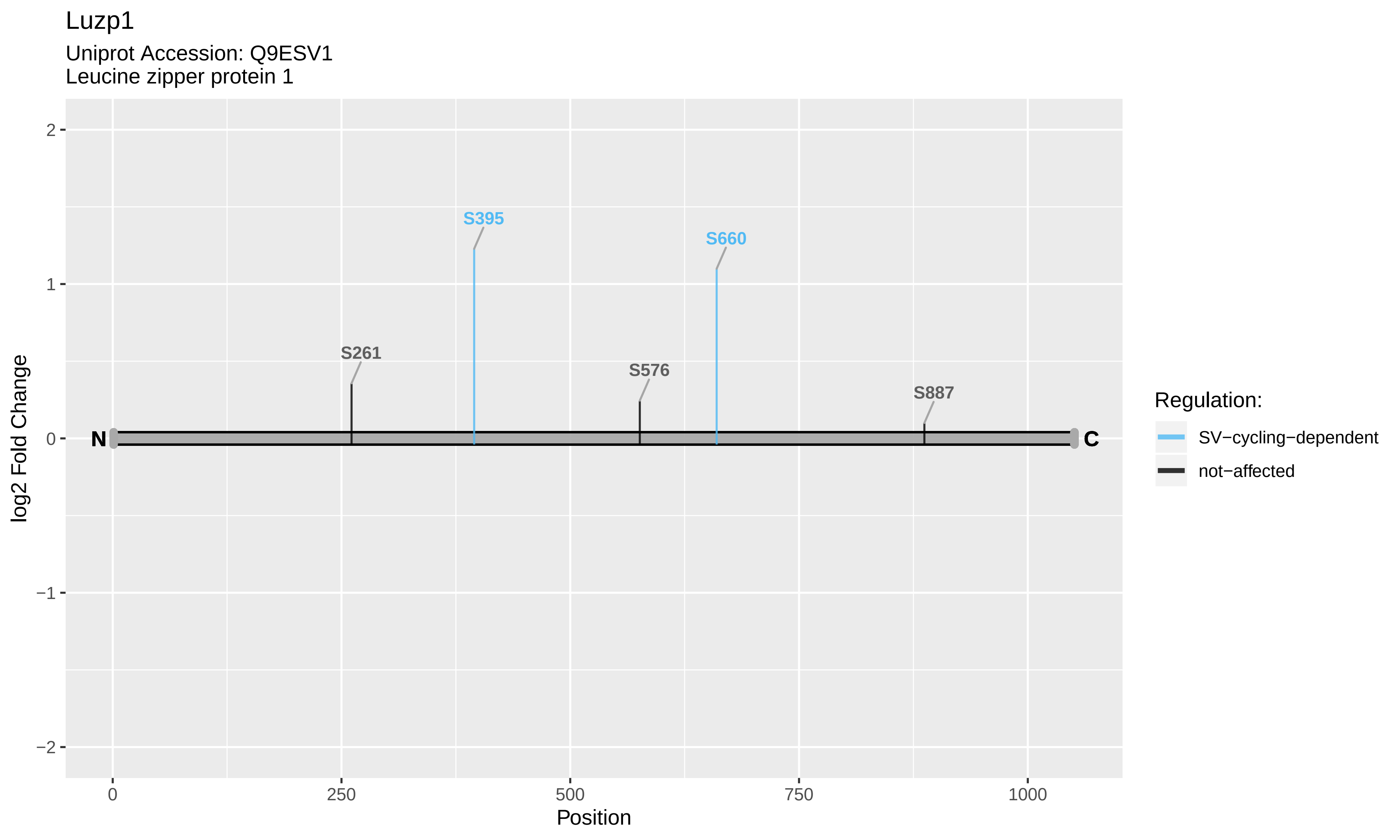
Luzp1
Uniprot Accession: Q9ESV1
Leucine zipper protein 1
S395
S660
S261
S576
S887
N
C
2
1
0
−1
−2
log2 Fold Change
0
250
500
750
1000
Position
Regulation:
SV−cycling−dependent
not−affected

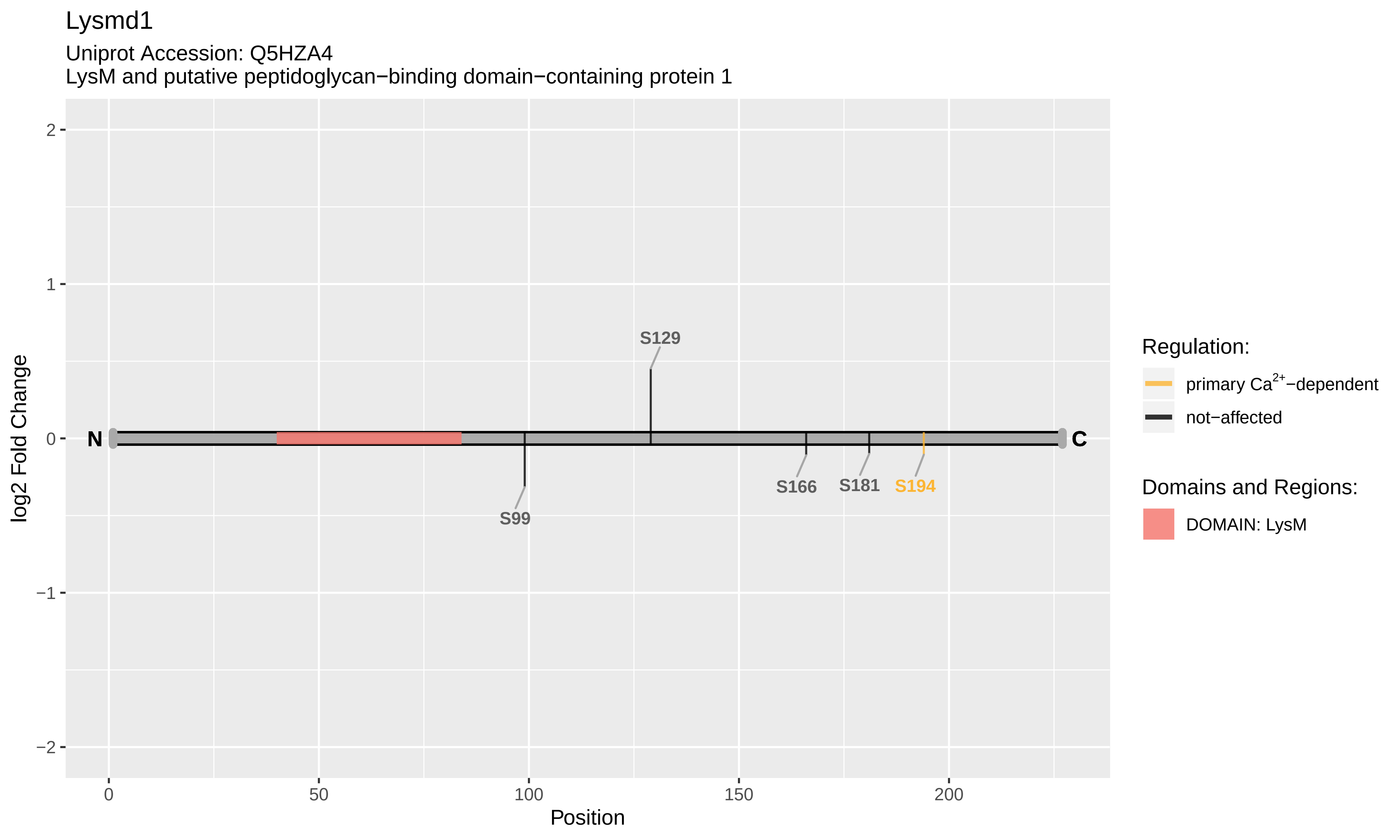
Lysmd1
Uniprot Accession: Q5HZA4
LysM and putative peptidoglycan−binding domain−containing protein 1
log2 Fold Change
Position
N
C
S99
S129
S166
S181
S194
Regulation:
primary Ca2+−dependent
not−affected
Domains and Regions:
DOMAIN: LysM
2
1
0
−1
−2
0
50
100
150
200

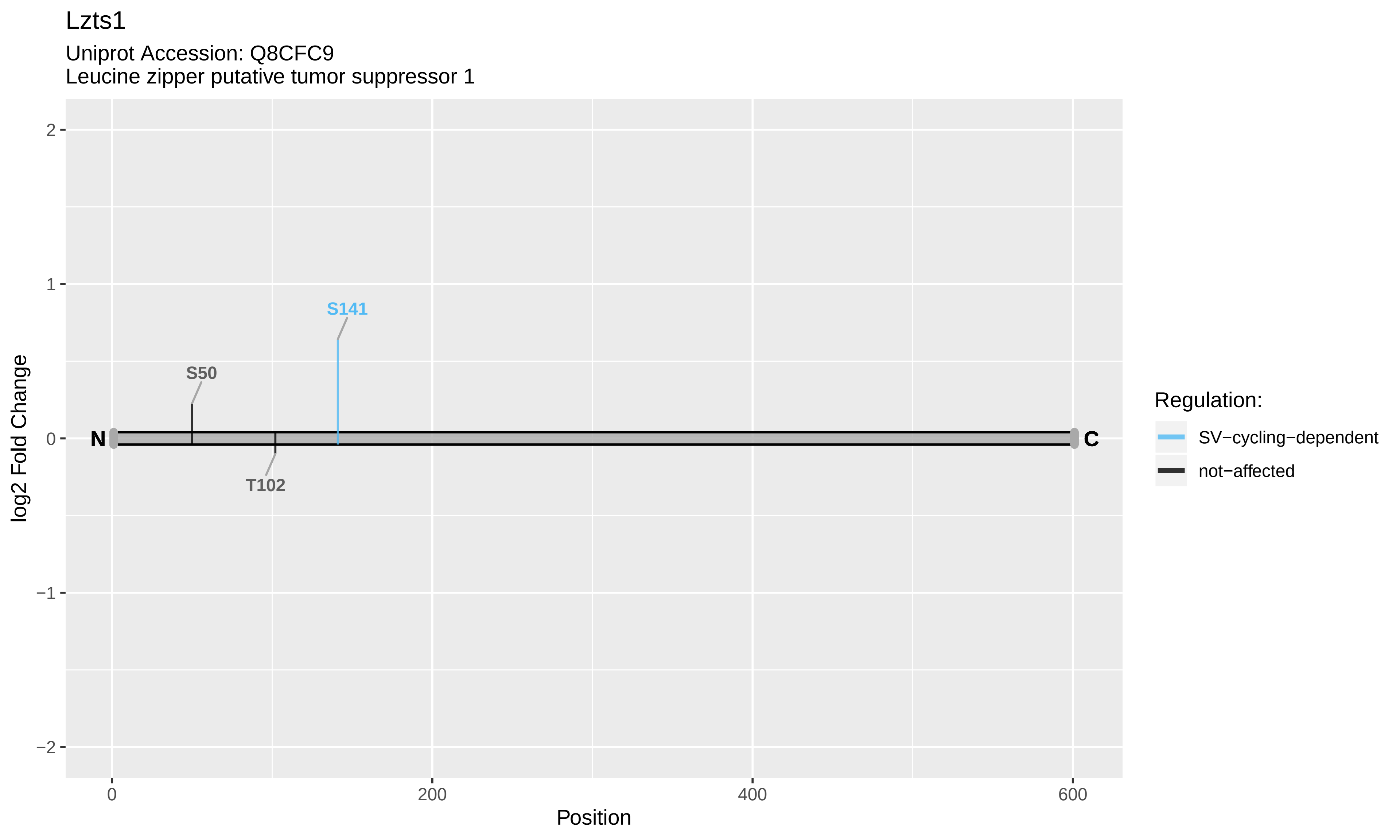

Lzts1
Uniprot Accession: Q8CFC9
Leucine zipper putative tumor suppressor 1
log2 Fold Change
2
1
0
−1
−2
0
200
400
600
Position
N
C
S141
S50
T102
Regulation:
SV−cycling−dependent
not−affected

Lzts3

Uniprot Accession: G3V8V8
Leucine zipper putative tumor suppressor 3

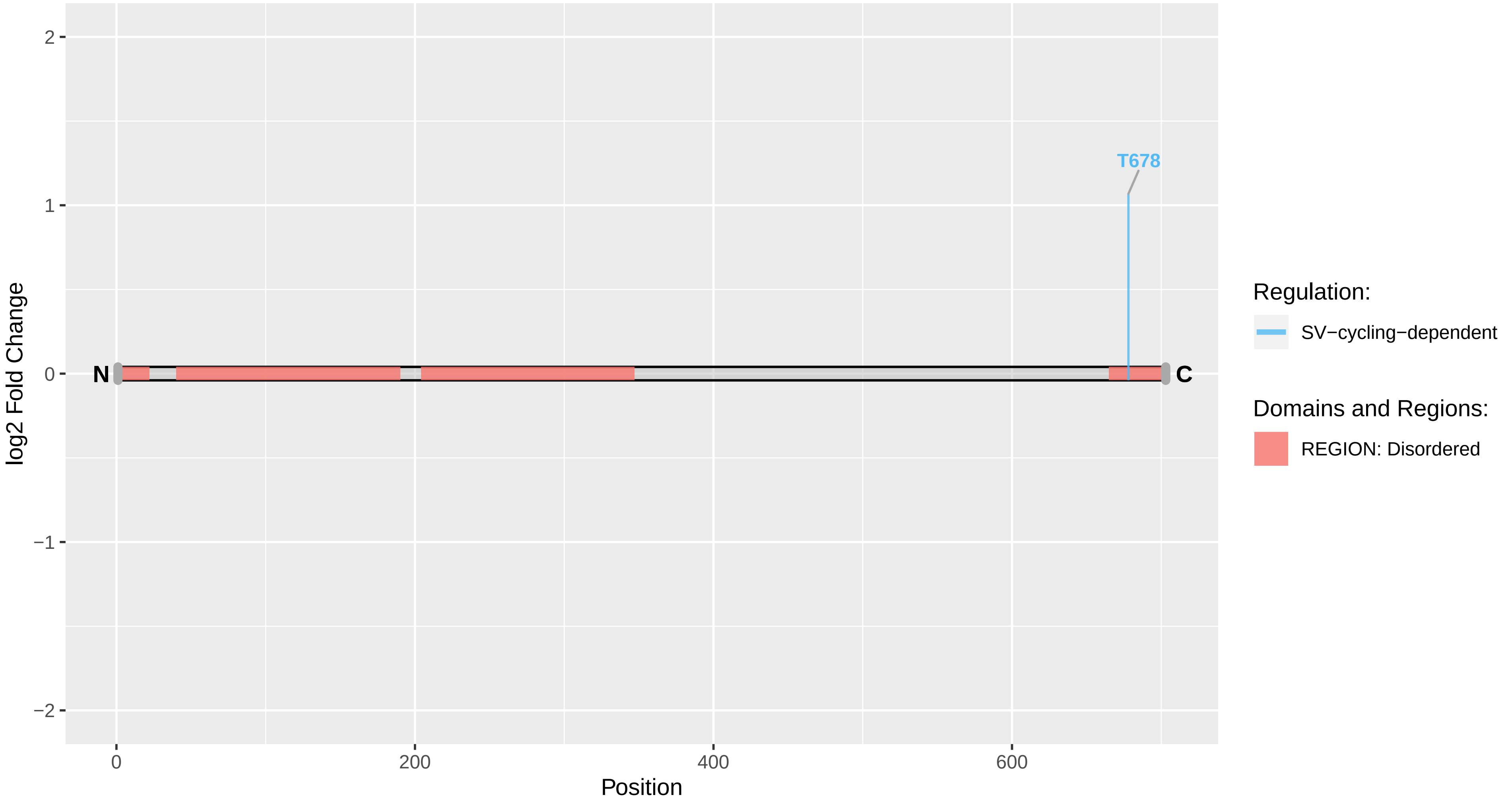

Maco1

Uniprot Accession: Q4V7D3
Macoilin

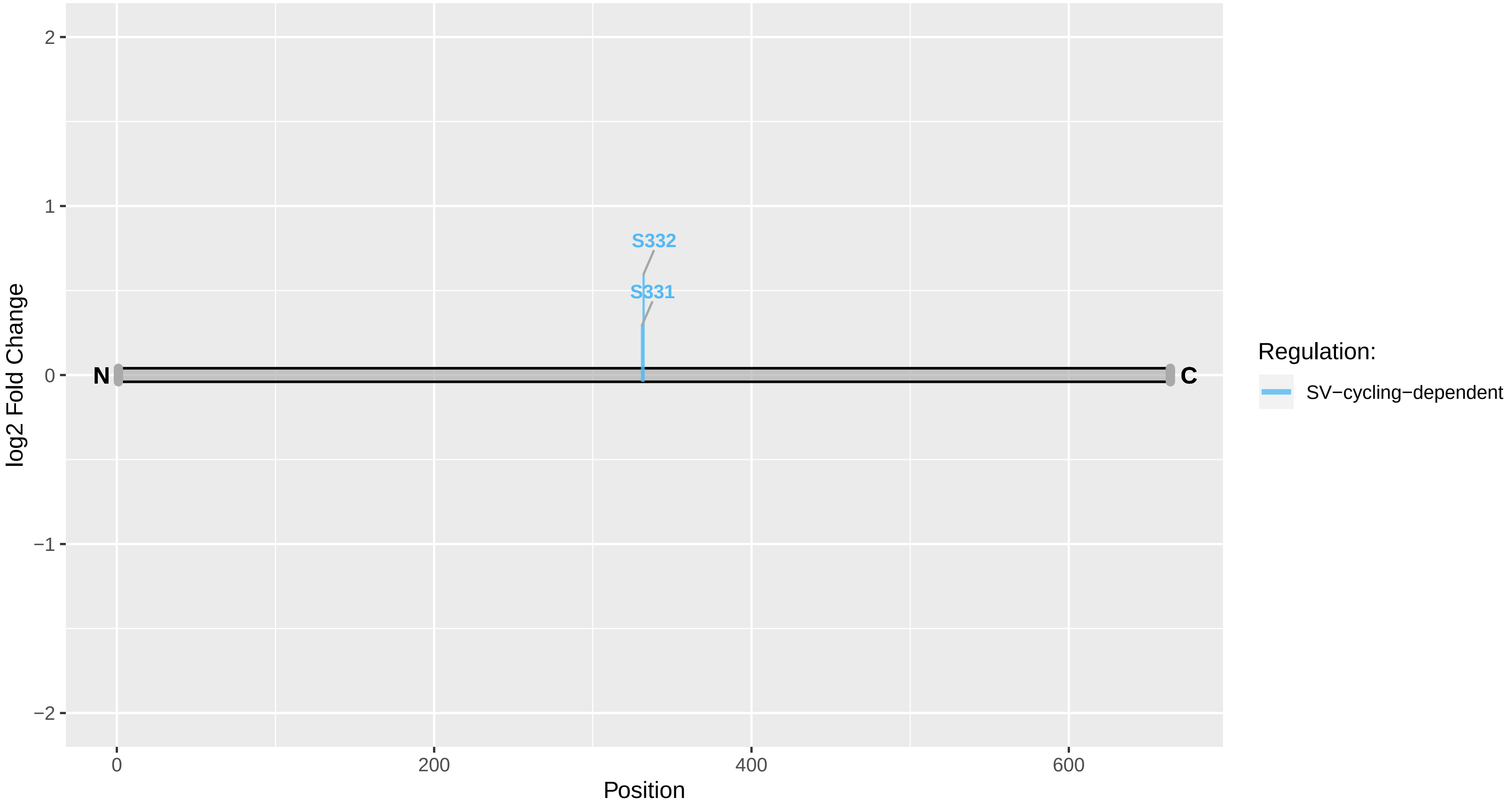

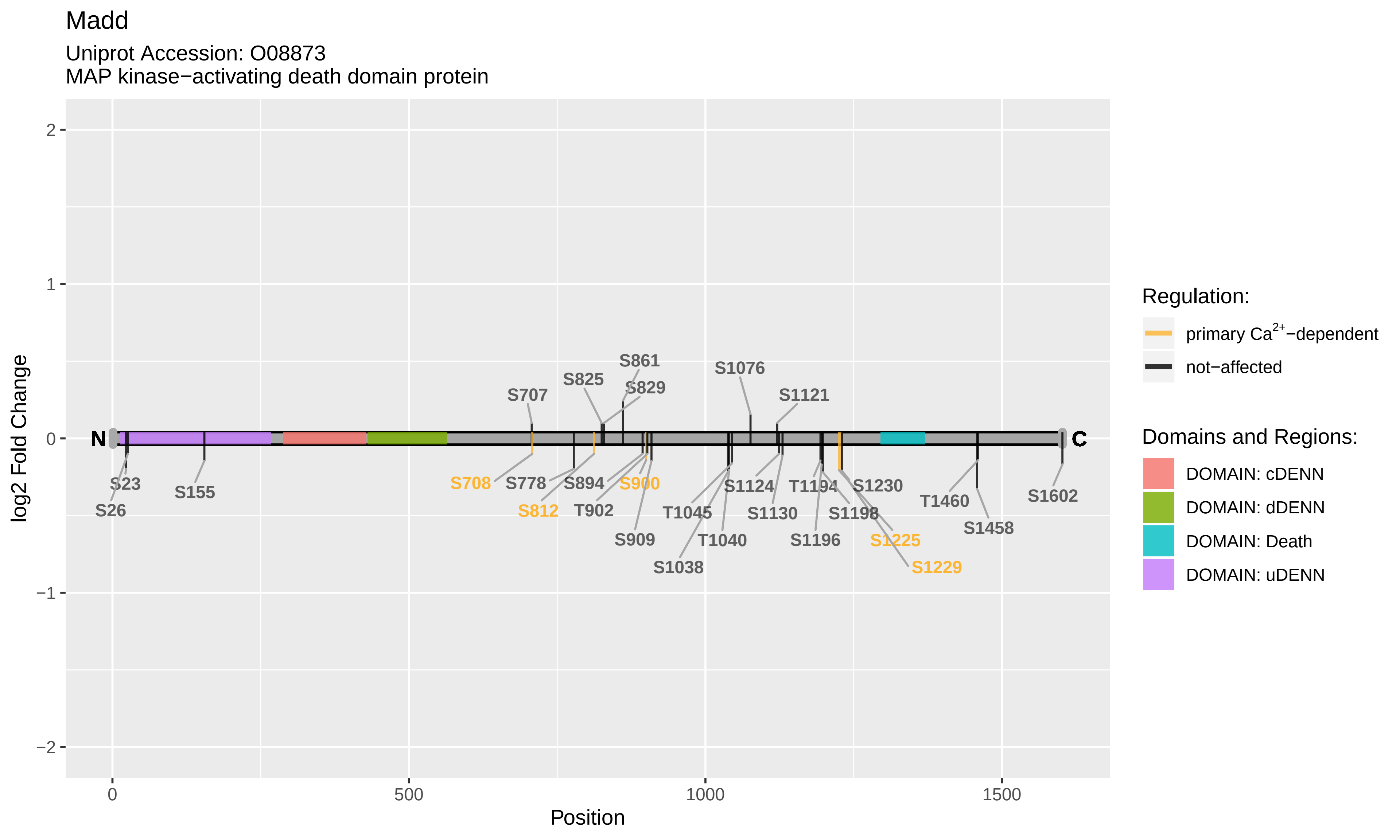
Madd
Uniprot Accession: O08873
MAP kinase−activating death domain protein
log2 Fold Change
Position
N
C
S23
S26
S155
S707
S708
S778
S812
S825
S829
S861
S894
T902
S900
S909
S1038
T1040
T1045
S1076
S1121
S1124
S1130
T1194
S1196
S1198
S1225
S1229
S1230
T1460
S1458
S1602
Regulation:
primary Ca2+−dependent
not−affected
Domains and Regions:
DOMAIN: cDENN
DOMAIN: dDENN
DOMAIN: Death
DOMAIN: uDENN

Mageh1

Uniprot Accession: Q5PPP4
Melanoma−associated antigen H1

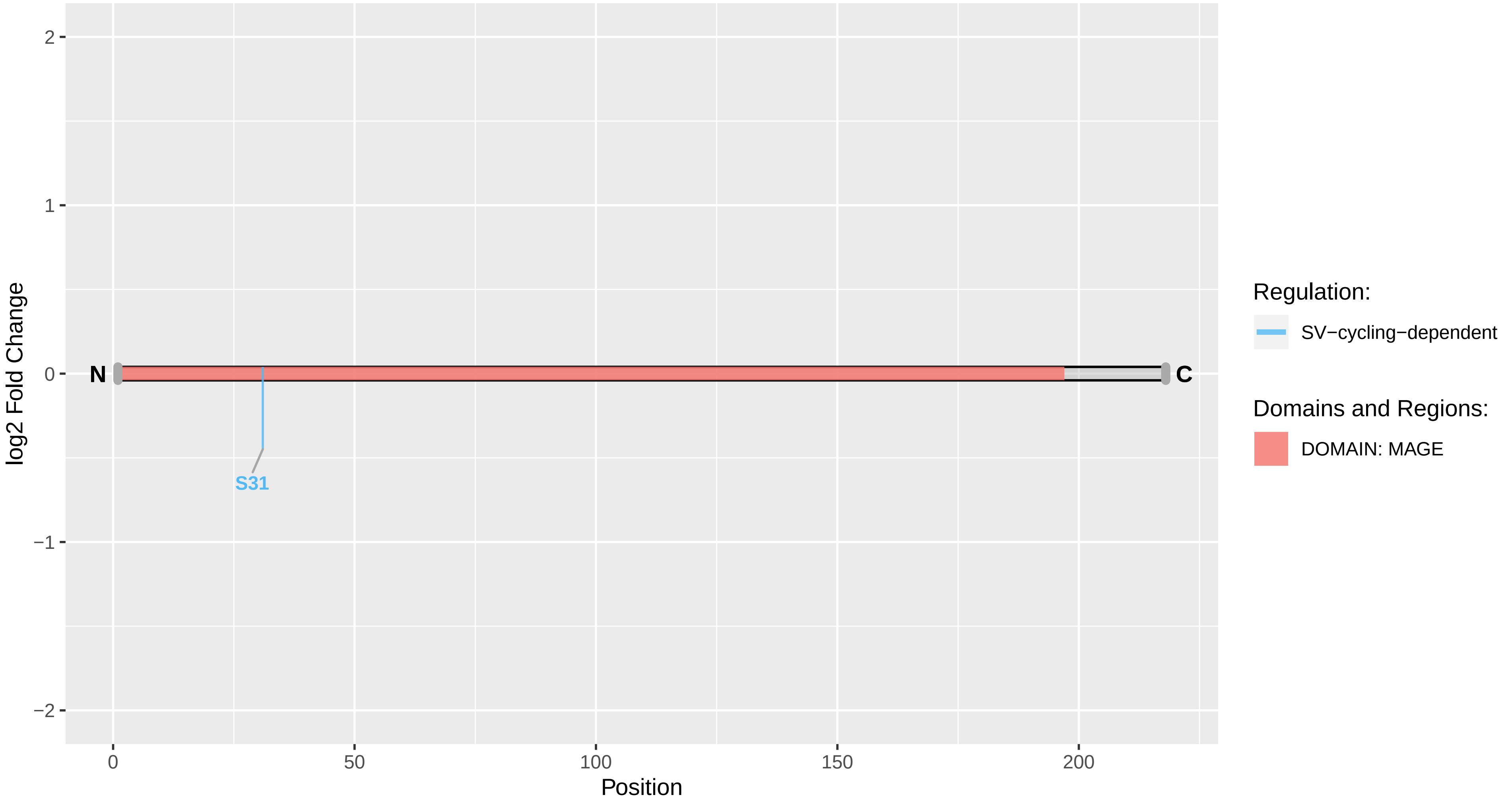

Magi1

Uniprot Accession: M0R8T1
Membrane−associated guanylate kinase, WW and PDZ domain−containing protein 1

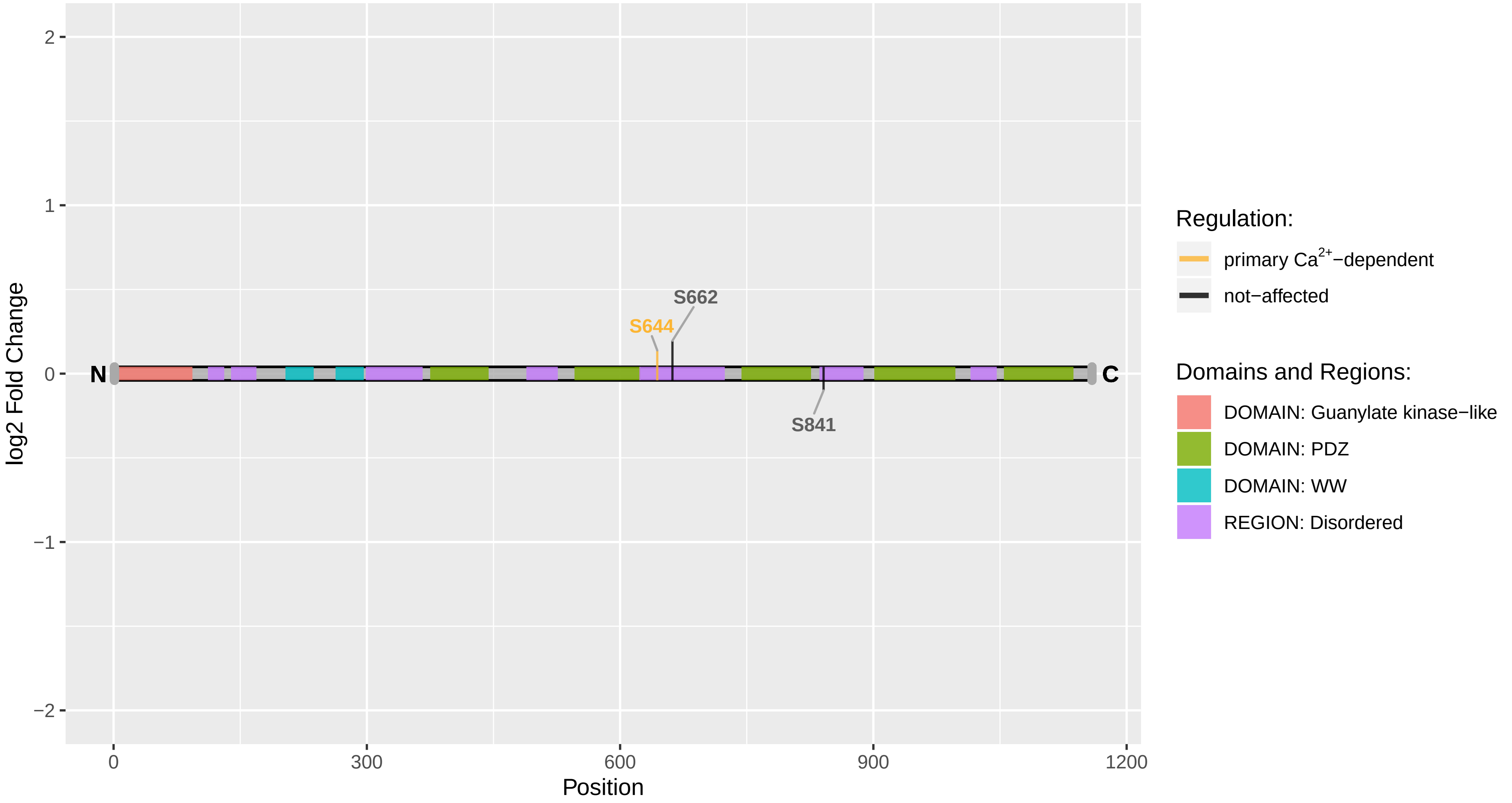

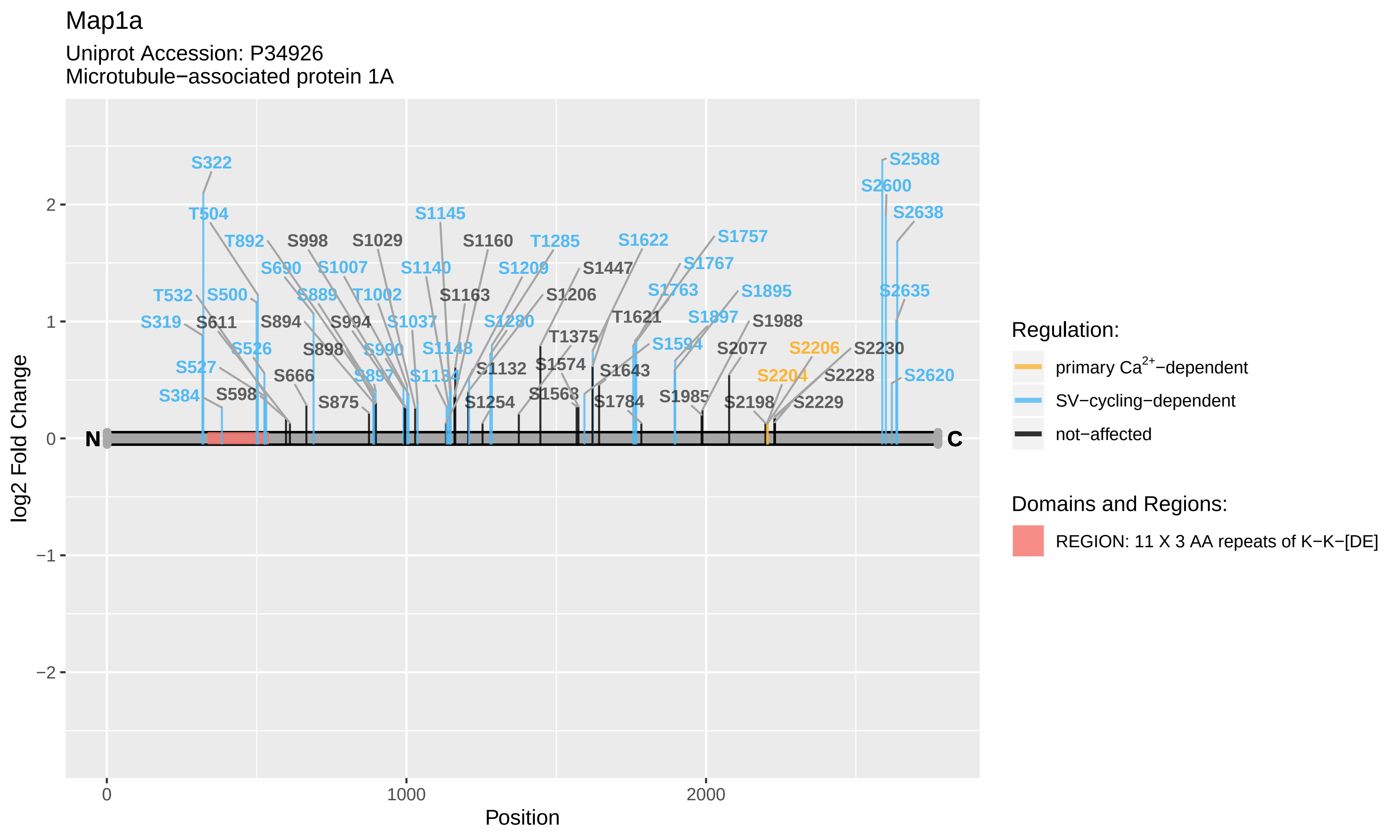

Map1a
Uniprot Accession: P34926
Microtubule−associated protein 1A
log2 Fold Change
Position
N
C
S322
T504
T892
S998
S1029
S1145
S1160
T1285
S1622
S1757
S2588
S2600
S2638
S690
S1007
S1140
S1209
S1447
S1767
T532
S500
S889
T1002
S1163
S1206
S1763
S1895
S2635
S319
S611
S894
S994
S1037
S1280
T1621
S1897
S1988
S526
S898
S990
S1148
T1375
S1594
S2077
S2206
S2230
S527
S666
S897
S1134
S1132
S1574
S1643
S2204
S2228
S2620
S384
S598
S875
S1254
S1568
S1784
S1985
S2198
S2229
Regulation:
primary Ca^{2+}−dependent
SV−cycling−dependent
not−affected
Domains and Regions:
REGION: 11 X 3 AA repeats of K−K−[DE]

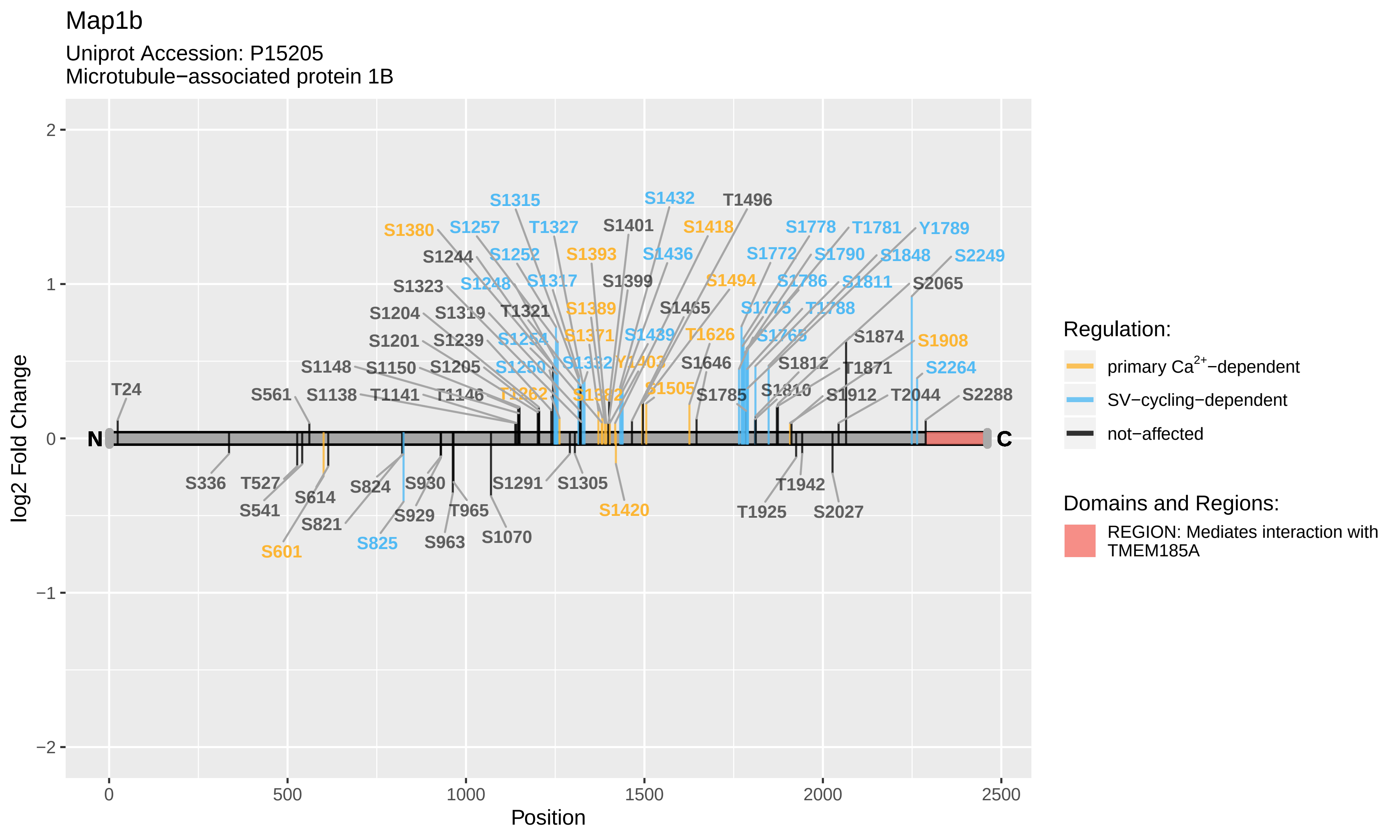

Map1b
Uniprot Accession: P15205
Microtubule−associated protein 1B
log2 Fold Change
Position
2
1
0
−1
−2
0
500
1000
1500
2000
2500
N
C
T24
S336
T527
S541
S561
S601
S614
S821
S824
S825
S929
S930
T965
S963
S1070
S1138
T1141
S1146
S1148
S1150
S1201
S1204
S1205
S1239
S1244
S1248
S1250
S1252
S1254
S1257
T1262
S1291
S1305
S1315
S1317
S1319
T1321
S1323
T1327
S1332
S1371
S1380
S1382
S1389
S1393
S1399
S1401
Y1403
S1418
S1420
S1432
S1436
S1439
S1465
S1494
T1496
S1505
T1626
S1646
S1765
S1772
S1775
S1778
T1781
S1785
S1786
T1788
Y1789
S1790
S1810
S1811
S1812
S1848
T1871
S1874
S1908
S1912
T1925
T1942
S2027
T2044
S2065
S2249
S2264
S2288
Regulation:
primary Ca2+−dependent
SV−cycling−dependent
not−affected
Domains and Regions:
REGION: Mediates interaction with TMEM185A

Map1s

Uniprot Accession: P0C5W1
Microtubule−associated protein 1S

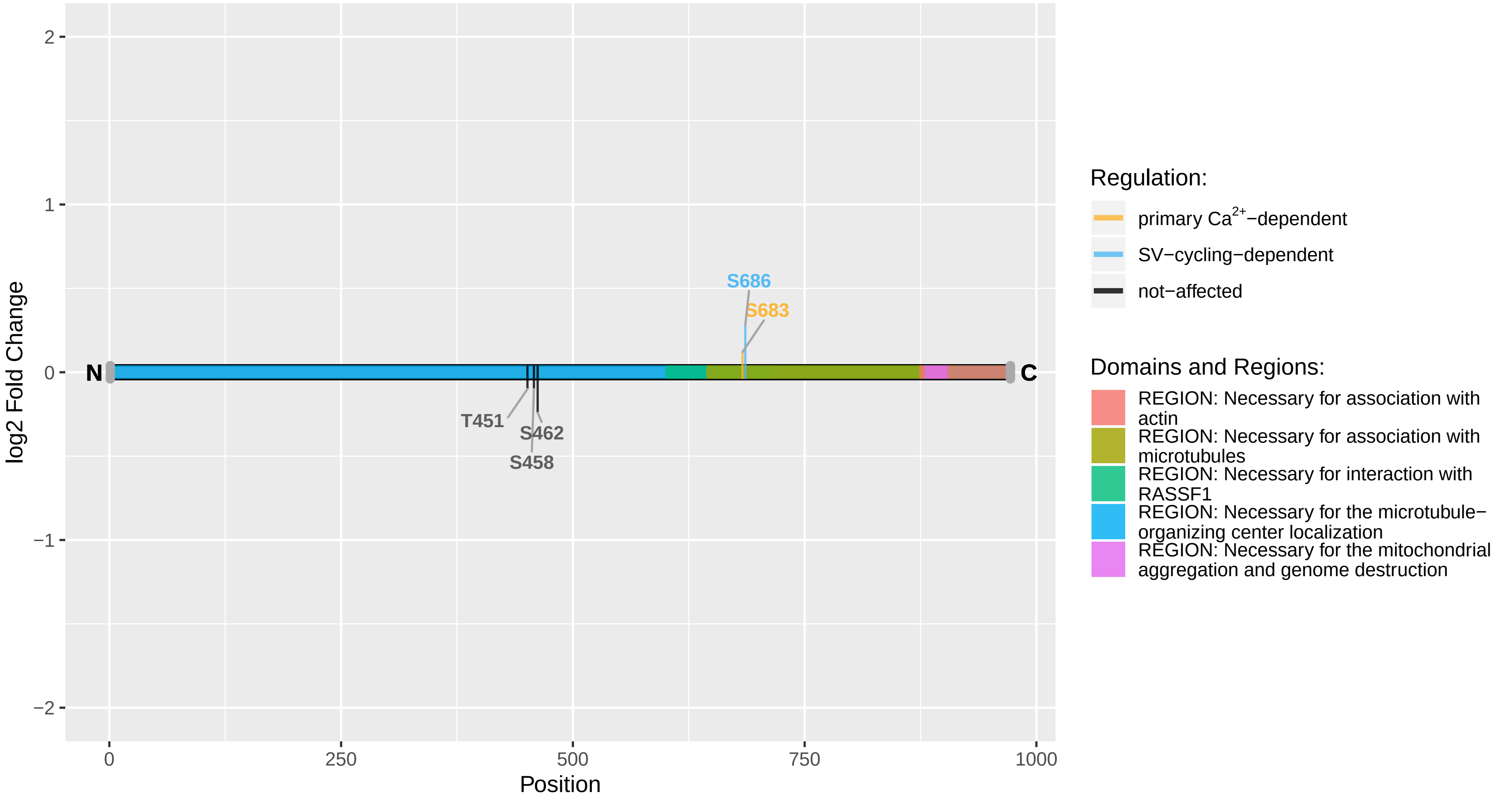

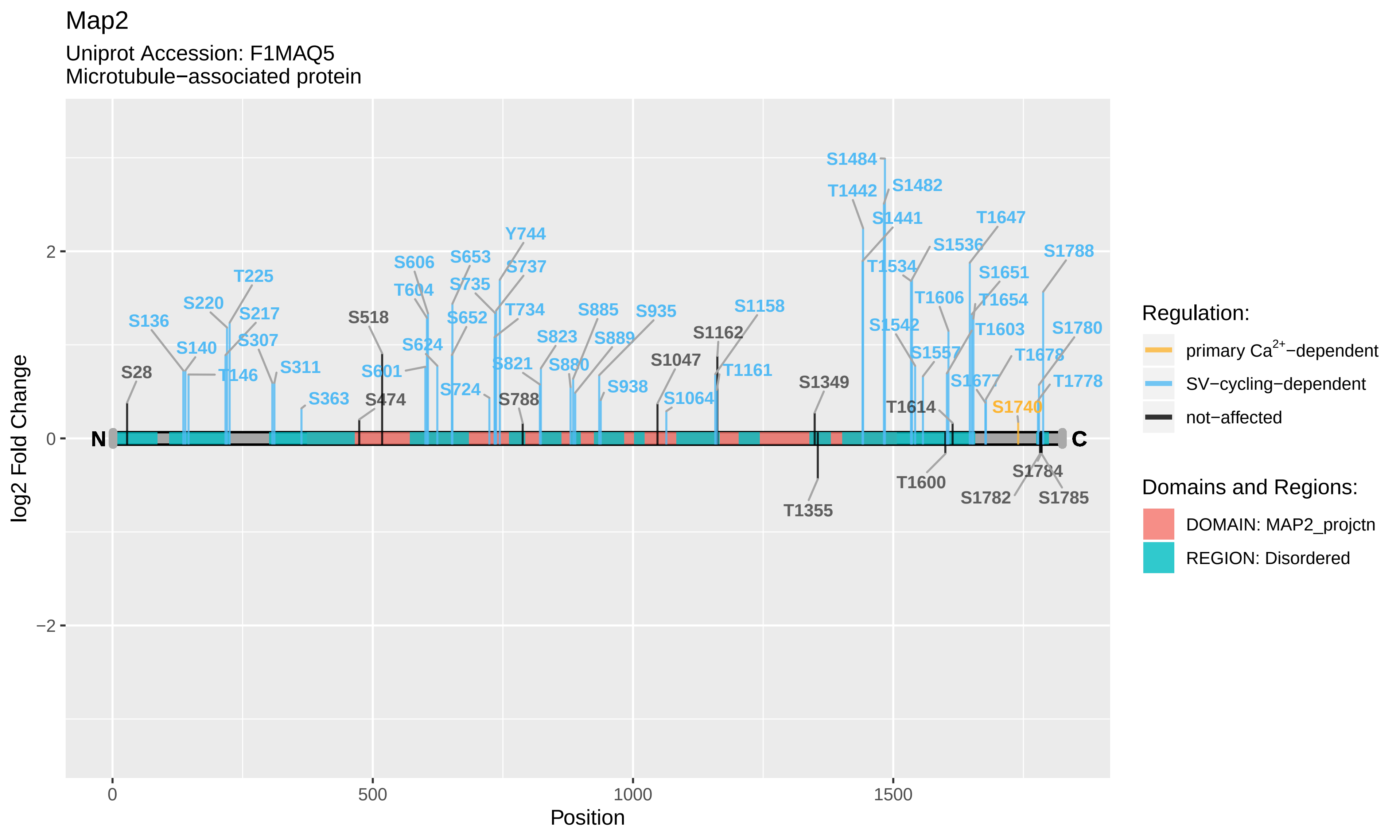
Map2
Uniprot Accession: F1MAQ5
Microtubule−associated protein
log2 Fold Change
Position
N
C
S1484
T1442
S1482
S1441
T1647
Y744
S1536
S1788
S606
S653
S737
T1534
S1651
T225
S735
T604
T1606
T1654
S220
S217
S652
T734
S885
S935
S1158
S136
S518
S1542
T1603
S1780
S307
S624
S823
S889
S1162
S140
S1557
T1678
S311
S601
S821
S880
S1047
T1161
S28
T146
S724
S938
S1349
S1677
T1778
S363
S474
S788
S1064
T1614
S1740
S1784
T1600
S1782
S1785
T1355
Regulation:
primary Ca^{2+}−dependent
SV−cycling−dependent
not−affected
Domains and Regions:
DOMAIN: MAP2_projctn
REGION: Disordered

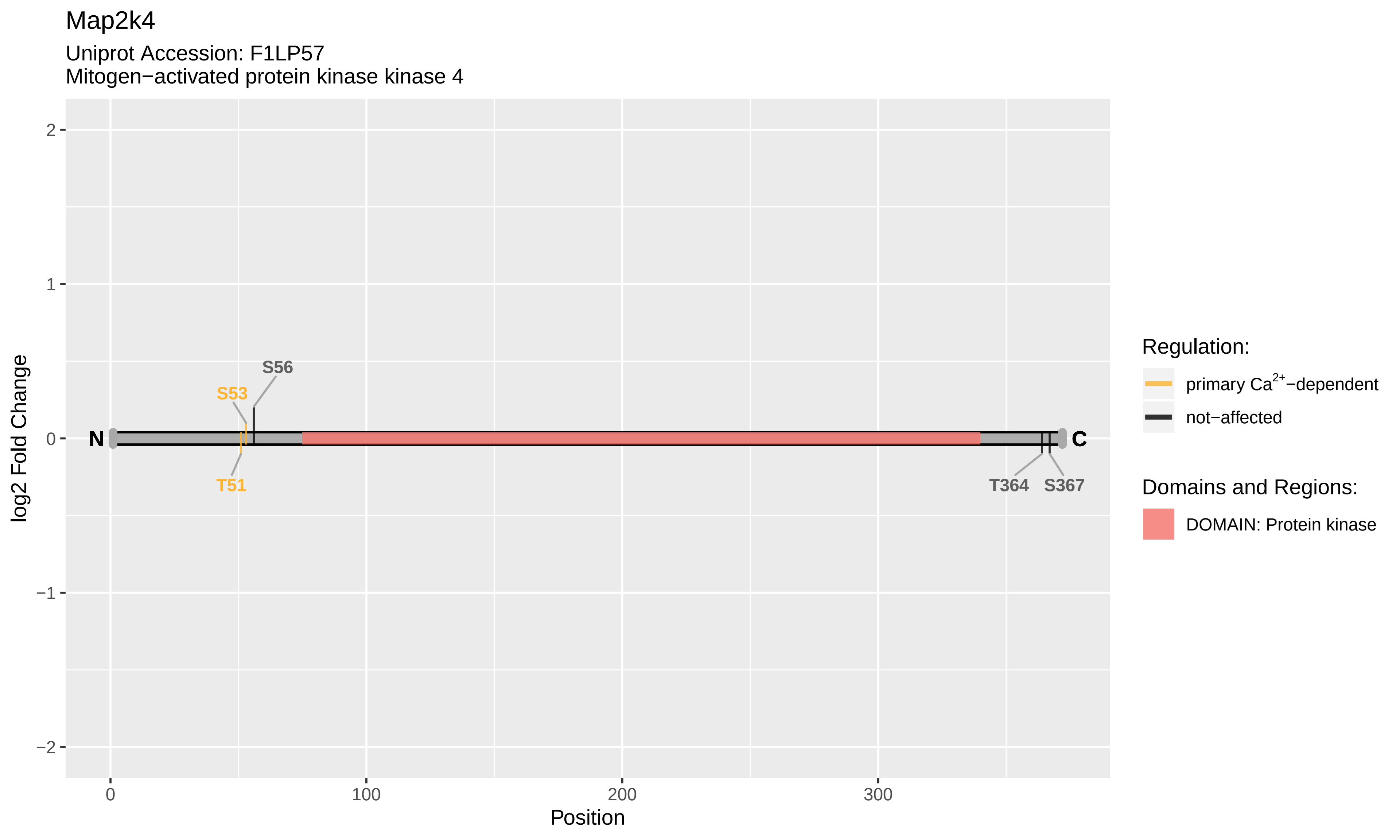

Map2k4
Uniprot Accession: F1LP57
Mitogen−activated protein kinase kinase 4
S56
S53
N
C
T51
T364
S367
2
1
0
−1
−2
log2 Fold Change
0
100
200
300
Position
Regulation:
primary Ca2+−dependent
not−affected
Domains and Regions:
DOMAIN: Protein kinase

Map2k7

Uniprot Accession: Q4KSH7
Dual specificity mitogen−activated protein kinase kinase 7

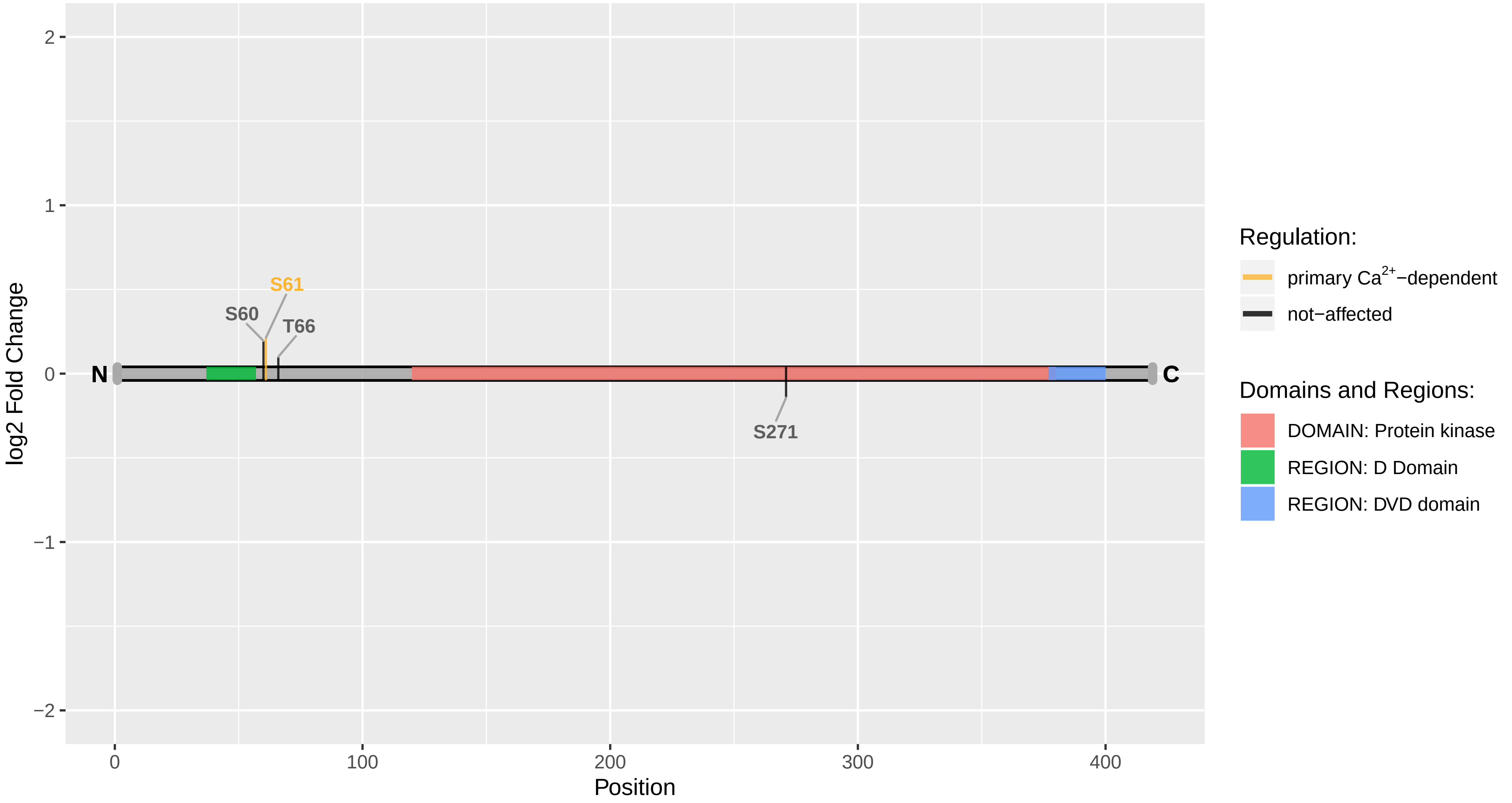

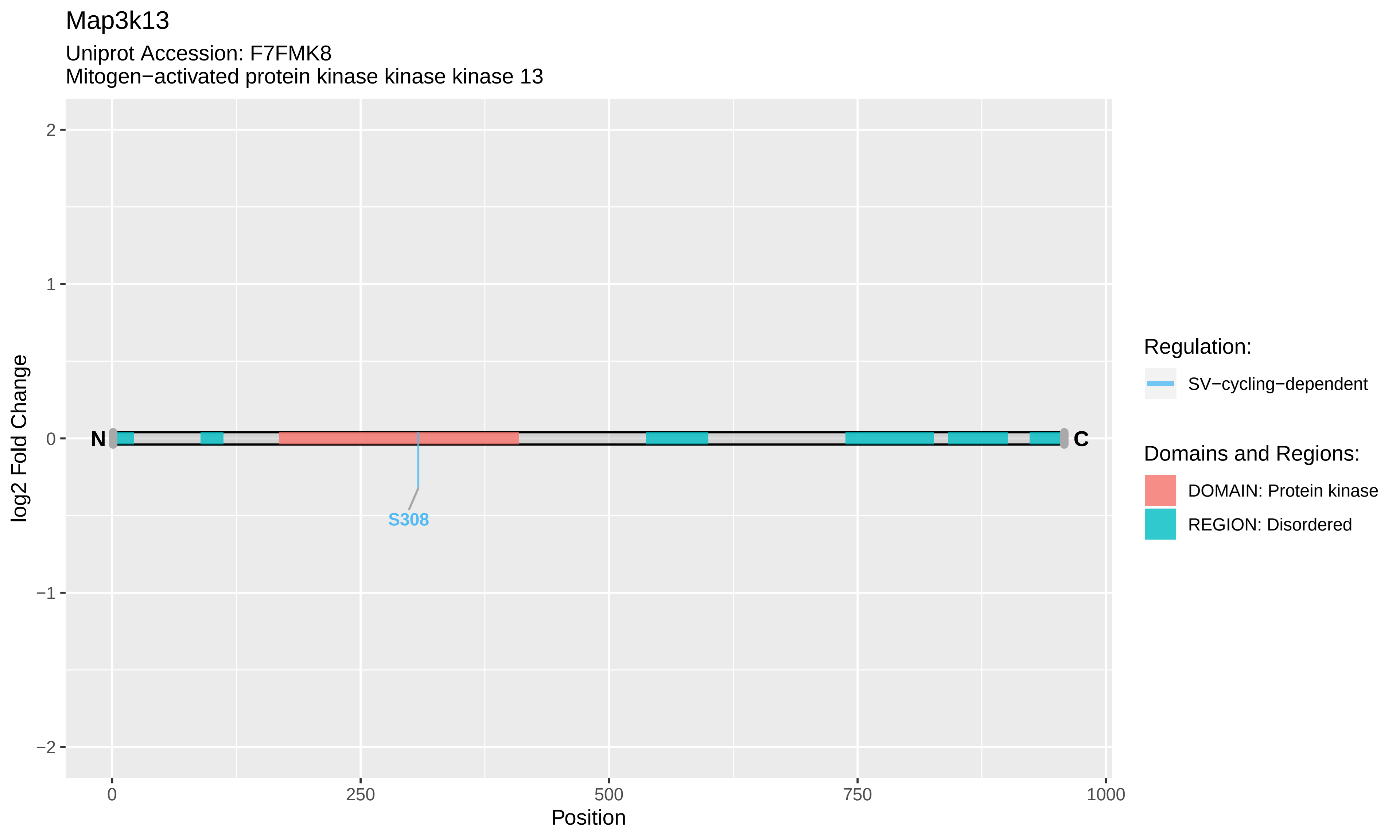
Map3k13
Uniprot Accession: F7FMK8
Mitogen−activated protein kinase kinase kinase 13
log2 Fold Change
Position
2
1
0
−1
−2
0
250
500
750
1000
N
C
S308
Regulation:
SV−cycling−dependent
Domains and Regions:
DOMAIN: Protein kinase
REGION: Disordered

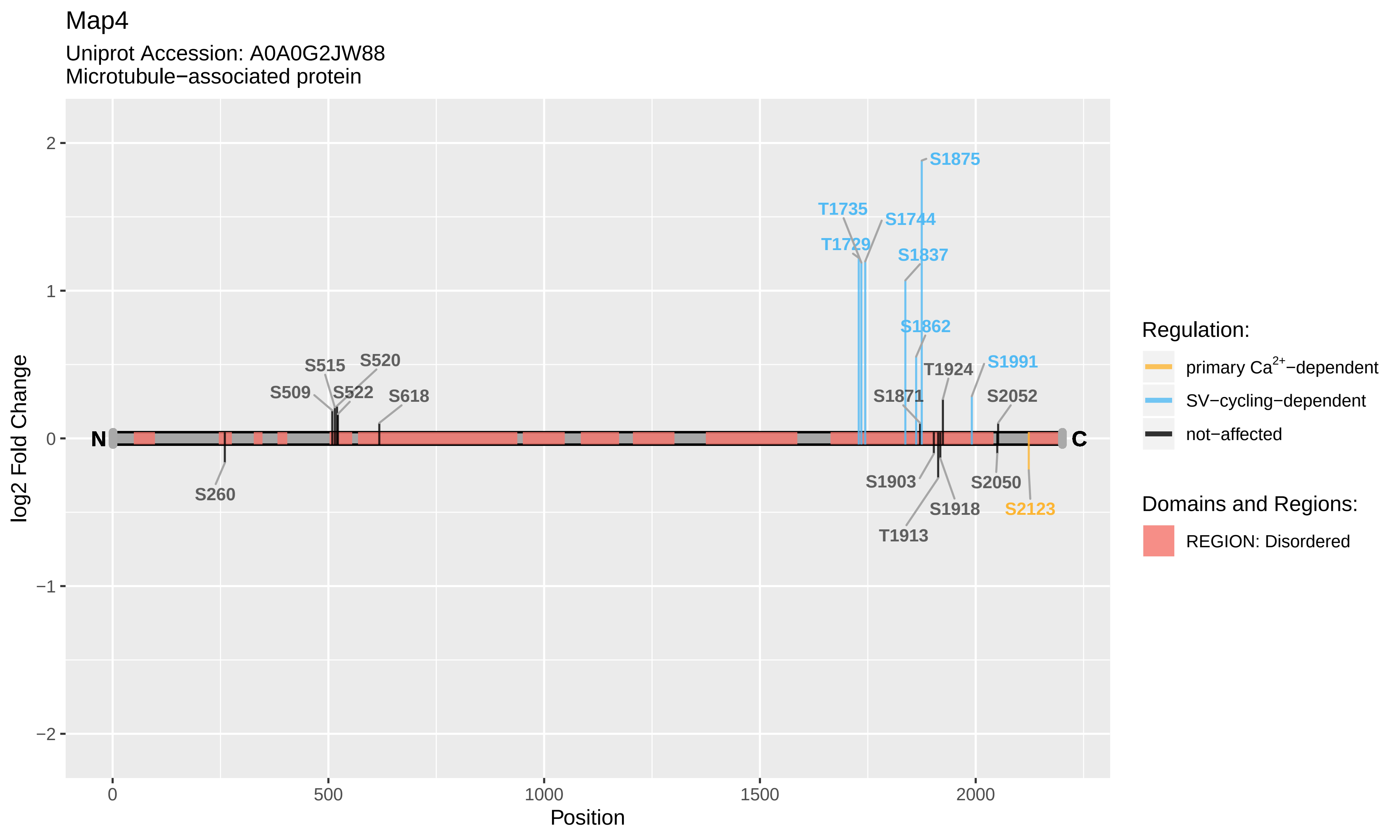

Map4
Uniprot Accession: A0A0G2JW88
Microtubule−associated protein
log2 Fold Change
Position
N
C
S1875
T1735
S1744
T1729
S1837
S1862
S1991
T1924
S1871
S2052
S515
S520
S509
S522
S618
S260
S1903
S2050
S1918
S2123
T1913
Regulation:
primary Ca2+−dependent
SV−cycling−dependent
not−affected
Domains and Regions:
REGION: Disordered

Map4k4

Uniprot Accession: A0A0G2K7W4
Mitogen−activated protein kinase kinase kinase kinase 4

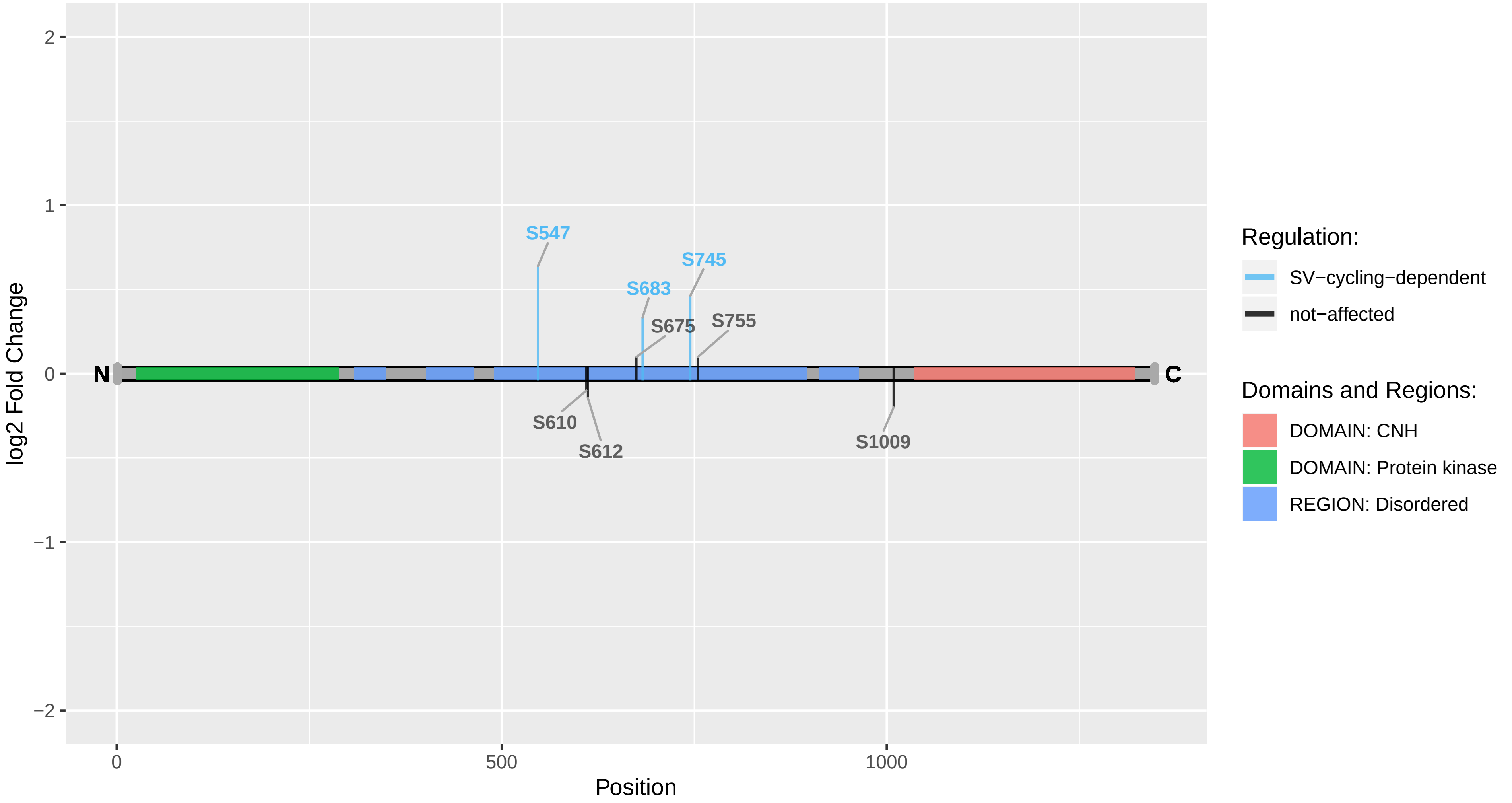

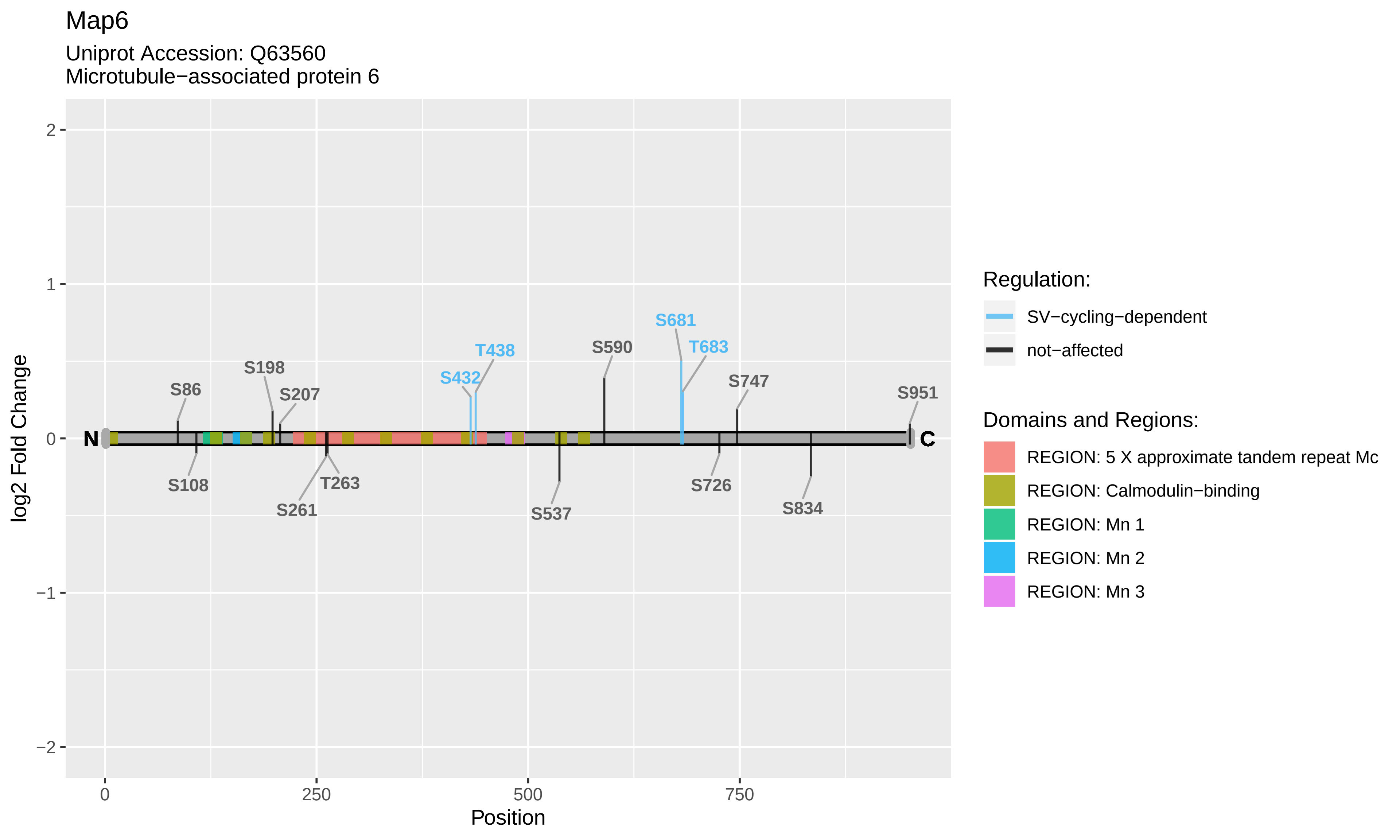
Map6
Uniprot Accession: Q63560
Microtubule−associated protein 6
log2 Fold Change
Position
2
1
0
−1
−2
0
250
500
750
N
C
S86
S108
S198
S207
S261
T263
S432
T438
S537
S590
S681
T683
S726
S747
S834
S951
Regulation:
SV−cycling−dependent
not−affected
Domains and Regions:
REGION: 5 X approximate tandem repeat Mc
REGION: Calmodulin−binding
REGION: Mn 1
REGION: Mn 2
REGION: Mn 3

Map7d1

Uniprot Accession: D4A644
MAP7 domain−containing 1

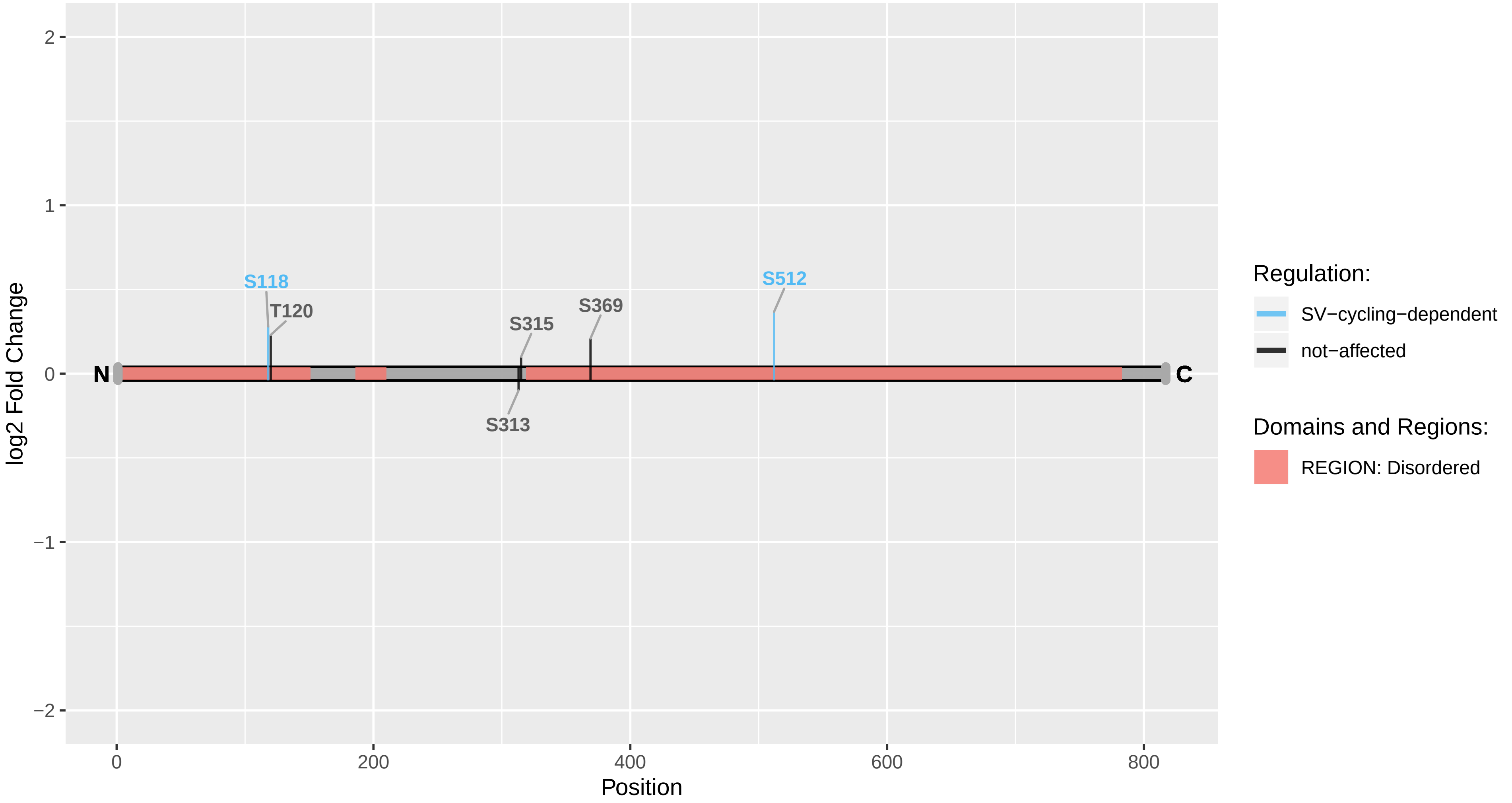

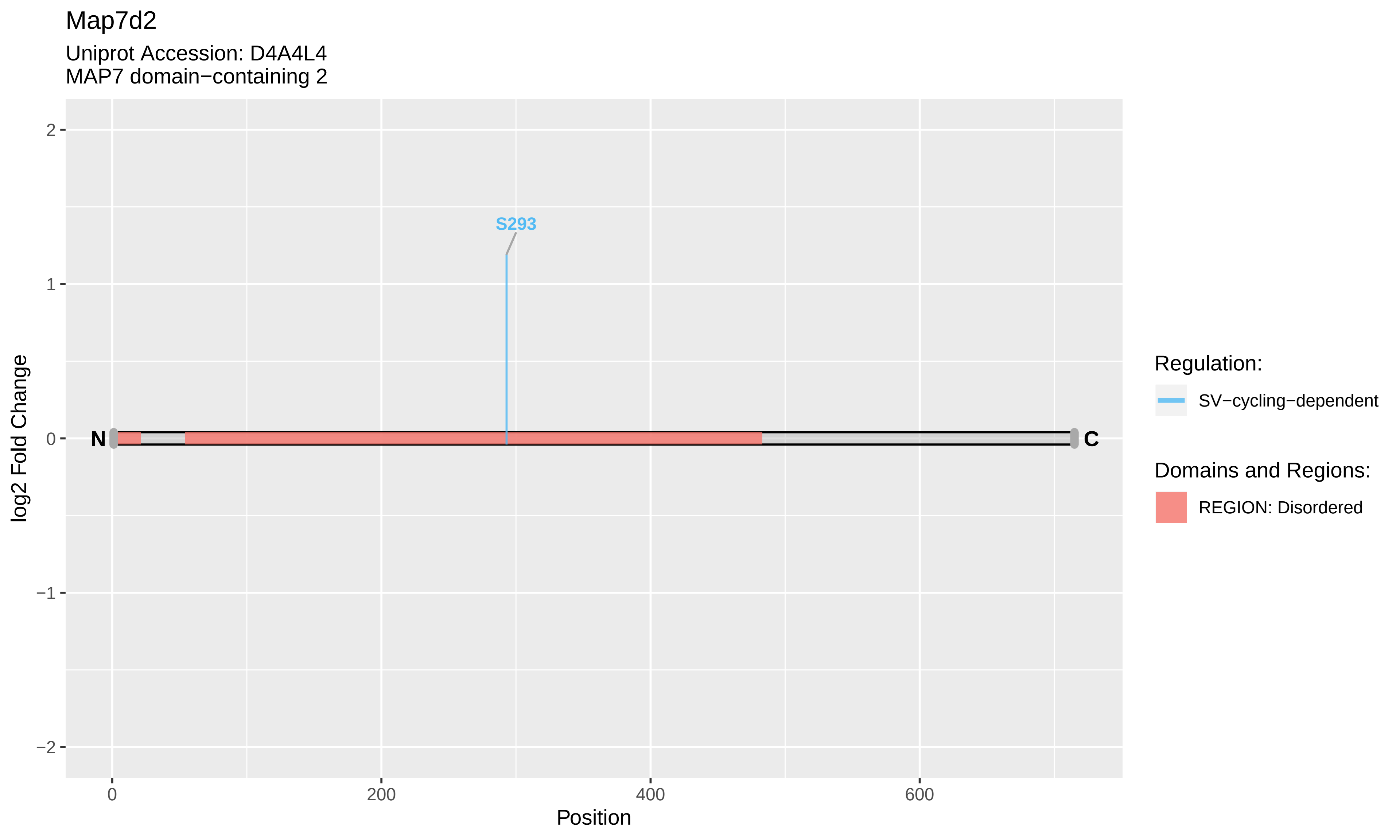
Map7d2
Uniprot Accession: D4A4L4
MAP7 domain−containing 2
S293
N
C
2
1
0
−1
−2
0
200
400
600
log2 Fold Change
Position
Regulation:
SV−cycling−dependent
Domains and Regions:
REGION: Disordered

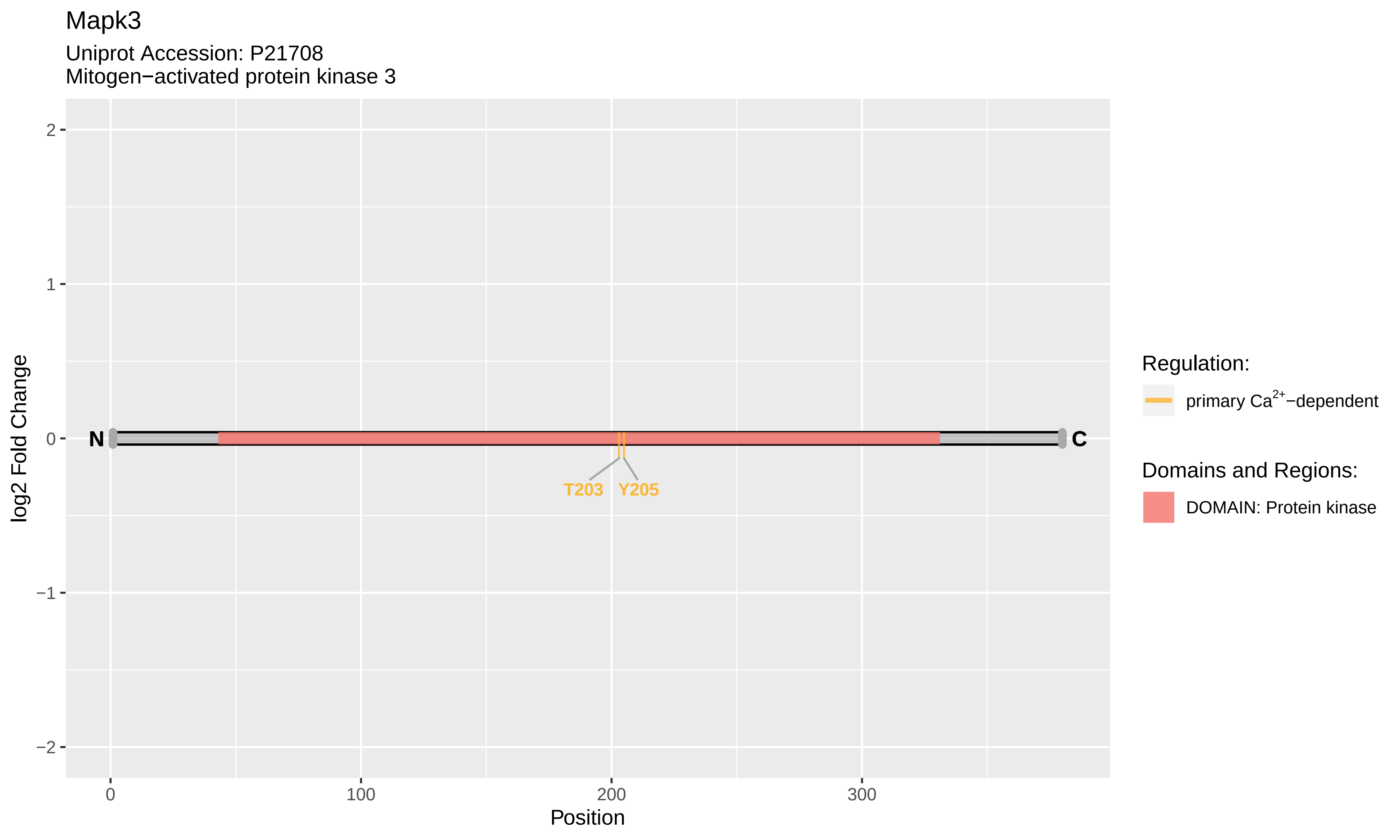

Mapk3
Uniprot Accession: P21708
Mitogen−activated protein kinase 3
log2 Fold Change
2
1
0
−1
−2
N
C
T203
Y205
0
100
200
300
Position
Regulation:
primary Ca2+−dependent
Domains and Regions:
DOMAIN: Protein kinase

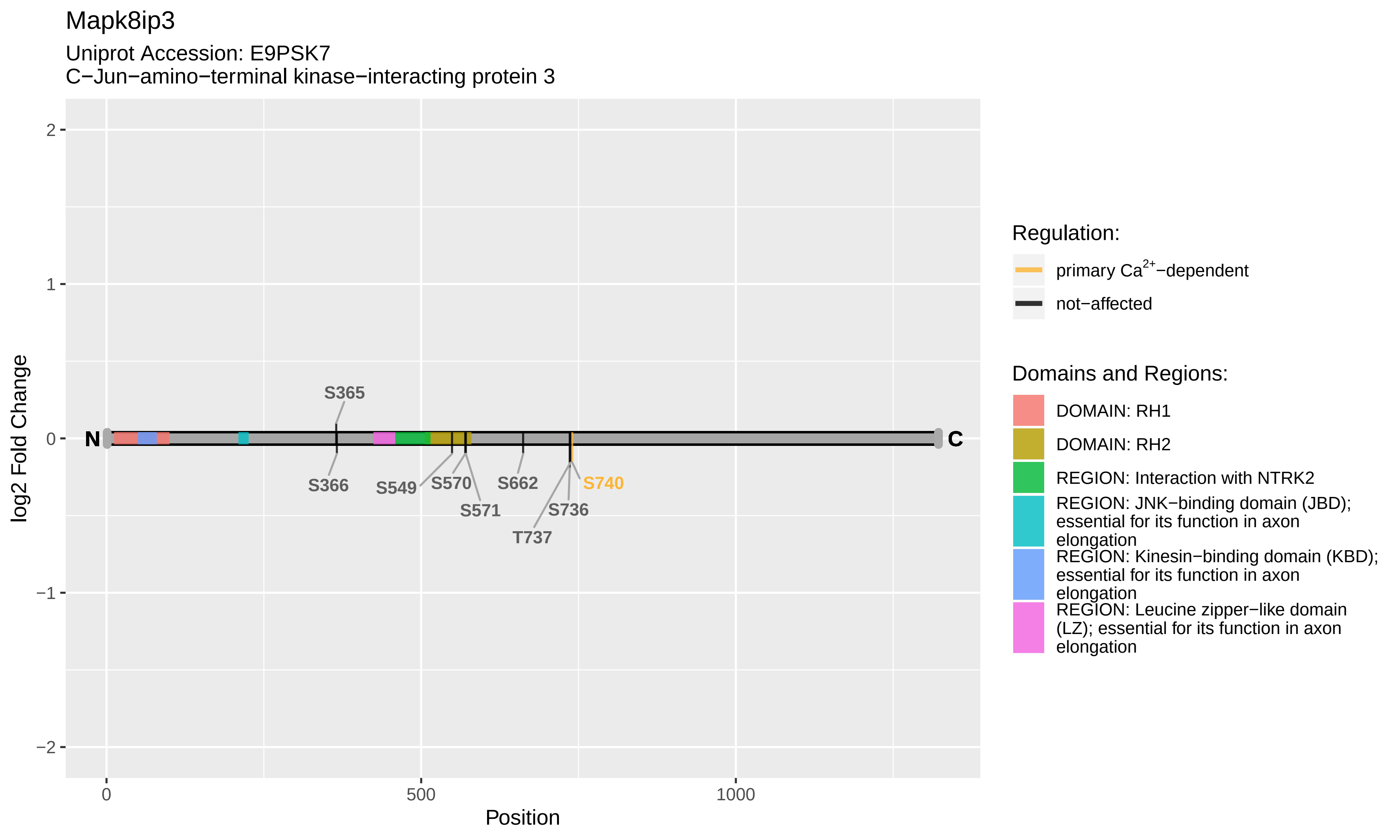

Mapk8ip3
Uniprot Accession: E9PSK7
C−Jun−amino−terminal kinase−interacting protein 3
log2 Fold Change
2
1
0
−1
−2
N
C
S365
S366
S549
S570
S571
S662
T737
S736
S740
0
500
1000
Position
Regulation:
primary Ca2+−dependent
not−affected
Domains and Regions:
DOMAIN: RH1
DOMAIN: RH2
REGION: Interaction with NTRK2
REGION: JNK−binding domain (JBD); essential for its function in axon elongation
REGION: Kinesin−binding domain (KBD); essential for its function in axon elongation
REGION: Leucine zipper−like domain (LZ); essential for its function in axon elongation

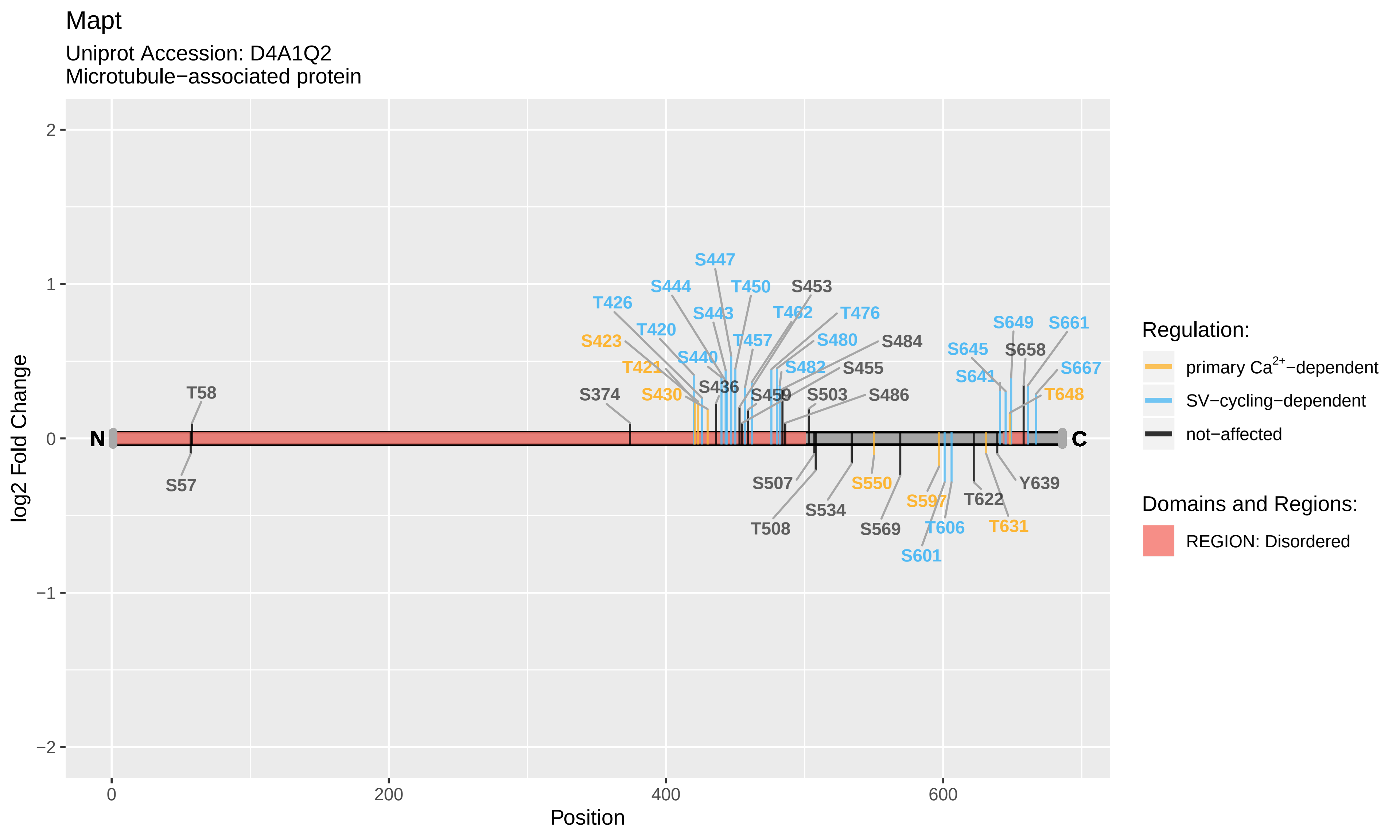
Mapt
Uniprot Accession: D4A1Q2
Microtubule−associated protein
log2 Fold Change
Position
2
1
0
−1
−2
0
200
400
600
N
C
T58
S57
S374
S423
T421
T426
T420
S430
S436
S440
S443
S444
S447
T450
S453
T457
S459
T462
T476
S480
S482
S455
S484
S503
S486
S507
T508
S534
S550
S569
S597
S601
T606
T622
T631
Y639
S641
S645
S649
S658
S661
S667
T648
Regulation:
primary Ca2+−dependent
SV−cycling−dependent
not−affected
Domains and Regions:
REGION: Disordered

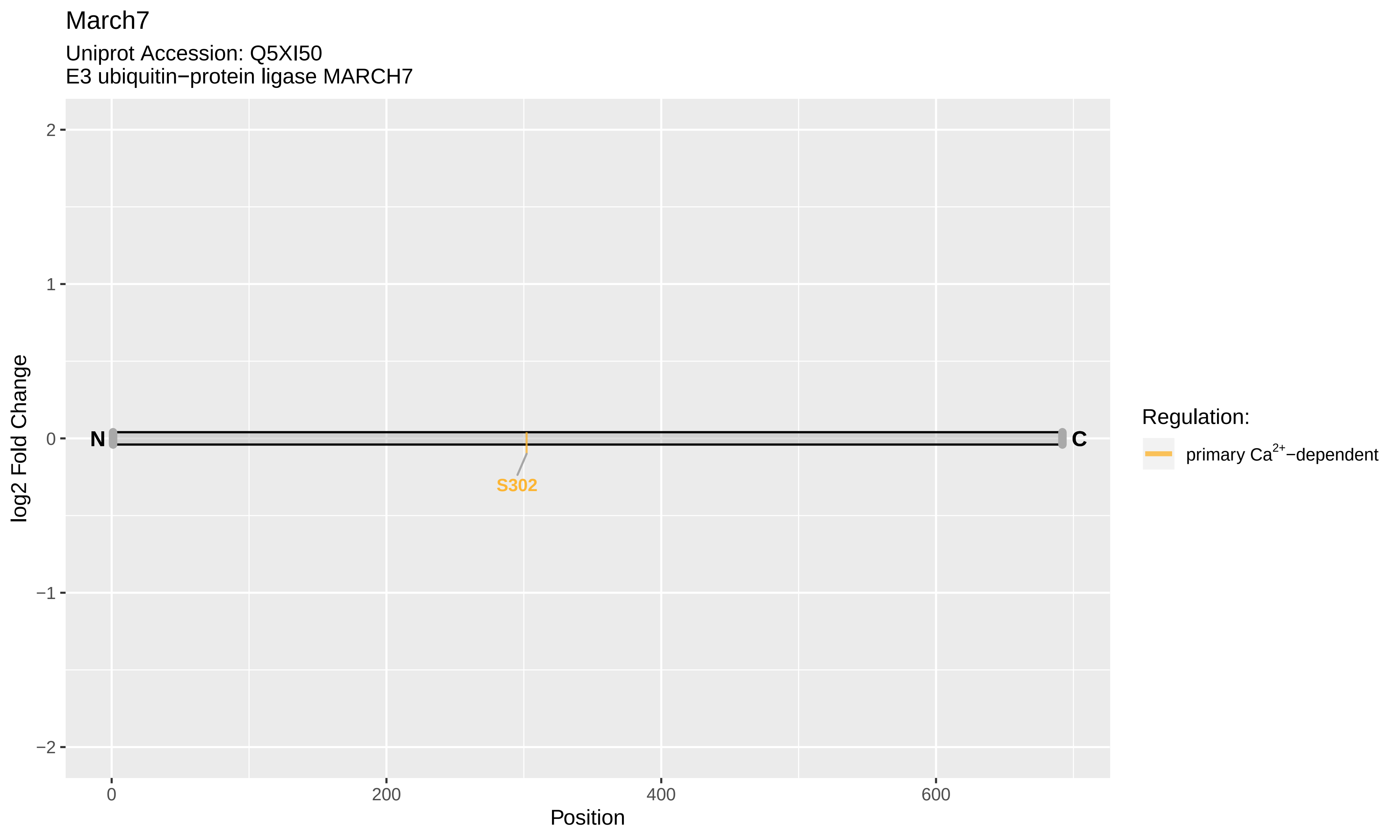

March7
Uniprot Accession: Q5XI50
E3 ubiquitin−protein ligase MARCH7
log2 Fold Change
2
1
0
−1
−2
N
C
S302
0
200
400
600
Position
Regulation:
primary Ca2+−dependent

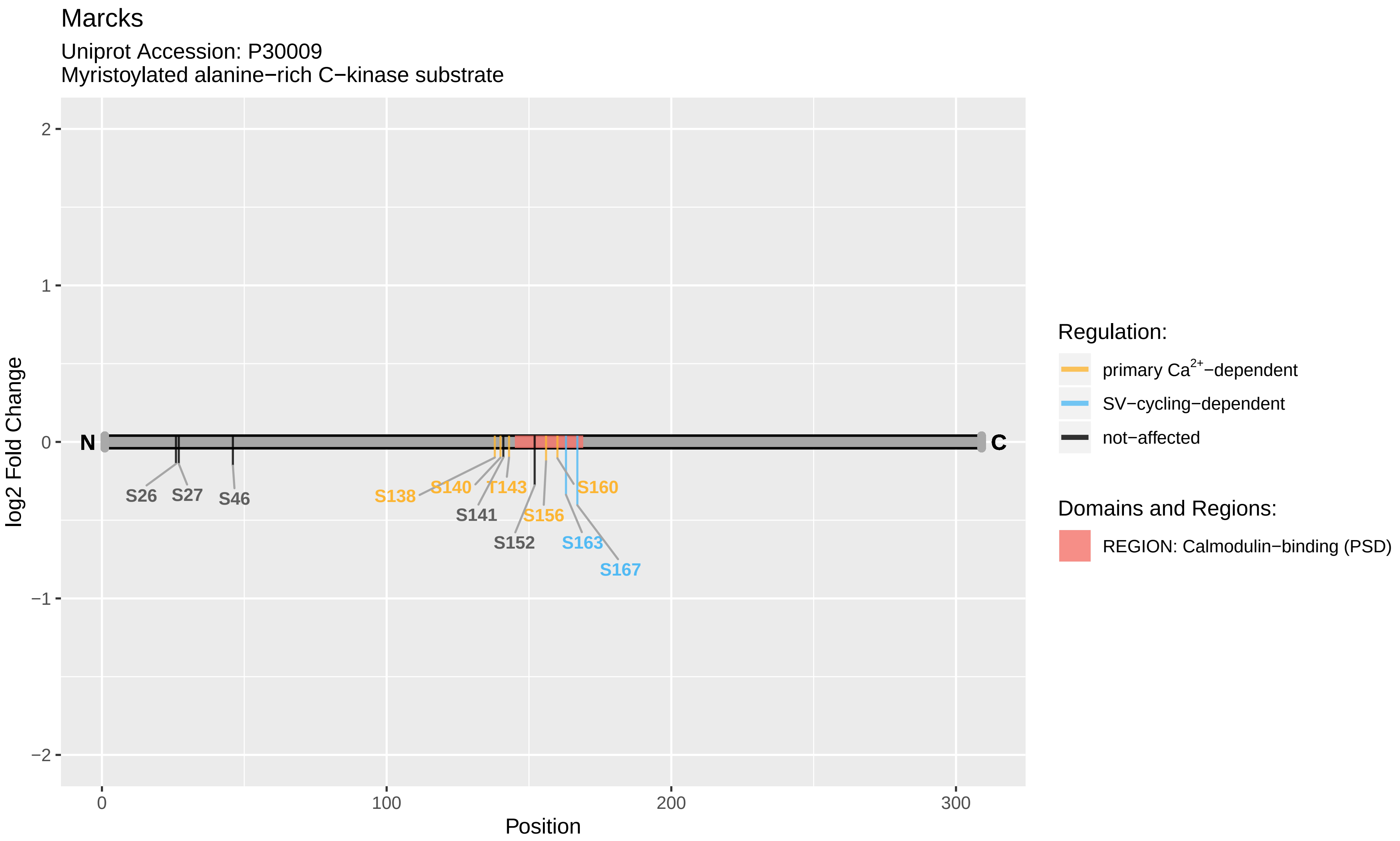
Marcks
Uniprot Accession: P30009
Myristoylated alanine−rich C−kinase substrate
log2 Fold Change
2
1
0
−1
−2
N
C
S26
S27
S46
S138
S140
T143
S141
S156
S160
S152
S163
S167
0
100
200
300
Position
Regulation:
primary Ca2+−dependent
SV−cycling−dependent
not−affected
Domains and Regions:
REGION: Calmodulin−binding (PSD)

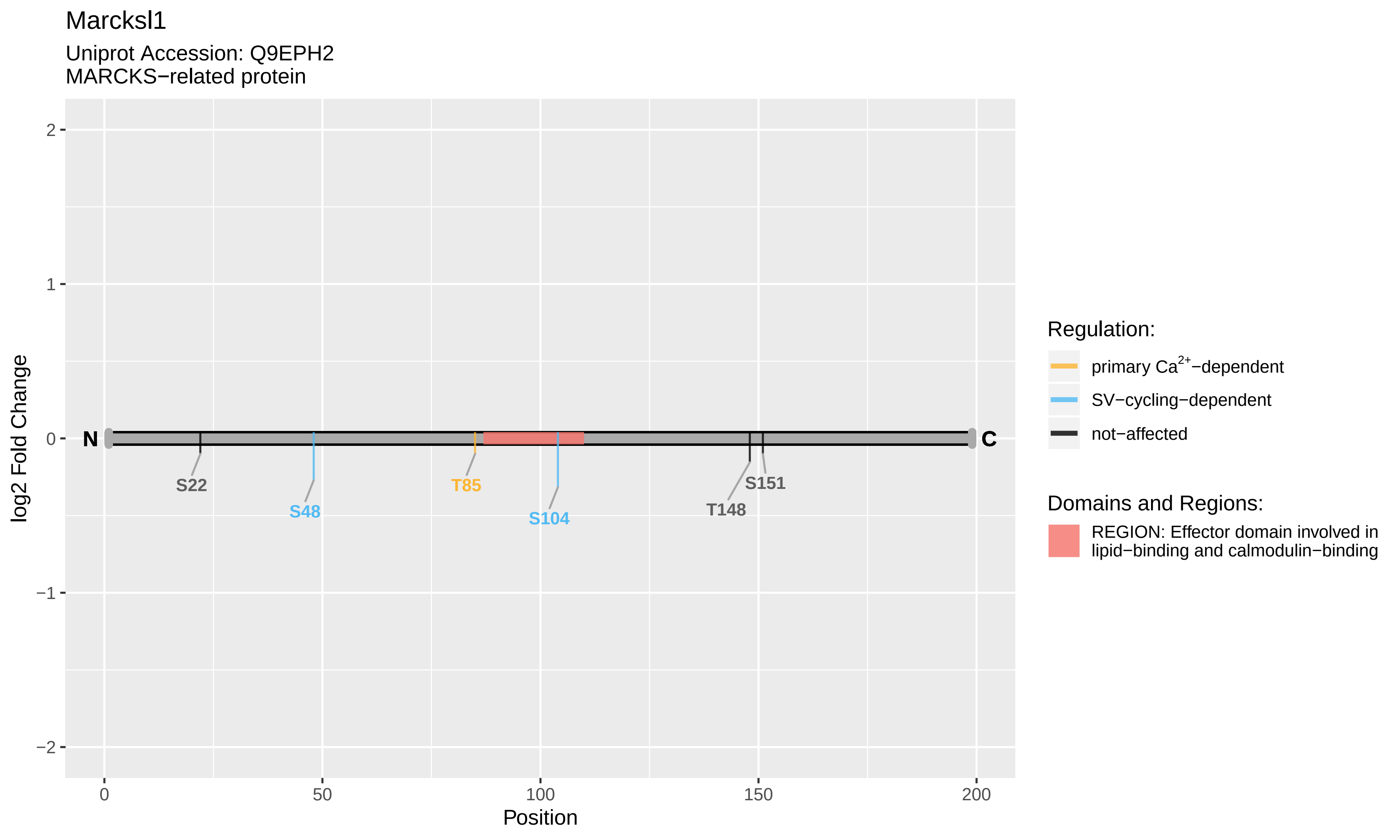
Marcksl1
Uniprot Accession: Q9EPH2
MARCKS−related protein
log2 Fold Change
2
1
0
−1
−2
N
C
S22
S48
T85
S104
T148
S151
0
50
100
150
200
Position
Regulation:
primary Ca2+−dependent
SV−cycling−dependent
not−affected
Domains and Regions:
REGION: Effector domain involved in lipid−binding and calmodulin−binding

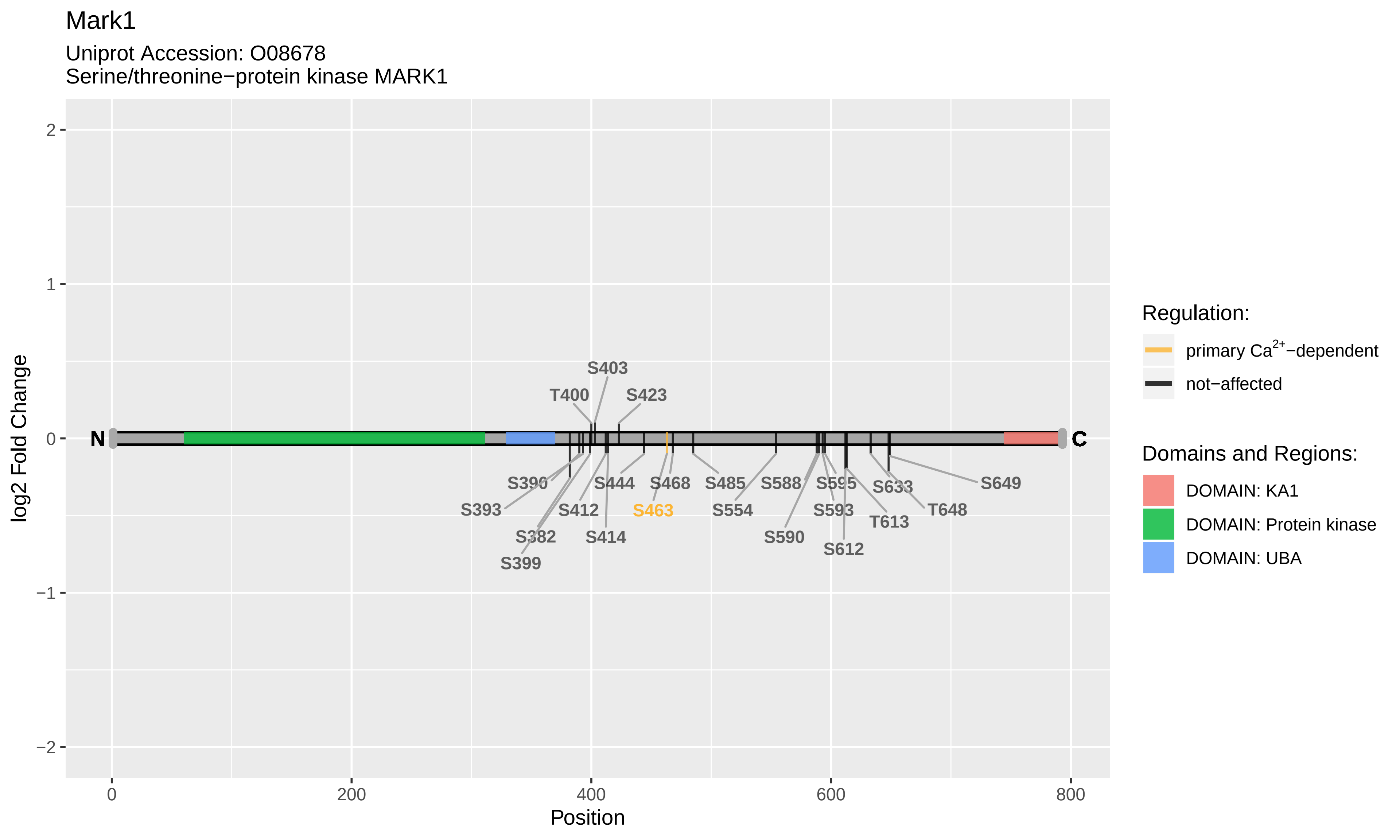

Mark1
Uniprot Accession: O08678
Serine/threonine−protein kinase MARK1
log2 Fold Change
Position
N
C
S403
T400
S423
S390
S444
S468
S485
S588
S595
S633
S649
S393
S412
S463
S554
S593
T648
S382
S414
S590
T613
S612
S399
Regulation:
primary Ca2+−dependent
not−affected
Domains and Regions:
DOMAIN: KA1
DOMAIN: Protein kinase
DOMAIN: UBA

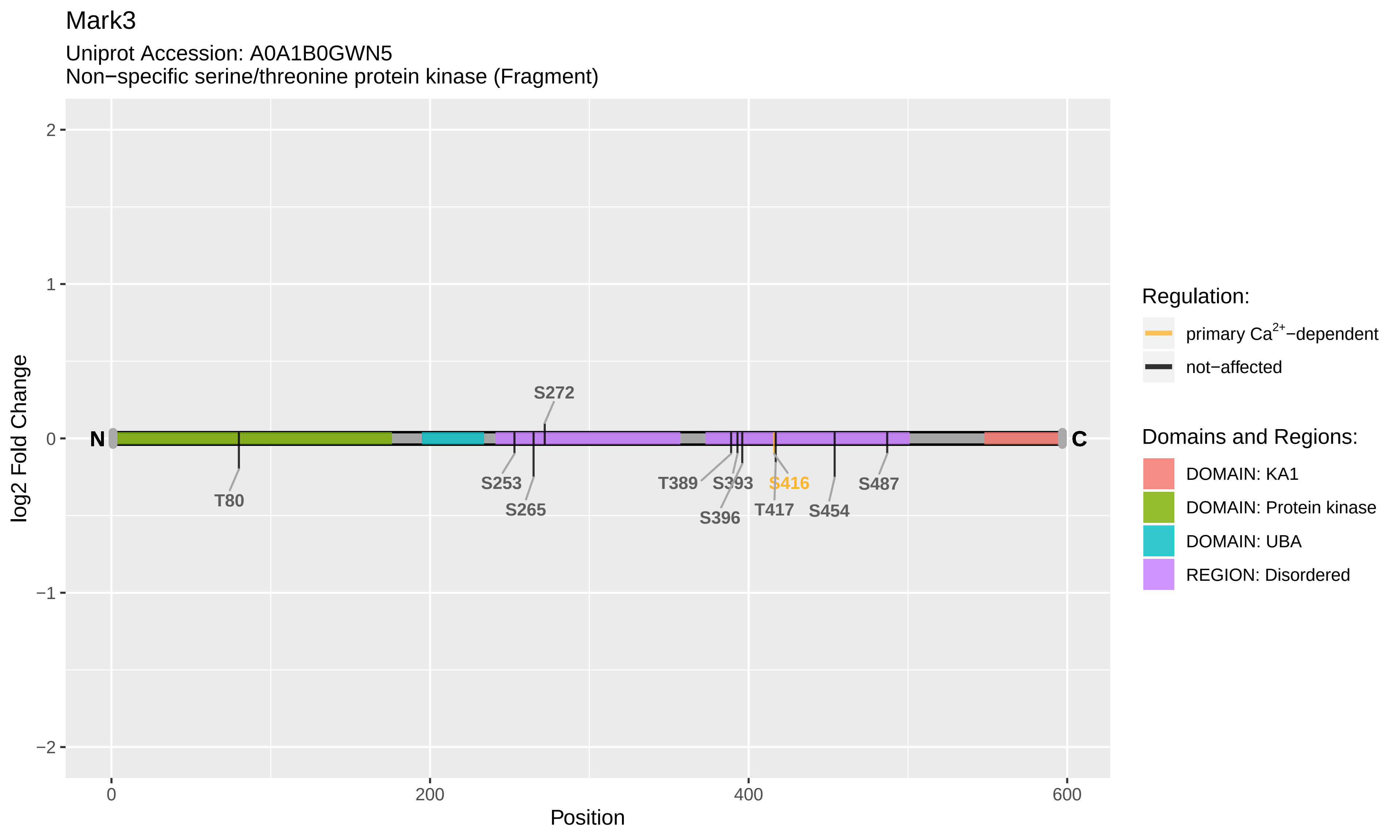

Mark3
Uniprot Accession: A0A1B0GWN5
Non−specific serine/threonine protein kinase (Fragment)
log2 Fold Change
Position
N
C
S272
T80
S253
S265
T389
S393
S396
S416
T417
S454
S487
Regulation:
primary Ca2+−dependent
not−affected
Domains and Regions:
DOMAIN: KA1
DOMAIN: Protein kinase
DOMAIN: UBA
REGION: Disordered

Mark3

Uniprot Accession: Q8VHF0
MAP/microtubule affinity−regulating kinase 3

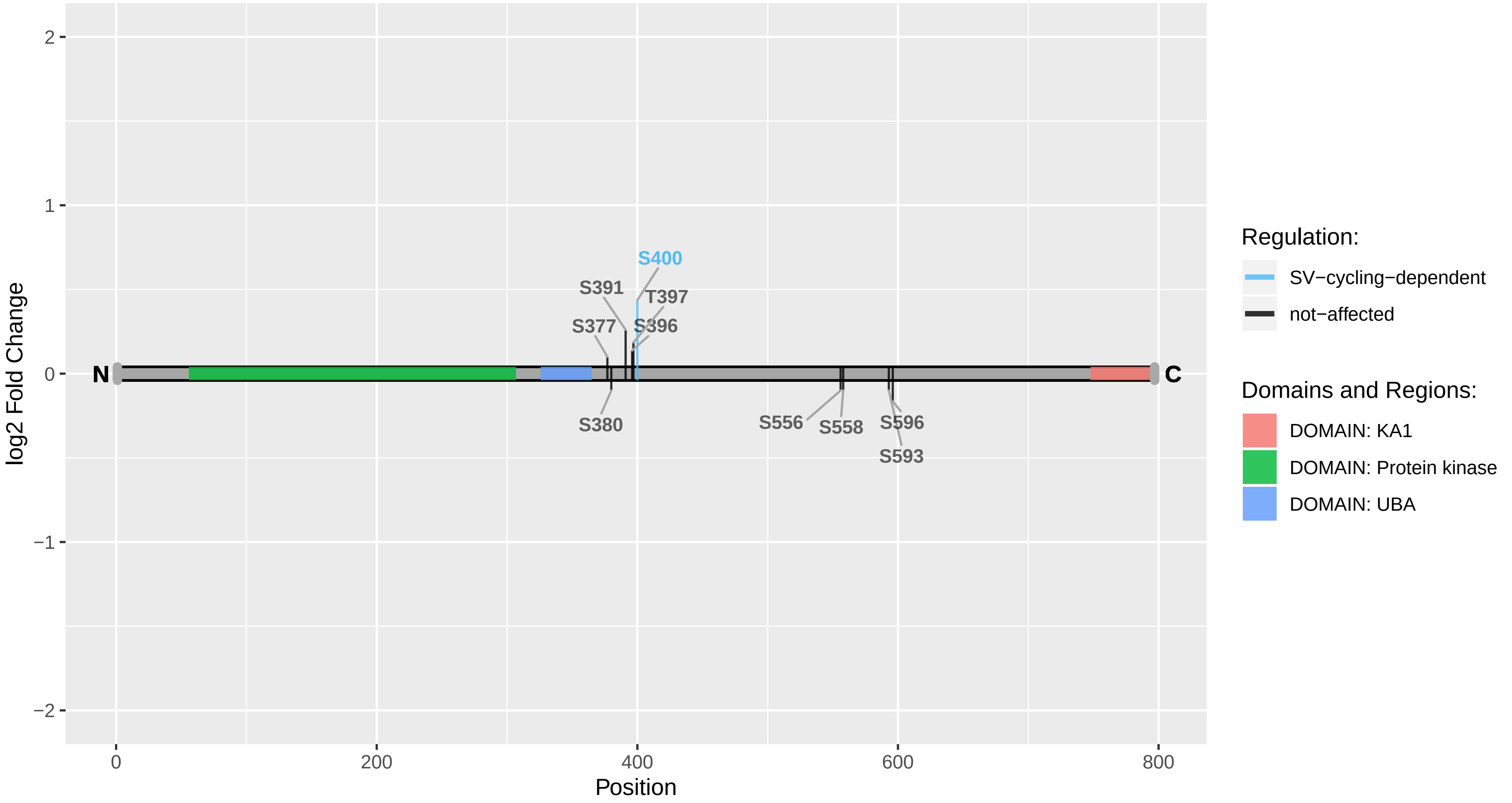

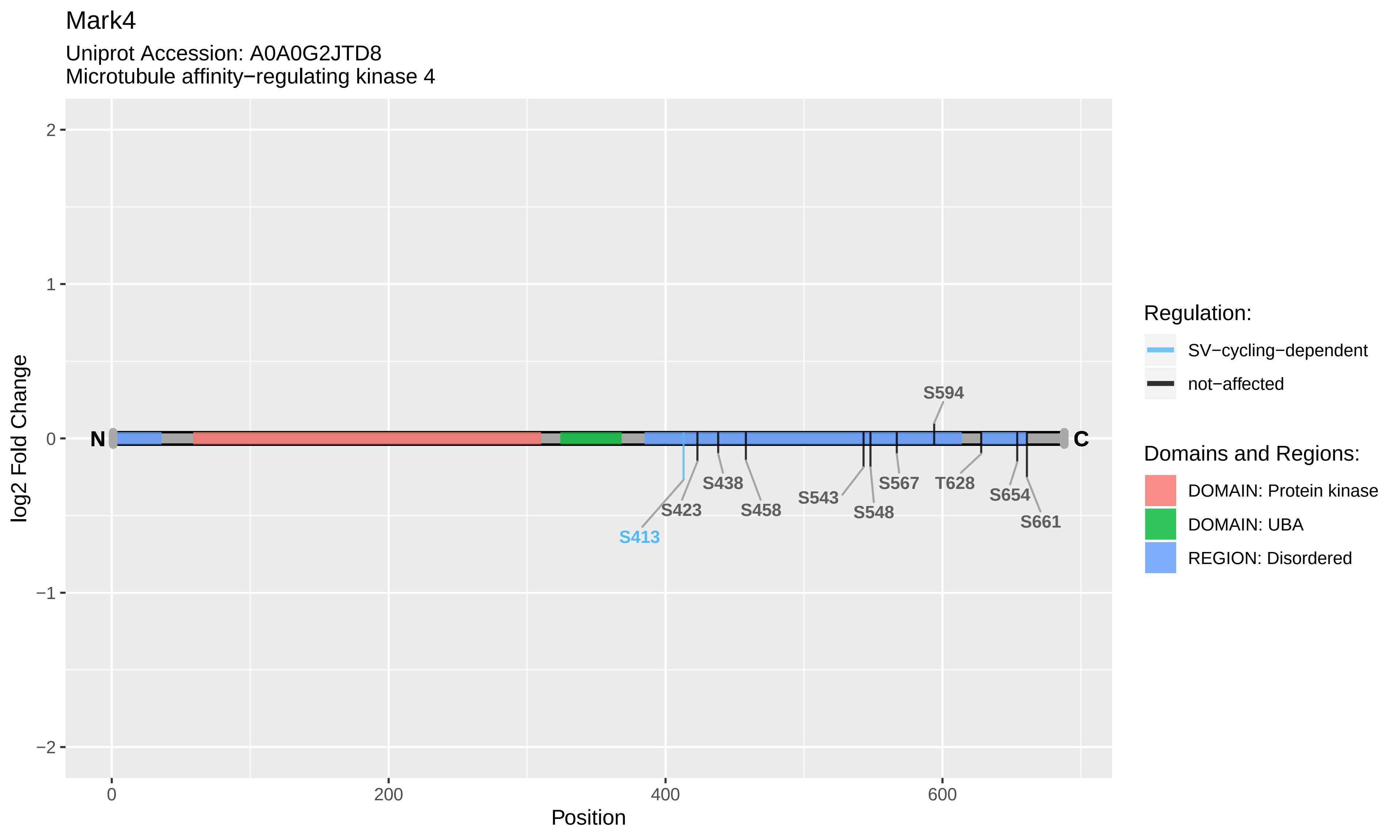

Mark4
Uniprot Accession: A0A0G2JTD8
Microtubule affinity−regulating kinase 4
log2 Fold Change
Position
2
1
0
−1
−2
0
200
400
600
N
C
S413
S423
S438
S458
S543
S548
S567
S594
T628
S654
S661
Regulation:
SV−cycling−dependent
not−affected
Domains and Regions:
DOMAIN: Protein kinase
DOMAIN: UBA
REGION: Disordered

Mast4

Uniprot Accession: M0R3L1
Microtubule−associated serine/threonine kinase family member 4

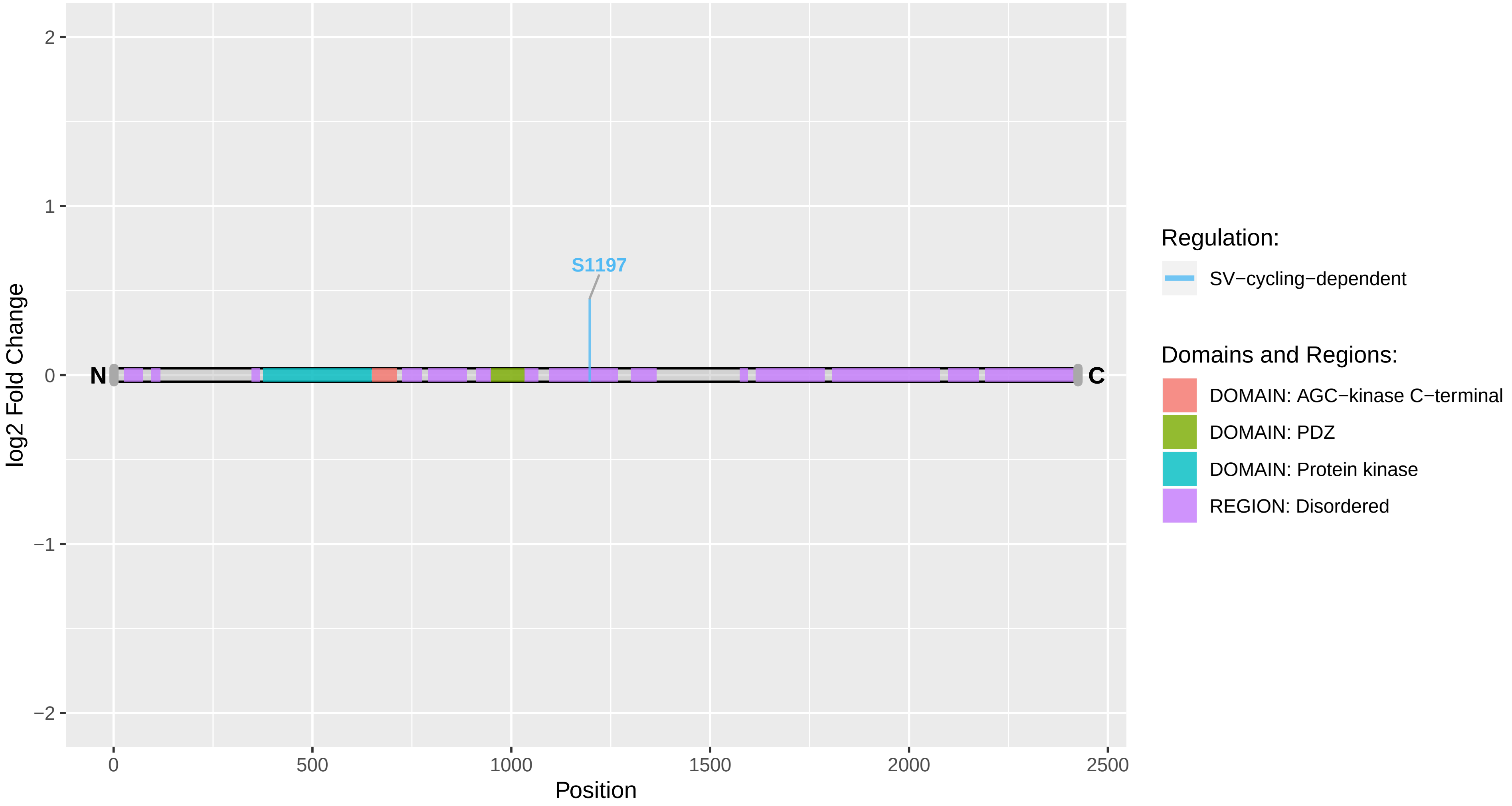

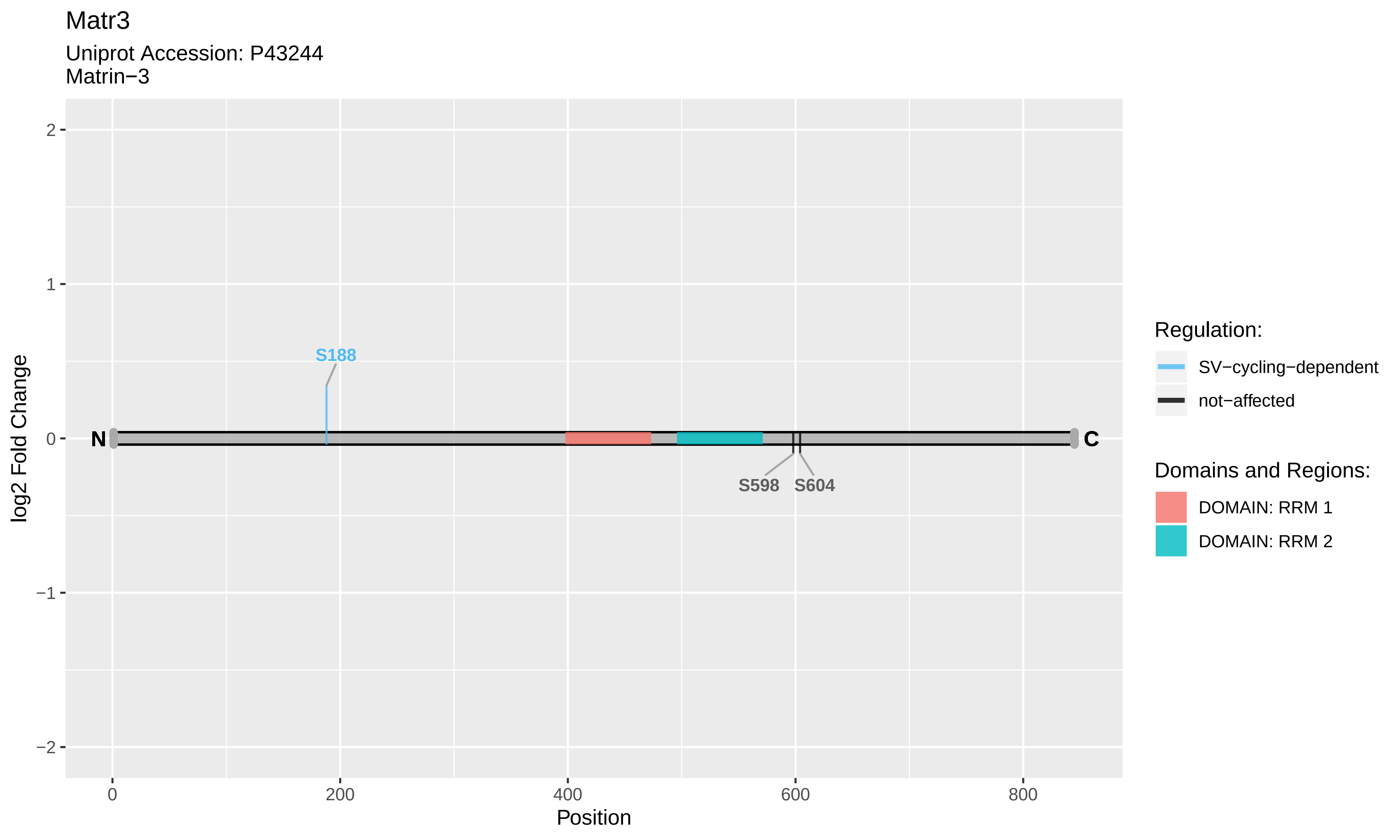

Matr3
Uniprot Accession: P43244
Matrin−3
log2 Fold Change
2
1
0
−1
−2
N
C
S188
S598
S604
0
200
400
600
800
Position
Regulation:
SV−cycling−dependent
not−affected
Domains and Regions:
DOMAIN: RRM 1
DOMAIN: RRM 2

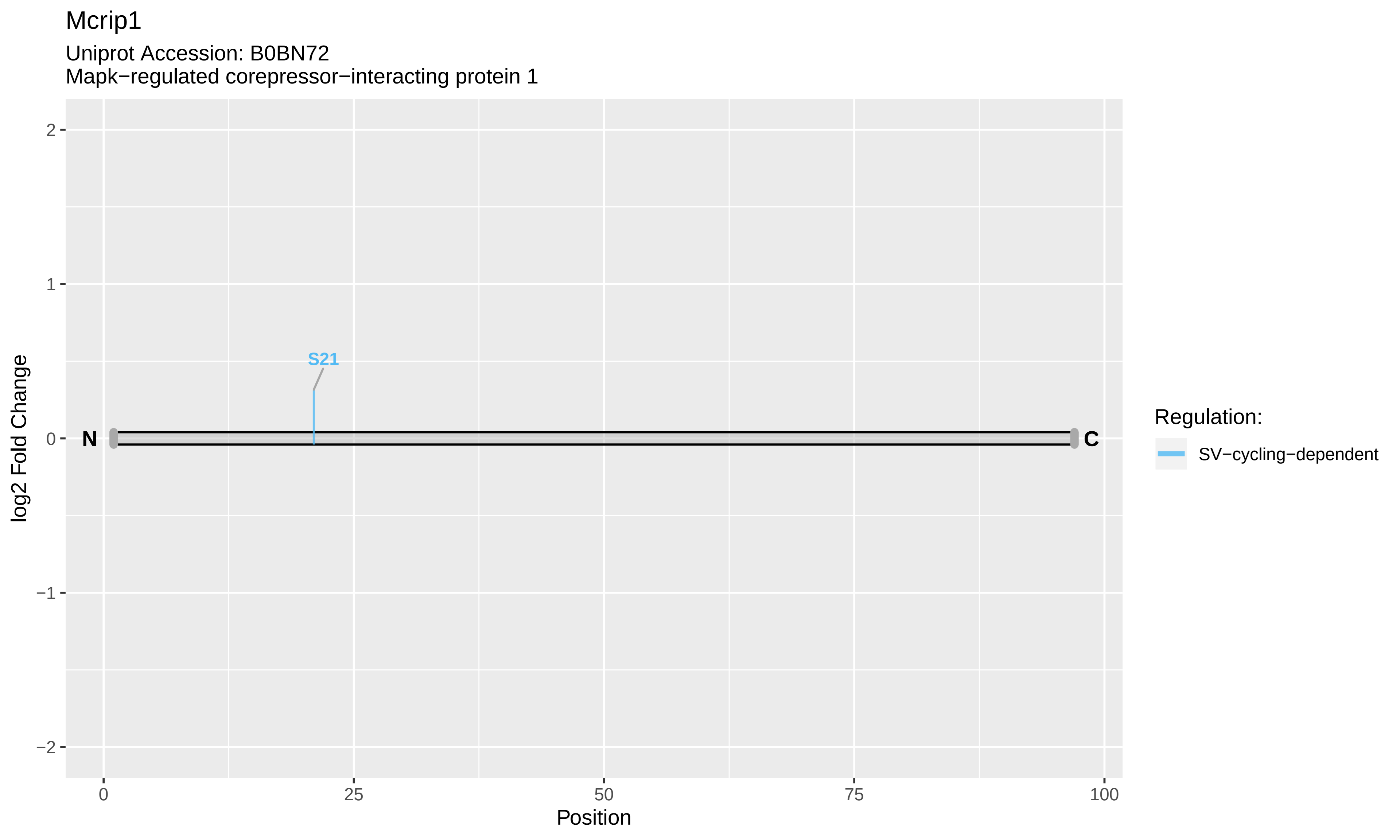
Mcrip1
Uniprot Accession: B0BN72
Mapk−regulated corepressor−interacting protein 1
S21
N
C
2
1
0
−1
−2
log2 Fold Change
0
25
50
75
100
Position
Regulation:
SV−cycling−dependent

Mdh2

Uniprot Accession: P04636
Malate dehydrogenase, mitochondrial

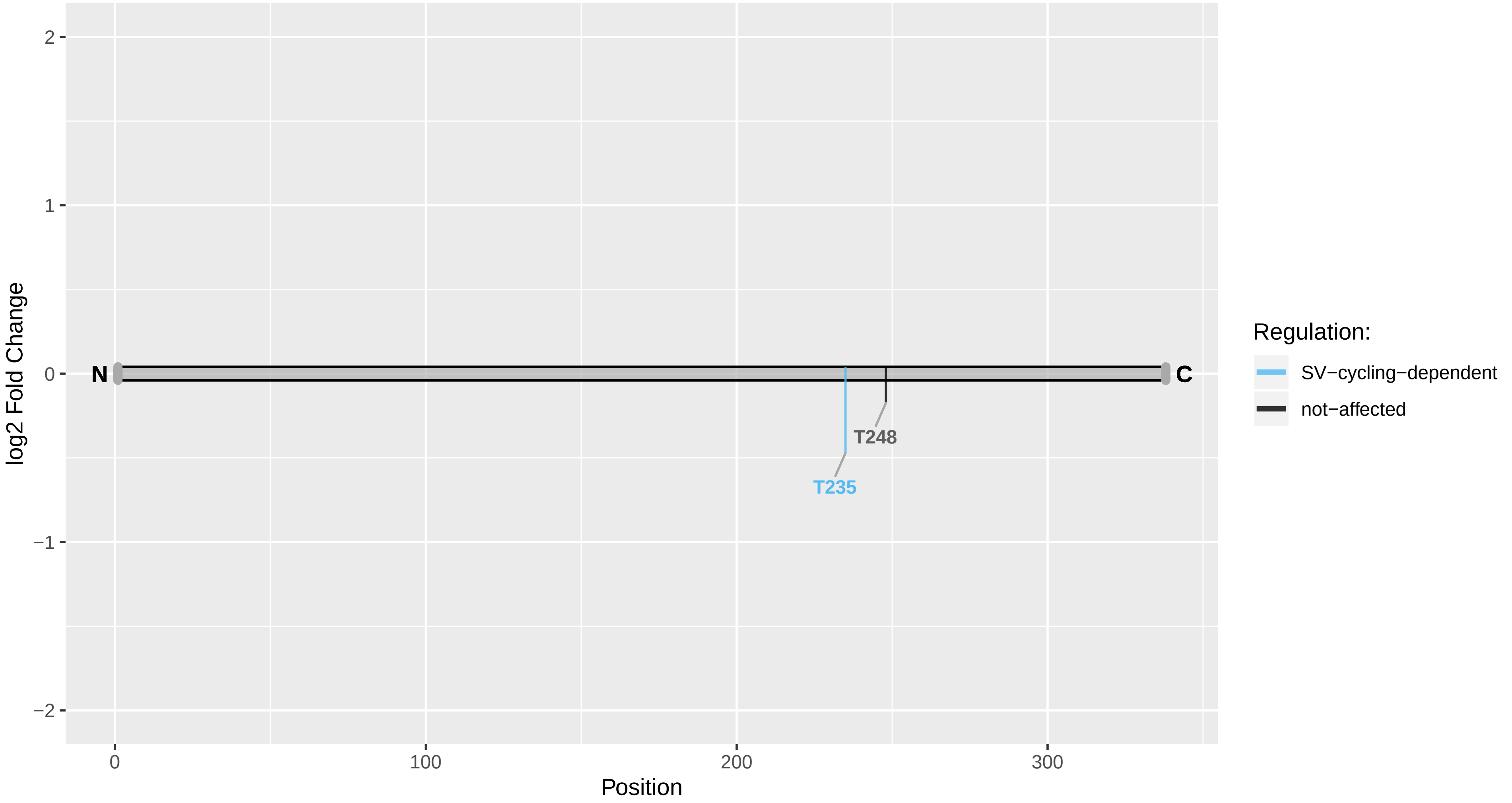

Mex3c

Uniprot Accession: D4A2R5
Mex−3 RNA−binding family member C

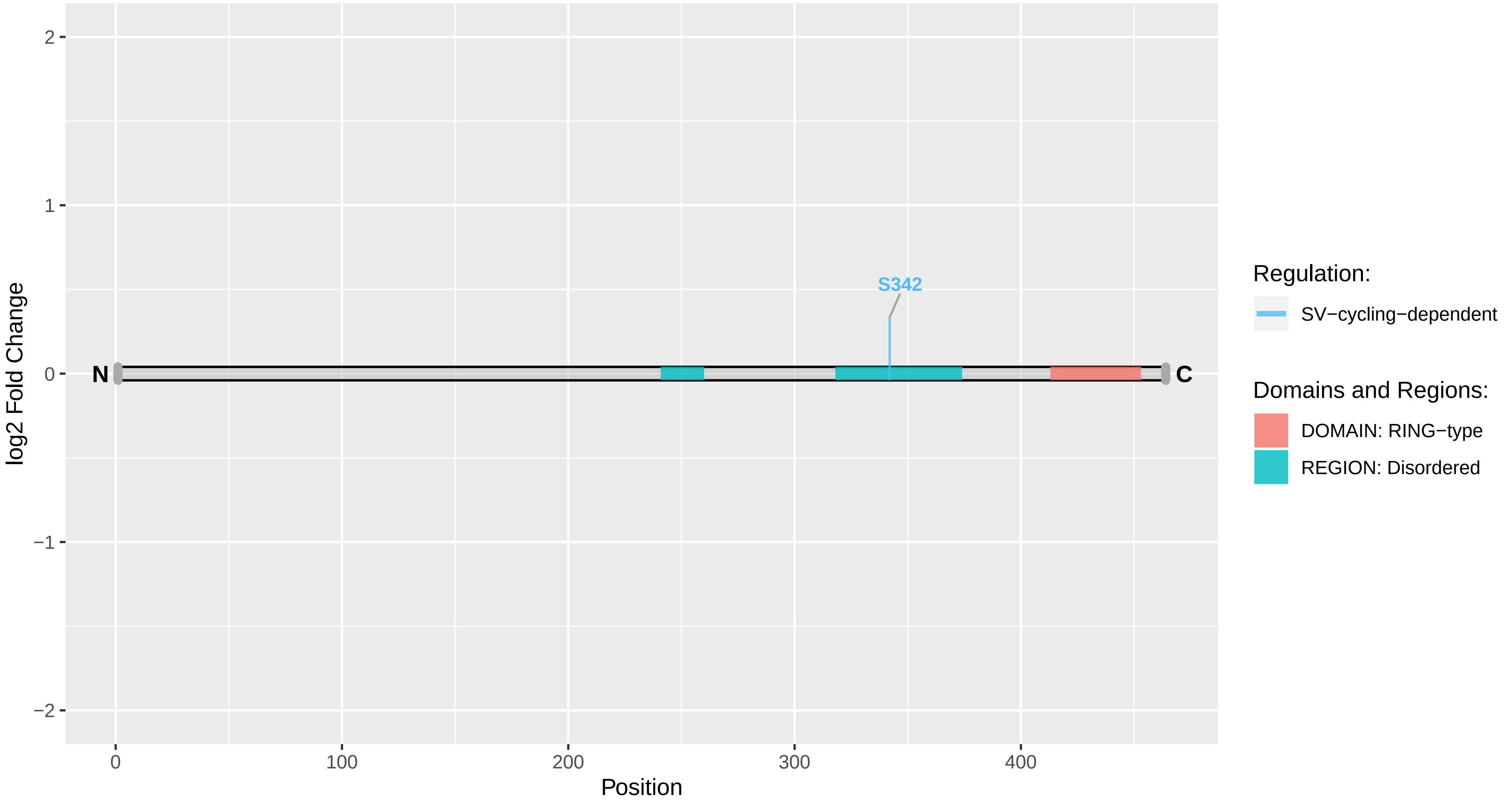

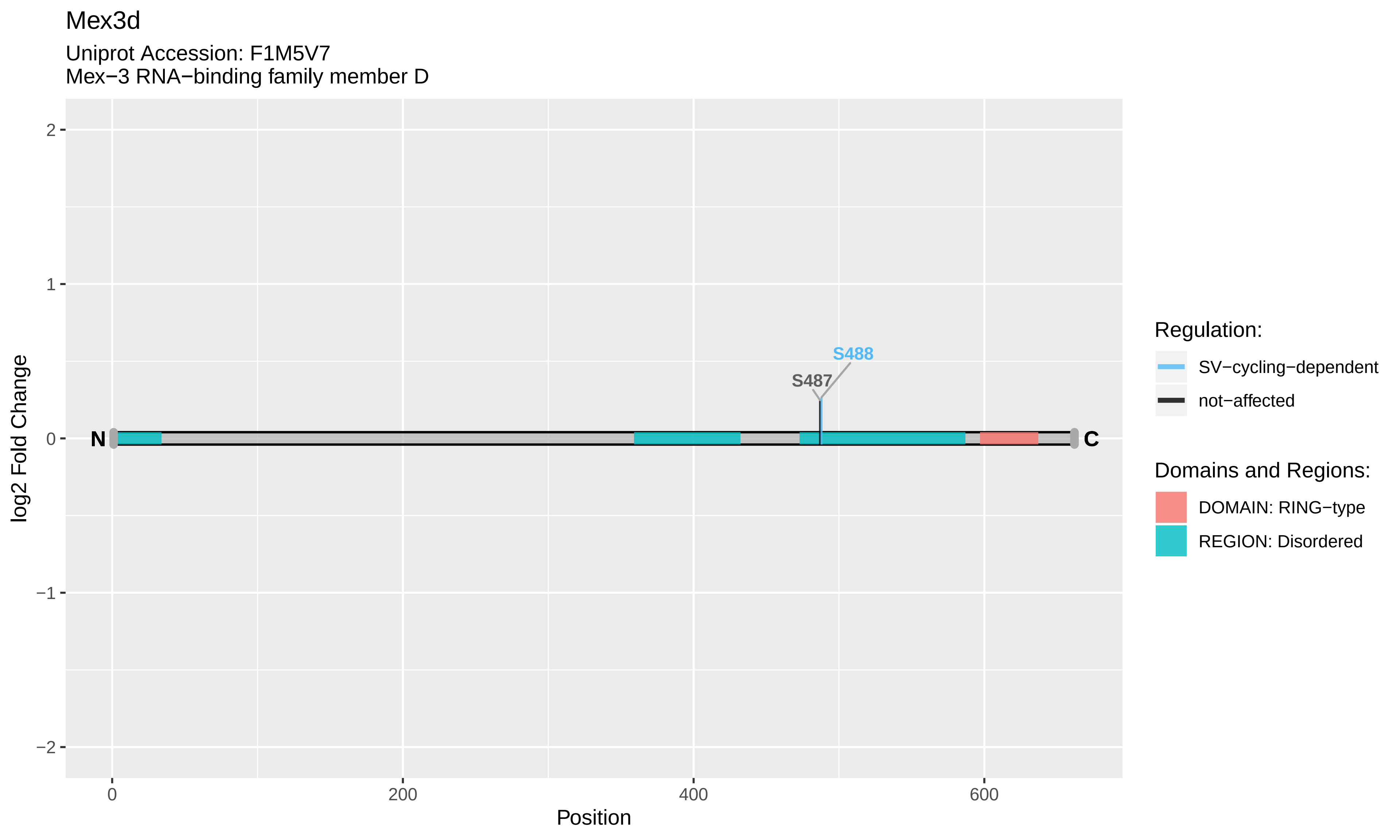

Mex3d
Uniprot Accession: F1M5V7
Mex−3 RNA−binding family member D
log2 Fold Change
2
1
0
−1
−2
N
C
S488
S487
0
200
400
600
Position
Regulation:
SV−cycling−dependent
not−affected
Domains and Regions:
DOMAIN: RING−type
REGION: Disordered

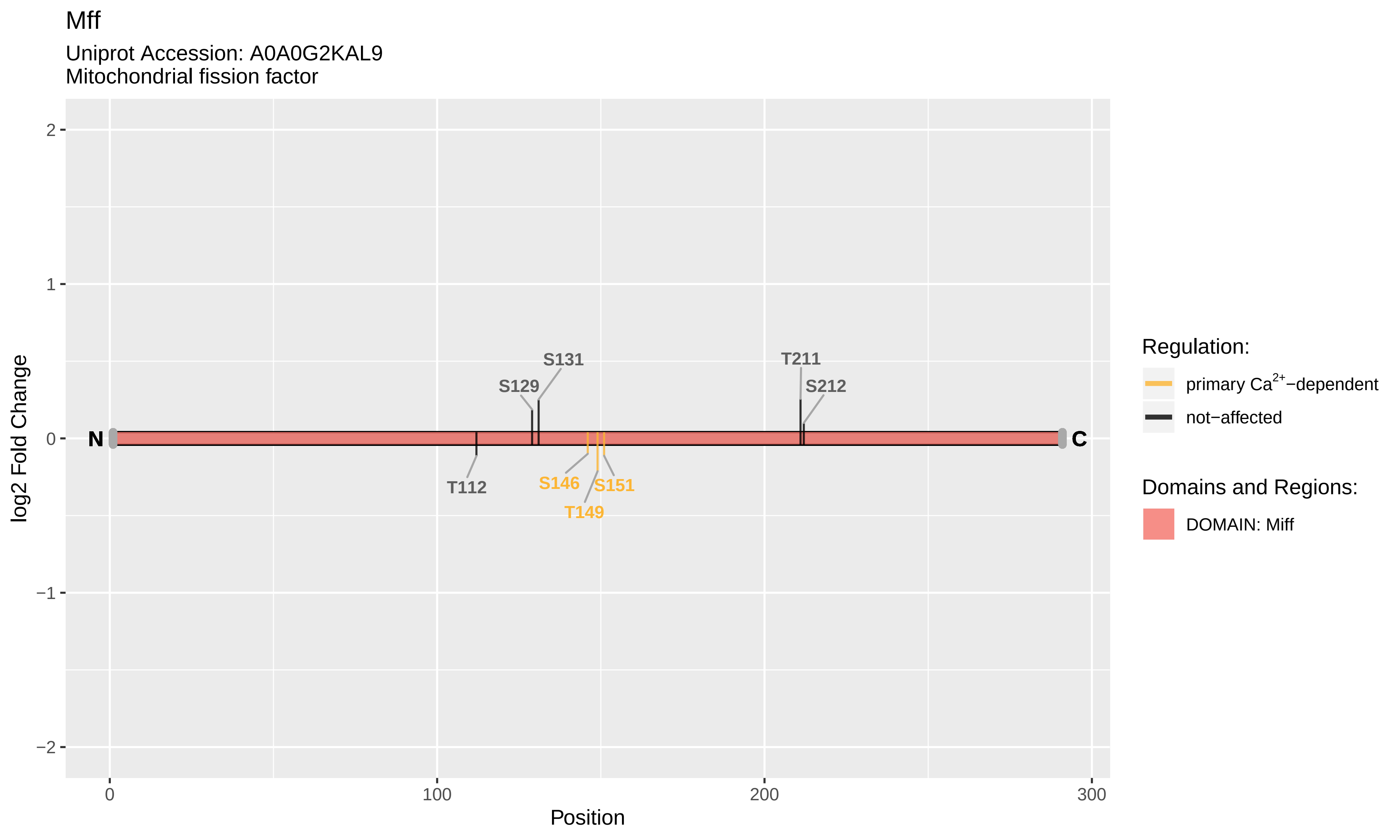
Mff
Uniprot Accession: A0A0G2KAL9
Mitochondrial fission factor
log2 Fold Change
2
1
0
−1
−2
N
C
S131
S129
T211
S212
T112
S146
S151
T149
0
100
200
300
Position
Regulation:
primary Ca2+−dependent
not−affected
Domains and Regions:
DOMAIN: Miff

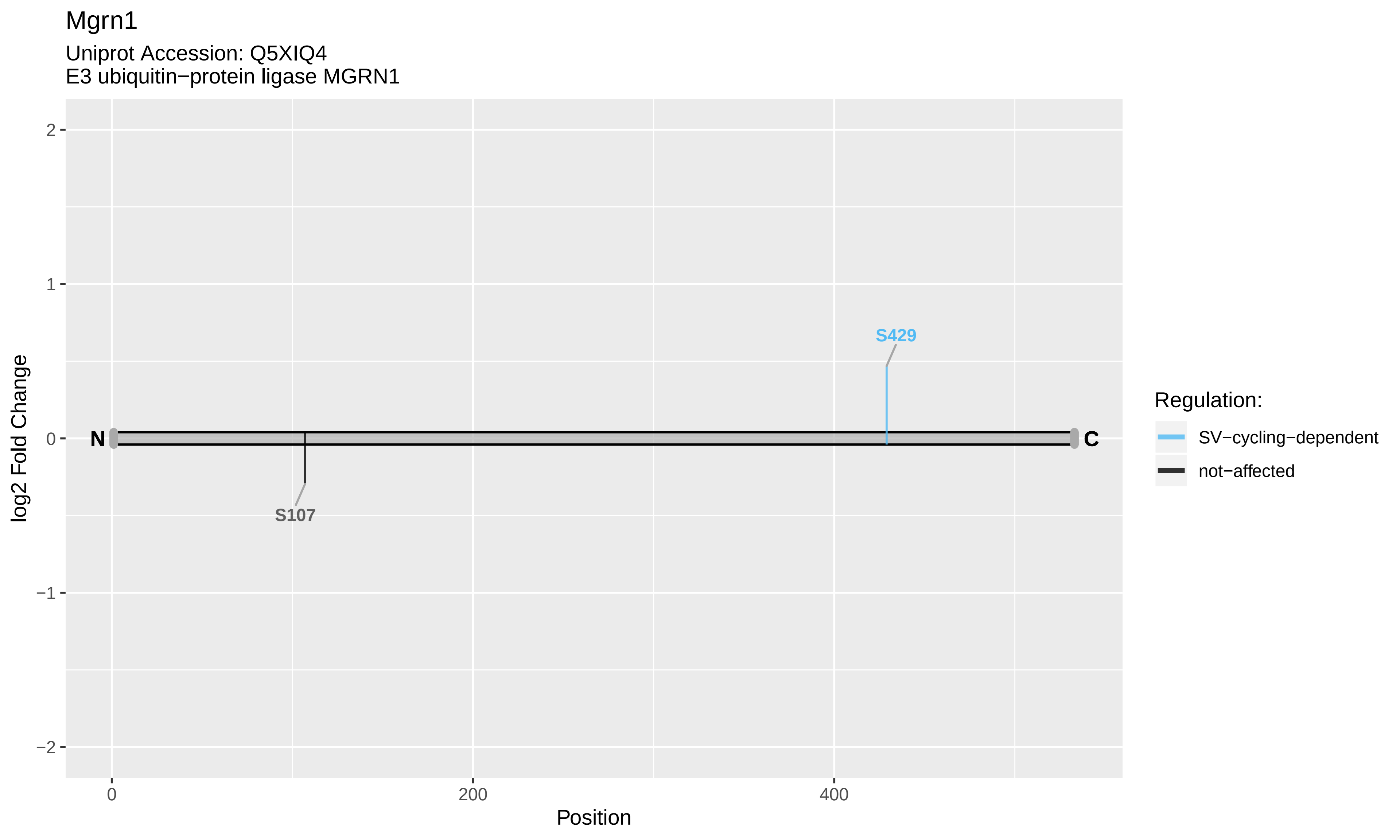
Mgrn1
Uniprot Accession: Q5XIQ4
E3 ubiquitin−protein ligase MGRN1
log2 Fold Change
2
1
0
−1
−2
N
C
S429
S107
0
200
400
Position
Regulation:
SV−cycling−dependent
not−affected

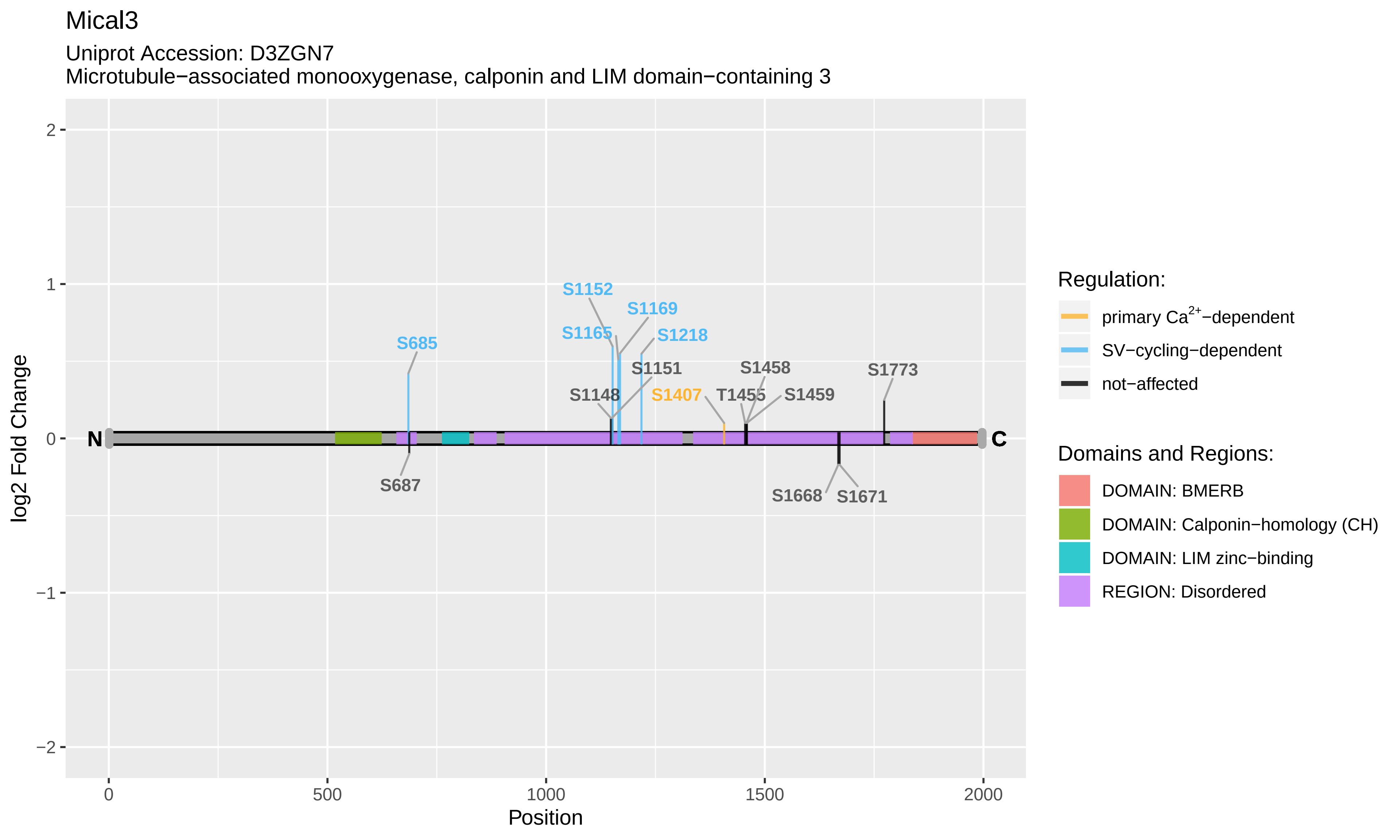

Mical3
Uniprot Accession: D3ZGN7
Microtubule−associated monooxygenase, calponin and LIM domain−containing 3
S1152
S1169
S1165
S1218
S685
S1151
S1458
S1773
S1148
S1407
T1455
S1459
N
C
S687
S1668
S1671
2
1
0
−1
−2
log2 Fold Change
0
500
1000
1500
2000
Position
Regulation:
primary Ca2+−dependent
SV−cycling−dependent
not−affected
Domains and Regions:
DOMAIN: BMERB
DOMAIN: Calponin−homology (CH)
DOMAIN: LIM zinc−binding
REGION: Disordered

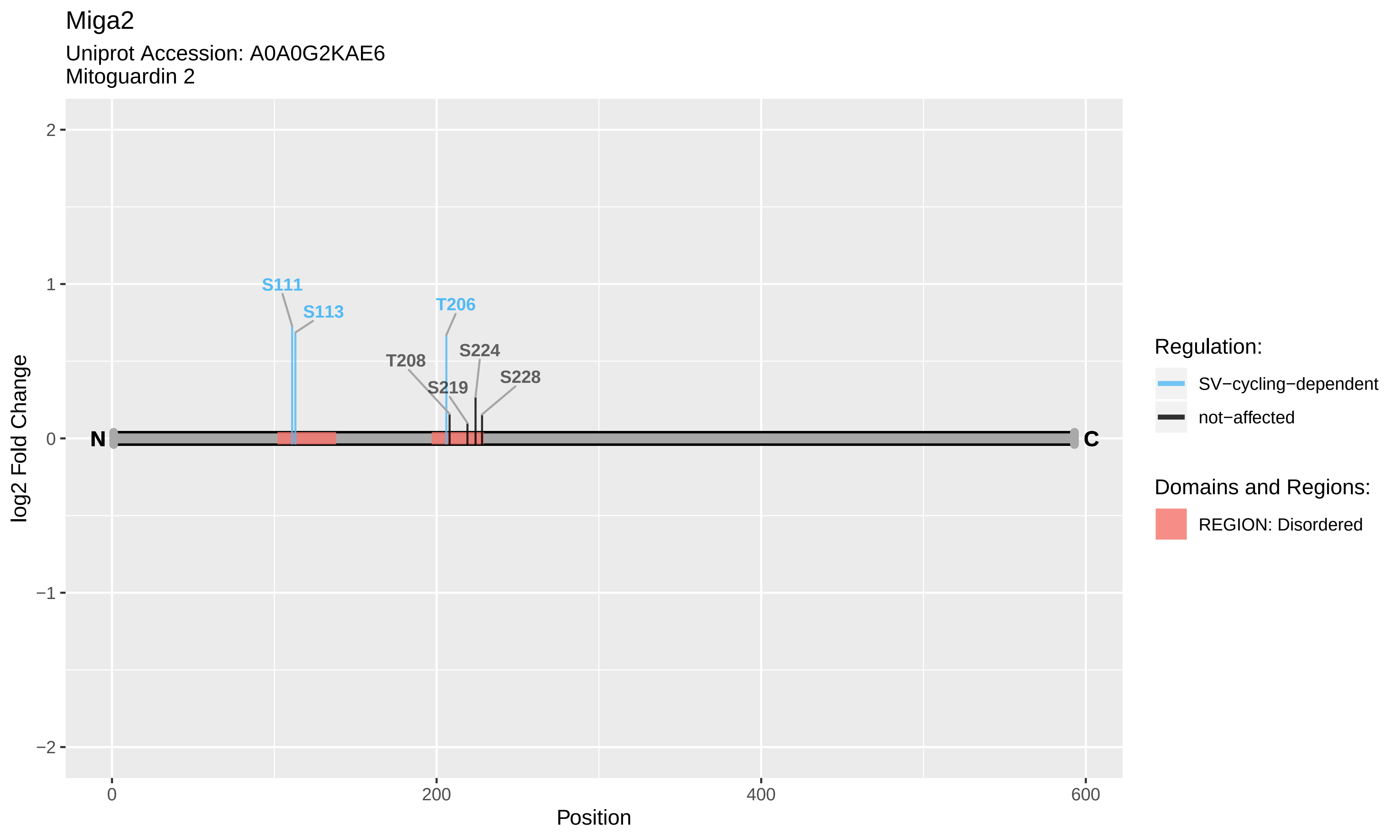
Miga2
Uniprot Accession: A0A0G2KAE6
Mitoguardin 2
S111
S113
T206
T208
S224
S219
S228
N
C
2
1
0
−1
−2
log2 Fold Change
0
200
400
600
Position
Regulation:
SV−cycling−dependent
not−affected
Domains and Regions:
REGION: Disordered

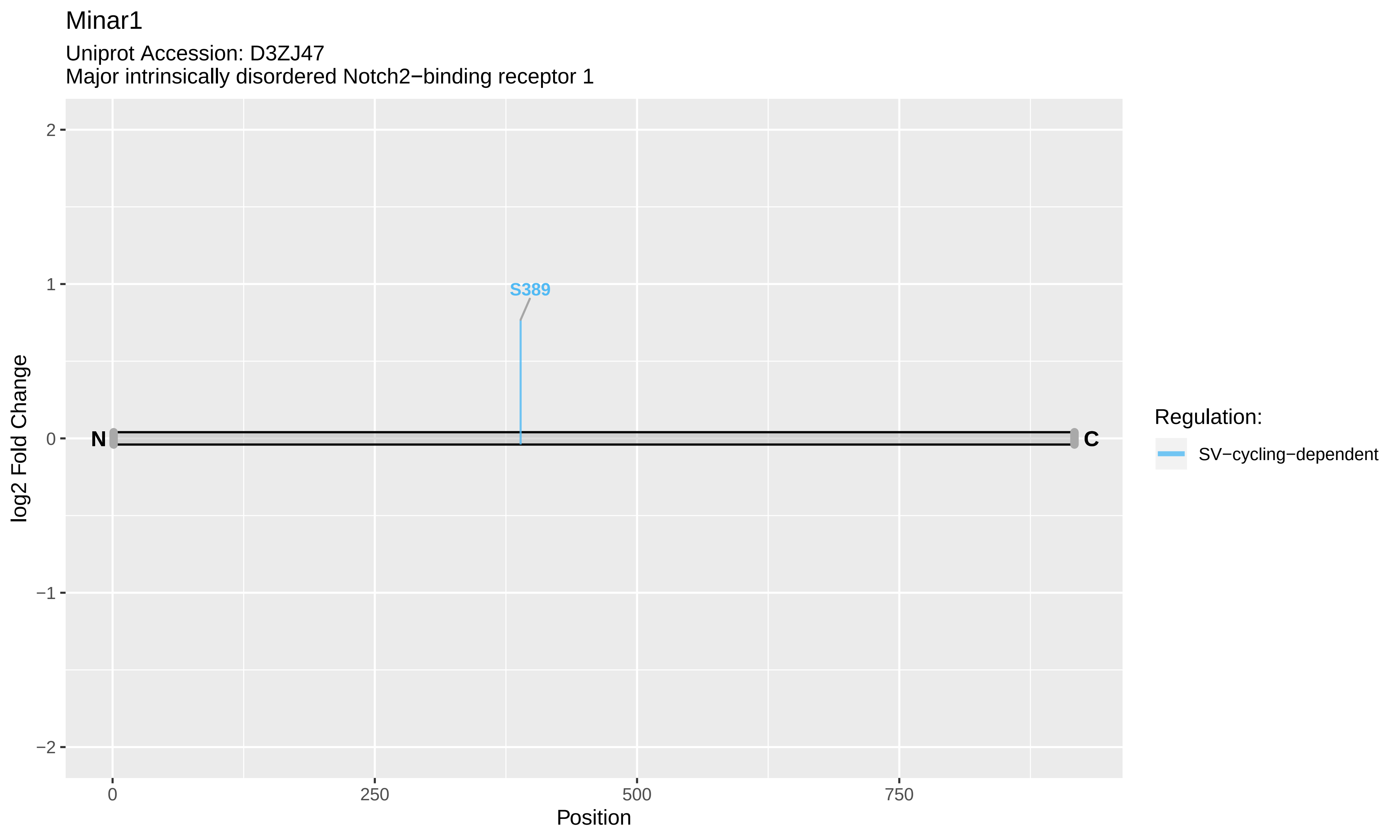
Minar1
Uniprot Accession: D3ZJ47
Major intrinsically disordered Notch2−binding receptor 1
S389
N
C
2
1
0
−1
−2
log2 Fold Change
0
250
500
750
Position
Regulation:
SV−cycling−dependent

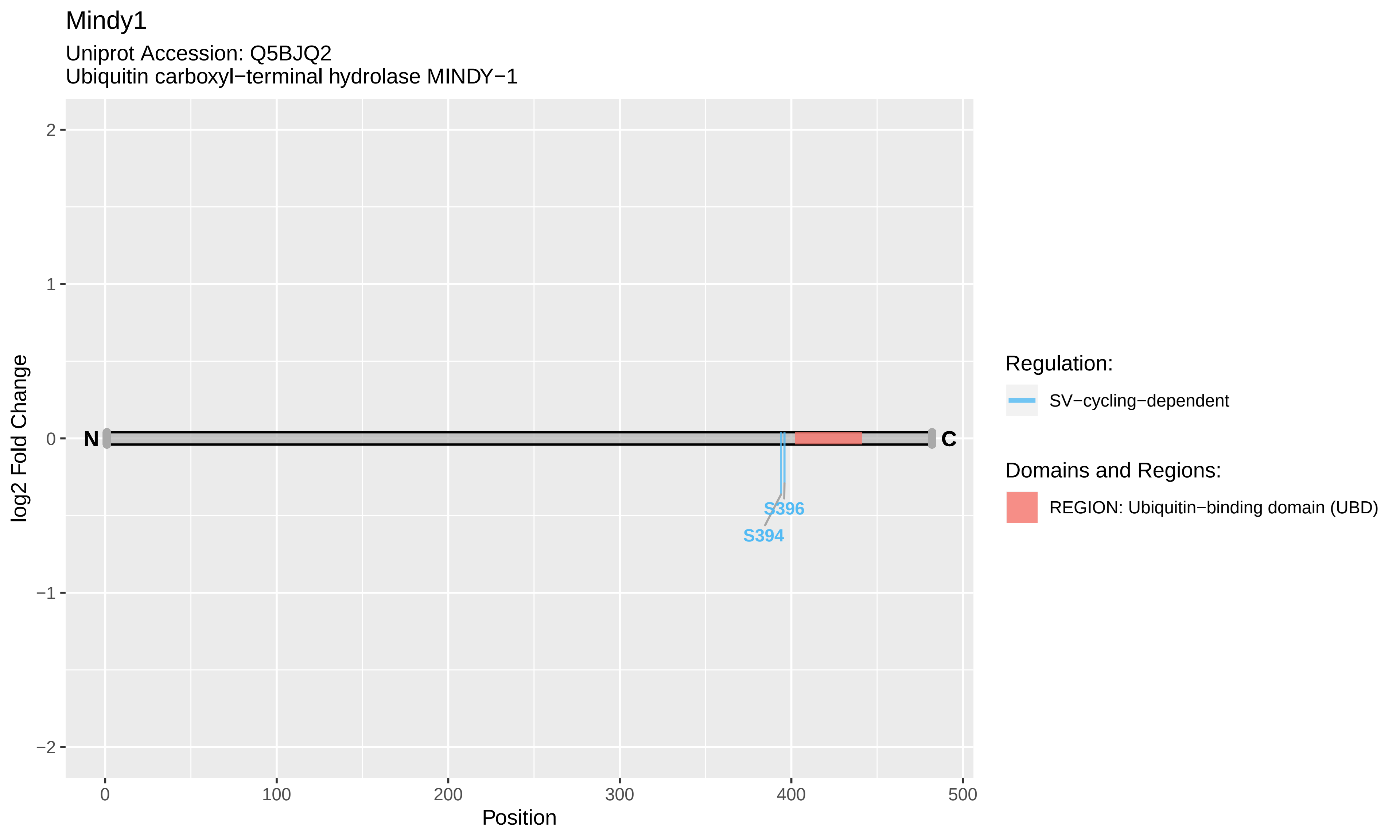

Mindy1
Uniprot Accession: Q5BJQ2
Ubiquitin carboxyl−terminal hydrolase MINDY−1
log2 Fold Change
2
1
0
−1
−2
N
C
S396
S394
0
100
200
300
400
500
Position
Regulation:
SV−cycling−dependent
Domains and Regions:
REGION: Ubiquitin−binding domain (UBD)

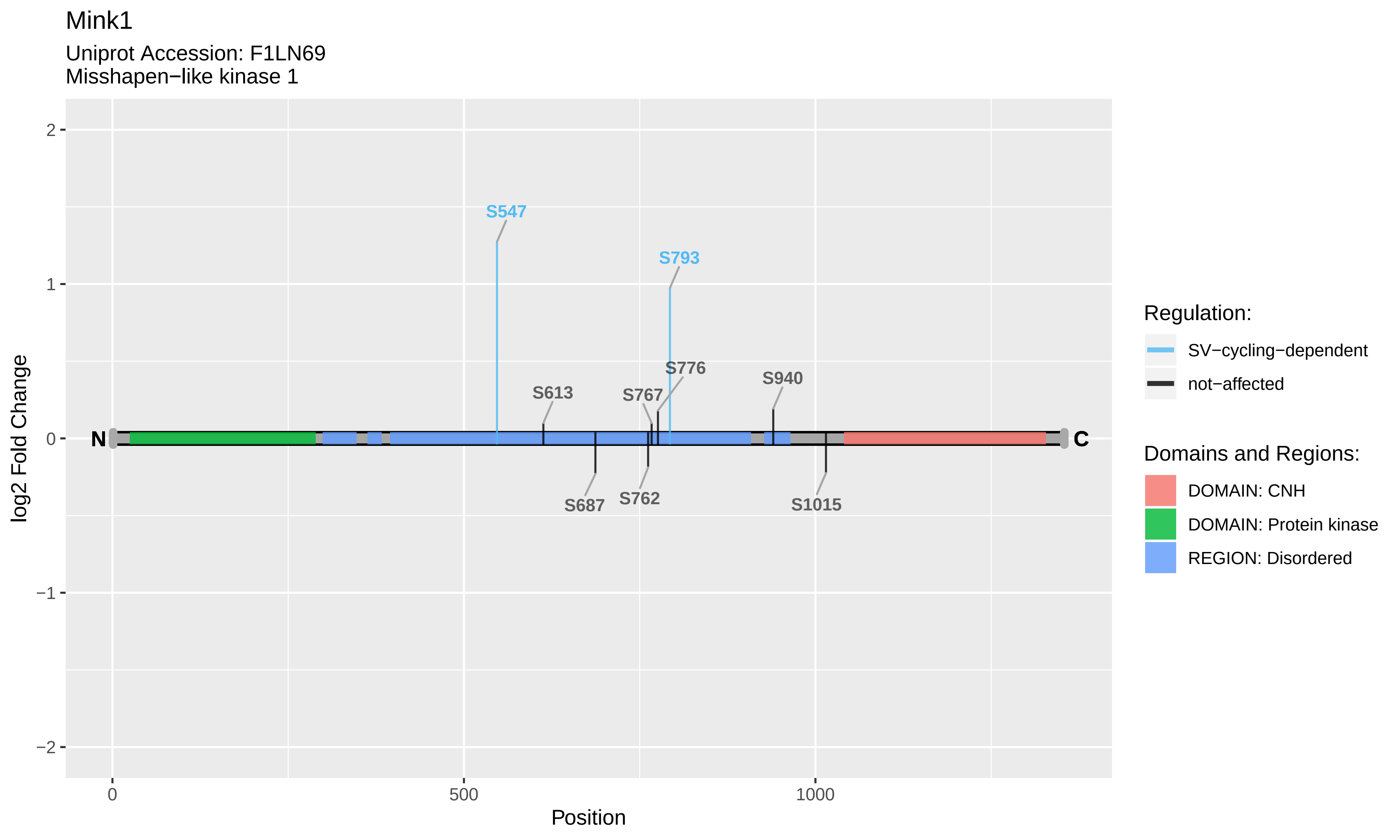

Mink1
Uniprot Accession: F1LN69
Misshapen−like kinase 1
S547
S793
S613
S767
S776
S940
S687
S762
S1015
N
C
2
1
0
−1
−2
0
500
1000
Position
log2 Fold Change
Regulation:
SV−cycling−dependent
not−affected
Domains and Regions:
DOMAIN: CNH
DOMAIN: Protein kinase
REGION: Disordered

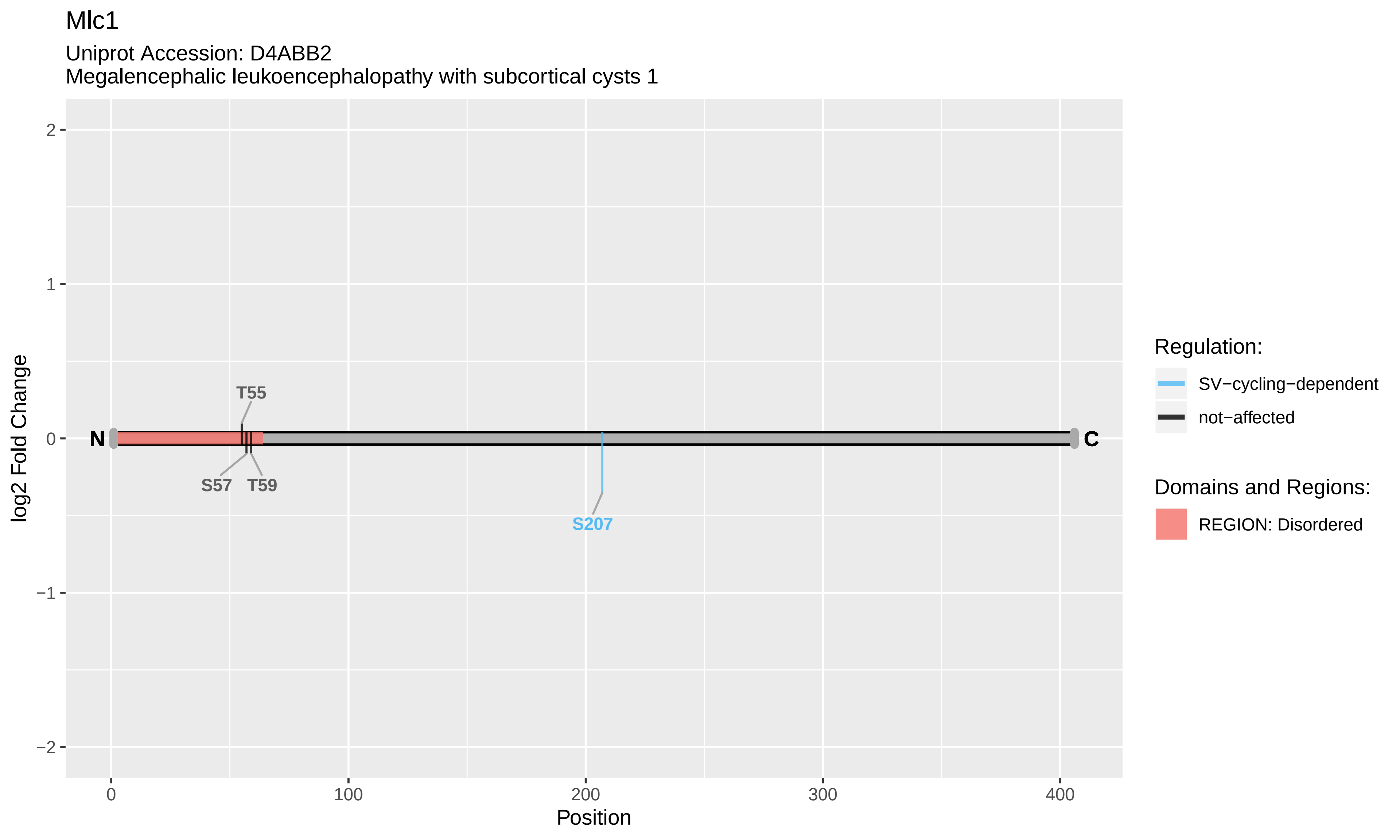

Mlc1
Uniprot Accession: D4ABB2
Megalencephalic leukoencephalopathy with subcortical cysts 1
2
1
0
−1
−2
log2 Fold Change
N
C
T55
S57
T59
S207
0
100
200
300
400
Position
Regulation:
SV−cycling−dependent
not−affected
Domains and Regions:
REGION: Disordered

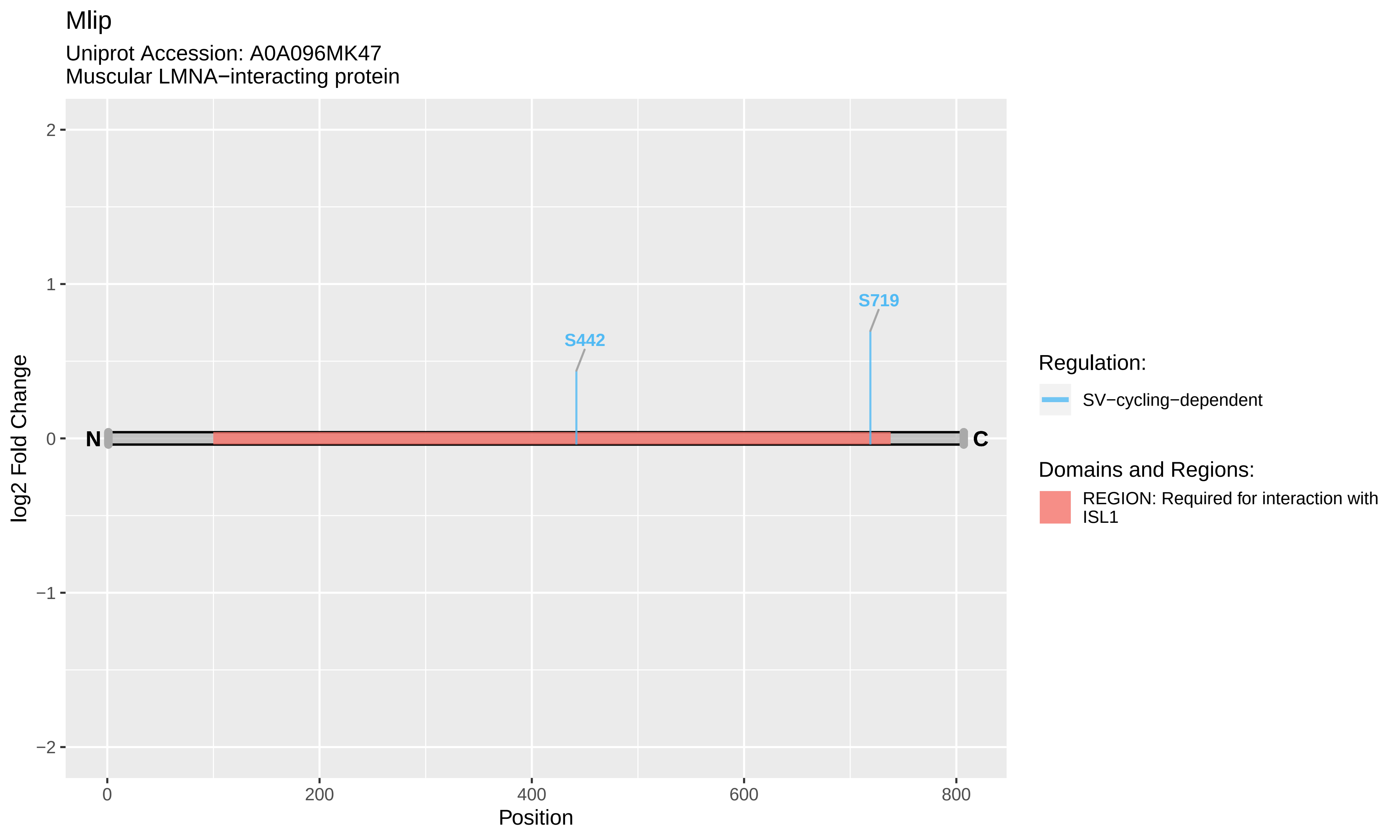

Mlip
Uniprot Accession: A0A096MK47
Muscular LMNA−interacting protein
log2 Fold Change
2
1
0
−1
−2
N
C
S442
S719
0
200
400
600
800
Position
Regulation:
SV−cycling−dependent
Domains and Regions:
REGION: Required for interaction with ISL1

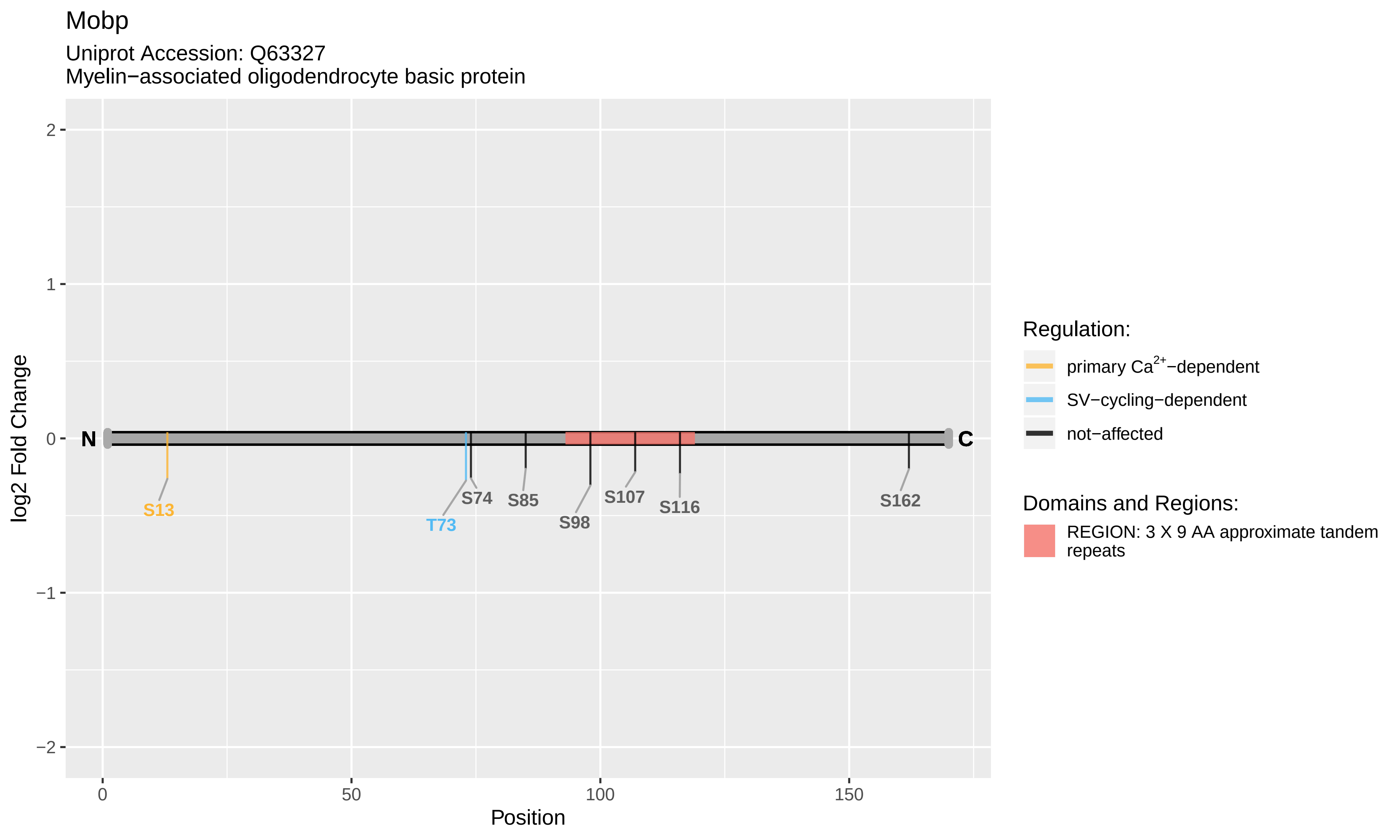

Mobp
Uniprot Accession: Q63327
Myelin−associated oligodendrocyte basic protein
log2 Fold Change
Position
N
C
S13
T73
S74
S85
S98
S107
S116
S162
2
1
0
−1
−2
0
50
100
150
Regulation:
primary Ca2+−dependent
SV−cycling−dependent
not−affected
Domains and Regions:
REGION: 3 X 9 AA approximate tandem repeats

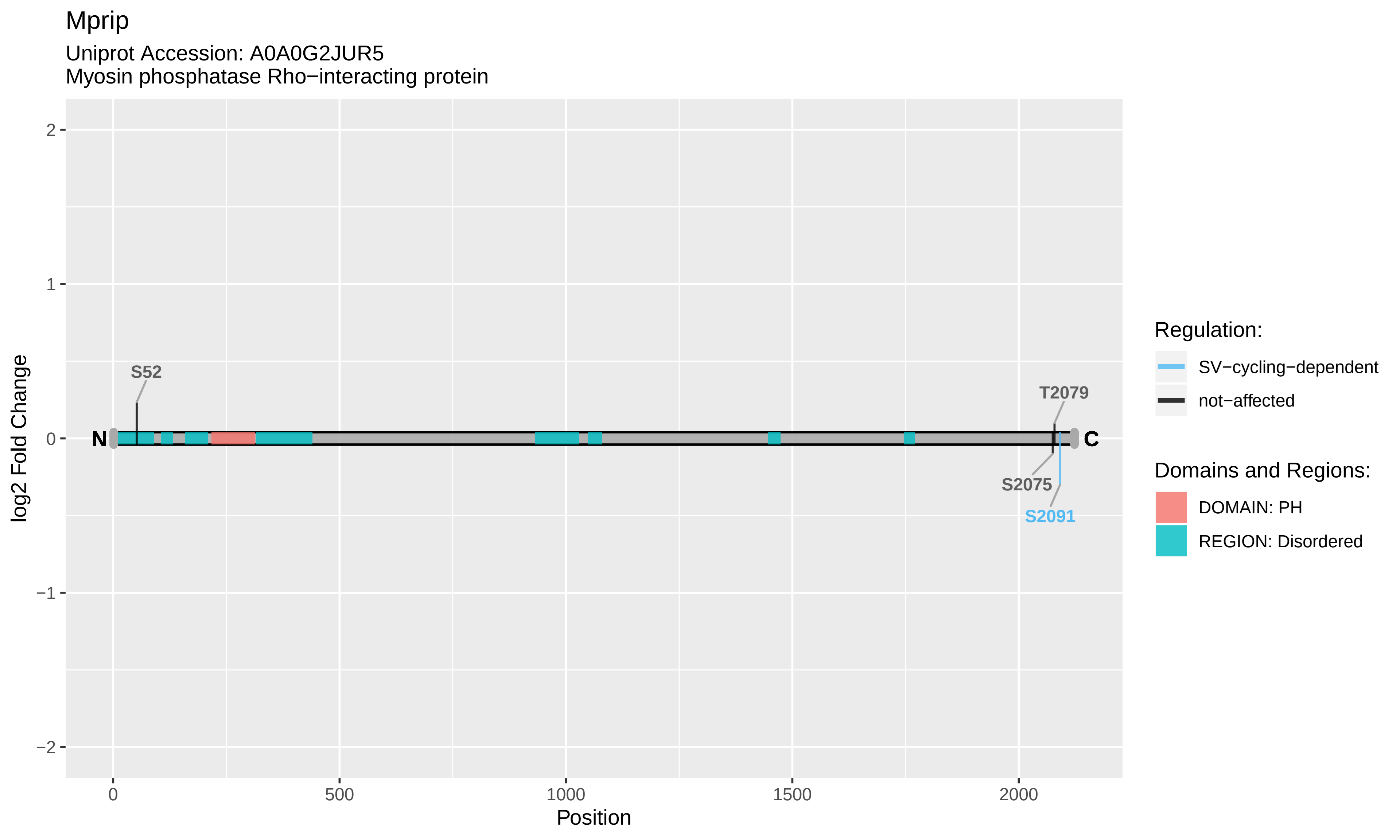

Mprip
Uniprot Accession: A0A0G2JUR5
Myosin phosphatase Rho−interacting protein
log2 Fold Change
2
1
0
−1
−2
S52
T2079
S2075
S2091
N
C
0
500
1000
1500
2000
Position
Regulation:
SV−cycling−dependent
not−affected
Domains and Regions:
DOMAIN: PH
REGION: Disordered

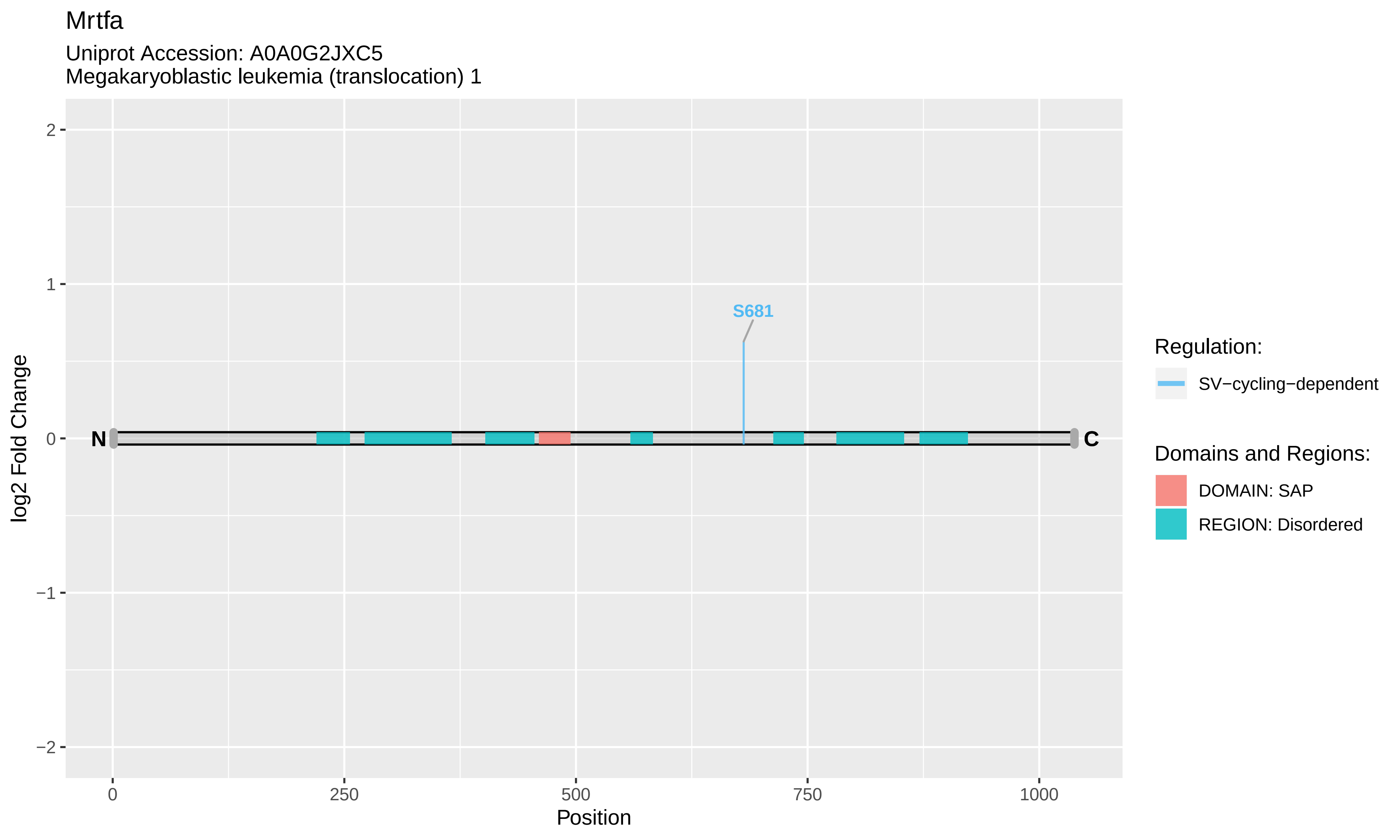

Mrtfa
Uniprot Accession: A0A0G2JXC5
Megakaryoblastic leukemia (translocation) 1
S681
N
C
log2 Fold Change
2
1
0
−1
−2
0
250
500
750
1000
Position
Regulation:
SV−cycling−dependent
Domains and Regions:
DOMAIN: SAP
REGION: Disordered

Mtch1

Uniprot Accession: B0BN30
Mitochondrial carrier 1

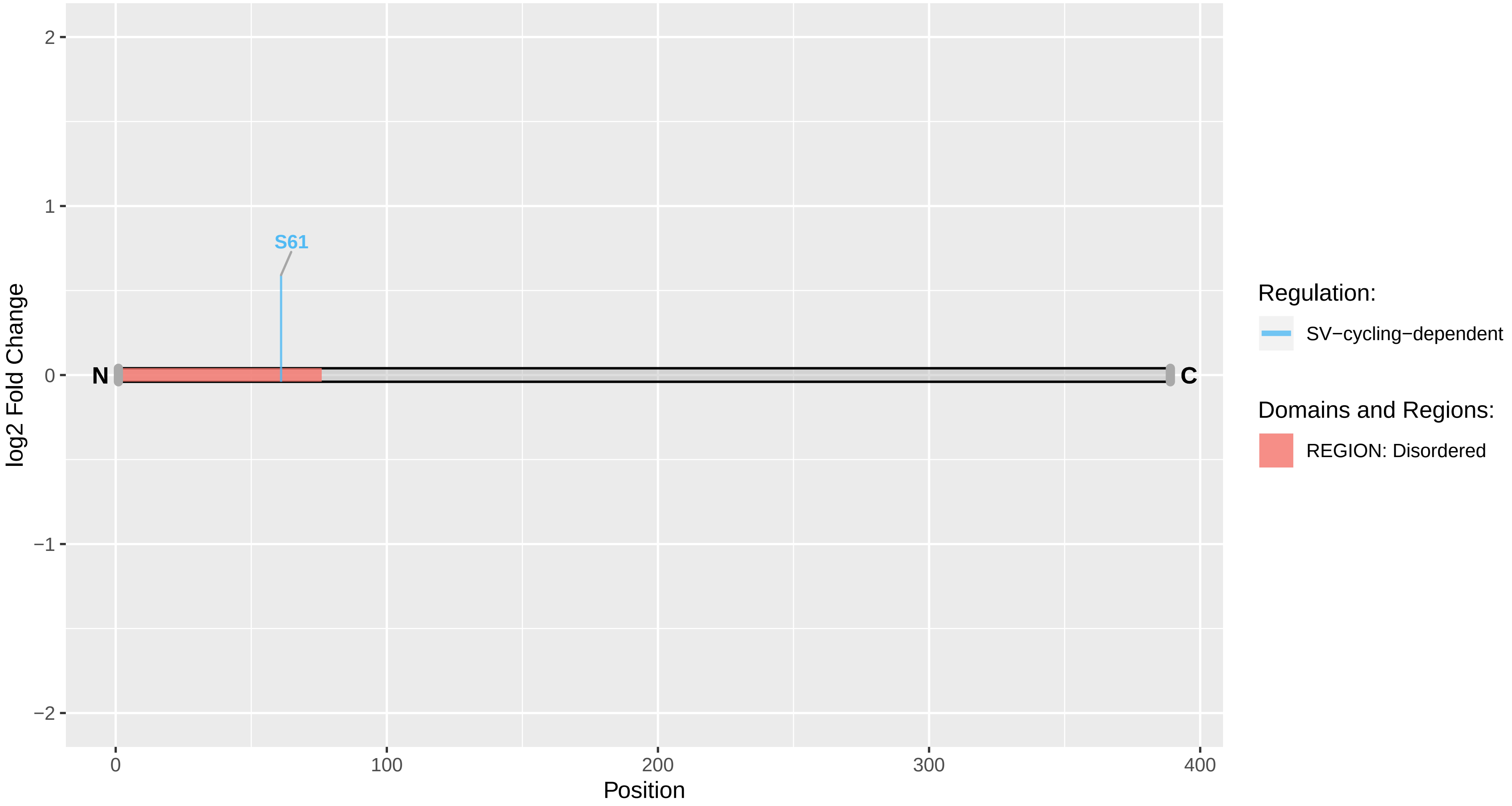

Mtcl1

Uniprot Accession: D4A5D4
Microtubule crosslinking factor 1

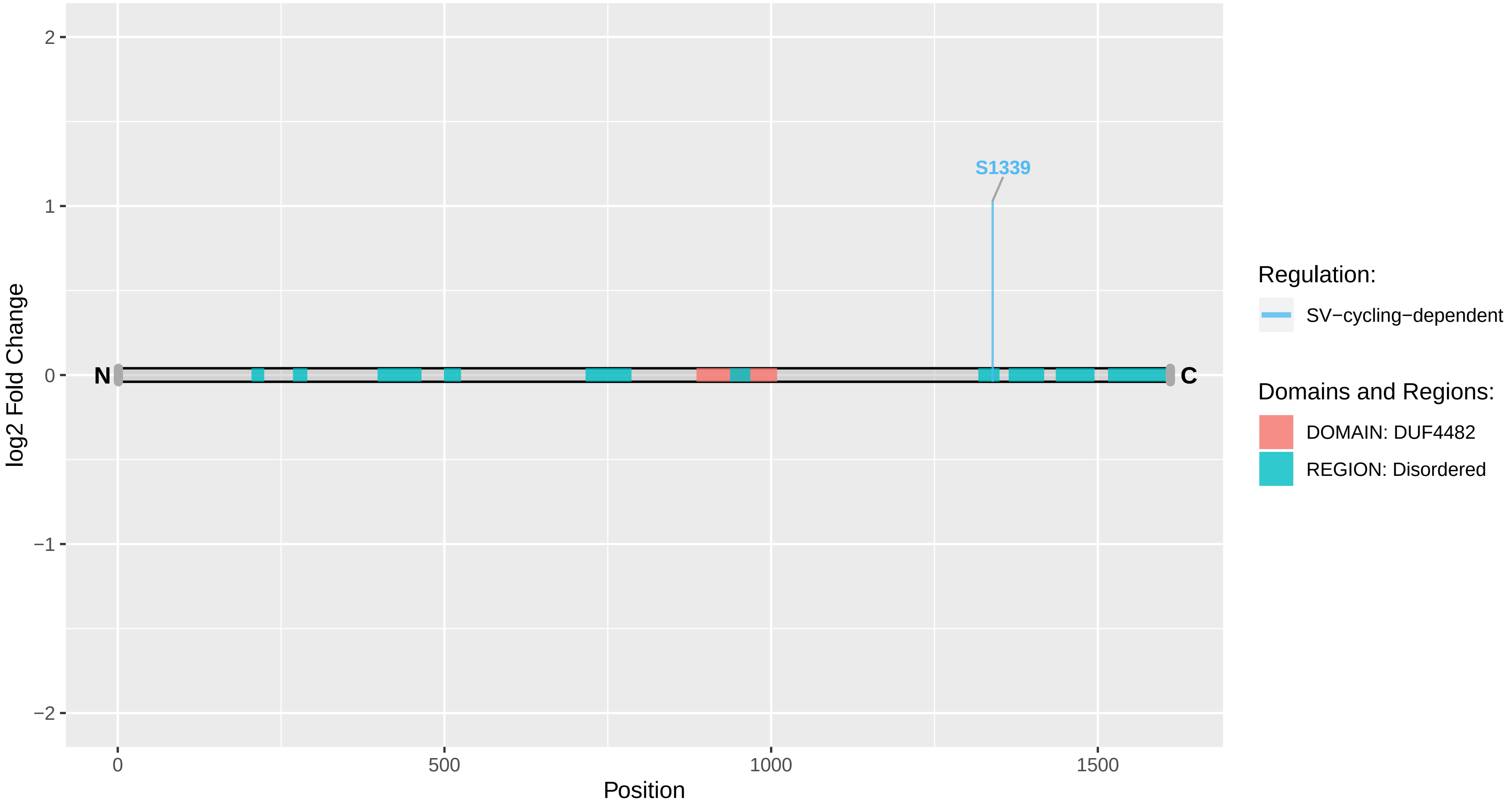

Mtss1

Uniprot Accession: D3ZYM5
MTSS1, I−BAR domain−containing

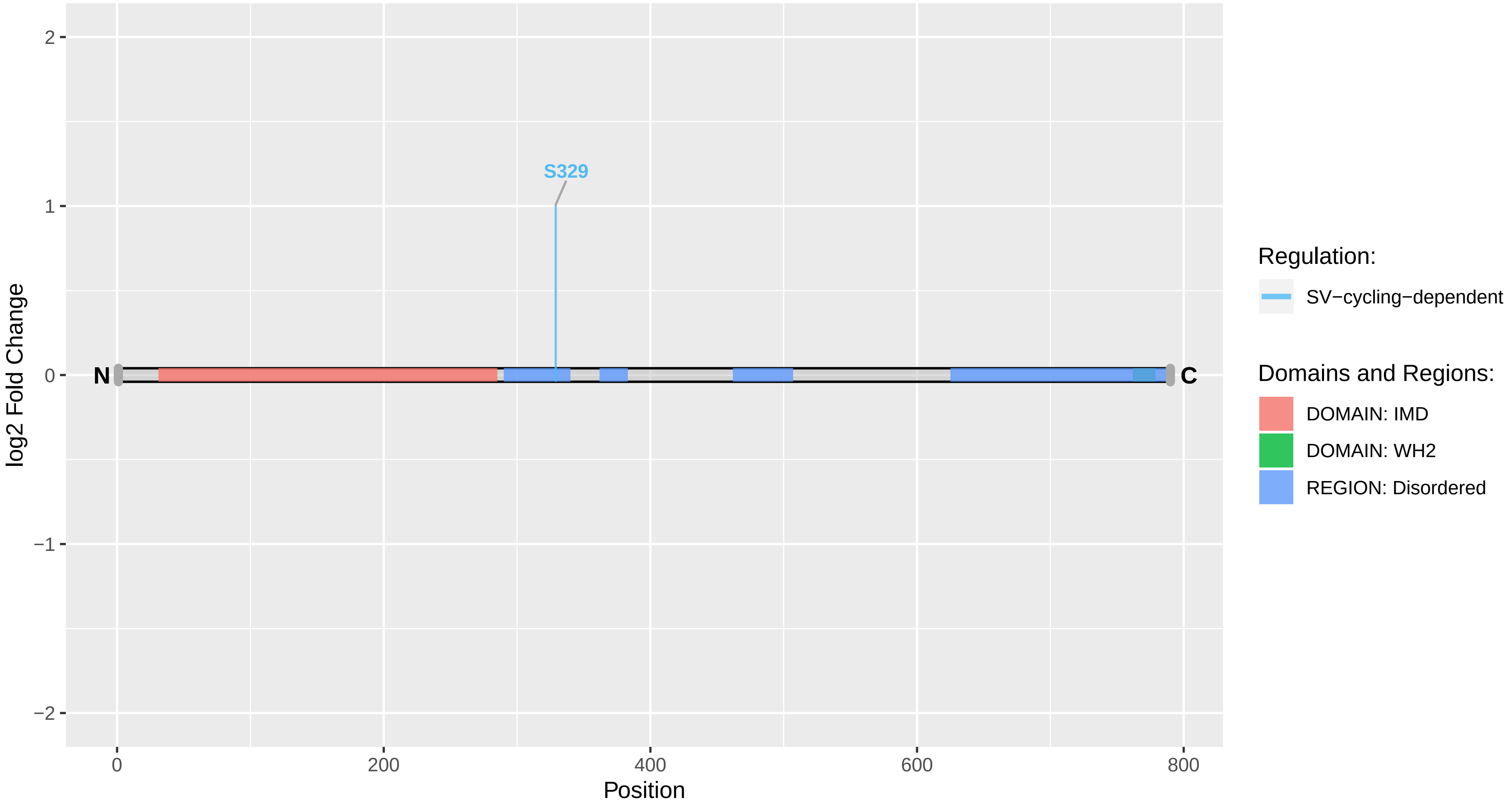

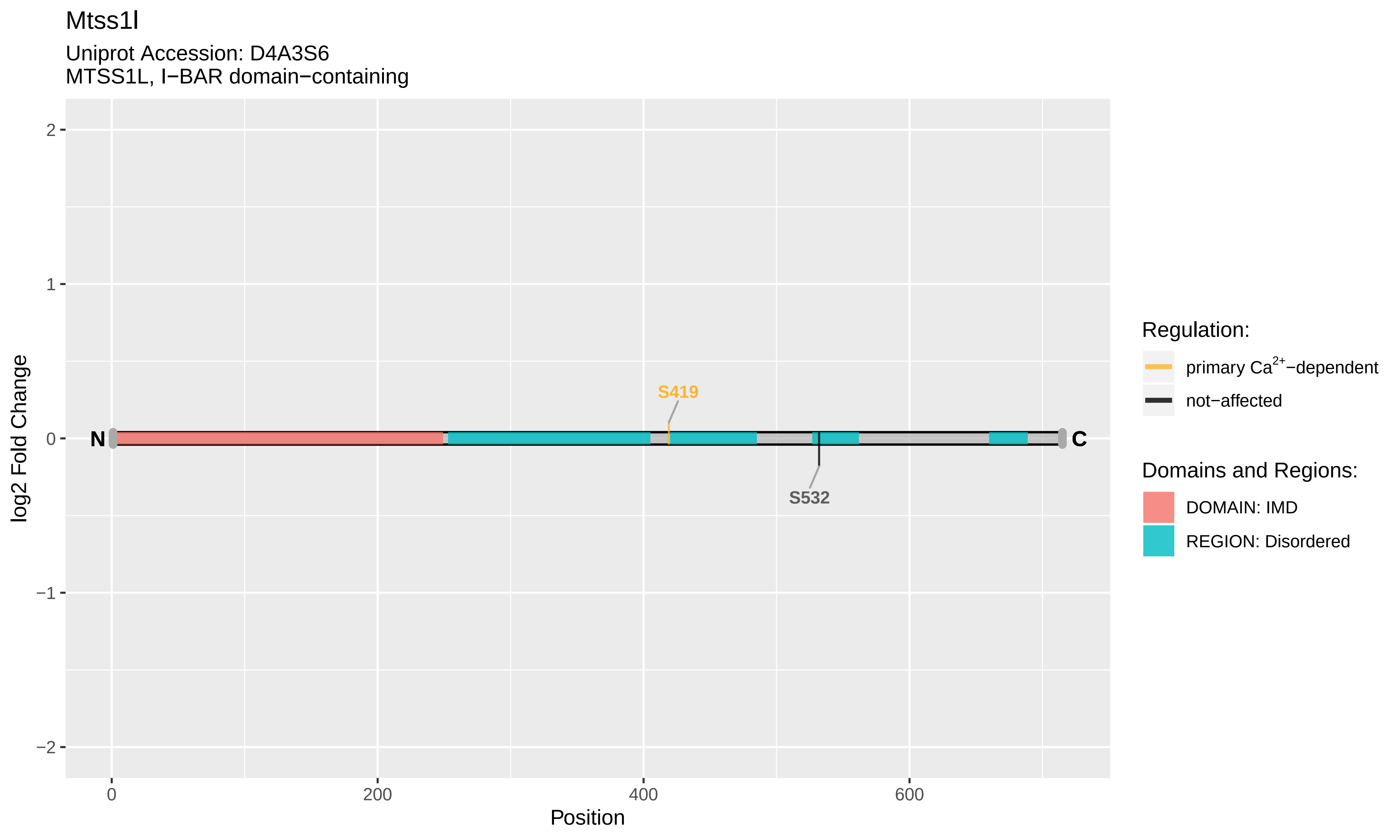

Mtss1l
Uniprot Accession: D4A3S6
MTSS1L, I−BAR domain−containing
log2 Fold Change
2
1
0
−1
−2
N
C
S419
S532
0
200
400
600
Position
Regulation:
primary Ca2+−dependent
not−affected
Domains and Regions:
DOMAIN: IMD
REGION: Disordered

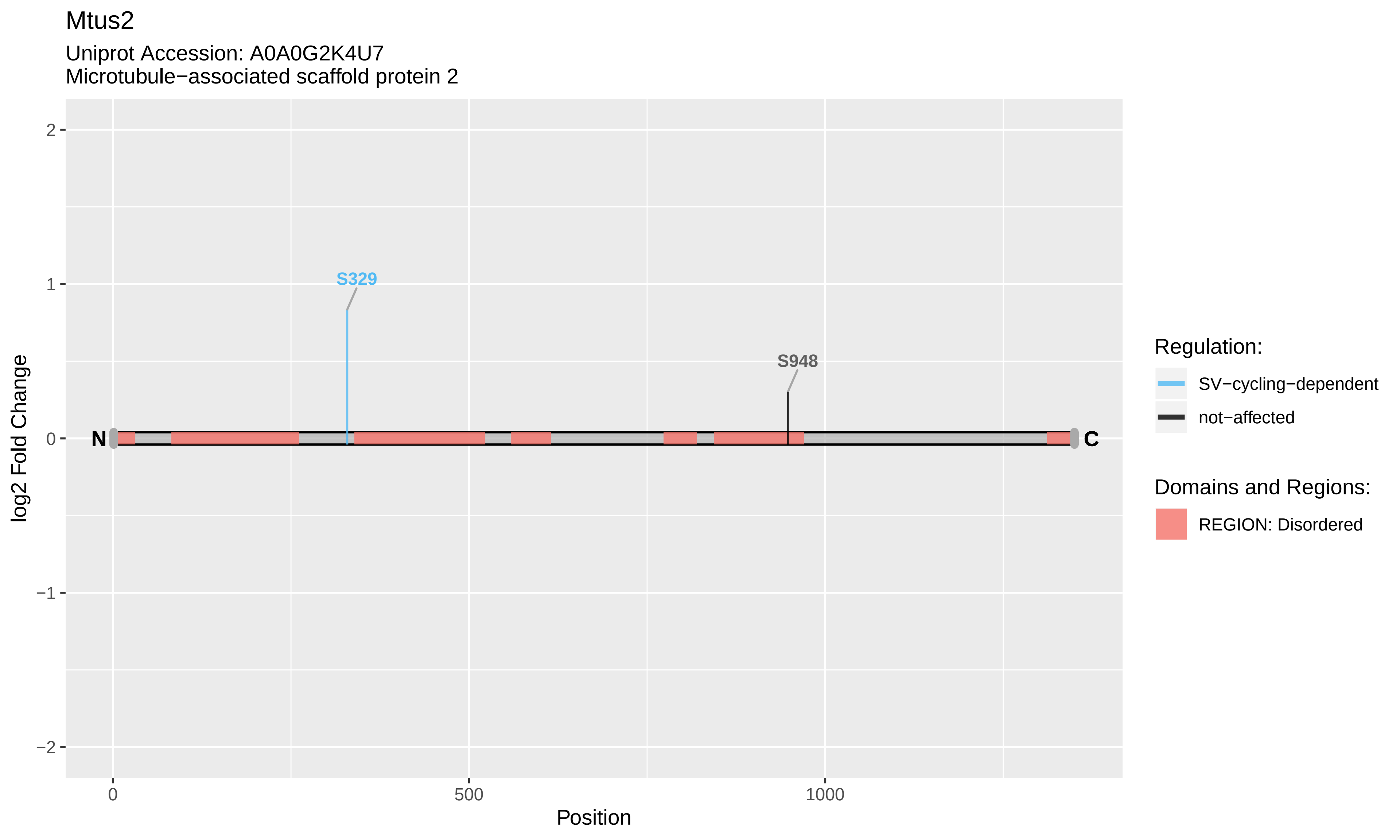

Mtus2
Uniprot Accession: A0A0G2K4U7
Microtubule−associated scaffold protein 2
S329
S948
N
C
2
1
0
−1
−2
log2 Fold Change
0
500
1000
Position
Regulation:
SV−cycling−dependent
not−affected
Domains and Regions:
REGION: Disordered

Mtx3

Uniprot Accession: D3ZNK1
Metaxin 3

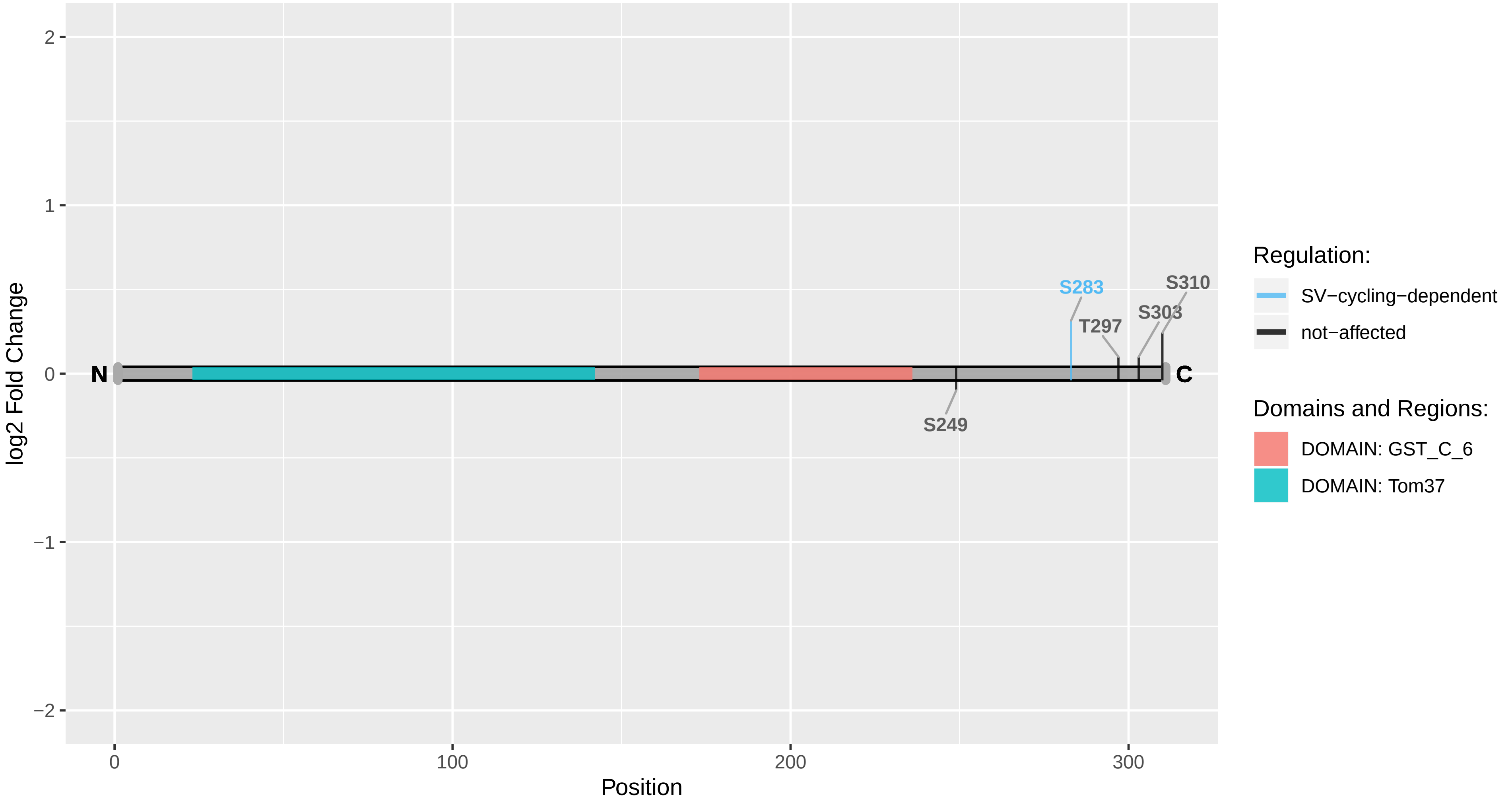

Mycbp2

Uniprot Accession: D4A2D3
MYC−binding protein 2, E3 ubiquitin protein ligase

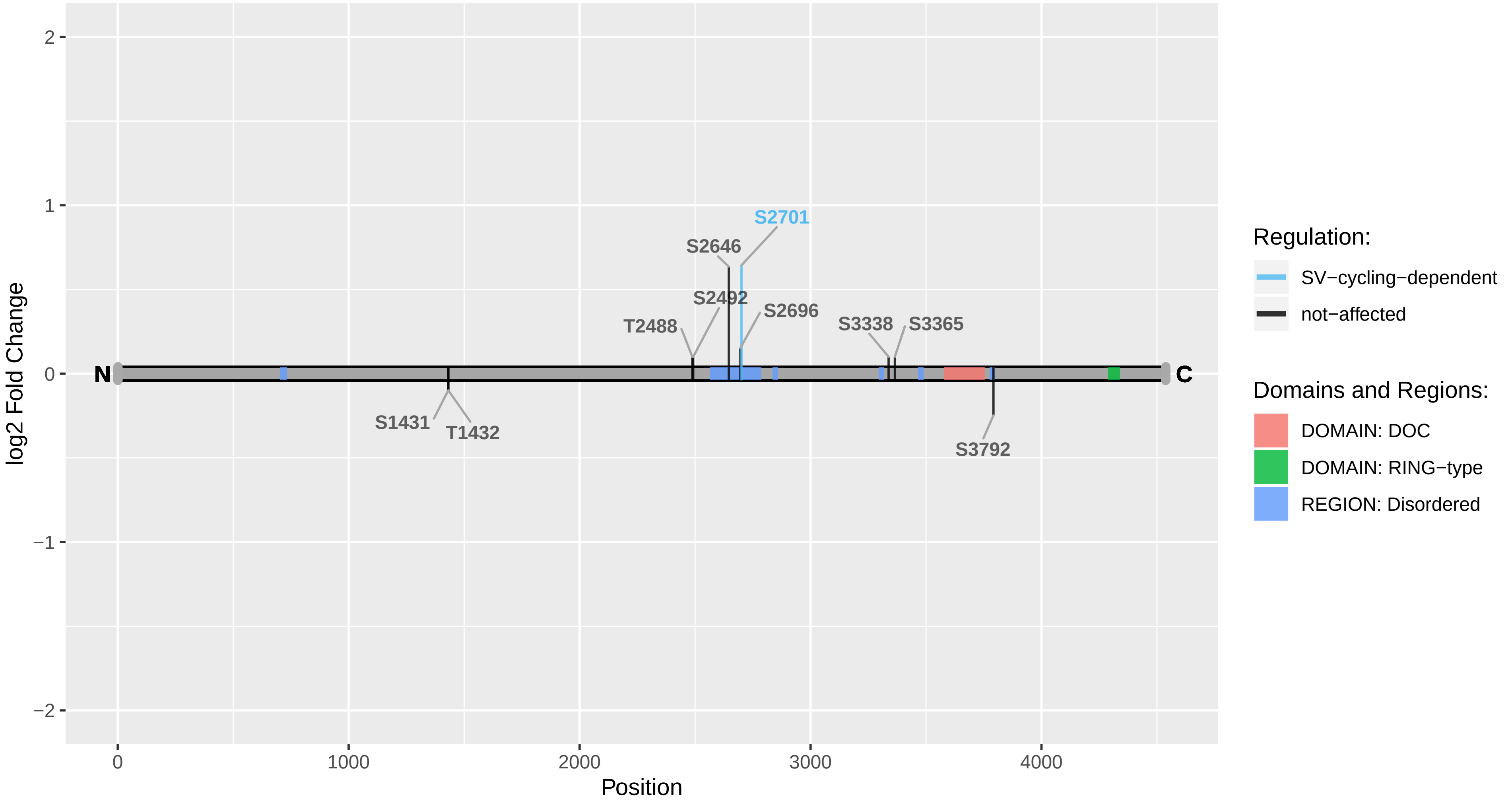

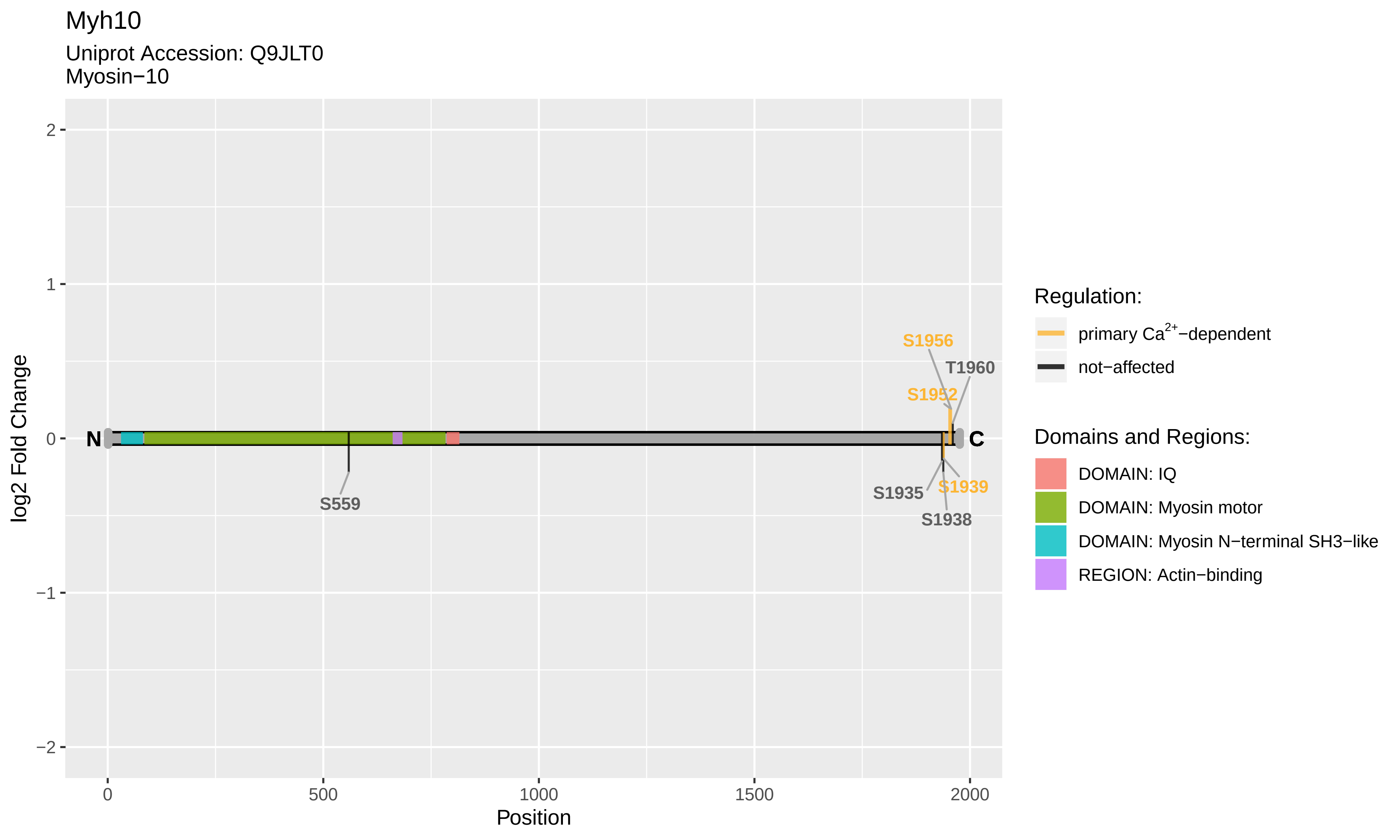
Myh10
Uniprot Accession: Q9JLT0
Myosin−10
log2 Fold Change
Position
2
1
0
−1
−2
0
500
1000
1500
2000
N
C
S559
S1956
T1960
S1952
S1935
S1939
S1938
Regulation:
primary Ca2+−dependent
not−affected
Domains and Regions:
DOMAIN: IQ
DOMAIN: Myosin motor
DOMAIN: Myosin N−terminal SH3−like
REGION: Actin−binding

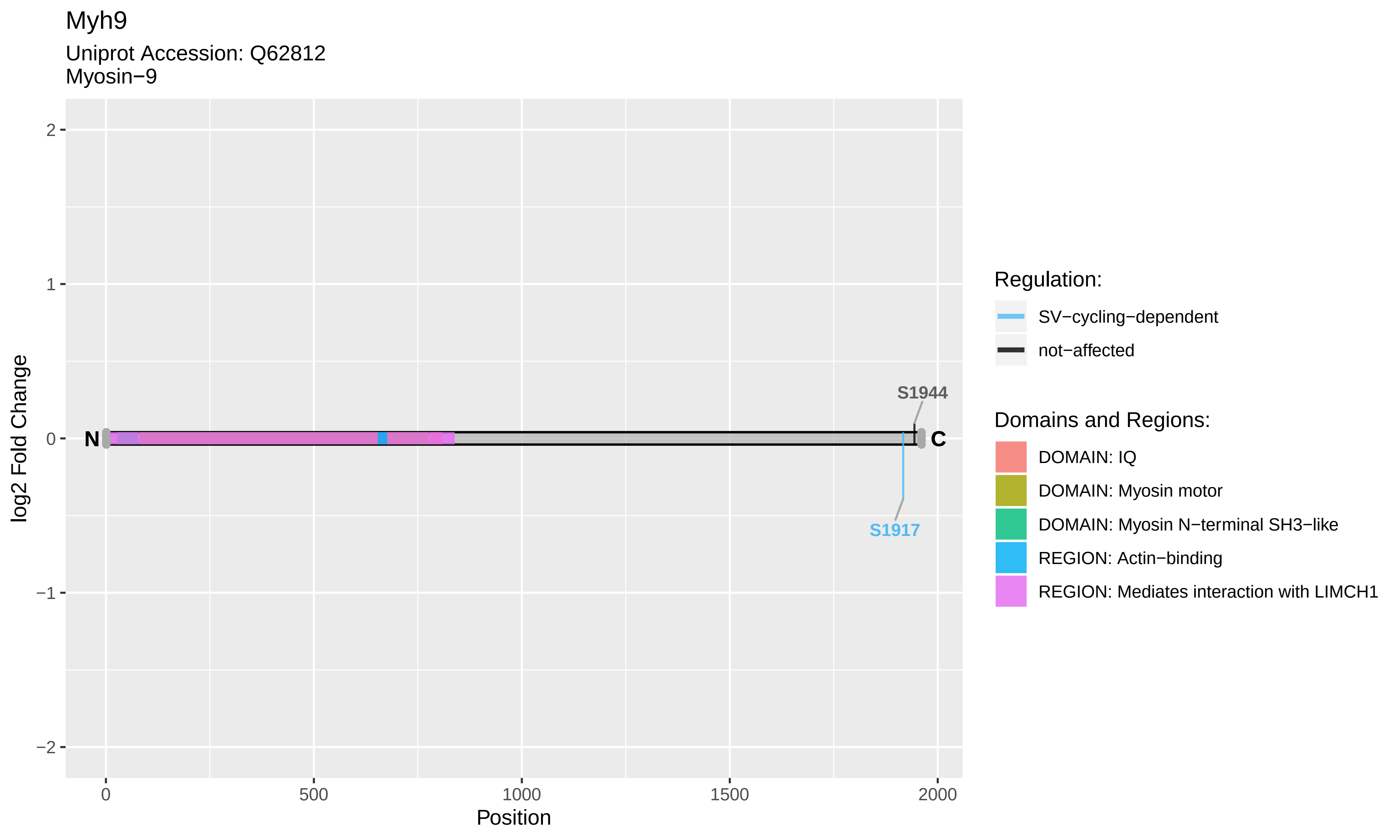

Myh9
Uniprot Accession: Q62812
Myosin−9
log2 Fold Change
2
1
0
−1
−2
N
C
S1944
S1917
0
500
1000
1500
2000
Position
Regulation:
SV−cycling−dependent
not−affected
Domains and Regions:
DOMAIN: IQ
DOMAIN: Myosin motor
DOMAIN: Myosin N−terminal SH3−like
REGION: Actin−binding
REGION: Mediates interaction with LIMCH1

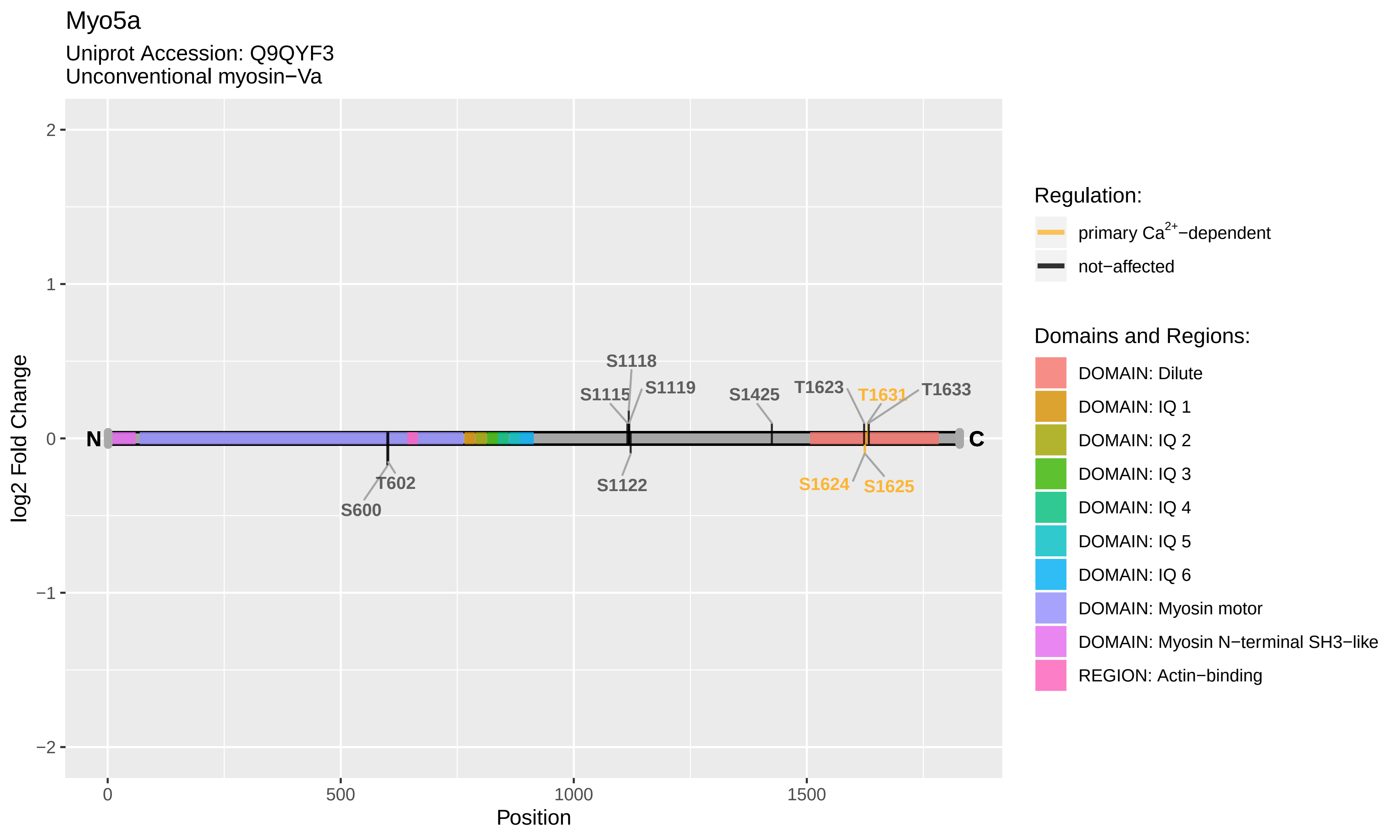
Myo5a
Uniprot Accession: Q9QYF3
Unconventional myosin−Va
log2 Fold Change
Position
N
C
S600
T602
S1115
S1118
S1119
S1122
S1425
T1623
S1624
S1625
T1631
T1633
Regulation:
primary Ca2+−dependent
not−affected
Domains and Regions:
DOMAIN: Dilute
DOMAIN: IQ 1
DOMAIN: IQ 2
DOMAIN: IQ 3
DOMAIN: IQ 4
DOMAIN: IQ 5
DOMAIN: IQ 6
DOMAIN: Myosin motor
DOMAIN: Myosin N−terminal SH3−like
REGION: Actin−binding

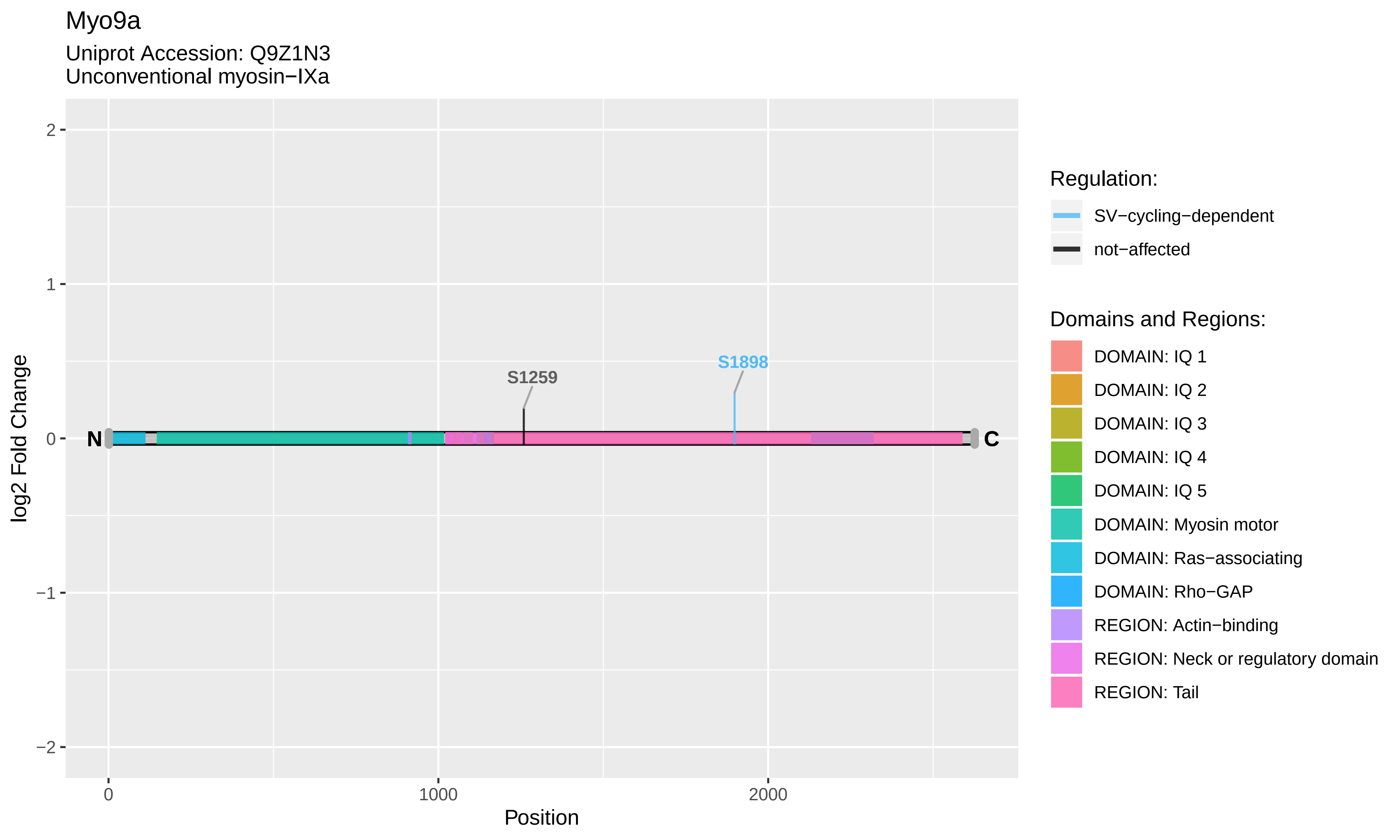

Myo9a
Uniprot Accession: Q9Z1N3
Unconventional myosin−IXa
log2 Fold Change
2
1
0
−1
−2
0
1000
2000
Position
N
C
S1259
S1898
Regulation:
SV−cycling−dependent
not−affected
Domains and Regions:
DOMAIN: IQ 1
DOMAIN: IQ 2
DOMAIN: IQ 3
DOMAIN: IQ 4
DOMAIN: IQ 5
DOMAIN: Myosin motor
DOMAIN: Ras−associating
DOMAIN: Rho−GAP
REGION: Actin−binding
REGION: Neck or regulatory domain
REGION: Tail

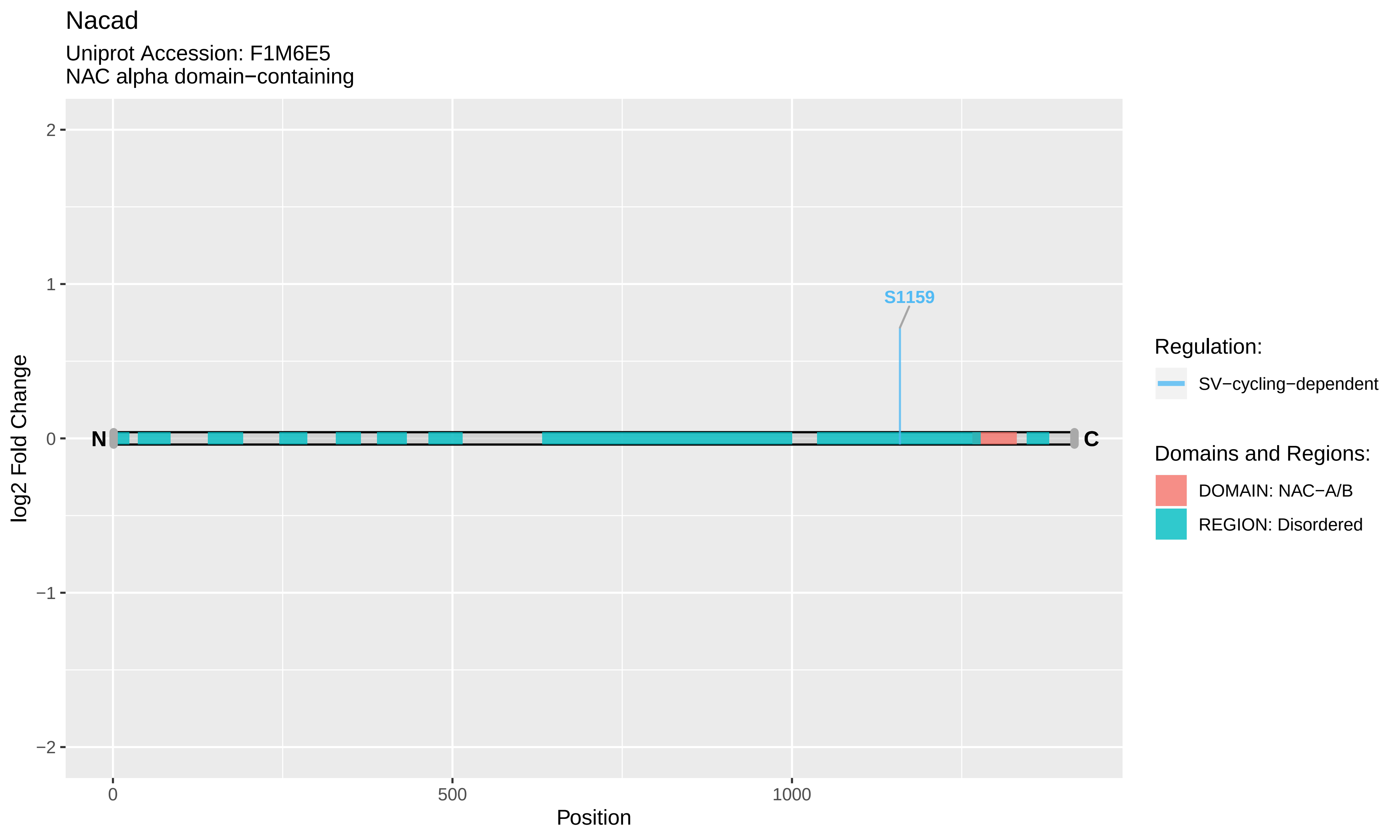

Nacad
Uniprot Accession: F1M6E5
NAC alpha domain−containing
S1159
N
C
2
1
0
−1
−2
log2 Fold Change
0
500
1000
Position
Regulation:
SV−cycling−dependent
Domains and Regions:
DOMAIN: NAC−A/B
REGION: Disordered

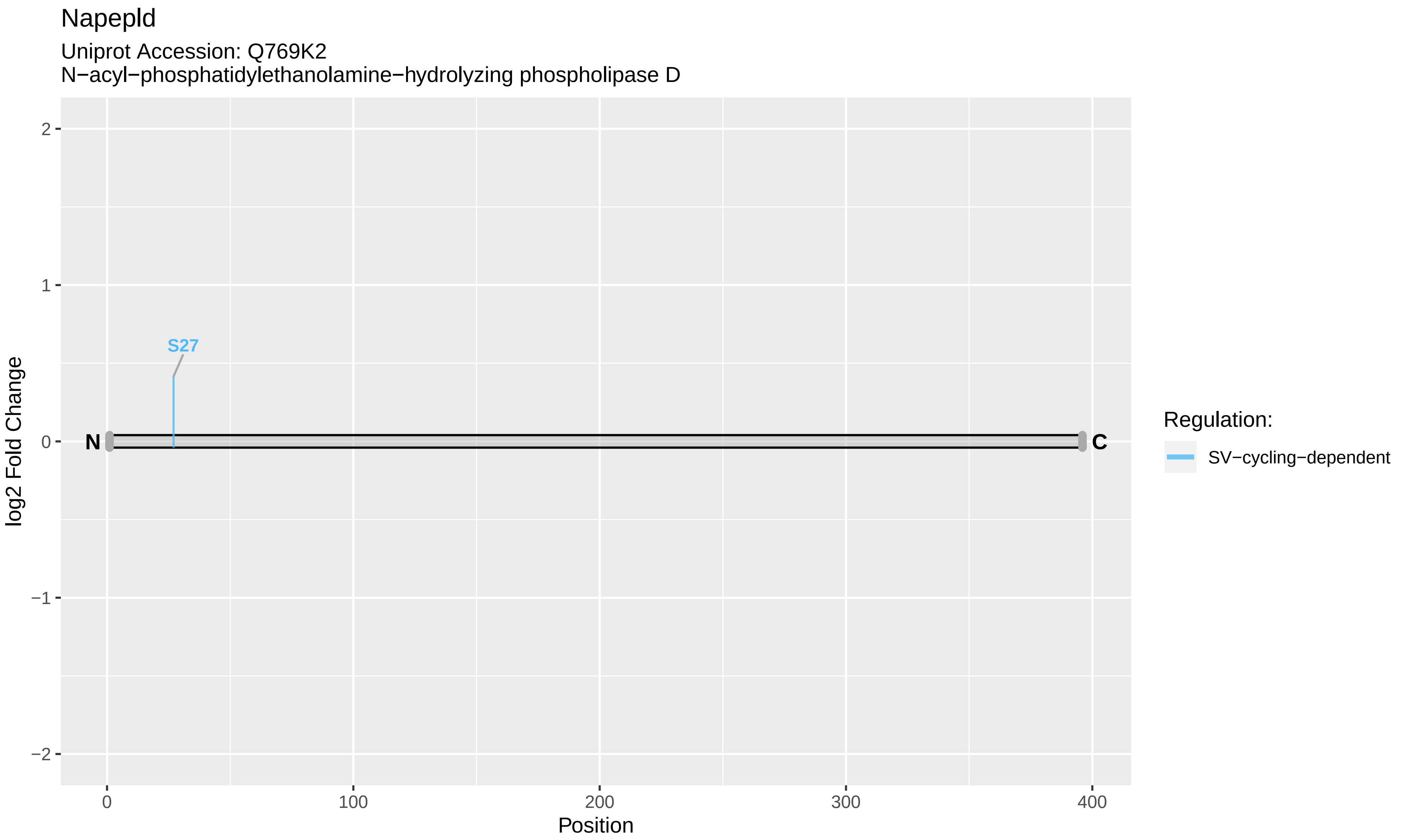

Napepld
Uniprot Accession: Q769K2
N−acyl−phosphatidylethanolamine−hydrolyzing phospholipase D
S27
N
C
log2 Fold Change
2
1
0
−1
−2
0
100
200
300
400
Position
Regulation:
SV−cycling−dependent

Nav1

Uniprot Accession: D4A5I4
Neuron navigator 1

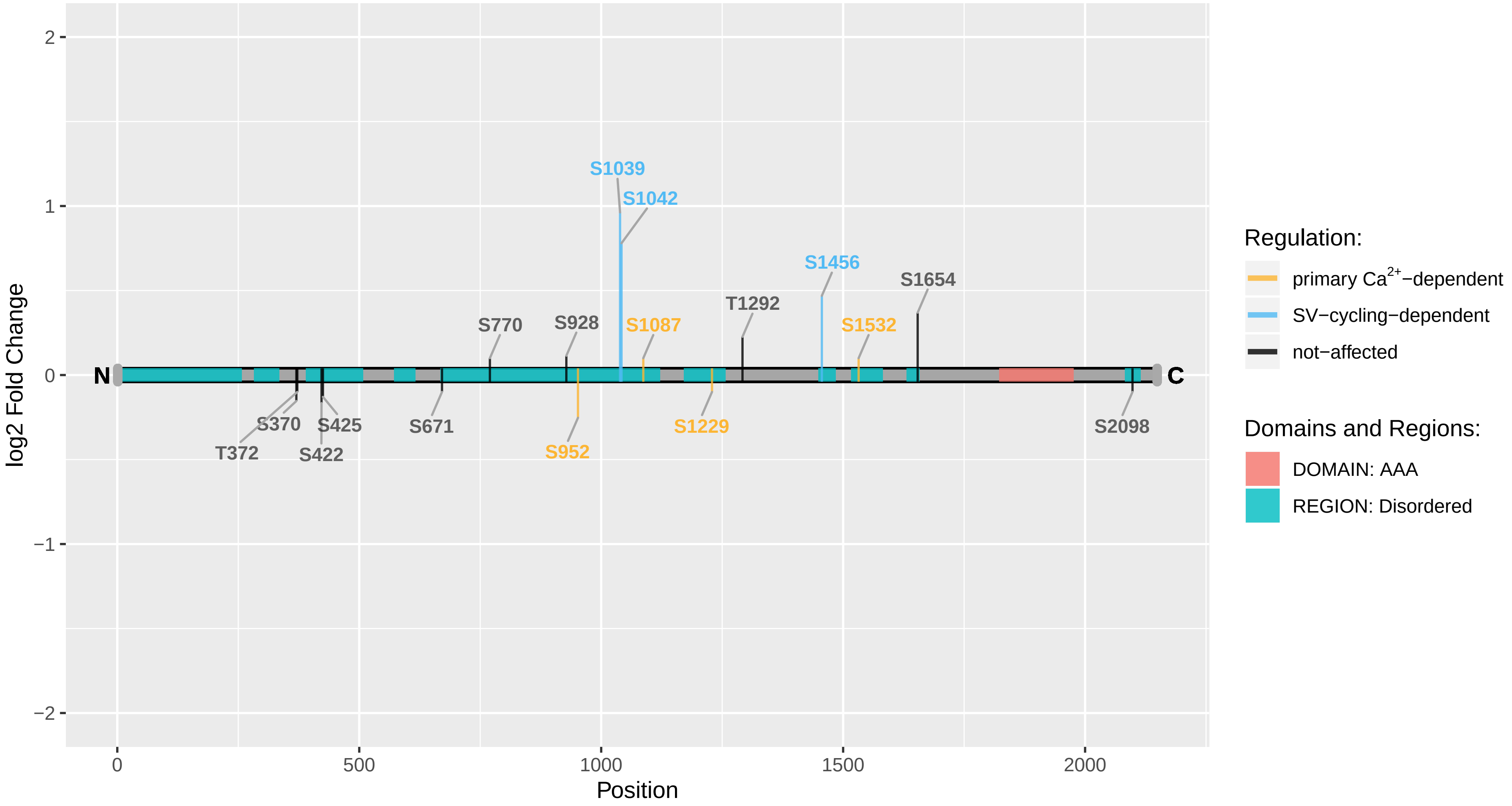

Ncam1

Uniprot Accession: A0A0G2K0M8
Neural cell adhesion molecule 1

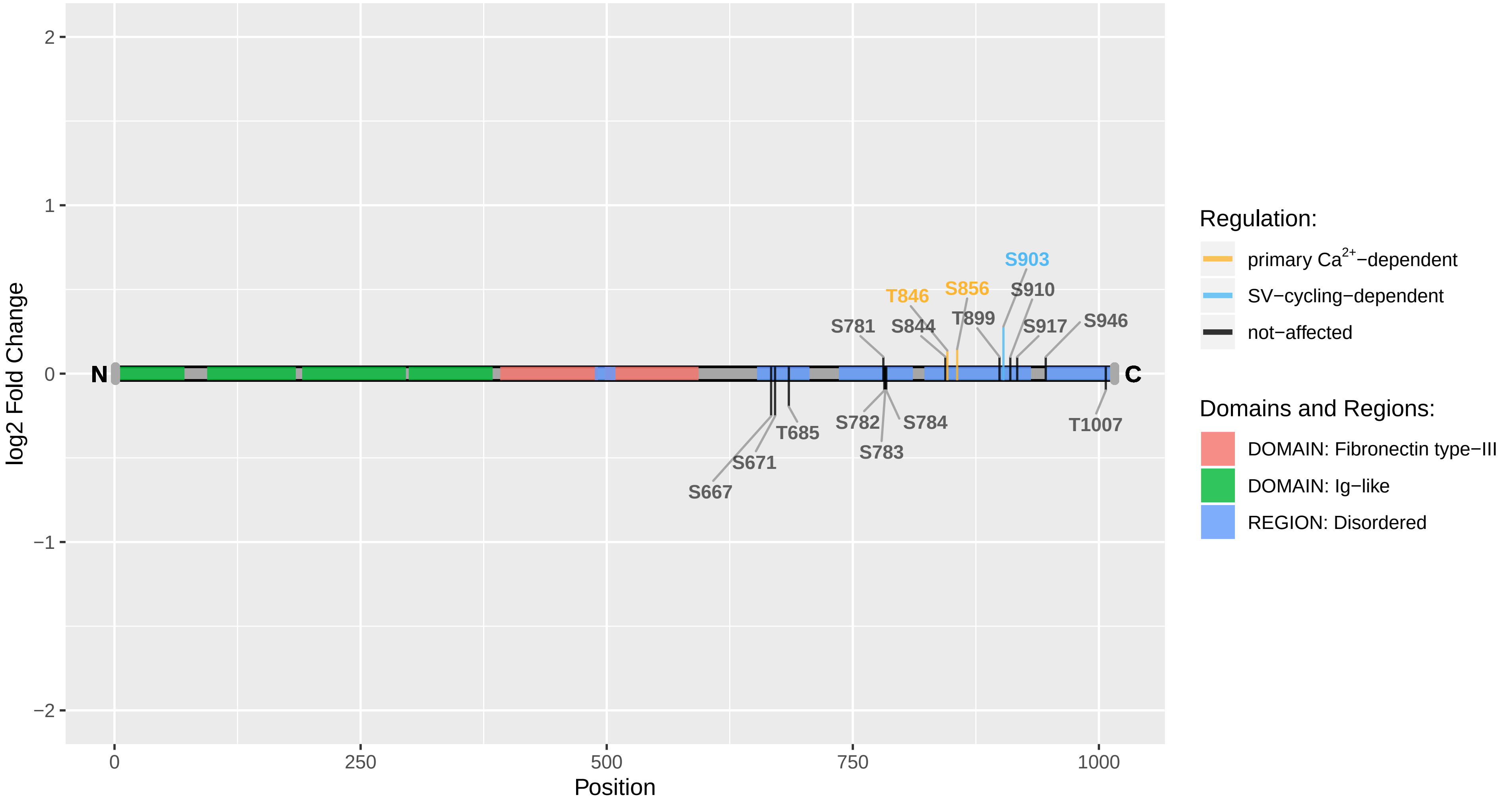

Ncoa7

Uniprot Accession: F1LWN1
Nuclear receptor coactivator 7

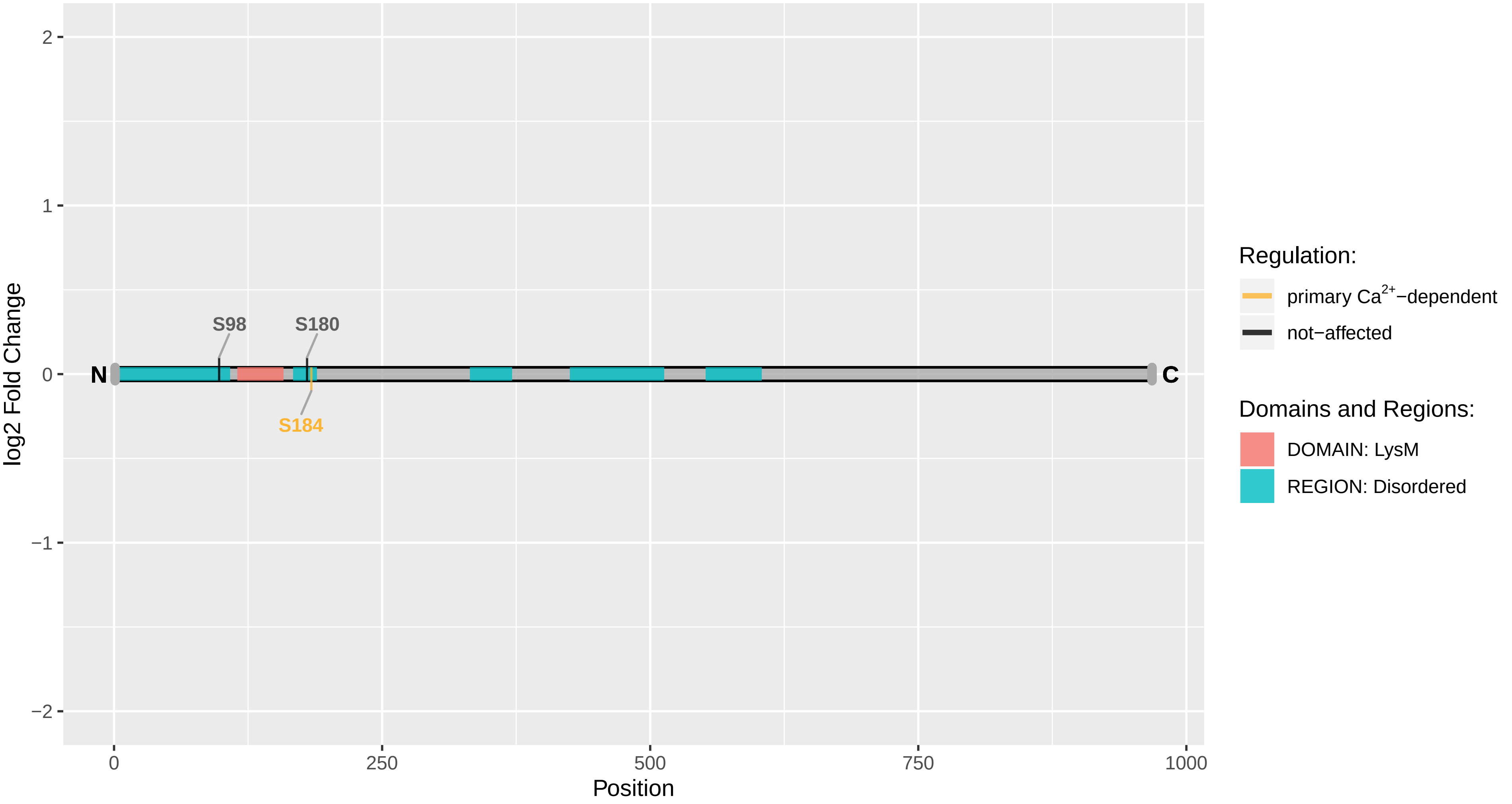

Ncor2

Uniprot Accession: D3ZXN9
Nuclear receptor co−repressor 2

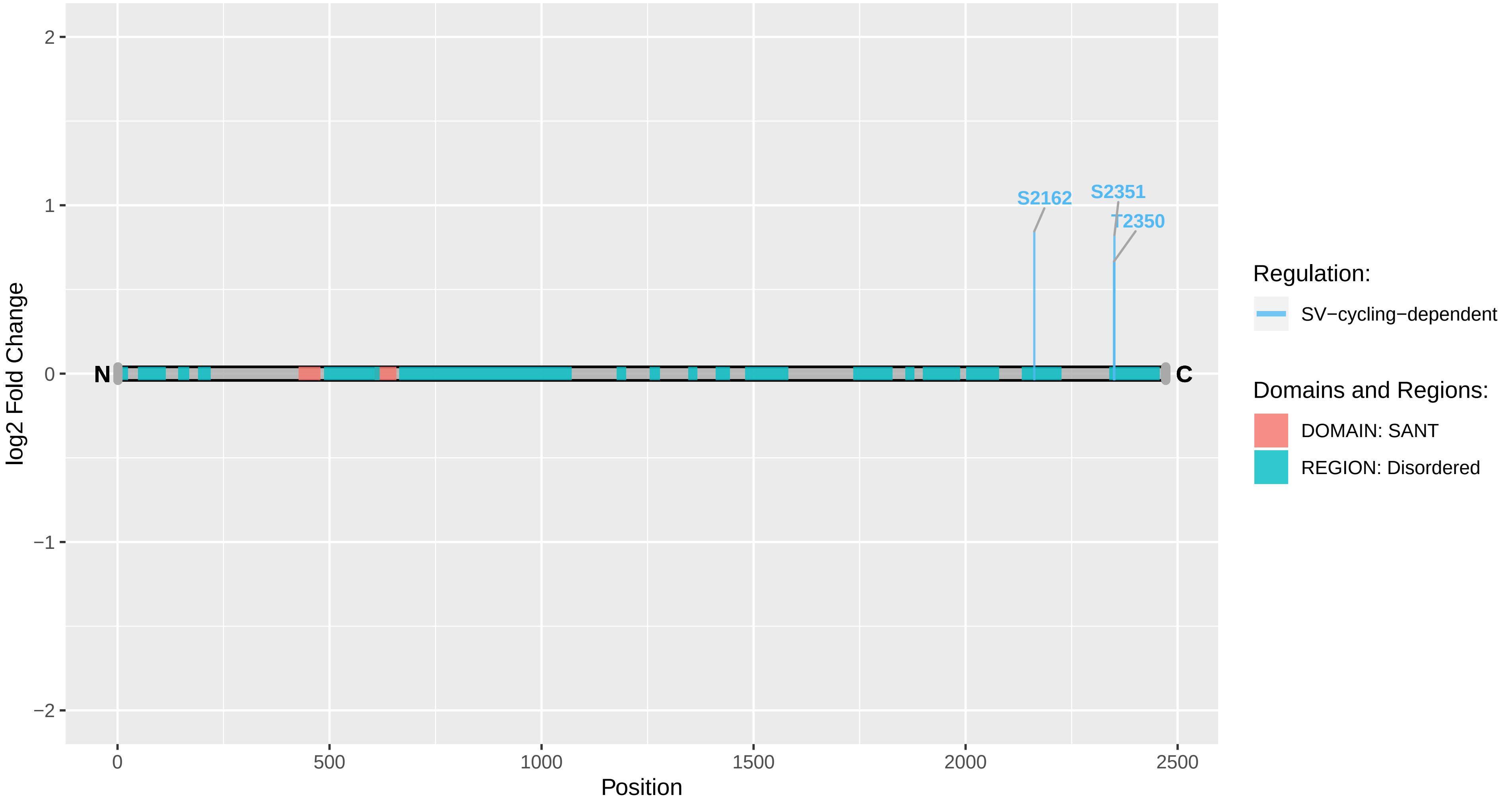

Ndrg2

Uniprot Accession: Q8VBU2
Protein NDRG2

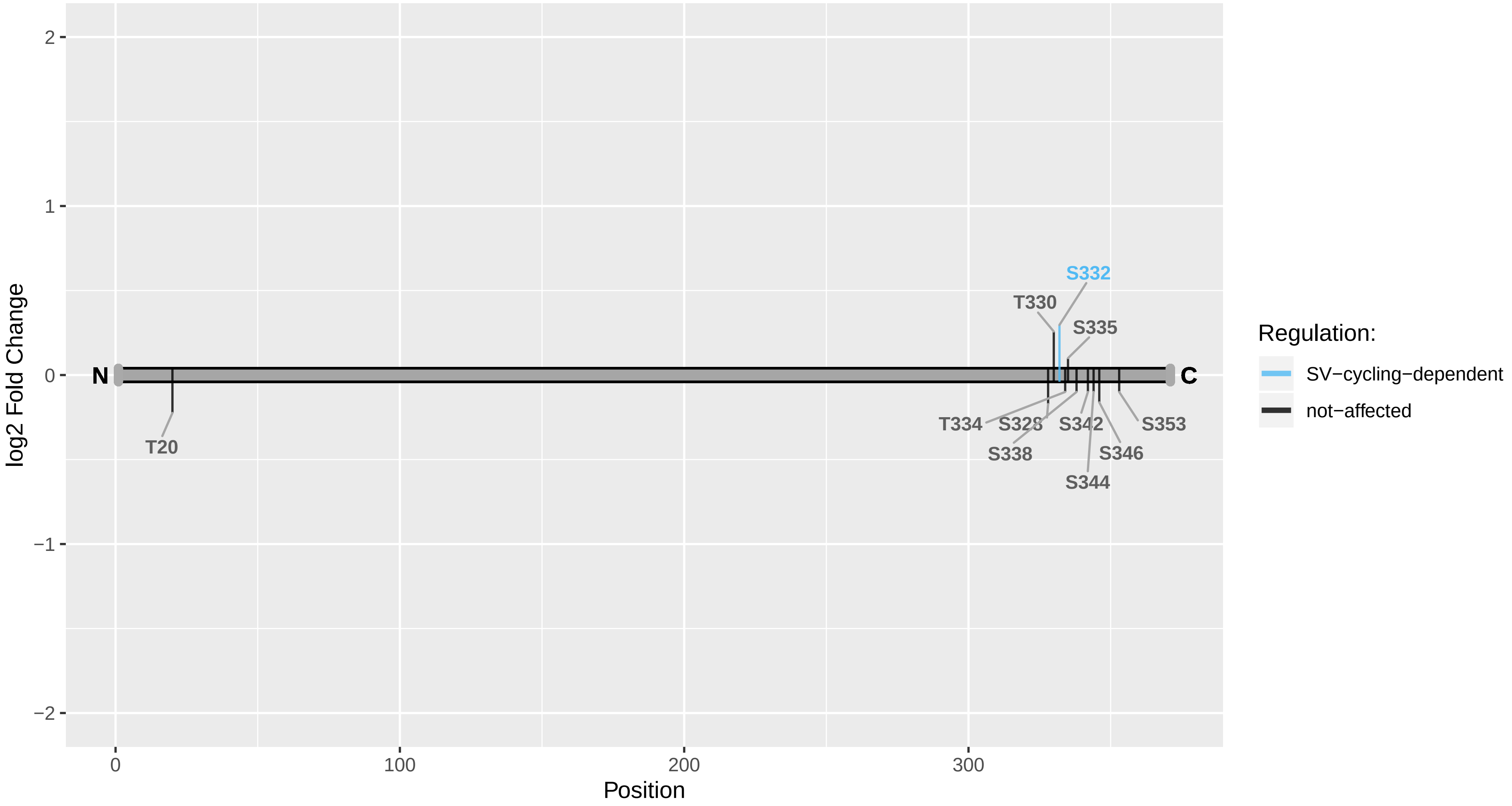

Ndufa2

Uniprot Accession: D3ZS58
NADH dehydrogenase [ubiquinone] 1 alpha subcomplex subunit 2

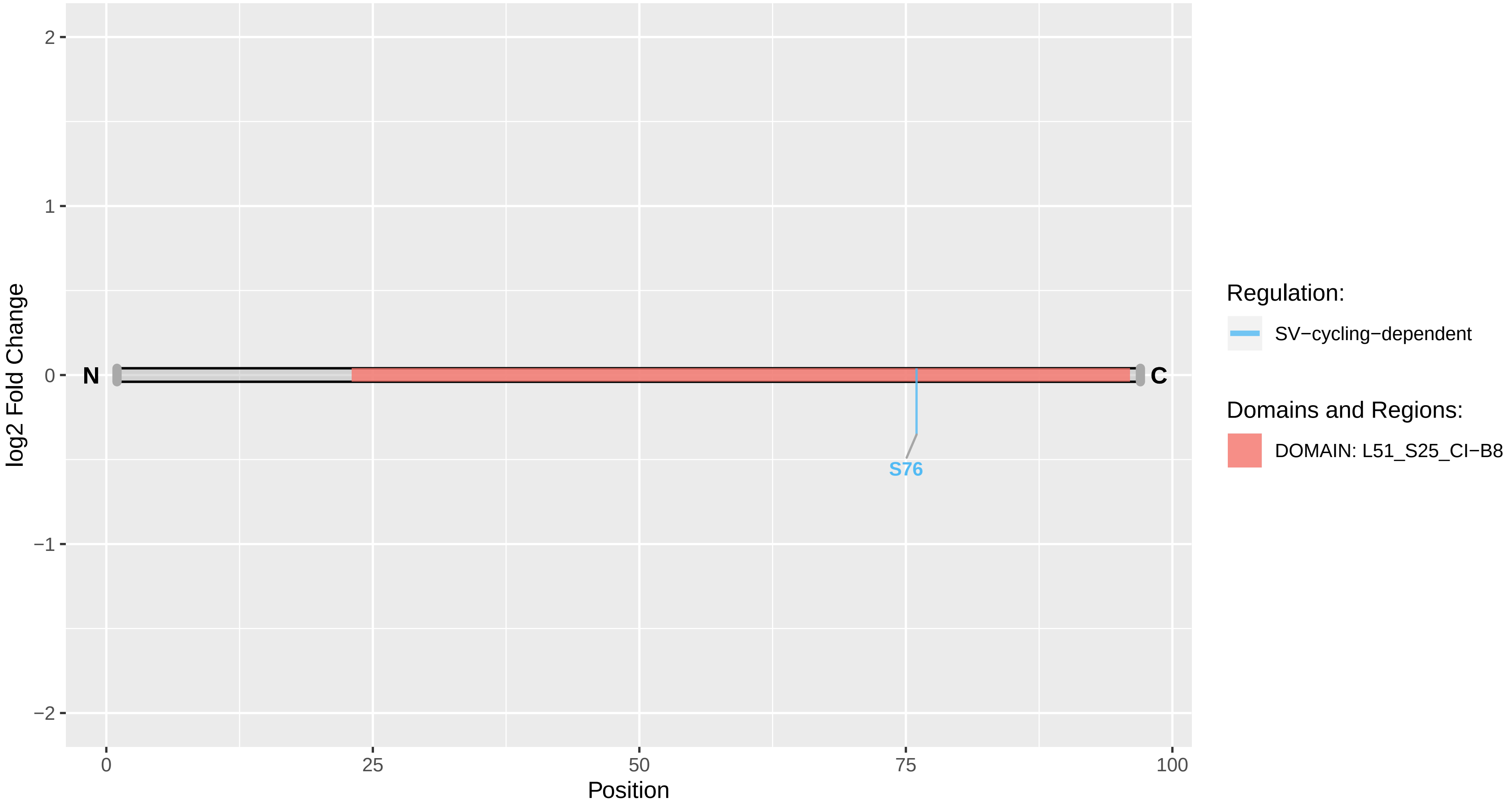

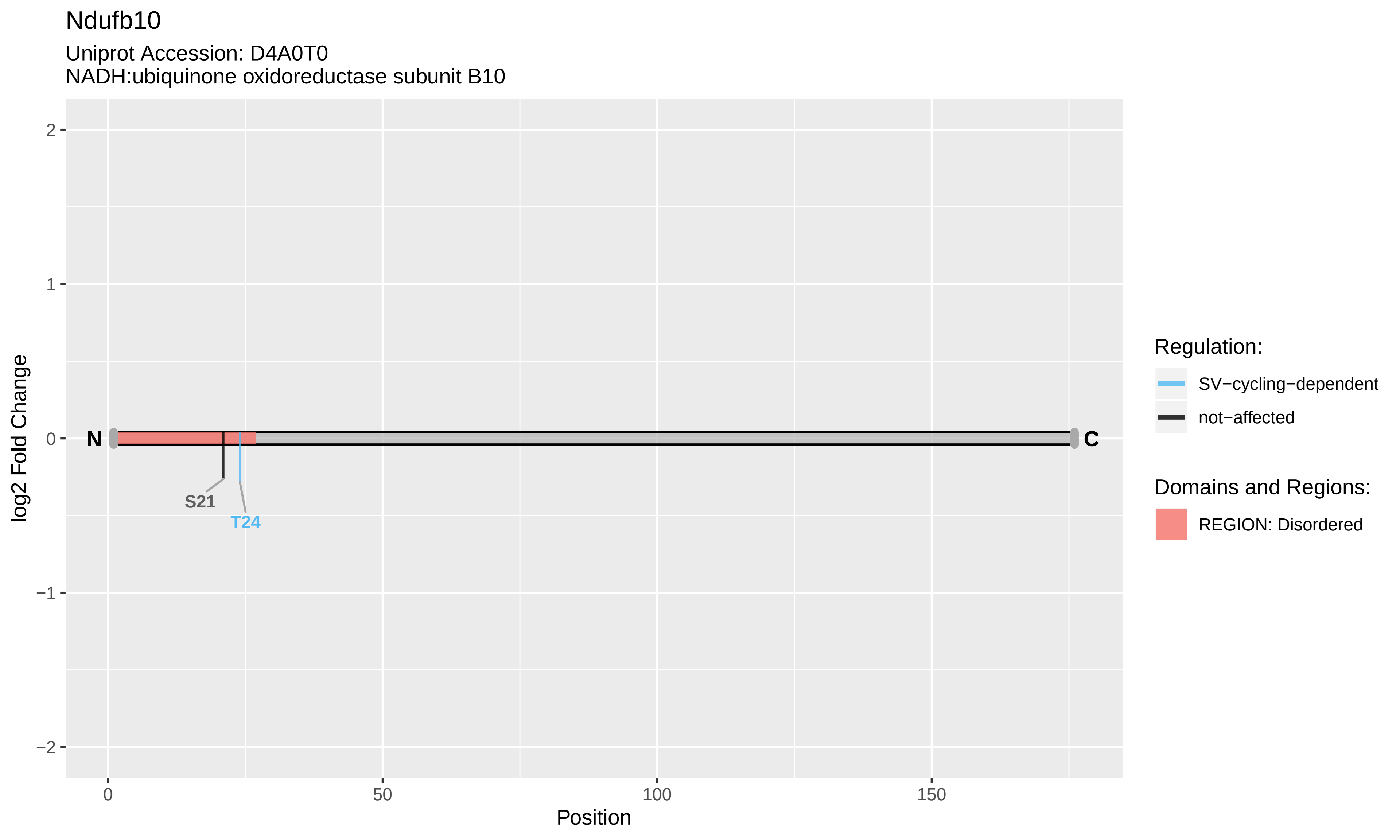

Ndufb10
Uniprot Accession: D4A0T0
NADH:ubiquinone oxidoreductase subunit B10
log2 Fold Change
2
1
0
−1
−2
N
C
S21
T24
0
50
100
150
Position
Regulation:
SV−cycling−dependent
not−affected
Domains and Regions:
REGION: Disordered

Ndufs5

Uniprot Accession: B5DEL8
NADH dehydrogenase (Ubiquinone) Fe−S protein 5

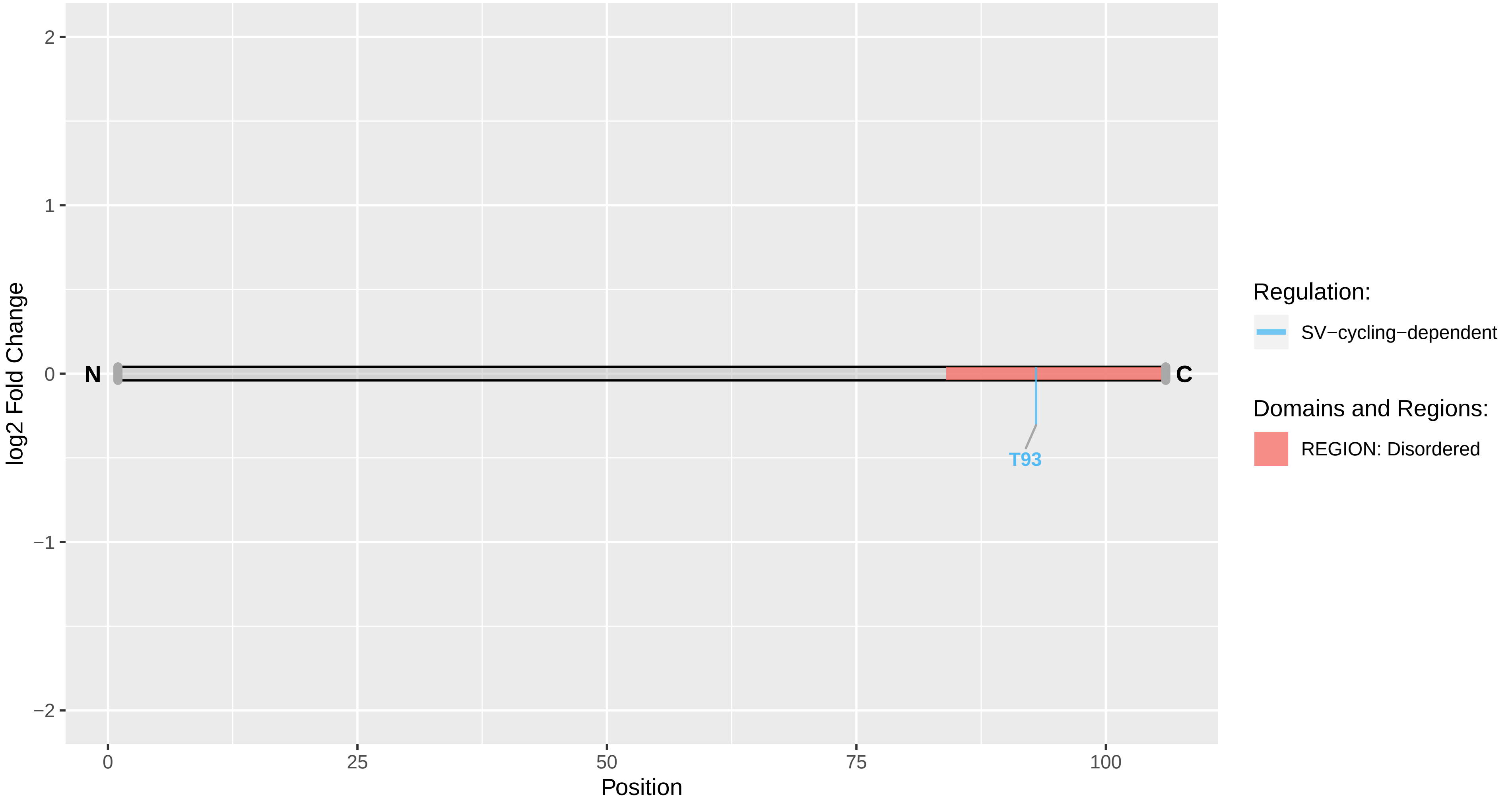

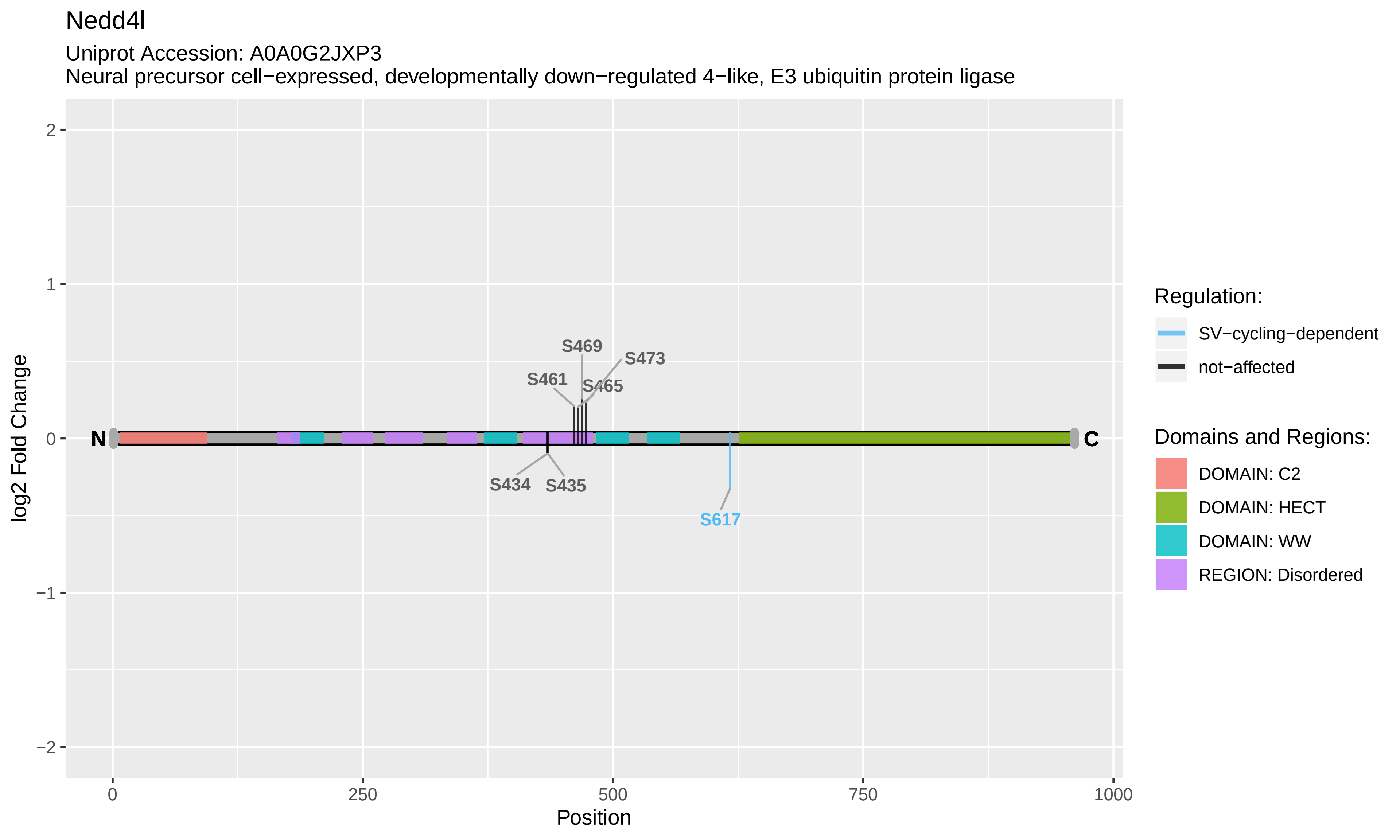

Nedd4l
Uniprot Accession: A0A0G2JXP3
Neural precursor cell−expressed, developmentally down−regulated 4−like, E3 ubiquitin protein ligase
log2 Fold Change
Position
N
C
S469
S473
S461
S465
S434
S435
S617
2
1
0
−1
−2
0
250
500
750
1000
Regulation:
SV−cycling−dependent
not−affected
Domains and Regions:
DOMAIN: C2
DOMAIN: HECT
DOMAIN: WW
REGION: Disordered

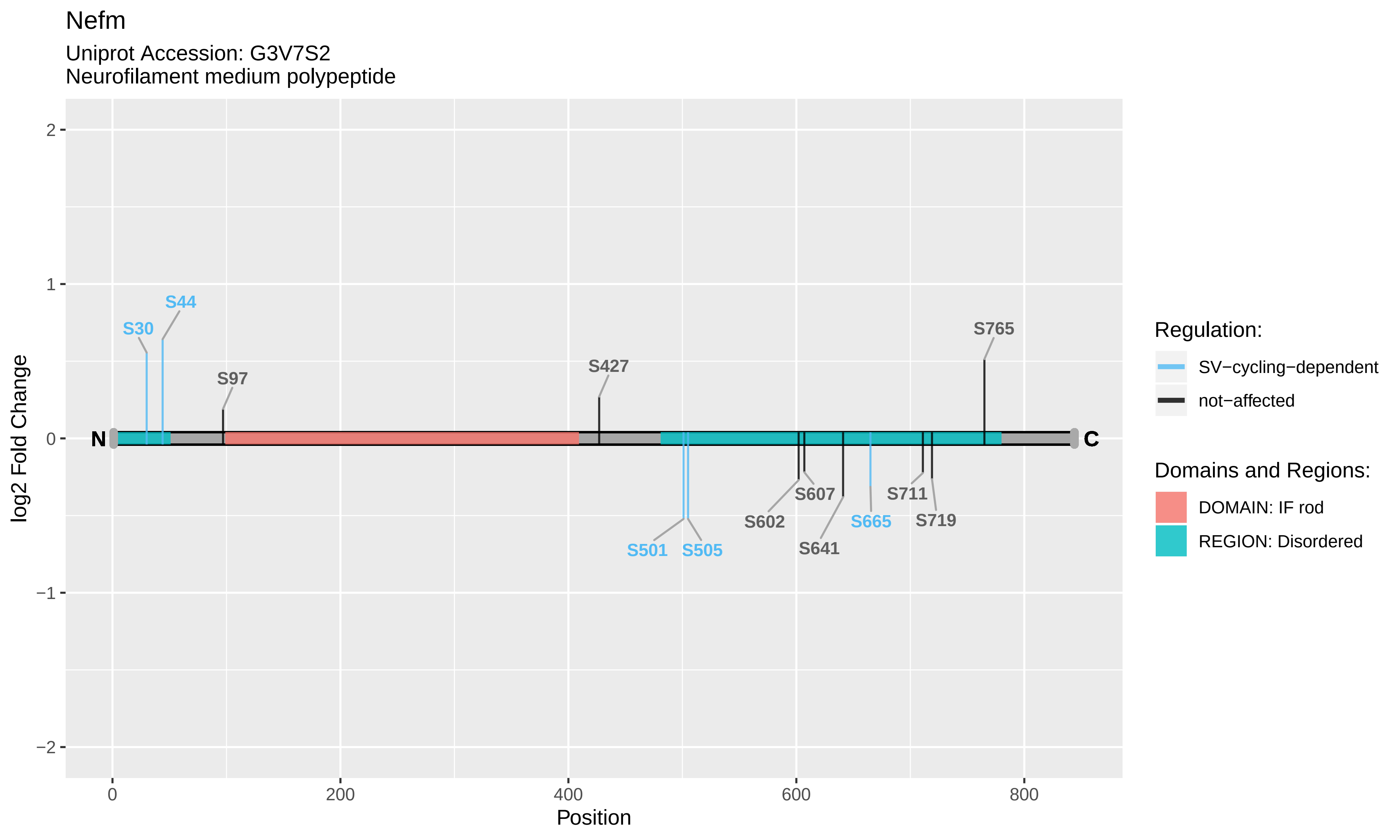

Nefm
Uniprot Accession: G3V7S2
Neurofilament medium polypeptide
S30
S44
S97
S427
S765
S501
S505
S602
S607
S641
S665
S711
S719
N
C
log2 Fold Change
Position
Regulation:
SV−cycling−dependent
not−affected
Domains and Regions:
DOMAIN: IF rod
REGION: Disordered

Nf1

Uniprot Accession: P97526
Neurofibromin

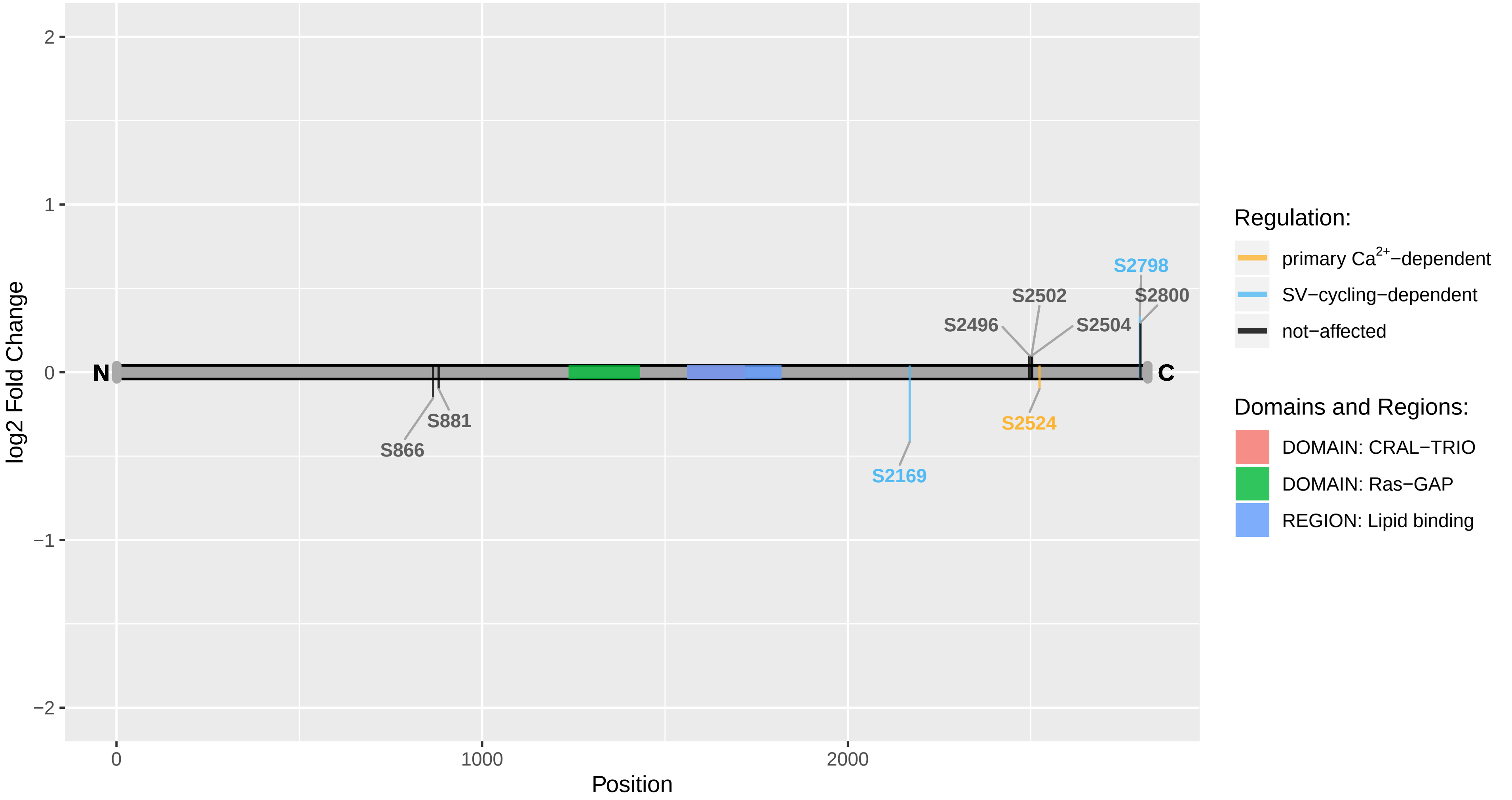

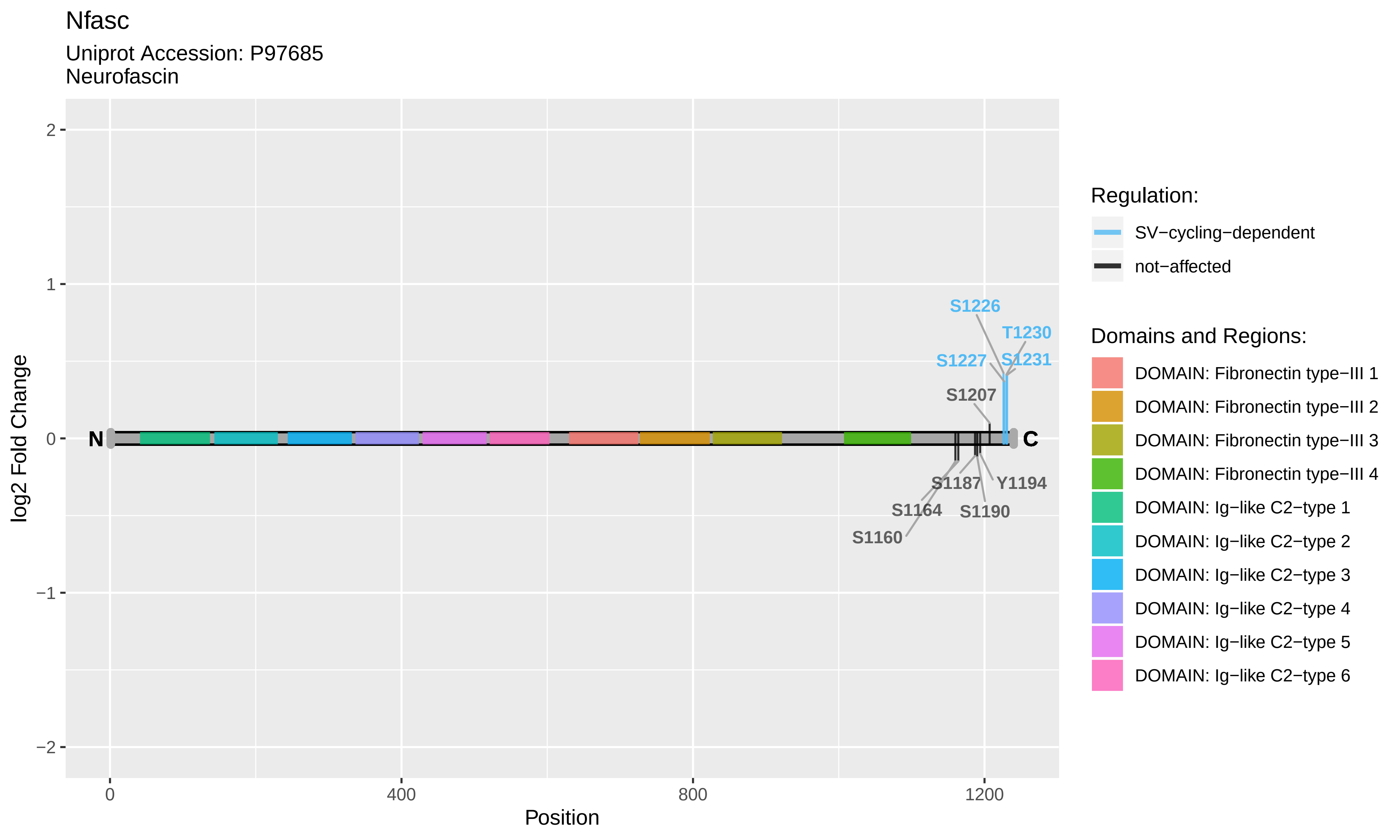
Nfasc
Uniprot Accession: P97685
Neurofascin
log2 Fold Change
2
1
0
−1
−2
0
400
800
1200
Position
N
C
S1226
T1230
S1227
S1231
S1207
S1187
Y1194
S1164
S1190
S1160
Regulation:
SV−cycling−dependent
not−affected
Domains and Regions:
DOMAIN: Fibronectin type−III 1
DOMAIN: Fibronectin type−III 2
DOMAIN: Fibronectin type−III 3
DOMAIN: Fibronectin type−III 4
DOMAIN: Ig−like C2−type 1
DOMAIN: Ig−like C2−type 2
DOMAIN: Ig−like C2−type 3
DOMAIN: Ig−like C2−type 4
DOMAIN: Ig−like C2−type 5
DOMAIN: Ig−like C2−type 6

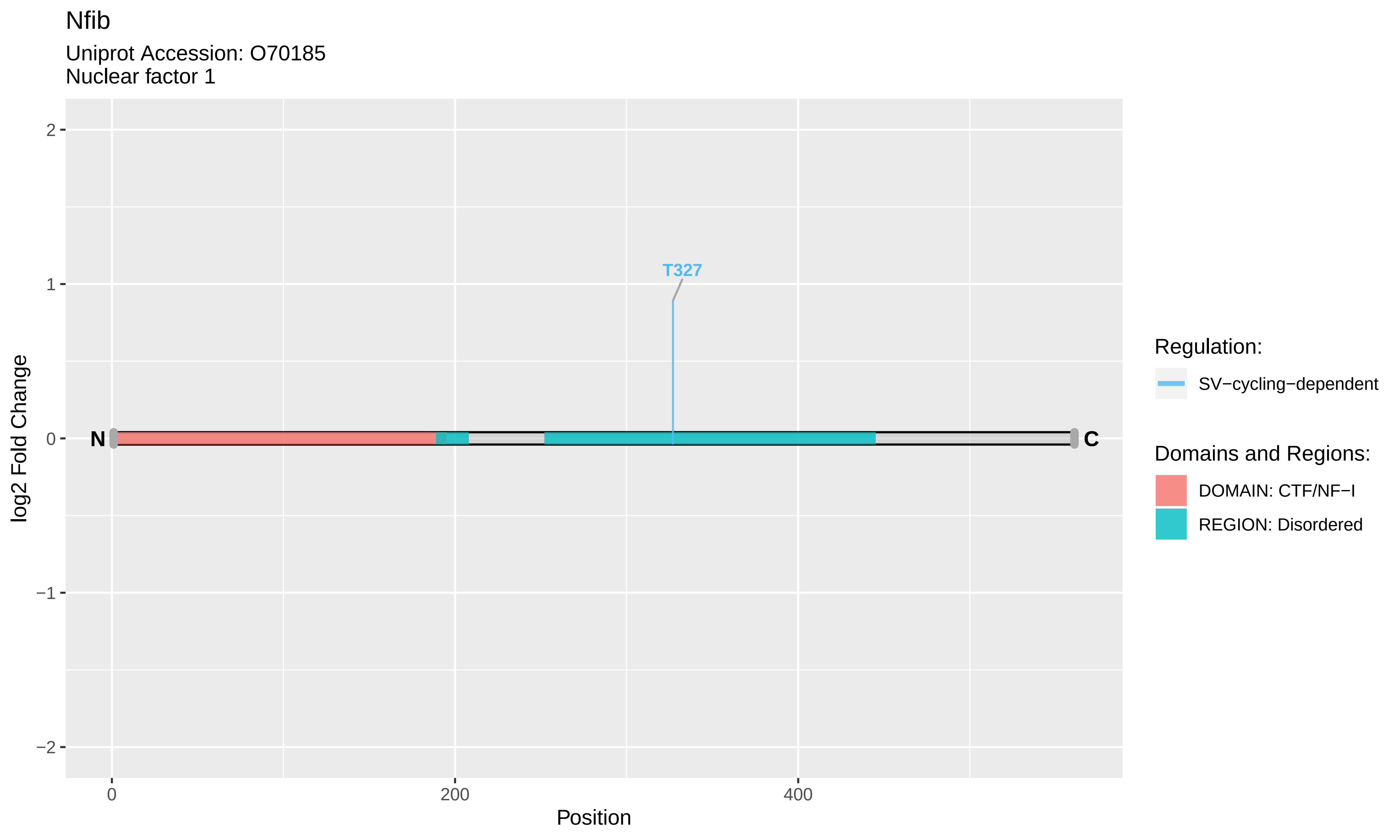

Nfib
Uniprot Accession: O70185
Nuclear factor 1
T327
N
C
2
1
0
−1
−2
log2 Fold Change
0
200
400
Position
Regulation:
SV−cycling−dependent
Domains and Regions:
DOMAIN: CTF/NF−I
REGION: Disordered

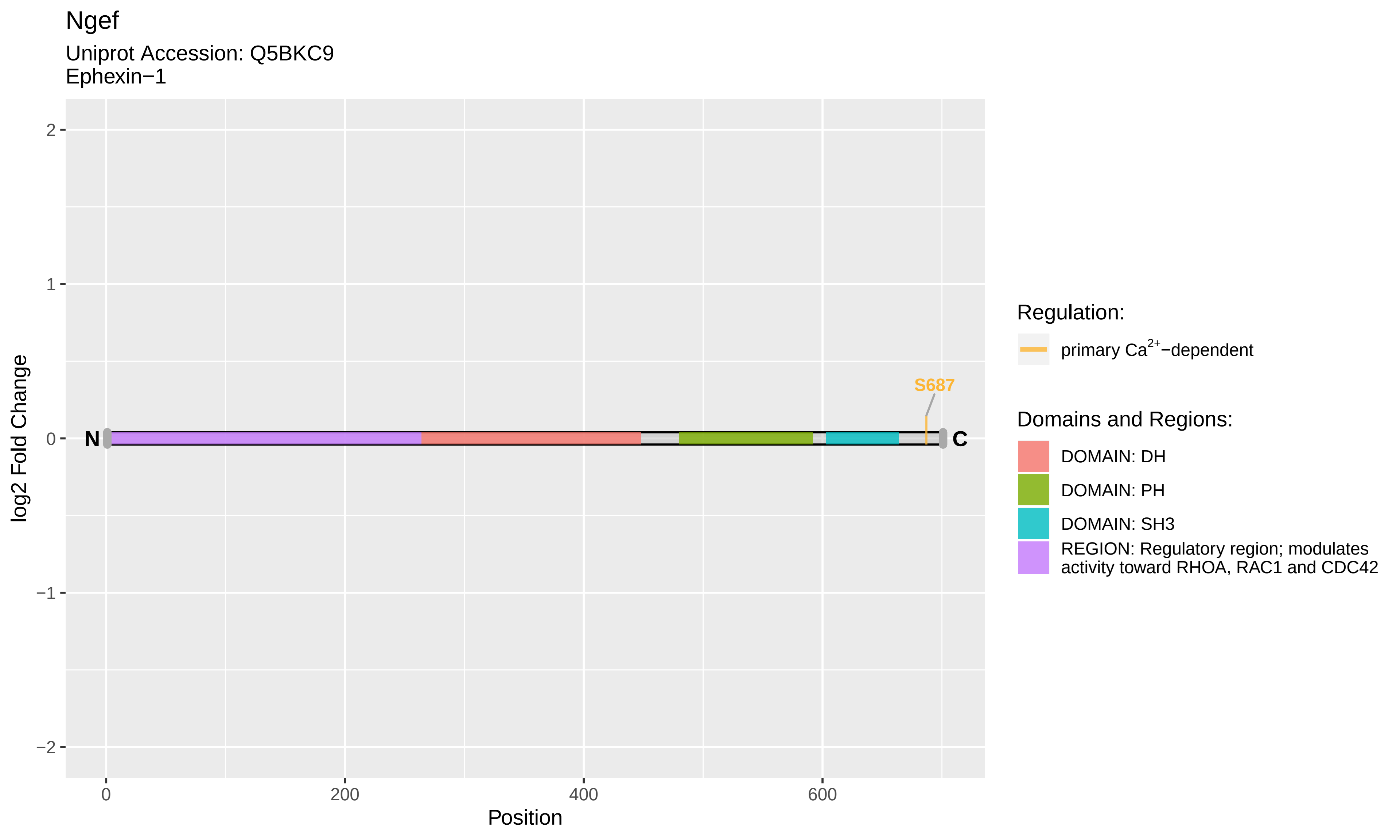

Ngef
Uniprot Accession: Q5BKC9
Ephexin−1
log2 Fold Change
2
1
0
−1
−2
N
C
S687
0
200
400
600
Position
Regulation:
primary Ca2+−dependent
Domains and Regions:
DOMAIN: DH
DOMAIN: PH
DOMAIN: SH3
REGION: Regulatory region; modulates activity toward RHOA, RAC1 and CDC42

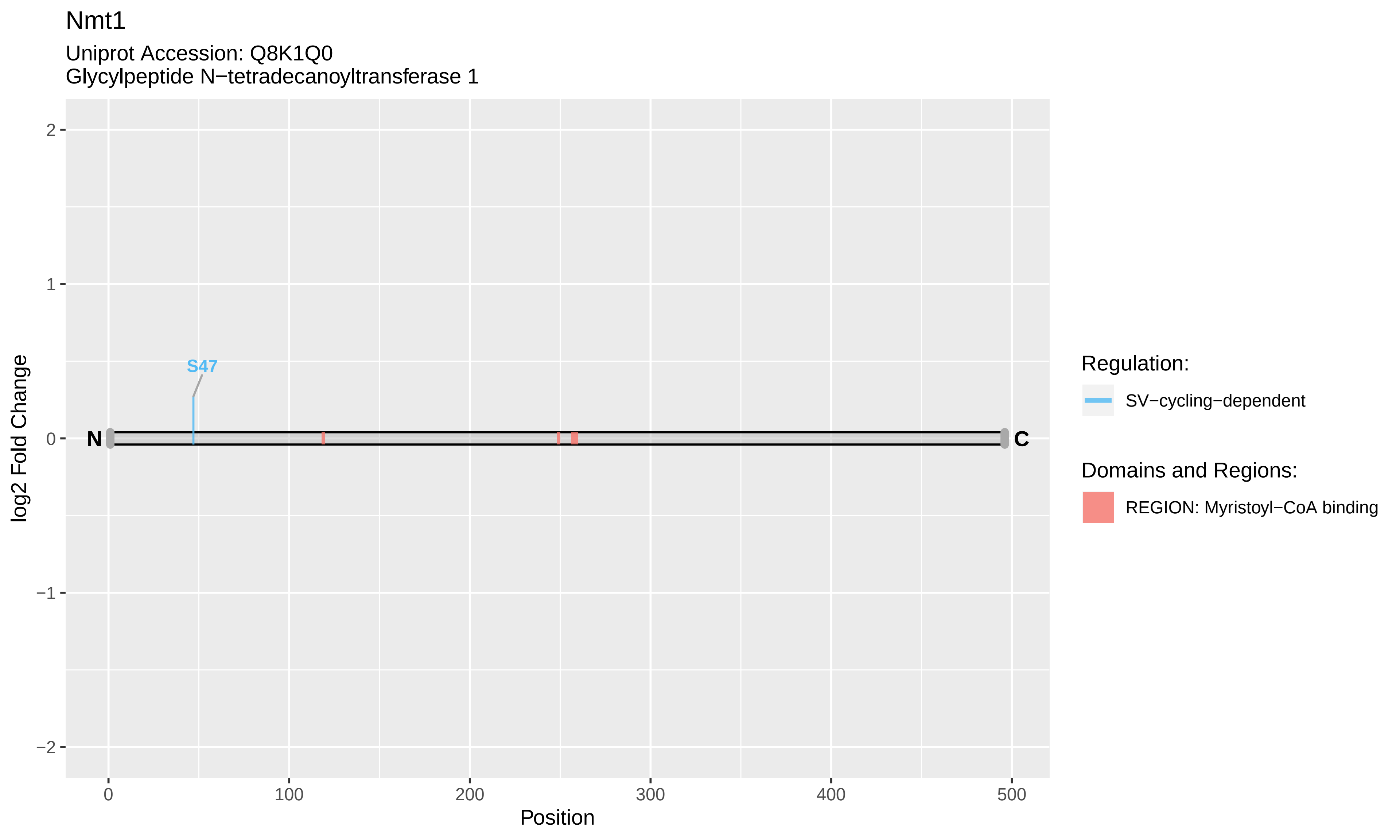

Nmt1
Uniprot Accession: Q8K1Q0
Glycylpeptide N−tetradecanoyltransferase 1
S47
N
C
log2 Fold Change
Position
2
1
0
−1
−2
0
100
200
300
400
500
Regulation:
SV−cycling−dependent
Domains and Regions:
REGION: Myristoyl−CoA binding

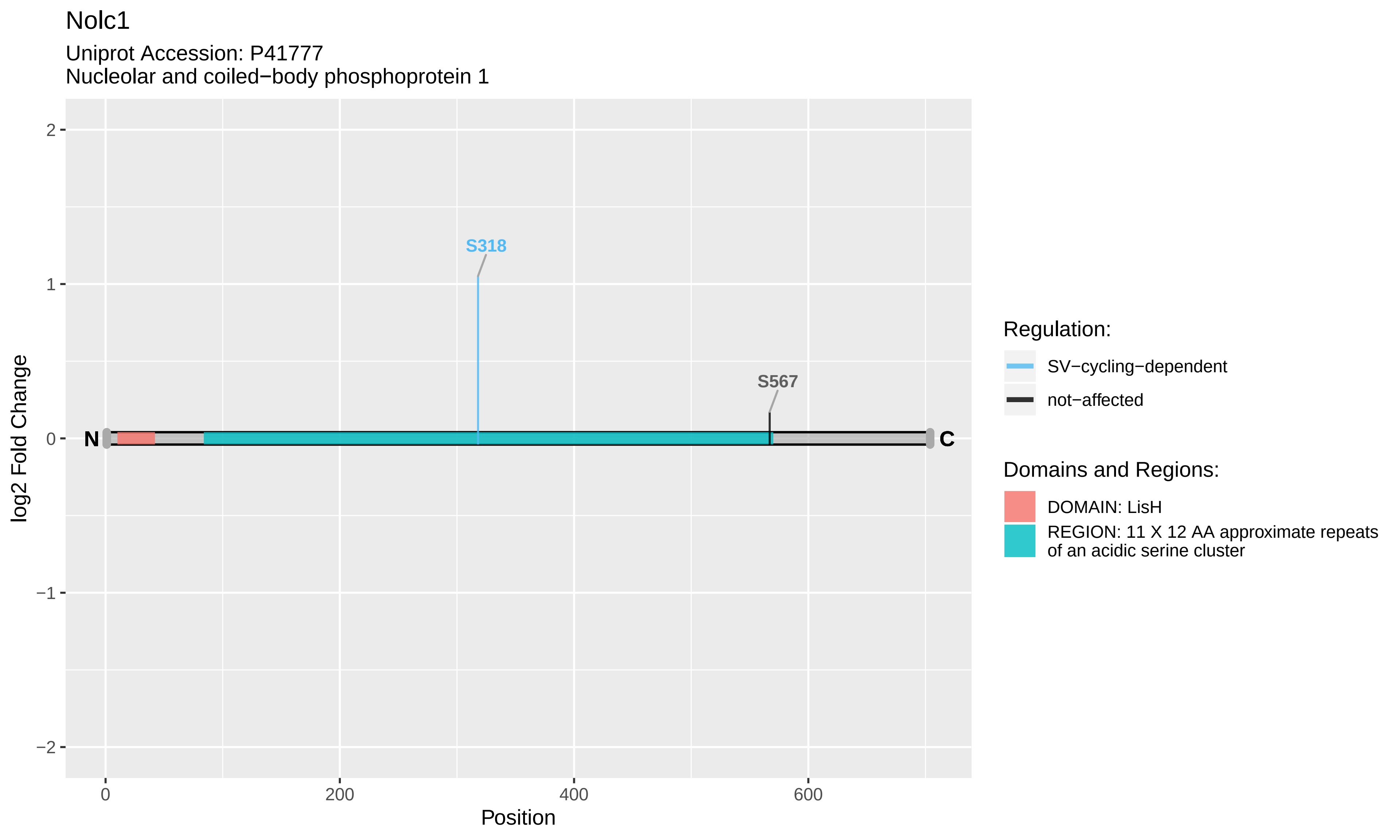
Nolc1
Uniprot Accession: P41777
Nucleolar and coiled−body phosphoprotein 1
S318
S567
N
C
2
1
0
−1
−2
log2 Fold Change
0
200
400
600
Position
Regulation:
SV−cycling−dependent
not−affected
Domains and Regions:
DOMAIN: LisH
REGION: 11 X 12 AA approximate repeats of an acidic serine cluster

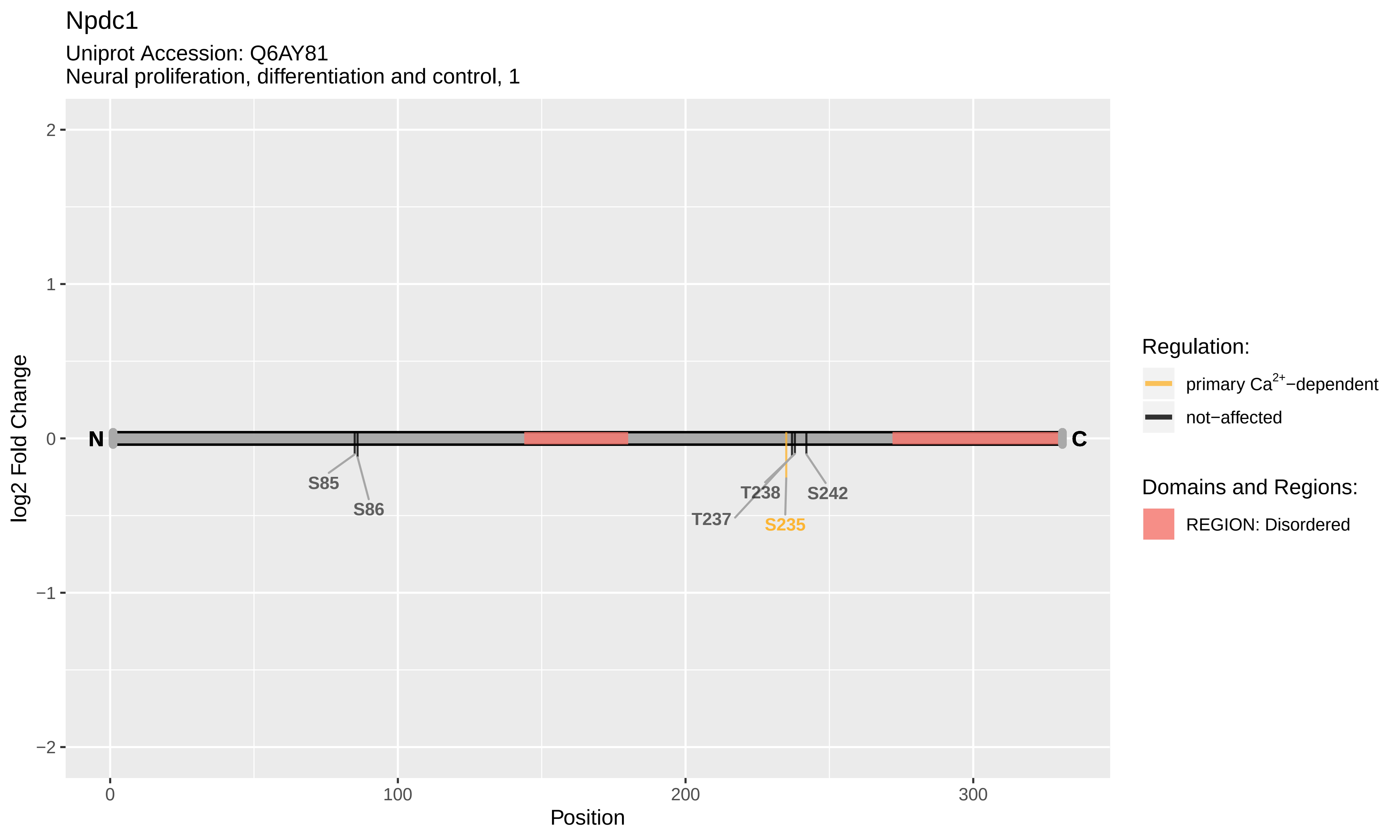
Npdc1
Uniprot Accession: Q6AY81
Neural proliferation, differentiation and control, 1
log2 Fold Change
Position
N
C
S85
S86
T238
T237
S235
S242
Regulation:
primary Ca2+−dependent
not−affected
Domains and Regions:
REGION: Disordered

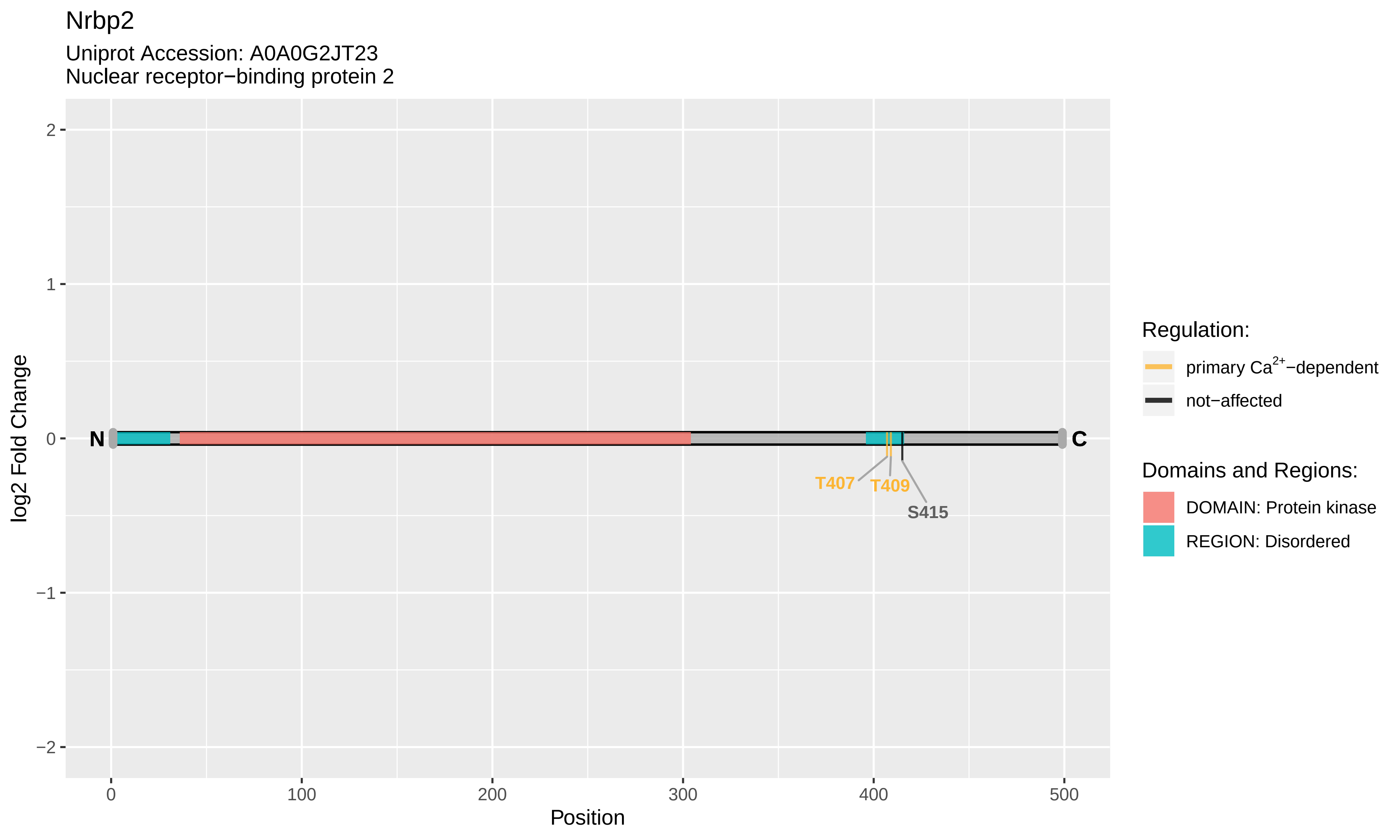
Nrbp2
Uniprot Accession: A0A0G2JT23
Nuclear receptor−binding protein 2
log2 Fold Change
Position
N
C
T407
T409
S415
2
1
0
−1
−2
0
100
200
300
400
500
Regulation:
primary Ca2+−dependent
not−affected
Domains and Regions:
DOMAIN: Protein kinase
REGION: Disordered

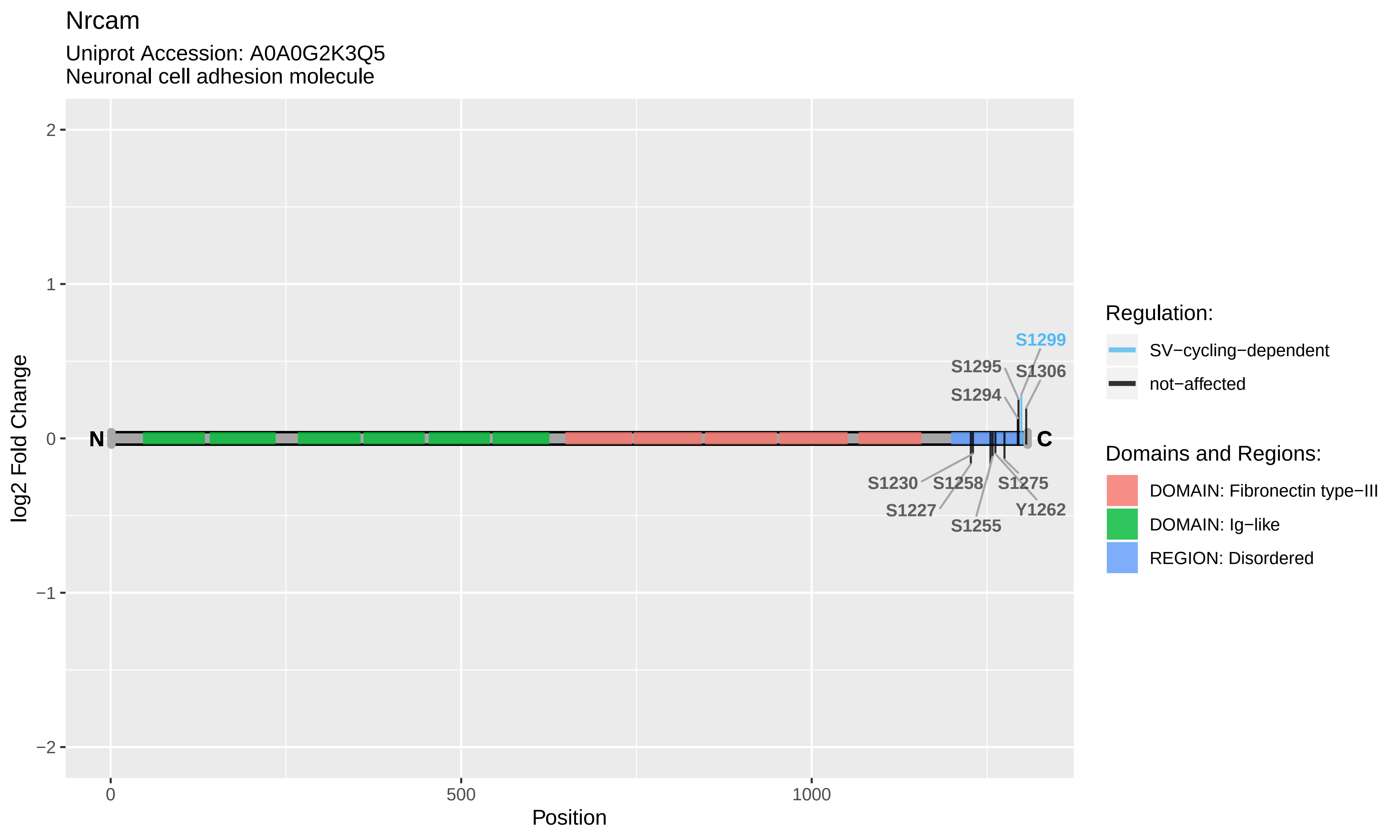
Nrcam
Uniprot Accession: A0A0G2K3Q5
Neuronal cell adhesion molecule
log2 Fold Change
2
1
0
−1
−2
N
C
S1299
S1295
S1306
S1294
S1230
S1258
S1275
S1227
Y1262
S1255
0
500
1000
Position
Regulation:
SV−cycling−dependent
not−affected
Domains and Regions:
DOMAIN: Fibronectin type−III
DOMAIN: Ig−like
REGION: Disordered

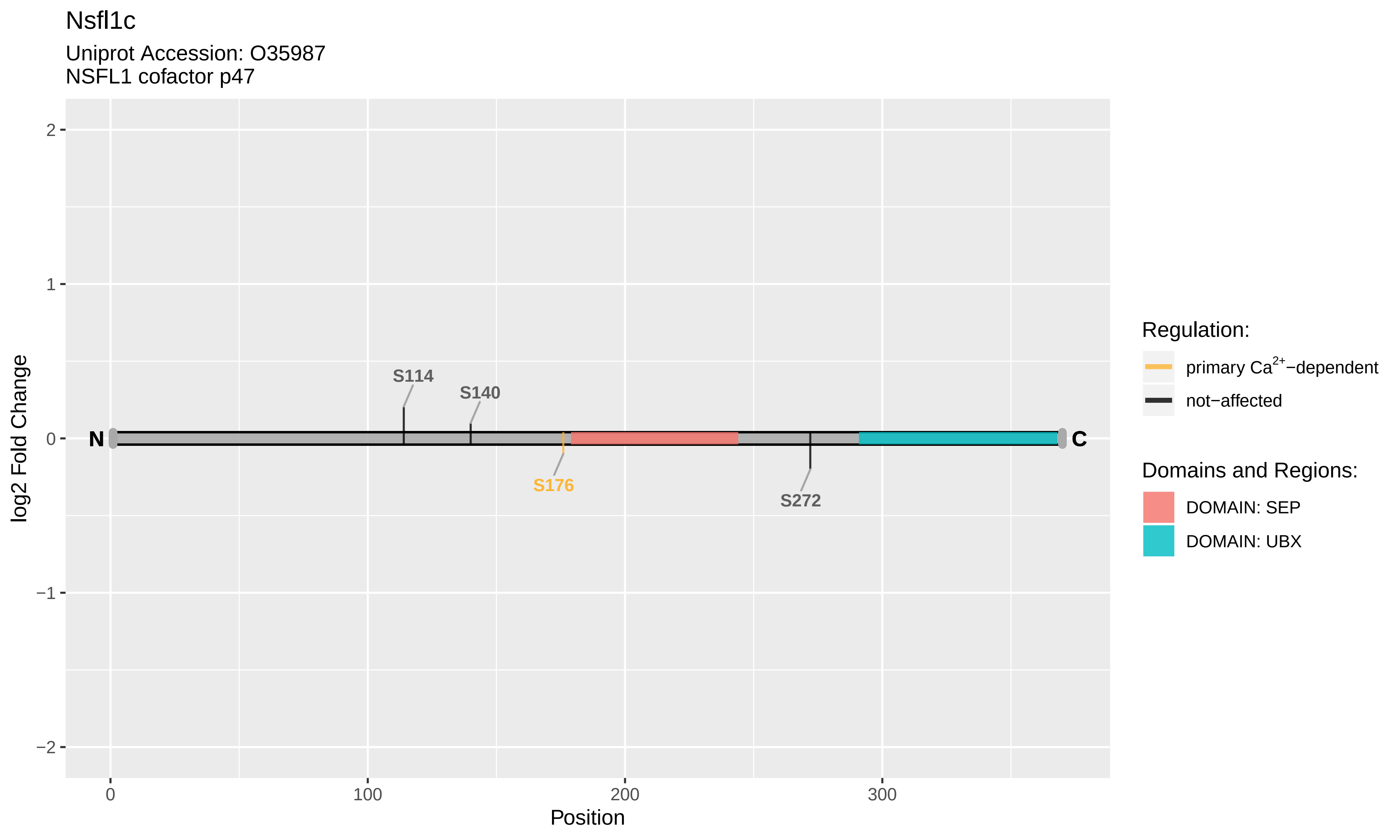

Nsfl1c
Uniprot Accession: O35987
NSFL1 cofactor p47
log2 Fold Change
Position
2
1
0
−1
−2
0
100
200
300
N
C
S114
S140
S176
S272
Regulation:
primary Ca2+−dependent
not−affected
Domains and Regions:
DOMAIN: SEP
DOMAIN: UBX

Nudt10

Uniprot Accession: D3ZYH3
Nudix hydrolase 11

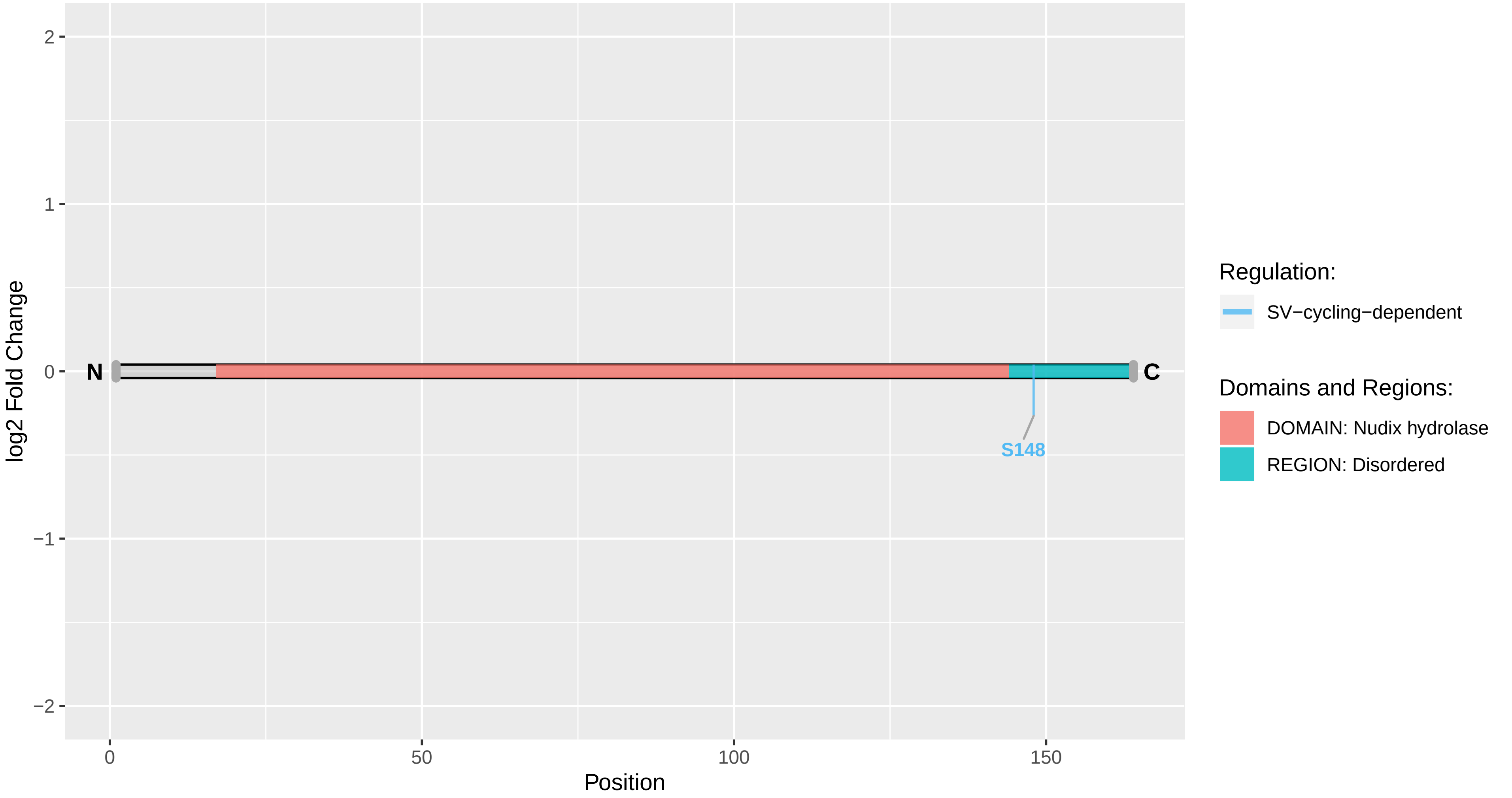

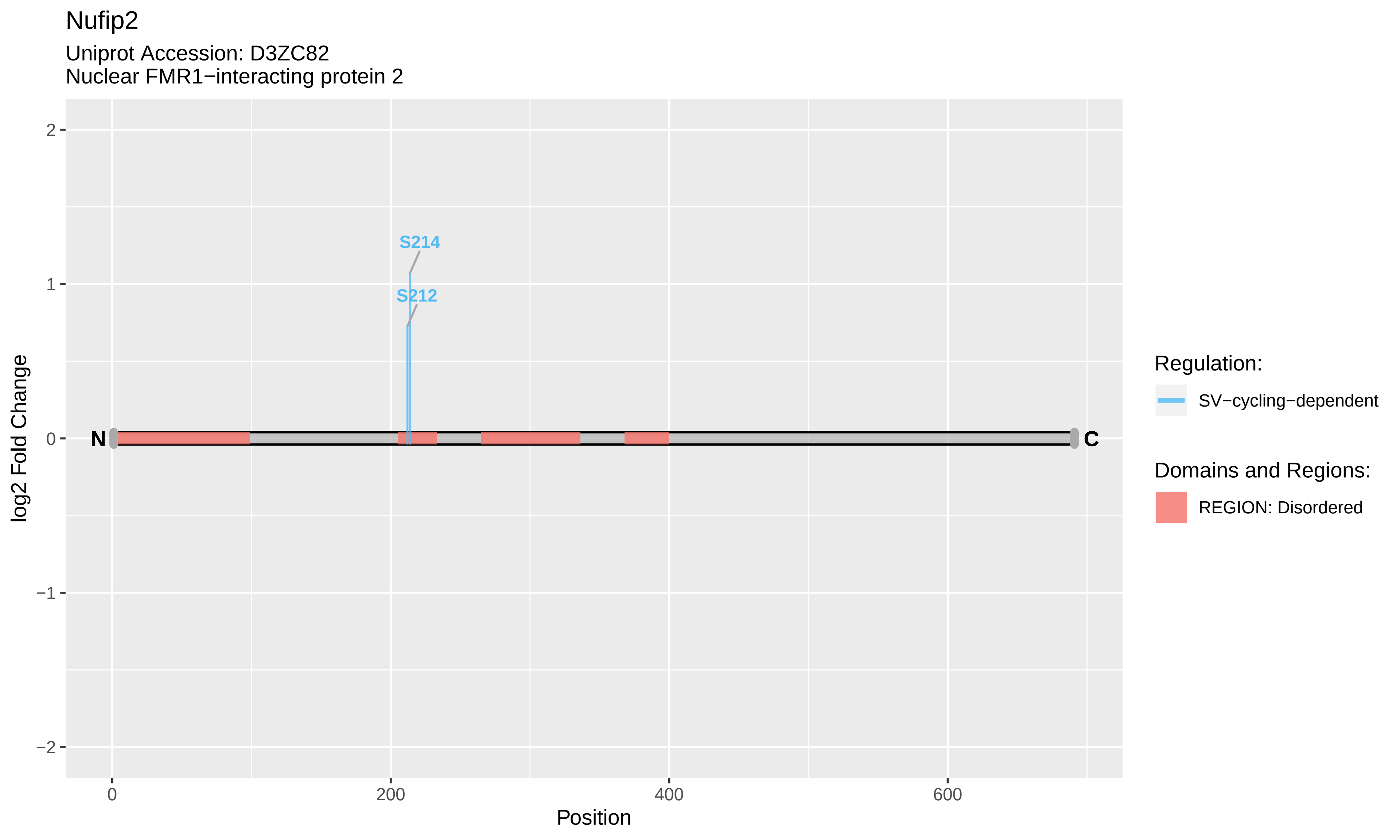

Nufip2
Uniprot Accession: D3ZC82
Nuclear FMR1−interacting protein 2
S214
S212
N
C
2
1
0
−1
−2
0
200
400
600
Position
log2 Fold Change
Regulation:
SV−cycling−dependent
Domains and Regions:
REGION: Disordered

Numb

Uniprot Accession: Q2LC84
Protein numb homolog

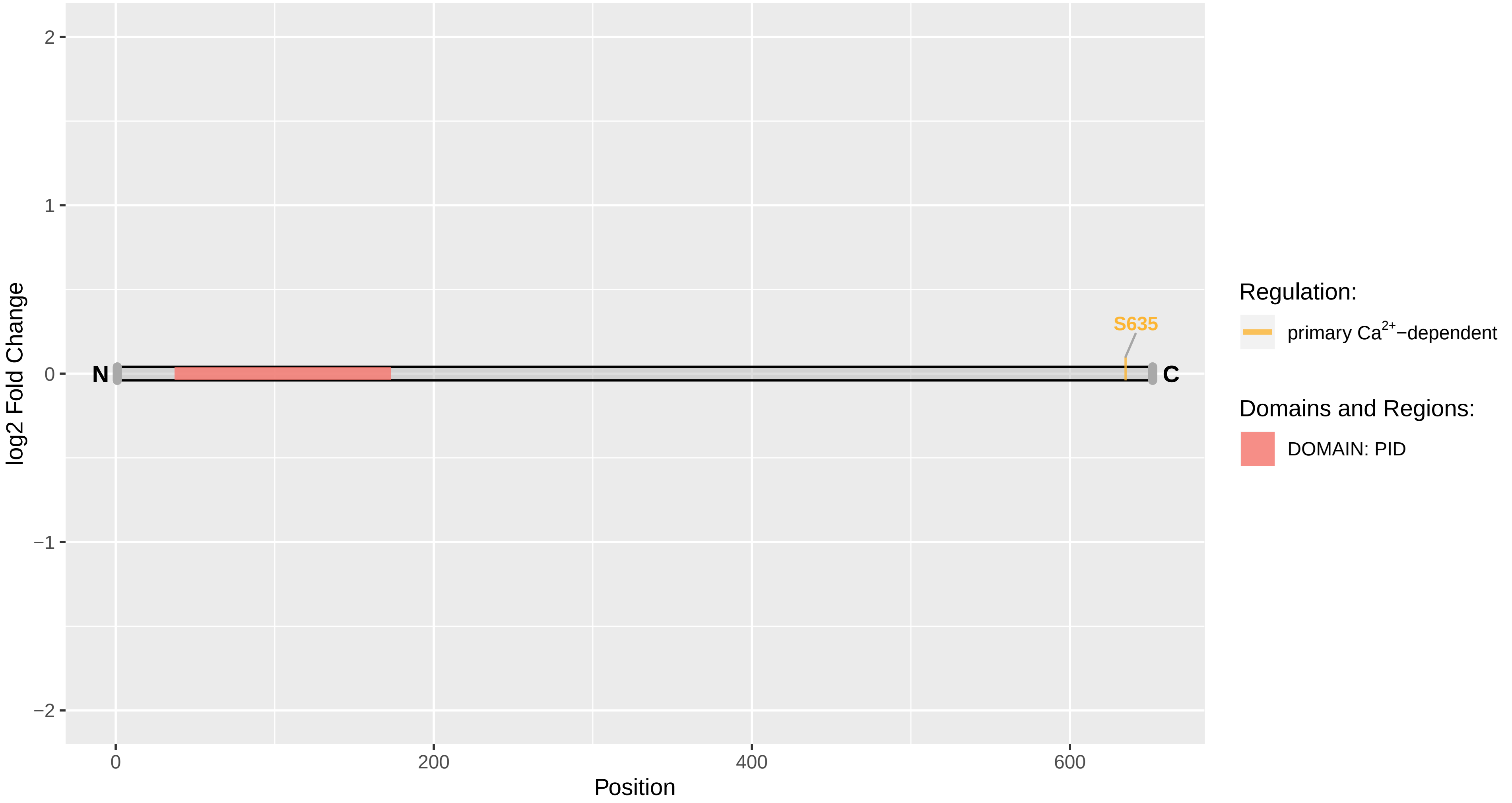

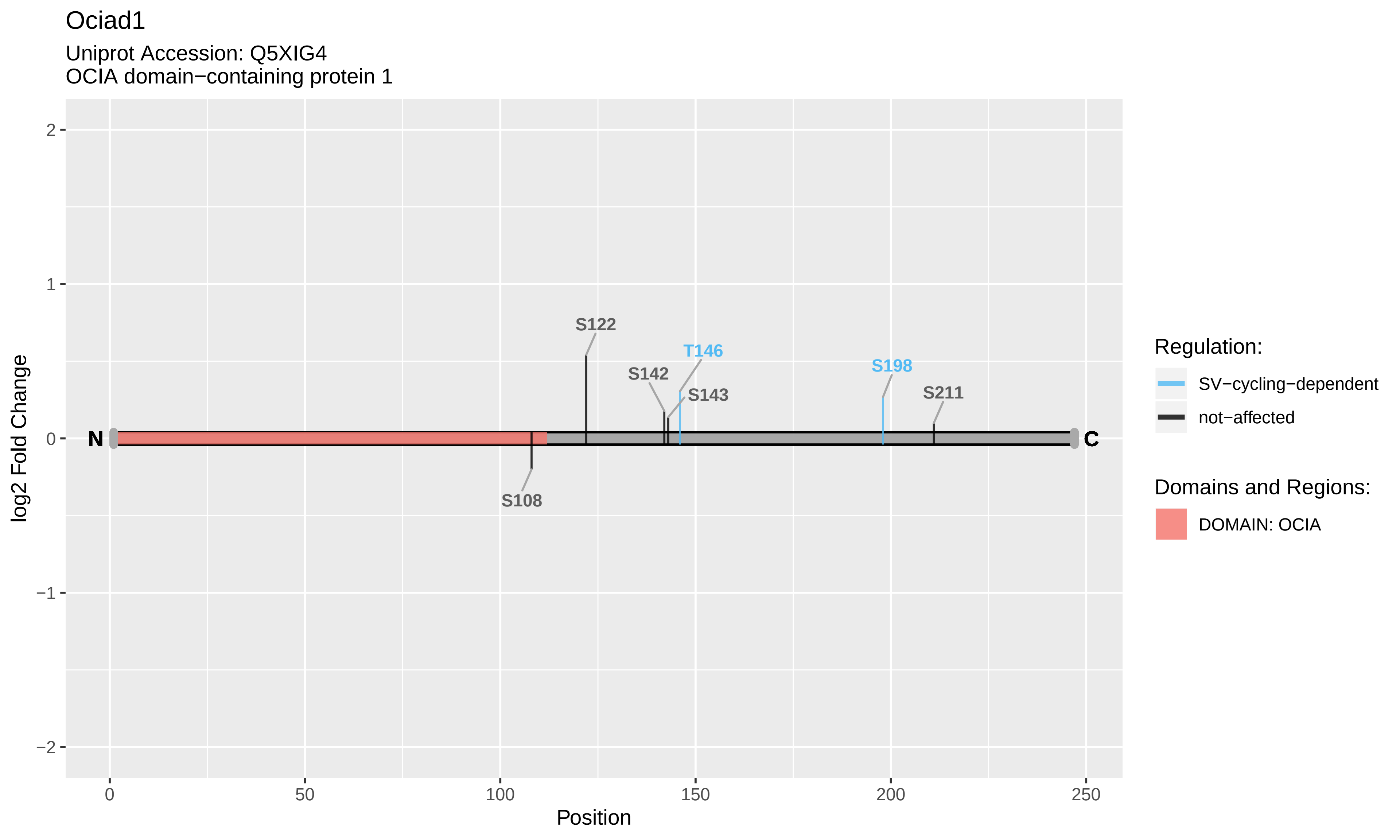
Ociad1
Uniprot Accession: Q5XIG4
OCIA domain−containing protein 1
log2 Fold Change
Position
N
C
S122
S142
T146
S143
S198
S211
S108
Regulation:
SV−cycling−dependent
not−affected
Domains and Regions:
DOMAIN: OCIA
2
1
0
−1
−2
0
50
100
150
200
250

Oprm1

Uniprot Accession: P33535
Mu−type opioid receptor

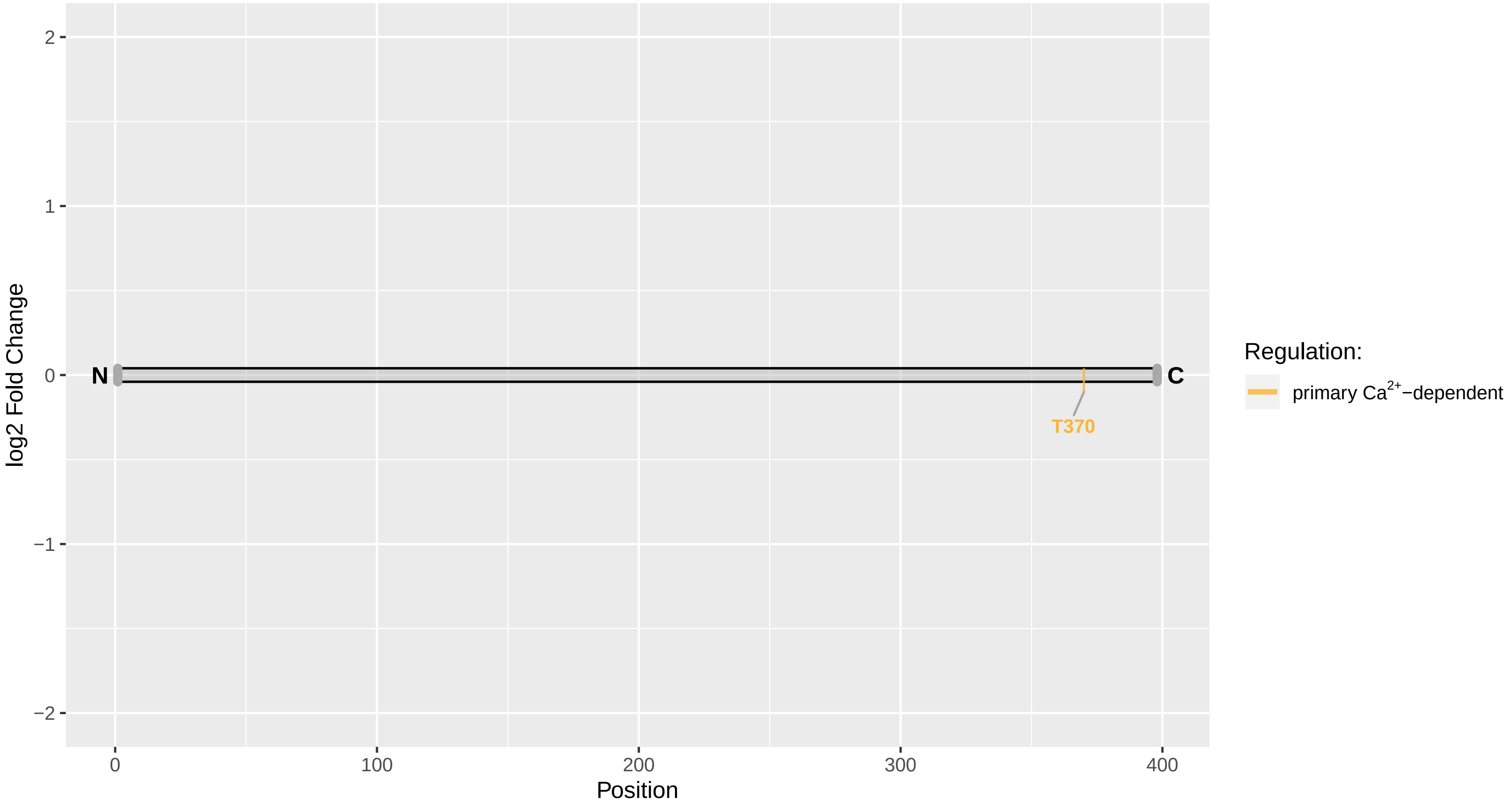

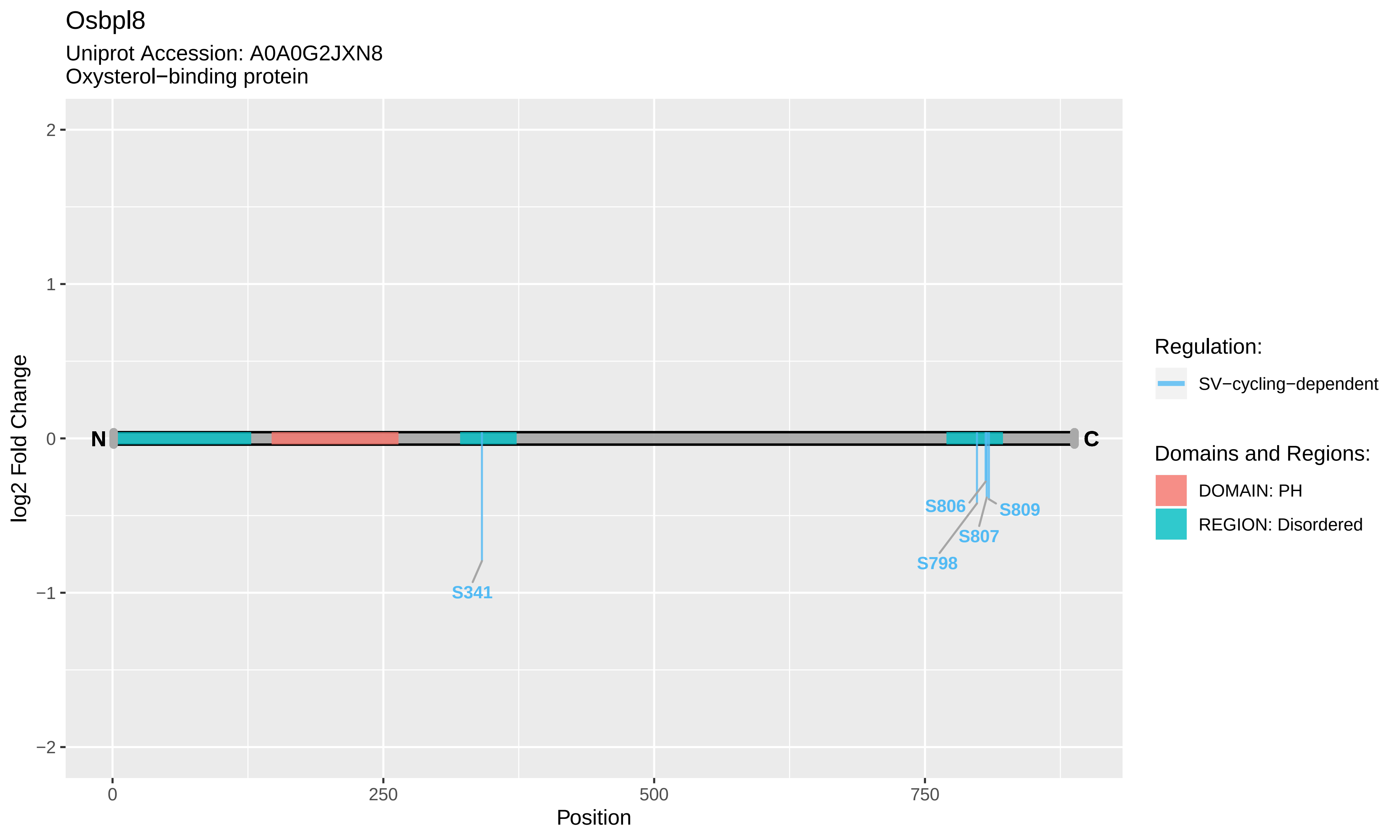

Osbpl8
Uniprot Accession: A0A0G2JXN8
Oxysterol−binding protein
log2 Fold Change
Position
2
1
0
−1
−2
0
250
500
750
N
C
S341
S806
S809
S807
S798
Regulation:
SV−cycling−dependent
Domains and Regions:
DOMAIN: PH
REGION: Disordered

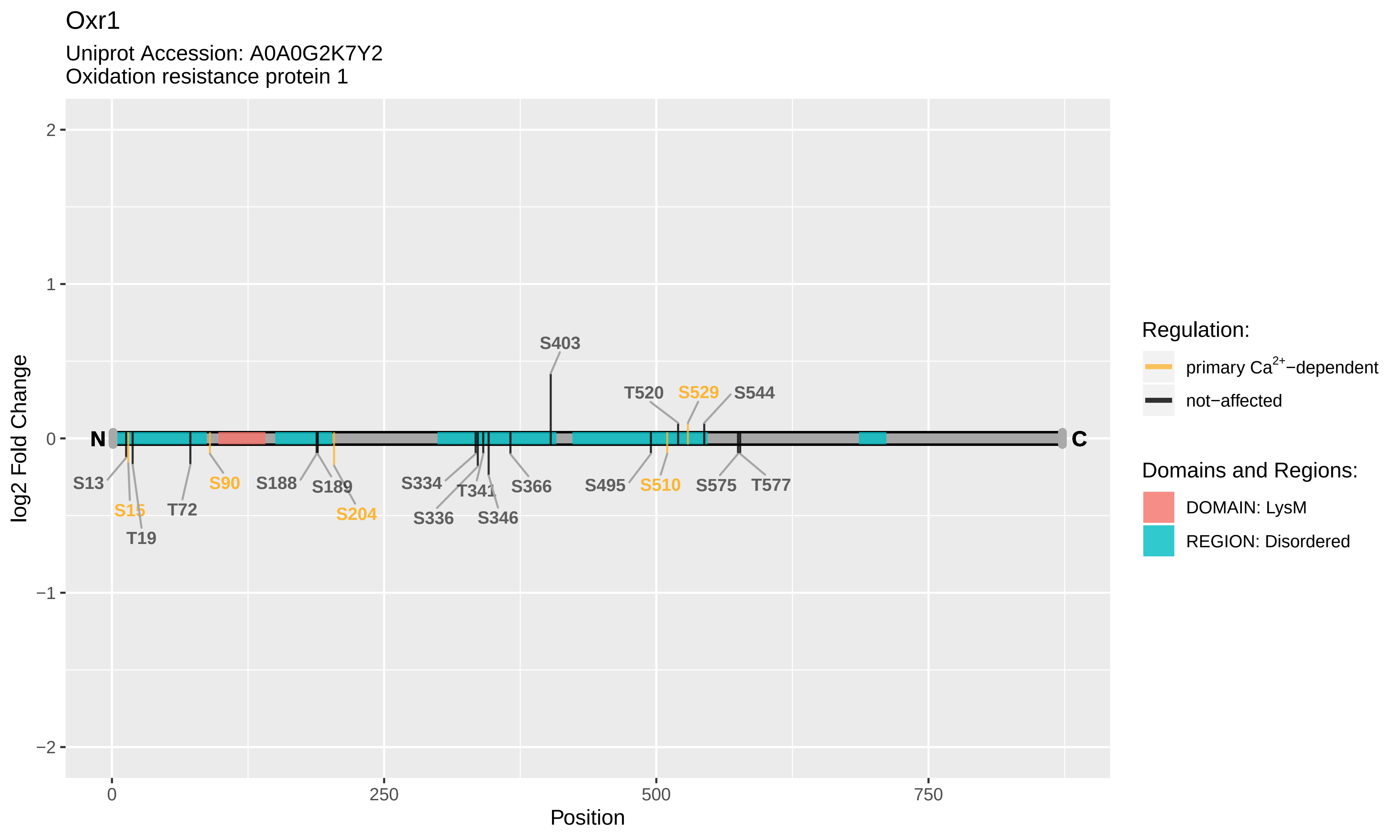

Oxr1
Uniprot Accession: A0A0G2K7Y2
Oxidation resistance protein 1
log2 Fold Change
2
1
0
−1
−2
N
C
S403
T520
S529
S544
S13
S15
T19
T72
S90
S188
S189
S204
S334
T341
S336
S346
S366
S495
S510
S575
T577
0
250
500
750
Position
Regulation:
primary Ca2+−dependent
not−affected
Domains and Regions:
DOMAIN: LysM
REGION: Disordered

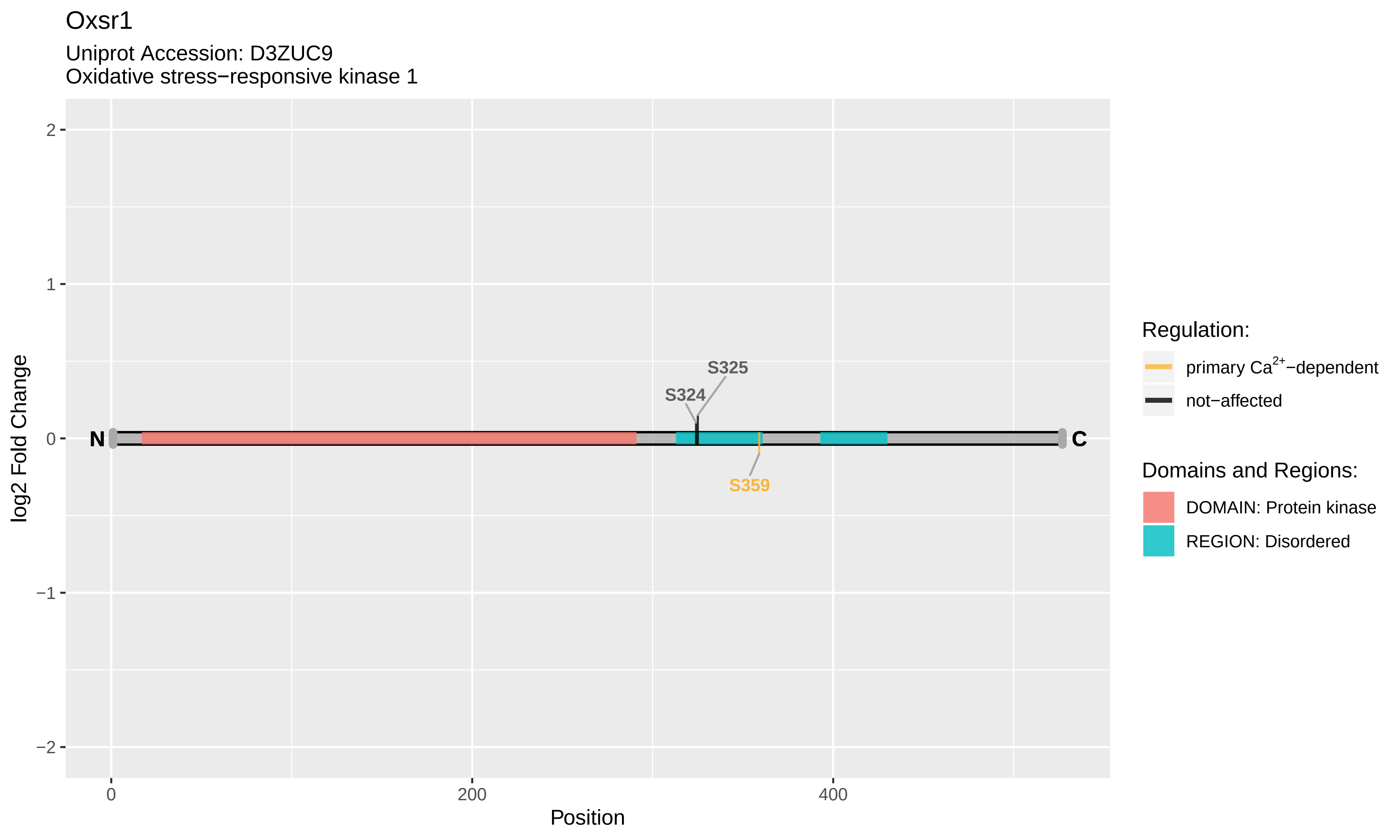

Oxsr1
Uniprot Accession: D3ZUC9
Oxidative stress−responsive kinase 1
2
1
0
−1
−2
log2 Fold Change
N
C
S325
S324
S359
0
200
400
Position
Regulation:
primary Ca2+−dependent
not−affected
Domains and Regions:
DOMAIN: Protein kinase
REGION: Disordered

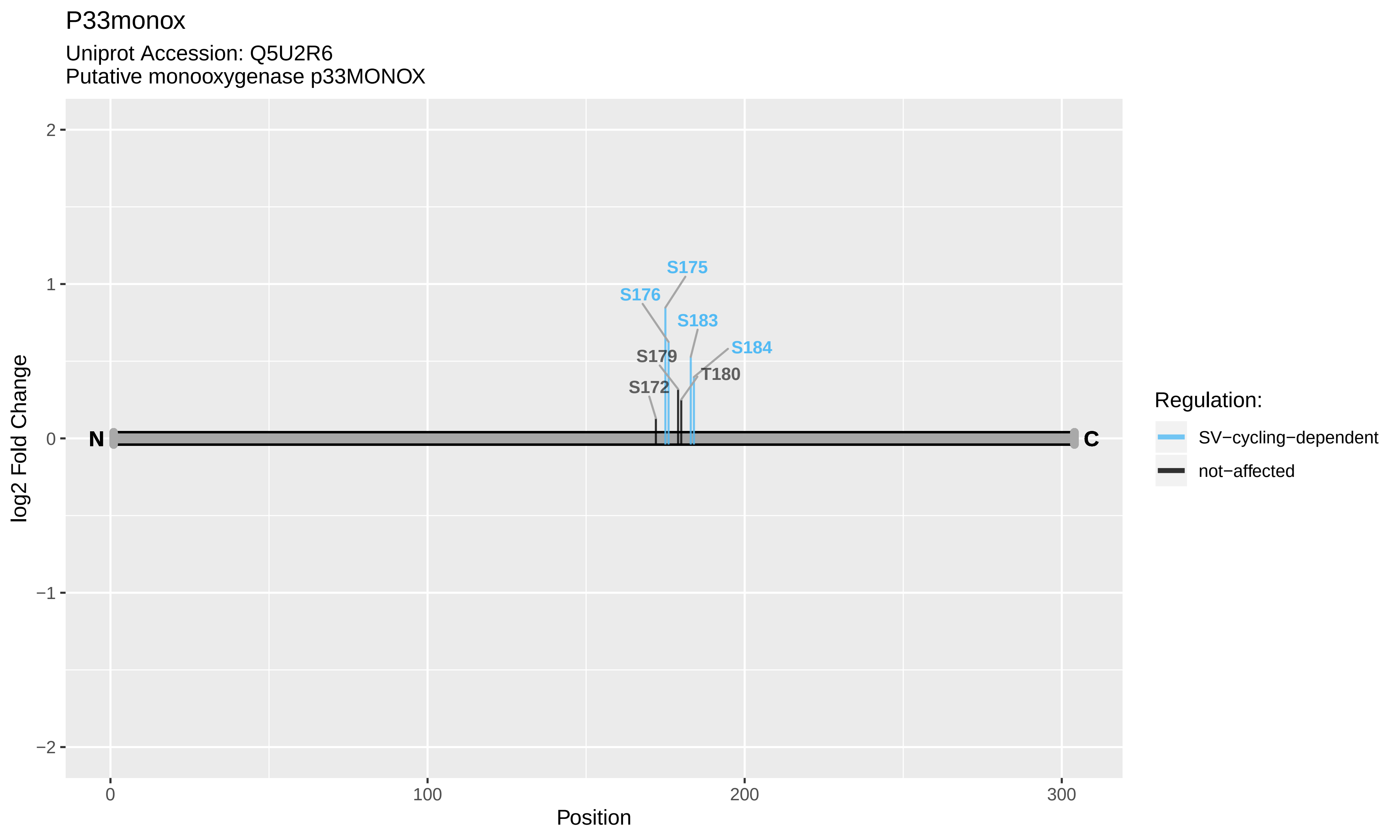
P33monox
Uniprot Accession: Q5U2R6
Putative monooxygenase p33MONOX
S175
S176
S183
S184
S179
T180
S172
N
C
2
1
0
−1
−2
0
100
200
300
Position
log2 Fold Change
Regulation:
SV−cycling−dependent
not−affected

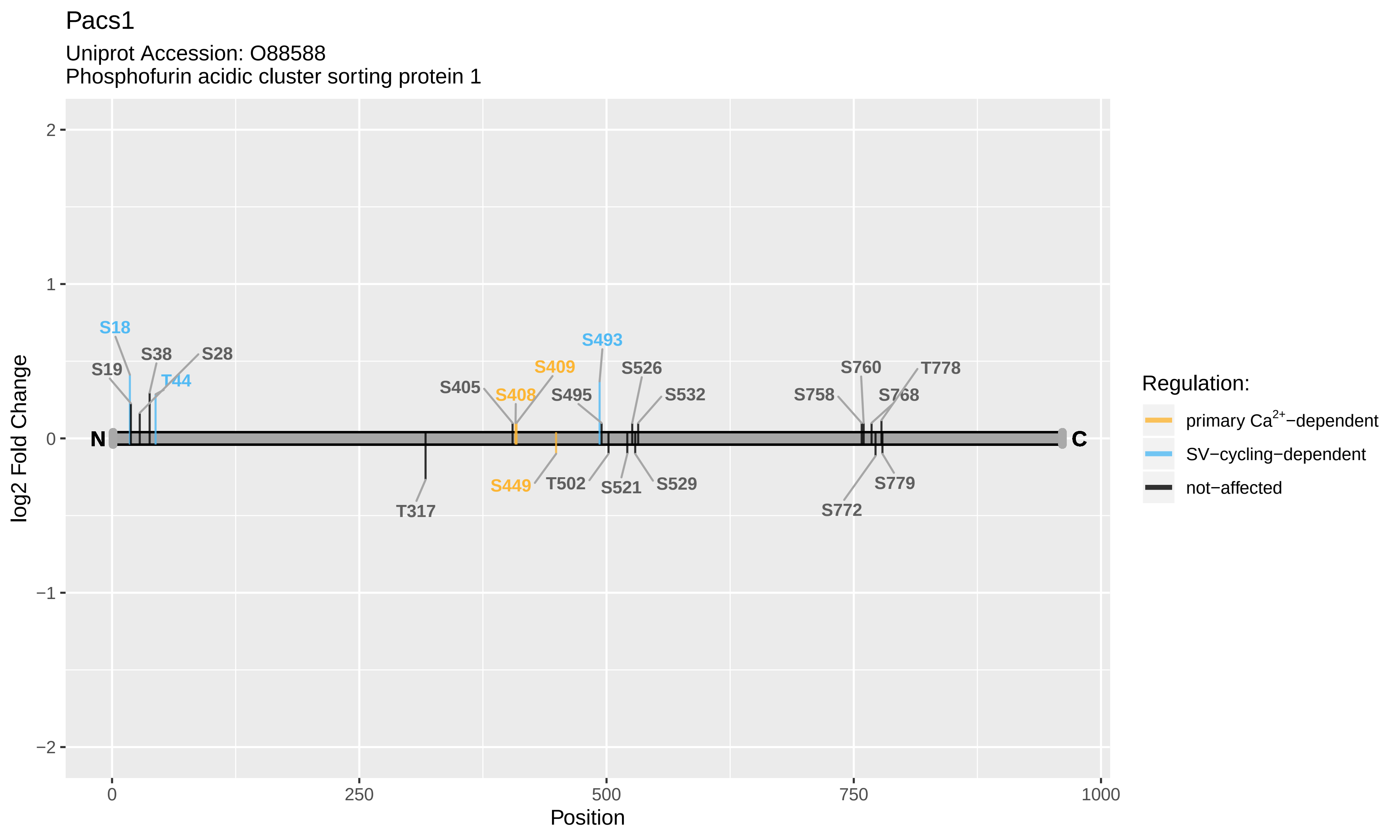

Pacs1
Uniprot Accession: O88588
Phosphofurin acidic cluster sorting protein 1
log2 Fold Change
Position
S18
S19
S38
S28
T44
S405
S408
S409
S493
S495
S526
S532
S760
T778
S758
S768
N
C
T317
S449
T502
S521
S529
S772
S779
Regulation:
primary Ca2+−dependent
SV−cycling−dependent
not−affected

Pacs2

Uniprot Accession: D3ZJG4
Phosphofurin acidic cluster sorting protein 2

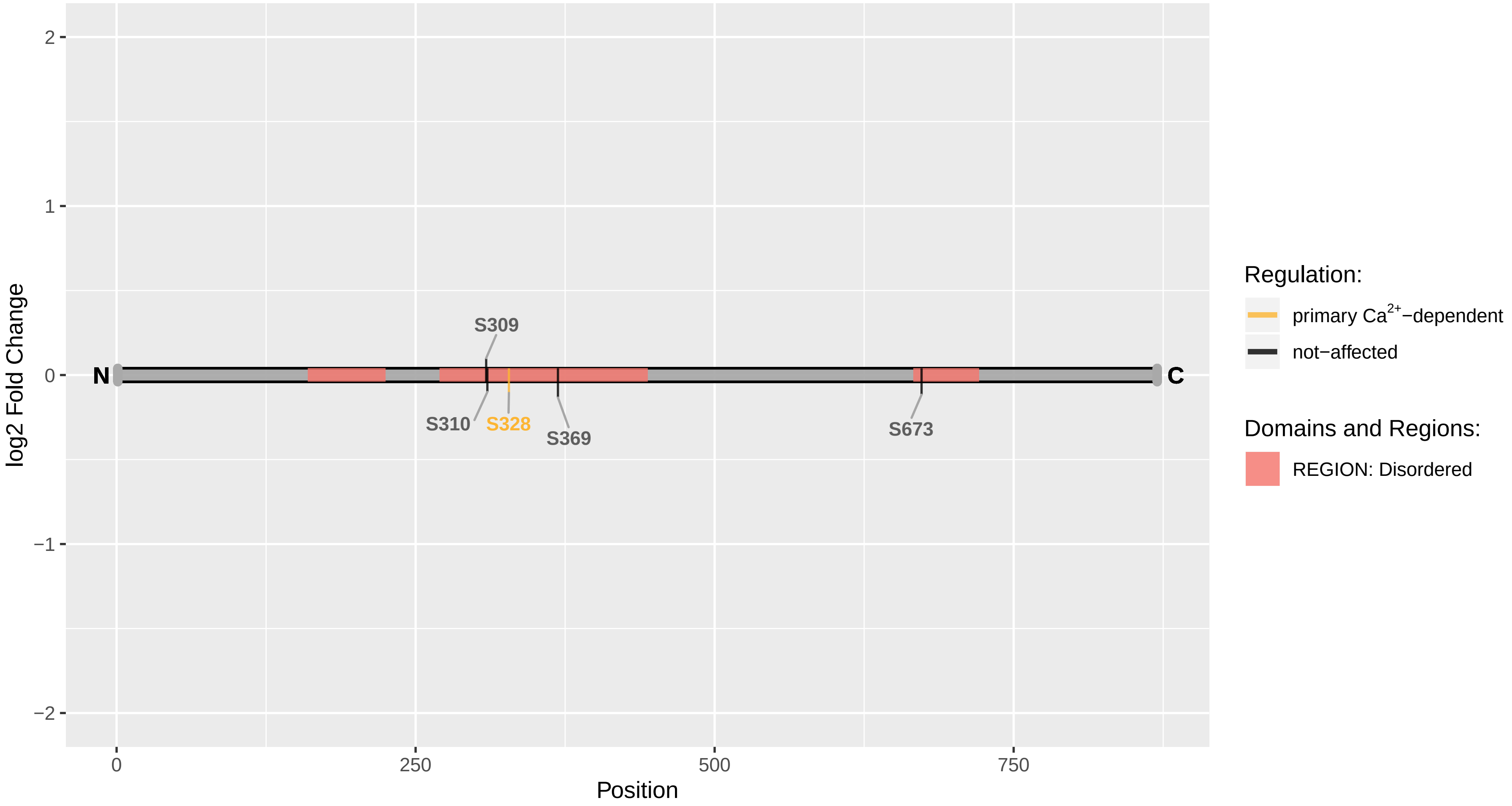

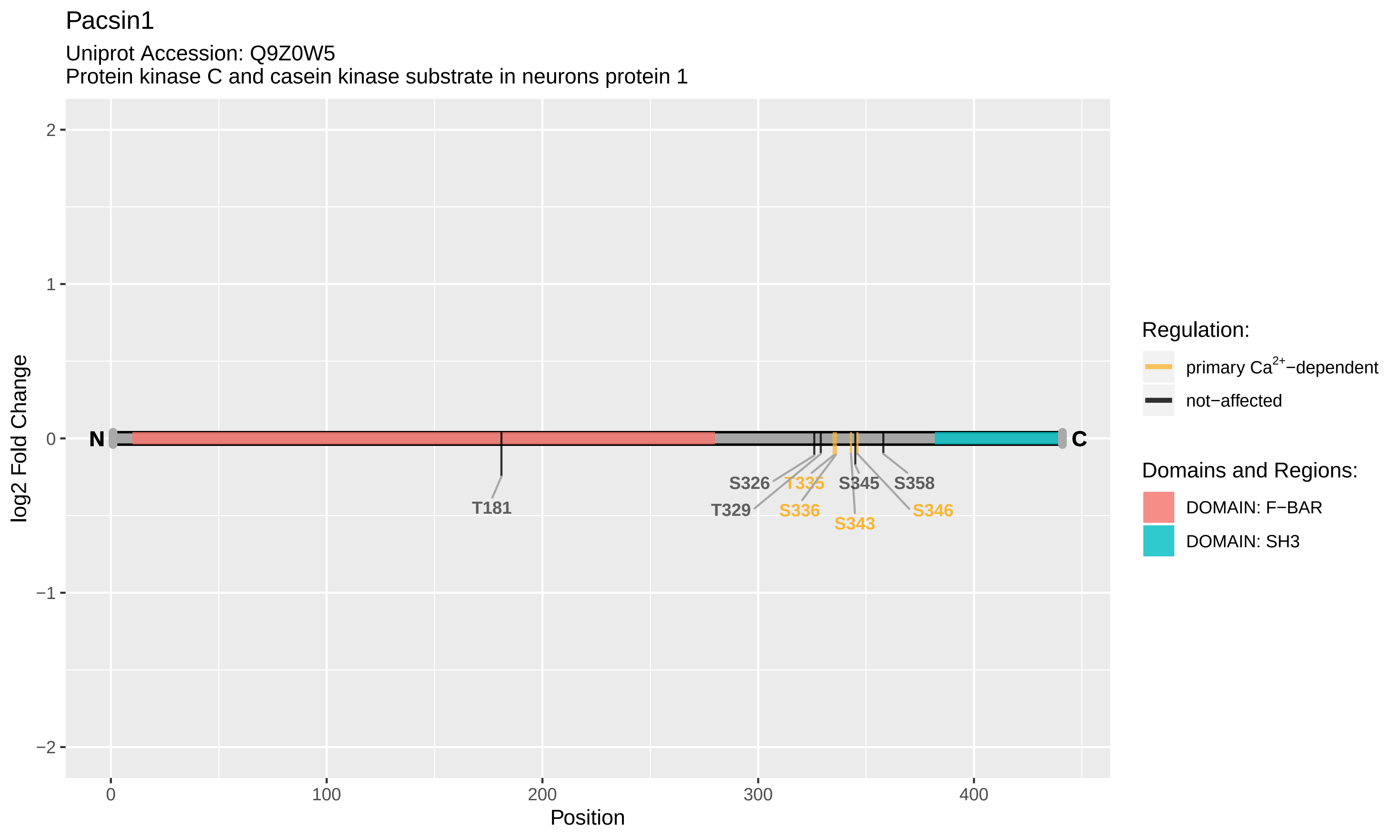
Pacsin1
Uniprot Accession: Q9Z0W5
Protein kinase C and casein kinase substrate in neurons protein 1
log2 Fold Change
Position
N
C
T181
S326
T329
T335
S336
S343
S345
S346
S358
Regulation:
primary Ca2+−dependent
not−affected
Domains and Regions:
DOMAIN: F−BAR
DOMAIN: SH3
2
1
0
−1
−2
0
100
200
300
400

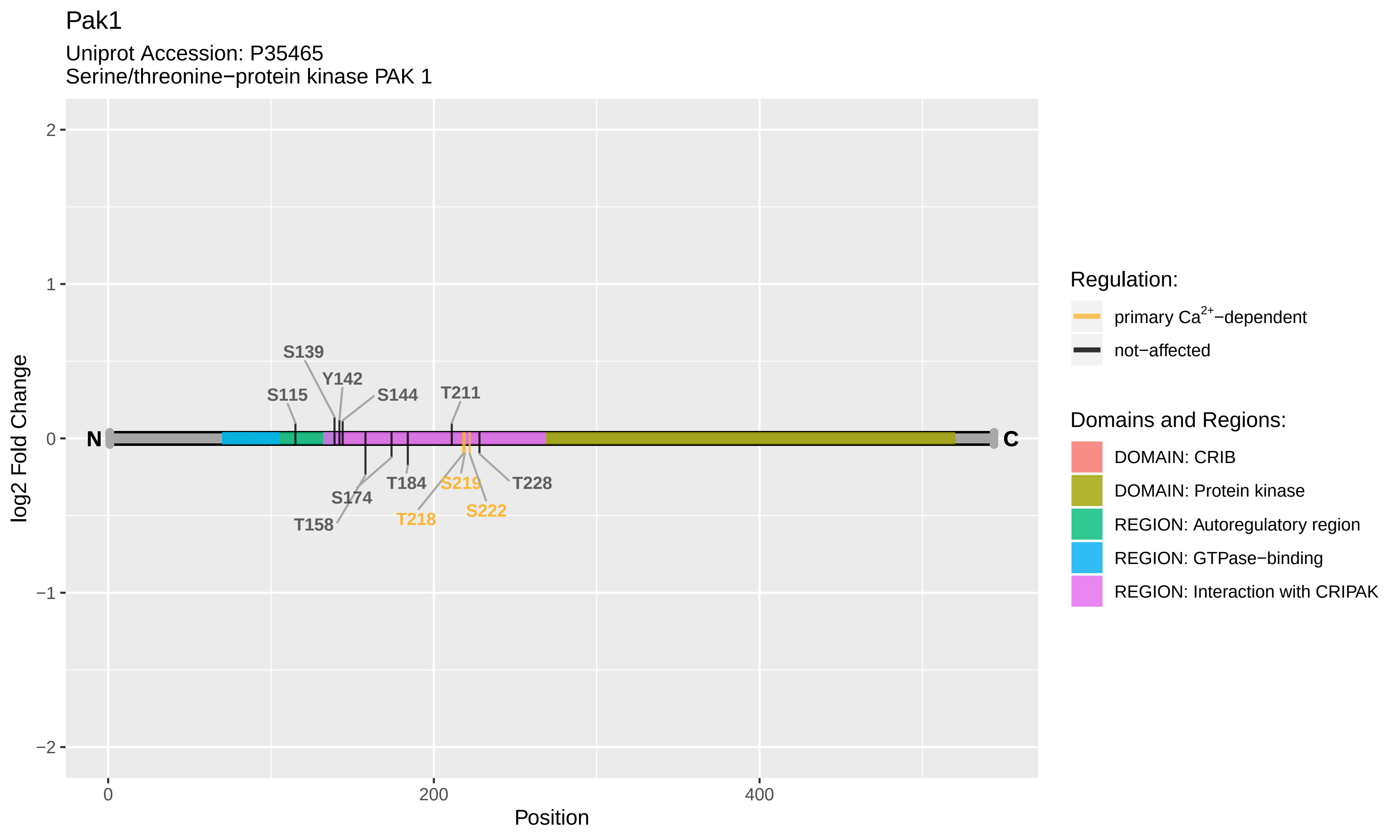
Pak1
Uniprot Accession: P35465
Serine/threonine−protein kinase PAK 1
log2 Fold Change
2
1
0
−1
−2
N
C
S139
Y142
S115
S144
T211
T184
S219
T228
S174
S222
T158
T218
0
200
400
Position
Regulation:
primary Ca2+−dependent
not−affected
Domains and Regions:
DOMAIN: CRIB
DOMAIN: Protein kinase
REGION: Autoregulatory region
REGION: GTPase−binding
REGION: Interaction with CRIPAK

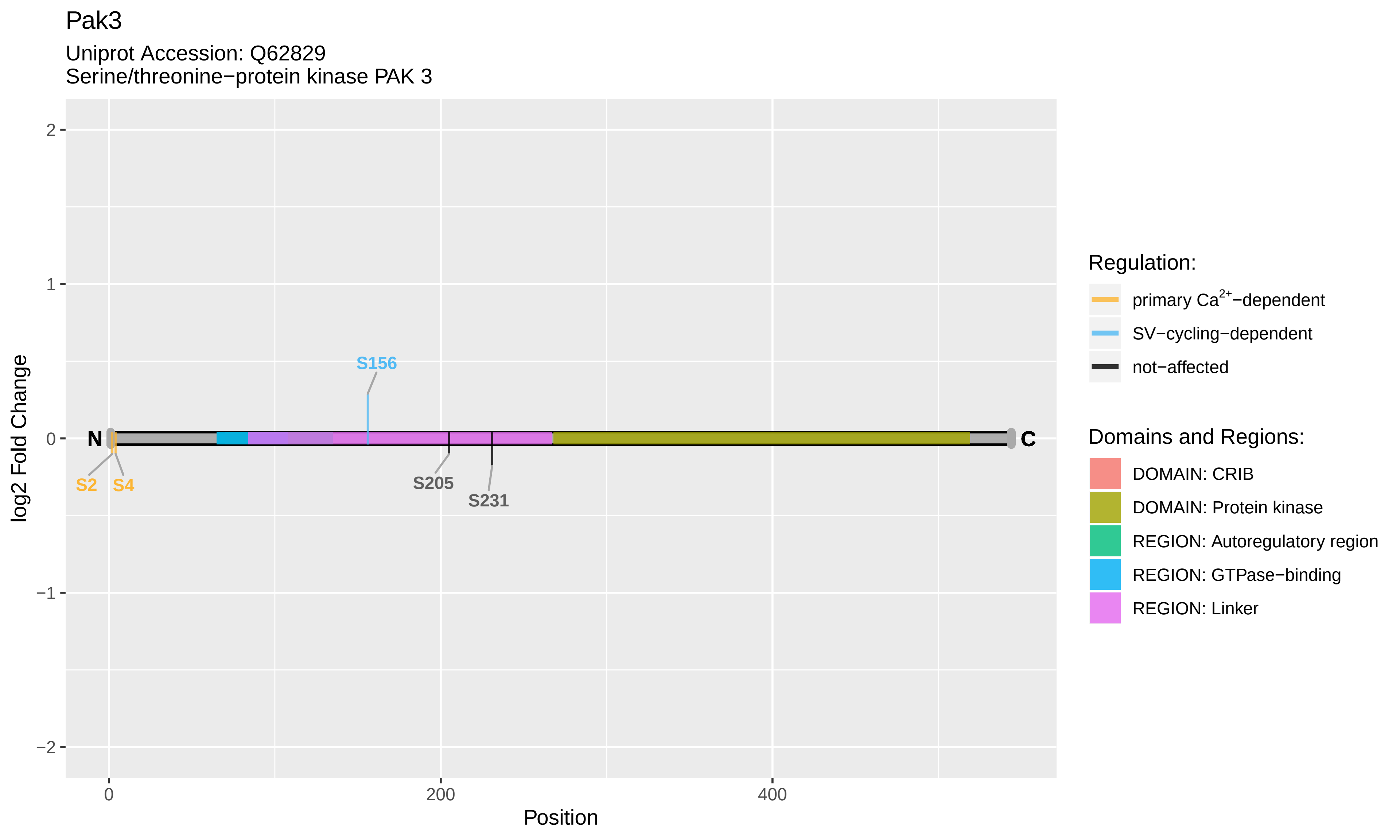

Pak3
Uniprot Accession: Q62829
Serine/threonine−protein kinase PAK 3
log2 Fold Change
2
1
0
−1
−2
Position
0
200
400
N
C
S156
S2
S4
S205
S231
Regulation:
primary Ca2+−dependent
SV−cycling−dependent
not−affected
Domains and Regions:
DOMAIN: CRIB
DOMAIN: Protein kinase
REGION: Autoregulatory region
REGION: GTPase−binding
REGION: Linker

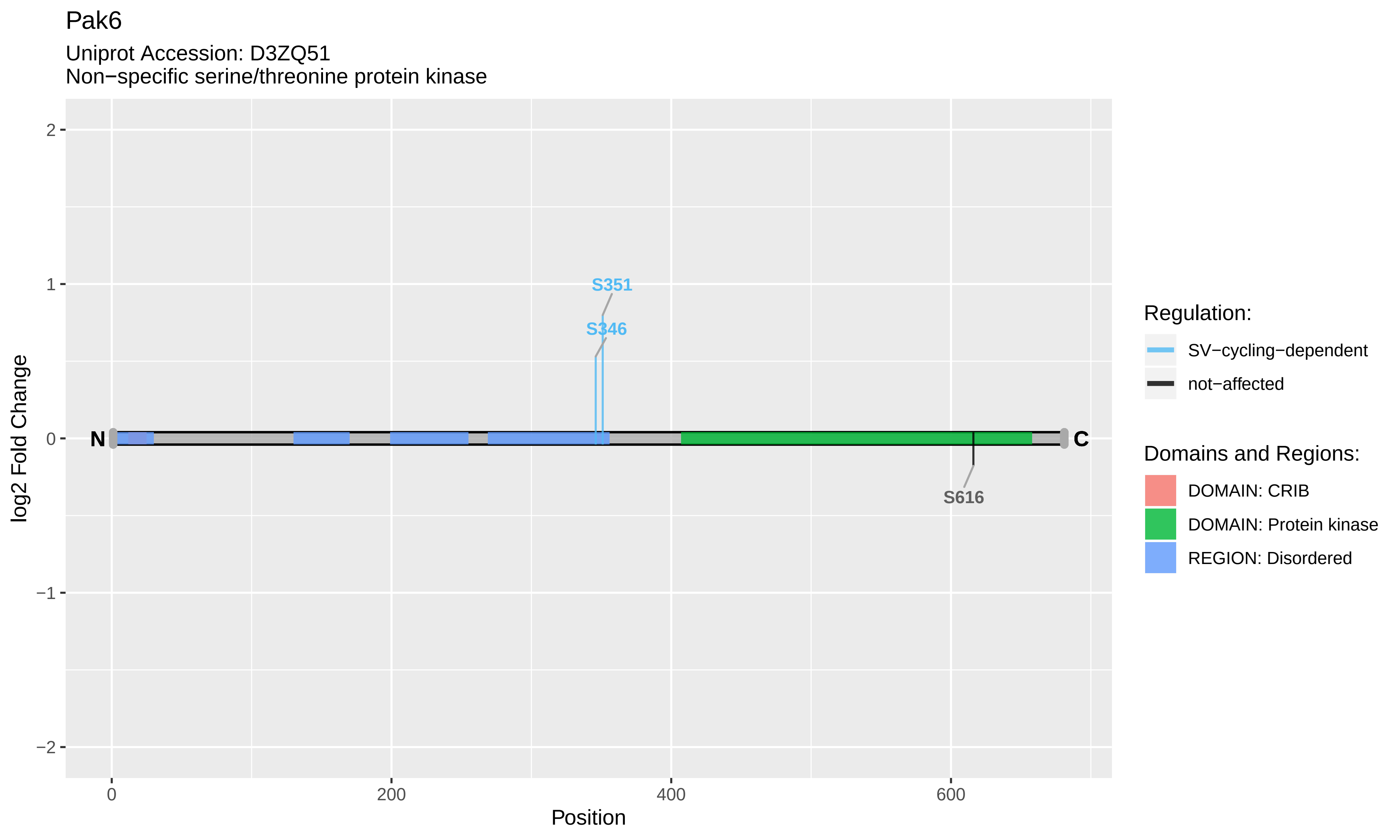

Pak6
Uniprot Accession: D3ZQ51
Non−specific serine/threonine protein kinase
S351
S346
S616
N
C
2
1
0
−1
−2
log2 Fold Change
0
200
400
600
Position
Regulation:
SV−cycling−dependent
not−affected
Domains and Regions:
DOMAIN: CRIB
DOMAIN: Protein kinase
REGION: Disordered

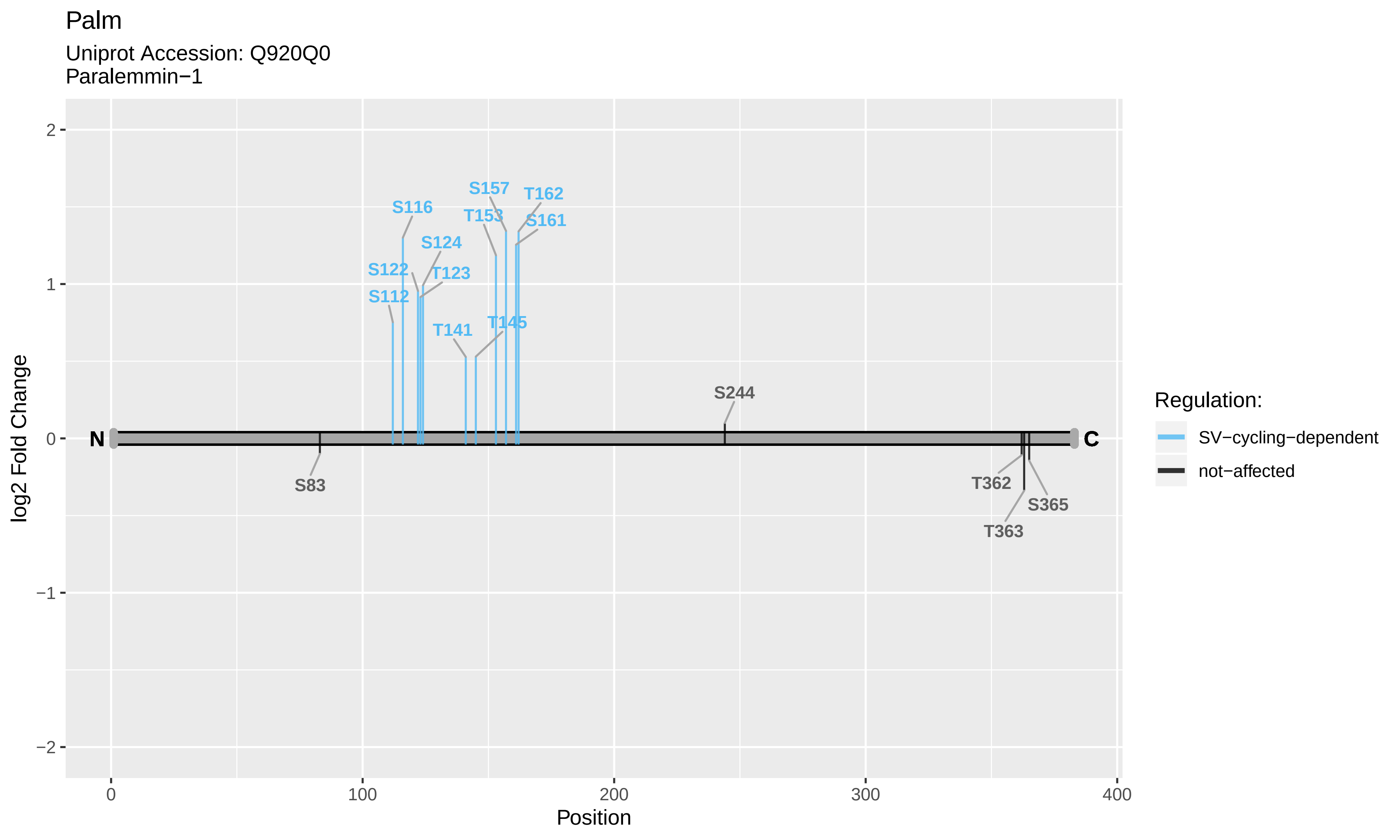

Palm
Uniprot Accession: Q920Q0
Paralemmin−1
log2 Fold Change
Position
N
C
S116
S157
T162
T153
S161
S124
S122
T123
S112
T141
T145
S244
S83
T362
S365
T363
Regulation:
SV−cycling−dependent
not−affected
2
1
0
−1
−2
0
100
200
300
400

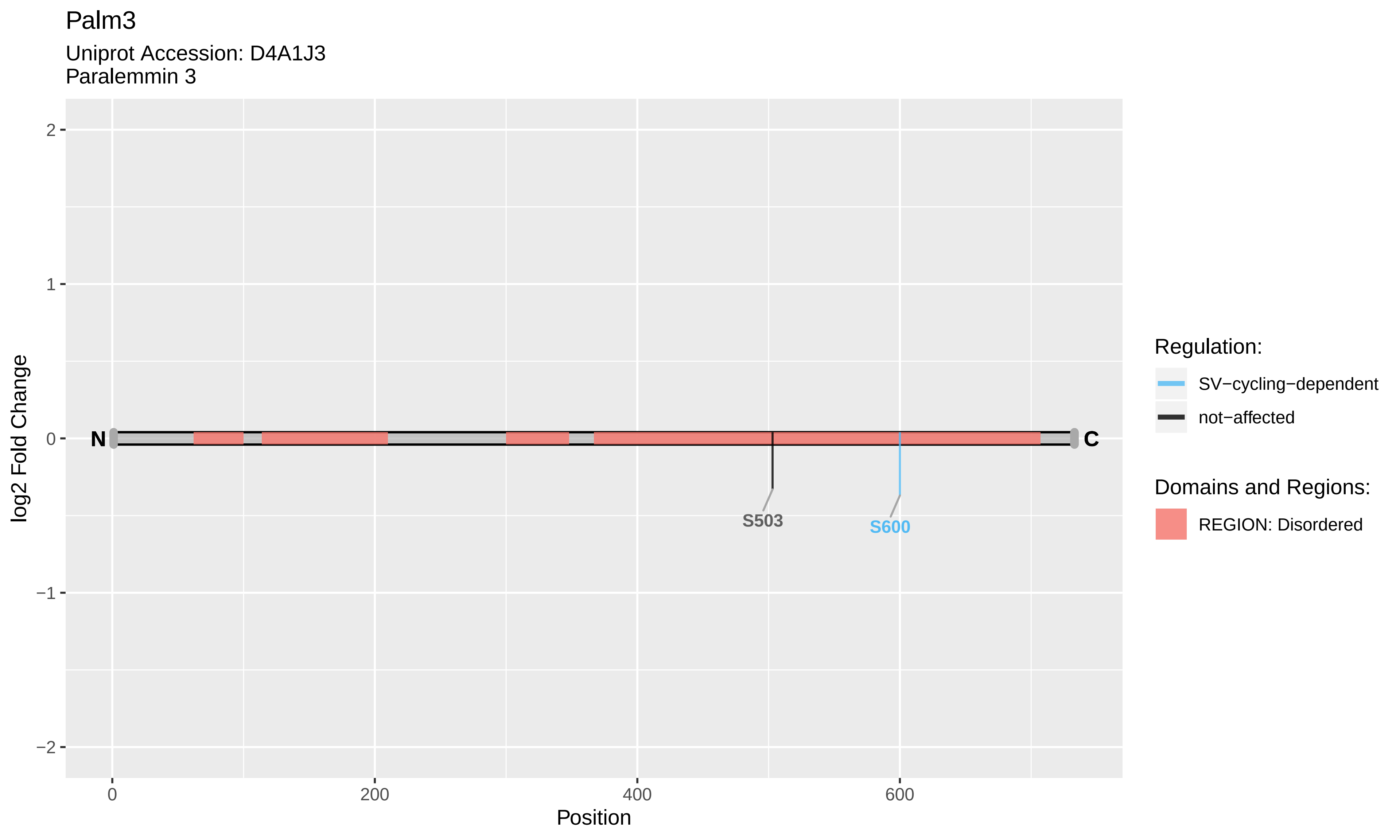

Palm3
Uniprot Accession: D4A1J3
Paralemmin 3
2
1
0
−1
−2
log2 Fold Change
N
C
S503
S600
0
200
400
600
Position
Regulation:
SV−cycling−dependent
not−affected
Domains and Regions:
REGION: Disordered

Palmd

Uniprot Accession: Q4KM62
Palmdelphin

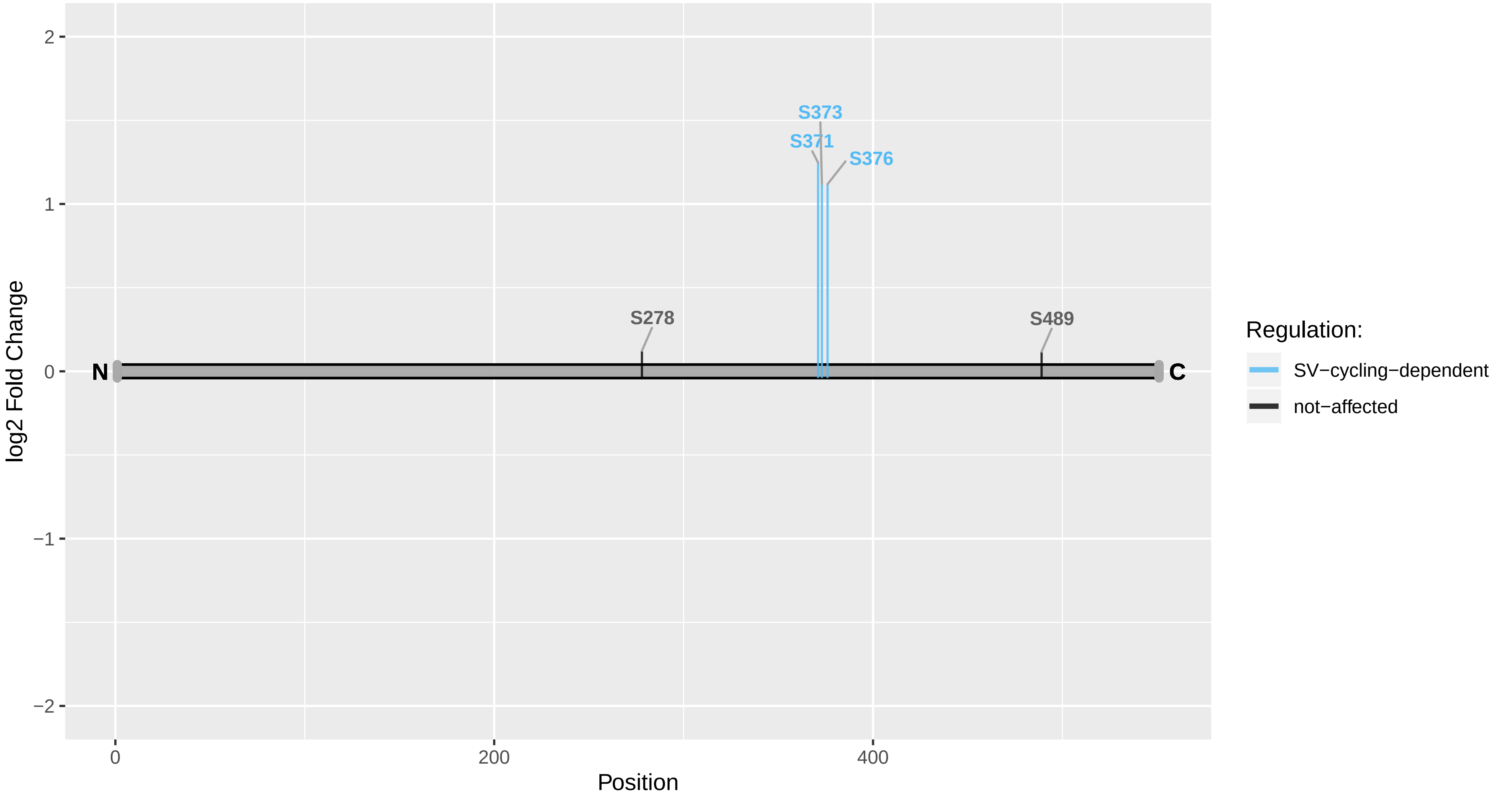

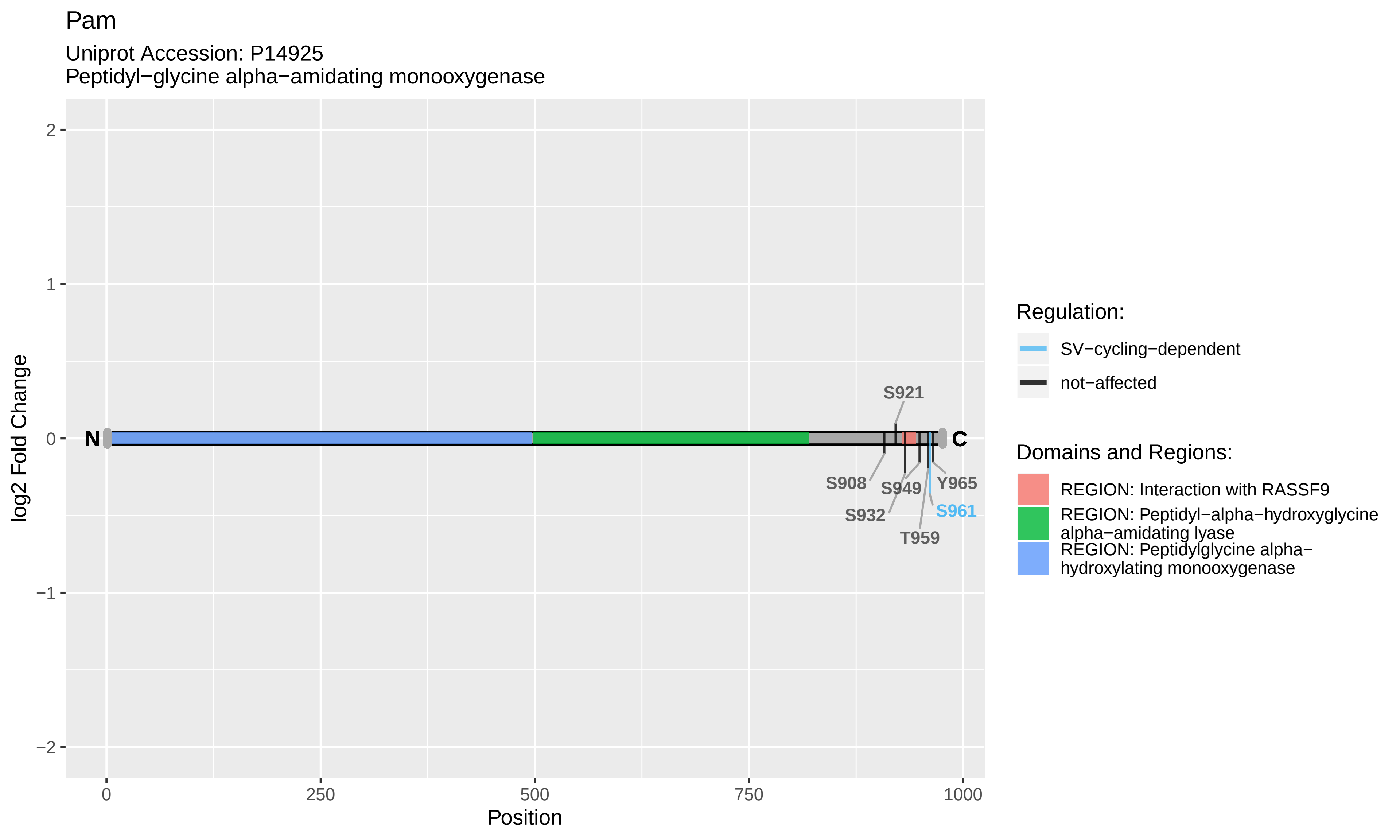

Pam
Uniprot Accession: P14925
Peptidyl−glycine alpha−amidating monooxygenase
log2 Fold Change
2
1
0
−1
−2
N
C
S921
S908
S949
Y965
S932
S961
T959
0
250
500
750
1000
Position
Regulation:
SV−cycling−dependent
not−affected
Domains and Regions:
REGION: Interaction with RASSF9
REGION: Peptidyl−alpha−hydroxyglycine alpha−amidating lyase
REGION: Peptidylglycine alpha−hydroxylating monooxygenase

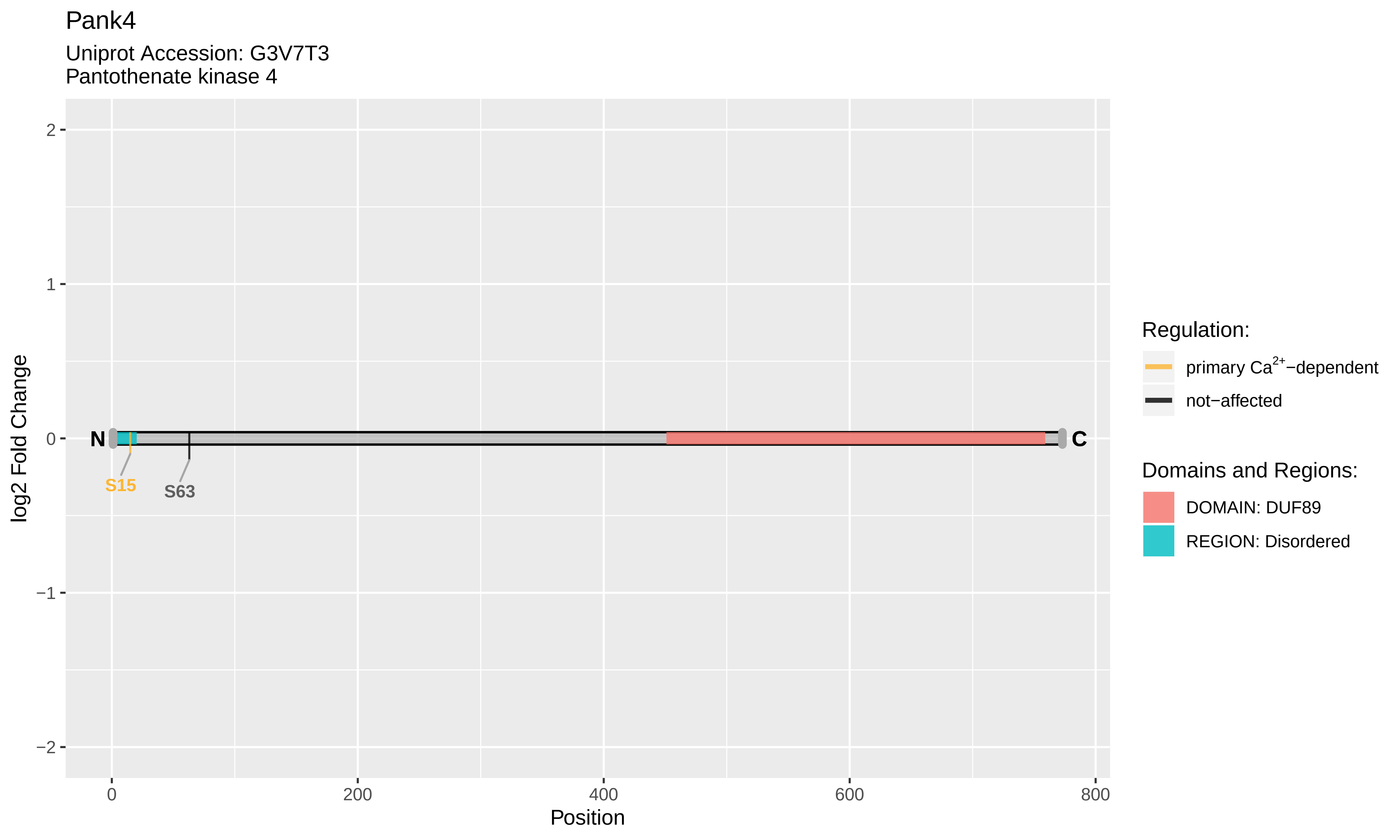
Pank4
Uniprot Accession: G3V7T3
Pantothenate kinase 4
log2 Fold Change
2
1
0
−1
−2
N
C
S15
S63
0
200
400
600
800
Position
Regulation:
primary Ca2+−dependent
not−affected
Domains and Regions:
DOMAIN: DUF89
REGION: Disordered

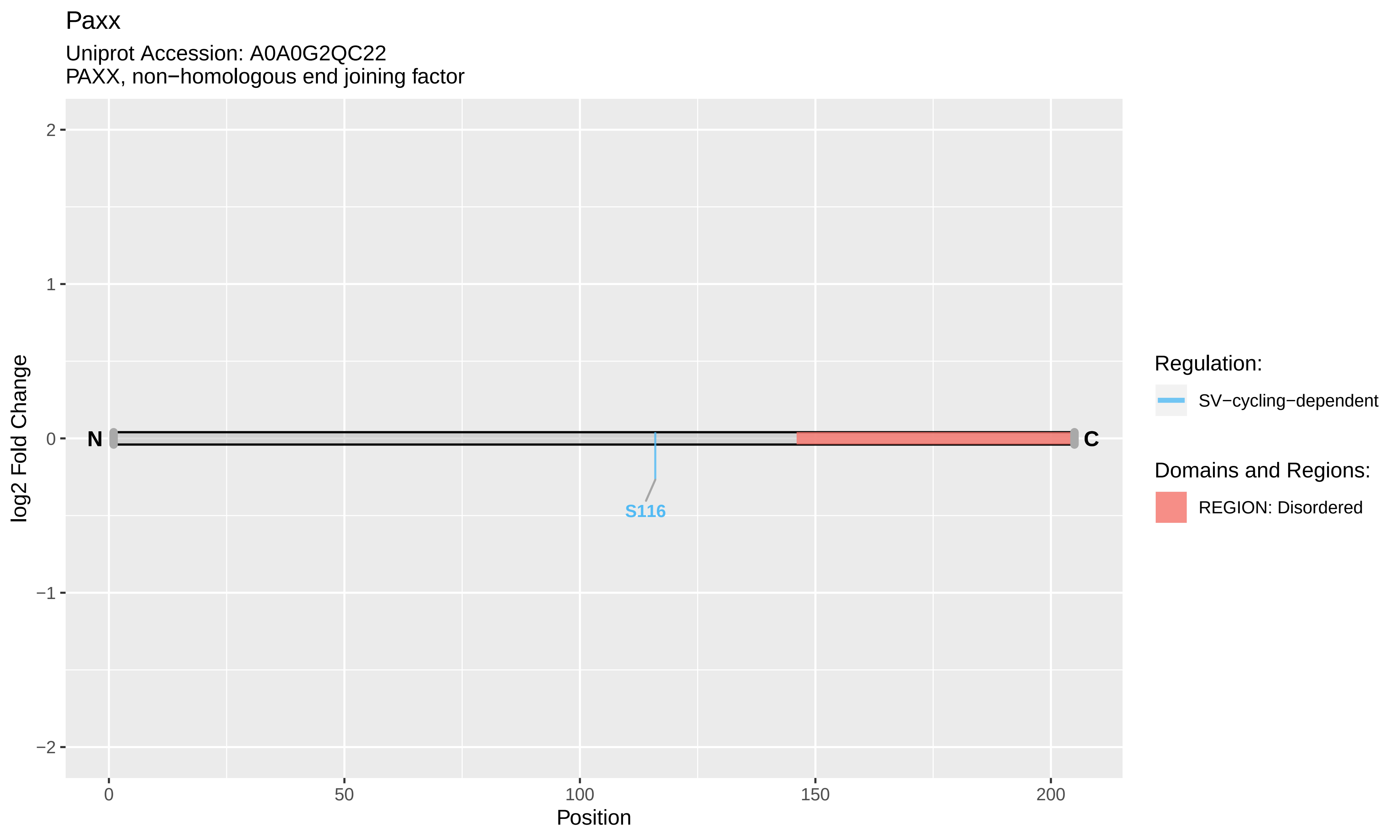

Paxx
Uniprot Accession: A0A0G2QC22
PAXX, non−homologous end joining factor
log2 Fold Change
2
1
0
−1
−2
N
C
S116
0
50
100
150
200
Position
Regulation:
SV−cycling−dependent
Domains and Regions:
REGION: Disordered

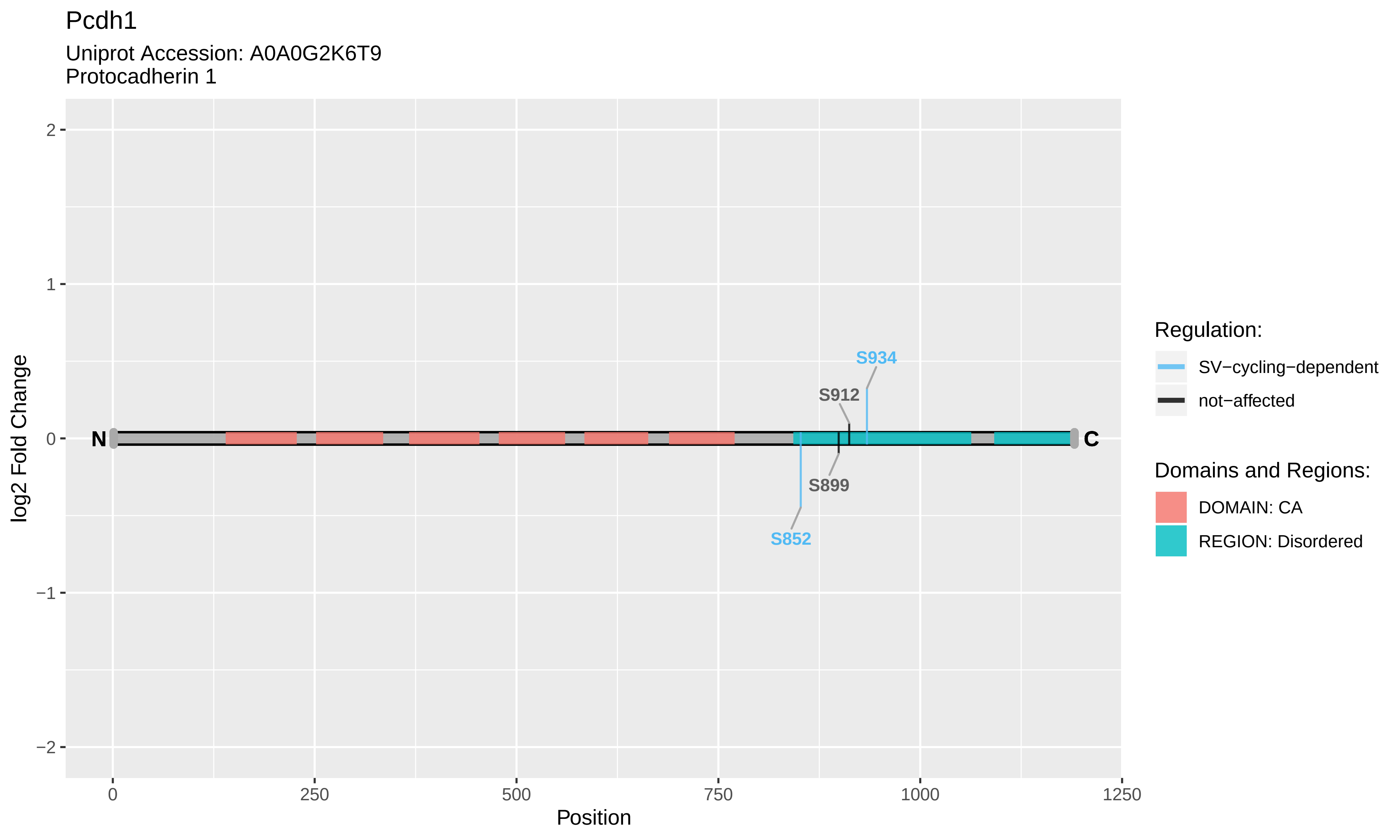

Pcdh1
Uniprot Accession: A0A0G2K6T9
Protocadherin 1
log2 Fold Change
Position
N
C
S934
S912
S899
S852
Regulation:
SV−cycling−dependent
not−affected
Domains and Regions:
DOMAIN: CA
REGION: Disordered

Pcdh17

Uniprot Accession: D3ZPN0
Protocadherin 17

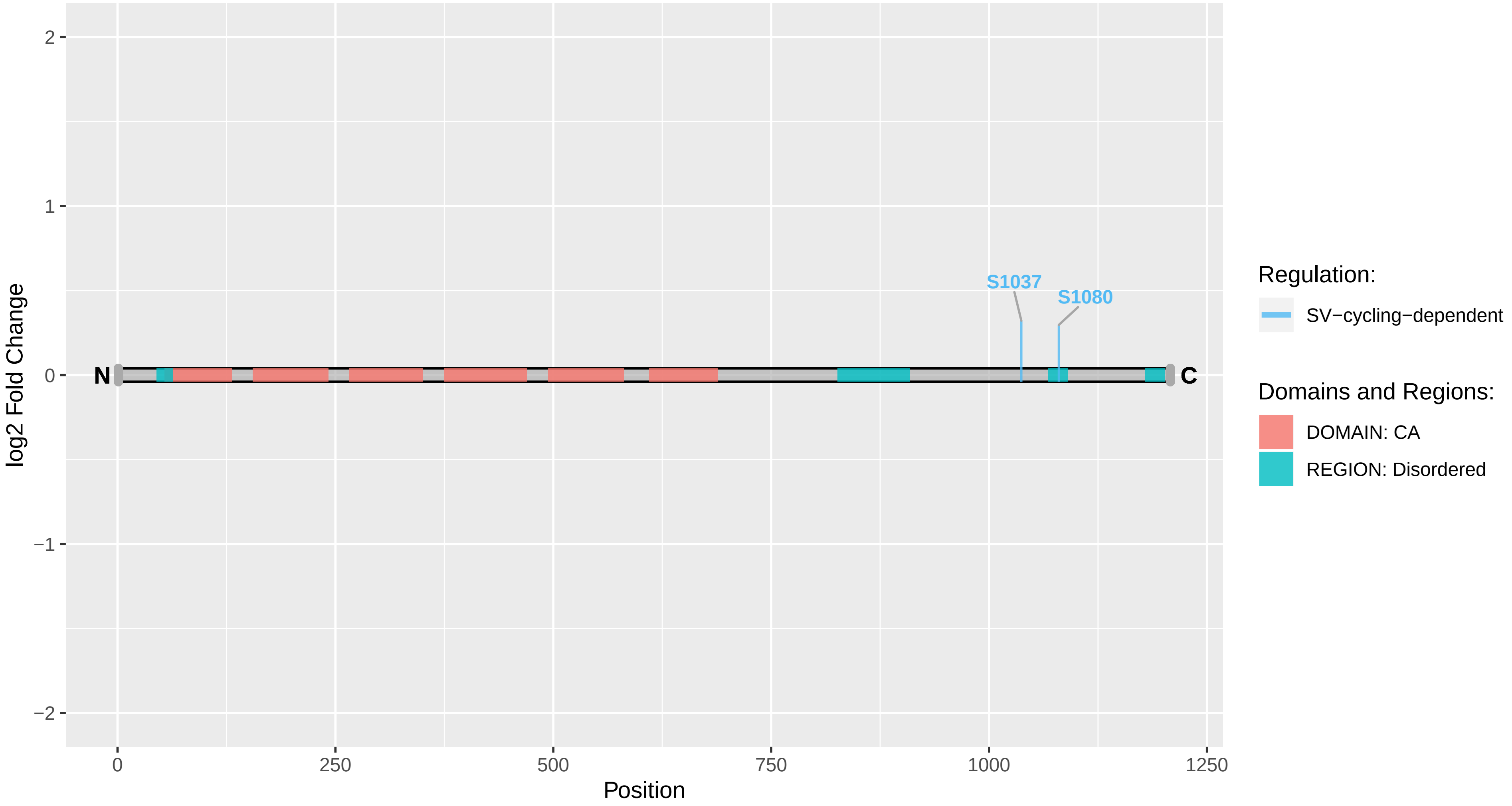

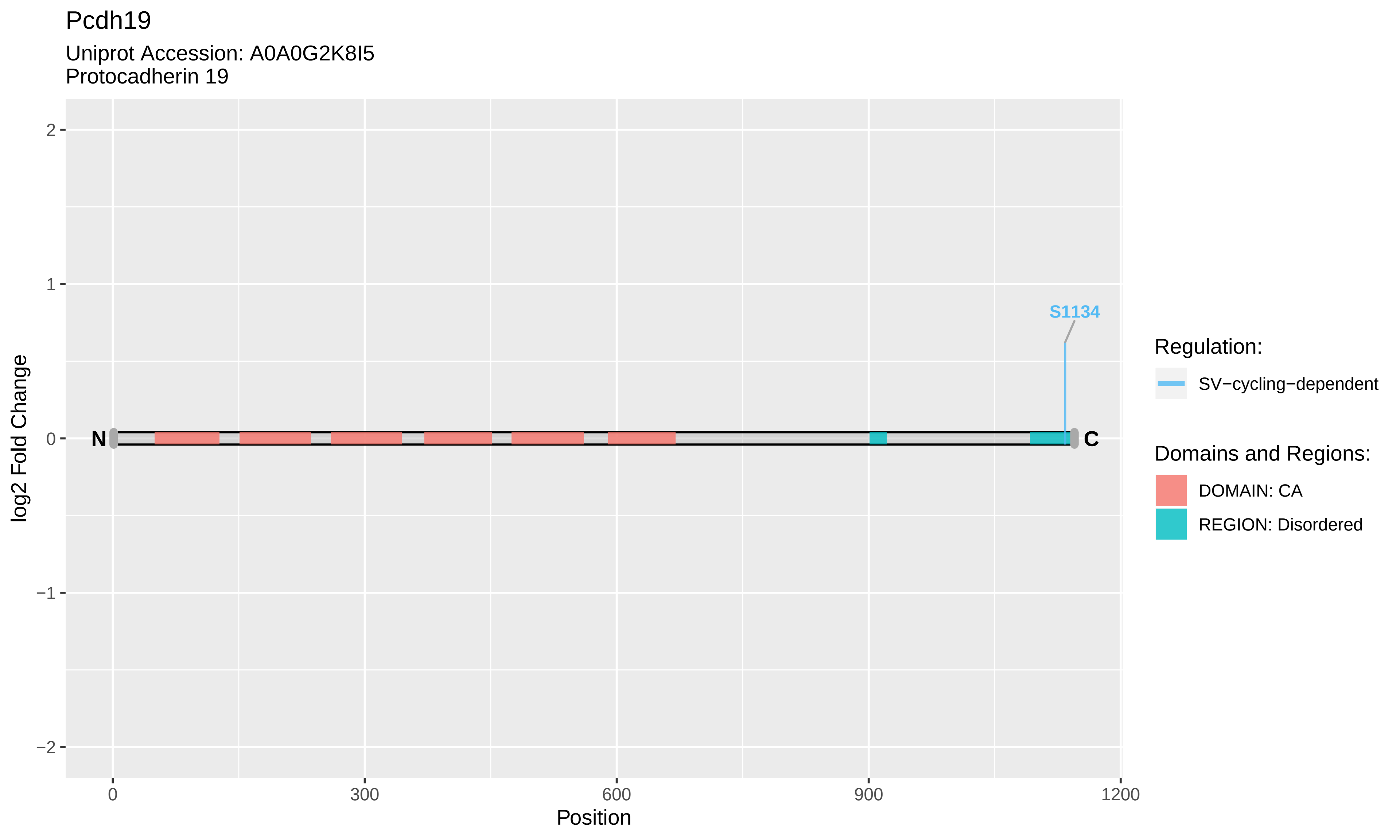
Pcdh19
Uniprot Accession: A0A0G2K8I5
Protocadherin 19
S1134
N
C
2
1
0
−1
−2
log2 Fold Change
0
300
600
900
1200
Position
Regulation:
SV−cycling−dependent
Domains and Regions:
DOMAIN: CA
REGION: Disordered

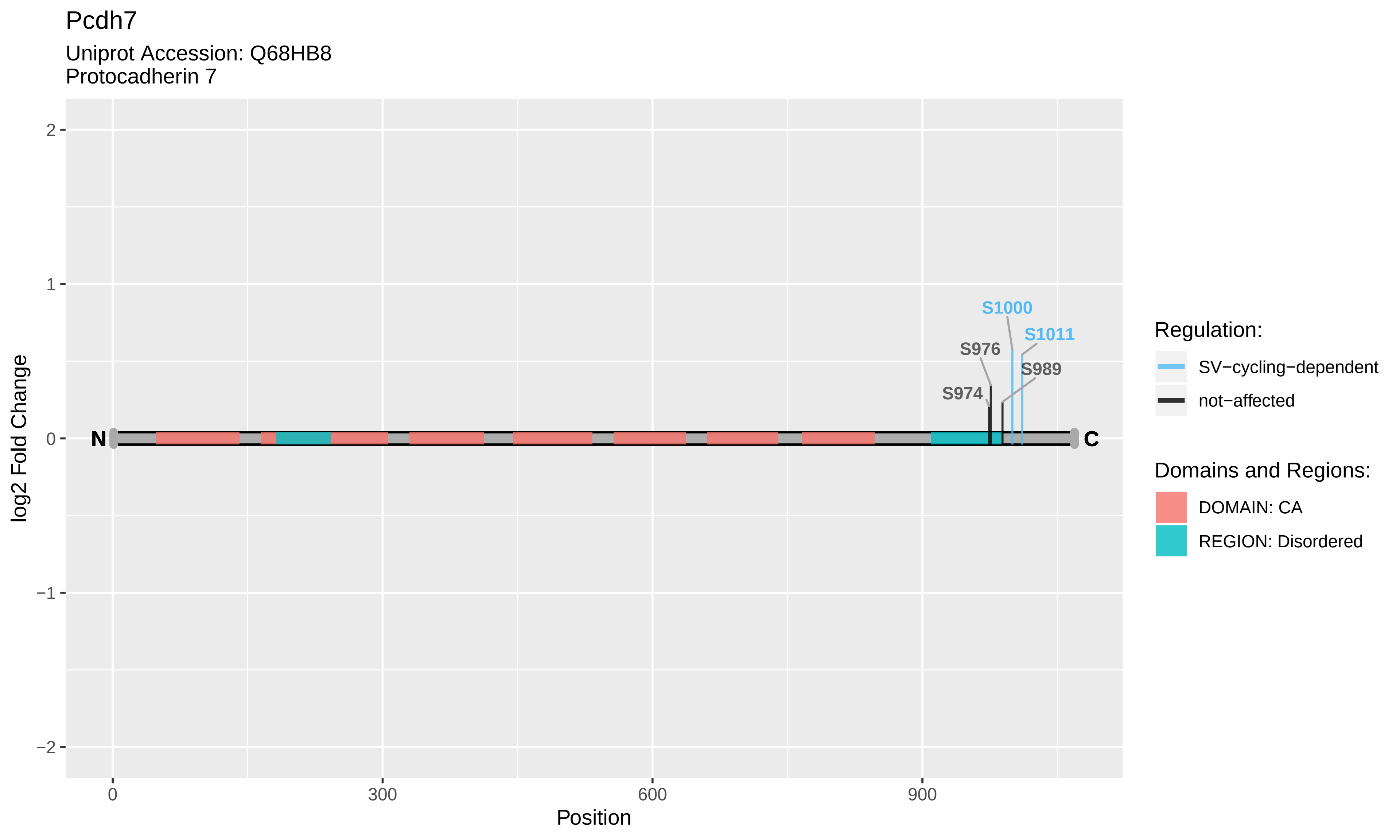

Pcdh7
Uniprot Accession: Q68HB8
Protocadherin 7
2
1
0
−1
−2
log2 Fold Change
0
300
600
900
Position
N
C
S1000
S1011
S976
S989
S974
Regulation:
SV−cycling−dependent
not−affected
Domains and Regions:
DOMAIN: CA
REGION: Disordered

Pcdh8

Uniprot Accession: D3ZE55
Protocadherin−8

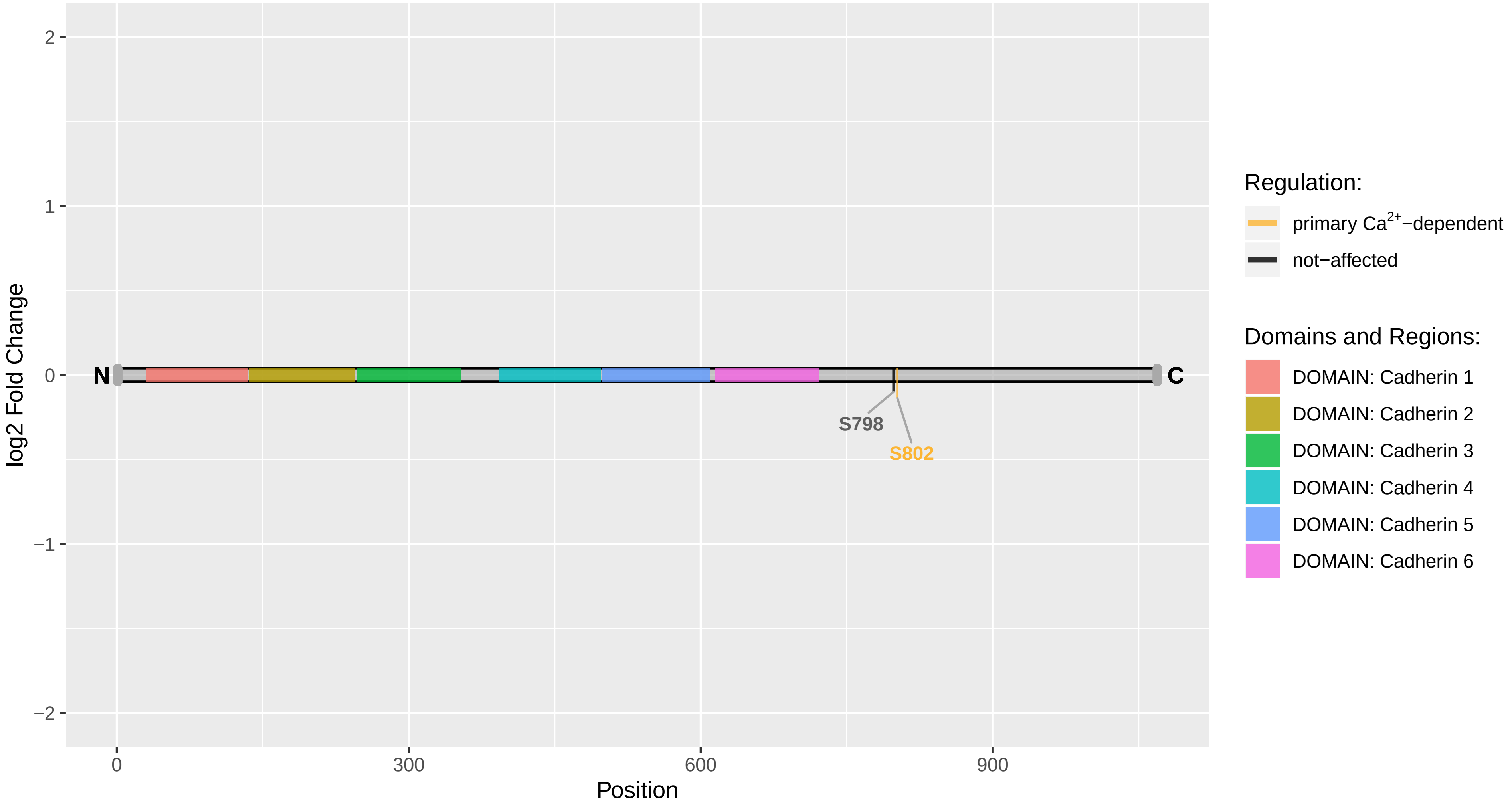

Pcdh9

Uniprot Accession: F1LS01
Protocadherin 9

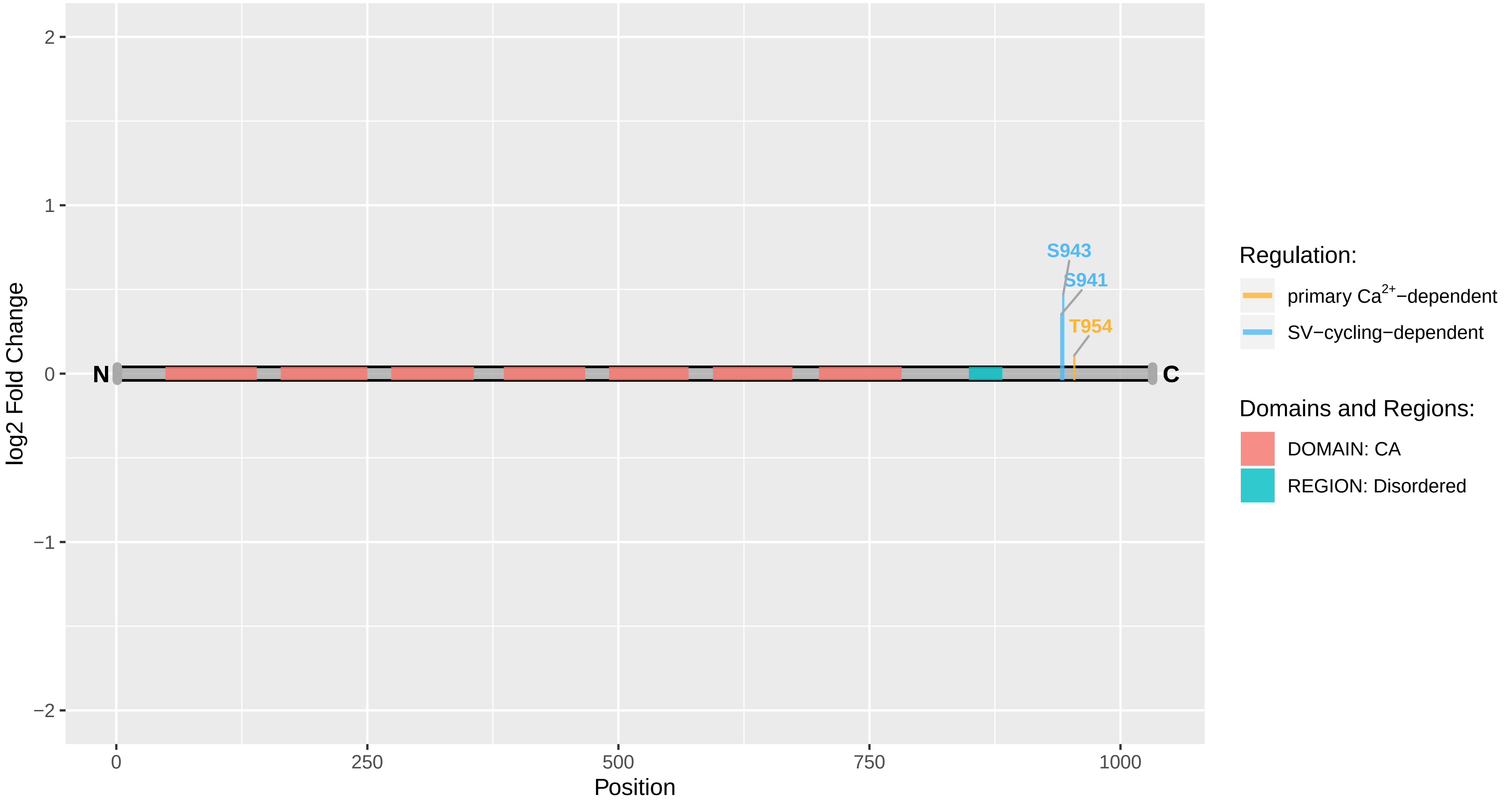

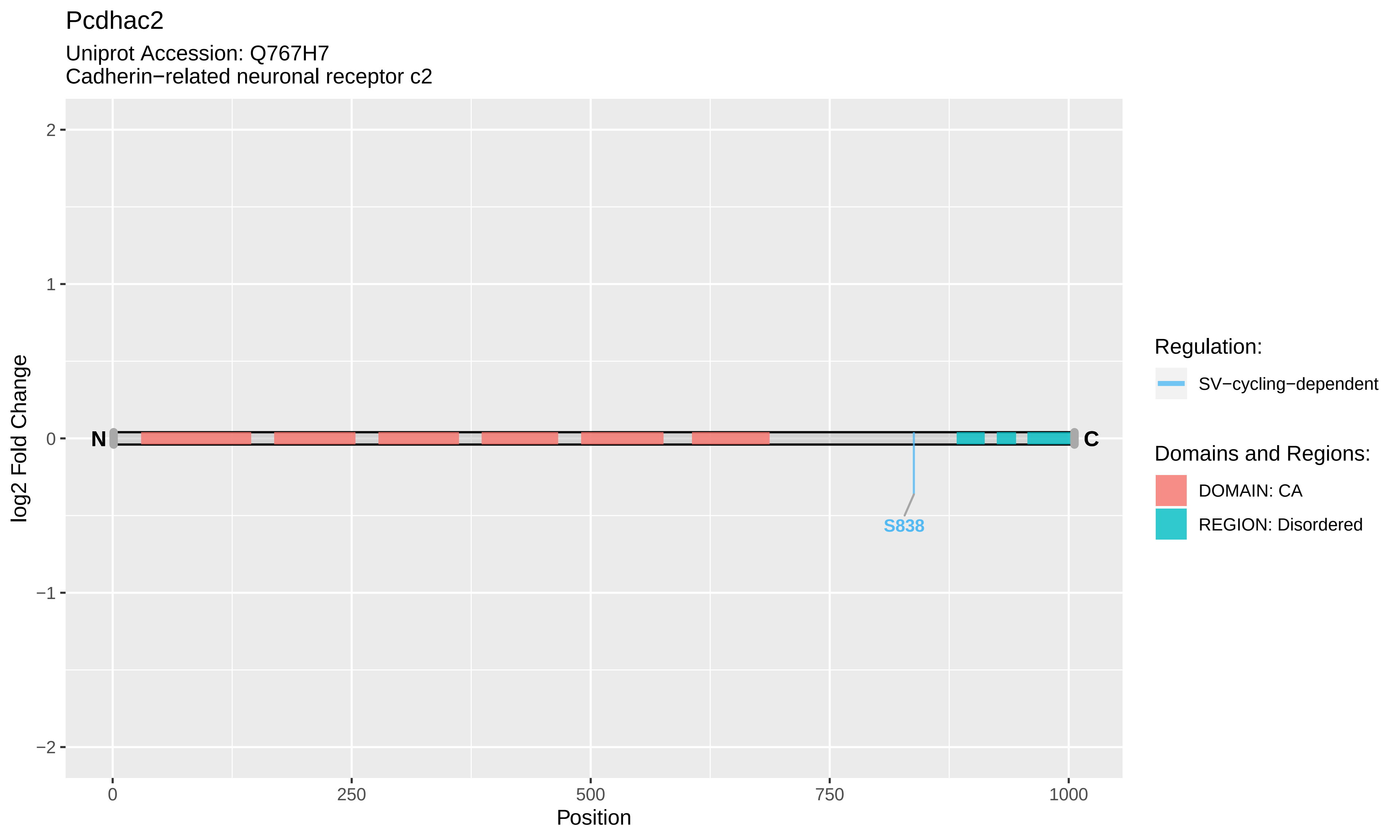

Pcdhac2
Uniprot Accession: Q767H7
Cadherin−related neuronal receptor c2
log2 Fold Change
2
1
0
−1
−2
N
C
S838
0
250
500
750
1000
Position
Regulation:
SV−cycling−dependent
Domains and Regions:
DOMAIN: CA
REGION: Disordered

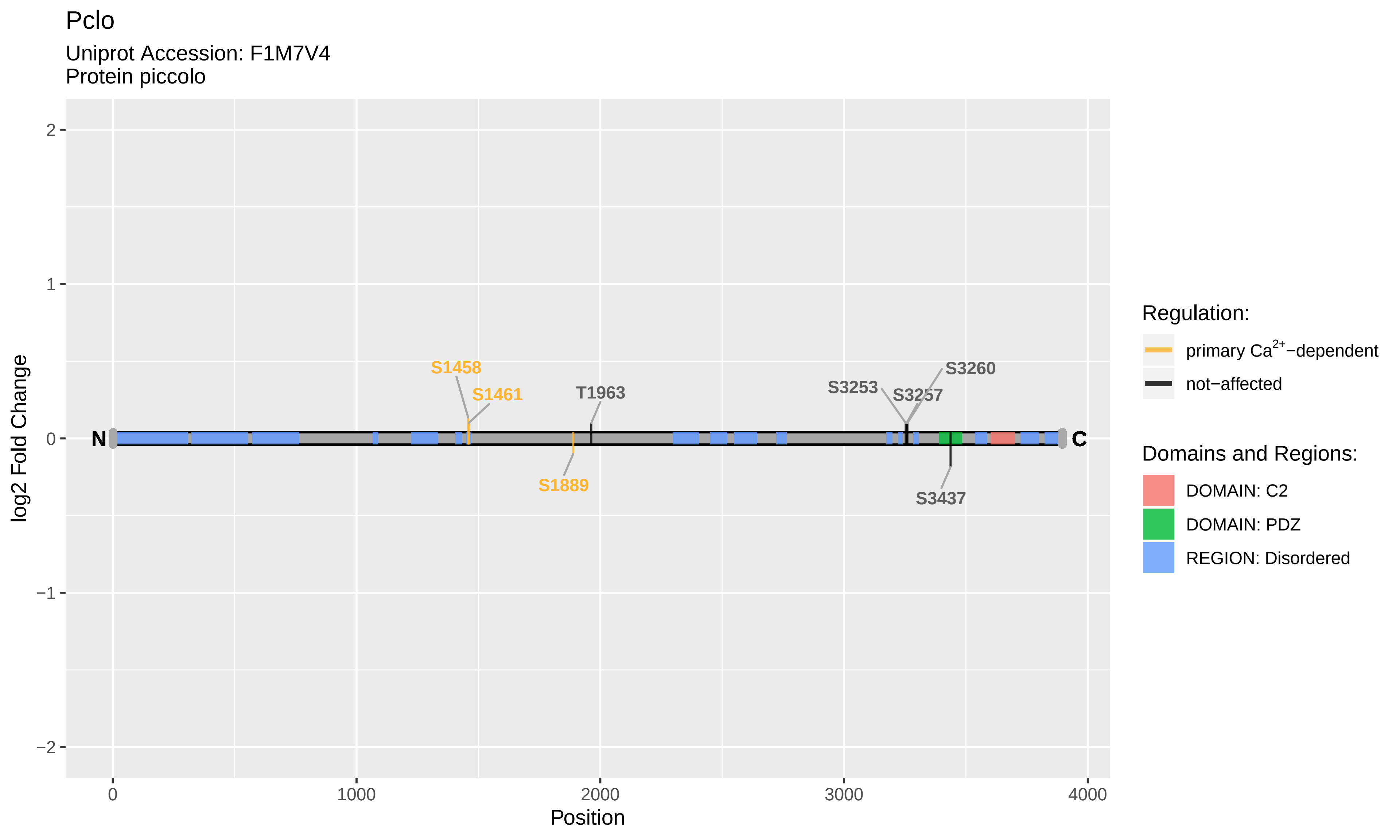
Pclo
Uniprot Accession: F1M7V4
Protein piccolo
S1458
S1461
T1963
S3253
S3257
S3260
S1889
S3437
N
C
log2 Fold Change
Position
2
1
0
−1
−2
0
1000
2000
3000
4000
Regulation:
primary Ca2+−dependent
not−affected
Domains and Regions:
DOMAIN: C2
DOMAIN: PDZ
REGION: Disordered

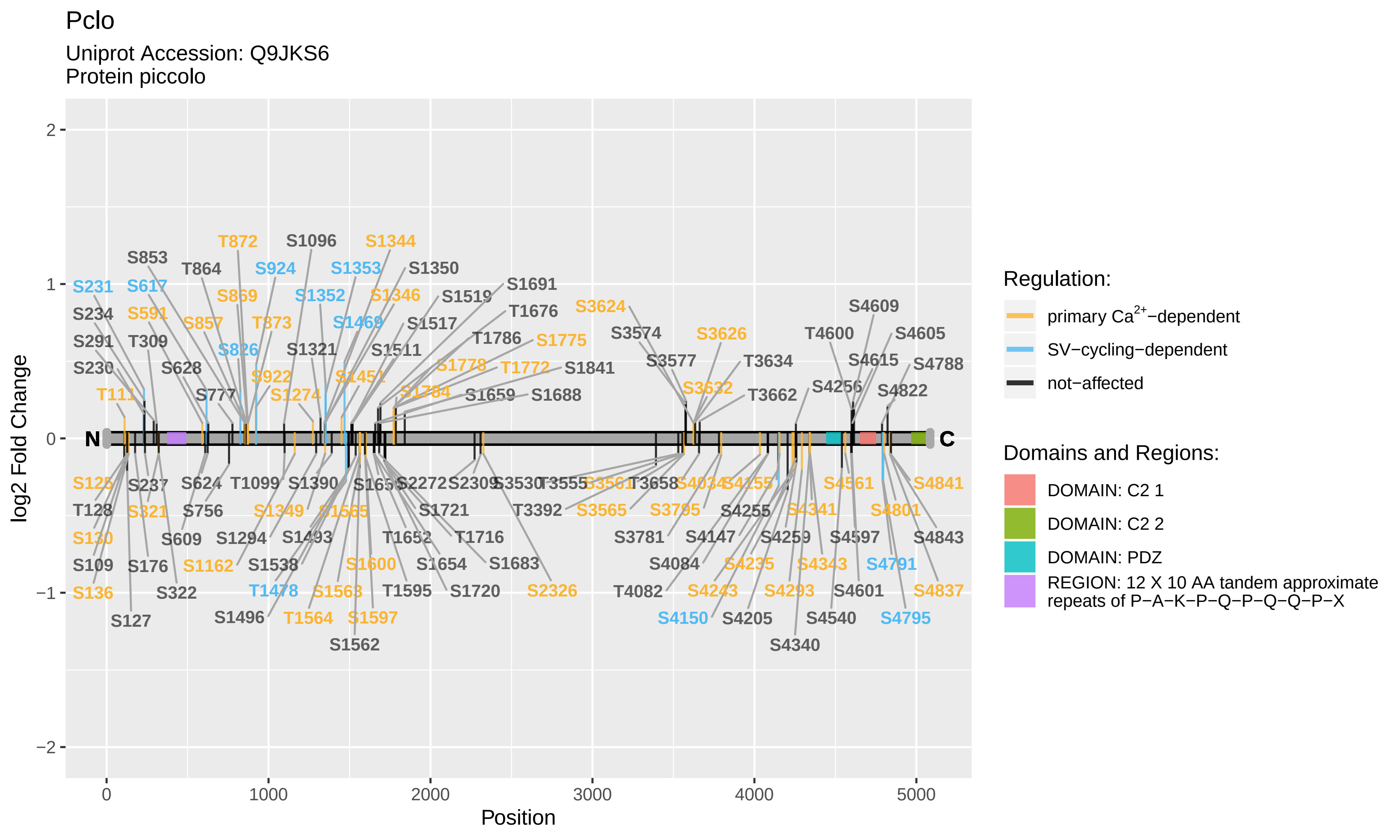

Pclo
Uniprot Accession: Q9JKS6
Protein piccolo
log2 Fold Change
Position
N
C
T872
S1096
S1344
S853
T864
S924
S1353
S1350
S231
S617
S869
S1352
S1346
S1519
S1691
S234
S591
S857
T873
S1469
S1517
T1676
S3624
S4609
S291
T309
S826
S1321
S1511
T1786
S1775
S3574
S3626
T4600
S4605
S230
S628
S922
S1451
S1778
T1772
S1841
S3577
T3634
S4615
S4788
T111
S777
S1274
S1784
S1659
S1688
S3632
T3662
S4256
S4822
S125
S237
S624
T1099
S1390
S2272
S2309
S3530
T3555
T3658
S4155
S4561
S4841
T128
S321
S756
S1349
S1565
S1721
T3392
S3565
S3795
S4255
S4341
S4801
S130
S609
S1294
S1493
T1652
T1716
S3781
S4147
S4259
S4597
S4843
S109
S176
S1162
S1538
S1600
S1654
S1683
S4084
S4235
S4343
S4791
S136
S322
T1478
S1563
T1595
S1720
S2326
T4082
S4243
S4293
S4601
S4837
S127
S1496
T1564
S1597
S4150
S4205
S4540
S4795
S1562
S4340
Regulation:
primary Ca2+−dependent
SV−cycling−dependent
not−affected
Domains and Regions:
DOMAIN: C2 1
DOMAIN: C2 2
DOMAIN: PDZ
REGION: 12 X 10 AA tandem approximate repeats of P−A−K−P−Q−P−Q−Q−P−X

Pcm1

Uniprot Accession: A0A0G2K0X1
Pericentriolar material 1

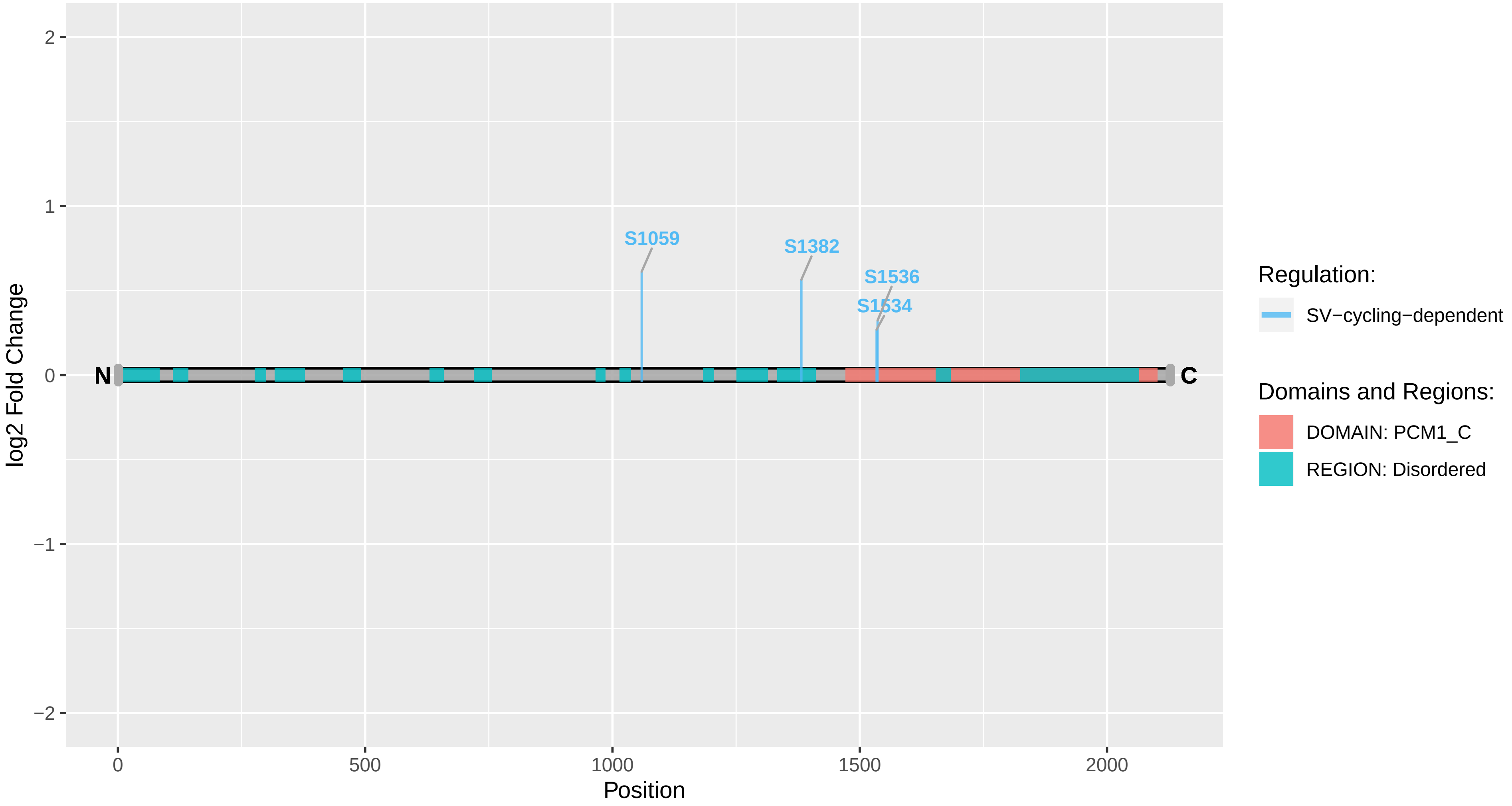

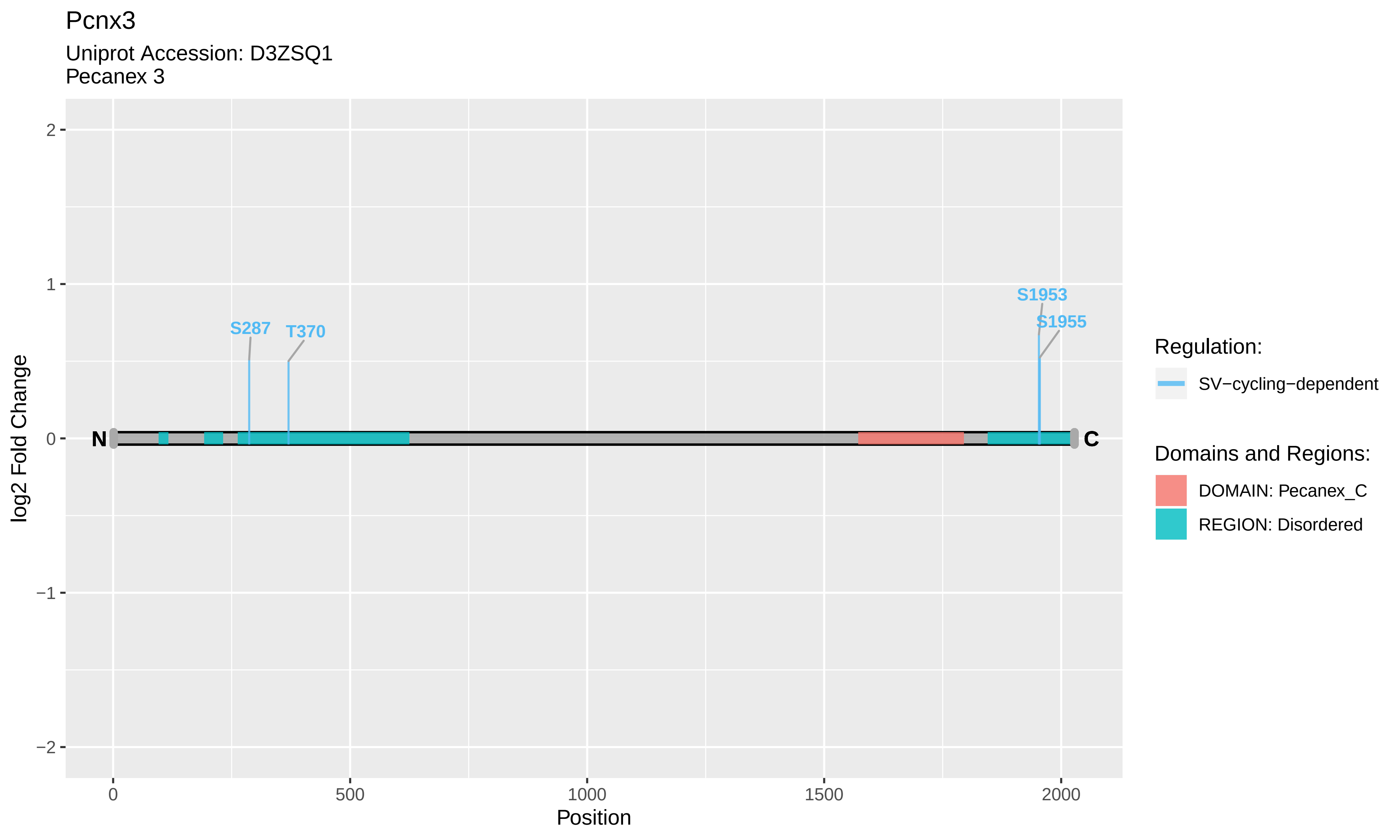

Pcnx3
Uniprot Accession: D3ZSQ1
Pecanex 3
S287
T370
S1953
S1955
N
C
log2 Fold Change
2
1
0
−1
−2
0
500
1000
1500
2000
Position
Regulation:
SV−cycling−dependent
Domains and Regions:
DOMAIN: Pecanex_C
REGION: Disordered

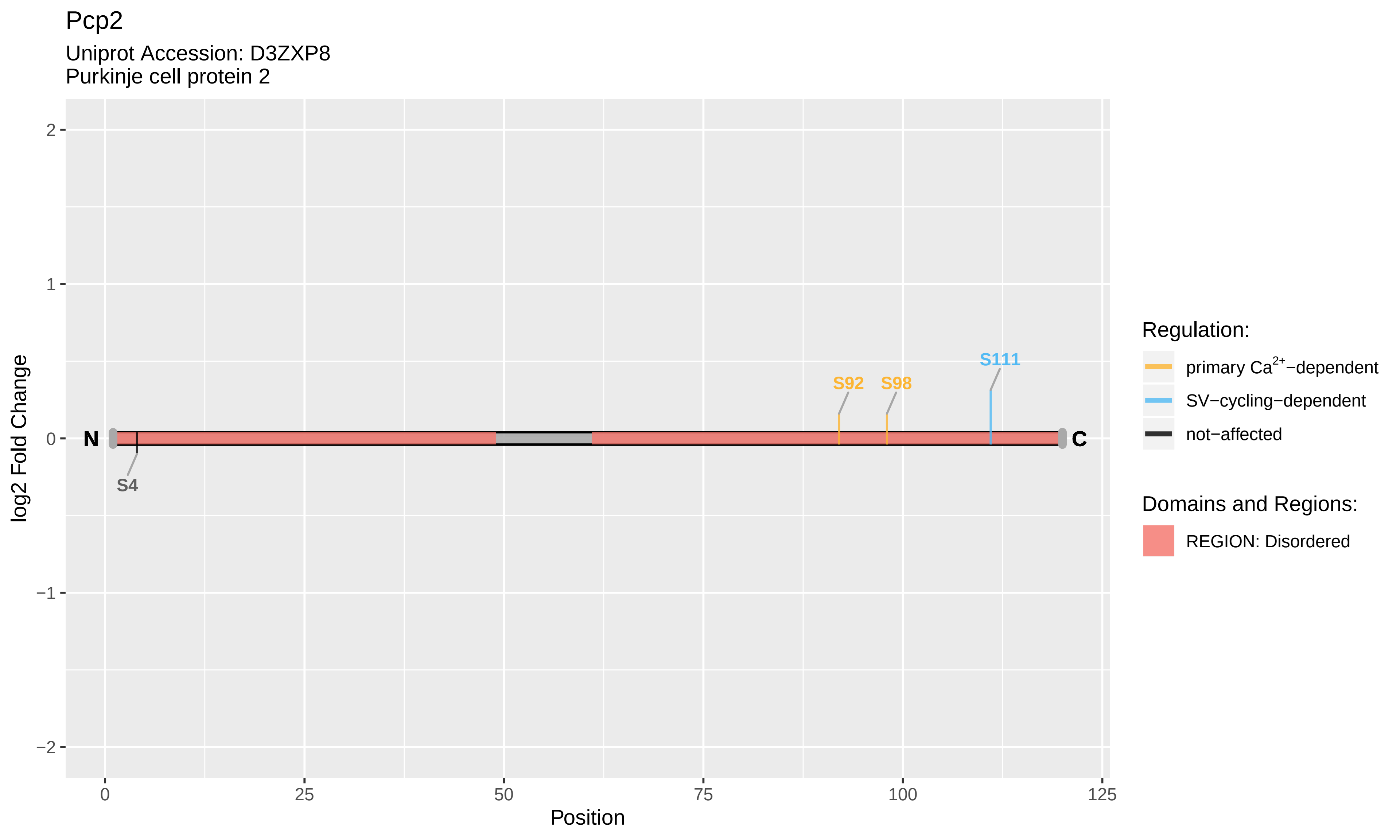
Pcp2
Uniprot Accession: D3ZXP8
Purkinje cell protein 2
log2 Fold Change
2
1
0
−1
−2
0
25
50
75
100
125
Position
N
C
S4
S92
S98
S111
Regulation:
primary Ca2+−dependent
SV−cycling−dependent
not−affected
Domains and Regions:
REGION: Disordered

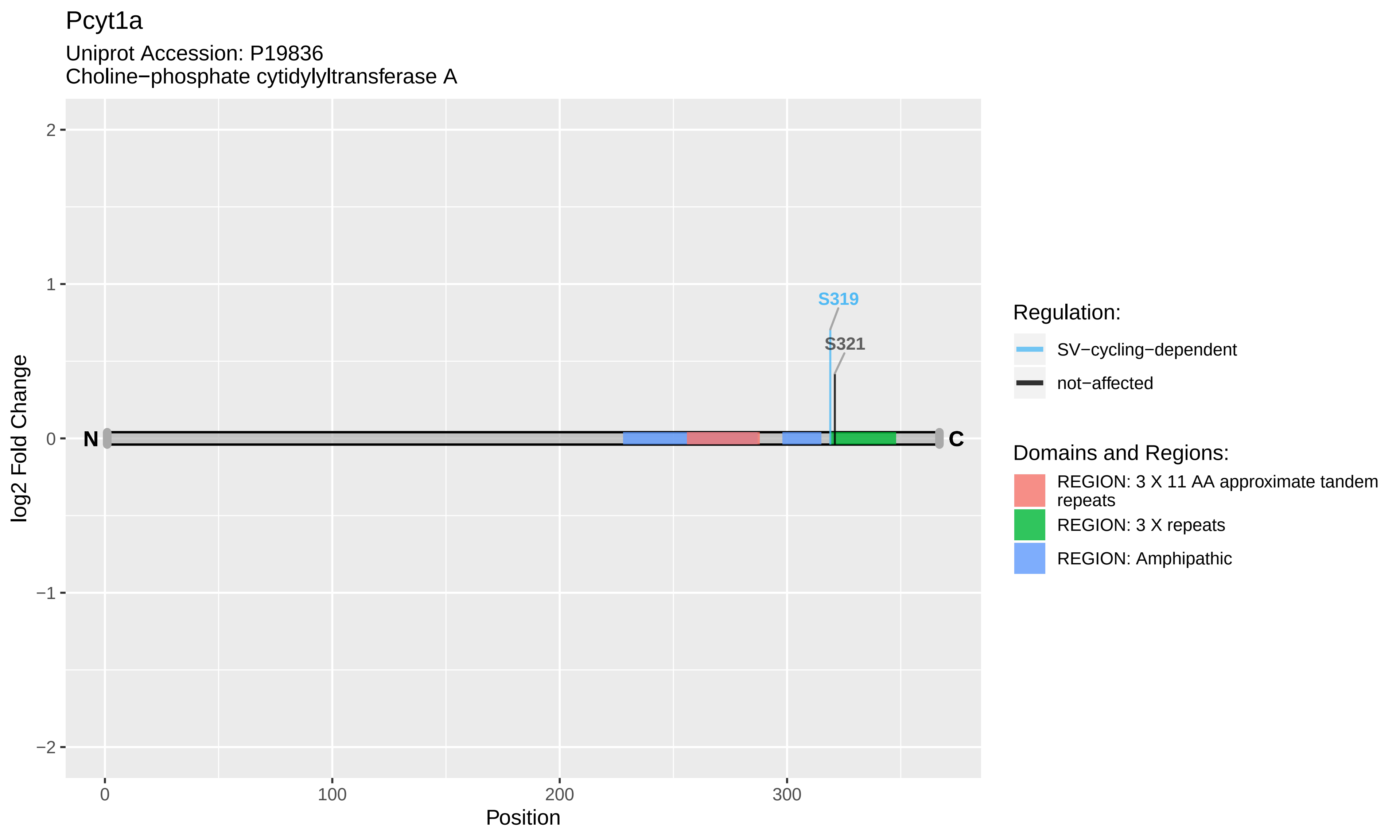

Pcyt1a
Uniprot Accession: P19836
Choline−phosphate cytidylyltransferase A
S319
S321
N
C
2
1
0
−1
−2
log2 Fold Change
0
100
200
300
Position
Regulation:
SV−cycling−dependent
not−affected
Domains and Regions:
REGION: 3 X 11 AA approximate tandem repeats
REGION: 3 X repeats
REGION: Amphipathic

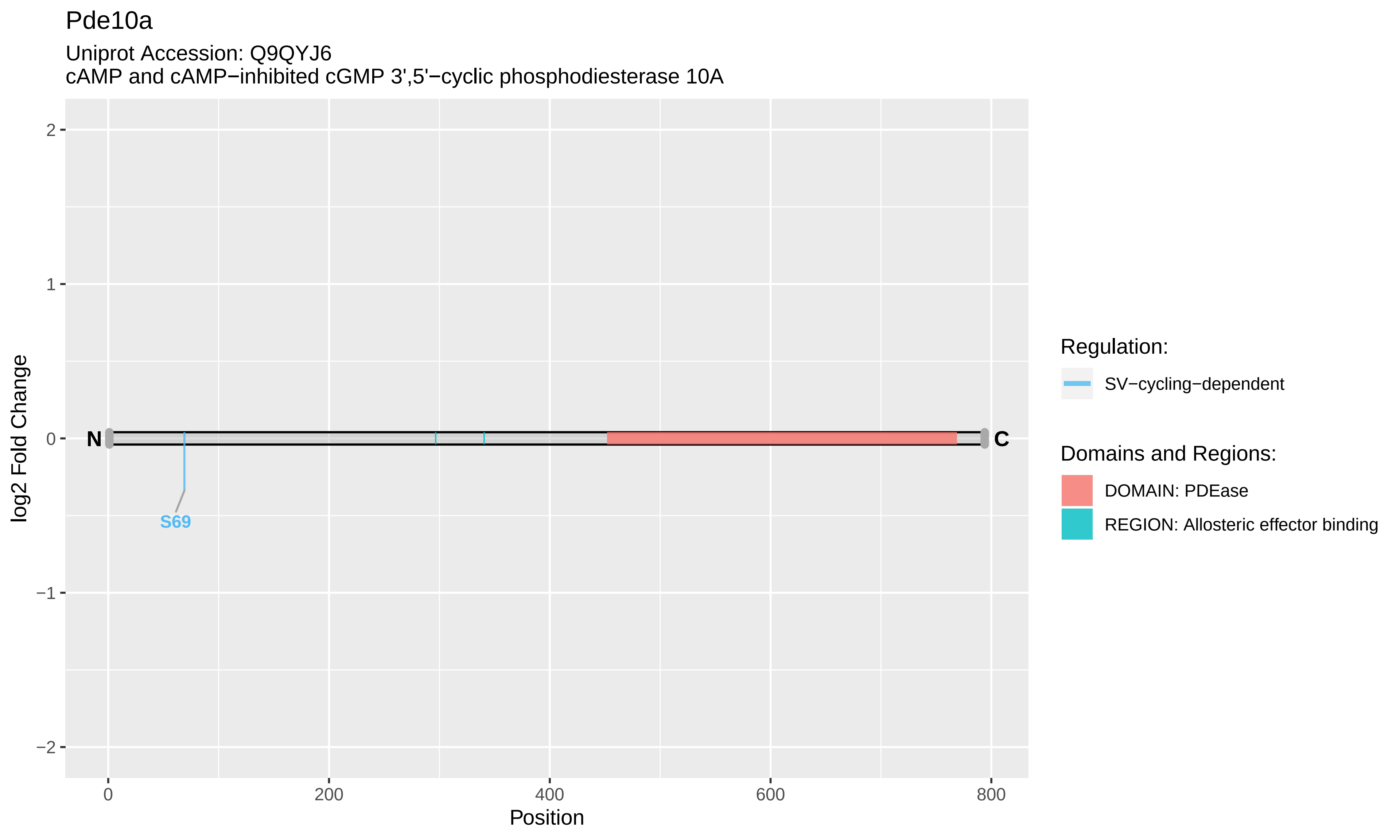

Pde10a
Uniprot Accession: Q9QYJ6
cAMP and cAMP−inhibited cGMP 3',5'−cyclic phosphodiesterase 10A
log2 Fold Change
2
1
0
−1
−2
N
C
S69
0
200
400
600
800
Position
Regulation:
SV−cycling−dependent
Domains and Regions:
DOMAIN: PDEase
REGION: Allosteric effector binding

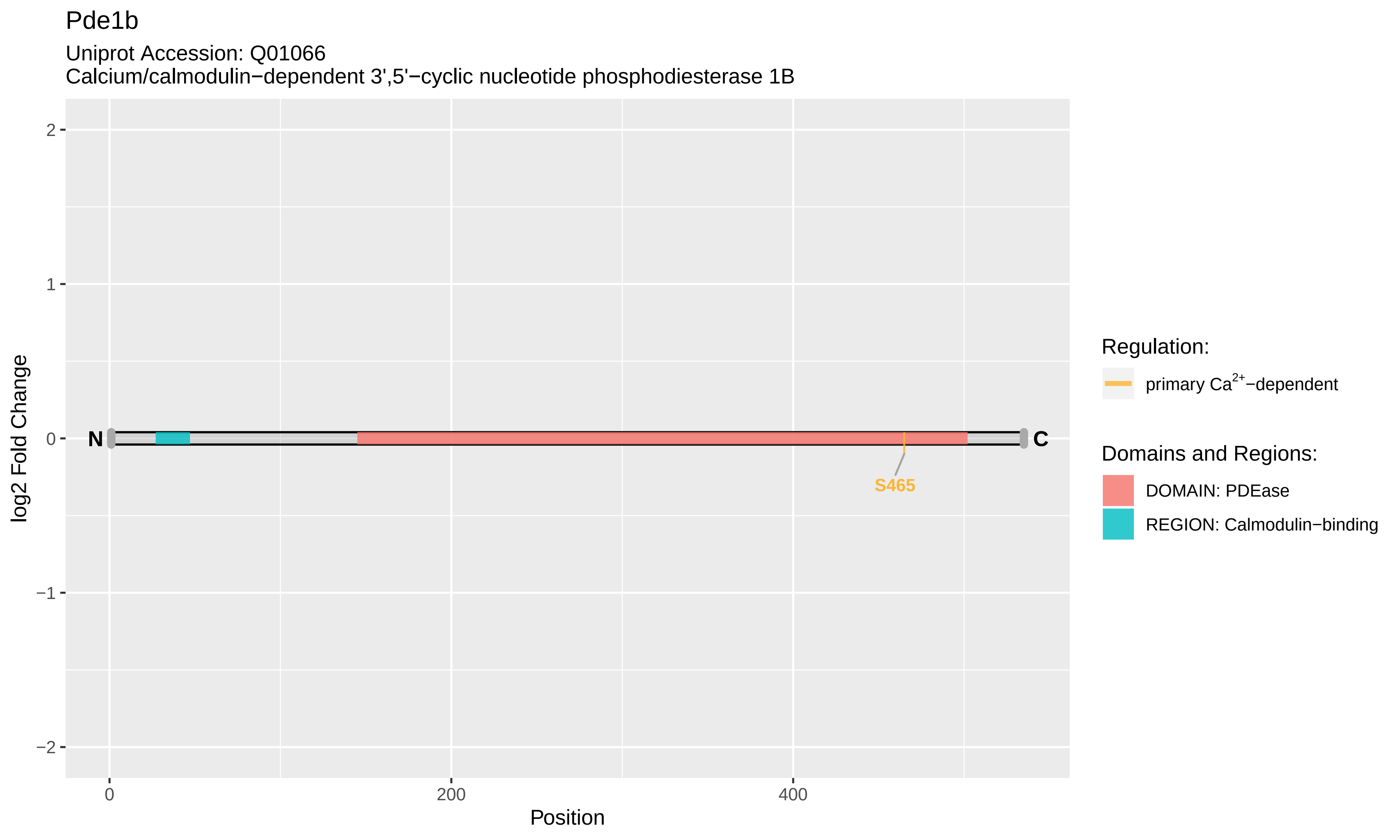

Pde1b
Uniprot Accession: Q01066
Calcium/calmodulin−dependent 3',5'−cyclic nucleotide phosphodiesterase 1B
log2 Fold Change
2
1
0
−1
−2
N
C
S465
0
200
400
Position
Regulation:
primary Ca2+−dependent
Domains and Regions:
DOMAIN: PDEase
REGION: Calmodulin−binding

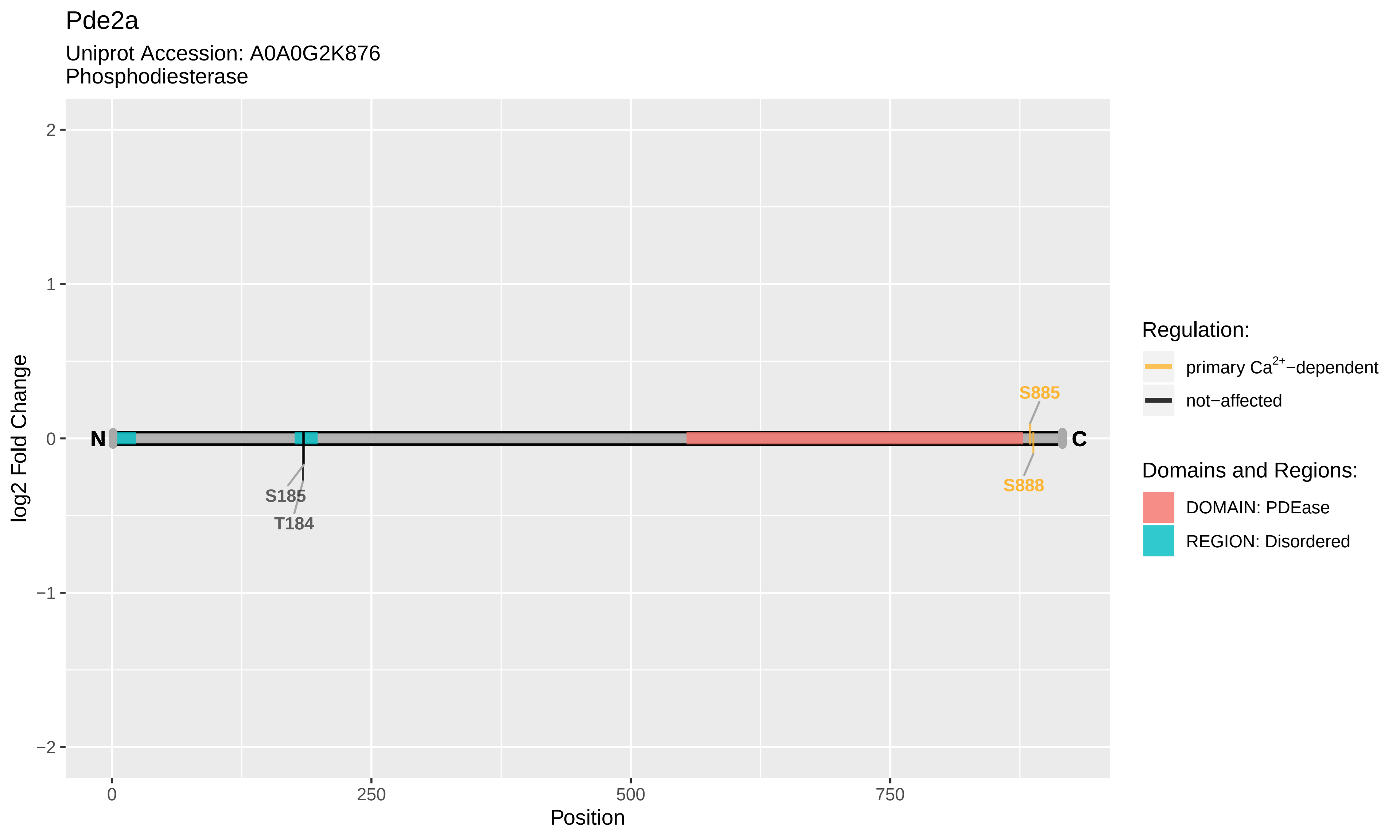
Pde2a
Uniprot Accession: A0A0G2K876
Phosphodiesterase
log2 Fold Change
2
1
0
−1
−2
N
C
S185
T184
S885
S888
0
250
500
750
Position
Regulation:
primary Ca2+−dependent
not−affected
Domains and Regions:
DOMAIN: PDEase
REGION: Disordered

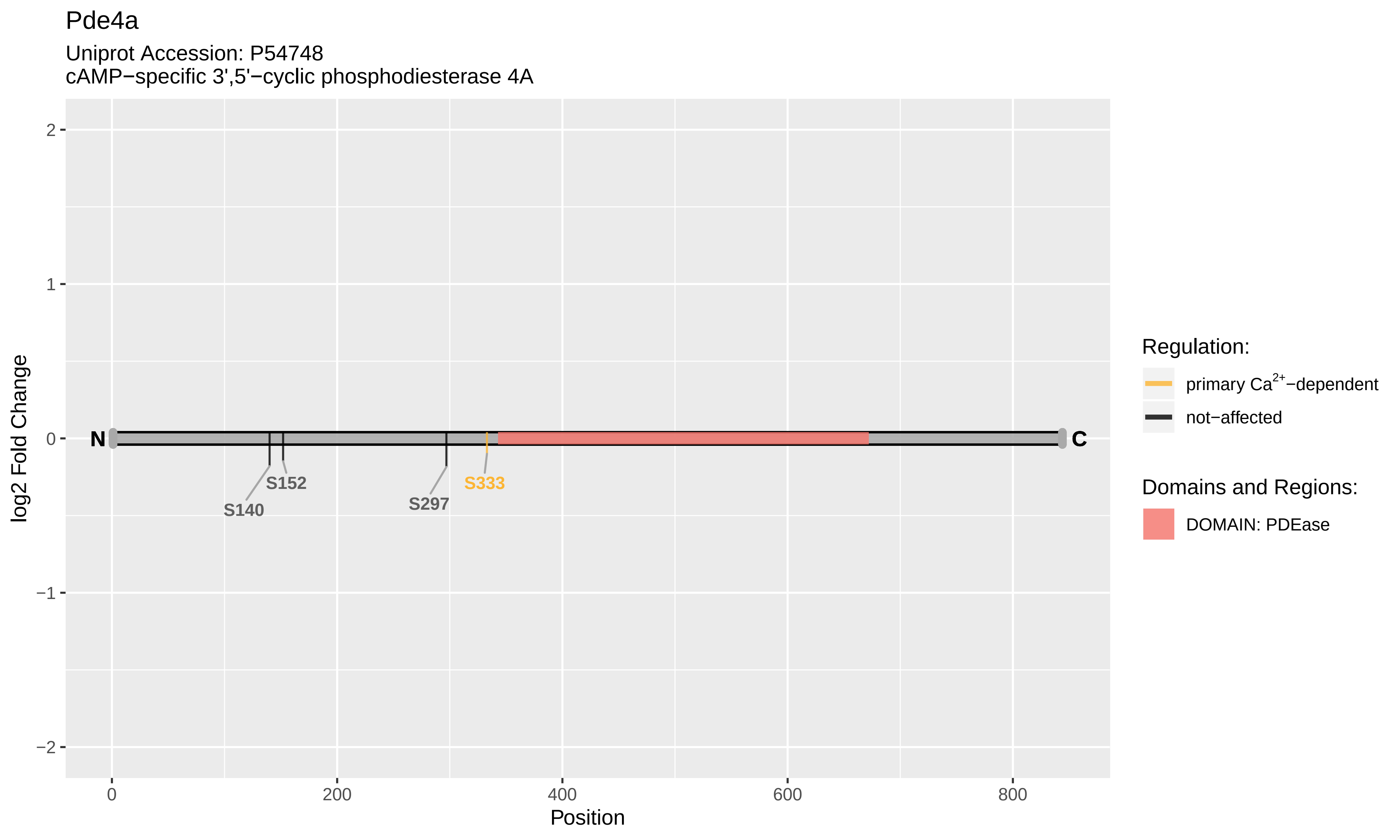
Pde4a
Uniprot Accession: P54748
cAMP−specific 3',5'−cyclic phosphodiesterase 4A
log2 Fold Change
Position
N
C
S140
S152
S297
S333
Regulation:
primary Ca2+−dependent
not−affected
Domains and Regions:
DOMAIN: PDEase

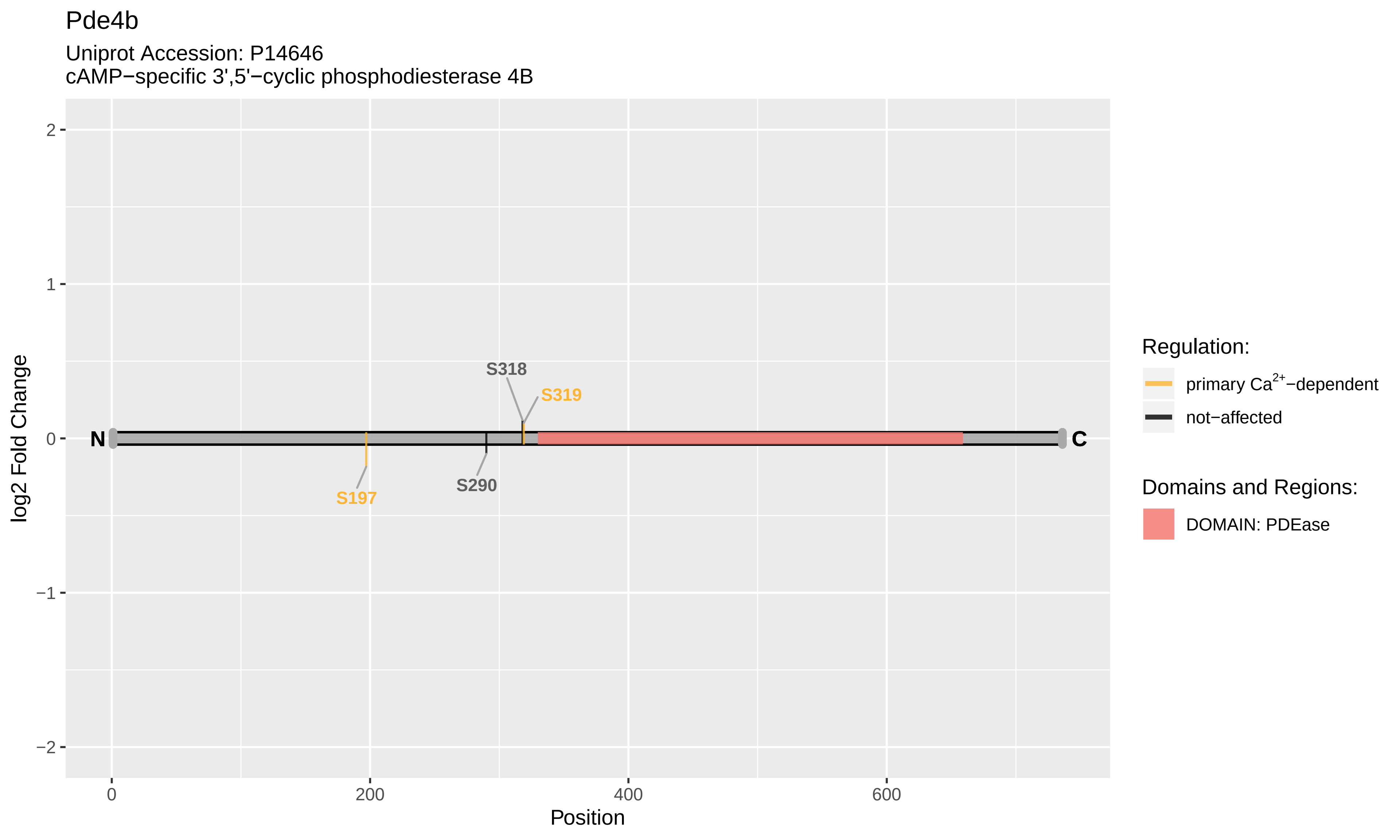
Pde4b
Uniprot Accession: P14646
cAMP−specific 3',5'−cyclic phosphodiesterase 4B
log2 Fold Change
Position
N
C
S318
S319
S197
S290
Regulation:
primary Ca2+−dependent
not−affected
Domains and Regions:
DOMAIN: PDEase
2
1
0
−1
−2
0
200
400
600

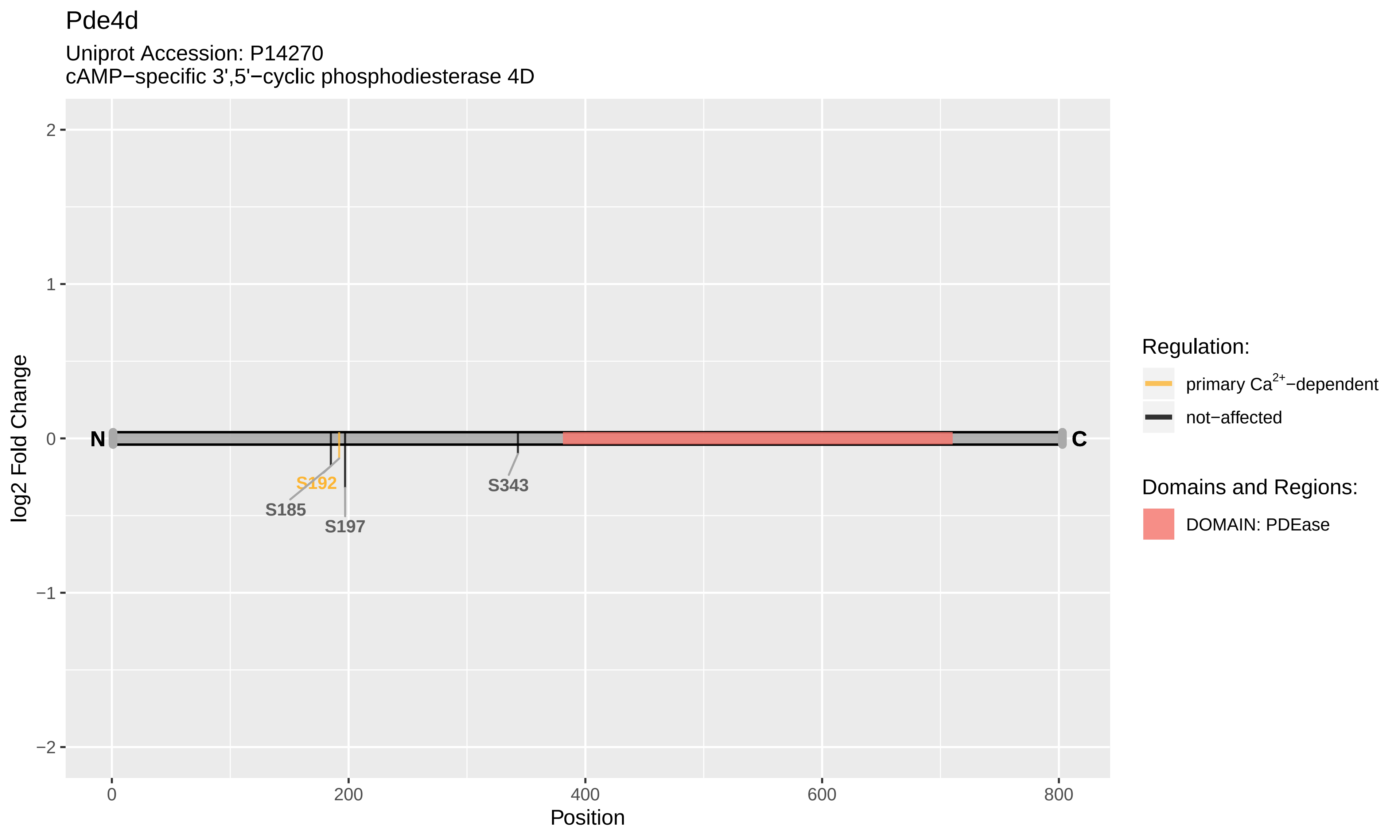

Pde4d
Uniprot Accession: P14270
cAMP−specific 3',5'−cyclic phosphodiesterase 4D
log2 Fold Change
Position
N
C
S192
S185
S197
S343
Regulation:
primary Ca2+−dependent
not−affected
Domains and Regions:
DOMAIN: PDEase

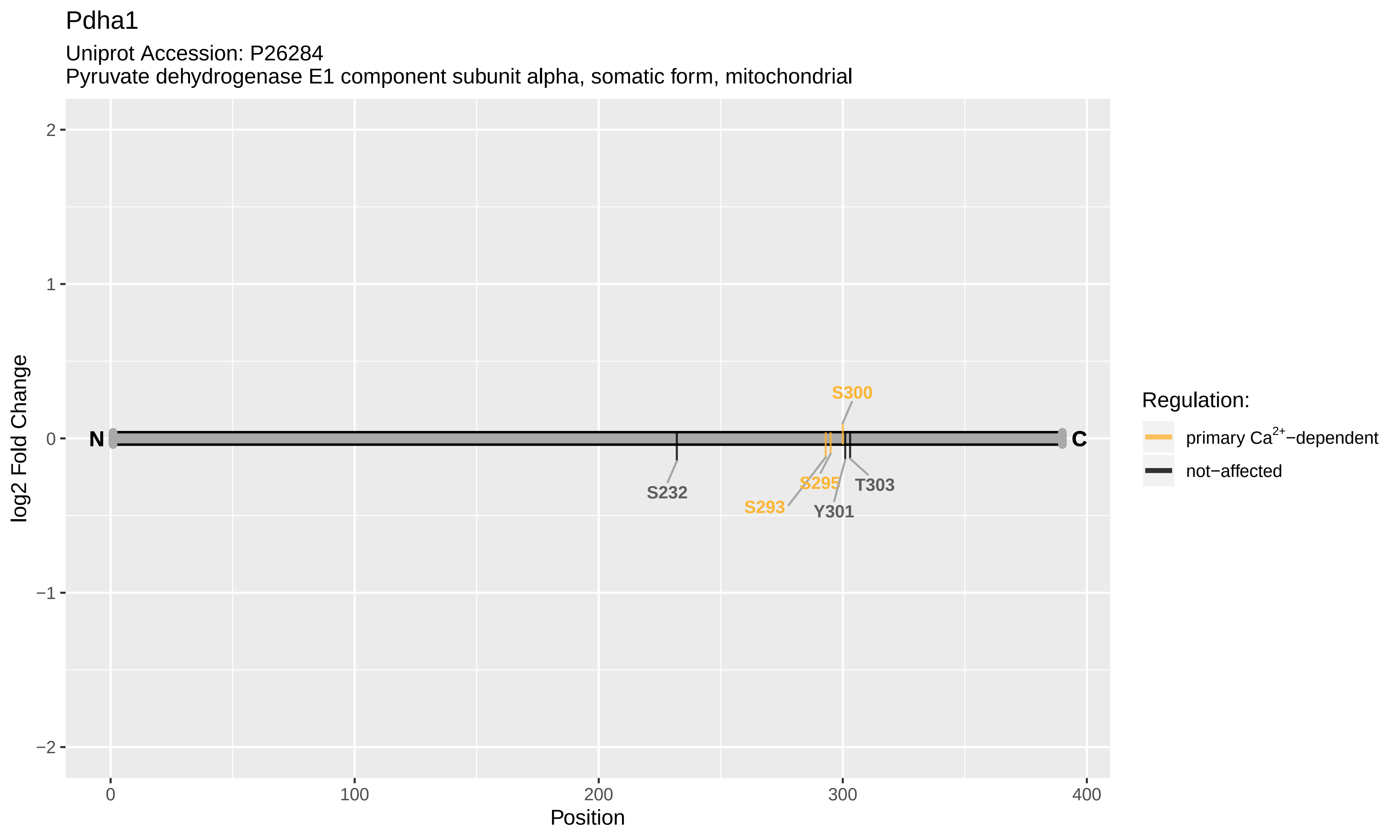

Pdha1
Uniprot Accession: P26284
Pyruvate dehydrogenase E1 component subunit alpha, somatic form, mitochondrial
log2 Fold Change
2
1
0
−1
−2
N
C
S300
S232
S295
T303
S293
Y301
0
100
200
300
400
Position
Regulation:
primary Ca2+−dependent
not−affected

Pdlim5

Uniprot Accession: Q62920
PDZ and LIM domain protein 5

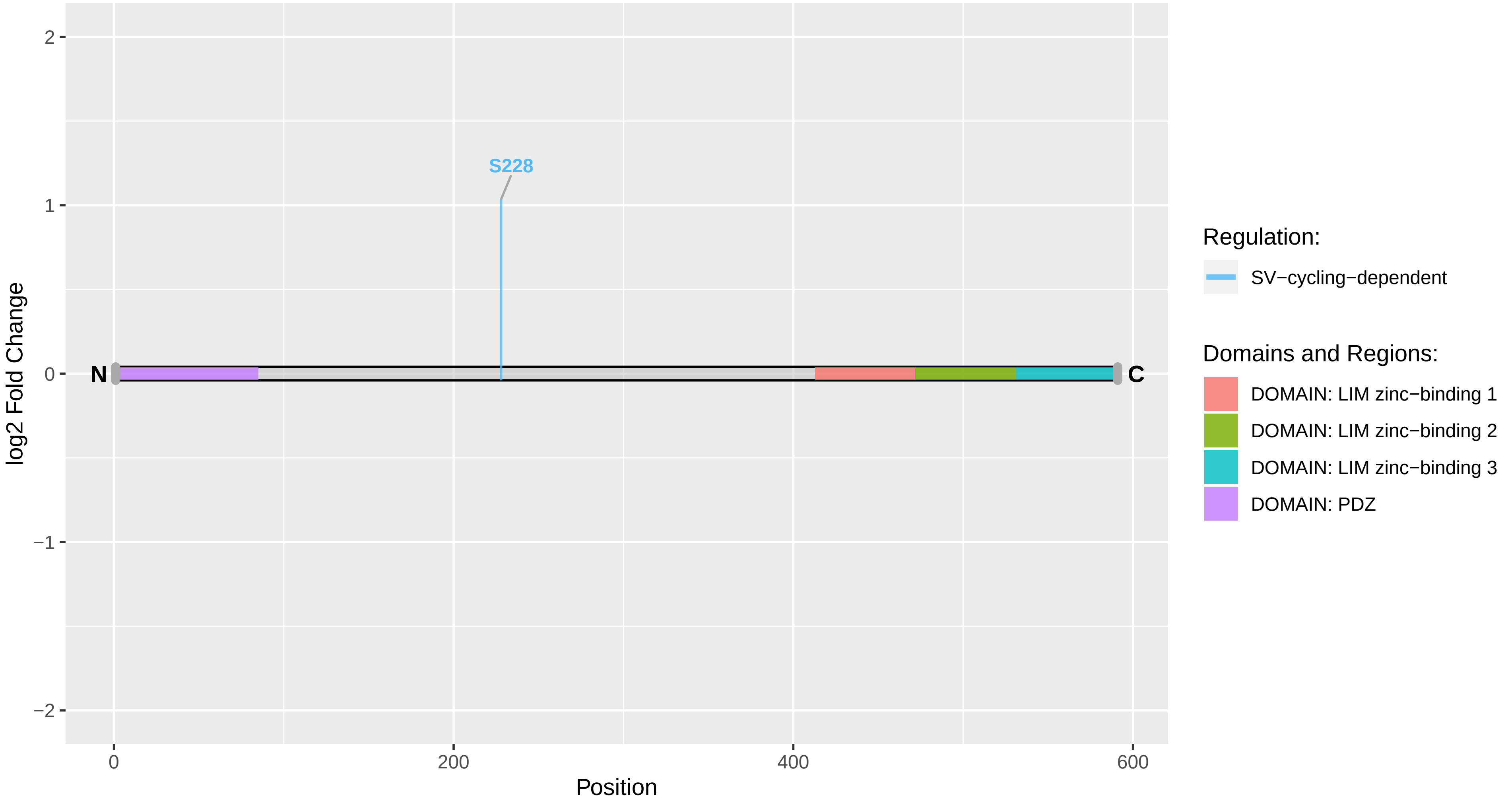

Pds5b

Uniprot Accession: Q6TRW4
Sister chromatid cohesion protein PDS5 homolog B

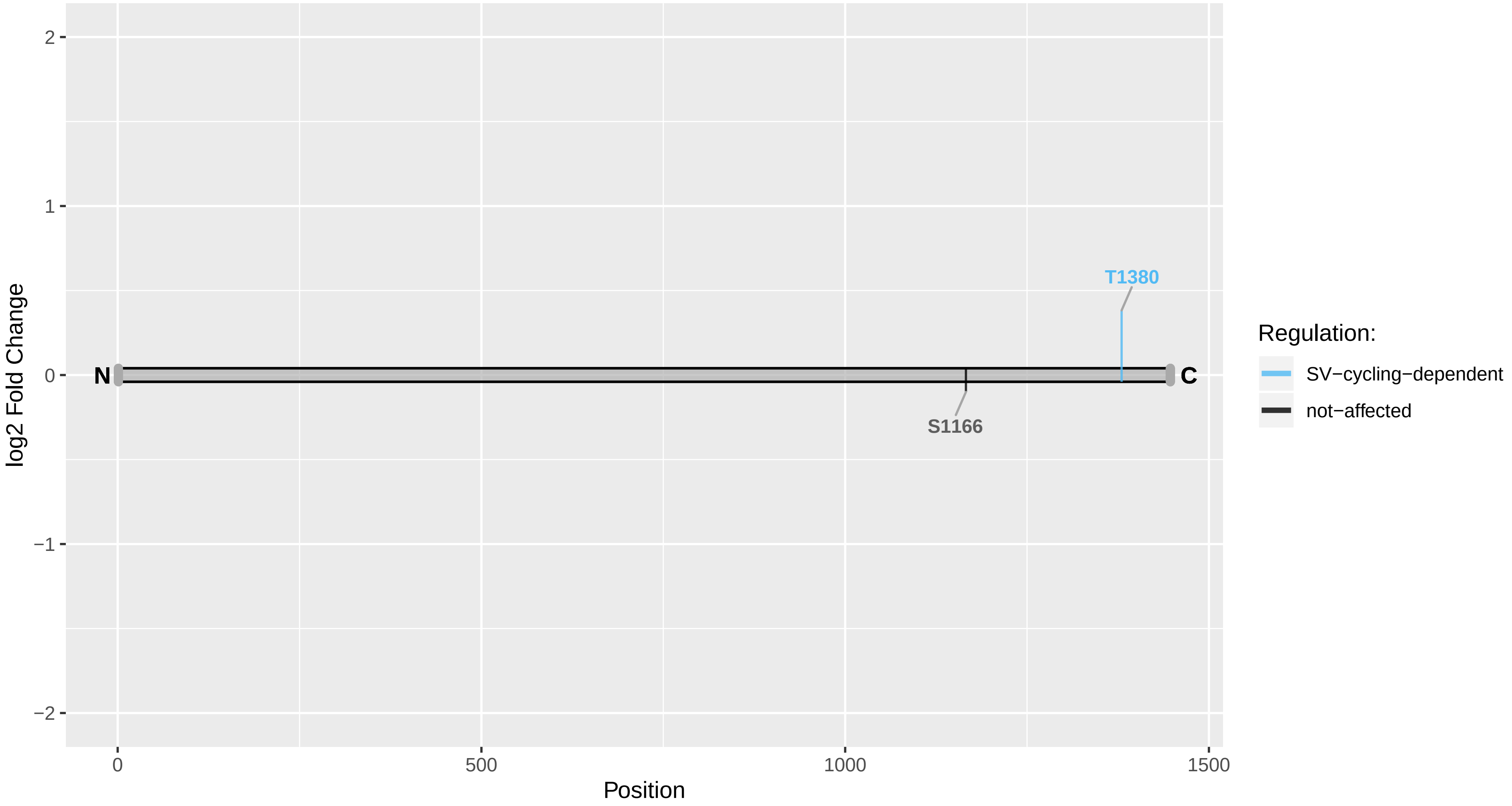

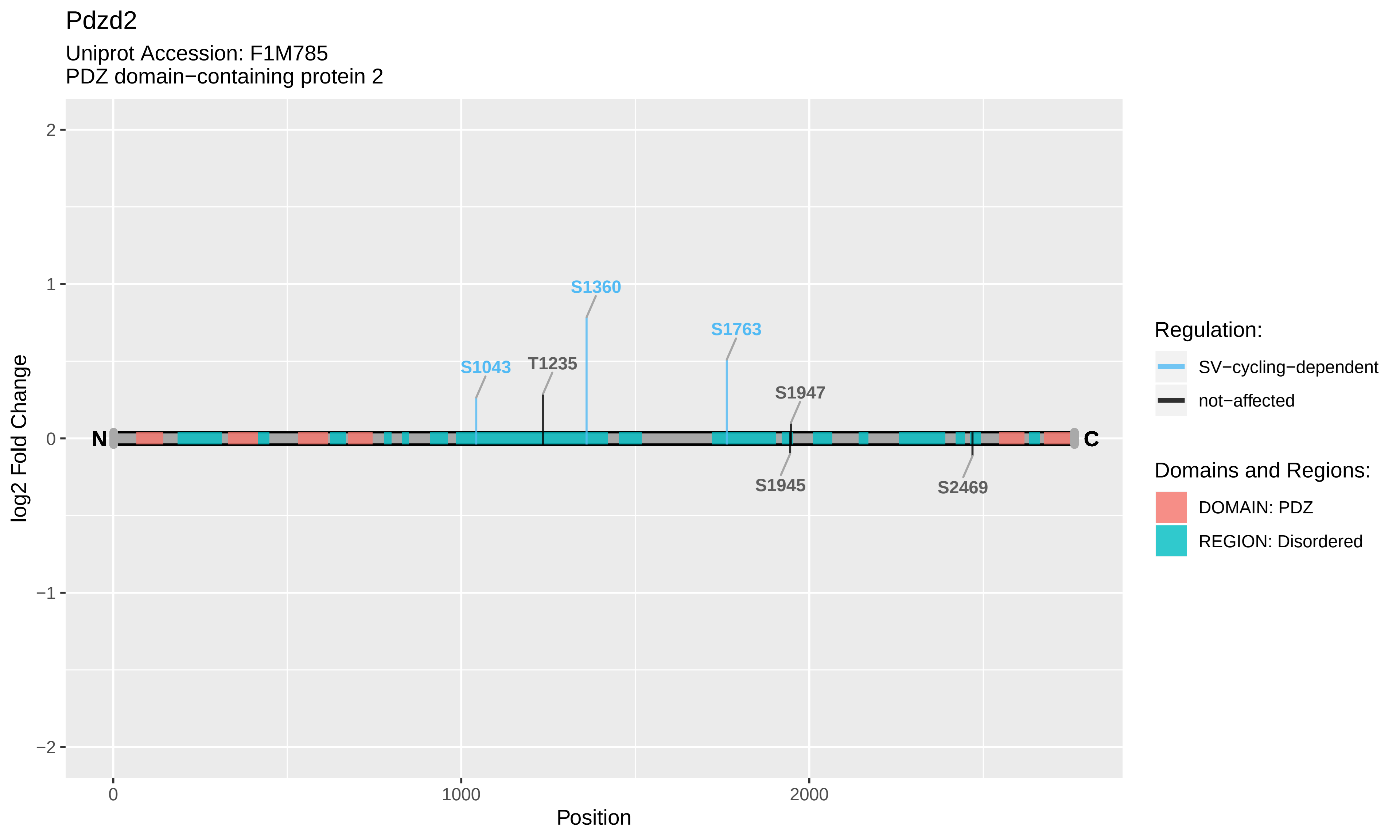

Pdzd2
Uniprot Accession: F1M785
PDZ domain−containing protein 2
S1360
S1763
S1043
T1235
S1947
N
C
S1945
S2469
2
1
0
−1
−2
0
1000
2000
Position
log2 Fold Change
Regulation:
SV−cycling−dependent
not−affected
Domains and Regions:
DOMAIN: PDZ
REGION: Disordered

Pdzd8

Uniprot Accession: D3ZXY2
PDZ domain containing 8 (Predicted)

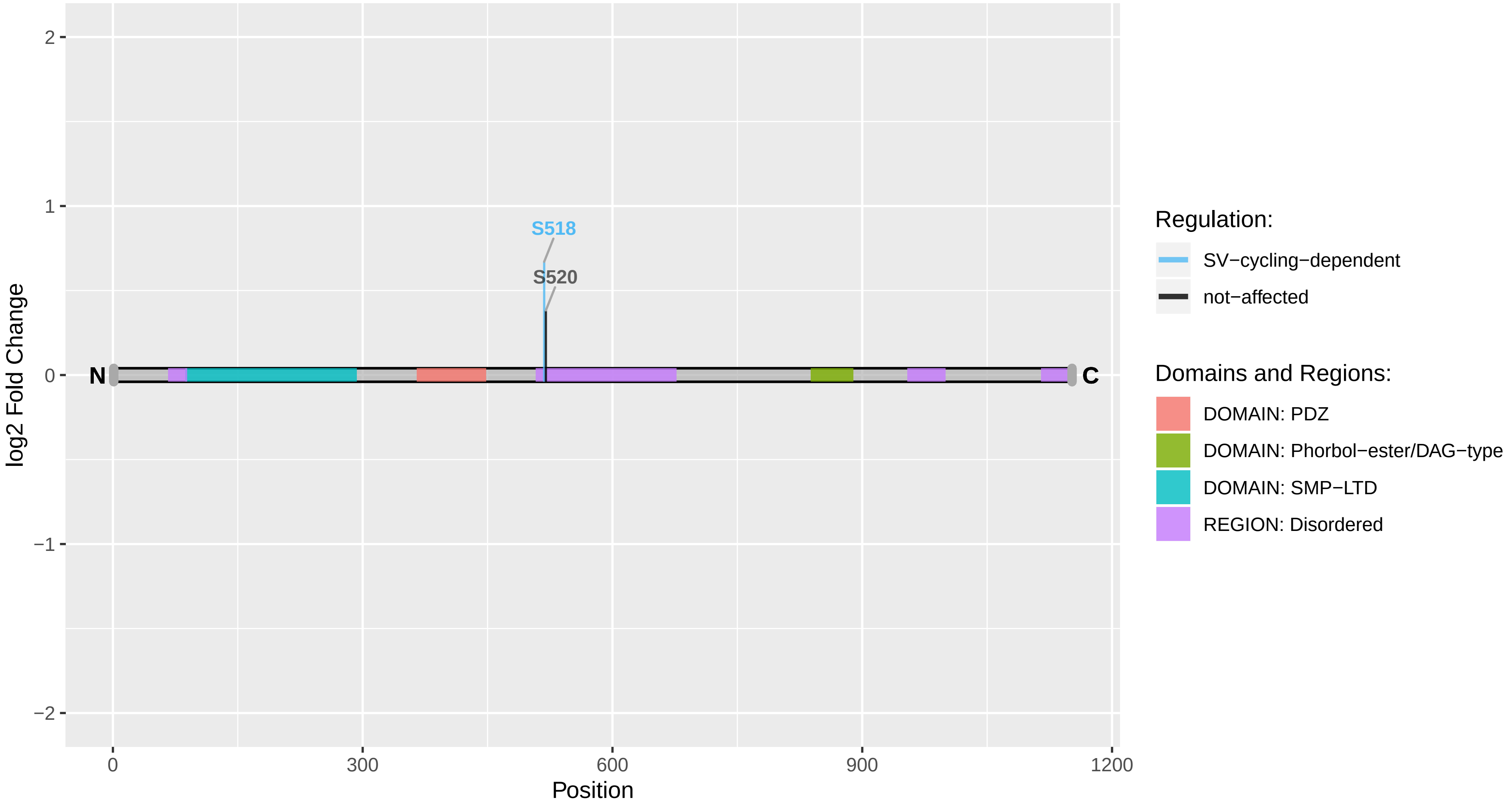

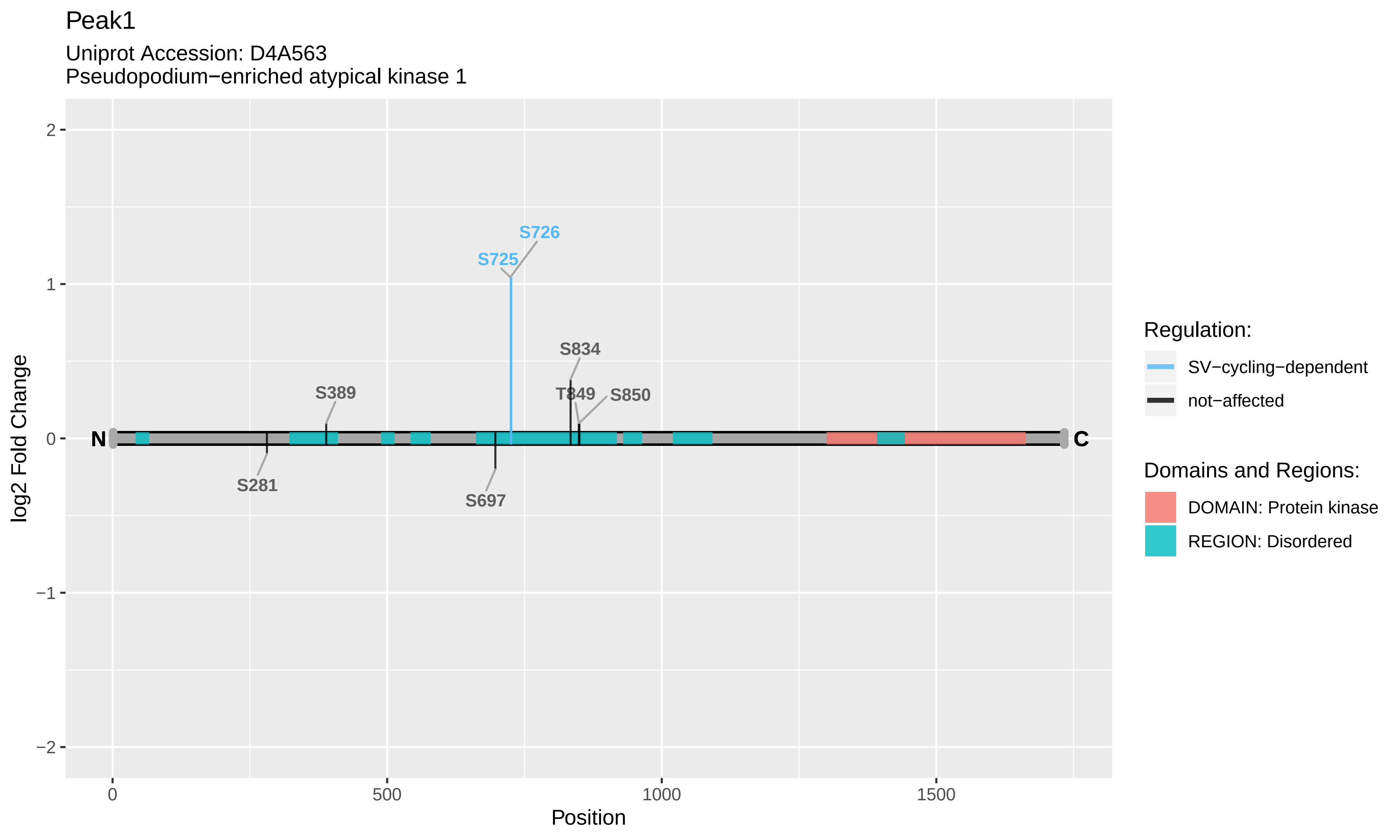
Peak1
Uniprot Accession: D4A563
Pseudopodium−enriched atypical kinase 1
S726
S725
S834
S389
T849
S850
N
C
S281
S697
log2 Fold Change
2
1
0
−1
−2
0
500
1000
1500
Position
Regulation:
SV−cycling−dependent
not−affected
Domains and Regions:
DOMAIN: Protein kinase
REGION: Disordered

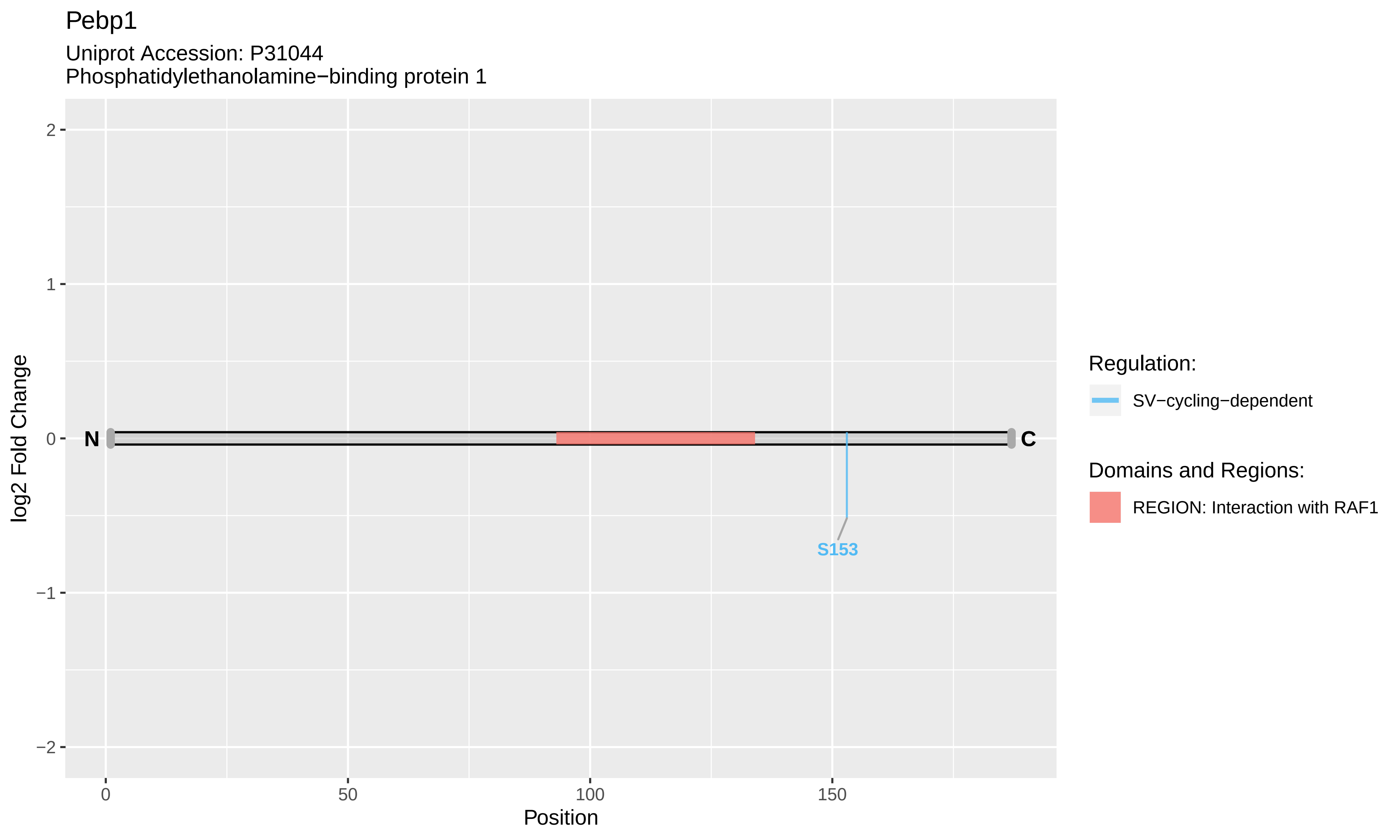
Pebp1
Uniprot Accession: P31044
Phosphatidylethanolamine−binding protein 1
log2 Fold Change
2
1
0
−1
−2
N
C
S153
0
50
100
150
Position
Regulation:
SV−cycling−dependent
Domains and Regions:
REGION: Interaction with RAF1

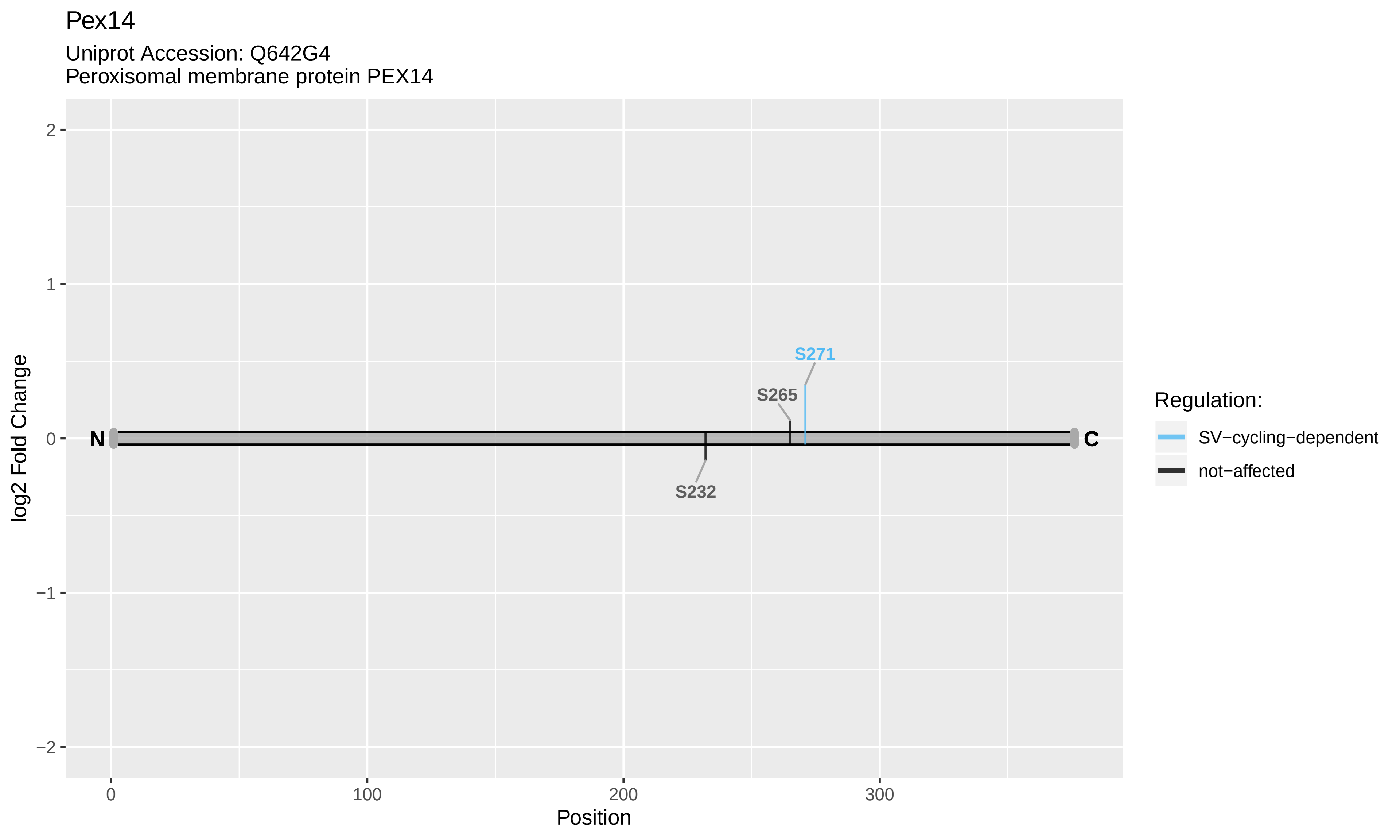
Pex14
Uniprot Accession: Q642G4
Peroxisomal membrane protein PEX14
log2 Fold Change
2
1
0
−1
−2
N
C
S271
S265
S232
0
100
200
300
Position
Regulation:
SV−cycling−dependent
not−affected

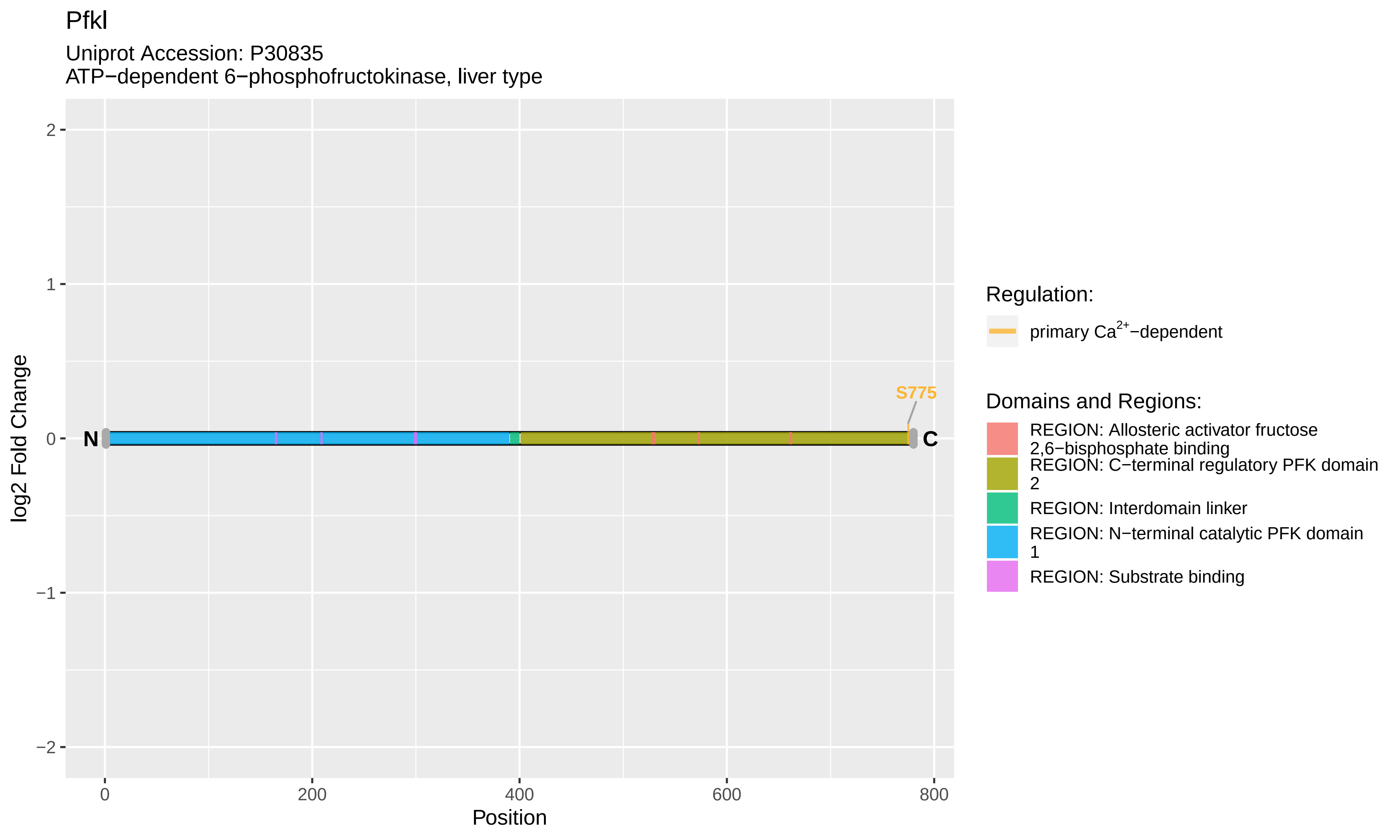

Pfkl
Uniprot Accession: P30835
ATP−dependent 6−phosphofructokinase, liver type
log2 Fold Change
Position
N
C
S775
Regulation:
primary Ca2+−dependent
Domains and Regions:
REGION: Allosteric activator fructose 2,6−bisphosphate binding
REGION: C−terminal regulatory PFK domain 2
REGION: Interdomain linker
REGION: N−terminal catalytic PFK domain 1
REGION: Substrate binding

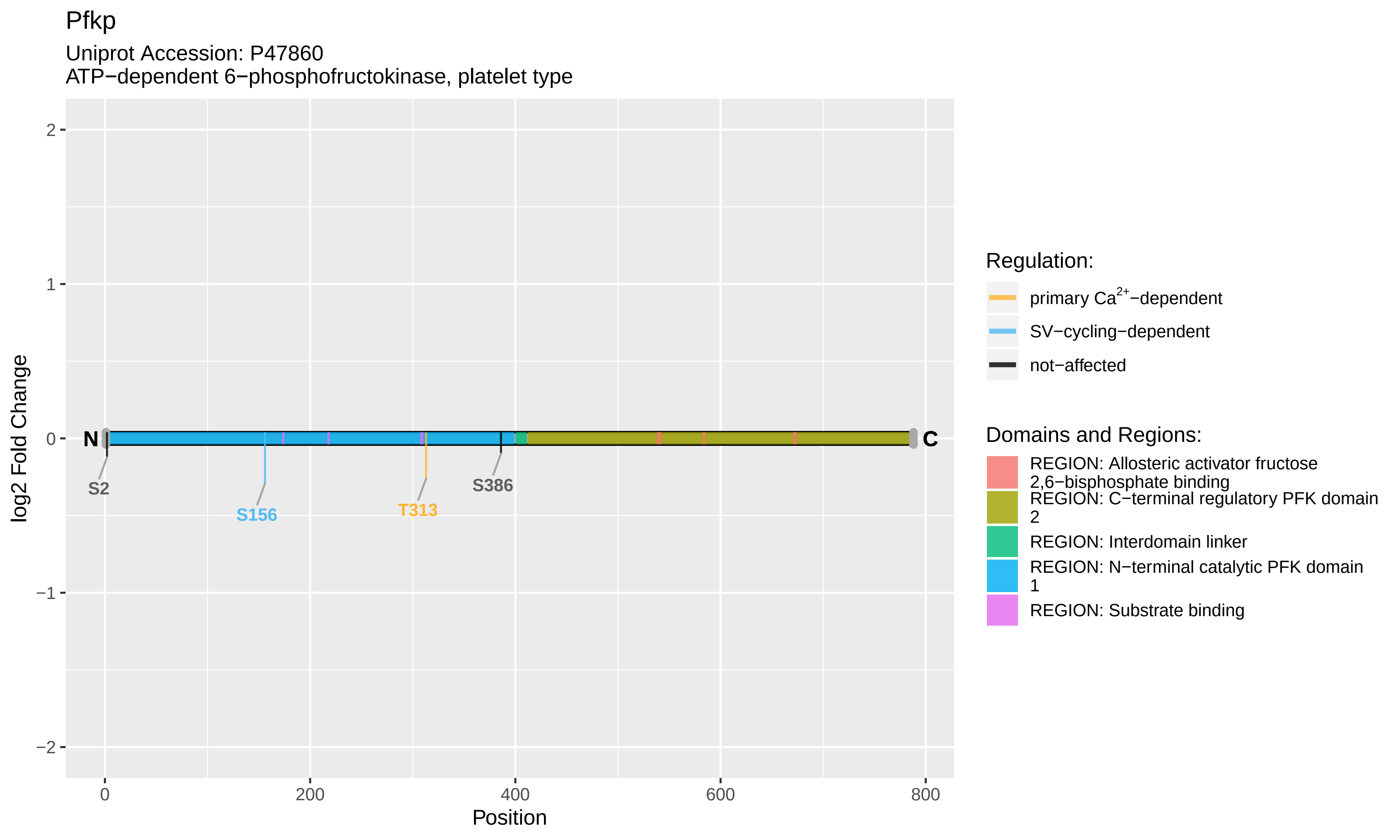
Pfkp
Uniprot Accession: P47860
ATP−dependent 6−phosphofructokinase, platelet type
log2 Fold Change
Position
N
C
S2
S156
T313
S386
Regulation:
primary Ca2+−dependent
SV−cycling−dependent
not−affected
Domains and Regions:
REGION: Allosteric activator fructose 2,6−bisphosphate binding
REGION: C−terminal regulatory PFK domain 2
REGION: Interdomain linker
REGION: N−terminal catalytic PFK domain 1
REGION: Substrate binding

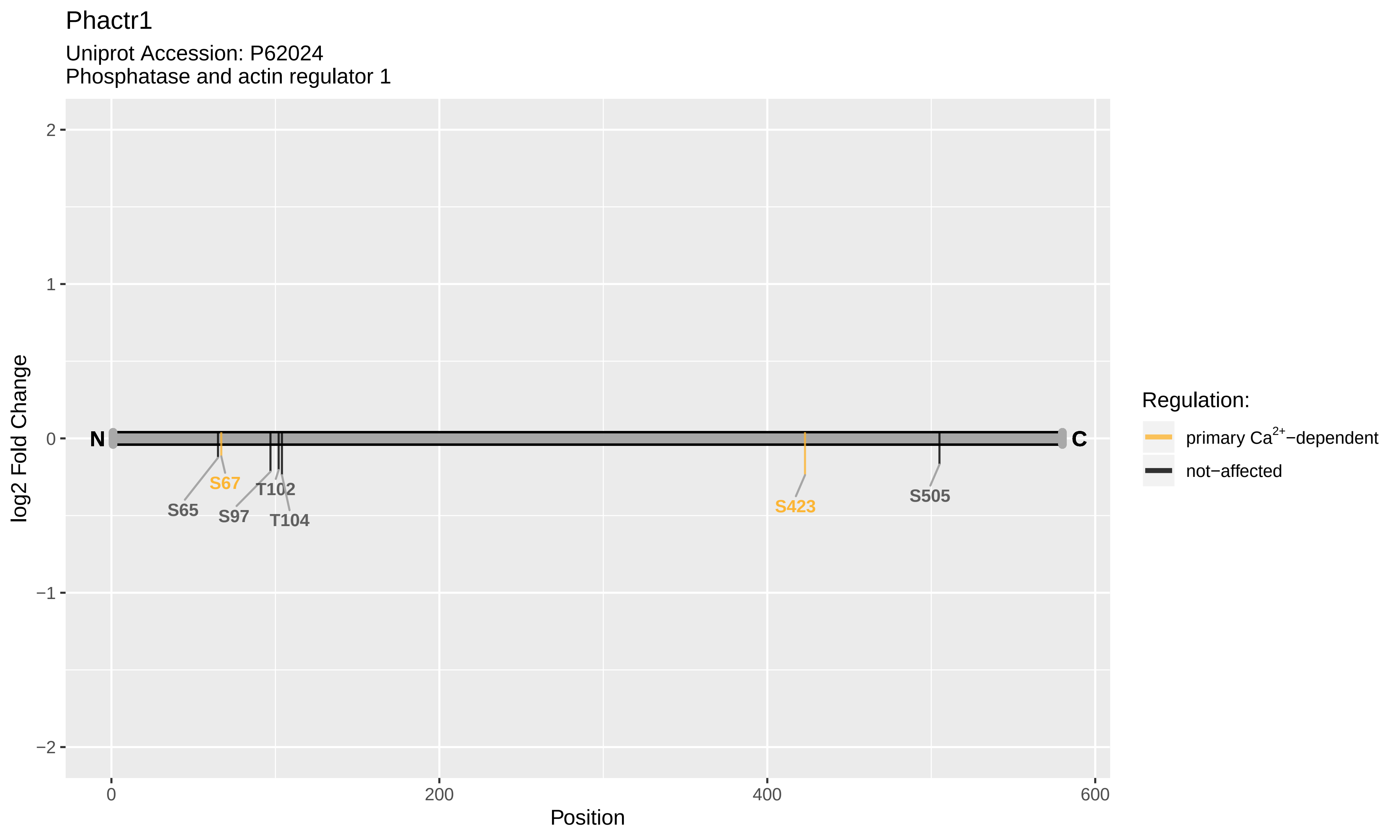
Phactr1
Uniprot Accession: P62024
Phosphatase and actin regulator 1
log2 Fold Change
Position
N
C
S65
S67
S97
T102
T104
S423
S505
Regulation:
primary Ca2+−dependent
not−affected
2
1
0
−1
−2
0
200
400
600

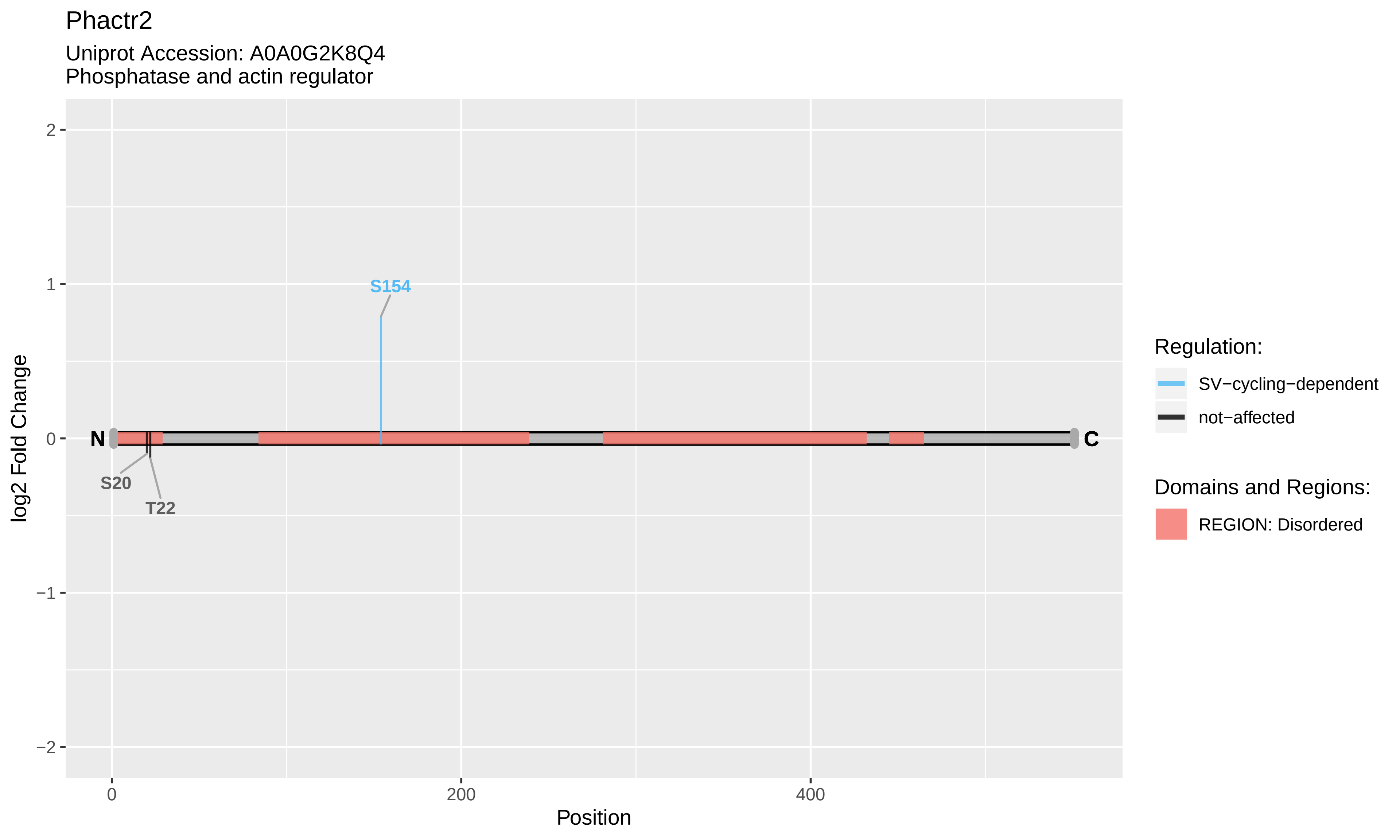

Phactr2
Uniprot Accession: A0A0G2K8Q4
Phosphatase and actin regulator
S154
S20
T22
N
C
2
1
0
−1
−2
0
200
400
log2 Fold Change
Position
Regulation:
SV−cycling−dependent
not−affected
Domains and Regions:
REGION: Disordered

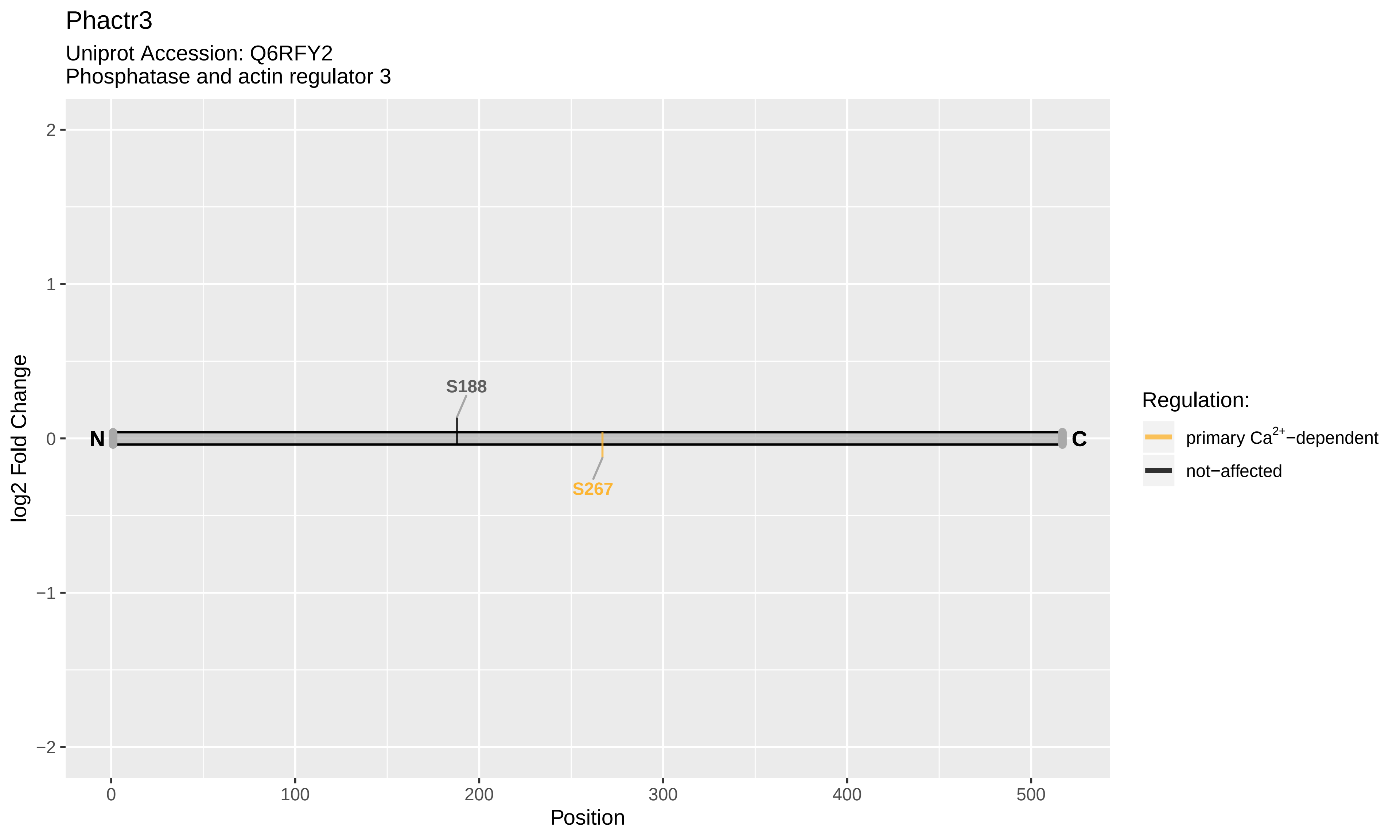

Phactr3
Uniprot Accession: Q6RFY2
Phosphatase and actin regulator 3
log2 Fold Change
2
1
0
−1
−2
N
C
S188
S267
0
100
200
300
400
500
Position
Regulation:
primary Ca2+−dependent
not−affected

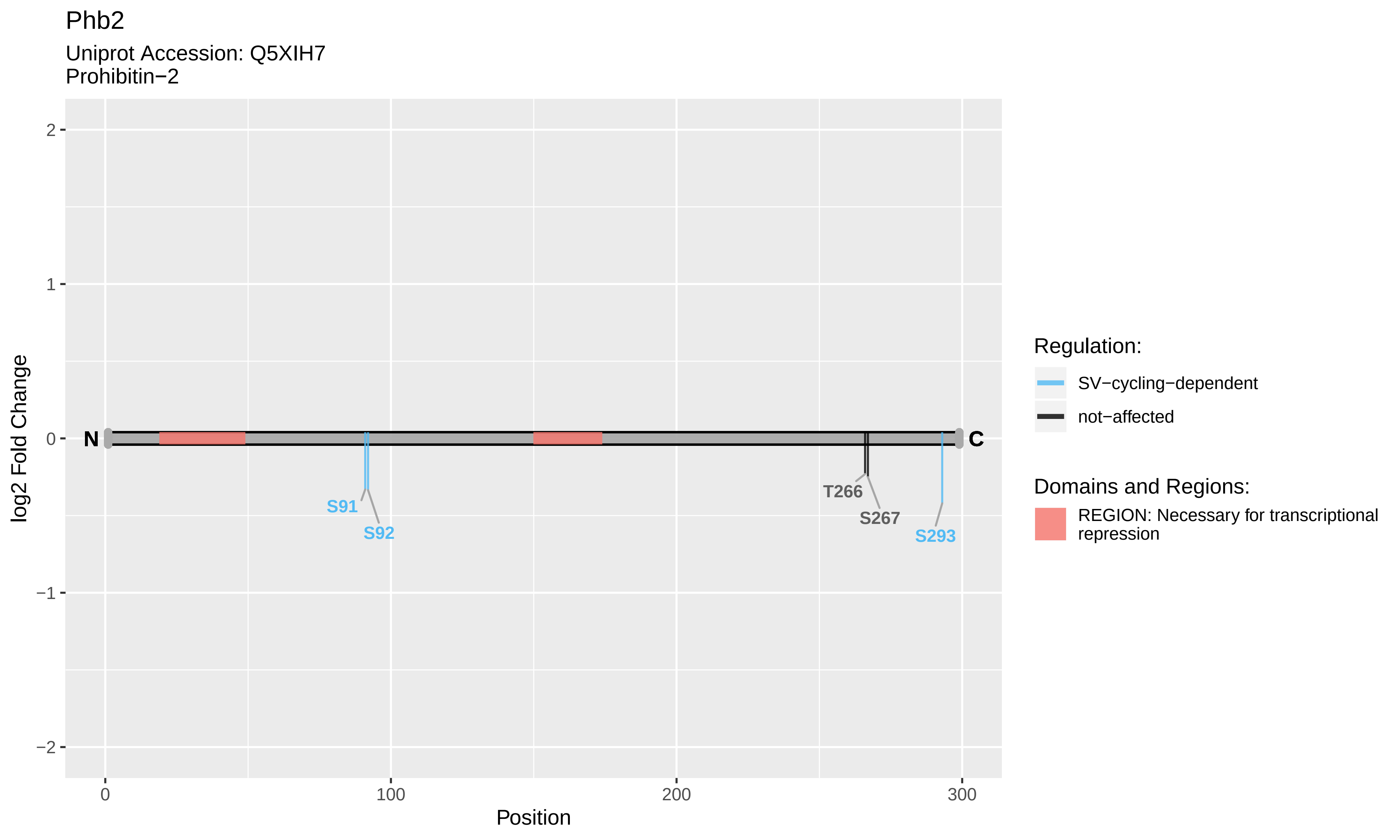
Phb2
Uniprot Accession: Q5XIH7
Prohibitin−2
log2 Fold Change
2
1
0
−1
−2
N
C
S91
S92
T266
S267
S293
0
100
200
300
Position
Regulation:
SV−cycling−dependent
not−affected
Domains and Regions:
REGION: Necessary for transcriptional repression

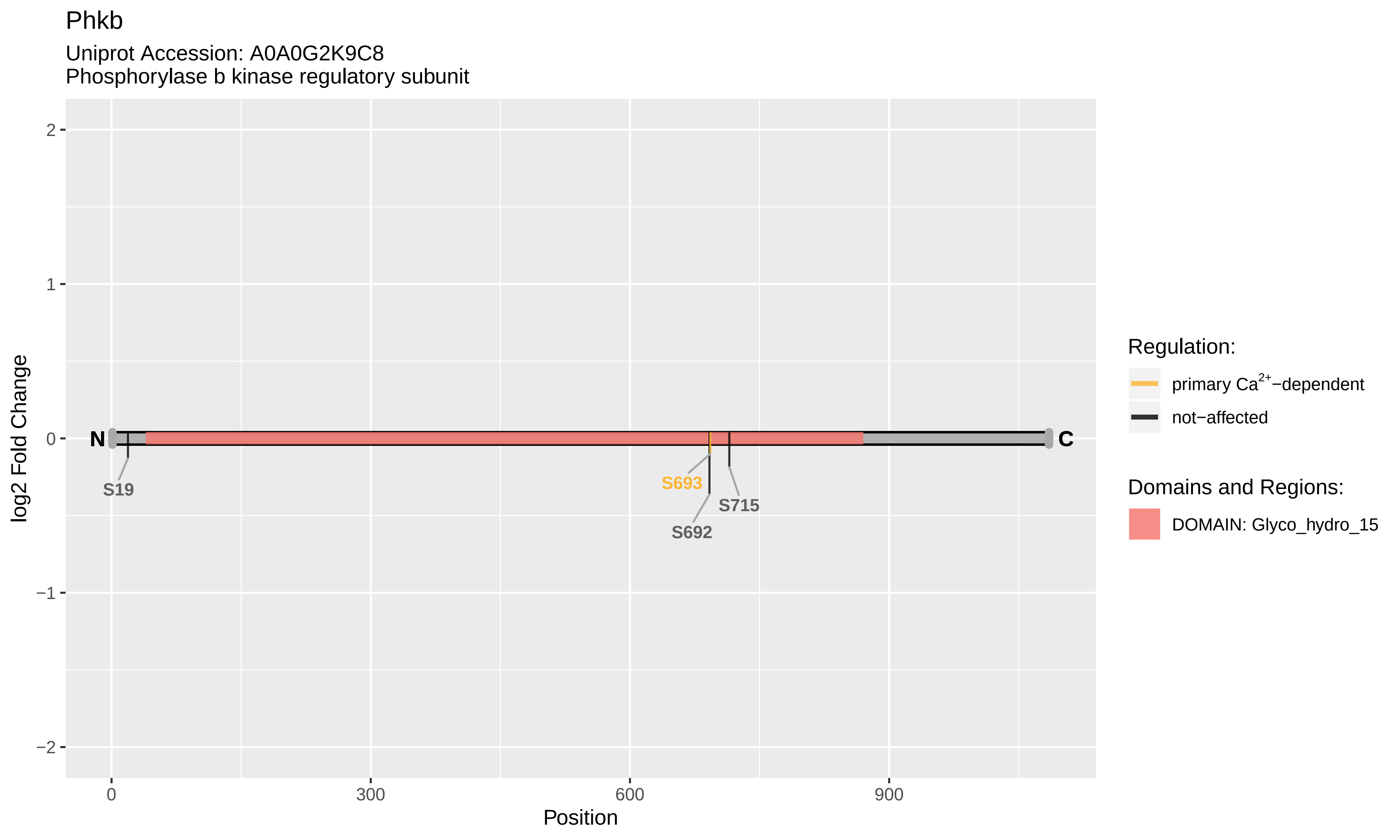
Phkb
Uniprot Accession: A0A0G2K9C8
Phosphorylase b kinase regulatory subunit
log2 Fold Change
2
1
0
−1
−2
N
C
S19
S693
S692
S715
0
300
600
900
Position
Regulation:
primary Ca2+−dependent
not−affected
Domains and Regions:
DOMAIN: Glyco_hydro_15

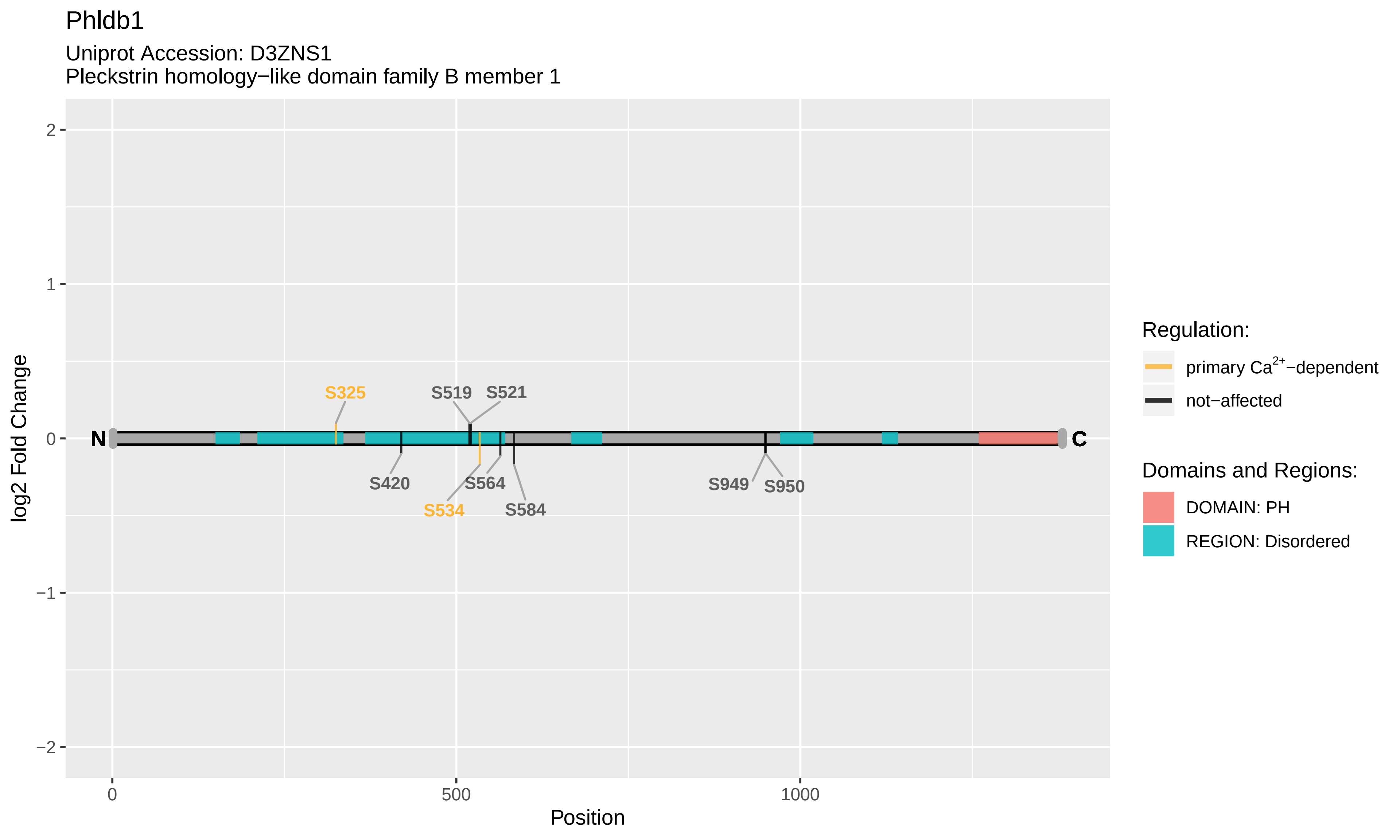
Phldb1
Uniprot Accession: D3ZNS1
Pleckstrin homology−like domain family B member 1
log2 Fold Change
2
1
0
−1
−2
N
C
S325
S519
S521
S420
S564
S534
S584
S949
S950
0
500
1000
Position
Regulation:
primary Ca2+−dependent
not−affected
Domains and Regions:
DOMAIN: PH
REGION: Disordered

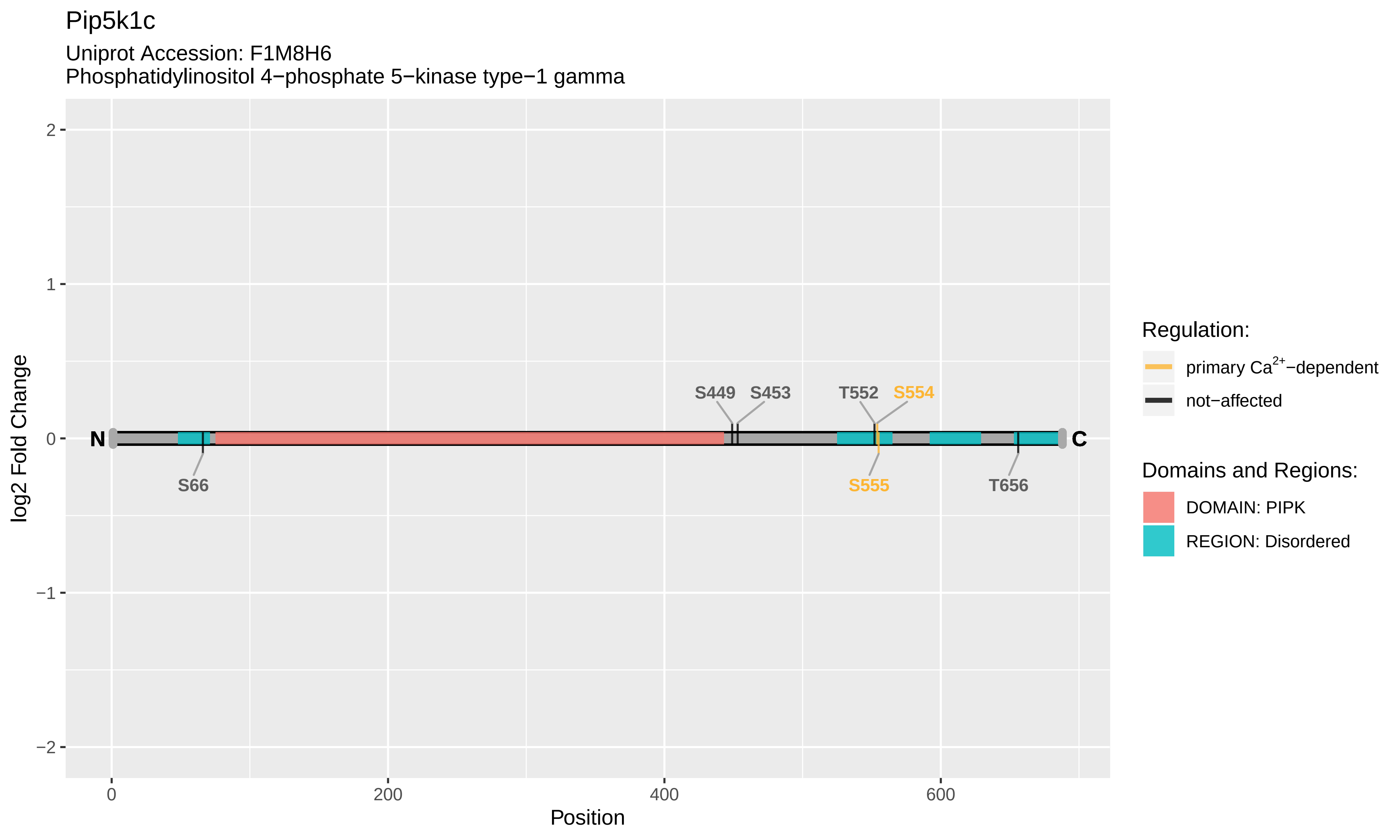

Pip5k1c
Uniprot Accession: F1M8H6
Phosphatidylinositol 4−phosphate 5−kinase type−1 gamma
2
1
0
−1
−2
log2 Fold Change
N
C
S449
S453
T552
S554
S66
S555
T656
0
200
400
600
Position
Regulation:
primary Ca2+−dependent
not−affected
Domains and Regions:
DOMAIN: PIPK
REGION: Disordered

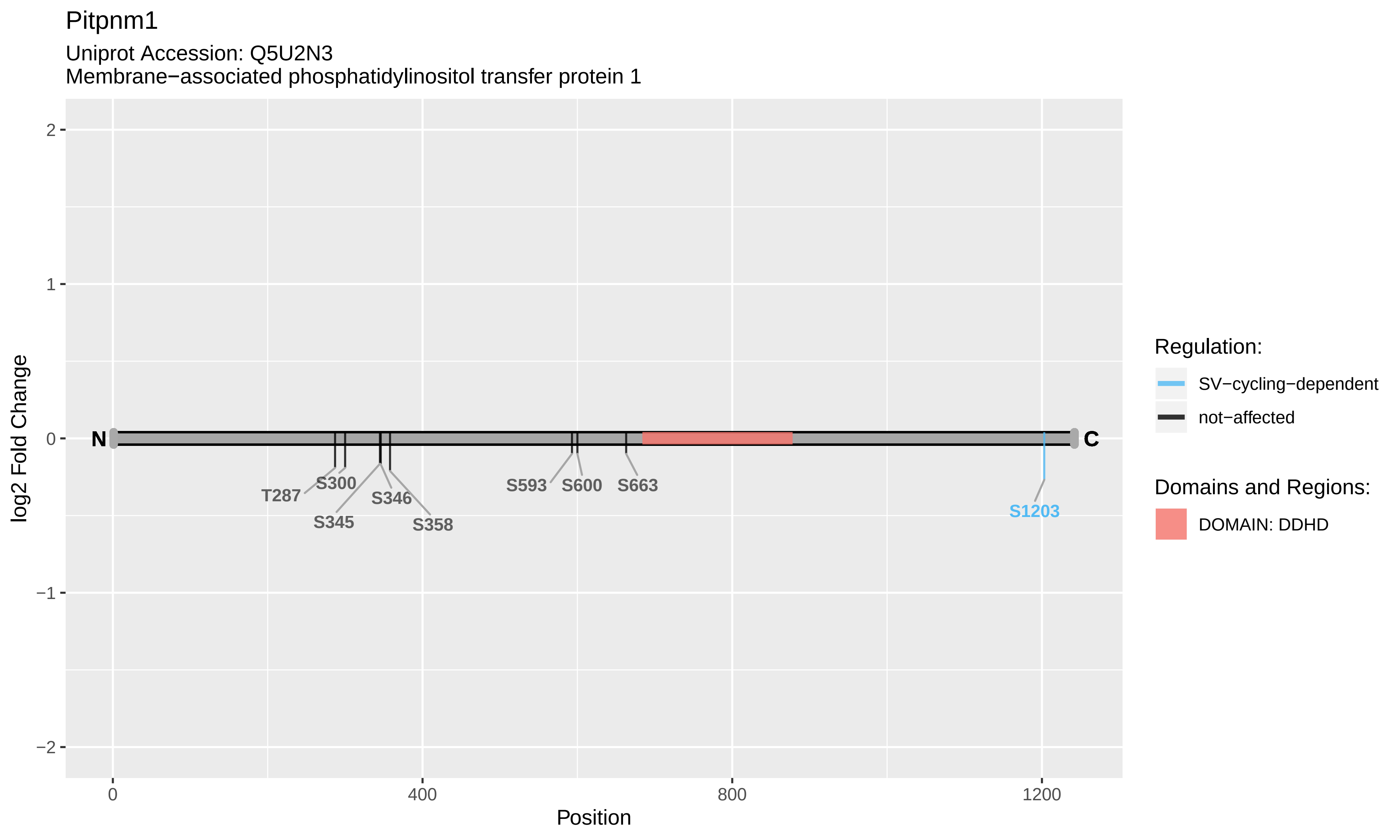

Pitpnm1
Uniprot Accession: Q5U2N3
Membrane−associated phosphatidylinositol transfer protein 1
log2 Fold Change
2
1
0
−1
−2
N
C
T287
S300
S345
S346
S358
S593
S600
S663
S1203
0
400
800
1200
Position
Regulation:
SV−cycling−dependent
not−affected
Domains and Regions:
DOMAIN: DDHD

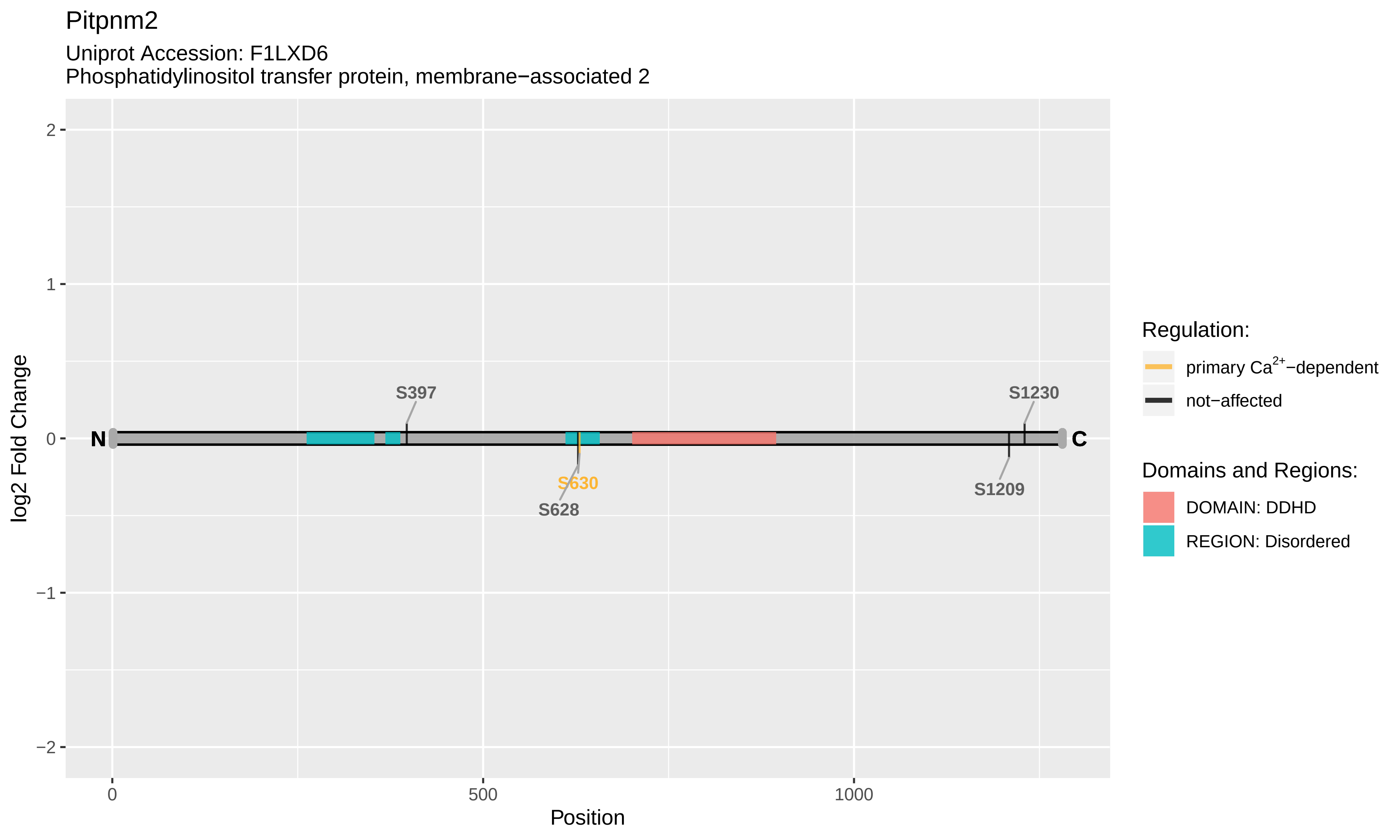
Pitpnm2
Uniprot Accession: F1LXD6
Phosphatidylinositol transfer protein, membrane−associated 2
log2 Fold Change
2
1
0
−1
−2
N
C
S397
S1230
S630
S628
S1209
0
500
1000
Position
Regulation:
primary Ca2+−dependent
not−affected
Domains and Regions:
DOMAIN: DDHD
REGION: Disordered

Pja1

Uniprot Accession: A0A0U1RRV0
Praja ring finger ubiquitin ligase 1 (Fragment)

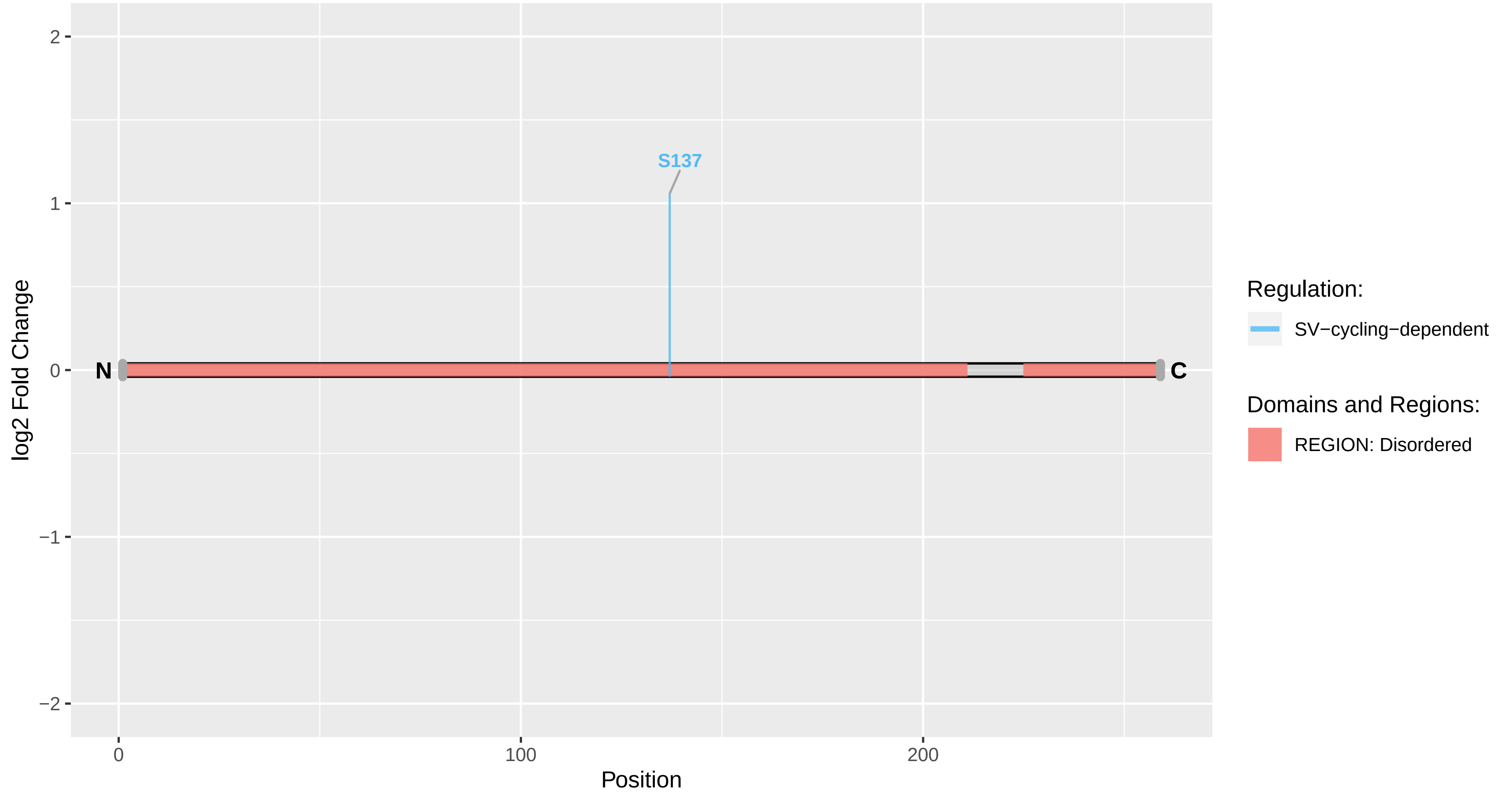

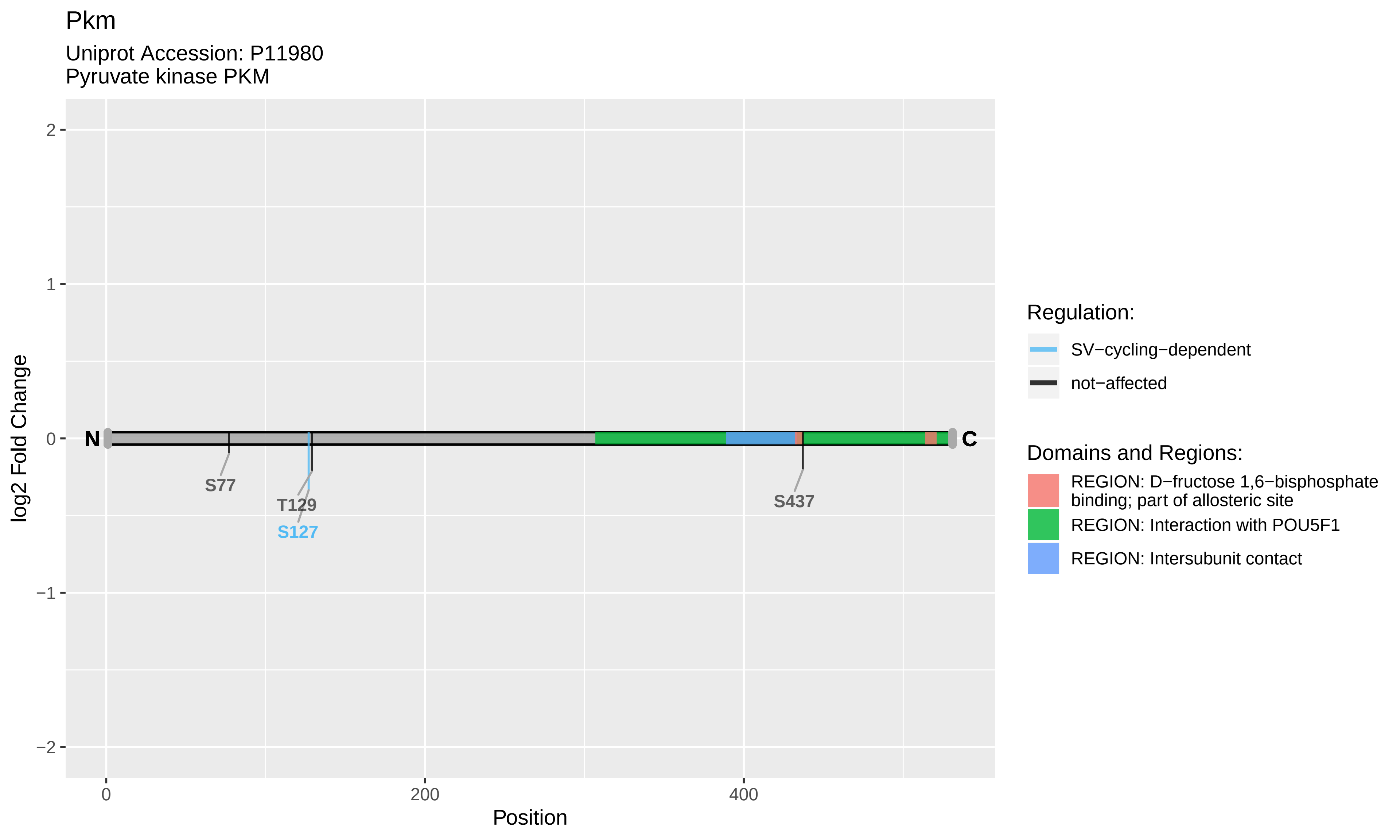
Pkm
Uniprot Accession: P11980
Pyruvate kinase PKM
log2 Fold Change
2
1
0
−1
−2
N
C
S77
T129
S127
S437
0
200
400
Position
Regulation:
SV−cycling−dependent
not−affected
Domains and Regions:
REGION: D−fructose 1,6−bisphosphate binding; part of allosteric site
REGION: Interaction with POU5F1
REGION: Intersubunit contact

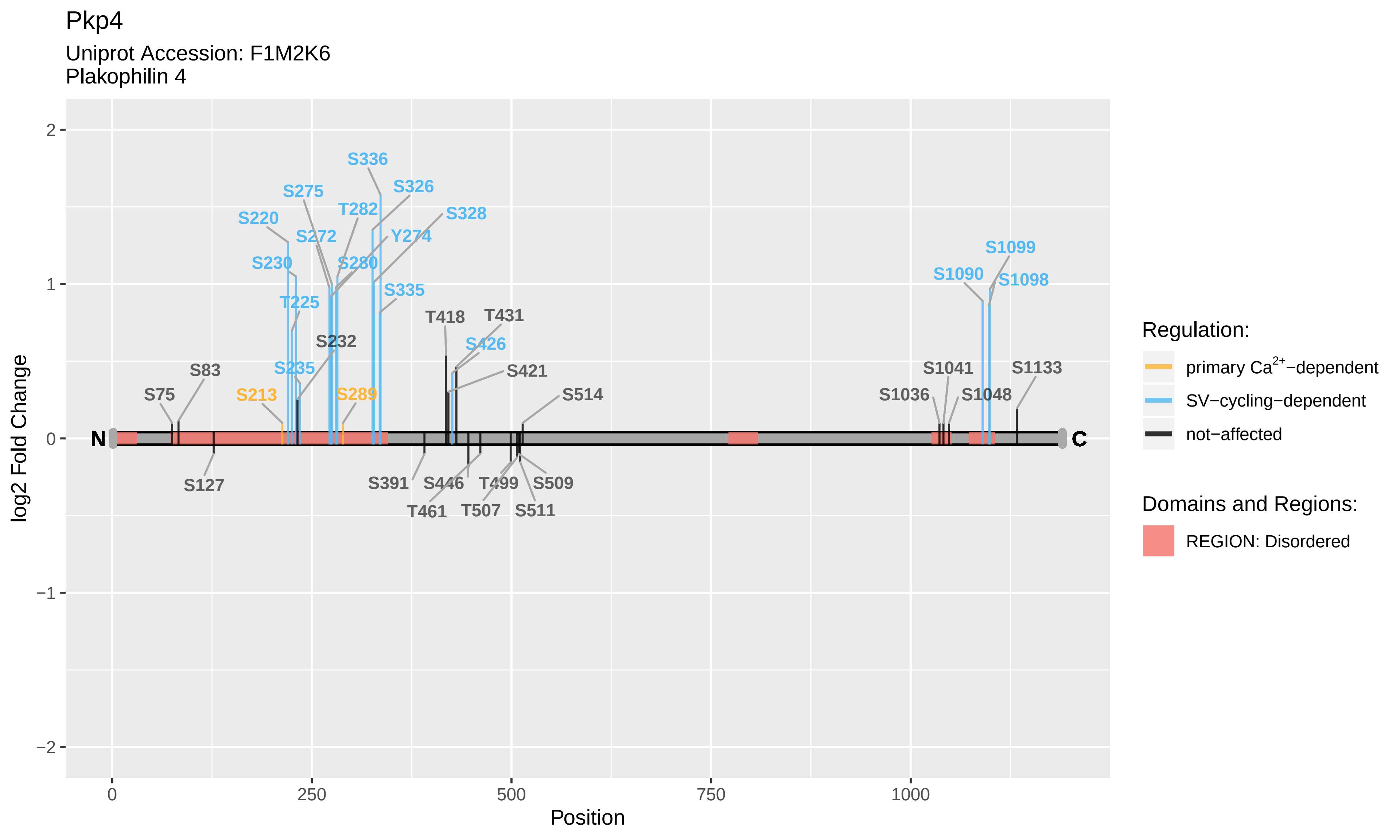

Pkp4
Uniprot Accession: F1M2K6
Plakophilin 4
log2 Fold Change
Position
N
C
S336
S326
S275
T282
S328
S220
S272
Y274
S230
S280
S335
T225
T418
T431
S232
S426
S235
S421
S83
S75
S213
S289
S514
S127
S391
S446
T499
S509
T461
T507
S511
S1099
S1090
S1098
S1041
S1133
S1036
S1048
Regulation:
primary Ca2+−dependent
SV−cycling−dependent
not−affected
Domains and Regions:
REGION: Disordered

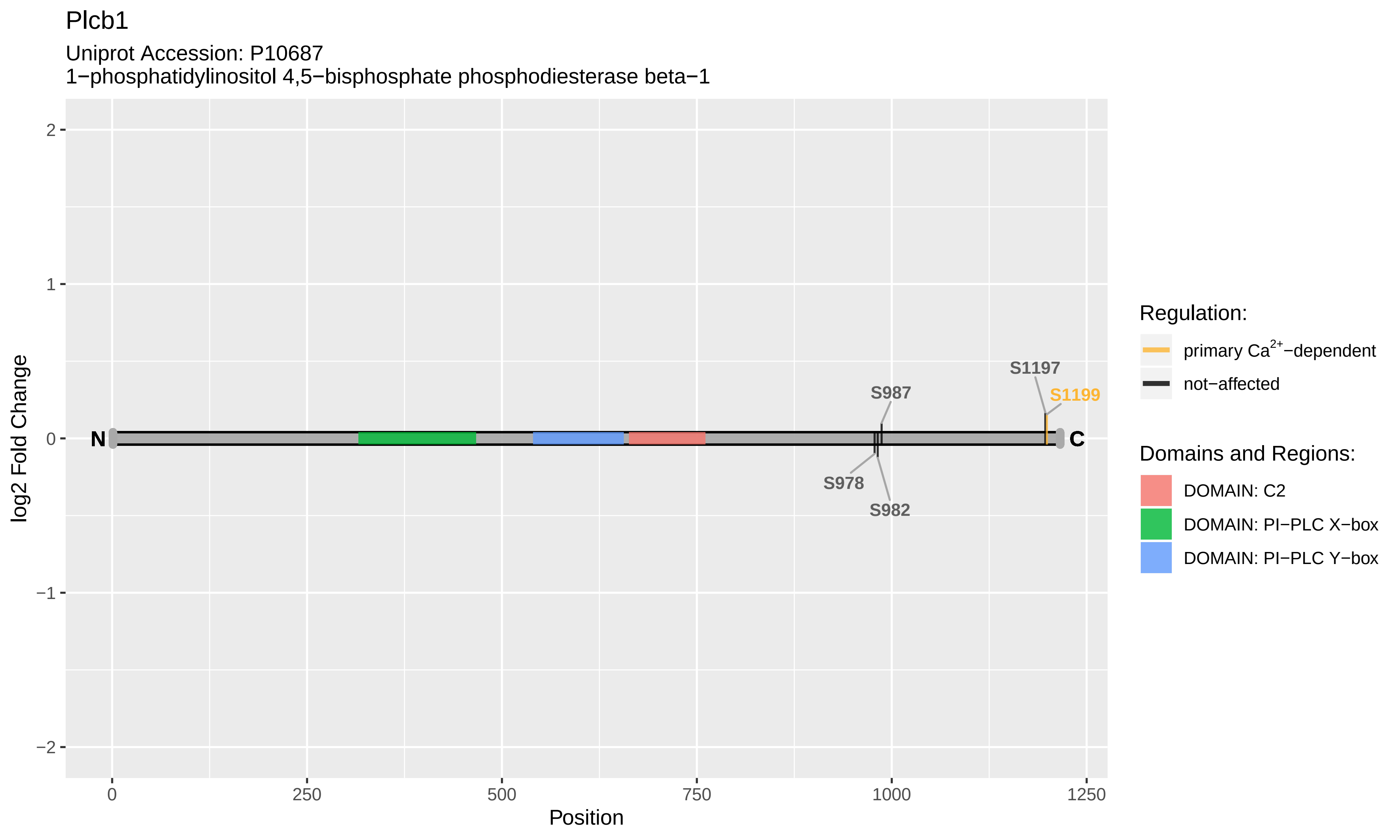

Plcb1
Uniprot Accession: P10687
1−phosphatidylinositol 4,5−bisphosphate phosphodiesterase beta−1
log2 Fold Change
Position
2
1
0
−1
−2
0
250
500
750
1000
1250
N
C
S987
S1197
S1199
S978
S982
Regulation:
primary Ca2+−dependent
not−affected
Domains and Regions:
DOMAIN: C2
DOMAIN: PI−PLC X−box
DOMAIN: PI−PLC Y−box

Plcb4

Uniprot Accession: D4A8C5
1−phosphatidylinositol 4,5−bisphosphate phosphodiesterase

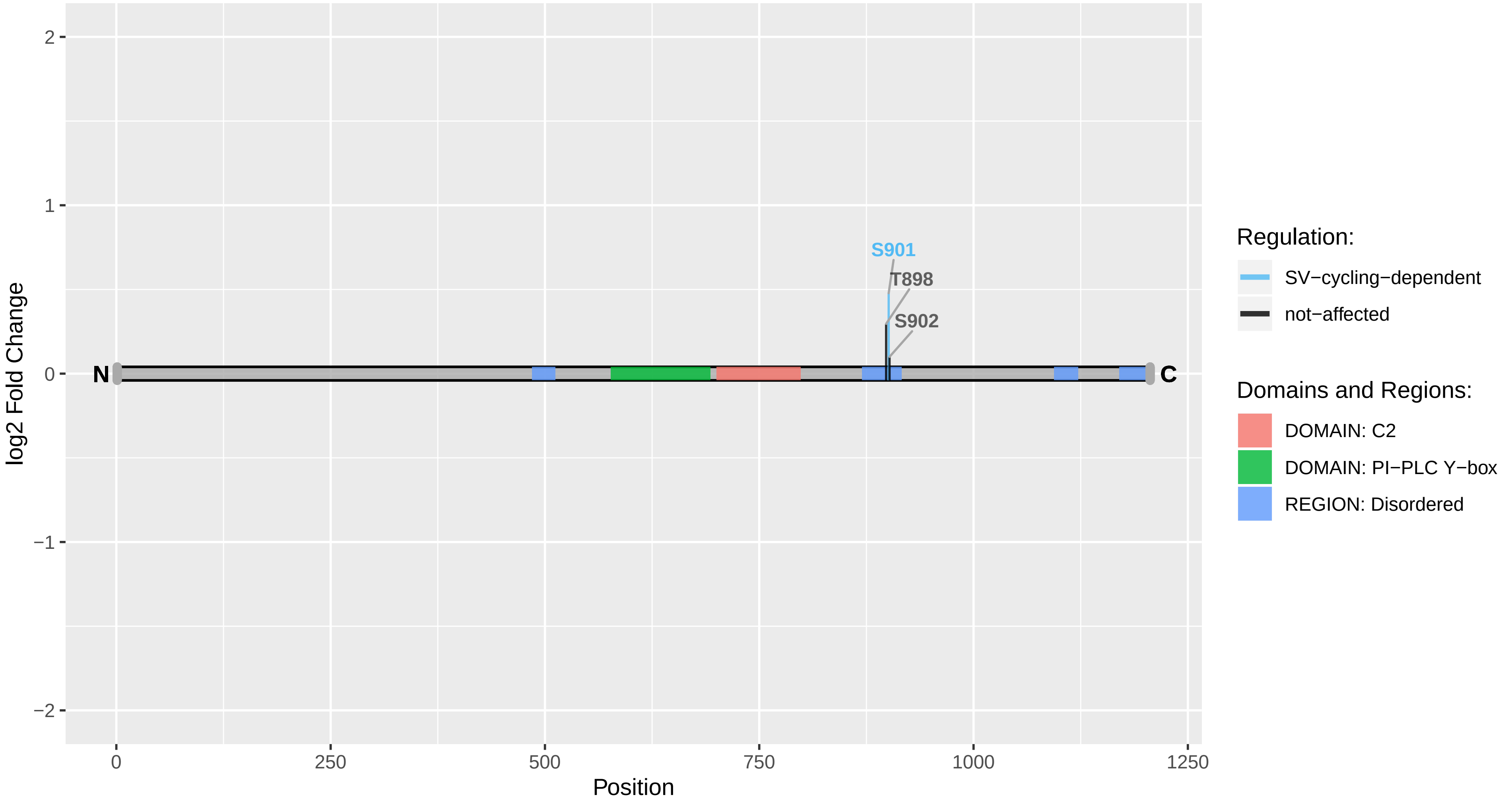

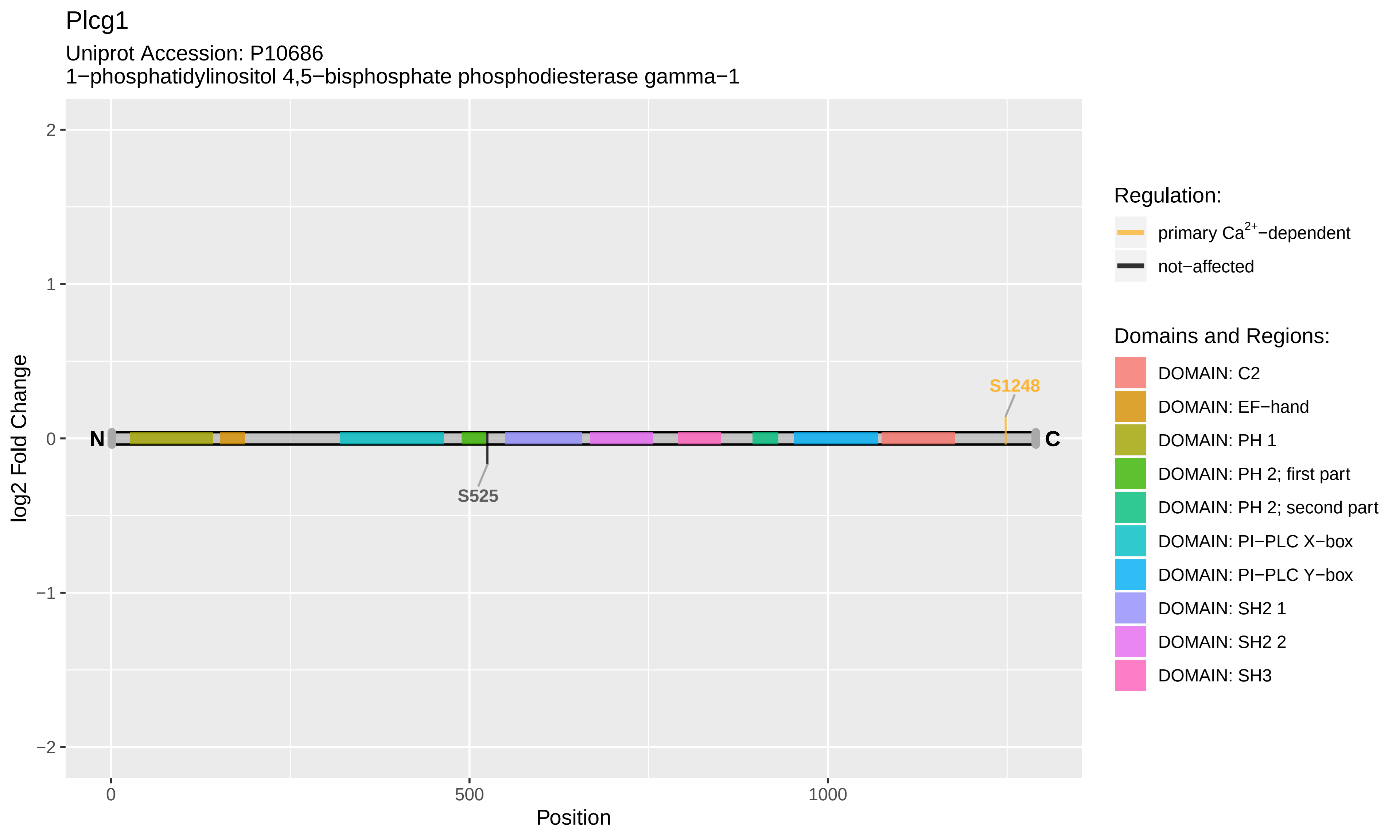

Plcg1
Uniprot Accession: P10686
1−phosphatidylinositol 4,5−bisphosphate phosphodiesterase gamma−1
log2 Fold Change
2
1
0
−1
−2
N
C
S1248
S525
0
500
1000
Position
Regulation:
primary Ca2+−dependent
not−affected
Domains and Regions:
DOMAIN: C2
DOMAIN: EF−hand
DOMAIN: PH 1
DOMAIN: PH 2; first part
DOMAIN: PH 2; second part
DOMAIN: PI−PLC X−box
DOMAIN: PI−PLC Y−box
DOMAIN: SH2 1
DOMAIN: SH2 2
DOMAIN: SH3

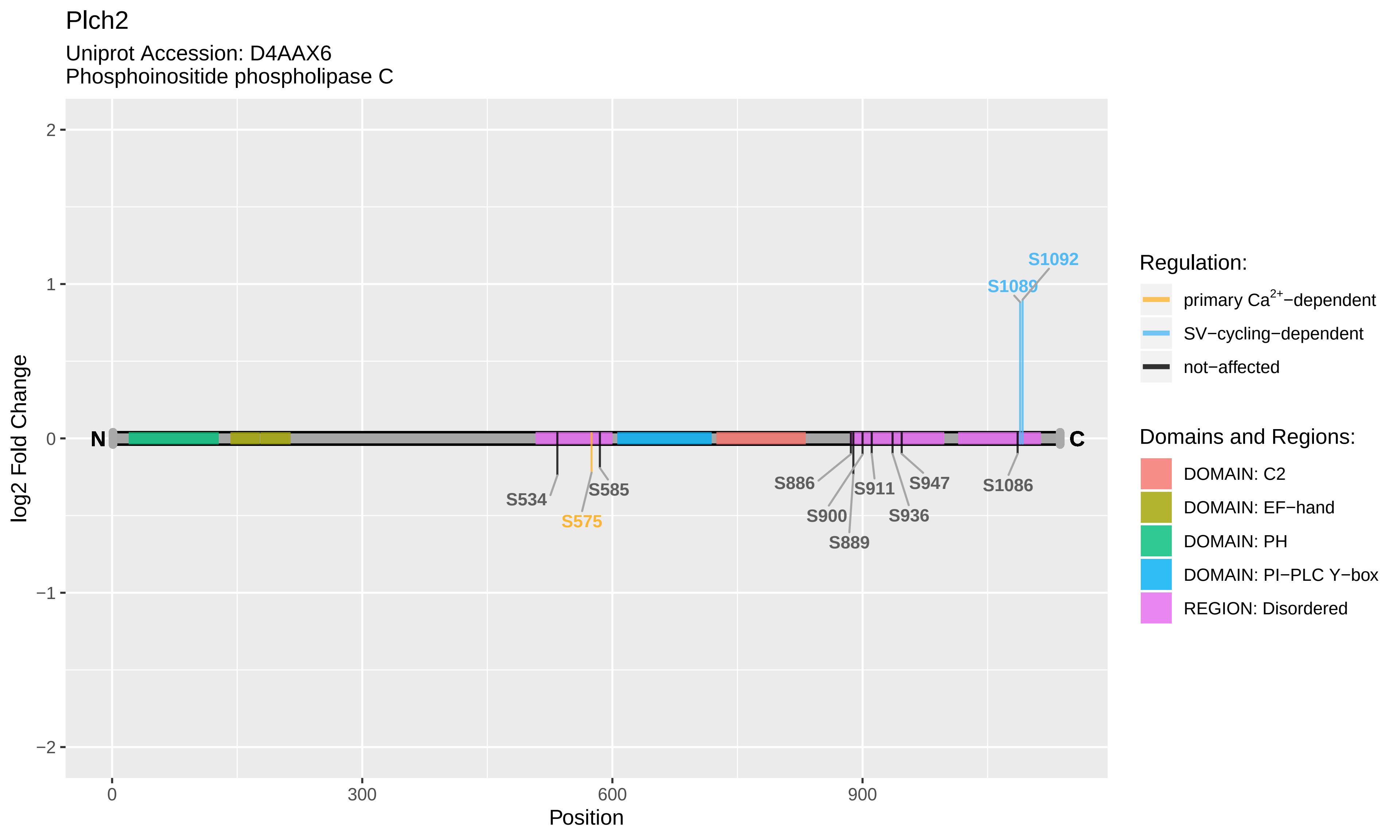

Plch2
Uniprot Accession: D4AAX6
Phosphoinositide phospholipase C
log2 Fold Change
Position
N
C
S1092
S1089
S534
S575
S585
S886
S900
S889
S911
S936
S947
S1086
Regulation:
primary Ca2+−dependent
SV−cycling−dependent
not−affected
Domains and Regions:
DOMAIN: C2
DOMAIN: EF−hand
DOMAIN: PH
DOMAIN: PI−PLC Y−box
REGION: Disordered

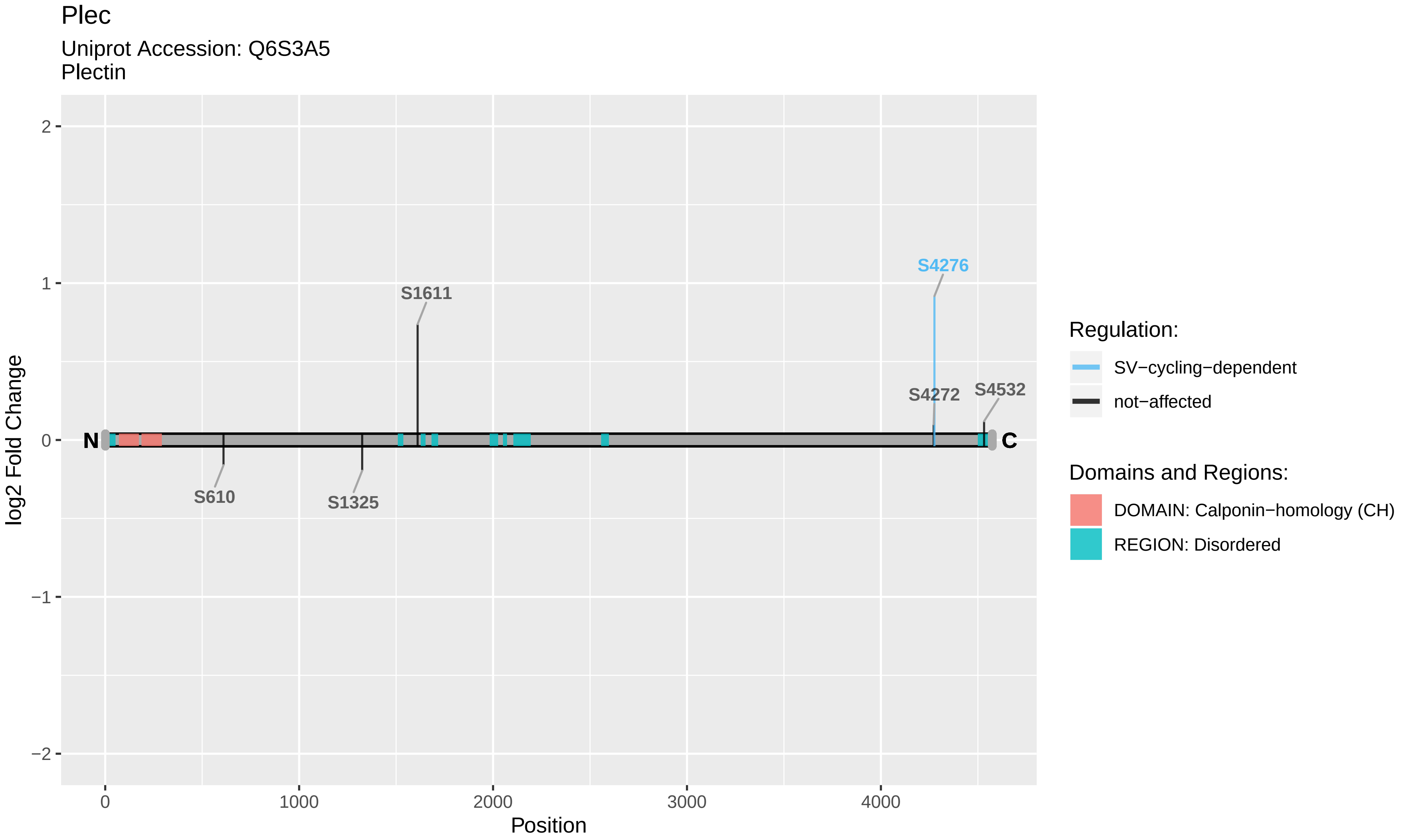
Plec
Uniprot Accession: Q6S3A5
Plectin
log2 Fold Change
Position
2
1
0
−1
−2
0
1000
2000
3000
4000
N
C
S1611
S4276
S4272
S4532
S610
S1325
Regulation:
SV−cycling−dependent
not−affected
Domains and Regions:
DOMAIN: Calponin−homology (CH)
REGION: Disordered

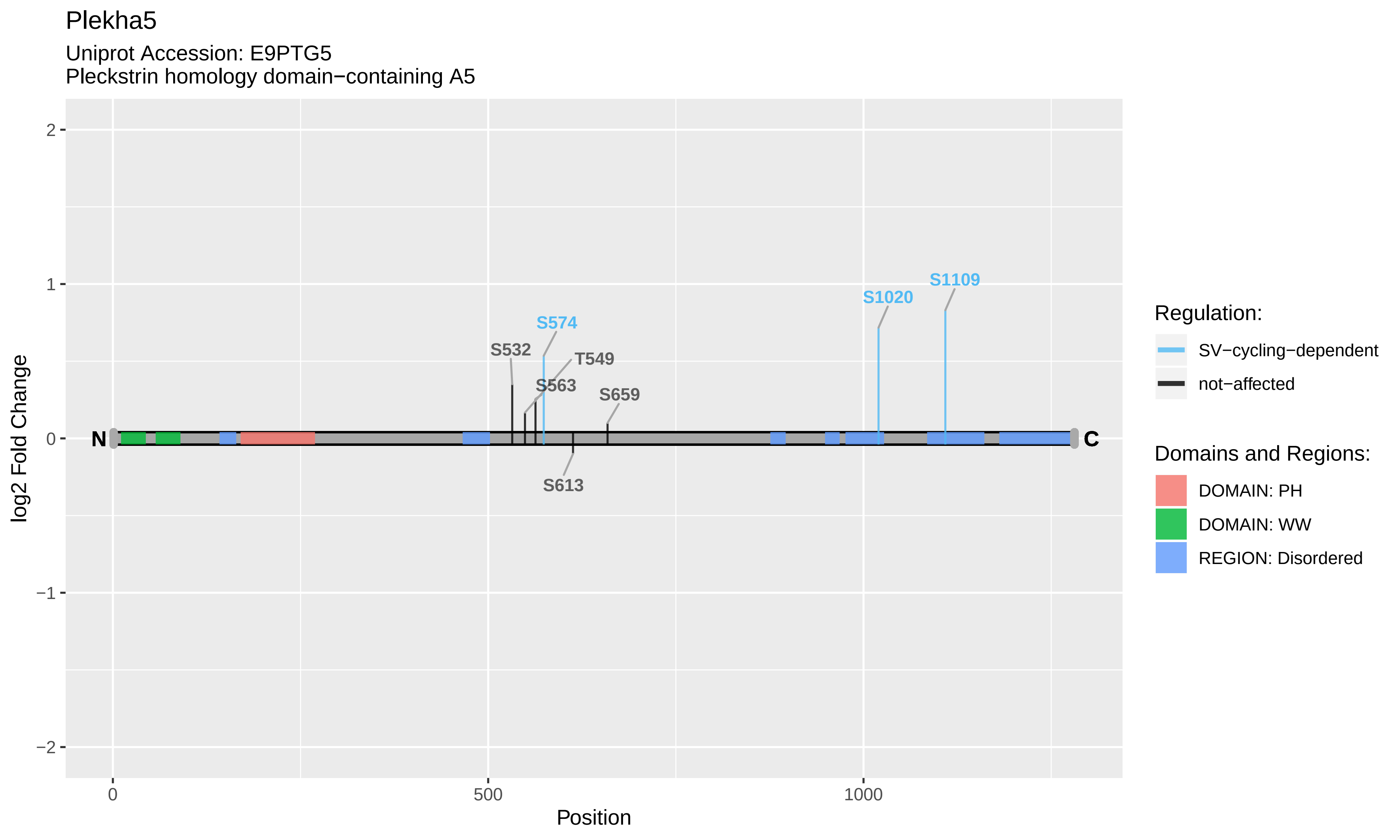
Plekha5
Uniprot Accession: E9PTG5
Pleckstrin homology domain−containing A5
log2 Fold Change
2
1
0
−1
−2
N
C
S532
S574
T549
S563
S659
S613
S1020
S1109
0
500
1000
Position
Regulation:
SV−cycling−dependent
not−affected
Domains and Regions:
DOMAIN: PH
DOMAIN: WW
REGION: Disordered

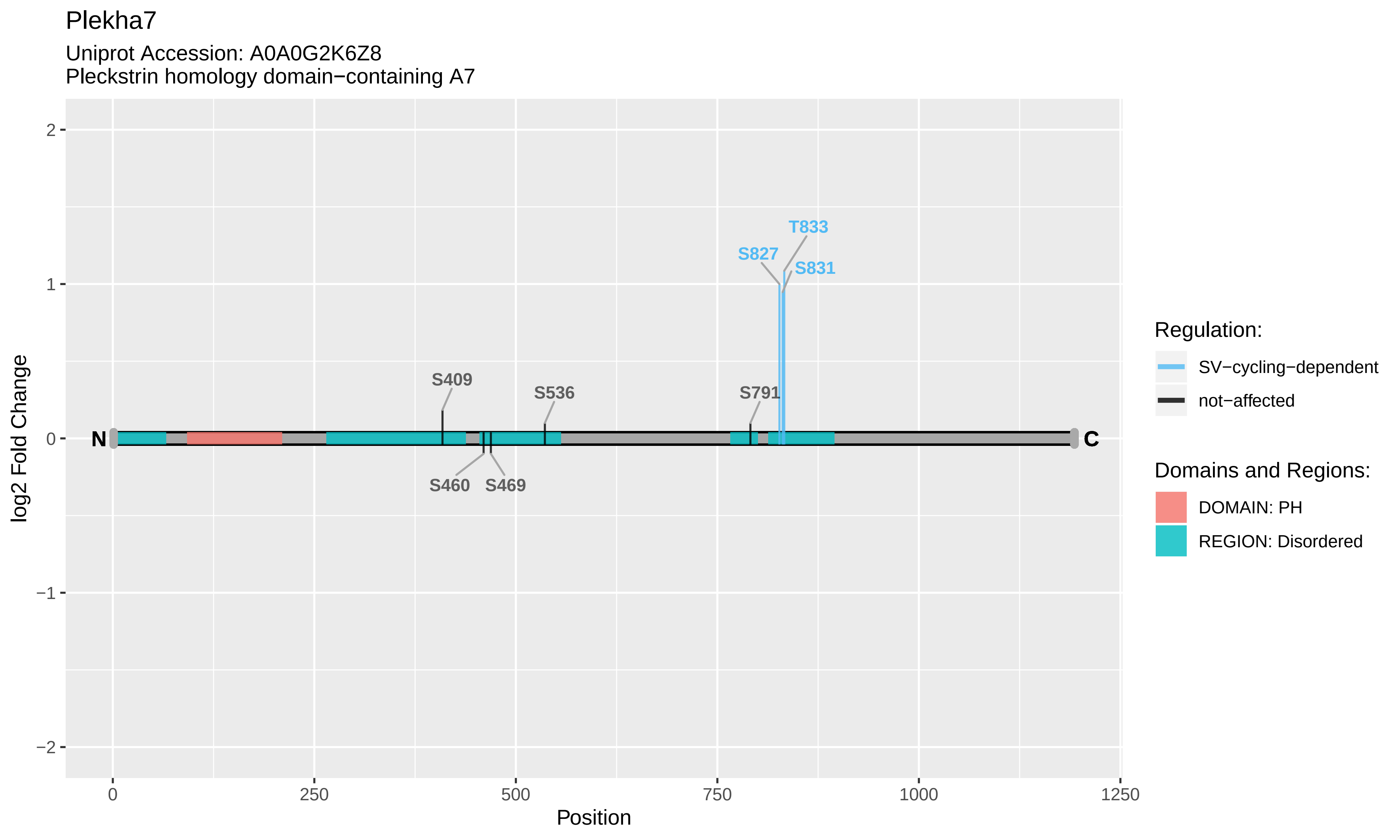

Plekha7
Uniprot Accession: A0A0G2K6Z8
Pleckstrin homology domain−containing A7
T833
S827
S831
S409
S536
S791
N
C
S460
S469
2
1
0
−1
−2
log2 Fold Change
0
250
500
750
1000
1250
Position
Regulation:
SV−cycling−dependent
not−affected
Domains and Regions:
DOMAIN: PH
REGION: Disordered

Plgrkt

Uniprot Accession: D4ACN8
Plasminogen receptor (KT)

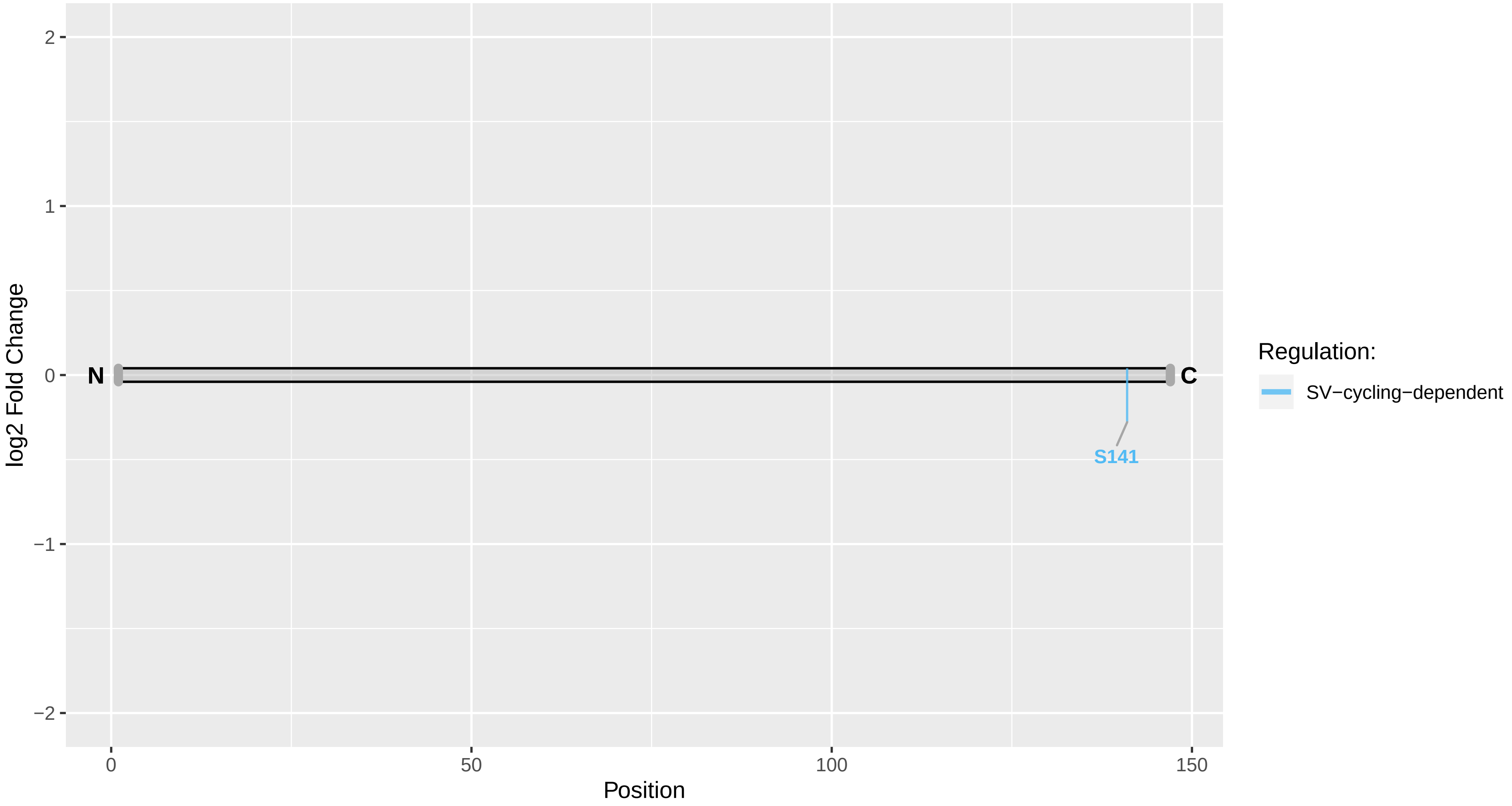

Plp1

Uniprot Accession: P60203
Myelin proteolipid protein

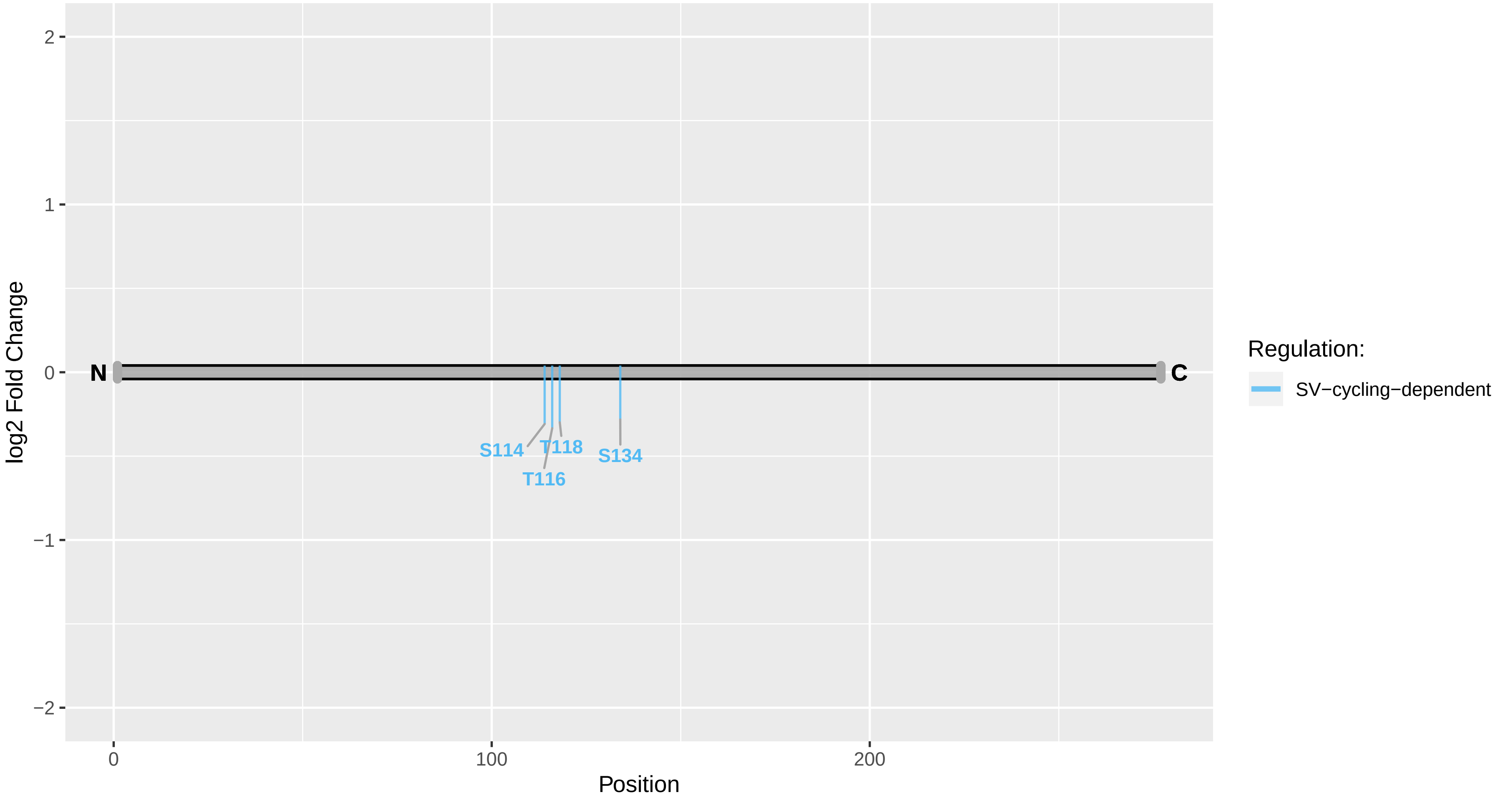

Plpp6

Uniprot Accession: Q66H88
Phospholipid phosphatase 6

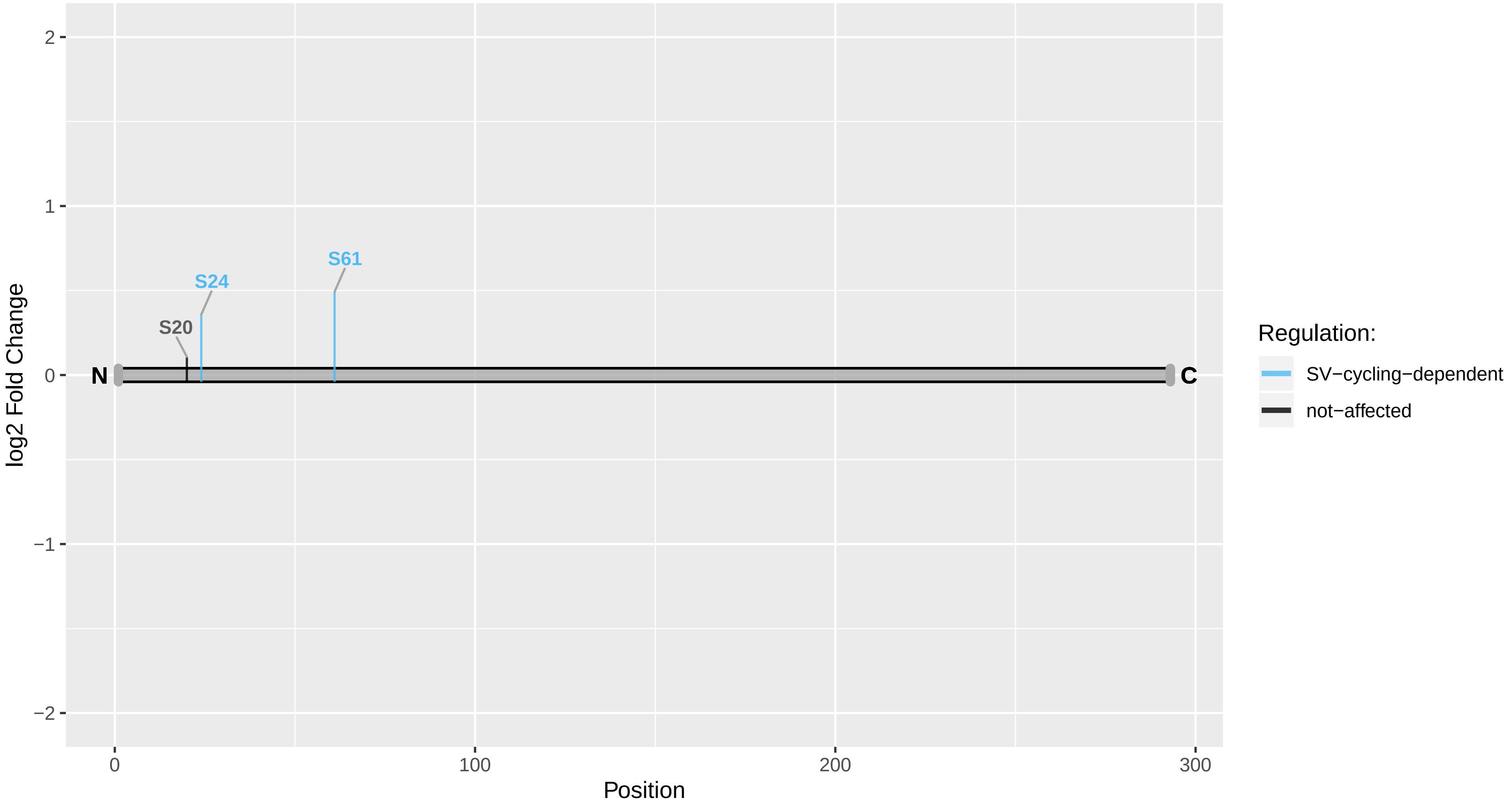

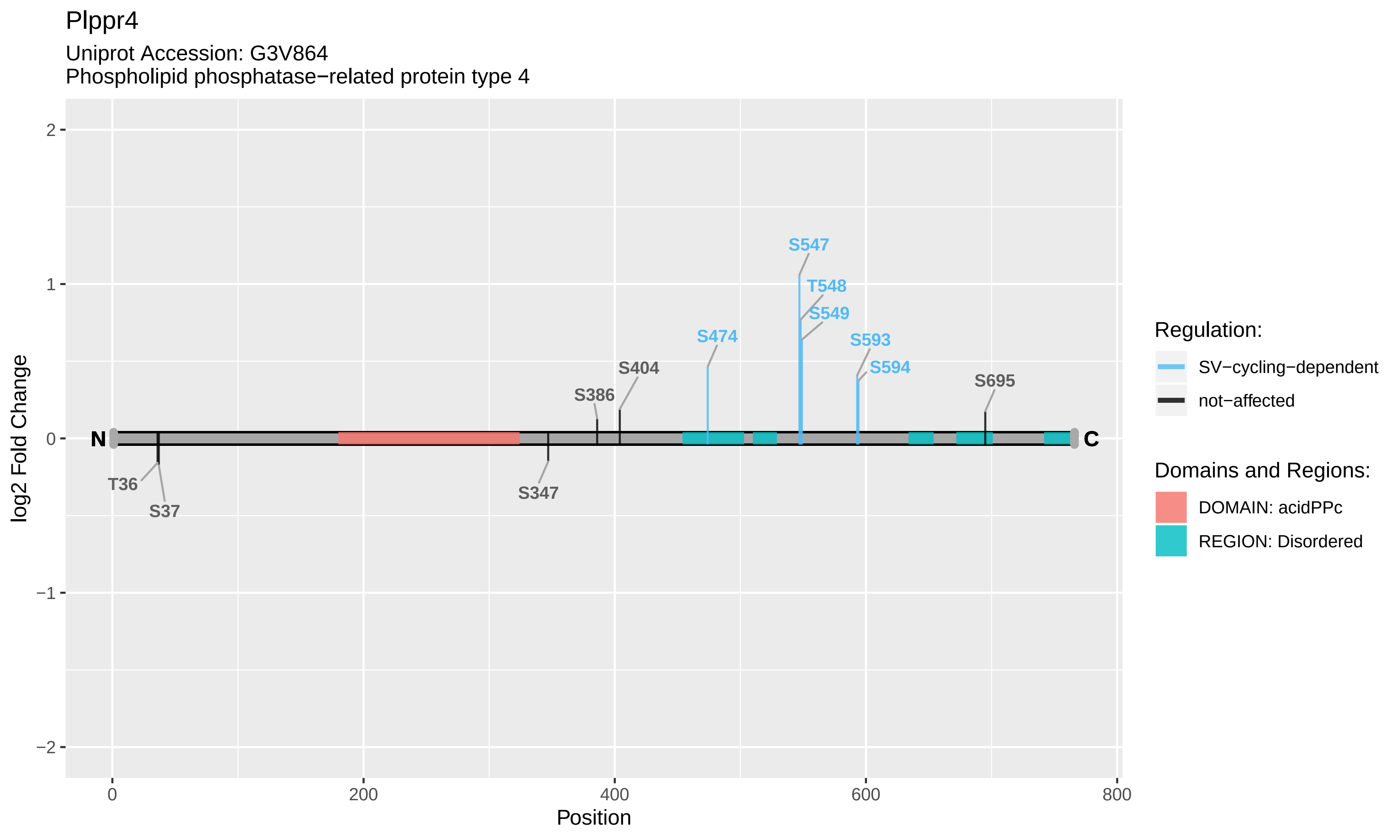

Plppr4
Uniprot Accession: G3V864
Phospholipid phosphatase−related protein type 4
log2 Fold Change
Position
N
C
T36
S37
S347
S386
S404
S474
S547
T548
S549
S593
S594
S695
Regulation:
SV−cycling−dependent
not−affected
Domains and Regions:
DOMAIN: acidPPc
REGION: Disordered

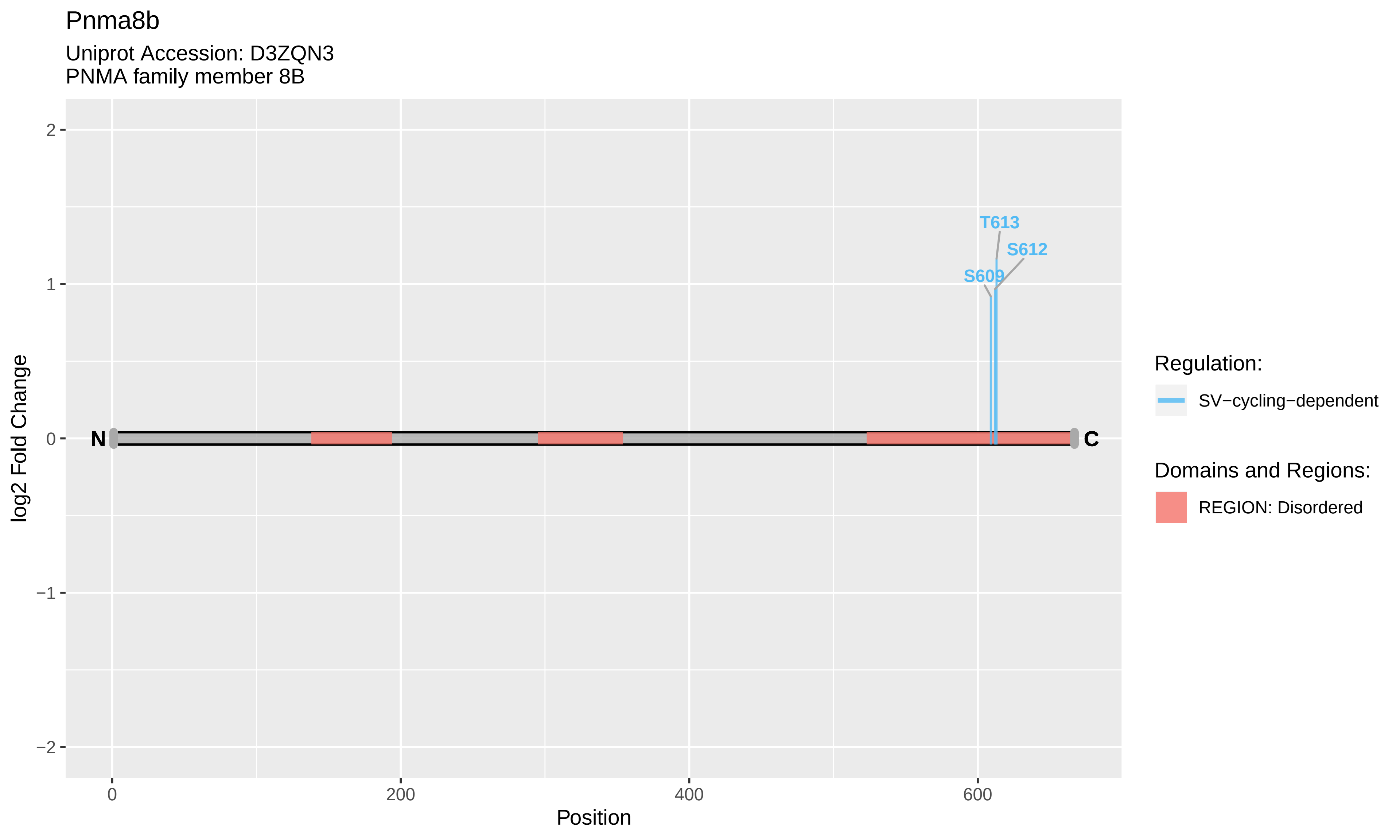

Pnma8b
Uniprot Accession: D3ZQN3
PNMA family member 8B
T613
S612
S609
N
C
log2 Fold Change
2
1
0
−1
−2
0
200
400
600
Position
Regulation:
SV−cycling−dependent
Domains and Regions:
REGION: Disordered

Pnpo

Uniprot Accession: O88794
Pyridoxine−5'−phosphate oxidase

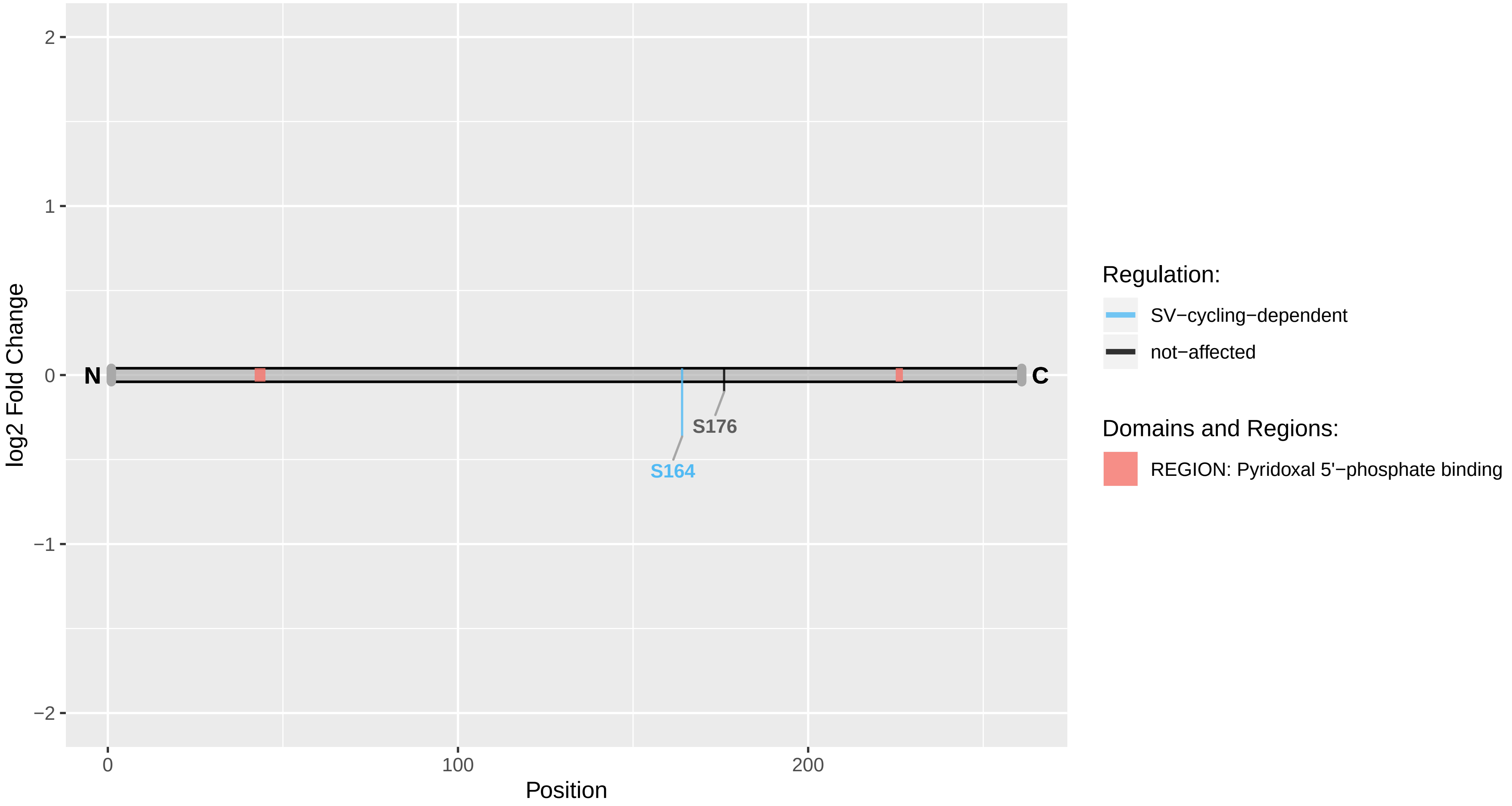

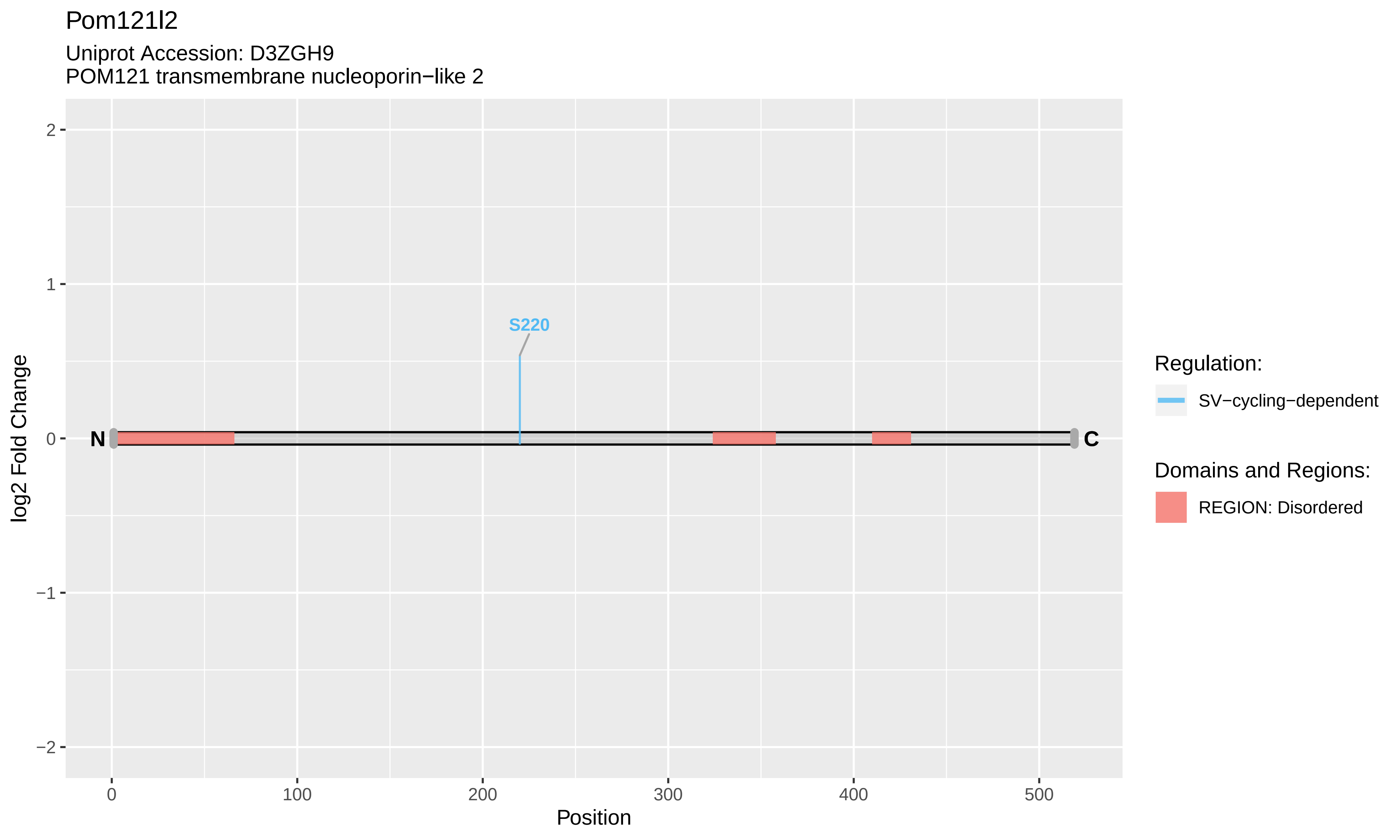

Pom121l2
Uniprot Accession: D3ZGH9
POM121 transmembrane nucleoporin−like 2
S220
N
C
log2 Fold Change
2
1
0
−1
−2
0
100
200
300
400
500
Position
Regulation:
SV−cycling−dependent
Domains and Regions:
REGION: Disordered

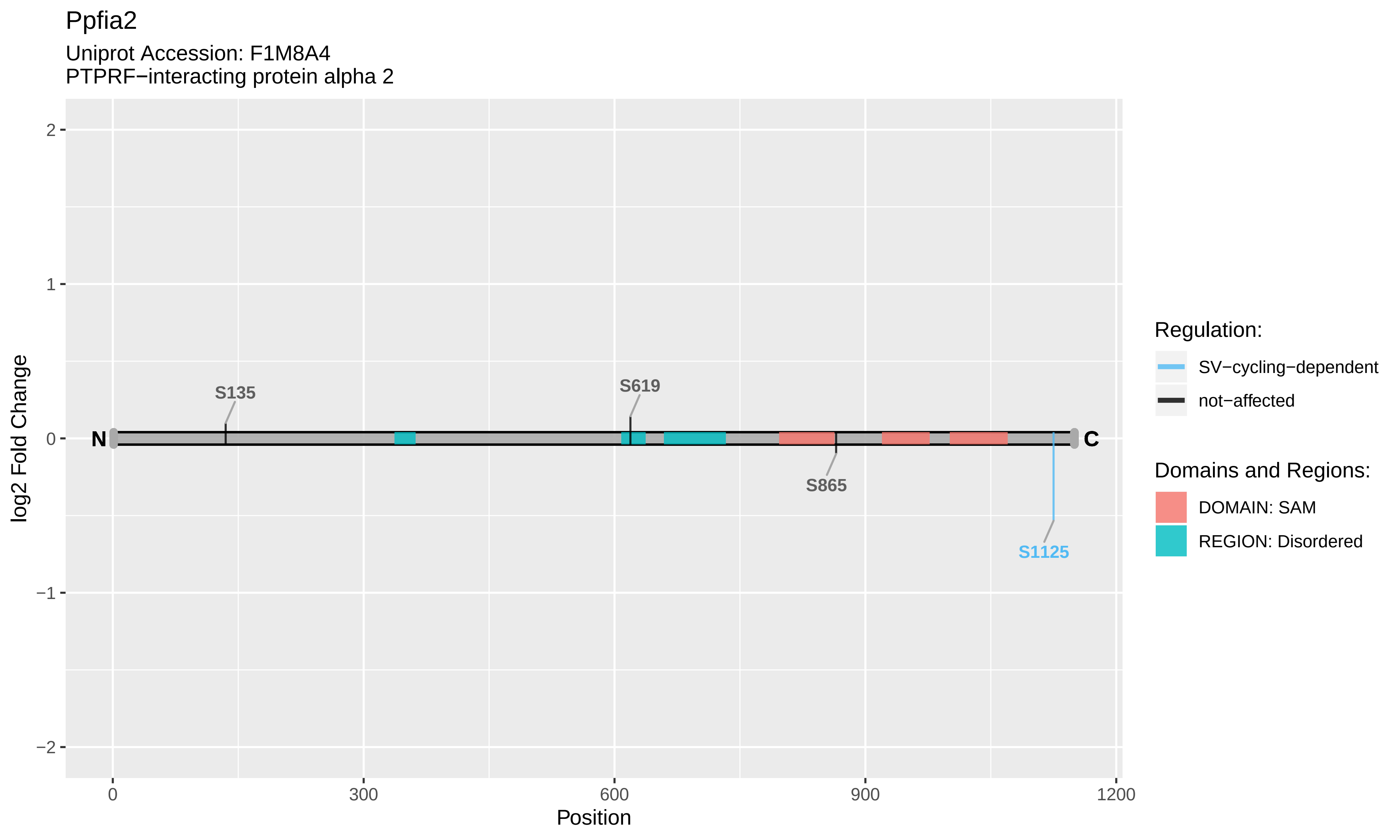
Ppfia2
Uniprot Accession: F1M8A4
PTPRF−interacting protein alpha 2
S135
S619
S865
S1125
N
C
2
1
0
−1
−2
log2 Fold Change
0
300
600
900
1200
Position
Regulation:
SV−cycling−dependent
not−affected
Domains and Regions:
DOMAIN: SAM
REGION: Disordered

Ppfia3

Uniprot Accession: Q91Z79
Liprin−alpha−3

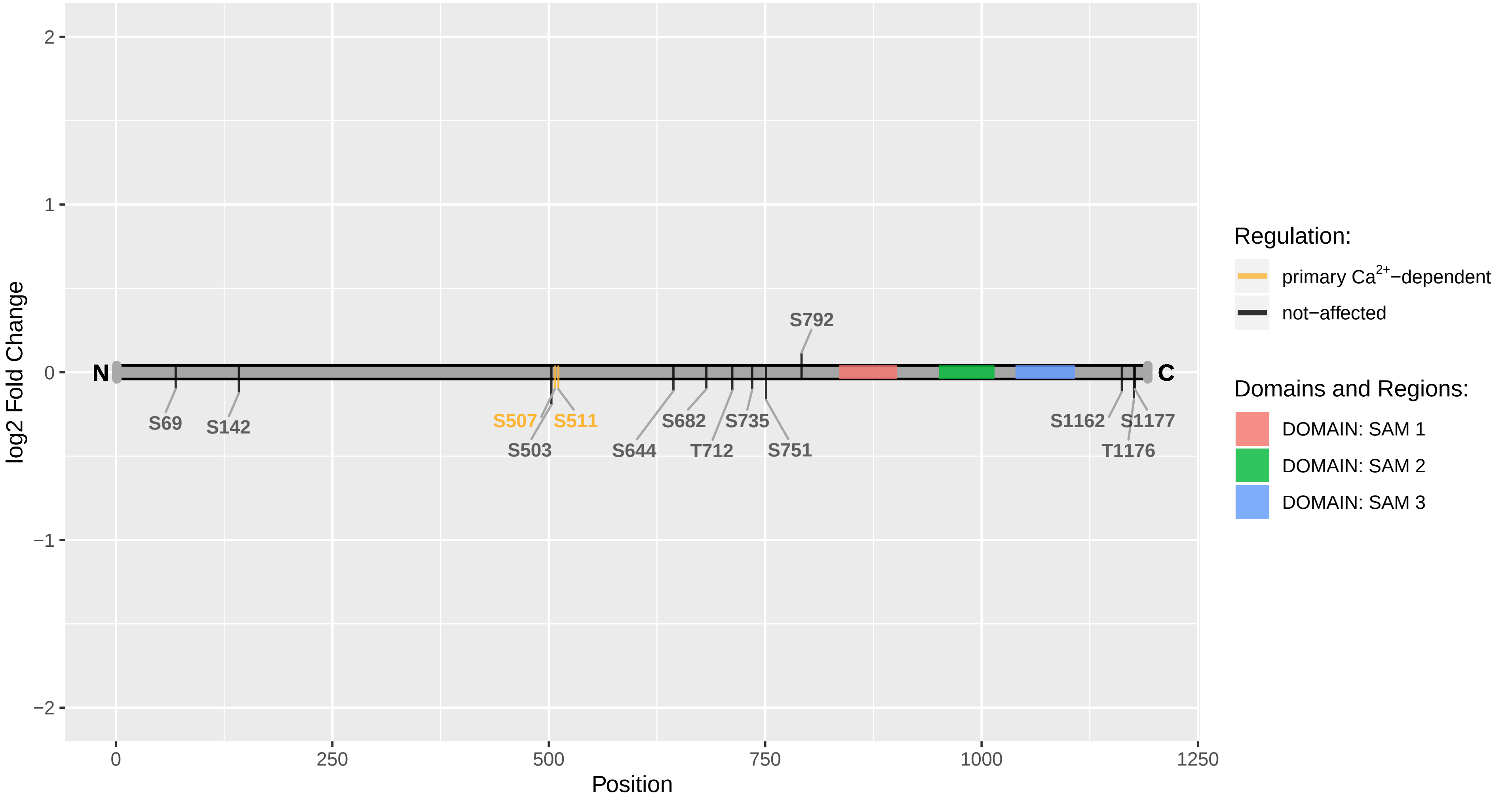

Ppfia4

Uniprot Accession: F1M863
Liprin−alpha−4

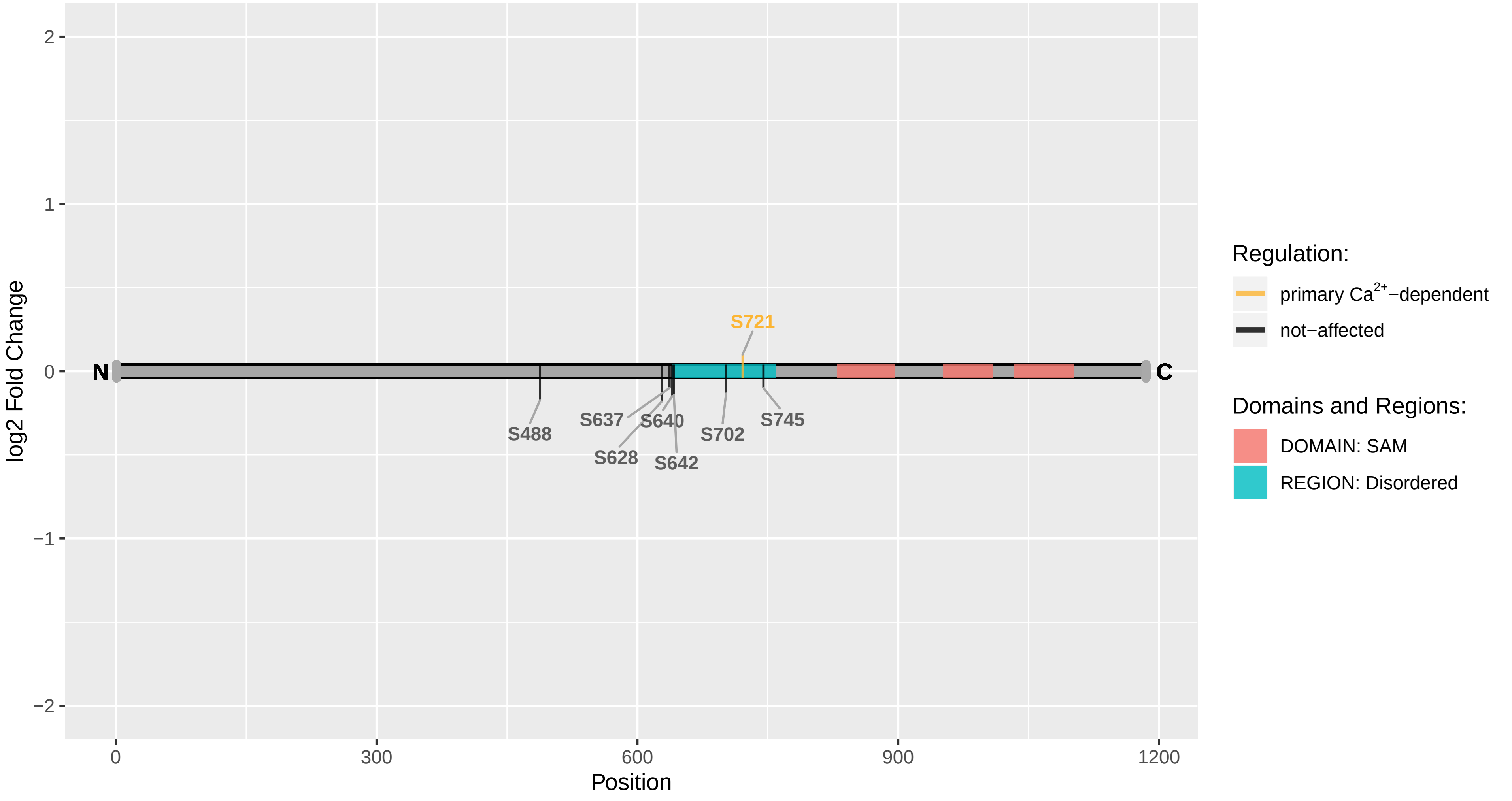

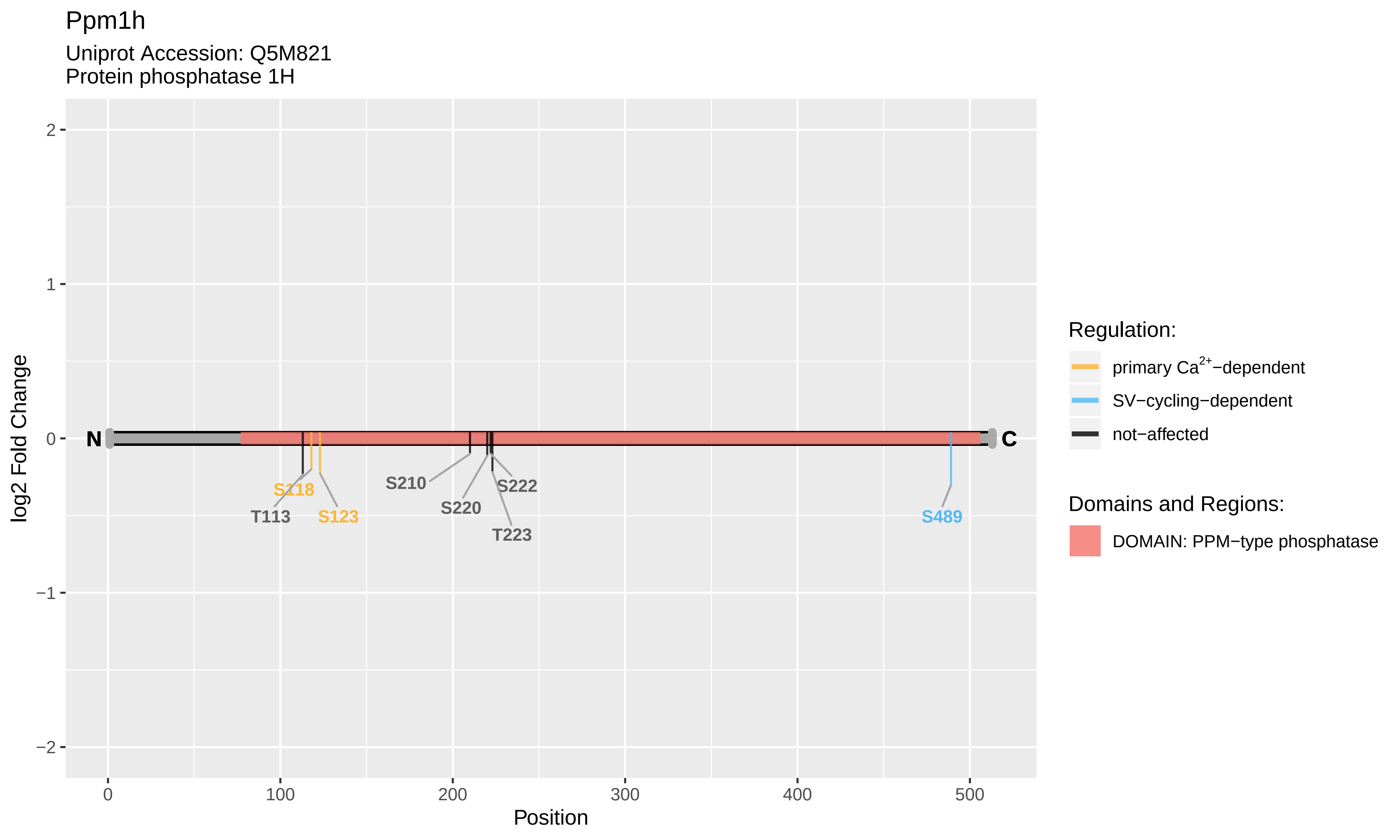

Ppm1h
Uniprot Accession: Q5M821
Protein phosphatase 1H
log2 Fold Change
Position
N
C
T113
S118
S123
S210
S220
S222
T223
S489
Regulation:
primary Ca2+−dependent
SV−cycling−dependent
not−affected
Domains and Regions:
DOMAIN: PPM−type phosphatase

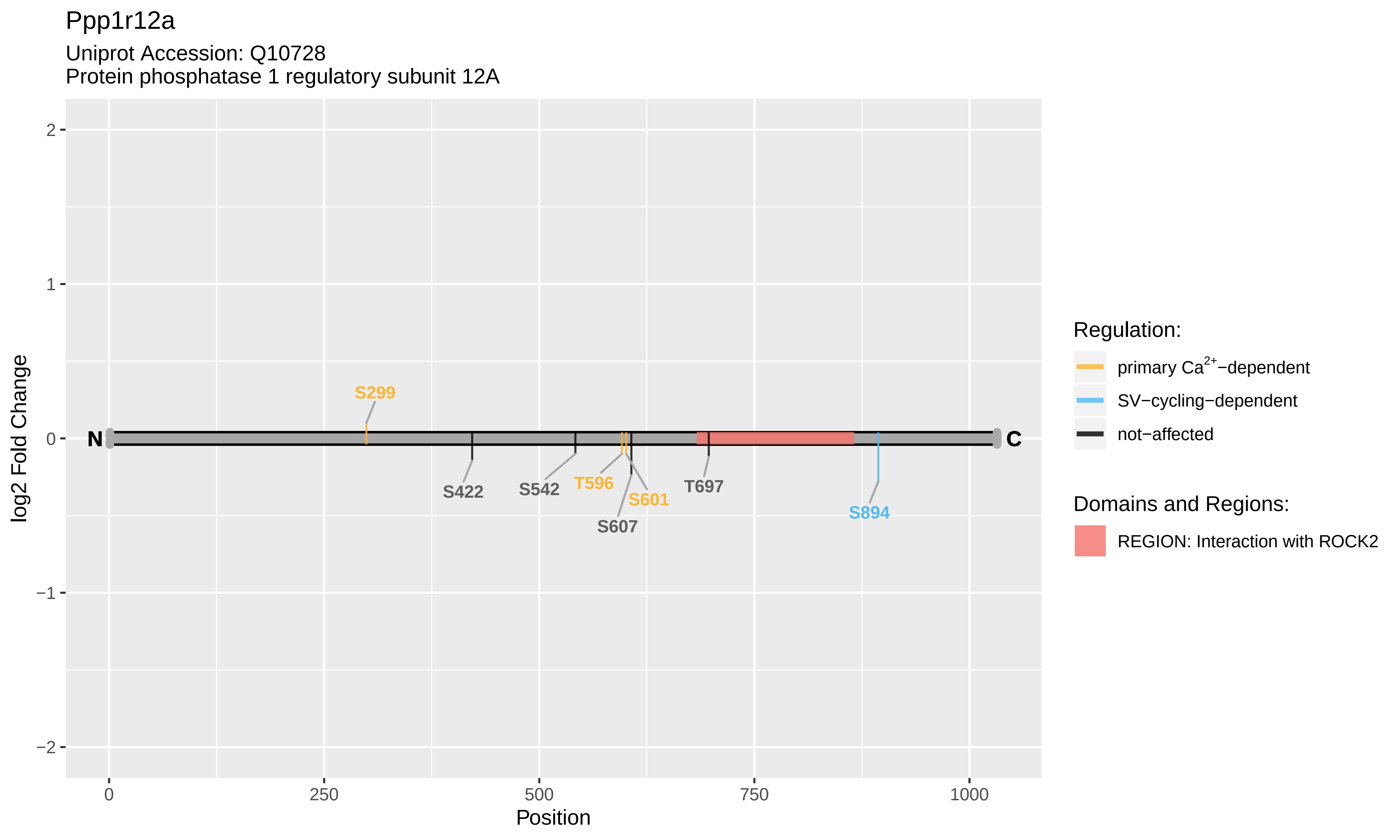

Ppp1r12a
Uniprot Accession: Q10728
Protein phosphatase 1 regulatory subunit 12A
log2 Fold Change
Position
2
1
0
−1
−2
0
250
500
750
1000
N
C
S299
S422
S542
T596
S601
S607
T697
S894
Regulation:
primary Ca2+−dependent
SV−cycling−dependent
not−affected
Domains and Regions:
REGION: Interaction with ROCK2

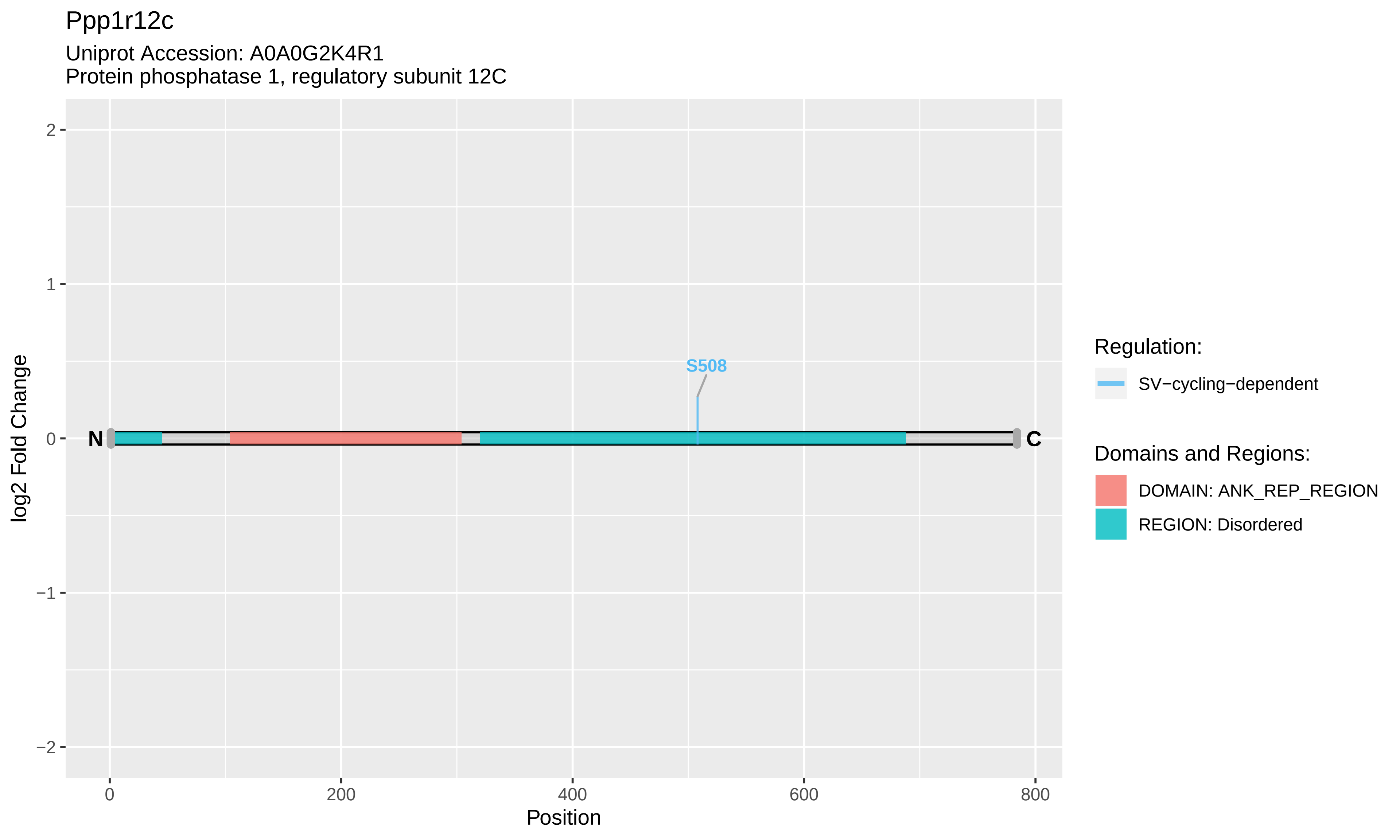

Ppp1r12c
Uniprot Accession: A0A0G2K4R1
Protein phosphatase 1, regulatory subunit 12C
S508
N
C
2
1
0
−1
−2
log2 Fold Change
0
200
400
600
800
Position
Regulation:
SV−cycling−dependent
Domains and Regions:
DOMAIN: ANK_REP_REGION
REGION: Disordered

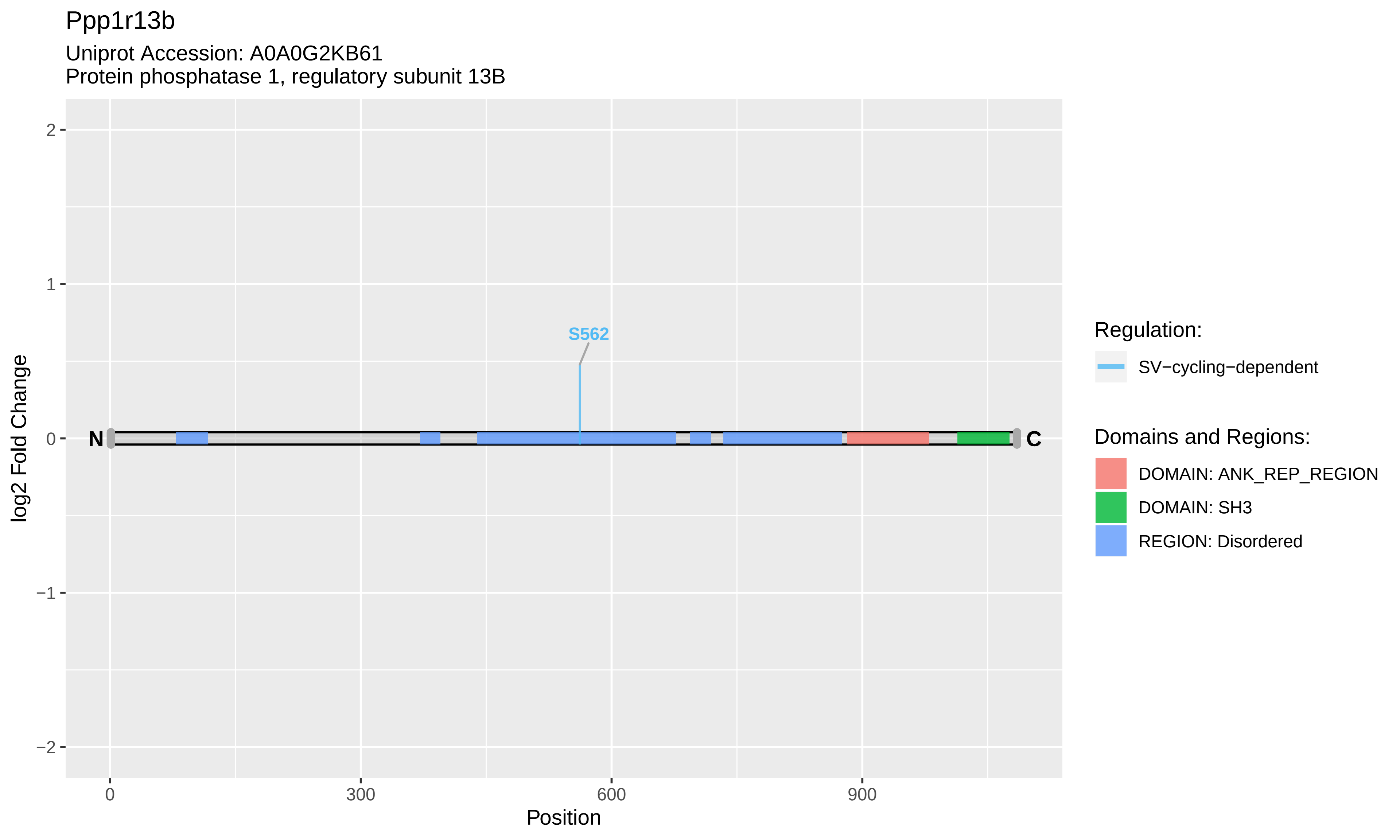
Ppp1r13b
Uniprot Accession: A0A0G2KB61
Protein phosphatase 1, regulatory subunit 13B
log2 Fold Change
2
1
0
−1
−2
N
C
S562
0
300
600
900
Position
Regulation:
SV−cycling−dependent
Domains and Regions:
DOMAIN: ANK_REP_REGION
DOMAIN: SH3
REGION: Disordered

Ppp1r16b

Uniprot Accession: D4AA72
Protein phosphatase 1, regulatory subunit 16B

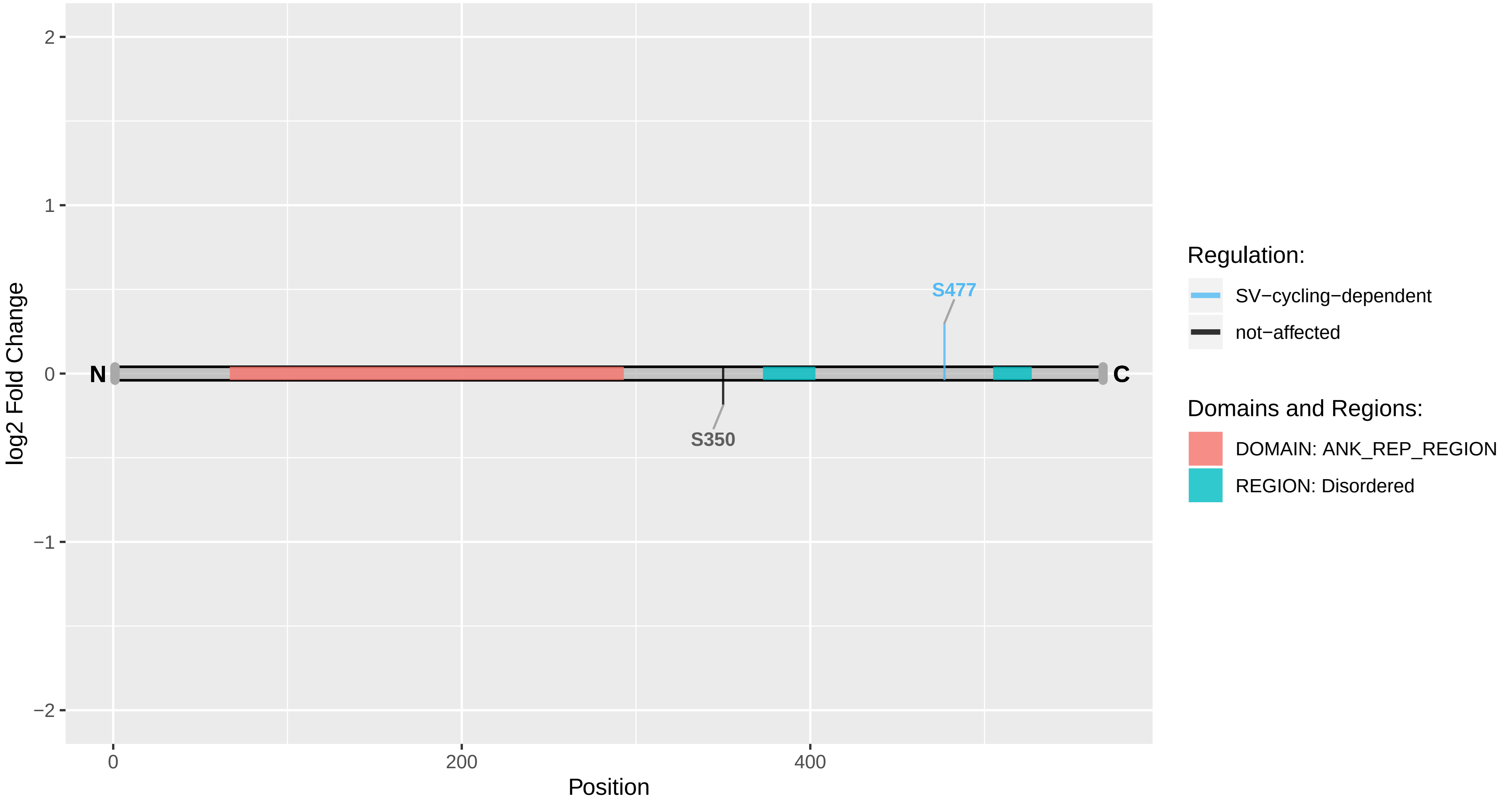

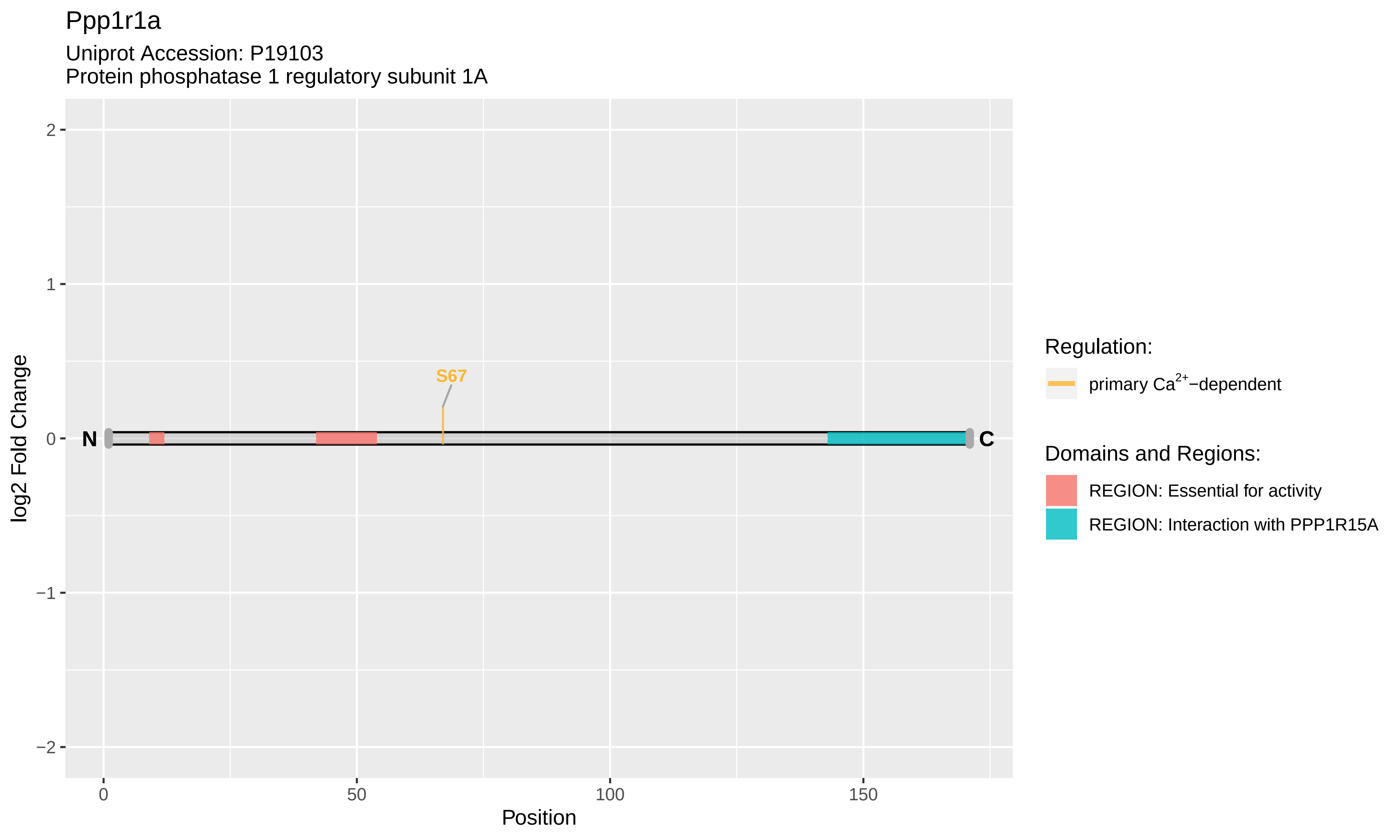

Ppp1r1a
Uniprot Accession: P19103
Protein phosphatase 1 regulatory subunit 1A
S67
N
C
2
1
0
−1
−2
log2 Fold Change
0
50
100
150
Position
Regulation:
primary Ca2+−dependent
Domains and Regions:
REGION: Essential for activity
REGION: Interaction with PPP1R15A

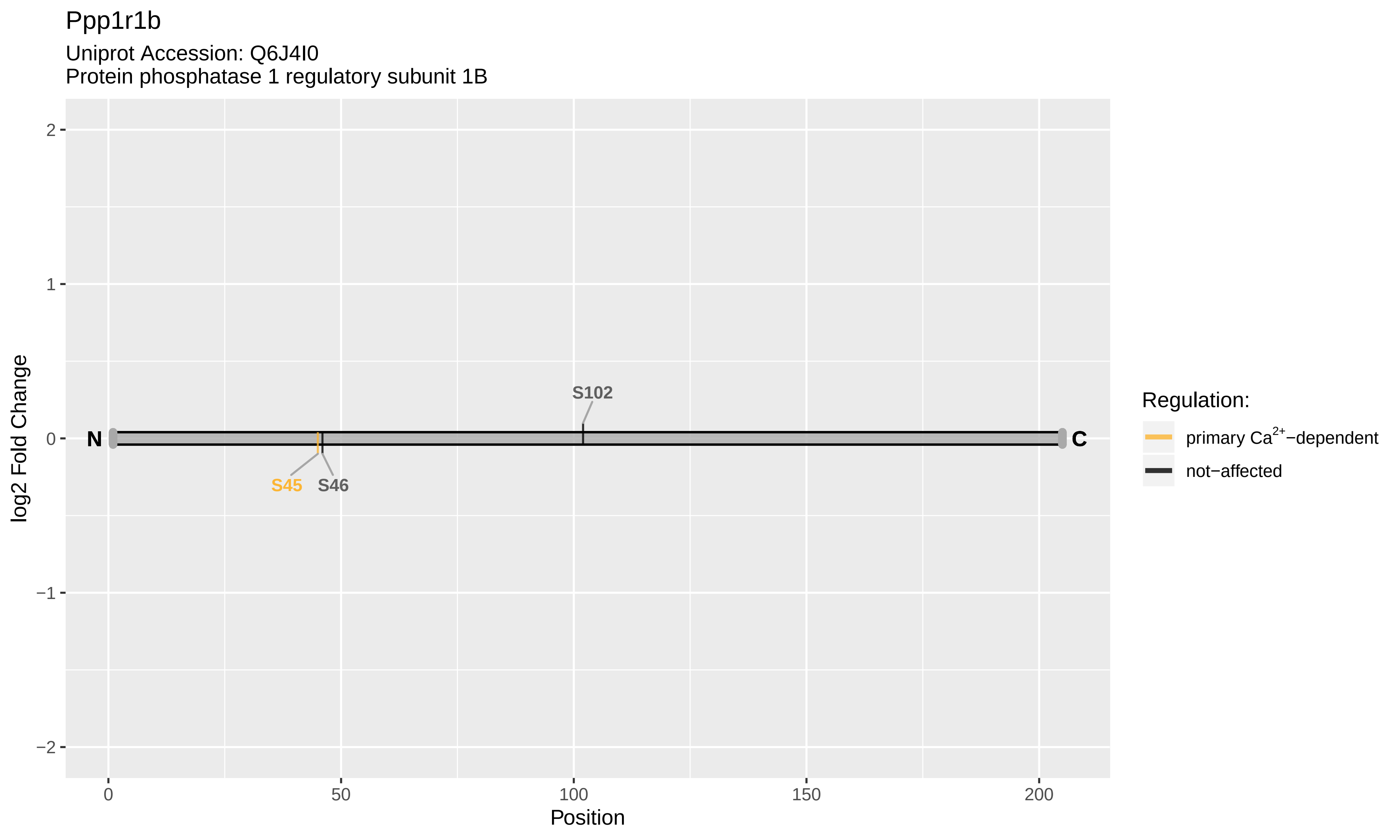

Ppp1r1b
Uniprot Accession: Q6J4I0
Protein phosphatase 1 regulatory subunit 1B
log2 Fold Change
2
1
0
−1
−2
N
C
S102
S45
S46
0
50
100
150
200
Position
Regulation:
primary Ca2+−dependent
not−affected

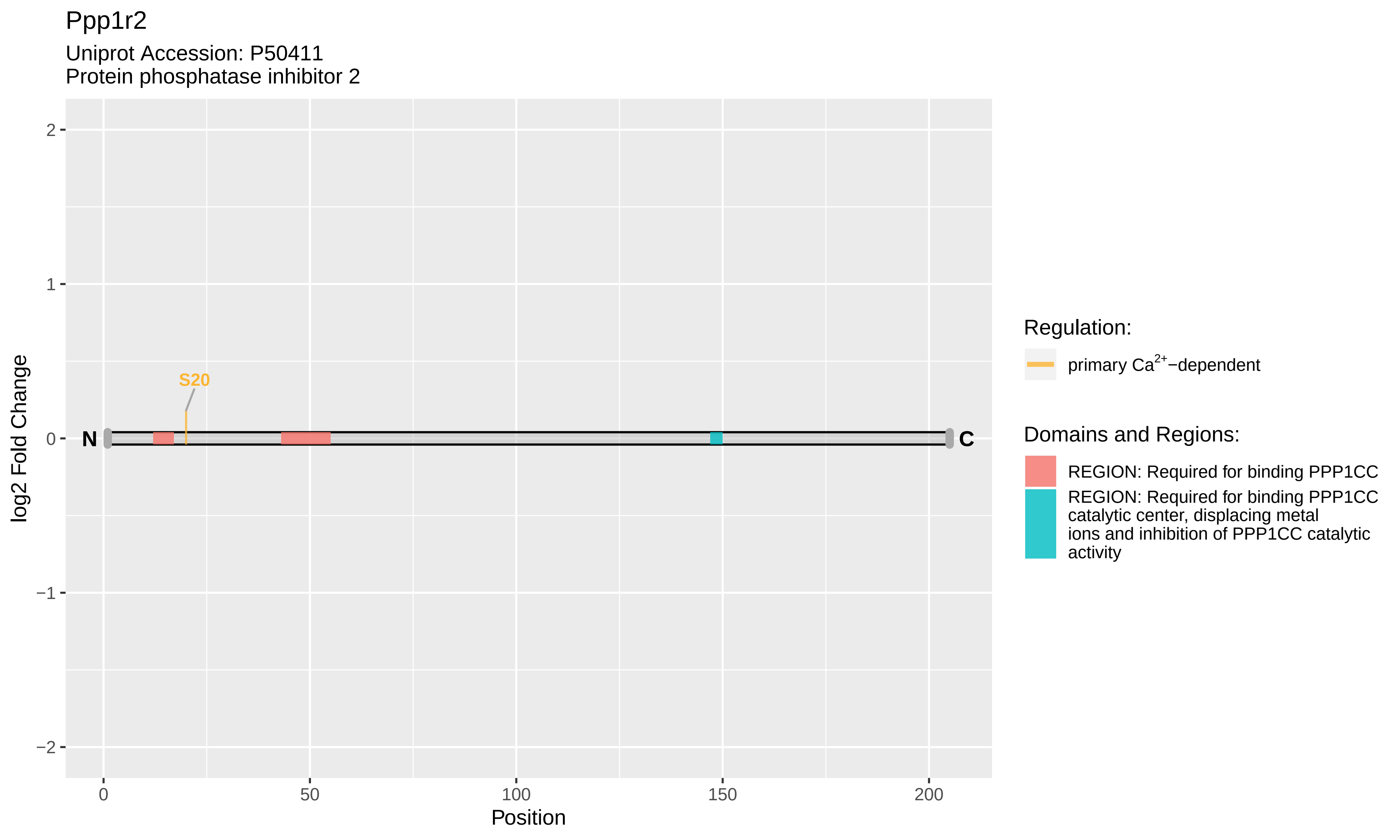

Ppp1r2
Uniprot Accession: P50411
Protein phosphatase inhibitor 2
log2 Fold Change
2
1
0
−1
−2
N
C
S20
0
50
100
150
200
Position
Regulation:
primary Ca2+−dependent
Domains and Regions:
REGION: Required for binding PPP1CC
REGION: Required for binding PPP1CC catalytic center, displacing metal ions and inhibition of PPP1CC catalytic activity

Ppp1r21

Uniprot Accession: F1LYZ8
Protein phosphatase 1, regulatory subunit 21

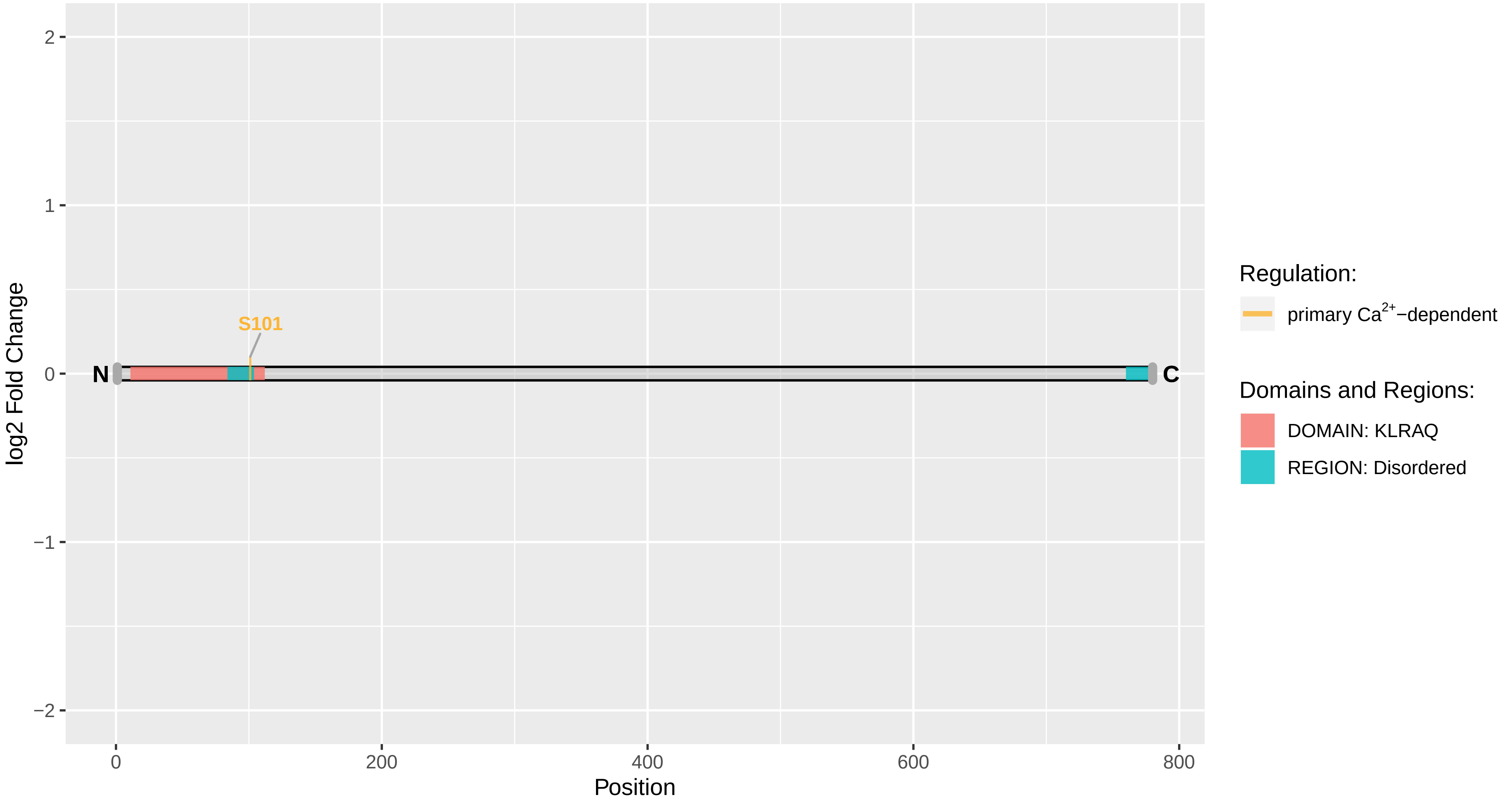

Ppp1r3d

Uniprot Accession: D3ZZ26
Protein phosphatase 1 regulatory subunit

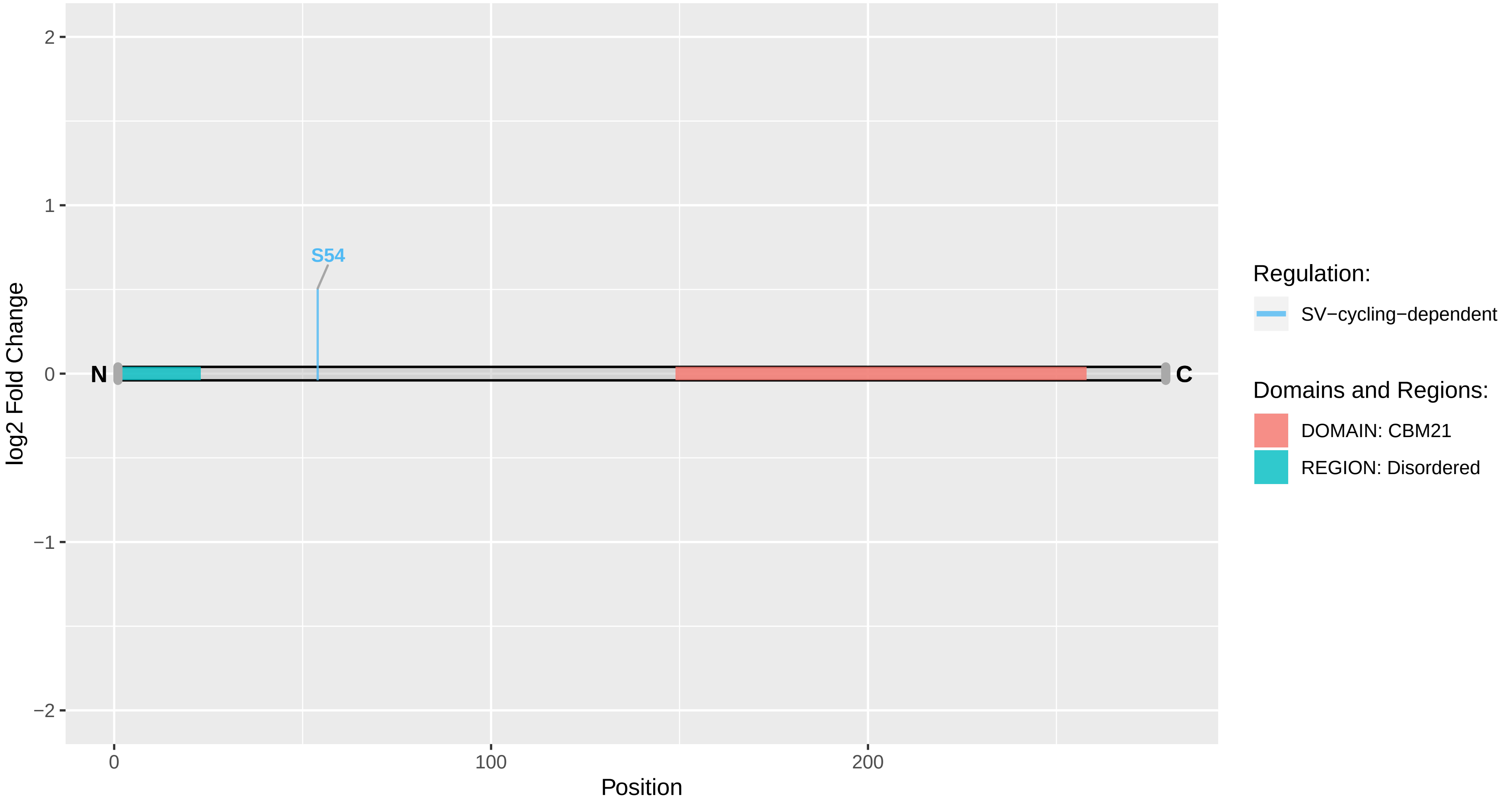

Ppp1r7

Uniprot Accession: Q5HZV9
Protein phosphatase 1 regulatory subunit 7

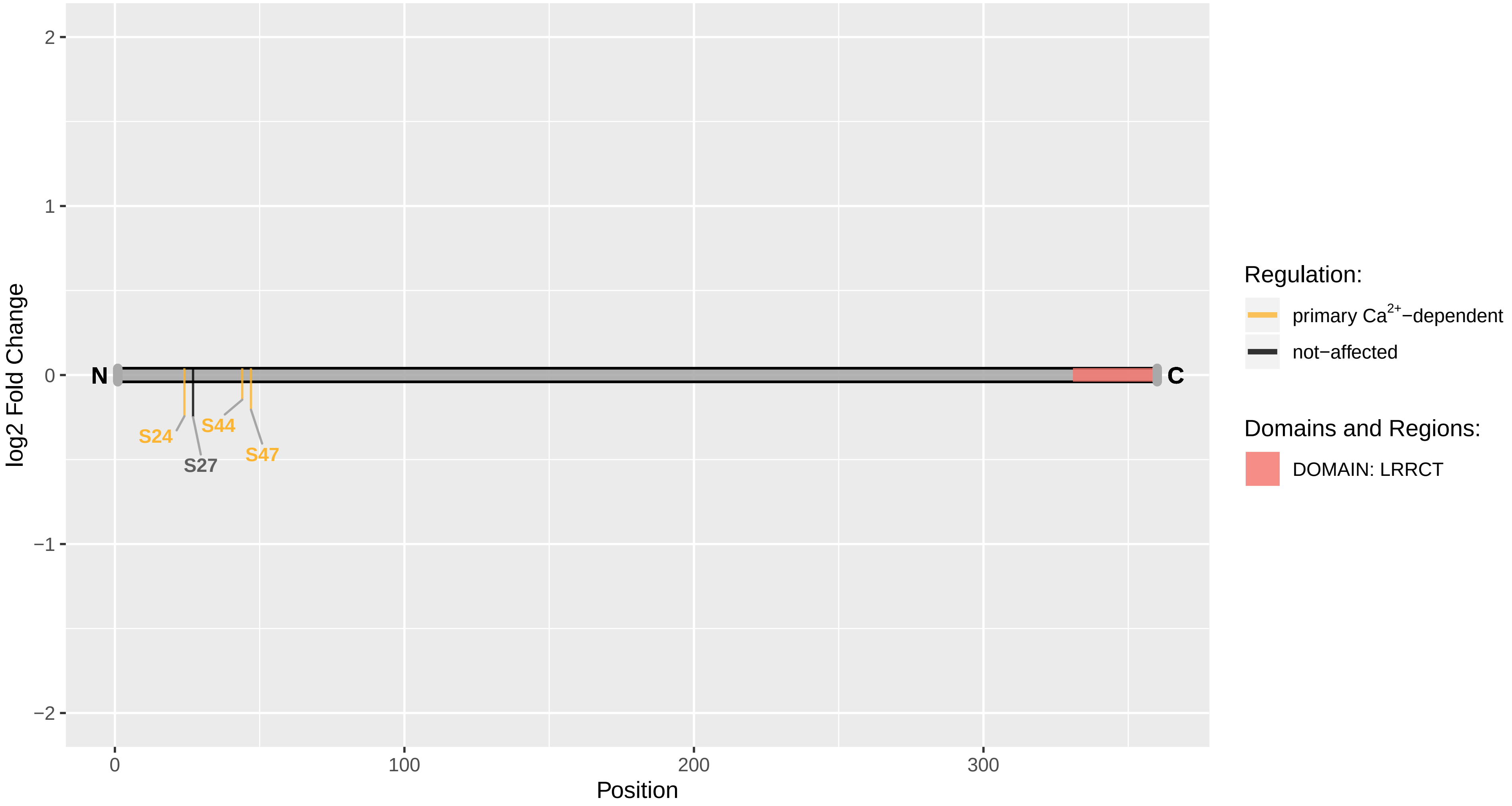

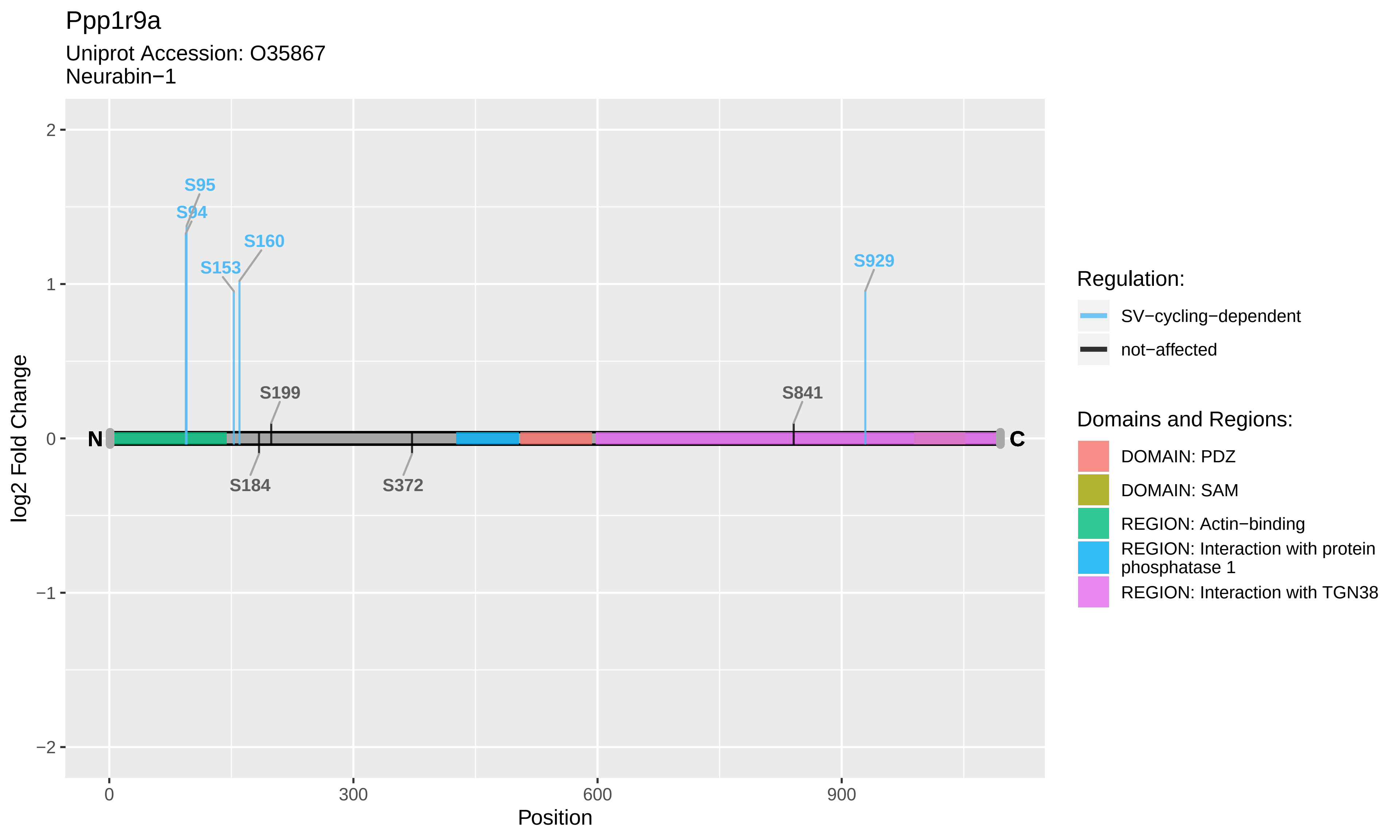

Ppp1r9a
Uniprot Accession: O35867
Neurabin−1
S95
S94
S160
S153
S929
S199
S841
S184
S372
N
C
2
1
0
−1
−2
log2 Fold Change
0
300
600
900
Position
Regulation:
SV−cycling−dependent
not−affected
Domains and Regions:
DOMAIN: PDZ
DOMAIN: SAM
REGION: Actin−binding
REGION: Interaction with protein phosphatase 1
REGION: Interaction with TGN38

Ppp1r9b

Uniprot Accession: O35274
Neurabin−2

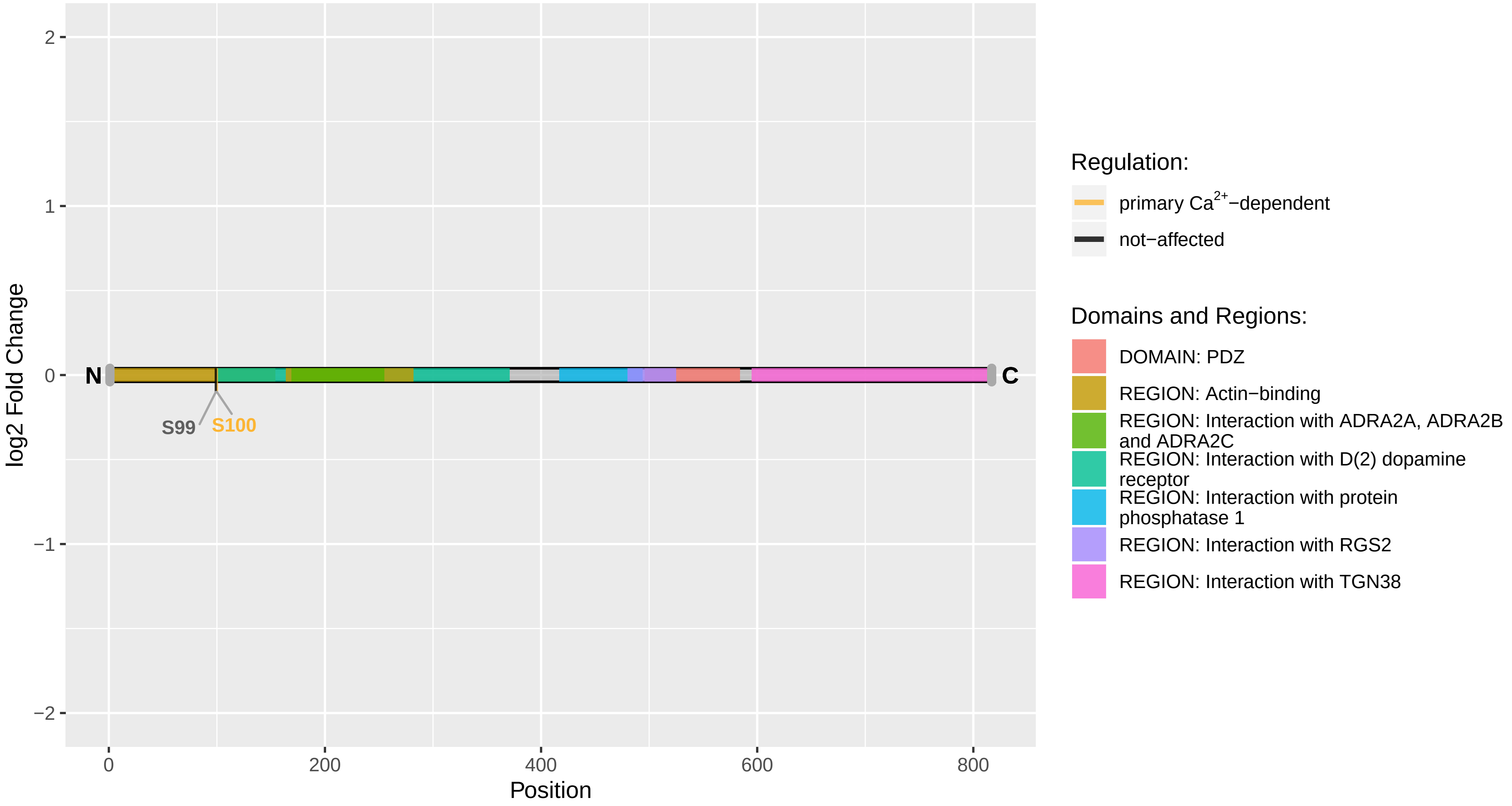

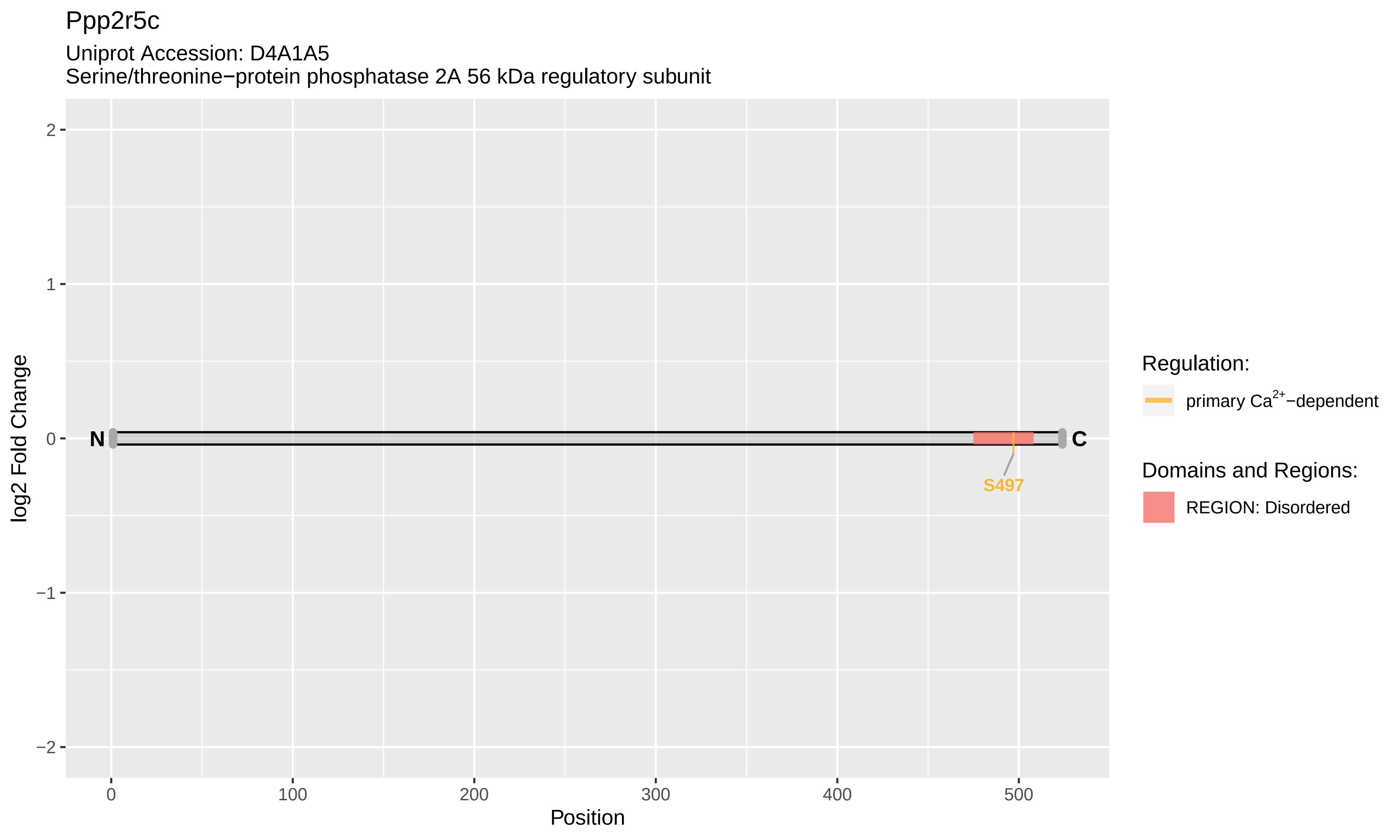

Ppp2r5c
Uniprot Accession: D4A1A5
Serine/threonine−protein phosphatase 2A 56 kDa regulatory subunit
log2 Fold Change
2
1
0
−1
−2
N
C
S497
0
100
200
300
400
500
Position
Regulation:
primary Ca2+−dependent
Domains and Regions:
REGION: Disordered

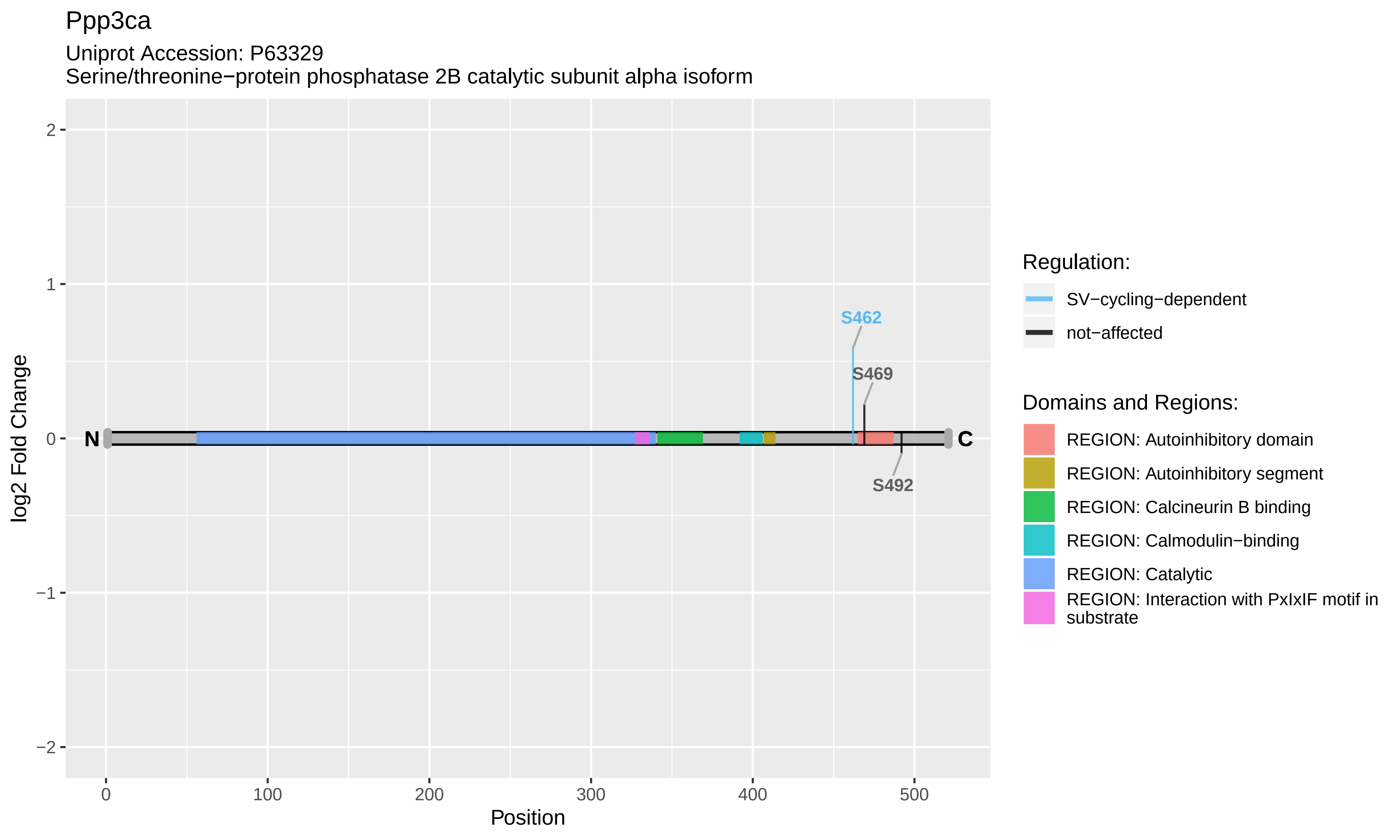

Ppp3ca
Uniprot Accession: P63329
Serine/threonine−protein phosphatase 2B catalytic subunit alpha isoform
log2 Fold Change
2
1
0
−1
−2
N
C
S462
S469
S492
0
100
200
300
400
500
Position
Regulation:
SV−cycling−dependent
not−affected
Domains and Regions:
REGION: Autoinhibitory domain
REGION: Autoinhibitory segment
REGION: Calcineurin B binding
REGION: Calmodulin−binding
REGION: Catalytic
REGION: Interaction with PxIxIF motif in substrate

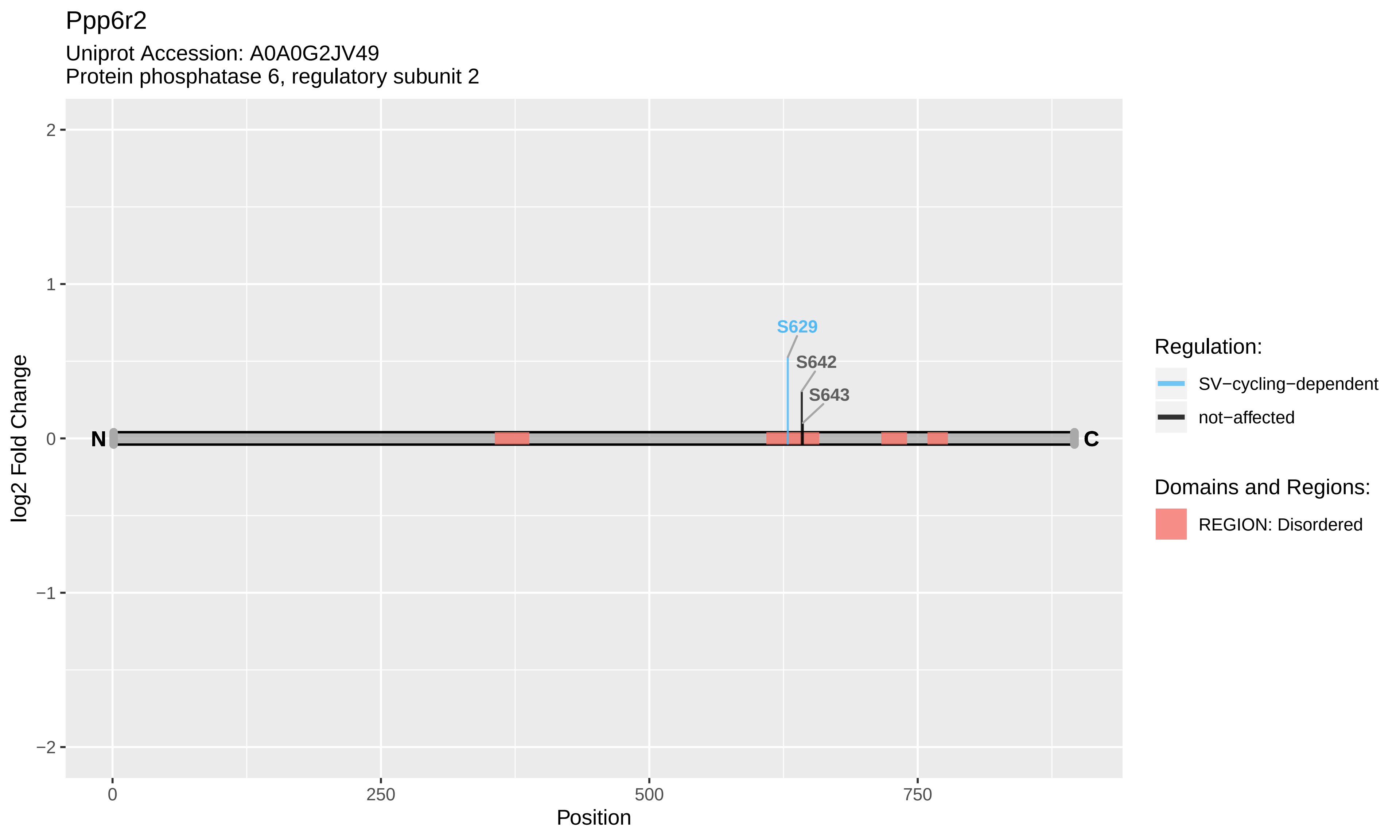

Ppp6r2
Uniprot Accession: A0A0G2JV49
Protein phosphatase 6, regulatory subunit 2
S629
S642
S643
N
C
2
1
0
−1
−2
0
250
500
750
log2 Fold Change
Position
Regulation:
SV−cycling−dependent
not−affected
Domains and Regions:
REGION: Disordered

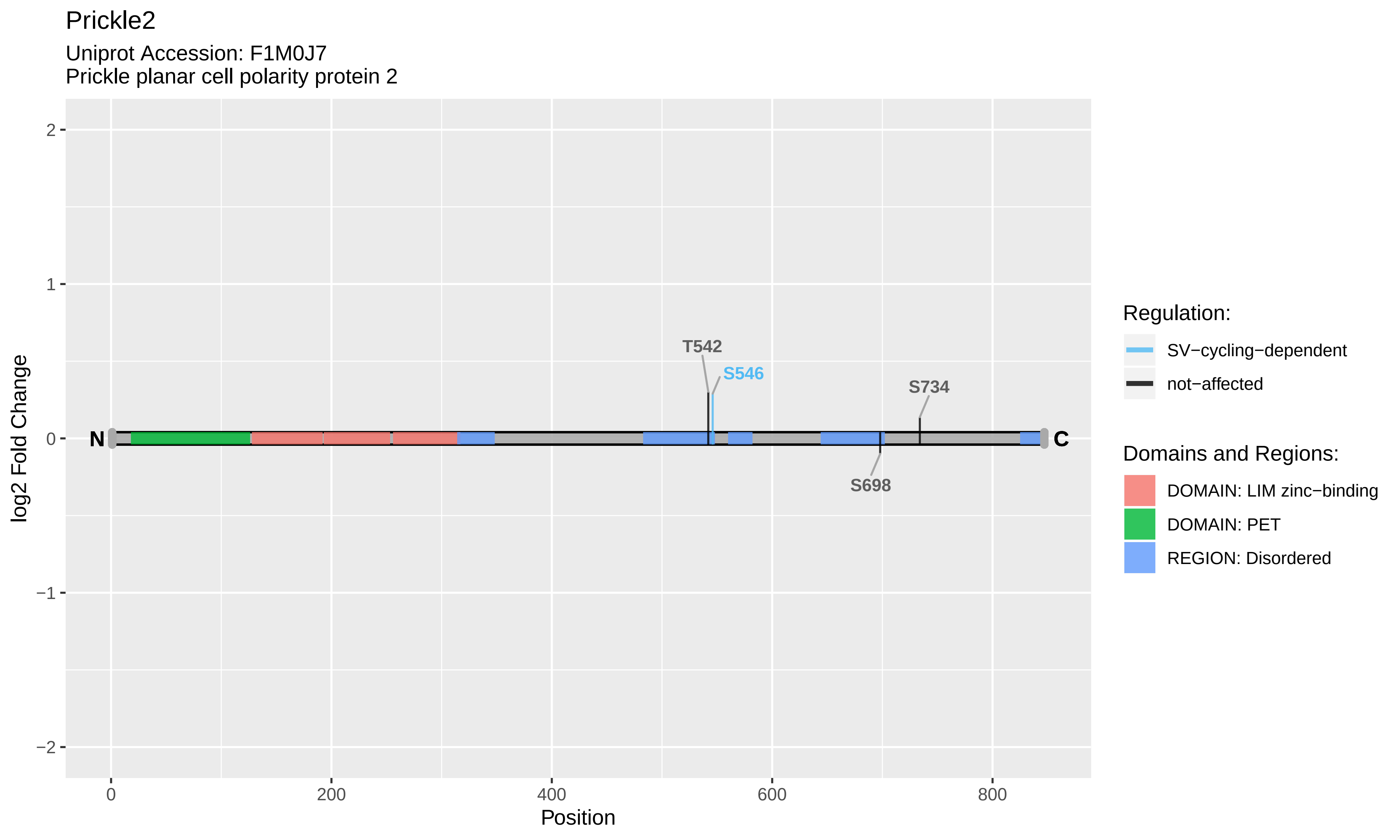
Prickle2
Uniprot Accession: F1M0J7
Prickle planar cell polarity protein 2
log2 Fold Change
2
1
0
−1
−2
N
C
T542
S546
S734
S698
0
200
400
600
800
Position
Regulation:
SV−cycling−dependent
not−affected
Domains and Regions:
DOMAIN: LIM zinc−binding
DOMAIN: PET
REGION: Disordered

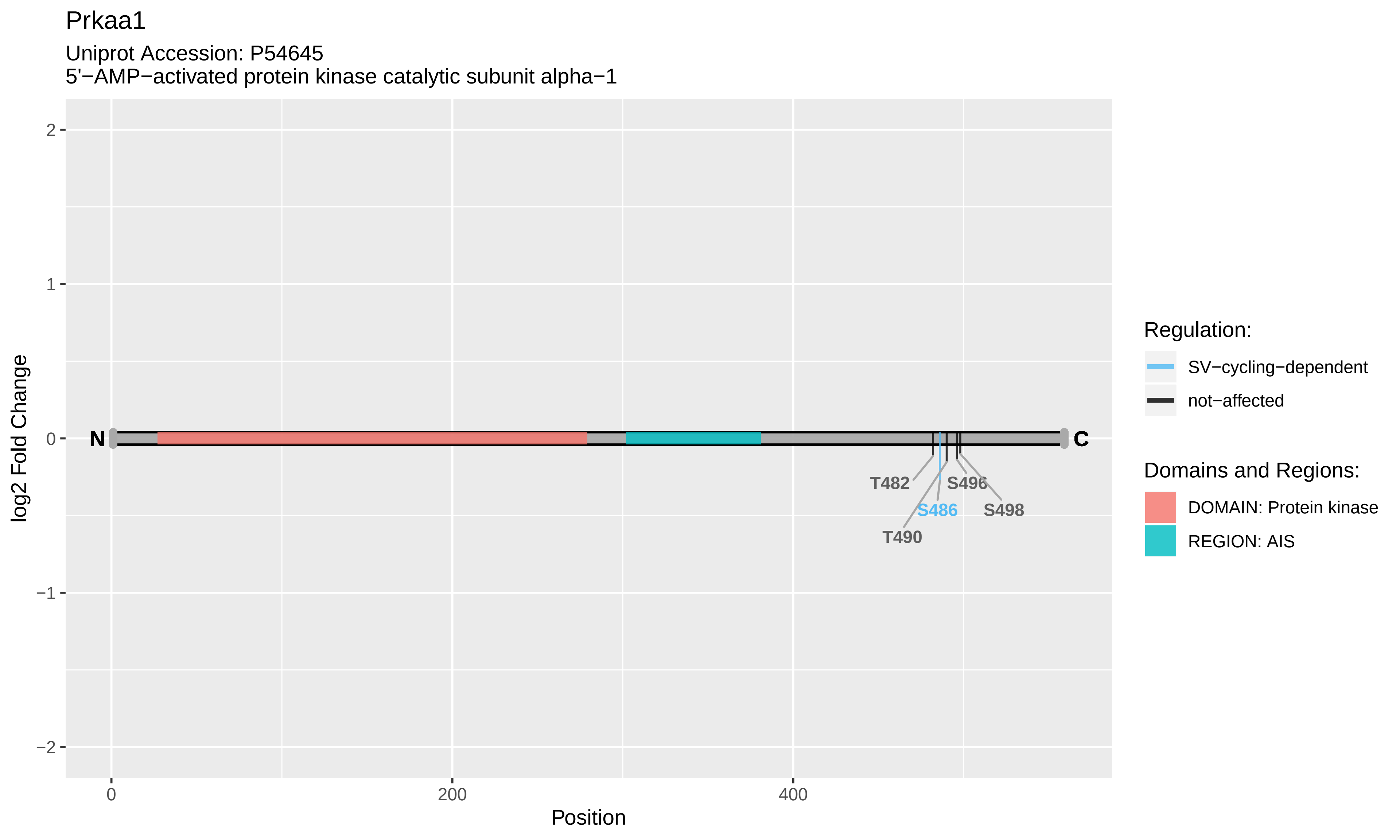
Prkaa1
Uniprot Accession: P54645
5'−AMP−activated protein kinase catalytic subunit alpha−1
2
1
0
−1
−2
log2 Fold Change
N
C
T482
S486
T490
S496
S498
0
200
400
Position
Regulation:
SV−cycling−dependent
not−affected
Domains and Regions:
DOMAIN: Protein kinase
REGION: AIS

Prkab1

Uniprot Accession: P80386
5'−AMP−activated protein kinase subunit beta−1

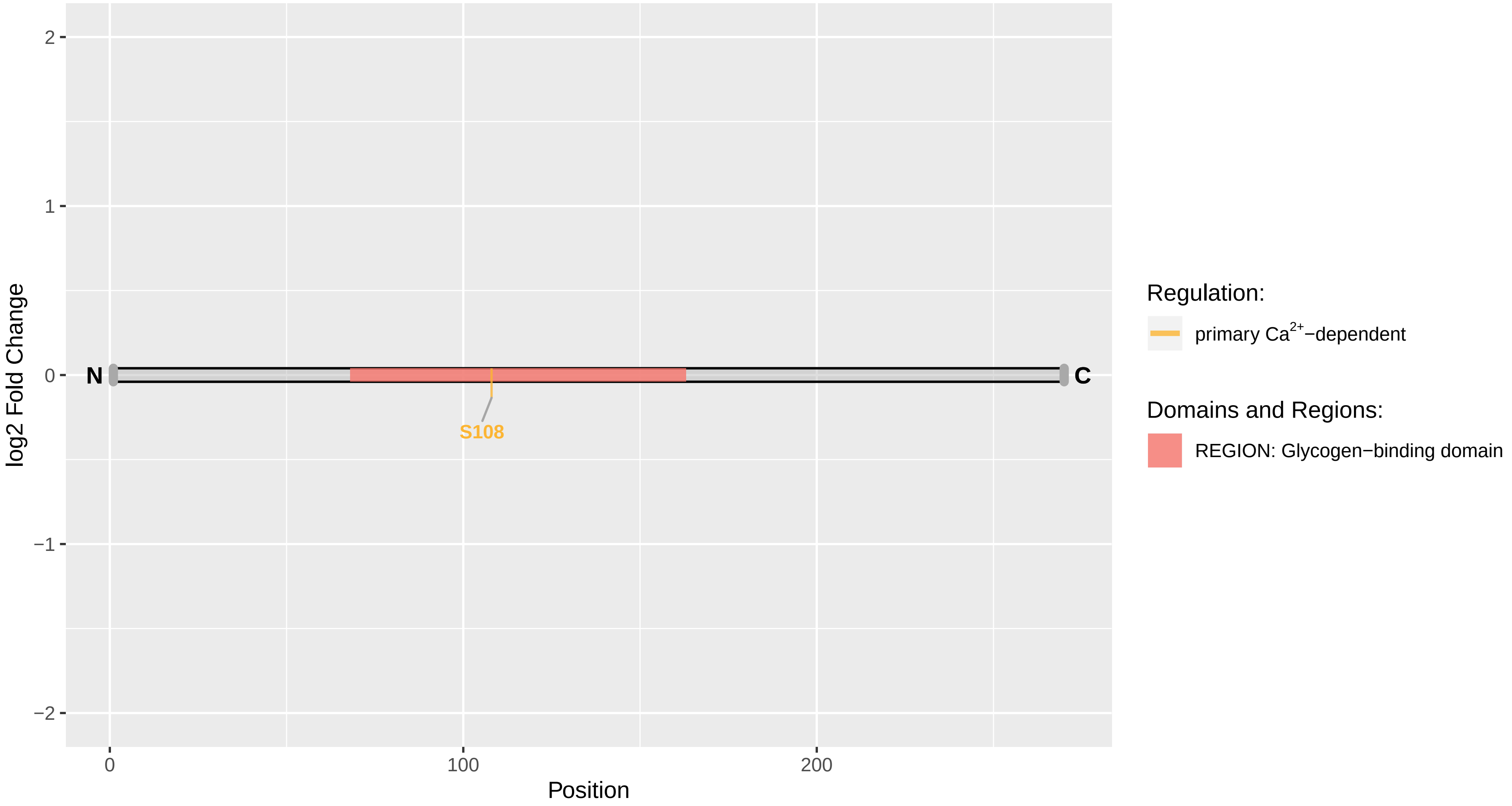

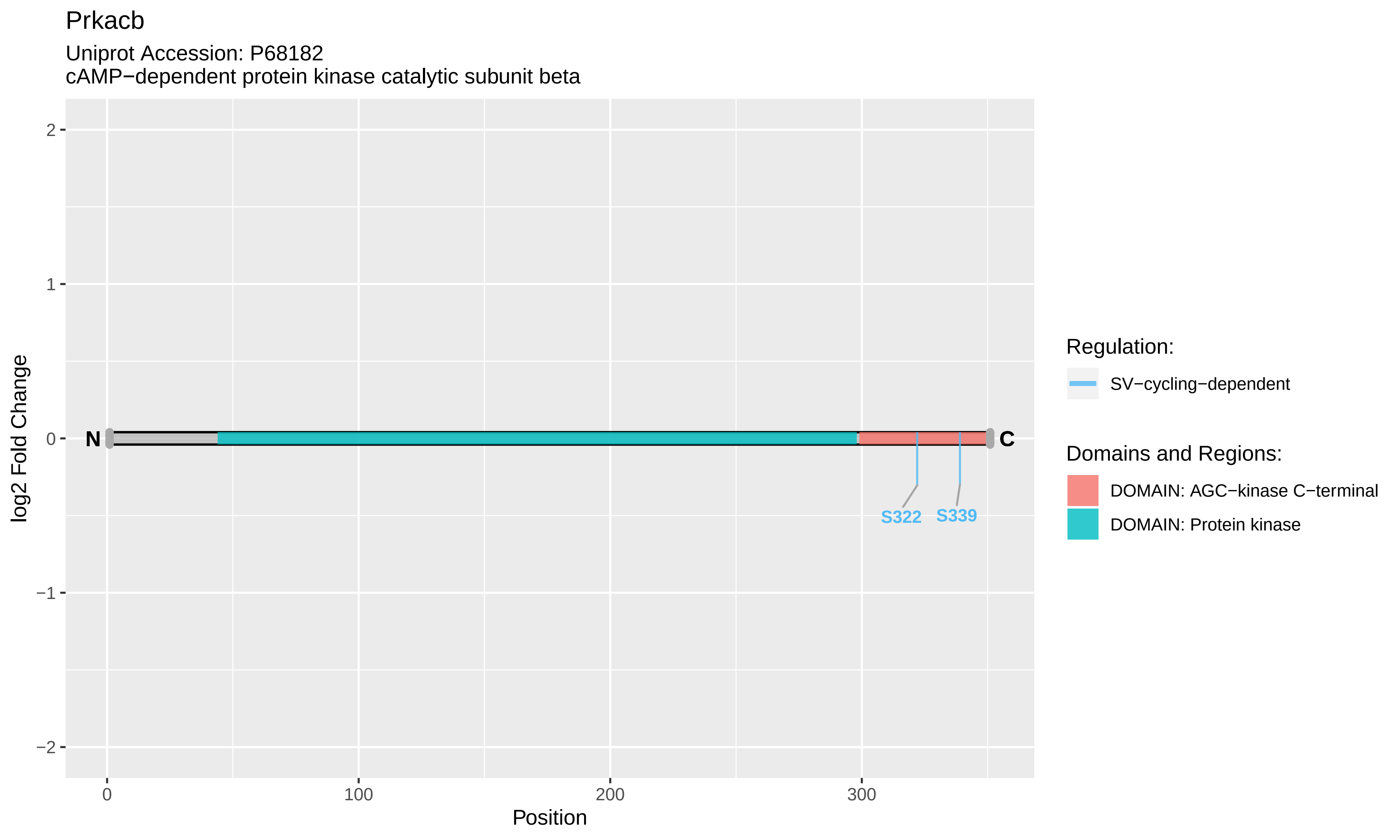

Prkacb
Uniprot Accession: P68182
cAMP−dependent protein kinase catalytic subunit beta
log2 Fold Change
2
1
0
−1
−2
N
C
S322
S339
0
100
200
300
Position
Regulation:
SV−cycling−dependent
Domains and Regions:
DOMAIN: AGC−kinase C−terminal
DOMAIN: Protein kinase

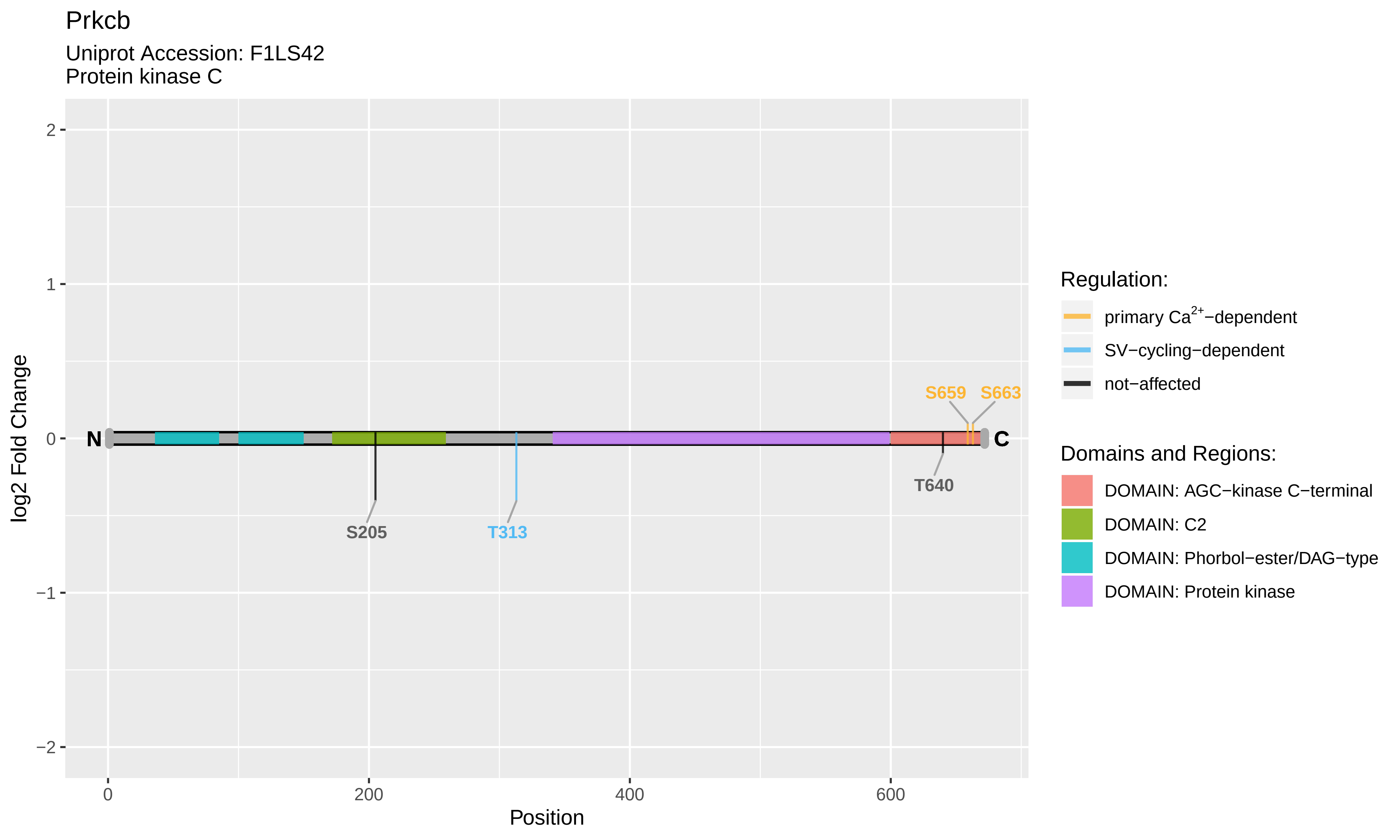

Prkcb
Uniprot Accession: F1LS42
Protein kinase C
log2 Fold Change
2
1
0
−1
−2
N
C
S659
S663
T640
S205
T313
0
200
400
600
Position
Regulation:
primary Ca2+−dependent
SV−cycling−dependent
not−affected
Domains and Regions:
DOMAIN: AGC−kinase C−terminal
DOMAIN: C2
DOMAIN: Phorbol−ester/DAG−type
DOMAIN: Protein kinase

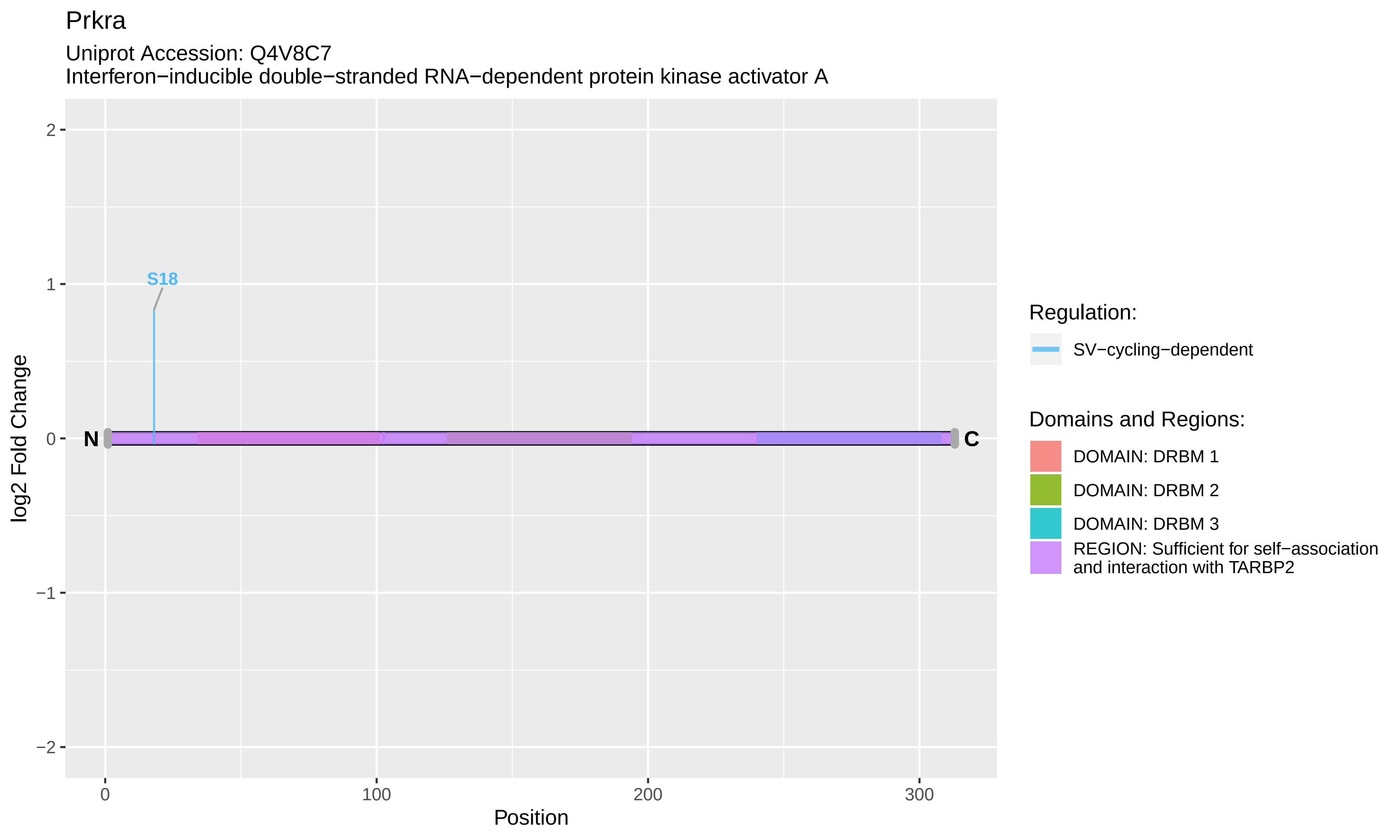

Prkra
Uniprot Accession: Q4V8C7
Interferon−inducible double−stranded RNA−dependent protein kinase activator A
S18
N
C
2
1
0
−1
−2
log2 Fold Change
0
100
200
300
Position
Regulation:
SV−cycling−dependent
Domains and Regions:
DOMAIN: DRBM 1
DOMAIN: DRBM 2
DOMAIN: DRBM 3
REGION: Sufficient for self−association and interaction with TARBP2

Prpf38b

Uniprot Accession: Q6AXY7
Pre−mRNA−splicing factor 38B

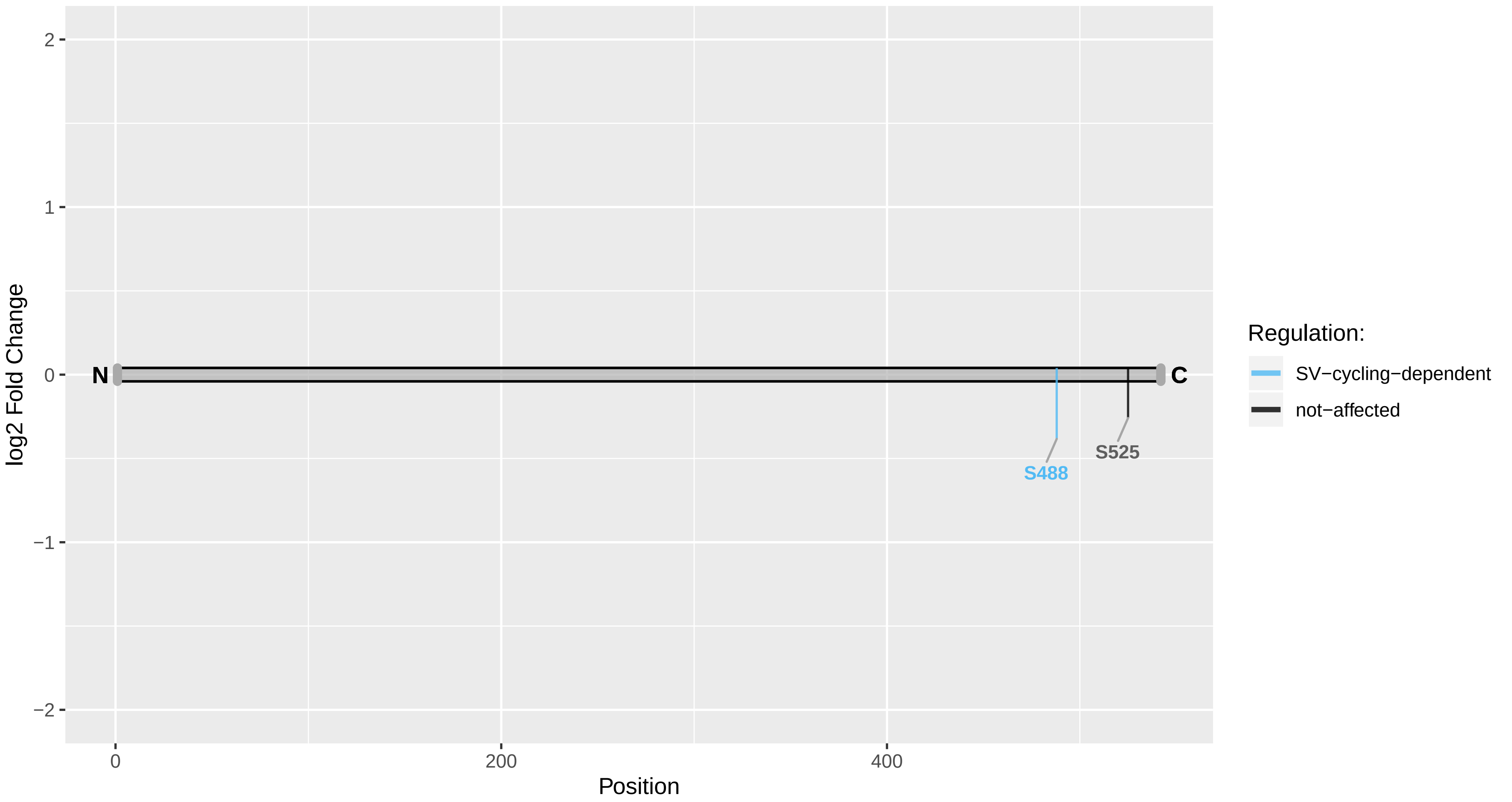

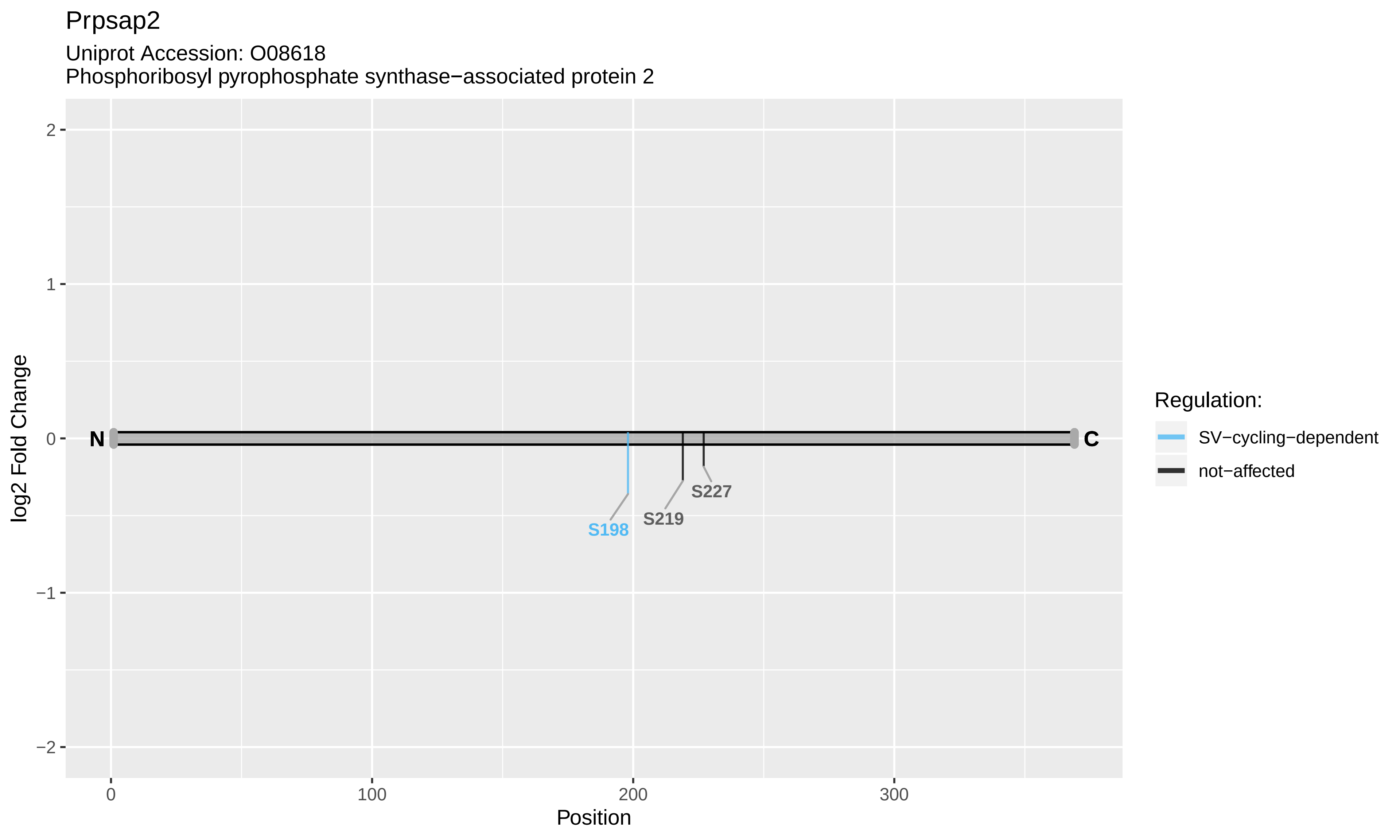
Prpsap2
Uniprot Accession: O08618
Phosphoribosyl pyrophosphate synthase−associated protein 2
log2 Fold Change
2
1
0
−1
−2
N
C
S198
S219
S227
0
100
200
300
Position
Regulation:
SV−cycling−dependent
not−affected

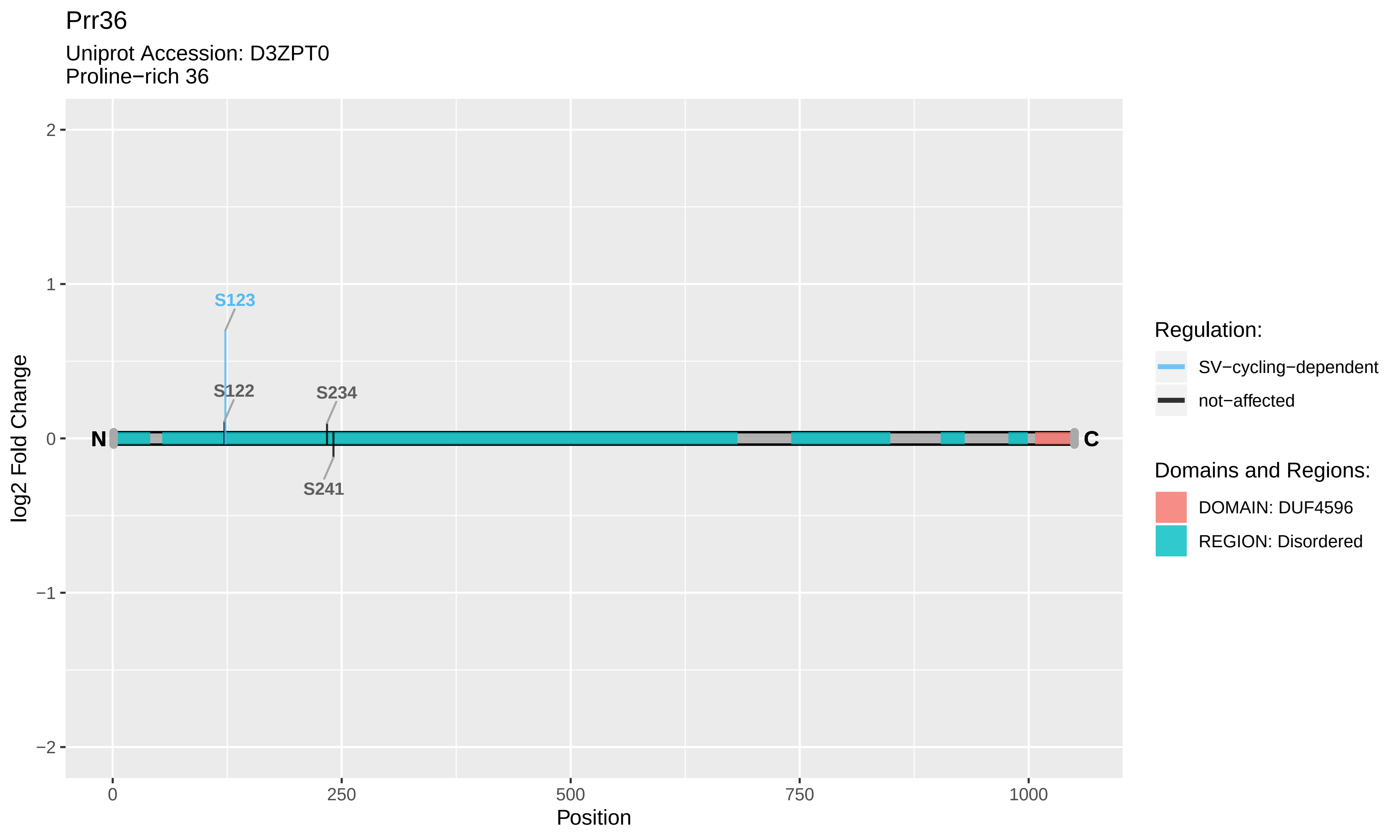

Prr36
Uniprot Accession: D3ZPT0
Proline−rich 36
S123
S122
S234
S241
N
C
log2 Fold Change
2
1
0
−1
−2
0
250
500
750
1000
Position
Regulation:
SV−cycling−dependent
not−affected
Domains and Regions:
DOMAIN: DUF4596
REGION: Disordered

Prrc2a

Uniprot Accession: Q6MG48
Protein PRRC2A

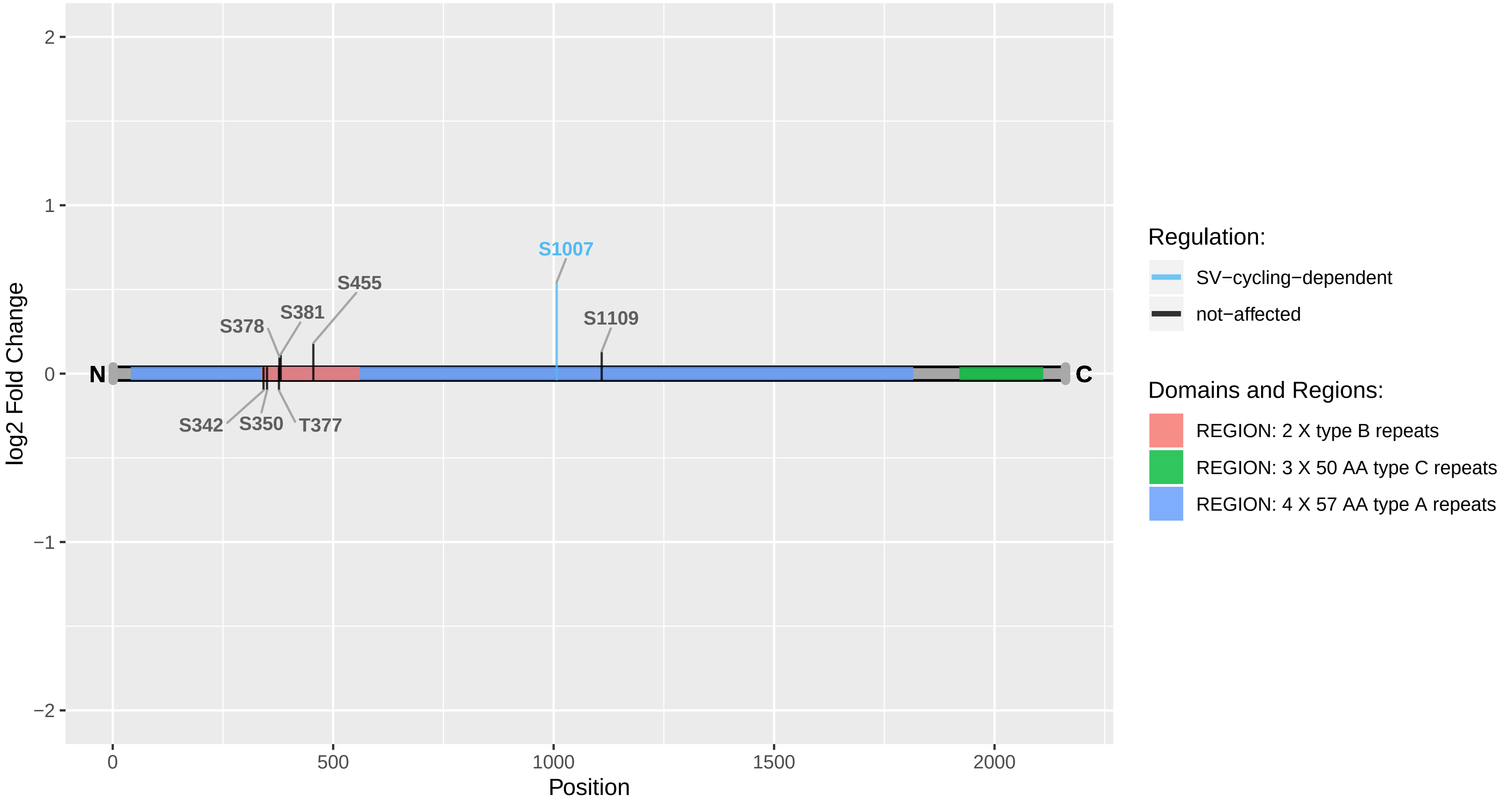

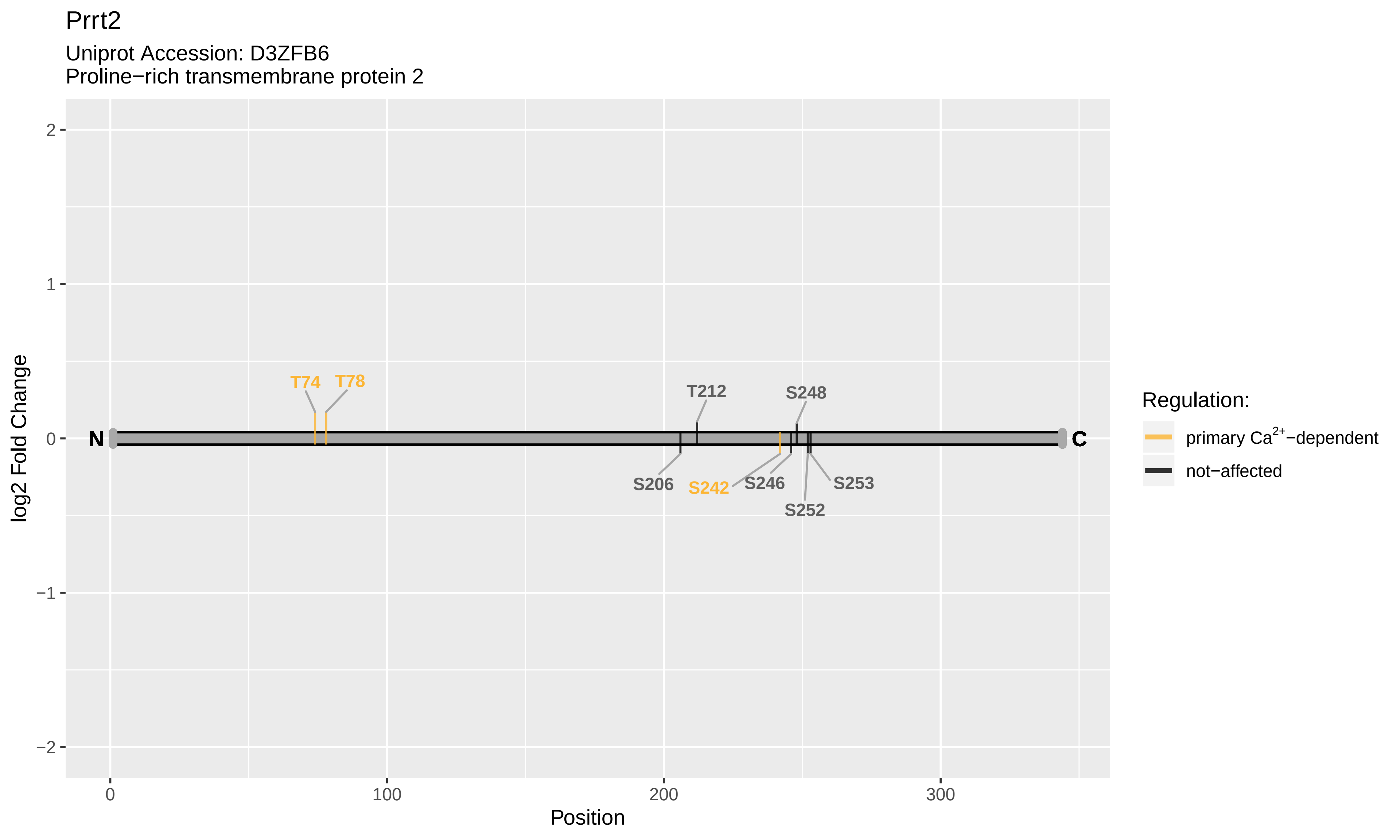
Prrt2
Uniprot Accession: D3ZFB6
Proline−rich transmembrane protein 2
log2 Fold Change
2
1
0
−1
−2
N
C
T74
T78
T212
S248
S206
S242
S246
S252
S253
0
100
200
300
Position
Regulation:
primary Ca2+−dependent
not−affected

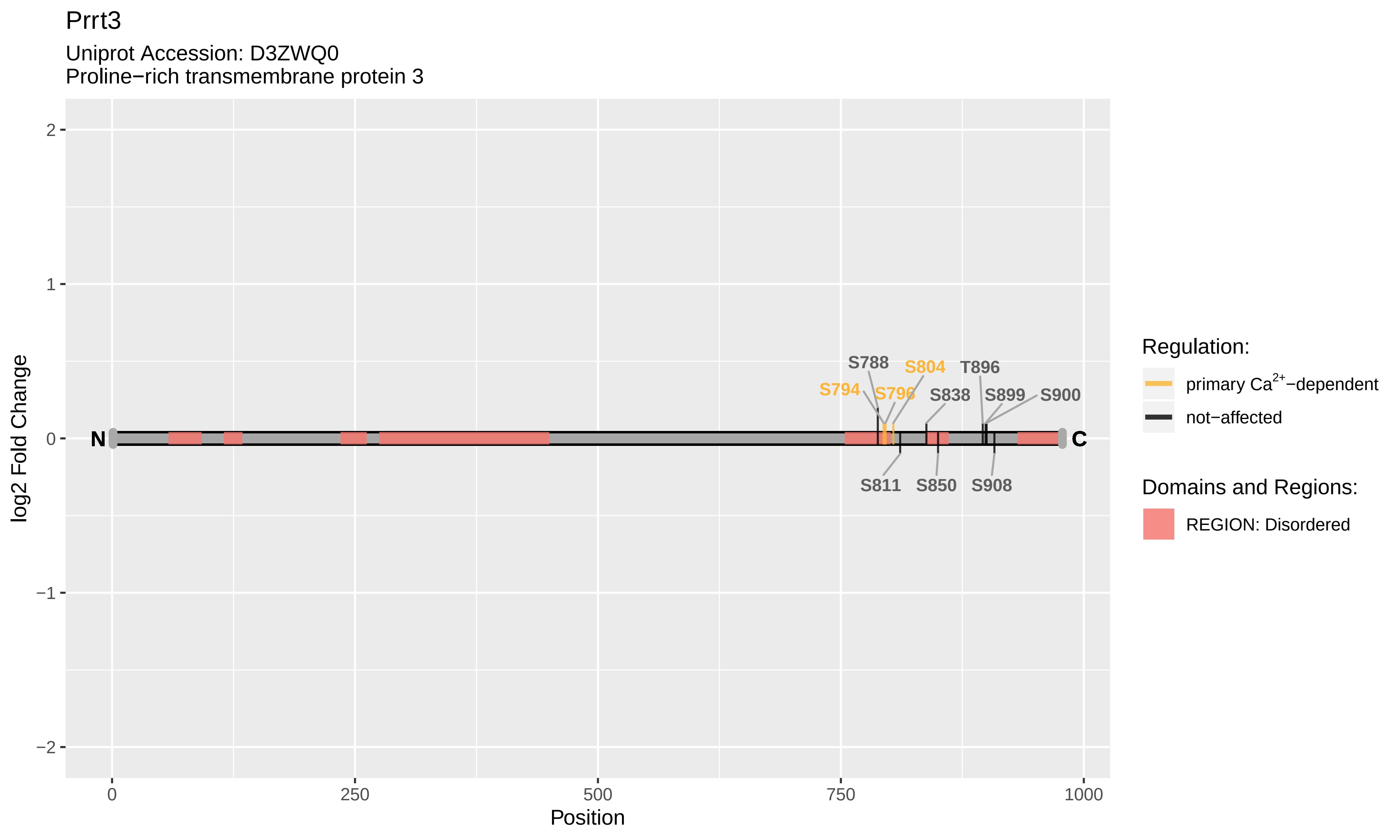

Prrt3
Uniprot Accession: D3ZWQ0
Proline−rich transmembrane protein 3
log2 Fold Change
Position
2
1
0
−1
−2
0
250
500
750
1000
N
C
S788
S804
T896
S794
S796
S838
S899
S900
S811
S850
S908
Regulation:
primary Ca2+−dependent
not−affected
Domains and Regions:
REGION: Disordered

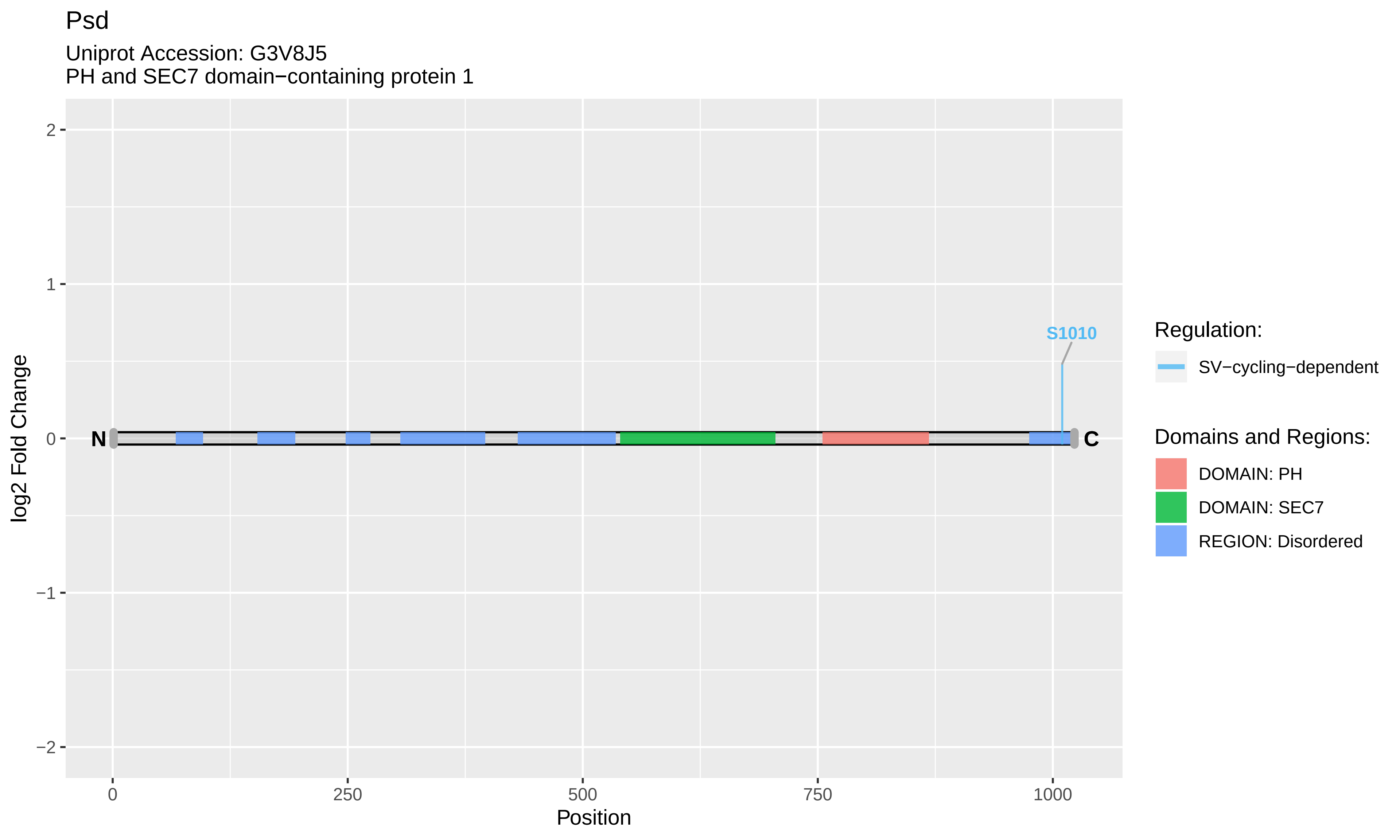

Psd
Uniprot Accession: G3V8J5
PH and SEC7 domain−containing protein 1
S1010
N
C
log2 Fold Change
Position
2
1
0
−1
−2
0
250
500
750
1000
Regulation:
SV−cycling−dependent
Domains and Regions:
DOMAIN: PH
DOMAIN: SEC7
REGION: Disordered

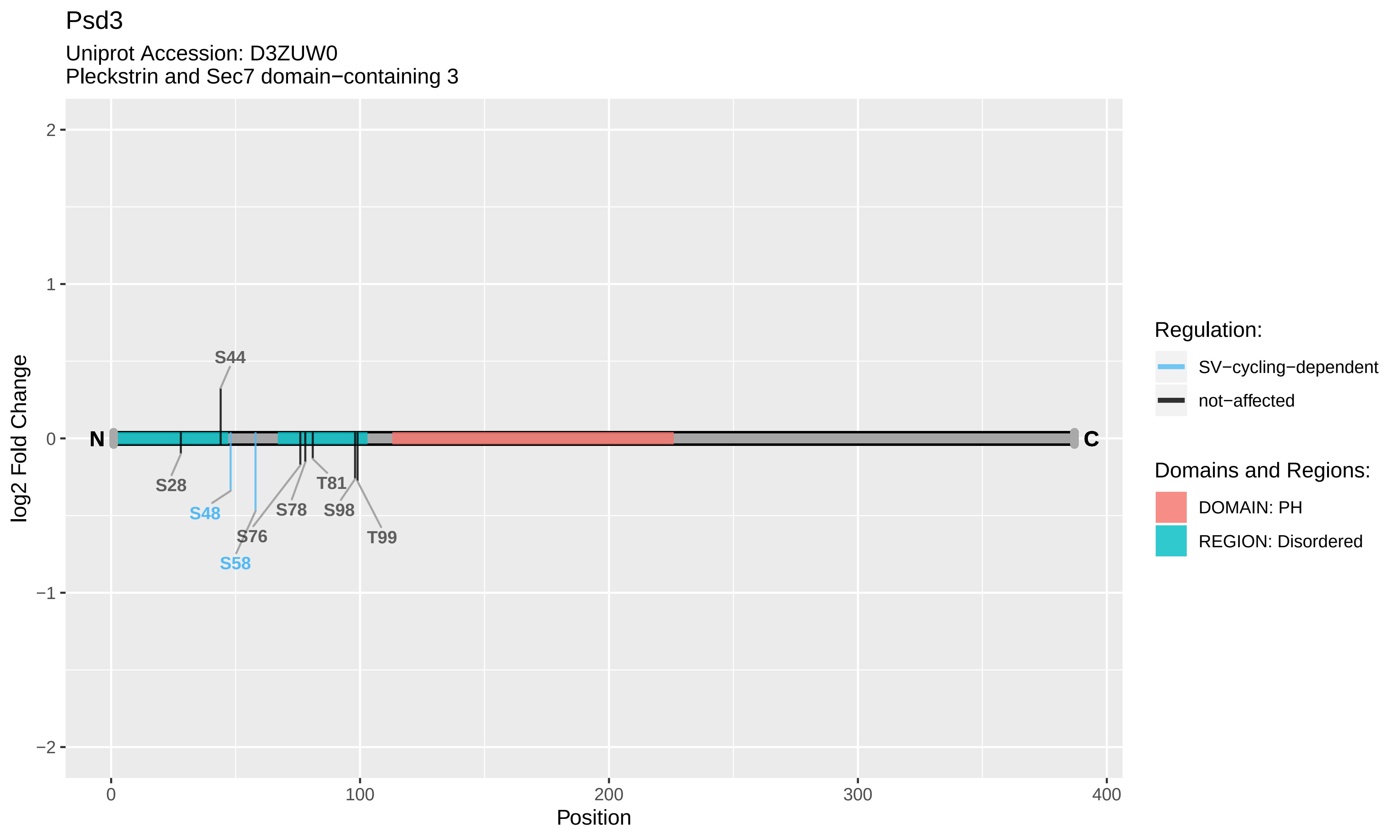

Psd3
Uniprot Accession: D3ZUW0
Pleckstrin and Sec7 domain−containing 3
log2 Fold Change
Position
N
C
S44
S28
S48
S76
S58
S78
T81
S98
T99
Regulation:
SV−cycling−dependent
not−affected
Domains and Regions:
DOMAIN: PH
REGION: Disordered

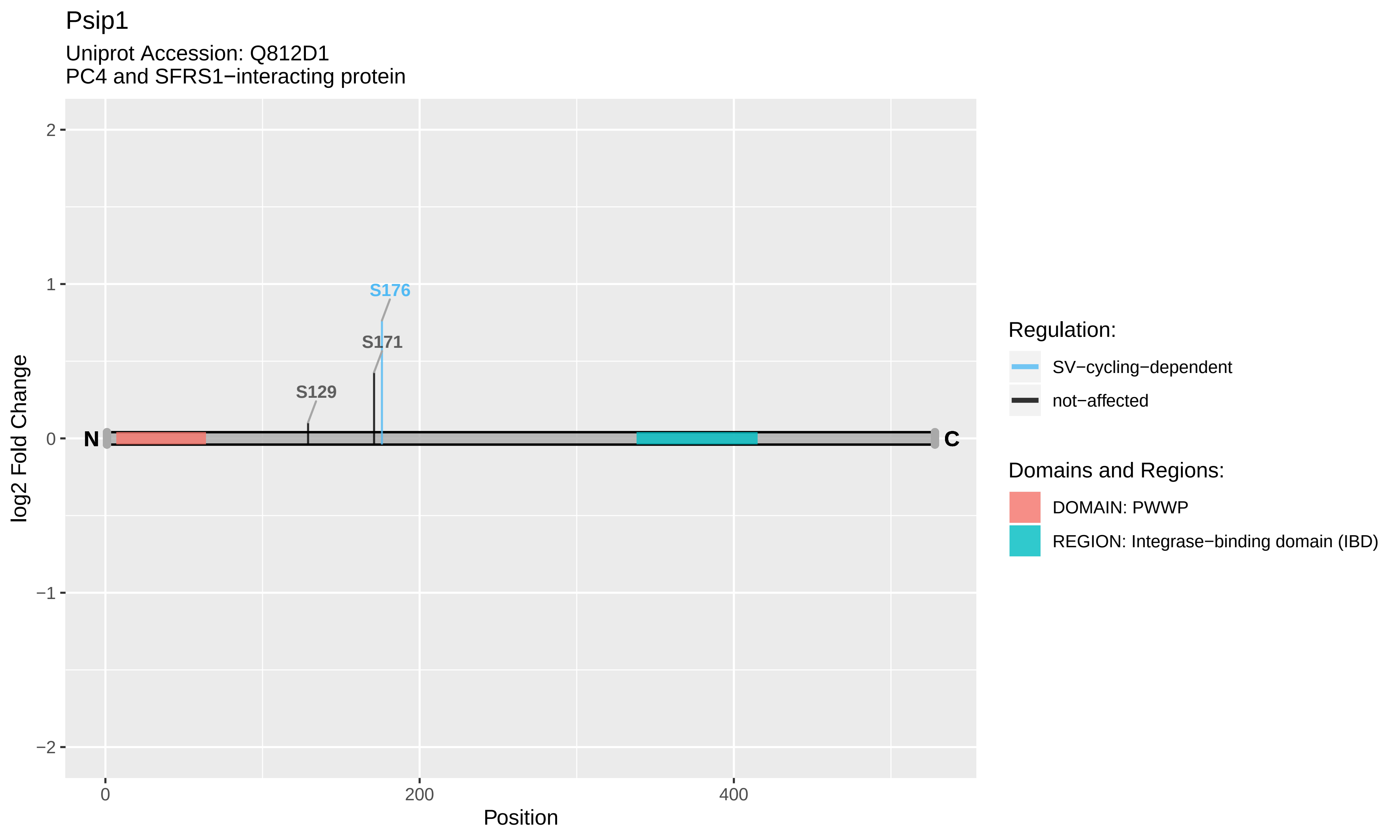

Psip1
Uniprot Accession: Q812D1
PC4 and SFRS1−interacting protein
log2 Fold Change
2
1
0
−1
−2
N
C
S176
S171
S129
0
200
400
Position
Regulation:
SV−cycling−dependent
not−affected
Domains and Regions:
DOMAIN: PWWP
REGION: Integrase−binding domain (IBD)

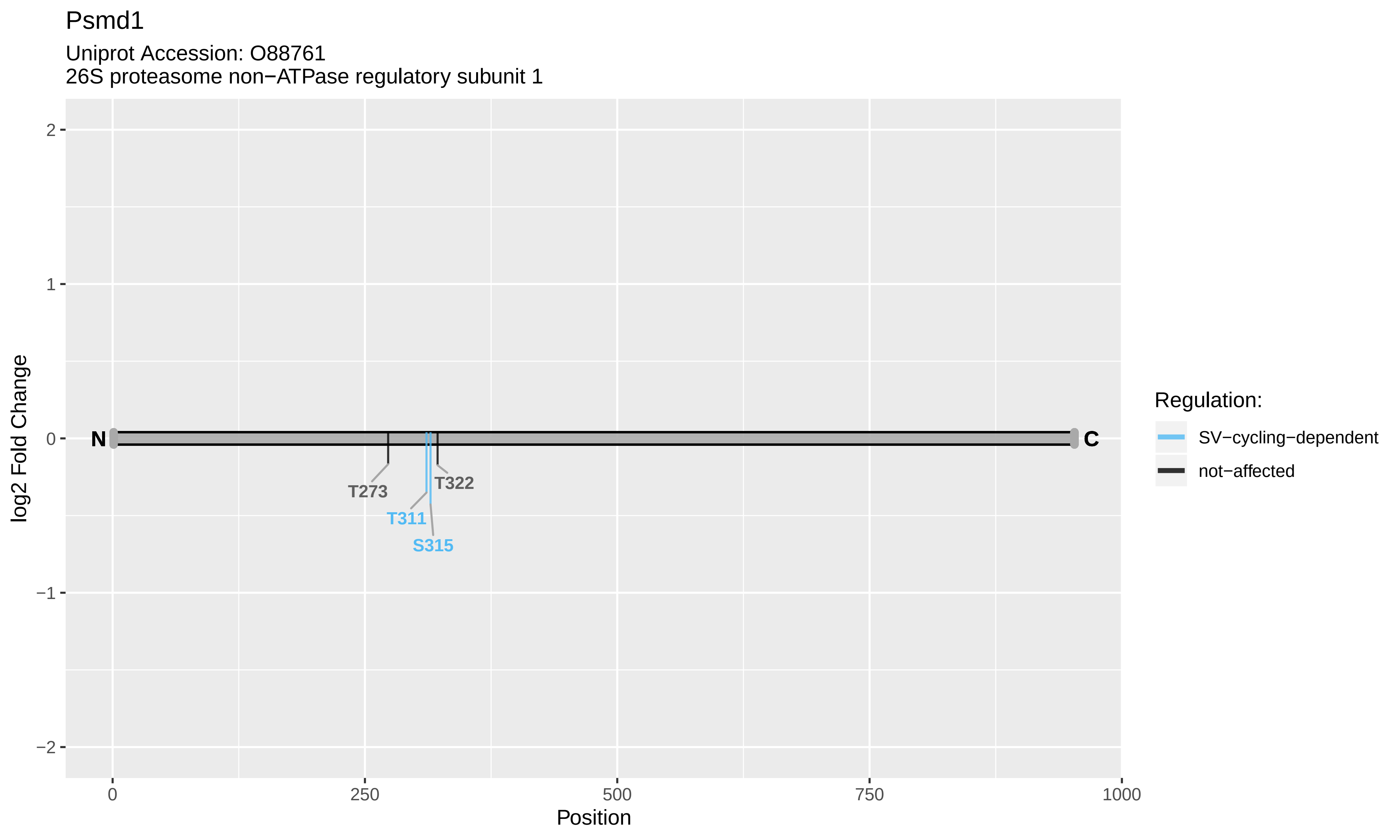

Psmd1
Uniprot Accession: O88761
26S proteasome non−ATPase regulatory subunit 1
log2 Fold Change
2
1
0
−1
−2
N
C
T273
T311
S315
T322
0
250
500
750
1000
Position
Regulation:
SV−cycling−dependent
not−affected

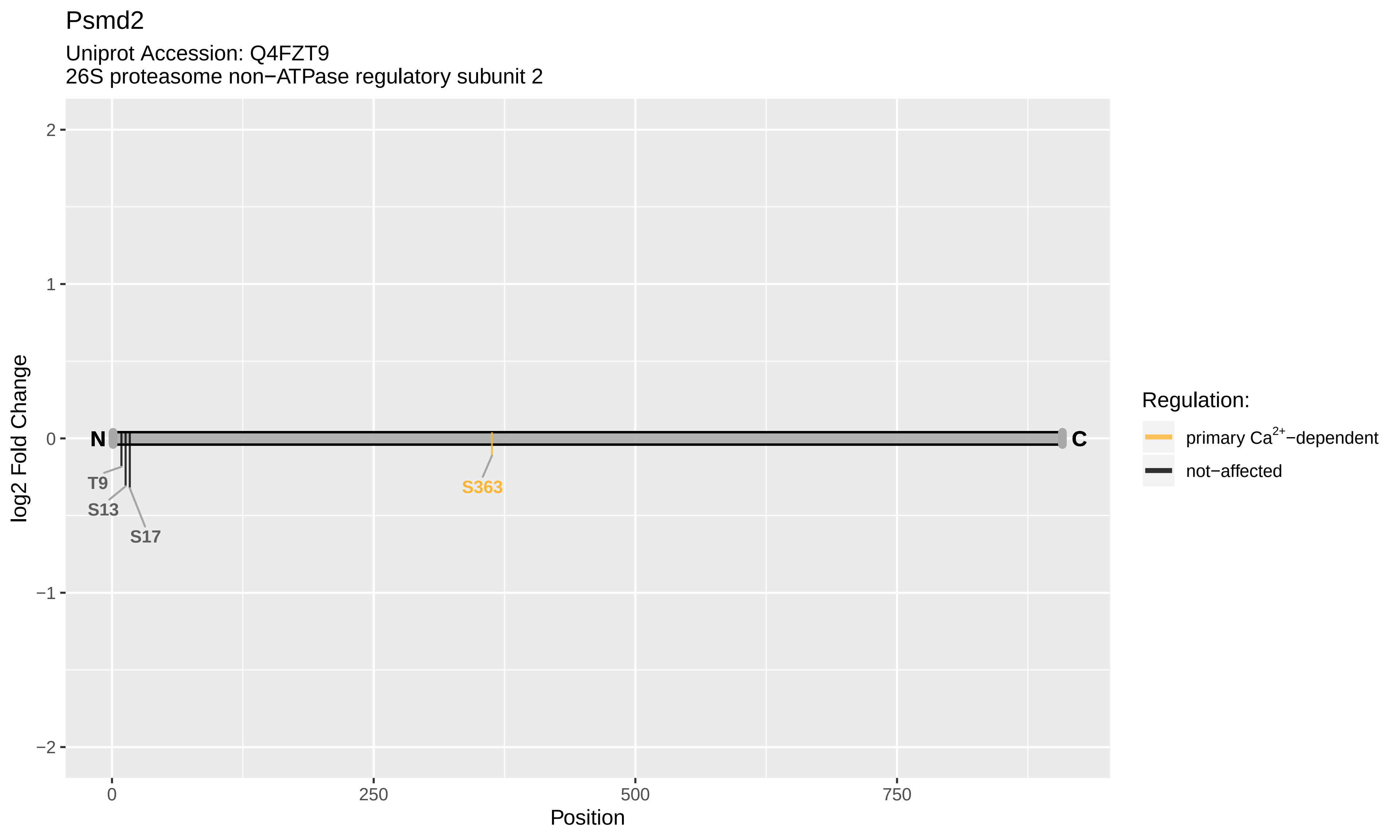
Psmd2
Uniprot Accession: Q4FZT9
26S proteasome non−ATPase regulatory subunit 2
log2 Fold Change
2
1
0
−1
−2
N
C
T9
S13
S17
S363
0
250
500
750
Position
Regulation:
primary Ca2+−dependent
not−affected

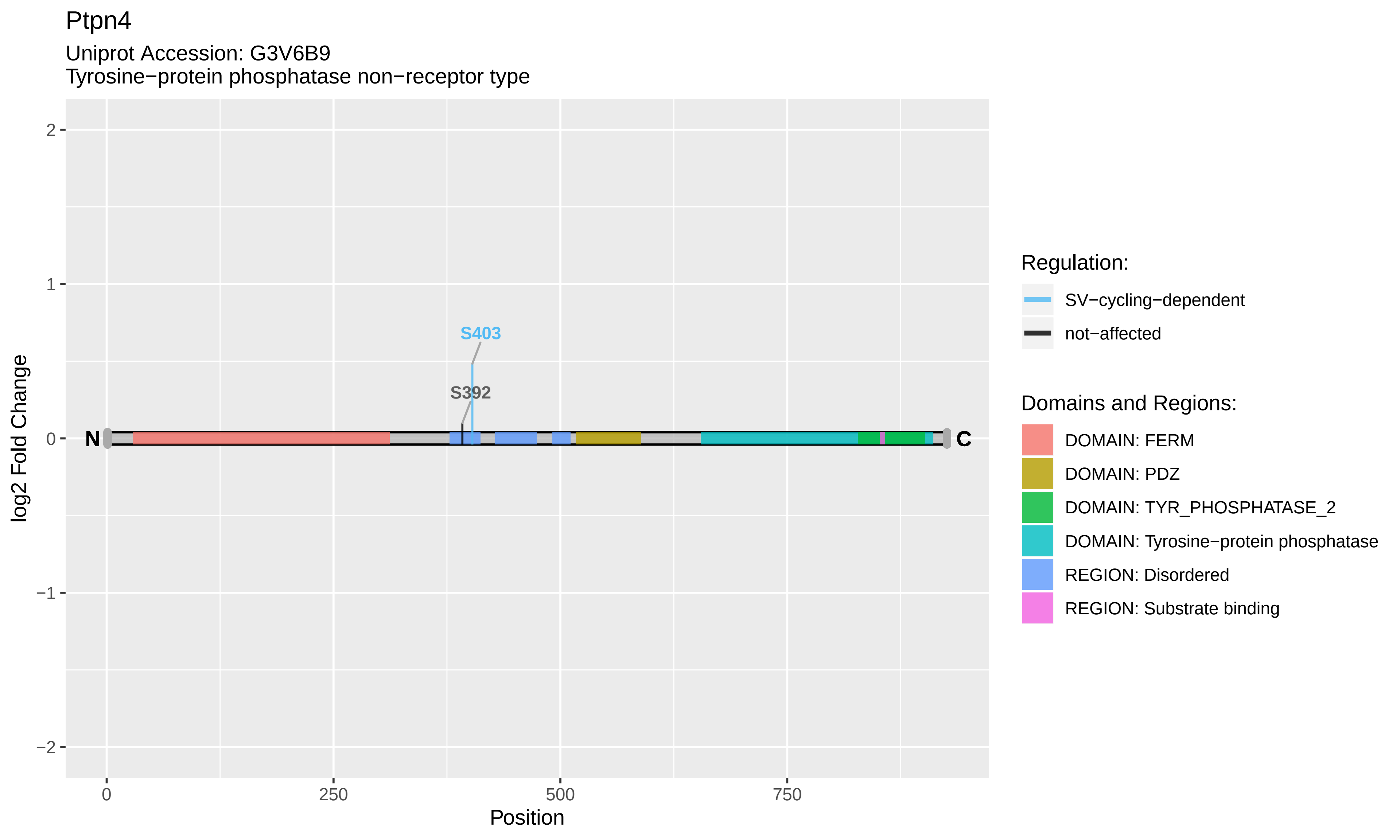

Ptpn4
Uniprot Accession: G3V6B9
Tyrosine−protein phosphatase non−receptor type
S403
S392
N
C
2
1
0
−1
−2
log2 Fold Change
0
250
500
750
Position
Regulation:
SV−cycling−dependent
not−affected
Domains and Regions:
DOMAIN: FERM
DOMAIN: PDZ
DOMAIN: TYR_PHOSPHATASE_2
DOMAIN: Tyrosine−protein phosphatase
REGION: Disordered
REGION: Substrate binding

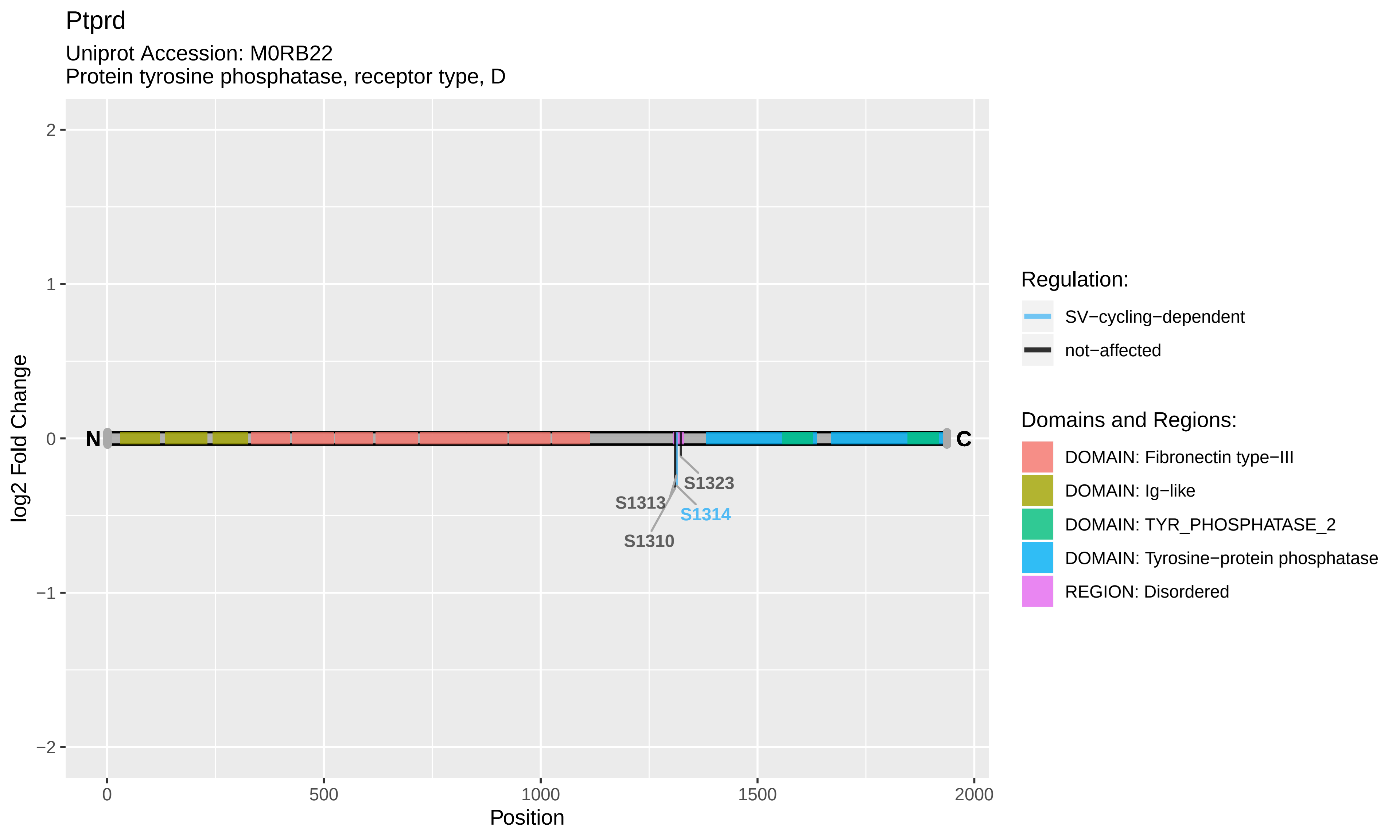

Ptprd
Uniprot Accession: M0RB22
Protein tyrosine phosphatase, receptor type, D
log2 Fold Change
2
1
0
−1
−2
N
C
S1323
S1313
S1314
S1310
0
500
1000
1500
2000
Position
Regulation:
SV−cycling−dependent
not−affected
Domains and Regions:
DOMAIN: Fibronectin type−III
DOMAIN: Ig−like
DOMAIN: TYR_PHOSPHATASE_2
DOMAIN: Tyrosine−protein phosphatase
REGION: Disordered

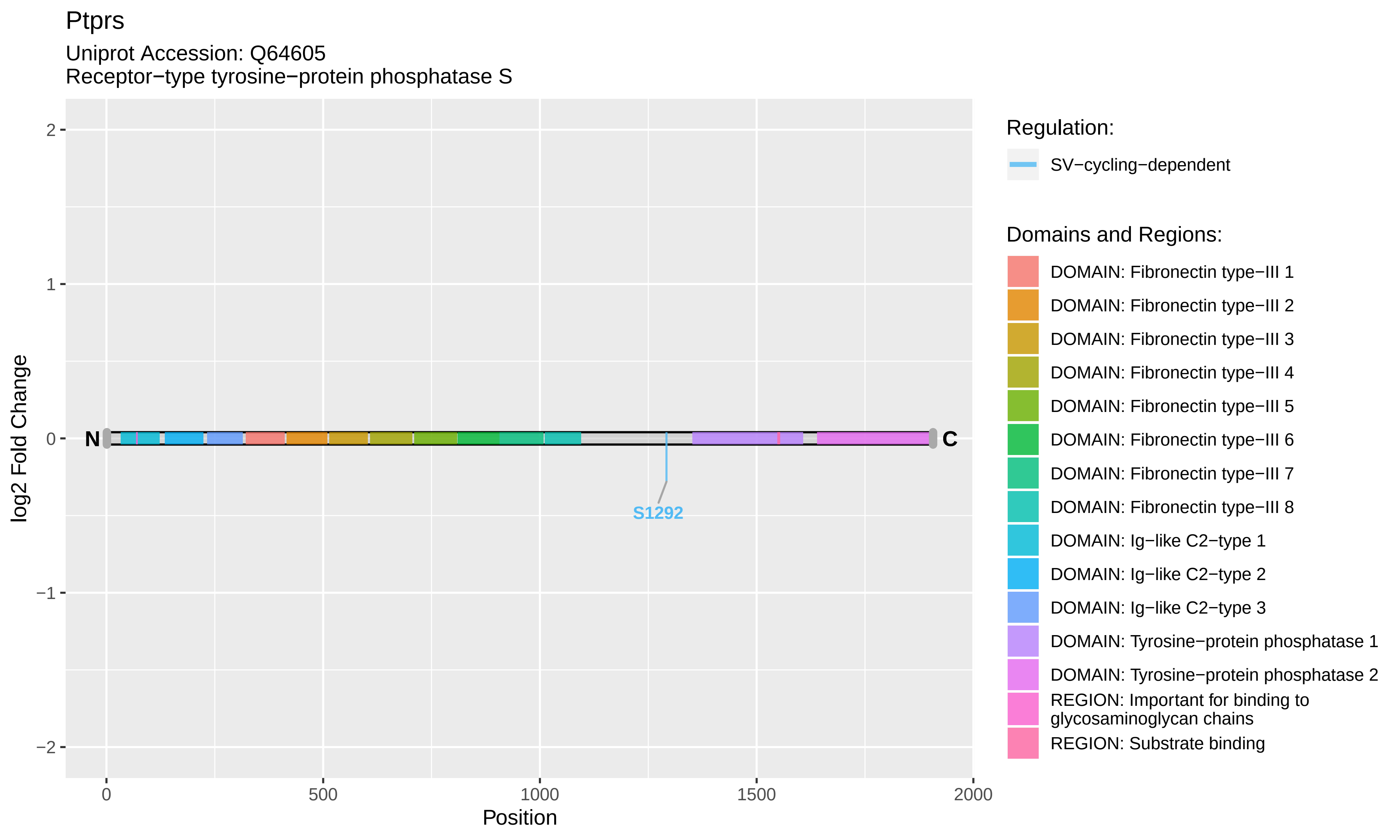
Ptprs
Uniprot Accession: Q64605
Receptor−type tyrosine−protein phosphatase S
log2 Fold Change
Position
N
C
S1292
Regulation:
SV−cycling−dependent
Domains and Regions:
DOMAIN: Fibronectin type−III 1
DOMAIN: Fibronectin type−III 2
DOMAIN: Fibronectin type−III 3
DOMAIN: Fibronectin type−III 4
DOMAIN: Fibronectin type−III 5
DOMAIN: Fibronectin type−III 6
DOMAIN: Fibronectin type−III 7
DOMAIN: Fibronectin type−III 8
DOMAIN: Ig−like C2−type 1
DOMAIN: Ig−like C2−type 2
DOMAIN: Ig−like C2−type 3
DOMAIN: Tyrosine−protein phosphatase 1
DOMAIN: Tyrosine−protein phosphatase 2
REGION: Important for binding to glycosaminoglycan chains
REGION: Substrate binding

Purg

Uniprot Accession: D3ZYS1
Purine−rich element−binding protein G

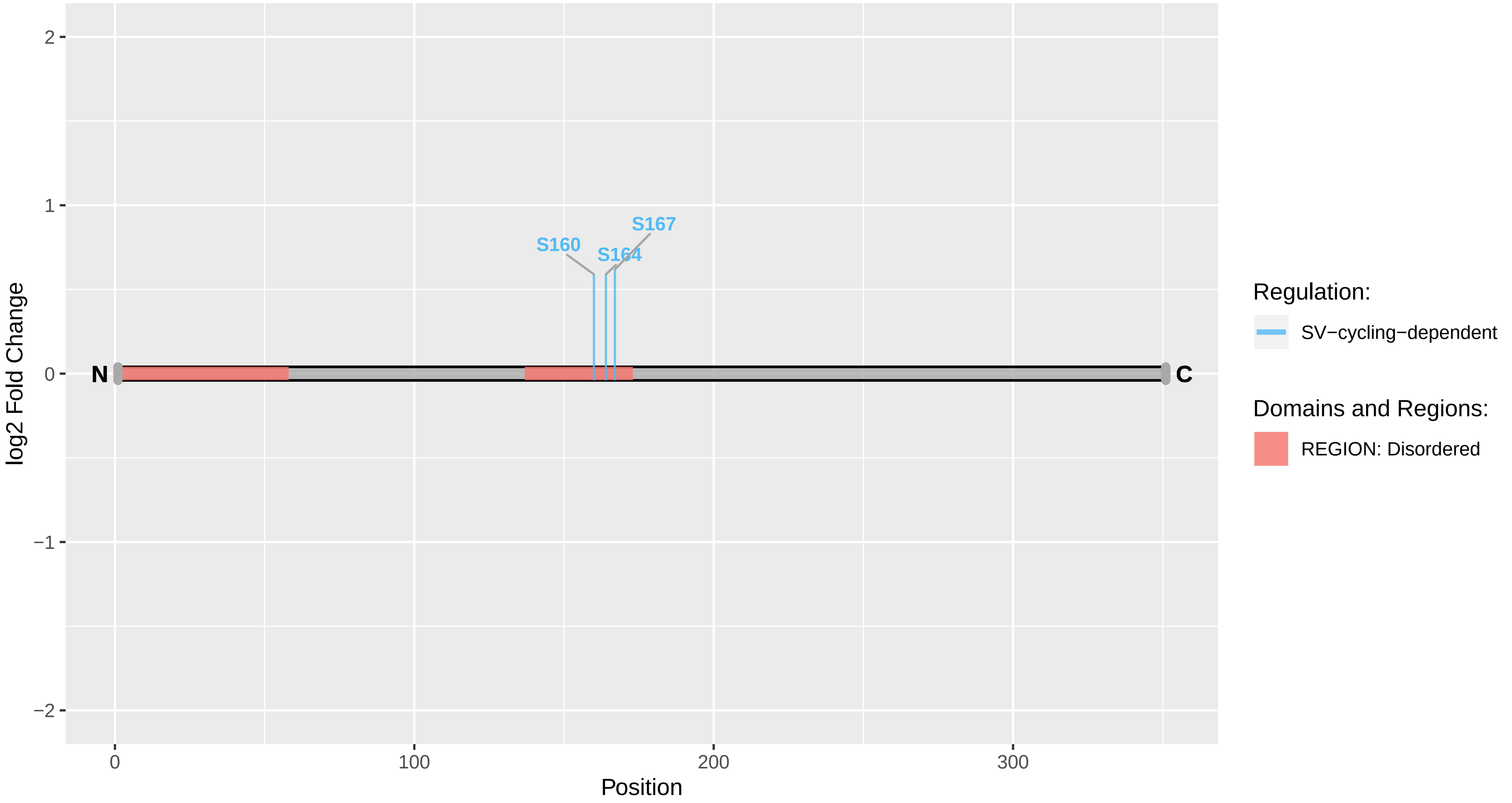

R3hdm2

Uniprot Accession: A0A0G2K4E8
R3H domain−containing 2

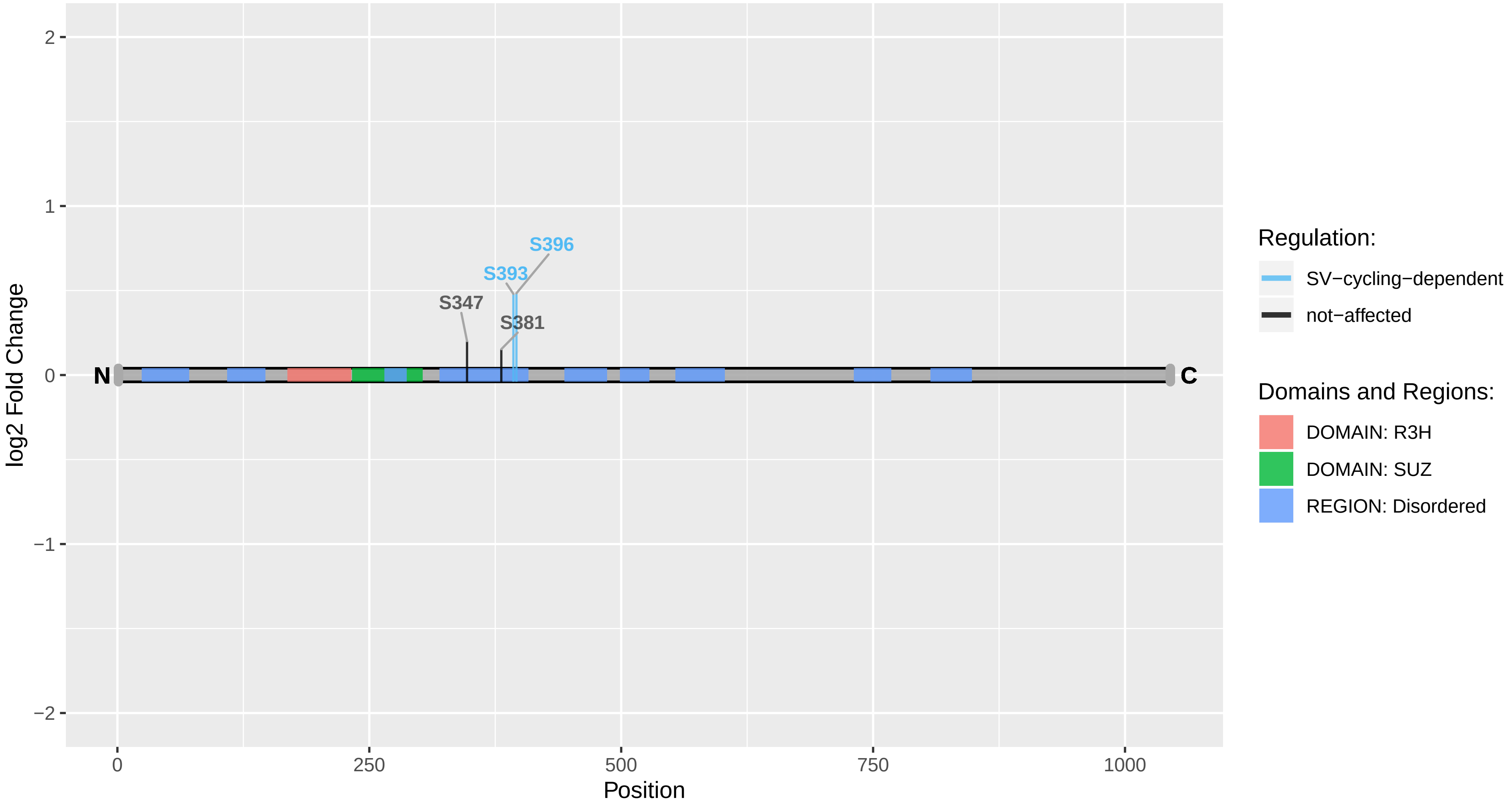

RGD1304884

Uniprot Accession: D4A3C2
Similar to RIKEN cDNA 6430548M08

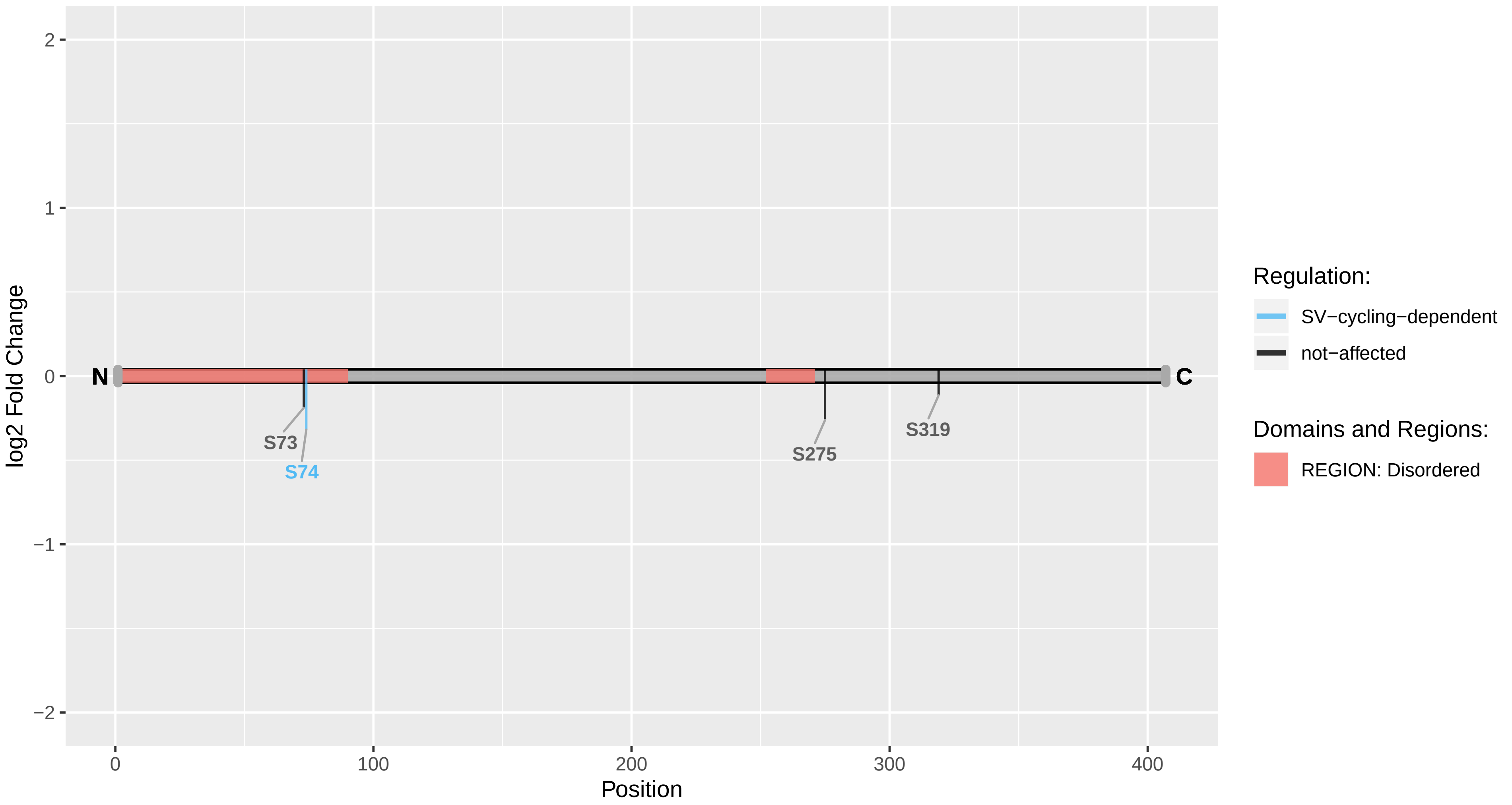

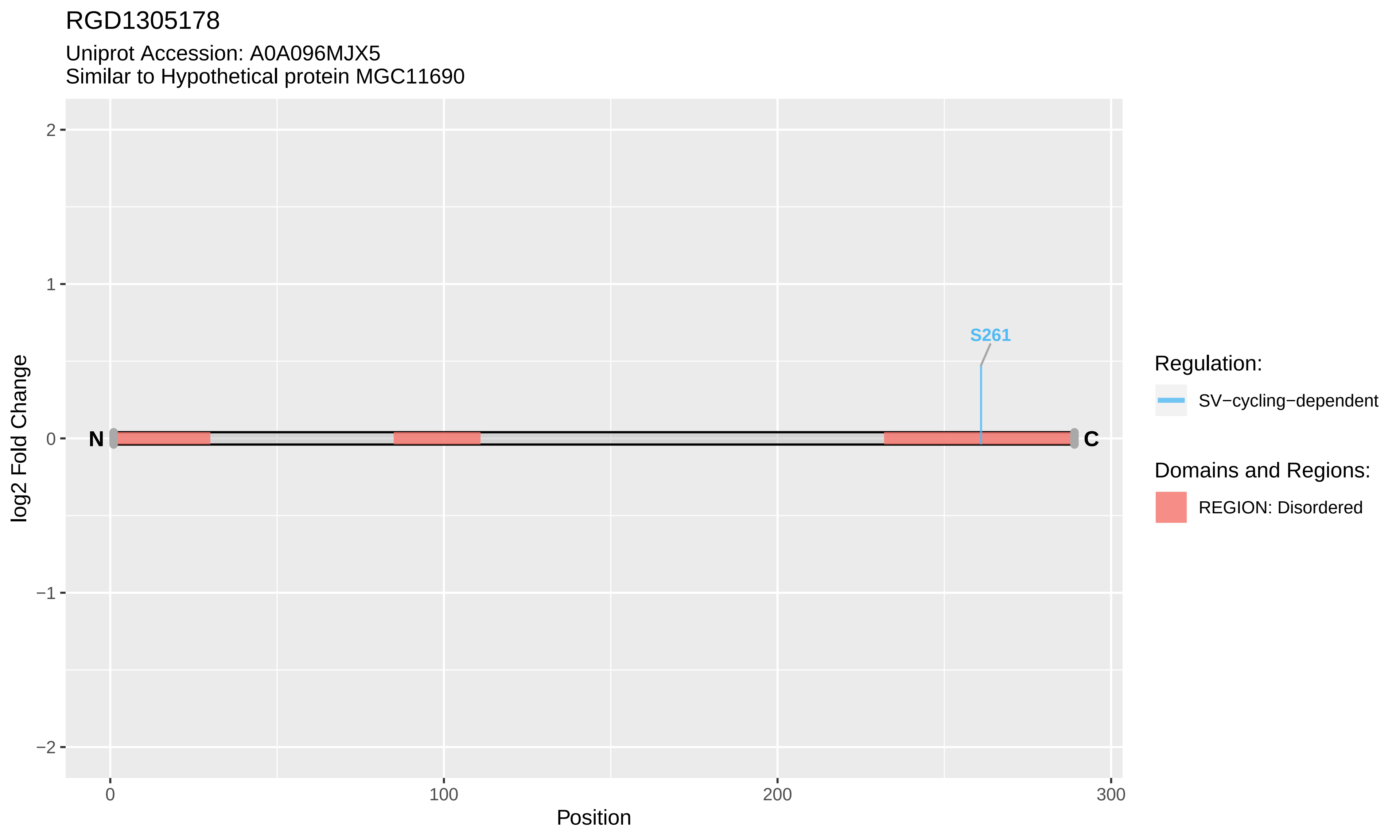
RGD1305178
Uniprot Accession: A0A096MJX5
Similar to Hypothetical protein MGC11690
S261
N
C
log2 Fold Change
Position
2
1
0
−1
−2
0
100
200
300
Regulation:
SV−cycling−dependent
Domains and Regions:
REGION: Disordered

RGD1305455

Uniprot Accession: A0A0G2KAX2
Similar to hypothetical protein FLJ10925

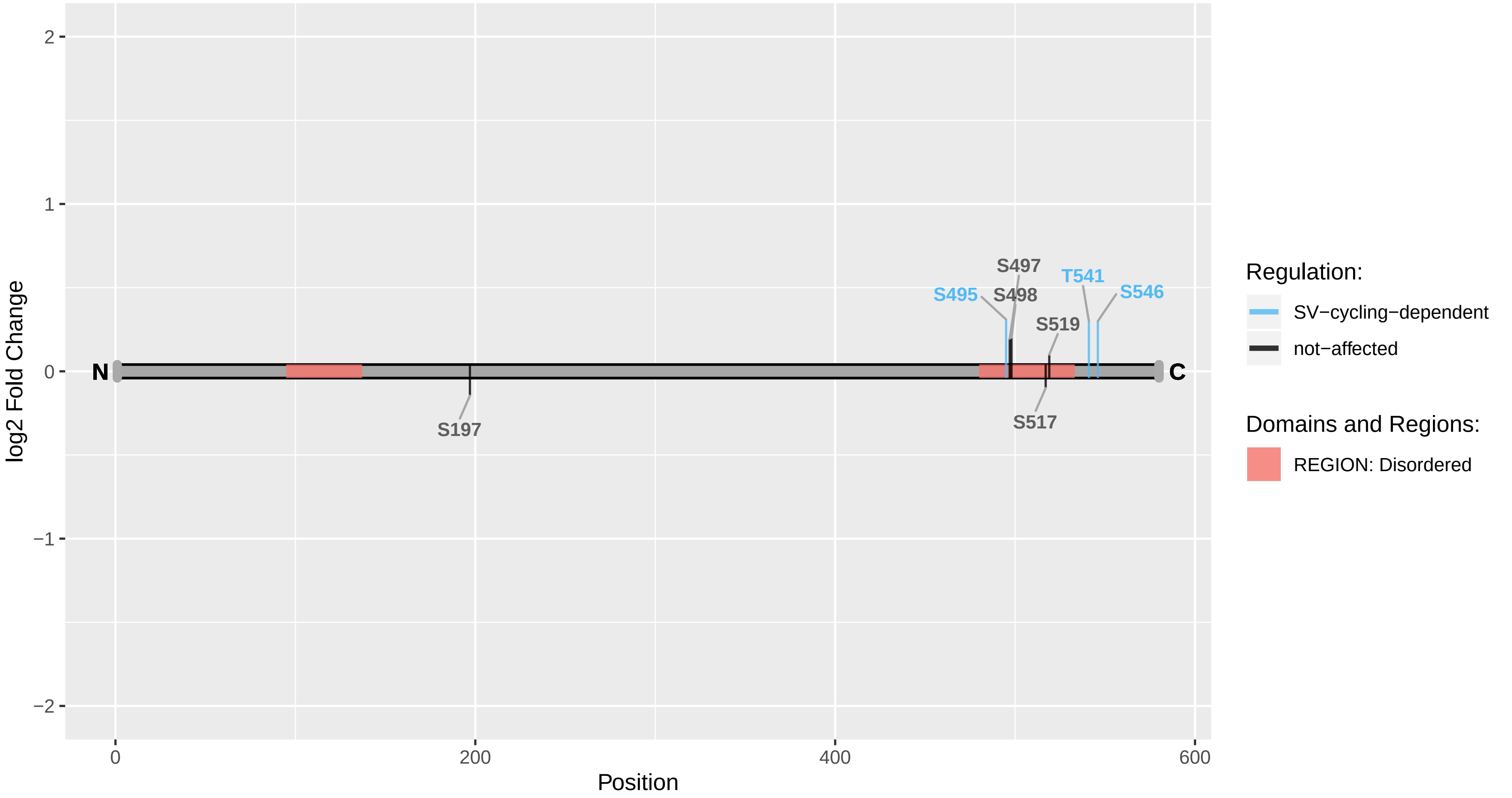

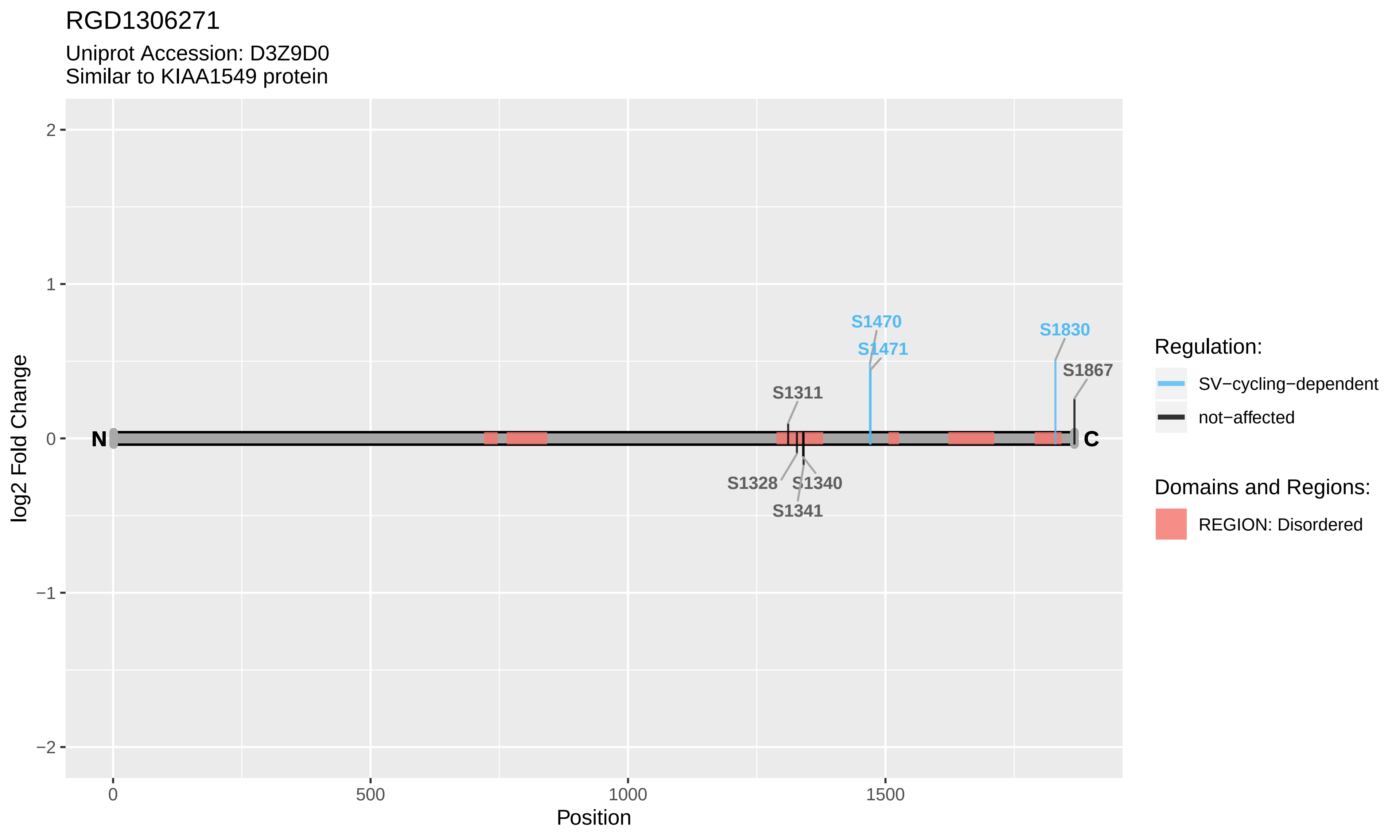
RGD1306271
Uniprot Accession: D3Z9D0
Similar to KIAA1549 protein
log2 Fold Change
Position
N
C
S1470
S1471
S1830
S1867
S1311
S1328
S1340
S1341
2
1
0
−1
−2
0
500
1000
1500
Regulation:
SV−cycling−dependent
not−affected
Domains and Regions:
REGION: Disordered

RGD1306556

Uniprot Accession: A0A0G2JZ88
Similar to hypothetical protein A530094D01

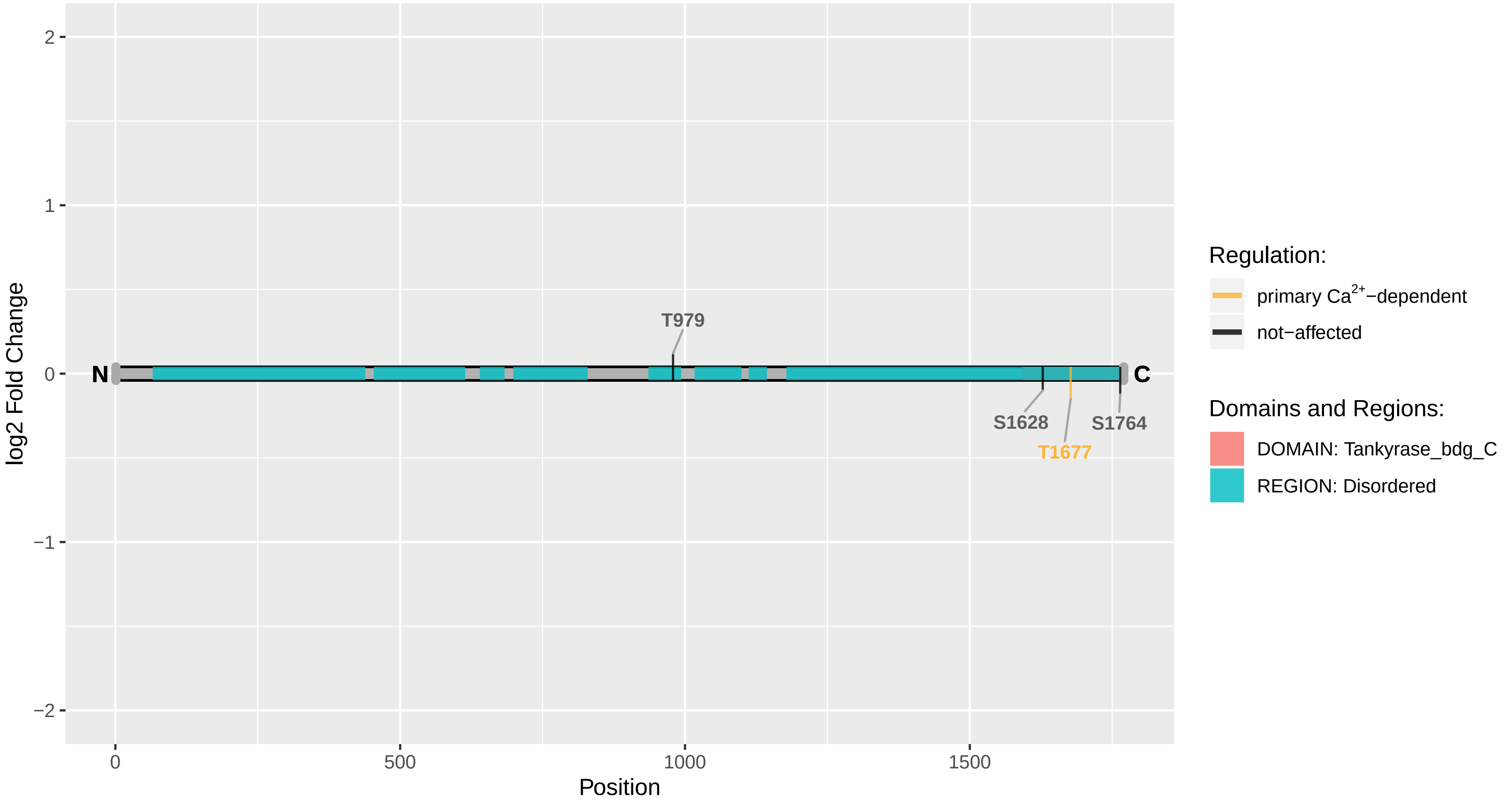

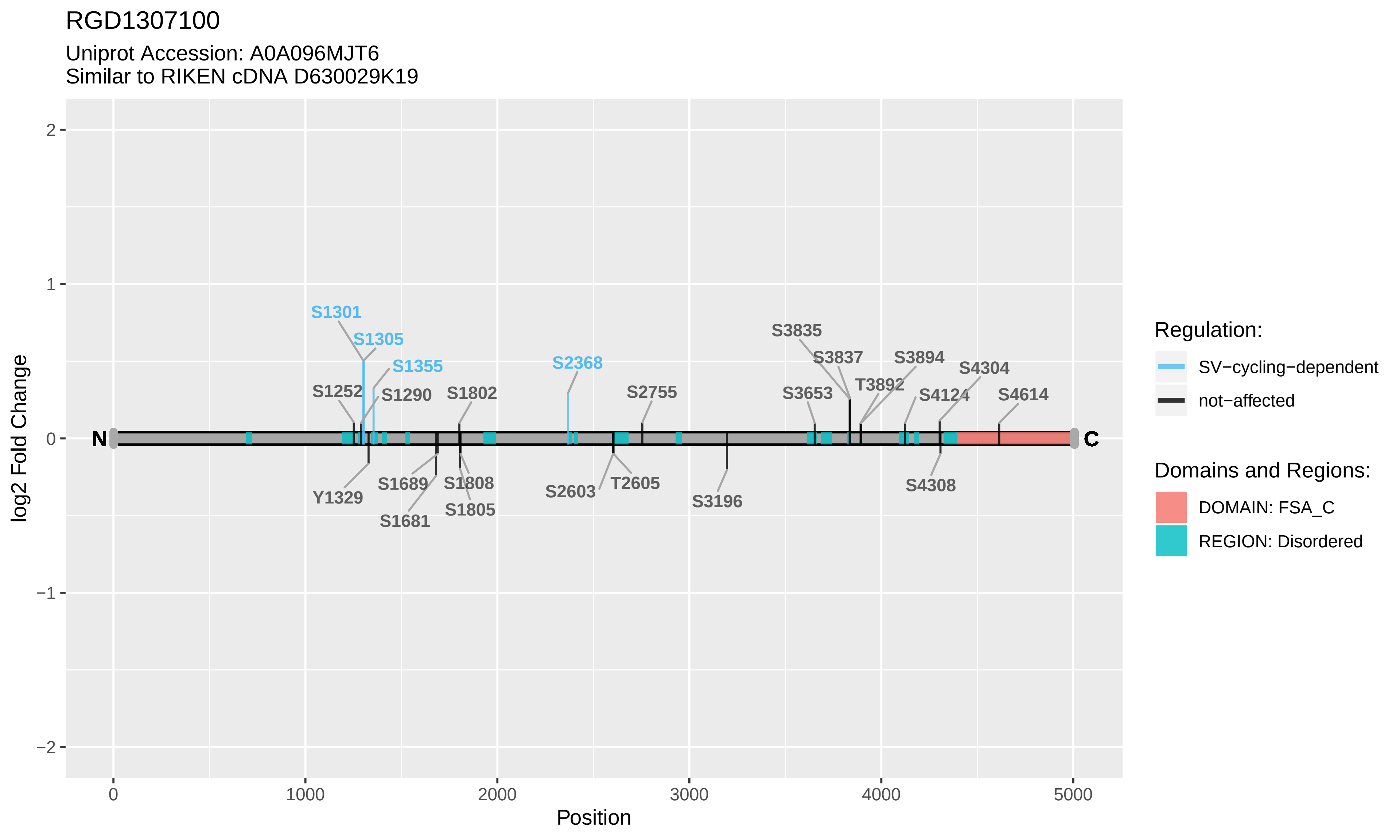
RGD1307100
Uniprot Accession: A0A096MJT6
Similar to RIKEN cDNA D630029K19
log2 Fold Change
Position
2
1
0
−1
−2
0
1000
2000
3000
4000
5000
N
C
S1301
S1305
S1355
S2368
S1252
S1290
S1802
S2755
S3835
S3837
S3894
T3892
S3653
S4304
S4124
S4614
Y1329
S1689
S1681
S1808
S1805
S2603
T2605
S3196
S4308
Regulation:
SV−cycling−dependent
not−affected
Domains and Regions:
DOMAIN: FSA_C
REGION: Disordered

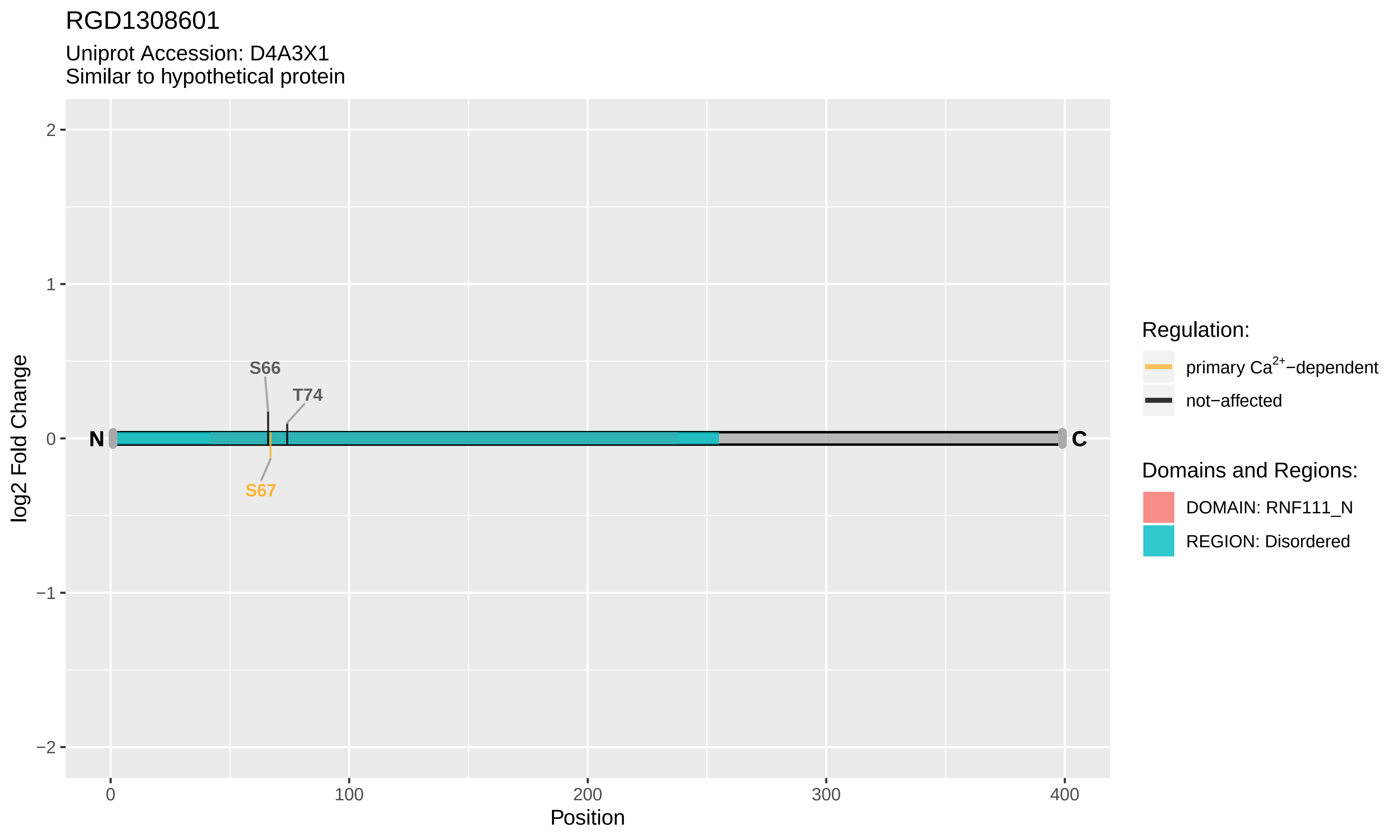
RGD1308601
Uniprot Accession: D4A3X1
Similar to hypothetical protein
log2 Fold Change
2
1
0
−1
−2
N
C
S66
T74
S67
0
100
200
300
400
Position
Regulation:
primary Ca2+−dependent
not−affected
Domains and Regions:
DOMAIN: RNF111_N
REGION: Disordered

RGD1310819

Uniprot Accession: D3ZBU7
Similar to putative protein (5S487)

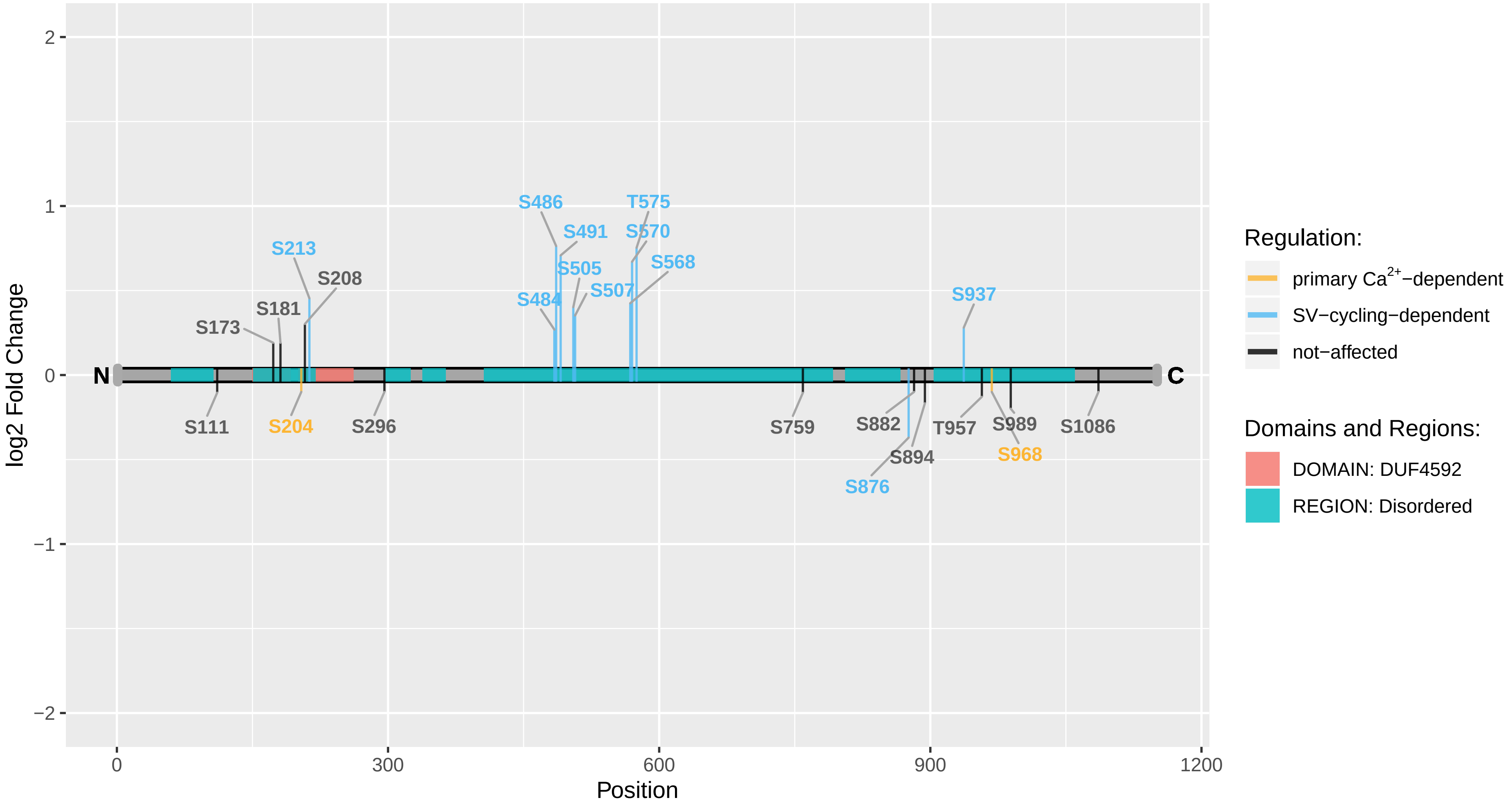

RGD1311575

Uniprot Accession: D4A5F4
Hypothetical LOC289568

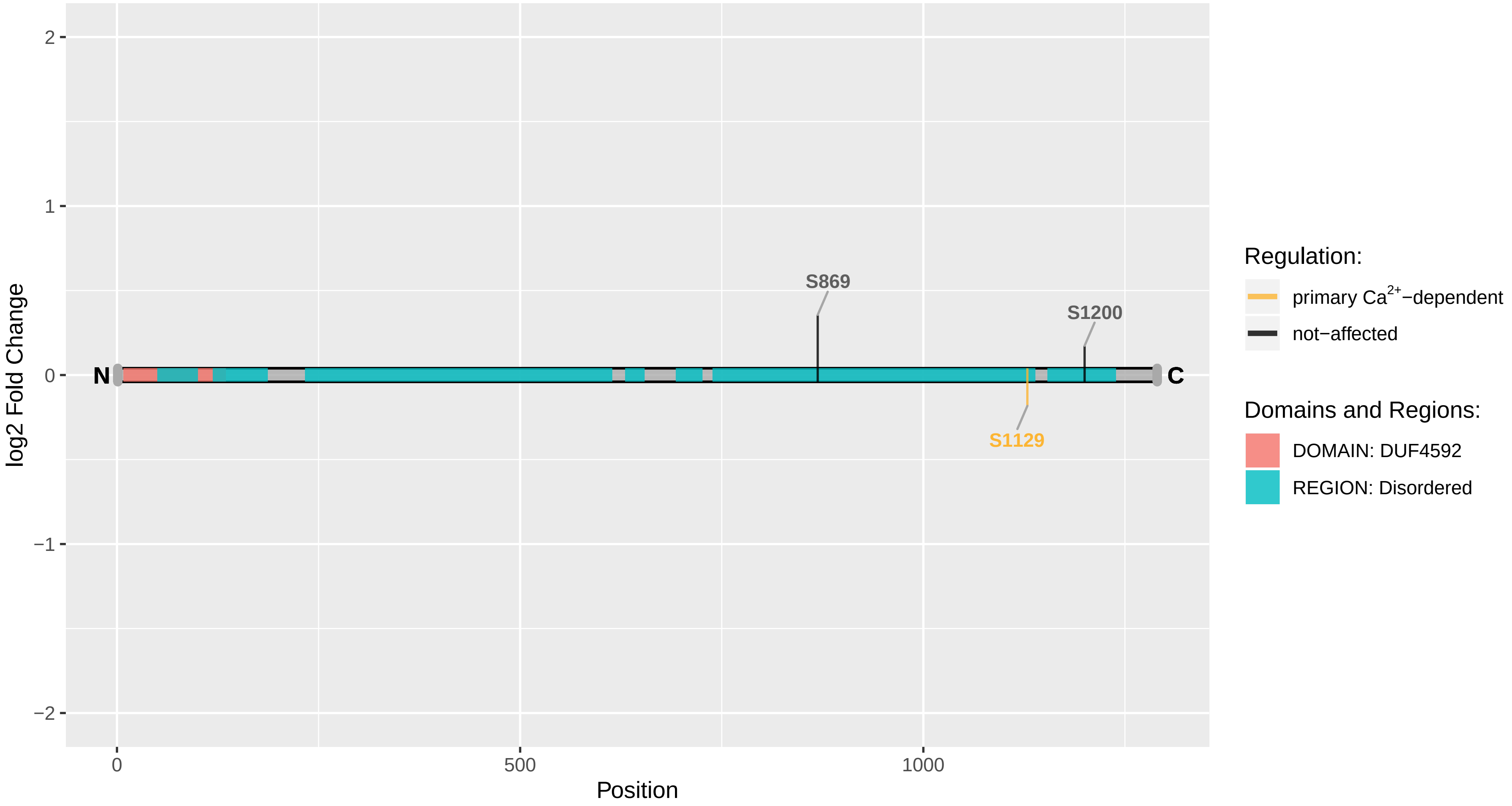

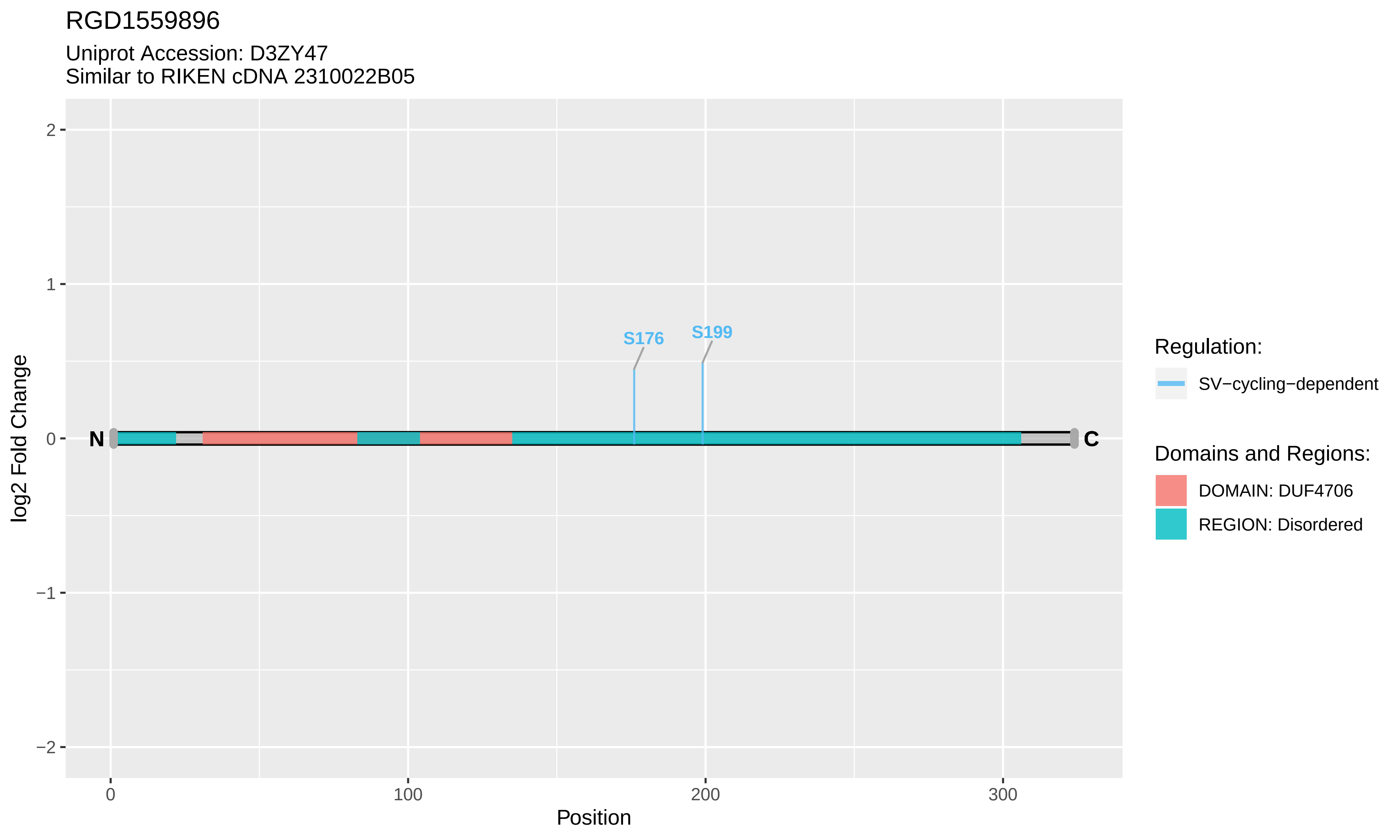

RGD1559896
Uniprot Accession: D3ZY47
Similar to RIKEN cDNA 2310022B05
S176
S199
N
C
log2 Fold Change
Position
Regulation:
SV−cycling−dependent
Domains and Regions:
DOMAIN: DUF4706
REGION: Disordered

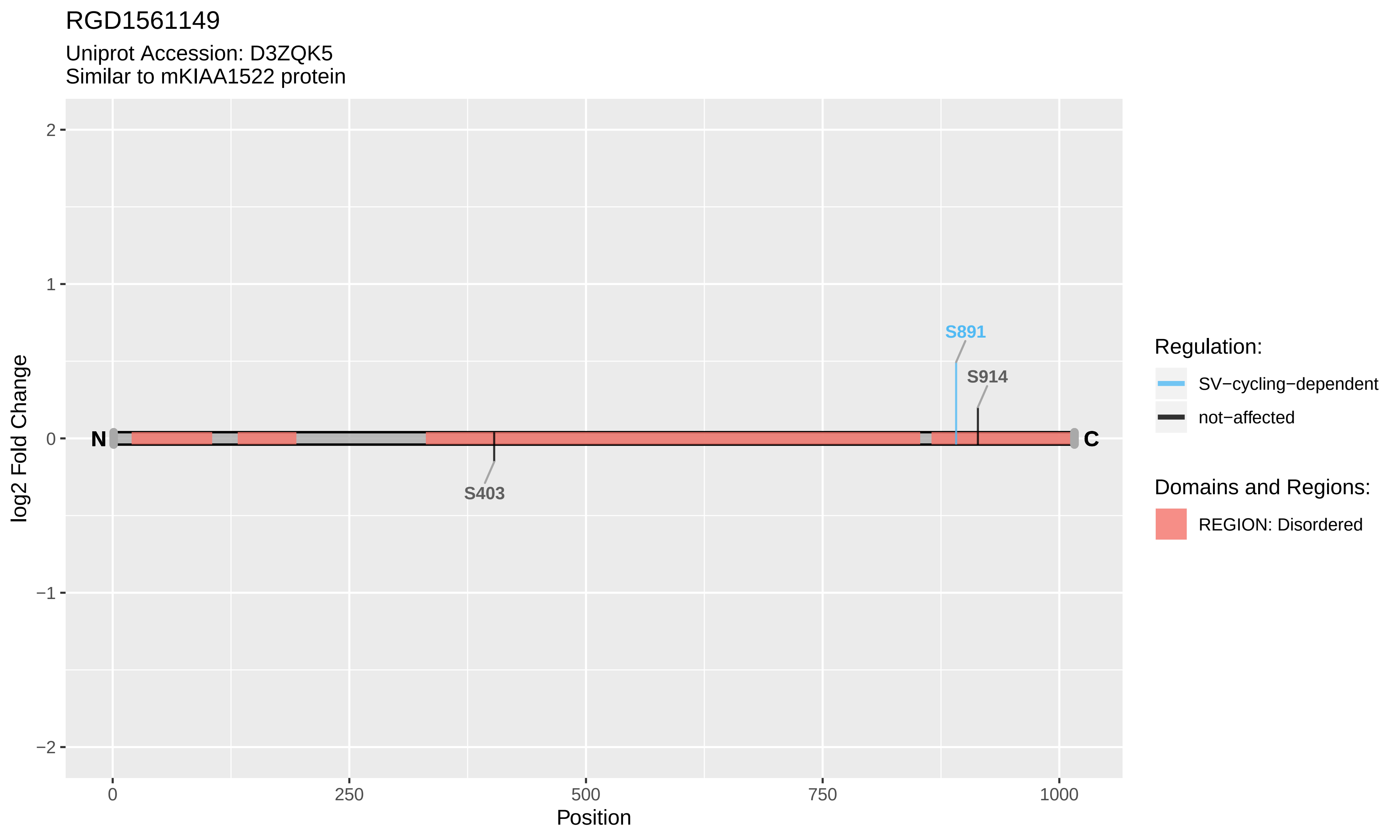
RGD1561149
Uniprot Accession: D3ZQK5
Similar to mKIAA1522 protein
log2 Fold Change
2
1
0
−1
−2
0
250
500
750
1000
Position
N
C
S891
S914
S403
Regulation:
SV−cycling−dependent
not−affected
Domains and Regions:
REGION: Disordered

Rab11fip5

Uniprot Accession: A0A0G2K1W1
RAB11 family−interacting protein 5

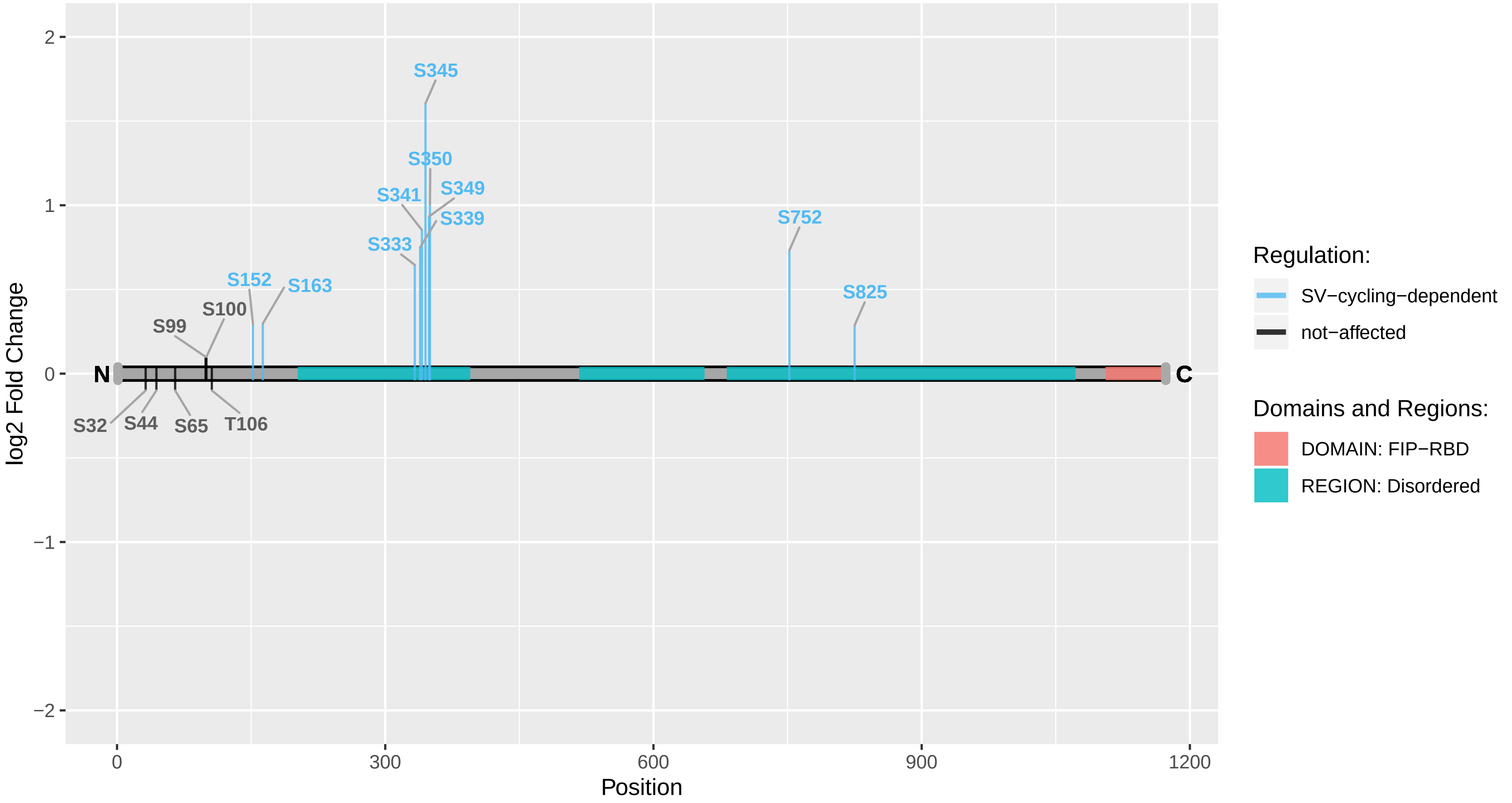

Rab11fip5

Uniprot Accession: A0A0G2K4A0
RAB11 family−interacting protein 5

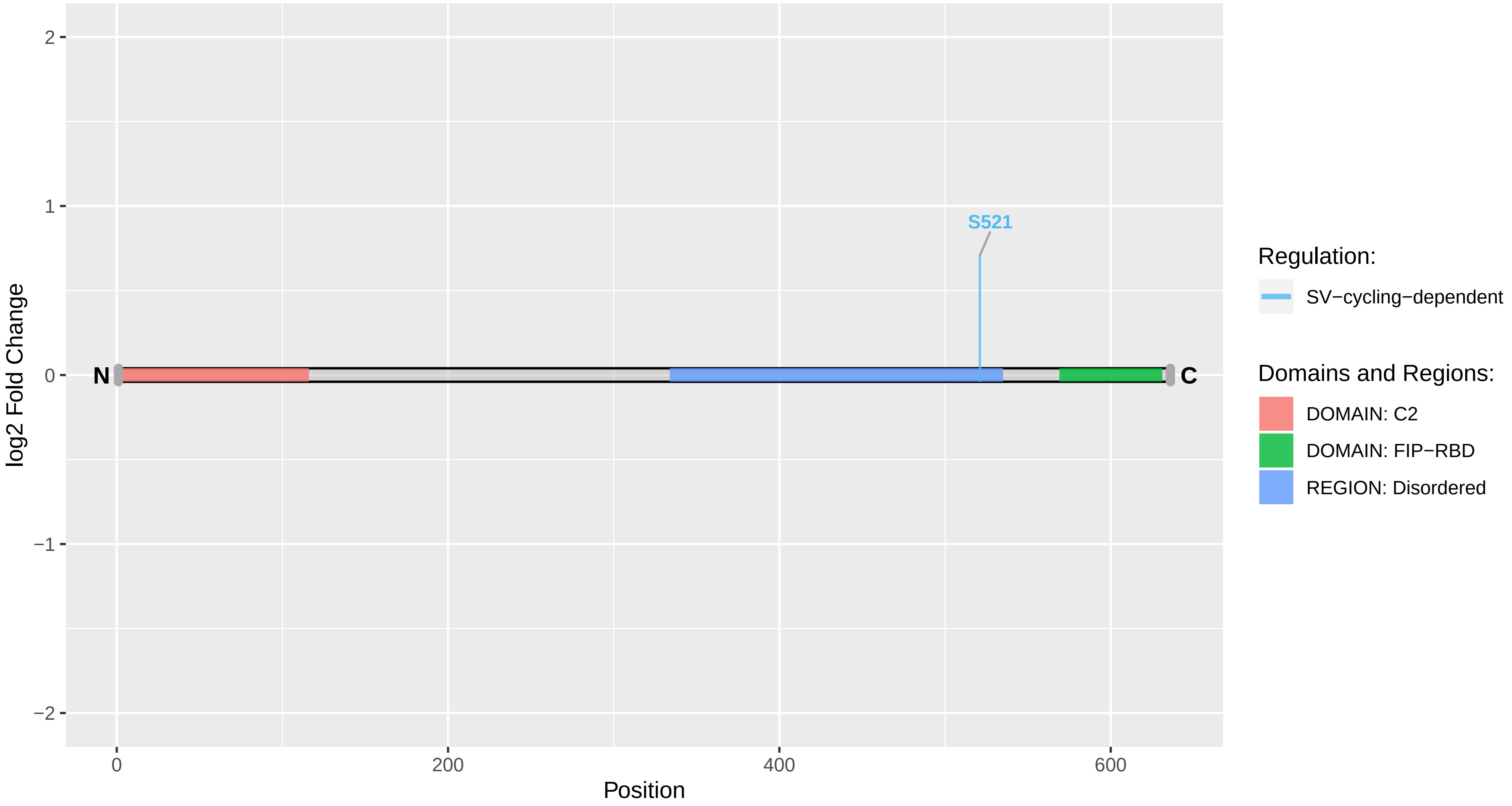

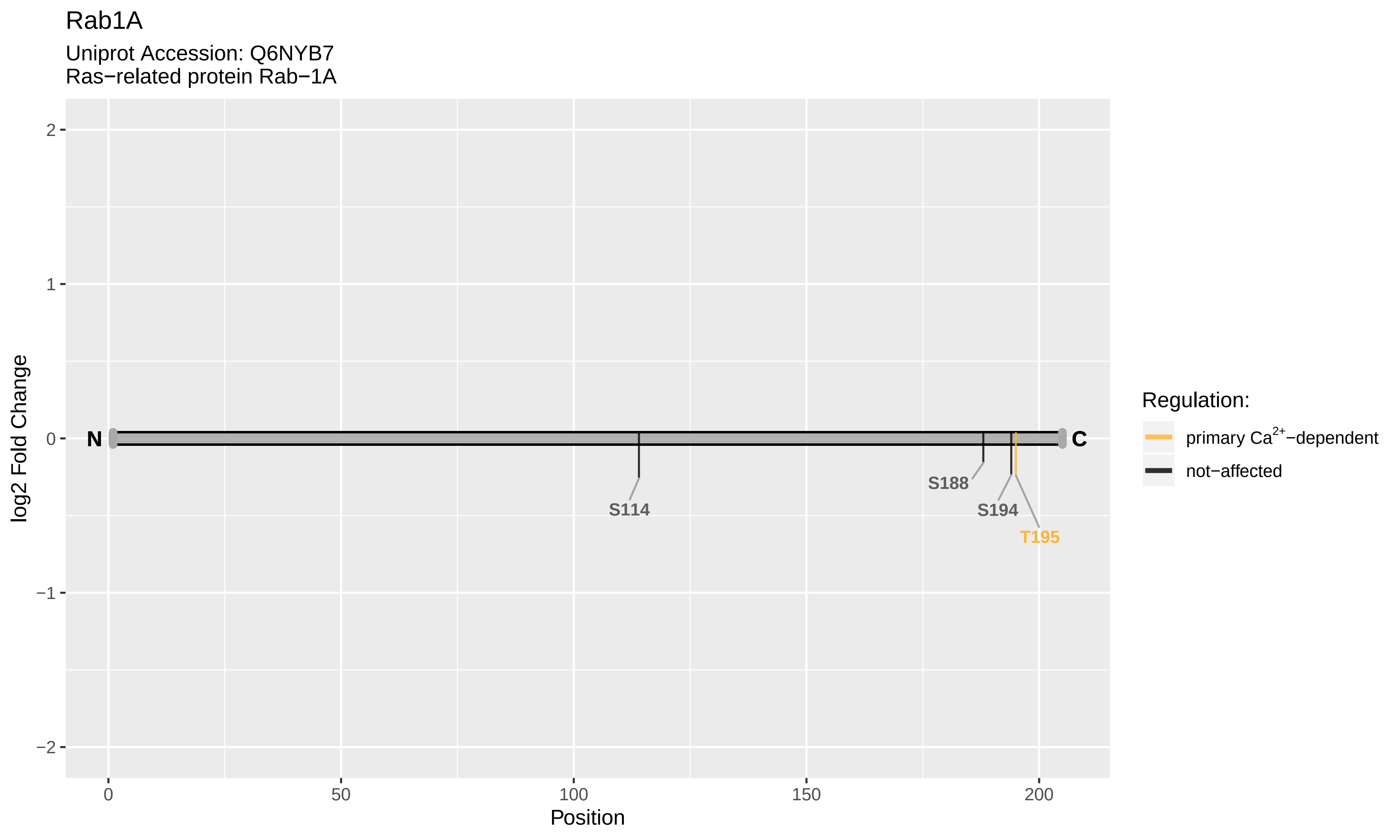
Rab1A
Uniprot Accession: Q6NYB7
Ras−related protein Rab−1A
log2 Fold Change
Position
N
C
S114
S188
S194
T195
Regulation:
primary Ca^{2+}−dependent
not−affected
2
1
0
−1
−2
0
50
100
150
200

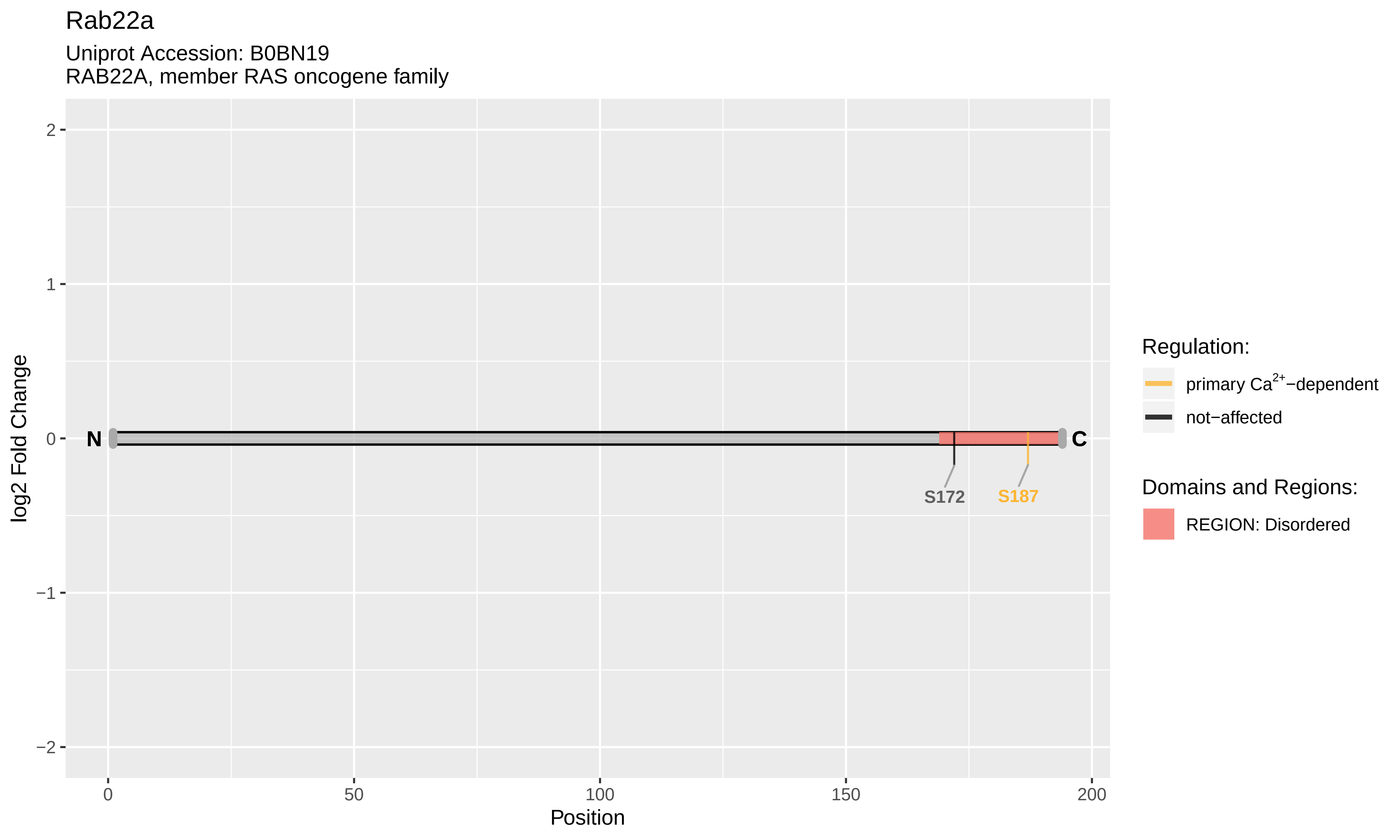

Rab22a
Uniprot Accession: B0BN19
RAB22A, member RAS oncogene family
log2 Fold Change
2
1
0
−1
−2
0
50
100
150
200
Position
N
C
S172
S187
Regulation:
primary Ca2+−dependent
not−affected
Domains and Regions:
REGION: Disordered

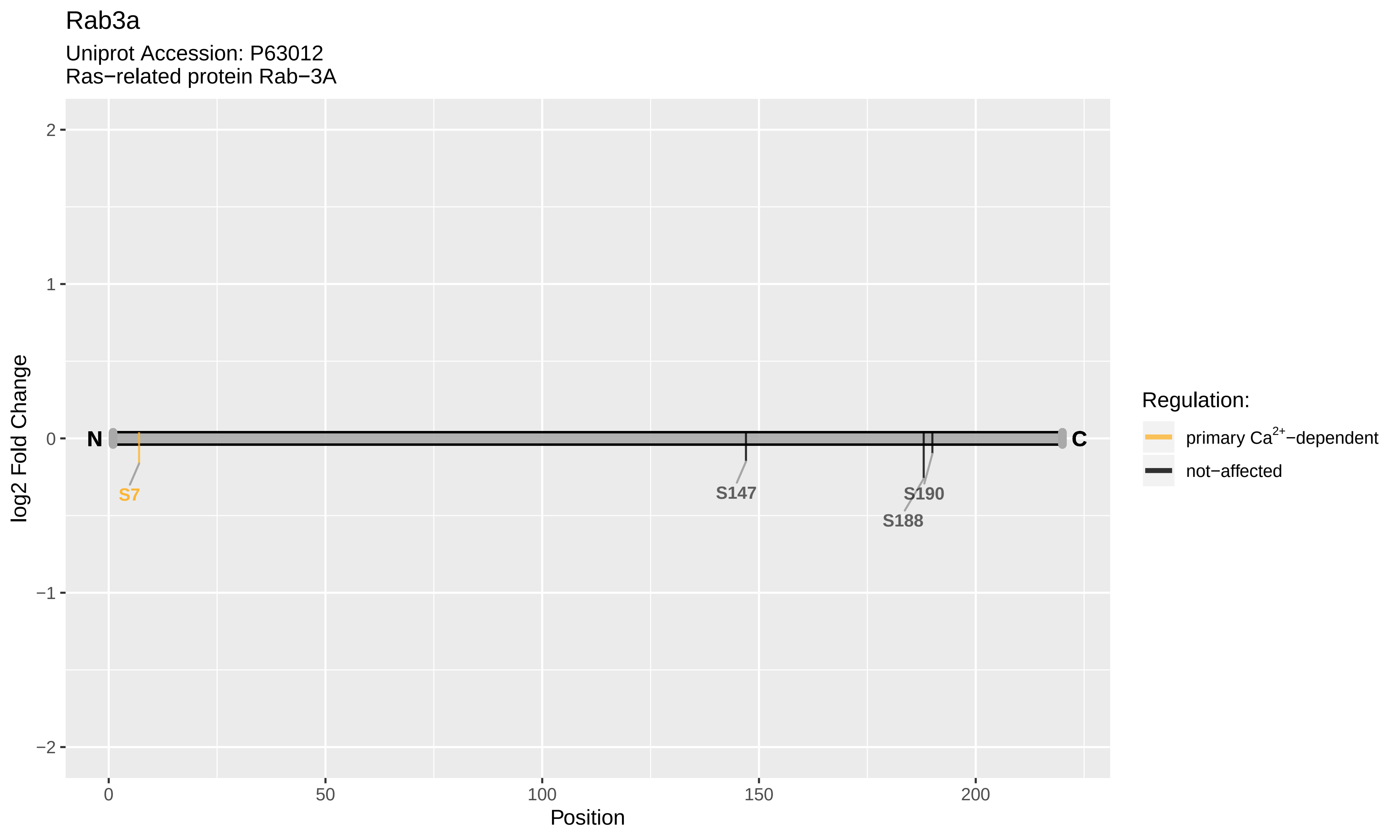
Rab3a
Uniprot Accession: P63012
Ras−related protein Rab−3A
log2 Fold Change
2
1
0
−1
−2
N
C
S7
S147
S190
S188
0
50
100
150
200
Position
Regulation:
primary Ca2+−dependent
not−affected

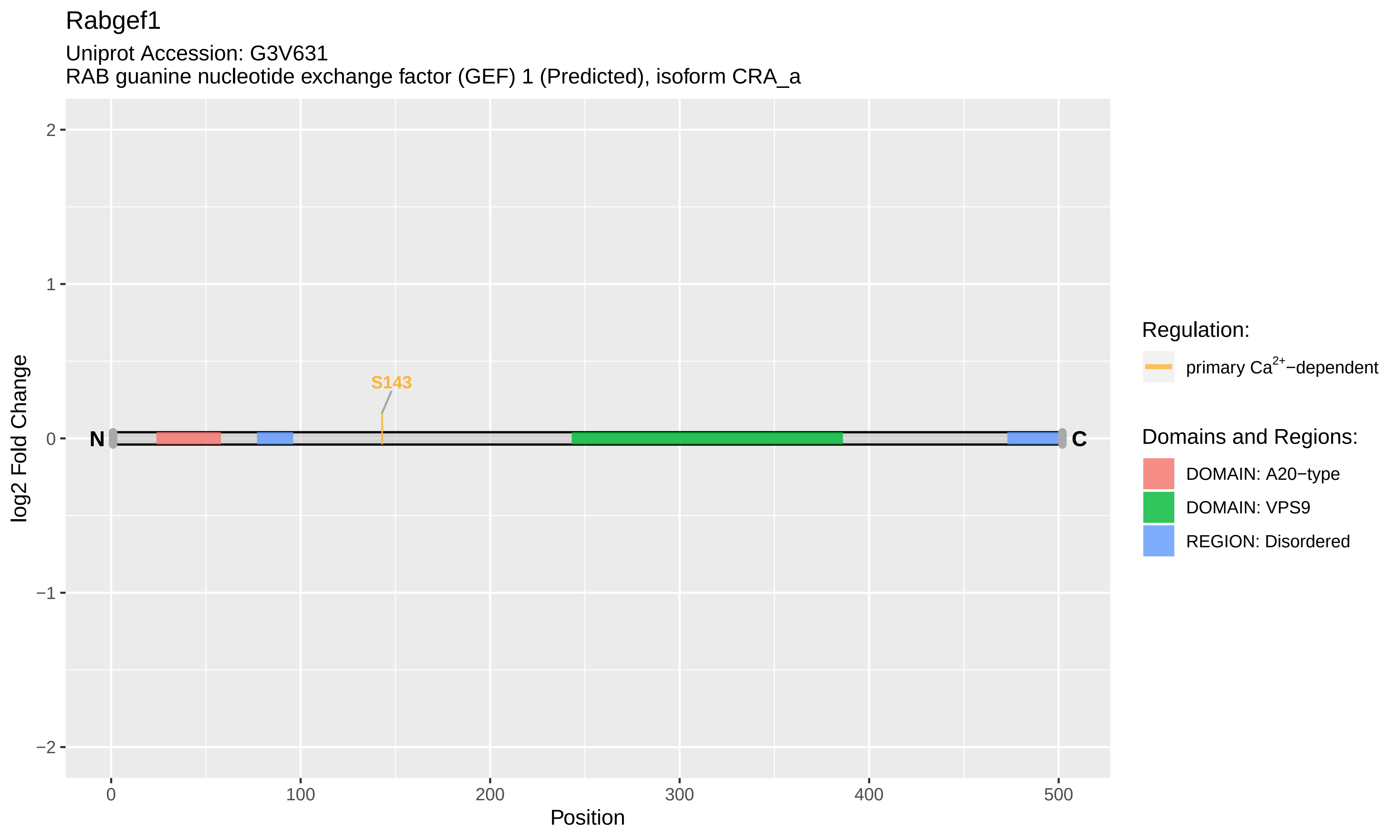

Rabgef1
Uniprot Accession: G3V631
RAB guanine nucleotide exchange factor (GEF) 1 (Predicted), isoform CRA_a
log2 Fold Change
2
1
0
−1
−2
N
C
S143
0
100
200
300
400
500
Position
Regulation:
primary Ca2+−dependent
Domains and Regions:
DOMAIN: A20−type
DOMAIN: VPS9
REGION: Disordered

Ralgapa1

Uniprot Accession: O55007
Ral GTPase−activating protein subunit alpha−1

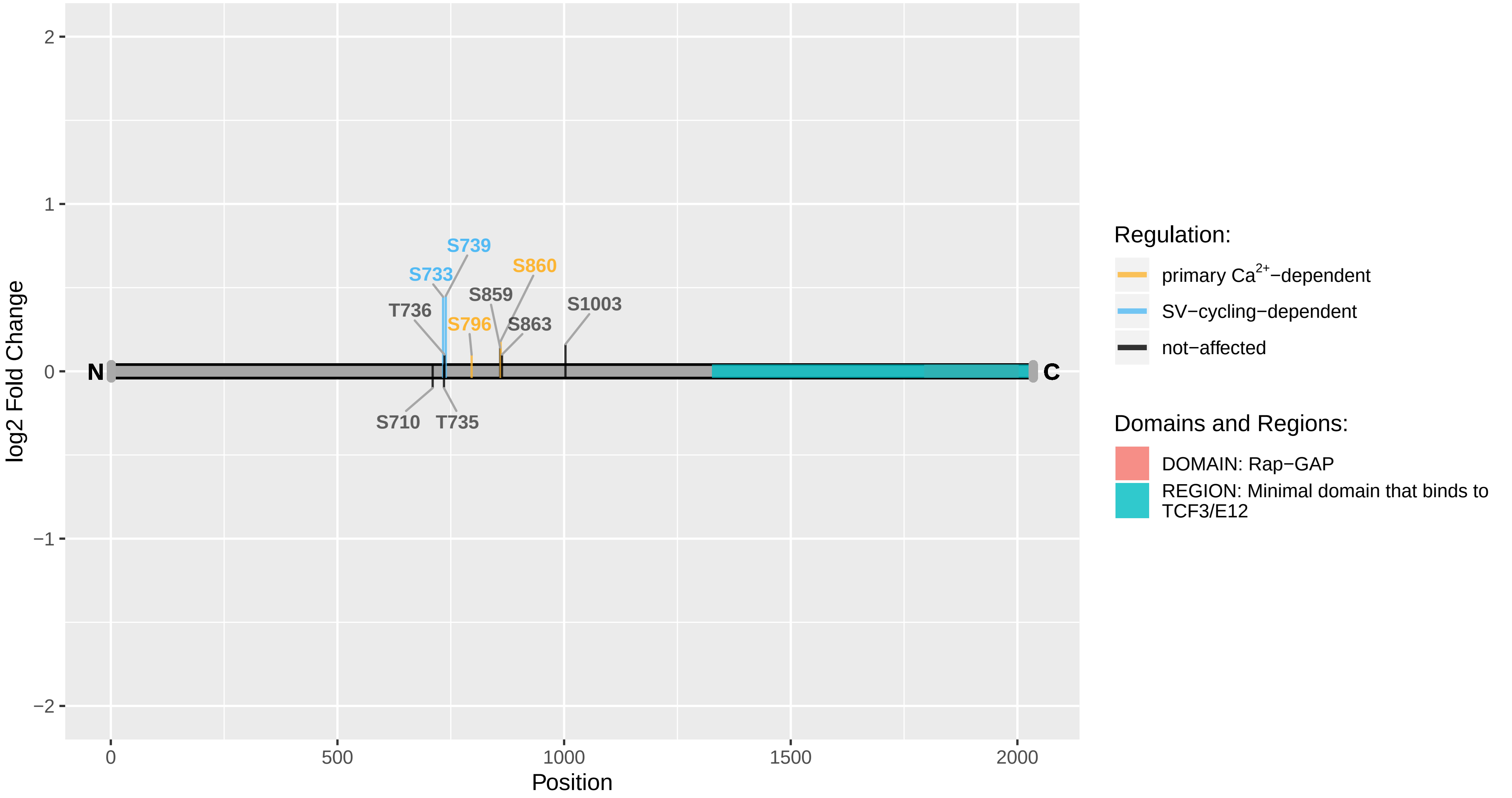

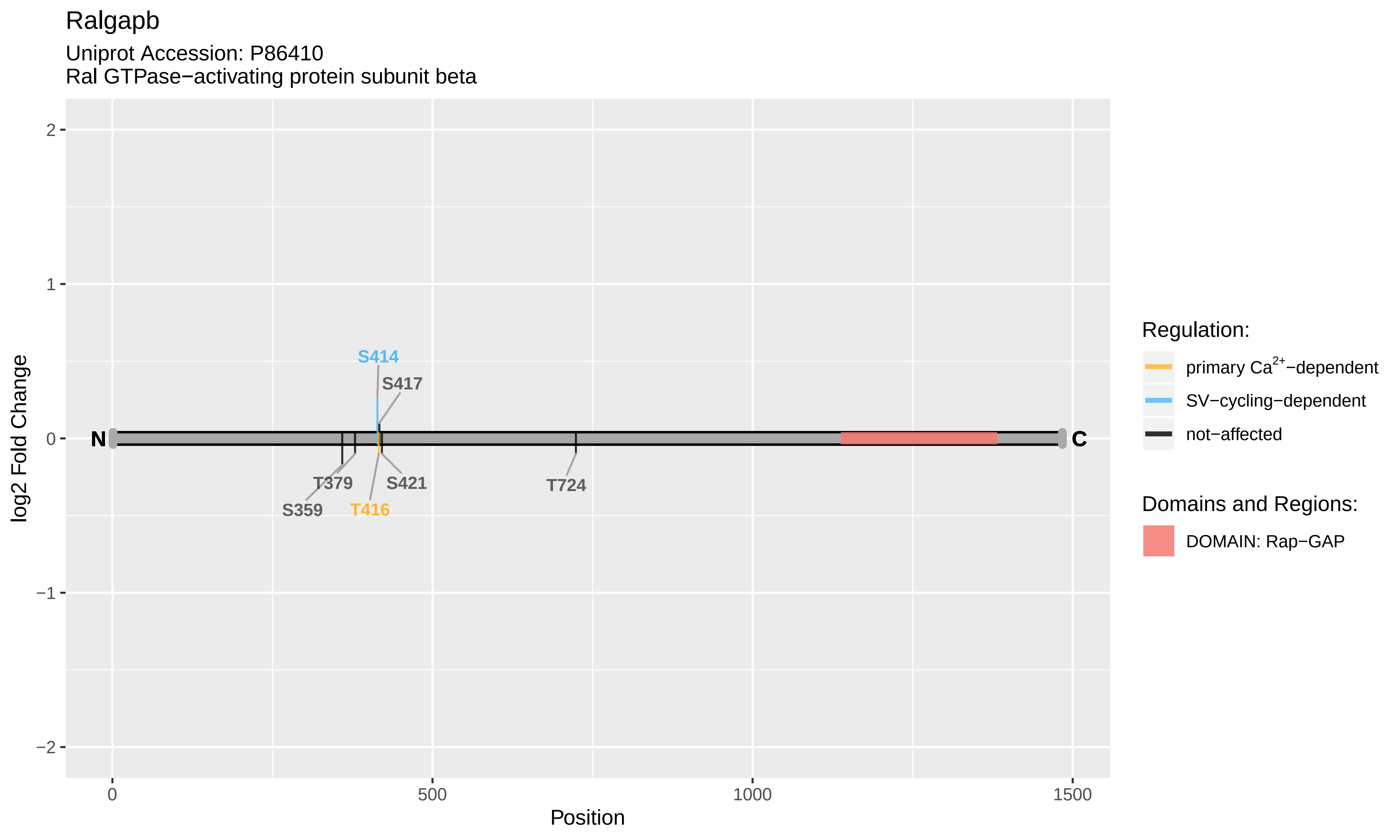

Ralgapb
Uniprot Accession: P86410
Ral GTPase−activating protein subunit beta
log2 Fold Change
2
1
0
−1
−2
N
C
S414
S417
T379
S421
S359
T416
T724
0
500
1000
1500
Position
Regulation:
primary Ca2+−dependent
SV−cycling−dependent
not−affected
Domains and Regions:
DOMAIN: Rap−GAP

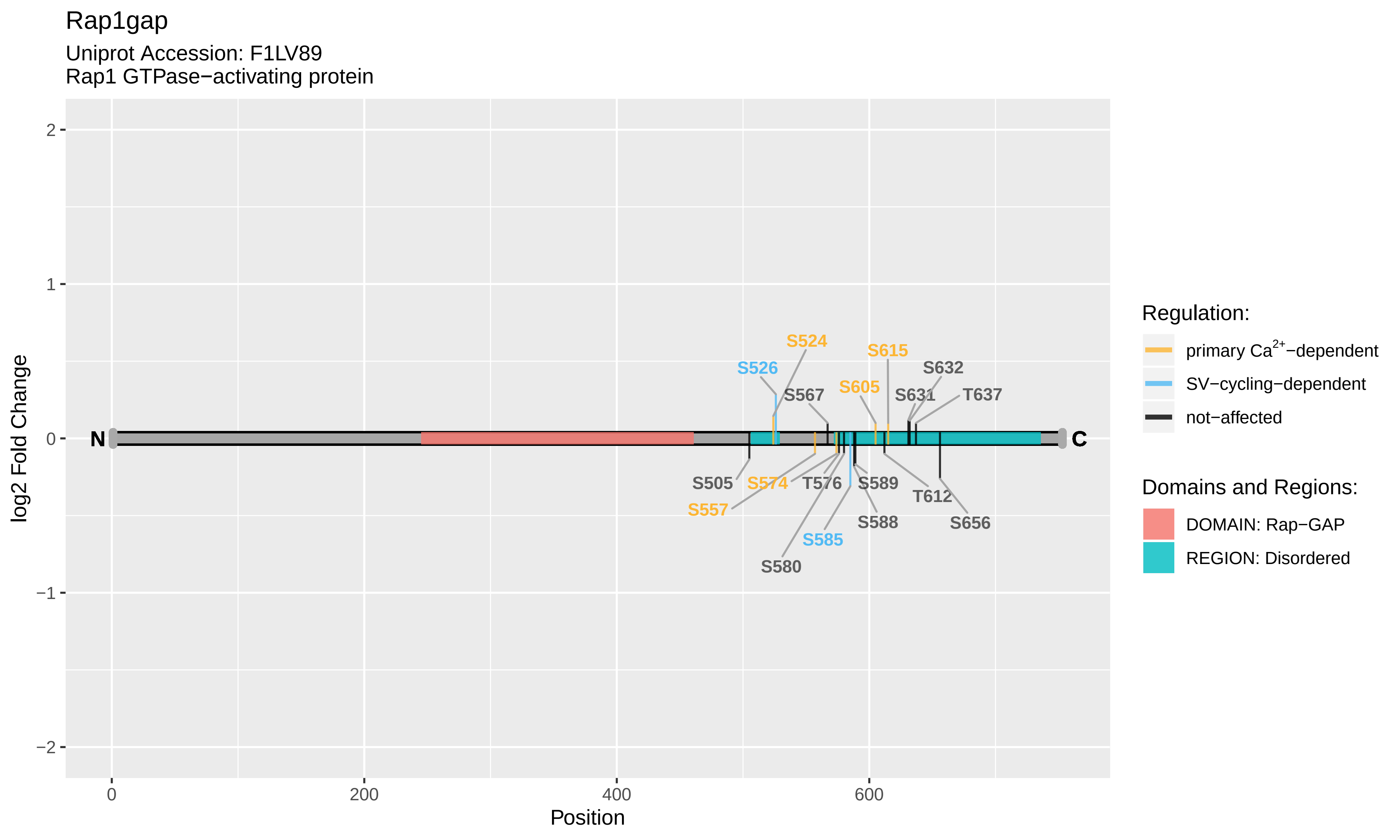
Rap1gap
Uniprot Accession: F1LV89
Rap1 GTPase−activating protein
log2 Fold Change
Position
N
C
S524
S526
S615
S632
S567
S605
S631
T637
S505
S574
T576
S589
T612
S557
S588
S656
S585
S580
Regulation:
primary Ca2+−dependent
SV−cycling−dependent
not−affected
Domains and Regions:
DOMAIN: Rap−GAP
REGION: Disordered

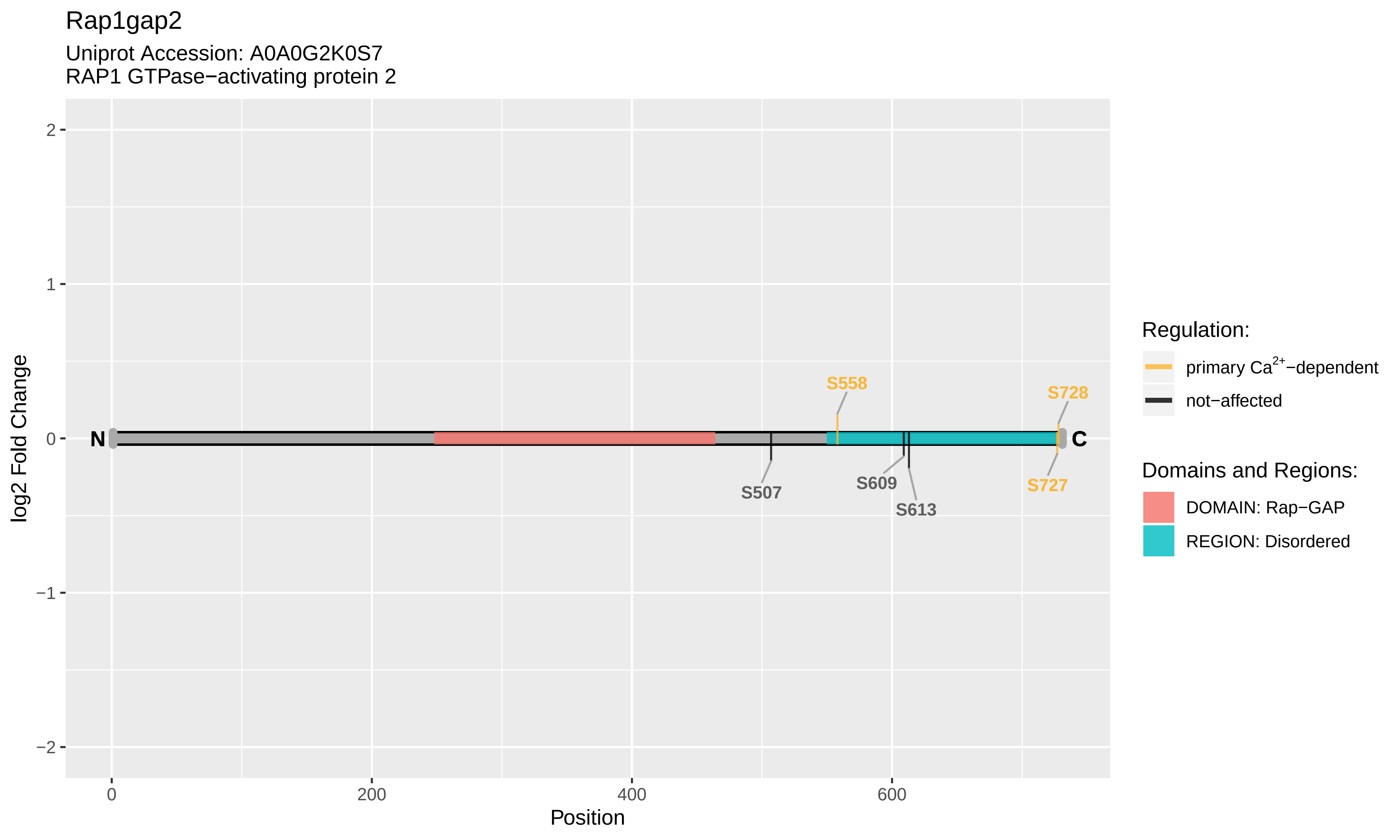

Rap1gap2
Uniprot Accession: A0A0G2K0S7
RAP1 GTPase−activating protein 2
log2 Fold Change
2
1
0
−1
−2
N
C
S558
S728
S507
S609
S613
S727
0
200
400
600
Position
Regulation:
primary Ca2+−dependent
not−affected
Domains and Regions:
DOMAIN: Rap−GAP
REGION: Disordered

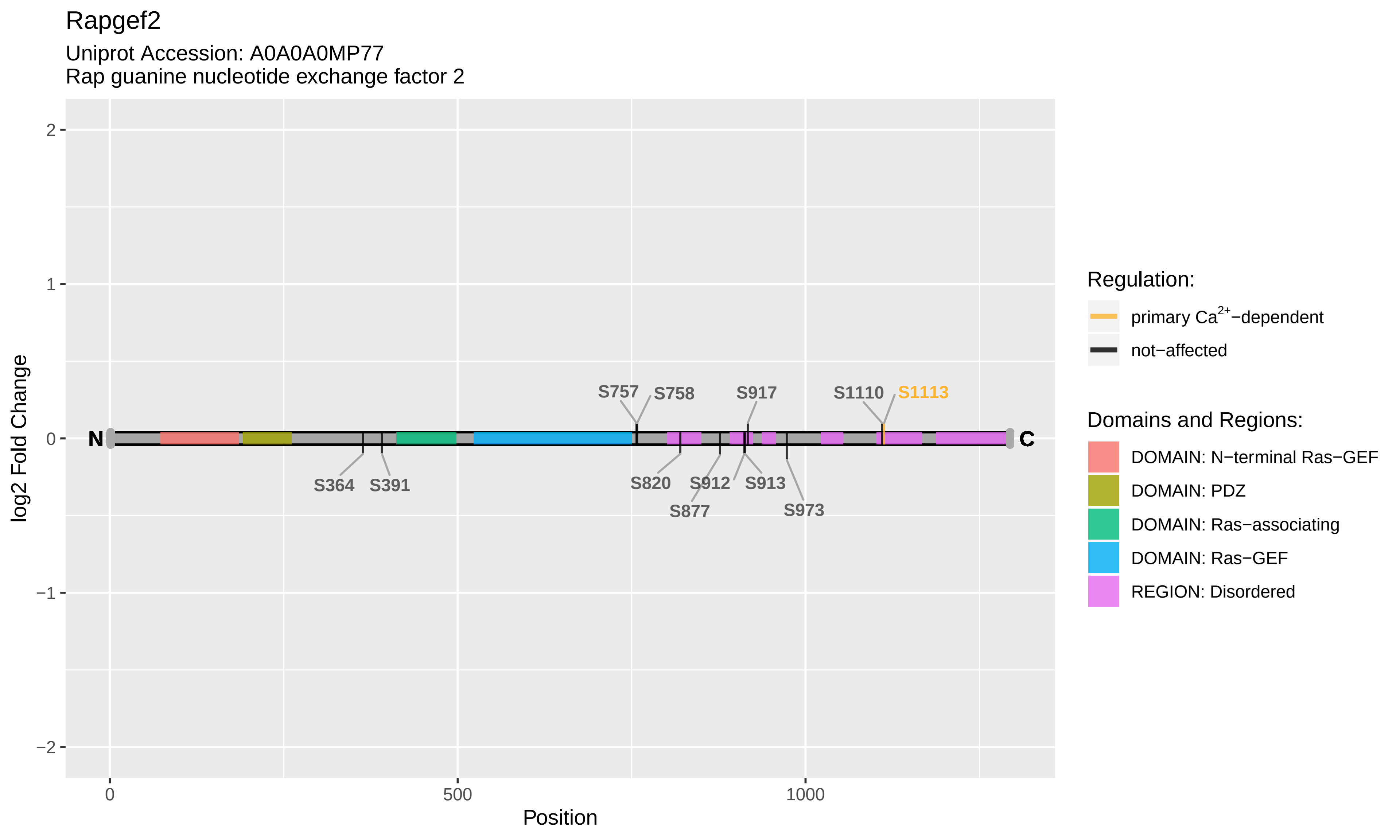

Rapgef2
Uniprot Accession: A0A0A0MP77
Rap guanine nucleotide exchange factor 2
log2 Fold Change
2
1
0
−1
−2
Position
0
500
1000
N
C
S757
S758
S917
S1110
S1113
S364
S391
S820
S912
S913
S877
S973
Regulation:
primary Ca2+−dependent
not−affected
Domains and Regions:
DOMAIN: N−terminal Ras−GEF
DOMAIN: PDZ
DOMAIN: Ras−associating
DOMAIN: Ras−GEF
REGION: Disordered

Rasal2

Uniprot Accession: A0A0G2JTA7
RAS protein activator−like 2

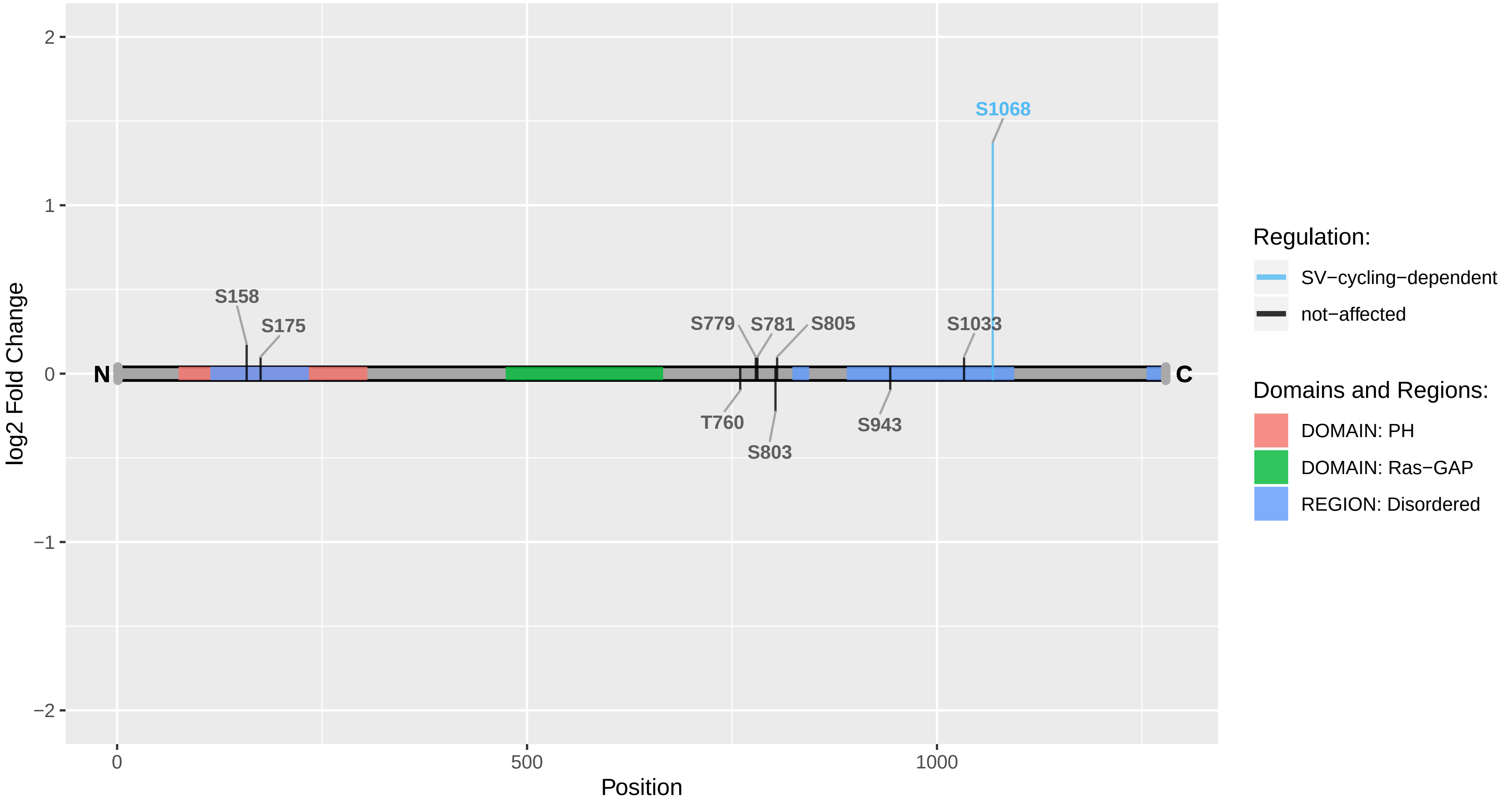

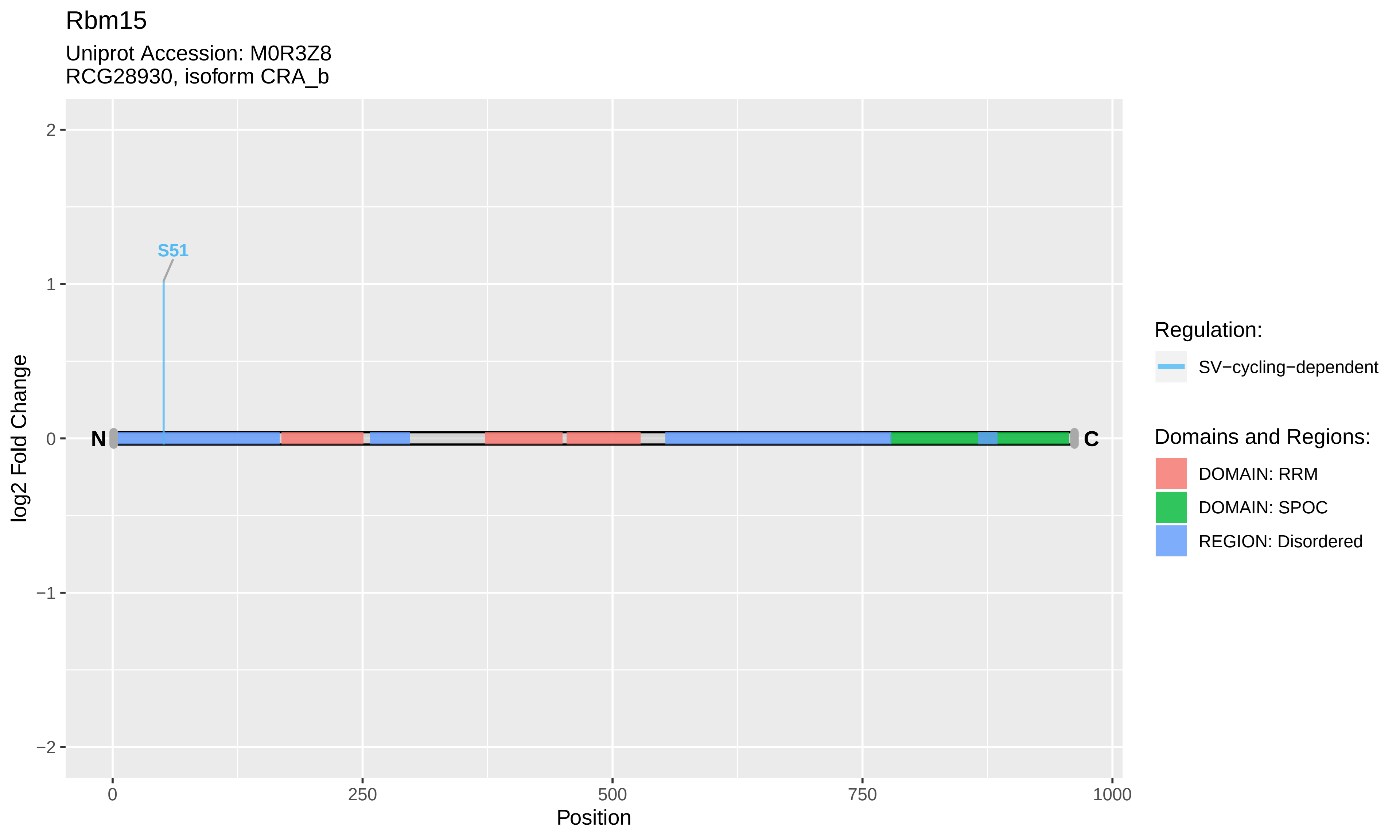

Rbm15
Uniprot Accession: M0R3Z8
RCG28930, isoform CRA_b
S51
N
C
2
1
0
−1
−2
log2 Fold Change
0
250
500
750
1000
Position
Regulation:
SV−cycling−dependent
Domains and Regions:
DOMAIN: RRM
DOMAIN: SPOC
REGION: Disordered

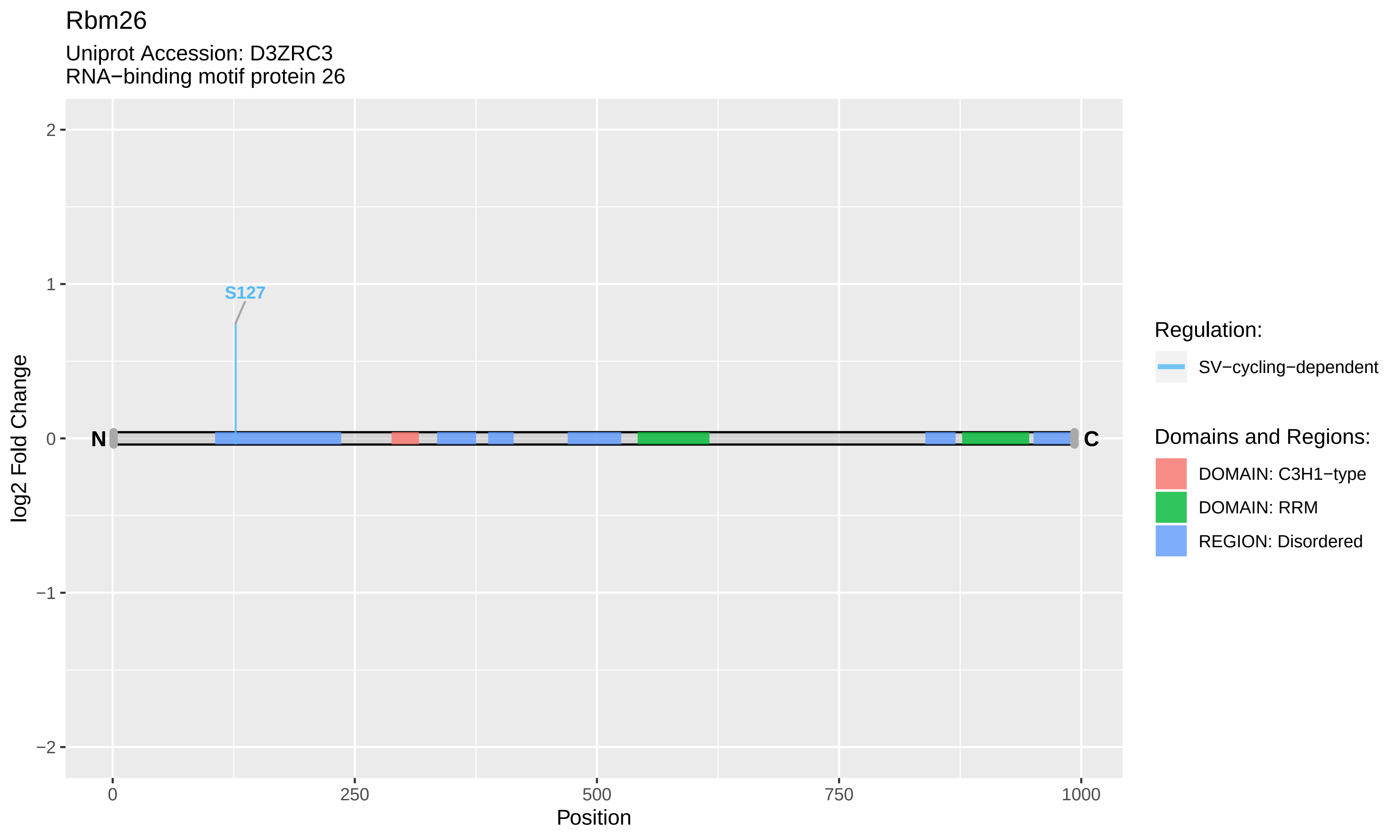

Rbm26
Uniprot Accession: D3ZRC3
RNA−binding motif protein 26
S127
N
C
log2 Fold Change
2
1
0
−1
−2
0
250
500
750
1000
Position
Regulation:
SV−cycling−dependent
Domains and Regions:
DOMAIN: C3H1−type
DOMAIN: RRM
REGION: Disordered

Rbm27

Uniprot Accession: F1M1R4
RNA−binding motif protein 27

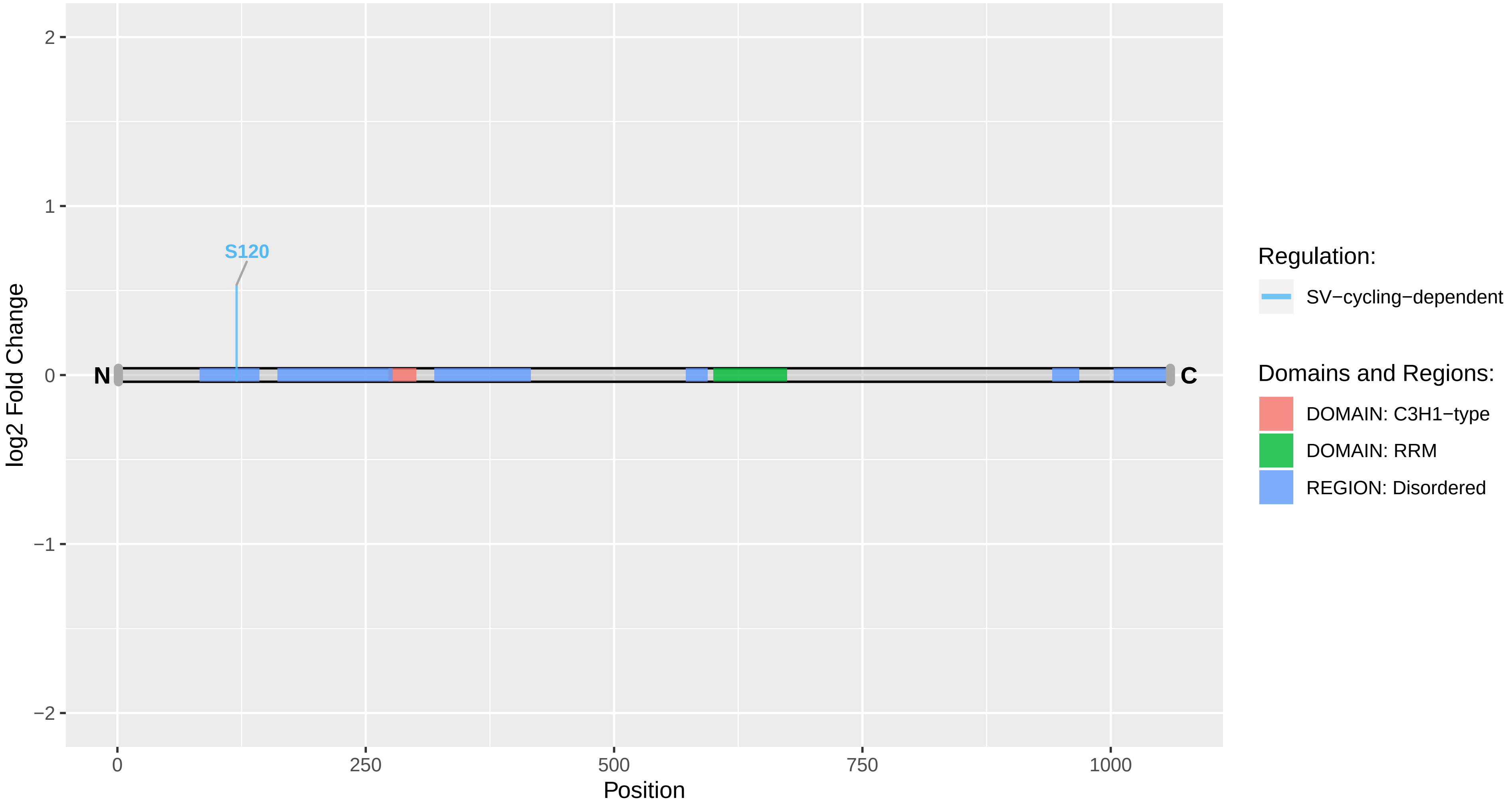

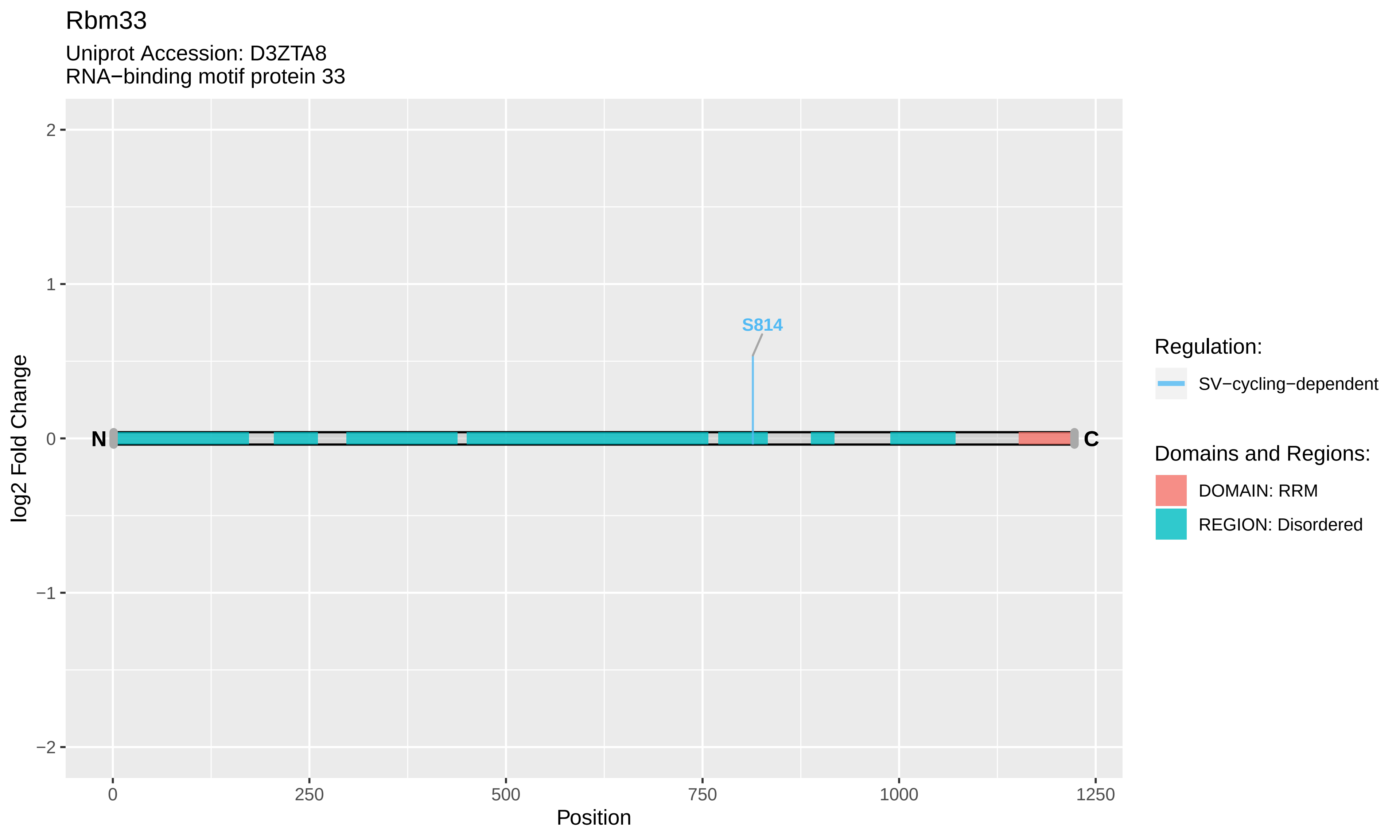

Rbm33
Uniprot Accession: D3ZTA8
RNA−binding motif protein 33
S814
N
C
2
1
0
−1
−2
0
250
500
750
1000
1250
Position
log2 Fold Change
Regulation:
SV−cycling−dependent
Domains and Regions:
DOMAIN: RRM
REGION: Disordered

Rbmxrtl

Uniprot Accession: P84586
RNA−binding motif protein, X chromosome retrogene−like

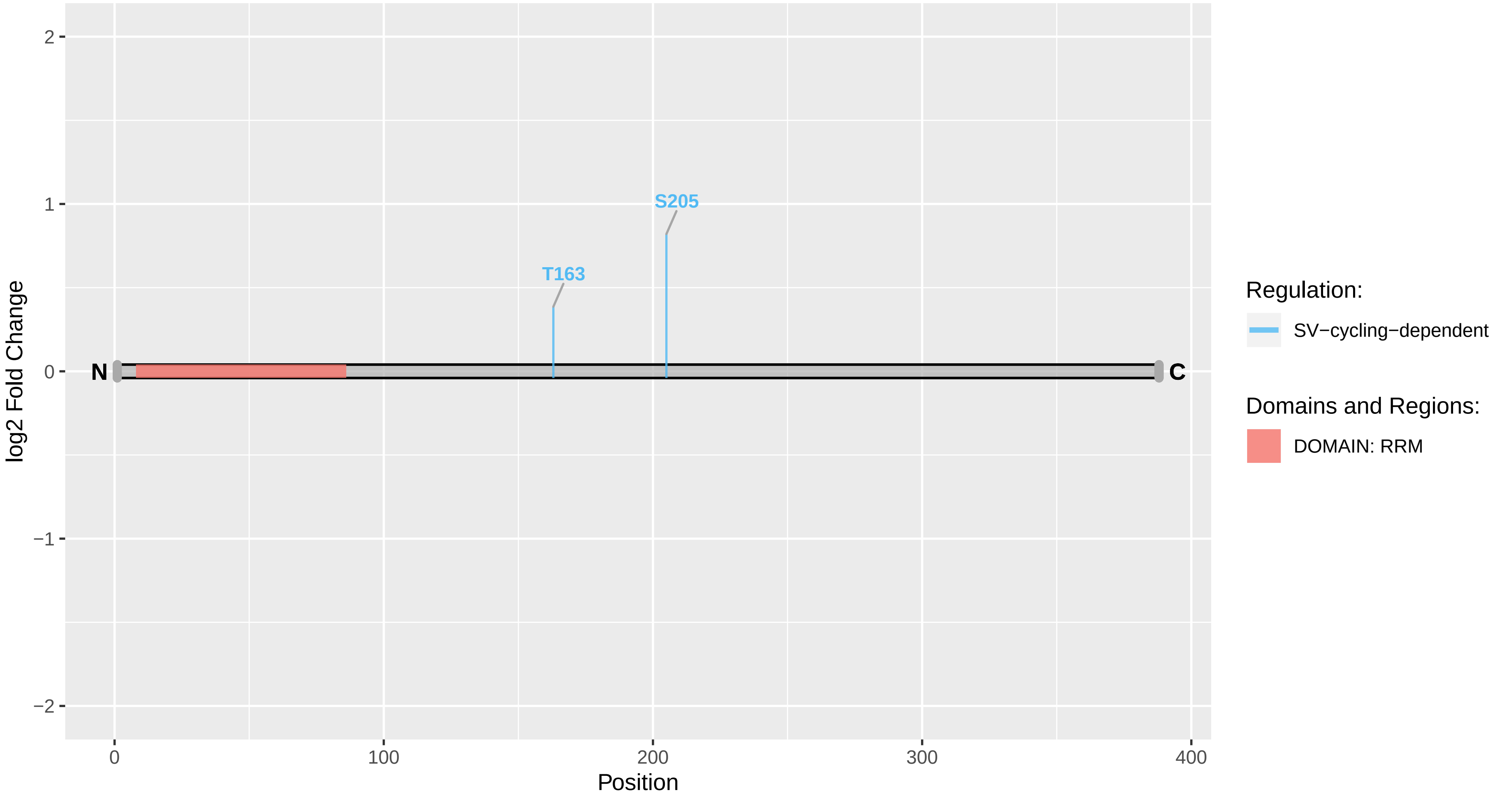

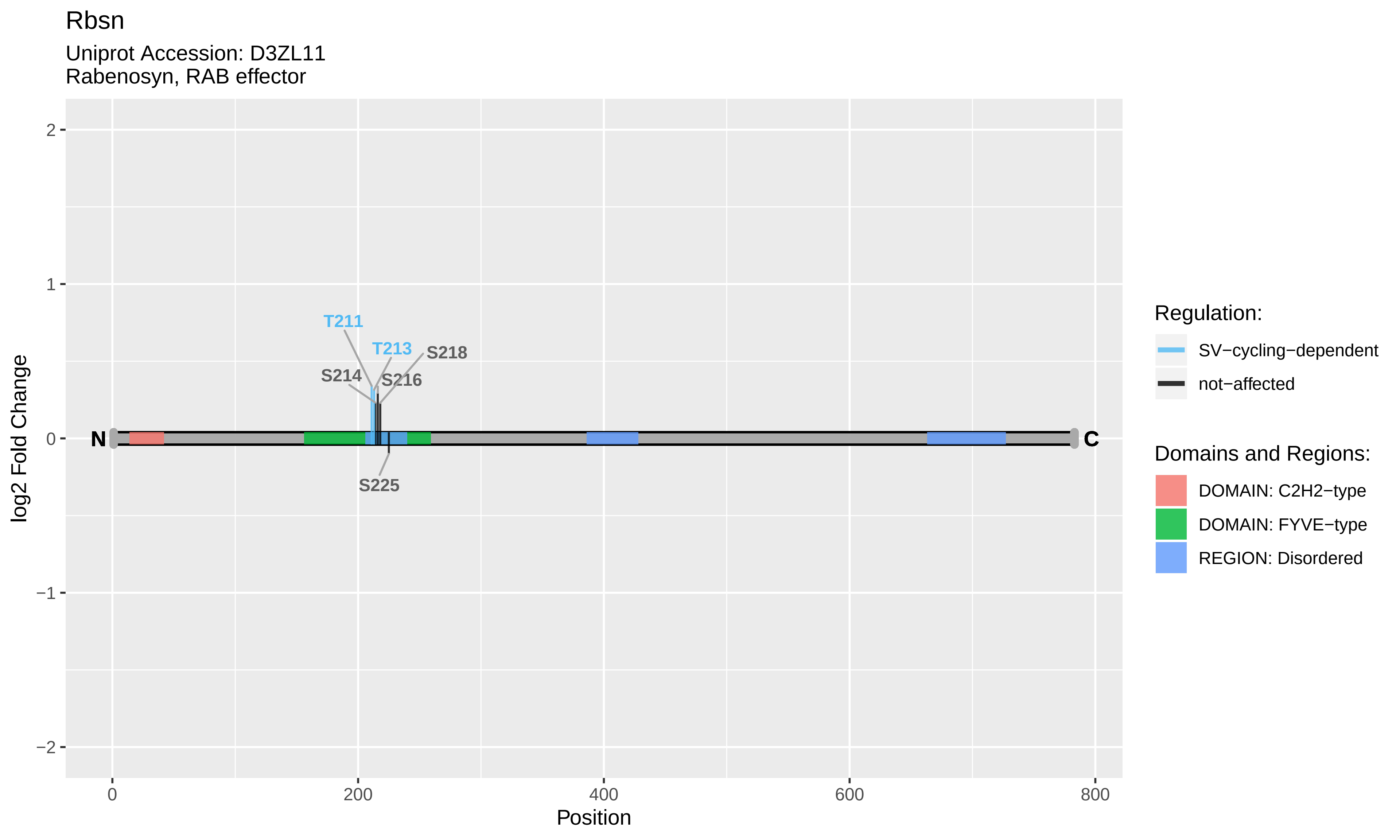

Rbsn
Uniprot Accession: D3ZL11
Rabenosyn, RAB effector
T211
T213
S218
S214
S216
S225
N
C
log2 Fold Change
Position
Regulation:
SV−cycling−dependent
not−affected
Domains and Regions:
DOMAIN: C2H2−type
DOMAIN: FYVE−type
REGION: Disordered

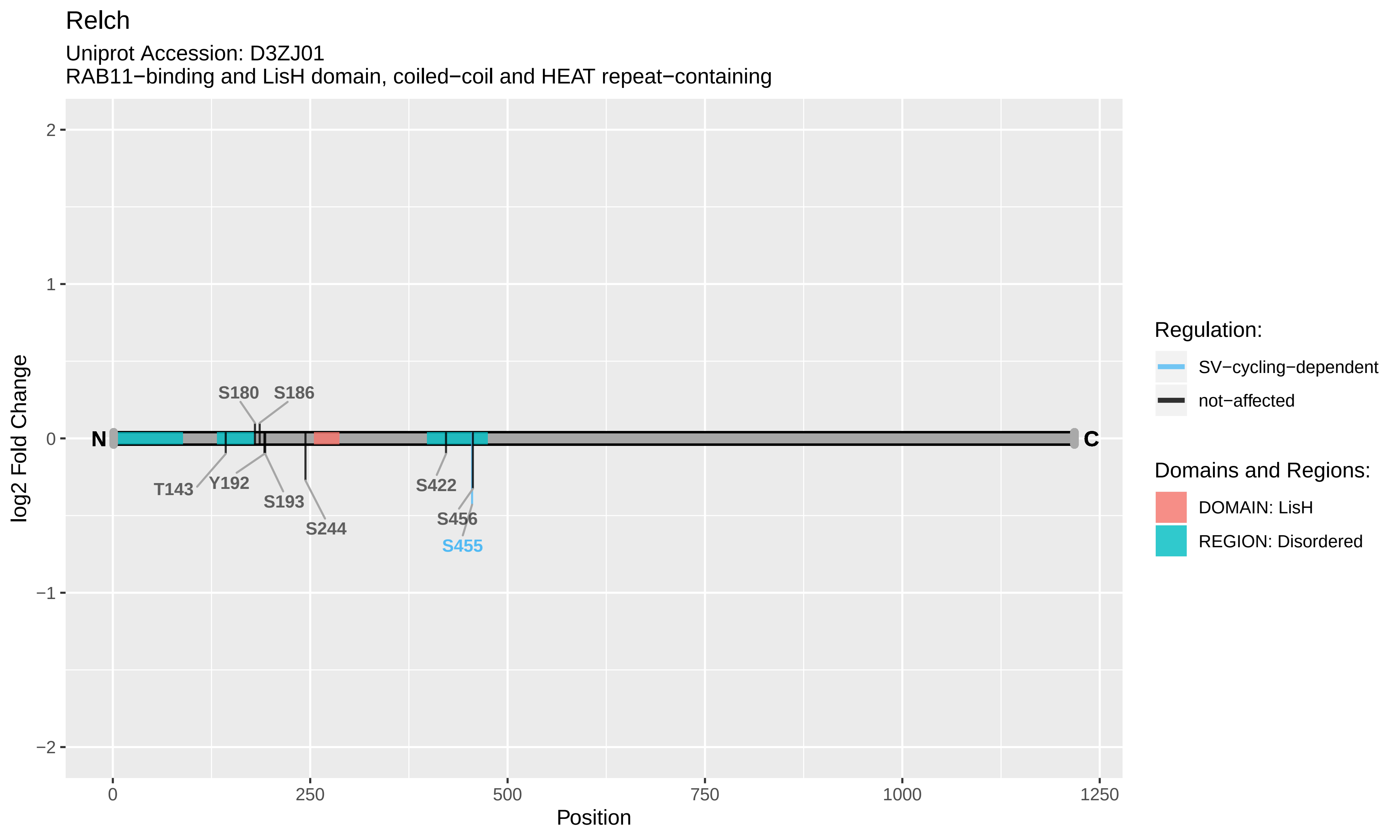
Relch
Uniprot Accession: D3ZJ01
RAB11−binding and LisH domain, coiled−coil and HEAT repeat−containing
log2 Fold Change
2
1
0
−1
−2
N
C
S180
S186
T143
Y192
S193
S244
S422
S456
S455
0
250
500
750
1000
1250
Position
Regulation:
SV−cycling−dependent
not−affected
Domains and Regions:
DOMAIN: LisH
REGION: Disordered

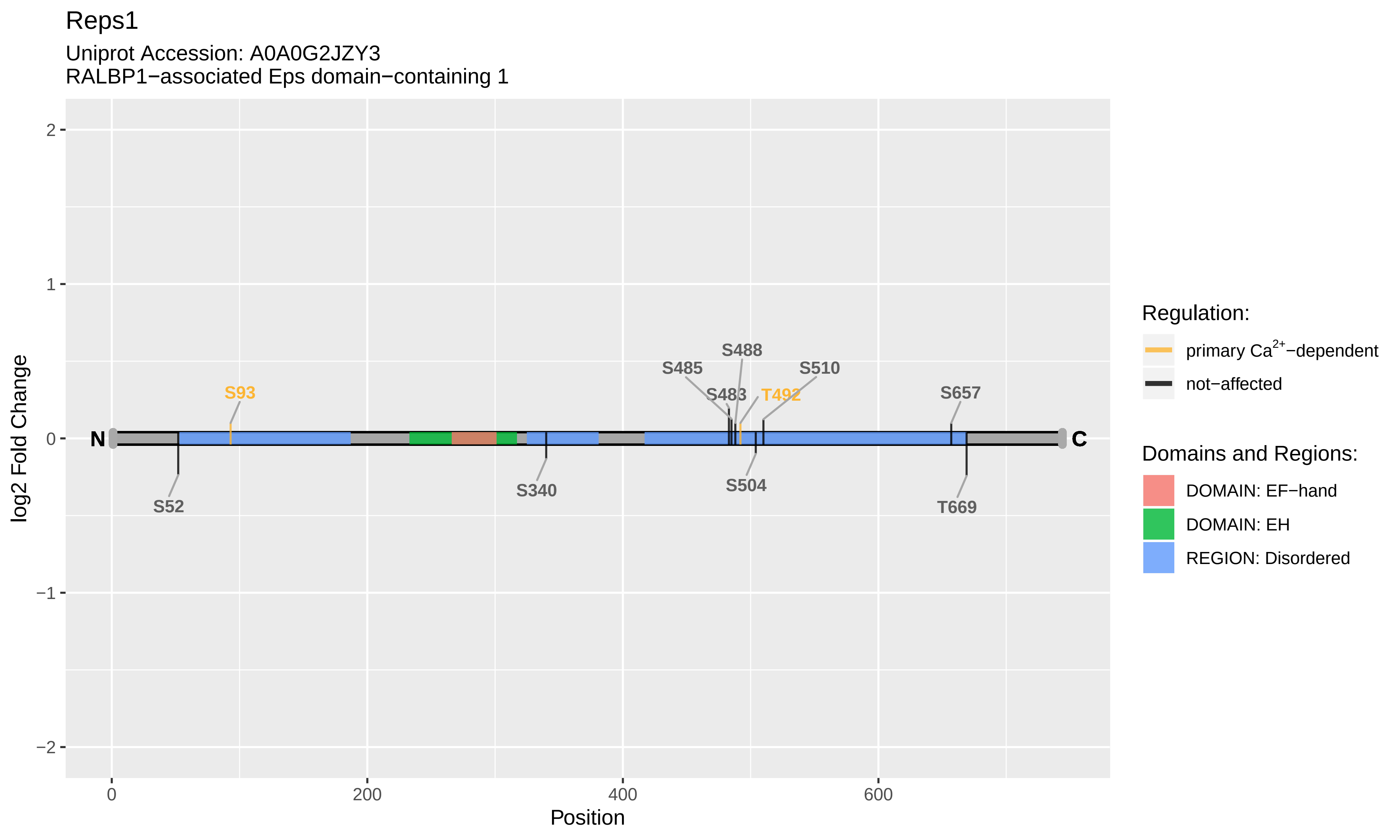

Reps1
Uniprot Accession: A0A0G2JZY3
RALBP1−associated Eps domain−containing 1
log2 Fold Change
2
1
0
−1
−2
Position
0
200
400
600
N
C
S93
S52
S340
S485
S488
S483
T492
S510
S504
S657
T669
Regulation:
primary Ca2+−dependent
not−affected
Domains and Regions:
DOMAIN: EF−hand
DOMAIN: EH
REGION: Disordered

Reps2

Uniprot Accession: A0A0G2K1L4
RALBP1 associated Eps domain containing protein 2

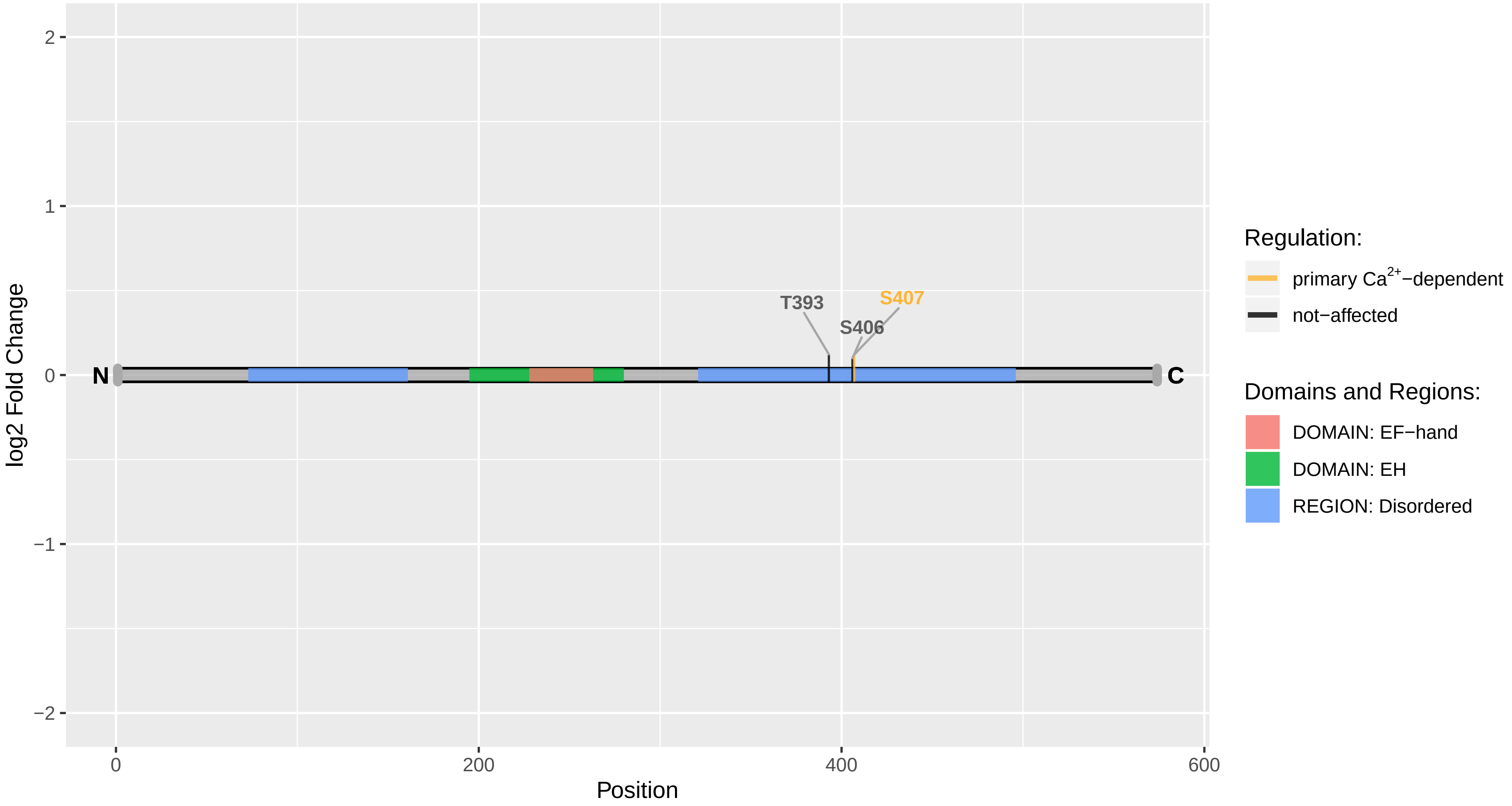

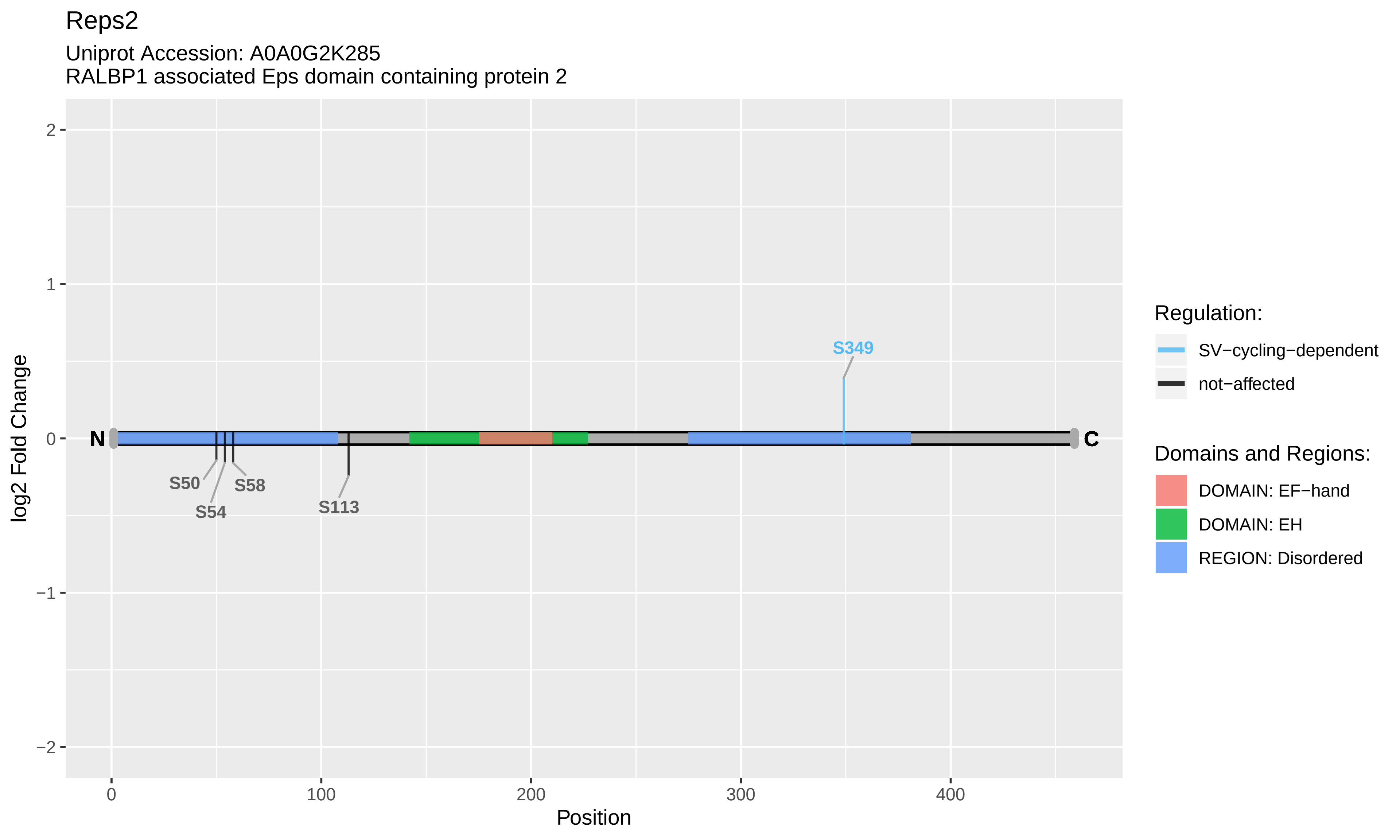
Reps2
Uniprot Accession: A0A0G2K285
RALBP1 associated Eps domain containing protein 2
log2 Fold Change
2
1
0
−1
−2
N
C
S50
S54
S58
S113
S349
0
100
200
300
400
Position
Regulation:
SV−cycling−dependent
not−affected
Domains and Regions:
DOMAIN: EF−hand
DOMAIN: EH
REGION: Disordered

Rgs12

Uniprot Accession: A0A0G2K326
Regulator of G−protein signaling 12, isoform CRA_b

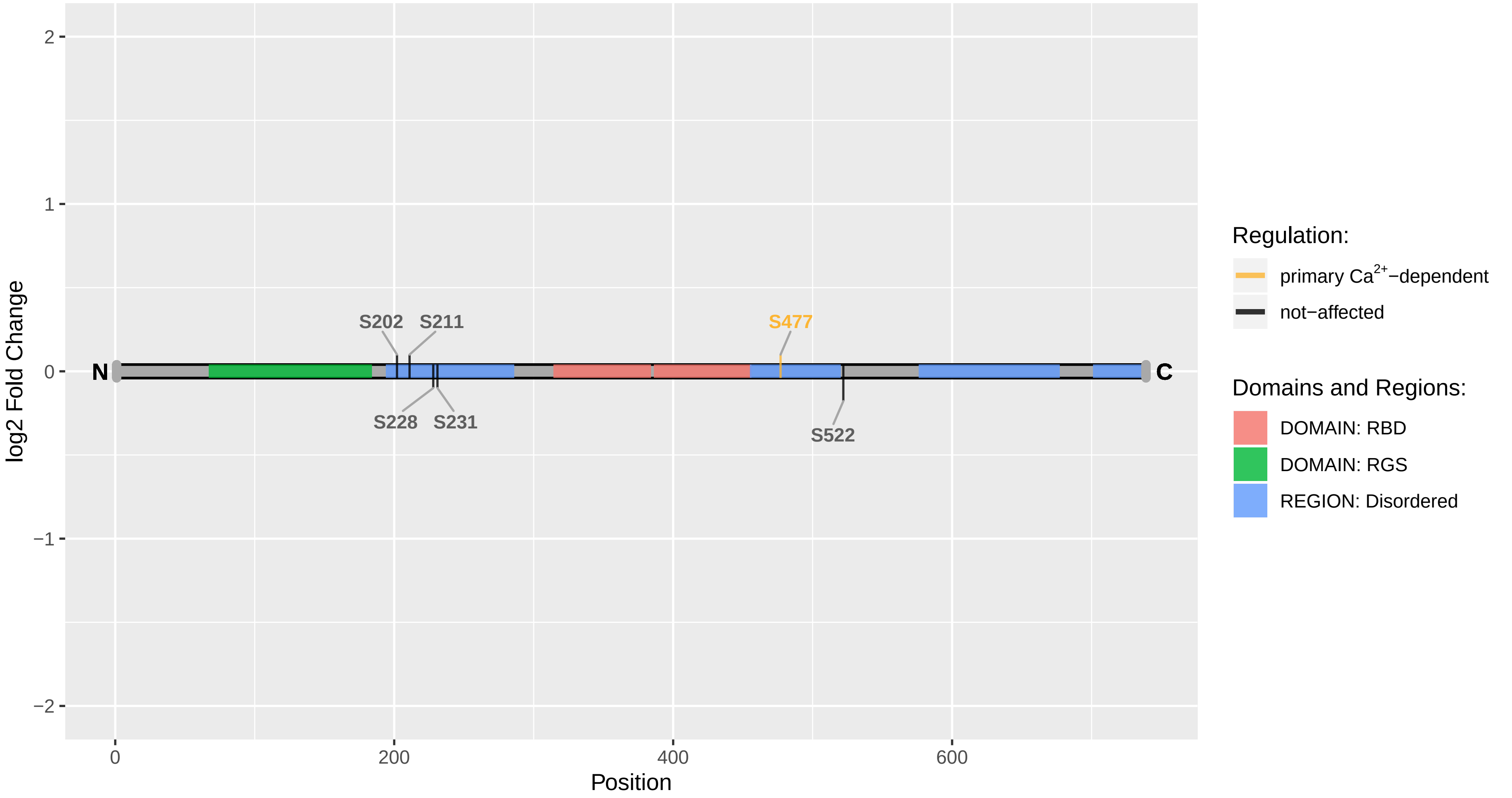

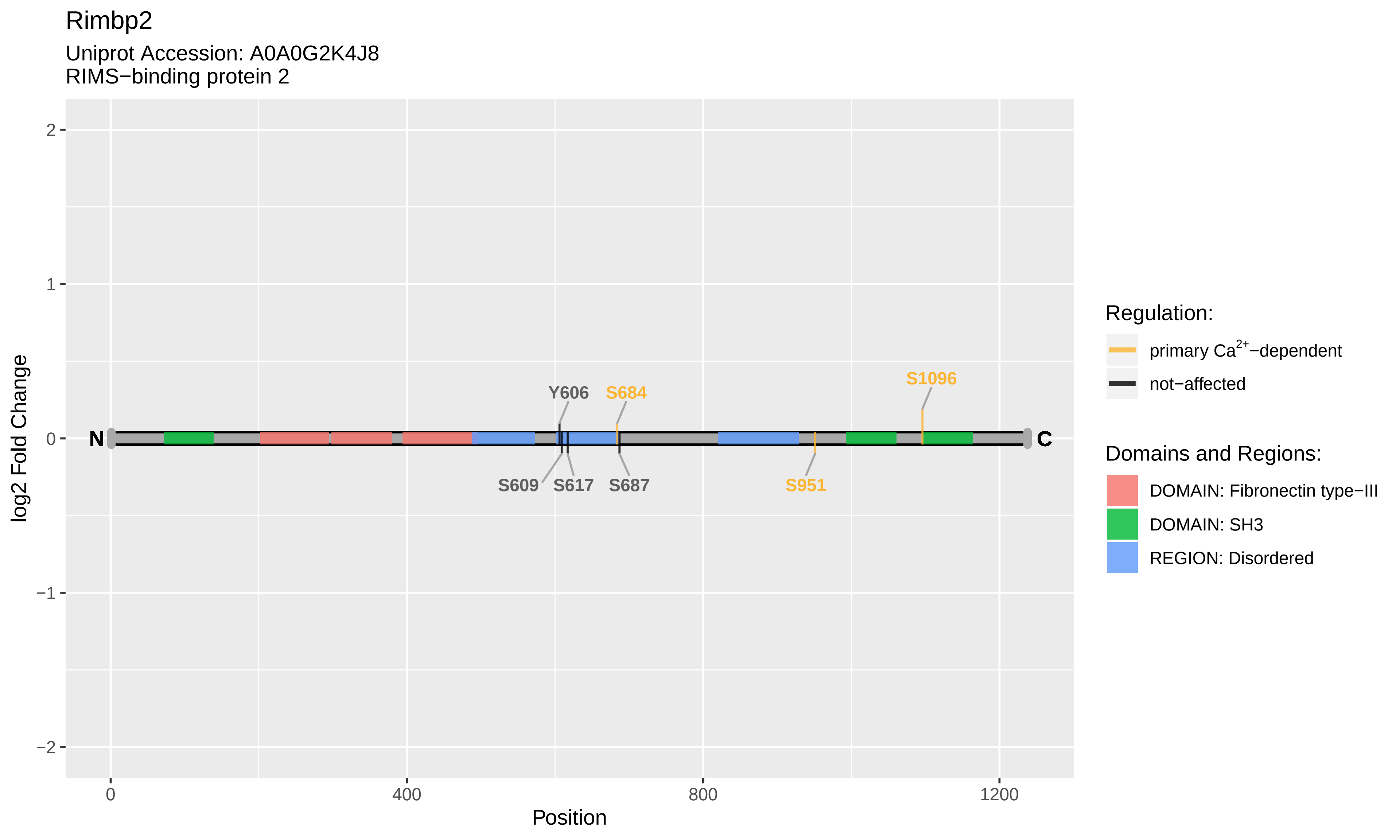

Rimbp2
Uniprot Accession: A0A0G2K4J8
RIMS−binding protein 2
log2 Fold Change
Position
N
C
Y606
S684
S1096
S609
S617
S687
S951
Regulation:
primary Ca2+−dependent
not−affected
Domains and Regions:
DOMAIN: Fibronectin type−III
DOMAIN: SH3
REGION: Disordered

Rims1

Uniprot Accession: A0A0G2JT77
Regulating synaptic membrane exocytosis protein 1

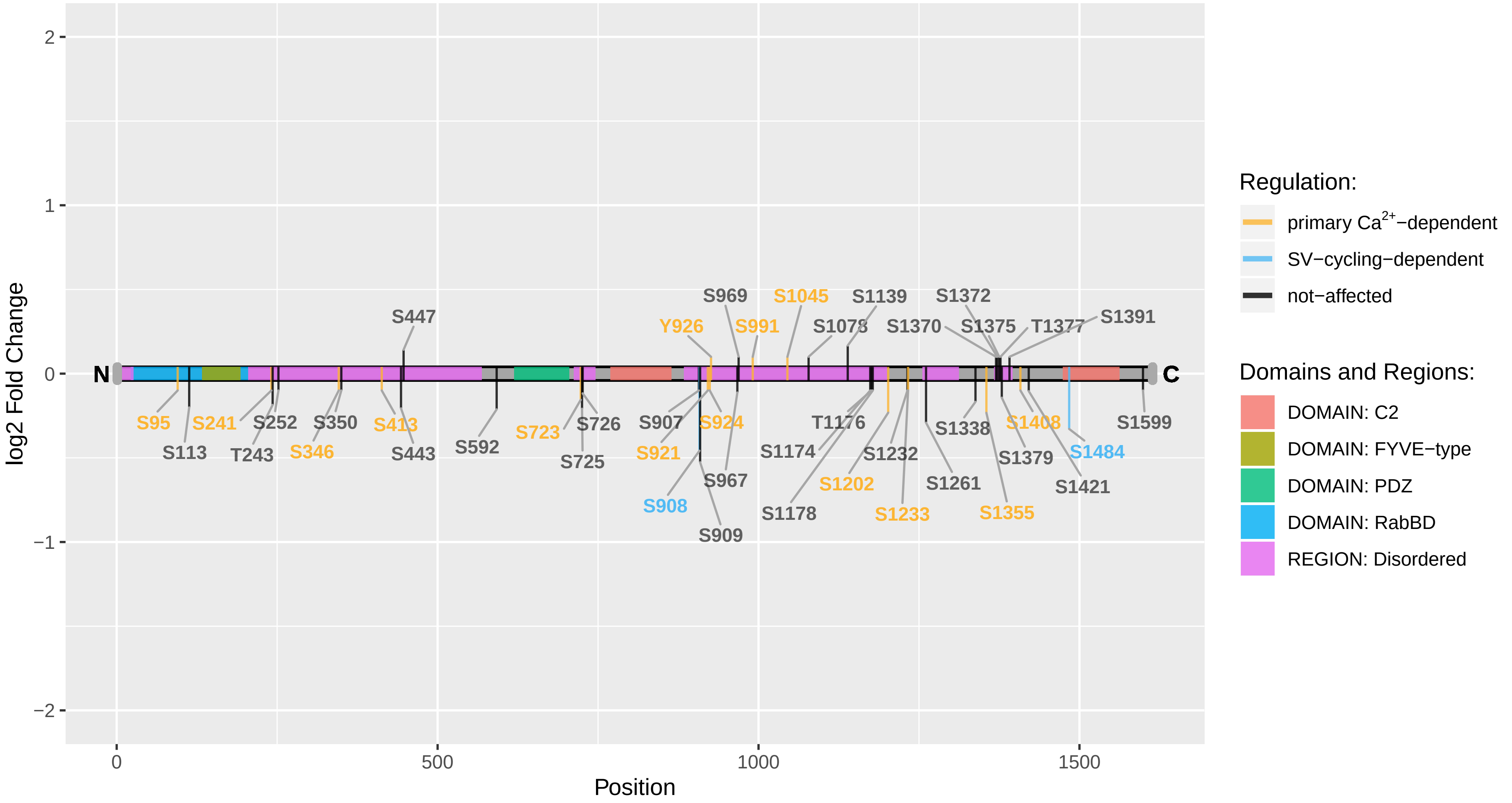

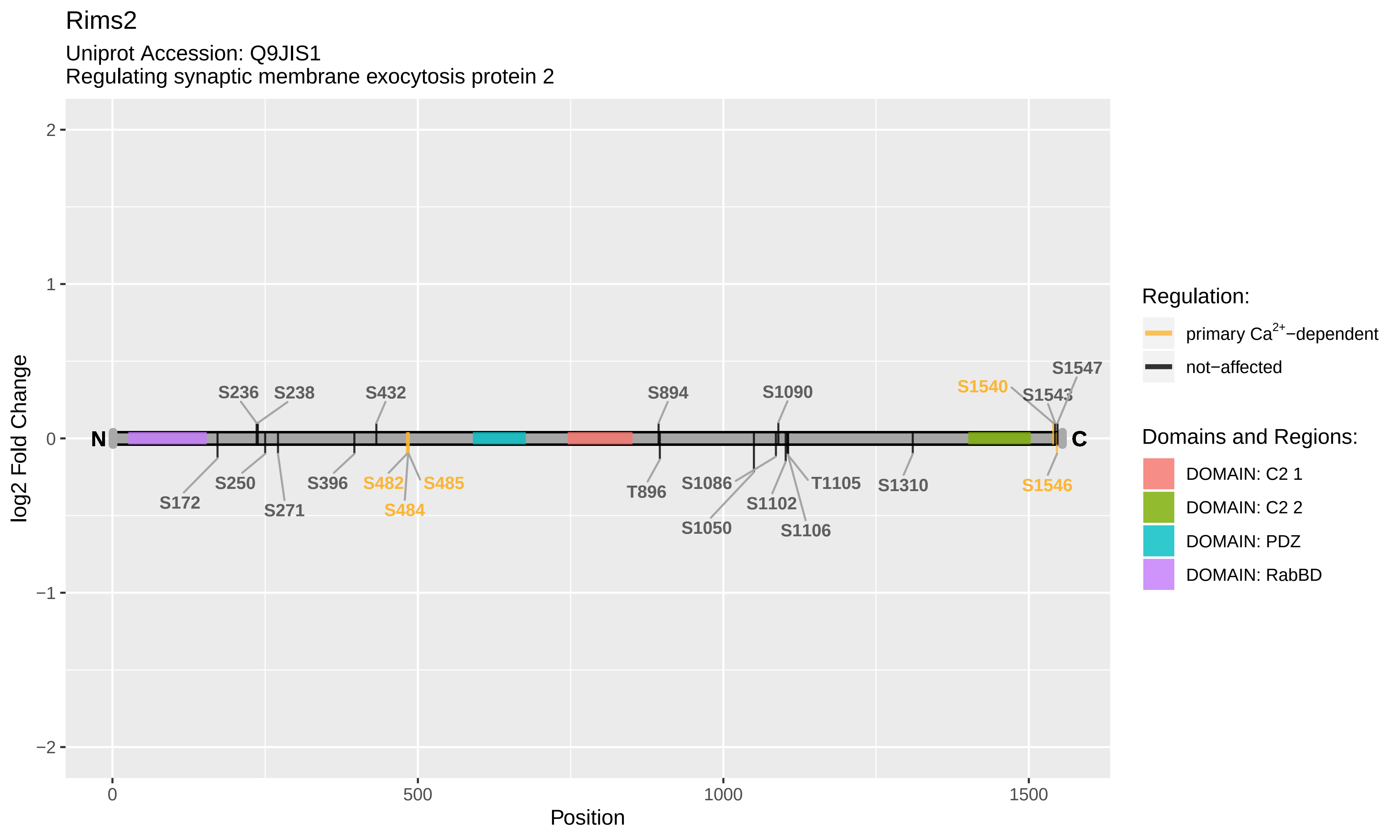
Rims2
Uniprot Accession: Q9JIS1
Regulating synaptic membrane exocytosis protein 2
log2 Fold Change
Position
N
C
S236
S238
S432
S894
S1090
S1540
S1543
S1547
S172
S250
S271
S396
S482
S484
S485
T896
S1086
S1050
S1102
T1105
S1106
S1310
S1546
Regulation:
primary Ca2+−dependent
not−affected
Domains and Regions:
DOMAIN: C2 1
DOMAIN: C2 2
DOMAIN: PDZ
DOMAIN: RabBD

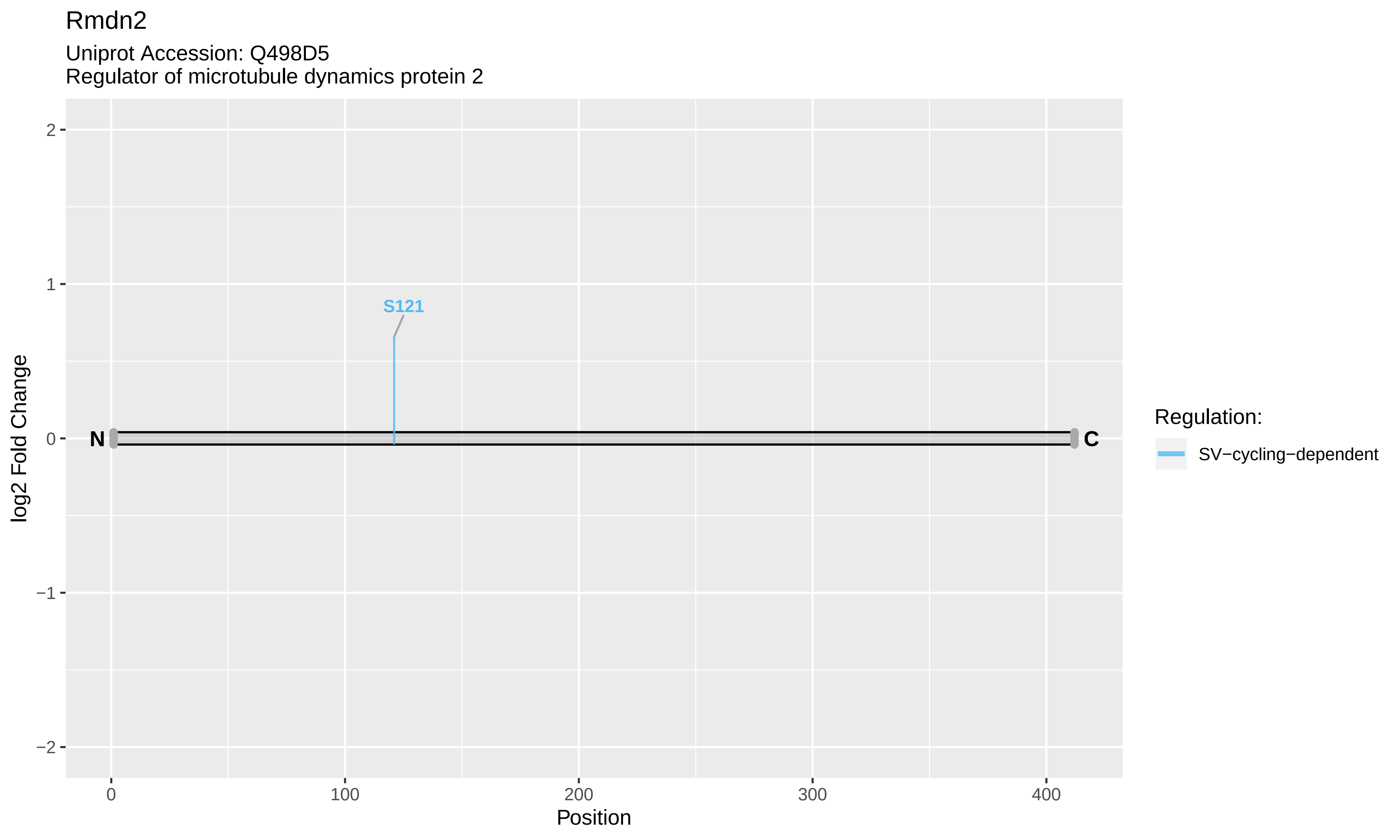

Rmdn2
Uniprot Accession: Q498D5
Regulator of microtubule dynamics protein 2
S121
N
C
2
1
0
−1
−2
log2 Fold Change
0
100
200
300
400
Position
Regulation:
SV−cycling−dependent

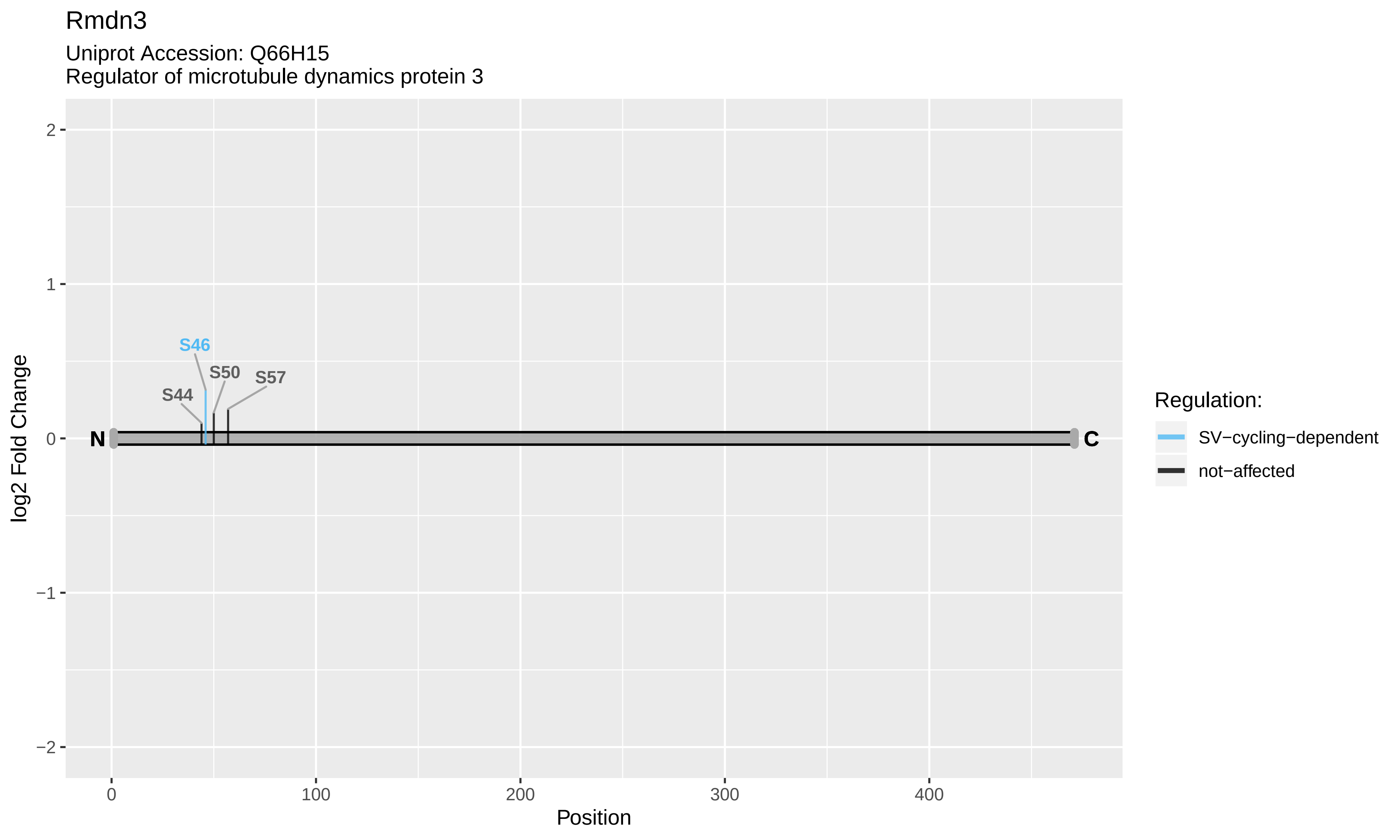
Rmdn3
Uniprot Accession: Q66H15
Regulator of microtubule dynamics protein 3
log2 Fold Change
Position
S46
S50
S57
S44
N
C
2
1
0
−1
−2
0
100
200
300
400
Regulation:
SV−cycling−dependent
not−affected

Rnf14

Uniprot Accession: Q3ZAU6
RBR−type E3 ubiquitin transferase

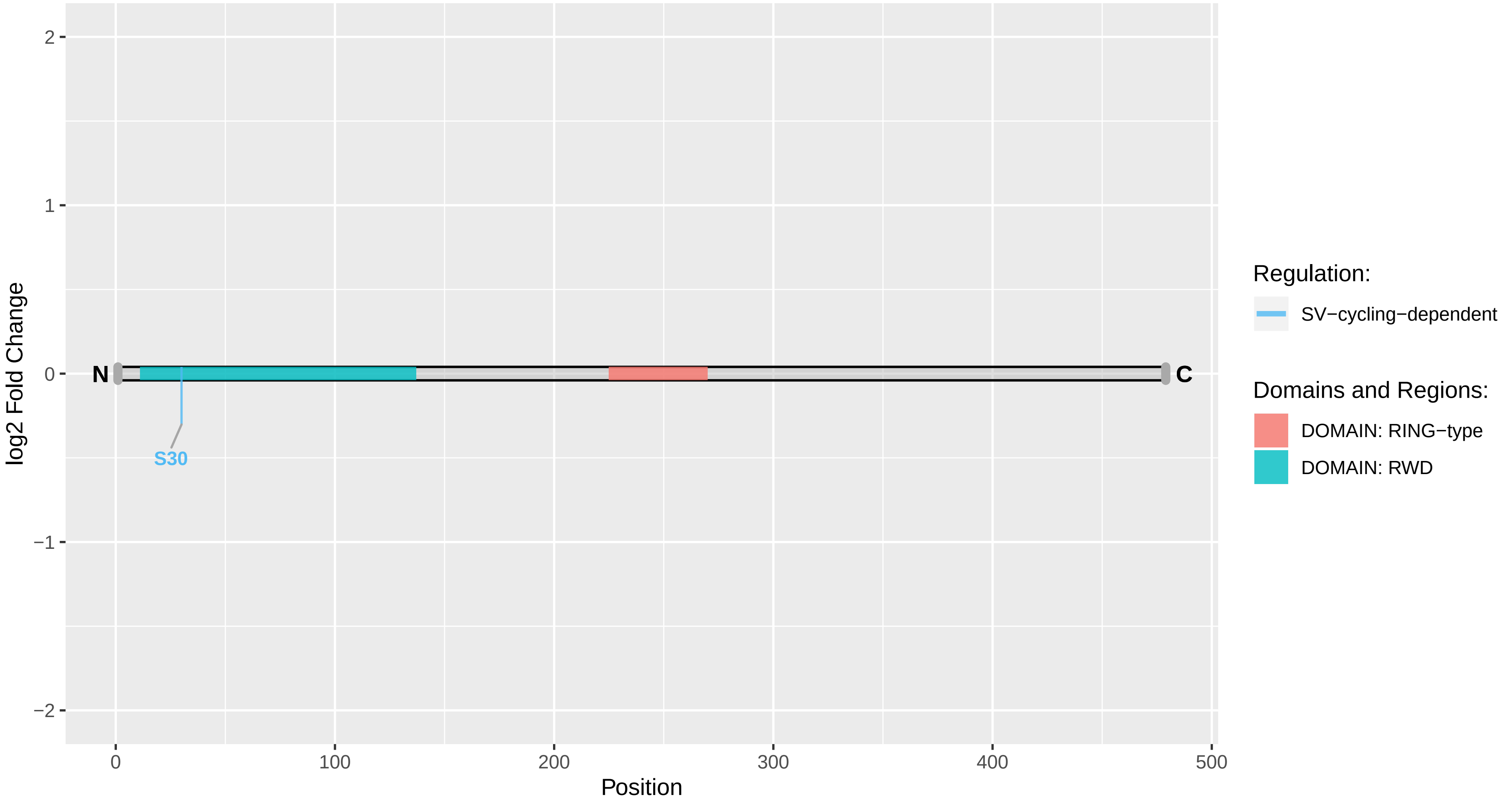

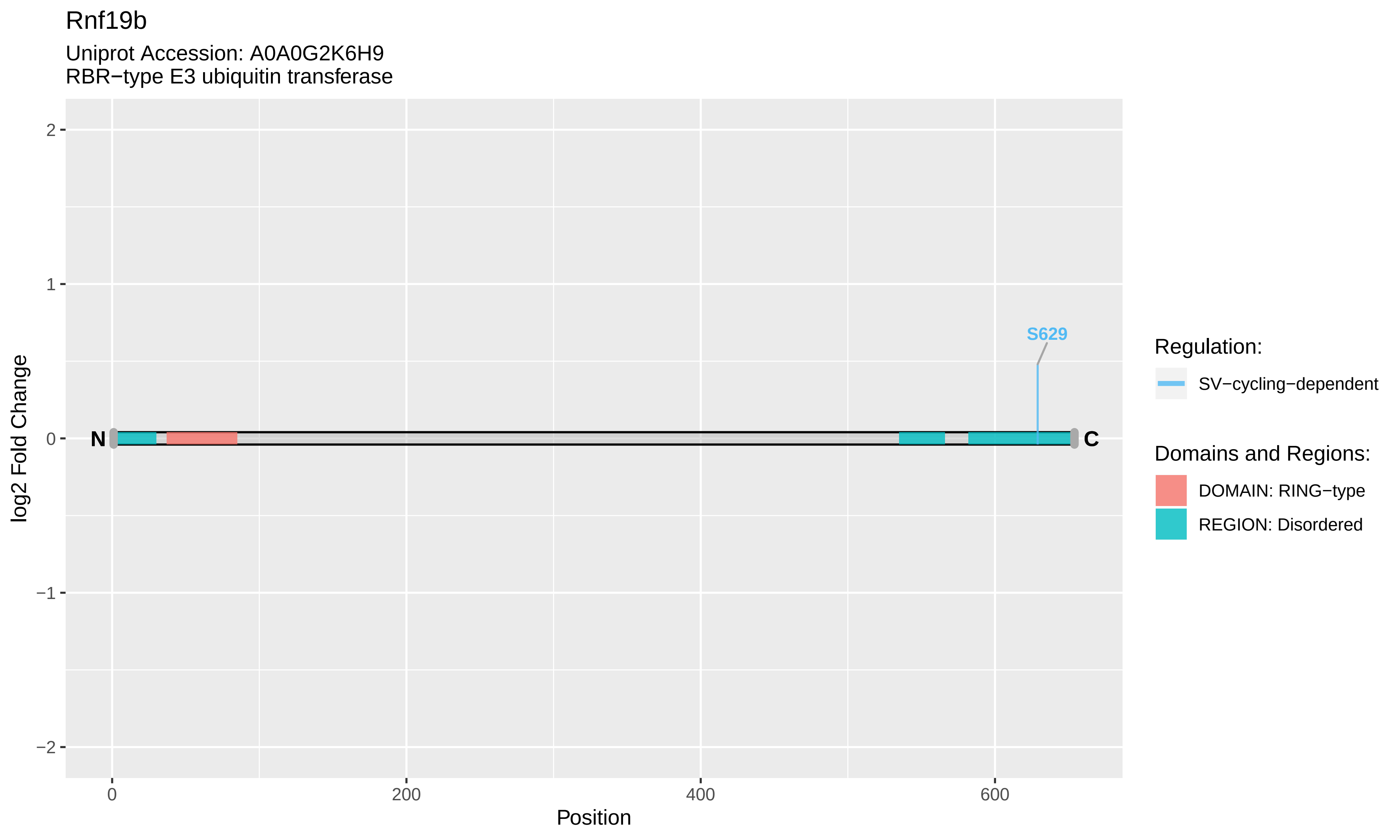

Rnf19b
Uniprot Accession: A0A0G2K6H9
RBR−type E3 ubiquitin transferase
S629
N
C
2
1
0
−1
−2
log2 Fold Change
0
200
400
600
Position
Regulation:
SV−cycling−dependent
Domains and Regions:
DOMAIN: RING−type
REGION: Disordered

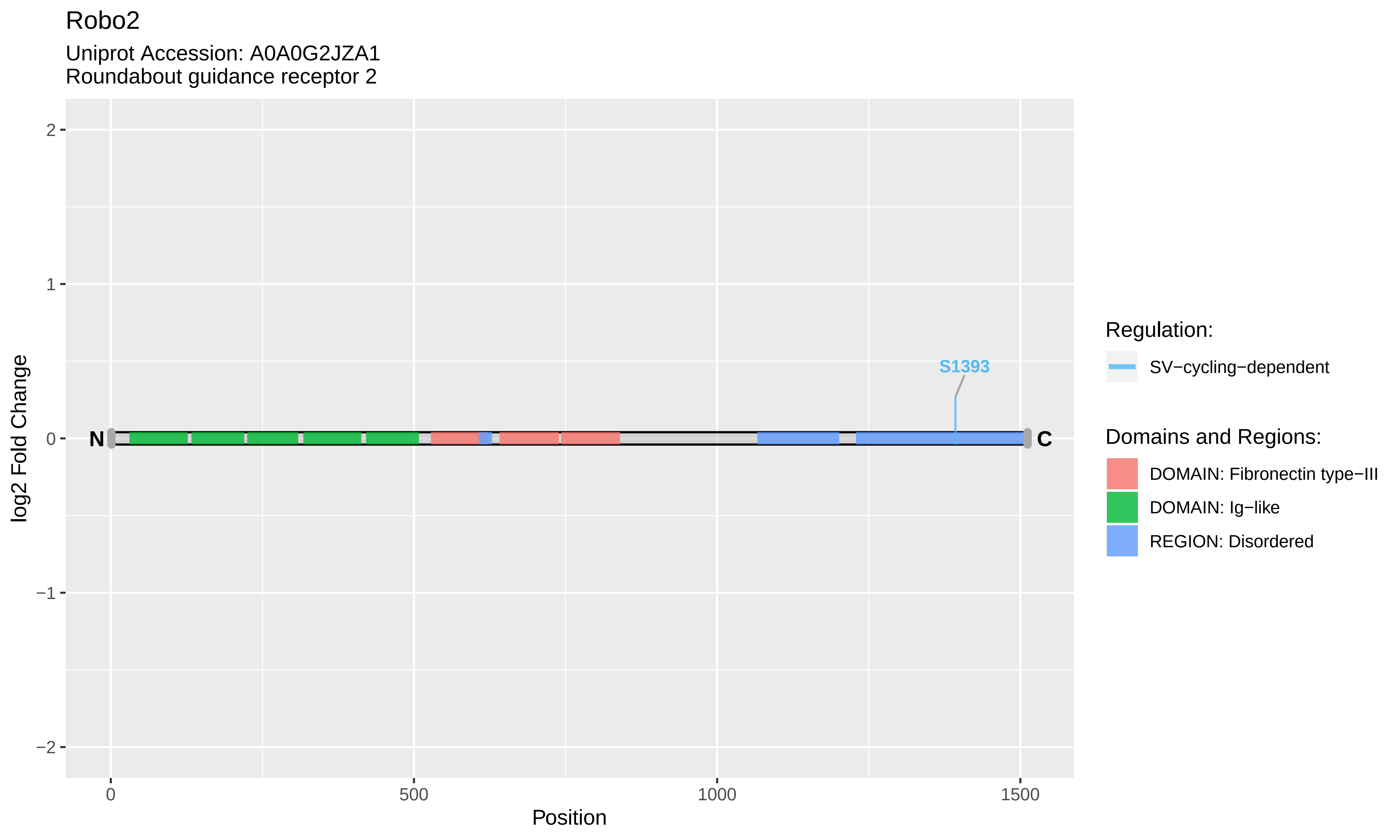

Robo2
Uniprot Accession: A0A0G2JZA1
Roundabout guidance receptor 2
log2 Fold Change
2
1
0
−1
−2
N
C
S1393
0
500
1000
1500
Position
Regulation:
SV−cycling−dependent
Domains and Regions:
DOMAIN: Fibronectin type−III
DOMAIN: Ig−like
REGION: Disordered

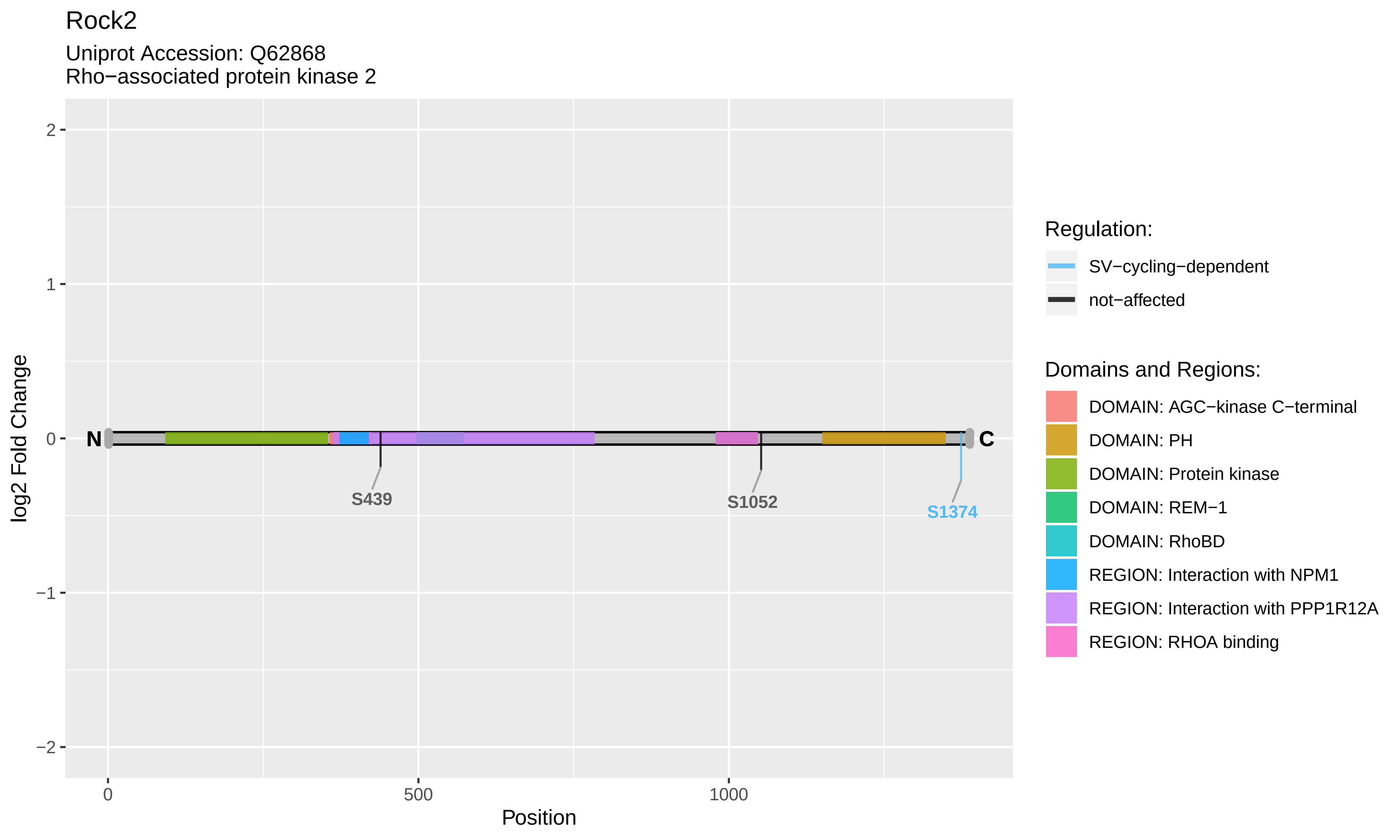

Rock2
Uniprot Accession: Q62868
Rho−associated protein kinase 2
log2 Fold Change
Position
2
1
0
−1
−2
0
500
1000
N
C
S439
S1052
S1374
Regulation:
SV−cycling−dependent
not−affected
Domains and Regions:
DOMAIN: AGC−kinase C−terminal
DOMAIN: PH
DOMAIN: Protein kinase
DOMAIN: REM−1
DOMAIN: RhoBD
REGION: Interaction with NPM1
REGION: Interaction with PPP1R12A
REGION: RHOA binding

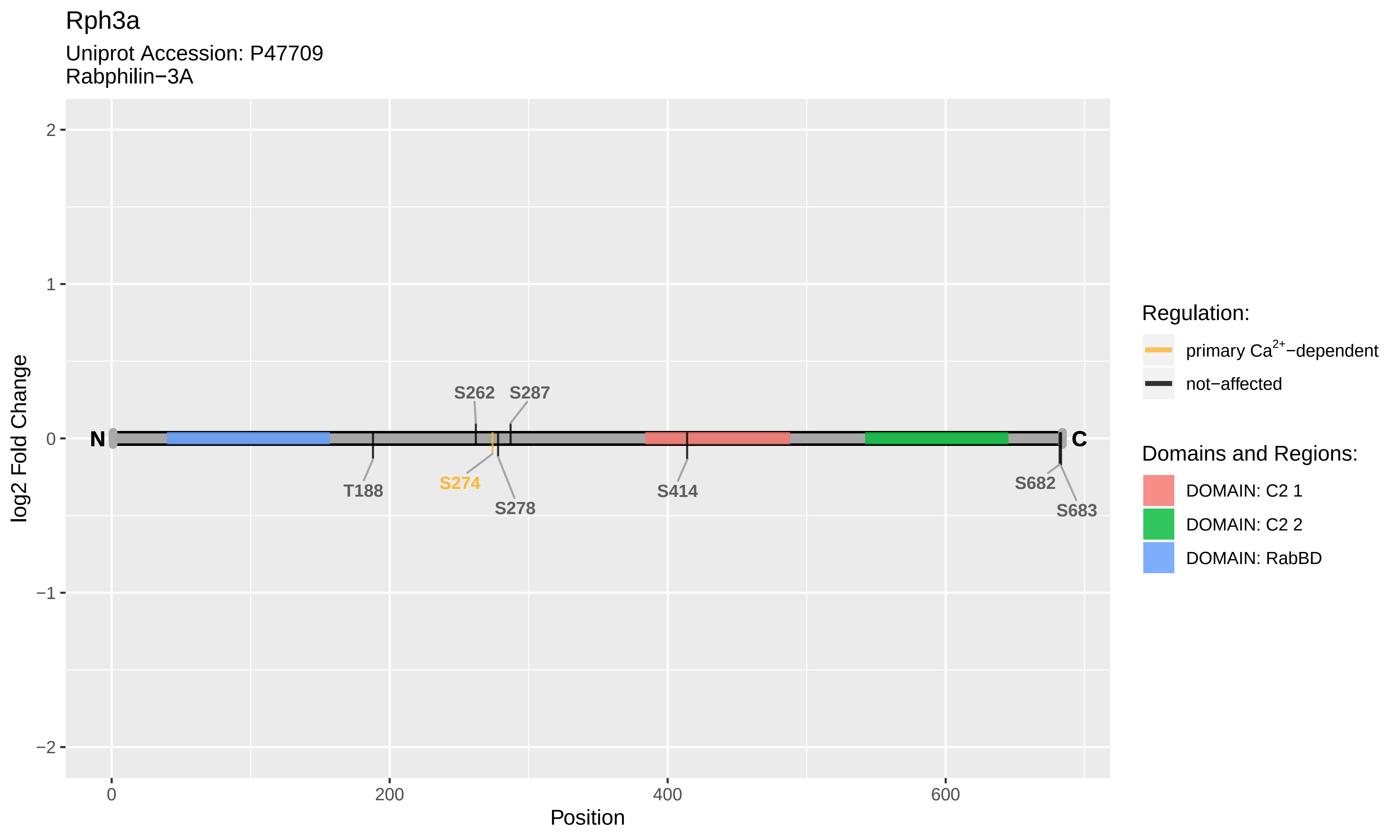

Rph3a
Uniprot Accession: P47709
Rabphilin−3A
2
1
0
−1
−2
log2 Fold Change
N
C
S262
S287
T188
S274
S278
S414
S682
S683
0
200
400
600
Position
Regulation:
primary Ca2+−dependent
not−affected
Domains and Regions:
DOMAIN: C2 1
DOMAIN: C2 2
DOMAIN: RabBD

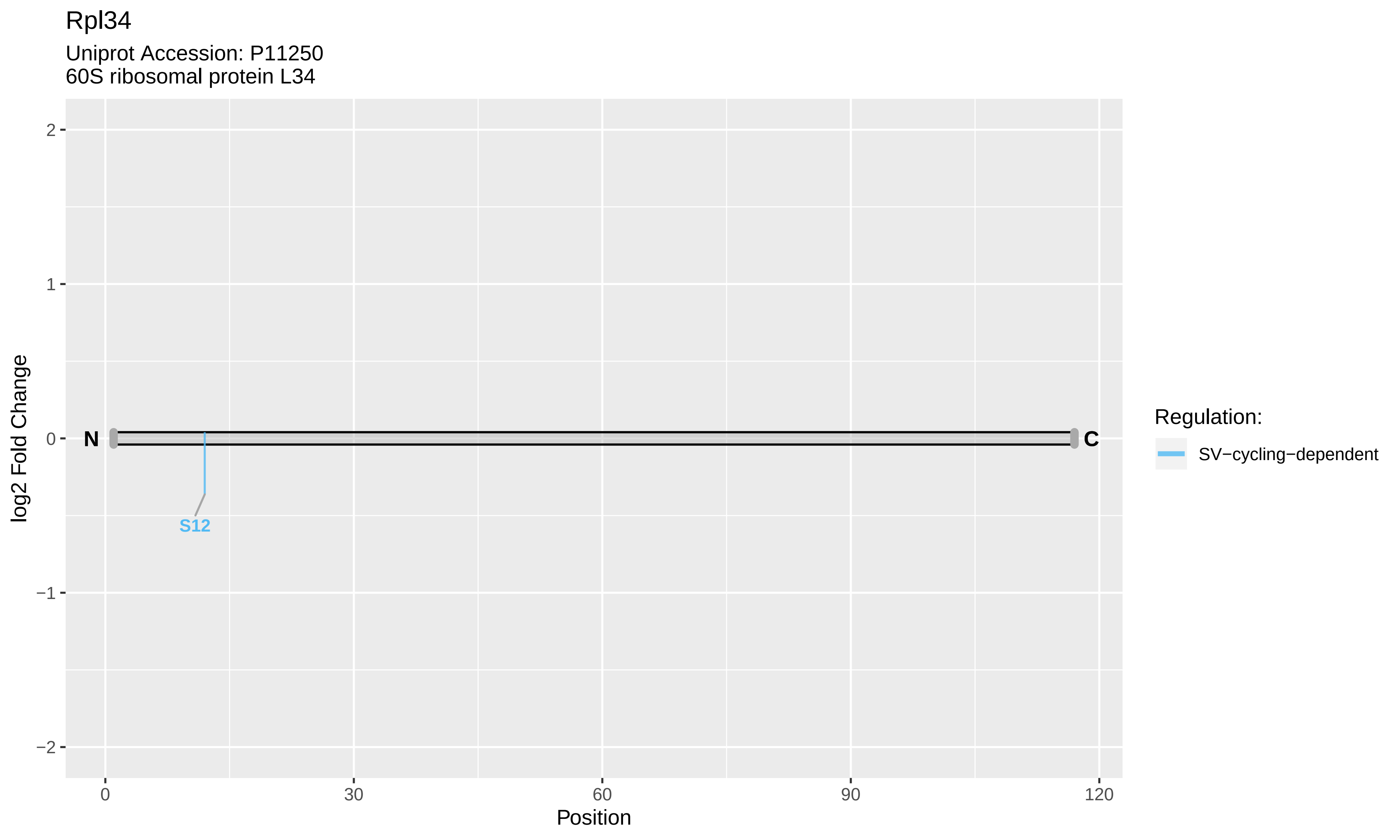
Rpl34
Uniprot Accession: P11250
60S ribosomal protein L34
2
1
0
−1
−2
log2 Fold Change
N
C
S12
0
30
60
90
120
Position
Regulation:
SV−cycling−dependent

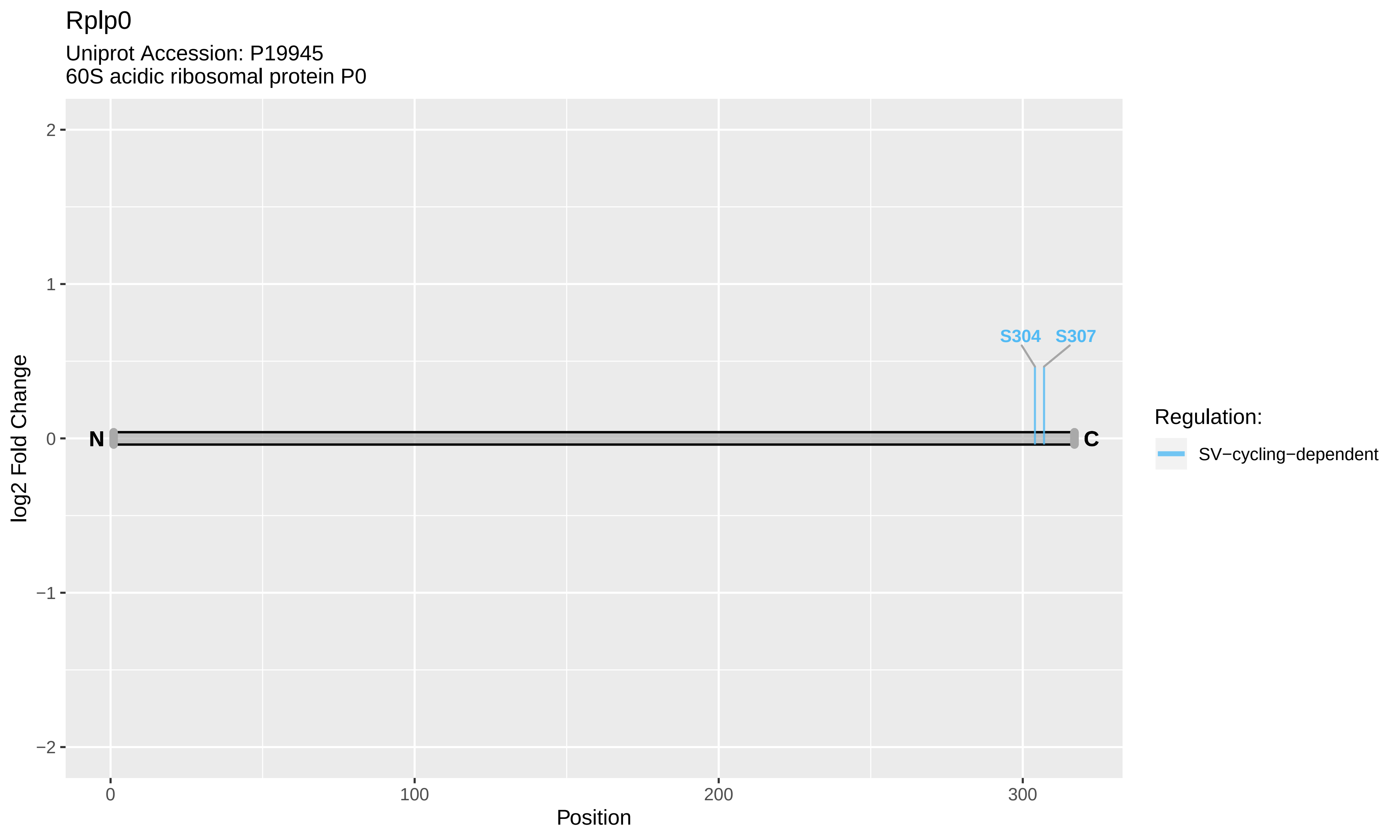
Rplp0
Uniprot Accession: P19945
60S acidic ribosomal protein P0
S304
S307
N
C
2
1
0
−1
−2
0
100
200
300
Position
log2 Fold Change
Regulation:
SV−cycling−dependent

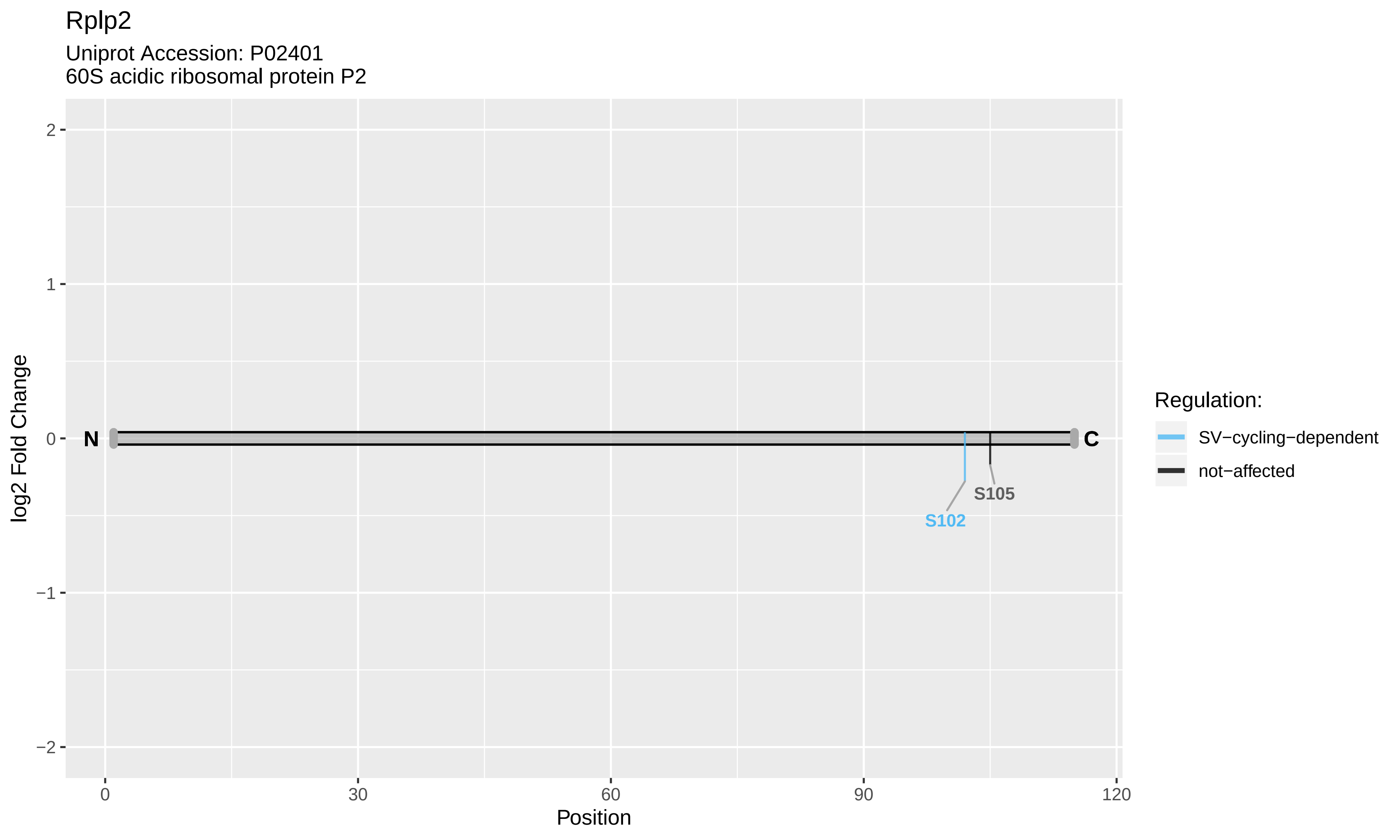
Rplp2
Uniprot Accession: P02401
60S acidic ribosomal protein P2
log2 Fold Change
2
1
0
−1
−2
N
C
S102
S105
0
30
60
90
120
Position
Regulation:
SV−cycling−dependent
not−affected

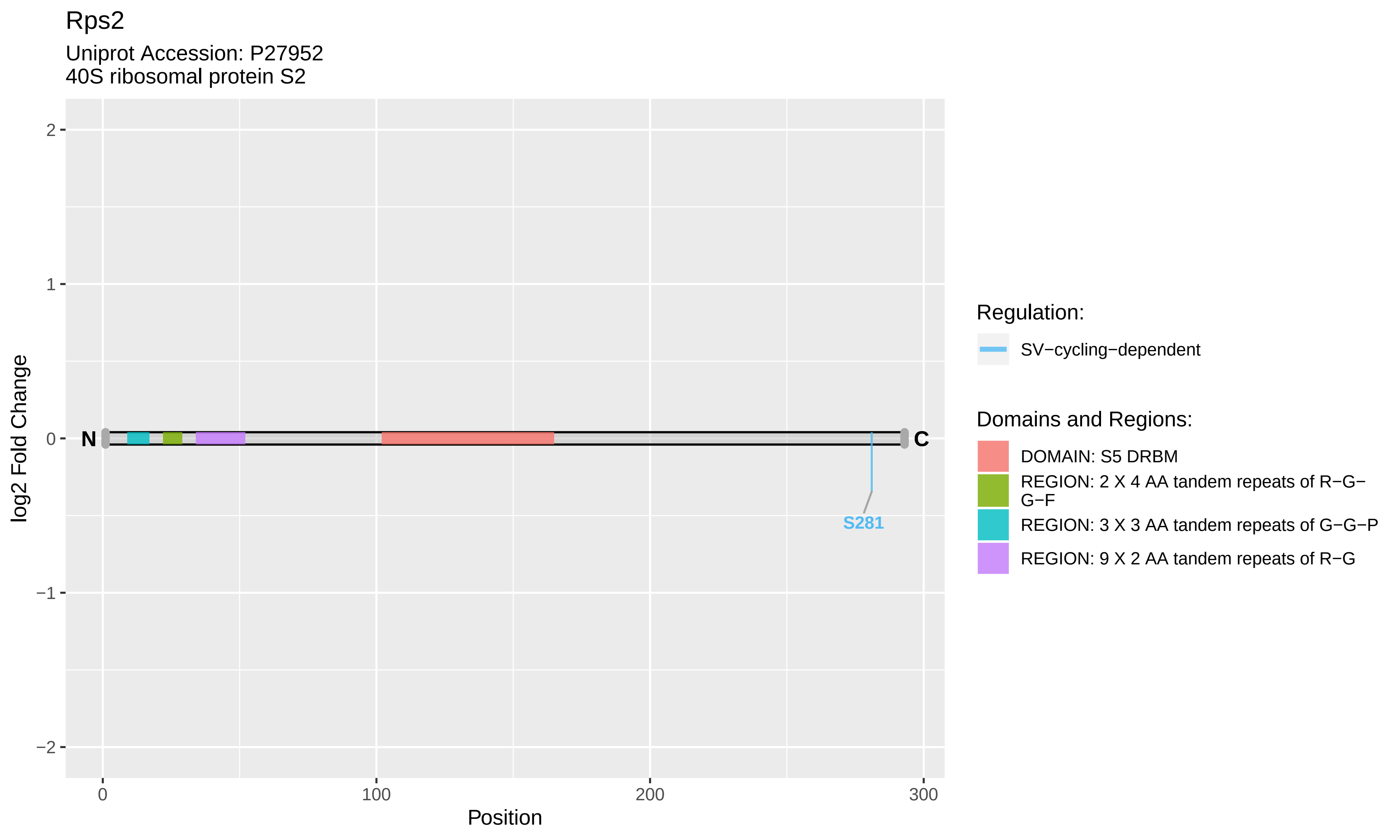

Rps2
Uniprot Accession: P27952
40S ribosomal protein S2
log2 Fold Change
2
1
0
−1
−2
N
C
S281
0
100
200
300
Position
Regulation:
SV−cycling−dependent
Domains and Regions:
DOMAIN: S5 DRBM
REGION: 2 X 4 AA tandem repeats of R−G−G−F
REGION: 3 X 3 AA tandem repeats of G−G−P
REGION: 9 X 2 AA tandem repeats of R−G

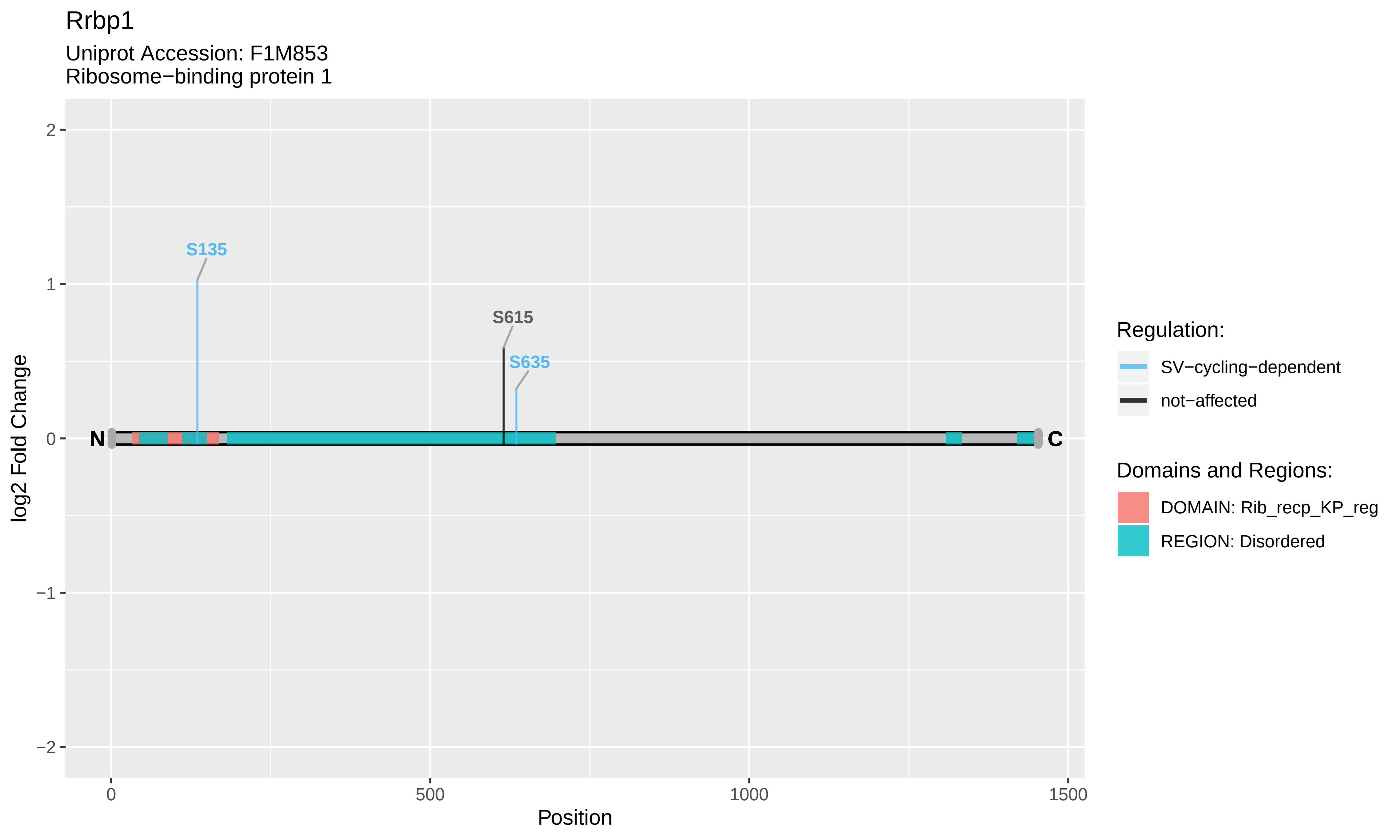
Rrbp1
Uniprot Accession: F1M853
Ribosome−binding protein 1
S135
S615
S635
N
C
log2 Fold Change
2
1
0
−1
−2
0
500
1000
1500
Position
Regulation:
SV−cycling−dependent
not−affected
Domains and Regions:
DOMAIN: Rib_recp_KP_reg
REGION: Disordered

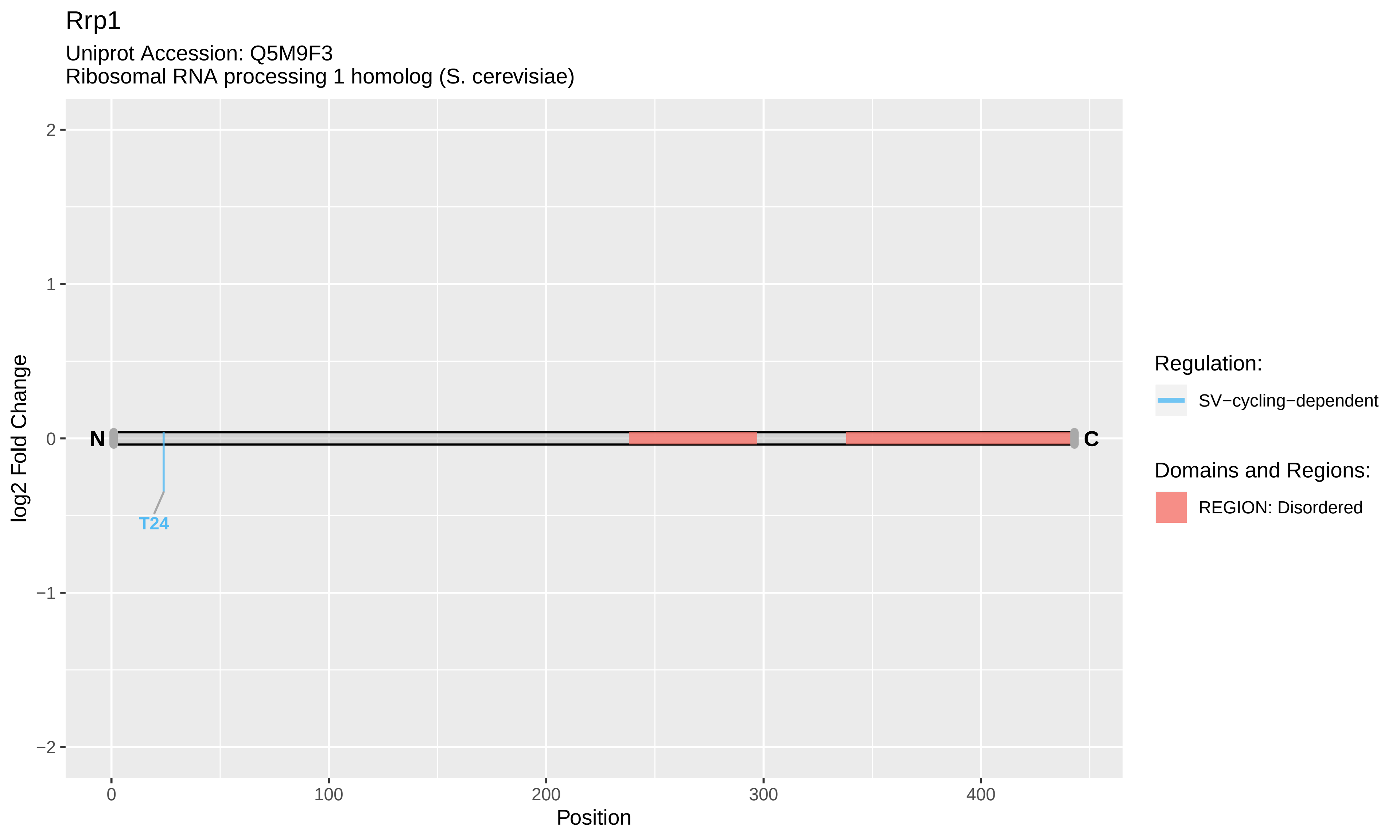

Rrp1
Uniprot Accession: Q5M9F3
Ribosomal RNA processing 1 homolog (S. cerevisiae)
log2 Fold Change
2
1
0
−1
−2
N
C
T24
0
100
200
300
400
Position
Regulation:
SV−cycling−dependent
Domains and Regions:
REGION: Disordered

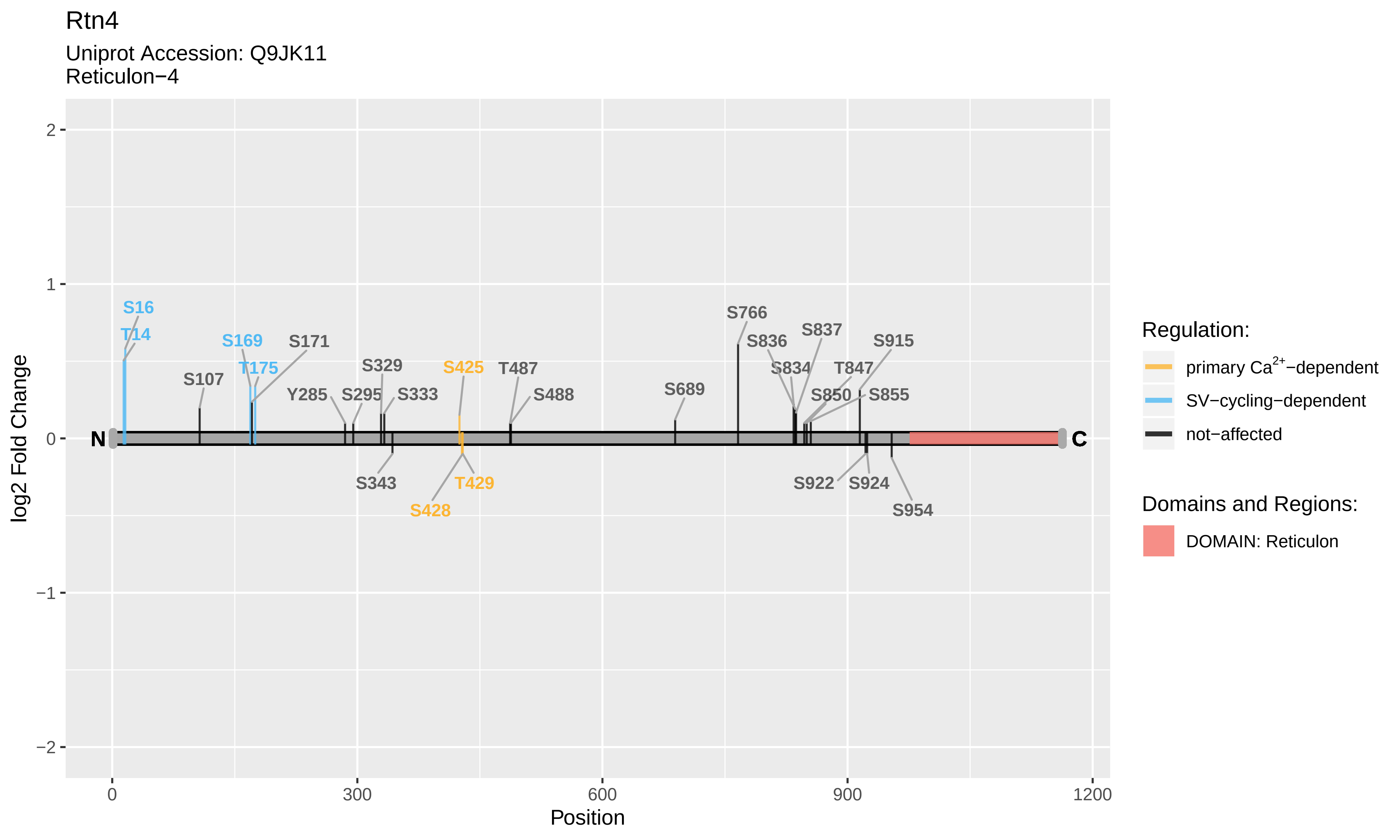

Rtn4
Uniprot Accession: Q9JK11
Reticulon−4
log2 Fold Change
Position
N
C
S16
T14
S169
T175
S171
S107
Y285
S295
S329
S333
S343
S425
S428
T429
T487
S488
S689
S766
S836
S837
S834
T847
S850
S855
S915
S922
S924
S954
2
1
0
−1
−2
0
300
600
900
1200
Regulation:
primary Ca2+−dependent
SV−cycling−dependent
not−affected
Domains and Regions:
DOMAIN: Reticulon

Rundc3a

Uniprot Accession: F1LRZ9
RUN domain−containing 3A

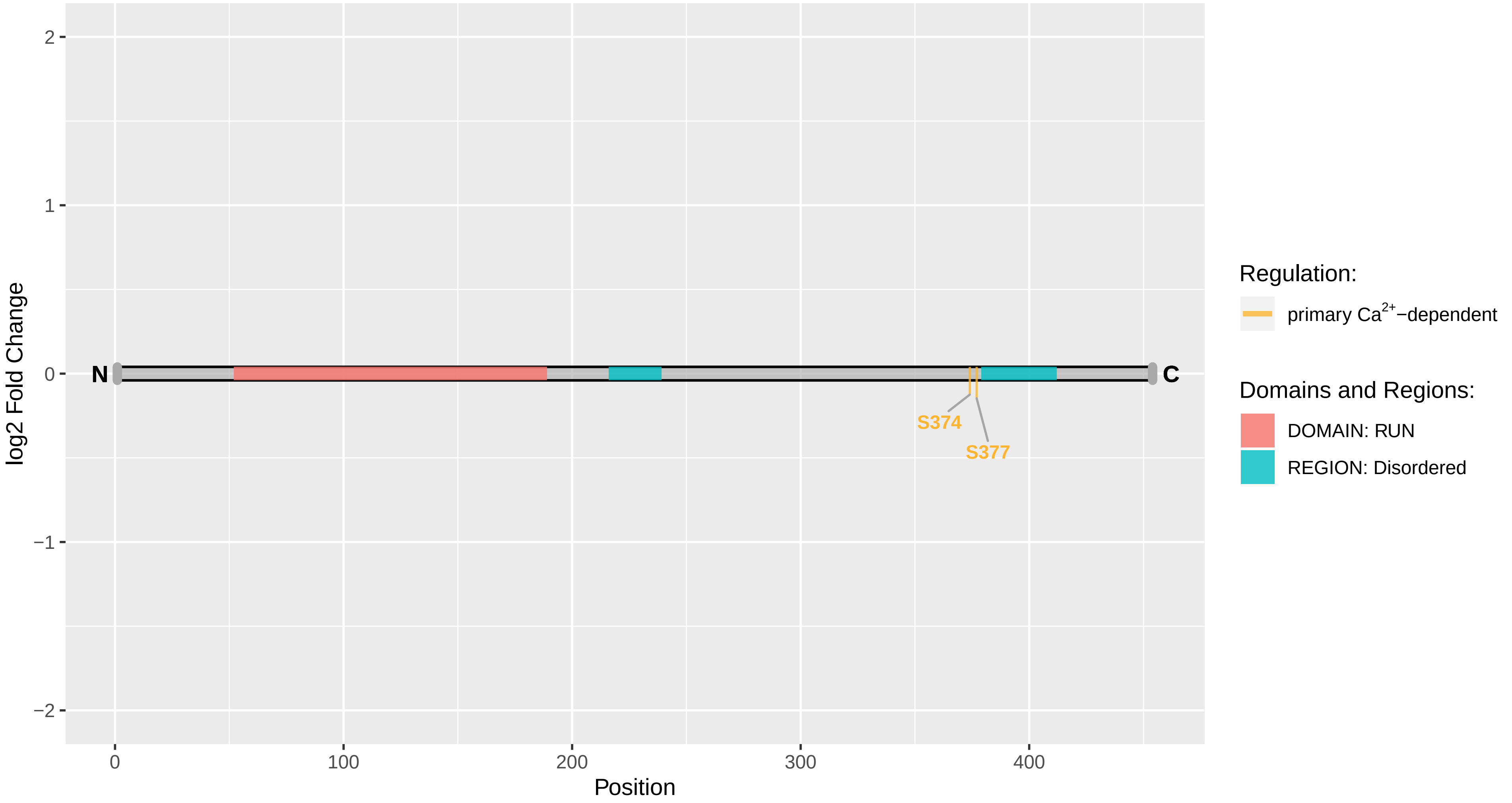

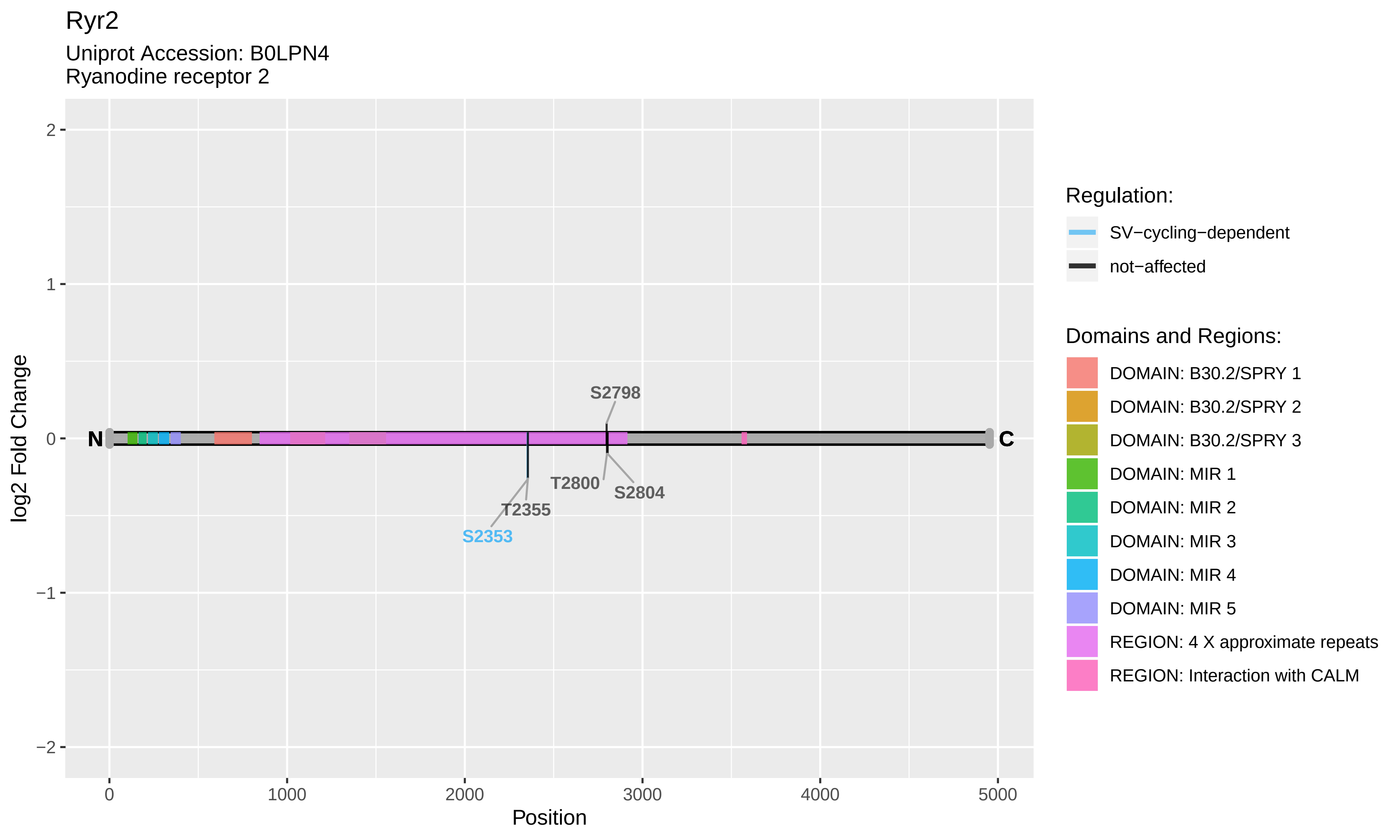

Ryr2
Uniprot Accession: B0LPN4
Ryanodine receptor 2
log2 Fold Change
2
1
0
−1
−2
N
C
S2798
T2800
S2804
T2355
S2353
0
1000
2000
3000
4000
5000
Position
Regulation:
SV−cycling−dependent
not−affected
Domains and Regions:
DOMAIN: B30.2/SPRY 1
DOMAIN: B30.2/SPRY 2
DOMAIN: B30.2/SPRY 3
DOMAIN: MIR 1
DOMAIN: MIR 2
DOMAIN: MIR 3
DOMAIN: MIR 4
DOMAIN: MIR 5
REGION: 4 X approximate repeats
REGION: Interaction with CALM

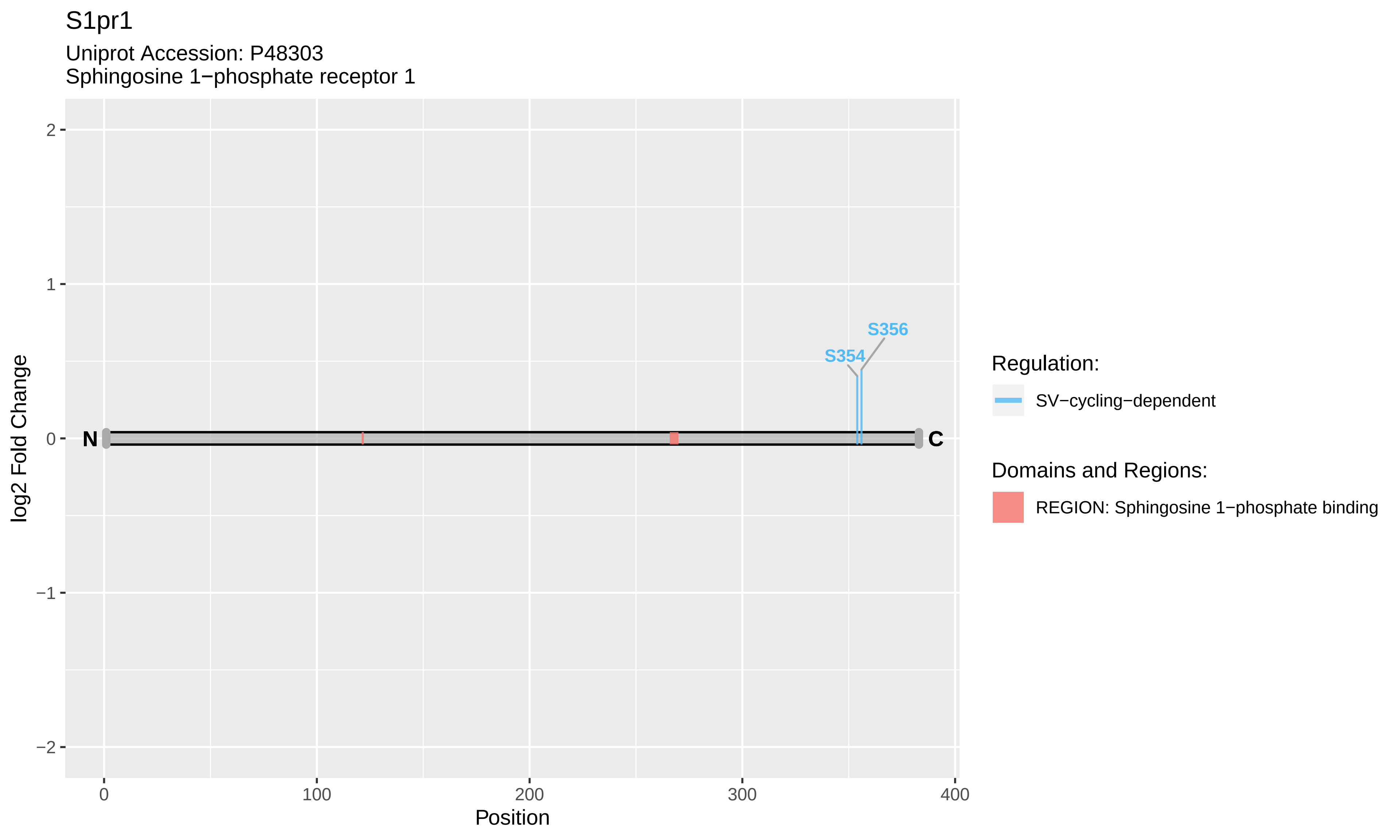
S1pr1
Uniprot Accession: P48303
Sphingosine 1−phosphate receptor 1
S356
S354
N
C
log2 Fold Change
Position
2
1
0
−1
−2
0
100
200
300
400
Regulation:
SV−cycling−dependent
Domains and Regions:
REGION: Sphingosine 1−phosphate binding

Safb

Uniprot Accession: O88453
Scaffold attachment factor B1

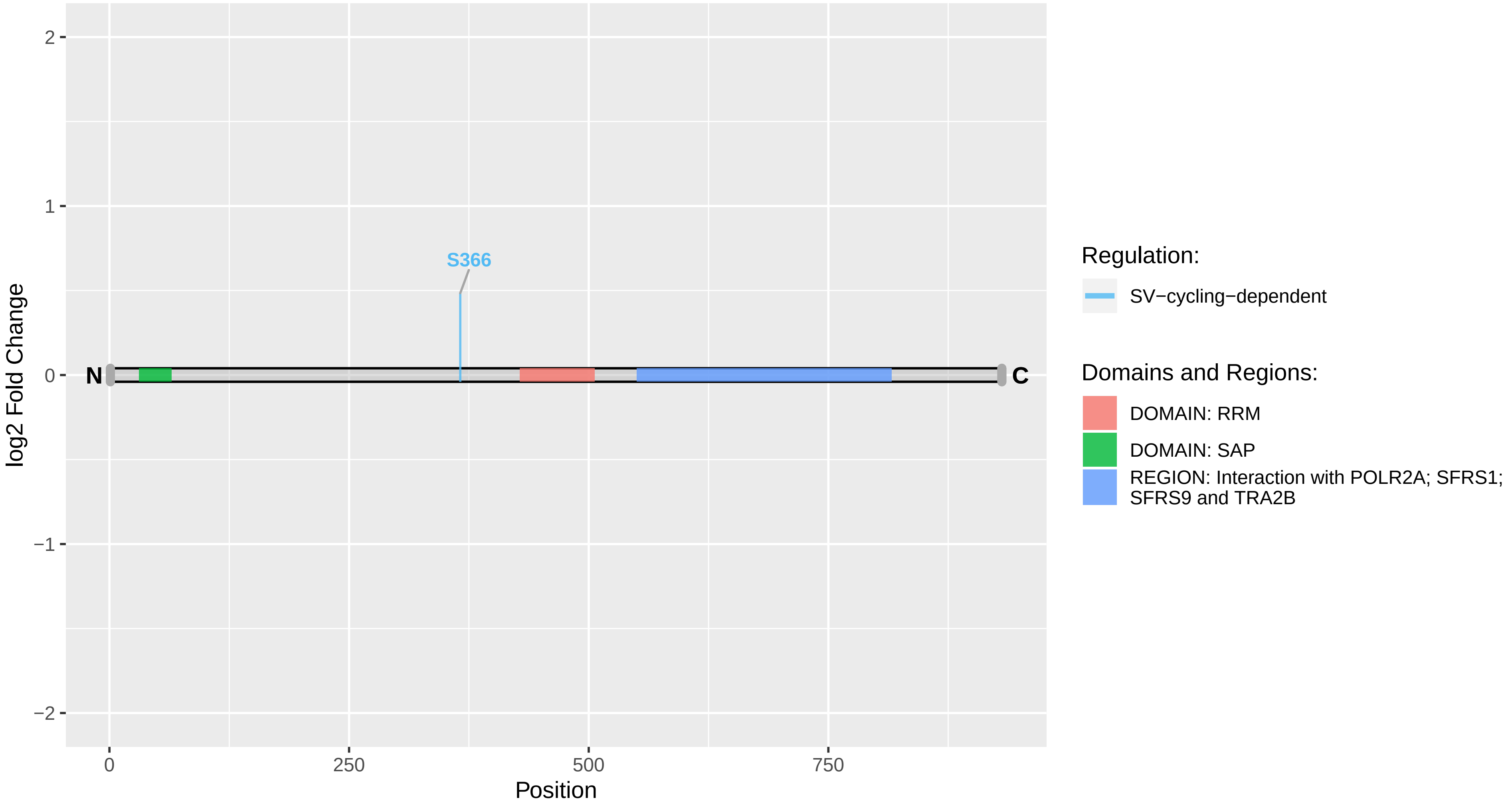

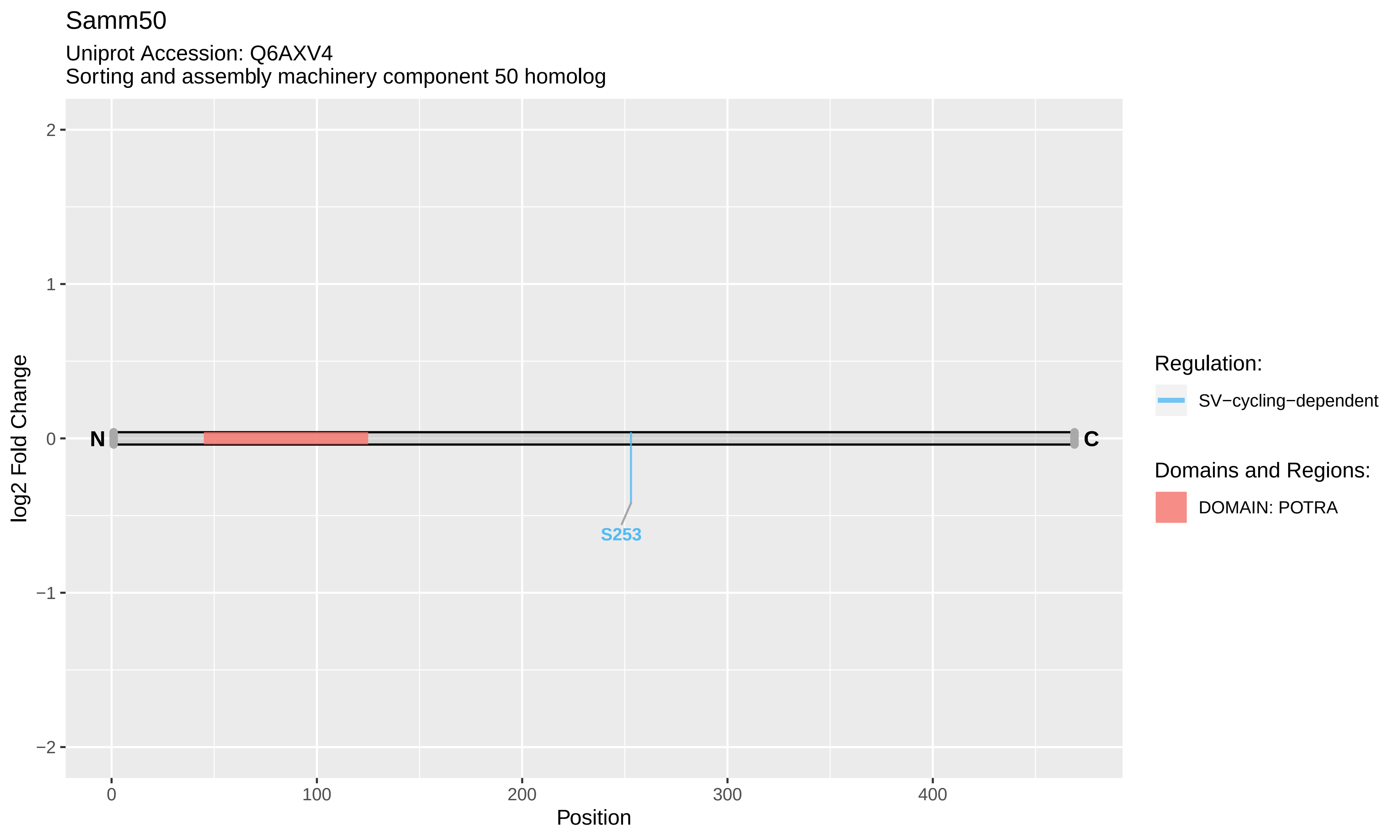

Samm50
Uniprot Accession: Q6AXV4
Sorting and assembly machinery component 50 homolog
log2 Fold Change
2
1
0
−1
−2
N
C
S253
0
100
200
300
400
Position
Regulation:
SV−cycling−dependent
Domains and Regions:
DOMAIN: POTRA

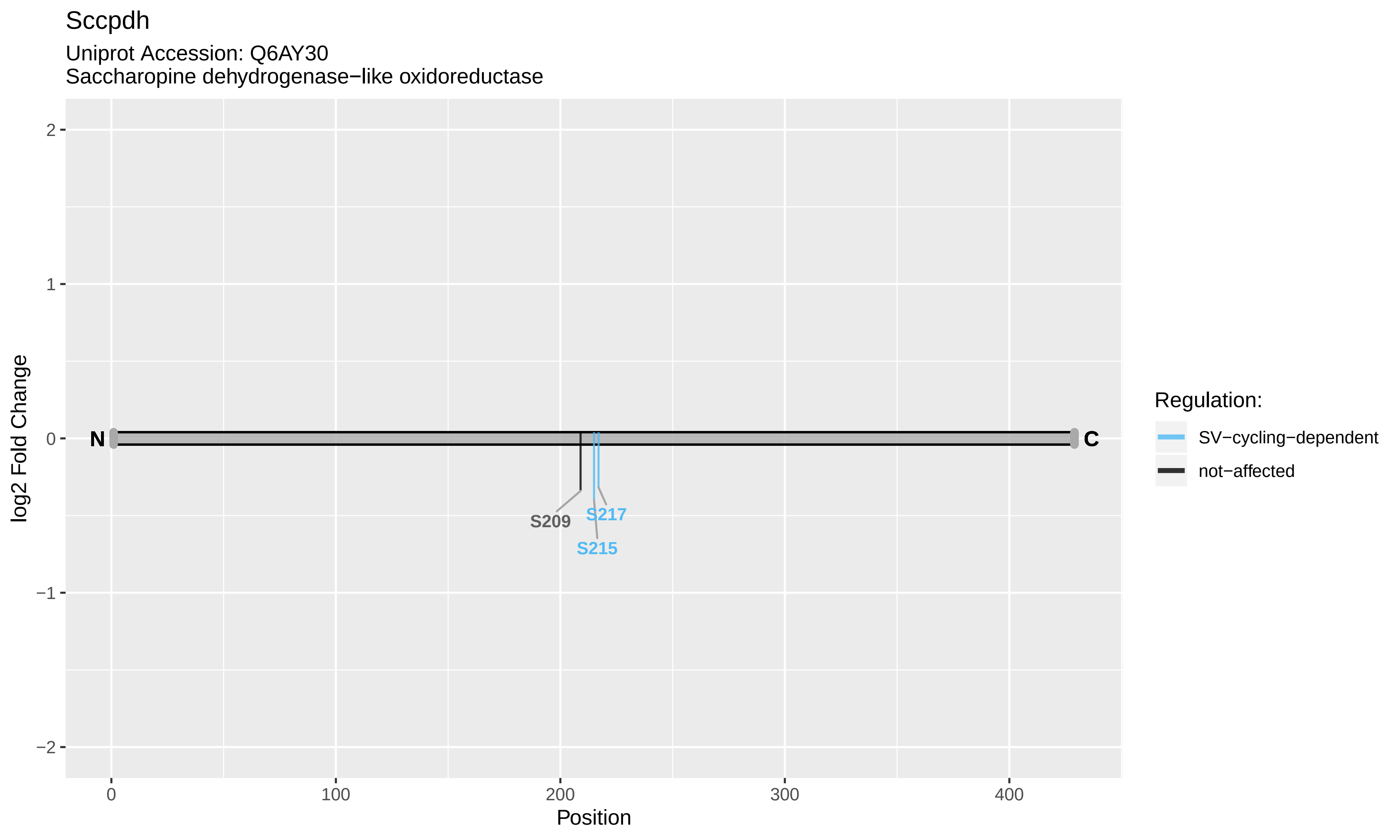

Sccpdh
Uniprot Accession: Q6AY30
Saccharopine dehydrogenase−like oxidoreductase
log2 Fold Change
Position
N
C
S209
S217
S215
Regulation:
SV−cycling−dependent
not−affected
2
1
0
−1
−2
0
100
200
300
400

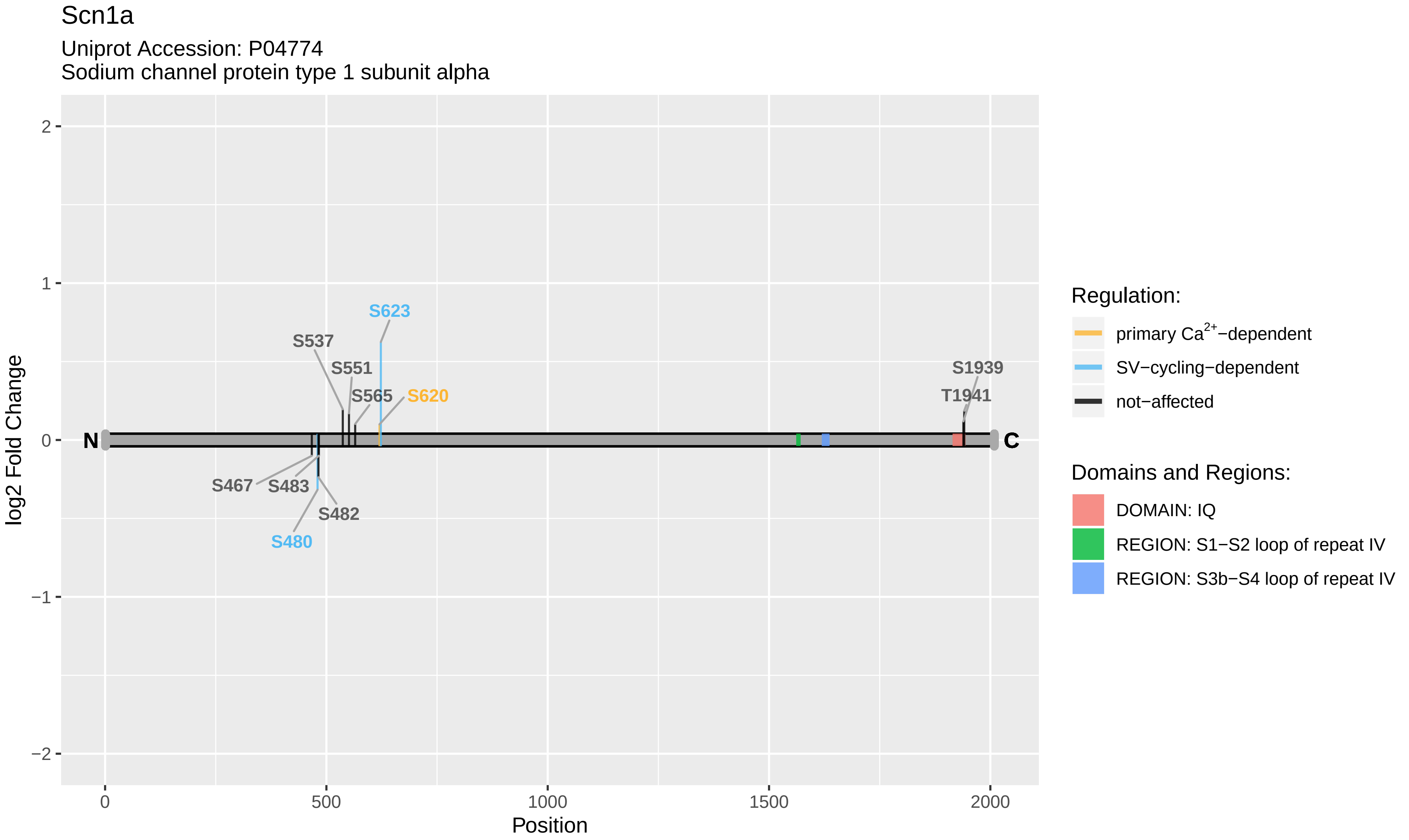

Scn1a
Uniprot Accession: P04774
Sodium channel protein type 1 subunit alpha
log2 Fold Change
Position
2
1
0
−1
−2
0
500
1000
1500
2000
N
C
S623
S537
S551
S565
S620
S1939
T1941
S467
S483
S482
S480
Regulation:
primary Ca2+−dependent
SV−cycling−dependent
not−affected
Domains and Regions:
DOMAIN: IQ
REGION: S1−S2 loop of repeat IV
REGION: S3b−S4 loop of repeat IV

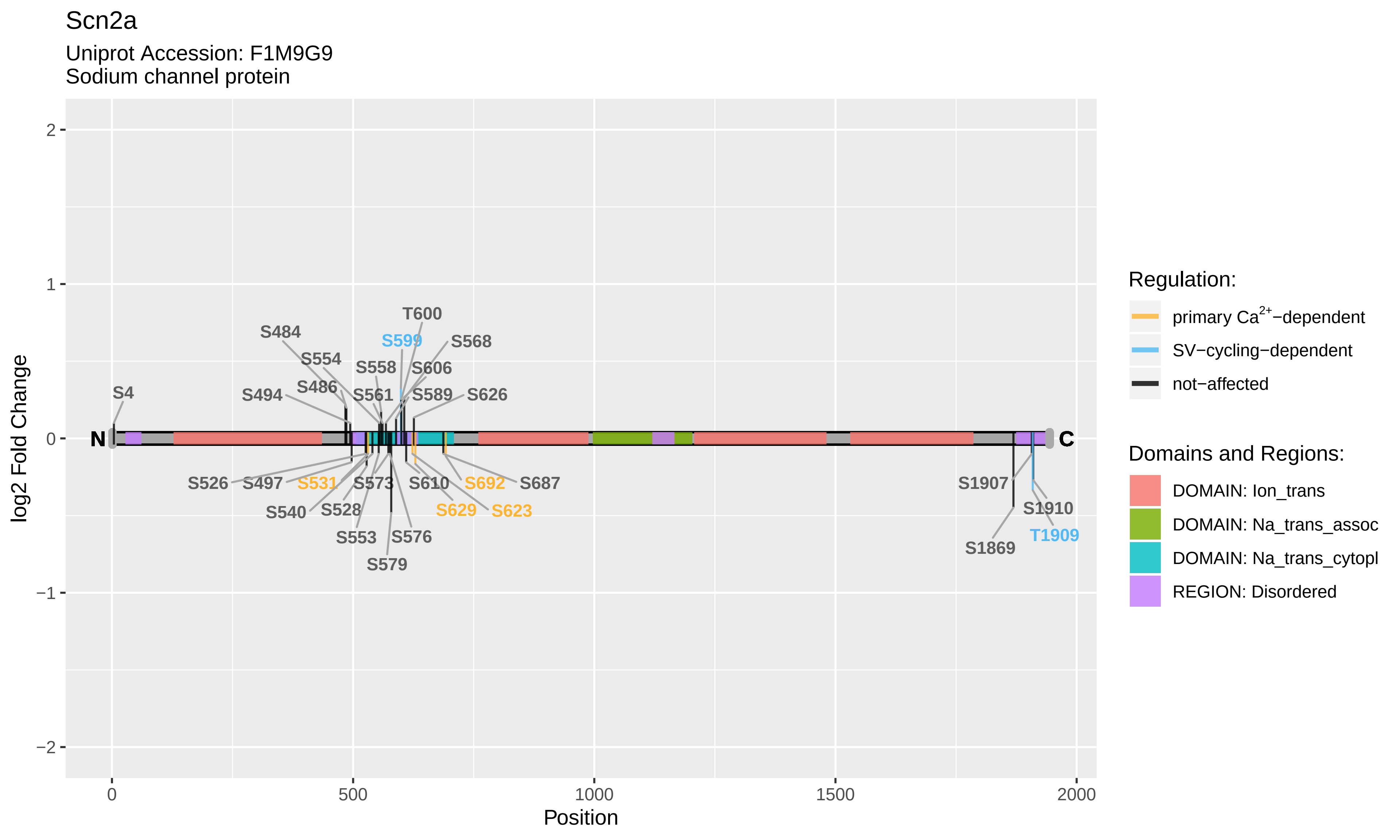
Scn2a
Uniprot Accession: F1M9G9
Sodium channel protein
log2 Fold Change
Position
N
C
S4
S484
S486
S494
S554
S558
S561
S599
T600
S568
S606
S589
S626
S526
S497
S531
S573
S610
S692
S687
S540
S528
S629
S623
S553
S576
S579
S1907
S1910
T1909
S1869
Regulation:
primary Ca2+−dependent
SV−cycling−dependent
not−affected
Domains and Regions:
DOMAIN: Ion_trans
DOMAIN: Na_trans_assoc
DOMAIN: Na_trans_cytopl
REGION: Disordered

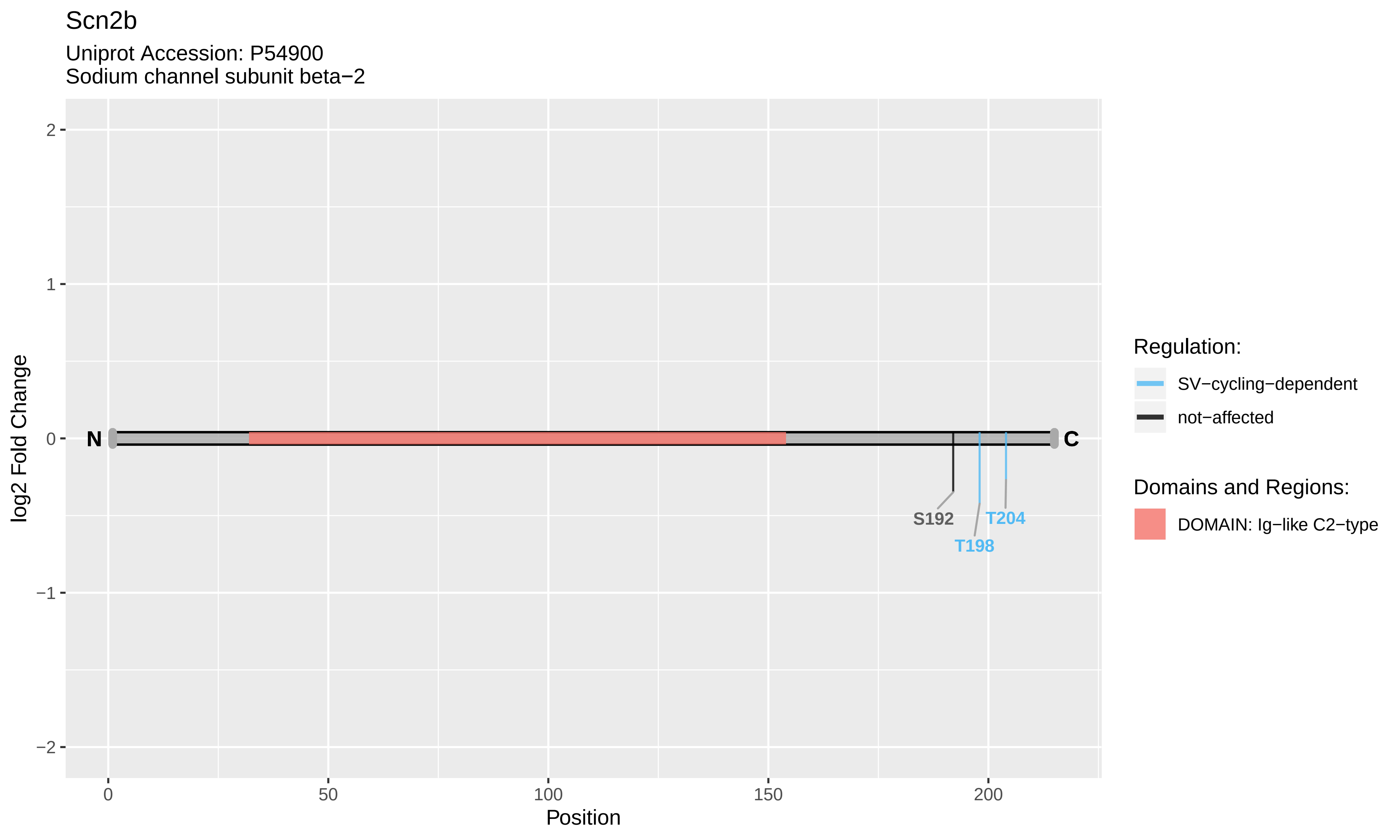
Scn2b
Uniprot Accession: P54900
Sodium channel subunit beta−2
log2 Fold Change
Position
N
C
S192
T198
T204
Regulation:
SV−cycling−dependent
not−affected
Domains and Regions:
DOMAIN: Ig−like C2−type
2
1
0
−1
−2
0
50
100
150
200

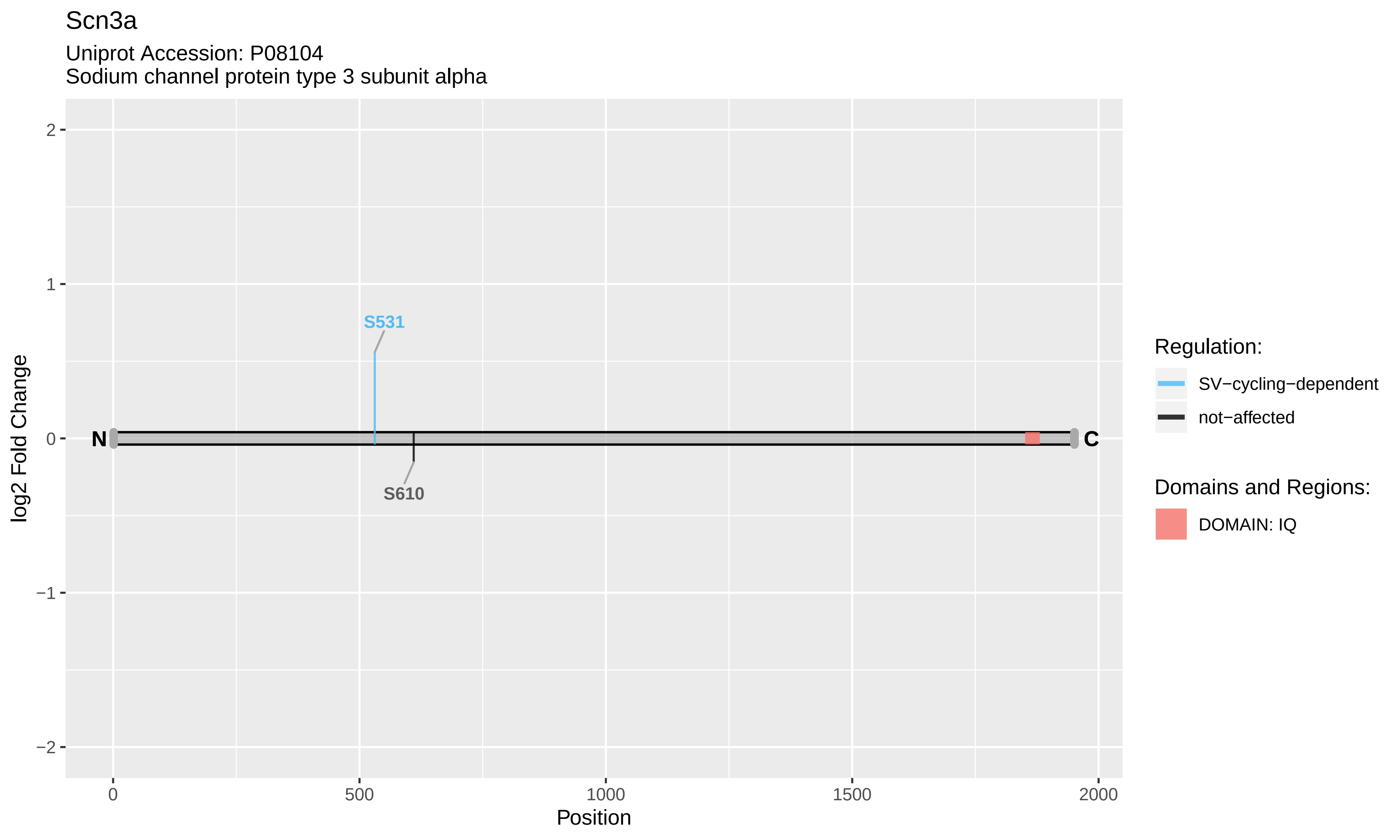
Scn3a
Uniprot Accession: P08104
Sodium channel protein type 3 subunit alpha
log2 Fold Change
2
1
0
−1
−2
N
C
S531
S610
0
500
1000
1500
2000
Position
Regulation:
SV−cycling−dependent
not−affected
Domains and Regions:
DOMAIN: IQ

Scyl2

Uniprot Accession: A0A0G2JVC2
SCY1−like pseudokinase 2

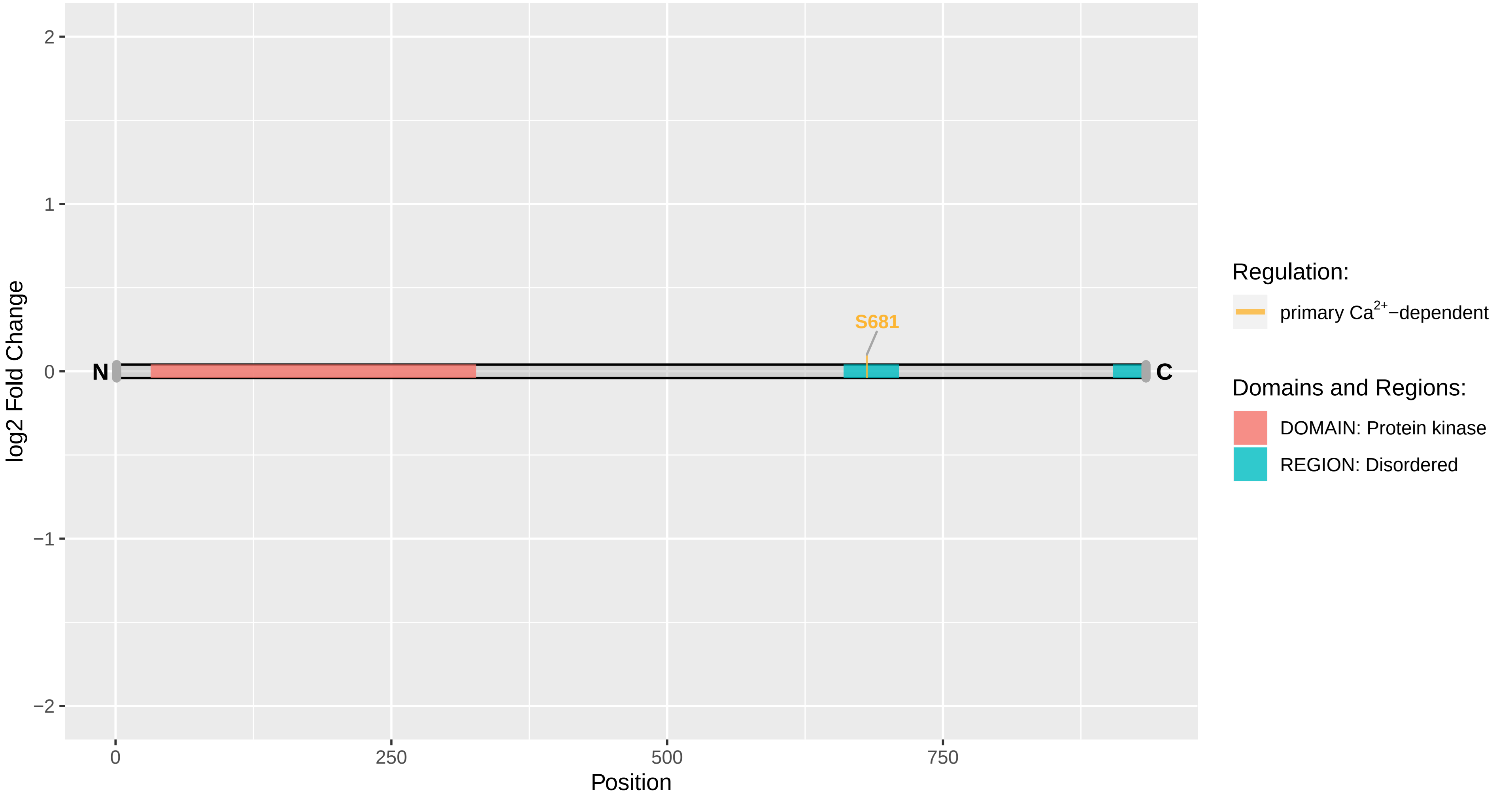

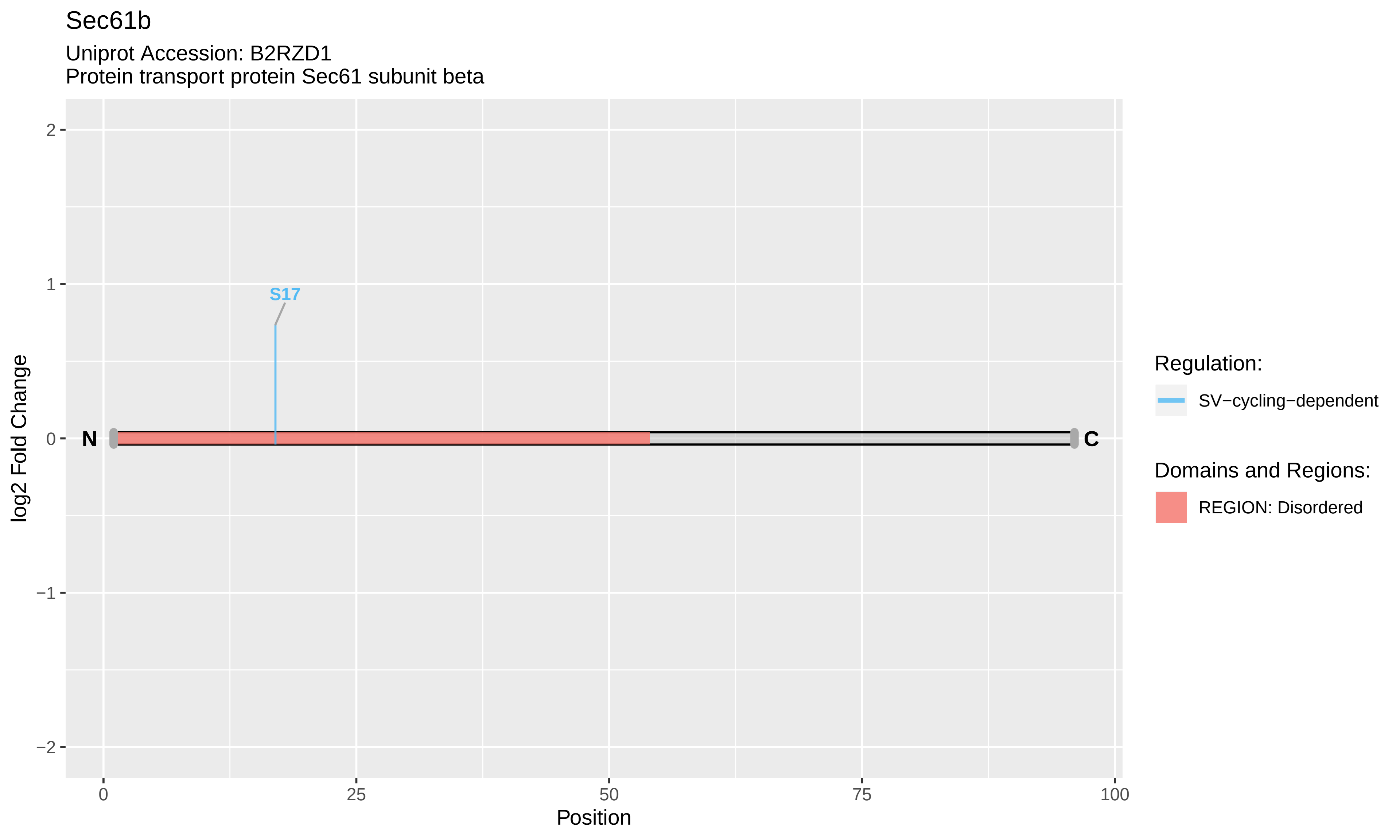
Sec61b
Uniprot Accession: B2RZD1
Protein transport protein Sec61 subunit beta
S17
N
C
log2 Fold Change
Position
2
1
0
−1
−2
0
25
50
75
100
Regulation:
SV−cycling−dependent
Domains and Regions:
REGION: Disordered

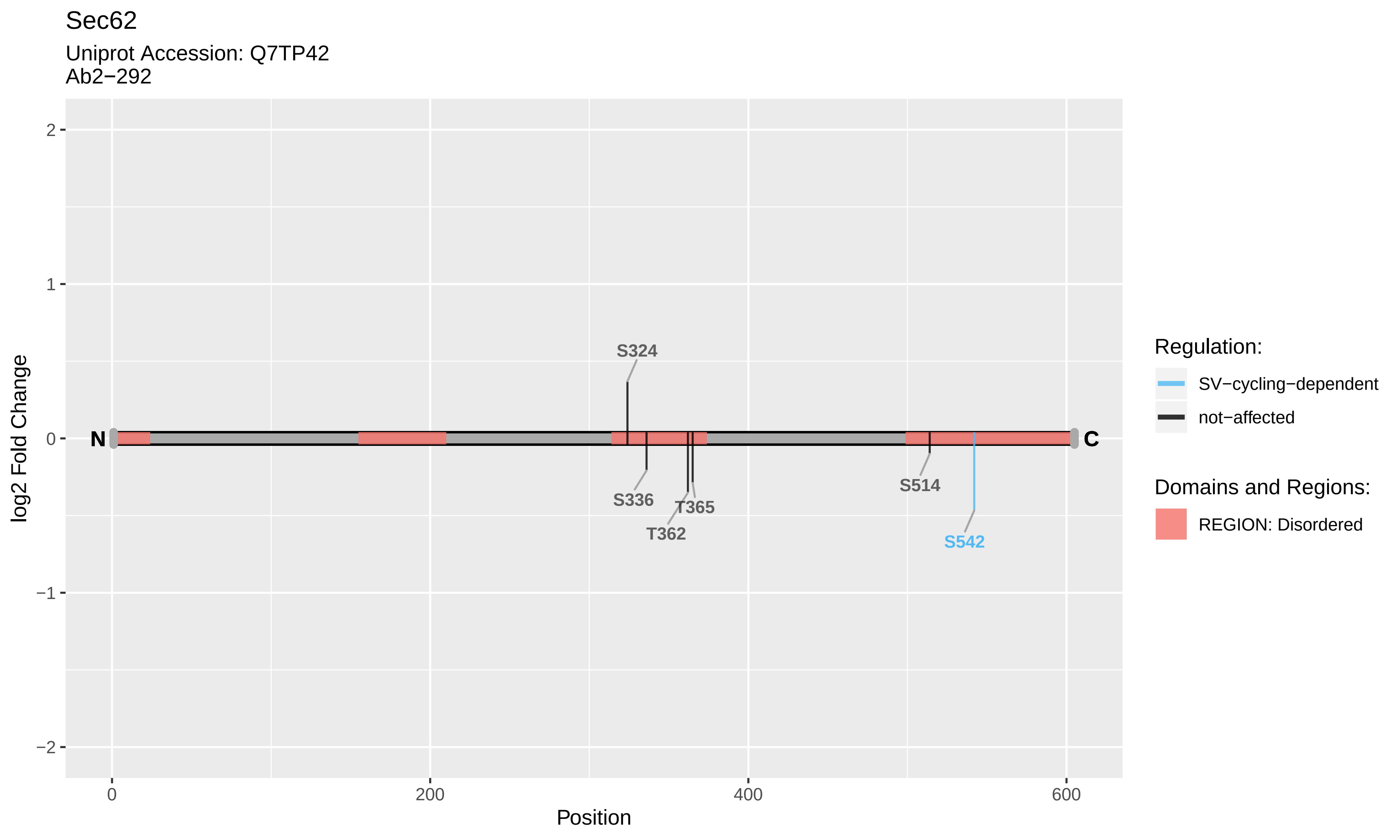

Sec62
Uniprot Accession: Q7TP42
Ab2−292
log2 Fold Change
2
1
0
−1
−2
N
C
S324
S336
T362
T365
S514
S542
0
200
400
600
Position
Regulation:
SV−cycling−dependent
not−affected
Domains and Regions:
REGION: Disordered

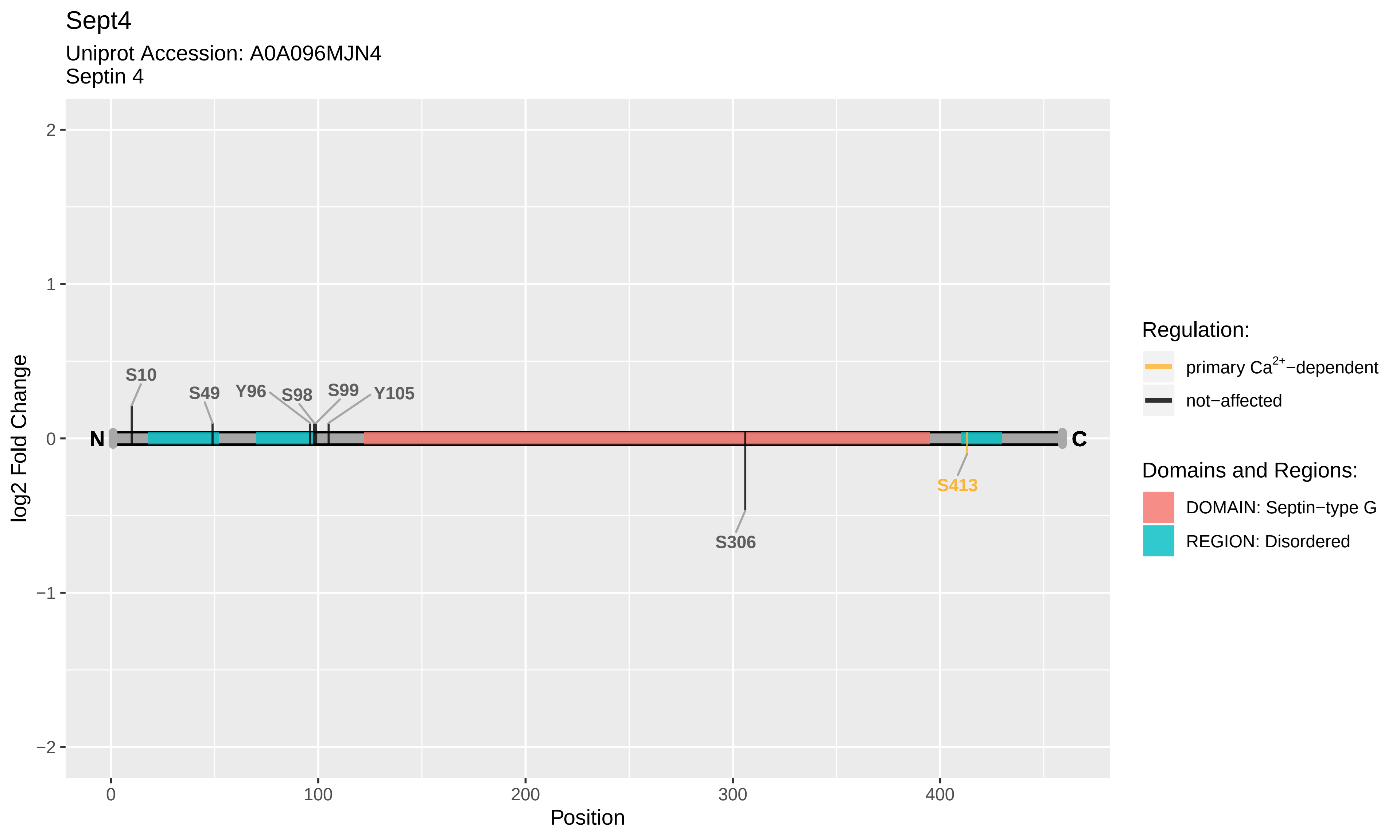

Sept4
Uniprot Accession: A0A096MJN4
Septin 4
log2 Fold Change
2
1
0
−1
−2
N
C
S10
S49
Y96
S98
S99
Y105
S306
S413
0
100
200
300
400
Position
Regulation:
primary Ca2+−dependent
not−affected
Domains and Regions:
DOMAIN: Septin−type G
REGION: Disordered

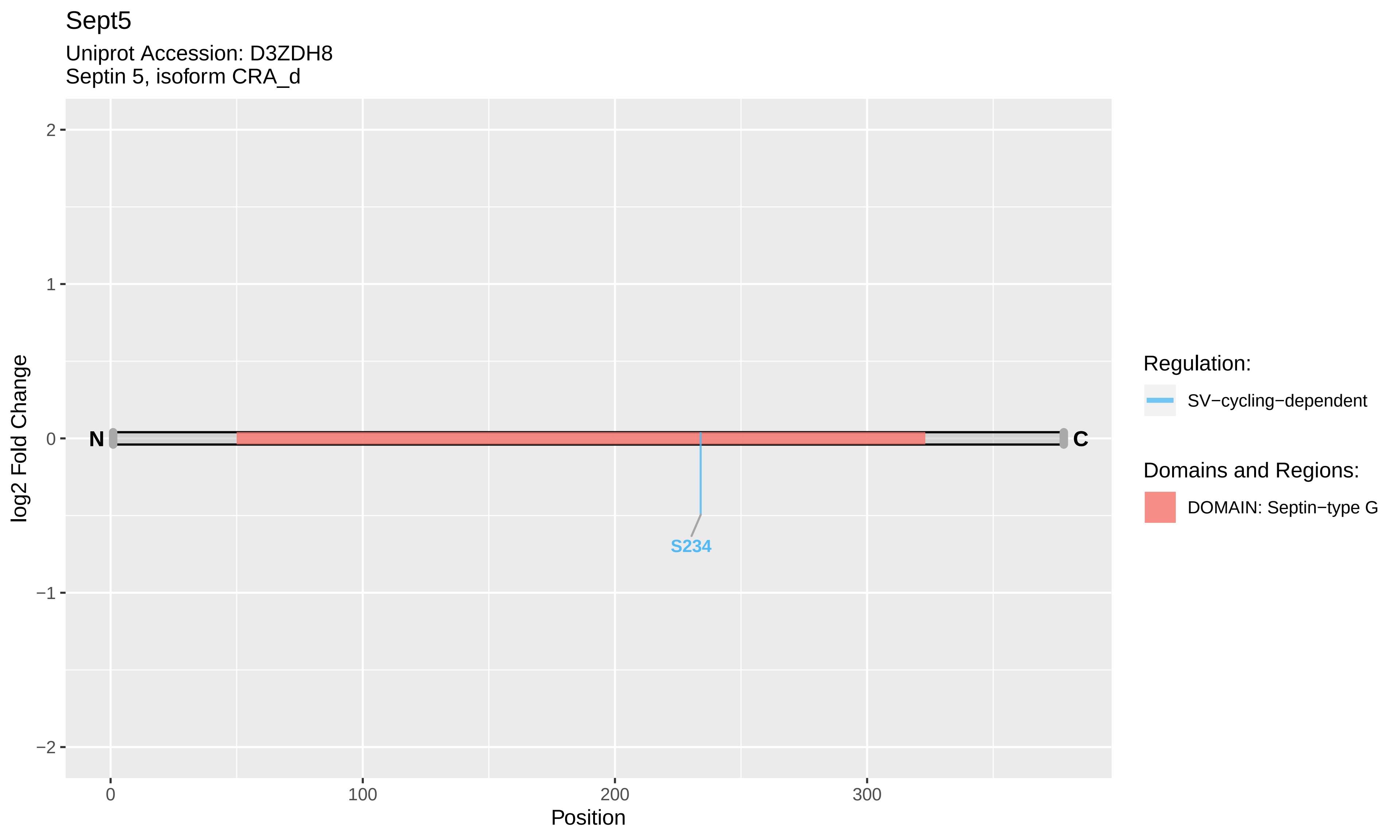
Sept5
Uniprot Accession: D3ZDH8
Septin 5, isoform CRA_d
log2 Fold Change
2
1
0
−1
−2
N
C
S234
0
100
200
300
Position
Regulation:
SV−cycling−dependent
Domains and Regions:
DOMAIN: Septin−type G

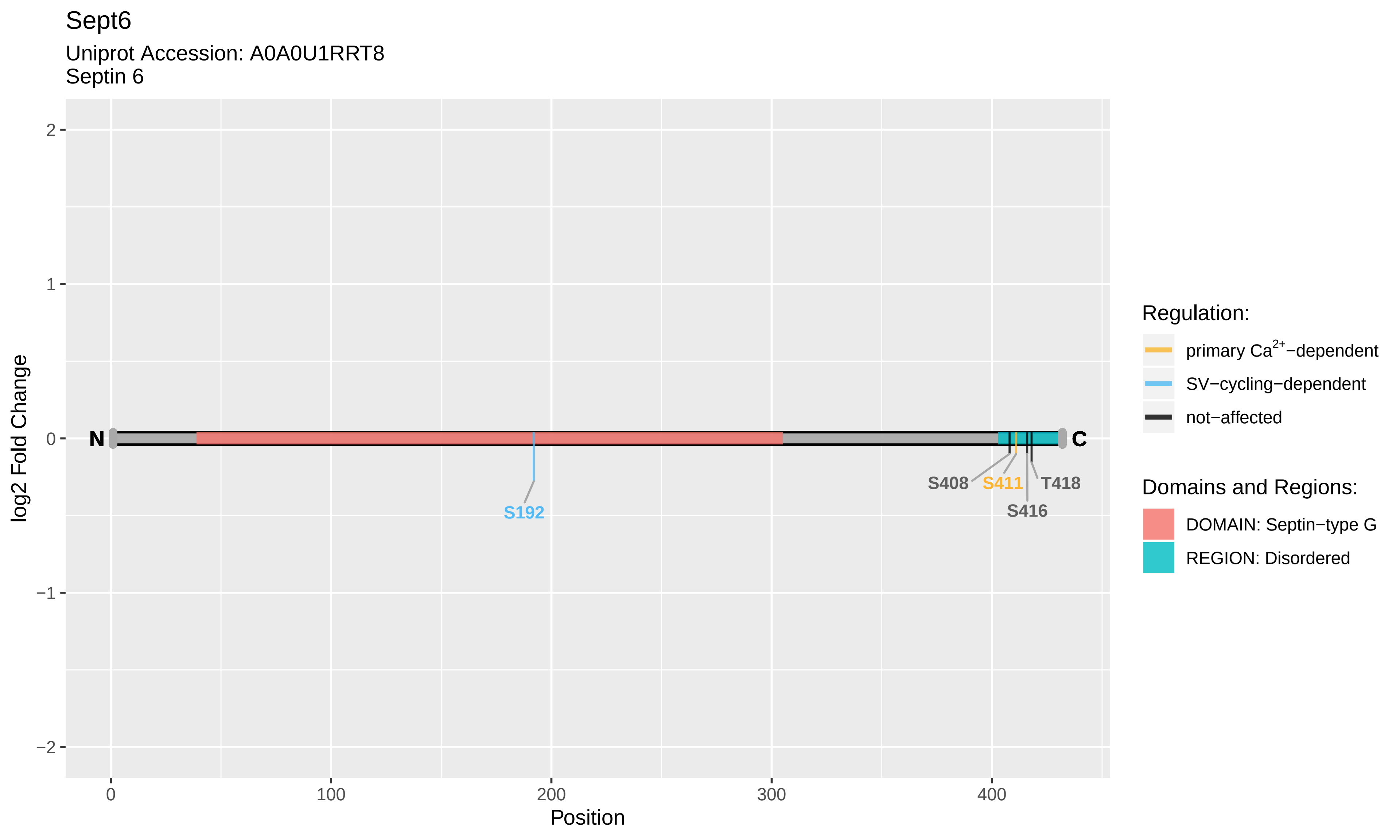
Sept6
Uniprot Accession: A0A0U1RRT8
Septin 6
log2 Fold Change
2
1
0
−1
−2
N
C
S192
S408
S411
T418
S416
0
100
200
300
400
Position
Regulation:
primary Ca2+−dependent
SV−cycling−dependent
not−affected
Domains and Regions:
DOMAIN: Septin−type G
REGION: Disordered

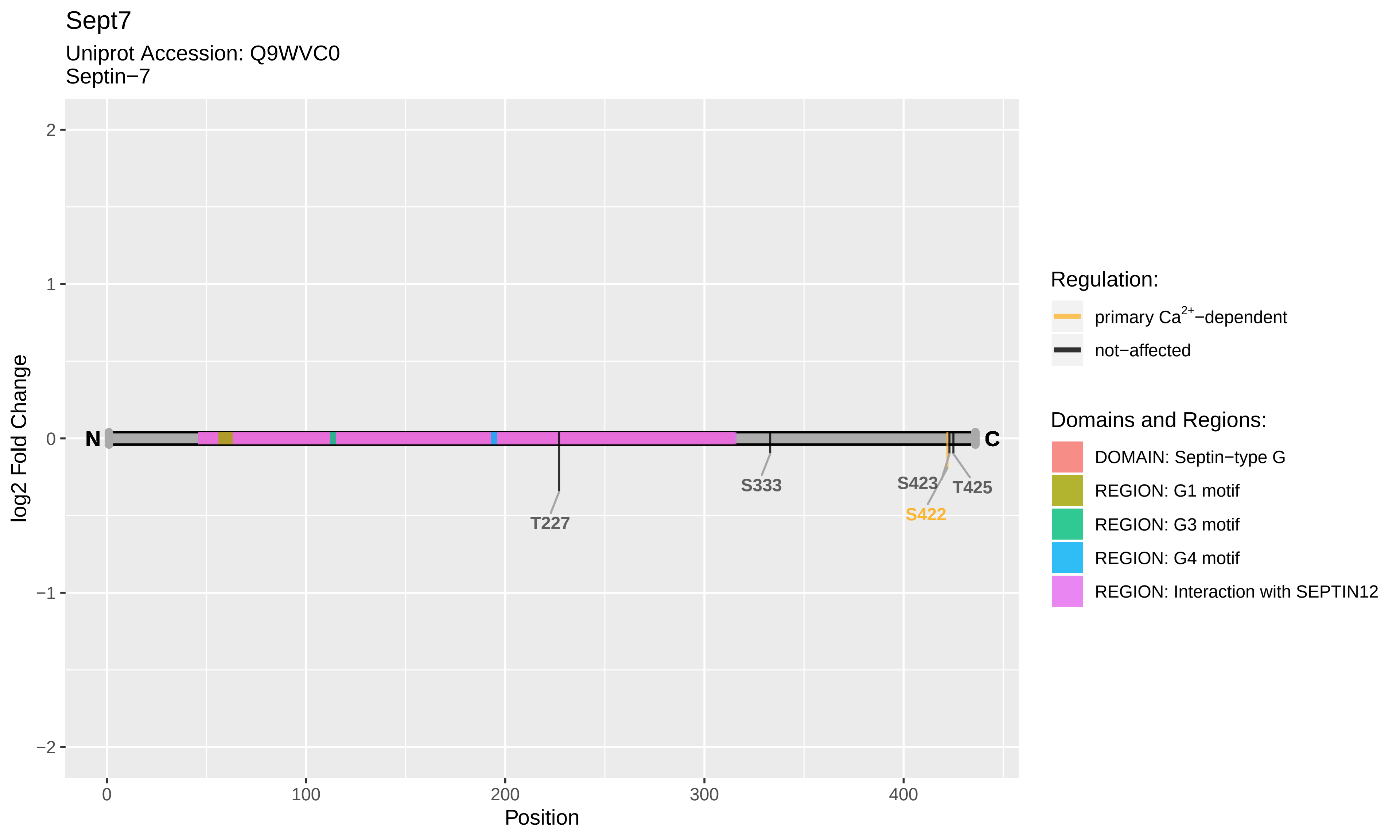

Sept7
Uniprot Accession: Q9WVC0
Septin−7
log2 Fold Change
2
1
0
−1
−2
N
C
T227
S333
S423
S422
T425
0
100
200
300
400
Position
Regulation:
primary Ca2+−dependent
not−affected
Domains and Regions:
DOMAIN: Septin−type G
REGION: G1 motif
REGION: G3 motif
REGION: G4 motif
REGION: Interaction with SEPTIN12

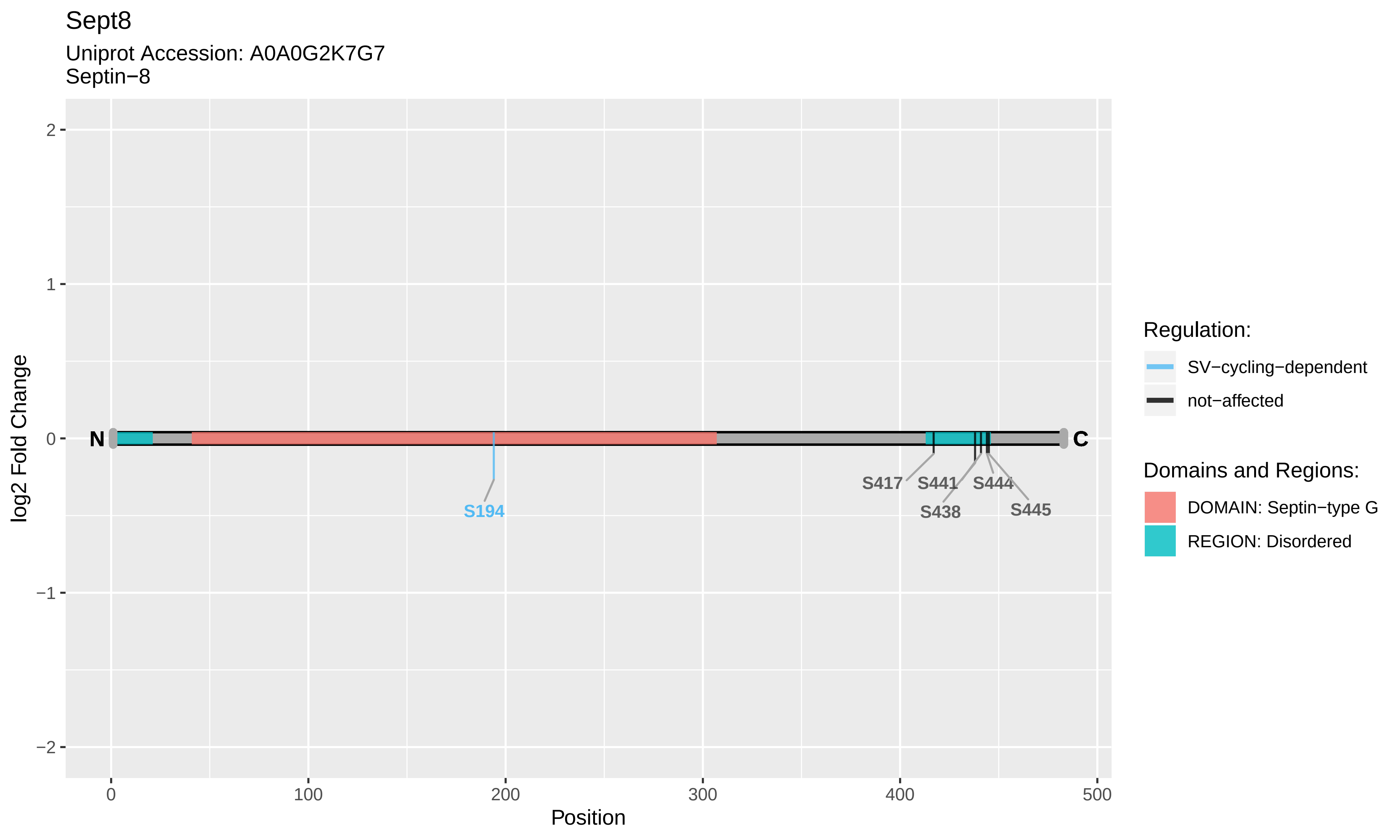
Sept8
Uniprot Accession: A0A0G2K7G7
Septin−8
2
1
0
−1
−2
log2 Fold Change
N
C
S194
S417
S441
S444
S438
S445
0
100
200
300
400
500
Position
Regulation:
SV−cycling−dependent
not−affected
Domains and Regions:
DOMAIN: Septin−type G
REGION: Disordered

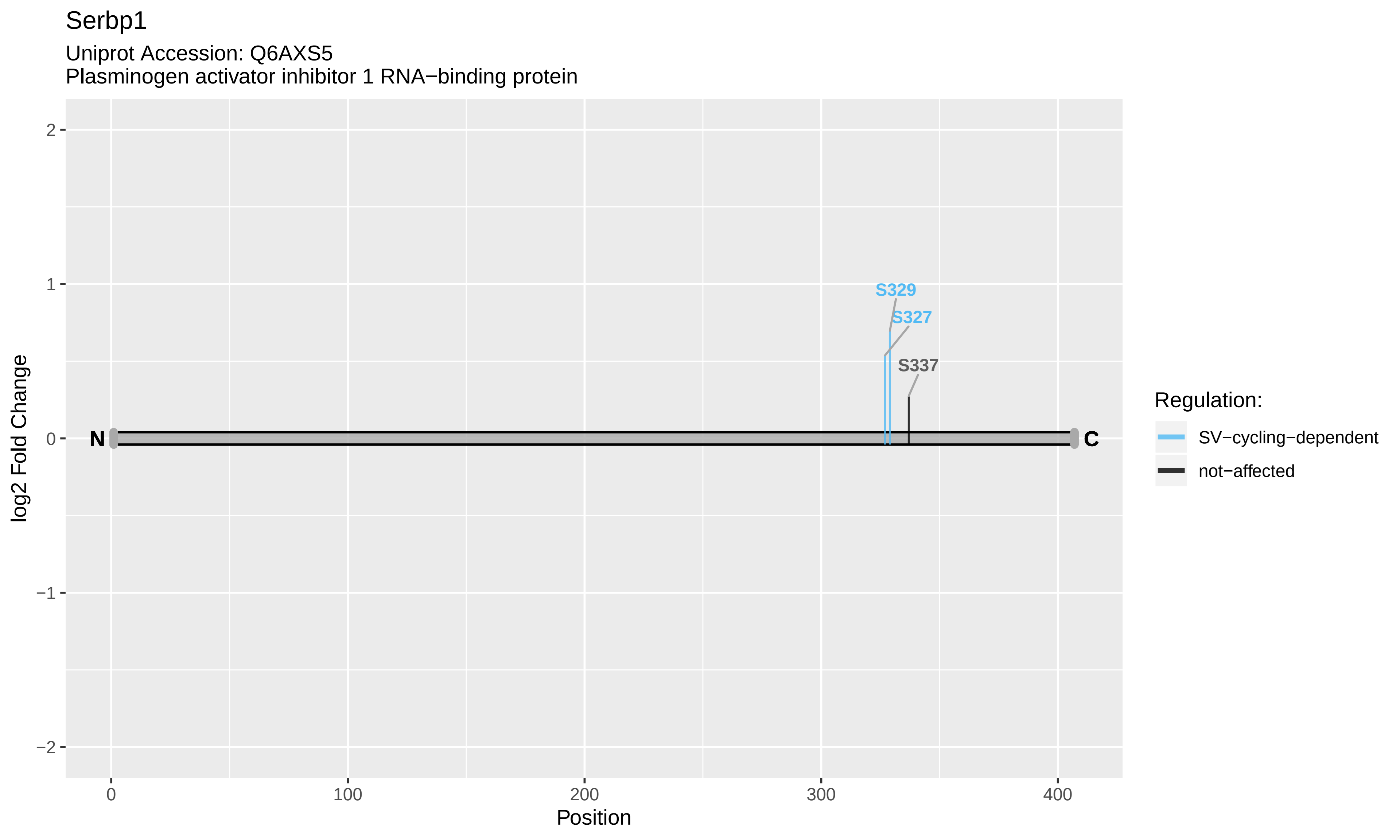
Serbp1
Uniprot Accession: Q6AXS5
Plasminogen activator inhibitor 1 RNA−binding protein
S329
S327
S337
N
C
2
1
0
−1
−2
0
100
200
300
400
log2 Fold Change
Position
Regulation:
SV−cycling−dependent
not−affected

Sergef

Uniprot Accession: D3ZN16
Secretion−regulating guanine nucleotide exchange factor

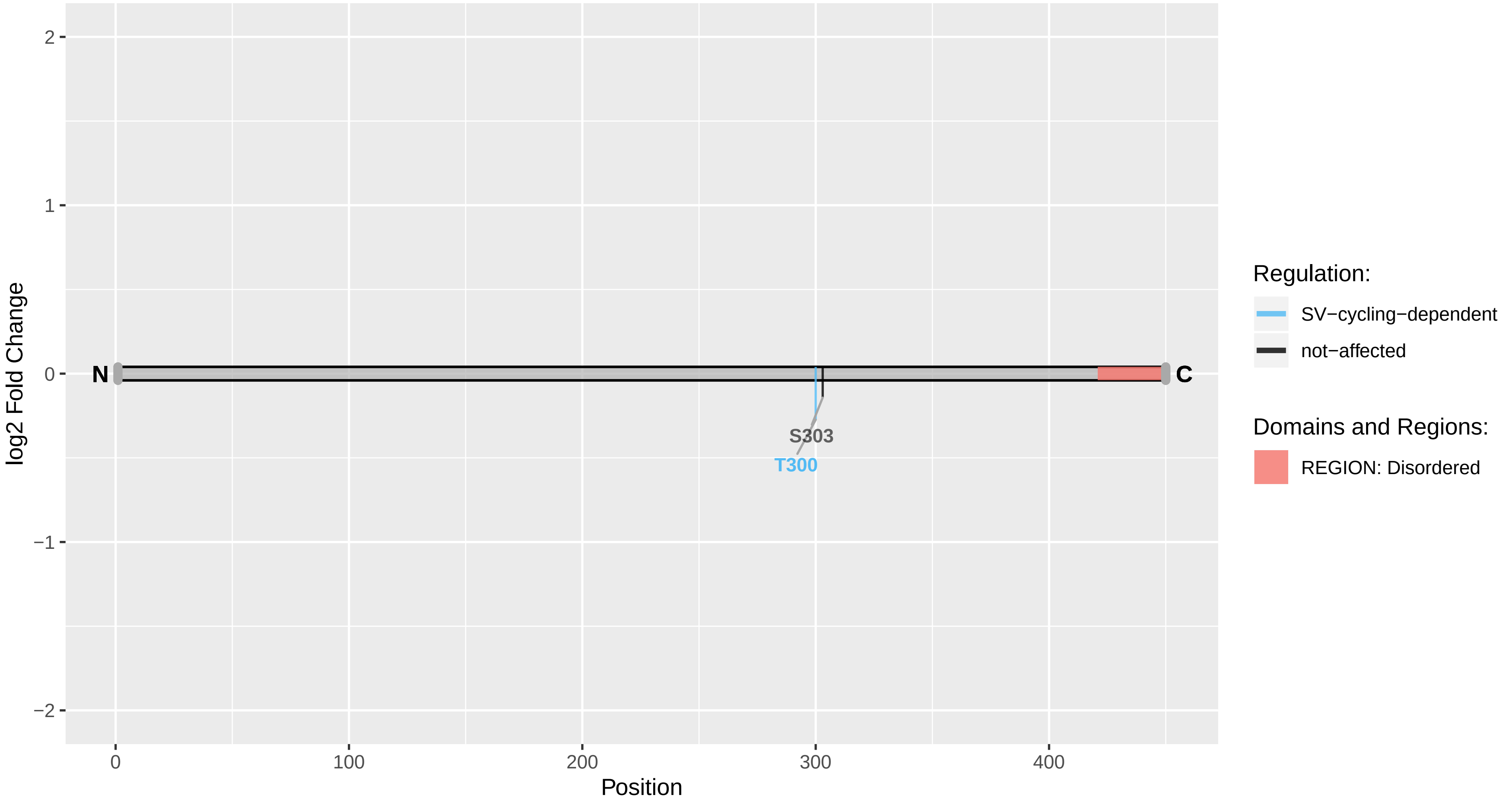

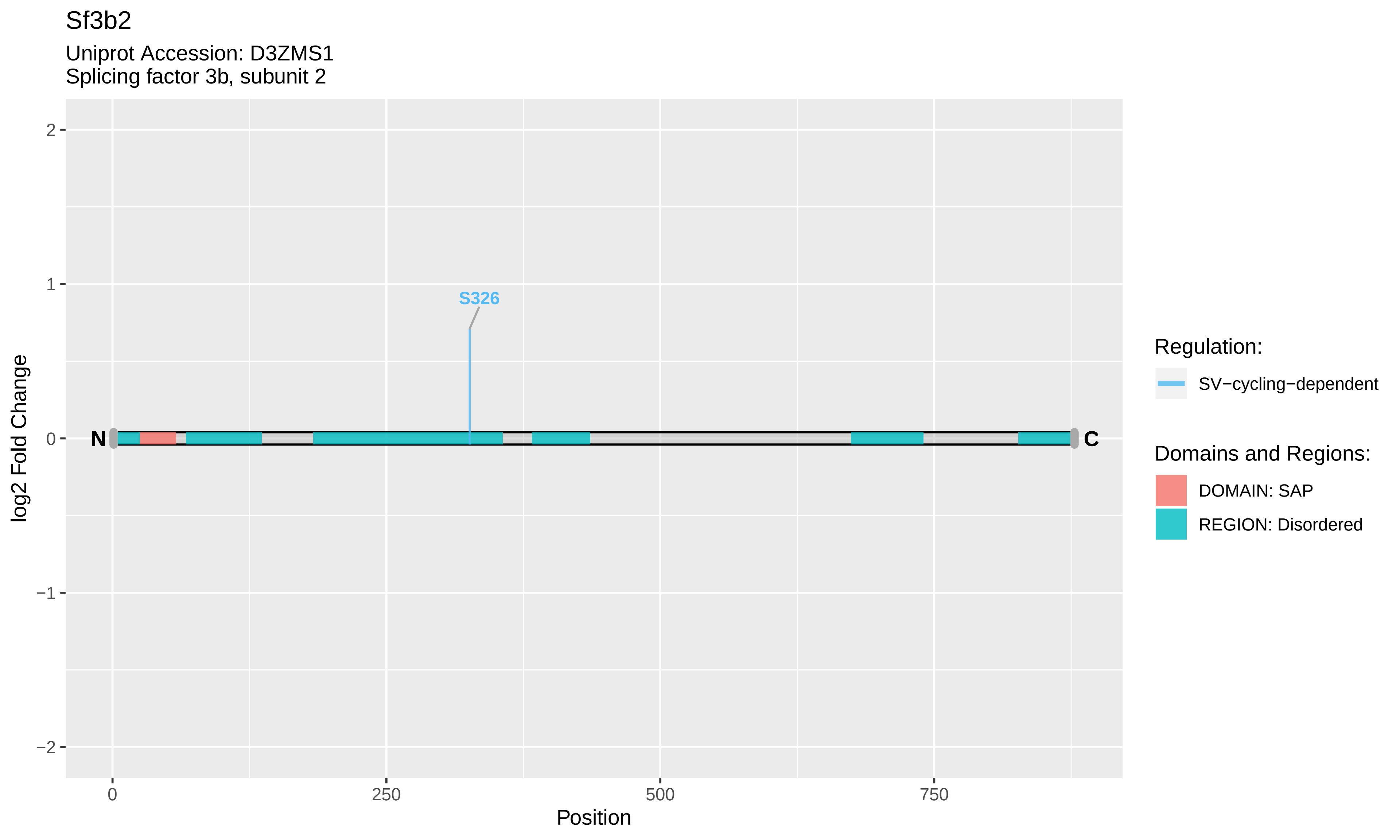

Sf3b2
Uniprot Accession: D3ZMS1
Splicing factor 3b, subunit 2
S326
N
C
2
1
0
−1
−2
log2 Fold Change
0
250
500
750
Position
Regulation:
SV−cycling−dependent
Domains and Regions:
DOMAIN: SAP
REGION: Disordered

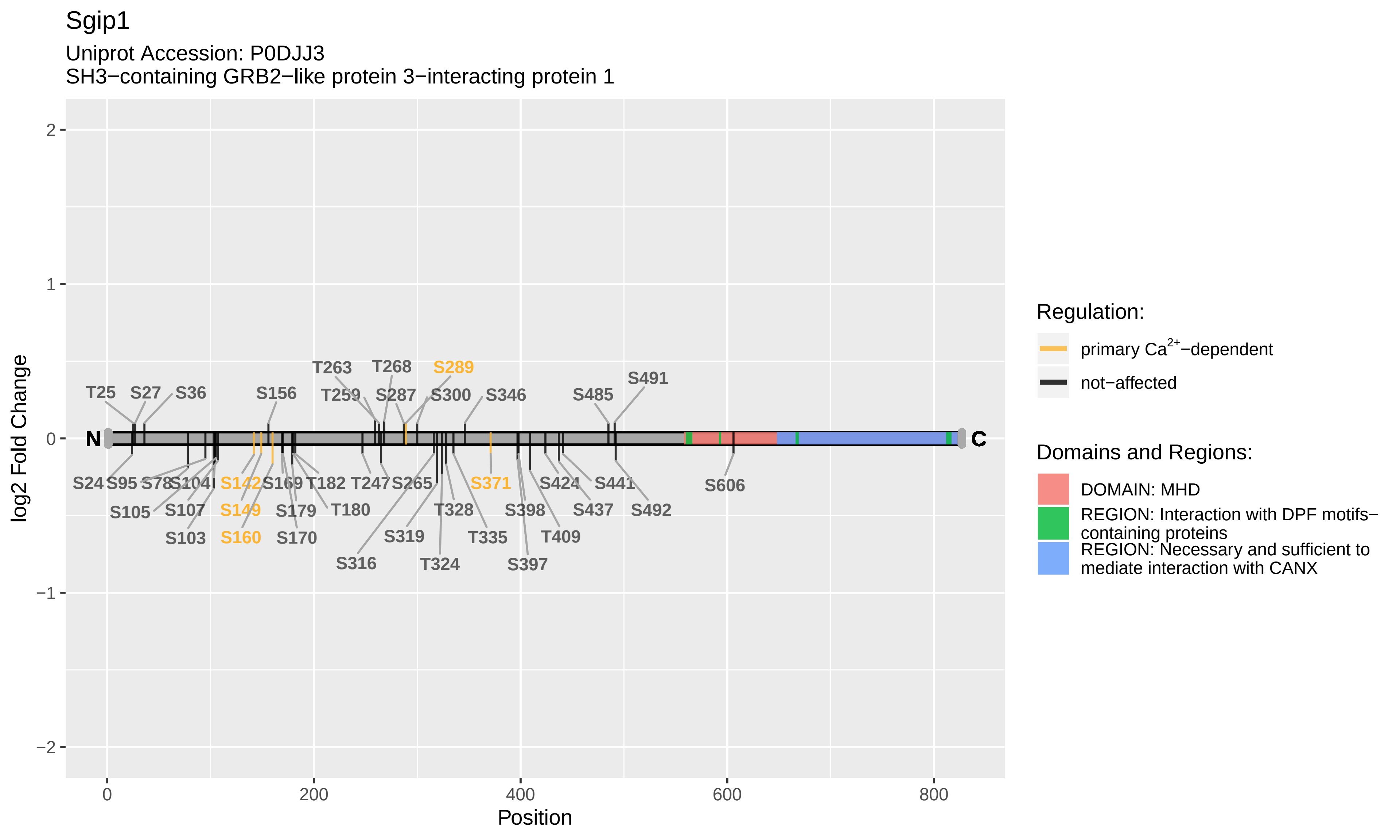

Sgip1
Uniprot Accession: P0DJJ3
SH3−containing GRB2−like protein 3−interacting protein 1
log2 Fold Change
Position
N
C
T25
S27
S36
S156
T263
T259
T268
S287
S289
S300
S346
S485
S491
S24
S95
S78
S104
S142
S169
T182
T247
S265
S371
S424
S441
S606
S105
S107
S149
S179
T180
T328
S398
S437
S492
S103
S160
S170
S319
T335
T409
S316
T324
S397
Regulation:
primary Ca2+−dependent
not−affected
Domains and Regions:
DOMAIN: MHD
REGION: Interaction with DPF motifs−containing proteins
REGION: Necessary and sufficient to mediate interaction with CANX

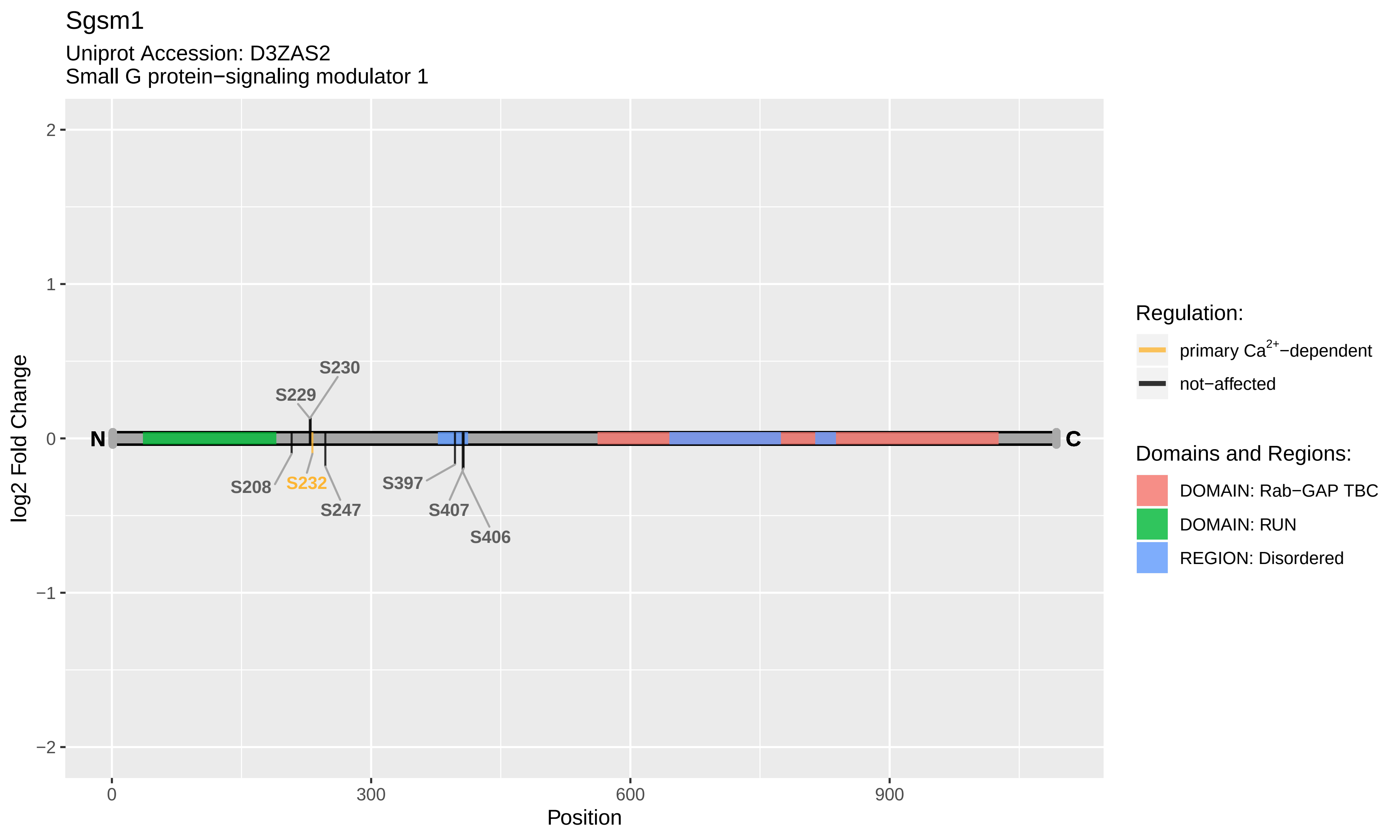

Sgsm1
Uniprot Accession: D3ZAS2
Small G protein−signaling modulator 1
log2 Fold Change
2
1
0
−1
−2
0
300
600
900
Position
N
C
S230
S229
S208
S232
S247
S397
S407
S406
Regulation:
primary Ca2+−dependent
not−affected
Domains and Regions:
DOMAIN: Rab−GAP TBC
DOMAIN: RUN
REGION: Disordered

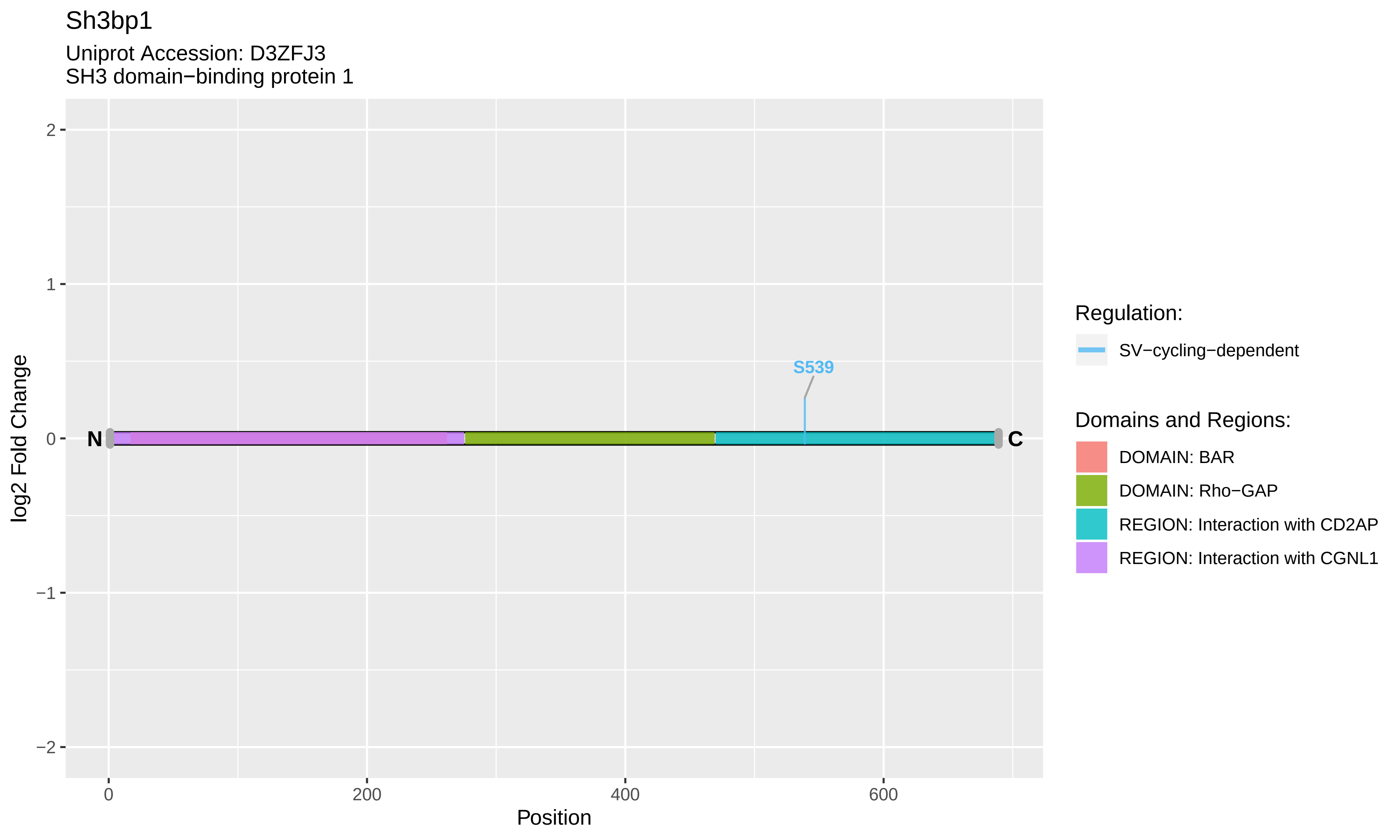
Sh3bp1
Uniprot Accession: D3ZFJ3
SH3 domain−binding protein 1
log2 Fold Change
2
1
0
−1
−2
0
200
400
600
Position
N
C
S539
Regulation:
SV−cycling−dependent
Domains and Regions:
DOMAIN: BAR
DOMAIN: Rho−GAP
REGION: Interaction with CD2AP
REGION: Interaction with CGNL1

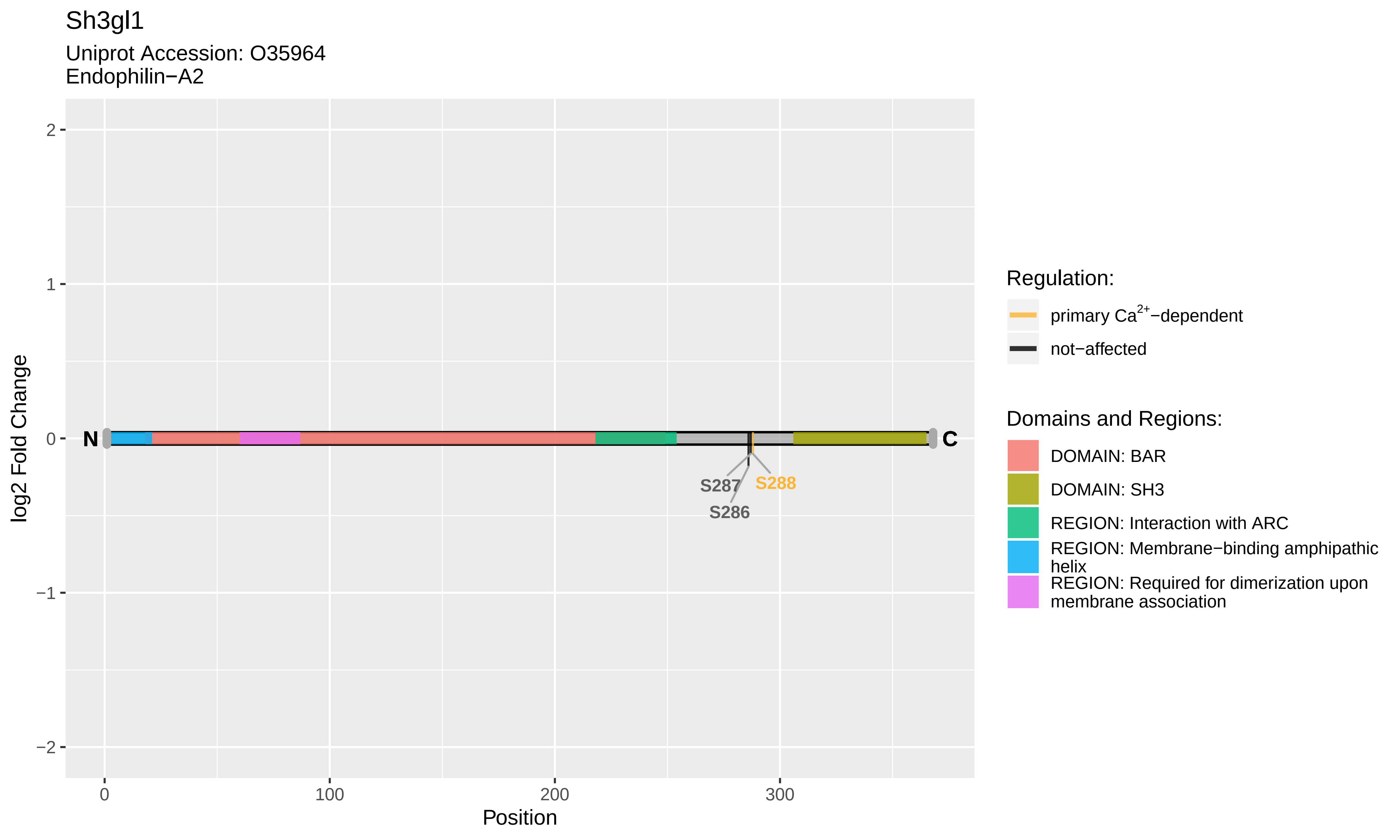
Sh3gl1
Uniprot Accession: O35964
Endophilin−A2
log2 Fold Change
2
1
0
−1
−2
N
C
S287
S288
S286
0
100
200
300
Position
Regulation:
primary Ca2+−dependent
not−affected
Domains and Regions:
DOMAIN: BAR
DOMAIN: SH3
REGION: Interaction with ARC
REGION: Membrane−binding amphipathic helix
REGION: Required for dimerization upon membrane association

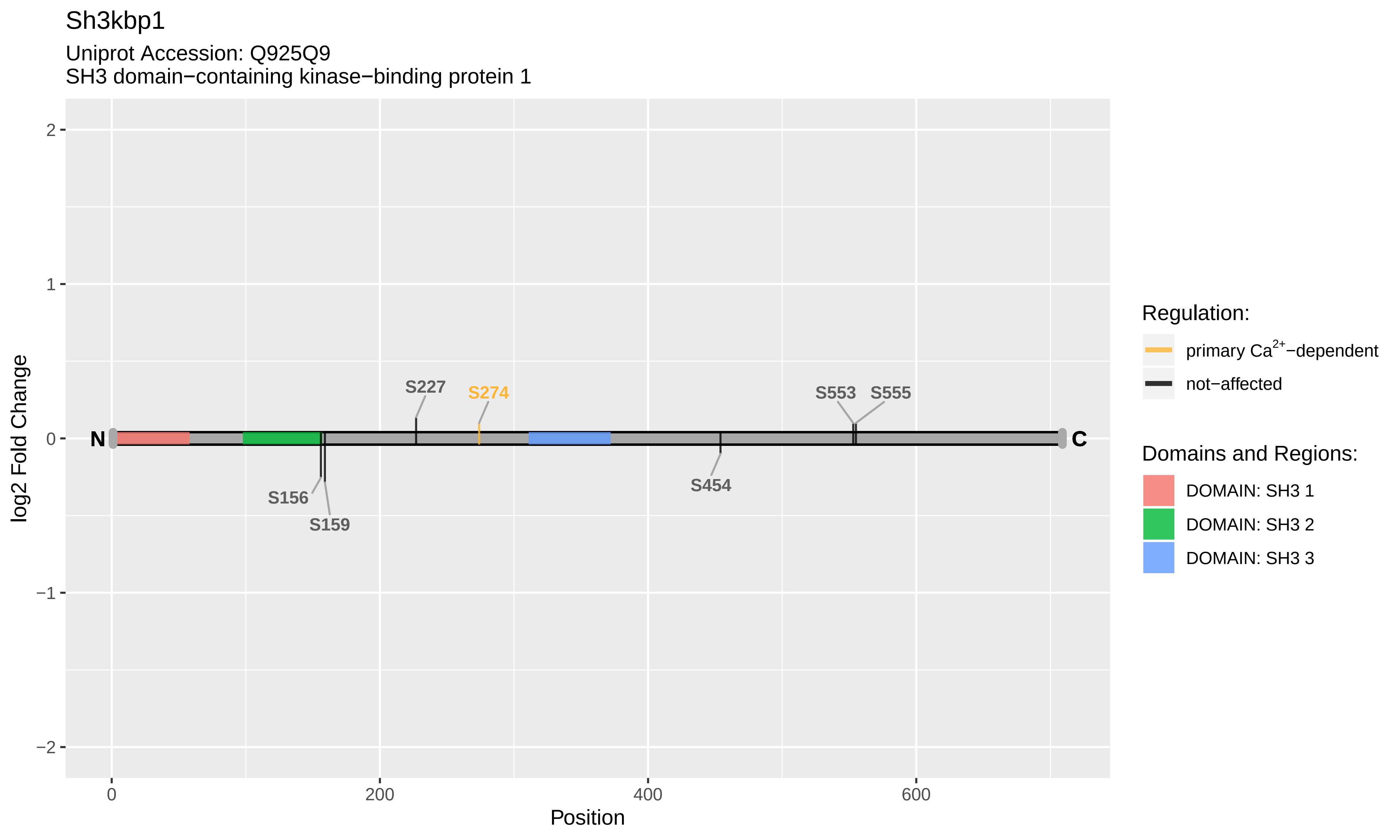
Sh3kbp1
Uniprot Accession: Q925Q9
SH3 domain−containing kinase−binding protein 1
log2 Fold Change
Position
N
C
S156
S159
S227
S274
S454
S553
S555
Regulation:
primary Ca2+−dependent
not−affected
Domains and Regions:
DOMAIN: SH3 1
DOMAIN: SH3 2
DOMAIN: SH3 3

Sh3pxd2a

Uniprot Accession: A0A0G2JX92
SH3 and PX domains 2A

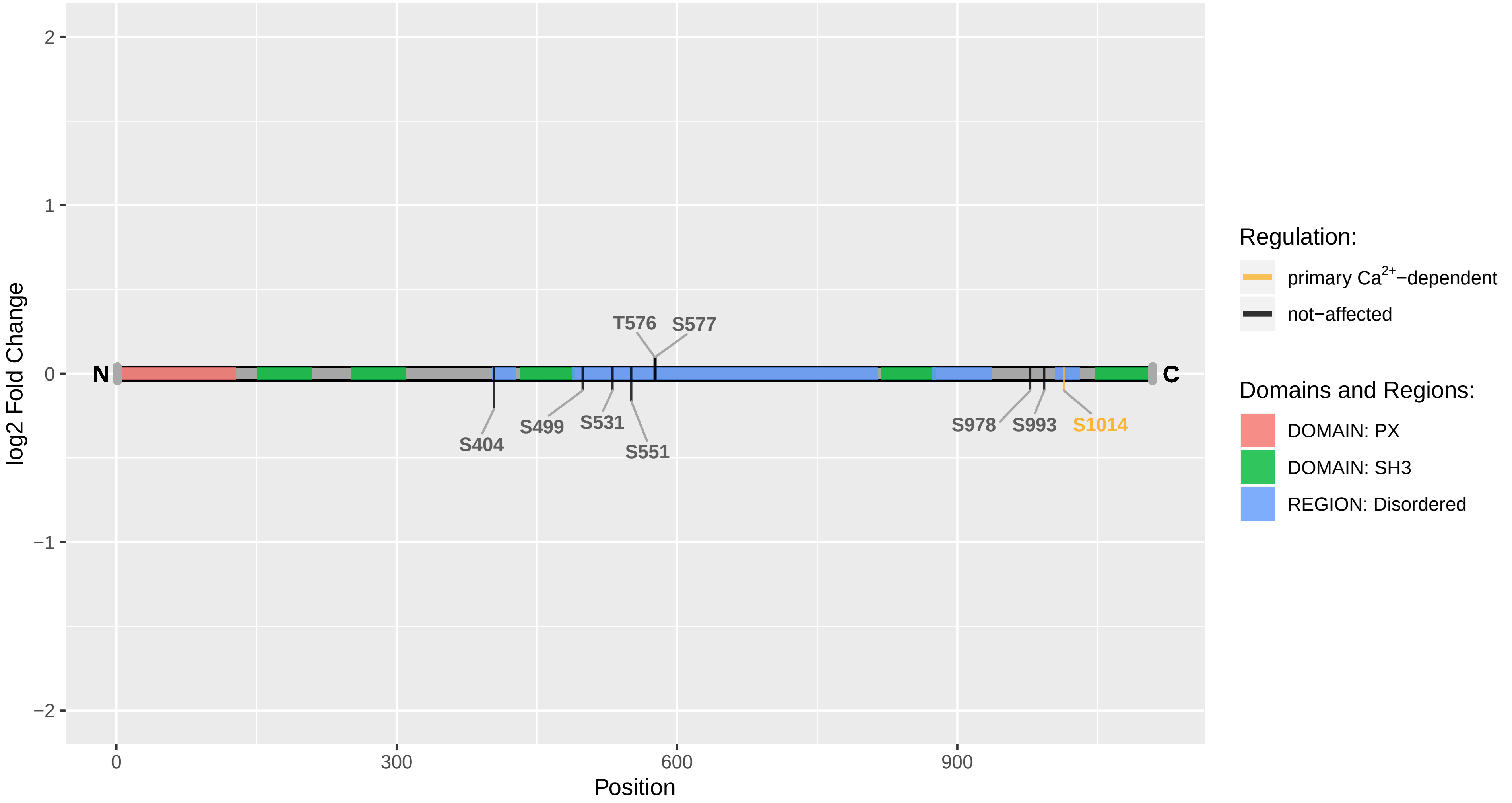

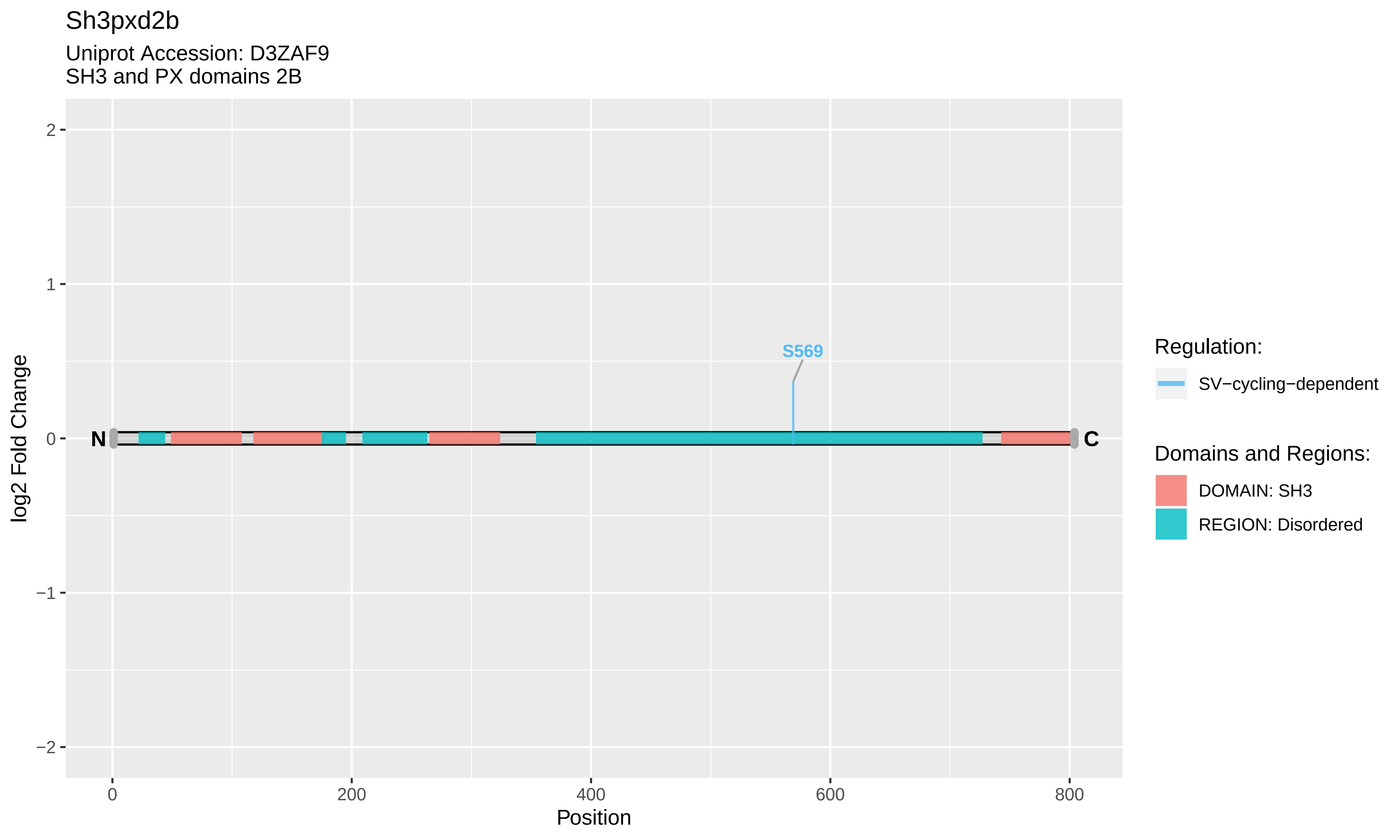
Sh3pxd2b
Uniprot Accession: D3ZAF9
SH3 and PX domains 2B
S569
N
C
log2 Fold Change
Position
2
1
0
−1
−2
0
200
400
600
800
Regulation:
SV−cycling−dependent
Domains and Regions:
DOMAIN: SH3
REGION: Disordered

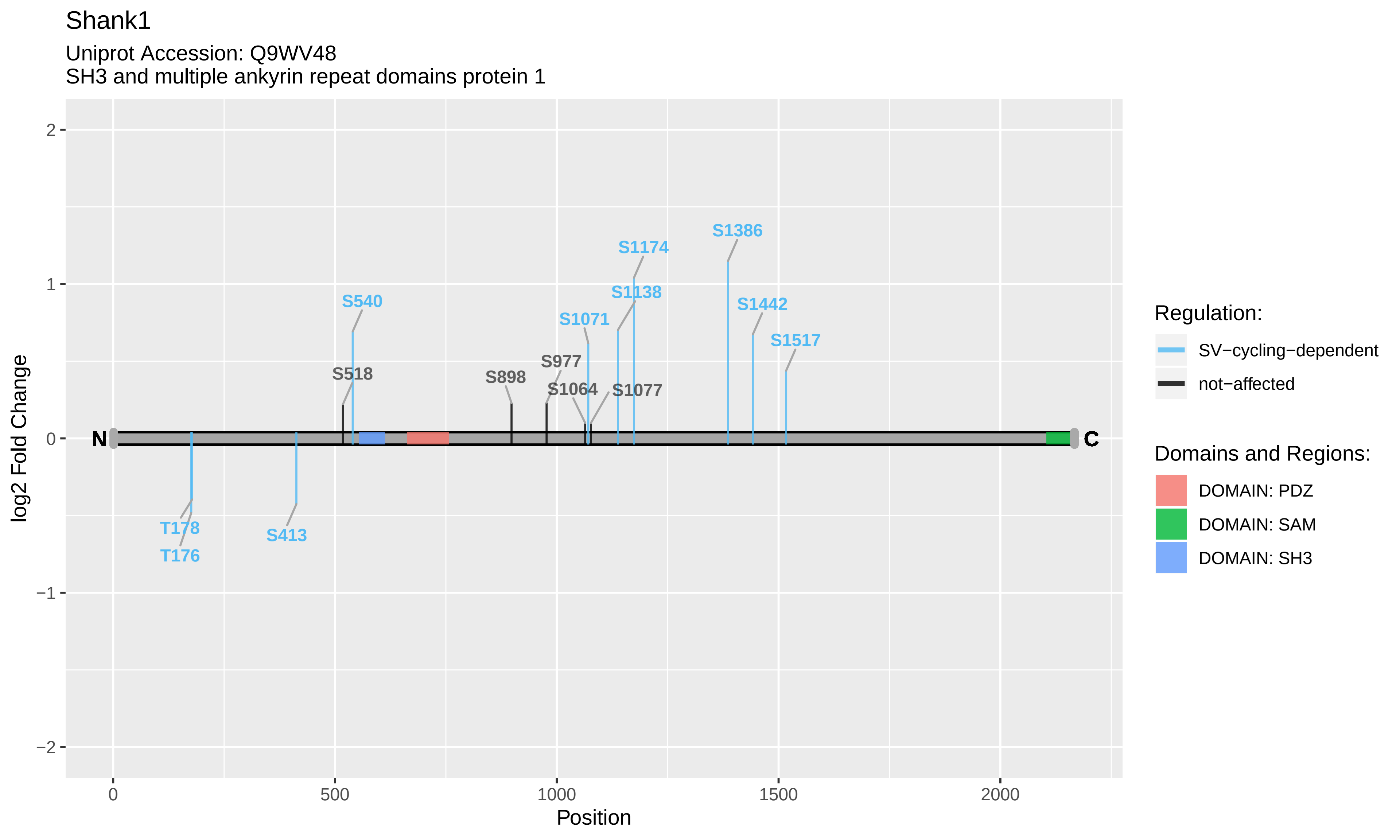

Shank1
Uniprot Accession: Q9WV48
SH3 and multiple ankyrin repeat domains protein 1
log2 Fold Change
2
1
0
−1
−2
N
C
S1386
S1174
S1138
S540
S1071
S1442
S1517
S977
S518
S898
S1064
S1077
T178
T176
S413
0
500
1000
1500
2000
Position
Regulation:
SV−cycling−dependent
not−affected
Domains and Regions:
DOMAIN: PDZ
DOMAIN: SAM
DOMAIN: SH3

Shank2

Uniprot Accession: Q9QX74
SH3 and multiple ankyrin repeat domains protein 2

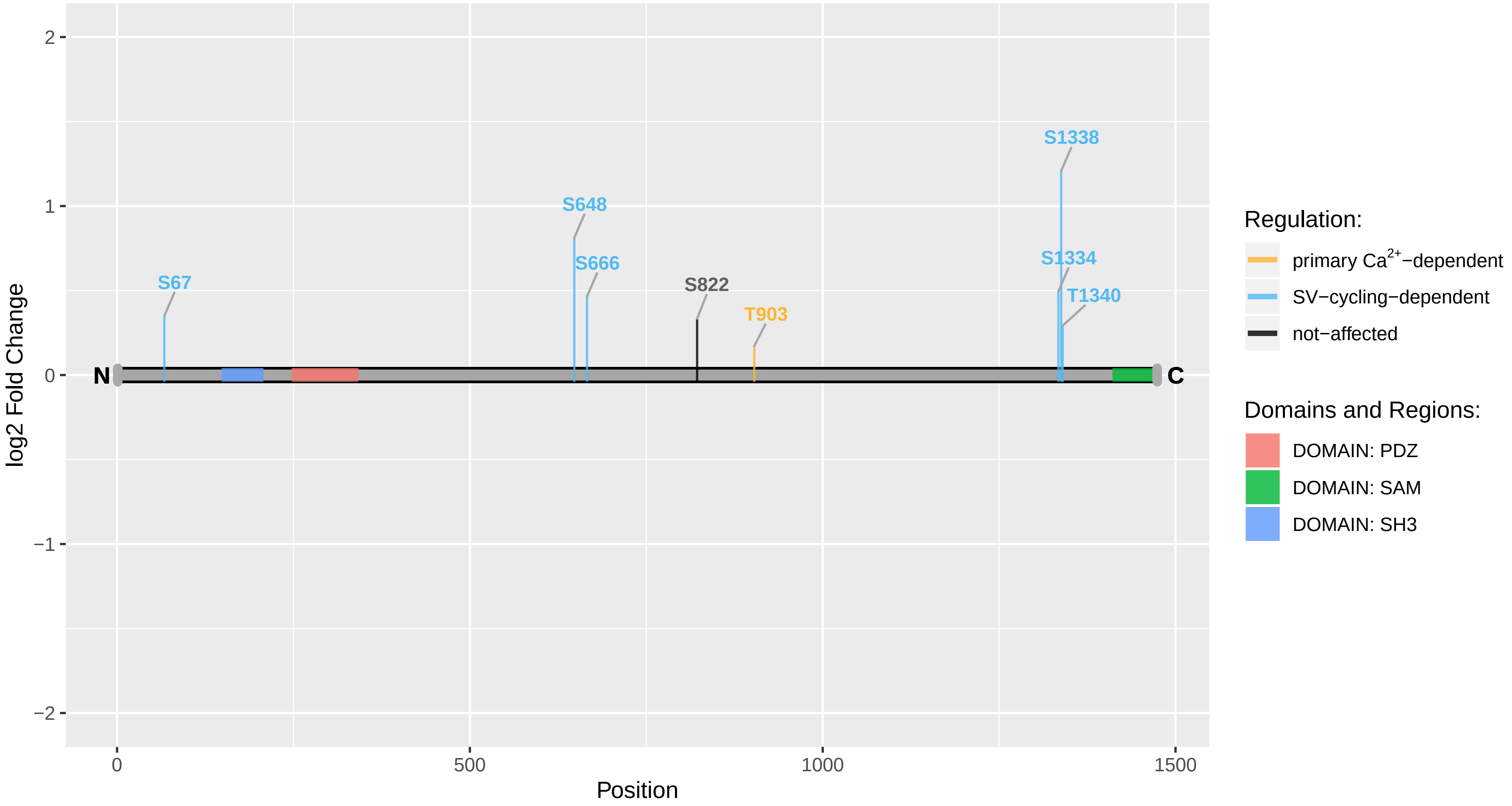

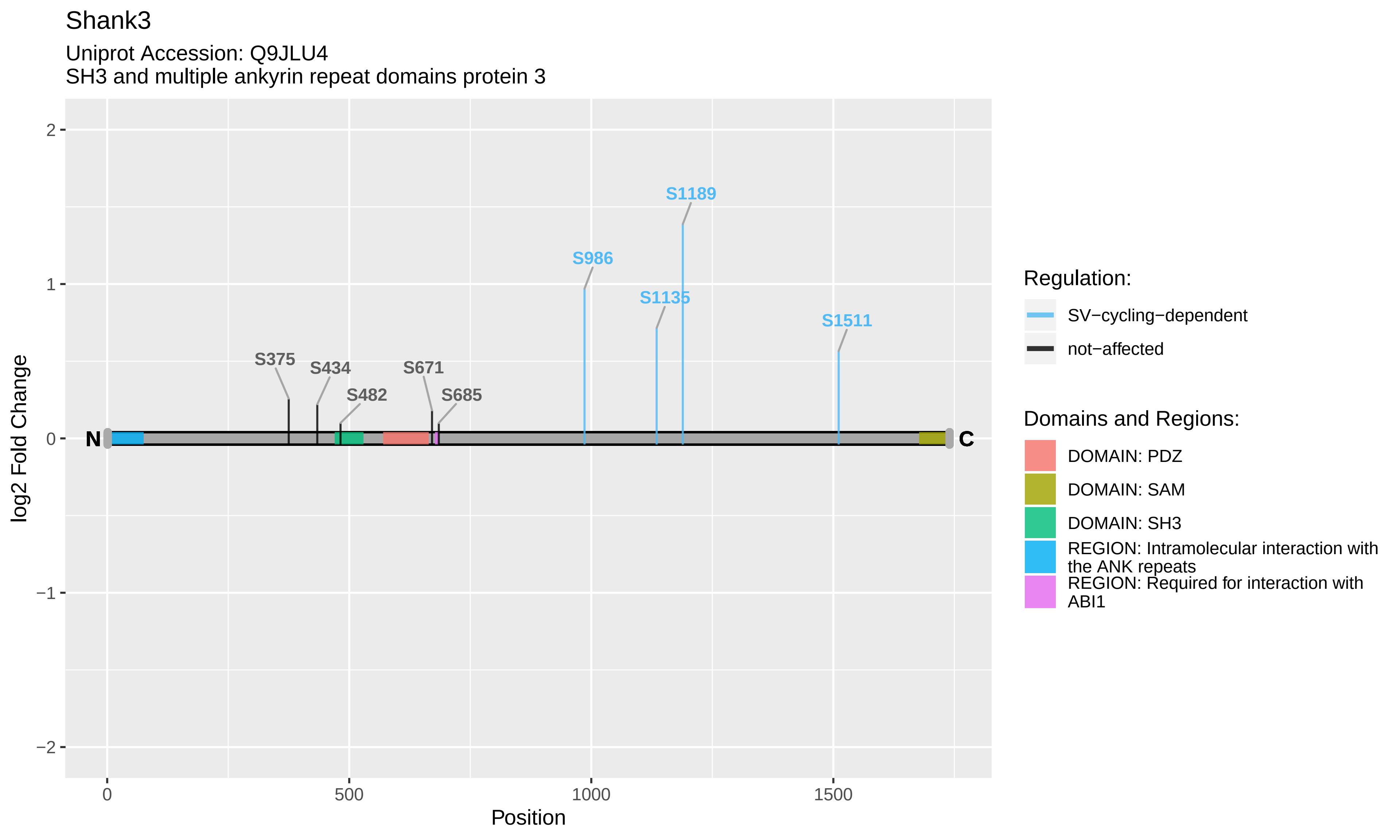

Shank3
Uniprot Accession: Q9JLU4
SH3 and multiple ankyrin repeat domains protein 3
log2 Fold Change
Position
2
1
0
−1
−2
0
500
1000
1500
N
C
S375
S434
S482
S671
S685
S986
S1135
S1189
S1511
Regulation:
SV−cycling−dependent
not−affected
Domains and Regions:
DOMAIN: PDZ
DOMAIN: SAM
DOMAIN: SH3
REGION: Intramolecular interaction with the ANK repeats
REGION: Required for interaction with ABI1

Shisa6

Uniprot Accession: D4A4M0
Shisa family member 6

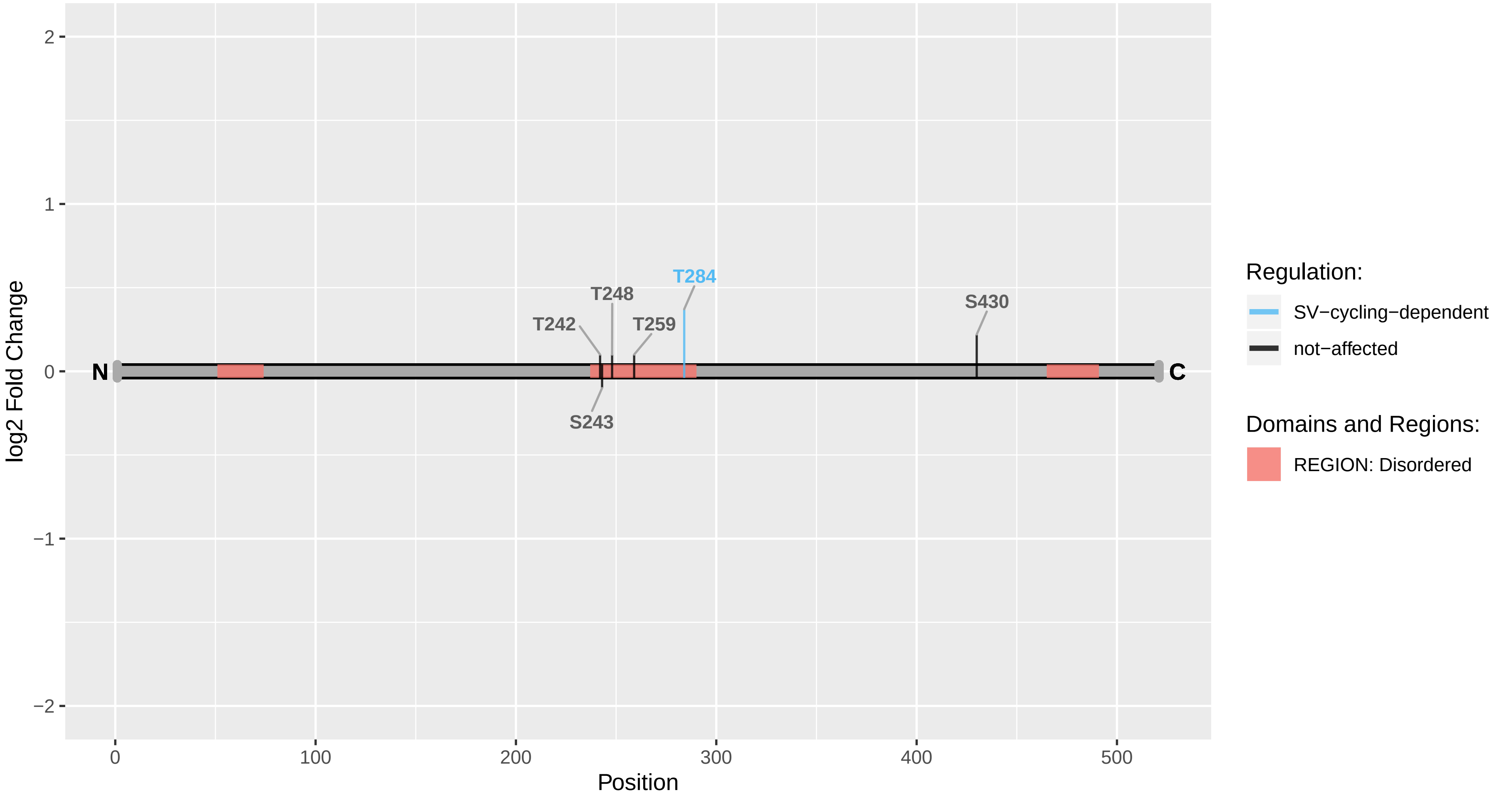

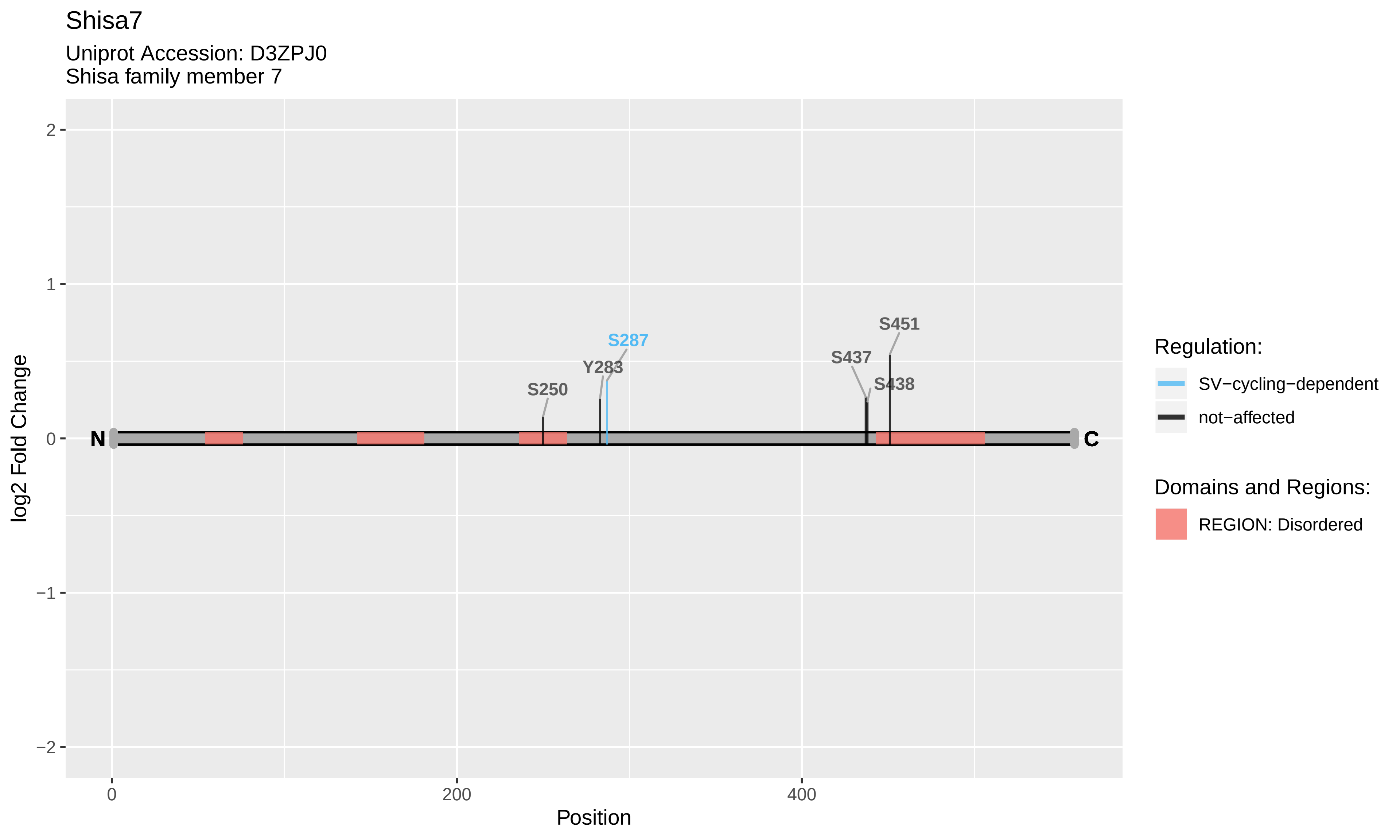

Shisa7
Uniprot Accession: D3ZPJ0
Shisa family member 7
log2 Fold Change
Position
N
C
S250
Y283
S287
S437
S438
S451
2
1
0
−1
−2
0
200
400
Regulation:
SV−cycling−dependent
not−affected
Domains and Regions:
REGION: Disordered

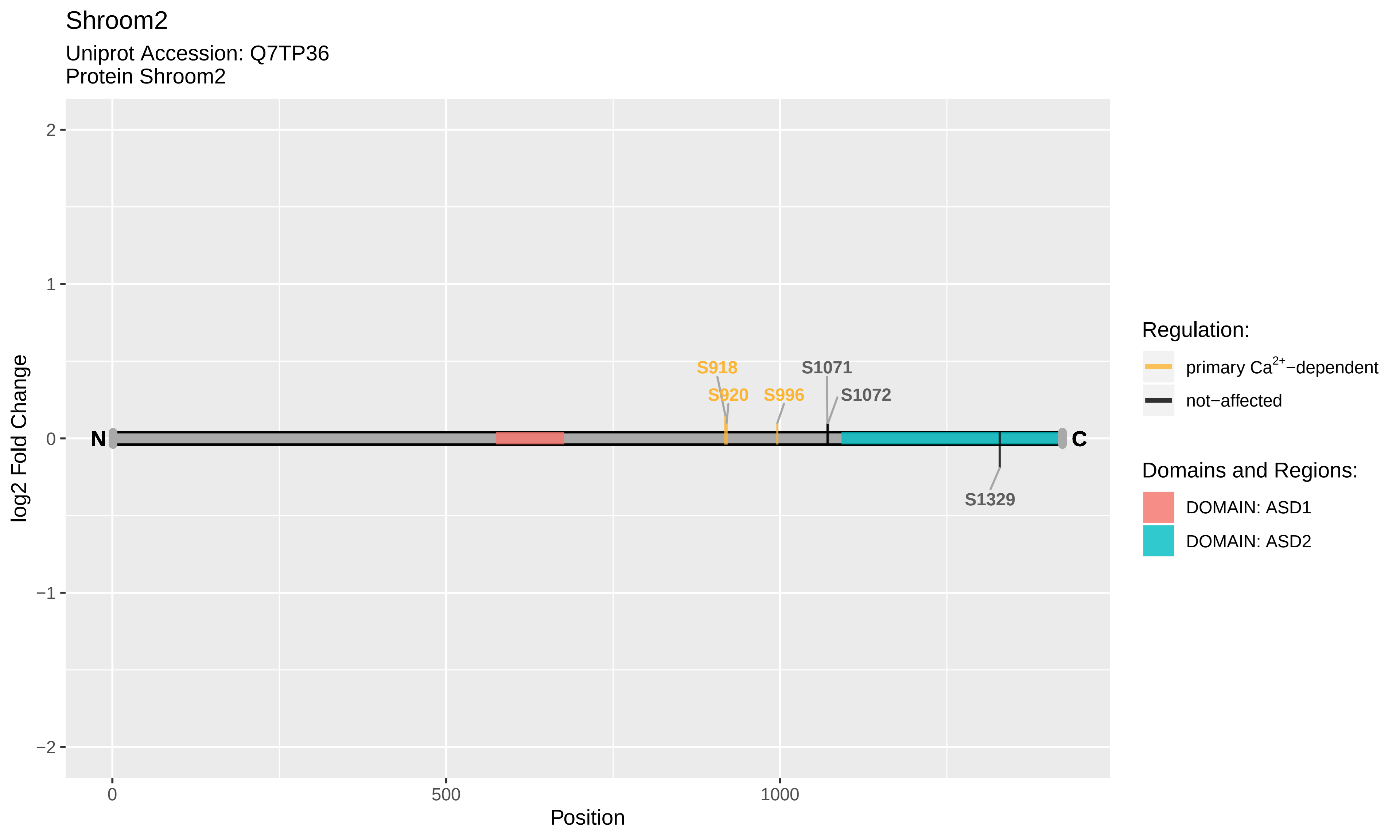
Shroom2
Uniprot Accession: Q7TP36
Protein Shroom2
log2 Fold Change
2
1
0
−1
−2
0
500
1000
Position
N
C
S918
S920
S996
S1071
S1072
S1329
Regulation:
primary Ca2+−dependent
not−affected
Domains and Regions:
DOMAIN: ASD1
DOMAIN: ASD2

Shtn1

Uniprot Accession: A0MZ67
Shootin−1

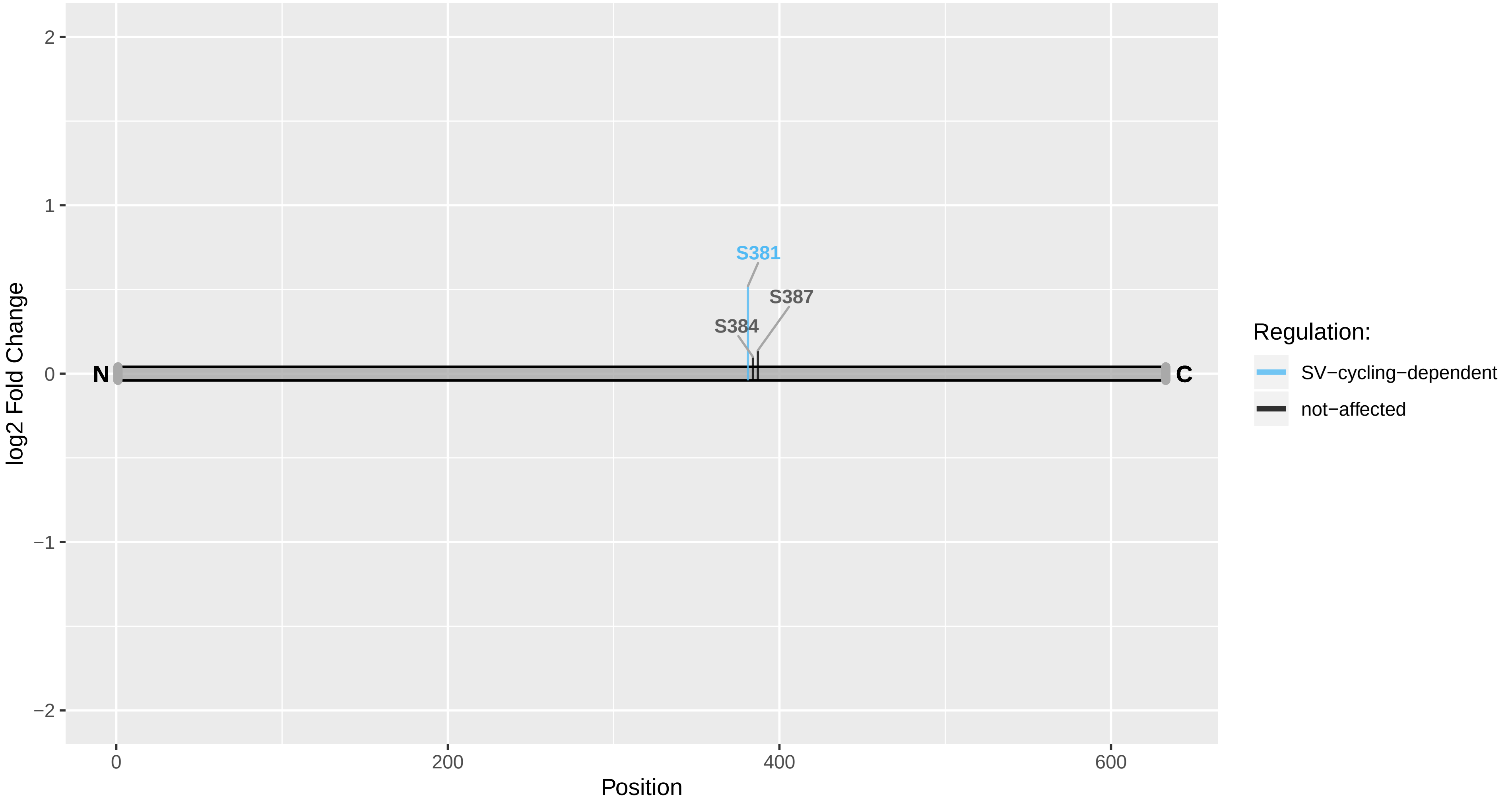

Sipa1l1

Uniprot Accession: A0A0G2KAW2
Signal−induced proliferation−associated 1 like 1, isoform CRA_b

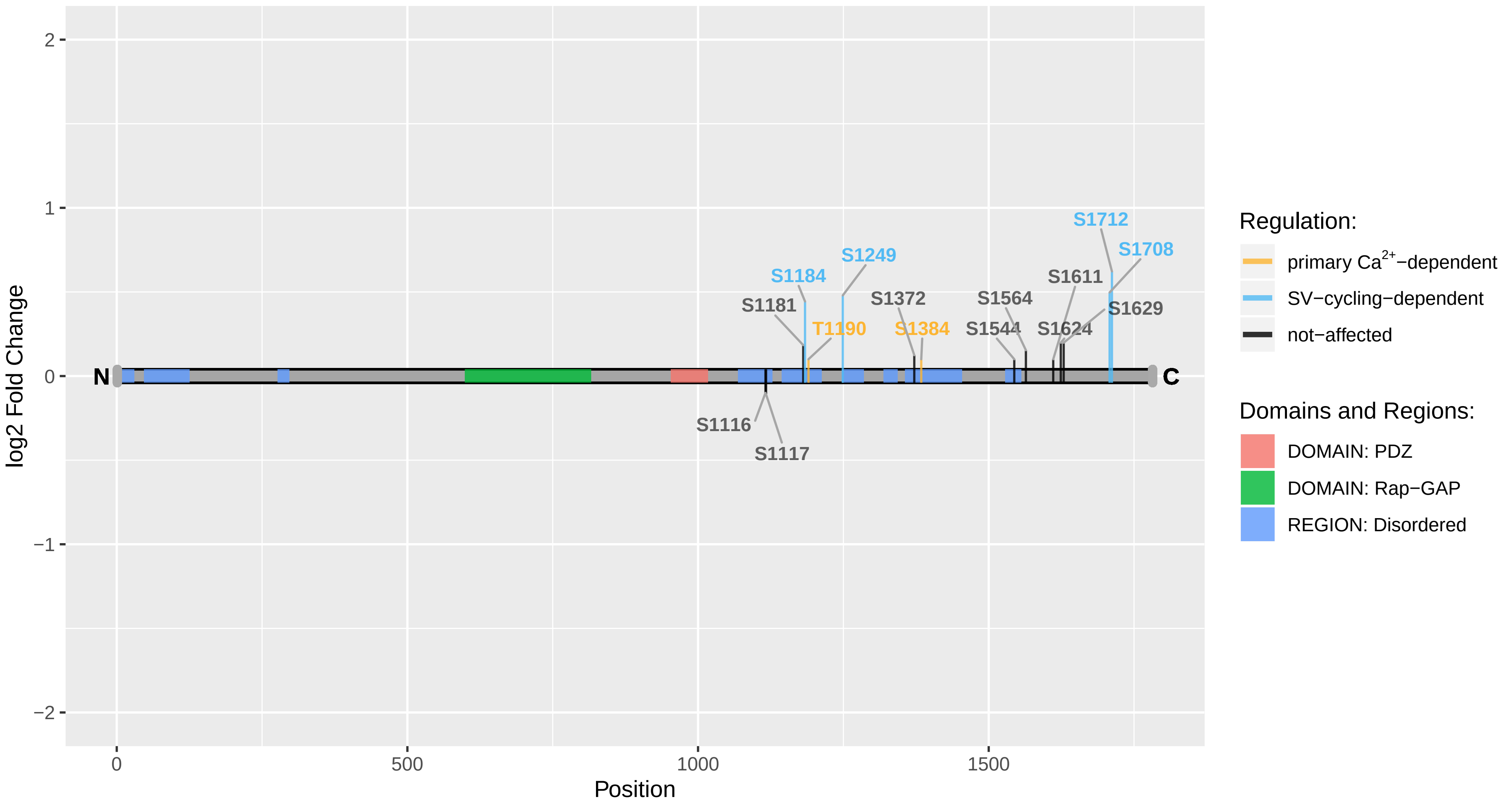

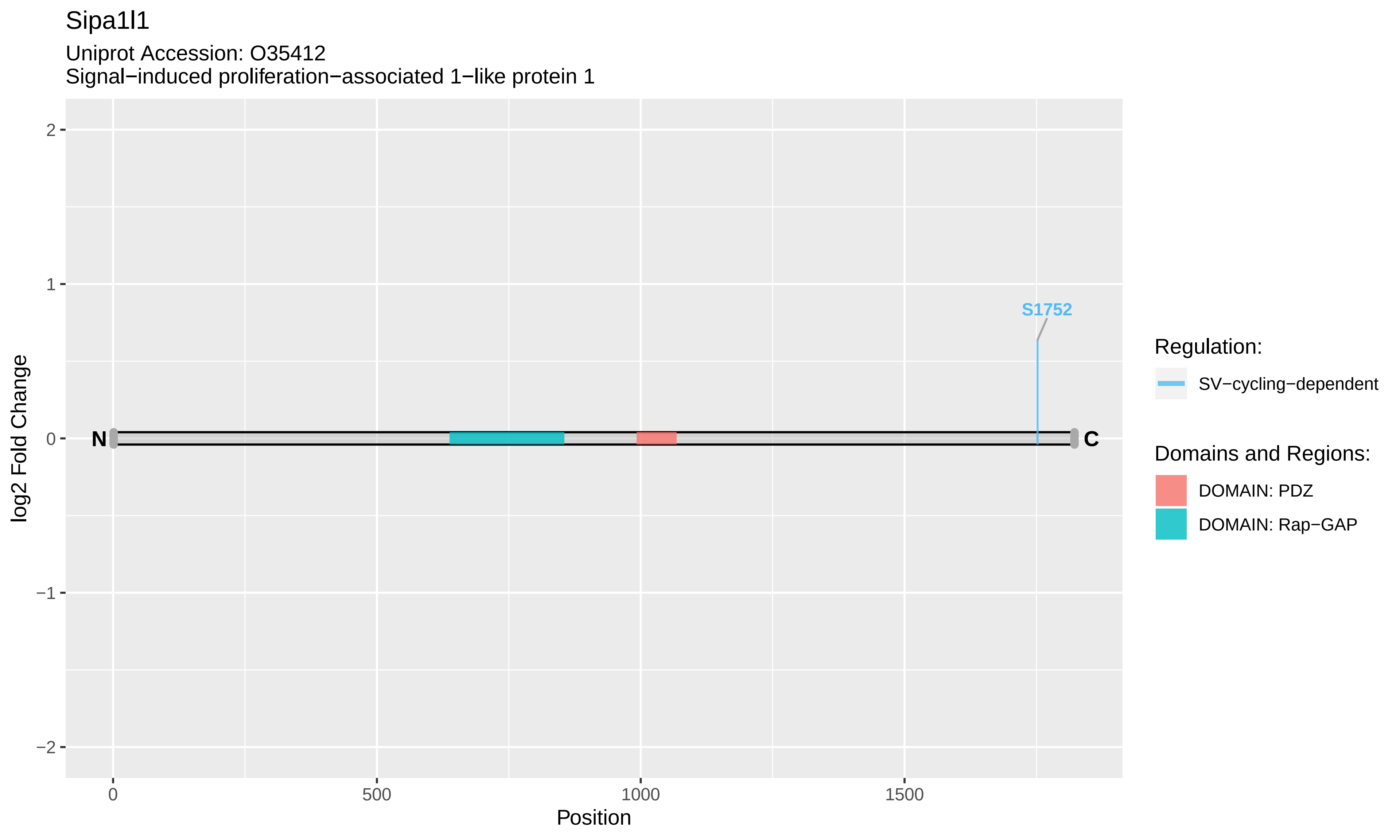
Sipa1l1
Uniprot Accession: O35412
Signal−induced proliferation−associated 1−like protein 1
S1752
N
C
log2 Fold Change
Position
2
1
0
−1
−2
0
500
1000
1500
Regulation:
SV−cycling−dependent
Domains and Regions:
DOMAIN: PDZ
DOMAIN: Rap−GAP

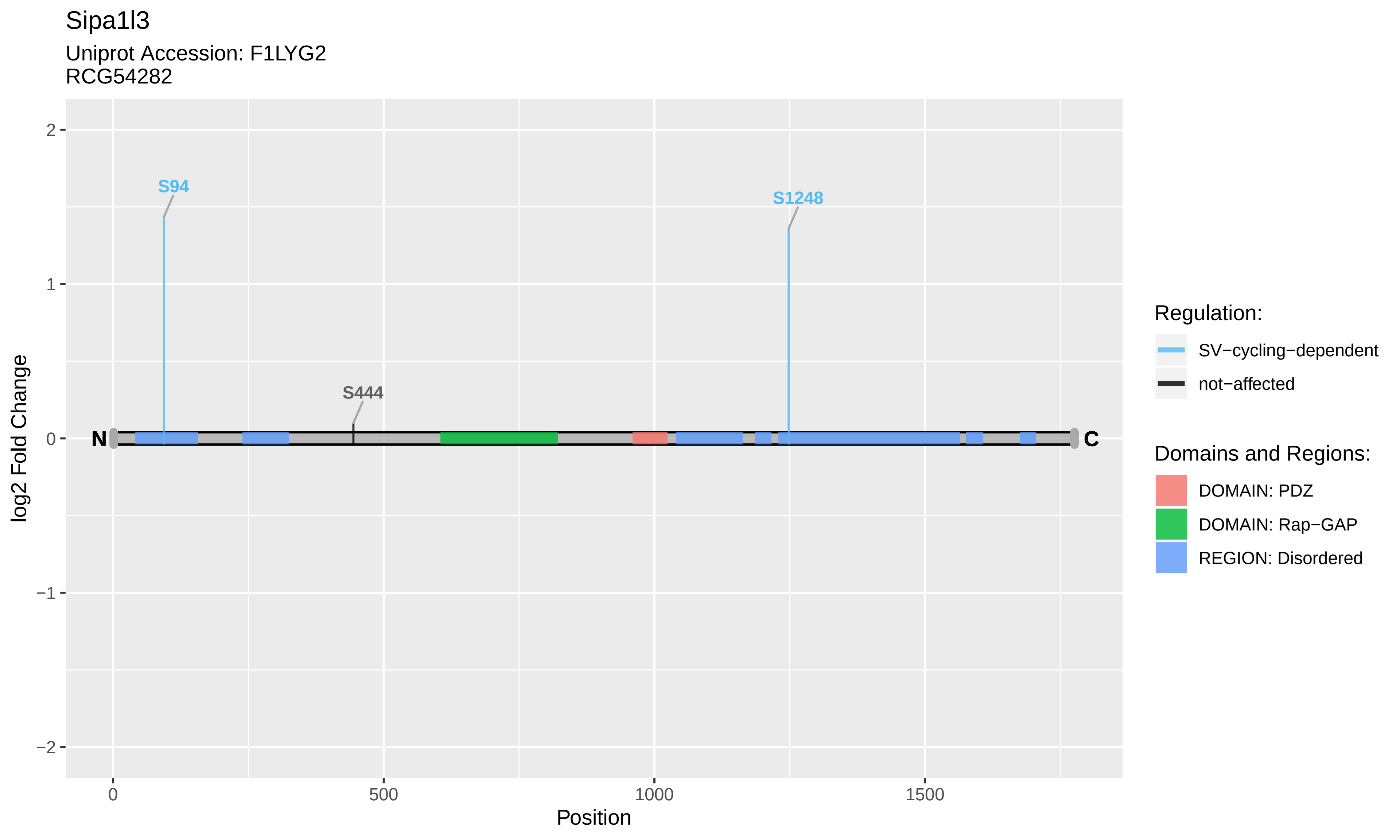
Sipa1l3
Uniprot Accession: F1LYG2
RCG54282
S94
S1248
S444
N
C
log2 Fold Change
Position
2
1
0
−1
−2
0
500
1000
1500
Regulation:
SV−cycling−dependent
not−affected
Domains and Regions:
DOMAIN: PDZ
DOMAIN: Rap−GAP
REGION: Disordered

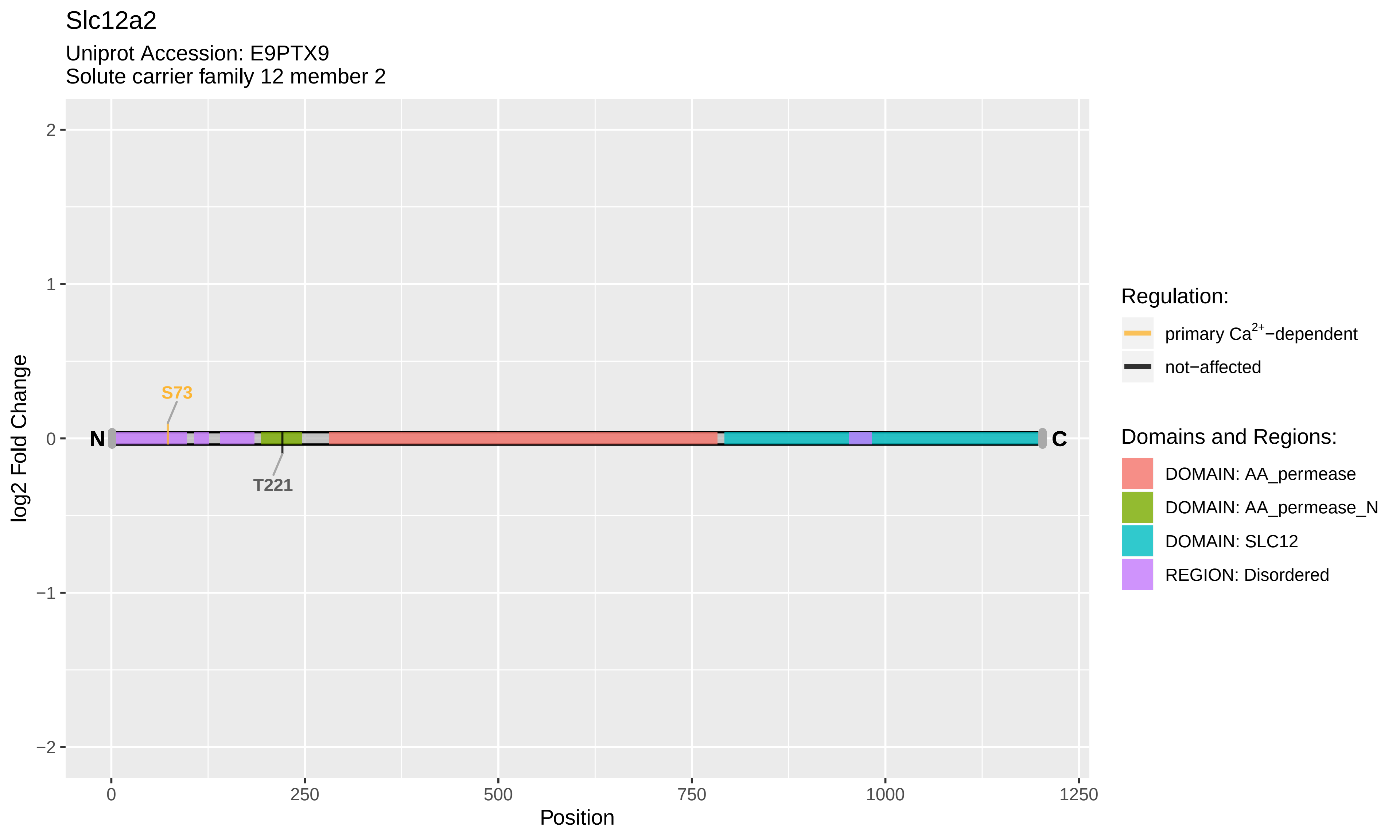

Slc12a2
Uniprot Accession: E9PTX9
Solute carrier family 12 member 2
log2 Fold Change
Position
S73
T221
N
C
2
1
0
−1
−2
0
250
500
750
1000
1250
Regulation:
primary Ca2+−dependent
not−affected
Domains and Regions:
DOMAIN: AA_permease
DOMAIN: AA_permease_N
DOMAIN: SLC12
REGION: Disordered

Slc12a5

Uniprot Accession: Q63633
Solute carrier family 12 member 5

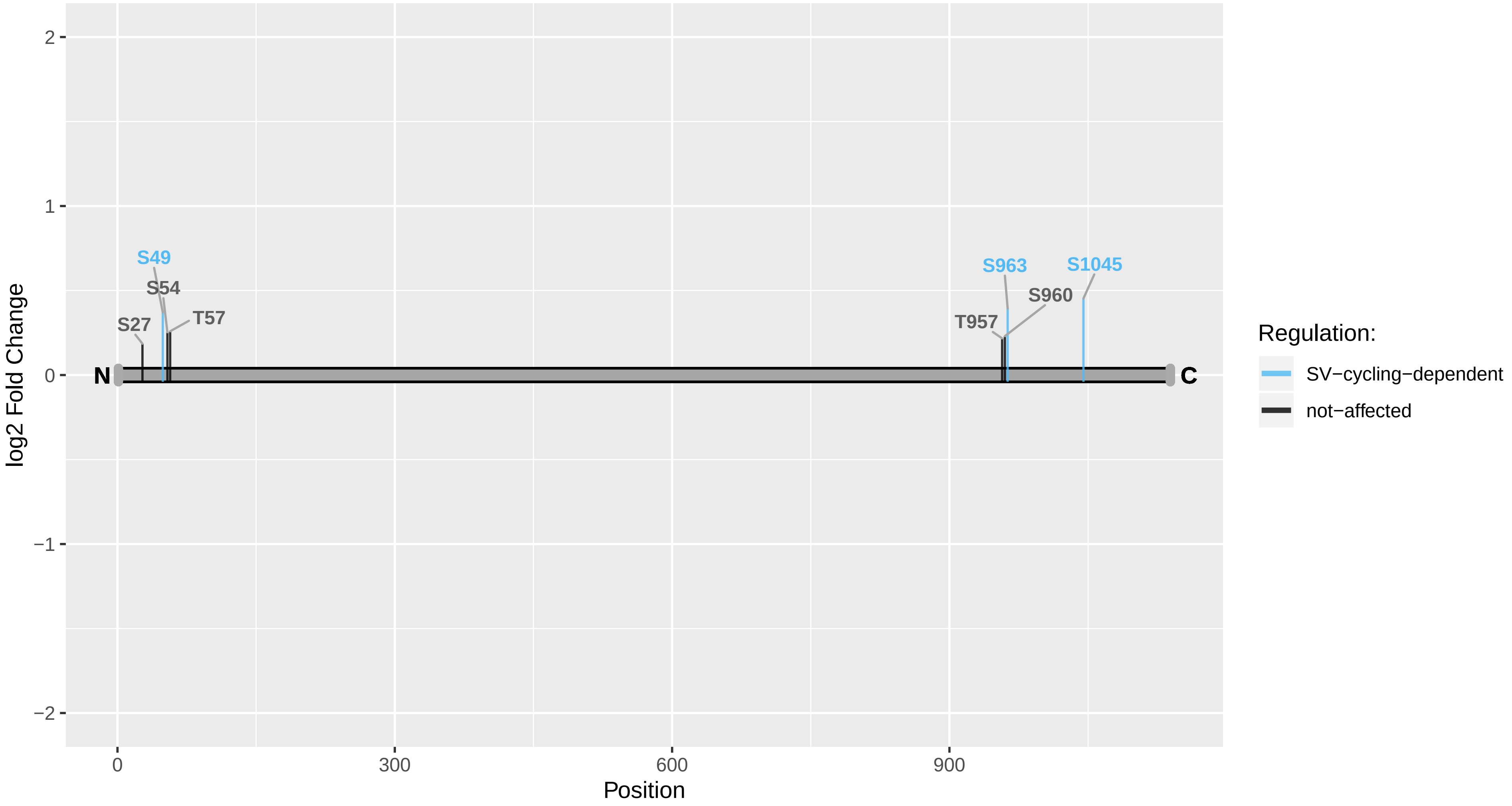

Slc12a6

Uniprot Accession: G3V6N7
RCG27287, isoform CRA_a

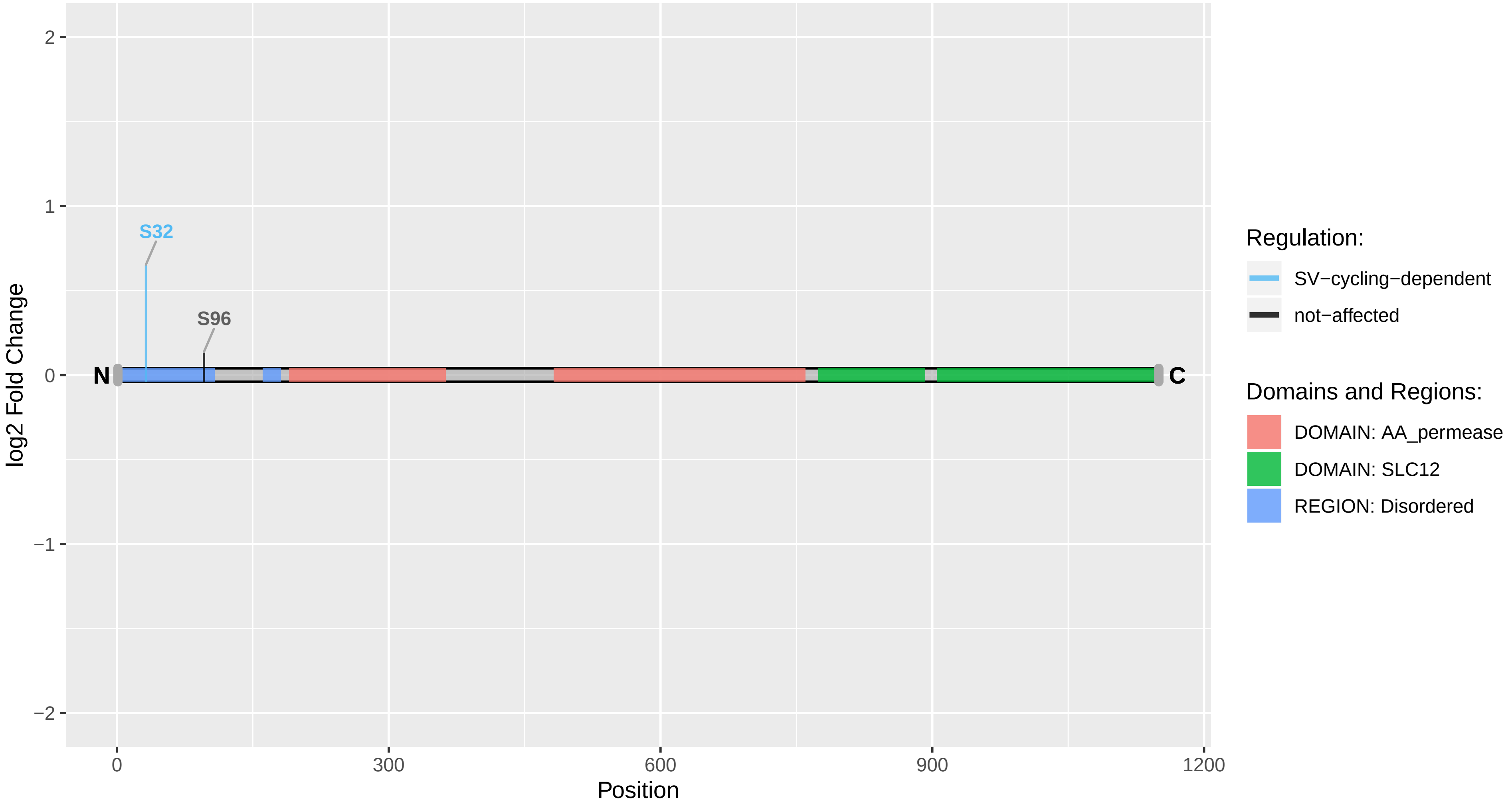

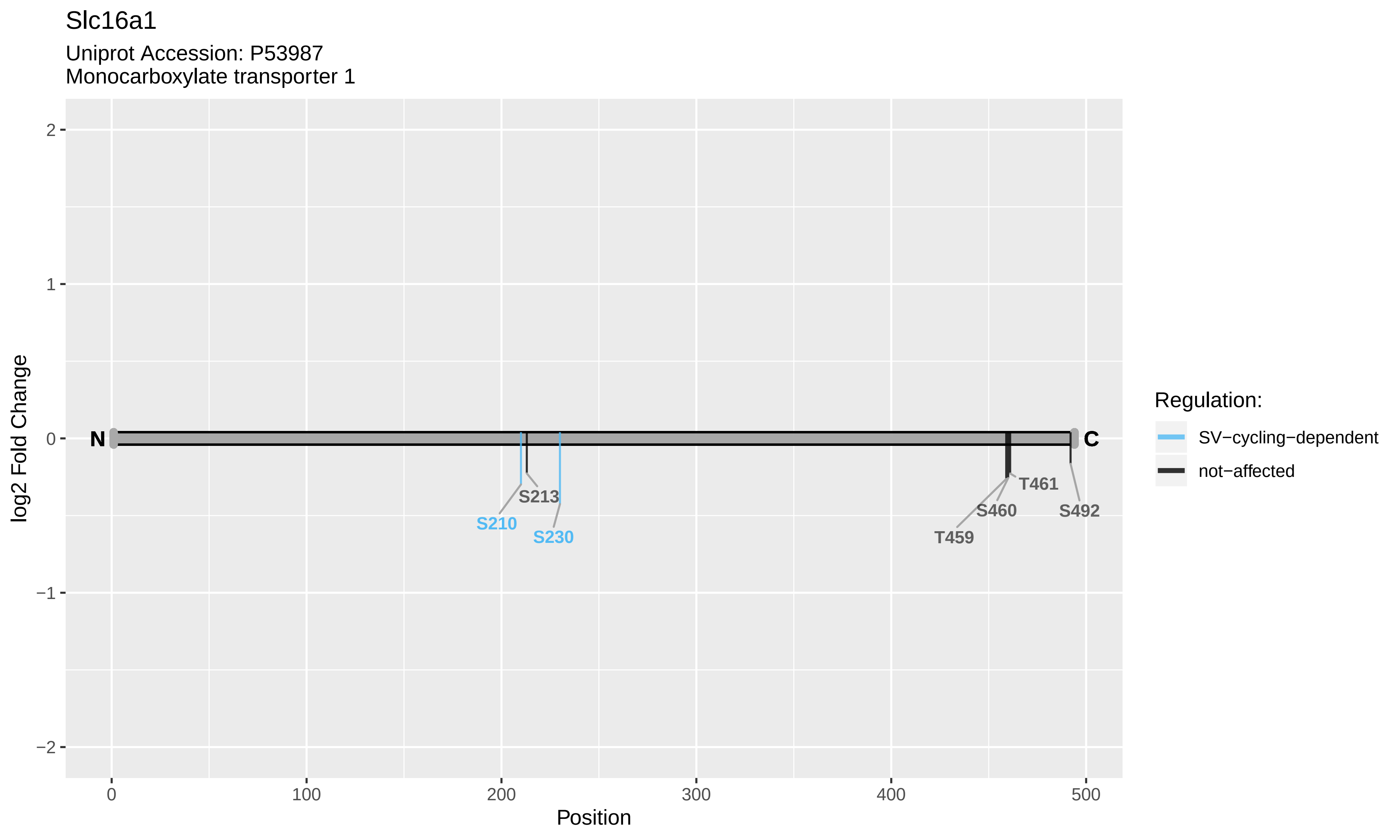

Slc16a1
Uniprot Accession: P53987
Monocarboxylate transporter 1
log2 Fold Change
2
1
0
−1
−2
N
C
S210
S213
S230
T459
S460
T461
S492
0
100
200
300
400
500
Position
Regulation:
SV−cycling−dependent
not−affected

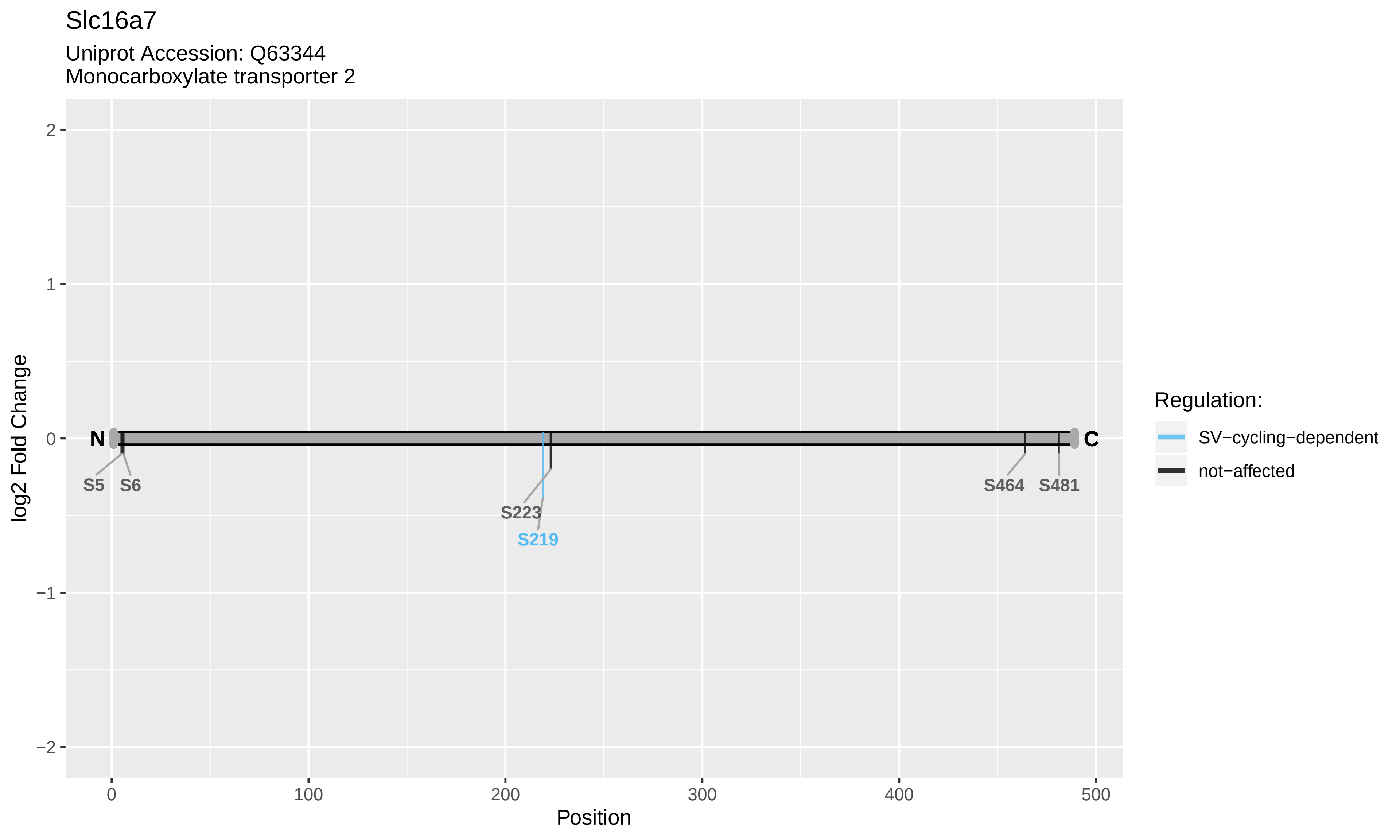
Slc16a7
Uniprot Accession: Q63344
Monocarboxylate transporter 2
log2 Fold Change
2
1
0
−1
−2
N
C
S5
S6
S223
S219
S464
S481
0
100
200
300
400
500
Position
Regulation:
SV−cycling−dependent
not−affected

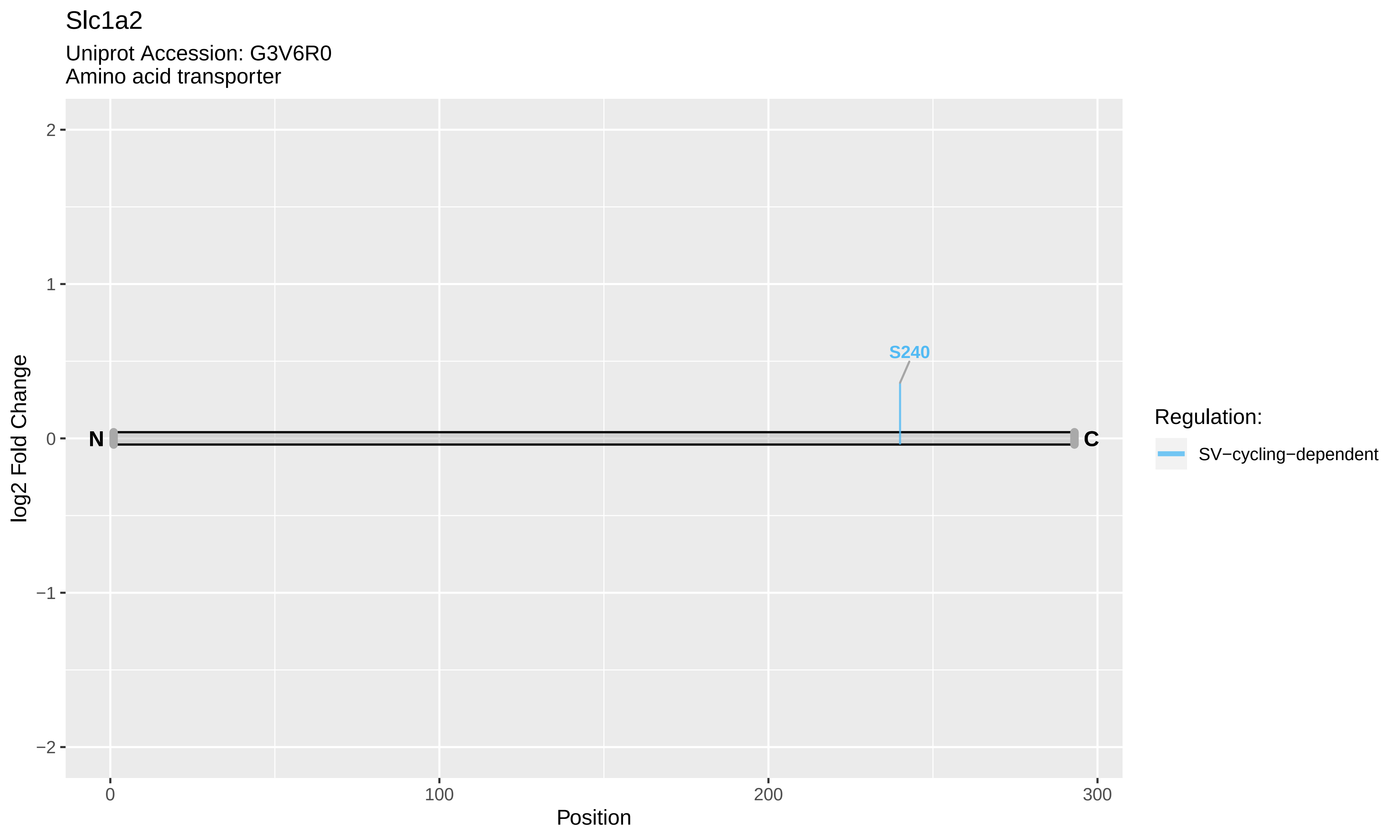
Slc1a2
Uniprot Accession: G3V6R0
Amino acid transporter
S240
N
C
2
1
0
−1
−2
0
100
200
300
Position
log2 Fold Change
Regulation:
SV−cycling−dependent

Slc20a2

Uniprot Accession: Q63488
Sodium−dependent phosphate transporter 2

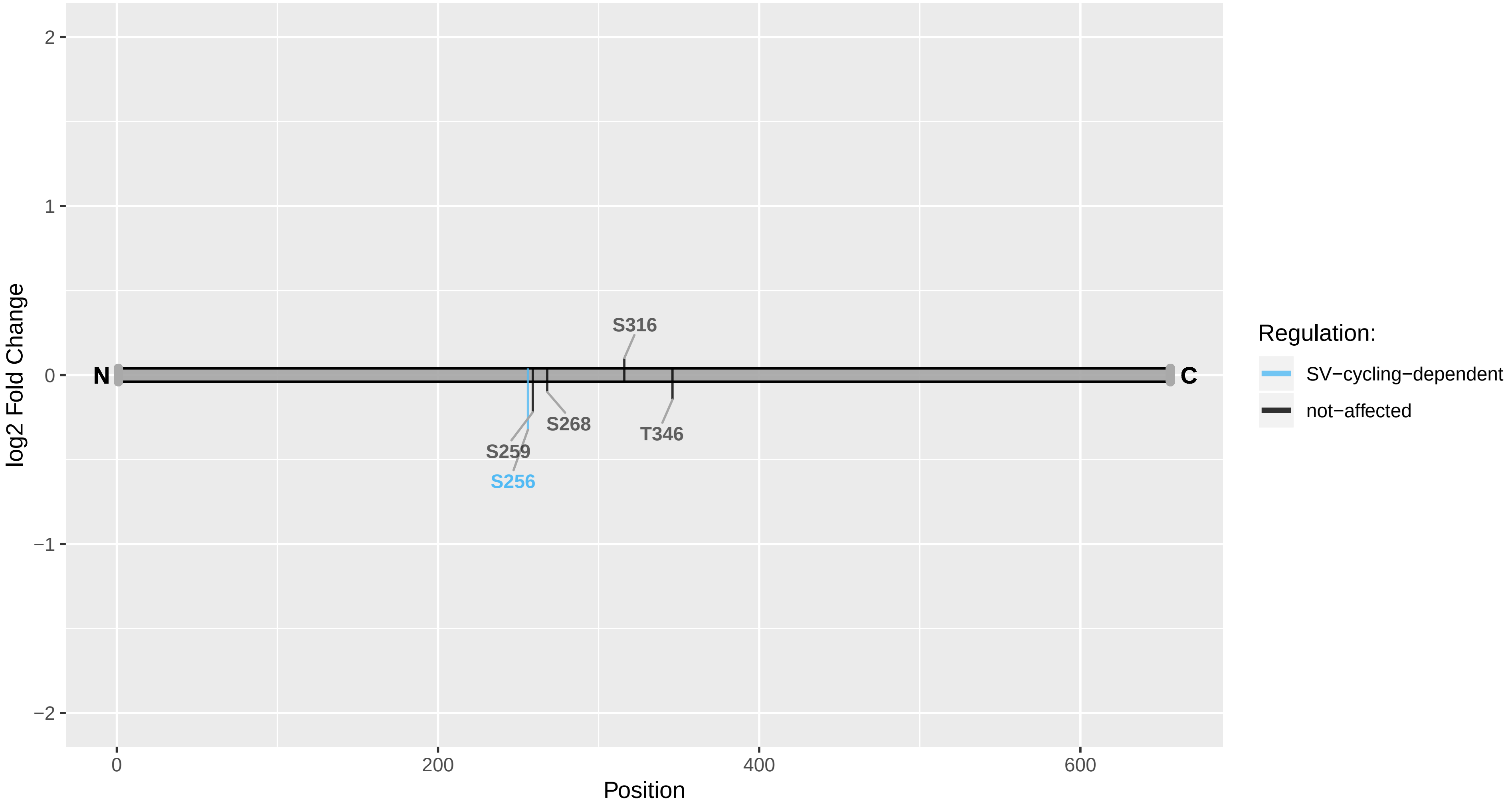

Slc22a5

Uniprot Accession: O70594
Solute carrier family 22 member 5

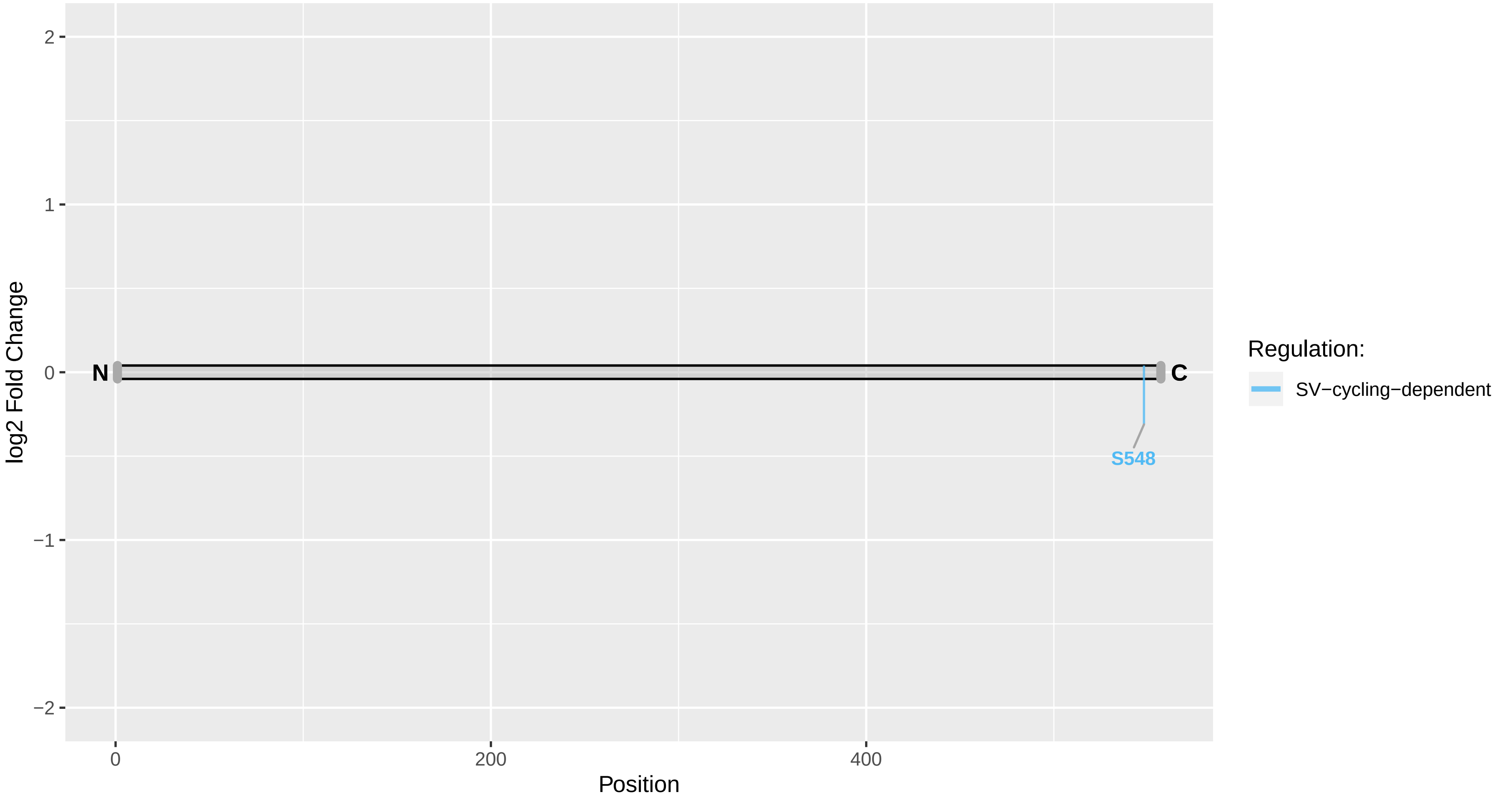

Slc23a2

Uniprot Accession: Q9WTW8
Solute carrier family 23 member 2

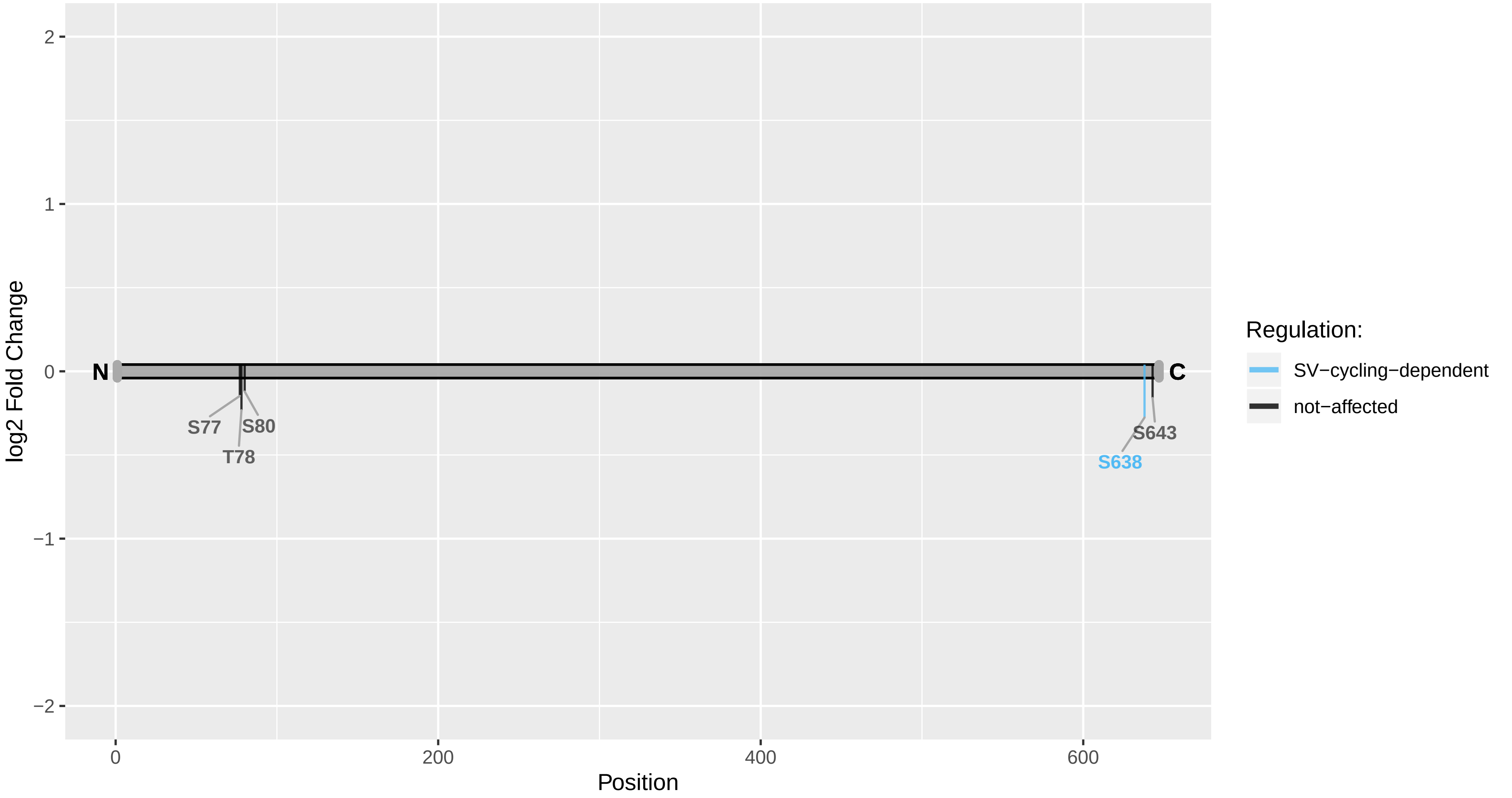

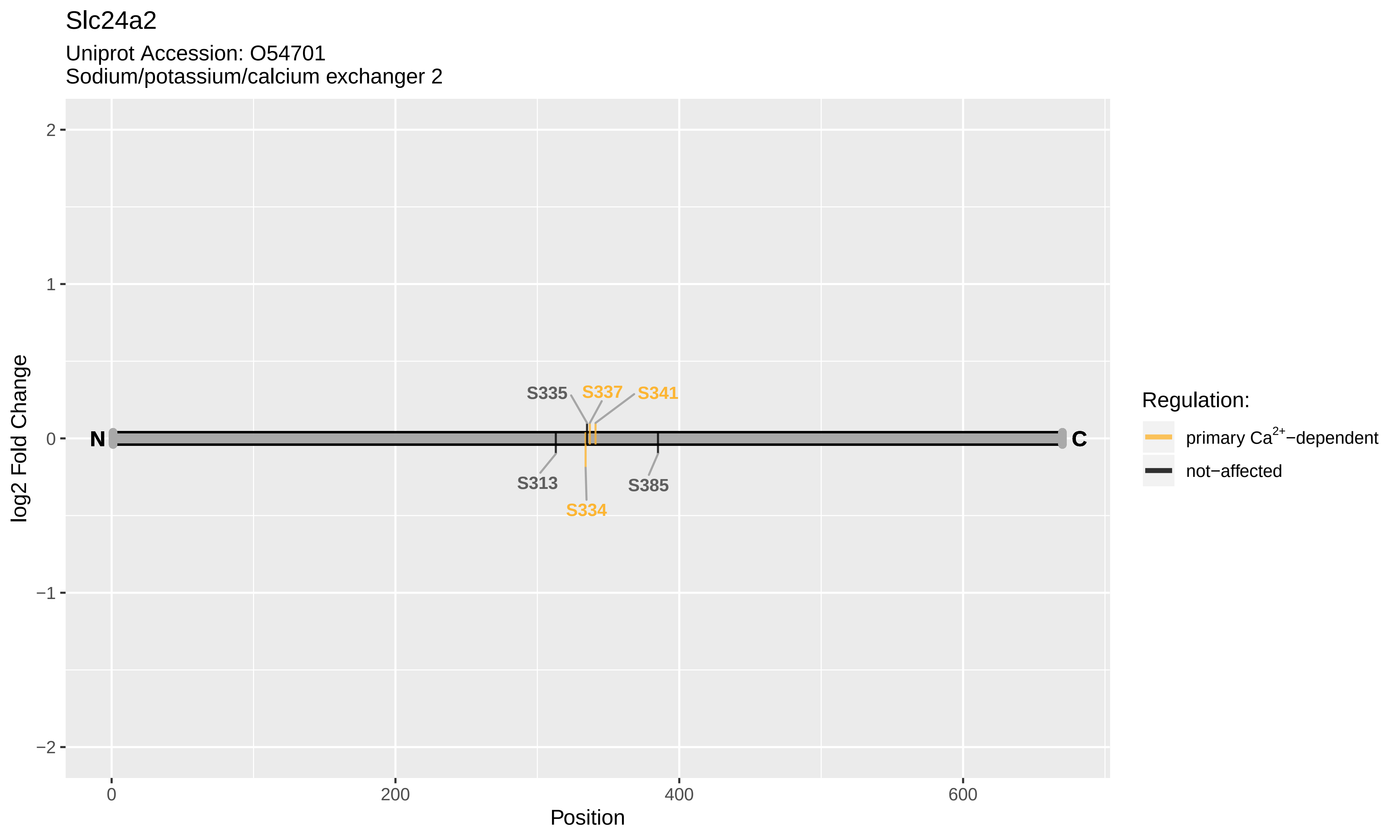

Slc24a2
Uniprot Accession: O54701
Sodium/potassium/calcium exchanger 2
log2 Fold Change
Position
N
C
S335
S337
S341
S313
S334
S385
Regulation:
primary Ca2+−dependent
not−affected

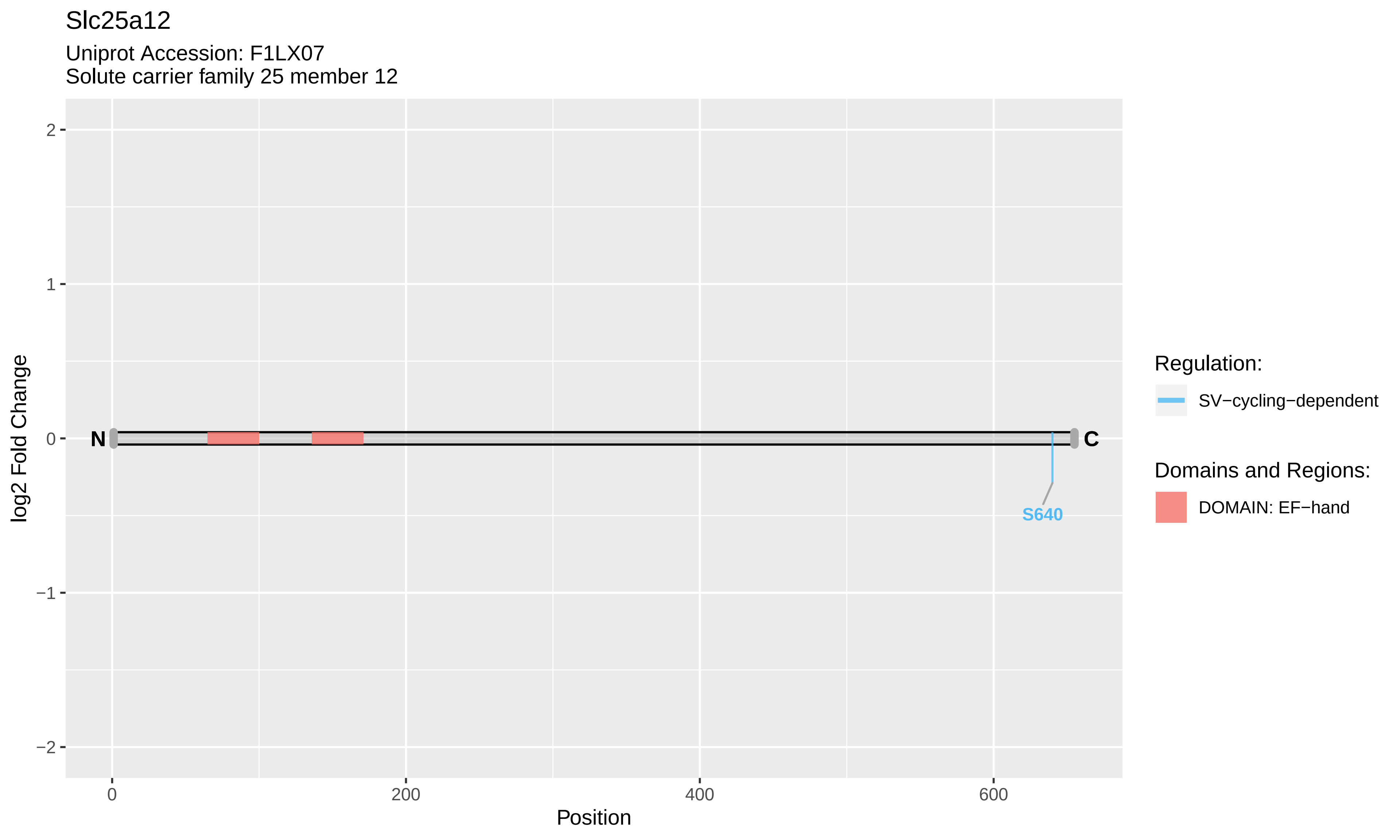

Slc25a12
Uniprot Accession: F1LX07
Solute carrier family 25 member 12
log2 Fold Change
Position
N
C
S640
Regulation:
SV−cycling−dependent
Domains and Regions:
DOMAIN: EF−hand
2
1
0
−1
−2
0
200
400
600

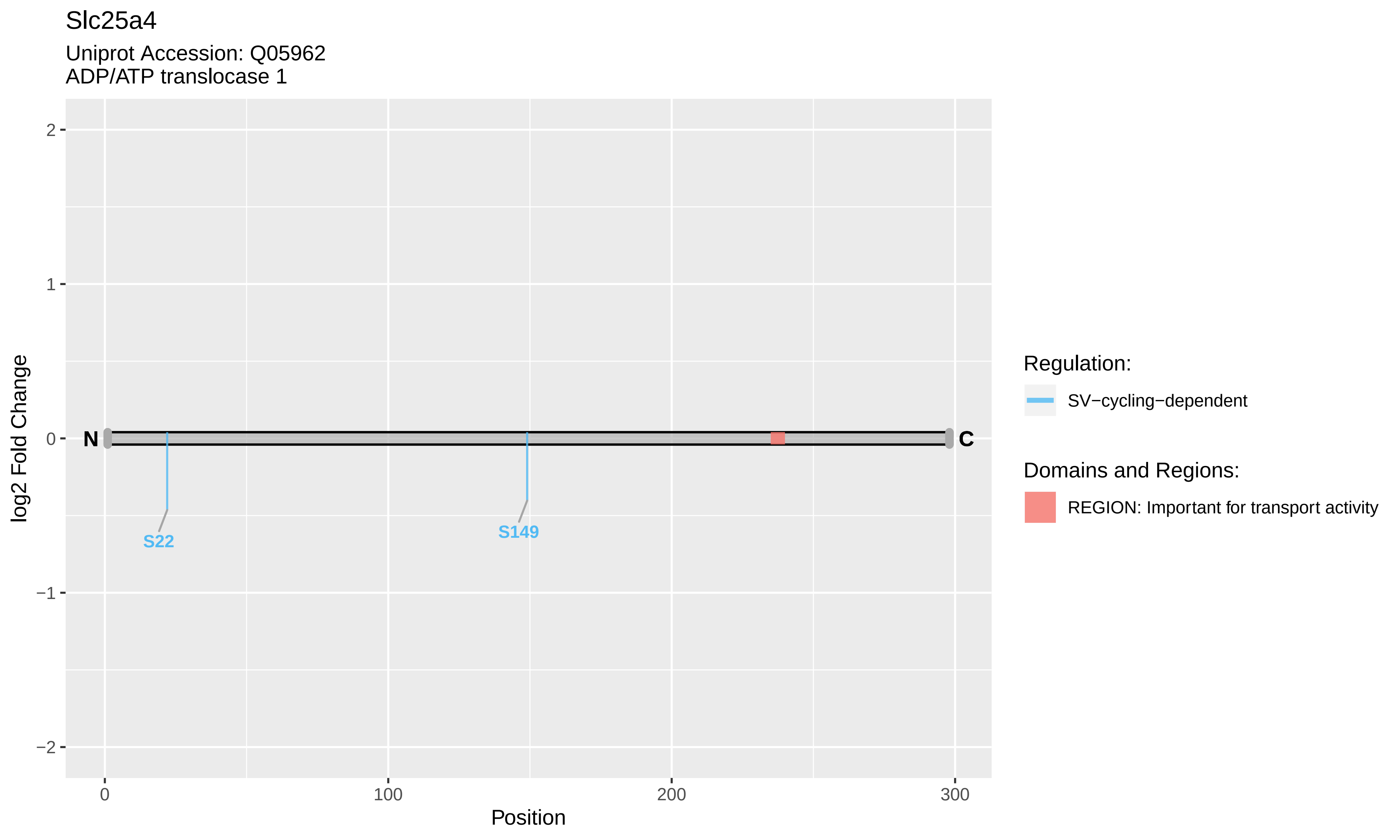
Slc25a4
Uniprot Accession: Q05962
ADP/ATP translocase 1
log2 Fold Change
2
1
0
−1
−2
N
C
S22
S149
0
100
200
300
Position
Regulation:
SV−cycling−dependent
Domains and Regions:
REGION: Important for transport activity

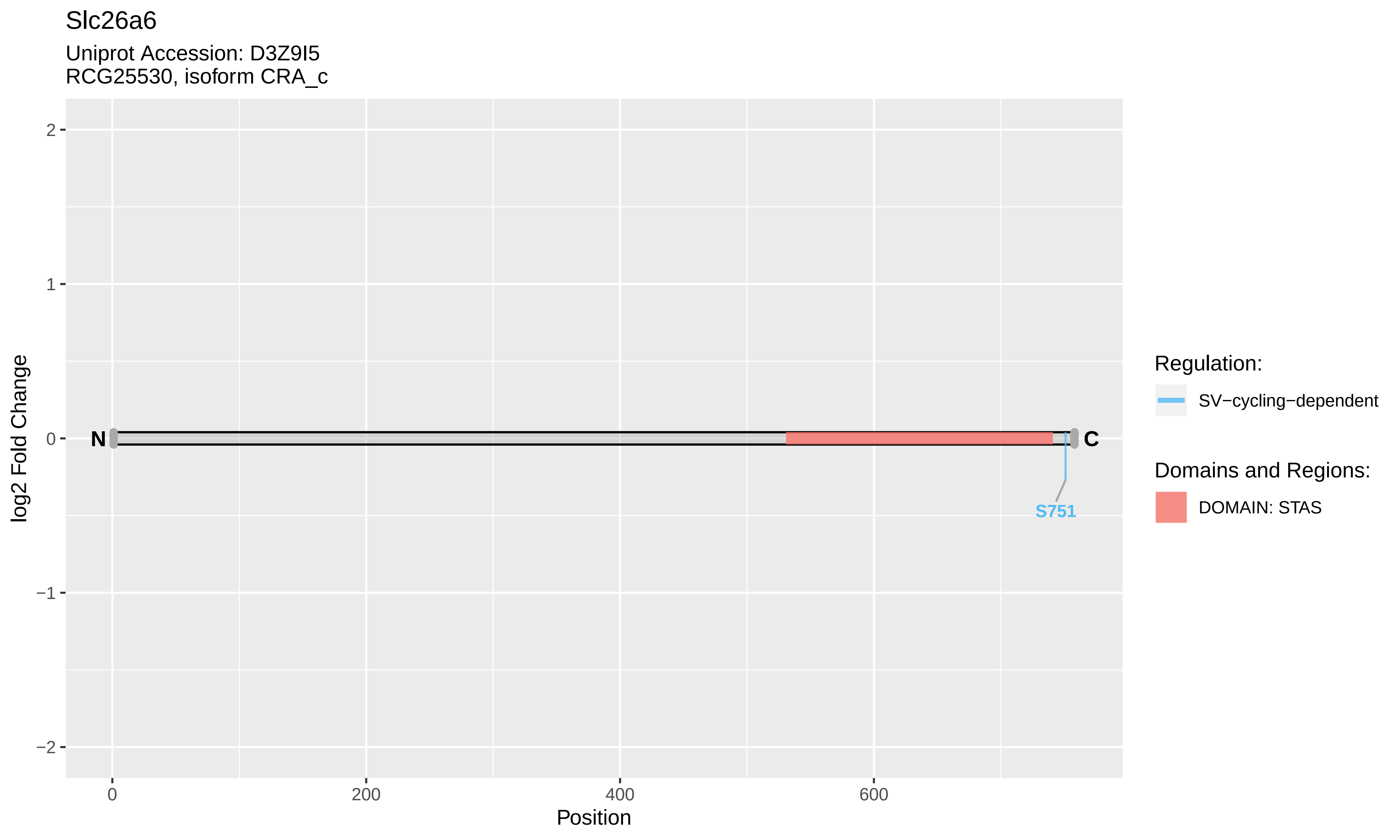

Slc26a6
Uniprot Accession: D3Z9I5
RCG25530, isoform CRA_c
log2 Fold Change
2
1
0
−1
−2
N
C
S751
0
200
400
600
Position
Regulation:
SV−cycling−dependent
Domains and Regions:
DOMAIN: STAS

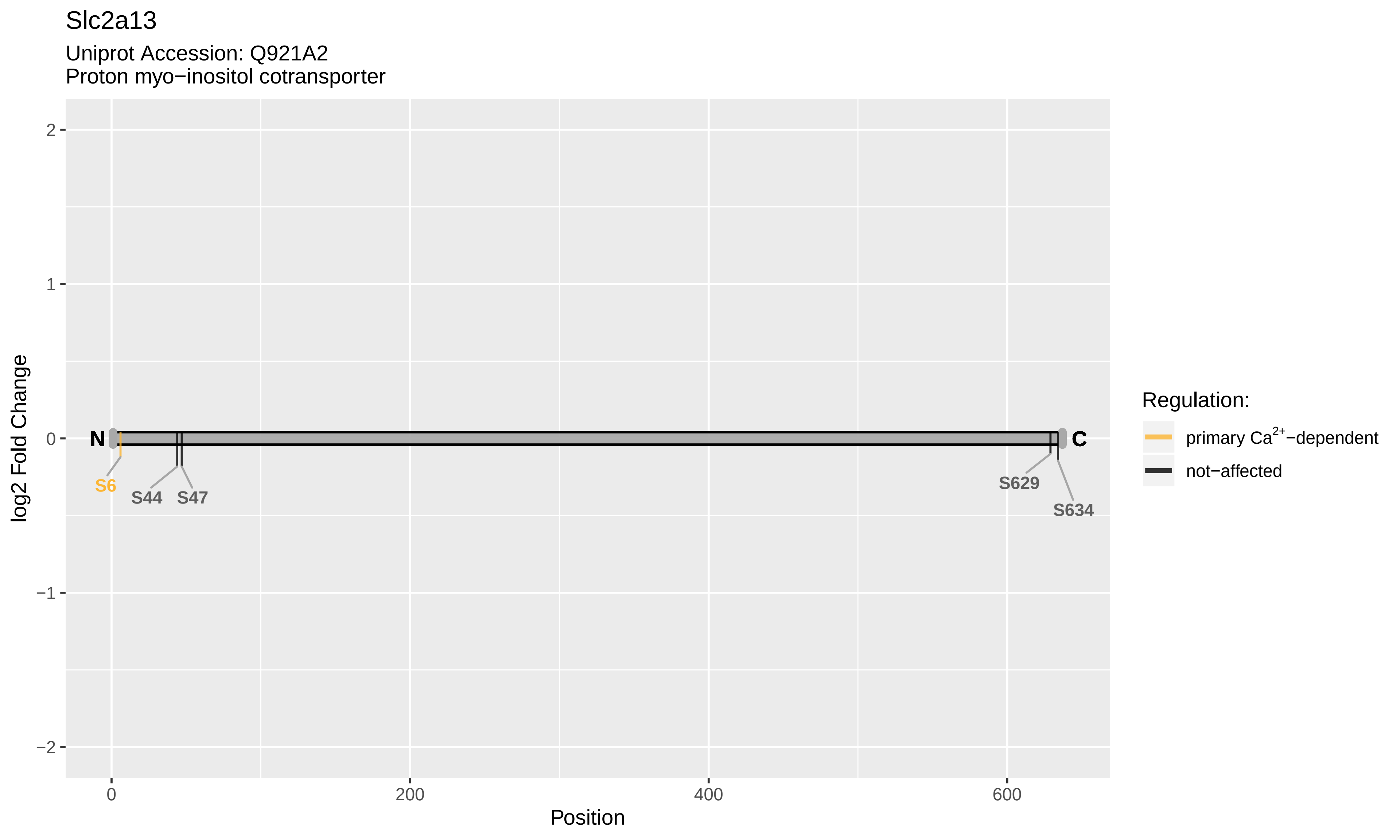

Slc2a13
Uniprot Accession: Q921A2
Proton myo−inositol cotransporter
log2 Fold Change
2
1
0
−1
−2
N
C
S6
S44
S47
S629
S634
0
200
400
600
Position
Regulation:
primary Ca2+−dependent
not−affected

Slc30a3

Uniprot Accession: Q6QIX3
Zinc transporter 3

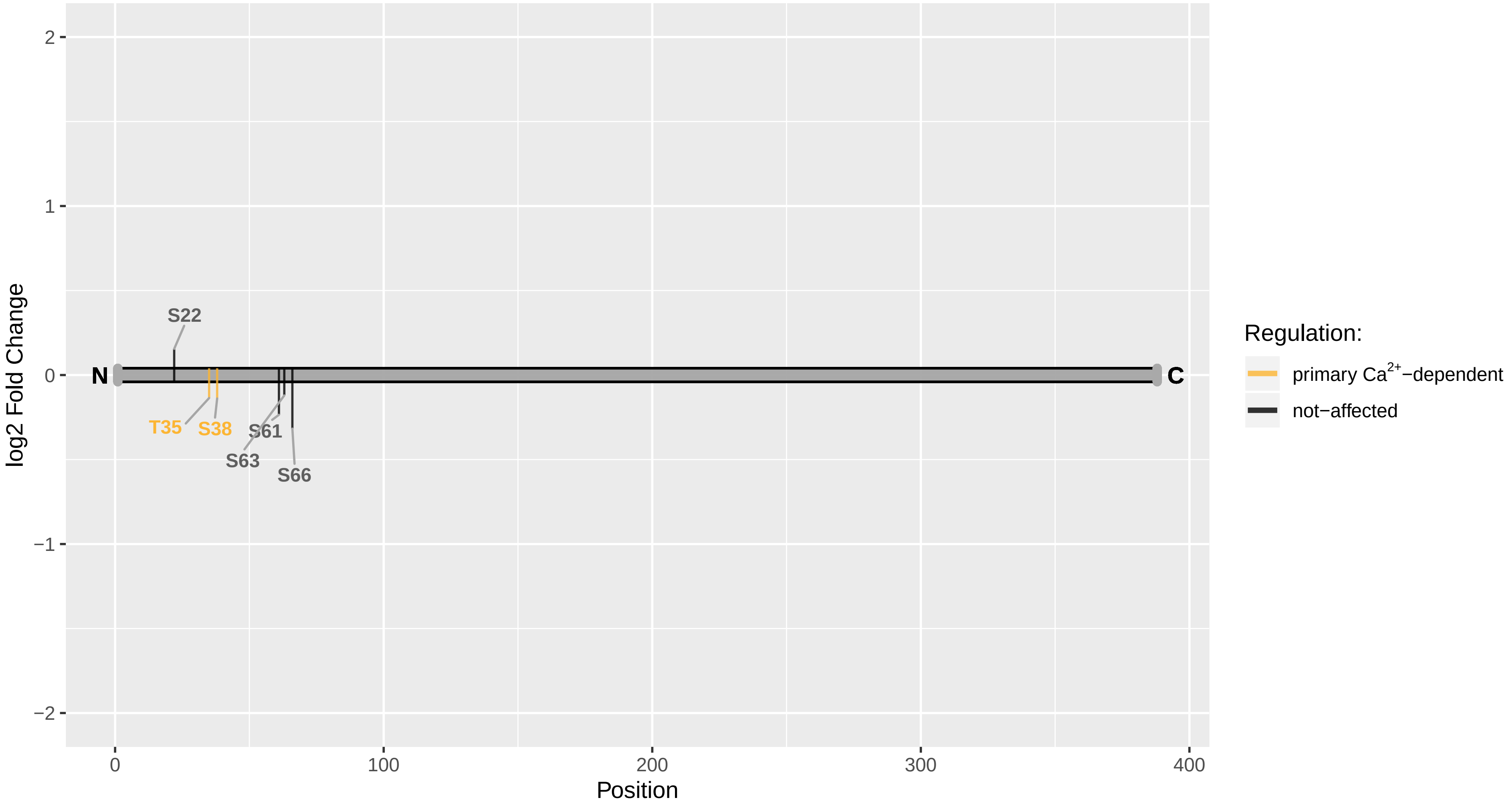

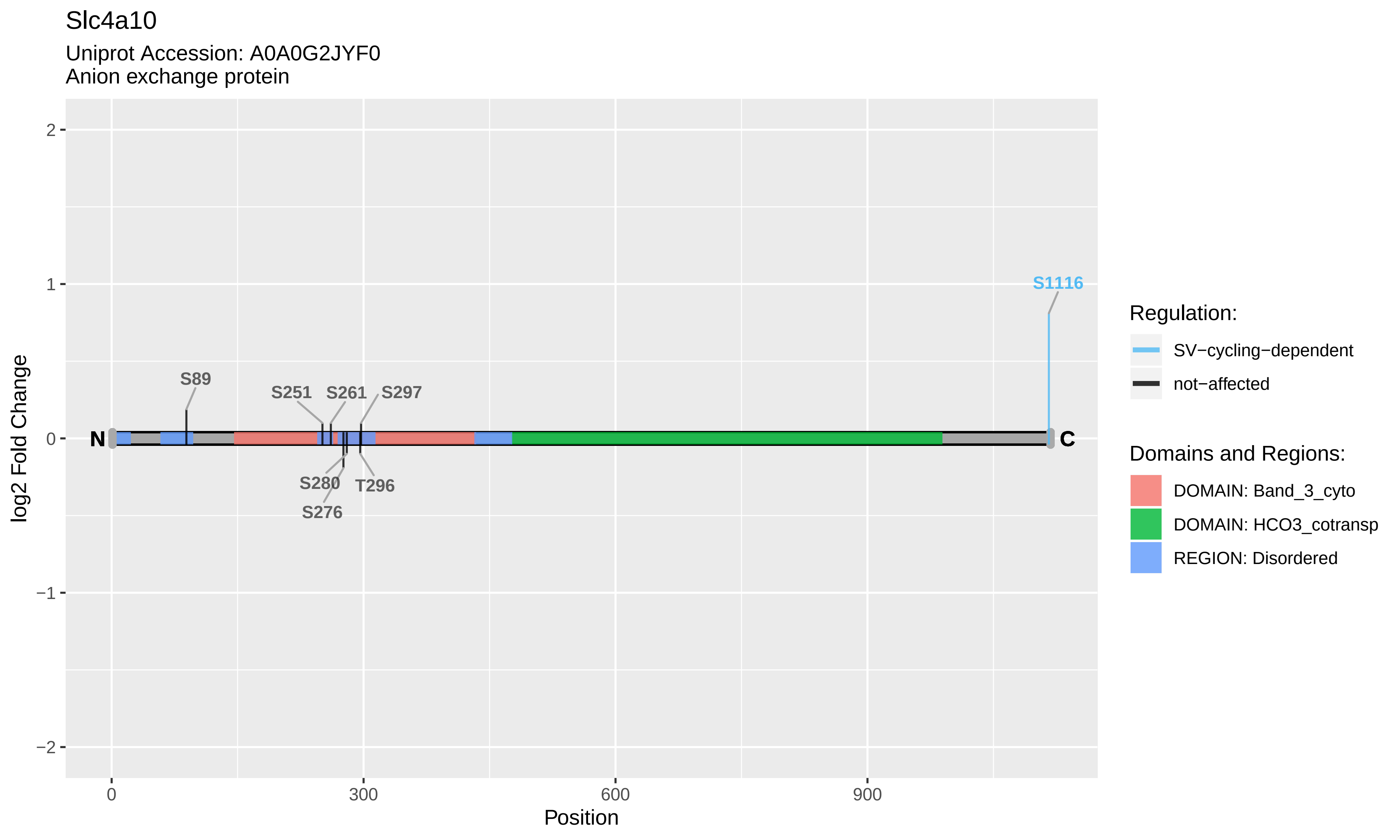
Slc4a10
Uniprot Accession: A0A0G2JYF0
Anion exchange protein
S1116
S89
S251
S261
S297
S280
T296
S276
N
C
2
1
0
−1
−2
0
300
600
900
Position
log2 Fold Change
Regulation:
SV−cycling−dependent
not−affected
Domains and Regions:
DOMAIN: Band_3_cyto
DOMAIN: HCO3_cotransp
REGION: Disordered

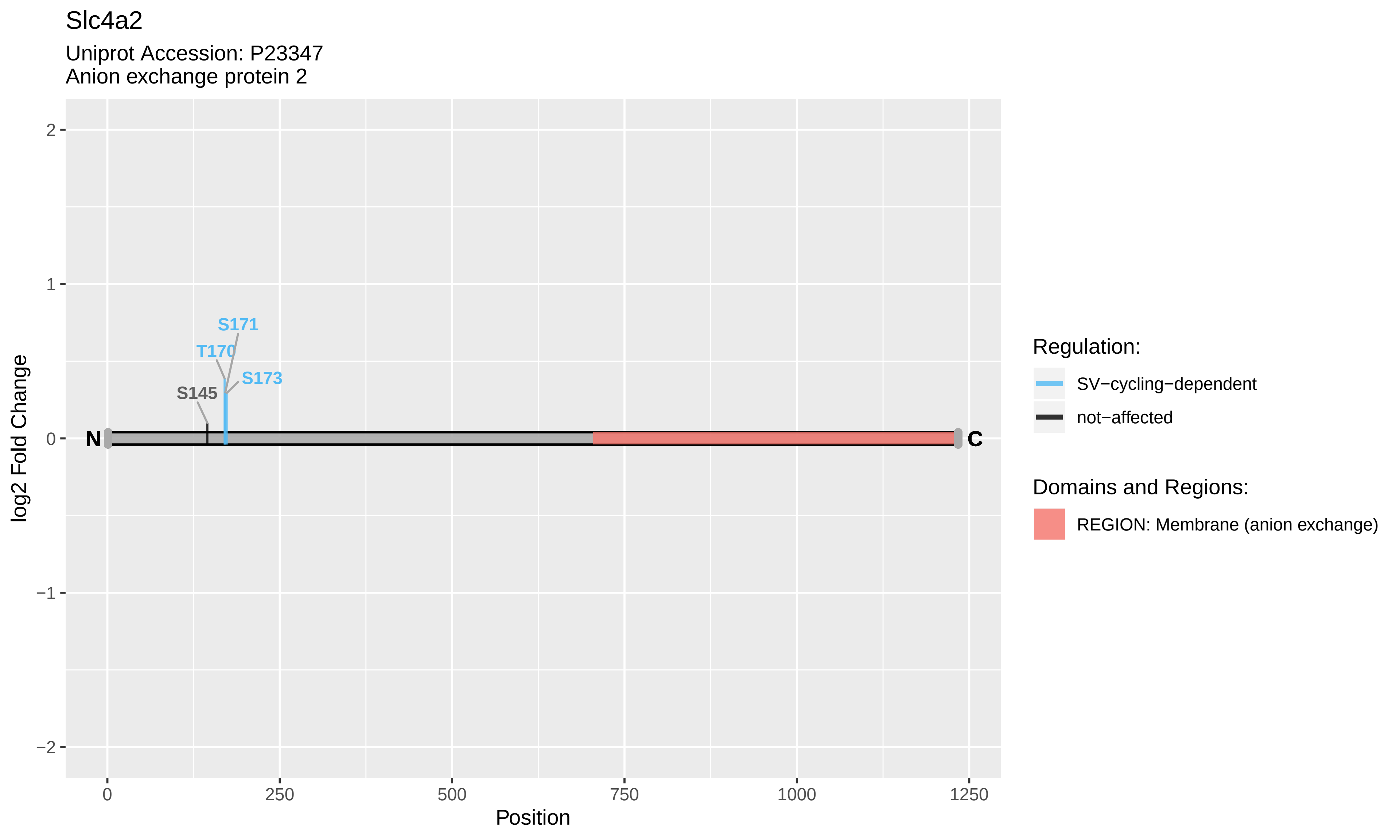

Slc4a2
Uniprot Accession: P23347
Anion exchange protein 2
log2 Fold Change
2
1
0
−1
−2
N
C
S171
T170
S173
S145
0
250
500
750
1000
1250
Position
Regulation:
SV−cycling−dependent
not−affected
Domains and Regions:
REGION: Membrane (anion exchange)

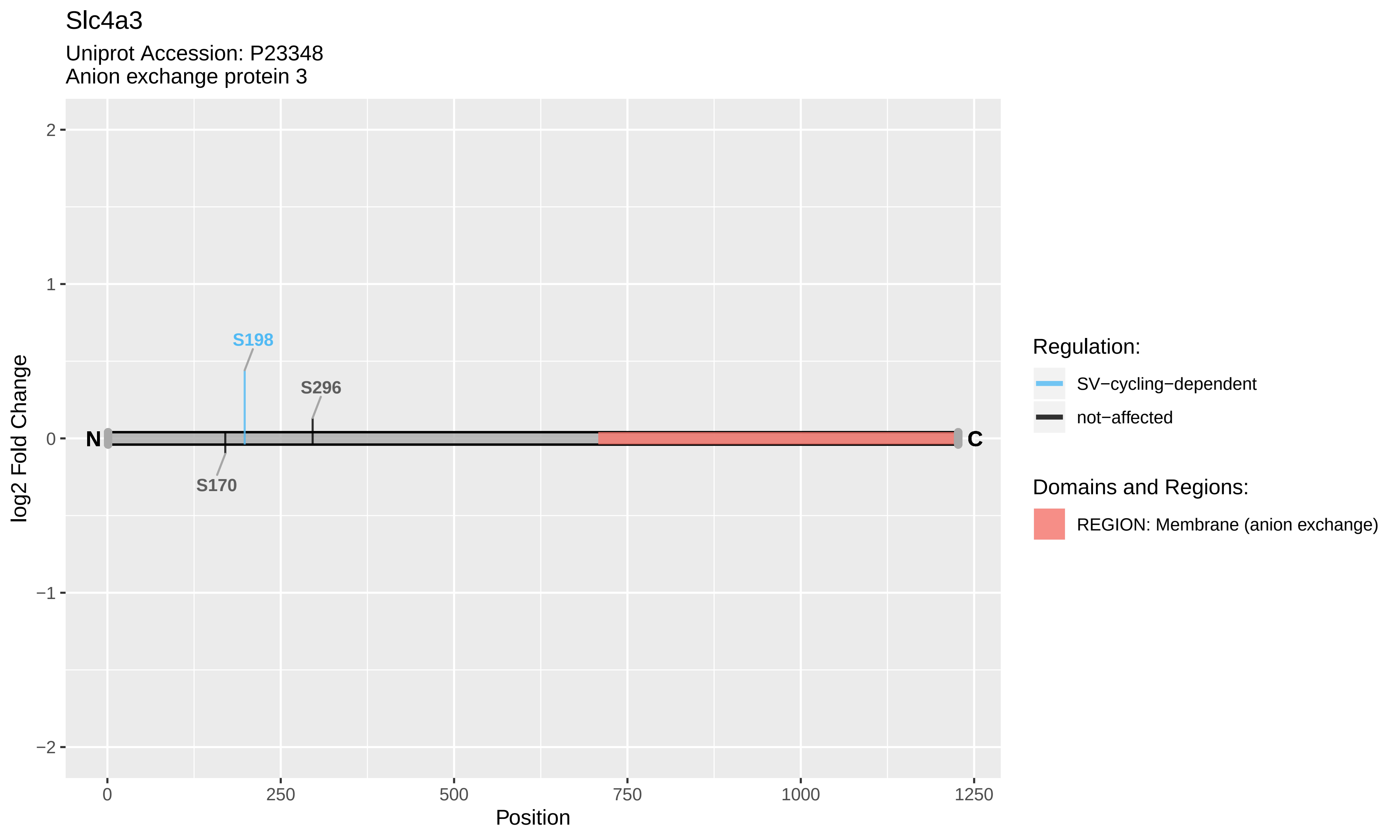
Slc4a3
Uniprot Accession: P23348
Anion exchange protein 3
log2 Fold Change
2
1
0
−1
−2
N
C
S198
S296
S170
0
250
500
750
1000
1250
Position
Regulation:
SV−cycling−dependent
not−affected
Domains and Regions:
REGION: Membrane (anion exchange)

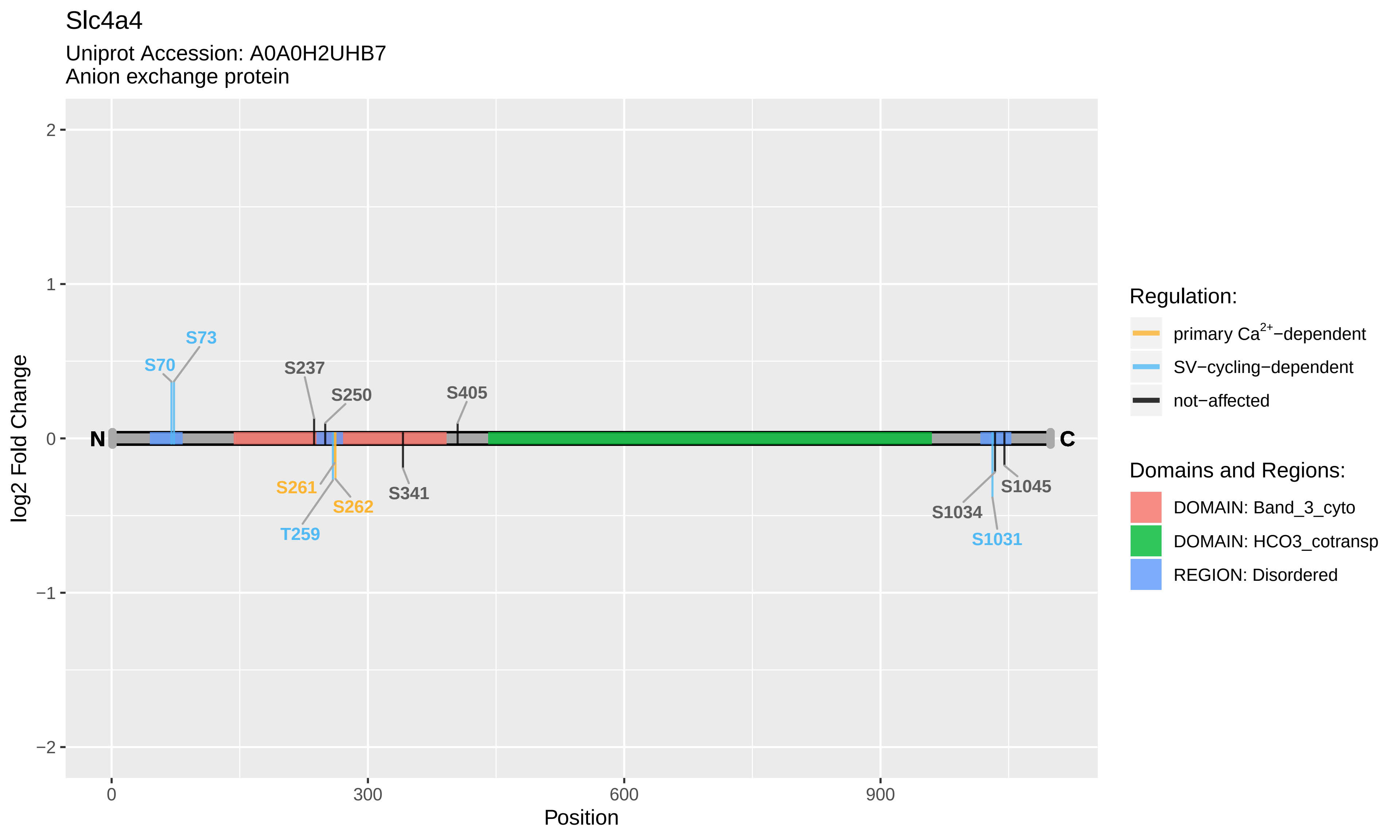

Slc4a4
Uniprot Accession: A0A0H2UHB7
Anion exchange protein
log2 Fold Change
Position
N
C
S73
S70
S237
S250
S405
S261
S262
T259
S341
S1045
S1034
S1031
Regulation:
primary Ca2+−dependent
SV−cycling−dependent
not−affected
Domains and Regions:
DOMAIN: Band_3_cyto
DOMAIN: HCO3_cotransp
REGION: Disordered

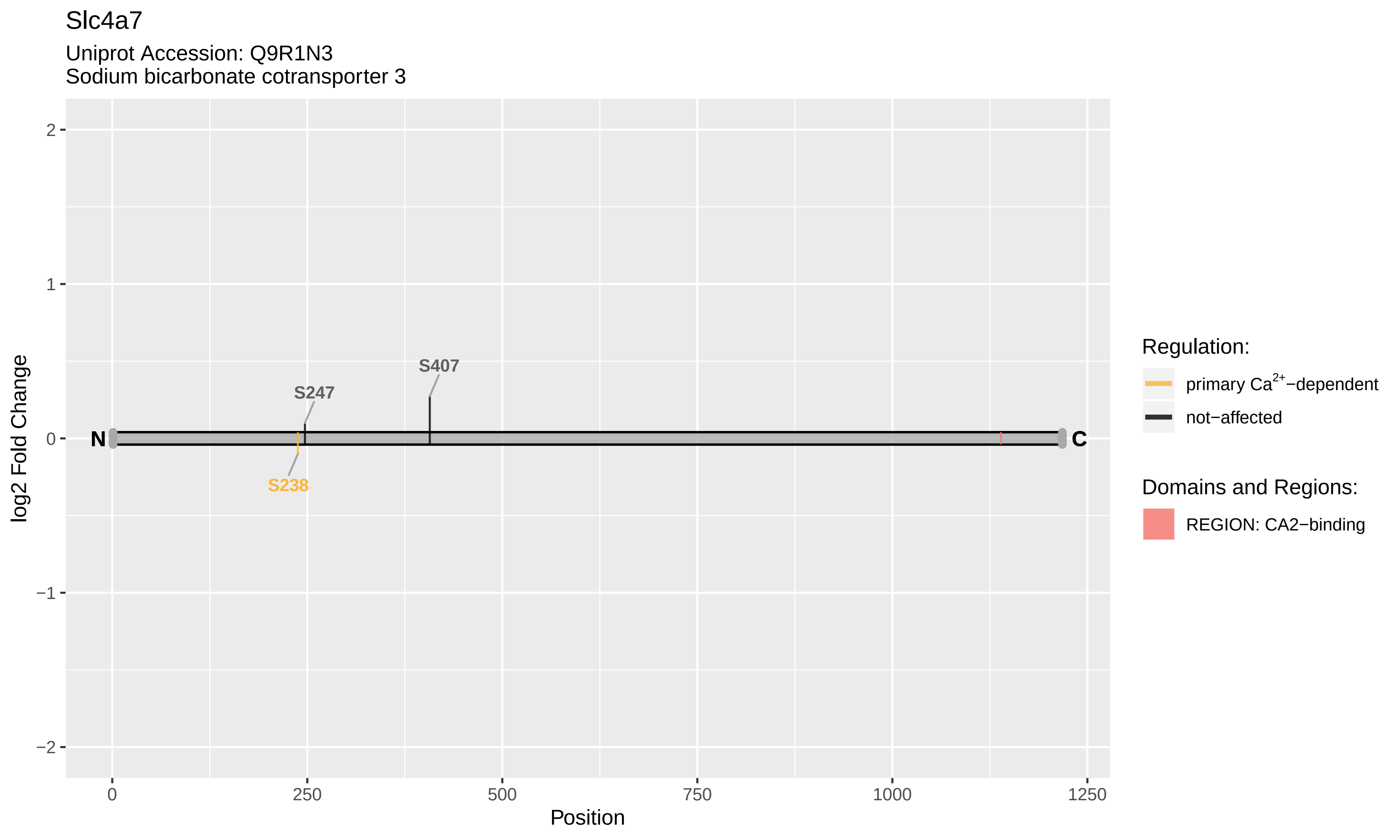
Slc4a7
Uniprot Accession: Q9R1N3
Sodium bicarbonate cotransporter 3
log2 Fold Change
Position
N
C
S407
S247
S238
2
1
0
−1
−2
0
250
500
750
1000
1250
Regulation:
primary Ca2+−dependent
not−affected
Domains and Regions:
REGION: CA2−binding

Slc6a15

Uniprot Accession: Q08469
Sodium−dependent neutral amino acid transporter B(0)AT2

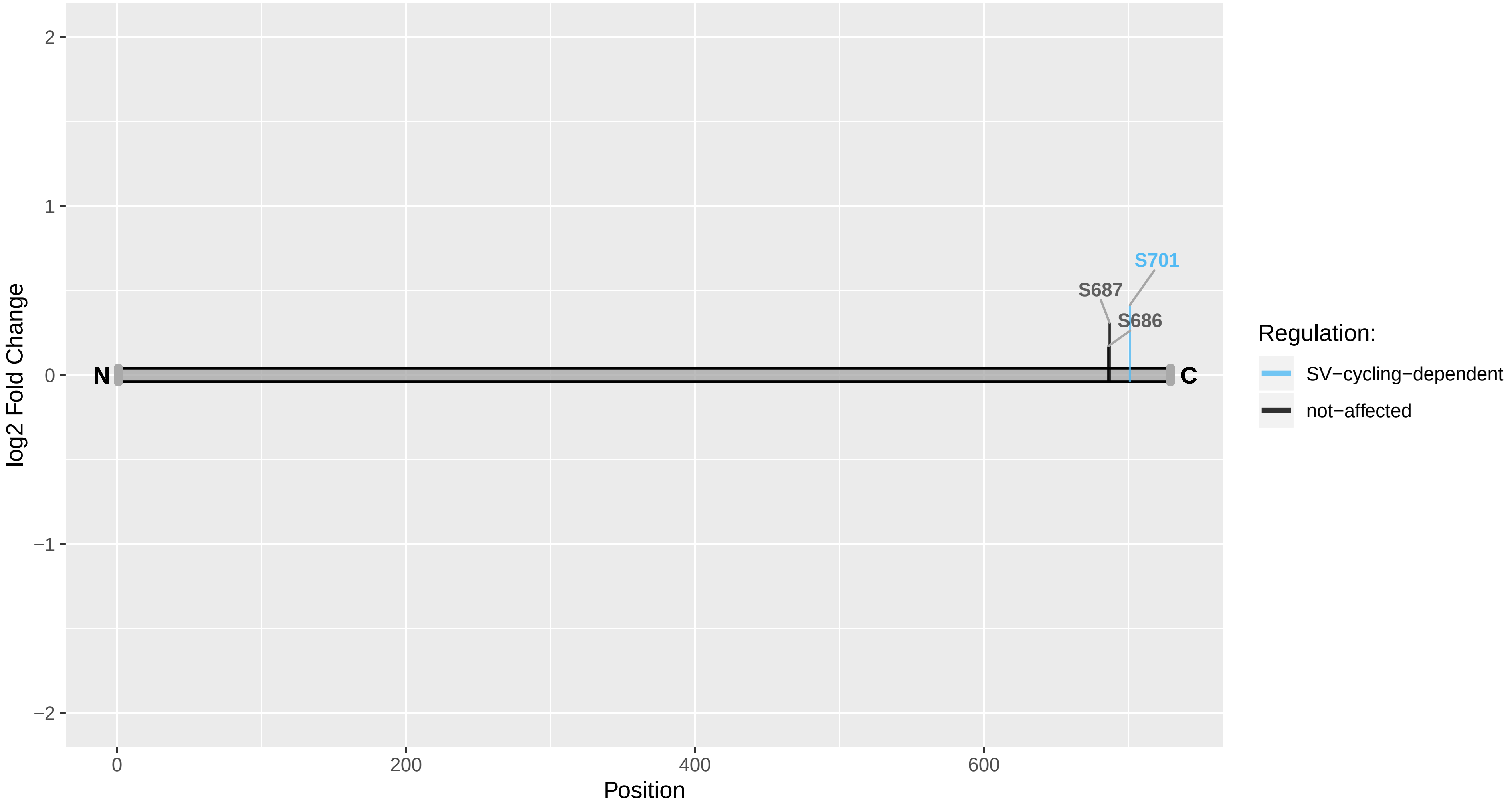

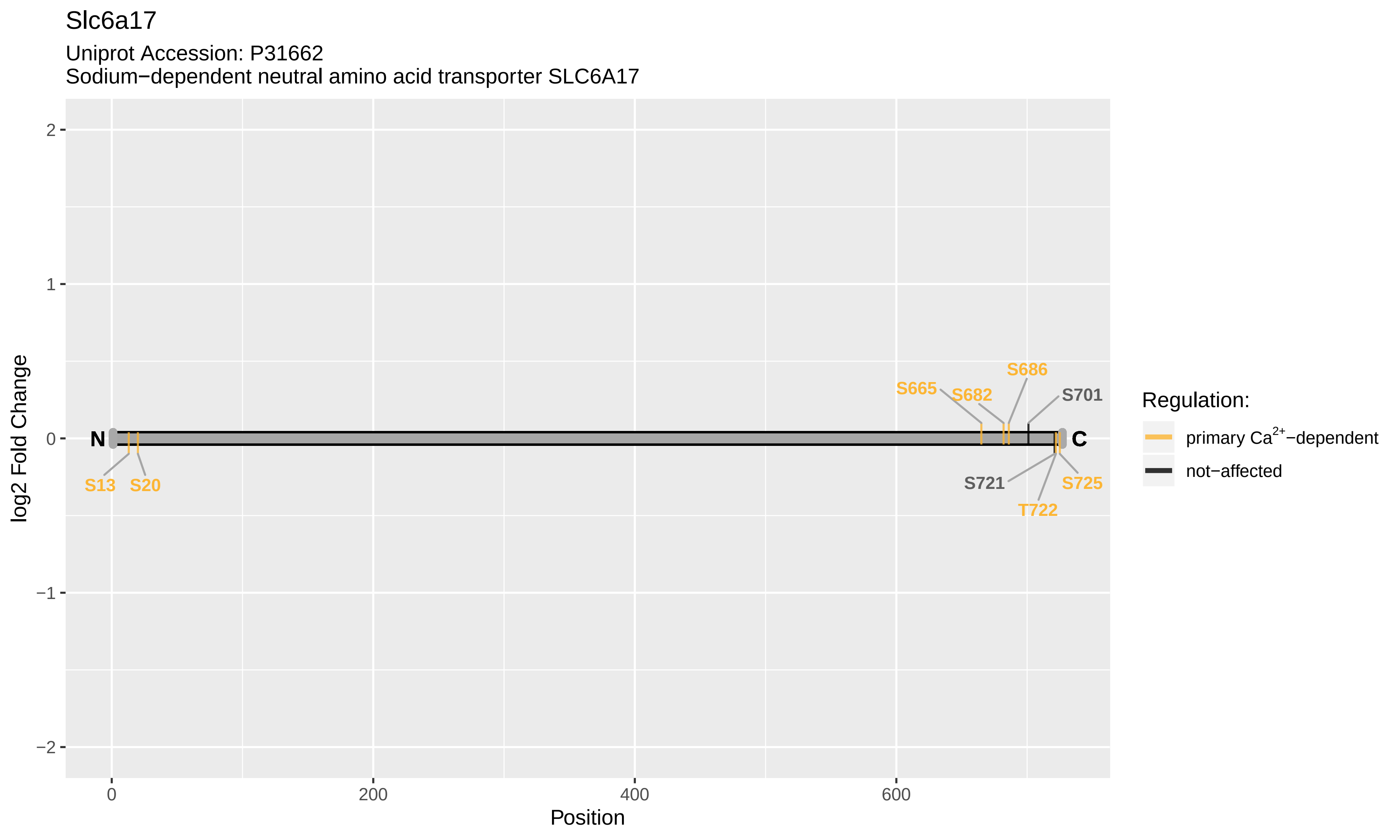

Slc6a17
Uniprot Accession: P31662
Sodium−dependent neutral amino acid transporter SLC6A17
log2 Fold Change
Position
N
C
S13
S20
S665
S682
S686
S701
S721
T722
S725
Regulation:
primary Ca2+−dependent
not−affected

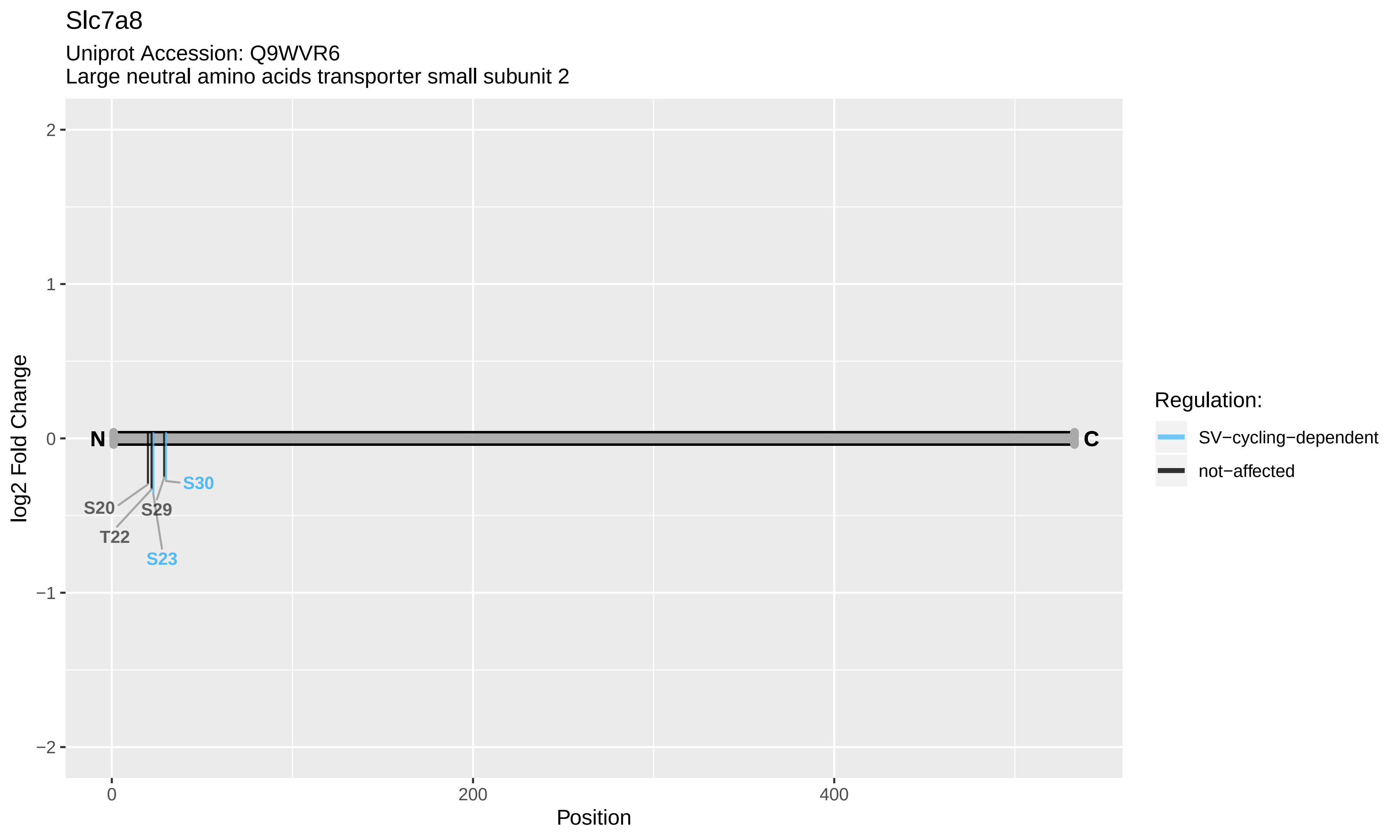

Slc7a8
Uniprot Accession: Q9WVR6
Large neutral amino acids transporter small subunit 2
log2 Fold Change
2
1
0
−1
−2
N
C
S20
T22
S23
S29
S30
0
200
400
Position
Regulation:
SV−cycling−dependent
not−affected

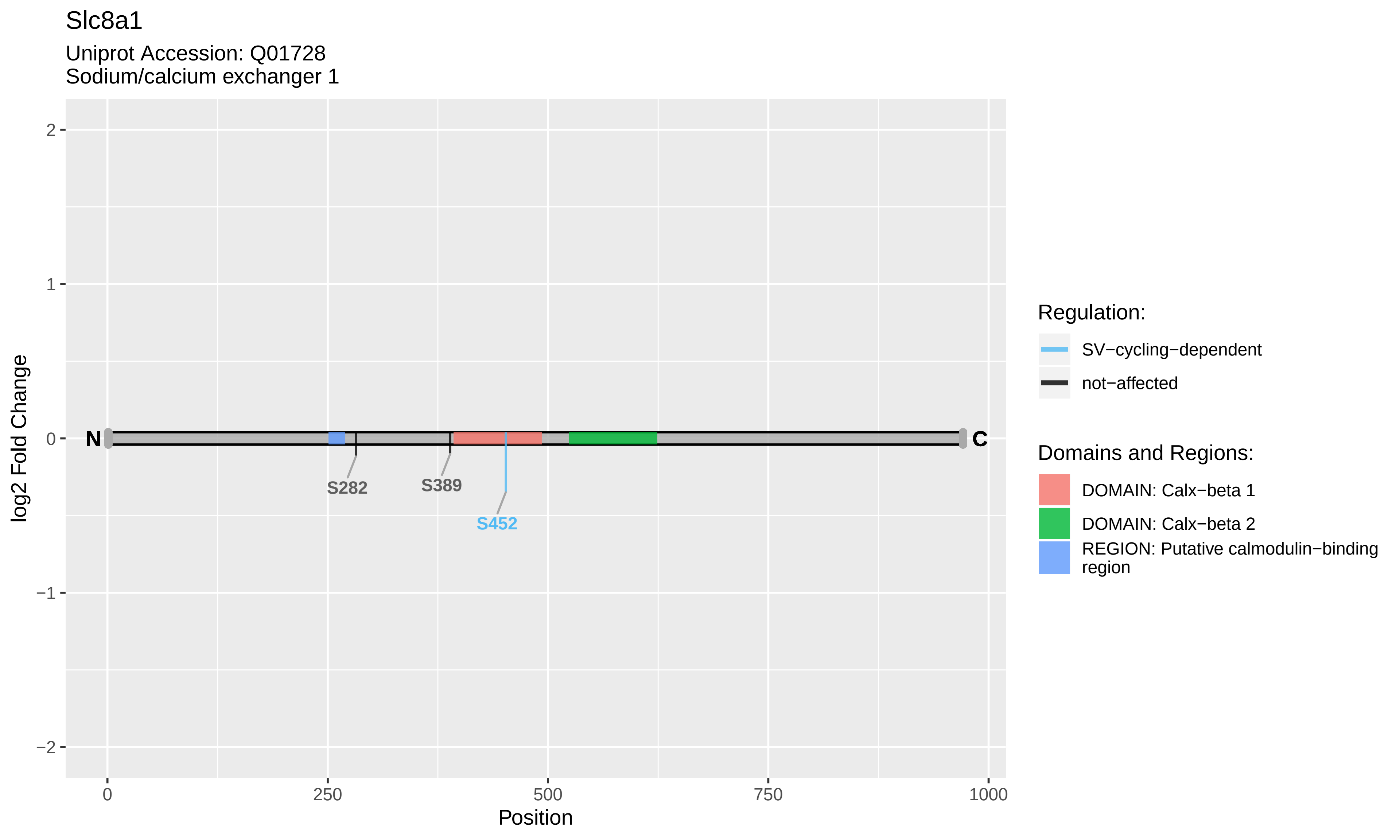

Slc8a1
Uniprot Accession: Q01728
Sodium/calcium exchanger 1
log2 Fold Change
2
1
0
−1
−2
N
C
S282
S389
S452
0
250
500
750
1000
Position
Regulation:
SV−cycling−dependent
not−affected
Domains and Regions:
DOMAIN: Calx−beta 1
DOMAIN: Calx−beta 2
REGION: Putative calmodulin−binding region

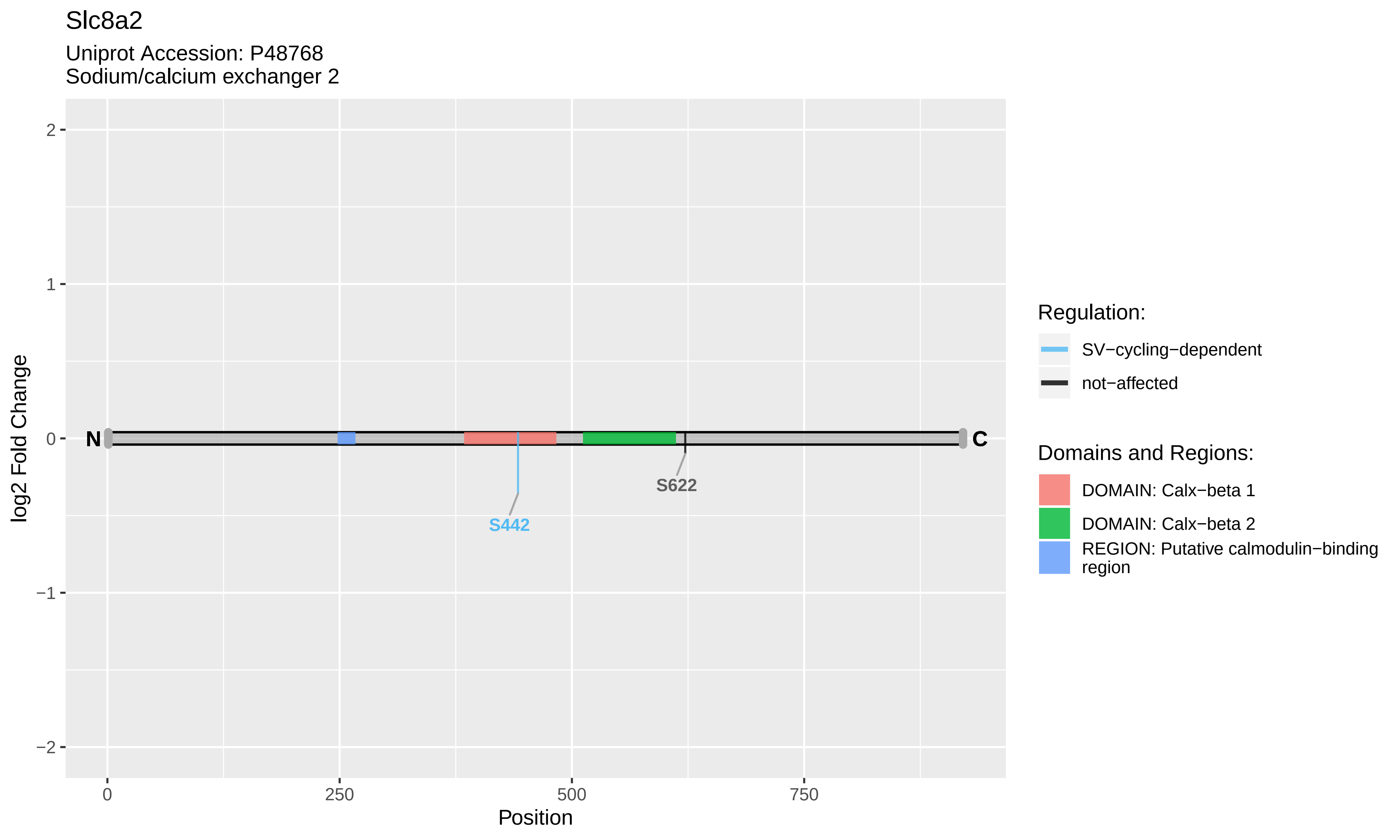
Slc8a2
Uniprot Accession: P48768
Sodium/calcium exchanger 2
log2 Fold Change
2
1
0
−1
−2
N
C
S442
S622
0
250
500
750
Position
Regulation:
SV−cycling−dependent
not−affected
Domains and Regions:
DOMAIN: Calx−beta 1
DOMAIN: Calx−beta 2
REGION: Putative calmodulin−binding region

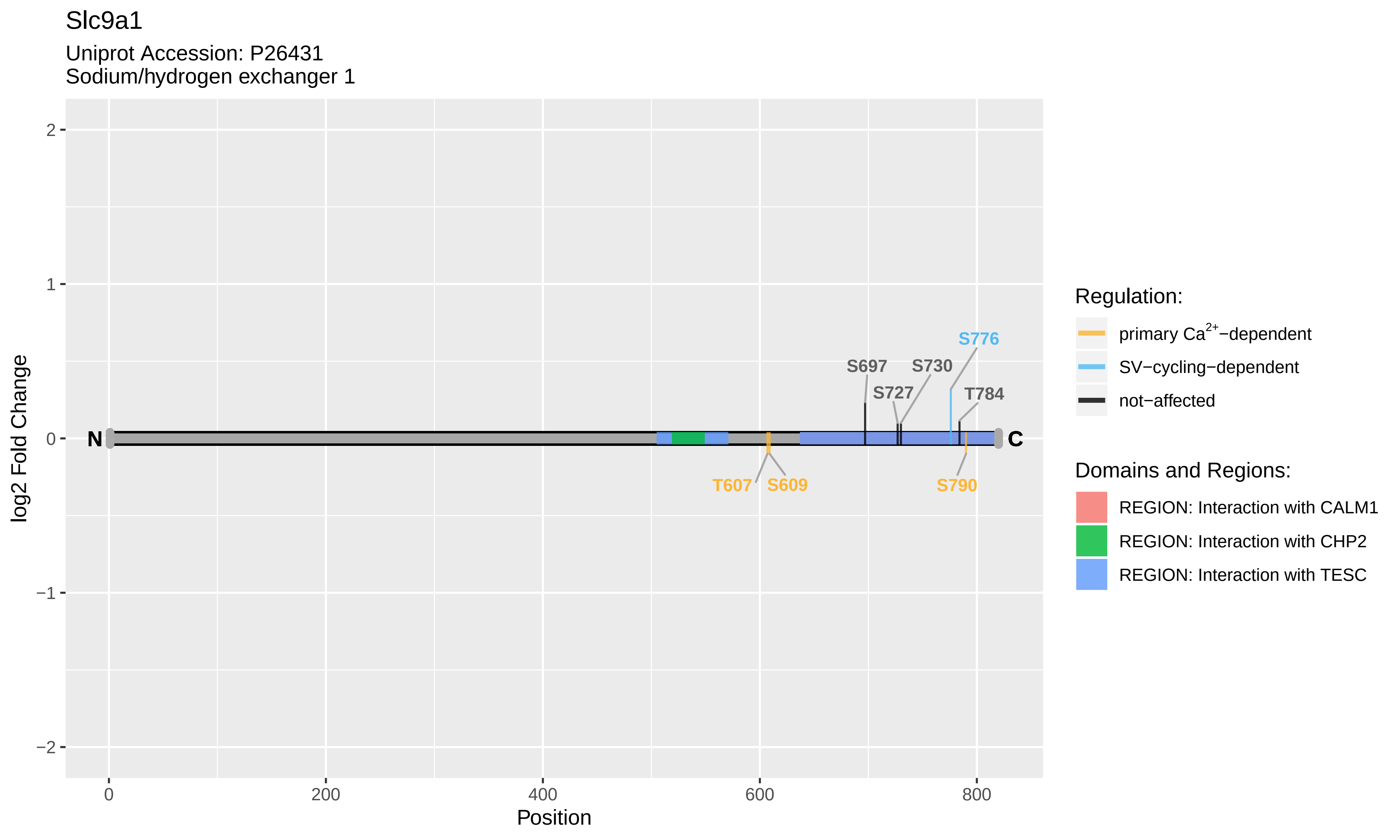

Slc9a1
Uniprot Accession: P26431
Sodium/hydrogen exchanger 1
log2 Fold Change
2
1
0
−1
−2
N
C
S776
S697
S730
S727
T784
T607
S609
S790
0
200
400
600
800
Position
Regulation:
primary Ca2+−dependent
SV−cycling−dependent
not−affected
Domains and Regions:
REGION: Interaction with CALM1
REGION: Interaction with CHP2
REGION: Interaction with TESC

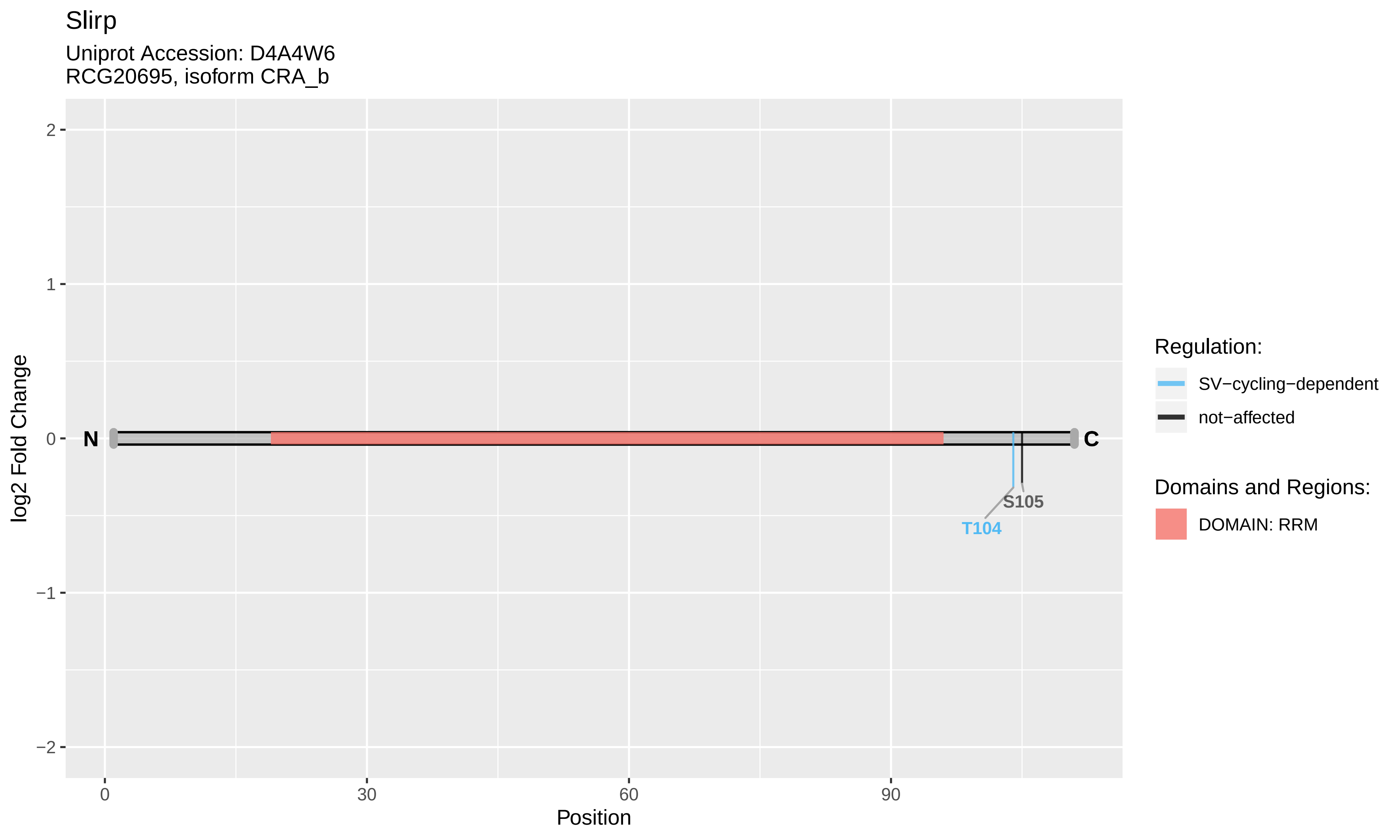

Slirp
Uniprot Accession: D4A4W6
RCG20695, isoform CRA_b
log2 Fold Change
2
1
0
−1
−2
N
C
S105
T104
0
30
60
90
Position
Regulation:
SV−cycling−dependent
not−affected
Domains and Regions:
DOMAIN: RRM

Slk

Uniprot Accession: G3V7I8
RCG57812, isoform CRA_b

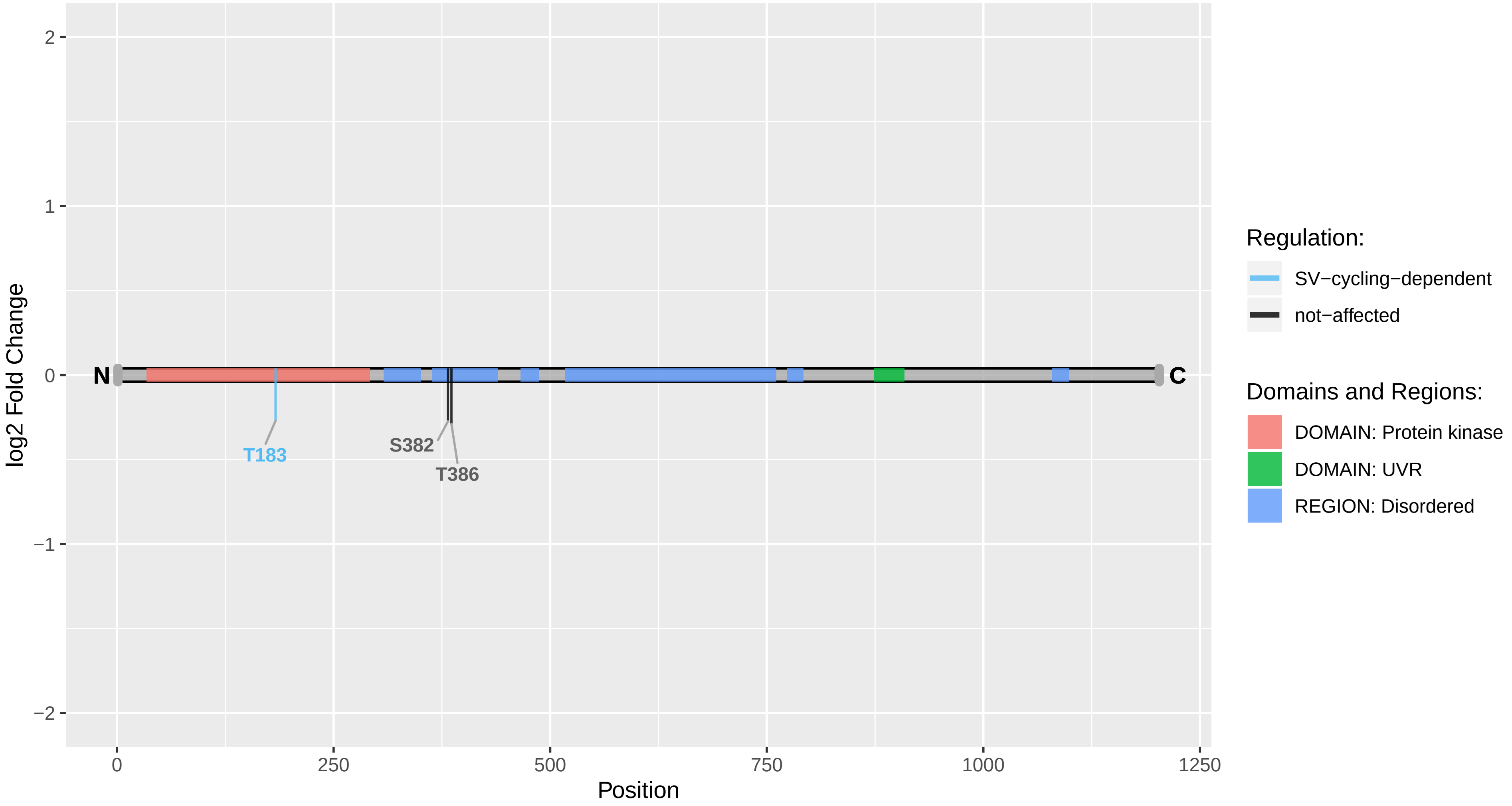

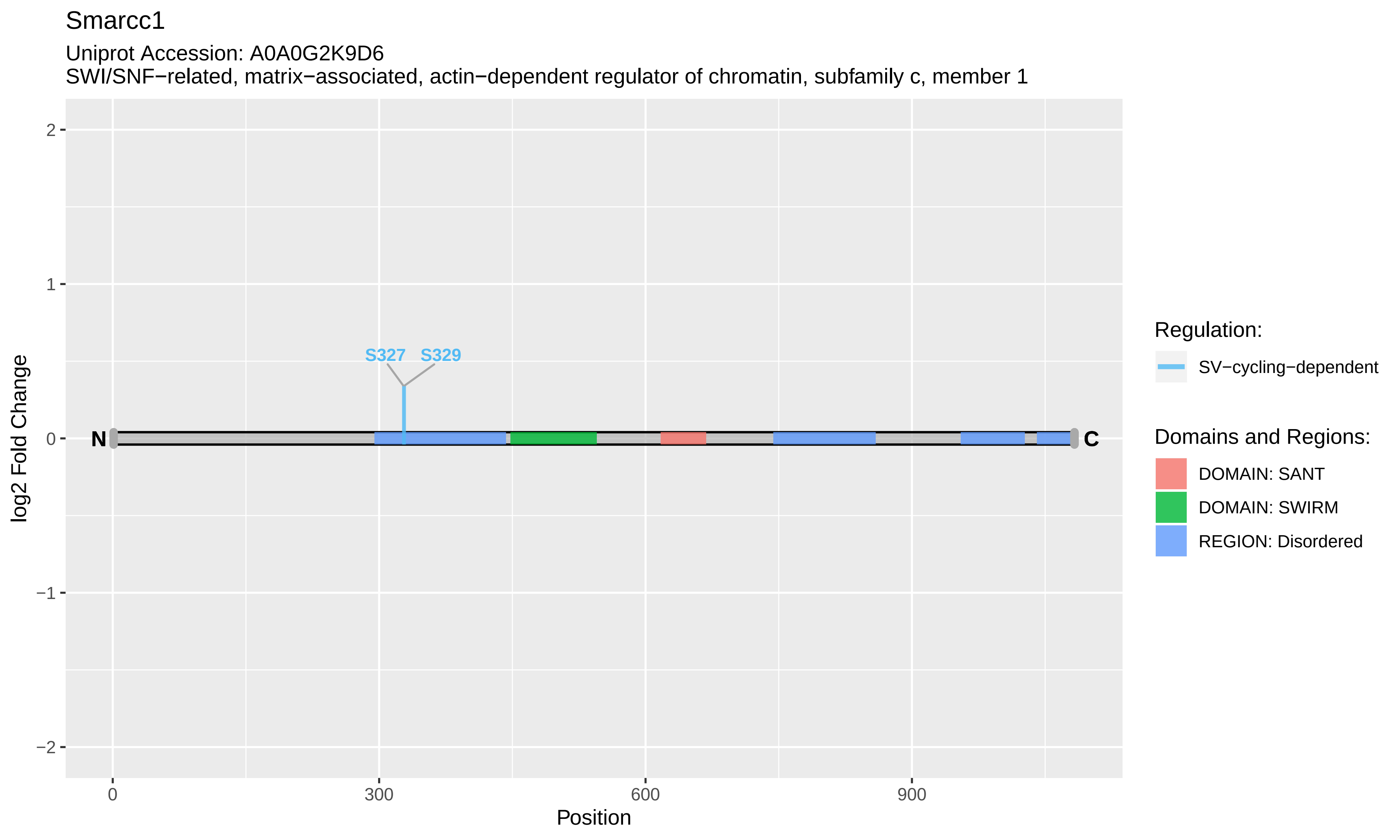
Smarcc1
Uniprot Accession: A0A0G2K9D6
SWI/SNF−related, matrix−associated, actin−dependent regulator of chromatin, subfamily c, member 1
S327
S329
N
C
log2 Fold Change
2
1
0
−1
−2
0
300
600
900
Position
Regulation:
SV−cycling−dependent
Domains and Regions:
DOMAIN: SANT
DOMAIN: SWIRM
REGION: Disordered

Smcr8

Uniprot Accession: D3ZJR6
Smith−Magenis syndrome chromosome region, candidate 8

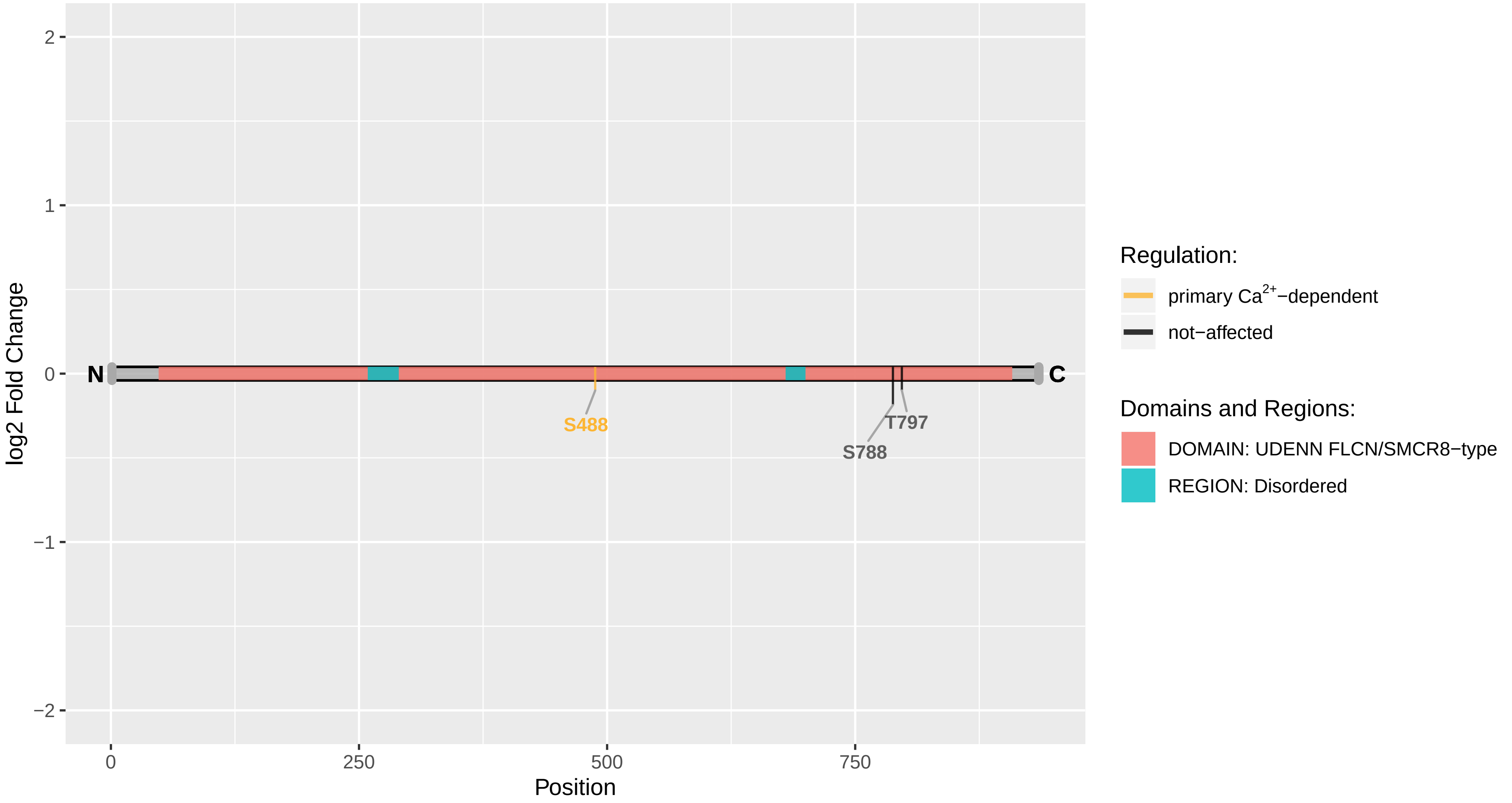

Smg7

Uniprot Accession: D4ACJ3
SMG7 nonsense mediated mRNA decay factor

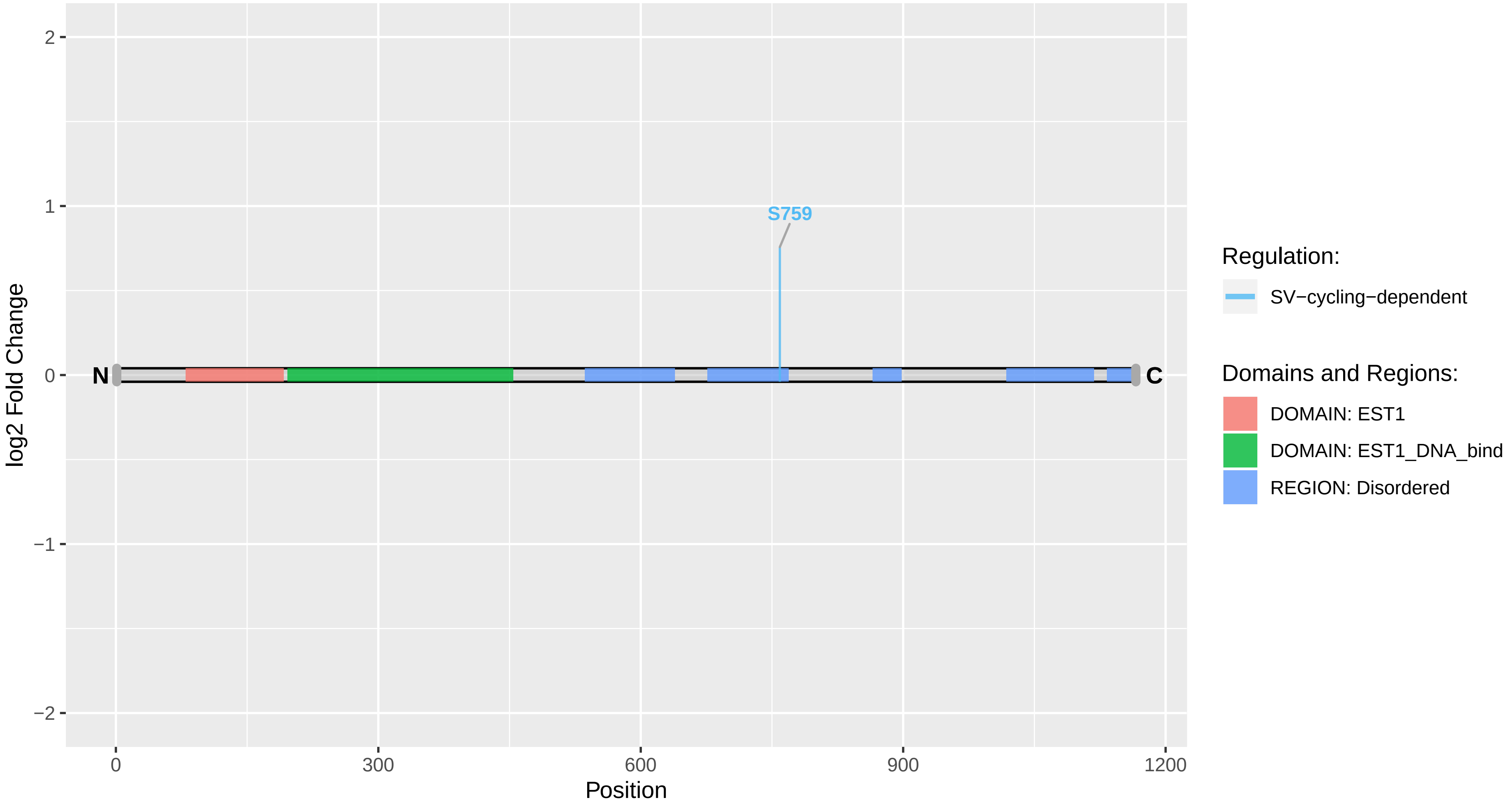

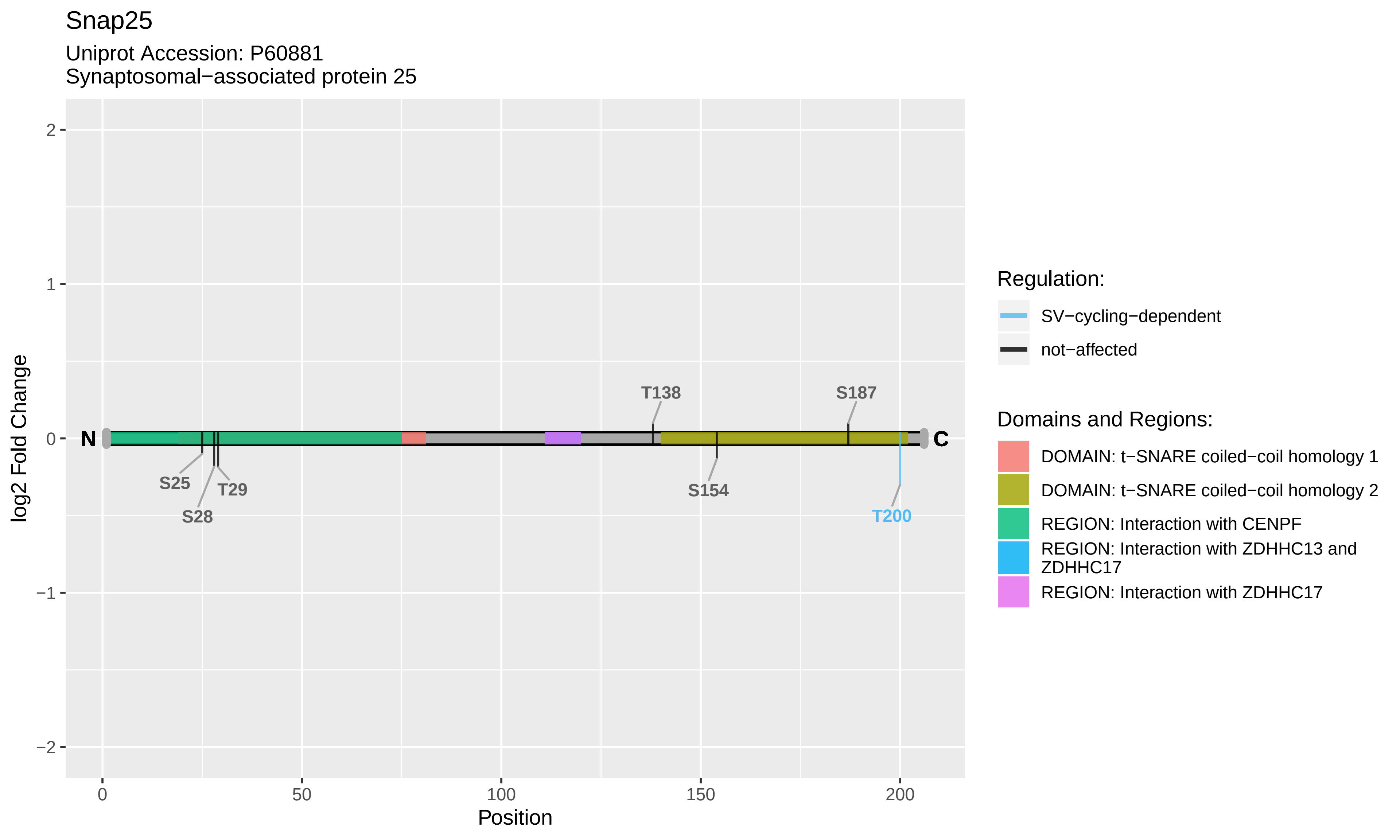
Snap25
Uniprot Accession: P60881
Synaptosomal−associated protein 25
log2 Fold Change
2
1
0
−1
−2
N
C
T138
S187
S25
T29
S28
S154
T200
0
50
100
150
200
Position
Regulation:
SV−cycling−dependent
not−affected
Domains and Regions:
DOMAIN: t−SNARE coiled−coil homology 1
DOMAIN: t−SNARE coiled−coil homology 2
REGION: Interaction with CENPF
REGION: Interaction with ZDHHC13 and ZDHHC17
REGION: Interaction with ZDHHC17

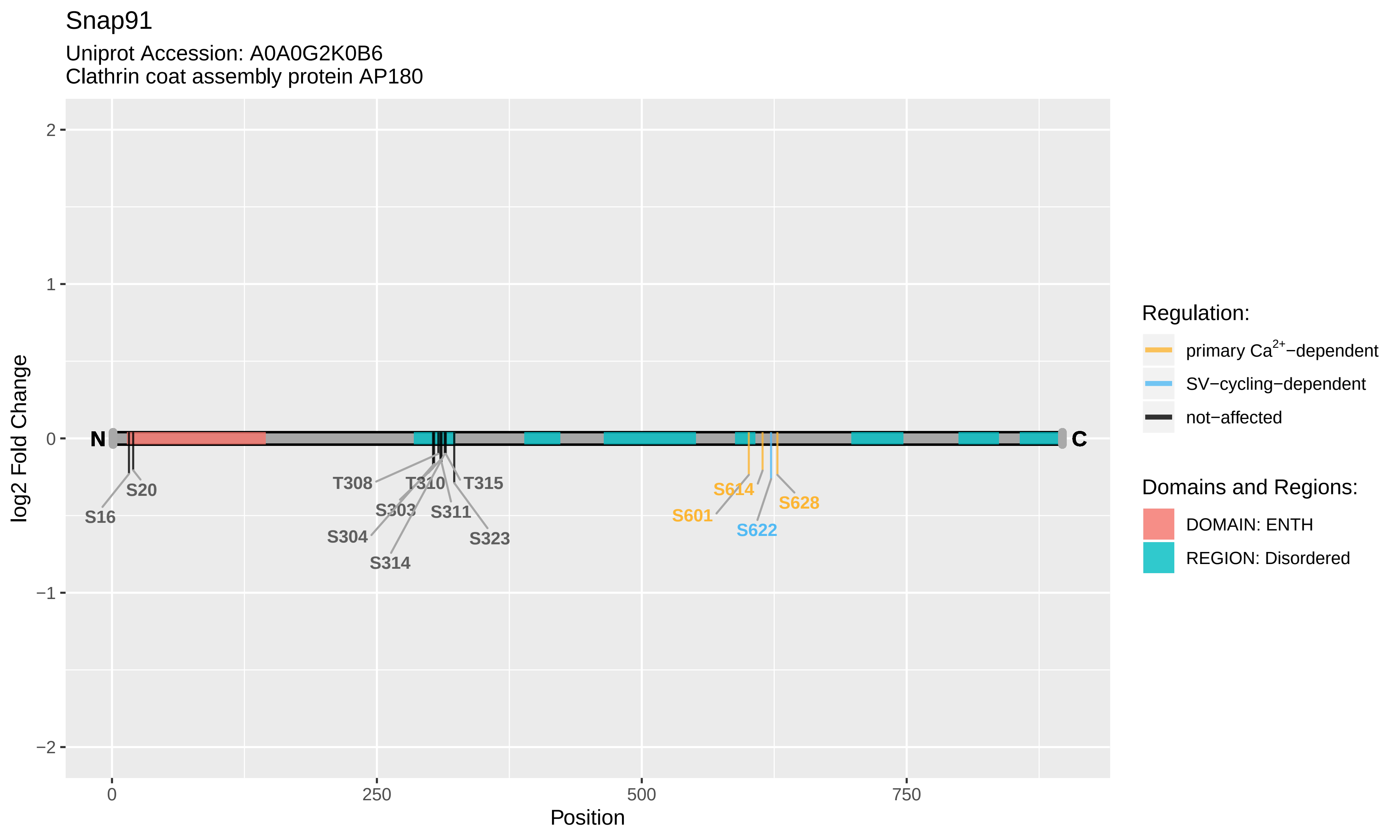

Snap91
Uniprot Accession: A0A0G2K0B6
Clathrin coat assembly protein AP180
log2 Fold Change
Position
2
1
0
−1
−2
0
250
500
750
N
C
S16
S20
T308
T310
T315
S303
S311
S304
S323
S314
S614
S601
S622
S628
Regulation:
primary Ca2+−dependent
SV−cycling−dependent
not−affected
Domains and Regions:
DOMAIN: ENTH
REGION: Disordered

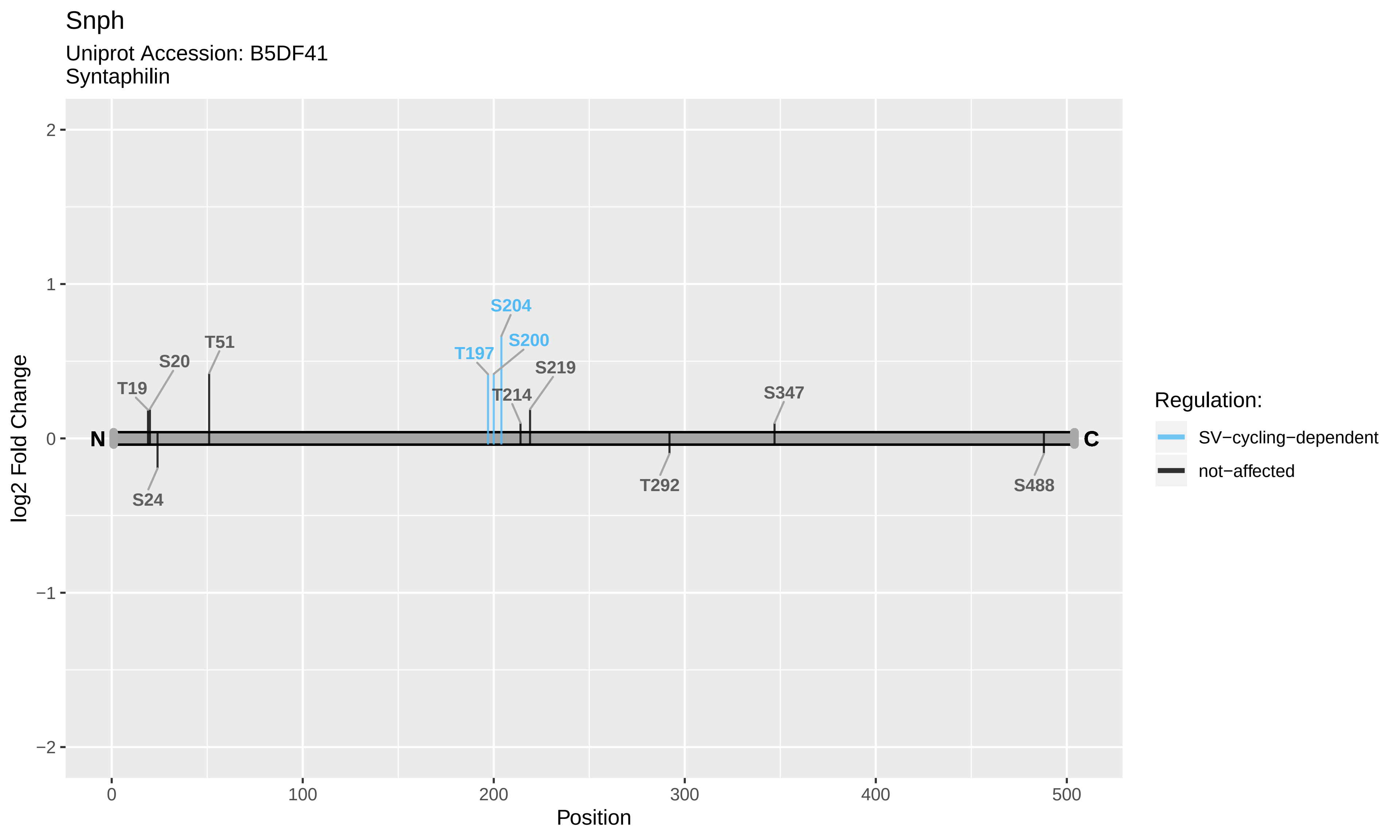

Snph
Uniprot Accession: B5DF41
Syntaphilin
log2 Fold Change
Position
2
1
0
−1
−2
0
100
200
300
400
500
N
C
T19
S20
S24
T51
T197
S200
S204
T214
S219
T292
S347
S488
Regulation:
SV−cycling−dependent
not−affected

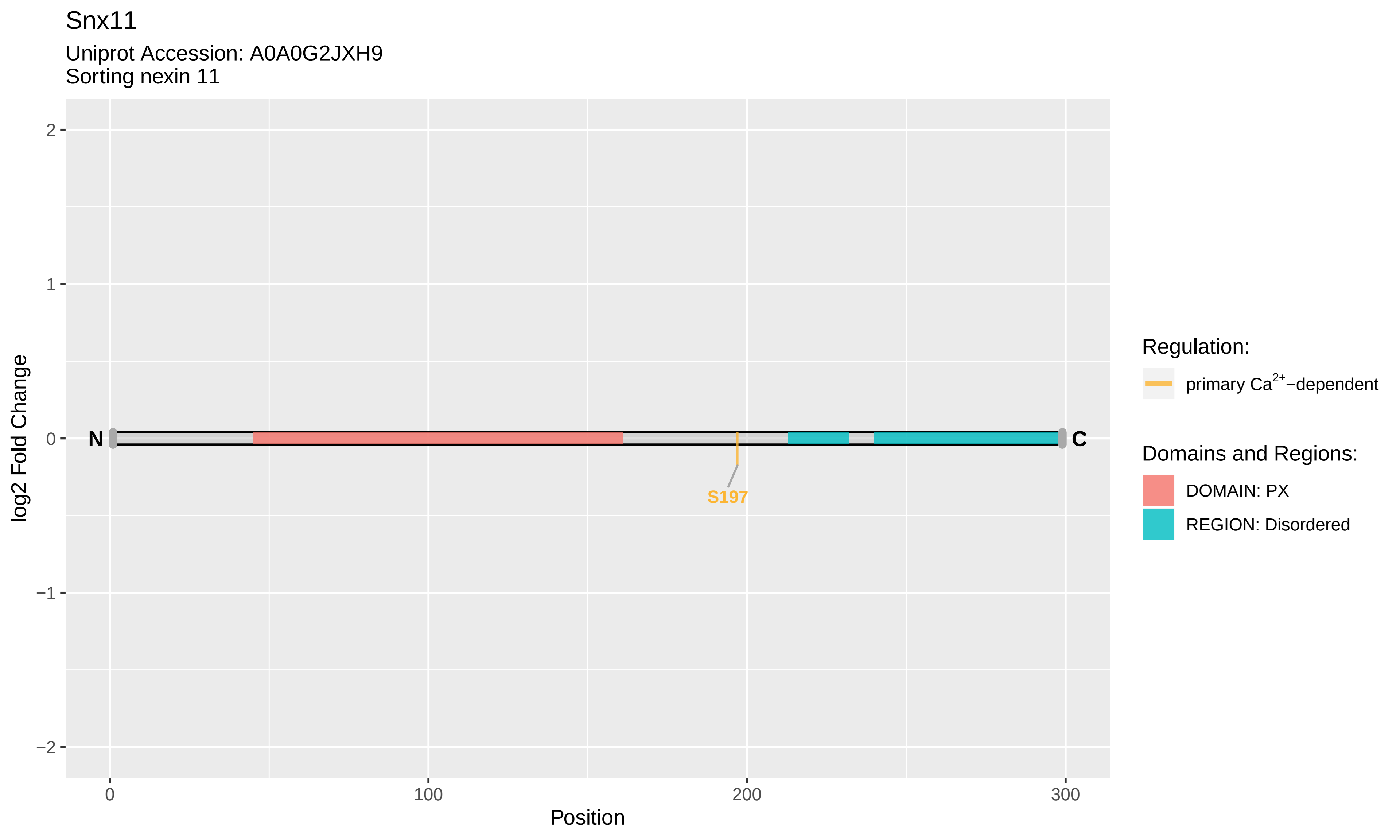
Snx11
Uniprot Accession: A0A0G2JXH9
Sorting nexin 11
log2 Fold Change
2
1
0
−1
−2
N
C
S197
0
100
200
300
Position
Regulation:
primary Ca2+−dependent
Domains and Regions:
DOMAIN: PX
REGION: Disordered

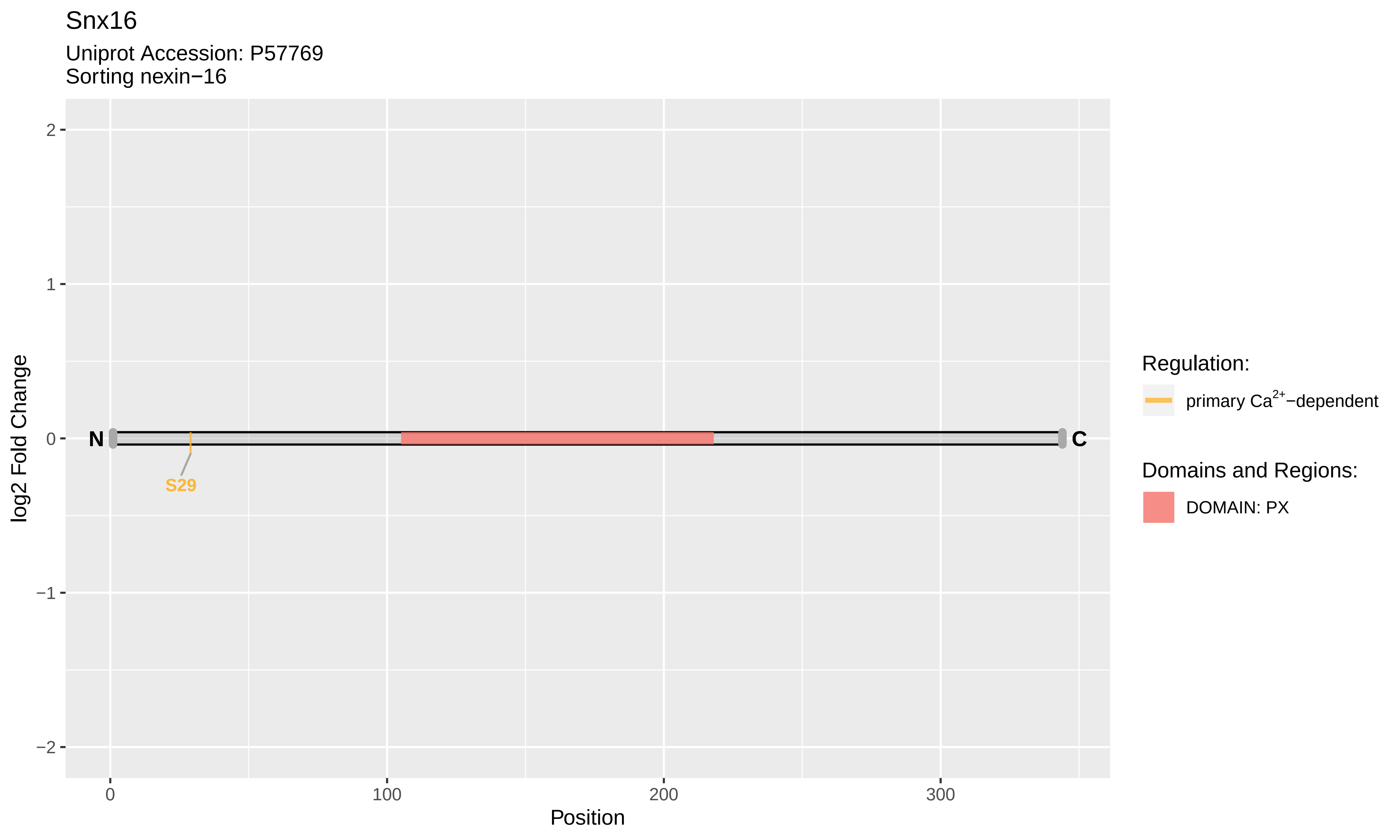

Snx16
Uniprot Accession: P57769
Sorting nexin−16
log2 Fold Change
2
1
0
−1
−2
N
C
S29
0
100
200
300
Position
Regulation:
primary Ca2+−dependent
Domains and Regions:
DOMAIN: PX

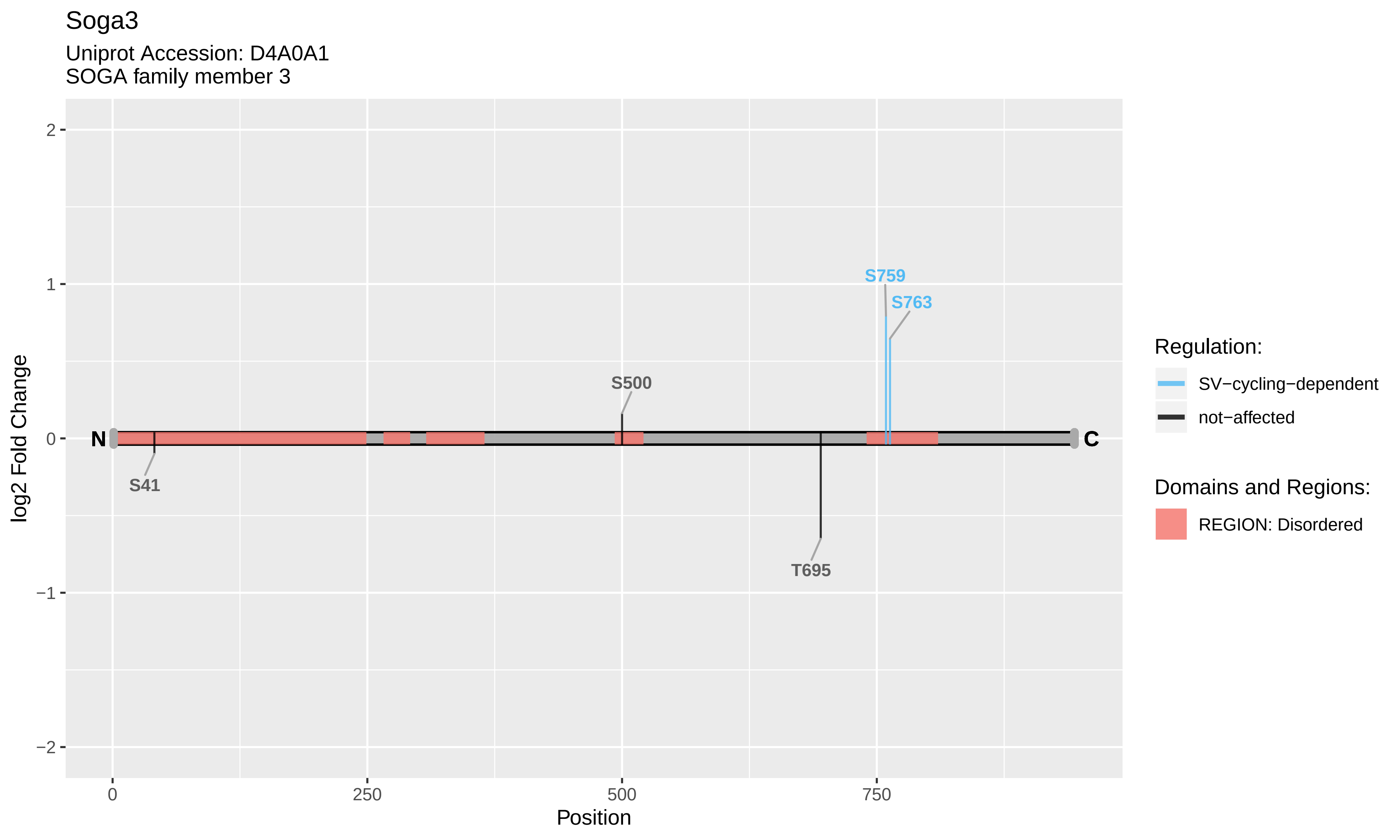

Soga3
Uniprot Accession: D4A0A1
SOGA family member 3
log2 Fold Change
2
1
0
−1
−2
N
C
S759
S763
S500
S41
T695
0
250
500
750
Position
Regulation:
SV−cycling−dependent
not−affected
Domains and Regions:
REGION: Disordered

Sorbs1

Uniprot Accession: F1M820
Sorbin and SH3 domain−containing protein 1

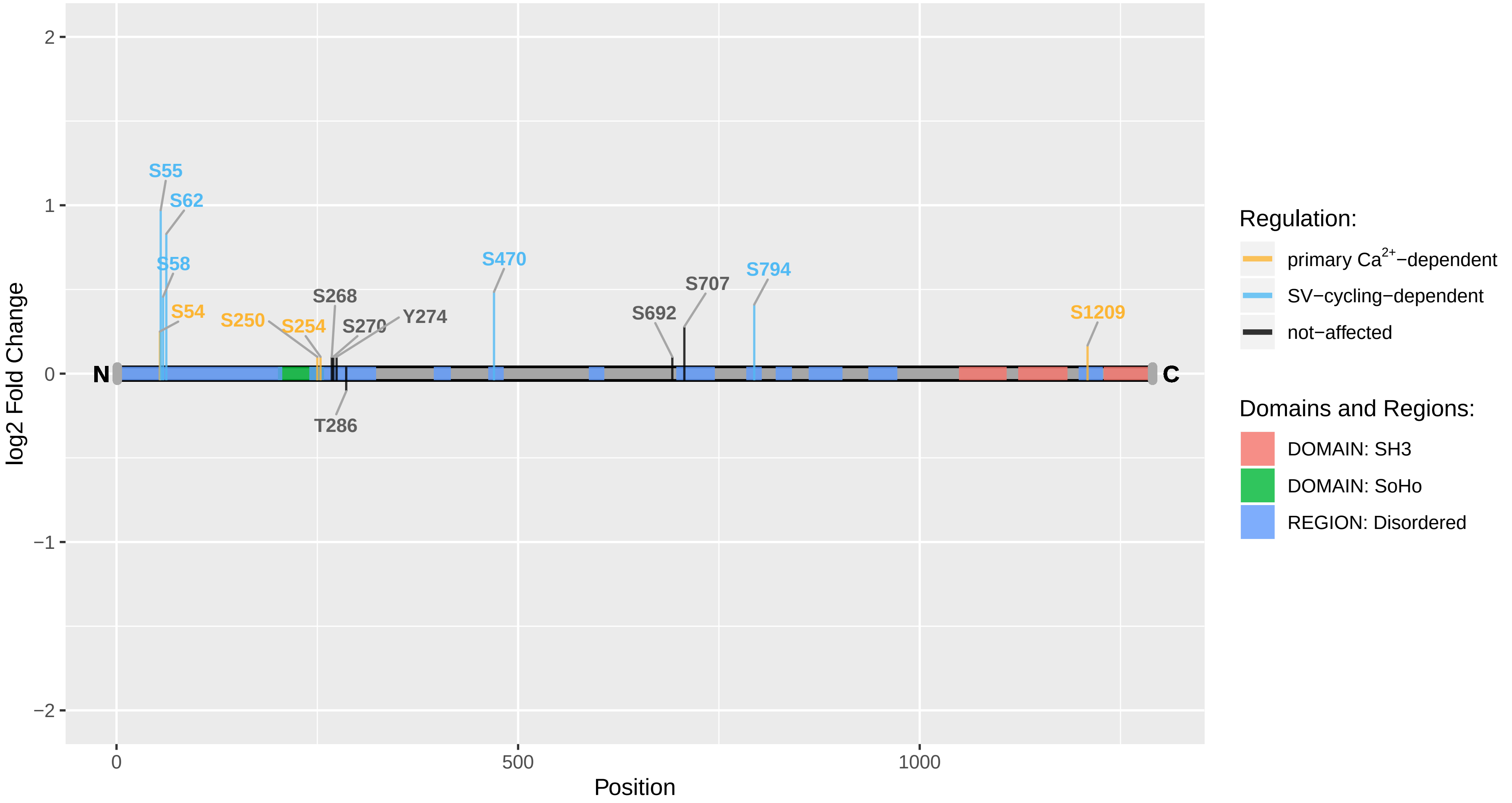

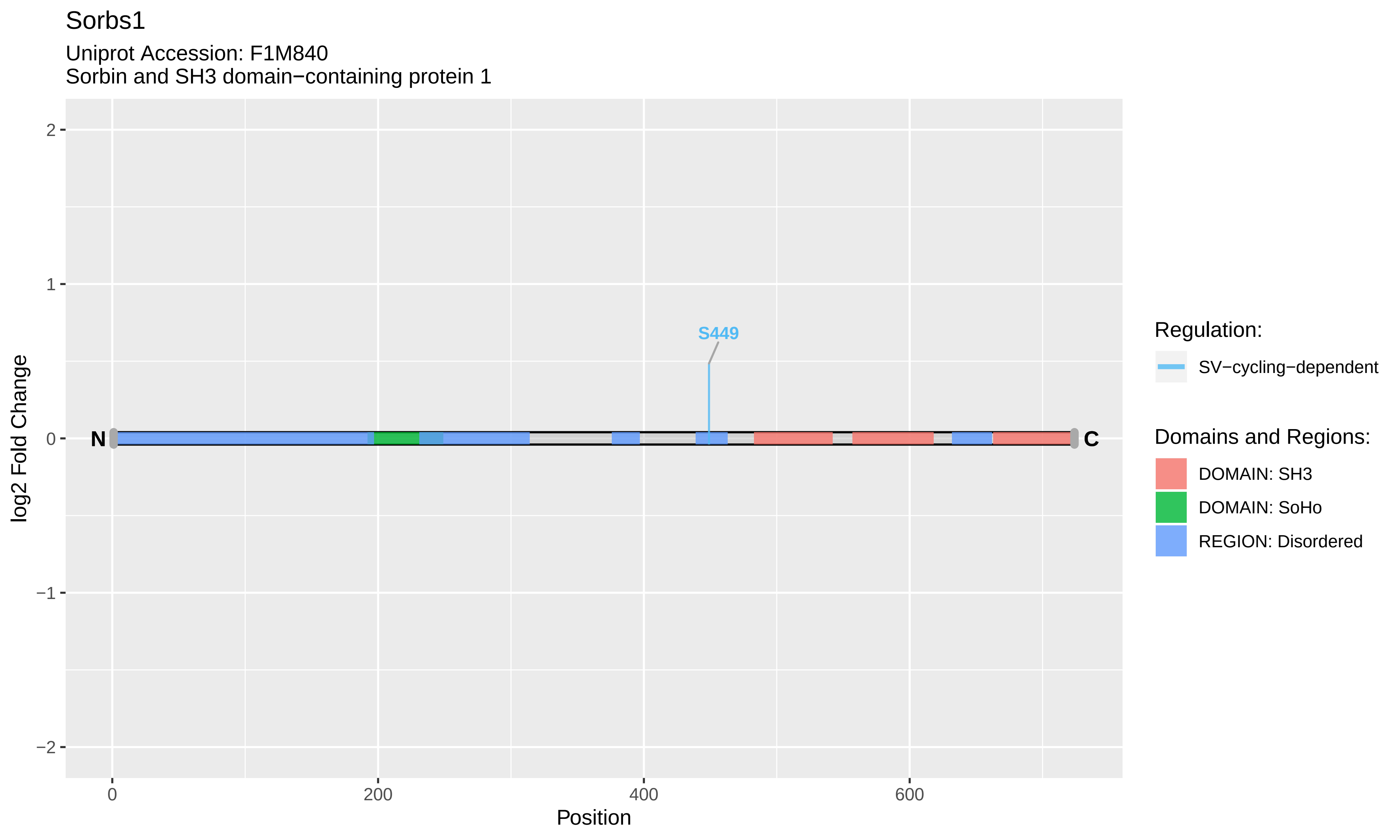

Sorbs1
Uniprot Accession: F1M840
Sorbin and SH3 domain−containing protein 1
S449
N
C
2
1
0
−1
−2
log2 Fold Change
0
200
400
600
Position
Regulation:
SV−cycling−dependent
Domains and Regions:
DOMAIN: SH3
DOMAIN: SoHo
REGION: Disordered

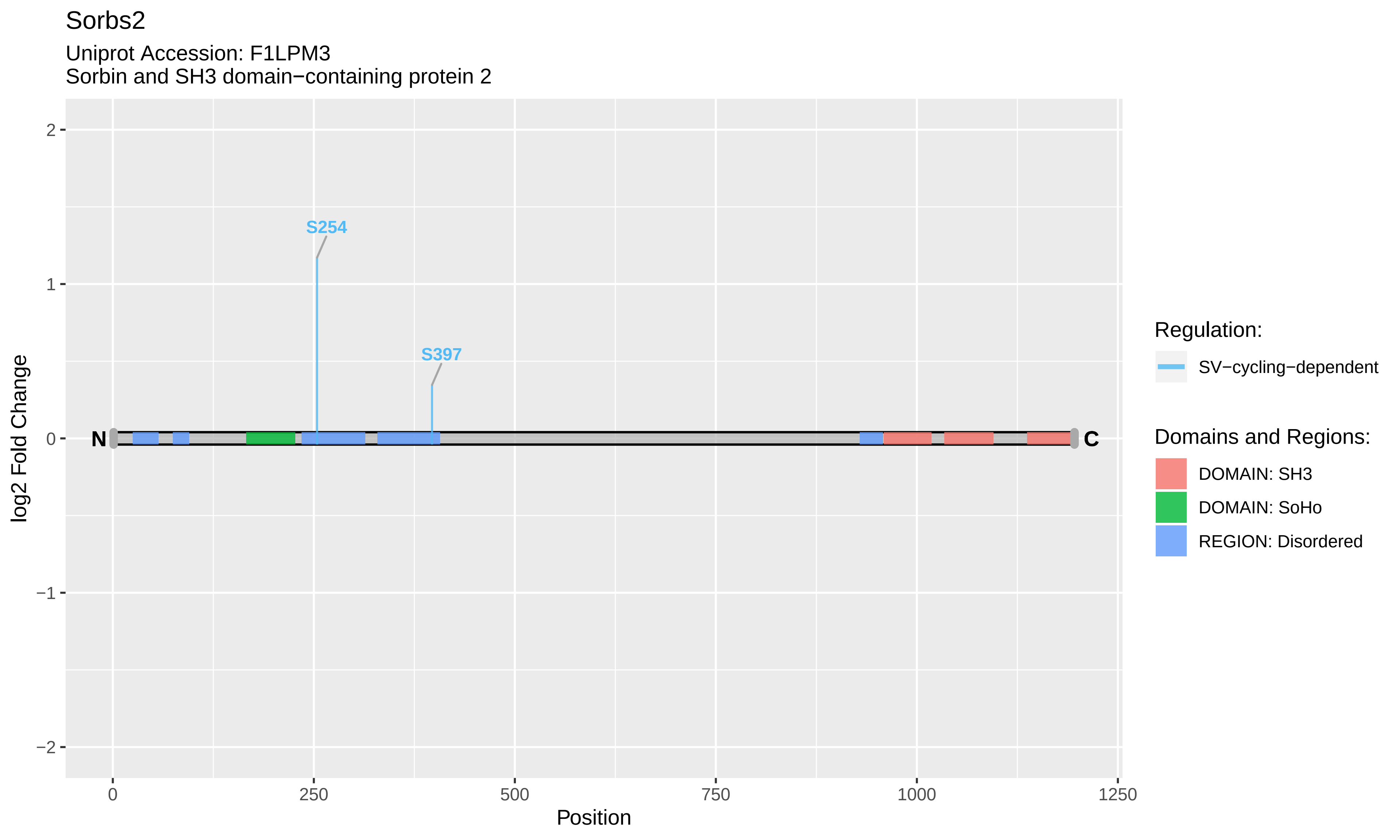
Sorbs2
Uniprot Accession: F1LPM3
Sorbin and SH3 domain−containing protein 2
S254
S397
N
C
log2 Fold Change
2
1
0
−1
−2
0
250
500
750
1000
1250
Position
Regulation:
SV−cycling−dependent
Domains and Regions:
DOMAIN: SH3
DOMAIN: SoHo
REGION: Disordered

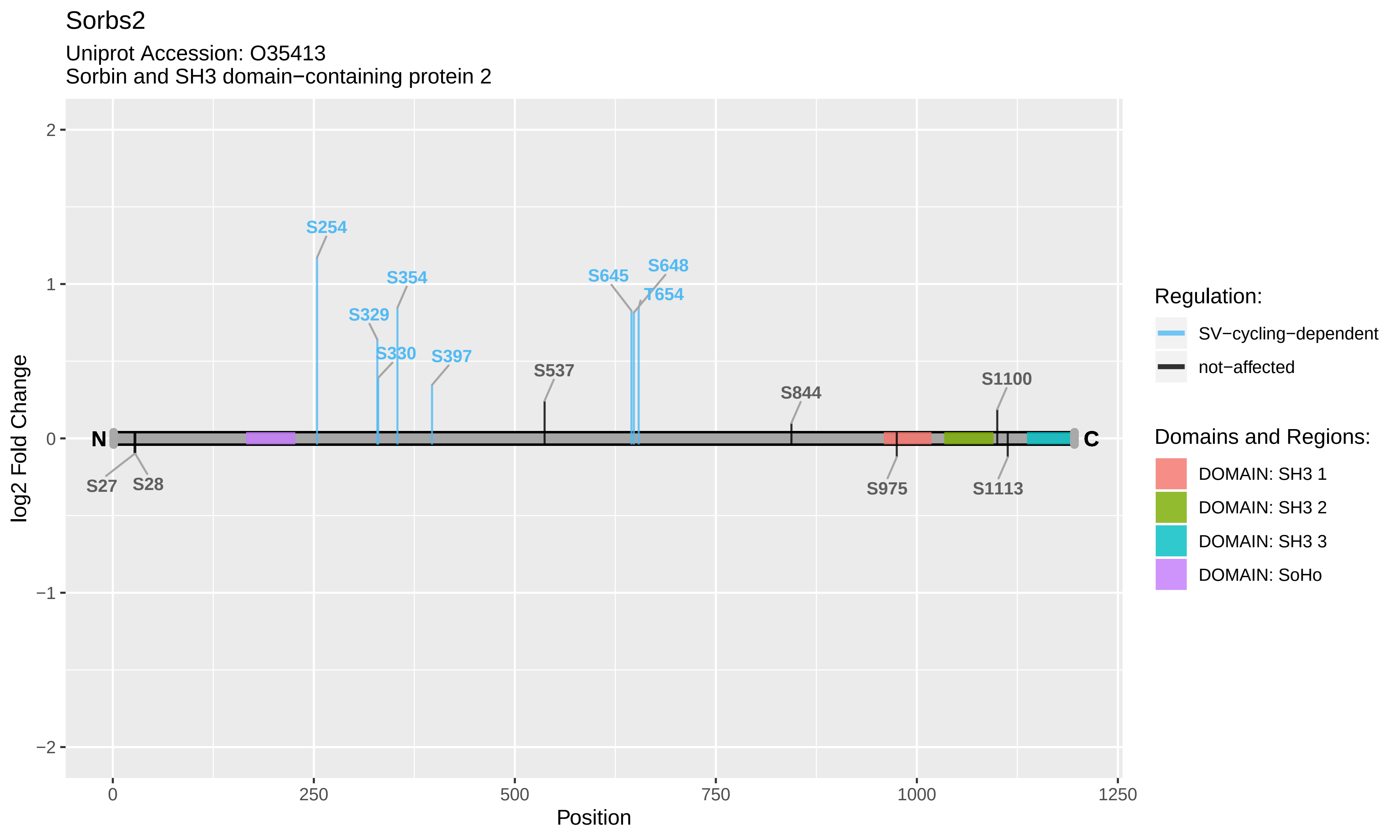
Sorbs2
Uniprot Accession: O35413
Sorbin and SH3 domain−containing protein 2
log2 Fold Change
Position
2
1
0
−1
−2
0
250
500
750
1000
1250
N
C
S254
S354
S329
S330
S397
S645
S648
T654
S537
S844
S1100
S27
S28
S975
S1113
Regulation:
SV−cycling−dependent
not−affected
Domains and Regions:
DOMAIN: SH3 1
DOMAIN: SH3 2
DOMAIN: SH3 3
DOMAIN: SoHo

Sord

Uniprot Accession: P27867
Sorbitol dehydrogenase

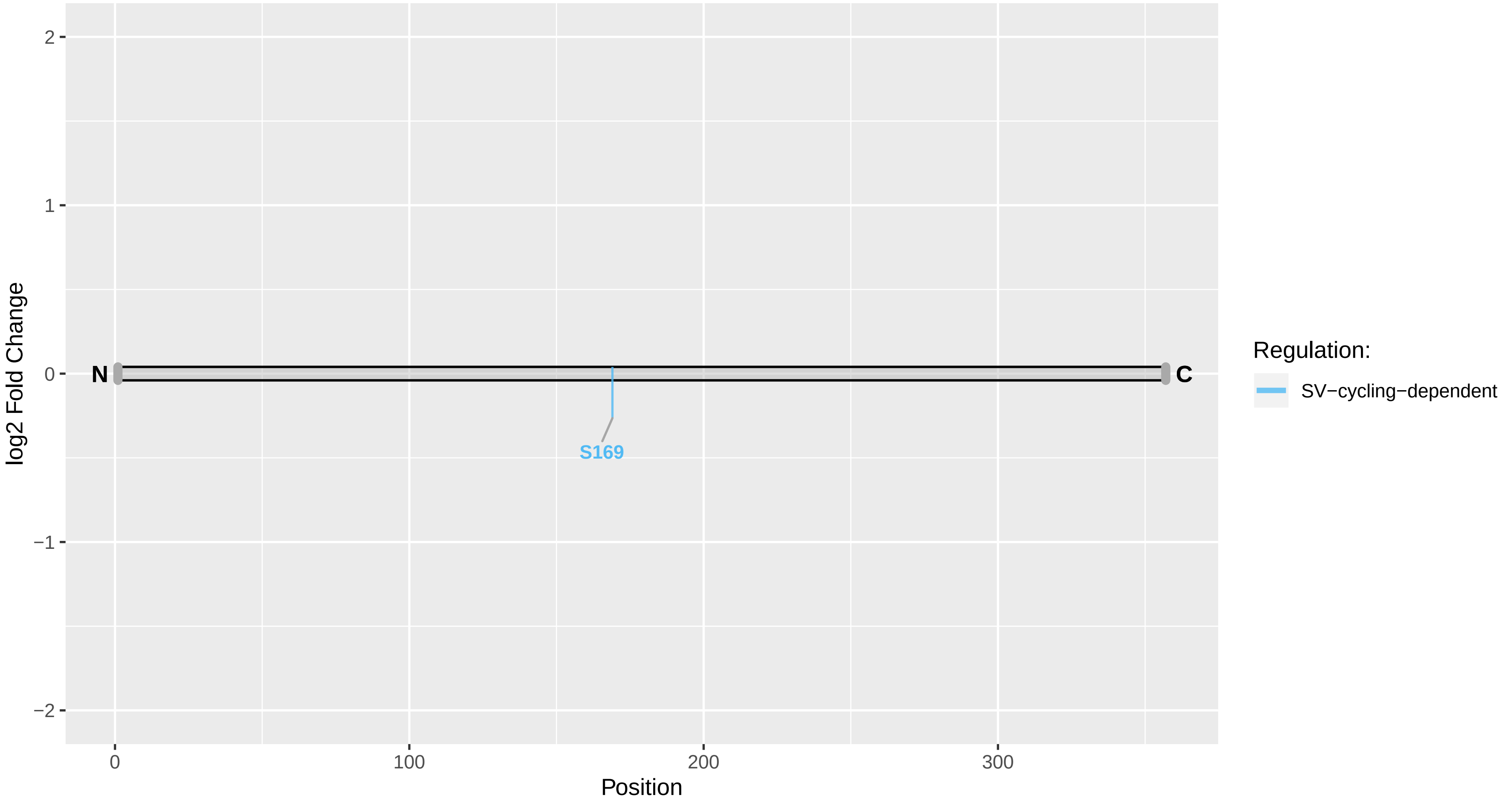

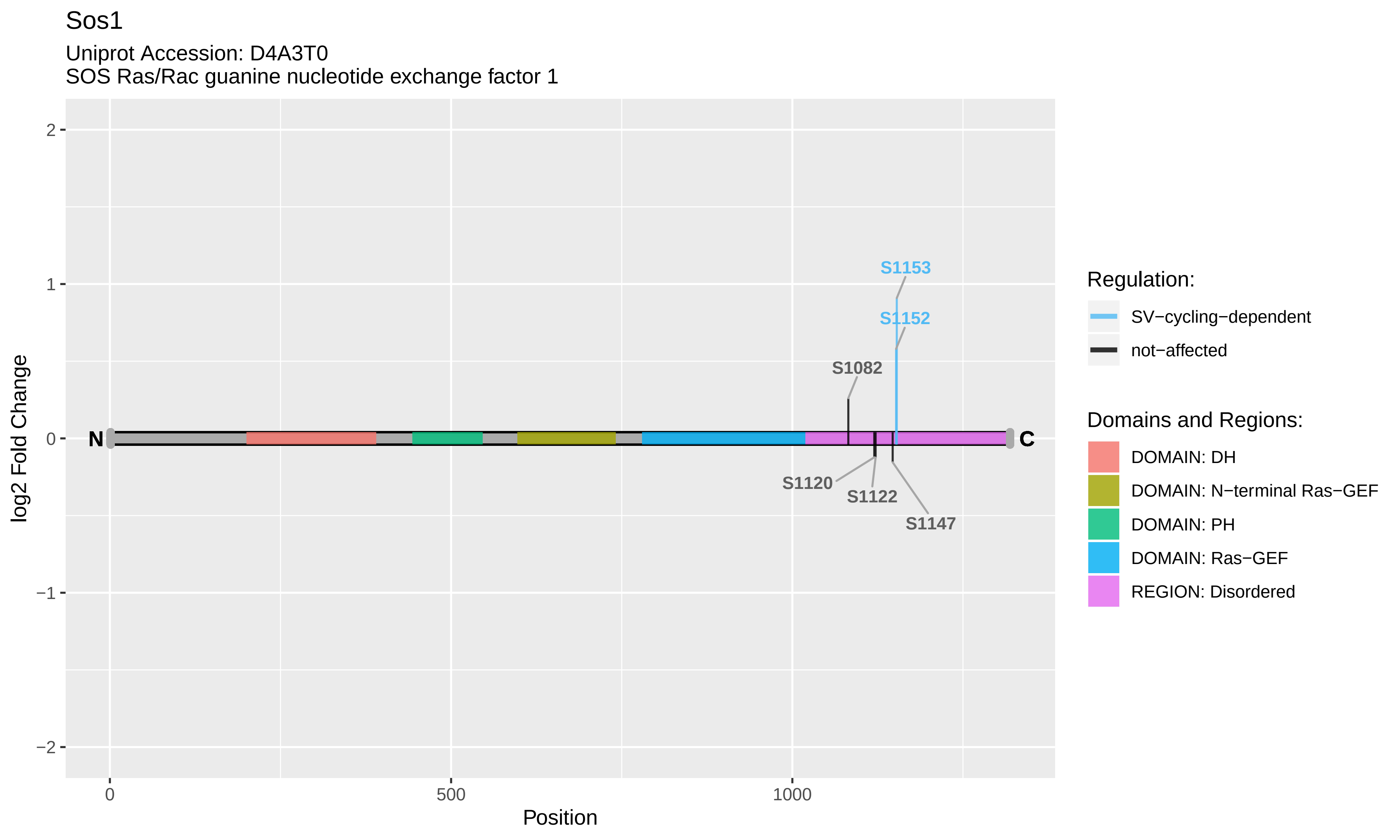

Sos1
Uniprot Accession: D4A3T0
SOS Ras/Rac guanine nucleotide exchange factor 1
log2 Fold Change
Position
N
C
S1153
S1152
S1082
S1120
S1122
S1147
Regulation:
SV−cycling−dependent
not−affected
Domains and Regions:
DOMAIN: DH
DOMAIN: N−terminal Ras−GEF
DOMAIN: PH
DOMAIN: Ras−GEF
REGION: Disordered

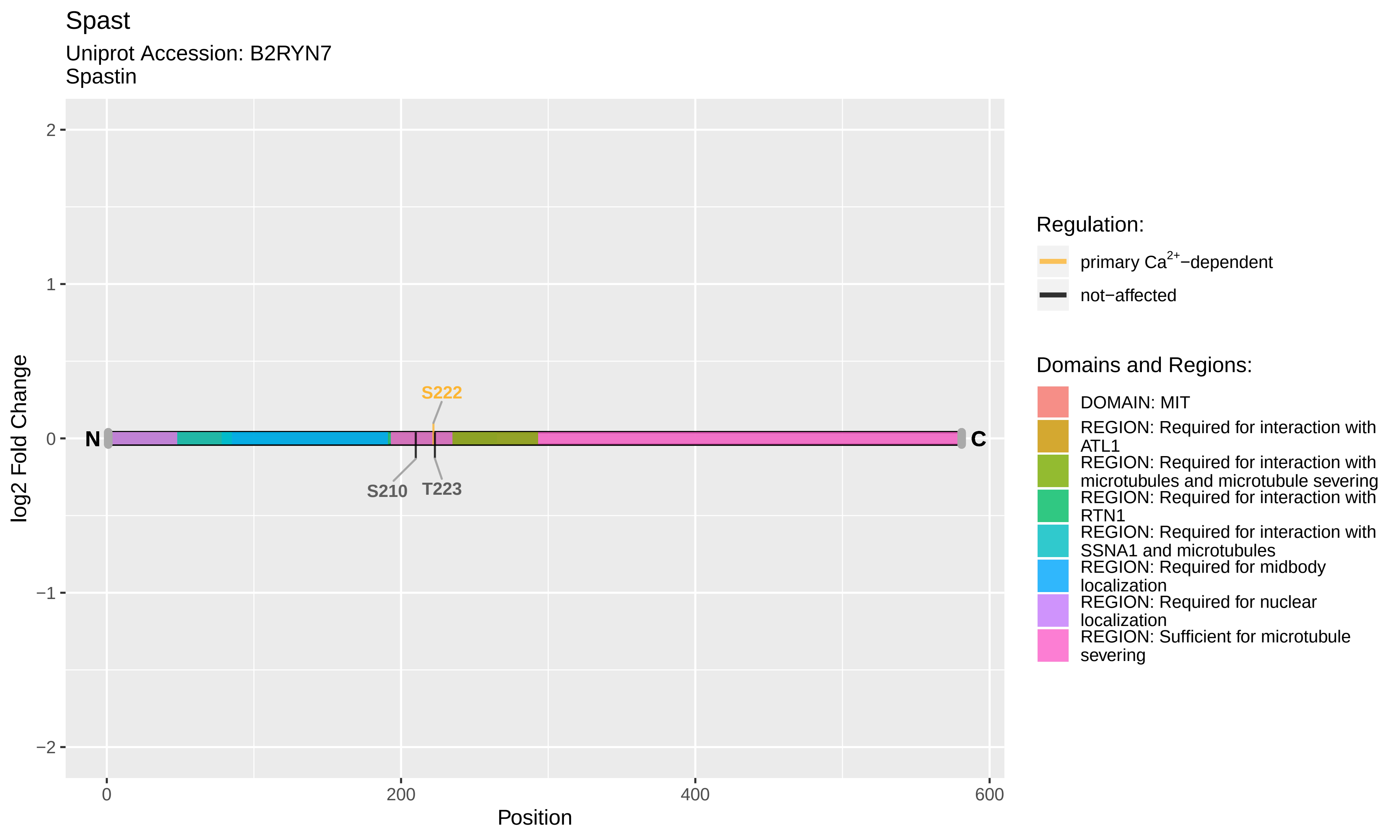
Spast
Uniprot Accession: B2RYN7
Spastin
log2 Fold Change
2
1
0
−1
−2
N
C
S222
S210
T223
0
200
400
600
Position
Regulation:
primary Ca2+−dependent
not−affected
Domains and Regions:
DOMAIN: MIT
REGION: Required for interaction with ATL1
REGION: Required for interaction with microtubules and microtubule severing
REGION: Required for interaction with RTN1
REGION: Required for interaction with SSNA1 and microtubules
REGION: Required for midbody localization
REGION: Required for nuclear localization
REGION: Sufficient for microtubule severing

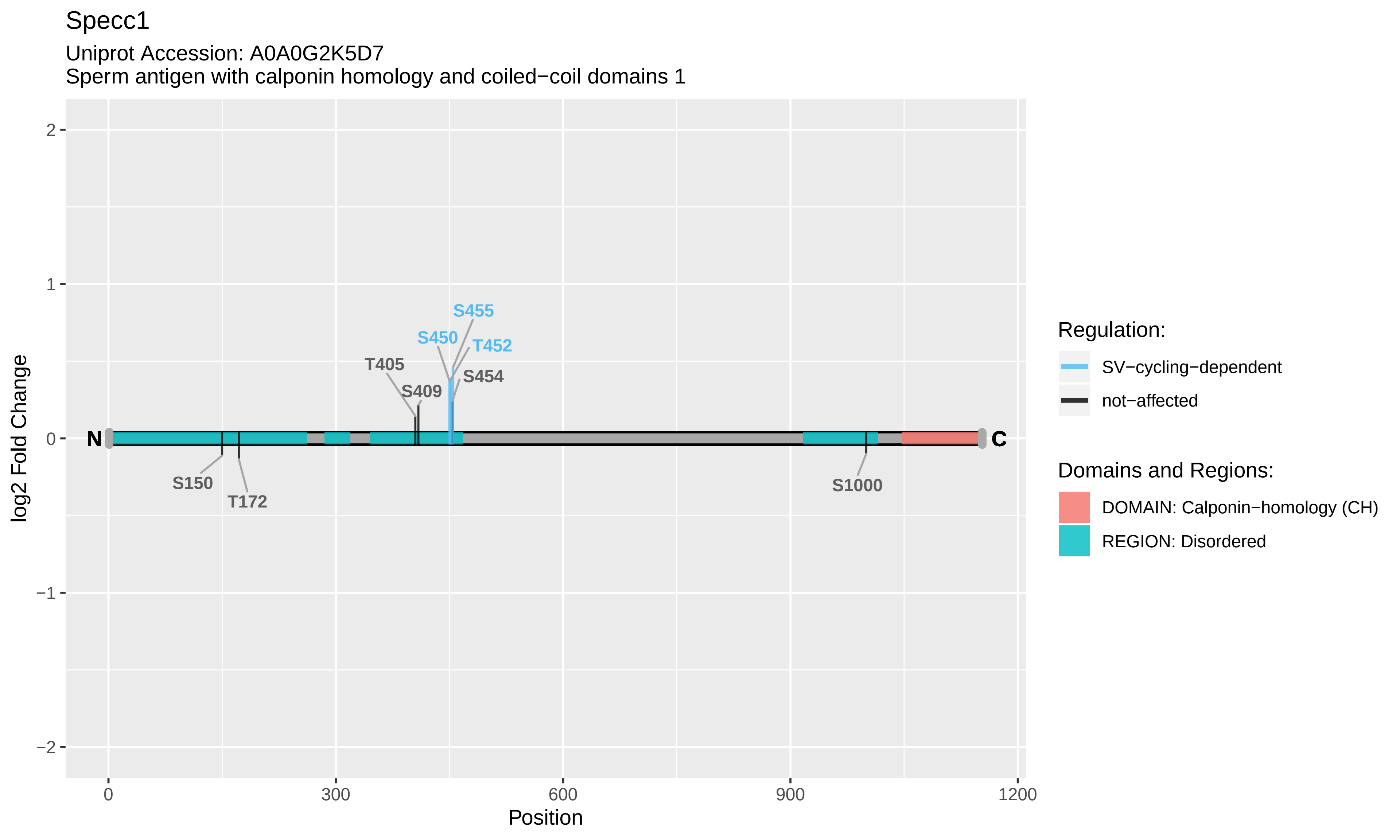

Specc1
Uniprot Accession: A0A0G2K5D7
Sperm antigen with calponin homology and coiled−coil domains 1
log2 Fold Change
Position
2
1
0
−1
−2
0
300
600
900
1200
N
C
S455
S450
T452
T405
S454
S409
S150
T172
S1000
Regulation:
SV−cycling−dependent
not−affected
Domains and Regions:
DOMAIN: Calponin−homology (CH)
REGION: Disordered

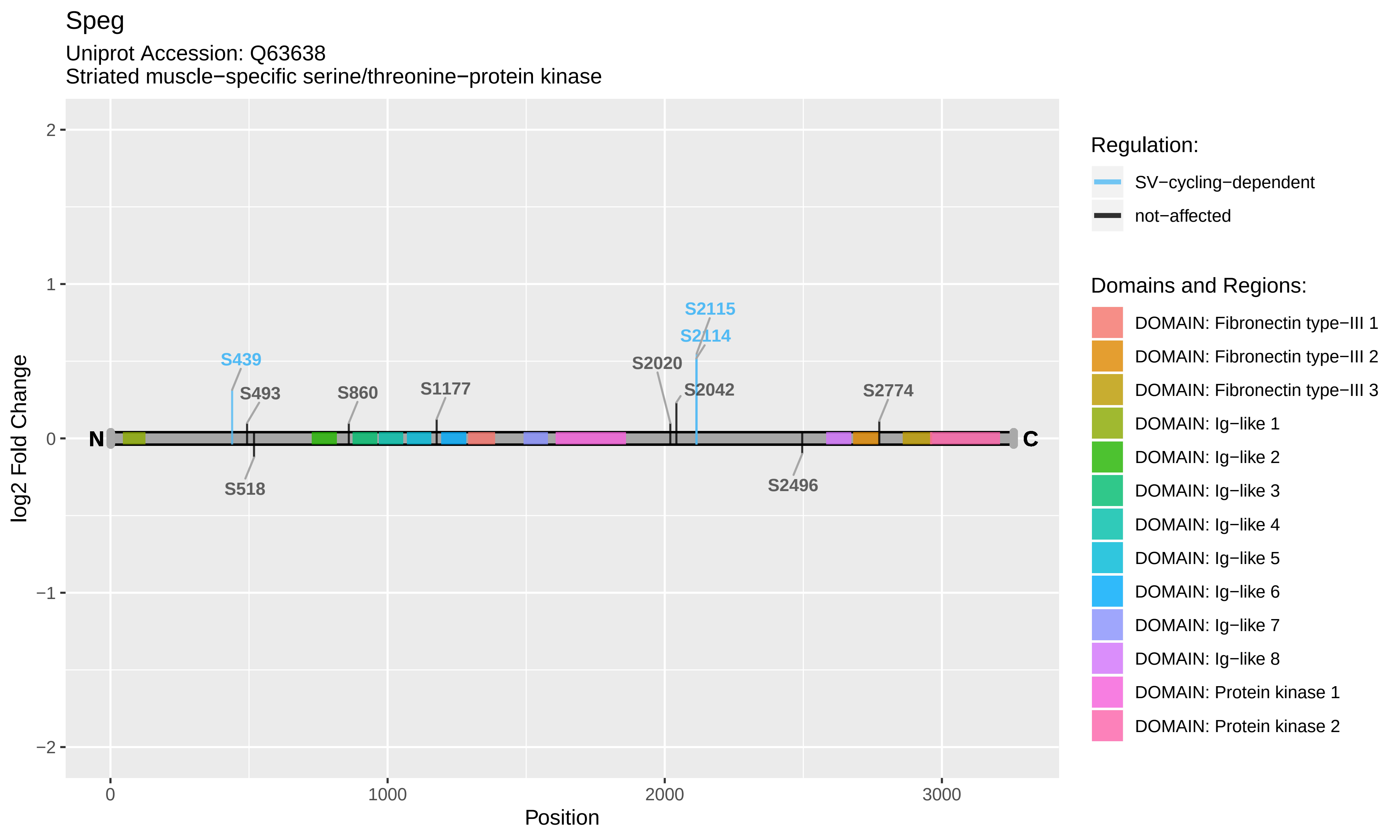

Speg
Uniprot Accession: Q63638
Striated muscle−specific serine/threonine−protein kinase
log2 Fold Change
Position
2
1
0
−1
−2
0
1000
2000
3000
N
C
S439
S493
S518
S860
S1177
S2020
S2042
S2114
S2115
S2496
S2774
Regulation:
SV−cycling−dependent
not−affected
Domains and Regions:
DOMAIN: Fibronectin type−III 1
DOMAIN: Fibronectin type−III 2
DOMAIN: Fibronectin type−III 3
DOMAIN: Ig−like 1
DOMAIN: Ig−like 2
DOMAIN: Ig−like 3
DOMAIN: Ig−like 4
DOMAIN: Ig−like 5
DOMAIN: Ig−like 6
DOMAIN: Ig−like 7
DOMAIN: Ig−like 8
DOMAIN: Protein kinase 1
DOMAIN: Protein kinase 2

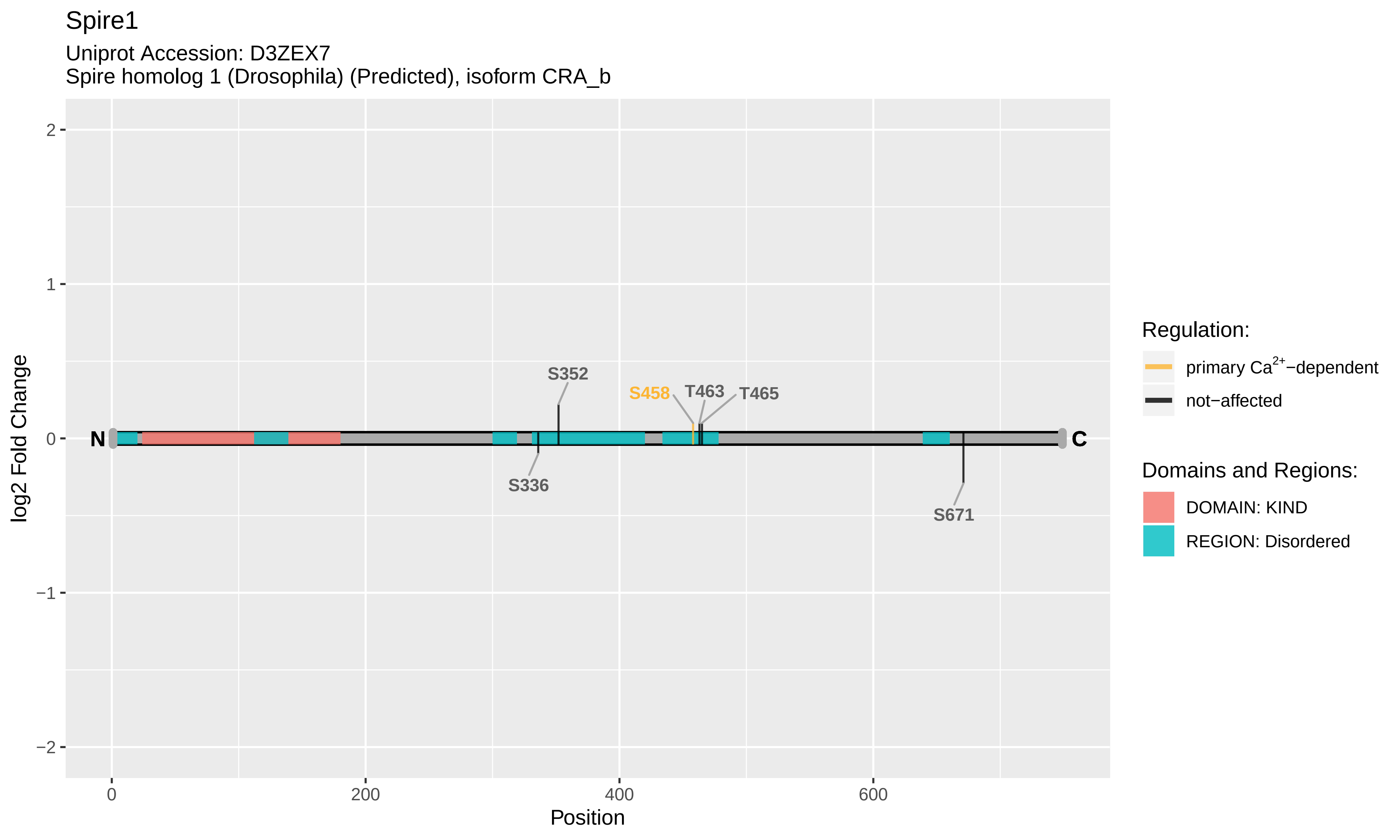

Spire1
Uniprot Accession: D3ZEX7
Spire homolog 1 (Drosophila) (Predicted), isoform CRA_b
log2 Fold Change
Position
N
C
S352
S458
T463
T465
S336
S671
2
1
0
−1
−2
0
200
400
600
Regulation:
primary Ca2+−dependent
not−affected
Domains and Regions:
DOMAIN: KIND
REGION: Disordered

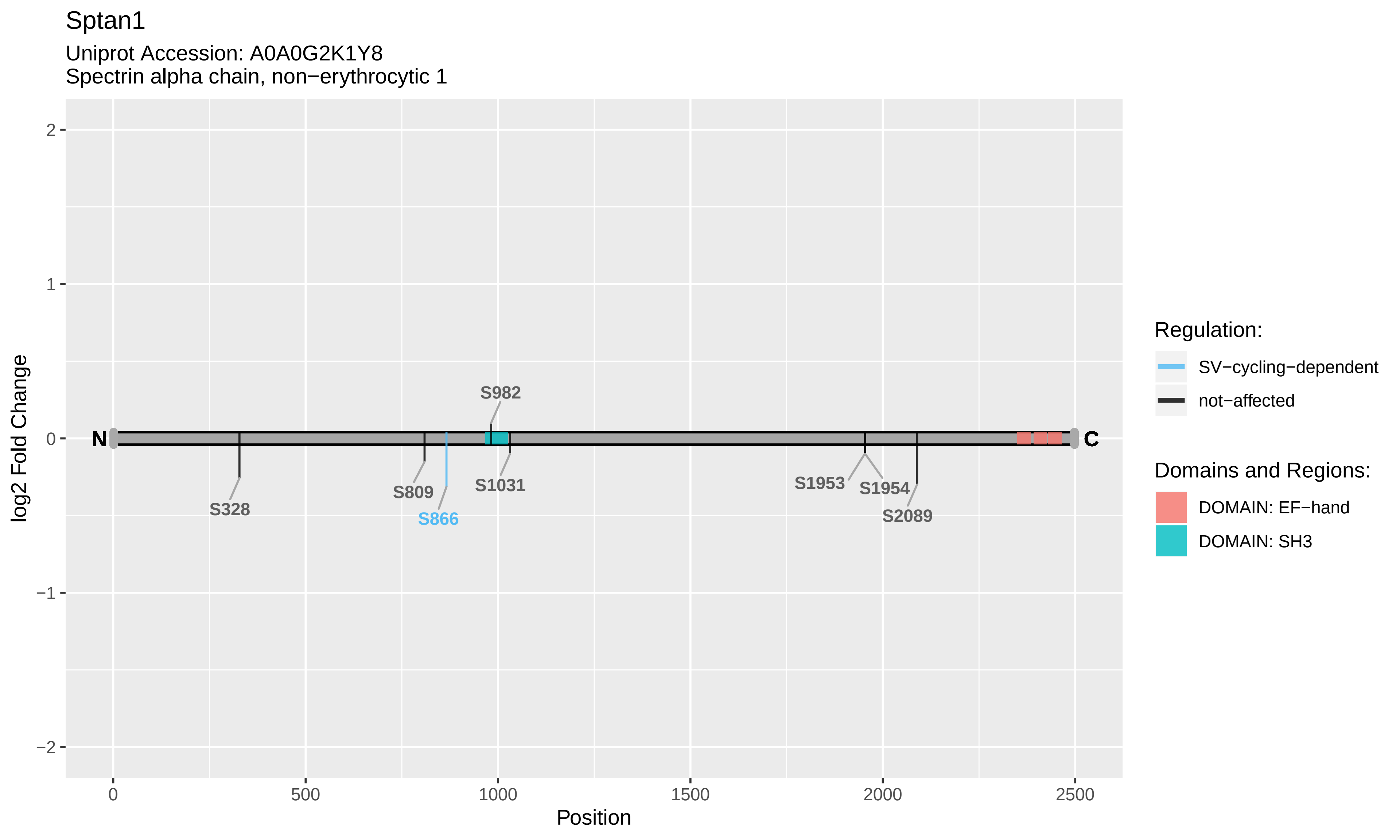

Sptan1
Uniprot Accession: A0A0G2K1Y8
Spectrin alpha chain, non−erythrocytic 1
log2 Fold Change
Position
N
C
S982
S328
S809
S866
S1031
S1953
S1954
S2089
Regulation:
SV−cycling−dependent
not−affected
Domains and Regions:
DOMAIN: EF−hand
DOMAIN: SH3
2
1
0
−1
−2
0
500
1000
1500
2000
2500

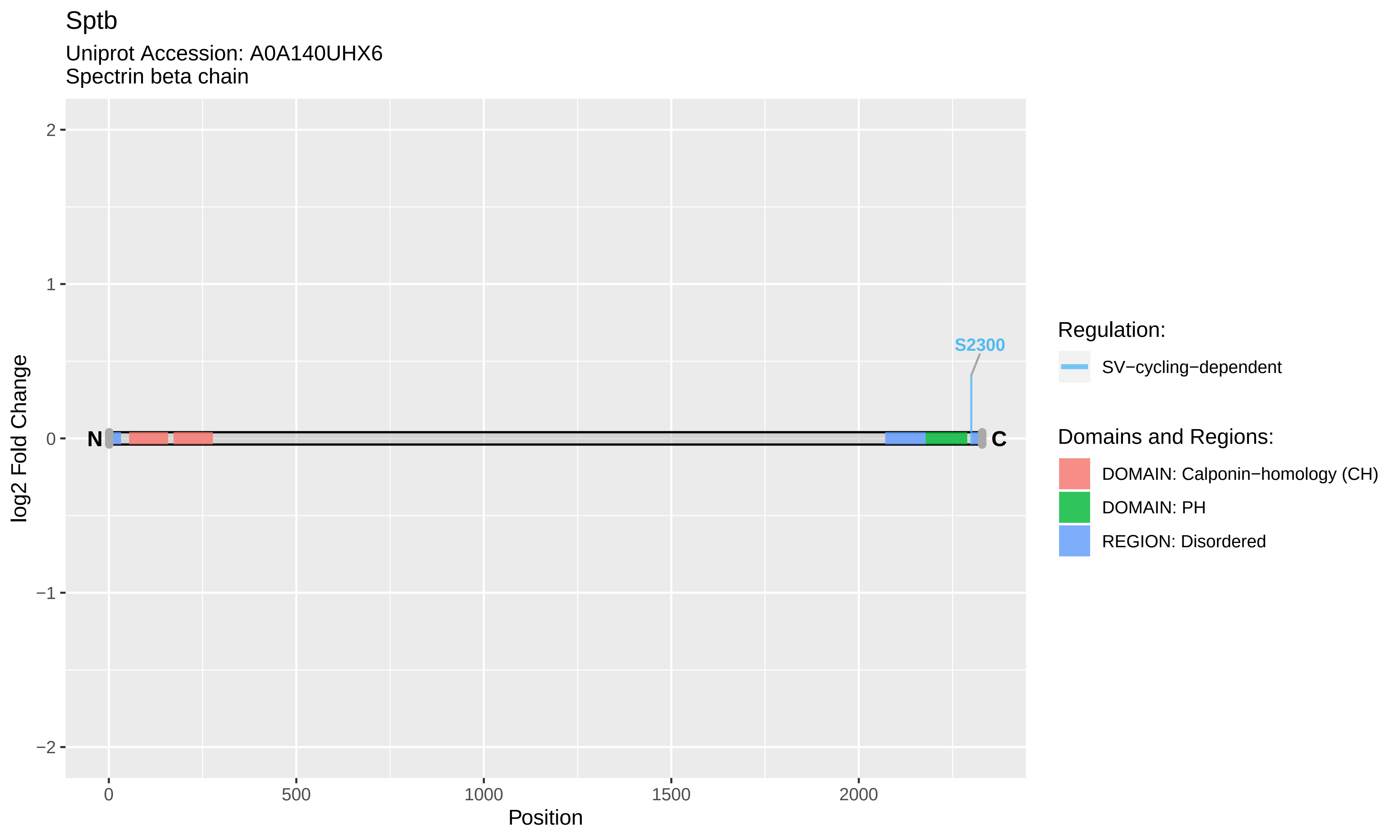
Sptb
Uniprot Accession: A0A140UHX6
Spectrin beta chain
log2 Fold Change
2
1
0
−1
−2
N
C
S2300
0
500
1000
1500
2000
Position
Regulation:
SV−cycling−dependent
Domains and Regions:
DOMAIN: Calponin−homology (CH)
DOMAIN: PH
REGION: Disordered

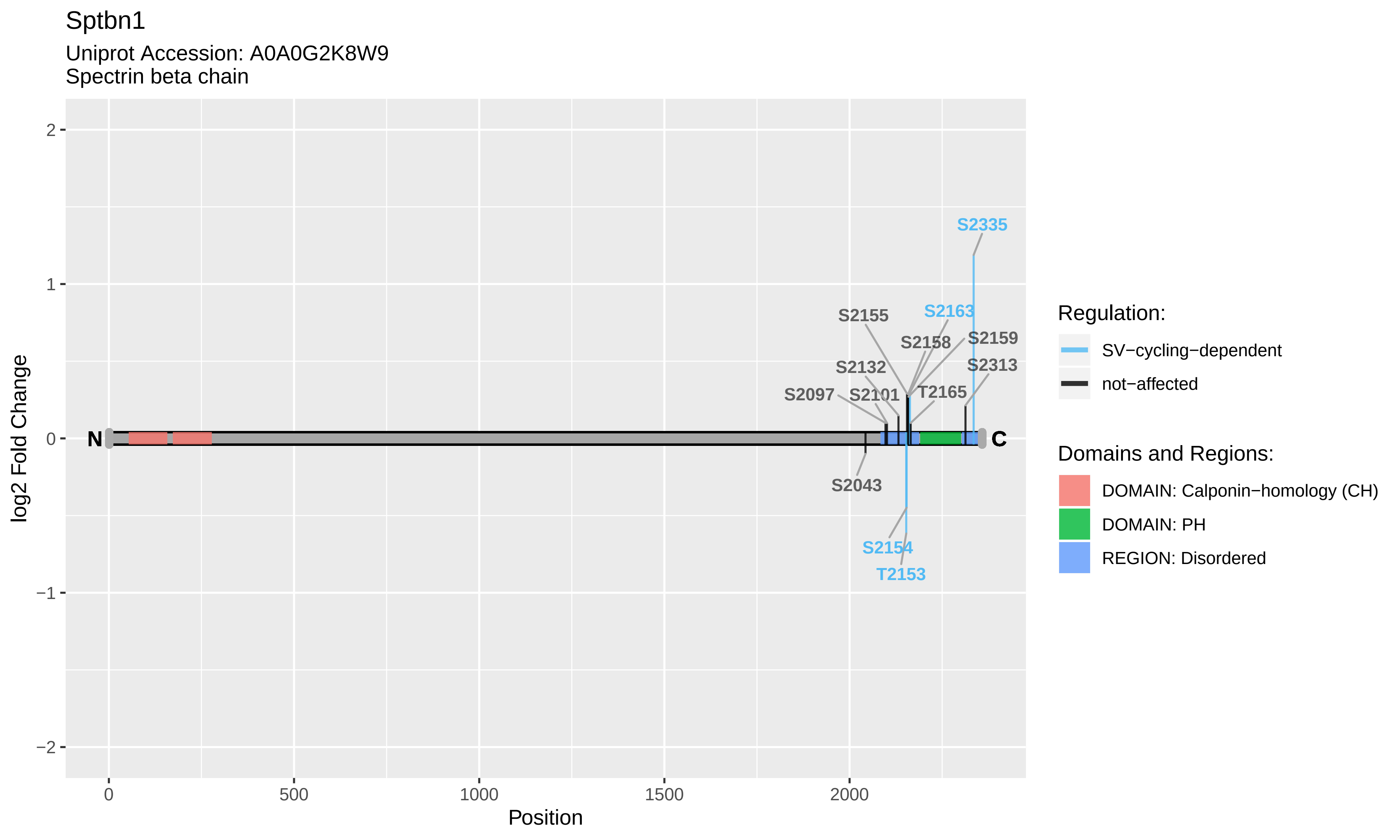

Sptbn1
Uniprot Accession: A0A0G2K8W9
Spectrin beta chain
log2 Fold Change
Position
2
1
0
−1
−2
0
500
1000
1500
2000
N
C
S2335
S2155
S2163
S2158
S2159
S2132
S2313
S2097
S2101
T2165
S2043
S2154
T2153
Regulation:
SV−cycling−dependent
not−affected
Domains and Regions:
DOMAIN: Calponin−homology (CH)
DOMAIN: PH
REGION: Disordered

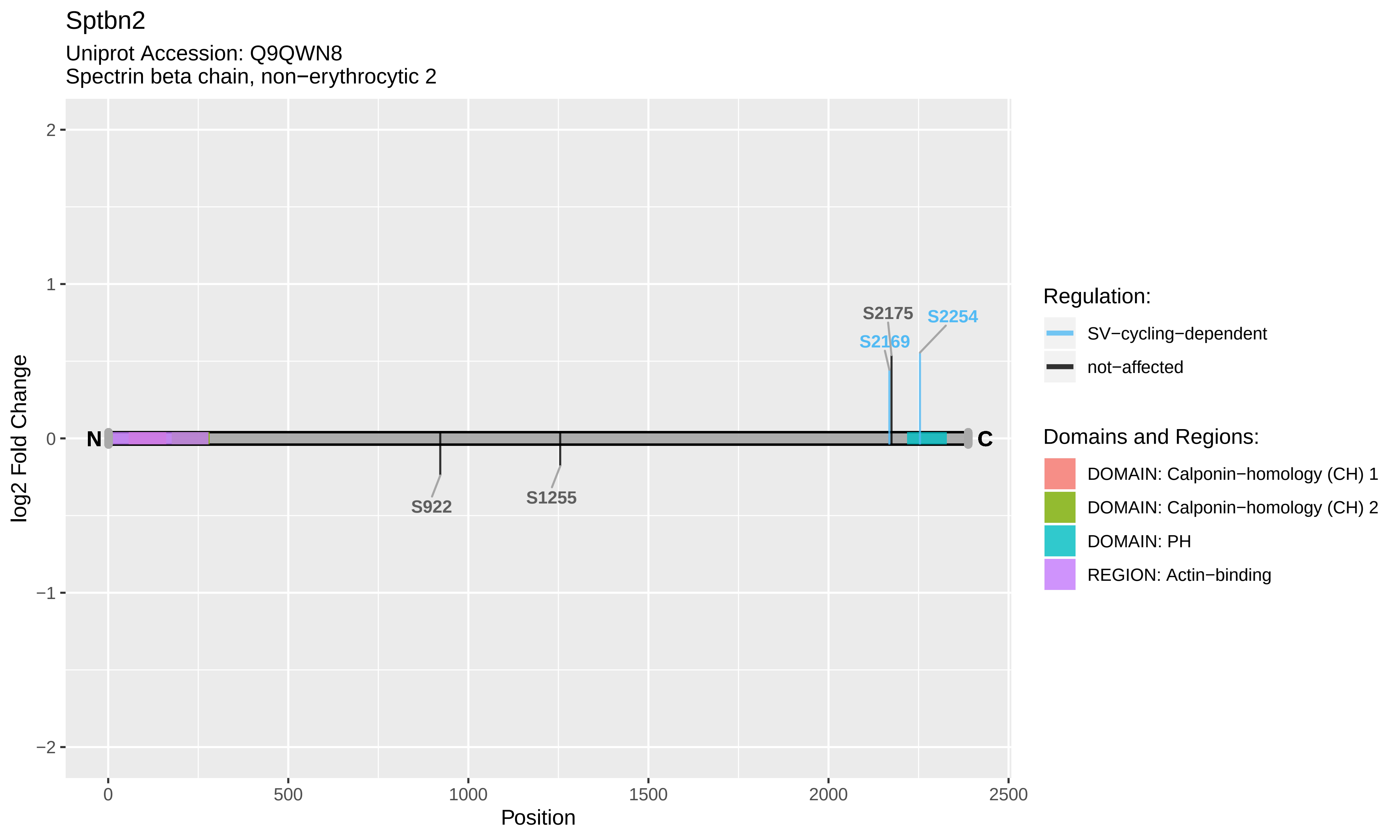

Sptbn2
Uniprot Accession: Q9QWN8
Spectrin beta chain, non−erythrocytic 2
log2 Fold Change
2
1
0
−1
−2
N
C
S2175
S2254
S2169
S922
S1255
0
500
1000
1500
2000
2500
Position
Regulation:
SV−cycling−dependent
not−affected
Domains and Regions:
DOMAIN: Calponin−homology (CH) 1
DOMAIN: Calponin−homology (CH) 2
DOMAIN: PH
REGION: Actin−binding

Sptbn4

Uniprot Accession: A0A0G2K677
Spectrin beta chain

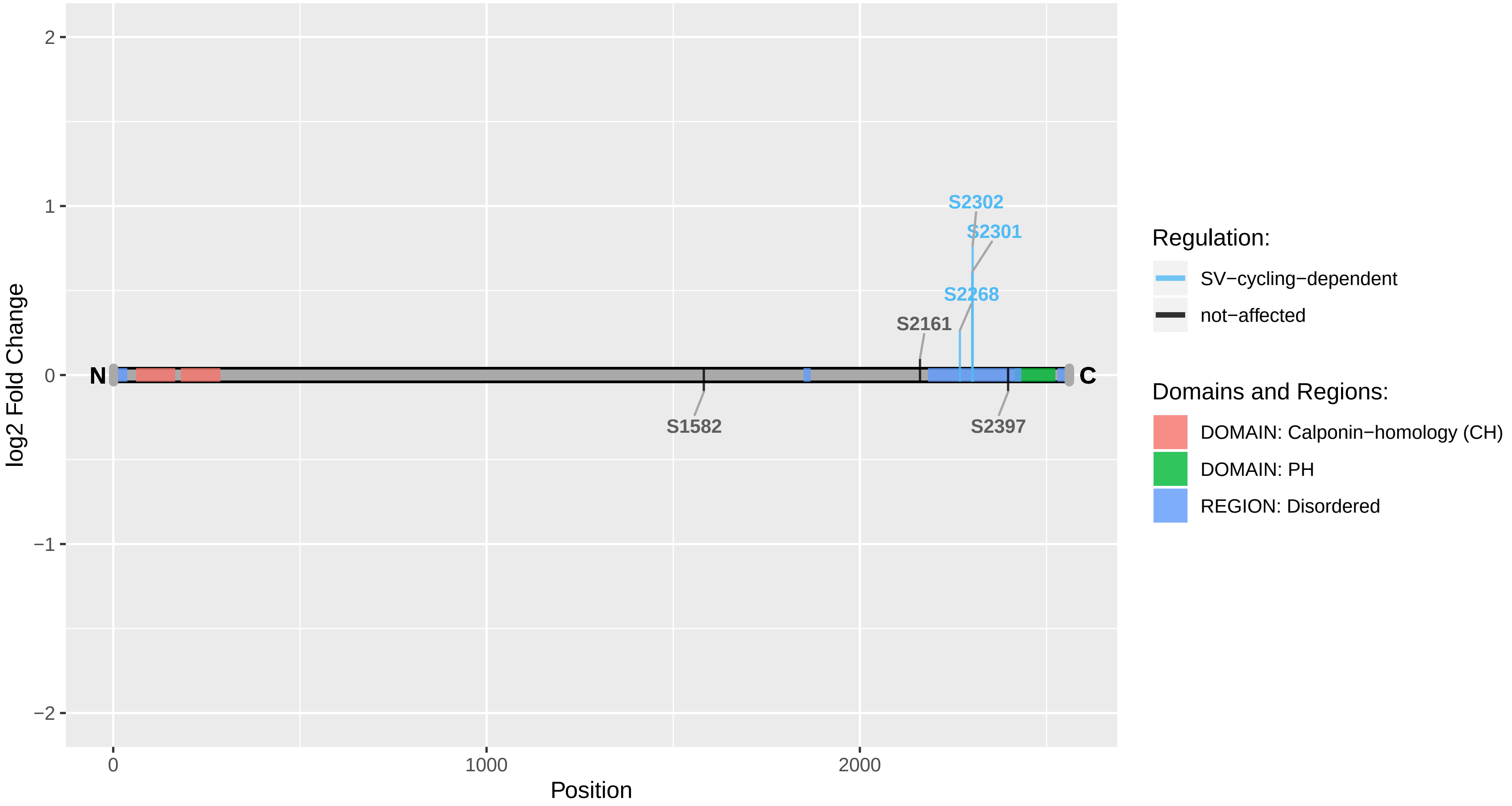

Srcin1

Uniprot Accession: Q9QXY2
SRC kinase signaling inhibitor 1

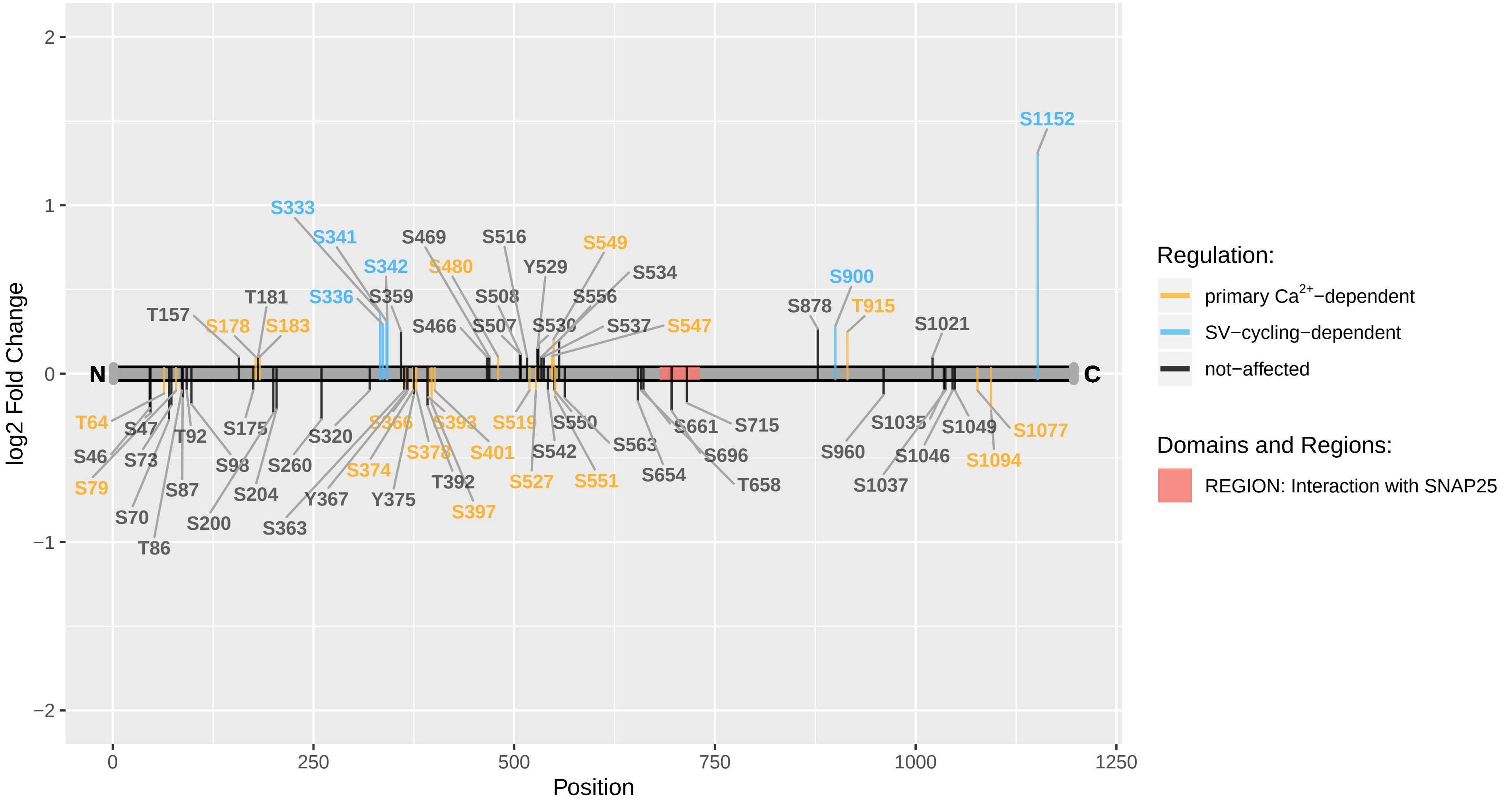

Srfbp1

Uniprot Accession: Q66H19
Serum response factor−binding protein 1

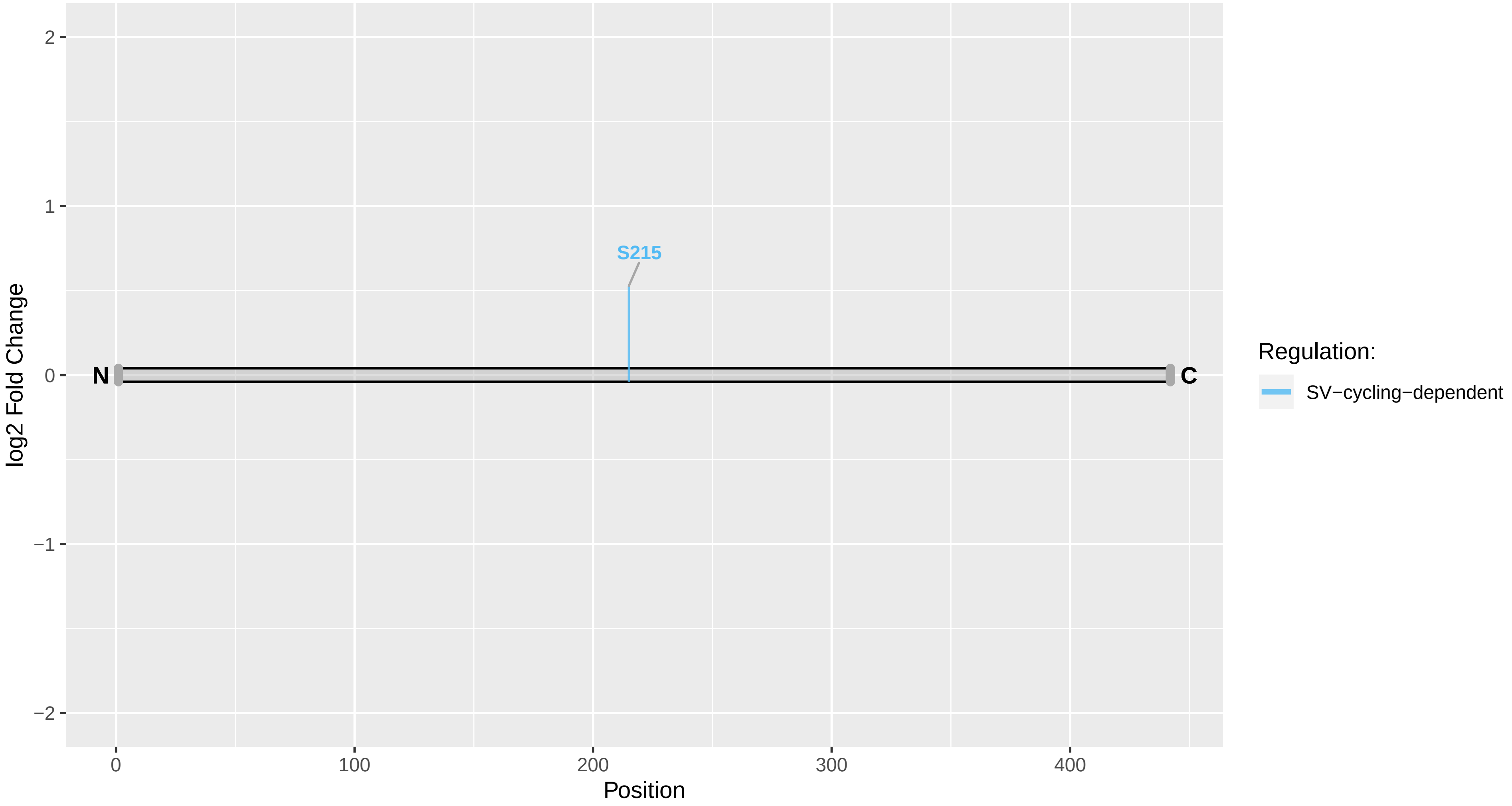

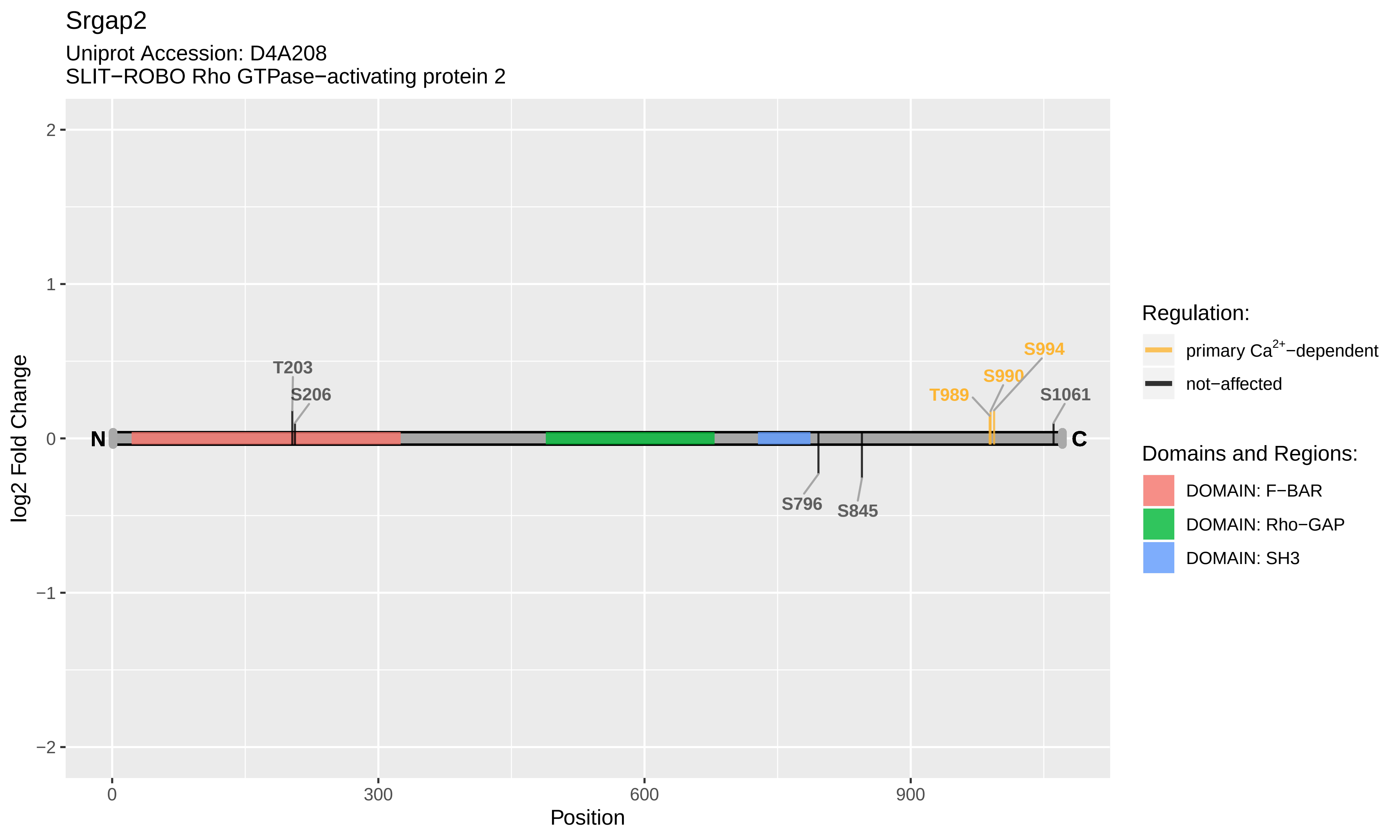

Srgap2
Uniprot Accession: D4A208
SLIT−ROBO Rho GTPase−activating protein 2
log2 Fold Change
Position
T203
S206
S796
S845
T989
S990
S994
S1061
N
C
Regulation:
primary Ca2+−dependent
not−affected
Domains and Regions:
DOMAIN: F−BAR
DOMAIN: Rho−GAP
DOMAIN: SH3

Srgap3

Uniprot Accession: F1M5M9
SLIT−ROBO Rho GTPase−activating protein 3

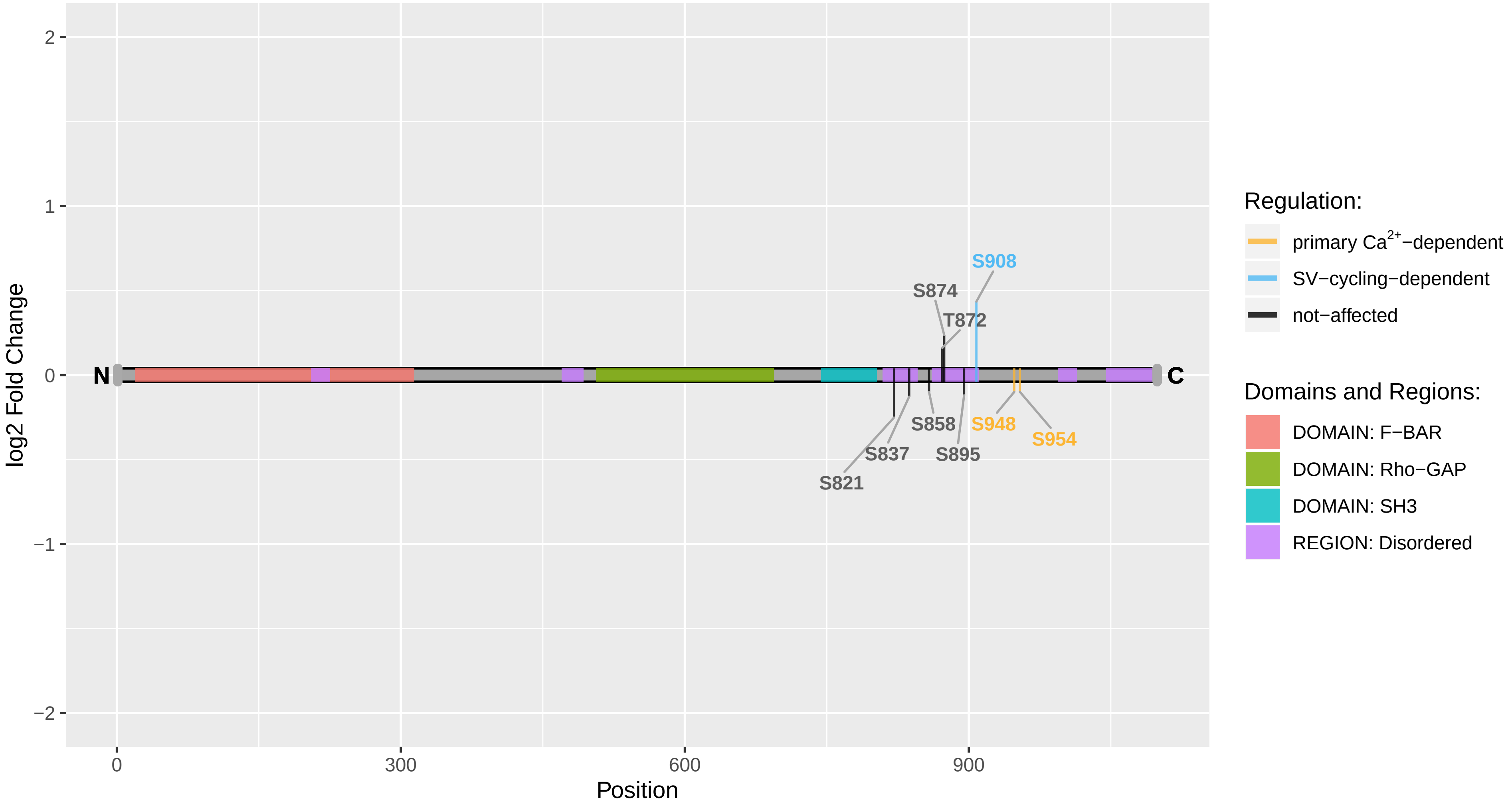

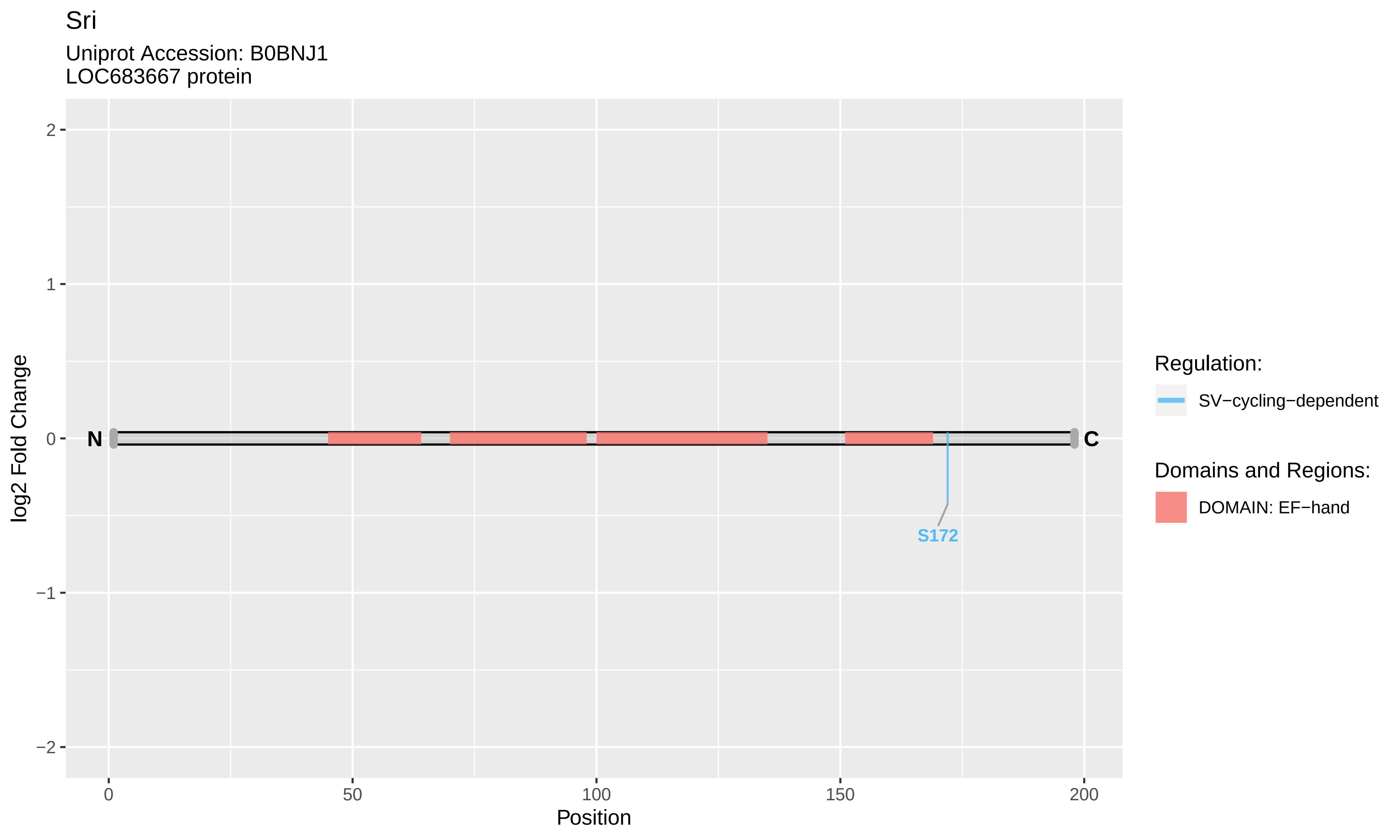
Sri
Uniprot Accession: B0BNJ1
LOC683667 protein
log2 Fold Change
2
1
0
−1
−2
N
C
S172
0
50
100
150
200
Position
Regulation:
SV−cycling−dependent
Domains and Regions:
DOMAIN: EF−hand

Srrm2

Uniprot Accession: A0A0G2K2M9
Serine/arginine repetitive matrix 2

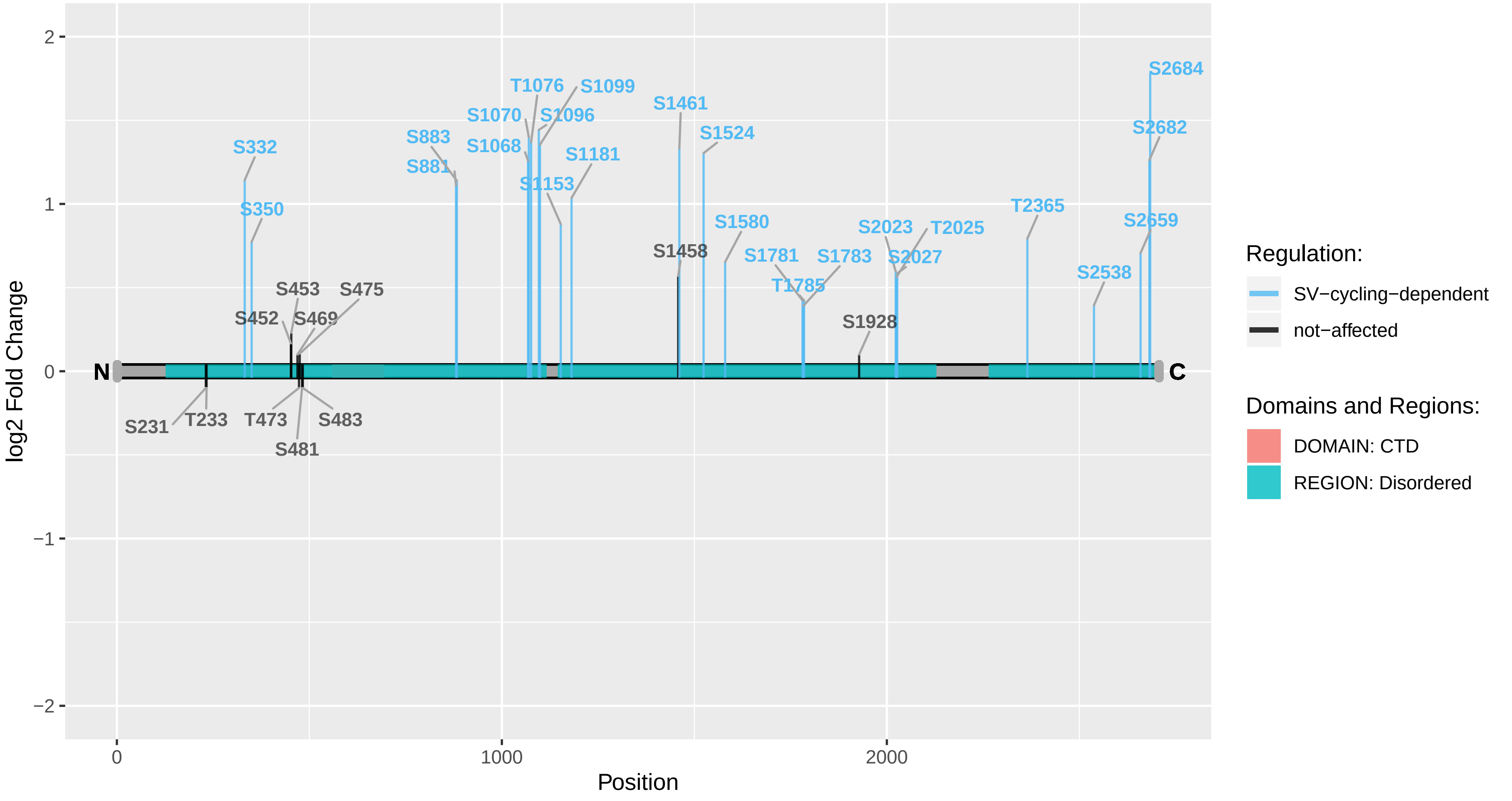

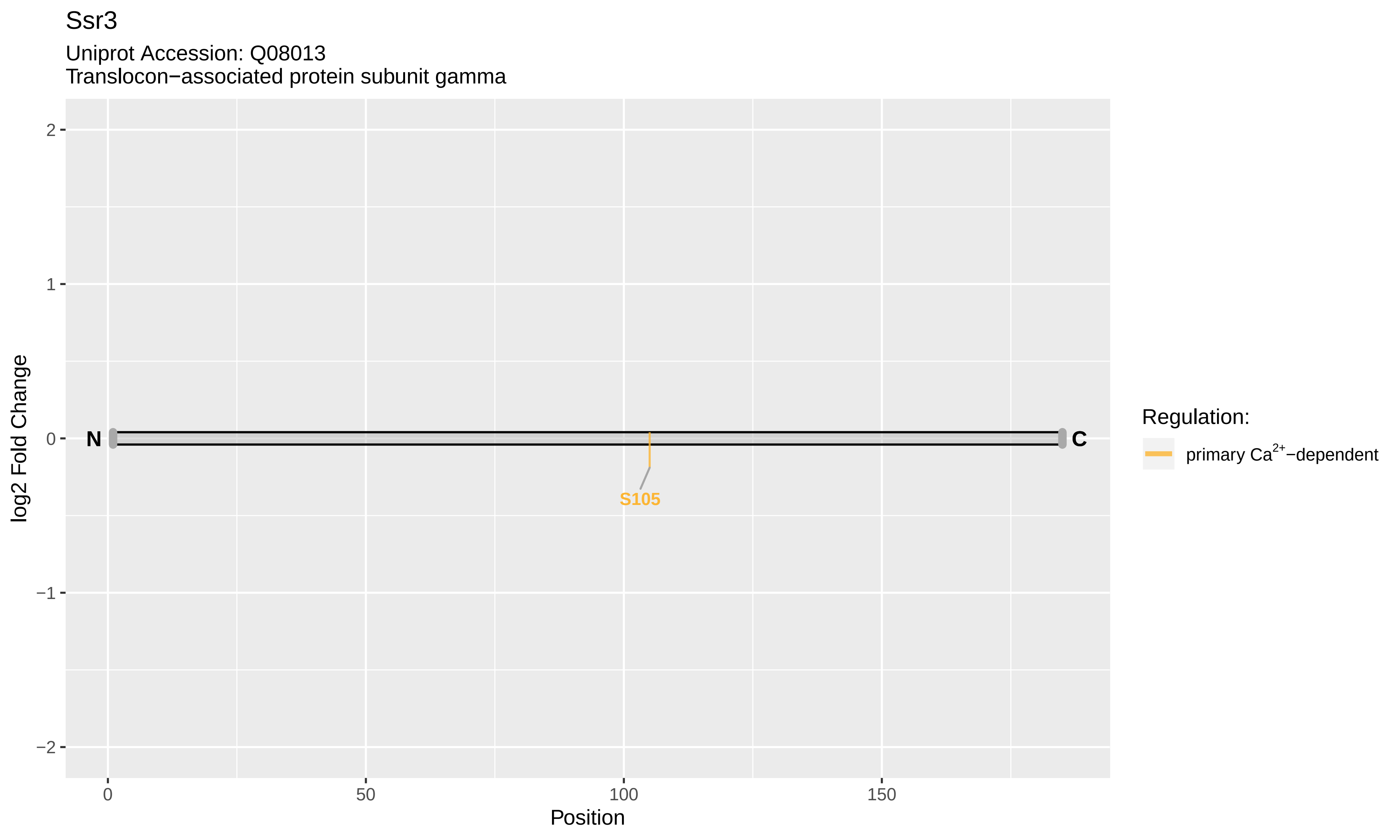

Ssr3
Uniprot Accession: Q08013
Translocon−associated protein subunit gamma
log2 Fold Change
2
1
0
−1
−2
N
C
S105
0
50
100
150
Position
Regulation:
primary Ca2+−dependent

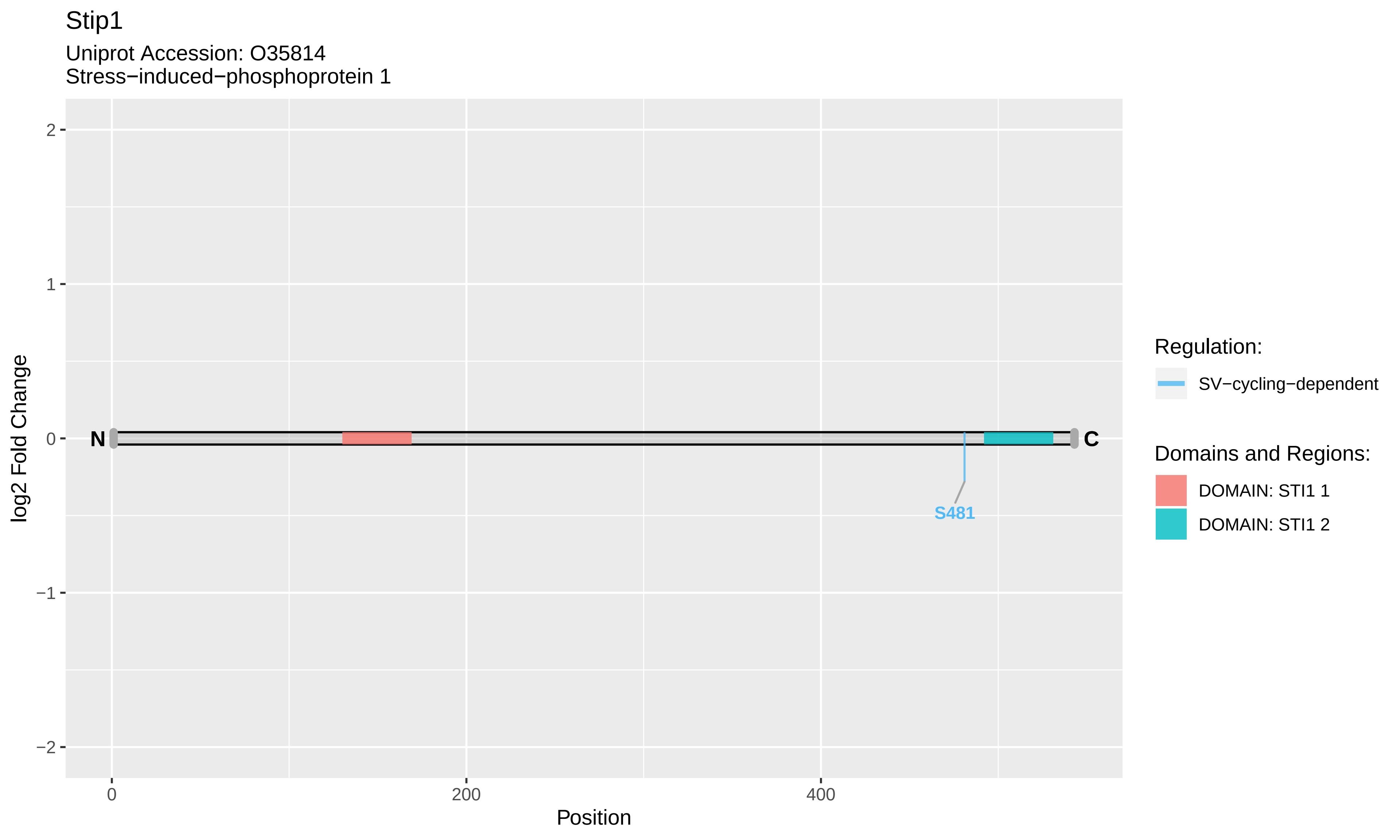

Stip1
Uniprot Accession: O35814
Stress−induced−phosphoprotein 1
log2 Fold Change
2
1
0
−1
−2
N
C
S481
0
200
400
Position
Regulation:
SV−cycling−dependent
Domains and Regions:
DOMAIN: STI1 1
DOMAIN: STI1 2

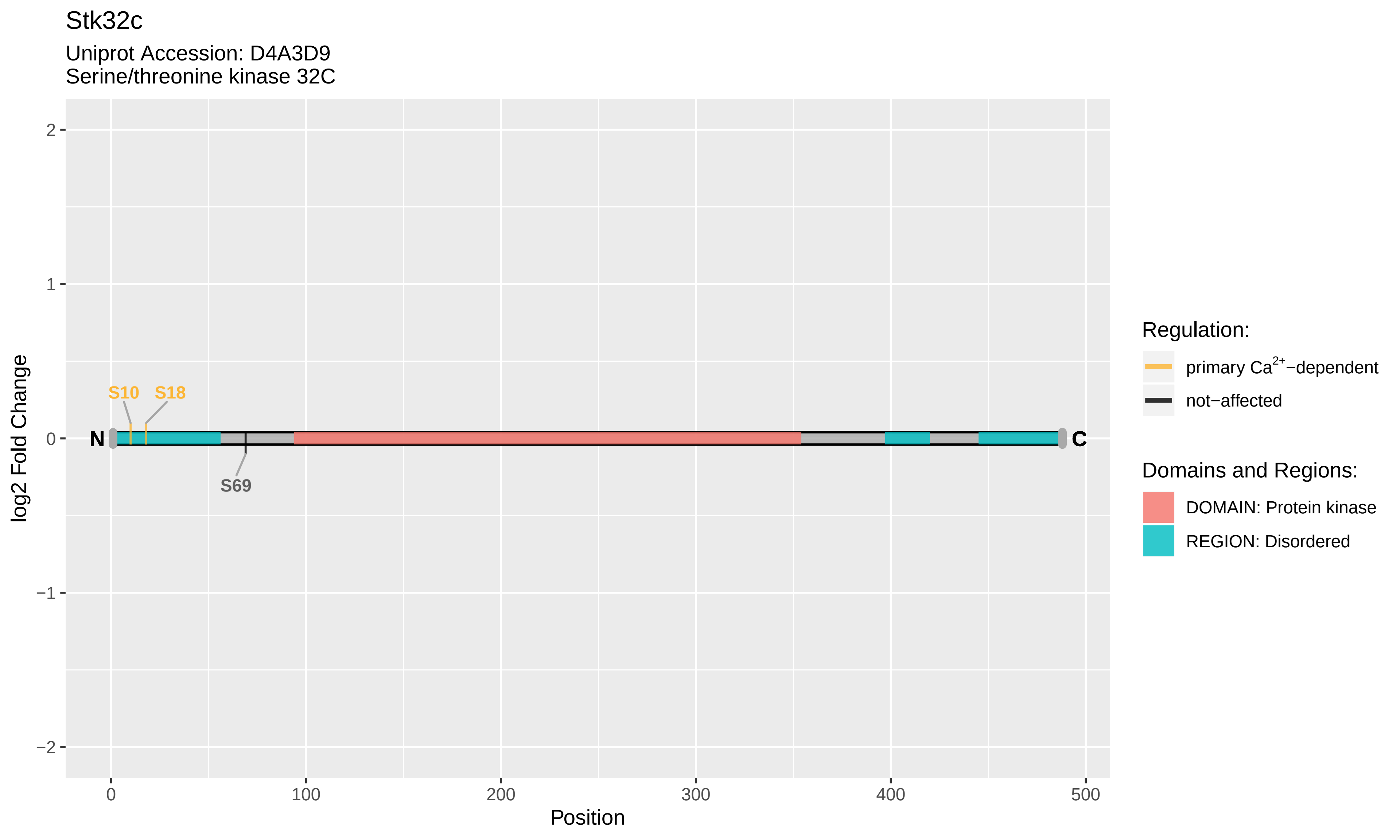
Stk32c
Uniprot Accession: D4A3D9
Serine/threonine kinase 32C
log2 Fold Change
Position
S10
S18
S69
N
C
2
1
0
−1
−2
0
100
200
300
400
500
Regulation:
primary Ca2+−dependent
not−affected
Domains and Regions:
DOMAIN: Protein kinase
REGION: Disordered

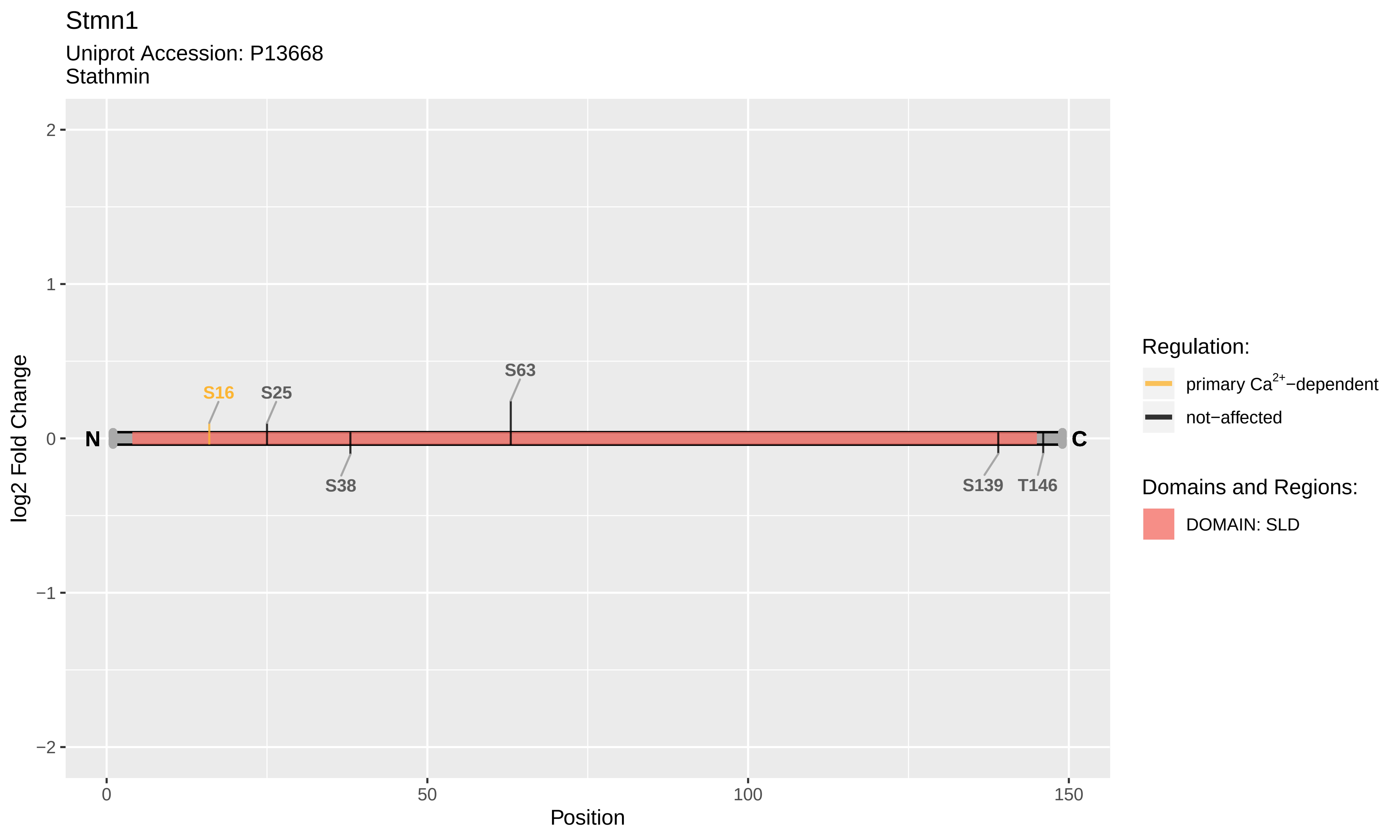

Stmn1
Uniprot Accession: P13668
Stathmin
log2 Fold Change
2
1
0
−1
−2
N
C
S16
S25
S63
S38
S139
T146
0
50
100
150
Position
Regulation:
primary Ca2+−dependent
not−affected
Domains and Regions:
DOMAIN: SLD

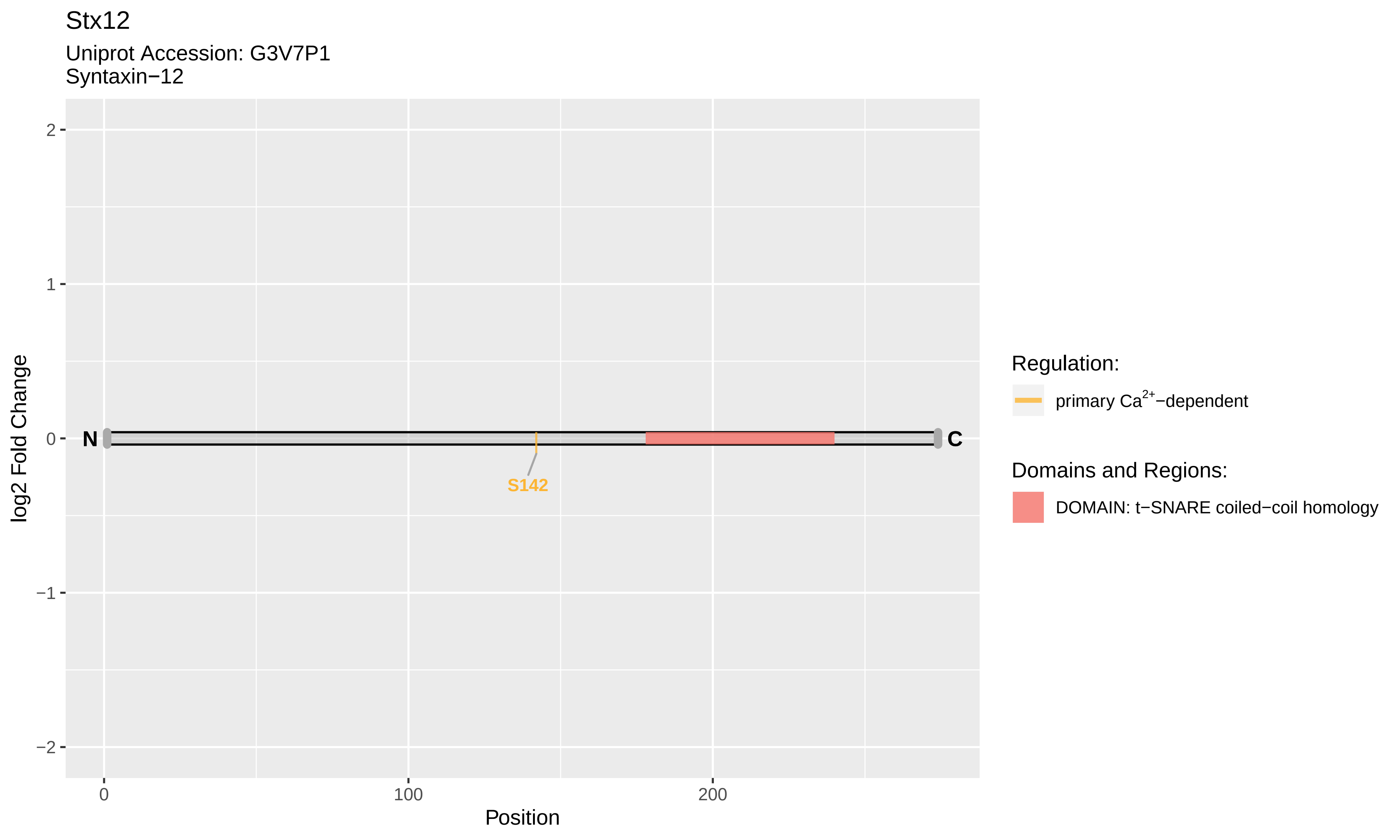

Stx12
Uniprot Accession: G3V7P1
Syntaxin−12
log2 Fold Change
2
1
0
−1
−2
N
C
S142
0
100
200
Position
Regulation:
primary Ca2+−dependent
Domains and Regions:
DOMAIN: t−SNARE coiled−coil homology

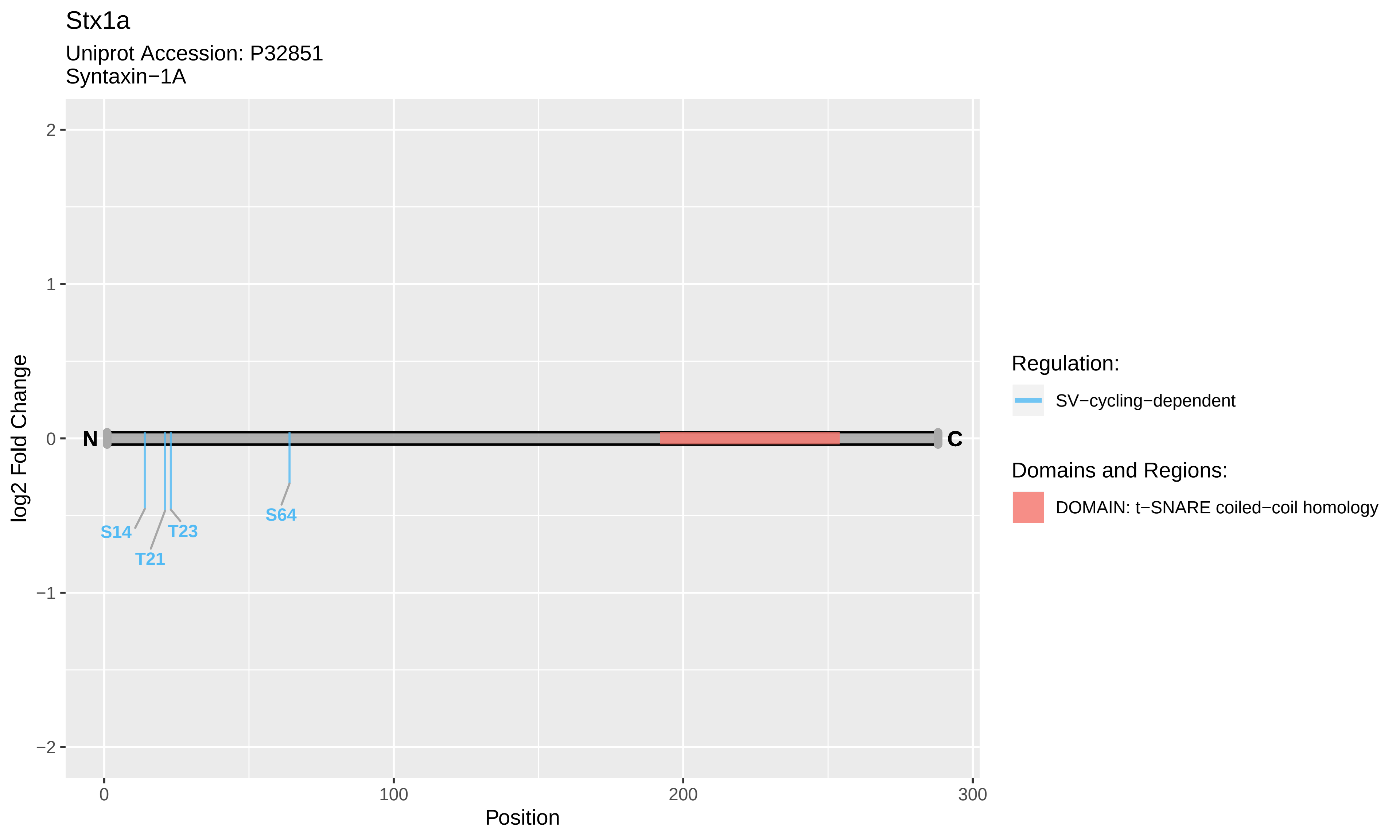
Stx1a
Uniprot Accession: P32851
Syntaxin−1A
log2 Fold Change
2
1
0
−1
−2
N
C
S14
T21
T23
S64
0
100
200
300
Position
Regulation:
SV−cycling−dependent
Domains and Regions:
DOMAIN: t−SNARE coiled−coil homology

Stxbp1

Uniprot Accession: P61765
Syntaxin−binding protein 1

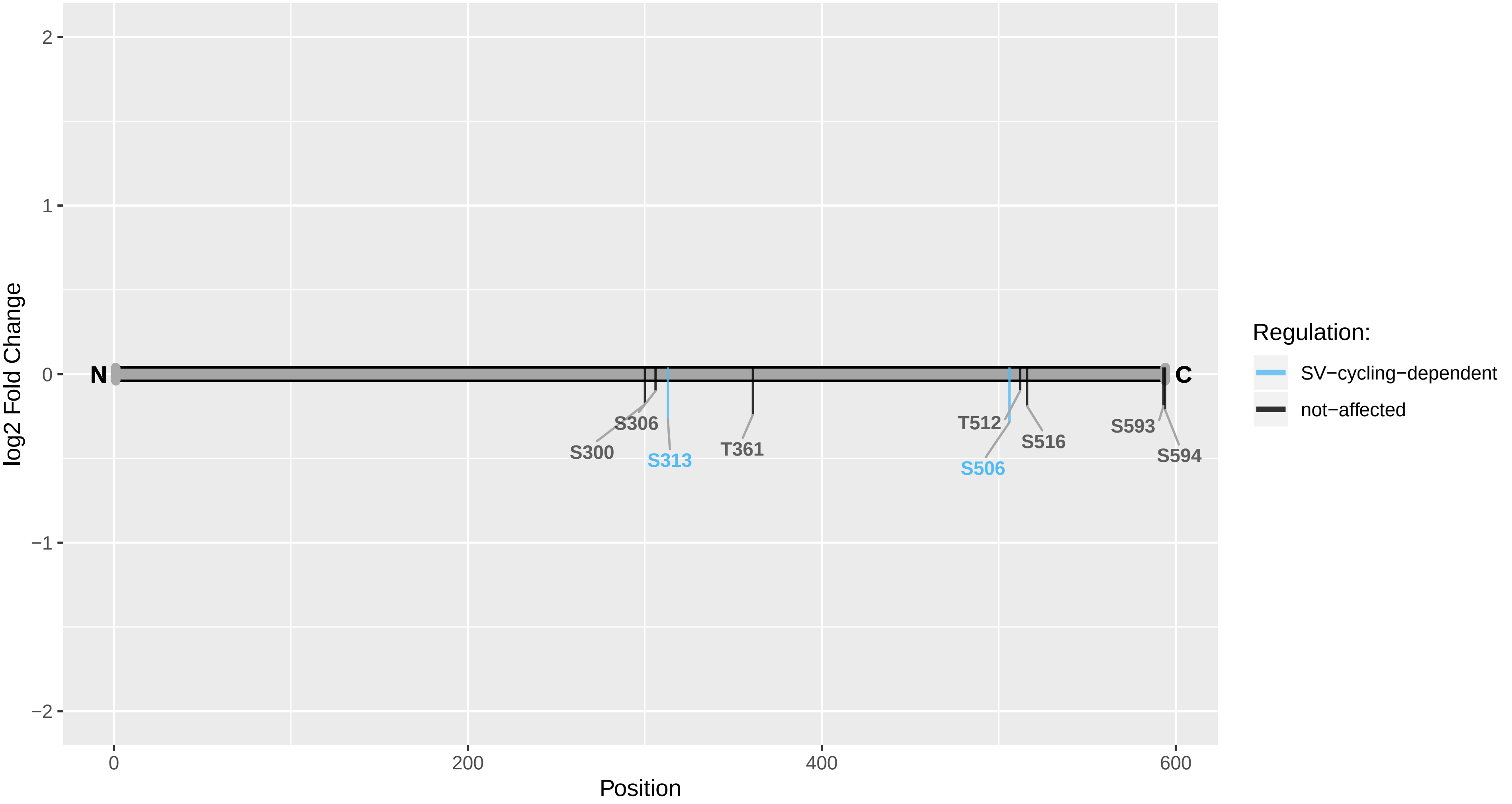

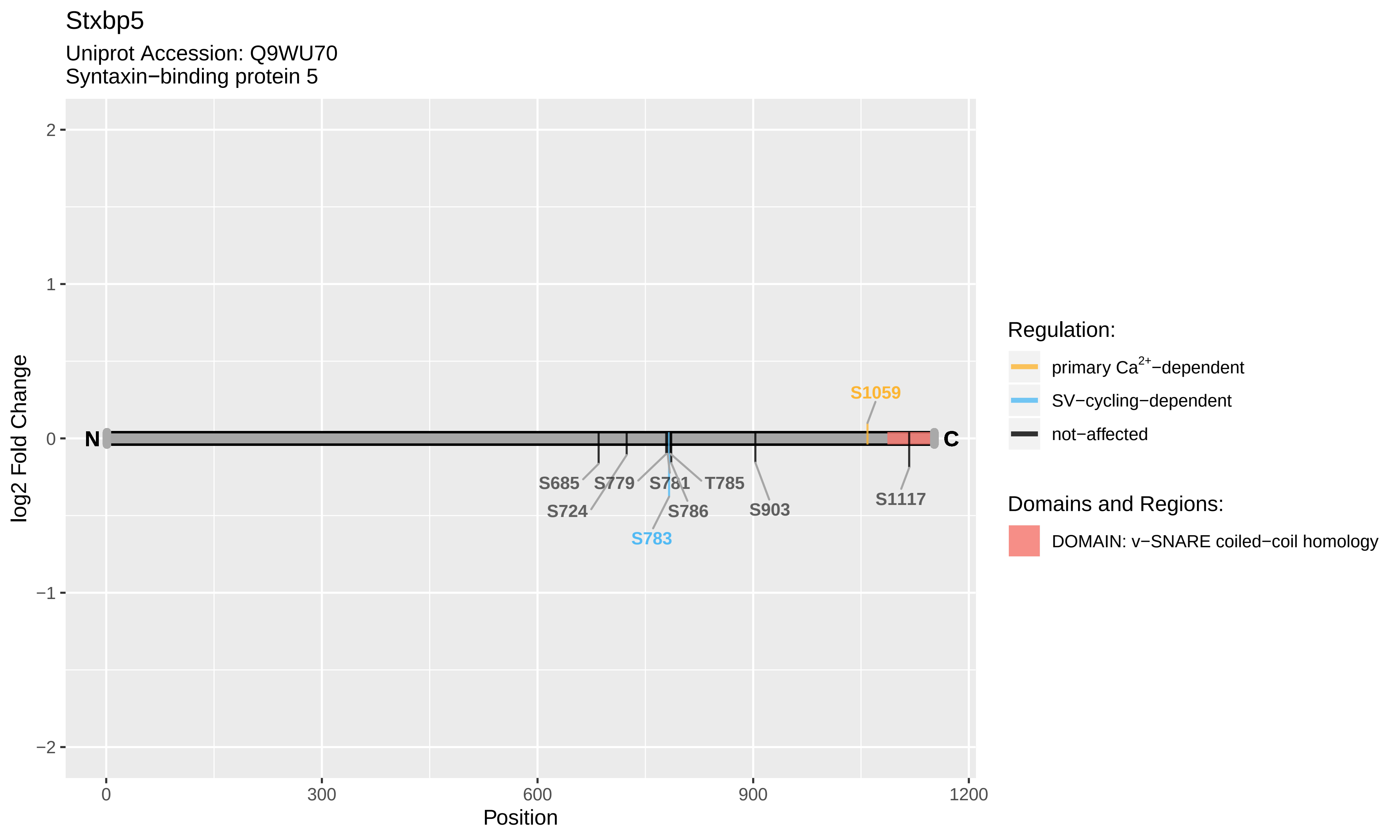

Stxbp5
Uniprot Accession: Q9WU70
Syntaxin−binding protein 5
log2 Fold Change
2
1
0
−1
−2
N
C
S1059
S685
S779
S781
T785
S724
S786
S783
S903
S1117
0
300
600
900
1200
Position
Regulation:
primary Ca2+−dependent
SV−cycling−dependent
not−affected
Domains and Regions:
DOMAIN: v−SNARE coiled−coil homology

Stxbp5l

Uniprot Accession: D3ZU84
Syntaxin−binding protein 5−like

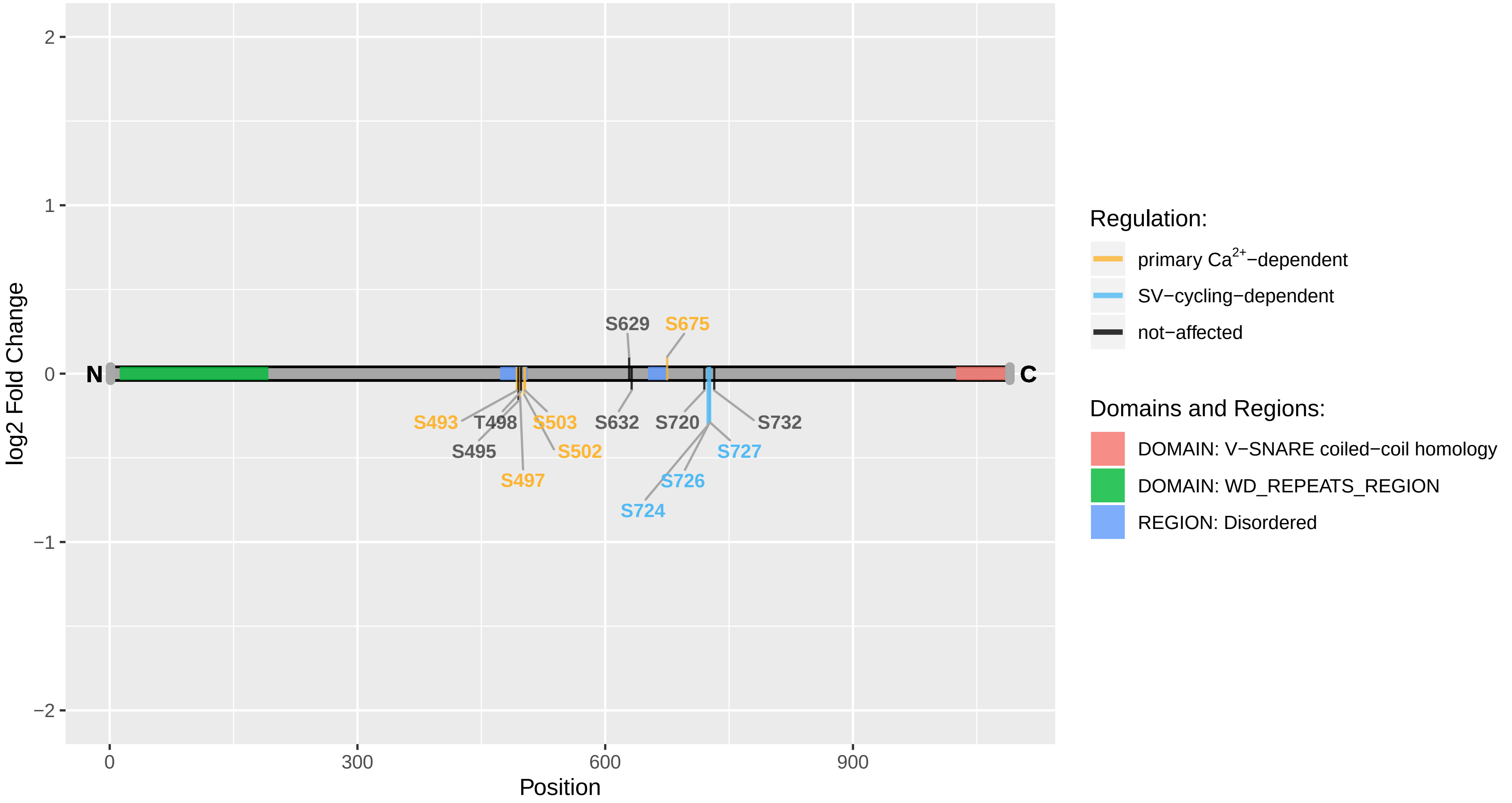

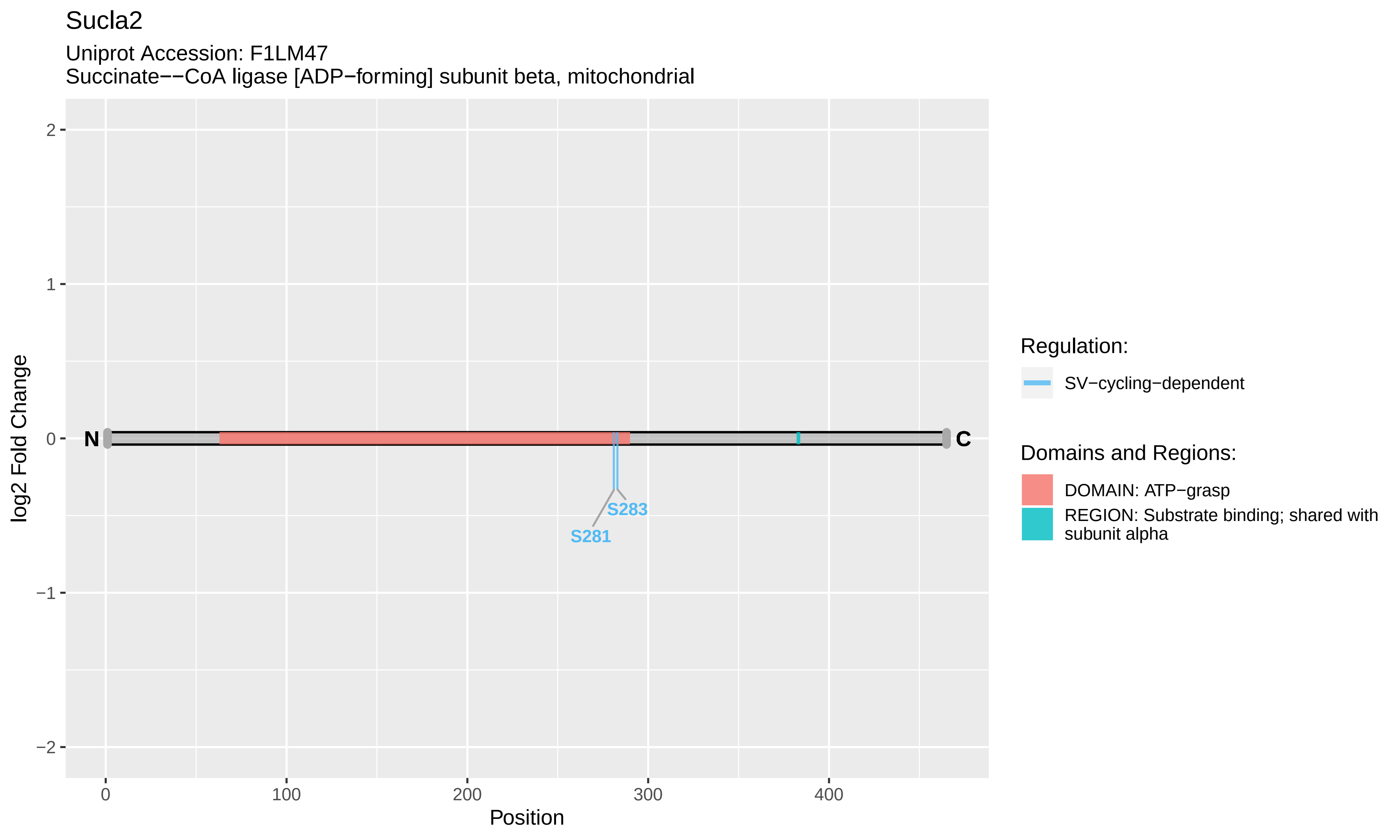
Sucla2
Uniprot Accession: F1LM47
Succinate−−CoA ligase [ADP−forming] subunit beta, mitochondrial
log2 Fold Change
2
1
0
−1
−2
N
C
S283
S281
0
100
200
300
400
Position
Regulation:
SV−cycling−dependent
Domains and Regions:
DOMAIN: ATP−grasp
REGION: Substrate binding; shared with subunit alpha

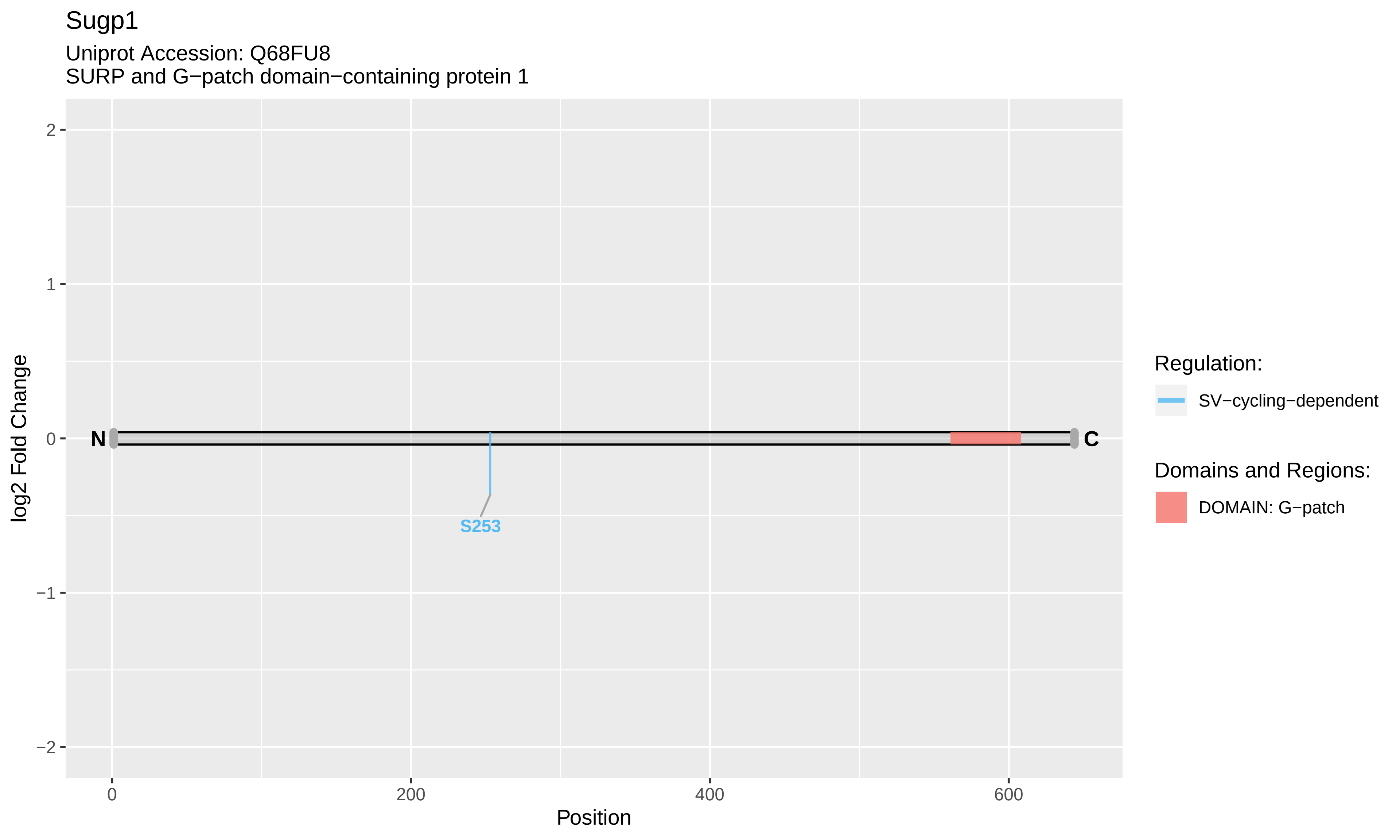

Sugp1
Uniprot Accession: Q68FU8
SURP and G−patch domain−containing protein 1
log2 Fold Change
2
1
0
−1
−2
N
C
S253
0
200
400
600
Position
Regulation:
SV−cycling−dependent
Domains and Regions:
DOMAIN: G−patch

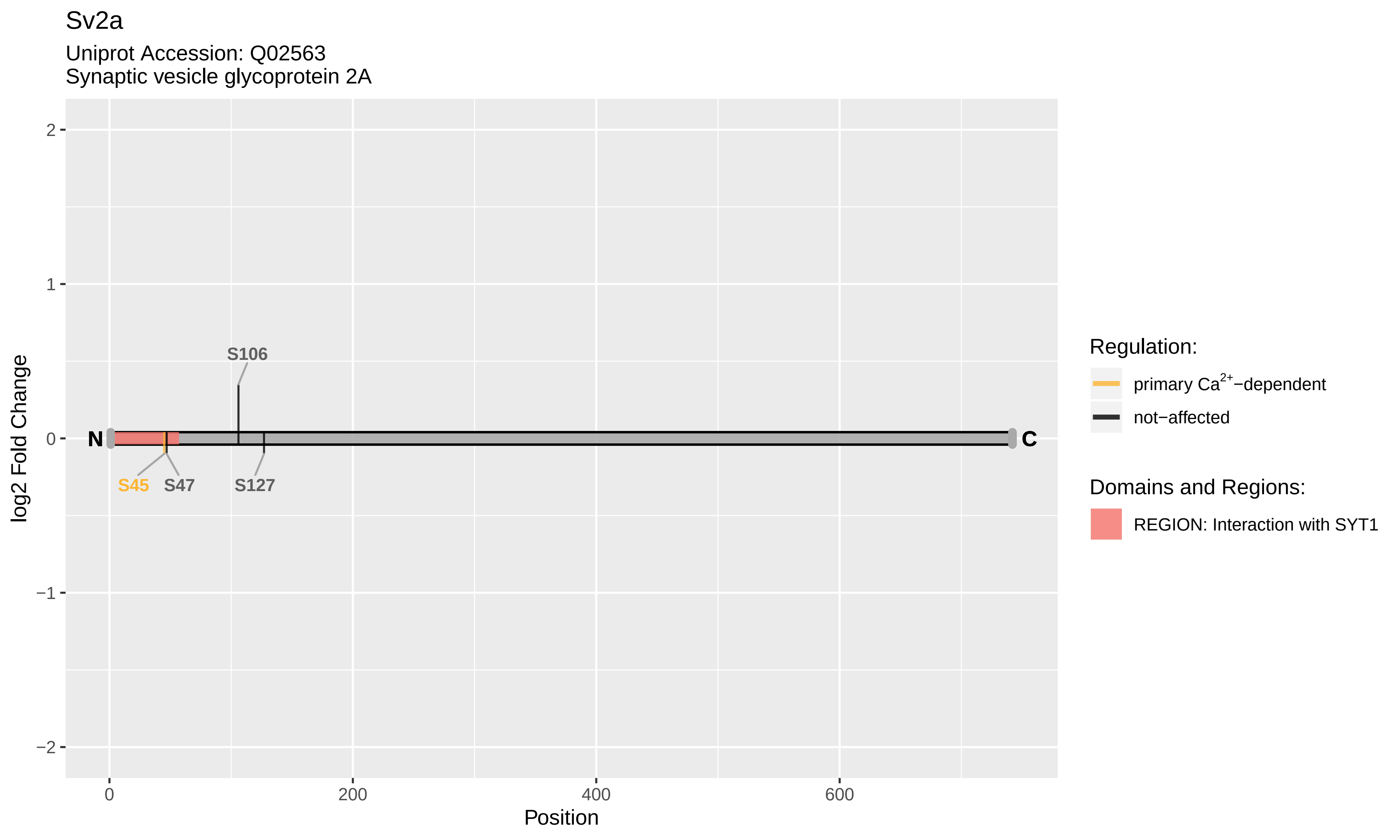

Sv2a
Uniprot Accession: Q02563
Synaptic vesicle glycoprotein 2A
S106
N
C
S45
S47
S127
log2 Fold Change
2
1
0
−1
−2
0
200
400
600
Position
Regulation:
primary Ca2+−dependent
not−affected
Domains and Regions:
REGION: Interaction with SYT1

Sv2b

Uniprot Accession: Q63564
Synaptic vesicle glycoprotein 2B

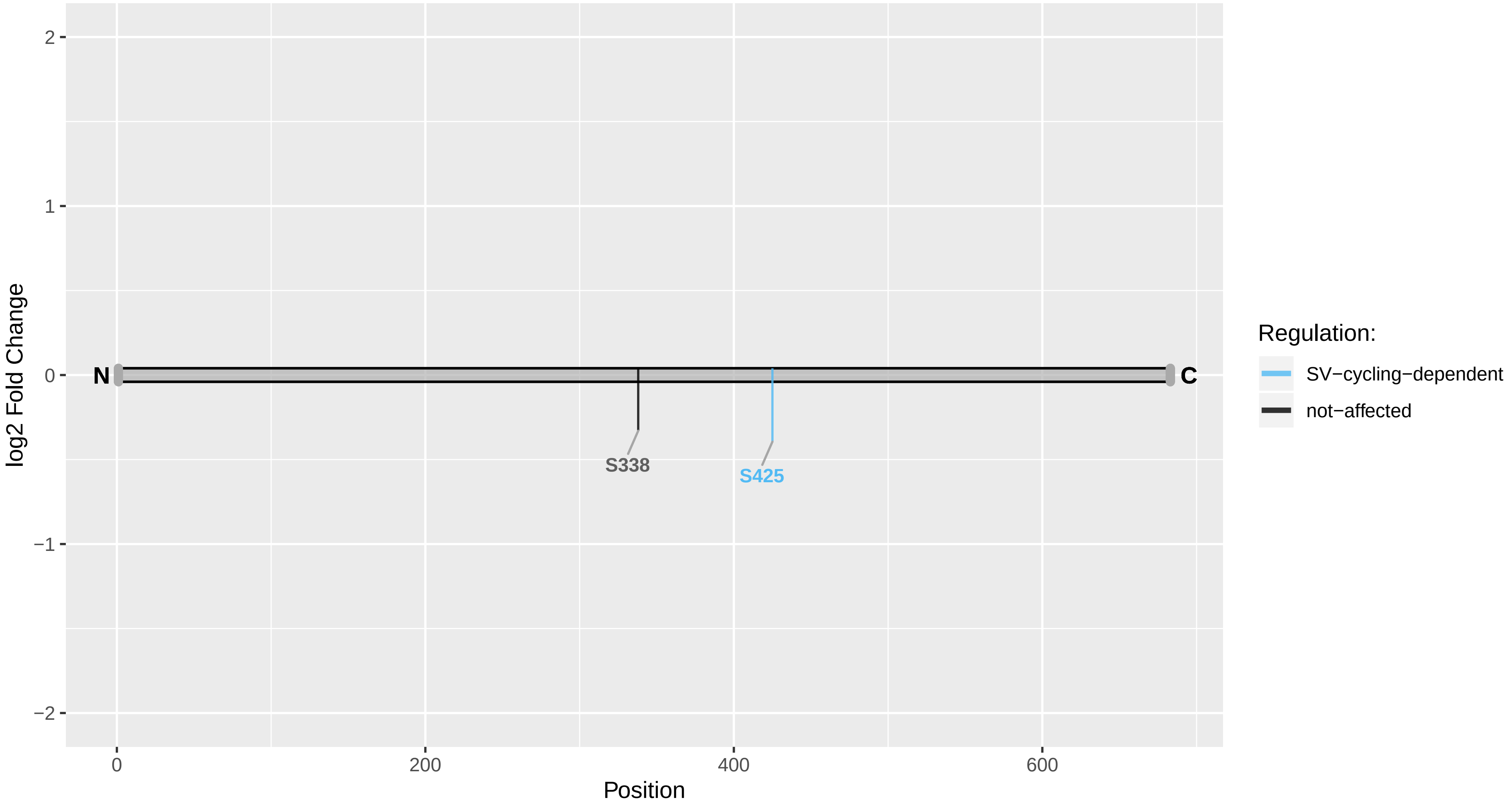

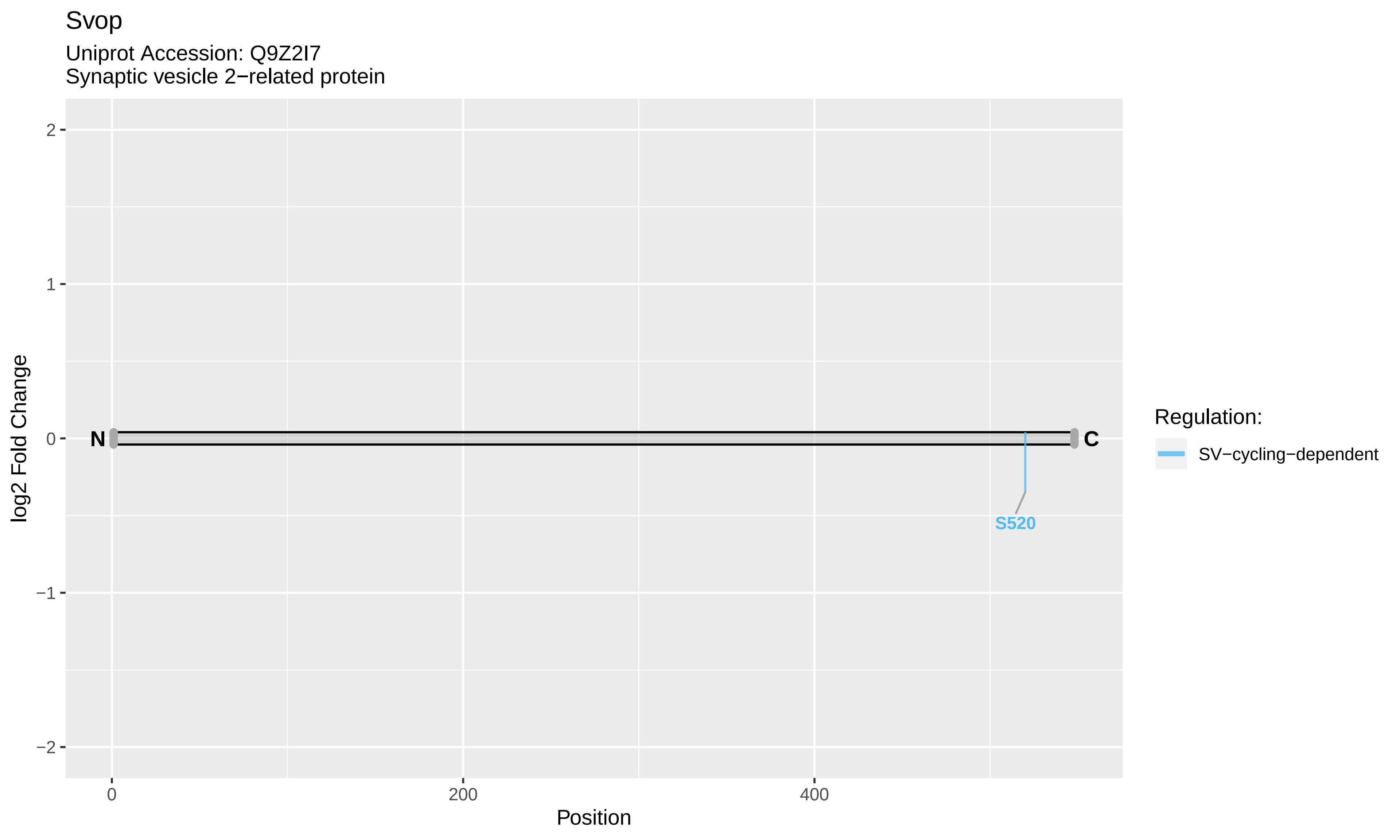

Svop
Uniprot Accession: Q9Z2I7
Synaptic vesicle 2−related protein
2
1
0
−1
−2
log2 Fold Change
N
C
S520
0
200
400
Position
Regulation:
SV−cycling−dependent

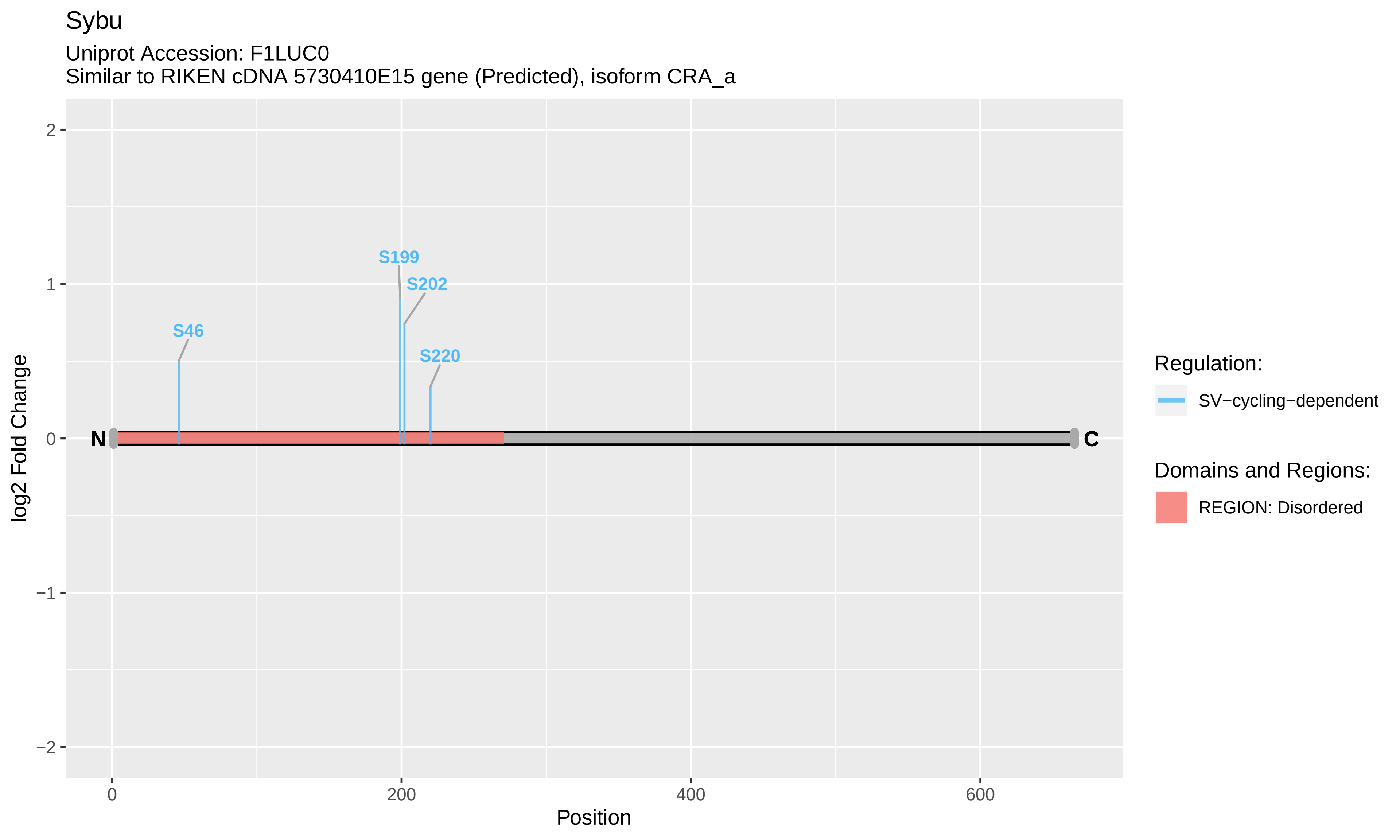

Sybu
Uniprot Accession: F1LUC0
Similar to RIKEN cDNA 5730410E15 gene (Predicted), isoform CRA_a
S199
S202
S46
S220
N
C
log2 Fold Change
Position
2
1
0
−1
−2
0
200
400
600
Regulation:
SV−cycling−dependent
Domains and Regions:
REGION: Disordered

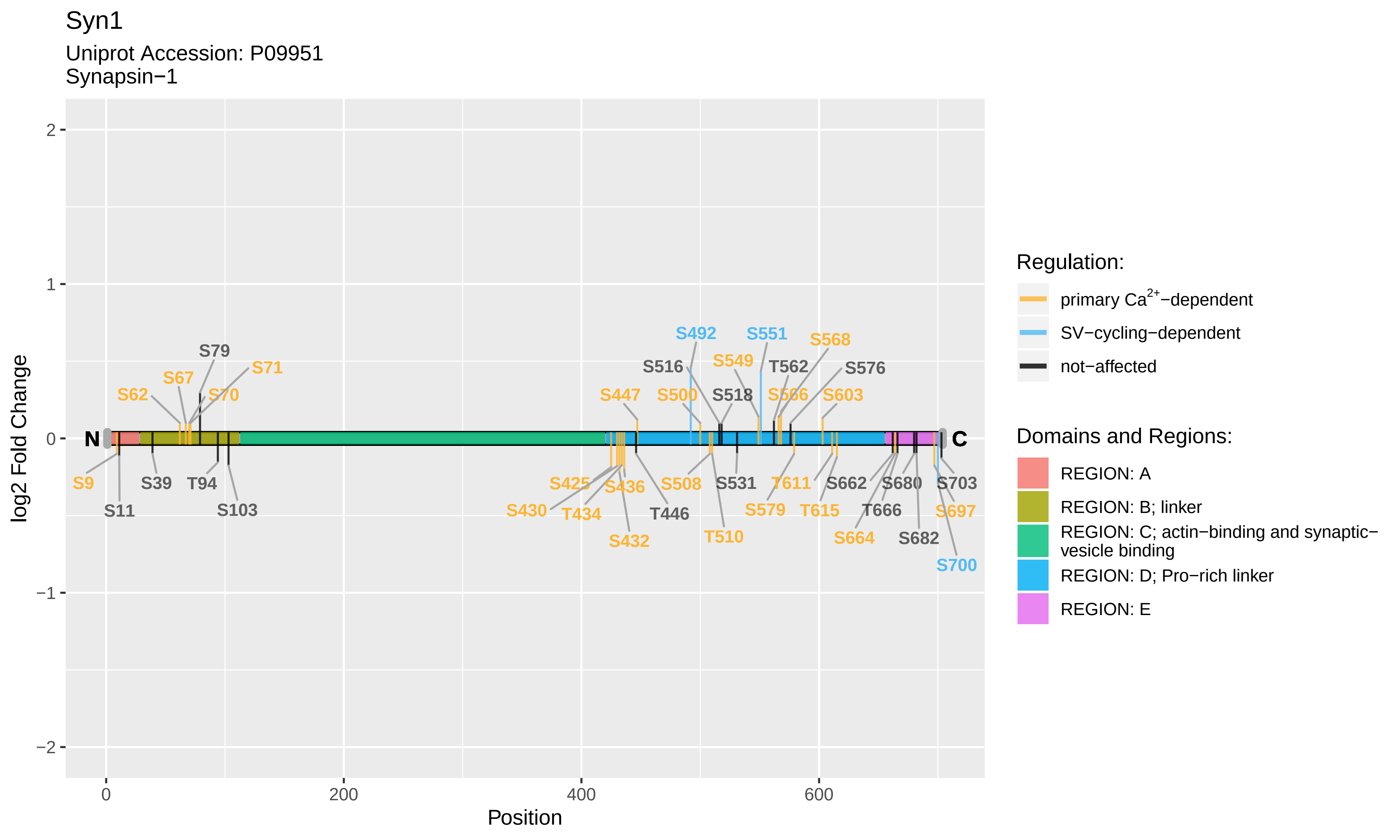

Syn1
Uniprot Accession: P09951
Synapsin−1
log2 Fold Change
Position
N
C
S79
S71
S67
S62
S70
S9
S11
S39
T94
S103
S492
S551
S568
S516
S549
T562
S576
S447
S500
S518
S566
S603
S425
S436
S430
T434
S432
T446
S508
S531
T510
T611
S662
S680
S703
S579
T615
T666
S697
S664
S682
S700
Regulation:
primary Ca2+−dependent
SV−cycling−dependent
not−affected
Domains and Regions:
REGION: A
REGION: B; linker
REGION: C; actin−binding and synaptic−vesicle binding
REGION: D; Pro−rich linker
REGION: E

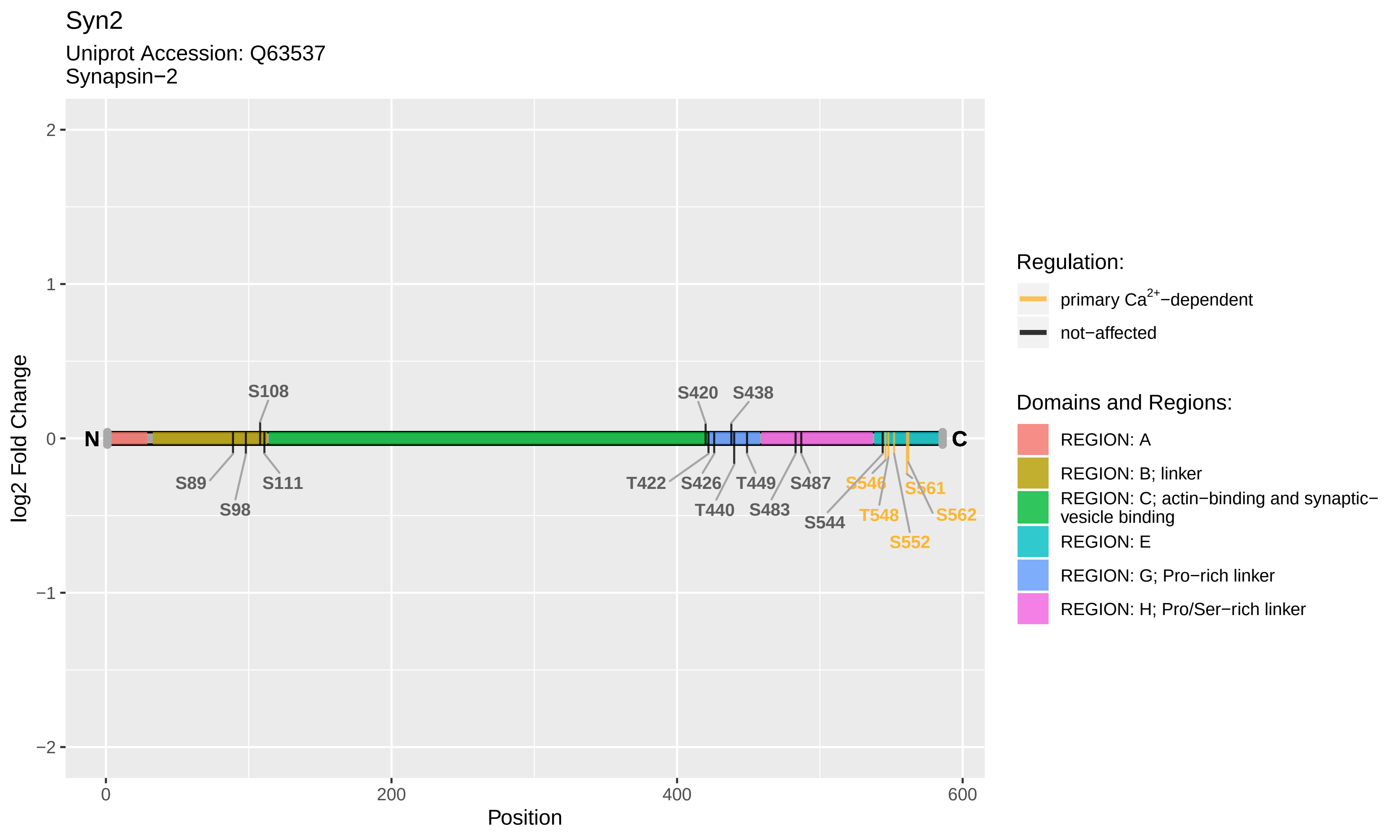

Syn2
Uniprot Accession: Q63537
Synapsin−2
log2 Fold Change
Position
N
C
S108
S420
S438
S89
S98
S111
T422
S426
T440
T449
S483
S487
S544
S546
T548
S552
S561
S562
Regulation:
primary Ca2+−dependent
not−affected
Domains and Regions:
REGION: A
REGION: B; linker
REGION: C; actin−binding and synaptic−vesicle binding
REGION: E
REGION: G; Pro−rich linker
REGION: H; Pro/Ser−rich linker

Syn3

Uniprot Accession: O70441
Synapsin−3

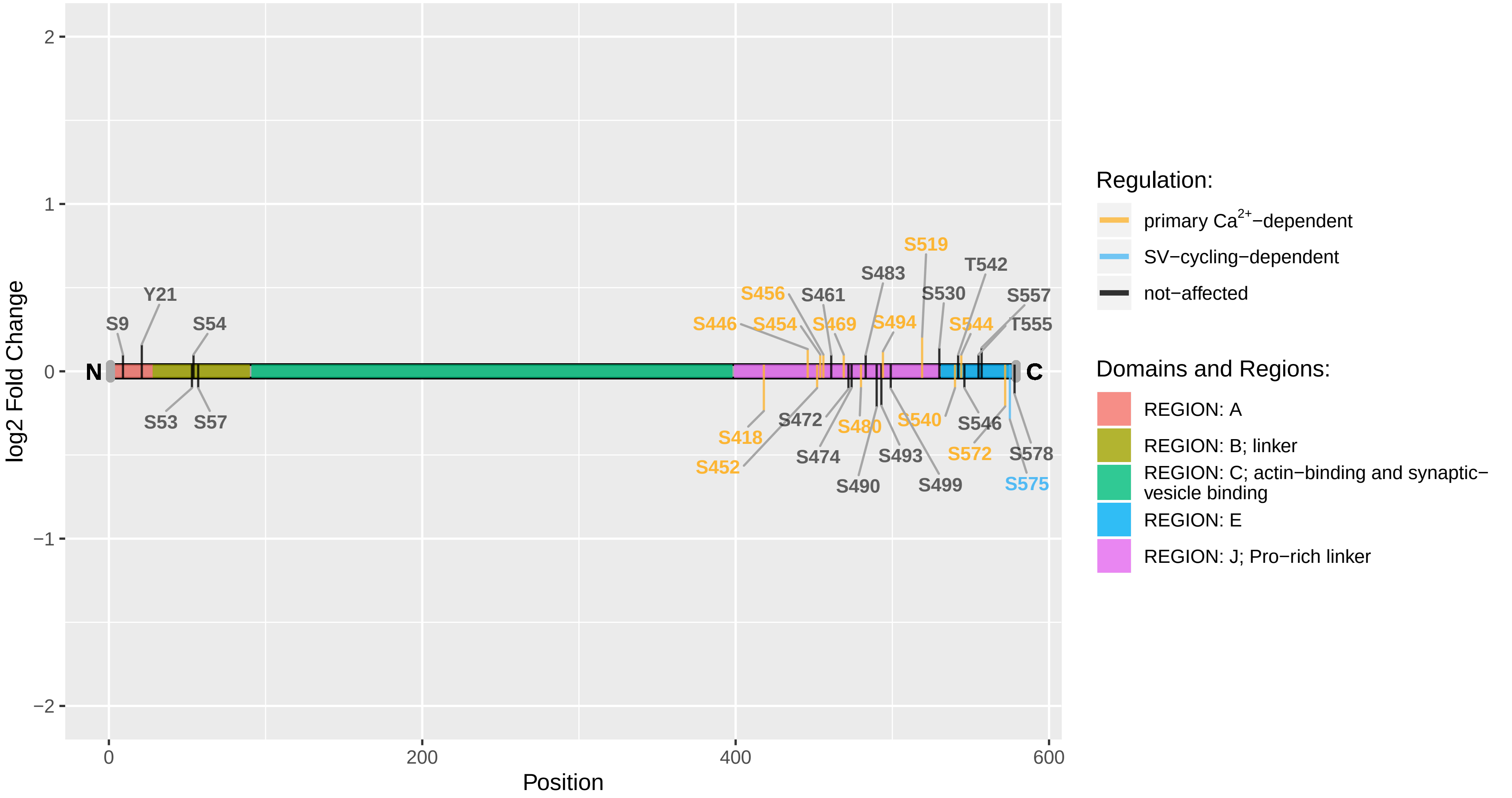

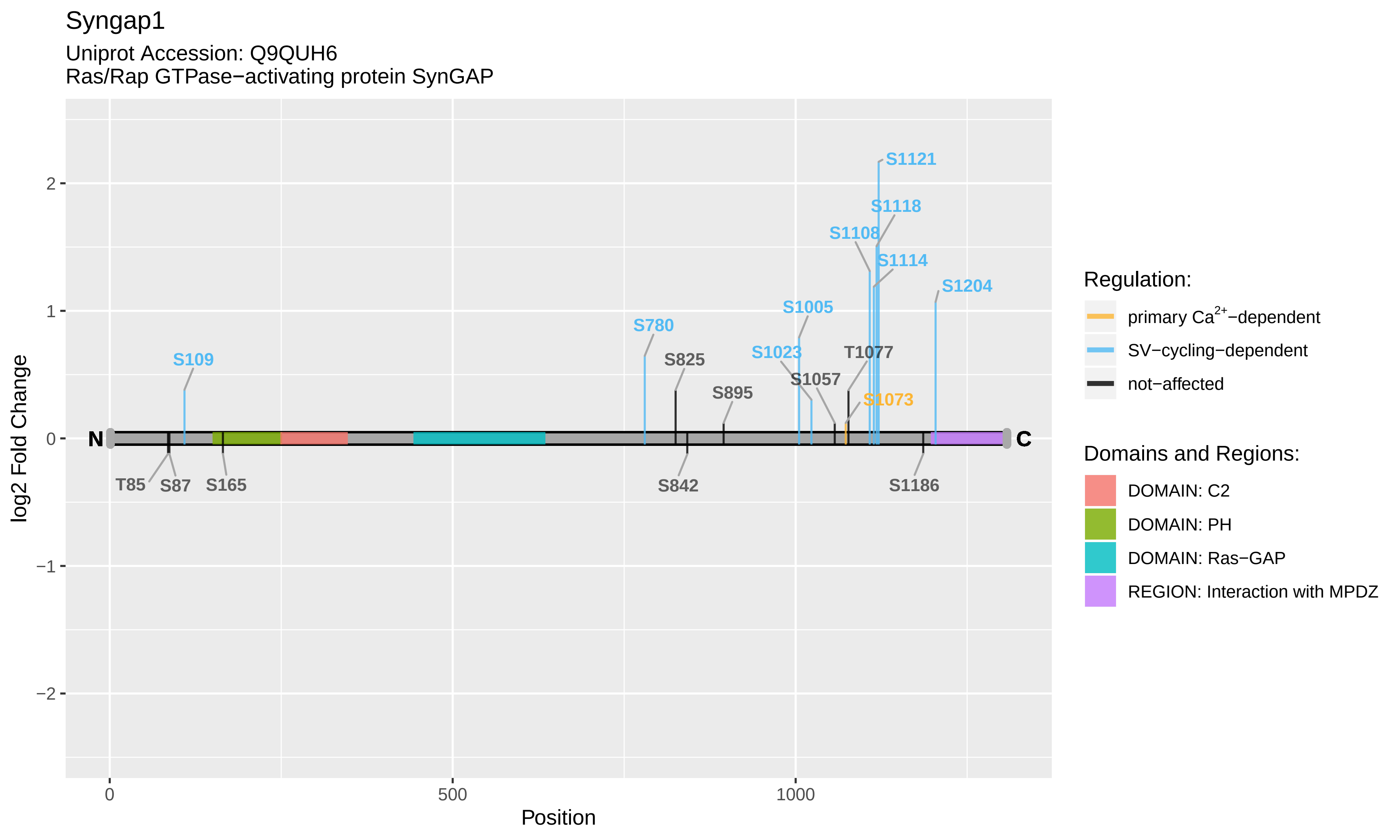

Syngap1
Uniprot Accession: Q9QUH6
Ras/Rap GTPase−activating protein SynGAP
log2 Fold Change
Position
N
C
S109
S780
S825
S895
S1005
S1023
S1057
T1077
S1073
S1108
S1118
S1121
S1114
S1204
T85
S87
S165
S842
S1186
2
1
0
−1
−2
0
500
1000
Regulation:
primary Ca2+−dependent
SV−cycling−dependent
not−affected
Domains and Regions:
DOMAIN: C2
DOMAIN: PH
DOMAIN: Ras−GAP
REGION: Interaction with MPDZ

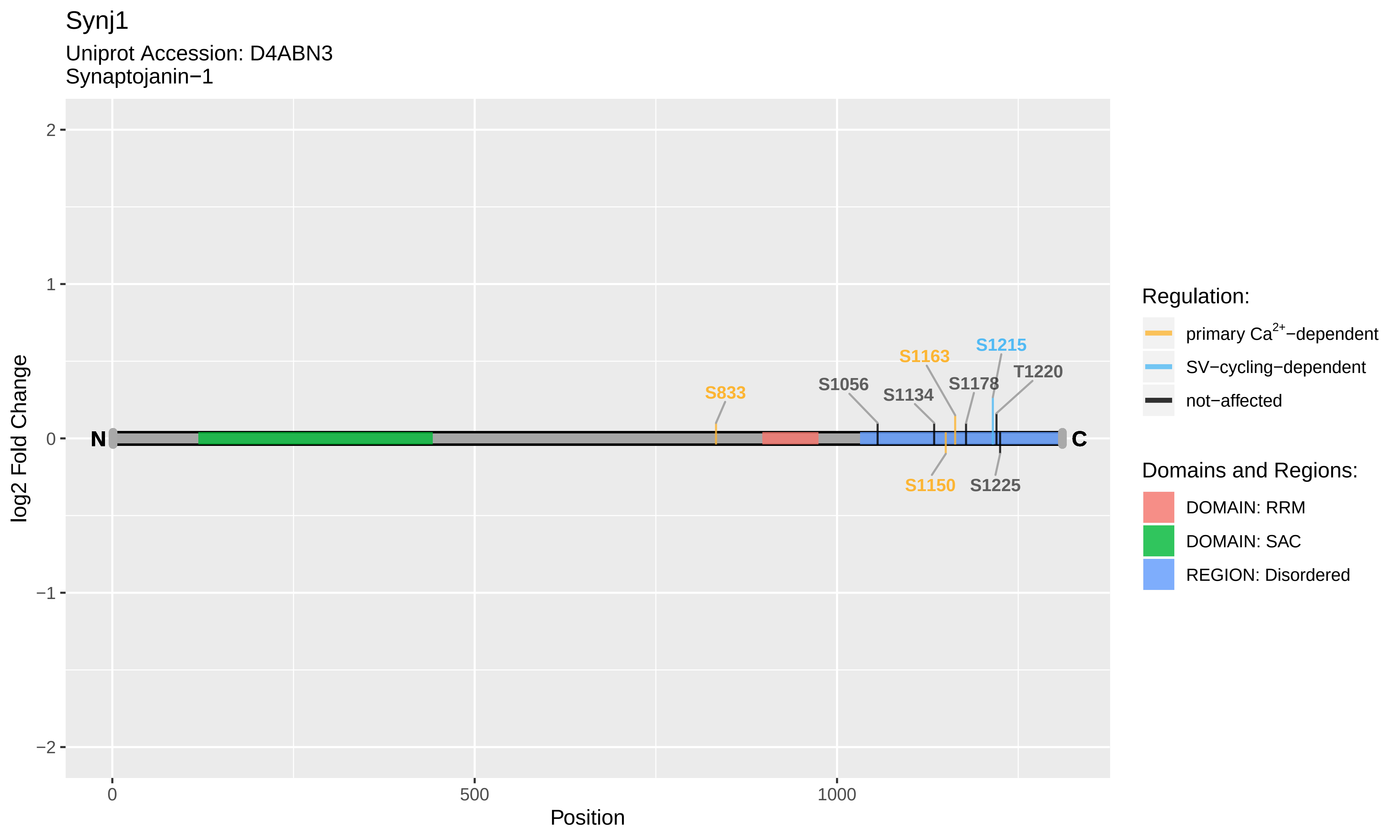
Synj1
Uniprot Accession: D4ABN3
Synaptojanin−1
log2 Fold Change
2
1
0
−1
−2
0
500
1000
Position
N
C
S833
S1056
S1134
S1163
S1178
S1215
T1220
S1150
S1225
Regulation:
primary Ca2+−dependent
SV−cycling−dependent
not−affected
Domains and Regions:
DOMAIN: RRM
DOMAIN: SAC
REGION: Disordered

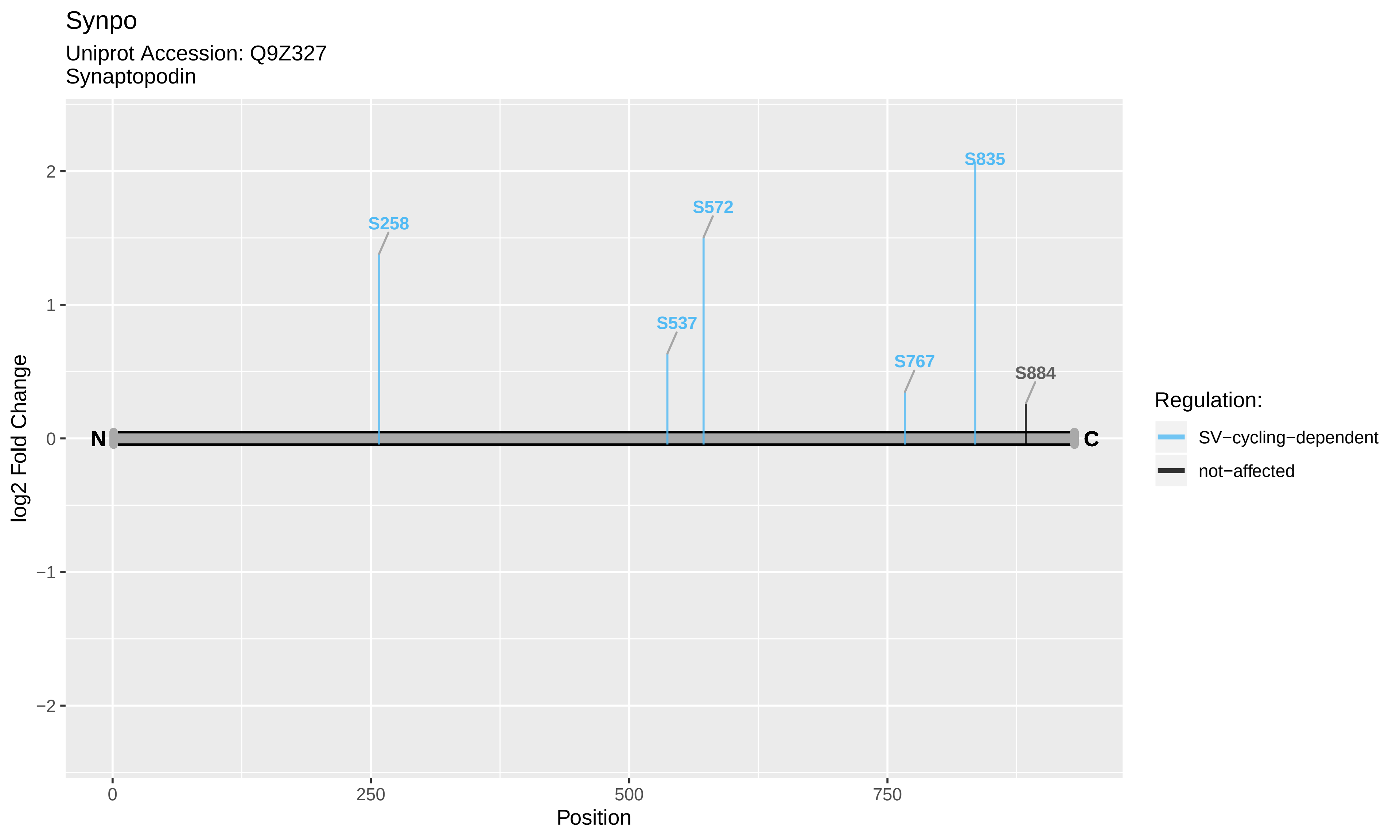

Synpo
Uniprot Accession: Q9Z327
Synaptopodin
S258
S537
S572
S767
S835
S884
N
C
log2 Fold Change
2
1
0
−1
−2
0
250
500
750
Position
Regulation:
SV−cycling−dependent
not−affected

Synrg

Uniprot Accession: Q9JKC9
Synergin gamma

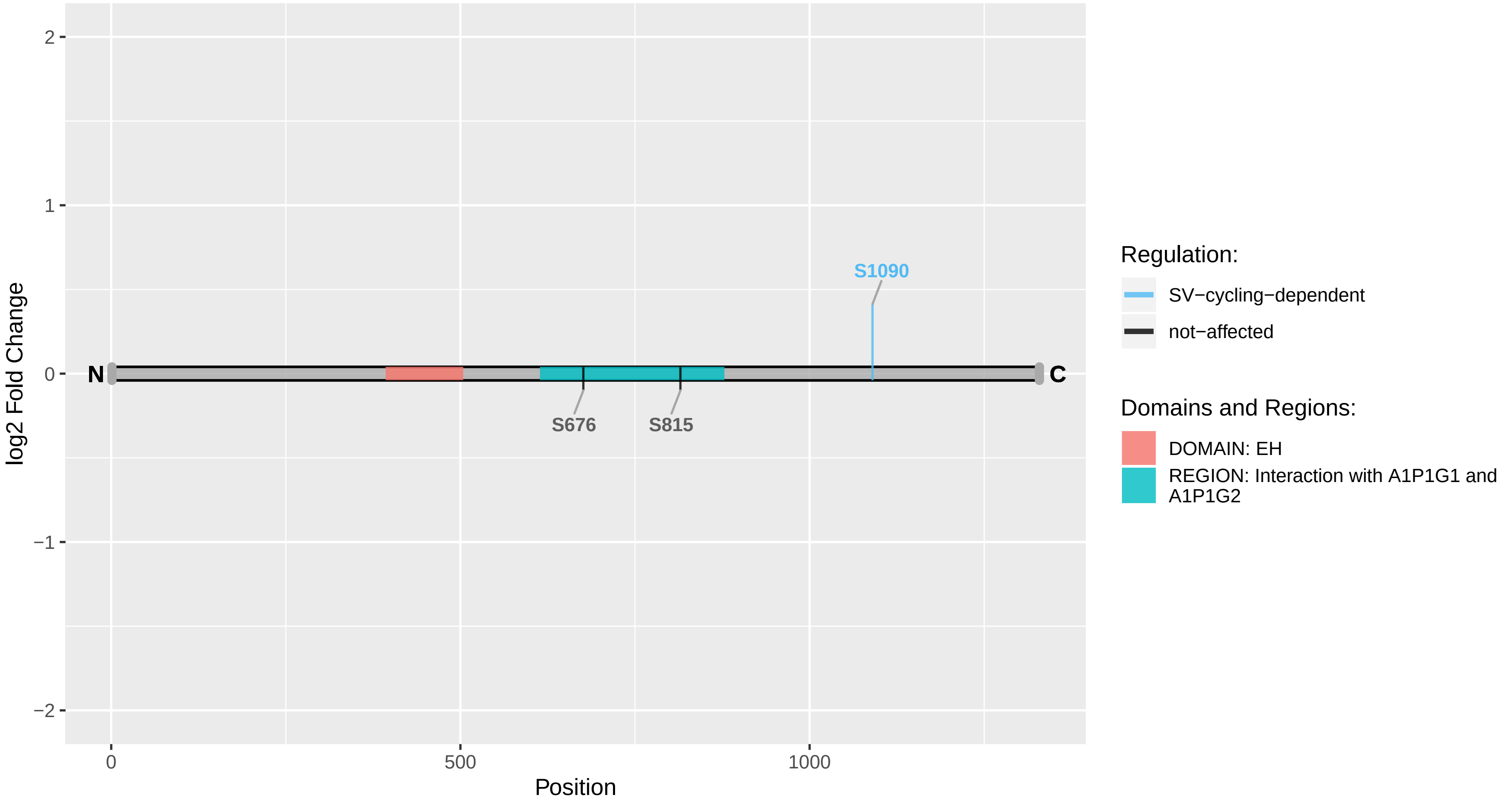

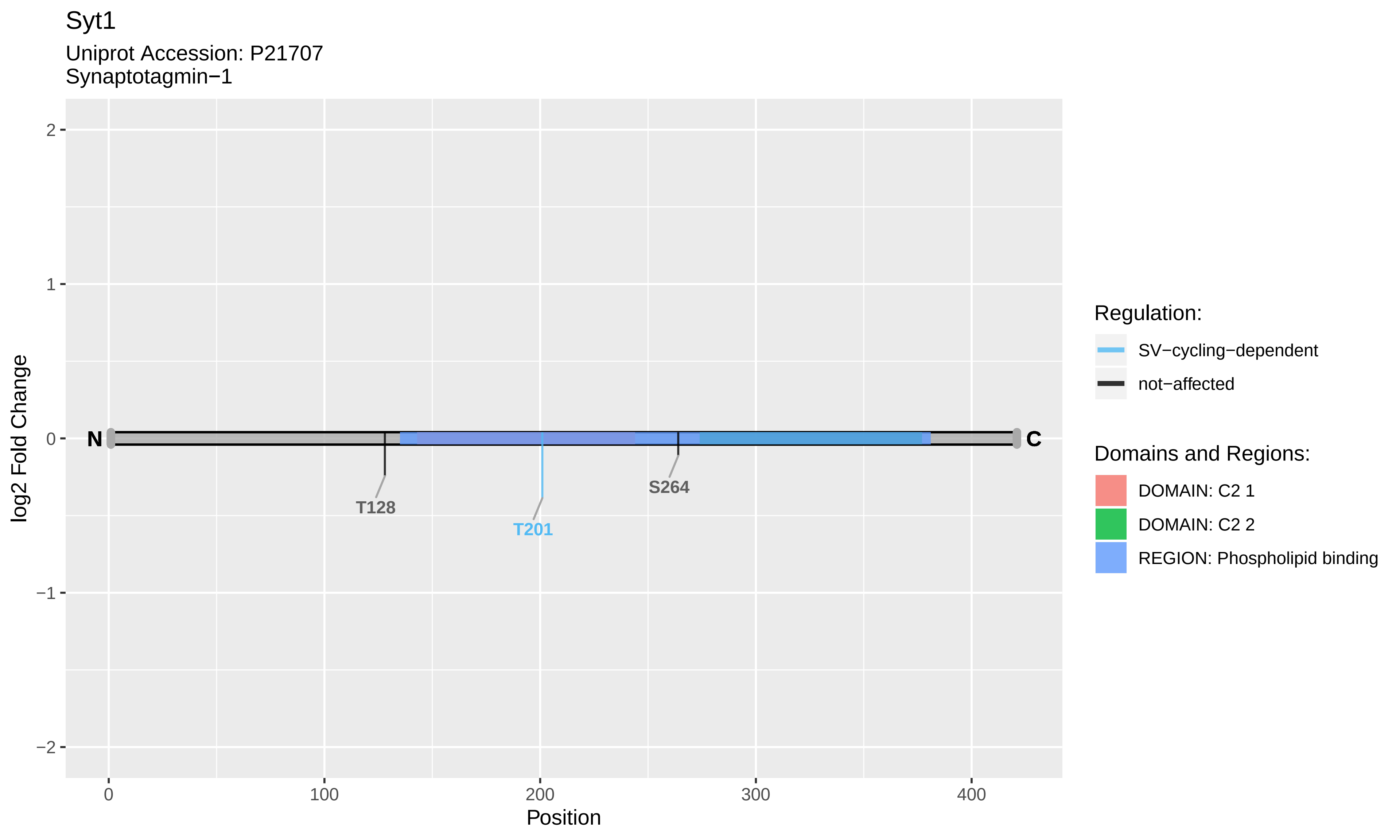

Syt1
Uniprot Accession: P21707
Synaptotagmin−1
log2 Fold Change
2
1
0
−1
−2
N
C
T128
T201
S264
0
100
200
300
400
Position
Regulation:
SV−cycling−dependent
not−affected
Domains and Regions:
DOMAIN: C2 1
DOMAIN: C2 2
REGION: Phospholipid binding

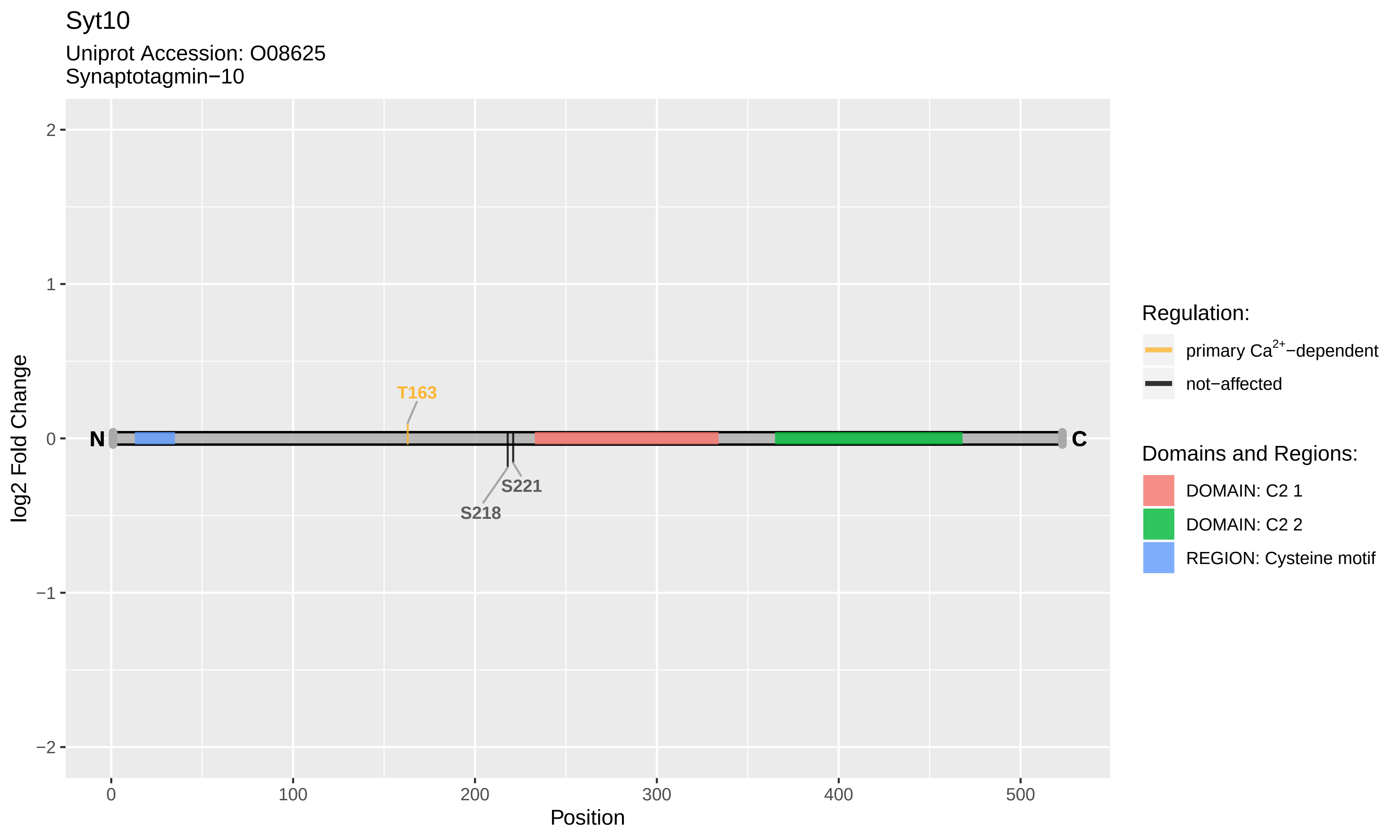

Syt10
Uniprot Accession: O08625
Synaptotagmin−10
T163
S221
S218
N
C
log2 Fold Change
2
1
0
−1
−2
0
100
200
300
400
500
Position
Regulation:
primary Ca2+−dependent
not−affected
Domains and Regions:
DOMAIN: C2 1
DOMAIN: C2 2
REGION: Cysteine motif

Syt17

Uniprot Accession: Q62807
Synaptotagmin−17

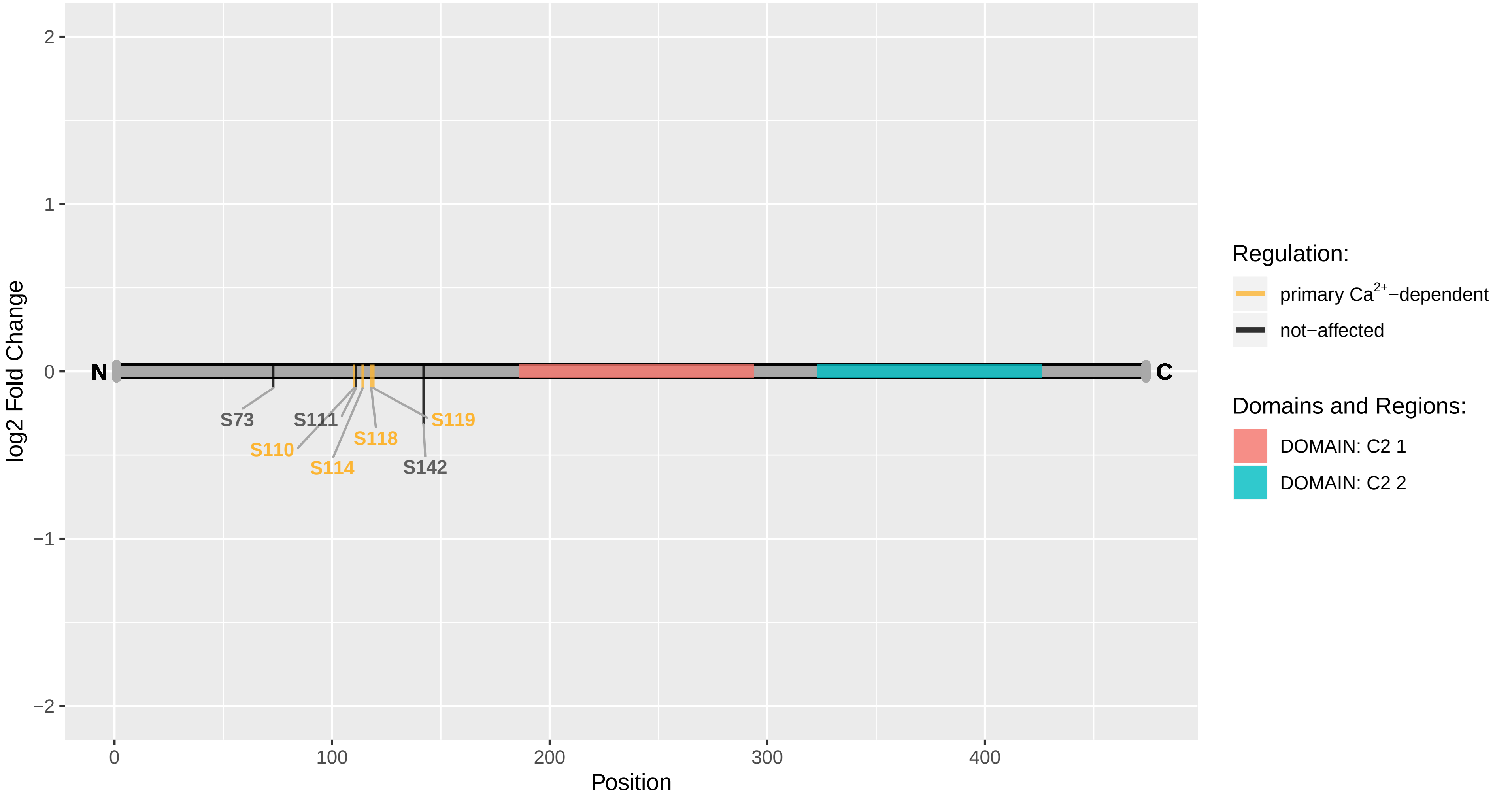

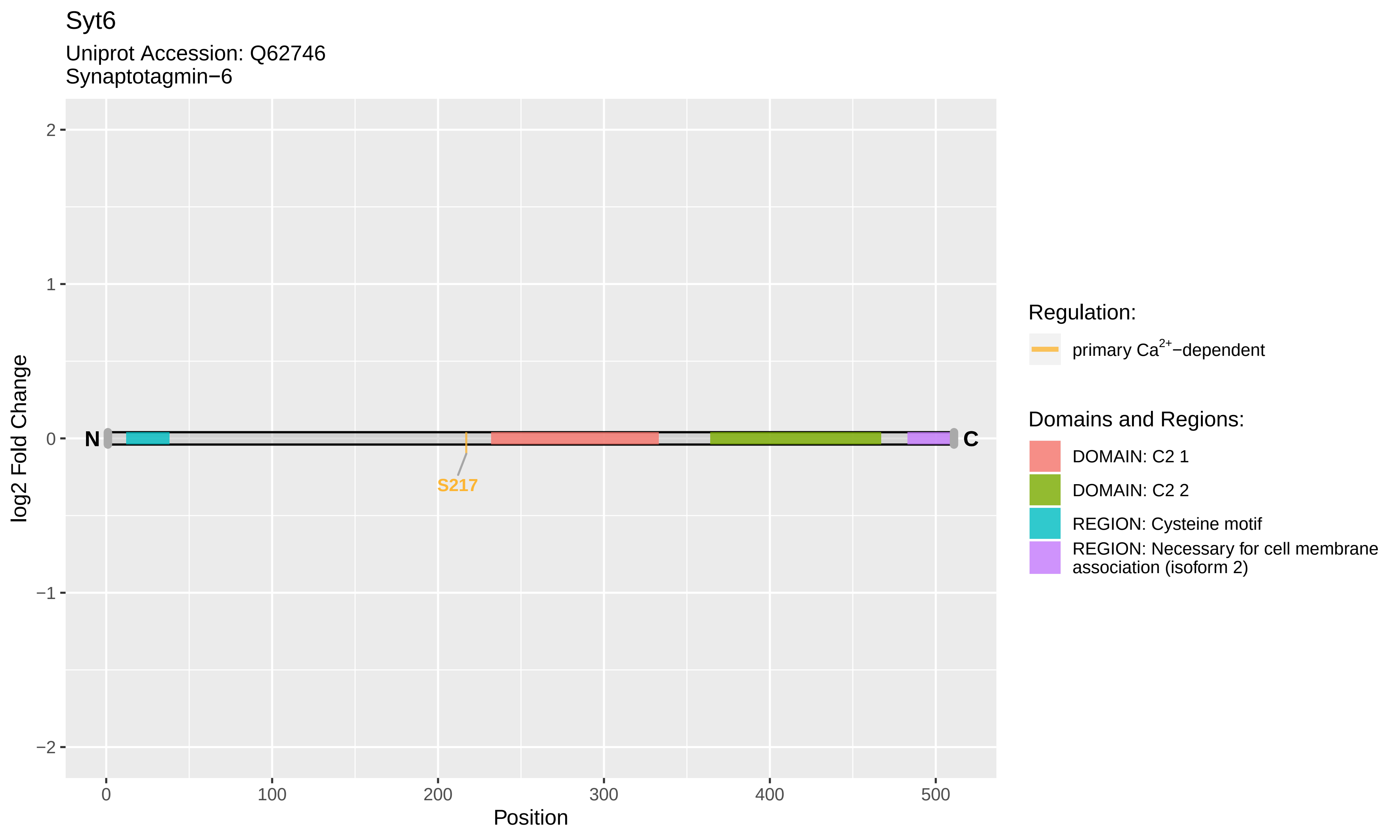
Syt6
Uniprot Accession: Q62746
Synaptotagmin−6
2
1
0
−1
−2
log2 Fold Change
N
C
S217
0
100
200
300
400
500
Position
Regulation:
primary Ca2+−dependent
Domains and Regions:
DOMAIN: C2 1
DOMAIN: C2 2
REGION: Cysteine motif
REGION: Necessary for cell membrane association (isoform 2)

Syt9

Uniprot Accession: Q925C0
Synaptotagmin−9

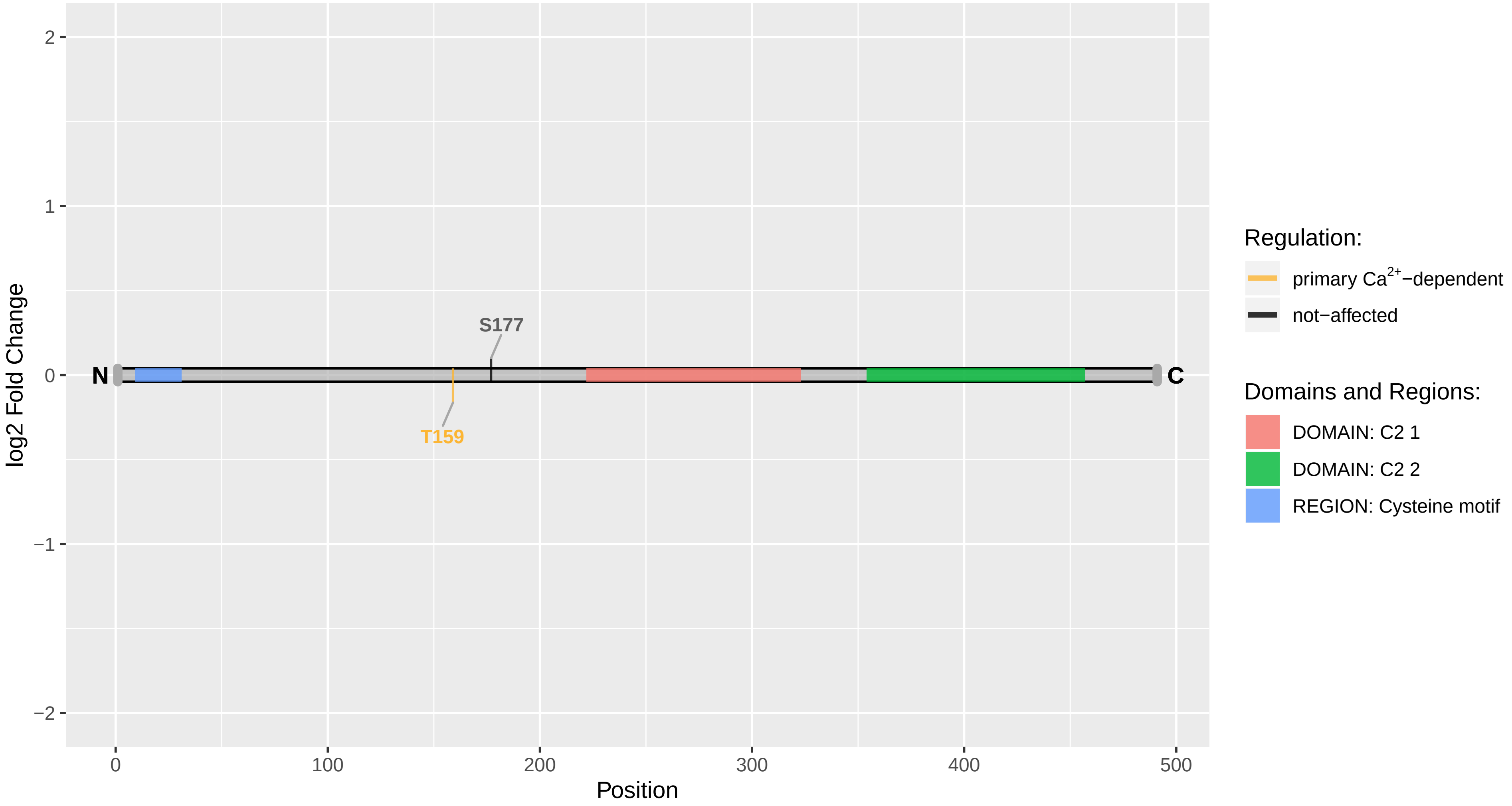

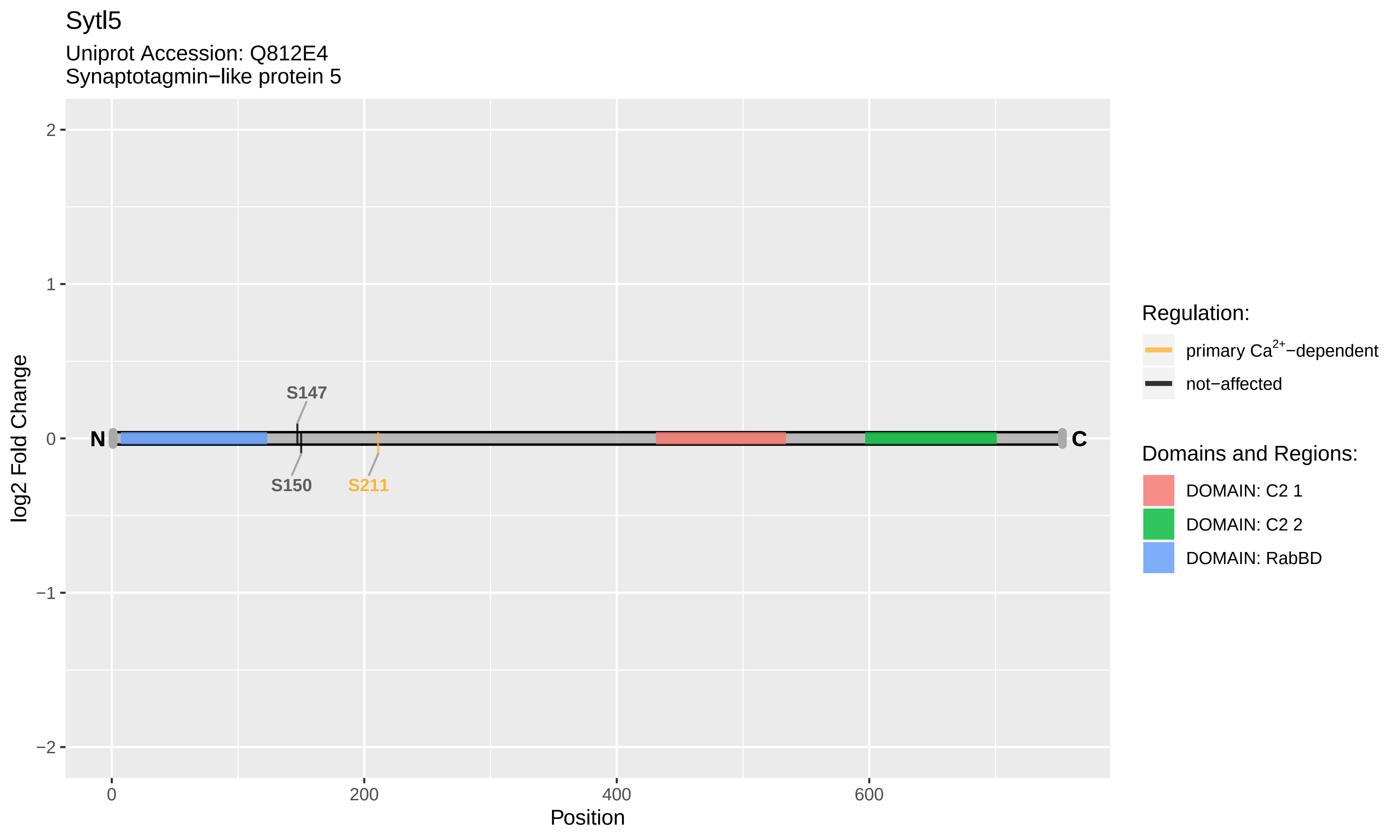

Sytl5
Uniprot Accession: Q812E4
Synaptotagmin−like protein 5
log2 Fold Change
2
1
0
−1
−2
N
C
S147
S150
S211
0
200
400
600
Position
Regulation:
primary Ca2+−dependent
not−affected
Domains and Regions:
DOMAIN: C2 1
DOMAIN: C2 2
DOMAIN: RabBD

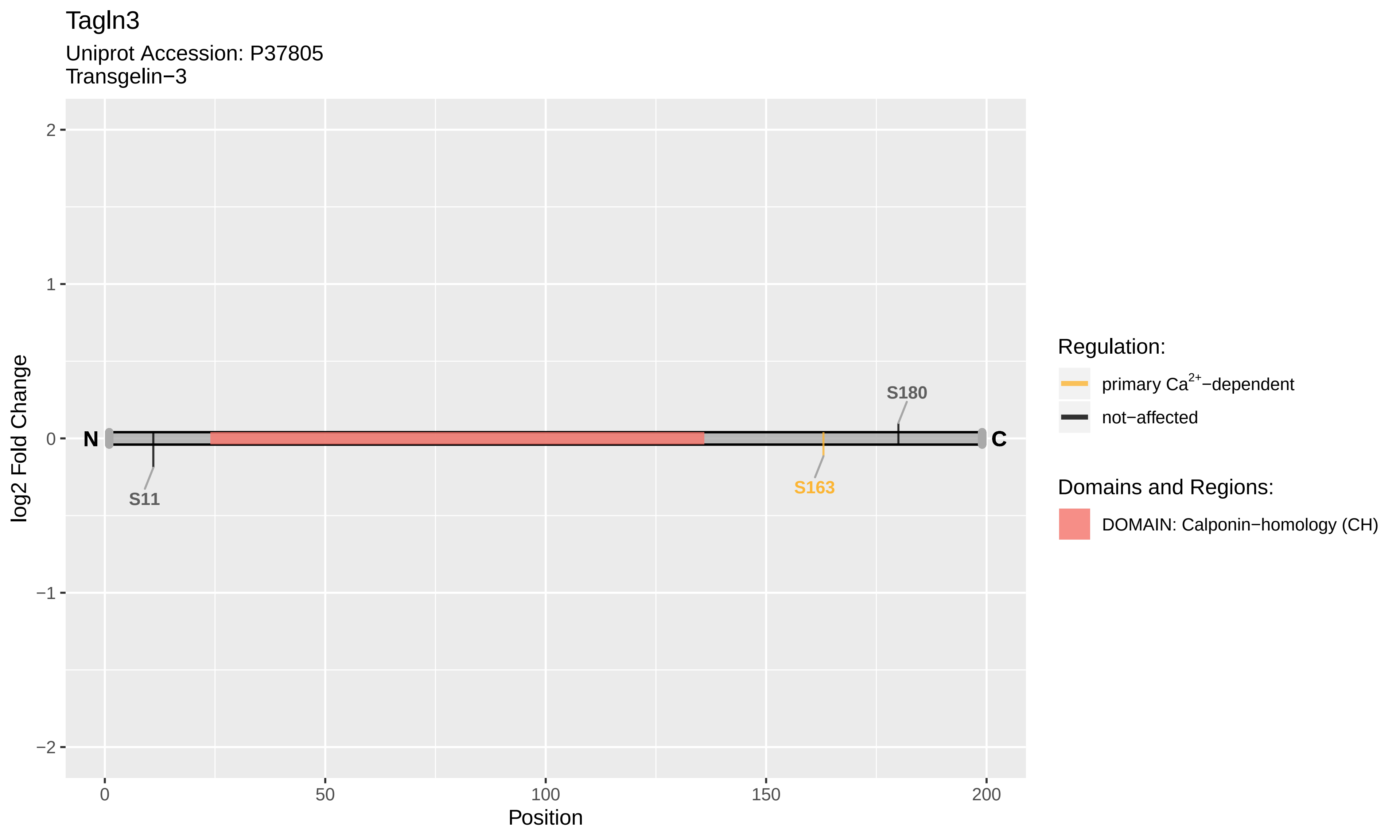
Tagln3
Uniprot Accession: P37805
Transgelin−3
log2 Fold Change
Position
N
C
S11
S163
S180
Regulation:
primary Ca2+−dependent
not−affected
Domains and Regions:
DOMAIN: Calponin−homology (CH)
2
1
0
−1
−2
0
50
100
150
200

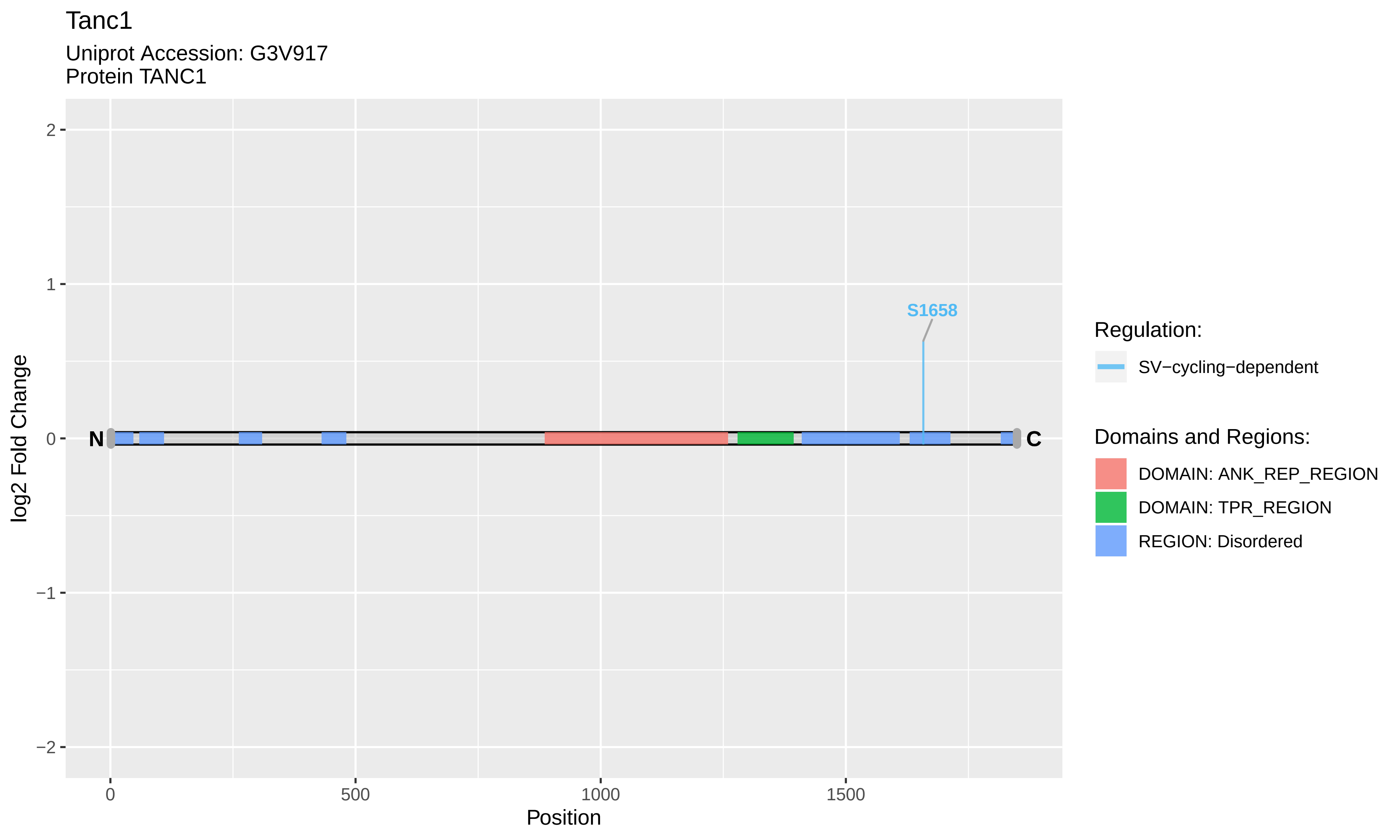

Tanc1
Uniprot Accession: G3V917
Protein TANC1
S1658
N
C
2
1
0
−1
−2
log2 Fold Change
0
500
1000
1500
Position
Regulation:
SV−cycling−dependent
Domains and Regions:
DOMAIN: ANK_REP_REGION
DOMAIN: TPR_REGION
REGION: Disordered

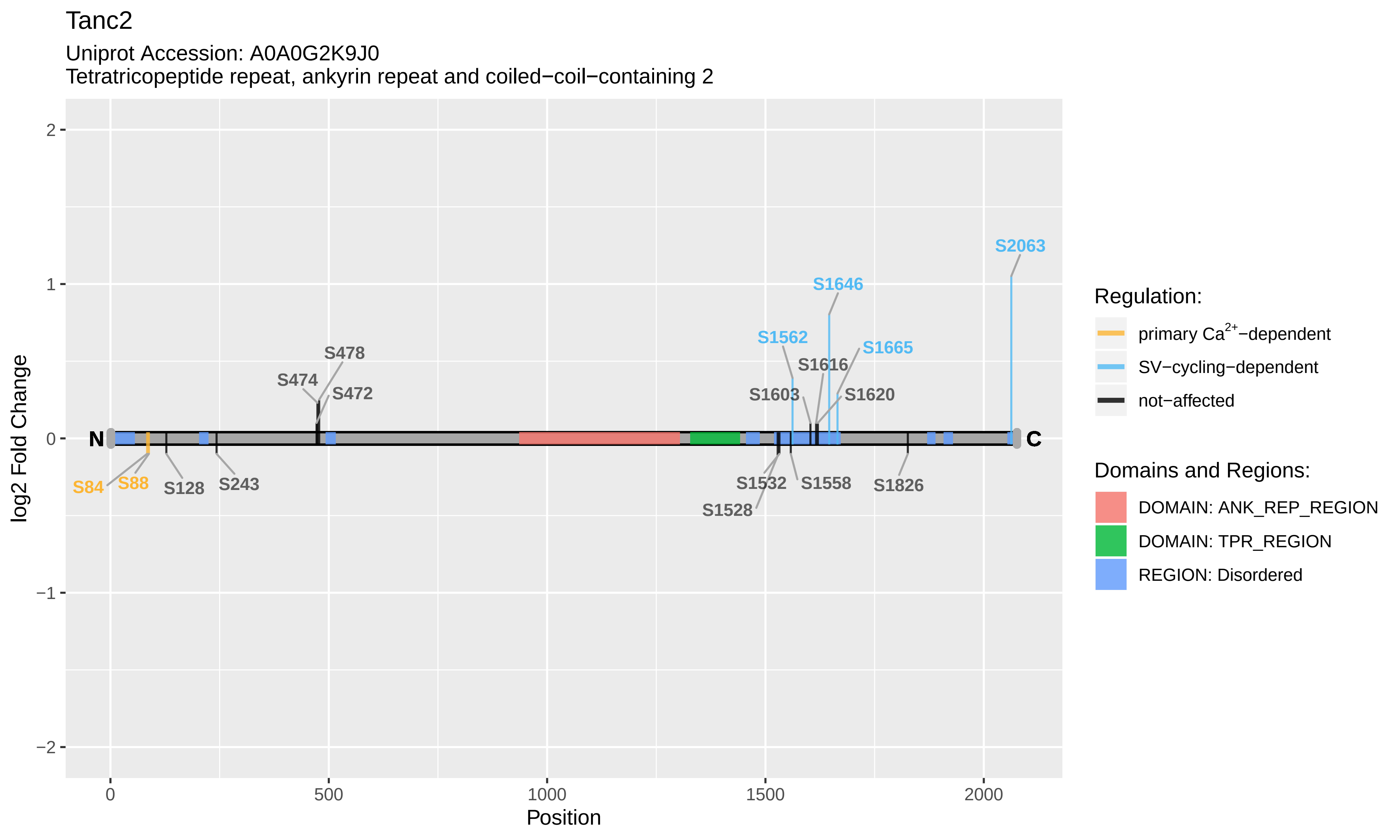

Tanc2
Uniprot Accession: A0A0G2K9J0
Tetratricopeptide repeat, ankyrin repeat and coiled−coil−containing 2
log2 Fold Change
Position
2
1
0
−1
−2
0
500
1000
1500
2000
N
C
S2063
S1646
S1562
S1665
S1616
S478
S474
S472
S1603
S1620
S84
S88
S128
S243
S1532
S1558
S1826
S1528
Regulation:
primary Ca2+−dependent
SV−cycling−dependent
not−affected
Domains and Regions:
DOMAIN: ANK_REP_REGION
DOMAIN: TPR_REGION
REGION: Disordered

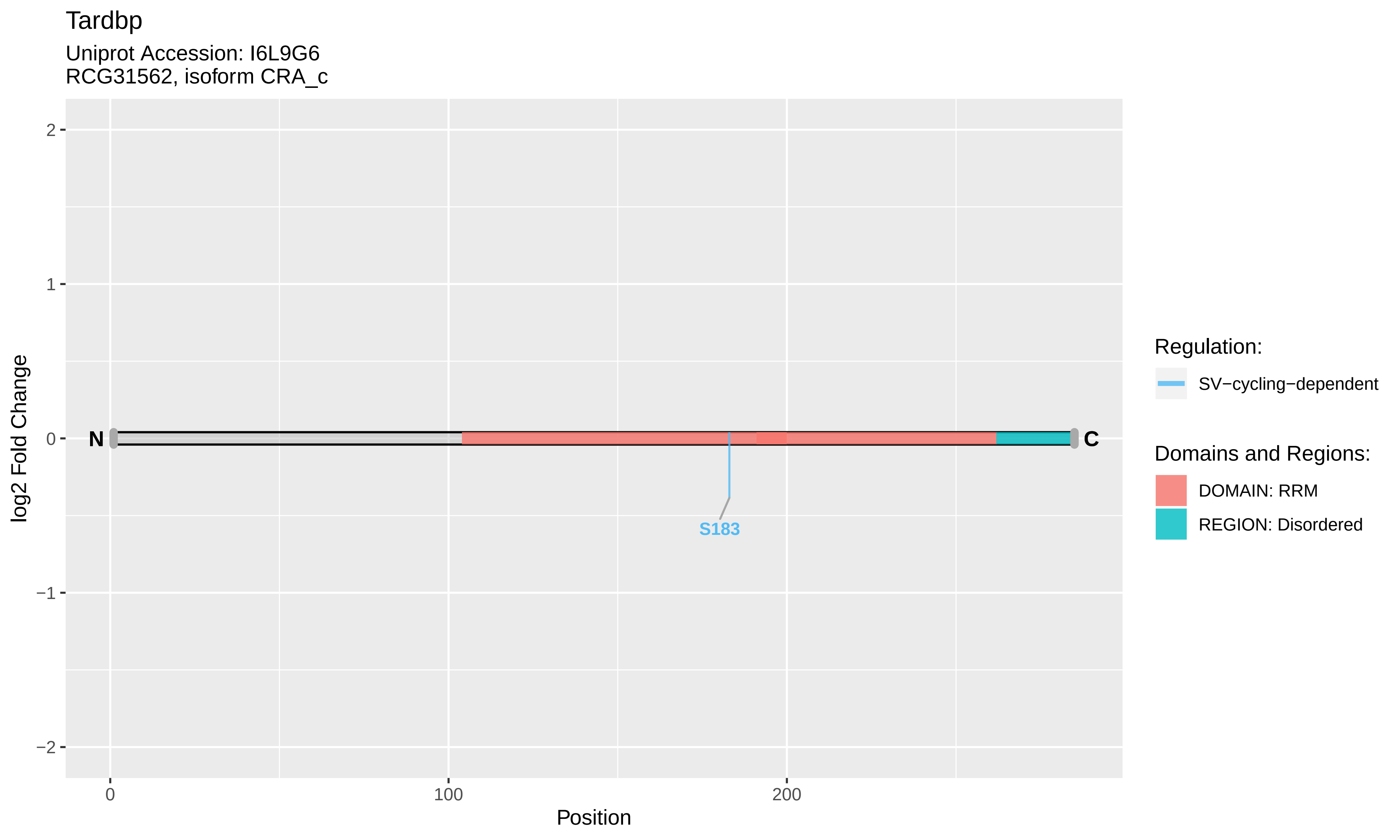

Tardbp
Uniprot Accession: I6L9G6
RCG31562, isoform CRA_c
log2 Fold Change
2
1
0
−1
−2
N
C
S183
0
100
200
Position
Regulation:
SV−cycling−dependent
Domains and Regions:
DOMAIN: RRM
REGION: Disordered

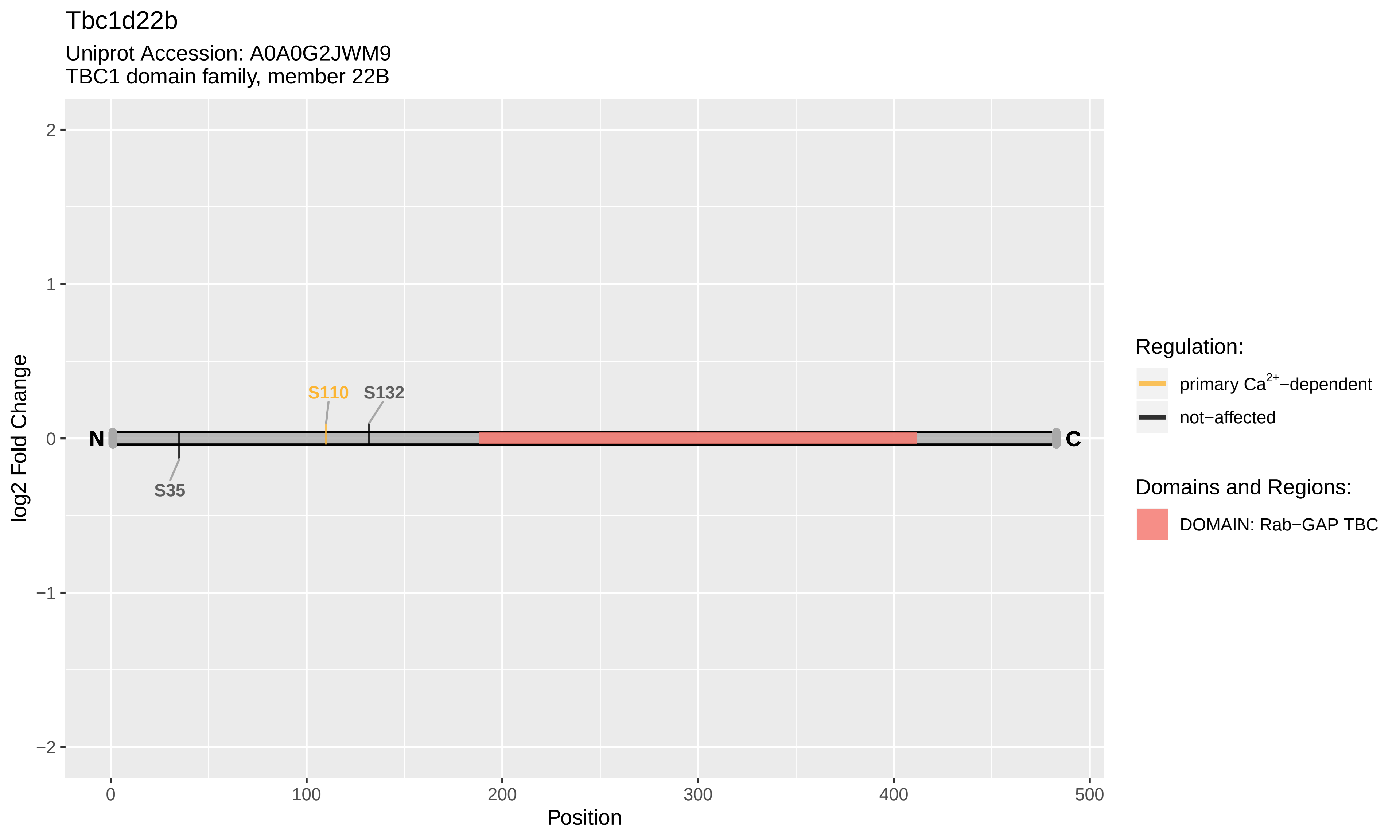

Tbc1d22b
Uniprot Accession: A0A0G2JWM9
TBC1 domain family, member 22B
S110
S132
S35
N
C
2
1
0
−1
−2
log2 Fold Change
0
100
200
300
400
500
Position
Regulation:
primary Ca2+−dependent
not−affected
Domains and Regions:
DOMAIN: Rab−GAP TBC

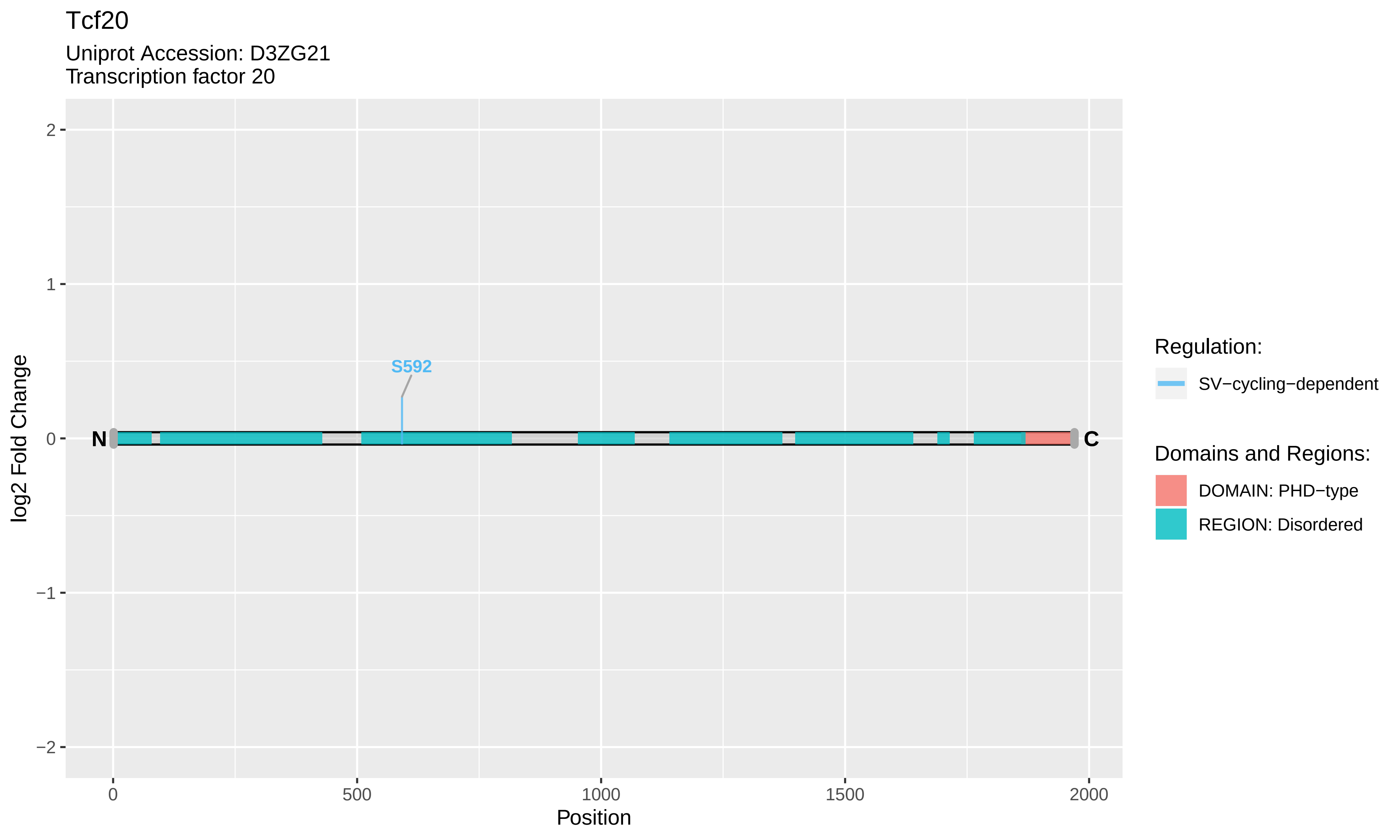

Tcf20
Uniprot Accession: D3ZG21
Transcription factor 20
S592
N
C
log2 Fold Change
Position
2
1
0
−1
−2
0
500
1000
1500
2000
Regulation:
SV−cycling−dependent
Domains and Regions:
DOMAIN: PHD−type
REGION: Disordered

Tcof1

Uniprot Accession: D4A206
Treacle ribosome biogenesis factor 1

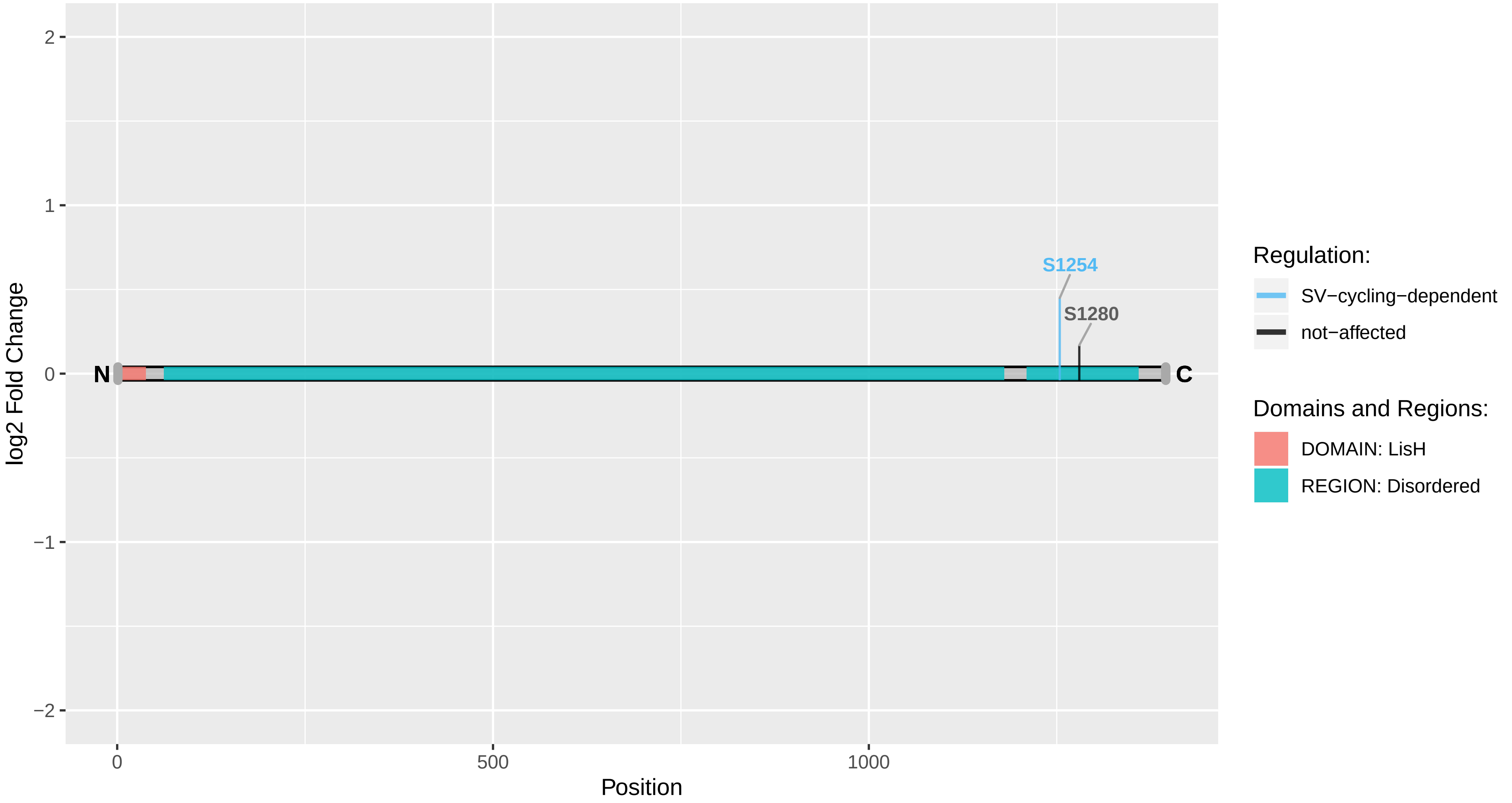

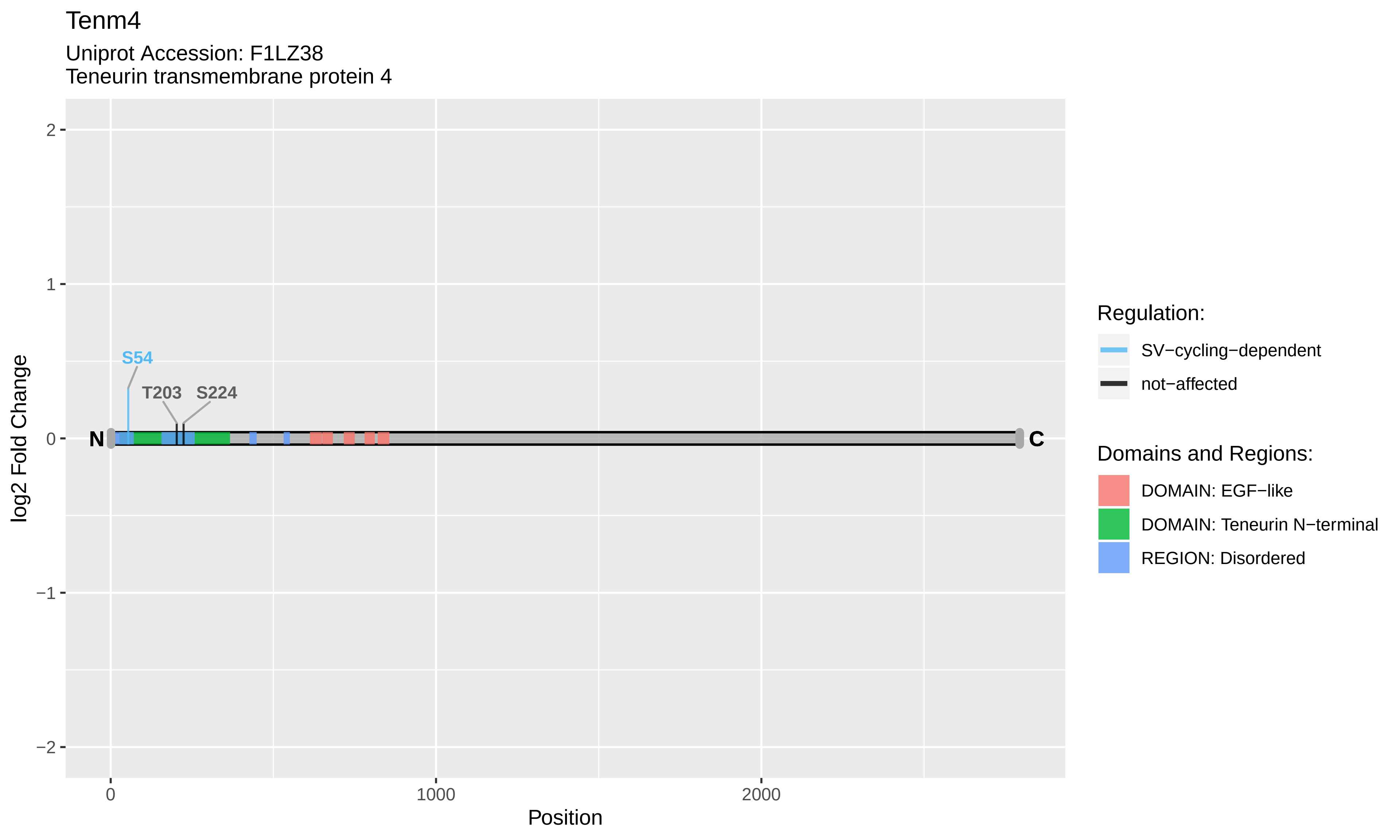

Tenm4
Uniprot Accession: F1LZ38
Teneurin transmembrane protein 4
log2 Fold Change
2
1
0
−1
−2
S54
T203
S224
N
C
0
1000
2000
Position
Regulation:
SV−cycling−dependent
not−affected
Domains and Regions:
DOMAIN: EGF−like
DOMAIN: Teneurin N−terminal
REGION: Disordered

Tex2

Uniprot Accession: G3V9L1
Testis expressed gene 2

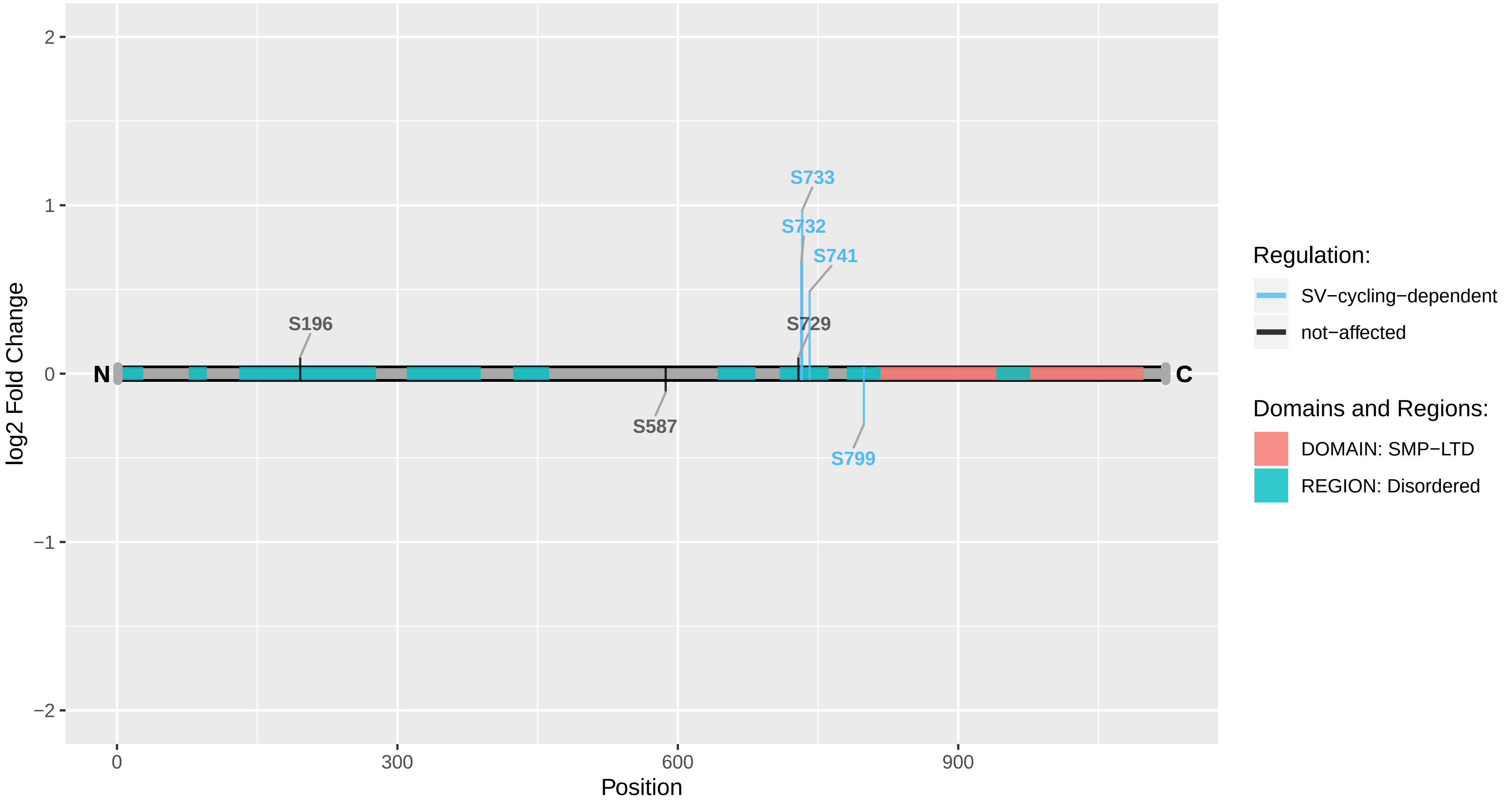

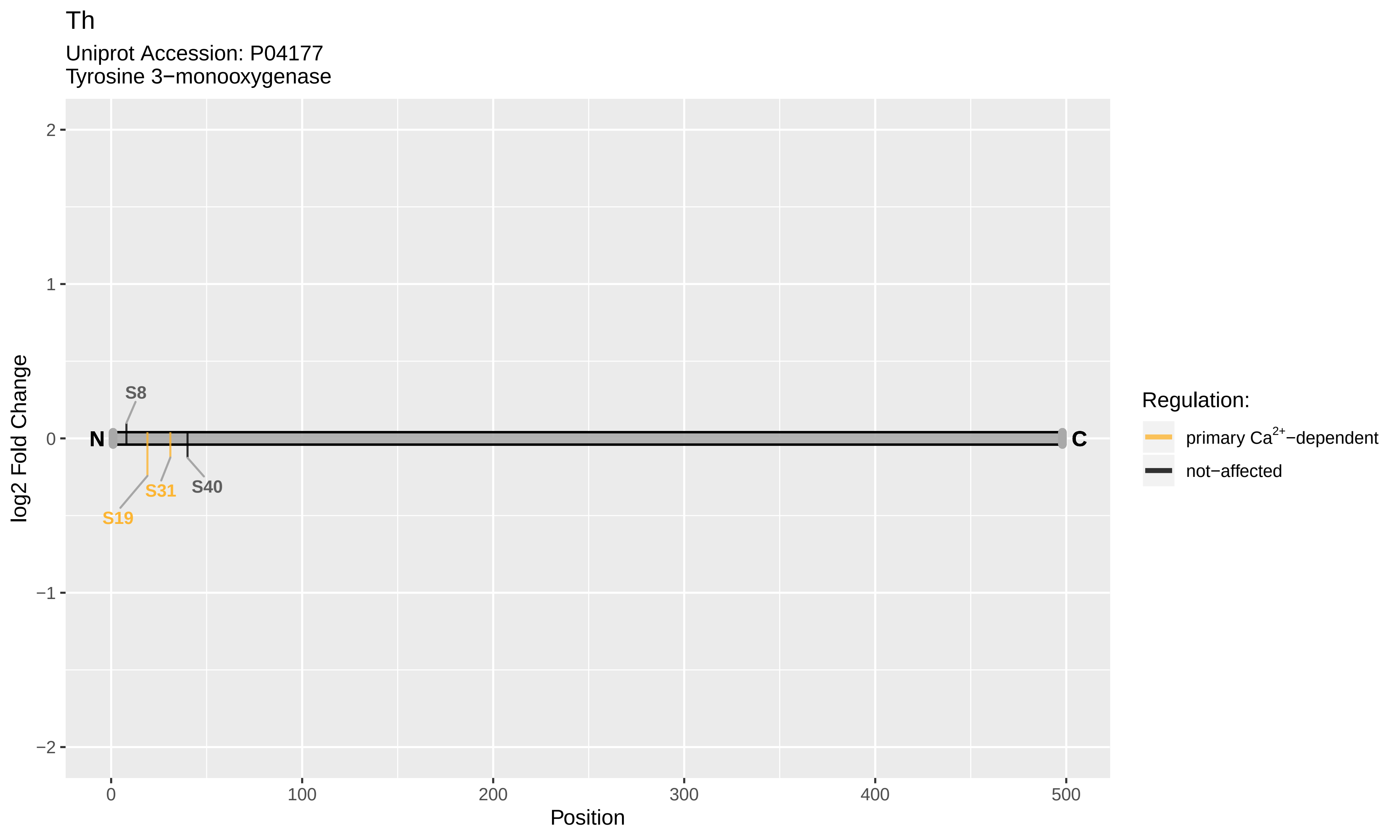

Th
Uniprot Accession: P04177
Tyrosine 3−monooxygenase
log2 Fold Change
Position
N
C
S8
S19
S31
S40
2
1
0
−1
−2
0
100
200
300
400
500
Regulation:
primary Ca2+−dependent
not−affected

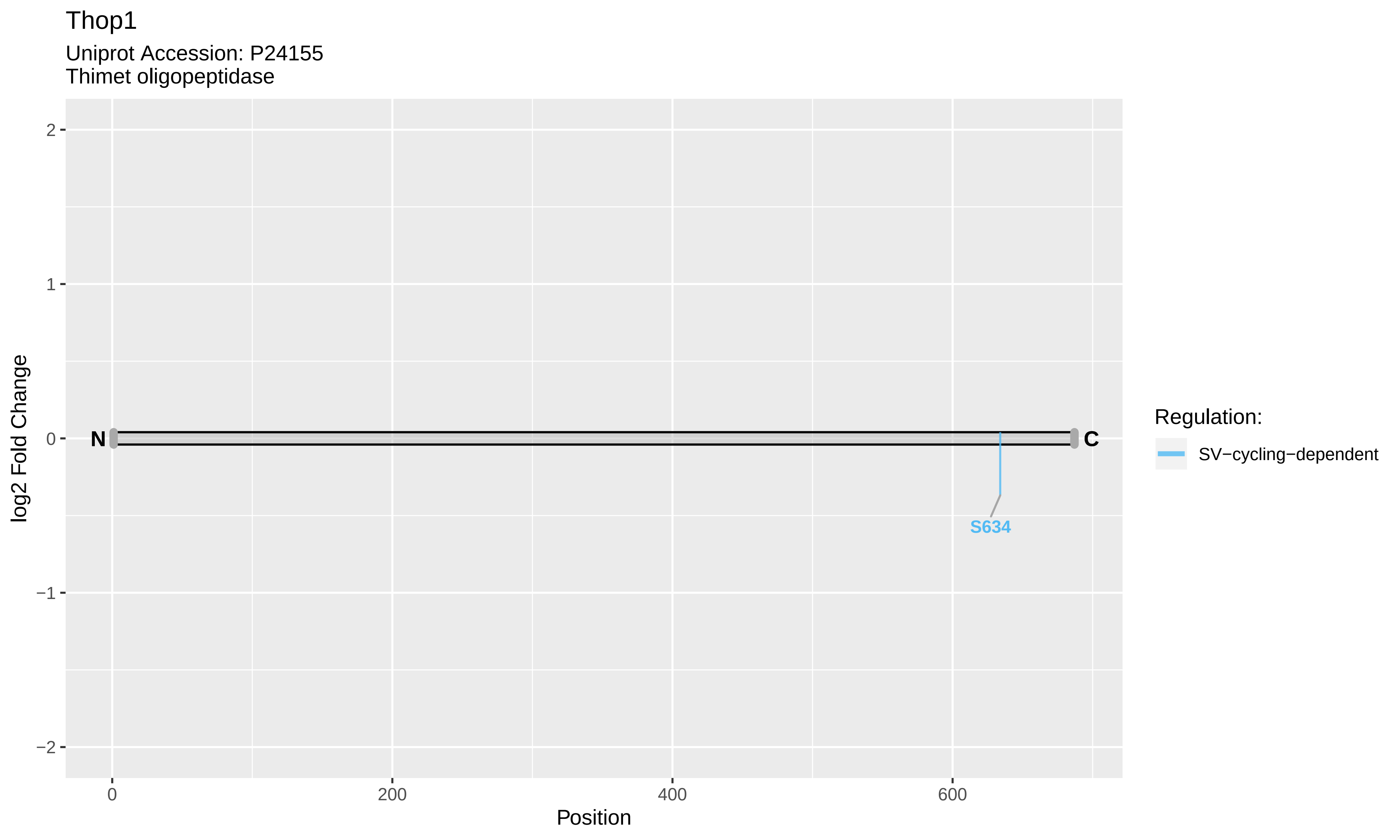
Thop1
Uniprot Accession: P24155
Thimet oligopeptidase
log2 Fold Change
Position
N
C
S634
Regulation:
SV−cycling−dependent
2
1
0
−1
−2
0
200
400
600

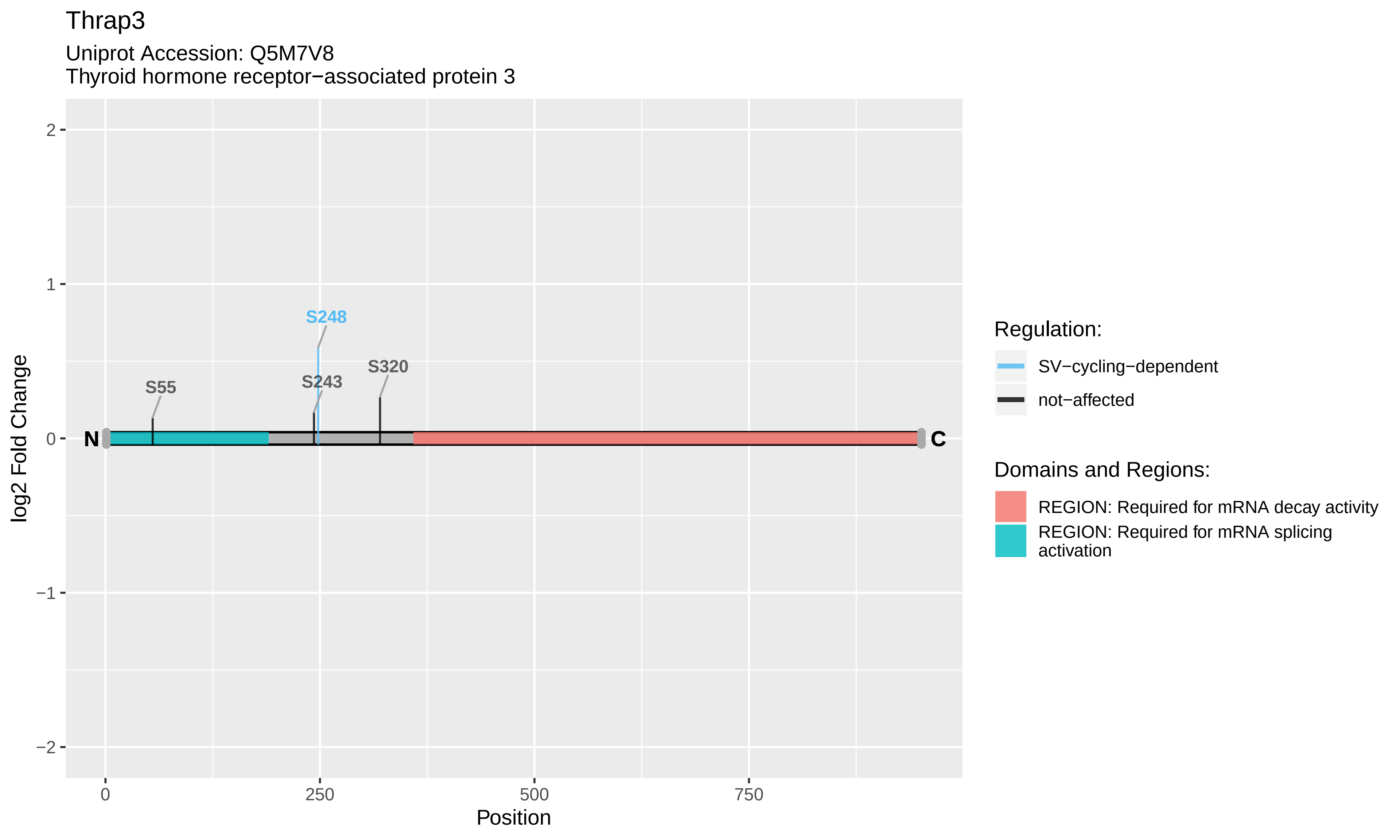
Thrap3
Uniprot Accession: Q5M7V8
Thyroid hormone receptor−associated protein 3
S248
S320
S243
S55
N
C
2
1
0
−1
−2
log2 Fold Change
0
250
500
750
Position
Regulation:
SV−cycling−dependent
not−affected
Domains and Regions:
REGION: Required for mRNA decay activity
REGION: Required for mRNA splicing activation

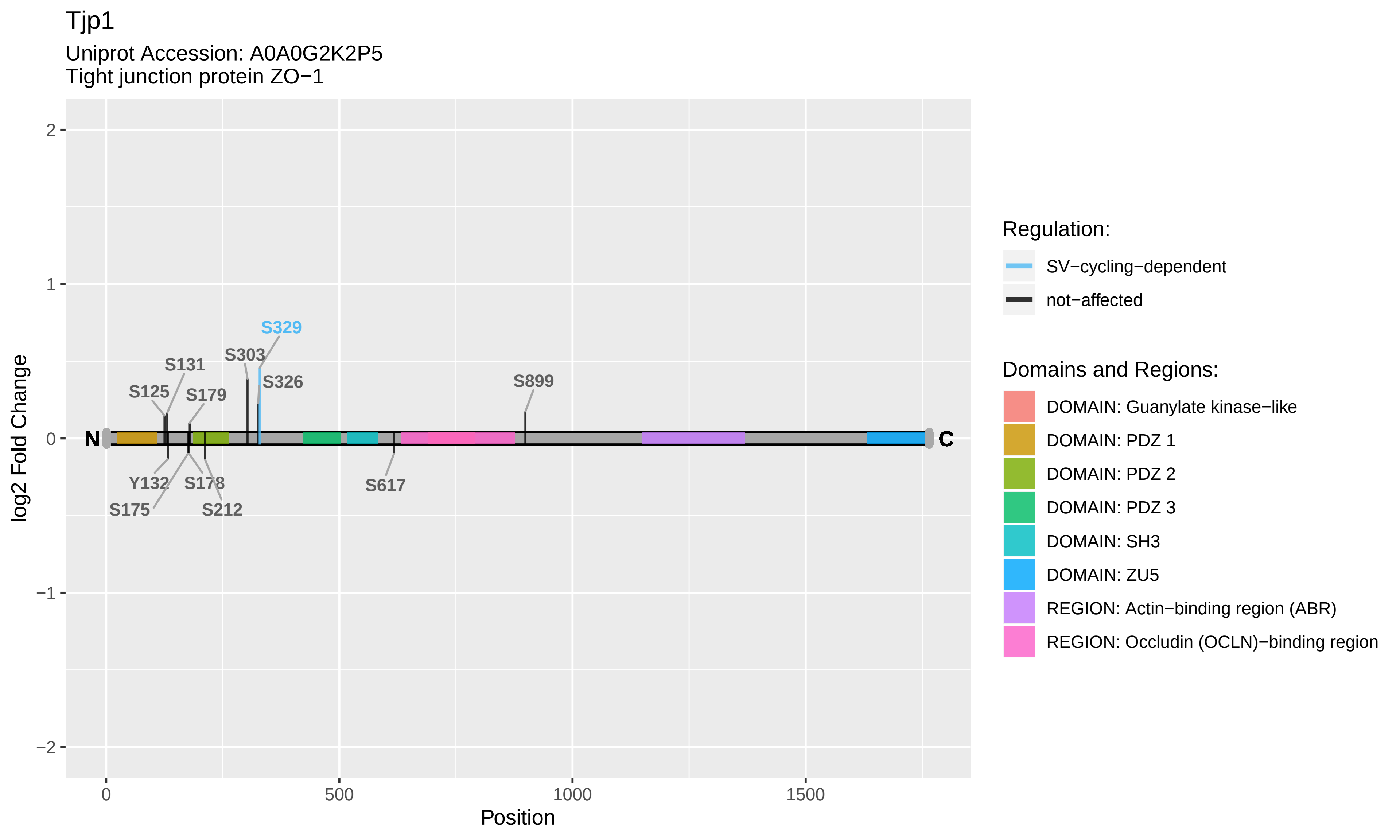

Tjp1
Uniprot Accession: A0A0G2K2P5
Tight junction protein ZO−1
S329
S303
S131
S326
S125
S179
S899
N
C
Y132
S178
S617
S175
S212
log2 Fold Change
2
1
0
−1
−2
0
500
1000
1500
Position
Regulation:
SV−cycling−dependent
not−affected
Domains and Regions:
DOMAIN: Guanylate kinase−like
DOMAIN: PDZ 1
DOMAIN: PDZ 2
DOMAIN: PDZ 3
DOMAIN: SH3
DOMAIN: ZU5
REGION: Actin−binding region (ABR)
REGION: Occludin (OCLN)−binding region

Tjp2

Uniprot Accession: Q3ZB99
Tight junction protein 2

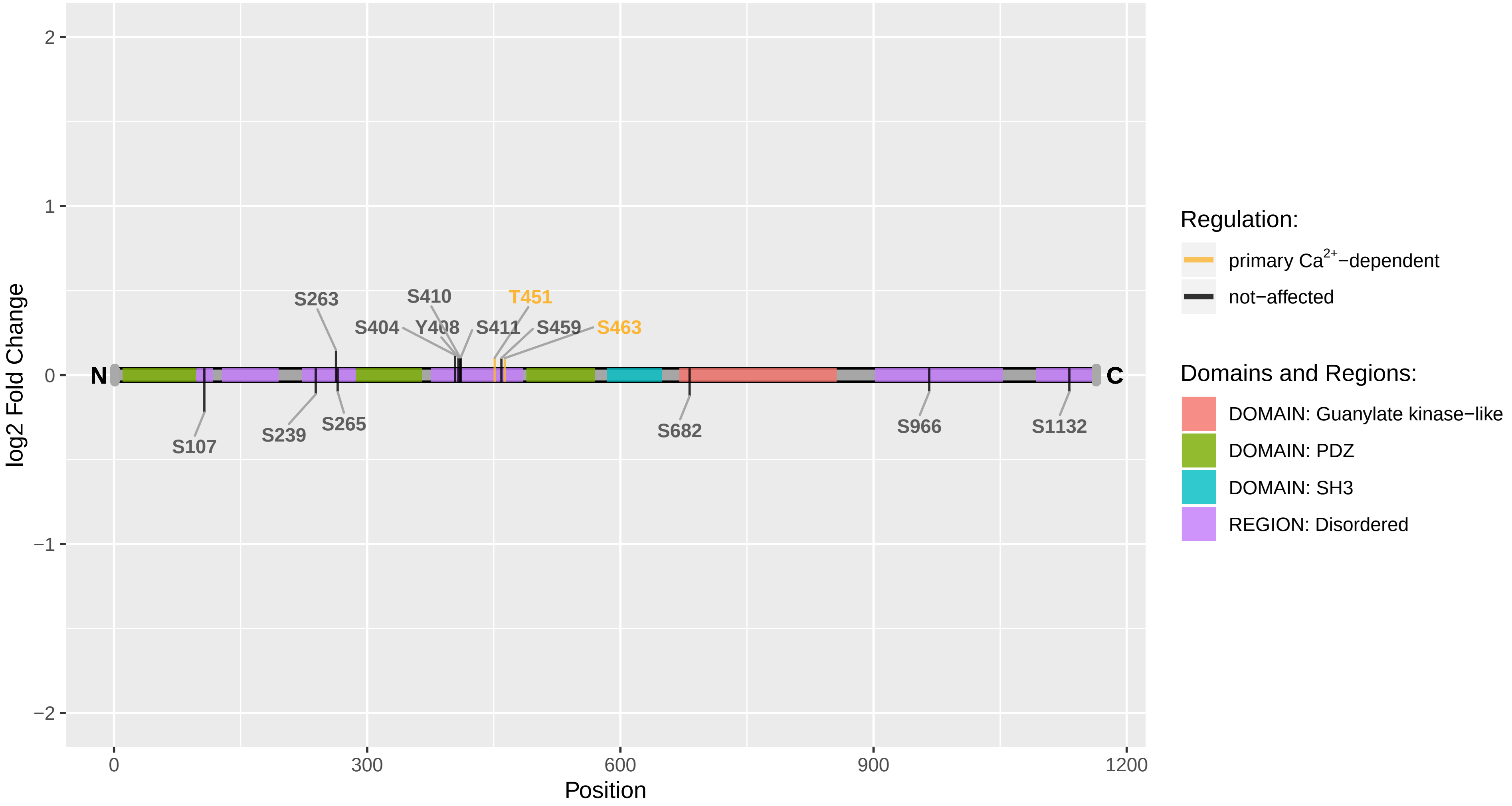

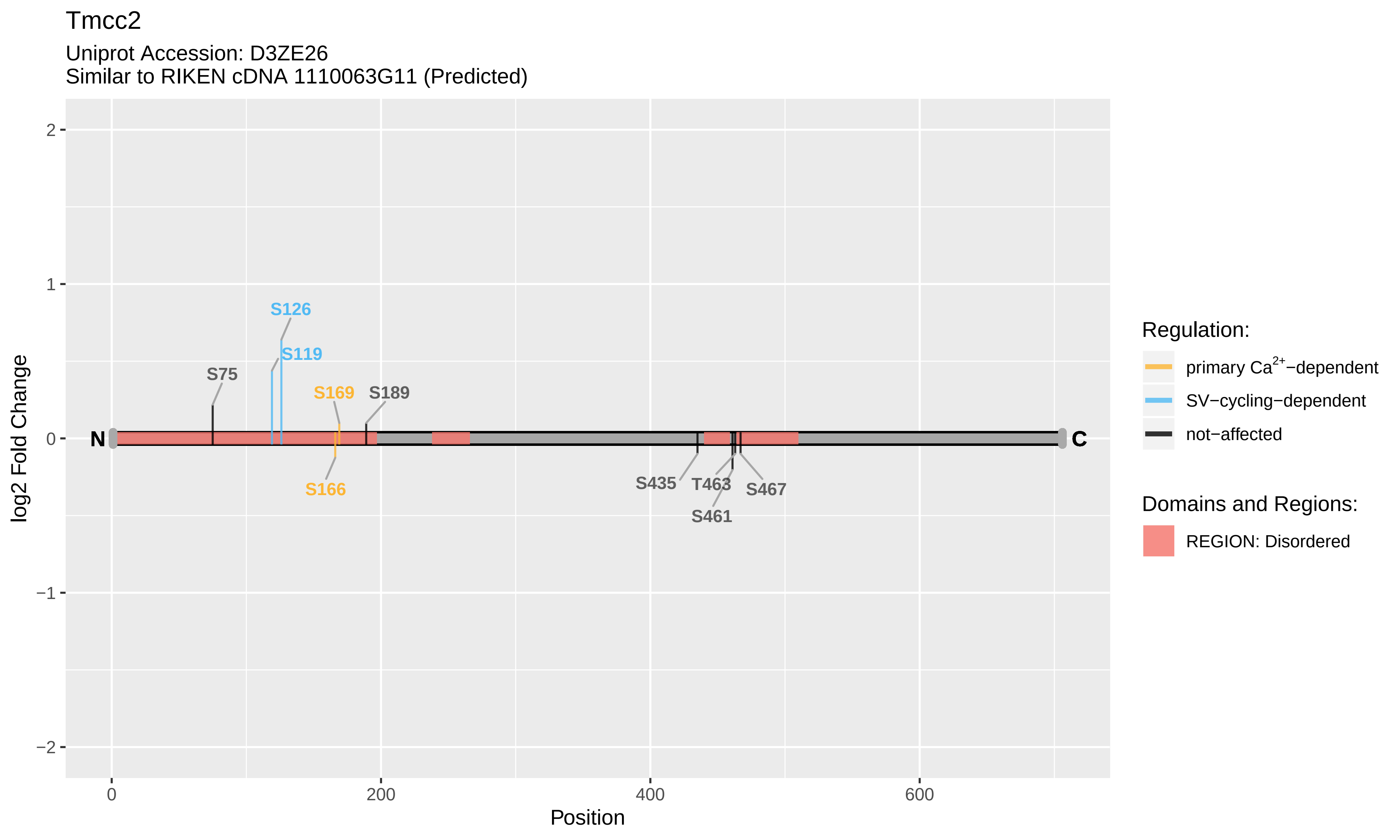

Tmcc2
Uniprot Accession: D3ZE26
Similar to RIKEN cDNA 1110063G11 (Predicted)
S126
S119
S75
S169
S189
S166
S435
T463
S461
S467
N
C
log2 Fold Change
Position
Regulation:
primary Ca2+−dependent
SV−cycling−dependent
not−affected
Domains and Regions:
REGION: Disordered

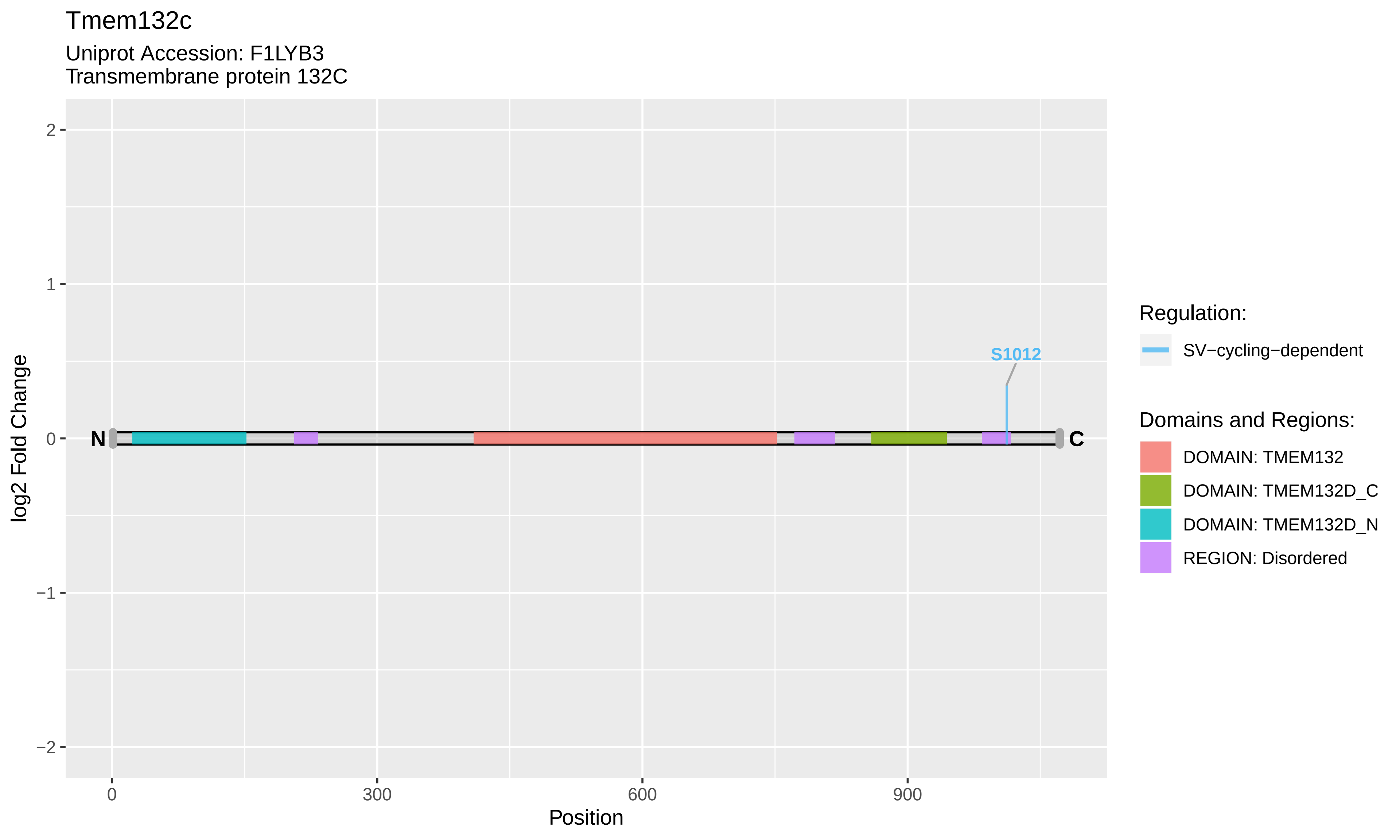

Tmem132c
Uniprot Accession: F1LYB3
Transmembrane protein 132C
S1012
N
C
2
1
0
−1
−2
log2 Fold Change
0
300
600
900
Position
Regulation:
SV−cycling−dependent
Domains and Regions:
DOMAIN: TMEM132
DOMAIN: TMEM132D_C
DOMAIN: TMEM132D_N
REGION: Disordered

Tmem163

Uniprot Accession: A9CMA6
Transmembrane protein 163

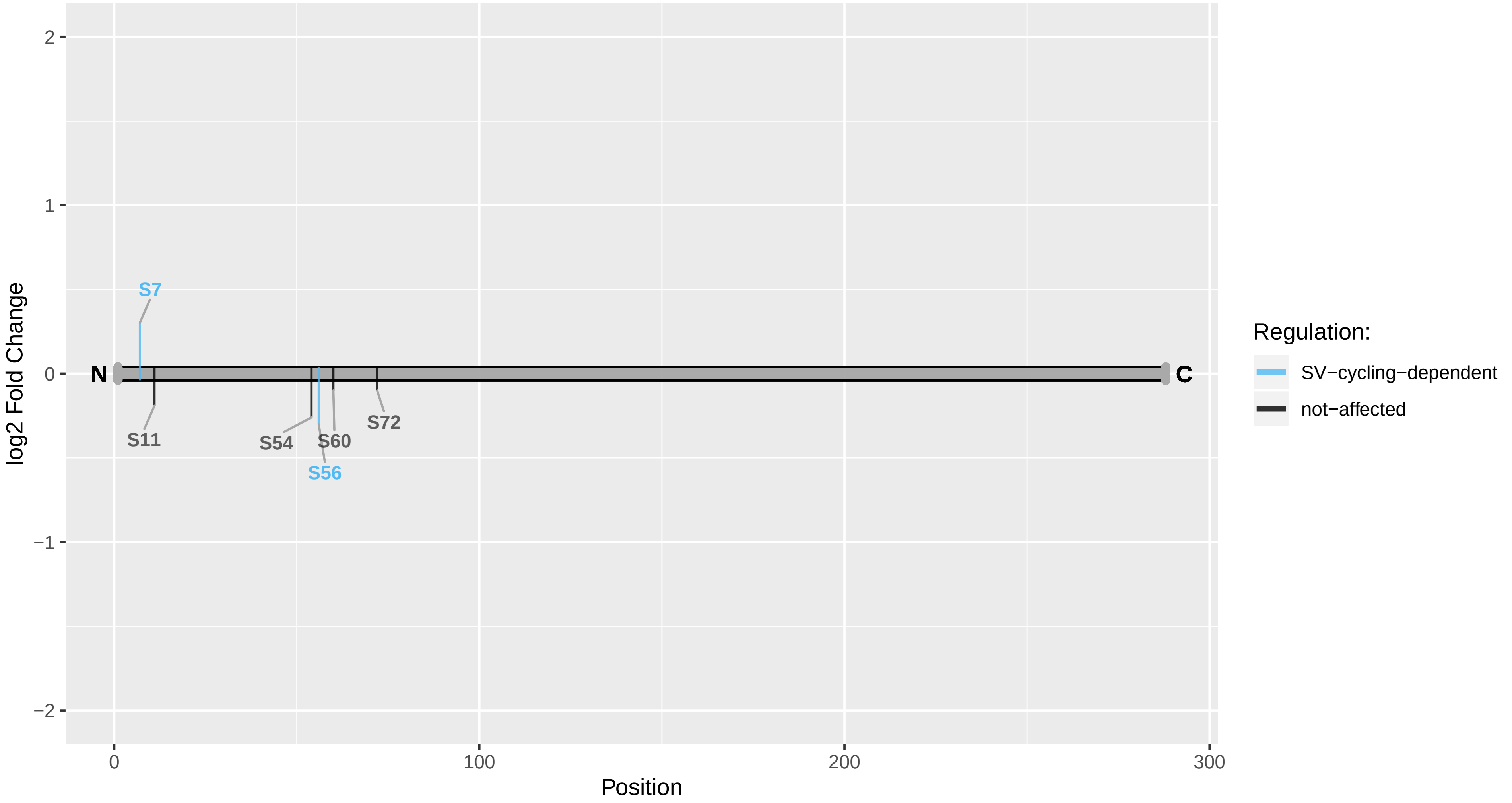

Tmem178a

Uniprot Accession: Q68FV0
Transmembrane protein 178A

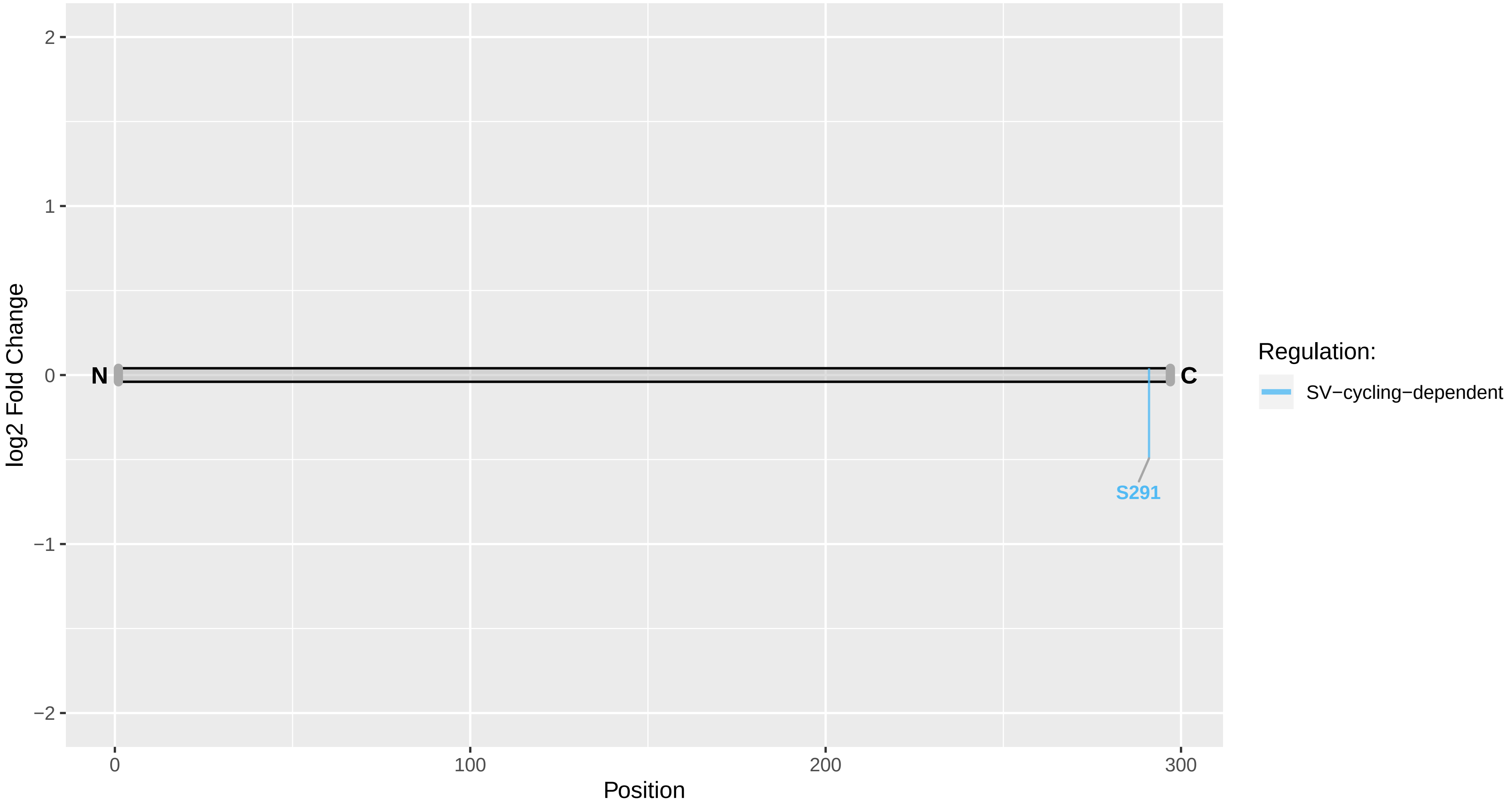

Tmem25

Uniprot Accession: D3Z9V8
RCG58455, isoform CRA_a

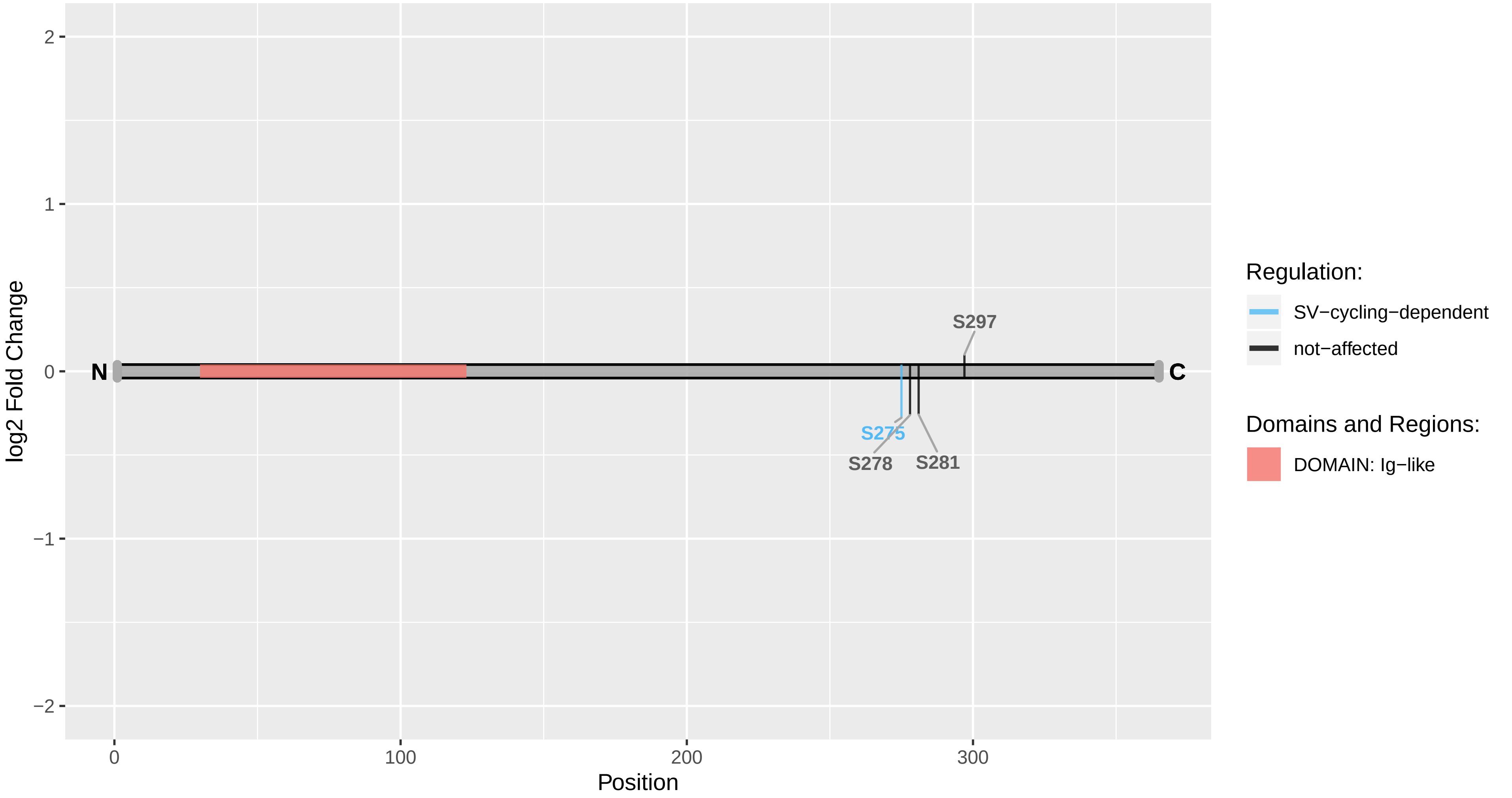

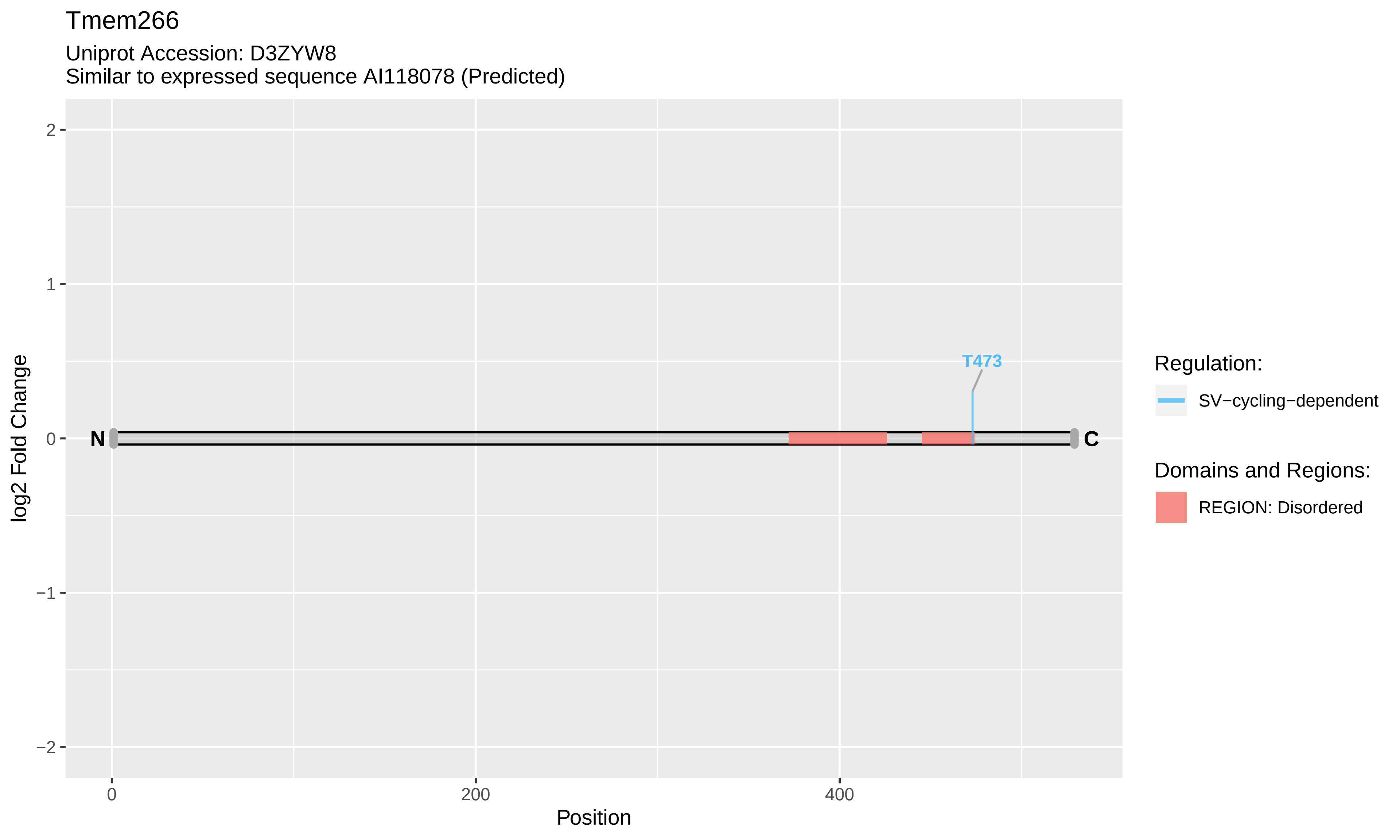
Tmem266
Uniprot Accession: D3ZYW8
Similar to expressed sequence AI118078 (Predicted)
T473
N
C
2
1
0
−1
−2
0
200
400
Position
log2 Fold Change
Regulation:
SV−cycling−dependent
Domains and Regions:
REGION: Disordered

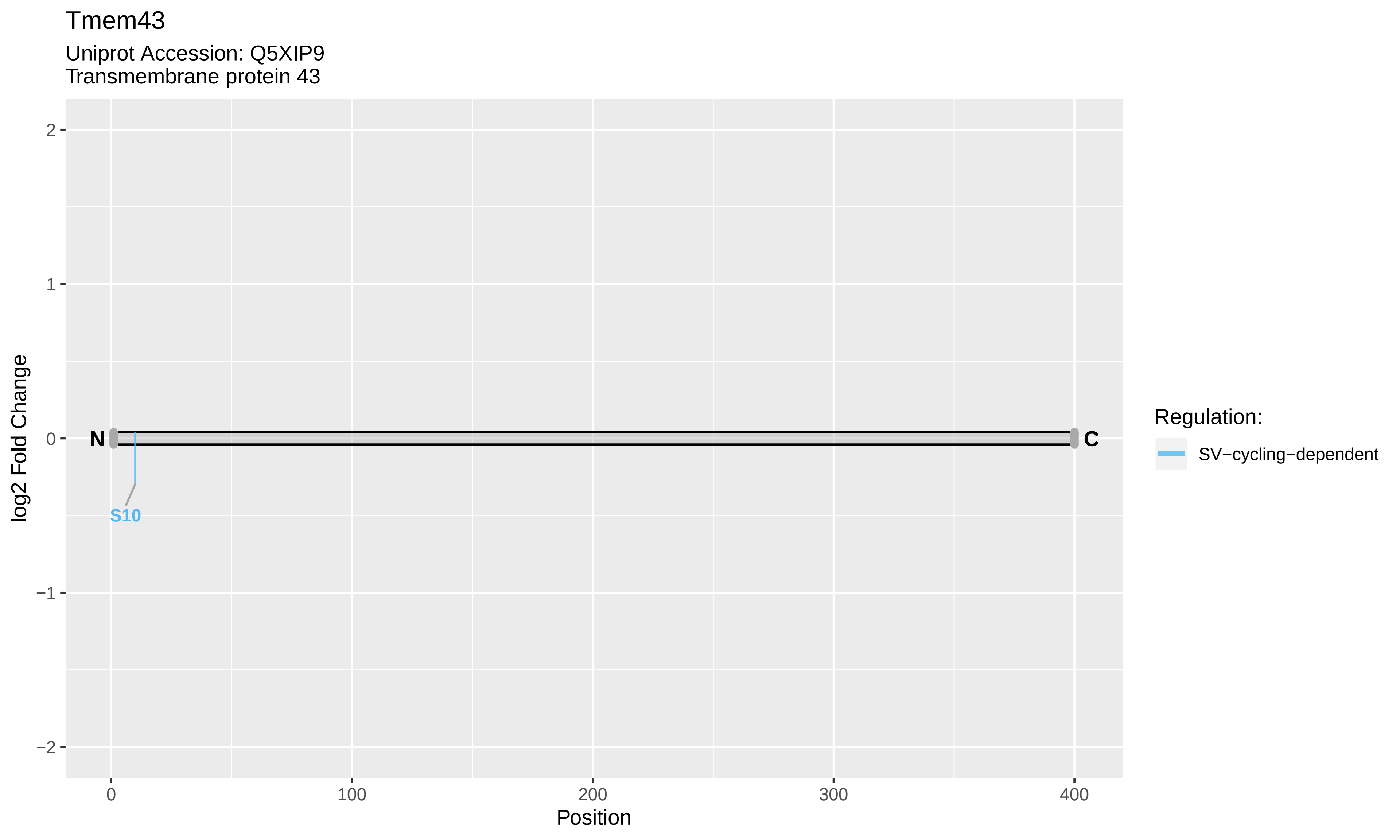
Tmem43
Uniprot Accession: Q5XIP9
Transmembrane protein 43
log2 Fold Change
2
1
0
−1
−2
N
C
S10
0
100
200
300
400
Position
Regulation:
SV−cycling−dependent

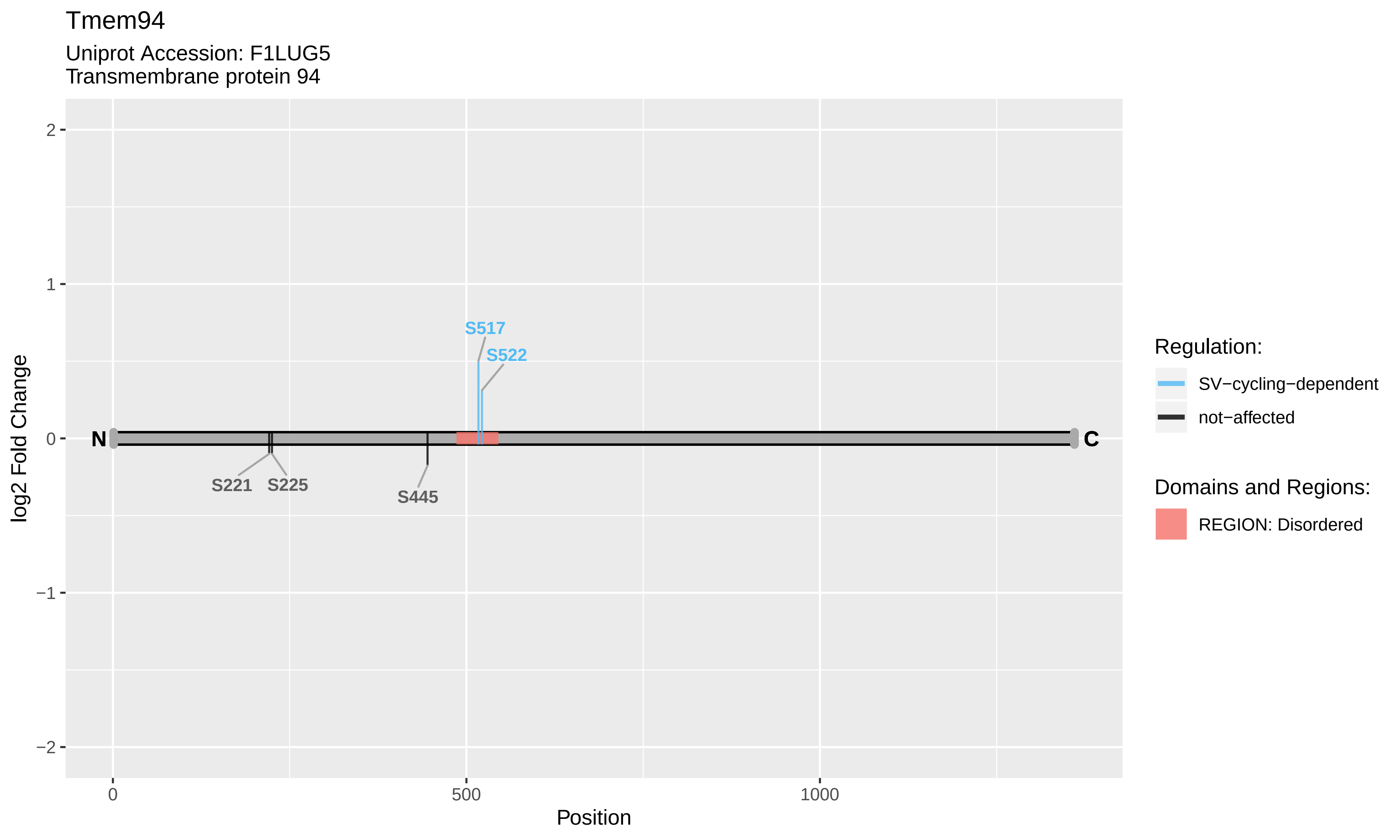

Tmem94
Uniprot Accession: F1LUG5
Transmembrane protein 94
log2 Fold Change
2
1
0
−1
−2
N
C
S517
S522
S221
S225
S445
0
500
1000
Position
Regulation:
SV−cycling−dependent
not−affected
Domains and Regions:
REGION: Disordered

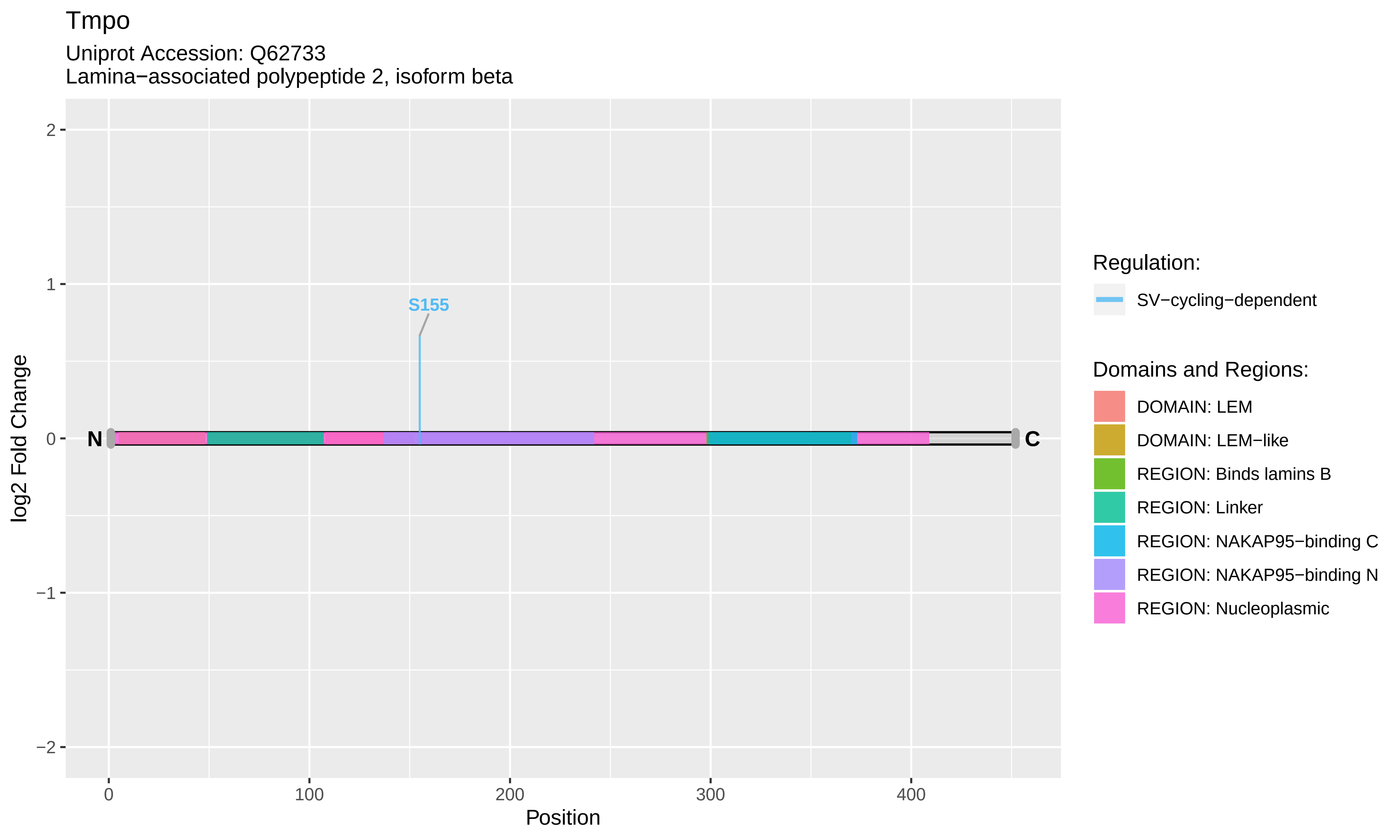
Tmpo
Uniprot Accession: Q62733
Lamina−associated polypeptide 2, isoform beta
S155
N
C
log2 Fold Change
Position
2
1
0
−1
−2
0
100
200
300
400
Regulation:
SV−cycling−dependent
Domains and Regions:
DOMAIN: LEM
DOMAIN: LEM−like
REGION: Binds lamins B
REGION: Linker
REGION: NAKAP95−binding C
REGION: NAKAP95−binding N
REGION: Nucleoplasmic

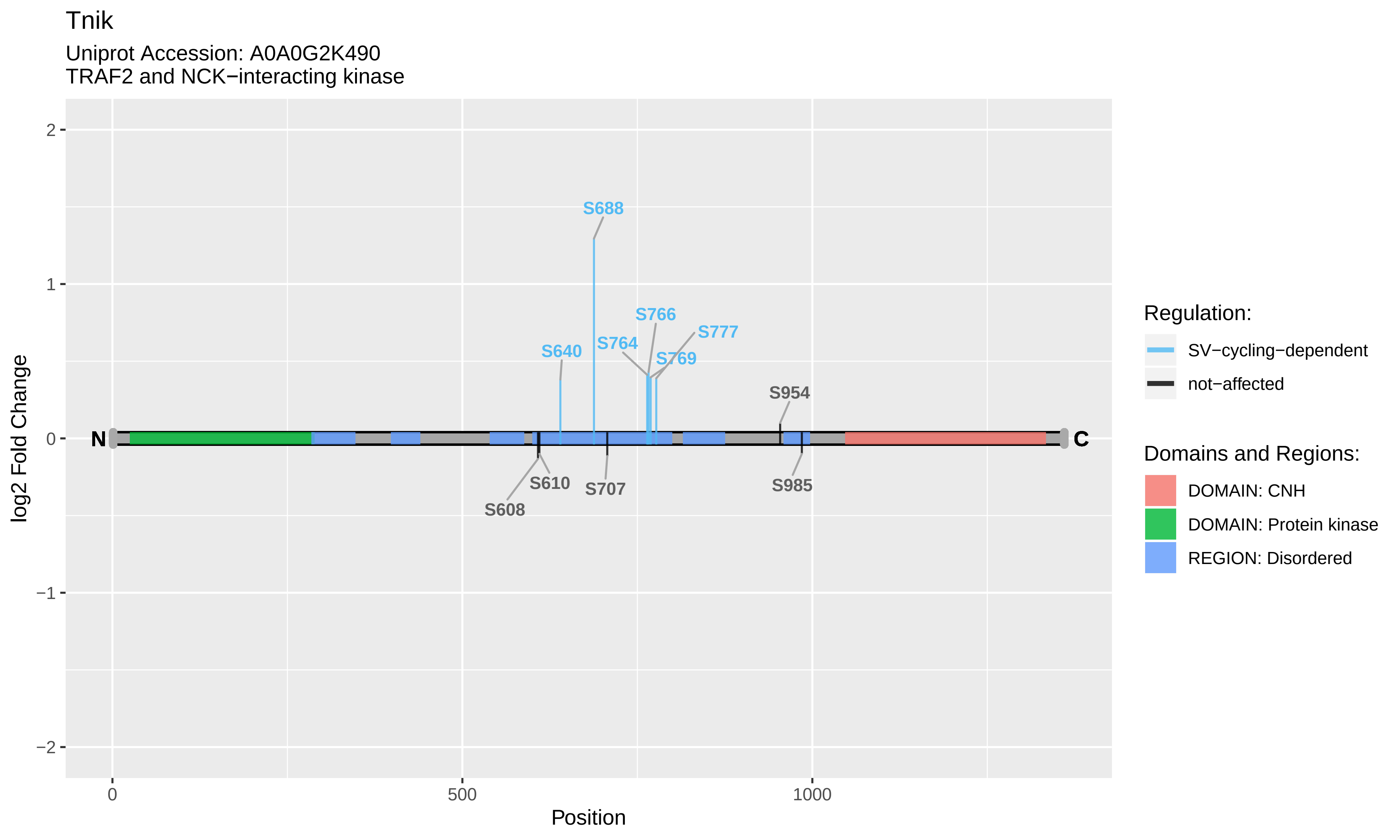

Tnik
Uniprot Accession: A0A0G2K490
TRAF2 and NCK−interacting kinase
S688
S766
S777
S764
S640
S769
S954
N
C
S610
S707
S985
S608
log2 Fold Change
2
1
0
−1
−2
0
500
1000
Position
Regulation:
SV−cycling−dependent
not−affected
Domains and Regions:
DOMAIN: CNH
DOMAIN: Protein kinase
REGION: Disordered

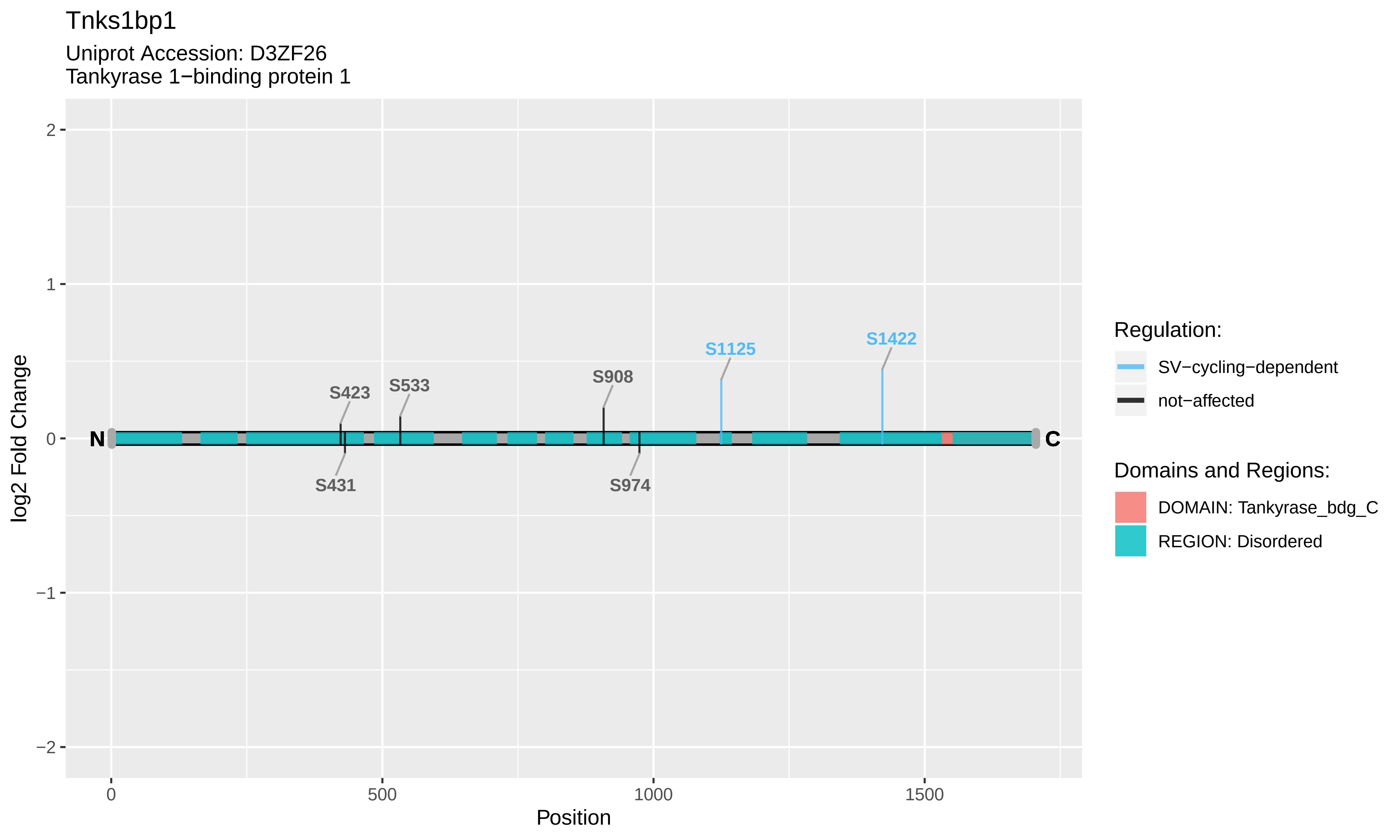

Tnks1bp1
Uniprot Accession: D3ZF26
Tankyrase 1−binding protein 1
log2 Fold Change
2
1
0
−1
−2
N
C
S423
S533
S908
S1125
S1422
S431
S974
0
500
1000
1500
Position
Regulation:
SV−cycling−dependent
not−affected
Domains and Regions:
DOMAIN: Tankyrase_bdg_C
REGION: Disordered

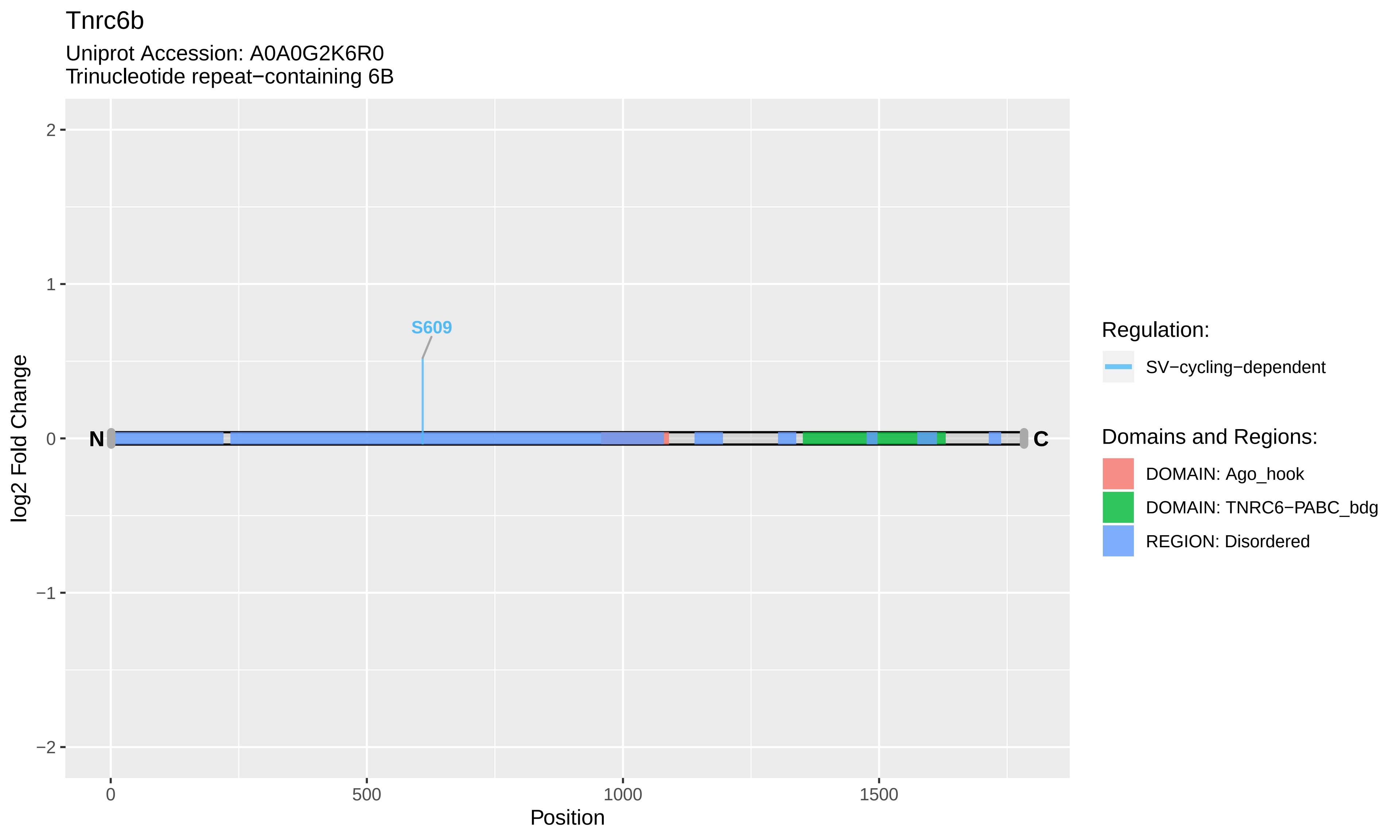

Tnrc6b
Uniprot Accession: A0A0G2K6R0
Trinucleotide repeat−containing 6B
S609
N
C
2
1
0
−1
−2
log2 Fold Change
0
500
1000
1500
Position
Regulation:
SV−cycling−dependent
Domains and Regions:
DOMAIN: Ago_hook
DOMAIN: TNRC6−PABC_bdg
REGION: Disordered

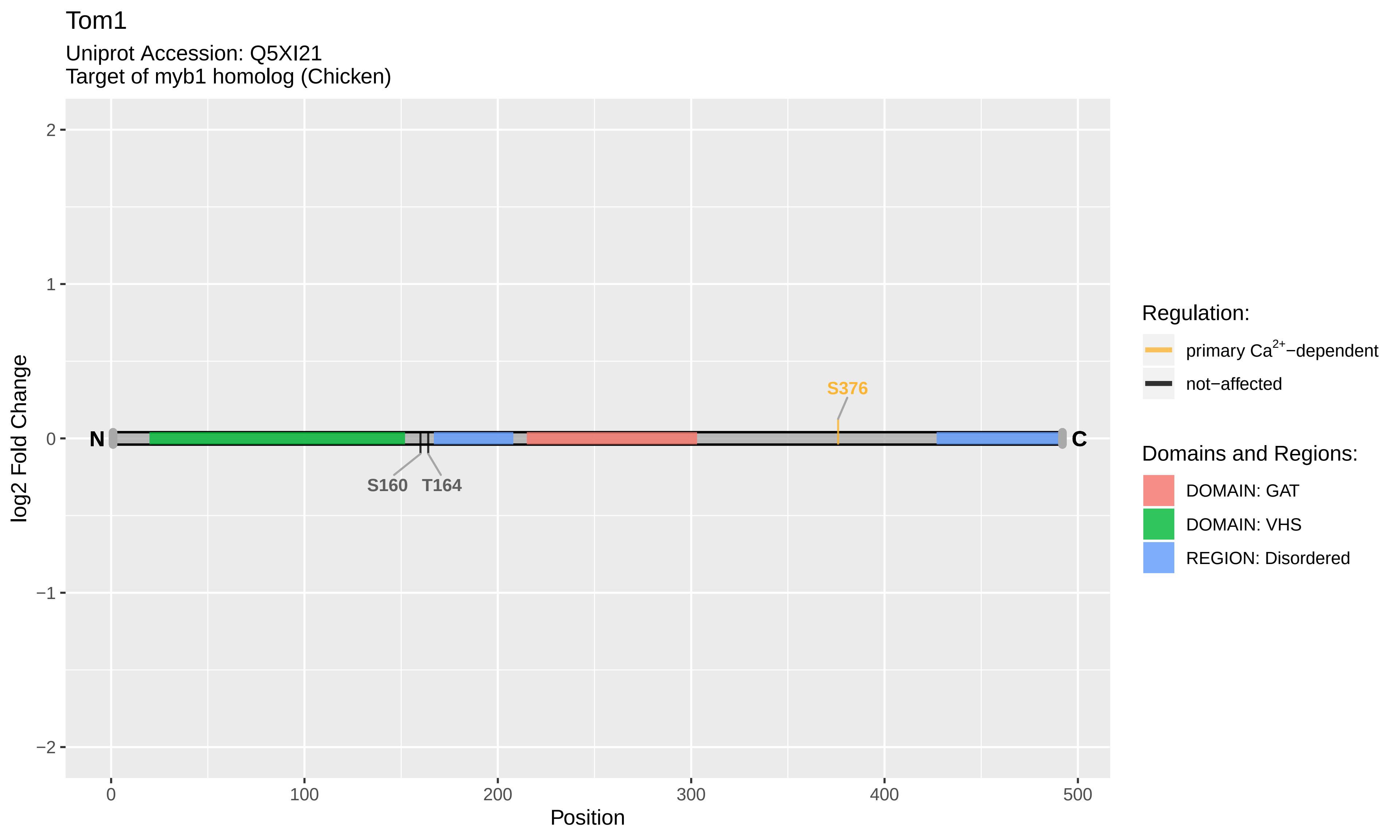
Tom1
Uniprot Accession: Q5XI21
Target of myb1 homolog (Chicken)
log2 Fold Change
2
1
0
−1
−2
N
C
S376
S160
T164
0
100
200
300
400
500
Position
Regulation:
primary Ca2+−dependent
not−affected
Domains and Regions:
DOMAIN: GAT
DOMAIN: VHS
REGION: Disordered

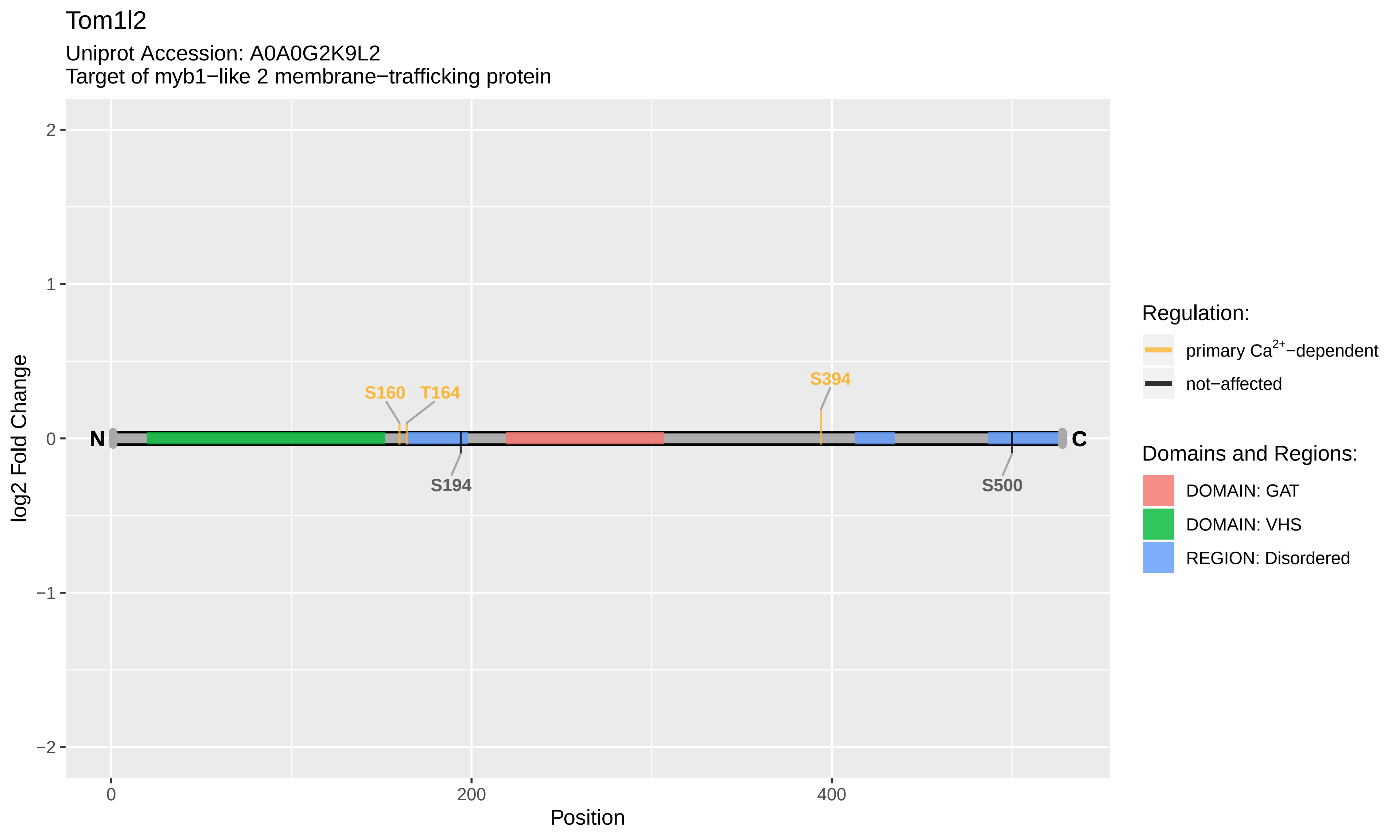
Tom1l2
Uniprot Accession: A0A0G2K9L2
Target of myb1−like 2 membrane−trafficking protein
log2 Fold Change
2
1
0
−1
−2
N
C
S160
T164
S394
S194
S500
0
200
400
Position
Regulation:
primary Ca2+−dependent
not−affected
Domains and Regions:
DOMAIN: GAT
DOMAIN: VHS
REGION: Disordered

Tomm70

Uniprot Accession: Q75Q39
Mitochondrial import receptor subunit TOM70

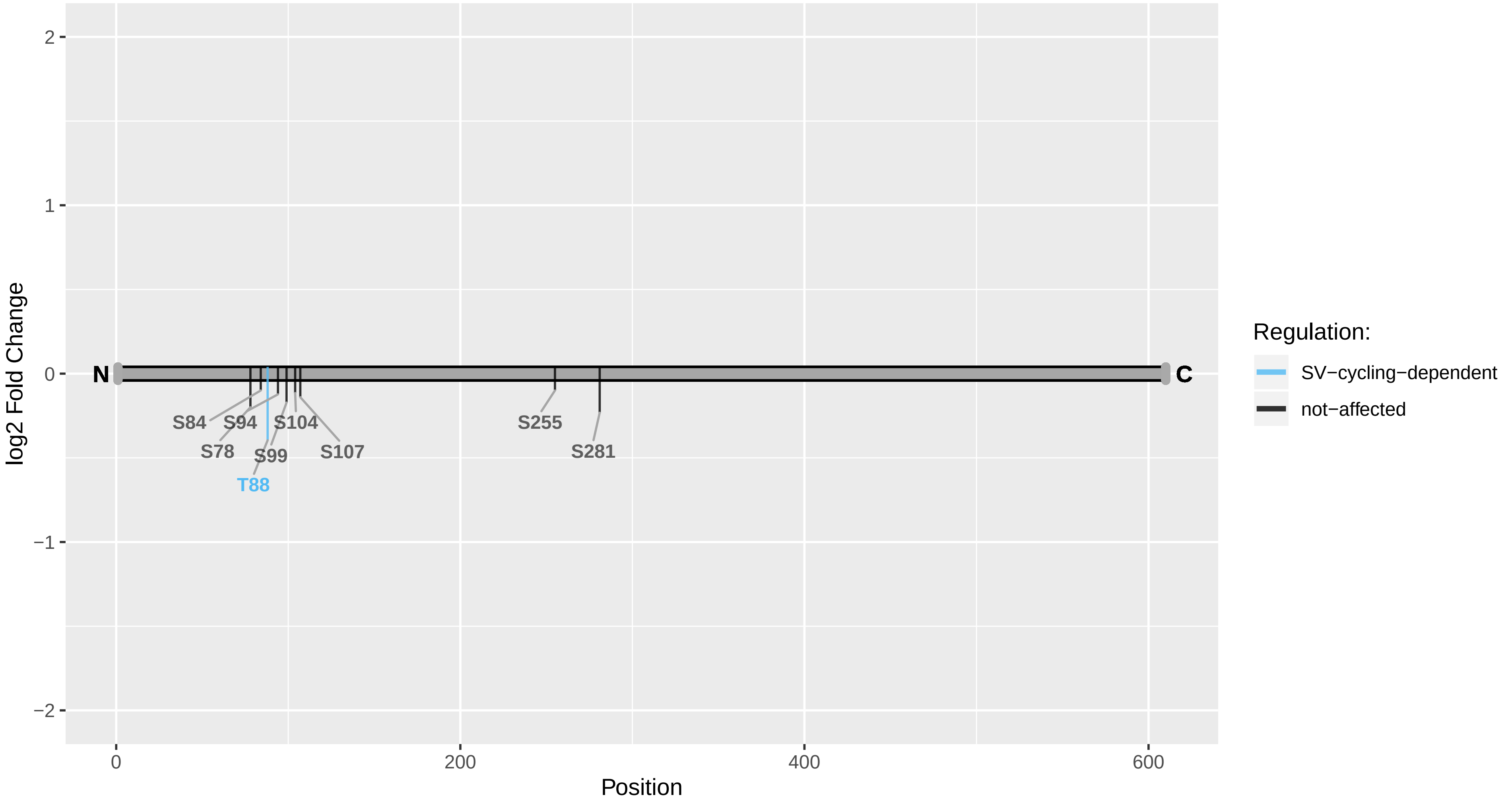

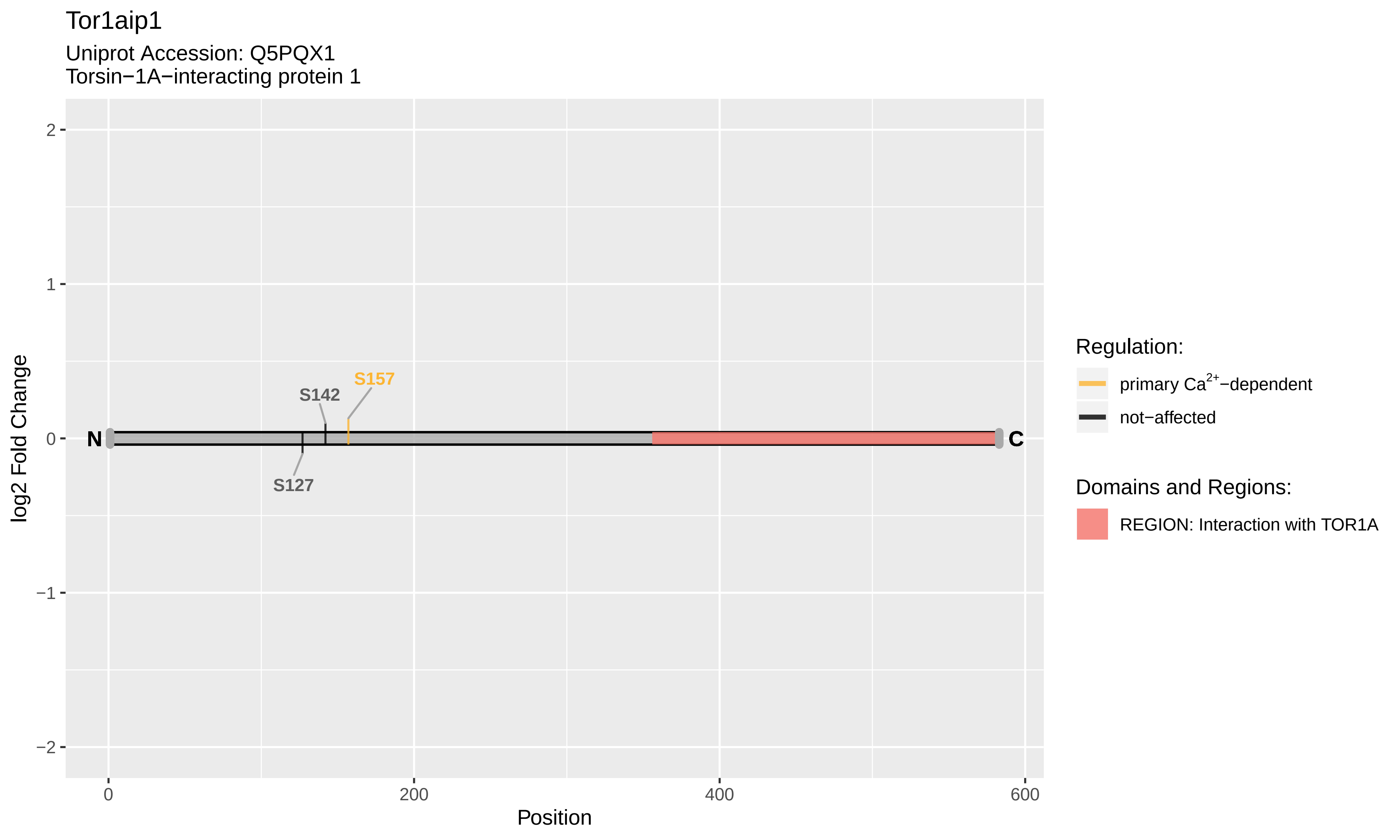
Tor1aip1
Uniprot Accession: Q5PQX1
Torsin−1A−interacting protein 1
2
1
0
−1
−2
log2 Fold Change
N
C
S157
S142
S127
0
200
400
600
Position
Regulation:
primary Ca2+−dependent
not−affected
Domains and Regions:
REGION: Interaction with TOR1A

Tp53i11

Uniprot Accession: B3DMA0
Tumor protein p53−inducible protein 11

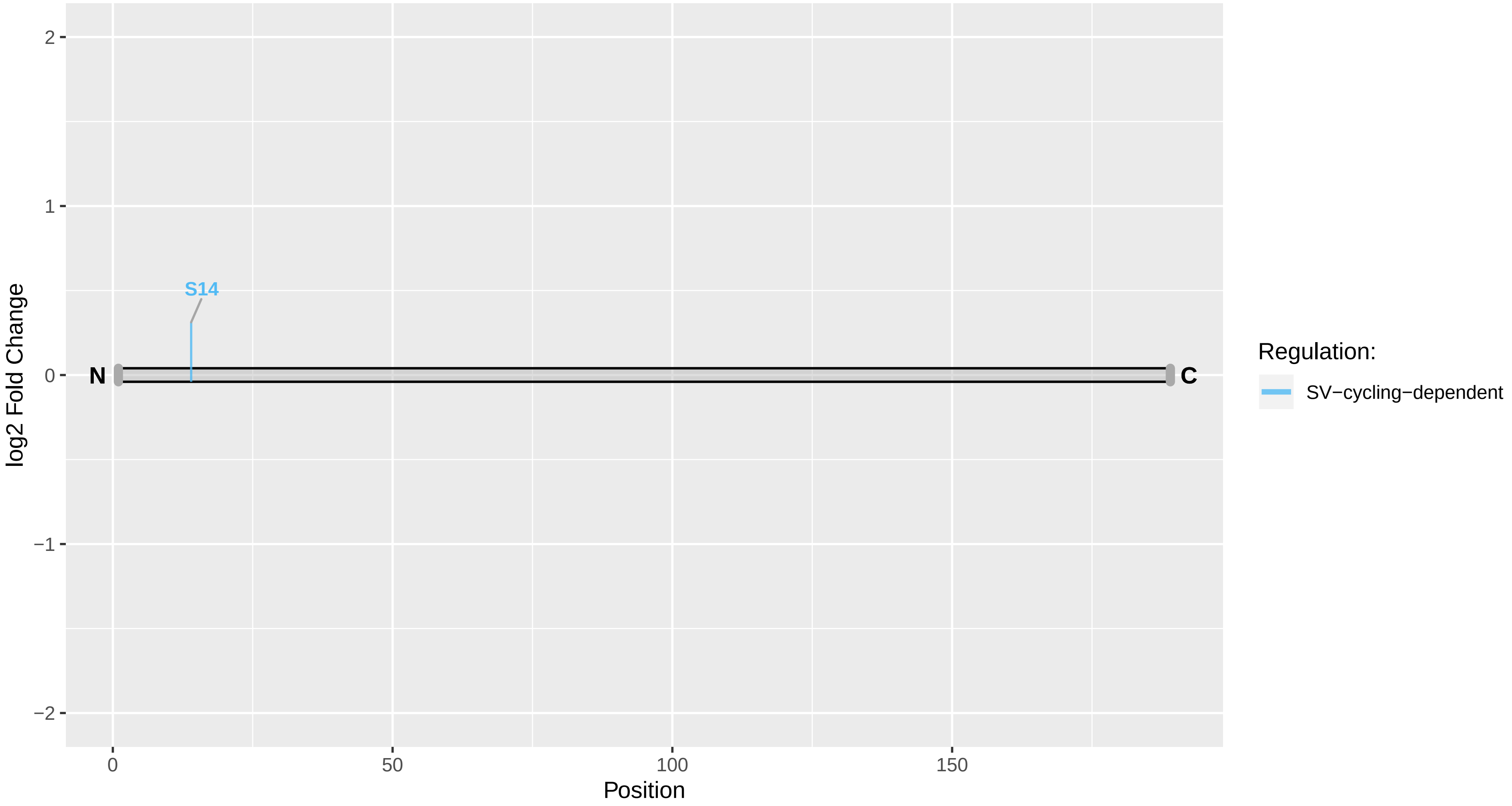

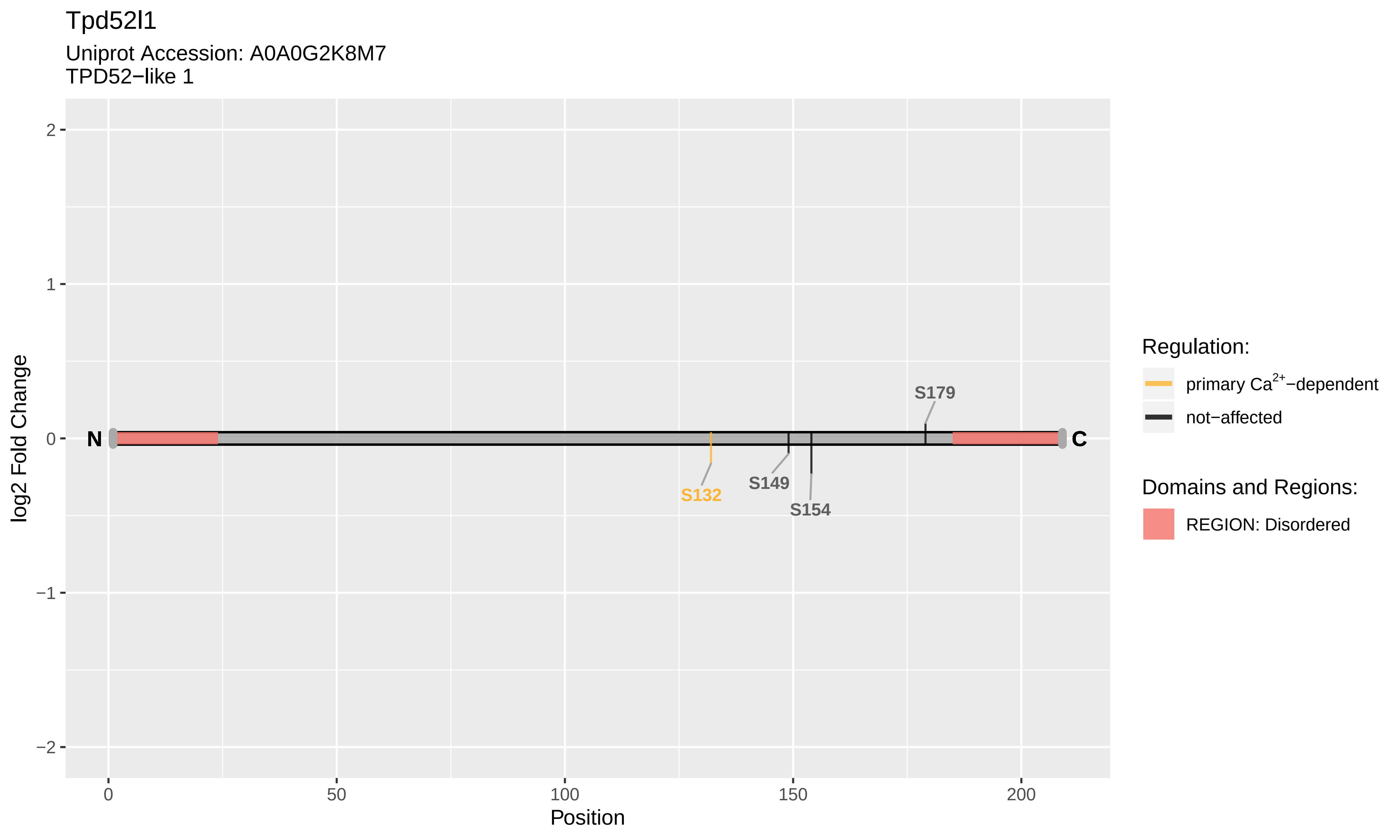

Tpd52l1
Uniprot Accession: A0A0G2K8M7
TPD52−like 1
log2 Fold Change
Position
N
C
S179
S132
S149
S154
Regulation:
primary Ca2+−dependent
not−affected
Domains and Regions:
REGION: Disordered

Tpd52l2

Uniprot Accession: Q6PCT3
Tumor protein D54

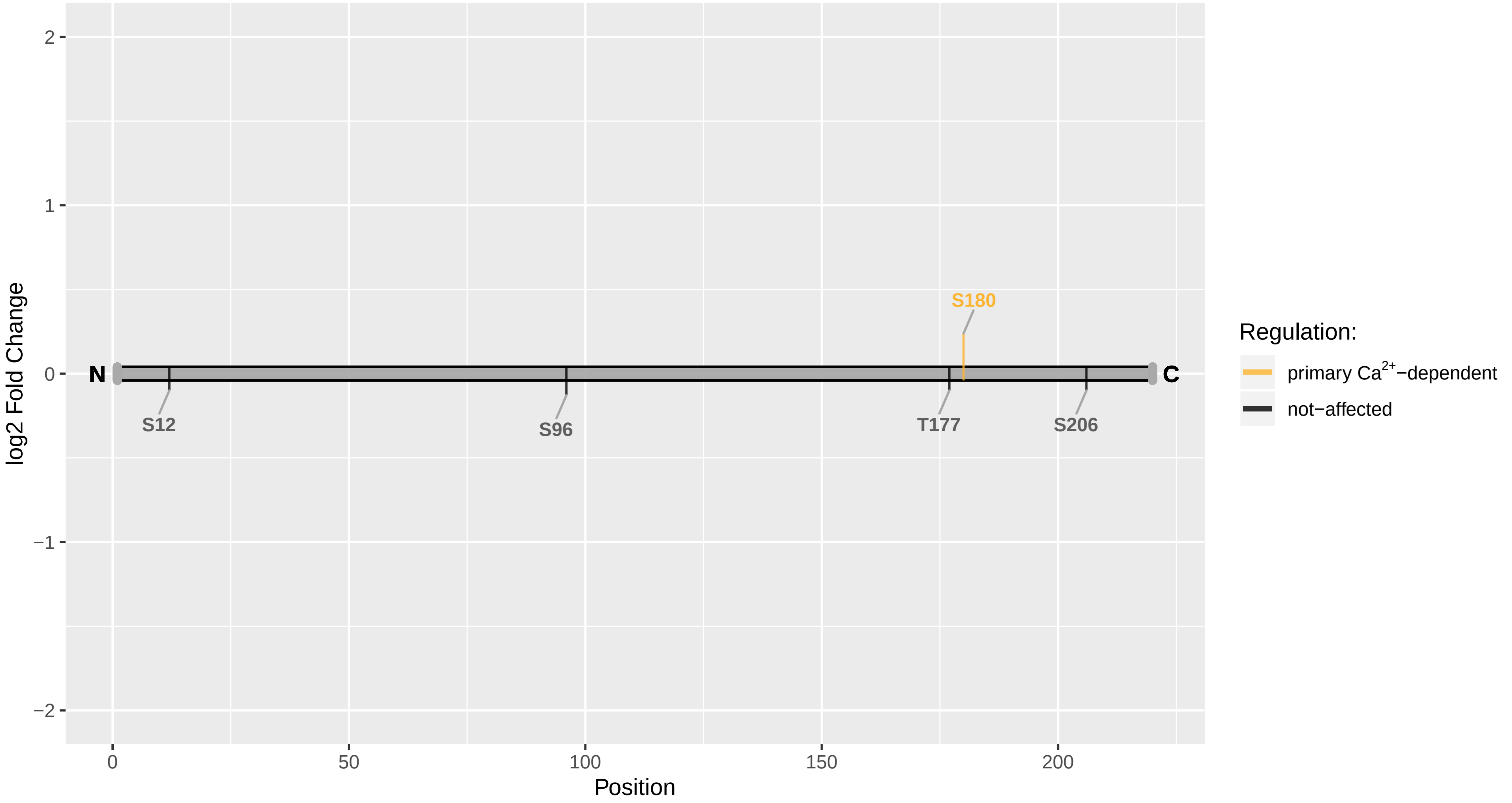

Tppp

Uniprot Accession: A0A0G2K2D6
Tubulin polymerization−promoting protein

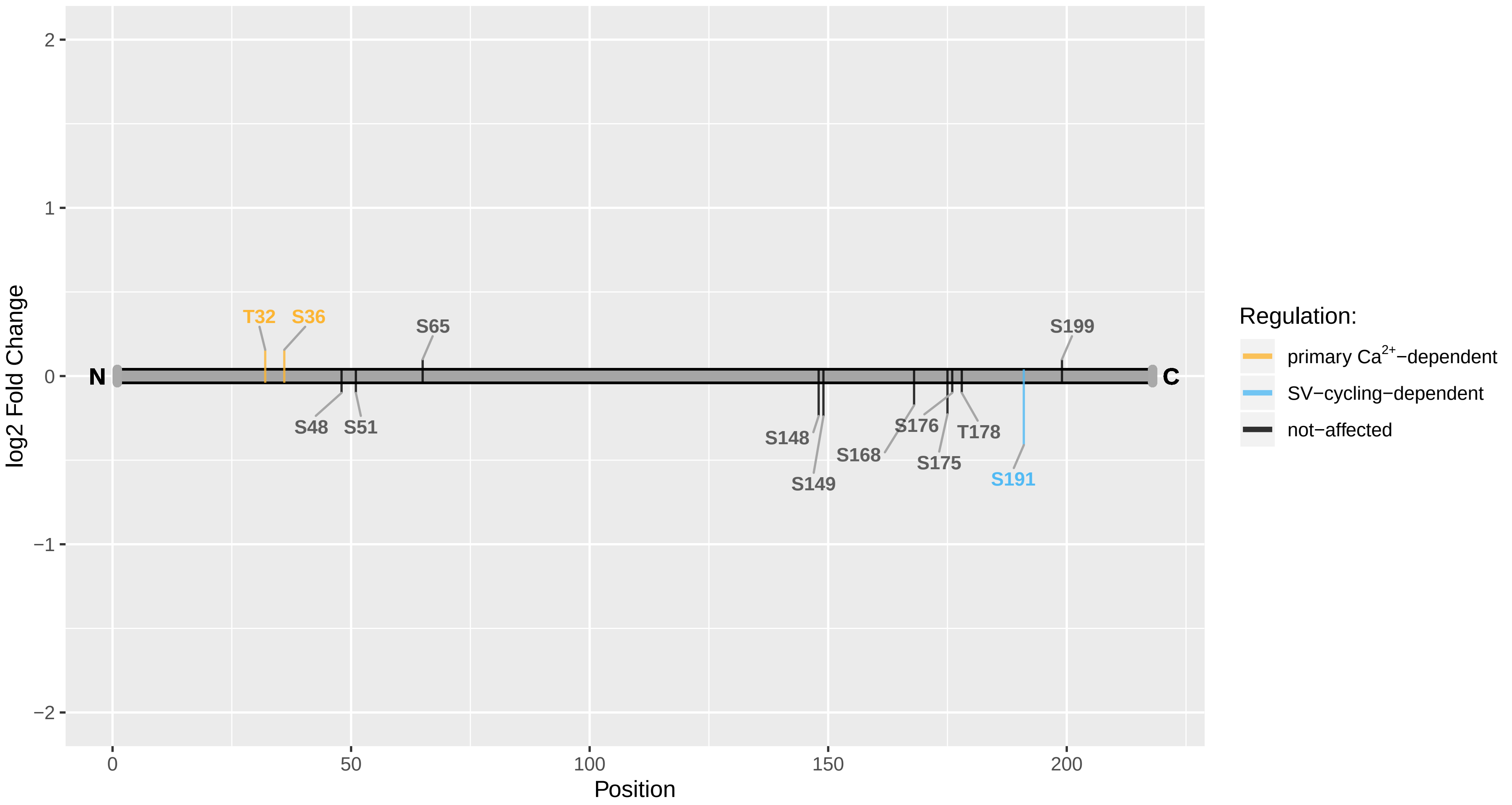

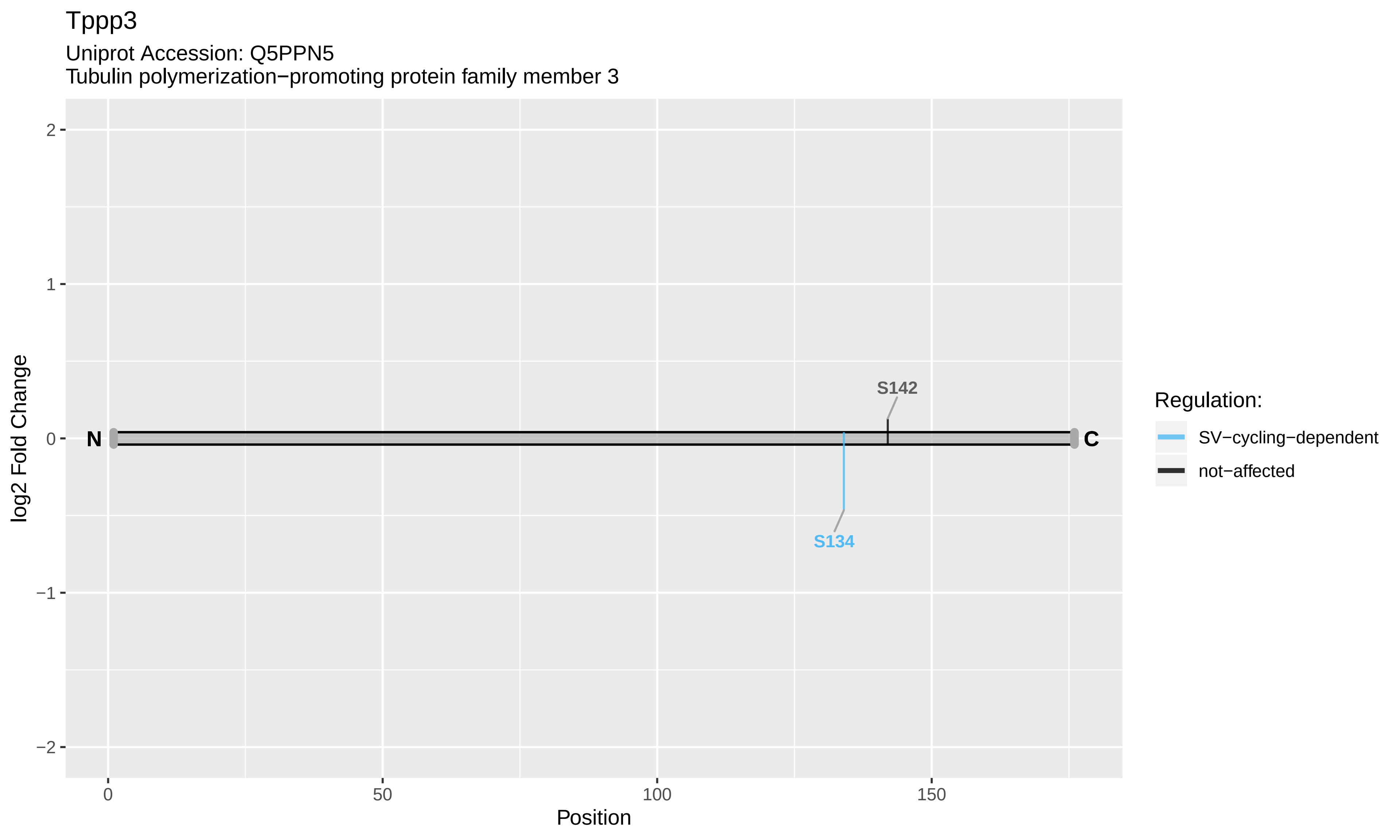

Tppp3
Uniprot Accession: Q5PPN5
Tubulin polymerization−promoting protein family member 3
log2 Fold Change
2
1
0
−1
−2
N
C
S142
S134
0
50
100
150
Position
Regulation:
SV−cycling−dependent
not−affected

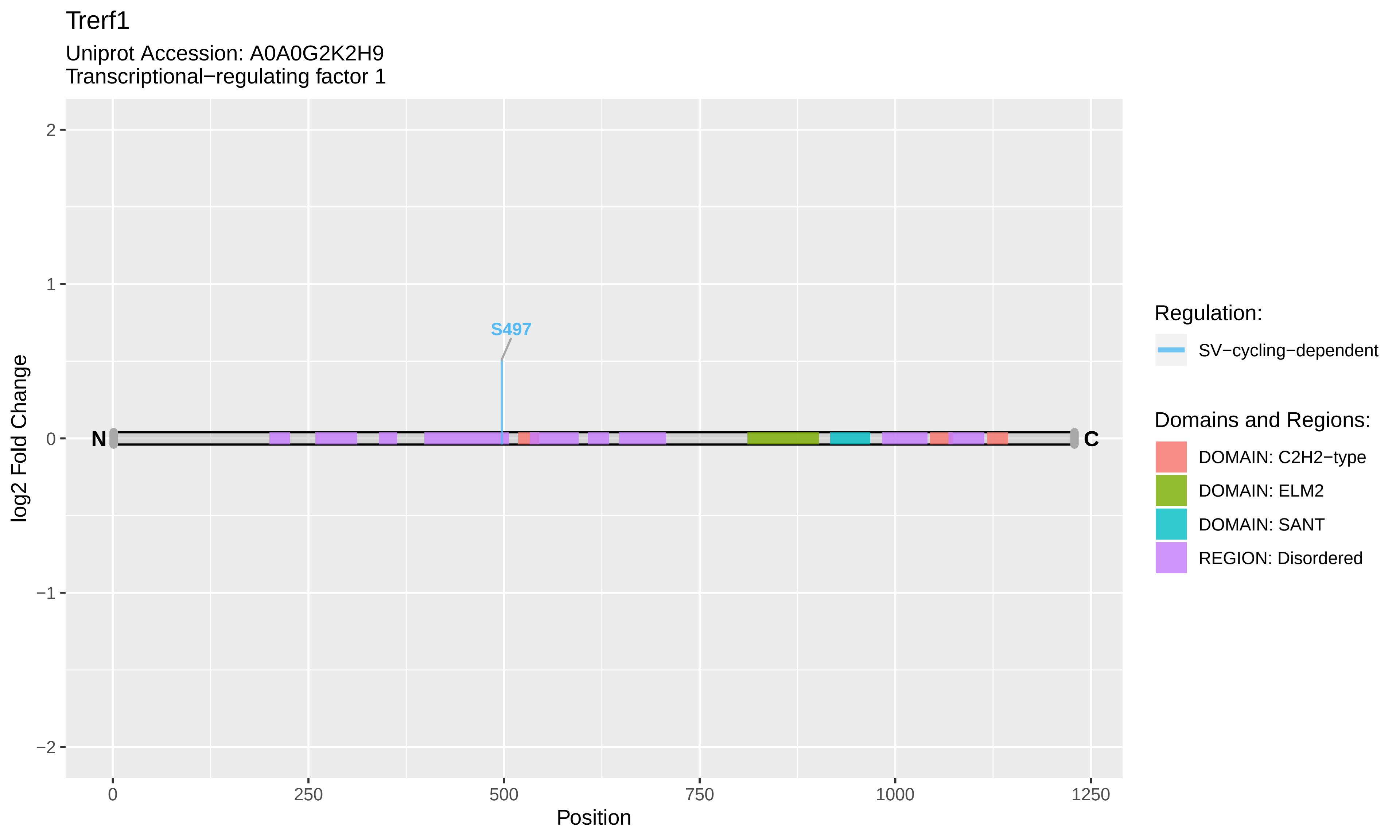
Trerf1
Uniprot Accession: A0A0G2K2H9
Transcriptional−regulating factor 1
S497
N
C
2
1
0
−1
−2
log2 Fold Change
0
250
500
750
1000
1250
Position
Regulation:
SV−cycling−dependent
Domains and Regions:
DOMAIN: C2H2−type
DOMAIN: ELM2
DOMAIN: SANT
REGION: Disordered

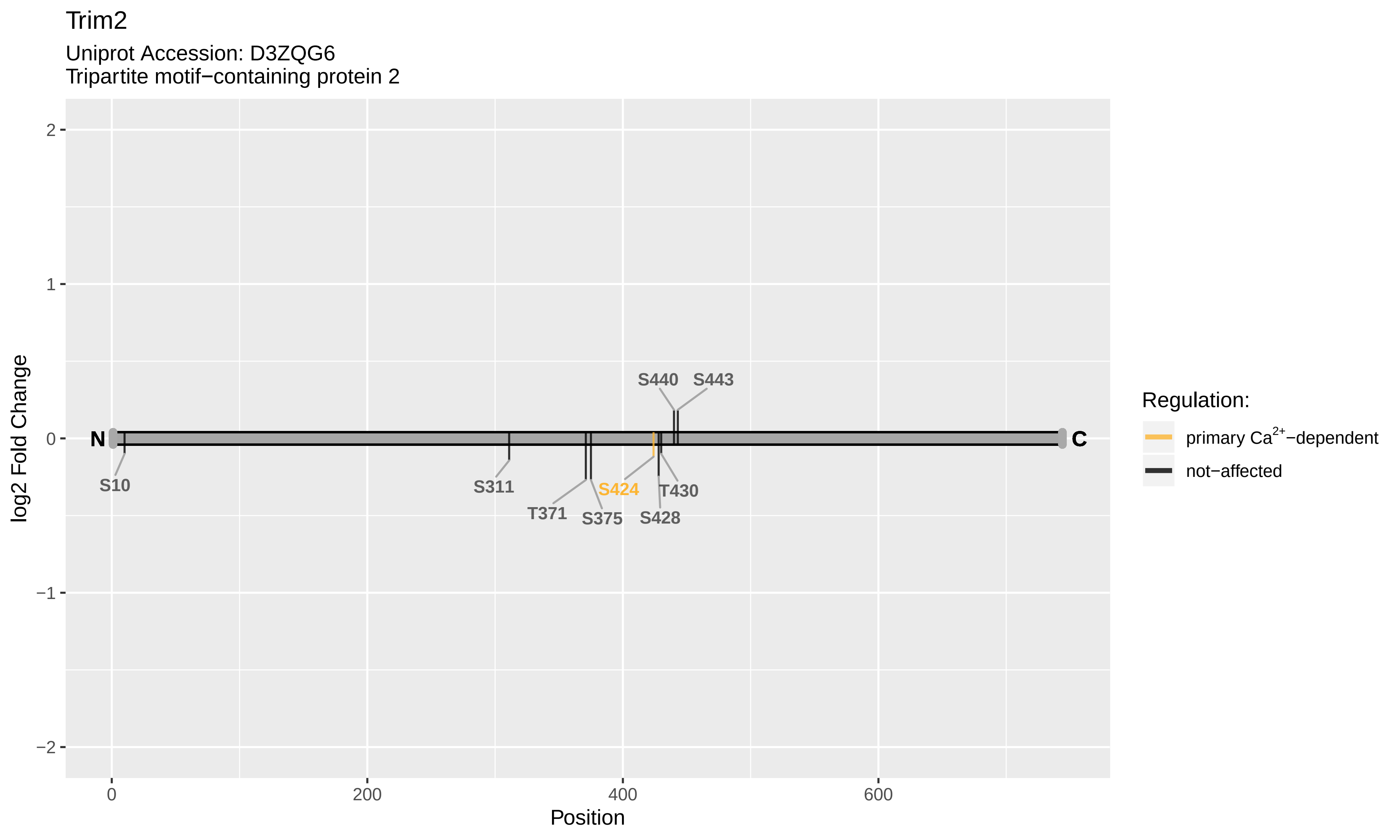

Trim2
Uniprot Accession: D3ZQG6
Tripartite motif−containing protein 2
log2 Fold Change
2
1
0
−1
−2
N
C
S10
S311
T371
S375
S424
S428
T430
S440
S443
0
200
400
600
Position
Regulation:
primary Ca2+−dependent
not−affected

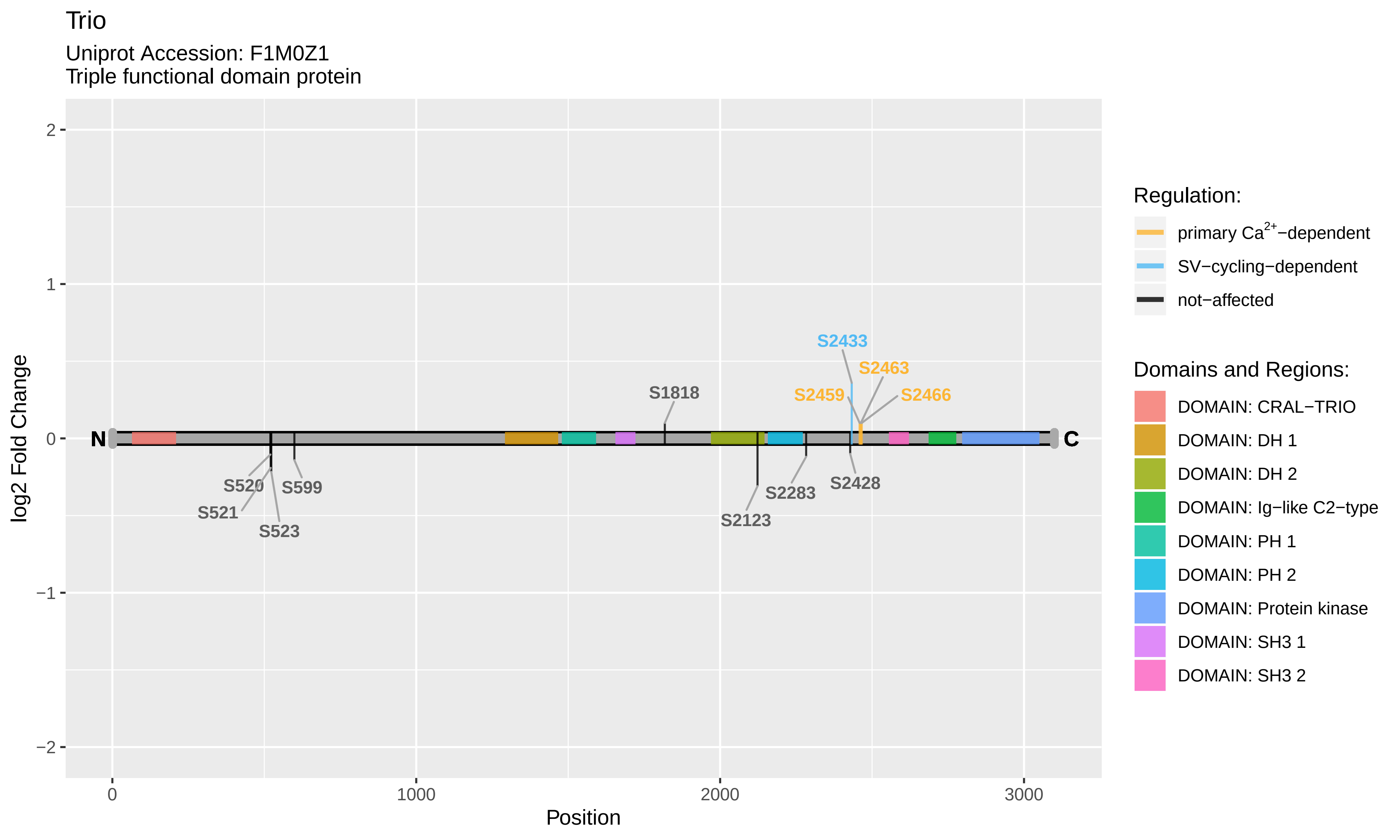

Trio
Uniprot Accession: F1M0Z1
Triple functional domain protein
log2 Fold Change
Position
N
C
S520
S521
S523
S599
S1818
S2123
S2283
S2428
S2433
S2459
S2463
S2466
Regulation:
primary Ca2+−dependent
SV−cycling−dependent
not−affected
Domains and Regions:
DOMAIN: CRAL−TRIO
DOMAIN: DH 1
DOMAIN: DH 2
DOMAIN: Ig−like C2−type
DOMAIN: PH 1
DOMAIN: PH 2
DOMAIN: Protein kinase
DOMAIN: SH3 1
DOMAIN: SH3 2

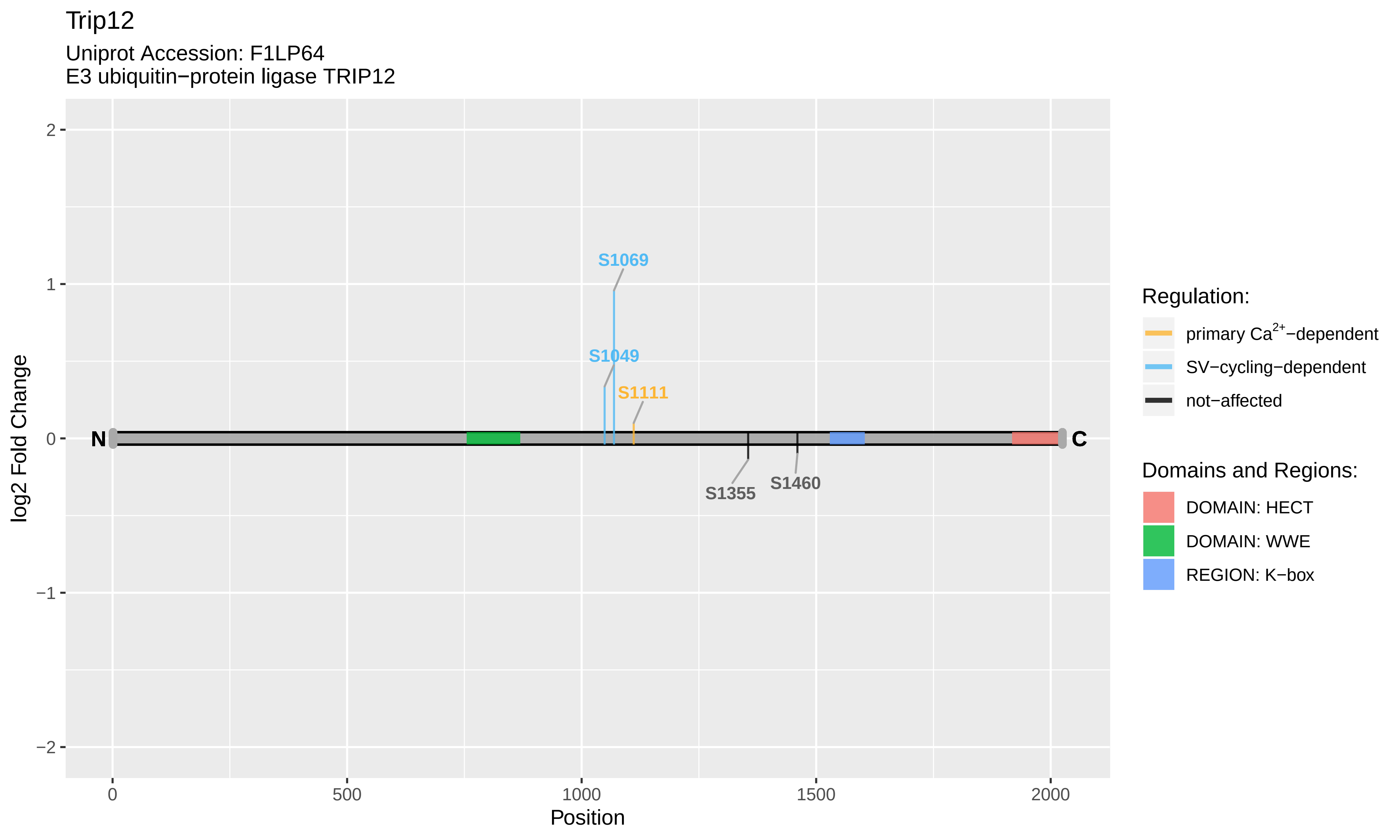
Trip12
Uniprot Accession: F1LP64
E3 ubiquitin−protein ligase TRIP12
log2 Fold Change
2
1
0
−1
−2
N
C
S1069
S1049
S1111
S1355
S1460
0
500
1000
1500
2000
Position
Regulation:
primary Ca2+−dependent
SV−cycling−dependent
not−affected
Domains and Regions:
DOMAIN: HECT
DOMAIN: WWE
REGION: K−box

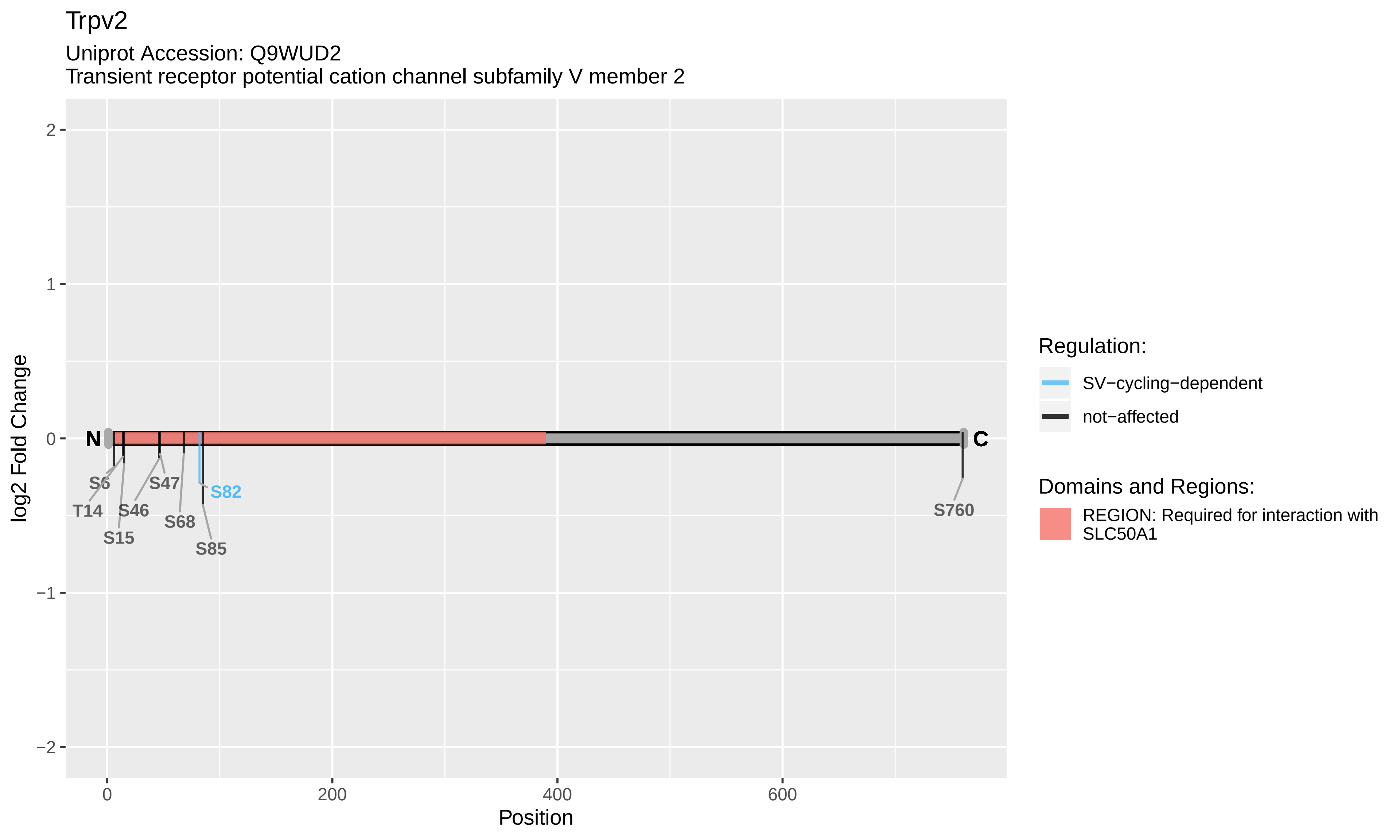

Trpv2
Uniprot Accession: Q9WUD2
Transient receptor potential cation channel subfamily V member 2
log2 Fold Change
Position
N
C
S6
T14
S15
S46
S47
S68
S82
S85
S760
Regulation:
SV−cycling−dependent
not−affected
Domains and Regions:
REGION: Required for interaction with SLC50A1

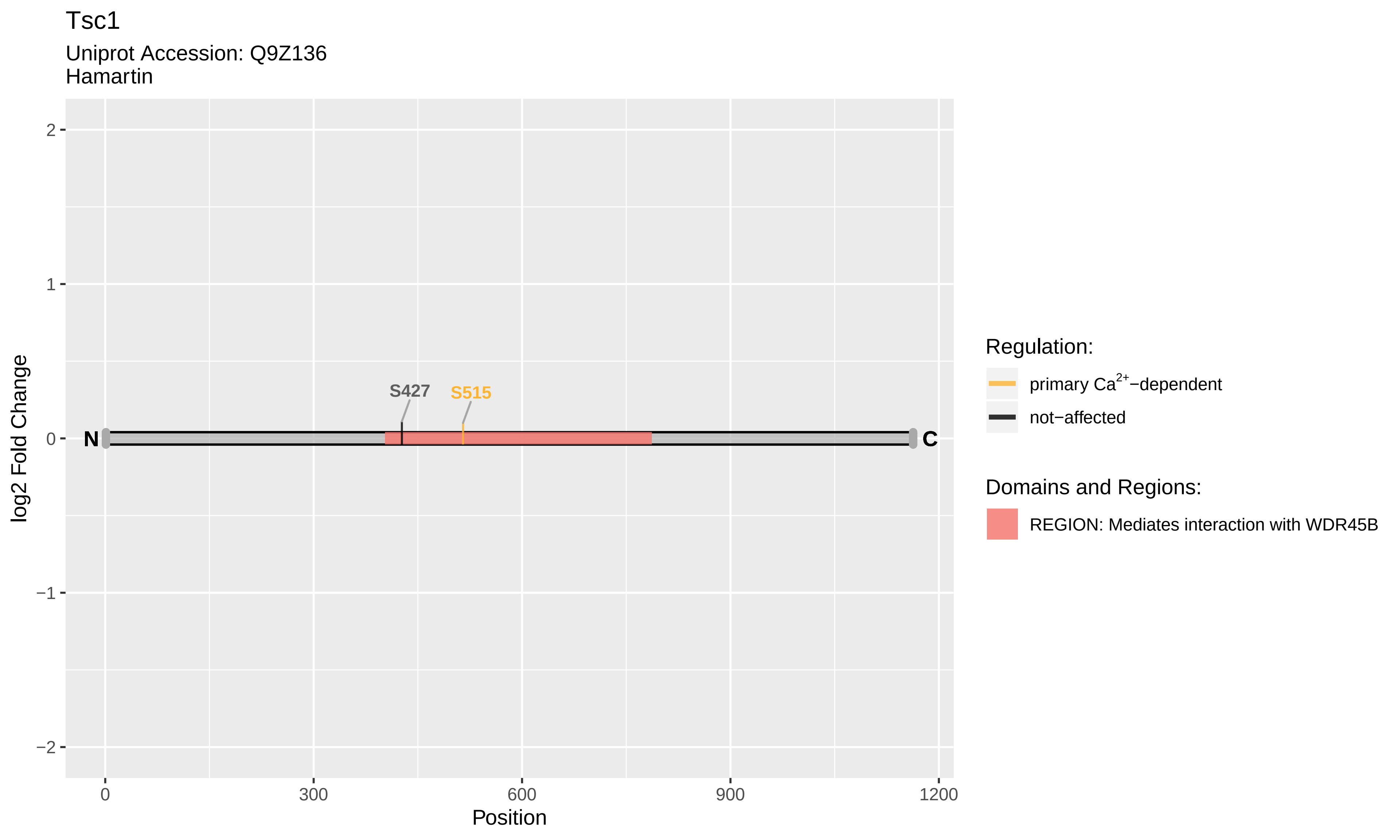

Tsc1
Uniprot Accession: Q9Z136
Hamartin
log2 Fold Change
2
1
0
−1
−2
0
300
600
900
1200
Position
N
C
S427
S515
Regulation:
primary Ca2+−dependent
not−affected
Domains and Regions:
REGION: Mediates interaction with WDR45B

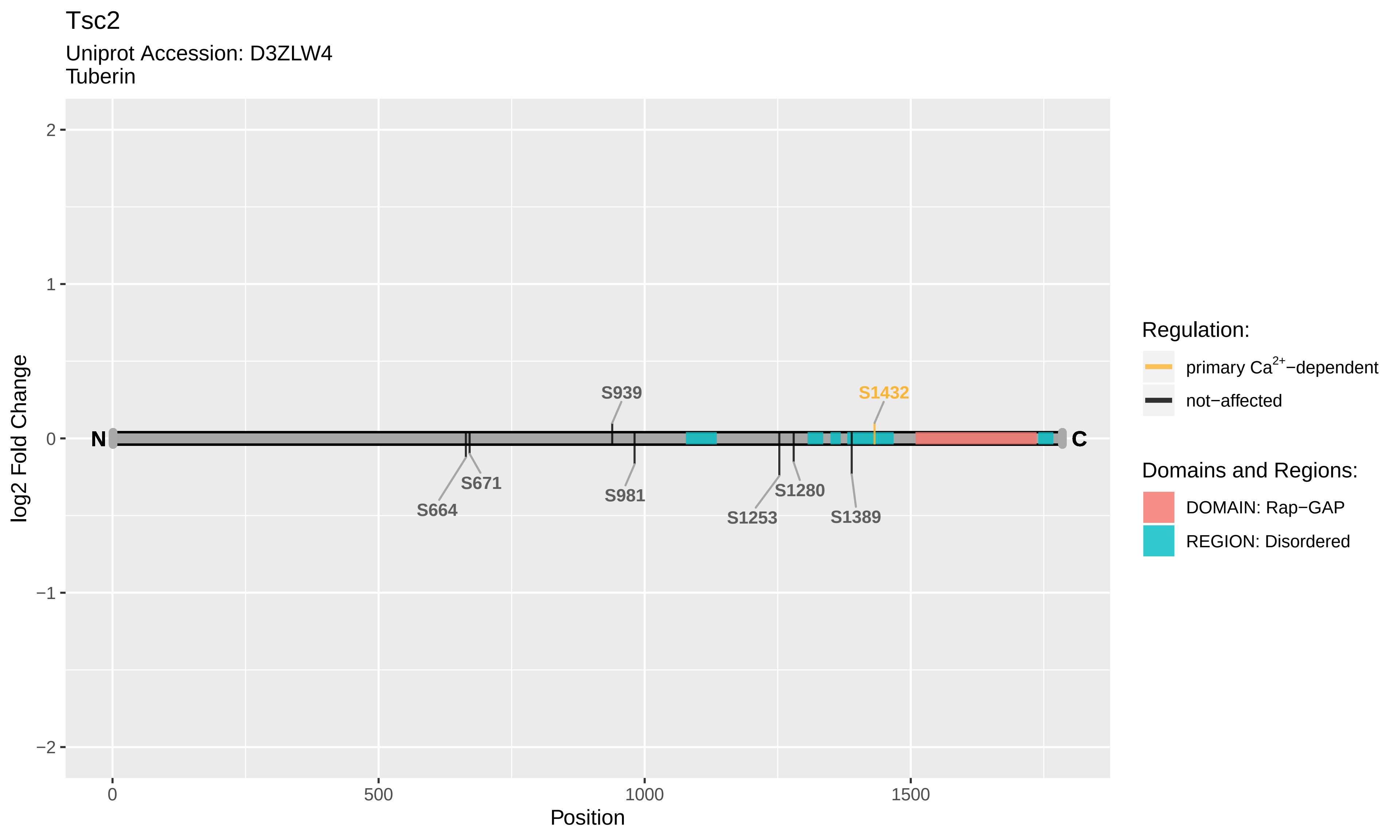

Tsc2
Uniprot Accession: D3ZLW4
Tuberin
log2 Fold Change
Position
N
C
S939
S1432
S664
S671
S981
S1253
S1280
S1389
Regulation:
primary Ca2+−dependent
not−affected
Domains and Regions:
DOMAIN: Rap−GAP
REGION: Disordered

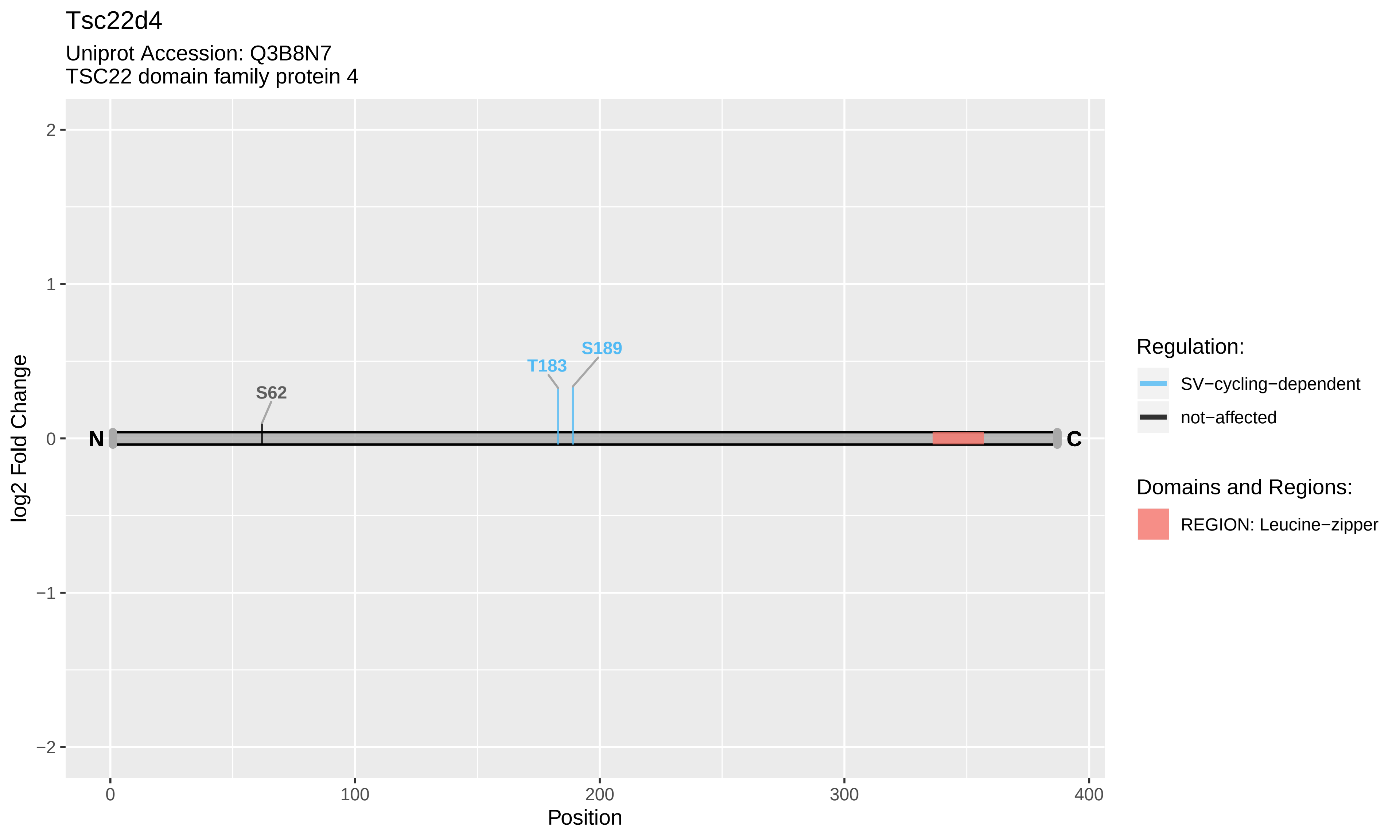
Tsc22d4
Uniprot Accession: Q3B8N7
TSC22 domain family protein 4
log2 Fold Change
2
1
0
−1
−2
N
C
S62
T183
S189
0
100
200
300
400
Position
Regulation:
SV−cycling−dependent
not−affected
Domains and Regions:
REGION: Leucine−zipper

Ttbk1

Uniprot Accession: D3ZAU7
Tau tubulin kinase 1

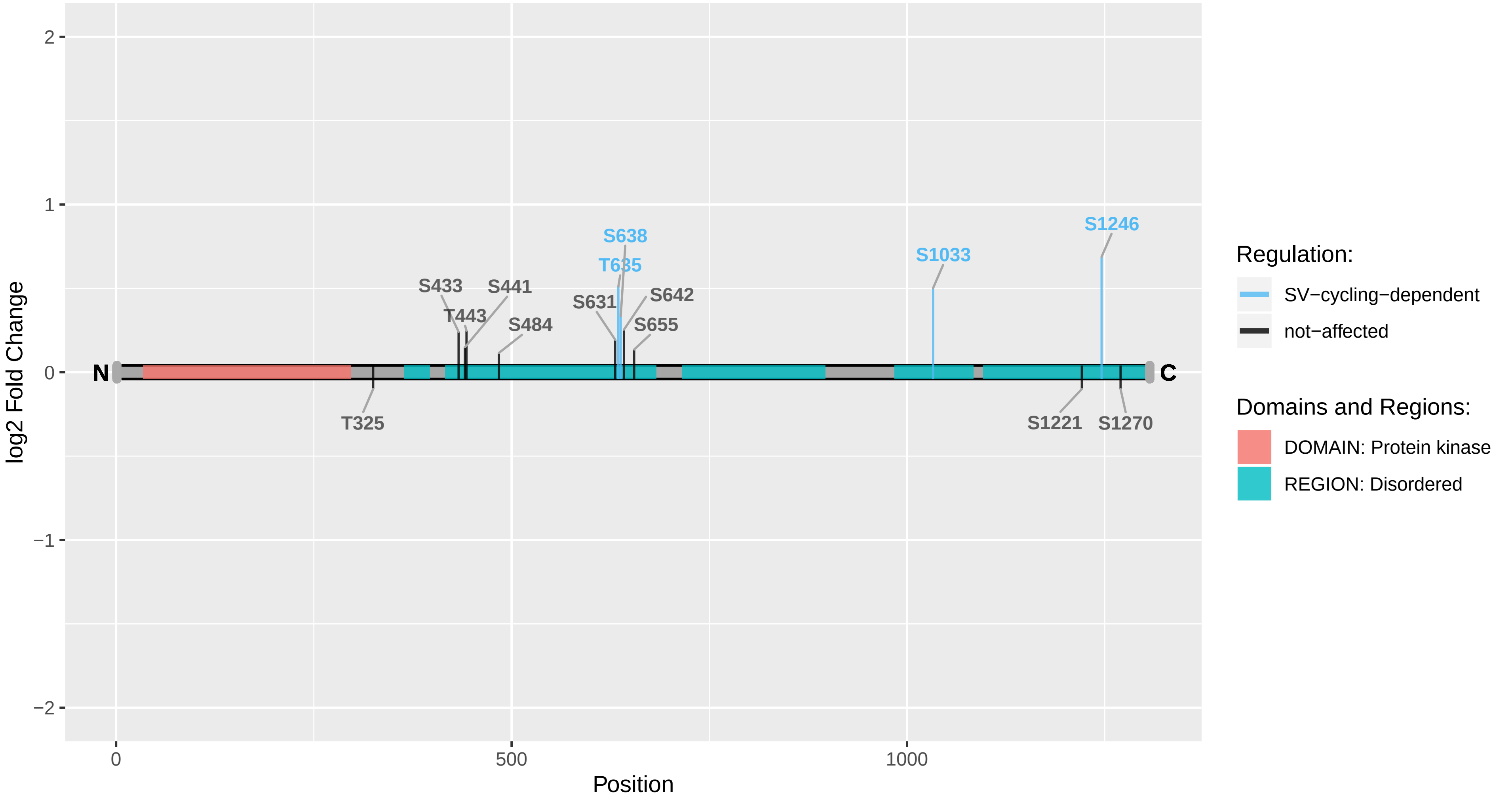

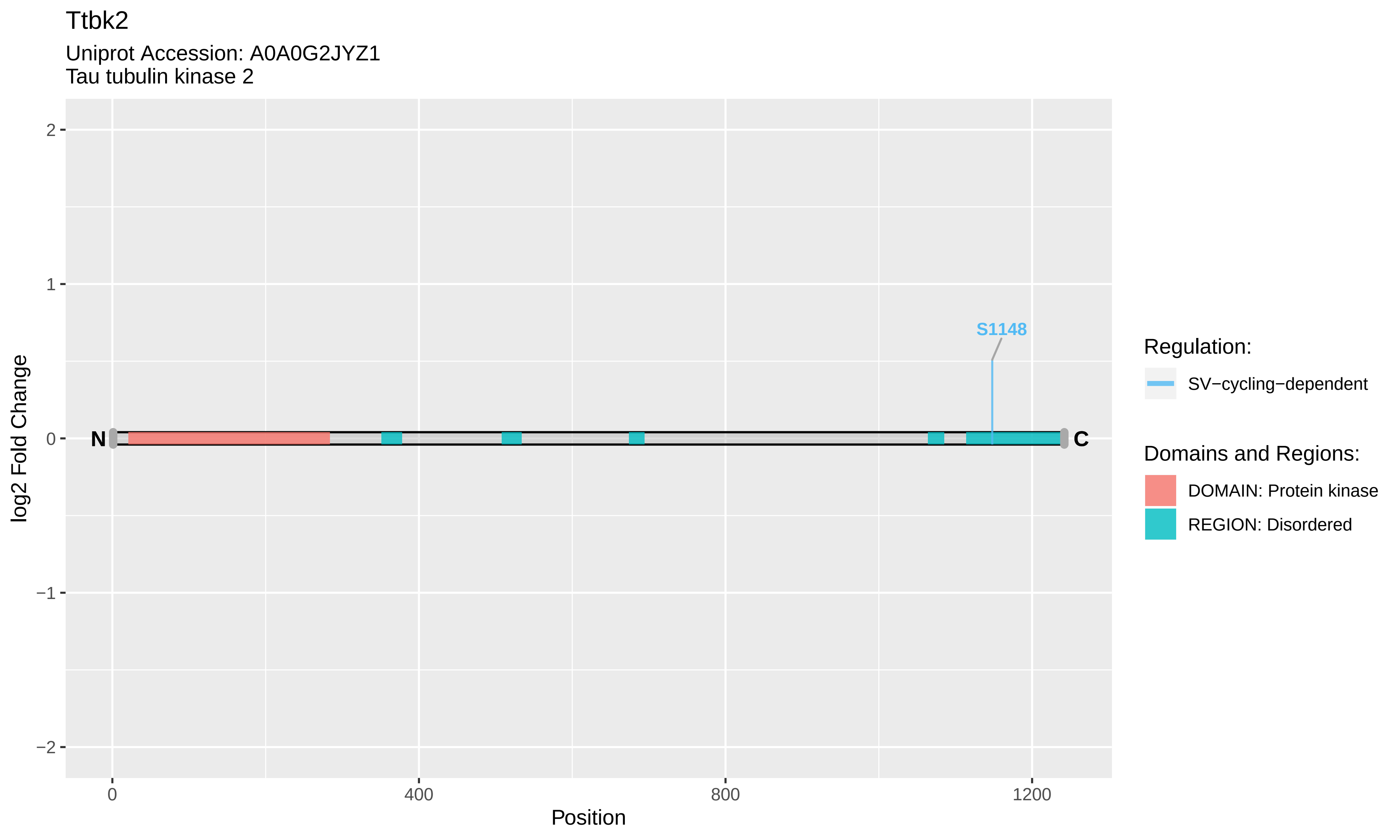
Ttbk2
Uniprot Accession: A0A0G2JYZ1
Tau tubulin kinase 2
S1148
N
C
log2 Fold Change
Position
Regulation:
SV−cycling−dependent
Domains and Regions:
DOMAIN: Protein kinase
REGION: Disordered

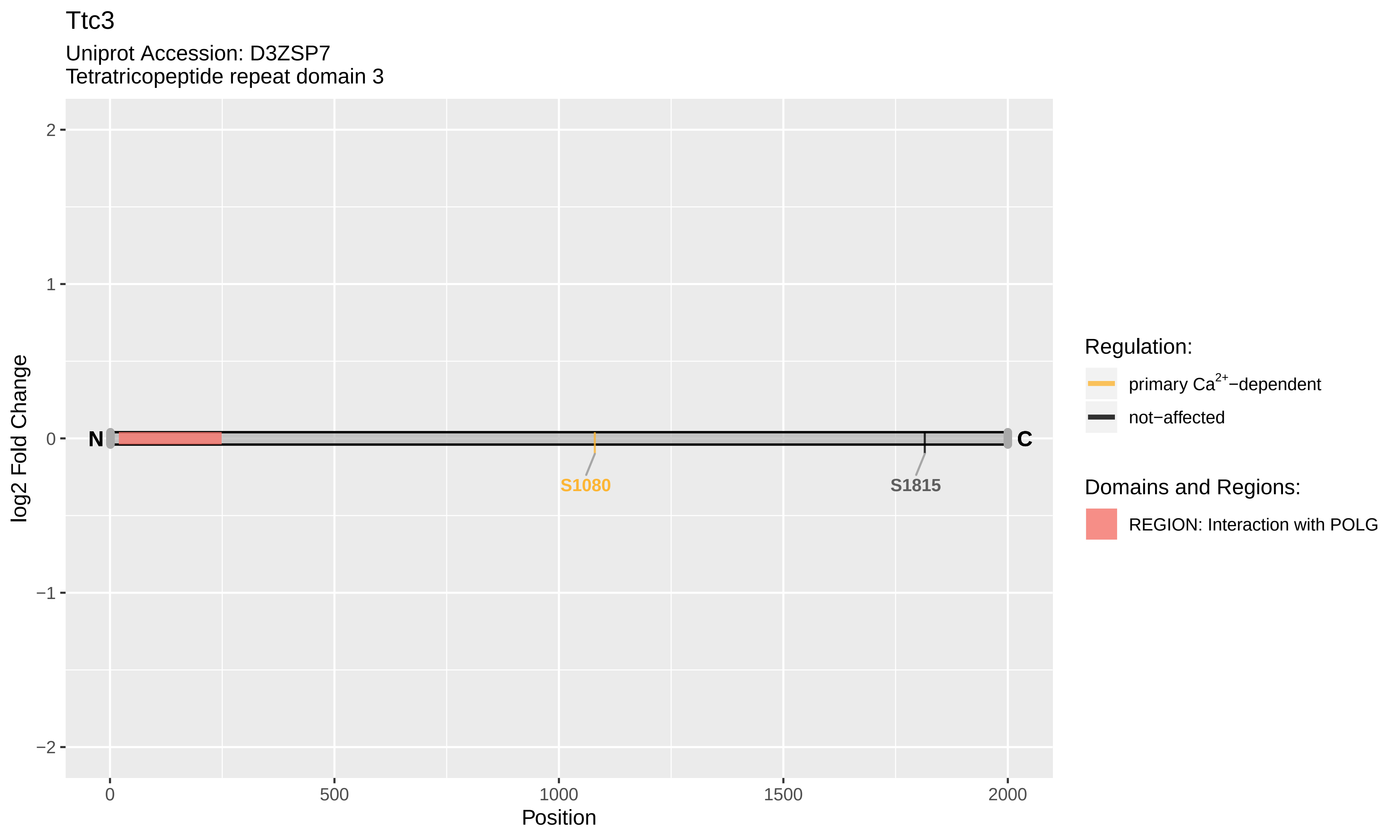

Ttc3
Uniprot Accession: D3ZSP7
Tetratricopeptide repeat domain 3
log2 Fold Change
2
1
0
−1
−2
N
C
S1080
S1815
0
500
1000
1500
2000
Position
Regulation:
primary Ca2+−dependent
not−affected
Domains and Regions:
REGION: Interaction with POLG

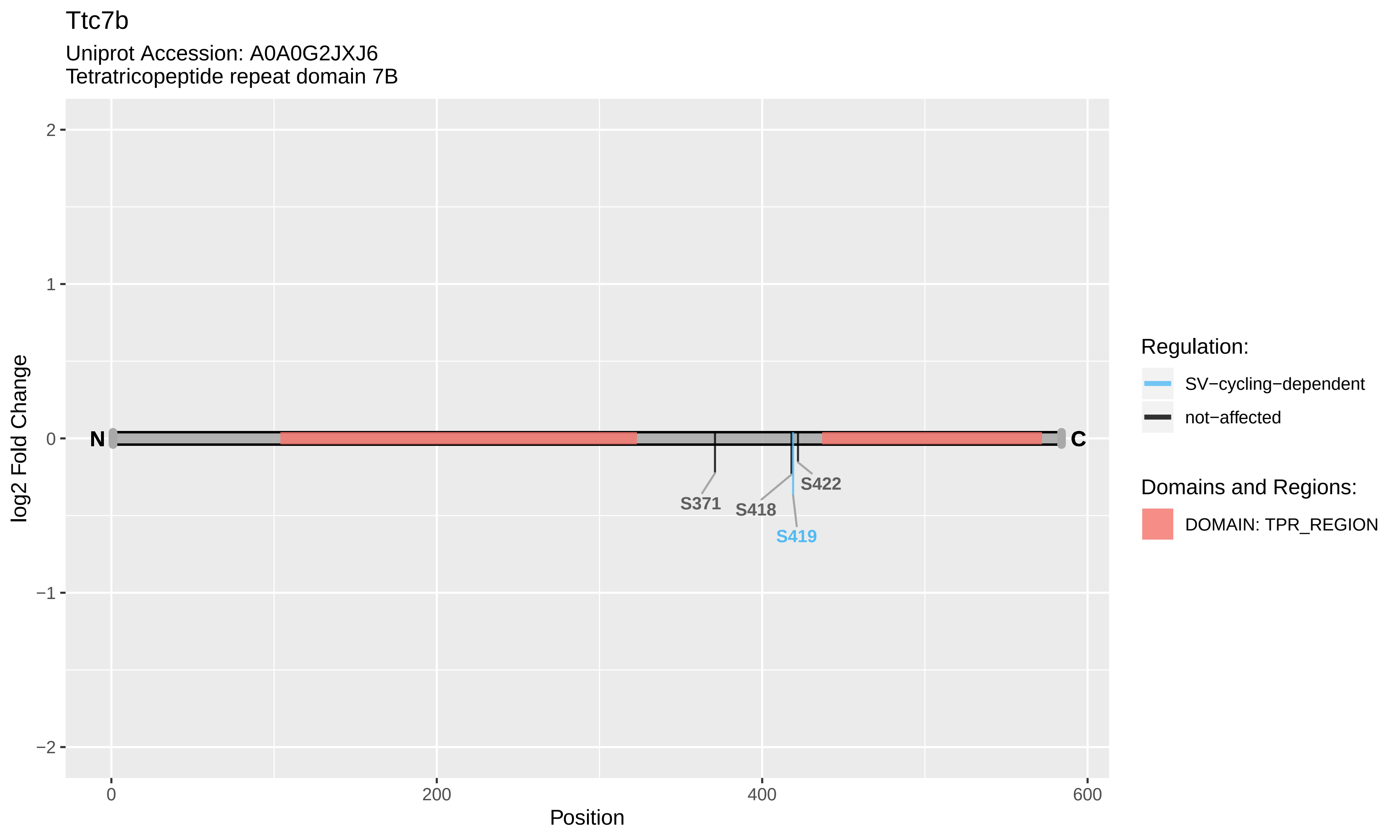
Ttc7b
Uniprot Accession: A0A0G2JXJ6
Tetratricopeptide repeat domain 7B
log2 Fold Change
2
1
0
−1
−2
N
C
S371
S418
S419
S422
0
200
400
600
Position
Regulation:
SV−cycling−dependent
not−affected
Domains and Regions:
DOMAIN: TPR_REGION

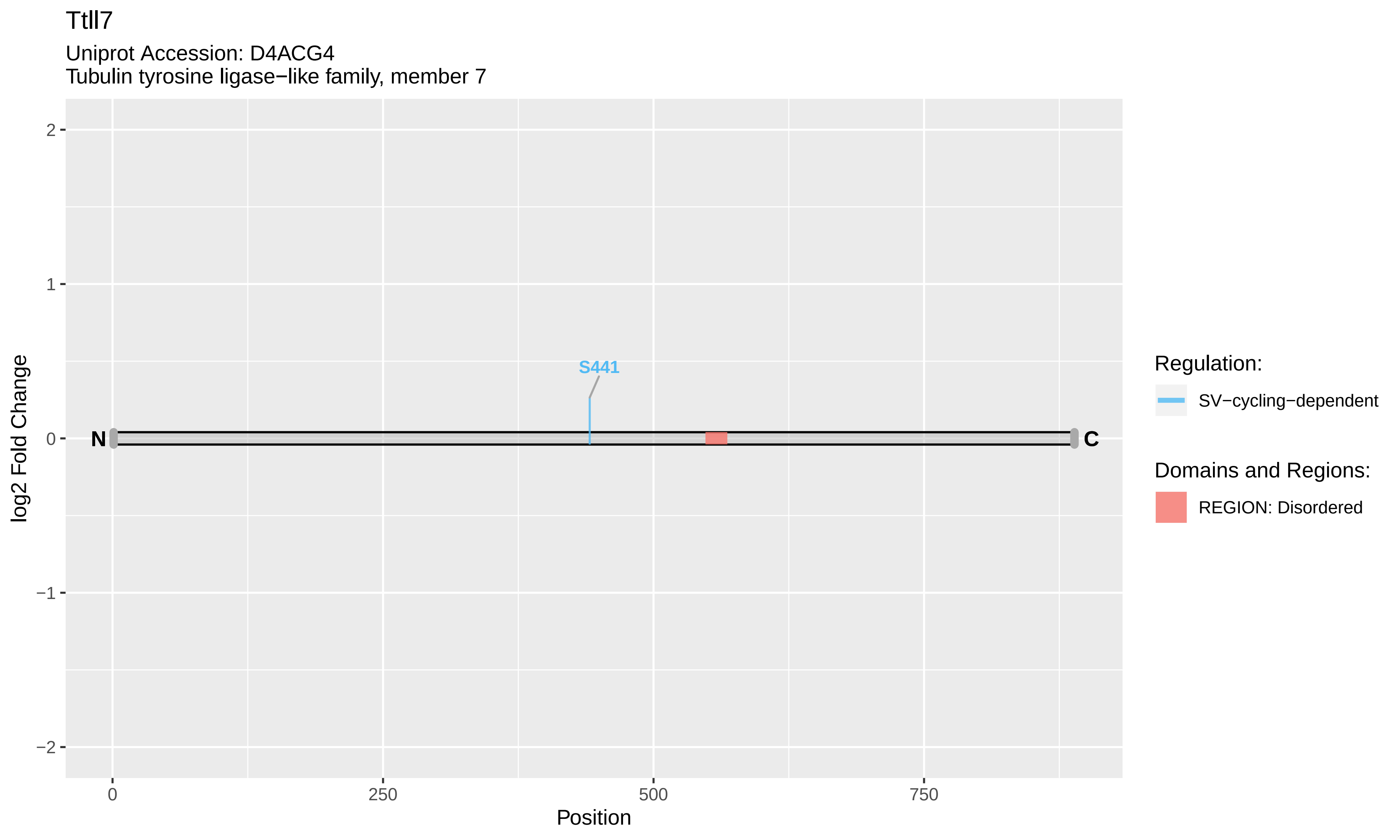
Ttll7
Uniprot Accession: D4ACG4
Tubulin tyrosine ligase−like family, member 7
2
1
0
−1
−2
log2 Fold Change
0
250
500
750
Position
N
C
S441
Regulation:
SV−cycling−dependent
Domains and Regions:
REGION: Disordered

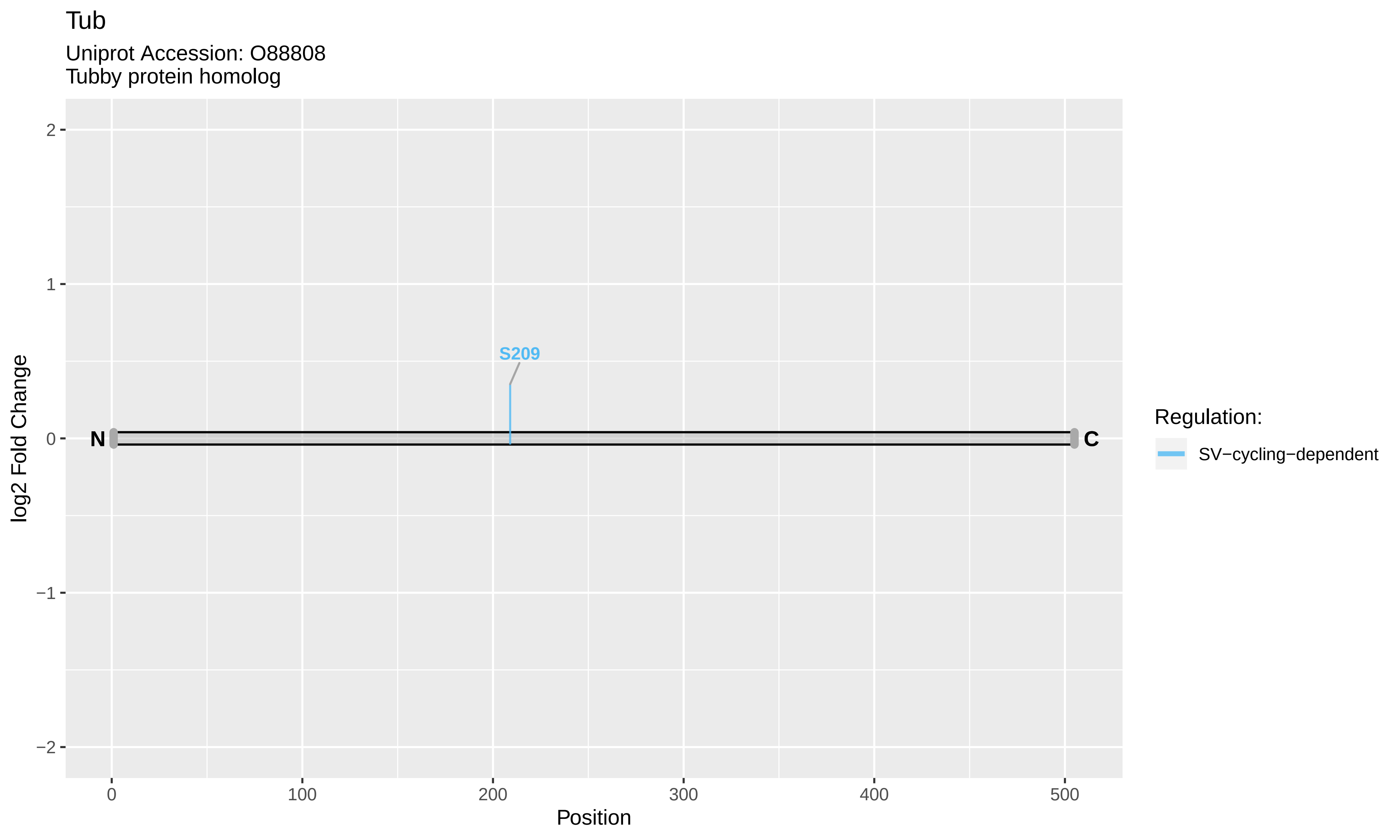
Tub
Uniprot Accession: O88808
Tubby protein homolog
S209
N
C
2
1
0
−1
−2
0
100
200
300
400
500
Position
log2 Fold Change
Regulation:
SV−cycling−dependent

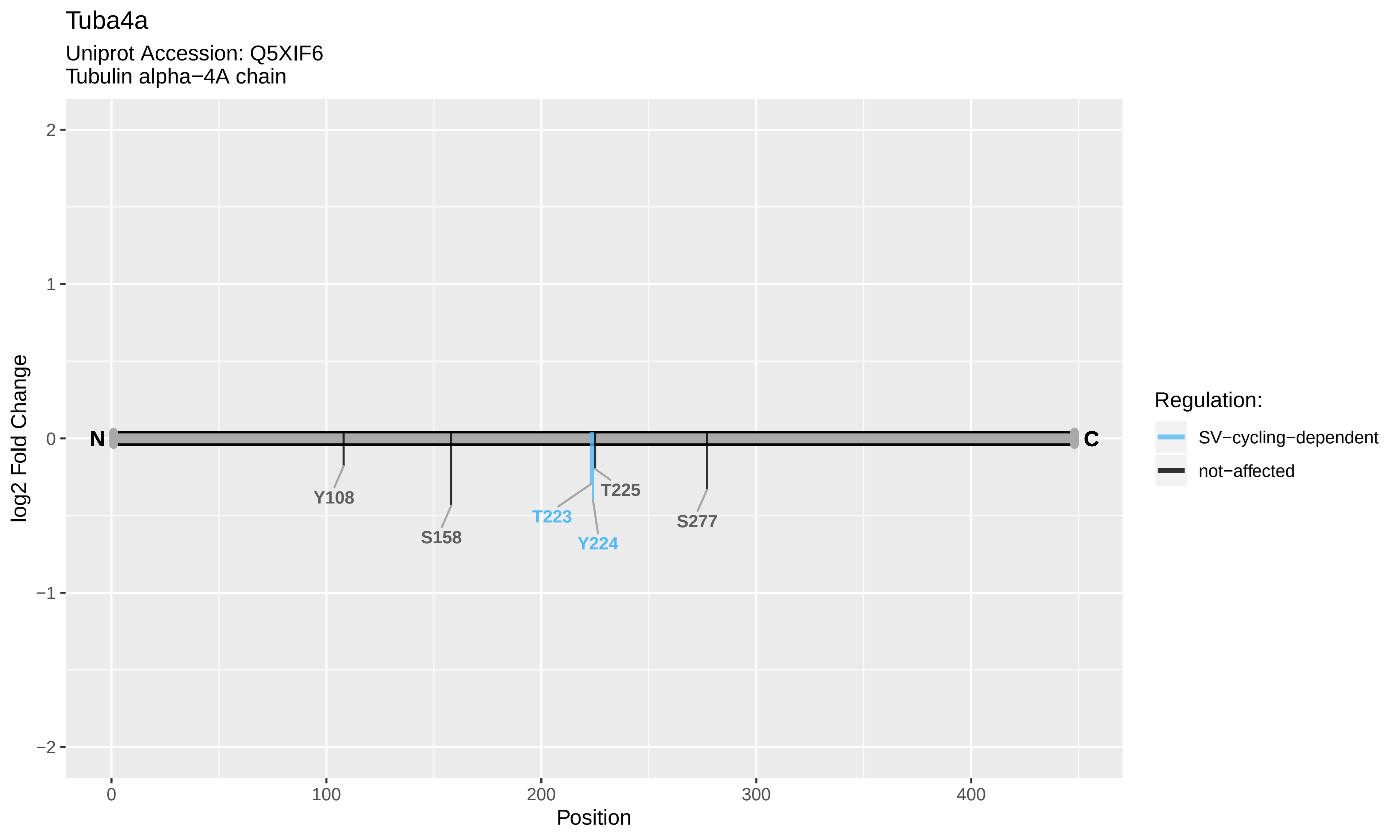
Tuba4a
Uniprot Accession: Q5XIF6
Tubulin alpha−4A chain
log2 Fold Change
2
1
0
−1
−2
N
C
Y108
S158
T223
Y224
T225
S277
0
100
200
300
400
Position
Regulation:
SV−cycling−dependent
not−affected

Tubb5

Uniprot Accession: P69897
Tubulin beta−5 chain

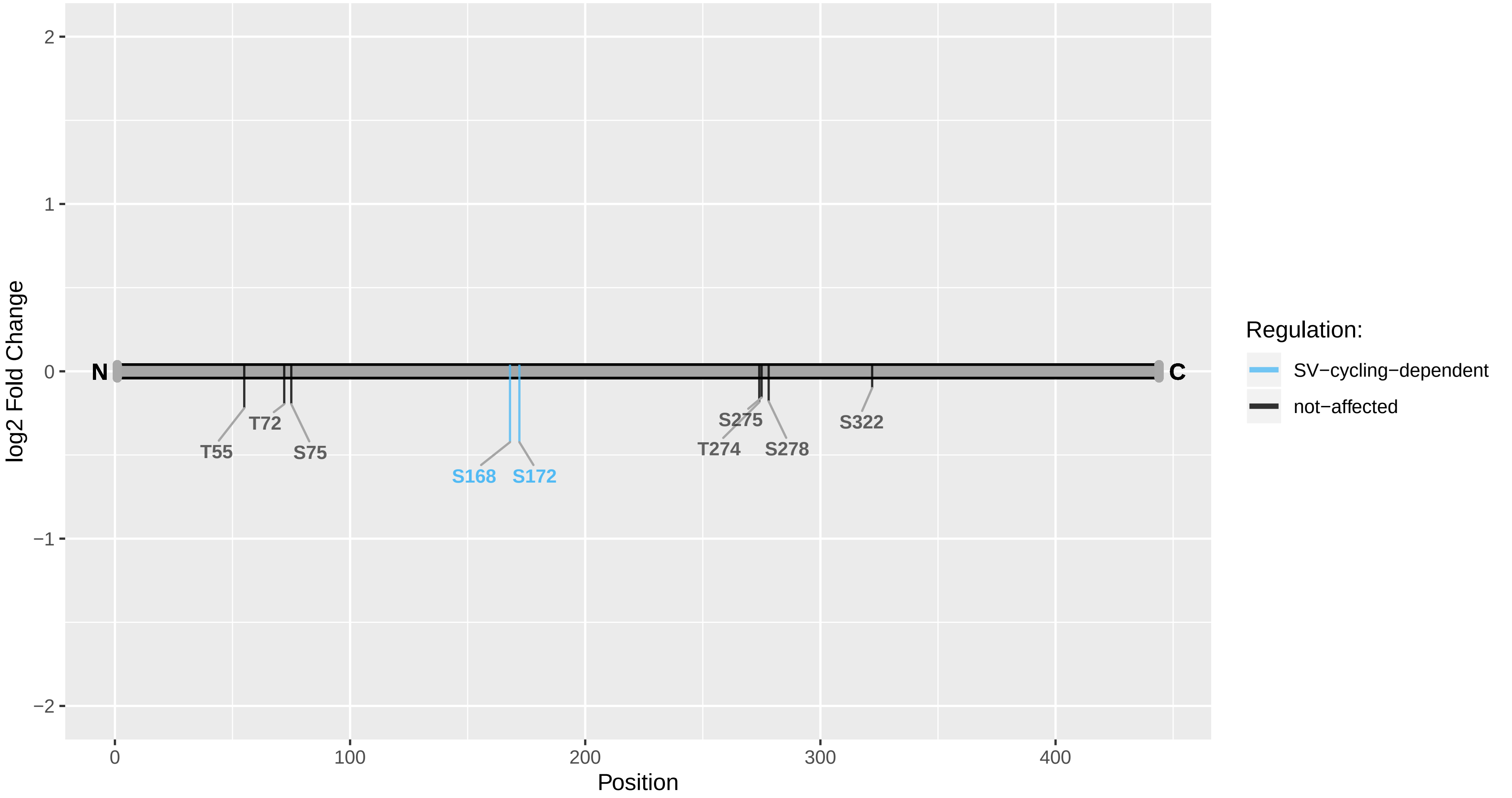

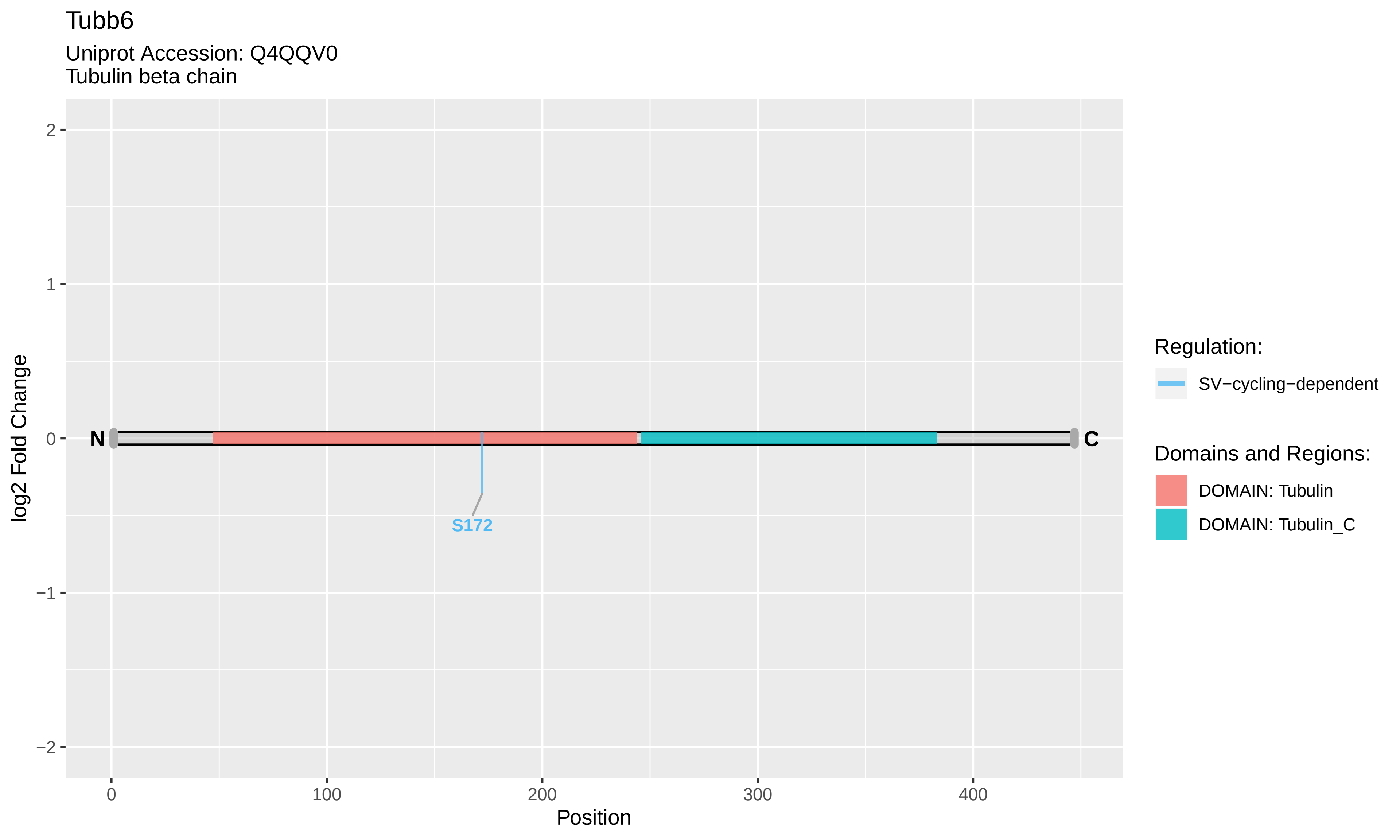
Tubb6
Uniprot Accession: Q4QQV0
Tubulin beta chain
log2 Fold Change
2
1
0
−1
−2
N
C
S172
0
100
200
300
400
Position
Regulation:
SV−cycling−dependent
Domains and Regions:
DOMAIN: Tubulin
DOMAIN: Tubulin_C

Uba1

Uniprot Accession: Q5U300
Ubiquitin−like modifier−activating enzyme 1

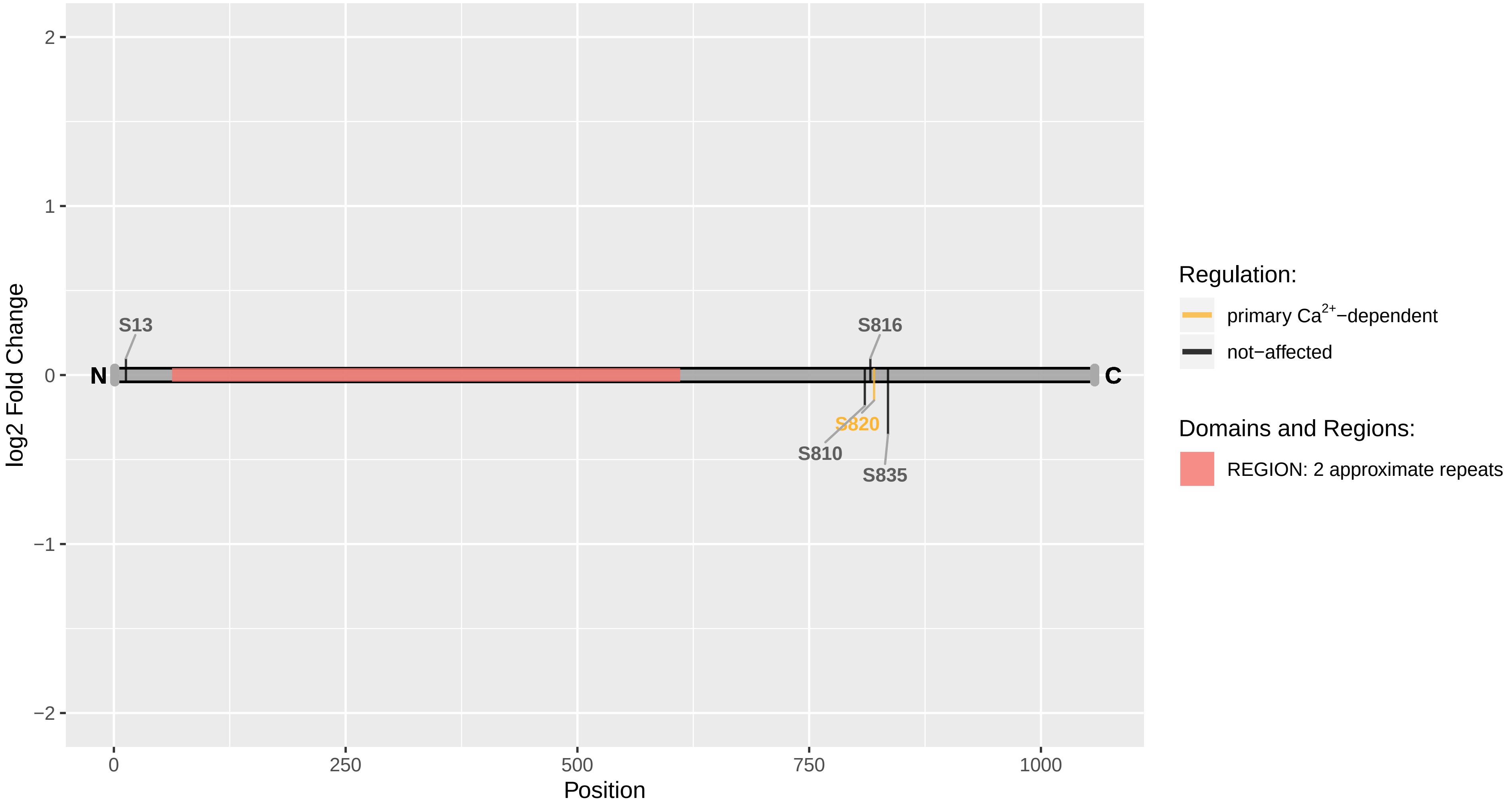

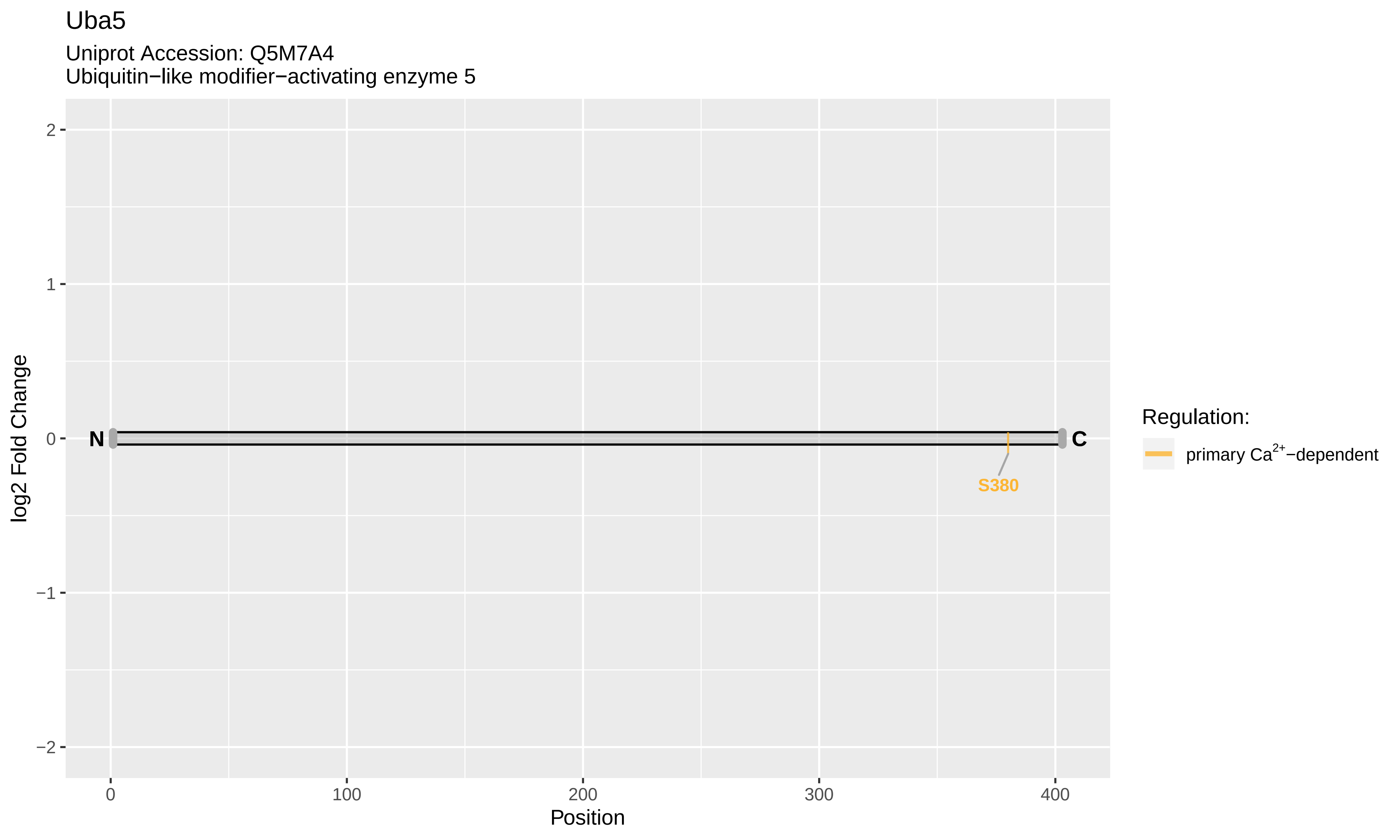

Uba5
Uniprot Accession: Q5M7A4
Ubiquitin−like modifier−activating enzyme 5
log2 Fold Change
2
1
0
−1
−2
N
C
S380
0
100
200
300
400
Position
Regulation:
primary Ca2+−dependent

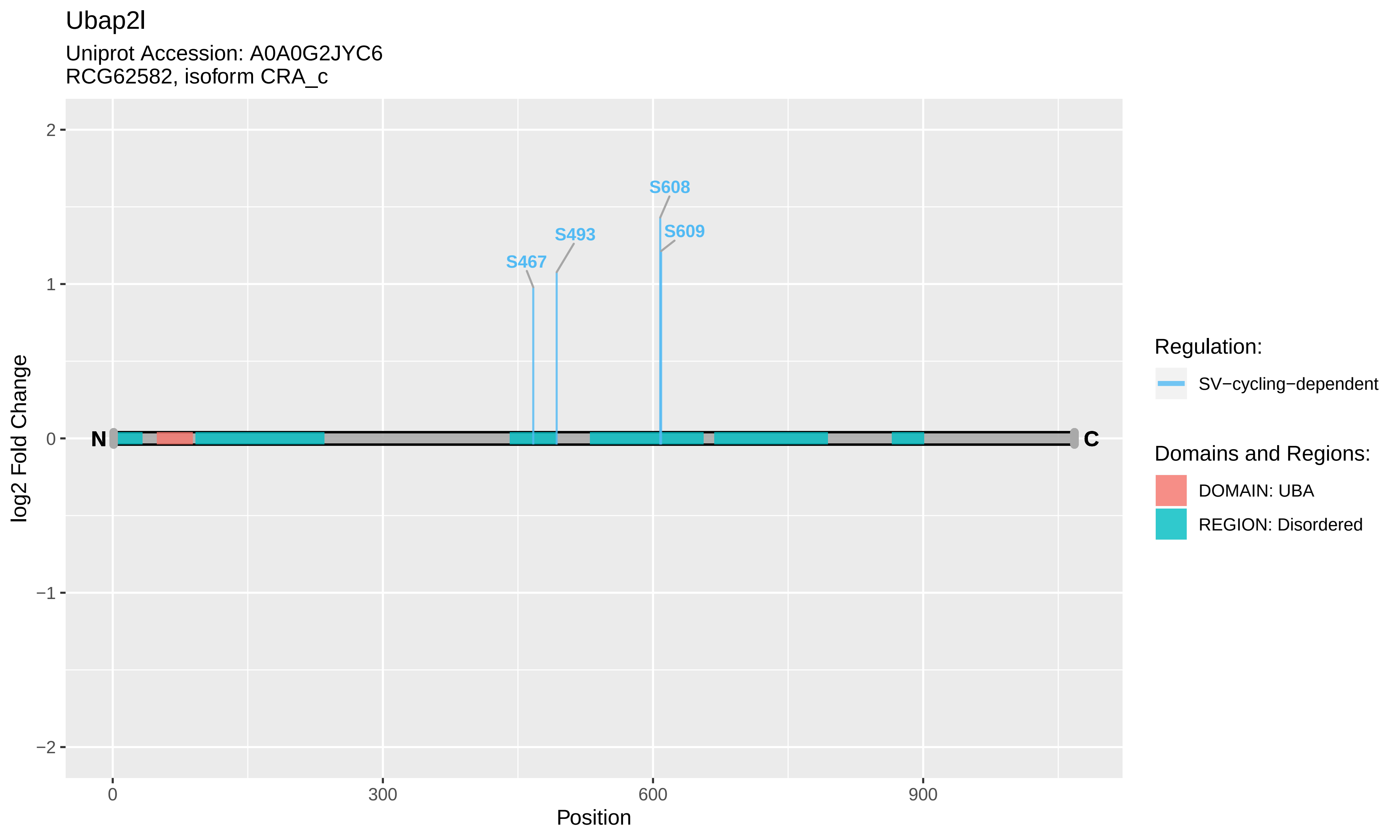
Ubap2l
Uniprot Accession: A0A0G2JYC6
RCG62582, isoform CRA_c
S608
S609
S493
S467
N
C
2
1
0
−1
−2
0
300
600
900
log2 Fold Change
Position
Regulation:
SV−cycling−dependent
Domains and Regions:
DOMAIN: UBA
REGION: Disordered

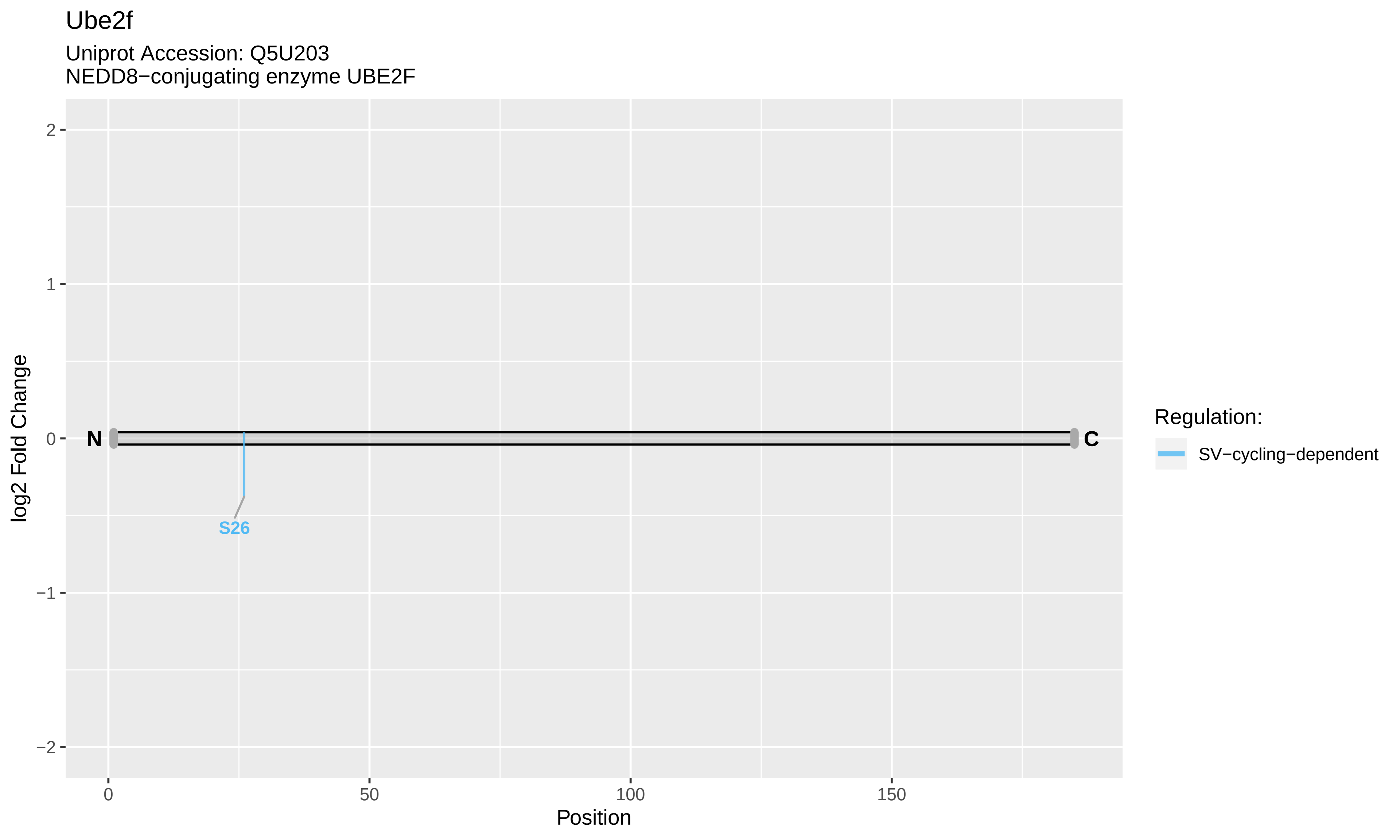
Ube2f
Uniprot Accession: Q5U203
NEDD8−conjugating enzyme UBE2F
log2 Fold Change
2
1
0
−1
−2
N
C
S26
0
50
100
150
Position
Regulation:
SV−cycling−dependent

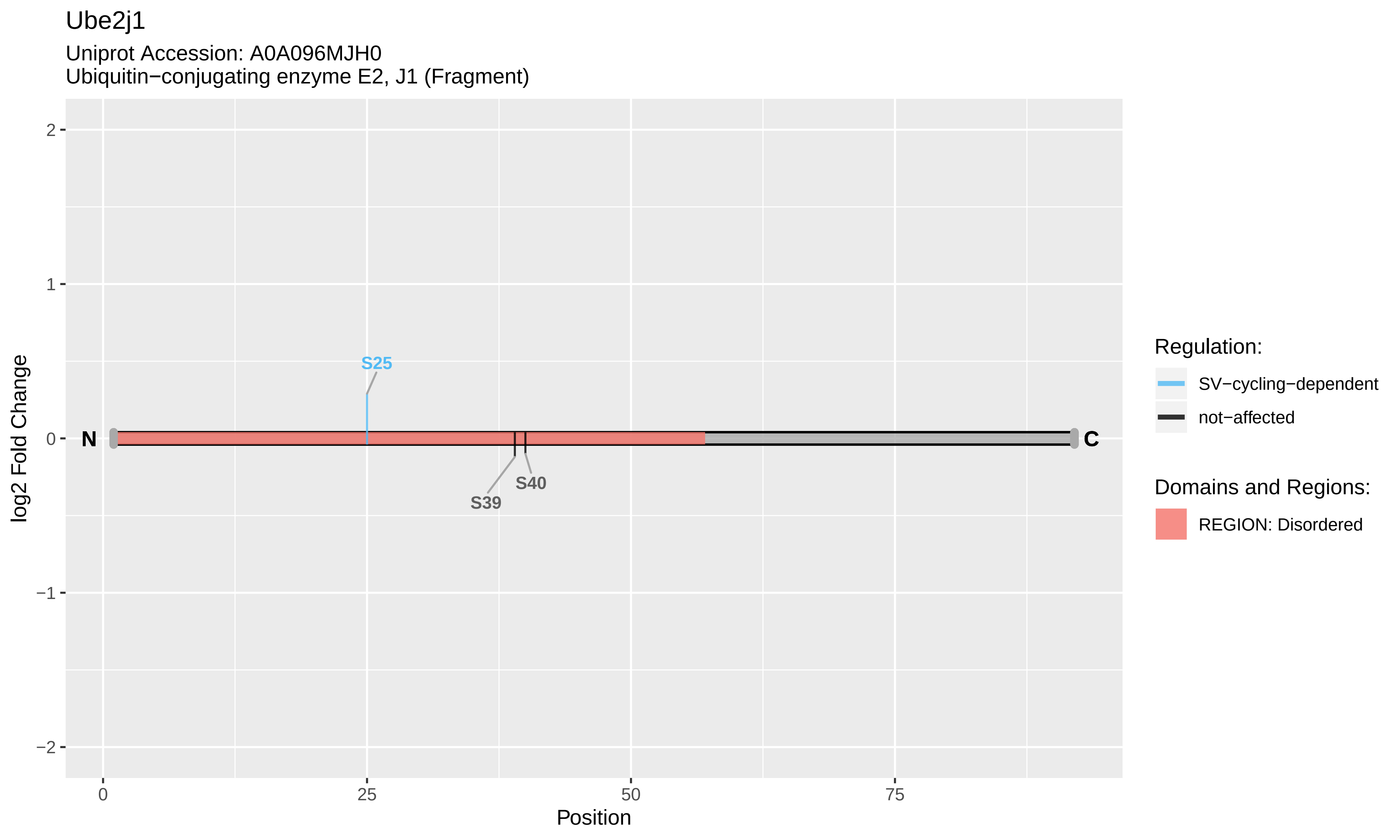

Ube2j1
Uniprot Accession: A0A096MJH0
Ubiquitin−conjugating enzyme E2, J1 (Fragment)
S25
N
C
S39
S40
log2 Fold Change
2
1
0
−1
−2
0
25
50
75
Position
Regulation:
SV−cycling−dependent
not−affected
Domains and Regions:
REGION: Disordered

Ube2m

Uniprot Accession: D3ZNQ6
Ubiquitin−conjugating enzyme E2M

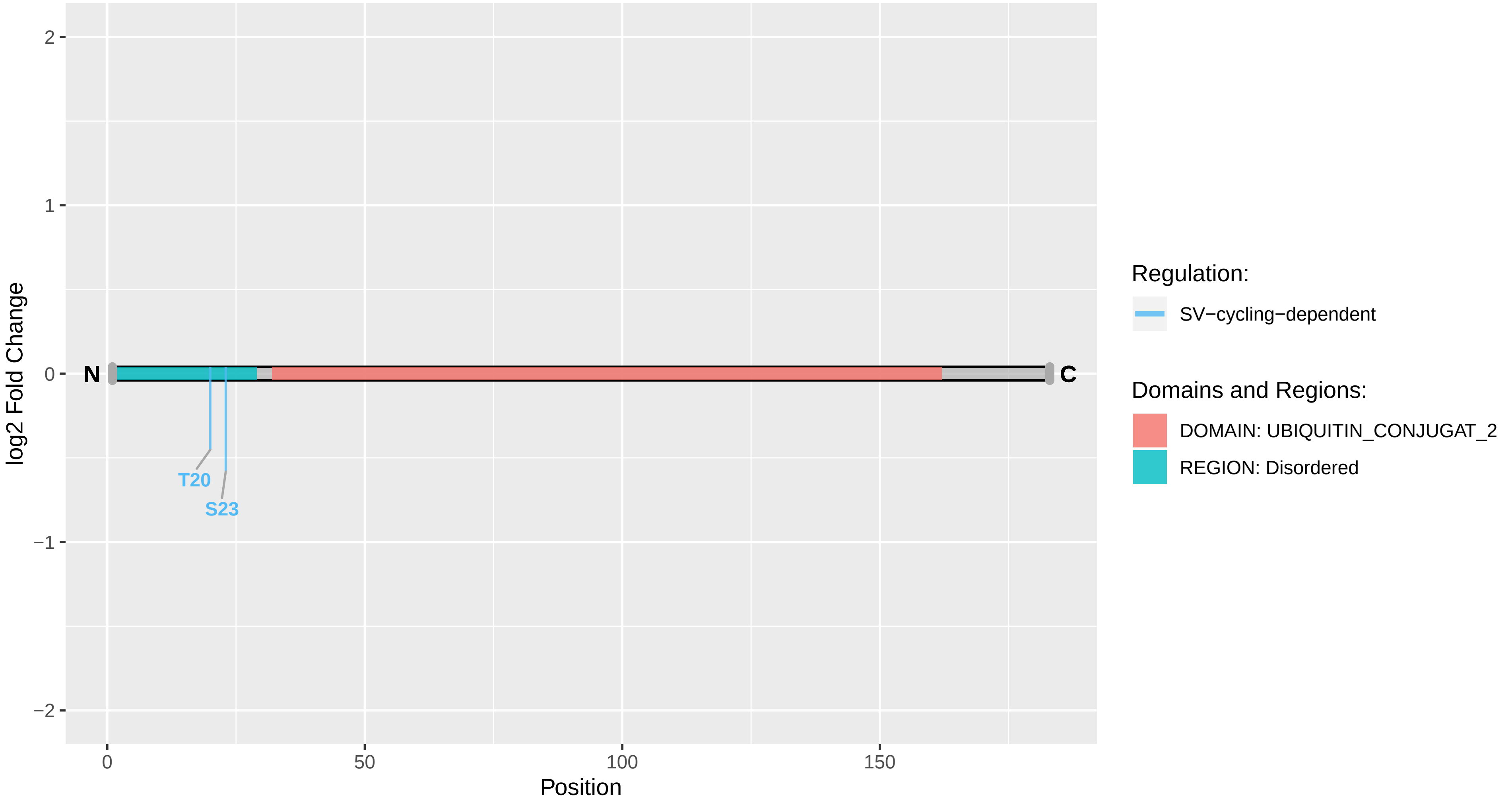

Ube2ql1

Uniprot Accession: D3ZXV0
Ubiquitin−conjugating enzyme E2Q family−like 1

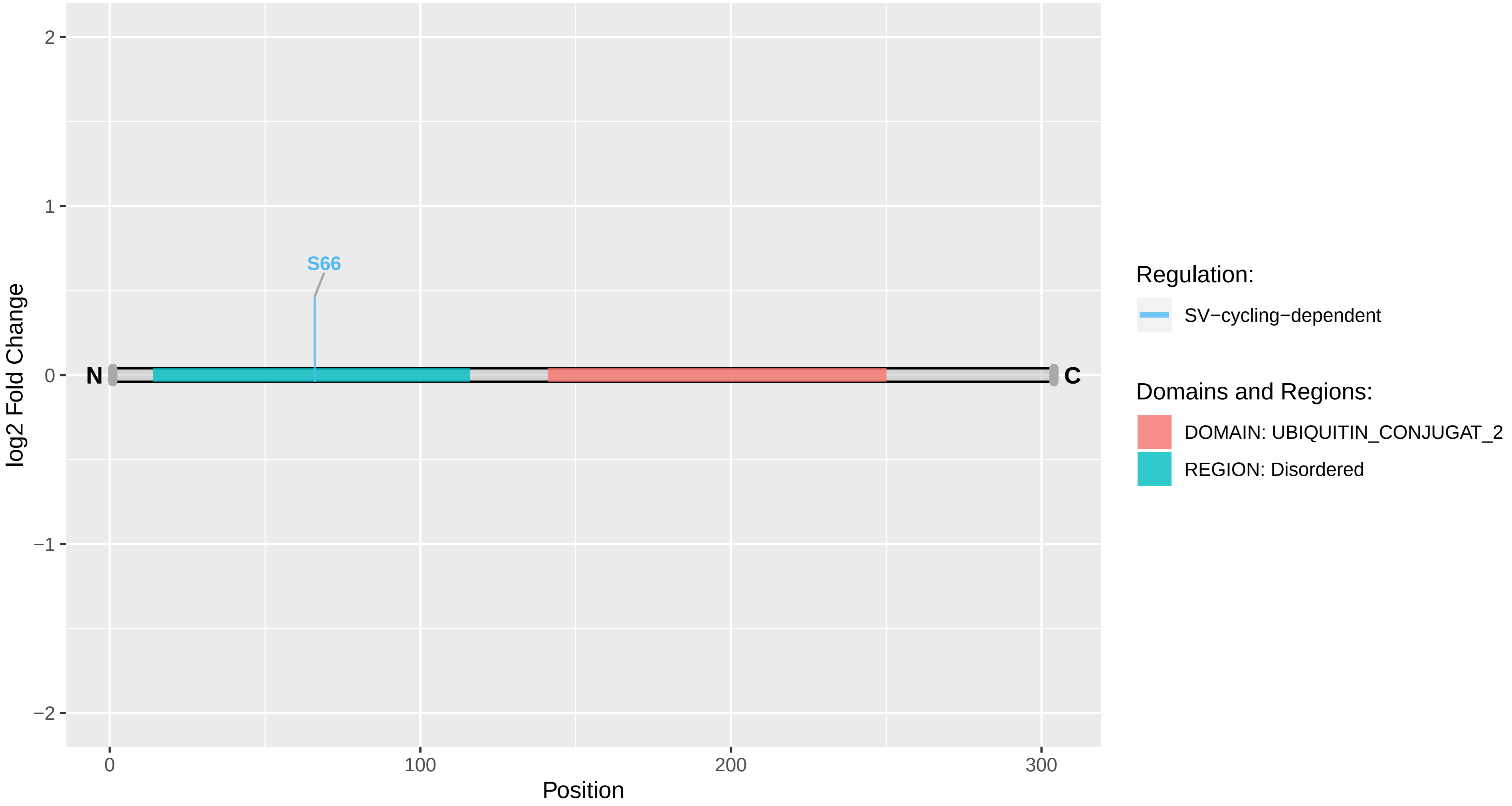

Ubxn2b

Uniprot Accession: P0C627
UBX domain−containing protein 2B

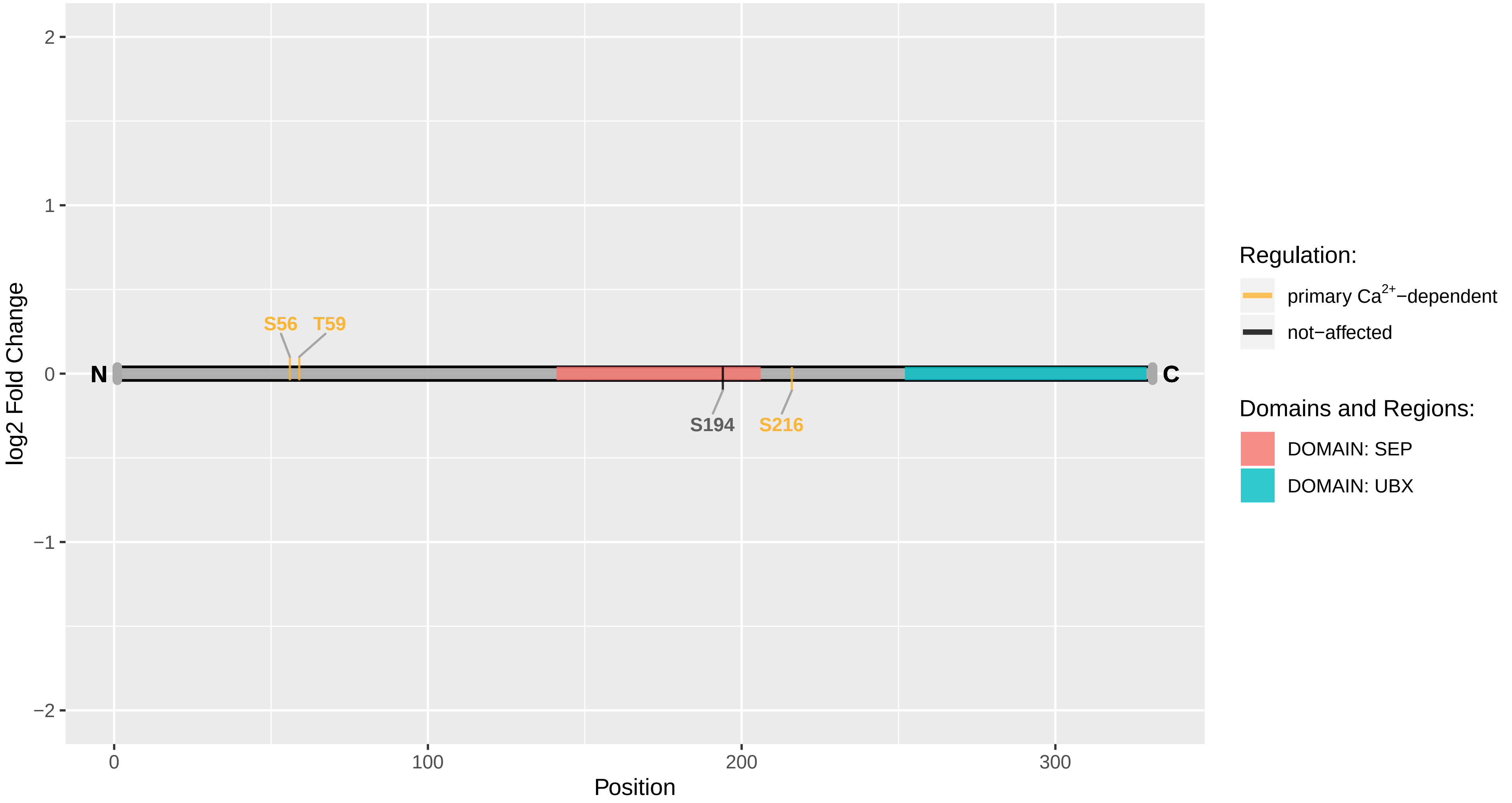

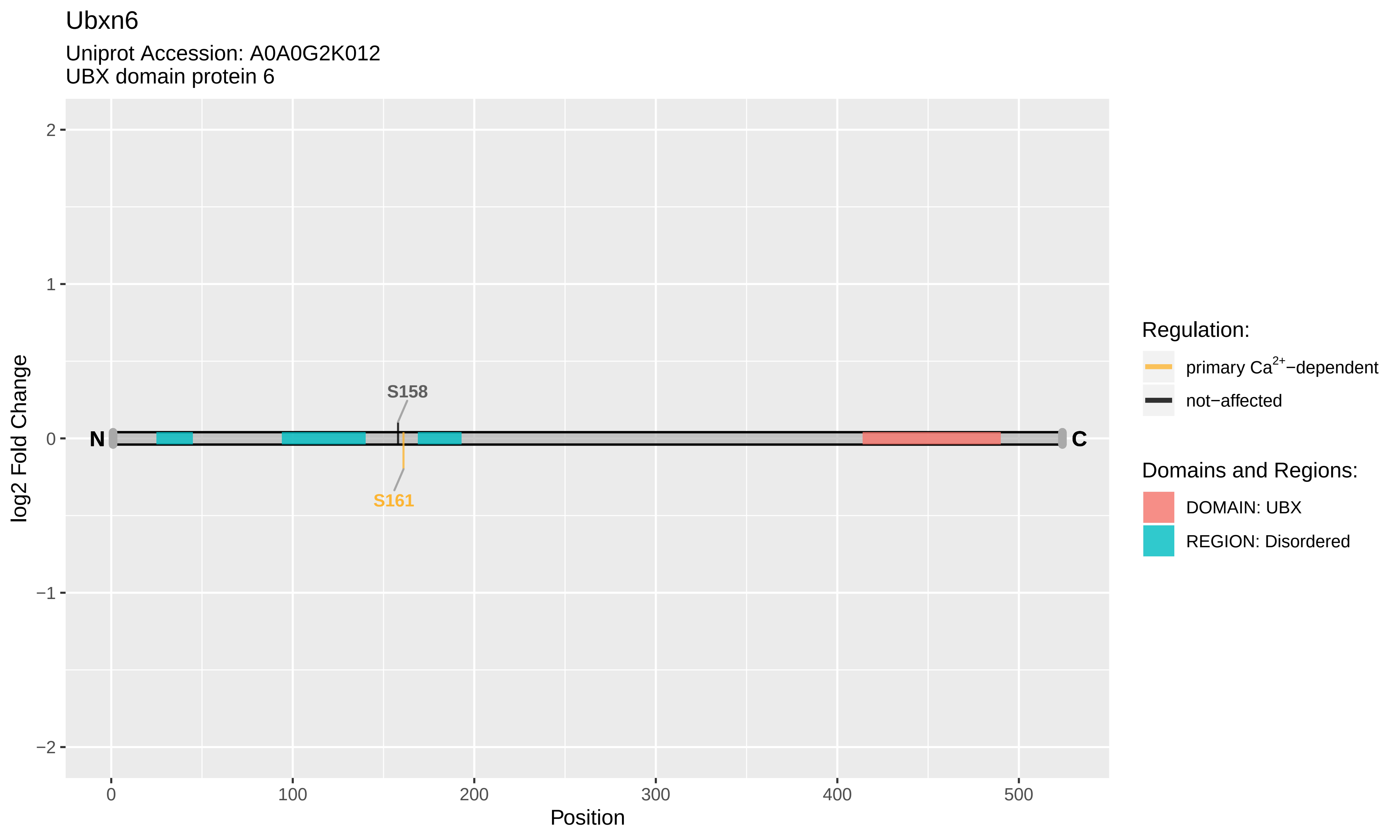
Ubxn6
Uniprot Accession: A0A0G2K012
UBX domain protein 6
log2 Fold Change
2
1
0
−1
−2
N
C
S158
S161
0
100
200
300
400
500
Position
Regulation:
primary Ca2+−dependent
not−affected
Domains and Regions:
DOMAIN: UBX
REGION: Disordered

Ufd1

Uniprot Accession: Q9ES53
Ubiquitin recognition factor in ER−associated degradation protein 1

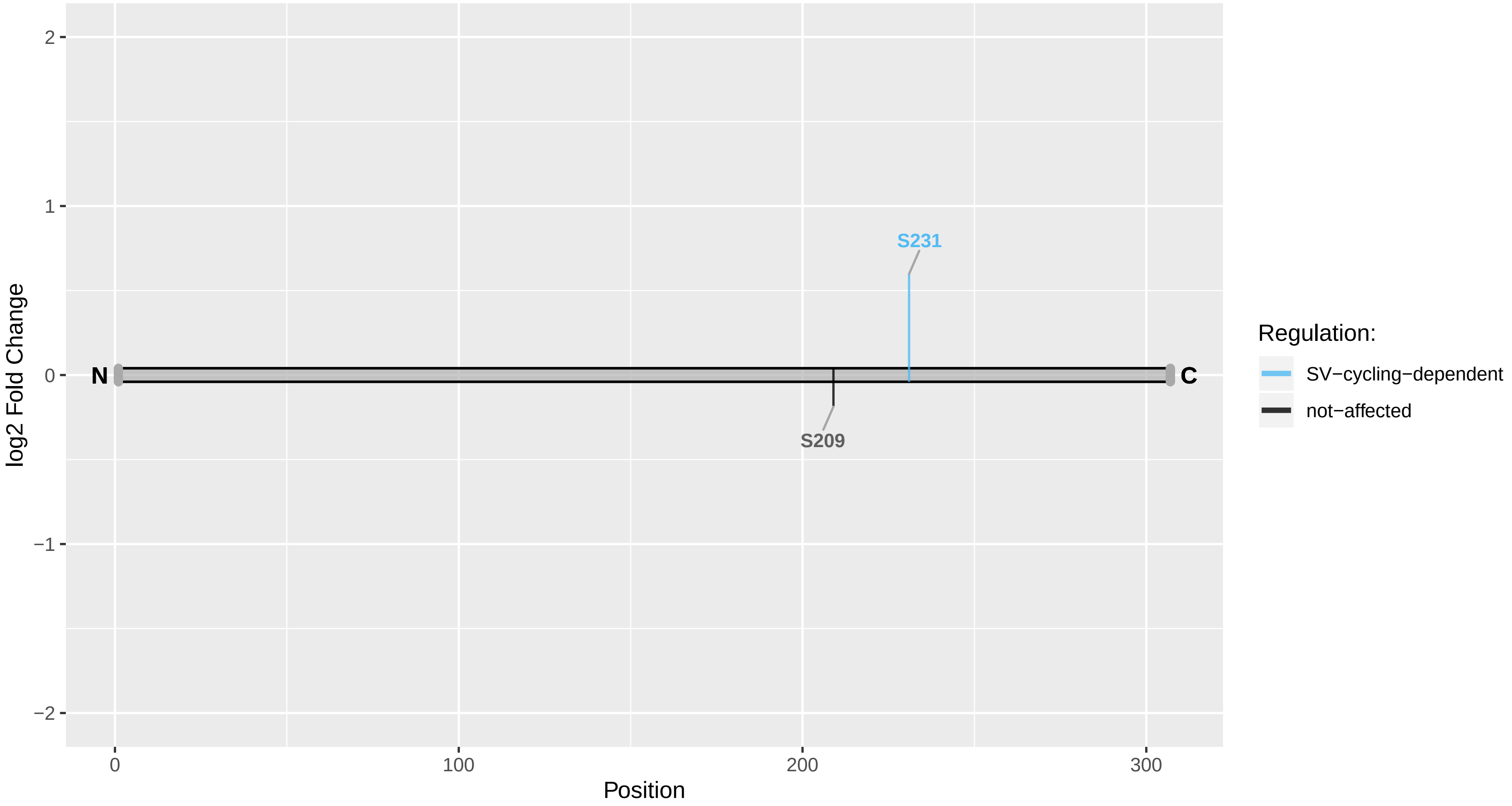

Uhrf1bp1

Uniprot Accession: D3ZMR2
UHRF1−binding protein 1

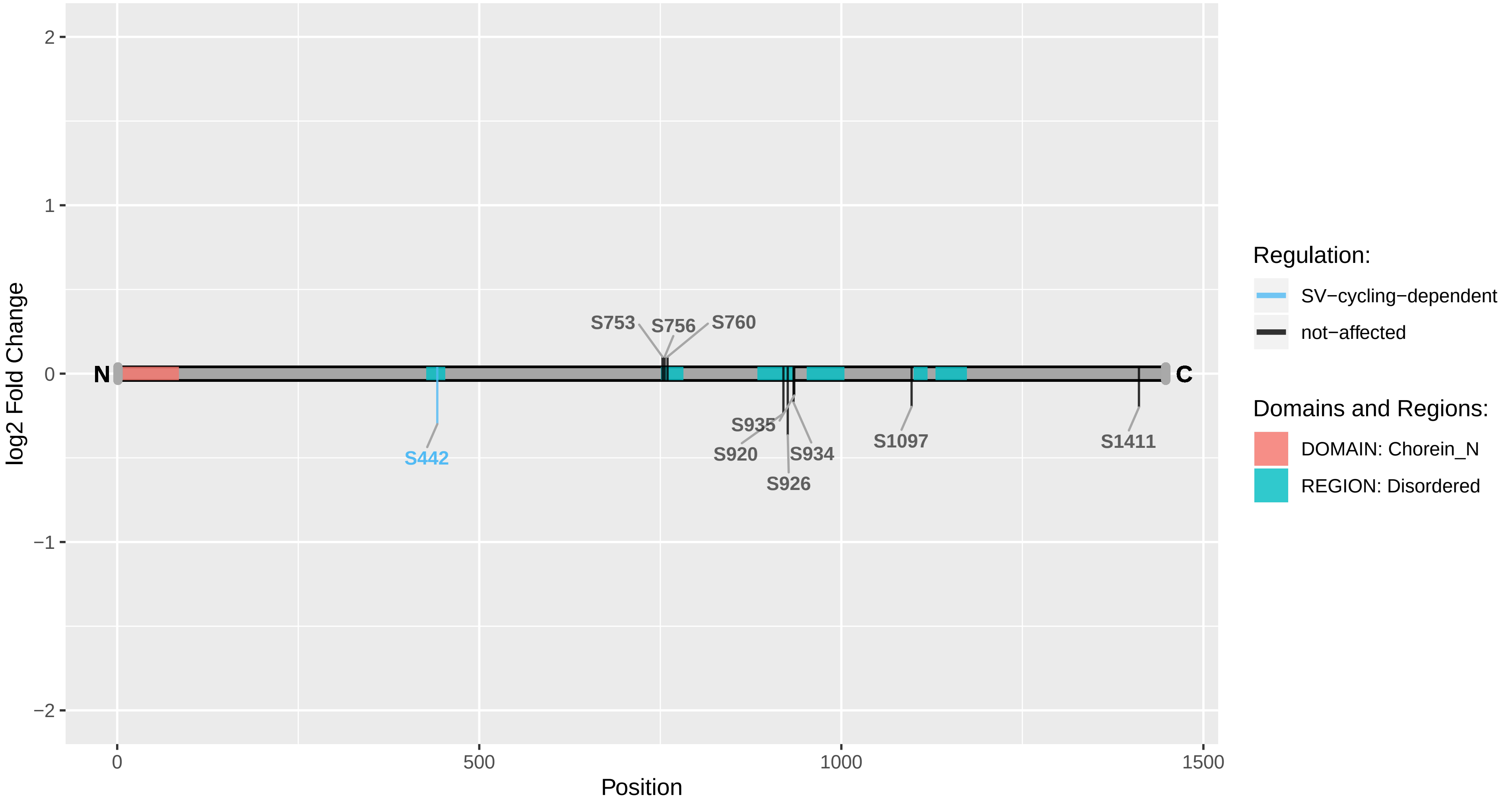

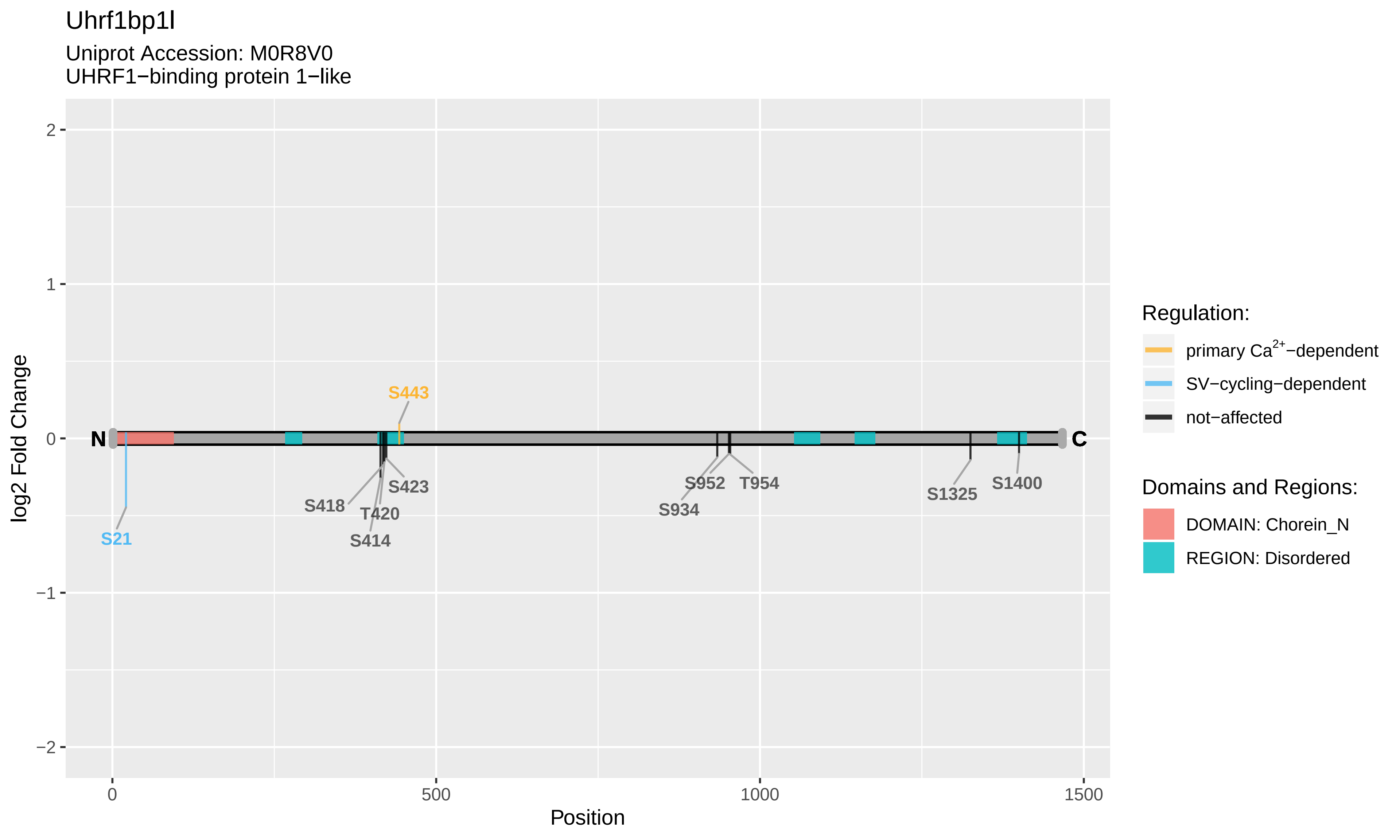
Uhrf1bp1l
Uniprot Accession: M0R8V0
UHRF1−binding protein 1−like
log2 Fold Change
Position
N
C
S443
S21
S418
T420
S414
S423
S952
T954
S934
S1325
S1400
Regulation:
primary Ca2+−dependent
SV−cycling−dependent
not−affected
Domains and Regions:
DOMAIN: Chorein_N
REGION: Disordered

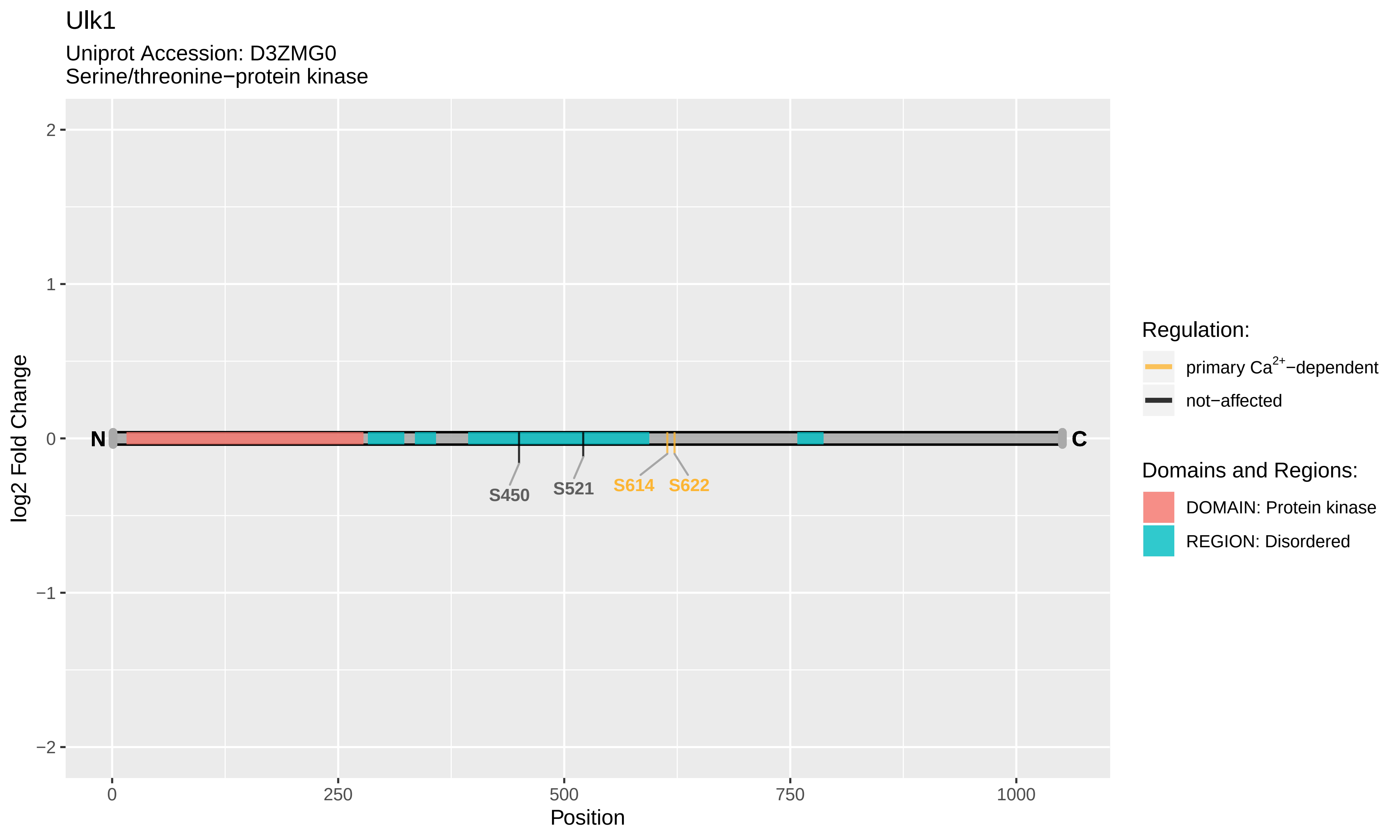
Ulk1
Uniprot Accession: D3ZMG0
Serine/threonine−protein kinase
log2 Fold Change
2
1
0
−1
−2
N
C
S450
S521
S614
S622
0
250
500
750
1000
Position
Regulation:
primary Ca2+−dependent
not−affected
Domains and Regions:
DOMAIN: Protein kinase
REGION: Disordered

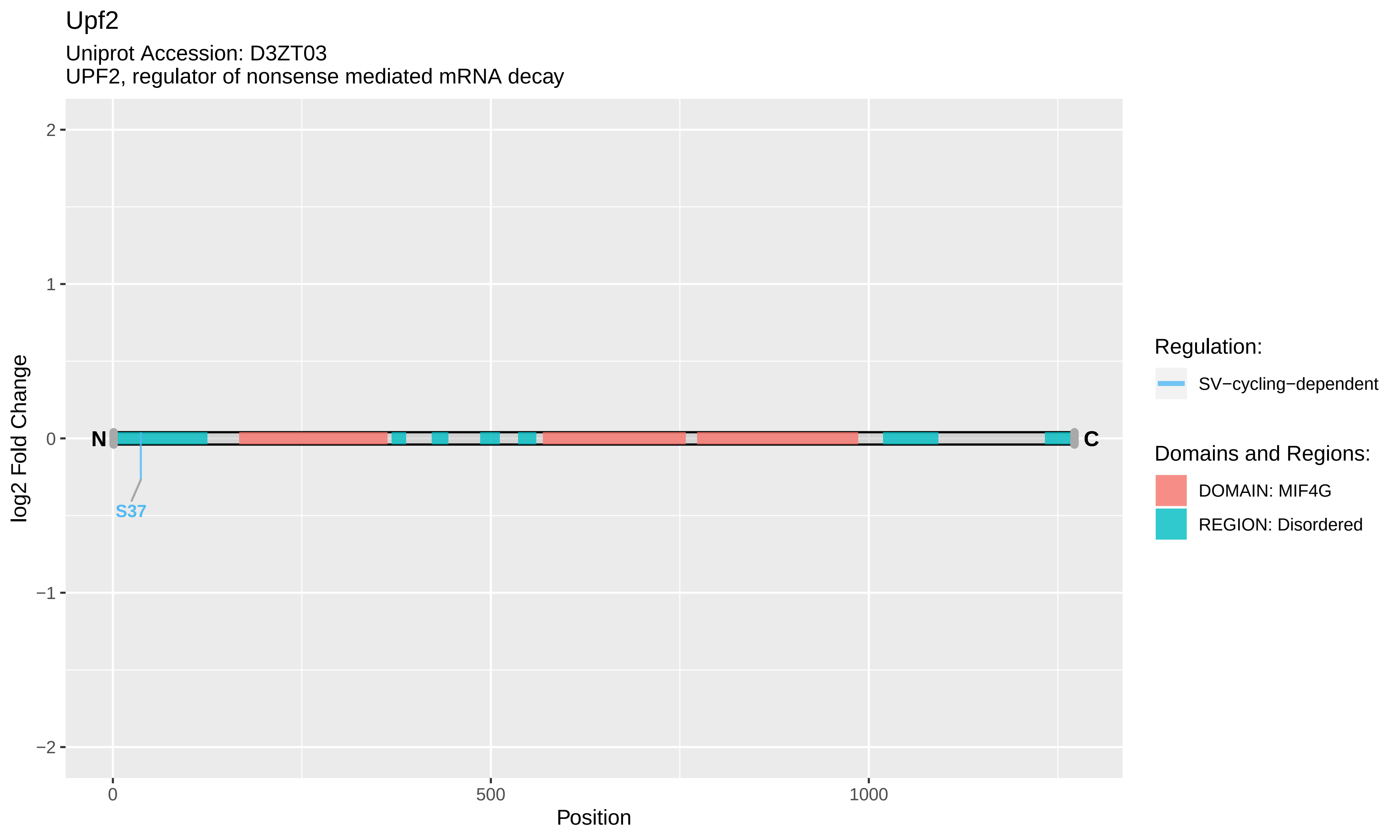

Upf2
Uniprot Accession: D3ZT03
UPF2, regulator of nonsense mediated mRNA decay
log2 Fold Change
2
1
0
−1
−2
N
C
S37
0
500
1000
Position
Regulation:
SV−cycling−dependent
Domains and Regions:
DOMAIN: MIF4G
REGION: Disordered

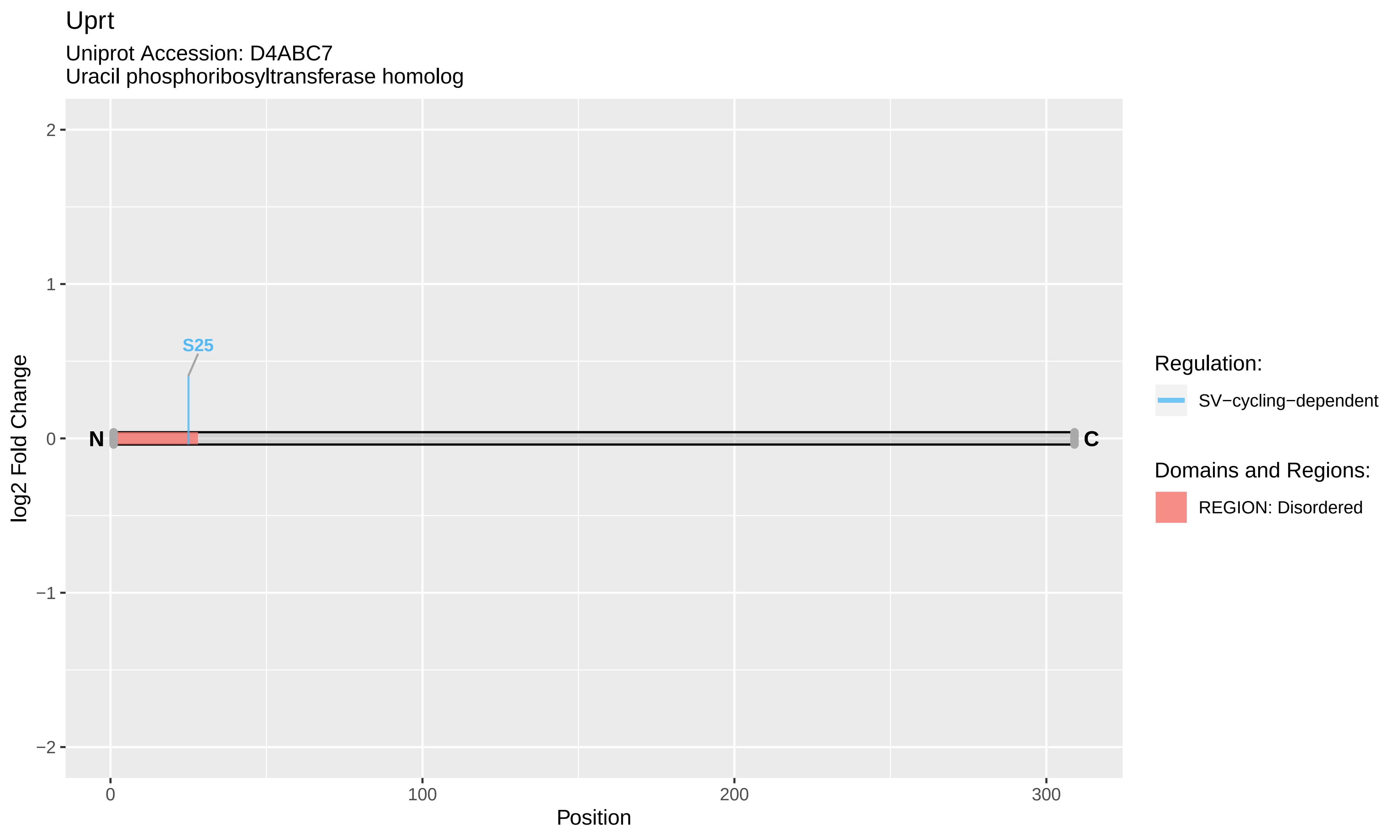
Uprt
Uniprot Accession: D4ABC7
Uracil phosphoribosyltransferase homolog
S25
N
C
2
1
0
−1
−2
log2 Fold Change
0
100
200
300
Position
Regulation:
SV−cycling−dependent
Domains and Regions:
REGION: Disordered

Uqcr10

Uniprot Accession: A0A0G2K8Q8
Ubiquinol−cytochrome c reductase, complex III subunit X

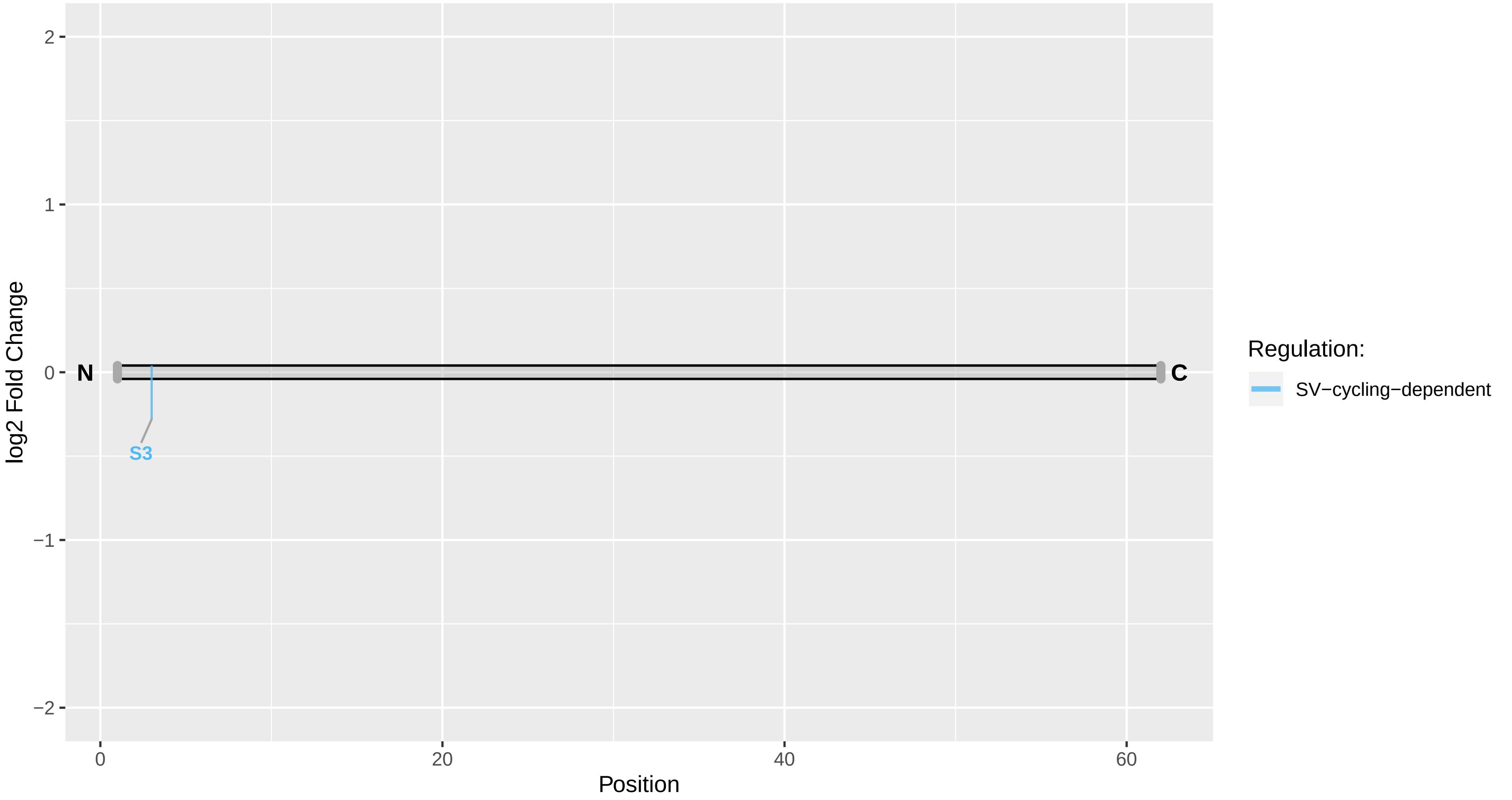

Uqcrc1

Uniprot Accession: Q68FY0
Cytochrome b−c1 complex subunit 1, mitochondrial

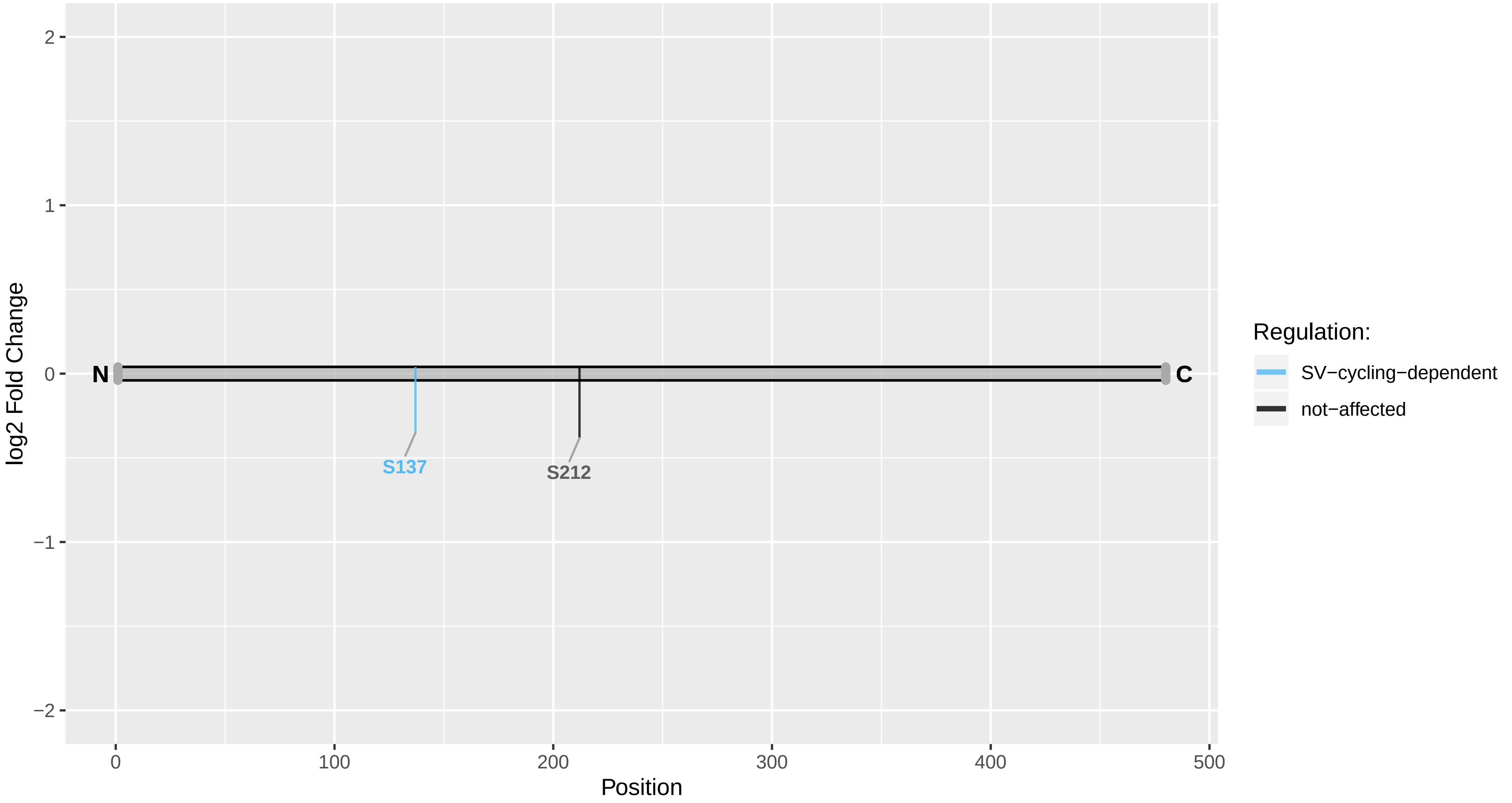

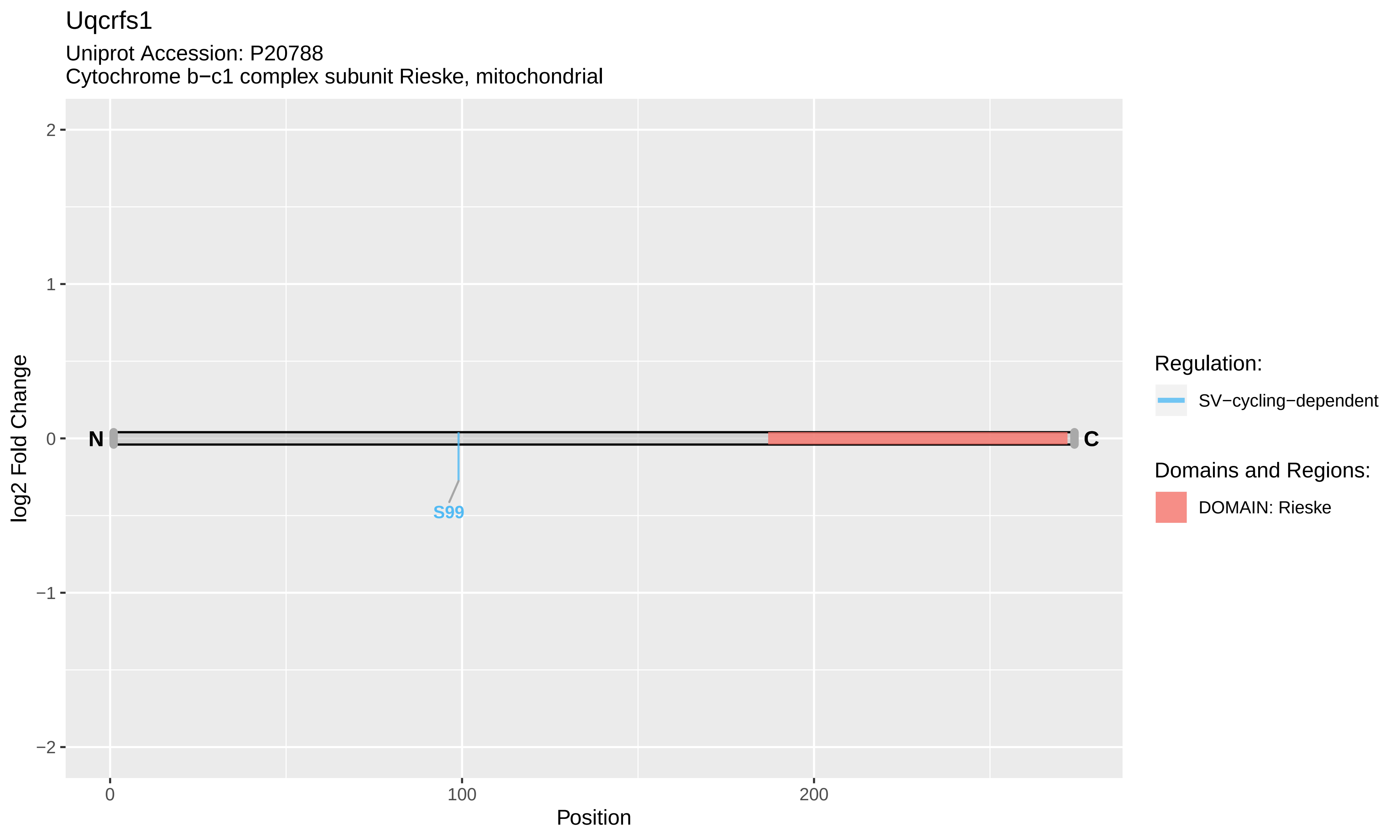
Uqcrfs1
Uniprot Accession: P20788
Cytochrome b−c1 complex subunit Rieske, mitochondrial
2
1
0
−1
−2
log2 Fold Change
N
C
S99
0
100
200
Position
Regulation:
SV−cycling−dependent
Domains and Regions:
DOMAIN: Rieske

Usp14

Uniprot Accession: Q5U2N2
Ubiquitin specific protease 14

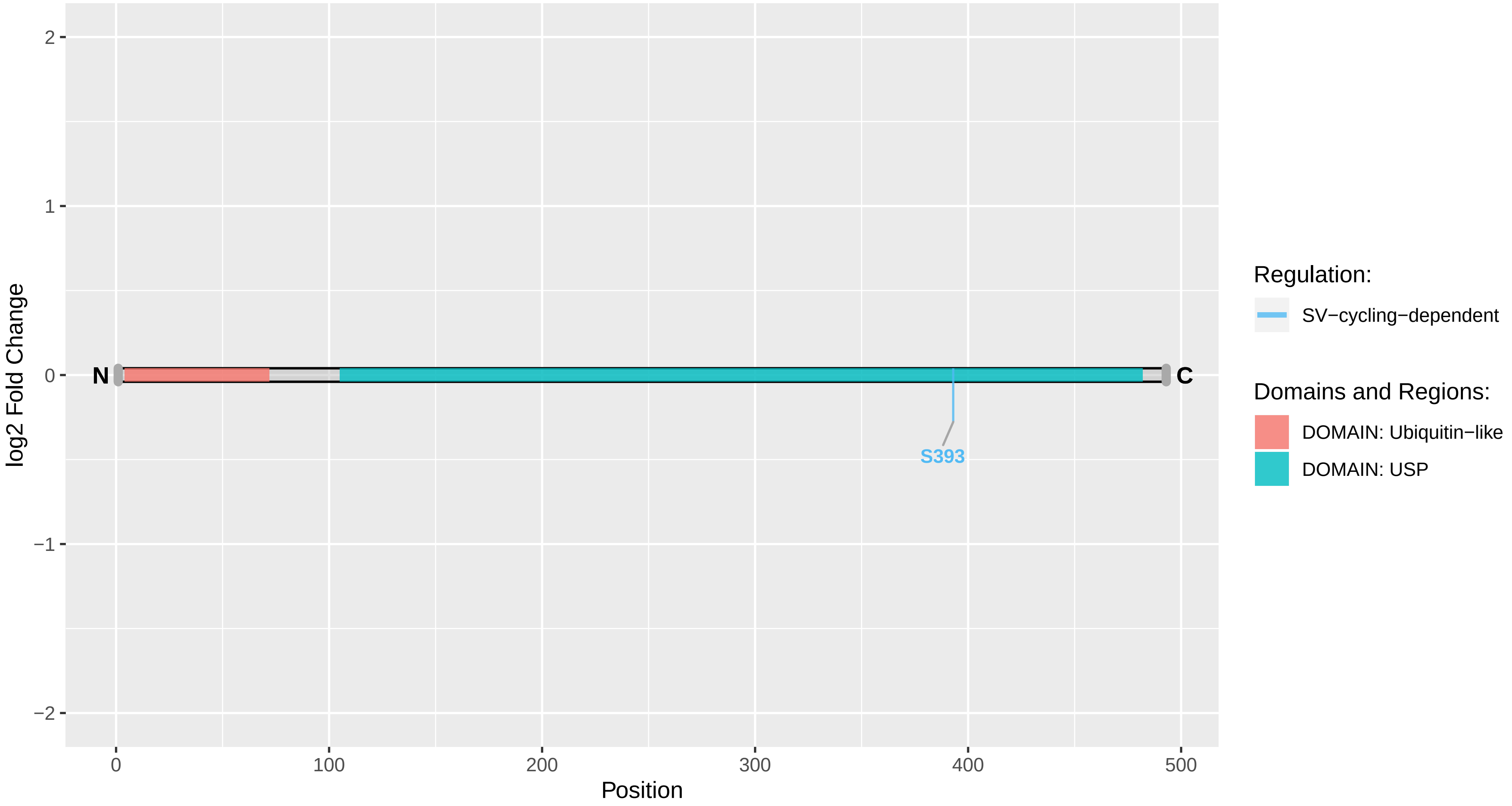

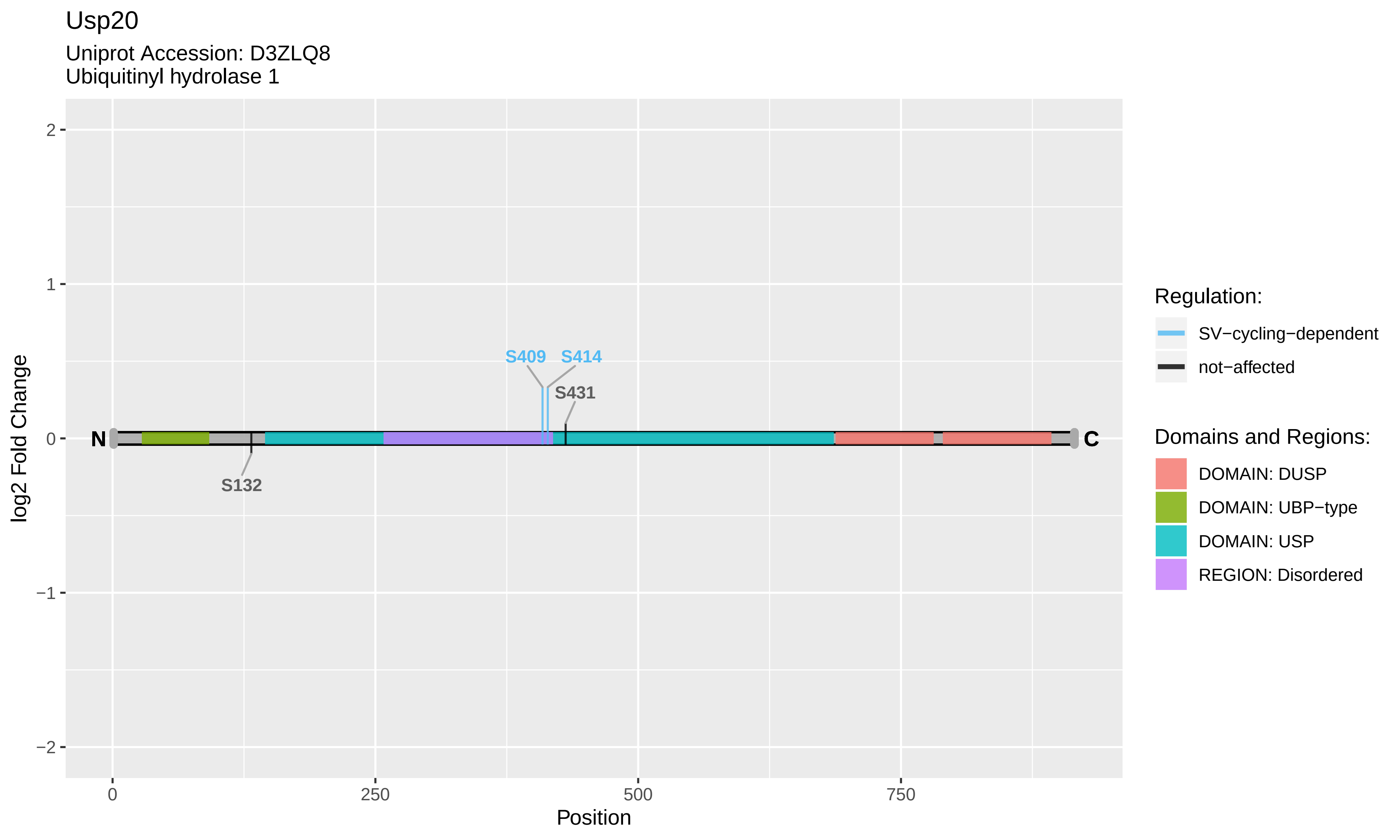

Usp20
Uniprot Accession: D3ZLQ8
Ubiquitinyl hydrolase 1
log2 Fold Change
Position
S409
S414
S431
S132
N
C
Regulation:
SV−cycling−dependent
not−affected
Domains and Regions:
DOMAIN: DUSP
DOMAIN: UBP−type
DOMAIN: USP
REGION: Disordered

Usp31

Uniprot Accession: A0A0G2K2V3
Ubiquitin−specific peptidase 31

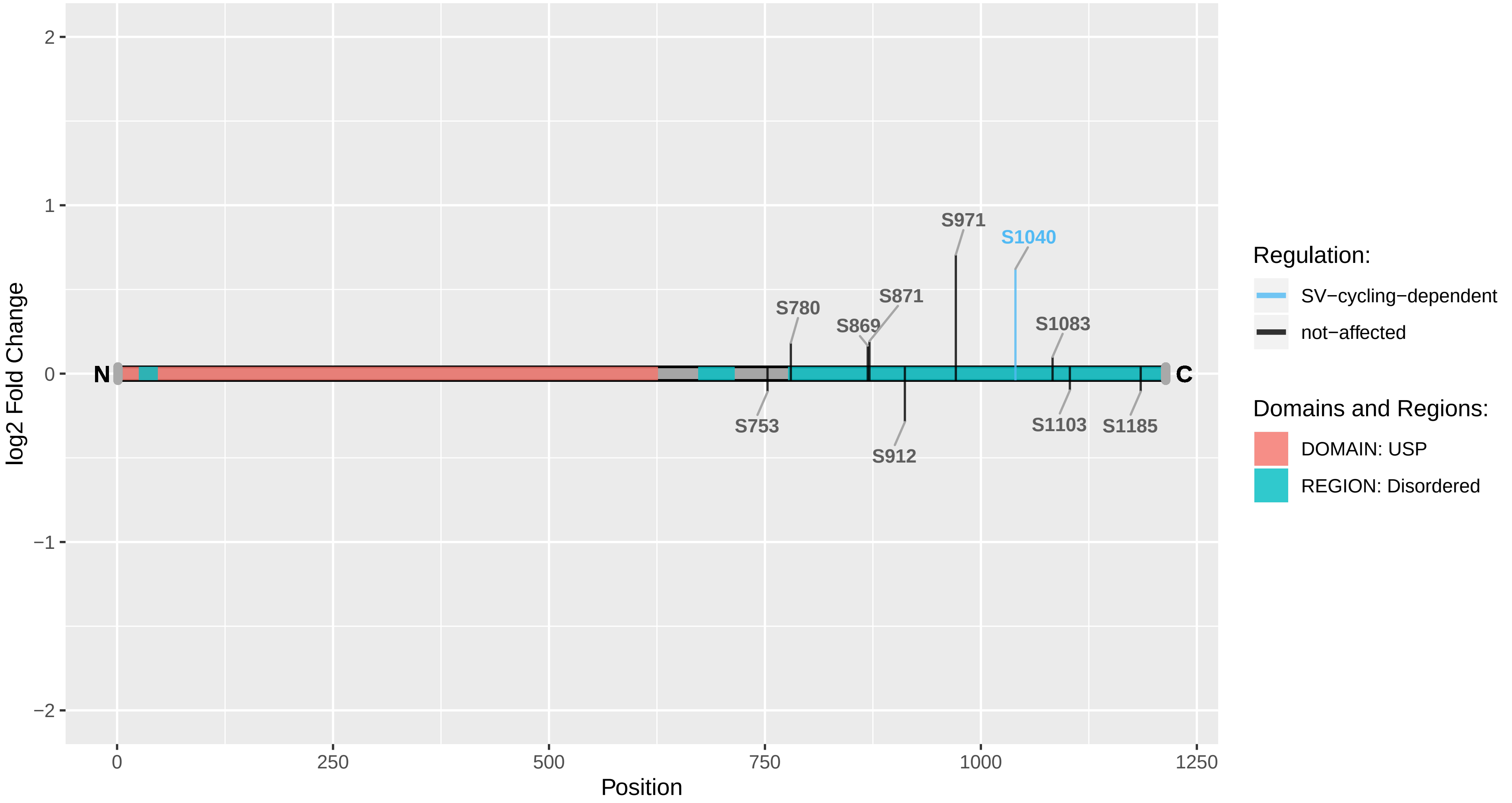

Usp32

Uniprot Accession: A0A0G2K952
Ubiquitin−specific peptidase 32

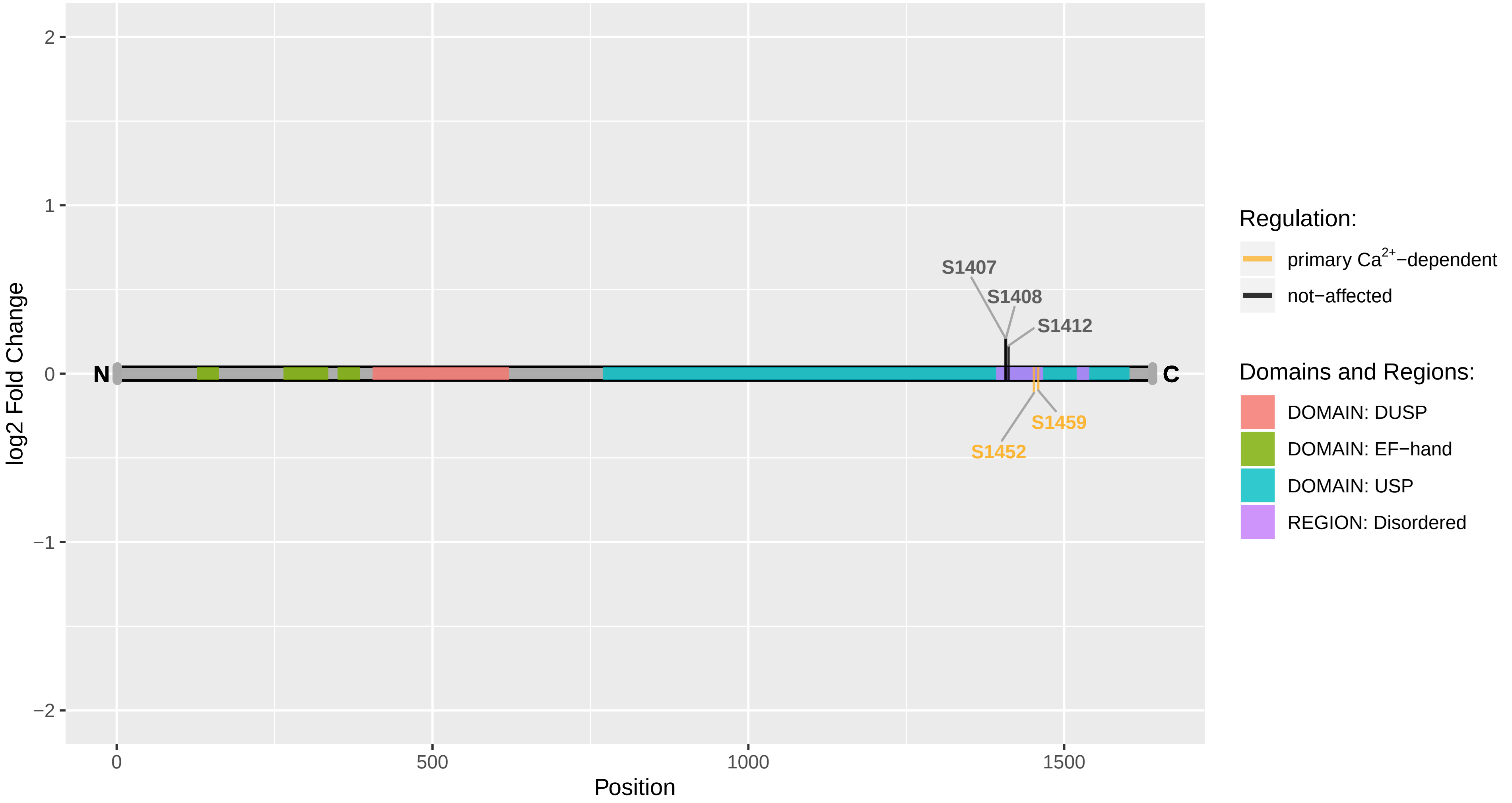

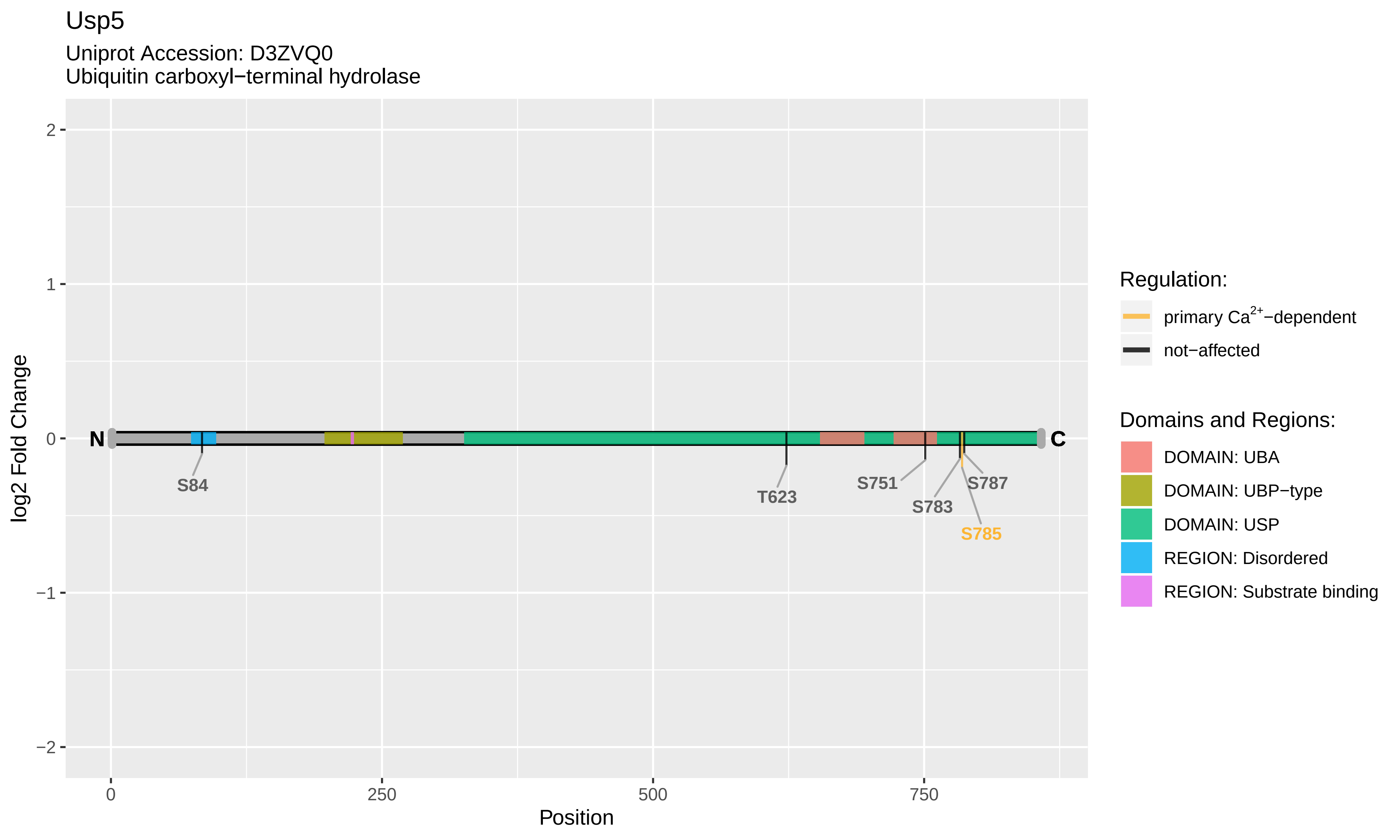
Usp5
Uniprot Accession: D3ZVQ0
Ubiquitin carboxyl−terminal hydrolase
log2 Fold Change
Position
N
C
S84
T623
S751
S783
S785
S787
Regulation:
primary Ca2+−dependent
not−affected
Domains and Regions:
DOMAIN: UBA
DOMAIN: UBP−type
DOMAIN: USP
REGION: Disordered
REGION: Substrate binding

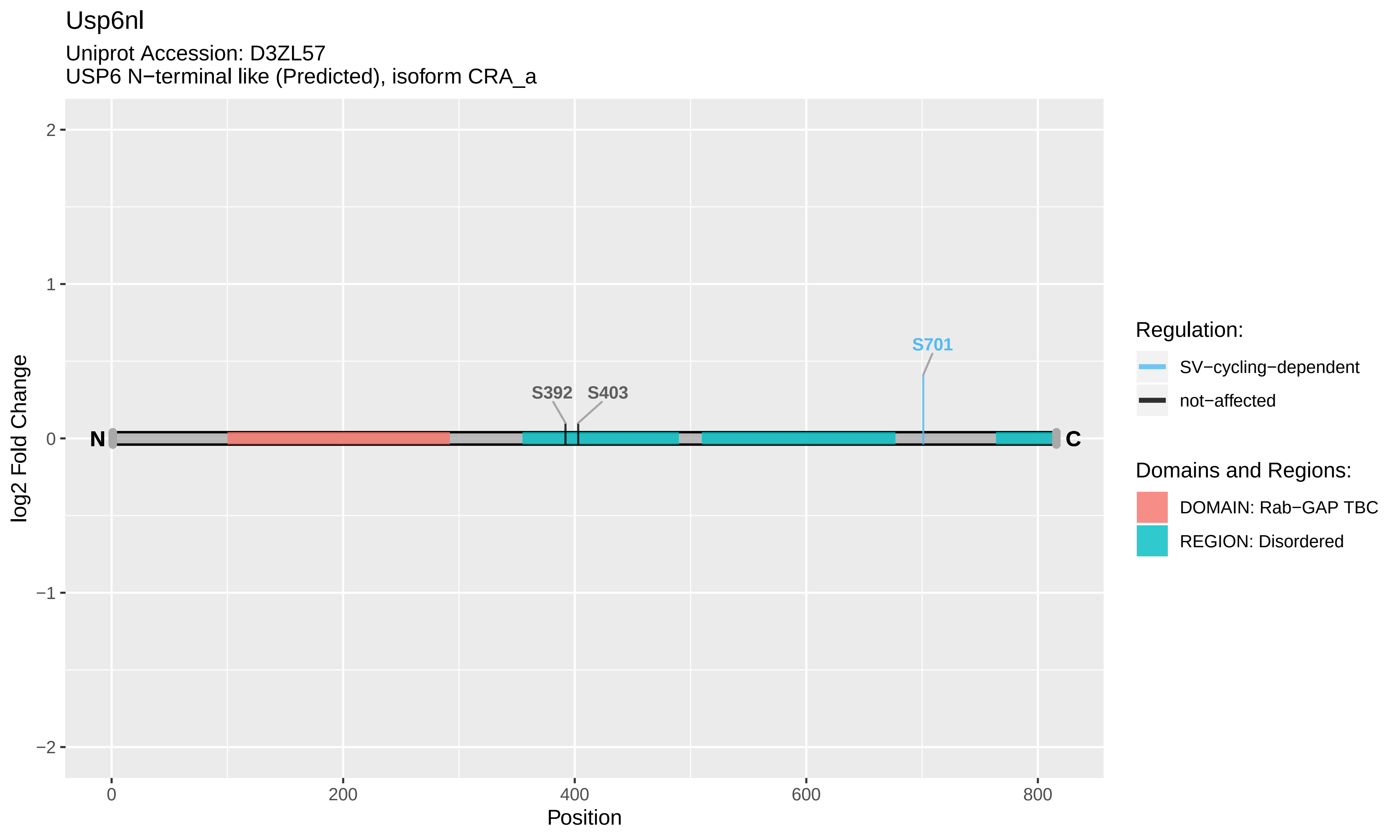

Usp6nl
Uniprot Accession: D3ZL57
USP6 N−terminal like (Predicted), isoform CRA_a
log2 Fold Change
Position
N
C
S392
S403
S701
Regulation:
SV−cycling−dependent
not−affected
Domains and Regions:
DOMAIN: Rab−GAP TBC
REGION: Disordered

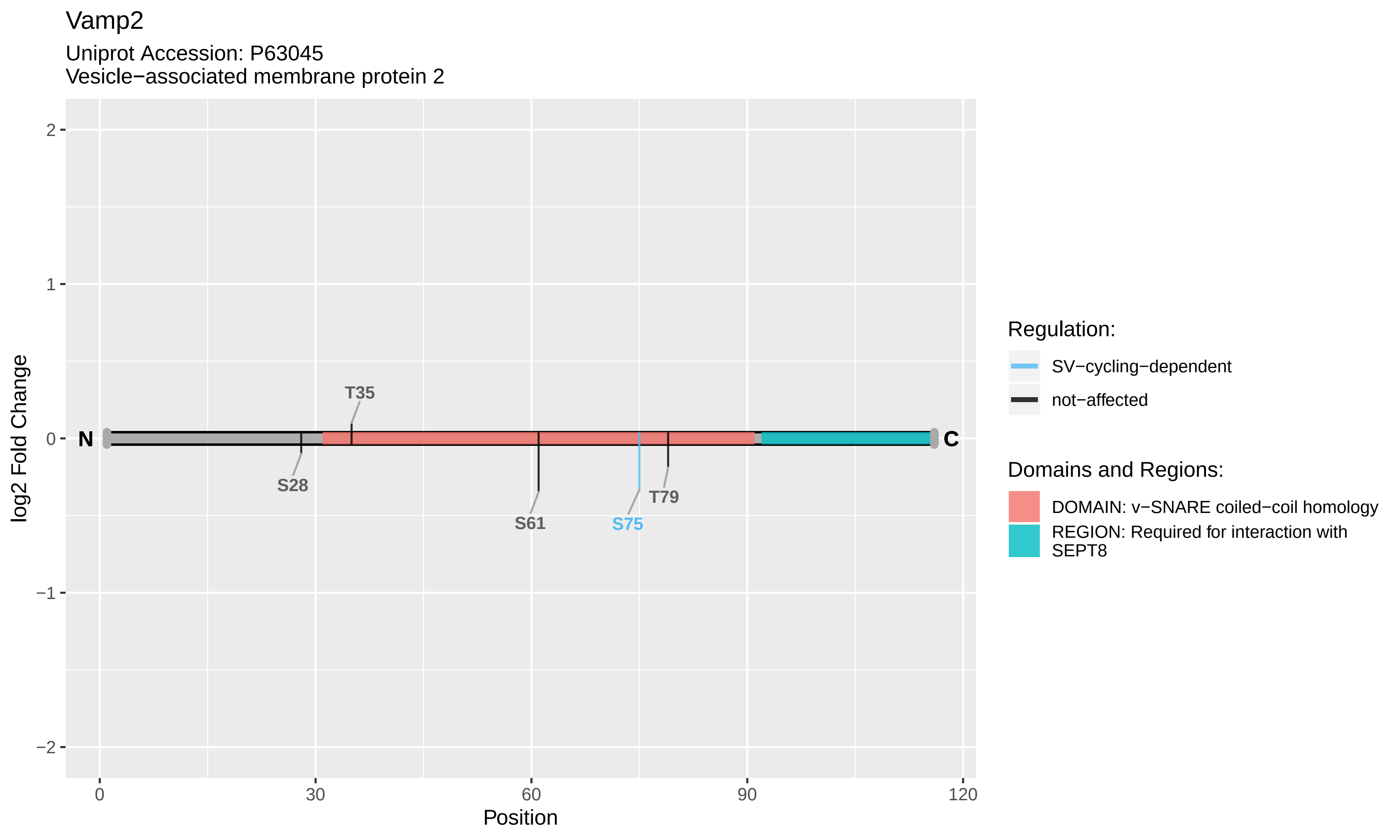

Vamp2
Uniprot Accession: P63045
Vesicle−associated membrane protein 2
log2 Fold Change
2
1
0
−1
−2
N
C
T35
S28
S61
S75
T79
0
30
60
90
120
Position
Regulation:
SV−cycling−dependent
not−affected
Domains and Regions:
DOMAIN: v−SNARE coiled−coil homology
REGION: Required for interaction with SEPT8

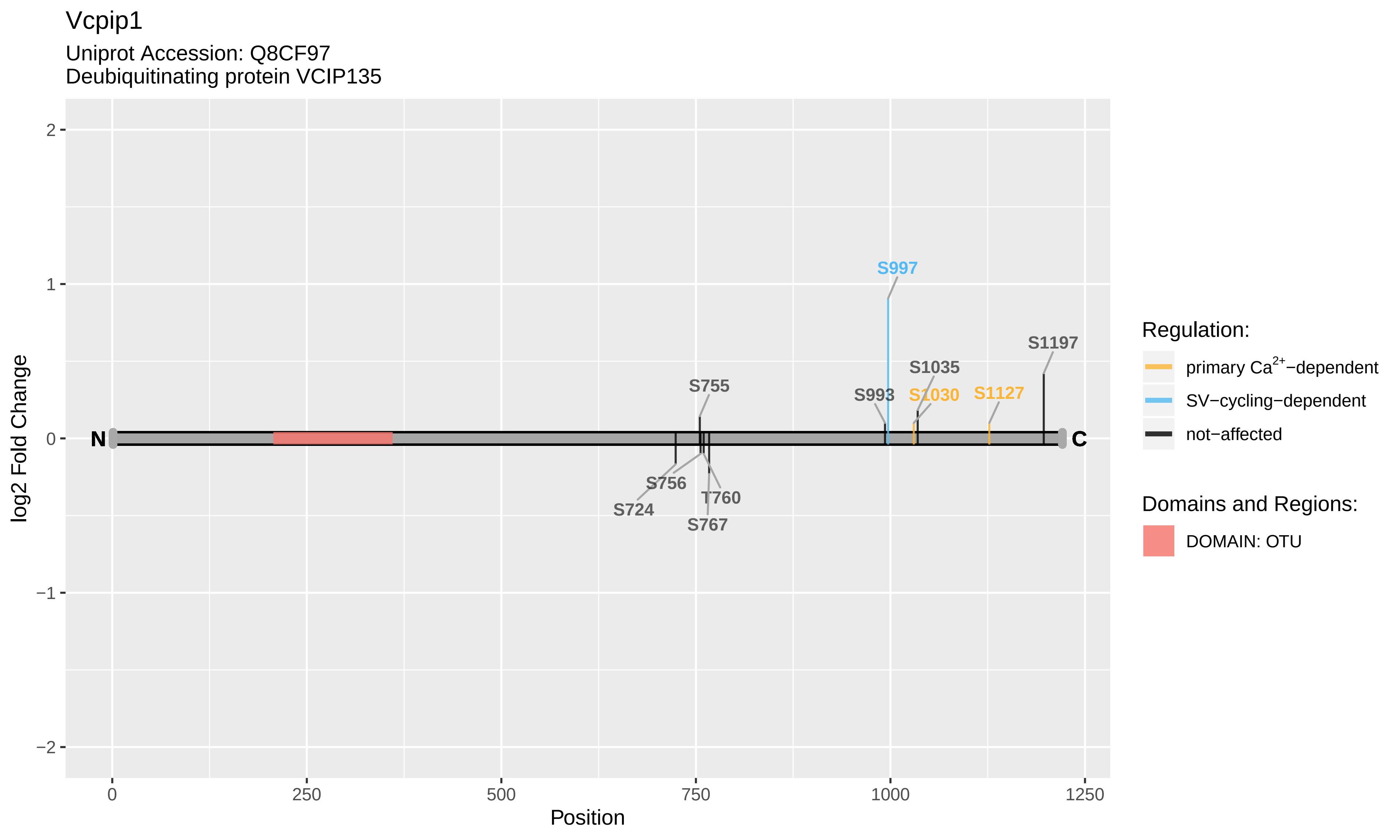
Vcpip1
Uniprot Accession: Q8CF97
Deubiquitinating protein VCIP135
log2 Fold Change
2
1
0
−1
−2
N
C
S997
S1197
S1035
S755
S993
S1030
S1127
S756
T760
S724
S767
0
250
500
750
1000
1250
Position
Regulation:
primary Ca2+−dependent
SV−cycling−dependent
not−affected
Domains and Regions:
DOMAIN: OTU

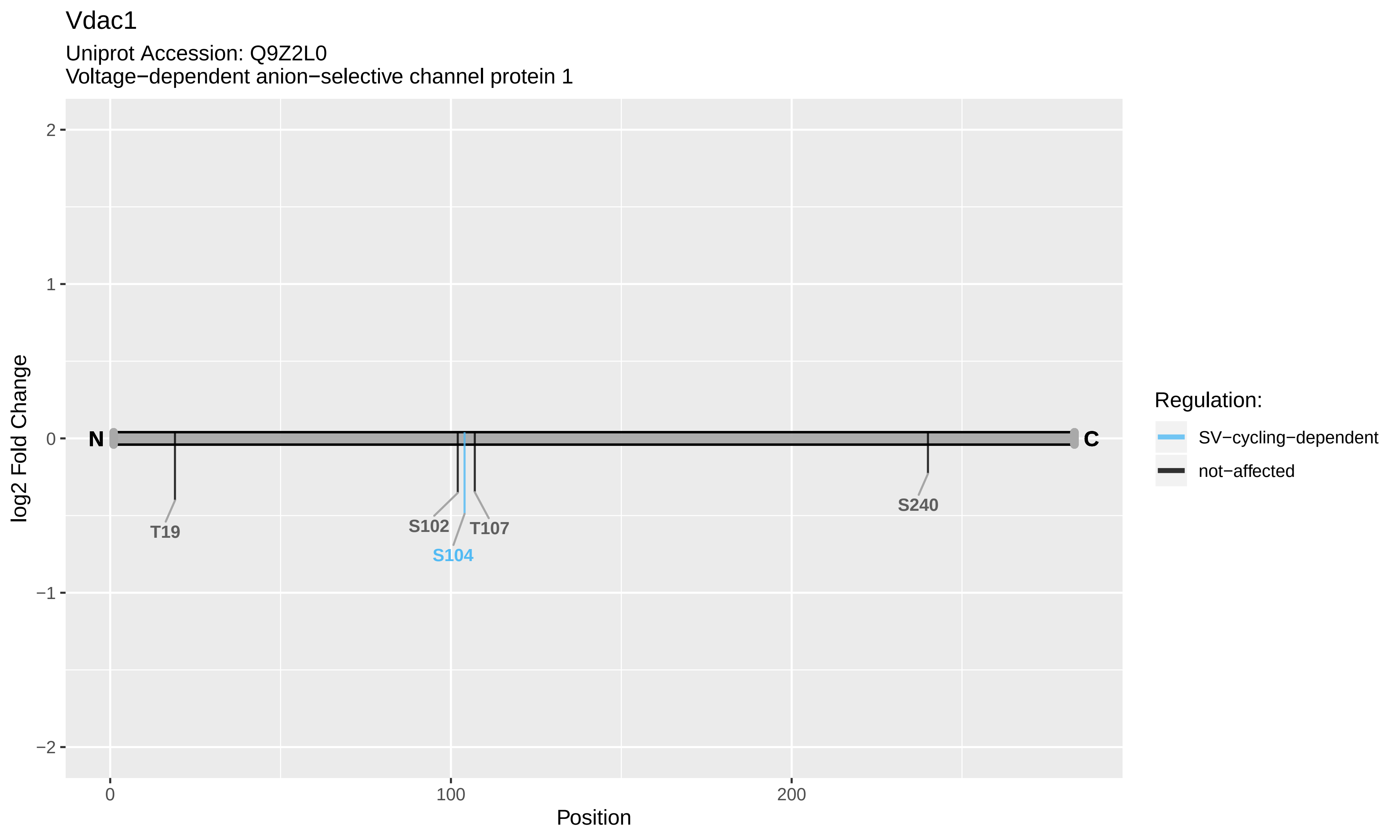
Vdac1
Uniprot Accession: Q9Z2L0
Voltage−dependent anion−selective channel protein 1
log2 Fold Change
2
1
0
−1
−2
N
C
T19
S102
S104
T107
S240
0
100
200
Position
Regulation:
SV−cycling−dependent
not−affected

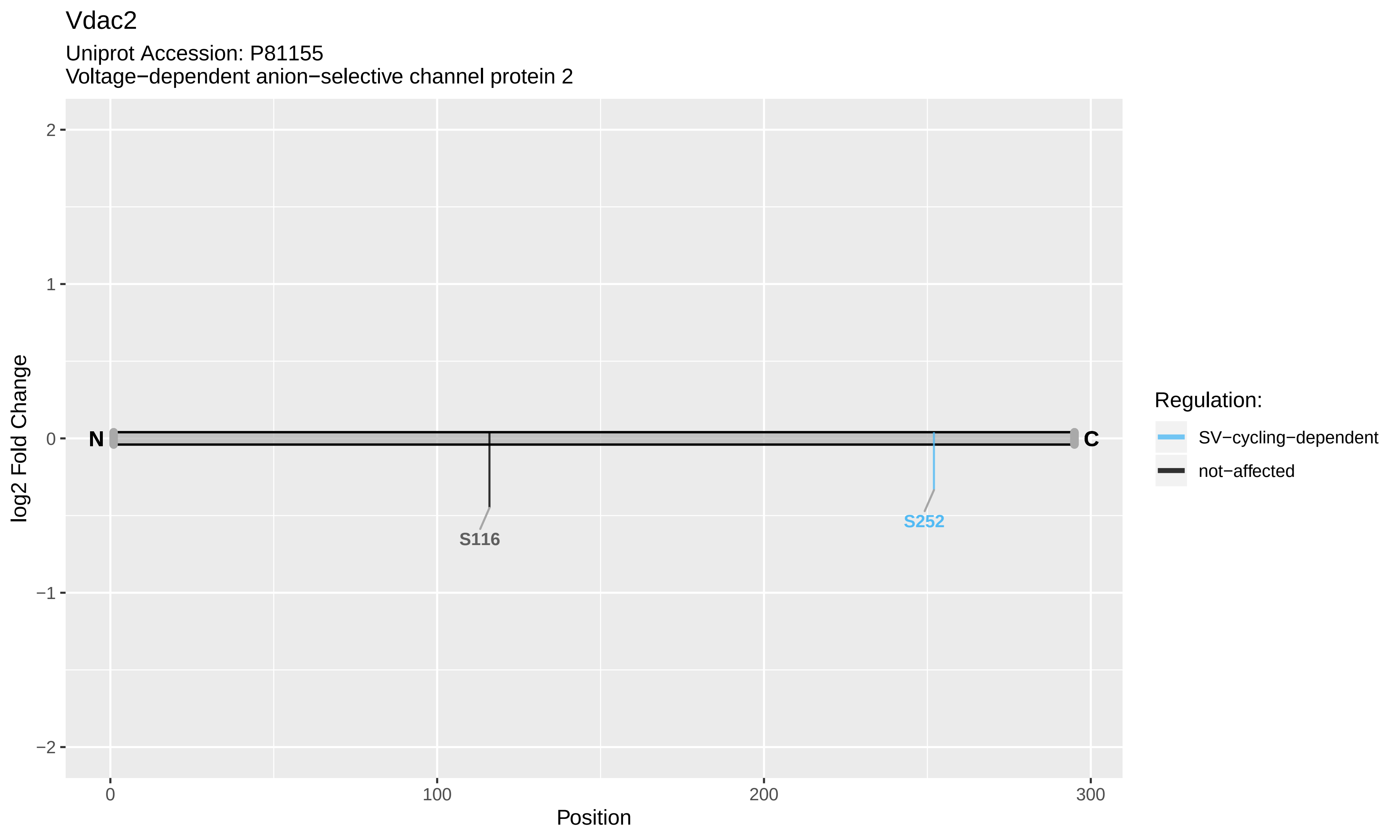

Vdac2
Uniprot Accession: P81155
Voltage−dependent anion−selective channel protein 2
log2 Fold Change
2
1
0
−1
−2
N
C
S116
S252
0
100
200
300
Position
Regulation:
SV−cycling−dependent
not−affected

Vps4a

Uniprot Accession: Q793F9
Vacuolar protein sorting−associated protein 4A

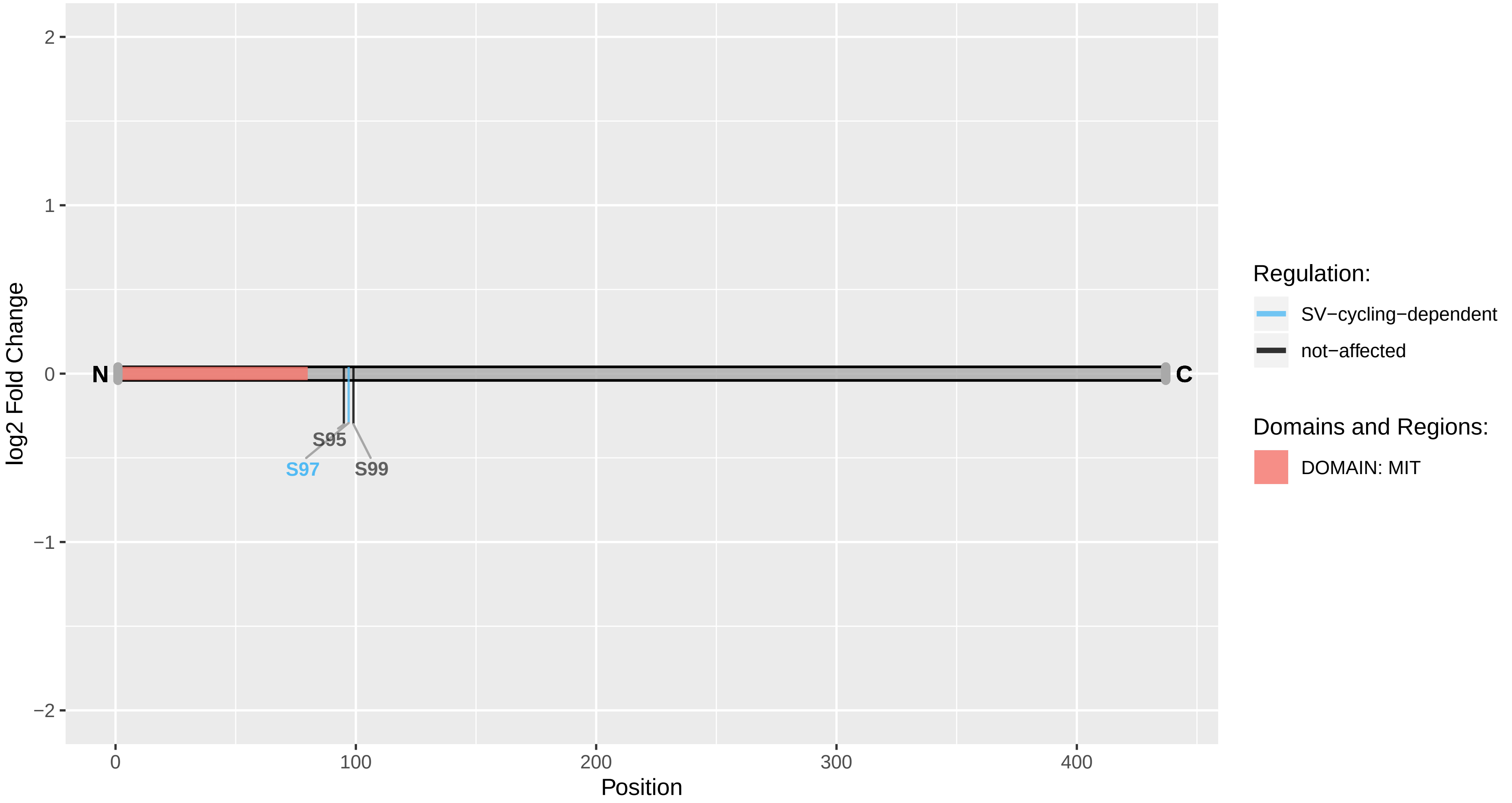

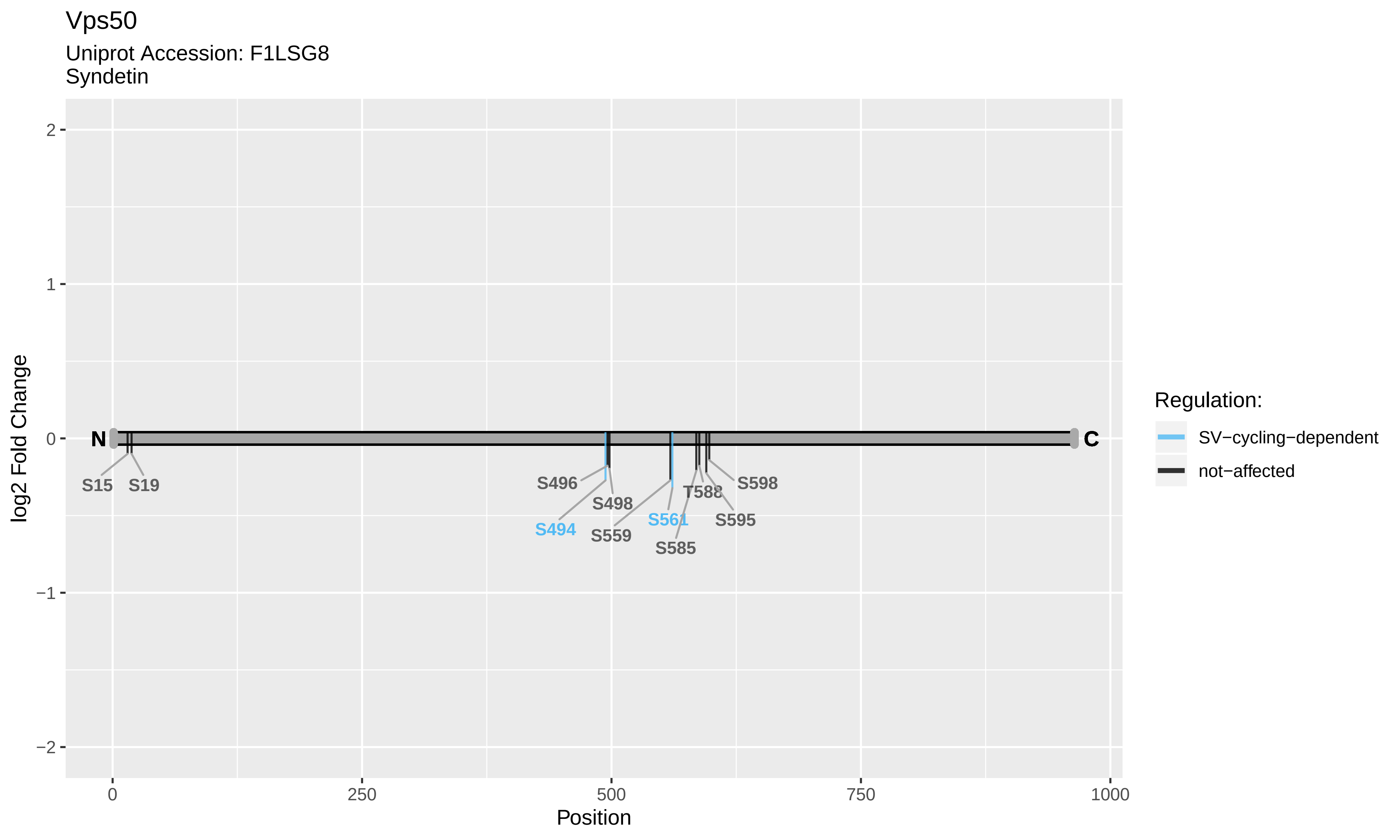

Vps50
Uniprot Accession: F1LSG8
Syndetin
log2 Fold Change
Position
N
C
S15
S19
S496
S498
S494
S559
S561
S585
T588
S595
S598
Regulation:
SV−cycling−dependent
not−affected

Vwa5b1

Uniprot Accession: D3ZP60
von Willebrand factor A domain−containing 5B1

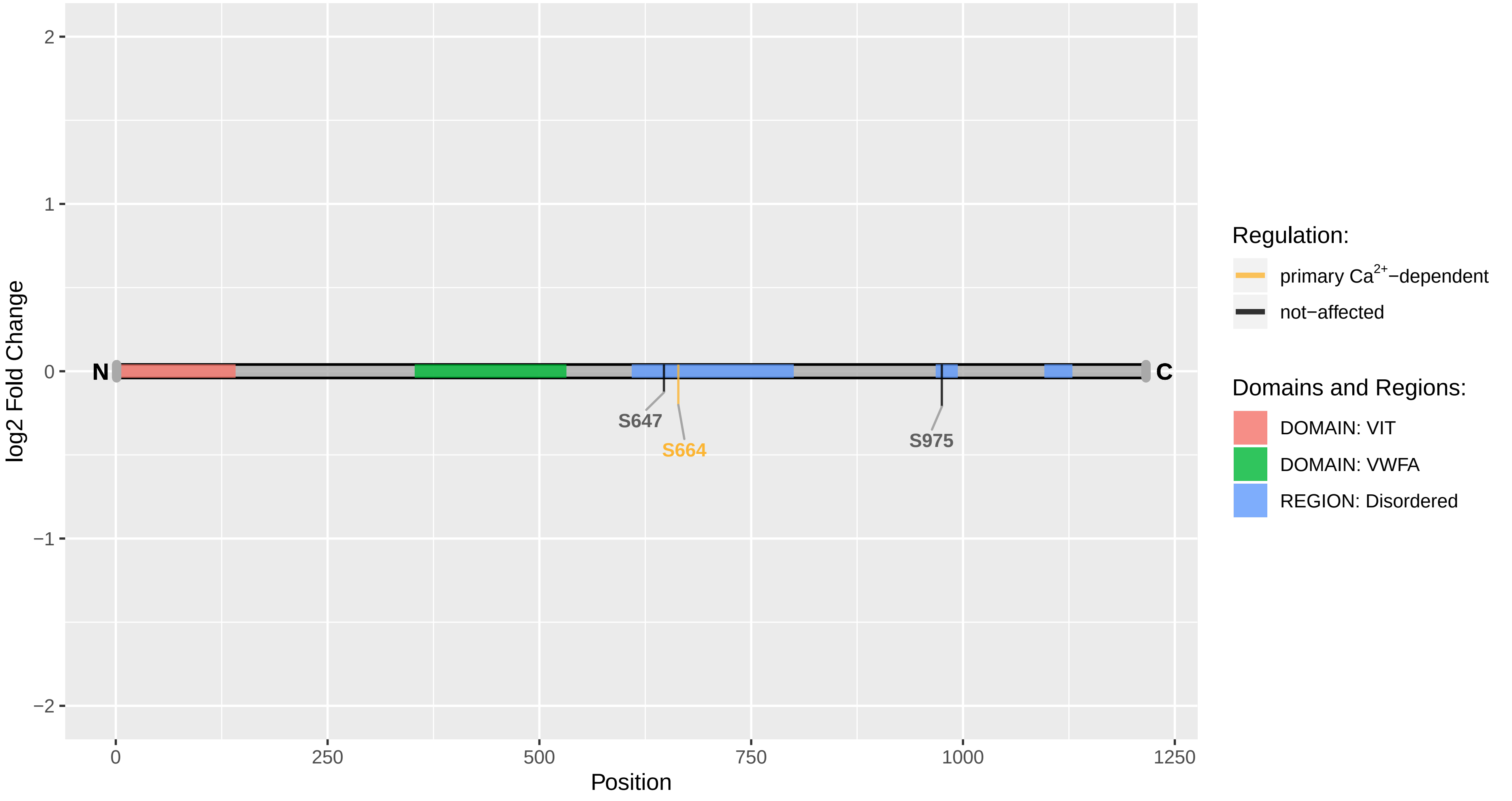

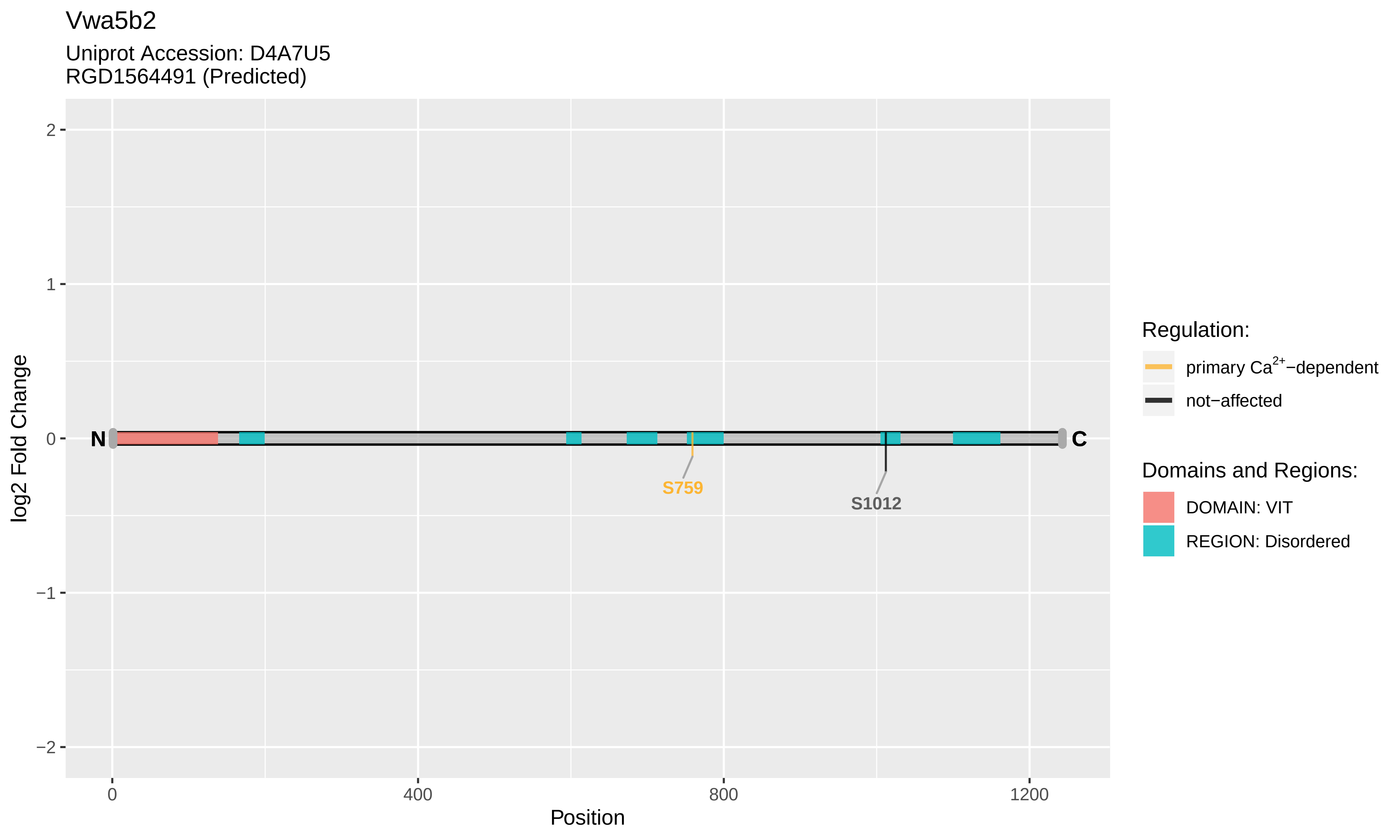

Vwa5b2
Uniprot Accession: D4A7U5
RGD1564491 (Predicted)
log2 Fold Change
2
1
0
−1
−2
N
C
S759
S1012
0
400
800
1200
Position
Regulation:
primary Ca2+−dependent
not−affected
Domains and Regions:
DOMAIN: VIT
REGION: Disordered

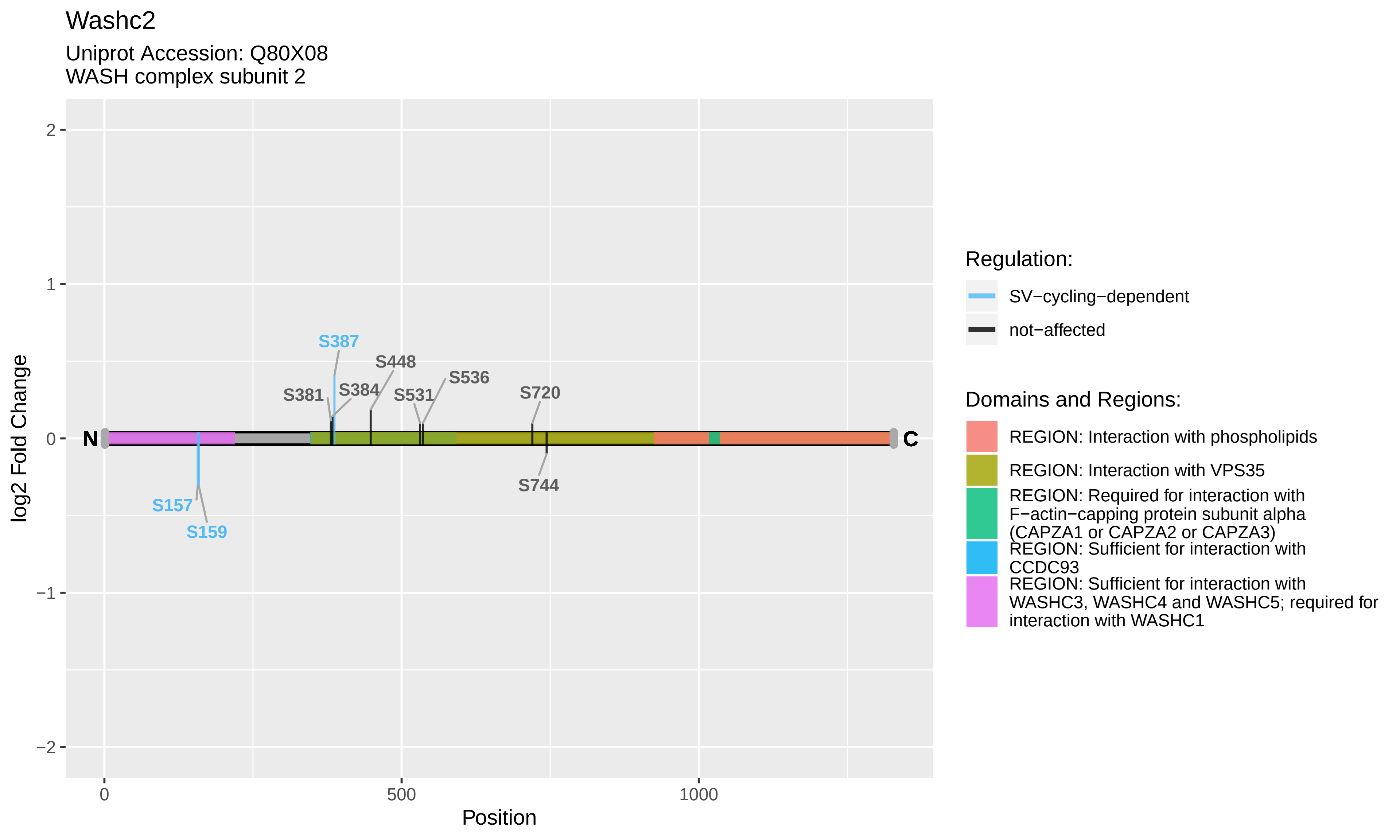
Washc2
Uniprot Accession: Q80X08
WASH complex subunit 2
log2 Fold Change
2
1
0
−1
−2
0
500
1000
Position
N
C
S387
S448
S381
S384
S531
S536
S720
S744
S157
S159
Regulation:
SV−cycling−dependent
not−affected
Domains and Regions:
REGION: Interaction with phospholipids
REGION: Interaction with VPS35
REGION: Required for interaction with F−actin−capping protein subunit alpha (CAPZA1 or CAPZA2 or CAPZA3)
REGION: Sufficient for interaction with CCDC93
REGION: Sufficient for interaction with WASHC3, WASHC4 and WASHC5; required for interaction with WASHC1

Wdfy3

Uniprot Accession: A0A0G2K9M4
WD repeat and FYVE domain−containing 3

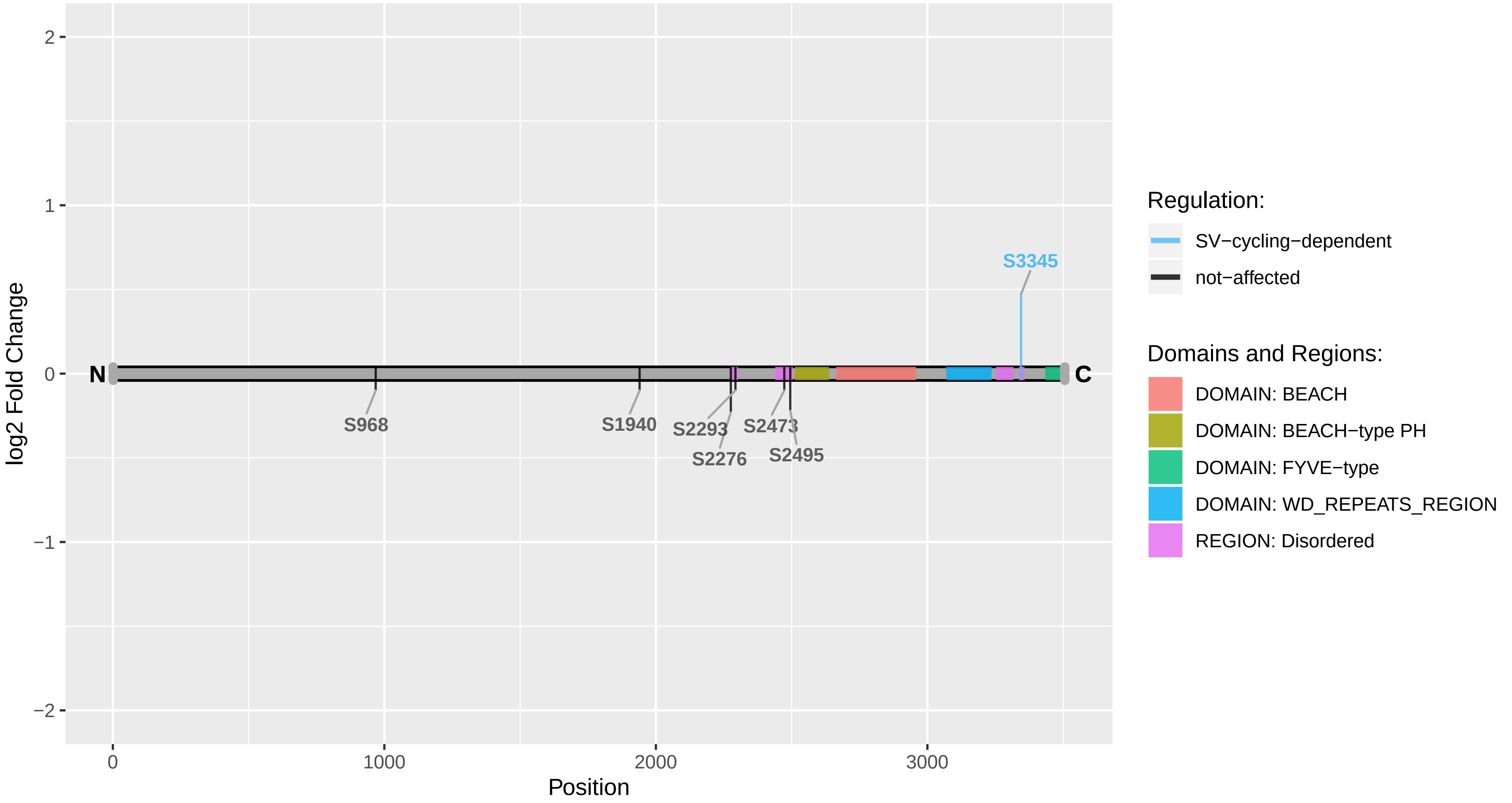

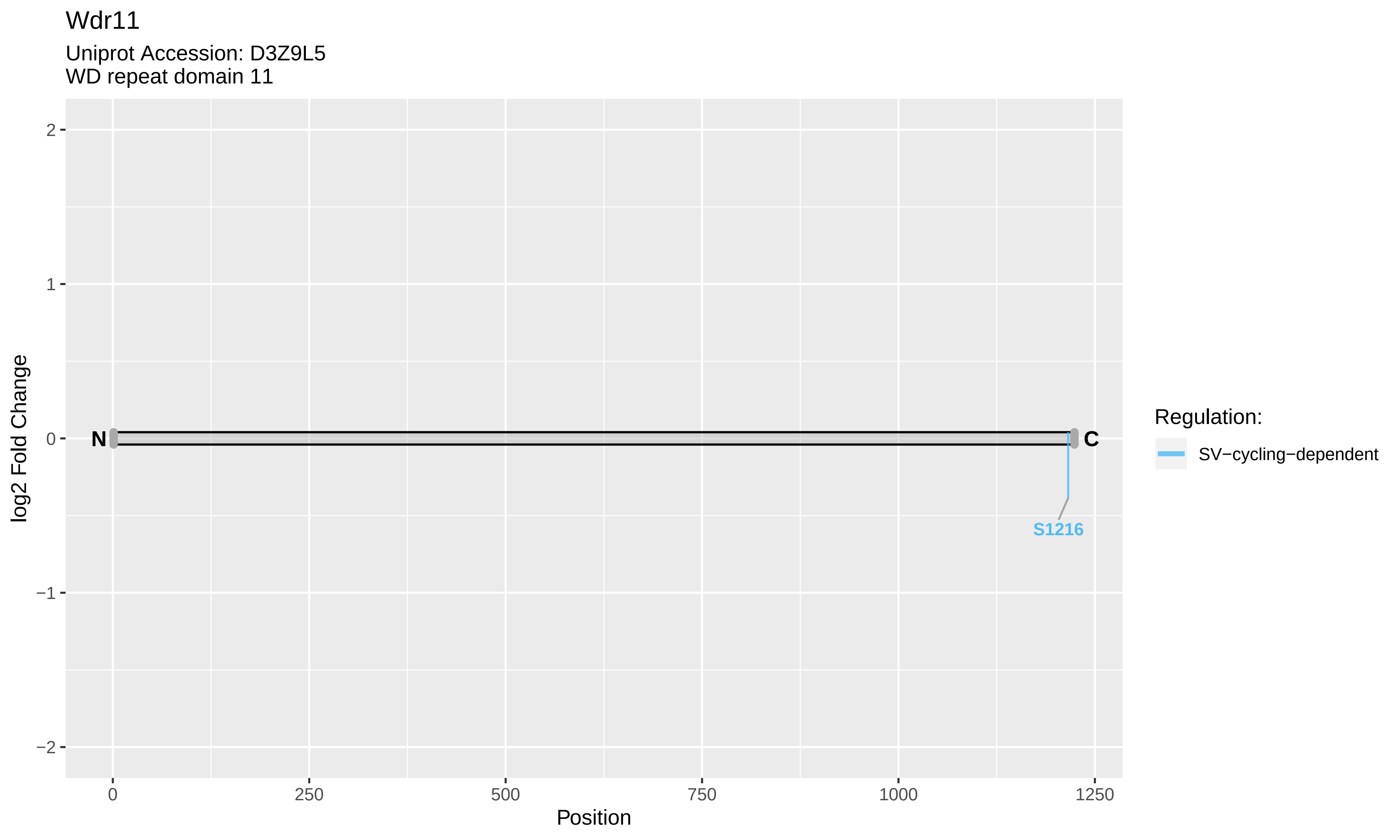
Wdr11
Uniprot Accession: D3Z9L5
WD repeat domain 11
log2 Fold Change
2
1
0
−1
−2
N
C
S1216
0
250
500
750
1000
1250
Position
Regulation:
SV−cycling−dependent

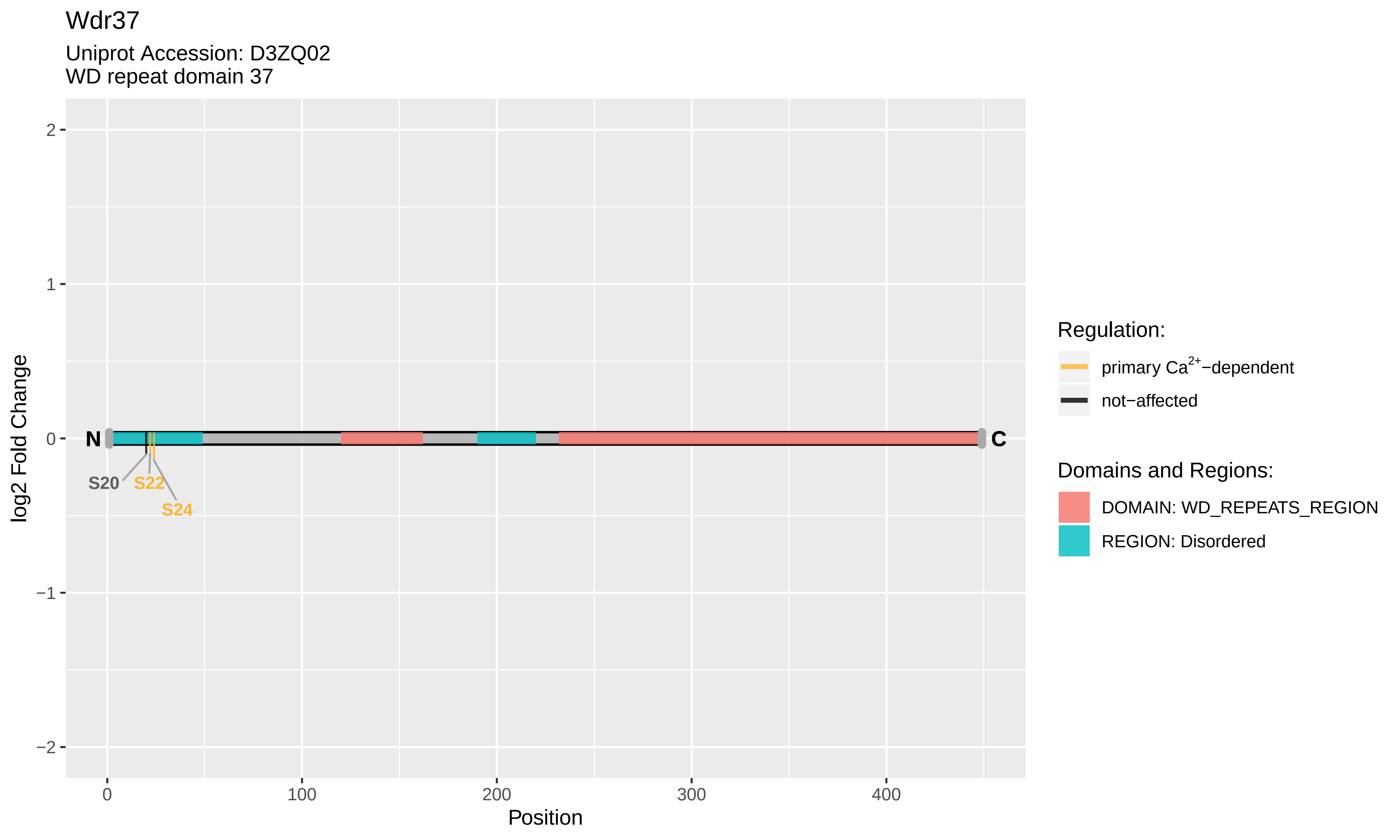
Wdr37
Uniprot Accession: D3ZQ02
WD repeat domain 37
log2 Fold Change
2
1
0
−1
−2
N
C
S20
S22
S24
0
100
200
300
400
Position
Regulation:
primary Ca2+−dependent
not−affected
Domains and Regions:
DOMAIN: WD_REPEATS_REGION
REGION: Disordered

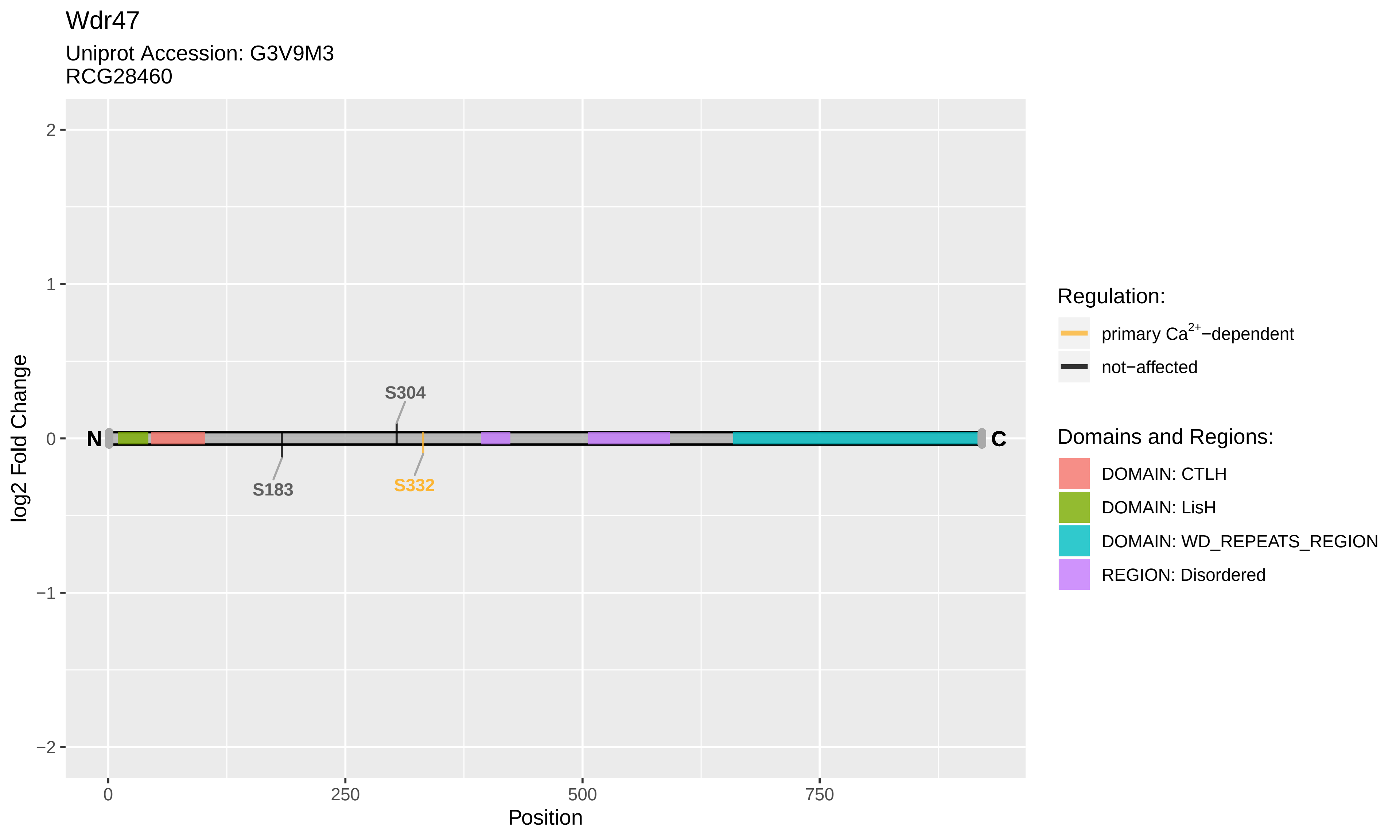
Wdr47
Uniprot Accession: G3V9M3
RCG28460
log2 Fold Change
2
1
0
−1
−2
N
C
S304
S183
S332
0
250
500
750
Position
Regulation:
primary Ca2+−dependent
not−affected
Domains and Regions:
DOMAIN: CTLH
DOMAIN: LisH
DOMAIN: WD_REPEATS_REGION
REGION: Disordered

Wdr48

Uniprot Accession: A0A0G2KAW5
WD repeat domain 48

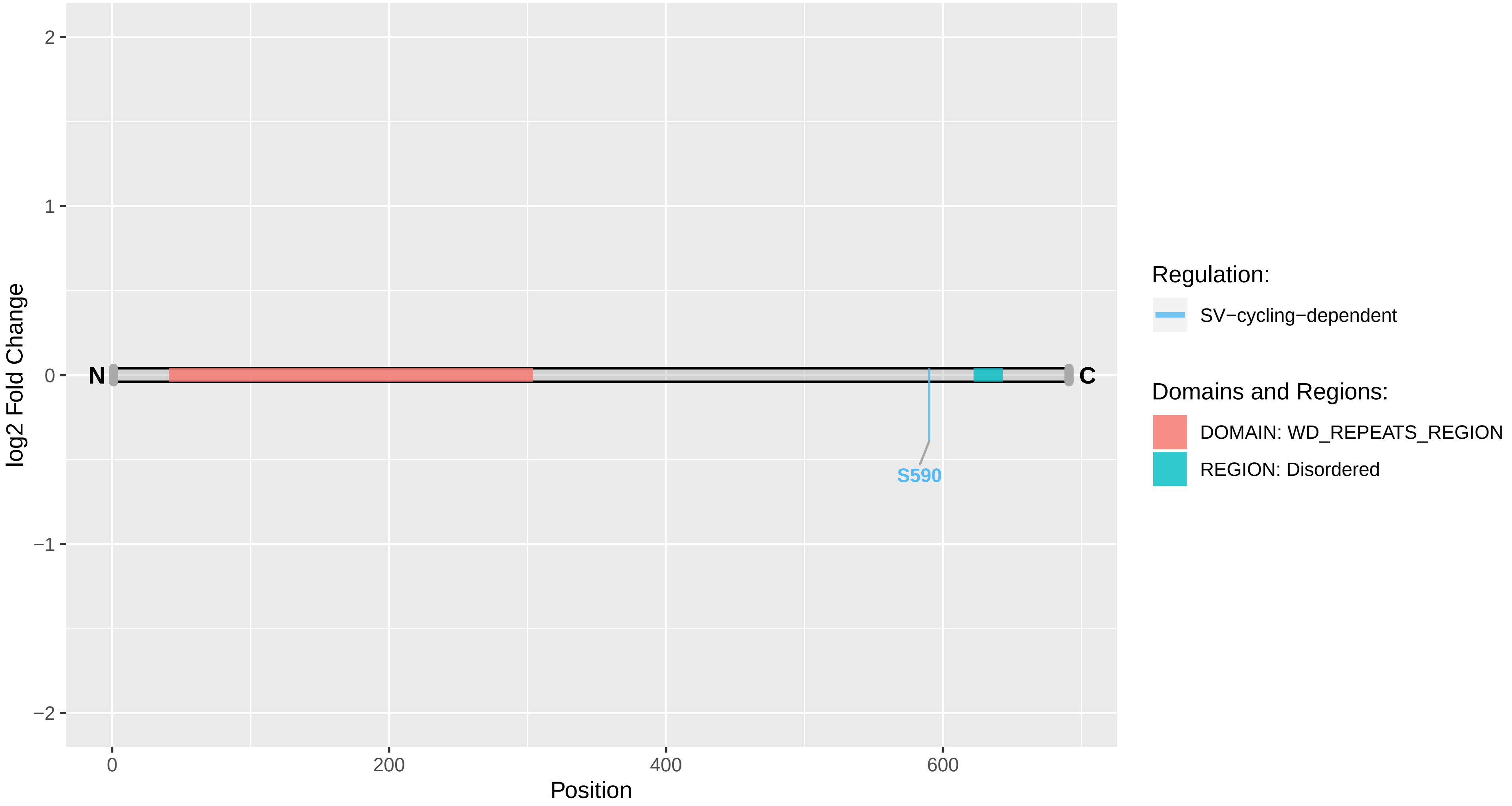

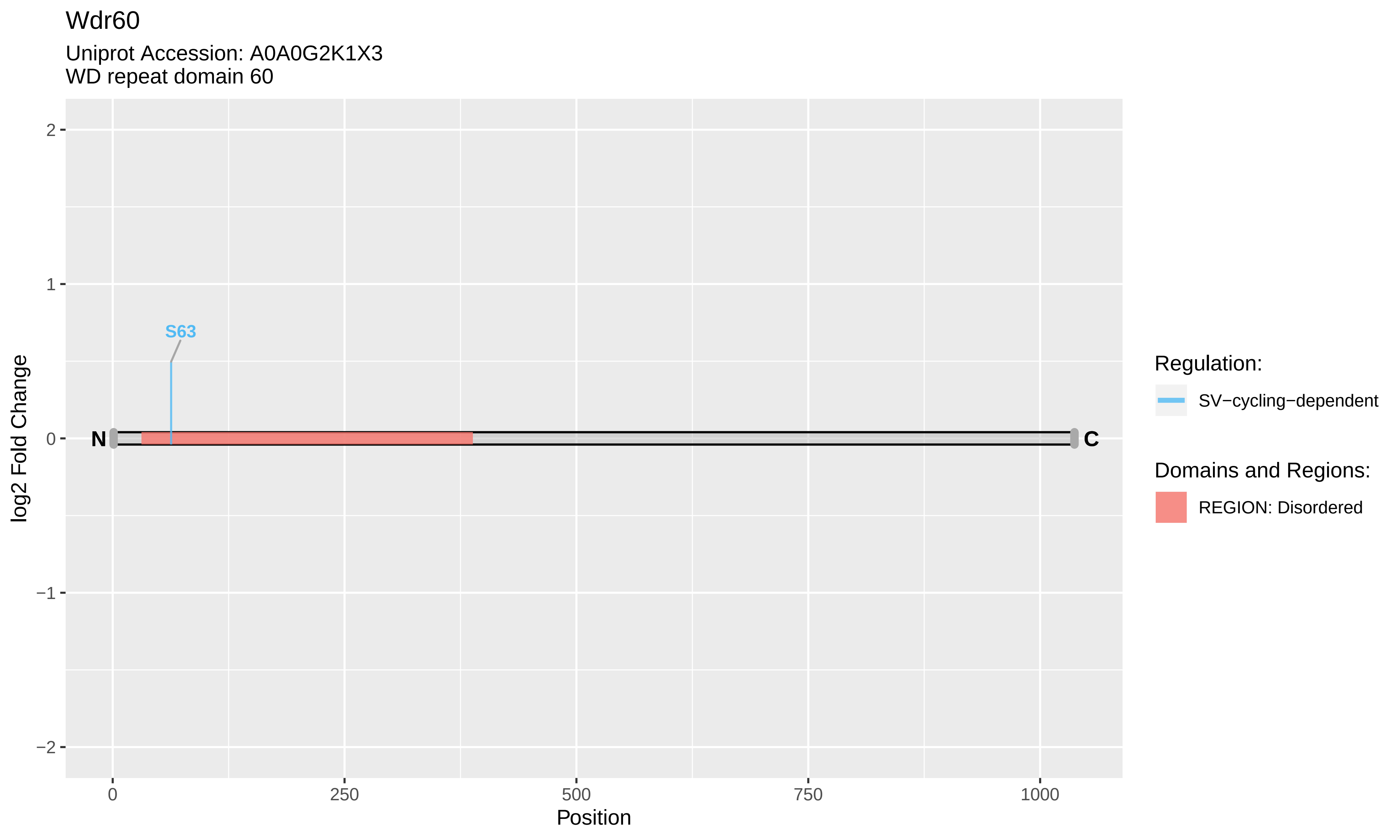

Wdr60
Uniprot Accession: A0A0G2K1X3
WD repeat domain 60
S63
N
C
2
1
0
−1
−2
log2 Fold Change
0
250
500
750
1000
Position
Regulation:
SV−cycling−dependent
Domains and Regions:
REGION: Disordered

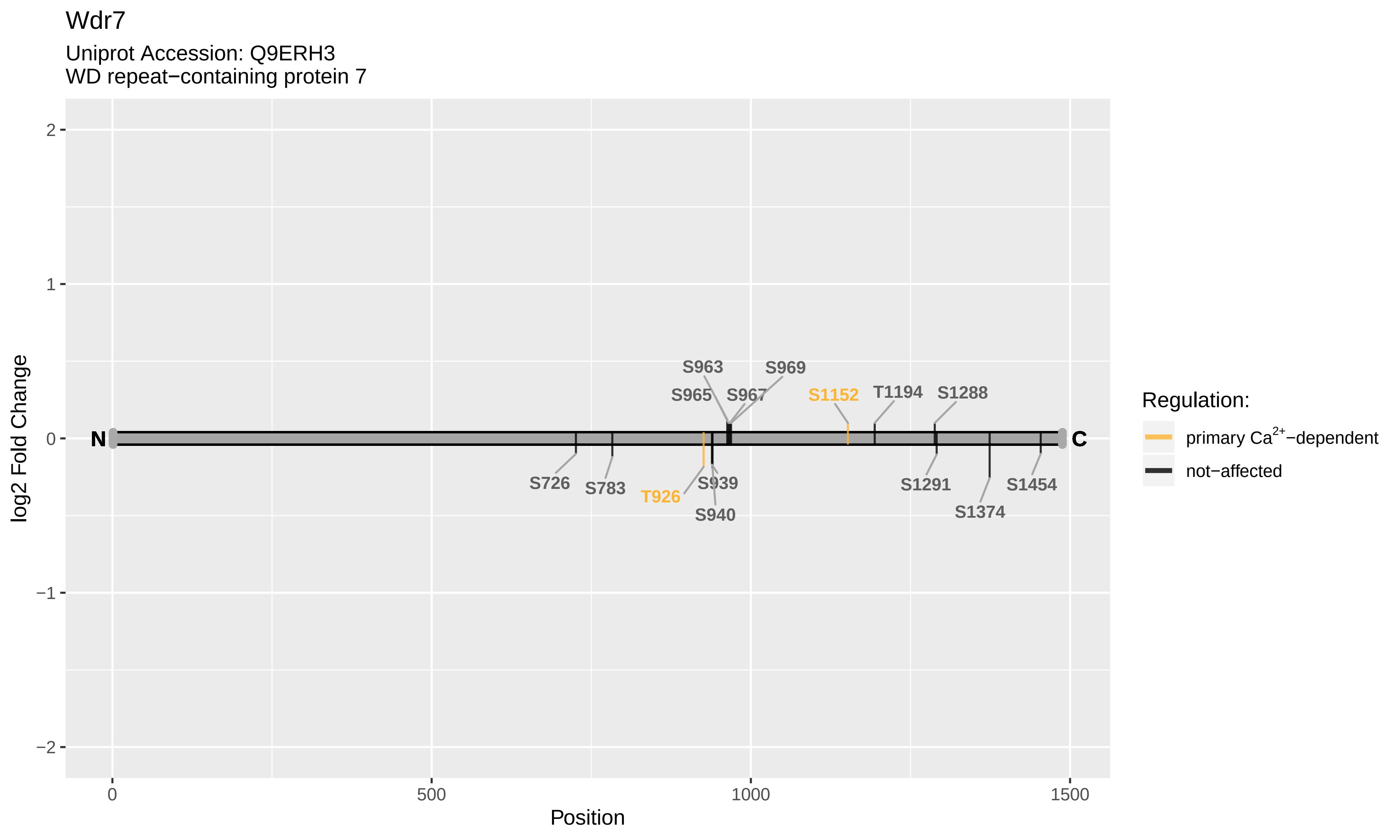
Wdr7
Uniprot Accession: Q9ERH3
WD repeat−containing protein 7
log2 Fold Change
2
1
0
−1
−2
N
C
S963
S969
S965
S967
S1152
T1194
S1288
S726
S783
T926
S939
S940
S1291
S1374
S1454
0
500
1000
1500
Position
Regulation:
primary Ca2+−dependent
not−affected

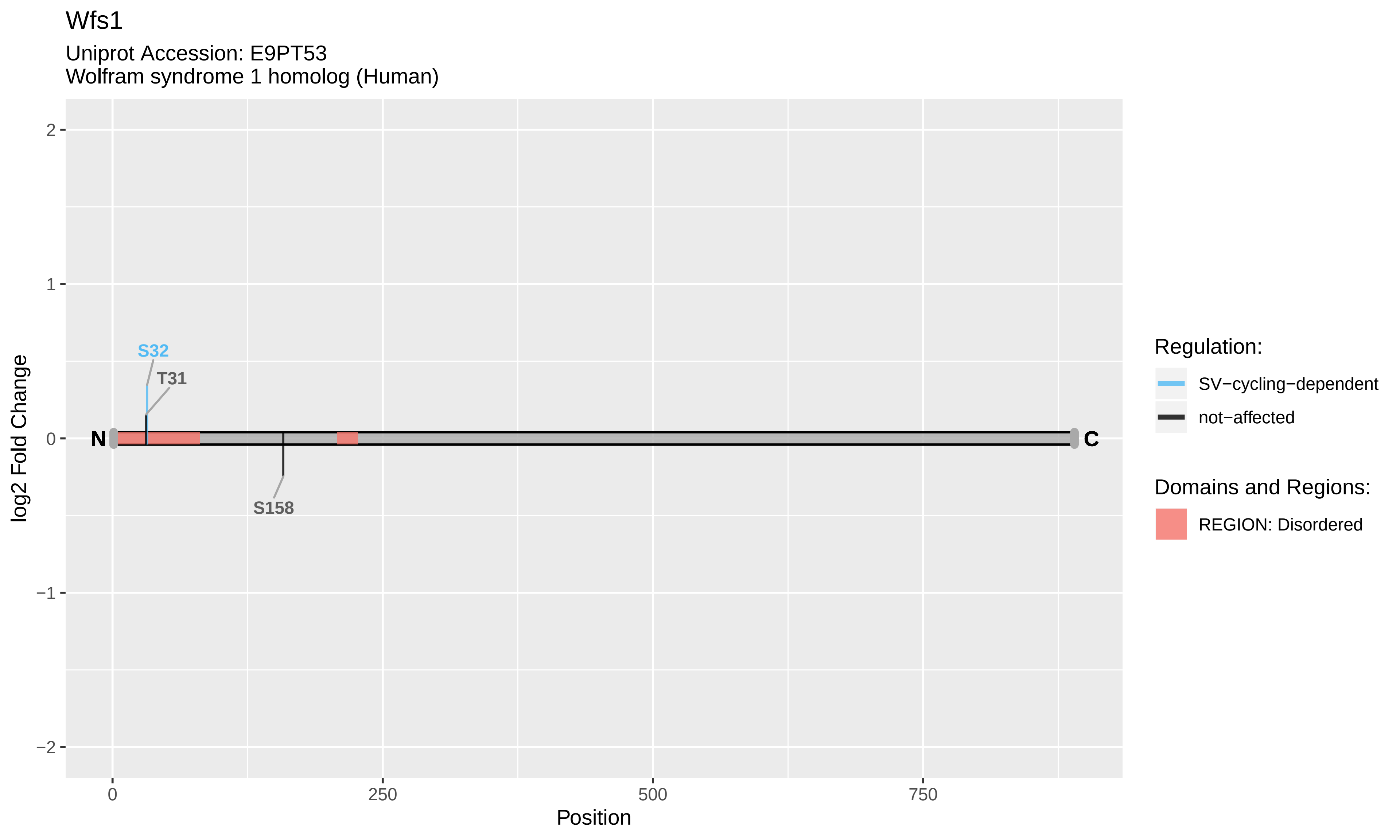
Wfs1
Uniprot Accession: E9PT53
Wolfram syndrome 1 homolog (Human)
S32
T31
S158
N
C
log2 Fold Change
2
1
0
−1
−2
0
250
500
750
Position
Regulation:
SV−cycling−dependent
not−affected
Domains and Regions:
REGION: Disordered

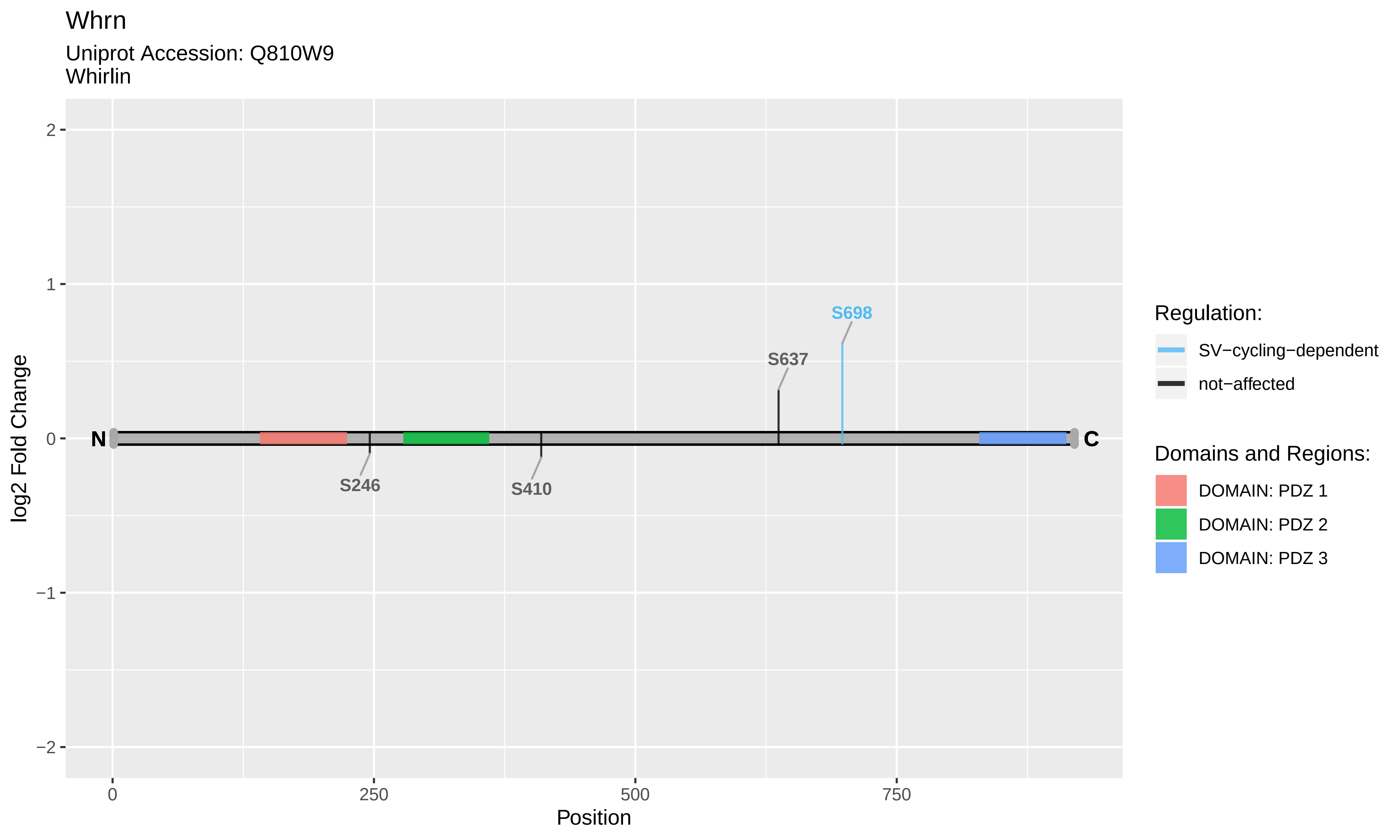

Whrn
Uniprot Accession: Q810W9
Whirlin
log2 Fold Change
Position
N
C
S246
S410
S637
S698
Regulation:
SV−cycling−dependent
not−affected
Domains and Regions:
DOMAIN: PDZ 1
DOMAIN: PDZ 2
DOMAIN: PDZ 3

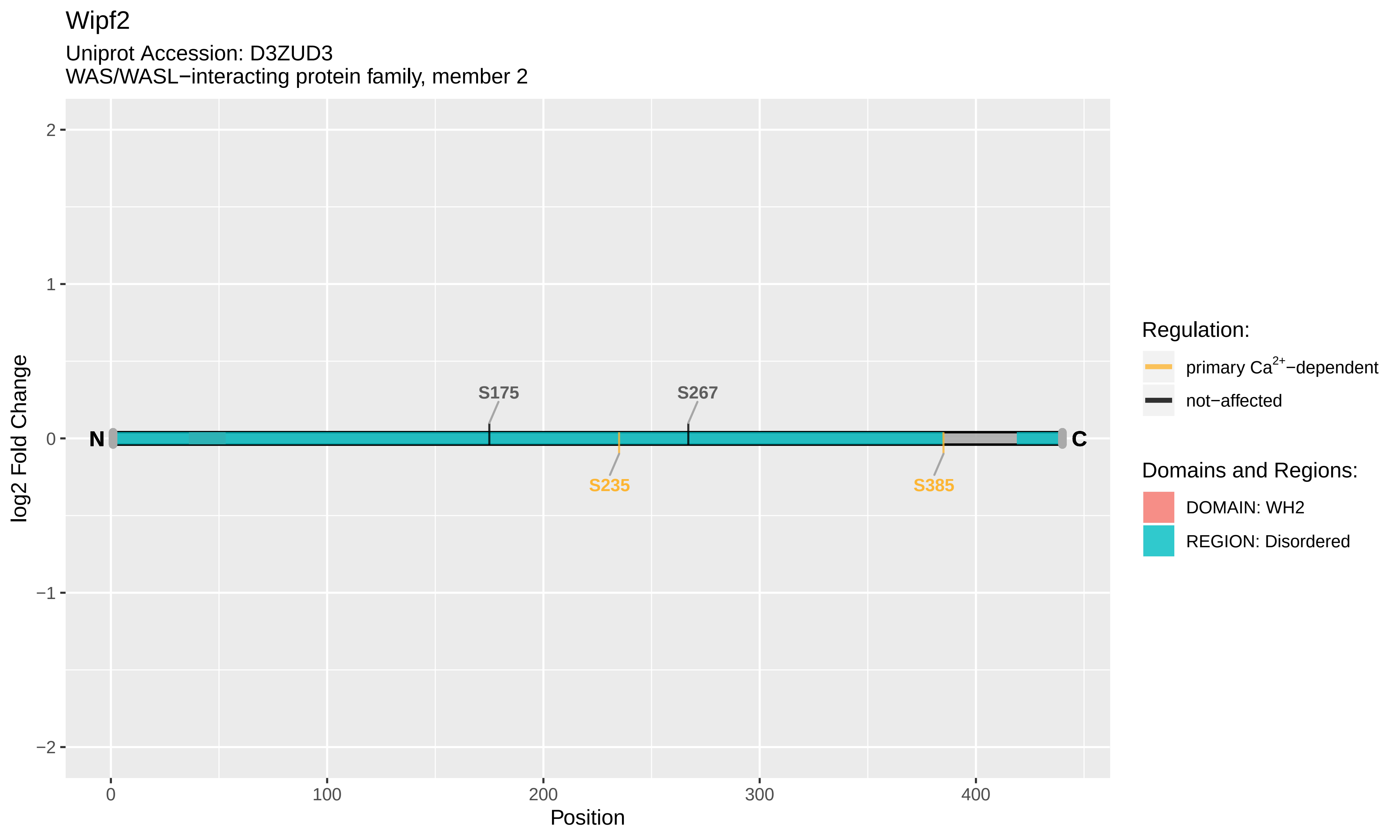

Wipf2
Uniprot Accession: D3ZUD3
WAS/WASL−interacting protein family, member 2
log2 Fold Change
2
1
0
−1
−2
N
C
S175
S267
S235
S385
0
100
200
300
400
Position
Regulation:
primary Ca2+−dependent
not−affected
Domains and Regions:
DOMAIN: WH2
REGION: Disordered

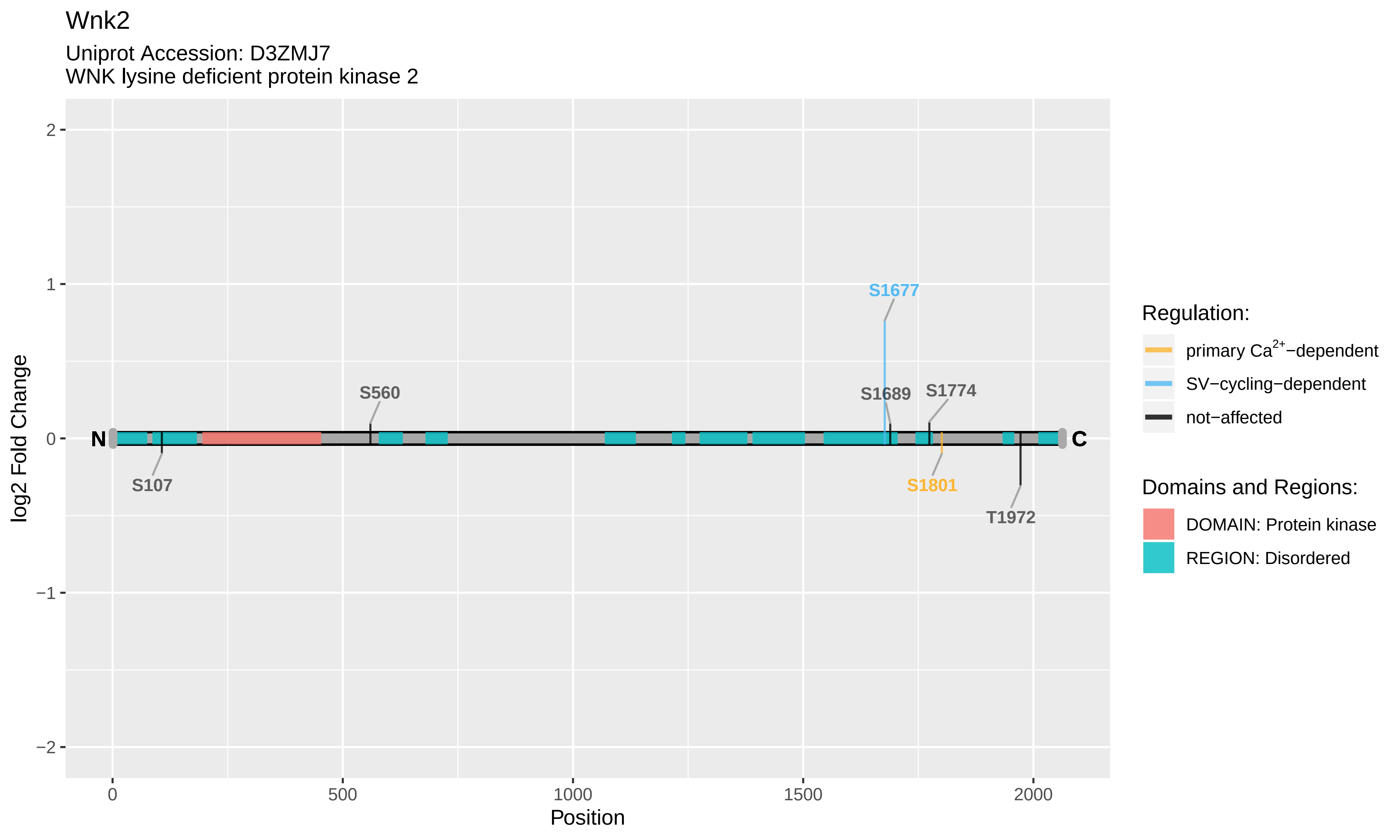

Wnk2
Uniprot Accession: D3ZMJ7
WNK lysine deficient protein kinase 2
log2 Fold Change
Position
N
C
S107
S560
S1677
S1689
S1774
S1801
T1972
Regulation:
primary Ca2+−dependent
SV−cycling−dependent
not−affected
Domains and Regions:
DOMAIN: Protein kinase
REGION: Disordered

Wrnip1

Uniprot Accession: Q8CG07
ATPase WRNIP1

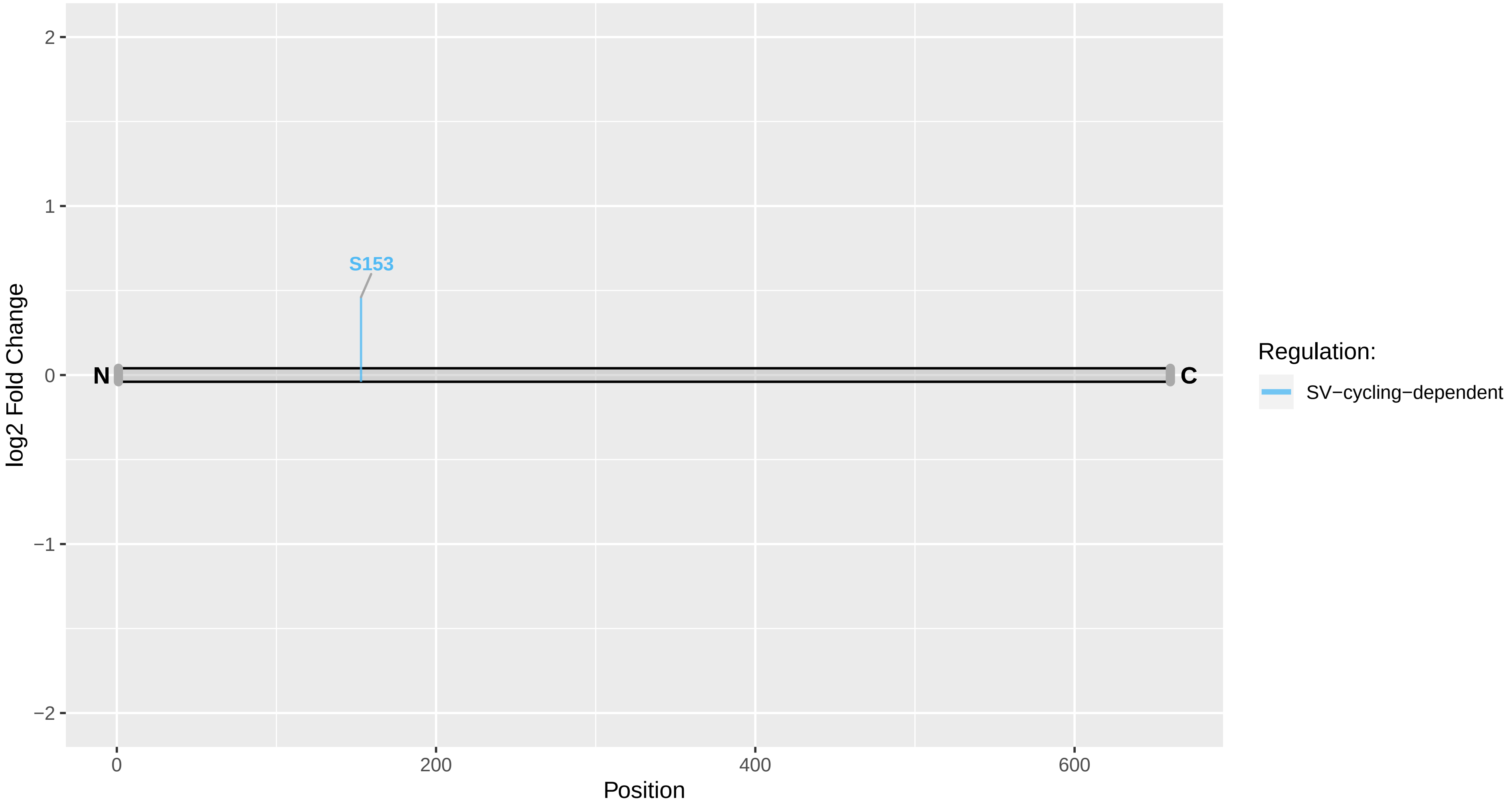

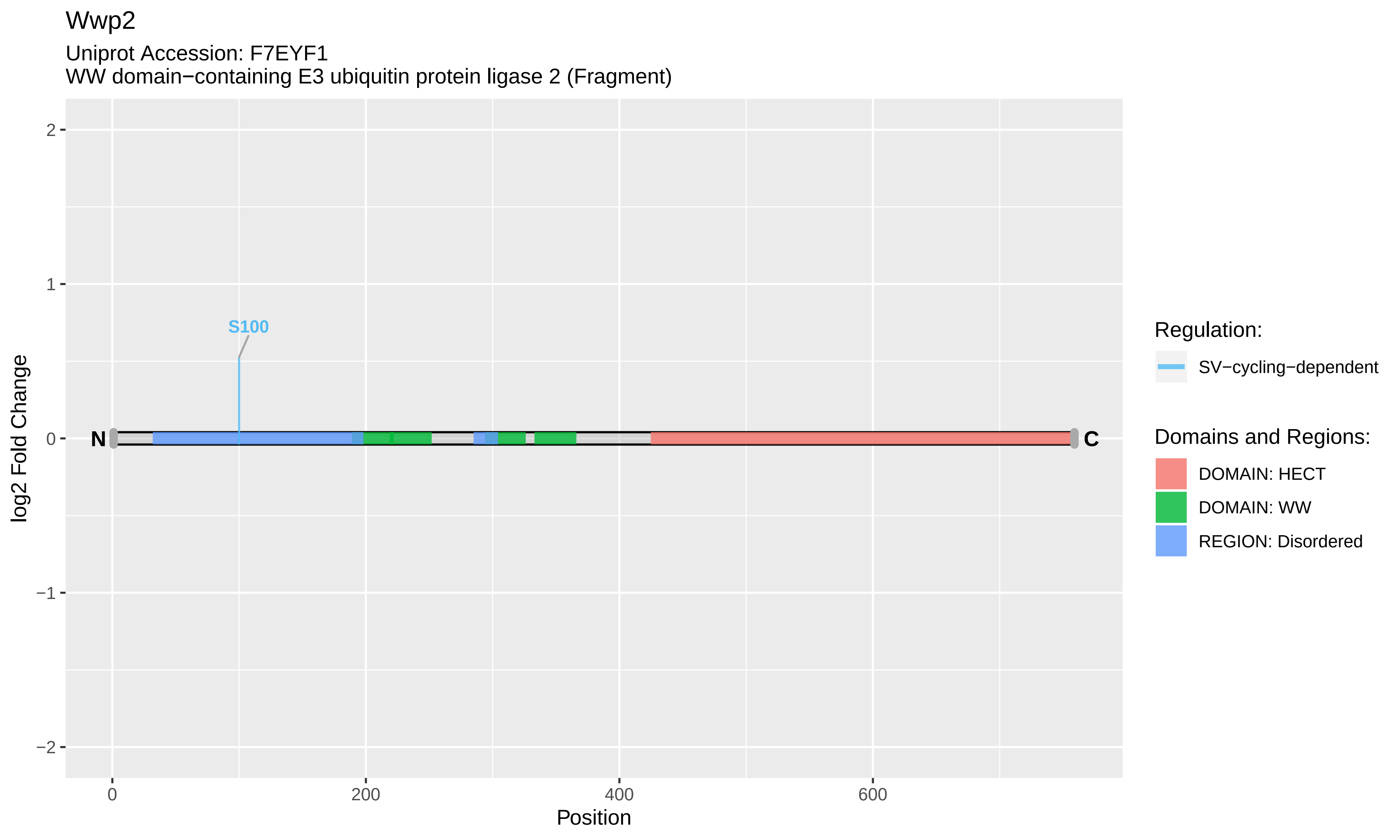
Wwp2
Uniprot Accession: F7EYF1
WW domain−containing E3 ubiquitin protein ligase 2 (Fragment)
S100
N
C
2
1
0
−1
−2
log2 Fold Change
0
200
400
600
Position
Regulation:
SV−cycling−dependent
Domains and Regions:
DOMAIN: HECT
DOMAIN: WW
REGION: Disordered

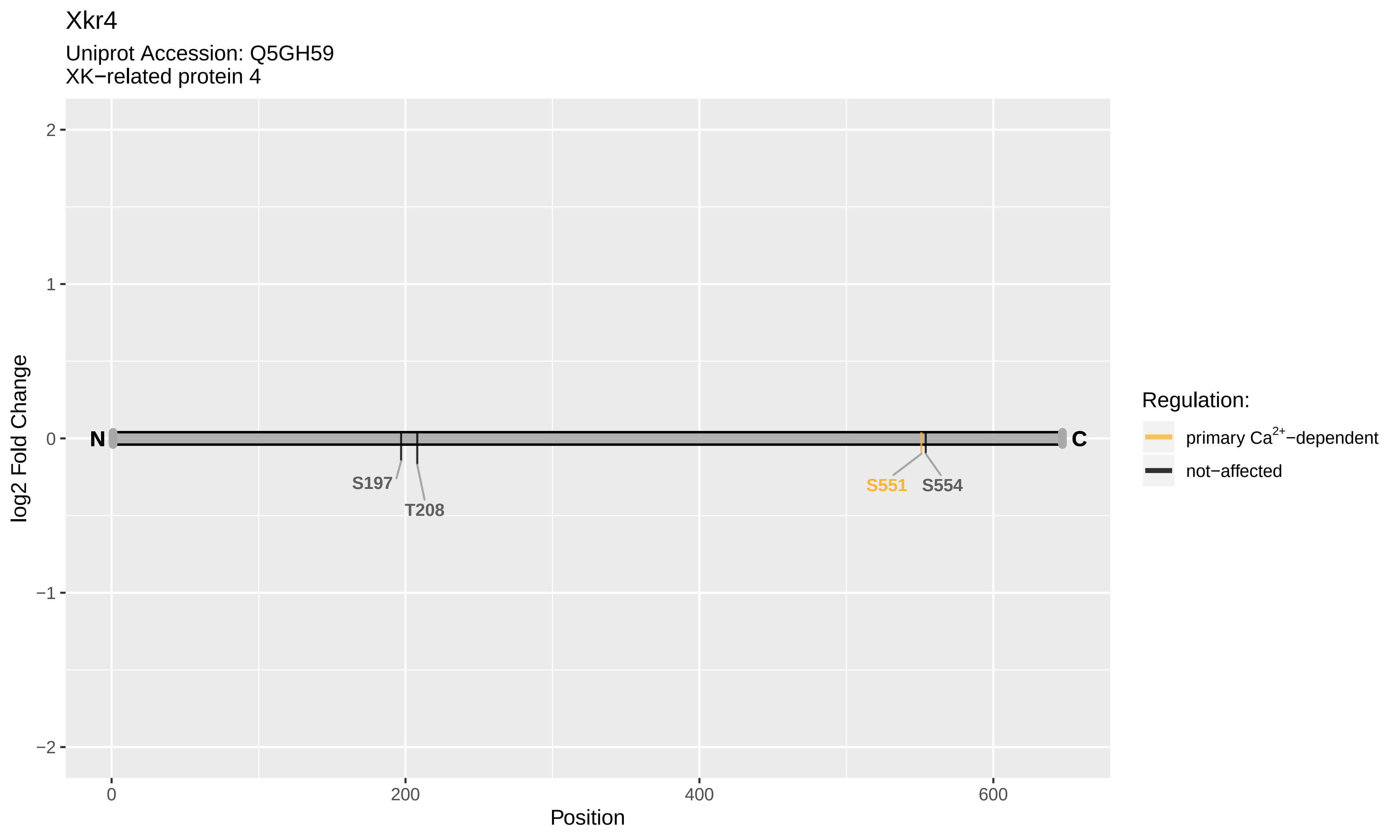

Xkr4
Uniprot Accession: Q5GH59
XK−related protein 4
log2 Fold Change
2
1
0
−1
−2
N
C
S197
T208
S551
S554
0
200
400
600
Position
Regulation:
primary Ca2+−dependent
not−affected

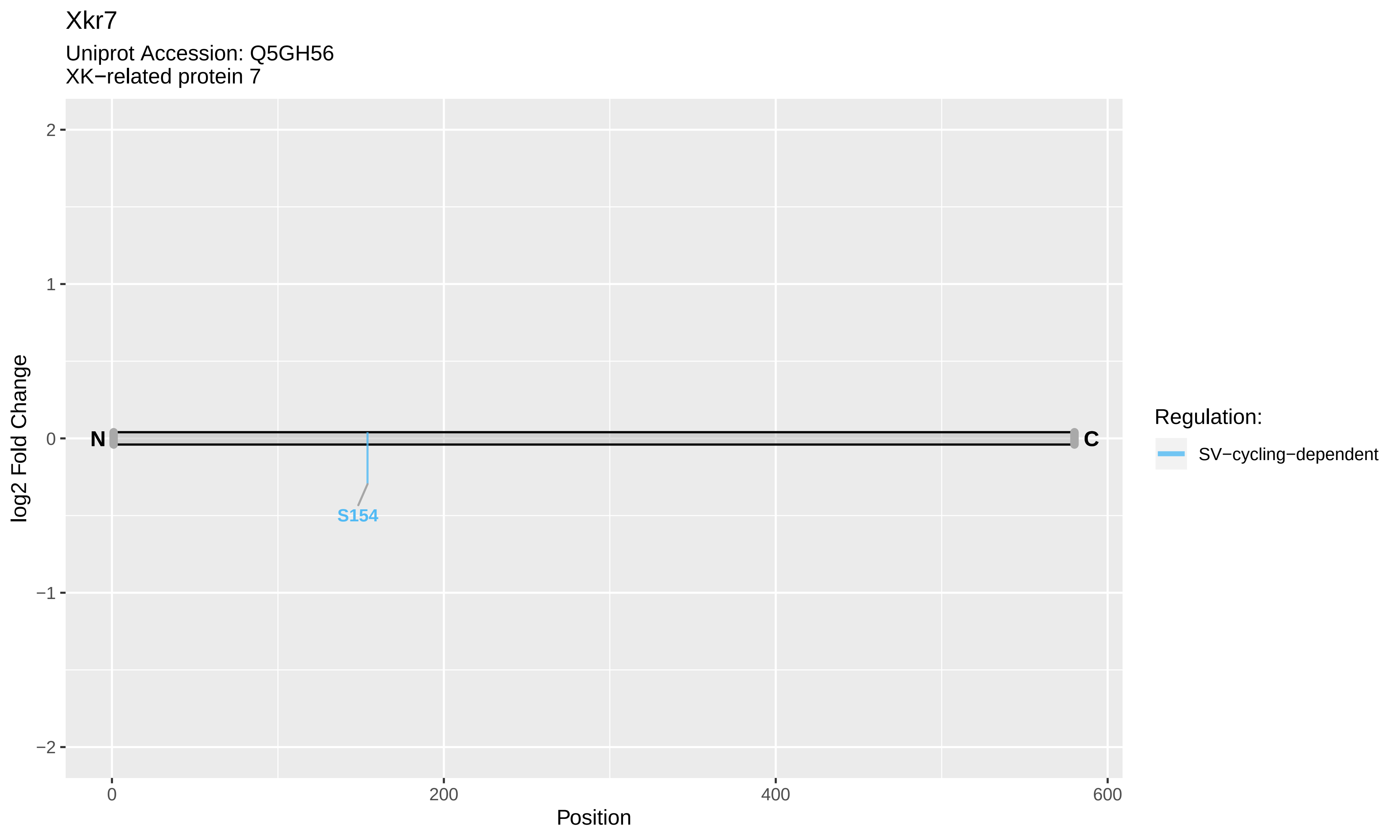
Xkr7
Uniprot Accession: Q5GH56
XK−related protein 7
2
1
0
−1
−2
log2 Fold Change
N
C
S154
0
200
400
600
Position
Regulation:
SV−cycling−dependent

Xrn1

Uniprot Accession: D4ABN8
5'−3' exoribonuclease 1

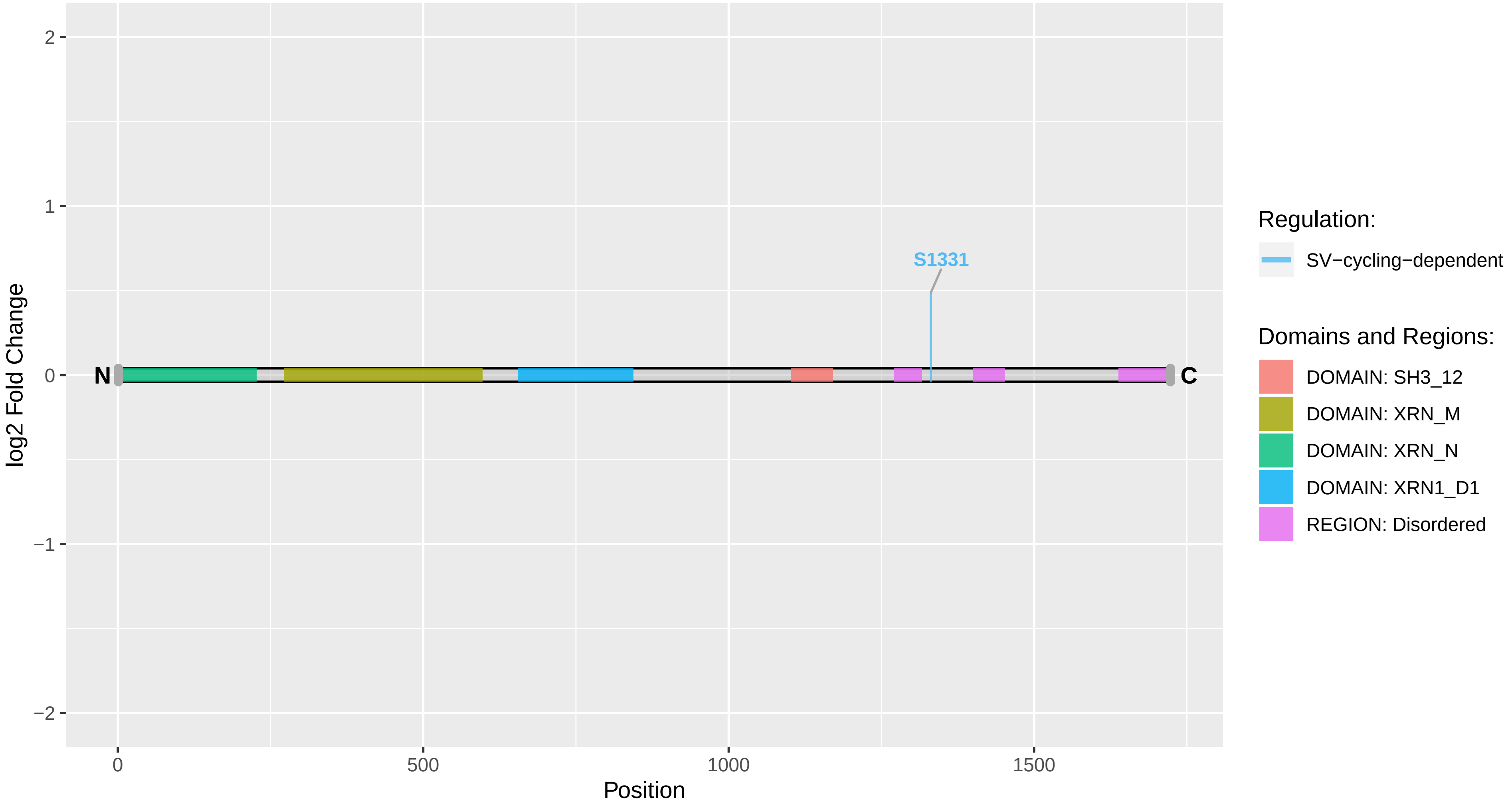

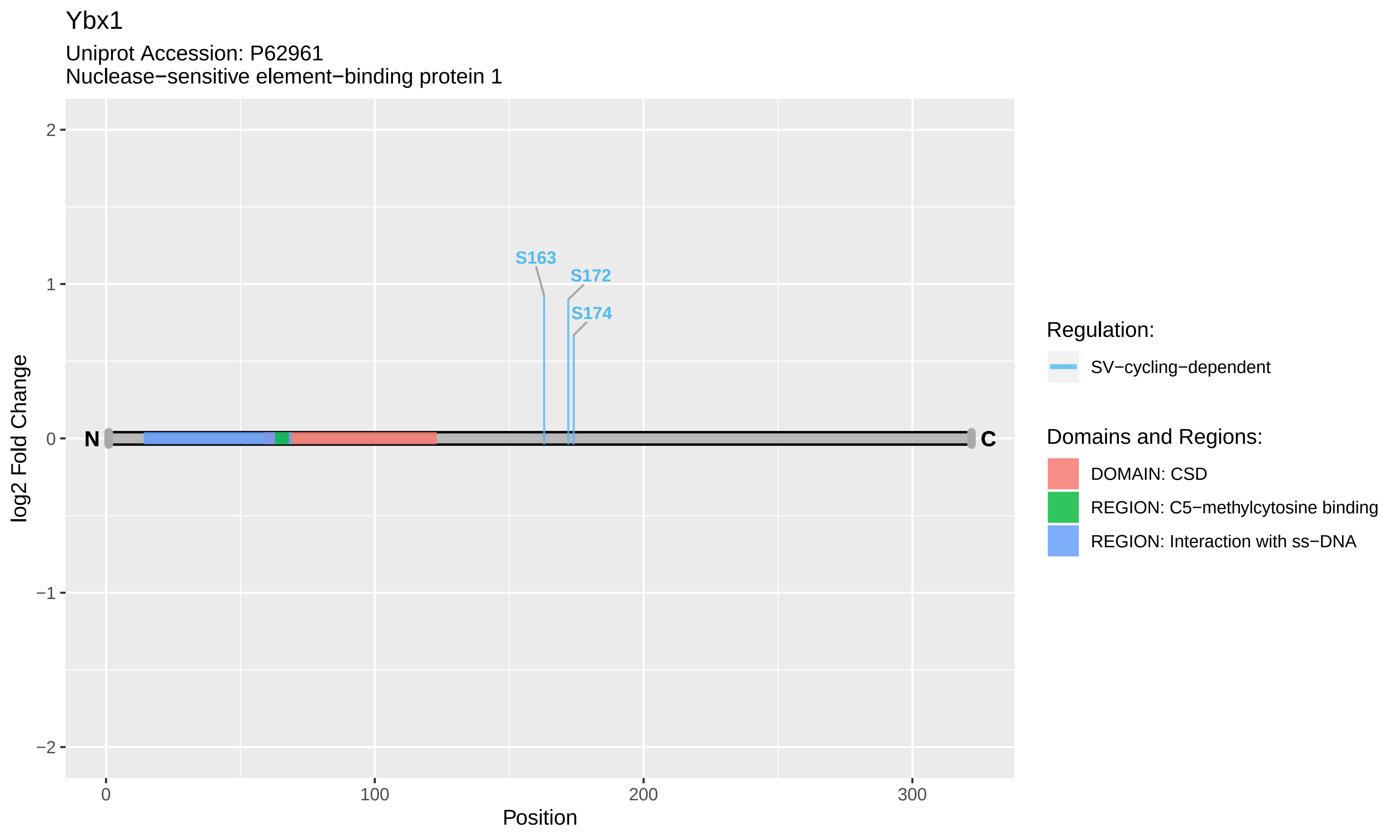

Ybx1
Uniprot Accession: P62961
Nuclease−sensitive element−binding protein 1
S163
S172
S174
N
C
2
1
0
−1
−2
log2 Fold Change
0
100
200
300
Position
Regulation:
SV−cycling−dependent
Domains and Regions:
DOMAIN: CSD
REGION: C5−methylcytosine binding
REGION: Interaction with ss−DNA

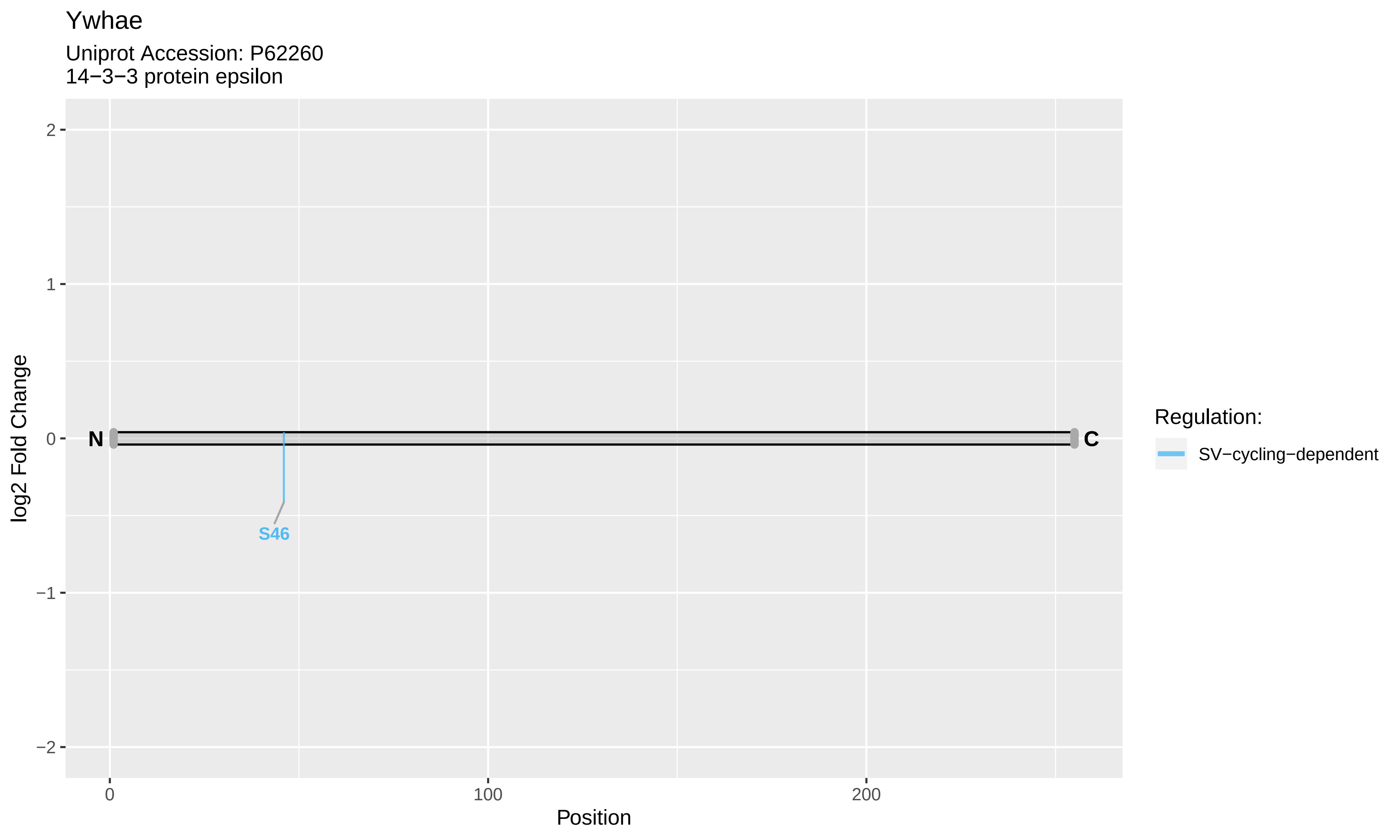
Ywhae
Uniprot Accession: P62260
14−3−3 protein epsilon
2
1
0
−1
−2
log2 Fold Change
N
C
S46
0
100
200
Position
Regulation:
SV−cycling−dependent

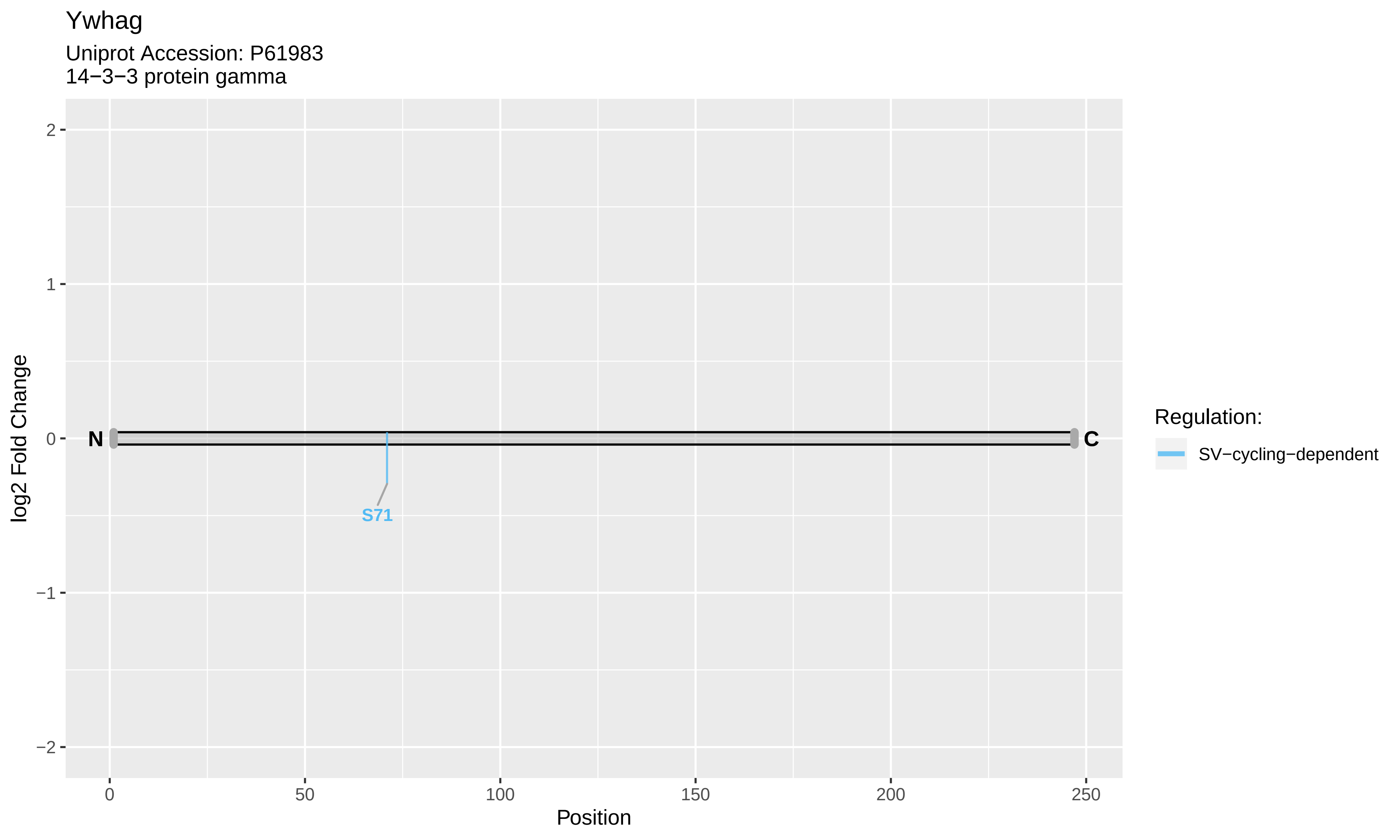

Ywhag
Uniprot Accession: P61983
14−3−3 protein gamma
log2 Fold Change
2
1
0
−1
−2
N
C
S71
0
50
100
150
200
250
Position
Regulation:
SV−cycling−dependent

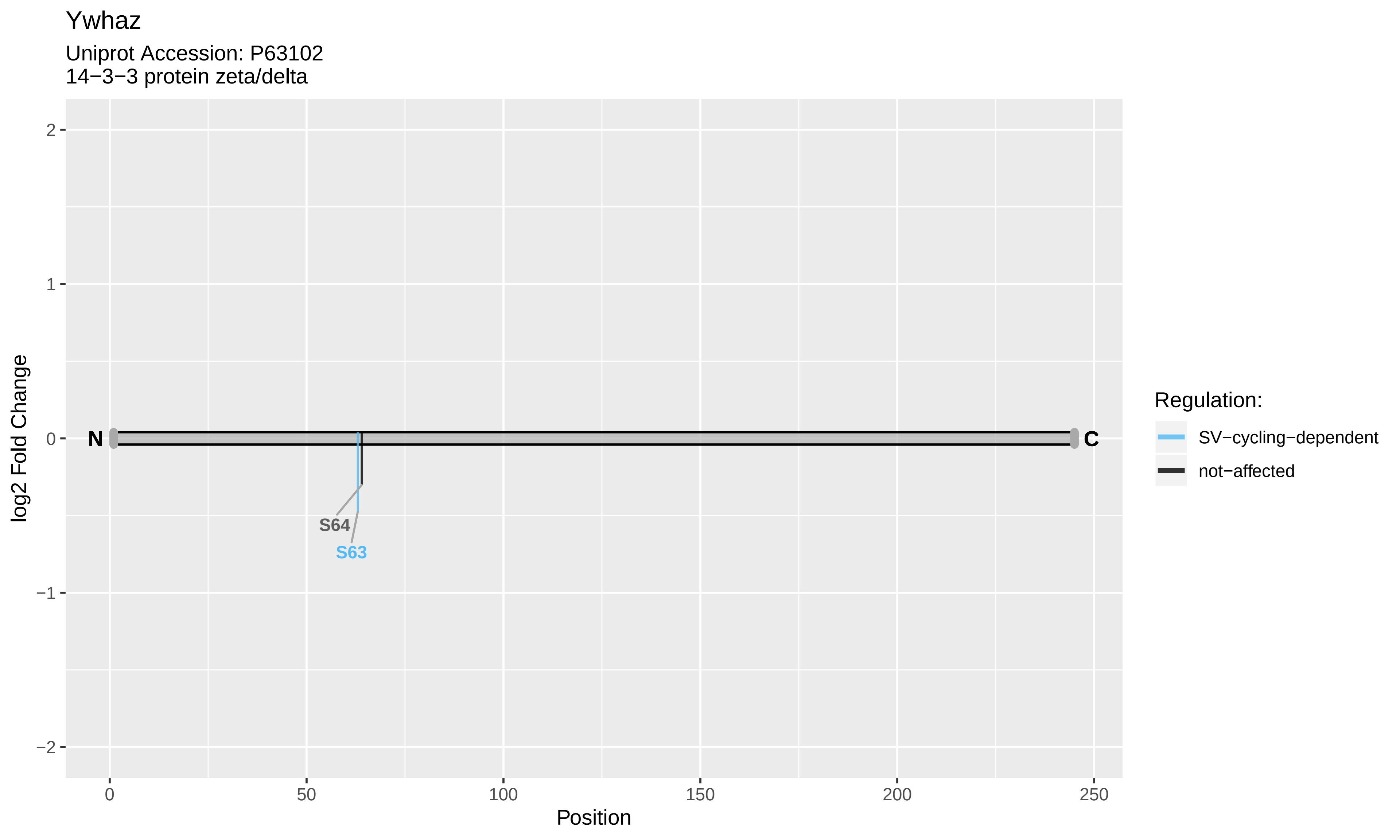

Ywhaz
Uniprot Accession: P63102
14−3−3 protein zeta/delta
log2 Fold Change
2
1
0
−1
−2
N
C
S64
S63
0
50
100
150
200
250
Position
Regulation:
SV−cycling−dependent
not−affected

Zbtb16

Uniprot Accession: Q5S3S9
Promyelocytic leukemia zinc finger protein

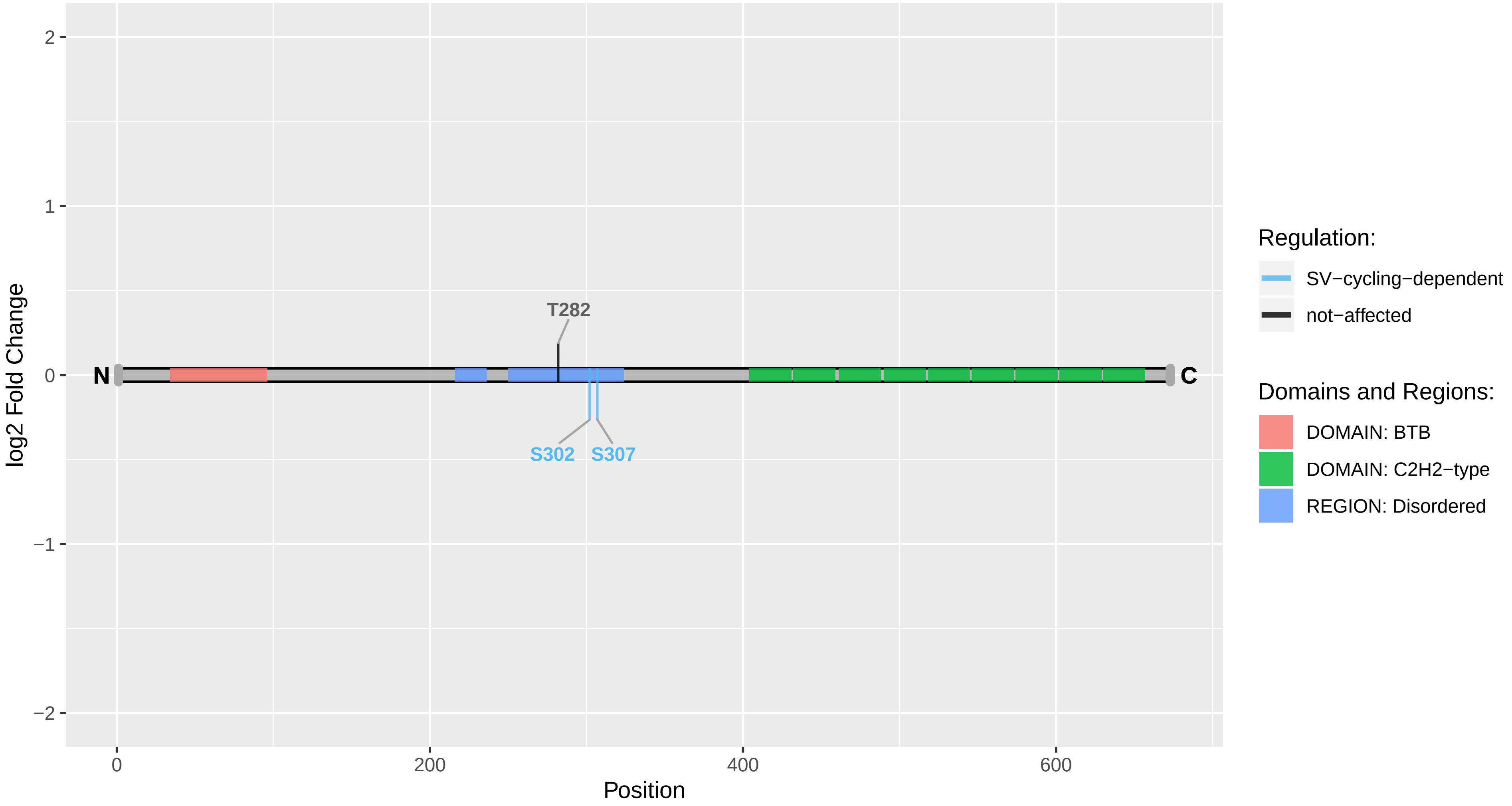

Zc2hc1a

Uniprot Accession: A0A0G2K782
Zinc finger, C2HC−type−containing 1A

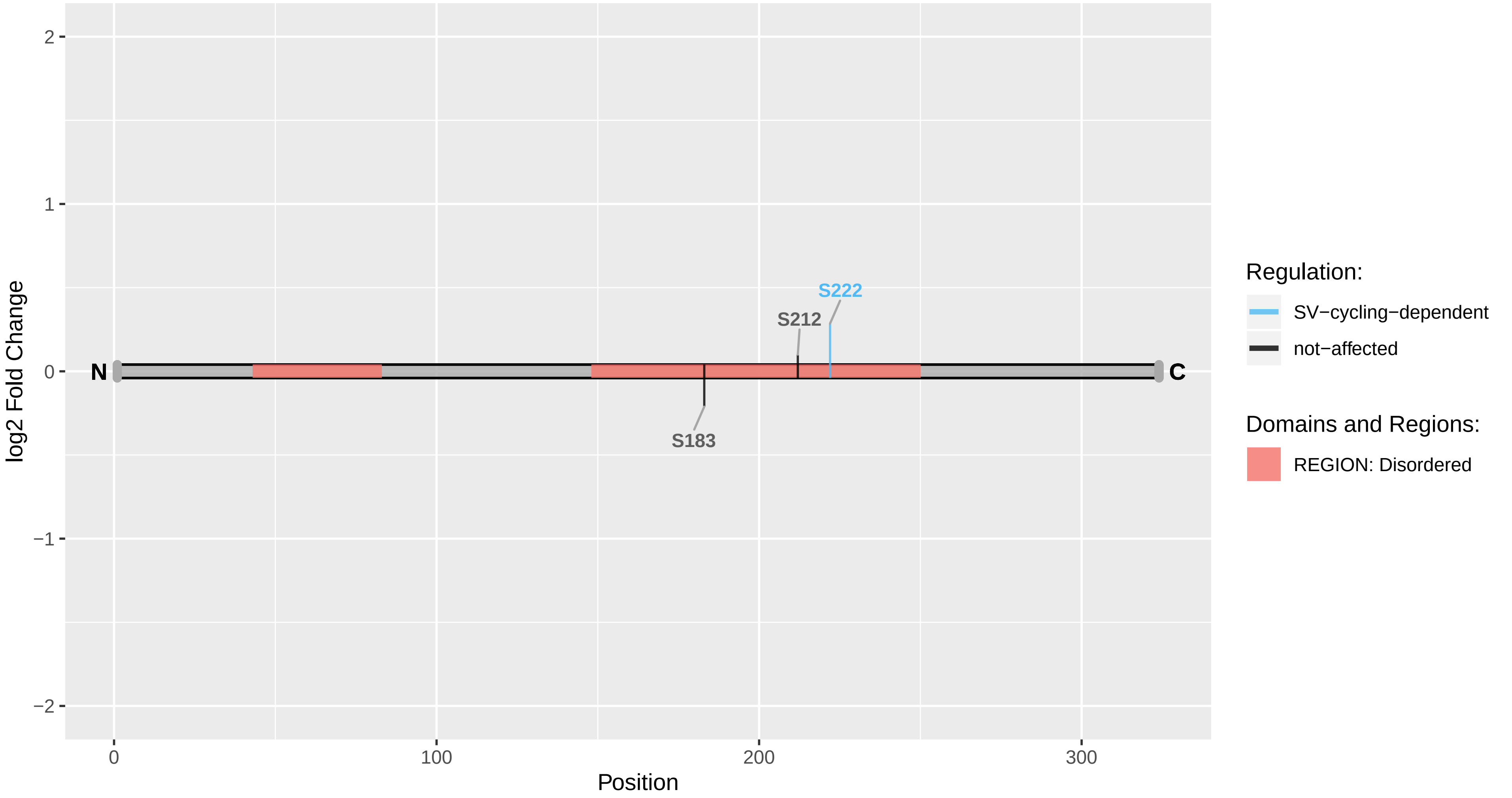

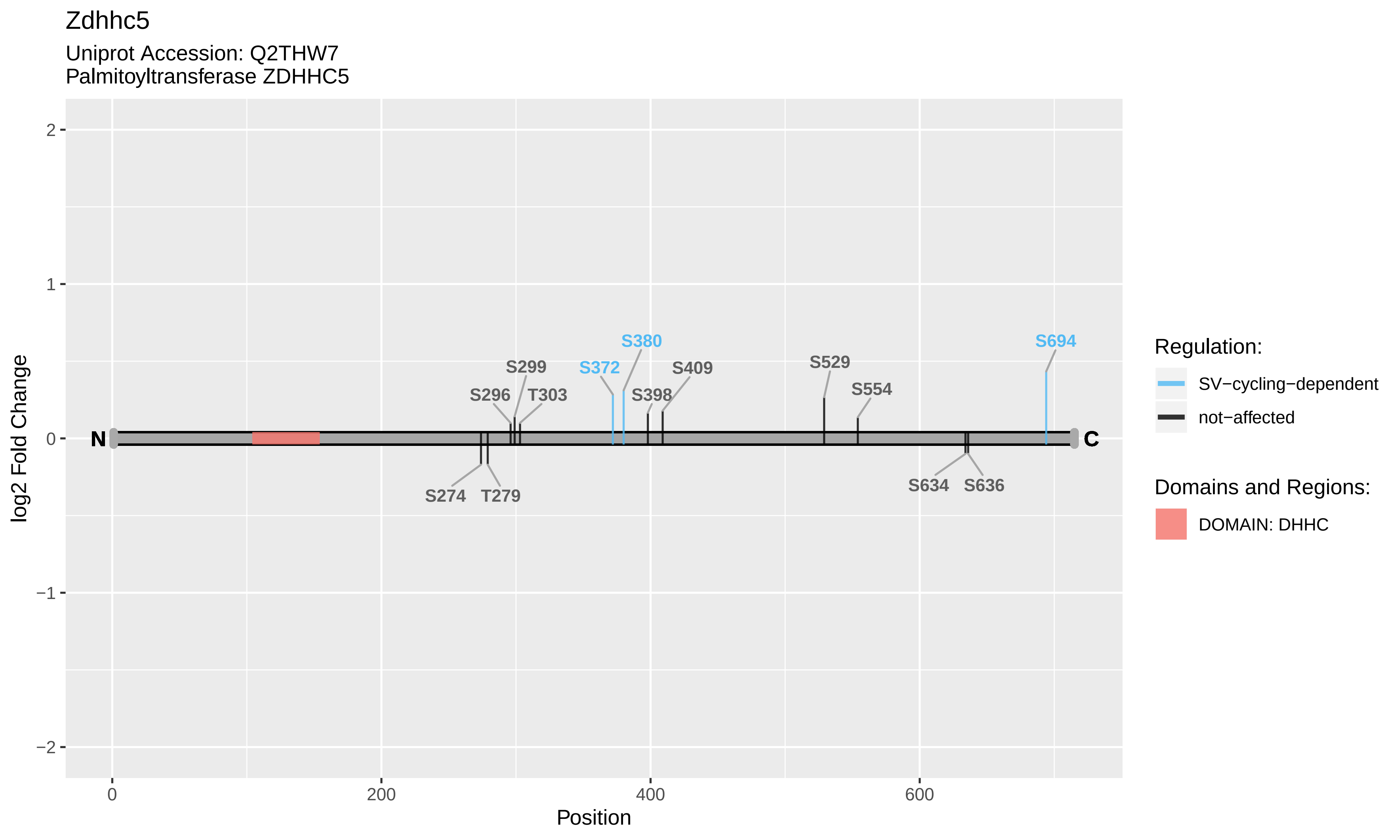

Zdhhc5
Uniprot Accession: Q2THW7
Palmitoyltransferase ZDHHC5
log2 Fold Change
Position
2
1
0
−1
−2
0
200
400
600
N
C
S299
S296
T303
S372
S380
S398
S409
S529
S554
S694
S274
T279
S634
S636
Regulation:
SV−cycling−dependent
not−affected
Domains and Regions:
DOMAIN: DHHC

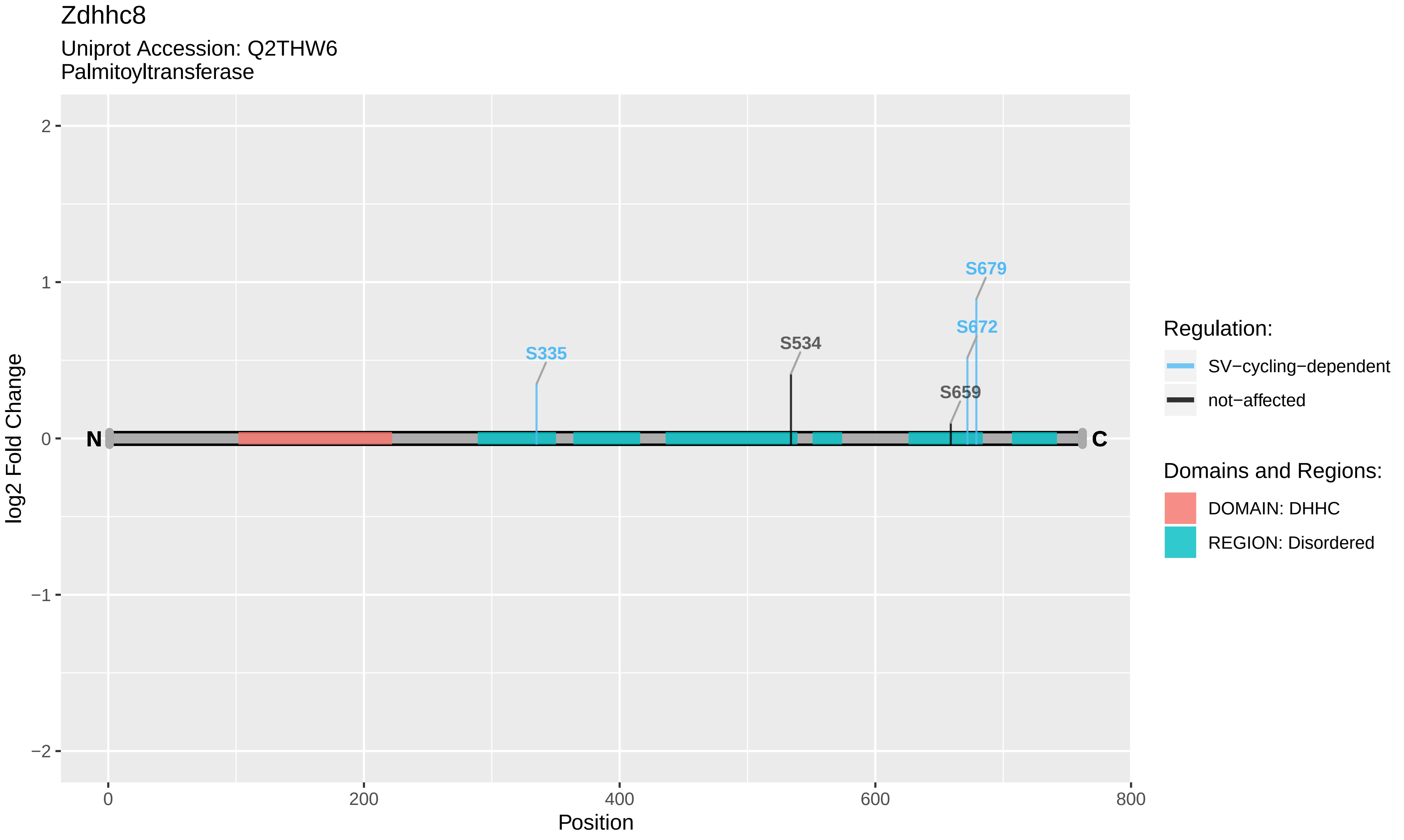

Zdhhc8
Uniprot Accession: Q2THW6
Palmitoyltransferase
log2 Fold Change
Position
N
C
S335
S534
S659
S672
S679
2
1
0
−1
−2
0
200
400
600
800
Regulation:
SV−cycling−dependent
not−affected
Domains and Regions:
DOMAIN: DHHC
REGION: Disordered

Zfp592

Uniprot Accession: D3ZJG8
Zinc finger protein 592

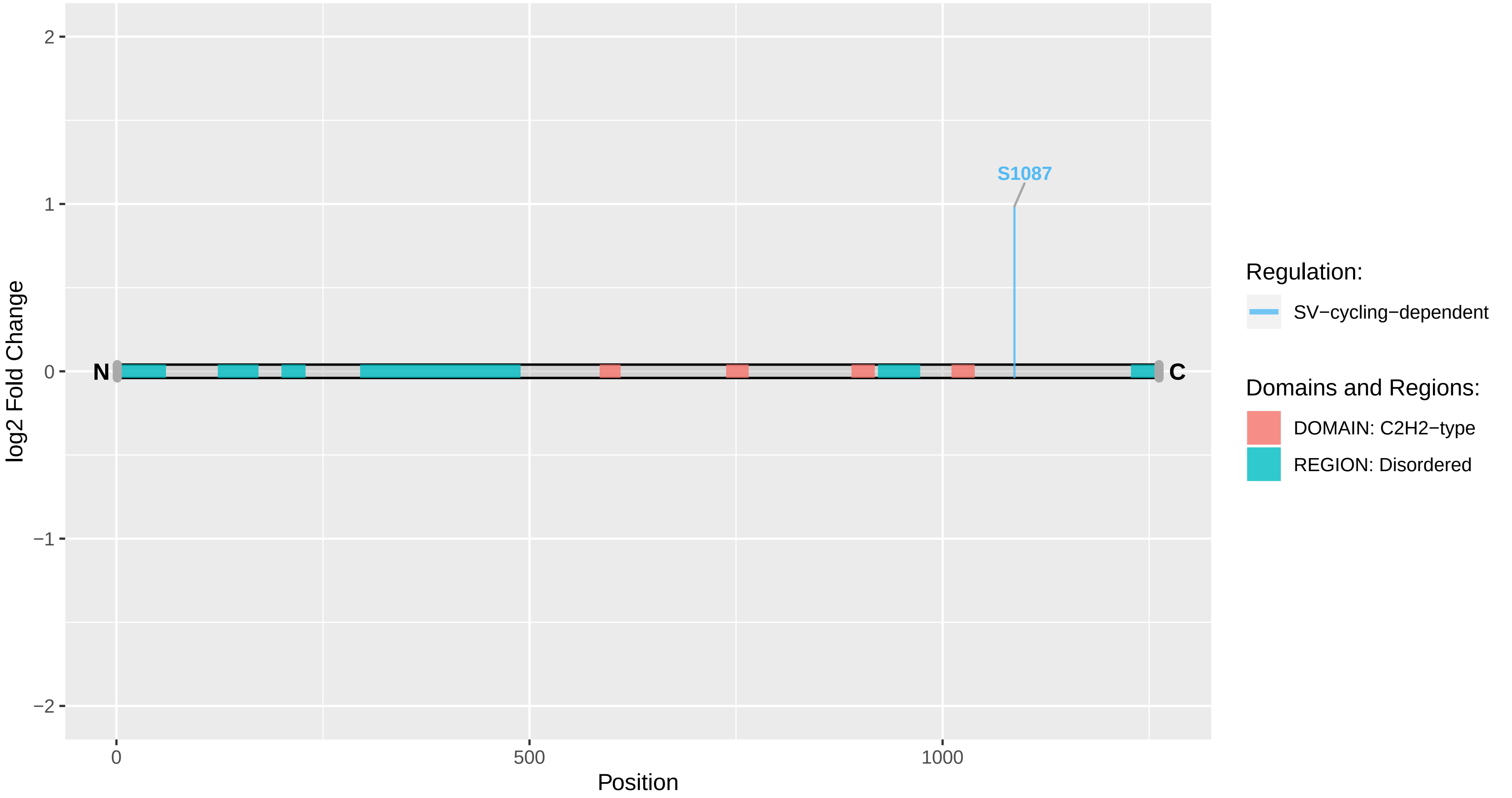

Zfp704

Uniprot Accession: A0A0G2K951
Zinc finger protein 704

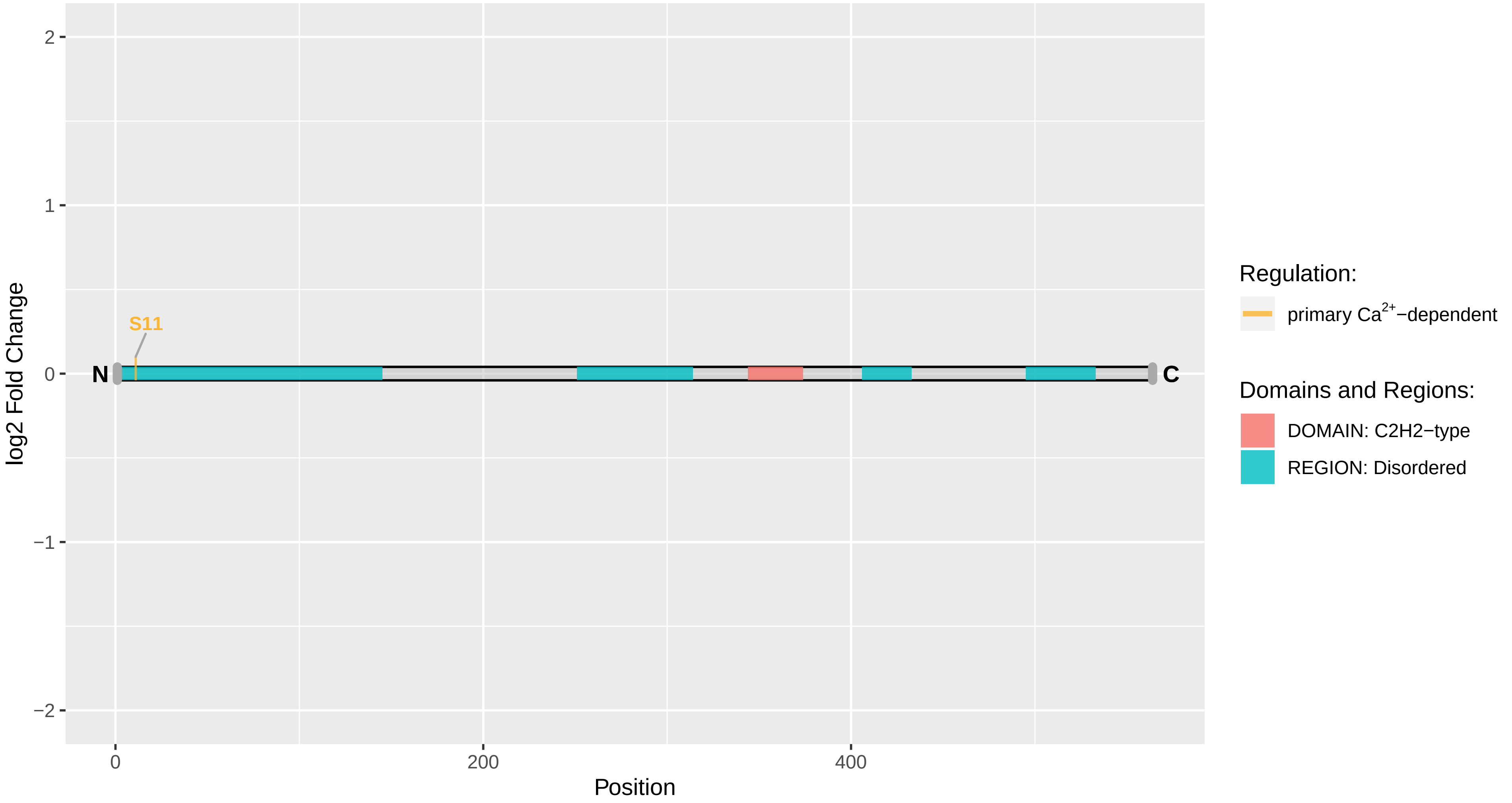

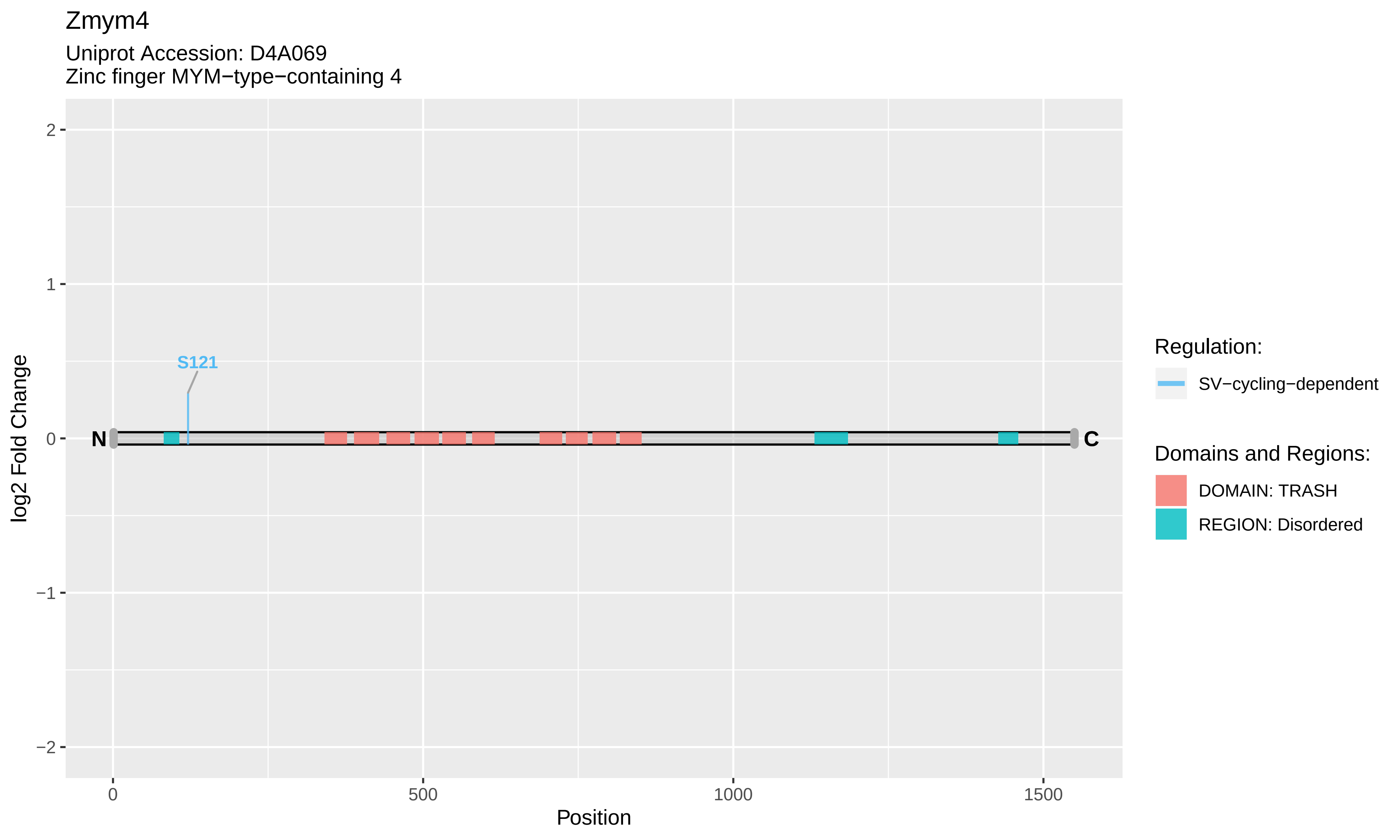
Zmym4
Uniprot Accession: D4A069
Zinc finger MYM−type−containing 4
log2 Fold Change
2
1
0
−1
−2
N
C
S121
0
500
1000
1500
Position
Regulation:
SV−cycling−dependent
Domains and Regions:
DOMAIN: TRASH
REGION: Disordered

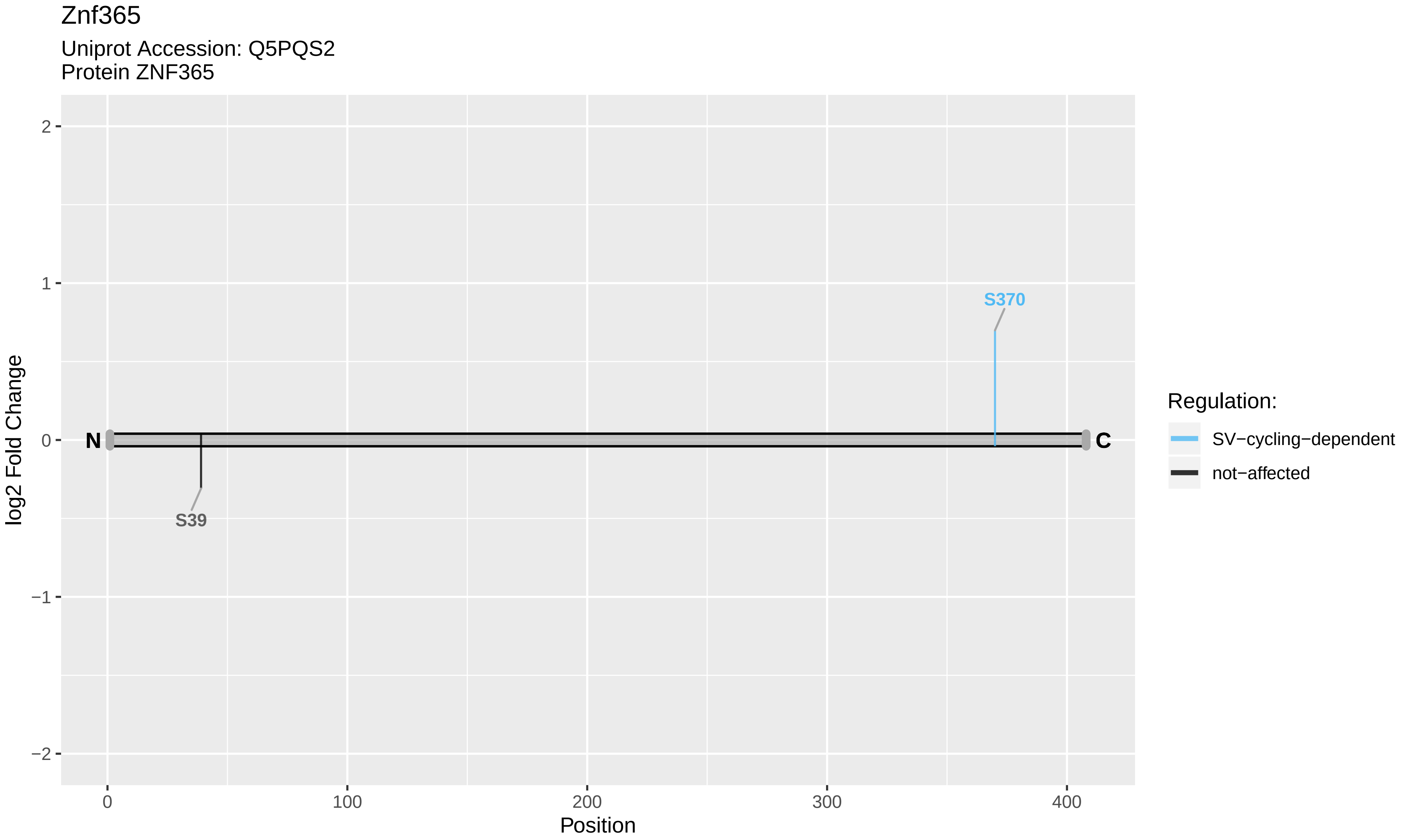

Znf365
Uniprot Accession: Q5PQS2
Protein ZNF365
log2 Fold Change
2
1
0
−1
−2
N
C
S370
S39
0
100
200
300
400
Position
Regulation:
SV−cycling−dependent
not−affected

Znf622

Uniprot Accession: Q7TM96
Zinc finger protein 622

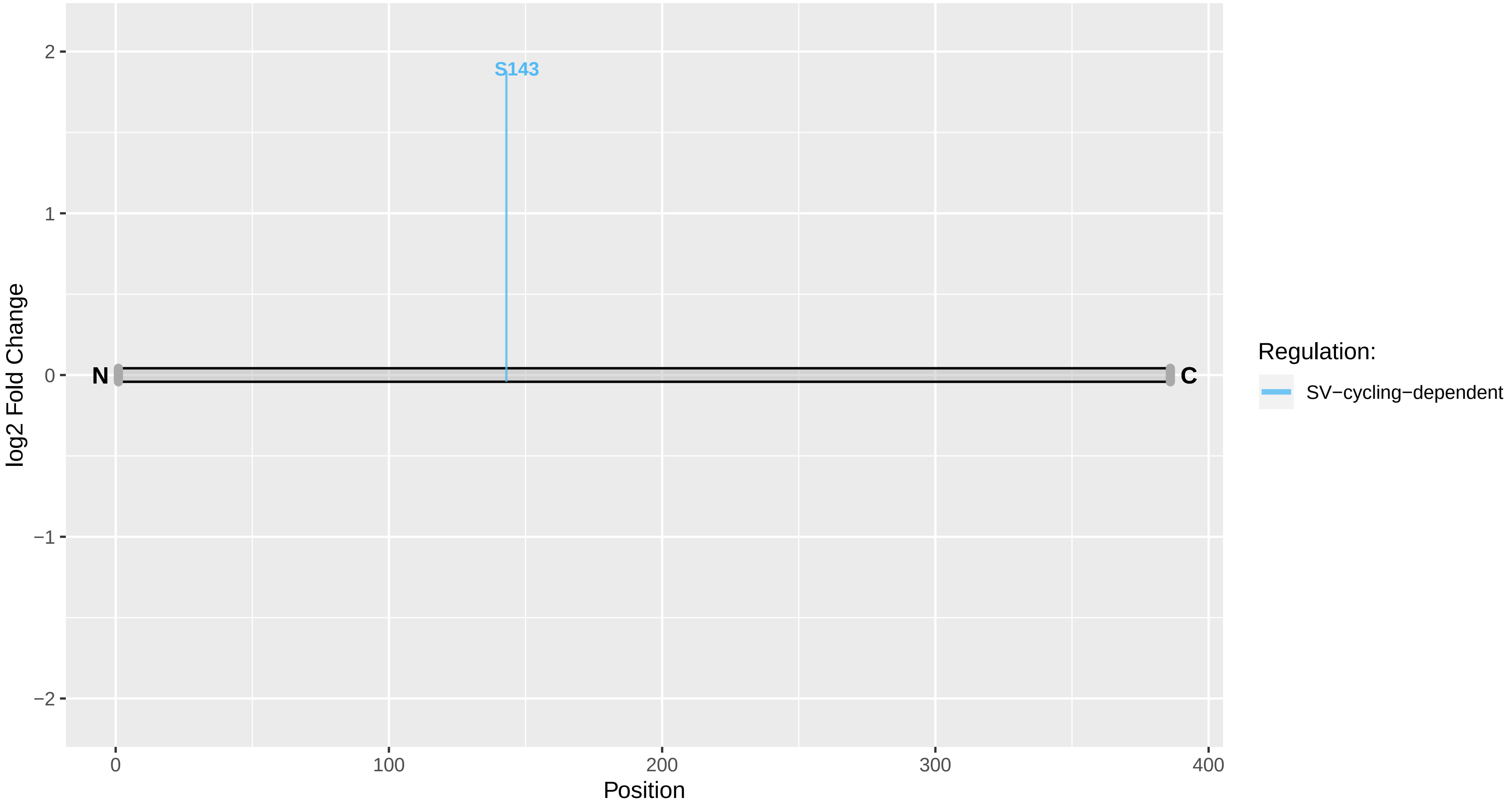

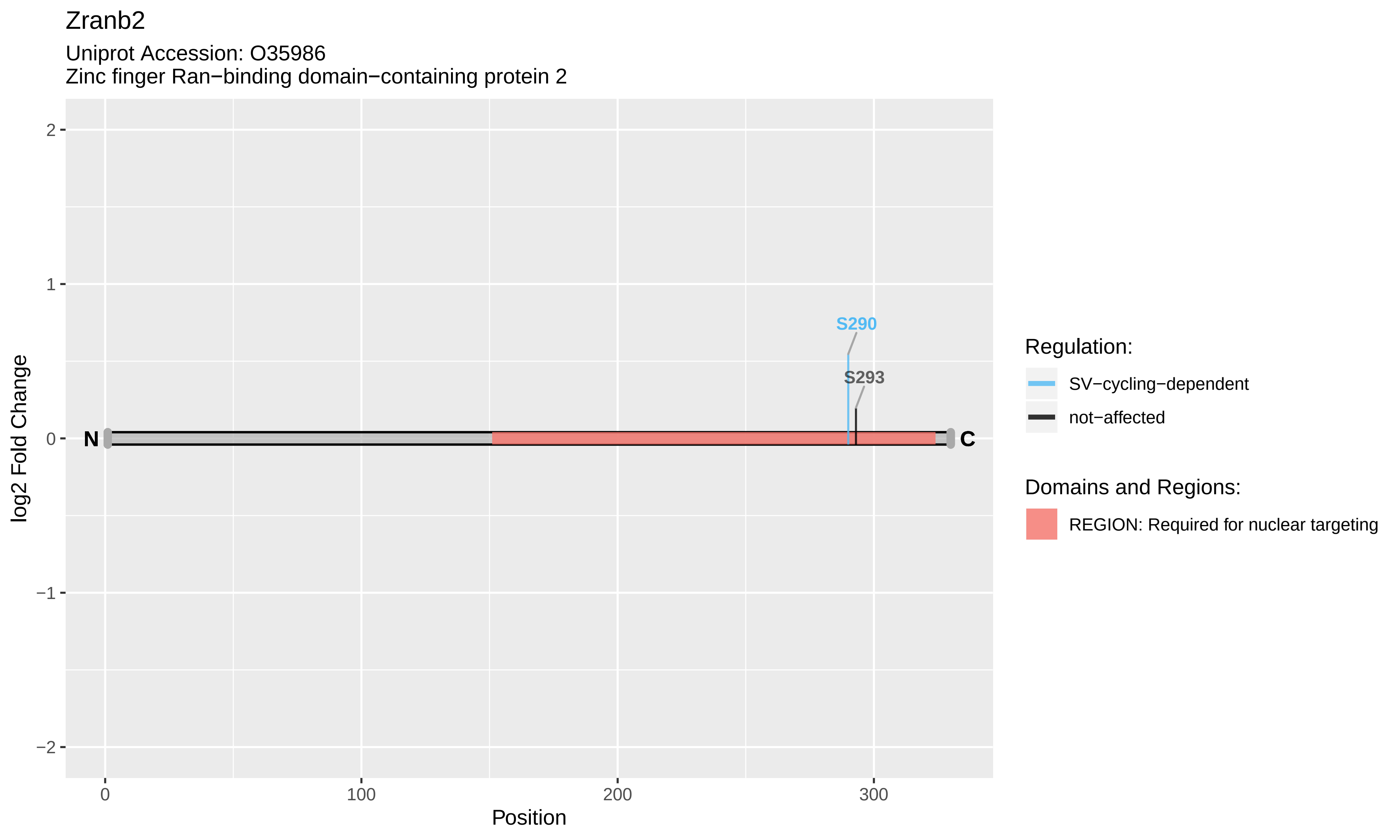
Zranb2
Uniprot Accession: O35986
Zinc finger Ran−binding domain−containing protein 2
log2 Fold Change
Position
N
C
S290
S293
Regulation:
SV−cycling−dependent
not−affected
Domains and Regions:
REGION: Required for nuclear targeting
2
1
0
−1
−2
0
100
200
300

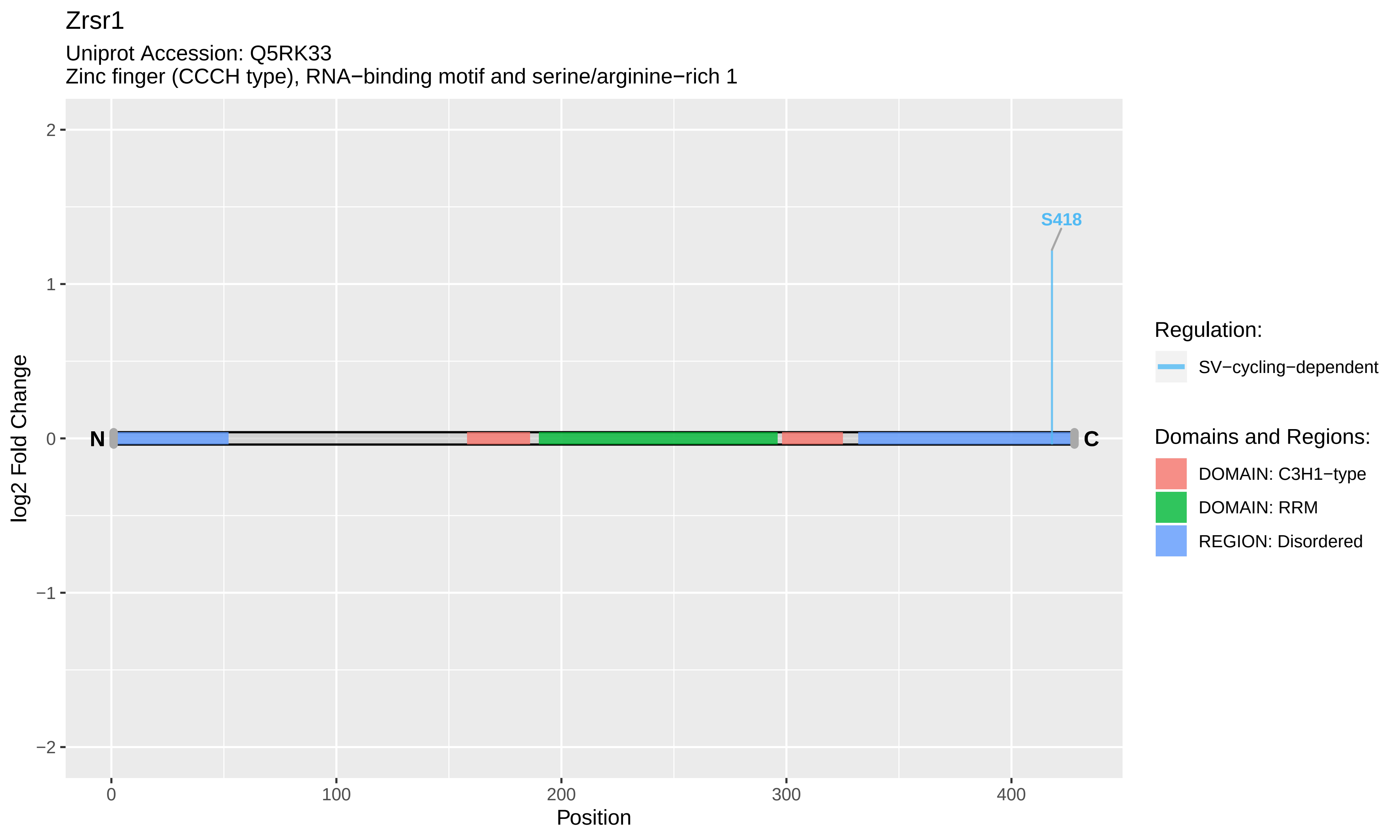
Zrsr1
Uniprot Accession: Q5RK33
Zinc finger (CCCH type), RNA−binding motif and serine/arginine−rich 1
S418
N
C
2
1
0
−1
−2
log2 Fold Change
0
100
200
300
400
Position
Regulation:
SV−cycling−dependent
Domains and Regions:
DOMAIN: C3H1−type
DOMAIN: RRM
REGION: Disordered
