# Supplementary material for: Protein Phosphorylation in Depolarized Synaptosomes: Dissecting Primary Effects of Calcium from Synaptic Vesicle Cycling
Source: Mol Cell Proteomics. 2021 Feb 12;20:100061. doi: 10.1016/j.mcpro.2021.100061 (PMC7995663; doi:10.1016/j.mcpro.2021.100061)
Supplement: Supplemental Material 5 [file mmc5.pdf]

Uniprot Accession: A0A0G2JW01
Uncharacterized protein

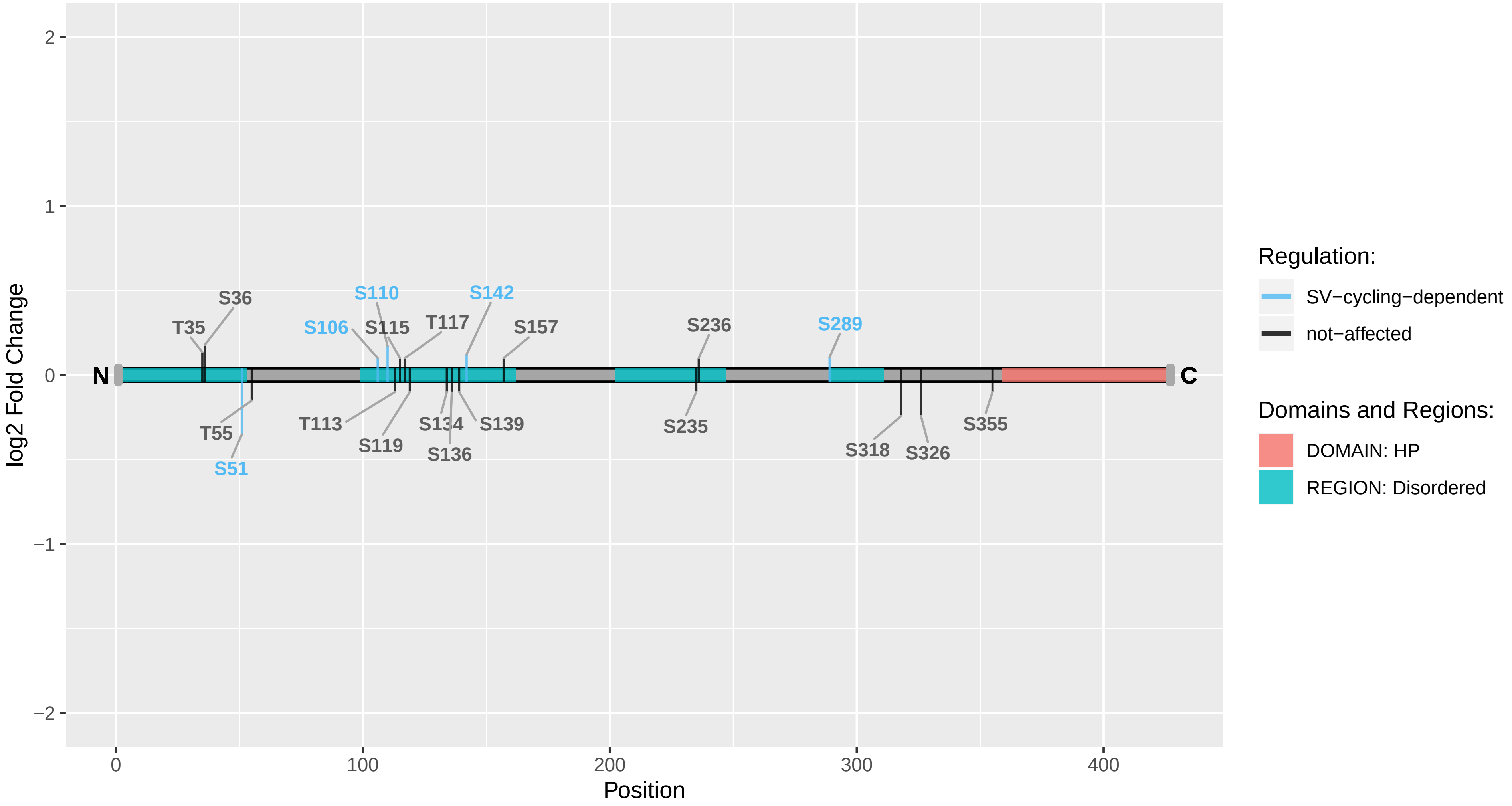

Uniprot Accession: A0A0G2JW65
Uncharacterized protein

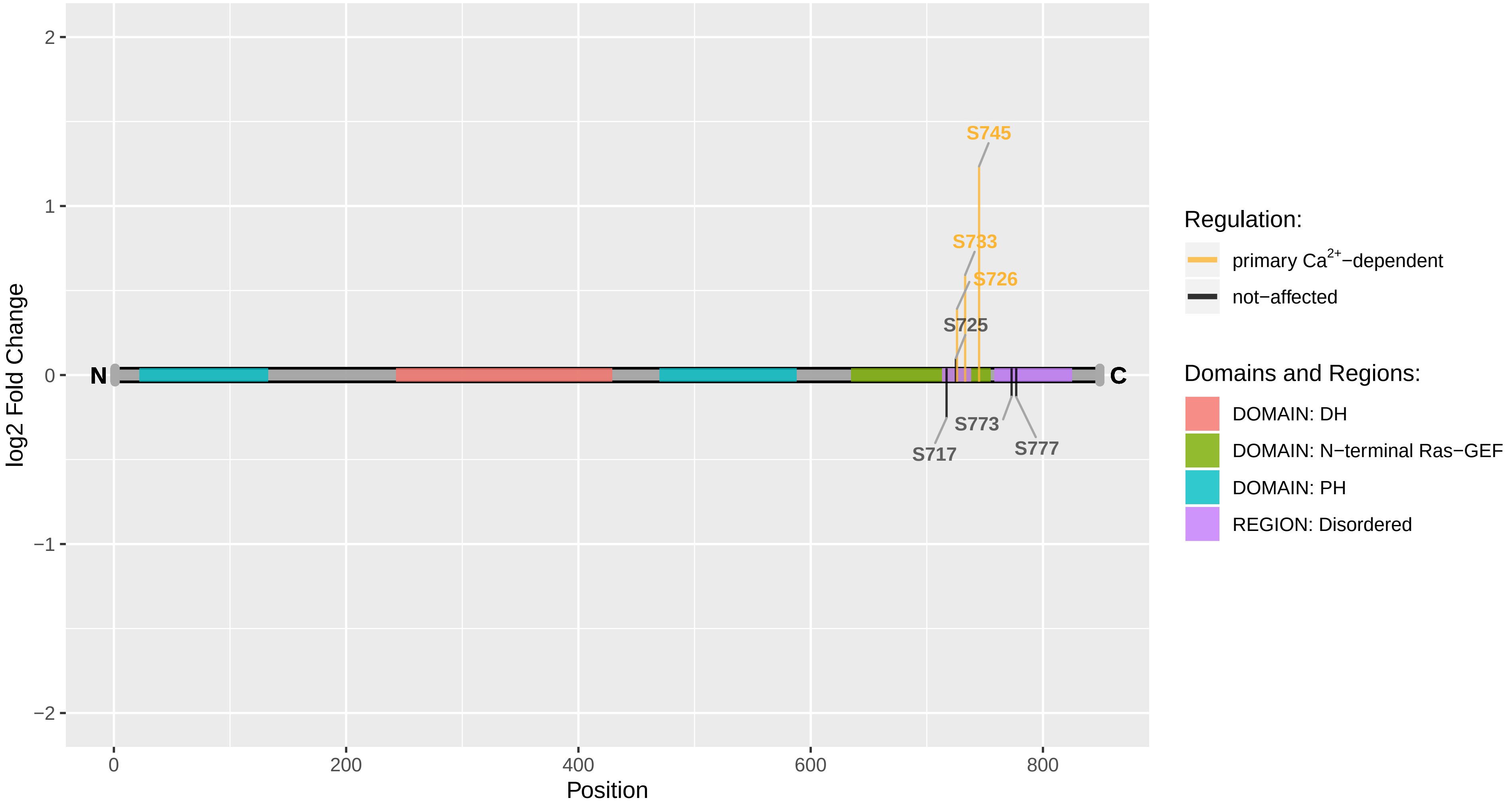

Uniprot Accession: A0A0G2JZ27
Uncharacterized protein

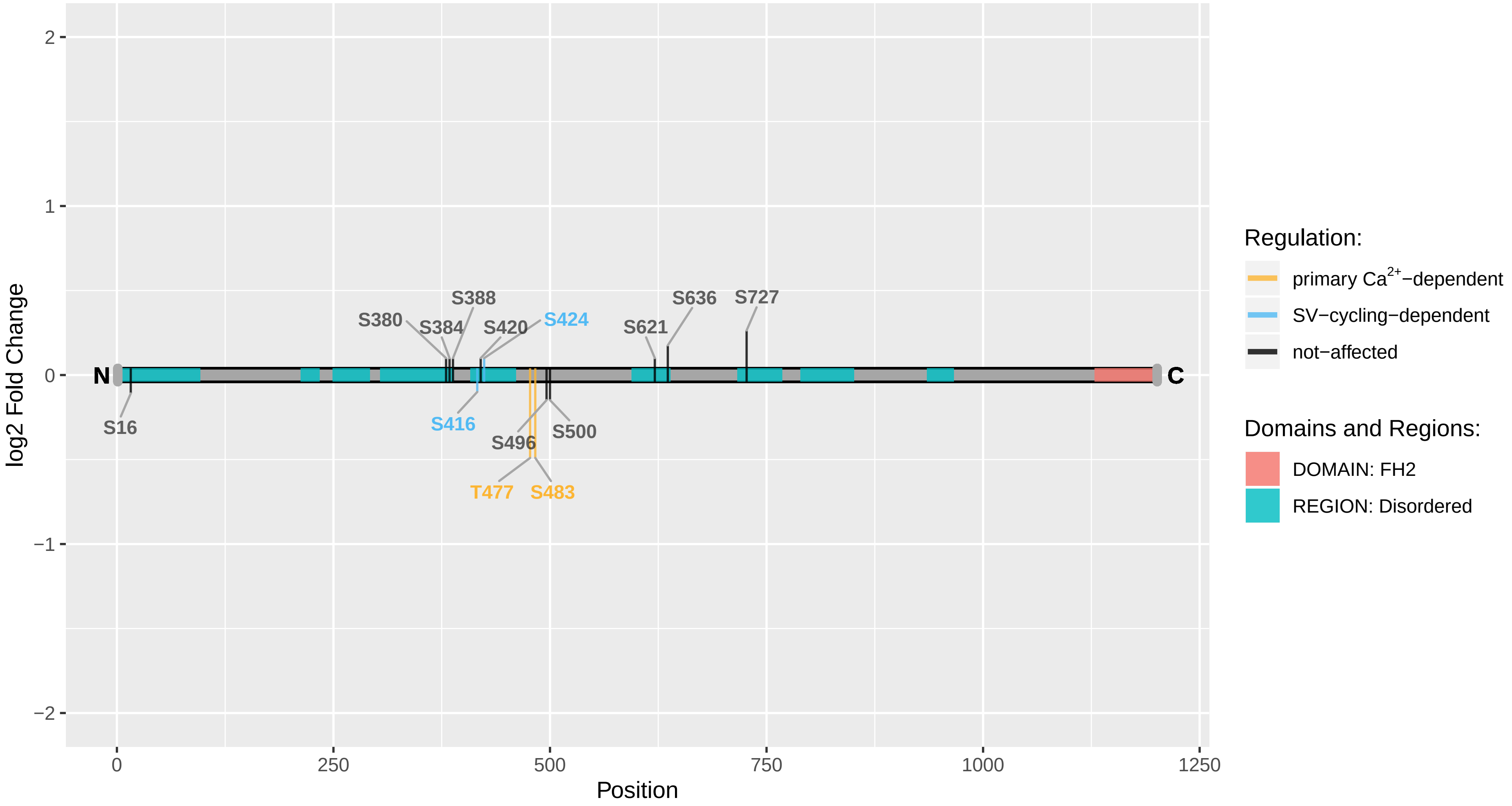

Uniprot Accession: A0A0G2K021
Thymosin beta

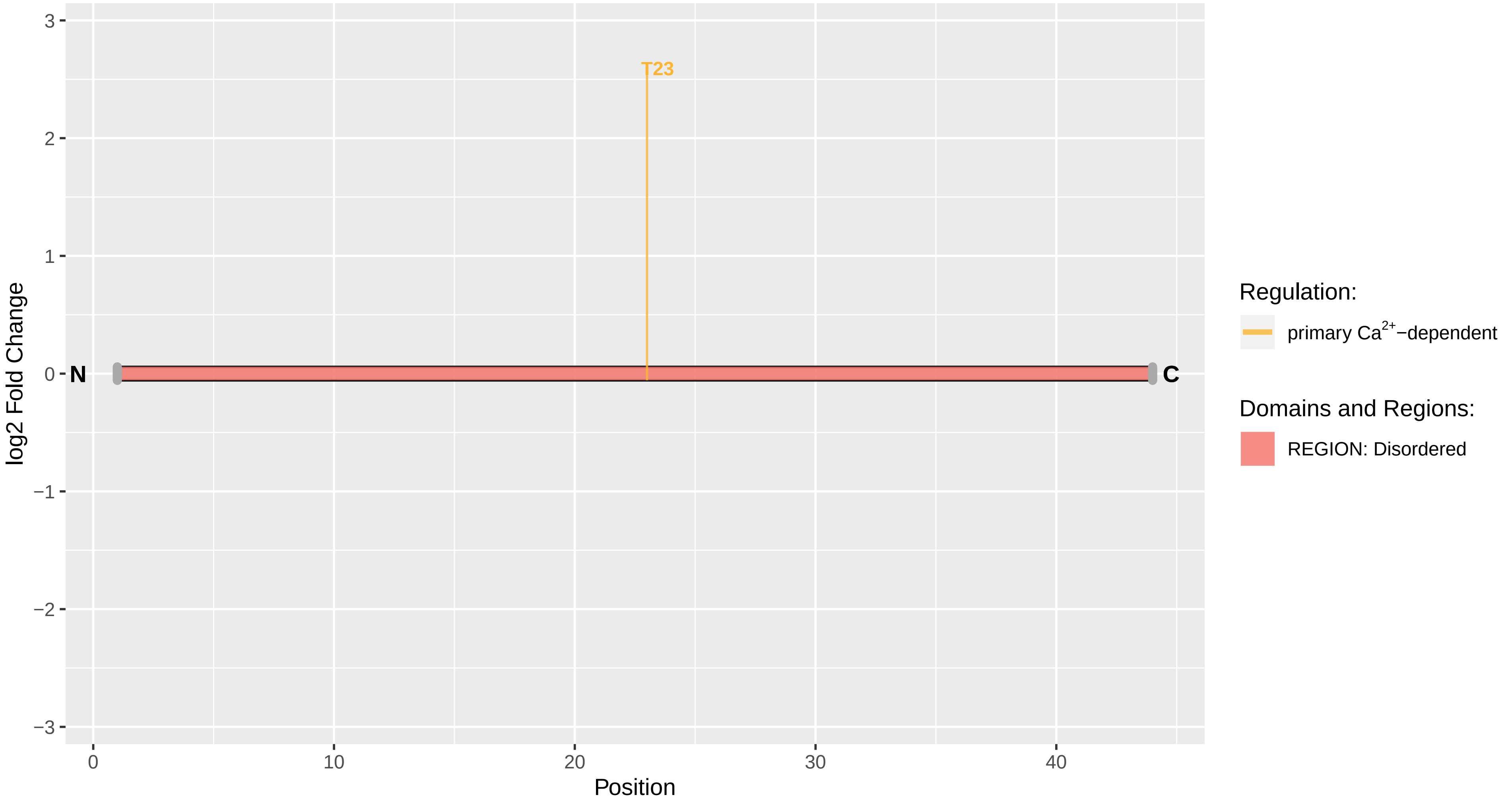

Uniprot Accession: D3ZJK8
Uncharacterized protein

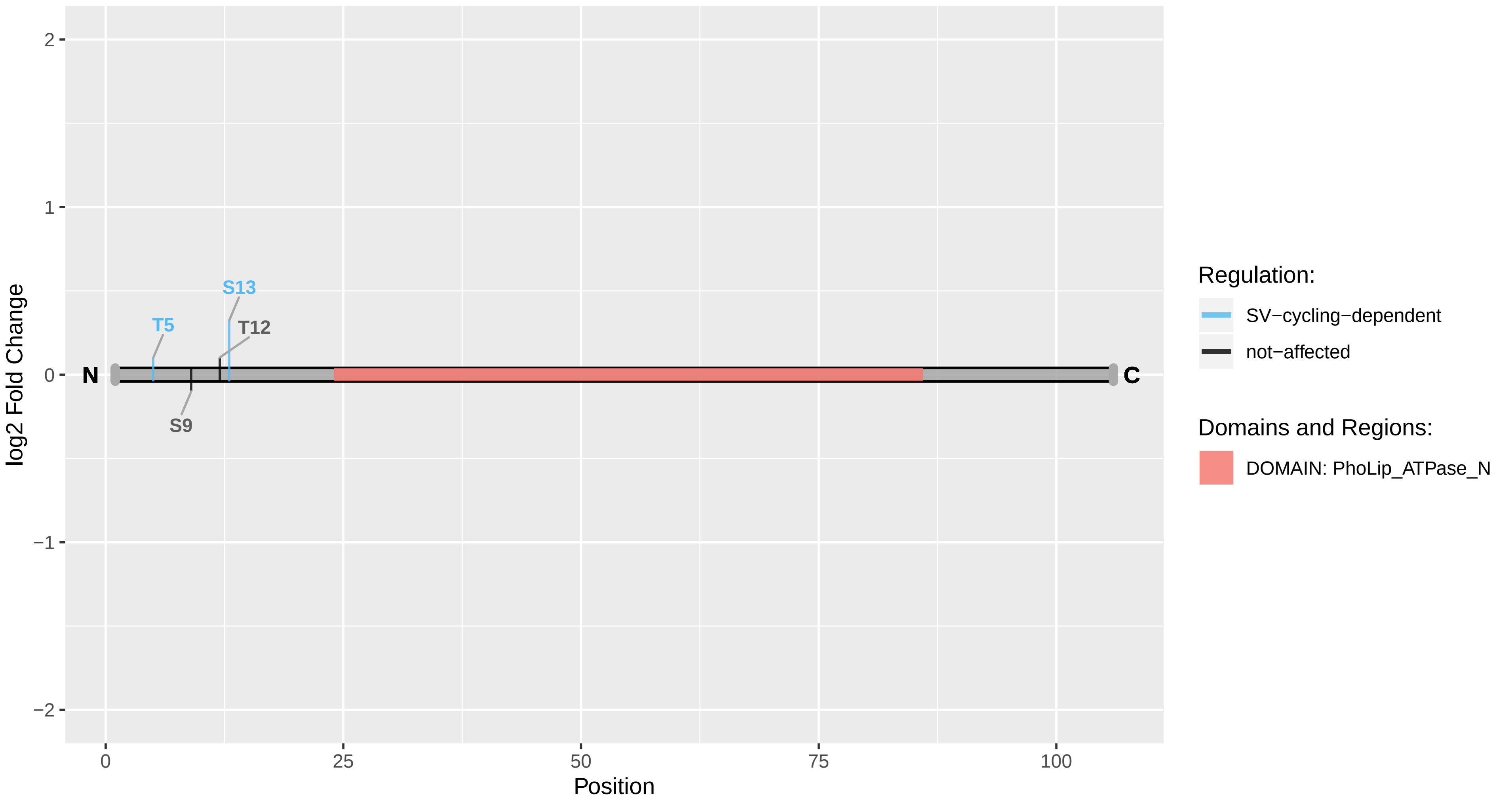

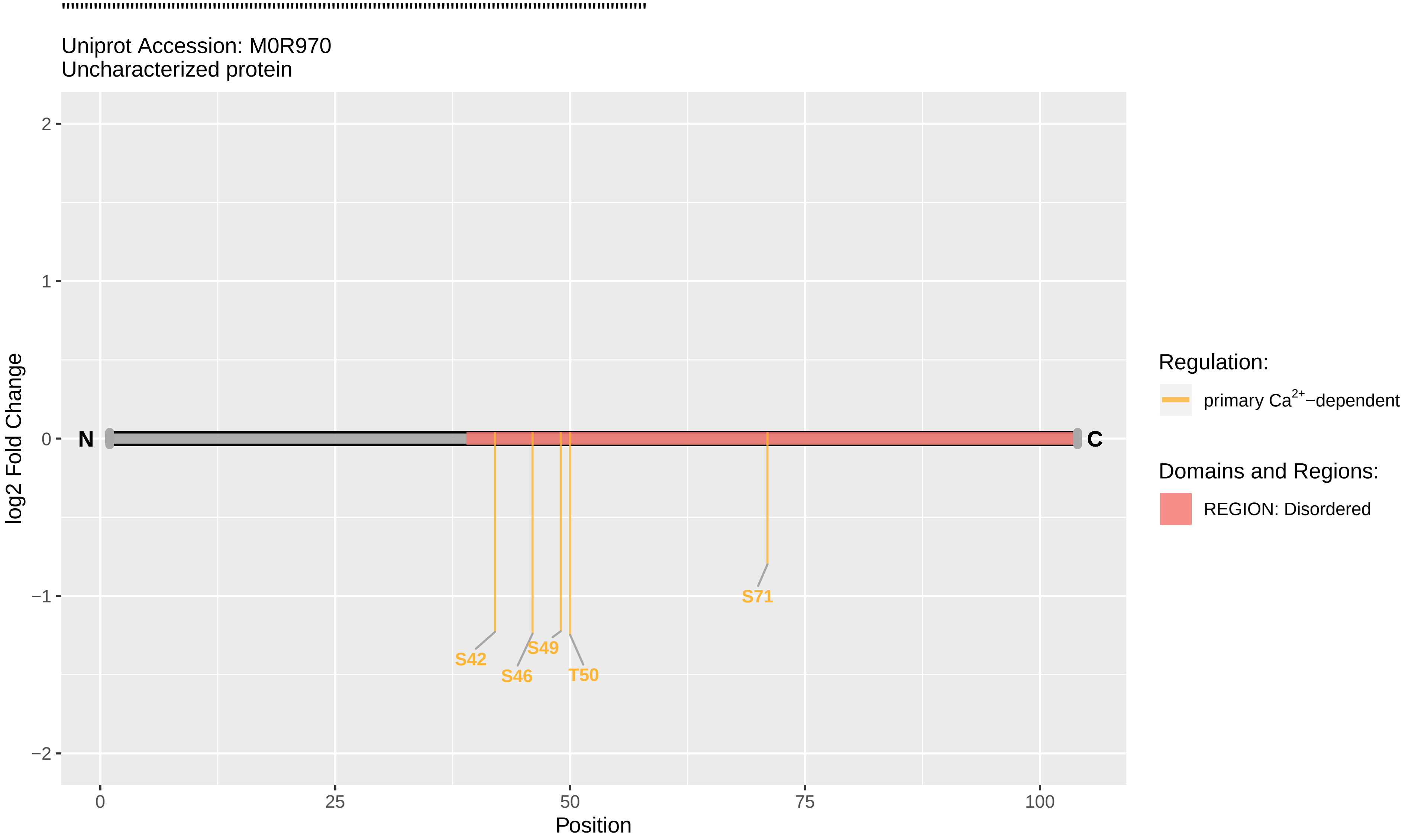

Uniprot Accession: M0R970
Uncharacterized protein
log2 Fold Change
2
1
0
−1
−2
N
C
S42
S46
S49
T50
S71
0
25
50
75
100
Position
Regulation:
primary Ca2+−dependent
Domains and Regions:
REGION: Disordered

Uniprot Accession: O08654
UPF0183 protein C16orf70 homolog

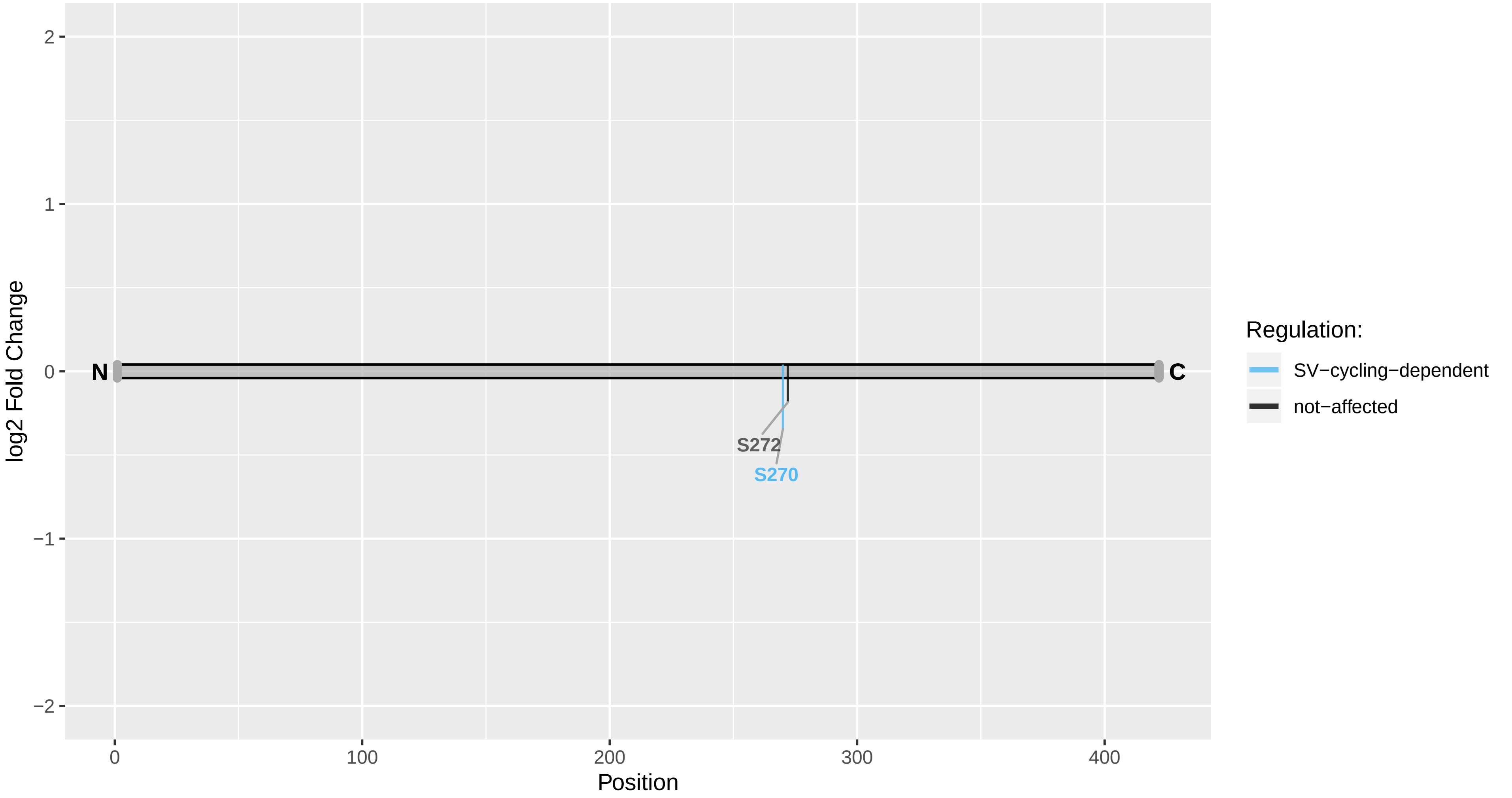

Uniprot Accession: P0CD96
Overexpressed in colon carcinoma 1 protein homolog

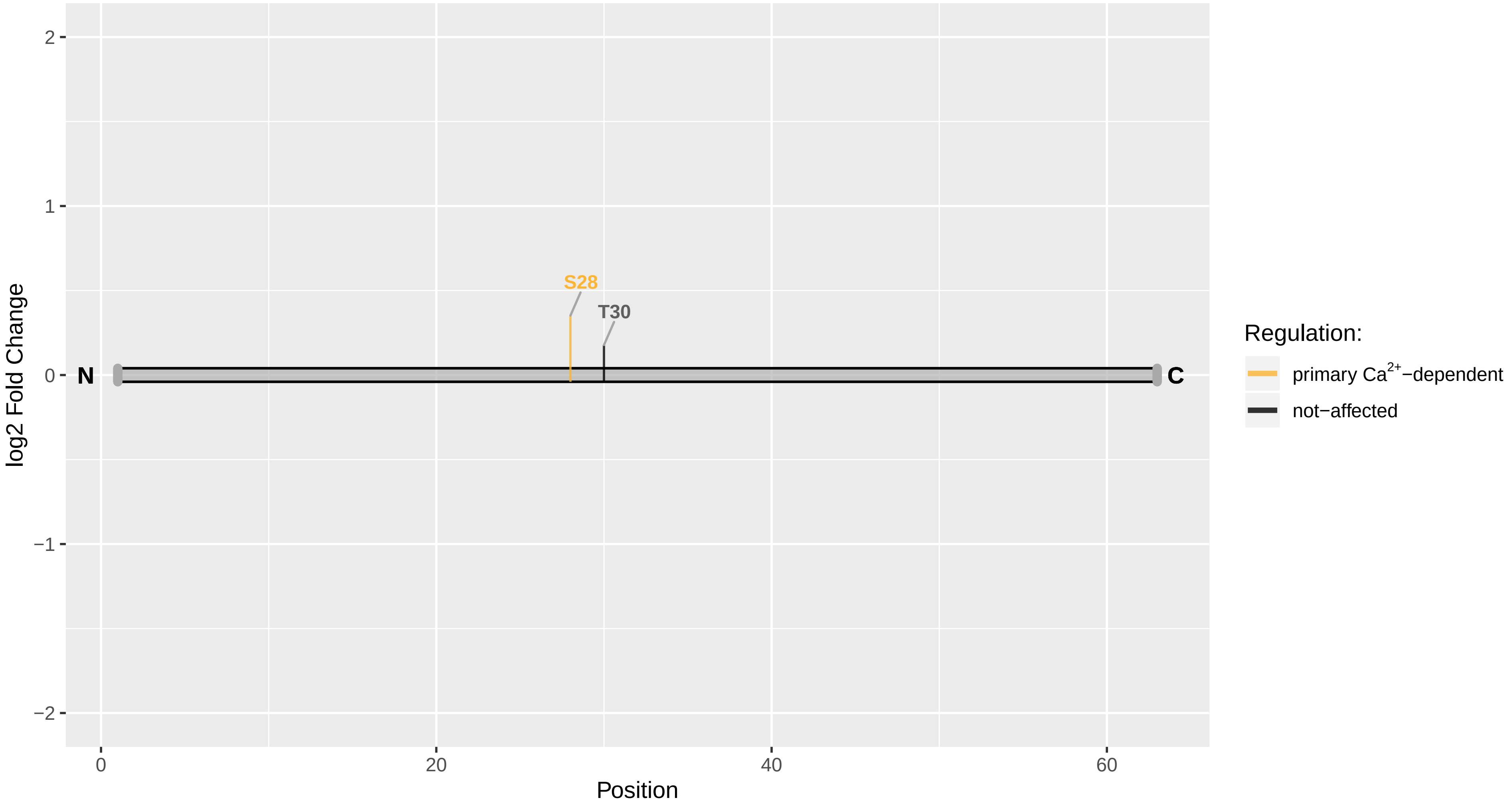

Uniprot Accession: Q63003
5E5 antigen

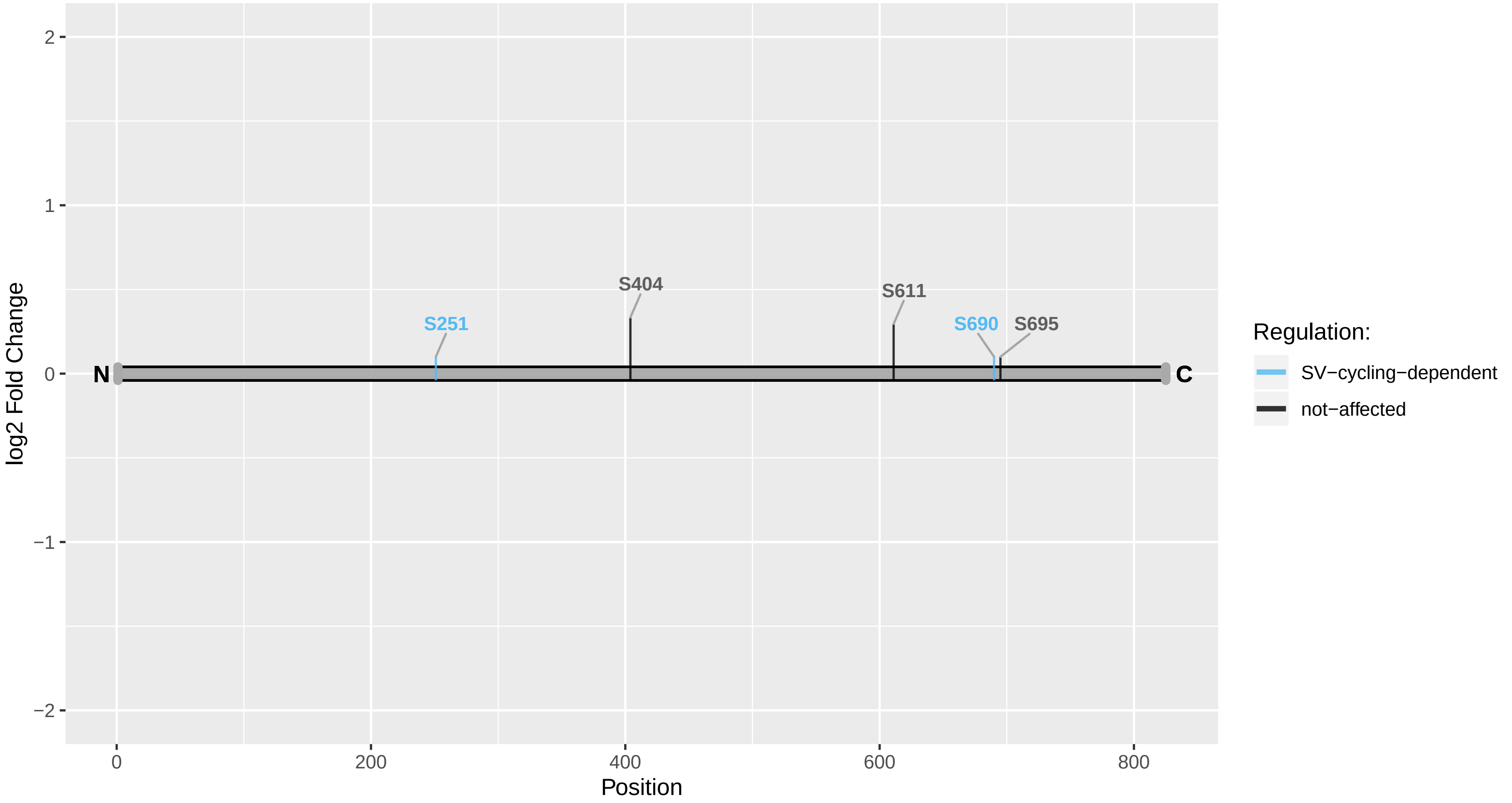

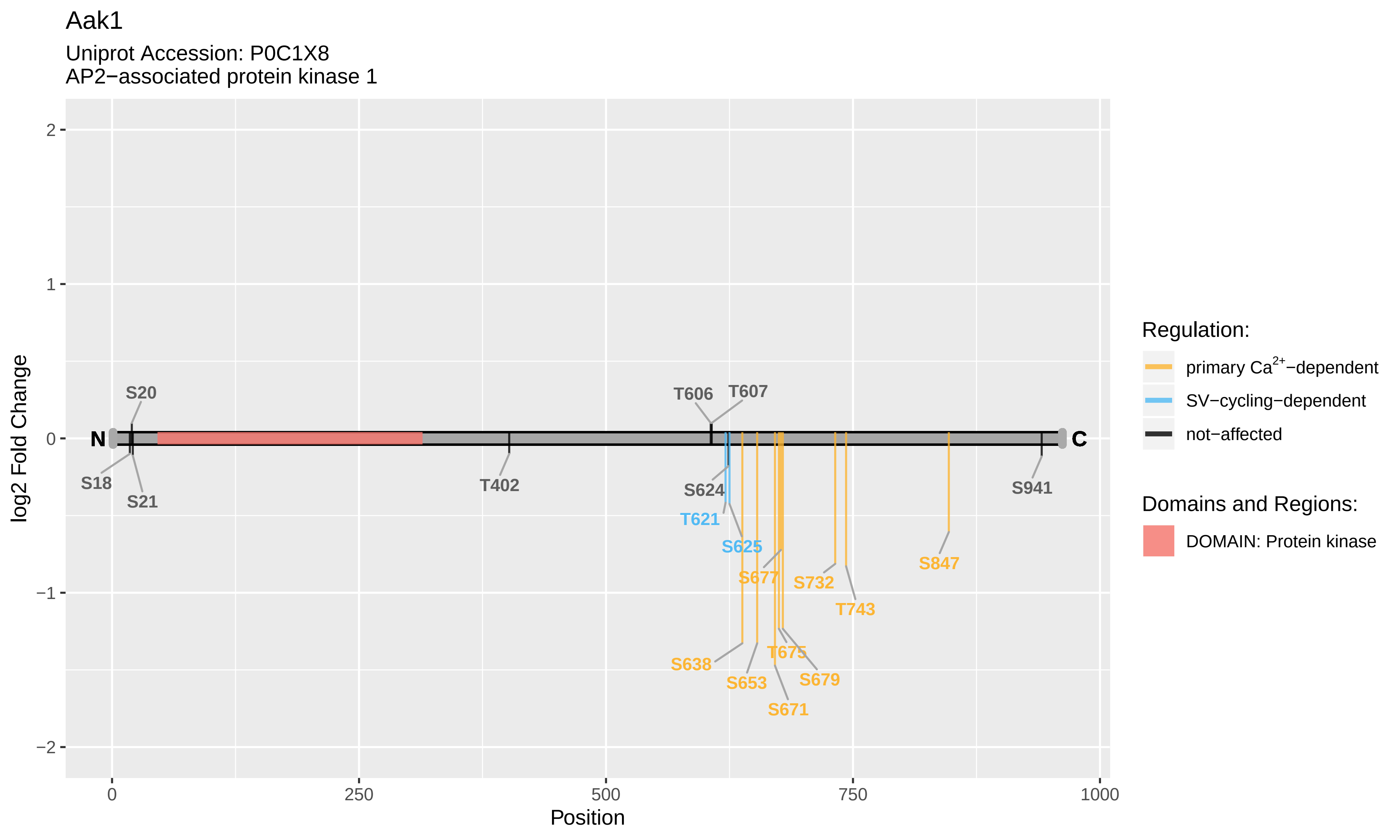

Aak1
Uniprot Accession: P0C1X8
AP2−associated protein kinase 1
log2 Fold Change
2
1
0
−1
−2
0
250
500
750
1000
Position
N
C
S20
S18
S21
T402
T606
T607
S624
T621
S625
S677
S732
T743
S847
S941
S638
S653
T675
S679
S671
Regulation:
primary Ca2+−dependent
SV−cycling−dependent
not−affected
Domains and Regions:
DOMAIN: Protein kinase

Abi1

Uniprot Accession: Q9QZM5
Abl interactor 1

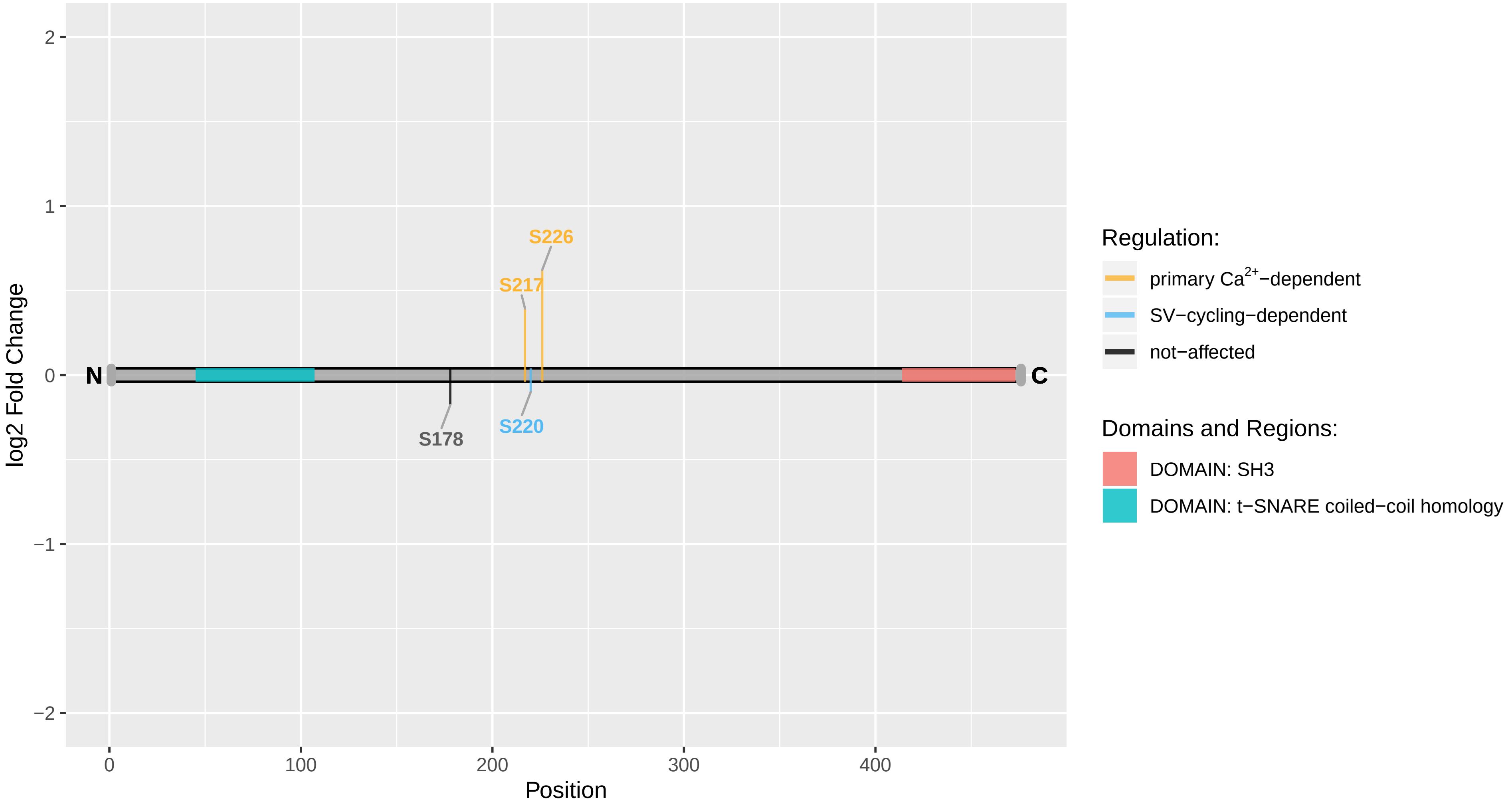

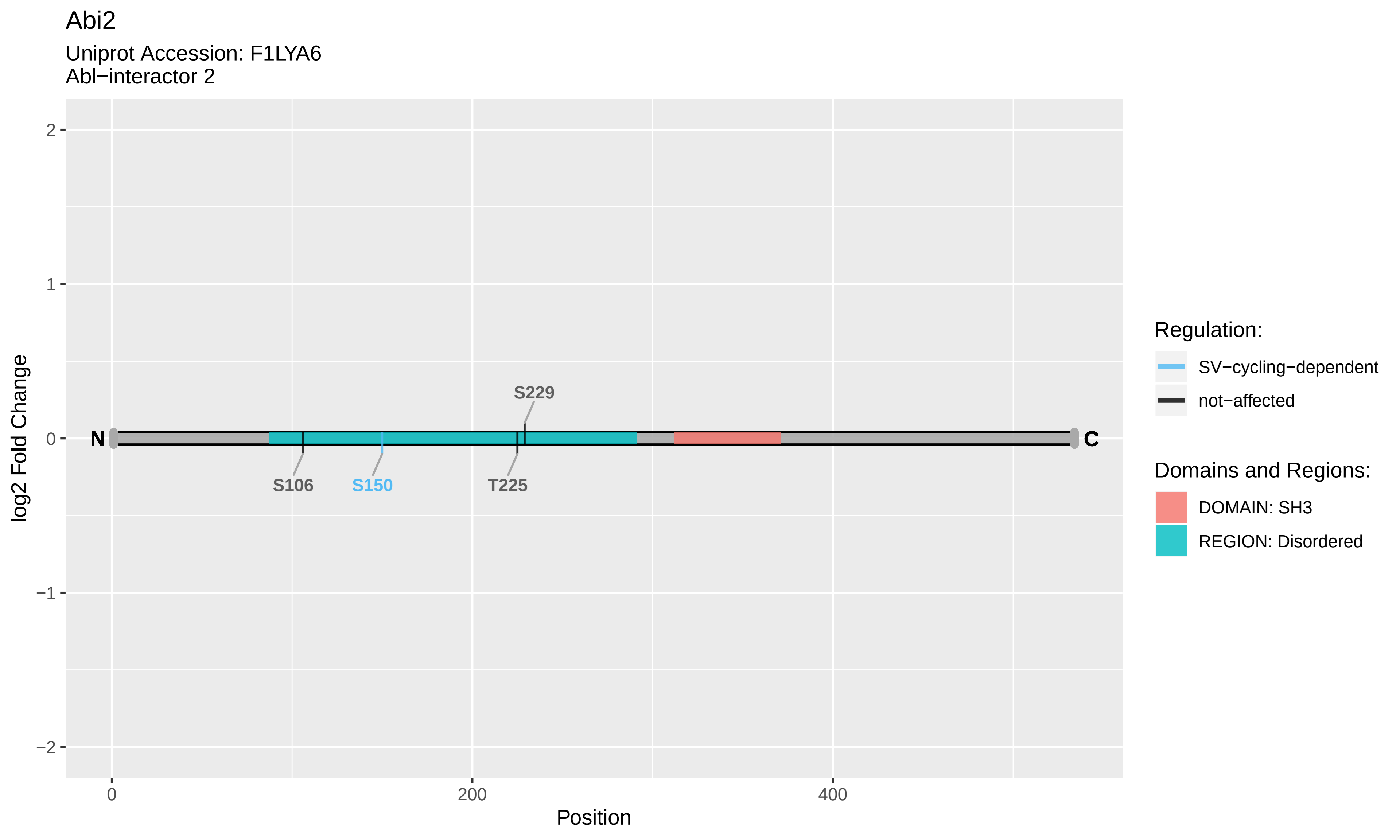
Abi2
Uniprot Accession: F1LYA6
Abl−interactor 2
2
1
0
−1
−2
log2 Fold Change
N
C
S229
S106
S150
T225
0
200
400
Position
Regulation:
SV−cycling−dependent
not−affected
Domains and Regions:
DOMAIN: SH3
REGION: Disordered

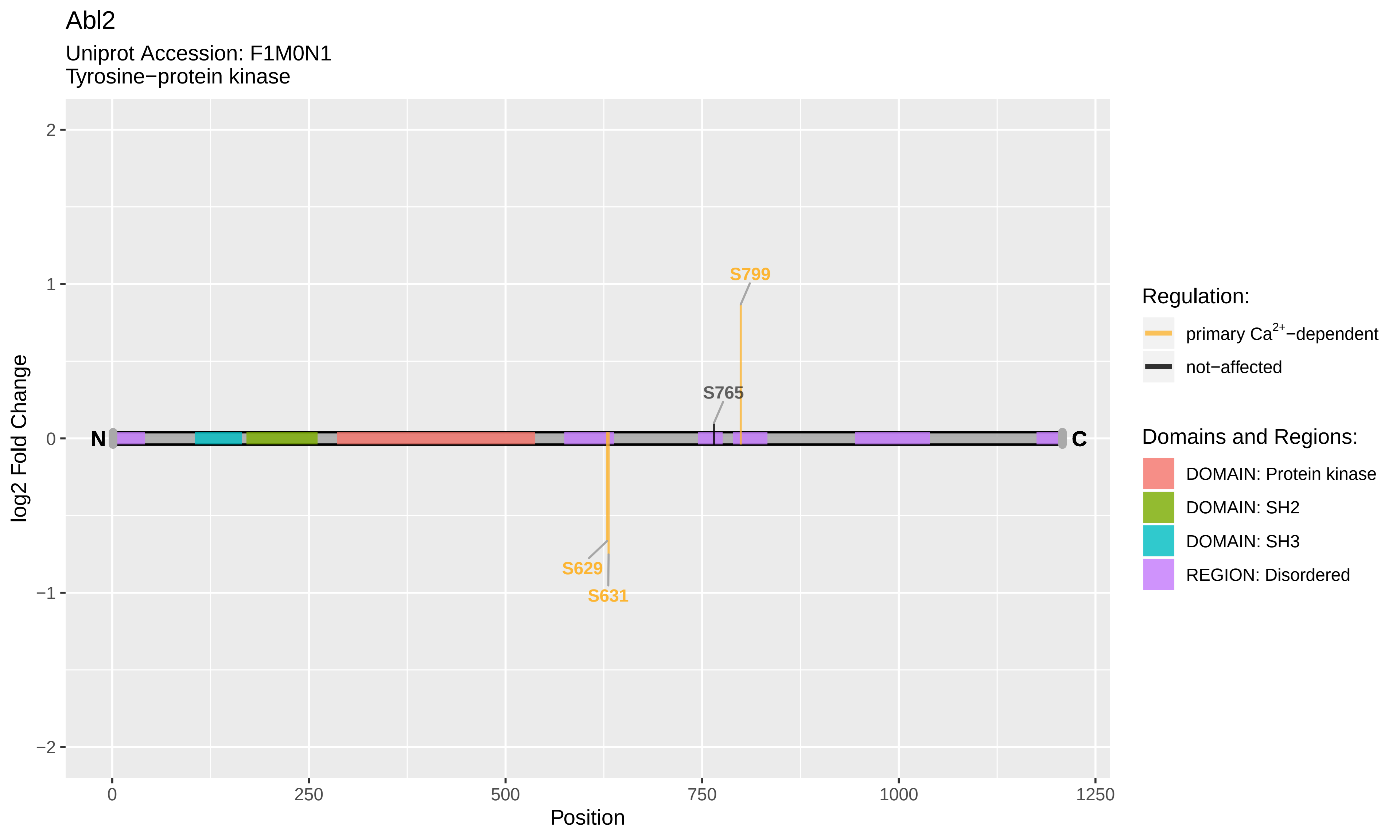

Abl2
Uniprot Accession: F1M0N1
Tyrosine−protein kinase
log2 Fold Change
Position
S799
S765
S629
S631
N
C
2
1
0
−1
−2
0
250
500
750
1000
1250
Regulation:
primary Ca2+−dependent
not−affected
Domains and Regions:
DOMAIN: Protein kinase
DOMAIN: SH2
DOMAIN: SH3
REGION: Disordered

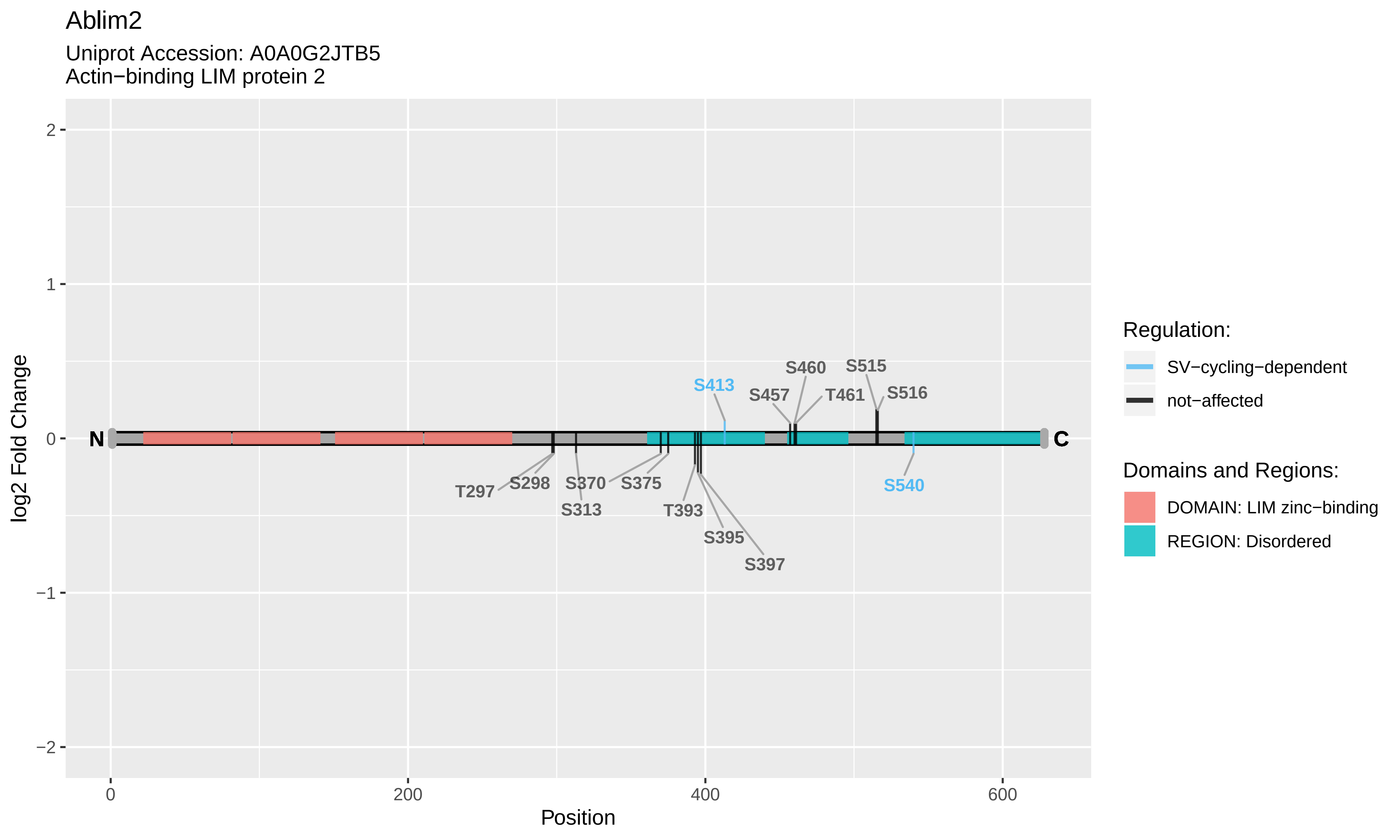
Ablim2
Uniprot Accession: A0A0G2JTB5
Actin−binding LIM protein 2
log2 Fold Change
2
1
0
−1
−2
N
C
S413
S457
S460
T461
S515
S516
T297
S298
S370
S375
S313
T393
S395
S397
S540
0
200
400
600
Position
Regulation:
SV−cycling−dependent
not−affected
Domains and Regions:
DOMAIN: LIM zinc−binding
REGION: Disordered

Ablim2

Uniprot Accession: Q6KC51
Actin−binding LIM protein 2

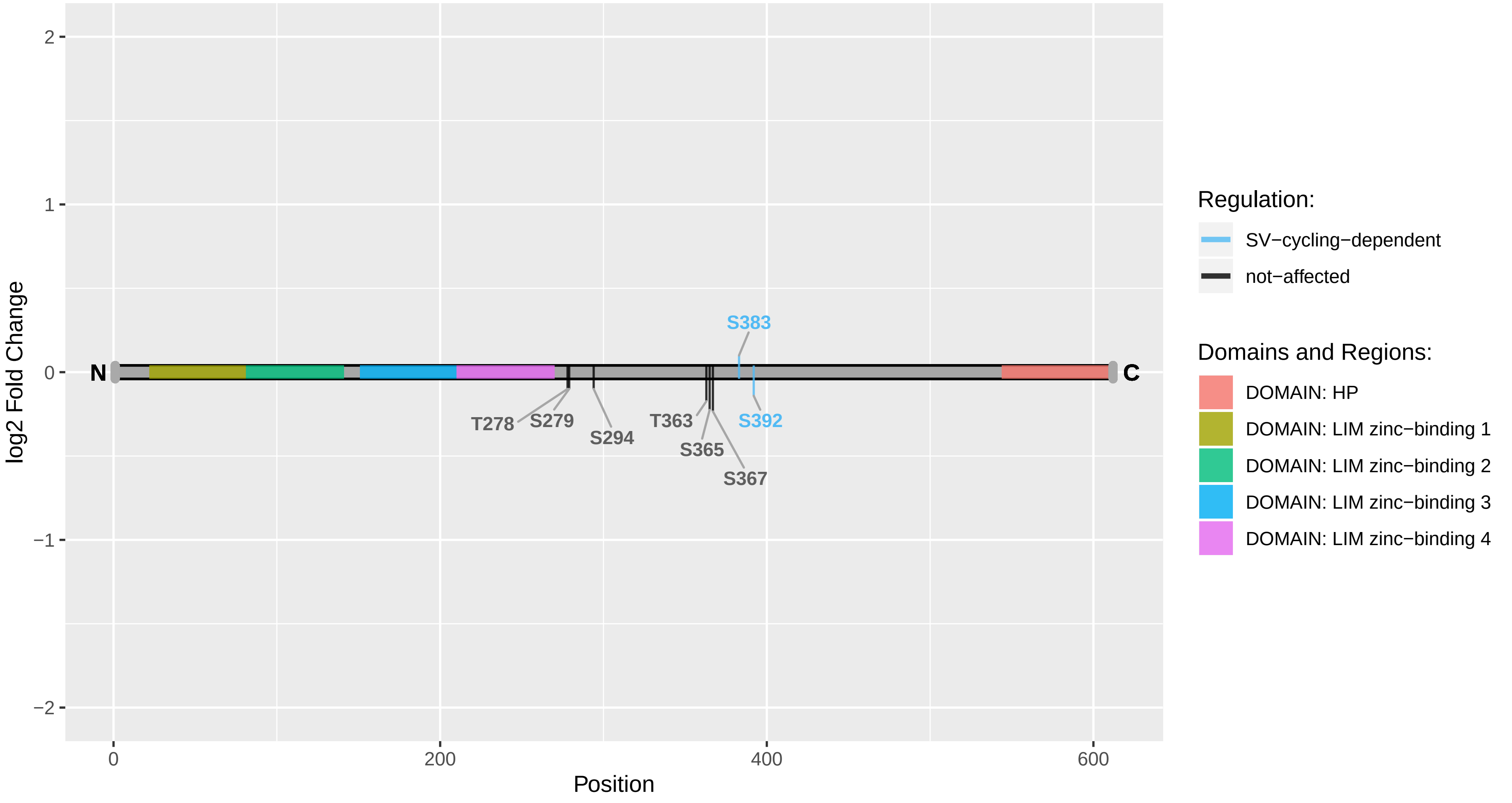

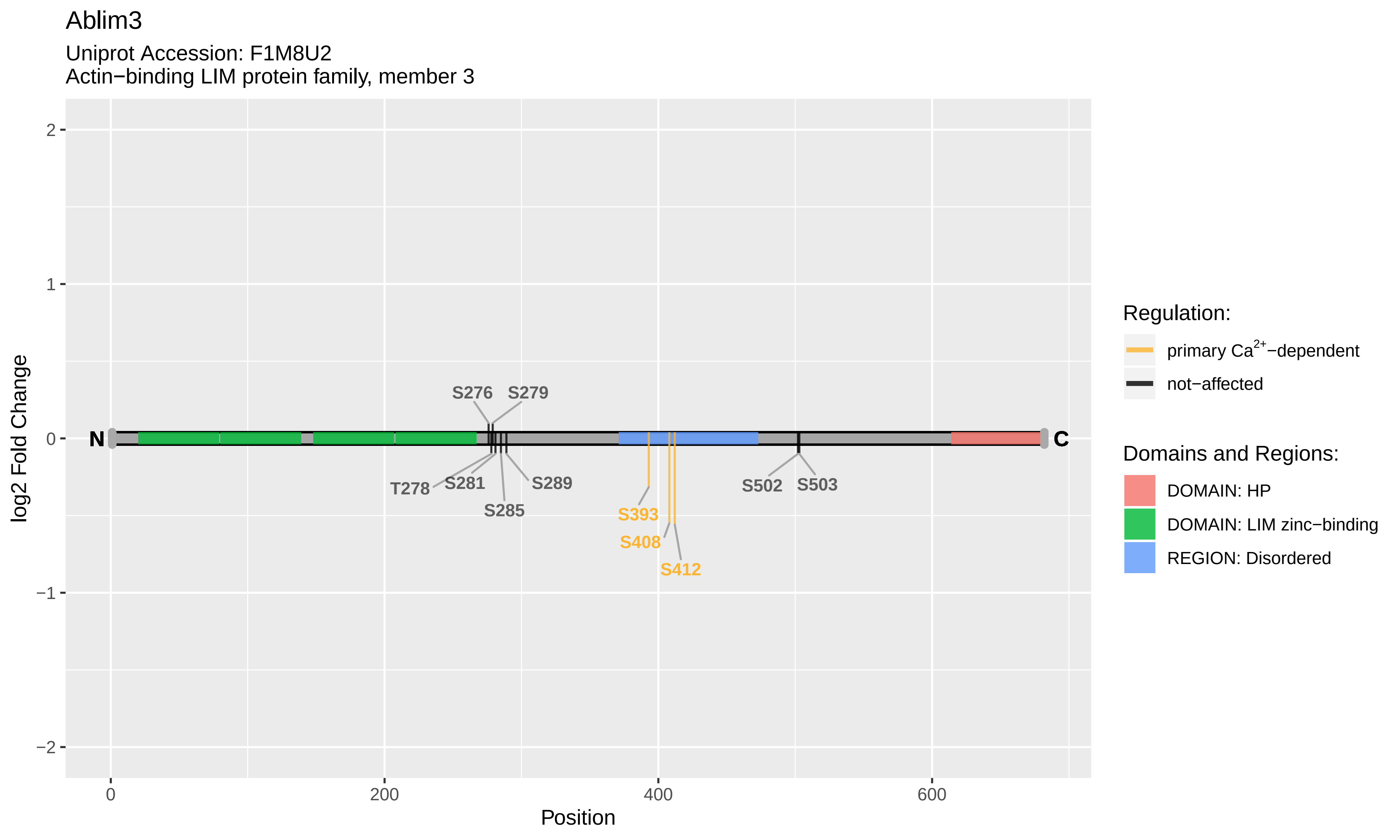

Ablim3
Uniprot Accession: F1M8U2
Actin−binding LIM protein family, member 3
log2 Fold Change
2
1
0
−1
−2
N
C
S276
S279
T278
S281
S285
S289
S393
S408
S412
S502
S503
0
200
400
600
Position
Regulation:
primary Ca2+−dependent
not−affected
Domains and Regions:
DOMAIN: HP
DOMAIN: LIM zinc−binding
REGION: Disordered

Acap2

Uniprot Accession: A0A0G2K4T3
Arf−GAP with coiled−coil, ANK repeat and PH domain−containing protein 2

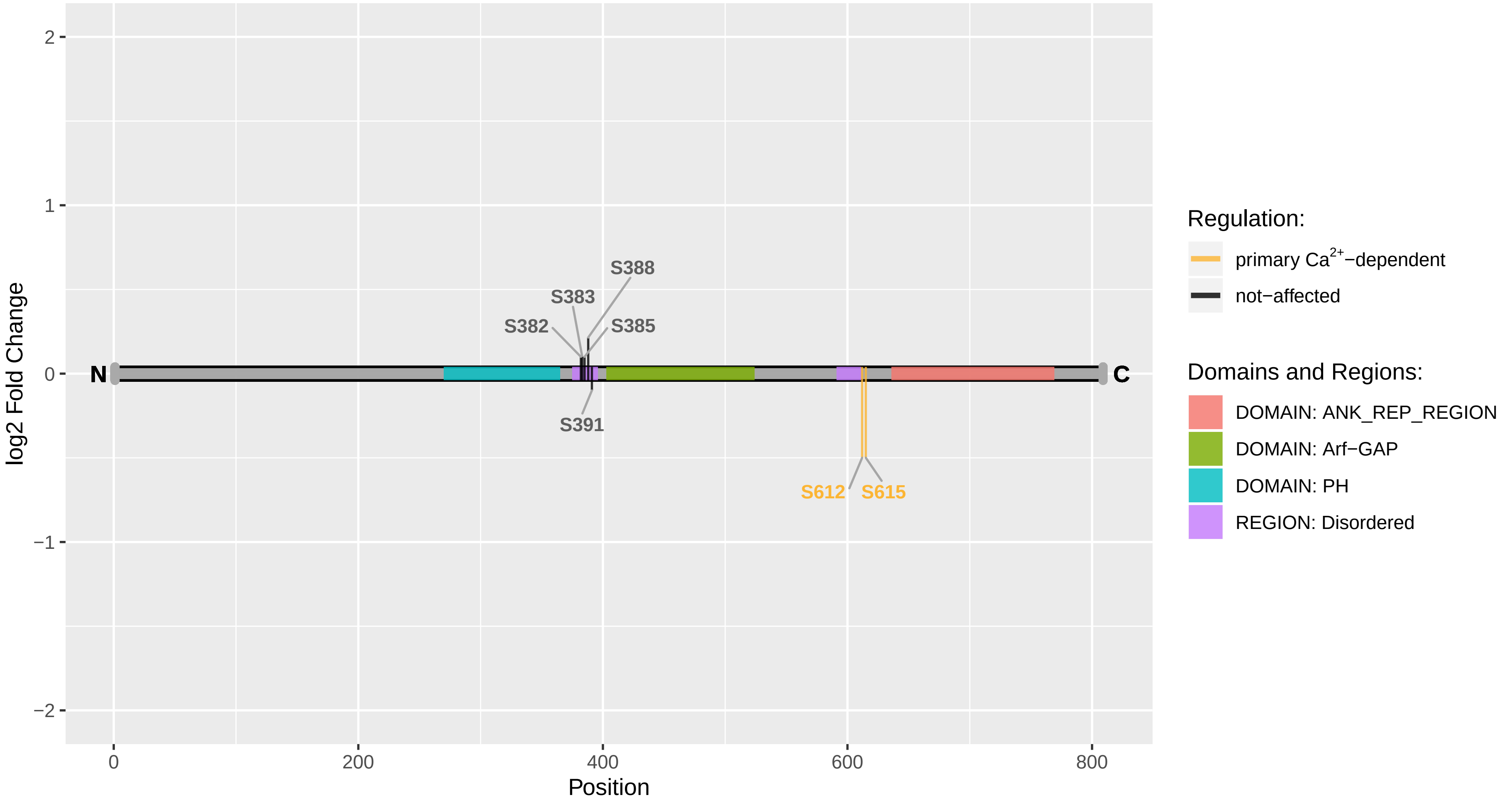

Adam22

Uniprot Accession: M0R5P8
ADAM metallopeptidase domain 22

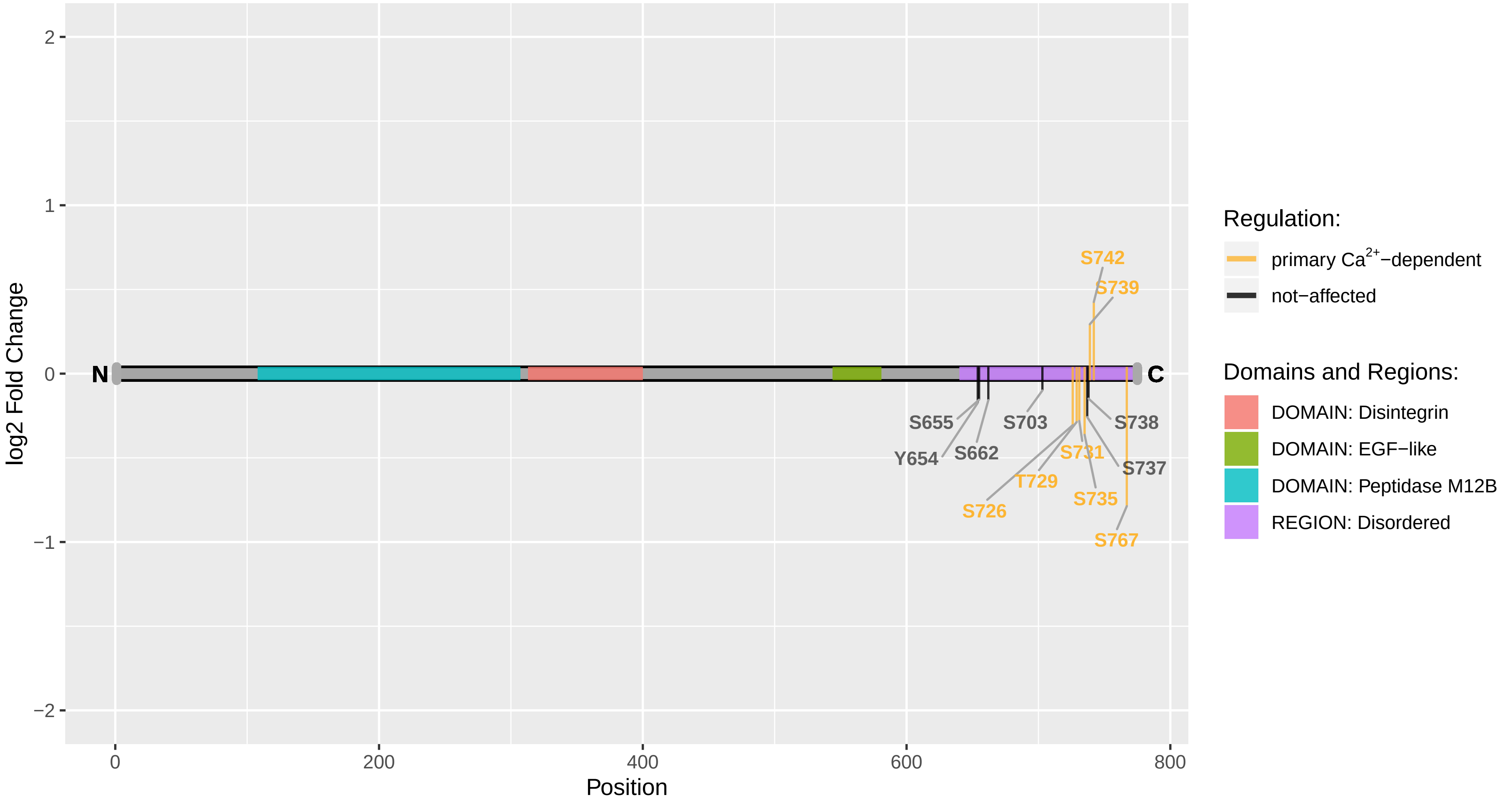

Adcy1

Uniprot Accession: D4A3N4
Adenylate cyclase

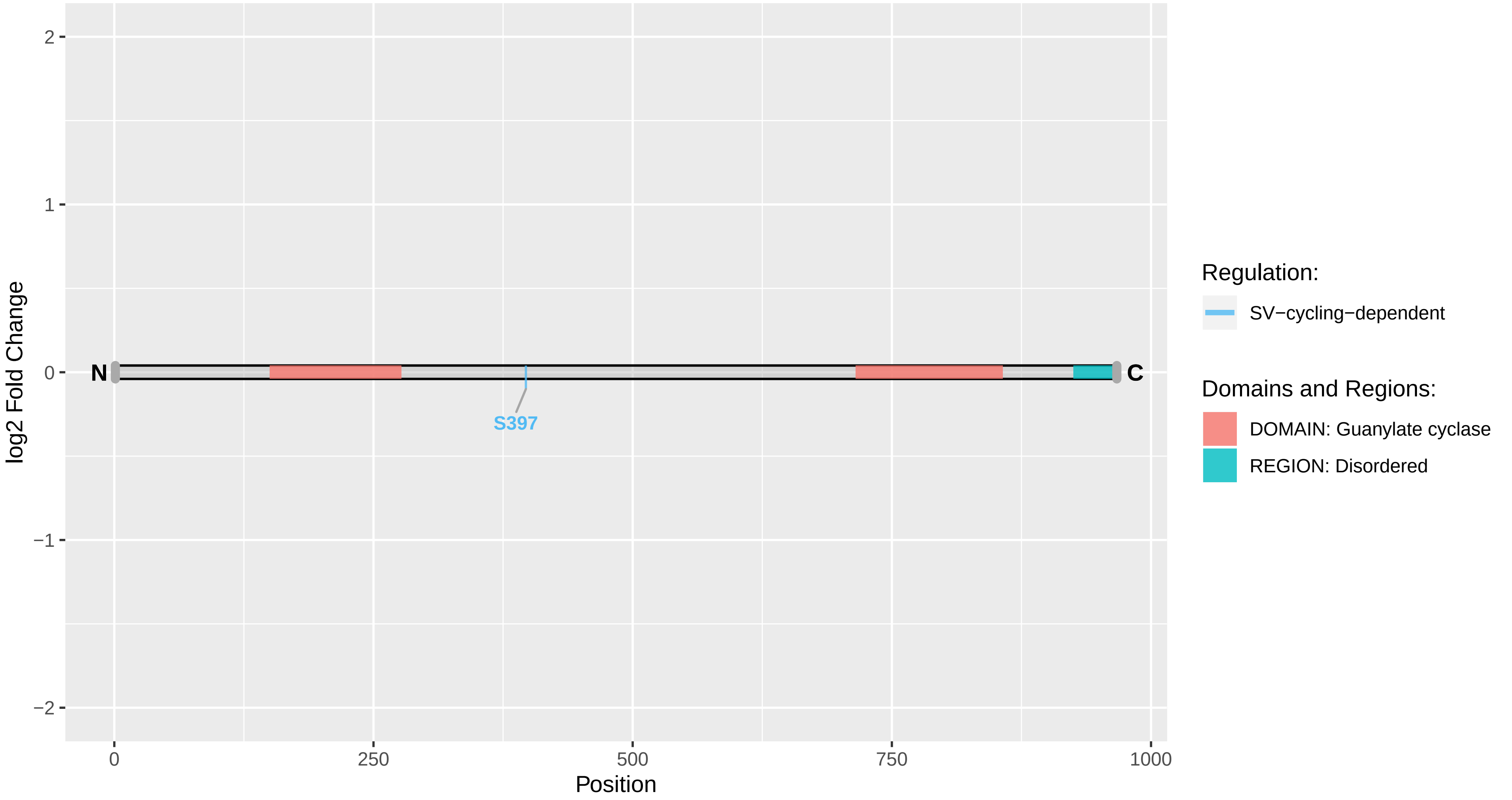

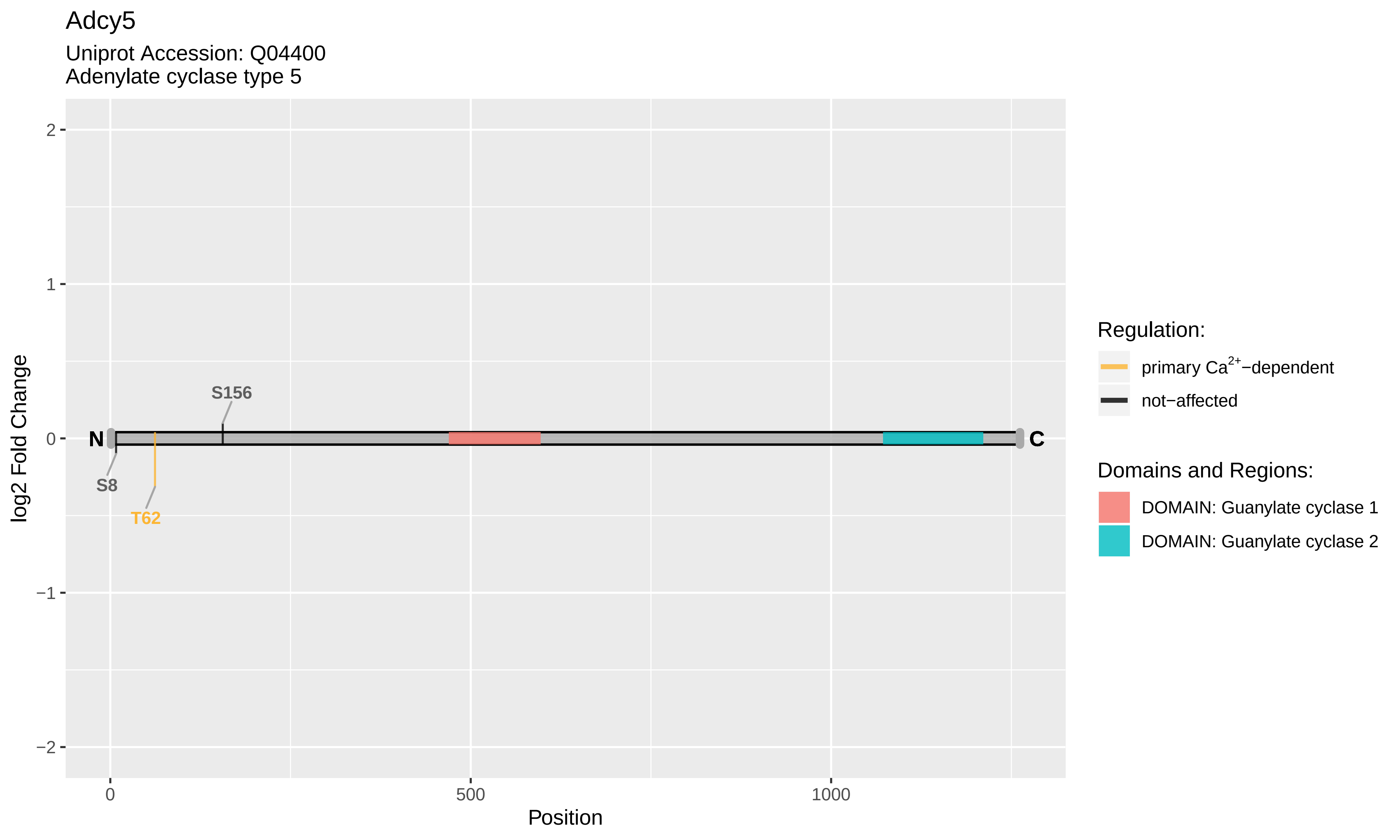

Adcy5
Uniprot Accession: Q04400
Adenylate cyclase type 5
S156
N
C
S8
T62
log2 Fold Change
Position
2
1
0
−1
−2
0
500
1000
Regulation:
primary Ca2+−dependent
not−affected
Domains and Regions:
DOMAIN: Guanylate cyclase 1
DOMAIN: Guanylate cyclase 2

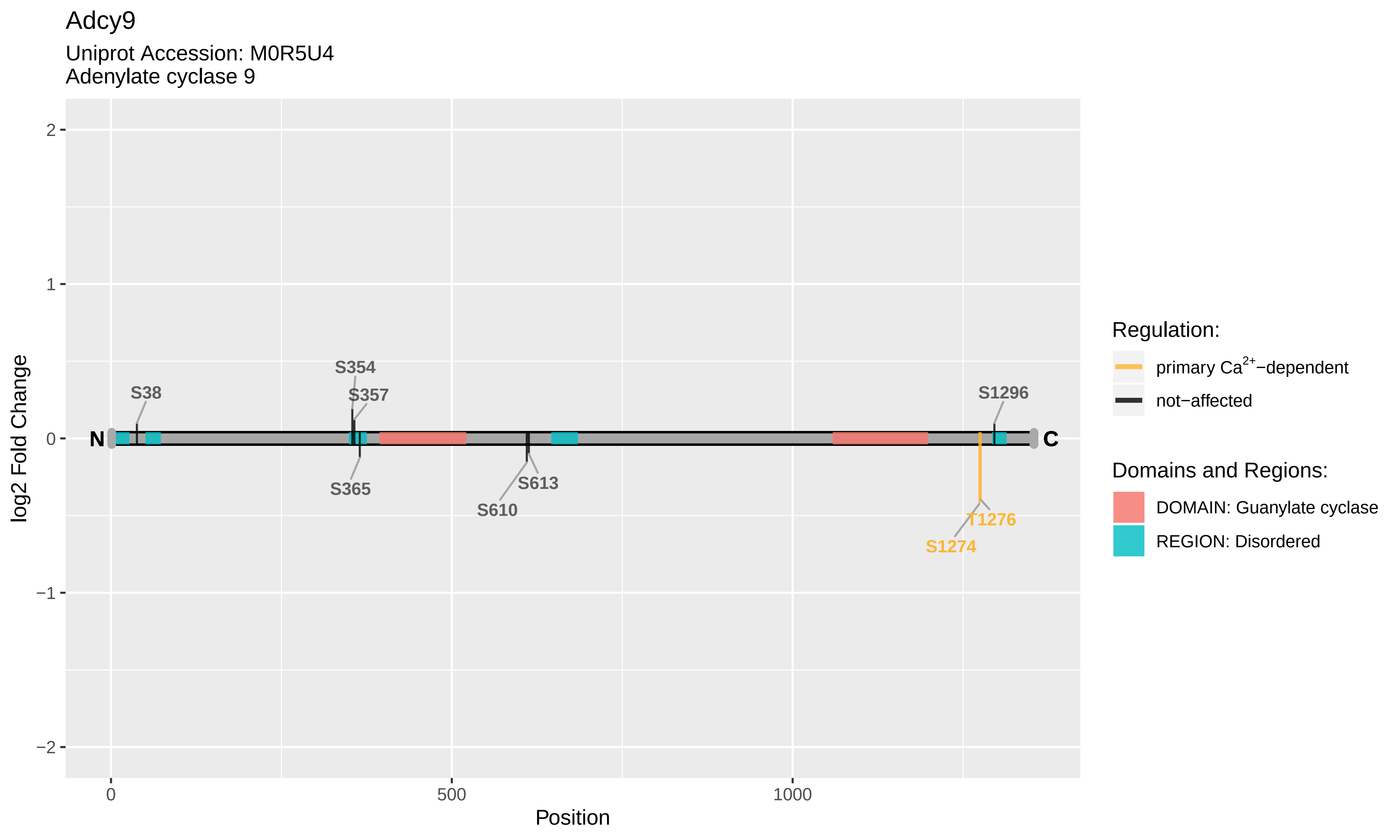

Adcy9
Uniprot Accession: M0R5U4
Adenylate cyclase 9
log2 Fold Change
Position
N
C
S38
S354
S357
S365
S610
S613
S1296
T1276
S1274
2
1
0
−1
−2
0
500
1000
Regulation:
primary Ca2+−dependent
not−affected
Domains and Regions:
DOMAIN: Guanylate cyclase
REGION: Disordered

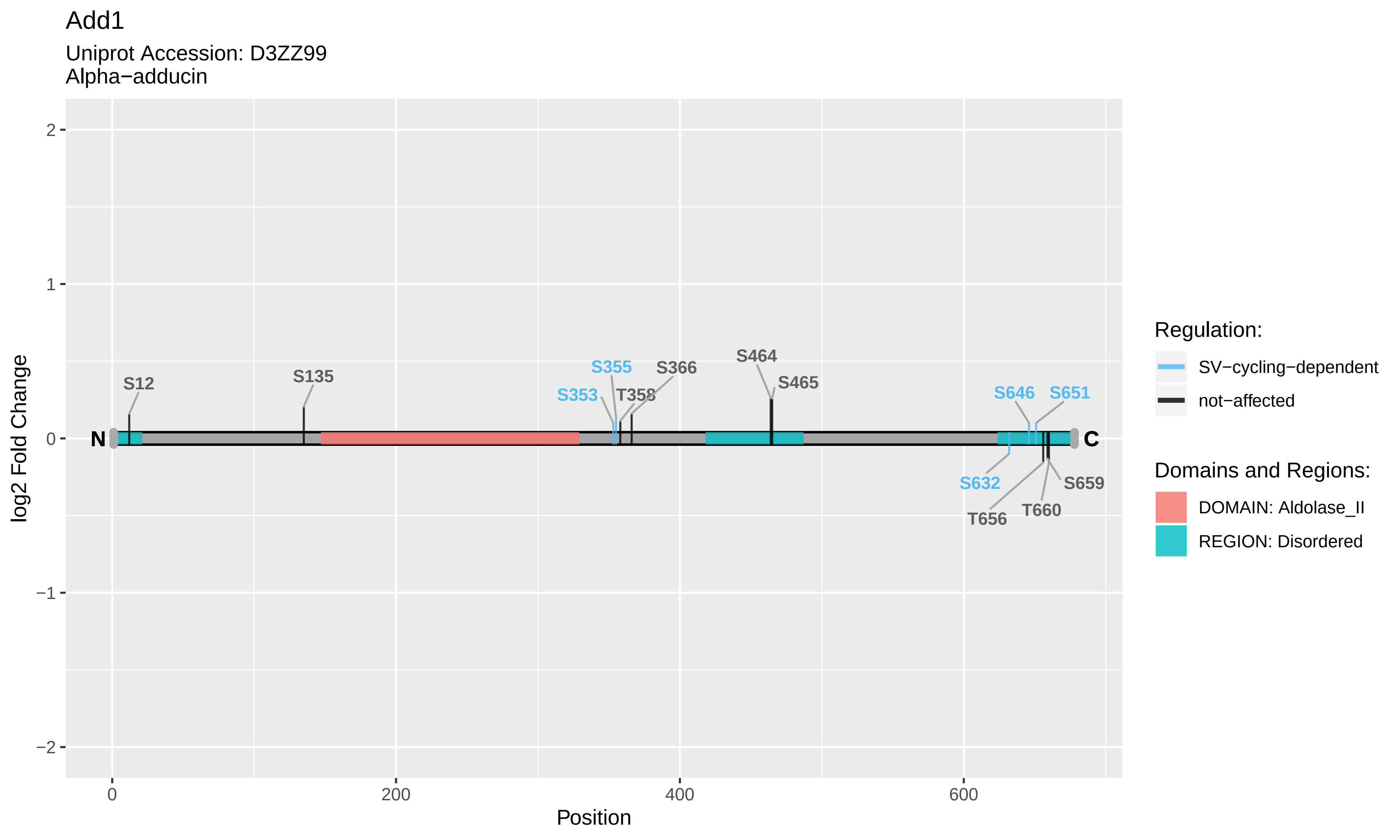

Add1
Uniprot Accession: D3ZZ99
Alpha−adducin
log2 Fold Change
Position
2
1
0
−1
−2
0
200
400
600
N
C
S12
S135
S353
S355
T358
S366
S464
S465
S646
S651
S632
T656
T660
S659
Regulation:
SV−cycling−dependent
not−affected
Domains and Regions:
DOMAIN: Aldolase_II
REGION: Disordered

Add1

Uniprot Accession: Q63028
Alpha−adducin

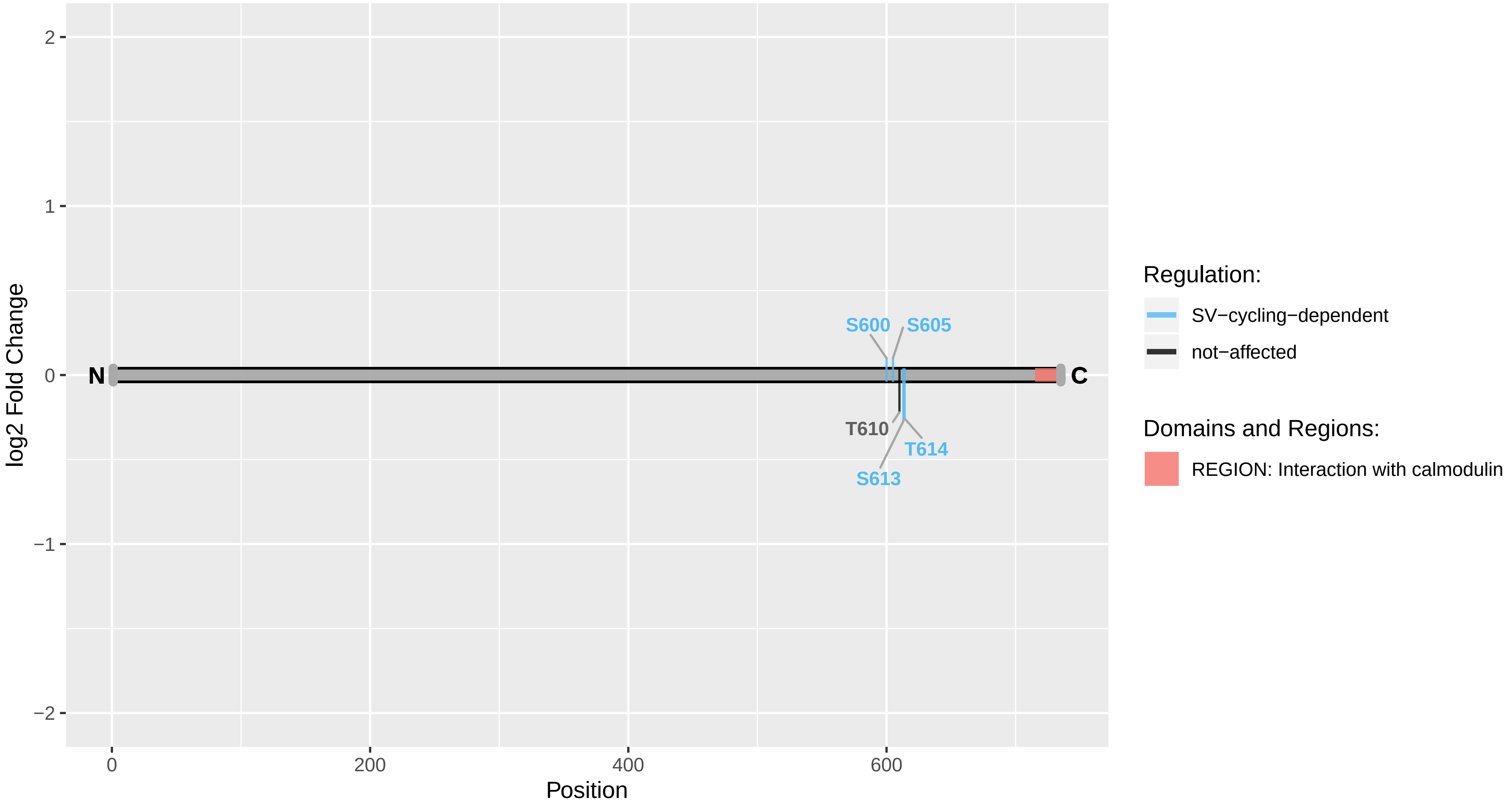

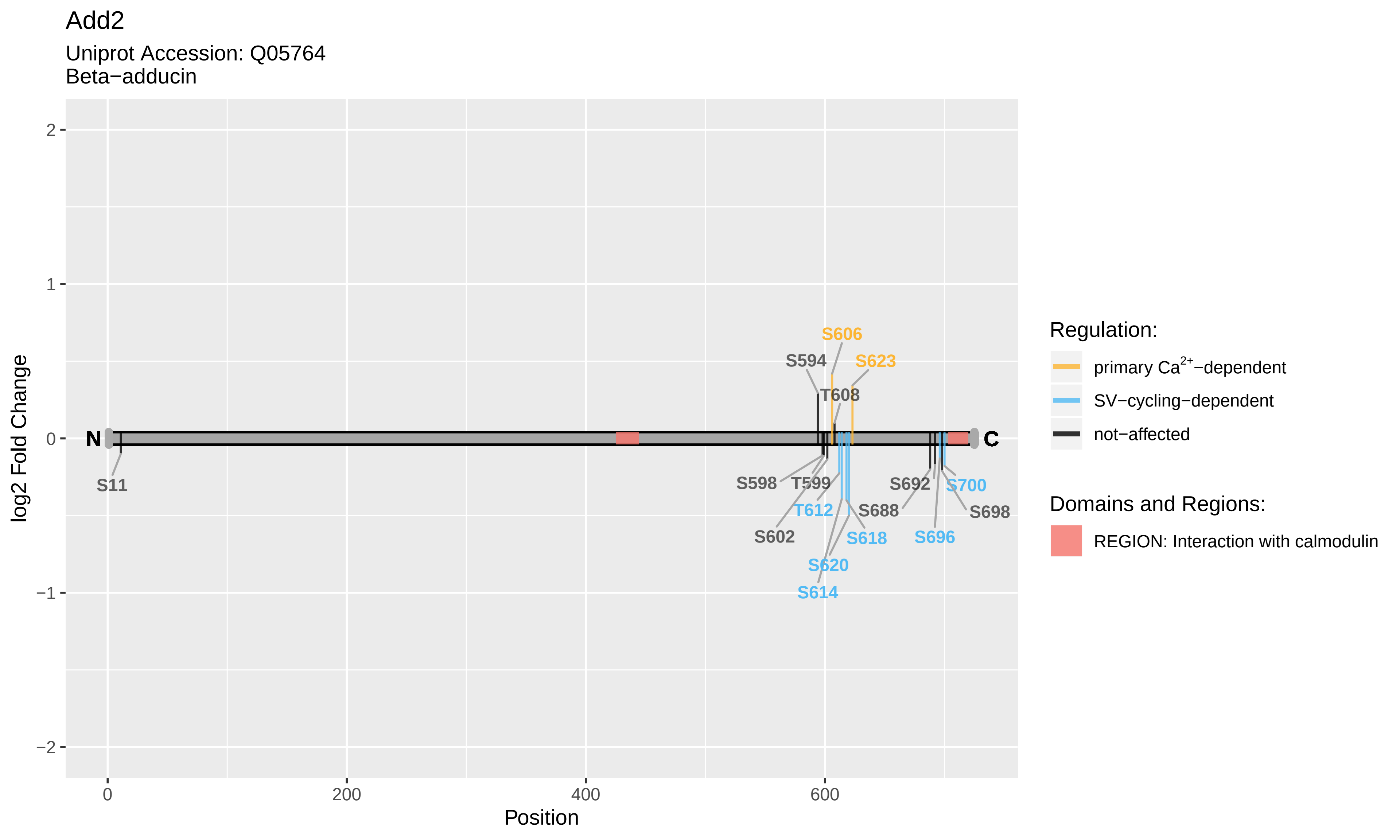

Add2
Uniprot Accession: Q05764
Beta−adducin
log2 Fold Change
Position
N
C
S606
S594
S623
T608
S11
S598
T599
T612
S602
S620
S614
S618
S692
S688
S700
S698
S696
Regulation:
primary Ca2+−dependent
SV−cycling−dependent
not−affected
Domains and Regions:
REGION: Interaction with calmodulin

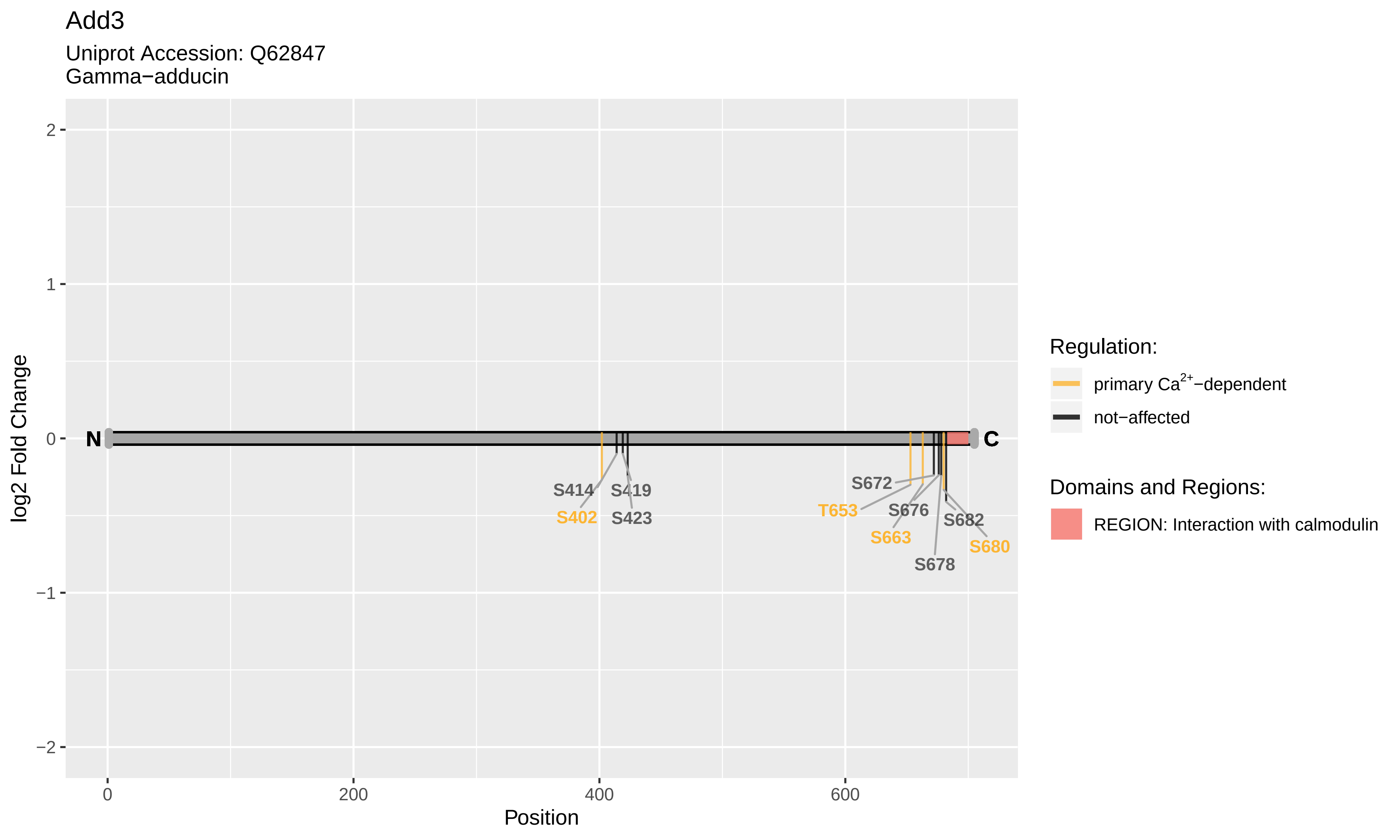

Add3
Uniprot Accession: Q62847
Gamma−adducin
log2 Fold Change
2
1
0
−1
−2
N
C
S414
S419
S402
S423
S672
T653
S676
S682
S663
S680
S678
0
200
400
600
Position
Regulation:
primary Ca2+−dependent
not−affected
Domains and Regions:
REGION: Interaction with calmodulin

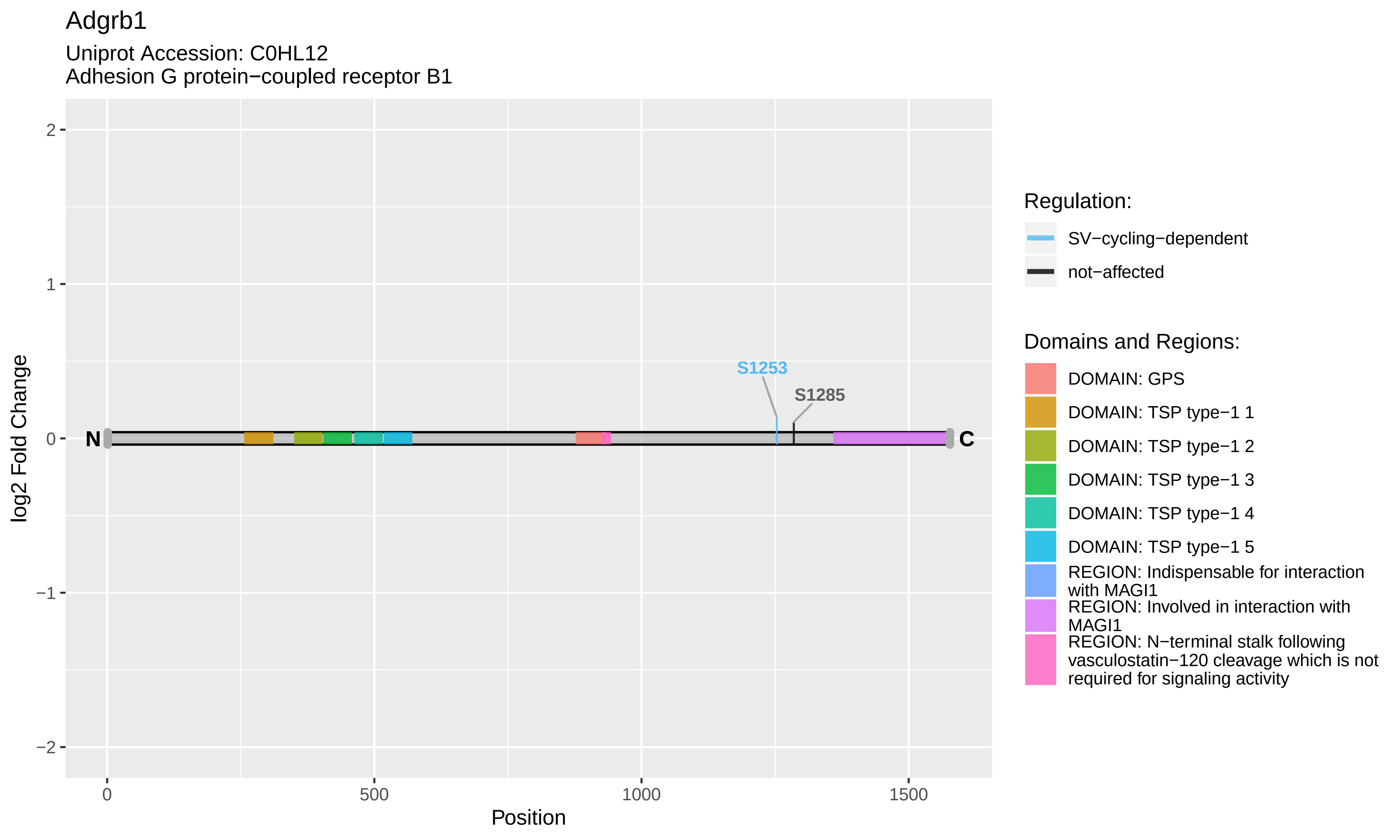

Adgrb1
Uniprot Accession: C0HL12
Adhesion G protein−coupled receptor B1
log2 Fold Change
2
1
0
−1
−2
N
C
S1253
S1285
0
500
1000
1500
Position
Regulation:
SV−cycling−dependent
not−affected
Domains and Regions:
DOMAIN: GPS
DOMAIN: TSP type−1 1
DOMAIN: TSP type−1 2
DOMAIN: TSP type−1 3
DOMAIN: TSP type−1 4
DOMAIN: TSP type−1 5
REGION: Indispensable for interaction with MAGI1
REGION: Involved in interaction with MAGI1
REGION: N−terminal stalk following vasculostatin−120 cleavage which is not required for signaling activity

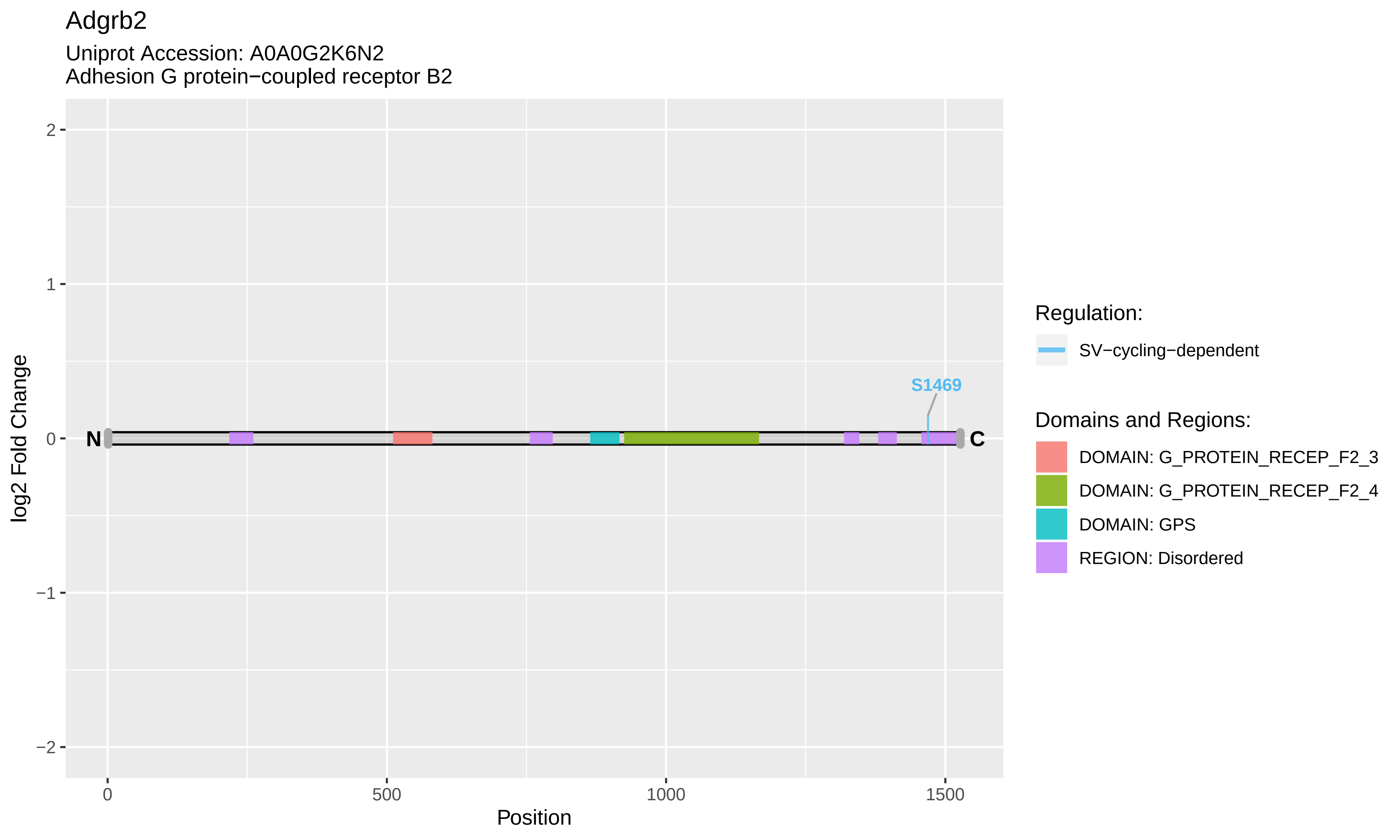
Adgrb2
Uniprot Accession: A0A0G2K6N2
Adhesion G protein−coupled receptor B2
log2 Fold Change
2
1
0
−1
−2
N
C
S1469
0
500
1000
1500
Position
Regulation:
SV−cycling−dependent
Domains and Regions:
DOMAIN: G_PROTEIN_RECEP_F2_3
DOMAIN: G_PROTEIN_RECEP_F2_4
DOMAIN: GPS
REGION: Disordered

Adgrl1

Uniprot Accession: O88917
Adhesion G protein−coupled receptor L1

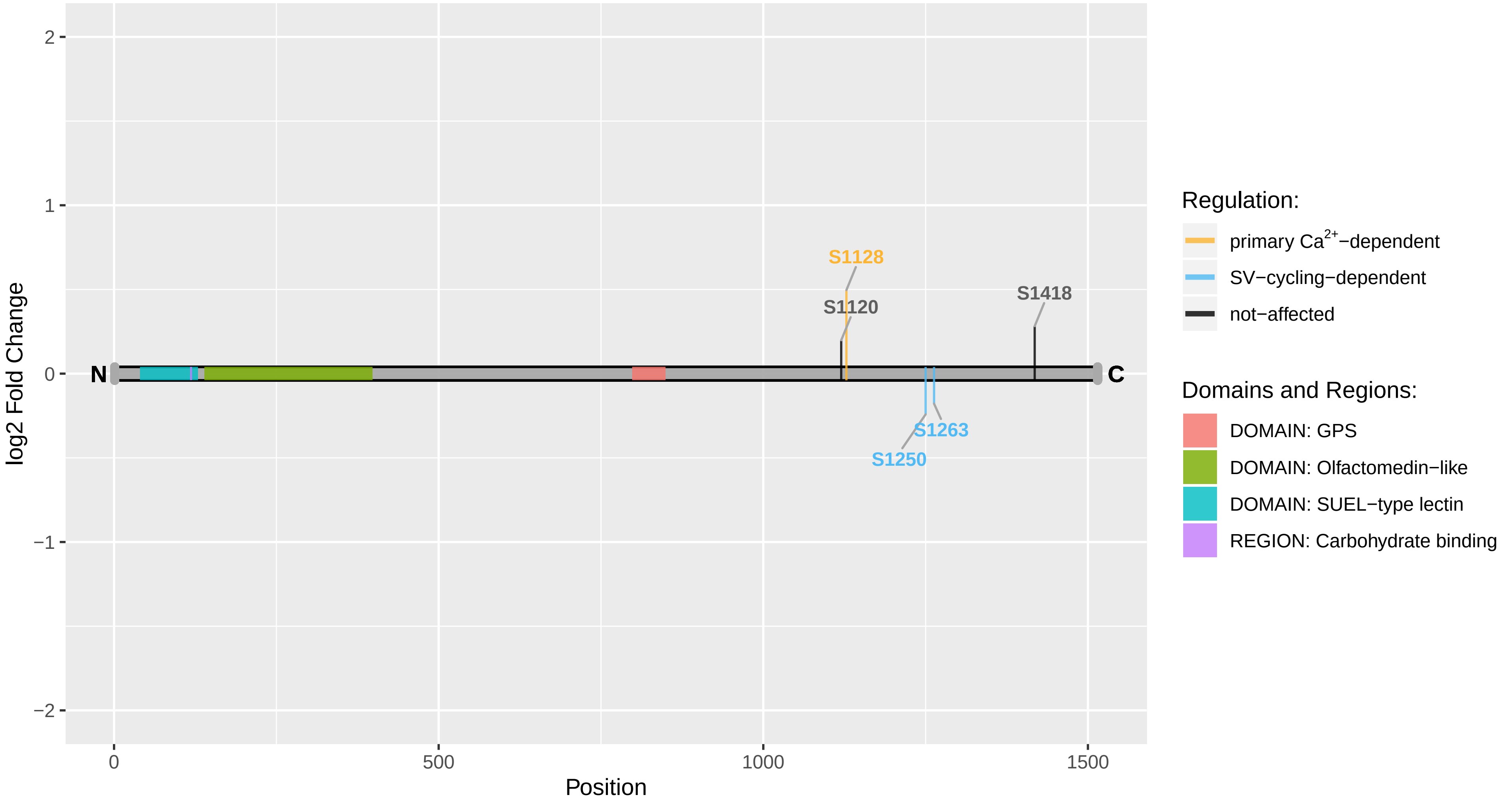

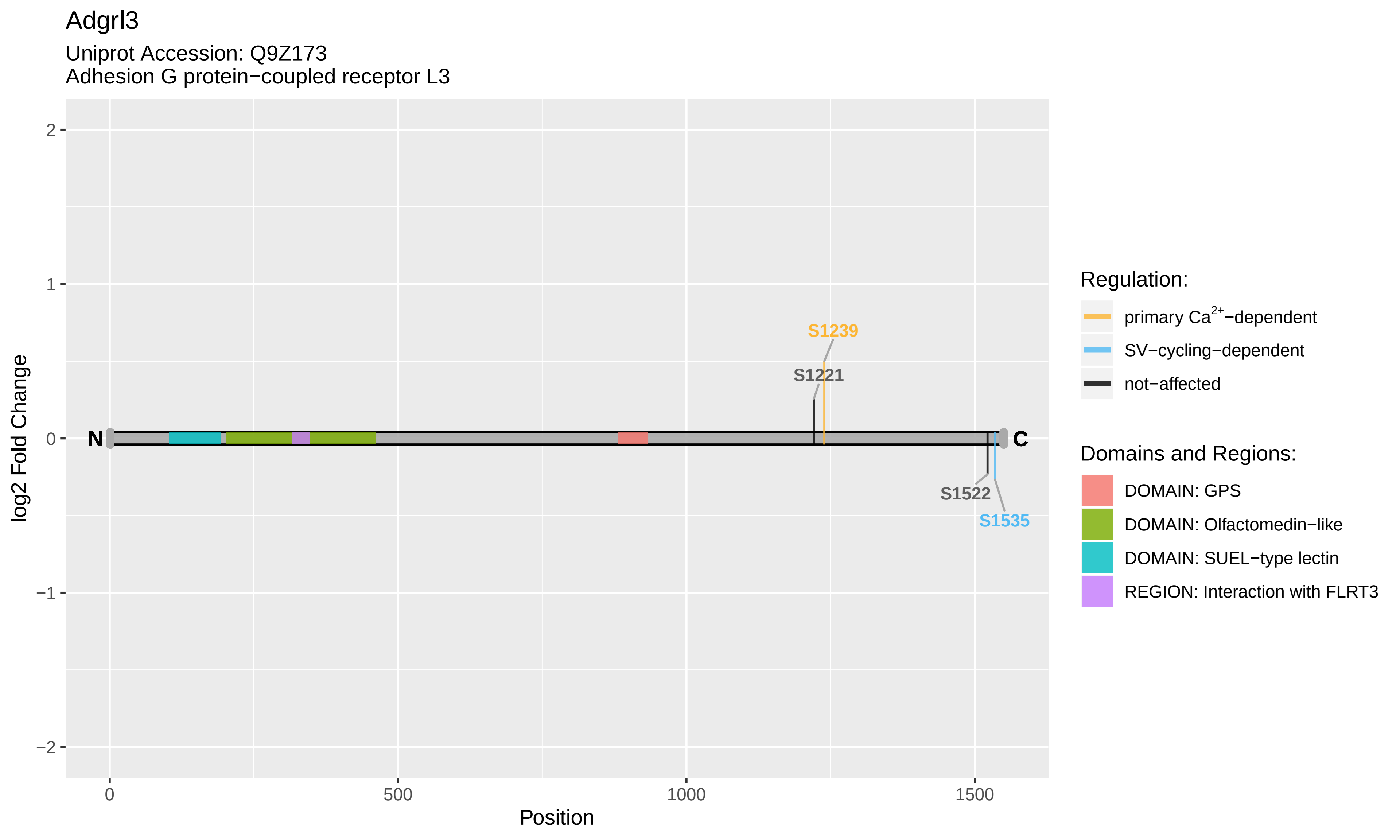
Adgrl3
Uniprot Accession: Q9Z173
Adhesion G protein−coupled receptor L3
log2 Fold Change
Position
N
C
S1239
S1221
S1522
S1535
Regulation:
primary Ca2+−dependent
SV−cycling−dependent
not−affected
Domains and Regions:
DOMAIN: GPS
DOMAIN: Olfactomedin−like
DOMAIN: SUEL−type lectin
REGION: Interaction with FLRT3

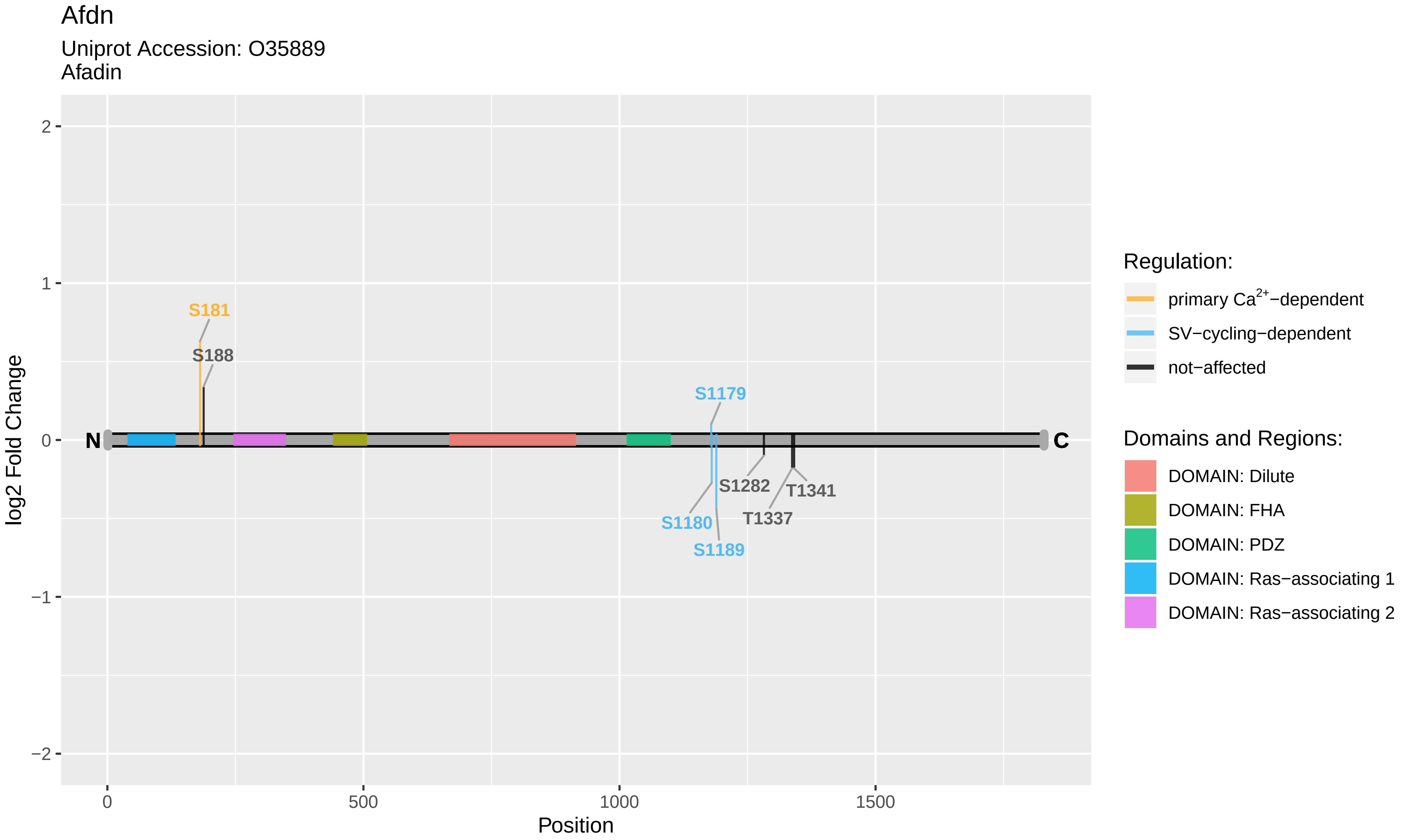

Afdn
Uniprot Accession: O35889
Afadin
S181
S188
S1179
S1180
S1189
S1282
T1337
T1341
N
C
2
1
0
−1
−2
log2 Fold Change
0
500
1000
1500
Position
Regulation:
primary Ca2+−dependent
SV−cycling−dependent
not−affected
Domains and Regions:
DOMAIN: Dilute
DOMAIN: FHA
DOMAIN: PDZ
DOMAIN: Ras−associating 1
DOMAIN: Ras−associating 2

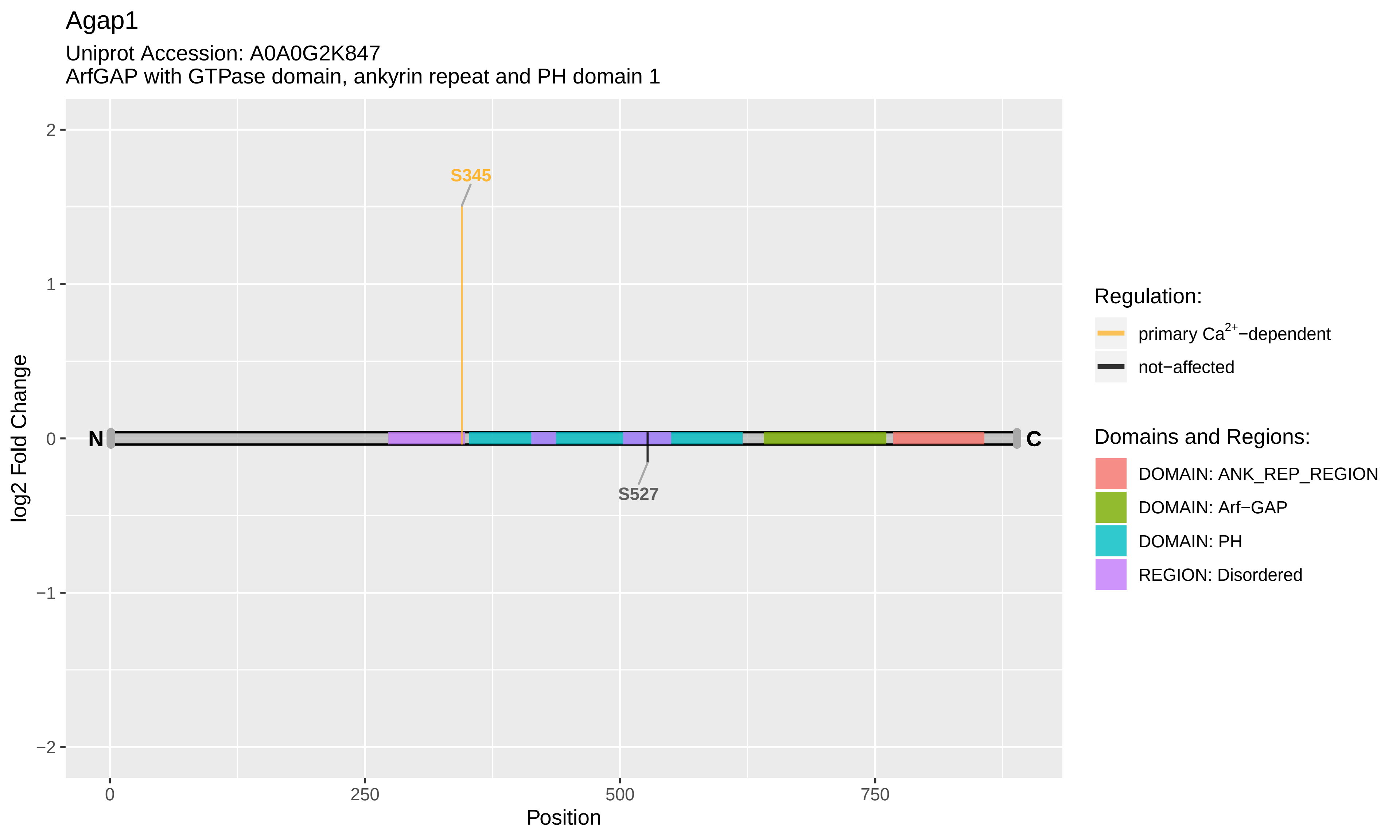

Agap1
Uniprot Accession: A0A0G2K847
ArfGAP with GTPase domain, ankyrin repeat and PH domain 1
S345
S527
N
C
2
1
0
−1
−2
log2 Fold Change
0
250
500
750
Position
Regulation:
primary Ca2+−dependent
not−affected
Domains and Regions:
DOMAIN: ANK_REP_REGION
DOMAIN: Arf−GAP
DOMAIN: PH
REGION: Disordered

Agap2

Uniprot Accession: Q8CGU4
Arf−GAP with GTPase, ANK repeat and PH domain−containing protein 2

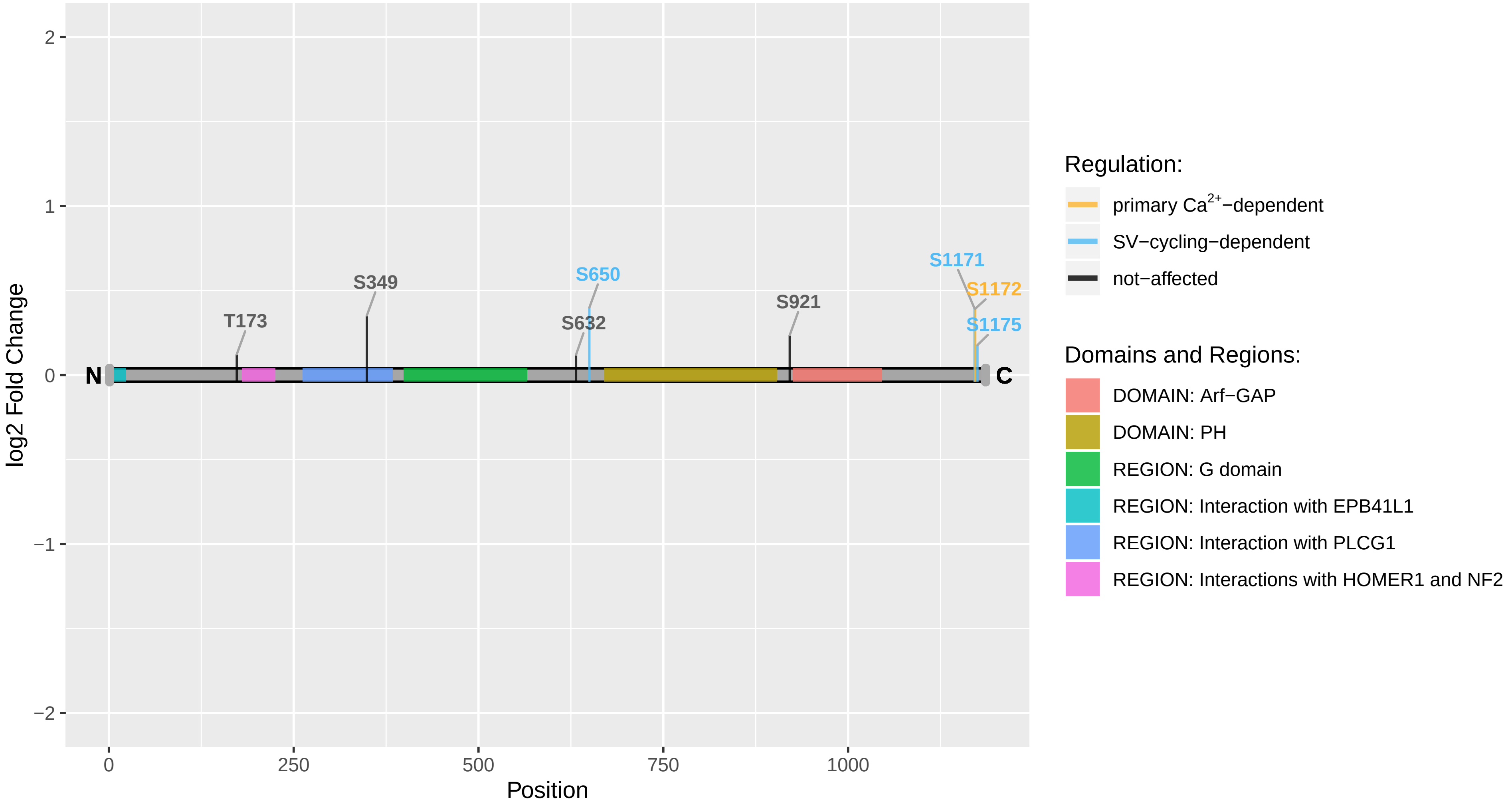

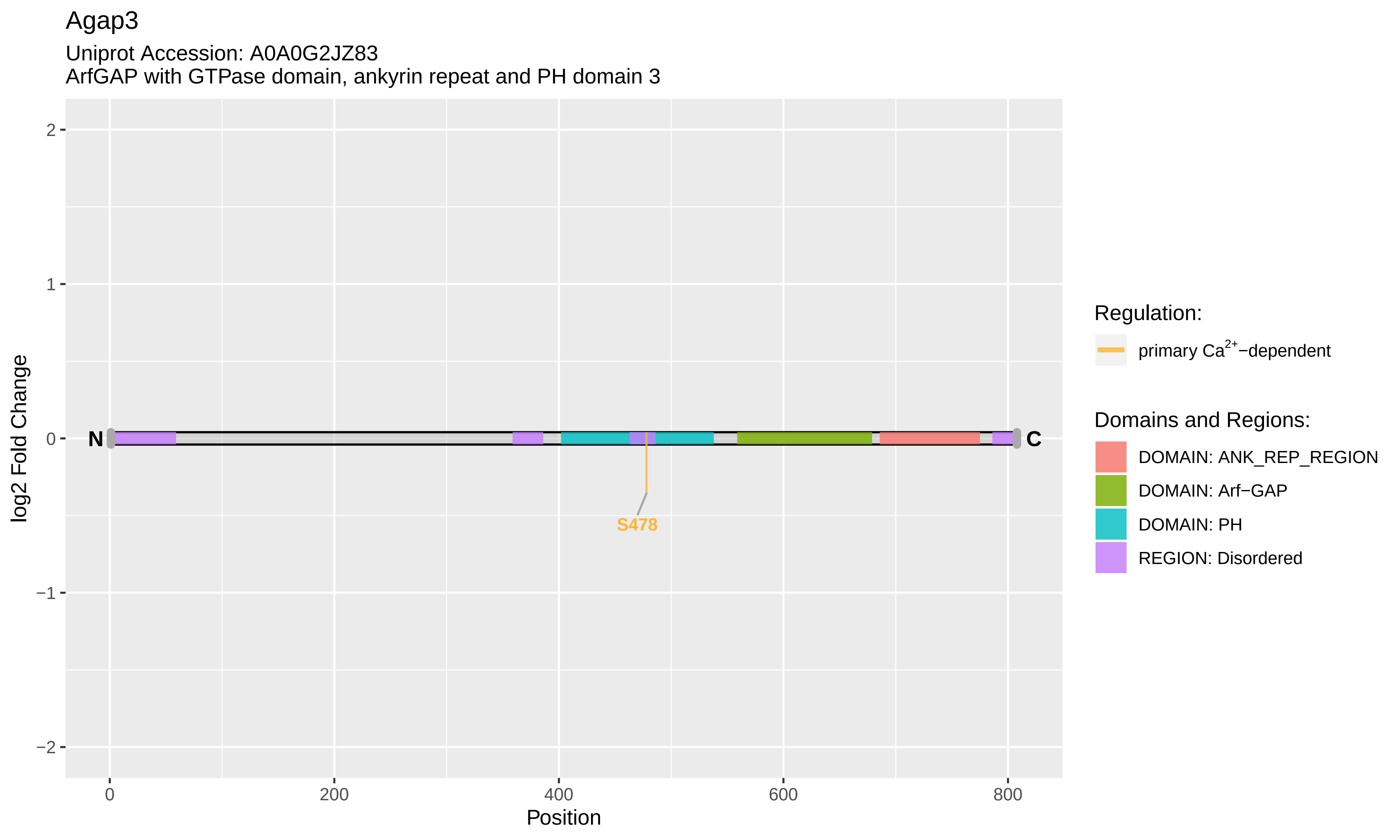

Agap3
Uniprot Accession: A0A0G2JZ83
ArfGAP with GTPase domain, ankyrin repeat and PH domain 3
log2 Fold Change
Position
N
C
S478
Regulation:
primary Ca2+−dependent
Domains and Regions:
DOMAIN: ANK_REP_REGION
DOMAIN: Arf−GAP
DOMAIN: PH
REGION: Disordered

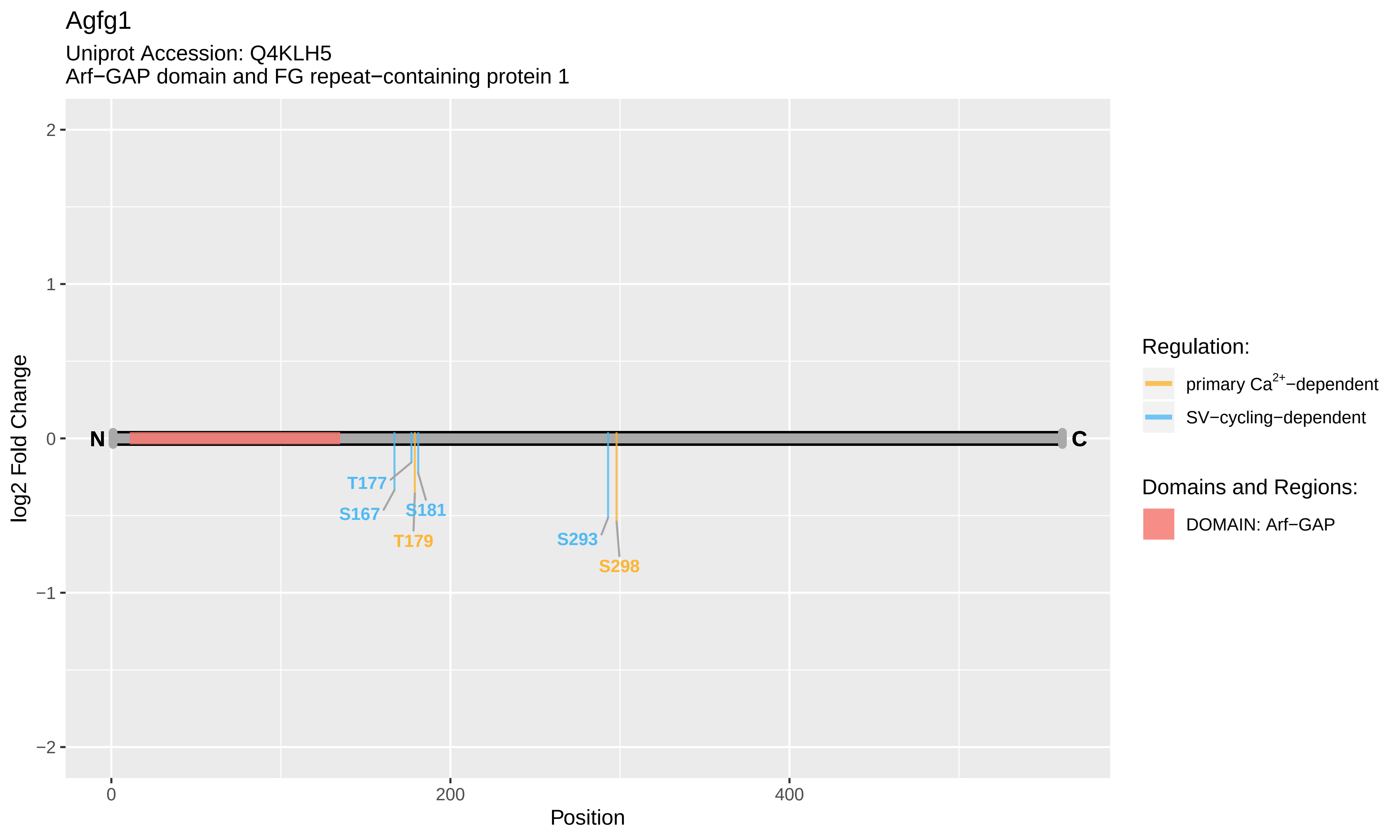

Agfg1
Uniprot Accession: Q4KLH5
Arf−GAP domain and FG repeat−containing protein 1
2
1
0
−1
−2
log2 Fold Change
0
200
400
Position
N
C
T177
S167
S181
T179
S293
S298
Regulation:
primary Ca2+−dependent
SV−cycling−dependent
Domains and Regions:
DOMAIN: Arf−GAP

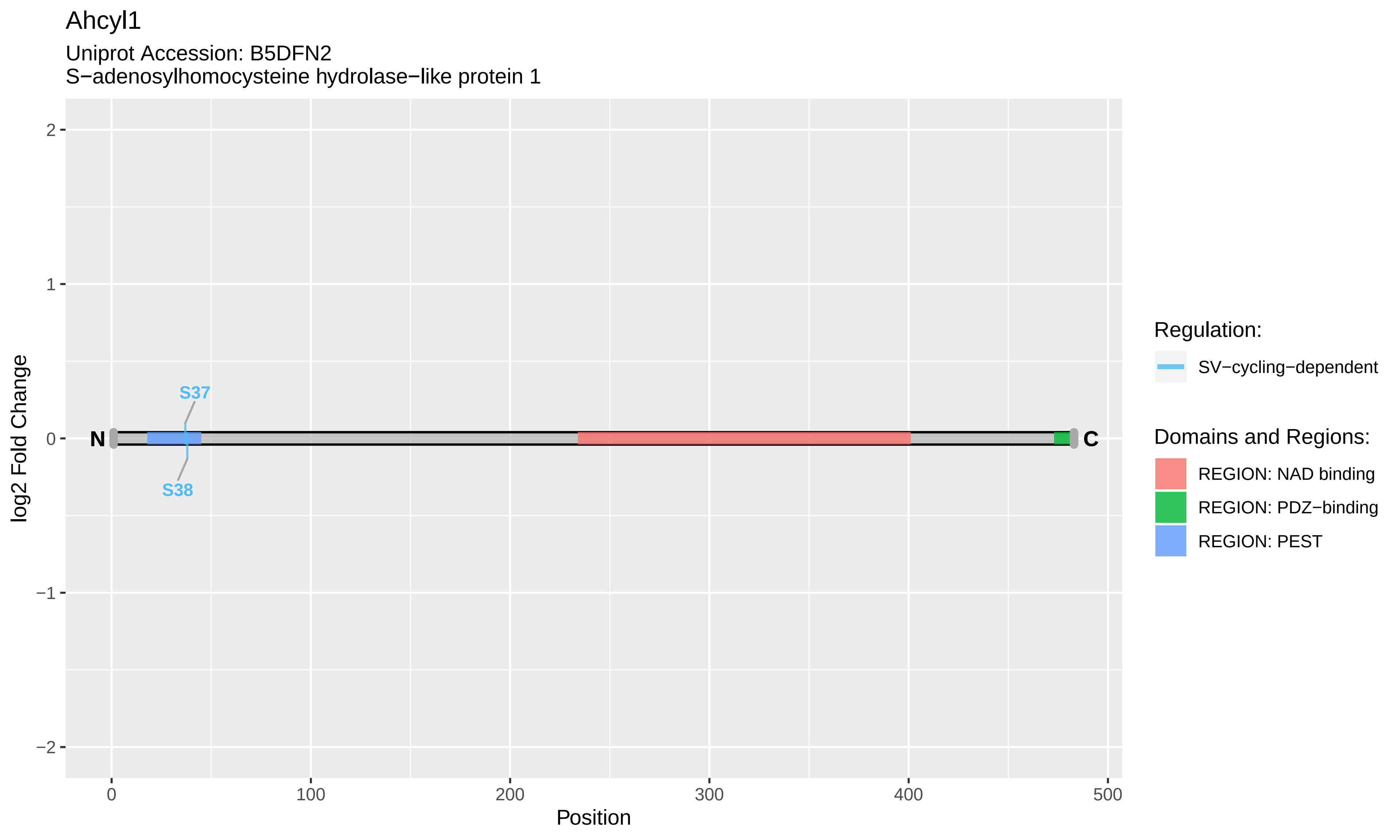
Ahcyl1
Uniprot Accession: B5DFN2
S−adenosylhomocysteine hydrolase−like protein 1
log2 Fold Change
Position
N
C
S37
S38
Regulation:
SV−cycling−dependent
Domains and Regions:
REGION: NAD binding
REGION: PDZ−binding
REGION: PEST

Ajm1

Uniprot Accession: D3ZTF0
Apical junction component 1 homolog

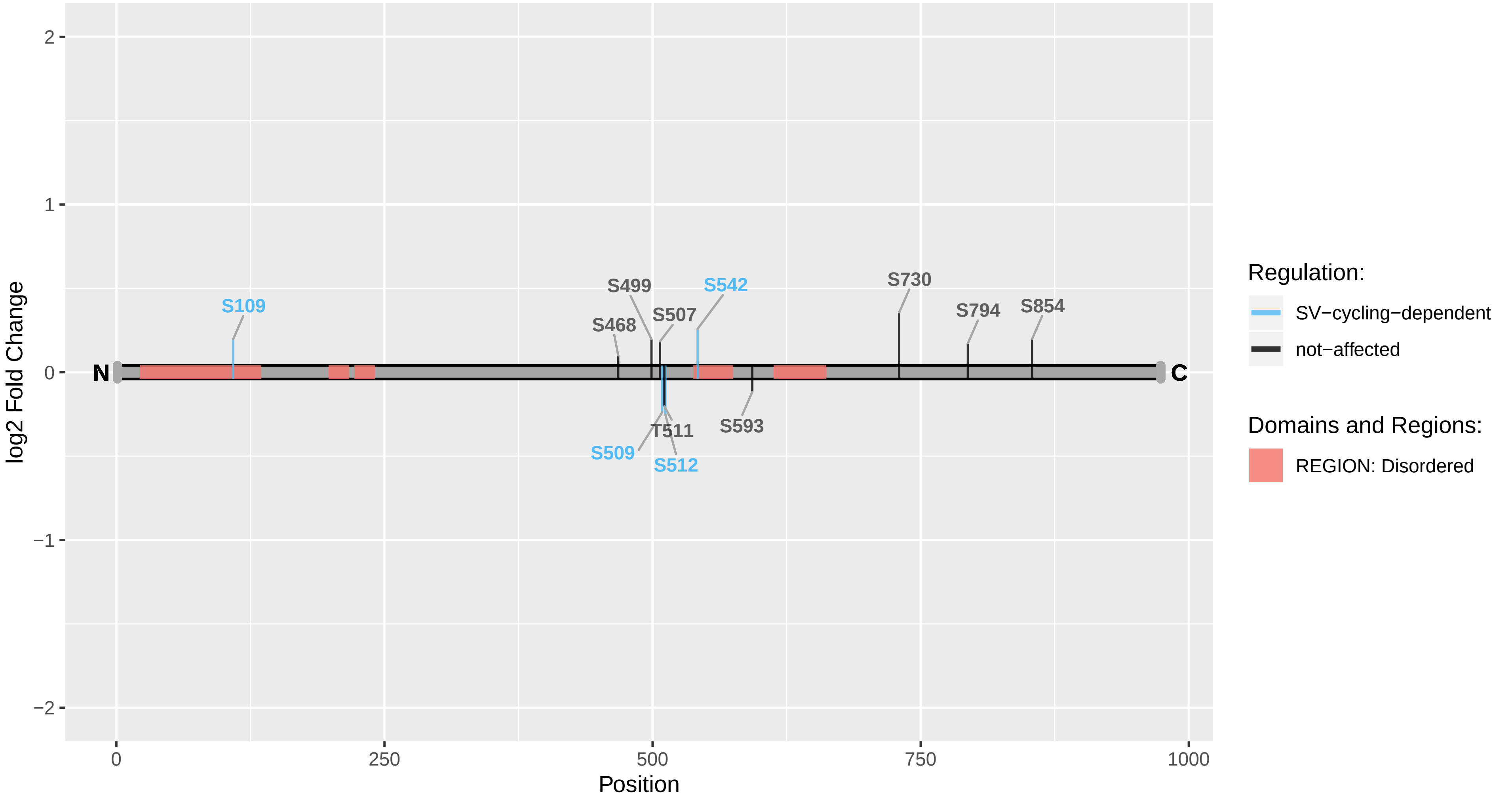

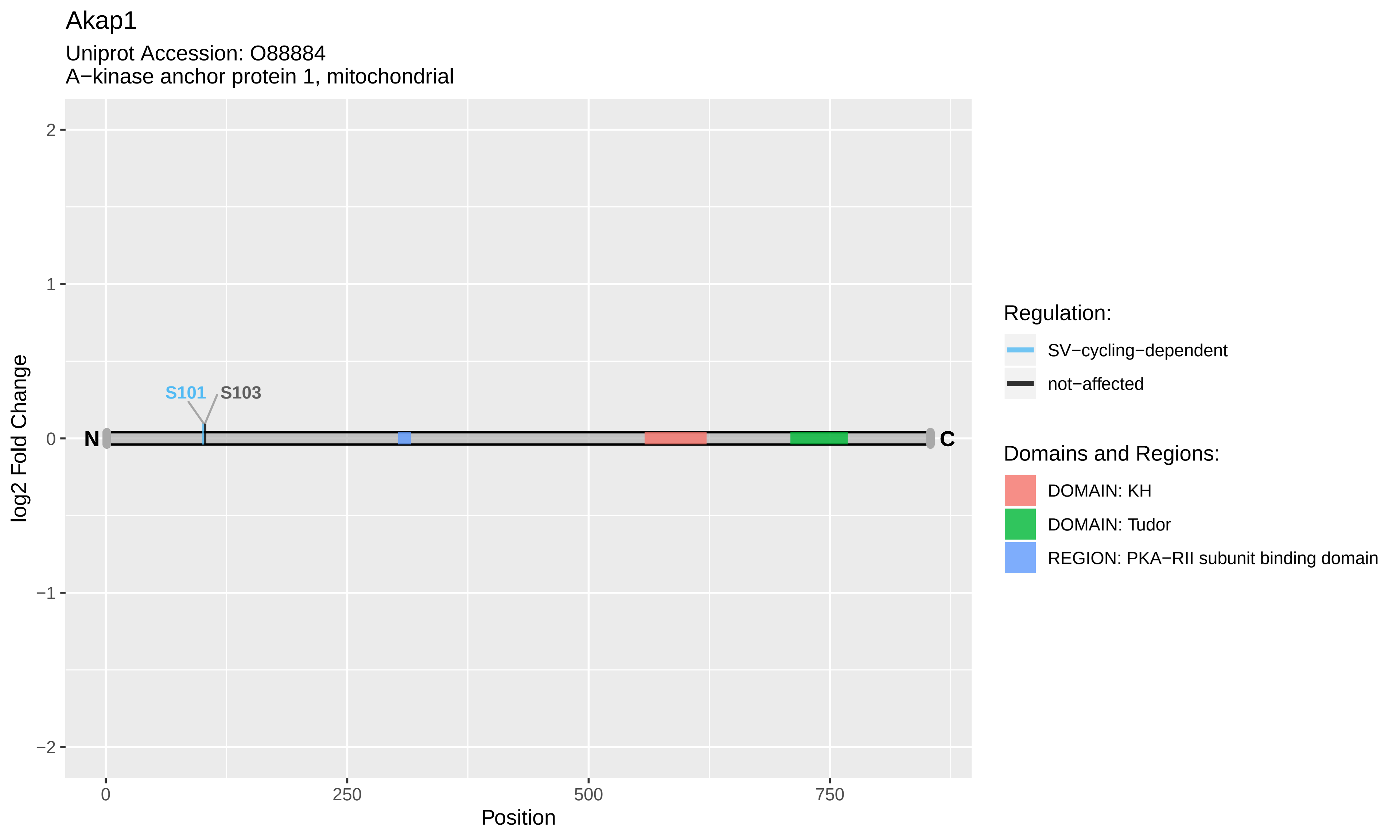
Akap1
Uniprot Accession: O88884
A−kinase anchor protein 1, mitochondrial
log2 Fold Change
2
1
0
−1
−2
S101
S103
N
C
0
250
500
750
Position
Regulation:
SV−cycling−dependent
not−affected
Domains and Regions:
DOMAIN: KH
DOMAIN: Tudor
REGION: PKA−RII subunit binding domain

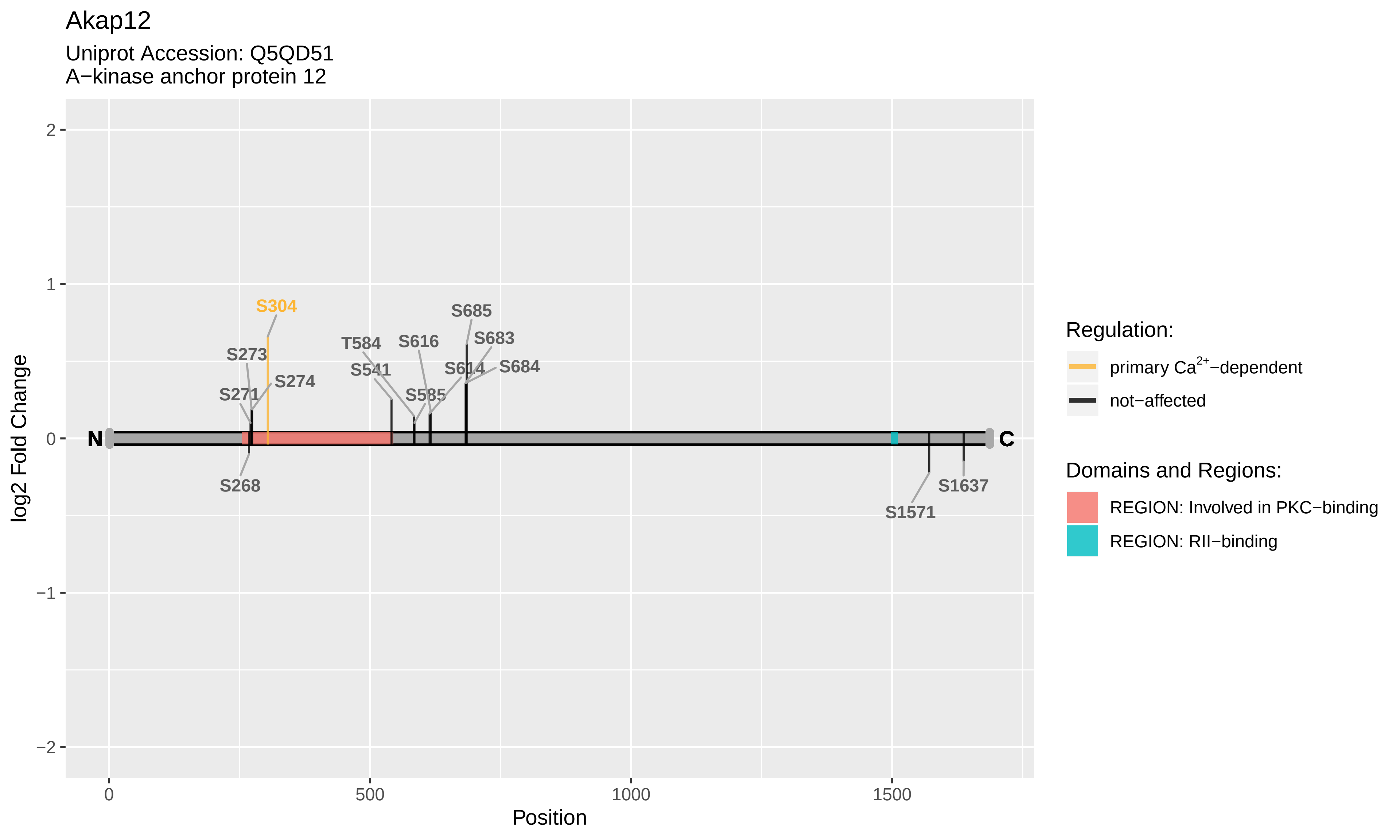

Akap12
Uniprot Accession: Q5QD51
A−kinase anchor protein 12
log2 Fold Change
Position
N
C
S304
S273
S274
S271
S268
T584
S541
S616
S585
S614
S685
S683
S684
S1571
S1637
Regulation:
primary Ca2+−dependent
not−affected
Domains and Regions:
REGION: Involved in PKC−binding
REGION: RII−binding

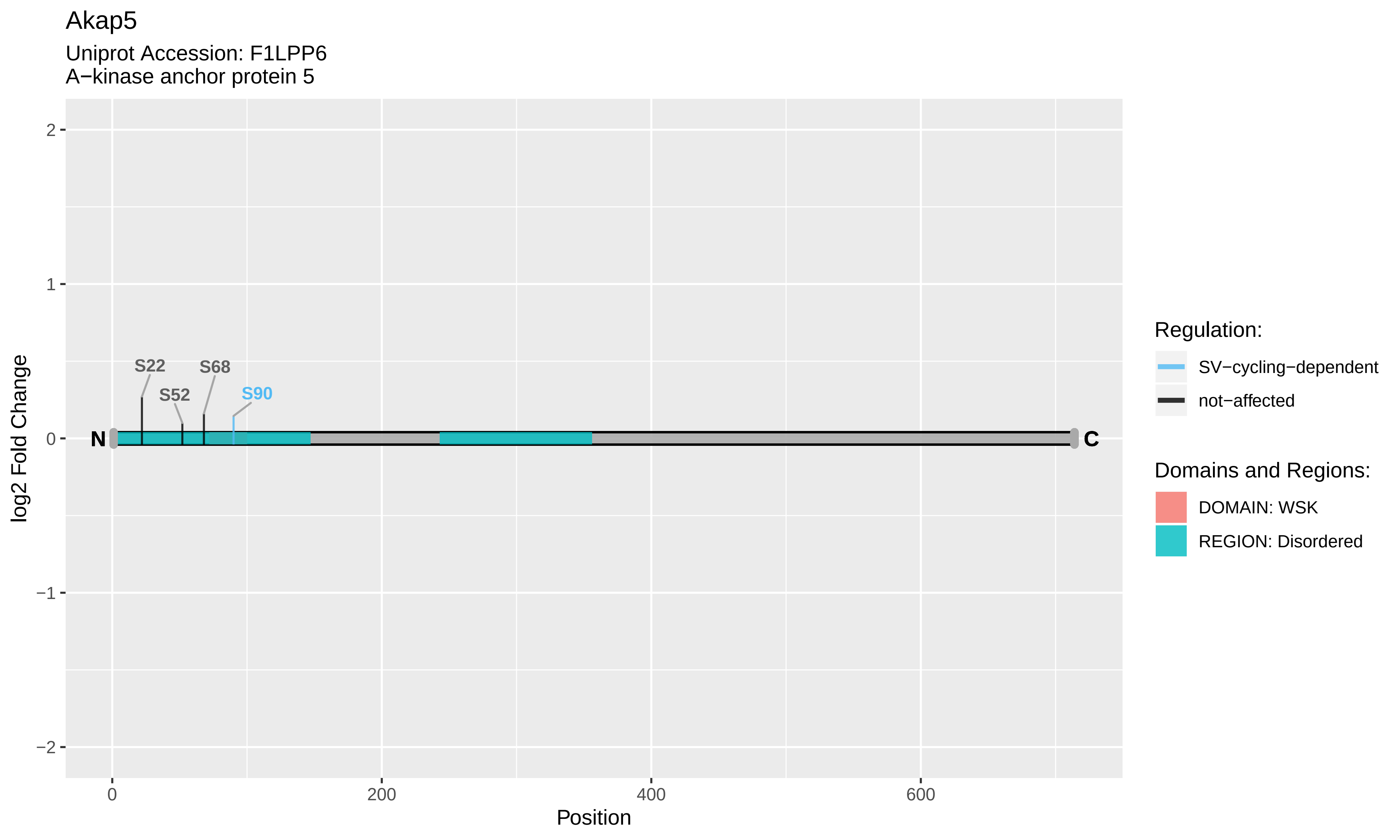

Akap5
Uniprot Accession: F1LPP6
A−kinase anchor protein 5
log2 Fold Change
Position
N
C
S22
S52
S68
S90
Regulation:
SV−cycling−dependent
not−affected
Domains and Regions:
DOMAIN: WSK
REGION: Disordered

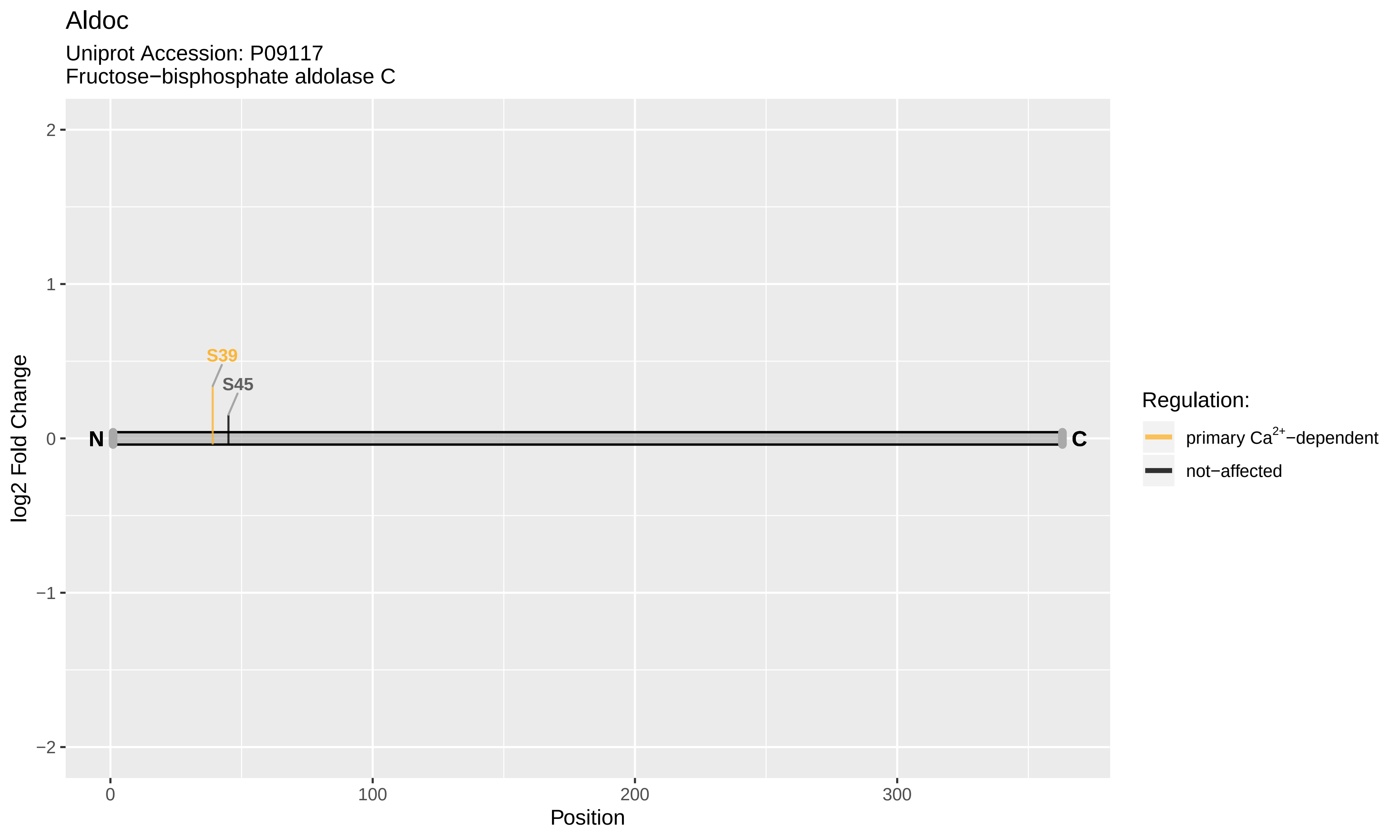
Aldoc
Uniprot Accession: P09117
Fructose−bisphosphate aldolase C
log2 Fold Change
2
1
0
−1
−2
S39
S45
N
C
0
100
200
300
Position
Regulation:
primary Ca2+−dependent
not−affected

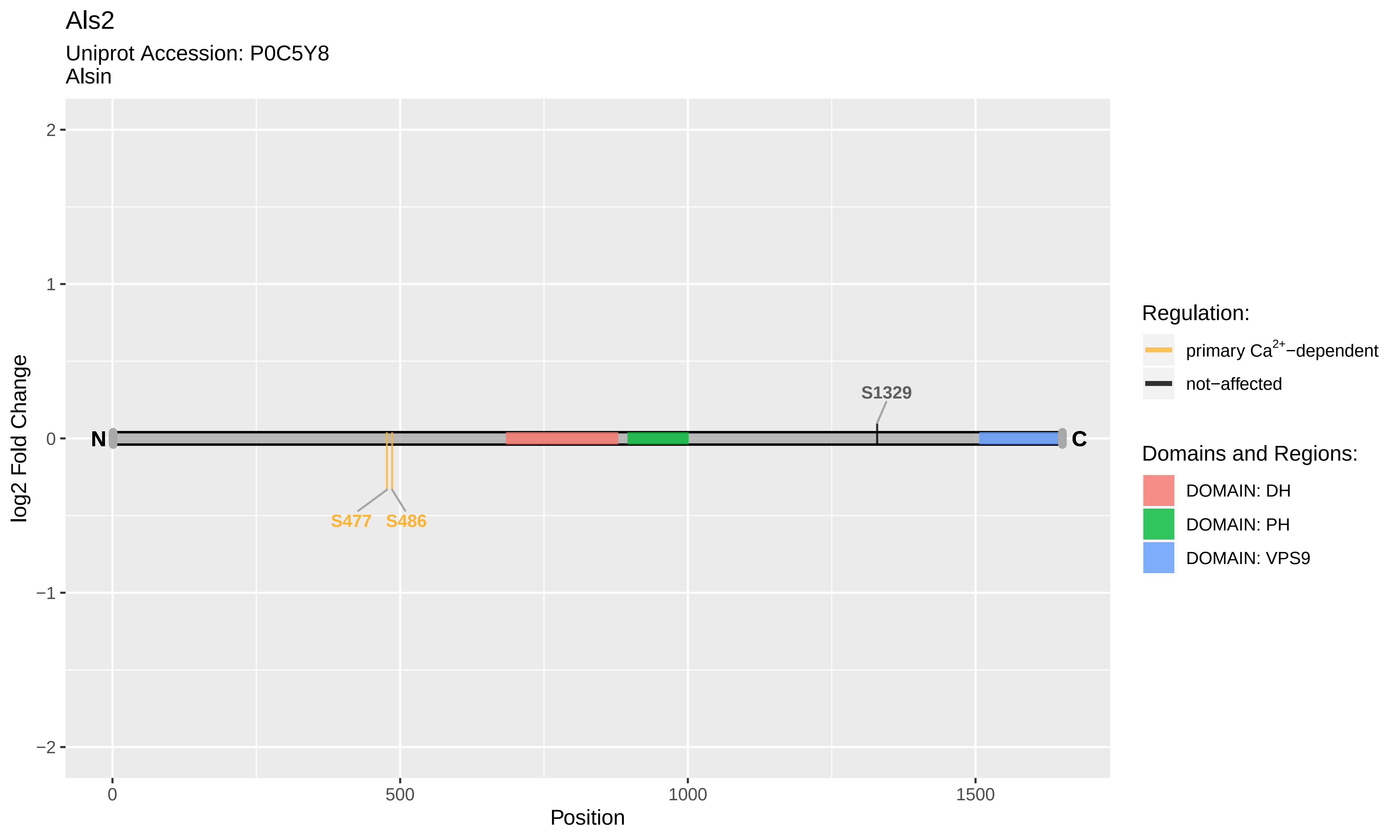

Als2
Uniprot Accession: P0C5Y8
Alsin
log2 Fold Change
2
1
0
−1
−2
N
C
S1329
S477
S486
0
500
1000
1500
Position
Regulation:
primary Ca^{2+}−dependent
not−affected
Domains and Regions:
DOMAIN: DH
DOMAIN: PH
DOMAIN: VPS9

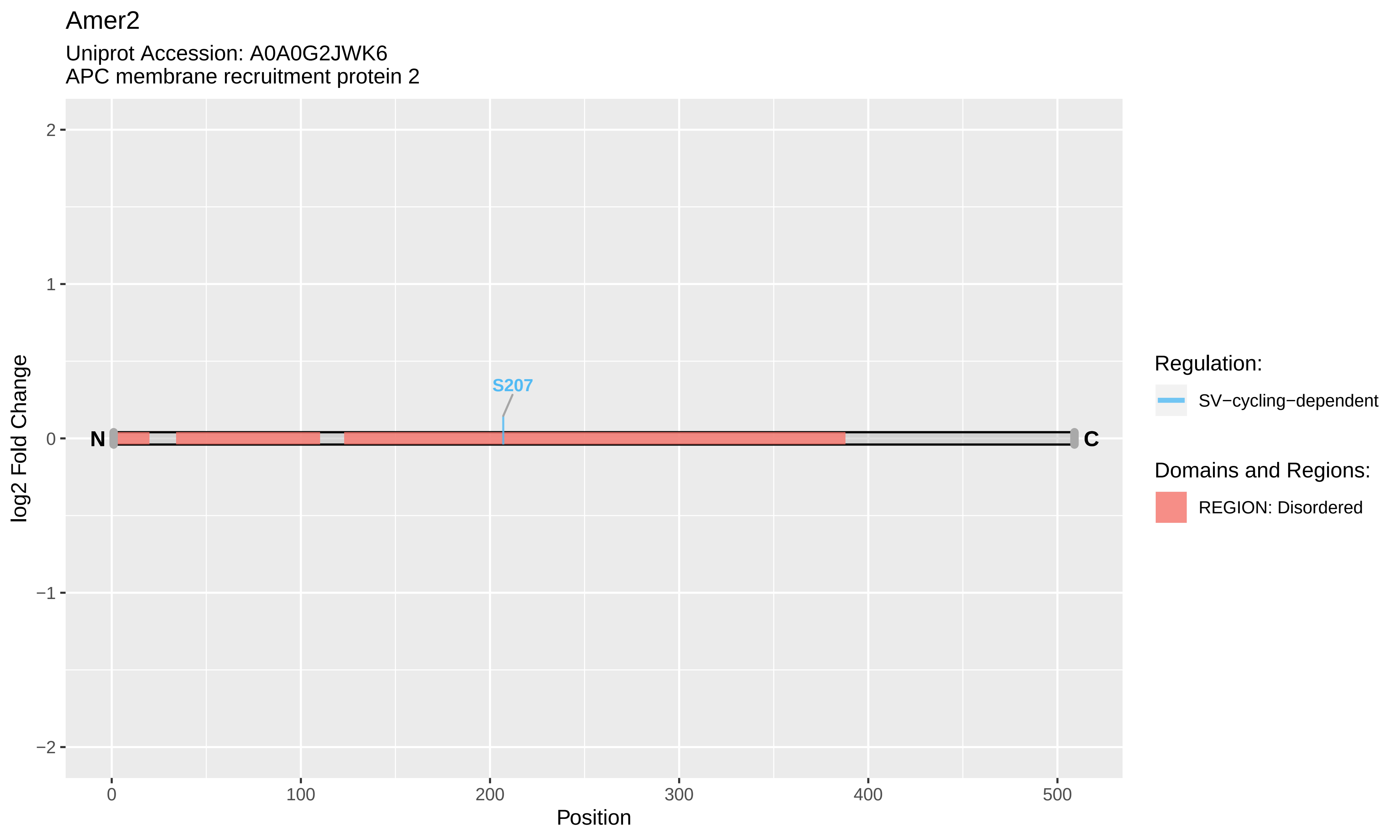

Amer2
Uniprot Accession: A0A0G2JWK6
APC membrane recruitment protein 2
log2 Fold Change
2
1
0
−1
−2
N
C
S207
0
100
200
300
400
500
Position
Regulation:
SV−cycling−dependent
Domains and Regions:
REGION: Disordered

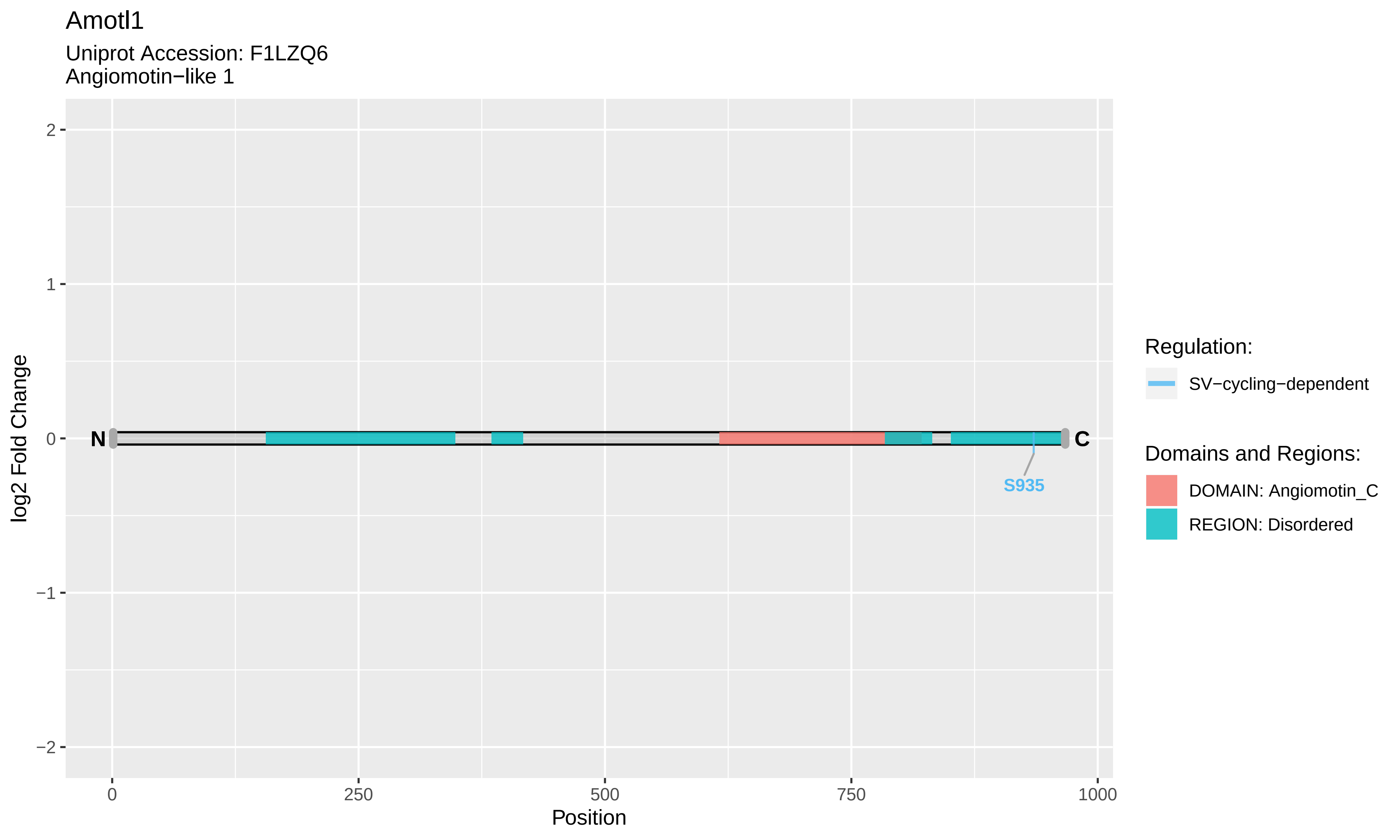

Amotl1
Uniprot Accession: F1LZQ6
Angiomotin−like 1
log2 Fold Change
2
1
0
−1
−2
N
C
S935
0
250
500
750
1000
Position
Regulation:
SV−cycling−dependent
Domains and Regions:
DOMAIN: Angiomotin_C
REGION: Disordered

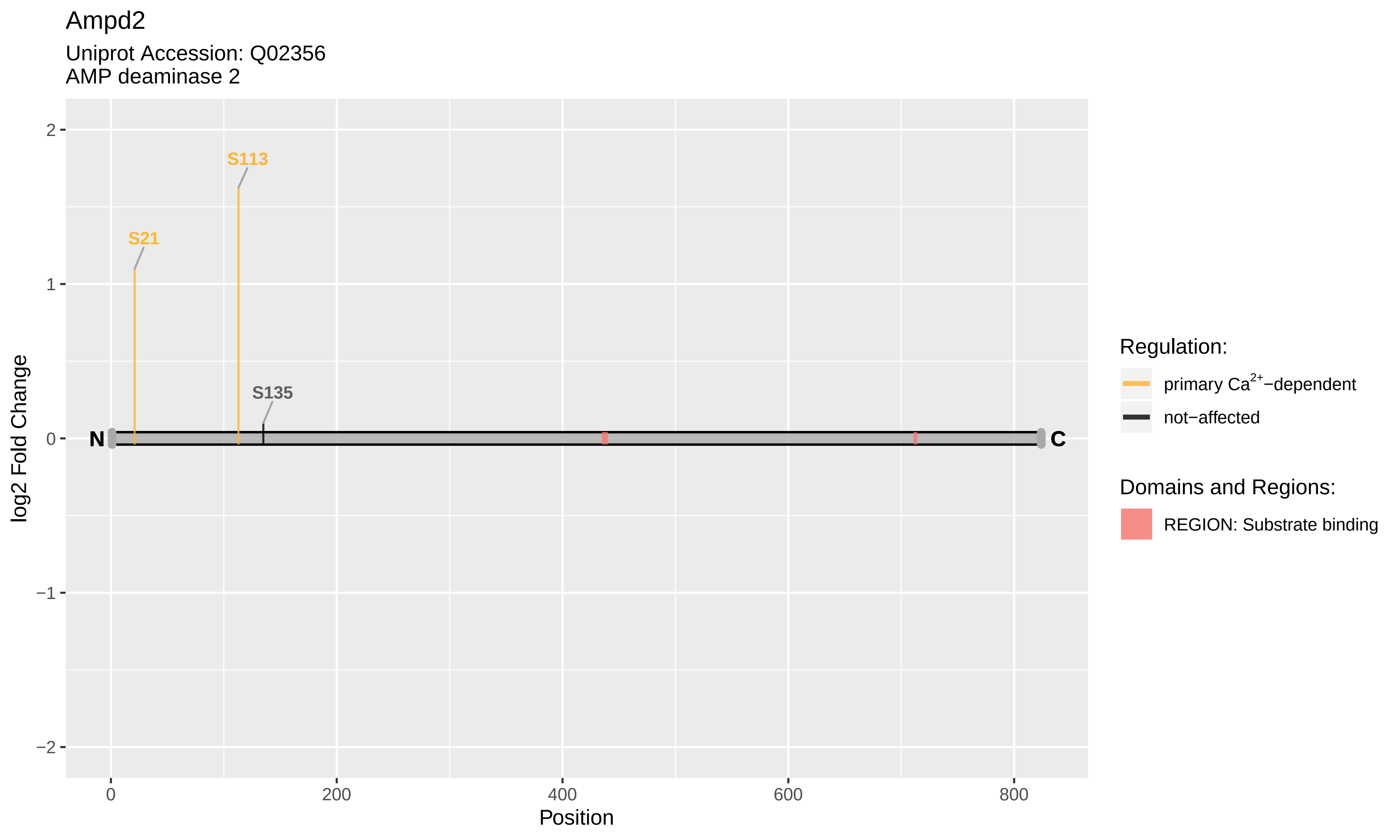

Ampd2
Uniprot Accession: Q02356
AMP deaminase 2
S113
S21
S135
N
C
2
1
0
−1
−2
0
200
400
600
800
Position
log2 Fold Change
Regulation:
primary Ca2+−dependent
not−affected
Domains and Regions:
REGION: Substrate binding

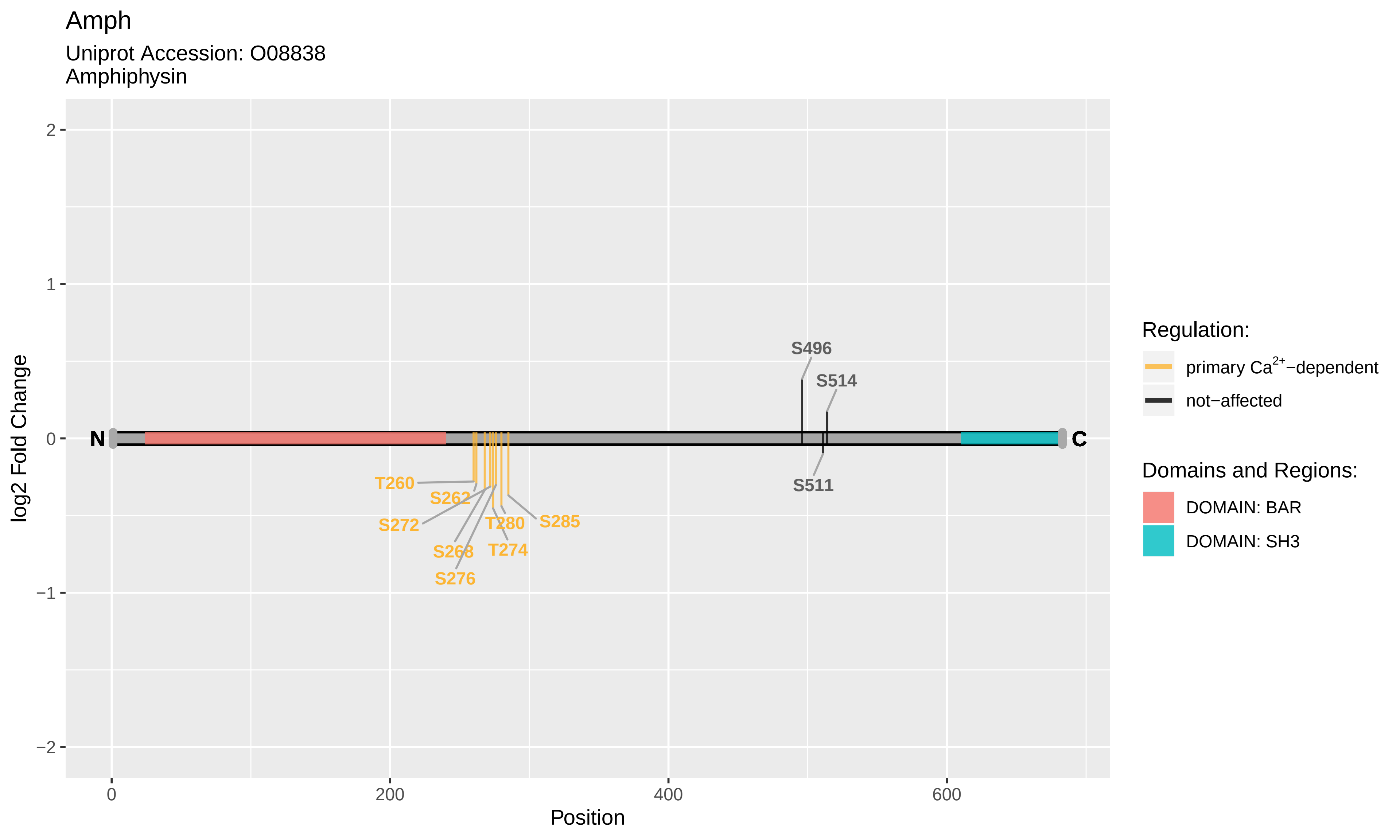

Amph
Uniprot Accession: O08838
Amphiphysin
log2 Fold Change
Position
2
1
0
−1
−2
0
200
400
600
N
C
T260
S262
S272
S268
S276
T280
T274
S285
S496
S514
S511
Regulation:
primary Ca2+−dependent
not−affected
Domains and Regions:
DOMAIN: BAR
DOMAIN: SH3

Ank2

Uniprot Accession: A0A0G2JZ56
Ankyrin 2

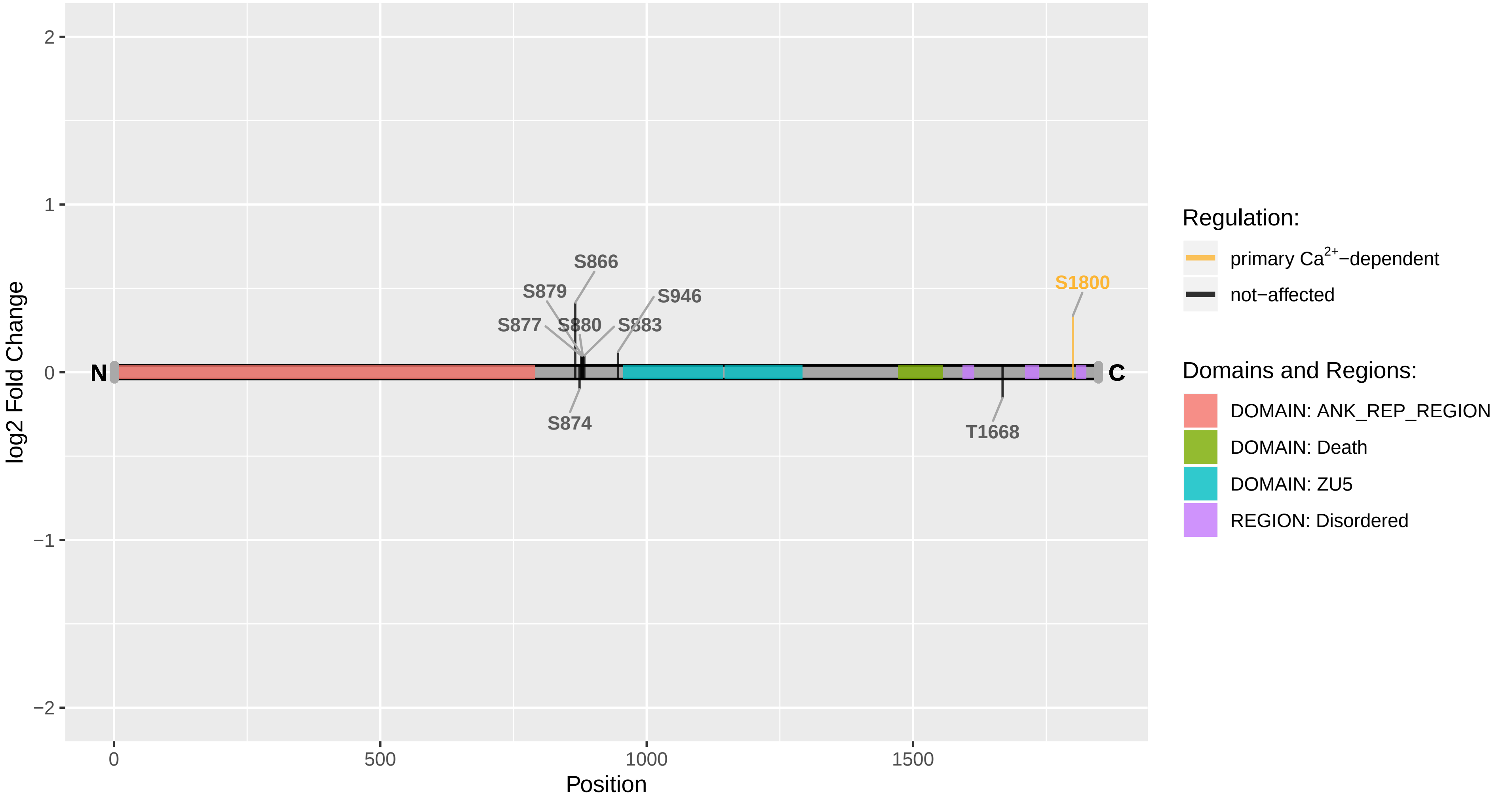

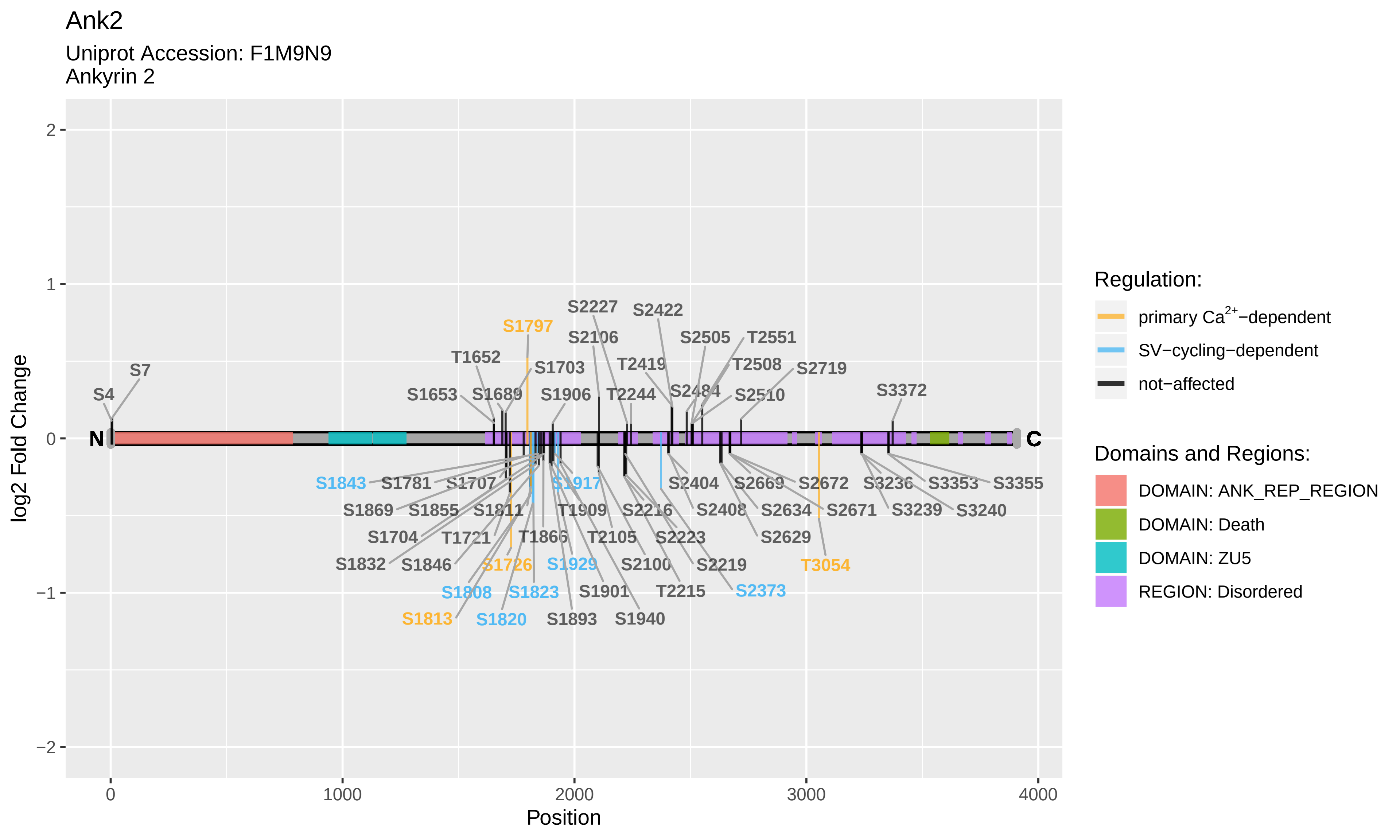

Ank2
Uniprot Accession: F1M9N9
Ankyrin 2
log2 Fold Change
Position
N
C
S4
S7
T1652
S1653
S1689
S1797
S1703
S1906
S2227
S2106
S2422
T2419
T2244
S2484
S2505
T2551
T2508
S2510
S2719
S3372
S1843
S1781
S1707
S1917
S2404
S2669
S2672
S3236
S3353
S3355
S1869
S1855
S1811
T1909
S2216
S2408
S2634
S2671
S3239
S3240
S1704
T1721
T1866
T2105
S2223
S2629
S1832
S1846
S1726
S1929
S2100
S2219
T3054
S1808
S1823
S1901
T2215
S2373
S1813
S1820
S1893
S1940
Regulation:
primary Ca^{2+}−dependent
SV−cycling−dependent
not−affected
Domains and Regions:
DOMAIN: ANK_REP_REGION
DOMAIN: Death
DOMAIN: ZU5
REGION: Disordered

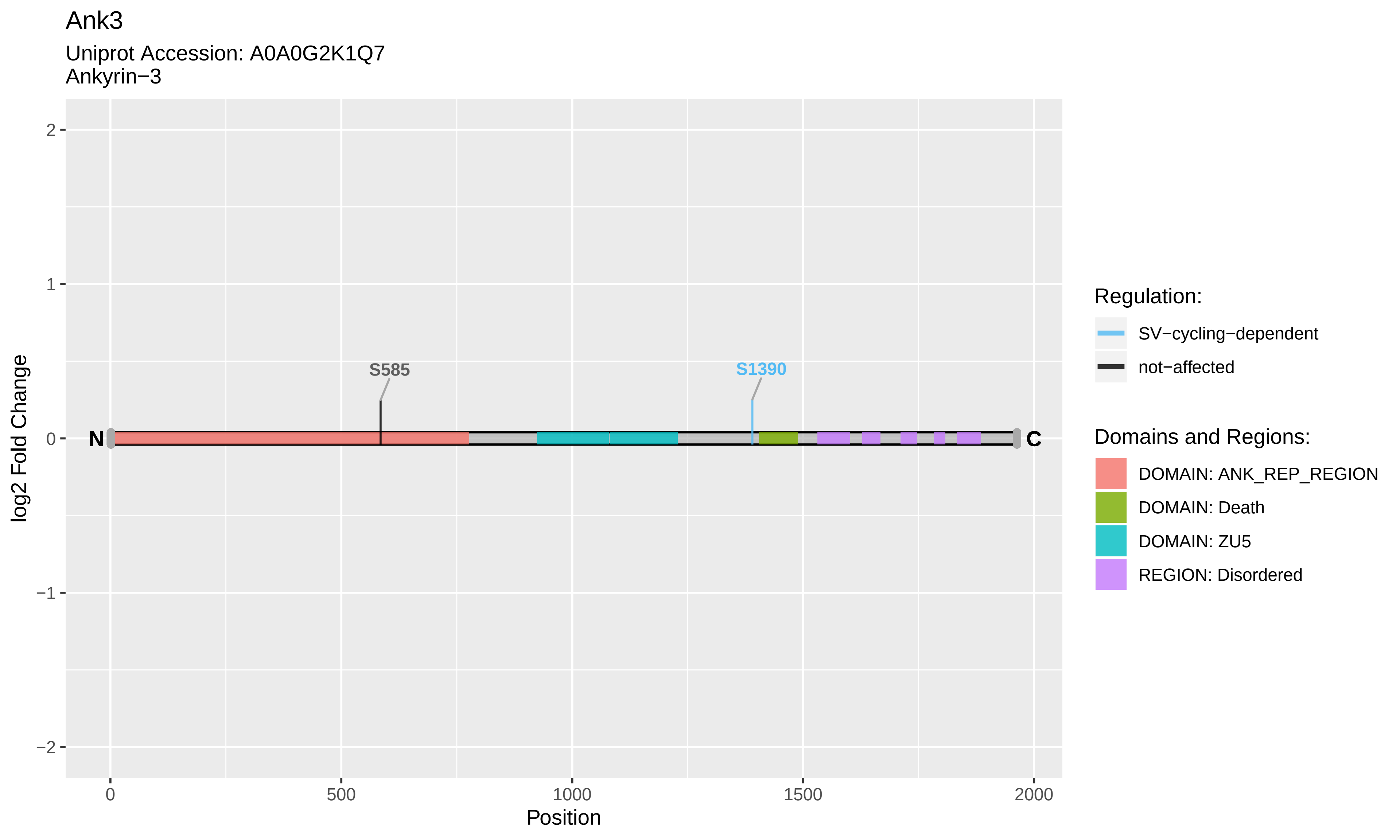

Ank3
Uniprot Accession: A0A0G2K1Q7
Ankyrin−3
log2 Fold Change
2
1
0
−1
−2
N
C
S585
S1390
0
500
1000
1500
2000
Position
Regulation:
SV−cycling−dependent
not−affected
Domains and Regions:
DOMAIN: ANK_REP_REGION
DOMAIN: Death
DOMAIN: ZU5
REGION: Disordered

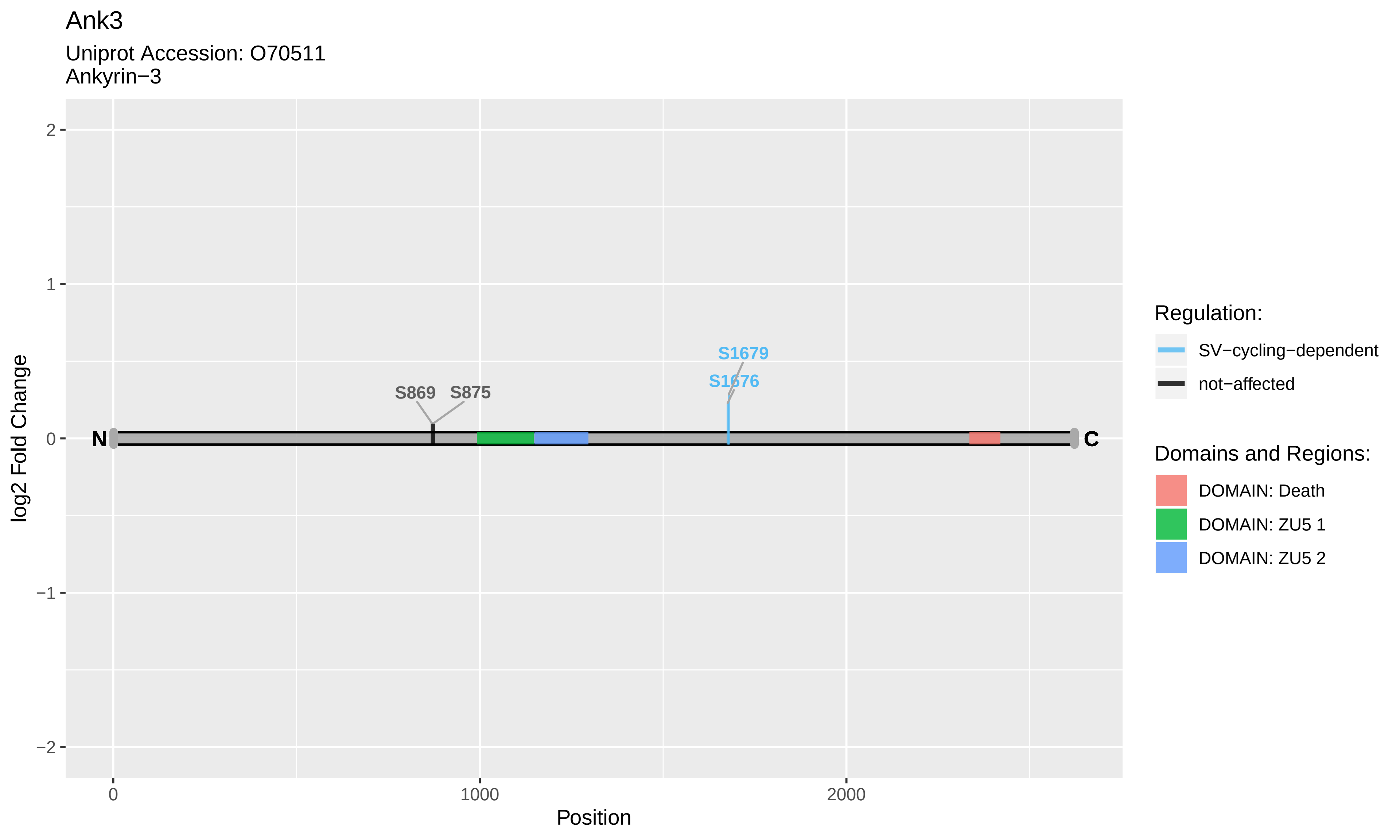

Ank3
Uniprot Accession: O70511
Ankyrin−3
log2 Fold Change
Position
2
1
0
−1
−2
0
1000
2000
N
C
S869
S875
S1679
S1676
Regulation:
SV−cycling−dependent
not−affected
Domains and Regions:
DOMAIN: Death
DOMAIN: ZU5 1
DOMAIN: ZU5 2

Ankrd34a

Uniprot Accession: Q5BJT1
Ankyrin repeat domain−containing protein 34A

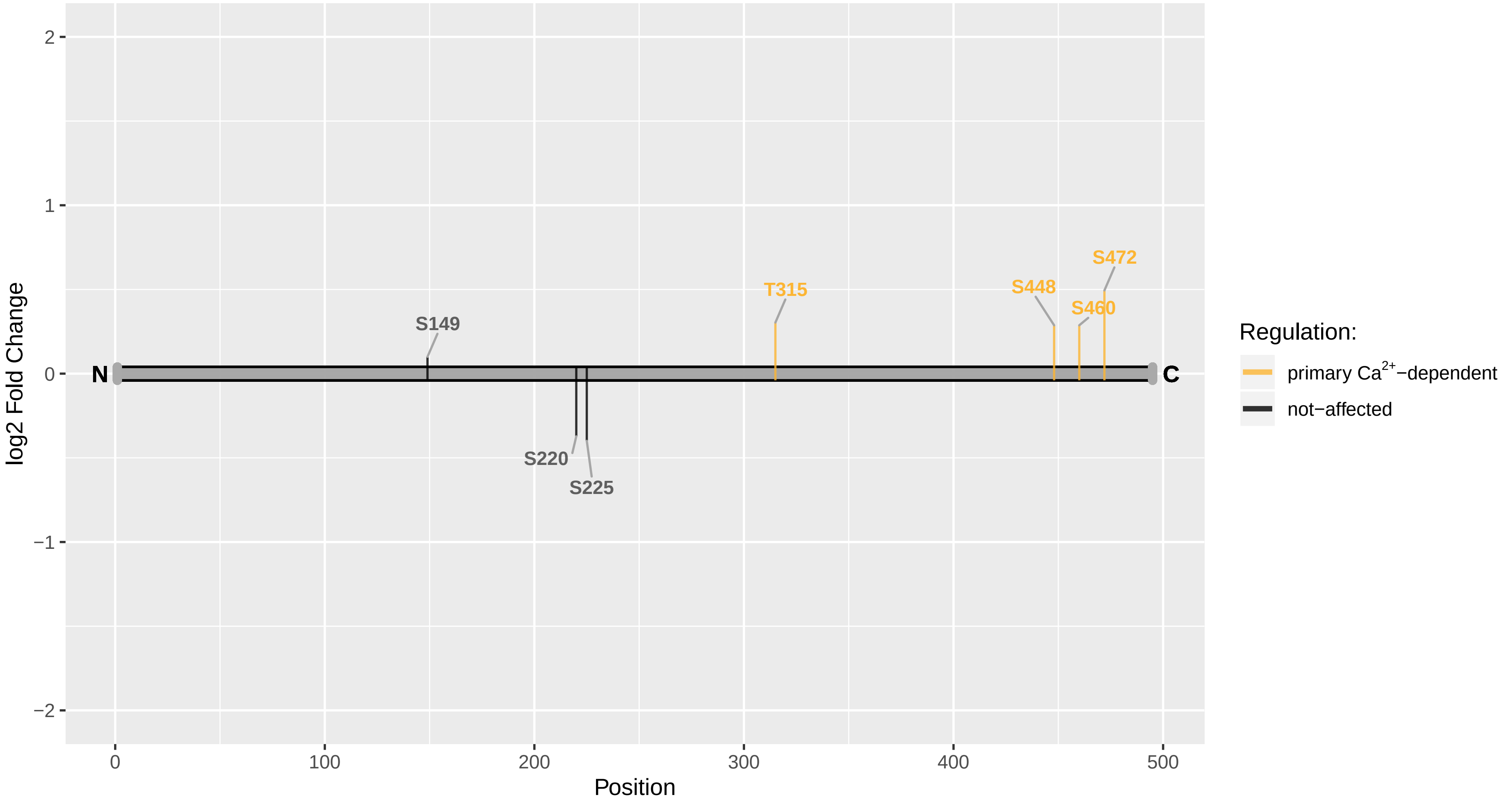

Ankrd34b

Uniprot Accession: D3ZKP1
Ankyrin repeat domain 34B

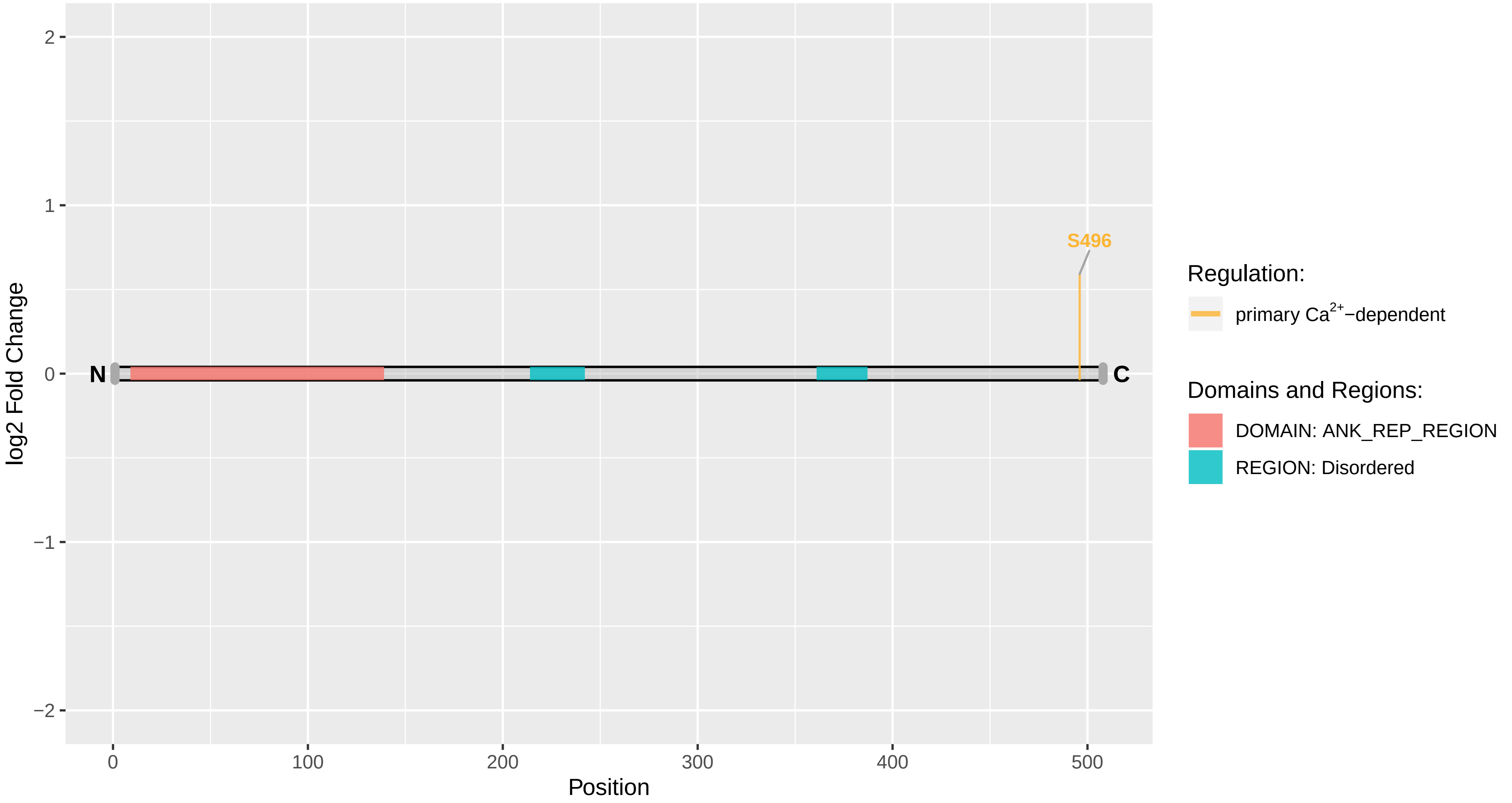

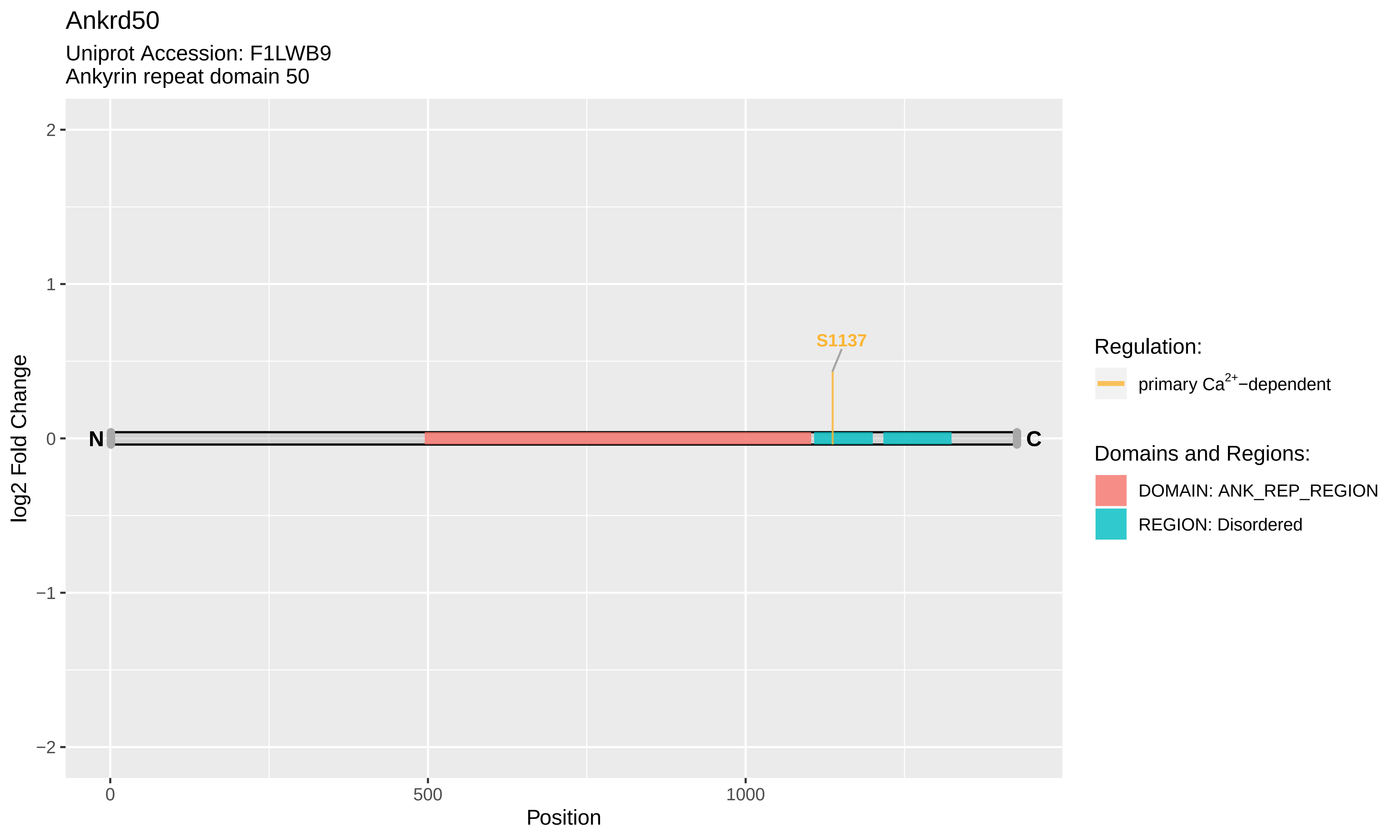

Ankrd50
Uniprot Accession: F1LWB9
Ankyrin repeat domain 50
S1137
N
C
2
1
0
−1
−2
0
500
1000
Position
log2 Fold Change
Regulation:
primary Ca2+−dependent
Domains and Regions:
DOMAIN: ANK_REP_REGION
REGION: Disordered

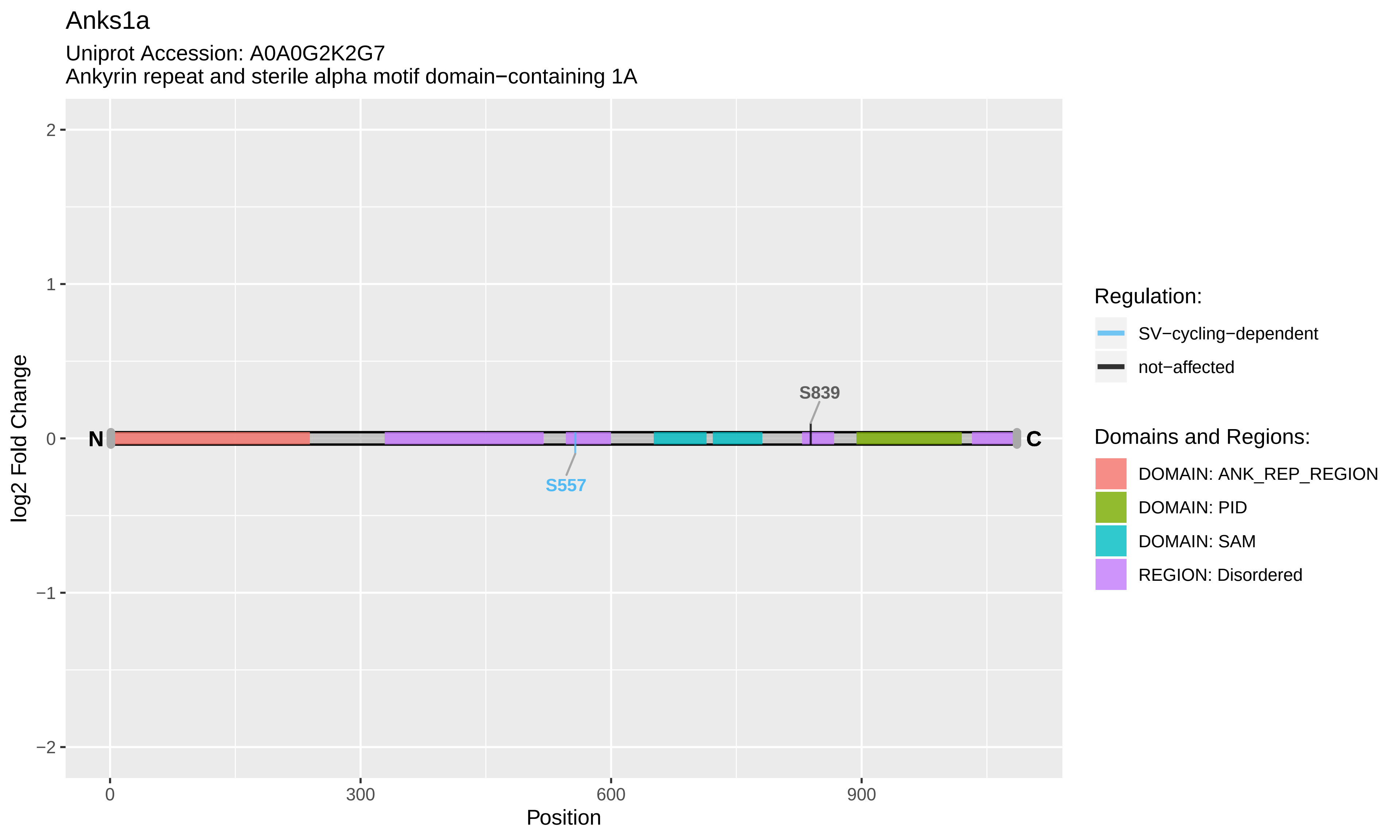

Anks1a
Uniprot Accession: A0A0G2K2G7
Ankyrin repeat and sterile alpha motif domain−containing 1A
log2 Fold Change
2
1
0
−1
−2
N
C
S839
S557
0
300
600
900
Position
Regulation:
SV−cycling−dependent
not−affected
Domains and Regions:
DOMAIN: ANK_REP_REGION
DOMAIN: PID
DOMAIN: SAM
REGION: Disordered

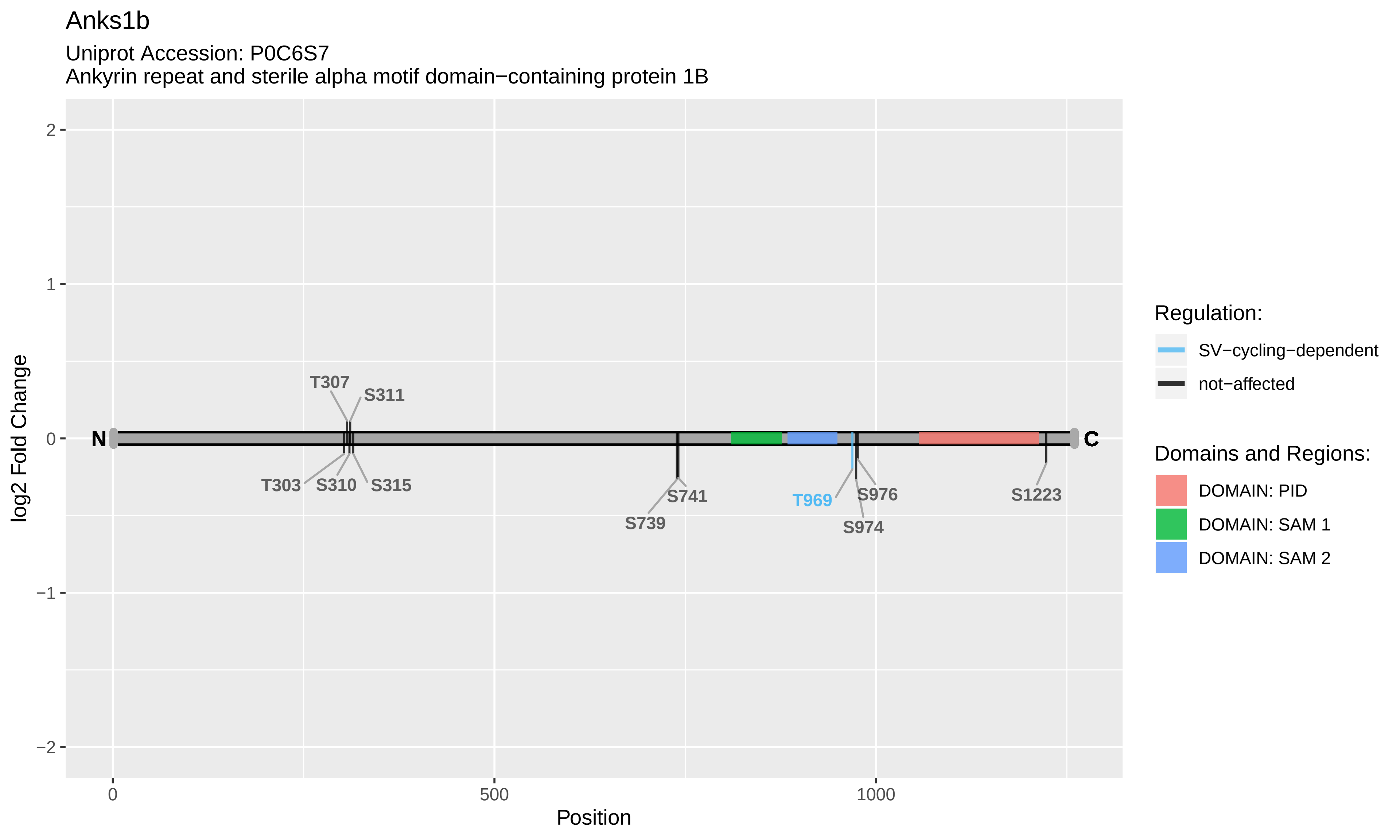

Anks1b
Uniprot Accession: P0C6S7
Ankyrin repeat and sterile alpha motif domain−containing protein 1B
log2 Fold Change
2
1
0
−1
−2
N
C
T307
S311
T303
S310
S315
S739
S741
T969
S976
S974
S1223
0
500
1000
Position
Regulation:
SV−cycling−dependent
not−affected
Domains and Regions:
DOMAIN: PID
DOMAIN: SAM 1
DOMAIN: SAM 2

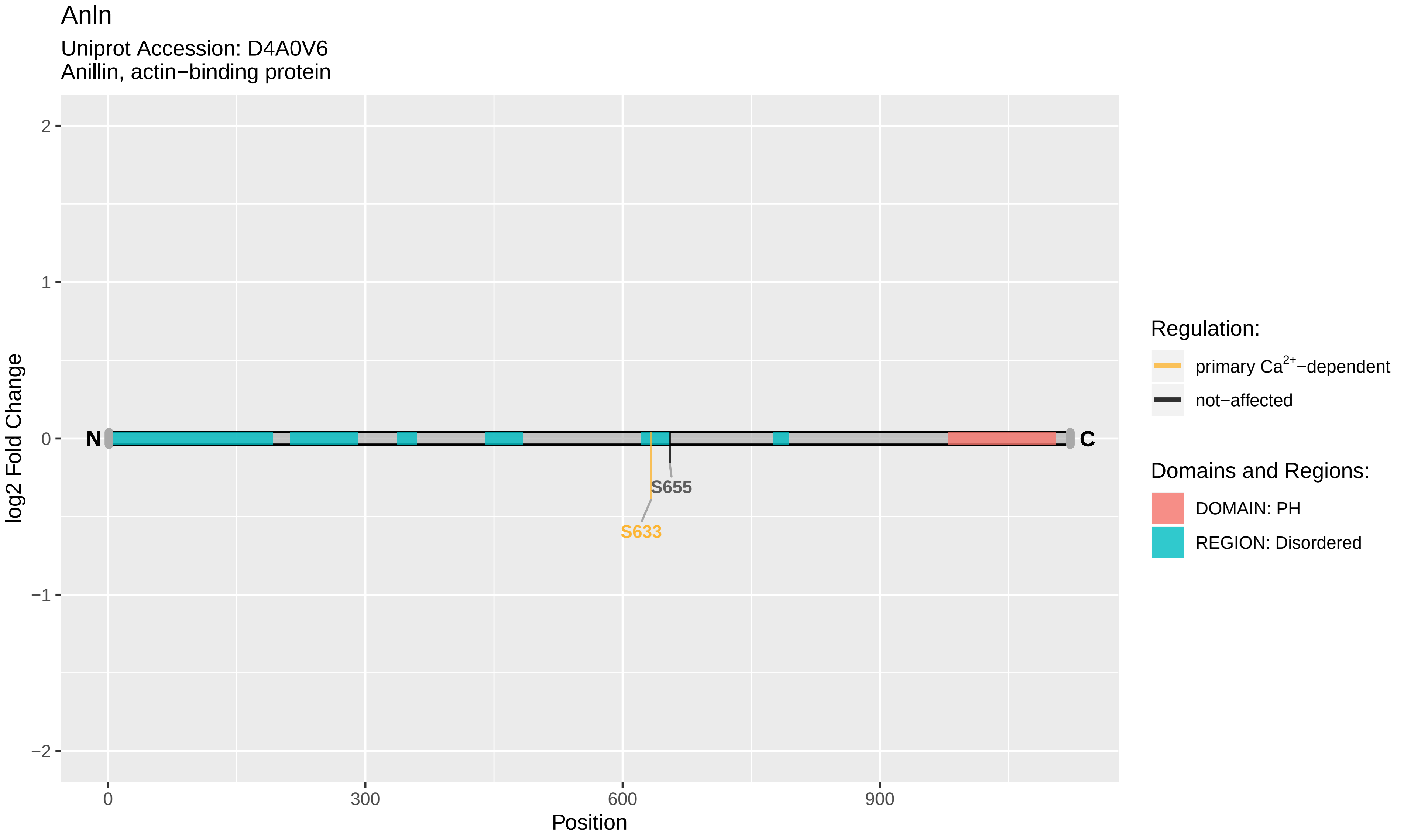
Anln
Uniprot Accession: D4A0V6
Anillin, actin−binding protein
log2 Fold Change
2
1
0
−1
−2
N
C
S655
S633
0
300
600
900
Position
Regulation:
primary Ca2+−dependent
not−affected
Domains and Regions:
DOMAIN: PH
REGION: Disordered

Ap2b1

Uniprot Accession: P62944
AP−2 complex subunit beta

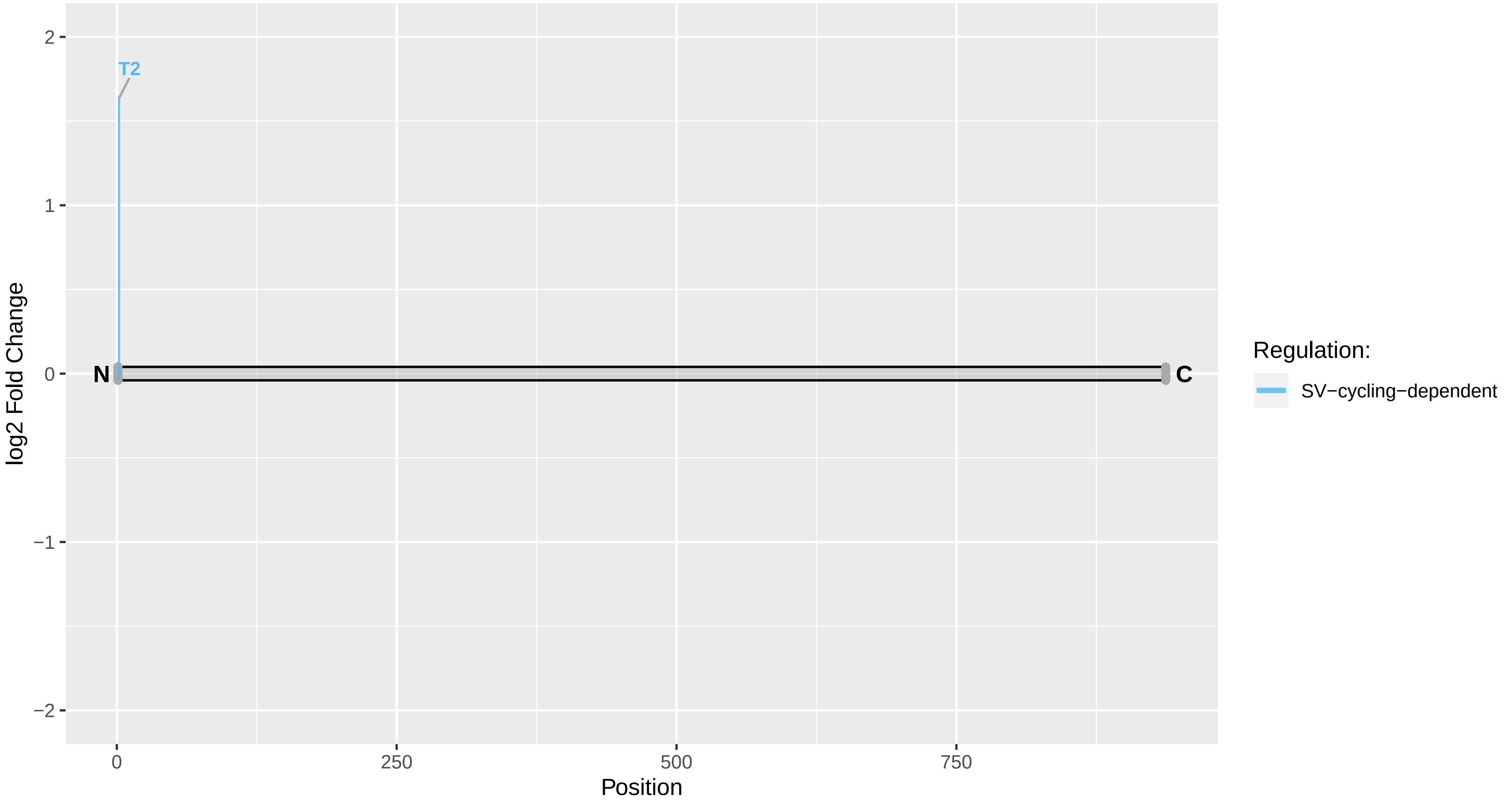

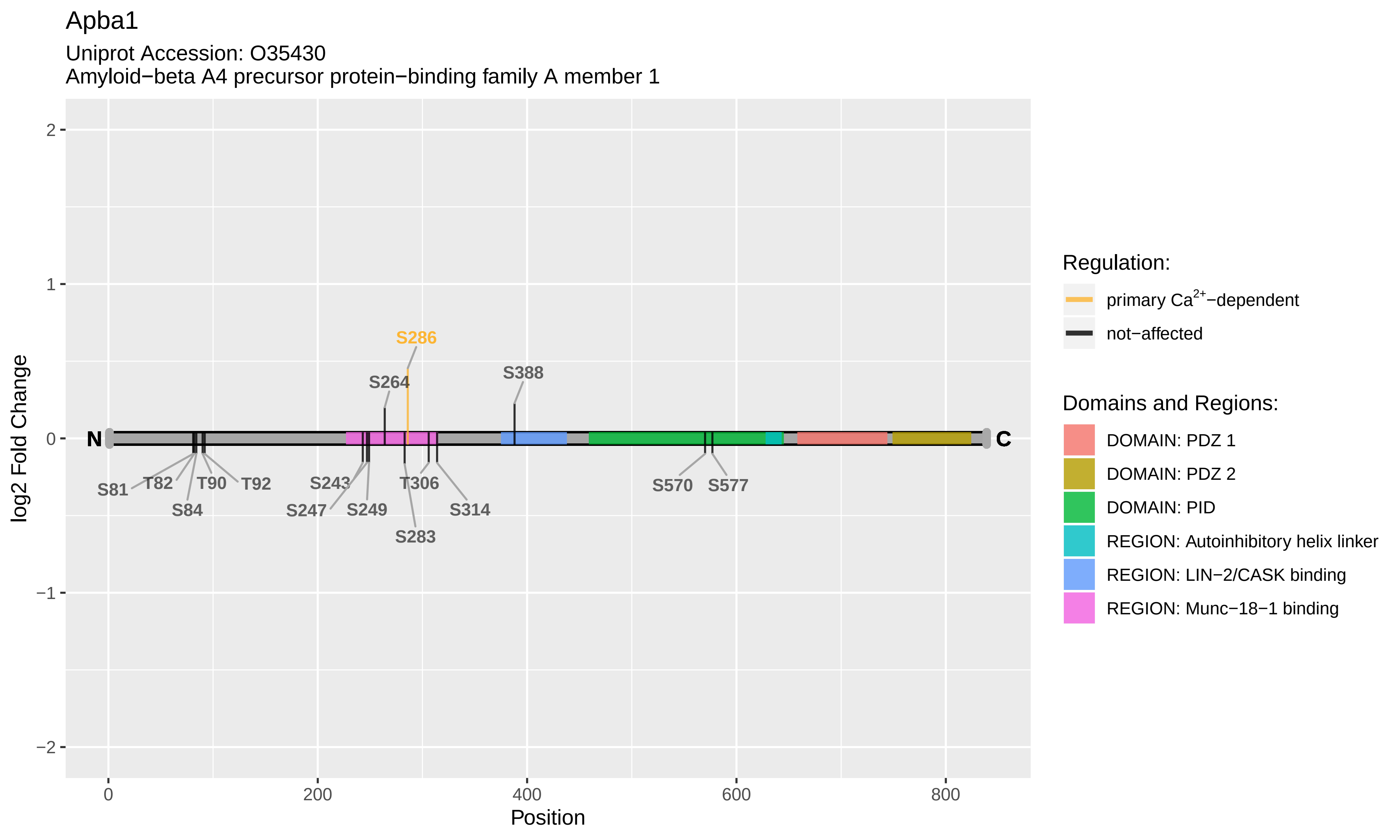

Apba1
Uniprot Accession: O35430
Amyloid−beta A4 precursor protein−binding family A member 1
S286
S264
S388
N
C
S81
T82
S84
T90
T92
S243
S247
S249
S283
T306
S314
S570
S577
log2 Fold Change
2
1
0
−1
−2
0
200
400
600
800
Position
Regulation:
primary Ca2+−dependent
not−affected
Domains and Regions:
DOMAIN: PDZ 1
DOMAIN: PDZ 2
DOMAIN: PID
REGION: Autoinhibitory helix linker
REGION: LIN−2/CASK binding
REGION: Munc−18−1 binding

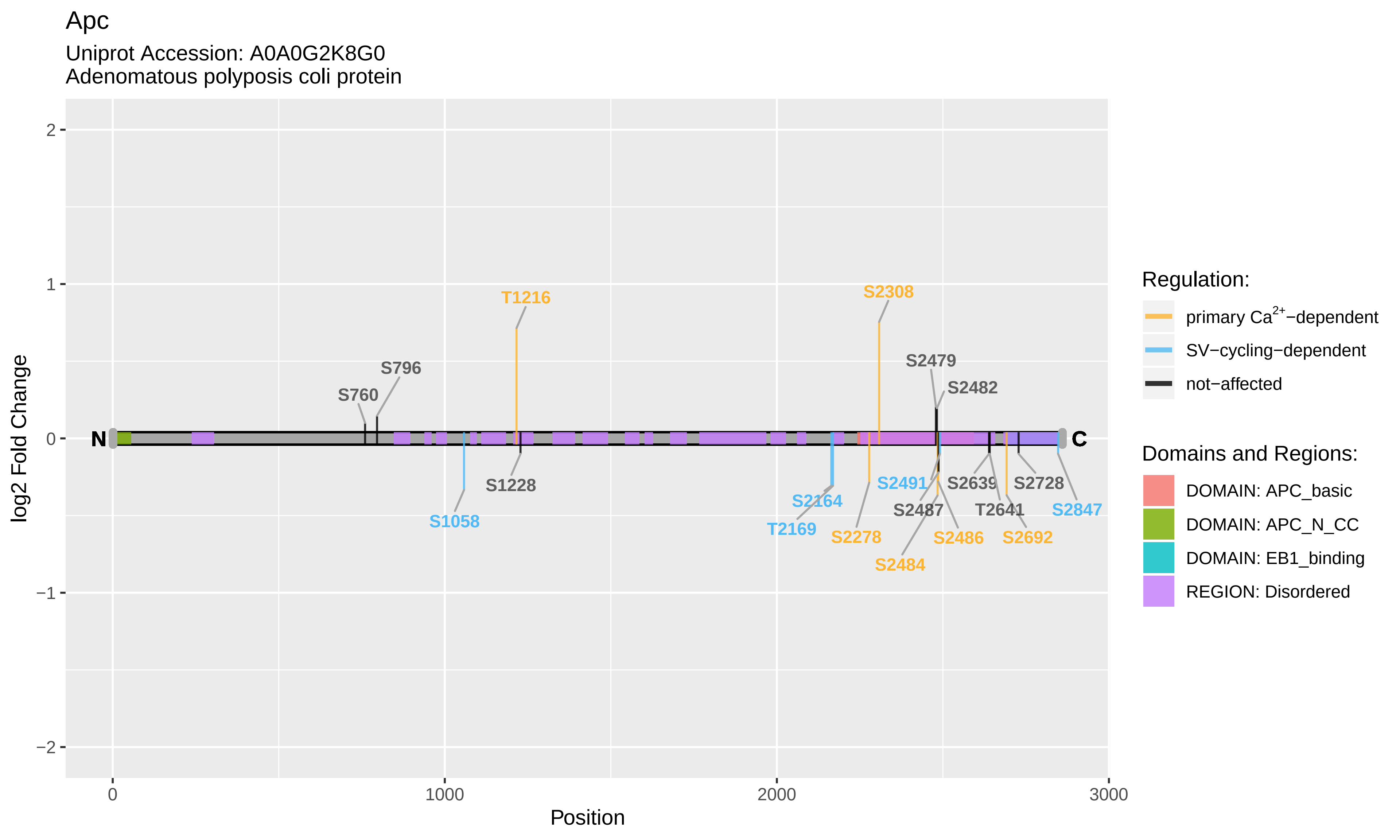
Apc
Uniprot Accession: A0A0G2K8G0
Adenomatous polyposis coli protein
log2 Fold Change
Position
N
C
T1216
S2308
S796
S760
S2479
S2482
S1228
S1058
S2164
T2169
S2278
S2484
S2491
S2487
S2486
S2639
T2641
S2692
S2728
S2847
Regulation:
primary Ca2+−dependent
SV−cycling−dependent
not−affected
Domains and Regions:
DOMAIN: APC_basic
DOMAIN: APC_N_CC
DOMAIN: EB1_binding
REGION: Disordered

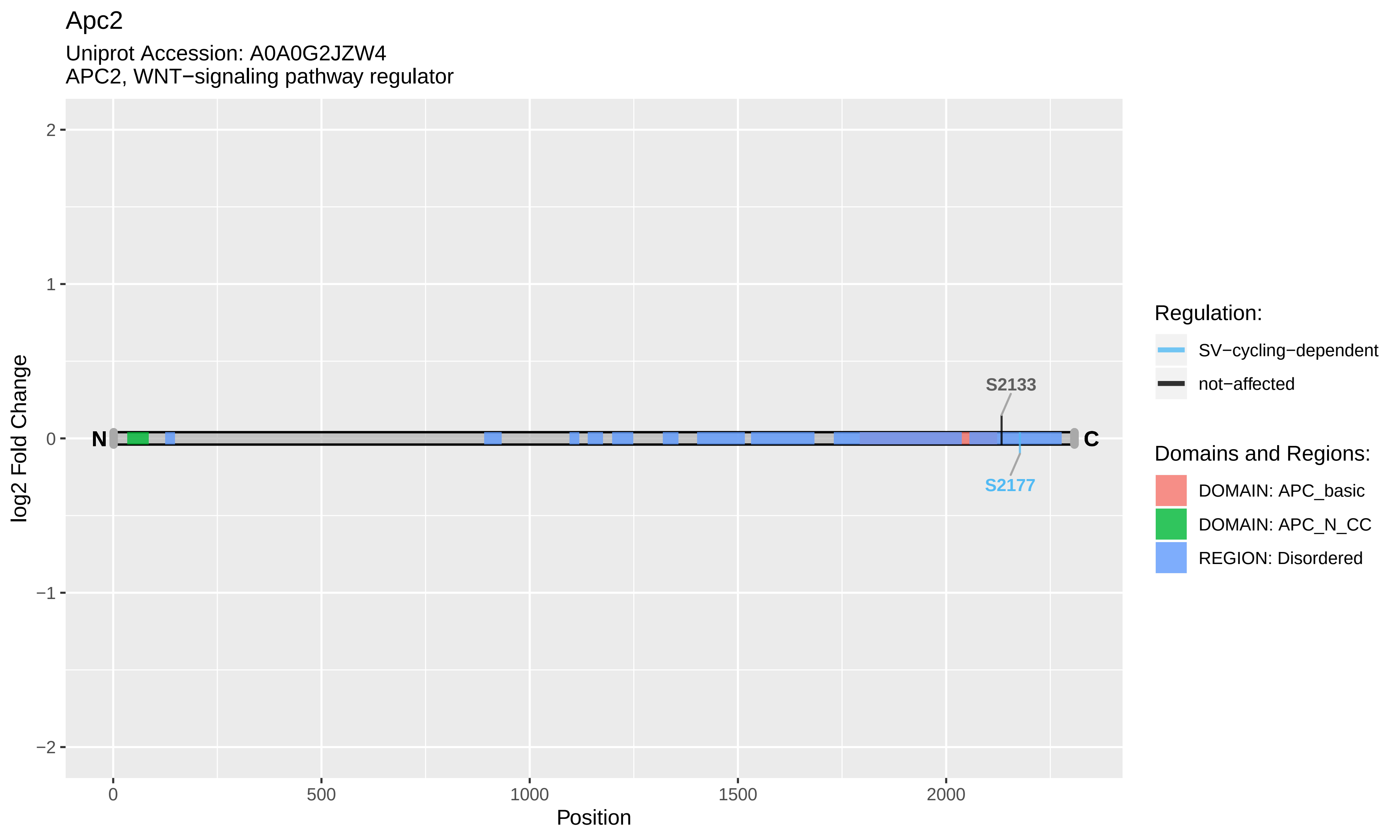

Apc2
Uniprot Accession: A0A0G2JZW4
APC2, WNT−signaling pathway regulator
log2 Fold Change
Position
N
C
S2133
S2177
Regulation:
SV−cycling−dependent
not−affected
Domains and Regions:
DOMAIN: APC_basic
DOMAIN: APC_N_CC
REGION: Disordered

Arfgap1

Uniprot Accession: Q3S4A4
ADP−ribosylation factor GTPase activating protein 1 heart isoform

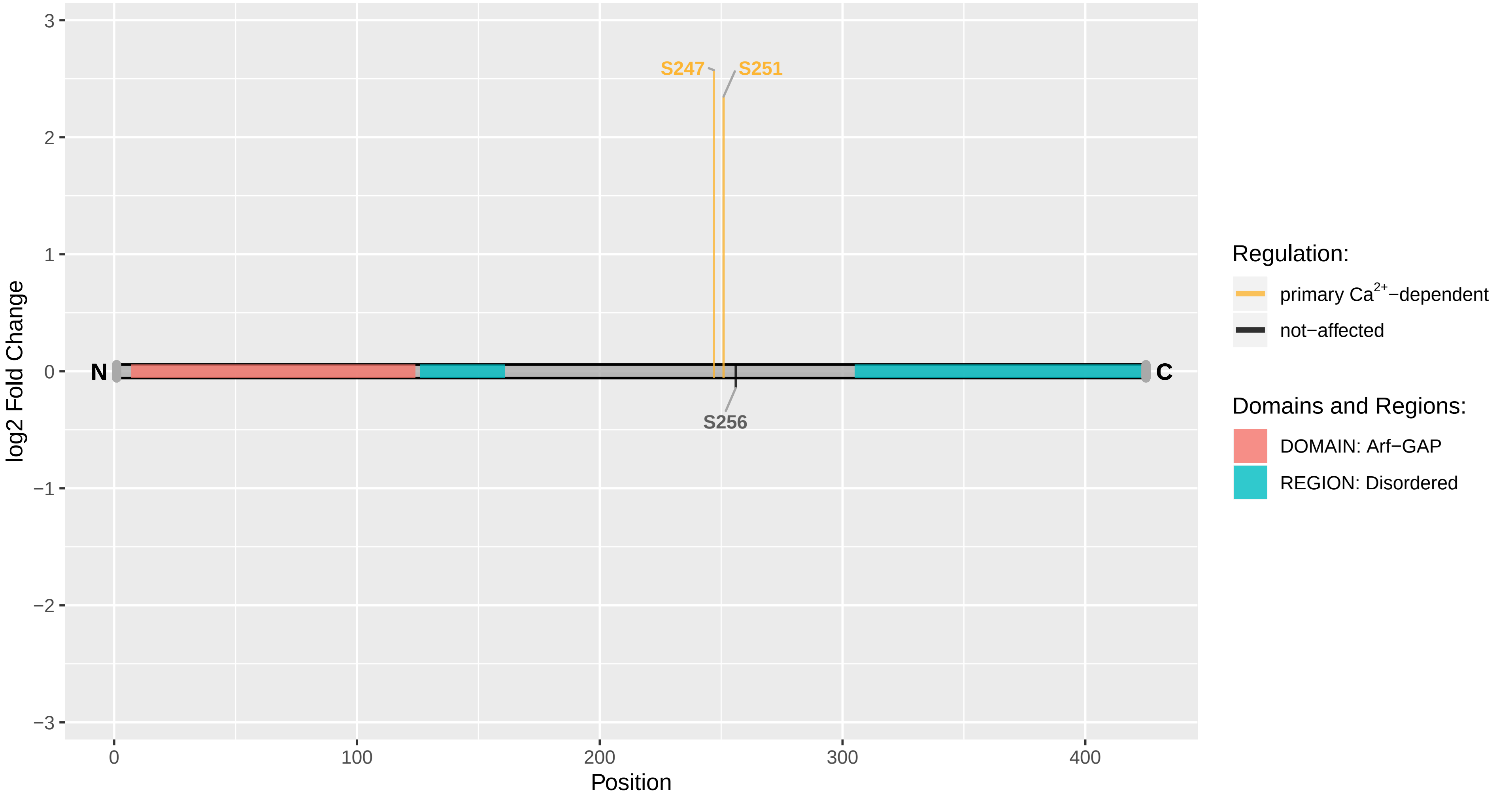

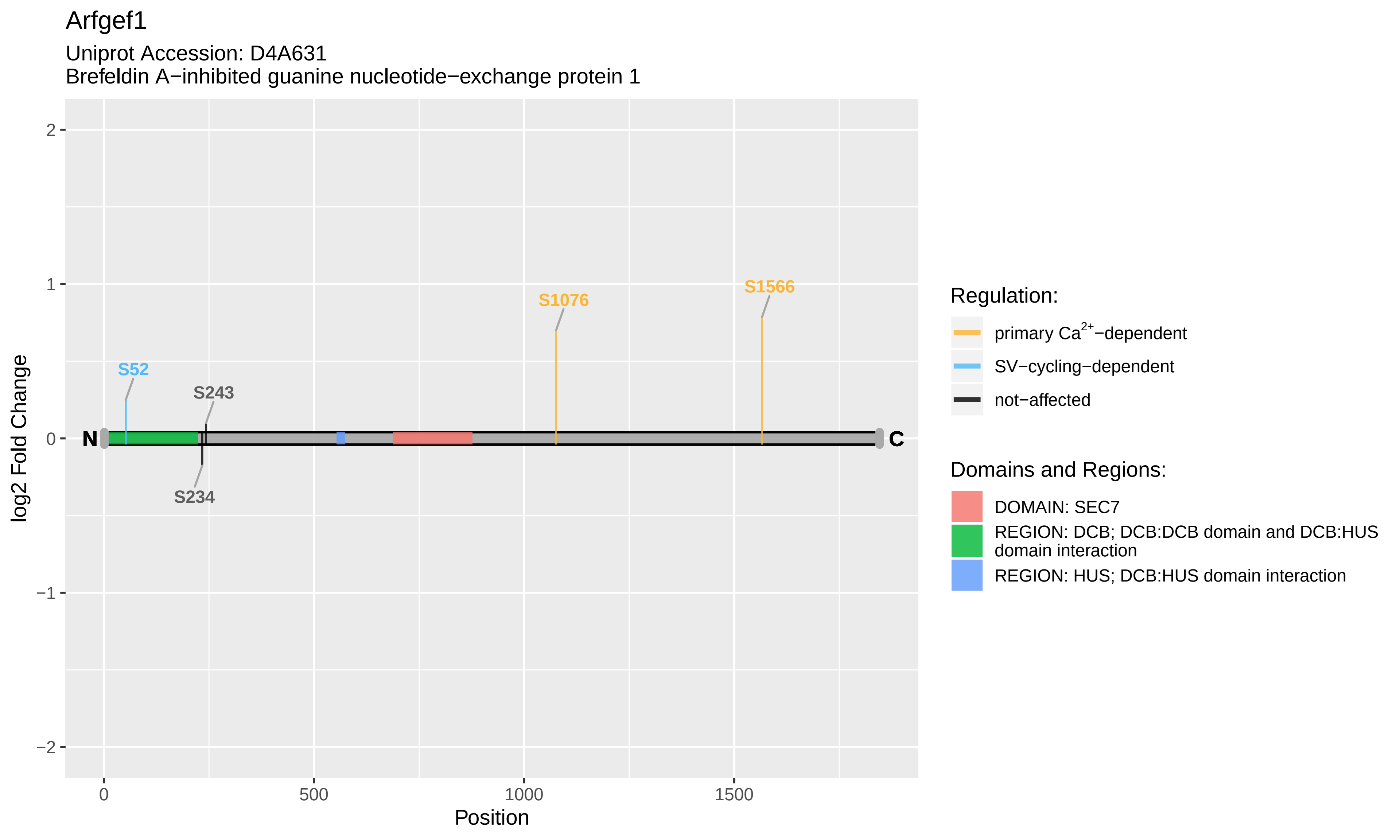

Arfgef1
Uniprot Accession: D4A631
Brefeldin A−inhibited guanine nucleotide−exchange protein 1
log2 Fold Change
2
1
0
−1
−2
0
500
1000
1500
Position
N
C
S52
S243
S234
S1076
S1566
Regulation:
primary Ca2+−dependent
SV−cycling−dependent
not−affected
Domains and Regions:
DOMAIN: SEC7
REGION: DCB; DCB:DCB domain and DCB:HUS domain interaction
REGION: HUS; DCB:HUS domain interaction

Arfip2

Uniprot Accession: Q6AY65
Arfaptin−2

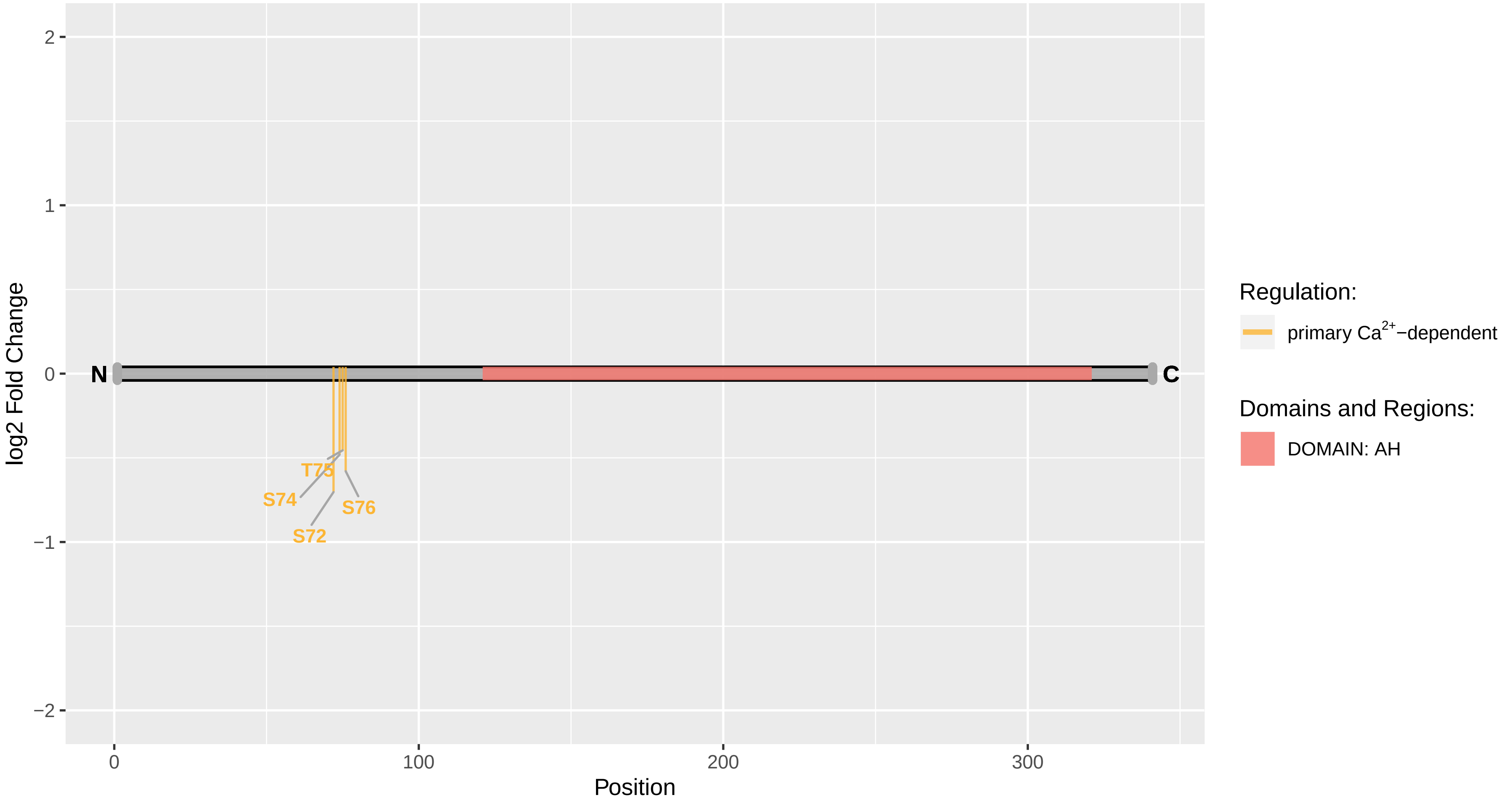

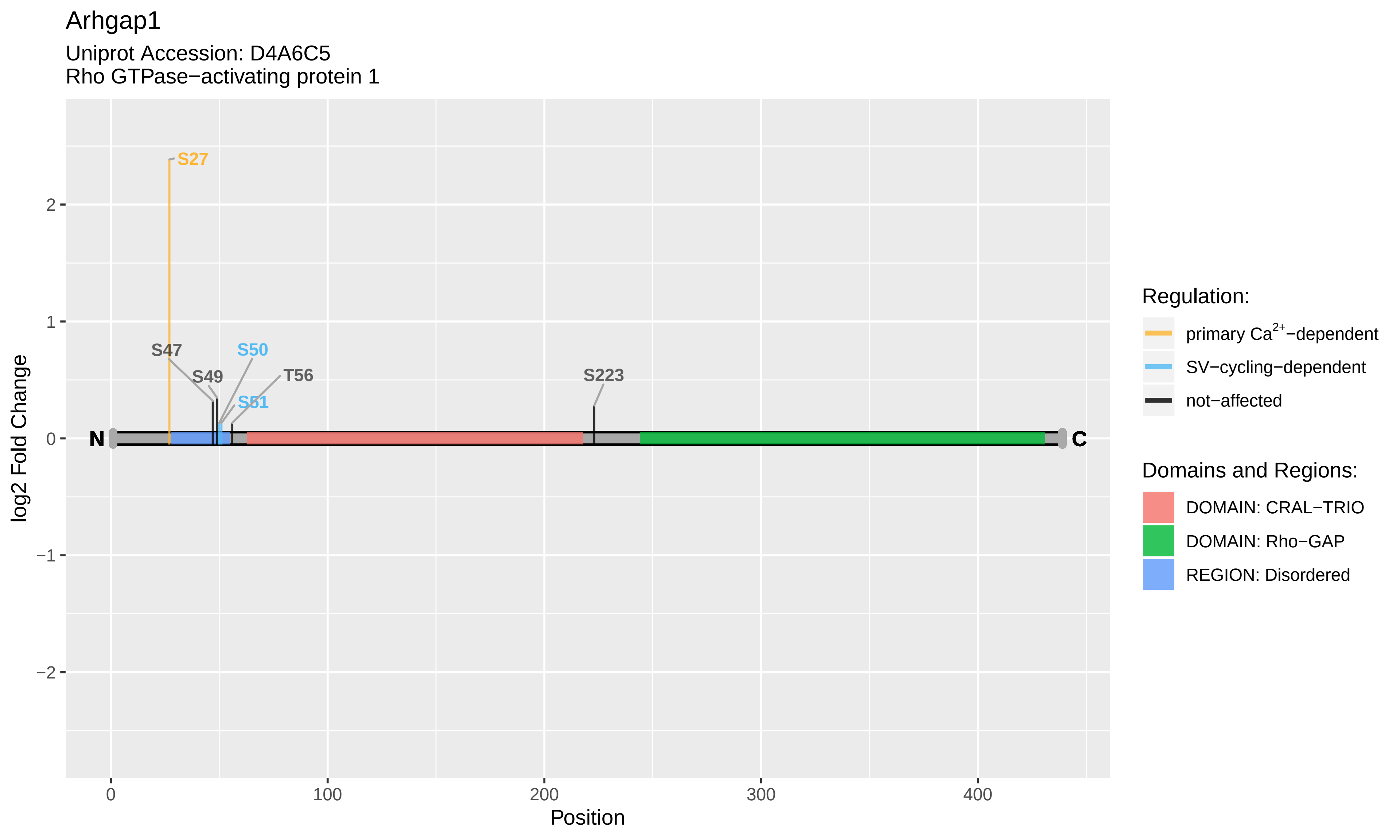
Arhgap1
Uniprot Accession: D4A6C5
Rho GTPase−activating protein 1
S27
S47
S49
S50
S51
T56
S223
N
C
2
1
0
−1
−2
log2 Fold Change
0
100
200
300
400
Position
Regulation:
primary Ca2+−dependent
SV−cycling−dependent
not−affected
Domains and Regions:
DOMAIN: CRAL−TRIO
DOMAIN: Rho−GAP
REGION: Disordered

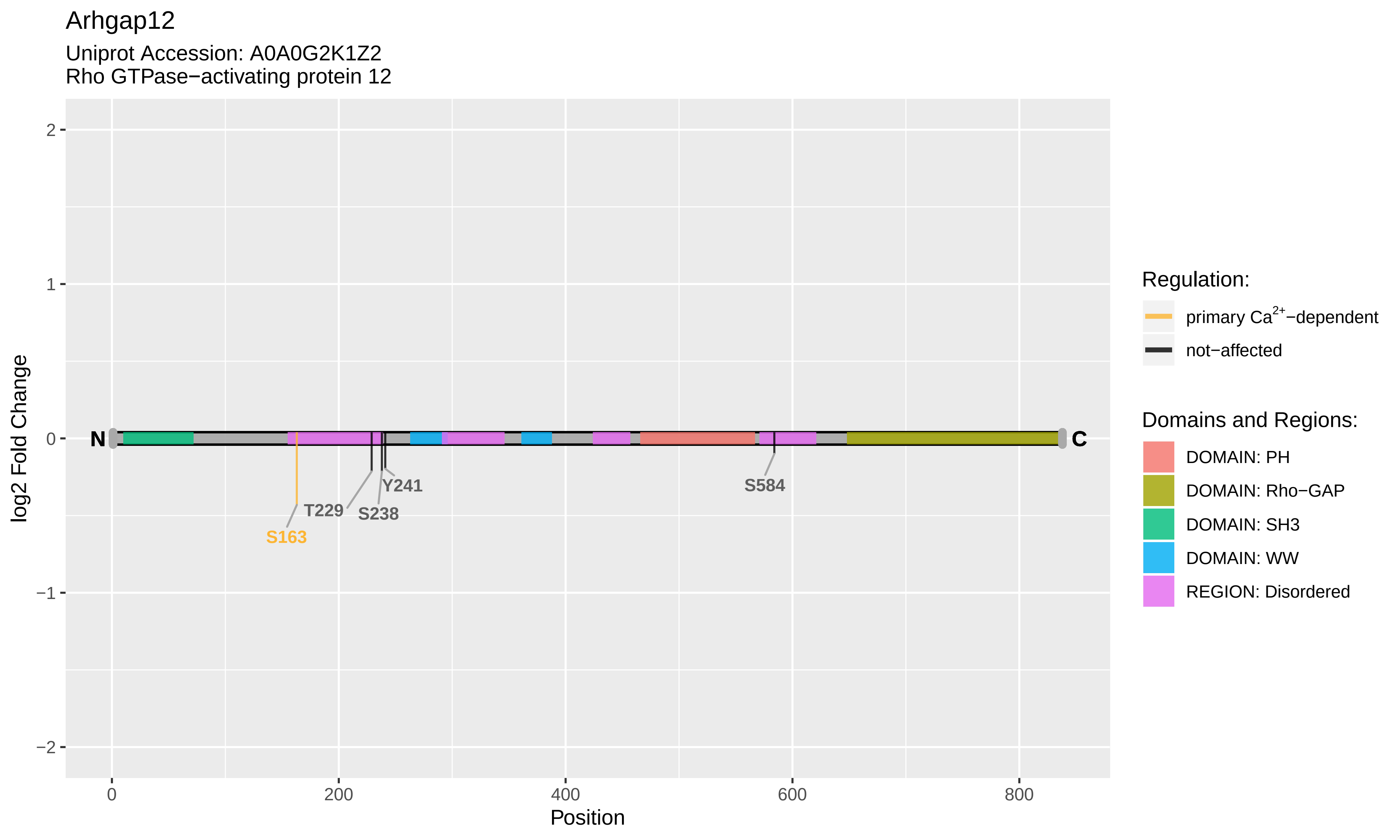

Arhgap12
Uniprot Accession: A0A0G2K1Z2
Rho GTPase−activating protein 12
log2 Fold Change
Position
N
C
S163
T229
S238
Y241
S584
Regulation:
primary Ca2+−dependent
not−affected
Domains and Regions:
DOMAIN: PH
DOMAIN: Rho−GAP
DOMAIN: SH3
DOMAIN: WW
REGION: Disordered

Arhgap21

Uniprot Accession: F1LXQ7
Rho GTPase−activating protein 21

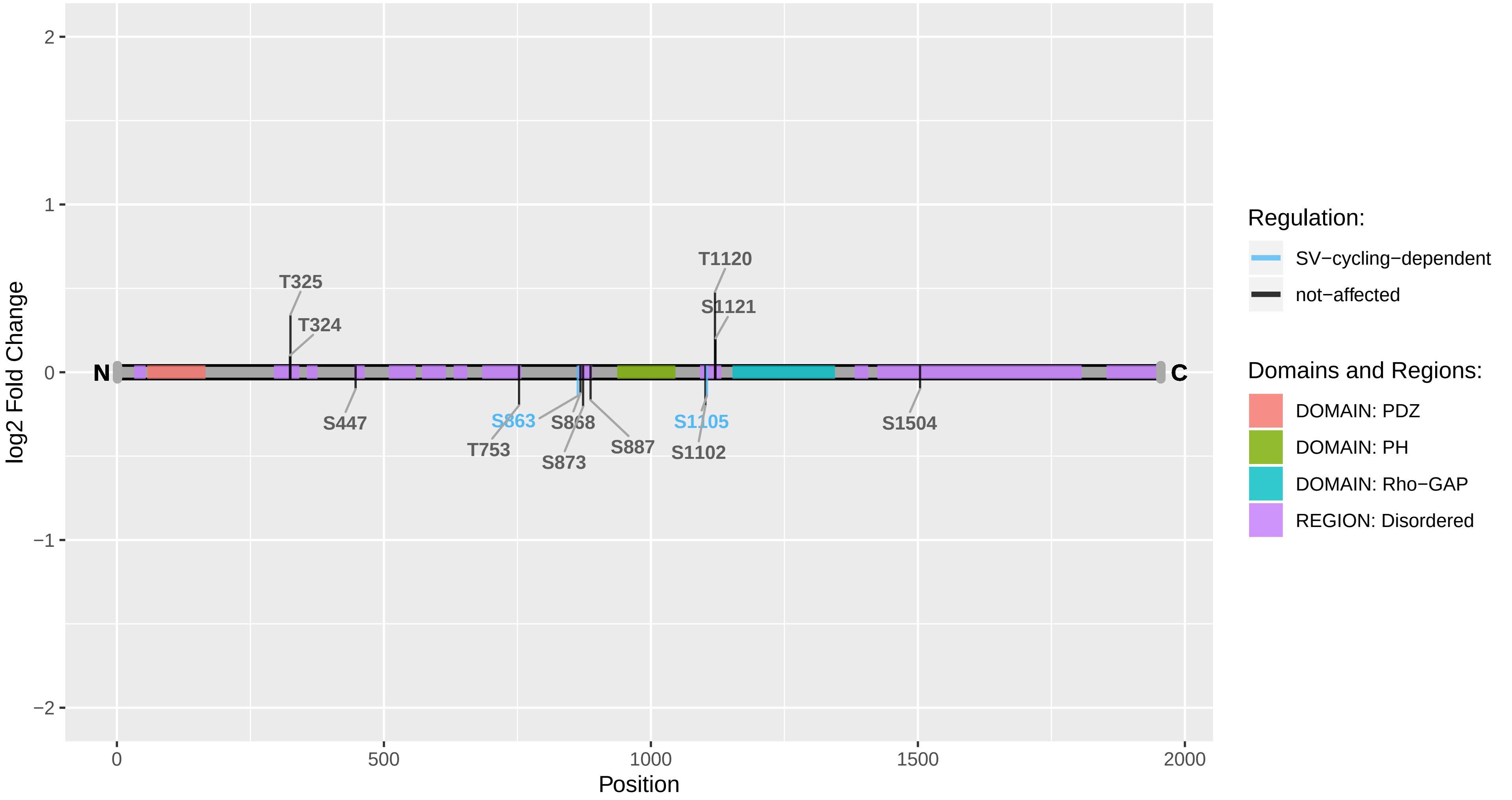

Arhgap32

Uniprot Accession: F1MAK3
Rho GTPase−activating protein 32

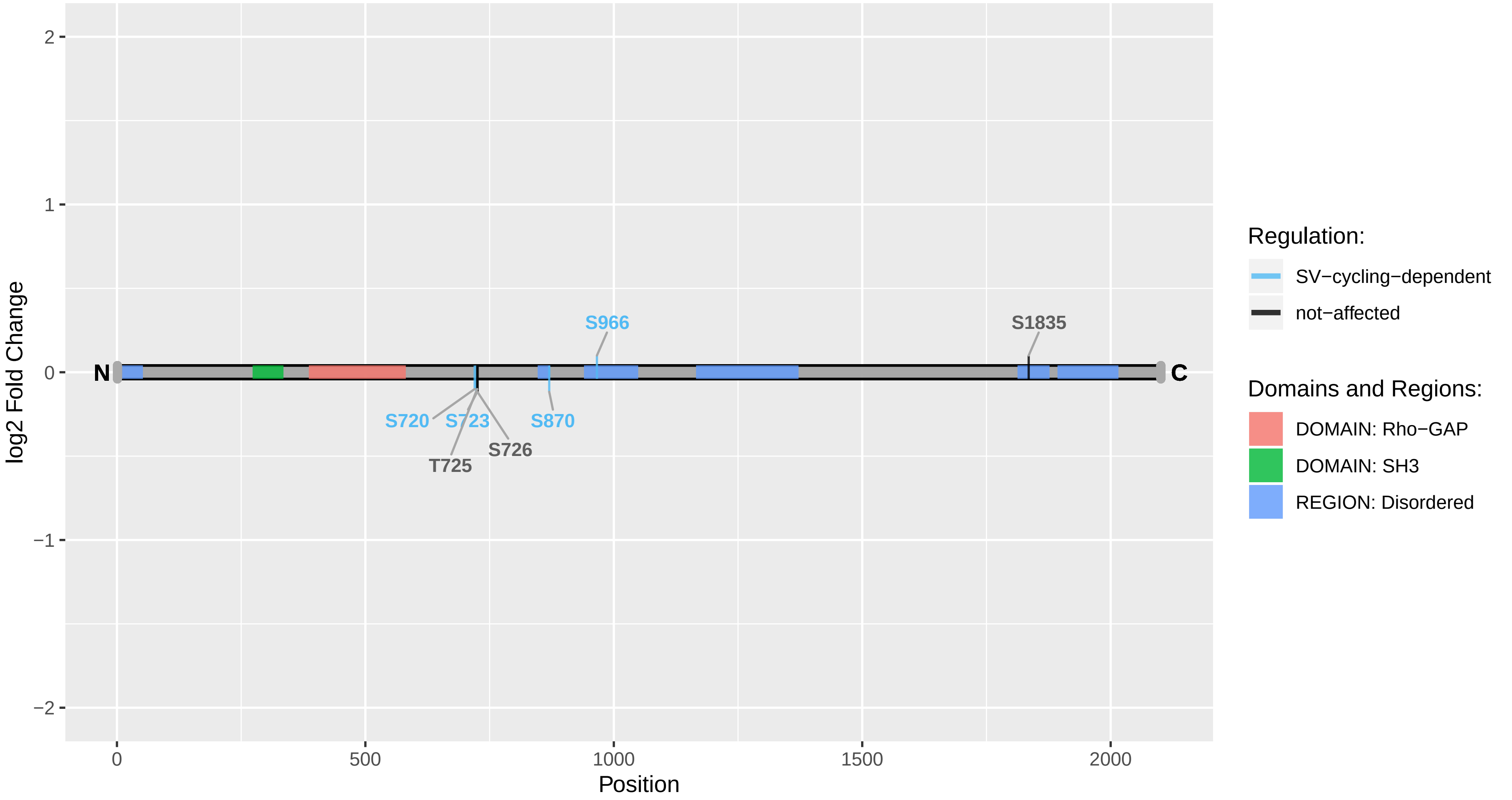

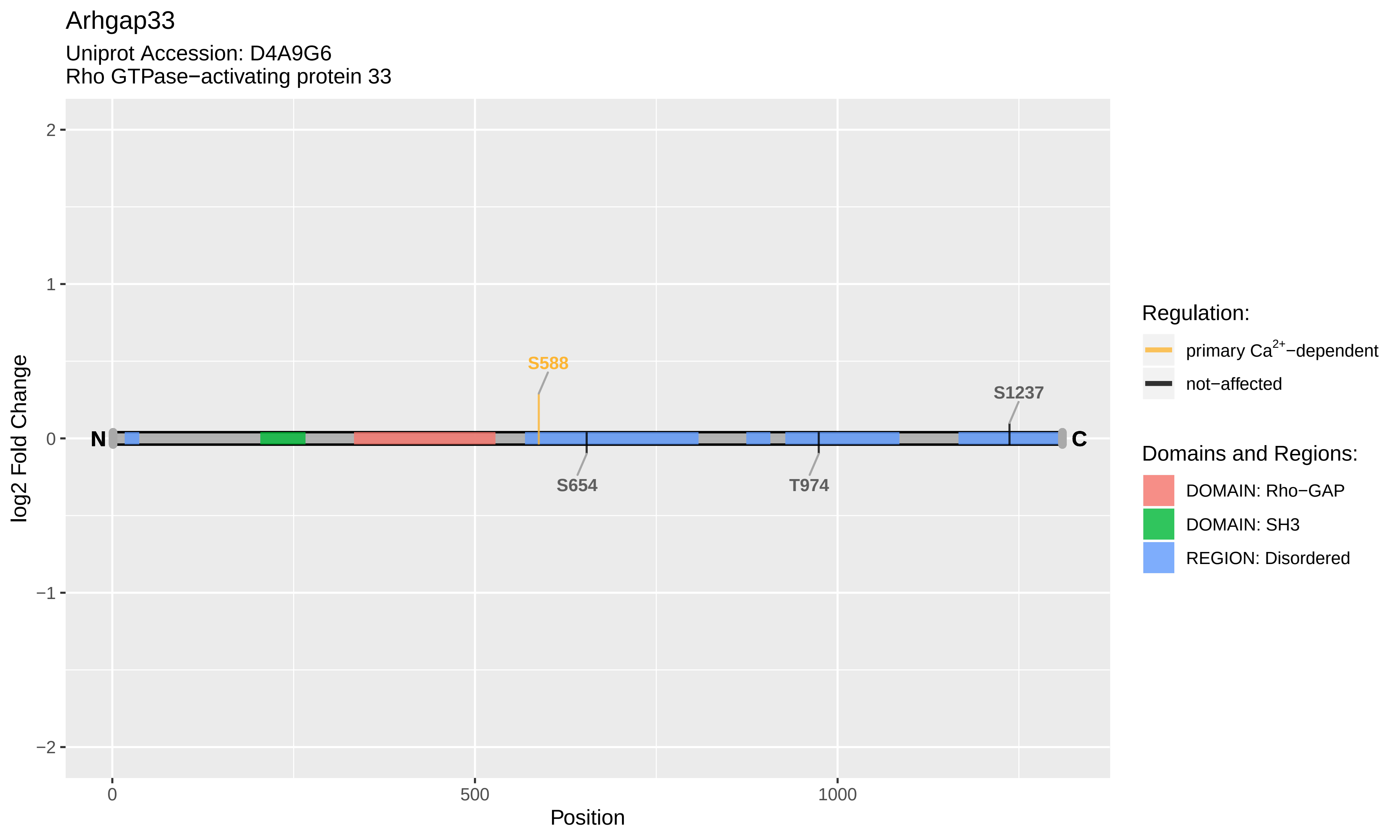
Arhgap33
Uniprot Accession: D4A9G6
Rho GTPase−activating protein 33
S588
S654
T974
S1237
N
C
2
1
0
−1
−2
log2 Fold Change
0
500
1000
Position
Regulation:
primary Ca2+−dependent
not−affected
Domains and Regions:
DOMAIN: Rho−GAP
DOMAIN: SH3
REGION: Disordered

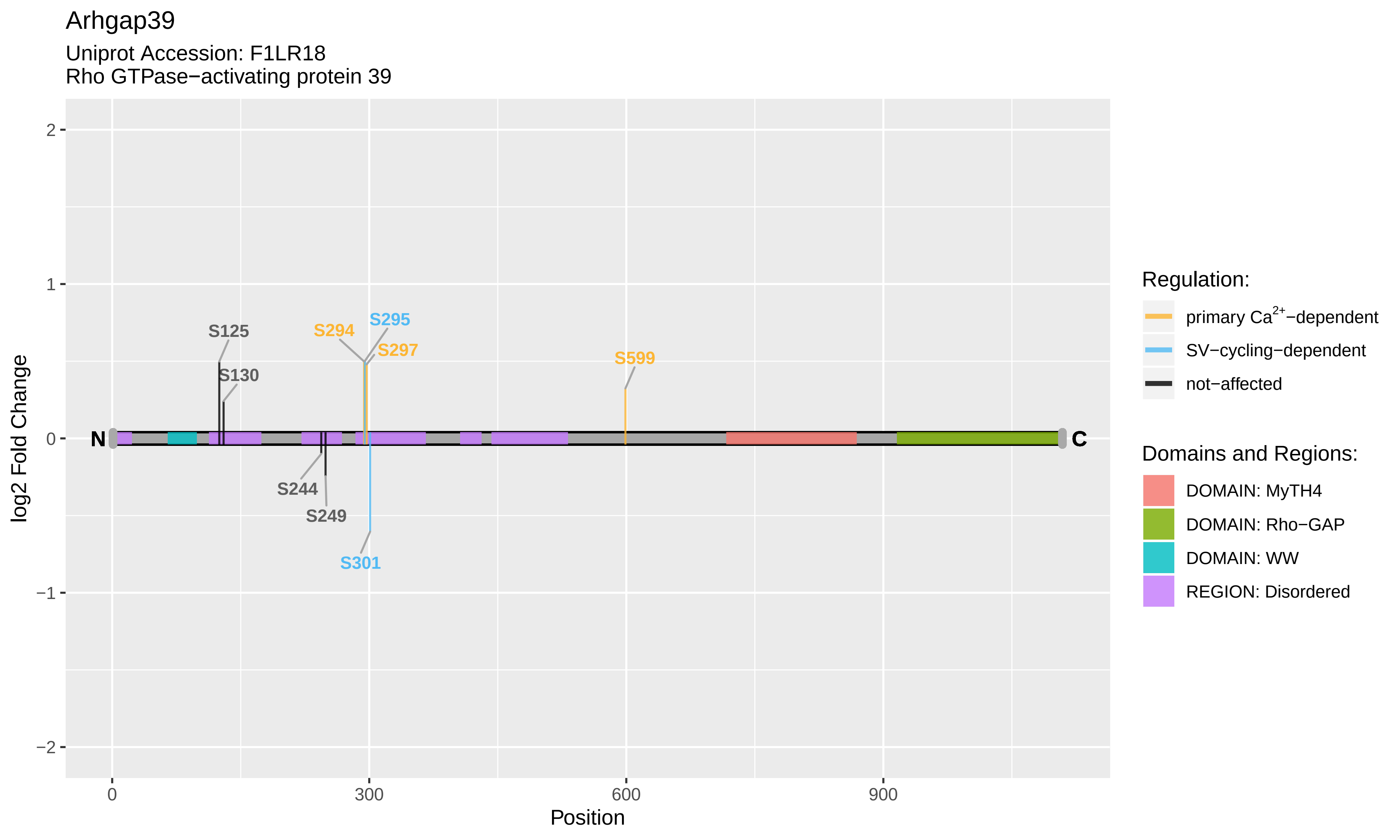

Arhgap39
Uniprot Accession: F1LR18
Rho GTPase−activating protein 39
log2 Fold Change
2
1
0
−1
−2
0
300
600
900
Position
N
C
S125
S130
S244
S249
S294
S295
S297
S301
S599
Regulation:
primary Ca2+−dependent
SV−cycling−dependent
not−affected
Domains and Regions:
DOMAIN: MyTH4
DOMAIN: Rho−GAP
DOMAIN: WW
REGION: Disordered

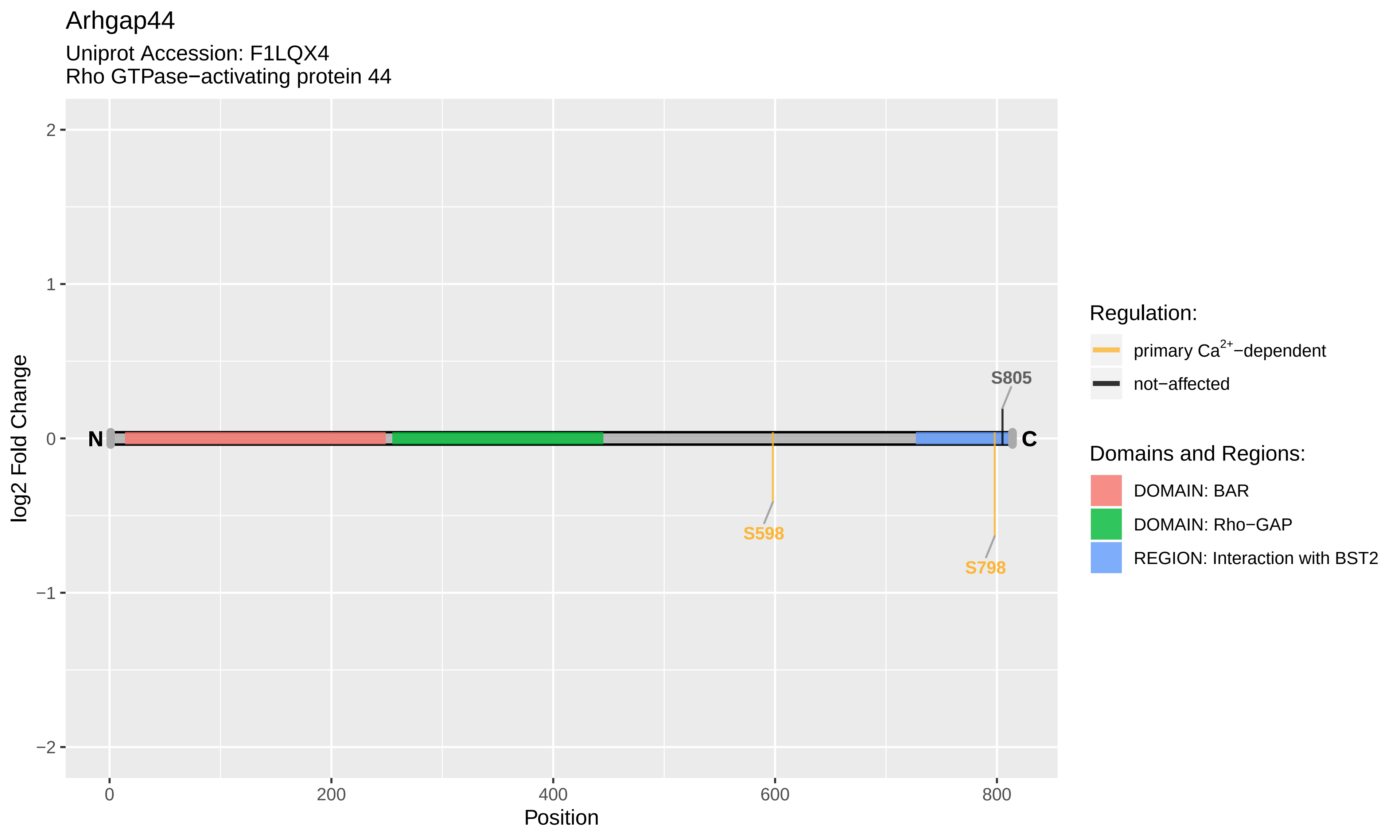

Arhgap44
Uniprot Accession: F1LQX4
Rho GTPase−activating protein 44
2
1
0
−1
−2
log2 Fold Change
N
C
S805
S598
S798
0
200
400
600
800
Position
Regulation:
primary Ca2+−dependent
not−affected
Domains and Regions:
DOMAIN: BAR
DOMAIN: Rho−GAP
REGION: Interaction with BST2

Arhgdia

Uniprot Accession: Q5XI73
Rho GDP−dissociation inhibitor 1

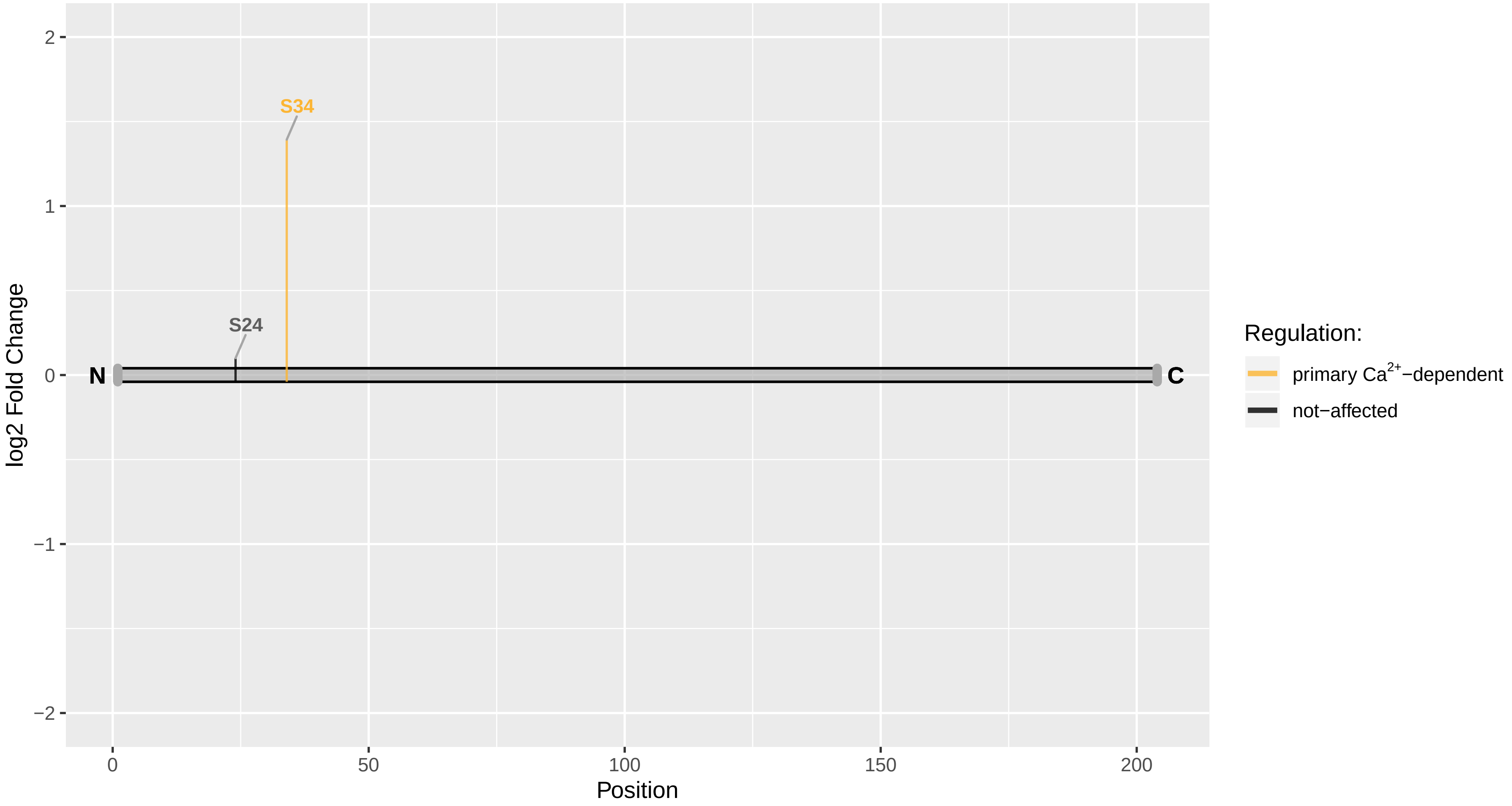

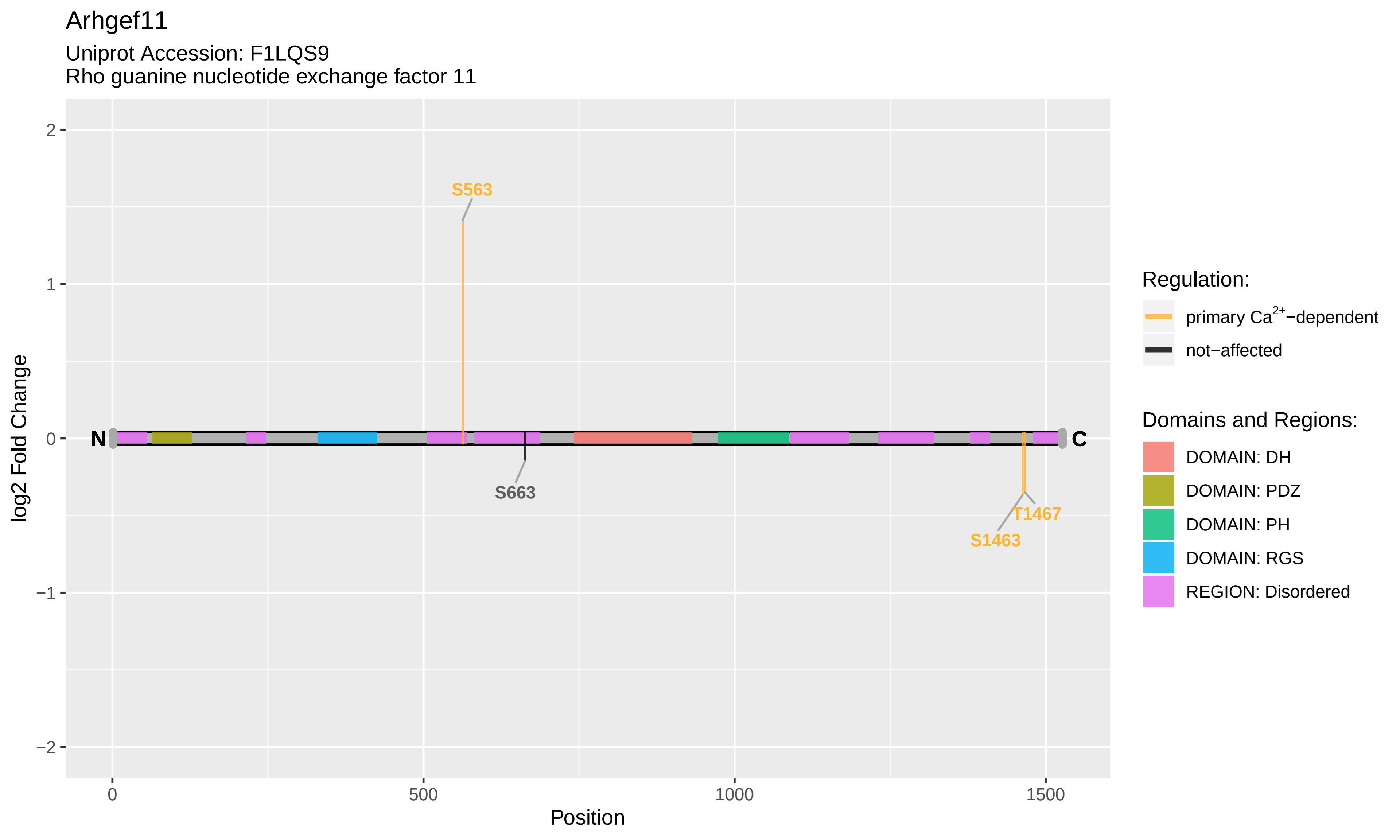

Arhgef11
Uniprot Accession: F1LQS9
Rho guanine nucleotide exchange factor 11
S563
S663
T1467
S1463
N
C
log2 Fold Change
Position
2
1
0
−1
−2
0
500
1000
1500
Regulation:
primary Ca2+−dependent
not−affected
Domains and Regions:
DOMAIN: DH
DOMAIN: PDZ
DOMAIN: PH
DOMAIN: RGS
REGION: Disordered

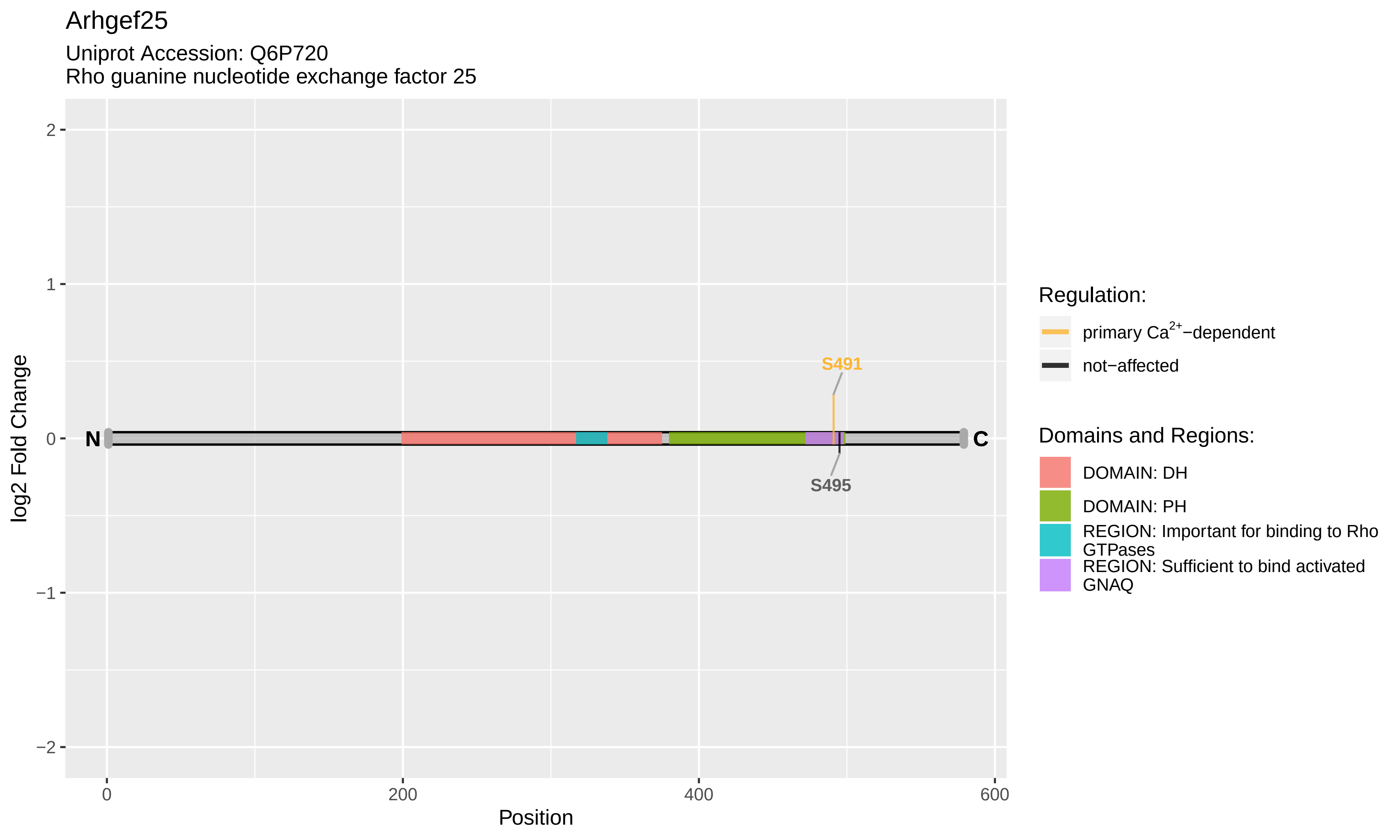

Arhgef25
Uniprot Accession: Q6P720
Rho guanine nucleotide exchange factor 25
log2 Fold Change
Position
N
C
S491
S495
Regulation:
primary Ca^{2+}−dependent
not−affected
Domains and Regions:
DOMAIN: DH
DOMAIN: PH
REGION: Important for binding to Rho GTPases
REGION: Sufficient to bind activated GNAQ

Arhgef33

Uniprot Accession: D3Z8W6
Rho guanine nucleotide exchange factor 33

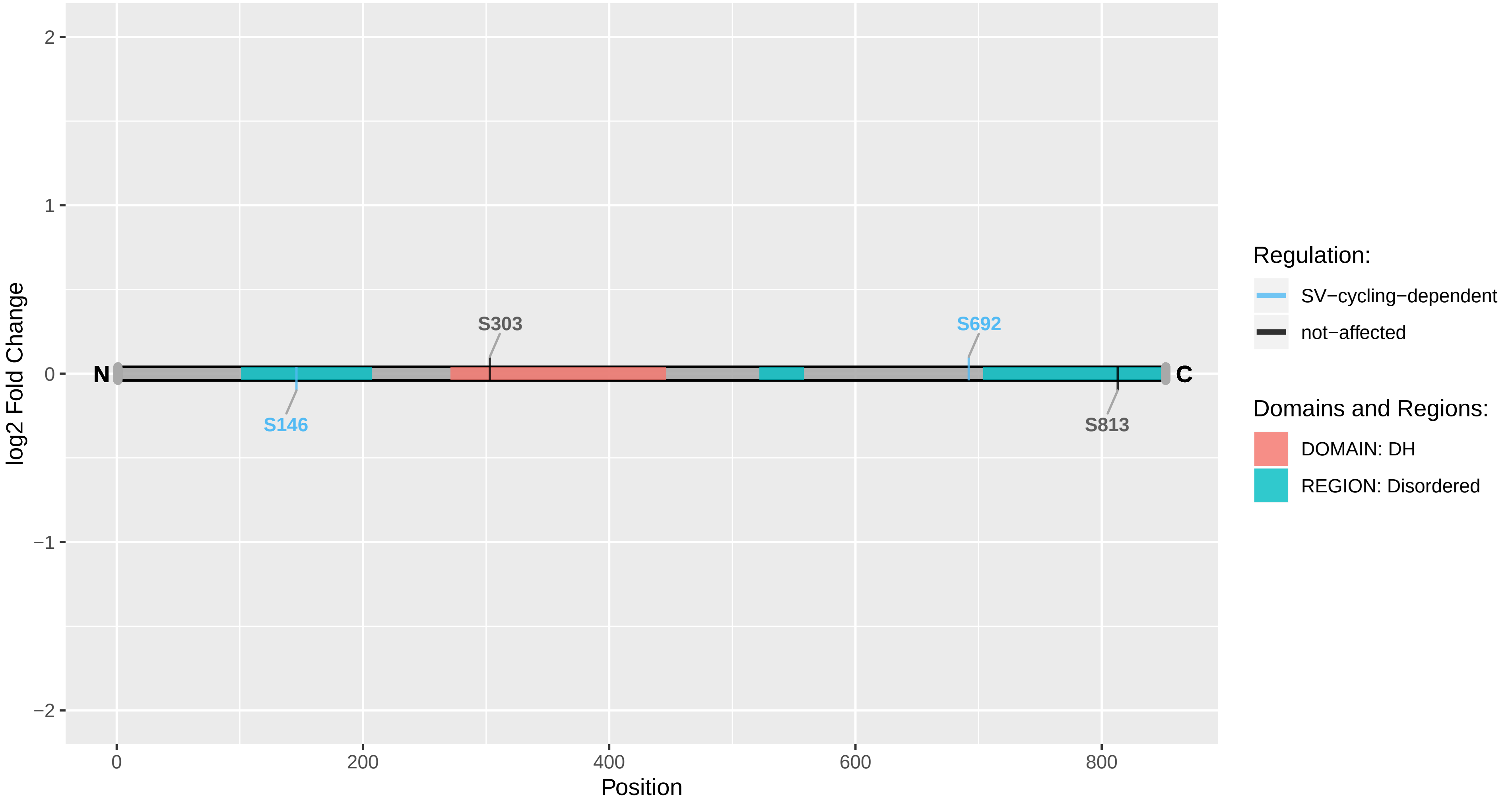

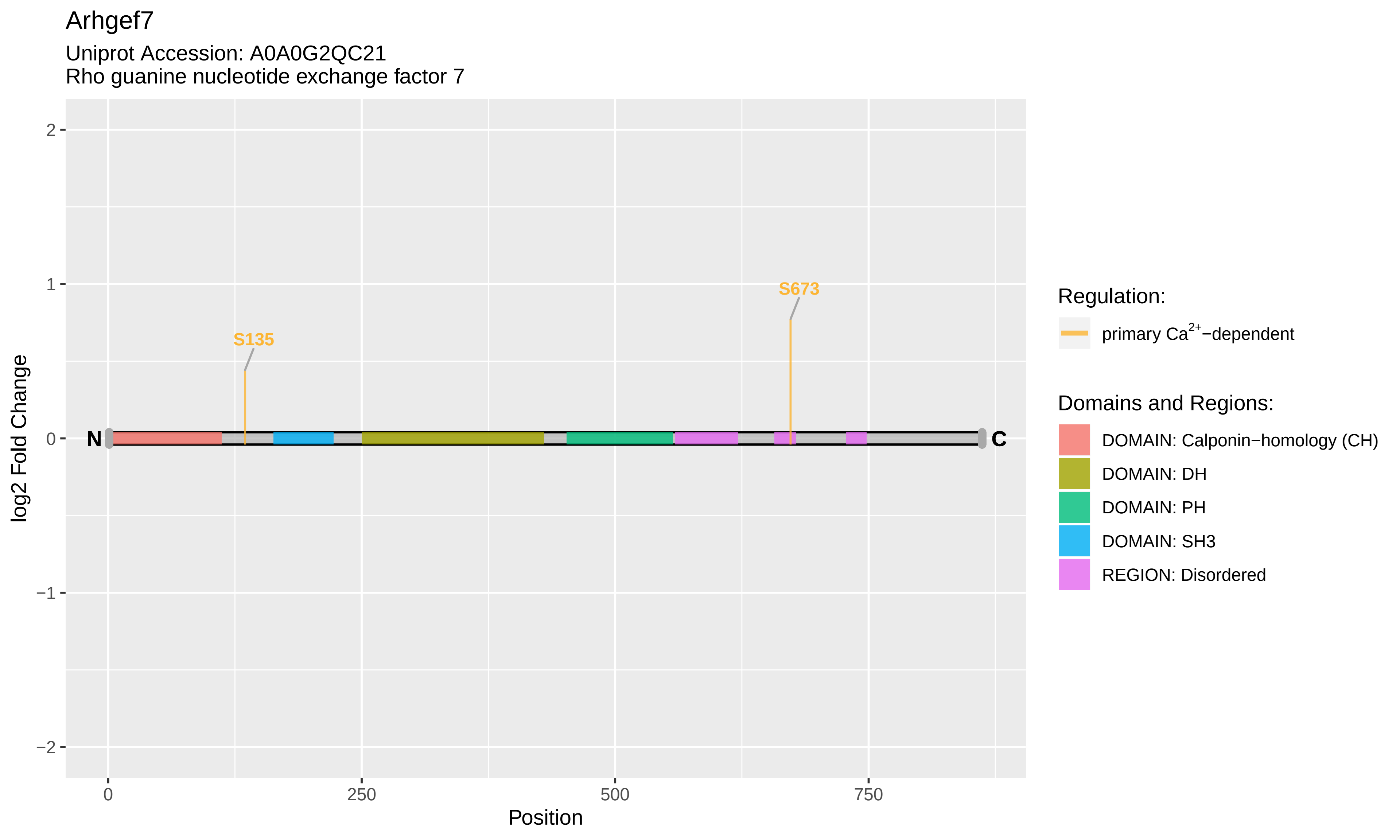
Arhgef7
Uniprot Accession: A0A0G2QC21
Rho guanine nucleotide exchange factor 7
S135
S673
N
C
2
1
0
−1
−2
0
250
500
750
log2 Fold Change
Position
Regulation:
primary Ca2+−dependent
Domains and Regions:
DOMAIN: Calponin−homology (CH)
DOMAIN: DH
DOMAIN: PH
DOMAIN: SH3
REGION: Disordered

Armc10

Uniprot Accession: B1WBW4
Armadillo repeat−containing protein 10

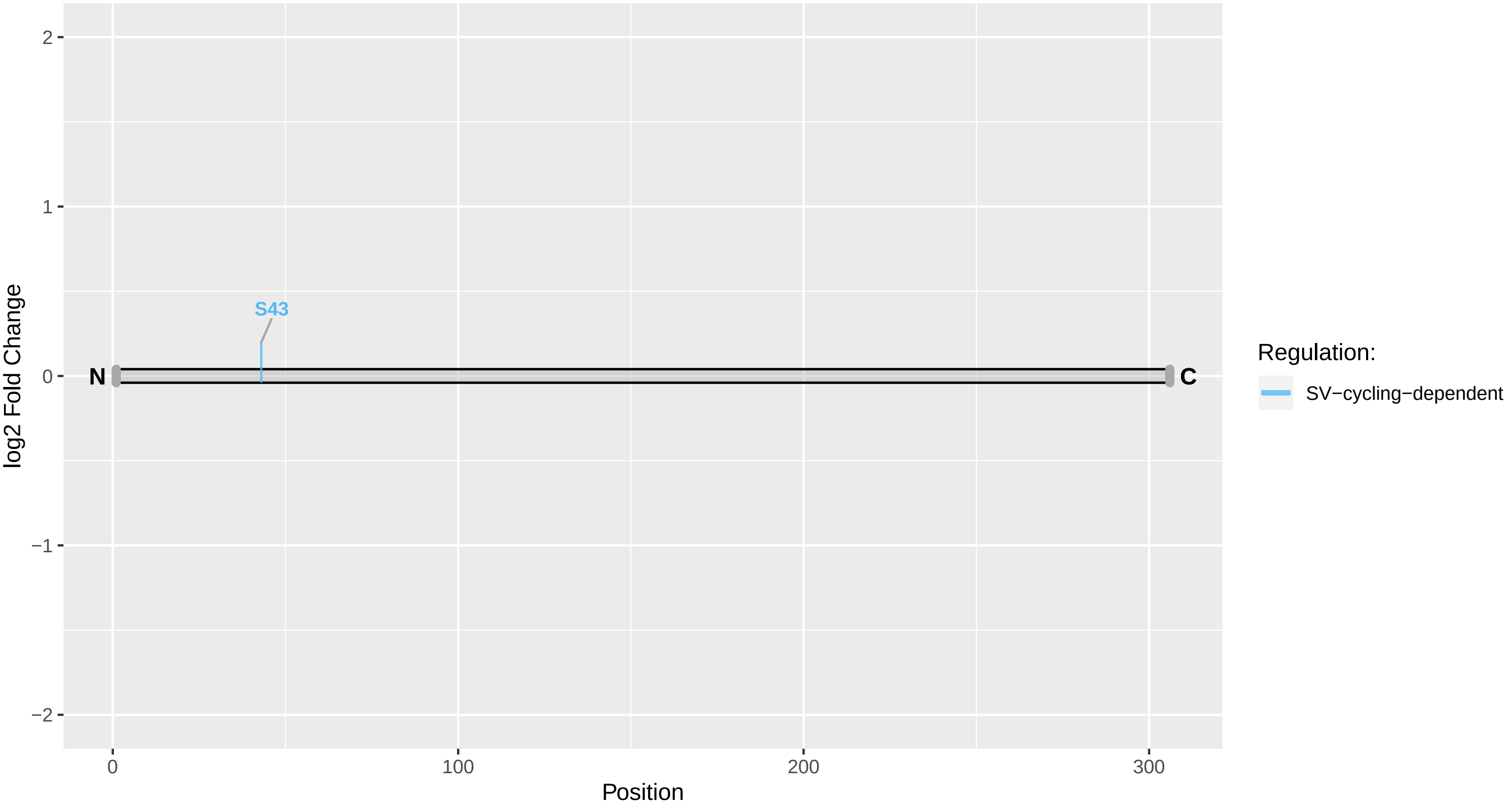

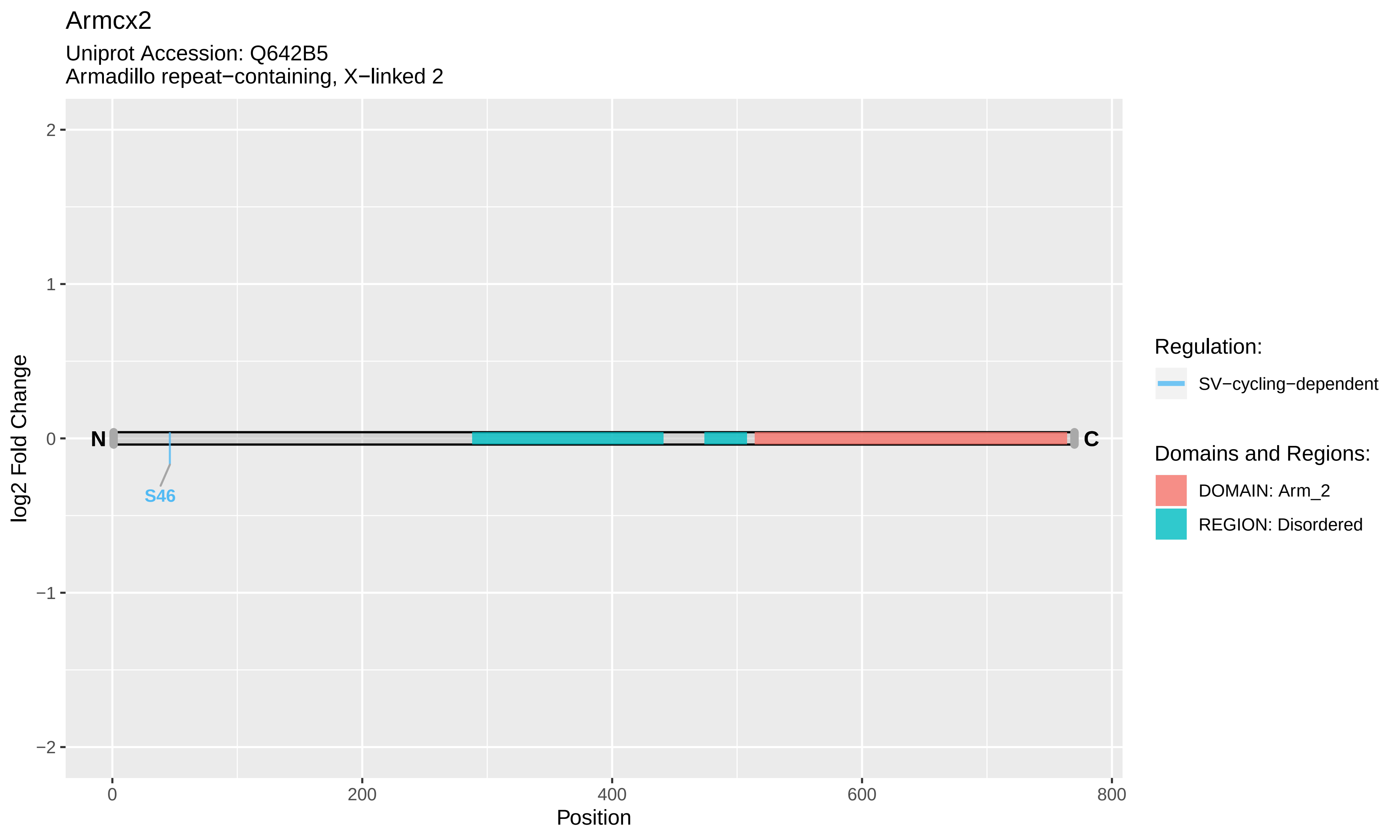
Armcx2
Uniprot Accession: Q642B5
Armadillo repeat−containing, X−linked 2
log2 Fold Change
2
1
0
−1
−2
N
C
S46
0
200
400
600
800
Position
Regulation:
SV−cycling−dependent
Domains and Regions:
DOMAIN: Arm_2
REGION: Disordered

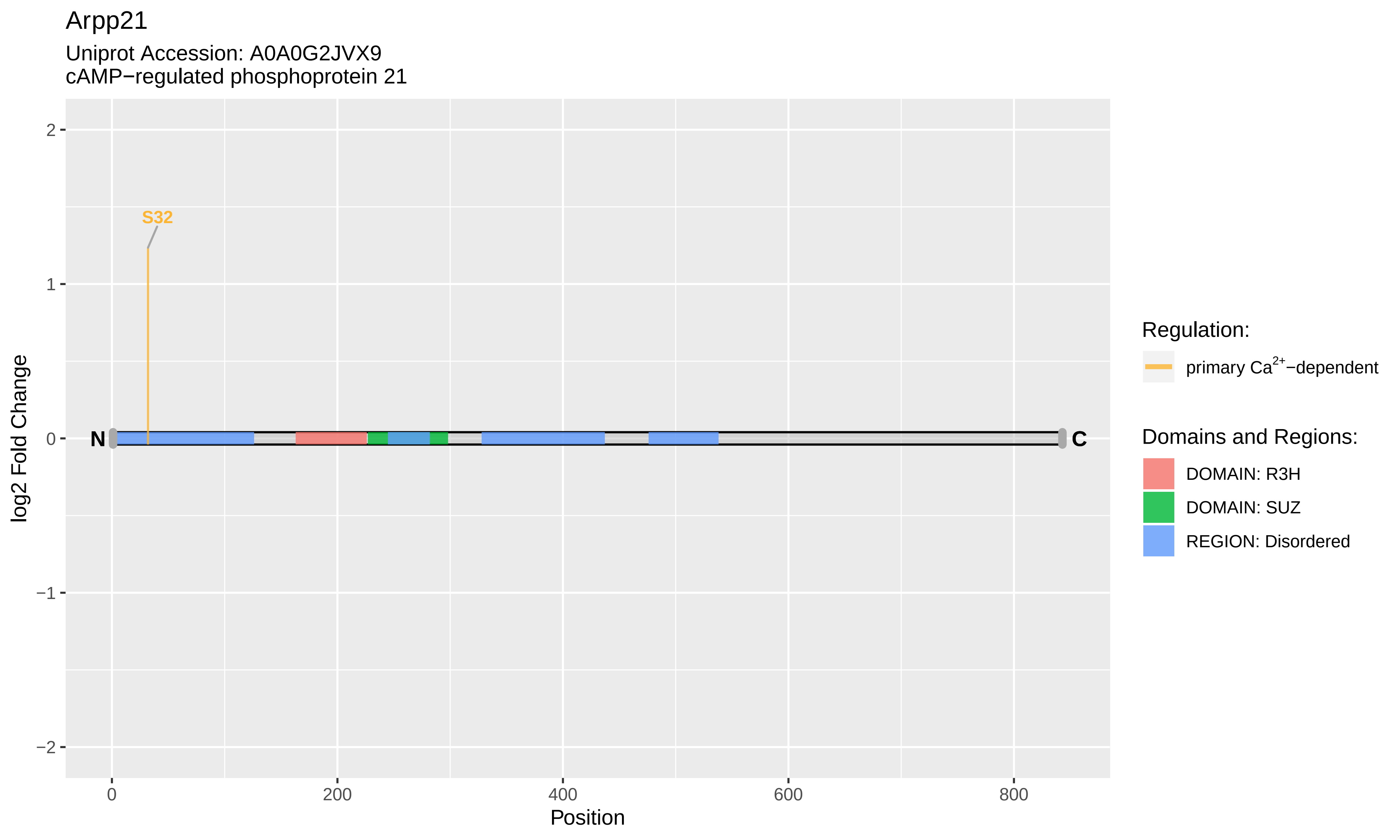
Arpp21
Uniprot Accession: A0A0G2JVX9
cAMP−regulated phosphoprotein 21
S32
N
C
2
1
0
−1
−2
log2 Fold Change
0
200
400
600
800
Position
Regulation:
primary Ca2+−dependent
Domains and Regions:
DOMAIN: R3H
DOMAIN: SUZ
REGION: Disordered

Arvcf

Uniprot Accession: B4F7F3
ARVCF, delta catenin family member

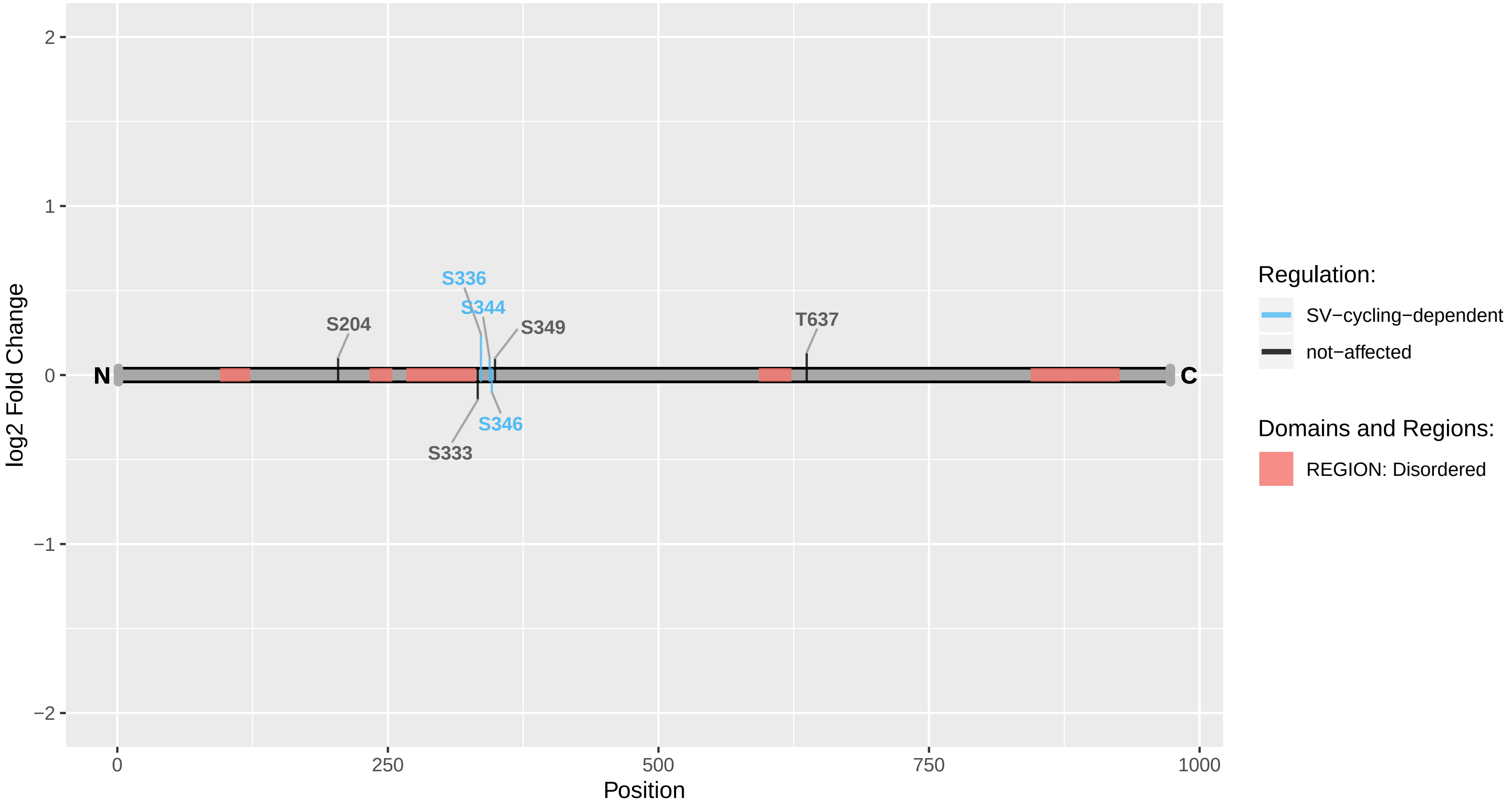

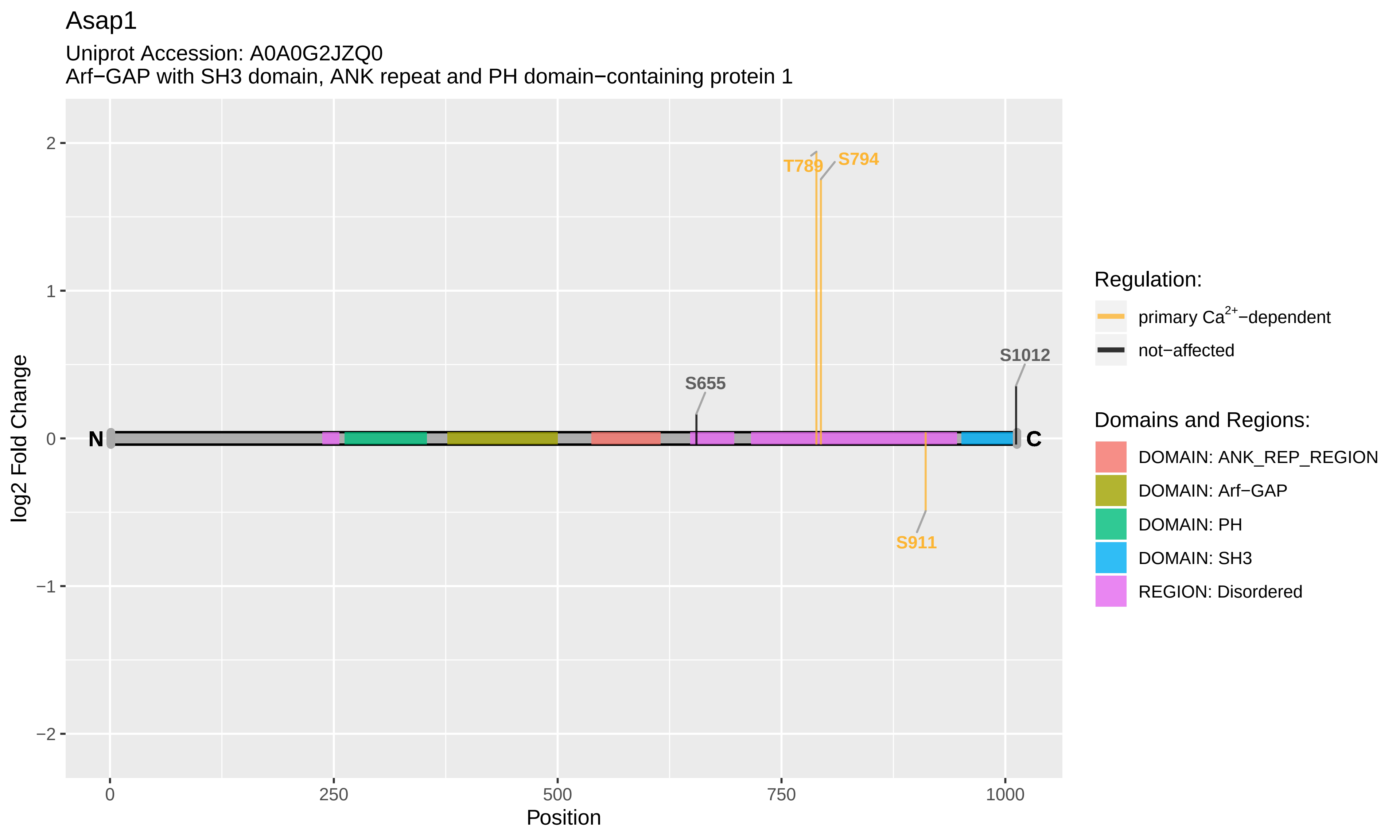

Asap1
Uniprot Accession: A0A0G2JZQ0
Arf−GAP with SH3 domain, ANK repeat and PH domain−containing protein 1
T789
S794
S1012
S655
S911
N
C
2
1
0
−1
−2
log2 Fold Change
0
250
500
750
1000
Position
Regulation:
primary Ca2+−dependent
not−affected
Domains and Regions:
DOMAIN: ANK_REP_REGION
DOMAIN: Arf−GAP
DOMAIN: PH
DOMAIN: SH3
REGION: Disordered

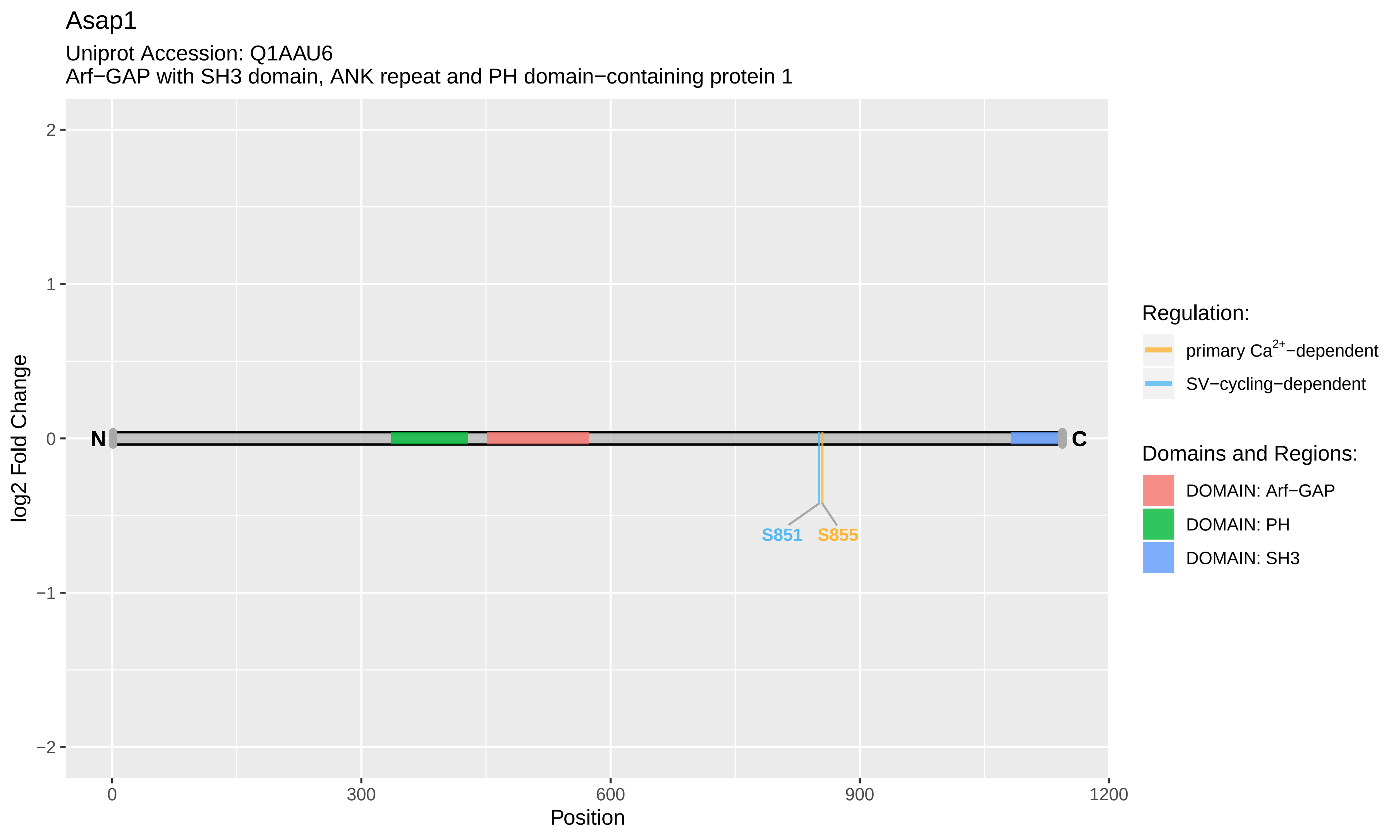
Asap1
Uniprot Accession: Q1AAU6
Arf−GAP with SH3 domain, ANK repeat and PH domain−containing protein 1
log2 Fold Change
2
1
0
−1
−2
N
C
S851
S855
0
300
600
900
1200
Position
Regulation:
primary Ca2+−dependent
SV−cycling−dependent
Domains and Regions:
DOMAIN: Arf−GAP
DOMAIN: PH
DOMAIN: SH3

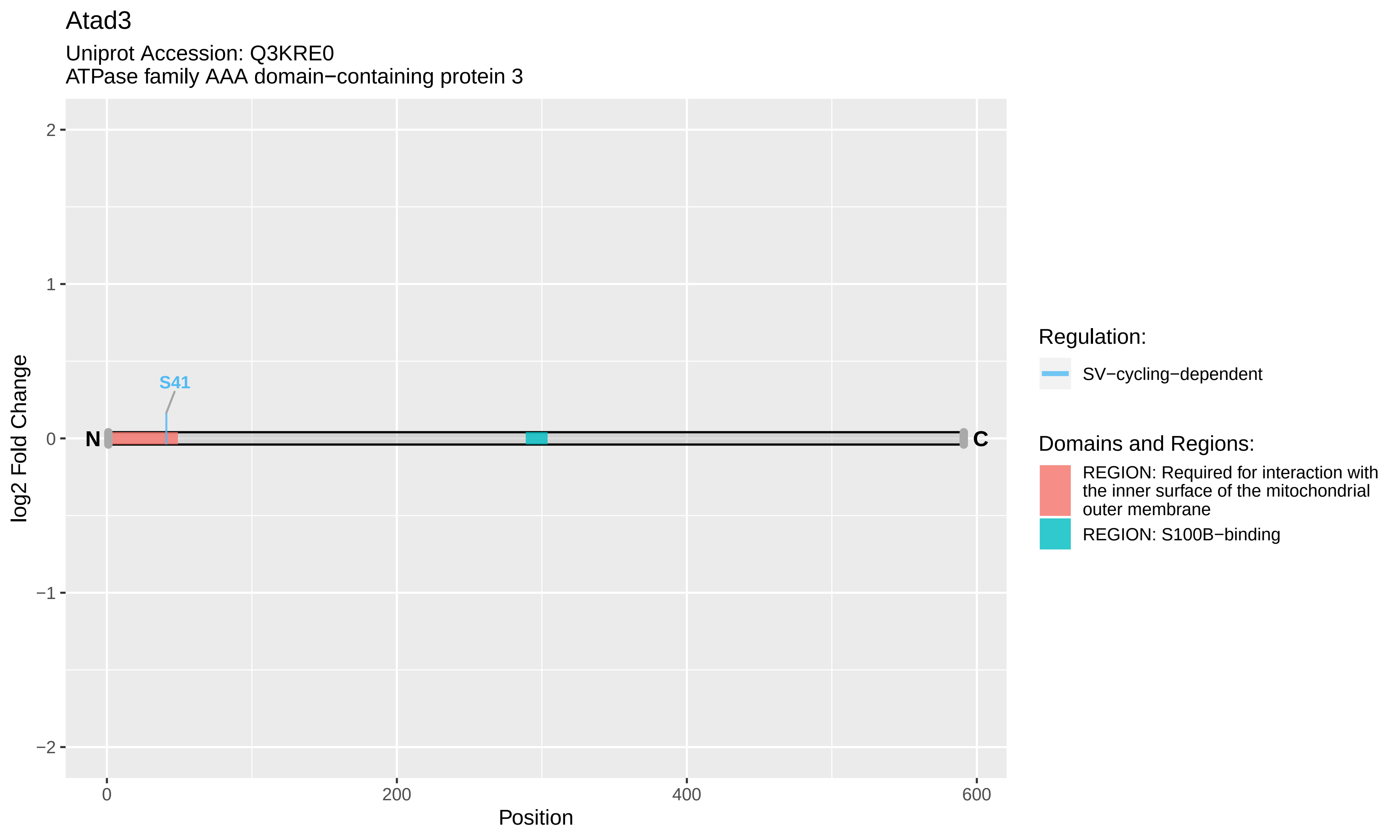
Atad3
Uniprot Accession: Q3KRE0
ATPase family AAA domain−containing protein 3
S41
N
C
2
1
0
−1
−2
log2 Fold Change
0
200
400
600
Position
Regulation:
SV−cycling−dependent
Domains and Regions:
REGION: Required for interaction with the inner surface of the mitochondrial outer membrane
REGION: S100B−binding

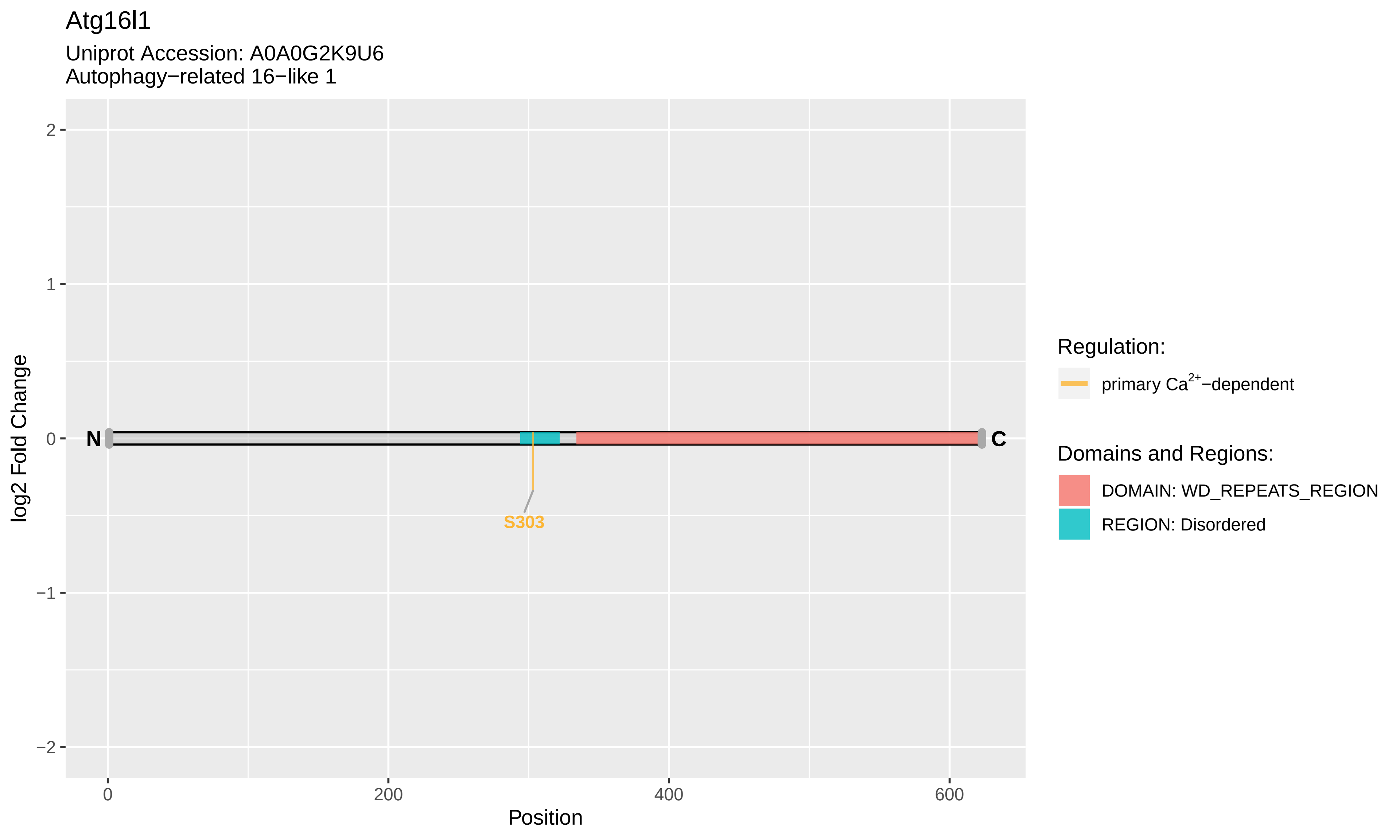

Atg16l1
Uniprot Accession: A0A0G2K9U6
Autophagy−related 16−like 1
log2 Fold Change
2
1
0
−1
−2
N
C
S303
0
200
400
600
Position
Regulation:
primary Ca2+−dependent
Domains and Regions:
DOMAIN: WD_REPEATS_REGION
REGION: Disordered

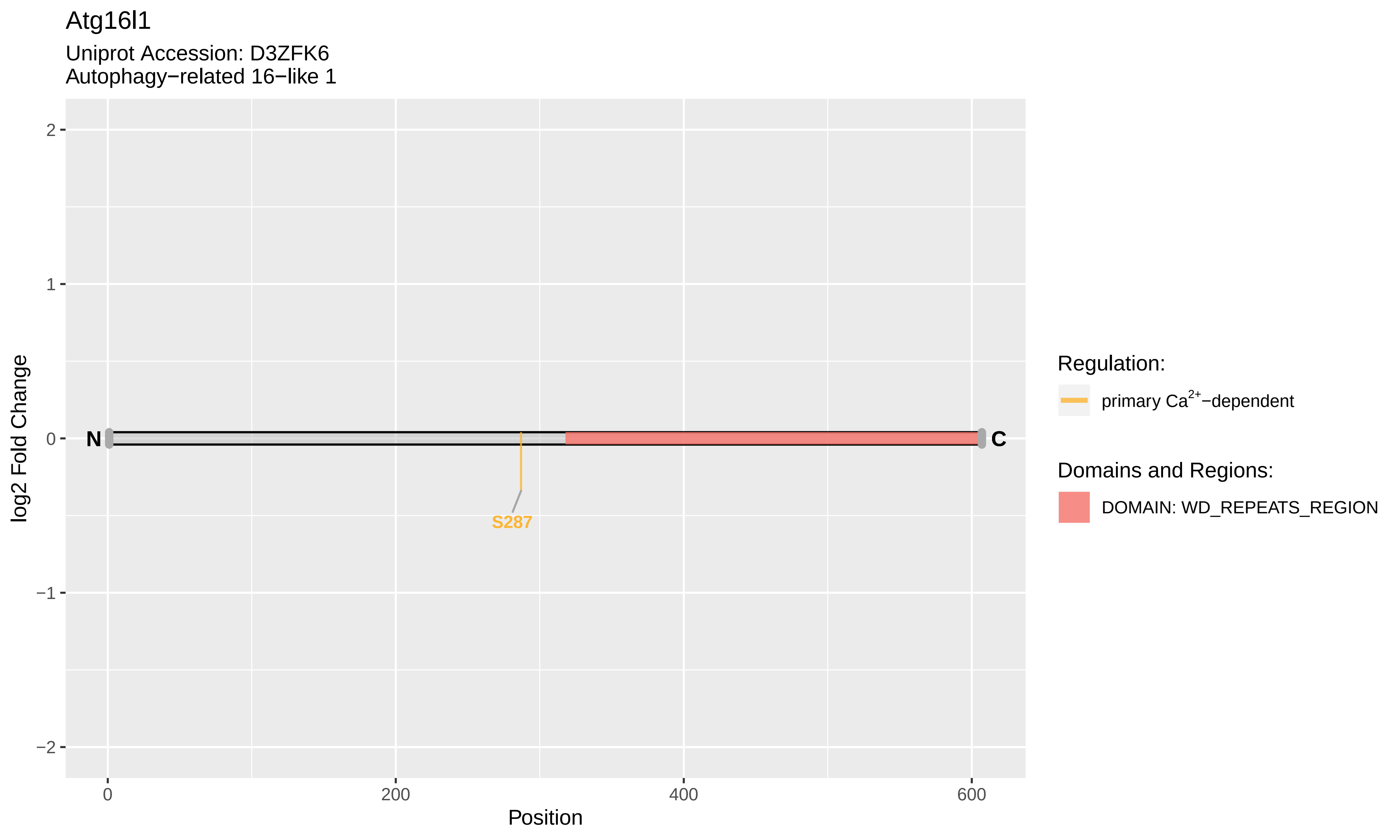

Atg16l1
Uniprot Accession: D3ZFK6
Autophagy−related 16−like 1
log2 Fold Change
2
1
0
−1
−2
N
C
S287
0
200
400
600
Position
Regulation:
primary Ca2+−dependent
Domains and Regions:
DOMAIN: WD_REPEATS_REGION

Atg2b

Uniprot Accession: F1MAF8
Autophagy related 2B

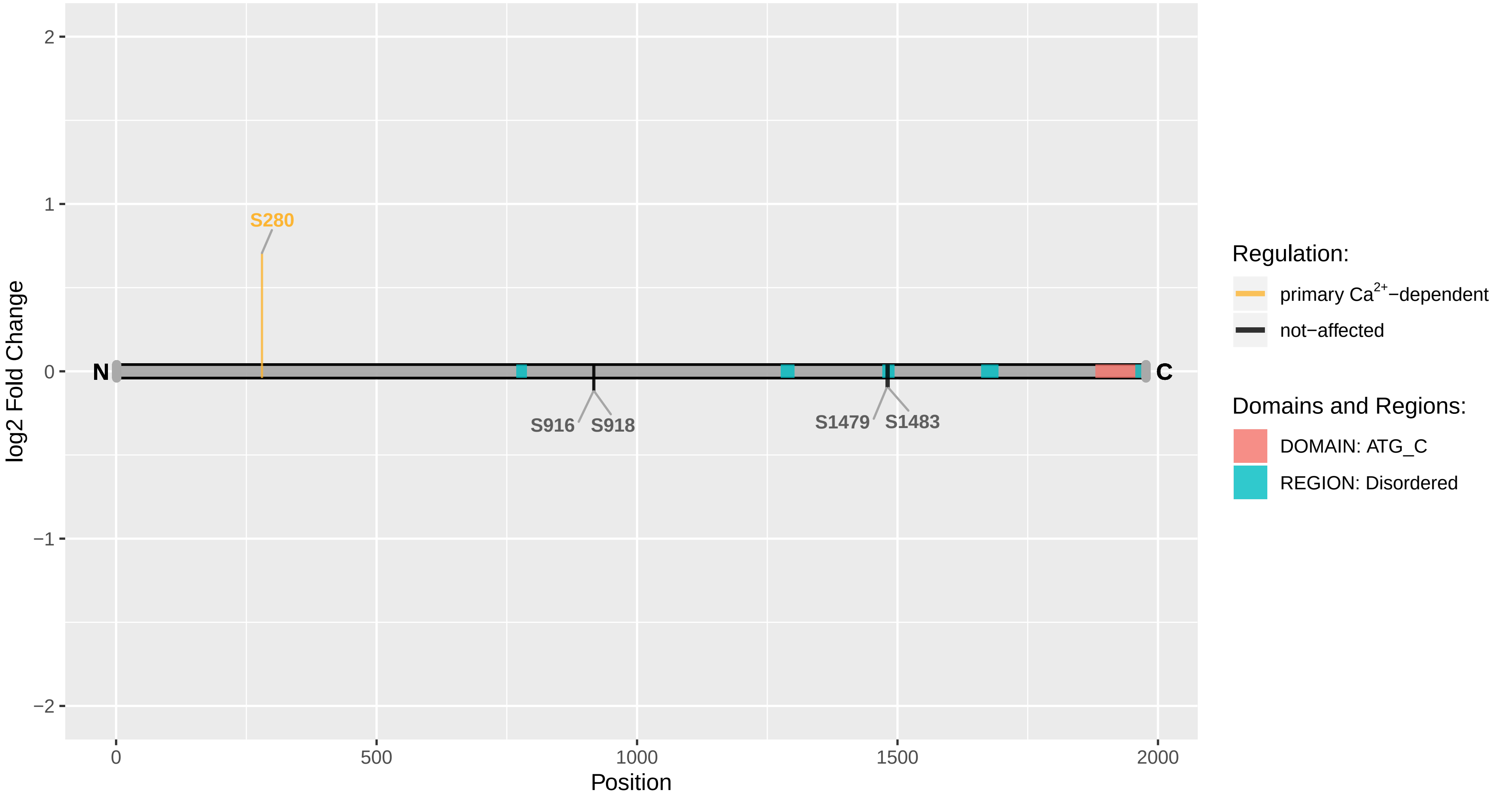

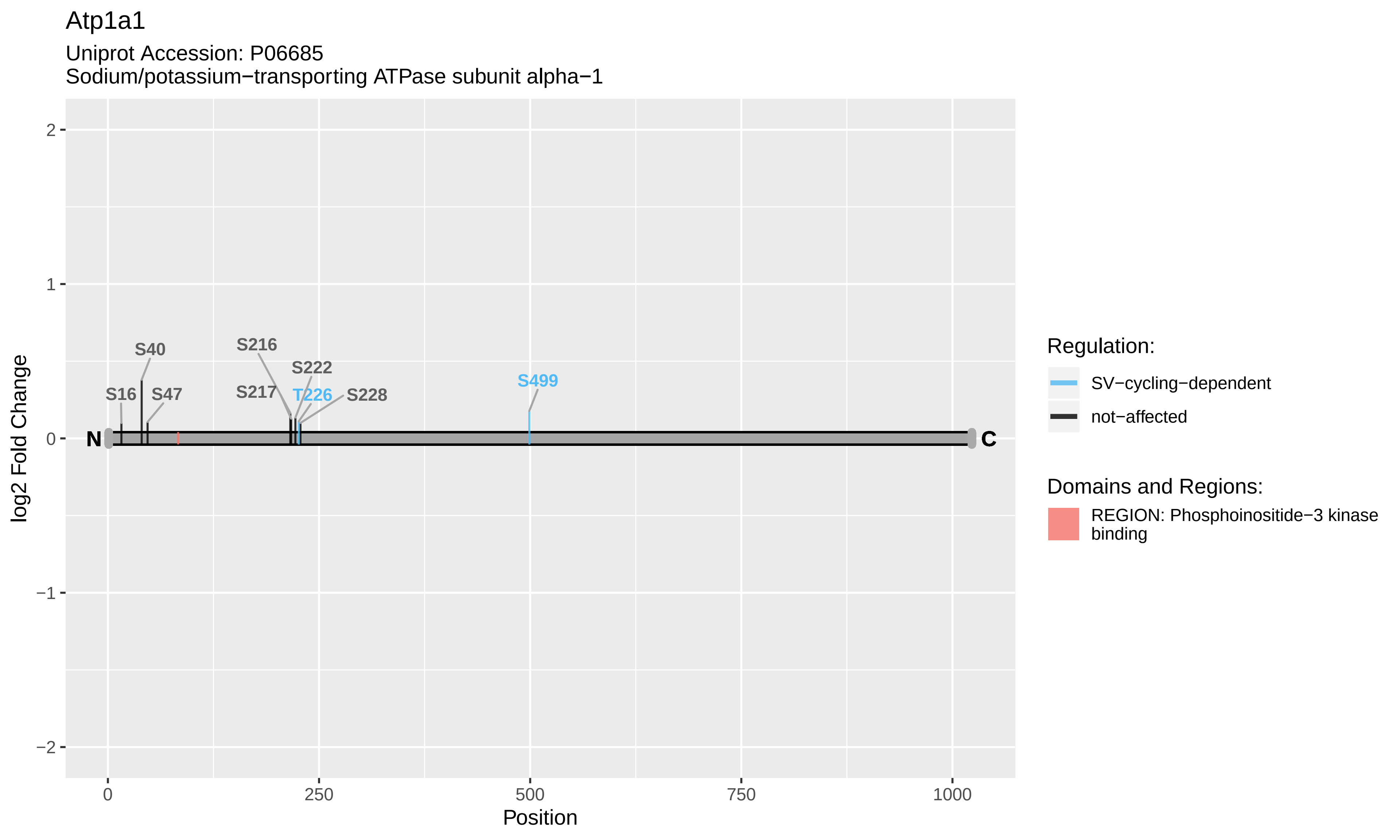

Atp1a1
Uniprot Accession: P06685
Sodium/potassium−transporting ATPase subunit alpha−1
log2 Fold Change
Position
2
1
0
−1
−2
0
250
500
750
1000
N
C
S16
S40
S47
S216
S217
S222
T226
S228
S499
Regulation:
SV−cycling−dependent
not−affected
Domains and Regions:
REGION: Phosphoinositide−3 kinase binding

Atp1a2

Uniprot Accession: P06686
Sodium/potassium−transporting ATPase subunit alpha−2

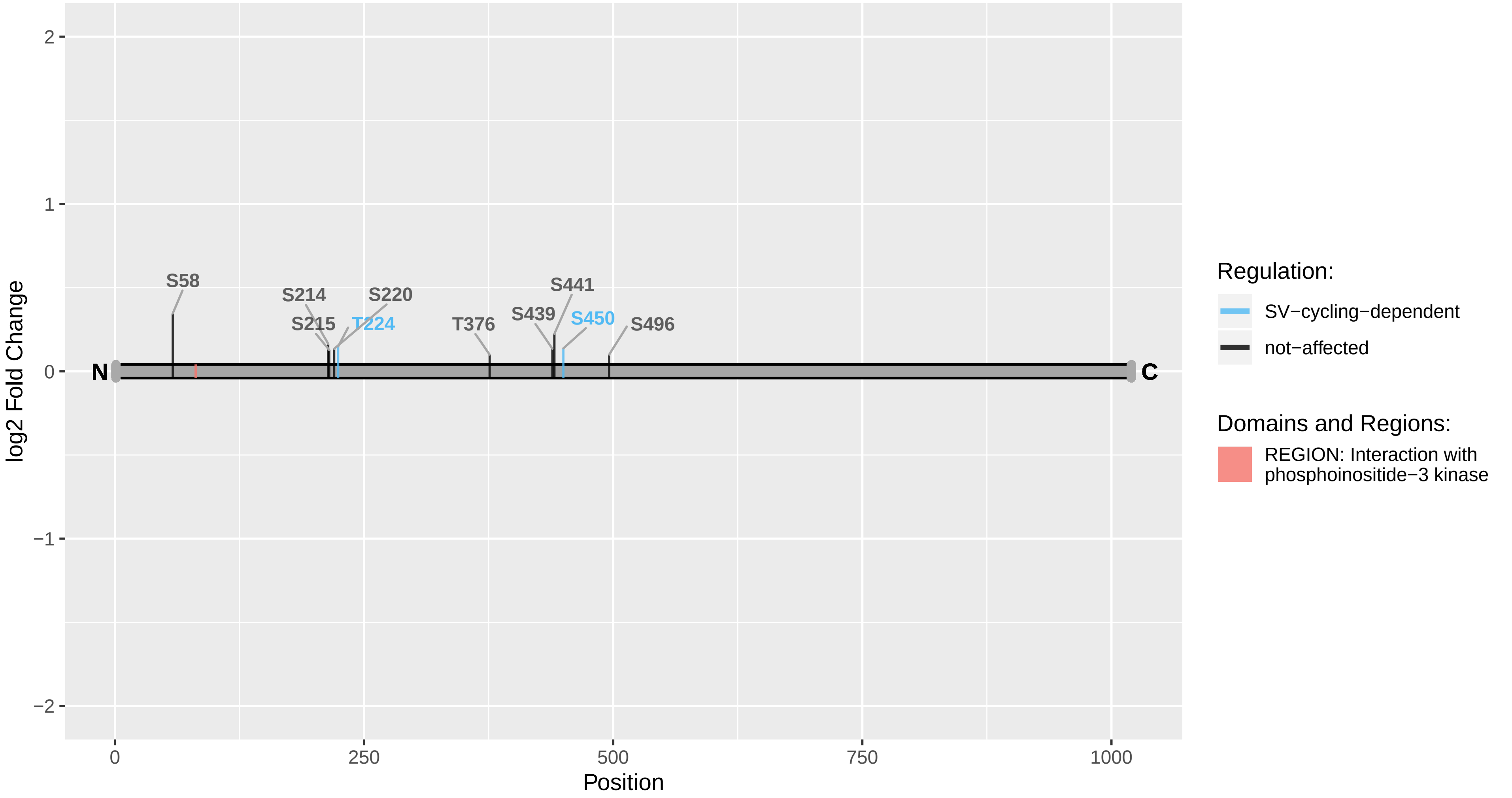

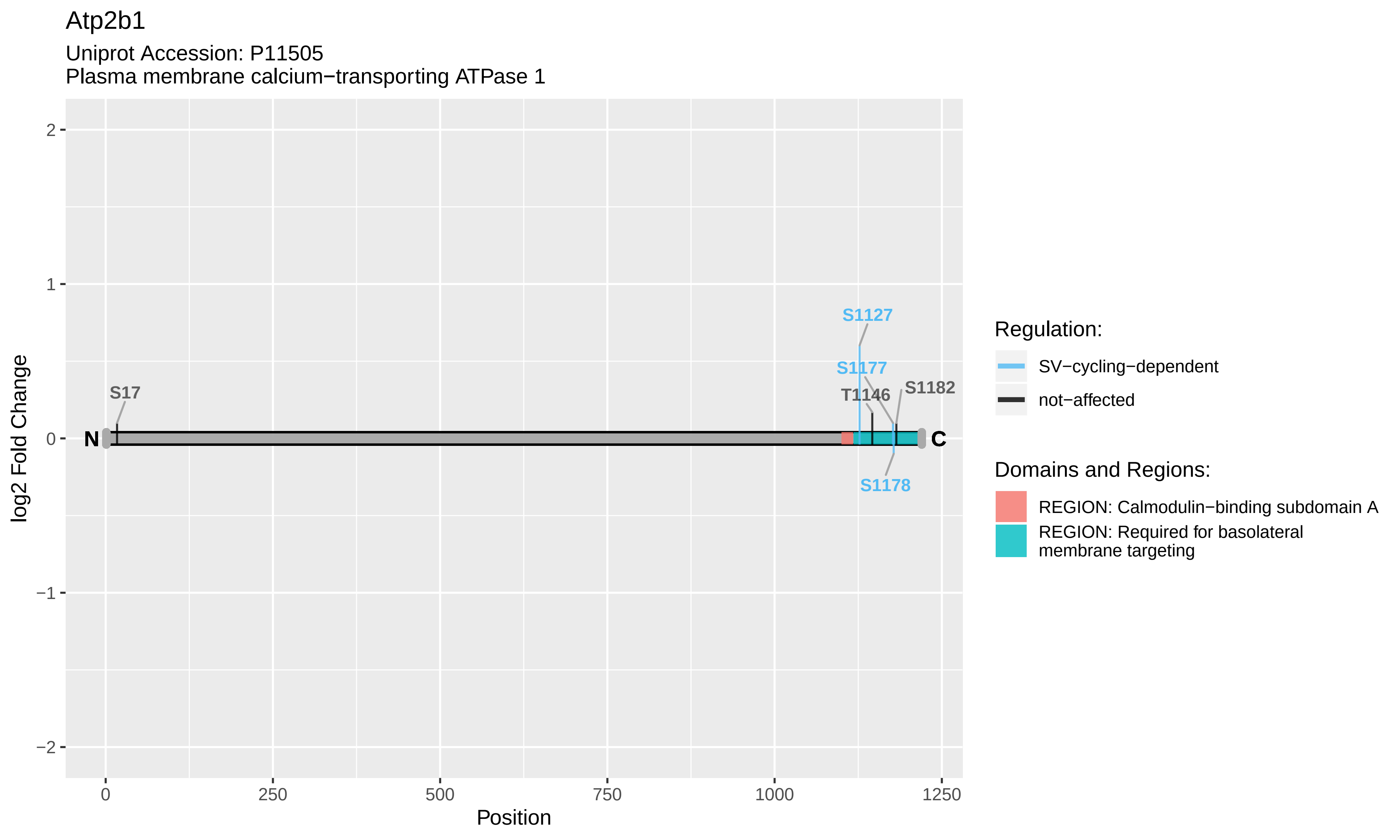

Atp2b1
Uniprot Accession: P11505
Plasma membrane calcium−transporting ATPase 1
log2 Fold Change
Position
2
1
0
−1
−2
0
250
500
750
1000
1250
N
C
S17
S1127
S1177
T1146
S1182
S1178
Regulation:
SV−cycling−dependent
not−affected
Domains and Regions:
REGION: Calmodulin−binding subdomain A
REGION: Required for basolateral membrane targeting

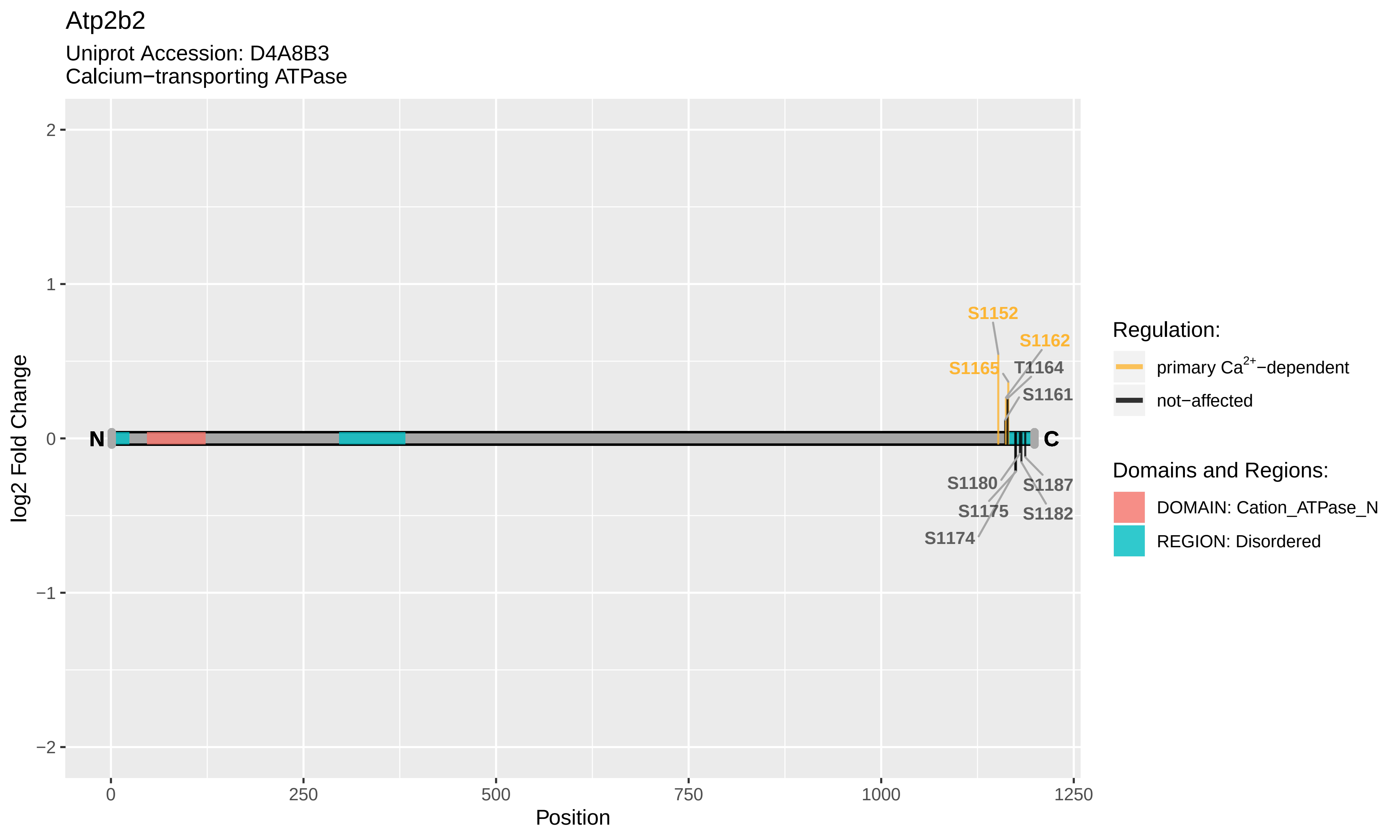

Atp2b2
Uniprot Accession: D4A8B3
Calcium−transporting ATPase
log2 Fold Change
2
1
0
−1
−2
N
C
S1152
S1162
S1165
T1164
S1161
S1180
S1187
S1175
S1182
S1174
0
250
500
750
1000
1250
Position
Regulation:
primary Ca2+−dependent
not−affected
Domains and Regions:
DOMAIN: Cation_ATPase_N
REGION: Disordered

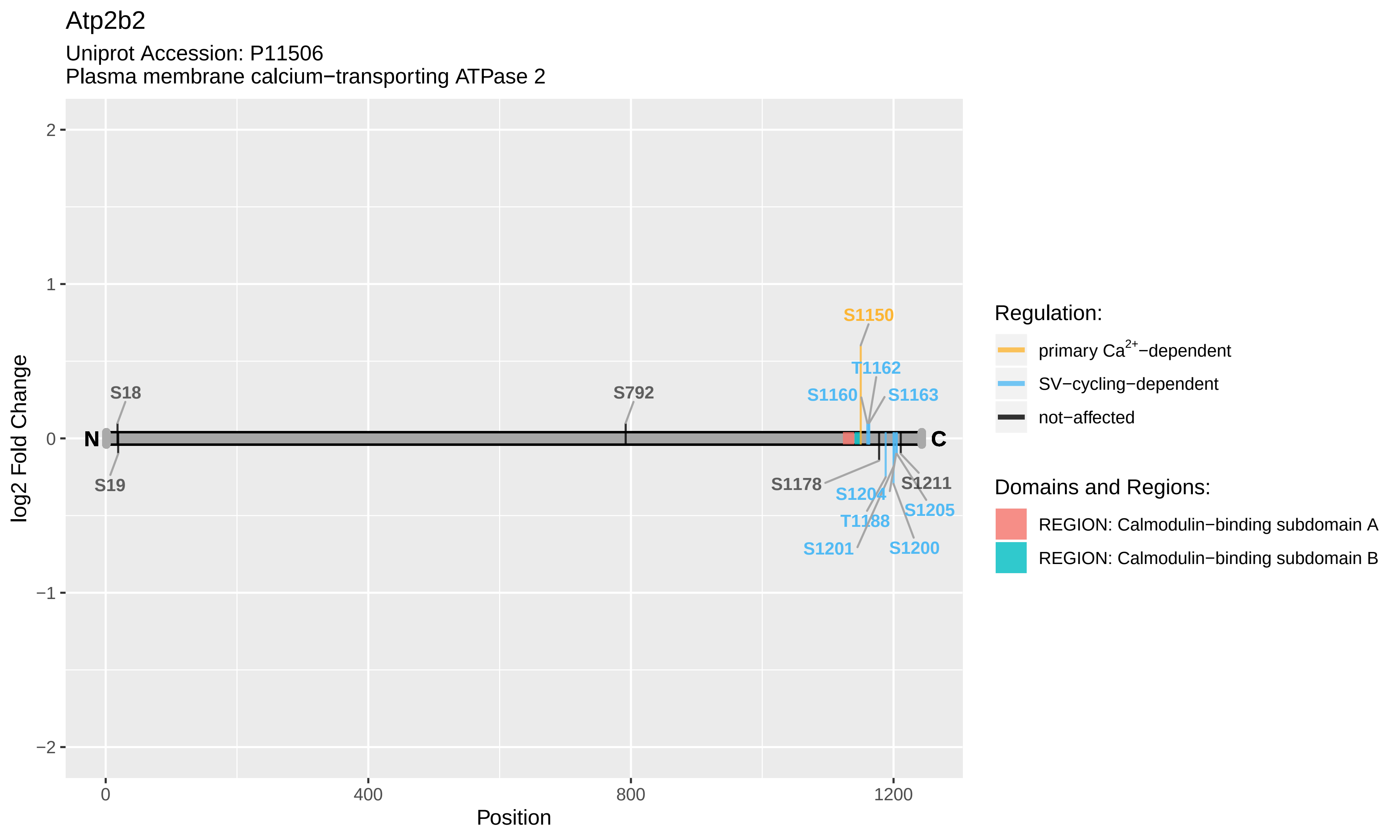

Atp2b2
Uniprot Accession: P11506
Plasma membrane calcium−transporting ATPase 2
log2 Fold Change
Position
N
C
S18
S19
S792
S1150
T1162
S1160
S1163
S1178
S1204
S1211
S1205
T1188
S1201
S1200
Regulation:
primary Ca2+−dependent
SV−cycling−dependent
not−affected
Domains and Regions:
REGION: Calmodulin−binding subdomain A
REGION: Calmodulin−binding subdomain B

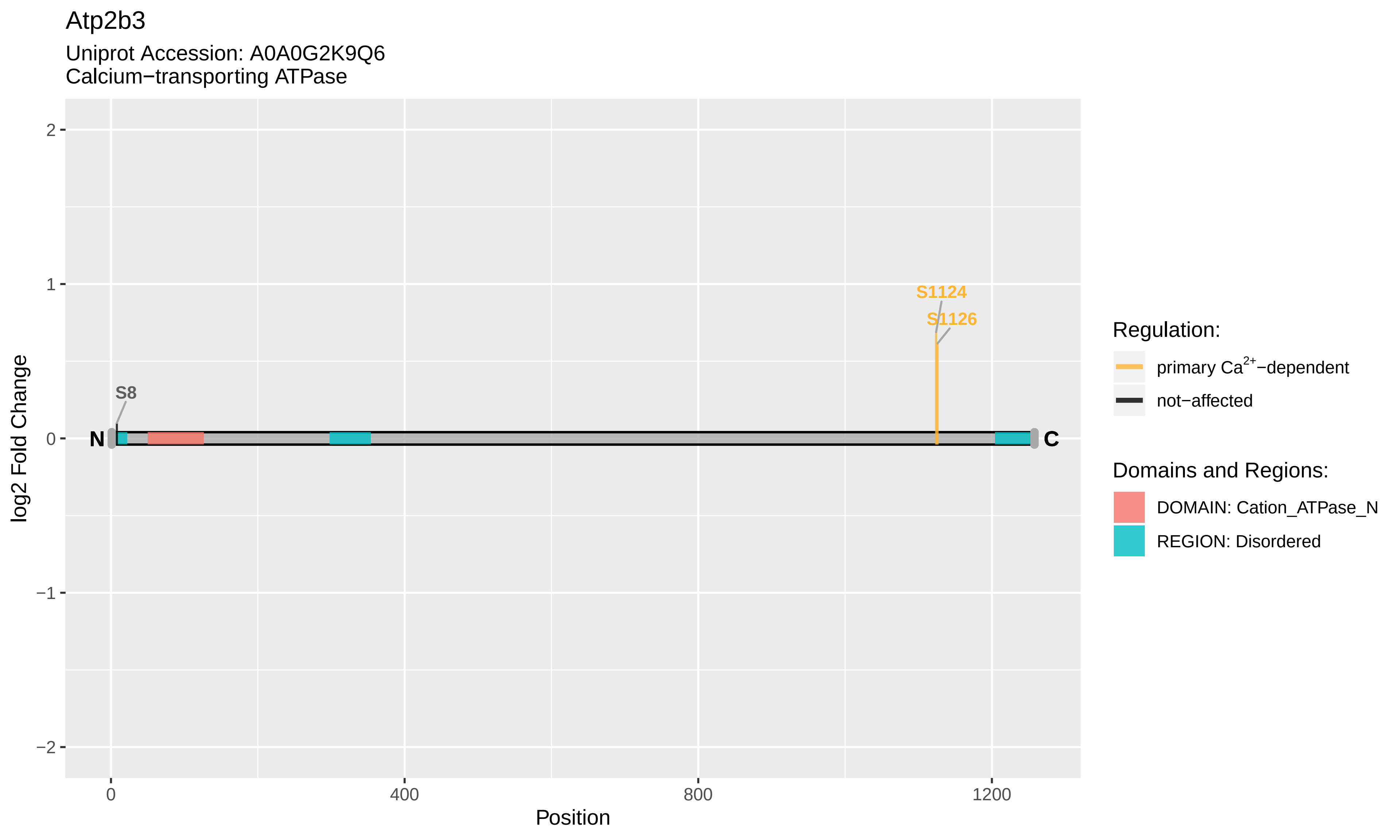
Atp2b3
Uniprot Accession: A0A0G2K9Q6
Calcium−transporting ATPase
S1124
S1126
S8
N
C
log2 Fold Change
Position
2
1
0
−1
−2
0
400
800
1200
Regulation:
primary Ca^{2+}−dependent
not−affected
Domains and Regions:
DOMAIN: Cation_ATPase_N
REGION: Disordered

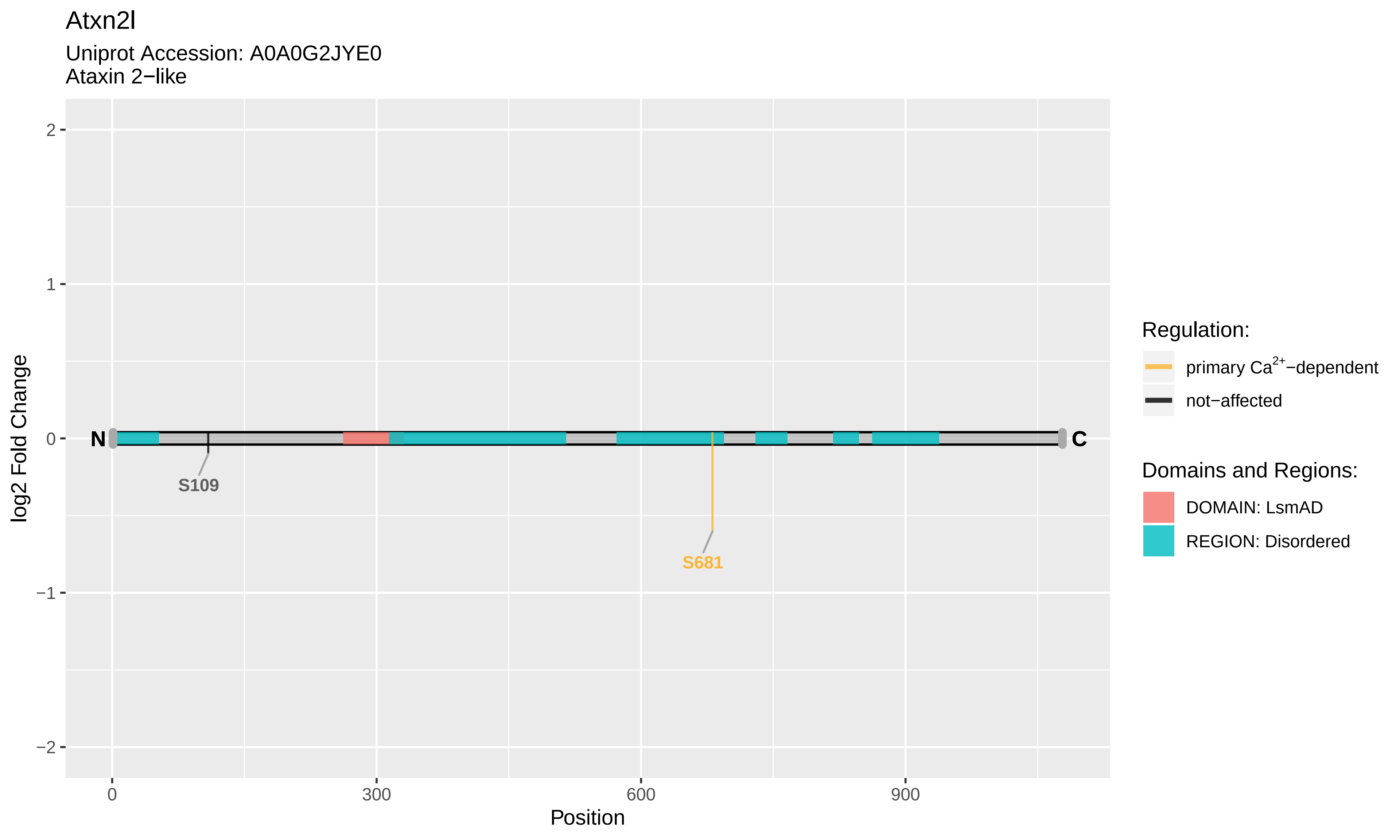
Atxn2l
Uniprot Accession: A0A0G2JYE0
Ataxin 2−like
log2 Fold Change
2
1
0
−1
−2
N
C
S109
S681
0
300
600
900
Position
Regulation:
primary Ca2+−dependent
not−affected
Domains and Regions:
DOMAIN: LsmAD
REGION: Disordered

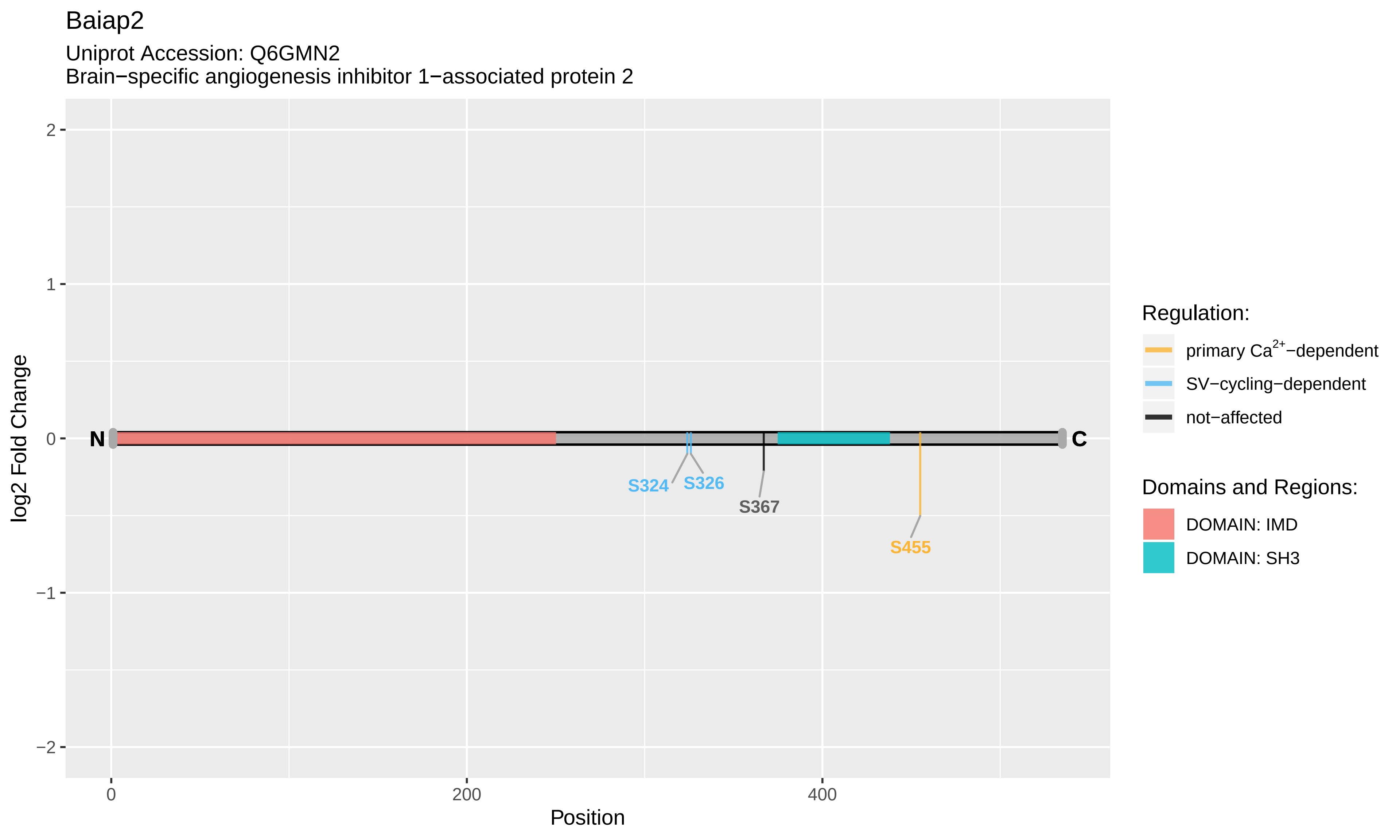

Baiap2
Uniprot Accession: Q6GMN2
Brain−specific angiogenesis inhibitor 1−associated protein 2
2
1
0
−1
−2
log2 Fold Change
N
C
S324
S326
S367
S455
0
200
400
Position
Regulation:
primary Ca2+−dependent
SV−cycling−dependent
not−affected
Domains and Regions:
DOMAIN: IMD
DOMAIN: SH3

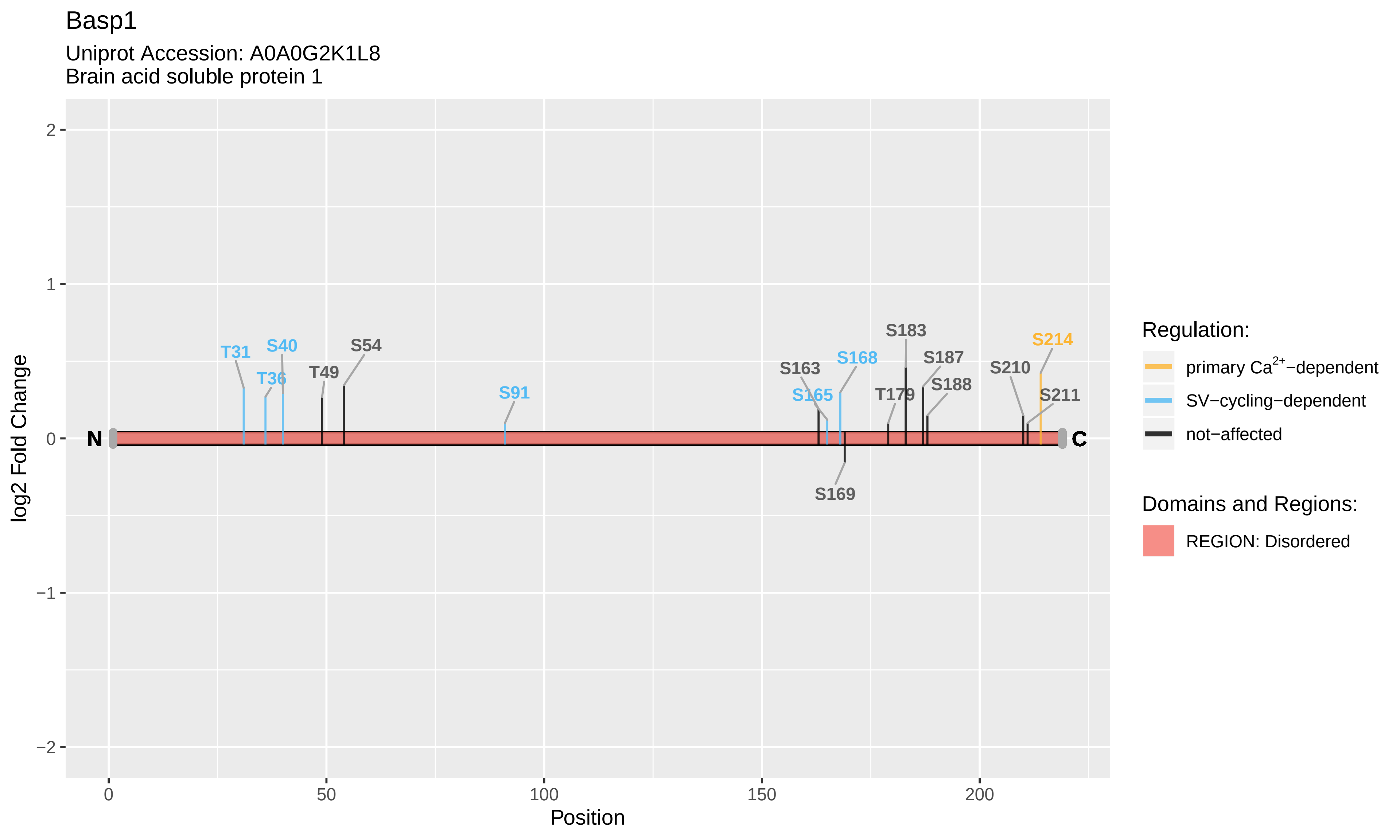
Basp1
Uniprot Accession: A0A0G2K1L8
Brain acid soluble protein 1
log2 Fold Change
Position
N
C
T31
S40
T36
T49
S54
S91
S163
S165
S168
S169
S183
T179
S187
S188
S210
S214
S211
2
1
0
−1
−2
0
50
100
150
200
Regulation:
primary Ca2+−dependent
SV−cycling−dependent
not−affected
Domains and Regions:
REGION: Disordered

Basp1

Uniprot Accession: Q05175
Brain acid soluble protein 1

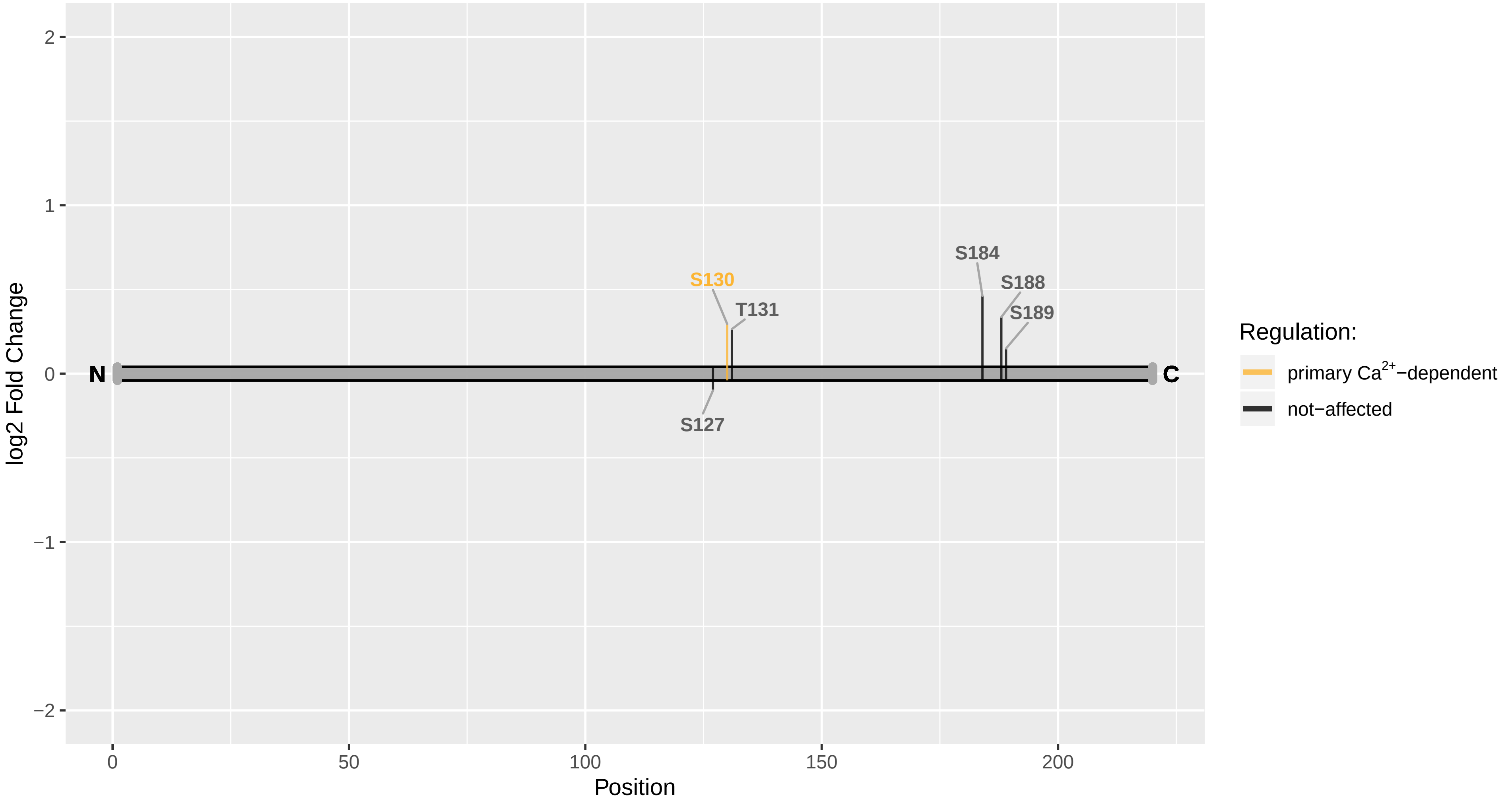

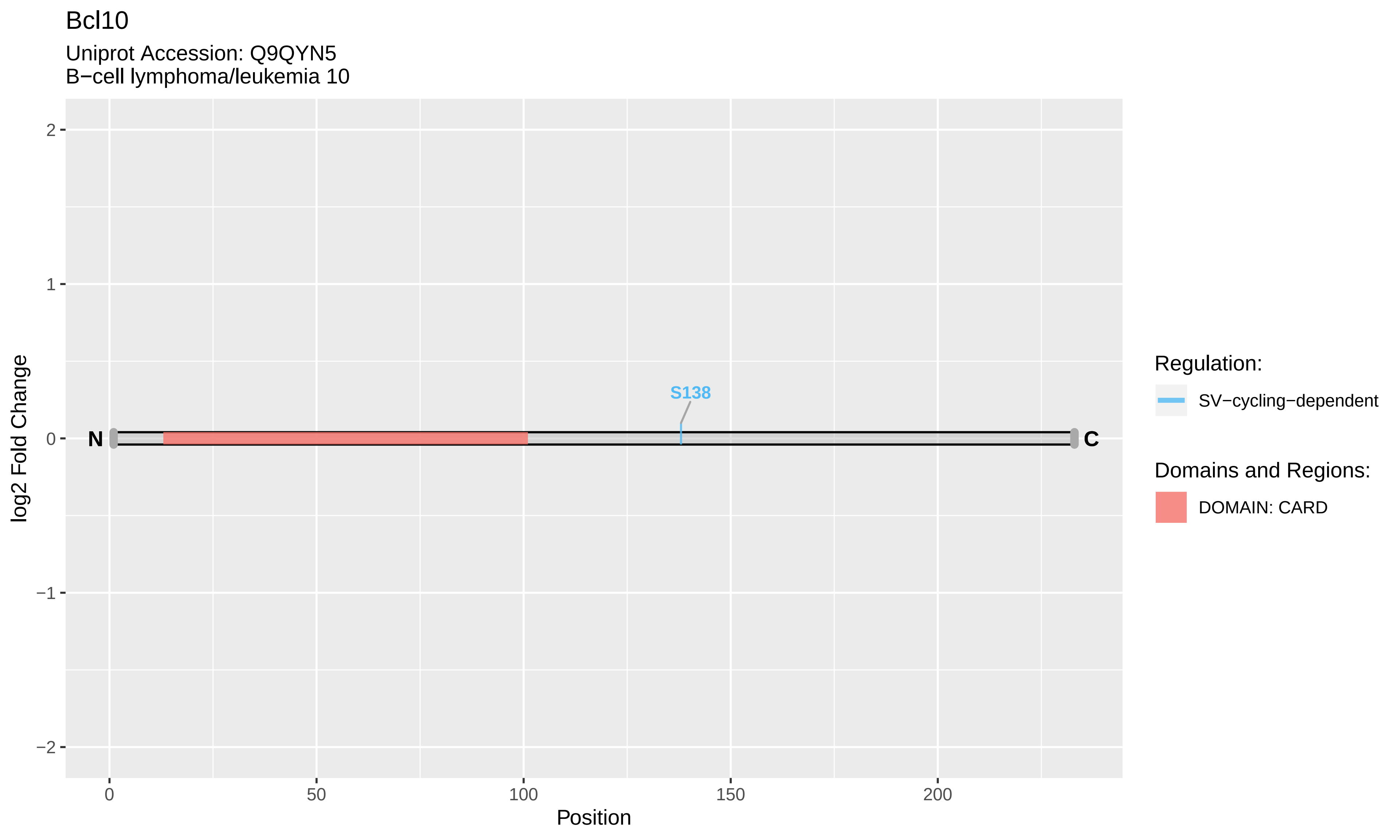

Bcl10
Uniprot Accession: Q9QYN5
B−cell lymphoma/leukemia 10
log2 Fold Change
2
1
0
−1
−2
N
C
S138
0
50
100
150
200
Position
Regulation:
SV−cycling−dependent
Domains and Regions:
DOMAIN: CARD

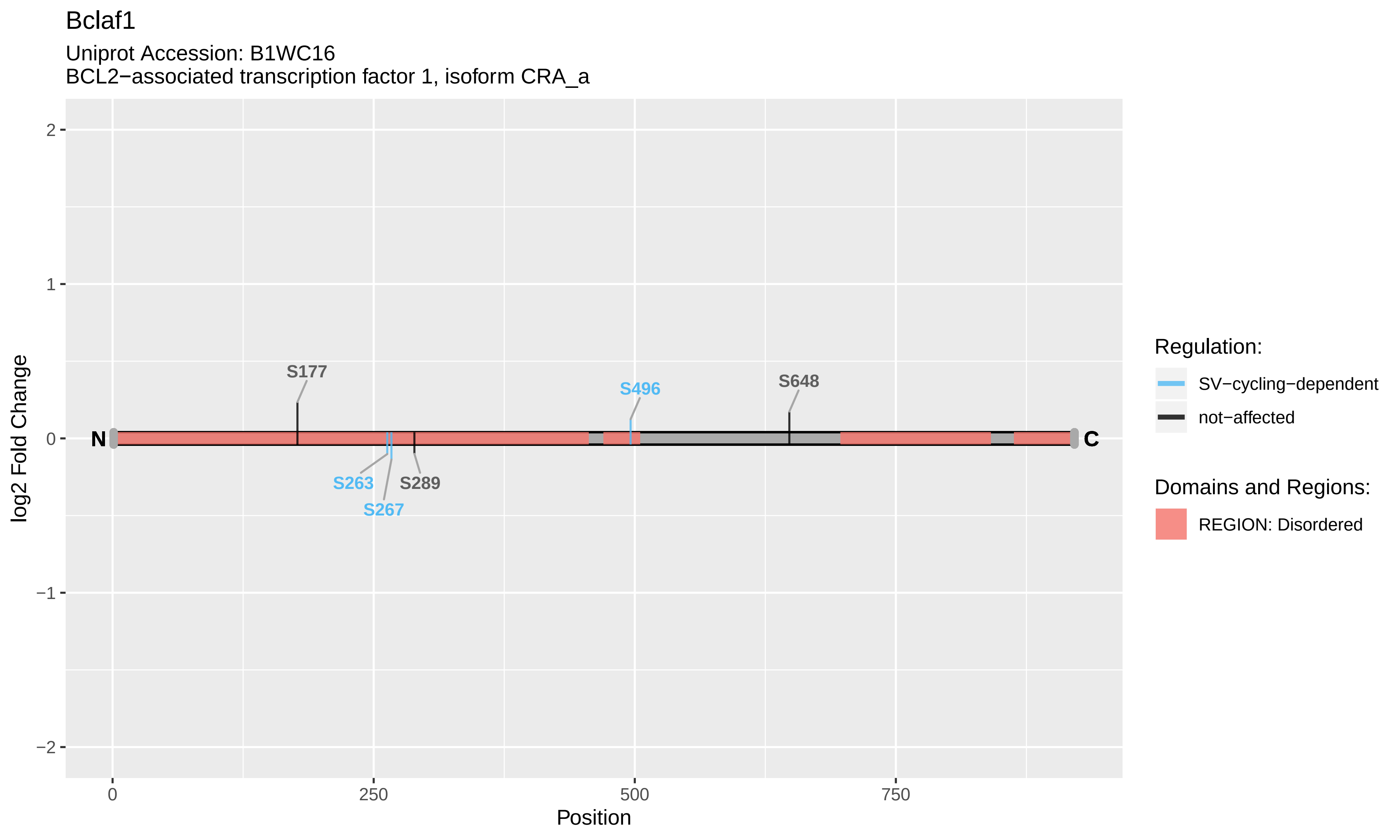

Bclaf1
Uniprot Accession: B1WC16
BCL2−associated transcription factor 1, isoform CRA_a
log2 Fold Change
2
1
0
−1
−2
N
C
S177
S496
S648
S263
S267
S289
0
250
500
750
Position
Regulation:
SV−cycling−dependent
not−affected
Domains and Regions:
REGION: Disordered

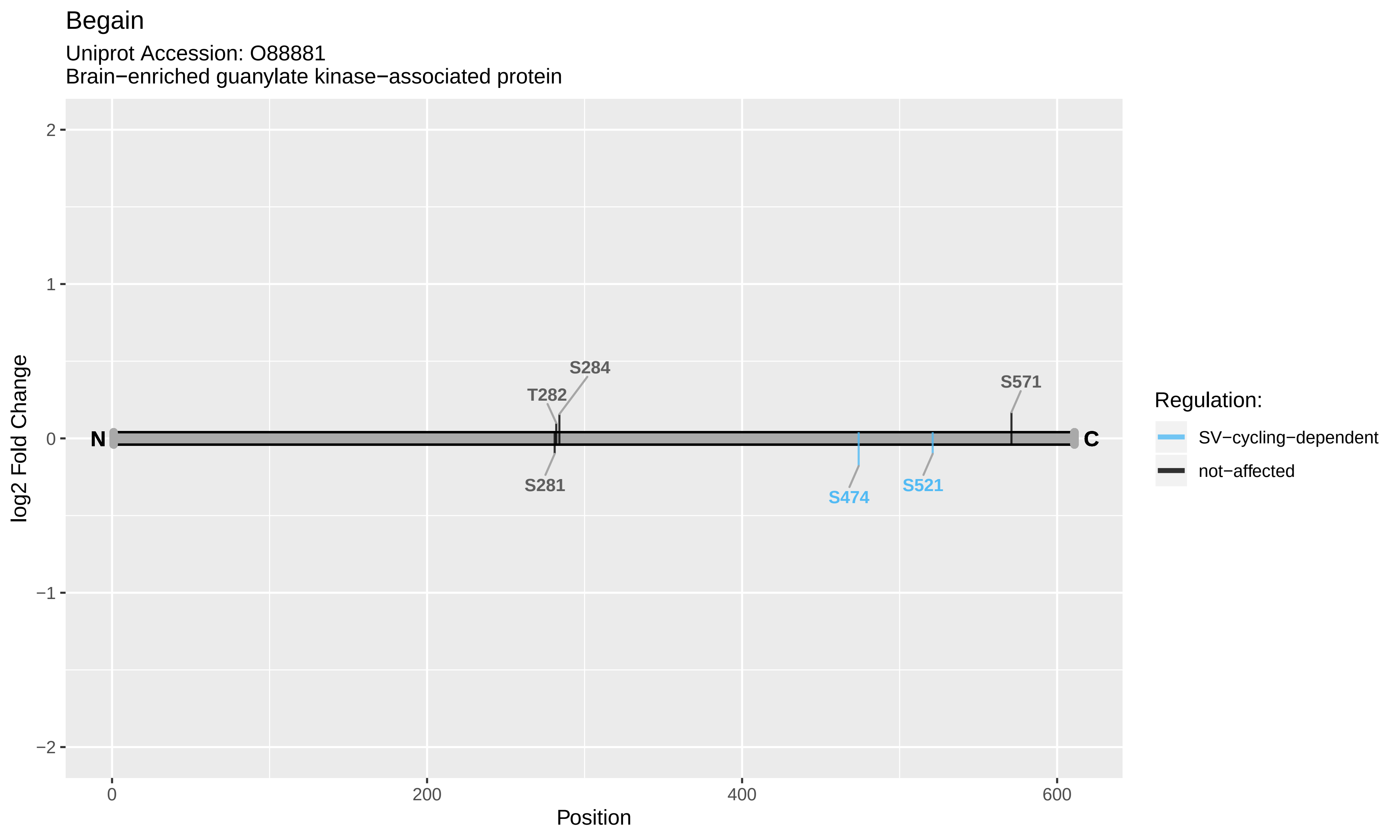
Begain
Uniprot Accession: O88881
Brain−enriched guanylate kinase−associated protein
log2 Fold Change
Position
N
C
S284
T282
S281
S474
S521
S571
Regulation:
SV−cycling−dependent
not−affected
2
1
0
−1
−2
0
200
400
600

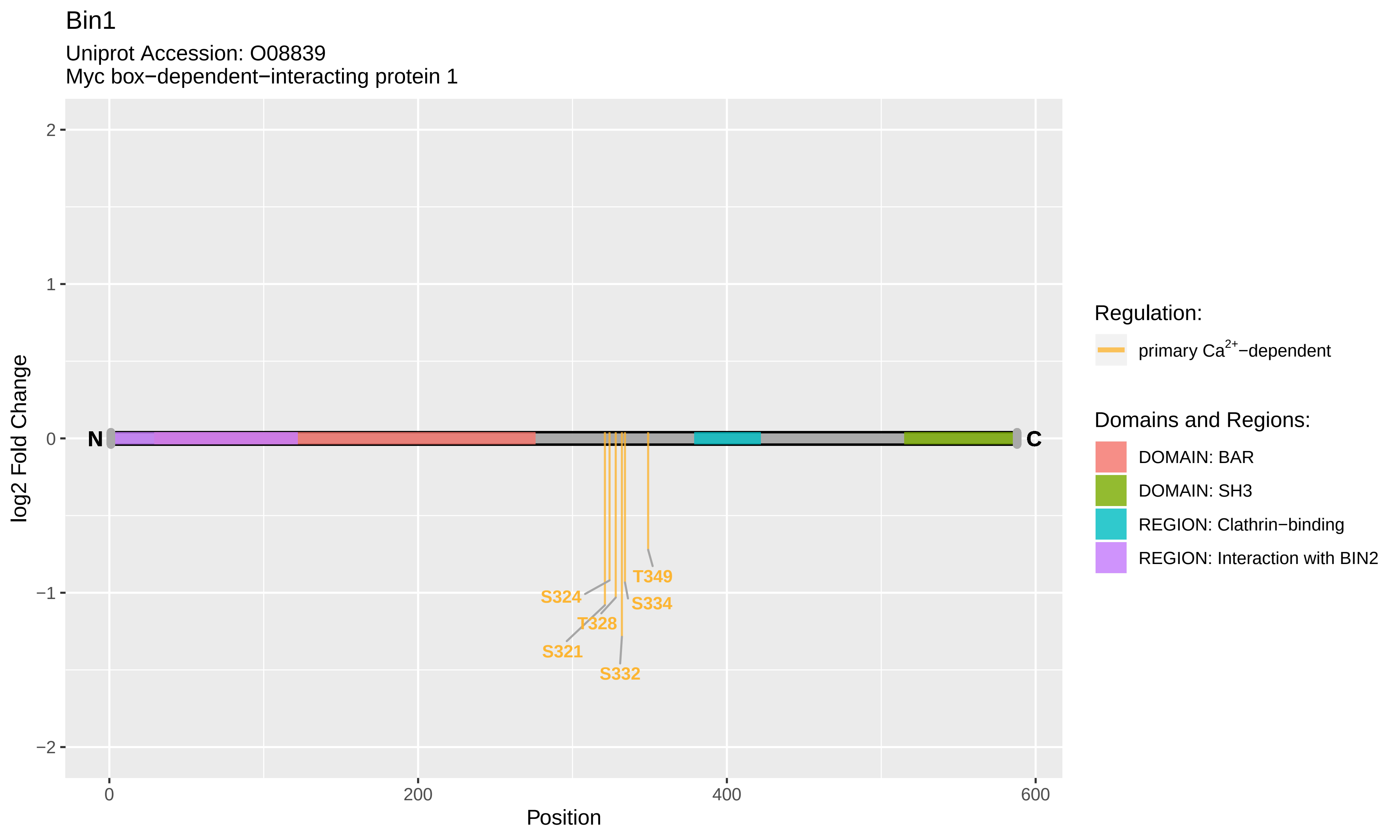

Bin1
Uniprot Accession: O08839
Myc box−dependent−interacting protein 1
log2 Fold Change
2
1
0
−1
−2
N
C
T349
S324
S334
T328
S321
S332
0
200
400
600
Position
Regulation:
primary Ca2+−dependent
Domains and Regions:
DOMAIN: BAR
DOMAIN: SH3
REGION: Clathrin−binding
REGION: Interaction with BIN2

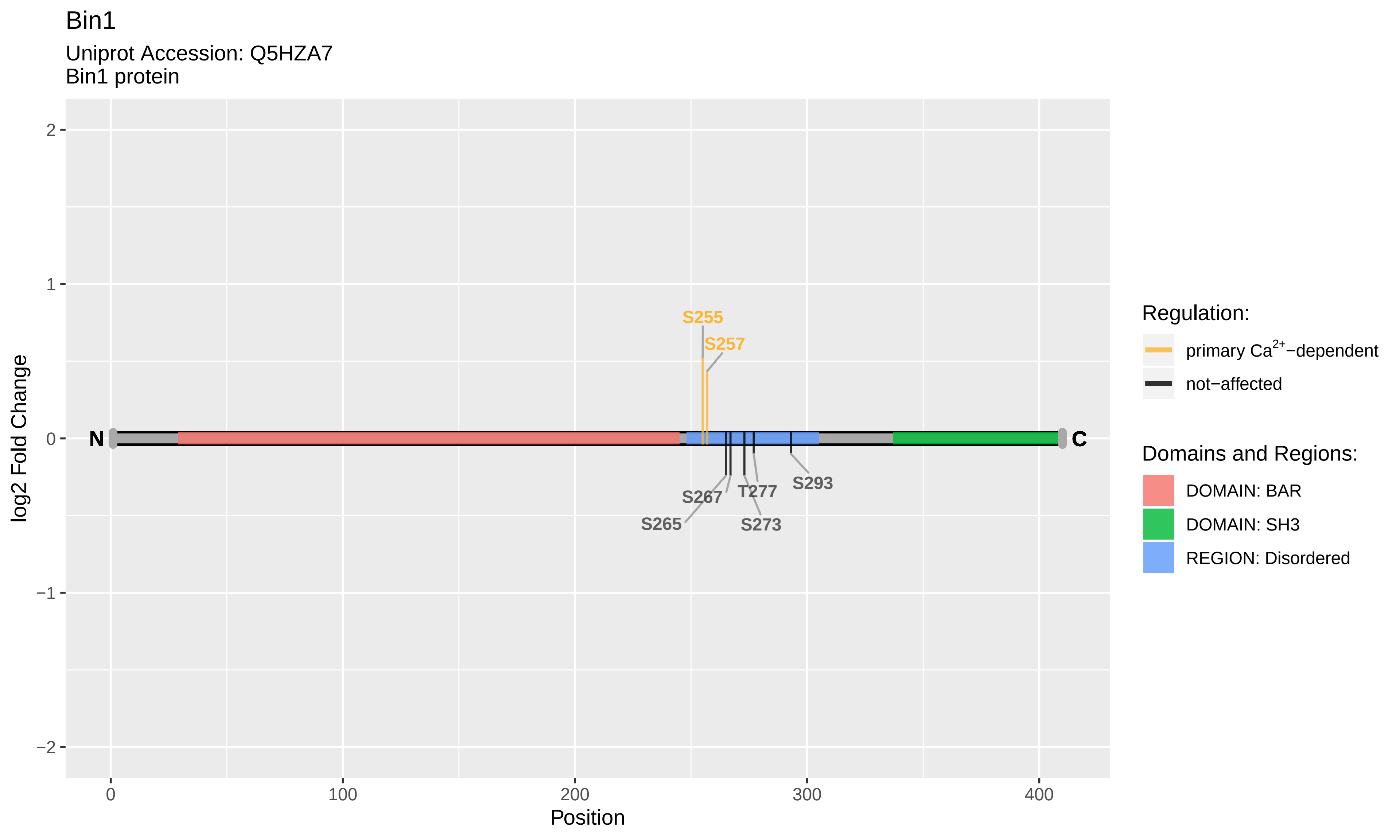
Bin1
Uniprot Accession: Q5HZA7
Bin1 protein
log2 Fold Change
Position
2
1
0
−1
−2
0
100
200
300
400
N
C
S255
S257
S267
S265
T277
S273
S293
Regulation:
primary Ca2+−dependent
not−affected
Domains and Regions:
DOMAIN: BAR
DOMAIN: SH3
REGION: Disordered

Bri3bp

Uniprot Accession: Q5U3Z5
BRI3−binding protein

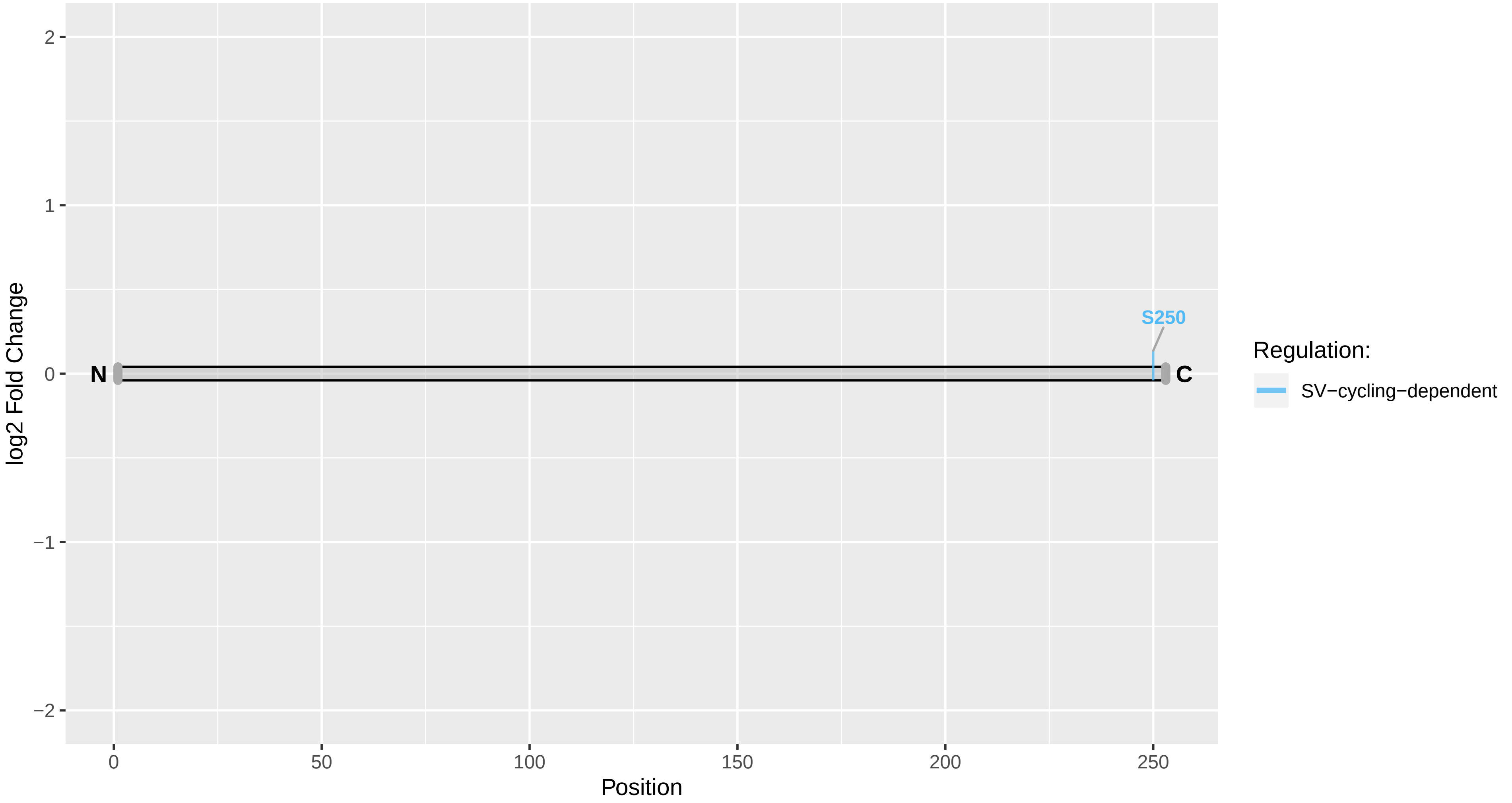

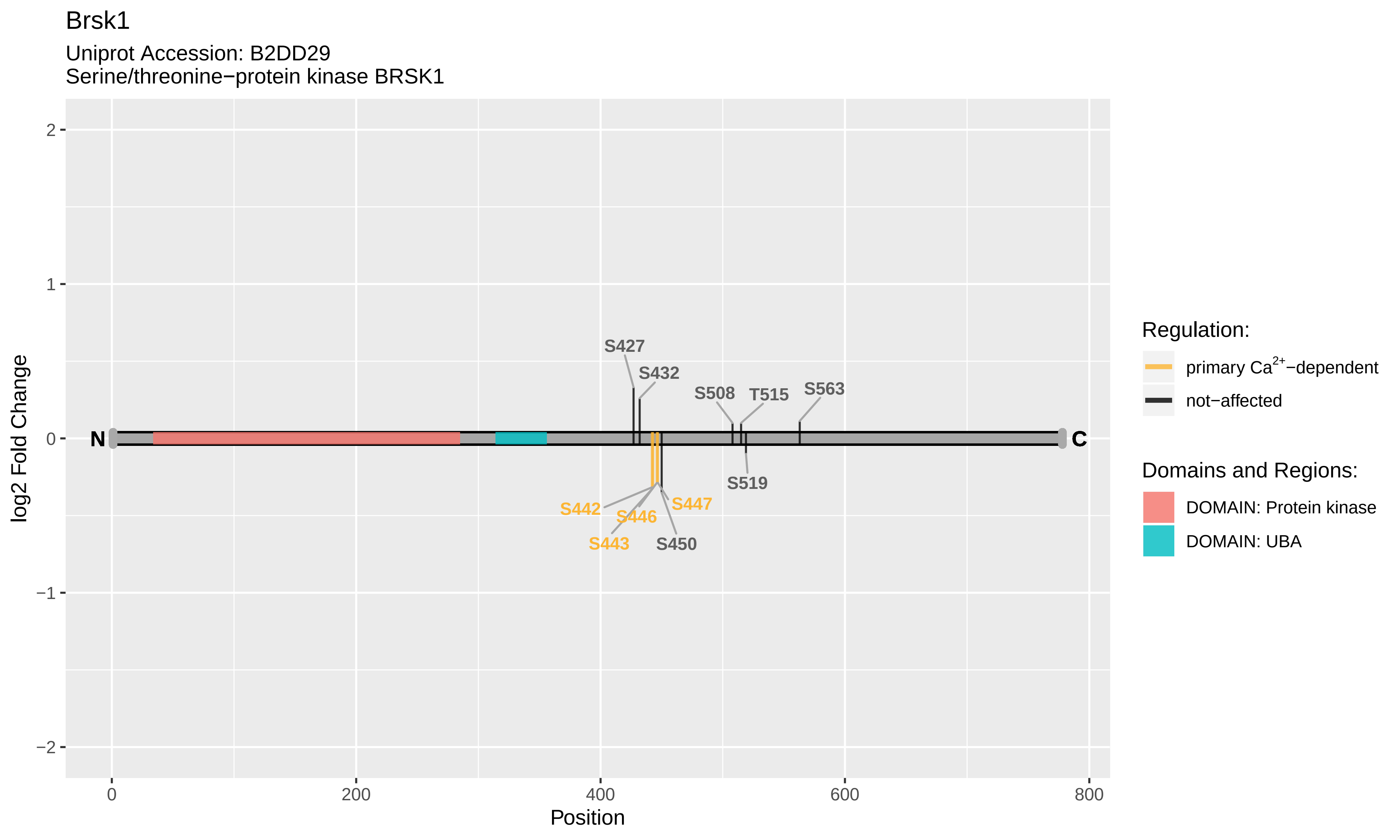

Brsk1
Uniprot Accession: B2DD29
Serine/threonine−protein kinase BRSK1
log2 Fold Change
2
1
0
−1
−2
N
C
S427
S432
S508
T515
S563
S519
S442
S446
S447
S443
S450
0
200
400
600
800
Position
Regulation:
primary Ca2+−dependent
not−affected
Domains and Regions:
DOMAIN: Protein kinase
DOMAIN: UBA

Brsk2

Uniprot Accession: D3ZML2
Serine/threonine−protein kinase BRSK2

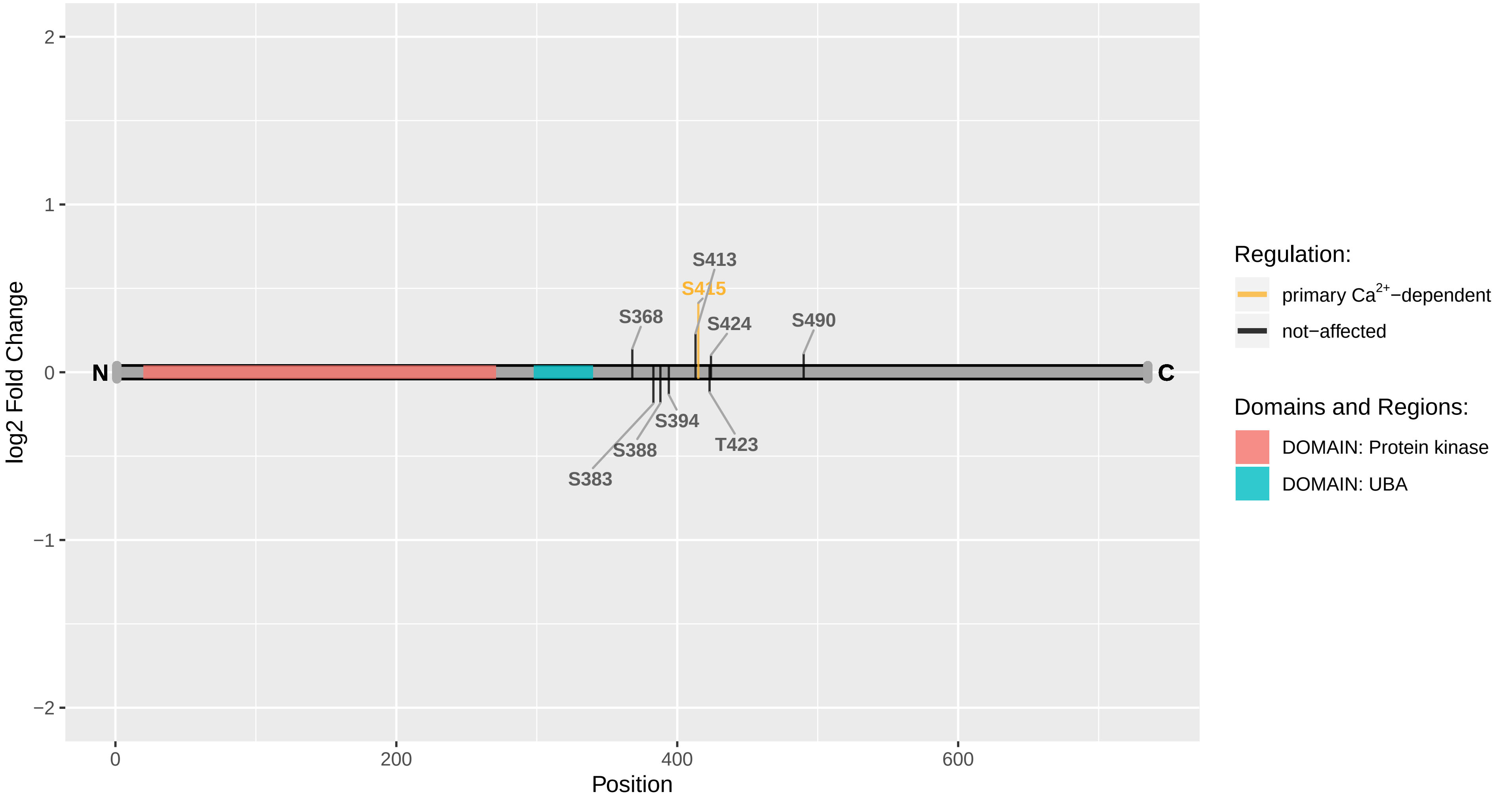

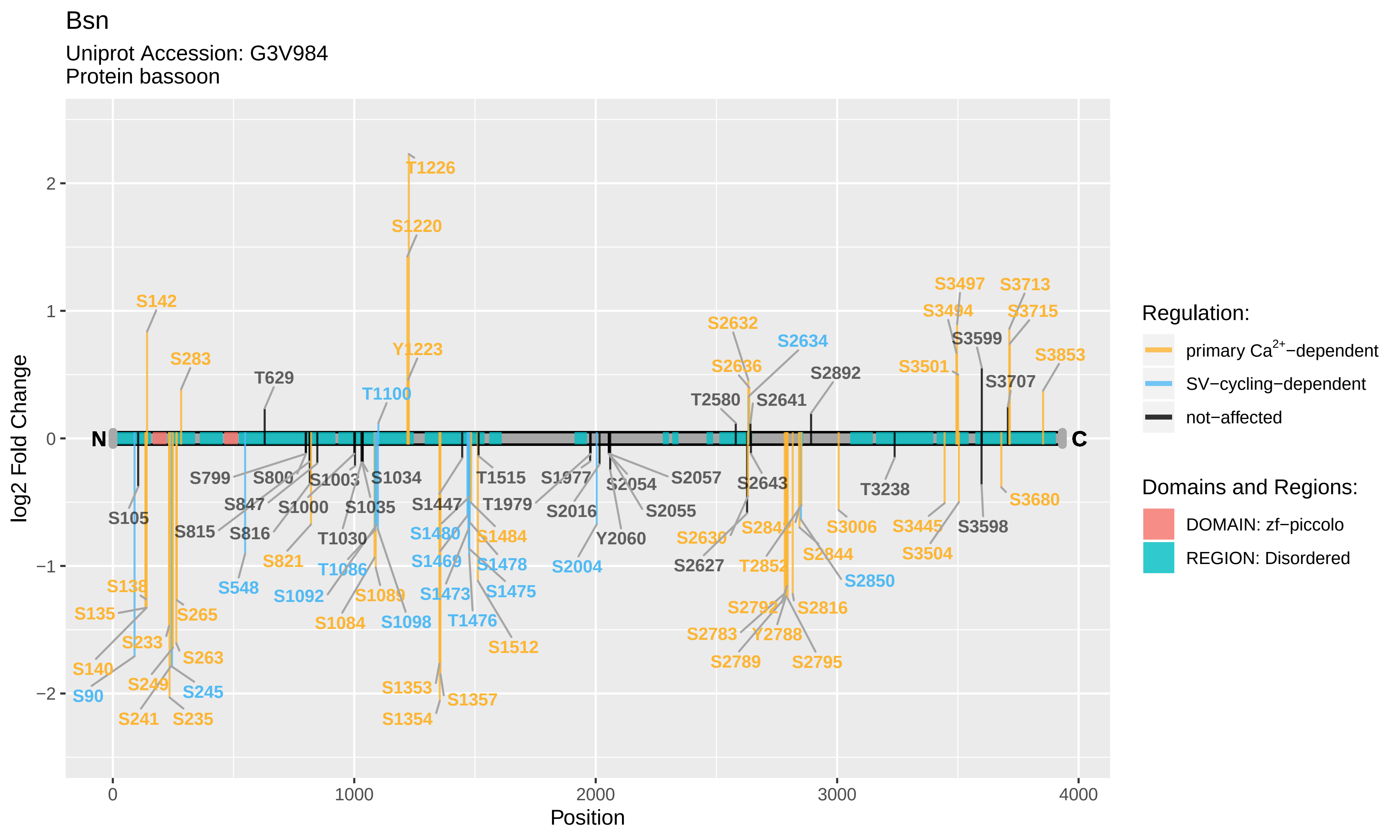
Bsn
Uniprot Accession: G3V984
Protein bassoon
log2 Fold Change
Position
Regulation:
primary Ca2+−dependent
SV−cycling−dependent
not−affected
Domains and Regions:
DOMAIN: zf−piccolo
REGION: Disordered
N
C
T1226
S1220
Y1223
T1100
S142
S283
T629
S3497
S3713
S3494
S3715
S2632
S2634
S3599
S3853
S2636
S3501
S2892
S3707
T2580
S2641
S799
S800
S1003
S1034
T1515
S1977
S2054
S2057
S2643
T3238
S3680
S105
S847
S1000
S1035
S1447
T1979
S2016
S2055
S815
S816
T1030
S1480
S1484
Y2060
S2630
S2841
S3006
S3445
S3598
S821
T1086
S1469
S1478
S2004
S2627
T2852
S2844
S3504
S138
S548
S1092
S1089
S1473
S1475
S2850
S135
S265
S1084
S1098
T1476
S2792
S2816
S233
S263
S1512
S2783
Y2788
S140
S249
S245
S2789
S2795
S90
S1353
S1357
S241
S235
S1354

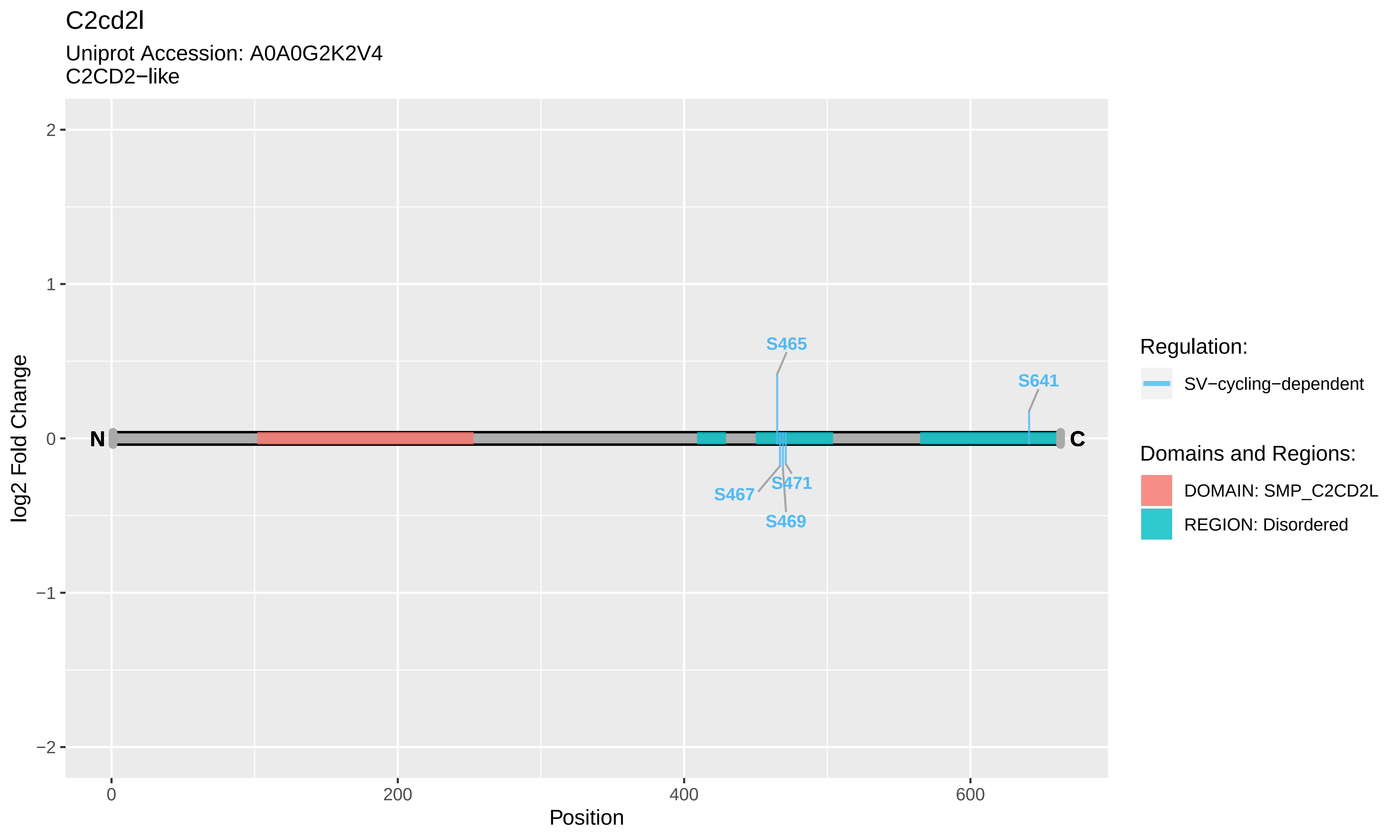
C2cd2l
Uniprot Accession: A0A0G2K2V4
C2CD2−like
log2 Fold Change
2
1
0
−1
−2
N
C
S465
S641
S467
S471
S469
0
200
400
600
Position
Regulation:
SV−cycling−dependent
Domains and Regions:
DOMAIN: SMP_C2CD2L
REGION: Disordered

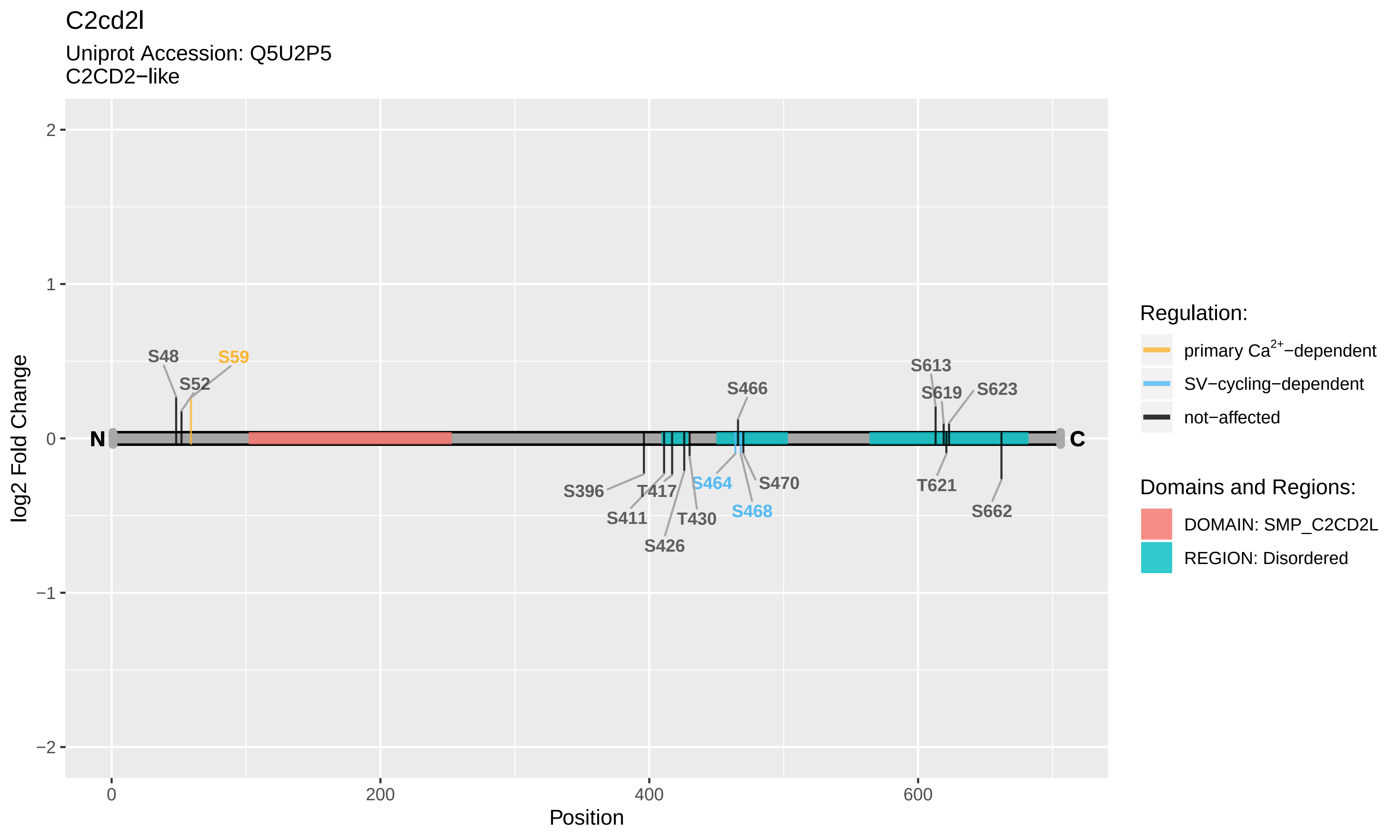

C2cd2l
Uniprot Accession: Q5U2P5
C2CD2−like
log2 Fold Change
Position
2
1
0
−1
−2
0
200
400
600
N
C
S48
S52
S59
S396
S411
T417
S426
T430
S464
S466
S468
S470
S613
S619
S623
T621
S662
Regulation:
primary Ca2+−dependent
SV−cycling−dependent
not−affected
Domains and Regions:
DOMAIN: SMP_C2CD2L
REGION: Disordered

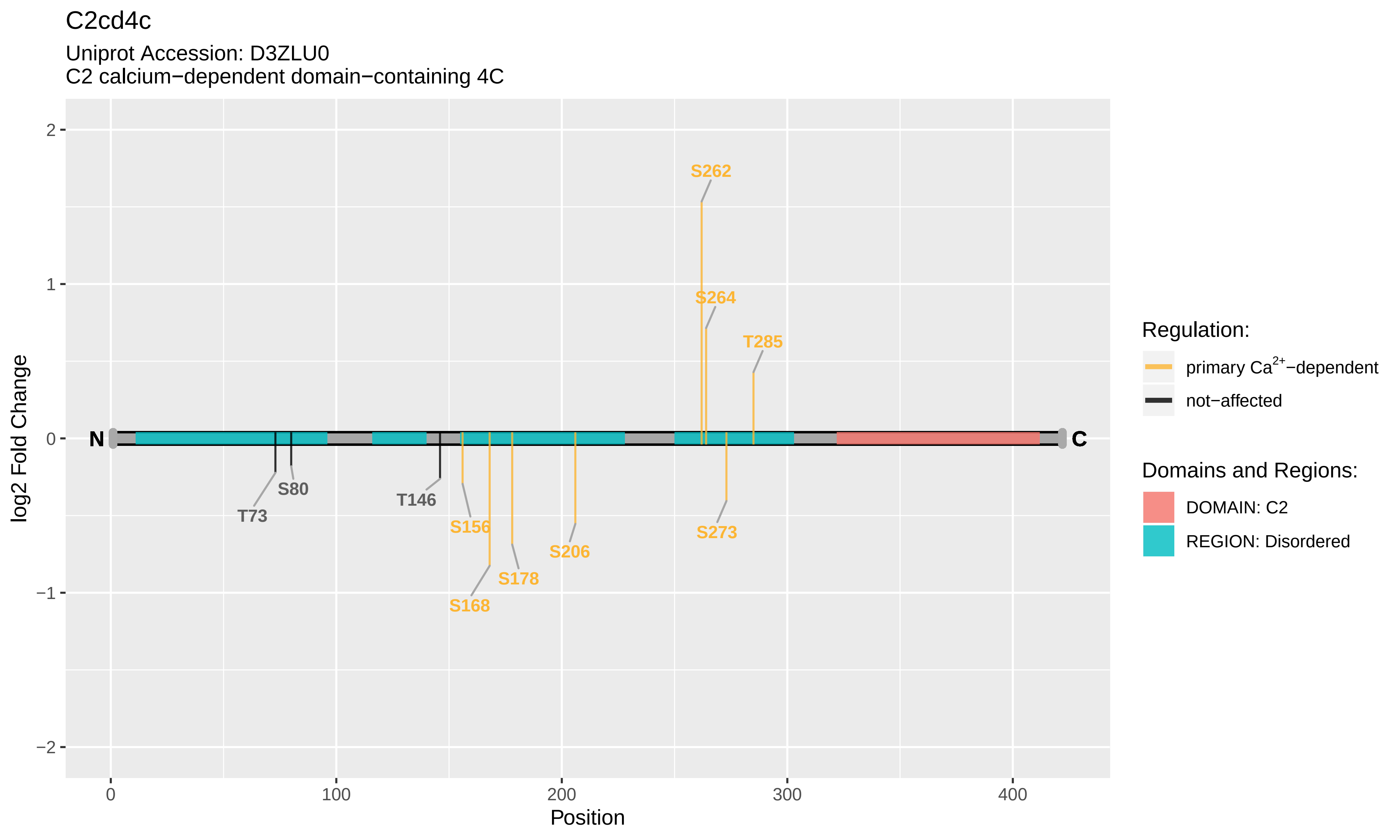
C2cd4c
Uniprot Accession: D3ZLU0
C2 calcium−dependent domain−containing 4C
log2 Fold Change
Position
N
C
S262
S264
T285
T73
S80
T146
S156
S168
S178
S206
S273
Regulation:
primary Ca2+−dependent
not−affected
Domains and Regions:
DOMAIN: C2
REGION: Disordered

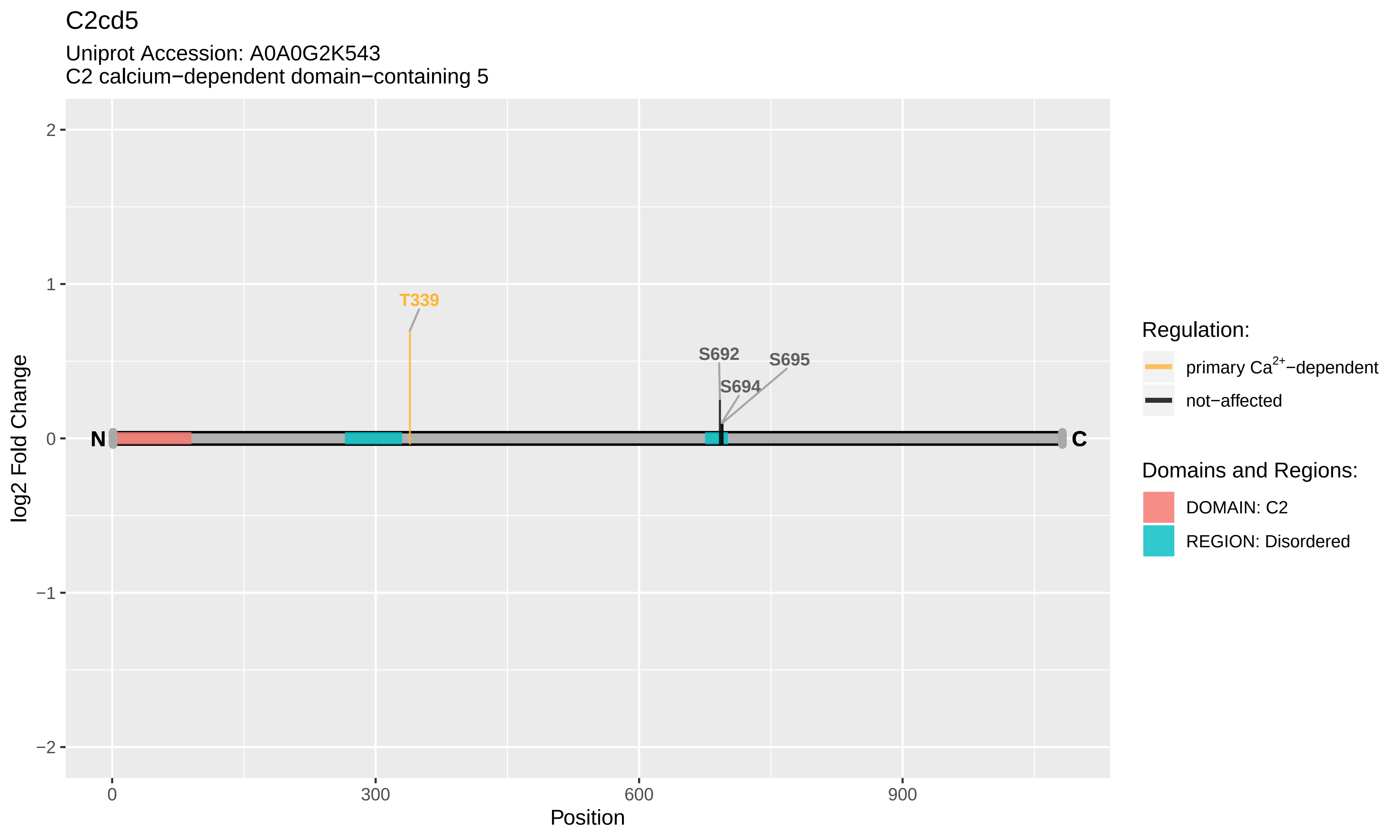

C2cd5
Uniprot Accession: A0A0G2K543
C2 calcium−dependent domain−containing 5
log2 Fold Change
2
1
0
−1
−2
T339
S692
S694
S695
N
C
0
300
600
900
Position
Regulation:
primary Ca2+−dependent
not−affected
Domains and Regions:
DOMAIN: C2
REGION: Disordered

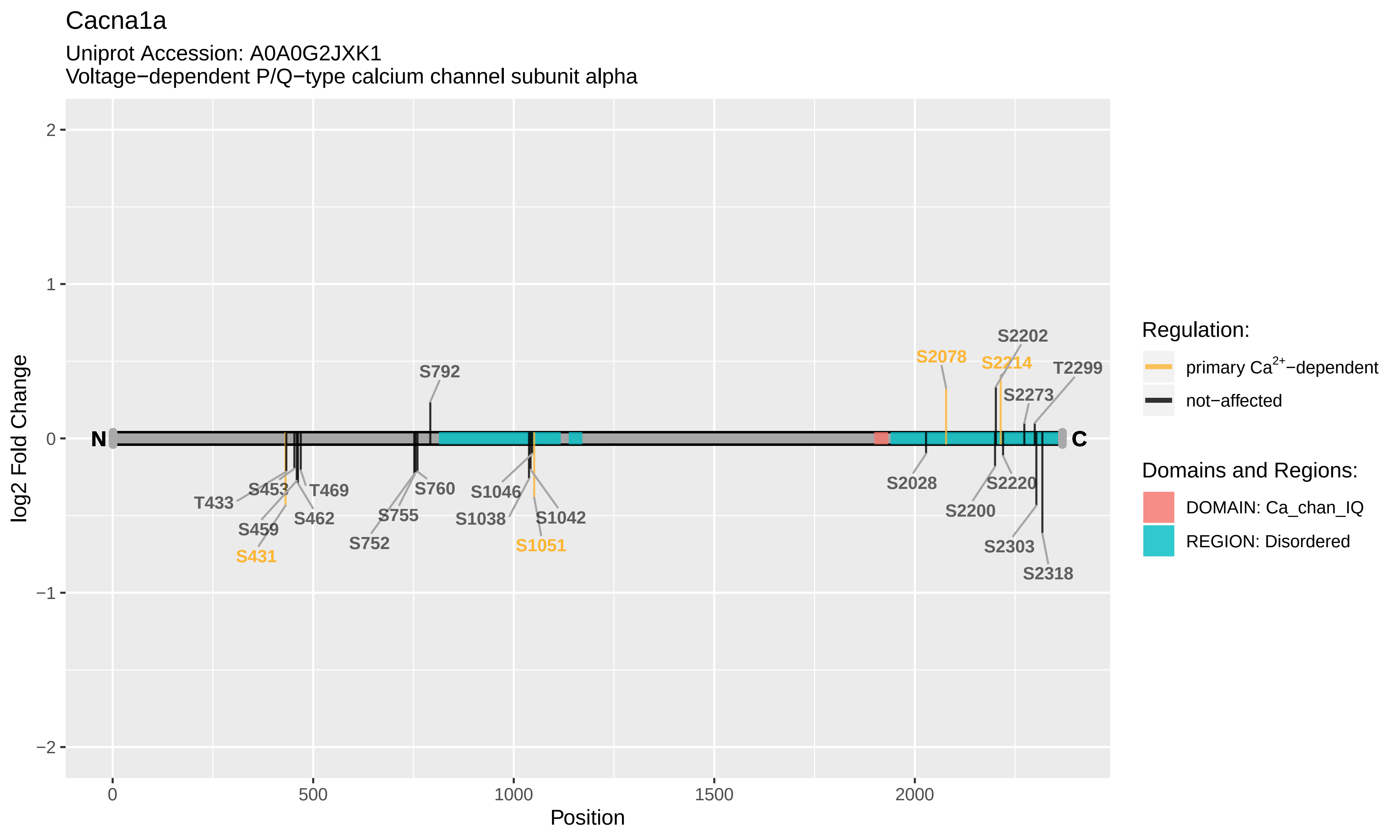
Cacna1a
Uniprot Accession: A0A0G2JXK1
Voltage−dependent P/Q−type calcium channel subunit alpha
log2 Fold Change
Position
N
C
S792
S2078
S2202
S2214
T2299
S2273
T433
S453
T469
S459
S462
S431
S760
S755
S752
S1046
S1038
S1042
S1051
S2028
S2220
S2200
S2303
S2318
Regulation:
primary Ca2+−dependent
not−affected
Domains and Regions:
DOMAIN: Ca_chan_IQ
REGION: Disordered

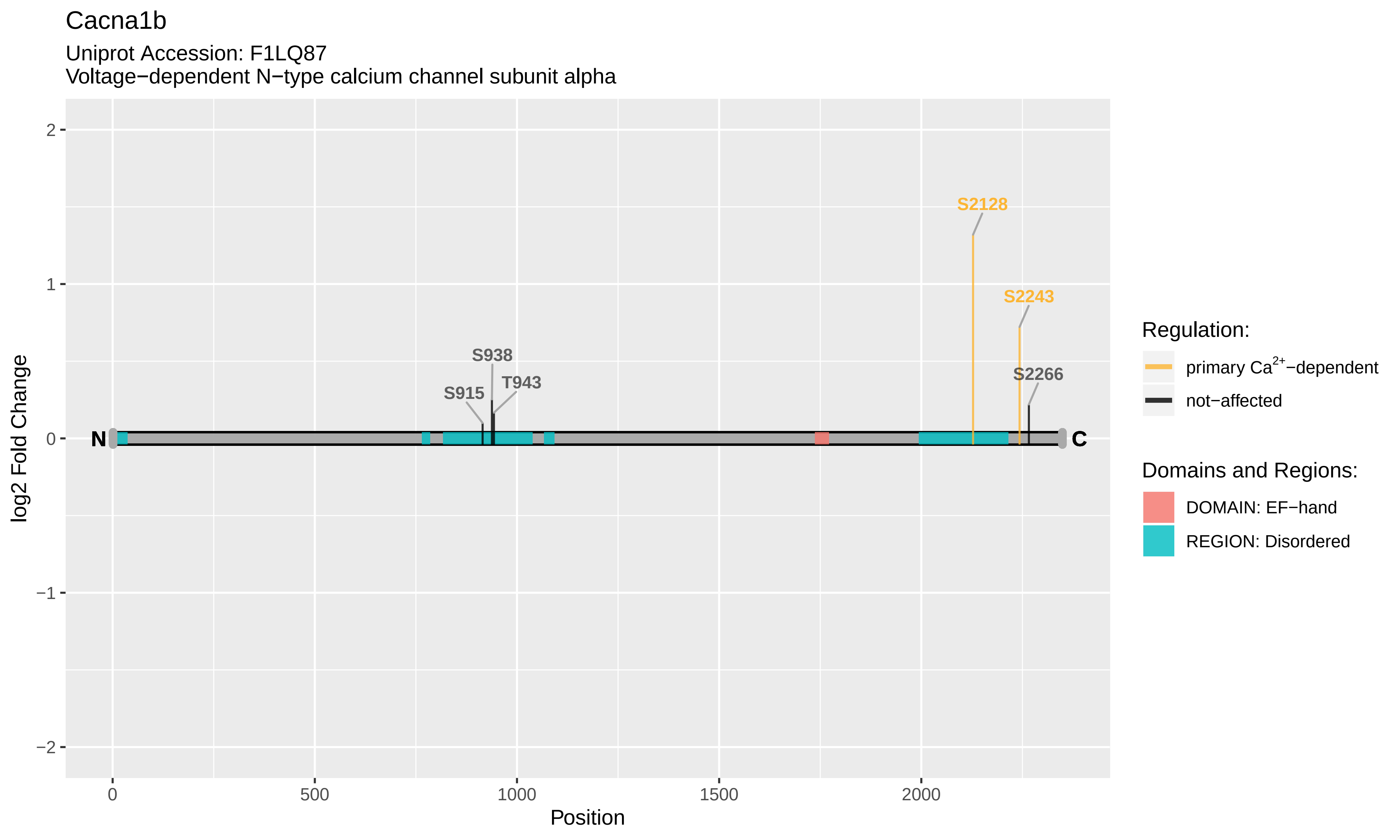

Cacna1b
Uniprot Accession: F1LQ87
Voltage−dependent N−type calcium channel subunit alpha
S2128
S2243
S938
T943
S915
S2266
N
C
2
1
0
−1
−2
0
500
1000
1500
2000
Position
log2 Fold Change
Regulation:
primary Ca2+−dependent
not−affected
Domains and Regions:
DOMAIN: EF−hand
REGION: Disordered

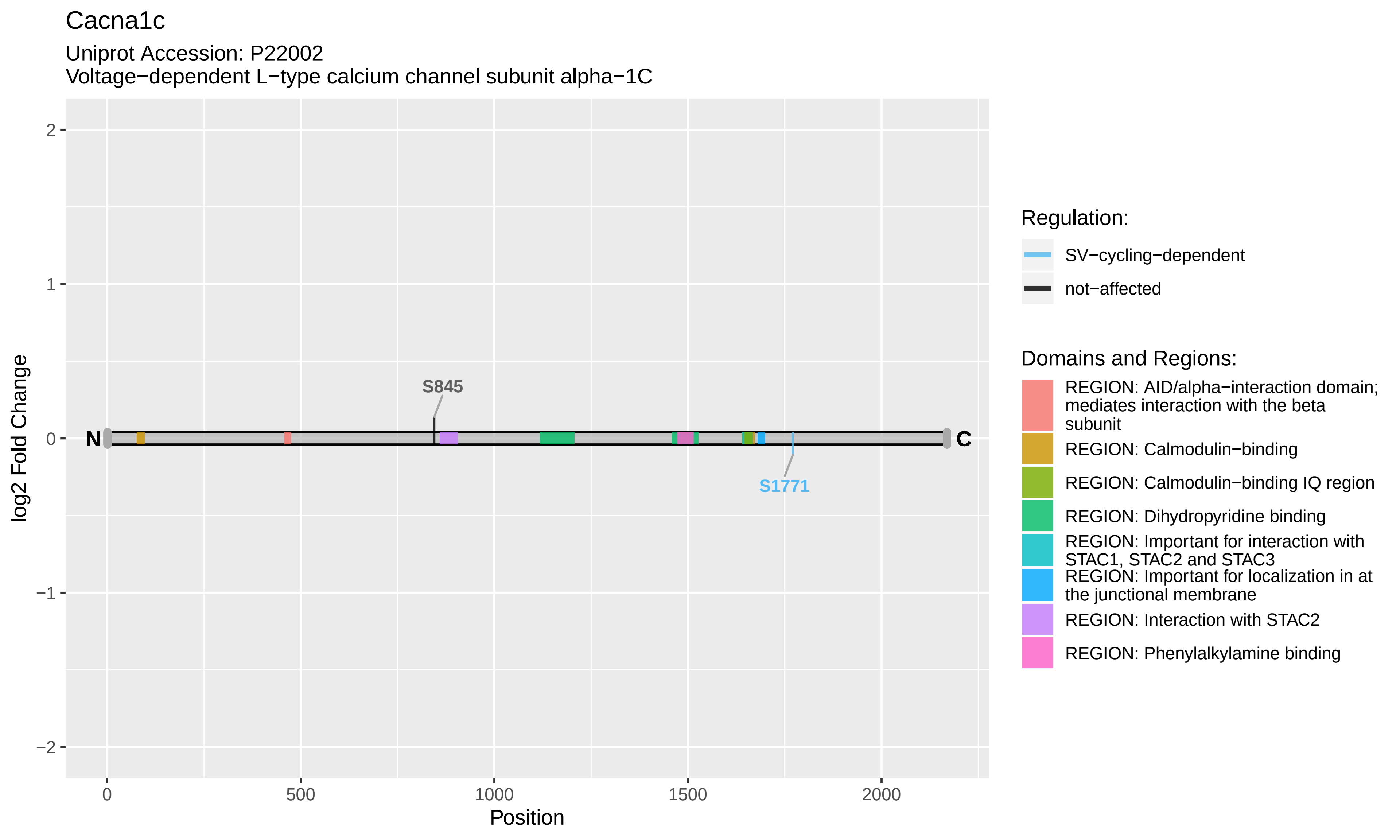
Cacna1c
Uniprot Accession: P22002
Voltage−dependent L−type calcium channel subunit alpha−1C
log2 Fold Change
Position
N
C
S845
S1771
Regulation:
SV−cycling−dependent
not−affected
Domains and Regions:
REGION: AID/alpha−interaction domain; mediates interaction with the beta subunit
REGION: Calmodulin−binding
REGION: Calmodulin−binding IQ region
REGION: Dihydropyridine binding
REGION: Important for interaction with STAC1, STAC2 and STAC3
REGION: Important for localization in at the junctional membrane
REGION: Interaction with STAC2
REGION: Phenylalkylamine binding

Cacna1e

Uniprot Accession: F1LMS1
Voltage−dependent R−type calcium channel subunit alpha

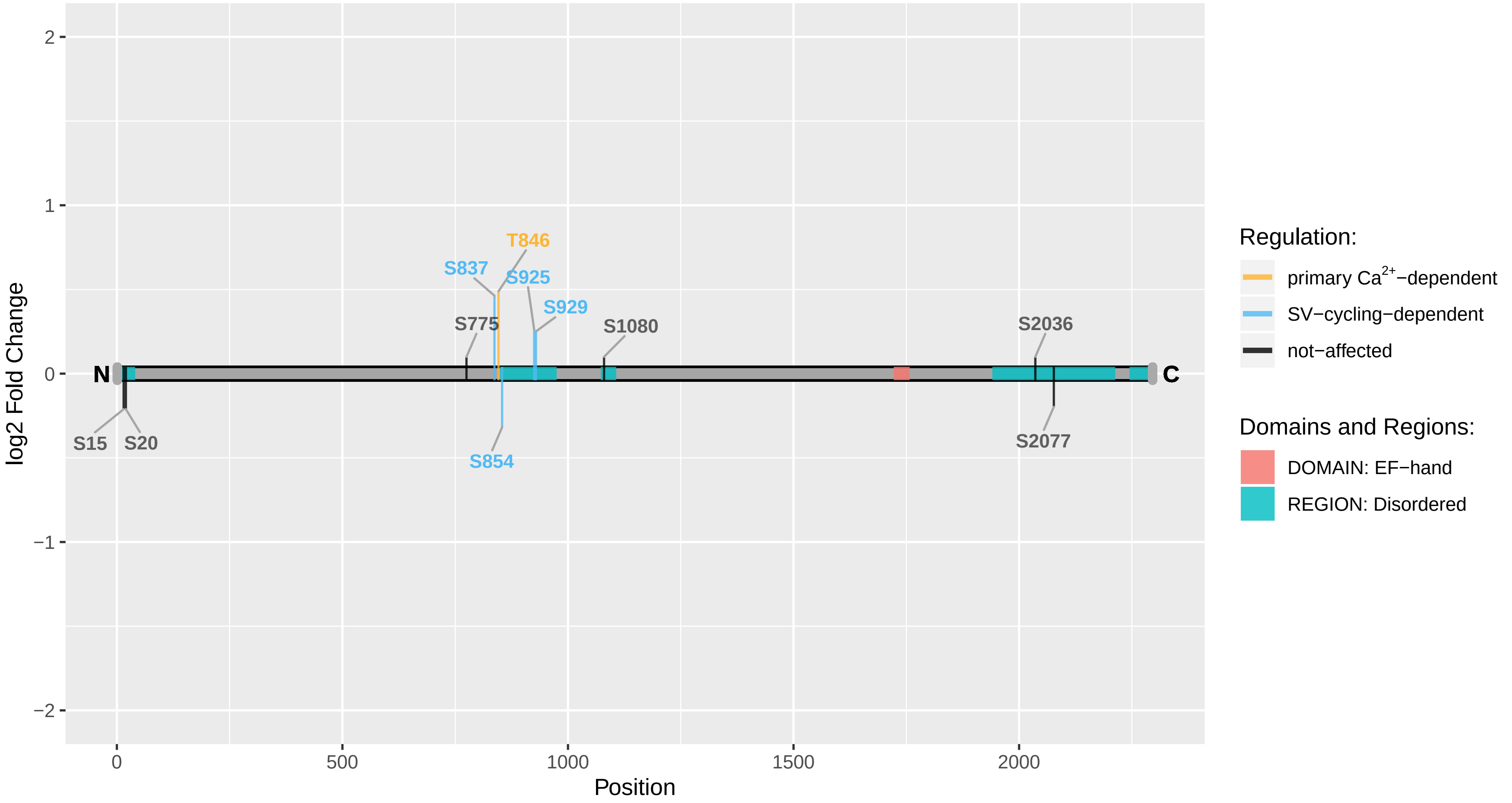

Cacna1g

Uniprot Accession: Q9WUB8
Voltage−dependent T−type calcium channel subunit alpha

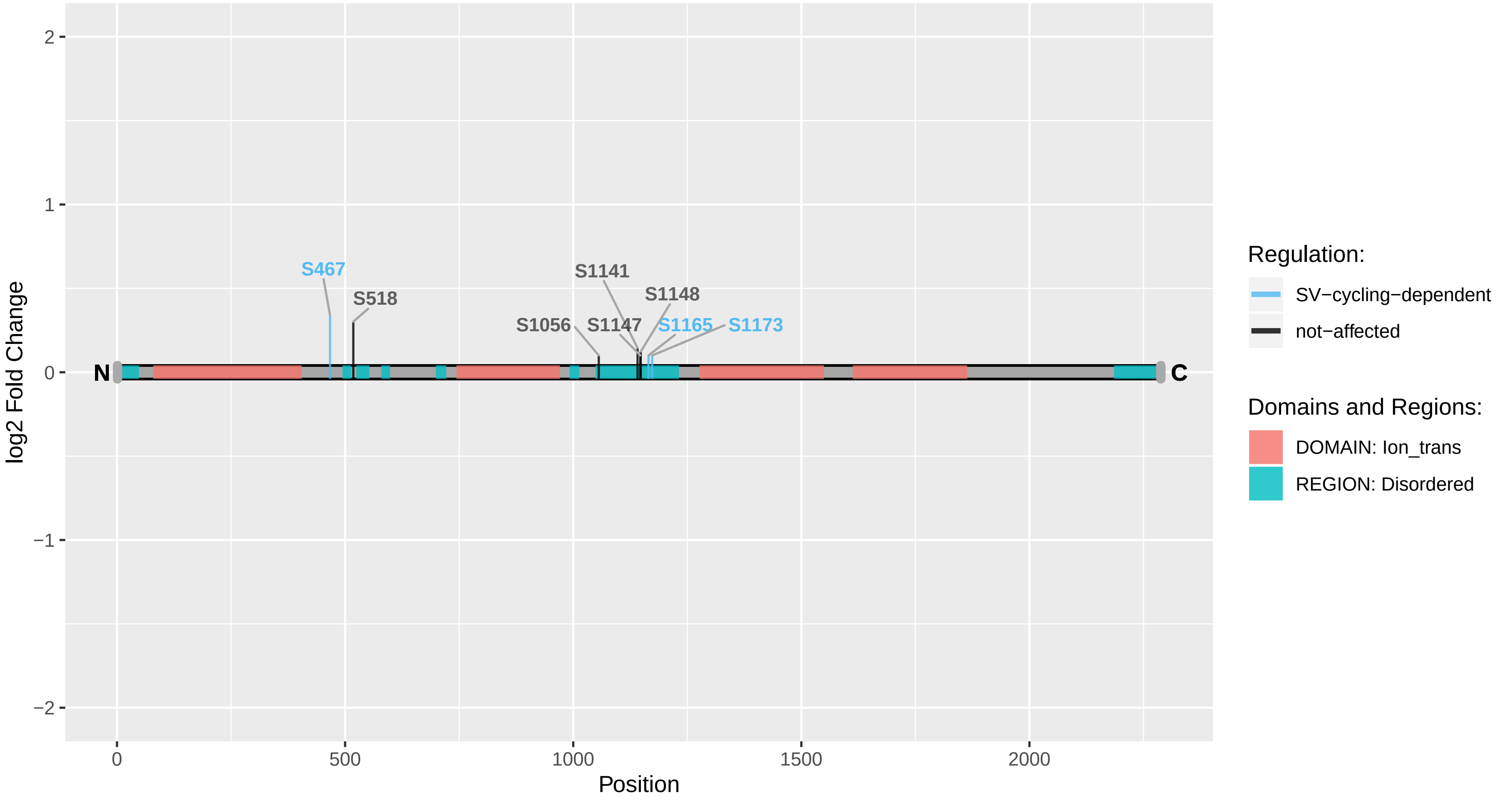

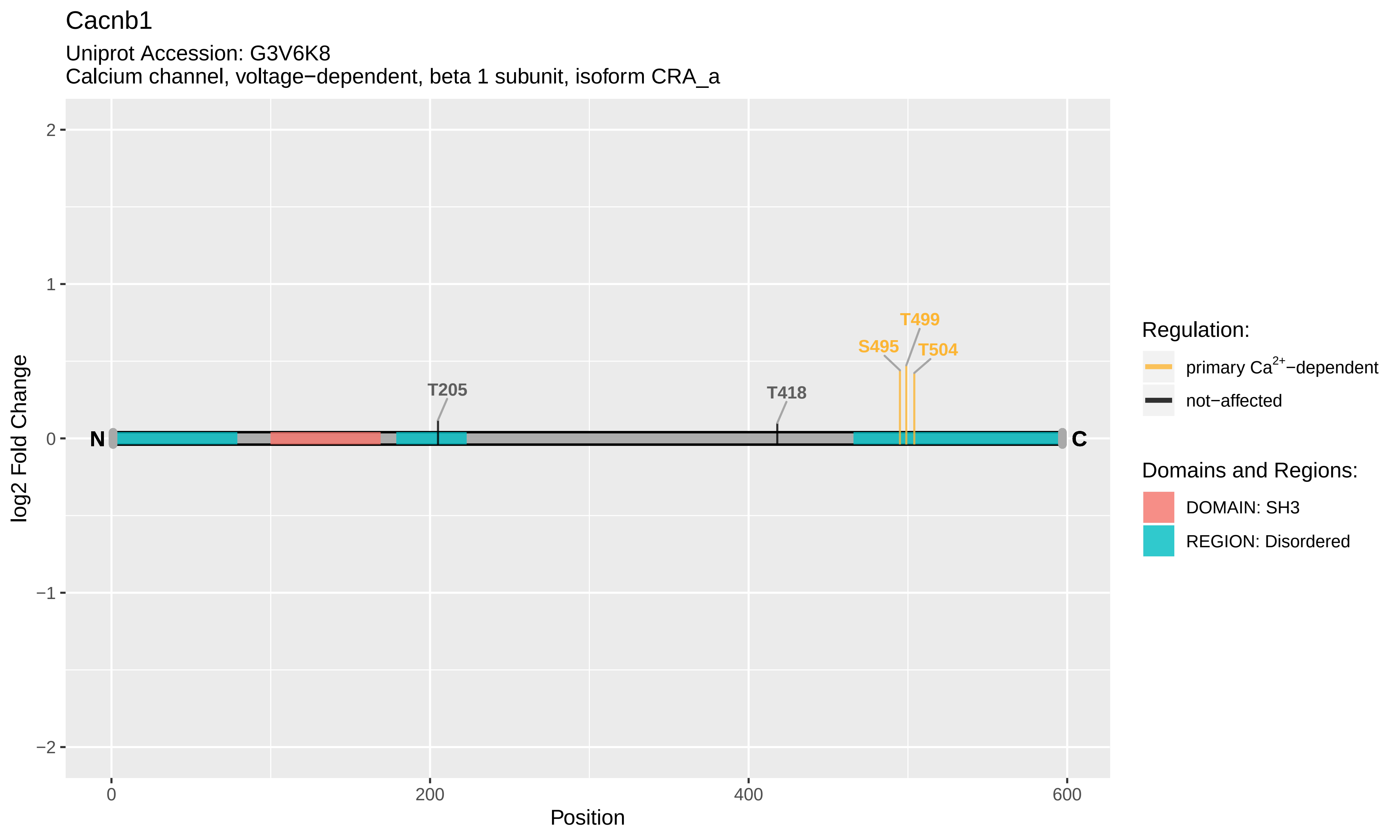

Cacnb1
Uniprot Accession: G3V6K8
Calcium channel, voltage−dependent, beta 1 subunit, isoform CRA_a
log2 Fold Change
Position
2
1
0
−1
−2
0
200
400
600
N
C
T205
T418
S495
T499
T504
Regulation:
primary Ca2+−dependent
not−affected
Domains and Regions:
DOMAIN: SH3
REGION: Disordered

Cacnb4

Uniprot Accession: D4A055
Voltage−dependent L−type calcium channel subunit beta−4

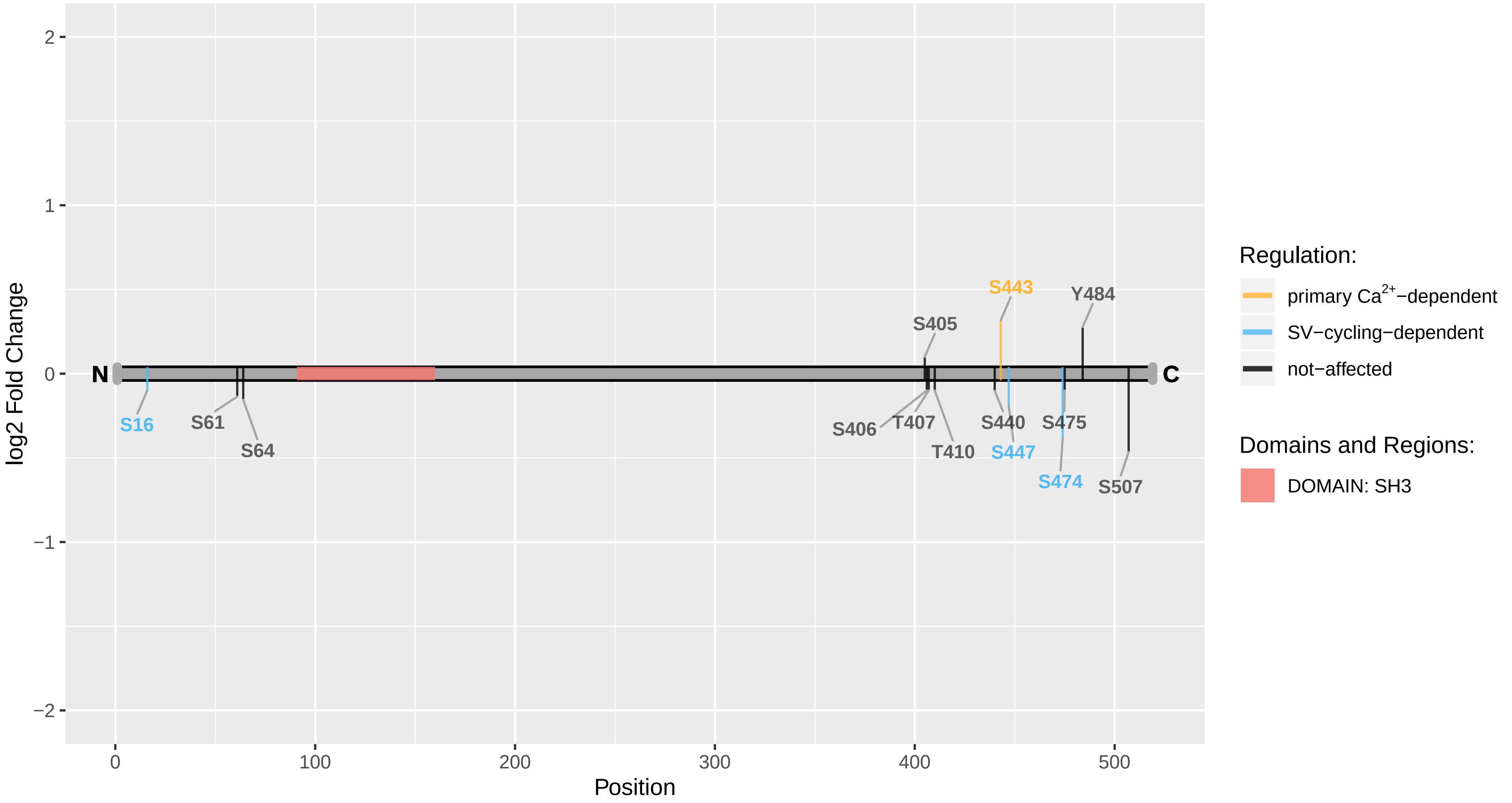

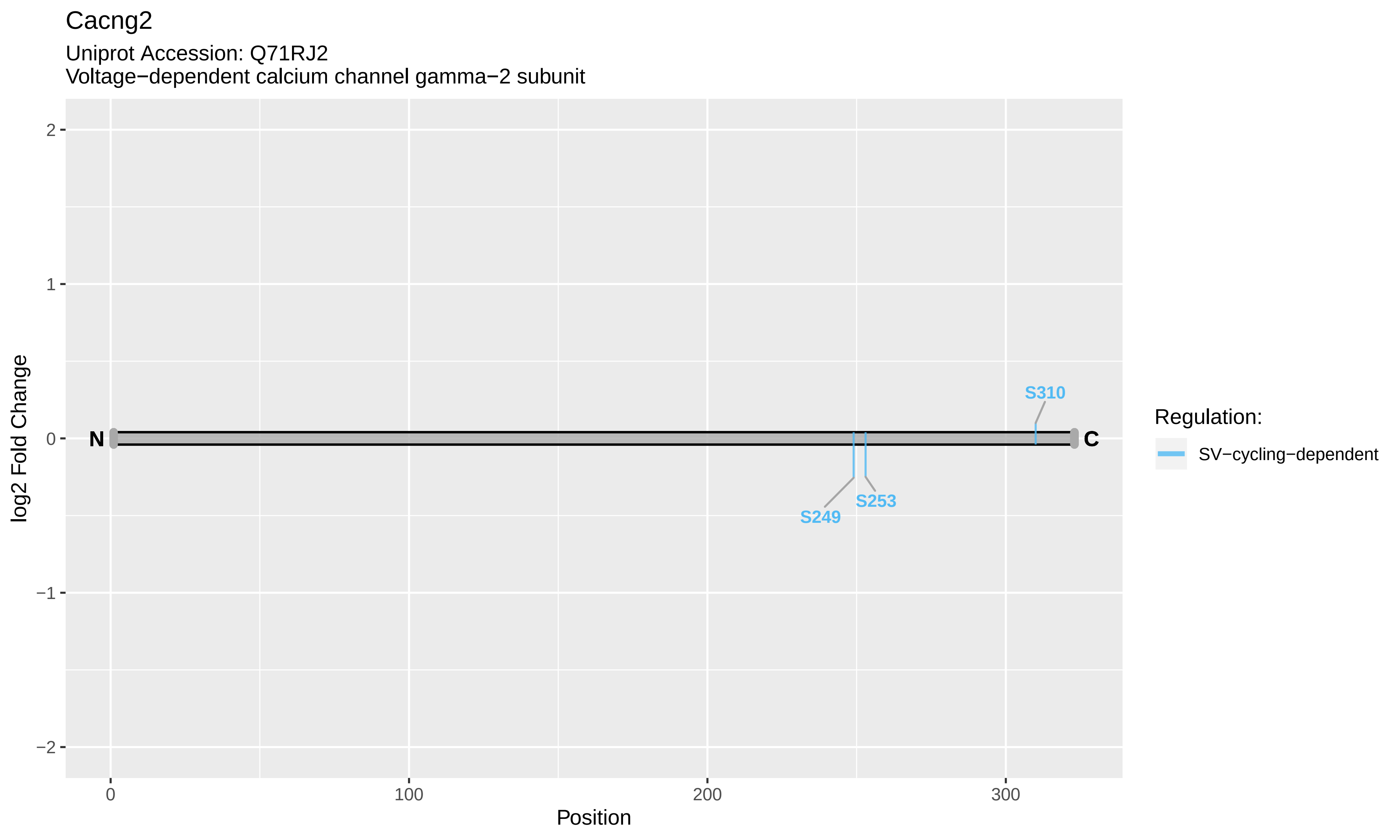

Cacng2
Uniprot Accession: Q71RJ2
Voltage−dependent calcium channel gamma−2 subunit
log2 Fold Change
2
1
0
−1
−2
N
C
S310
S249
S253
0
100
200
300
Position
Regulation:
SV−cycling−dependent

Cacng8

Uniprot Accession: Q8VHW5
Voltage−dependent calcium channel gamma−8 subunit

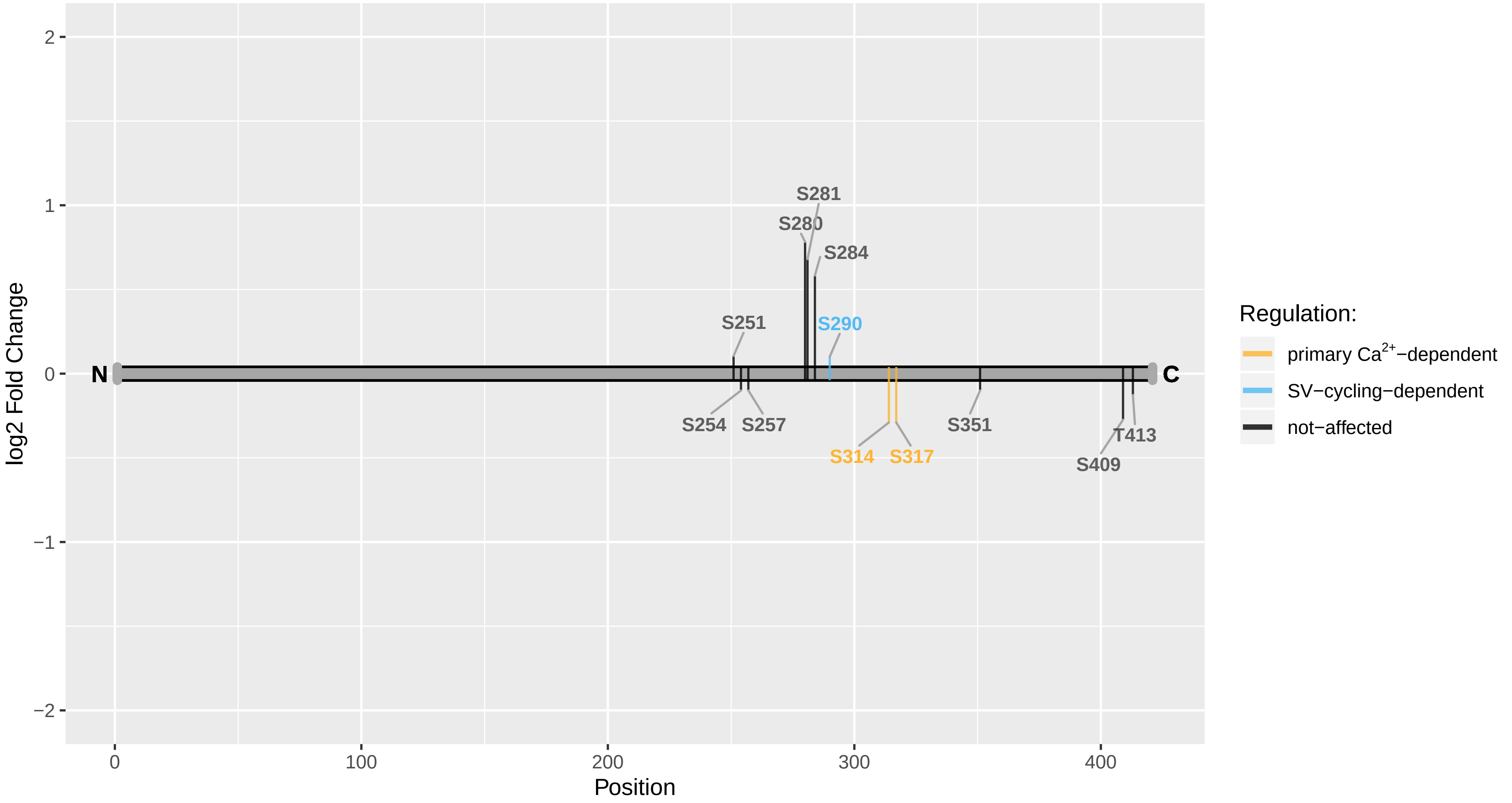

Cadps

Uniprot Accession: F1LLX6
Calcium−dependent secretion activator 1

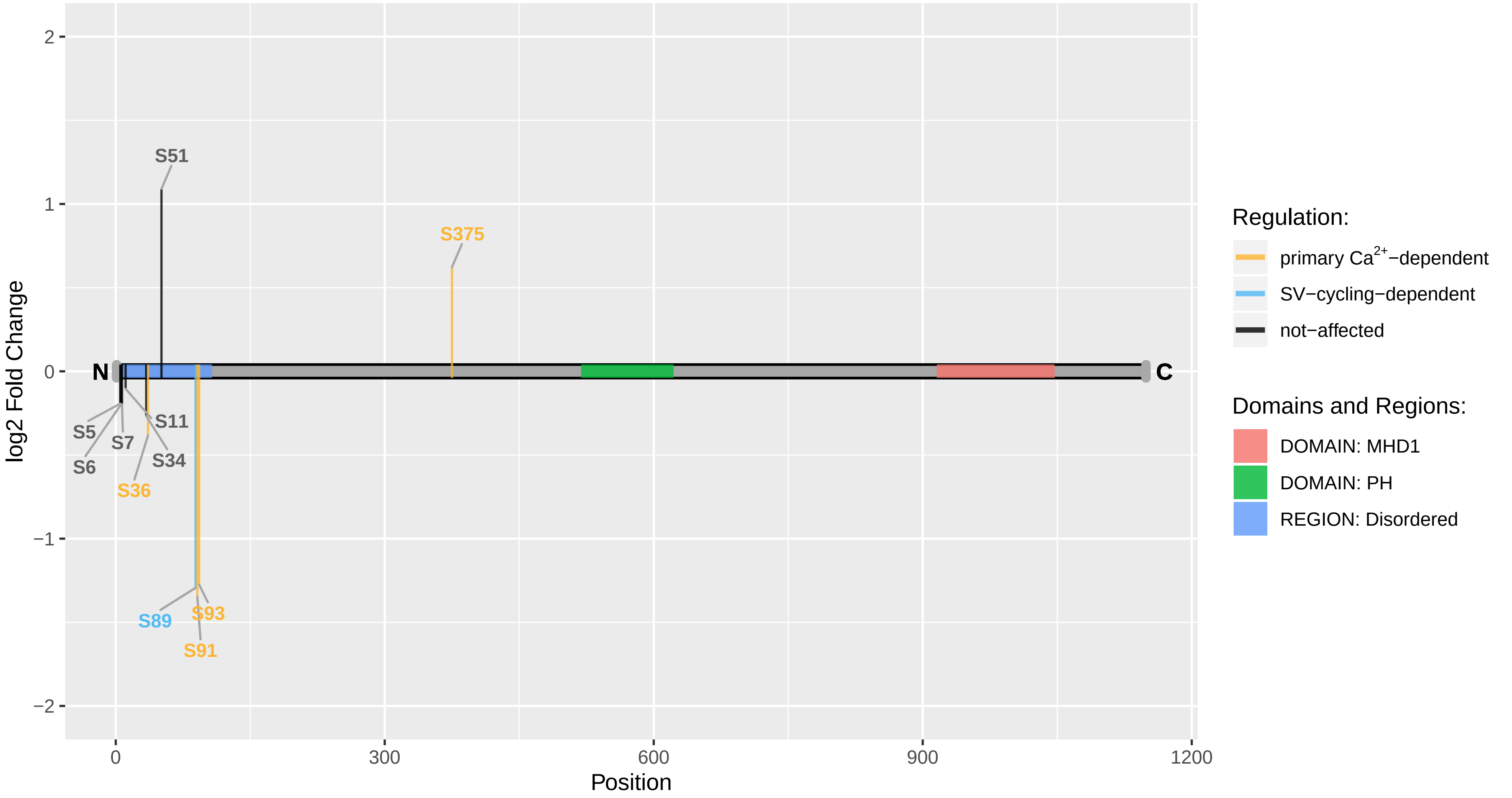

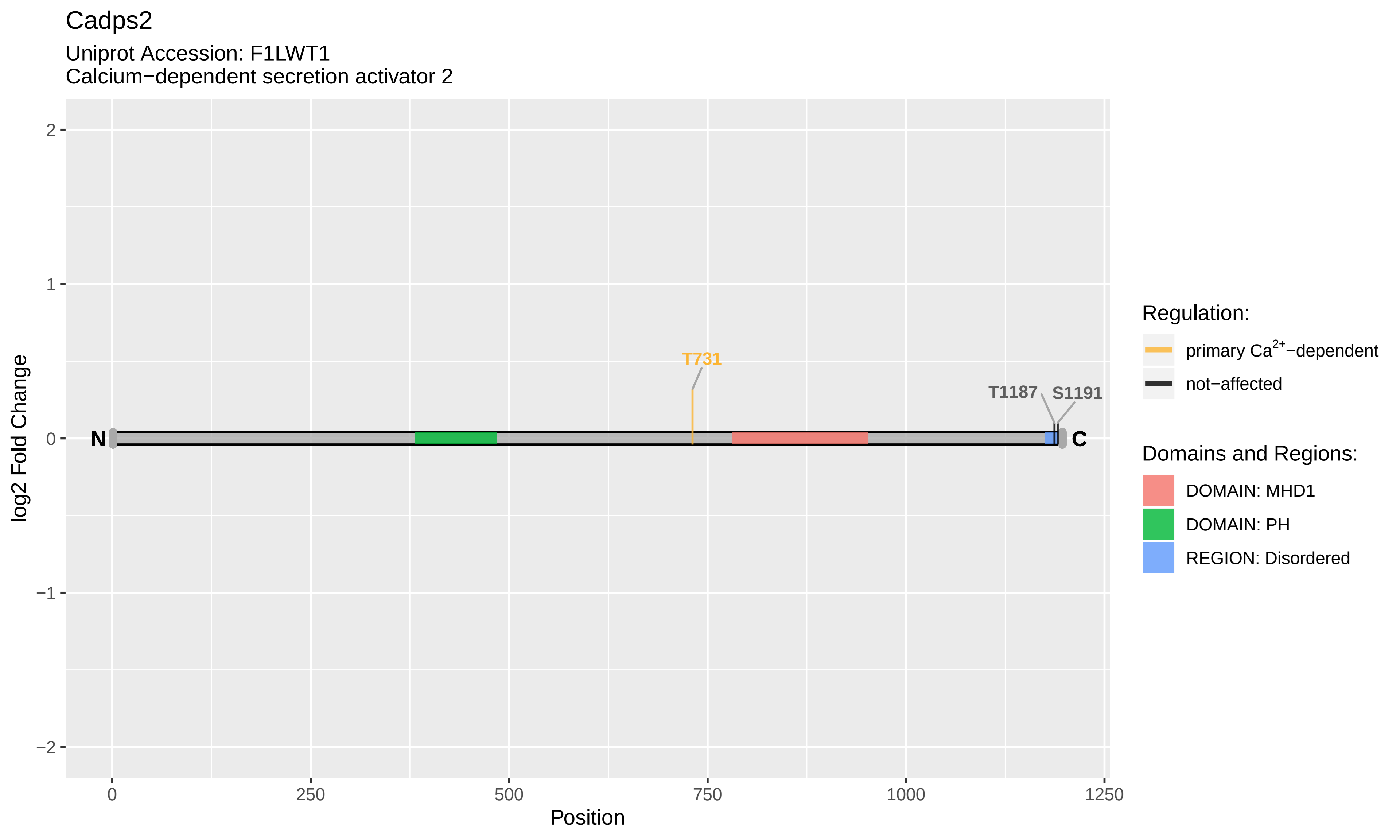

Cadps2
Uniprot Accession: F1LWT1
Calcium−dependent secretion activator 2
T731
T1187
S1191
N
C
log2 Fold Change
Position
Regulation:
primary Ca2+−dependent
not−affected
Domains and Regions:
DOMAIN: MHD1
DOMAIN: PH
REGION: Disordered

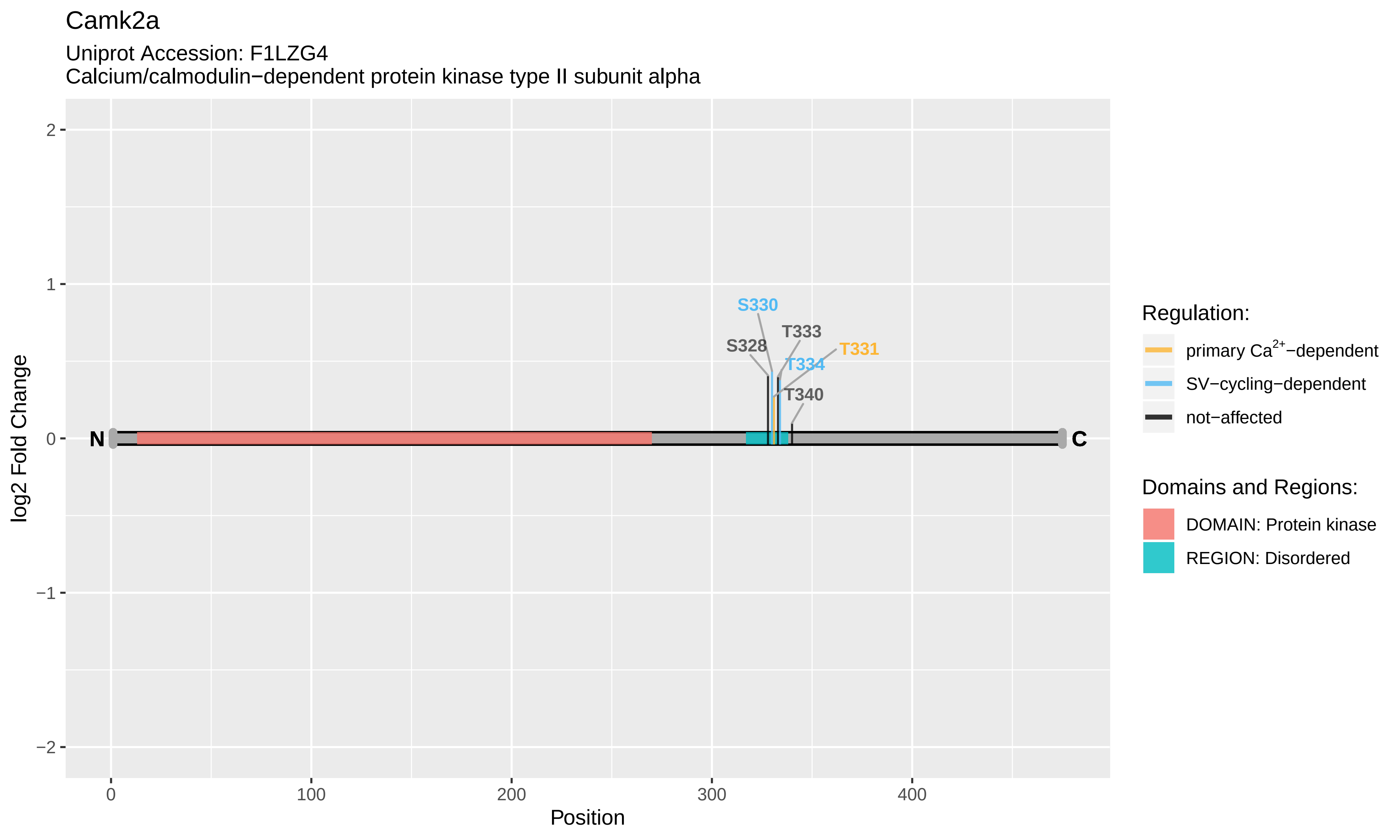

Camk2a
Uniprot Accession: F1LZG4
Calcium/calmodulin−dependent protein kinase type II subunit alpha
S330
T333
S328
T331
T334
T340
N
C
2
1
0
−1
−2
log2 Fold Change
0
100
200
300
400
Position
Regulation:
primary Ca^{2+}−dependent
SV−cycling−dependent
not−affected
Domains and Regions:
DOMAIN: Protein kinase
REGION: Disordered

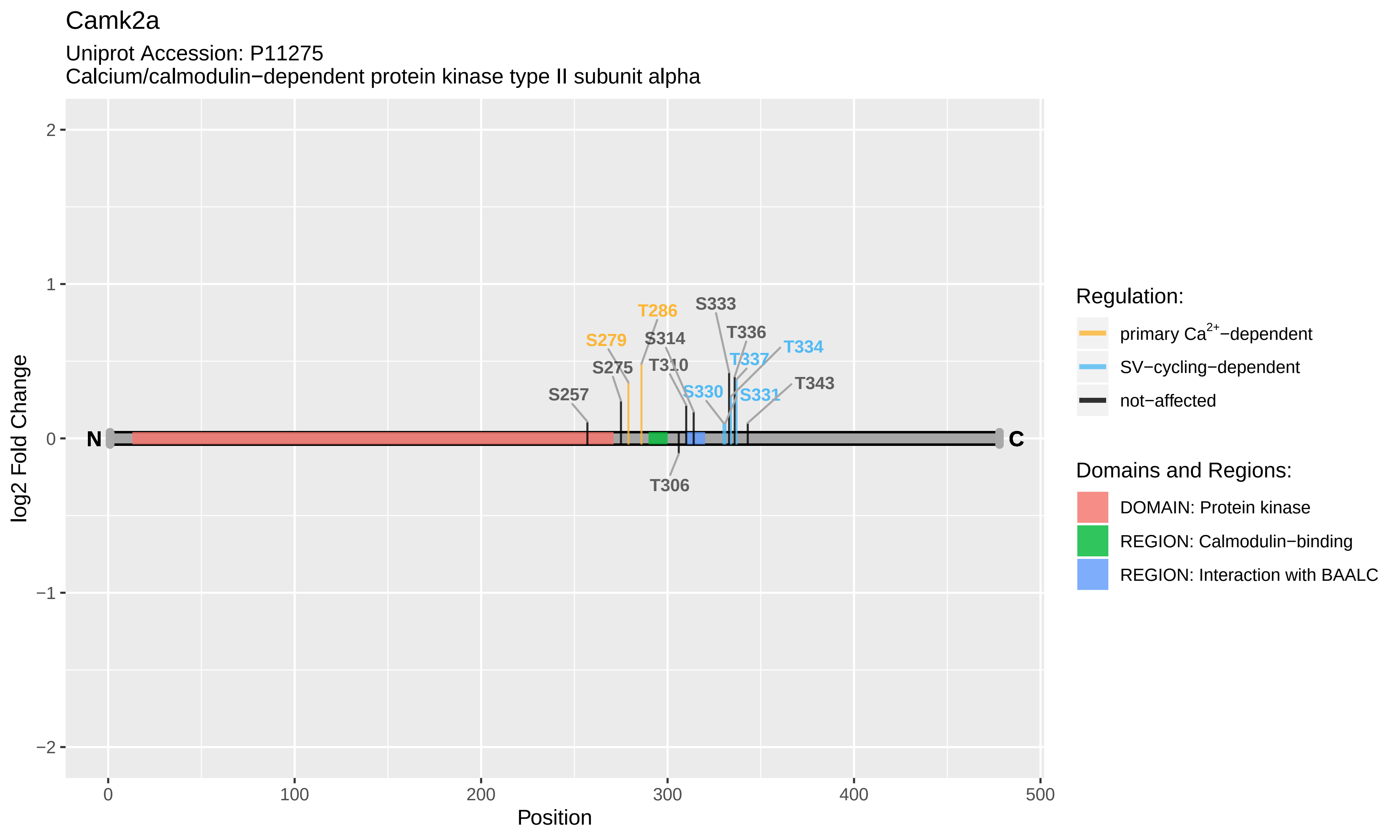

Camk2a
Uniprot Accession: P11275
Calcium/calmodulin−dependent protein kinase type II subunit alpha
log2 Fold Change
Position
2
1
0
−1
−2
0
100
200
300
400
500
N
C
T286
S333
T336
S279
S314
T334
T337
S275
T310
T343
S330
S331
S257
T306
Regulation:
primary Ca2+−dependent
SV−cycling−dependent
not−affected
Domains and Regions:
DOMAIN: Protein kinase
REGION: Calmodulin−binding
REGION: Interaction with BAALC

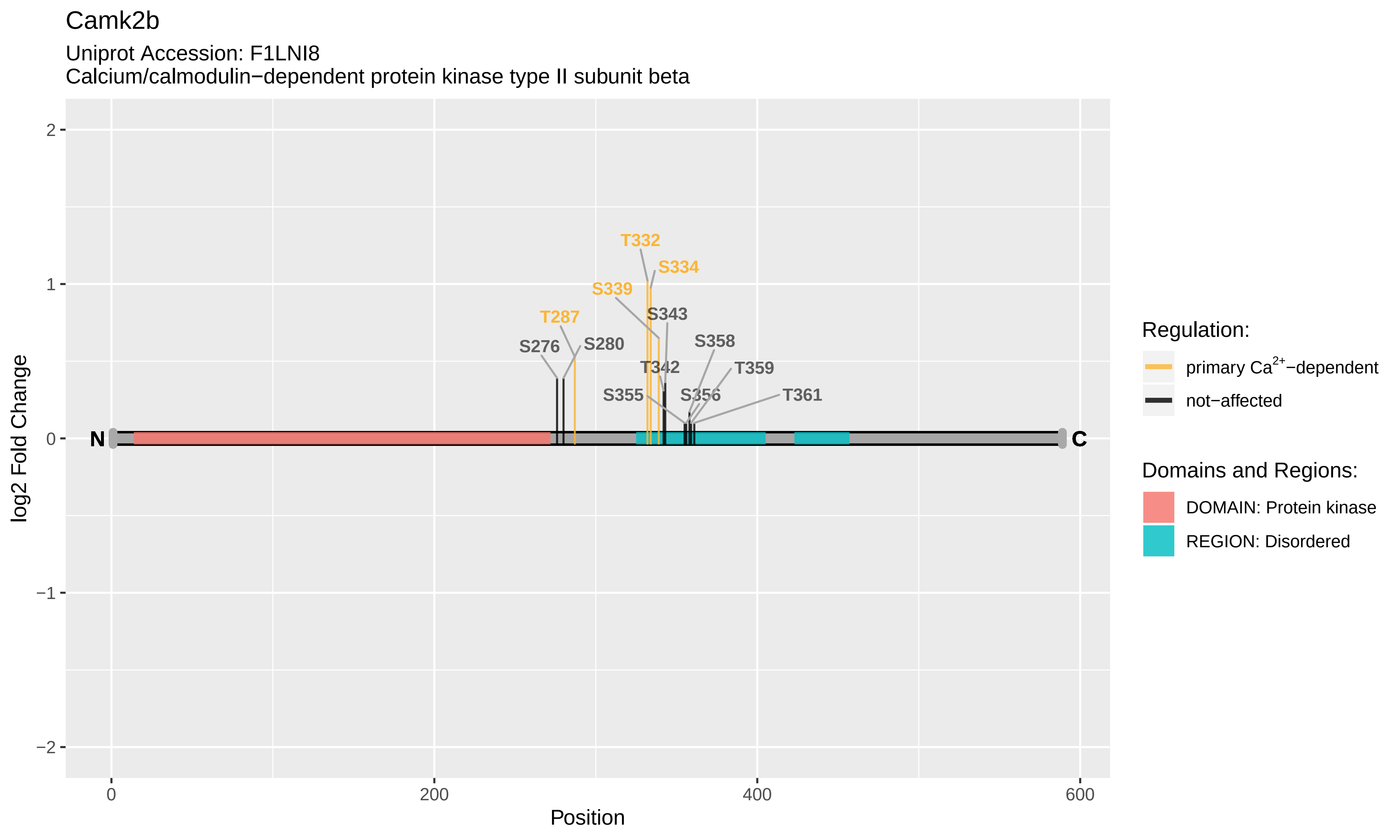

Camk2b
Uniprot Accession: F1LNI8
Calcium/calmodulin−dependent protein kinase type II subunit beta
T332
S334
S339
S343
T287
S276
S280
S358
T342
T359
S355
S356
T361
N
C
log2 Fold Change
Position
Regulation:
primary Ca2+−dependent
not−affected
Domains and Regions:
DOMAIN: Protein kinase
REGION: Disordered

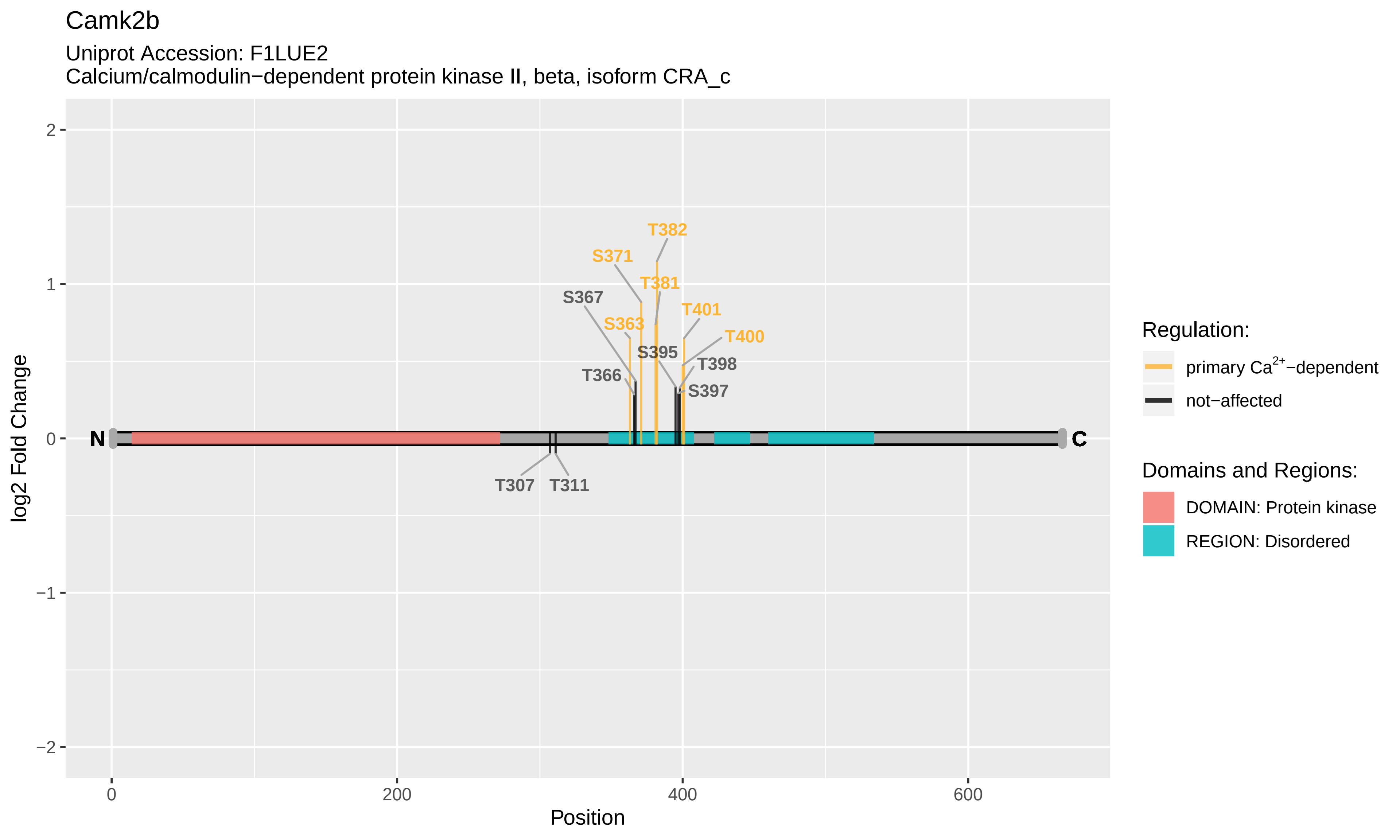
Camk2b
Uniprot Accession: F1LUE2
Calcium/calmodulin−dependent protein kinase II, beta, isoform CRA_c
T382
S371
T381
S367
T401
S363
T400
S395
T398
T366
S397
N
C
T307
T311
log2 Fold Change
Position
2
1
0
−1
−2
0
200
400
600
Regulation:
primary Ca2+−dependent
not−affected
Domains and Regions:
DOMAIN: Protein kinase
REGION: Disordered

Camk2b

Uniprot Accession: P08413
Calcium/calmodulin−dependent protein kinase type II subunit beta

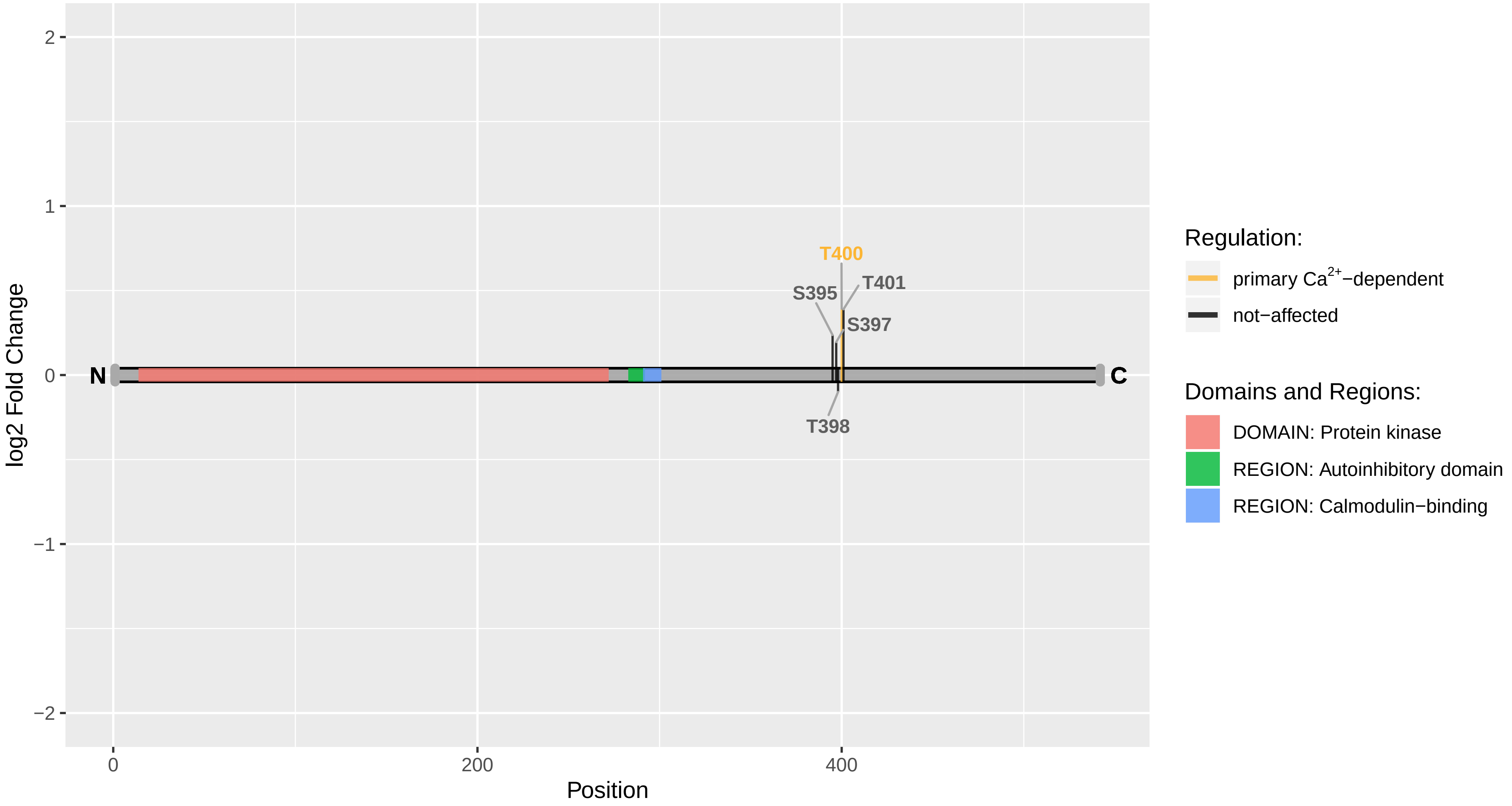

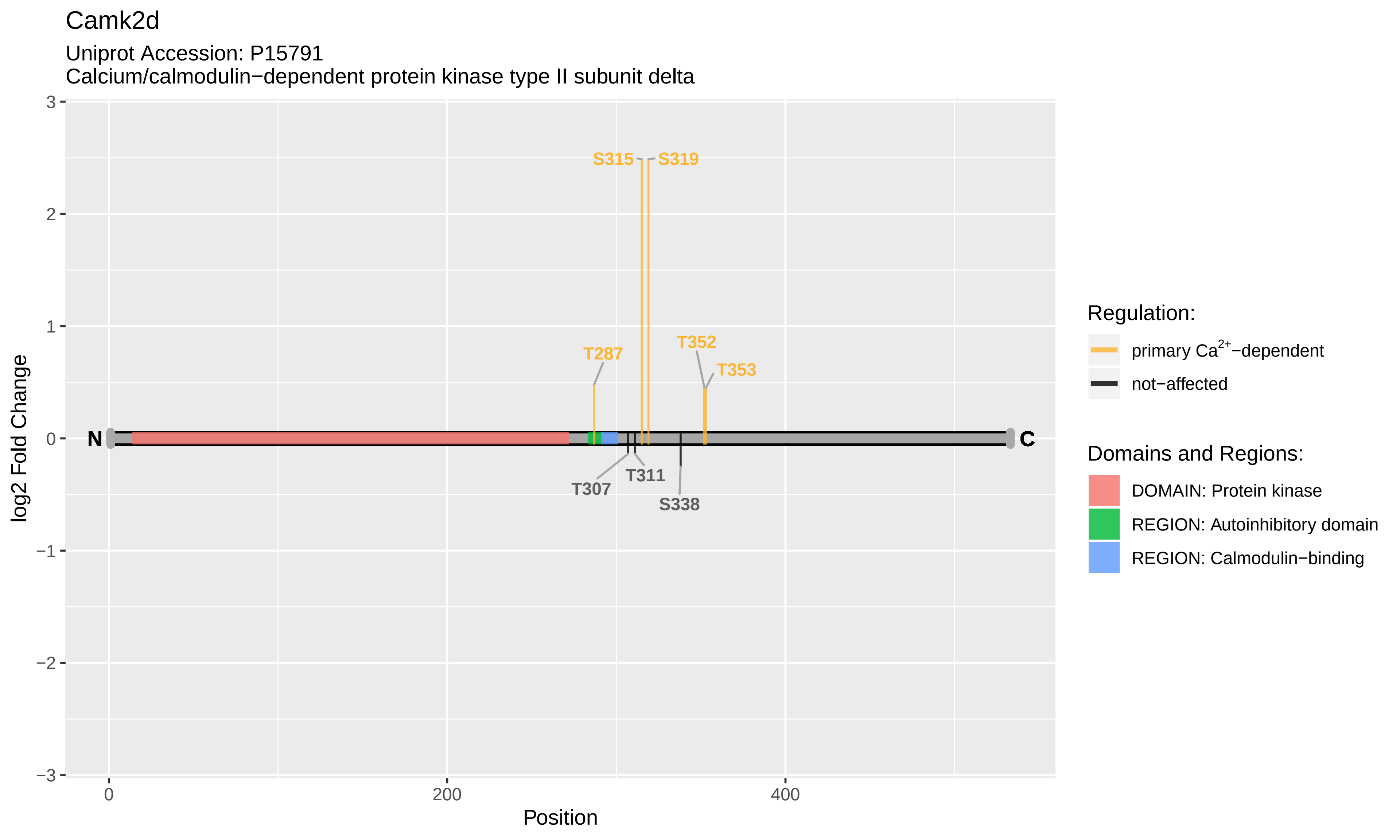

Camk2d
Uniprot Accession: P15791
Calcium/calmodulin−dependent protein kinase type II subunit delta
S315
S319
T287
T352
T353
N
C
T307
T311
S338
log2 Fold Change
3
2
1
0
−1
−2
−3
0
200
400
Position
Regulation:
primary Ca2+−dependent
not−affected
Domains and Regions:
DOMAIN: Protein kinase
REGION: Autoinhibitory domain
REGION: Calmodulin−binding

Camk2g

Uniprot Accession: F1M3F8
Calcium/calmodulin−dependent protein kinase type II subunit gamma

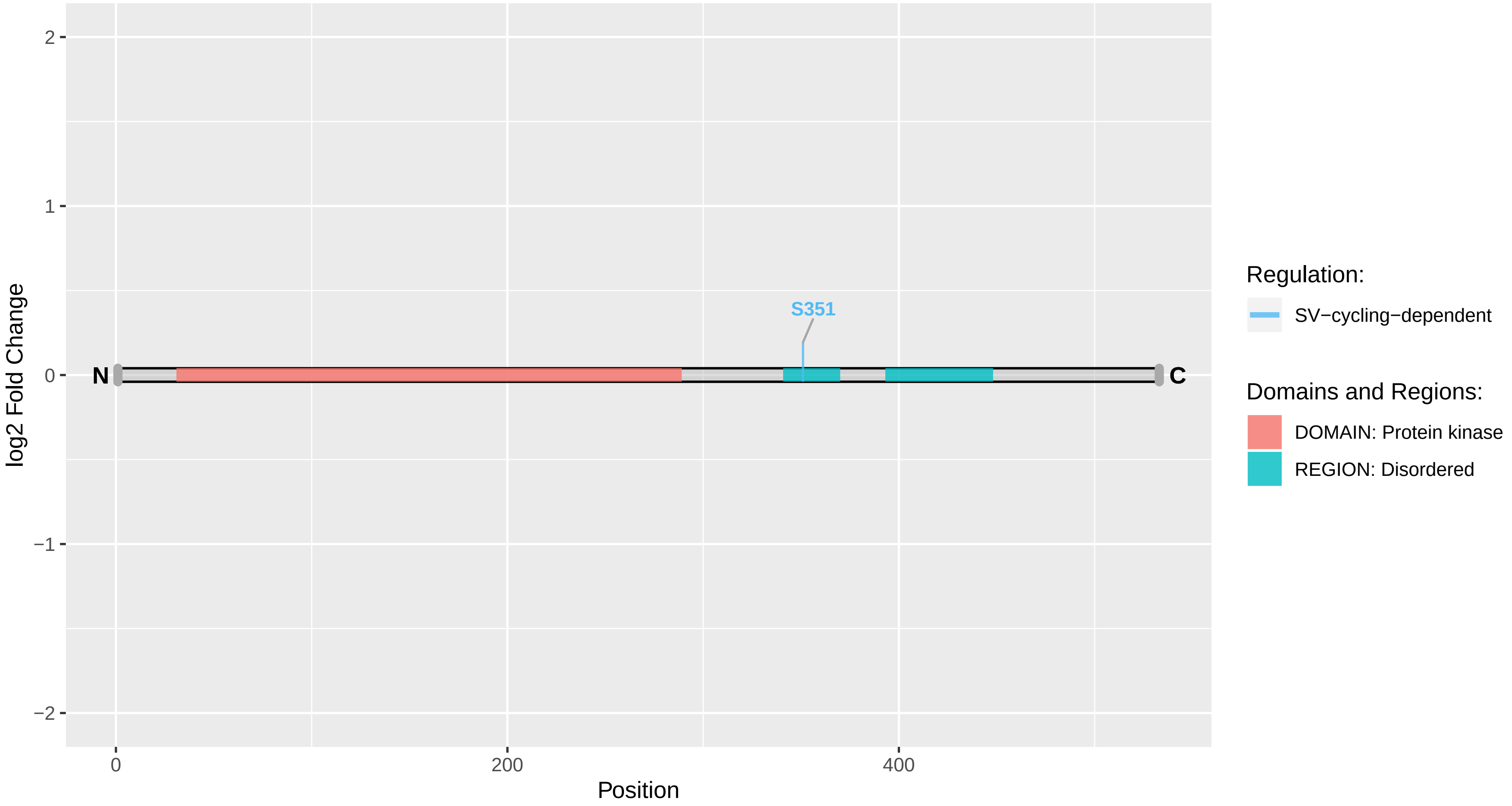

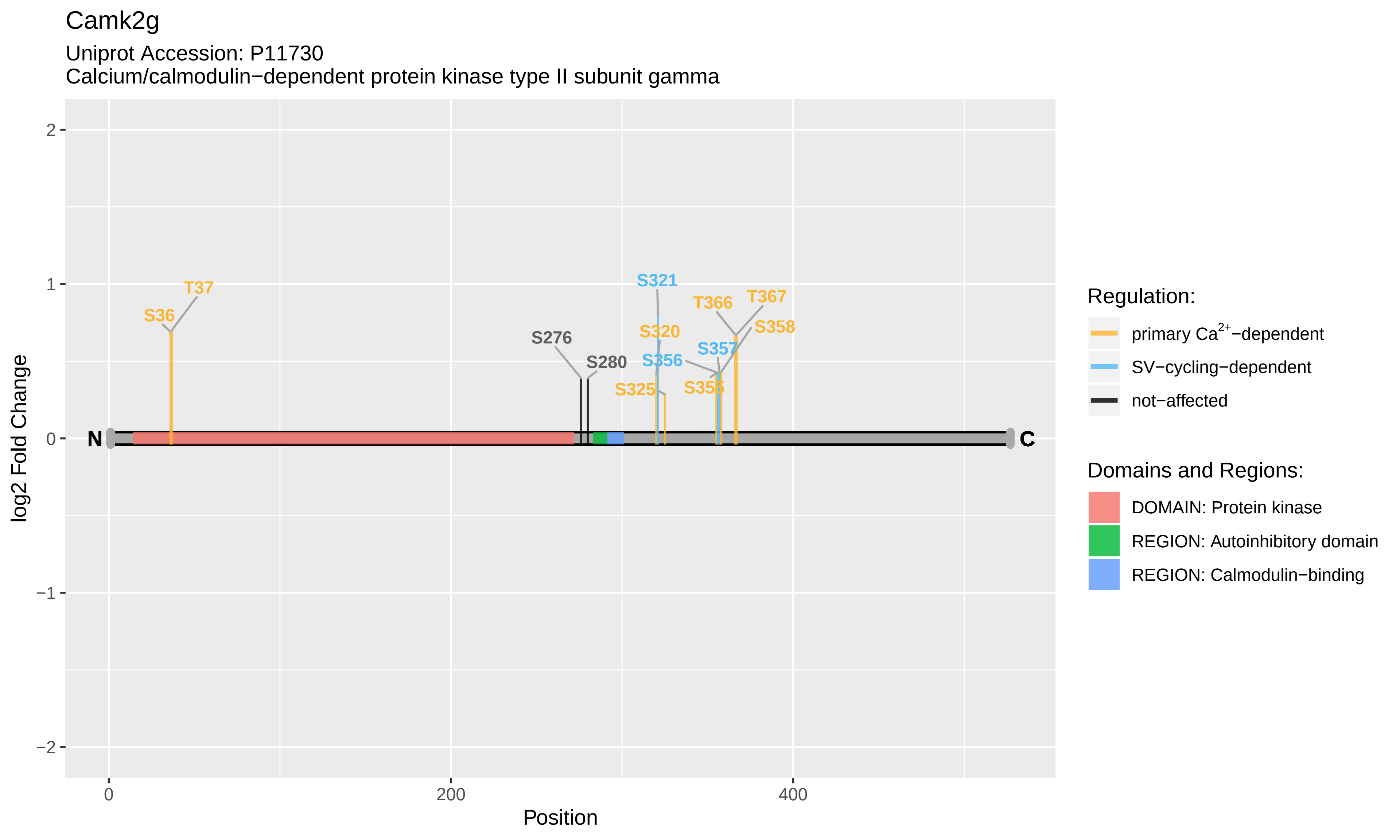

Camk2g
Uniprot Accession: P11730
Calcium/calmodulin−dependent protein kinase type II subunit gamma
log2 Fold Change
2
1
0
−1
−2
Position
0
200
400
N
C
S36
T37
S276
S280
S321
S320
S356
S325
S357
S355
T366
T367
S358
Regulation:
primary Ca^{2+}−dependent
SV−cycling−dependent
not−affected
Domains and Regions:
DOMAIN: Protein kinase
REGION: Autoinhibitory domain
REGION: Calmodulin−binding

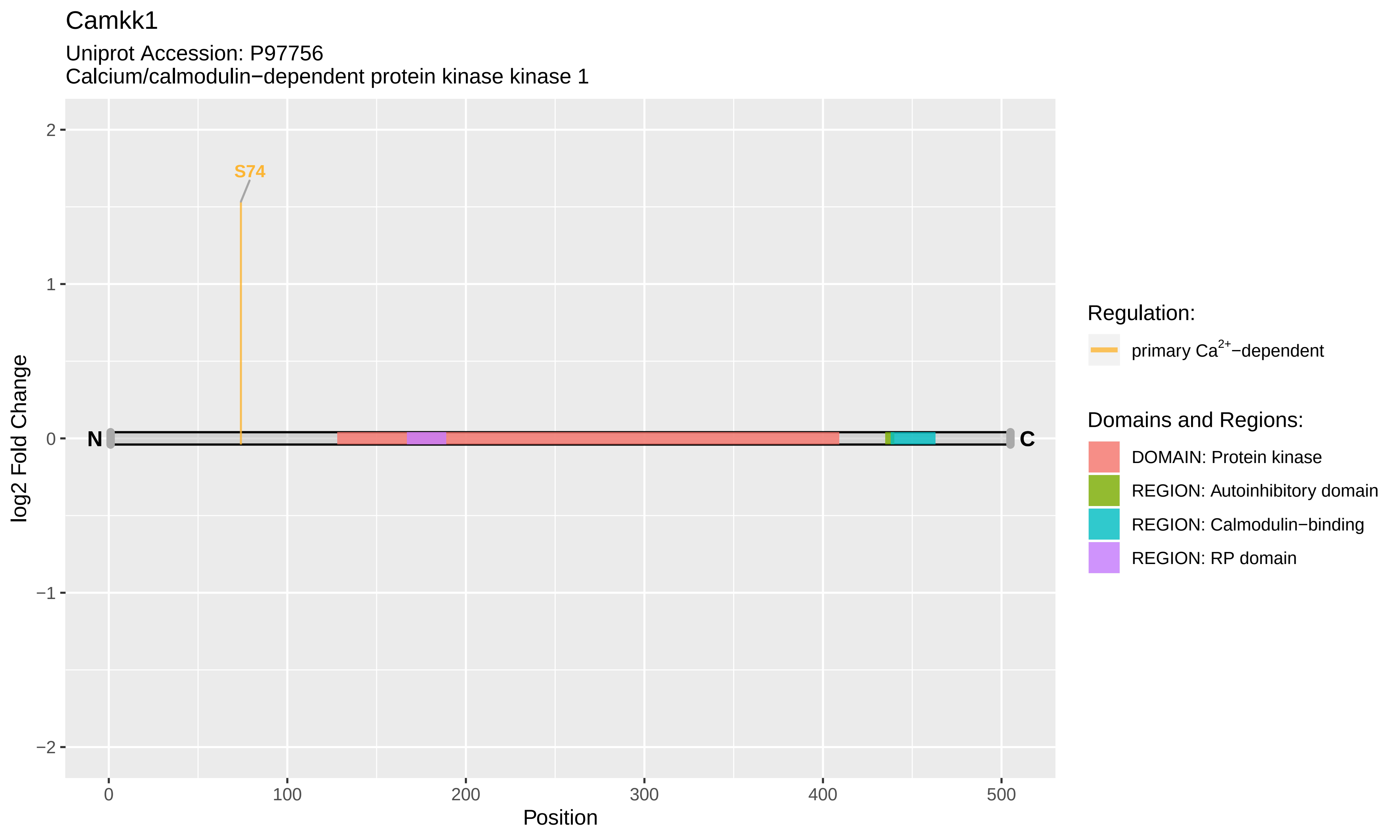

Camkk1
Uniprot Accession: P97756
Calcium/calmodulin−dependent protein kinase kinase 1
S74
N
C
2
1
0
−1
−2
log2 Fold Change
0
100
200
300
400
500
Position
Regulation:
primary Ca2+−dependent
Domains and Regions:
DOMAIN: Protein kinase
REGION: Autoinhibitory domain
REGION: Calmodulin−binding
REGION: RP domain

Camkv

Uniprot Accession: Q63092
CaM kinase−like vesicle−associated protein

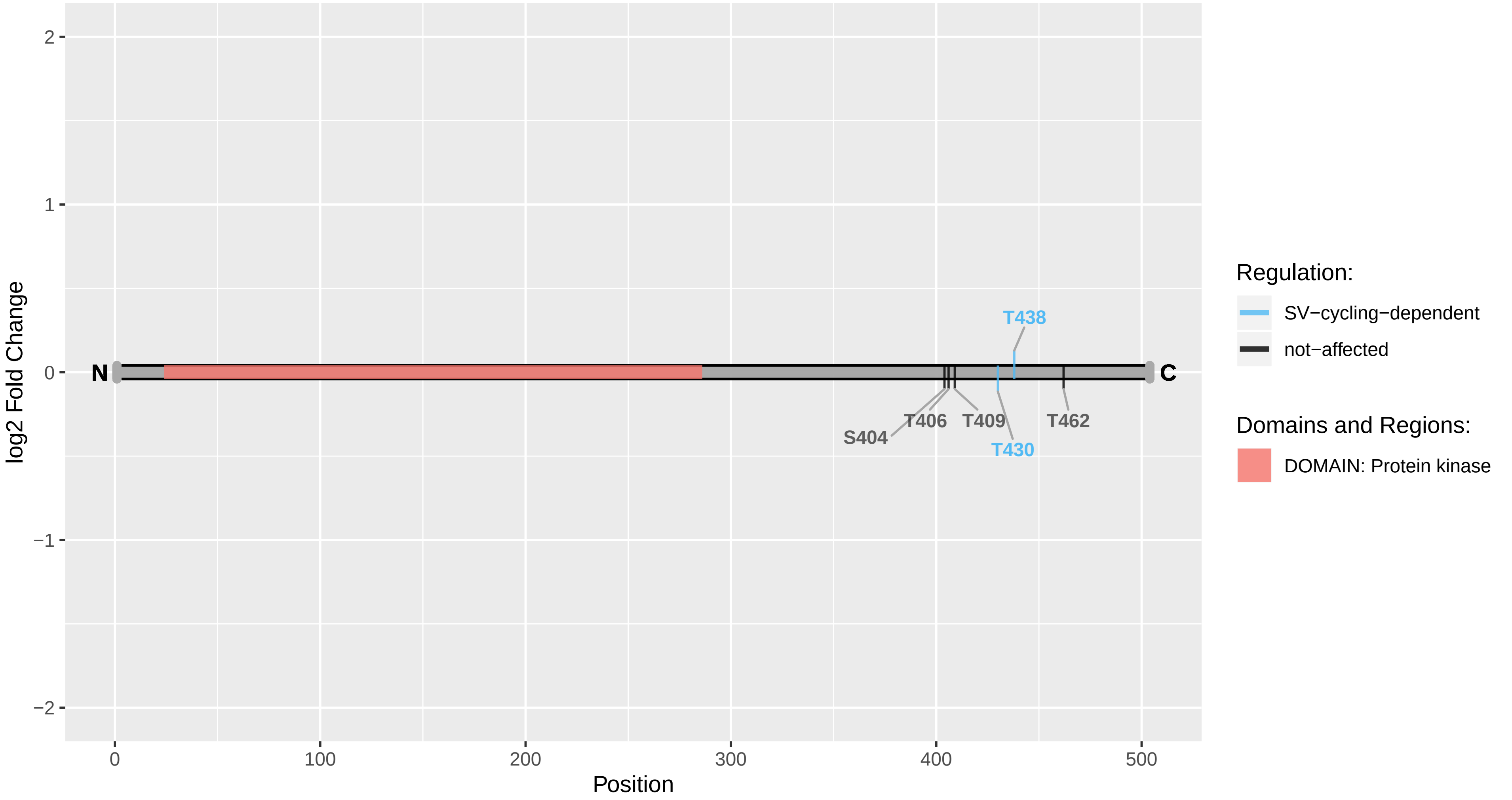

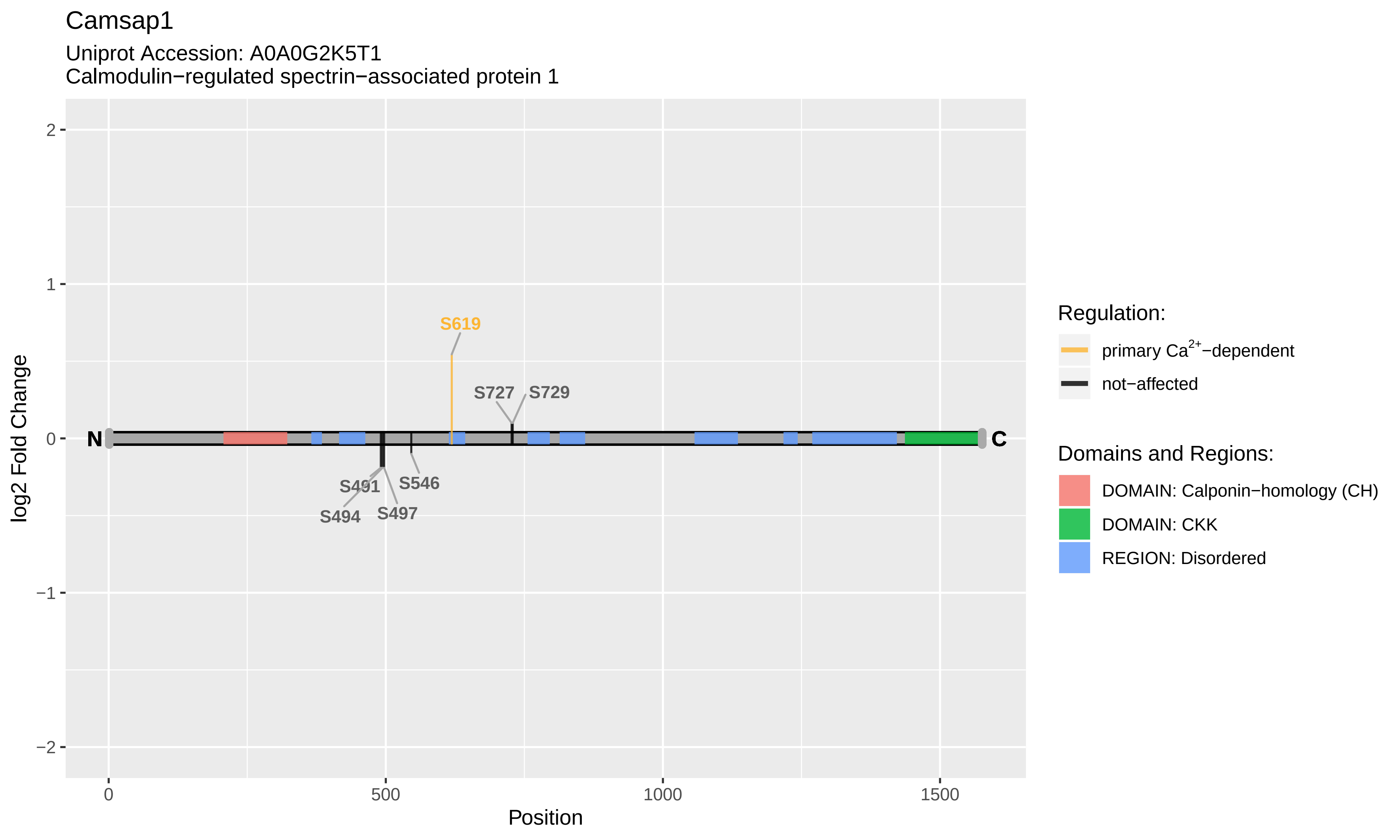

Camsap1
Uniprot Accession: A0A0G2K5T1
Calmodulin−regulated spectrin−associated protein 1
S619
S727
S729
S491
S494
S497
S546
N
C
2
1
0
−1
−2
0
500
1000
1500
log2 Fold Change
Position
Regulation:
primary Ca2+−dependent
not−affected
Domains and Regions:
DOMAIN: Calponin−homology (CH)
DOMAIN: CKK
REGION: Disordered

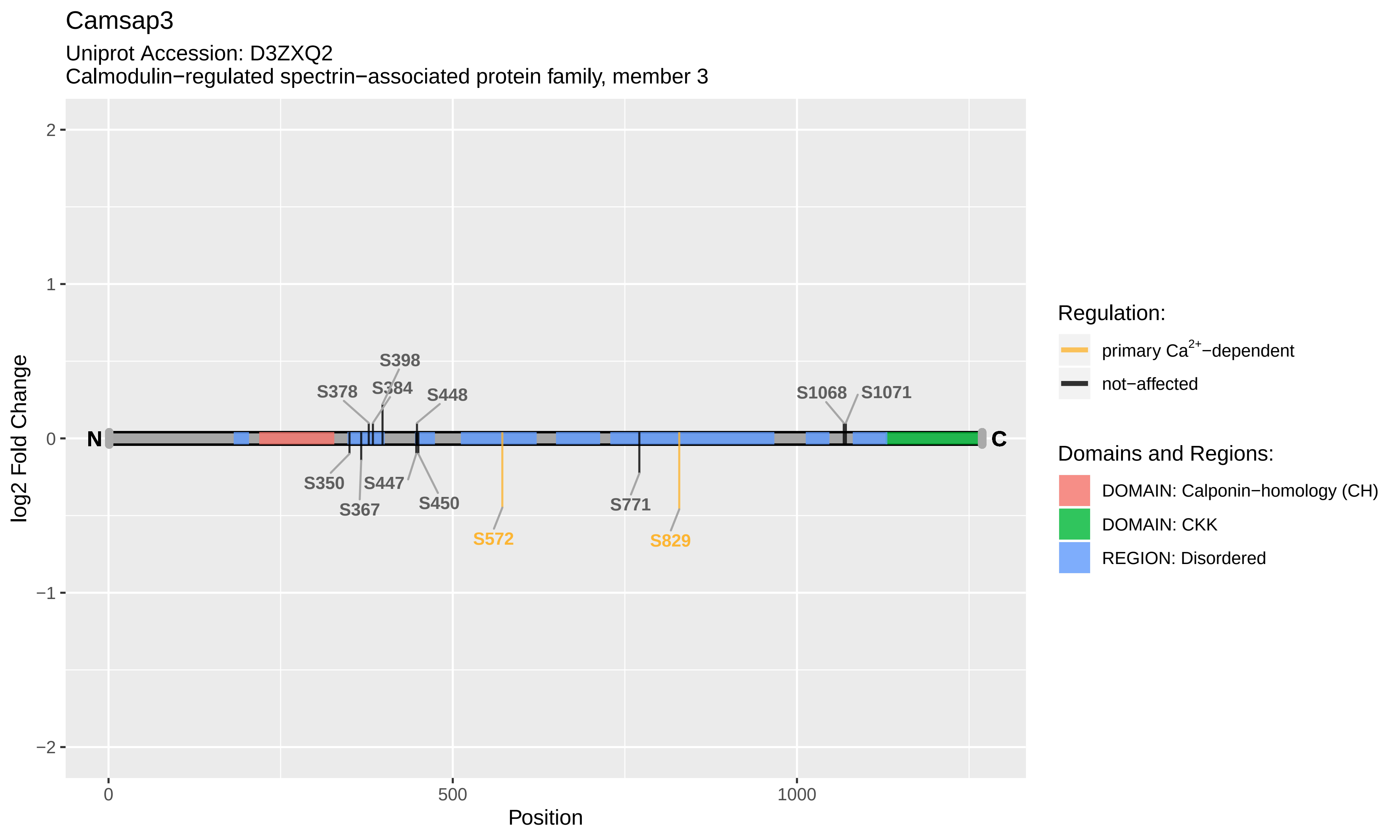

Camsap3
Uniprot Accession: D3ZXQ2
Calmodulin−regulated spectrin−associated protein family, member 3
log2 Fold Change
Position
N
C
S398
S378
S384
S448
S1068
S1071
S350
S447
S367
S450
S771
S572
S829
Regulation:
primary Ca2+−dependent
not−affected
Domains and Regions:
DOMAIN: Calponin−homology (CH)
DOMAIN: CKK
REGION: Disordered

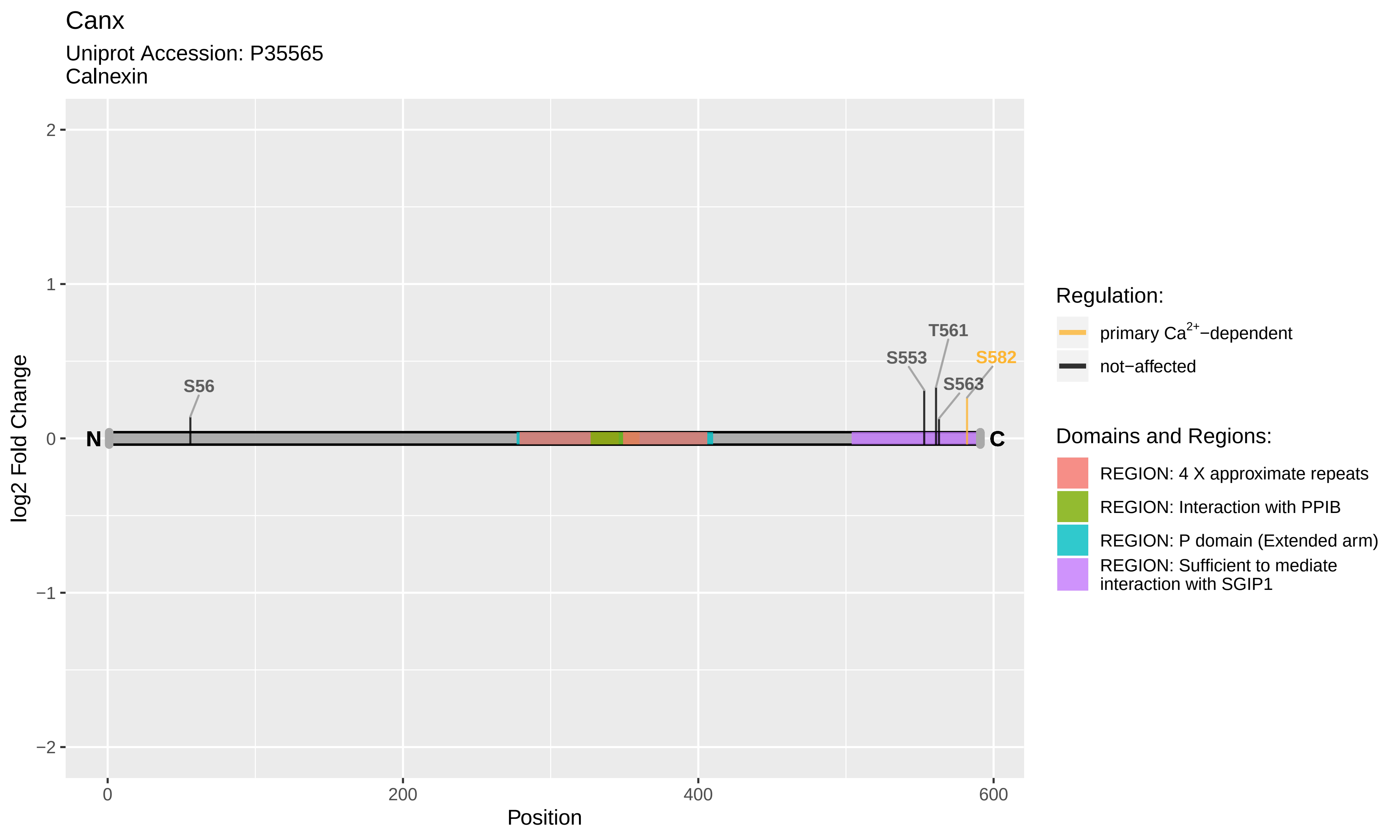

Canx
Uniprot Accession: P35565
Calnexin
log2 Fold Change
Position
N
C
S56
S553
T561
S563
S582
Regulation:
primary Ca2+−dependent
not−affected
Domains and Regions:
REGION: 4 X approximate repeats
REGION: Interaction with PPIB
REGION: P domain (Extended arm)
REGION: Sufficient to mediate interaction with SGIP1

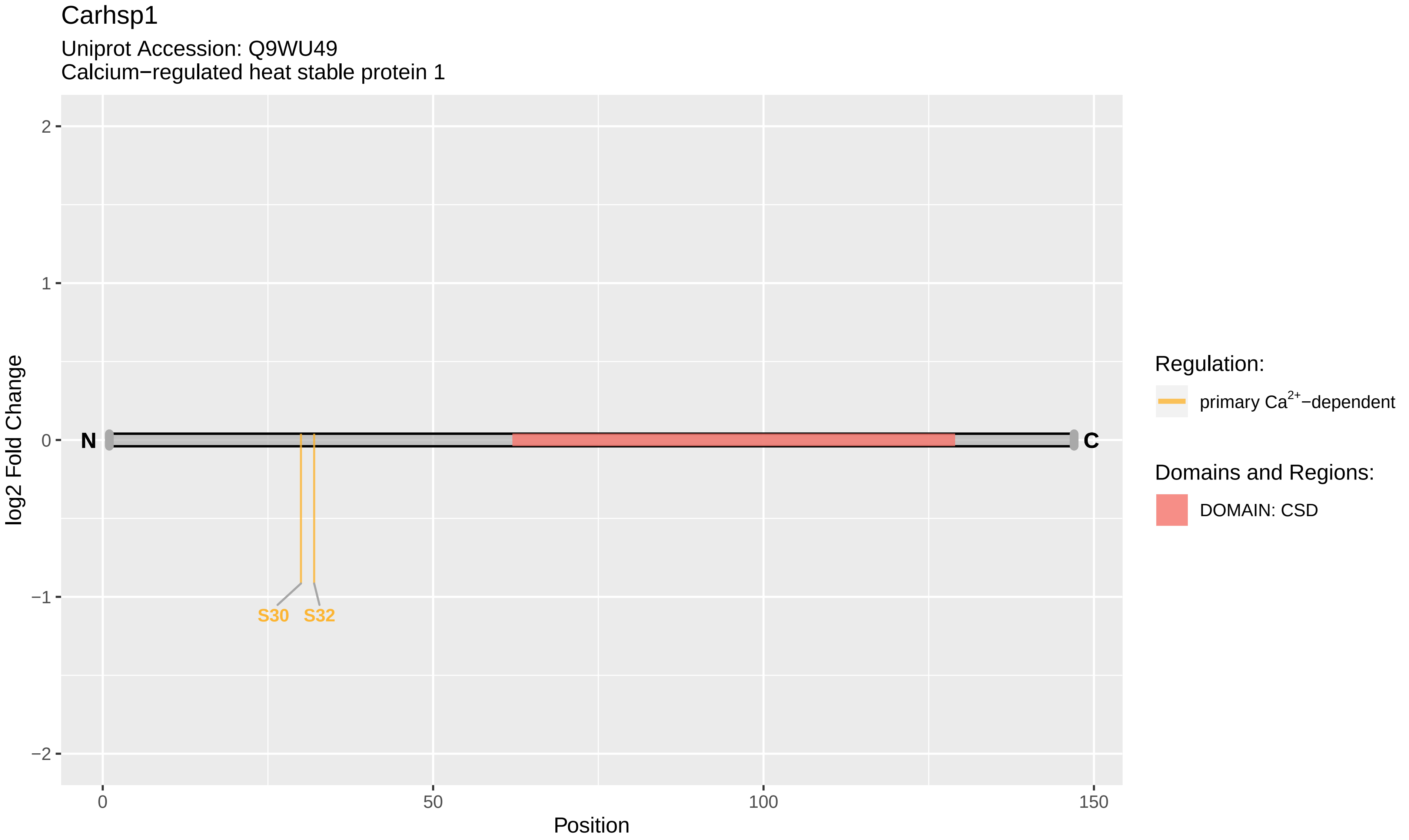
Carhsp1
Uniprot Accession: Q9WU49
Calcium−regulated heat stable protein 1
log2 Fold Change
2
1
0
−1
−2
N
C
S30
S32
0
50
100
150
Position
Regulation:
primary Ca2+−dependent
Domains and Regions:
DOMAIN: CSD

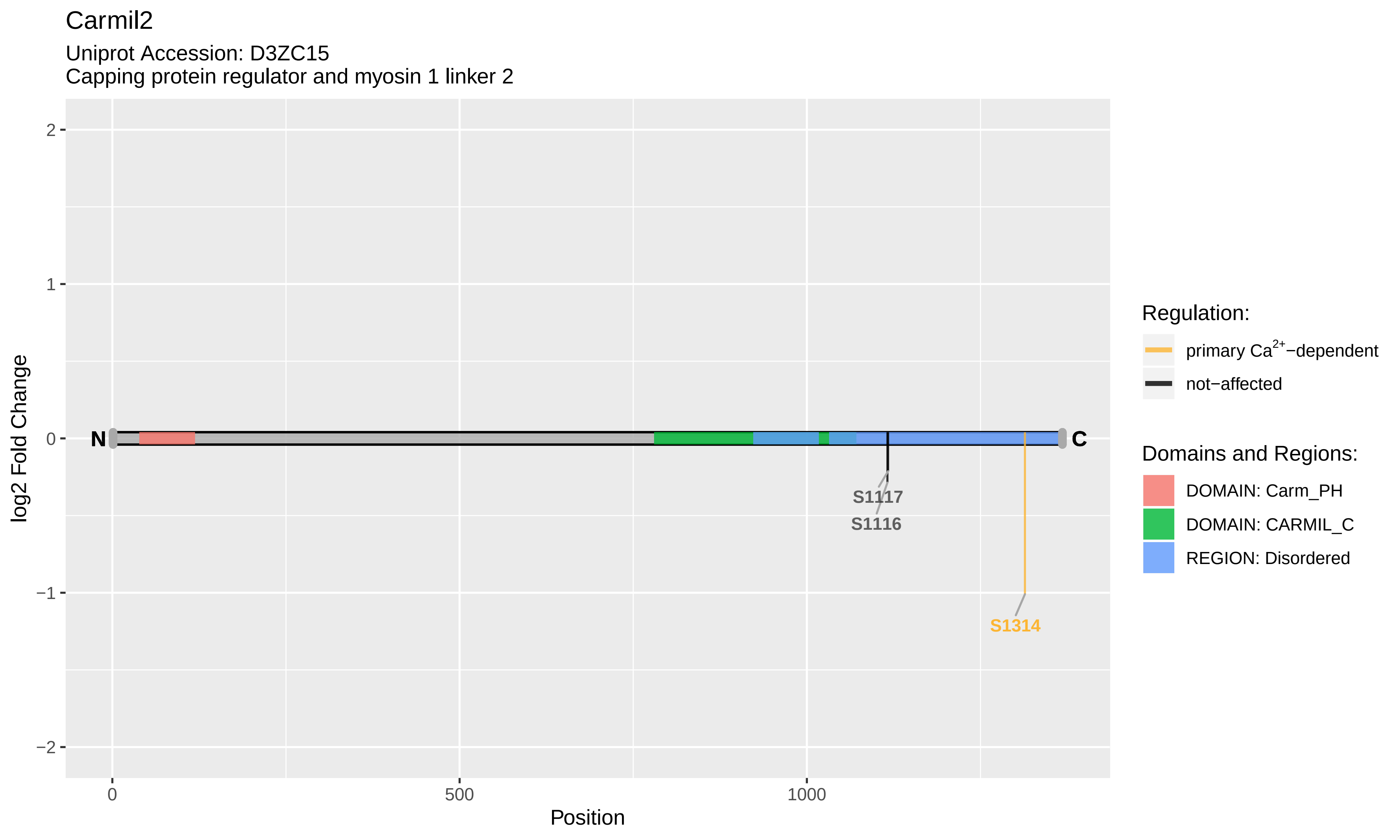

Carmil2
Uniprot Accession: D3ZC15
Capping protein regulator and myosin 1 linker 2
log2 Fold Change
2
1
0
−1
−2
N
C
S1117
S1116
S1314
0
500
1000
Position
Regulation:
primary Ca2+−dependent
not−affected
Domains and Regions:
DOMAIN: Carm_PH
DOMAIN: CARMIL_C
REGION: Disordered

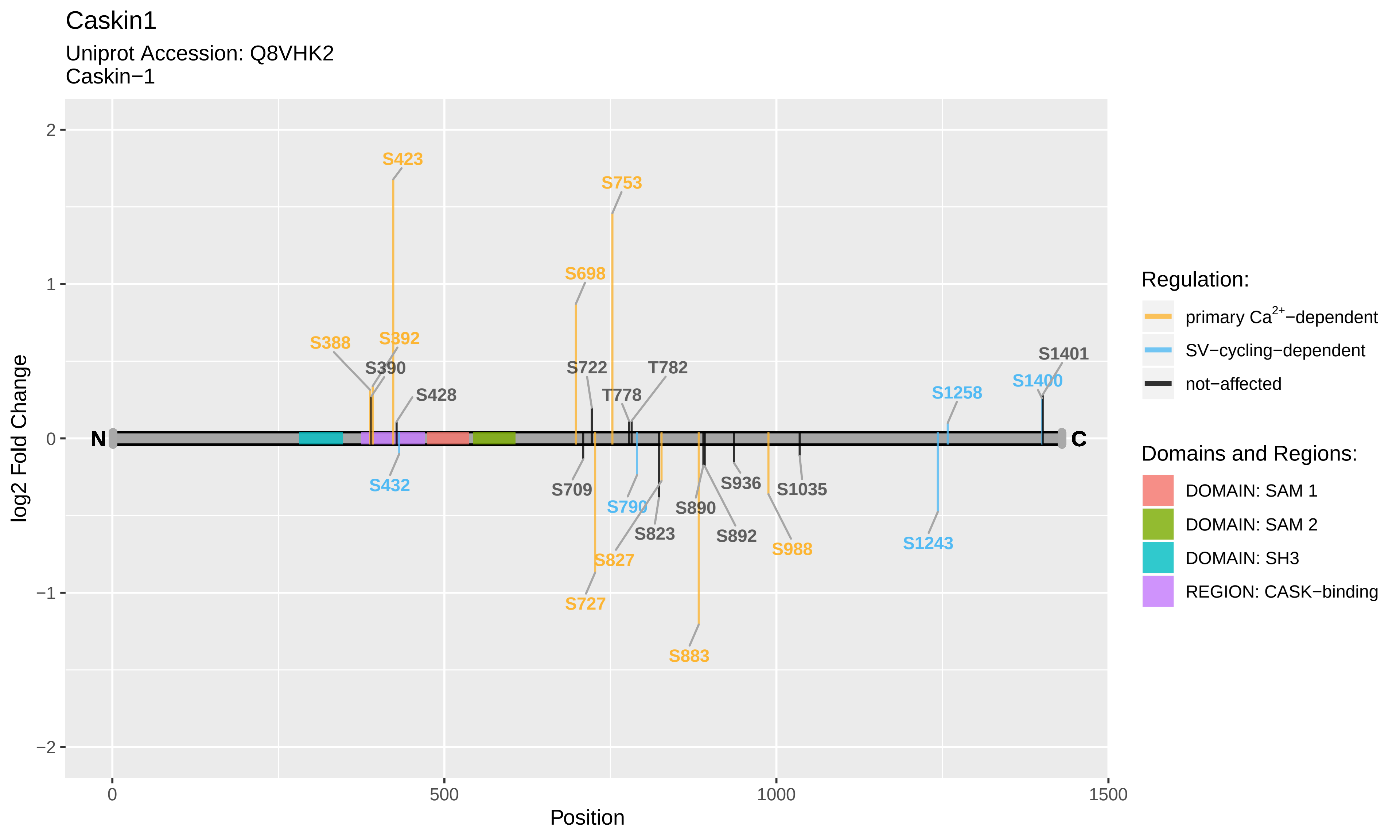
Caskin1
Uniprot Accession: Q8VHK2
Caskin−1
log2 Fold Change
Position
2
1
0
−1
−2
0
500
1000
1500
N
C
S423
S753
S698
S388
S892
S390
S428
S722
T782
T778
S1401
S1400
S1258
S432
S709
S790
S823
S827
S727
S890
S892
S936
S1035
S988
S883
S1243
Regulation:
primary Ca2+−dependent
SV−cycling−dependent
not−affected
Domains and Regions:
DOMAIN: SAM 1
DOMAIN: SAM 2
DOMAIN: SH3
REGION: CASK−binding

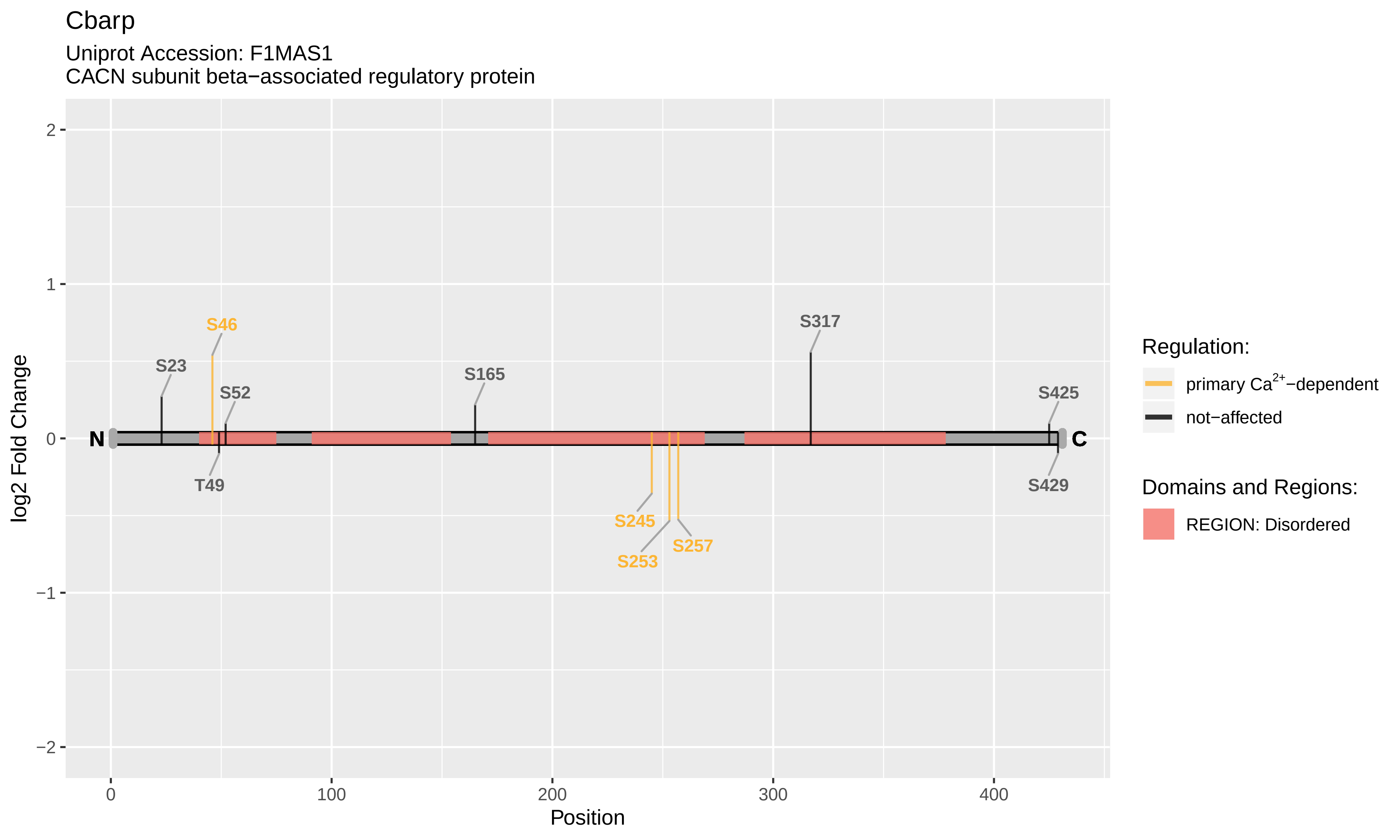
Cbarp
Uniprot Accession: F1MAS1
CACN subunit beta−associated regulatory protein
log2 Fold Change
Position
2
1
0
−1
−2
0
100
200
300
400
N
C
S23
S46
S52
T49
S165
S245
S253
S257
S317
S425
S429
Regulation:
primary Ca2+−dependent
not−affected
Domains and Regions:
REGION: Disordered

Ccdc120

Uniprot Accession: A0A0G2JUY5
Coiled−coil domain−containing 120

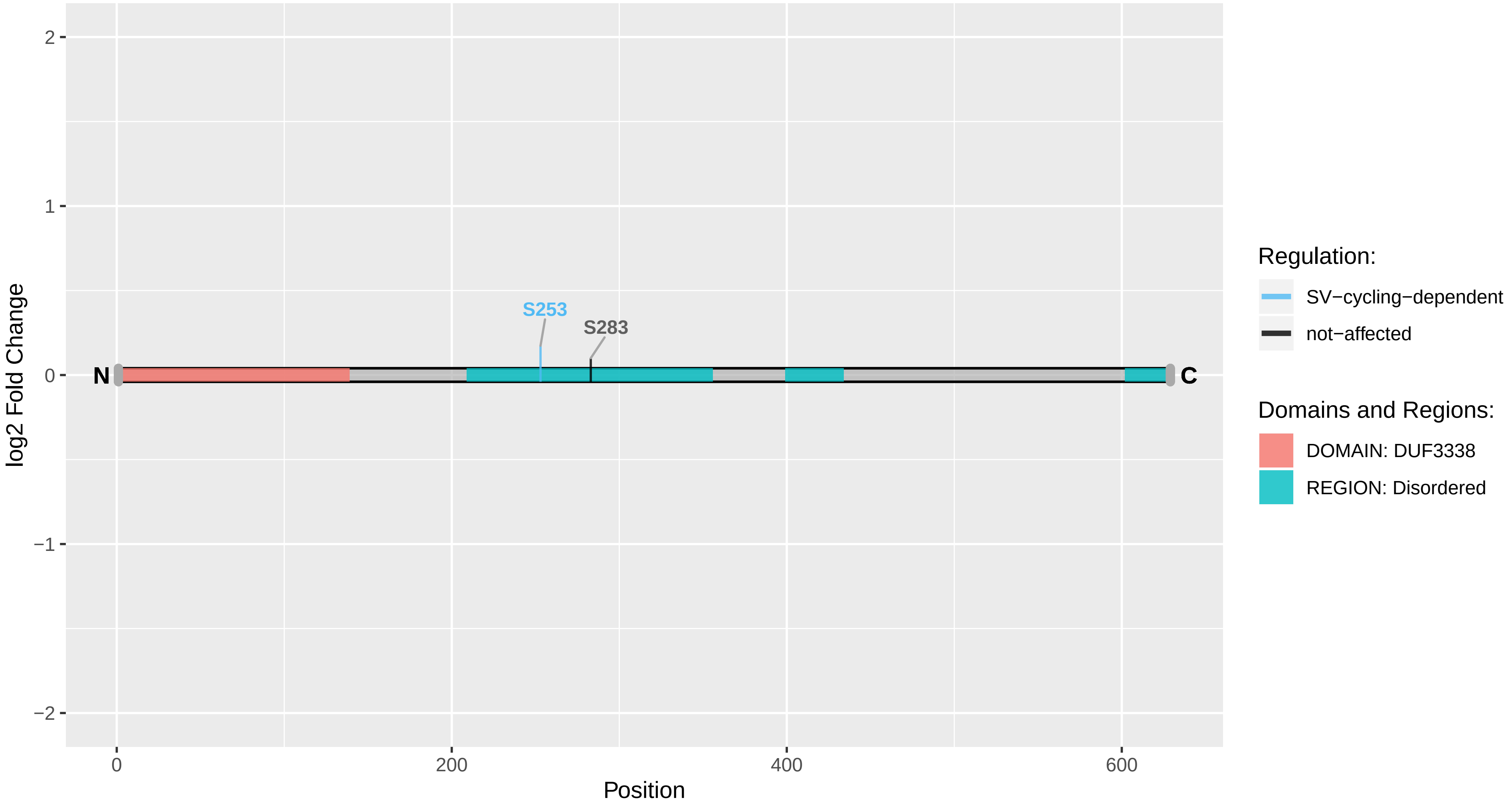

Ccdc177

Uniprot Accession: D4A8V2
Coiled−coil domain−containing 177

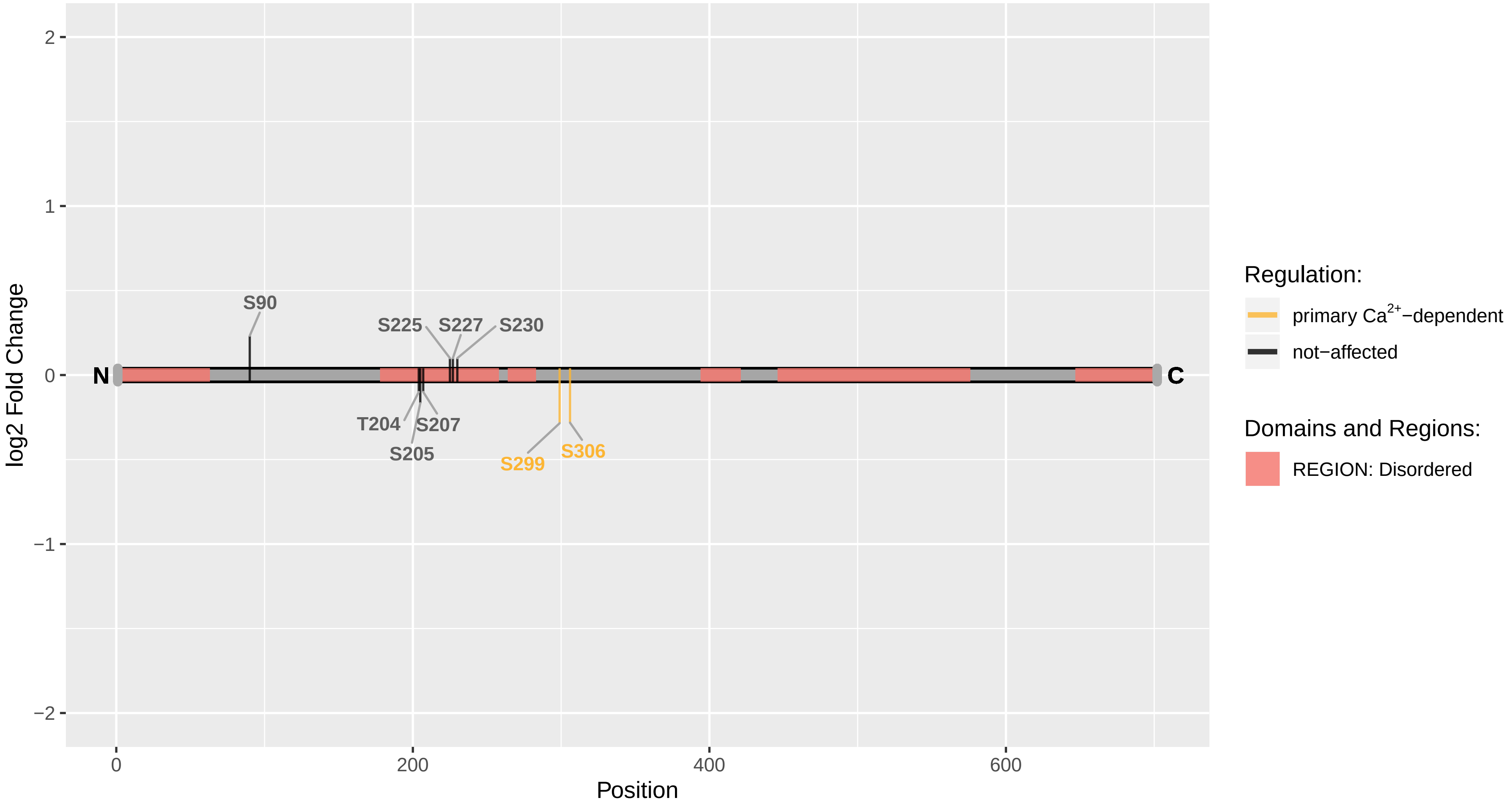

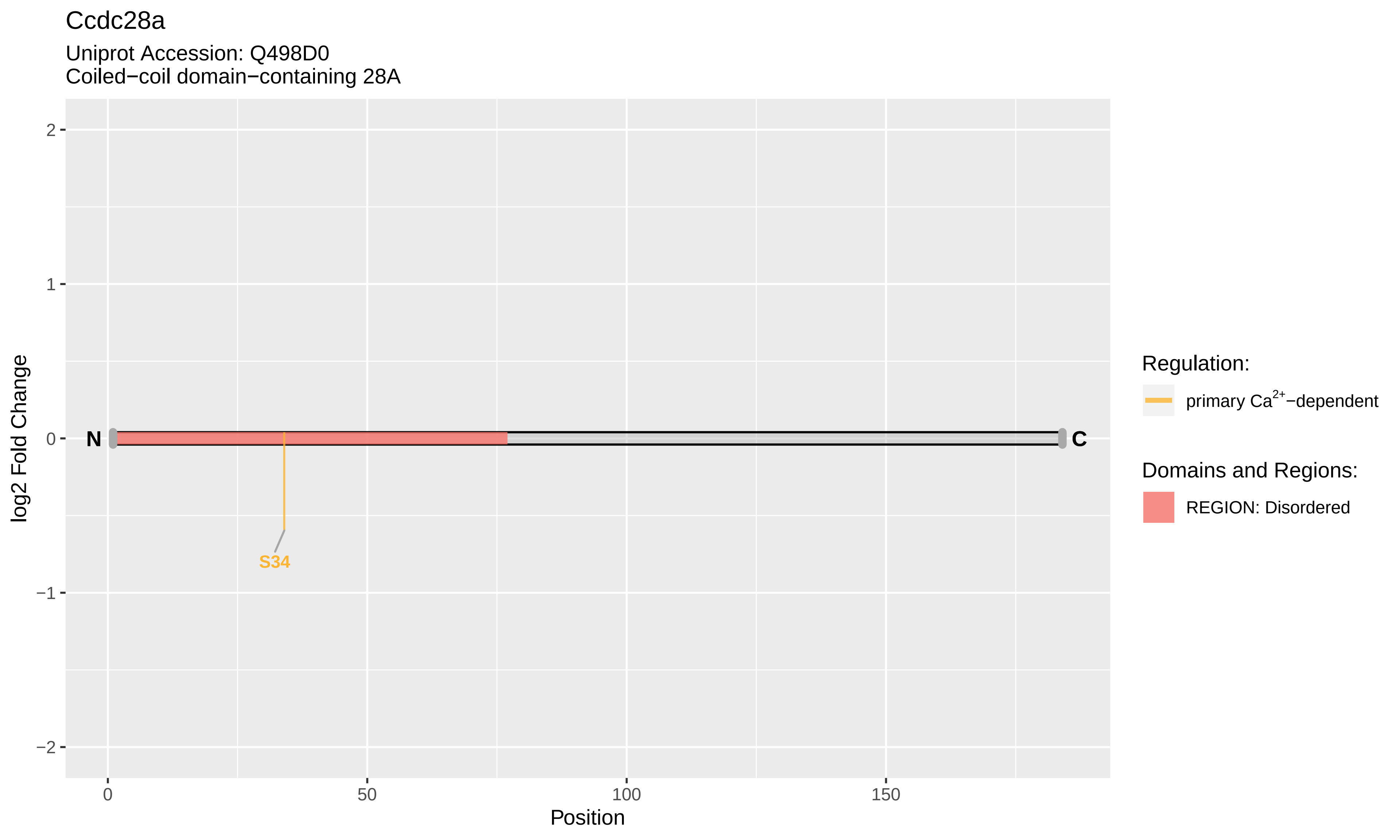

Ccdc28a
Uniprot Accession: Q498D0
Coiled−coil domain−containing 28A
log2 Fold Change
Position
2
1
0
−1
−2
0
50
100
150
N
C
S34
Regulation:
primary Ca2+−dependent
Domains and Regions:
REGION: Disordered

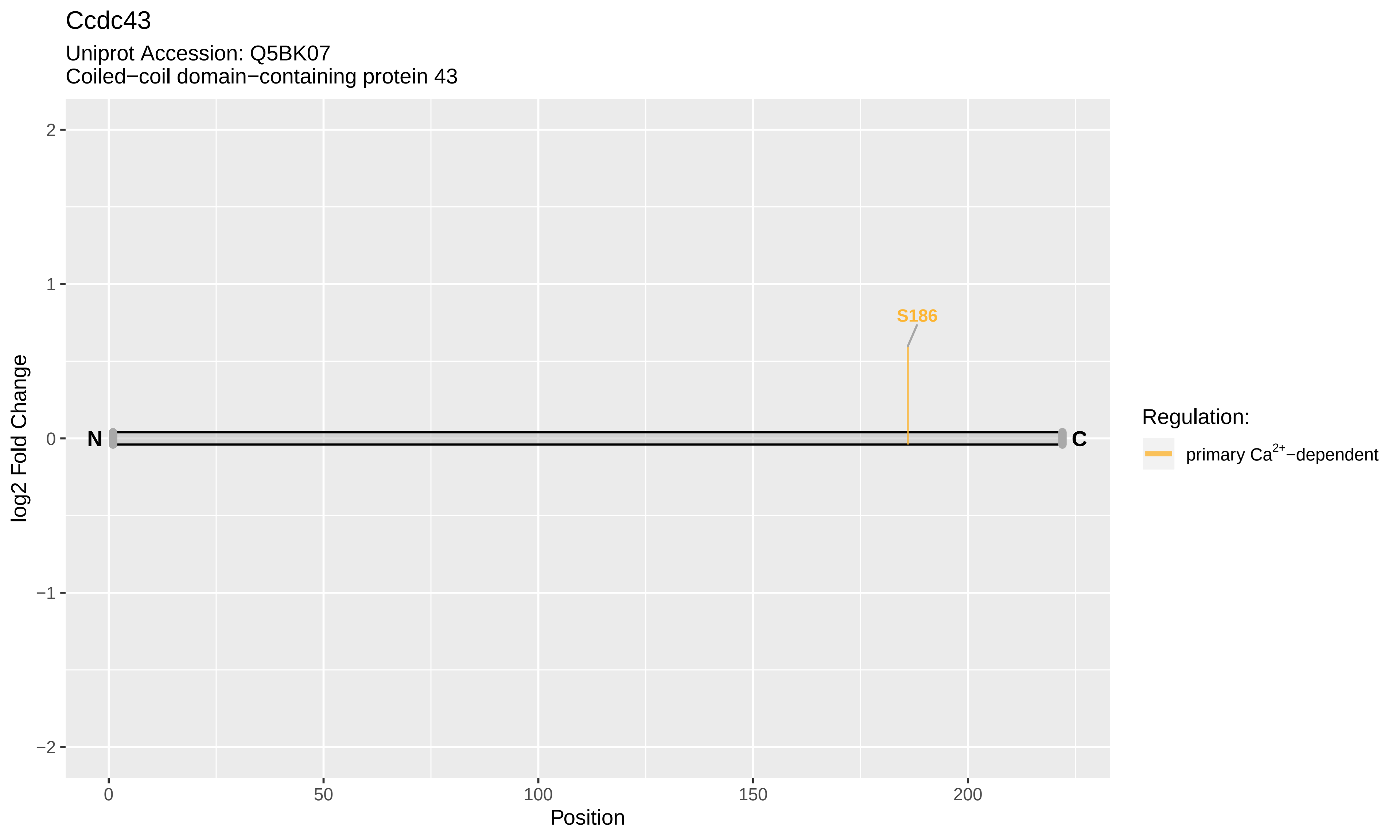
Ccdc43
Uniprot Accession: Q5BK07
Coiled−coil domain−containing protein 43
log2 Fold Change
2
1
0
−1
−2
N
C
S186
0
50
100
150
200
Position
Regulation:
primary Ca2+−dependent

Ccdc6

Uniprot Accession: D4AEK9
Coiled−coil domain−containing 6

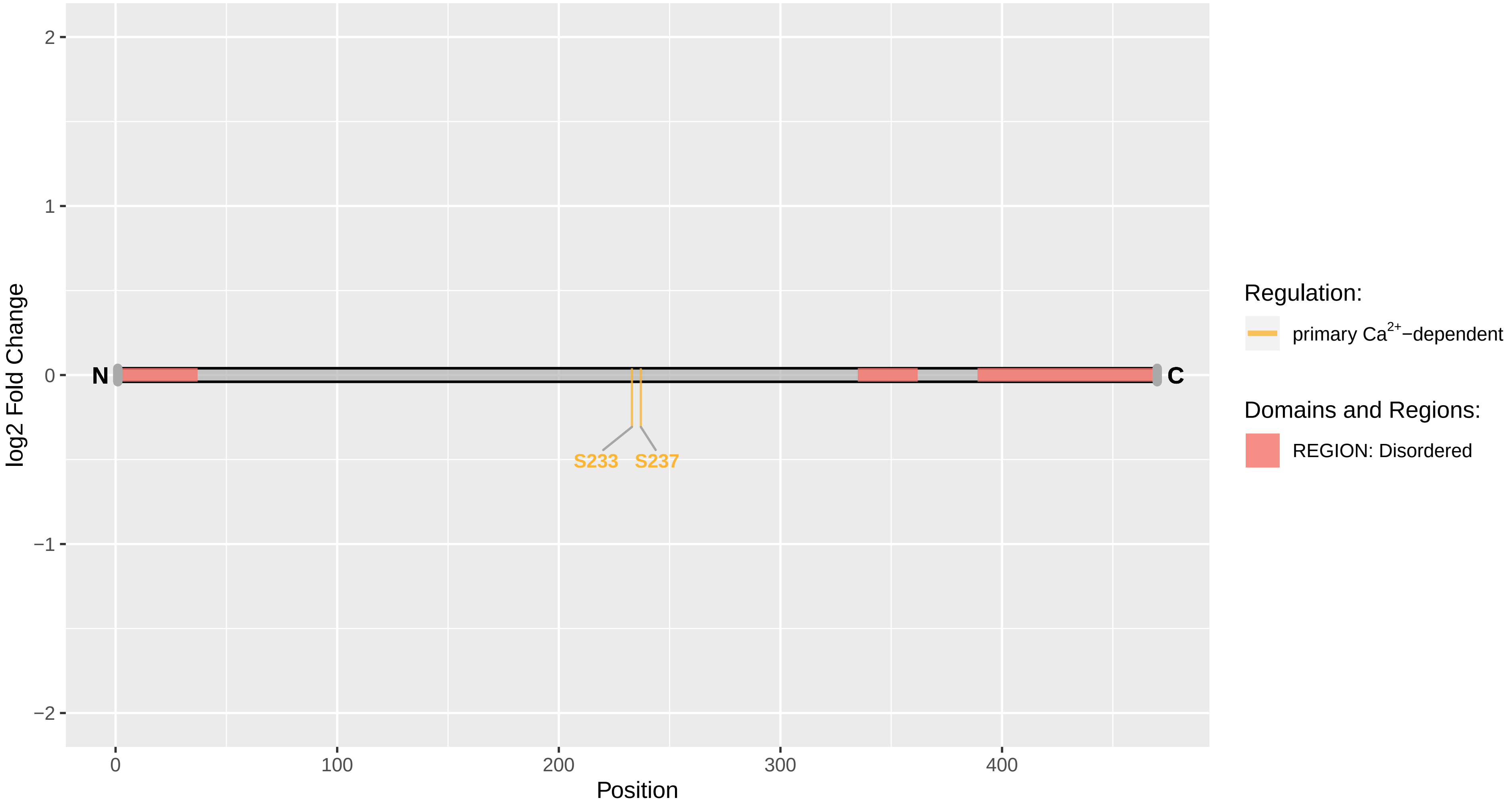

Ccs

Uniprot Accession: Q9JK72
Copper chaperone for superoxide dismutase

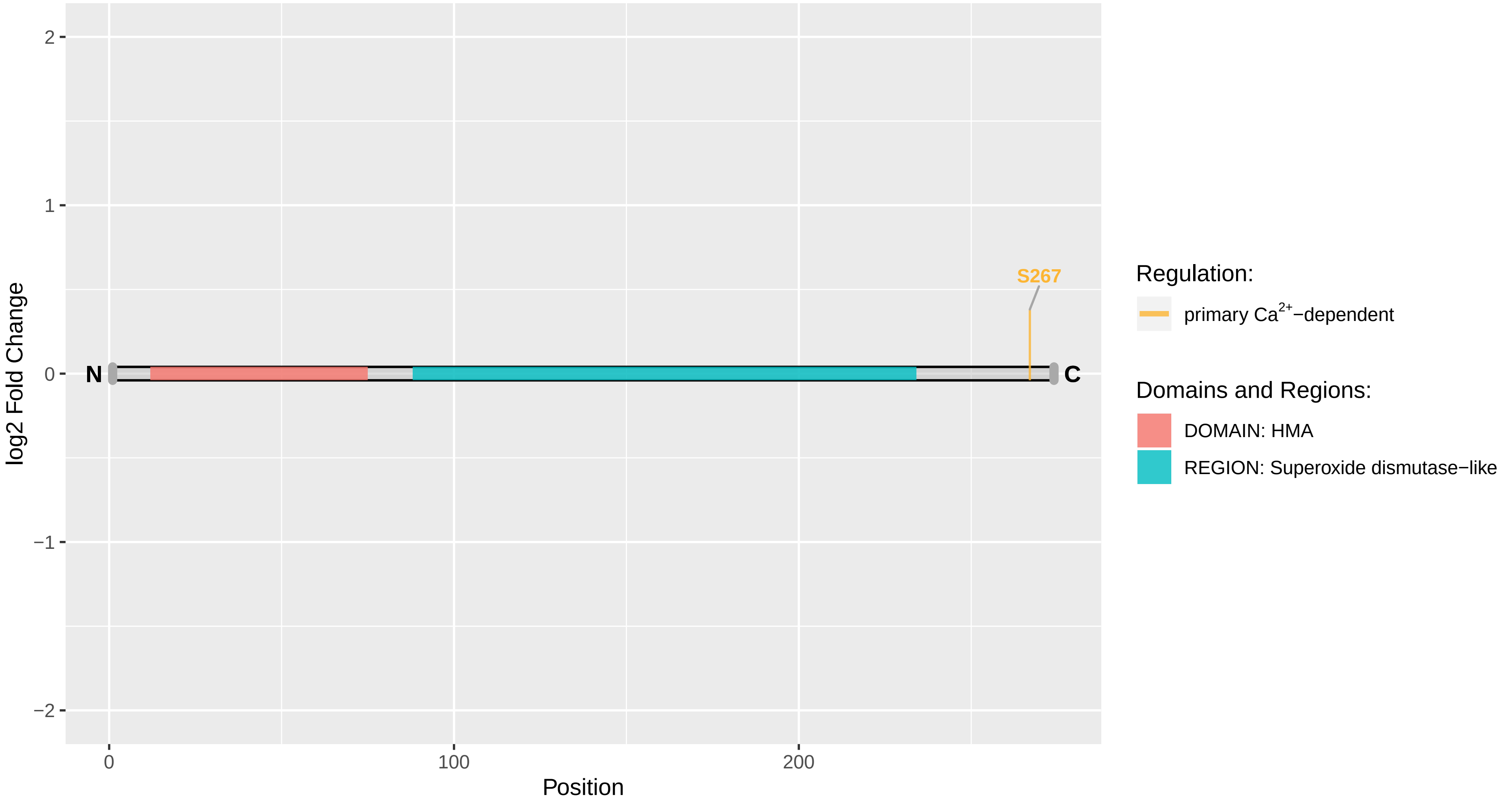

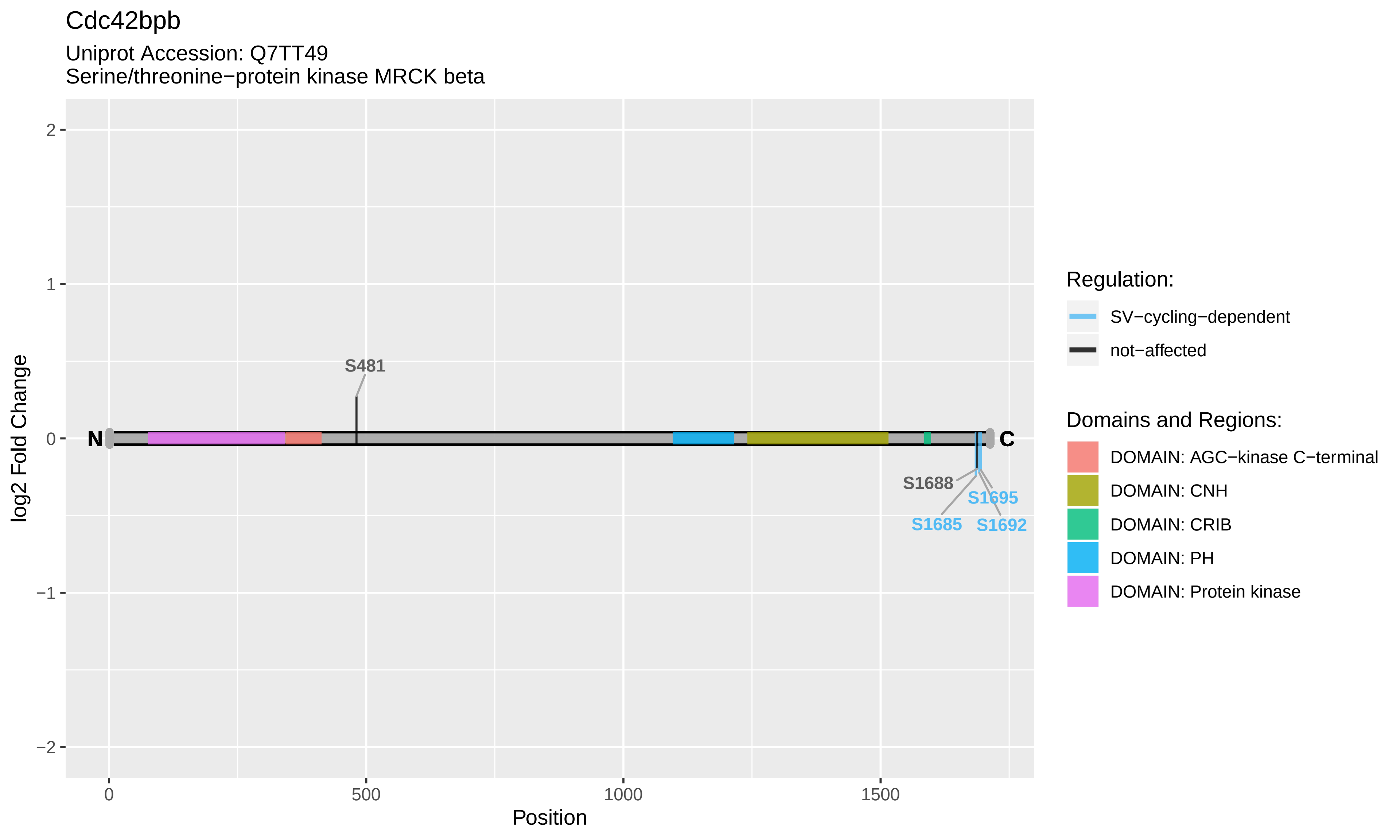

Cdc42bpb
Uniprot Accession: Q7TT49
Serine/threonine−protein kinase MRCK beta
log2 Fold Change
2
1
0
−1
−2
N
C
S481
S1688
S1695
S1685
S1692
0
500
1000
1500
Position
Regulation:
SV−cycling−dependent
not−affected
Domains and Regions:
DOMAIN: AGC−kinase C−terminal
DOMAIN: CNH
DOMAIN: CRIB
DOMAIN: PH
DOMAIN: Protein kinase

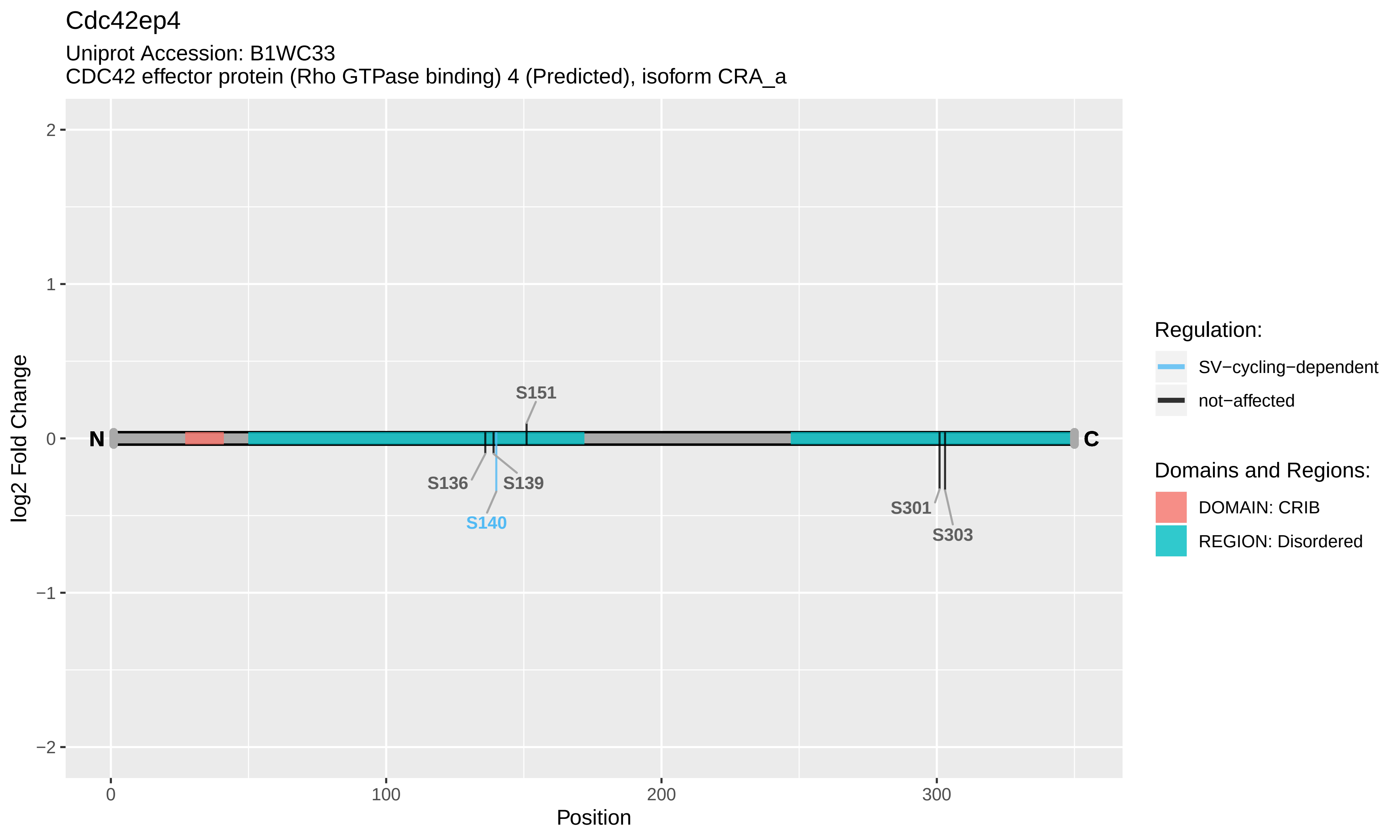
Cdc42ep4
Uniprot Accession: B1WC33
CDC42 effector protein (Rho GTPase binding) 4 (Predicted), isoform CRA_a
log2 Fold Change
Position
N
C
S136
S139
S140
S151
S301
S303
Regulation:
SV−cycling−dependent
not−affected
Domains and Regions:
DOMAIN: CRIB
REGION: Disordered
2
1
0
−1
−2
0
100
200
300

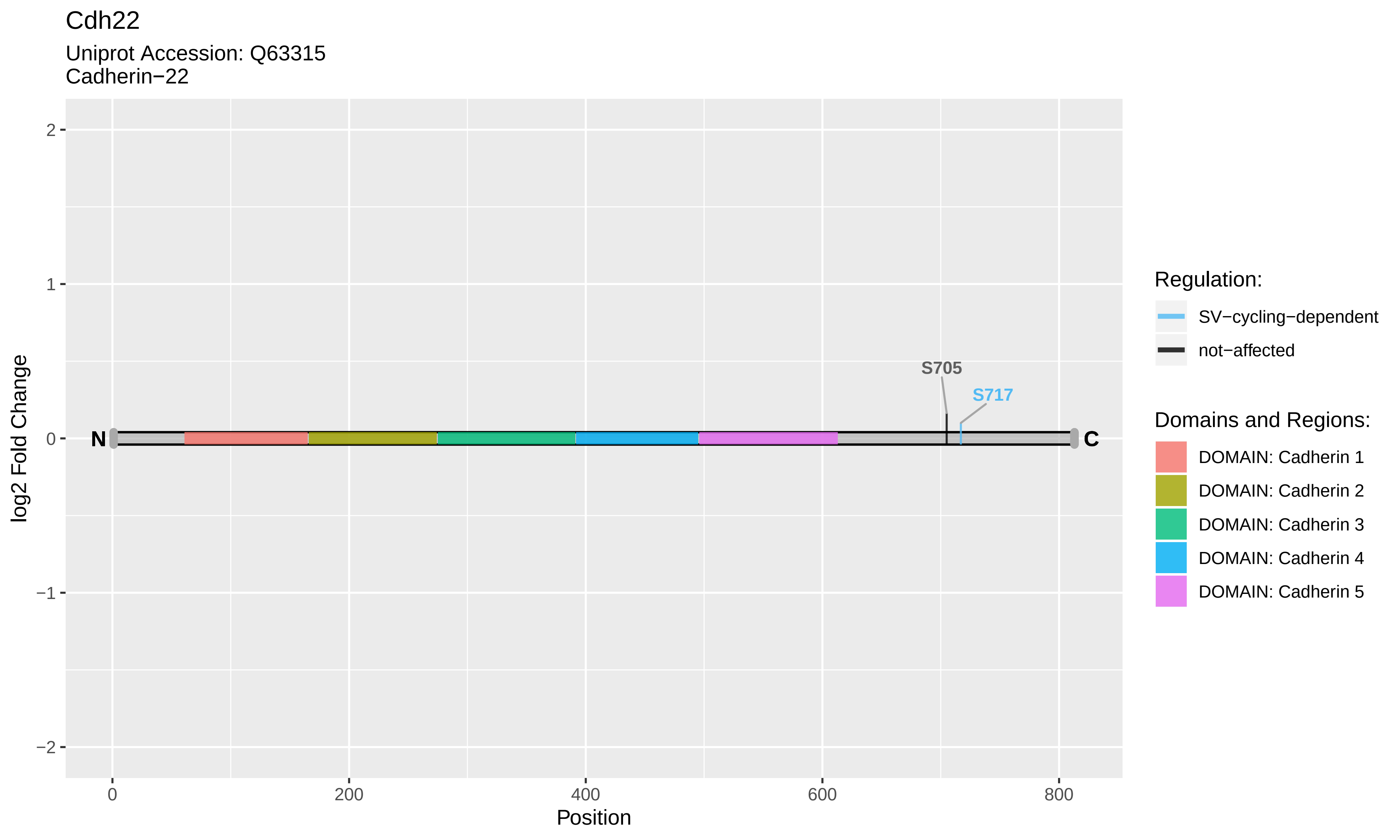

Cdh22
Uniprot Accession: Q63315
Cadherin−22
log2 Fold Change
Position
N
C
S705
S717
2
1
0
−1
−2
0
200
400
600
800
Regulation:
SV−cycling−dependent
not−affected
Domains and Regions:
DOMAIN: Cadherin 1
DOMAIN: Cadherin 2
DOMAIN: Cadherin 3
DOMAIN: Cadherin 4
DOMAIN: Cadherin 5

Cdk18

Uniprot Accession: O35832
Cyclin−dependent kinase 18

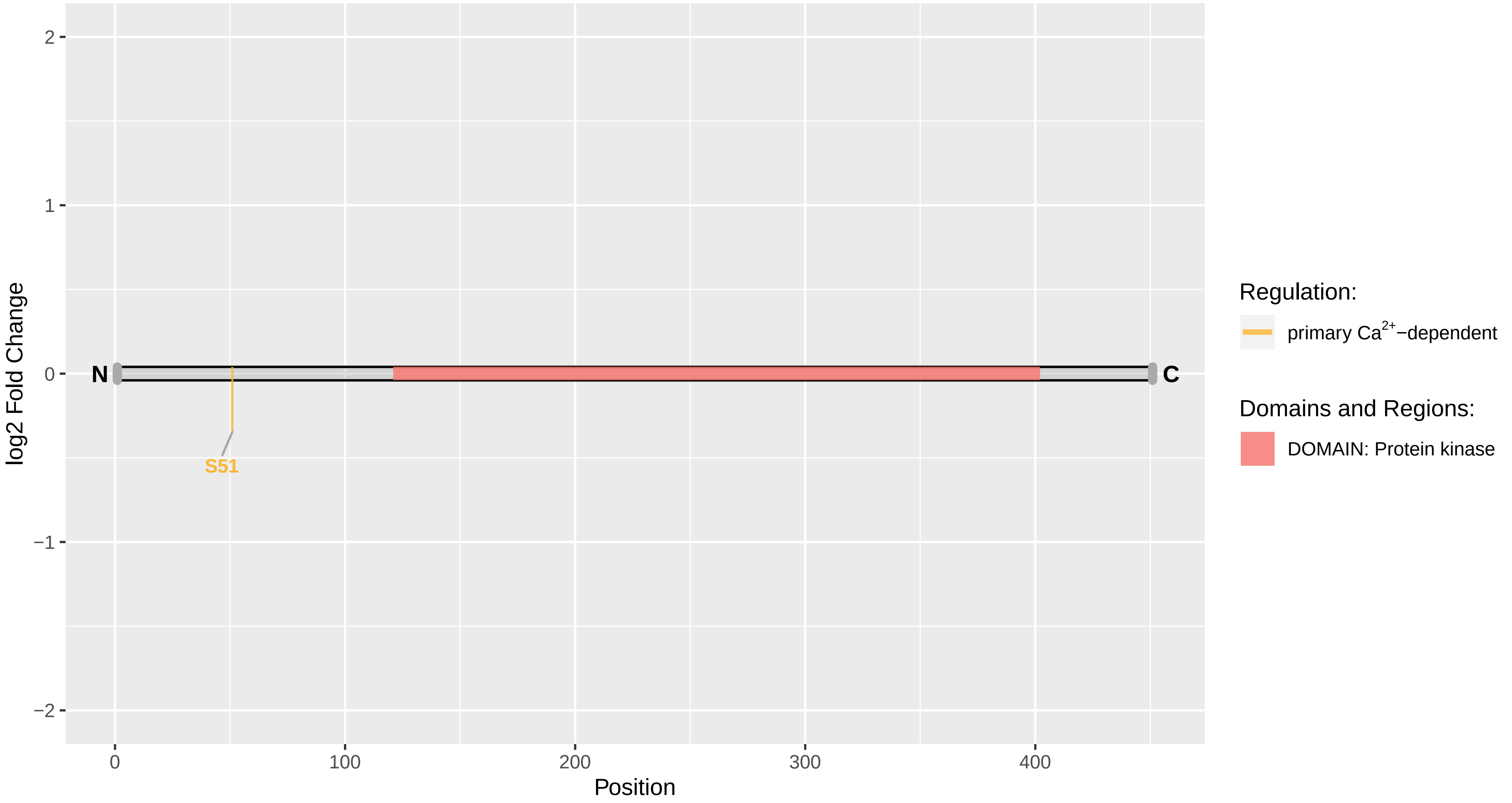

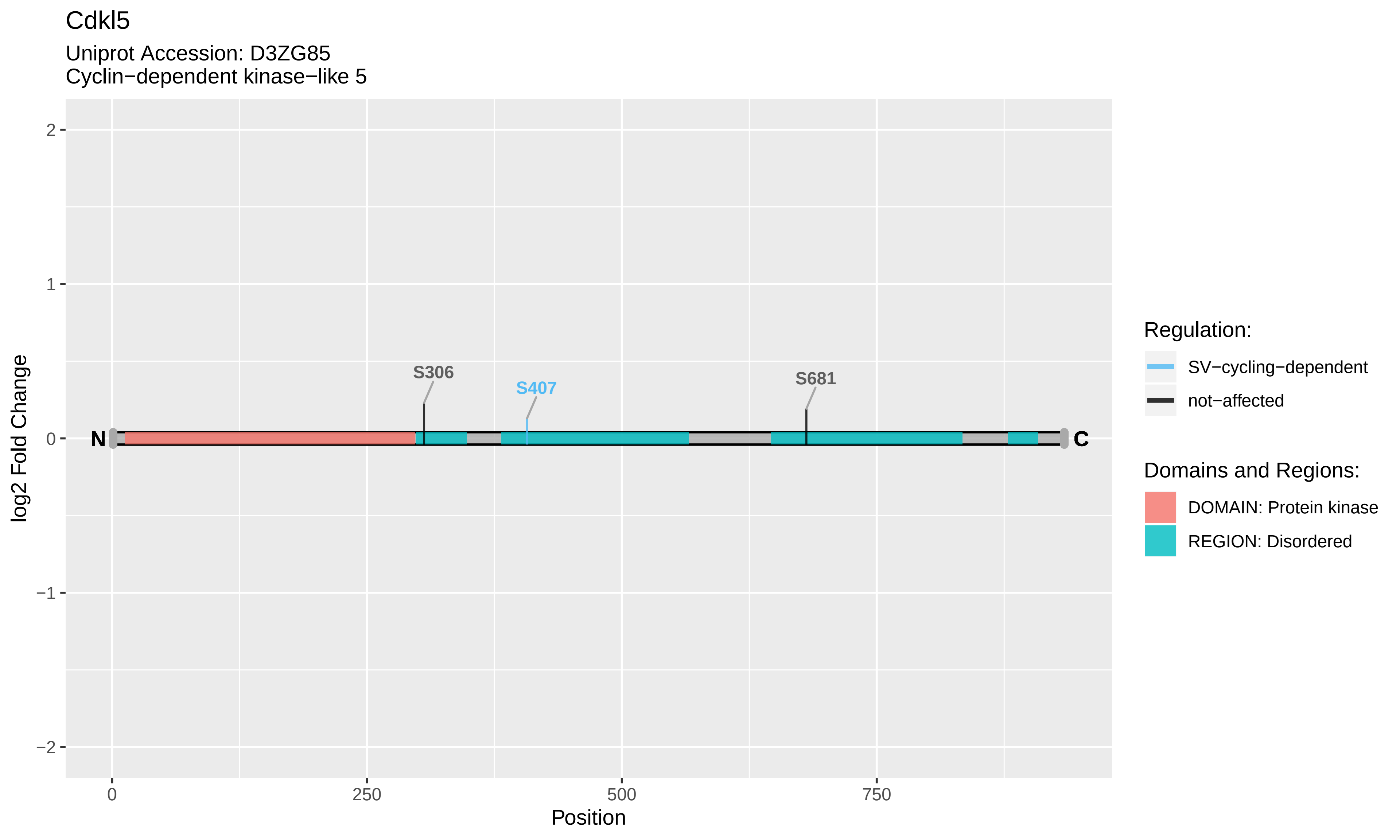

Cdkl5
Uniprot Accession: D3ZG85
Cyclin−dependent kinase−like 5
log2 Fold Change
2
1
0
−1
−2
N
C
S306
S407
S681
0
250
500
750
Position
Regulation:
SV−cycling−dependent
not−affected
Domains and Regions:
DOMAIN: Protein kinase
REGION: Disordered

Cds2

Uniprot Accession: Q91XU8
Phosphatidate cytidylyltransferase 2

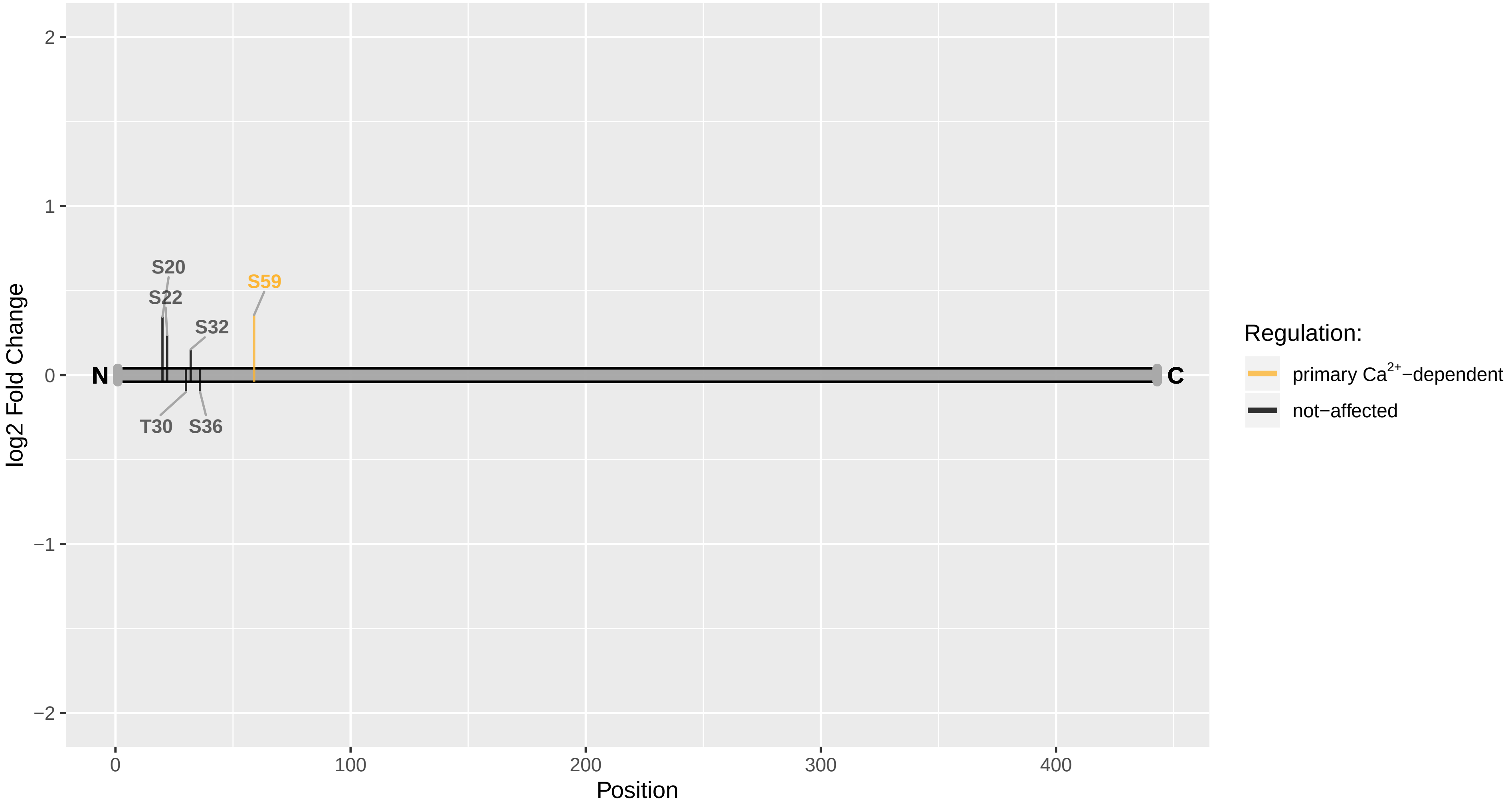

Cdv3

Uniprot Accession: Q5XIM5
Protein CDV3 homolog

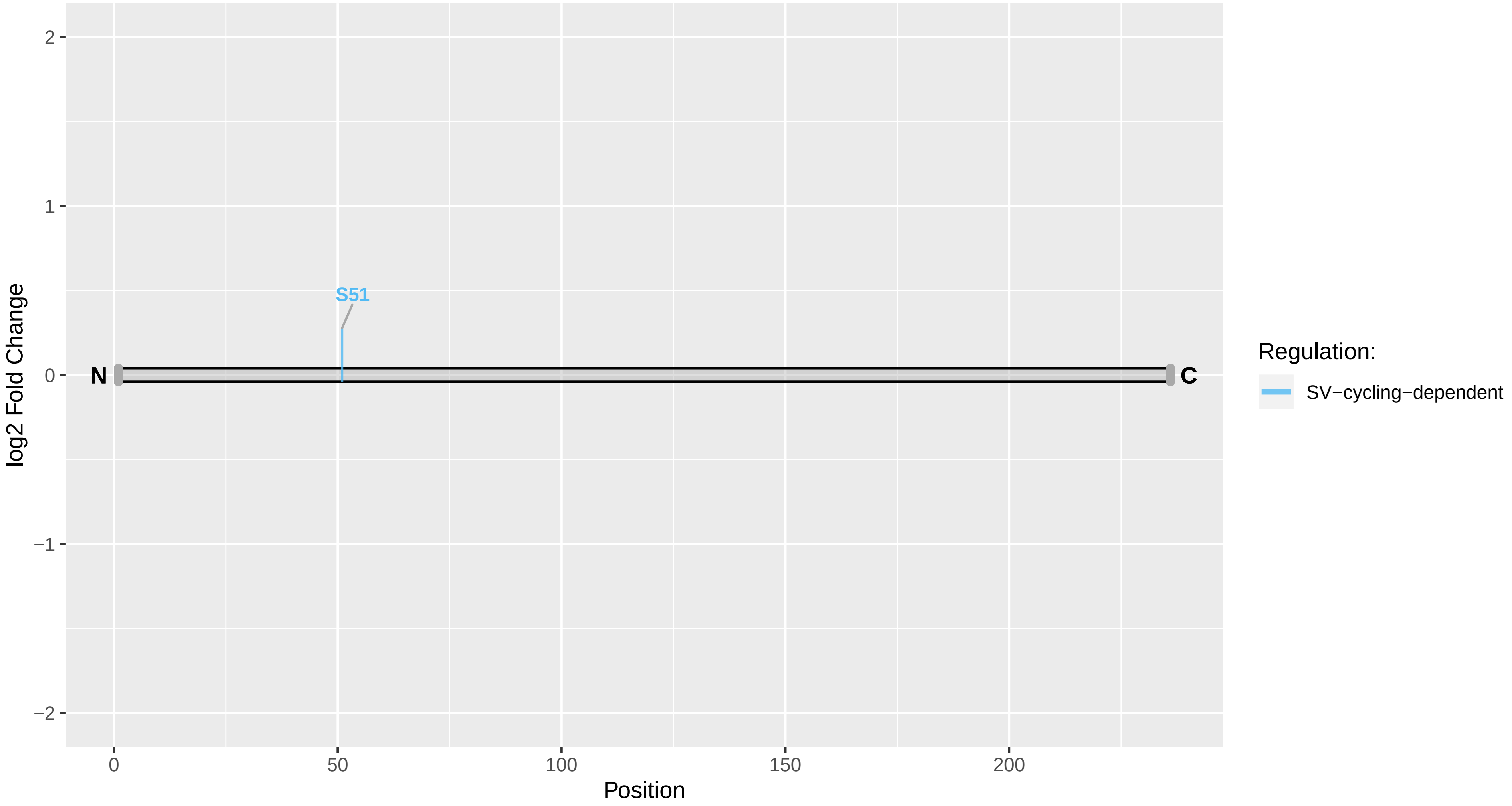

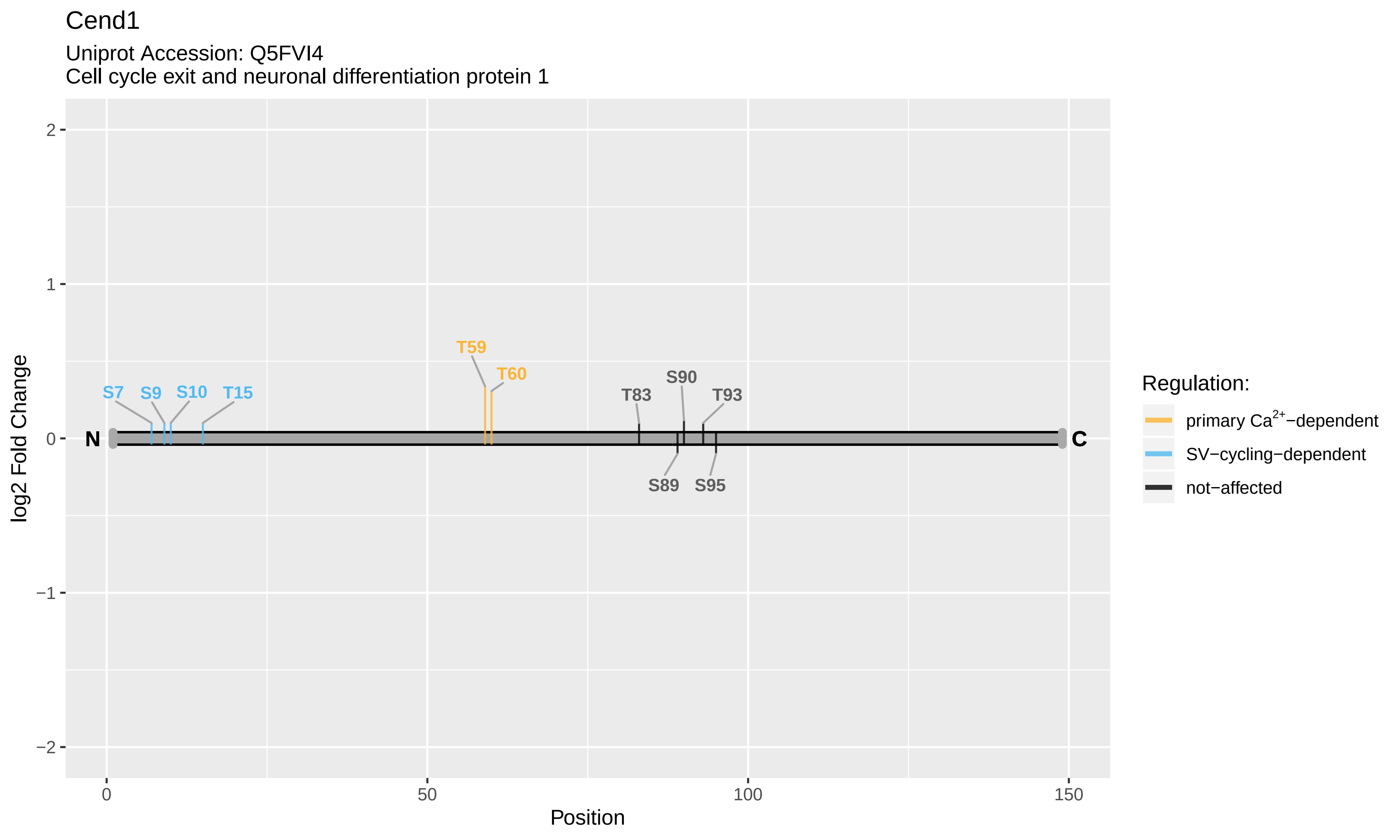

Cend1
Uniprot Accession: Q5FVI4
Cell cycle exit and neuronal differentiation protein 1
log2 Fold Change
2
1
0
−1
−2
N
C
S7
S9
S10
T15
T59
T60
T83
S90
T93
S89
S95
0
50
100
150
Position
Regulation:
primary Ca2+−dependent
SV−cycling−dependent
not−affected

Cep170

Uniprot Accession: A0A0G2K315
Uncharacterized protein

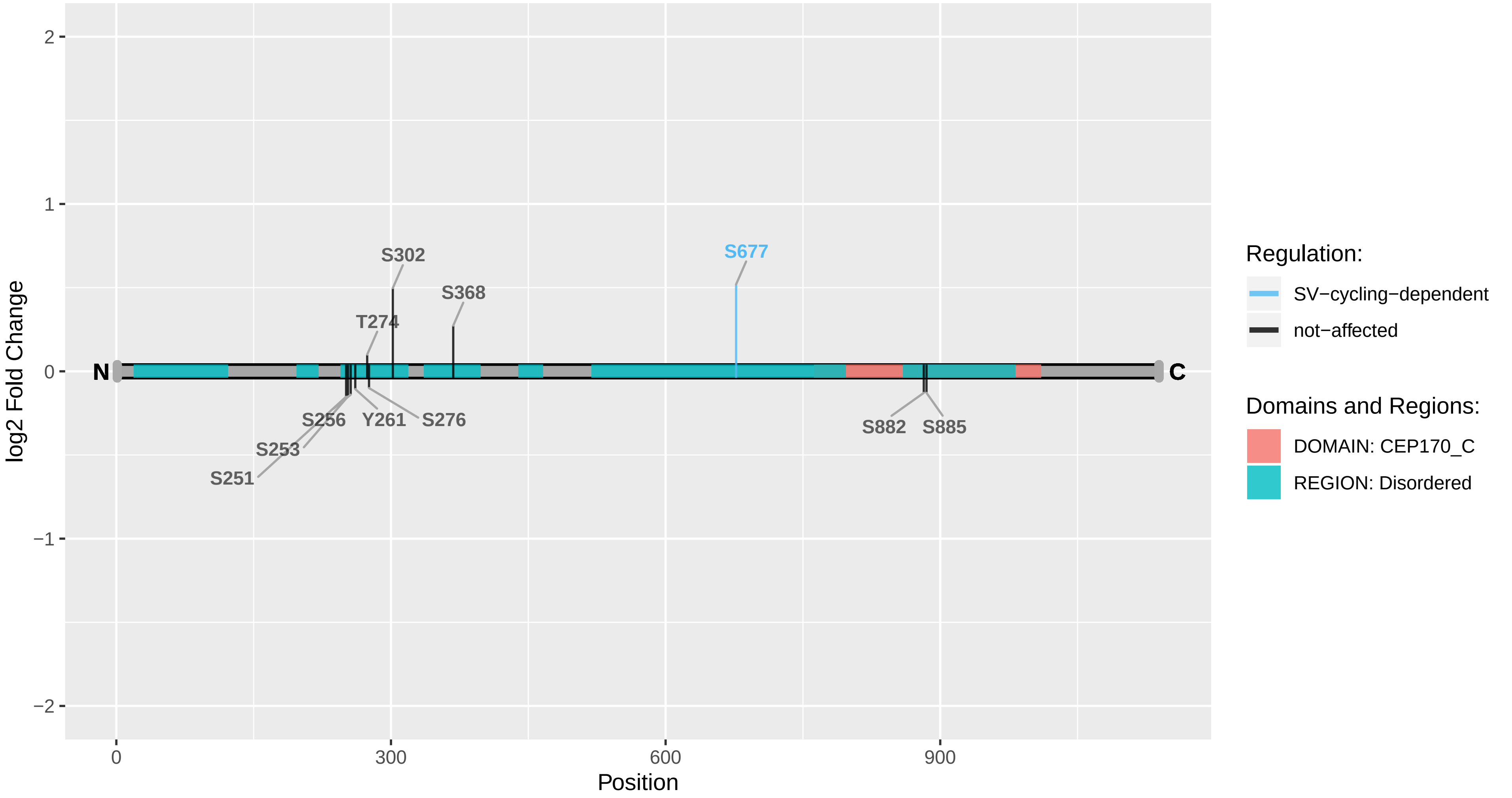

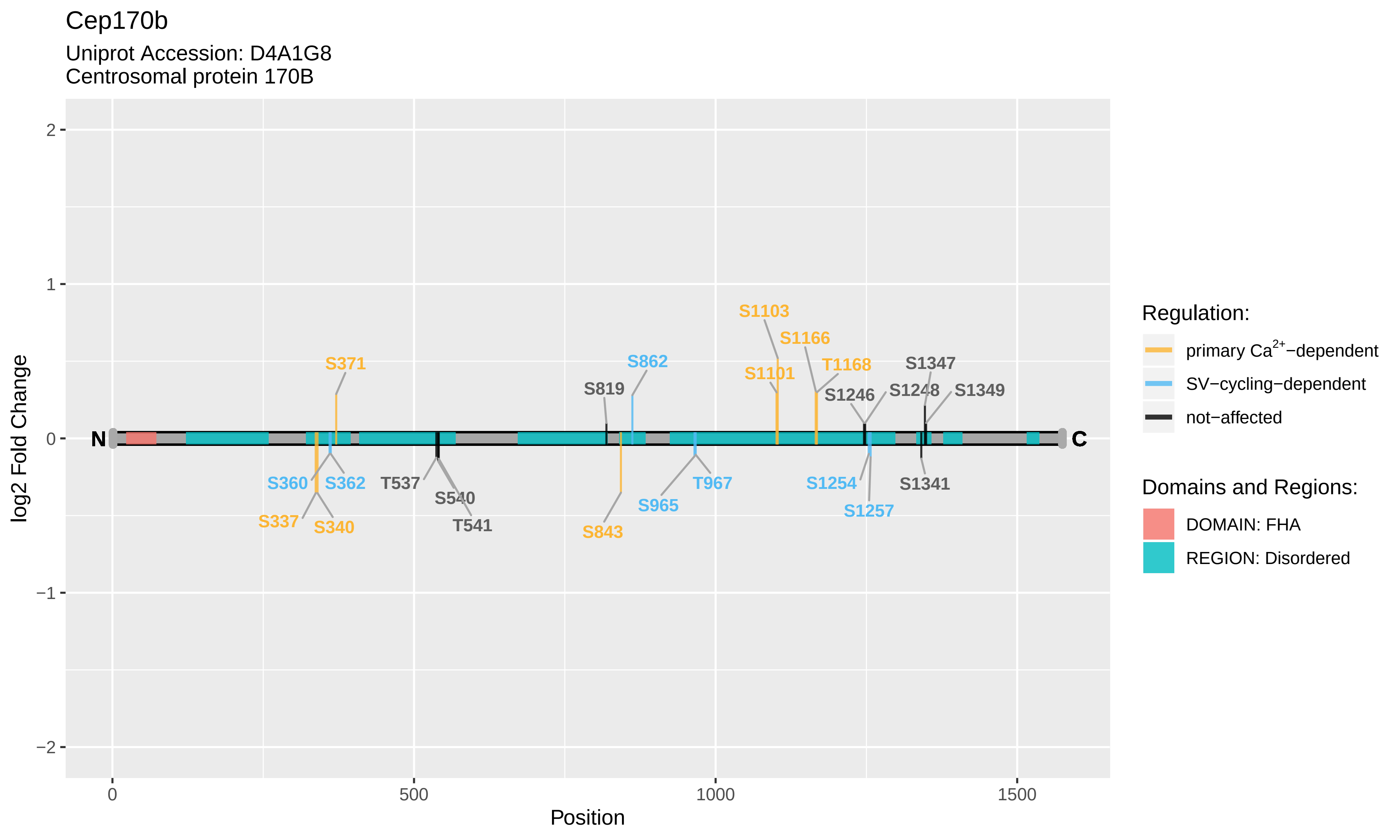
Cep170b
Uniprot Accession: D4A1G8
Centrosomal protein 170B
log2 Fold Change
2
1
0
−1
−2
Position
0
500
1000
1500
N
C
S371
S360
S362
S337
S340
T537
S540
T541
S819
S862
S843
S965
T967
S1103
S1101
S1166
T1168
S1246
S1248
S1347
S1349
S1254
S1257
S1341
Regulation:
primary Ca^{2+}−dependent
SV−cycling−dependent
not−affected
Domains and Regions:
DOMAIN: FHA
REGION: Disordered

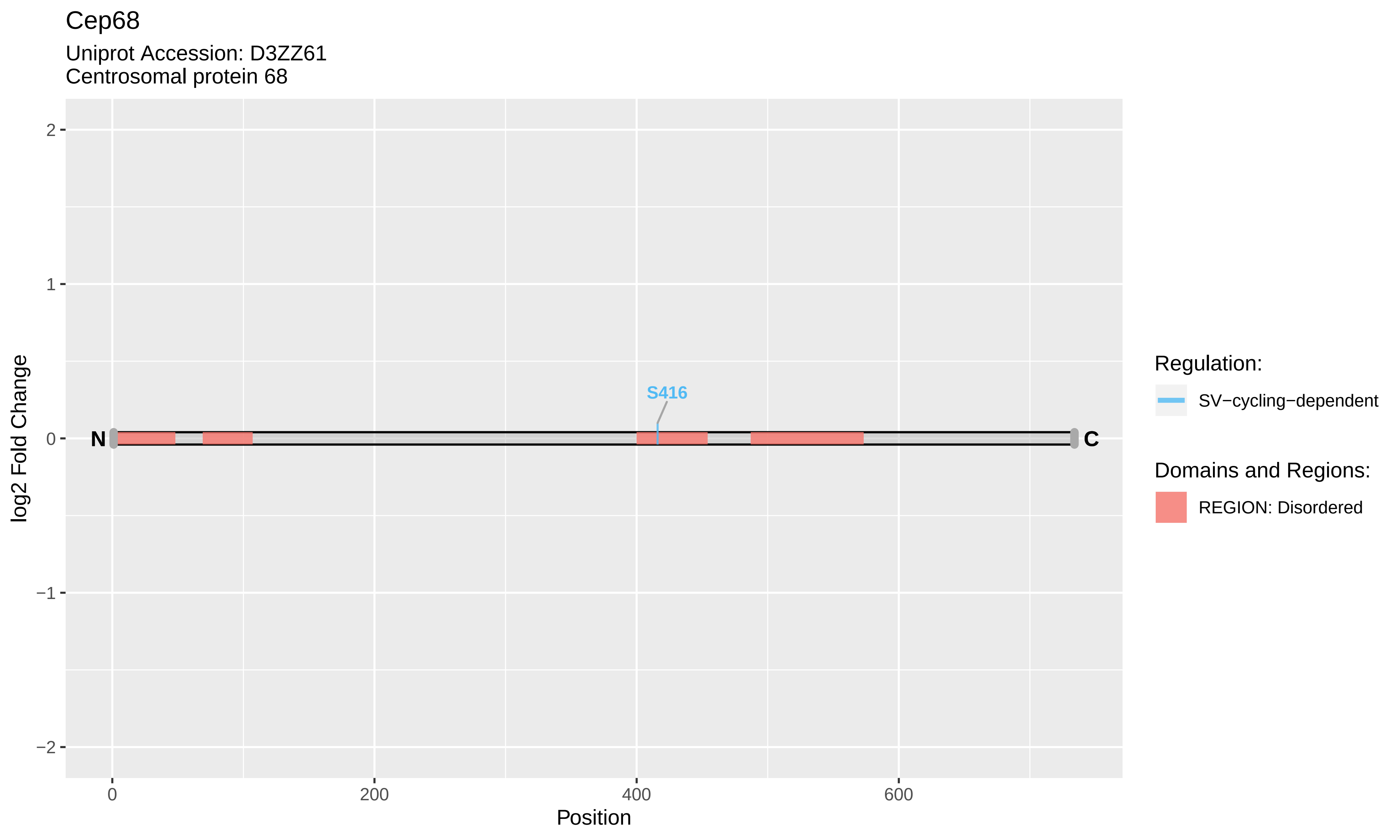

Cep68
Uniprot Accession: D3ZZ61
Centrosomal protein 68
S416
N
C
log2 Fold Change
Position
2
1
0
−1
−2
0
200
400
600
Regulation:
SV−cycling−dependent
Domains and Regions:
REGION: Disordered

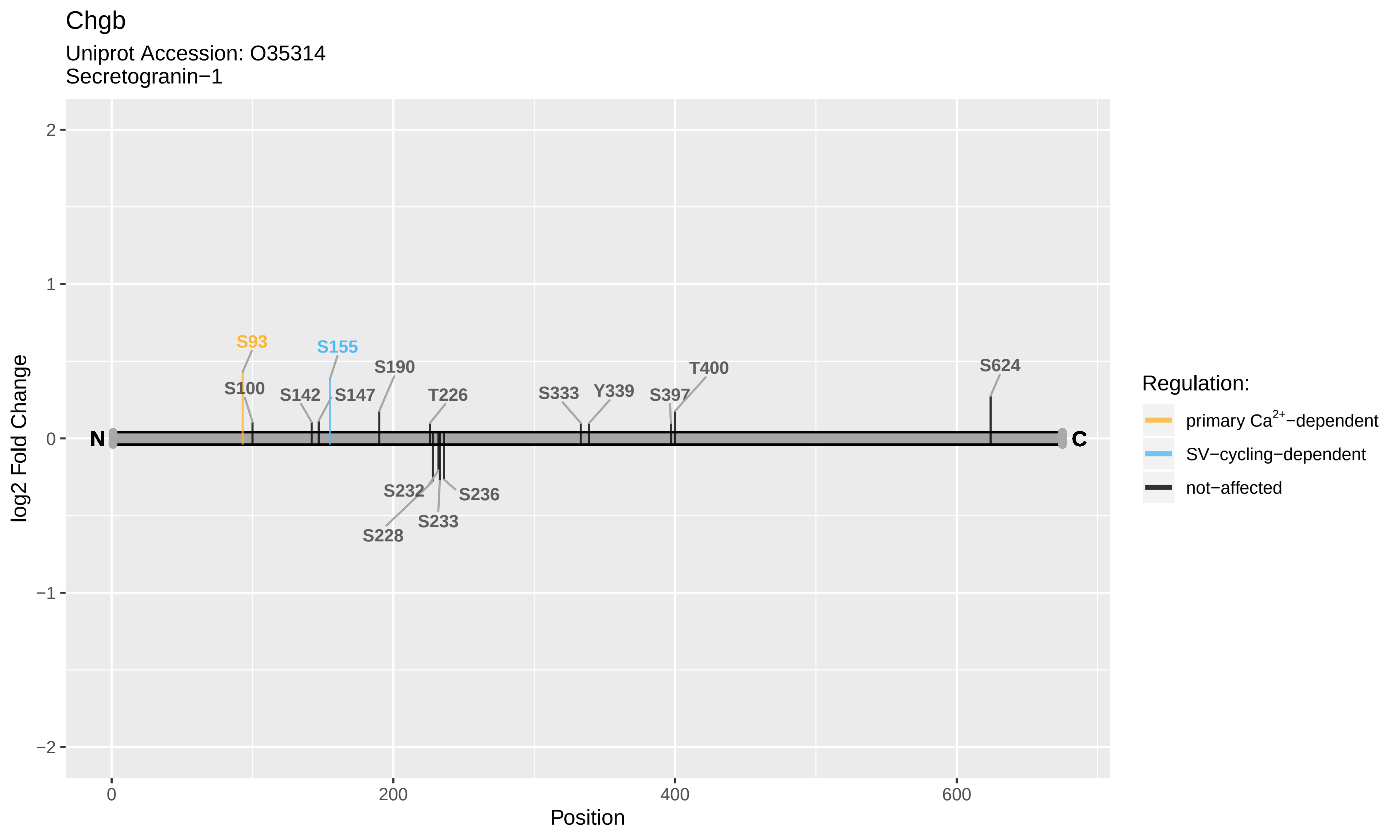

Chgb
Uniprot Accession: O35314
Secretogranin−1
log2 Fold Change
Position
N
C
S93
S155
S100
S142
S147
S190
T226
S333
Y339
S397
T400
S624
S232
S236
S233
S228
Regulation:
primary Ca2+−dependent
SV−cycling−dependent
not−affected

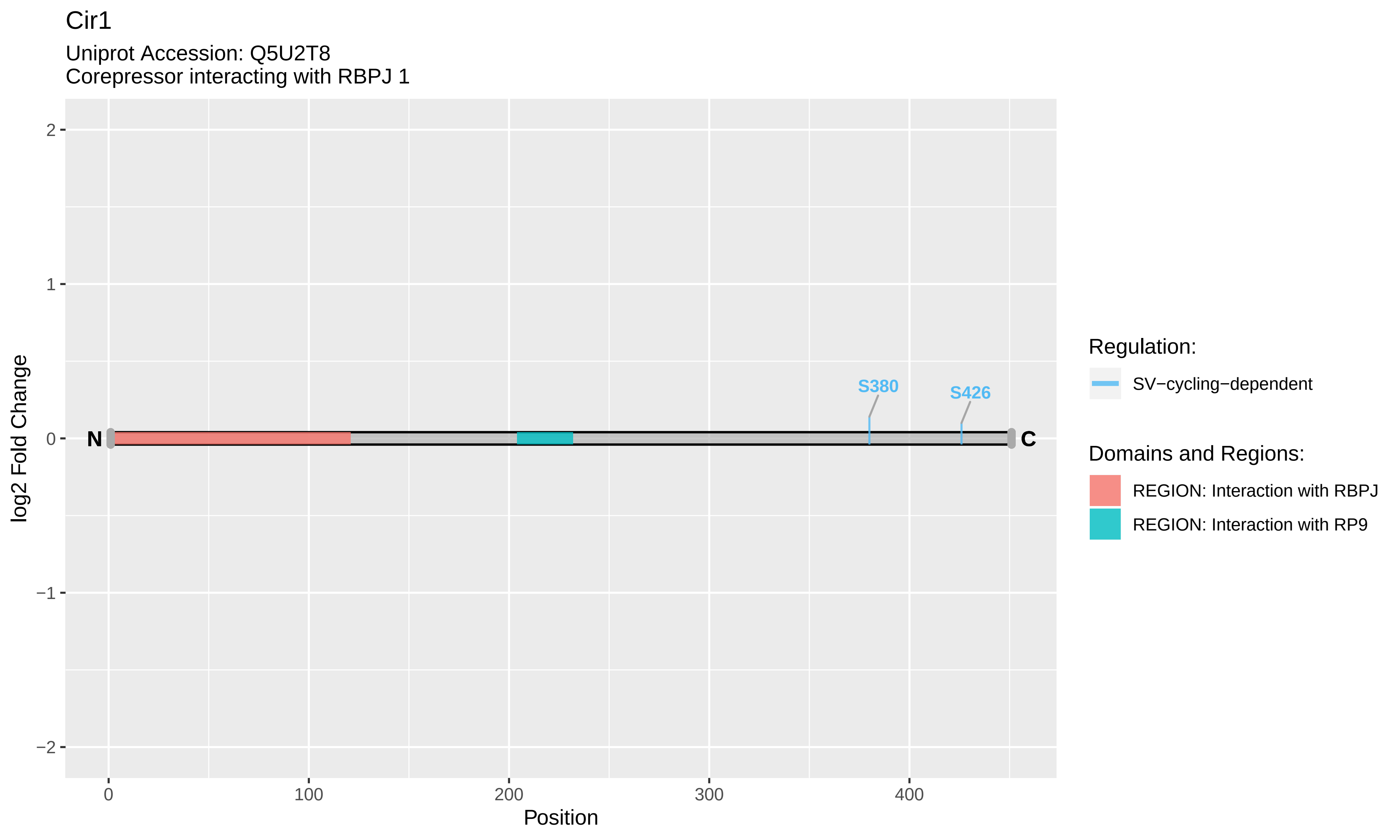

Cir1
Uniprot Accession: Q5U2T8
Corepressor interacting with RBPJ 1
log2 Fold Change
2
1
0
−1
−2
N
C
S380
S426
0
100
200
300
400
Position
Regulation:
SV−cycling−dependent
Domains and Regions:
REGION: Interaction with RBPJ
REGION: Interaction with RP9

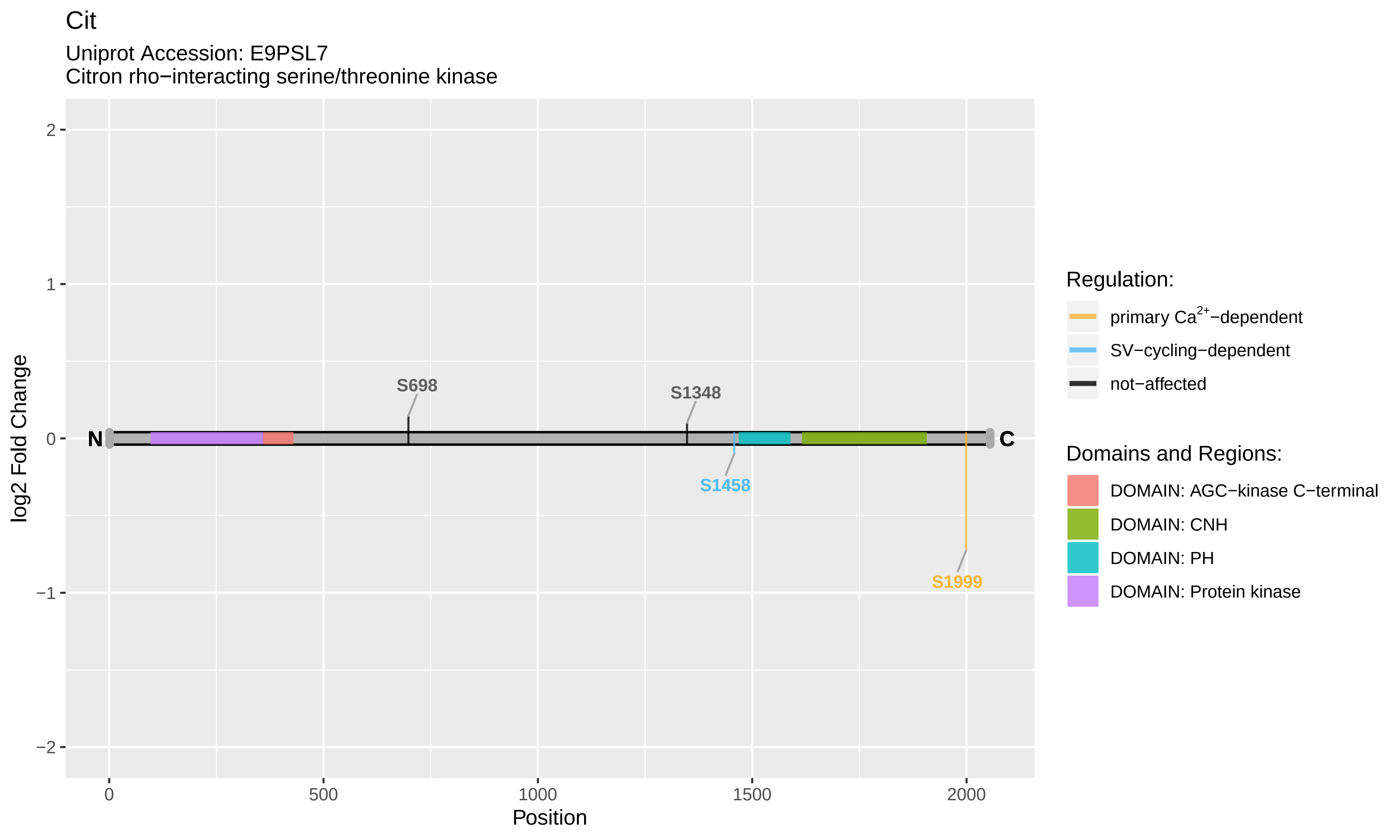
Cit
Uniprot Accession: E9PSL7
Citron rho−interacting serine/threonine kinase
log2 Fold Change
2
1
0
−1
−2
N
C
S698
S1348
S1458
S1999
0
500
1000
1500
2000
Position
Regulation:
primary Ca2+−dependent
SV−cycling−dependent
not−affected
Domains and Regions:
DOMAIN: AGC−kinase C−terminal
DOMAIN: CNH
DOMAIN: PH
DOMAIN: Protein kinase

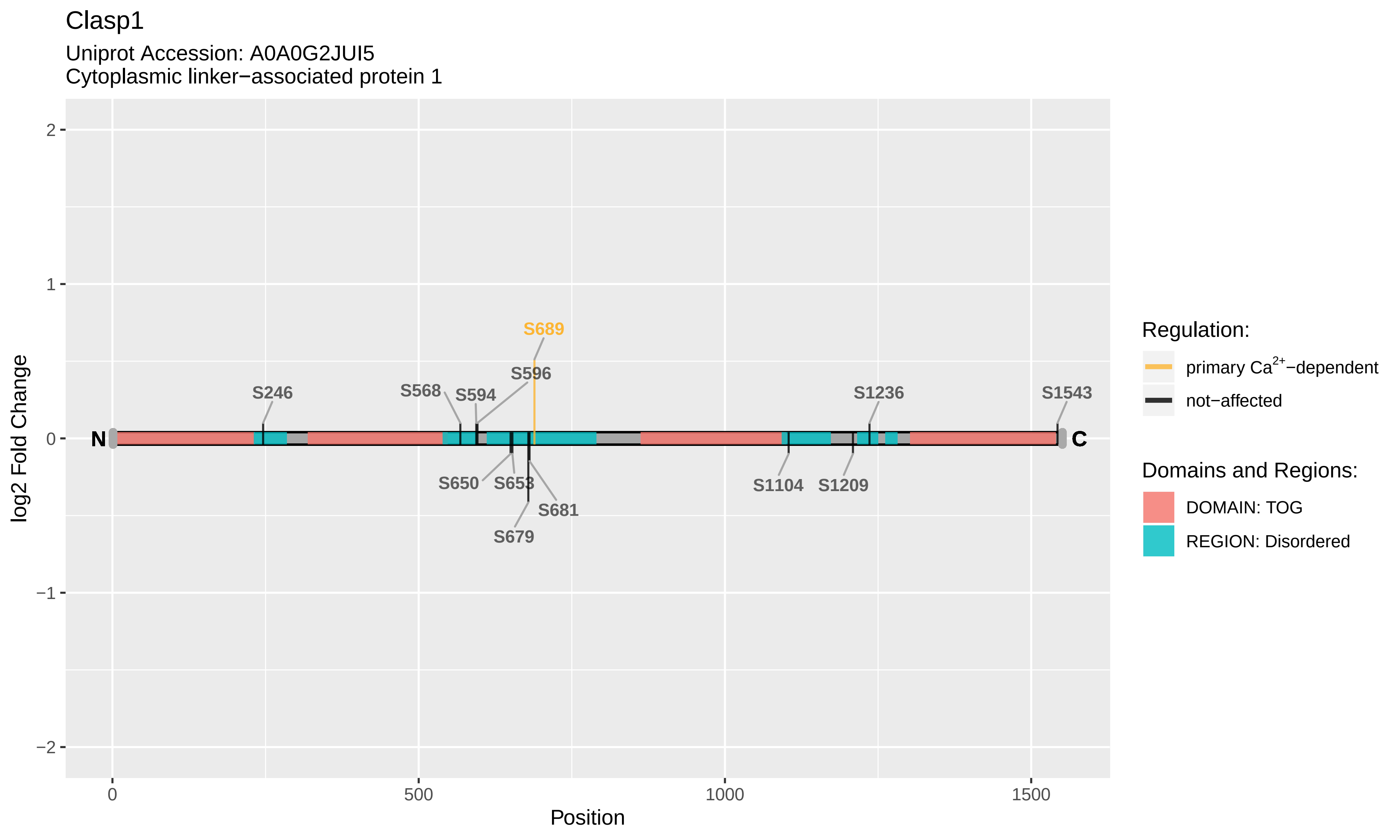

Clasp1
Uniprot Accession: A0A0G2JUI5
Cytoplasmic linker−associated protein 1
log2 Fold Change
2
1
0
−1
−2
N
C
S689
S596
S246
S568
S594
S1236
S1543
S650
S653
S681
S679
S1104
S1209
0
500
1000
1500
Position
Regulation:
primary Ca2+−dependent
not−affected
Domains and Regions:
DOMAIN: TOG
REGION: Disordered

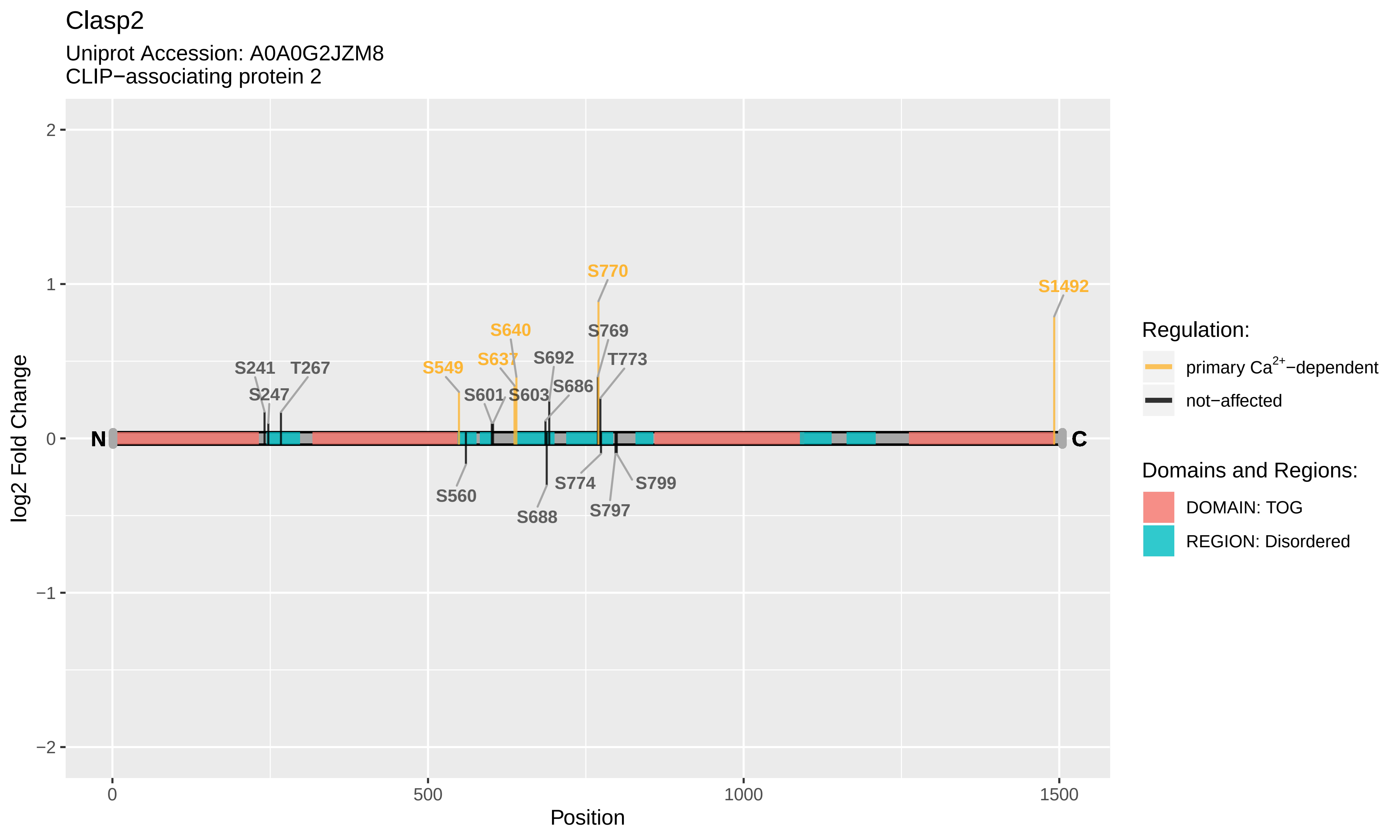

Clasp2
Uniprot Accession: A0A0G2JZM8
CLIP−associating protein 2
log2 Fold Change
Position
N
C
S241
S247
T267
S549
S560
S601
S603
S637
S640
S686
S688
S692
S769
S770
T773
S774
S797
S799
S1492
Regulation:
primary Ca2+−dependent
not−affected
Domains and Regions:
DOMAIN: TOG
REGION: Disordered

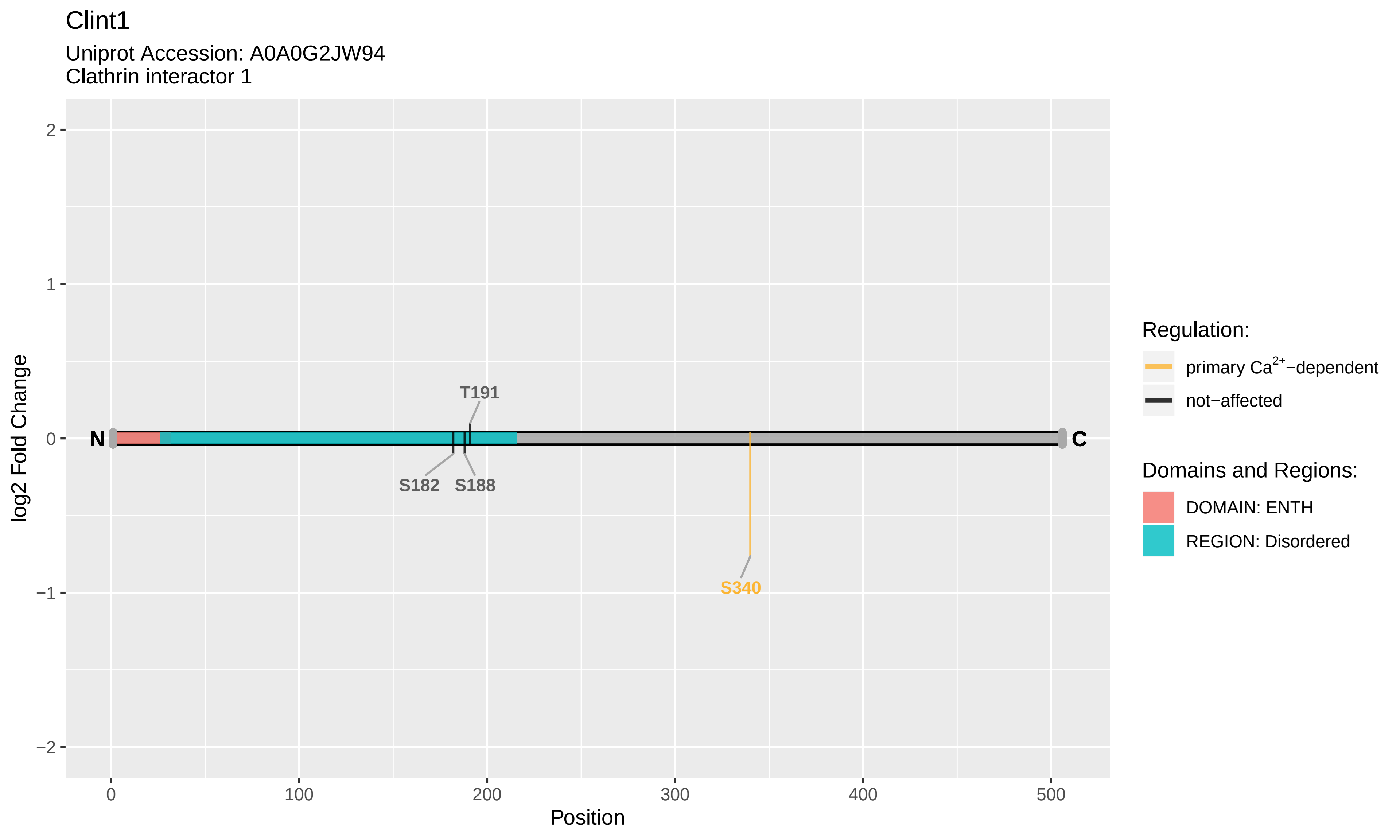

Clint1
Uniprot Accession: A0A0G2JW94
Clathrin interactor 1
log2 Fold Change
Position
N
C
T191
S182
S188
S340
Regulation:
primary Ca2+−dependent
not−affected
Domains and Regions:
DOMAIN: ENTH
REGION: Disordered

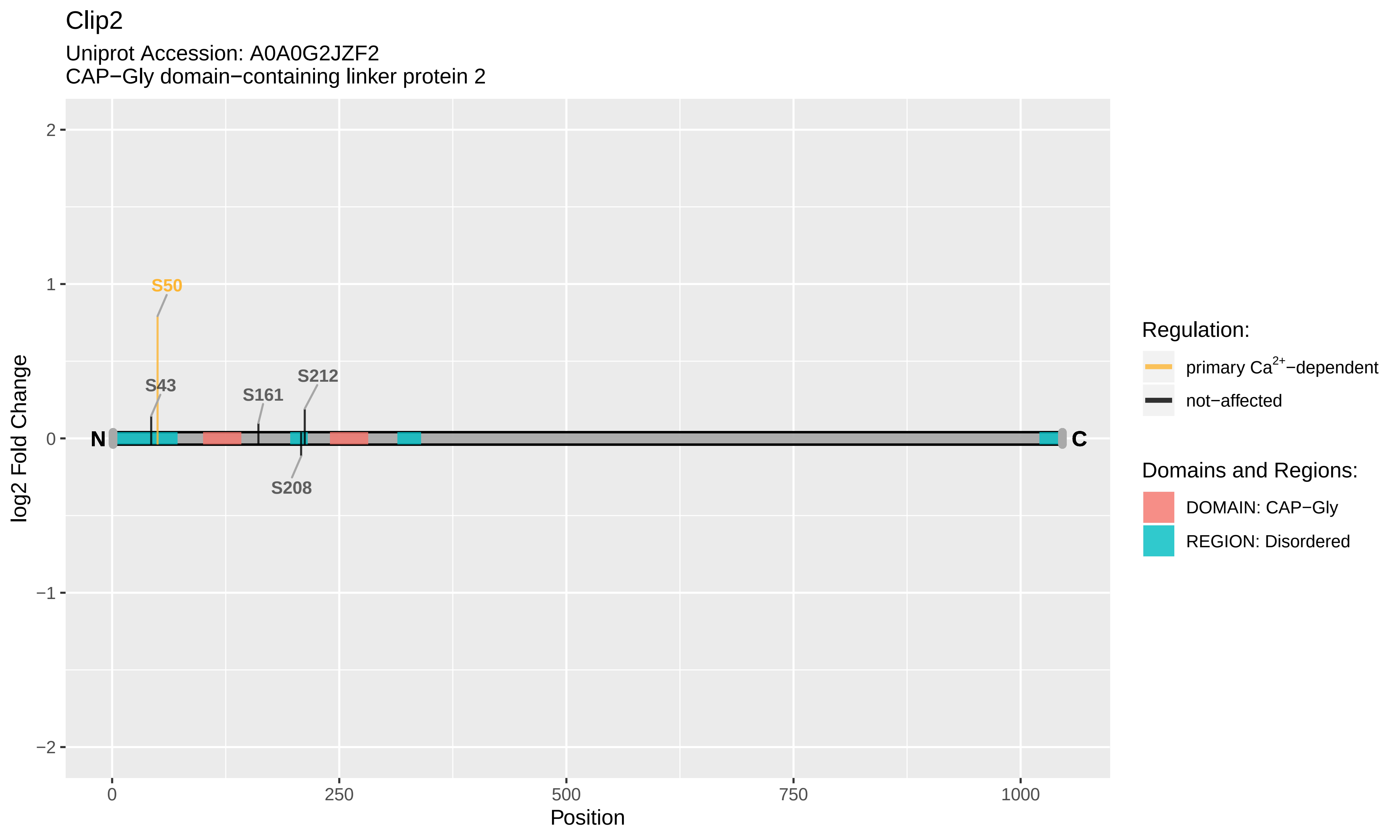

Clip2
Uniprot Accession: A0A0G2JZF2
CAP−Gly domain−containing linker protein 2
S50
S43
S161
S212
S208
N
C
log2 Fold Change
2
1
0
−1
−2
0
250
500
750
1000
Position
Regulation:
primary Ca2+−dependent
not−affected
Domains and Regions:
DOMAIN: CAP−Gly
REGION: Disordered

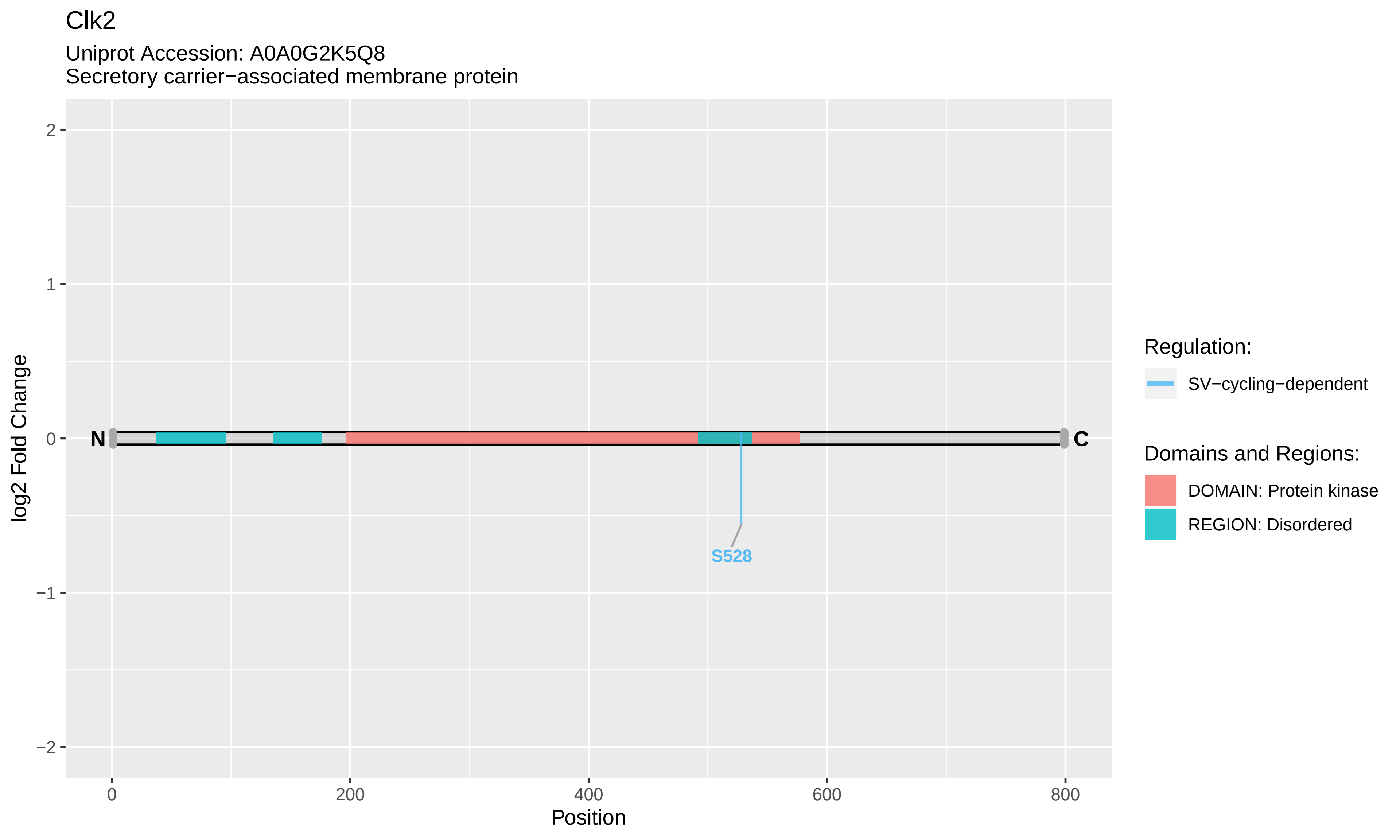

Clk2
Uniprot Accession: A0A0G2K5Q8
Secretory carrier−associated membrane protein
log2 Fold Change
2
1
0
−1
−2
N
C
S528
0
200
400
600
800
Position
Regulation:
SV−cycling−dependent
Domains and Regions:
DOMAIN: Protein kinase
REGION: Disordered

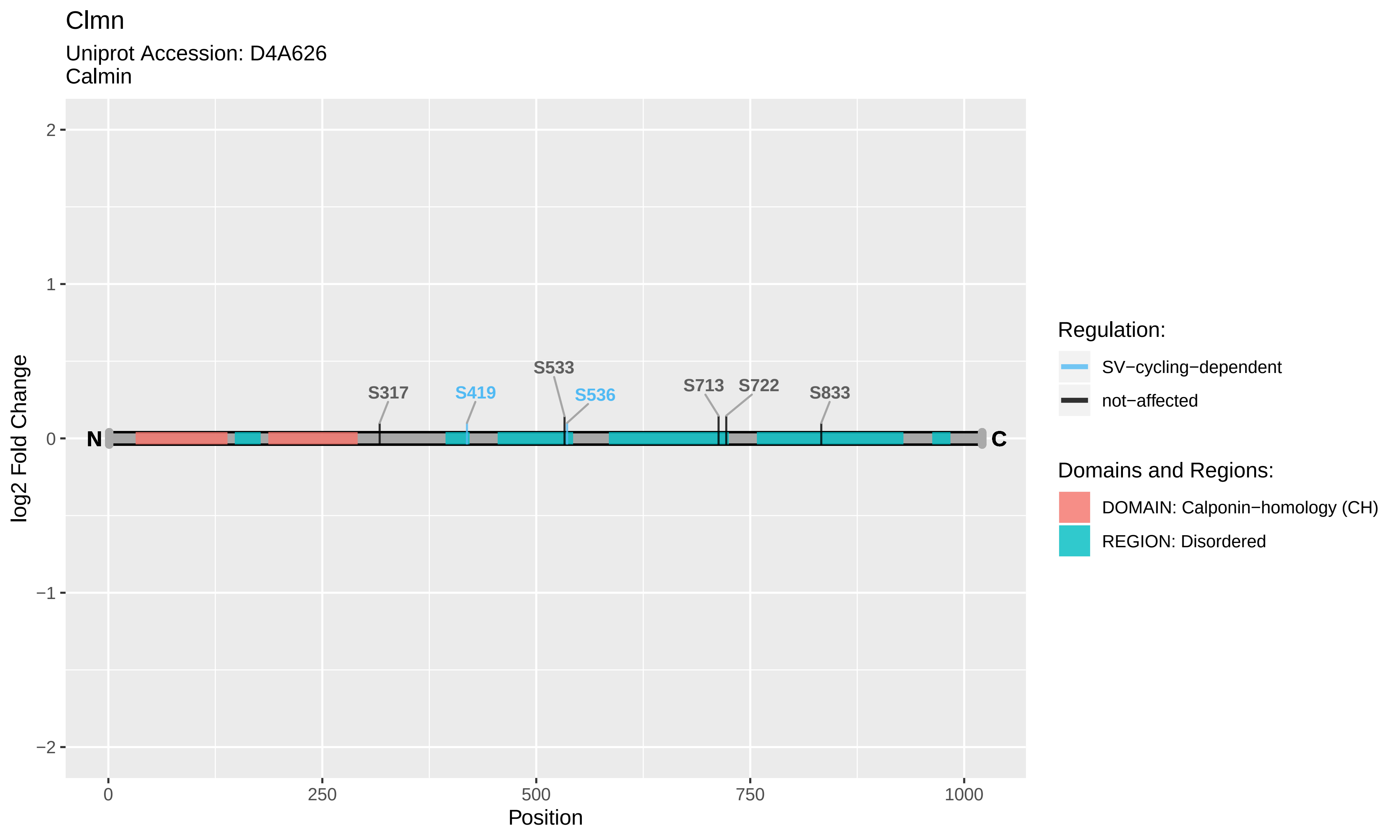

Clmn
Uniprot Accession: D4A626
Calmin
log2 Fold Change
2
1
0
−1
−2
N
C
S317
S419
S533
S536
S713
S722
S833
0
250
500
750
1000
Position
Regulation:
SV−cycling−dependent
not−affected
Domains and Regions:
DOMAIN: Calponin−homology (CH)
REGION: Disordered

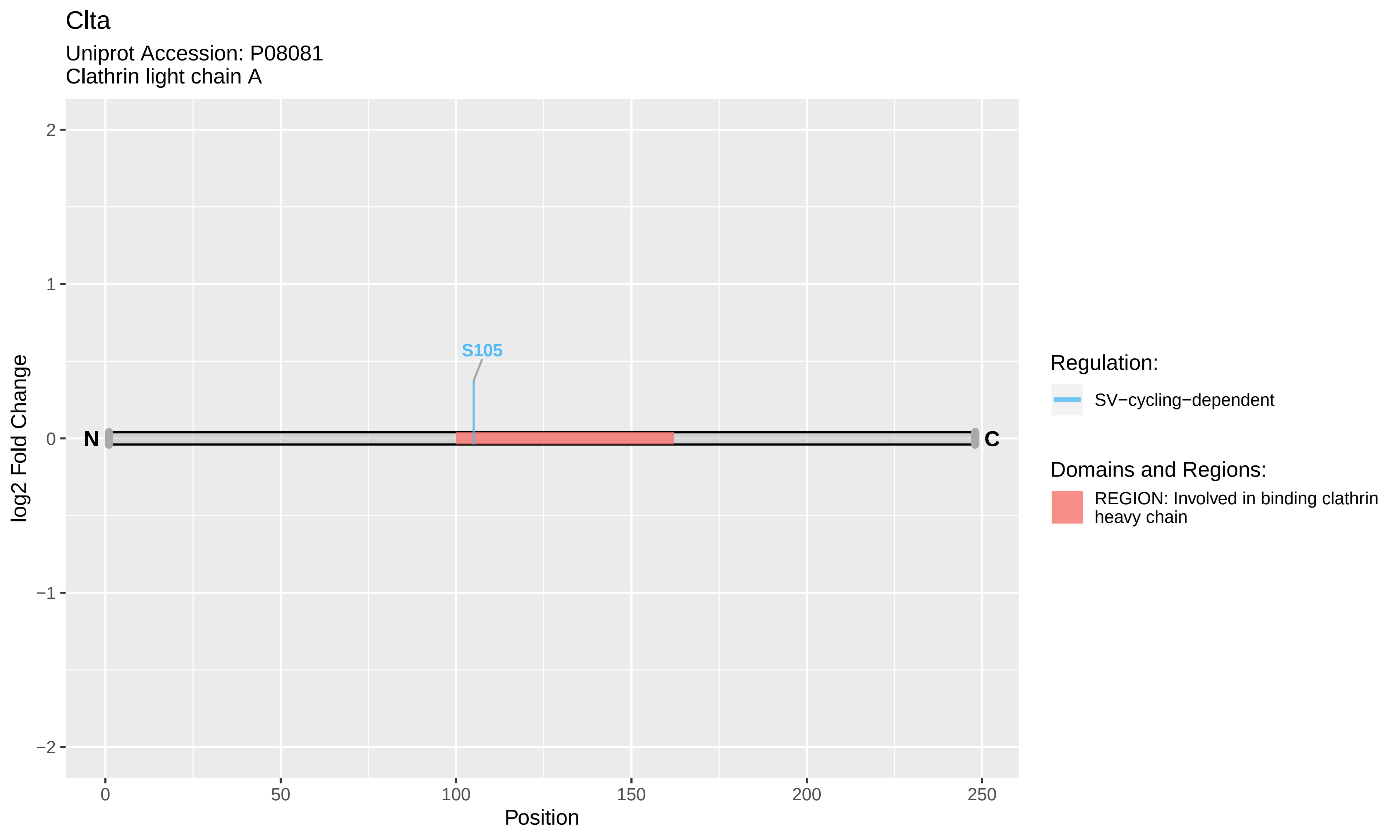

Clta
Uniprot Accession: P08081
Clathrin light chain A
S105
N
C
2
1
0
−1
−2
log2 Fold Change
0
50
100
150
200
250
Position
Regulation:
SV−cycling−dependent
Domains and Regions:
REGION: Involved in binding clathrin heavy chain

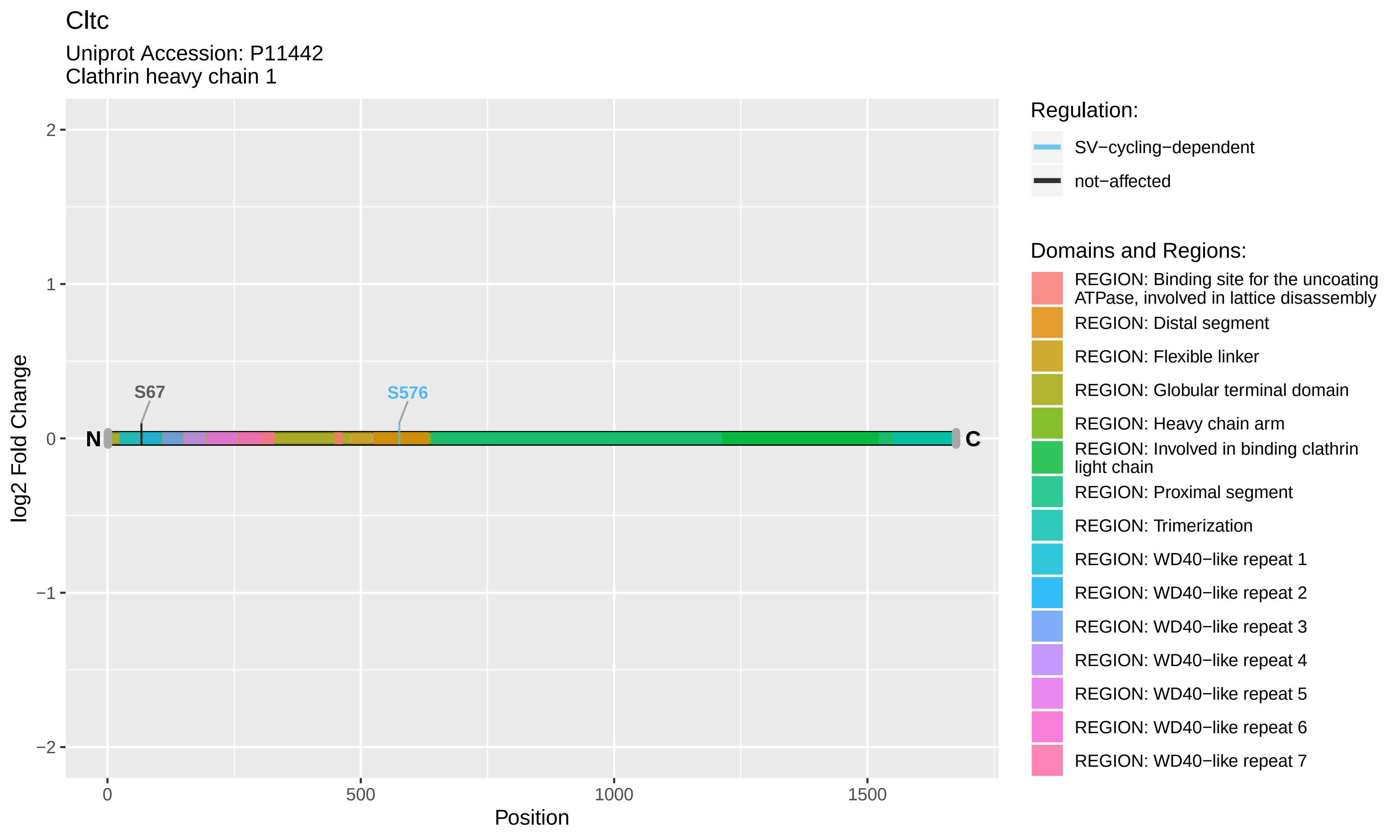

Cltc
Uniprot Accession: P11442
Clathrin heavy chain 1
S67
S576
N
C
2
1
0
−1
−2
log2 Fold Change
0
500
1000
1500
Position
Regulation:
SV−cycling−dependent
not−affected
Domains and Regions:
REGION: Binding site for the uncoating ATPase, involved in lattice disassembly
REGION: Distal segment
REGION: Flexible linker
REGION: Globular terminal domain
REGION: Heavy chain arm
REGION: Involved in binding clathrin light chain
REGION: Proximal segment
REGION: Trimerization
REGION: WD40−like repeat 1
REGION: WD40−like repeat 2
REGION: WD40−like repeat 3
REGION: WD40−like repeat 4
REGION: WD40−like repeat 5
REGION: WD40−like repeat 6
REGION: WD40−like repeat 7

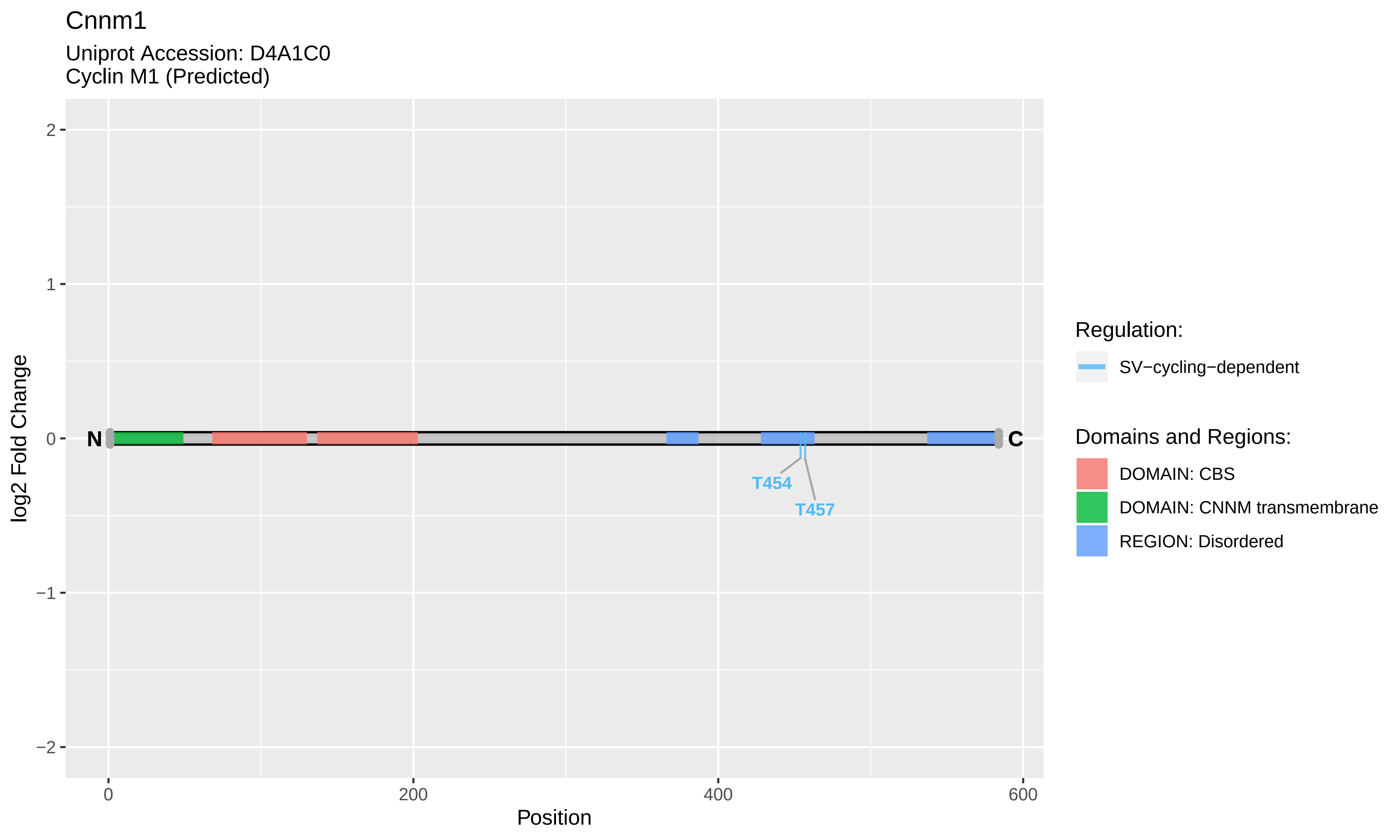

Cnnm1
Uniprot Accession: D4A1C0
Cyclin M1 (Predicted)
log2 Fold Change
Position
N
C
T454
T457
Regulation:
SV−cycling−dependent
Domains and Regions:
DOMAIN: CBS
DOMAIN: CNNM transmembrane
REGION: Disordered

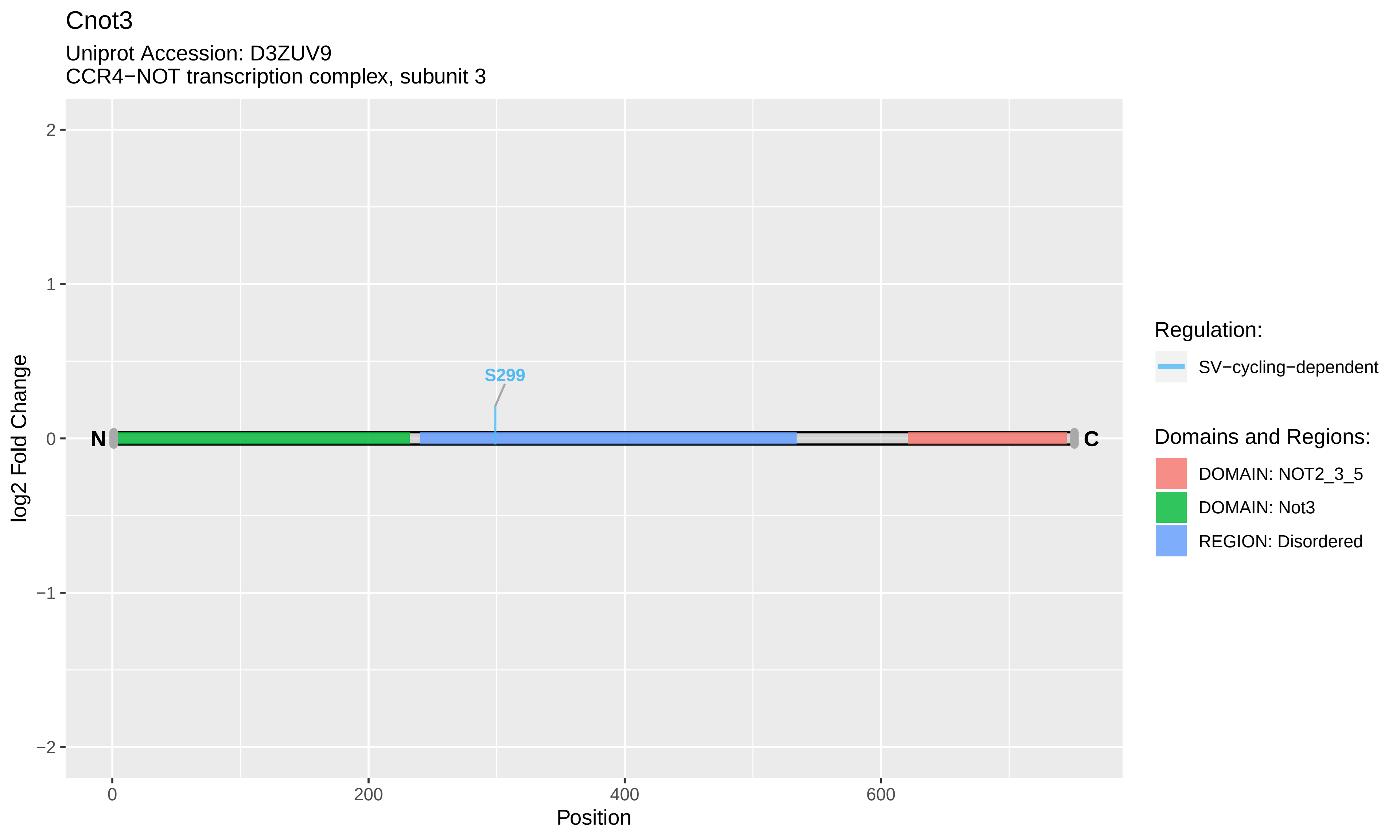
Cnot3
Uniprot Accession: D3ZUV9
CCR4−NOT transcription complex, subunit 3
S299
N
C
2
1
0
−1
−2
log2 Fold Change
0
200
400
600
Position
Regulation:
SV−cycling−dependent
Domains and Regions:
DOMAIN: NOT2_3_5
DOMAIN: Not3
REGION: Disordered

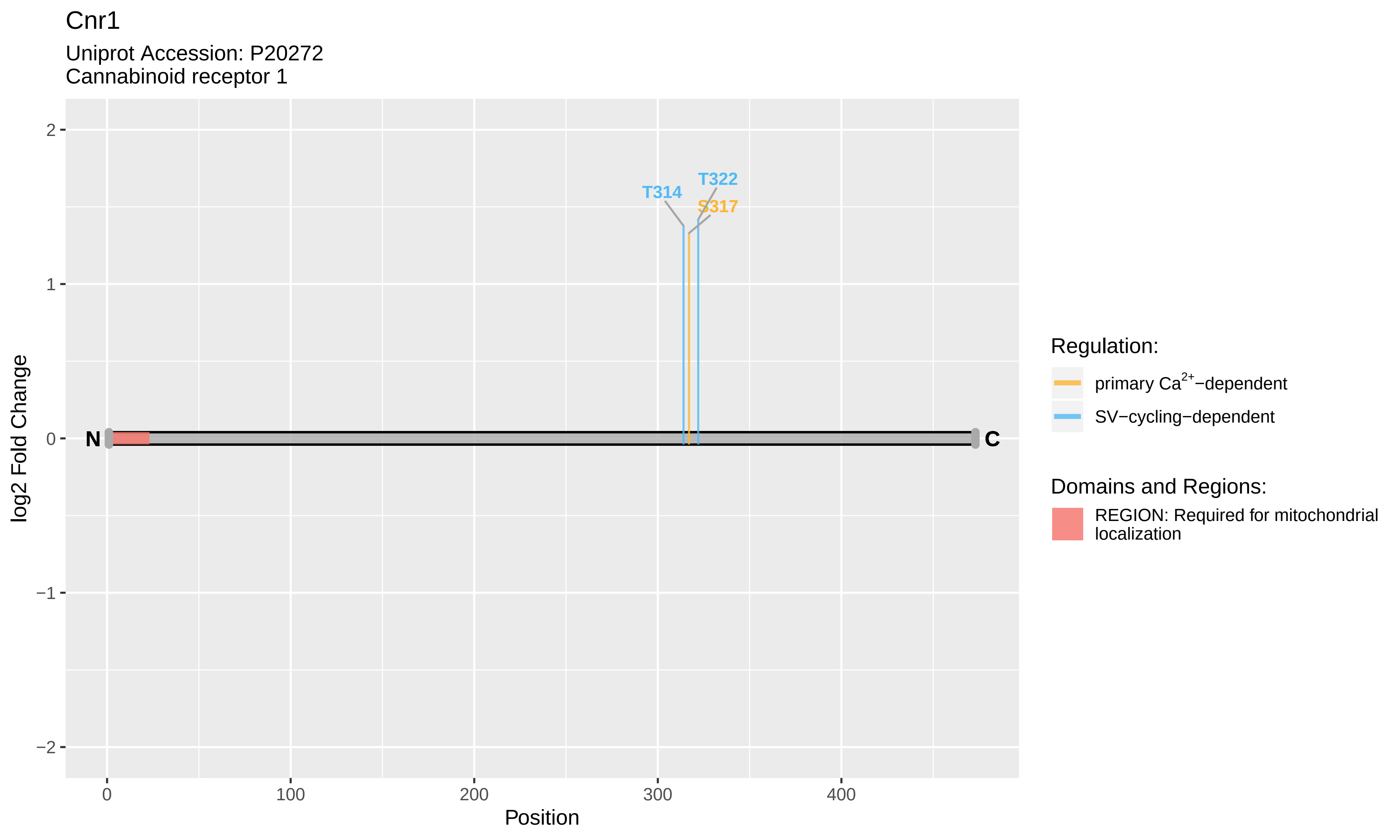

Cnr1
Uniprot Accession: P20272
Cannabinoid receptor 1
T314
T322
S317
N
C
log2 Fold Change
2
1
0
−1
−2
0
100
200
300
400
Position
Regulation:
primary Ca2+−dependent
SV−cycling−dependent
Domains and Regions:
REGION: Required for mitochondrial localization

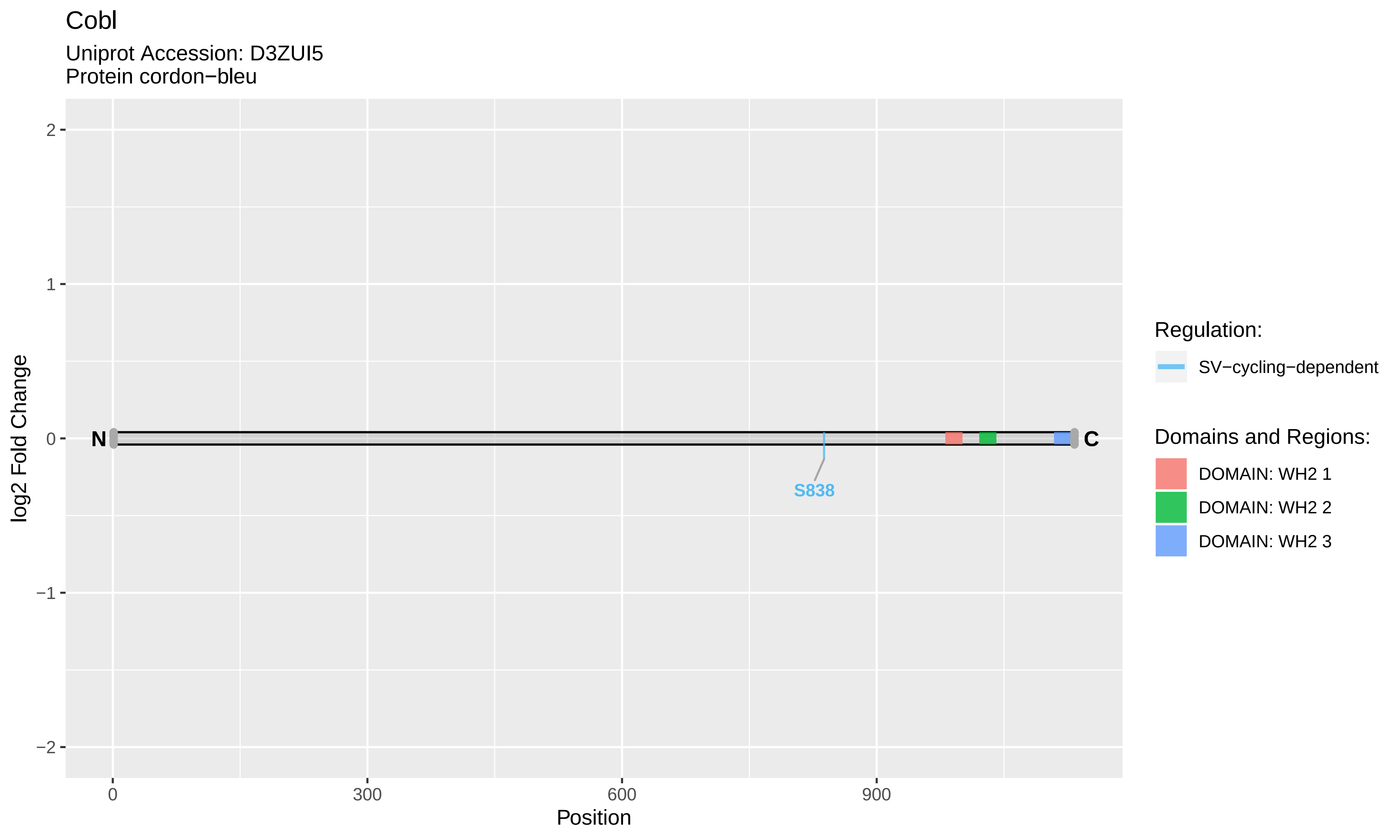

Cobl
Uniprot Accession: D3ZUI5
Protein cordon−bleu
log2 Fold Change
2
1
0
−1
−2
N
C
S838
0
300
600
900
Position
Regulation:
SV−cycling−dependent
Domains and Regions:
DOMAIN: WH2 1
DOMAIN: WH2 2
DOMAIN: WH2 3

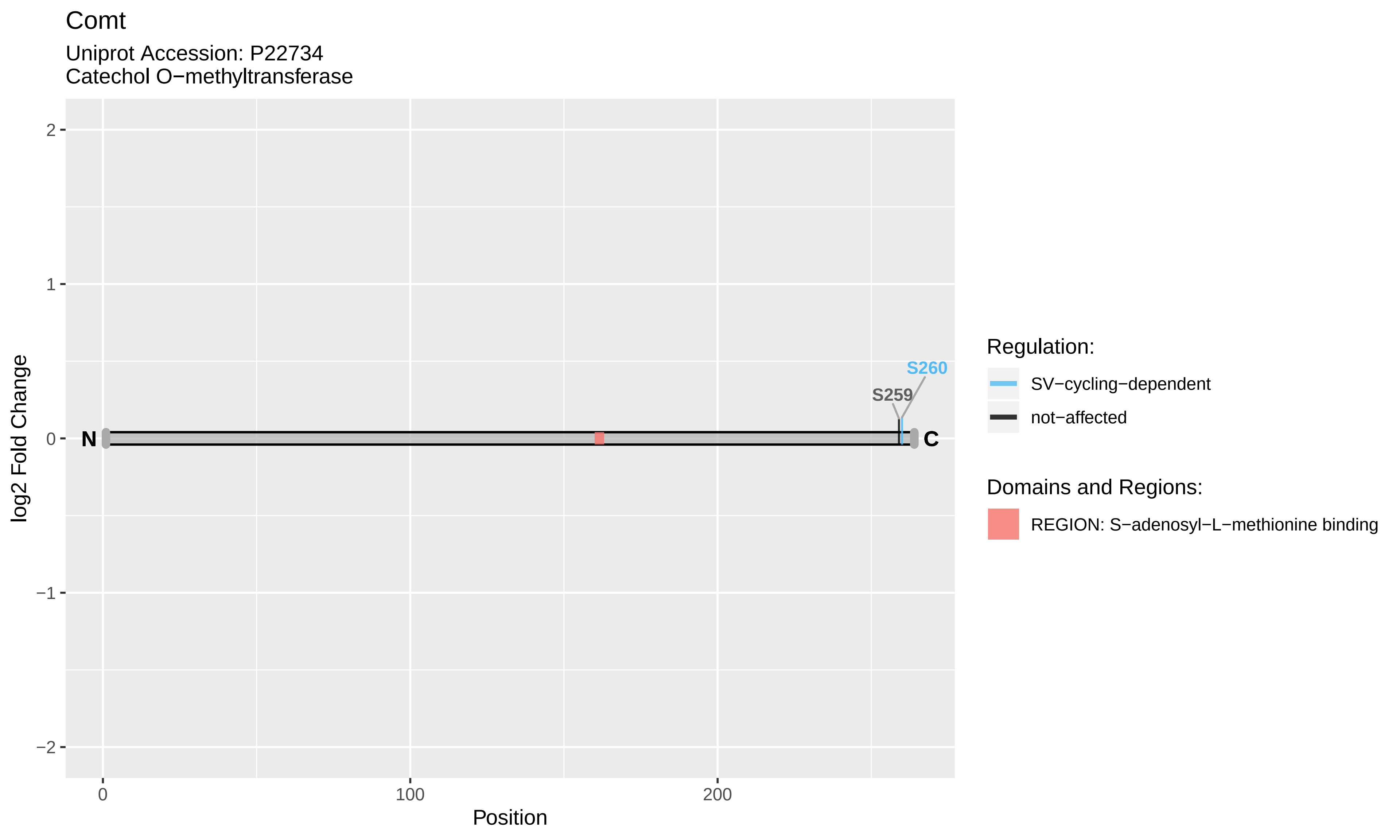

Comt
Uniprot Accession: P22734
Catechol O−methyltransferase
log2 Fold Change
2
1
0
−1
−2
N
C
S259
S260
0
100
200
Position
Regulation:
SV−cycling−dependent
not−affected
Domains and Regions:
REGION: S−adenosyl−L−methionine binding

Coro1a

Uniprot Accession: Q91ZN1
Coronin−1A

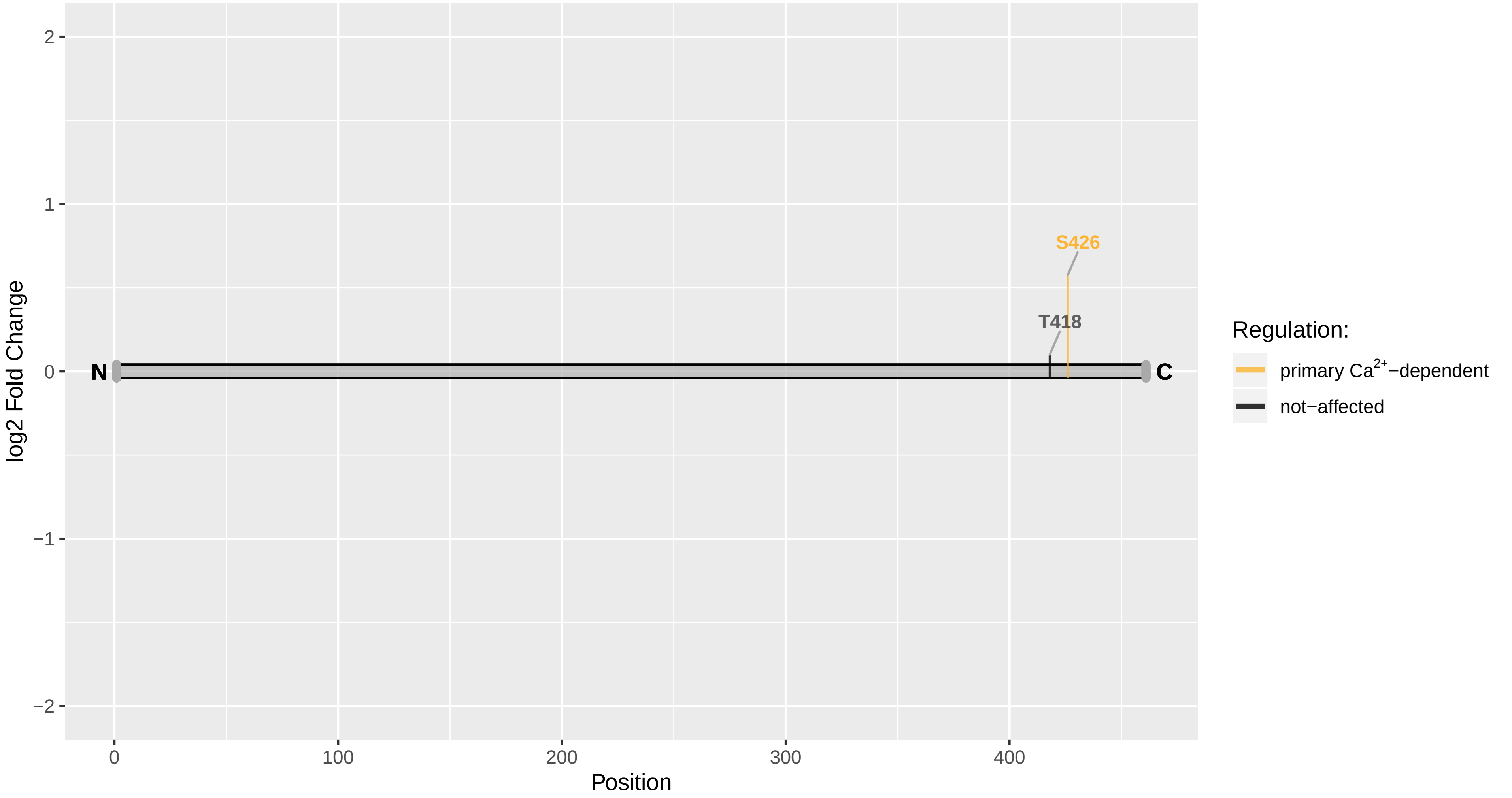

Cox4i1

Uniprot Accession: P10888
Cytochrome c oxidase subunit 4 isoform 1, mitochondrial

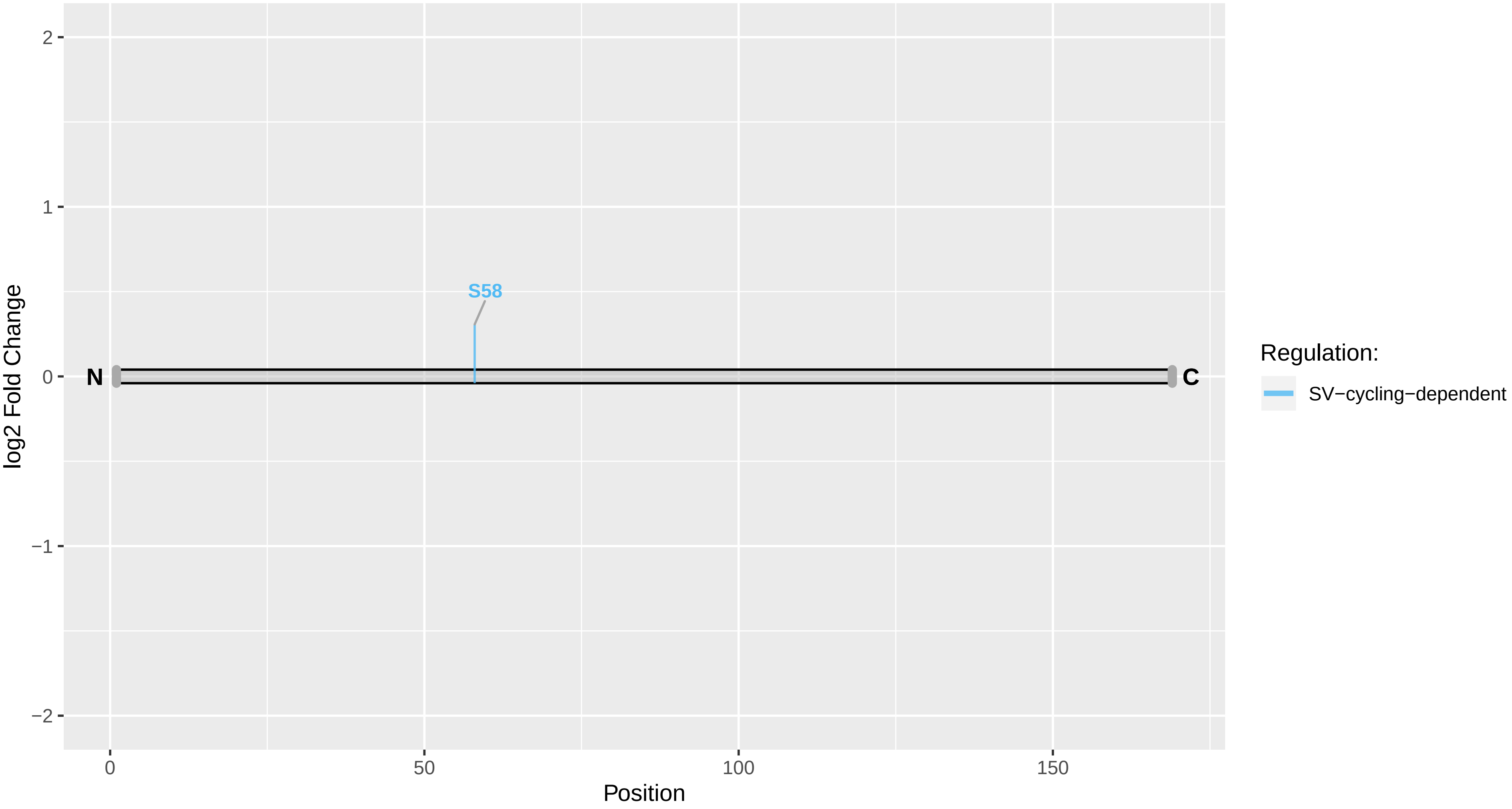

Cox7c

Uniprot Accession: P80432
Cytochrome c oxidase subunit 7C, mitochondrial

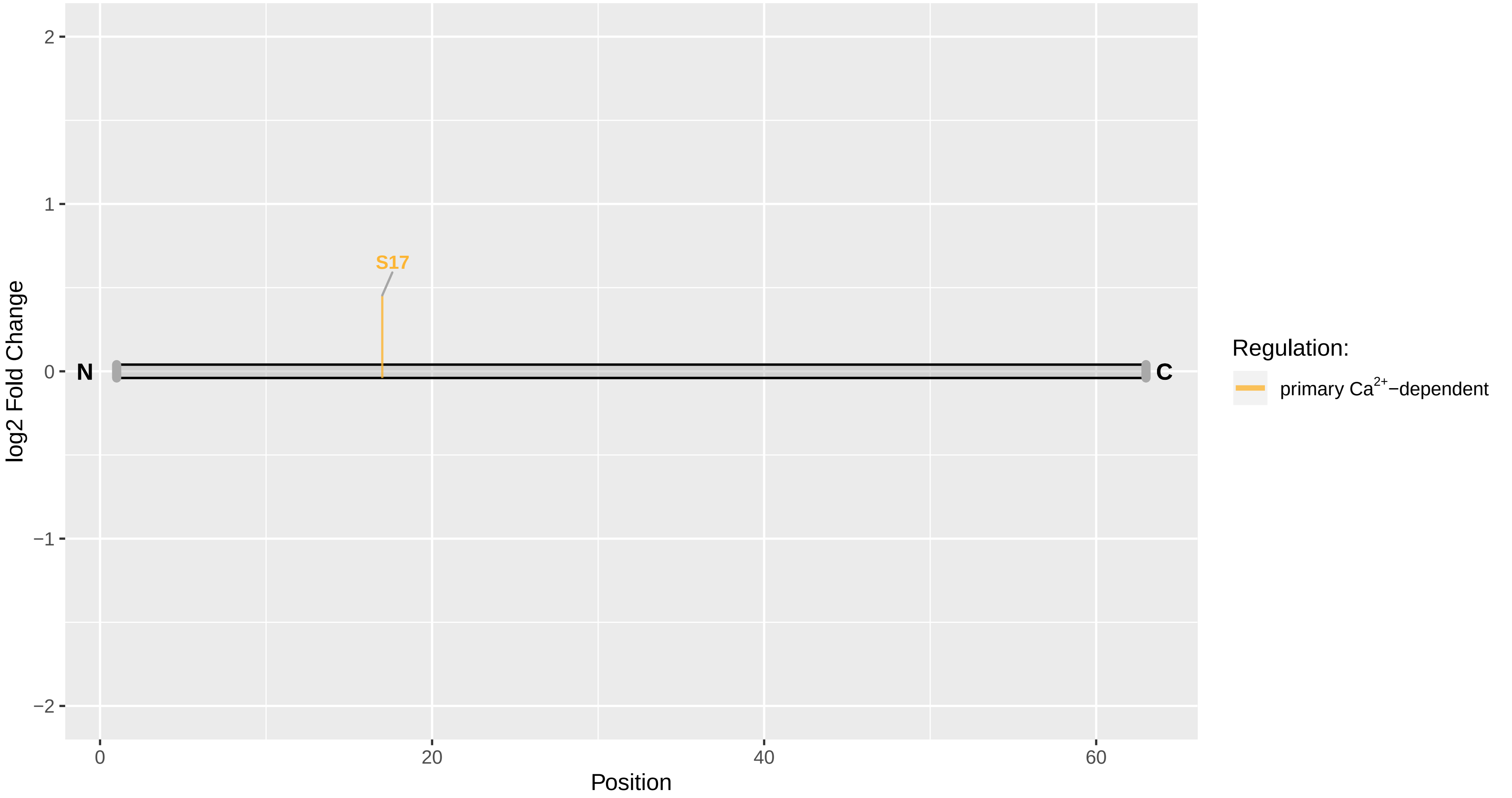

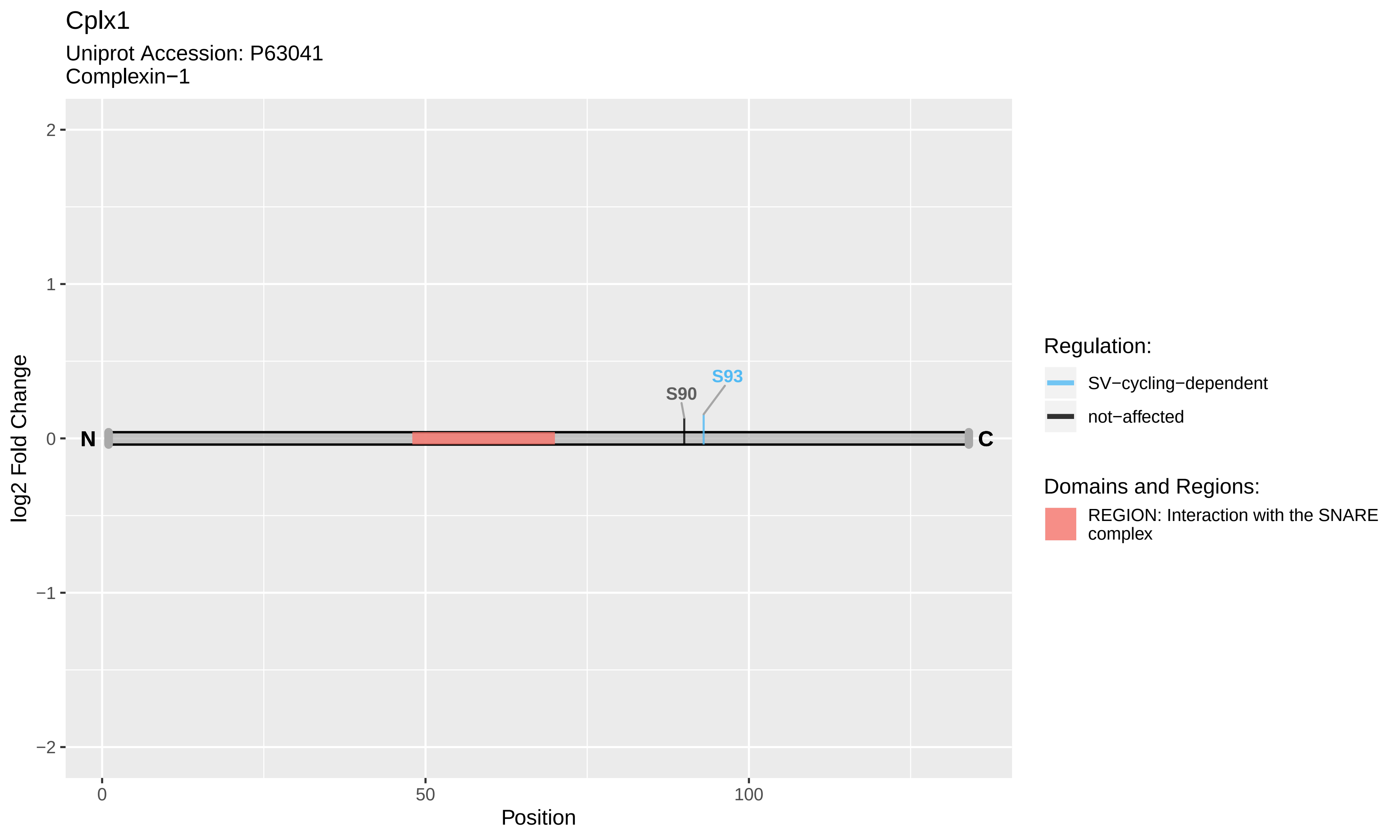

Cplx1
Uniprot Accession: P63041
Complexin−1
log2 Fold Change
2
1
0
−1
−2
N
C
S90
S93
0
50
100
Position
Regulation:
SV−cycling−dependent
not−affected
Domains and Regions:
REGION: Interaction with the SNARE complex

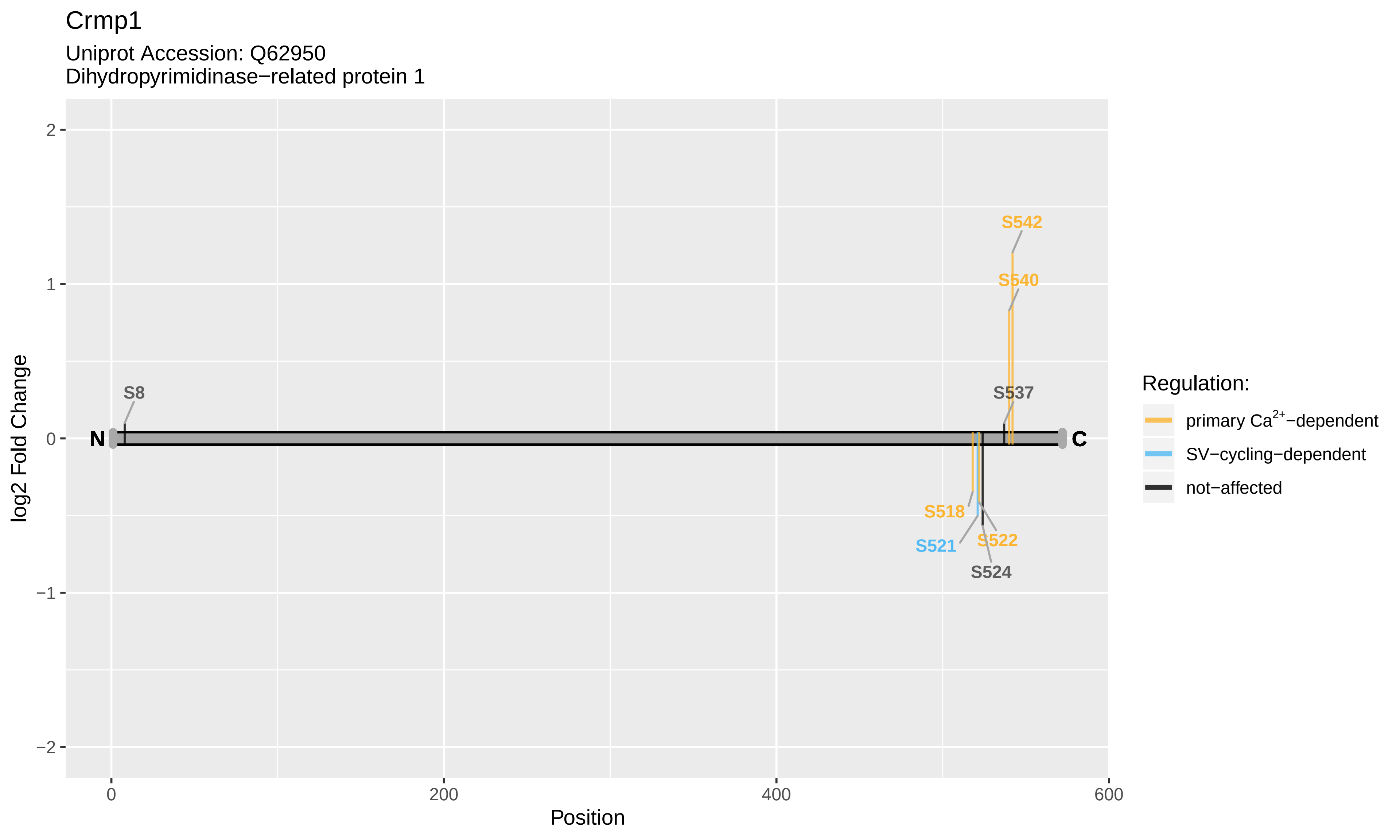

Crmp1
Uniprot Accession: Q62950
Dihydropyrimidinase−related protein 1
log2 Fold Change
Position
N
C
S8
S537
S540
S542
S518
S521
S522
S524
Regulation:
primary Ca2+−dependent
SV−cycling−dependent
not−affected

Crtc1

Uniprot Accession: Q157S1
CREB−regulated transcription coactivator 1

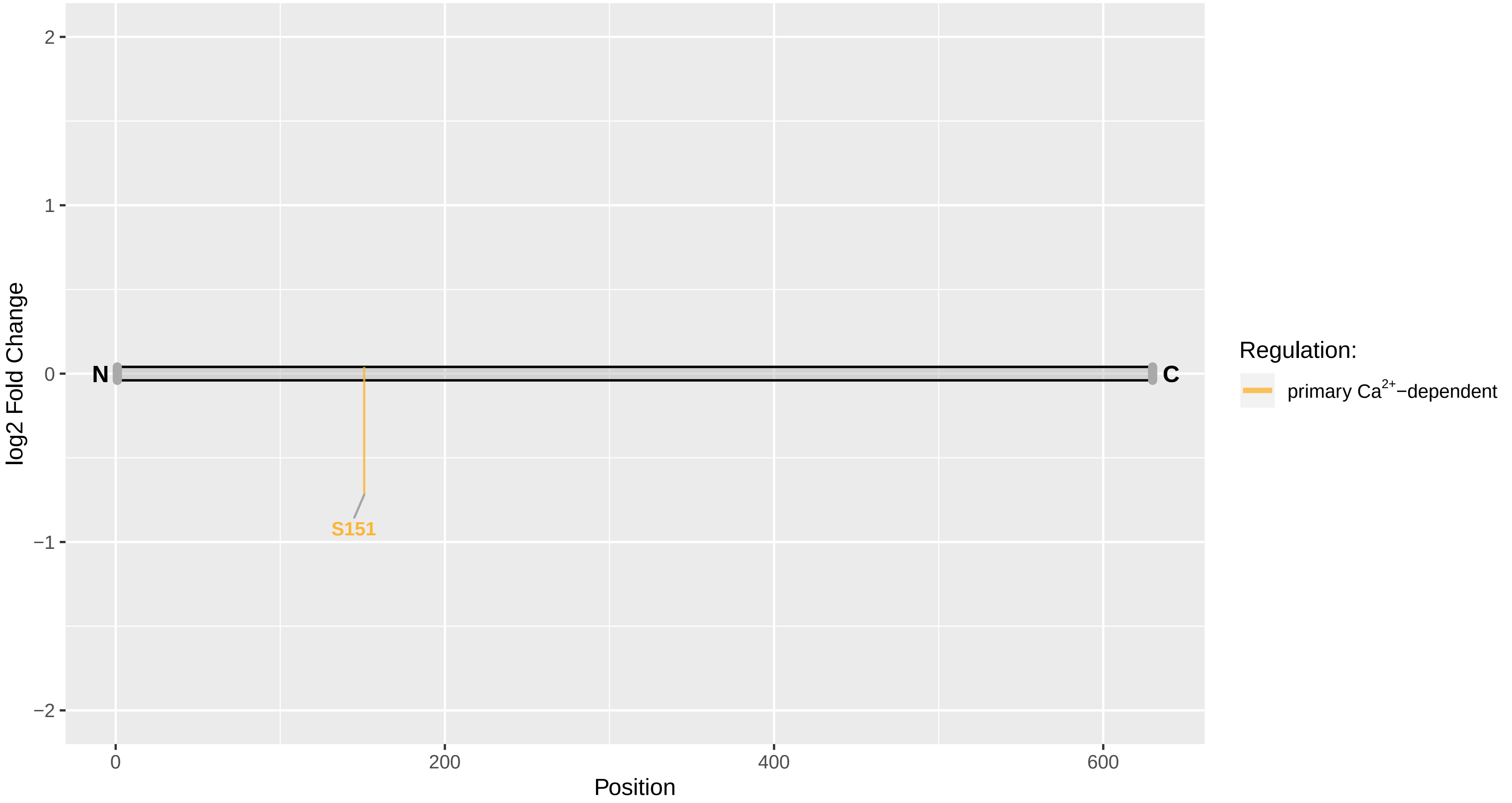

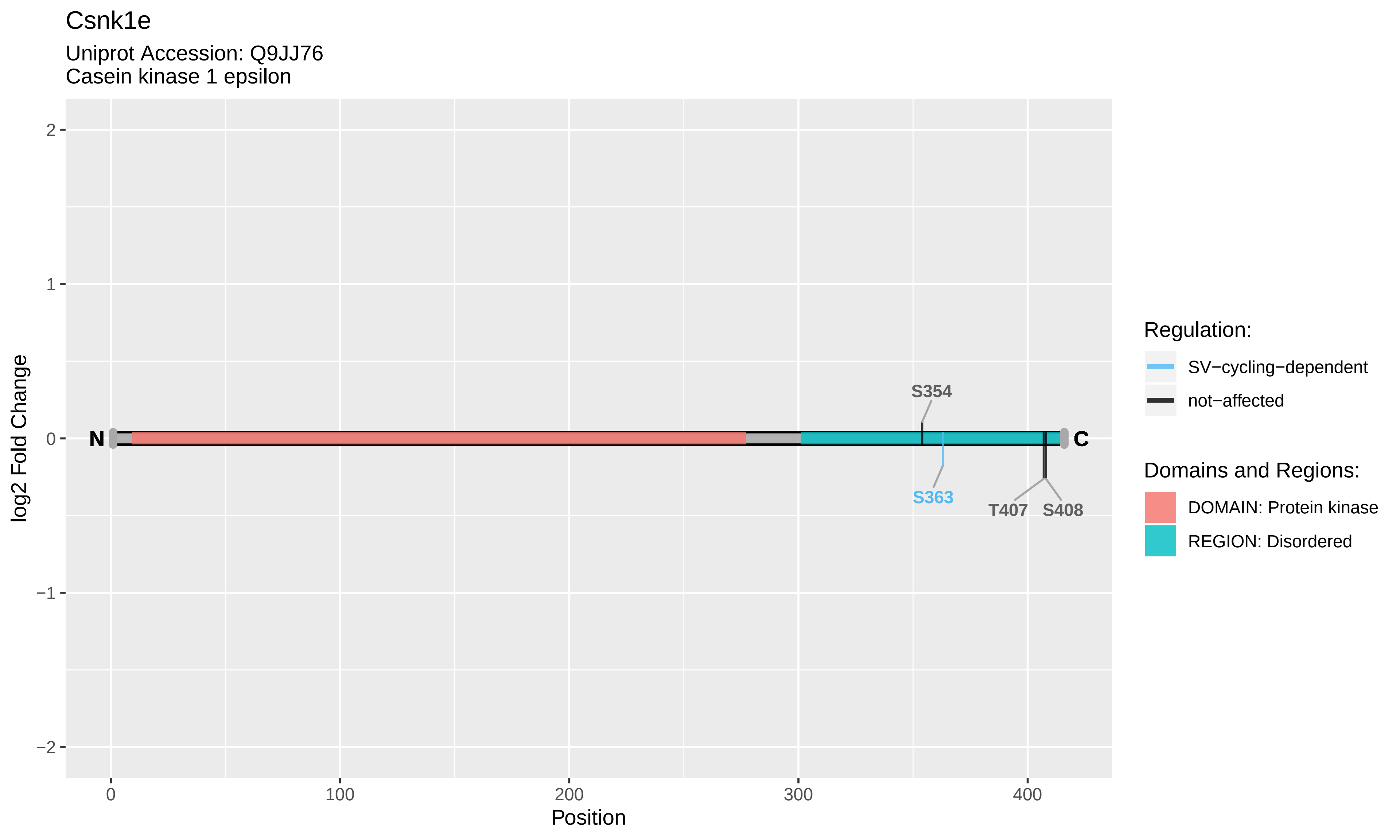

Csnk1e
Uniprot Accession: Q9JJ76
Casein kinase 1 epsilon
log2 Fold Change
2
1
0
−1
−2
N
C
S354
S363
T407
S408
0
100
200
300
400
Position
Regulation:
SV−cycling−dependent
not−affected
Domains and Regions:
DOMAIN: Protein kinase
REGION: Disordered

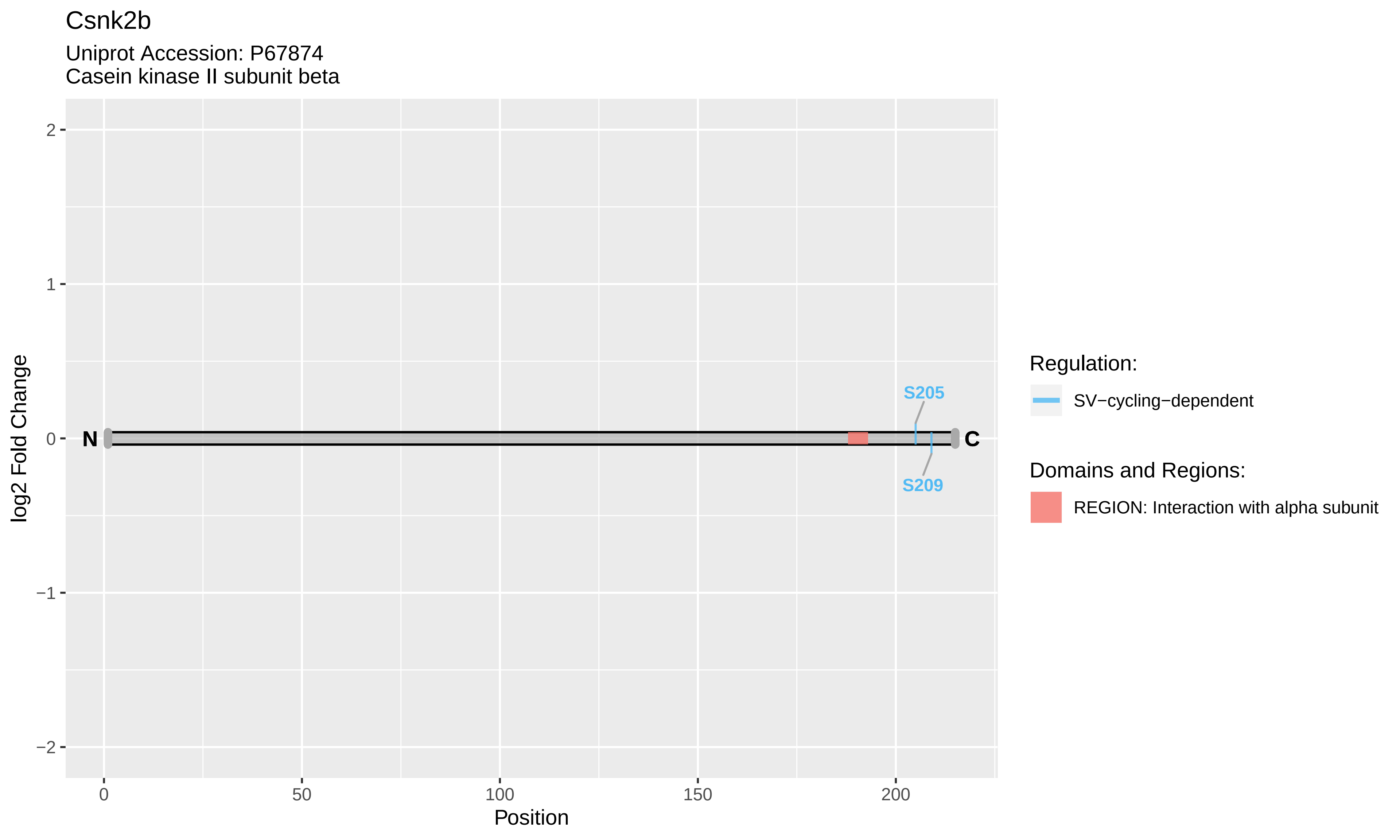

Csnk2b
Uniprot Accession: P67874
Casein kinase II subunit beta
log2 Fold Change
2
1
0
−1
−2
N
C
S205
S209
0
50
100
150
200
Position
Regulation:
SV−cycling−dependent
Domains and Regions:
REGION: Interaction with alpha subunit

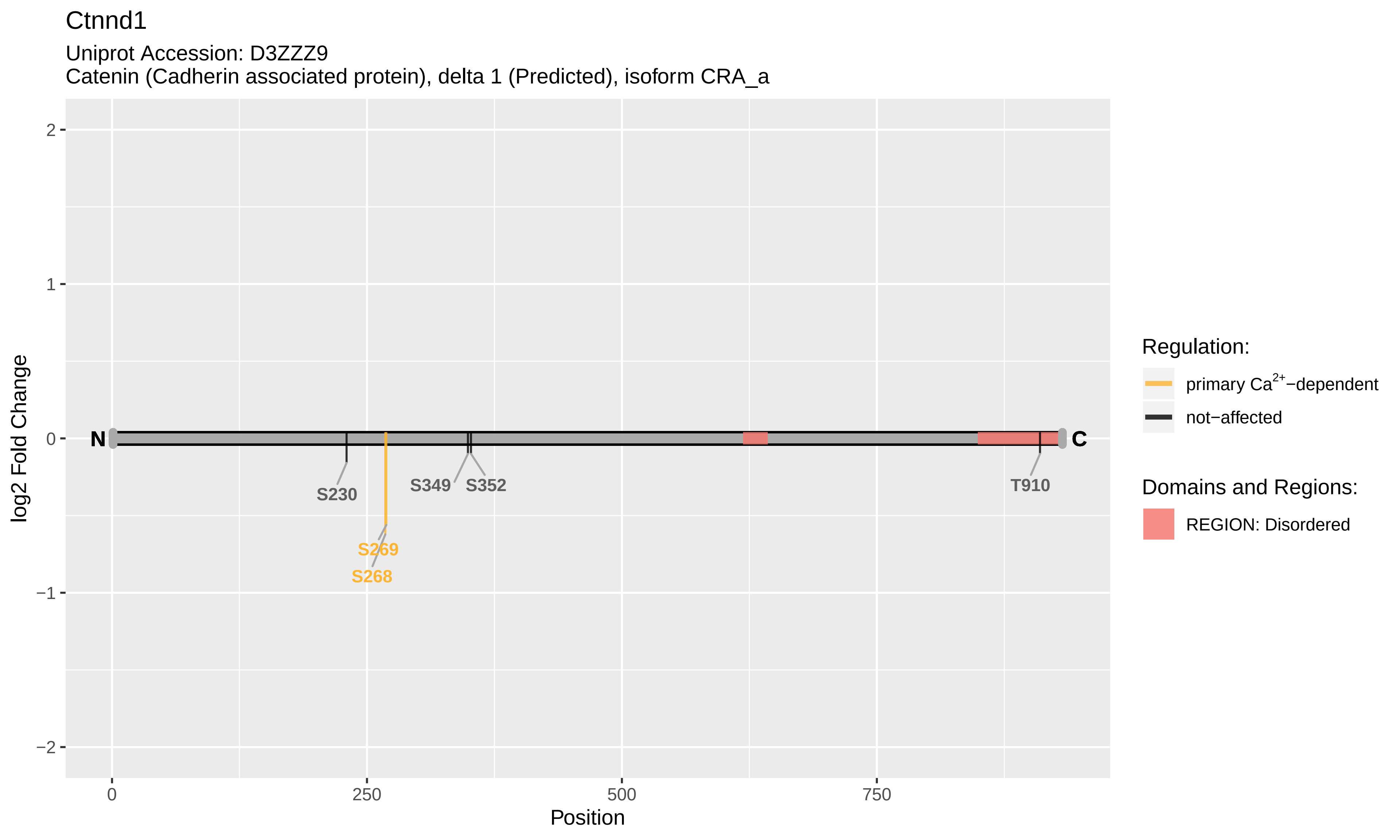
Ctnnd1
Uniprot Accession: D3ZZZ9
Catenin (Cadherin associated protein), delta 1 (Predicted), isoform CRA_a
log2 Fold Change
2
1
0
−1
−2
N
C
S230
S269
S268
S349
S352
T910
0
250
500
750
Position
Regulation:
primary Ca2+−dependent
not−affected
Domains and Regions:
REGION: Disordered

Ctnnd2

Uniprot Accession: F1M787
Catenin delta−2

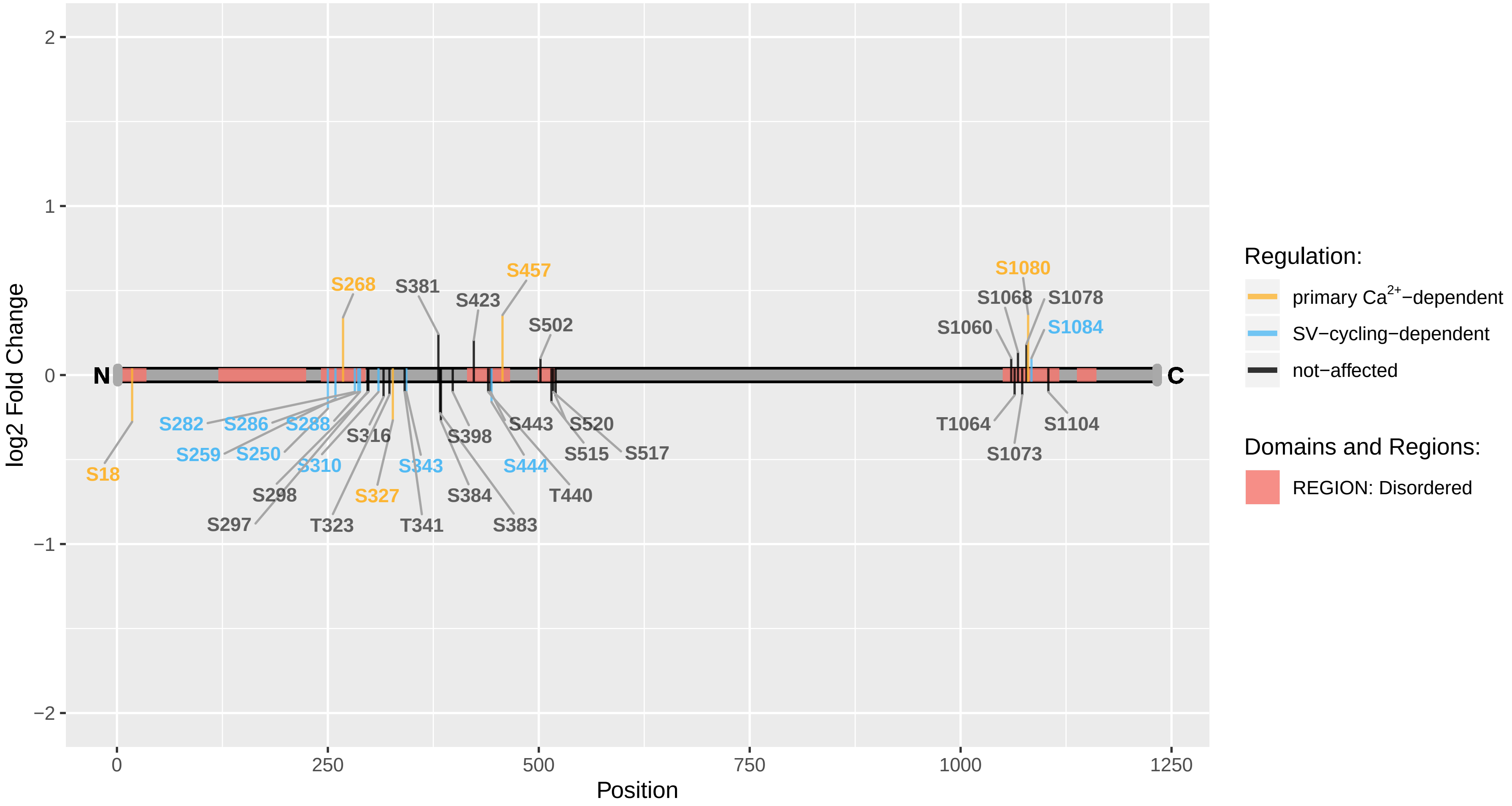

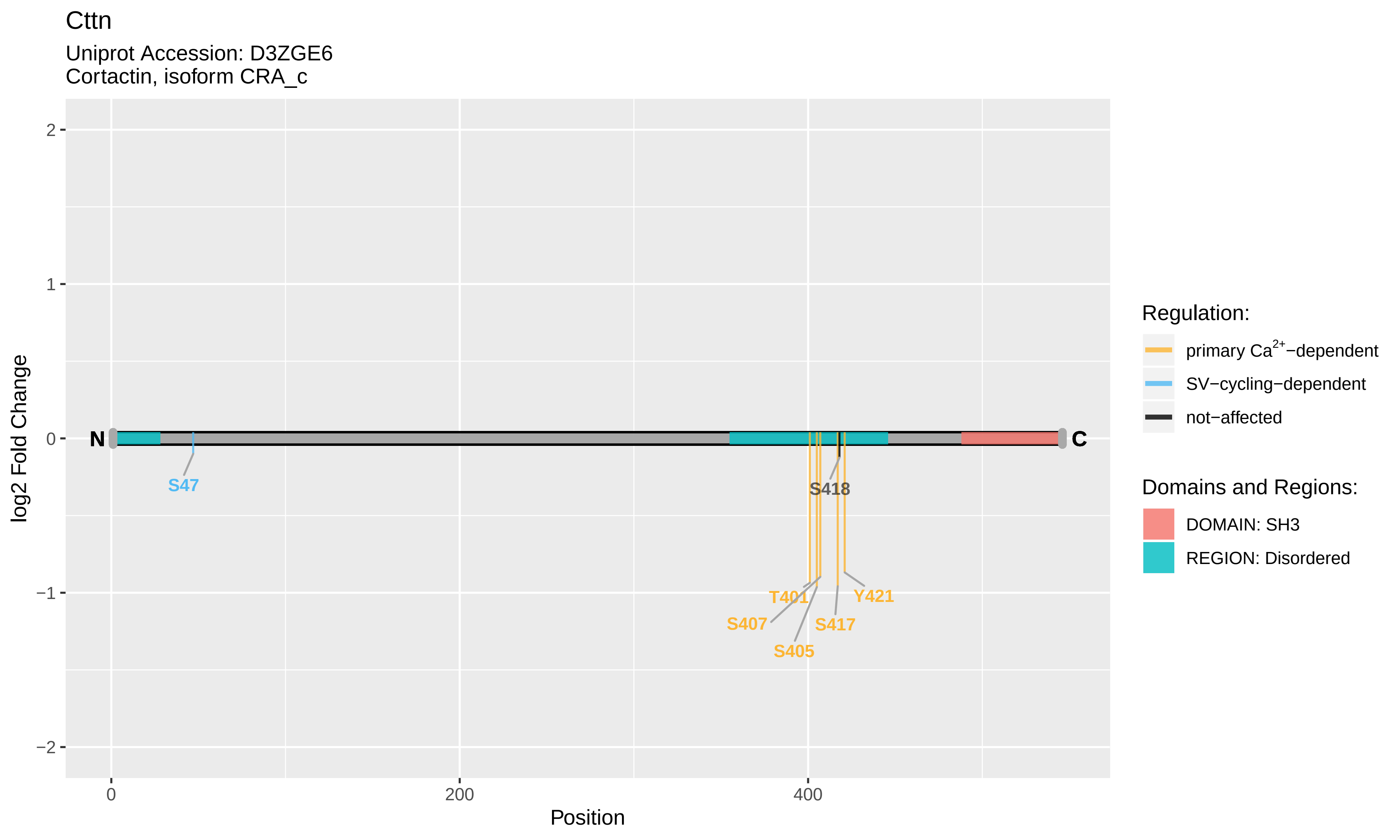

Cttn
Uniprot Accession: D3ZGE6
Cortactin, isoform CRA_c
log2 Fold Change
2
1
0
−1
−2
0
200
400
Position
N
C
S47
S418
T401
S407
S405
S417
Y421
Regulation:
primary Ca2+−dependent
SV−cycling−dependent
not−affected
Domains and Regions:
DOMAIN: SH3
REGION: Disordered

Cttnbp2

Uniprot Accession: Q2IBD4
Cortactin−binding protein 2

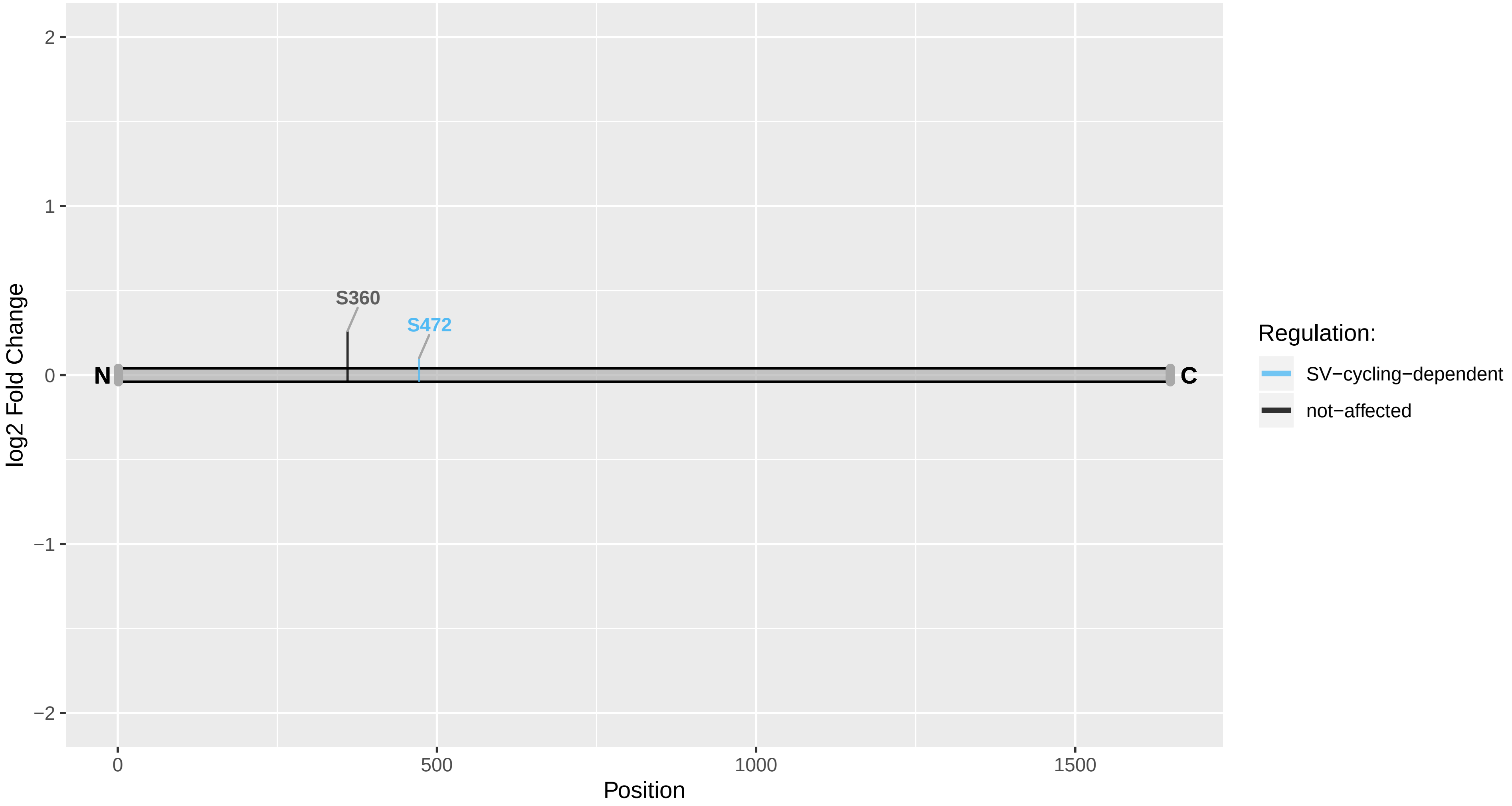

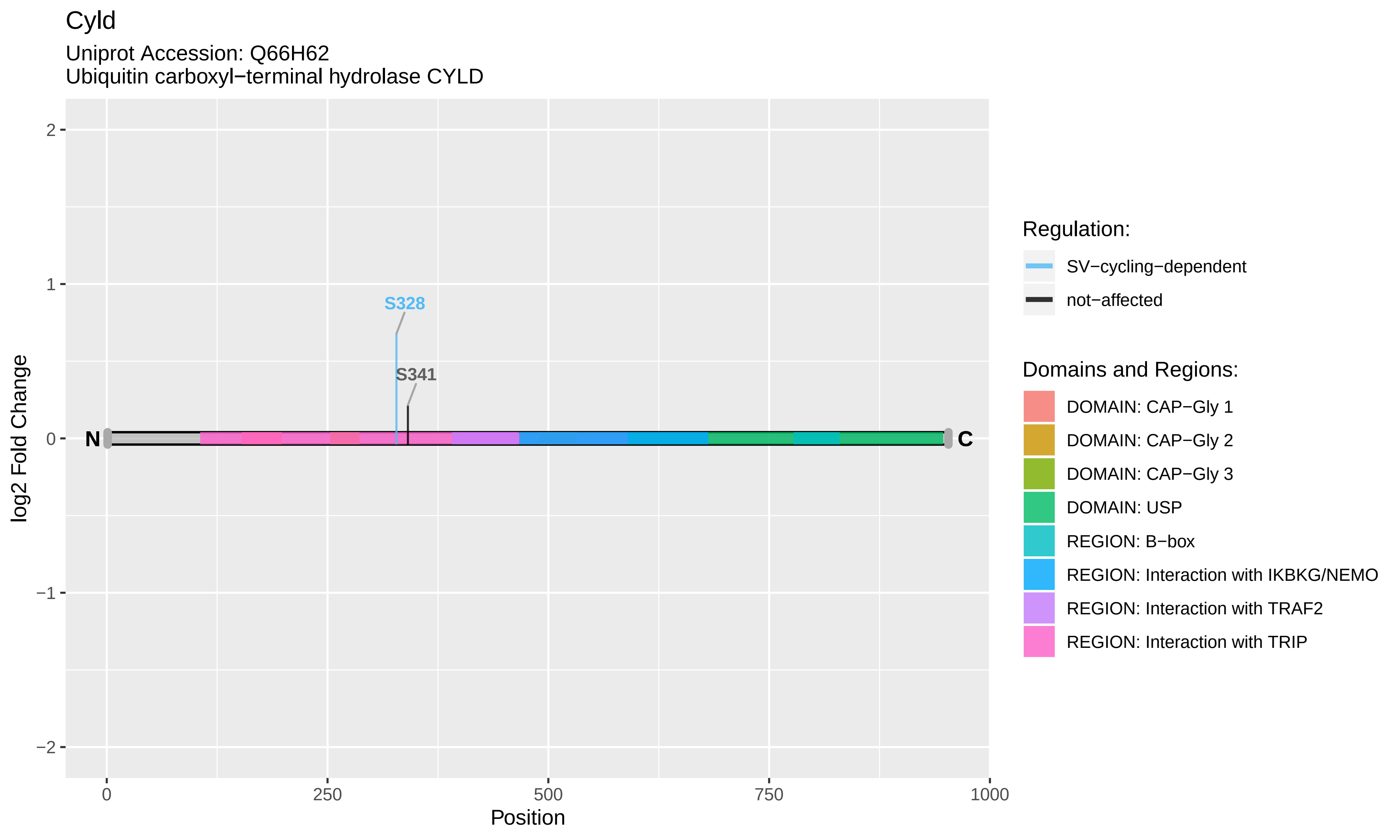
Cyld
Uniprot Accession: Q66H62
Ubiquitin carboxyl−terminal hydrolase CYLD
S328
S341
N
C
log2 Fold Change
2
1
0
−1
−2
0
250
500
750
1000
Position
Regulation:
SV−cycling−dependent
not−affected
Domains and Regions:
DOMAIN: CAP−Gly 1
DOMAIN: CAP−Gly 2
DOMAIN: CAP−Gly 3
DOMAIN: USP
REGION: B−box
REGION: Interaction with IKBKG/NEMO
REGION: Interaction with TRAF2
REGION: Interaction with TRIP

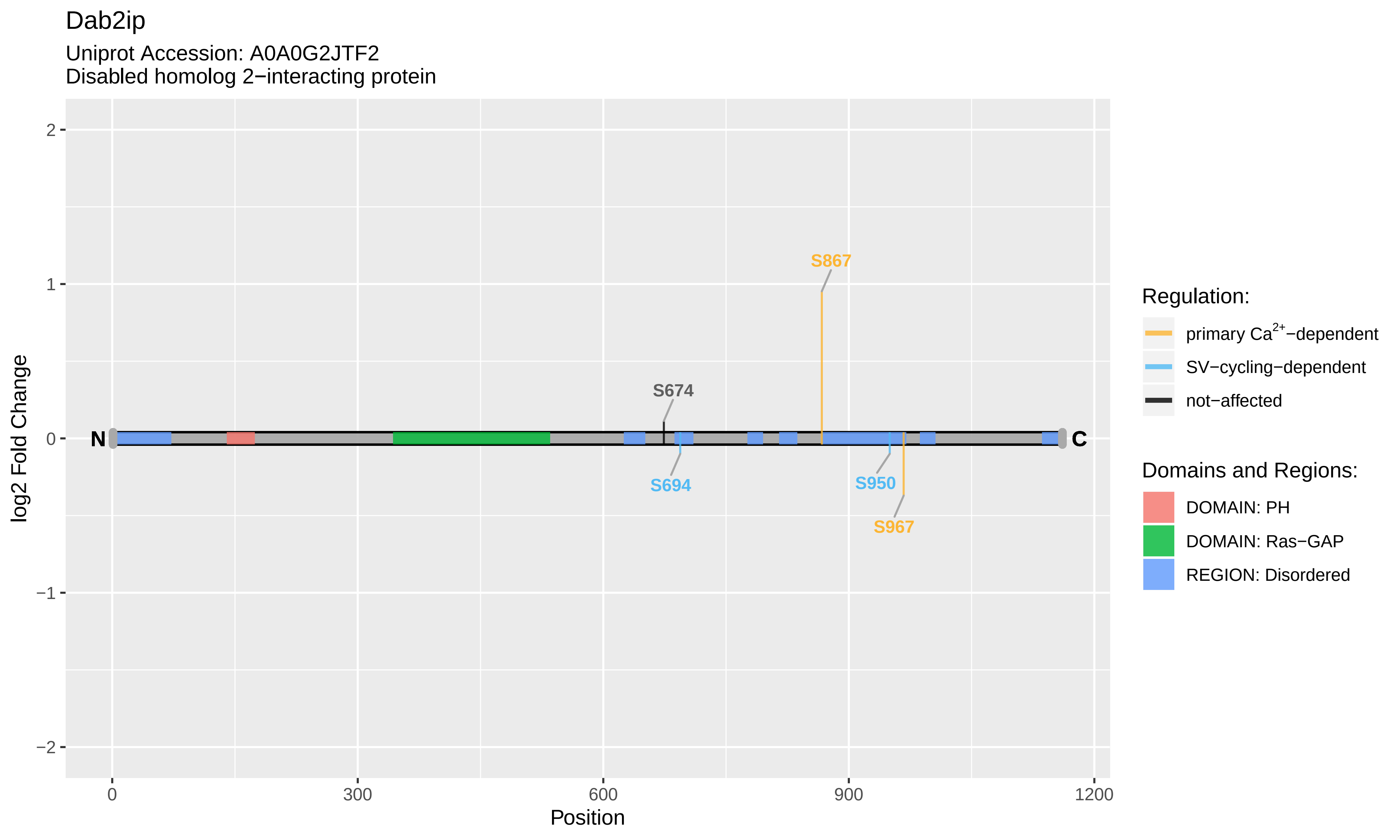

Dab2ip
Uniprot Accession: A0A0G2JTF2
Disabled homolog 2−interacting protein
log2 Fold Change
2
1
0
−1
−2
N
C
S867
S674
S694
S950
S967
0
300
600
900
1200
Position
Regulation:
primary Ca2+−dependent
SV−cycling−dependent
not−affected
Domains and Regions:
DOMAIN: PH
DOMAIN: Ras−GAP
REGION: Disordered

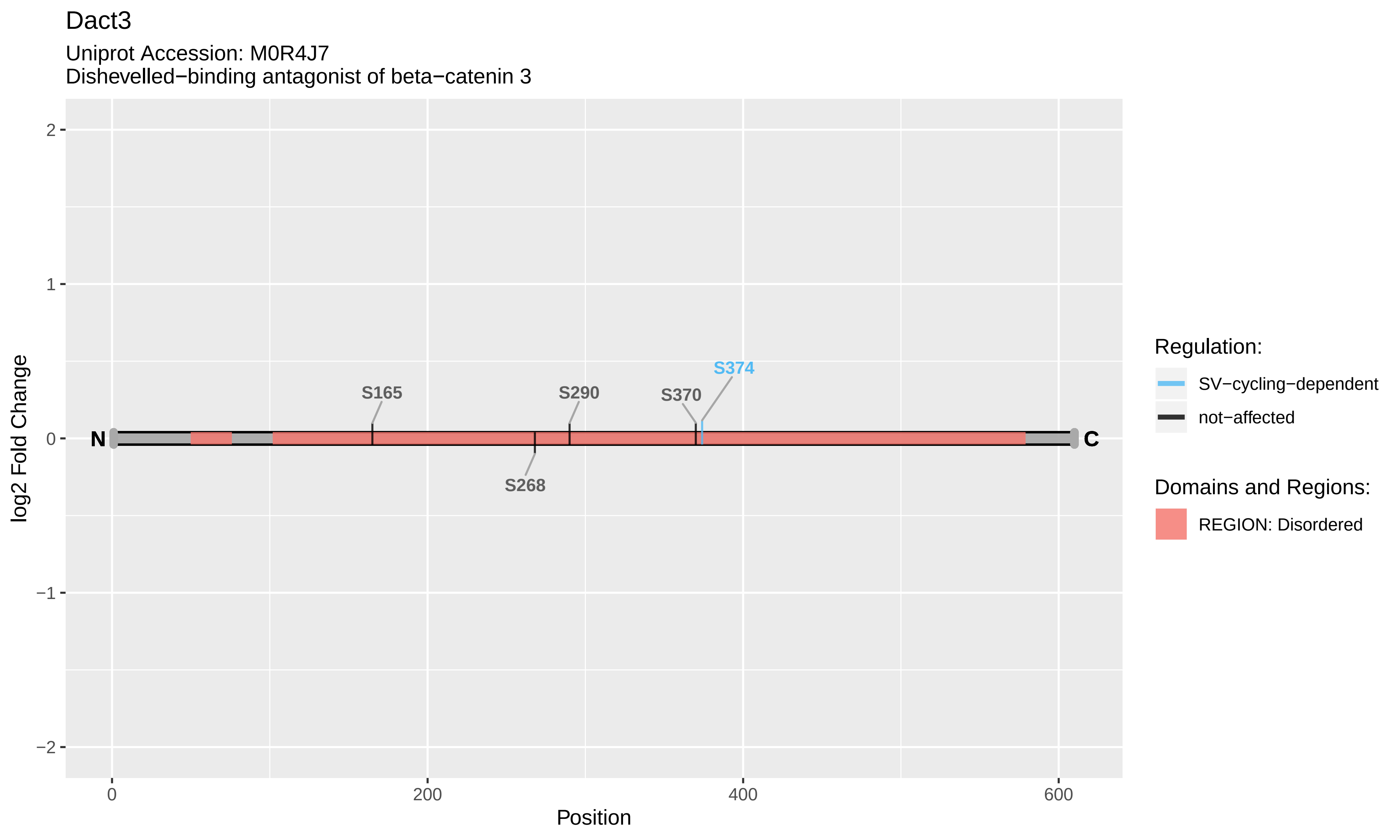

Dact3
Uniprot Accession: M0R4J7
Dishevelled−binding antagonist of beta−catenin 3
log2 Fold Change
2
1
0
−1
−2
N
C
S165
S290
S370
S374
S268
0
200
400
600
Position
Regulation:
SV−cycling−dependent
not−affected
Domains and Regions:
REGION: Disordered

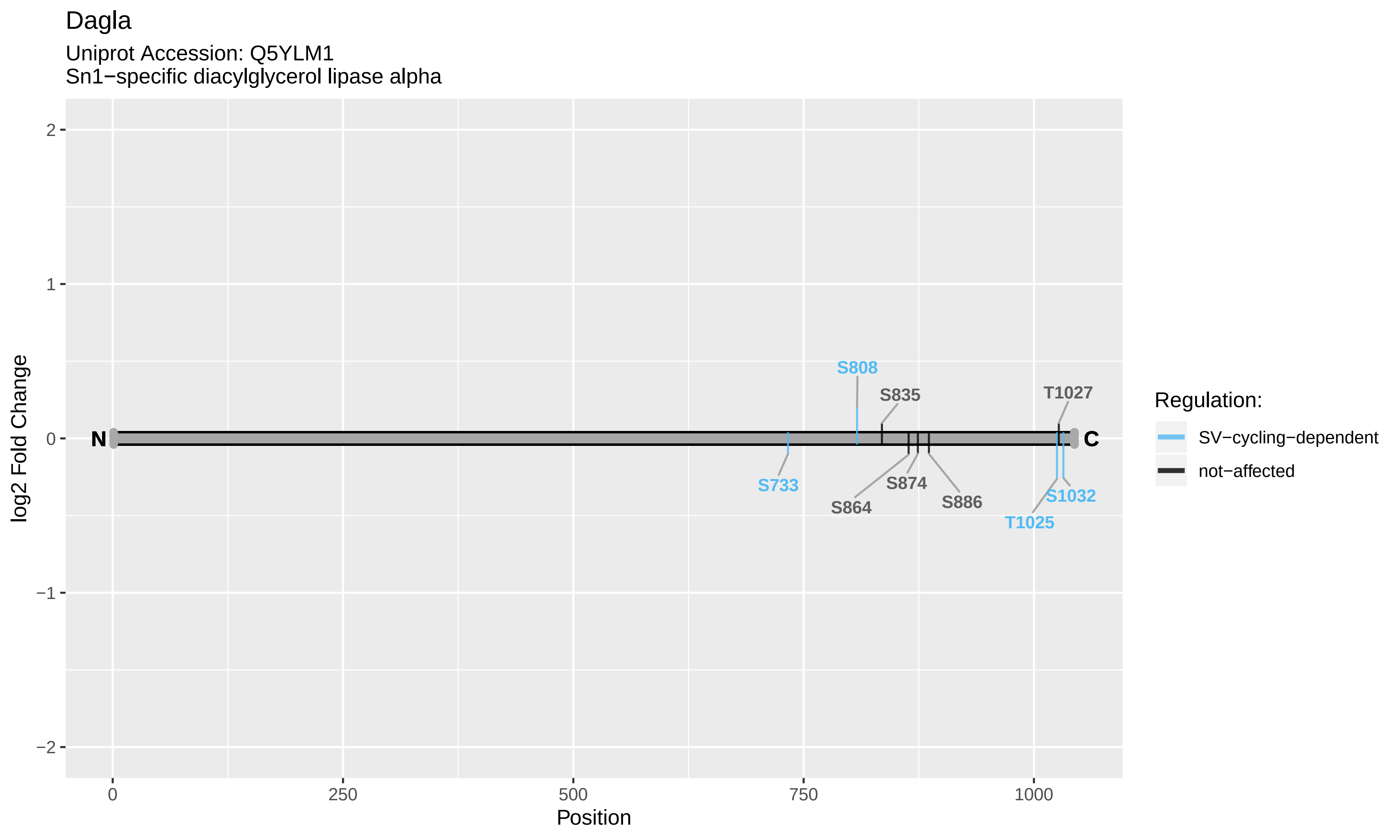
Dagla
Uniprot Accession: Q5YLM1
Sn1−specific diacylglycerol lipase alpha
log2 Fold Change
Position
N
C
S808
S835
T1027
S733
S864
S874
S886
S1032
T1025
2
1
0
−1
−2
0
250
500
750
1000
Regulation:
SV−cycling−dependent
not−affected

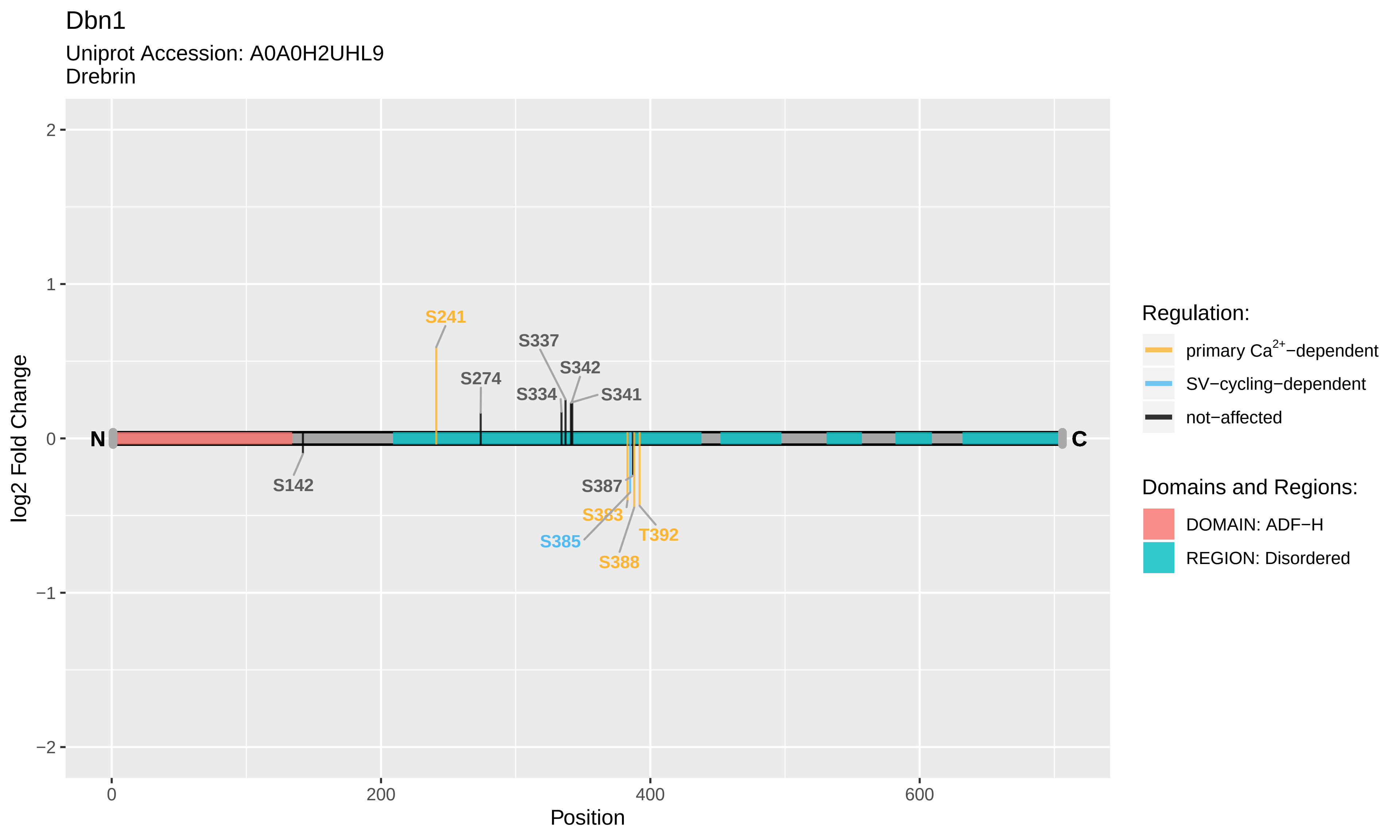

Dbn1
Uniprot Accession: A0A0H2UHL9
Drebrin
S241
S337
S342
S274
S334
S341
S142
S387
S383
S385
T392
S388
N
C
2
1
0
−1
−2
0
200
400
600
log2 Fold Change
Position
Regulation:
primary Ca2+−dependent
SV−cycling−dependent
not−affected
Domains and Regions:
DOMAIN: ADF−H
REGION: Disordered

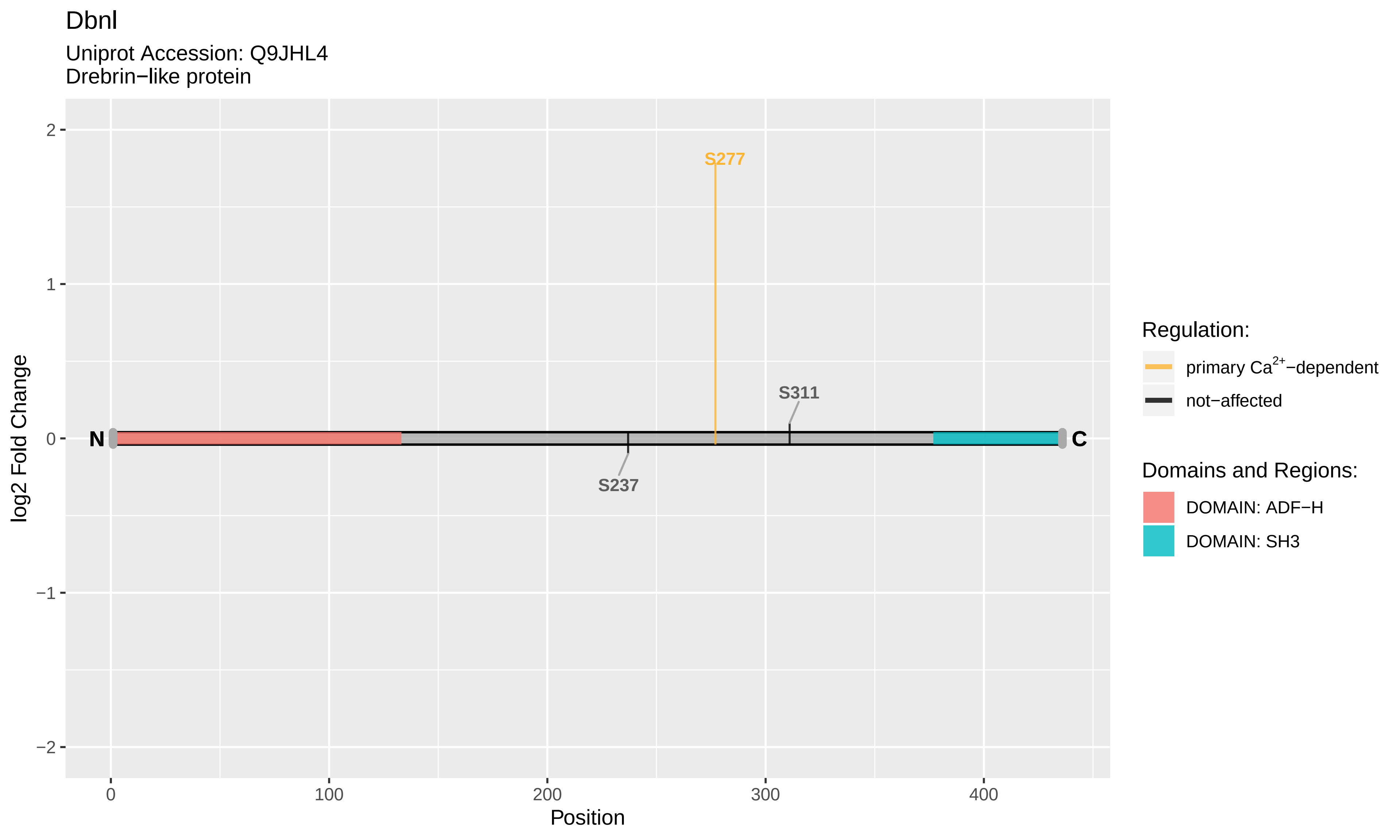

Dbnl
Uniprot Accession: Q9JHL4
Drebrin−like protein
S277
S311
S237
N
C
log2 Fold Change
Position
2
1
0
−1
−2
0
100
200
300
400
Regulation:
primary Ca2+−dependent
not−affected
Domains and Regions:
DOMAIN: ADF−H
DOMAIN: SH3

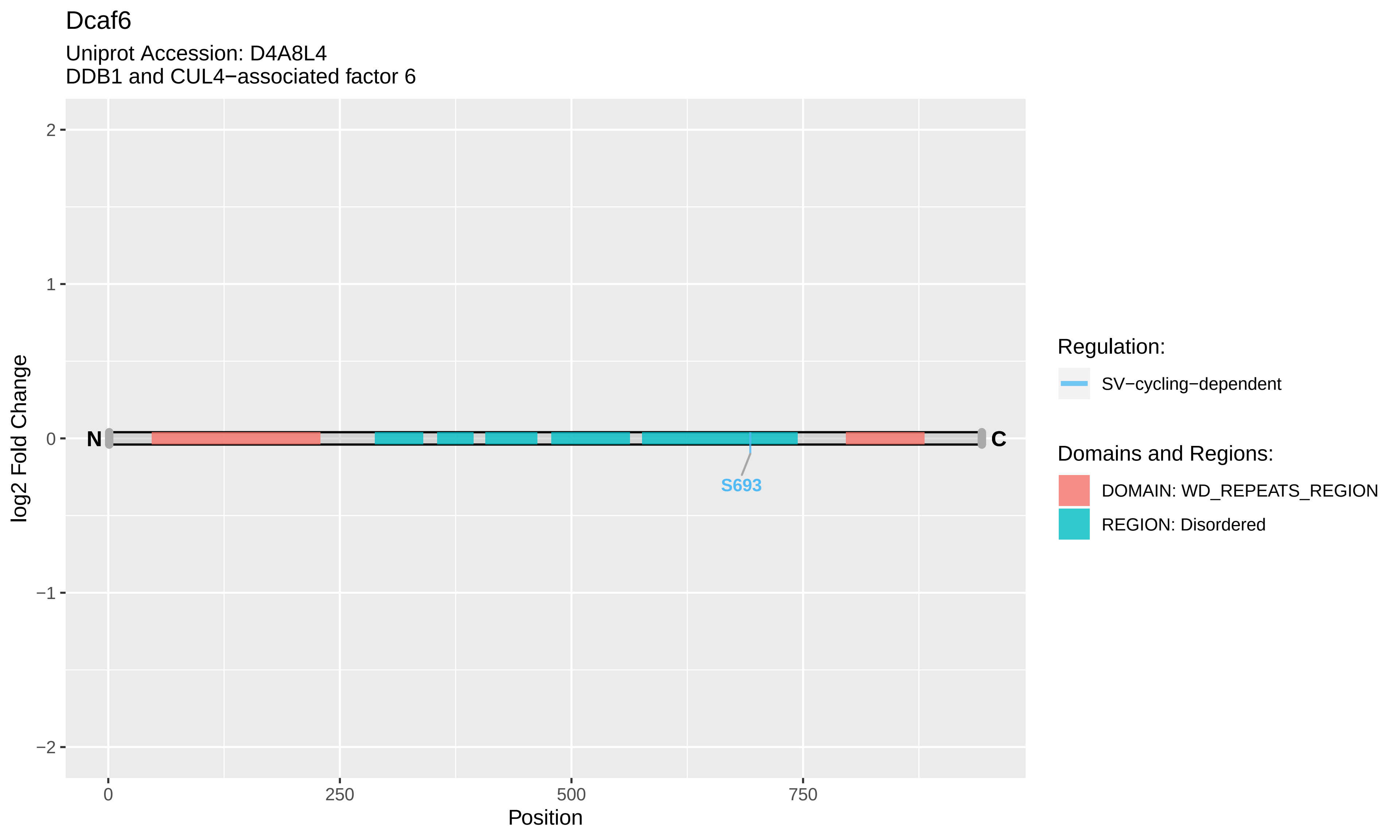
Dcaf6
Uniprot Accession: D4A8L4
DDB1 and CUL4−associated factor 6
log2 Fold Change
2
1
0
−1
−2
N
C
S693
0
250
500
750
Position
Regulation:
SV−cycling−dependent
Domains and Regions:
DOMAIN: WD_REPEATS_REGION
REGION: Disordered

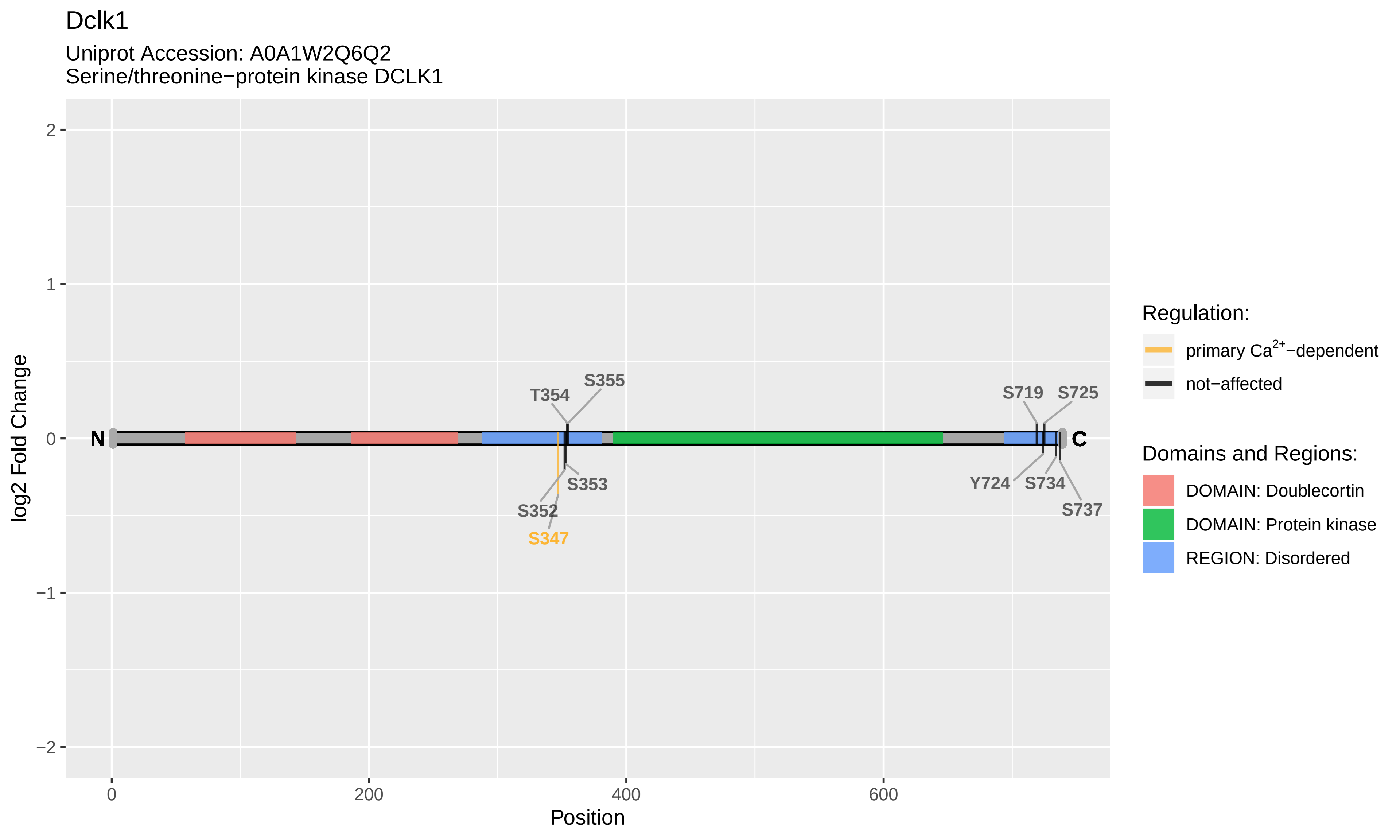

Dclk1
Uniprot Accession: A0A1W2Q6Q2
Serine/threonine−protein kinase DCLK1
log2 Fold Change
Position
N
C
T354
S355
S353
S352
S347
S719
S725
Y724
S734
S737
Regulation:
primary Ca2+−dependent
not−affected
Domains and Regions:
DOMAIN: Doublecortin
DOMAIN: Protein kinase
REGION: Disordered

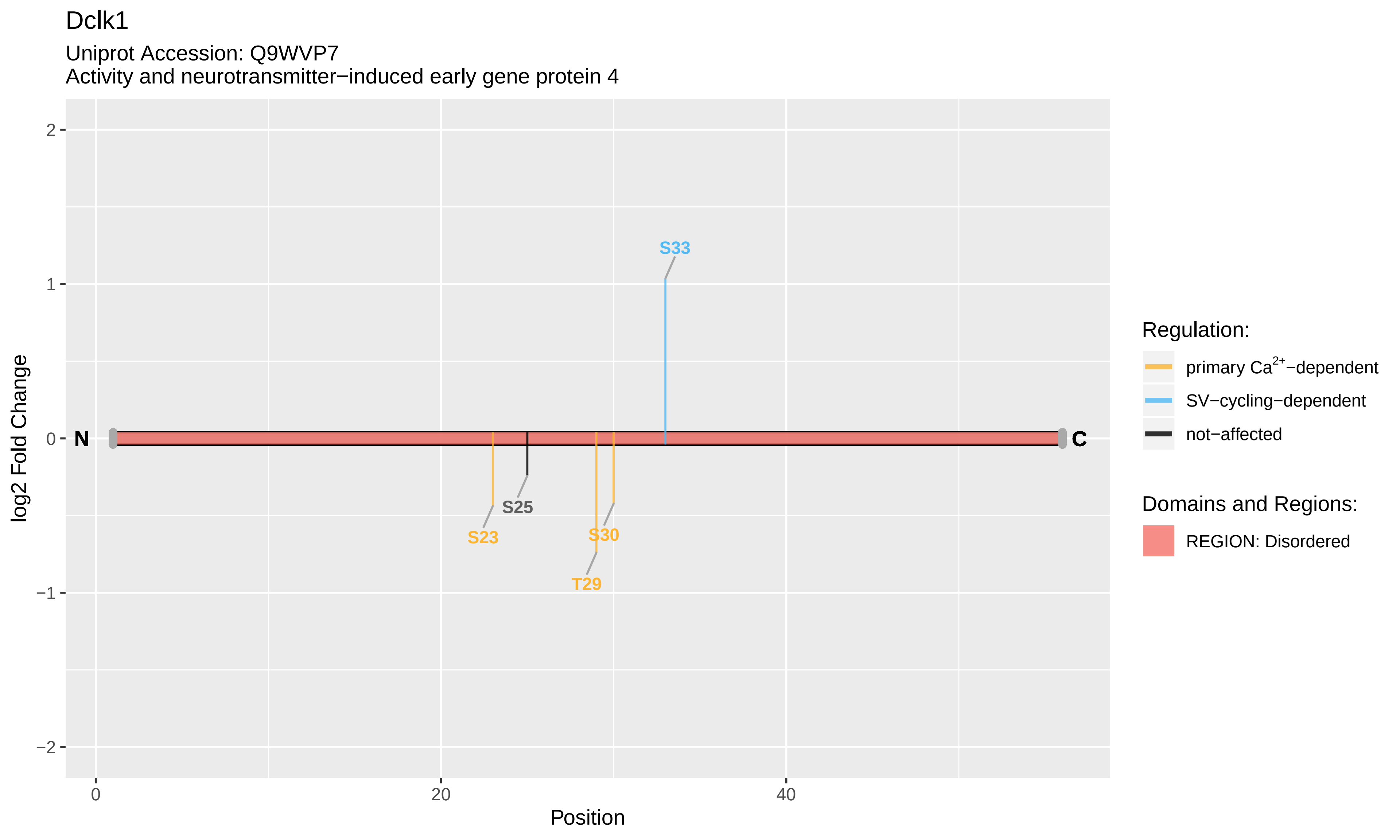

Dclk1
Uniprot Accession: Q9WVP7
Activity and neurotransmitter−induced early gene protein 4
S33
S25
S23
S30
T29
N
C
2
1
0
−1
−2
0
20
40
log2 Fold Change
Position
Regulation:
primary Ca2+−dependent
SV−cycling−dependent
not−affected
Domains and Regions:
REGION: Disordered

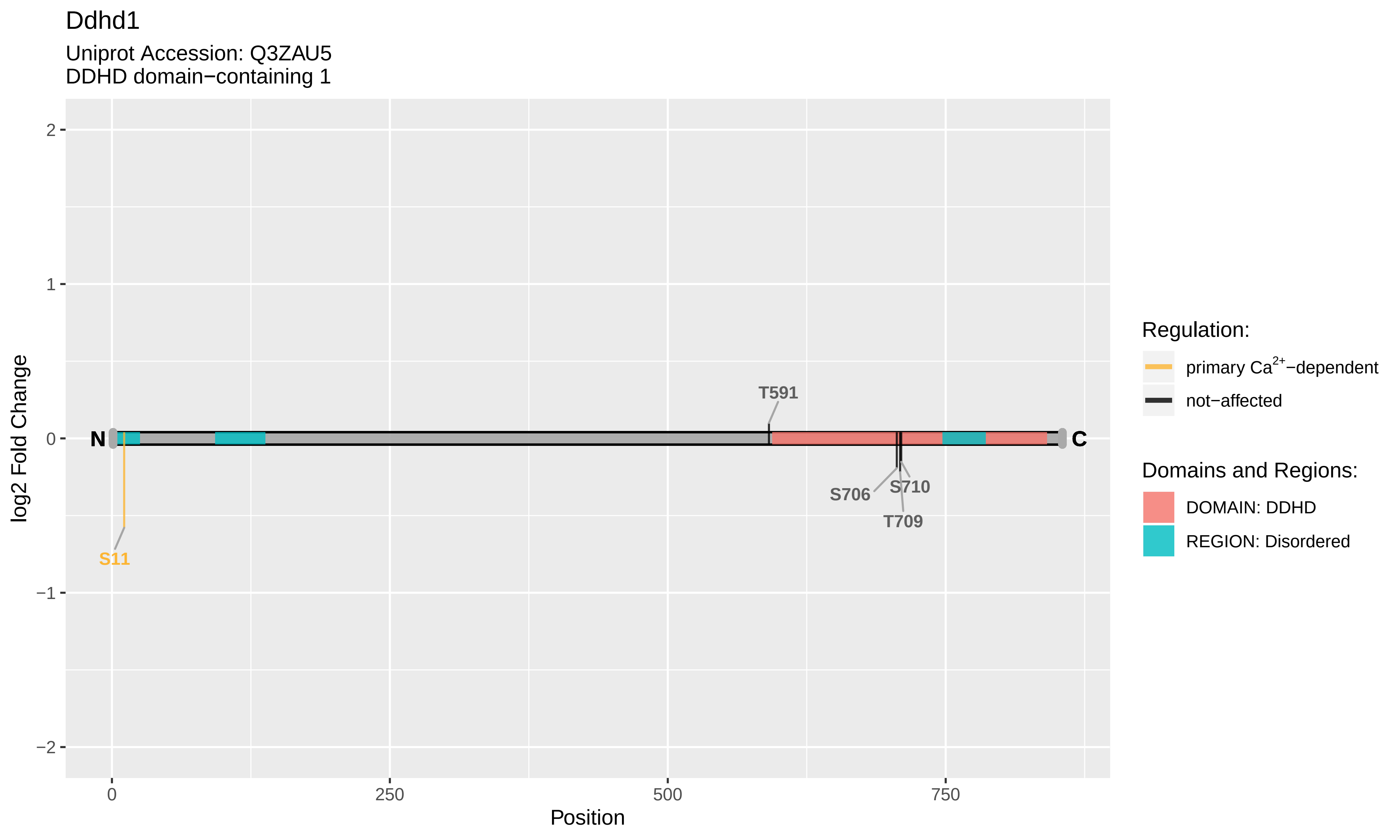

Ddhd1
Uniprot Accession: Q3ZAU5
DDHD domain−containing 1
log2 Fold Change
2
1
0
−1
−2
Position
0
250
500
750
N
C
T591
S706
S710
T709
S11
Regulation:
primary Ca2+−dependent
not−affected
Domains and Regions:
DOMAIN: DDHD
REGION: Disordered

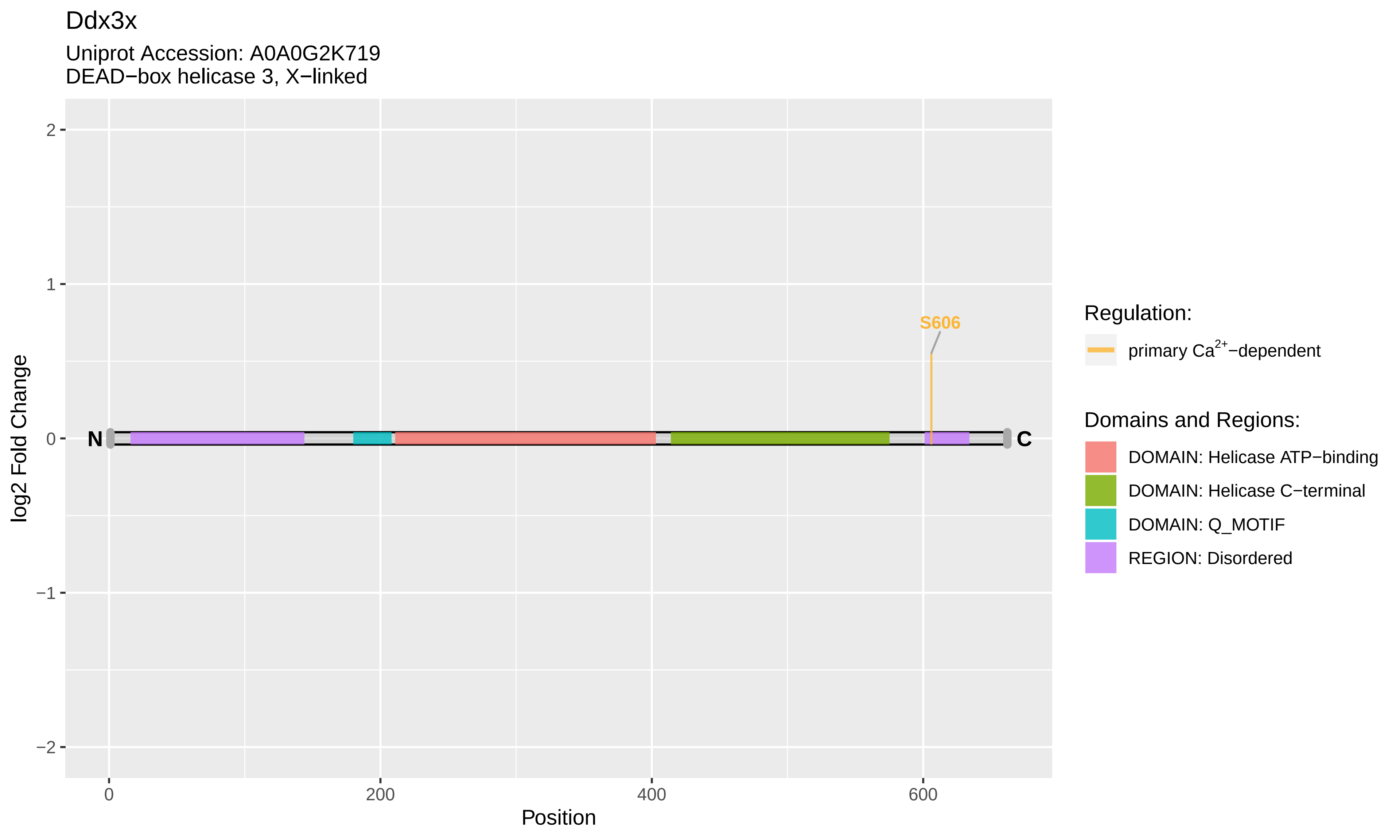
Ddx3x
Uniprot Accession: A0A0G2K719
DEAD−box helicase 3, X−linked
S606
N
C
2
1
0
−1
−2
0
200
400
600
Position
log2 Fold Change
Regulation:
primary Ca2+−dependent
Domains and Regions:
DOMAIN: Helicase ATP−binding
DOMAIN: Helicase C−terminal
DOMAIN: Q_MOTIF
REGION: Disordered

Dgkb

Uniprot Accession: P49621
Diacylglycerol kinase beta

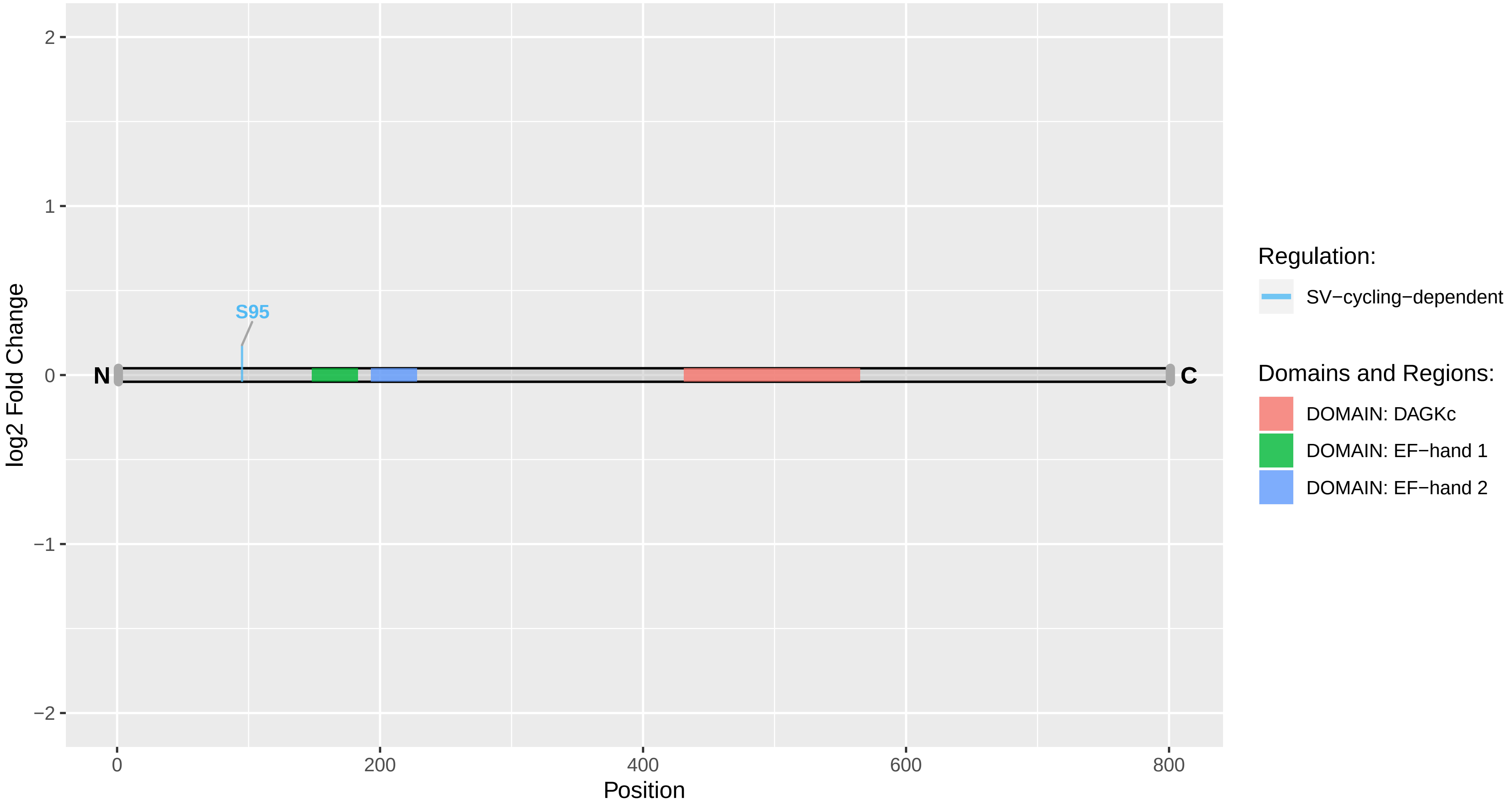

Dip2b

Uniprot Accession: A0A0G2JY22
Disco−interacting protein 2 homolog B

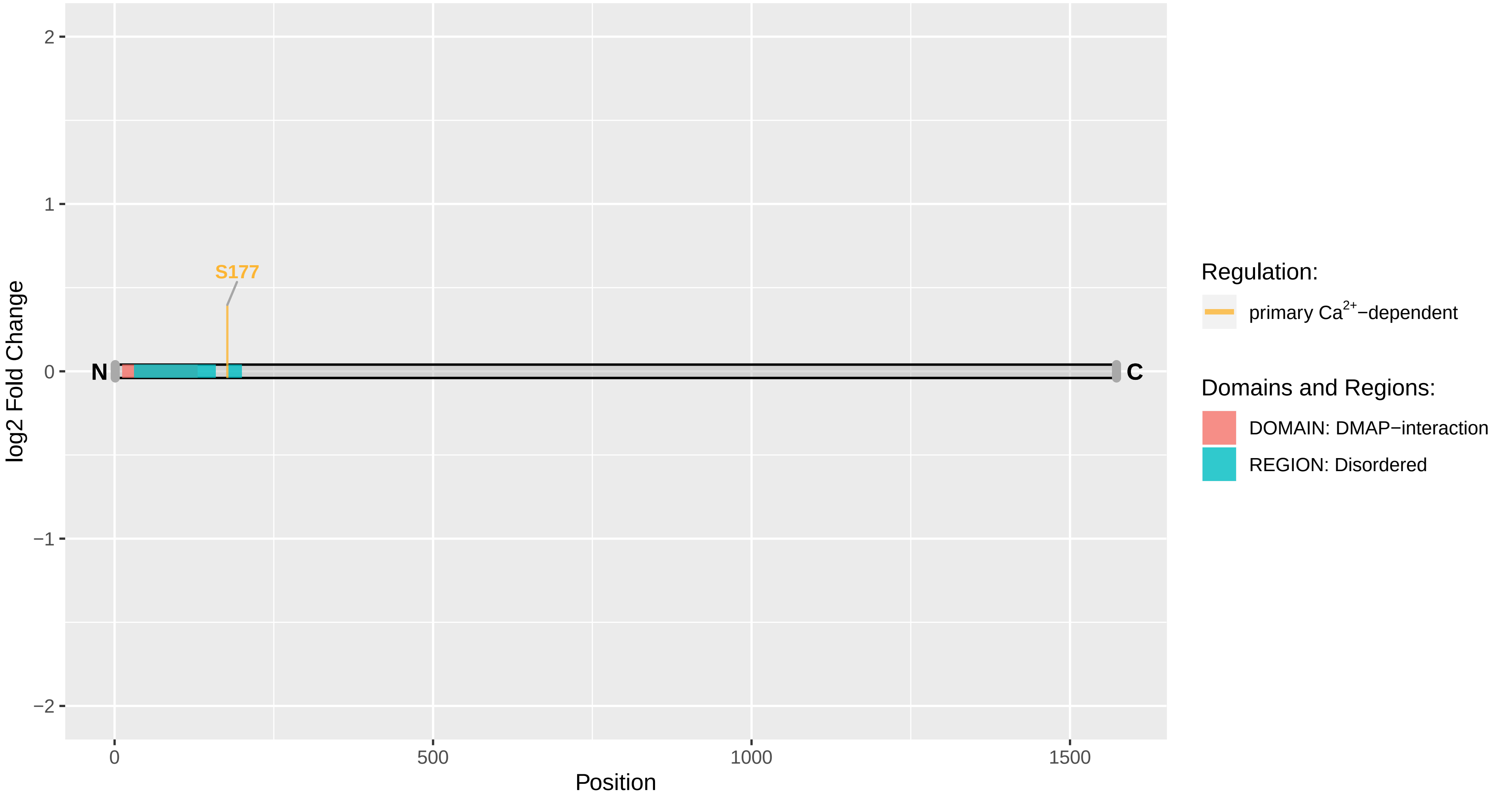

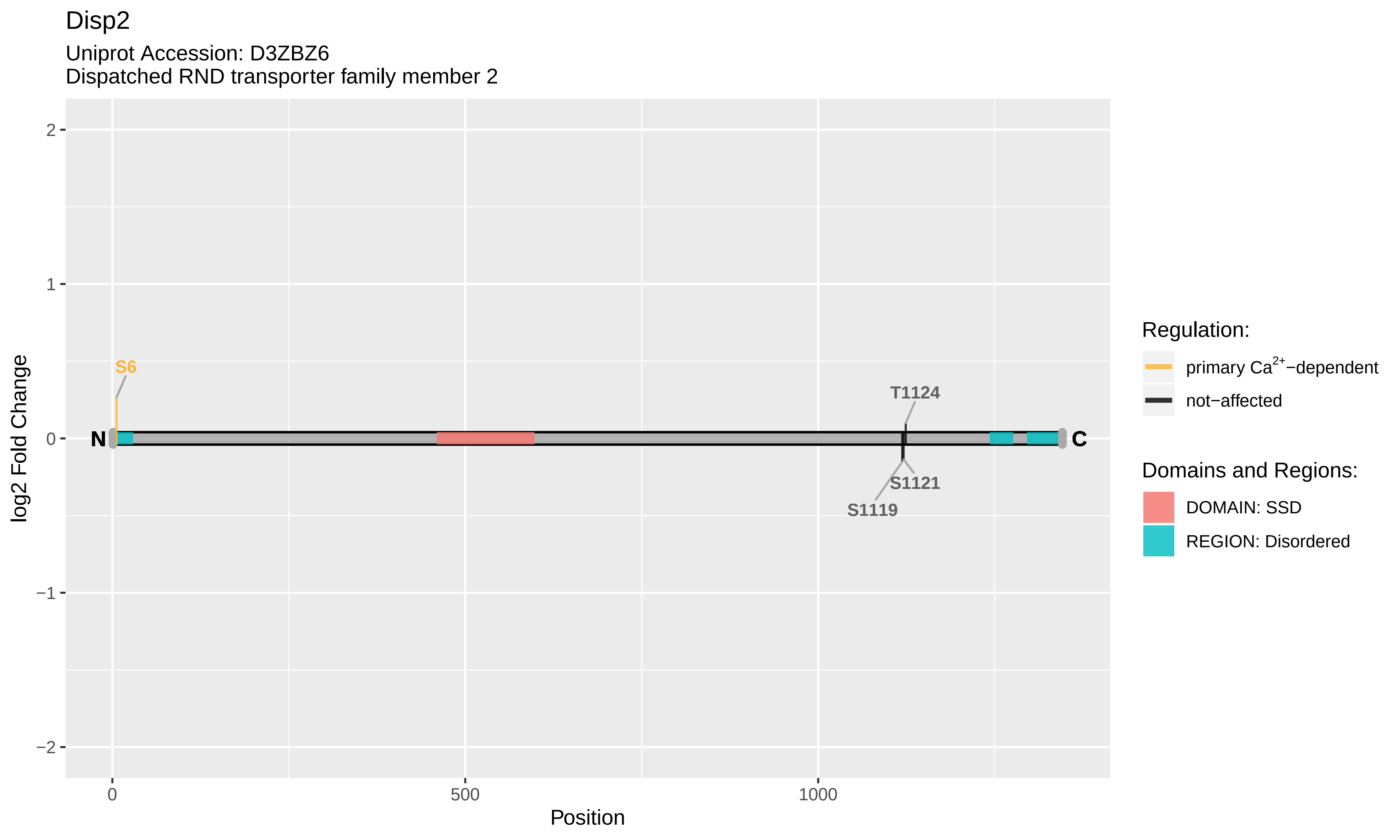

Disp2
Uniprot Accession: D3ZBZ6
Dispatched RND transporter family member 2
S6
T1124
S1121
S1119
N
C
log2 Fold Change
Position
2
1
0
−1
−2
0
500
1000
Regulation:
primary Ca2+−dependent
not−affected
Domains and Regions:
DOMAIN: SSD
REGION: Disordered

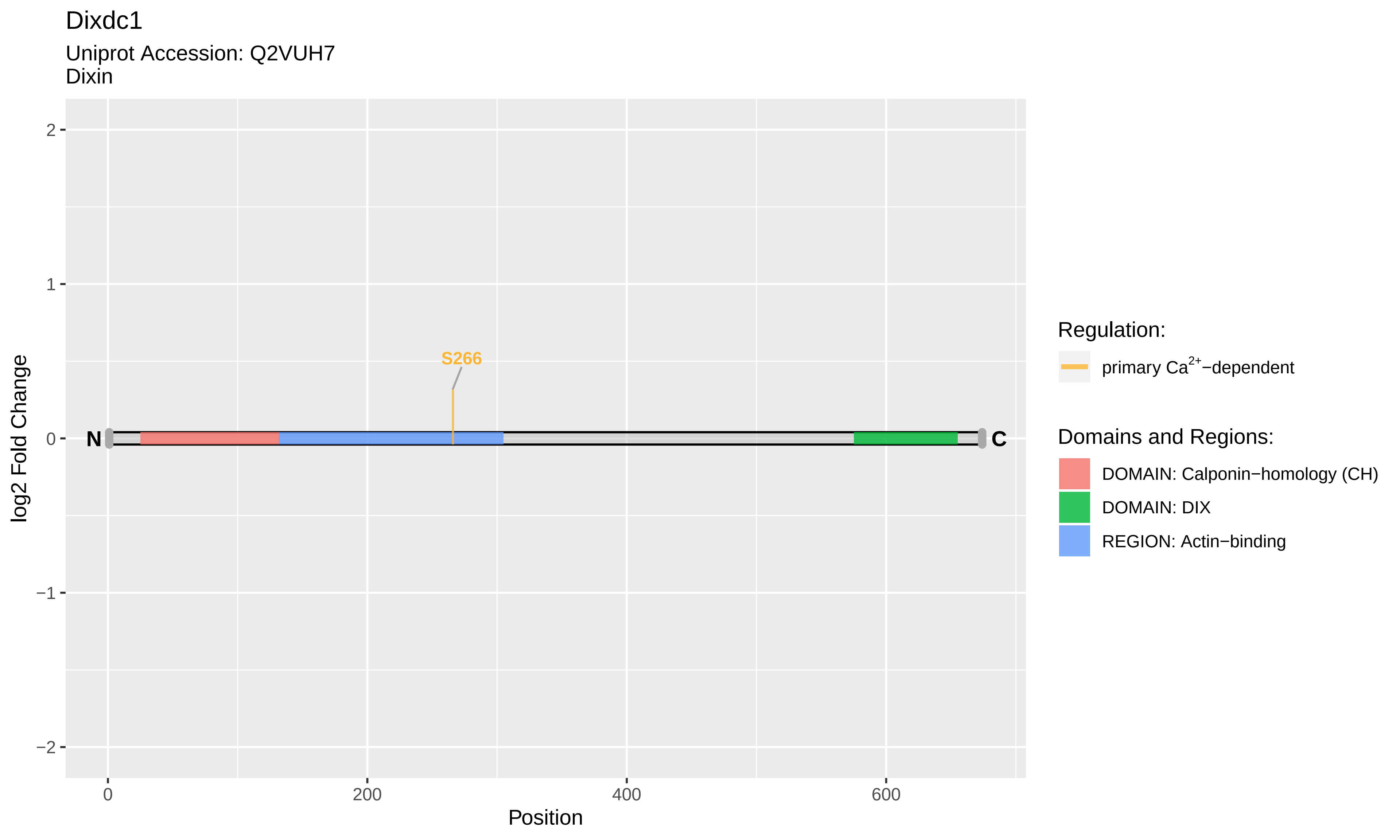

Dixdc1
Uniprot Accession: Q2VUH7
Dixin
S266
N
C
log2 Fold Change
2
1
0
−1
−2
0
200
400
600
Position
Regulation:
primary Ca2+−dependent
Domains and Regions:
DOMAIN: Calponin−homology (CH)
DOMAIN: DIX
REGION: Actin−binding

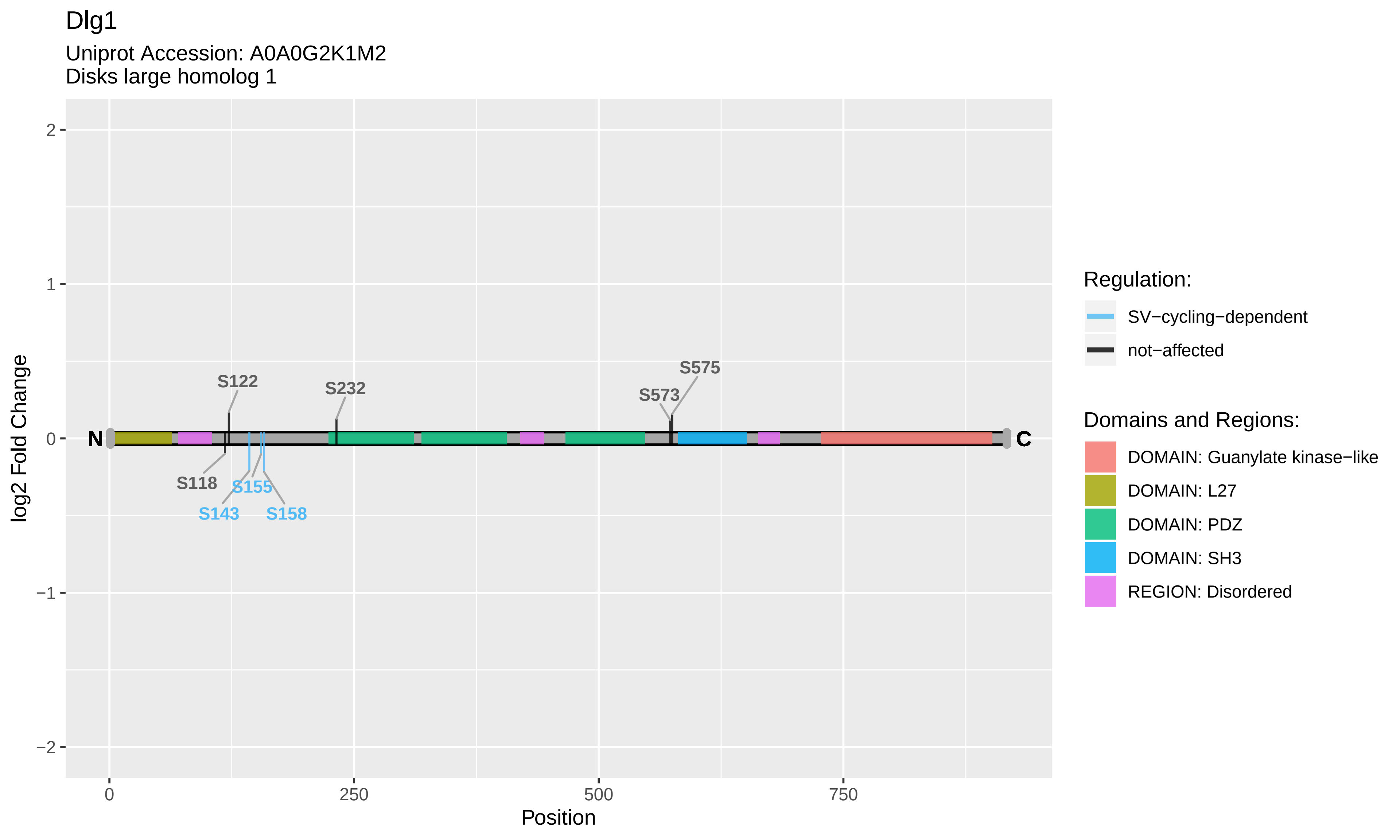
Dlg1
Uniprot Accession: A0A0G2K1M2
Disks large homolog 1
log2 Fold Change
2
1
0
−1
−2
N
C
S122
S232
S575
S573
S118
S155
S143
S158
0
250
500
750
Position
Regulation:
SV−cycling−dependent
not−affected
Domains and Regions:
DOMAIN: Guanylate kinase−like
DOMAIN: L27
DOMAIN: PDZ
DOMAIN: SH3
REGION: Disordered

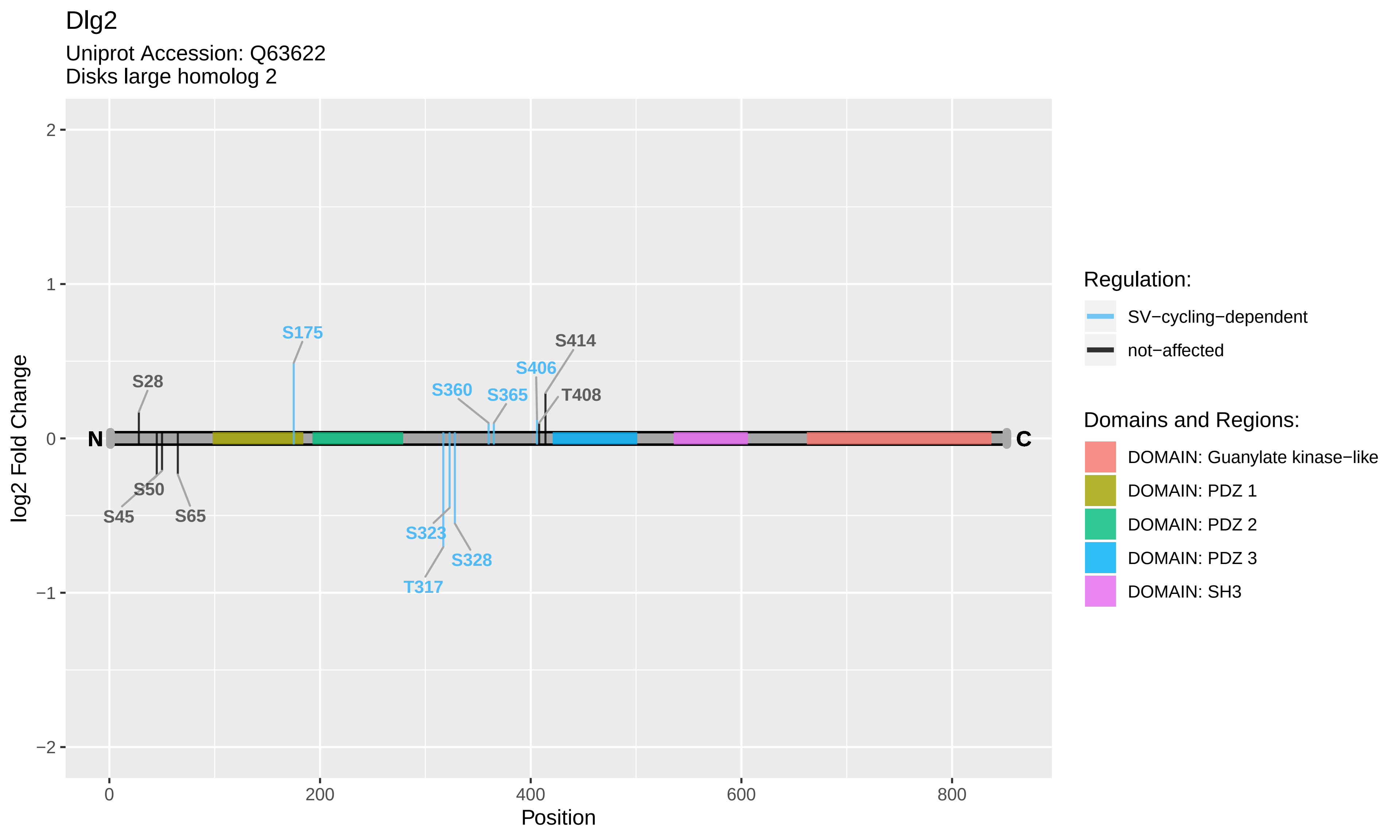

Dlg2
Uniprot Accession: Q63622
Disks large homolog 2
log2 Fold Change
Position
2
1
0
−1
−2
0
200
400
600
800
N
C
S28
S175
S360
S365
S406
S414
T408
S50
S45
S65
S323
S328
T317
Regulation:
SV−cycling−dependent
not−affected
Domains and Regions:
DOMAIN: Guanylate kinase−like
DOMAIN: PDZ 1
DOMAIN: PDZ 2
DOMAIN: PDZ 3
DOMAIN: SH3

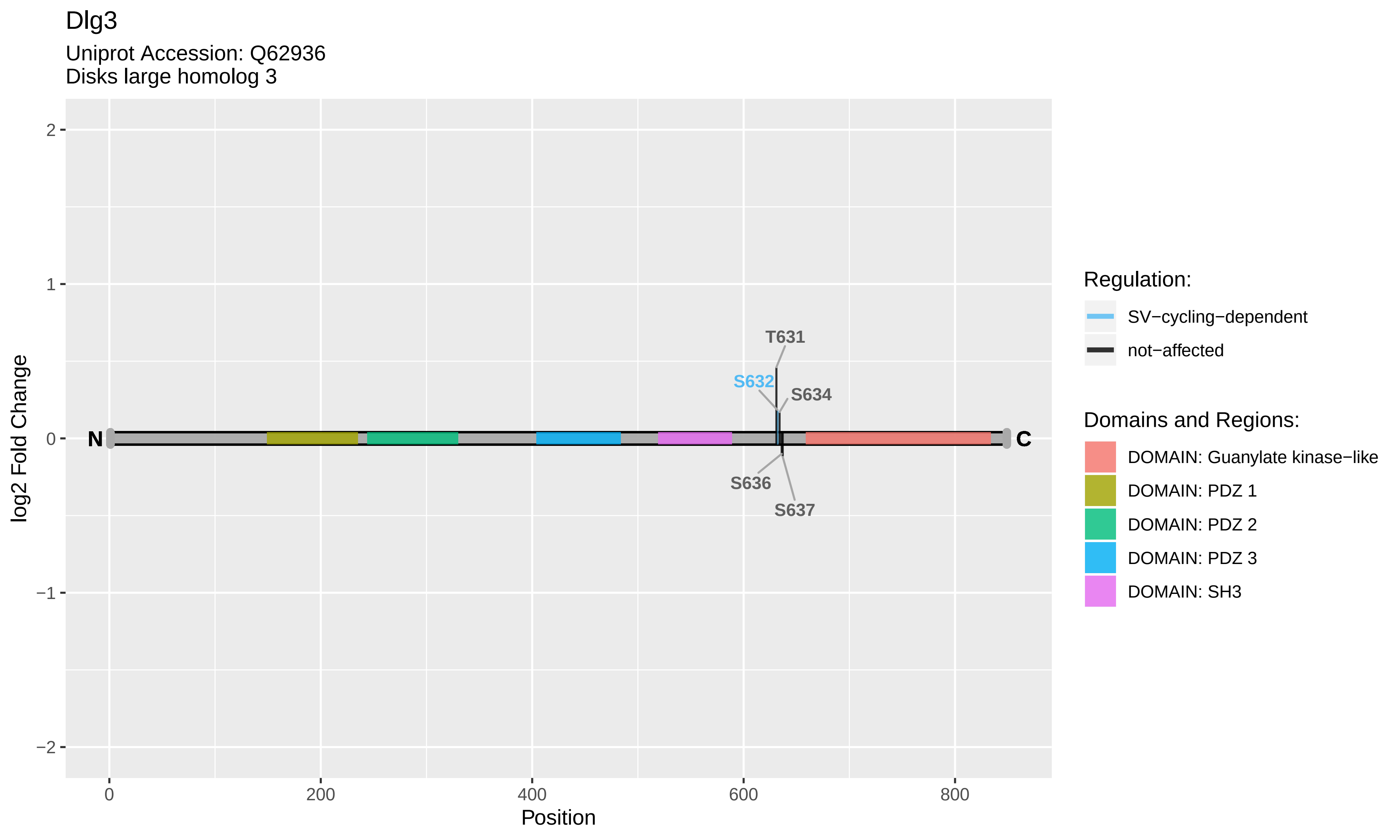

Dlg3
Uniprot Accession: Q62936
Disks large homolog 3
log2 Fold Change
Position
N
C
T631
S632
S634
S636
S637
Regulation:
SV−cycling−dependent
not−affected
Domains and Regions:
DOMAIN: Guanylate kinase−like
DOMAIN: PDZ 1
DOMAIN: PDZ 2
DOMAIN: PDZ 3
DOMAIN: SH3

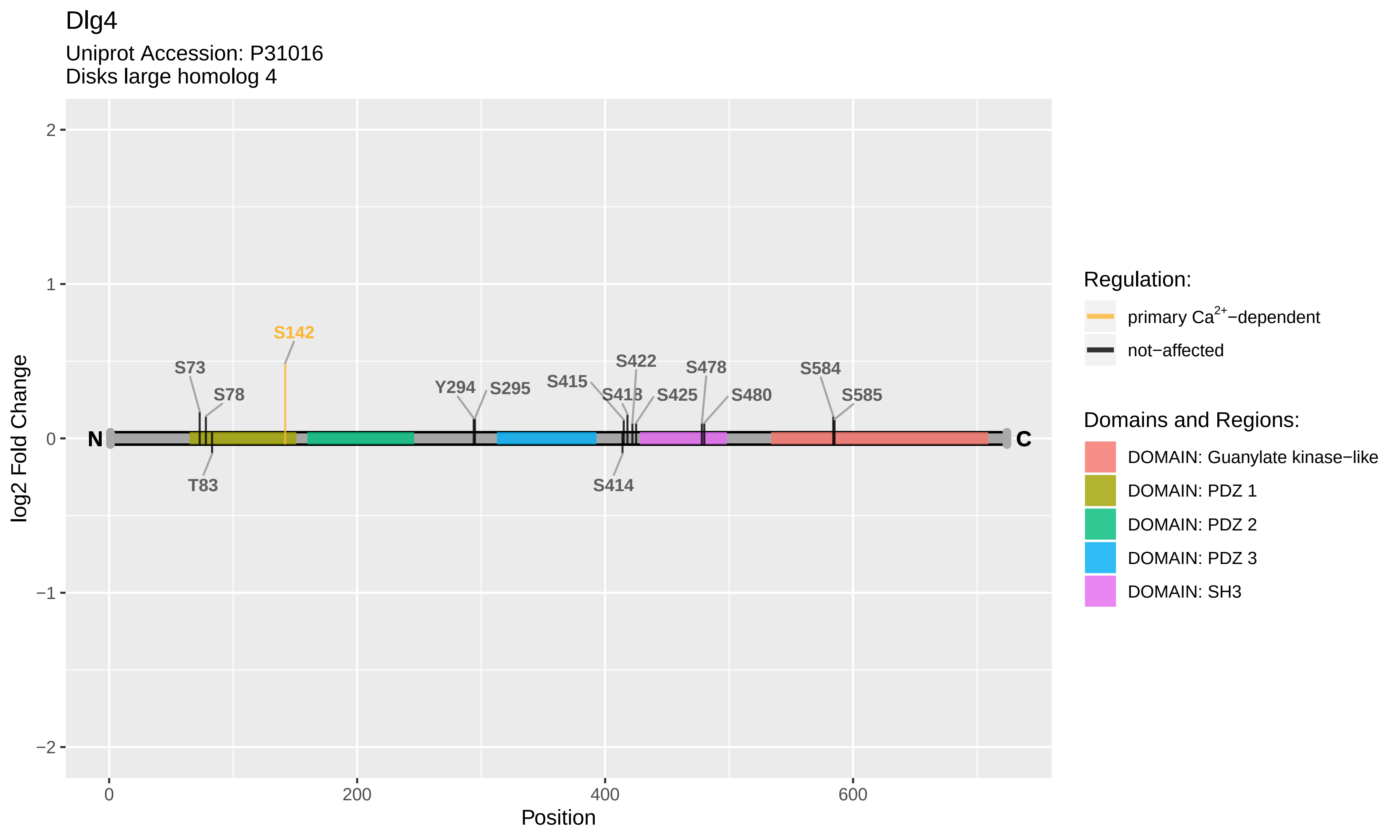
Dlg4
Uniprot Accession: P31016
Disks large homolog 4
log2 Fold Change
Position
N
C
S142
S73
S78
T83
Y294
S295
S415
S418
S422
S425
S414
S478
S480
S584
S585
2
1
0
−1
−2
0
200
400
600
Regulation:
primary Ca2+−dependent
not−affected
Domains and Regions:
DOMAIN: Guanylate kinase−like
DOMAIN: PDZ 1
DOMAIN: PDZ 2
DOMAIN: PDZ 3
DOMAIN: SH3

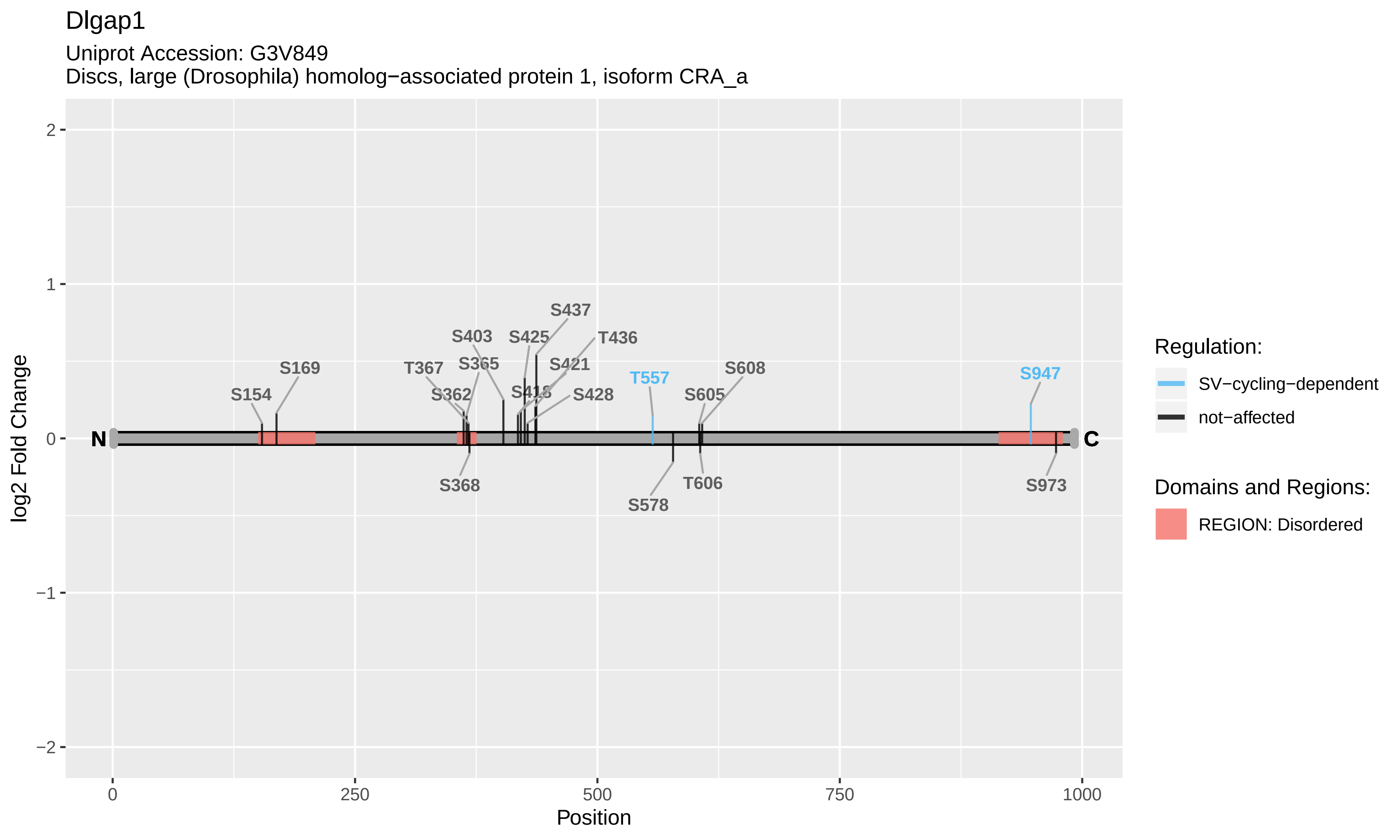

Dlgap1
Uniprot Accession: G3V849
Discs, large (Drosophila) homolog−associated protein 1, isoform CRA_a
log2 Fold Change
Position
N
C
S154
S169
T367
S362
S365
S403
S368
S425
S418
S437
T436
S421
S428
T557
S578
S605
S608
T606
S947
S973
Regulation:
SV−cycling−dependent
not−affected
Domains and Regions:
REGION: Disordered

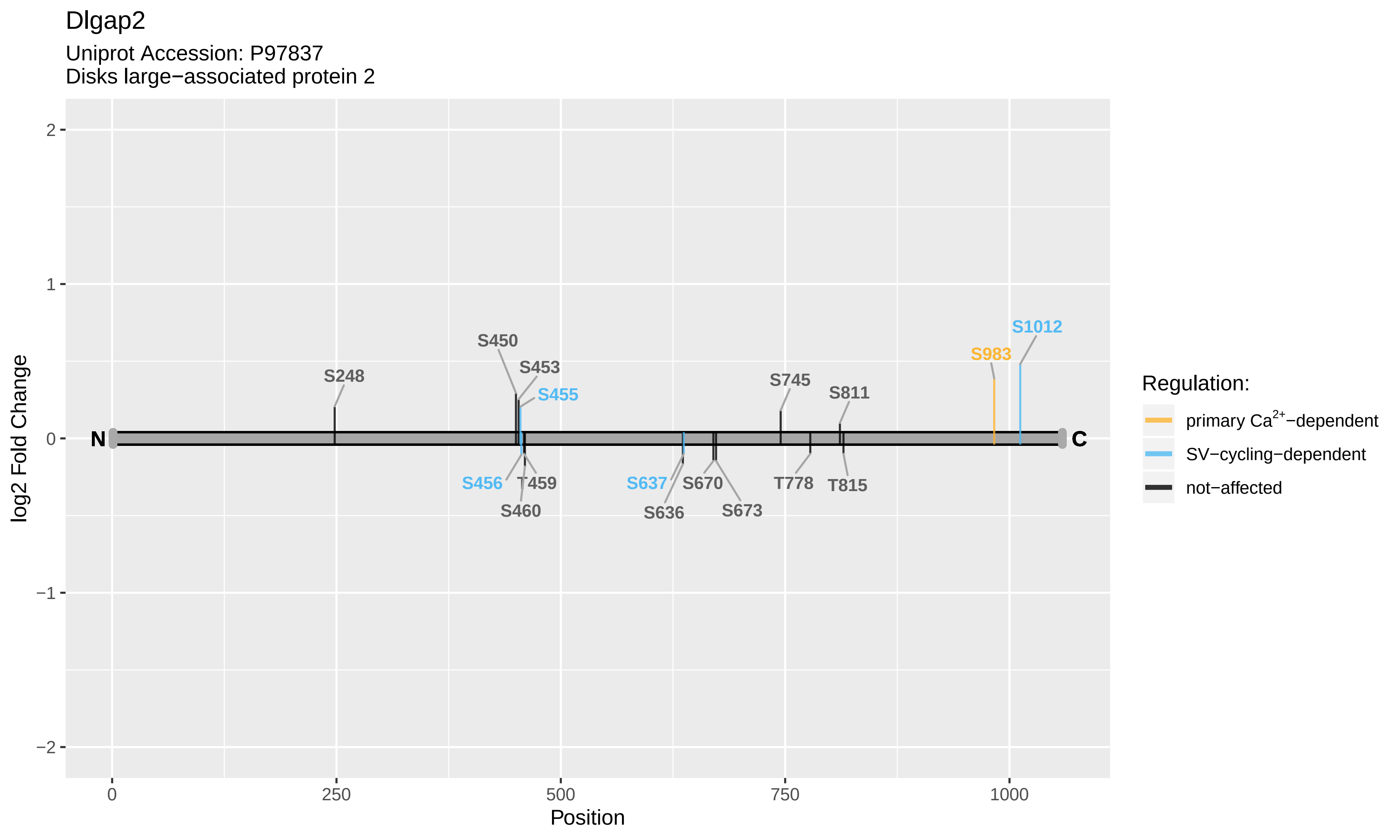

Dlgap2
Uniprot Accession: P97837
Disks large−associated protein 2
log2 Fold Change
Position
2
1
0
−1
−2
0
250
500
750
1000
N
C
S248
S450
S453
S455
S456
T459
S460
S637
S636
S670
S673
S745
T778
S811
T815
S983
S1012
Regulation:
primary Ca2+−dependent
SV−cycling−dependent
not−affected

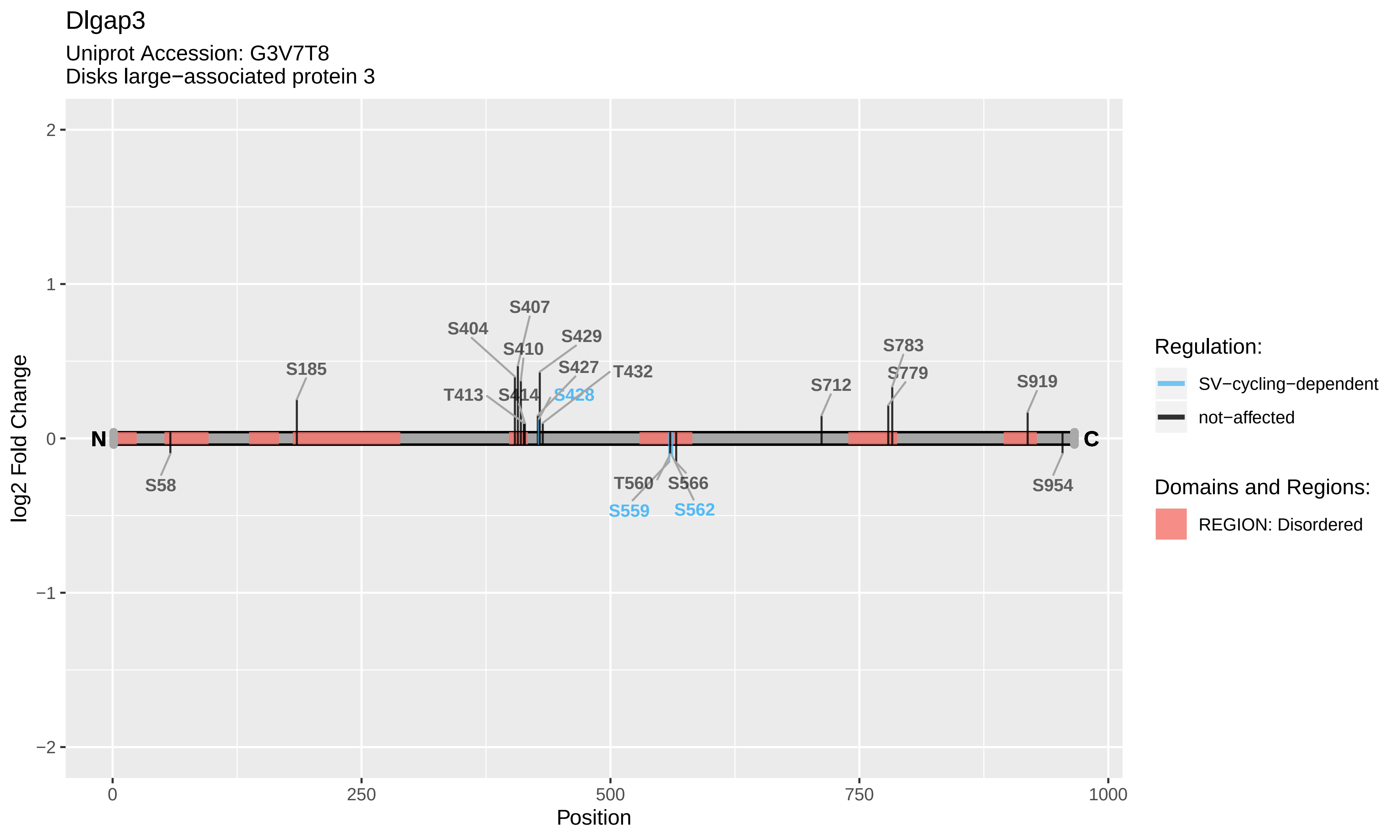

Dlgap3
Uniprot Accession: G3V7T8
Disks large−associated protein 3
log2 Fold Change
Position
S407
S404
S410
S429
S427
T432
S185
T413
S414
S428
S783
S779
S712
S919
S58
T560
S566
S559
S562
S954
N
C
Regulation:
SV−cycling−dependent
not−affected
Domains and Regions:
REGION: Disordered

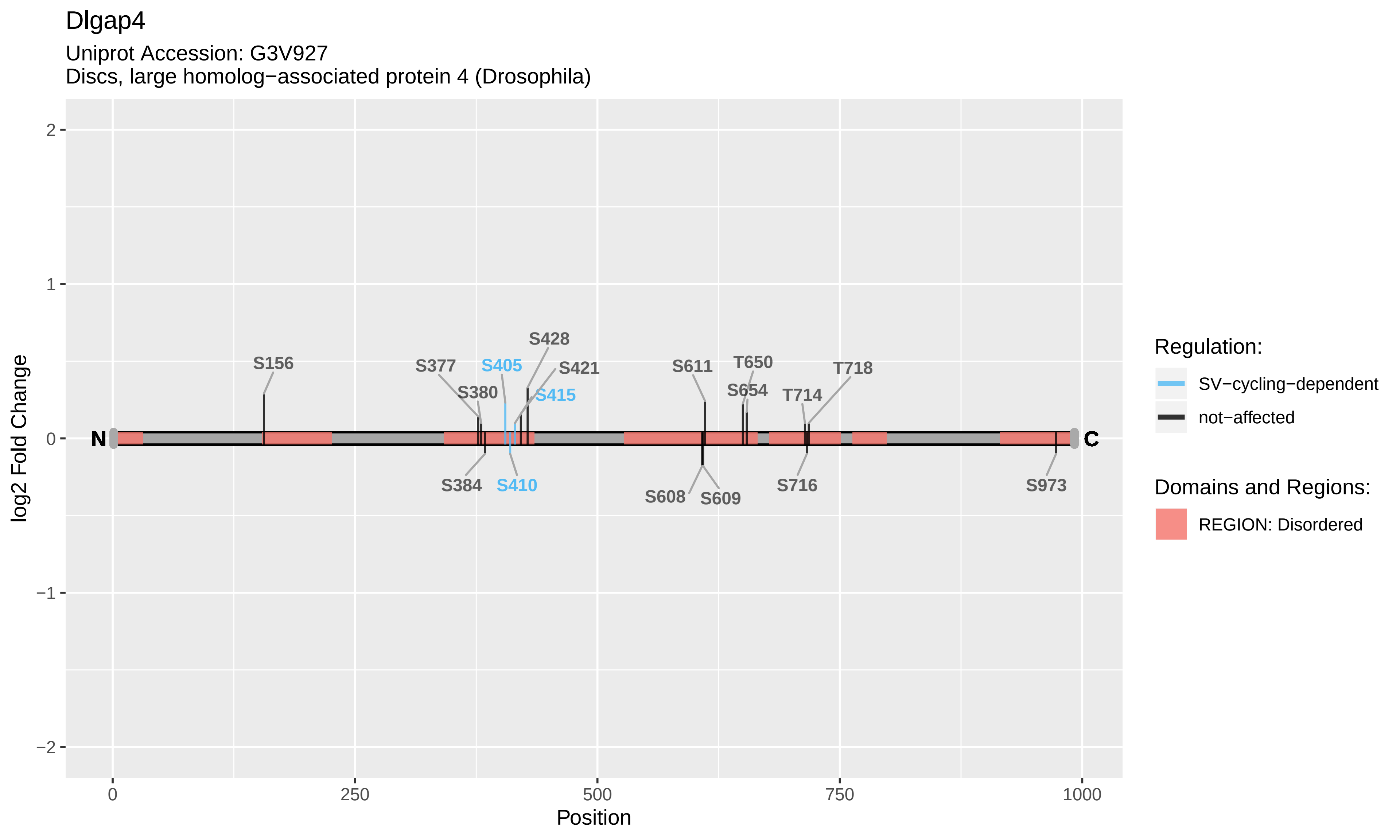
Dlgap4
Uniprot Accession: G3V927
Discs, large homolog−associated protein 4 (Drosophila)
log2 Fold Change
Position
2
1
0
−1
−2
0
250
500
750
1000
N
C
S156
S377
S380
S384
S405
S410
S415
S421
S428
S608
S609
S611
T650
S654
T714
S716
T718
S973
Regulation:
SV−cycling−dependent
not−affected
Domains and Regions:
REGION: Disordered

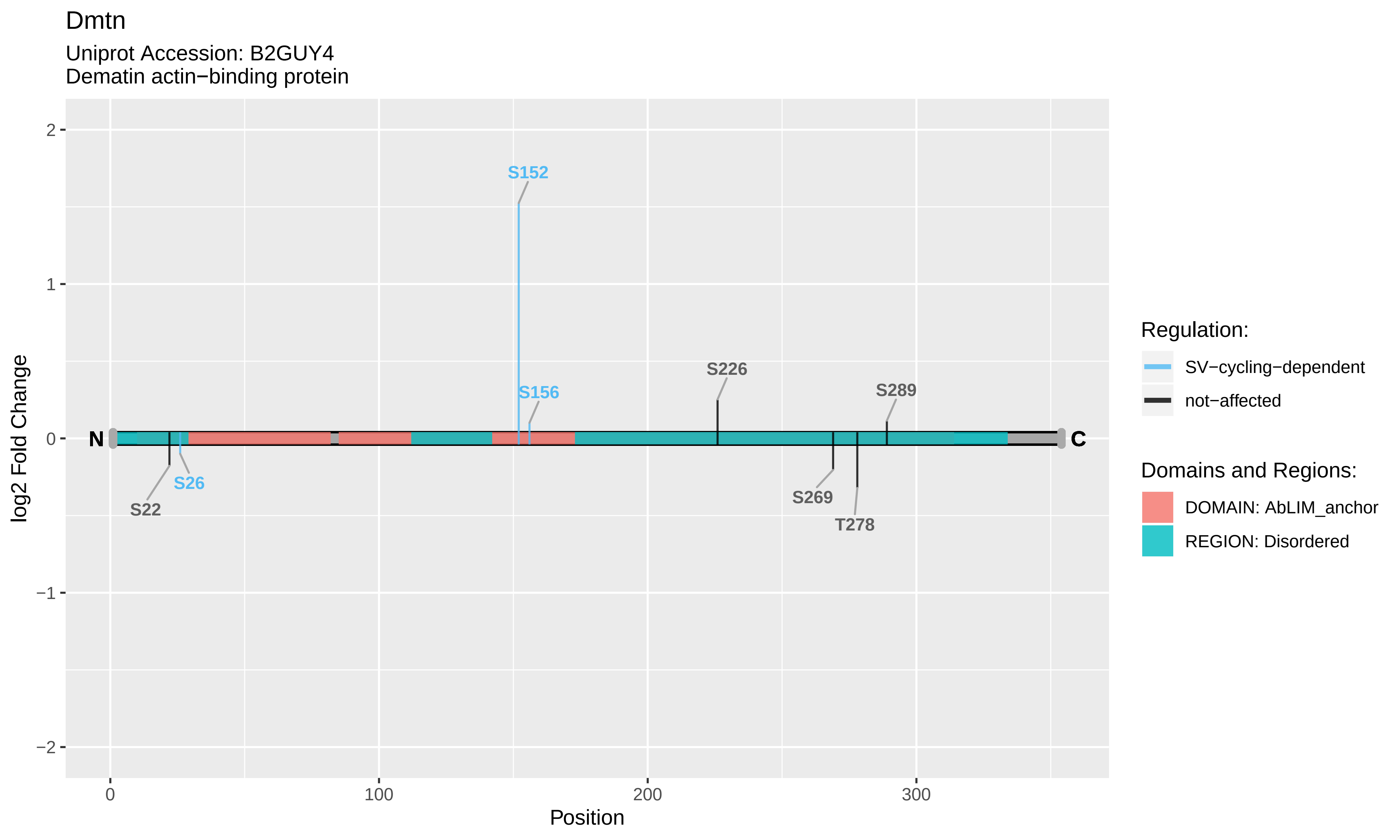

Dmtn
Uniprot Accession: B2GUY4
Dematin actin−binding protein
log2 Fold Change
Position
2
1
0
−1
−2
0
100
200
300
N
C
S152
S156
S226
S289
S22
S26
S269
T278
Regulation:
SV−cycling−dependent
not−affected
Domains and Regions:
DOMAIN: AbLIM_anchor
REGION: Disordered

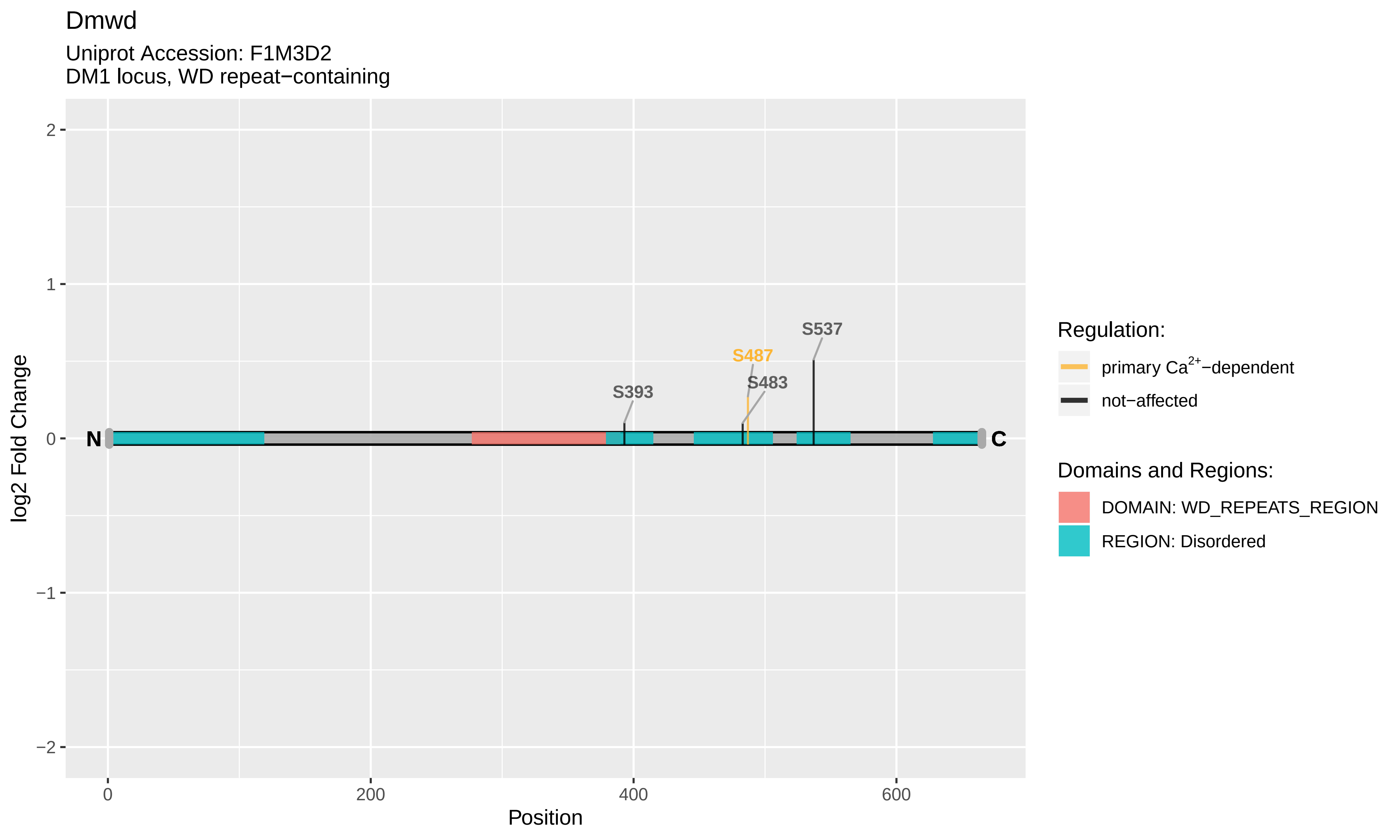

Dmwd
Uniprot Accession: F1M3D2
DM1 locus, WD repeat−containing
log2 Fold Change
Position
N
C
S393
S487
S483
S537
Regulation:
primary Ca2+−dependent
not−affected
Domains and Regions:
DOMAIN: WD_REPEATS_REGION
REGION: Disordered
2
1
0
−1
−2
0
200
400
600

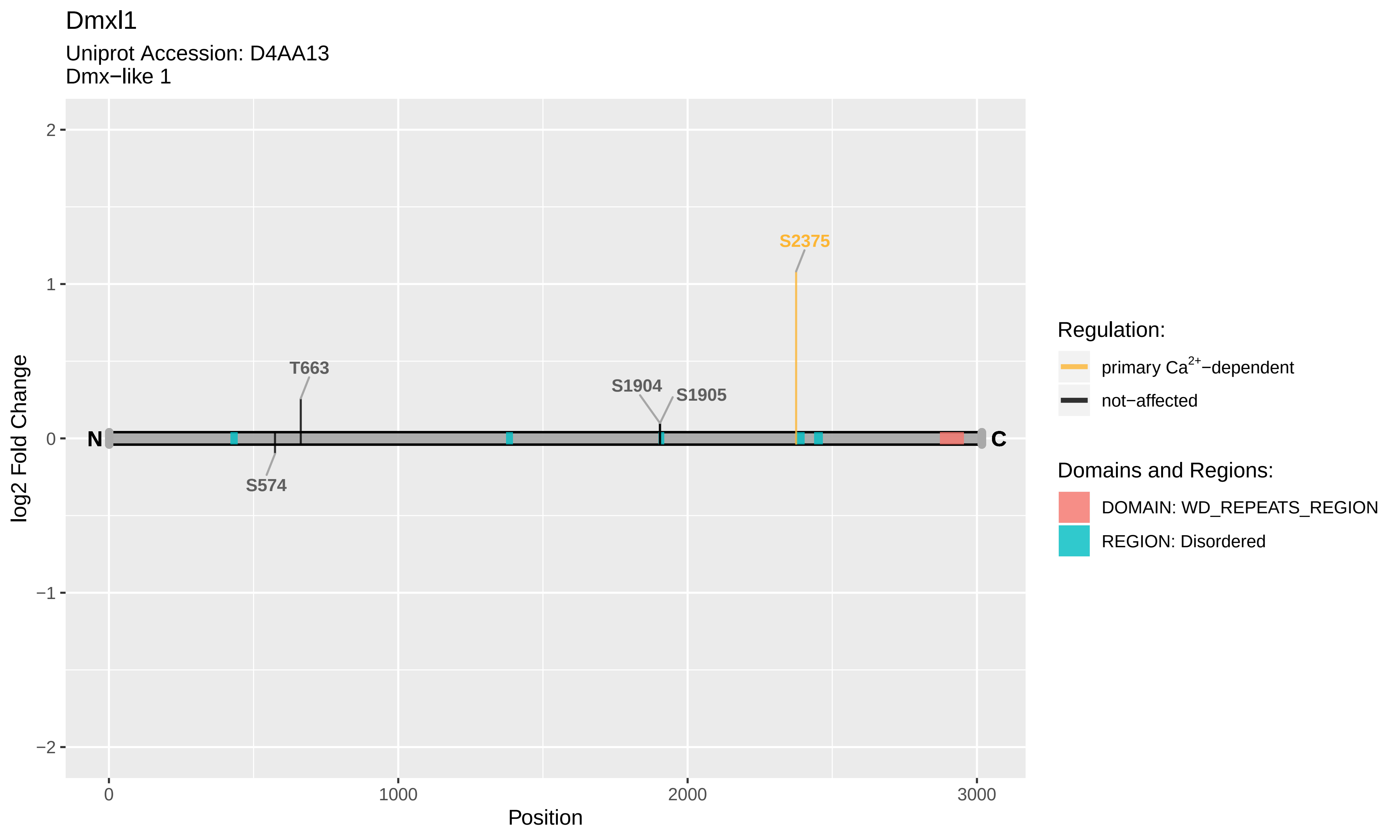

Dmxl1
Uniprot Accession: D4AA13
Dmx−like 1
log2 Fold Change
2
1
0
−1
−2
N
C
T663
S574
S1904
S1905
S2375
0
1000
2000
3000
Position
Regulation:
primary Ca2+−dependent
not−affected
Domains and Regions:
DOMAIN: WD_REPEATS_REGION
REGION: Disordered

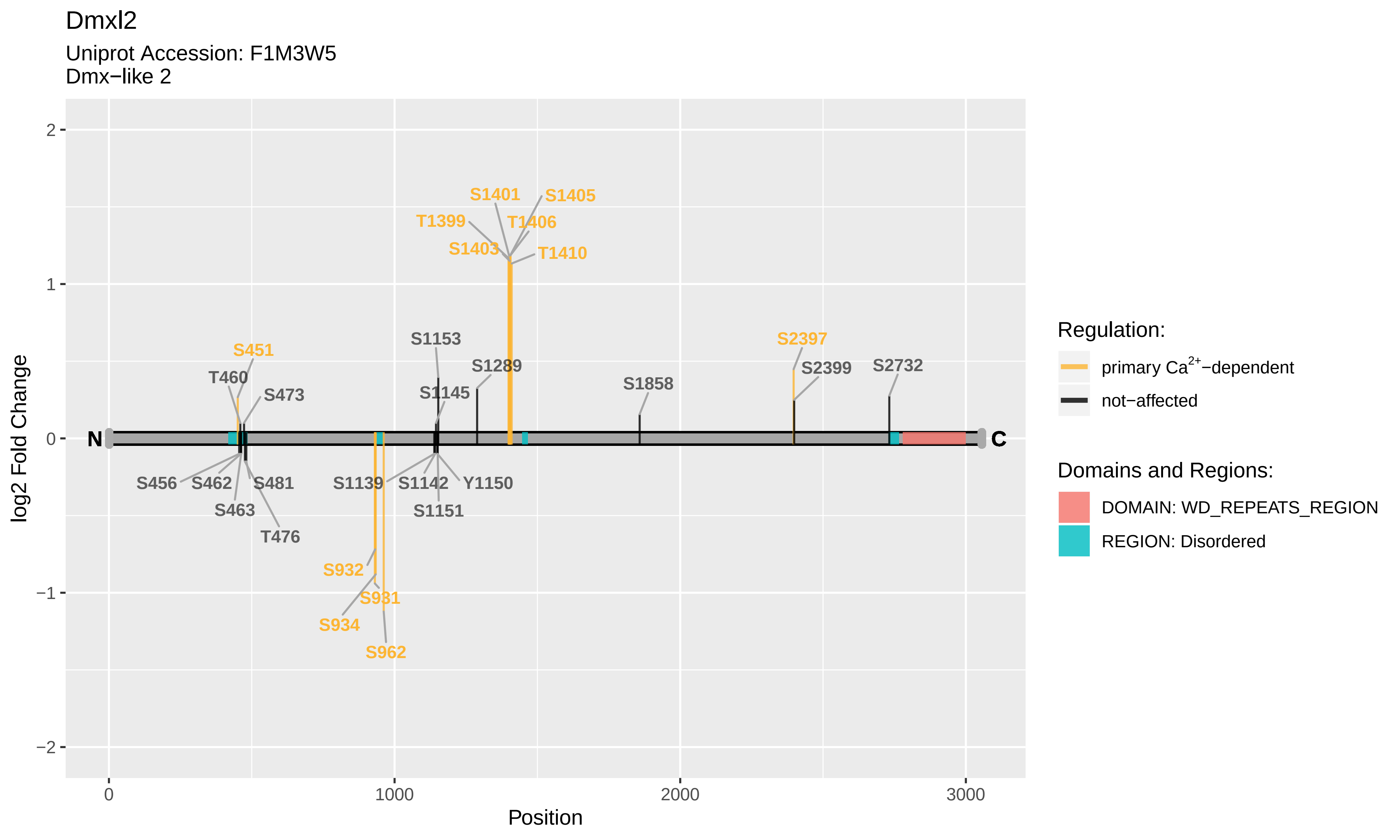

Dmxl2
Uniprot Accession: F1M3W5
Dmx−like 2
log2 Fold Change
Position
N
C
S1401
S1405
T1399
T1406
S1403
T1410
S451
T460
S473
S1153
S1145
S1289
S1858
S2397
S2399
S2732
S456
S462
S481
S463
T476
S1139
S1142
Y1150
S1151
S932
S981
S934
S962
2
1
0
−1
−2
0
1000
2000
3000
Regulation:
primary Ca2+−dependent
not−affected
Domains and Regions:
DOMAIN: WD_REPEATS_REGION
REGION: Disordered

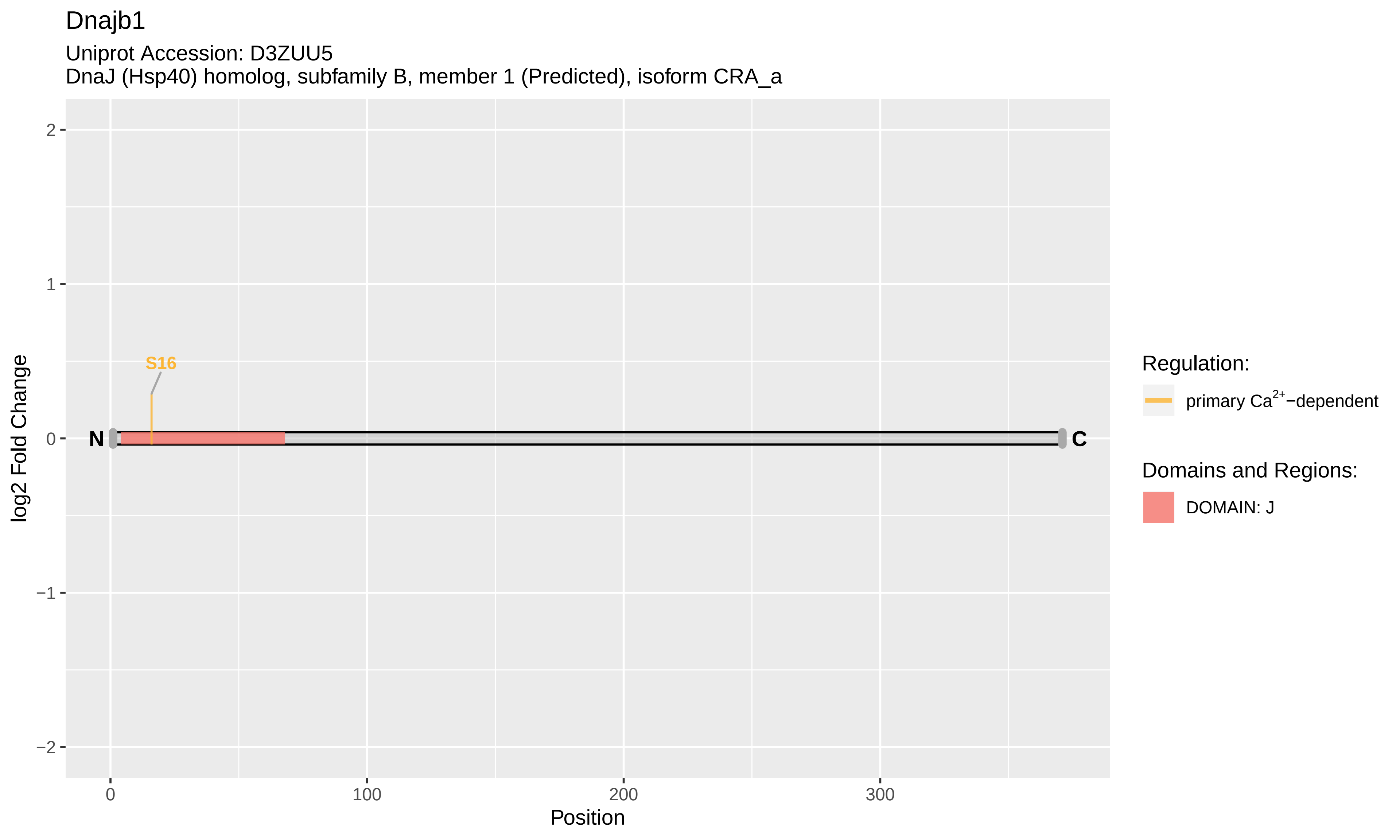

Dnajb1
Uniprot Accession: D3ZUU5
DnaJ (Hsp40) homolog, subfamily B, member 1 (Predicted), isoform CRA_a
S16
N
C
2
1
0
−1
−2
log2 Fold Change
0
100
200
300
Position
Regulation:
primary Ca2+−dependent
Domains and Regions:
DOMAIN: J

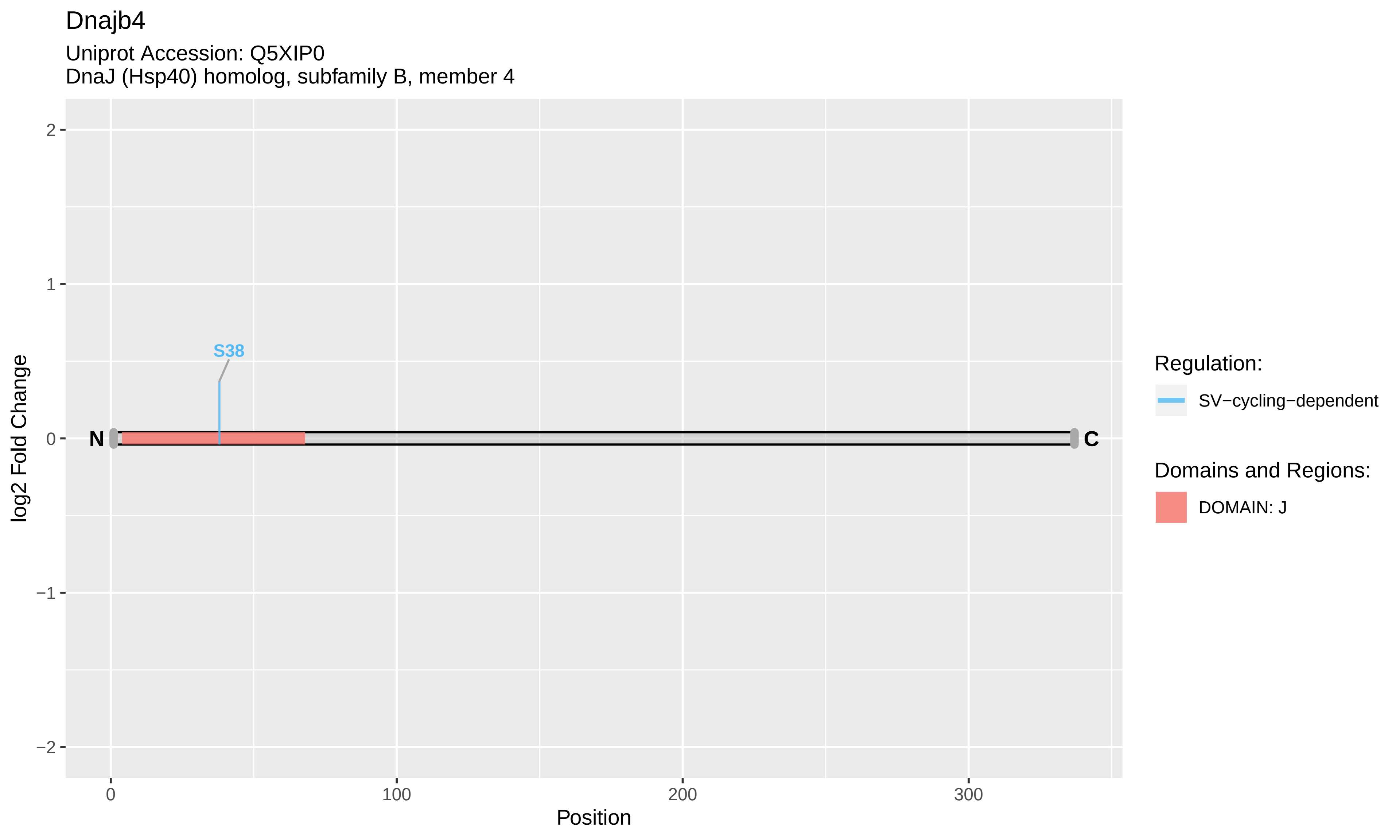

Dnajb4
Uniprot Accession: Q5XIP0
DnaJ (Hsp40) homolog, subfamily B, member 4
S38
N
C
2
1
0
−1
−2
log2 Fold Change
0
100
200
300
Position
Regulation:
SV−cycling−dependent
Domains and Regions:
DOMAIN: J

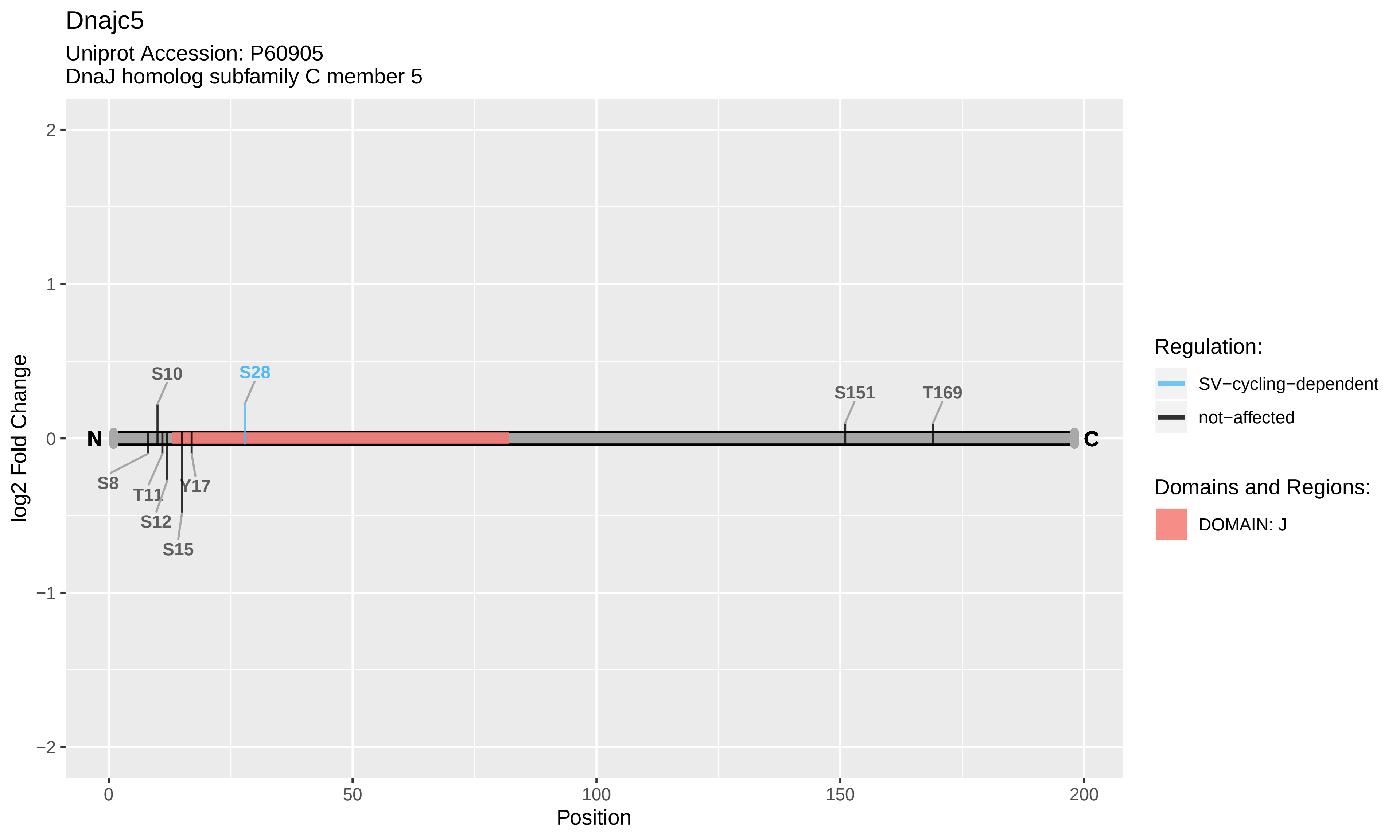
Dnajc5
Uniprot Accession: P60905
DnaJ homolog subfamily C member 5
log2 Fold Change
Position
N
C
S10
S28
S151
T169
S8
T11
Y17
S12
S15
Regulation:
SV−cycling−dependent
not−affected
Domains and Regions:
DOMAIN: J
2
1
0
−1
−2
0
50
100
150
200

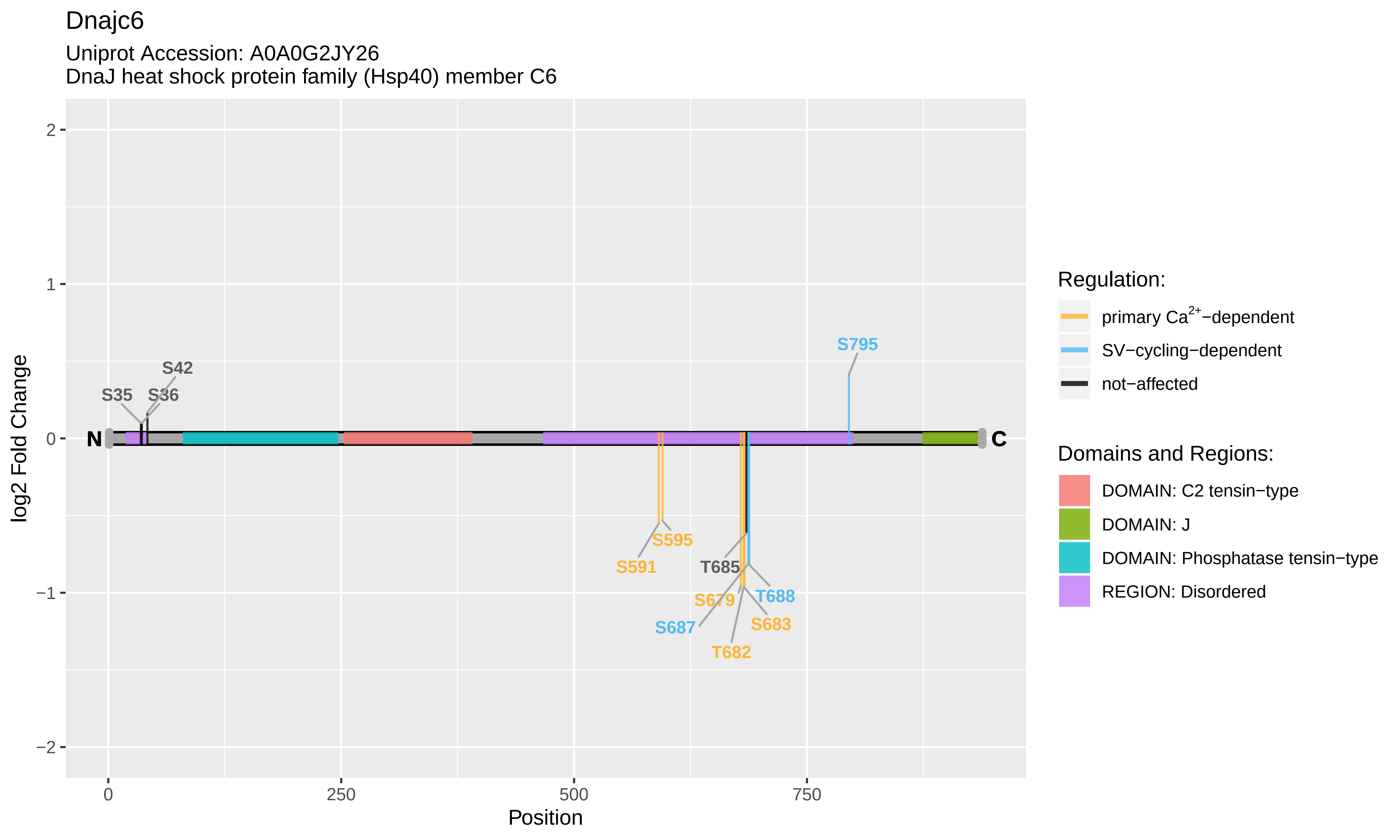

Dnajc6
Uniprot Accession: A0A0G2JY26
DnaJ heat shock protein family (Hsp40) member C6
log2 Fold Change
Position
N
C
S35
S36
S42
S591
S595
T685
S679
S687
T682
S683
T688
S795
Regulation:
primary Ca2+−dependent
SV−cycling−dependent
not−affected
Domains and Regions:
DOMAIN: C2 tensin−type
DOMAIN: J
DOMAIN: Phosphatase tensin−type
REGION: Disordered

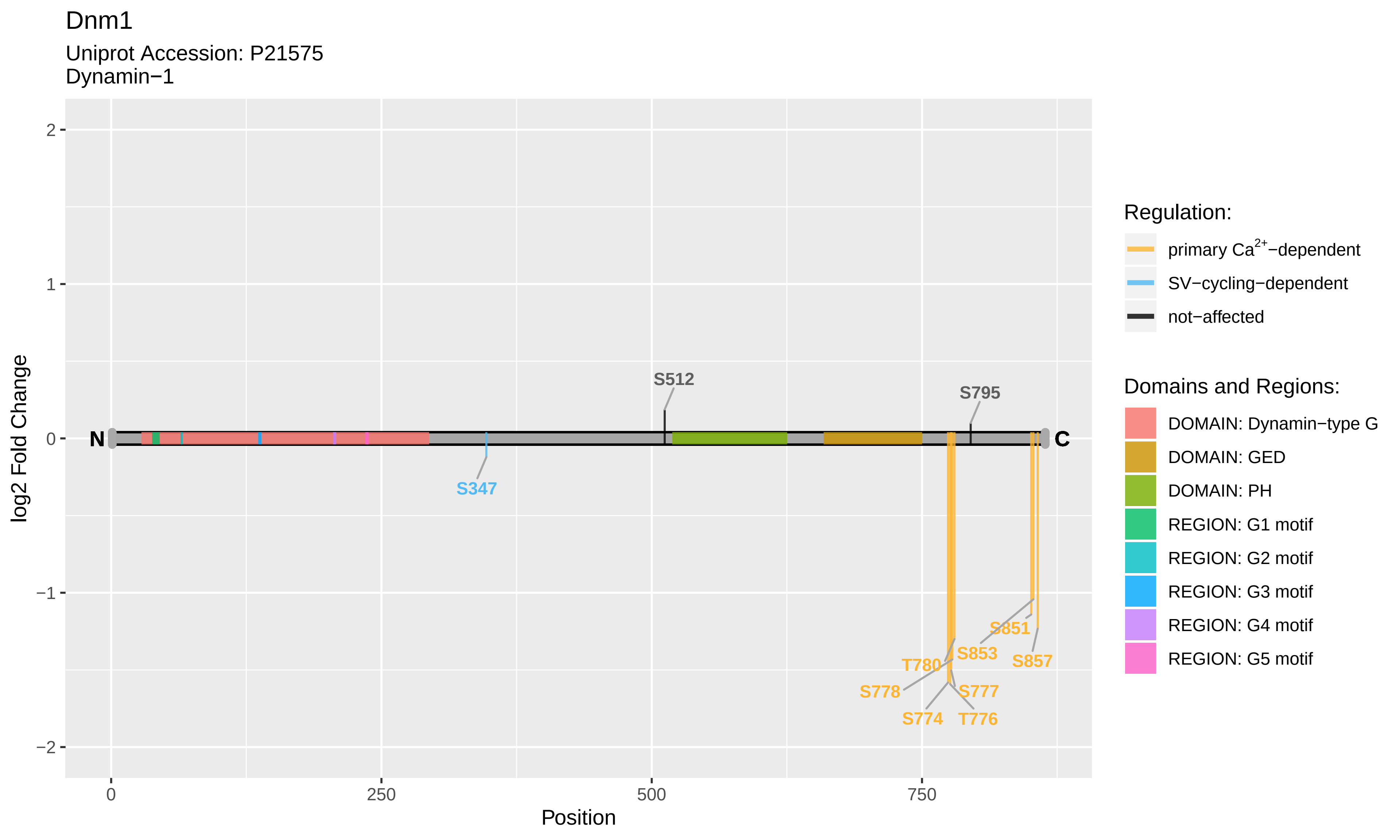

Dnm1
Uniprot Accession: P21575
Dynamin−1
log2 Fold Change
Position
N
C
S512
S795
S347
S851
S853
T780
S857
S778
S777
S774
T776
Regulation:
primary Ca2+−dependent
SV−cycling−dependent
not−affected
Domains and Regions:
DOMAIN: Dynamin−type G
DOMAIN: GED
DOMAIN: PH
REGION: G1 motif
REGION: G2 motif
REGION: G3 motif
REGION: G4 motif
REGION: G5 motif

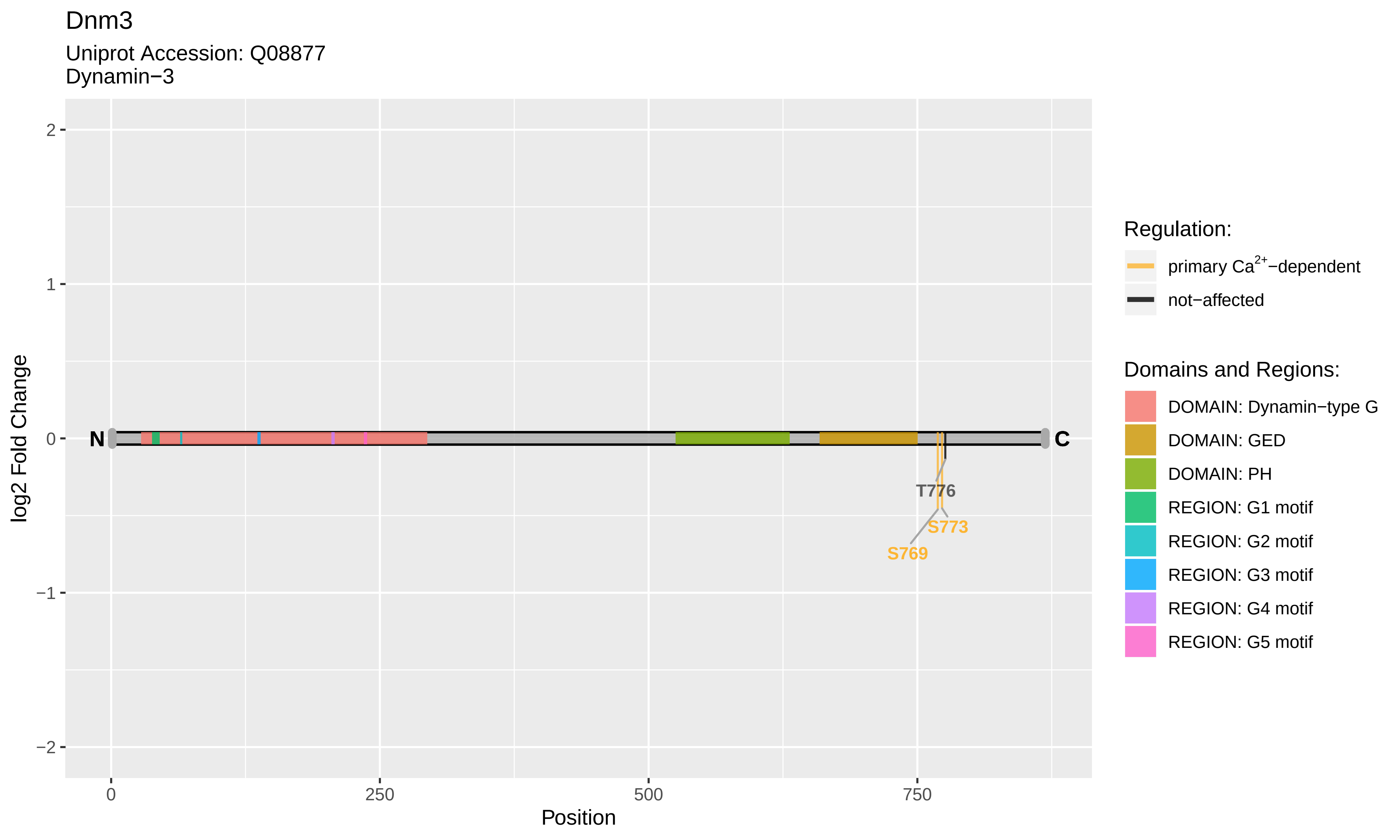
Dnm3
Uniprot Accession: Q08877
Dynamin−3
log2 Fold Change
Position
N
C
T776
S773
S769
2
1
0
−1
−2
0
250
500
750
Regulation:
primary Ca2+−dependent
not−affected
Domains and Regions:
DOMAIN: Dynamin−type G
DOMAIN: GED
DOMAIN: PH
REGION: G1 motif
REGION: G2 motif
REGION: G3 motif
REGION: G4 motif
REGION: G5 motif

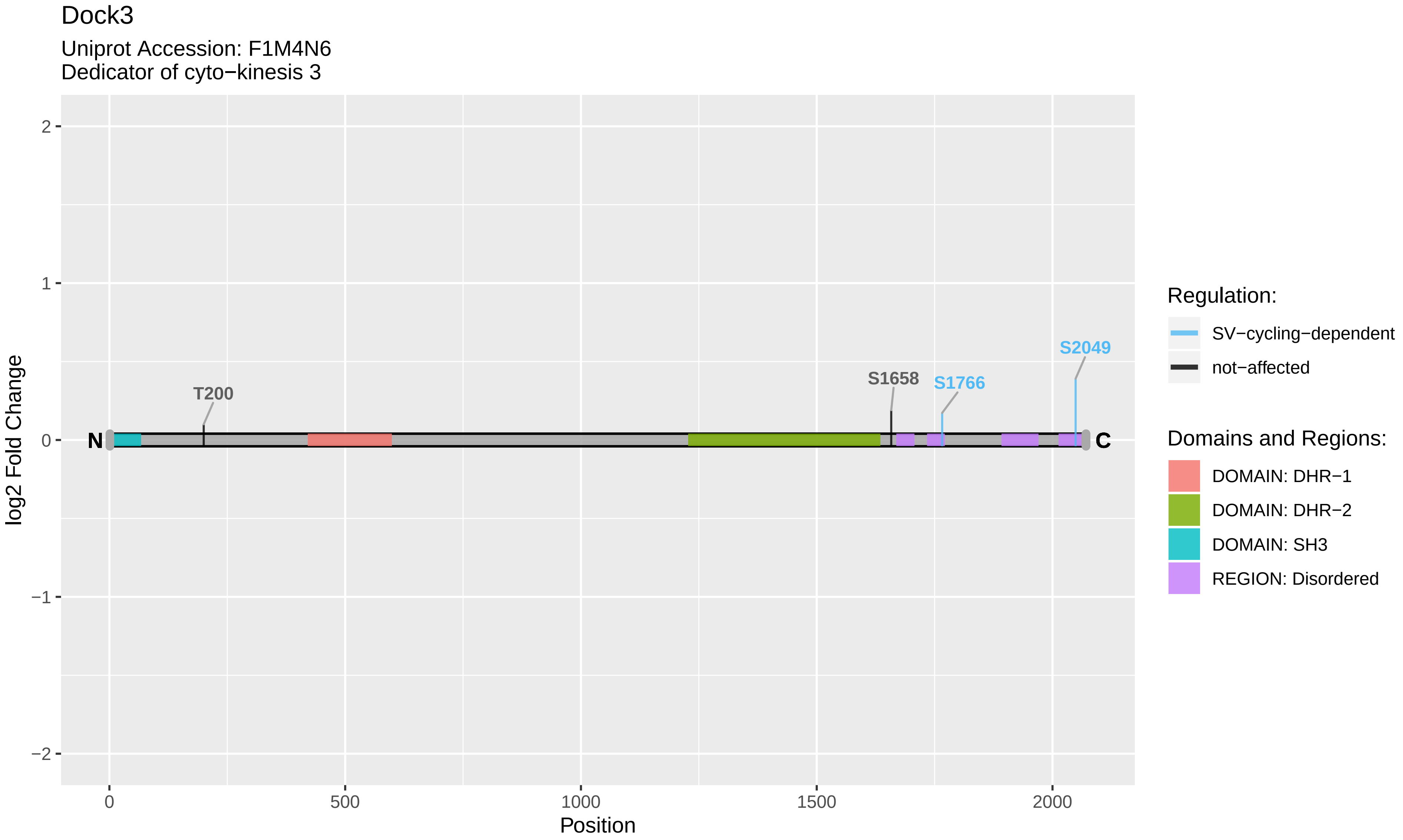
Dock3
Uniprot Accession: F1M4N6
Dedicator of cyto−kinesis 3
log2 Fold Change
Position
N
C
T200
S1658
S1766
S2049
Regulation:
SV−cycling−dependent
not−affected
Domains and Regions:
DOMAIN: DHR−1
DOMAIN: DHR−2
DOMAIN: SH3
REGION: Disordered

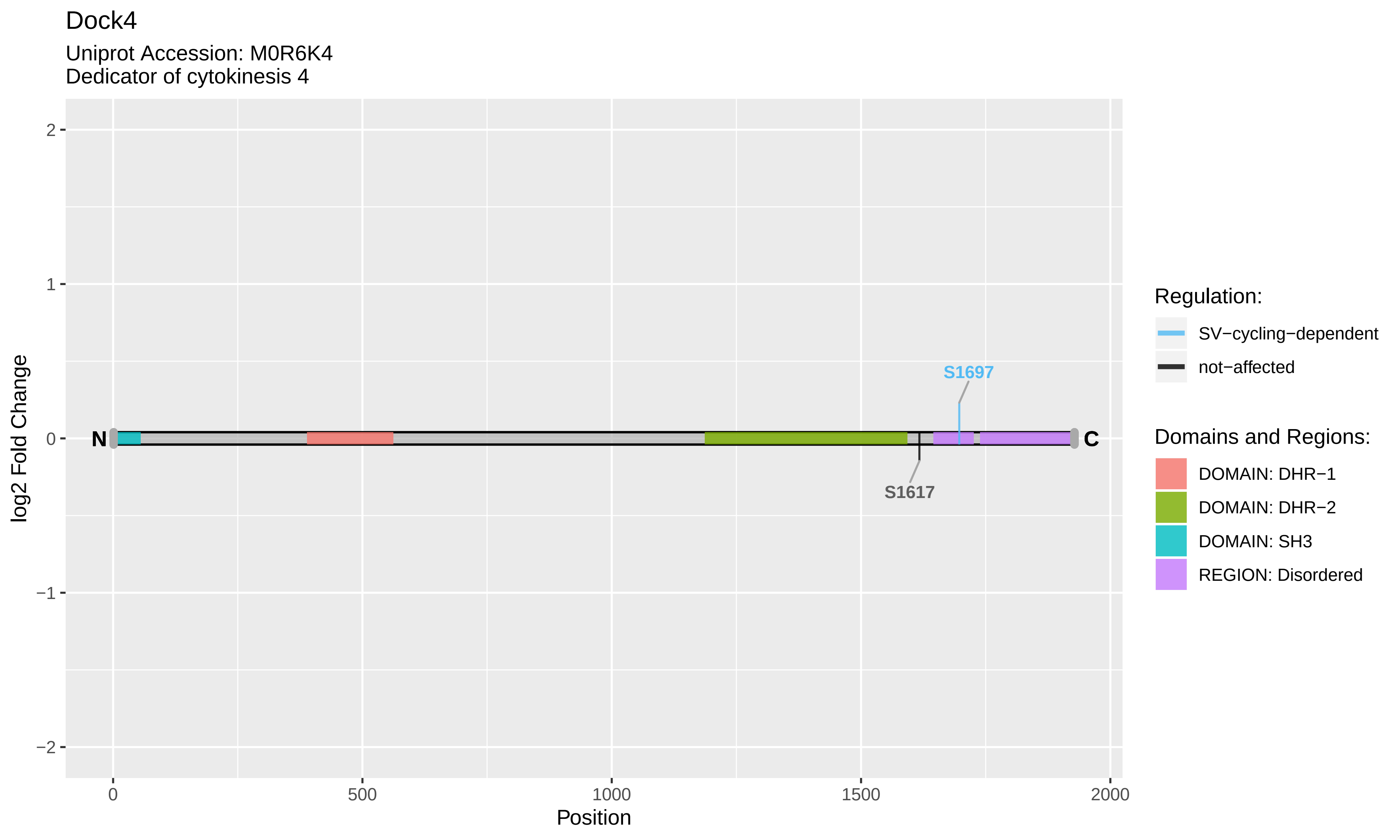

Dock4
Uniprot Accession: M0R6K4
Dedicator of cytokinesis 4
log2 Fold Change
Position
N
C
S1697
S1617
2
1
0
−1
−2
0
500
1000
1500
2000
Regulation:
SV−cycling−dependent
not−affected
Domains and Regions:
DOMAIN: DHR−1
DOMAIN: DHR−2
DOMAIN: SH3
REGION: Disordered

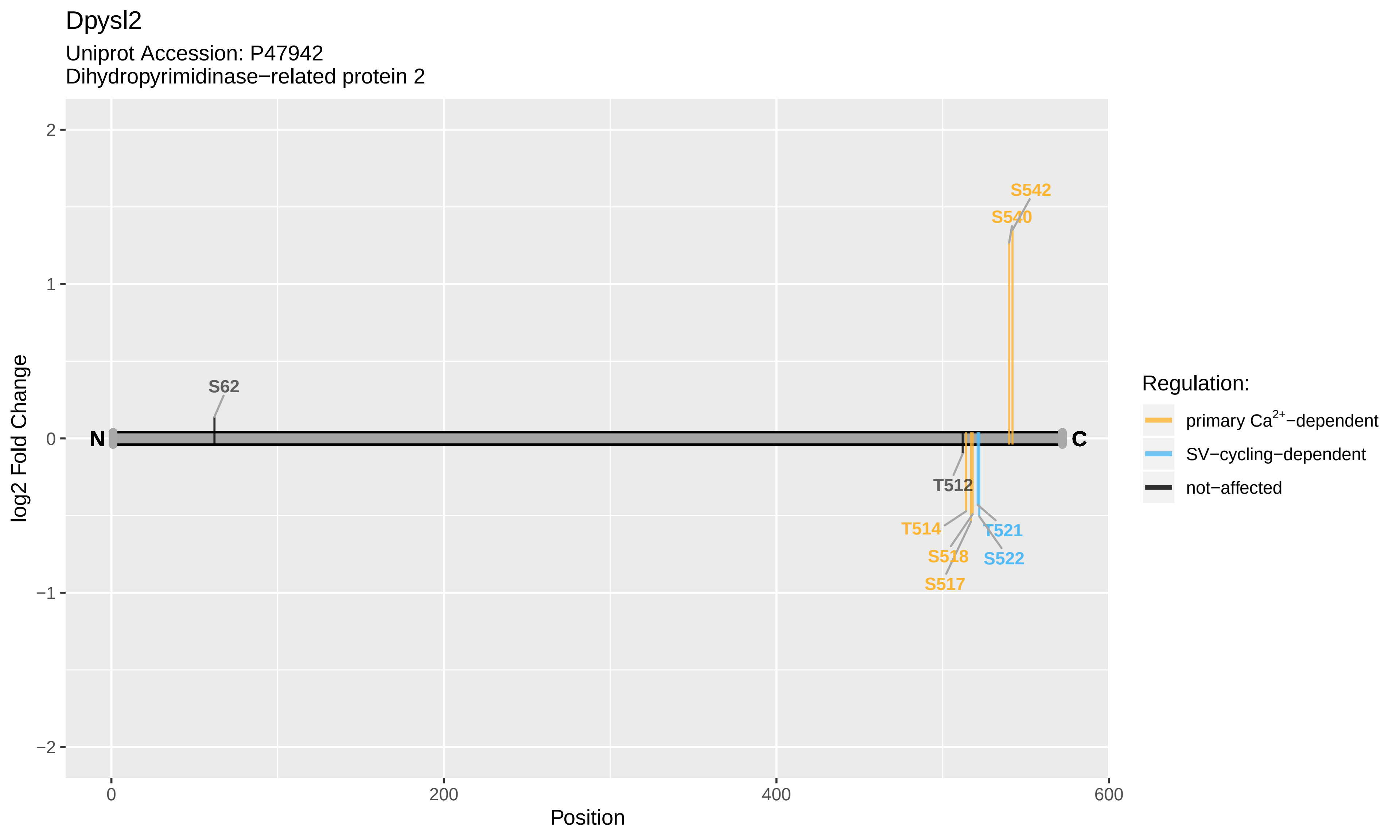
Dpysl2
Uniprot Accession: P47942
Dihydropyrimidinase−related protein 2
log2 Fold Change
Position
2
1
0
−1
−2
0
200
400
600
N
C
S62
S542
S540
T512
T514
S518
S517
T521
S522
Regulation:
primary Ca2+−dependent
SV−cycling−dependent
not−affected

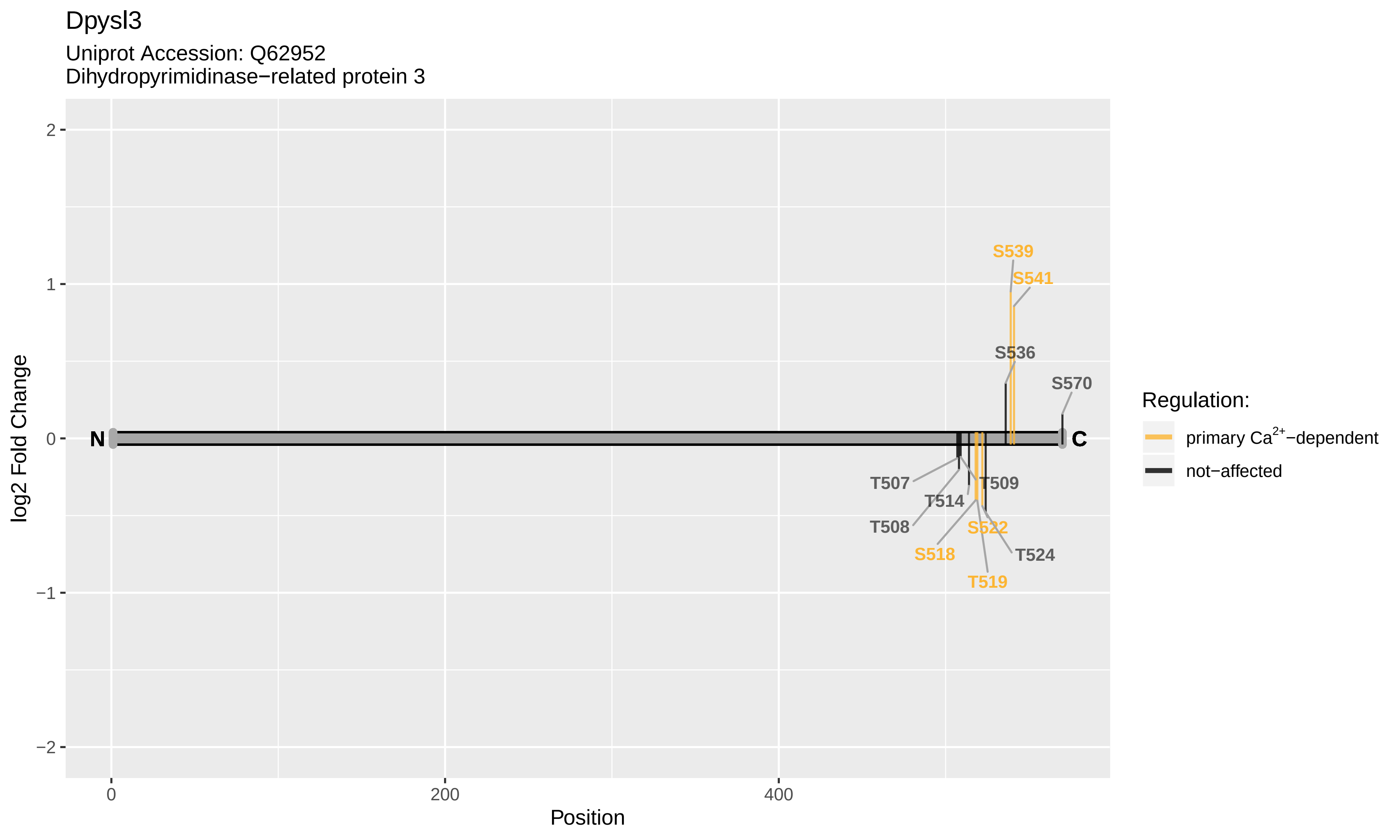

Dpysl3
Uniprot Accession: Q62952
Dihydropyrimidinase−related protein 3
log2 Fold Change
Position
N
C
S539
S541
S536
S570
T507
T509
T514
T508
S522
S518
T524
T519
2
1
0
−1
−2
0
200
400
Regulation:
primary Ca2+−dependent
not−affected

Dpysl4

Uniprot Accession: Q62951
Dihydropyrimidinase−related protein 4 (Fragment)

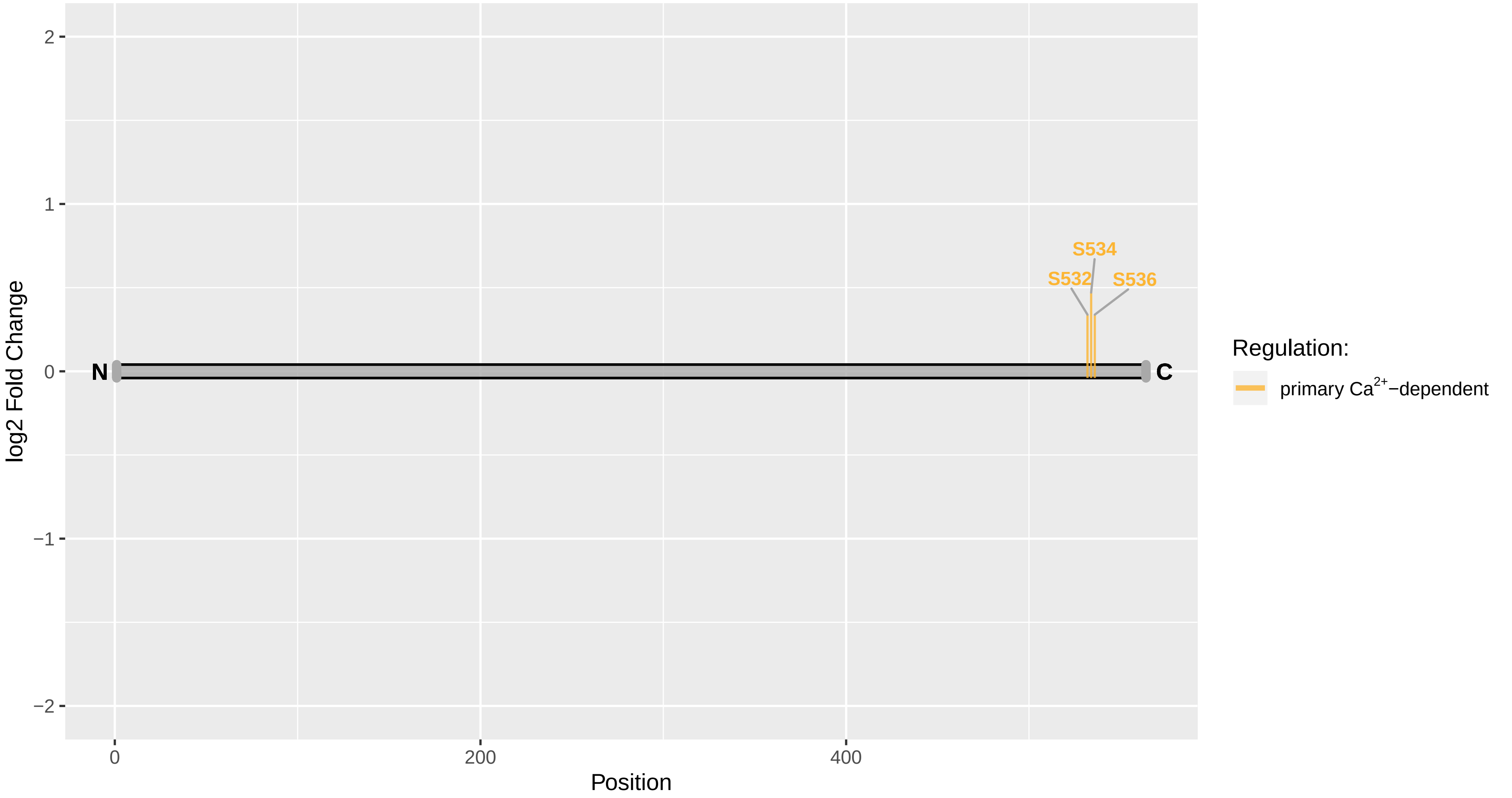

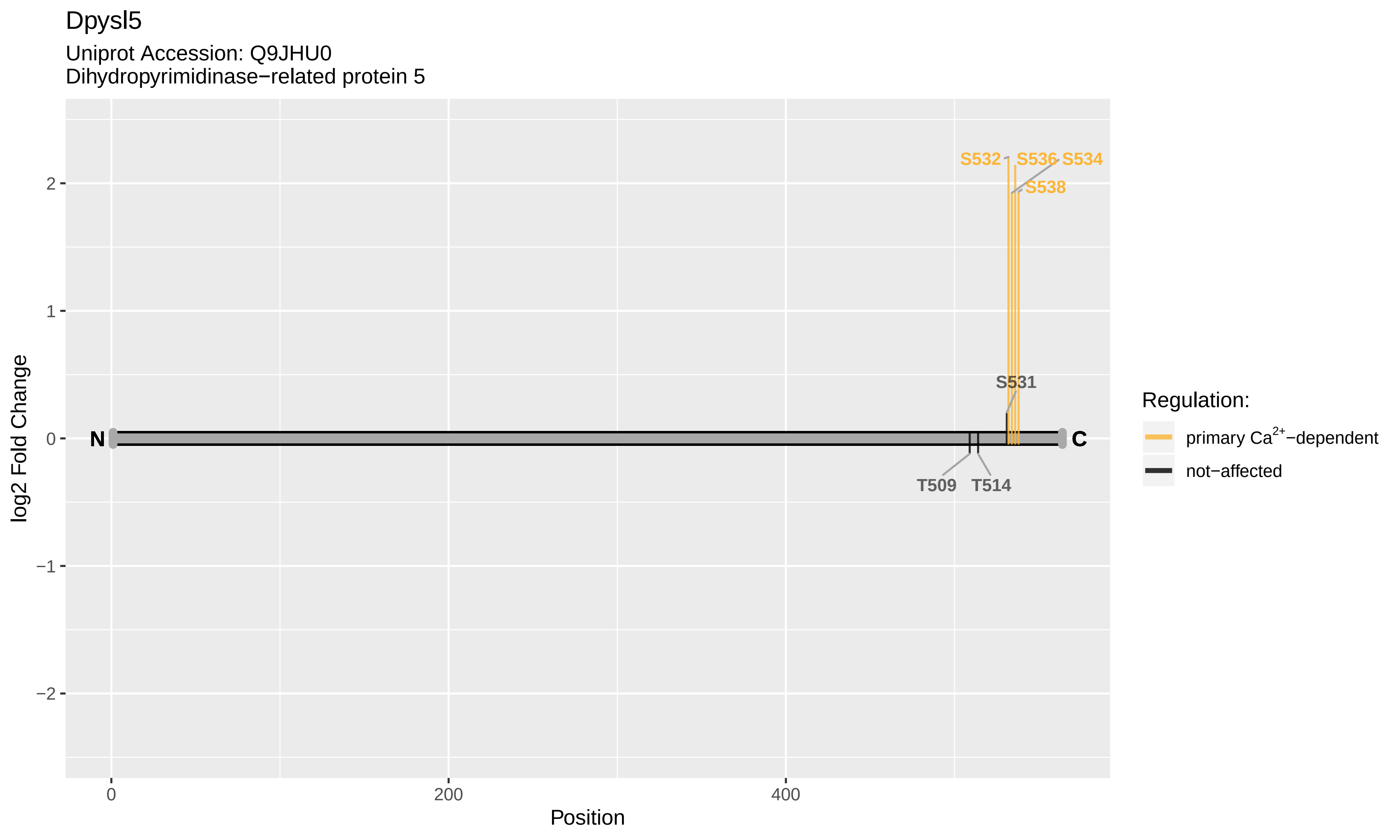

Dpysl5
Uniprot Accession: Q9JHU0
Dihydropyrimidinase−related protein 5
S532
S536
S534
S538
S531
T509
T514
N
C
2
1
0
−1
−2
log2 Fold Change
0
200
400
Position
Regulation:
primary Ca2+−dependent
not−affected

Drd1

Uniprot Accession: P18901
D(1A) dopamine receptor

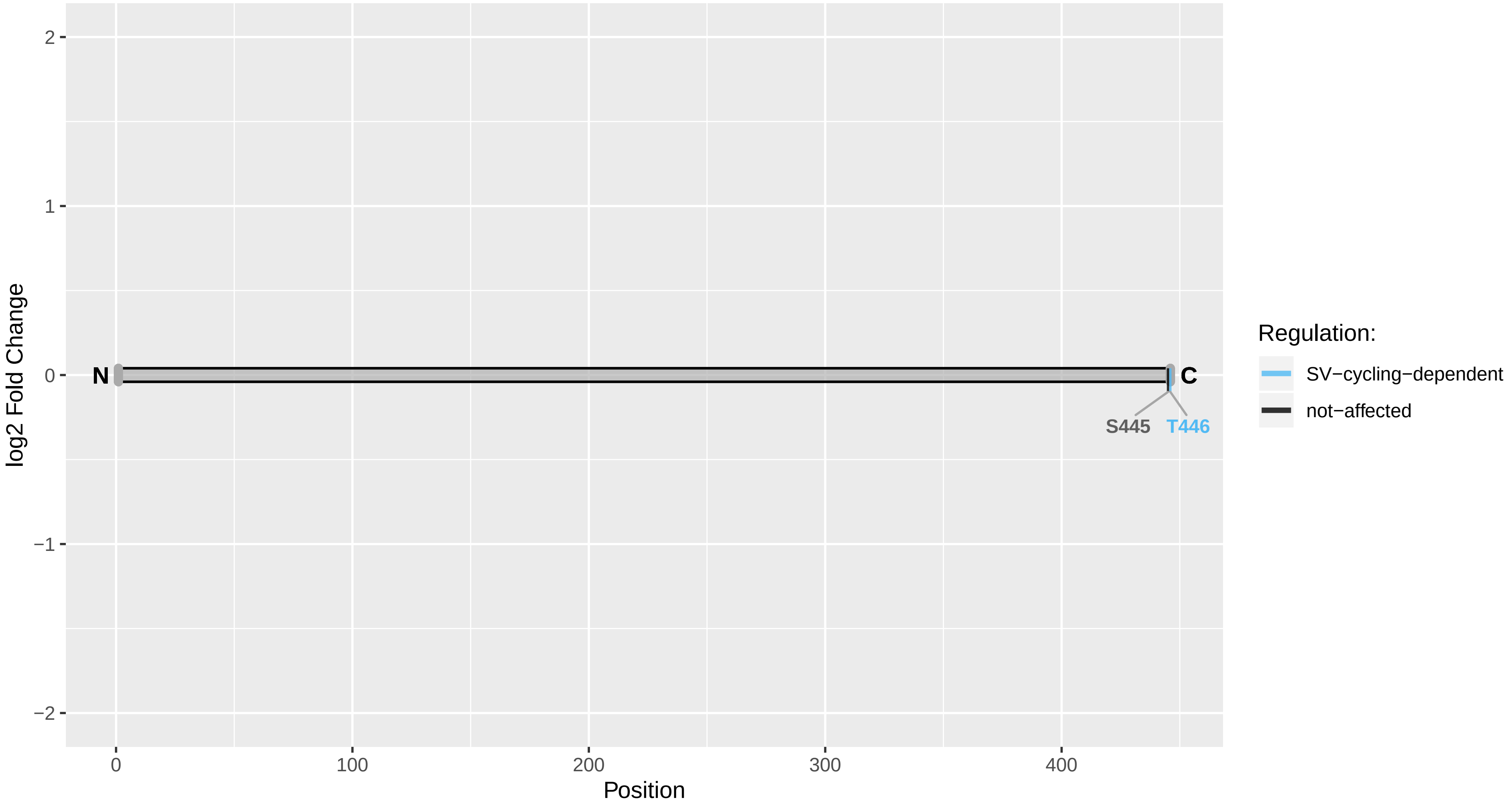

Dtd1

Uniprot Accession: B0K014
D−aminoacyl−tRNA deacylase

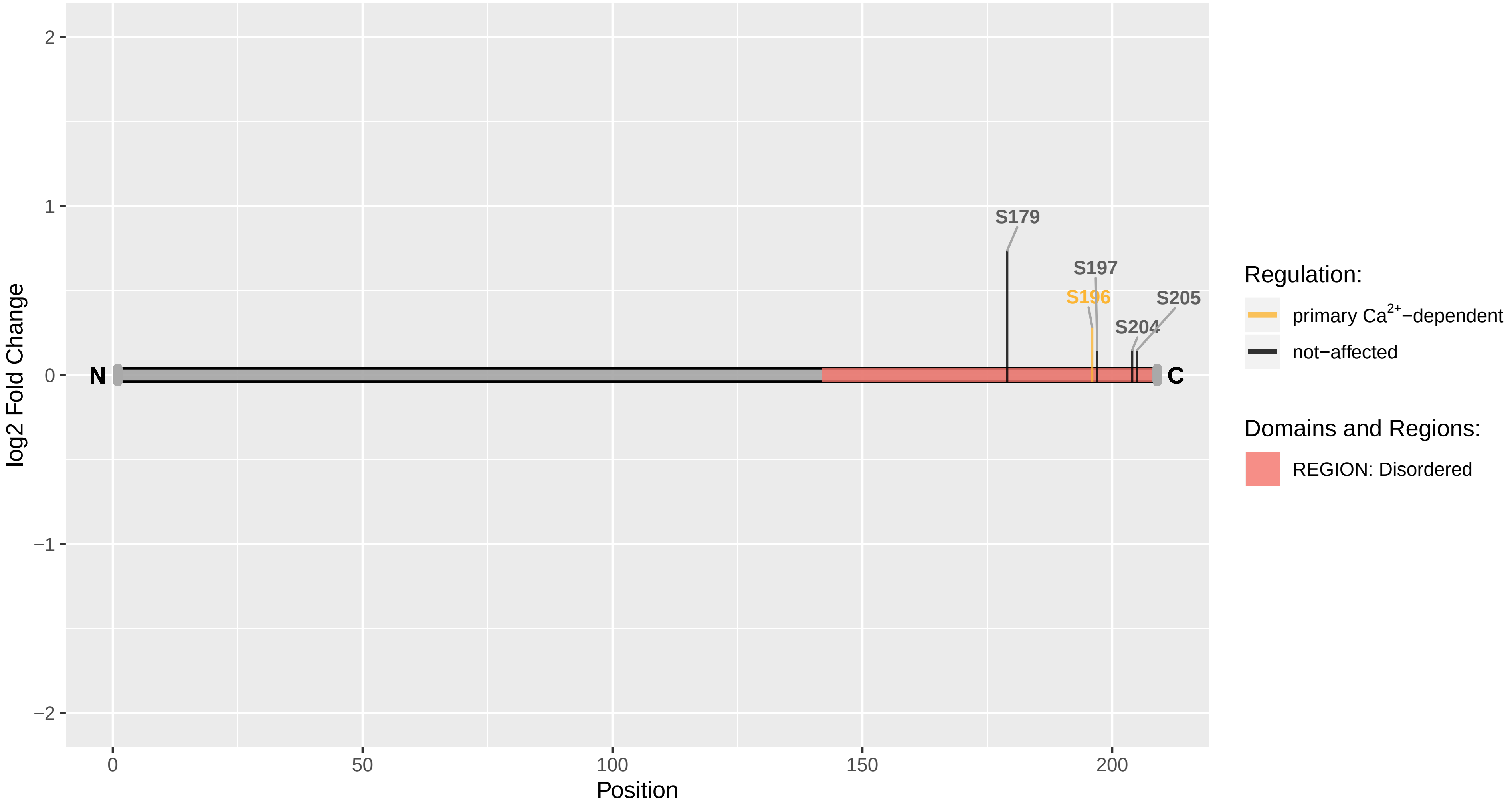

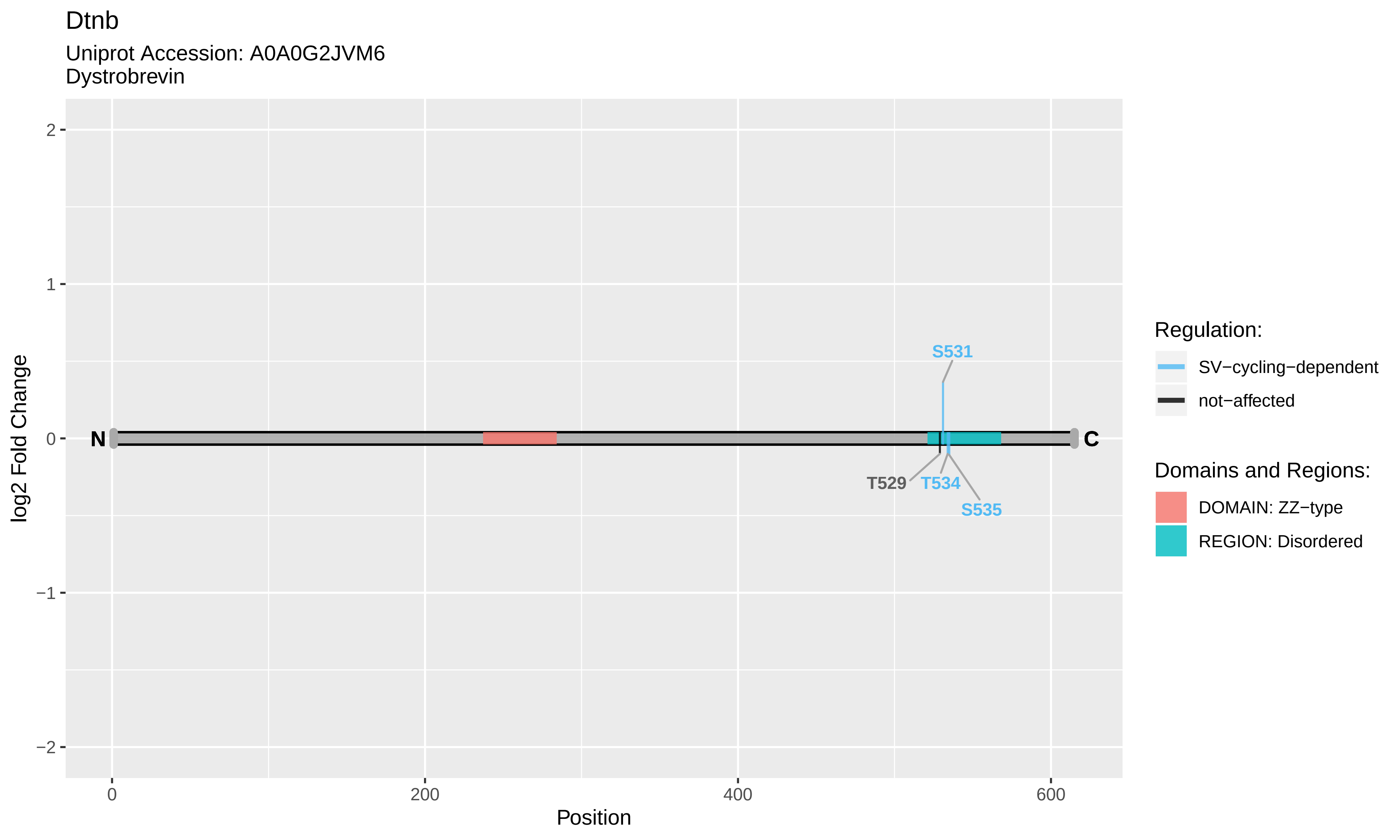

Dtnb
Uniprot Accession: A0A0G2JVM6
Dystrobrevin
2
1
0
−1
−2
log2 Fold Change
N
C
S531
T529
T534
S535
0
200
400
600
Position
Regulation:
SV−cycling−dependent
not−affected
Domains and Regions:
DOMAIN: ZZ−type
REGION: Disordered

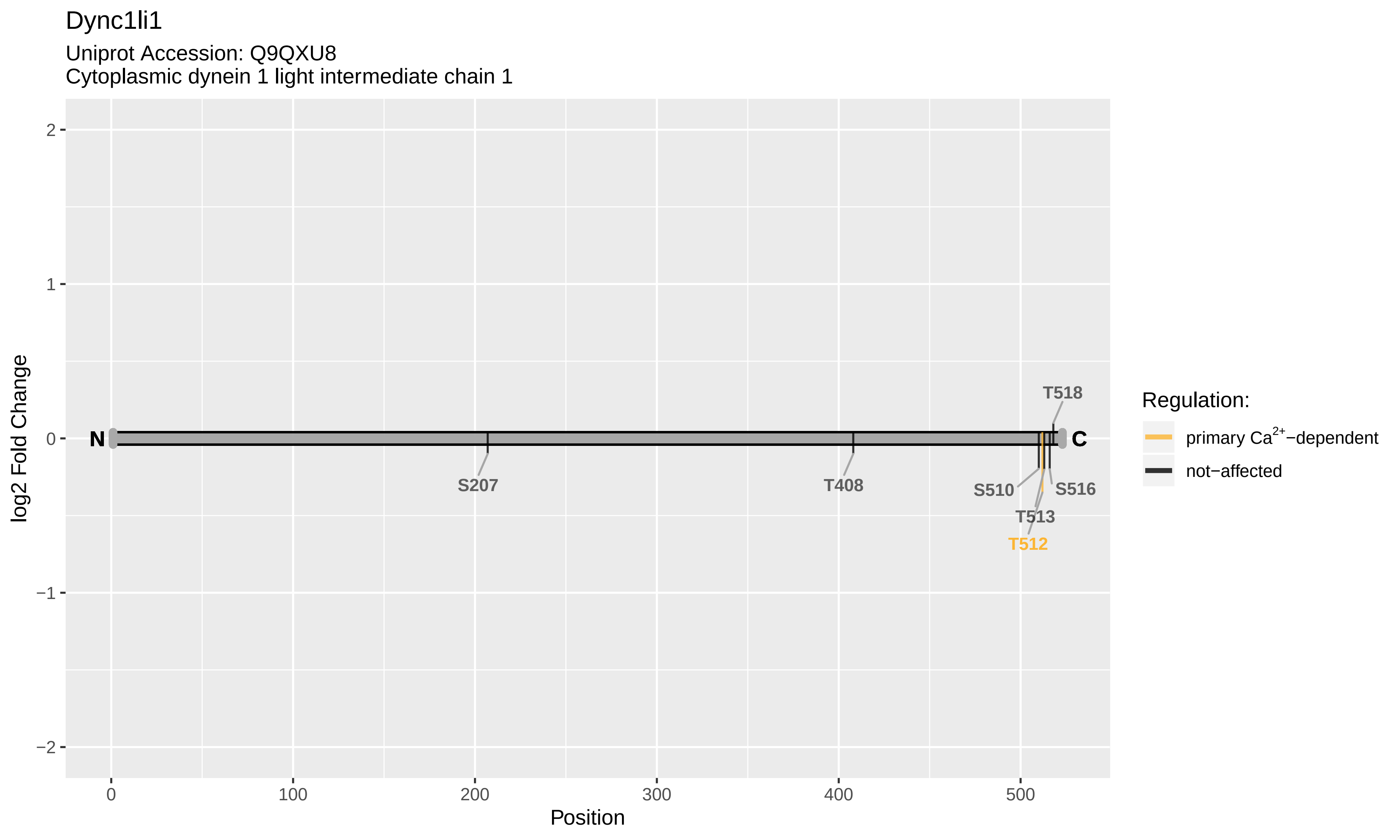

Dync1li1
Uniprot Accession: Q9QXU8
Cytoplasmic dynein 1 light intermediate chain 1
log2 Fold Change
Position
N
C
S207
T408
S510
T513
T512
S516
T518
Regulation:
primary Ca2+−dependent
not−affected

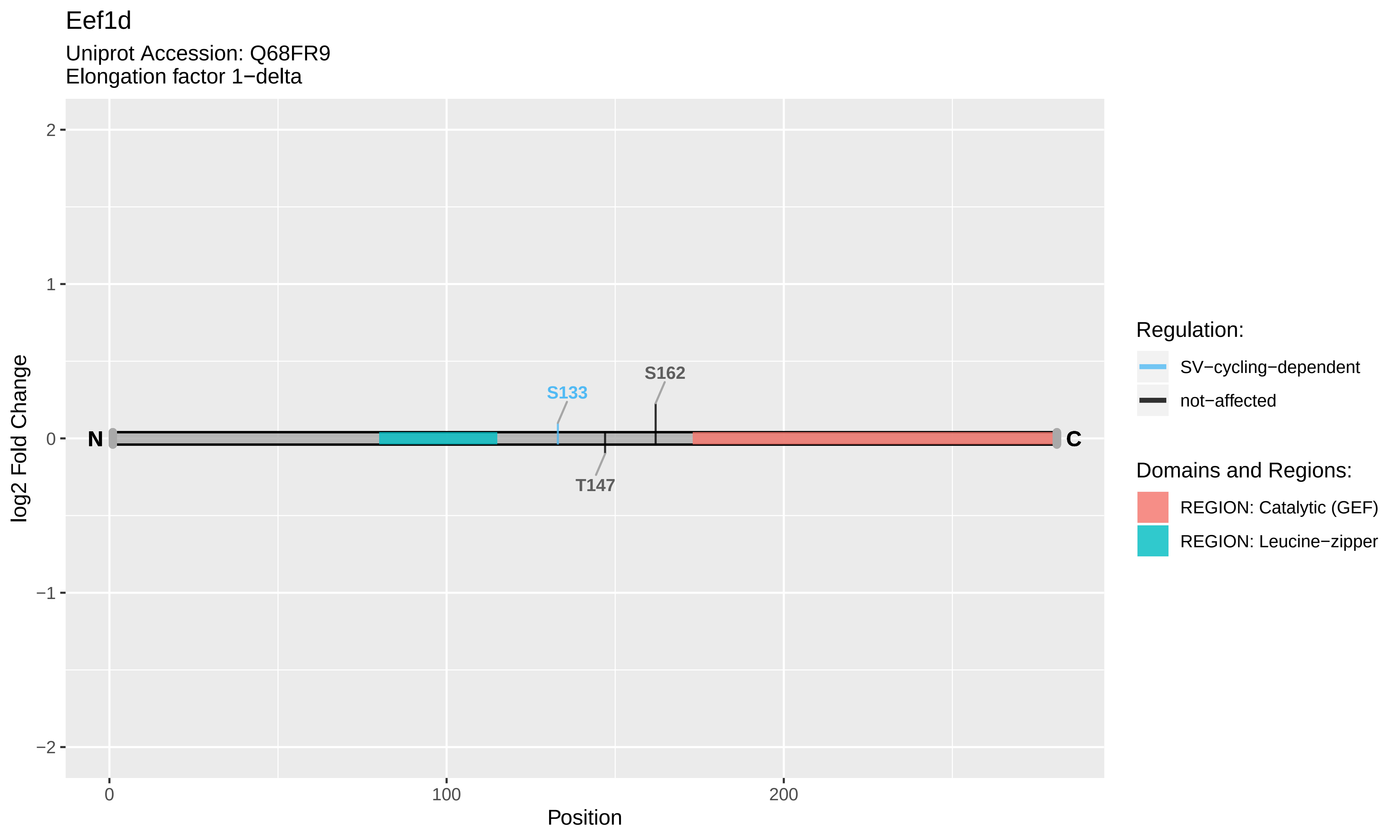

Eef1d
Uniprot Accession: Q68FR9
Elongation factor 1−delta
log2 Fold Change
2
1
0
−1
−2
N
C
S133
S162
T147
0
100
200
Position
Regulation:
SV−cycling−dependent
not−affected
Domains and Regions:
REGION: Catalytic (GEF)
REGION: Leucine−zipper

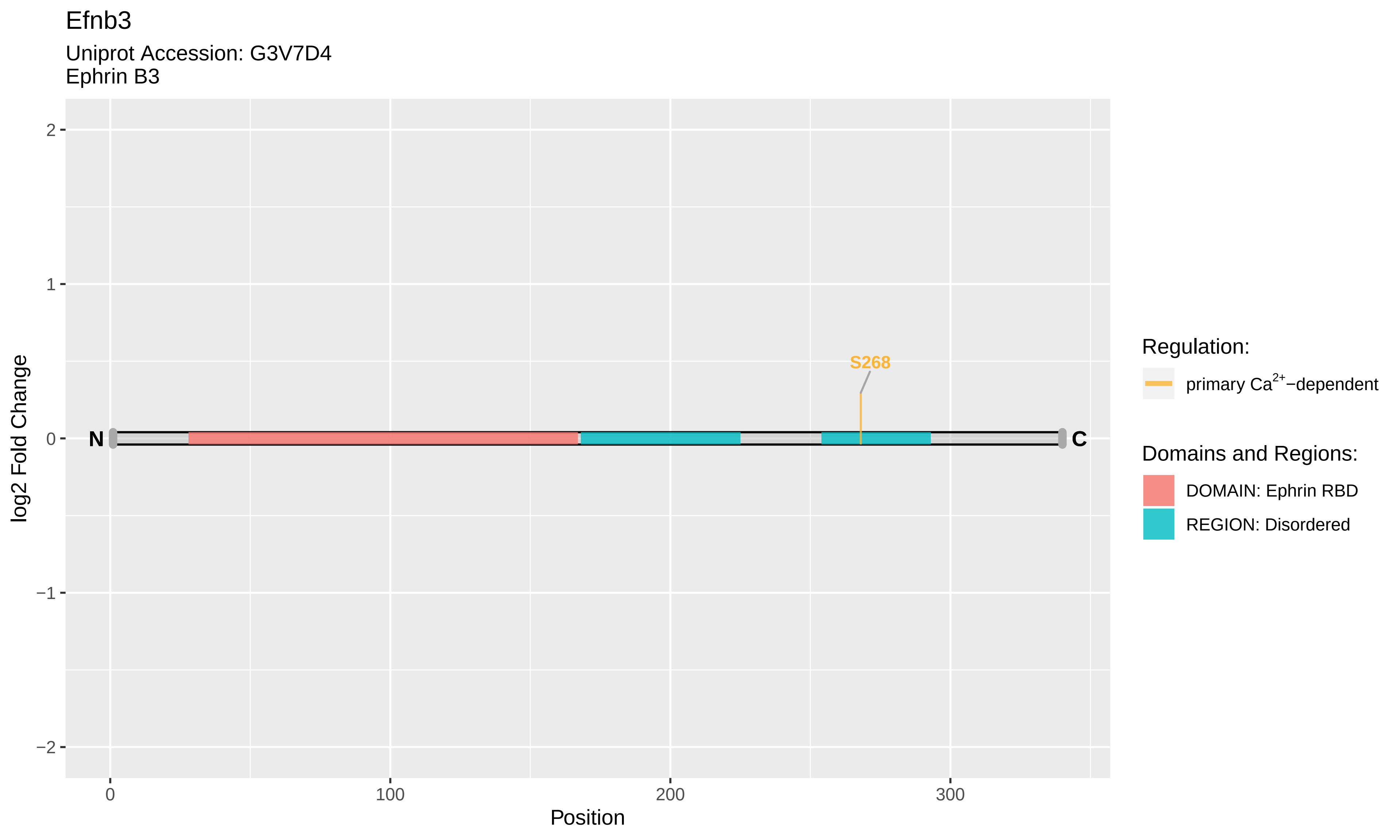

Efnb3
Uniprot Accession: G3V7D4
Ephrin B3
S268
N
C
2
1
0
−1
−2
log2 Fold Change
0
100
200
300
Position
Regulation:
primary Ca2+−dependent
Domains and Regions:
DOMAIN: Ephrin RBD
REGION: Disordered

Efr3b

Uniprot Accession: F1LTW9
EFR3 homolog B

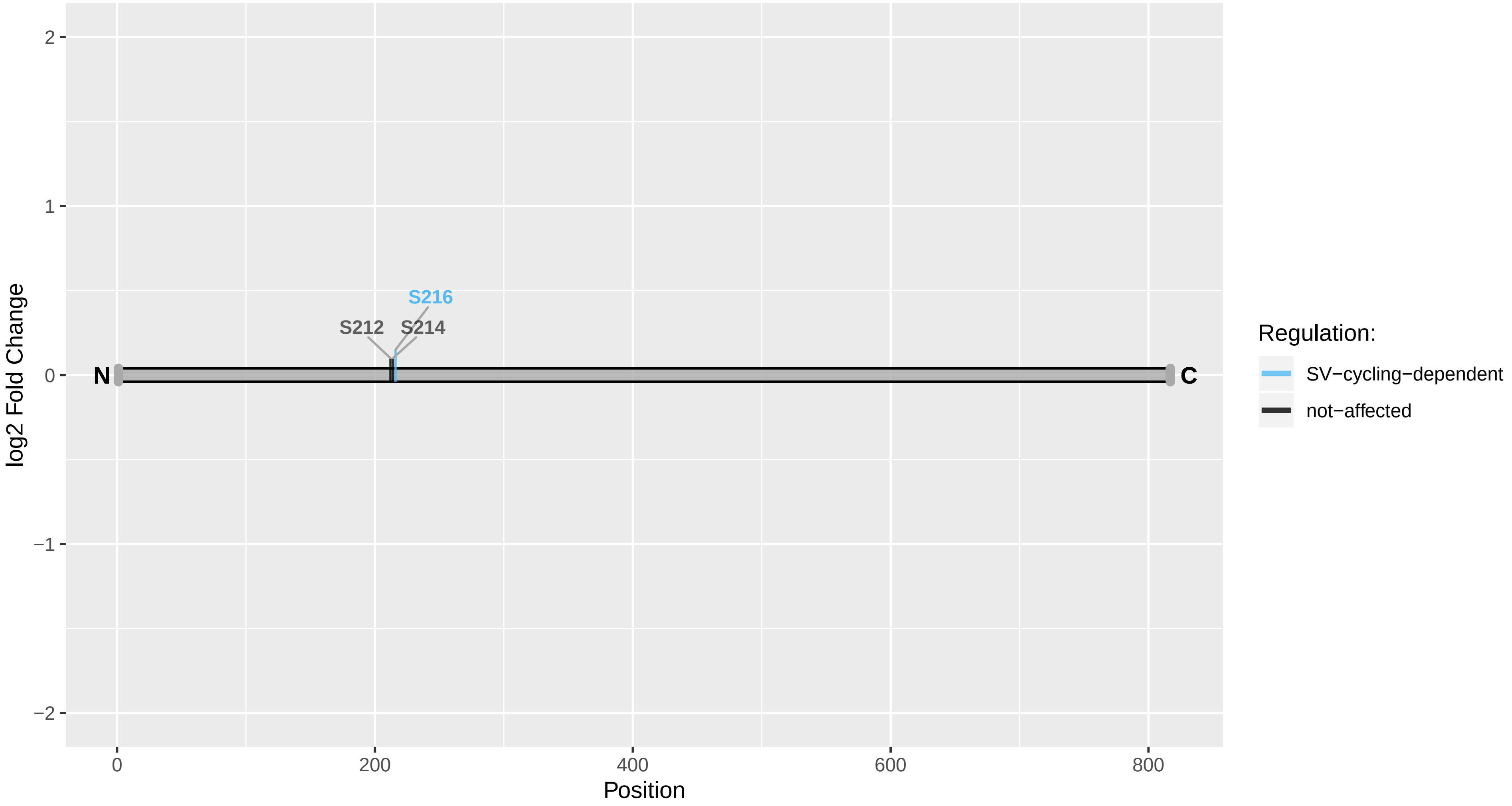

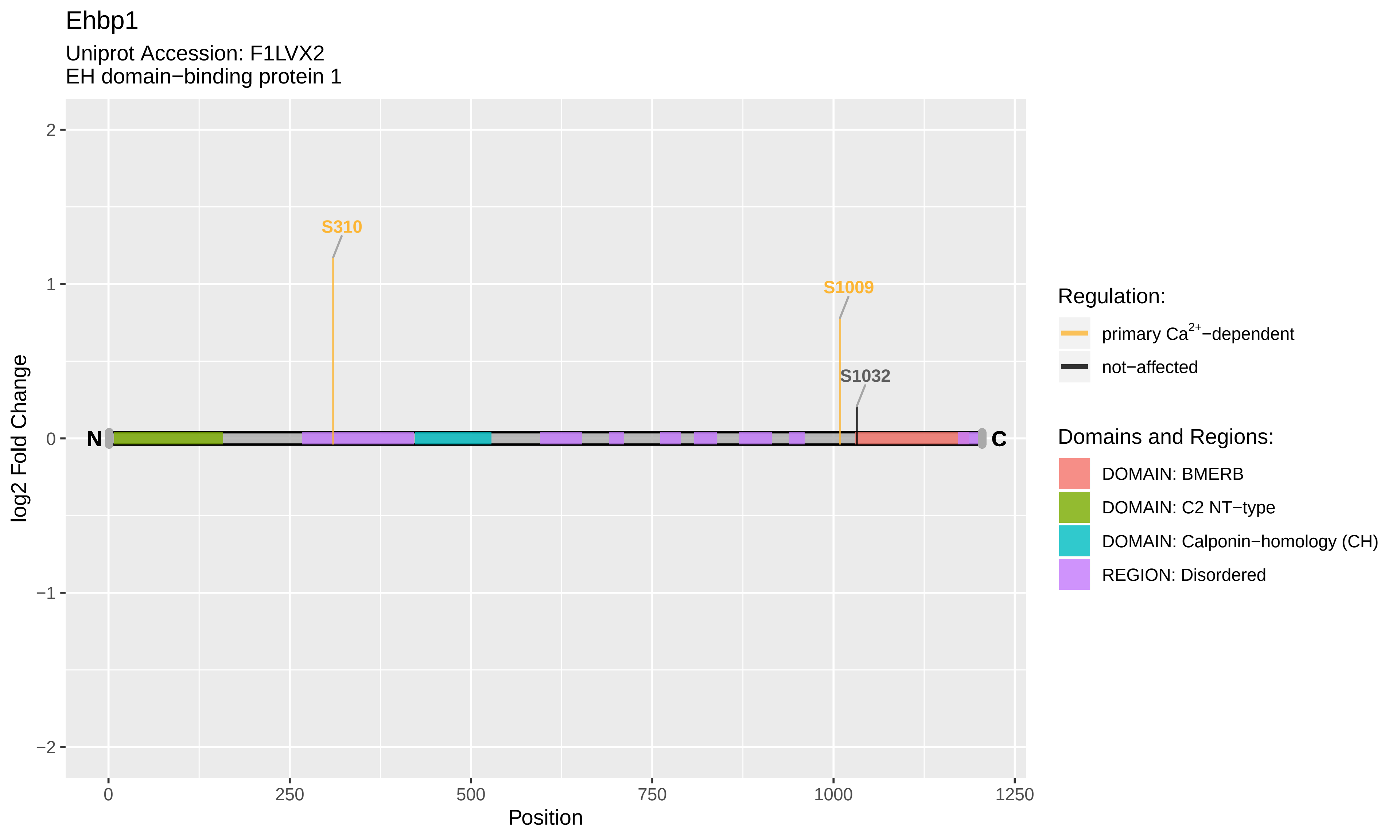
Ehbp1
Uniprot Accession: F1LVX2
EH domain−binding protein 1
S310
S1009
S1032
N
C
log2 Fold Change
2
1
0
−1
−2
0
250
500
750
1000
1250
Position
Regulation:
primary Ca2+−dependent
not−affected
Domains and Regions:
DOMAIN: BMERB
DOMAIN: C2 NT−type
DOMAIN: Calponin−homology (CH)
REGION: Disordered

Eif3d

Uniprot Accession: Q6AYK8
Eukaryotic translation initiation factor 3 subunit D

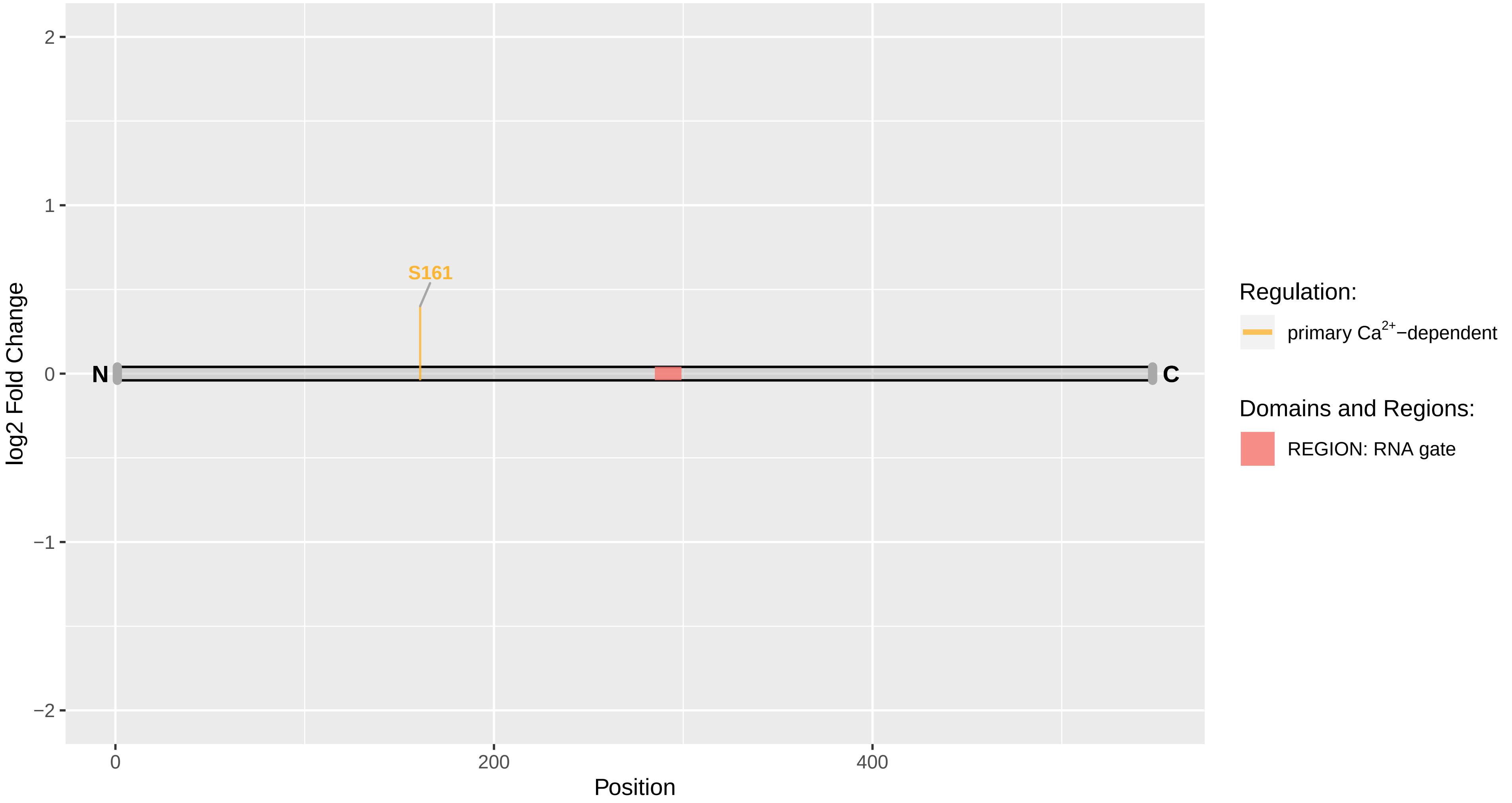

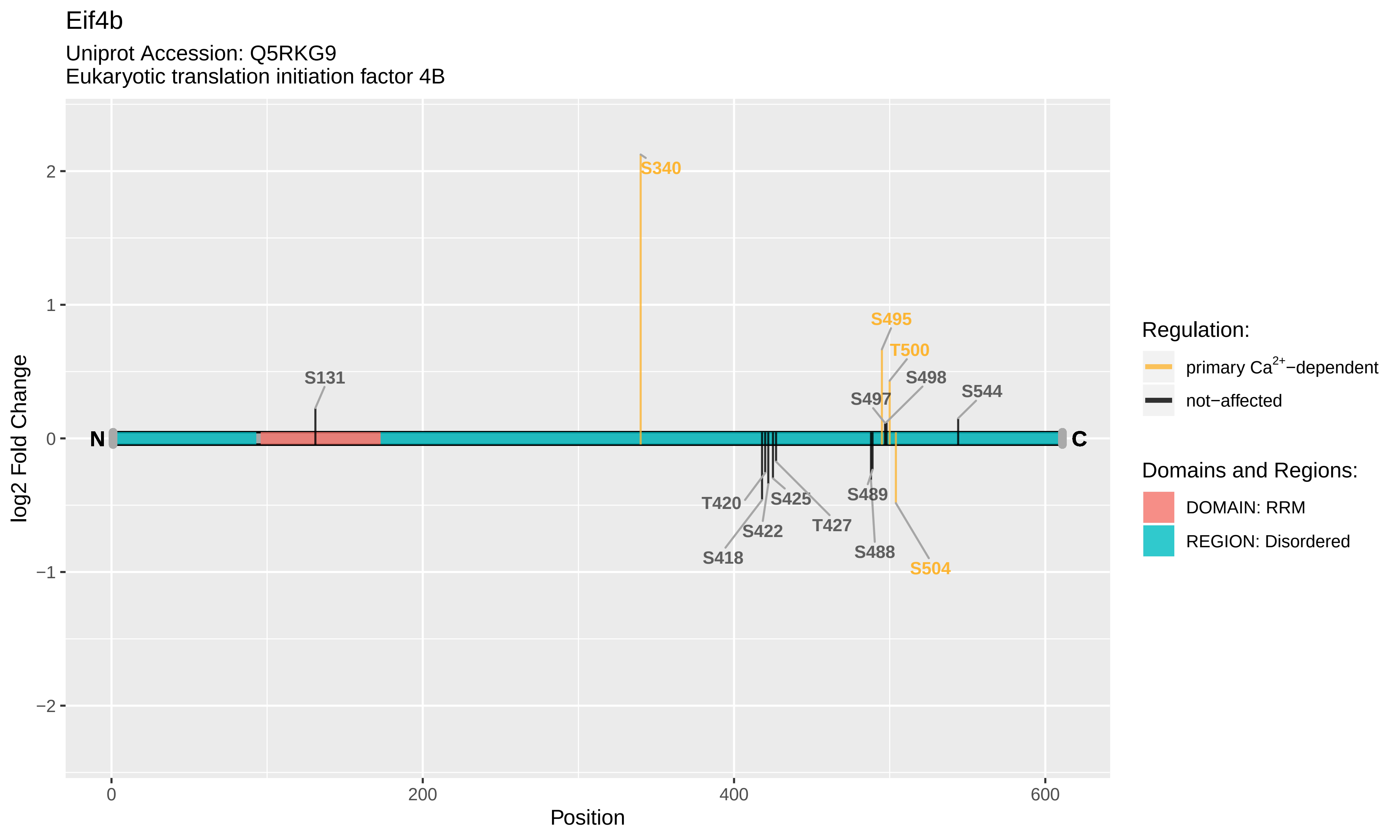

Eif4b
Uniprot Accession: Q5RKG9
Eukaryotic translation initiation factor 4B
log2 Fold Change
Position
2
1
0
−1
−2
0
200
400
600
N
C
S340
S495
T500
S498
S131
S497
S544
T420
S425
S489
S422
T427
S418
S488
S504
Regulation:
primary Ca2+−dependent
not−affected
Domains and Regions:
DOMAIN: RRM
REGION: Disordered

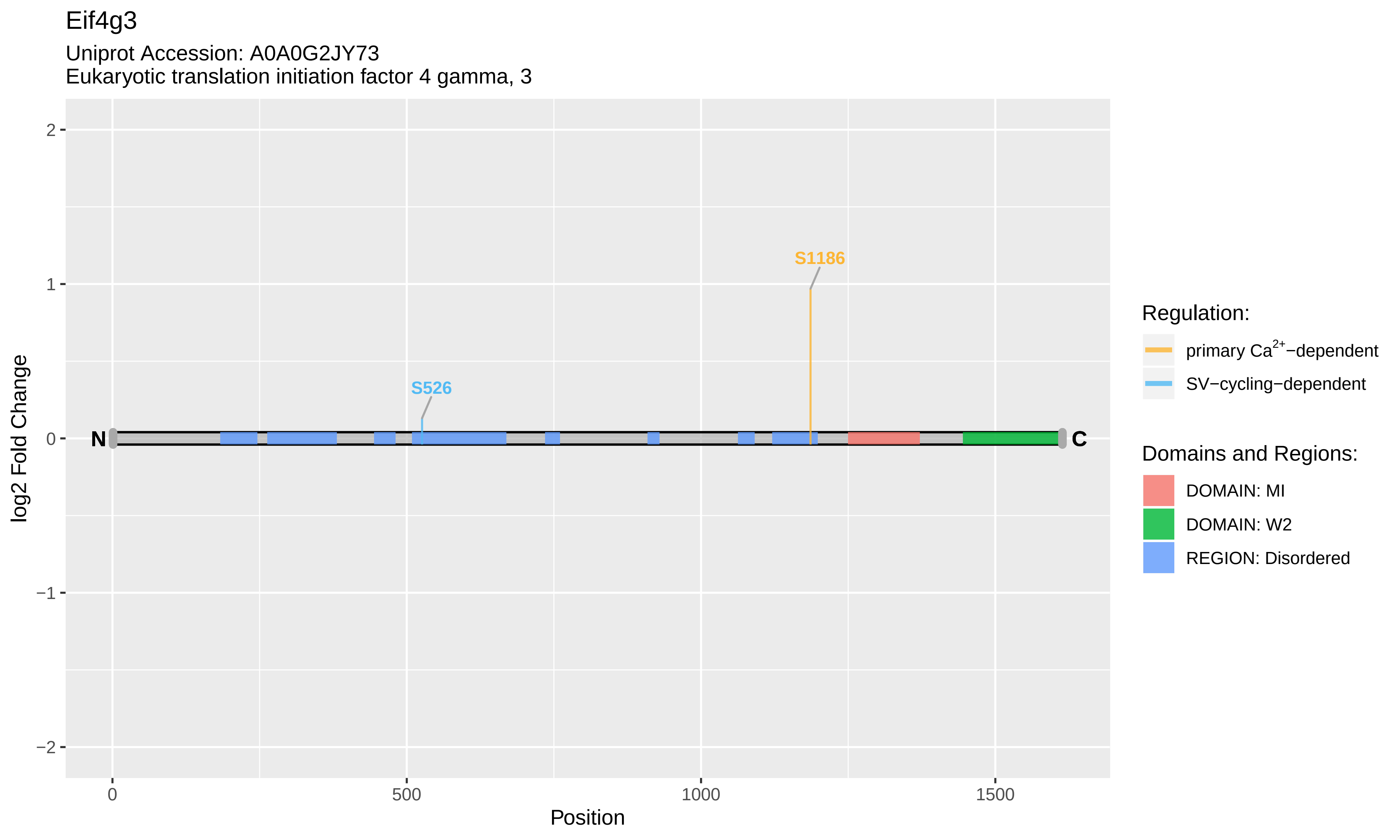

Eif4g3
Uniprot Accession: A0A0G2JY73
Eukaryotic translation initiation factor 4 gamma, 3
S1186
S526
N
C
2
1
0
−1
−2
log2 Fold Change
0
500
1000
1500
Position
Regulation:
primary Ca2+−dependent
SV−cycling−dependent
Domains and Regions:
DOMAIN: MI
DOMAIN: W2
REGION: Disordered

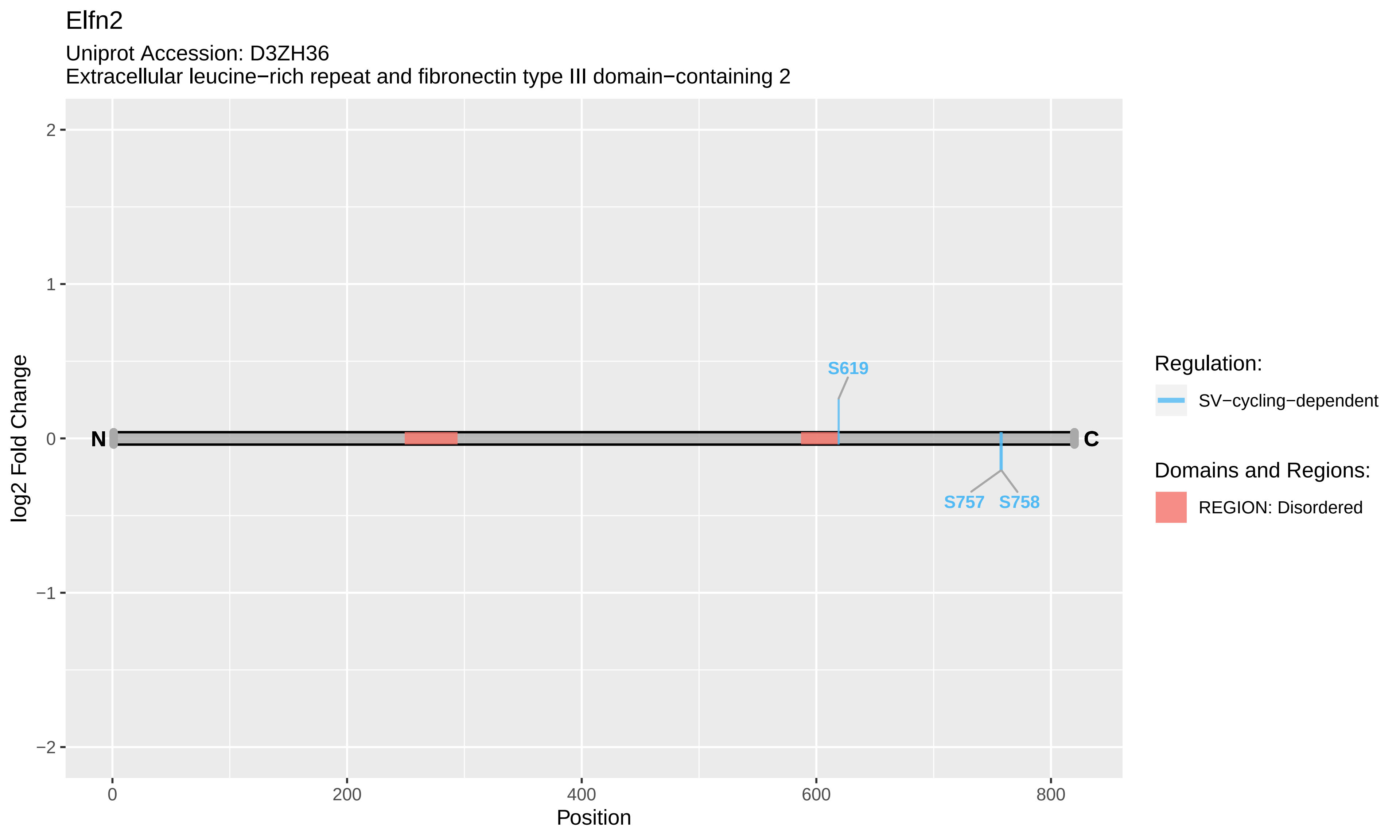
Elfn2
Uniprot Accession: D3ZH36
Extracellular leucine−rich repeat and fibronectin type III domain−containing 2
S619
S757
S758
N
C
2
1
0
−1
−2
log2 Fold Change
0
200
400
600
800
Position
Regulation:
SV−cycling−dependent
Domains and Regions:
REGION: Disordered

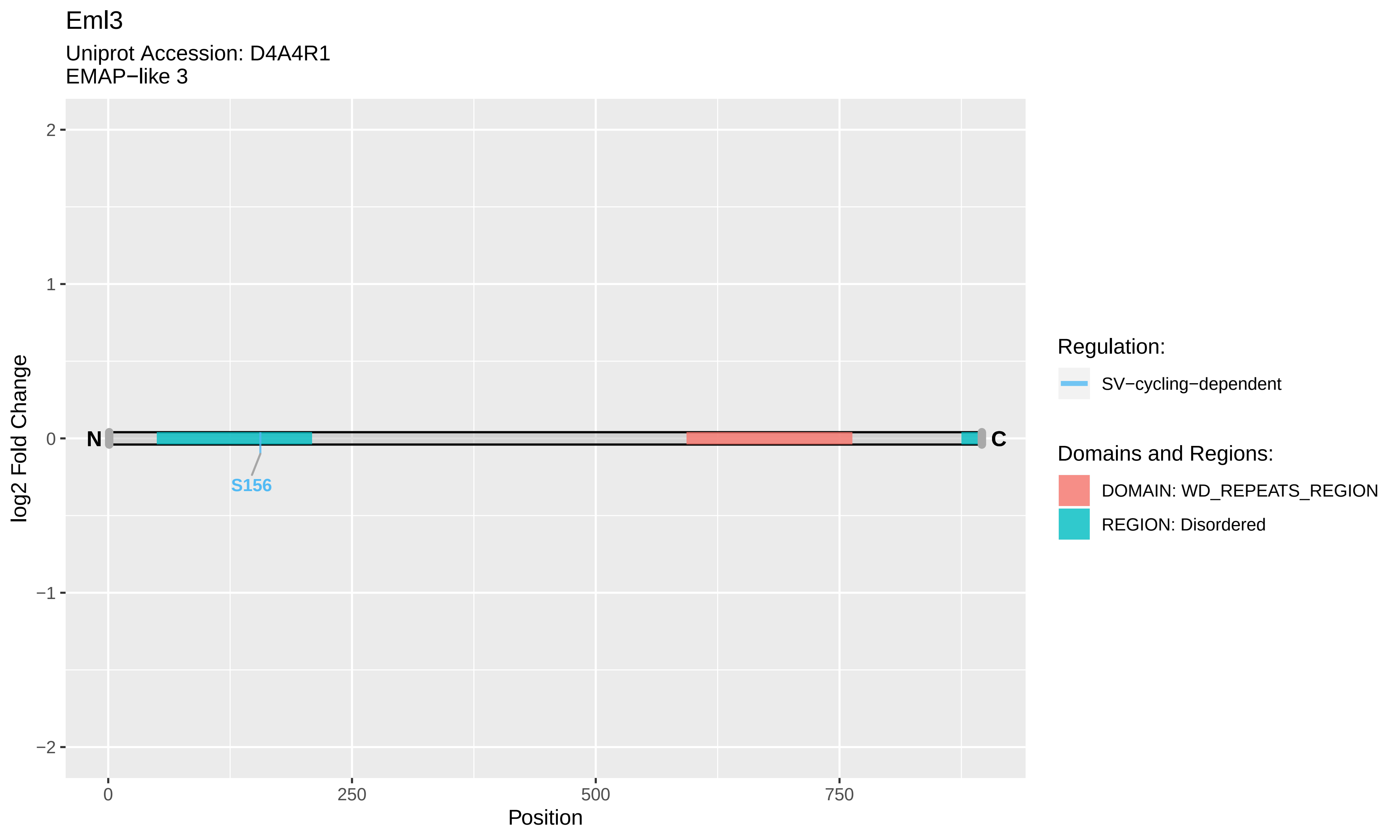

Eml3
Uniprot Accession: D4A4R1
EMAP−like 3
log2 Fold Change
2
1
0
−1
−2
N
C
S156
0
250
500
750
Position
Regulation:
SV−cycling−dependent
Domains and Regions:
DOMAIN: WD_REPEATS_REGION
REGION: Disordered

Eno1

Uniprot Accession: P04764
Alpha−enolase

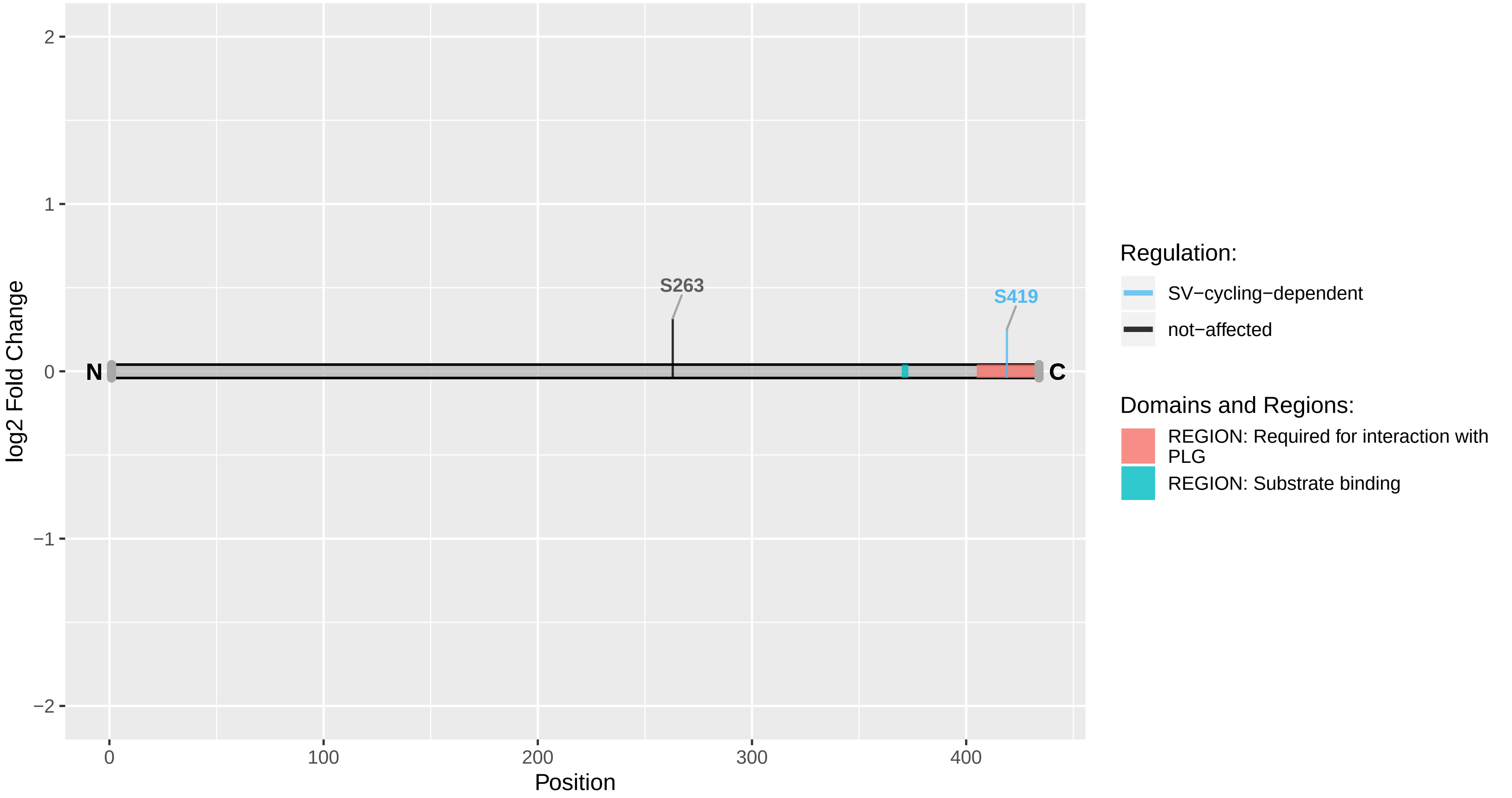

Ensa

Uniprot Accession: P60841
Alpha−endosulfine

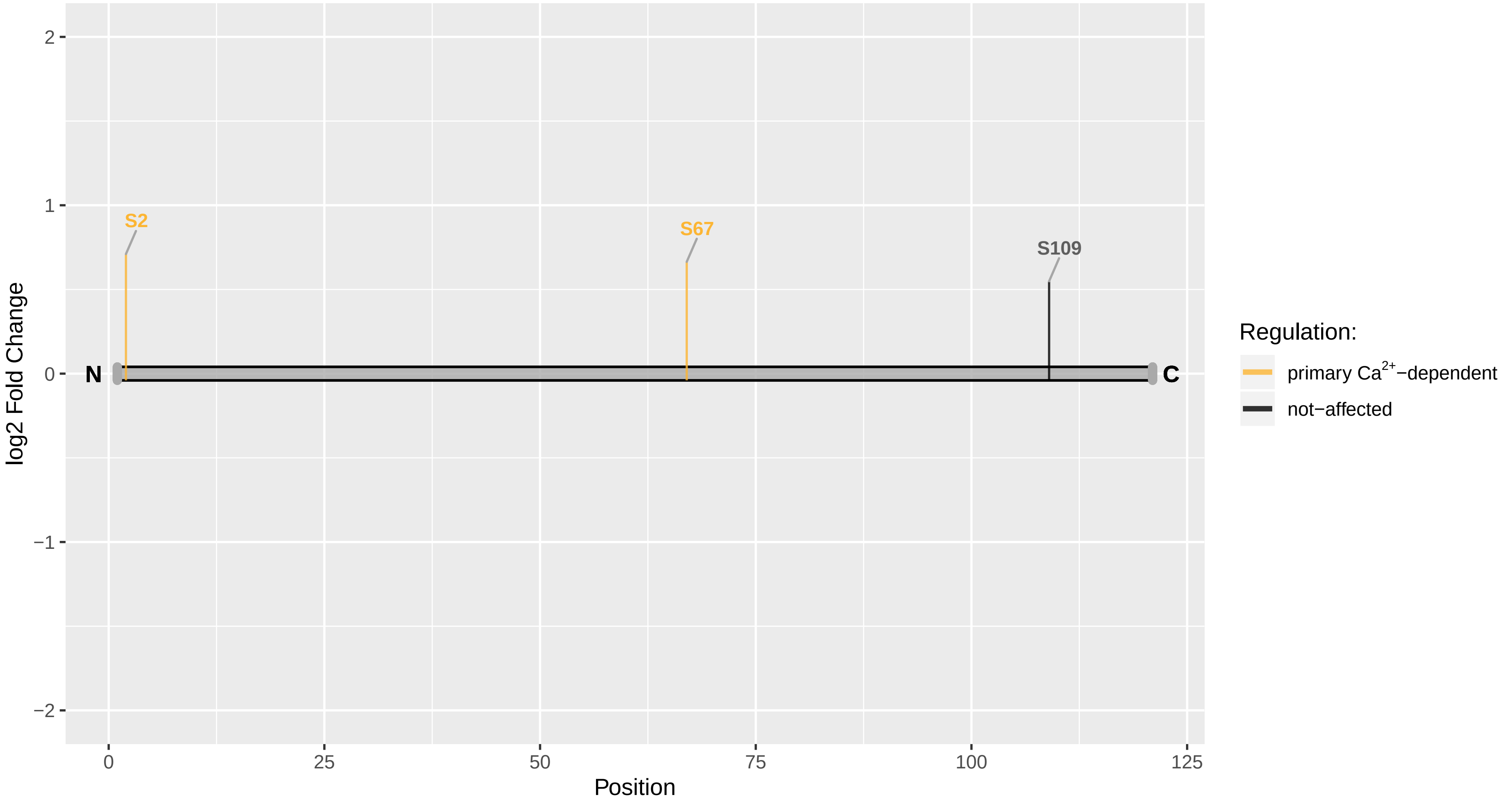

Epb41l1

Uniprot Accession: A0A0G2K0F3
Band 4.1−like protein 1−like

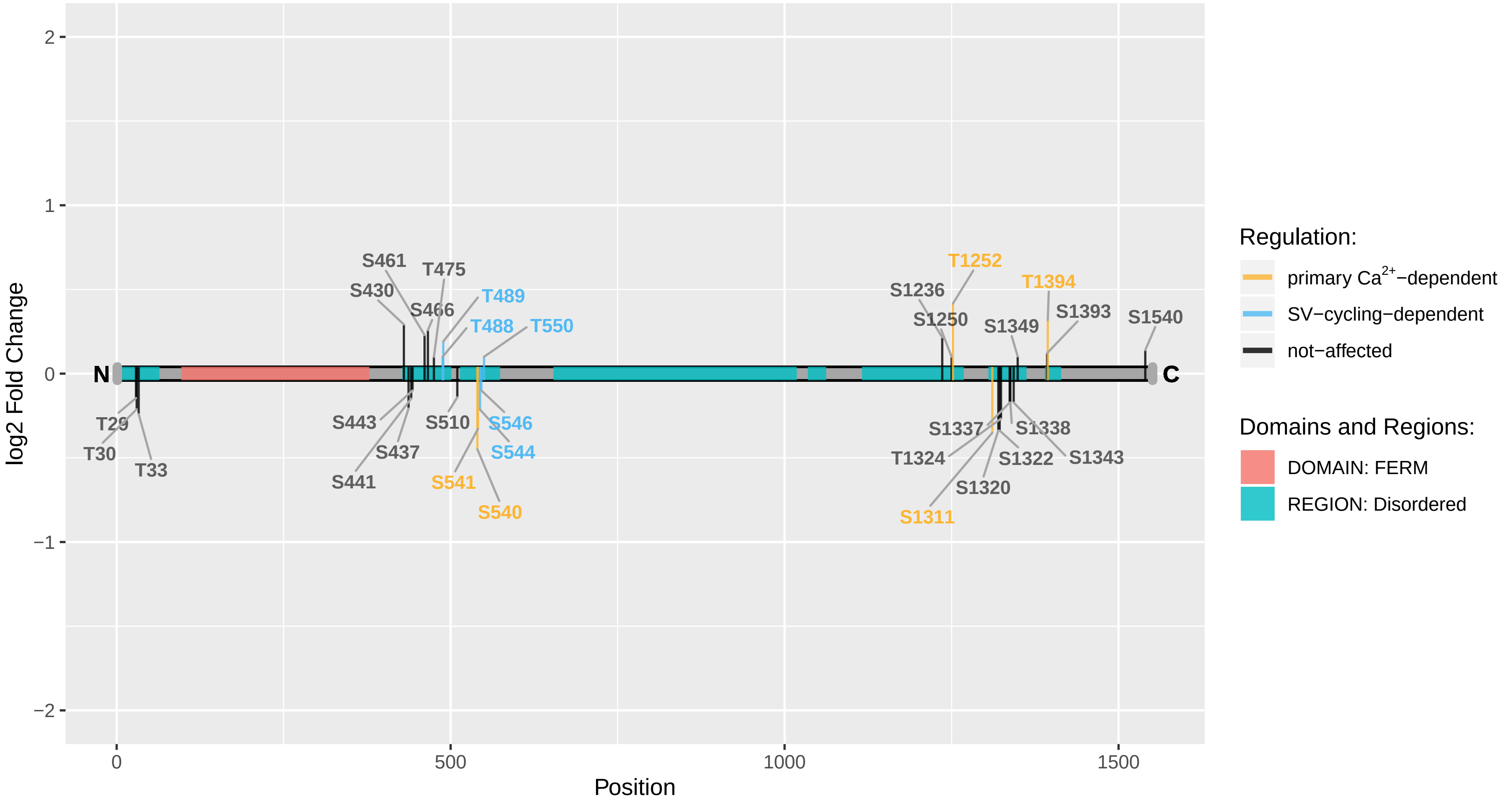

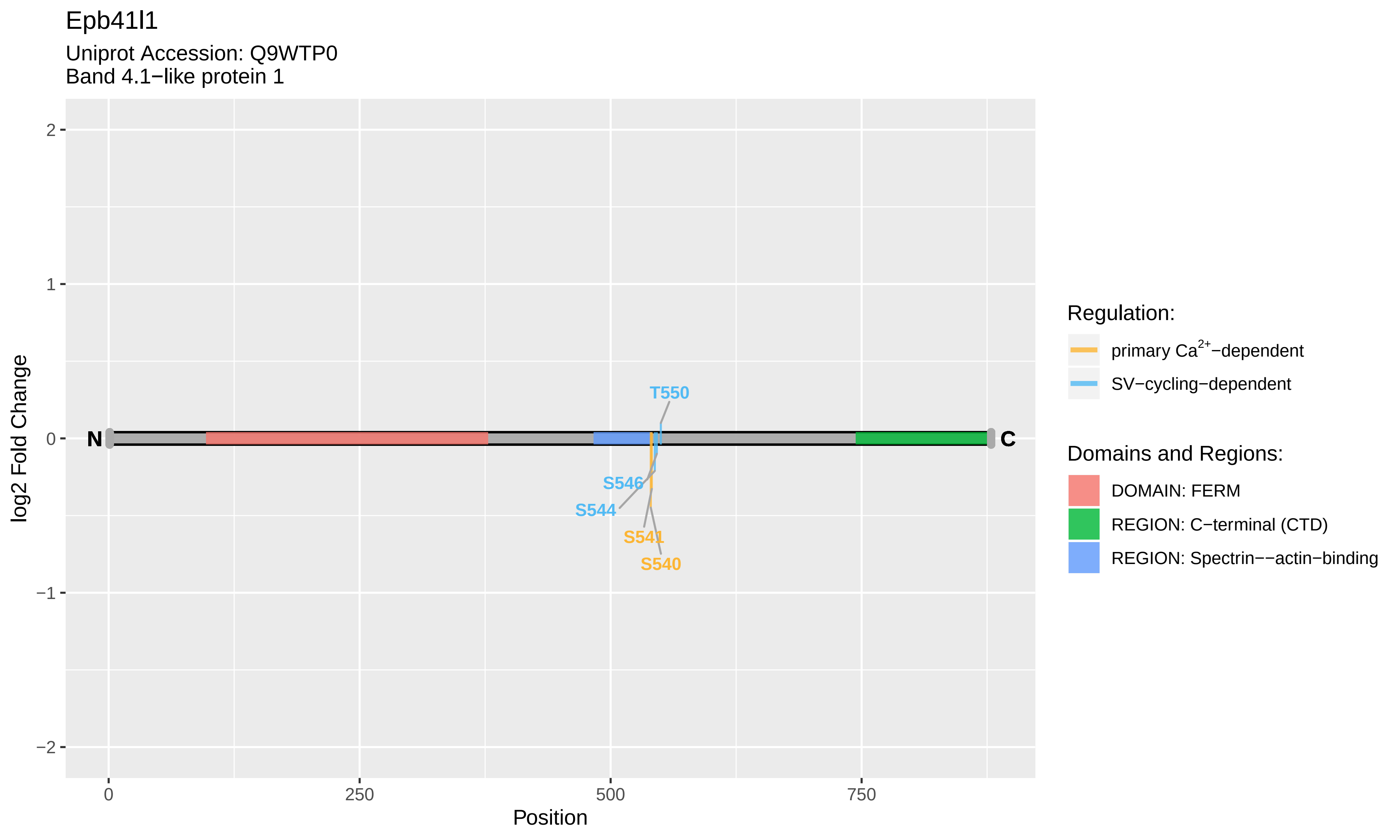

Epb41l1
Uniprot Accession: Q9WTP0
Band 4.1−like protein 1
log2 Fold Change
Position
N
C
T550
S546
S544
S541
S540
Regulation:
primary Ca2+−dependent
SV−cycling−dependent
Domains and Regions:
DOMAIN: FERM
REGION: C−terminal (CTD)
REGION: Spectrin−−actin−binding

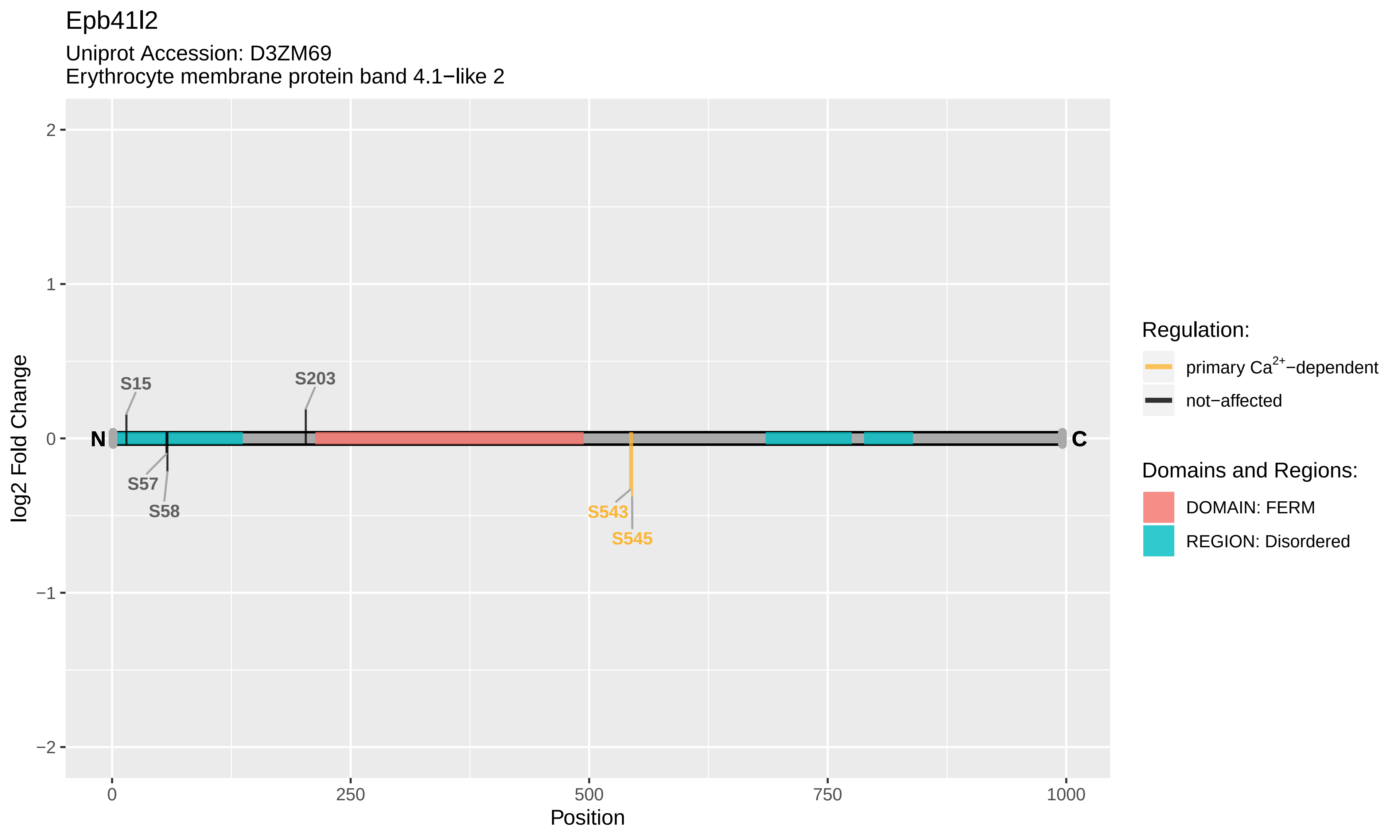

Epb41l2
Uniprot Accession: D3ZM69
Erythrocyte membrane protein band 4.1−like 2
log2 Fold Change
Position
2
1
0
−1
−2
0
250
500
750
1000
N
C
S15
S203
S57
S58
S543
S545
Regulation:
primary Ca2+−dependent
not−affected
Domains and Regions:
DOMAIN: FERM
REGION: Disordered

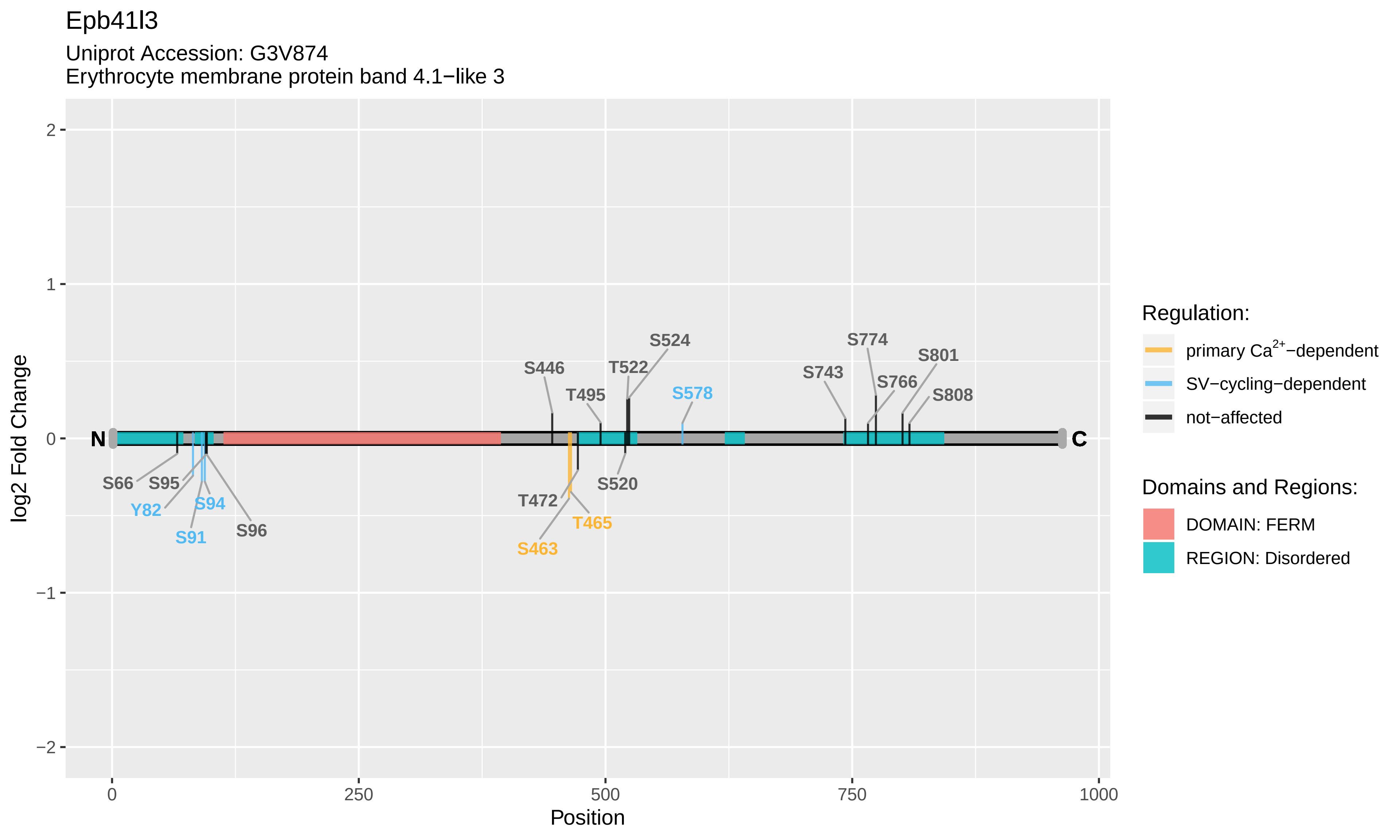

Epb41l3
Uniprot Accession: G3V874
Erythrocyte membrane protein band 4.1−like 3
log2 Fold Change
Position
2
1
0
−1
−2
0
250
500
750
1000
N
C
S66
S95
Y82
S94
S91
S96
S446
T495
T522
S524
S578
S520
T472
T465
S463
S743
S774
S766
S801
S808
Regulation:
primary Ca2+−dependent
SV−cycling−dependent
not−affected
Domains and Regions:
DOMAIN: FERM
REGION: Disordered

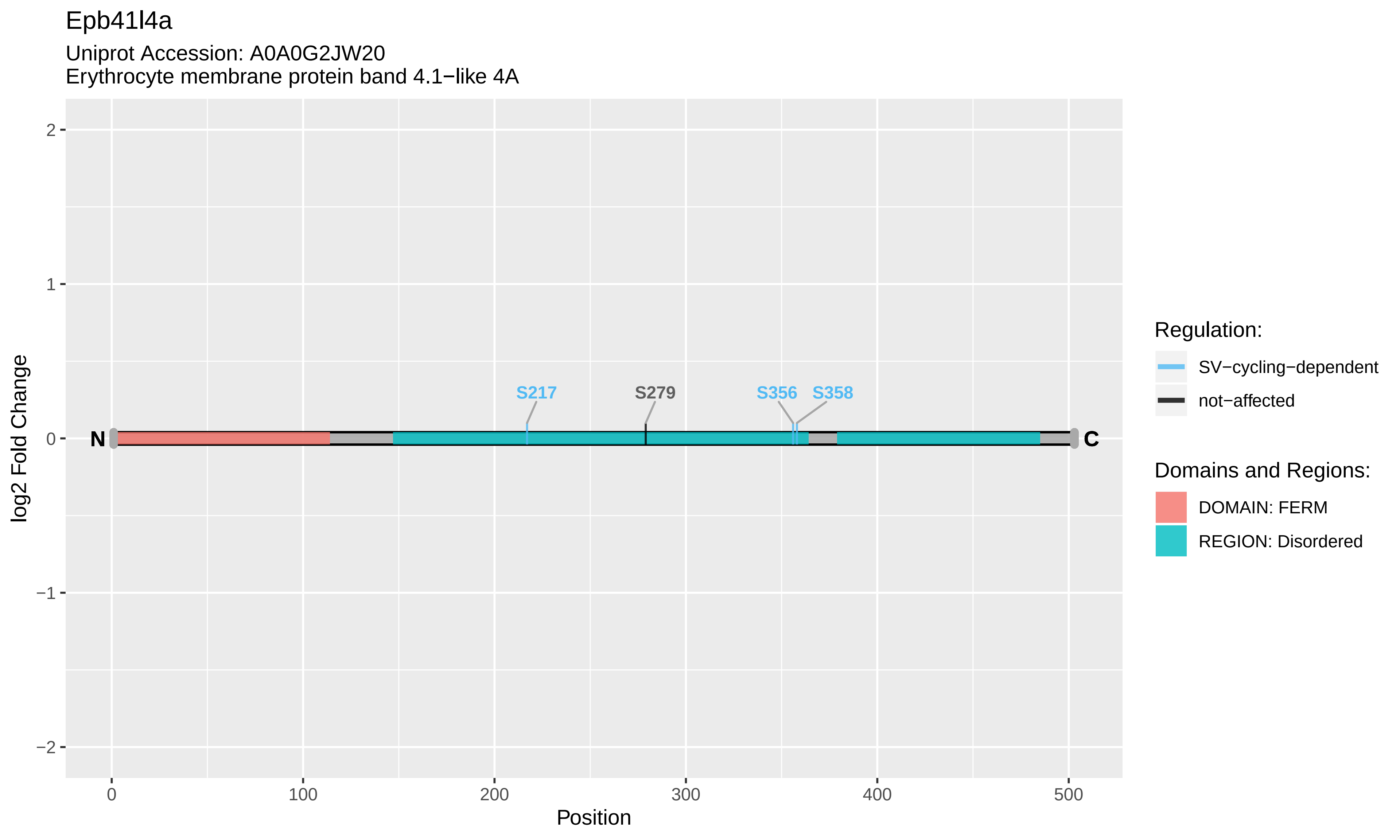

Epb41l4a
Uniprot Accession: A0A0G2JW20
Erythrocyte membrane protein band 4.1−like 4A
S217
S279
S356
S358
N
C
2
1
0
−1
−2
log2 Fold Change
0
100
200
300
400
500
Position
Regulation:
SV−cycling−dependent
not−affected
Domains and Regions:
DOMAIN: FERM
REGION: Disordered

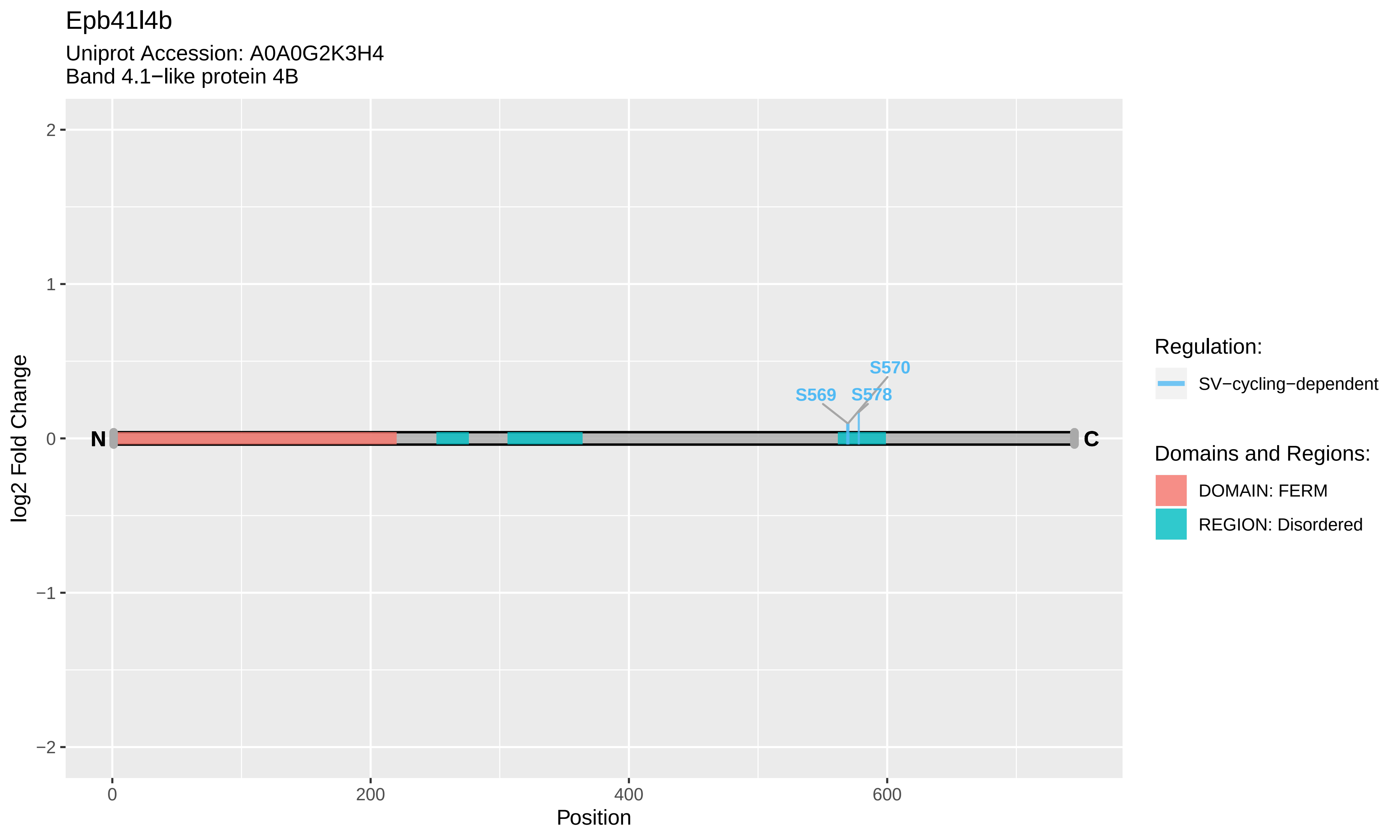

Epb41l4b
Uniprot Accession: A0A0G2K3H4
Band 4.1−like protein 4B
log2 Fold Change
Position
N
C
S569
S570
S578
2
1
0
−1
−2
0
200
400
600
Regulation:
SV−cycling−dependent
Domains and Regions:
DOMAIN: FERM
REGION: Disordered

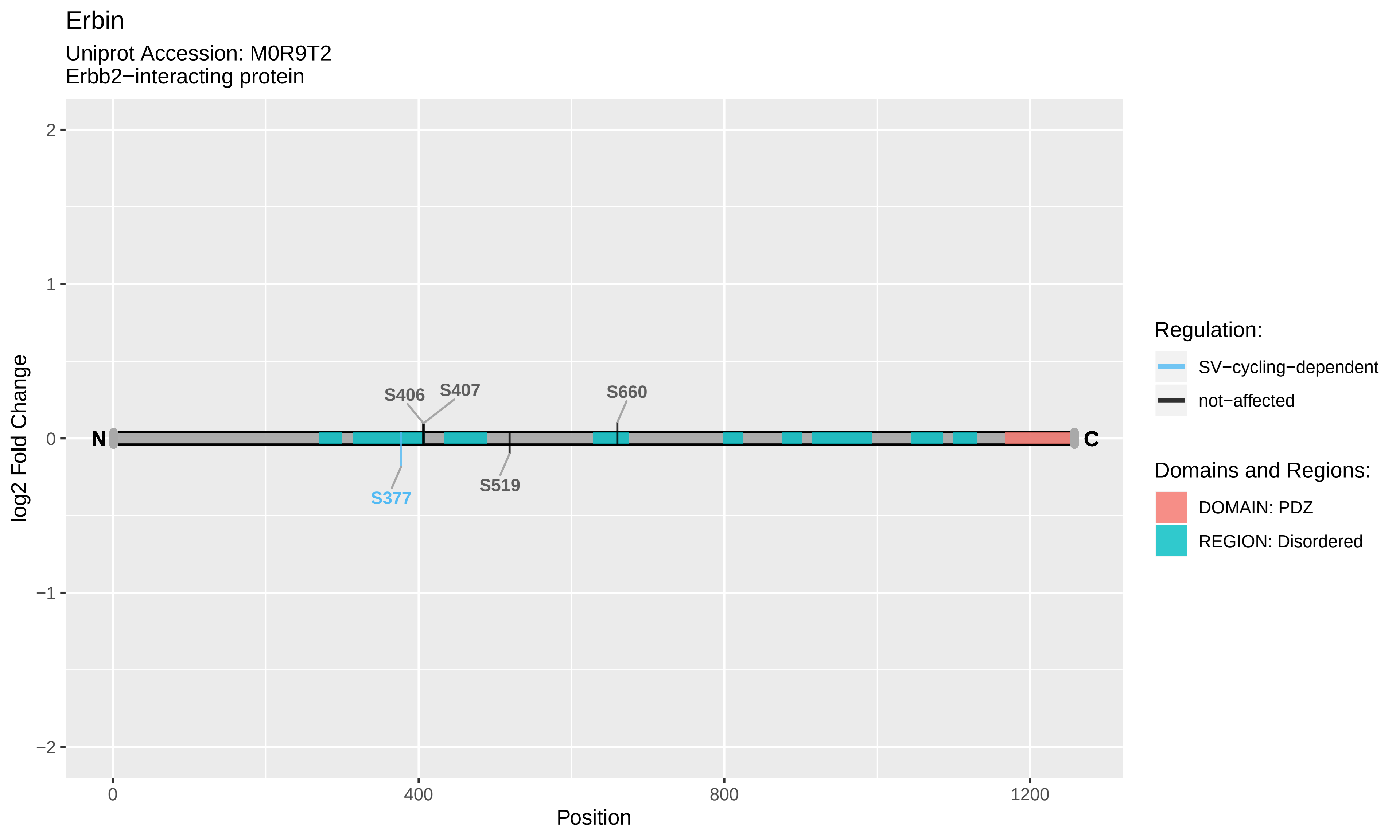

Erbin
Uniprot Accession: M0R9T2
Erbb2−interacting protein
S406
S407
S660
S377
S519
N
C
2
1
0
−1
−2
log2 Fold Change
0
400
800
1200
Position
Regulation:
SV−cycling−dependent
not−affected
Domains and Regions:
DOMAIN: PDZ
REGION: Disordered

Erc1

Uniprot Accession: A0A0G2JYT1
ELKS/Rab6−interacting/CAST family member 1

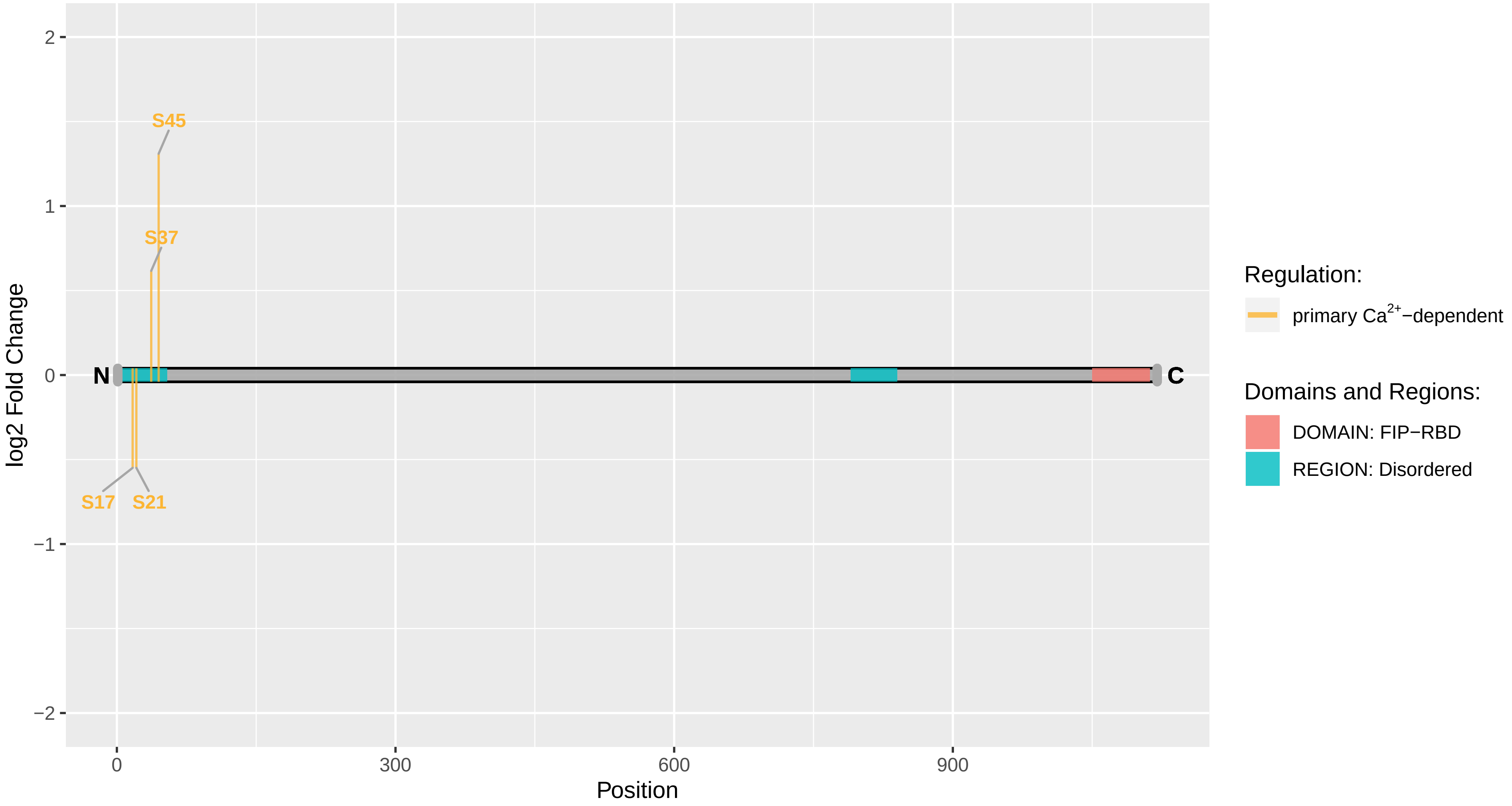

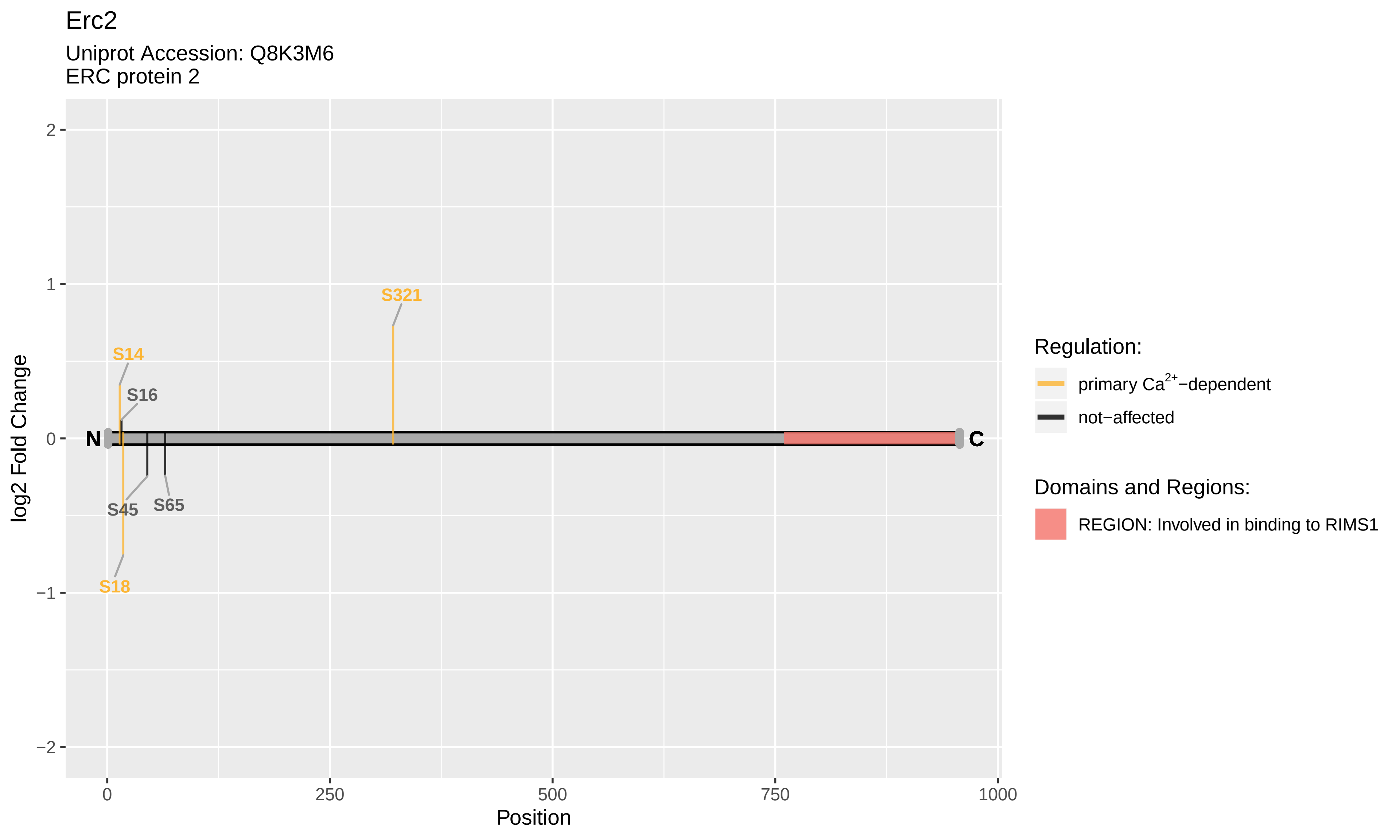
Erc2
Uniprot Accession: Q8K3M6
ERC protein 2
log2 Fold Change
Position
N
C
S321
S14
S16
S45
S65
S18
2
1
0
−1
−2
0
250
500
750
1000
Regulation:
primary Ca^{2+}−dependent
not−affected
Domains and Regions:
REGION: Involved in binding to RIMS1

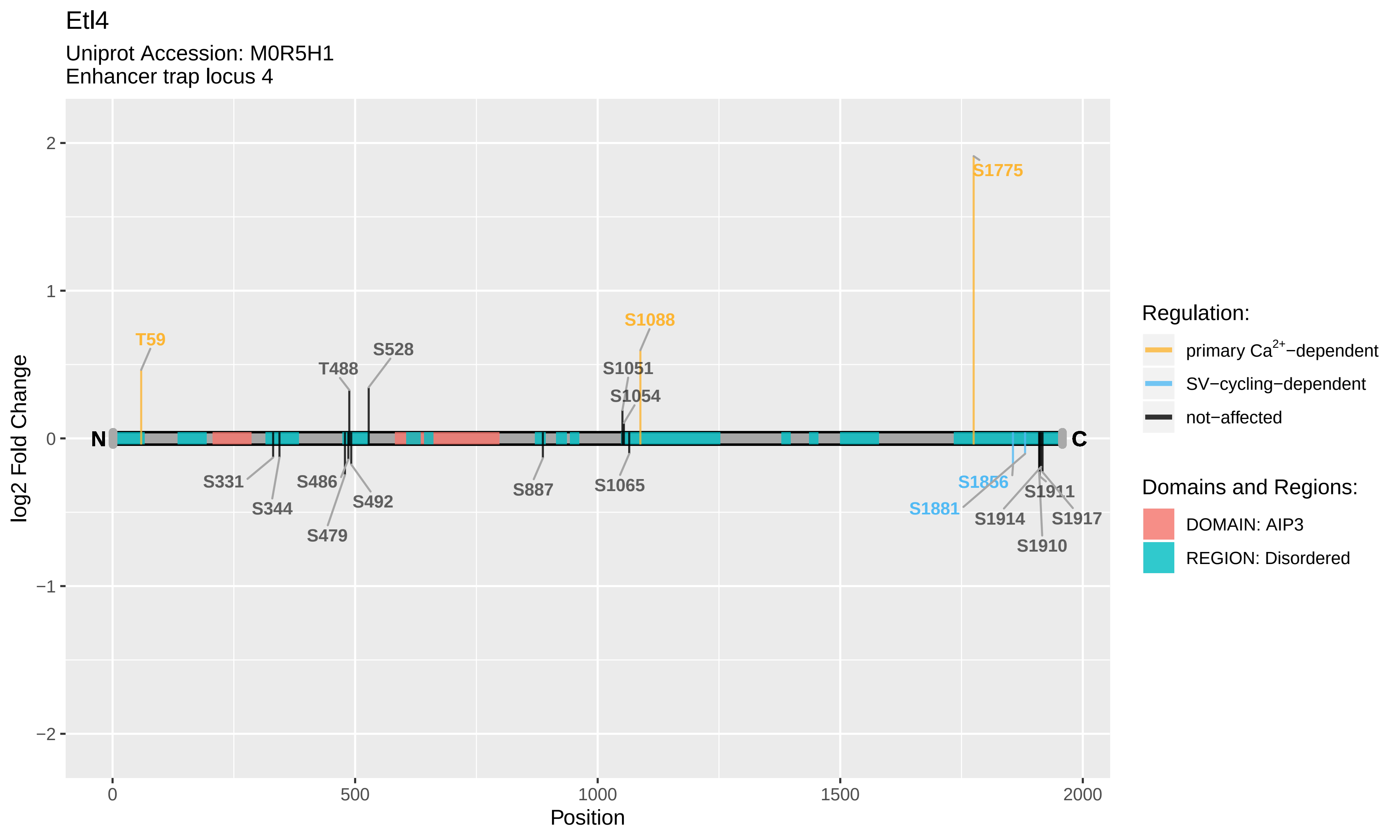

Etl4
Uniprot Accession: M0R5H1
Enhancer trap locus 4
log2 Fold Change
Position
2
1
0
−1
−2
0
500
1000
1500
2000
N
C
T59
S528
T488
S1088
S1051
S1054
S1775
S331
S344
S486
S479
S492
S887
S1065
S1856
S1881
S1911
S1914
S1917
S1910
Regulation:
primary Ca2+−dependent
SV−cycling−dependent
not−affected
Domains and Regions:
DOMAIN: AIP3
REGION: Disordered

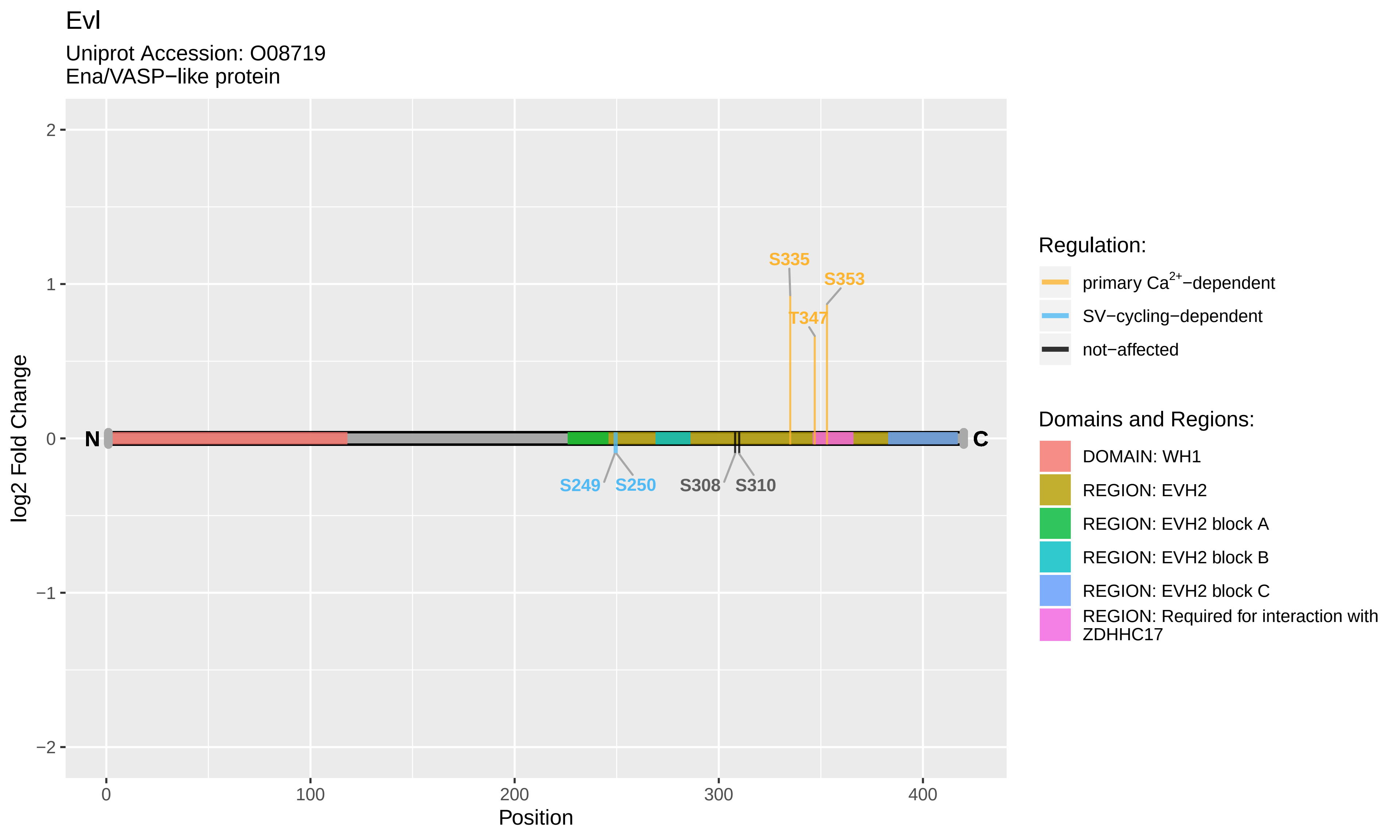

Evl
Uniprot Accession: O08719
Ena/VASP−like protein
log2 Fold Change
Position
2
1
0
−1
−2
0
100
200
300
400
N
C
S335
S353
T347
S249
S250
S308
S310
Regulation:
primary Ca2+−dependent
SV−cycling−dependent
not−affected
Domains and Regions:
DOMAIN: WH1
REGION: EVH2
REGION: EVH2 block A
REGION: EVH2 block B
REGION: EVH2 block C
REGION: Required for interaction with ZDHHC17

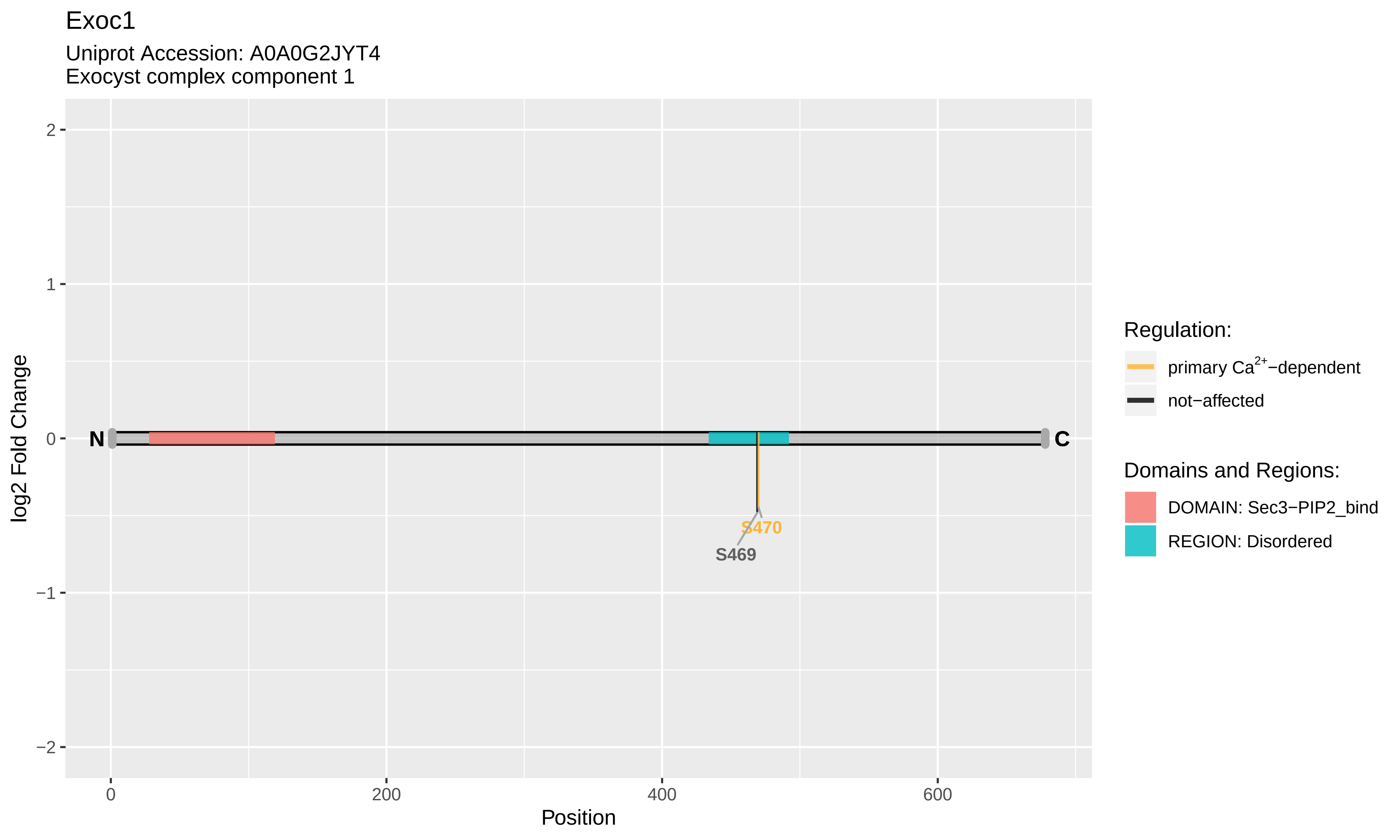
Exoc1
Uniprot Accession: A0A0G2JYT4
Exocyst complex component 1
log2 Fold Change
2
1
0
−1
−2
N
C
S470
S469
0
200
400
600
Position
Regulation:
primary Ca2+−dependent
not−affected
Domains and Regions:
DOMAIN: Sec3−PIP2_bind
REGION: Disordered

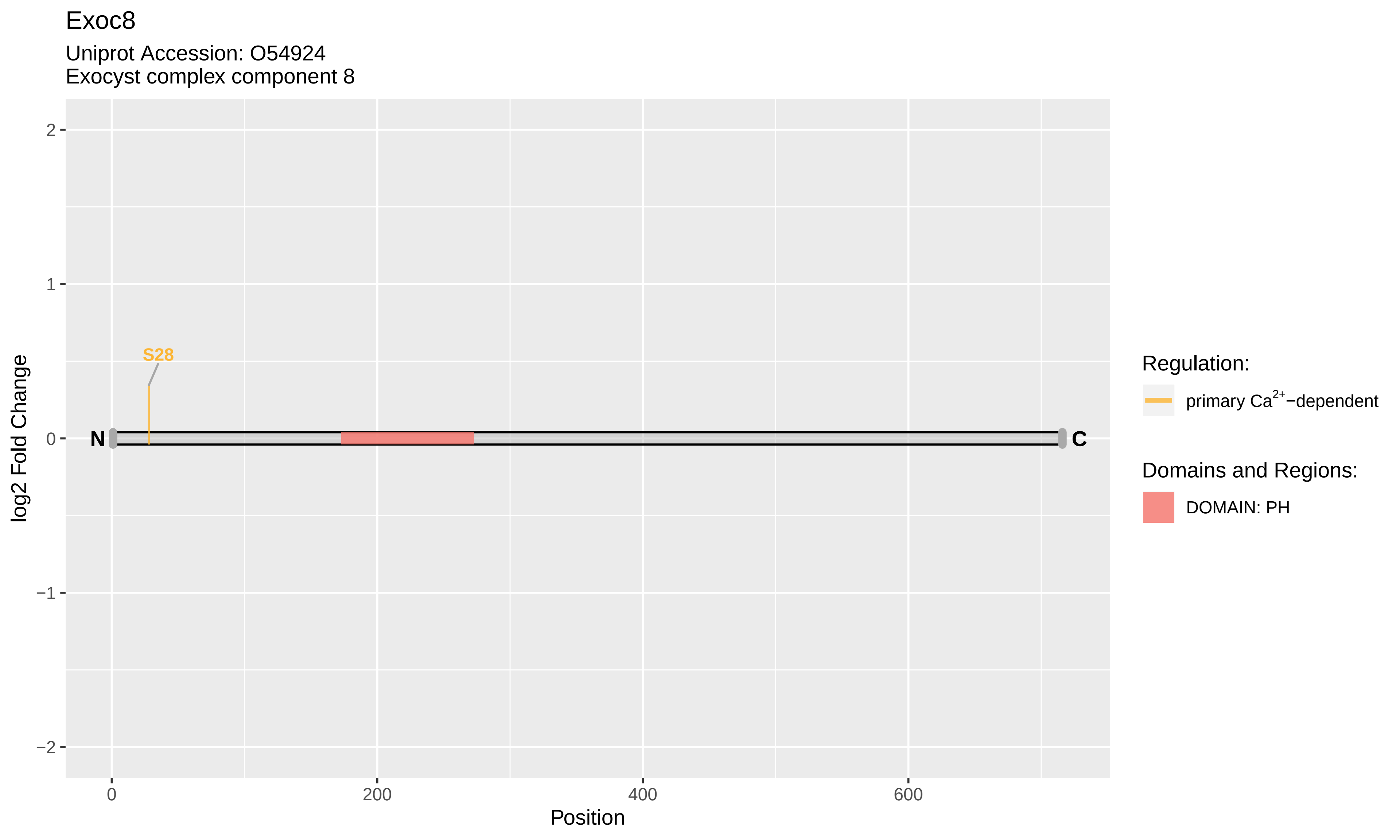

Exoc8
Uniprot Accession: O54924
Exocyst complex component 8
S28
N
C
log2 Fold Change
2
1
0
−1
−2
0
200
400
600
Position
Regulation:
primary Ca2+−dependent
Domains and Regions:
DOMAIN: PH

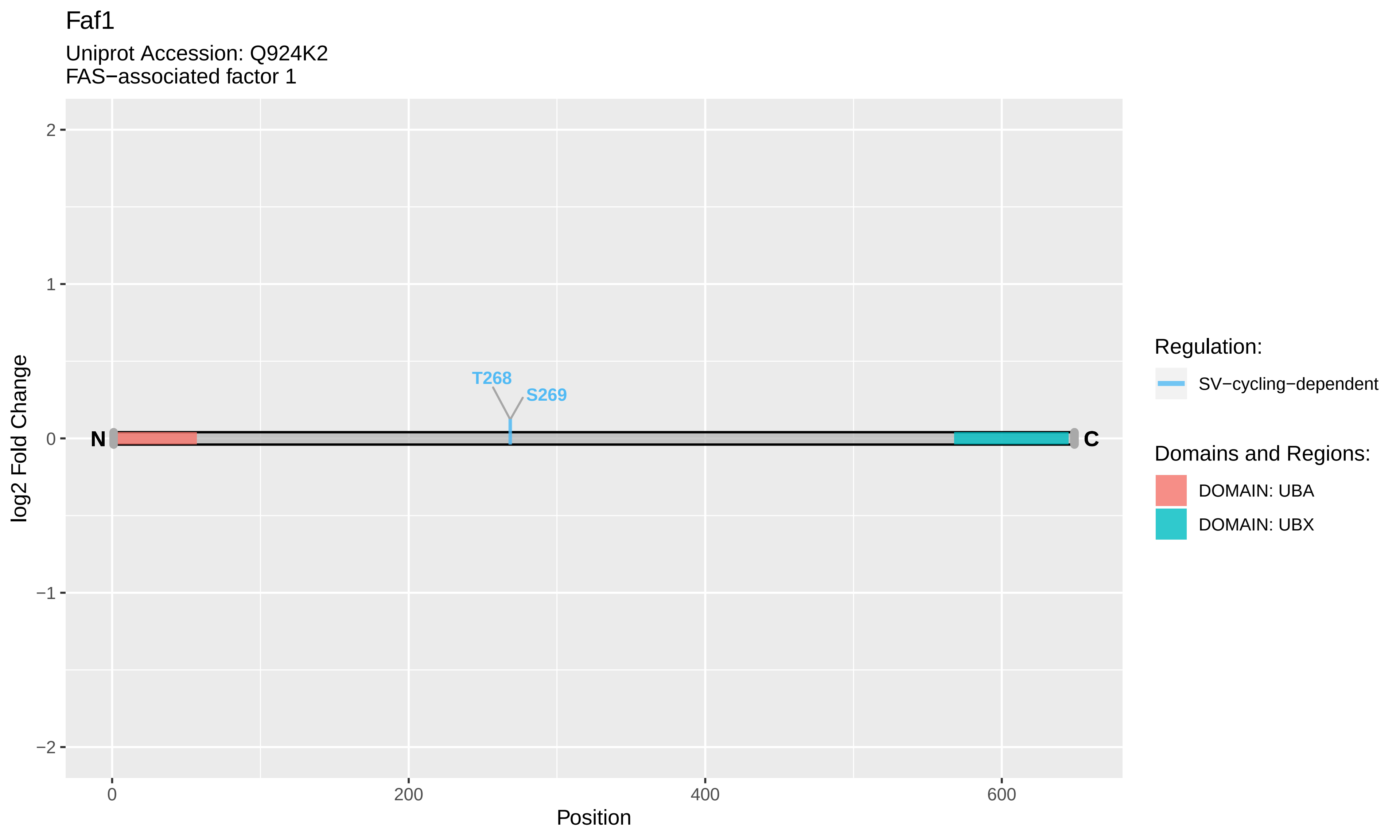
Faf1
Uniprot Accession: Q924K2
FAS−associated factor 1
log2 Fold Change
Position
2
1
0
−1
−2
0
200
400
600
N
C
T268
S269
Regulation:
SV−cycling−dependent
Domains and Regions:
DOMAIN: UBA
DOMAIN: UBX

Fam126b

Uniprot Accession: Q4V7D4
Family with sequence similarity 126, member B

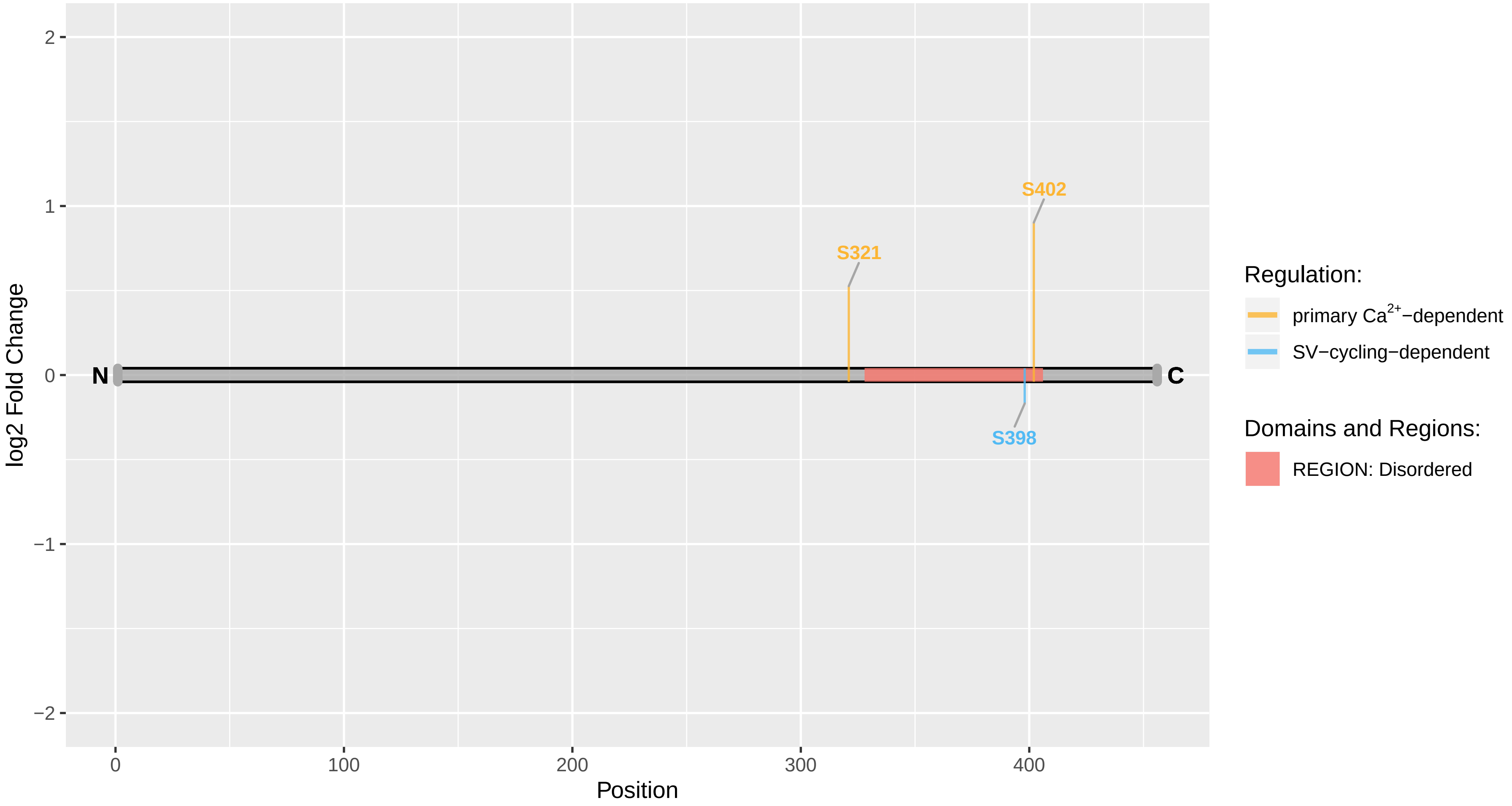

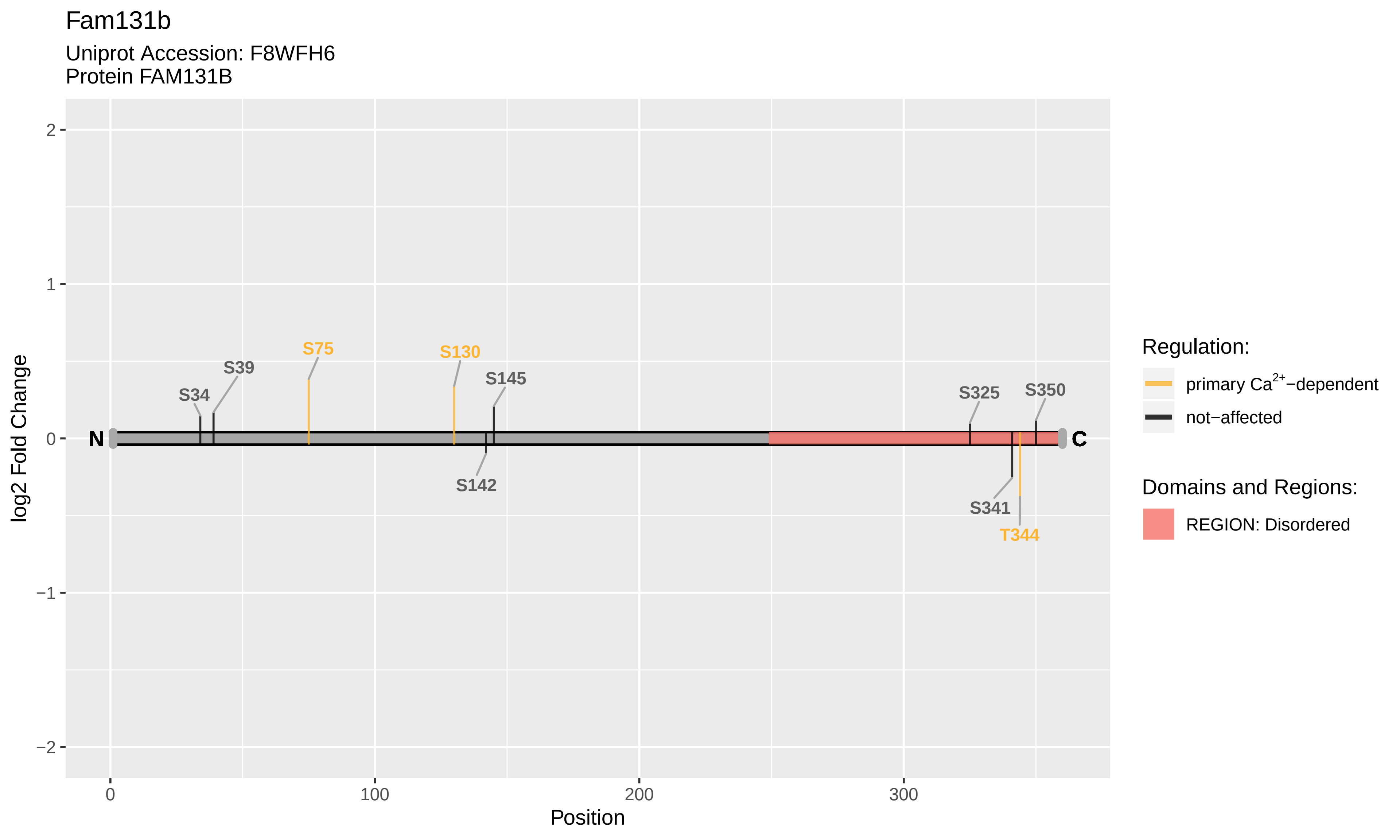
Fam131b
Uniprot Accession: F8WFH6
Protein FAM131B
log2 Fold Change
2
1
0
−1
−2
N
C
S34
S39
S75
S130
S145
S142
S325
S350
S341
T344
0
100
200
300
Position
Regulation:
primary Ca2+−dependent
not−affected
Domains and Regions:
REGION: Disordered

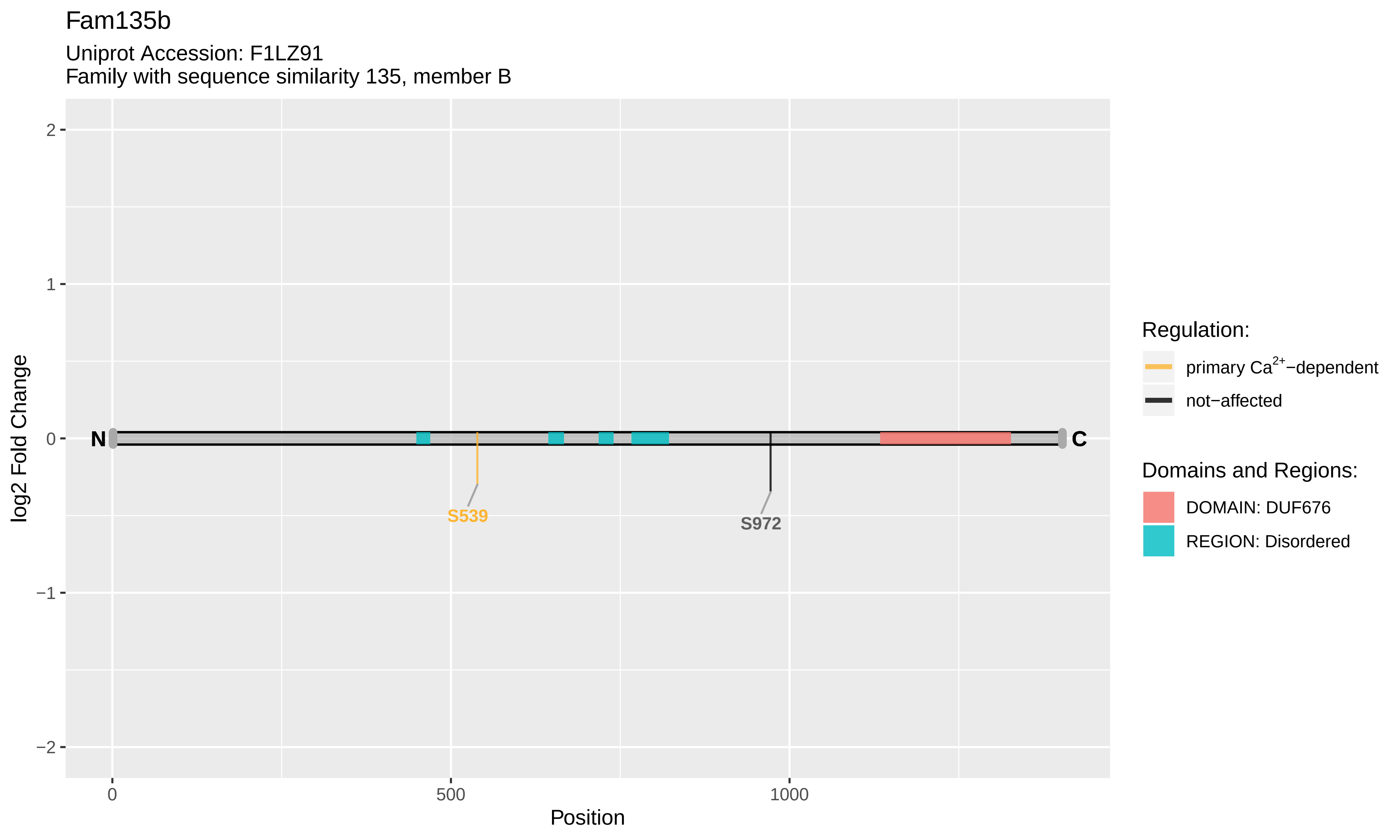
Fam135b
Uniprot Accession: F1LZ91
Family with sequence similarity 135, member B
log2 Fold Change
2
1
0
−1
−2
N
C
S539
S972
0
500
1000
Position
Regulation:
primary Ca2+−dependent
not−affected
Domains and Regions:
DOMAIN: DUF676
REGION: Disordered

Fam171b

Uniprot Accession: D3ZTG3
Family with sequence similarity 171, member B

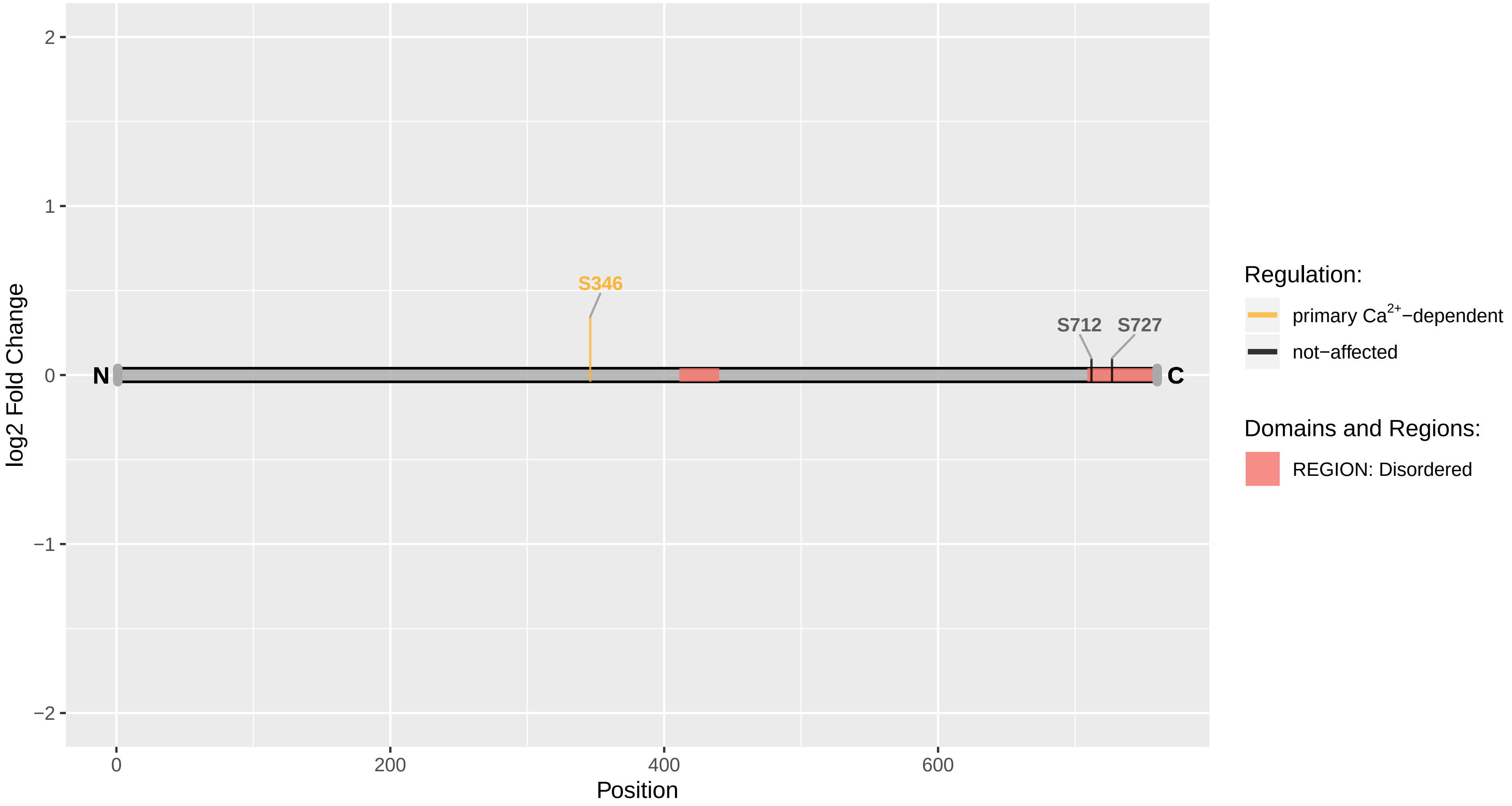

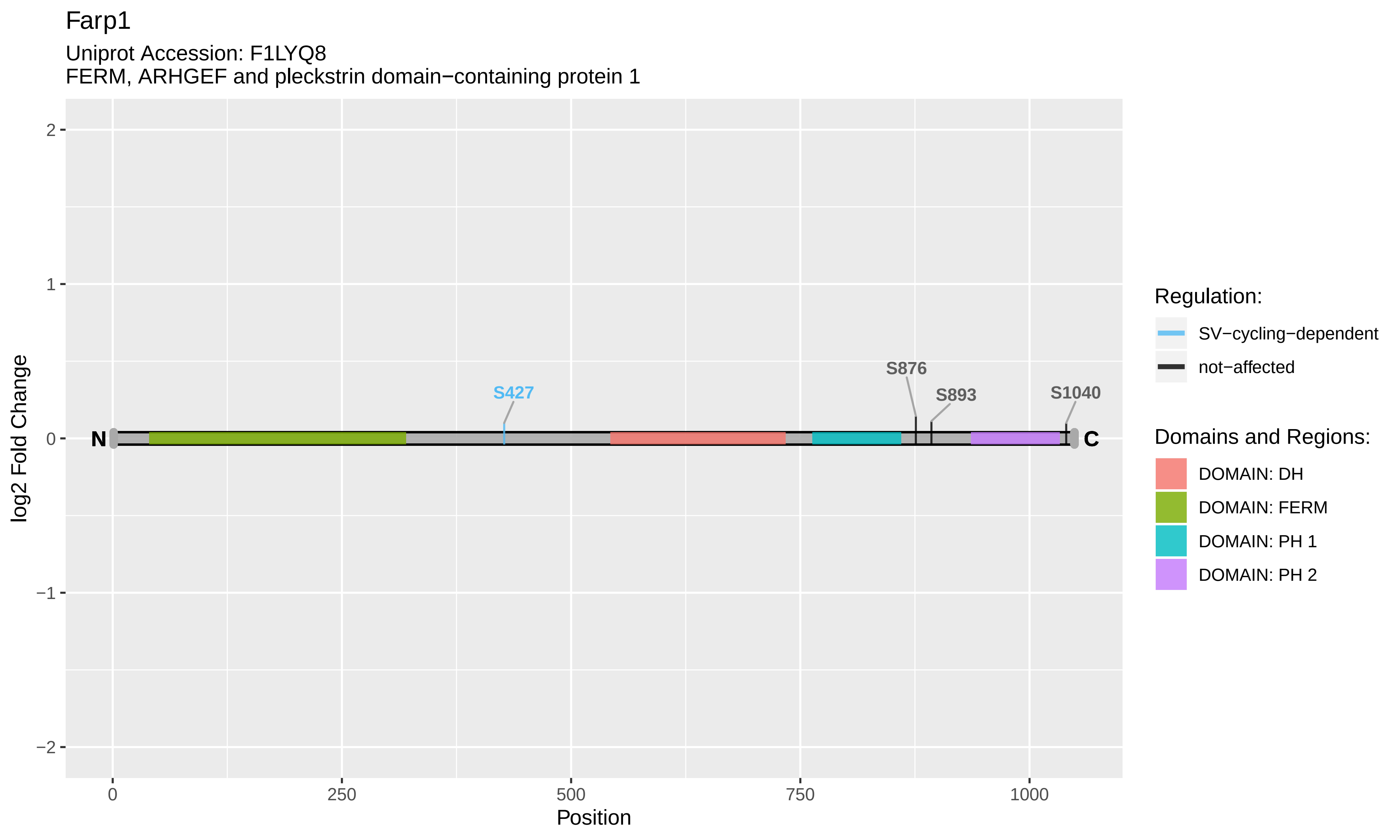

Farp1
Uniprot Accession: F1LYQ8
FERM, ARHGEF and pleckstrin domain−containing protein 1
log2 Fold Change
Position
S427
S876
S893
S1040
N
C
Regulation:
SV−cycling−dependent
not−affected
Domains and Regions:
DOMAIN: DH
DOMAIN: FERM
DOMAIN: PH 1
DOMAIN: PH 2

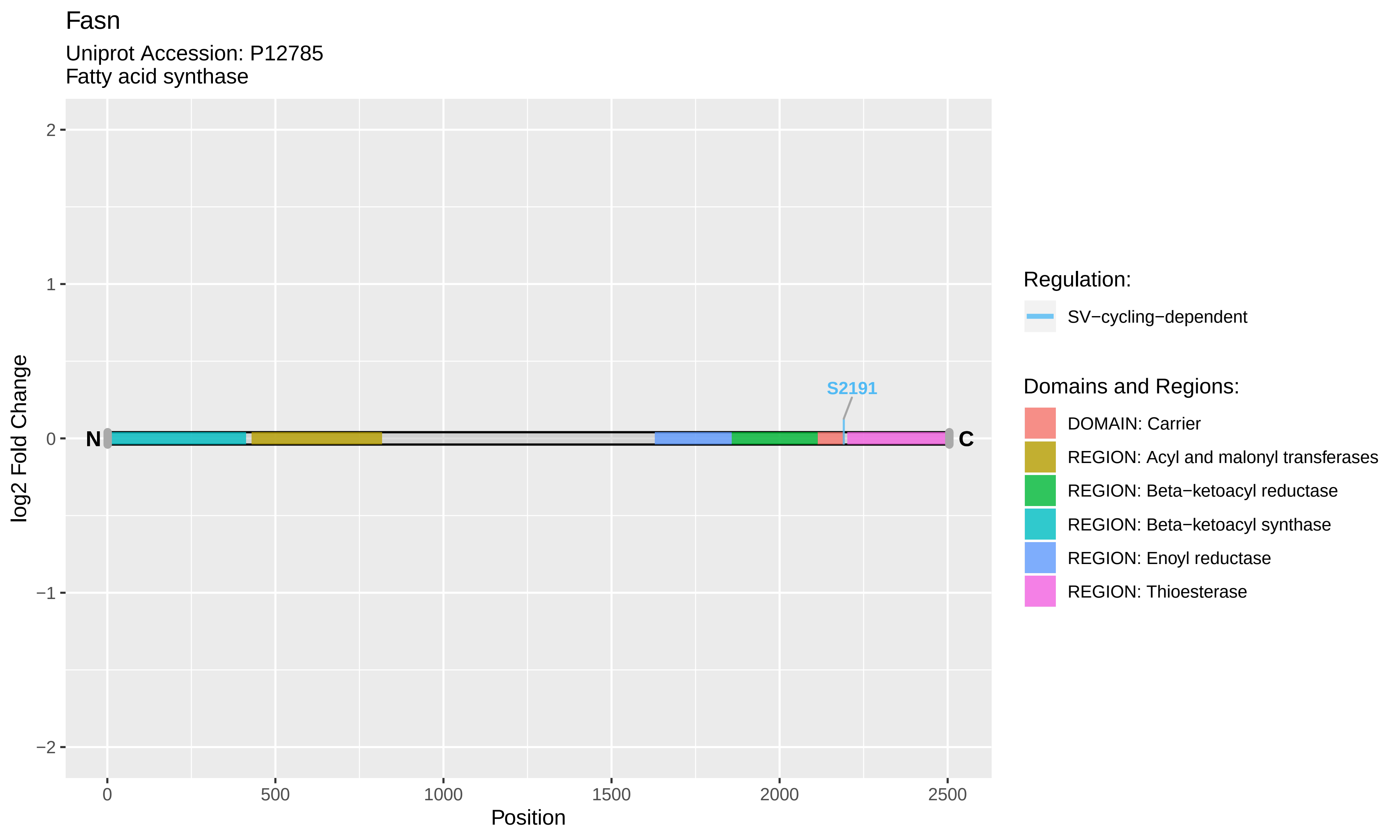
Fasn
Uniprot Accession: P12785
Fatty acid synthase
log2 Fold Change
2
1
0
−1
−2
N
C
S2191
0
500
1000
1500
2000
2500
Position
Regulation:
SV−cycling−dependent
Domains and Regions:
DOMAIN: Carrier
REGION: Acyl and malonyl transferases
REGION: Beta−ketoacyl reductase
REGION: Beta−ketoacyl synthase
REGION: Enoyl reductase
REGION: Thioesterase

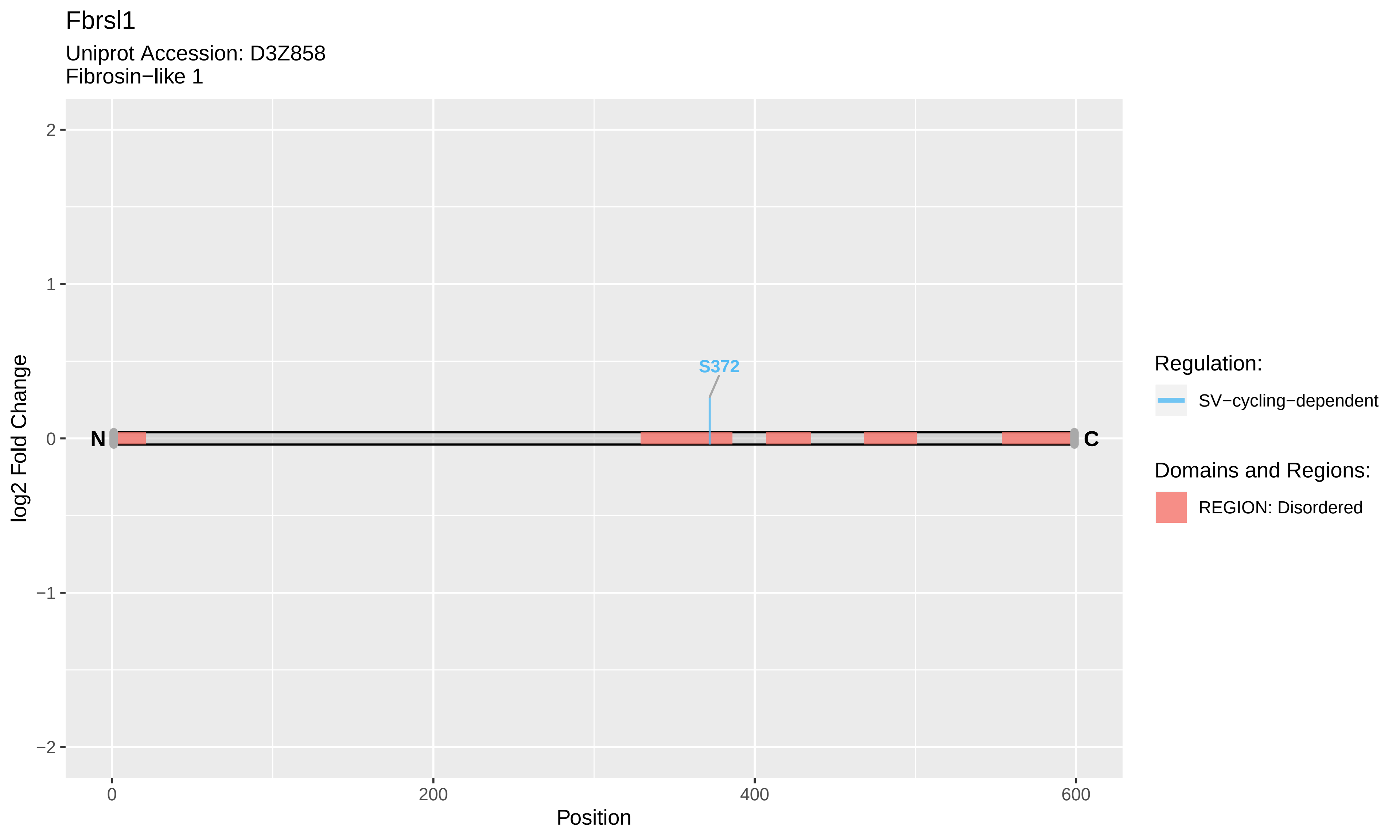

Fbrsl1
Uniprot Accession: D3Z858
Fibrosin−like 1
S372
N
C
2
1
0
−1
−2
0
200
400
600
Position
log2 Fold Change
Regulation:
SV−cycling−dependent
Domains and Regions:
REGION: Disordered

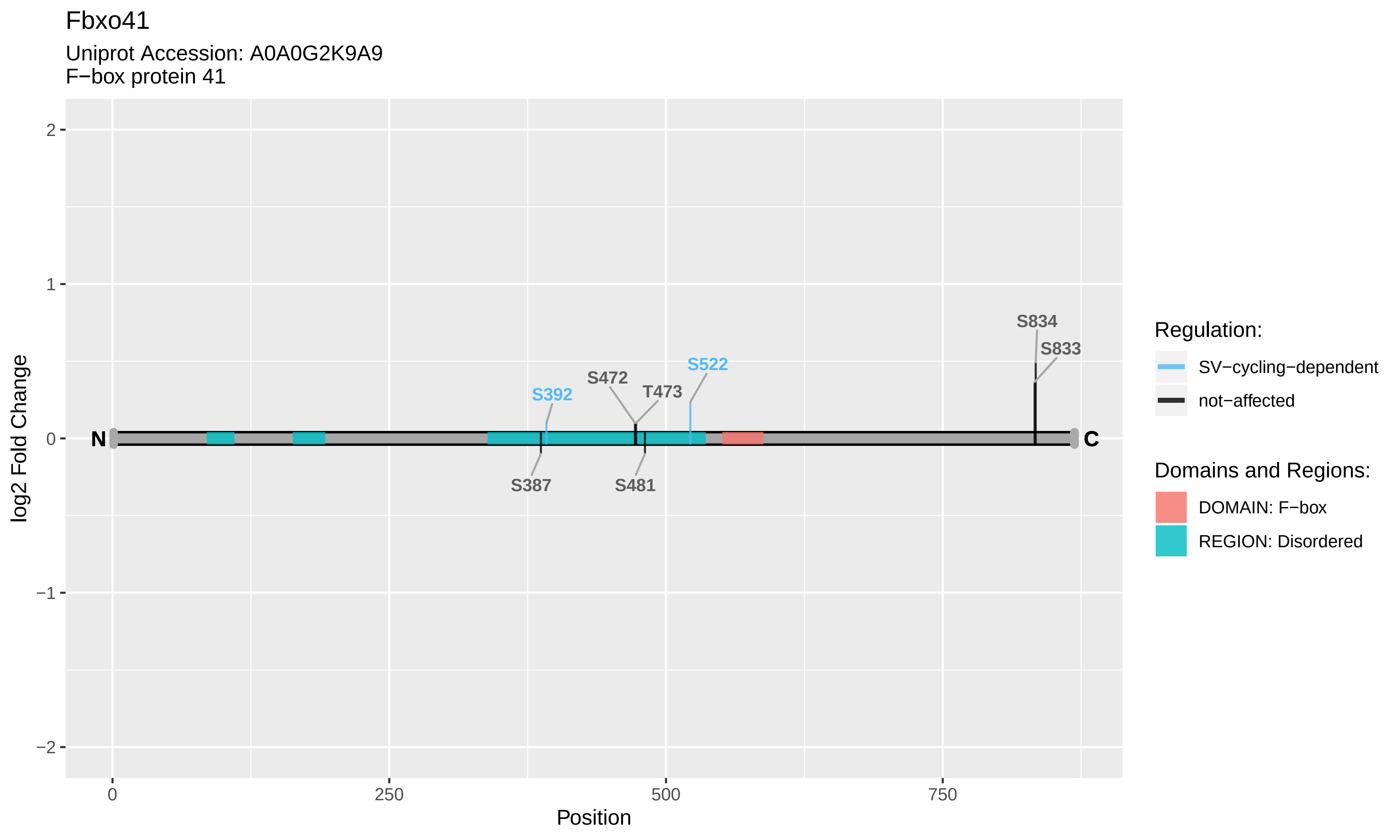
Fbxo41
Uniprot Accession: A0A0G2K9A9
F−box protein 41
log2 Fold Change
Position
N
C
S392
S387
S472
T473
S481
S522
S834
S833
Regulation:
SV−cycling−dependent
not−affected
Domains and Regions:
DOMAIN: F−box
REGION: Disordered

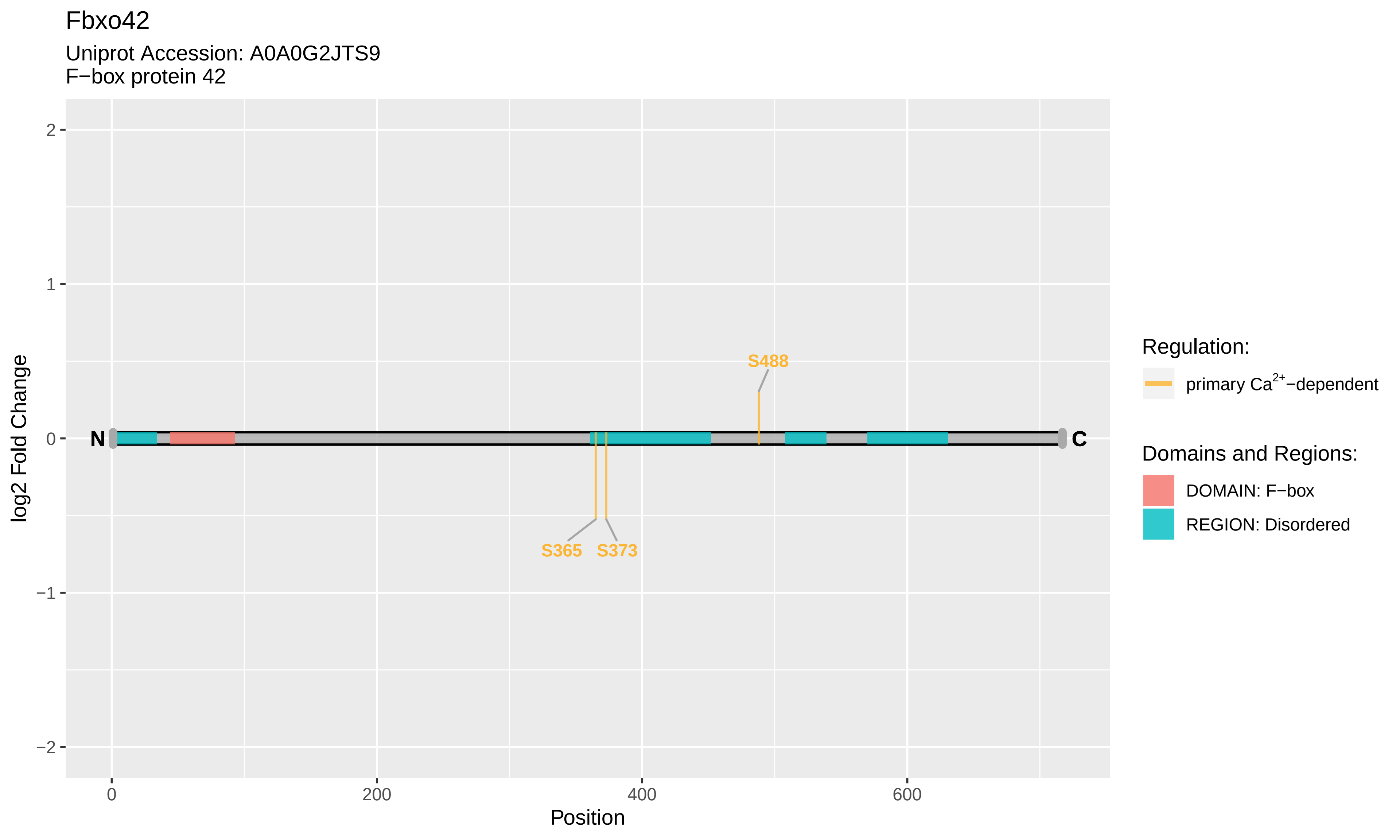
Fbxo42
Uniprot Accession: A0A0G2JTS9
F−box protein 42
S488
S365
S373
N
C
log2 Fold Change
Position
2
1
0
−1
−2
0
200
400
600
Regulation:
primary Ca2+−dependent
Domains and Regions:
DOMAIN: F−box
REGION: Disordered

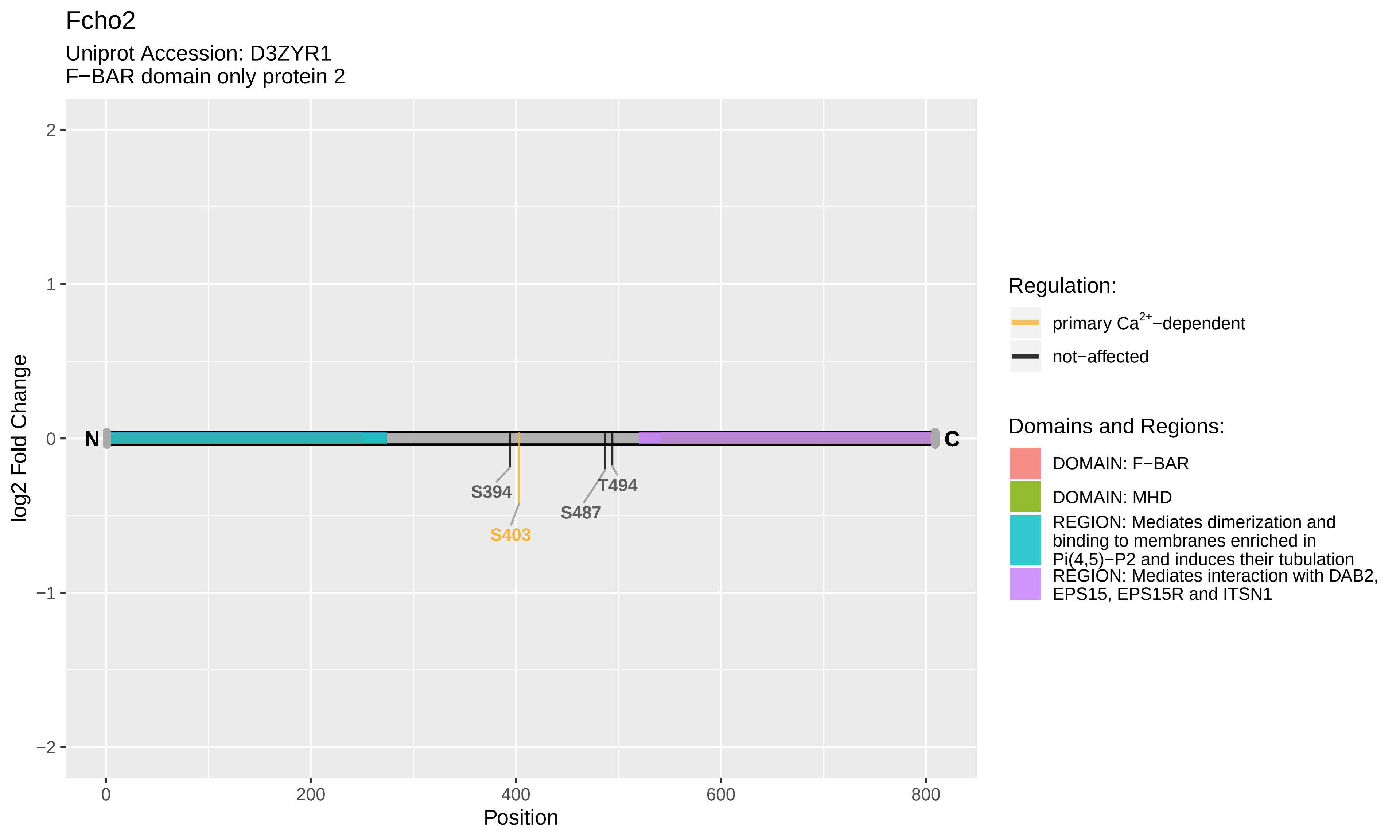

Fcho2
Uniprot Accession: D3ZYR1
F−BAR domain only protein 2
log2 Fold Change
2
1
0
−1
−2
N
C
S394
S403
S487
T494
0
200
400
600
800
Position
Regulation:
primary Ca2+−dependent
not−affected
Domains and Regions:
DOMAIN: F−BAR
DOMAIN: MHD
REGION: Mediates dimerization and binding to membranes enriched in Pi(4,5)−P2 and induces their tubulation
REGION: Mediates interaction with DAB2, EPS15, EPS15R and ITSN1

Fgf14

Uniprot Accession: Q8R5L7
Fibroblast growth factor 14

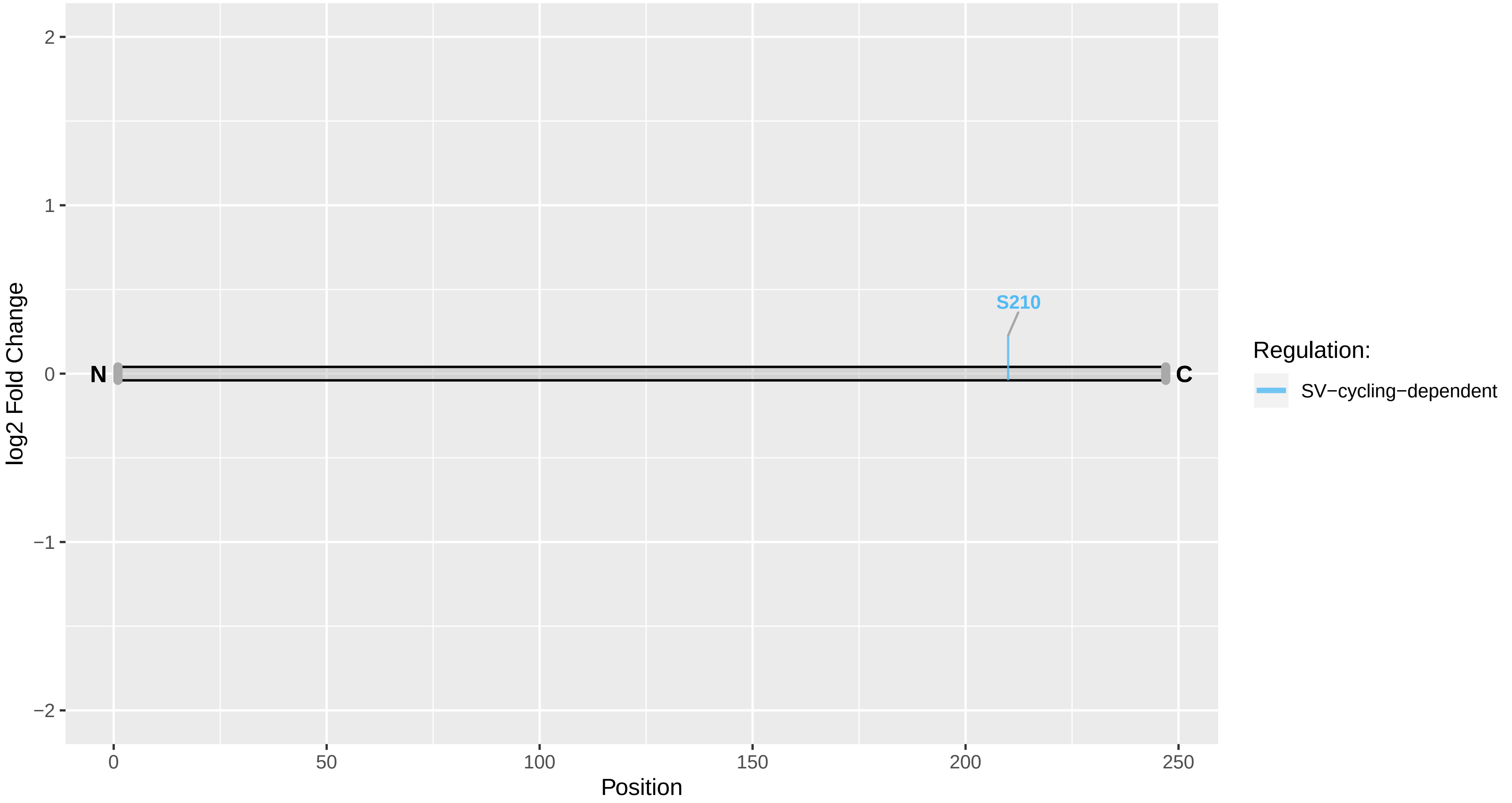

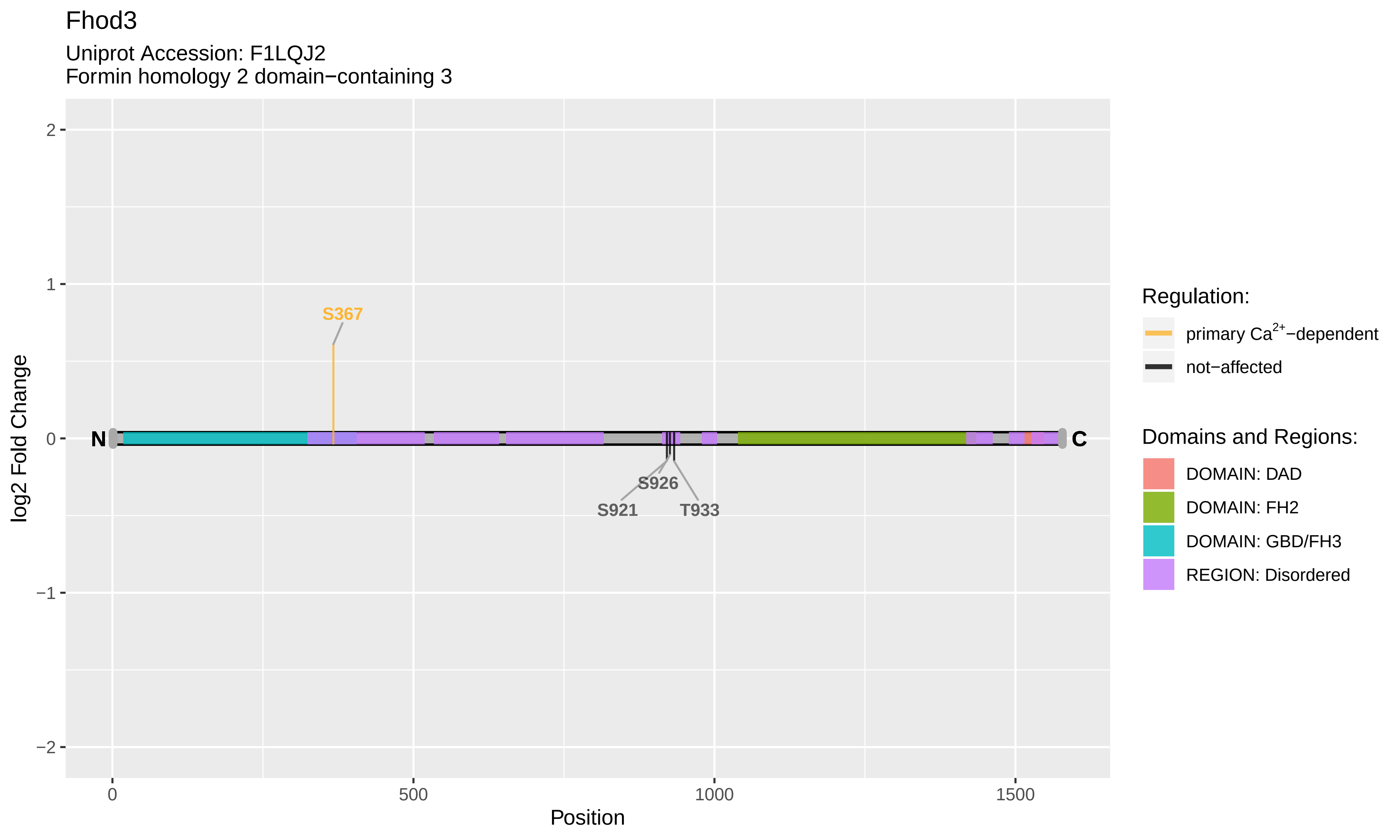
Fhod3
Uniprot Accession: F1LQJ2
Formin homology 2 domain−containing 3
log2 Fold Change
Position
N
C
S367
S921
S926
T933
Regulation:
primary Ca2+−dependent
not−affected
Domains and Regions:
DOMAIN: DAD
DOMAIN: FH2
DOMAIN: GBD/FH3
REGION: Disordered
2
1
0
−1
−2
0
500
1000
1500

Flii

Uniprot Accession: Q5RKI5
FLII, actin−remodeling protein

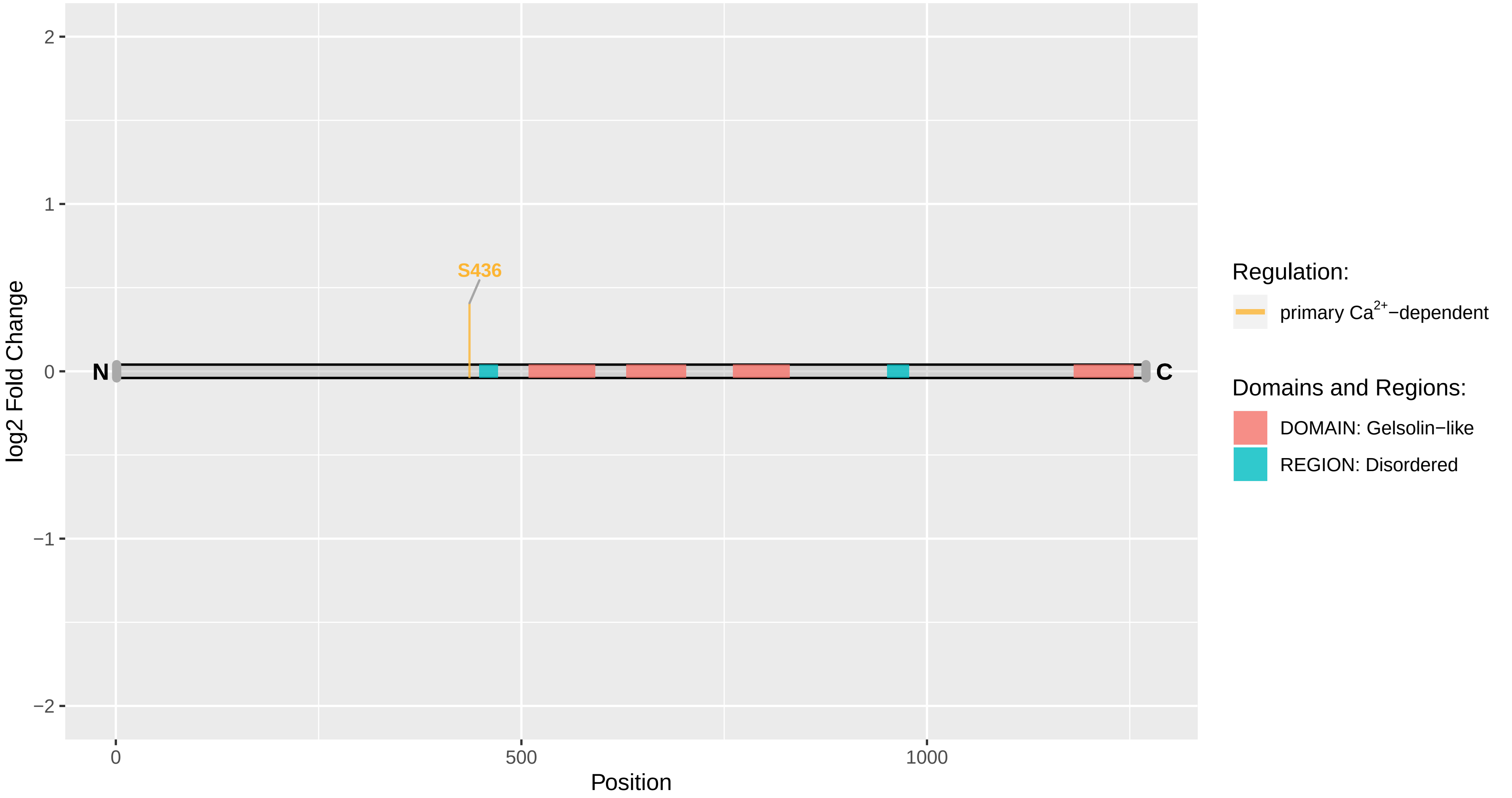

Frs2

Uniprot Accession: A0A0G2JZZ5
Fibroblast growth factor receptor substrate 2

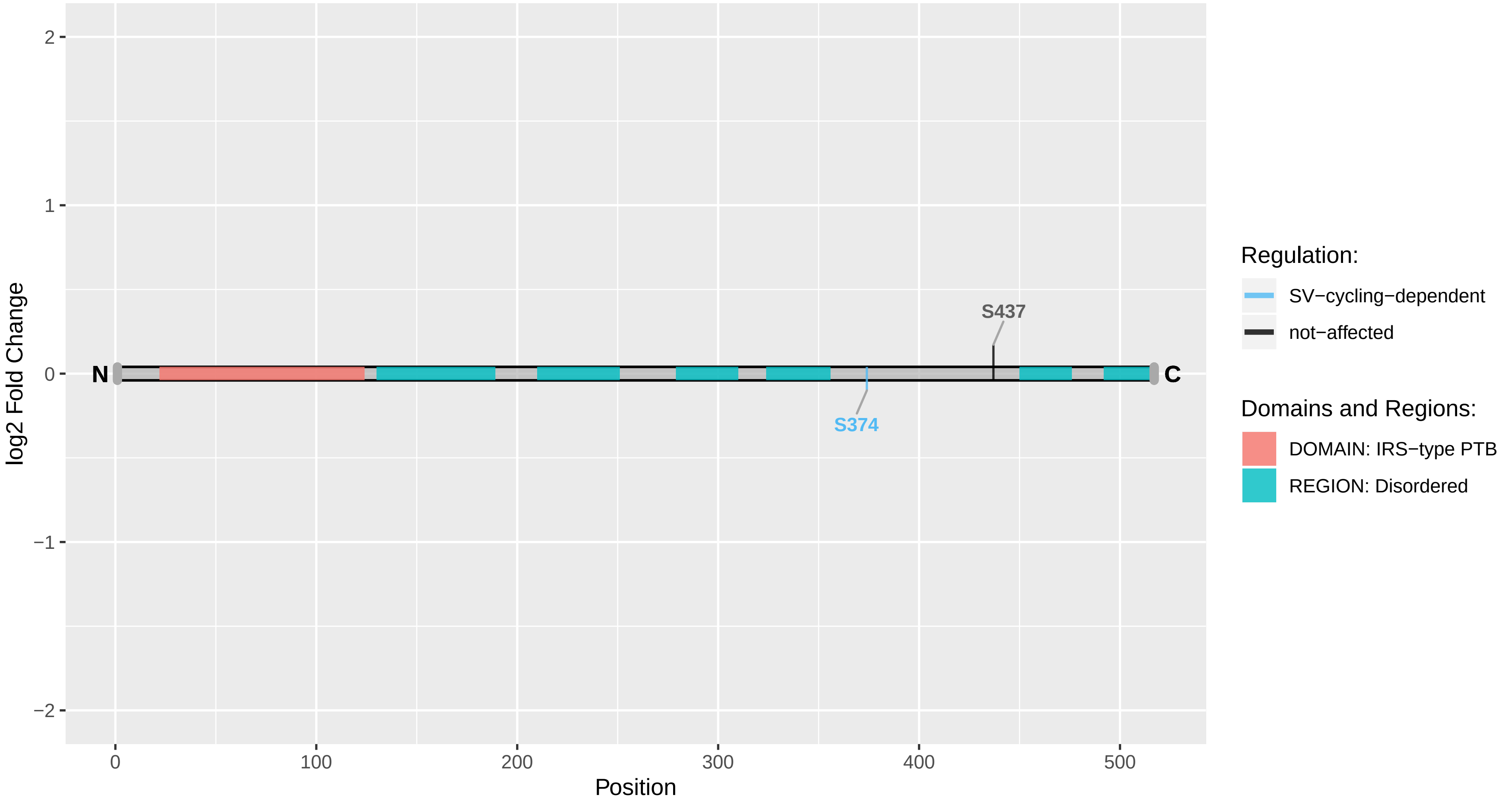

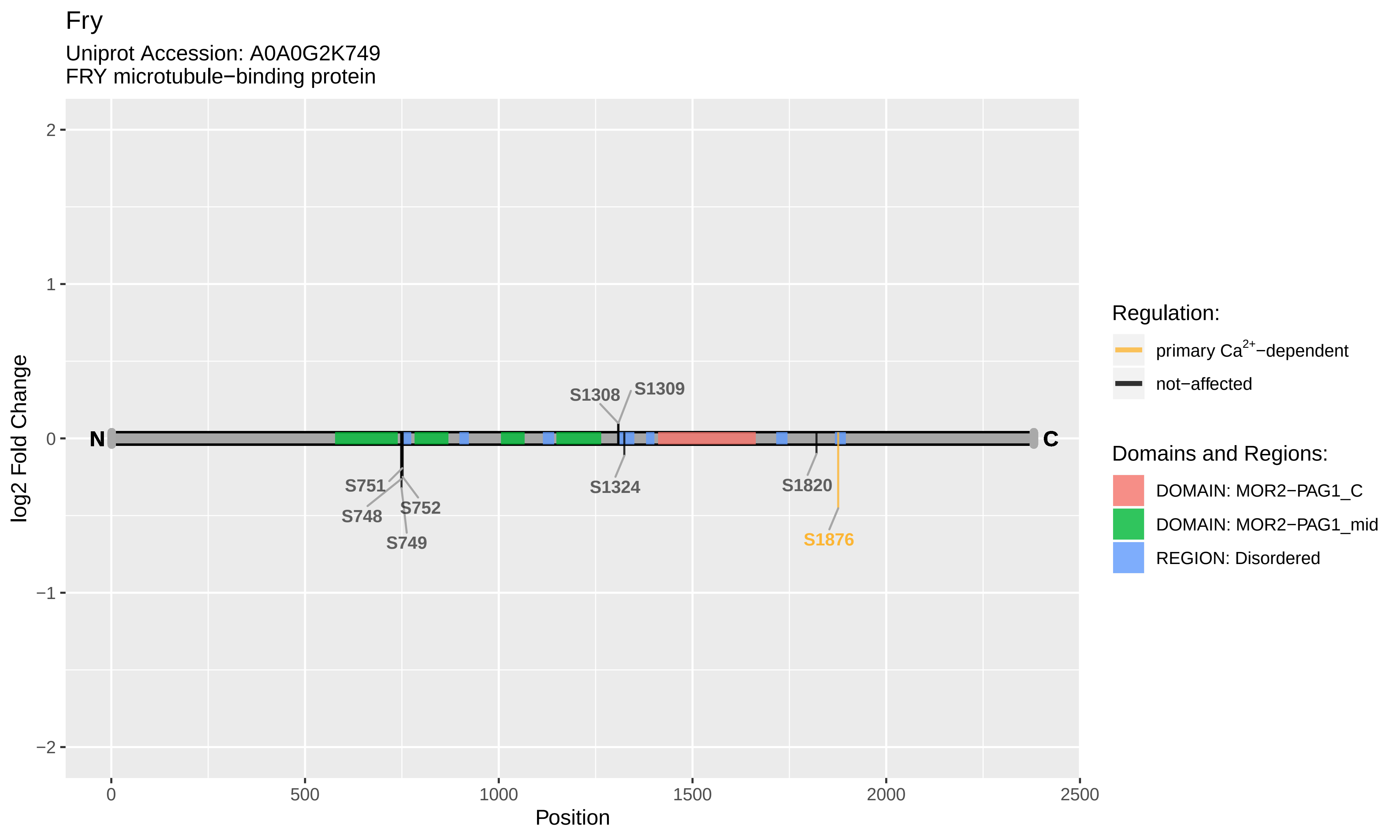

Fry
Uniprot Accession: A0A0G2K749
FRY microtubule−binding protein
log2 Fold Change
Position
N
C
S1308
S1309
S751
S748
S752
S749
S1324
S1820
S1876
Regulation:
primary Ca2+−dependent
not−affected
Domains and Regions:
DOMAIN: MOR2−PAG1_C
DOMAIN: MOR2−PAG1_mid
REGION: Disordered

Fscn1

Uniprot Accession: P85845
Fascin

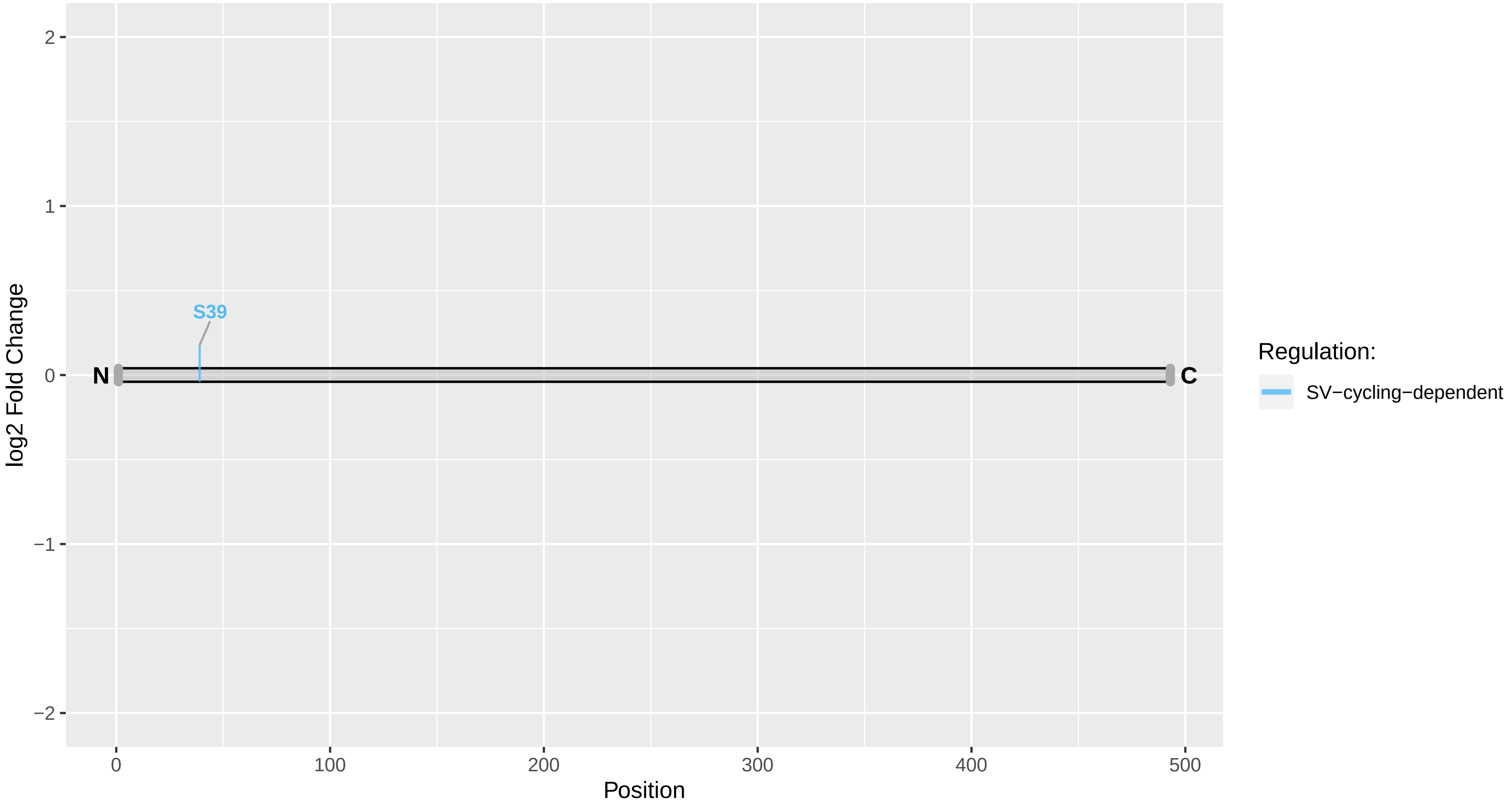

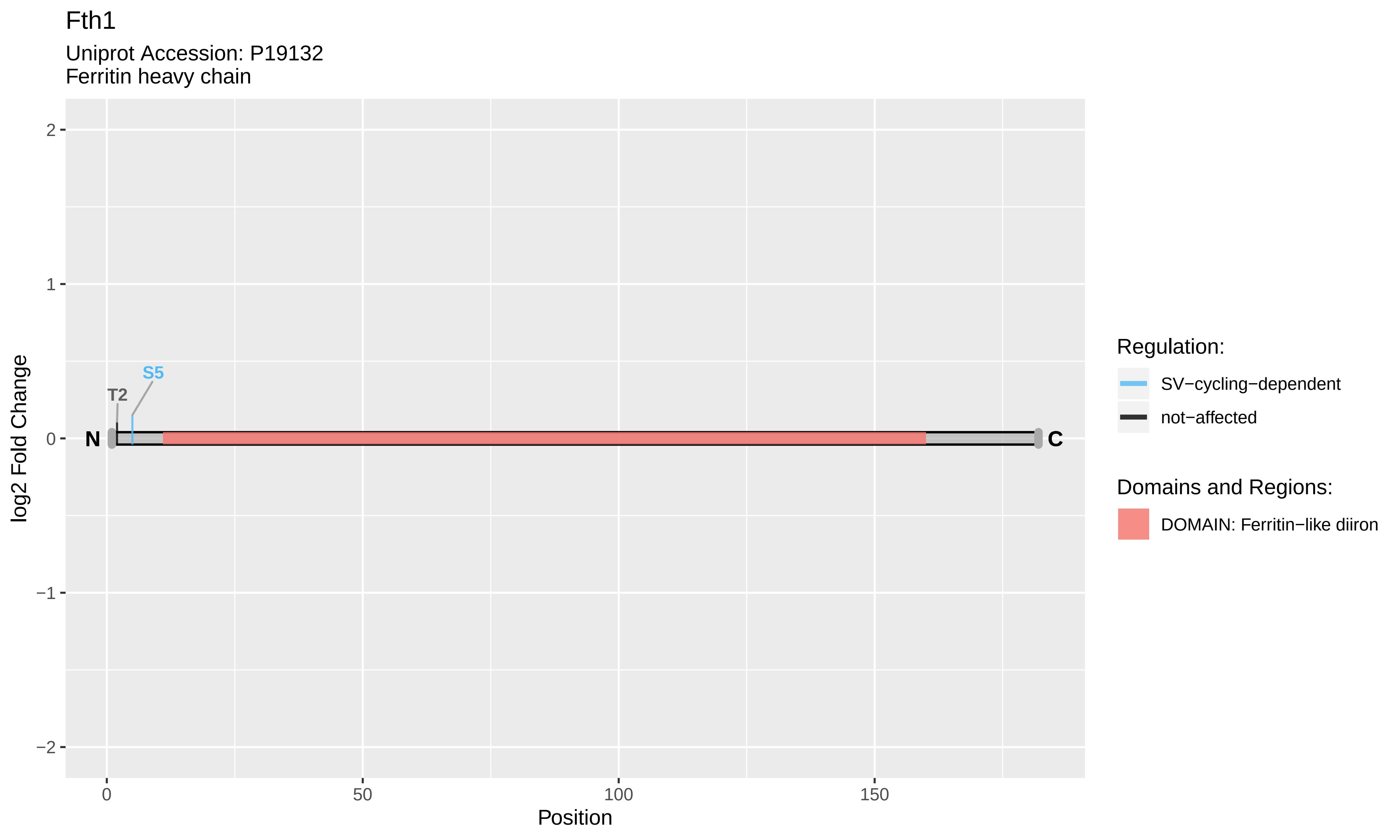

Fth1
Uniprot Accession: P19132
Ferritin heavy chain
log2 Fold Change
2
1
0
−1
−2
N
C
T2
S5
0
50
100
150
Position
Regulation:
SV−cycling−dependent
not−affected
Domains and Regions:
DOMAIN: Ferritin−like diiron

G3bp2

Uniprot Accession: Q6AY21
G3BP stress granule assembly factor 2

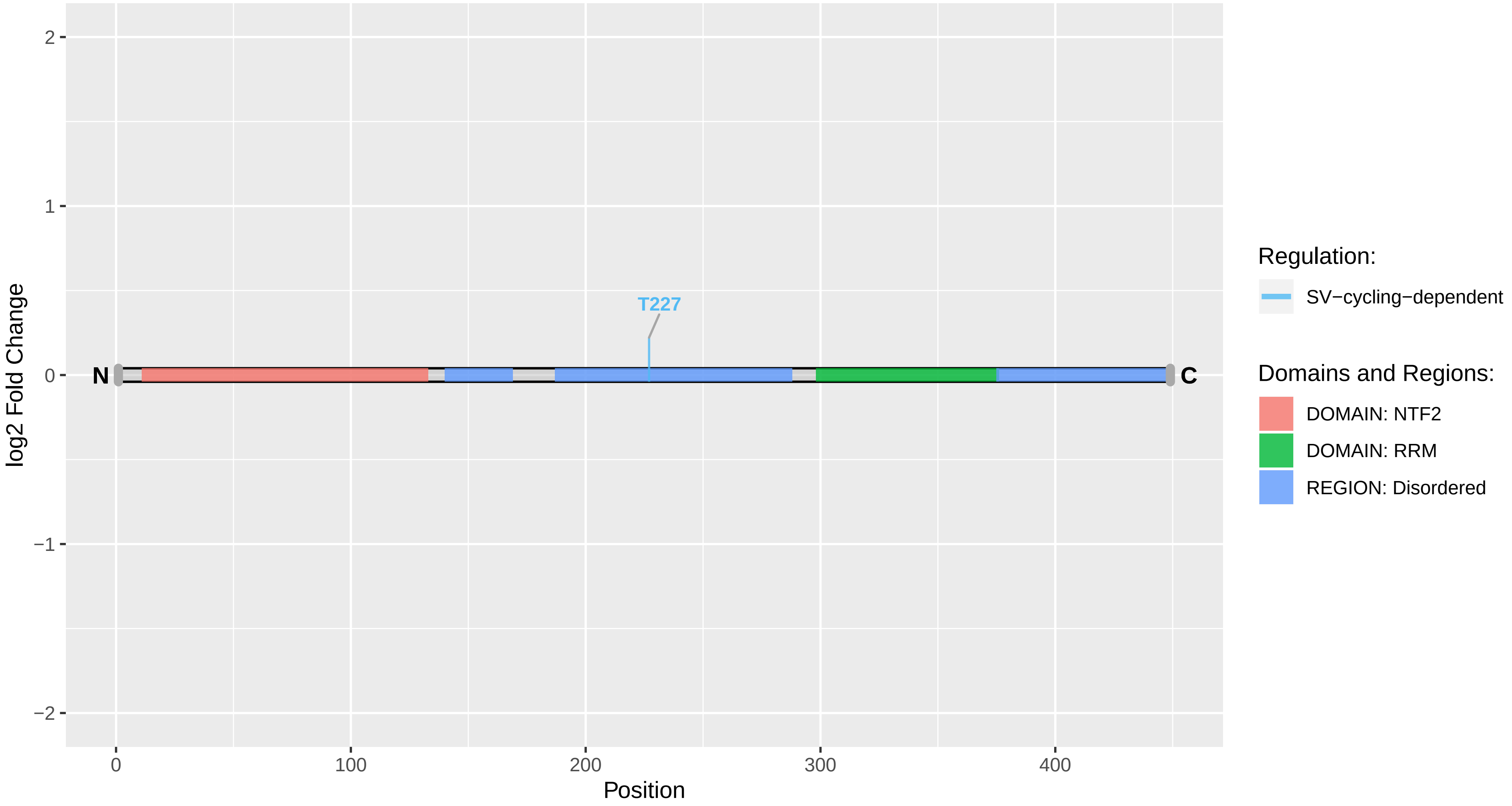

Gabbr2

Uniprot Accession: O88871
Gamma−aminobutyric acid type B receptor subunit 2

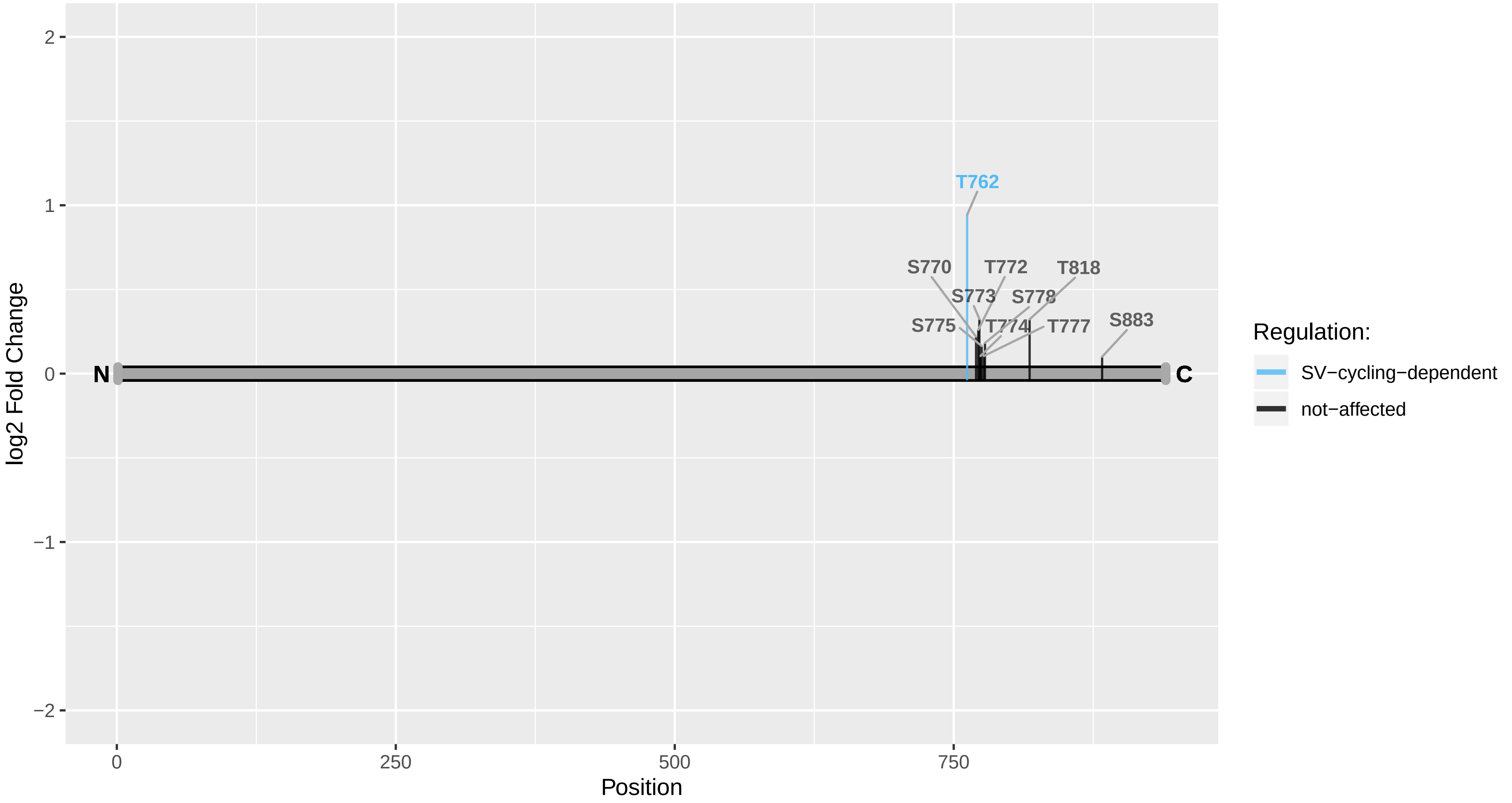

Gad1

Uniprot Accession: P18088
Glutamate decarboxylase 1

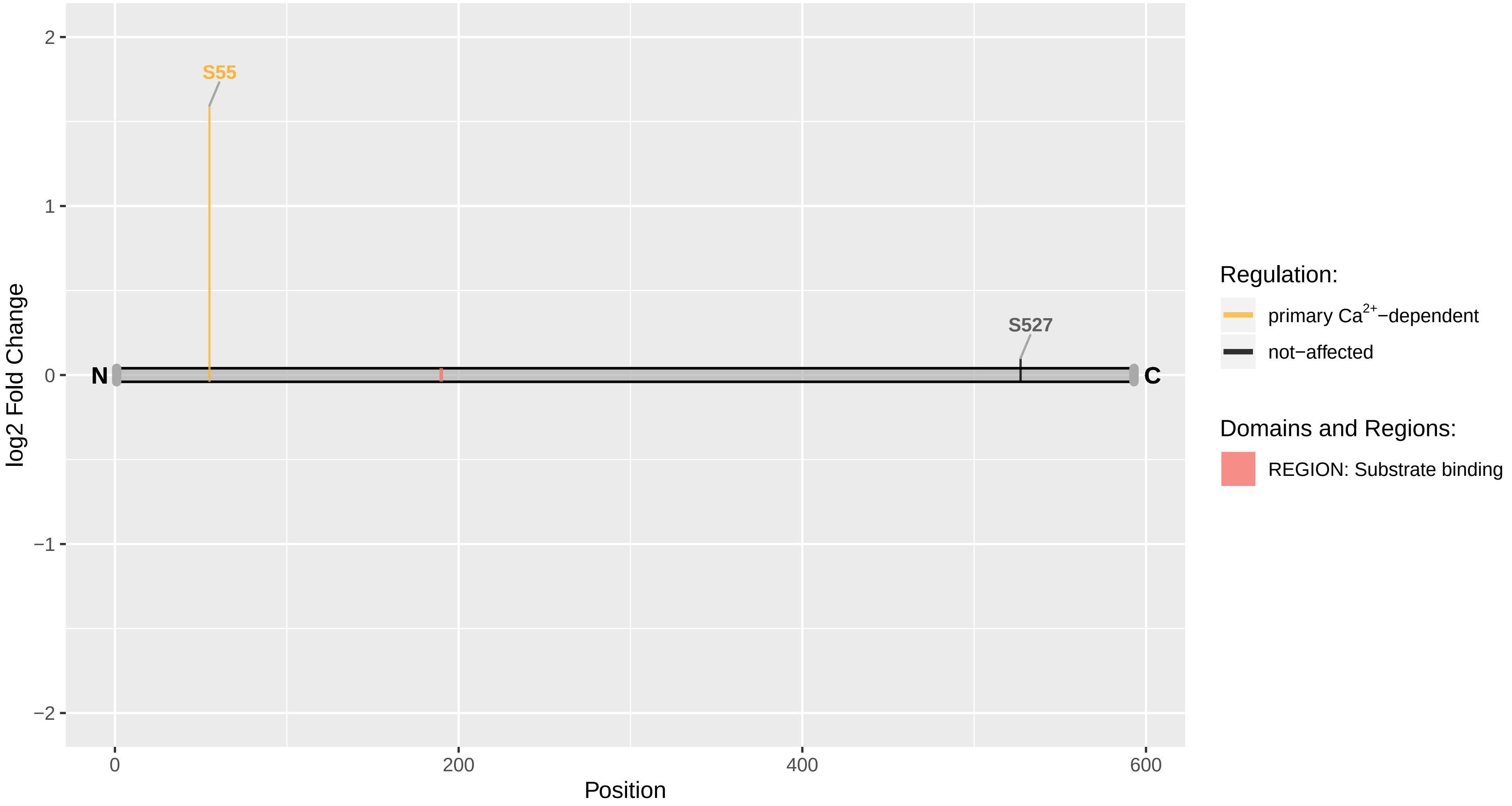

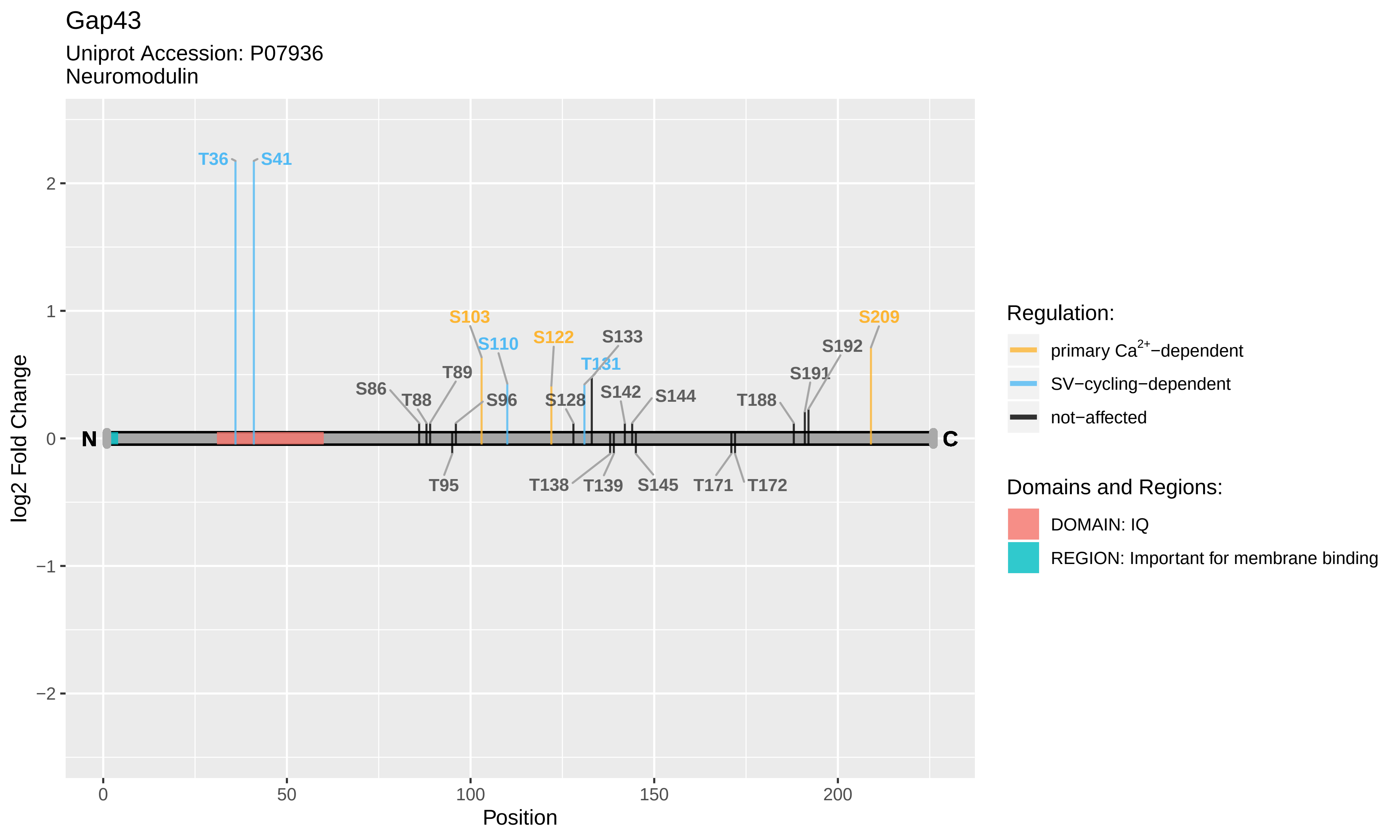
Gap43
Uniprot Accession: P07936
Neuromodulin
T36
S41
S103
S110
S122
S133
T131
T89
S86
T88
S96
S128
S142
S144
T188
S191
S192
S209
N
C
T95
T138
T139
S145
T171
T172
log2 Fold Change
Position
Regulation:
primary Ca^{2+}−dependent
SV−cycling−dependent
not−affected
Domains and Regions:
DOMAIN: IQ
REGION: Important for membrane binding

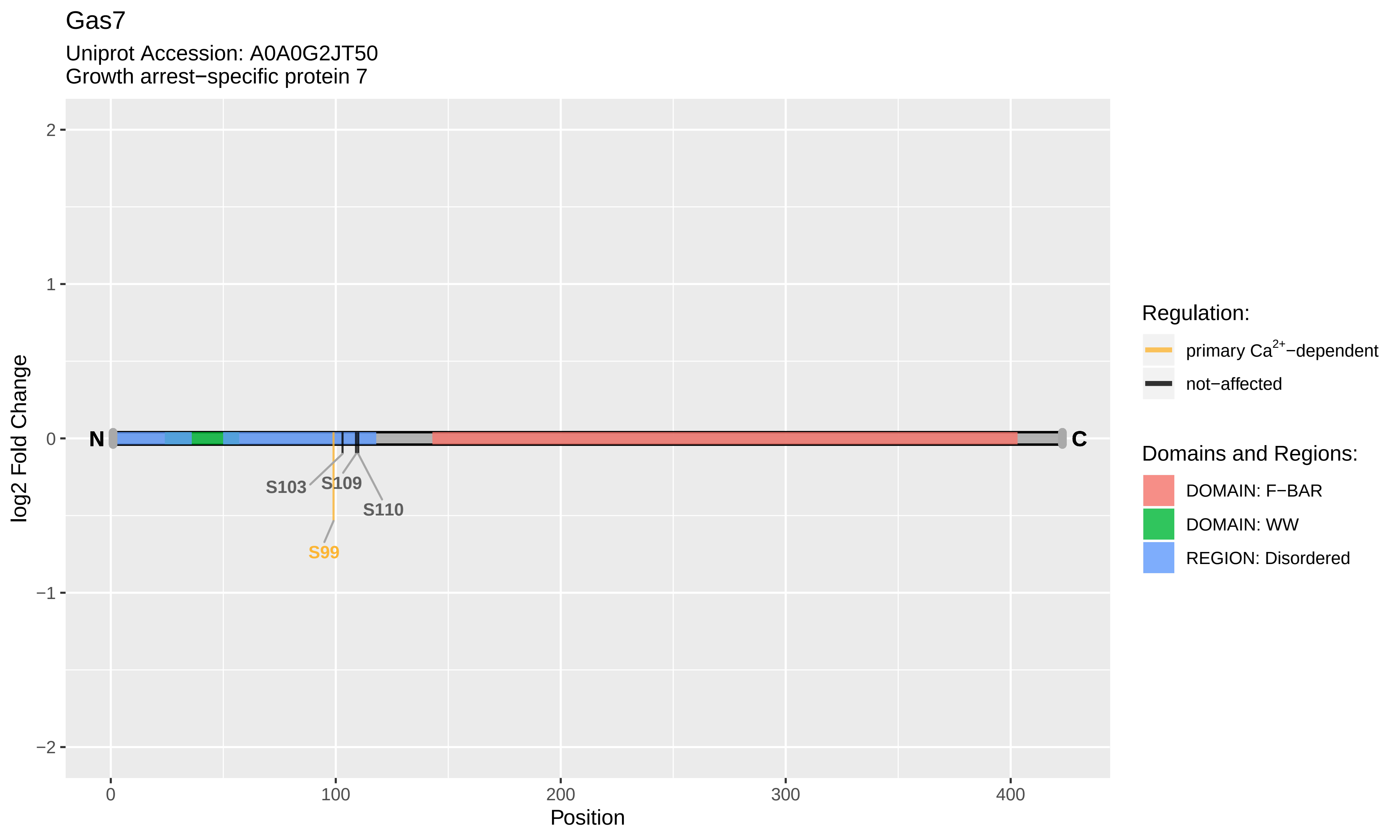
Gas7
Uniprot Accession: A0A0G2JT50
Growth arrest−specific protein 7
log2 Fold Change
Position
N
C
S103
S109
S110
S99
2
1
0
−1
−2
0
100
200
300
400
Regulation:
primary Ca2+−dependent
not−affected
Domains and Regions:
DOMAIN: F−BAR
DOMAIN: WW
REGION: Disordered

Gipc1

Uniprot Accession: Q9Z254
PDZ domain−containing protein GIPC1

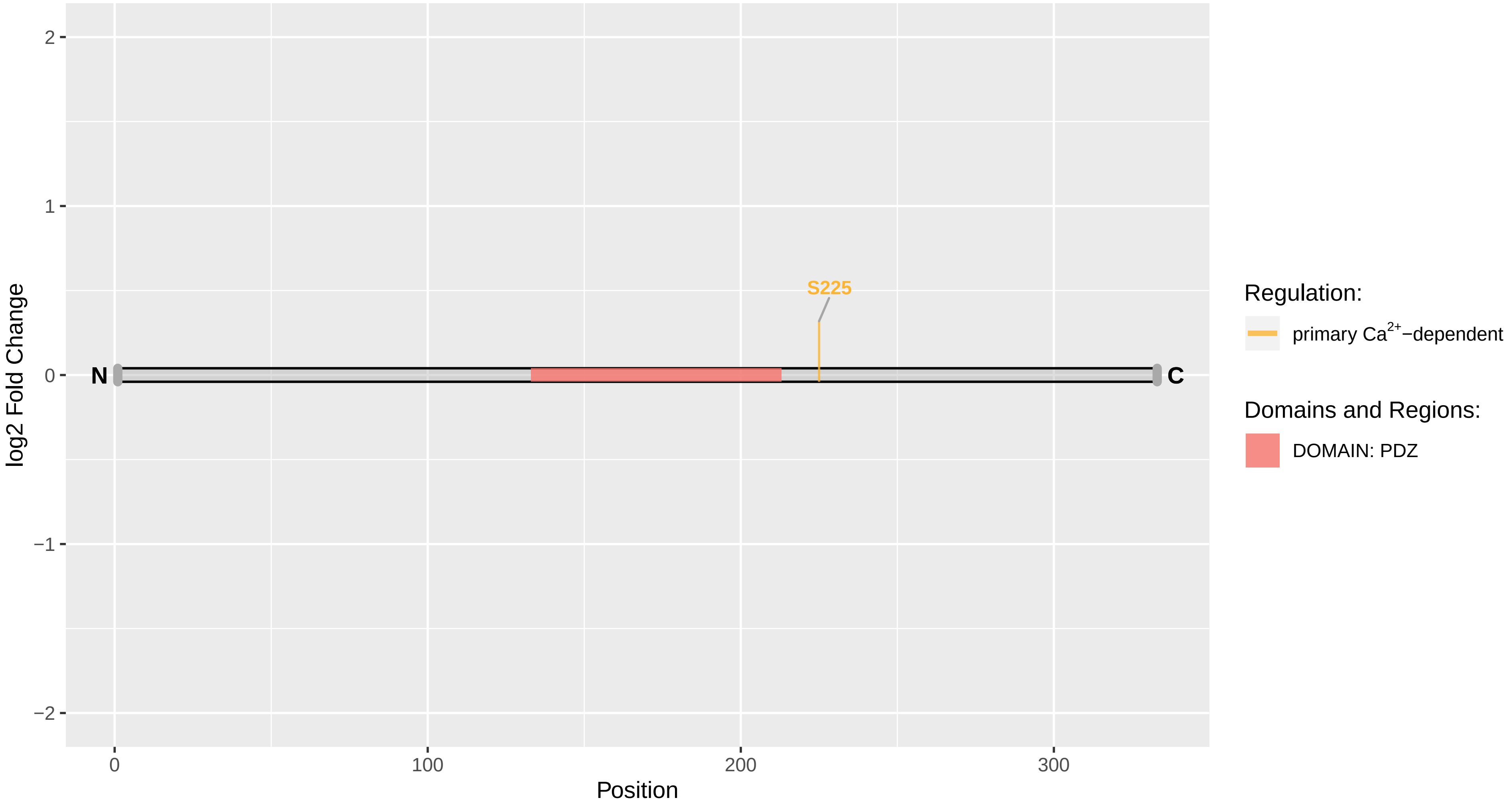

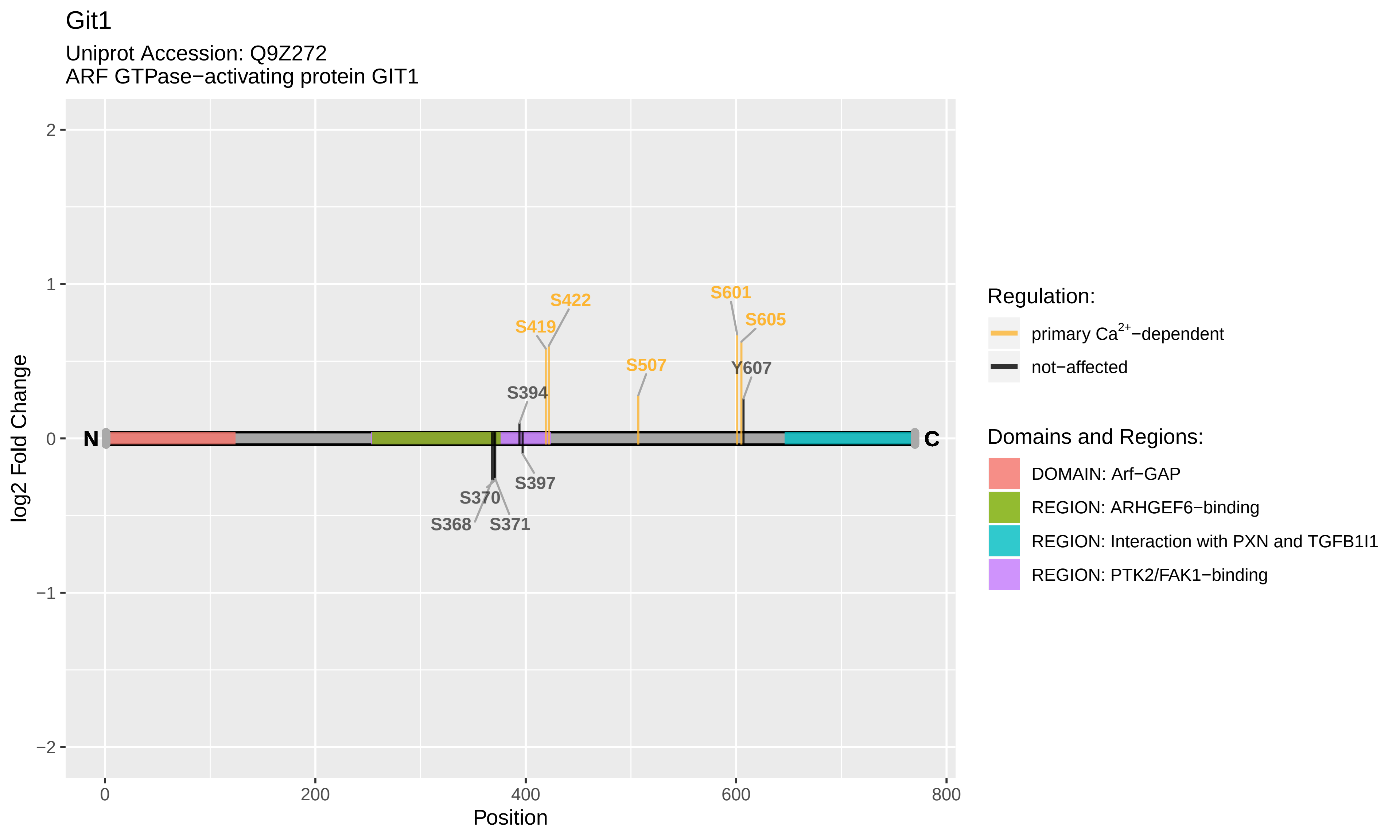

Git1
Uniprot Accession: Q9Z272
ARF GTPase−activating protein GIT1
log2 Fold Change
Position
N
C
S422
S419
S601
S605
S507
Y607
S394
S397
S370
S368
S371
Regulation:
primary Ca2+−dependent
not−affected
Domains and Regions:
DOMAIN: Arf−GAP
REGION: ARHGEF6−binding
REGION: Interaction with PXN and TGFB1I1
REGION: PTK2/FAK1−binding

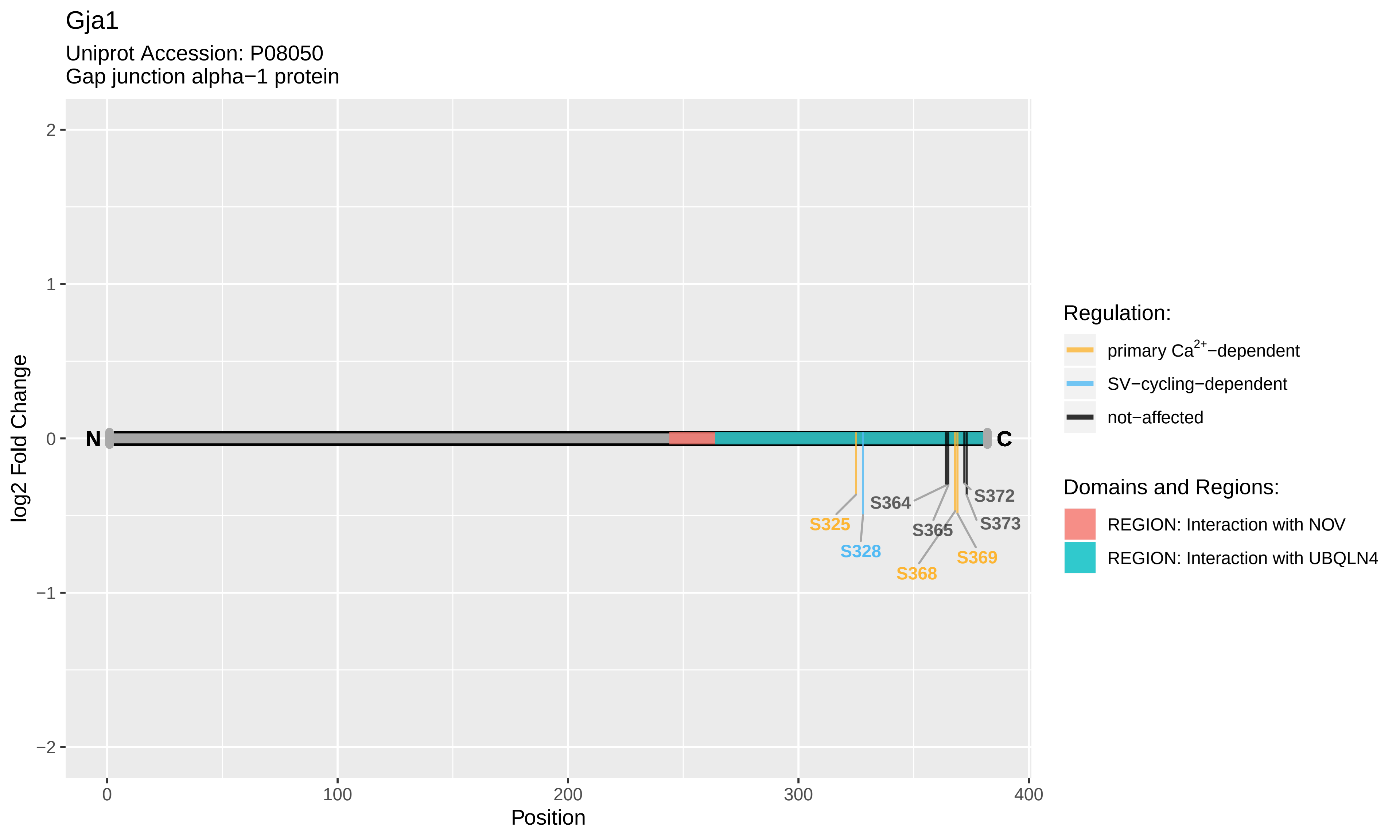

Gja1
Uniprot Accession: P08050
Gap junction alpha−1 protein
log2 Fold Change
2
1
0
−1
−2
N
C
S325
S328
S364
S365
S368
S369
S372
S373
0
100
200
300
400
Position
Regulation:
primary Ca2+−dependent
SV−cycling−dependent
not−affected
Domains and Regions:
REGION: Interaction with NOV
REGION: Interaction with UBQLN4

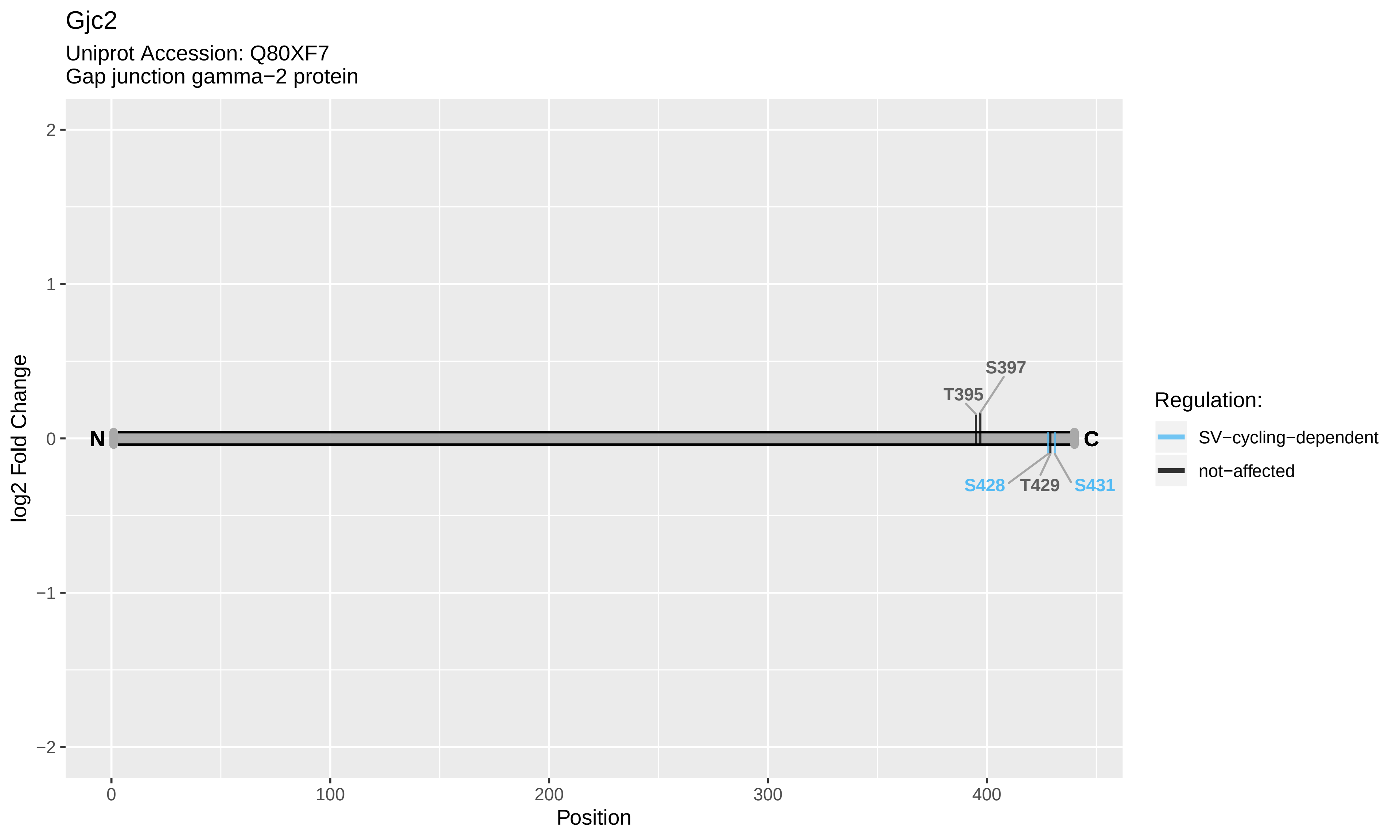

Gjc2
Uniprot Accession: Q80XF7
Gap junction gamma−2 protein
log2 Fold Change
2
1
0
−1
−2
N
C
T395
S397
S428
T429
S431
0
100
200
300
400
Position
Regulation:
SV−cycling−dependent
not−affected

Glrx5

Uniprot Accession: D4ADD7
Glutaredoxin 5

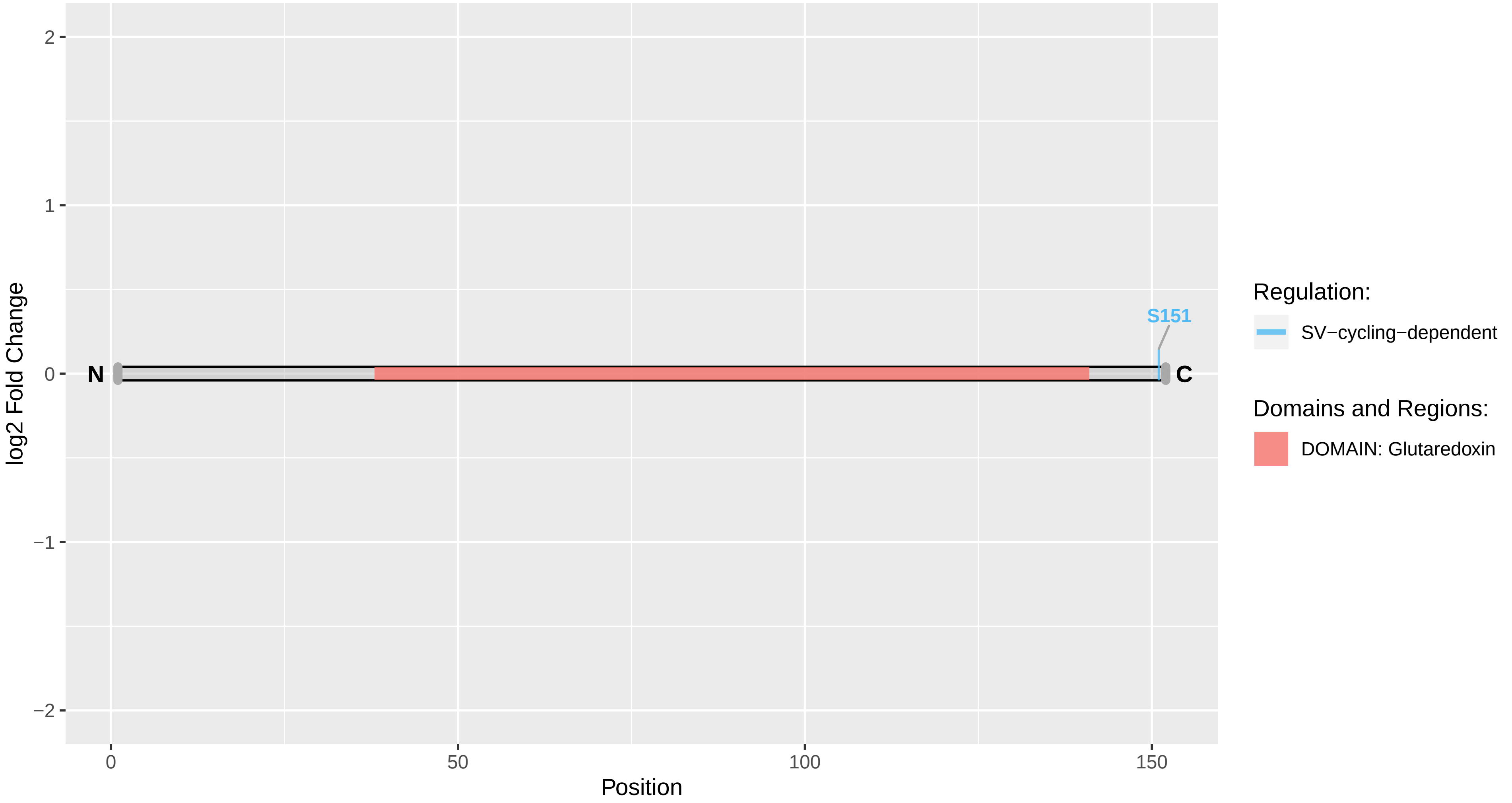

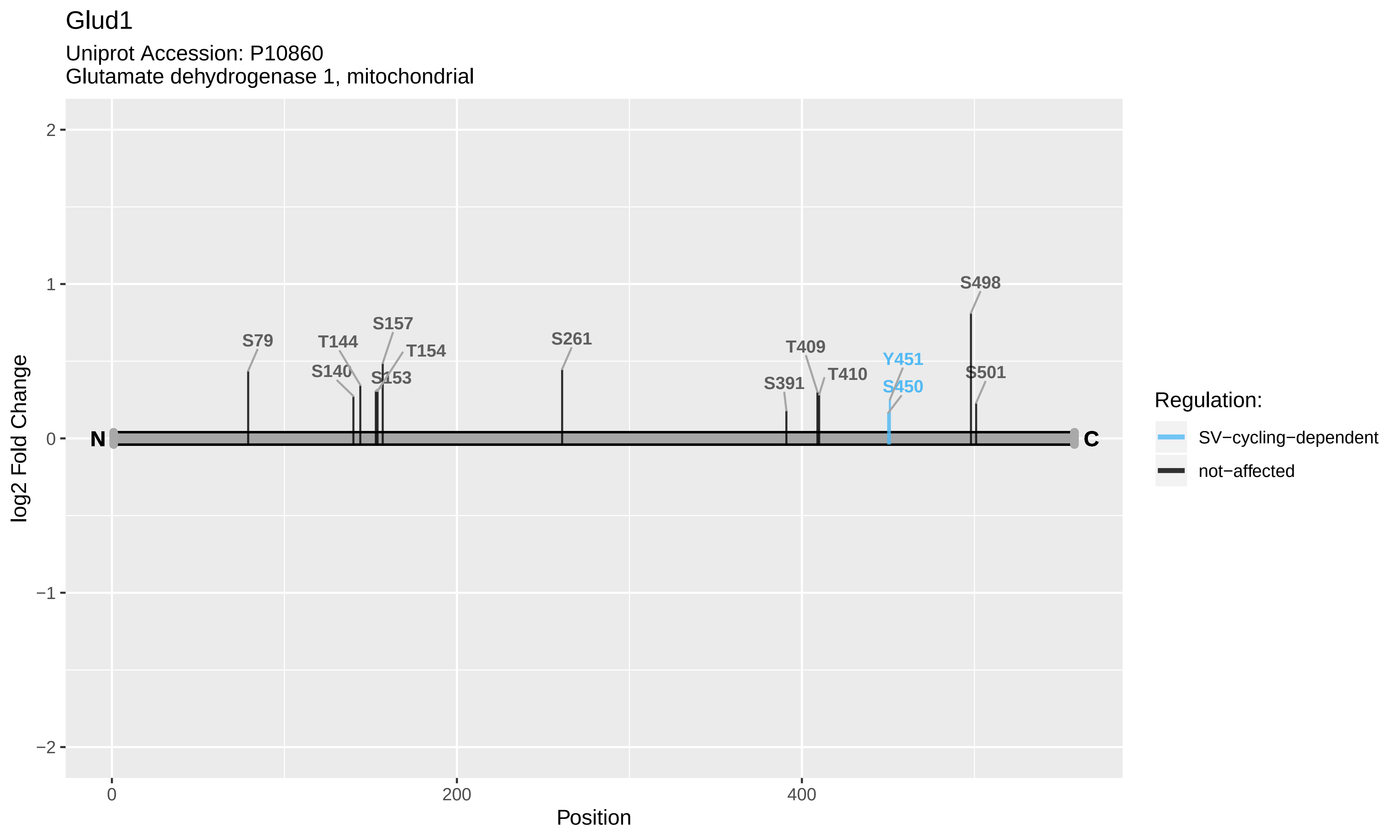

Glud1
Uniprot Accession: P10860
Glutamate dehydrogenase 1, mitochondrial
log2 Fold Change
Position
N
C
S79
T144
S140
S157
T154
S153
S261
T409
S391
T410
Y451
S450
S498
S501
Regulation:
SV−cycling−dependent
not−affected

Gnb1

Uniprot Accession: P54311
Guanine nucleotide−binding protein G(I)/G(S)/G(T) subunit beta−1

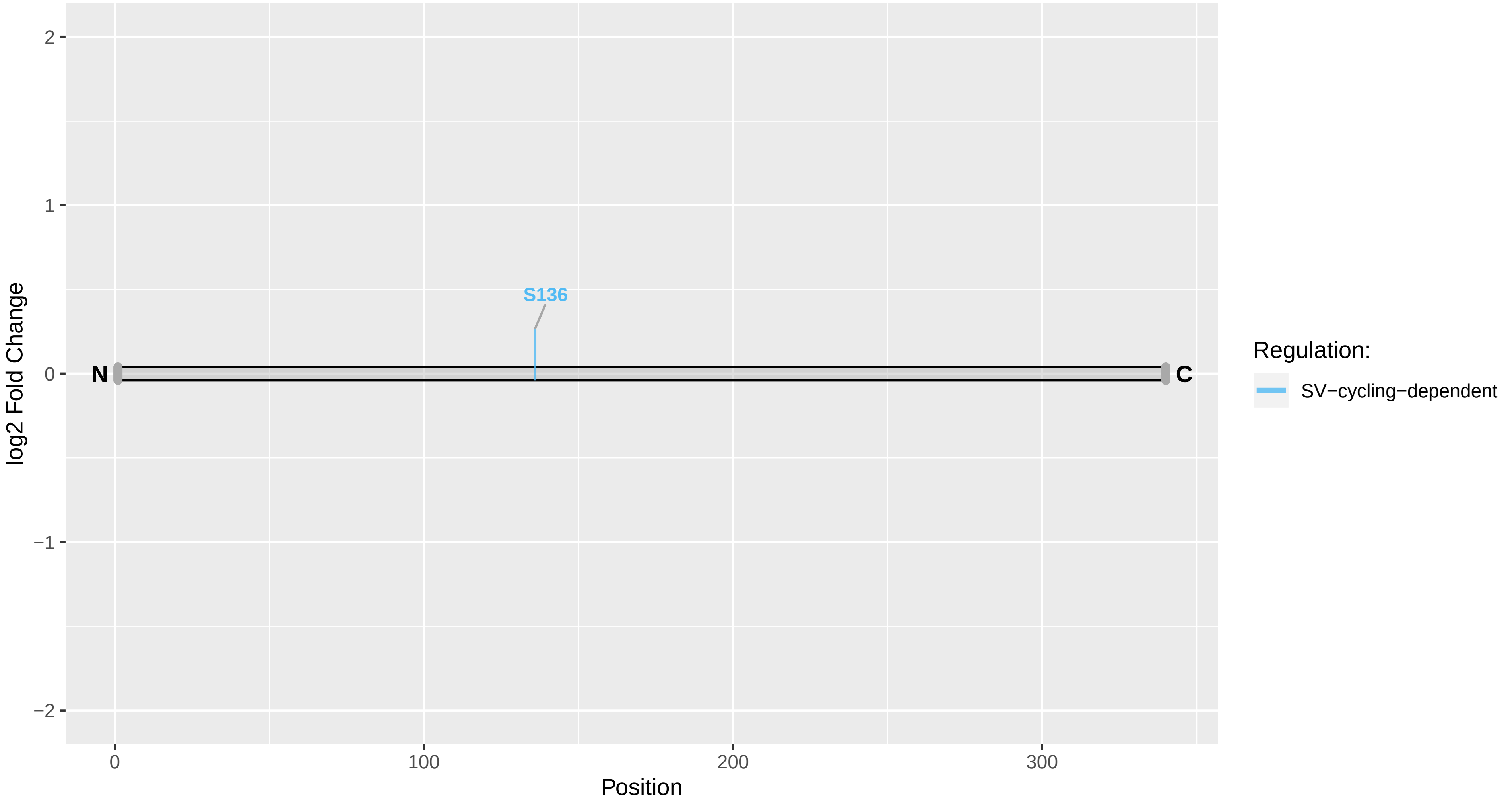

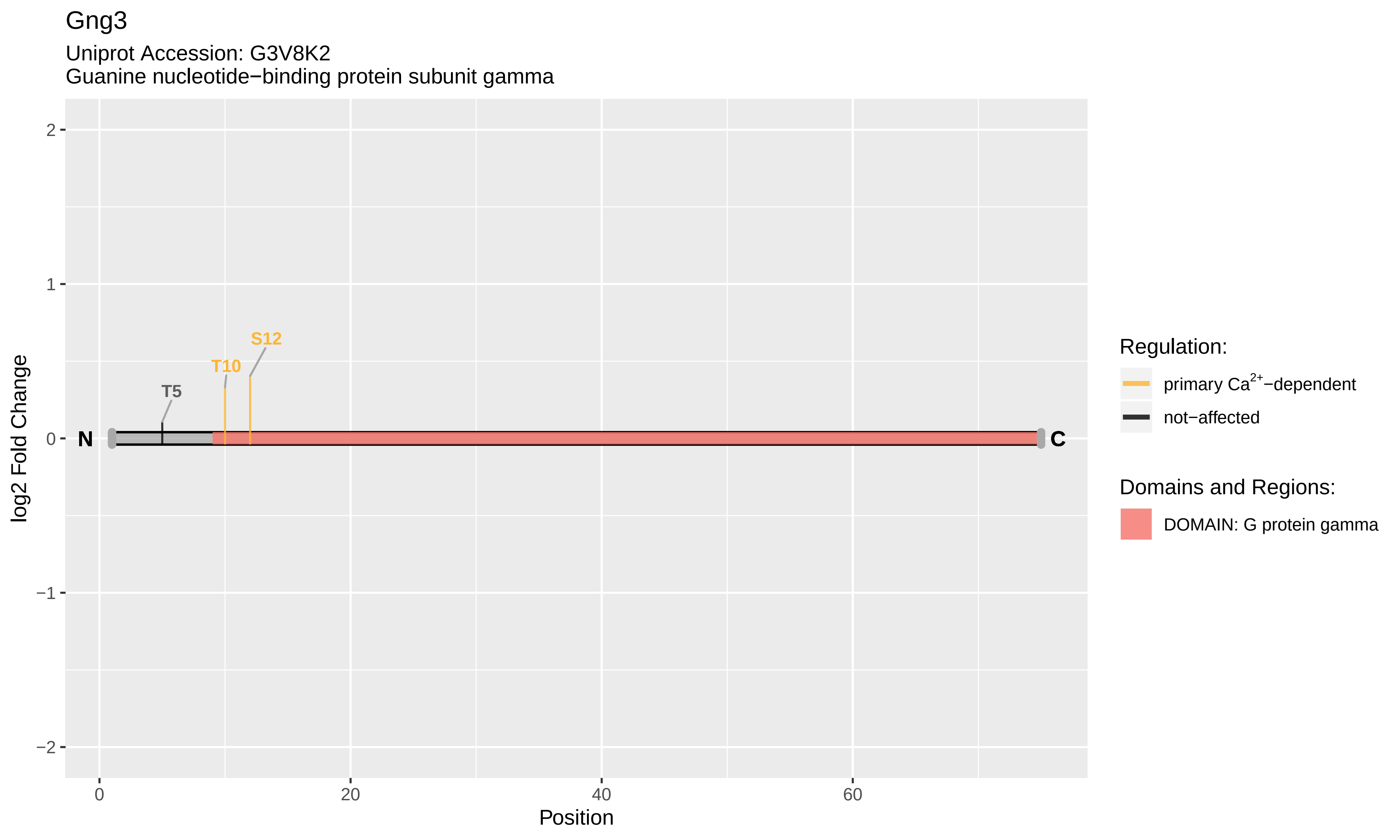
Gng3
Uniprot Accession: G3V8K2
Guanine nucleotide−binding protein subunit gamma
log2 Fold Change
Position
2
1
0
−1
−2
0
20
40
60
N
C
T5
T10
S12
Regulation:
primary Ca2+−dependent
not−affected
Domains and Regions:
DOMAIN: G protein gamma

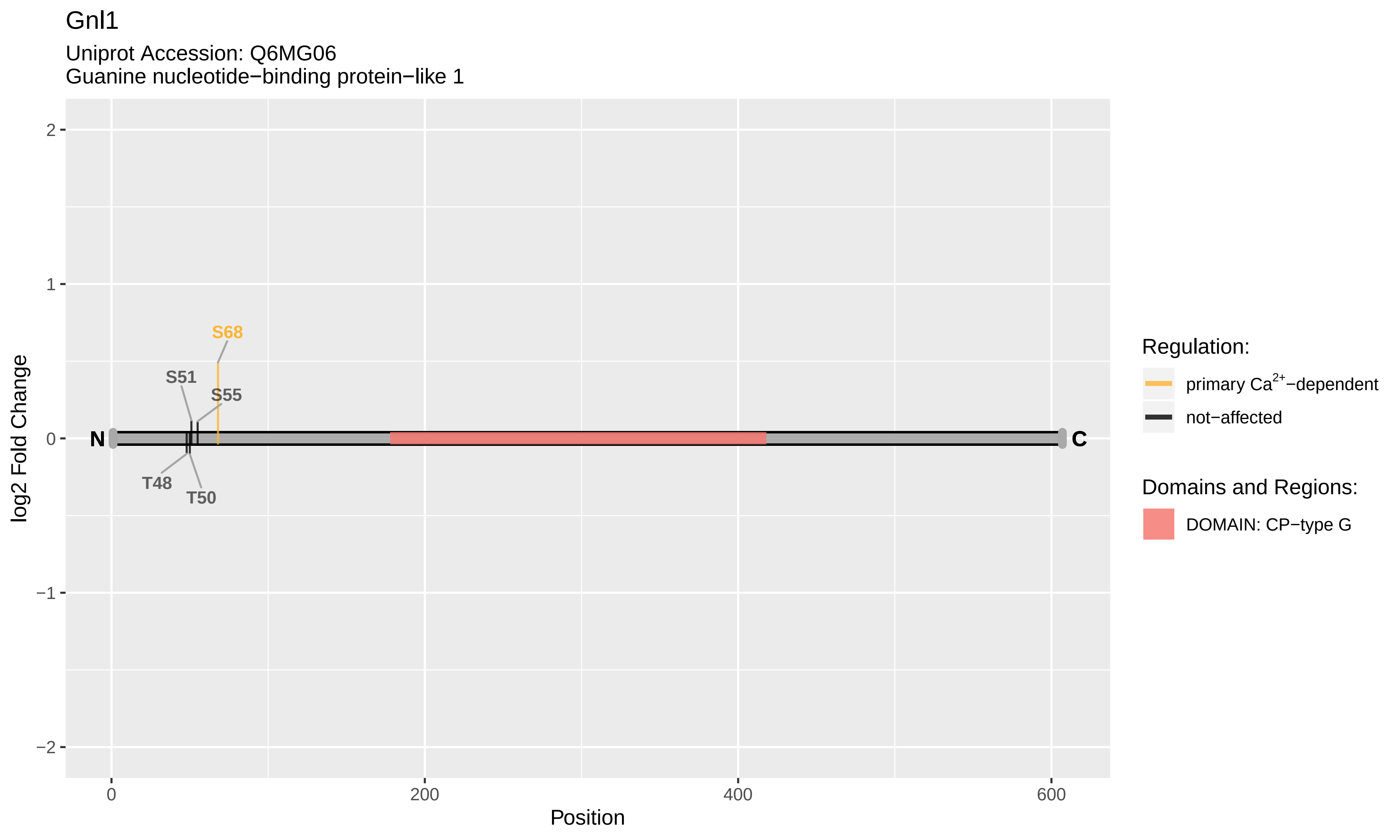

Gnl1
Uniprot Accession: Q6MG06
Guanine nucleotide−binding protein−like 1
S68
S51
S55
T48
T50
N
C
2
1
0
−1
−2
log2 Fold Change
0
200
400
600
Position
Regulation:
primary Ca2+−dependent
not−affected
Domains and Regions:
DOMAIN: CP−type G

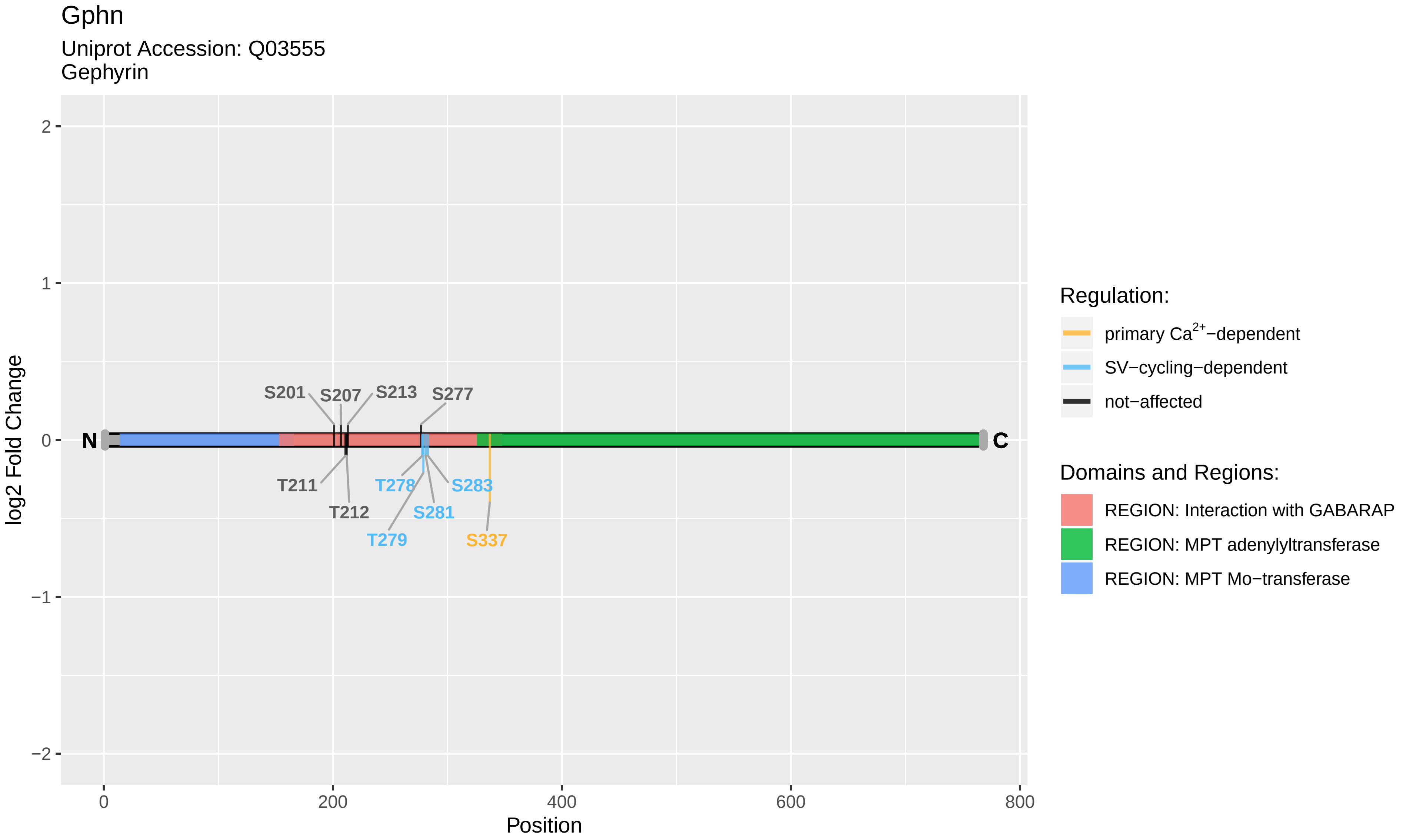

Gphn
Uniprot Accession: Q03555
Gephyrin
S201
S207
S213
S277
N
C
T211
T212
T278
T279
S281
S283
S337
2
1
0
−1
−2
log2 Fold Change
0
200
400
600
800
Position
Regulation:
primary Ca2+−dependent
SV−cycling−dependent
not−affected
Domains and Regions:
REGION: Interaction with GABARAP
REGION: MPT adenylyltransferase
REGION: MPT Mo−transferase

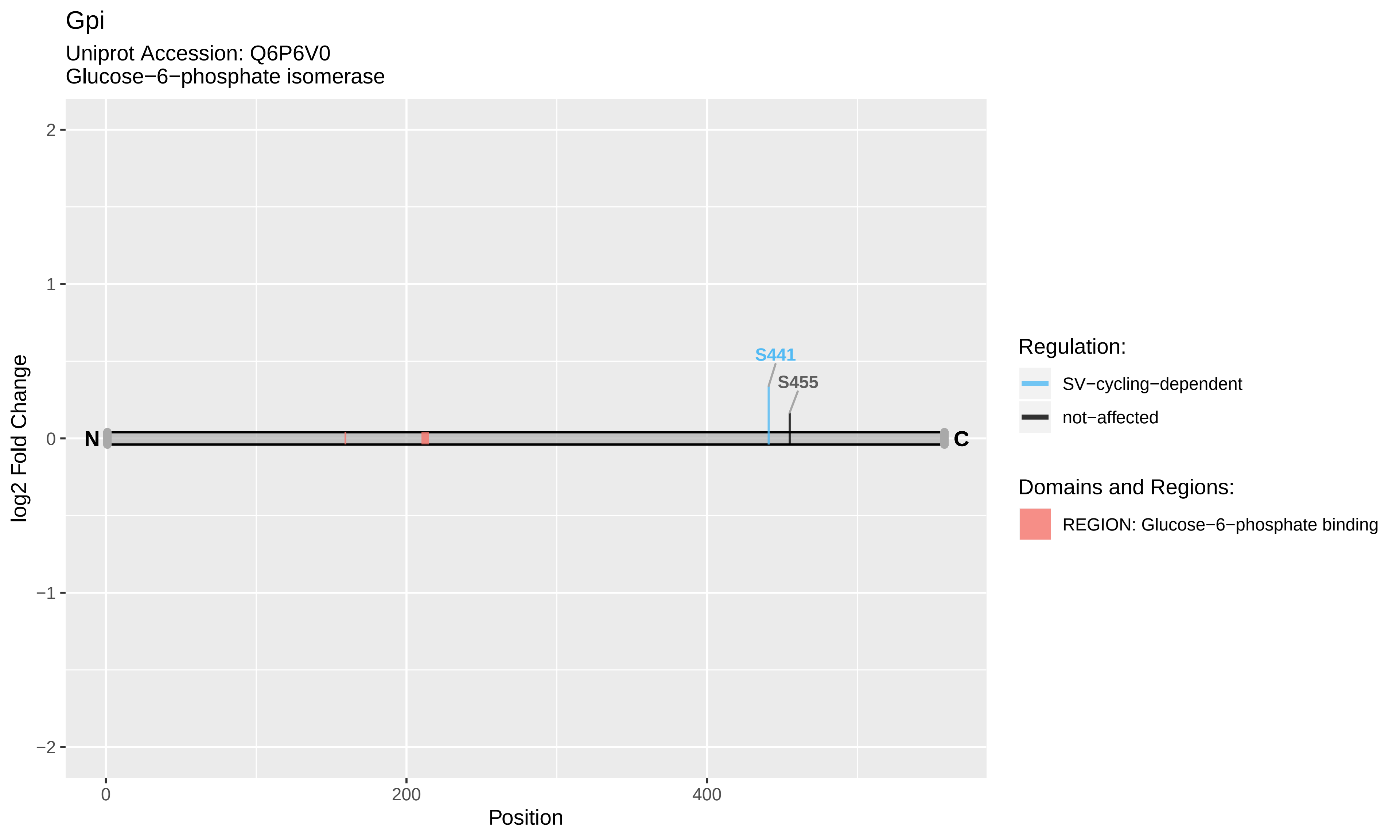

Gpi
Uniprot Accession: Q6P6V0
Glucose−6−phosphate isomerase
log2 Fold Change
2
1
0
−1
−2
N
C
S441
S455
0
200
400
Position
Regulation:
SV−cycling−dependent
not−affected
Domains and Regions:
REGION: Glucose−6−phosphate binding

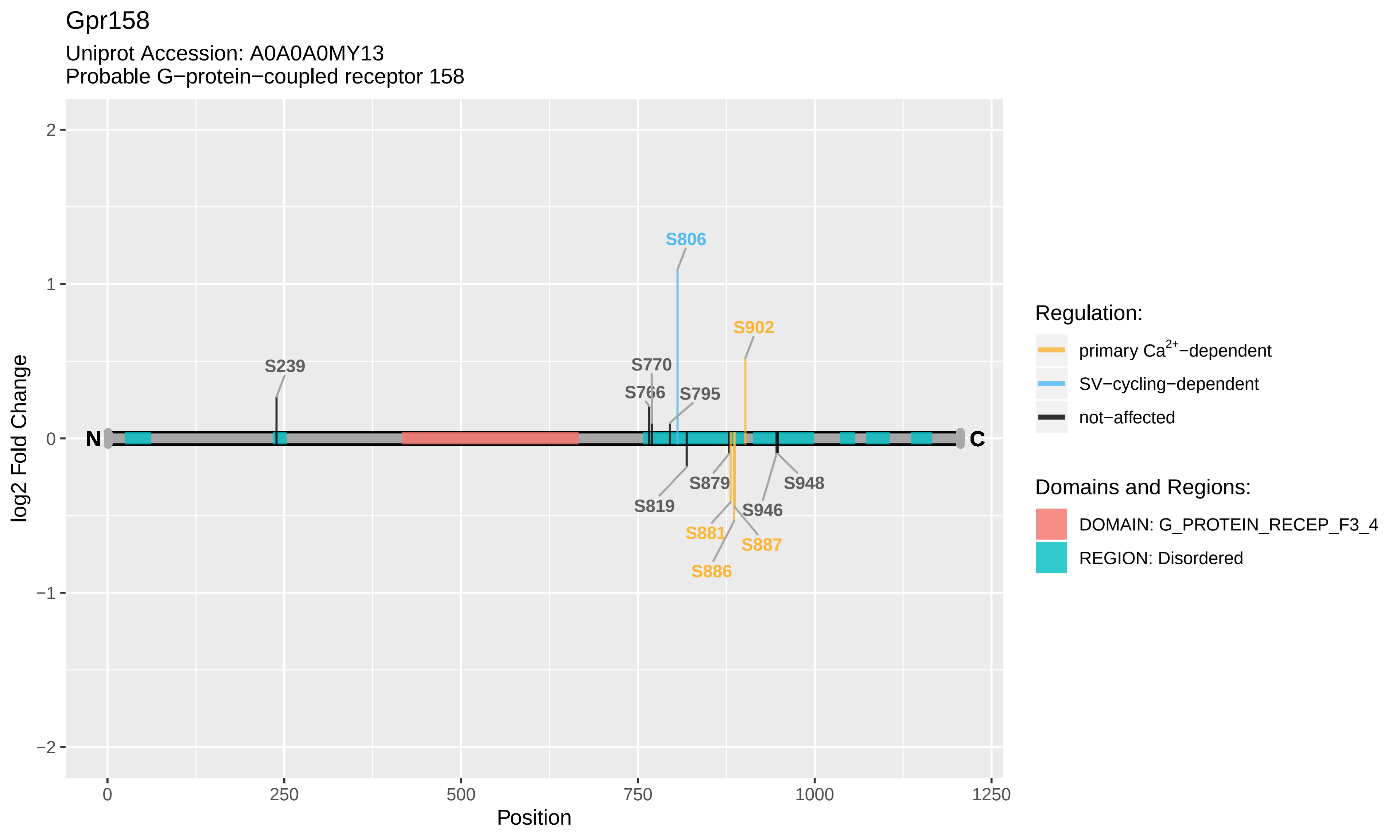

Gpr158
Uniprot Accession: A0A0A0MY13
Probable G−protein−coupled receptor 158
log2 Fold Change
Position
2
1
0
−1
−2
0
250
500
750
1000
1250
N
C
S806
S902
S239
S770
S766
S795
S879
S948
S819
S946
S881
S887
S886
Regulation:
primary Ca2+−dependent
SV−cycling−dependent
not−affected
Domains and Regions:
DOMAIN: G_PROTEIN_RECEP_F3_4
REGION: Disordered

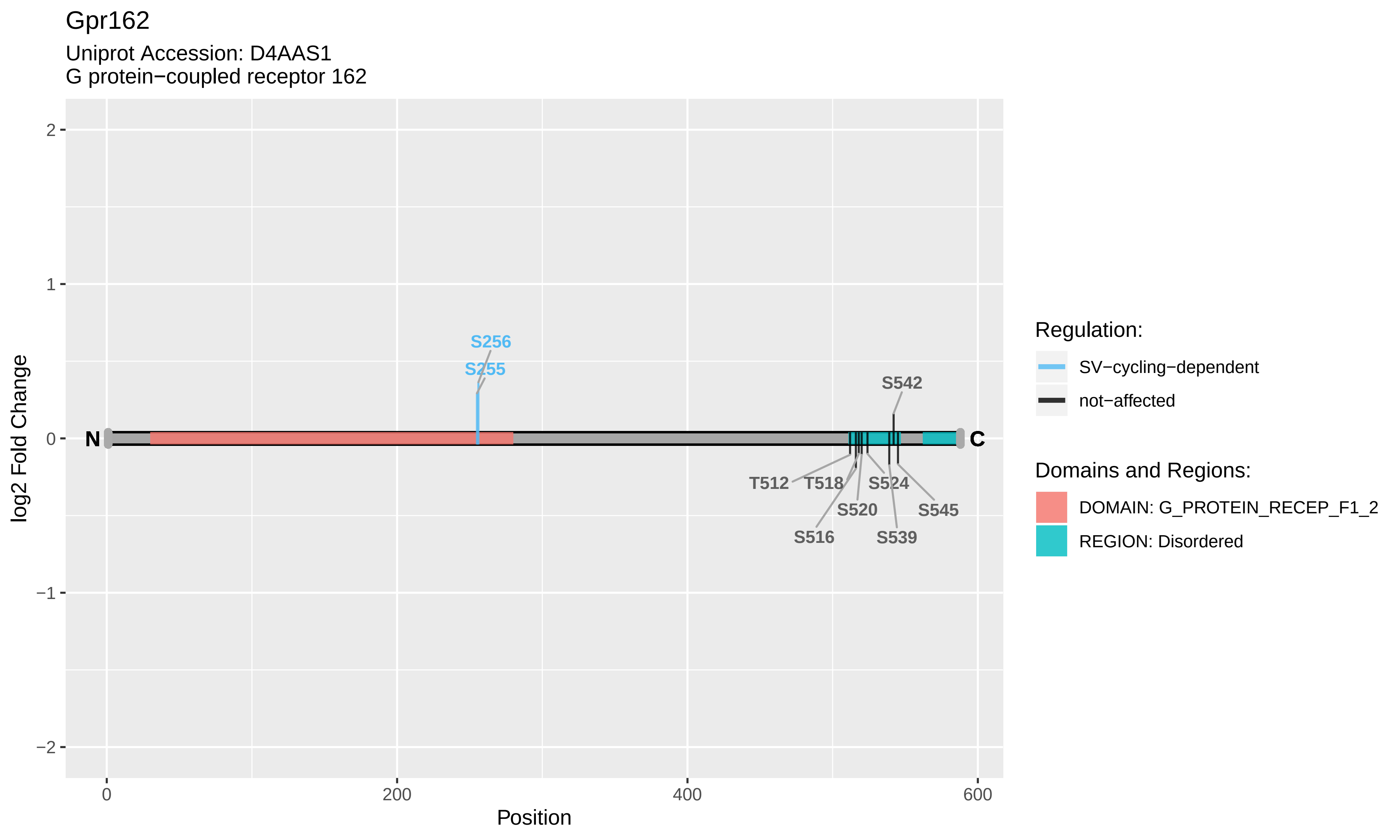

Gpr162
Uniprot Accession: D4AAS1
G protein−coupled receptor 162
log2 Fold Change
Position
N
C
S256
S255
S542
T512
T518
S524
S520
S545
S516
S539
Regulation:
SV−cycling−dependent
not−affected
Domains and Regions:
DOMAIN: G_PROTEIN_RECEP_F1_2
REGION: Disordered

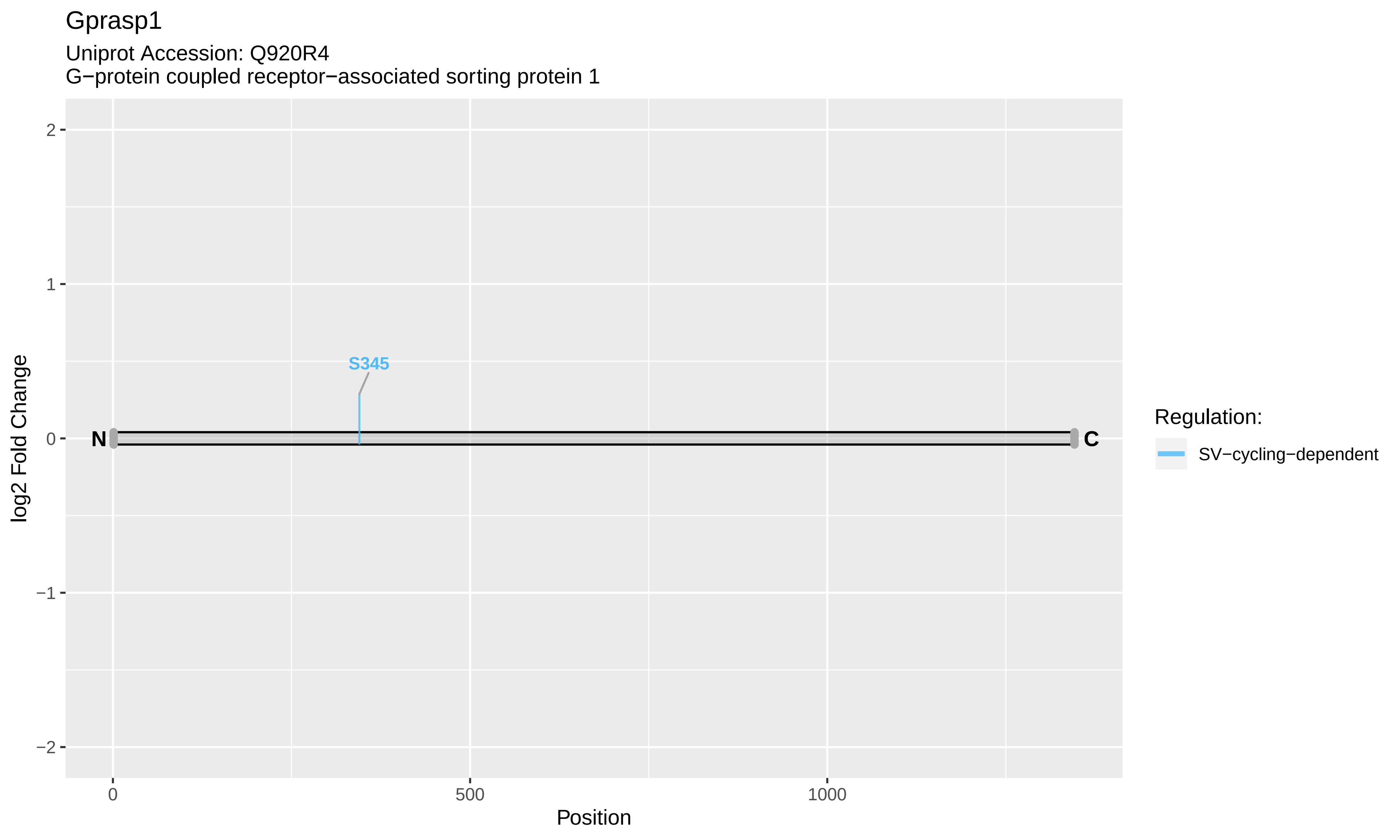
Gprasp1
Uniprot Accession: Q920R4
G−protein coupled receptor−associated sorting protein 1
S345
N
C
log2 Fold Change
2
1
0
−1
−2
0
500
1000
Position
Regulation:
SV−cycling−dependent

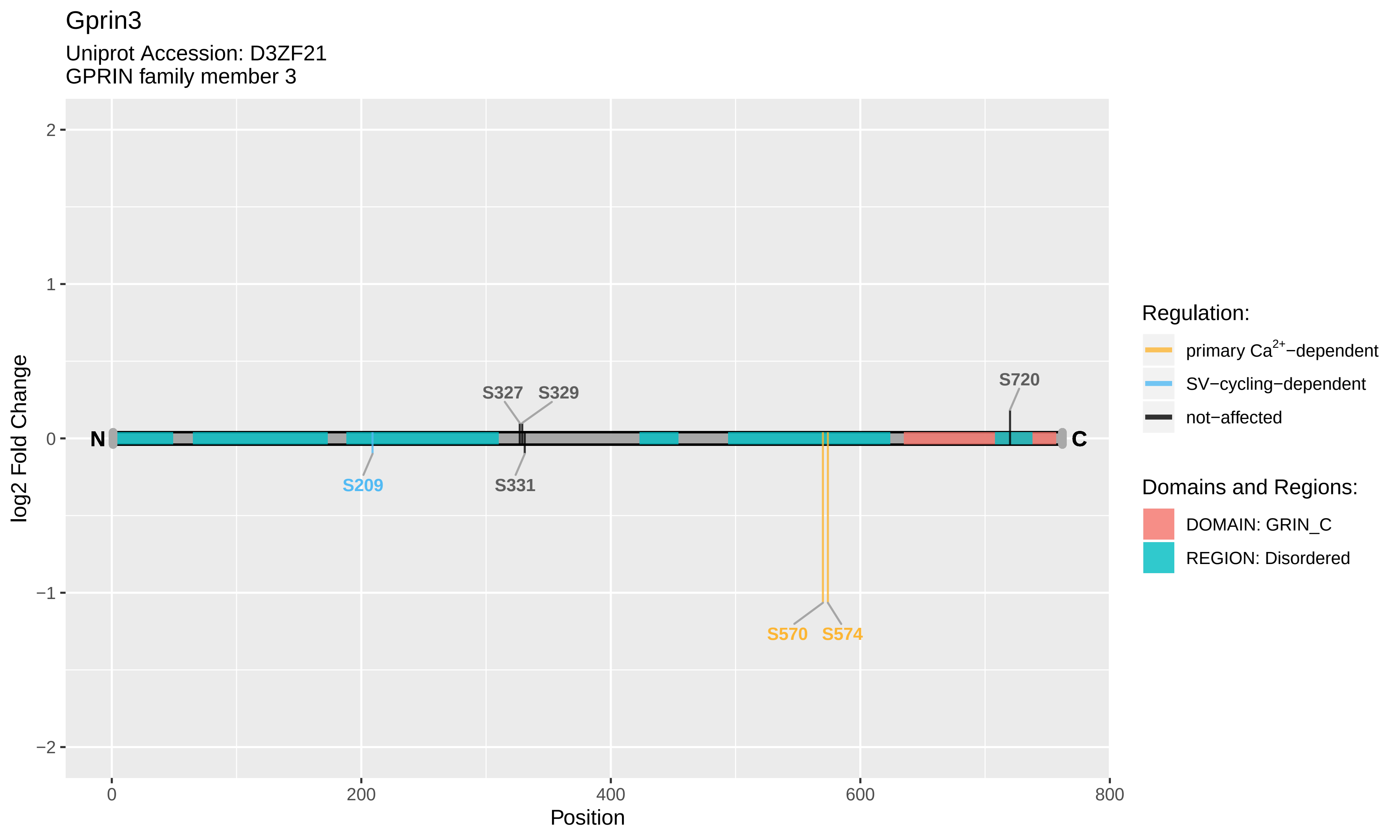

Gprin3
Uniprot Accession: D3ZF21
GPRIN family member 3
log2 Fold Change
Position
N
C
S209
S327
S329
S331
S570
S574
S720
Regulation:
primary Ca2+−dependent
SV−cycling−dependent
not−affected
Domains and Regions:
DOMAIN: GRIN_C
REGION: Disordered

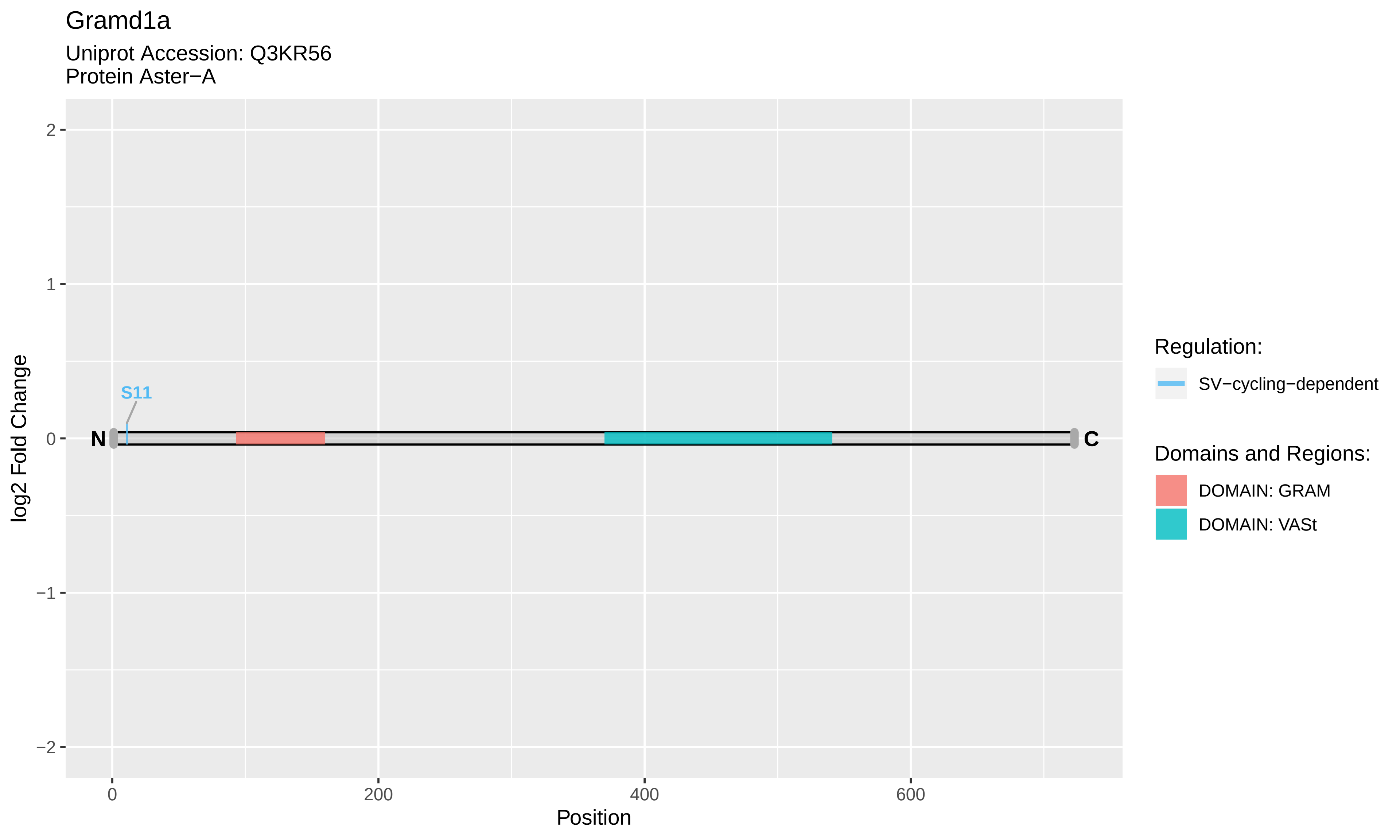

Gramd1a
Uniprot Accession: Q3KR56
Protein Aster−A
log2 Fold Change
2
1
0
−1
−2
N
C
S11
0
200
400
600
Position
Regulation:
SV−cycling−dependent
Domains and Regions:
DOMAIN: GRAM
DOMAIN: VASt

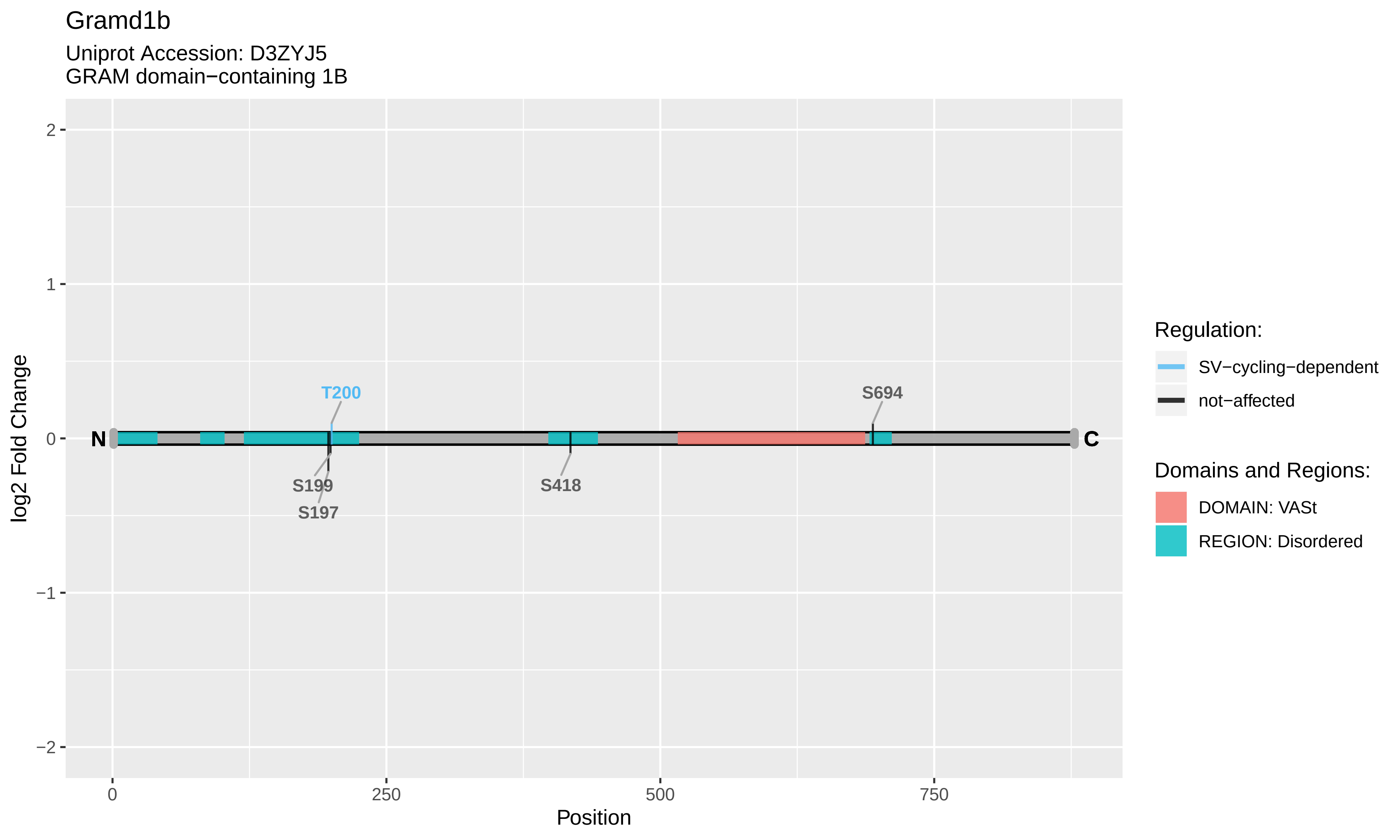

Gramd1b
Uniprot Accession: D3ZYJ5
GRAM domain−containing 1B
log2 Fold Change
Position
T200
S694
S199
S197
S418
N
C
Regulation:
SV−cycling−dependent
not−affected
Domains and Regions:
DOMAIN: VASt
REGION: Disordered

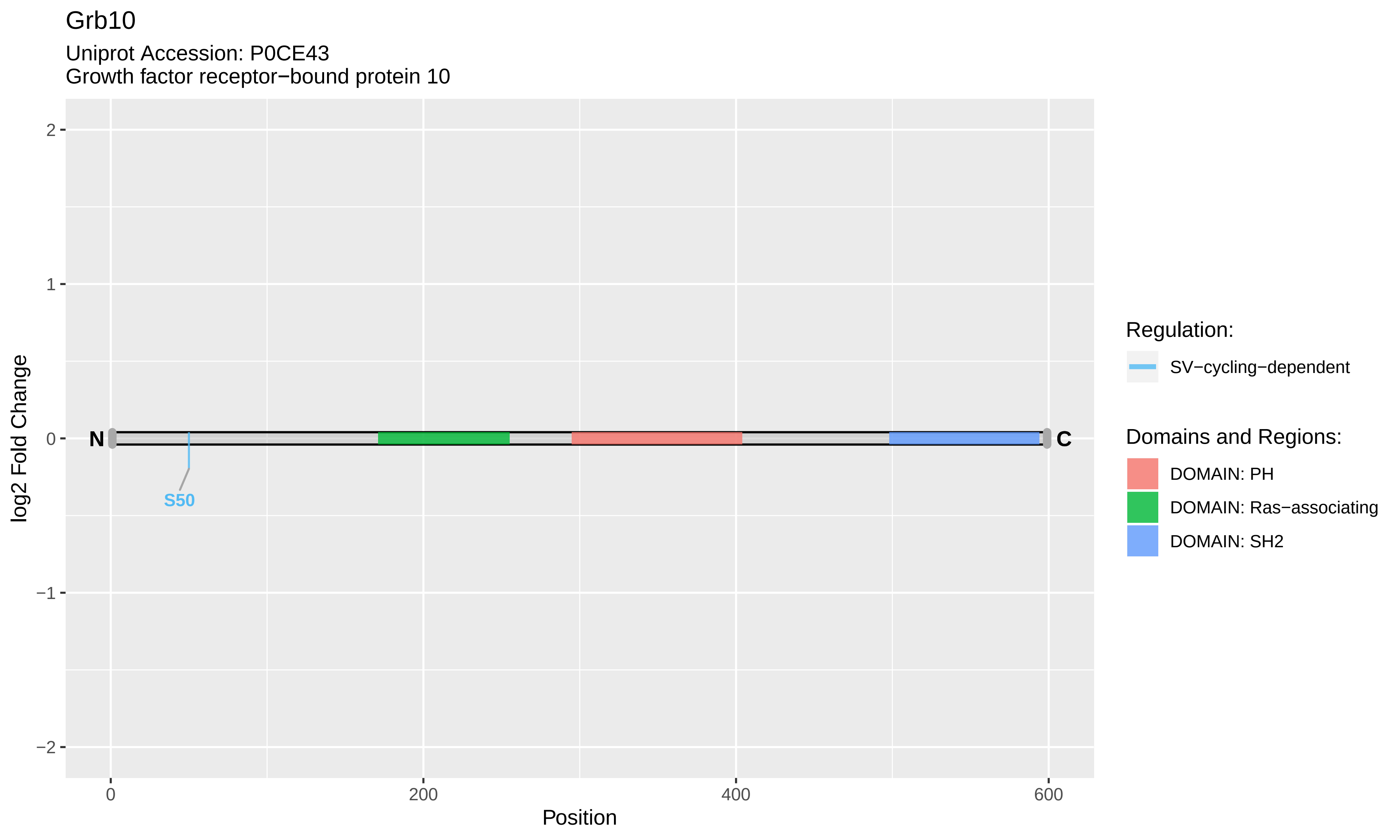

Grb10
Uniprot Accession: P0CE43
Growth factor receptor−bound protein 10
log2 Fold Change
2
1
0
−1
−2
N
C
S50
0
200
400
600
Position
Regulation:
SV−cycling−dependent
Domains and Regions:
DOMAIN: PH
DOMAIN: Ras−associating
DOMAIN: SH2

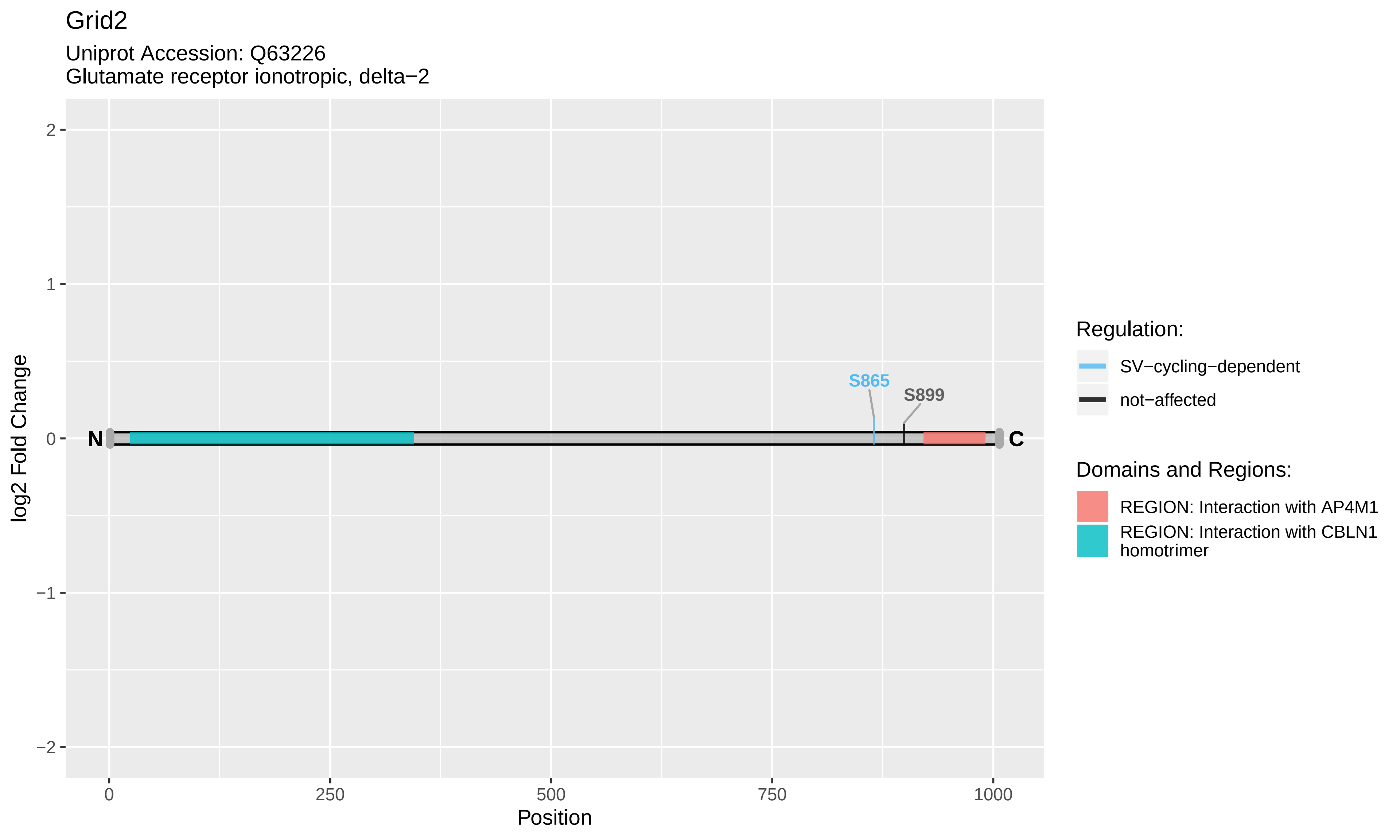

Grid2
Uniprot Accession: Q63226
Glutamate receptor ionotropic, delta−2
log2 Fold Change
2
1
0
−1
−2
N
C
S865
S899
0
250
500
750
1000
Position
Regulation:
SV−cycling−dependent
not−affected
Domains and Regions:
REGION: Interaction with AP4M1
REGION: Interaction with CBLN1 homotrimer

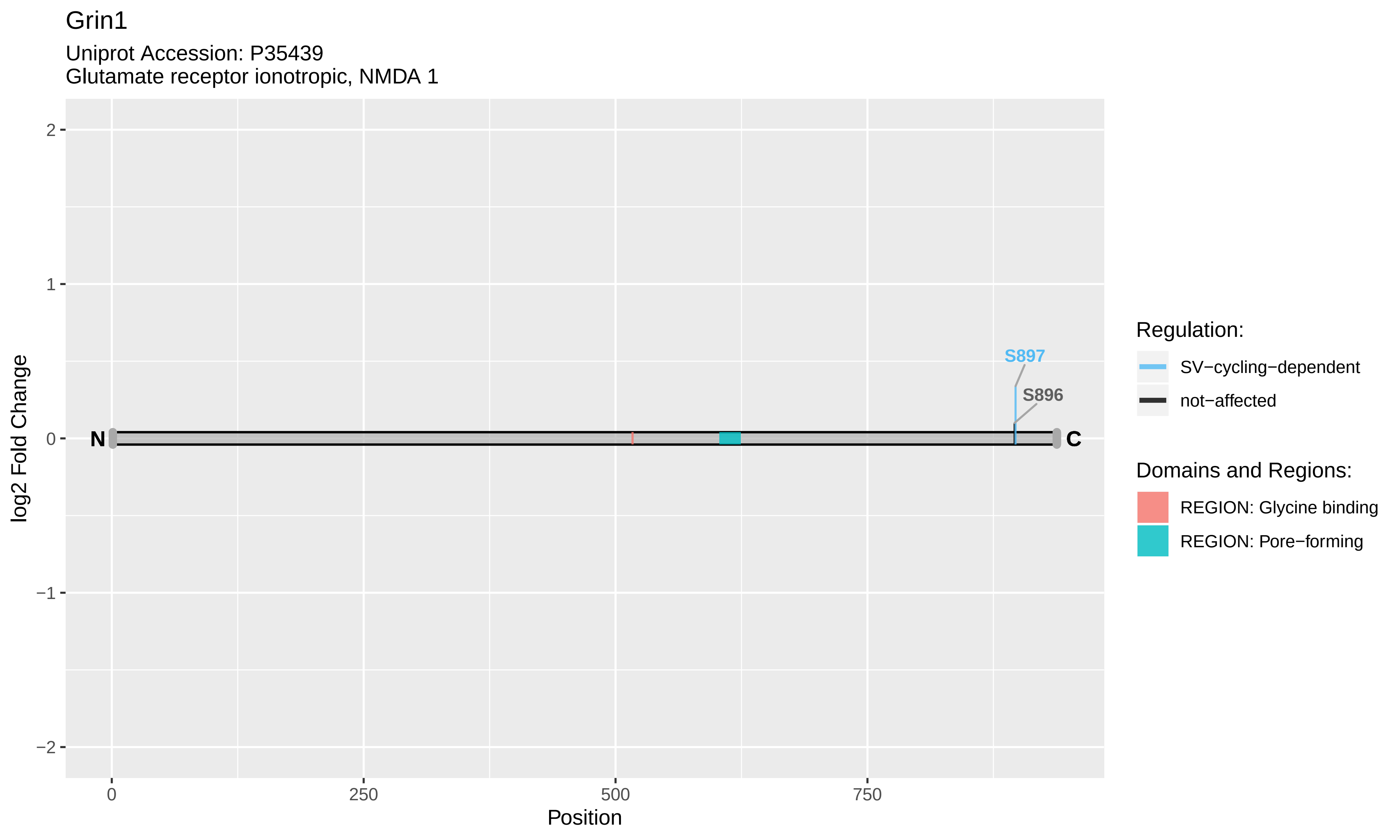
Grin1
Uniprot Accession: P35439
Glutamate receptor ionotropic, NMDA 1
log2 Fold Change
2
1
0
−1
−2
N
C
S897
S896
0
250
500
750
Position
Regulation:
SV−cycling−dependent
not−affected
Domains and Regions:
REGION: Glycine binding
REGION: Pore−forming

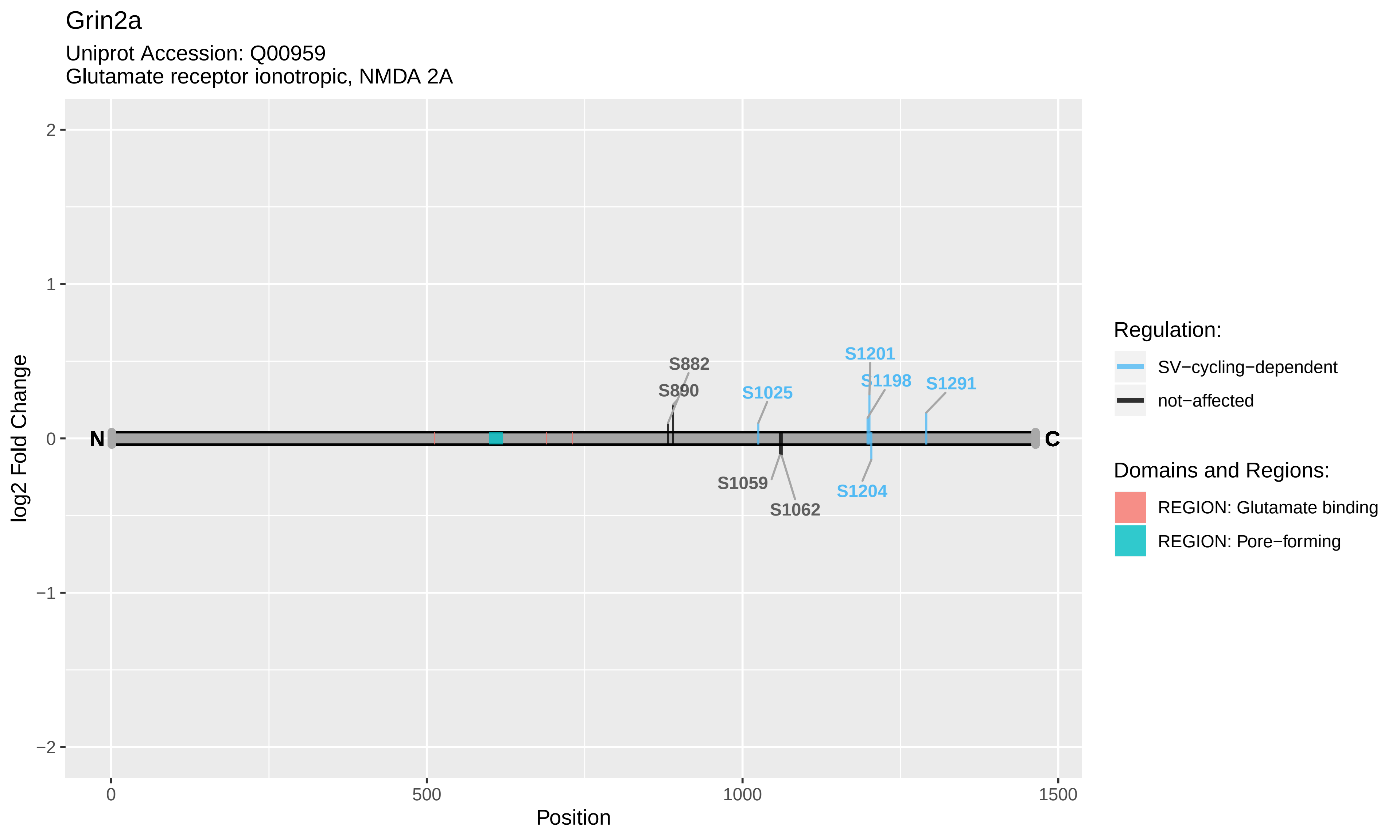

Grin2a
Uniprot Accession: Q00959
Glutamate receptor ionotropic, NMDA 2A
2
1
0
−1
−2
log2 Fold Change
0
500
1000
1500
Position
N
C
S882
S890
S1025
S1201
S1198
S1291
S1059
S1062
S1204
Regulation:
SV−cycling−dependent
not−affected
Domains and Regions:
REGION: Glutamate binding
REGION: Pore−forming

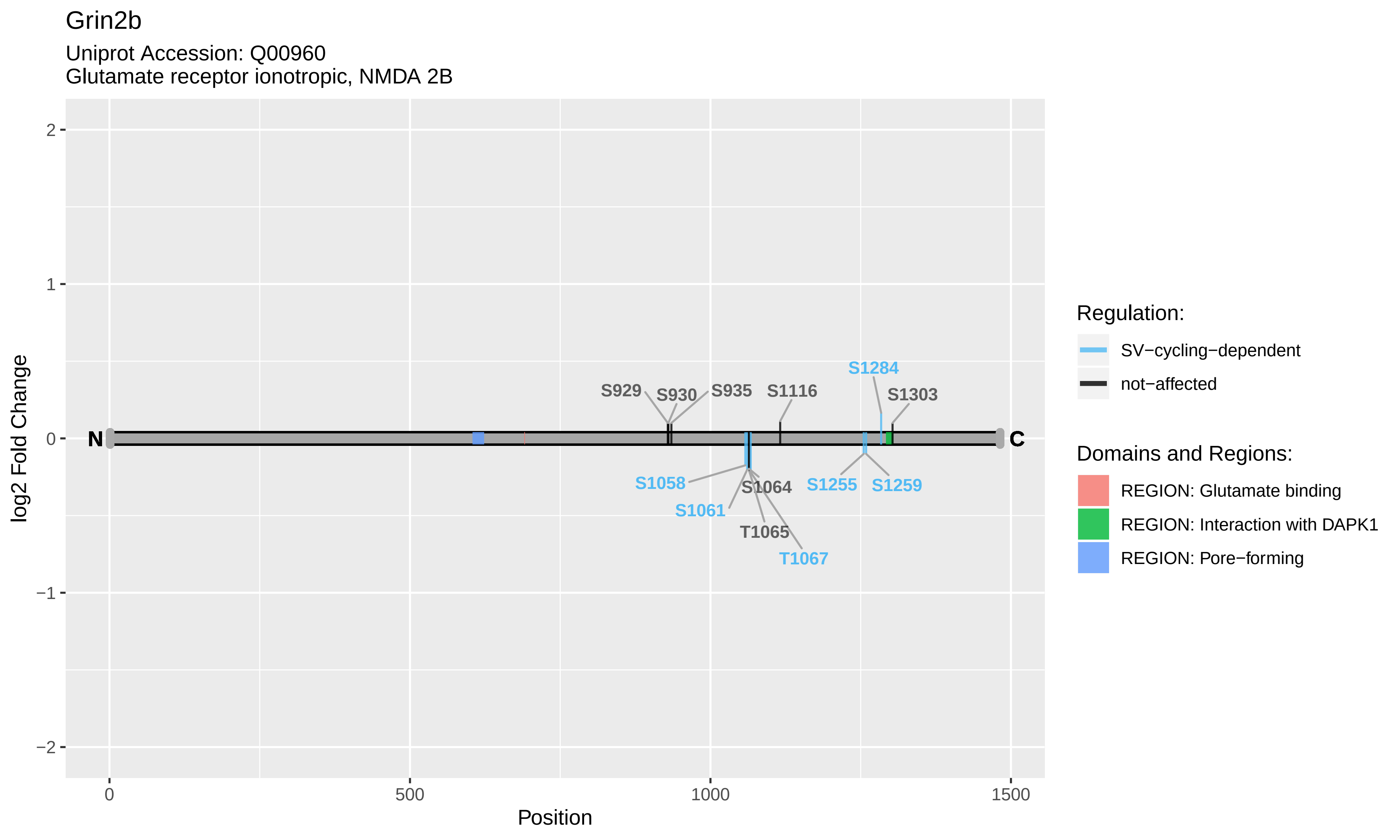

Grin2b
Uniprot Accession: Q00960
Glutamate receptor ionotropic, NMDA 2B
log2 Fold Change
Position
N
C
S929
S930
S935
S1116
S1284
S1303
S1058
S1061
S1064
T1065
T1067
S1255
S1259
Regulation:
SV−cycling−dependent
not−affected
Domains and Regions:
REGION: Glutamate binding
REGION: Interaction with DAPK1
REGION: Pore−forming

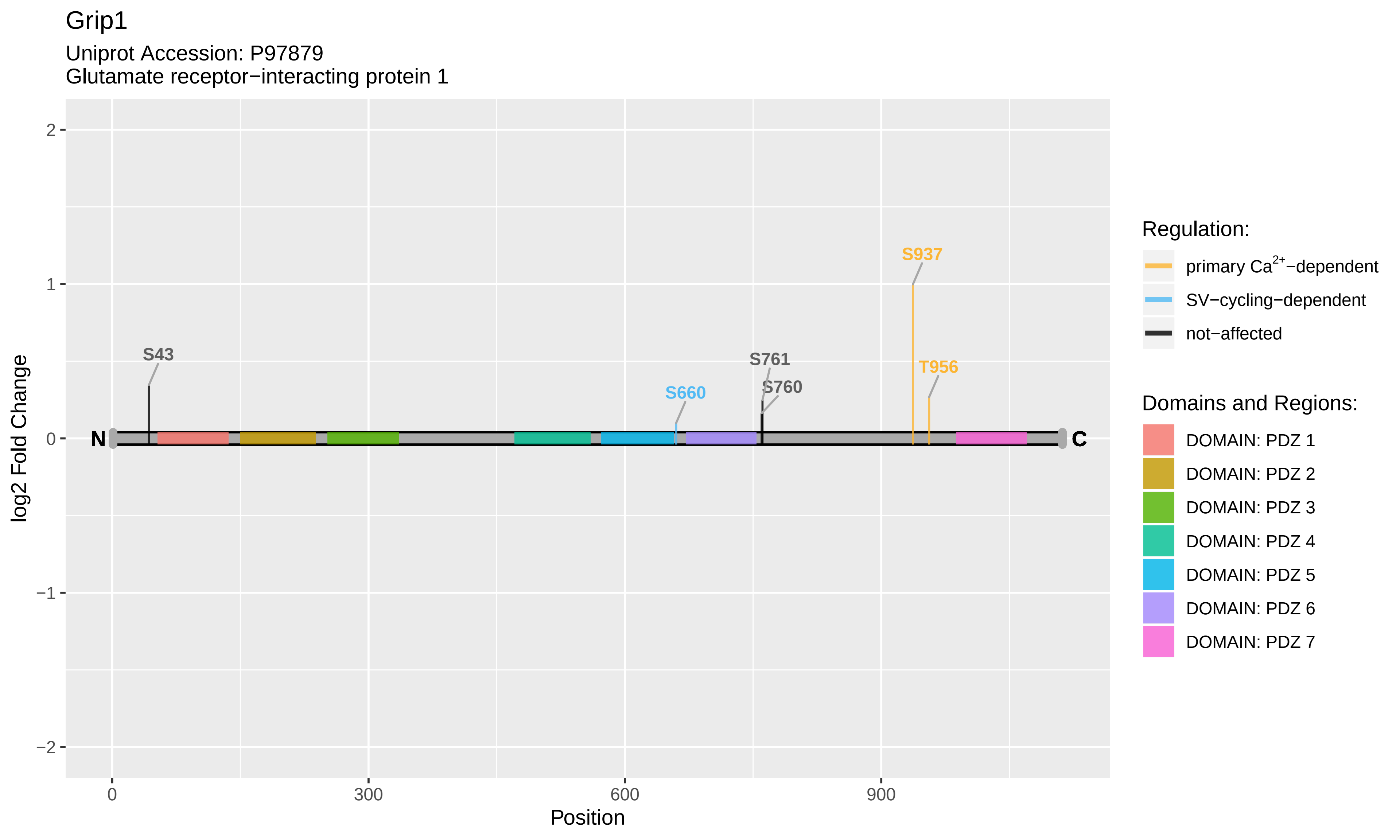

Grip1
Uniprot Accession: P97879
Glutamate receptor−interacting protein 1
S43
S660
S761
S760
S937
T956
N
C
2
1
0
−1
−2
log2 Fold Change
0
300
600
900
Position
Regulation:
primary Ca2+−dependent
SV−cycling−dependent
not−affected
Domains and Regions:
DOMAIN: PDZ 1
DOMAIN: PDZ 2
DOMAIN: PDZ 3
DOMAIN: PDZ 4
DOMAIN: PDZ 5
DOMAIN: PDZ 6
DOMAIN: PDZ 7

Grk2

Uniprot Accession: P26817
Beta−adrenergic receptor kinase 1

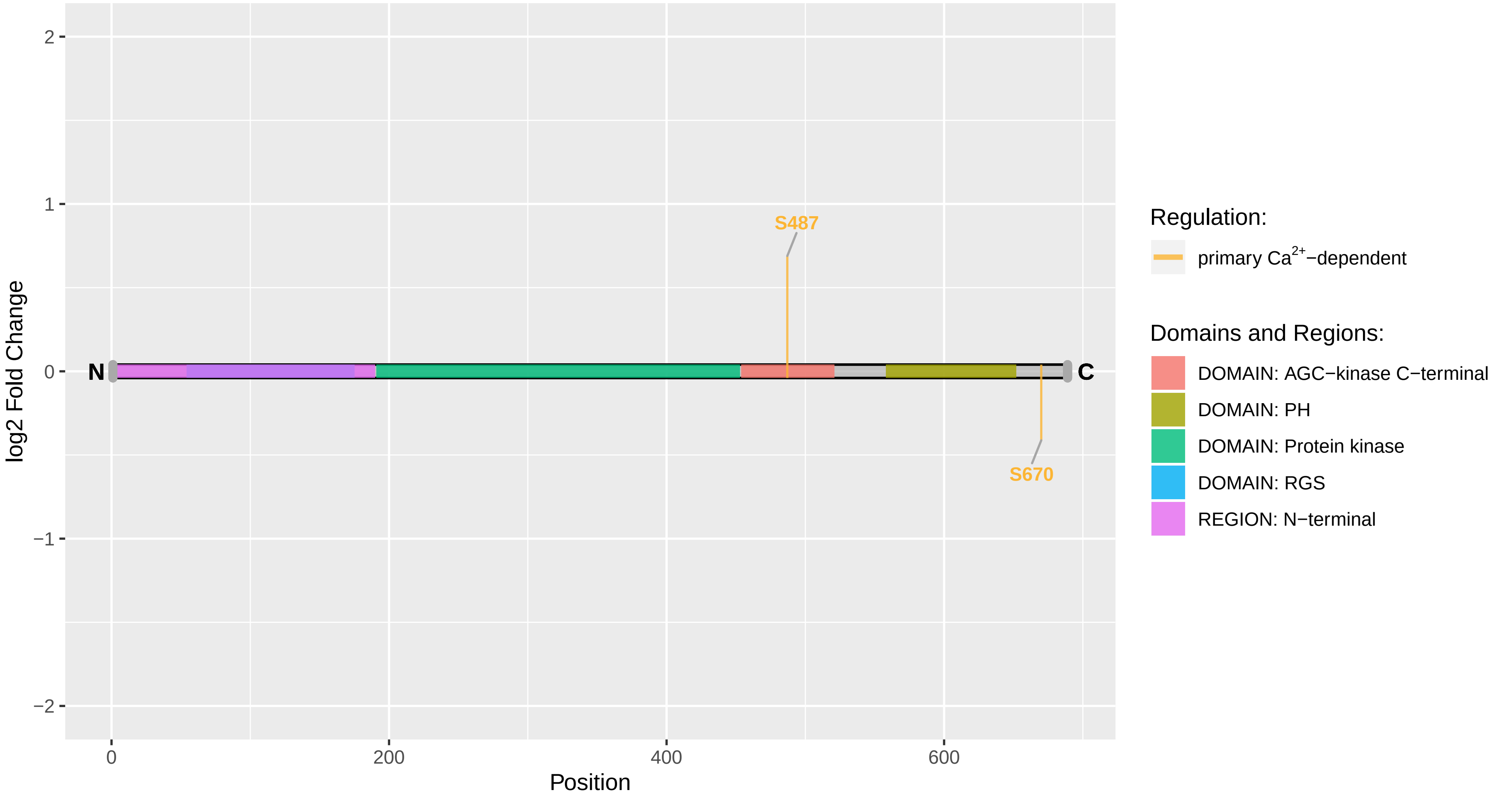

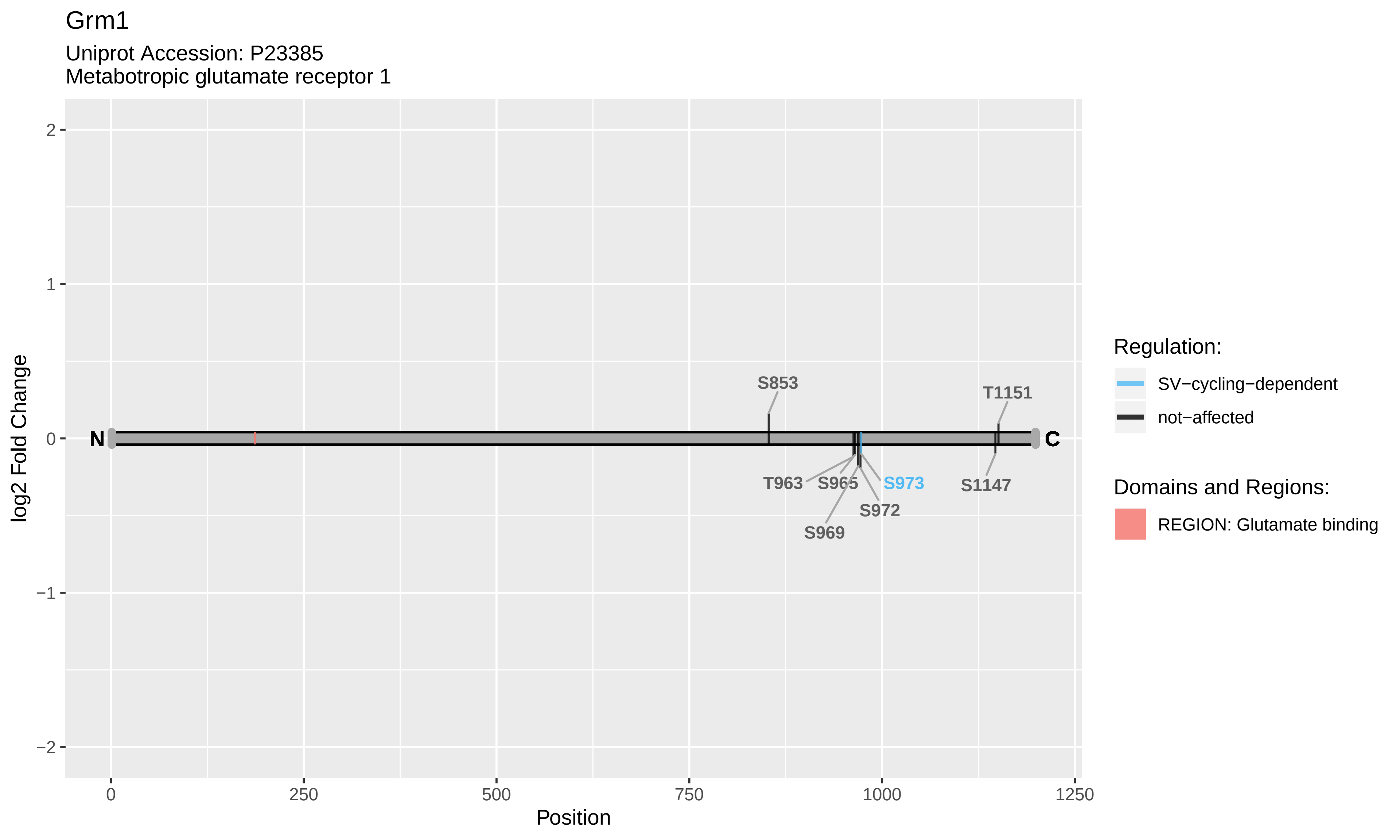

Grm1
Uniprot Accession: P23385
Metabotropic glutamate receptor 1
log2 Fold Change
Position
0
250
500
750
1000
1250
2
1
0
−1
−2
N
C
S853
T1151
T963
S965
S973
S972
S969
S1147
Regulation:
SV−cycling−dependent
not−affected
Domains and Regions:
REGION: Glutamate binding

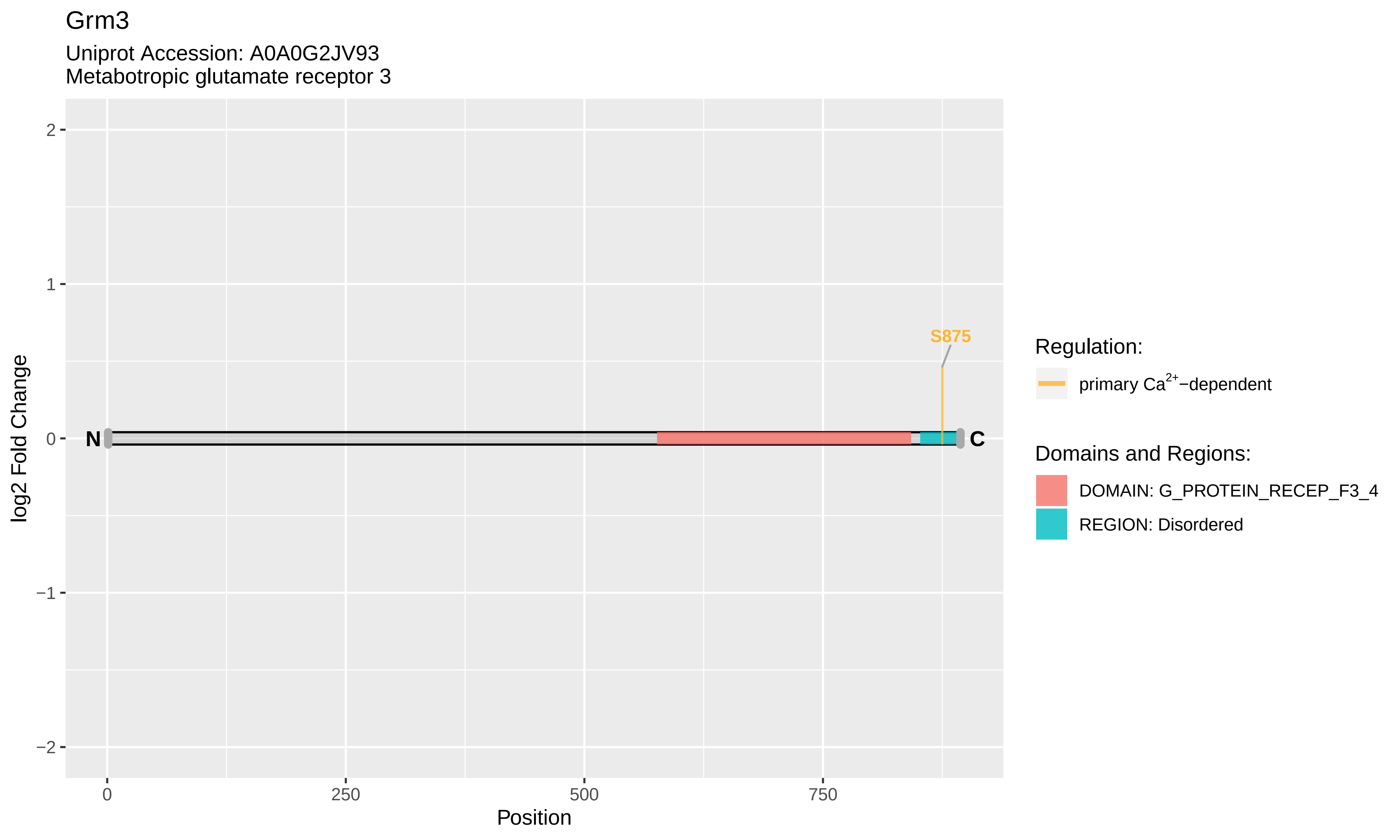

Grm3
Uniprot Accession: A0A0G2JV93
Metabotropic glutamate receptor 3
S875
N
C
2
1
0
−1
−2
log2 Fold Change
0
250
500
750
Position
Regulation:
primary Ca2+−dependent
Domains and Regions:
DOMAIN: G_PROTEIN_RECEP_F3_4
REGION: Disordered

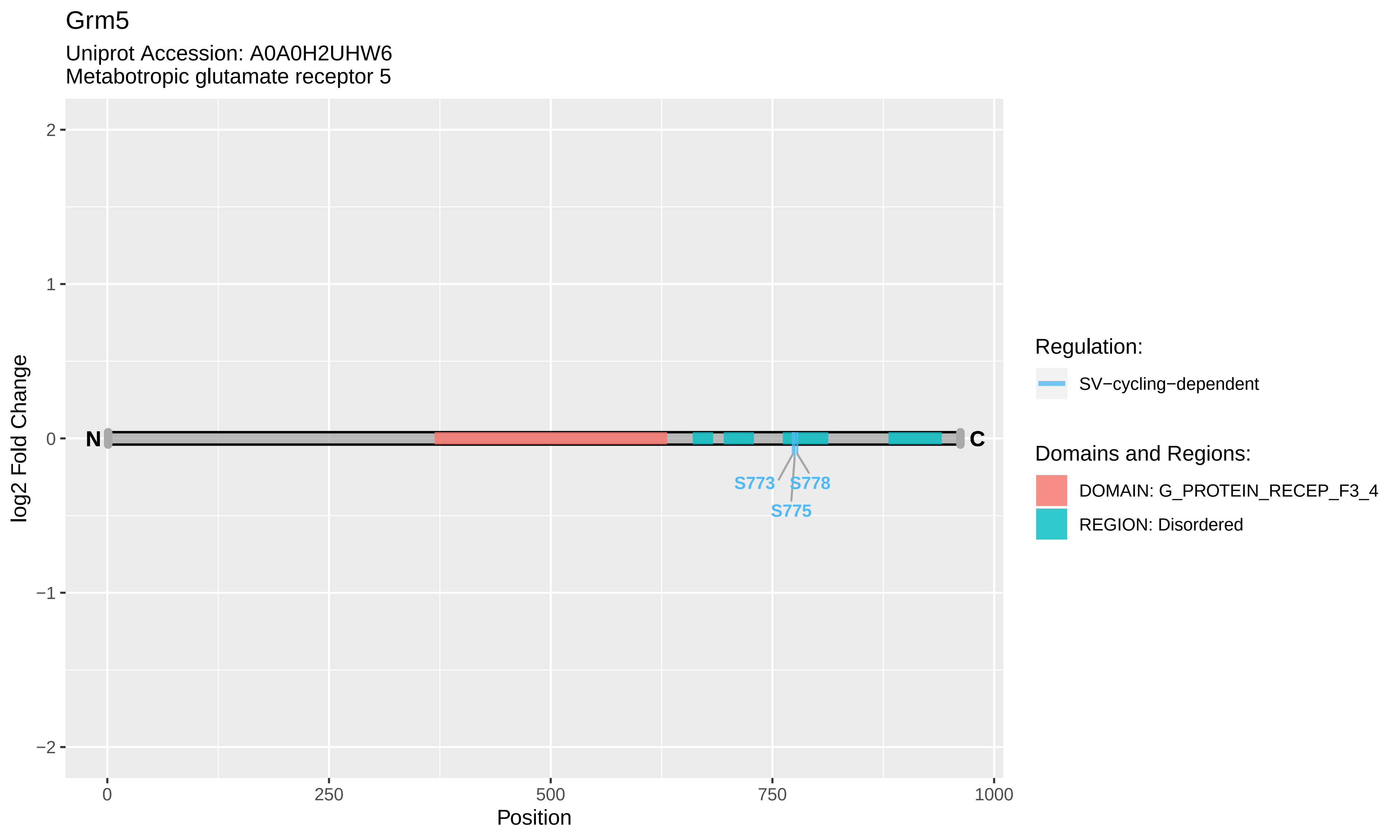

Grm5
Uniprot Accession: A0A0H2UHW6
Metabotropic glutamate receptor 5
log2 Fold Change
Position
N
C
S773
S778
S775
Regulation:
SV−cycling−dependent
Domains and Regions:
DOMAIN: G_PROTEIN_RECEP_F3_4
REGION: Disordered

Gucy1a2

Uniprot Accession: Q9WVI4
Guanylate cyclase soluble subunit alpha−2

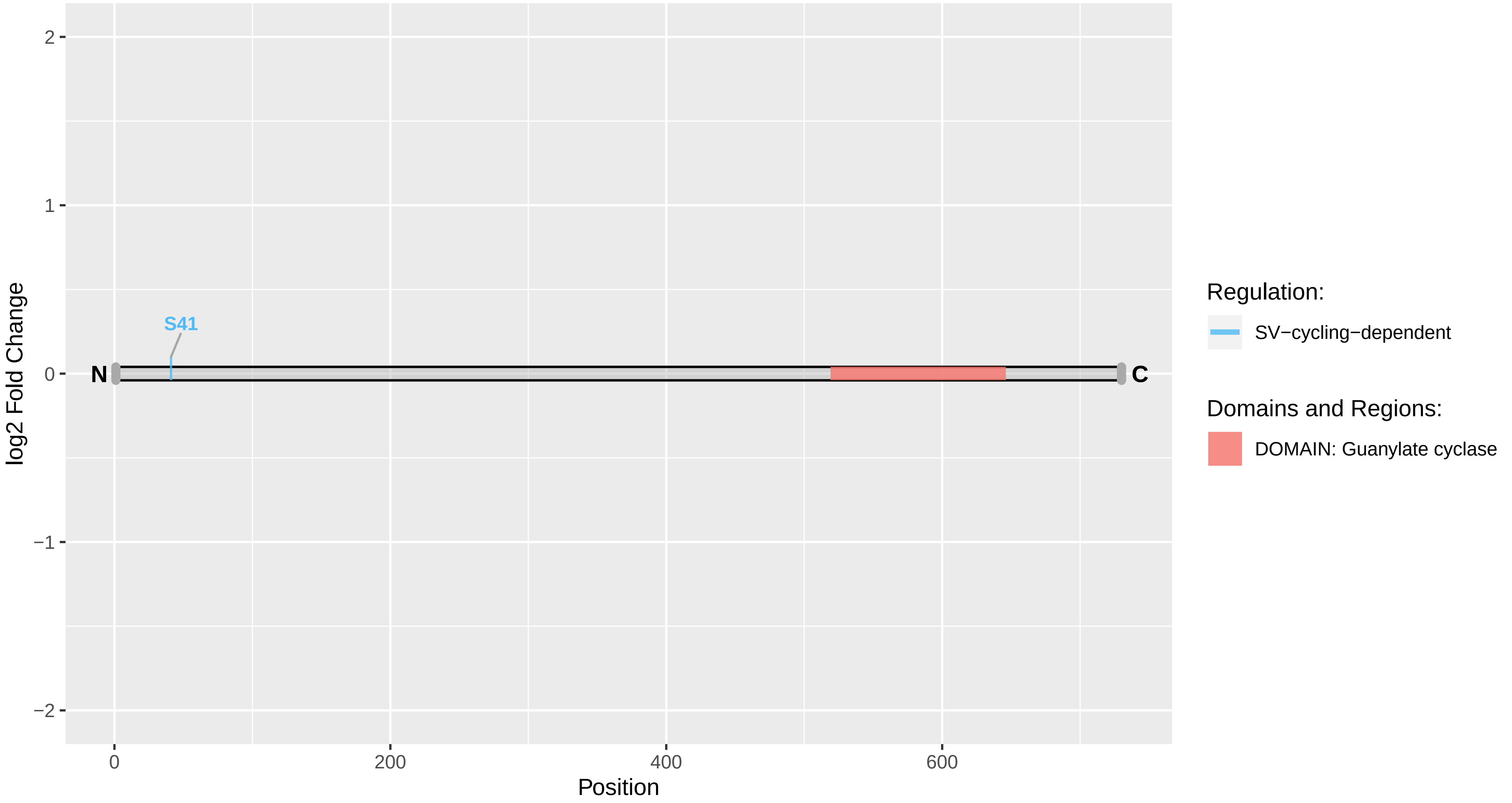

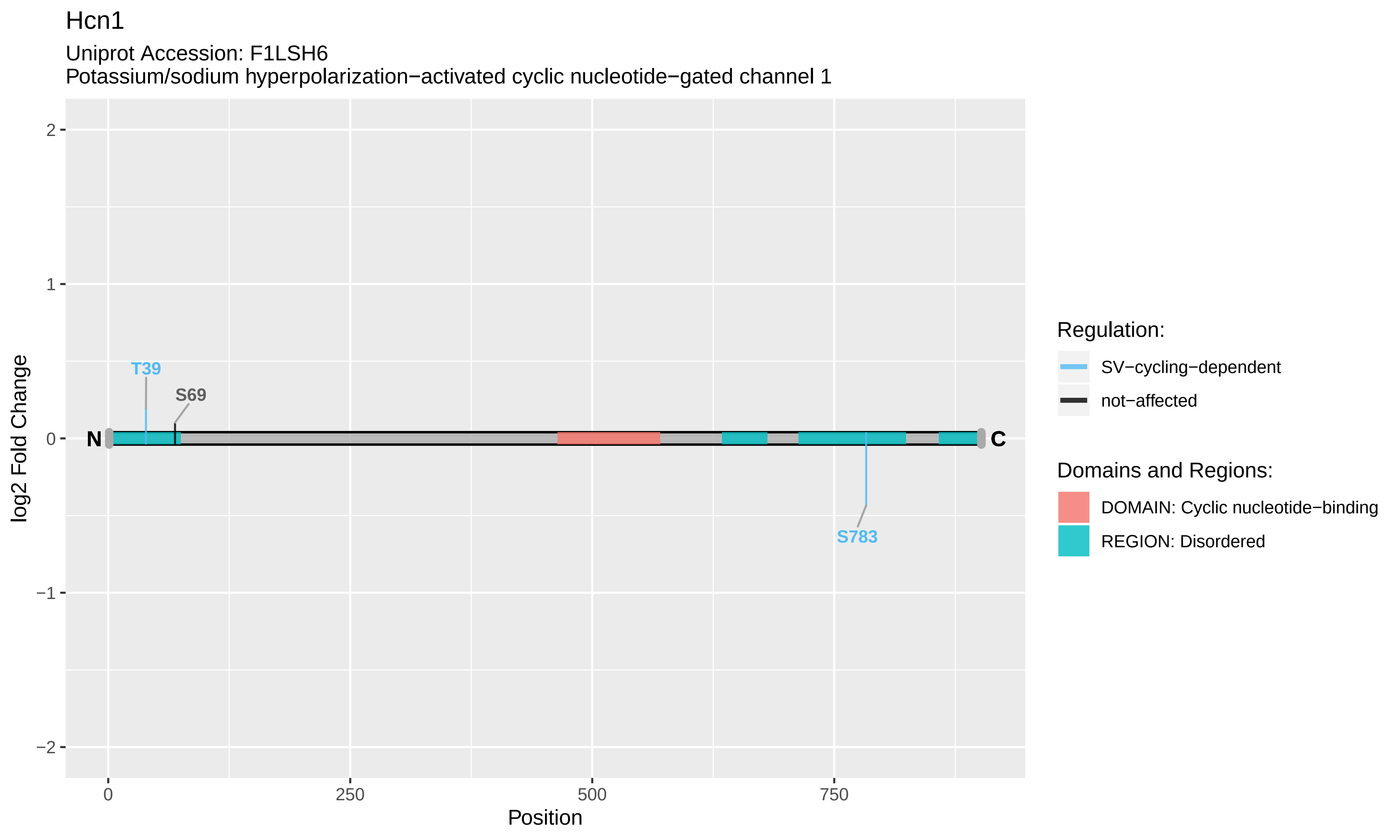

Hcn1
Uniprot Accession: F1LSH6
Potassium/sodium hyperpolarization−activated cyclic nucleotide−gated channel 1
log2 Fold Change
2
1
0
−1
−2
T39
S69
S783
N
C
0
250
500
750
Position
Regulation:
SV−cycling−dependent
not−affected
Domains and Regions:
DOMAIN: Cyclic nucleotide−binding
REGION: Disordered

Hcn2

Uniprot Accession: Q9JKA9
Potassium/sodium hyperpolarization−activated cyclic nucleotide−gated channel 2

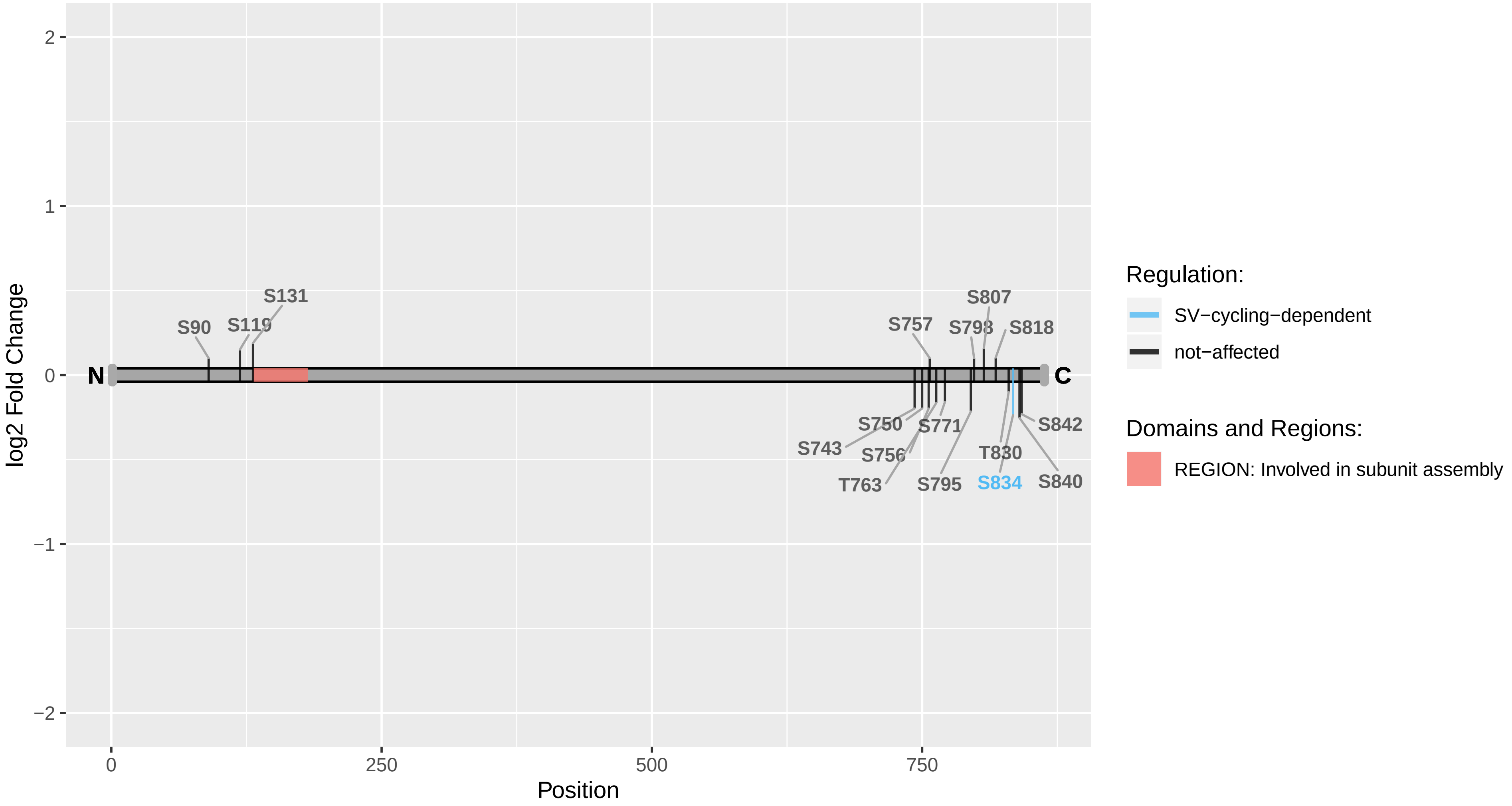

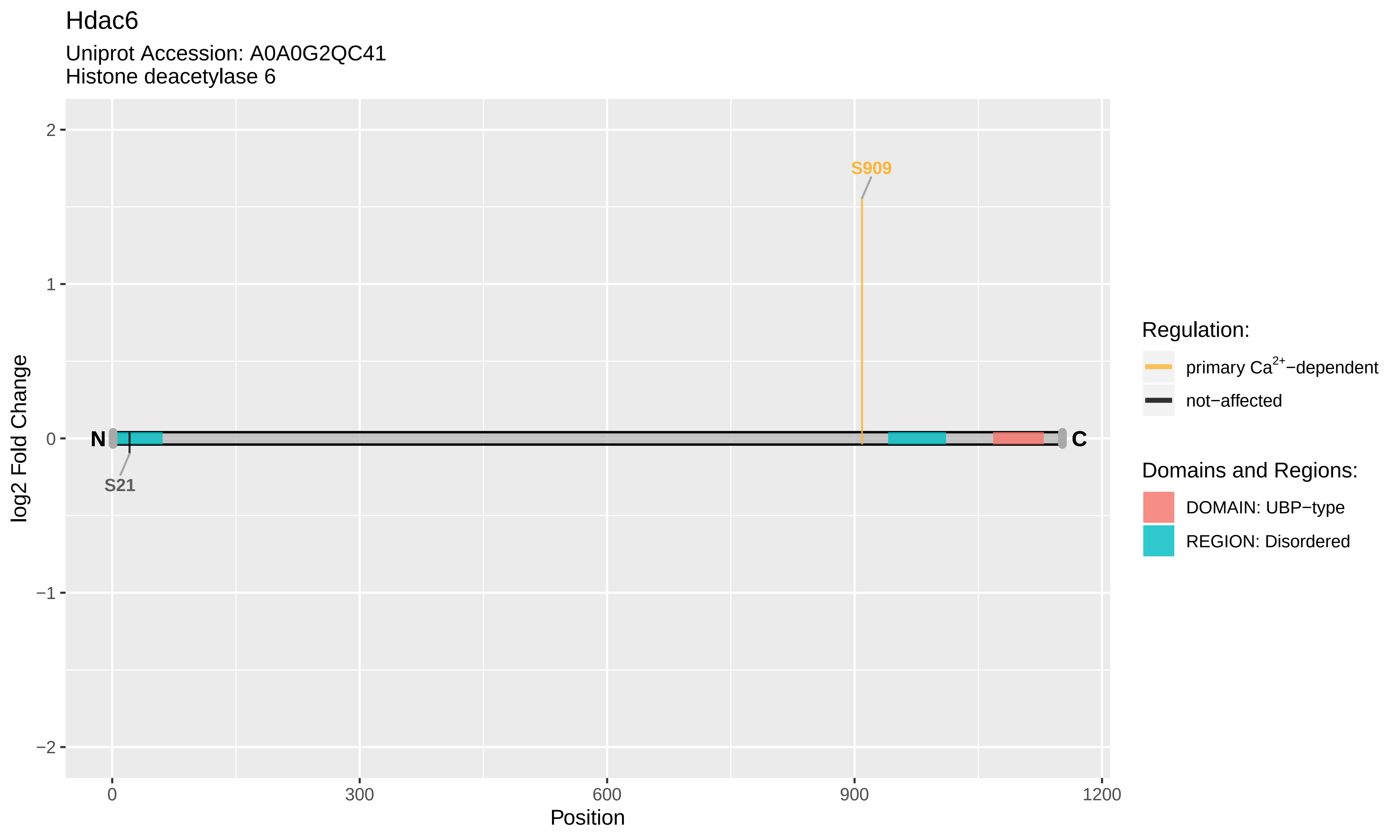

Hdac6
Uniprot Accession: A0A0G2QC41
Histone deacetylase 6
S909
N
C
S21
log2 Fold Change
Position
2
1
0
−1
−2
0
300
600
900
1200
Regulation:
primary Ca2+−dependent
not−affected
Domains and Regions:
DOMAIN: UBP−type
REGION: Disordered

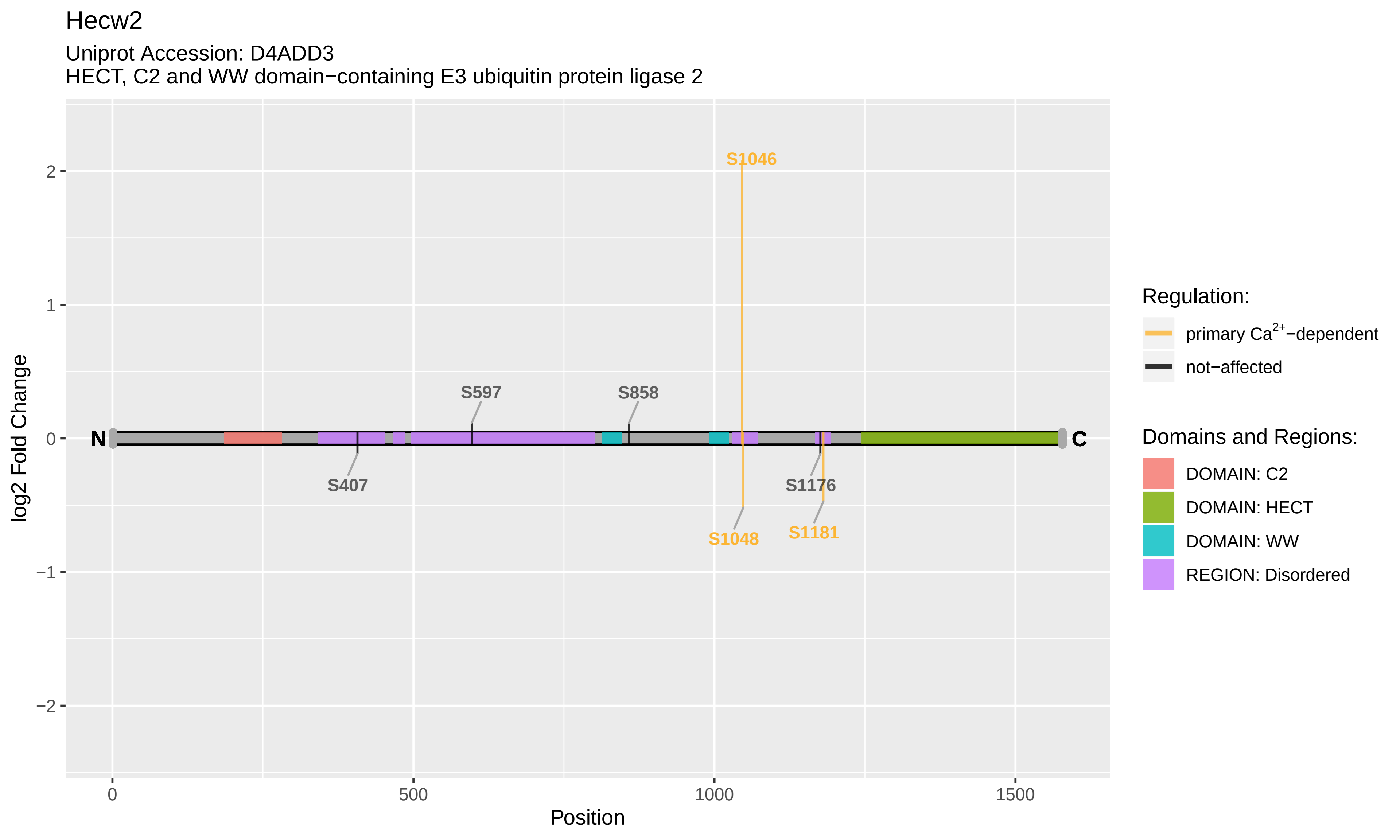

Hecw2
Uniprot Accession: D4ADD3
HECT, C2 and WW domain−containing E3 ubiquitin protein ligase 2
S1046
S597
S858
N
C
S407
S1176
S1048
S1181
log2 Fold Change
2
1
0
−1
−2
0
500
1000
1500
Position
Regulation:
primary Ca2+−dependent
not−affected
Domains and Regions:
DOMAIN: C2
DOMAIN: HECT
DOMAIN: WW
REGION: Disordered

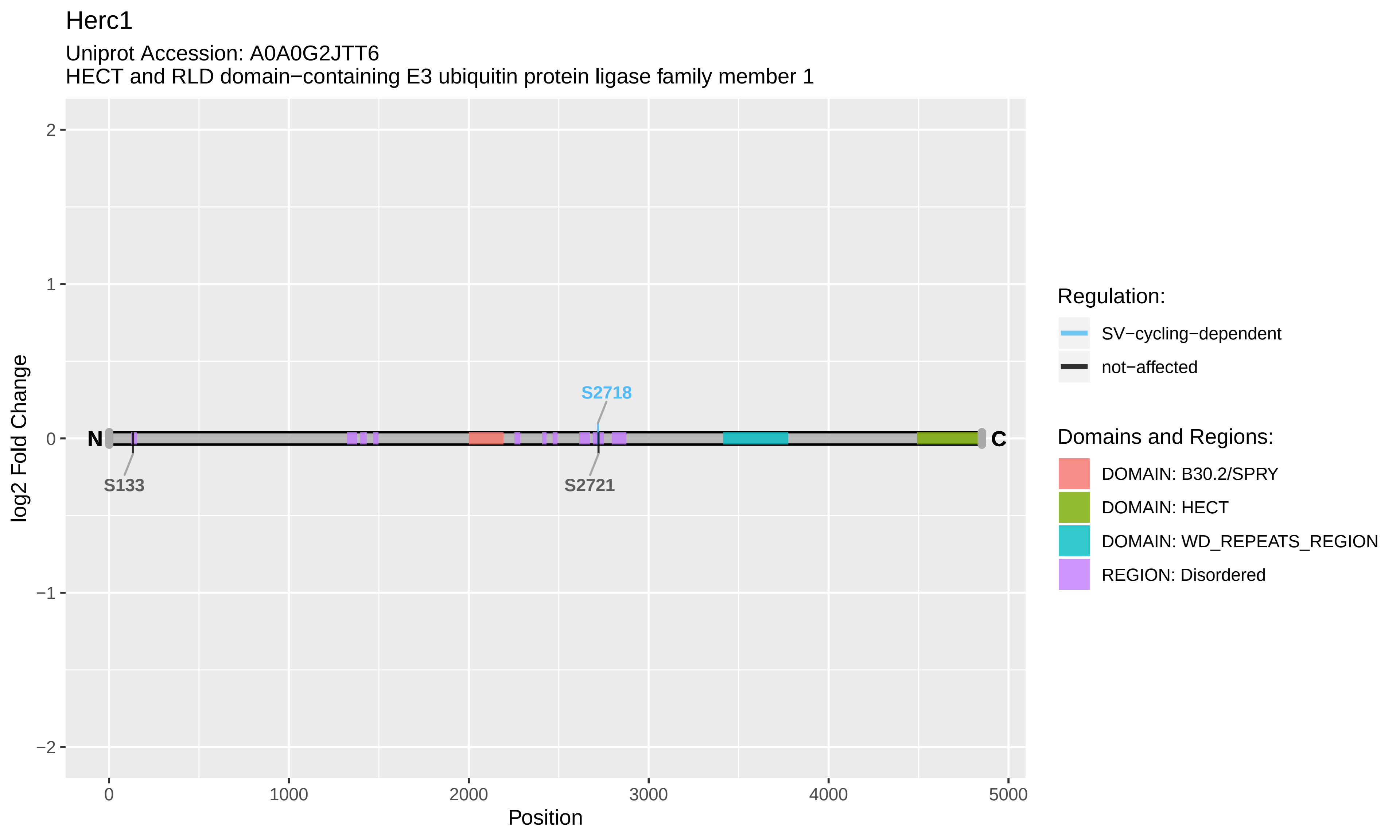

Herc1
Uniprot Accession: A0A0G2JTT6
HECT and RLD domain−containing E3 ubiquitin protein ligase family member 1
log2 Fold Change
Position
S2718
S133
S2721
N
C
Regulation:
SV−cycling−dependent
not−affected
Domains and Regions:
DOMAIN: B30.2/SPRY
DOMAIN: HECT
DOMAIN: WD_REPEATS_REGION
REGION: Disordered

Hmgn3

Uniprot Accession: Q66H40
High mobility group nucleosome−binding domain−containing protein 3

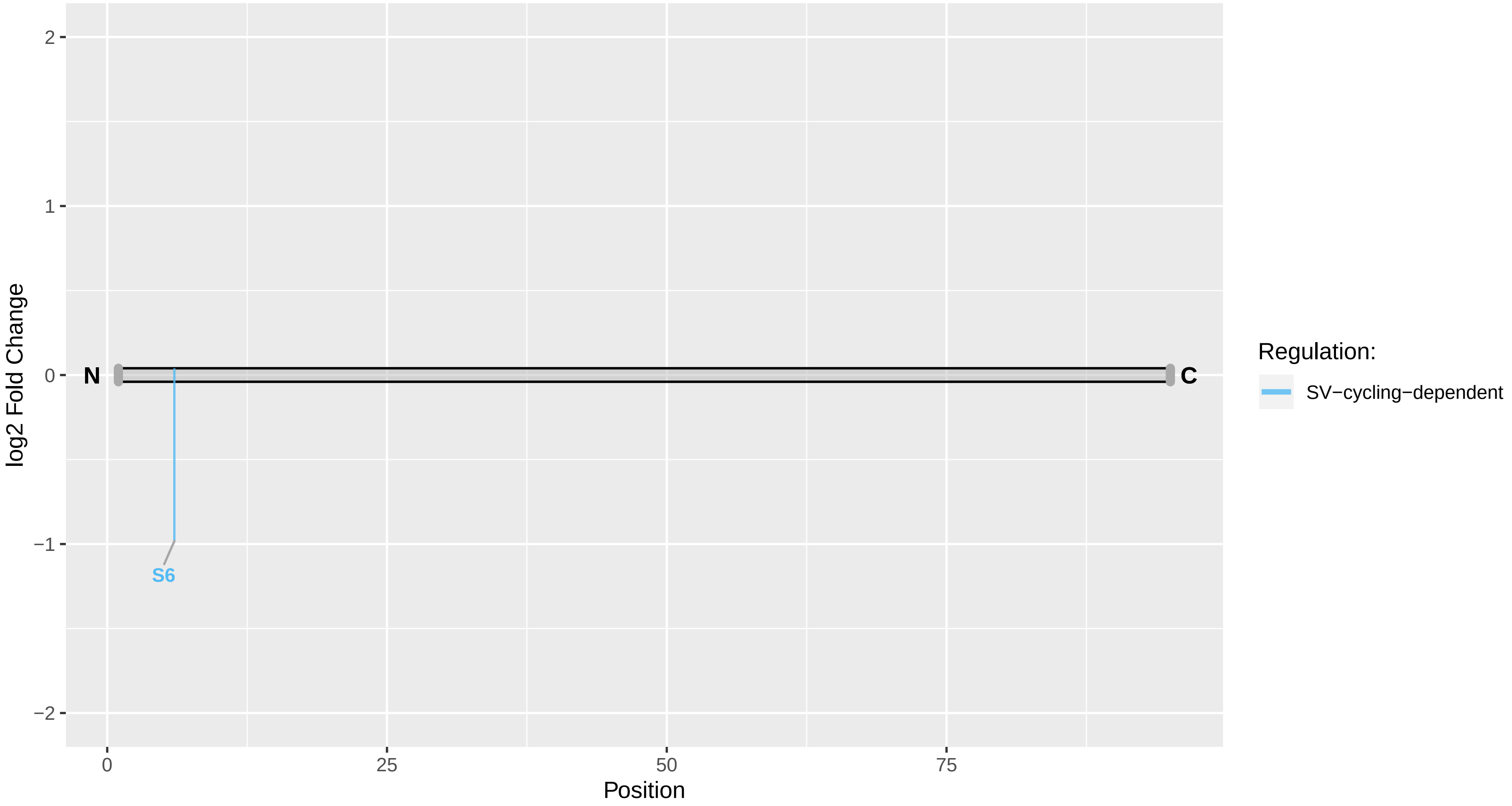

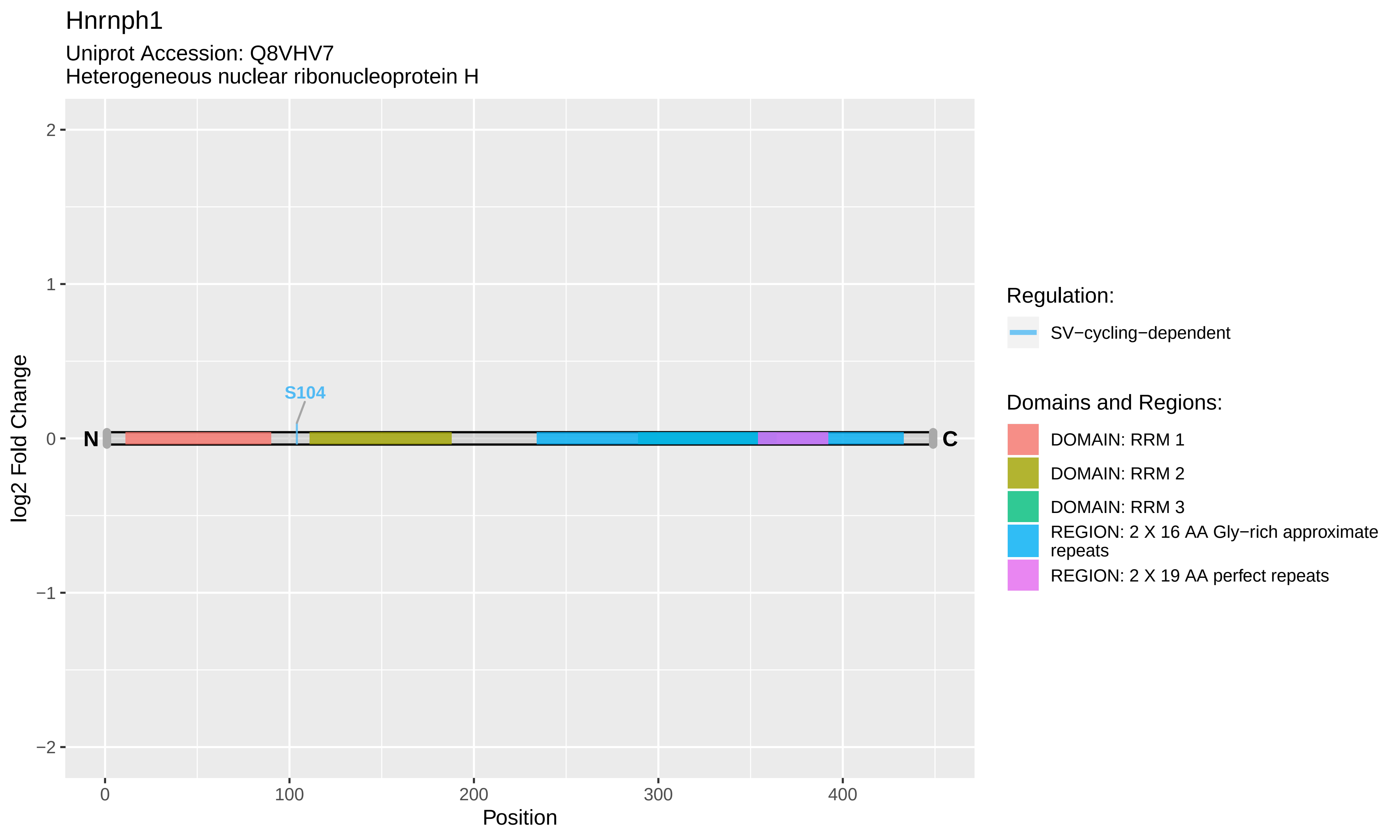
Hnrnph1
Uniprot Accession: Q8VHV7
Heterogeneous nuclear ribonucleoprotein H
S104
N
C
2
1
0
−1
−2
log2 Fold Change
0
100
200
300
400
Position
Regulation:
SV−cycling−dependent
Domains and Regions:
DOMAIN: RRM 1
DOMAIN: RRM 2
DOMAIN: RRM 3
REGION: 2 X 16 AA Gly−rich approximate repeats
REGION: 2 X 19 AA perfect repeats

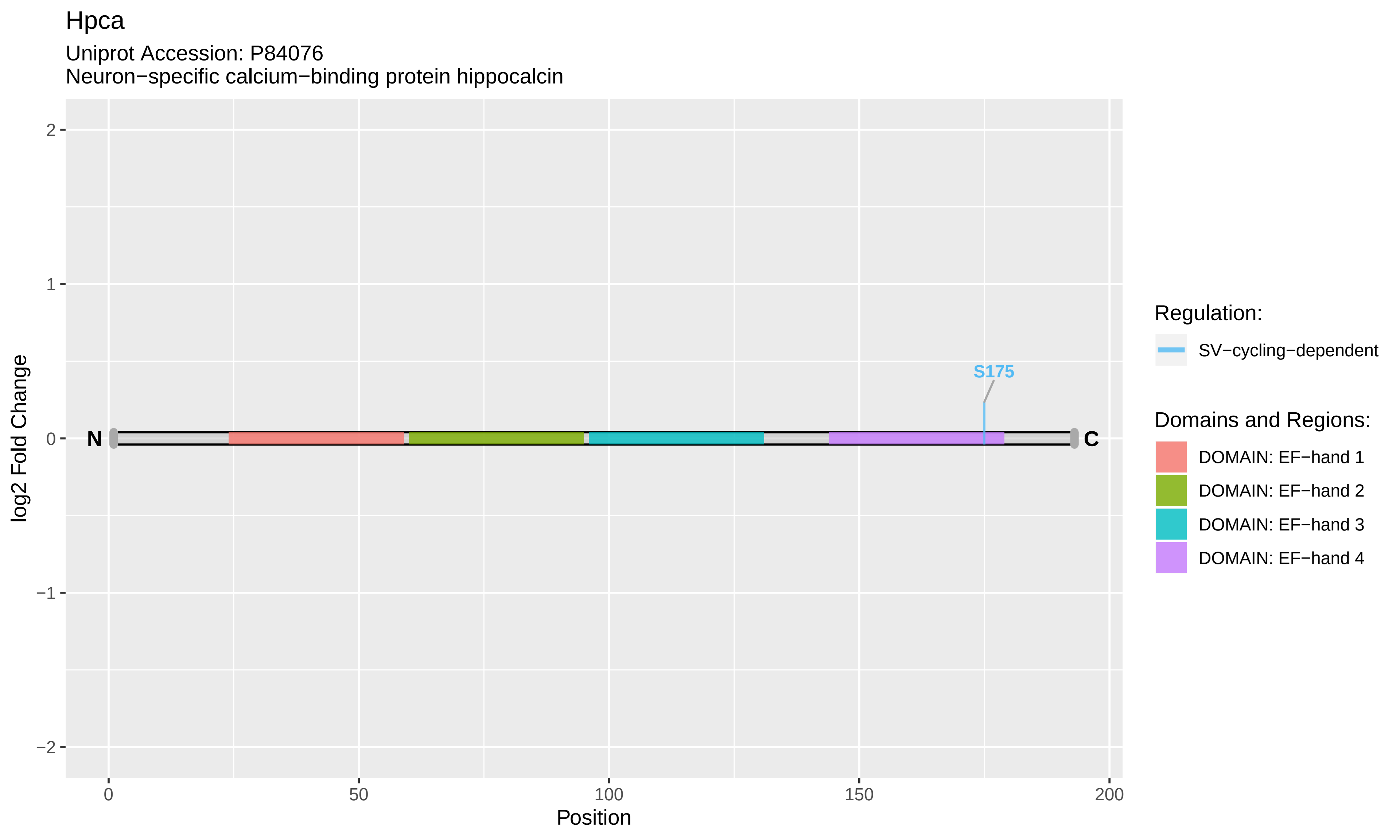

Hpca
Uniprot Accession: P84076
Neuron−specific calcium−binding protein hippocalcin
log2 Fold Change
2
1
0
−1
−2
N
C
S175
0
50
100
150
200
Position
Regulation:
SV−cycling−dependent
Domains and Regions:
DOMAIN: EF−hand 1
DOMAIN: EF−hand 2
DOMAIN: EF−hand 3
DOMAIN: EF−hand 4

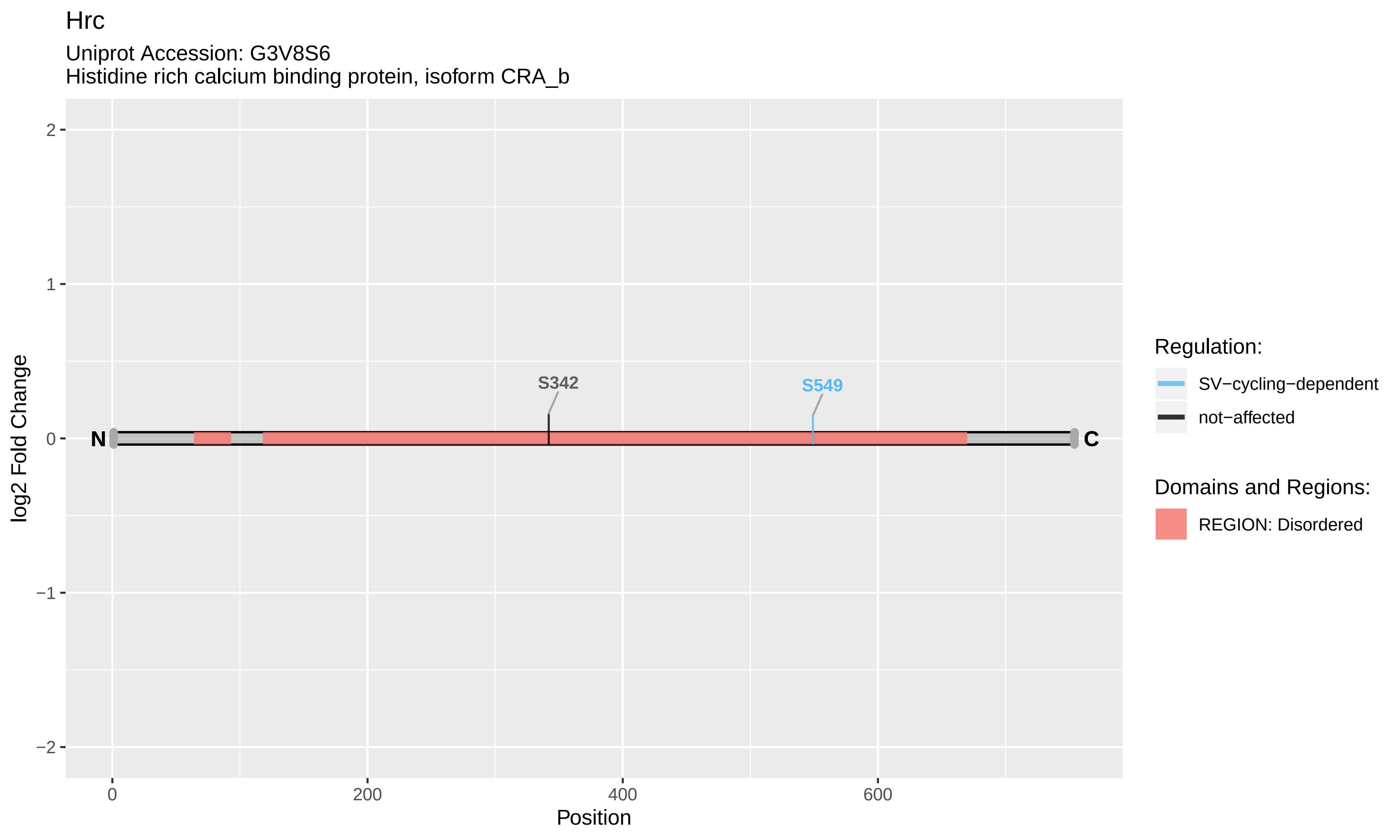
Hrc
Uniprot Accession: G3V8S6
Histidine rich calcium binding protein, isoform CRA_b
log2 Fold Change
2
1
0
−1
−2
N
C
S342
S549
0
200
400
600
Position
Regulation:
SV−cycling−dependent
not−affected
Domains and Regions:
REGION: Disordered

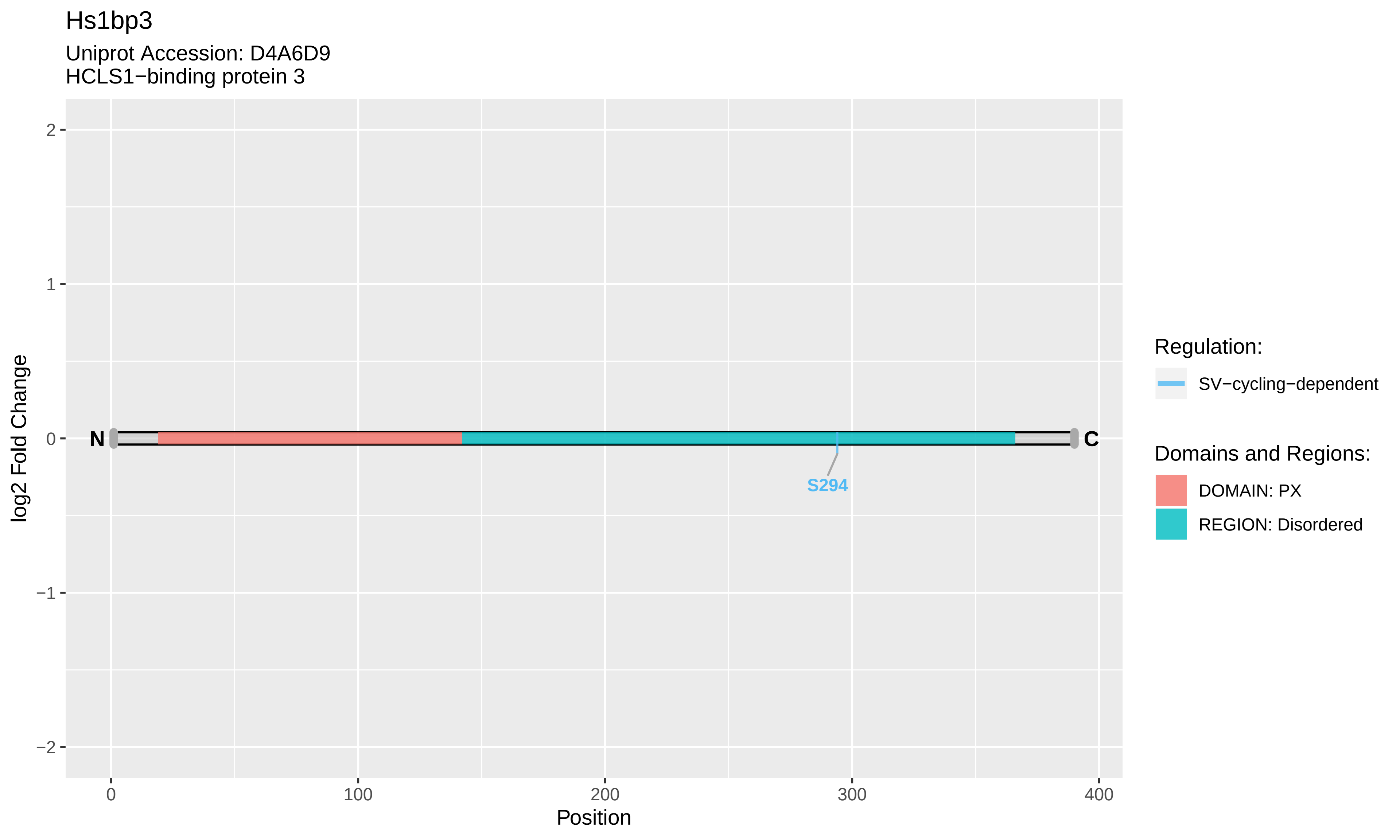

Hs1bp3
Uniprot Accession: D4A6D9
HCLS1−binding protein 3
log2 Fold Change
2
1
0
−1
−2
N
C
S294
0
100
200
300
400
Position
Regulation:
SV−cycling−dependent
Domains and Regions:
DOMAIN: PX
REGION: Disordered

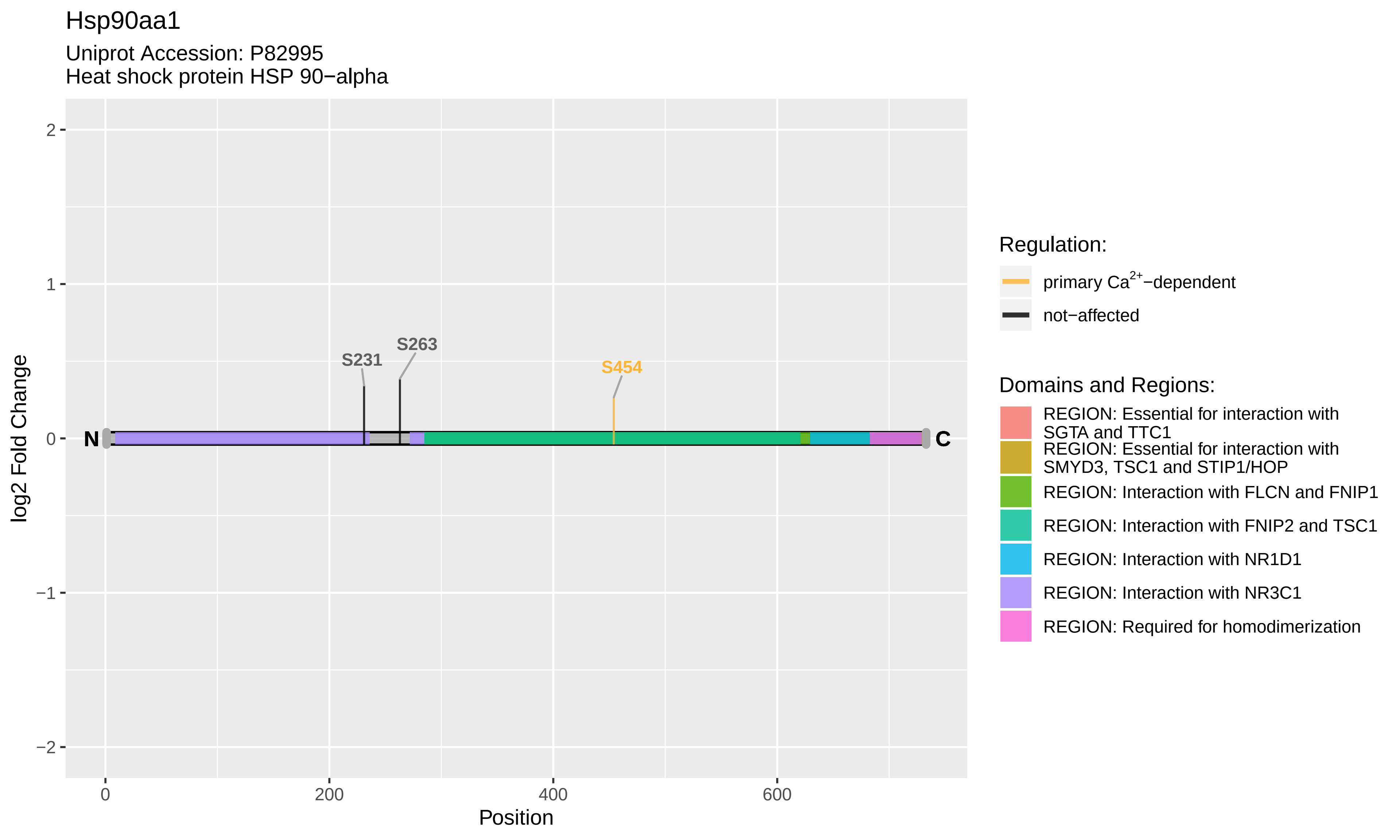

Hsp90aa1
Uniprot Accession: P82995
Heat shock protein HSP 90−alpha
log2 Fold Change
Position
S231
S263
S454
N
C
2
1
0
−1
−2
0
200
400
600
Regulation:
primary Ca2+−dependent
not−affected
Domains and Regions:
REGION: Essential for interaction with SGTA and TTC1
REGION: Essential for interaction with SMYD3, TSC1 and STIP1/HOP
REGION: Interaction with FLCN and FNIP1
REGION: Interaction with FNIP2 and TSC1
REGION: Interaction with NR1D1
REGION: Interaction with NR3C1
REGION: Required for homodimerization

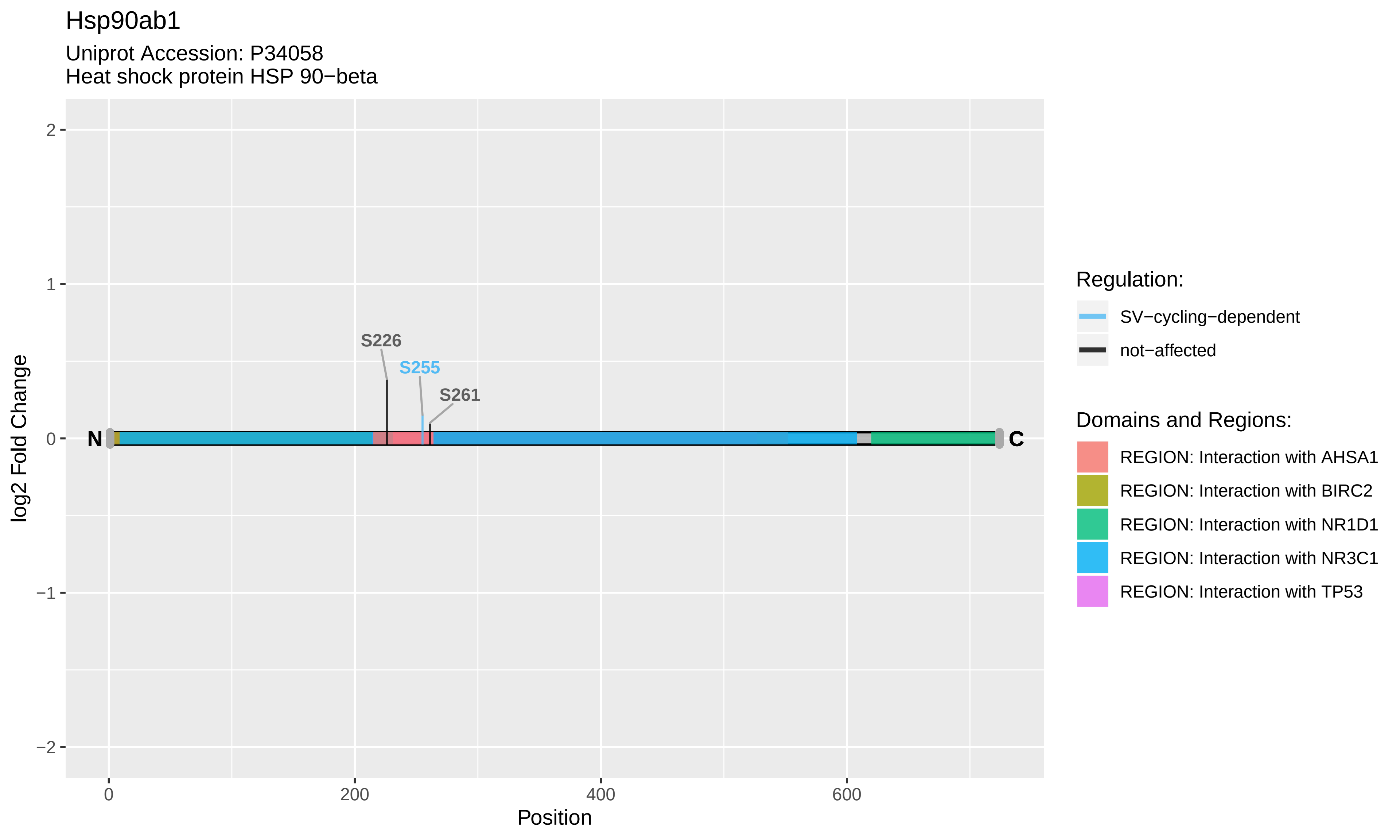

Hsp90ab1
Uniprot Accession: P34058
Heat shock protein HSP 90−beta
S226
S255
S261
N
C
2
1
0
−1
−2
log2 Fold Change
0
200
400
600
Position
Regulation:
SV−cycling−dependent
not−affected
Domains and Regions:
REGION: Interaction with AHSA1
REGION: Interaction with BIRC2
REGION: Interaction with NR1D1
REGION: Interaction with NR3C1
REGION: Interaction with TP53

Idh3a

Uniprot Accession: Q99NA5
Isocitrate dehydrogenase [NAD] subunit alpha, mitochondrial

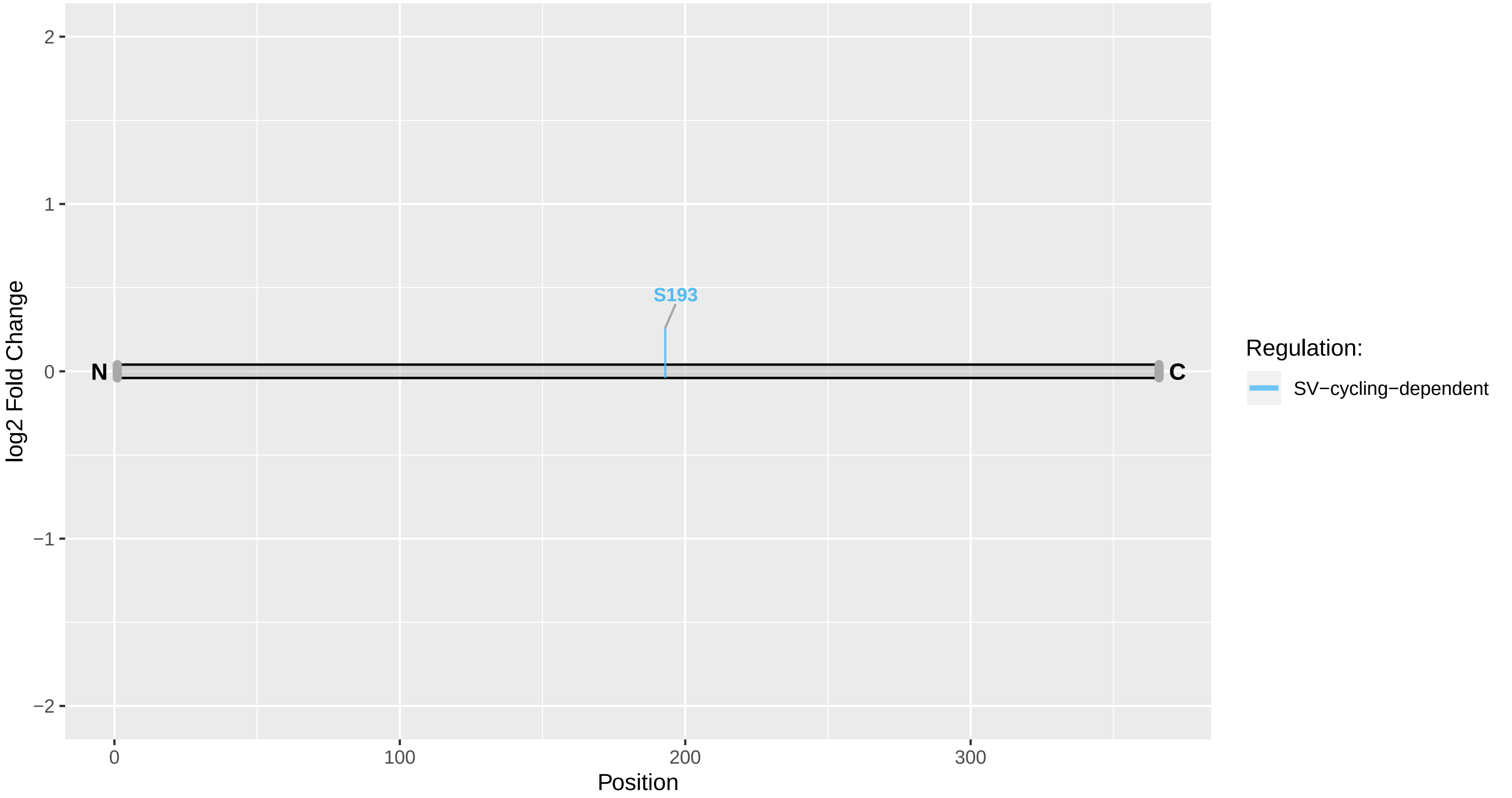

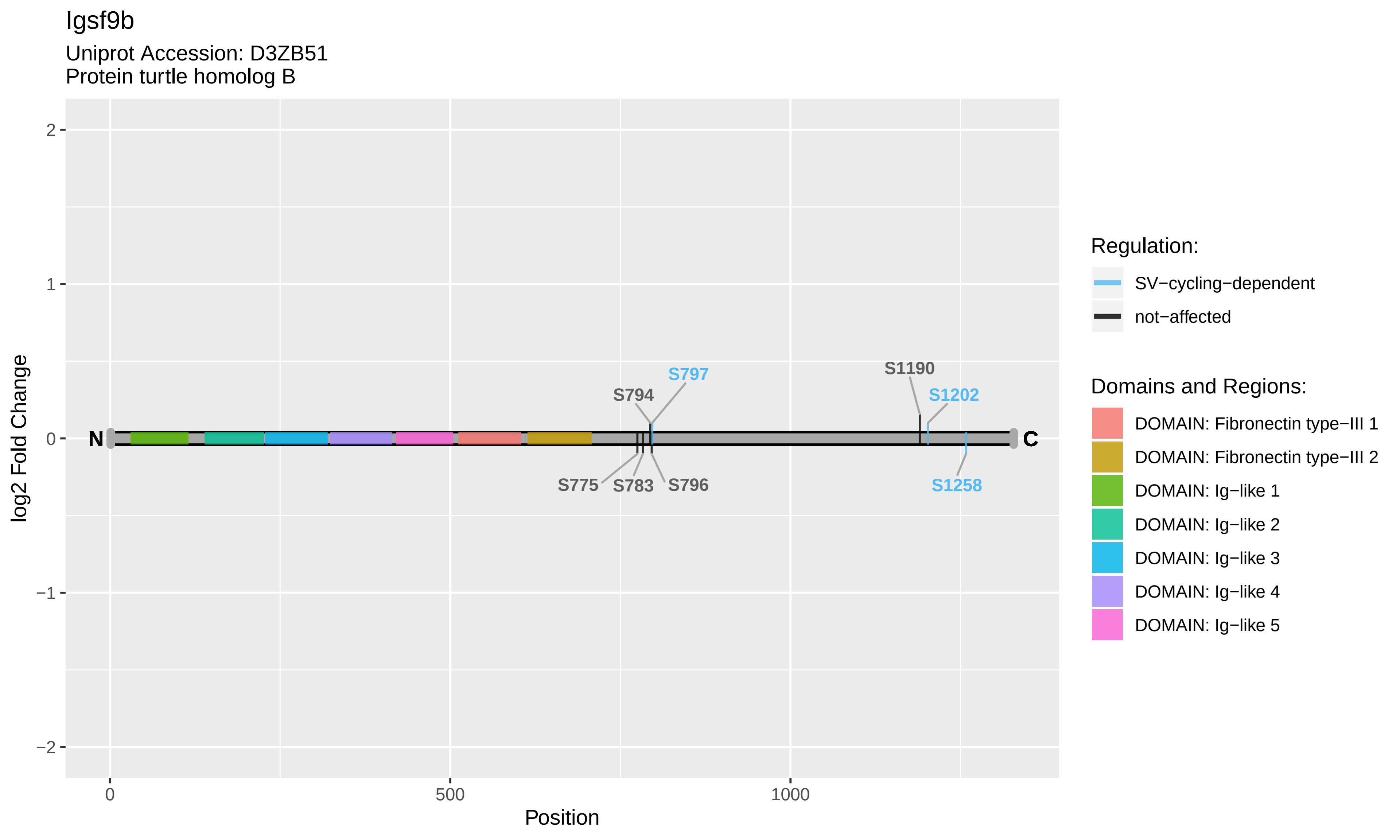
Igsf9b
Uniprot Accession: D3ZB51
Protein turtle homolog B
log2 Fold Change
2
1
0
−1
−2
N
C
S797
S794
S1190
S1202
S775
S783
S796
S1258
0
500
1000
Position
Regulation:
SV−cycling−dependent
not−affected
Domains and Regions:
DOMAIN: Fibronectin type−III 1
DOMAIN: Fibronectin type−III 2
DOMAIN: Ig−like 1
DOMAIN: Ig−like 2
DOMAIN: Ig−like 3
DOMAIN: Ig−like 4
DOMAIN: Ig−like 5

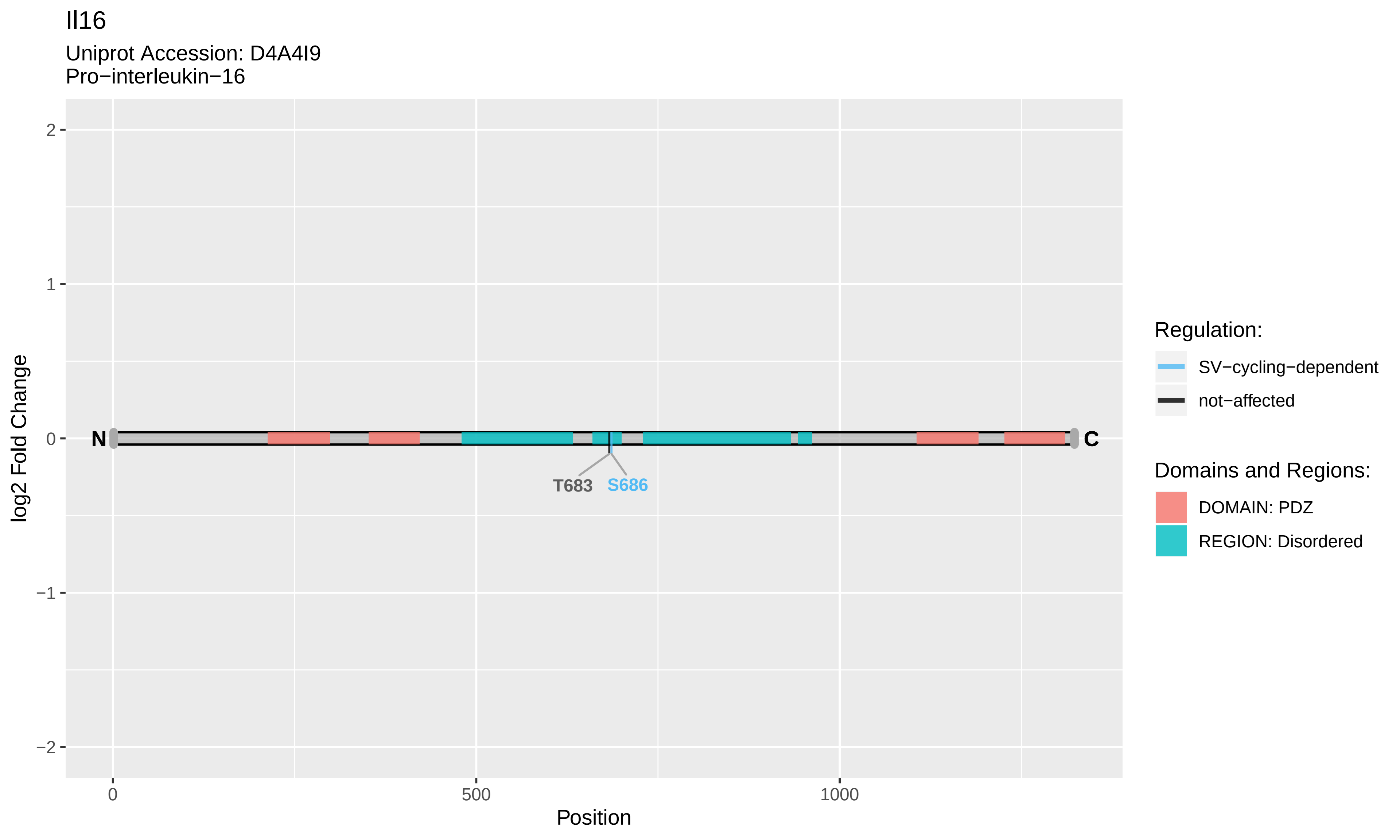

Il16
Uniprot Accession: D4A4I9
Pro−interleukin−16
log2 Fold Change
2
1
0
−1
−2
N
C
T683
S686
0
500
1000
Position
Regulation:
SV−cycling−dependent
not−affected
Domains and Regions:
DOMAIN: PDZ
REGION: Disordered

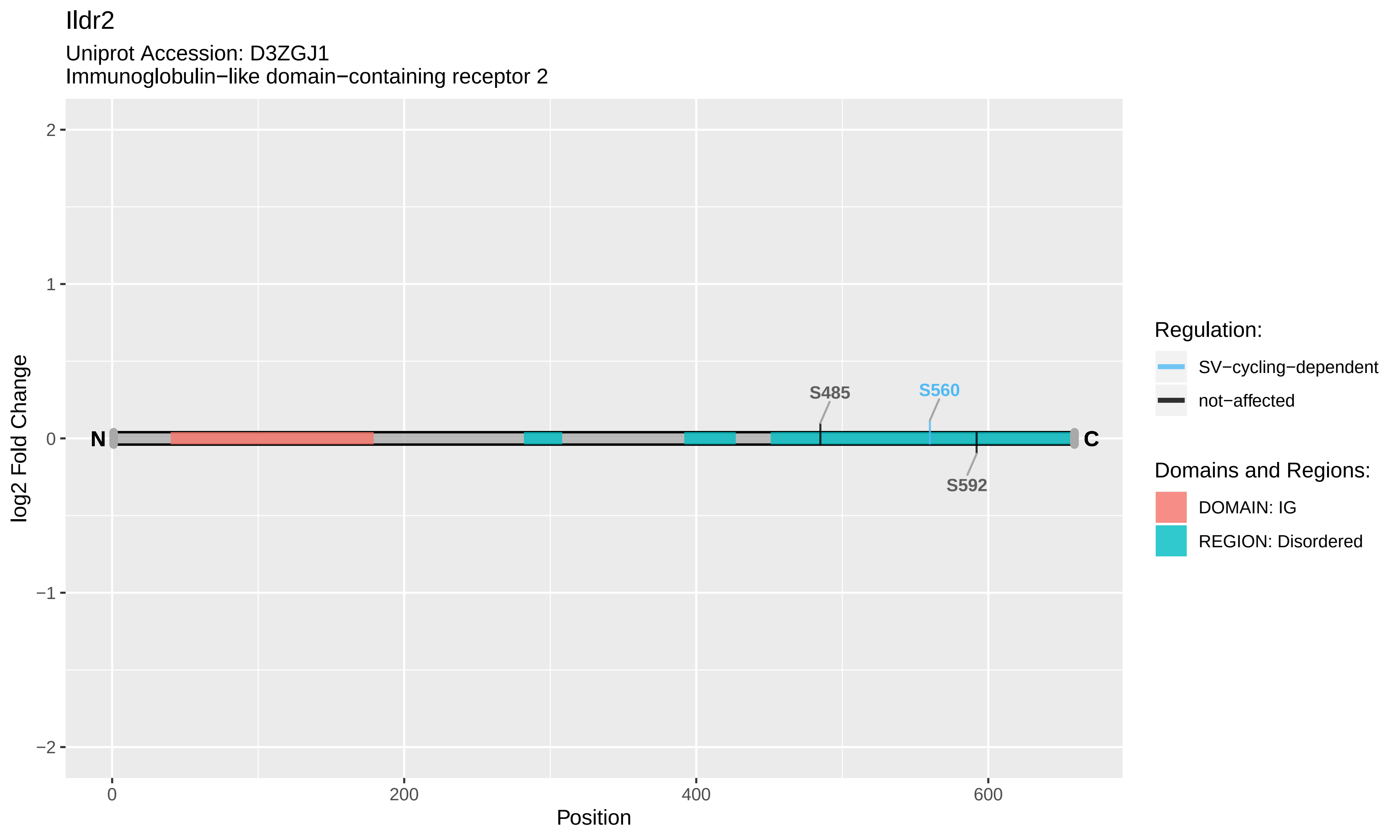

Ildr2
Uniprot Accession: D3ZGJ1
Immunoglobulin−like domain−containing receptor 2
log2 Fold Change
2
1
0
−1
−2
N
C
S485
S560
S592
0
200
400
600
Position
Regulation:
SV−cycling−dependent
not−affected
Domains and Regions:
DOMAIN: IG
REGION: Disordered

Inpp5e

Uniprot Accession: Q9WVR1
72 kDa inositol polyphosphate 5−phosphatase

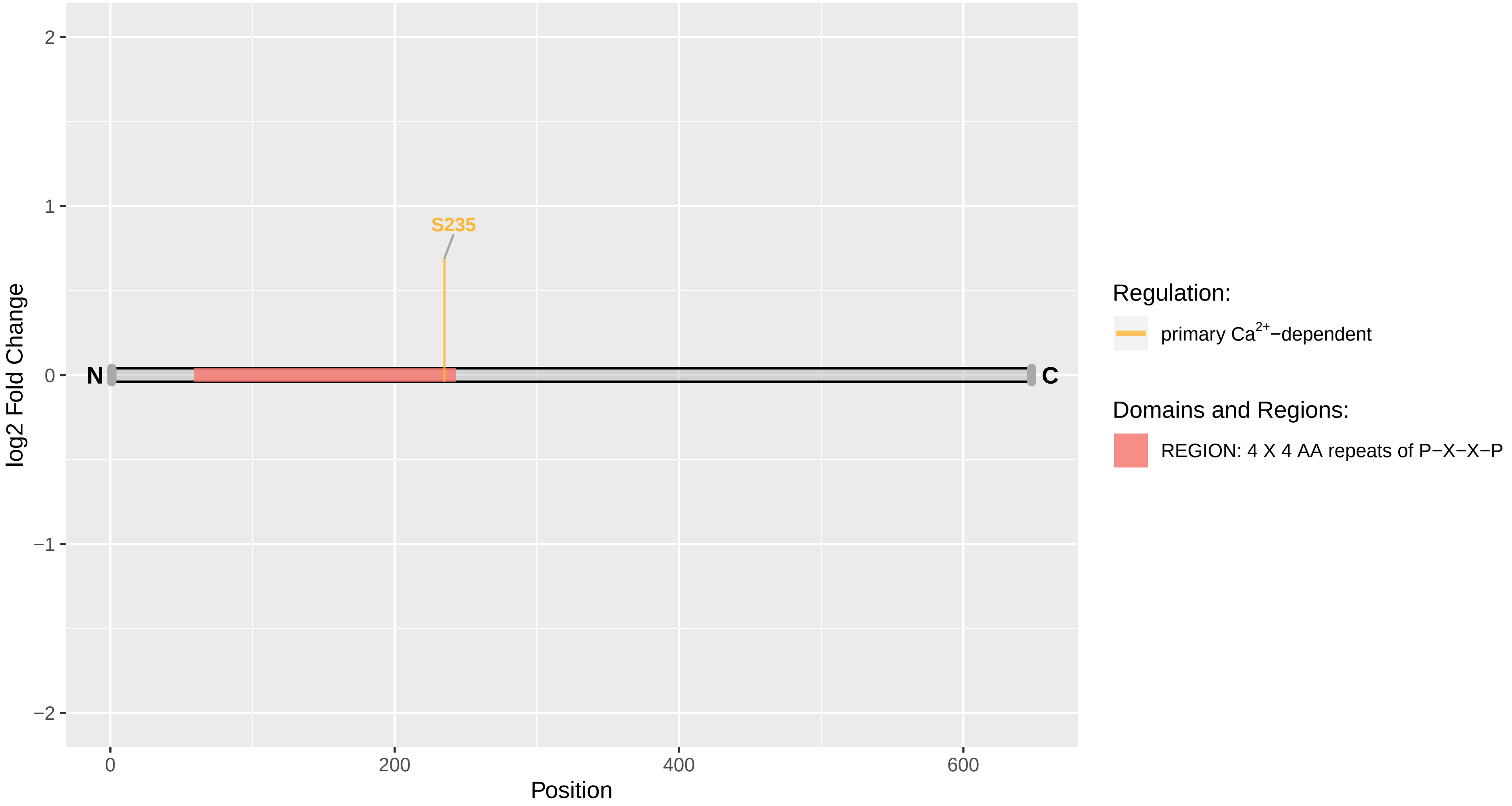

Inpp5f

Uniprot Accession: D3ZKG7
Inositol polyphosphate−5−phosphatase F

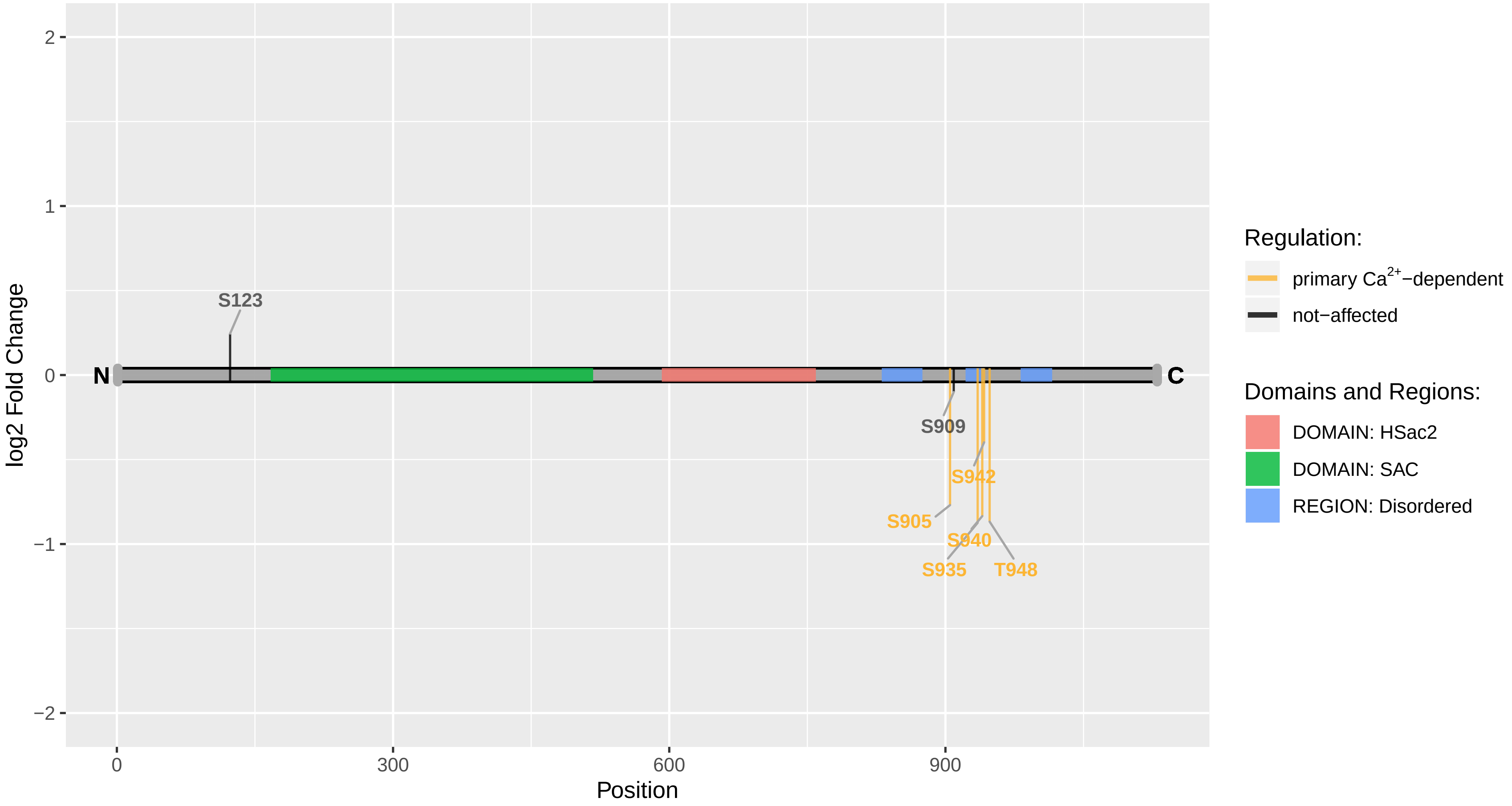

Inpp5j

Uniprot Accession: Q9JMC1
Phosphatidylinositol 4,5−bisphosphate 5−phosphatase A

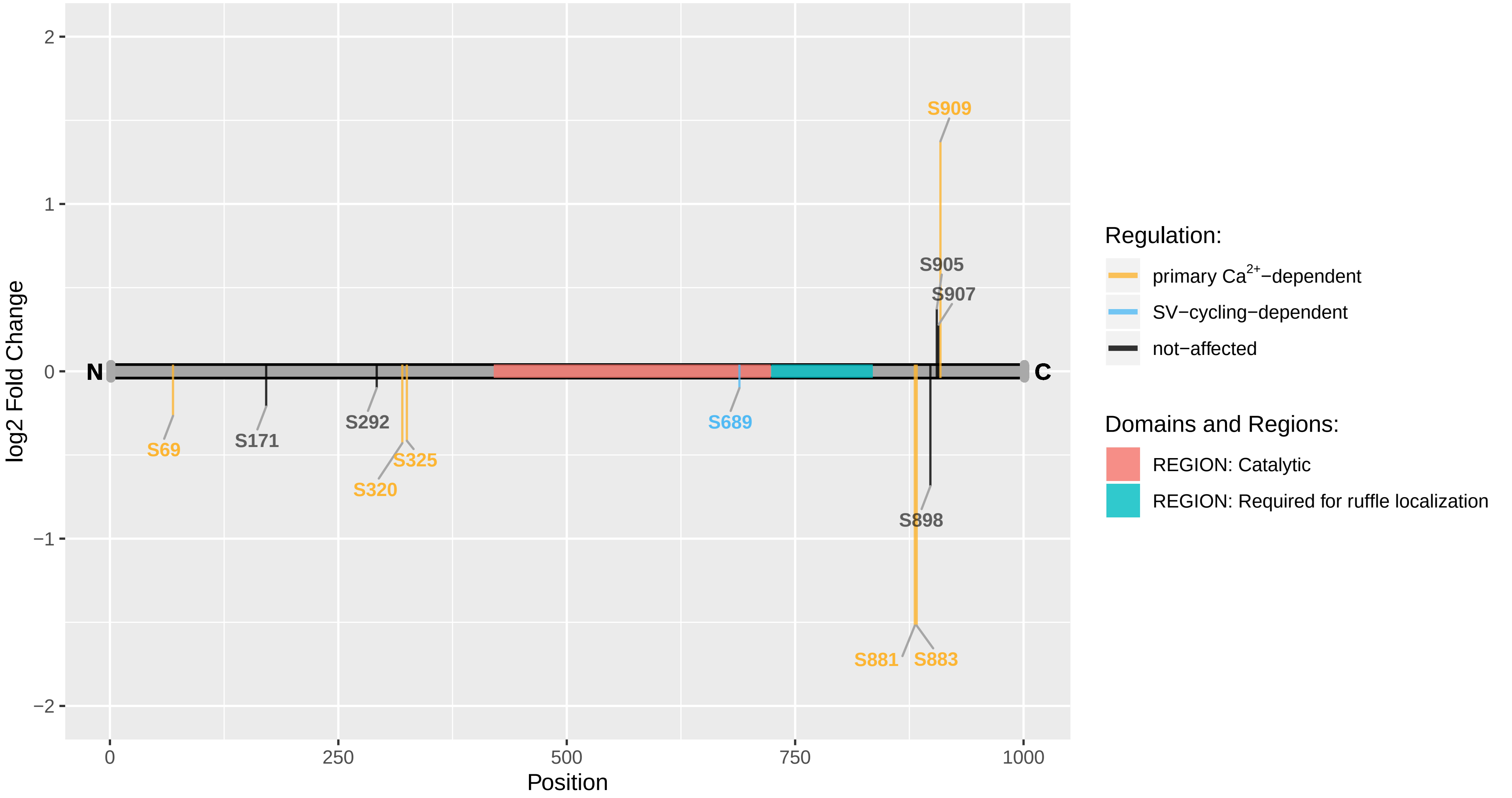

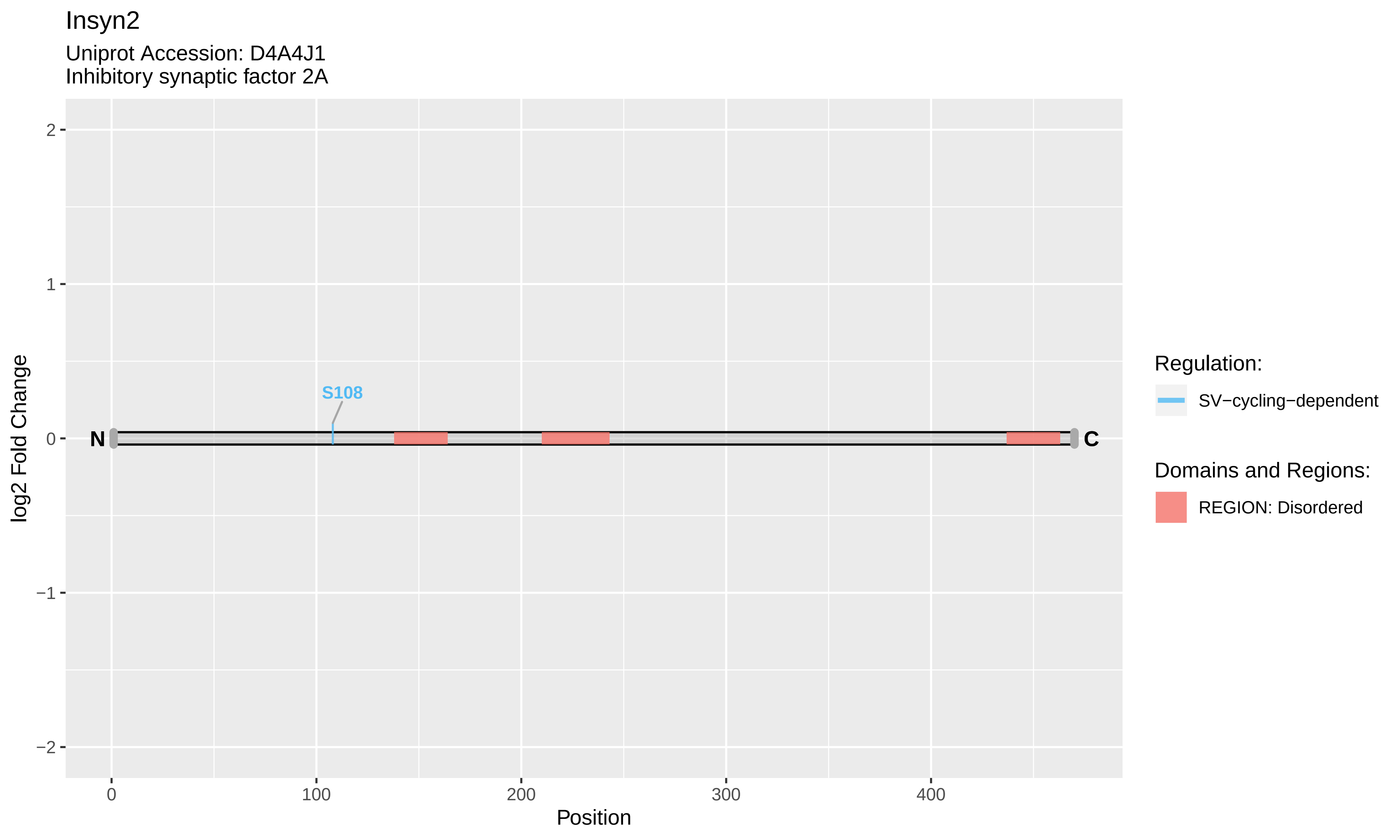

Insyn2
Uniprot Accession: D4A4J1
Inhibitory synaptic factor 2A
S108
N
C
log2 Fold Change
2
1
0
−1
−2
0
100
200
300
400
Position
Regulation:
SV−cycling−dependent
Domains and Regions:
REGION: Disordered

Iqsec1

Uniprot Accession: A0A0G2JUG7
IQ motif and Sec7 domain 1

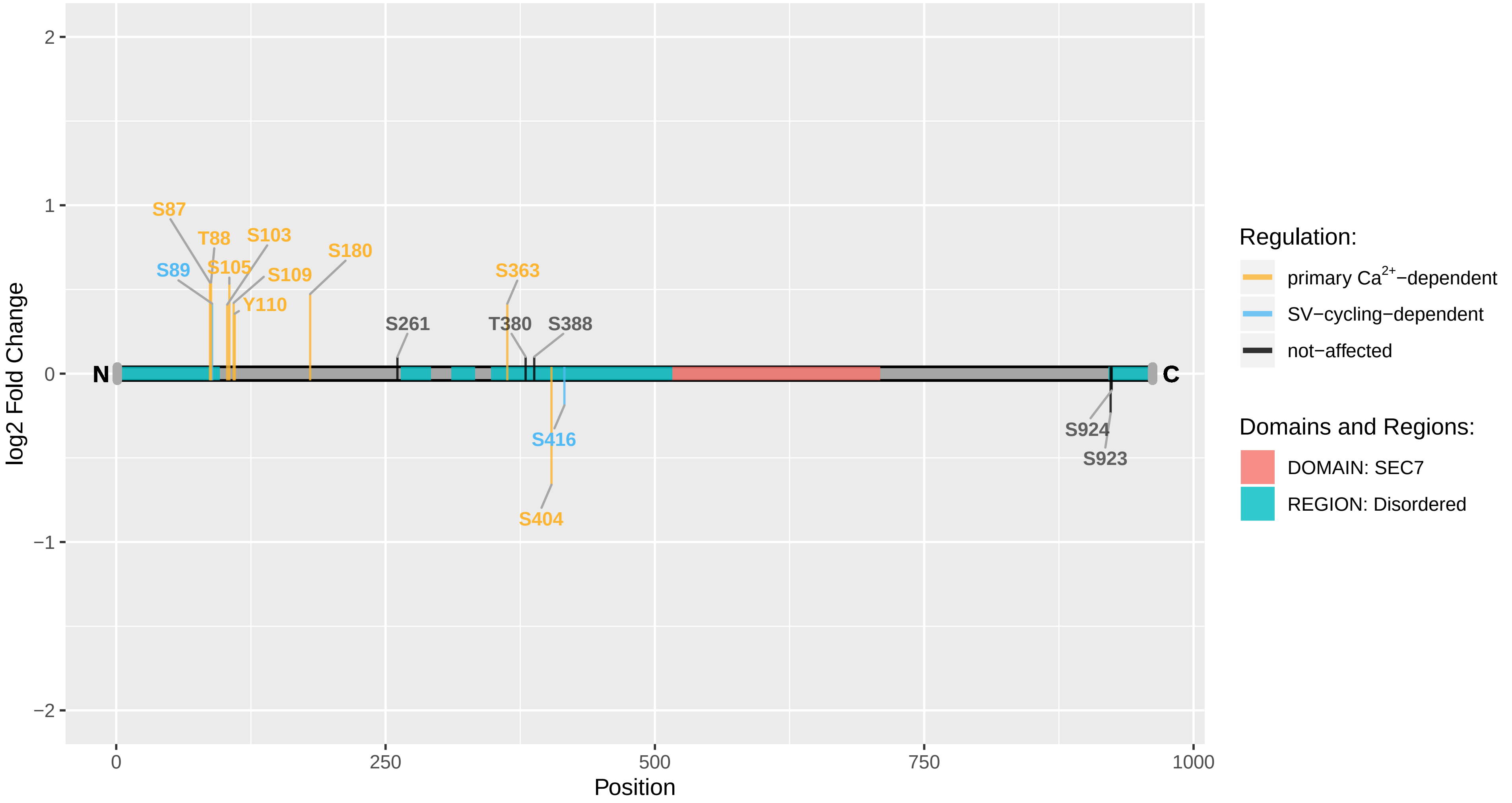

Iqsec2

Uniprot Accession: A0A0G2JZX5
IQ motif and Sec7 domain 2

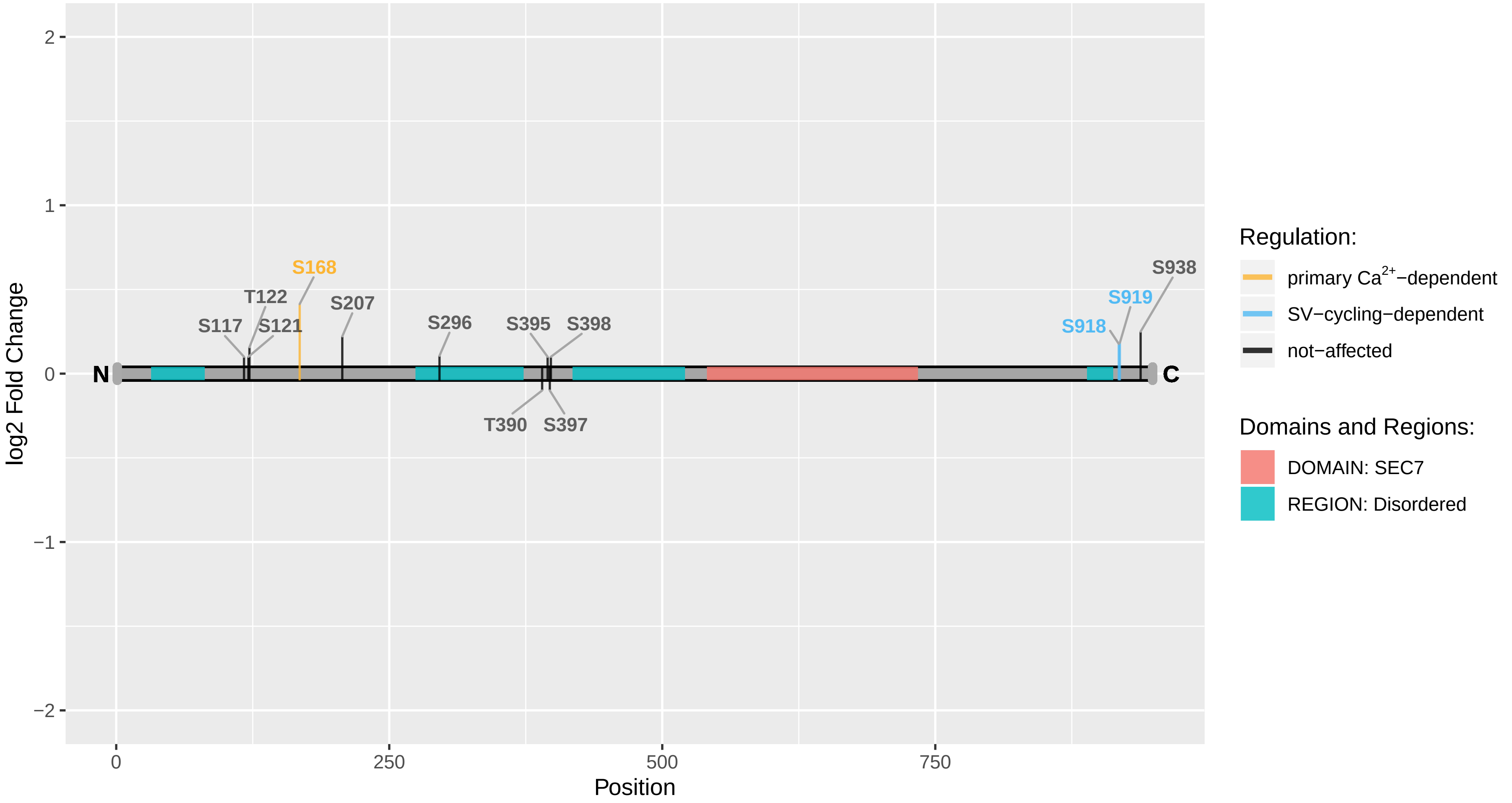

Iqsec3

Uniprot Accession: Q76M68
IQ motif and SEC7 domain−containing protein 3

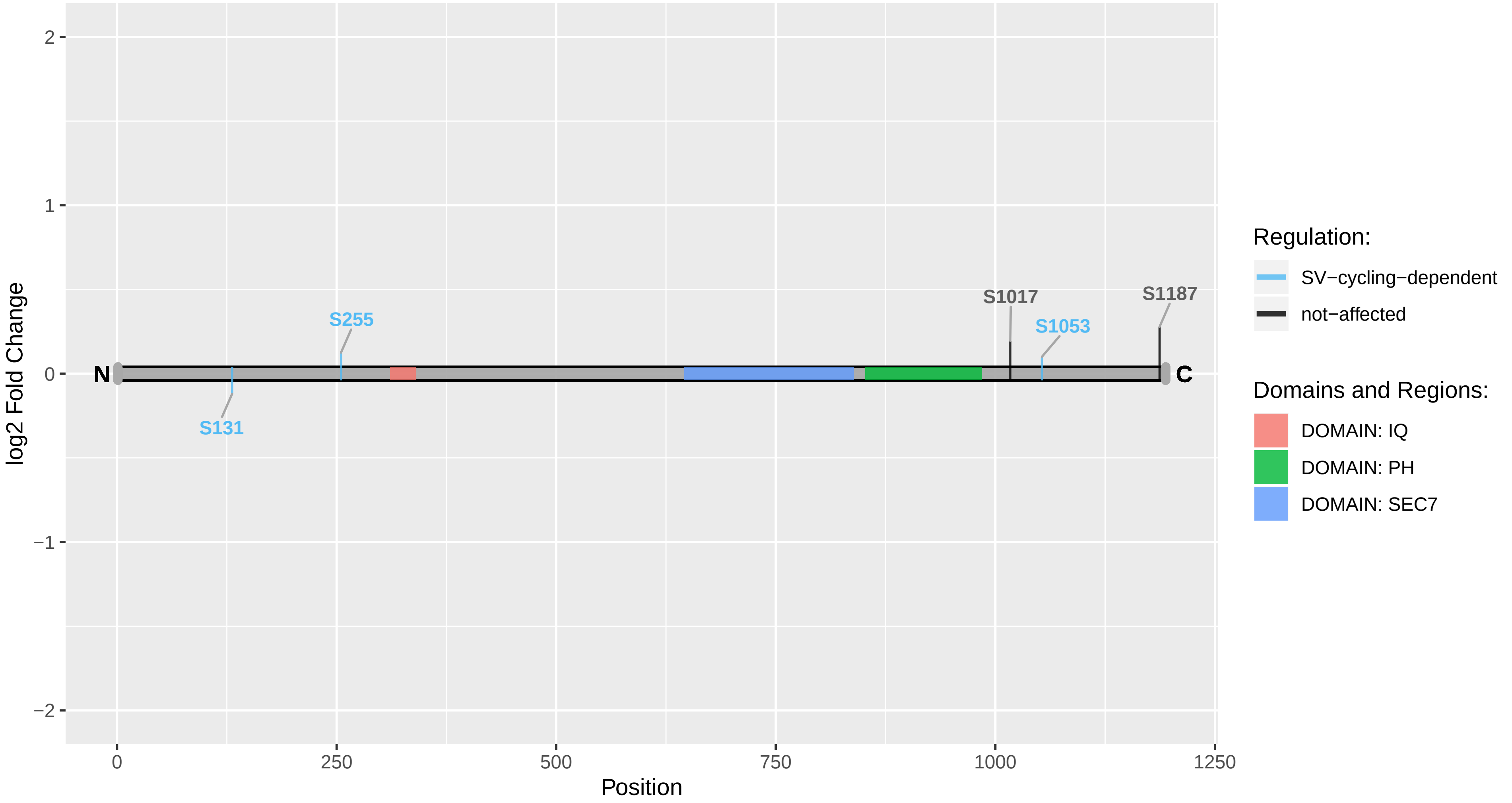

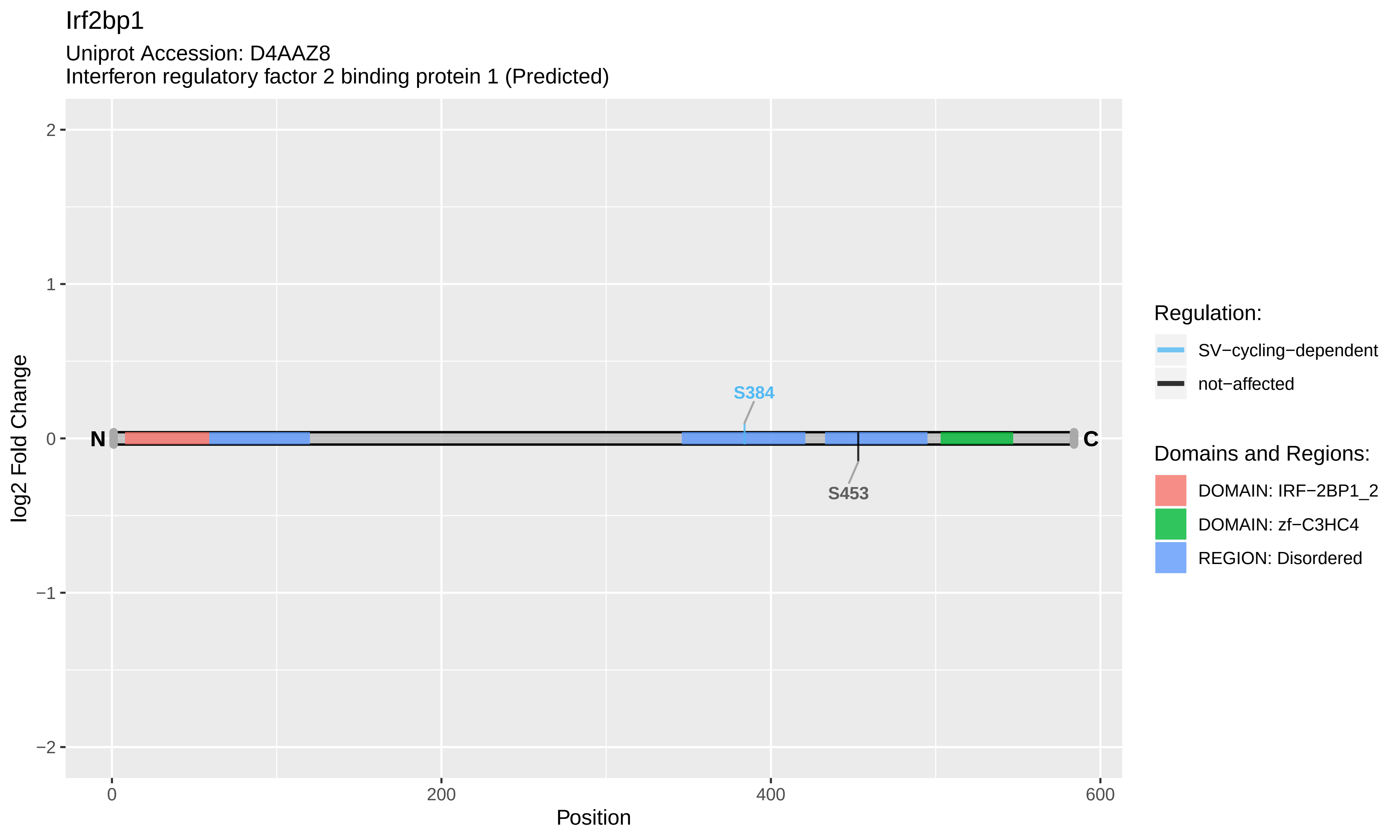
Irf2bp1
Uniprot Accession: D4AAZ8
Interferon regulatory factor 2 binding protein 1 (Predicted)
log2 Fold Change
2
1
0
−1
−2
N
C
S384
S453
0
200
400
600
Position
Regulation:
SV−cycling−dependent
not−affected
Domains and Regions:
DOMAIN: IRF−2BP1_2
DOMAIN: zf−C3HC4
REGION: Disordered

Irs1

Uniprot Accession: P35570
Insulin receptor substrate 1

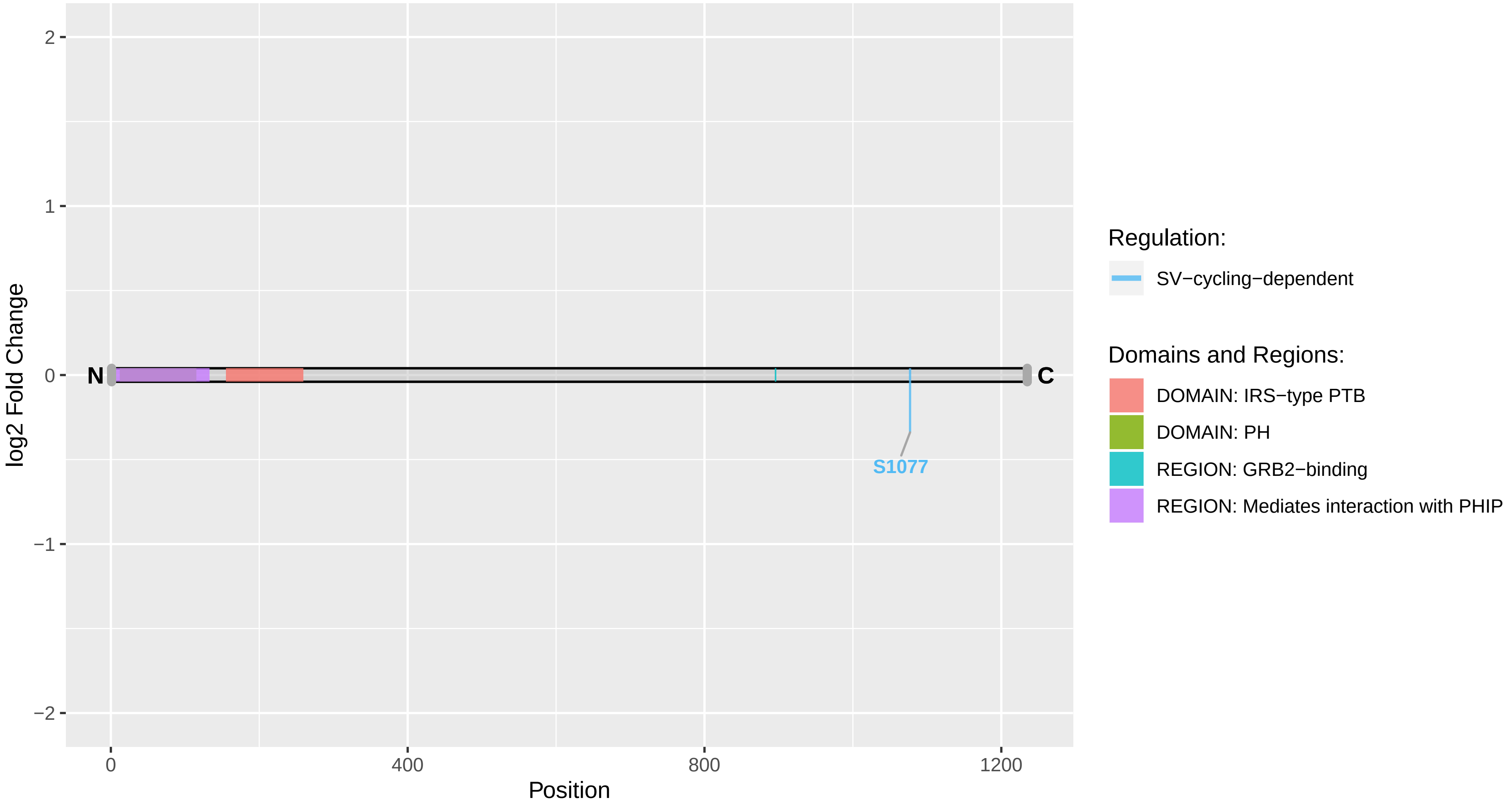

Itfg2

Uniprot Accession: A0A0G2JZW3
Integrin alpha FG−GAP repeat−containing 2

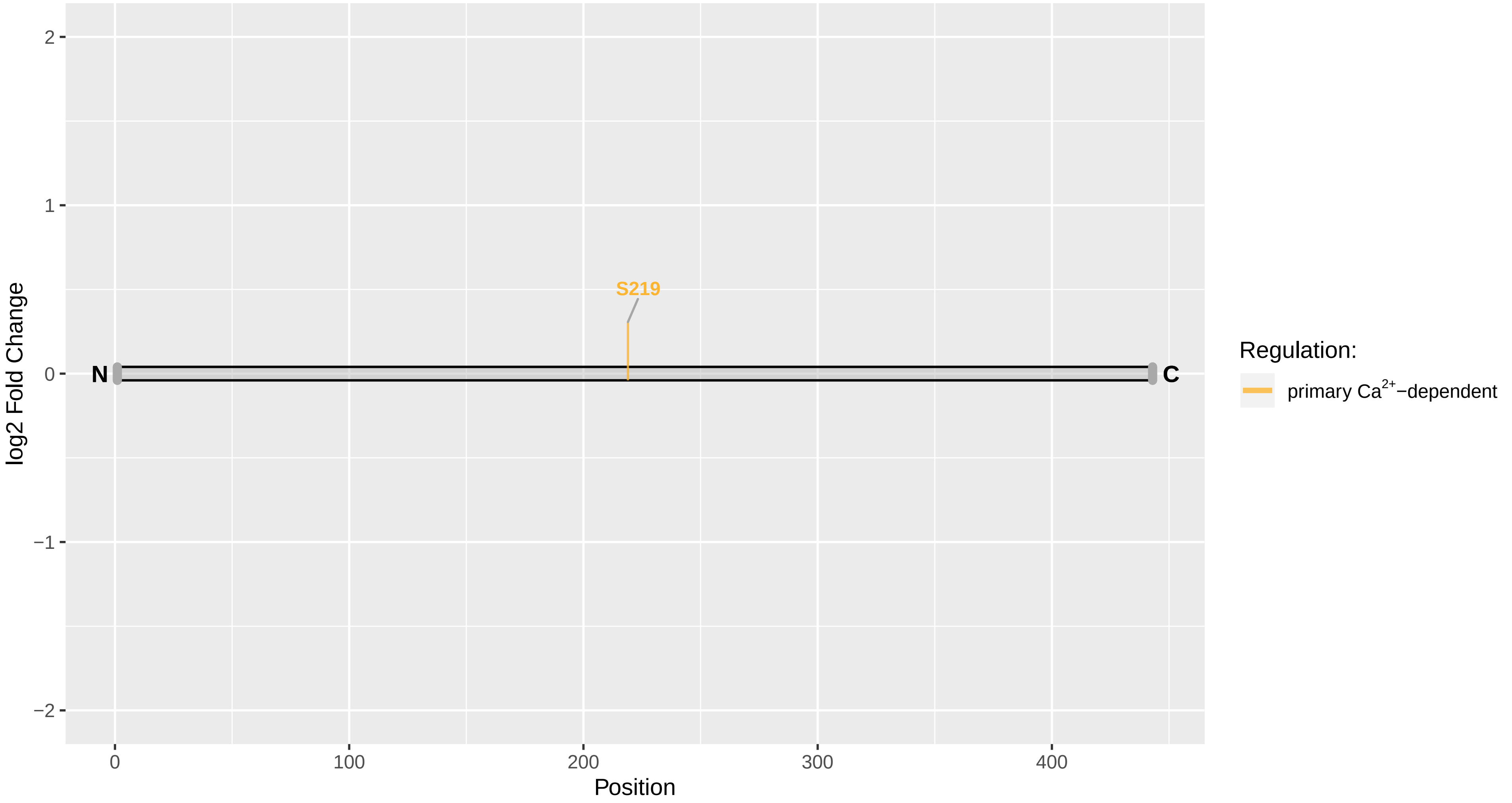

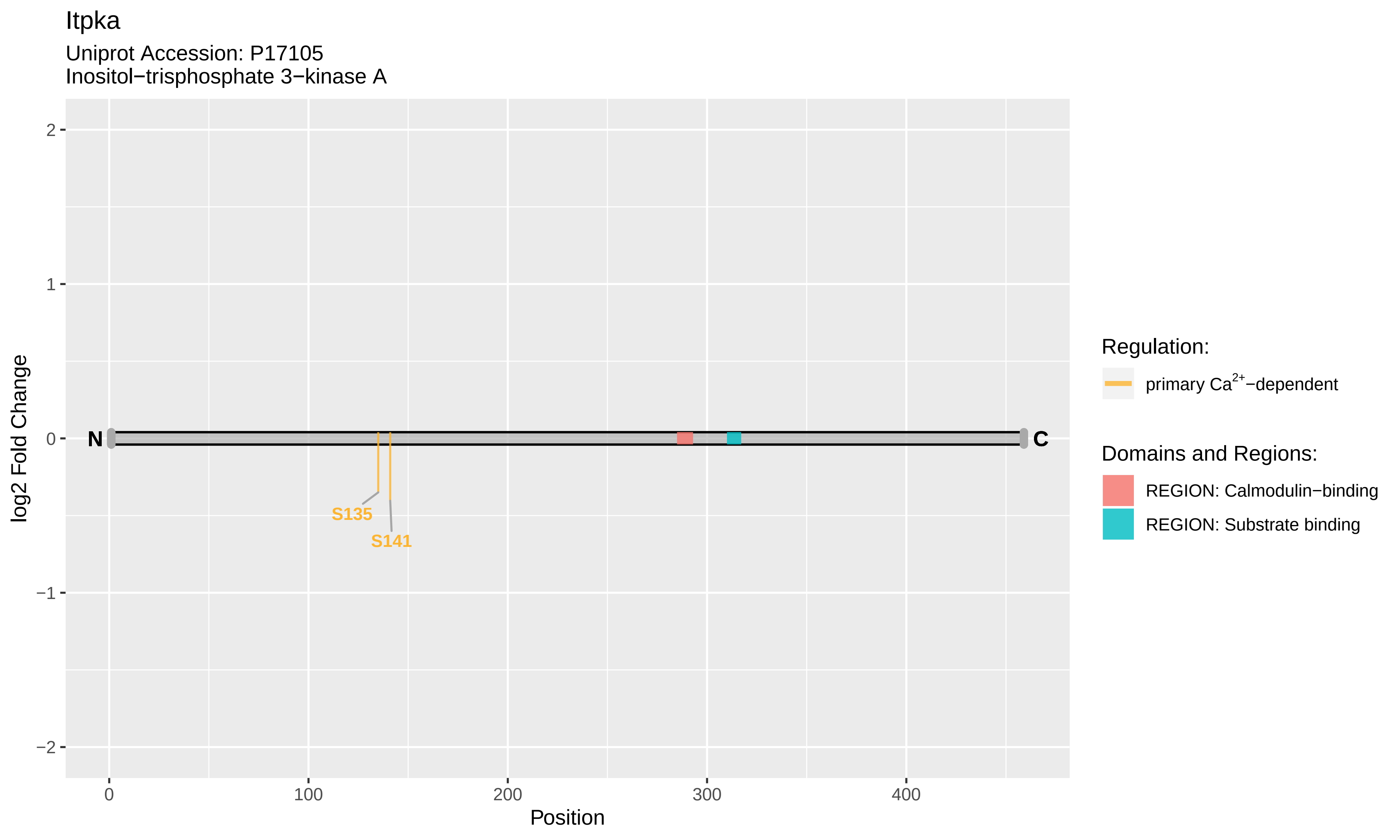

Itpka
Uniprot Accession: P17105
Inositol−trisphosphate 3−kinase A
log2 Fold Change
2
1
0
−1
−2
N
C
S135
S141
0
100
200
300
400
Position
Regulation:
primary Ca2+−dependent
Domains and Regions:
REGION: Calmodulin−binding
REGION: Substrate binding

Itpr1

Uniprot Accession: F1LNT1
Inositol 1,4,5−trisphosphate receptor type 1

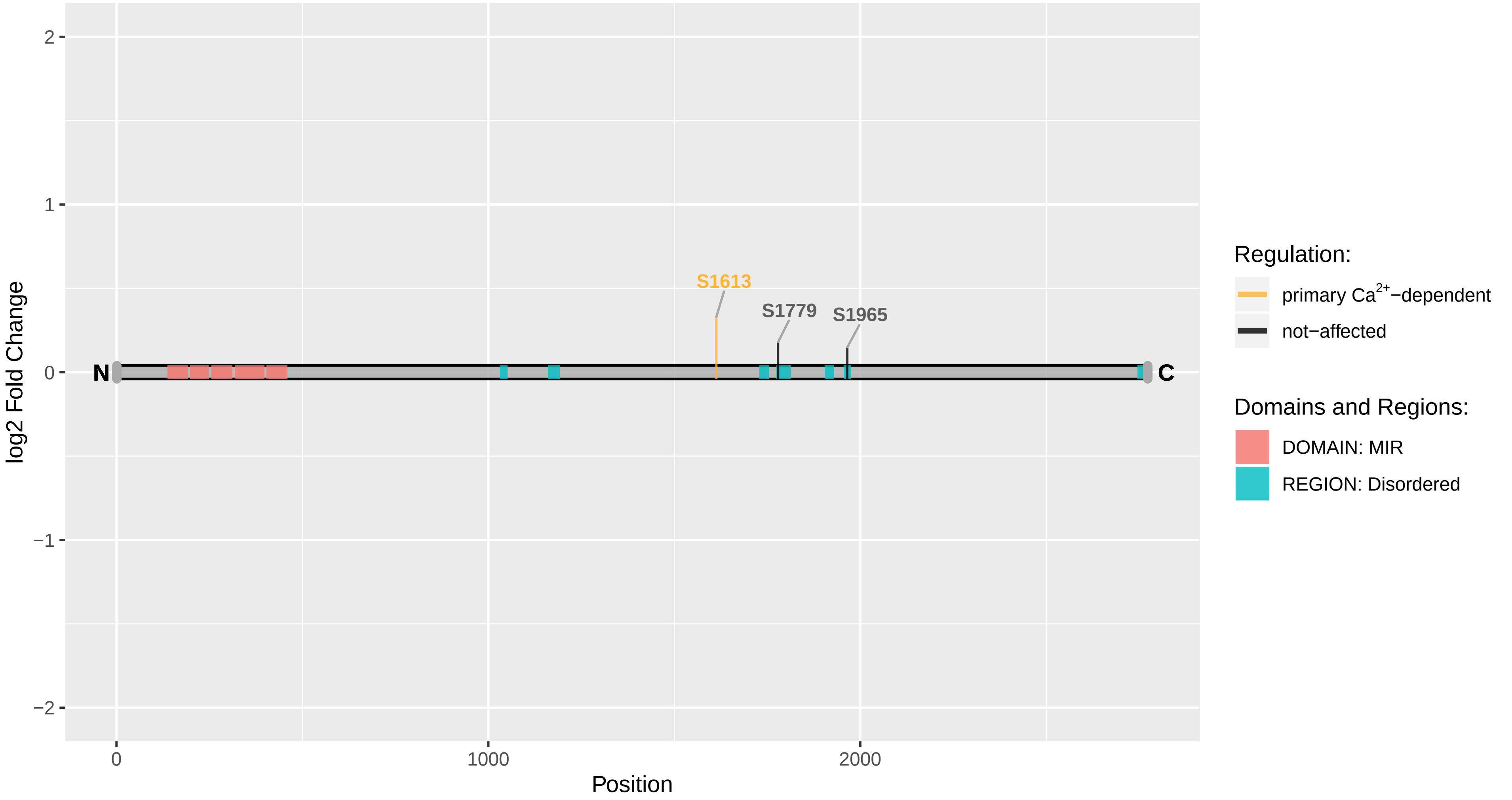

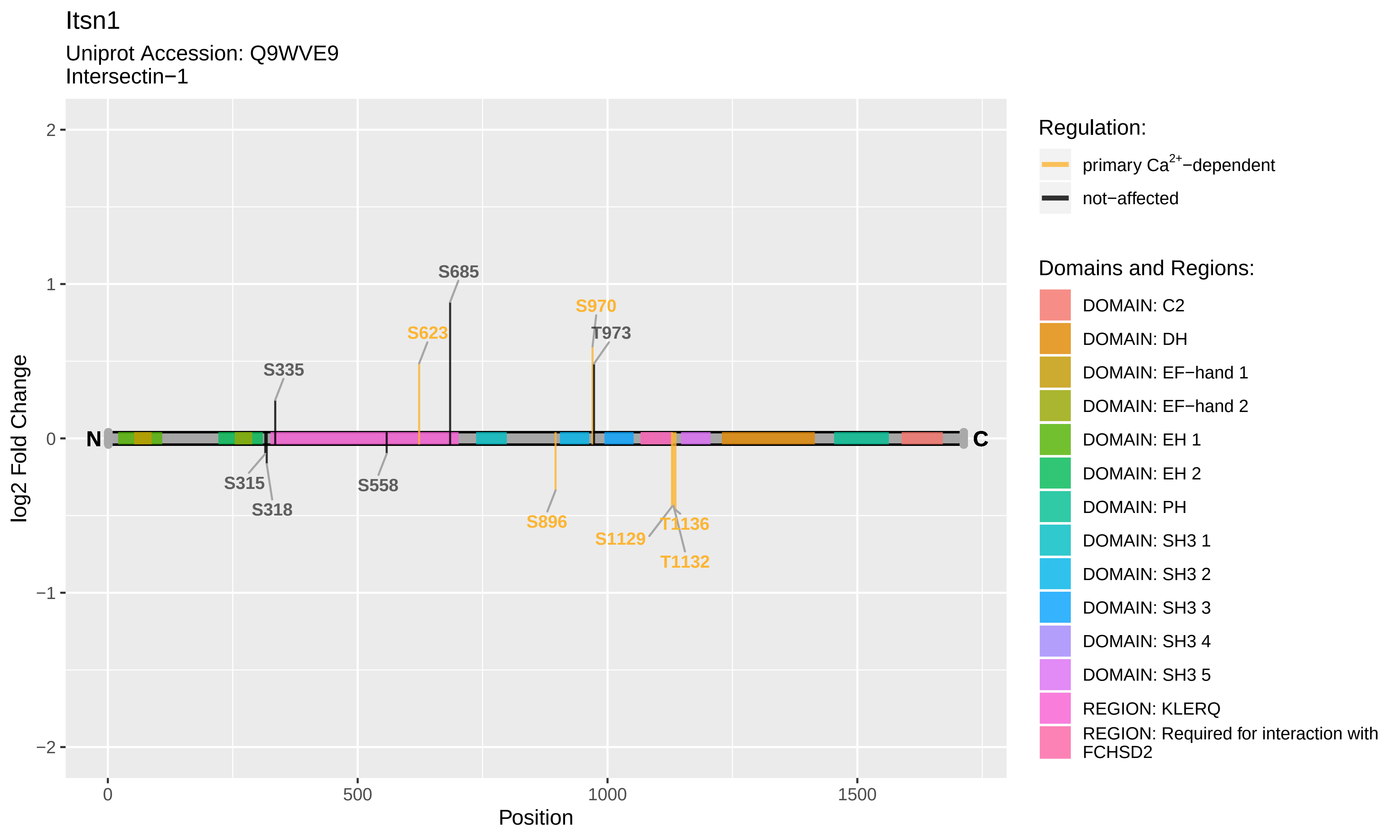

Itsn1
Uniprot Accession: Q9WVE9
Intersectin−1
log2 Fold Change
Position
2
1
0
−1
−2
0
500
1000
1500
N
C
S685
S970
T973
S623
S335
S315
S318
S558
S896
S1129
T1136
T1132
Regulation:
primary Ca2+−dependent
not−affected
Domains and Regions:
DOMAIN: C2
DOMAIN: DH
DOMAIN: EF−hand 1
DOMAIN: EF−hand 2
DOMAIN: EH 1
DOMAIN: EH 2
DOMAIN: PH
DOMAIN: SH3 1
DOMAIN: SH3 2
DOMAIN: SH3 3
DOMAIN: SH3 4
DOMAIN: SH3 5
REGION: KLERQ
REGION: Required for interaction with FCHSD2

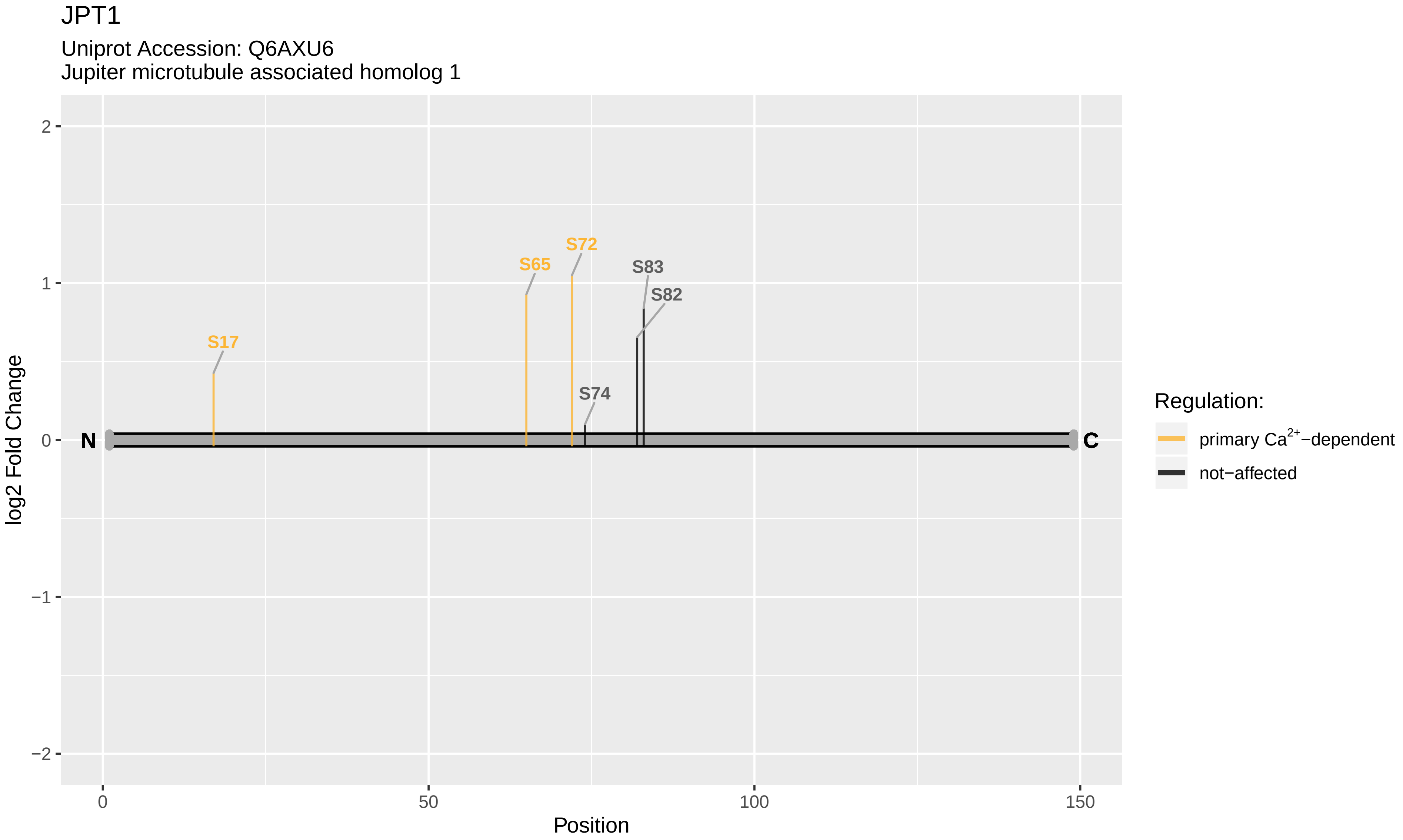
JPT1
Uniprot Accession: Q6AXU6
Jupiter microtubule associated homolog 1
log2 Fold Change
2
1
0
−1
−2
N
C
S17
S65
S72
S74
S83
S82
0
50
100
150
Position
Regulation:
primary Ca2+−dependent
not−affected

Jph1

Uniprot Accession: D3ZQ55
Junctophilin 1

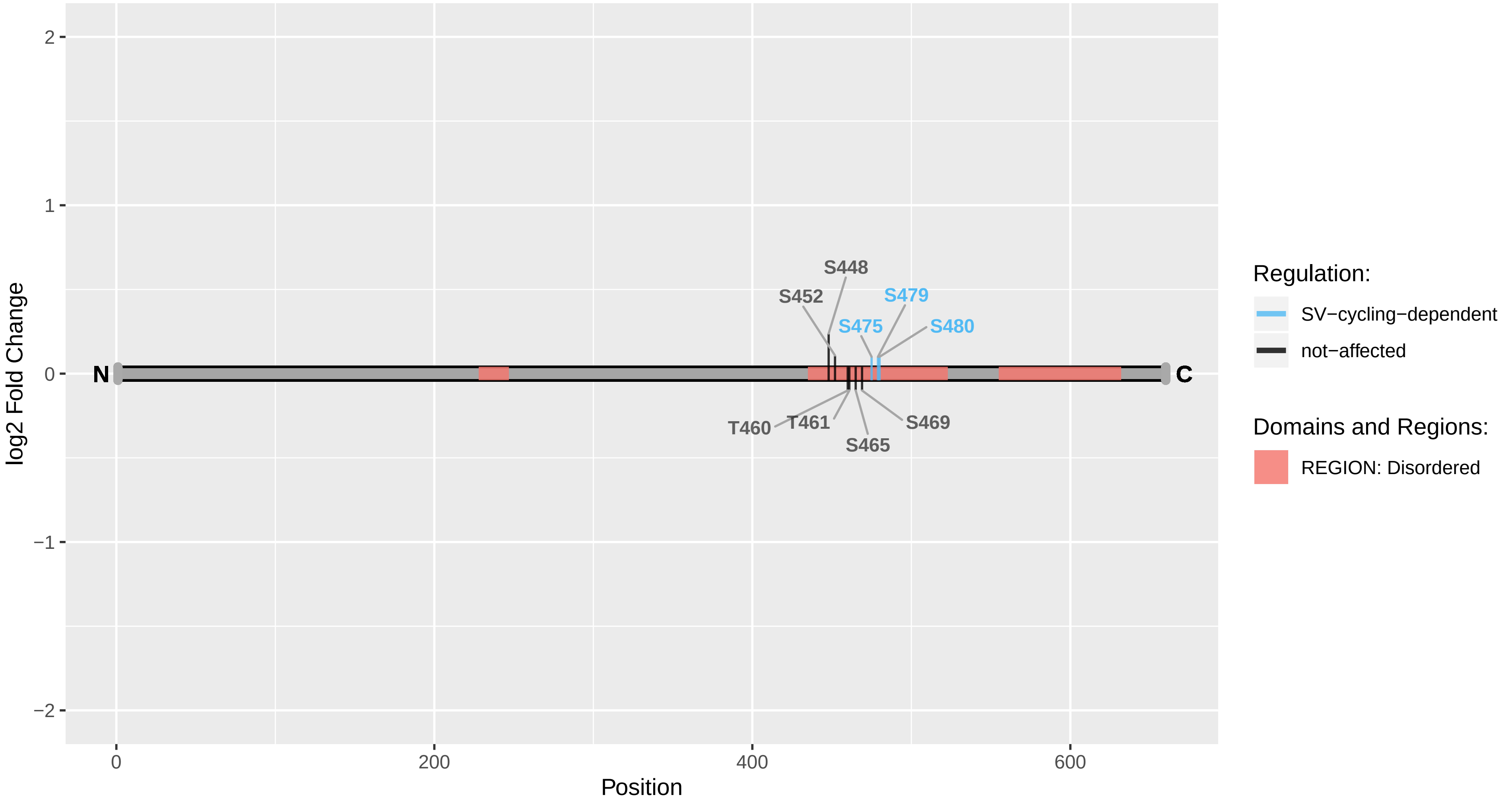

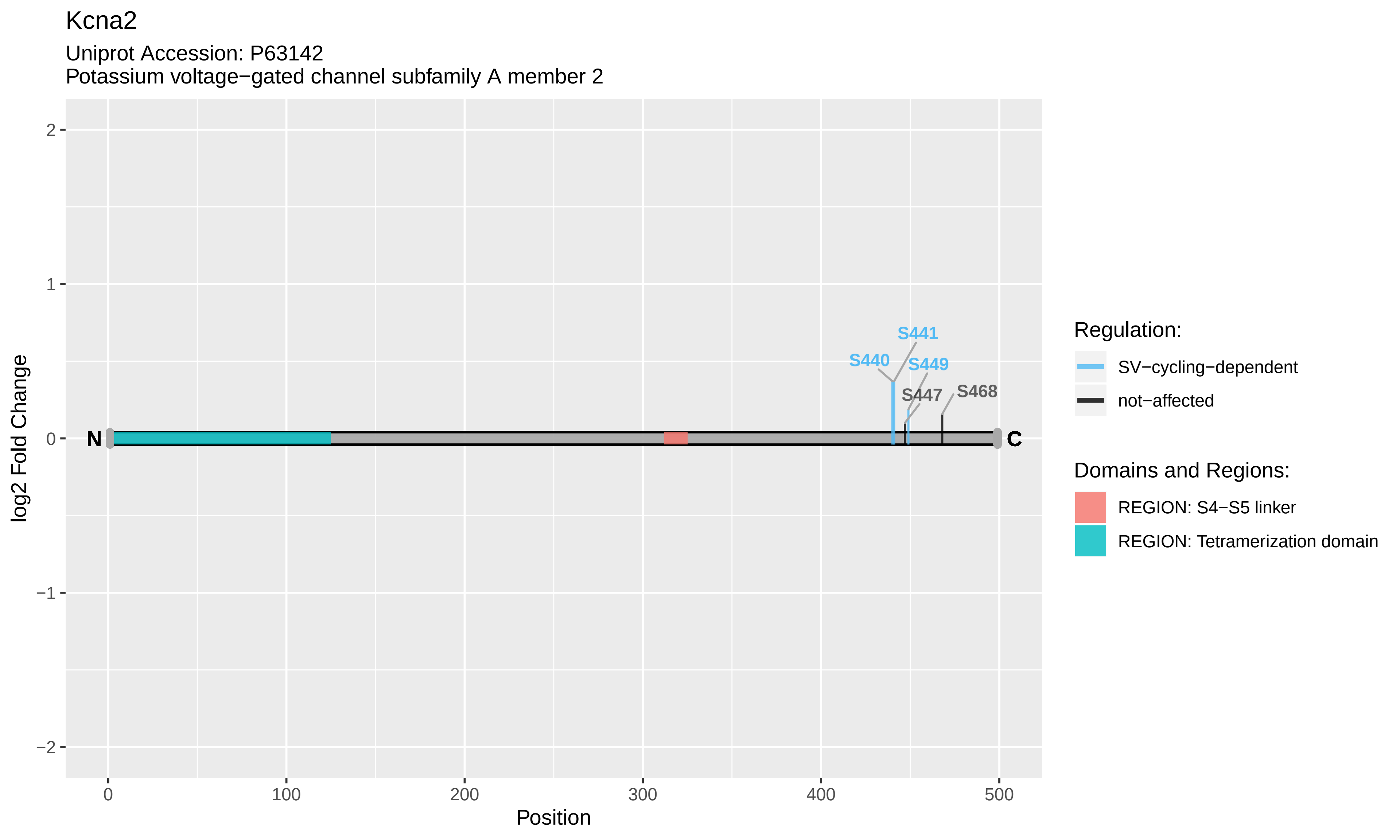
Kcna2
Uniprot Accession: P63142
Potassium voltage−gated channel subfamily A member 2
S441
S440
S449
S447
S468
N
C
2
1
0
−1
−2
log2 Fold Change
0
100
200
300
400
500
Position
Regulation:
SV−cycling−dependent
not−affected
Domains and Regions:
REGION: S4−S5 linker
REGION: Tetramerization domain

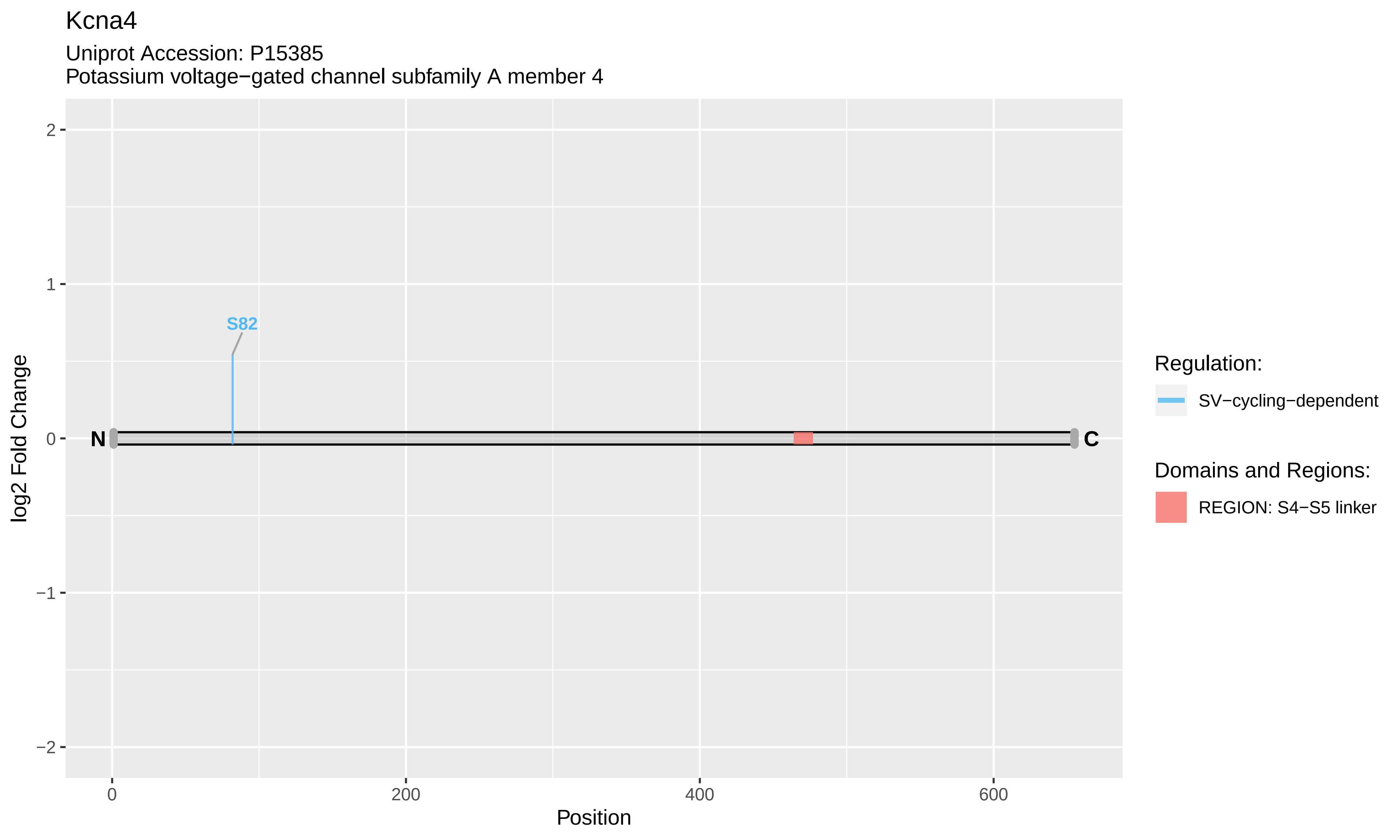

Kcna4
Uniprot Accession: P15385
Potassium voltage−gated channel subfamily A member 4
log2 Fold Change
2
1
0
−1
−2
S82
N
C
0
200
400
600
Position
Regulation:
SV−cycling−dependent
Domains and Regions:
REGION: S4−S5 linker

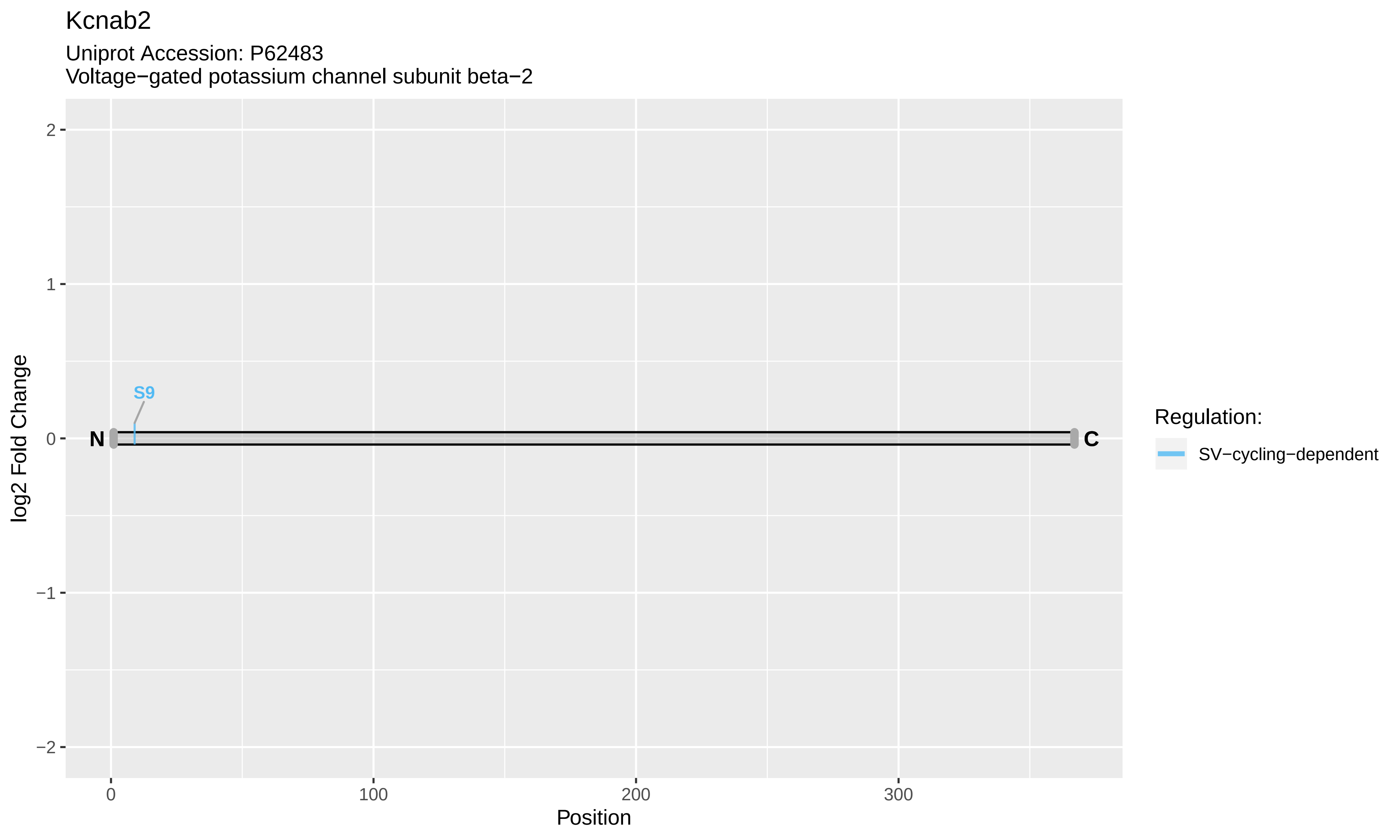
Kcnab2
Uniprot Accession: P62483
Voltage−gated potassium channel subunit beta−2
2
1
0
−1
−2
log2 Fold Change
N
C
S9
0
100
200
300
Position
Regulation:
SV−cycling−dependent

Kcnb1

Uniprot Accession: P15387
Potassium voltage−gated channel subfamily B member 1

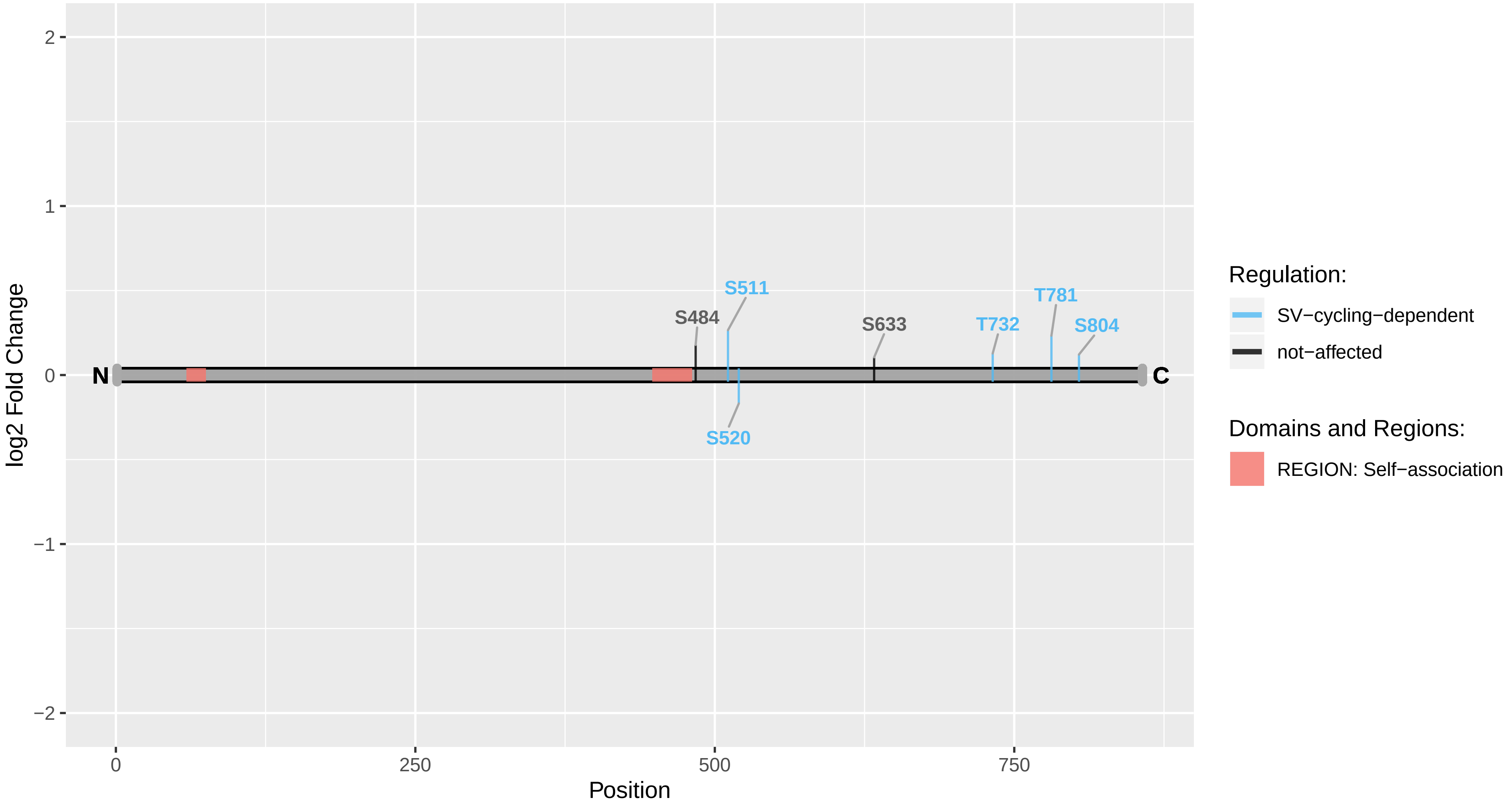

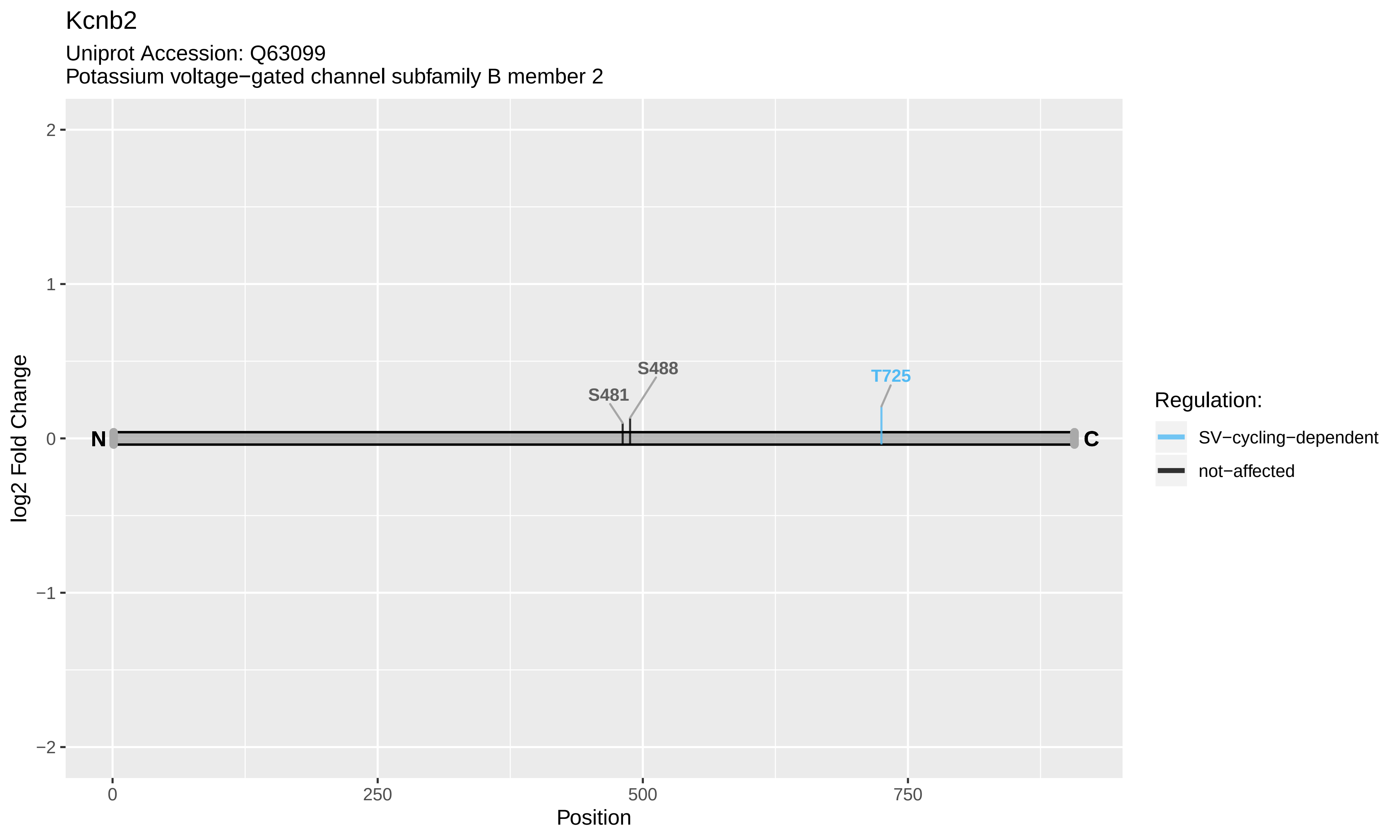

Kcnb2
Uniprot Accession: Q63099
Potassium voltage−gated channel subfamily B member 2
log2 Fold Change
Position
2
1
0
−1
−2
0
250
500
750
N
C
S481
S488
T725
Regulation:
SV−cycling−dependent
not−affected

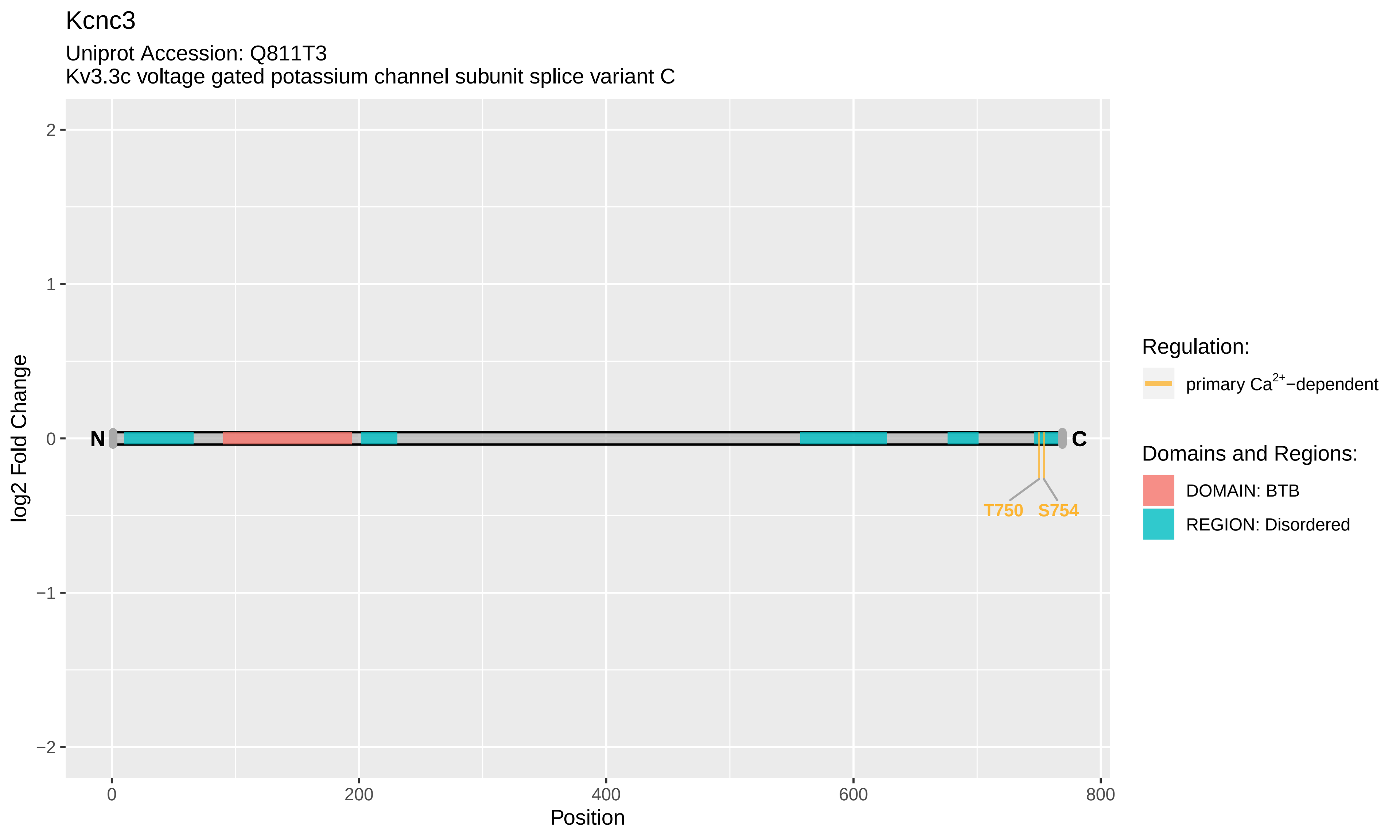
Kcnc3
Uniprot Accession: Q811T3
Kv3.3c voltage gated potassium channel subunit splice variant C
log2 Fold Change
2
1
0
−1
−2
N
C
T750
S754
0
200
400
600
800
Position
Regulation:
primary Ca2+−dependent
Domains and Regions:
DOMAIN: BTB
REGION: Disordered

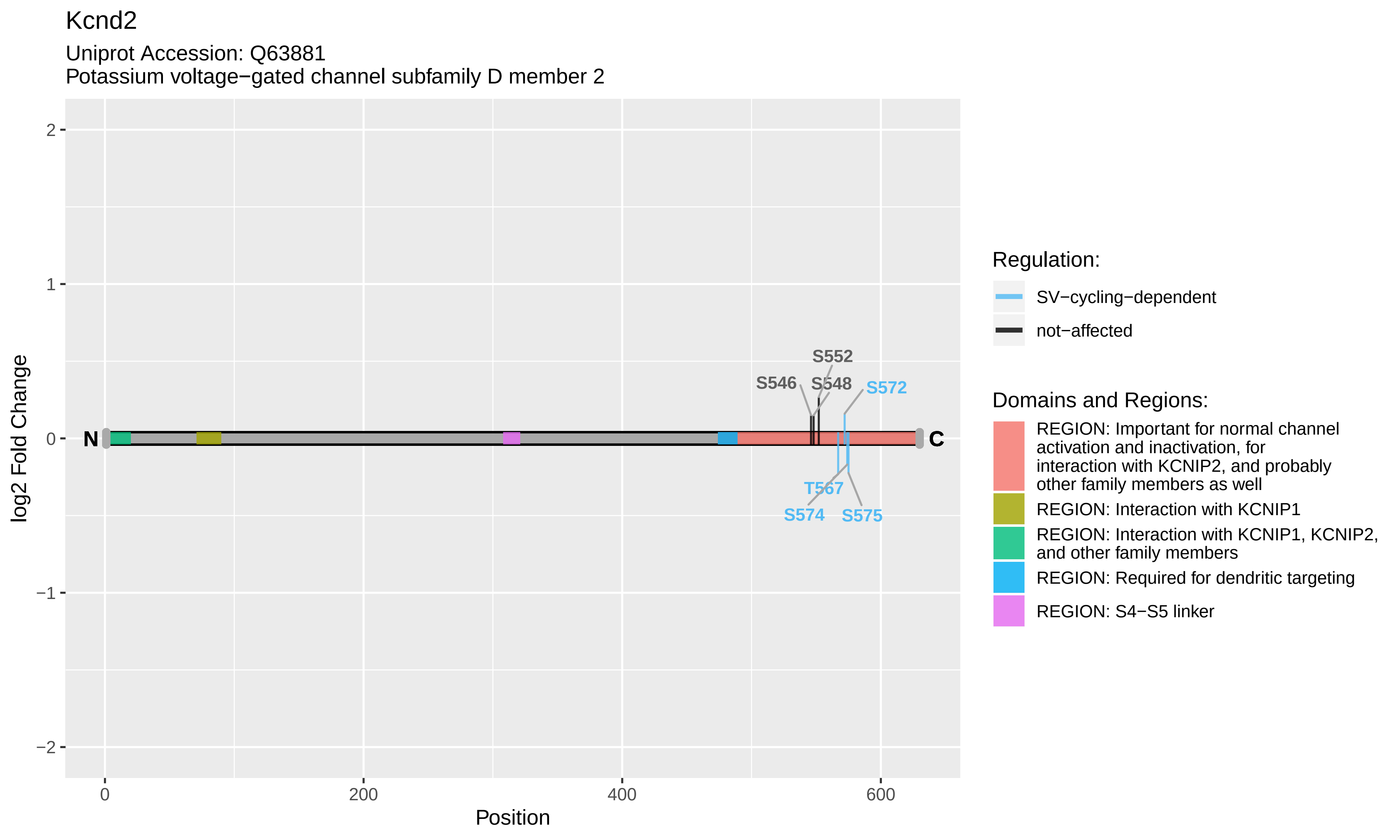

Kcnd2
Uniprot Accession: Q63881
Potassium voltage−gated channel subfamily D member 2
2
1
0
−1
−2
log2 Fold Change
N
C
S552
S546
S548
S572
T567
S574
S575
0
200
400
600
Position
Regulation:
SV−cycling−dependent
not−affected
Domains and Regions:
REGION: Important for normal channel activation and inactivation, for interaction with KCNIP2, and probably other family members as well
REGION: Interaction with KCNIP1
REGION: Interaction with KCNIP1, KCNIP2, and other family members
REGION: Required for dendritic targeting
REGION: S4−S5 linker

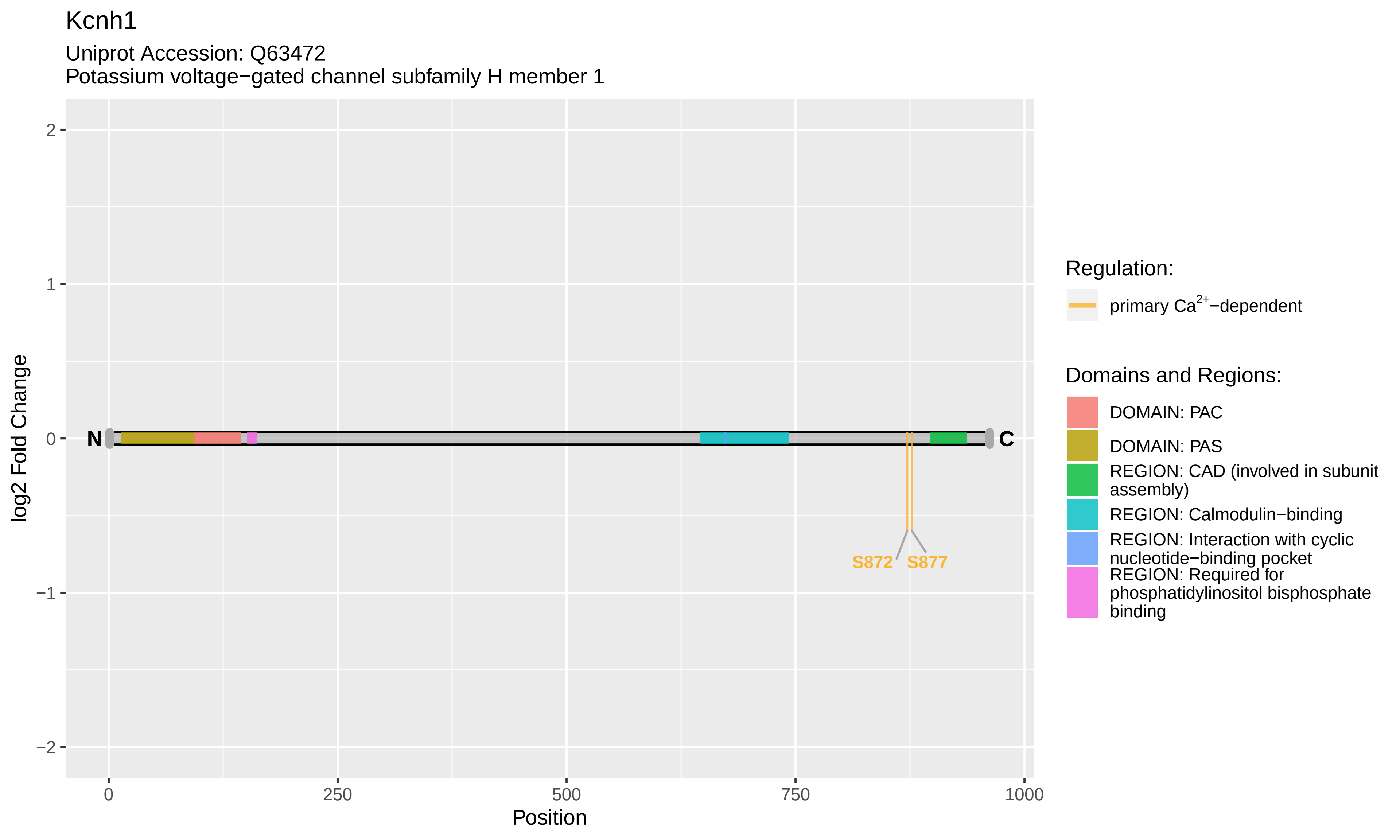

Kcnh1
Uniprot Accession: Q63472
Potassium voltage−gated channel subfamily H member 1
log2 Fold Change
2
1
0
−1
−2
N
C
S872
S877
0
250
500
750
1000
Position
Regulation:
primary Ca2+−dependent
Domains and Regions:
DOMAIN: PAC
DOMAIN: PAS
REGION: CAD (involved in subunit assembly)
REGION: Calmodulin−binding
REGION: Interaction with cyclic nucleotide−binding pocket
REGION: Required for phosphatidylinositol bisphosphate binding

Kcnma1

Uniprot Accession: F1LNC7
Calcium−activated potassium channel subunit alpha−1

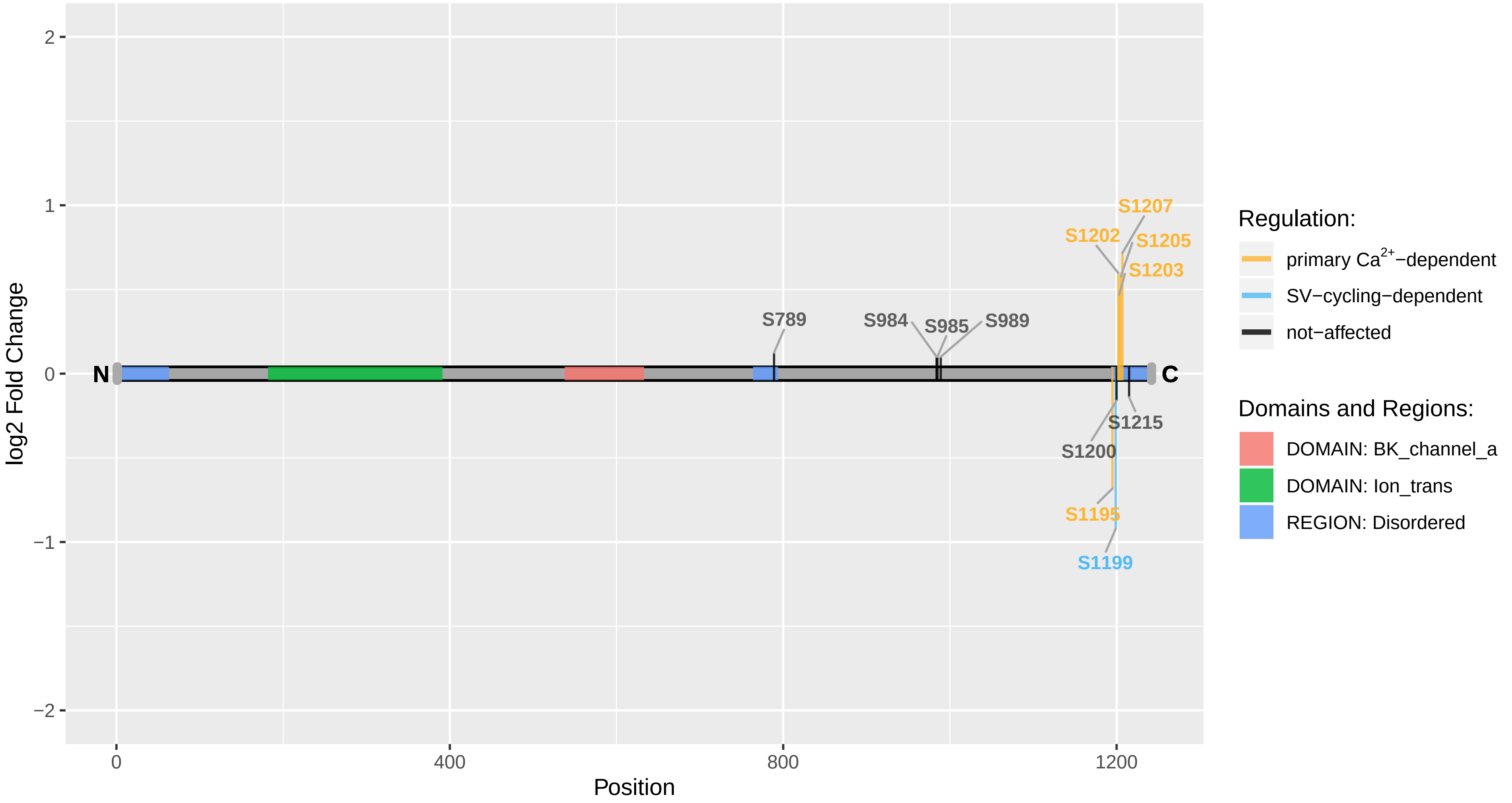

Kcnq2

Uniprot Accession: O88943
Potassium voltage−gated channel subfamily KQT member 2

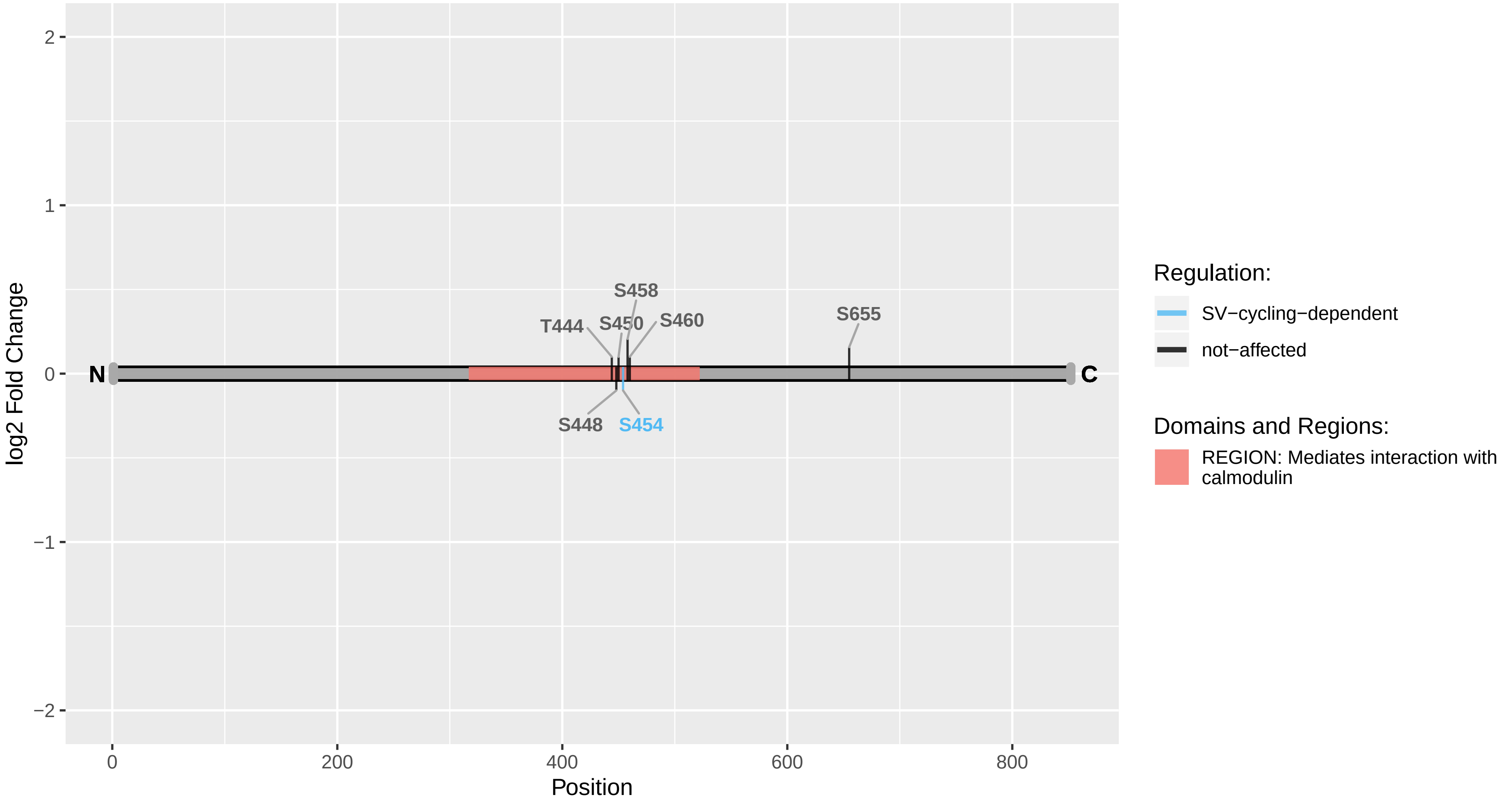

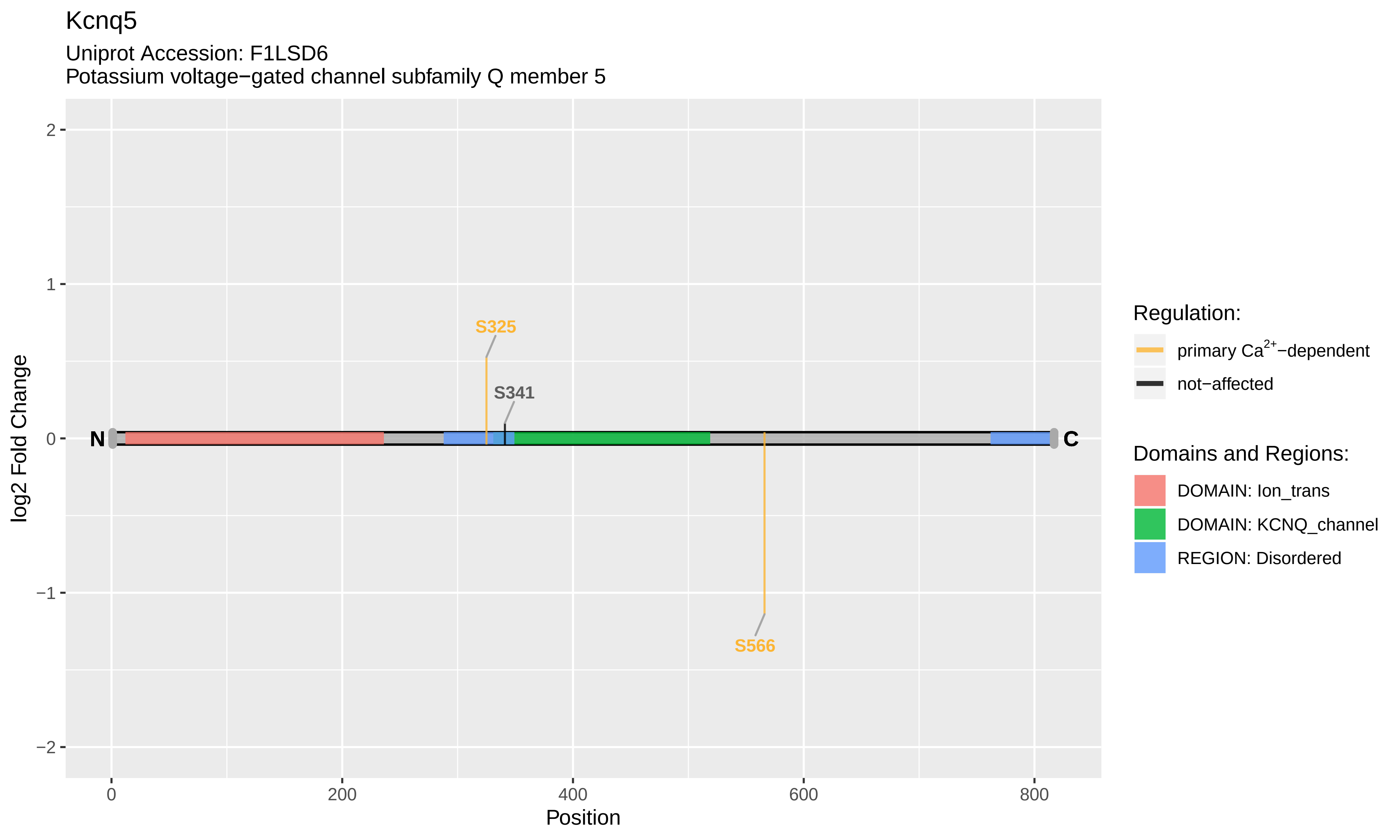
Kcnq5
Uniprot Accession: F1LSD6
Potassium voltage−gated channel subfamily Q member 5
log2 Fold Change
Position
2
1
0
−1
−2
0
200
400
600
800
N
C
S325
S341
S566
Regulation:
primary Ca2+−dependent
not−affected
Domains and Regions:
DOMAIN: Ion_trans
DOMAIN: KCNQ_channel
REGION: Disordered

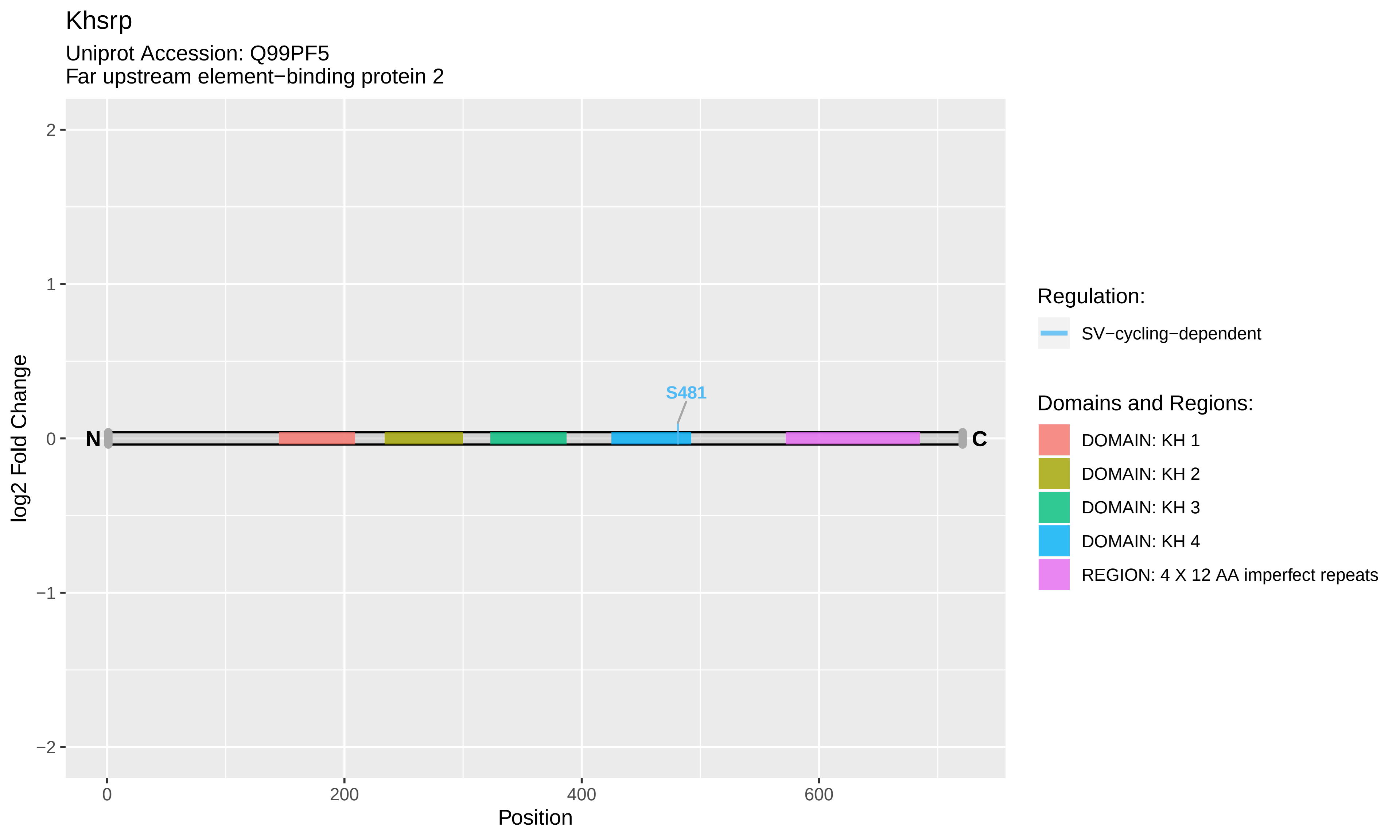
Khsrp
Uniprot Accession: Q99PF5
Far upstream element−binding protein 2
log2 Fold Change
2
1
0
−1
−2
N
C
S481
0
200
400
600
Position
Regulation:
SV−cycling−dependent
Domains and Regions:
DOMAIN: KH 1
DOMAIN: KH 2
DOMAIN: KH 3
DOMAIN: KH 4
REGION: 4 X 12 AA imperfect repeats

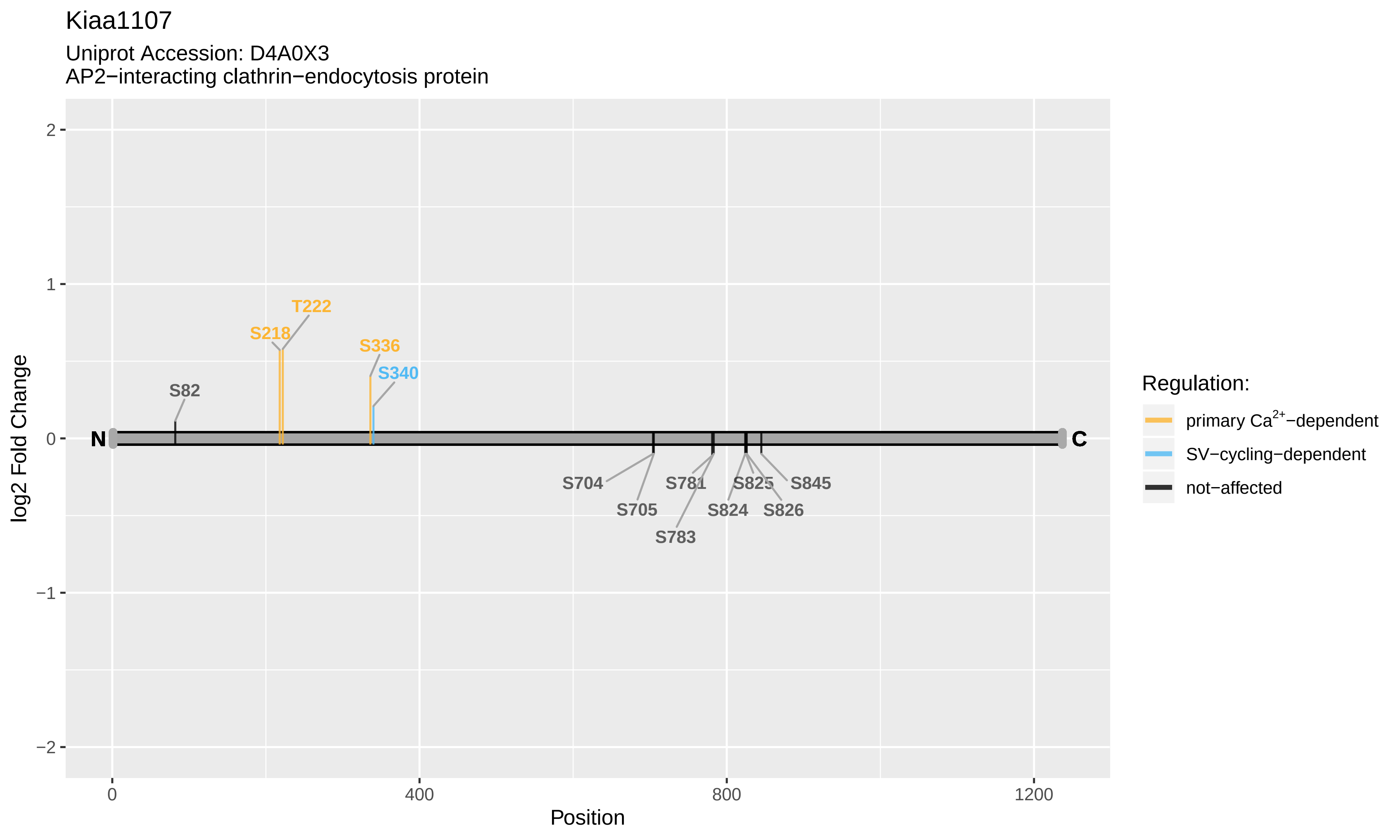

Kiaa1107
Uniprot Accession: D4A0X3
AP2−interacting clathrin−endocytosis protein
log2 Fold Change
2
1
0
−1
−2
Position
0
400
800
1200
N
C
S82
S218
T222
S336
S340
S704
S705
S781
S783
S824
S825
S826
S845
Regulation:
primary Ca2+−dependent
SV−cycling−dependent
not−affected

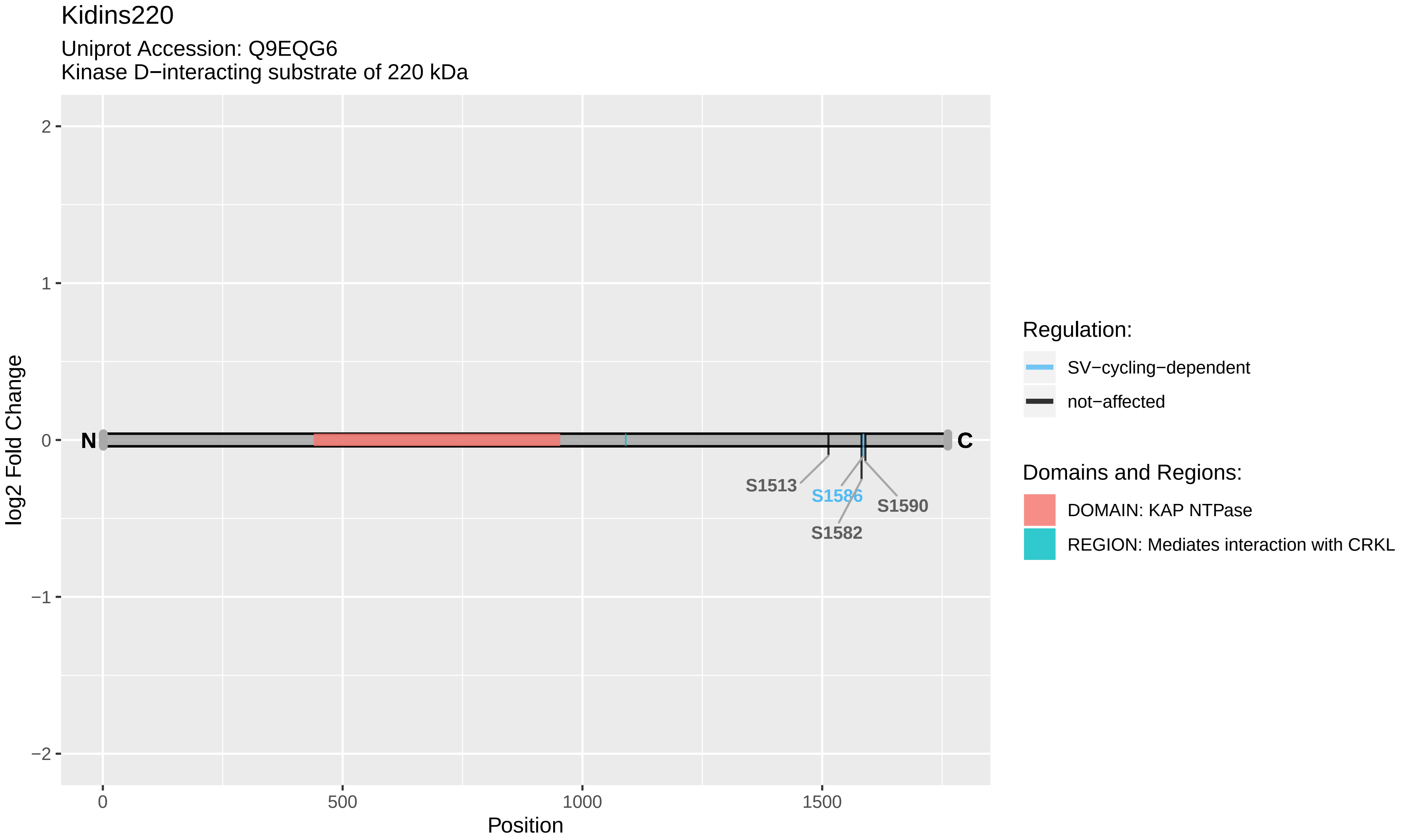
Kidins220
Uniprot Accession: Q9EQG6
Kinase D−interacting substrate of 220 kDa
log2 Fold Change
2
1
0
−1
−2
N
C
S1513
S1586
S1590
S1582
0
500
1000
1500
Position
Regulation:
SV−cycling−dependent
not−affected
Domains and Regions:
DOMAIN: KAP NTPase
REGION: Mediates interaction with CRKL

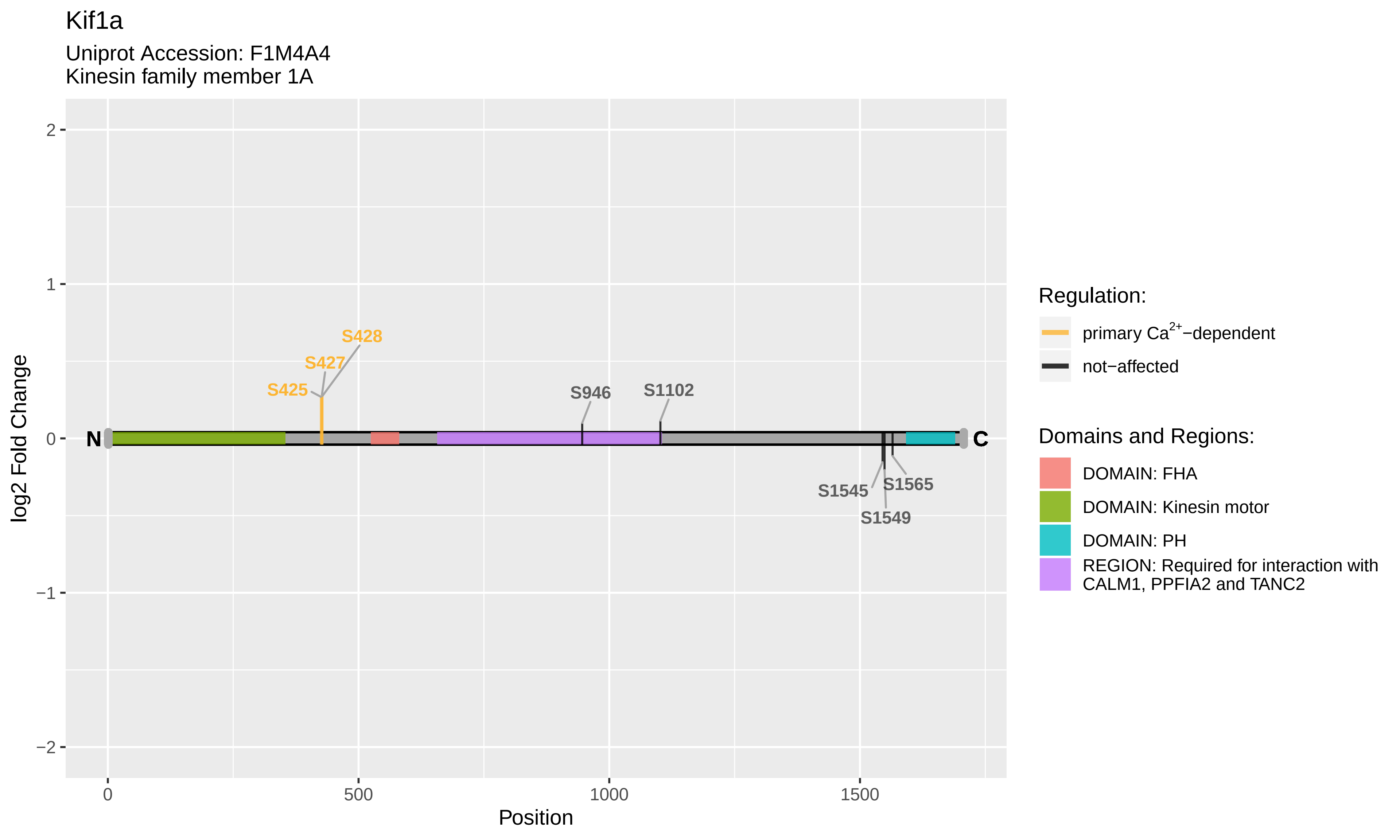

Kif1a
Uniprot Accession: F1M4A4
Kinesin family member 1A
log2 Fold Change
Position
N
C
S425
S427
S428
S946
S1102
S1545
S1549
S1565
2
1
0
−1
−2
0
500
1000
1500
Regulation:
primary Ca2+−dependent
not−affected
Domains and Regions:
DOMAIN: FHA
DOMAIN: Kinesin motor
DOMAIN: PH
REGION: Required for interaction with CALM1, PPFIA2 and TANC2

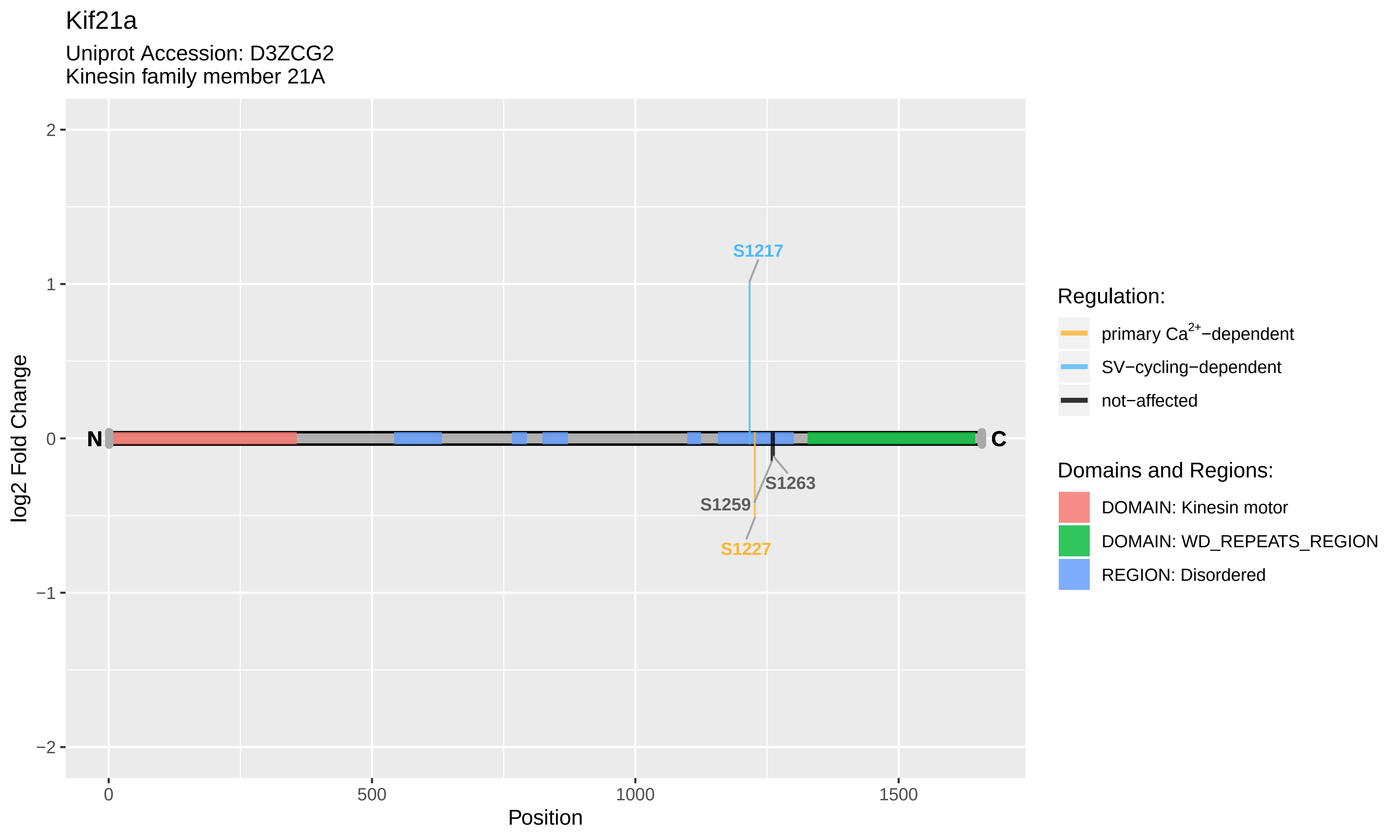
Kif21a
Uniprot Accession: D3ZCG2
Kinesin family member 21A
log2 Fold Change
Position
2
1
0
−1
−2
0
500
1000
1500
N
C
S1217
S1263
S1259
S1227
Regulation:
primary Ca2+−dependent
SV−cycling−dependent
not−affected
Domains and Regions:
DOMAIN: Kinesin motor
DOMAIN: WD_REPEATS_REGION
REGION: Disordered

Kif2a

Uniprot Accession: Q9WV63
Kinesin−like protein KIF2A

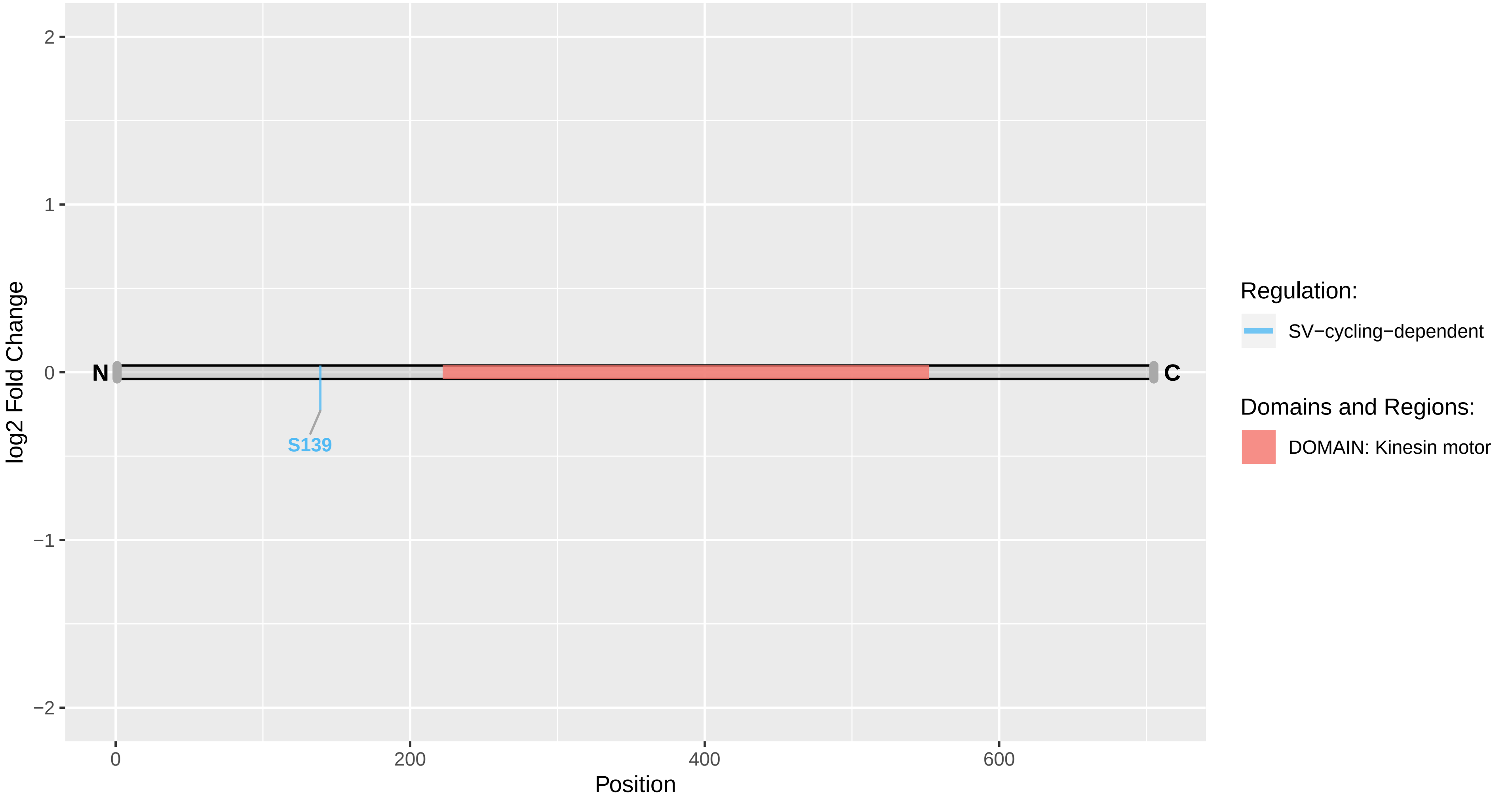

Kif5c

Uniprot Accession: G3V6L4
Kinesin−like protein

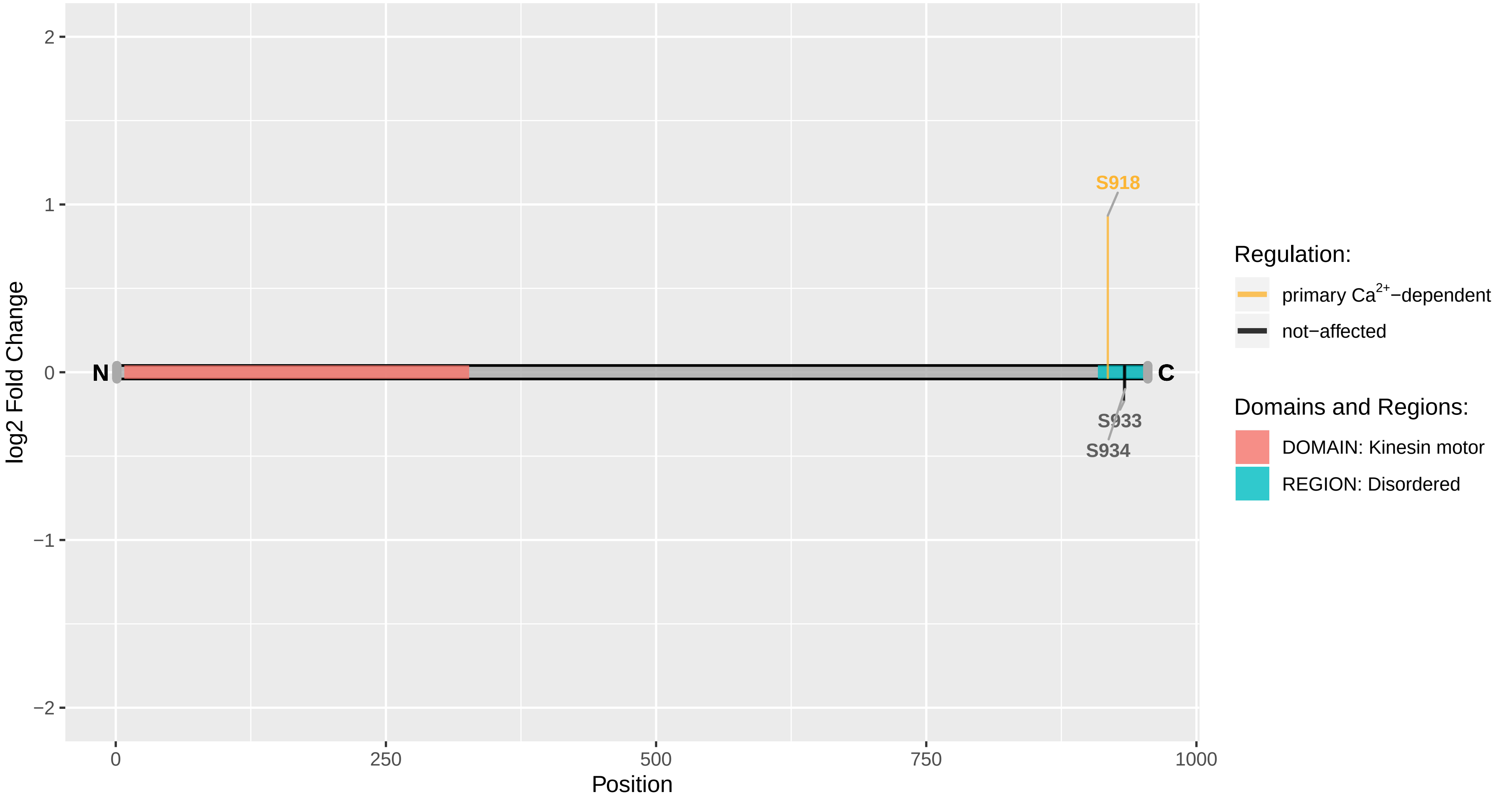

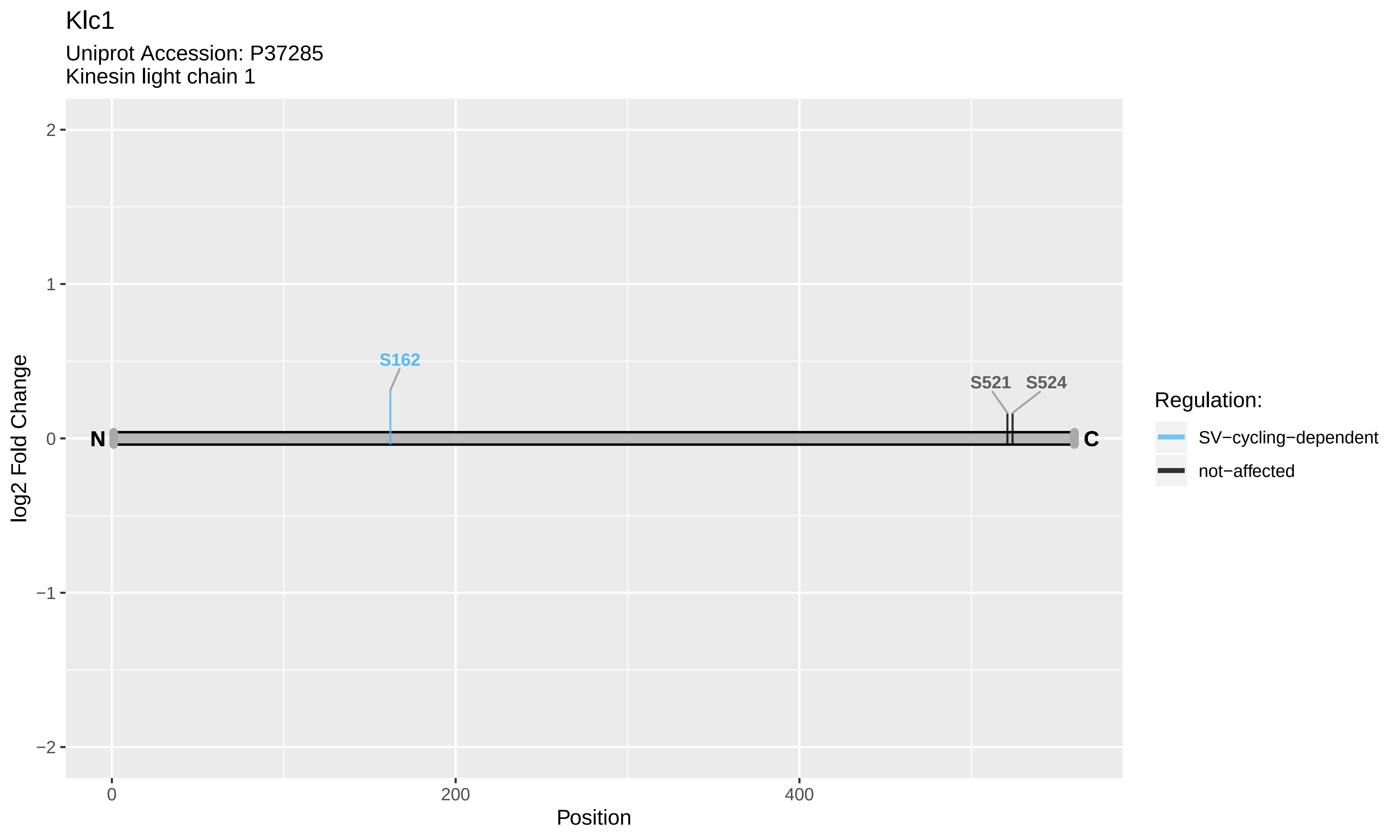

Klc1
Uniprot Accession: P37285
Kinesin light chain 1
S162
S521
S524
N
C
log2 Fold Change
2
1
0
−1
−2
0
200
400
Position
Regulation:
SV−cycling−dependent
not−affected

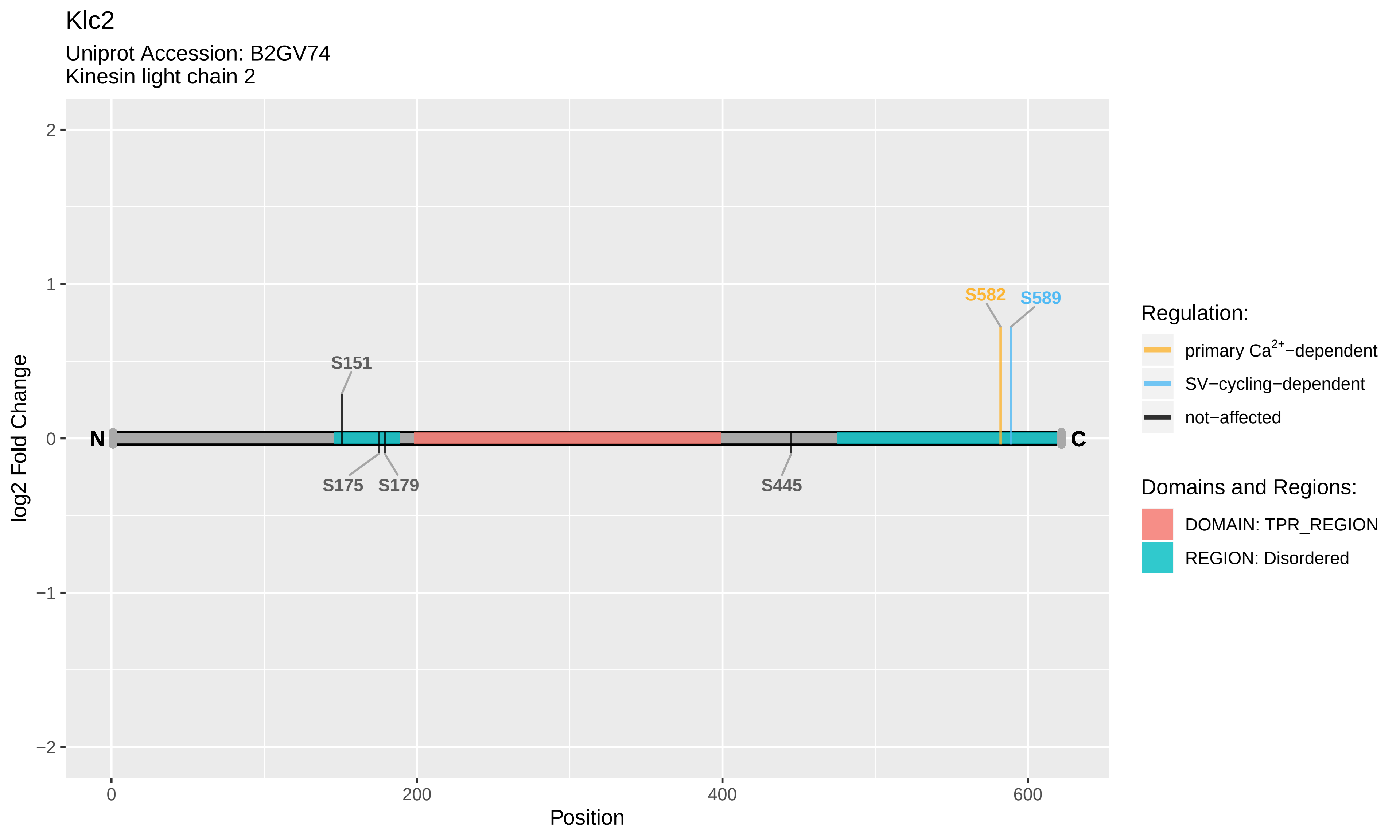
Klc2
Uniprot Accession: B2GV74
Kinesin light chain 2
log2 Fold Change
Position
N
C
S151
S175
S179
S445
S582
S589
Regulation:
primary Ca2+−dependent
SV−cycling−dependent
not−affected
Domains and Regions:
DOMAIN: TPR_REGION
REGION: Disordered

Klc4

Uniprot Accession: Q5PQM2
Kinesin light chain 4

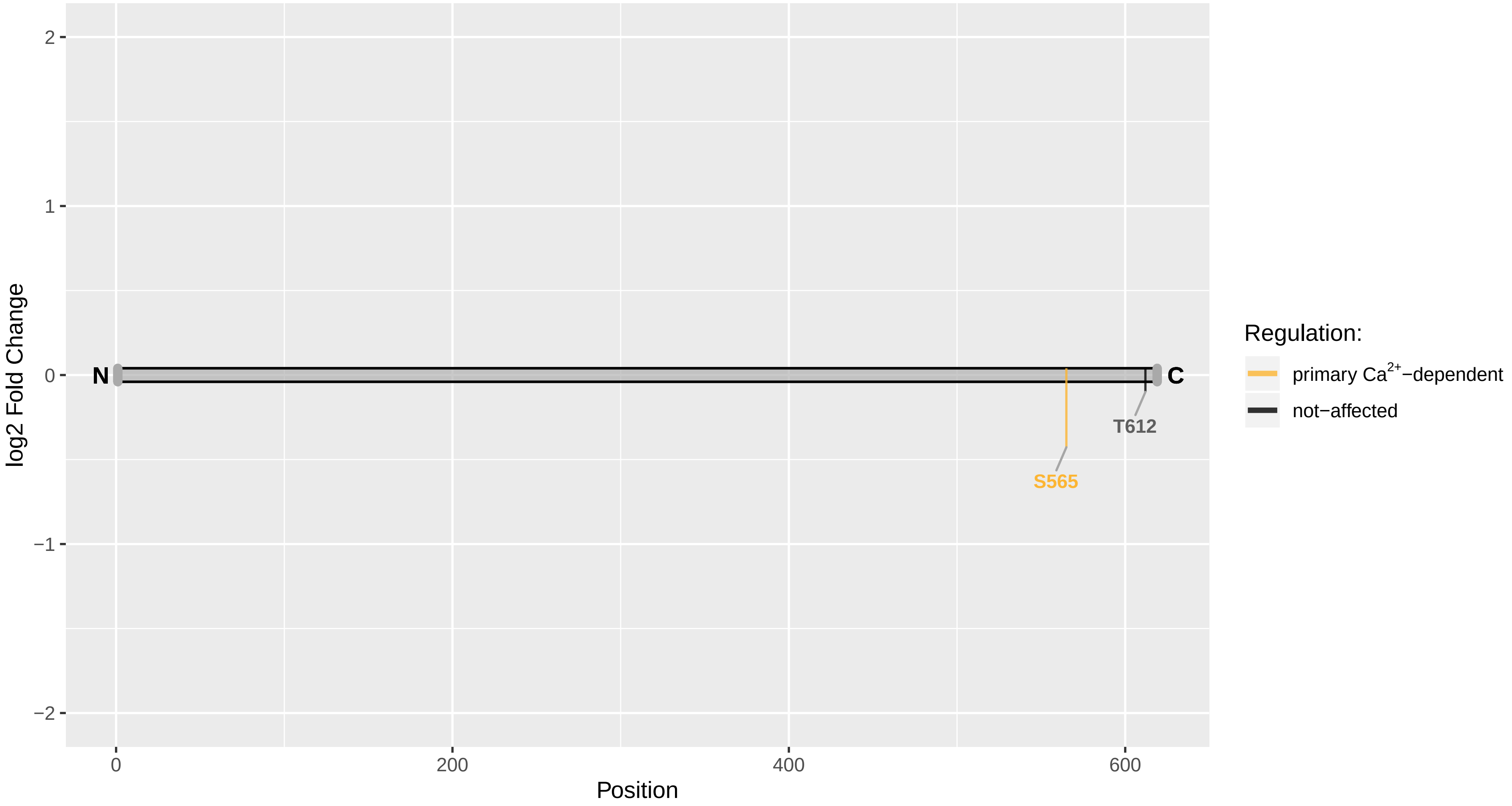

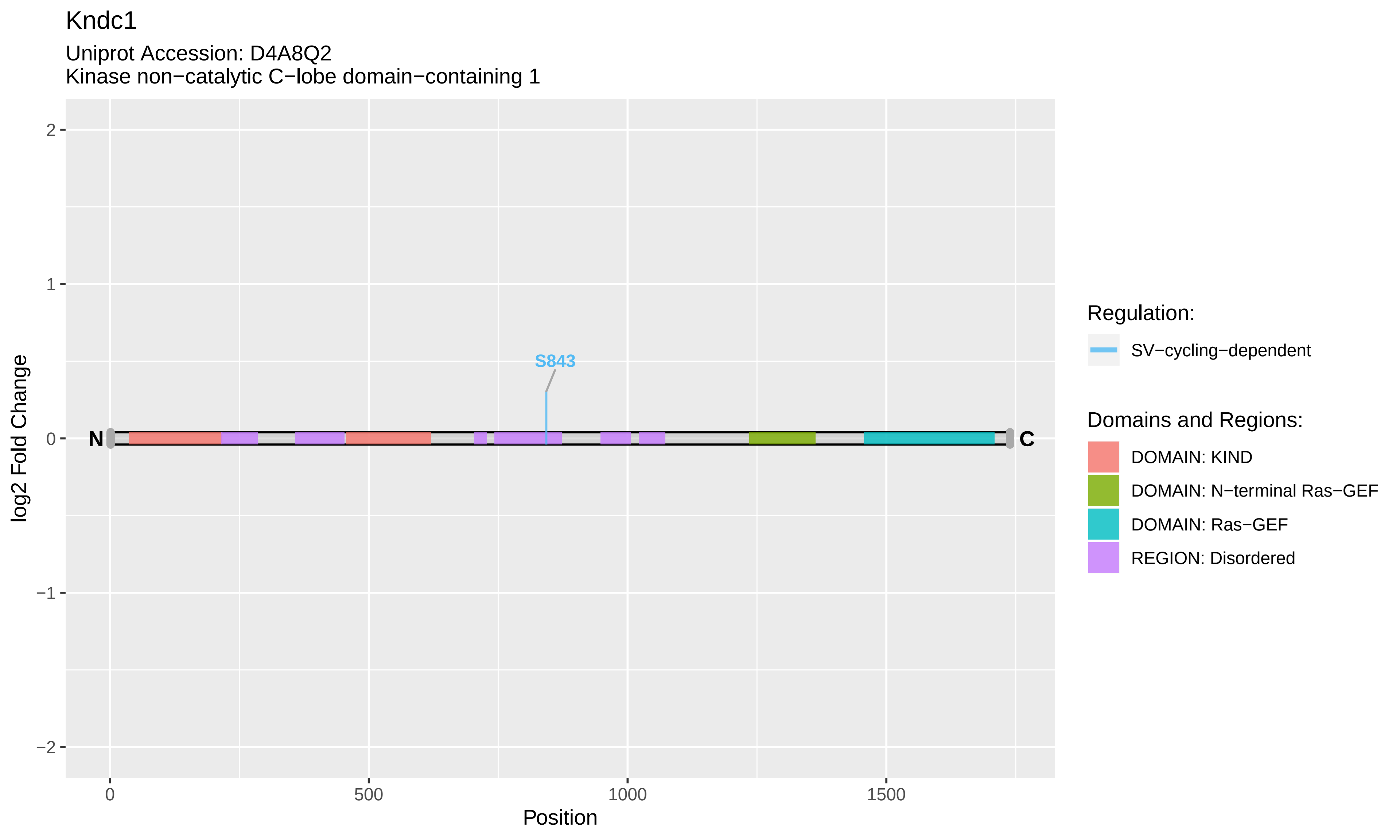
Kndc1
Uniprot Accession: D4A8Q2
Kinase non−catalytic C−lobe domain−containing 1
S843
N
C
log2 Fold Change
Position
2
1
0
−1
−2
0
500
1000
1500
Regulation:
SV−cycling−dependent
Domains and Regions:
DOMAIN: KIND
DOMAIN: N−terminal Ras−GEF
DOMAIN: Ras−GEF
REGION: Disordered

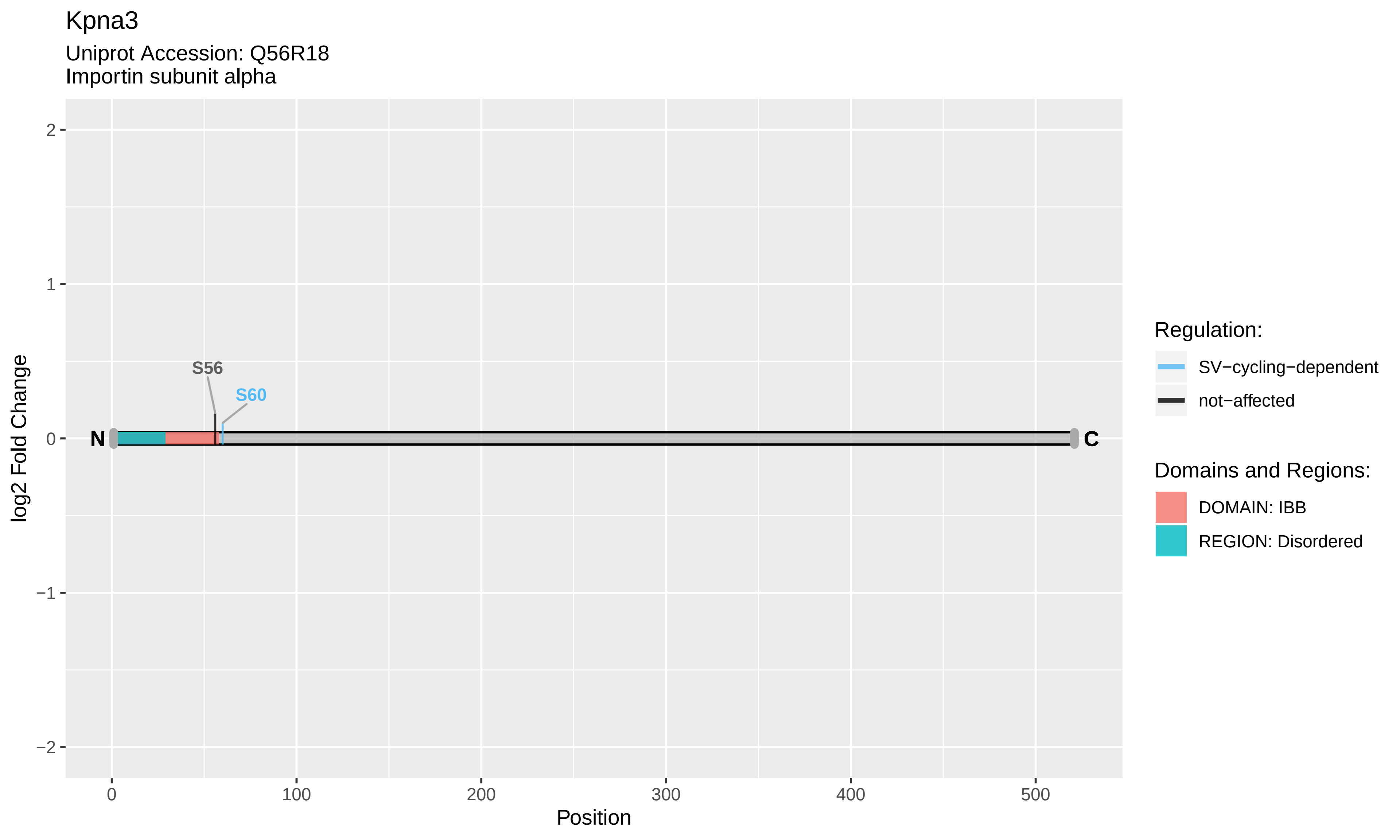

Kpna3
Uniprot Accession: Q56R18
Importin subunit alpha
log2 Fold Change
Position
S56
S60
N
C
Regulation:
SV−cycling−dependent
not−affected
Domains and Regions:
DOMAIN: IBB
REGION: Disordered

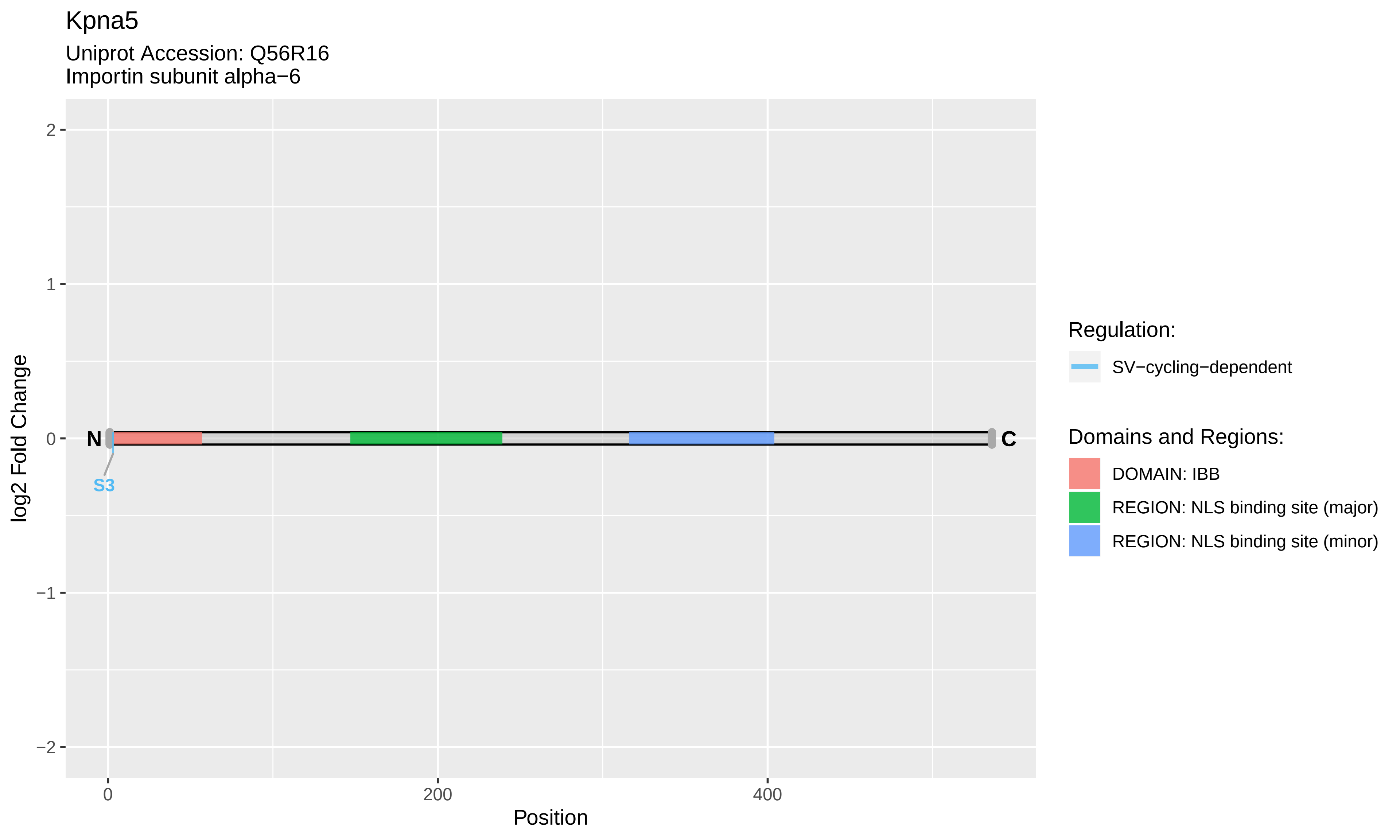

Kpna5
Uniprot Accession: Q56R16
Importin subunit alpha−6
log2 Fold Change
2
1
0
−1
−2
N
C
S3
0
200
400
Position
Regulation:
SV−cycling−dependent
Domains and Regions:
DOMAIN: IBB
REGION: NLS binding site (major)
REGION: NLS binding site (minor)

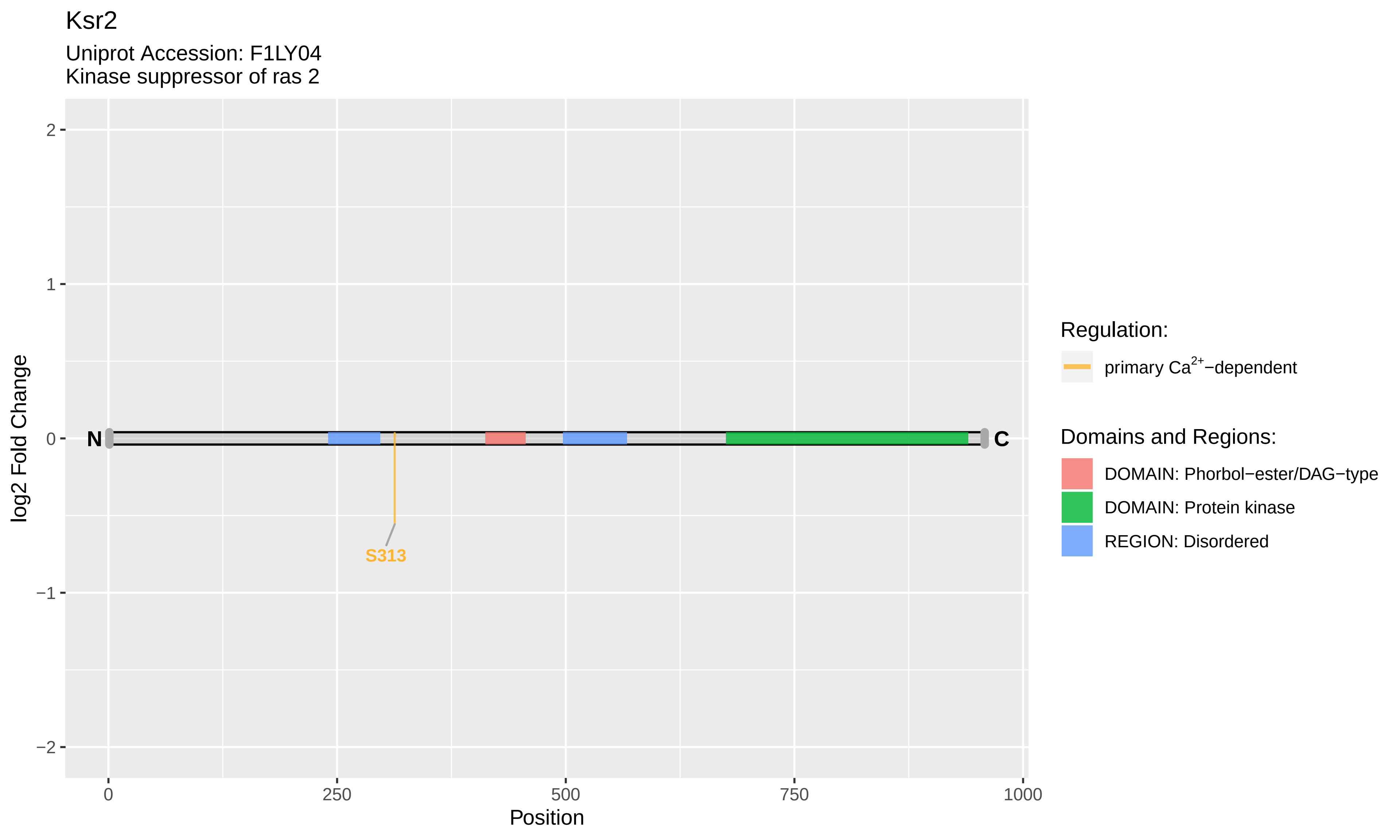

Ksr2
Uniprot Accession: F1LY04
Kinase suppressor of ras 2
log2 Fold Change
2
1
0
−1
−2
N
C
S313
0
250
500
750
1000
Position
Regulation:
primary Ca2+−dependent
Domains and Regions:
DOMAIN: Phorbol−ester/DAG−type
DOMAIN: Protein kinase
REGION: Disordered

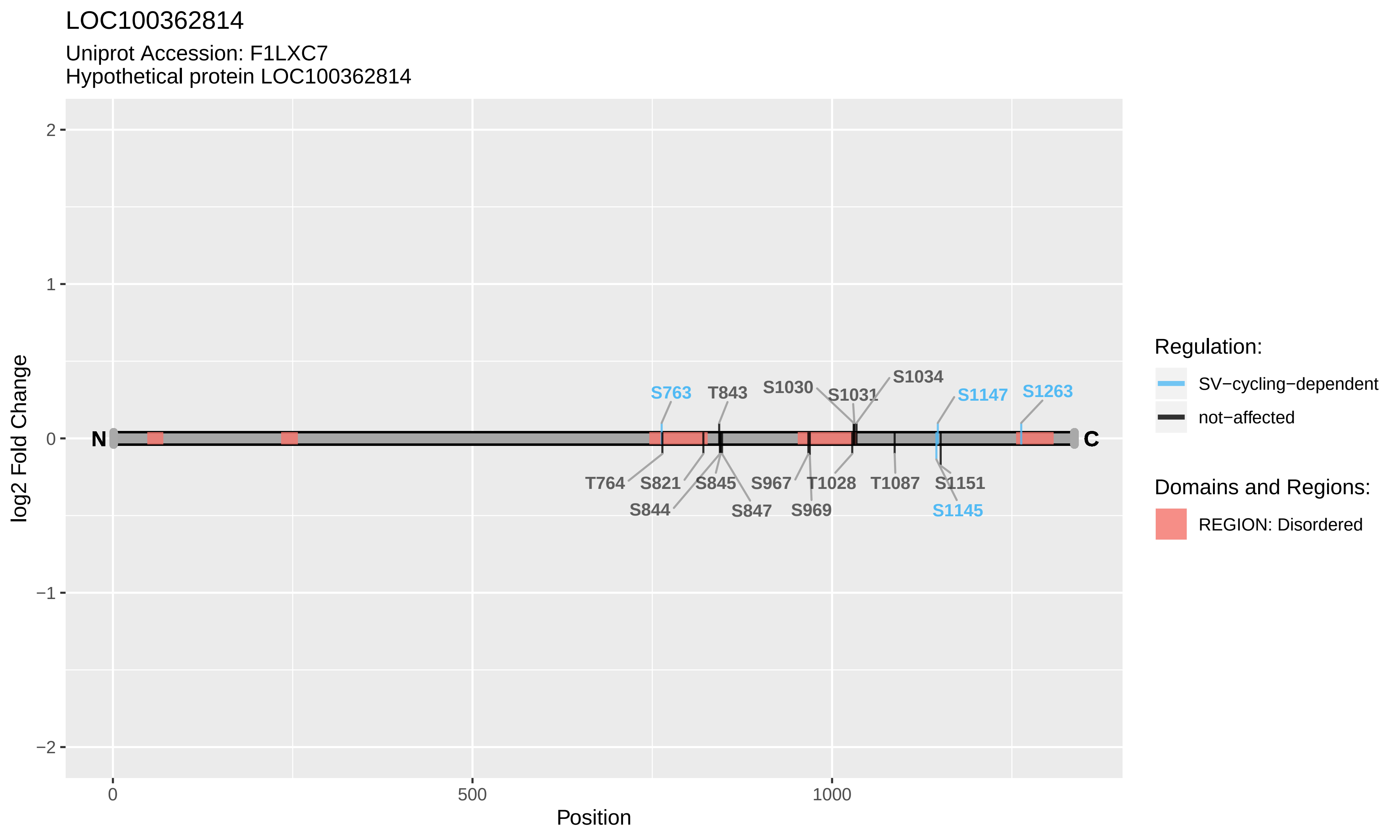

LOC100362814
Uniprot Accession: F1LXC7
Hypothetical protein LOC100362814
log2 Fold Change
2
1
0
−1
−2
Position
0
500
1000
N
C
S763
T843
S1030
S1031
S1034
S1147
S1263
T764
S821
S845
S967
T1028
T1087
S1151
S844
S847
S969
S1145
Regulation:
SV−cycling−dependent
not−affected
Domains and Regions:
REGION: Disordered

LOC100909750

Uniprot Accession: D3ZGM3
Tyrosine−protein kinase

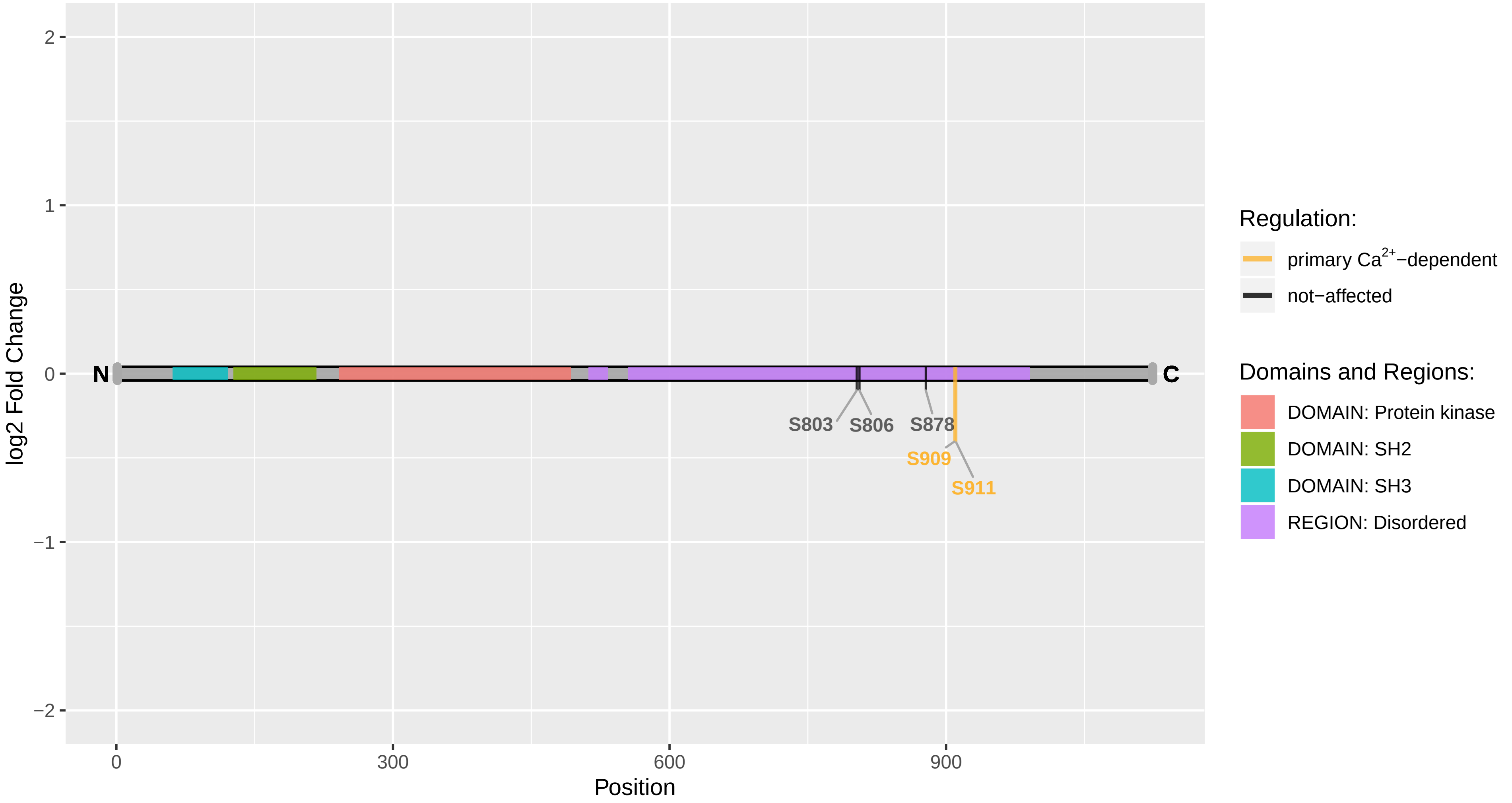

LOC100911440

Uniprot Accession: A0A0G2K5L2
RCG47744, isoform CRA_c

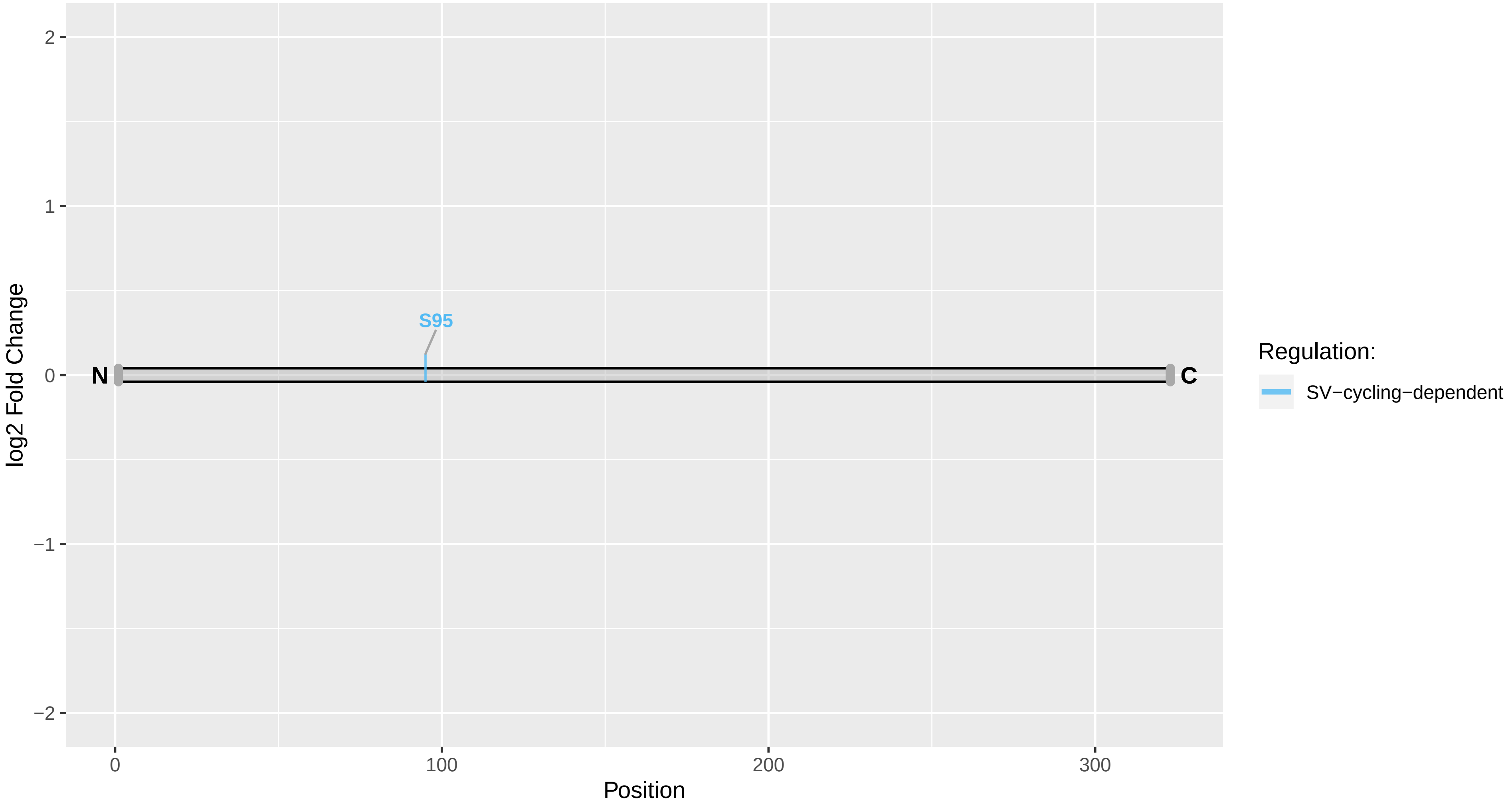

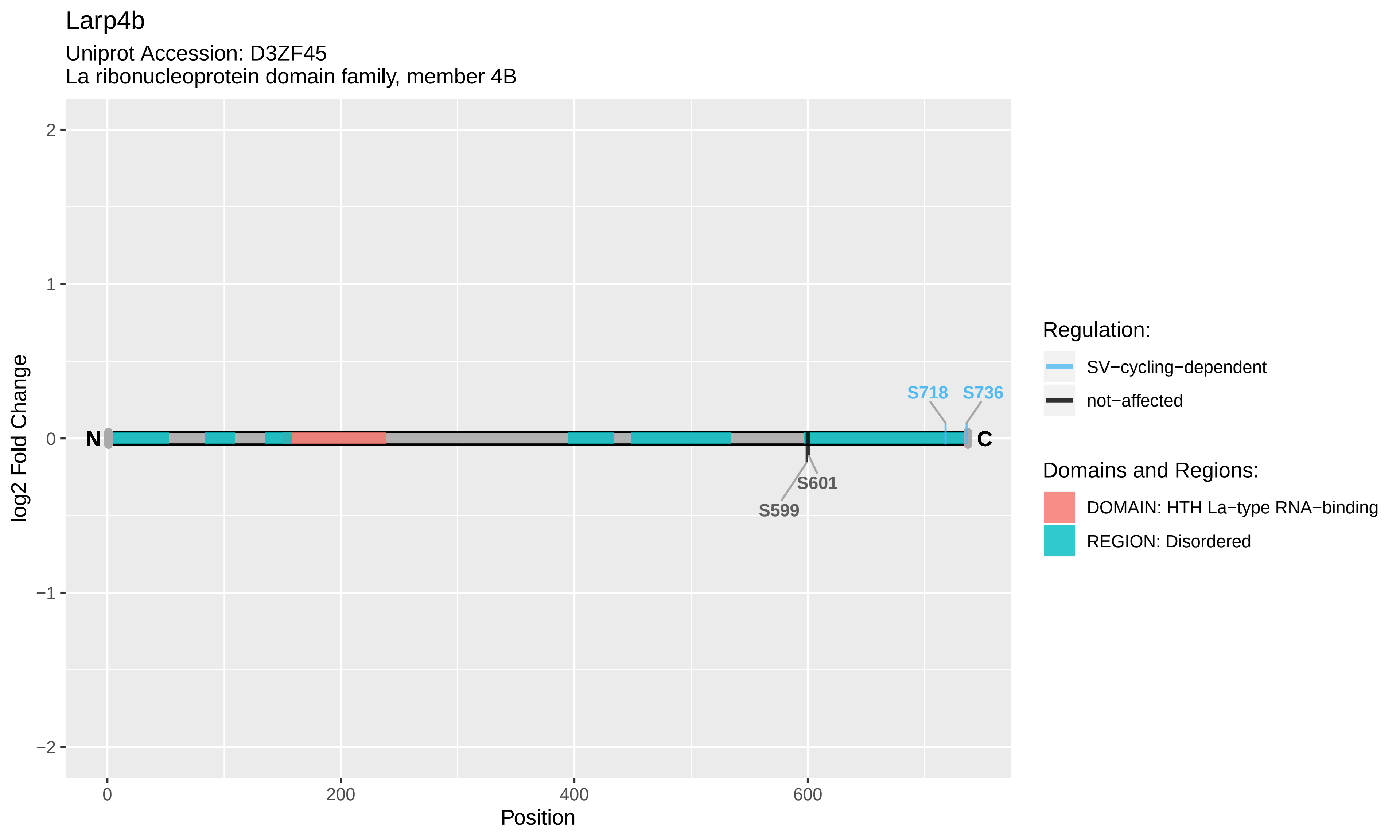

Larp4b
Uniprot Accession: D3ZF45
La ribonucleoprotein domain family, member 4B
log2 Fold Change
Position
N
C
S718
S736
S599
S601
Regulation:
SV−cycling−dependent
not−affected
Domains and Regions:
DOMAIN: HTH La−type RNA−binding
REGION: Disordered
2
1
0
−1
−2
0
200
400
600

Lasp1

Uniprot Accession: Q99MZ8
LIM and SH3 domain protein 1

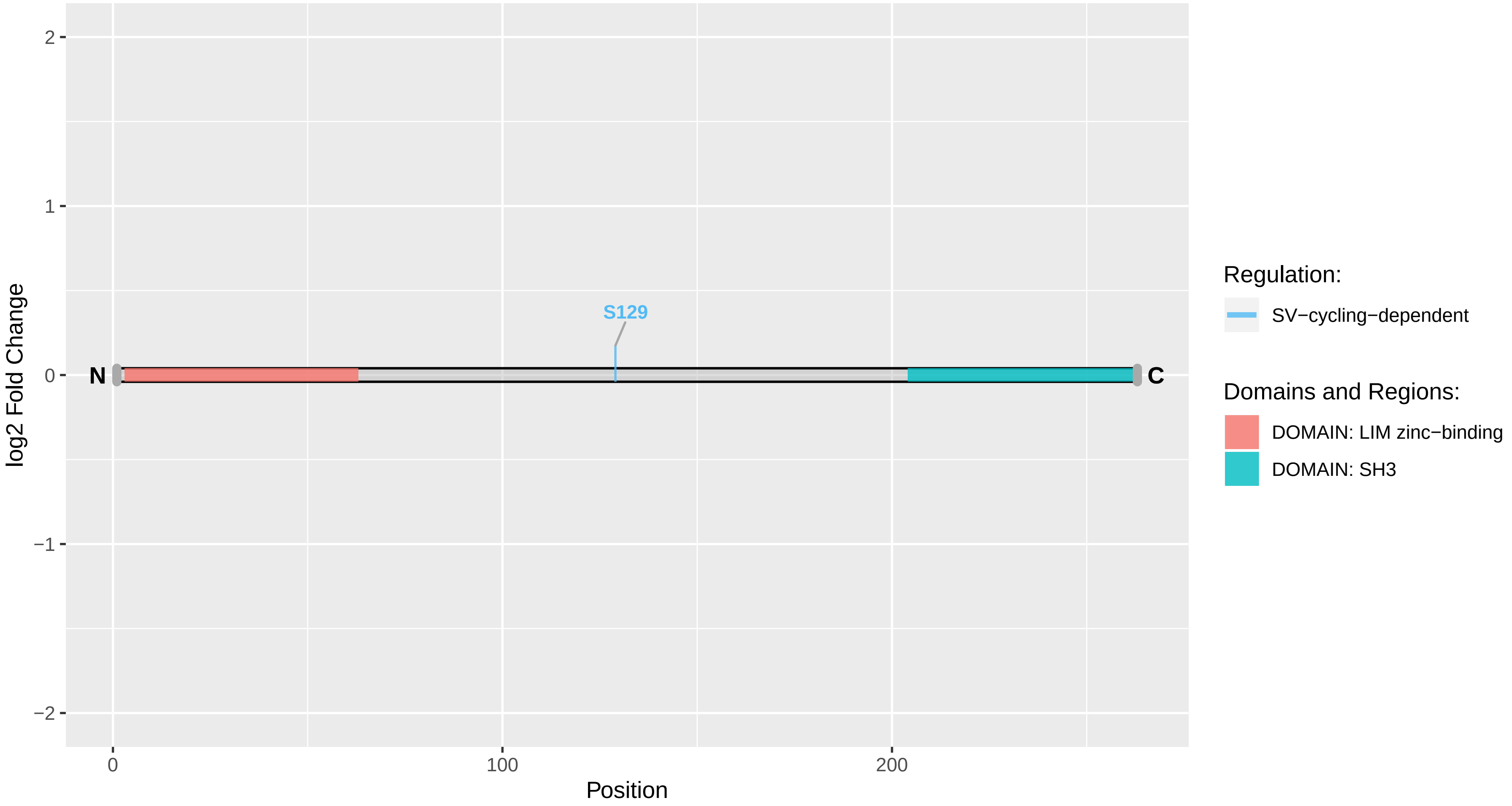

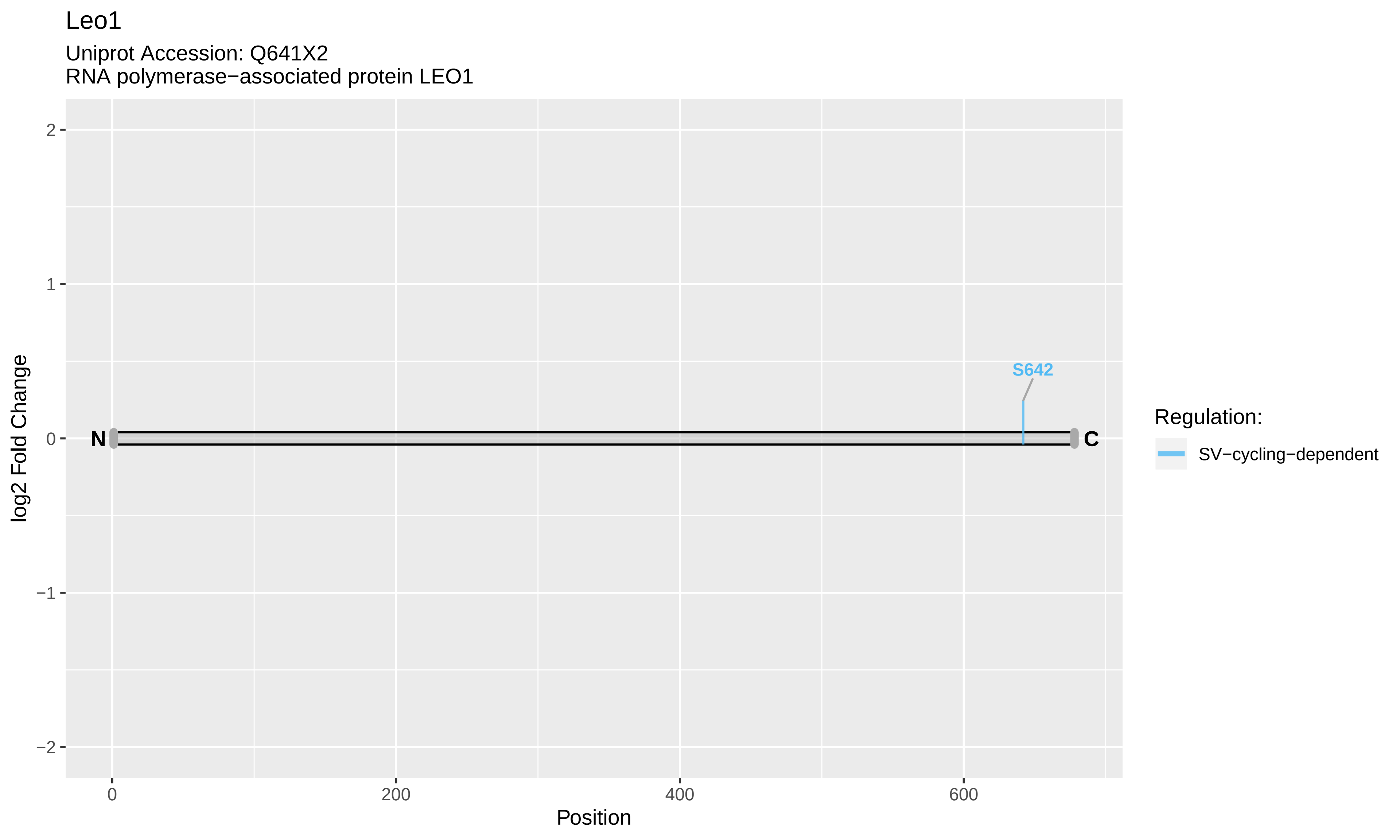
Leo1
Uniprot Accession: Q641X2
RNA polymerase−associated protein LEO1
S642
N
C
2
1
0
−1
−2
log2 Fold Change
0
200
400
600
Position
Regulation:
SV−cycling−dependent

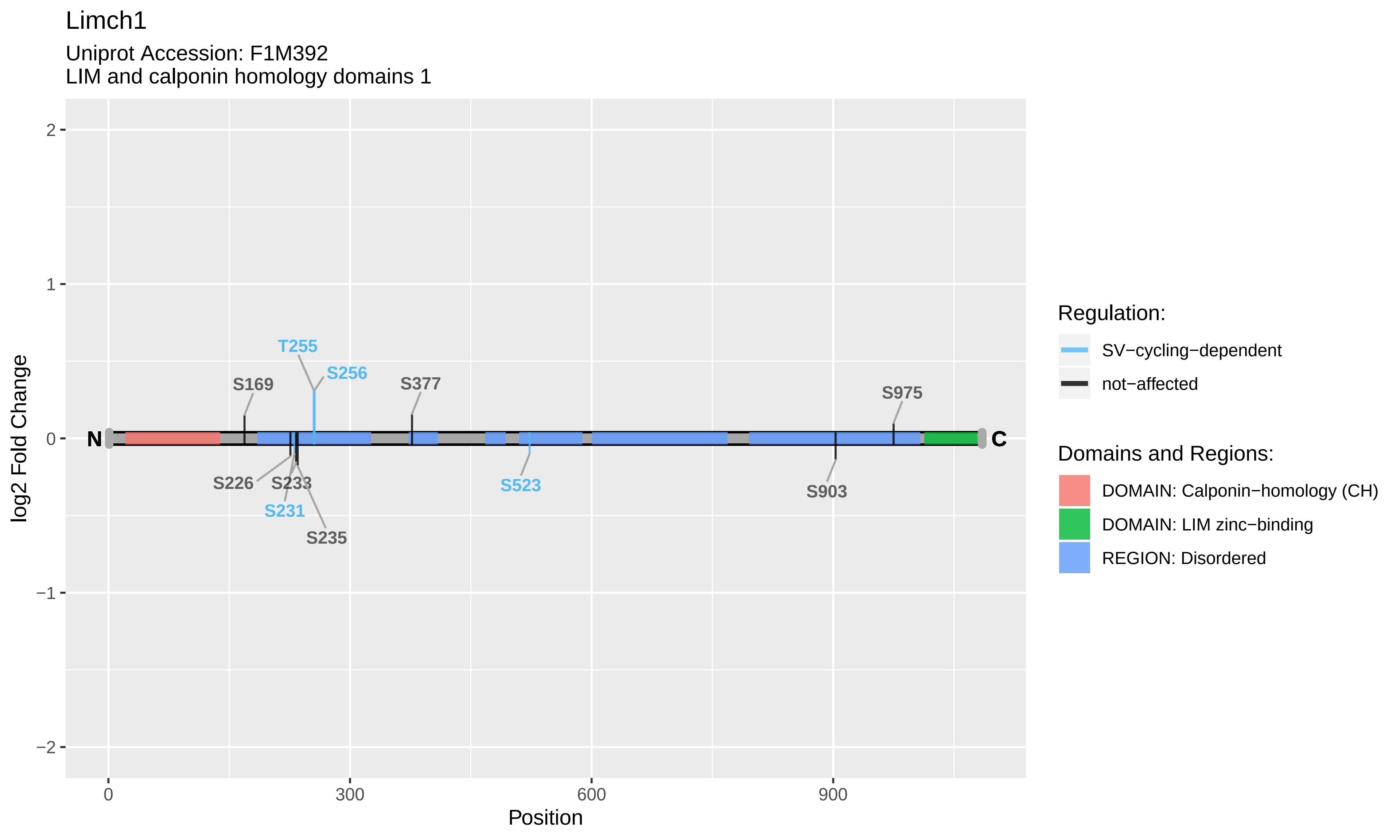

Limch1
Uniprot Accession: F1M392
LIM and calponin homology domains 1
log2 Fold Change
2
1
0
−1
−2
N
C
T255
S256
S169
S377
S975
S226
S233
S231
S235
S523
S903
0
300
600
900
Position
Regulation:
SV−cycling−dependent
not−affected
Domains and Regions:
DOMAIN: Calponin−homology (CH)
DOMAIN: LIM zinc−binding
REGION: Disordered

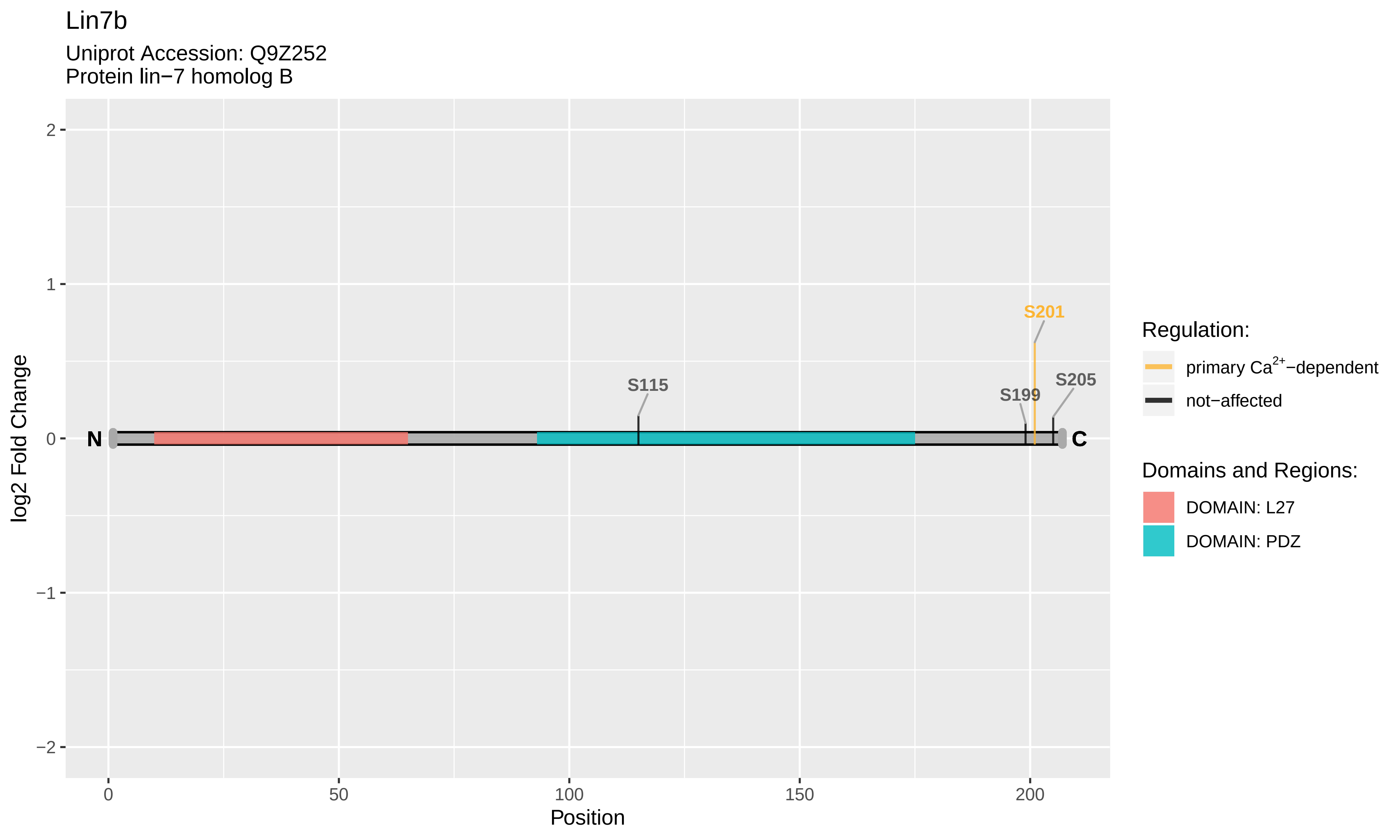

Lin7b
Uniprot Accession: Q9Z252
Protein lin−7 homolog B
log2 Fold Change
2
1
0
−1
−2
N
C
S115
S199
S201
S205
0
50
100
150
200
Position
Regulation:
primary Ca2+−dependent
not−affected
Domains and Regions:
DOMAIN: L27
DOMAIN: PDZ

Lmo7

Uniprot Accession: A0A0G2K8R3
LIM domain 7

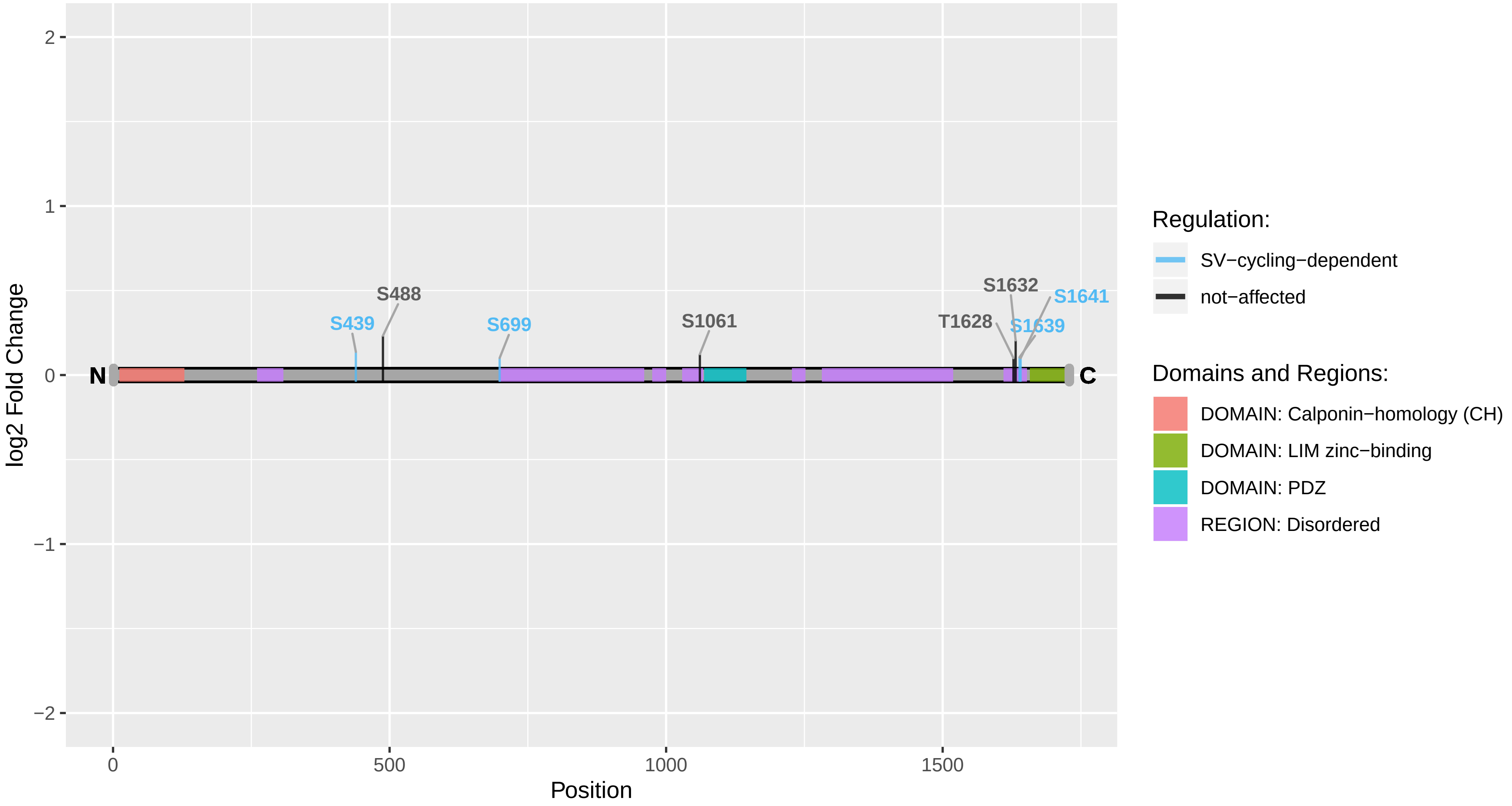

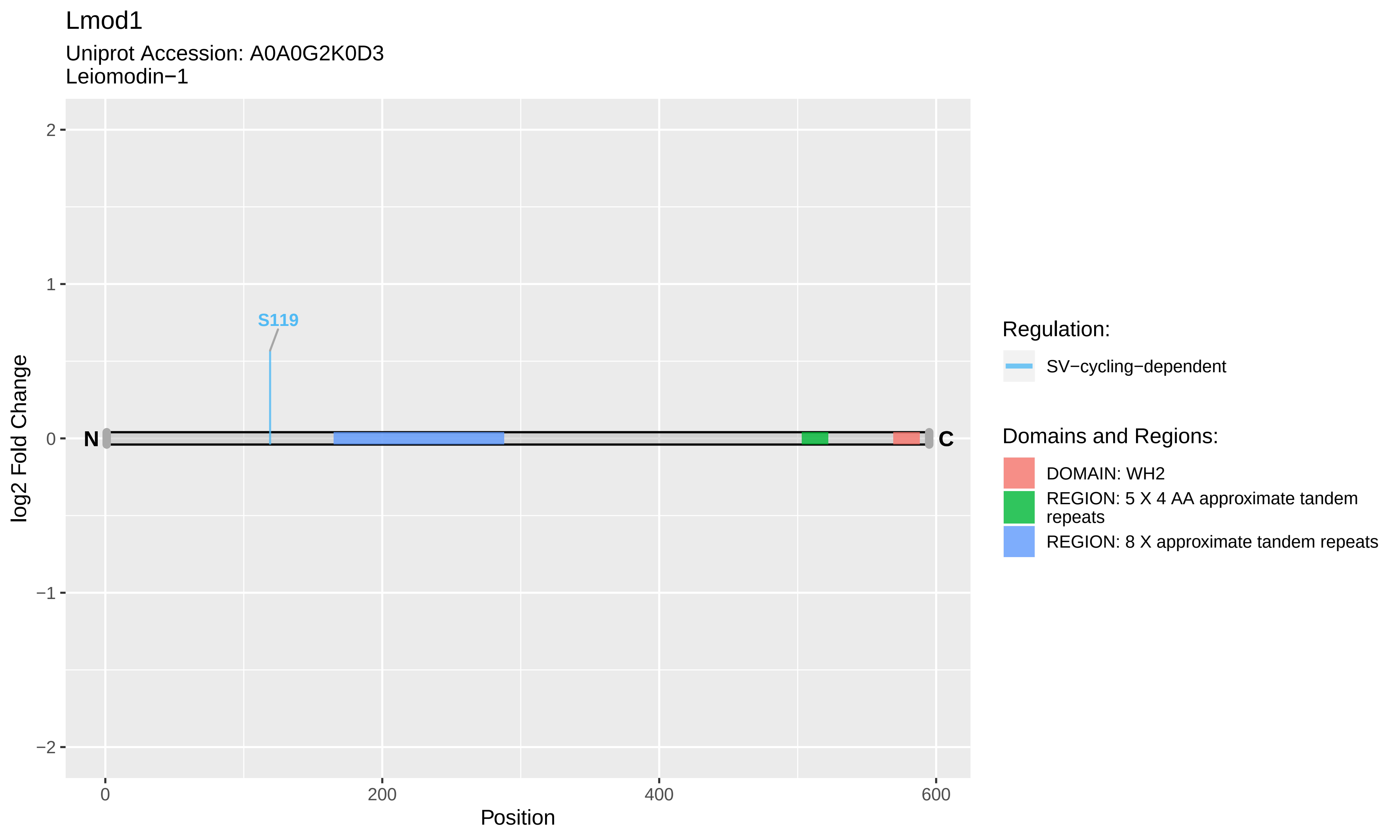

Lmod1
Uniprot Accession: A0A0G2K0D3
Leiomodin−1
S119
N
C
2
1
0
−1
−2
log2 Fold Change
0
200
400
600
Position
Regulation:
SV−cycling−dependent
Domains and Regions:
DOMAIN: WH2
REGION: 5 X 4 AA approximate tandem repeats
REGION: 8 X approximate tandem repeats

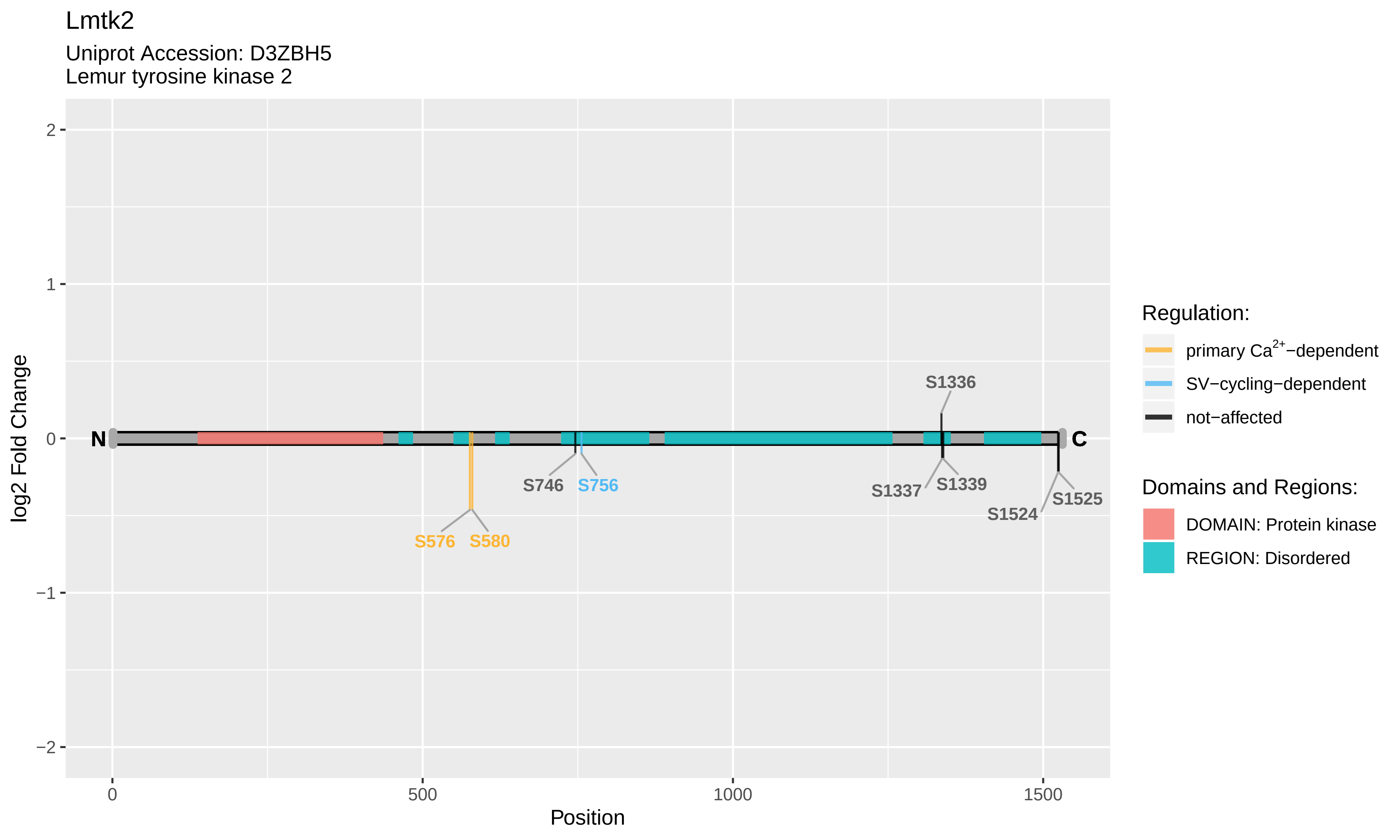

Lmtk2
Uniprot Accession: D3ZBH5
Lemur tyrosine kinase 2
log2 Fold Change
Position
2
1
0
−1
−2
0
500
1000
1500
N
C
S576
S580
S746
S756
S1336
S1337
S1339
S1524
S1525
Regulation:
primary Ca2+−dependent
SV−cycling−dependent
not−affected
Domains and Regions:
DOMAIN: Protein kinase
REGION: Disordered

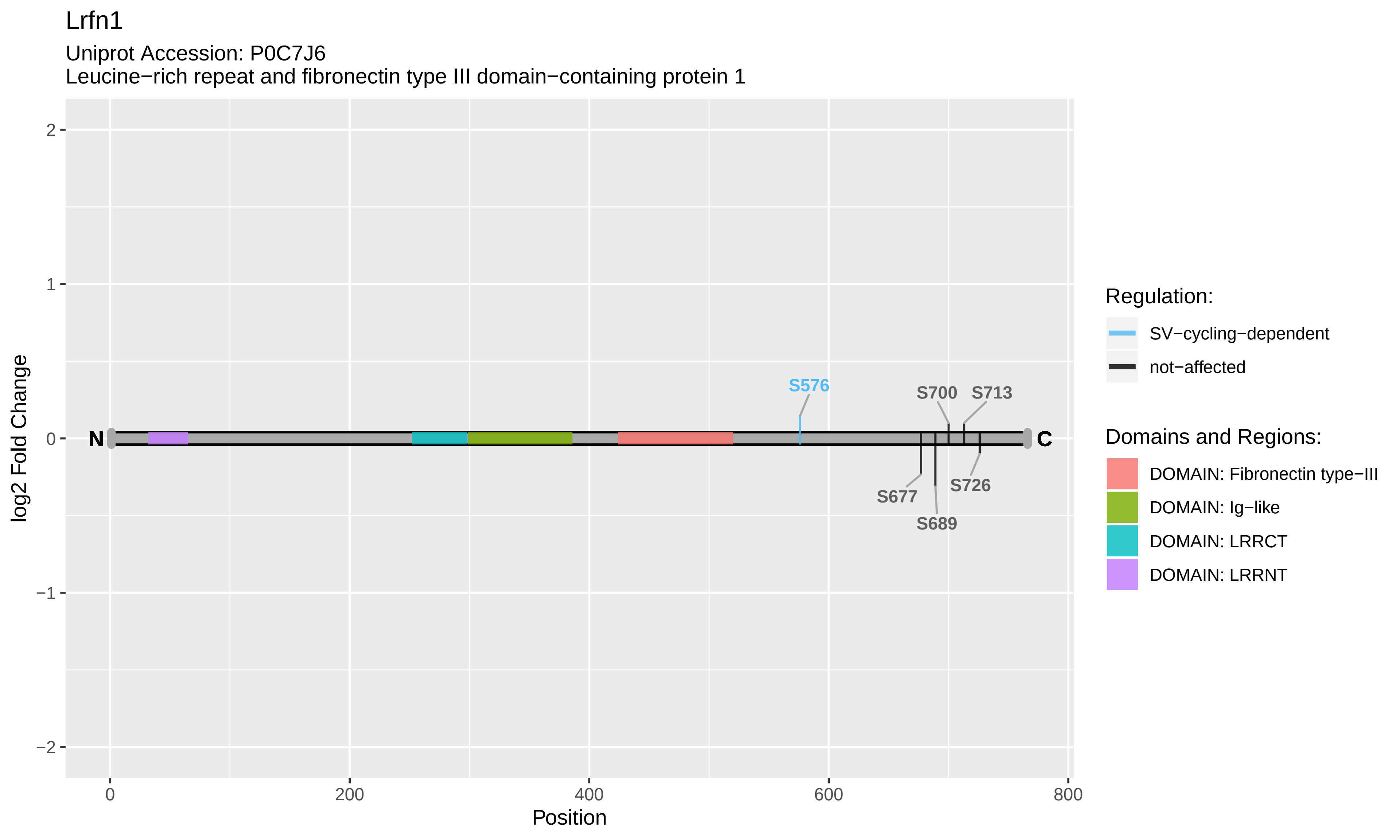

Lrfn1
Uniprot Accession: P0C7J6
Leucine−rich repeat and fibronectin type III domain−containing protein 1
log2 Fold Change
2
1
0
−1
−2
N
C
S576
S700
S713
S677
S689
S726
0
200
400
600
800
Position
Regulation:
SV−cycling−dependent
not−affected
Domains and Regions:
DOMAIN: Fibronectin type−III
DOMAIN: Ig−like
DOMAIN: LRRCT
DOMAIN: LRRNT

Lrrc47

Uniprot Accession: F1LT49
Leucine−rich repeat−containing 47

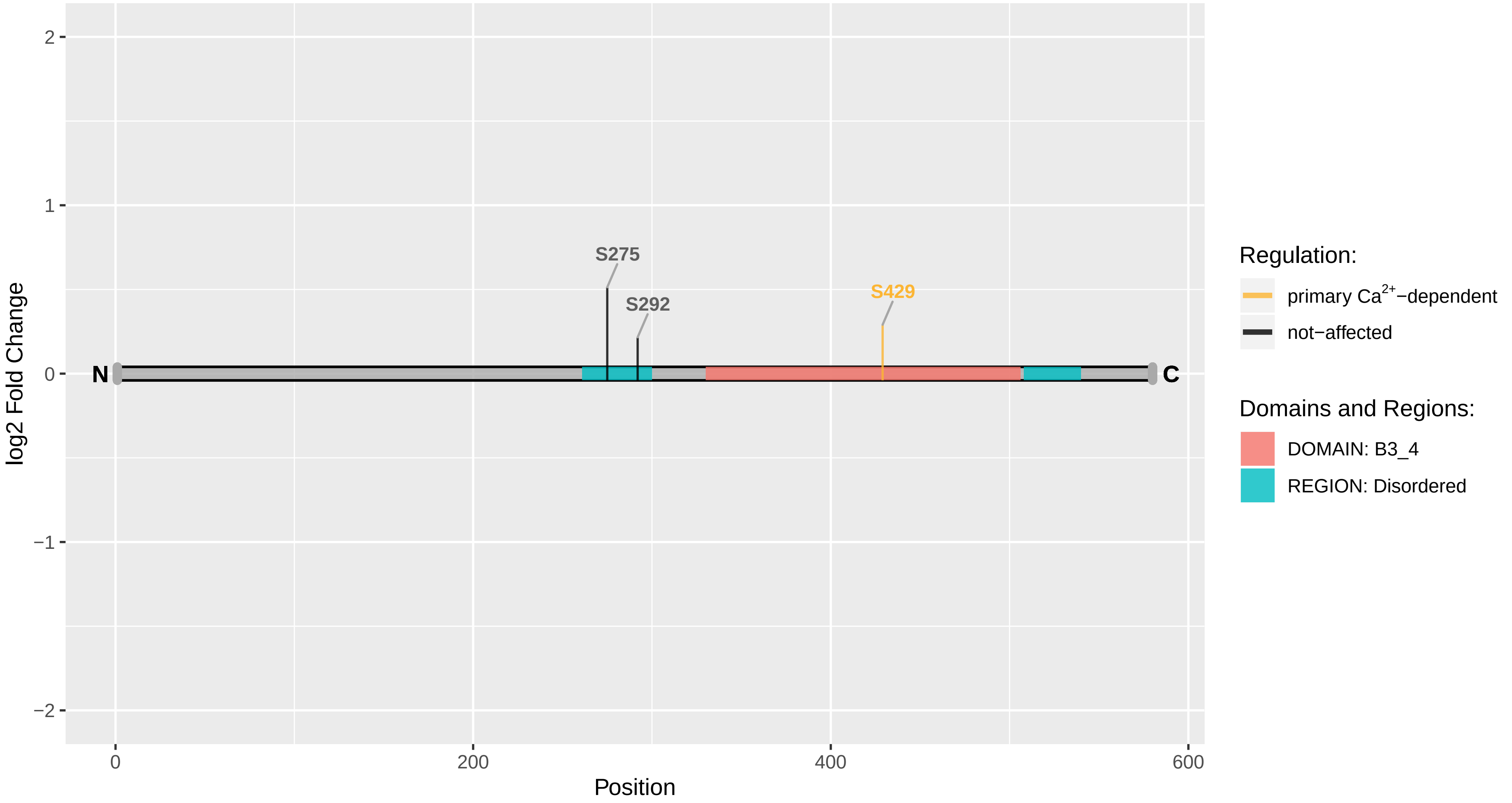

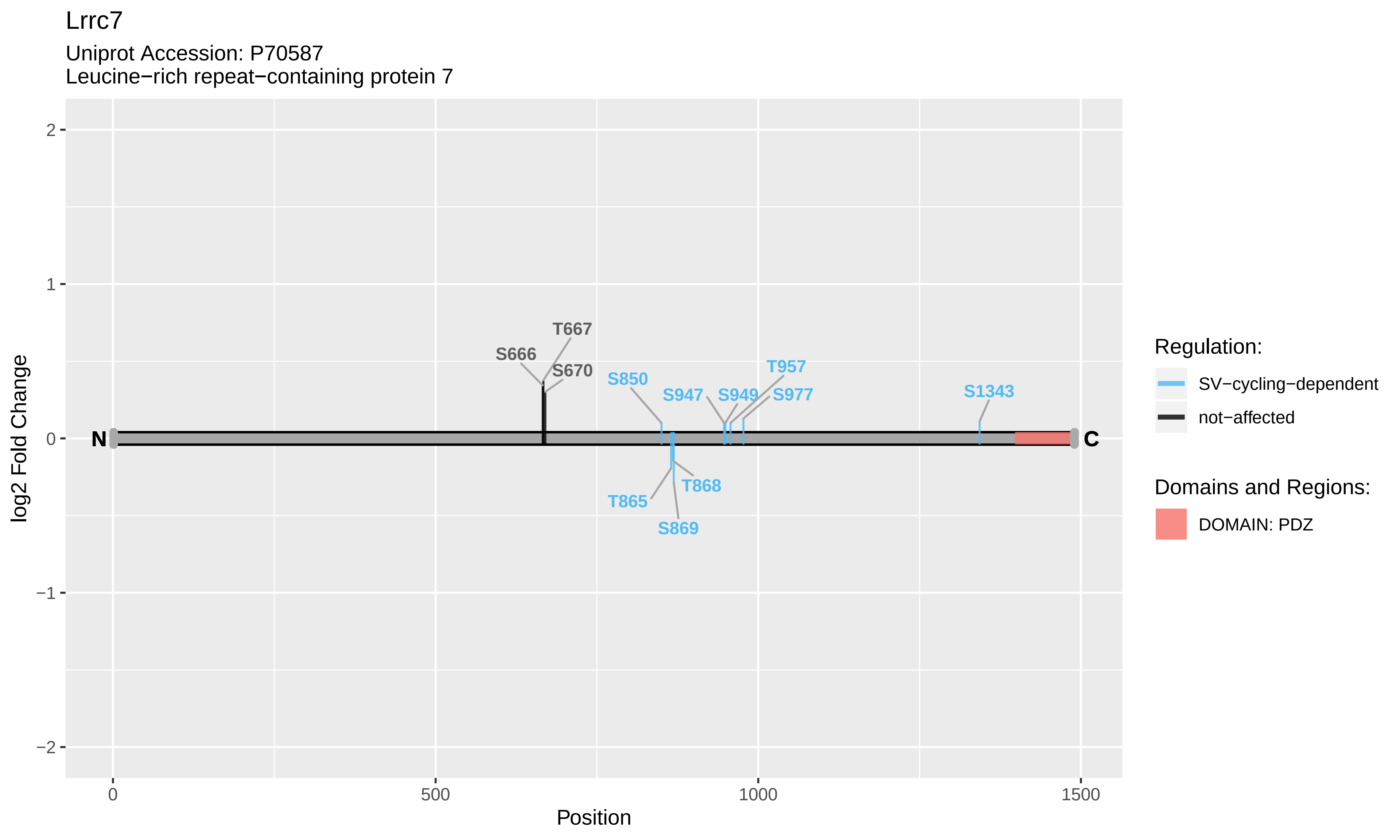

Lrrc7
Uniprot Accession: P70587
Leucine−rich repeat−containing protein 7
T667
S666
S670
S850
S947
S949
T957
S977
S1343
N
C
T868
T865
S869
log2 Fold Change
2
1
0
−1
−2
0
500
1000
1500
Position
Regulation:
SV−cycling−dependent
not−affected
Domains and Regions:
DOMAIN: PDZ

Lrrfip2

Uniprot Accession: Q4V7E8
Leucine−rich repeat flightless−interacting protein 2

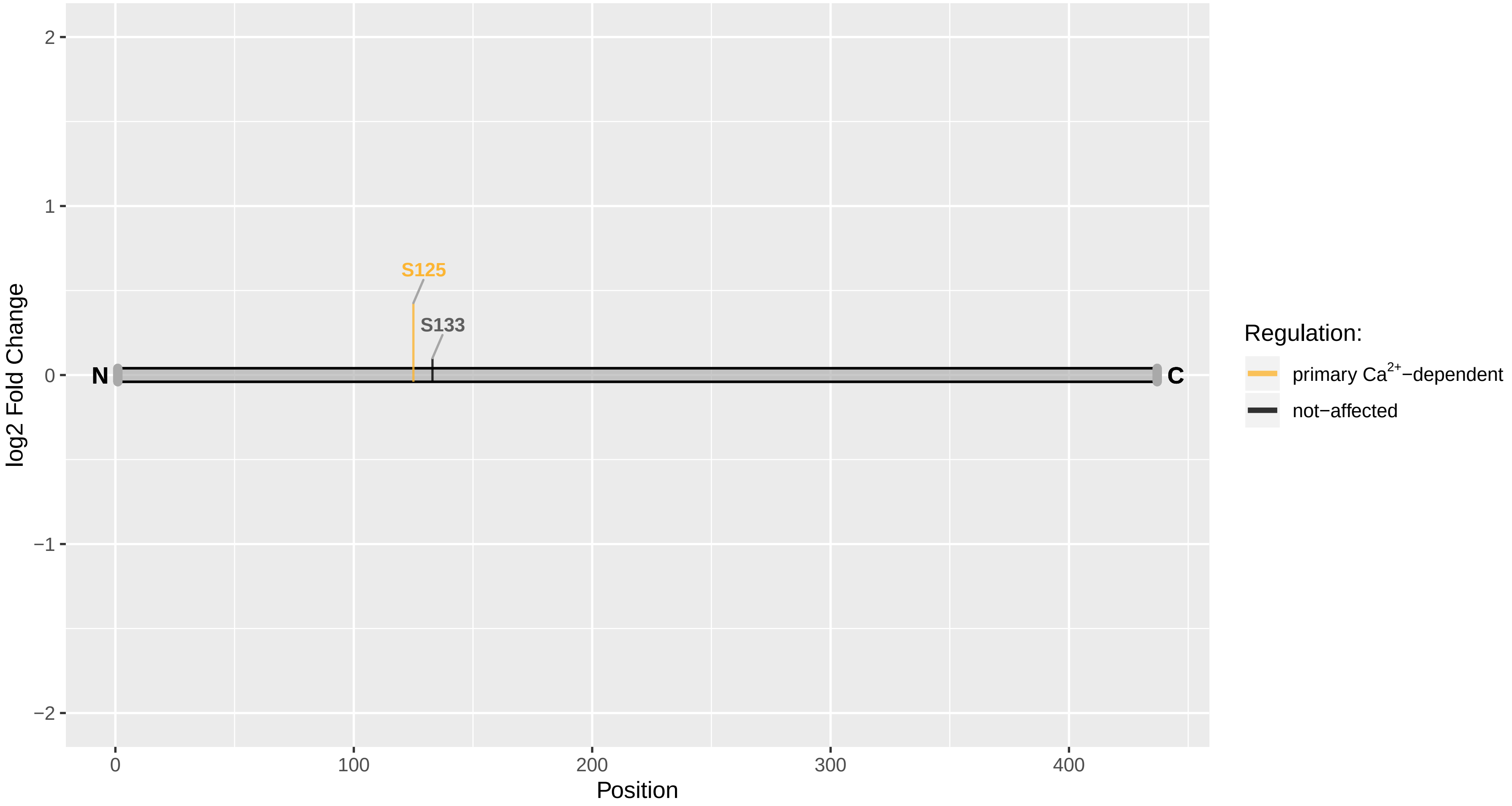

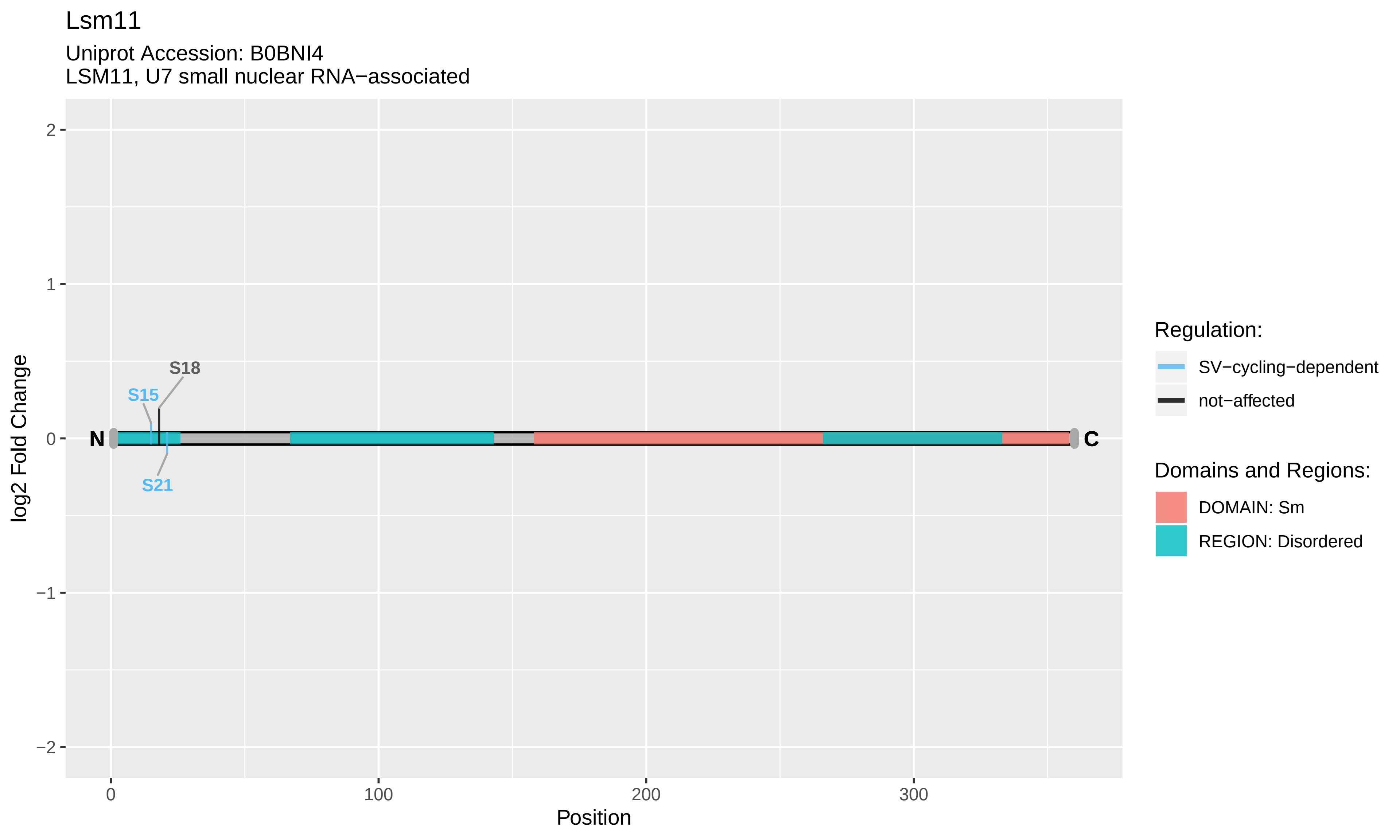

Lsm11
Uniprot Accession: B0BNI4
LSM11, U7 small nuclear RNA−associated
log2 Fold Change
2
1
0
−1
−2
N
C
S18
S15
S21
0
100
200
300
Position
Regulation:
SV−cycling−dependent
not−affected
Domains and Regions:
DOMAIN: Sm
REGION: Disordered

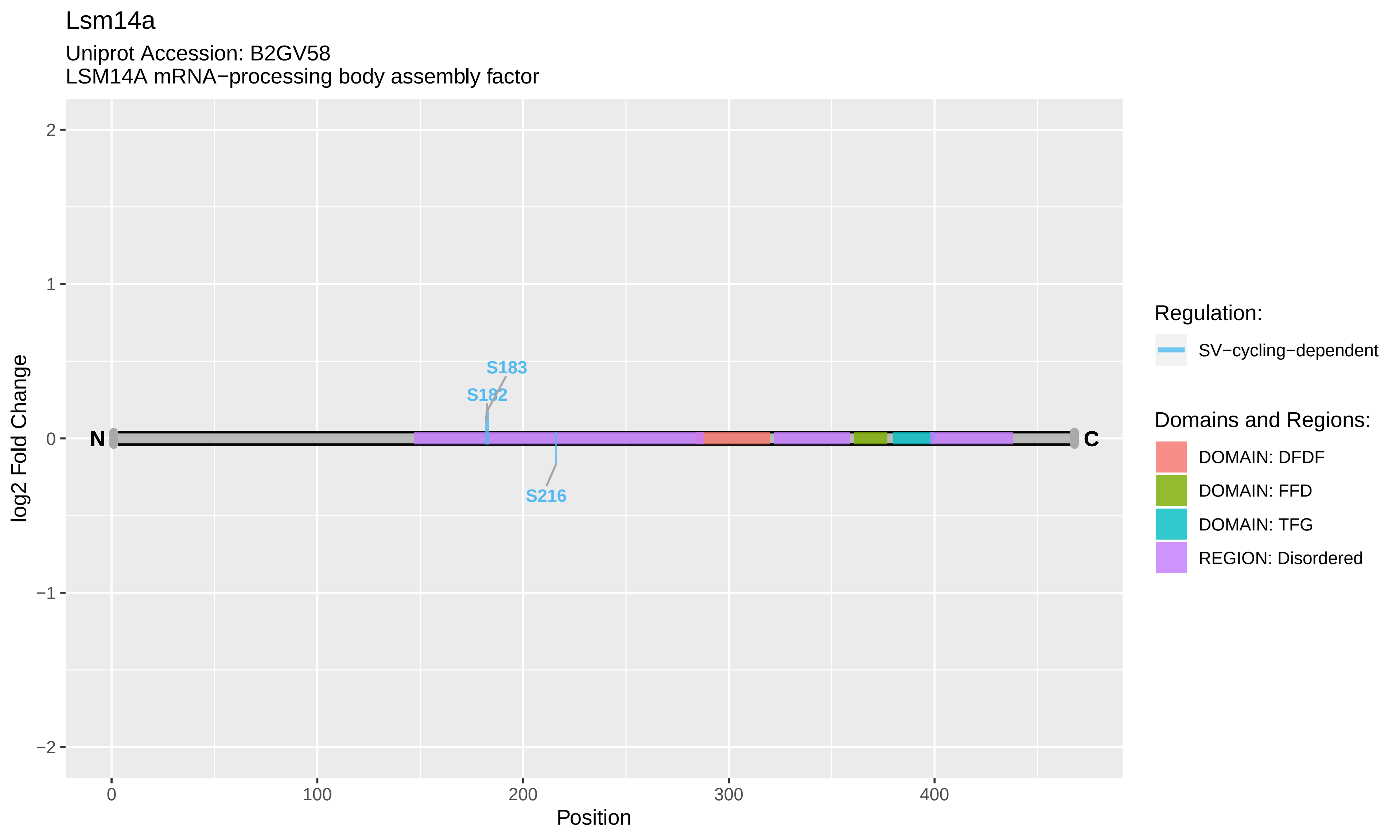
Lsm14a
Uniprot Accession: B2GV58
LSM14A mRNA−processing body assembly factor
log2 Fold Change
2
1
0
−1
−2
N
C
S183
S182
S216
0
100
200
300
400
Position
Regulation:
SV−cycling−dependent
Domains and Regions:
DOMAIN: DFDF
DOMAIN: FFD
DOMAIN: TFG
REGION: Disordered

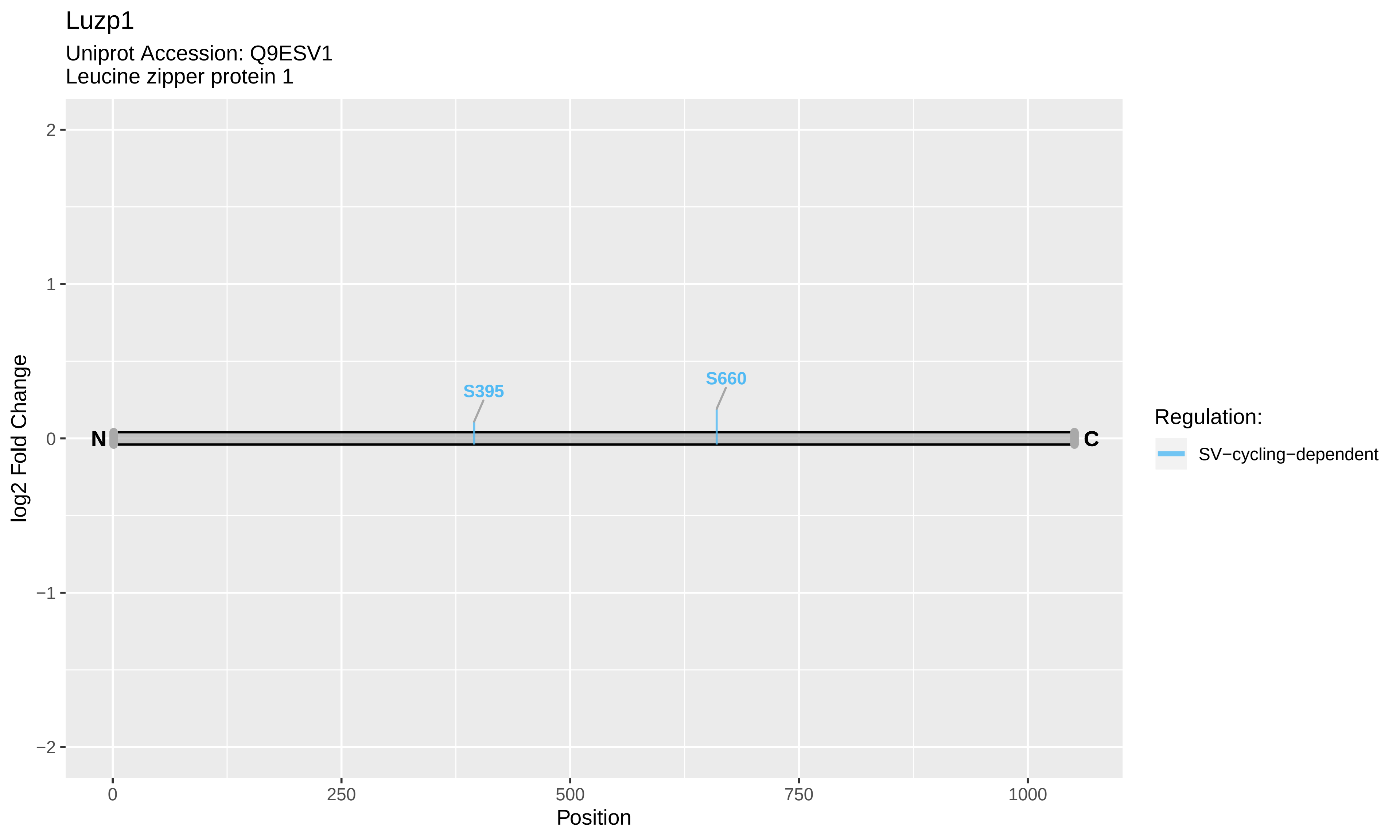

Luzp1
Uniprot Accession: Q9ESV1
Leucine zipper protein 1
log2 Fold Change
2
1
0
−1
−2
N
C
S395
S660
0
250
500
750
1000
Position
Regulation:
SV−cycling−dependent

Lysmd1

Uniprot Accession: Q5HZA4
LysM and putative peptidoglycan−binding domain−containing protein 1

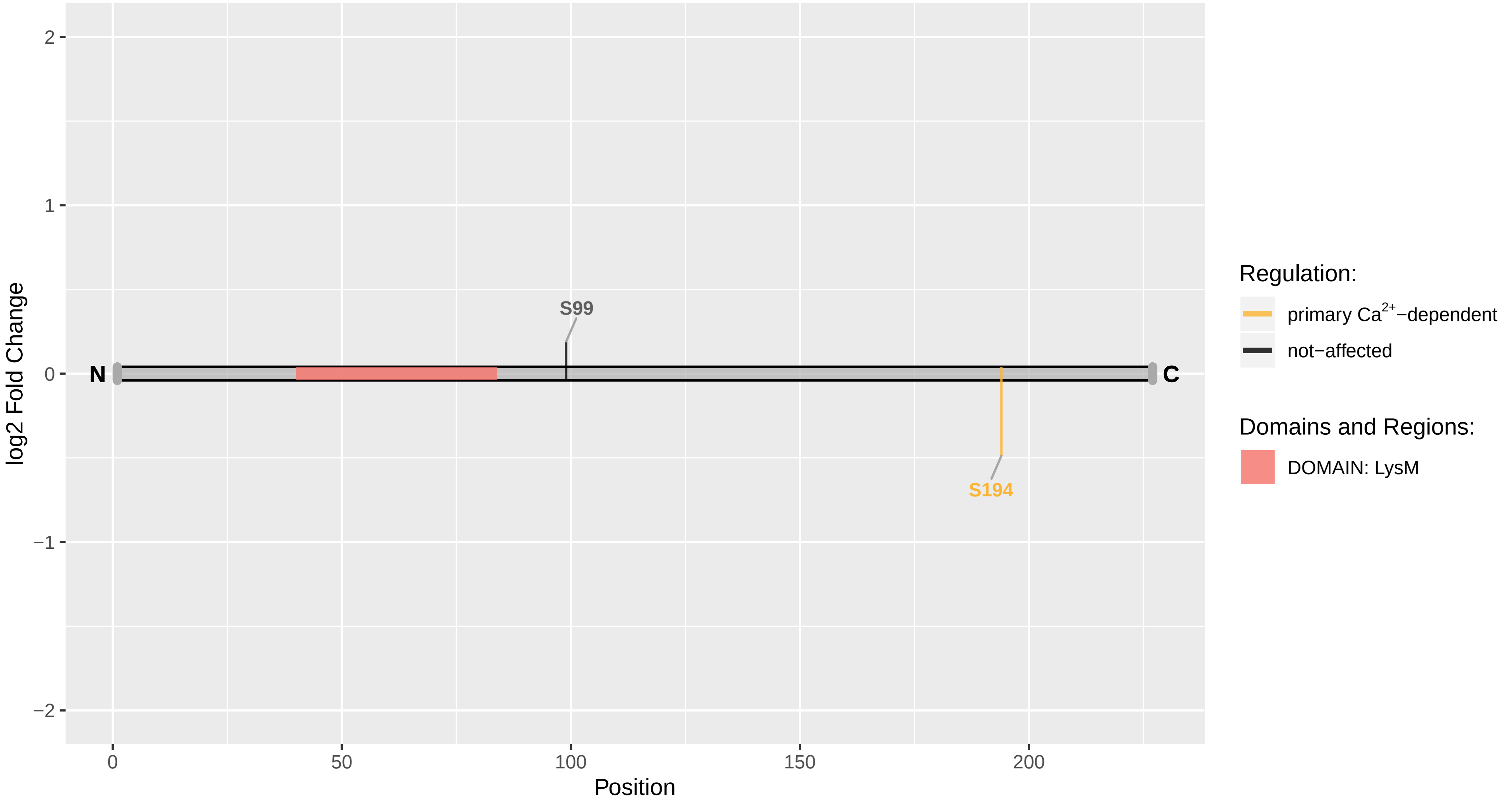

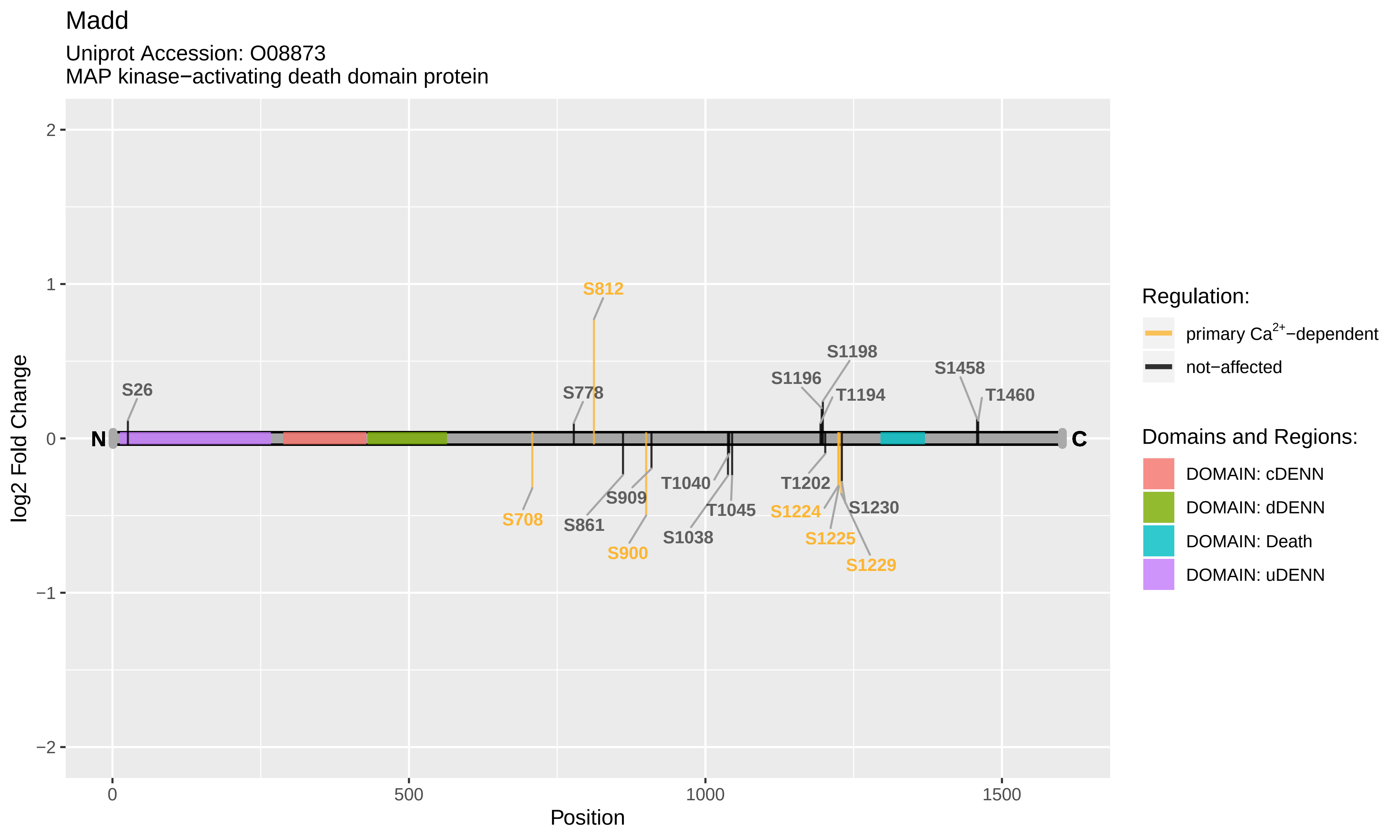

Madd
Uniprot Accession: O08873
MAP kinase−activating death domain protein
log2 Fold Change
Position
2
1
0
−1
−2
0
500
1000
1500
N
C
S812
S26
S778
S1198
S1196
T1194
S1458
T1460
S708
S861
S909
S900
T1040
S1038
T1045
T1202
S1224
S1225
S1229
S1230
Regulation:
primary Ca^{2+}−dependent
not−affected
Domains and Regions:
DOMAIN: cDENN
DOMAIN: dDENN
DOMAIN: Death
DOMAIN: uDENN

Magi1

Uniprot Accession: M0R8T1
Membrane−associated guanylate kinase, WW and PDZ domain−containing protein 1

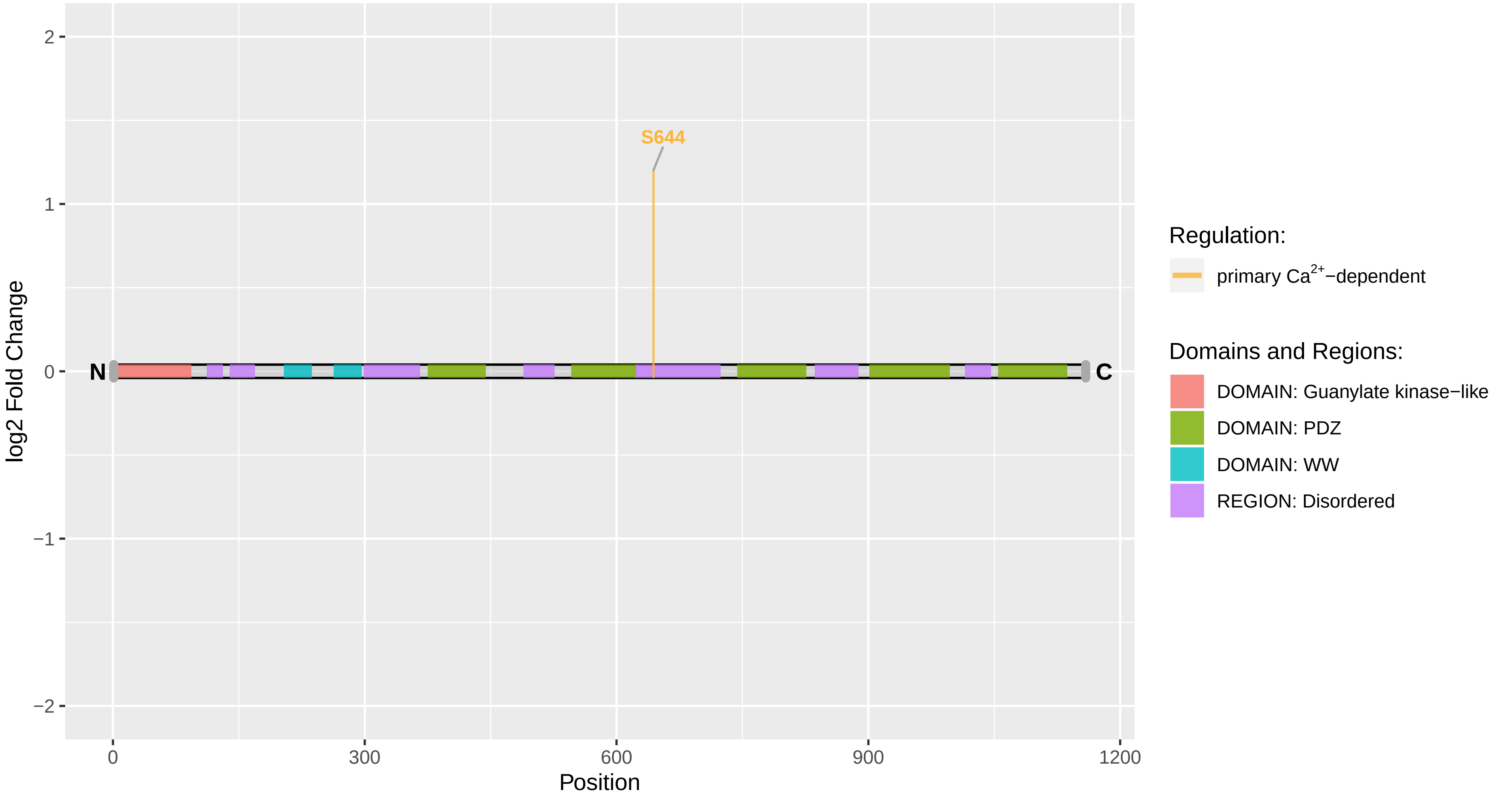

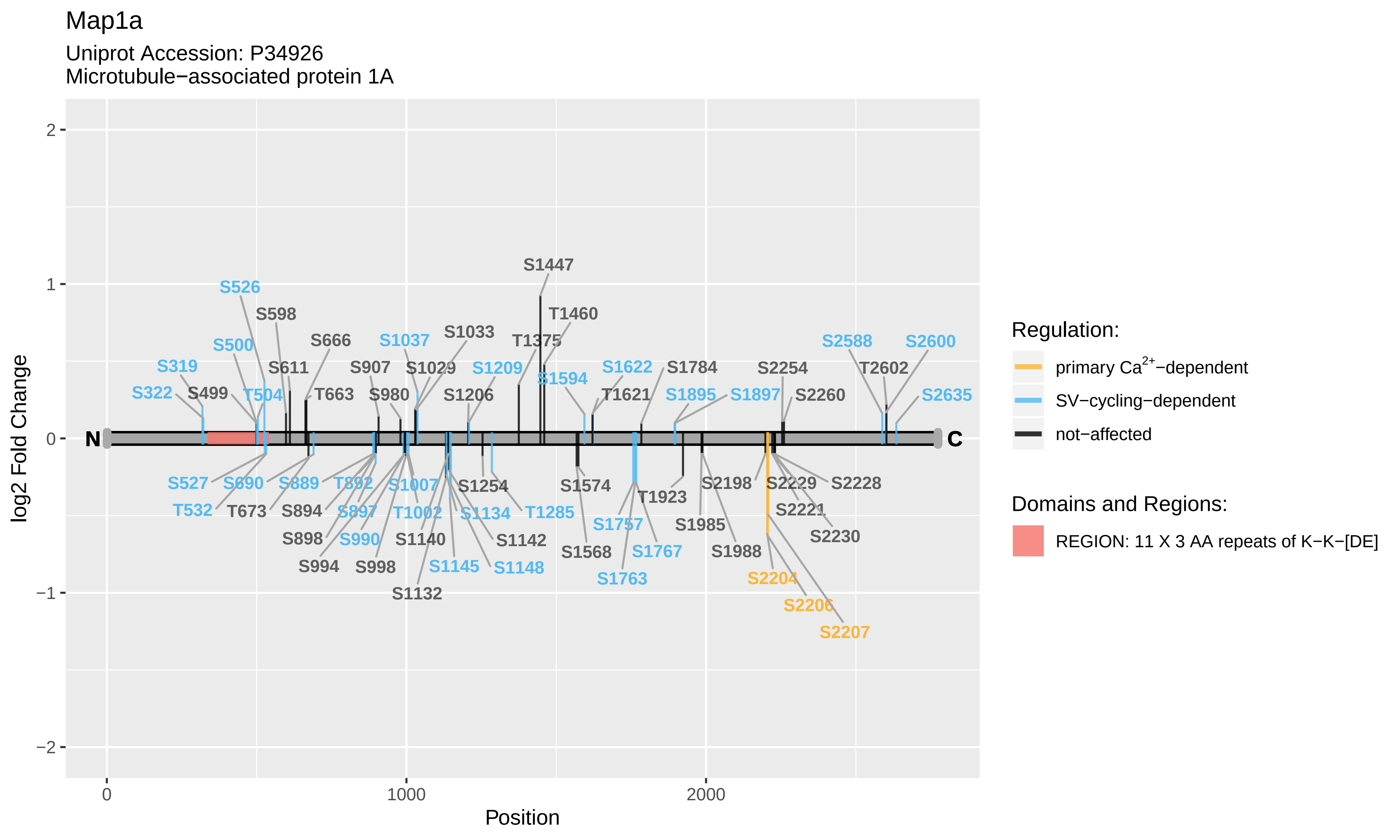
Map1a
Uniprot Accession: P34926
Microtubule−associated protein 1A
log2 Fold Change
2
1
0
−1
−2
Position
0
1000
2000
N
C
S1447
S526
S598
T1460
S666
S1037
S1033
T1375
S500
S2588
S2600
S319
S611
S907
S1029
S1209
S1622
S1784
S2254
T2602
S1594
S322
S499
T504
T663
S980
S1206
T1621
S1895
S1897
S2260
S2635
S527
S690
S889
T892
S1007
S1254
S1574
T1923
S2198
S2229
S2228
T532
T673
S894
S897
T1002
S1134
T1285
S2221
S1757
S1985
S898
S990
S1140
S1142
S2230
S1568
S1767
S1988
S994
S998
S1145
S1148
S1763
S2204
S1132
S2206
S2207
Regulation:
primary Ca^{2+}−dependent
SV−cycling−dependent
not−affected
Domains and Regions:
REGION: 11 X 3 AA repeats of K−K−[DE]

Map1b

Uniprot Accession: P15205
Microtubule−associated protein 1B

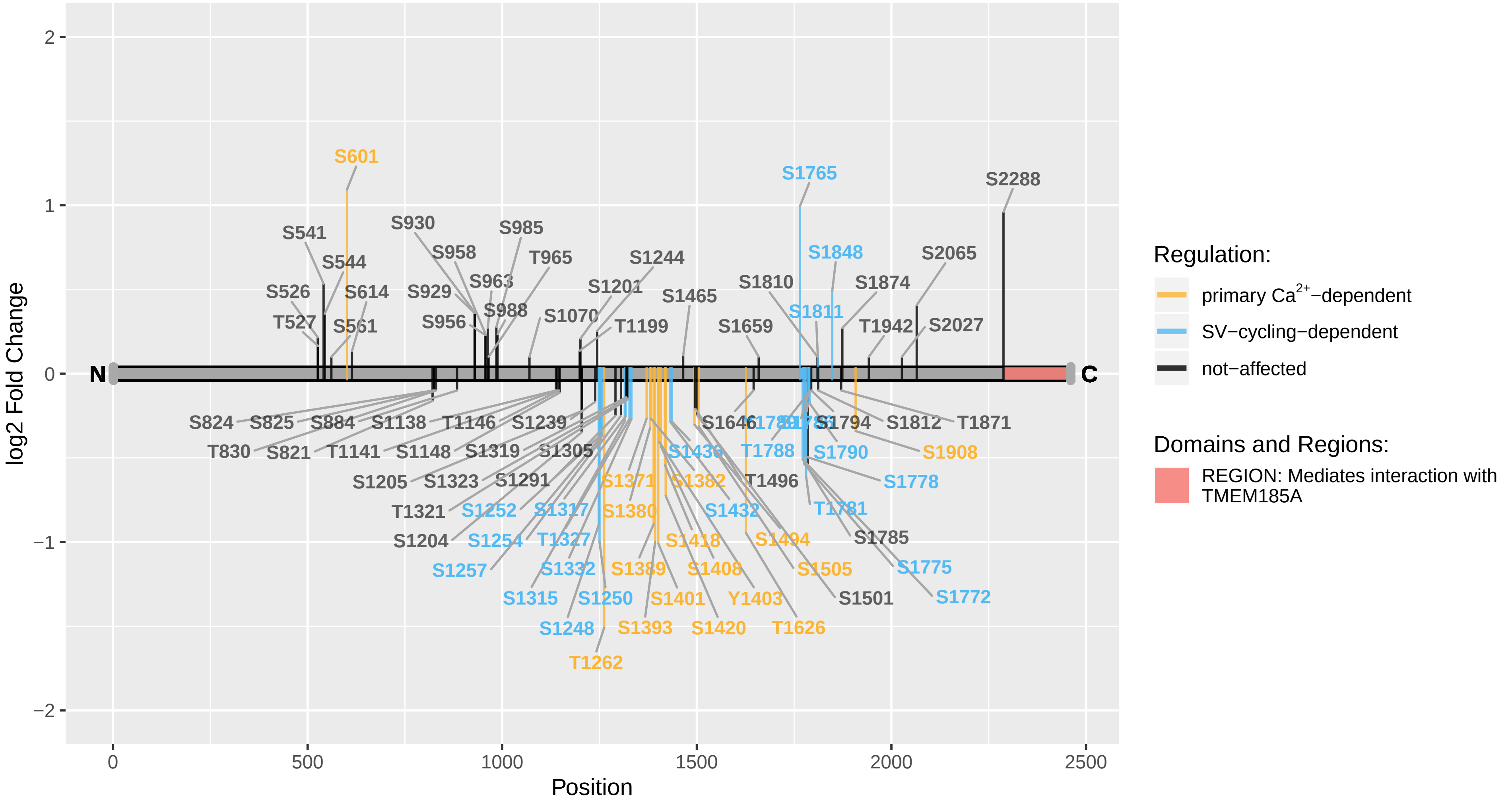

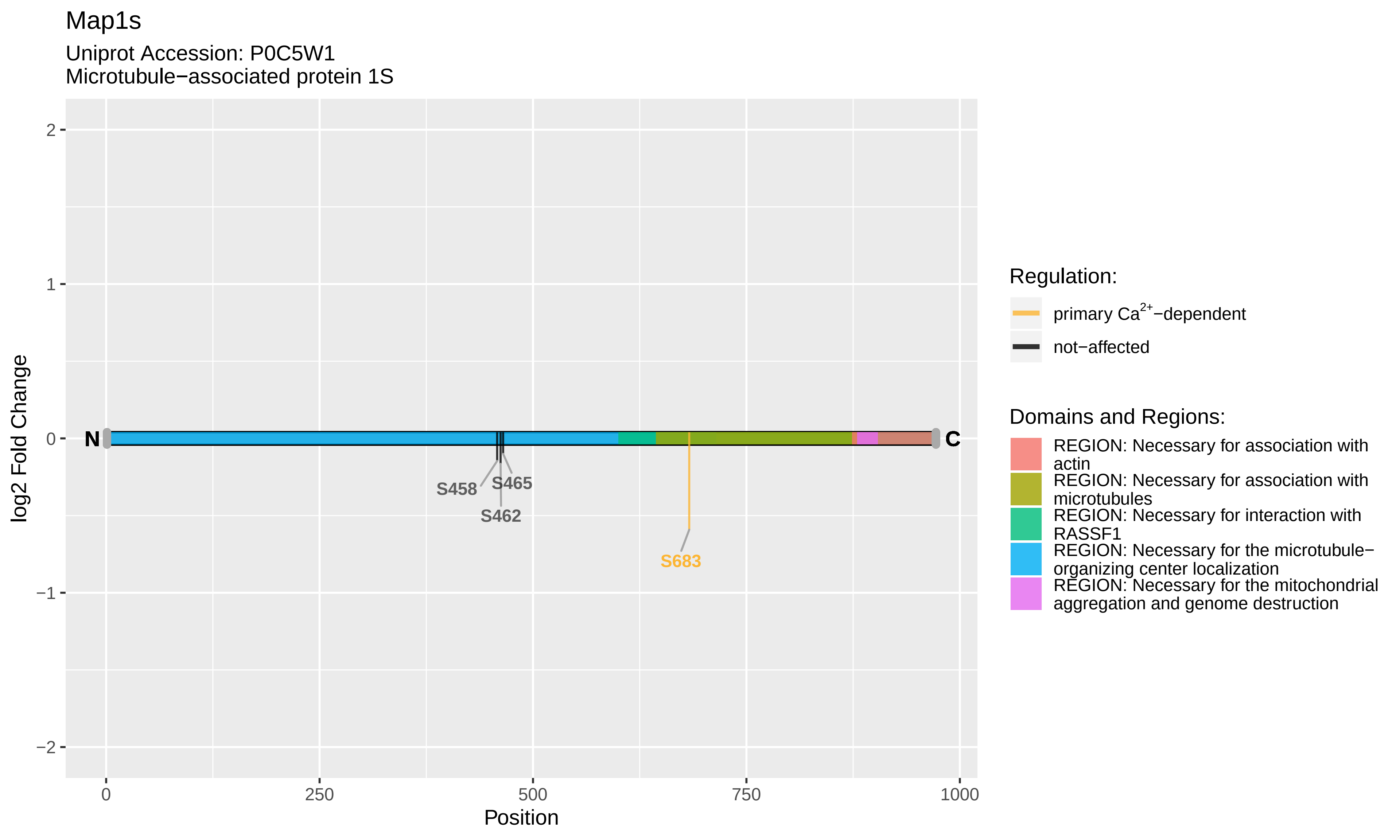

Map1s
Uniprot Accession: P0C5W1
Microtubule−associated protein 1S
log2 Fold Change
Position
N
C
S458
S465
S462
S683
2
1
0
−1
−2
0
250
500
750
1000
Regulation:
primary Ca2+−dependent
not−affected
Domains and Regions:
REGION: Necessary for association with actin
REGION: Necessary for association with microtubules
REGION: Necessary for interaction with RASSF1
REGION: Necessary for the microtubule−organizing center localization
REGION: Necessary for the mitochondrial aggregation and genome destruction

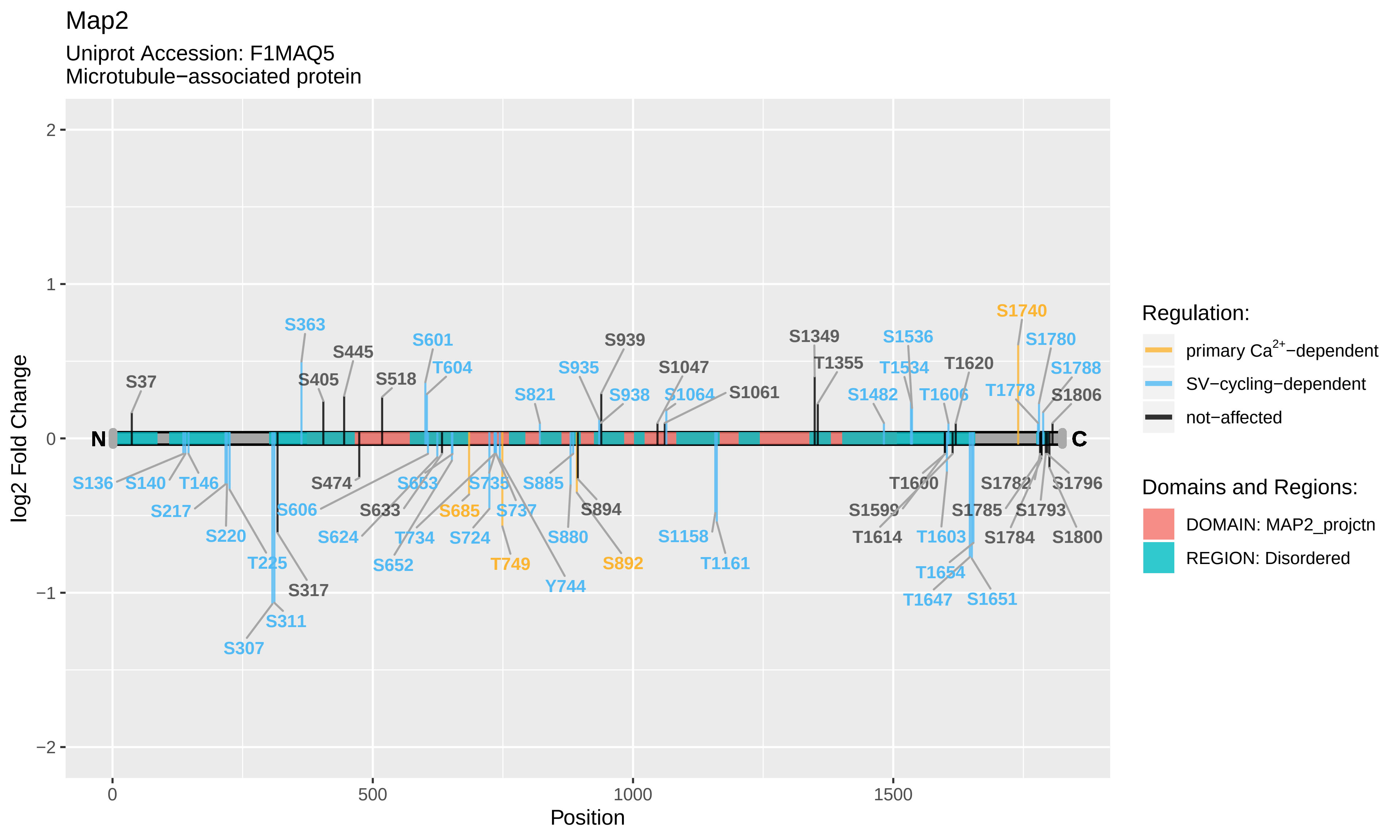

Map2
Uniprot Accession: F1MAQ5
Microtubule−associated protein
log2 Fold Change
Position
N
C
S37
S363
S405
S445
S518
S601
T604
S939
S935
S938
S821
S1047
S1064
S1061
S1349
T1355
S1536
T1534
S1482
T1606
T1620
S1740
S1780
S1788
T1778
S1806
S136
S140
T146
S217
S220
T225
S307
S311
S317
S474
S606
S633
S624
S652
S653
T734
S685
S735
S737
S724
T749
Y744
S885
S880
S894
S892
S1158
T1161
T1600
S1599
T1614
T1603
T1654
T1647
S1651
S1782
S1785
S1784
S1793
S1796
S1800
Regulation:
primary Ca^{2+}−dependent
SV−cycling−dependent
not−affected
Domains and Regions:
DOMAIN: MAP2_projctn
REGION: Disordered

Map2k4

Uniprot Accession: F1LP57
Mitogen−activated protein kinase kinase 4

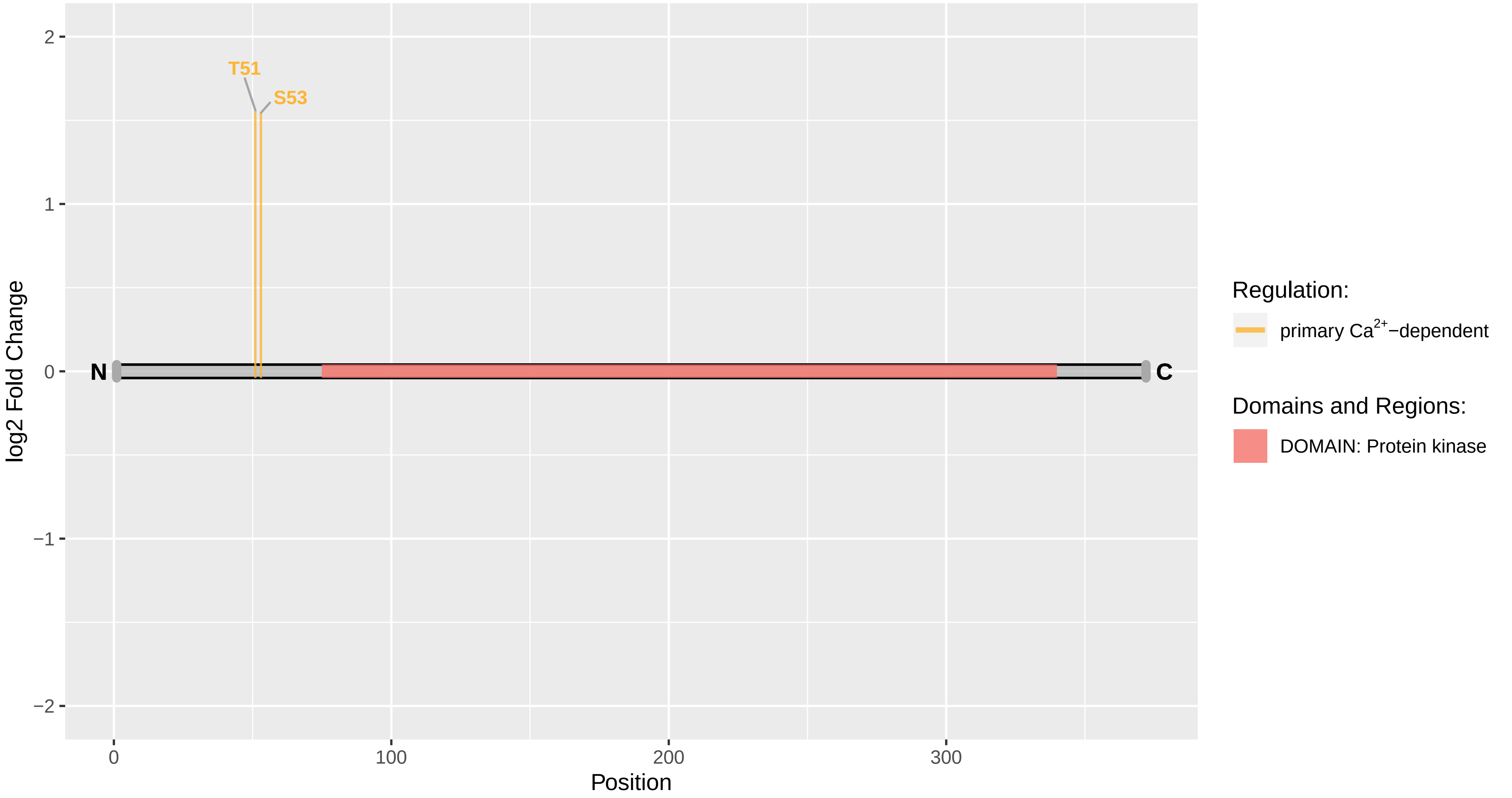

Map2k7

Uniprot Accession: Q4KSH7
Dual specificity mitogen−activated protein kinase kinase 7

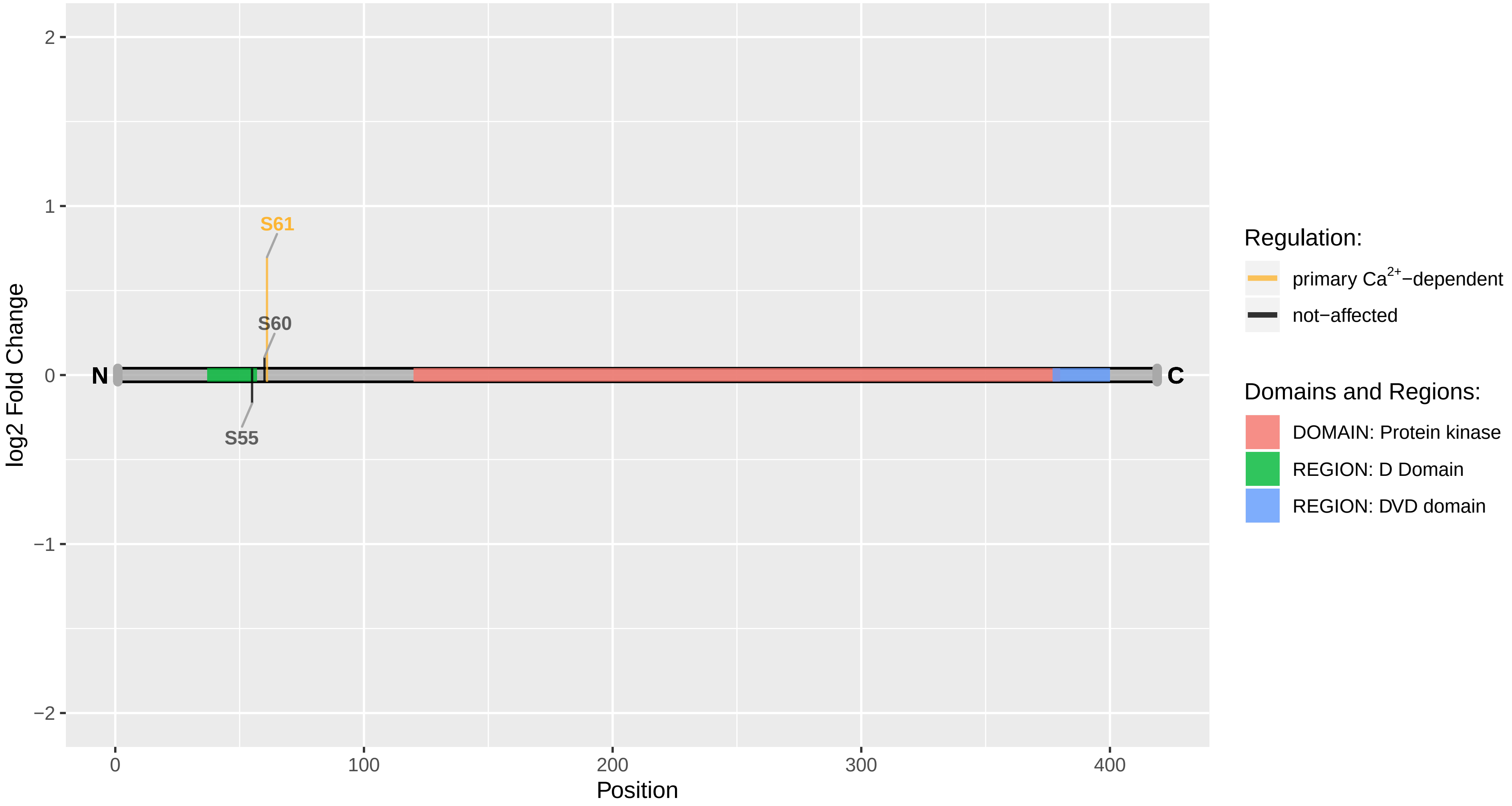

Map3k10

Uniprot Accession: D3ZG83
Mitogen−activated protein kinase kinase kinase 10

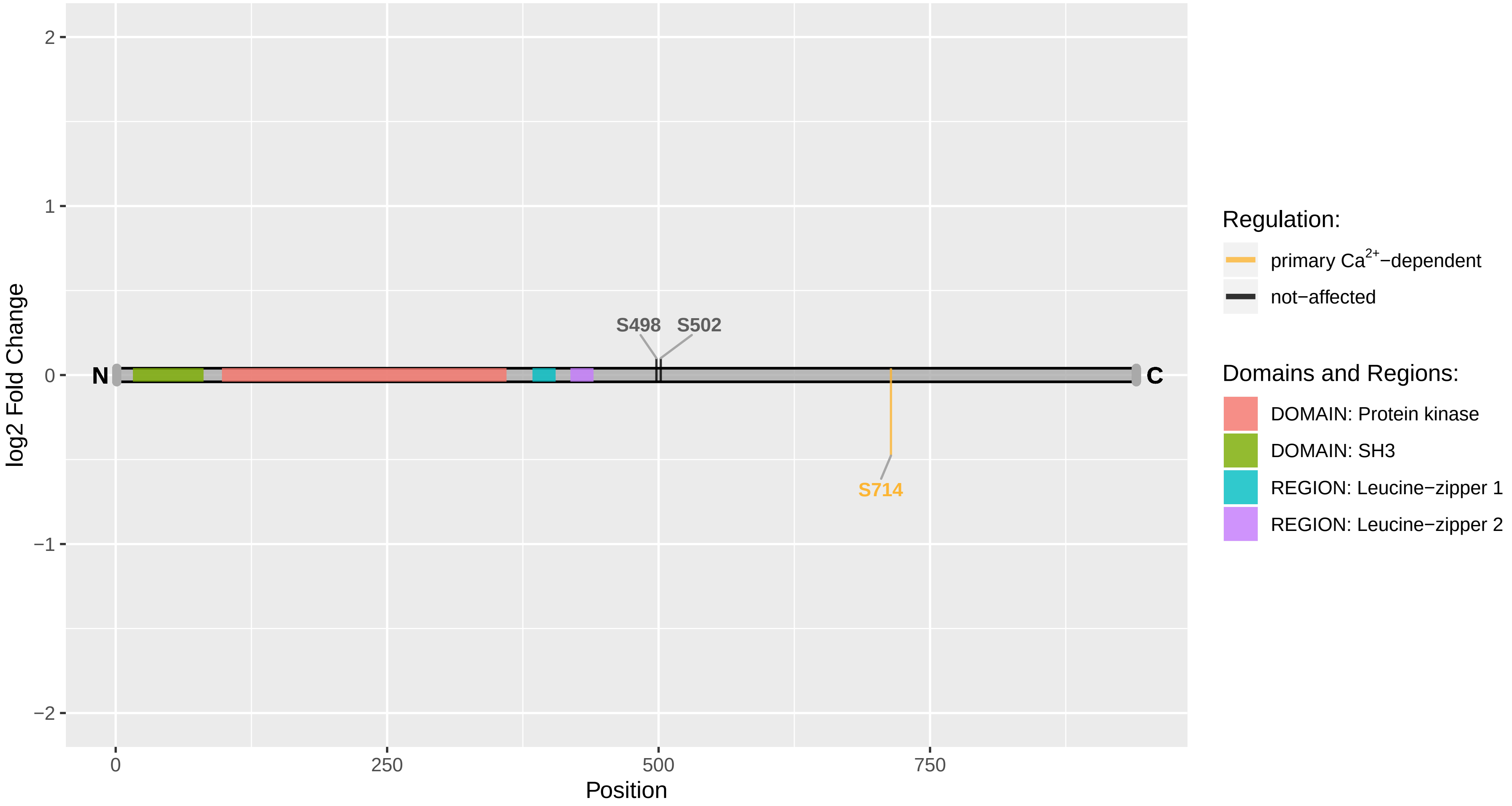

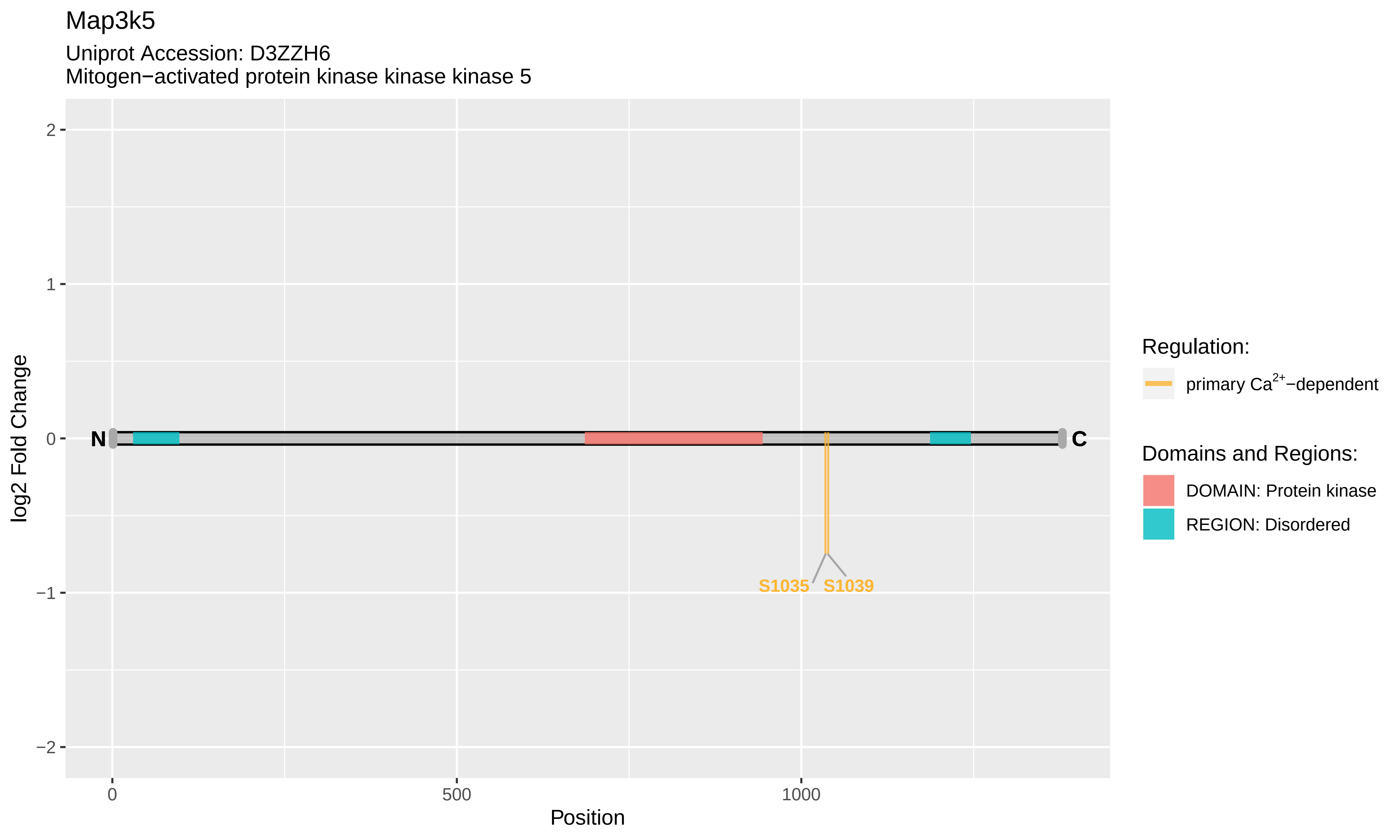

Map3k5
Uniprot Accession: D3ZZH6
Mitogen−activated protein kinase kinase kinase 5
2
1
0
−1
−2
log2 Fold Change
N
C
S1035
S1039
0
500
1000
Position
Regulation:
primary Ca2+−dependent
Domains and Regions:
DOMAIN: Protein kinase
REGION: Disordered

Map4

Uniprot Accession: A0A0G2JW88
Microtubule−associated protein

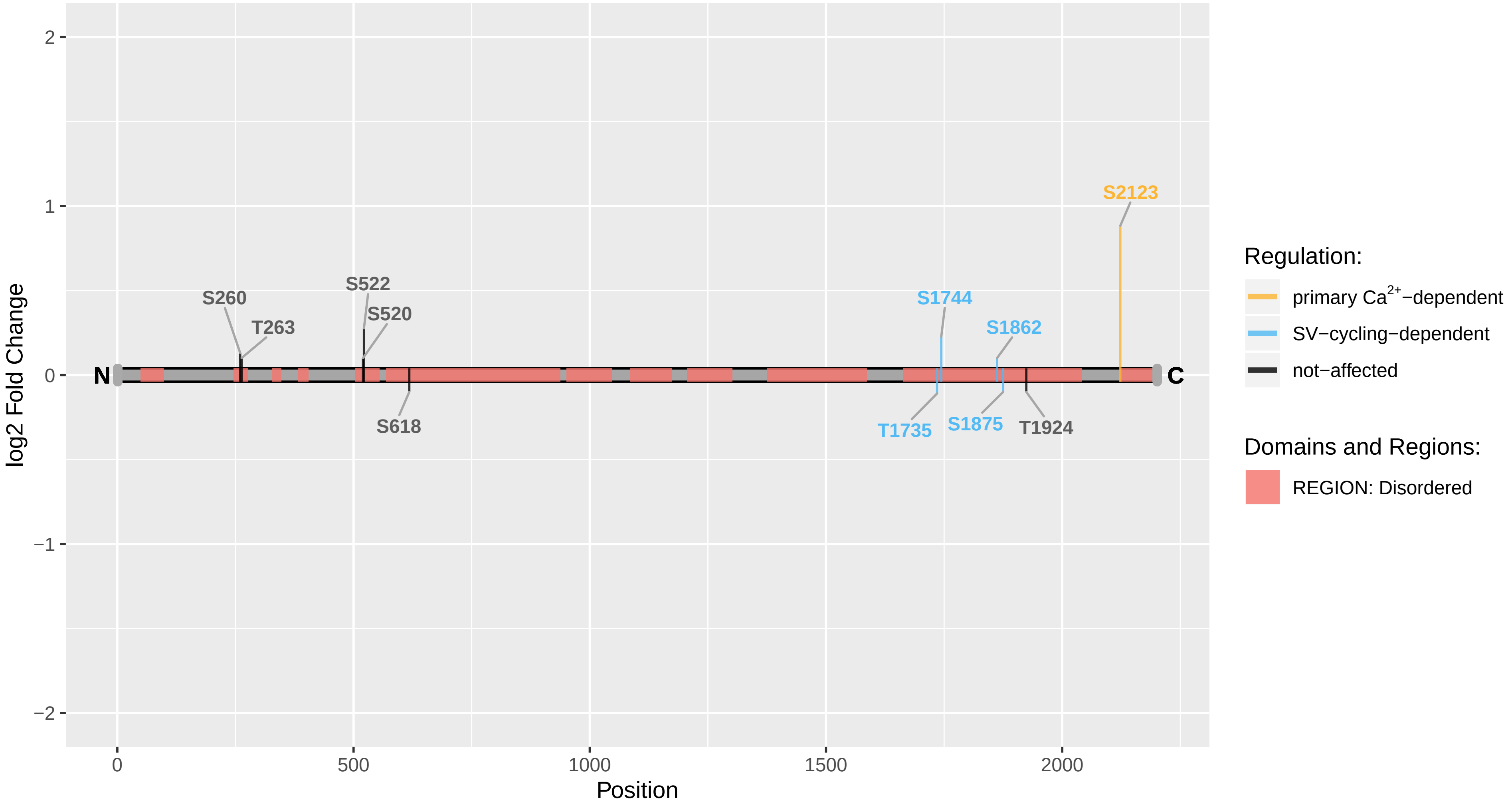

Map4k4

Uniprot Accession: A0A0G2K7W4
Mitogen−activated protein kinase kinase kinase kinase 4

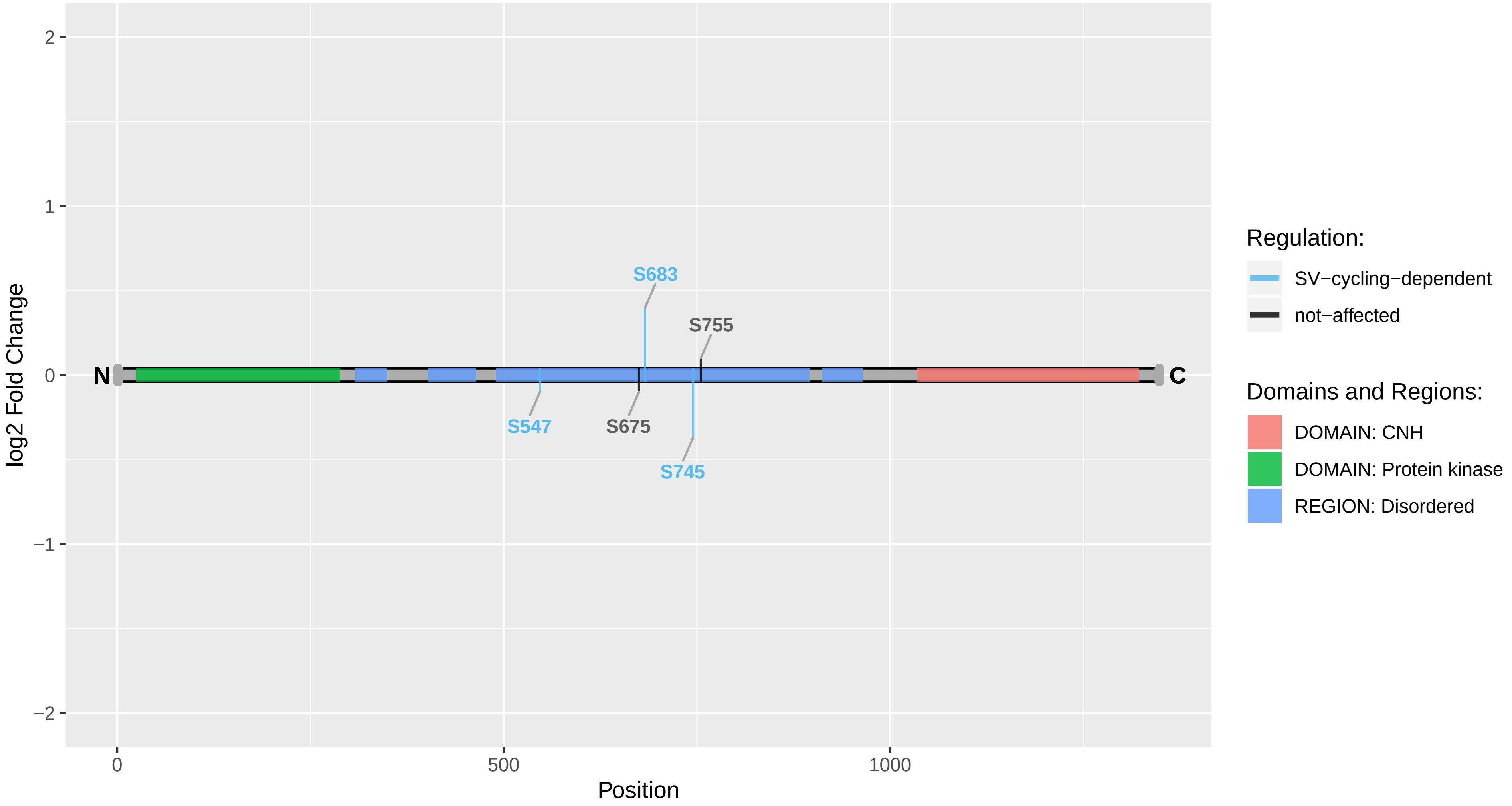

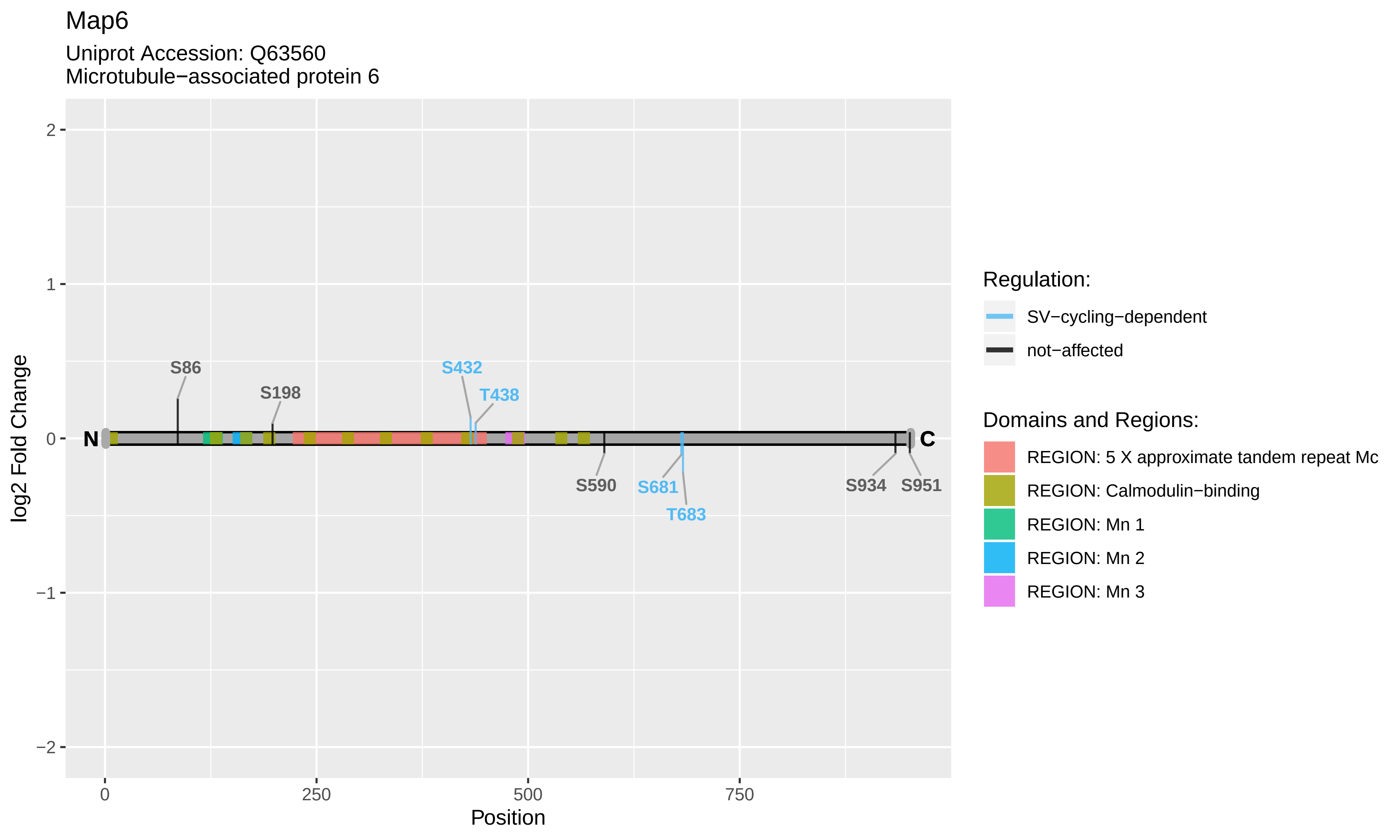

Map6
Uniprot Accession: Q63560
Microtubule−associated protein 6
log2 Fold Change
2
1
0
−1
−2
0
250
500
750
Position
N
C
S86
S198
S432
T438
S590
S681
T683
S934
S951
Regulation:
SV−cycling−dependent
not−affected
Domains and Regions:
REGION: 5 X approximate tandem repeat Mc
REGION: Calmodulin−binding
REGION: Mn 1
REGION: Mn 2
REGION: Mn 3

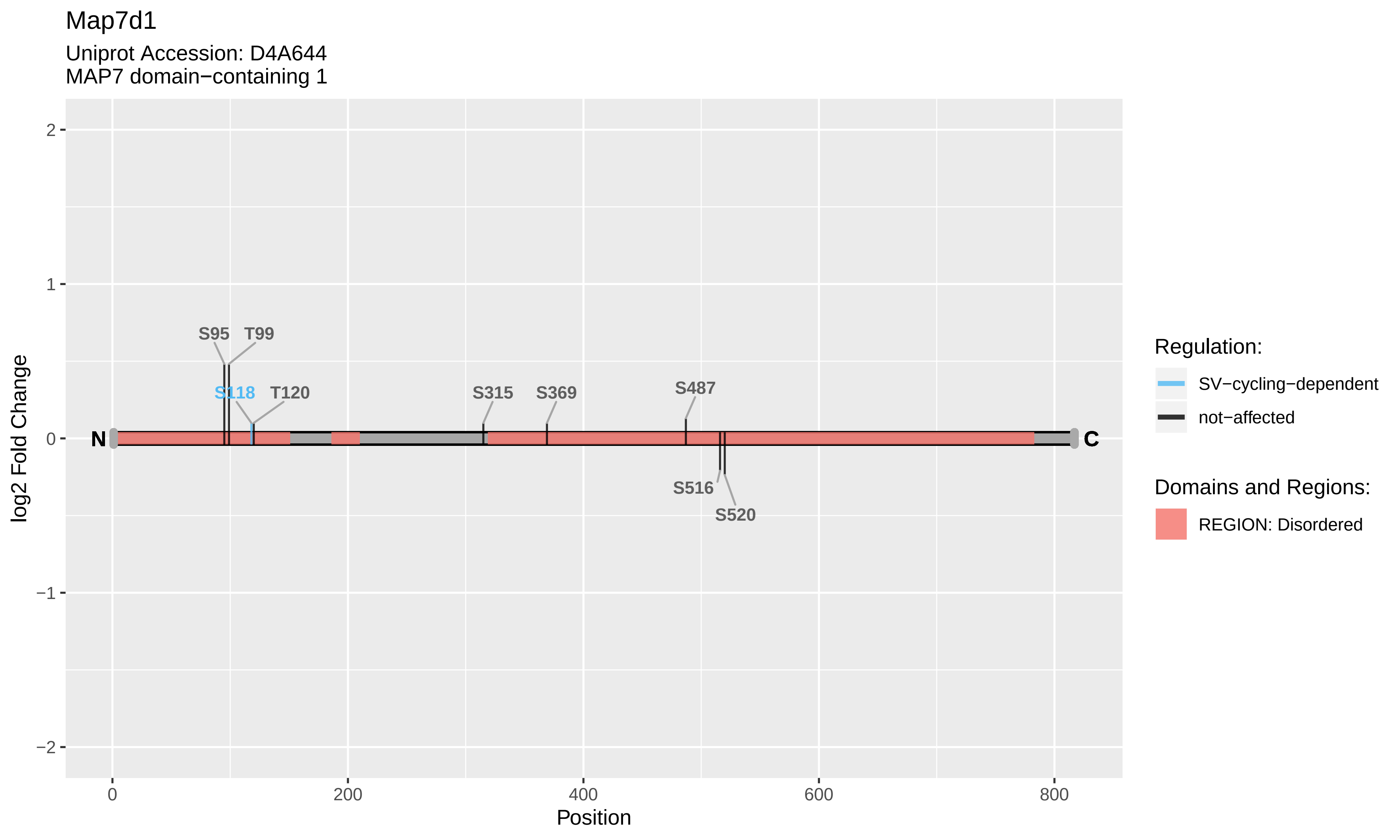

Map7d1
Uniprot Accession: D4A644
MAP7 domain−containing 1
log2 Fold Change
Position
2
1
0
−1
−2
0
200
400
600
800
N
C
S95
T99
S118
T120
S315
S369
S487
S516
S520
Regulation:
SV−cycling−dependent
not−affected
Domains and Regions:
REGION: Disordered

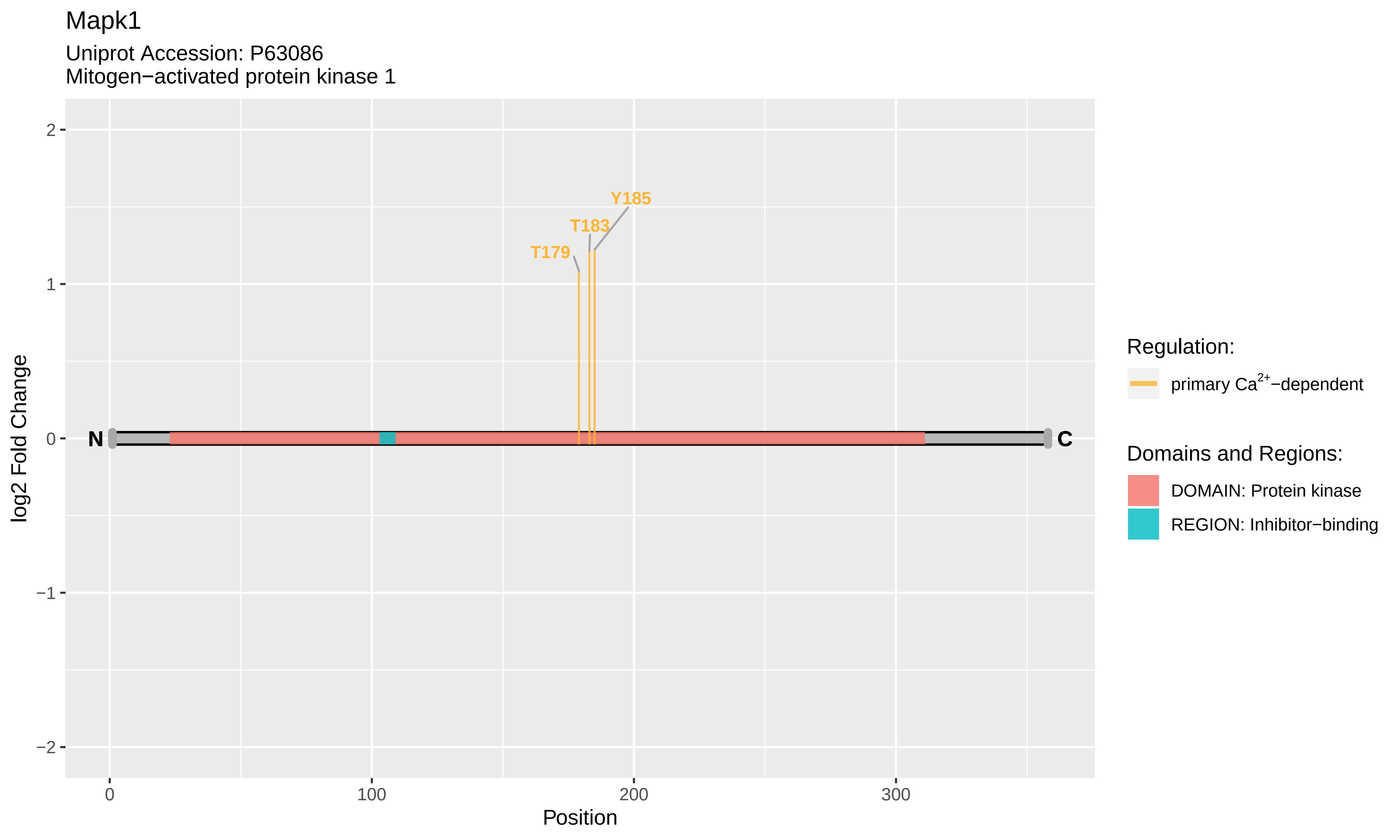

Mapk1
Uniprot Accession: P63086
Mitogen−activated protein kinase 1
Y185
T183
T179
N
C
2
1
0
−1
−2
log2 Fold Change
0
100
200
300
Position
Regulation:
primary Ca2+−dependent
Domains and Regions:
DOMAIN: Protein kinase
REGION: Inhibitor−binding

Mapk3

Uniprot Accession: P21708
Mitogen−activated protein kinase 3

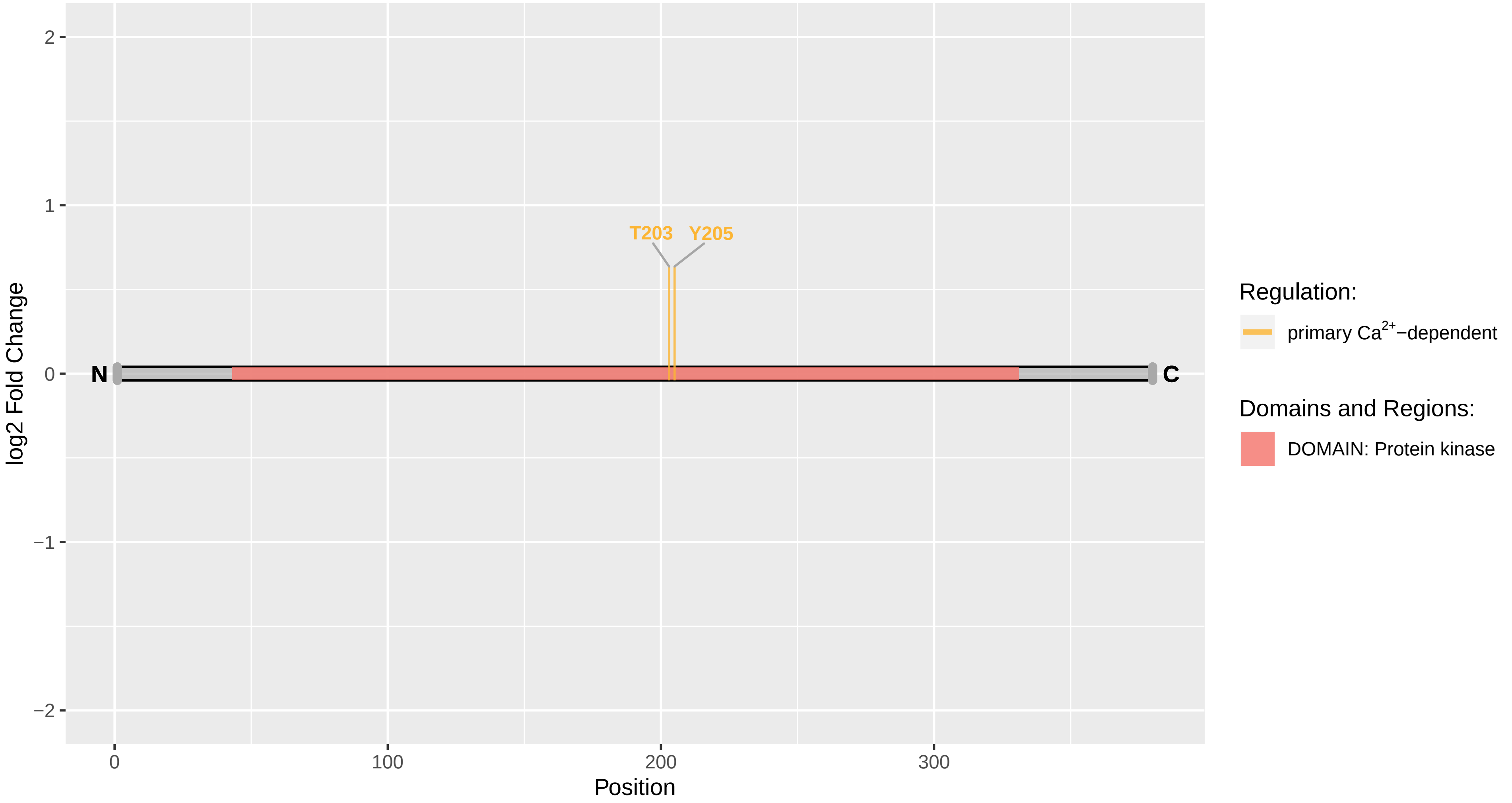

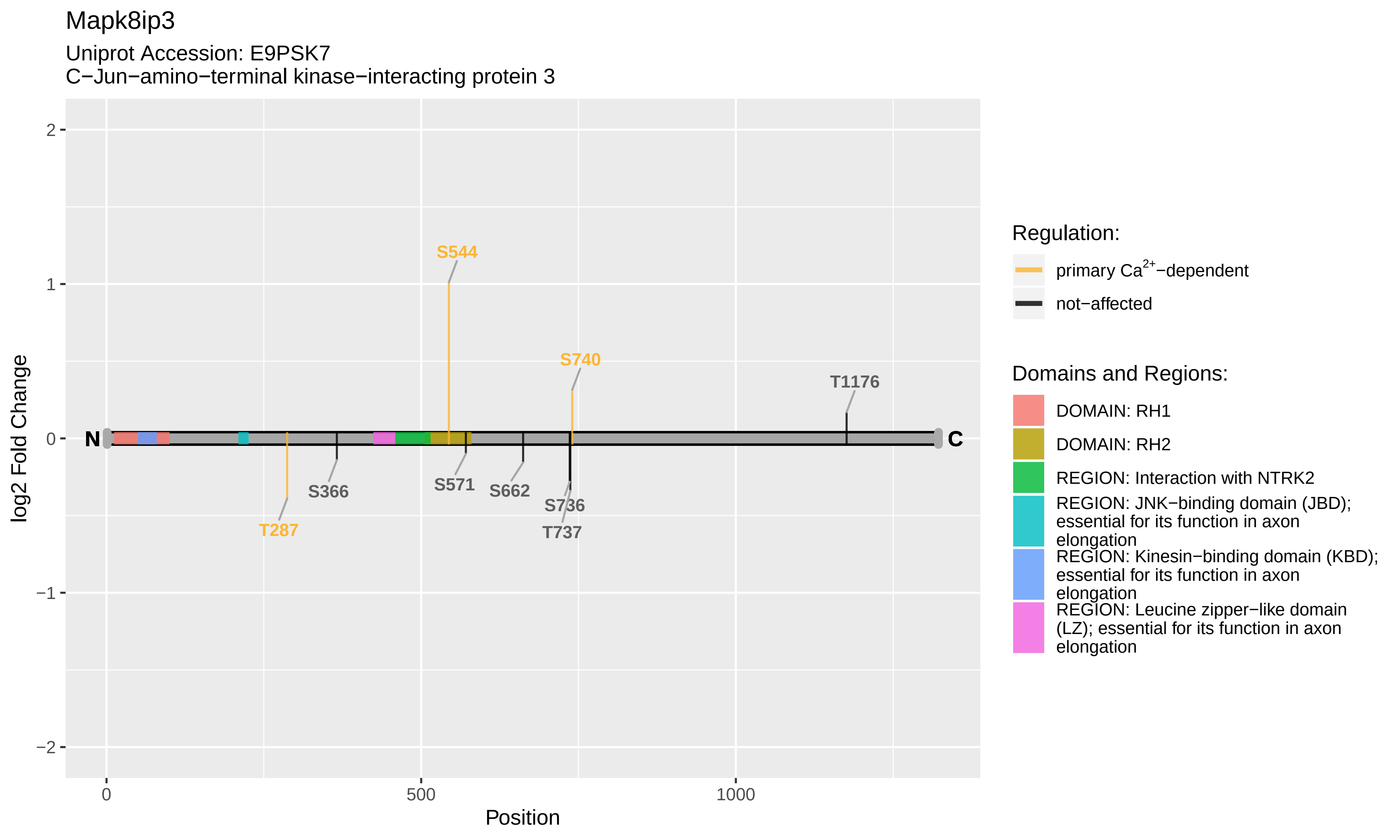

Mapk8ip3
Uniprot Accession: E9PSK7
C−Jun−amino−terminal kinase−interacting protein 3
log2 Fold Change
Position
2
1
0
−1
−2
0
500
1000
N
C
S544
S740
T1176
T287
S366
S571
S662
S736
T737
Regulation:
primary Ca2+−dependent
not−affected
Domains and Regions:
DOMAIN: RH1
DOMAIN: RH2
REGION: Interaction with NTRK2
REGION: JNK−binding domain (JBD); essential for its function in axon elongation
REGION: Kinesin−binding domain (KBD); essential for its function in axon elongation
REGION: Leucine zipper−like domain (LZ); essential for its function in axon elongation

Mapt

Uniprot Accession: D4A1Q2
Microtubule−associated protein

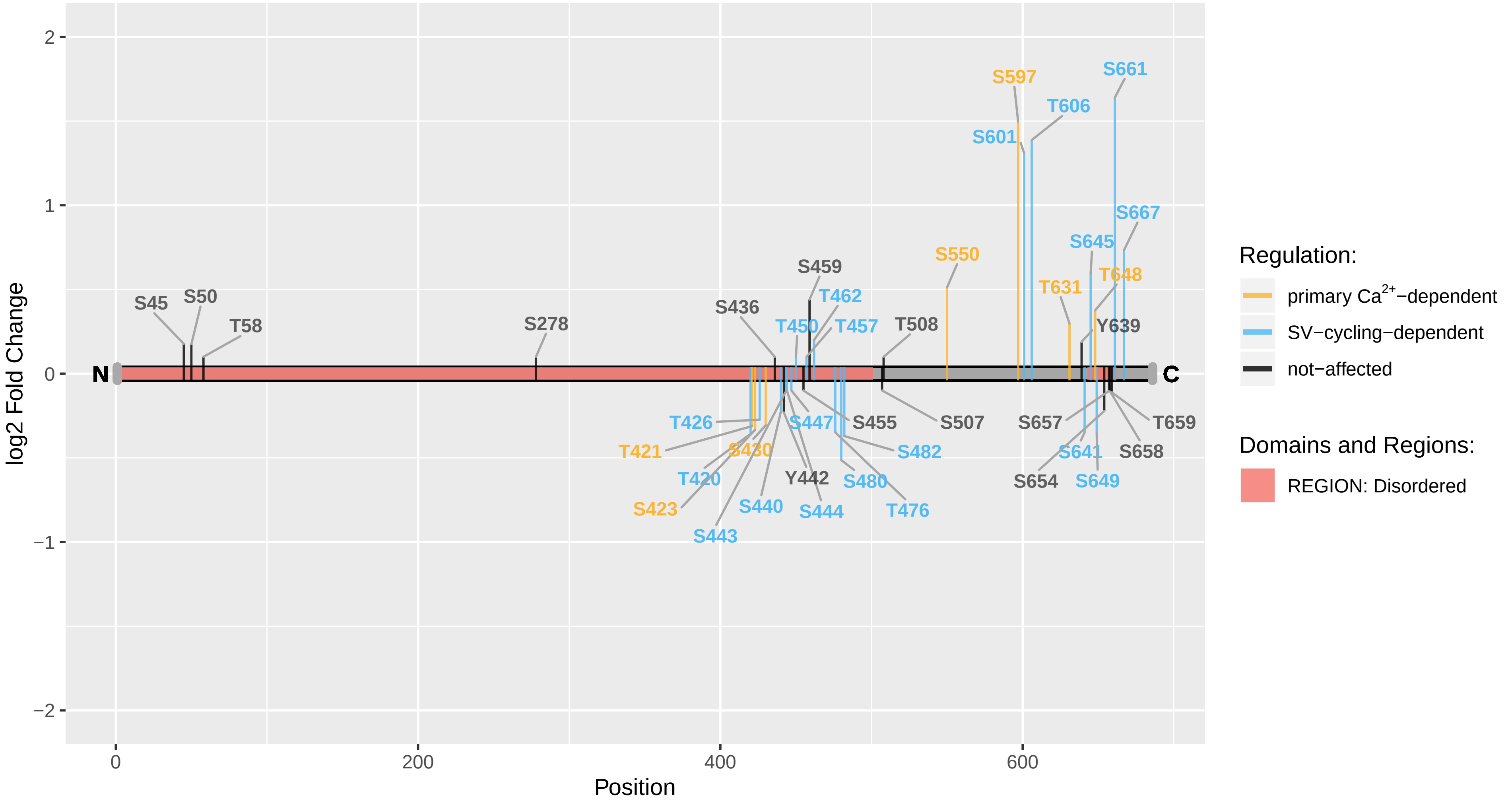

March7

Uniprot Accession: Q5XI50
E3 ubiquitin−protein ligase MARCH7

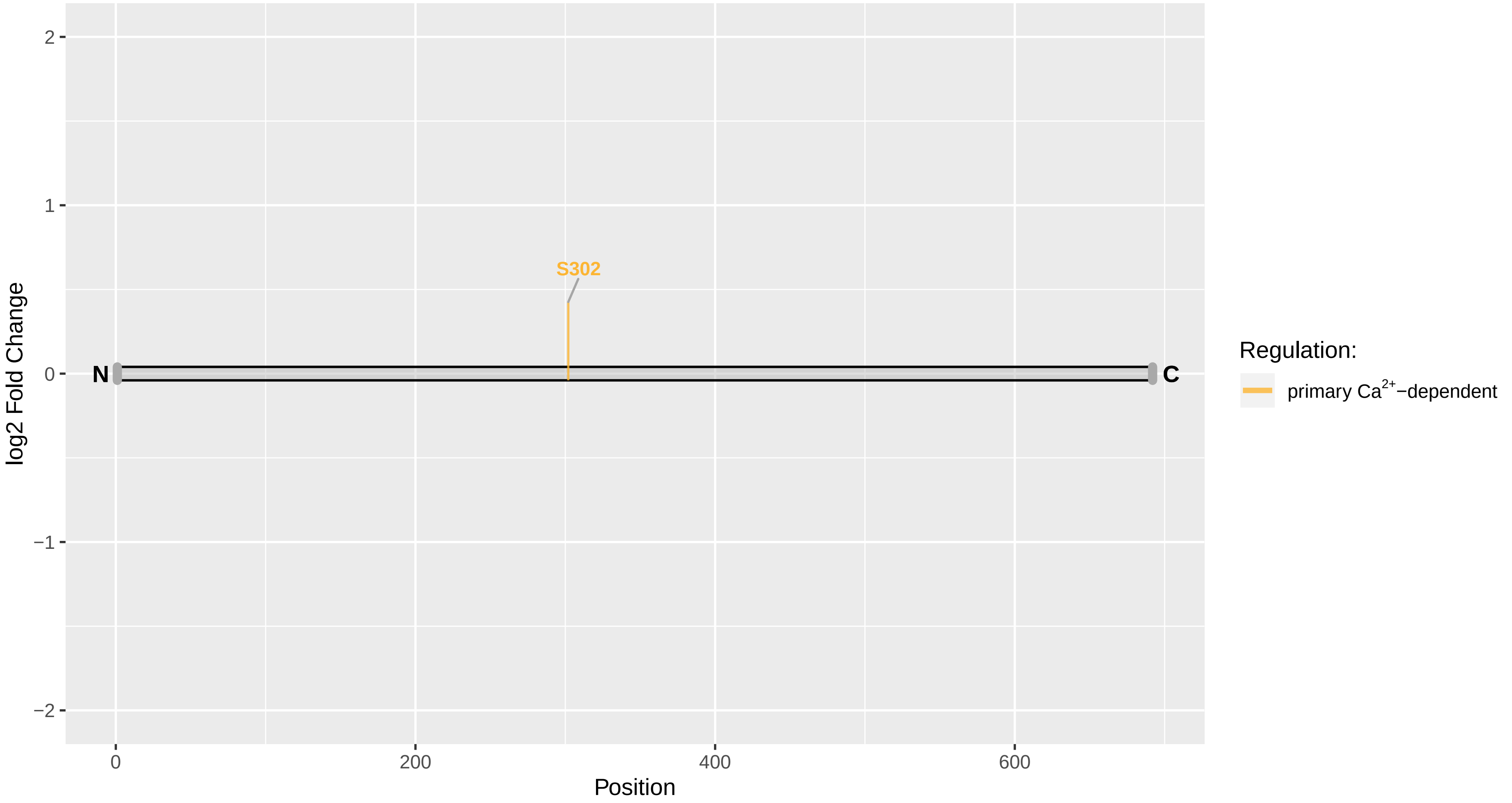

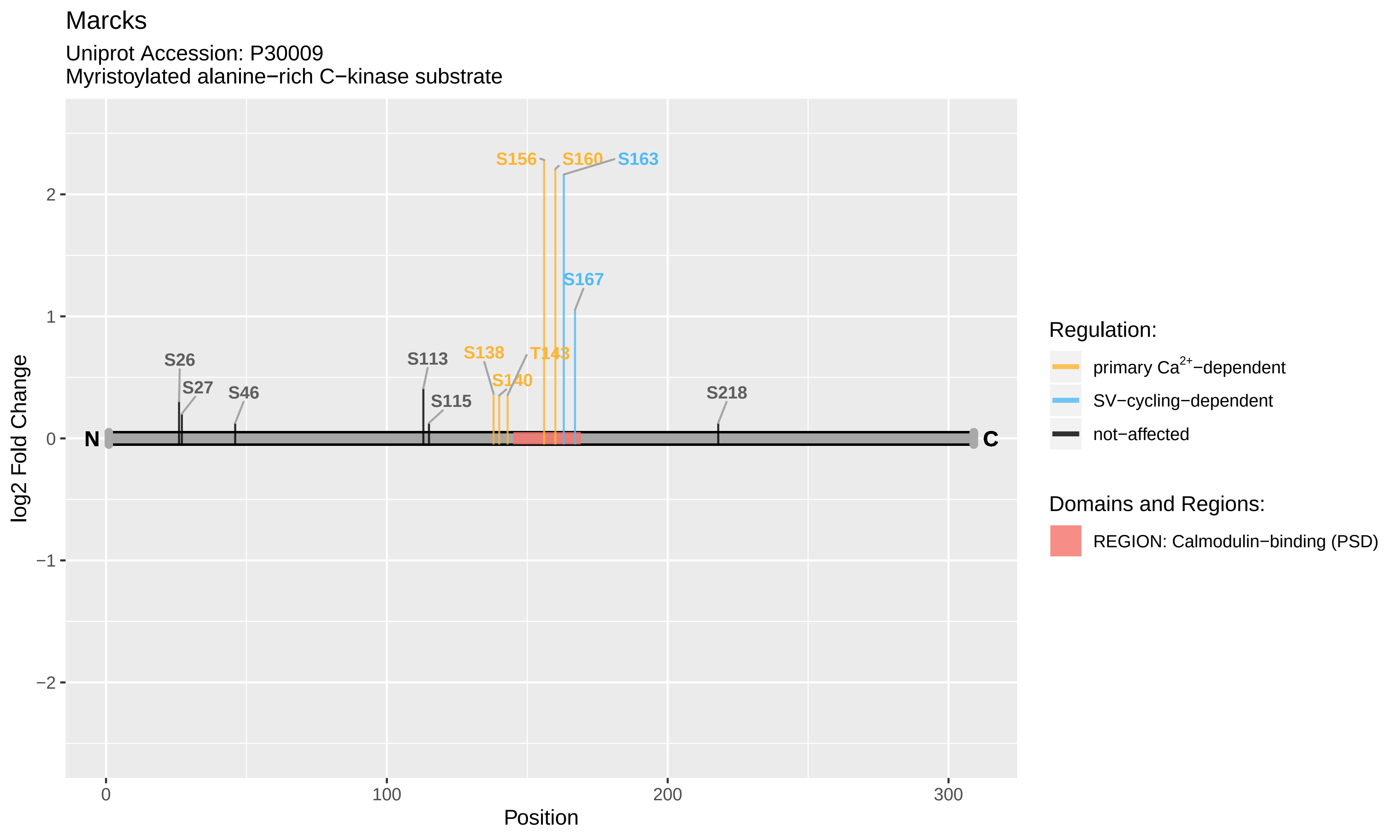

Marcks
Uniprot Accession: P30009
Myristoylated alanine−rich C−kinase substrate
S156
S160
S163
S167
S26
S27
S46
S113
S115
S138
T143
S140
S218
N
C
2
1
0
−1
−2
log2 Fold Change
0
100
200
300
Position
Regulation:
primary Ca2+−dependent
SV−cycling−dependent
not−affected
Domains and Regions:
REGION: Calmodulin−binding (PSD)

Marcksl1

Uniprot Accession: Q9EPH2
MARCKS−related protein

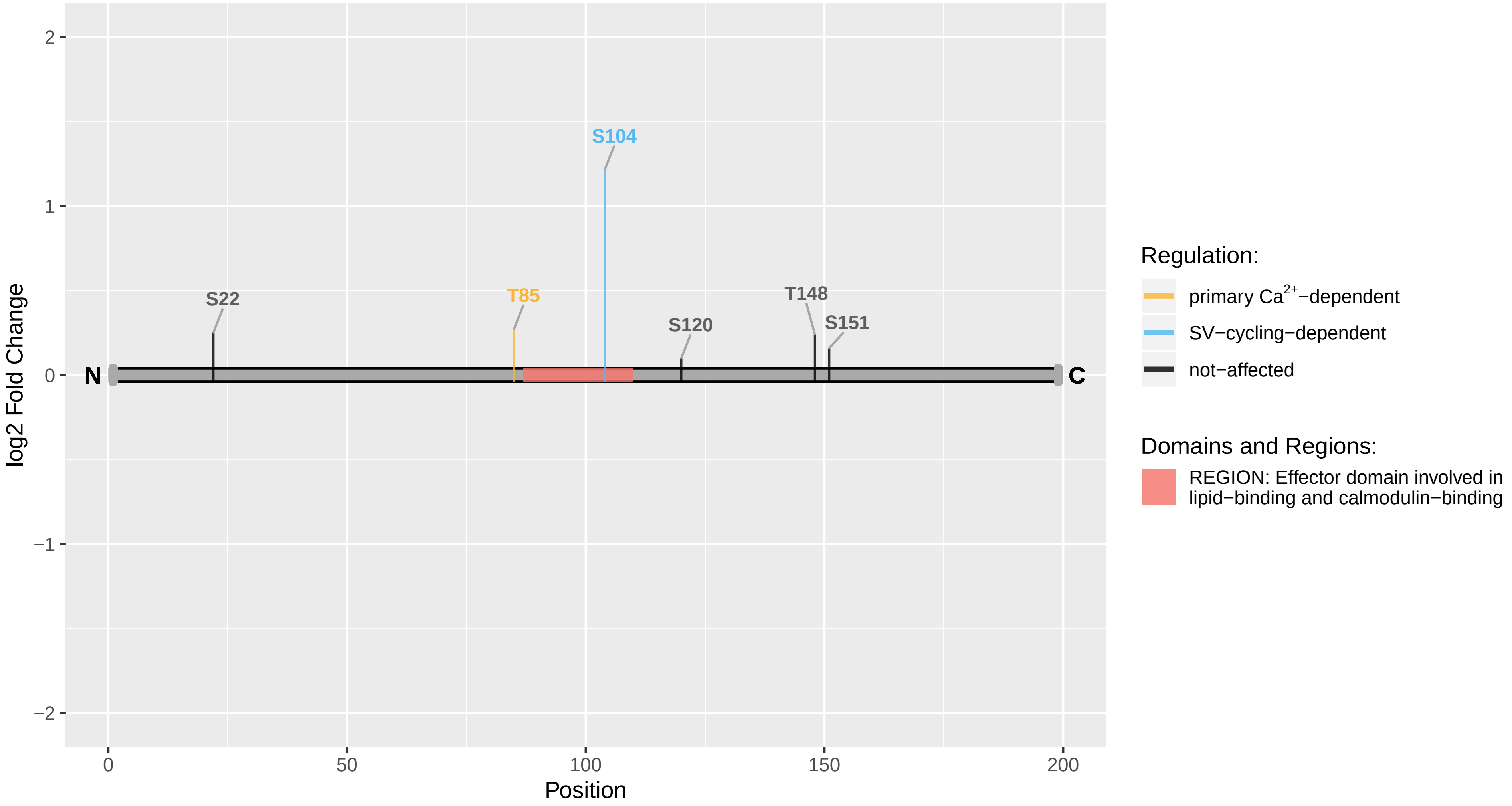

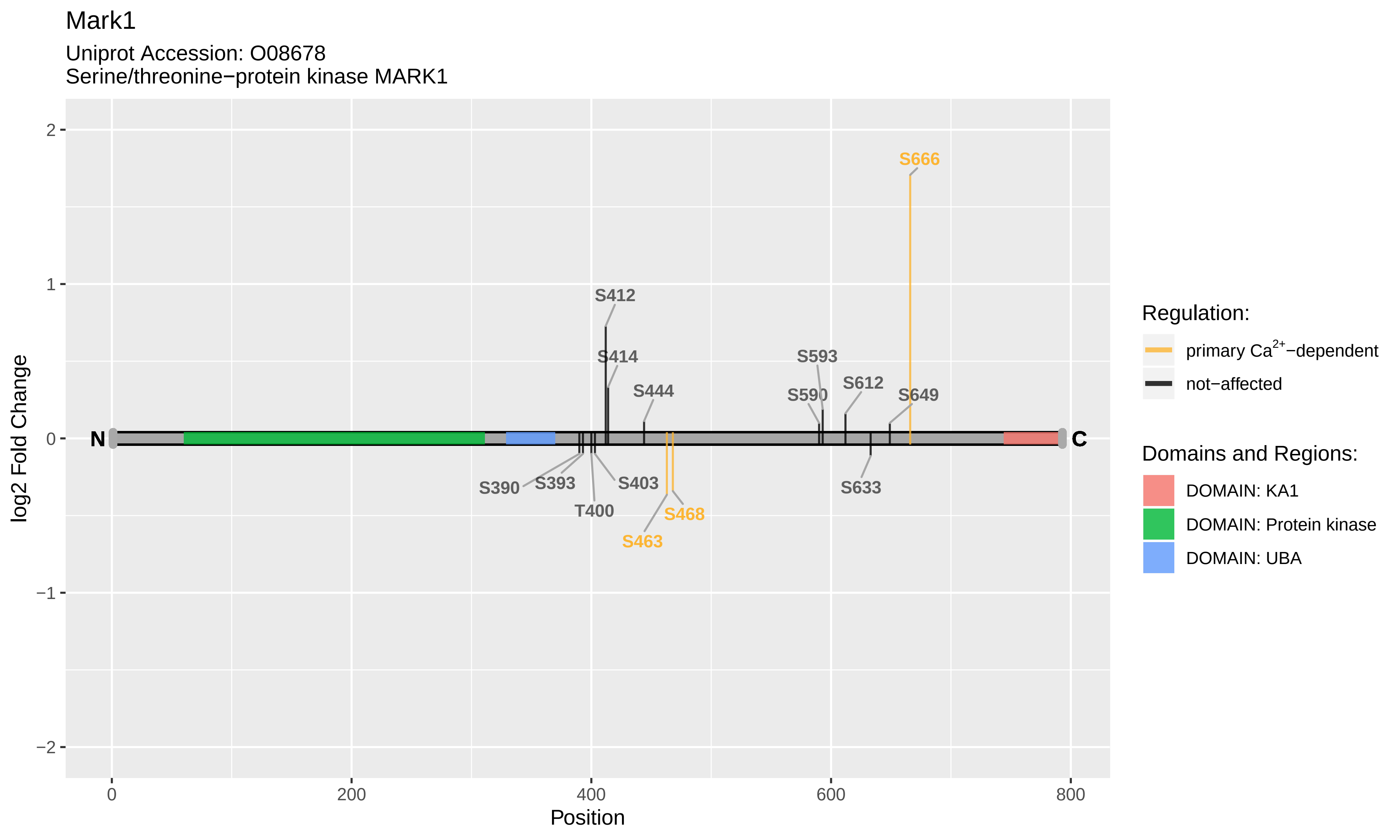

Mark1
Uniprot Accession: O08678
Serine/threonine−protein kinase MARK1
log2 Fold Change
Position
N
C
S666
S412
S414
S444
S593
S590
S612
S649
S390
S393
T400
S403
S463
S468
S633
Regulation:
primary Ca2+−dependent
not−affected
Domains and Regions:
DOMAIN: KA1
DOMAIN: Protein kinase
DOMAIN: UBA

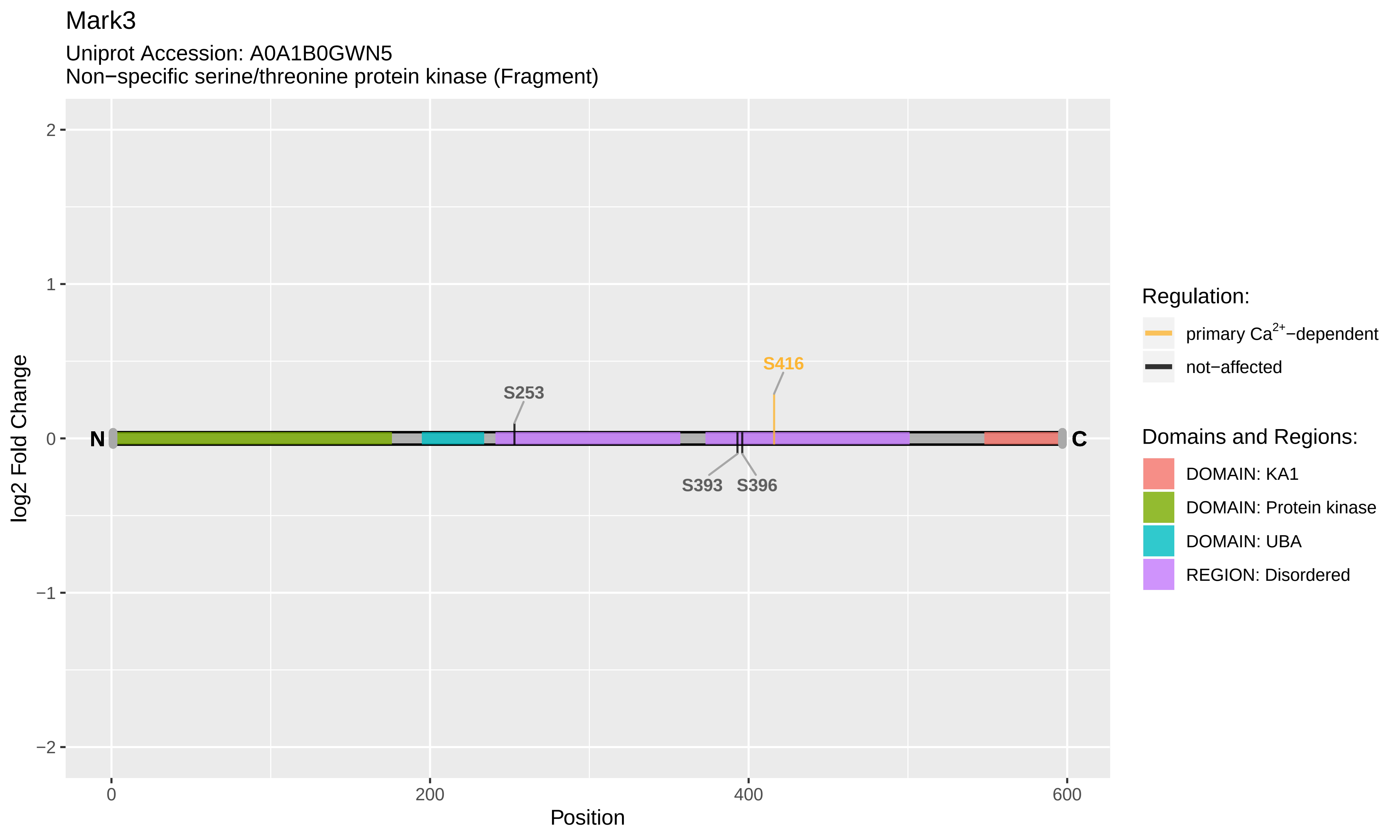

Mark3
Uniprot Accession: A0A1B0GWN5
Non−specific serine/threonine protein kinase (Fragment)
log2 Fold Change
Position
2
1
0
−1
−2
0
200
400
600
N
C
S253
S416
S393
S396
Regulation:
primary Ca2+−dependent
not−affected
Domains and Regions:
DOMAIN: KA1
DOMAIN: Protein kinase
DOMAIN: UBA
REGION: Disordered

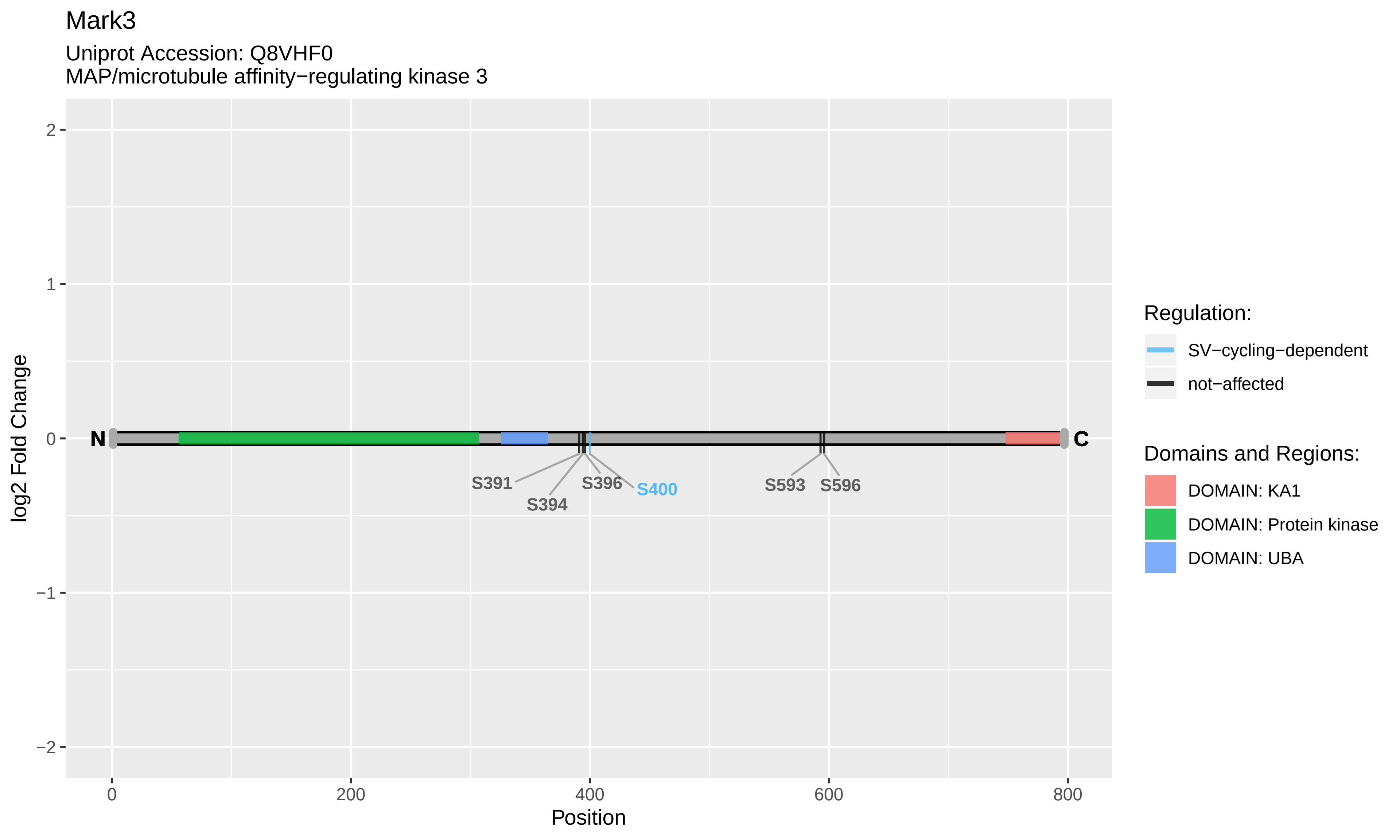

Mark3
Uniprot Accession: Q8VHF0
MAP/microtubule affinity−regulating kinase 3
log2 Fold Change
Position
N
C
S391
S394
S396
S400
S593
S596
Regulation:
SV−cycling−dependent
not−affected
Domains and Regions:
DOMAIN: KA1
DOMAIN: Protein kinase
DOMAIN: UBA

Mast1

Uniprot Accession: Q810W7
Microtubule−associated serine/threonine−protein kinase 1

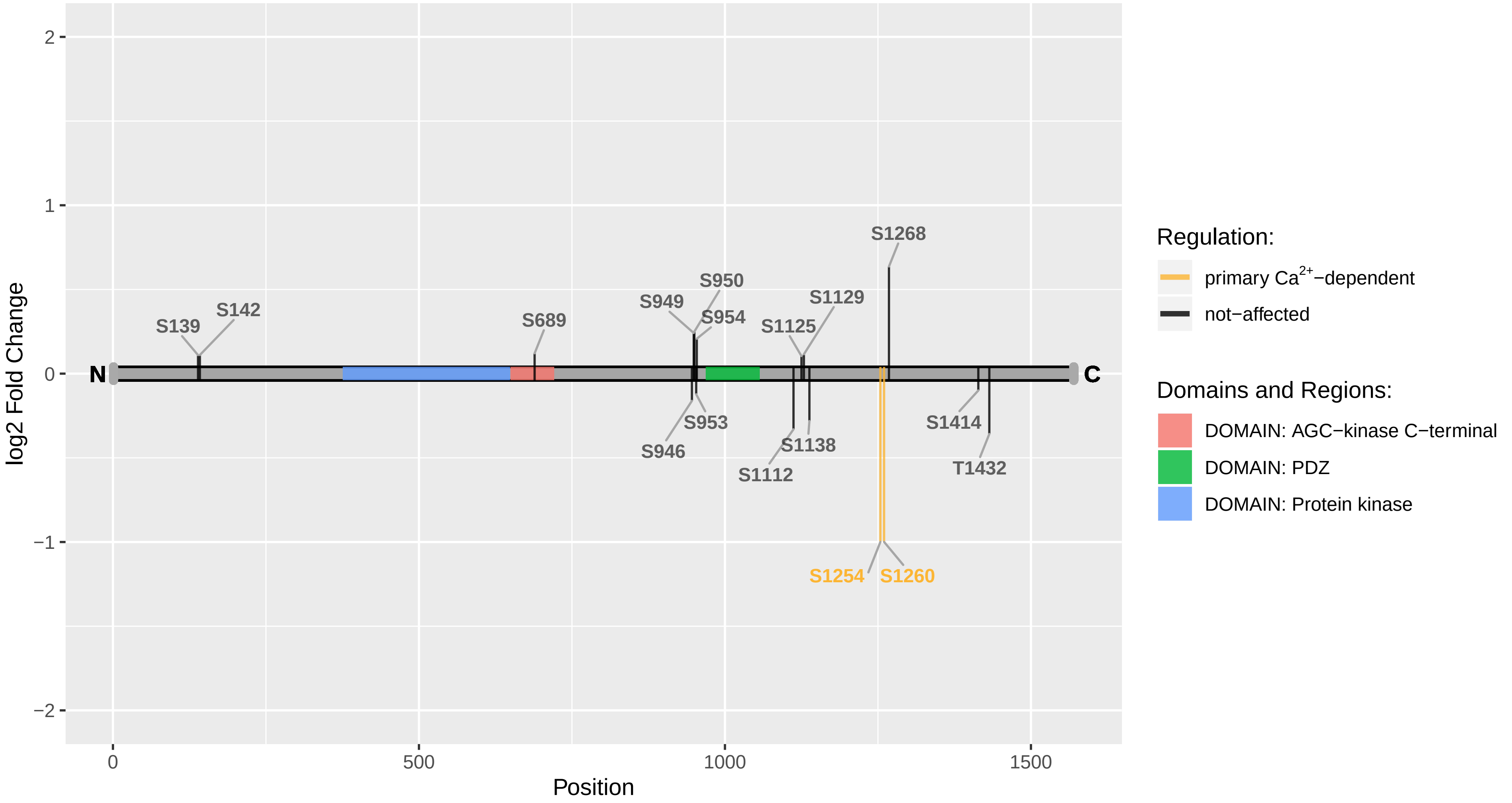

Mcf2l

Uniprot Accession: Q63406
Guanine nucleotide exchange factor DBS

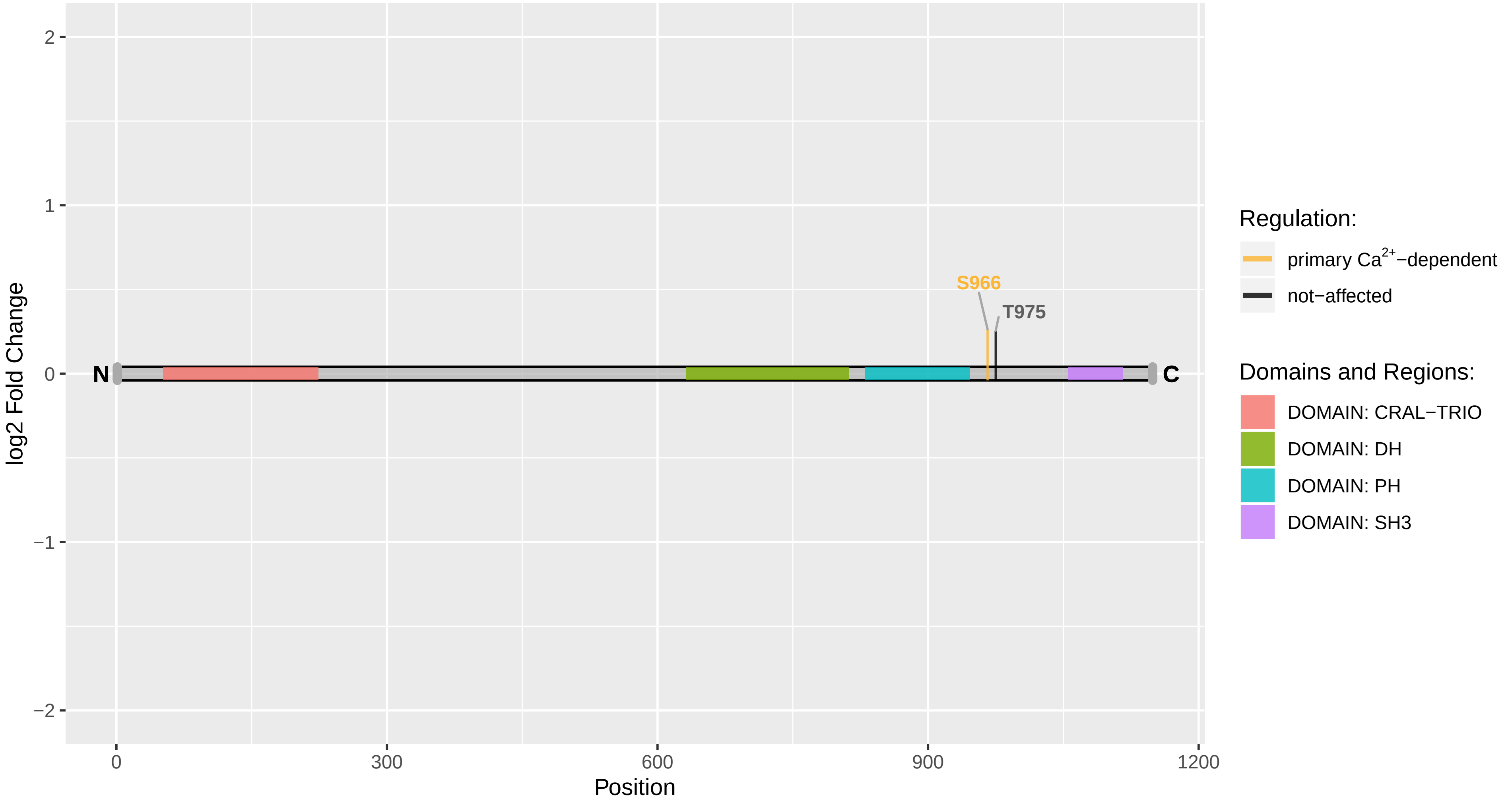

Mcrip1

Uniprot Accession: B0BN72
Mapk−regulated corepressor−interacting protein 1

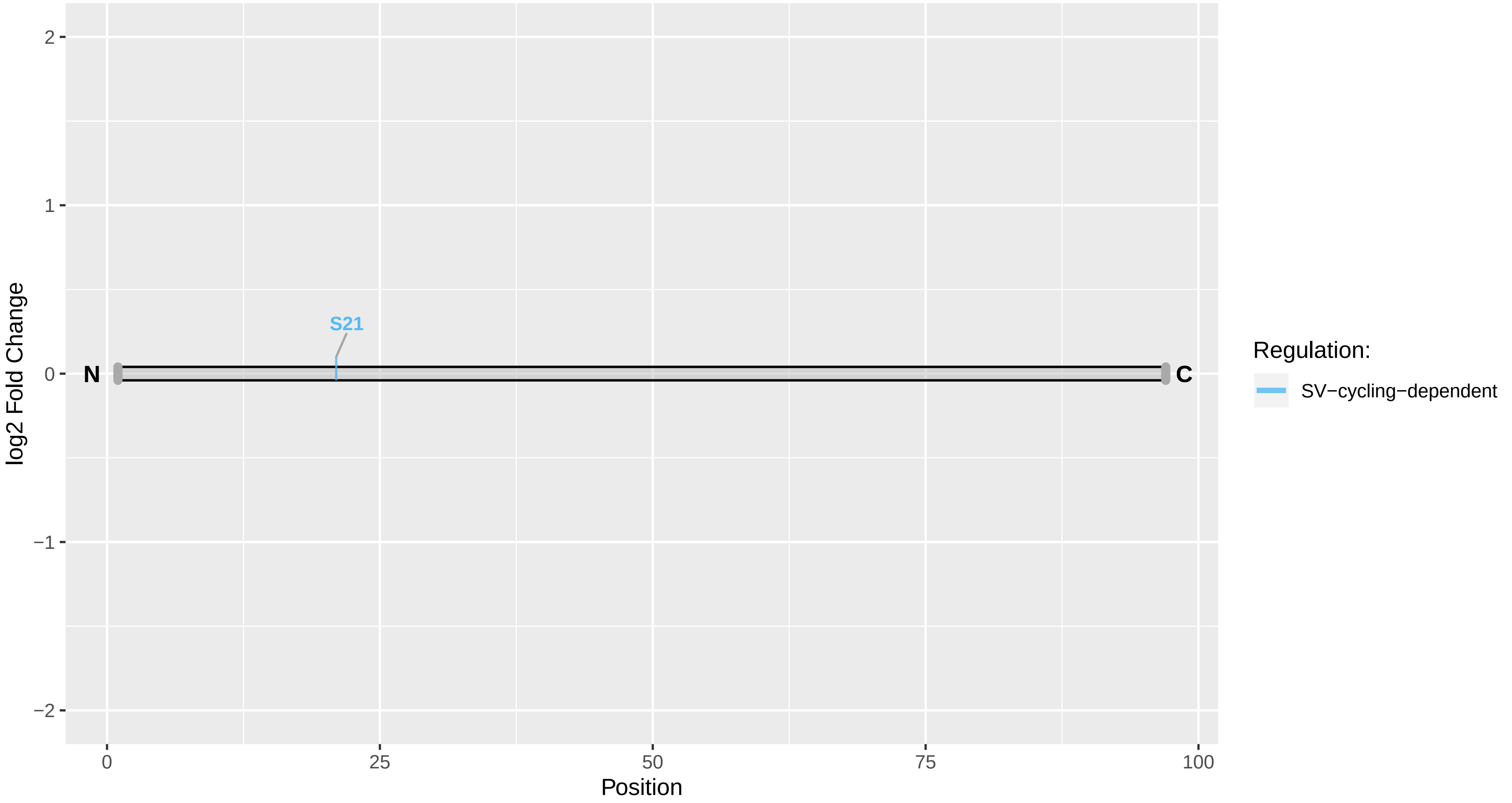

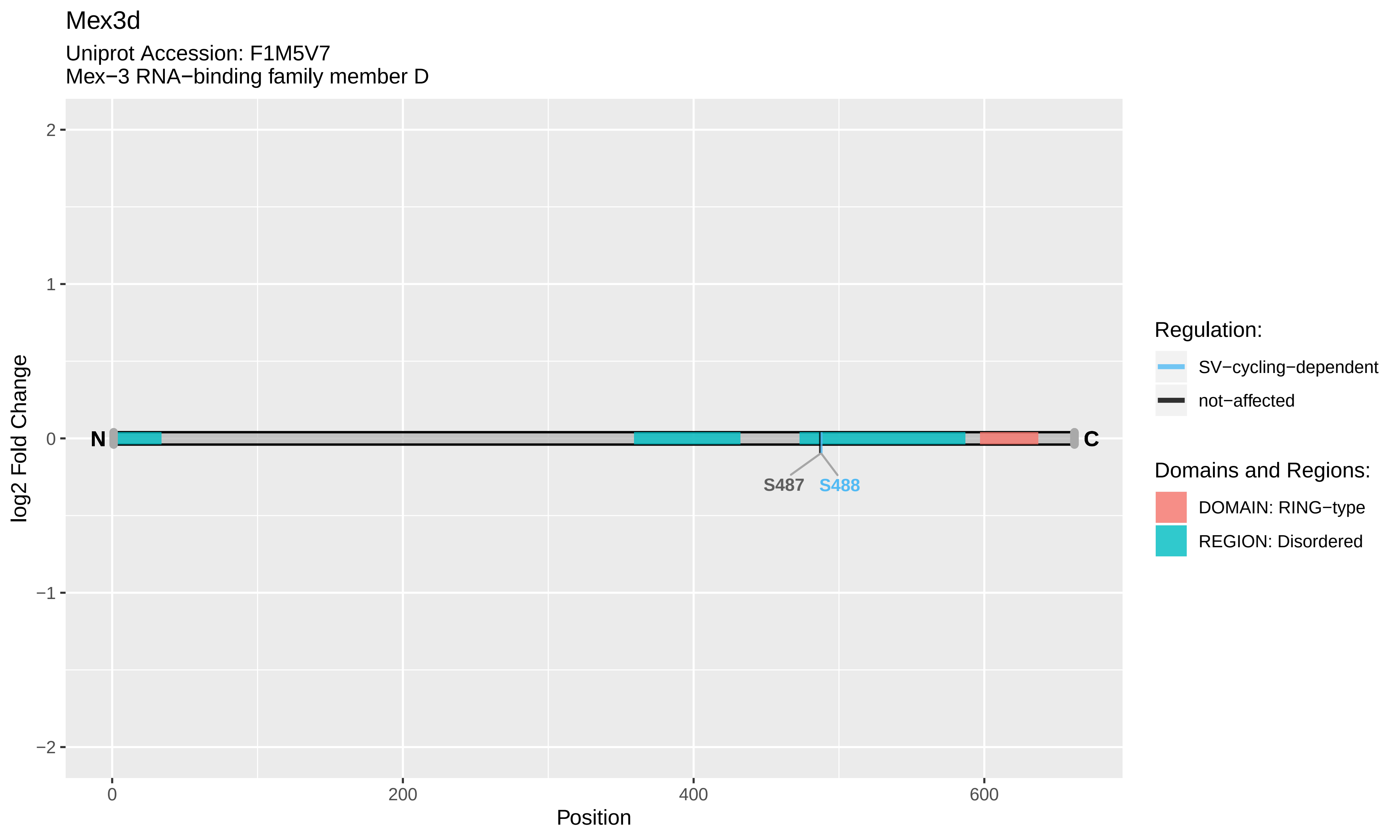

Mex3d
Uniprot Accession: F1M5V7
Mex−3 RNA−binding family member D
log2 Fold Change
2
1
0
−1
−2
N
C
S487
S488
0
200
400
600
Position
Regulation:
SV−cycling−dependent
not−affected
Domains and Regions:
DOMAIN: RING−type
REGION: Disordered

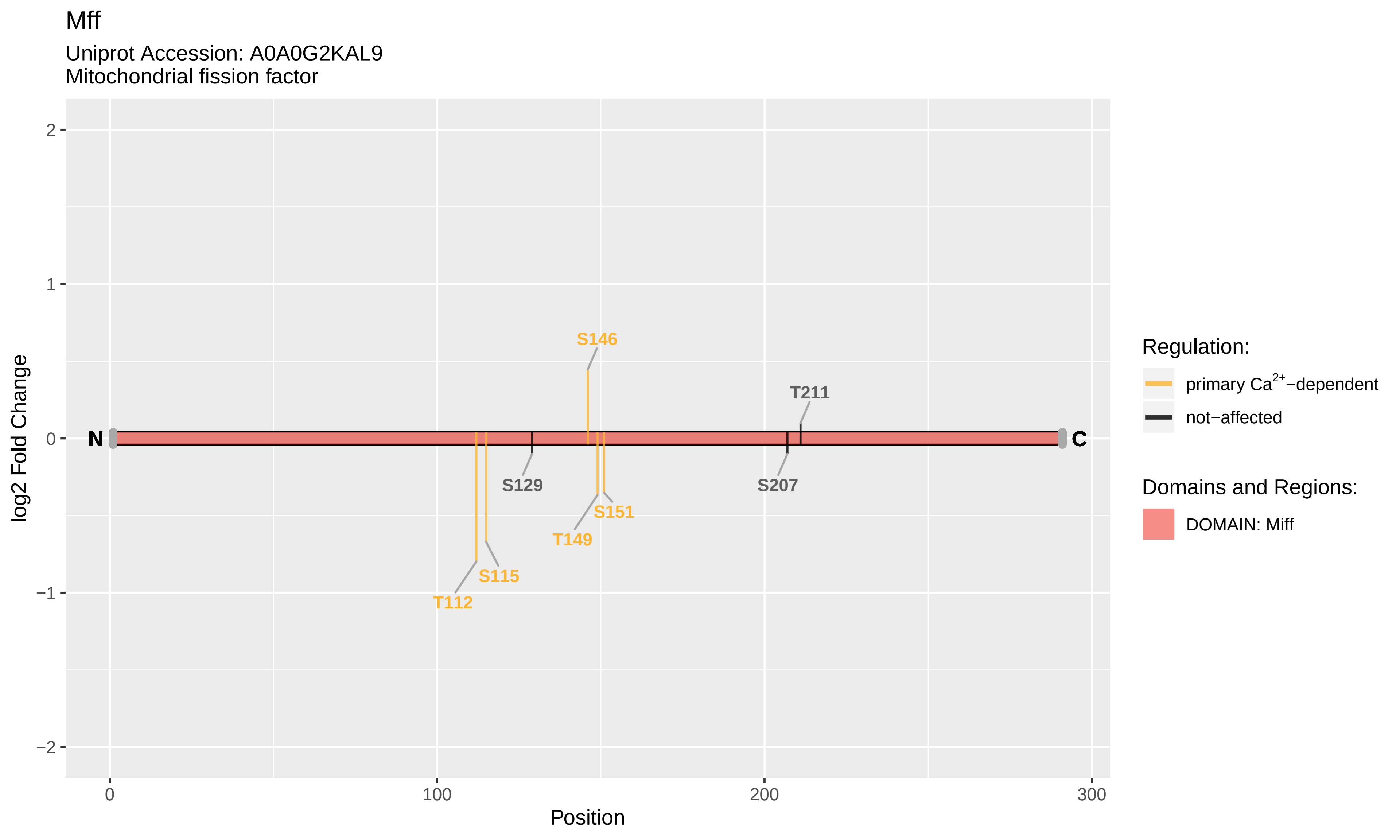

Mff
Uniprot Accession: A0A0G2KAL9
Mitochondrial fission factor
log2 Fold Change
Position
N
C
S146
T211
S129
S207
S151
T149
S115
T112
Regulation:
primary Ca2+−dependent
not−affected
Domains and Regions:
DOMAIN: Miff

Mfsd4a

Uniprot Accession: A0A0G2K6P5
Major facilitator superfamily domain−containing 4A

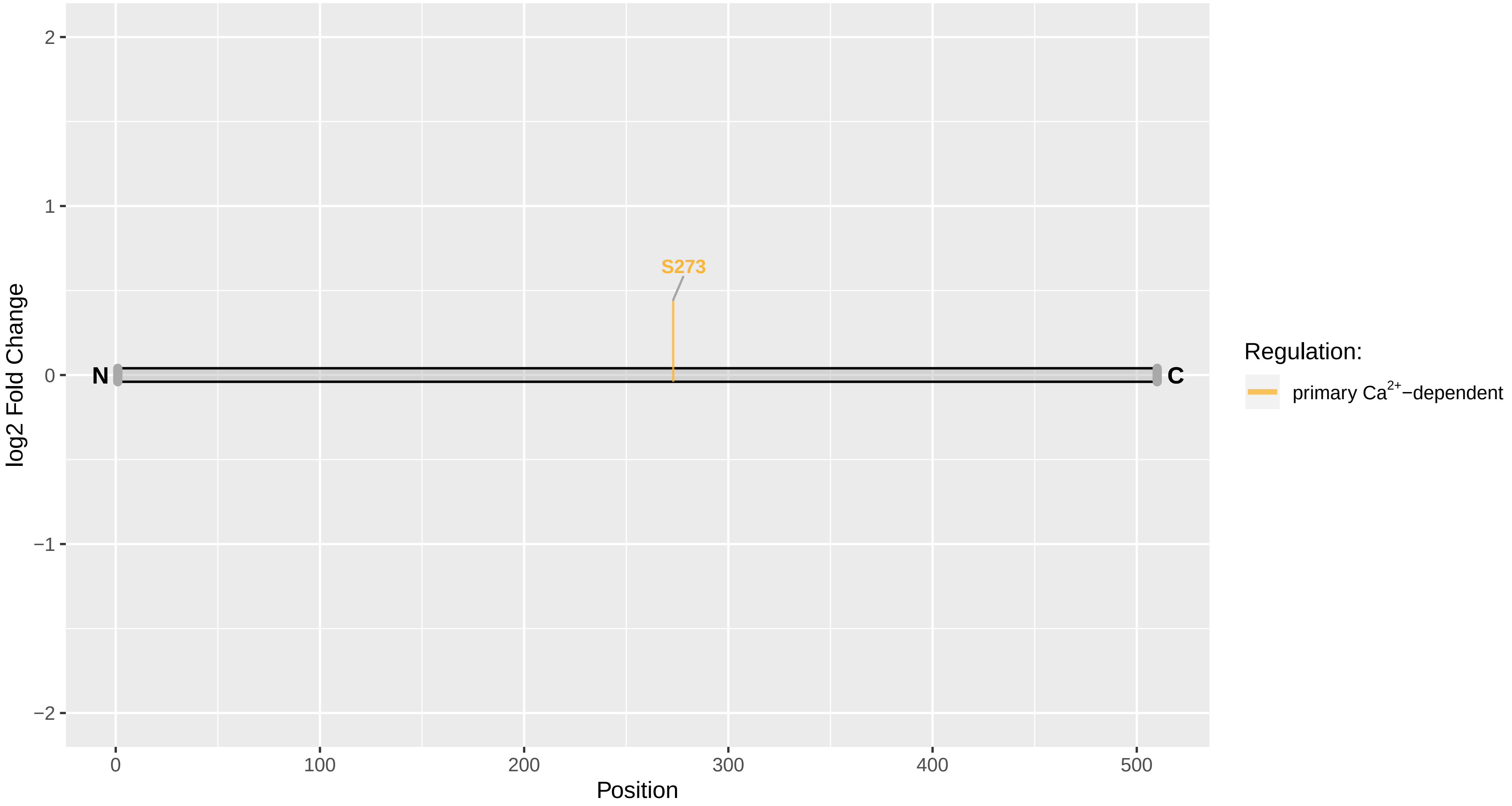

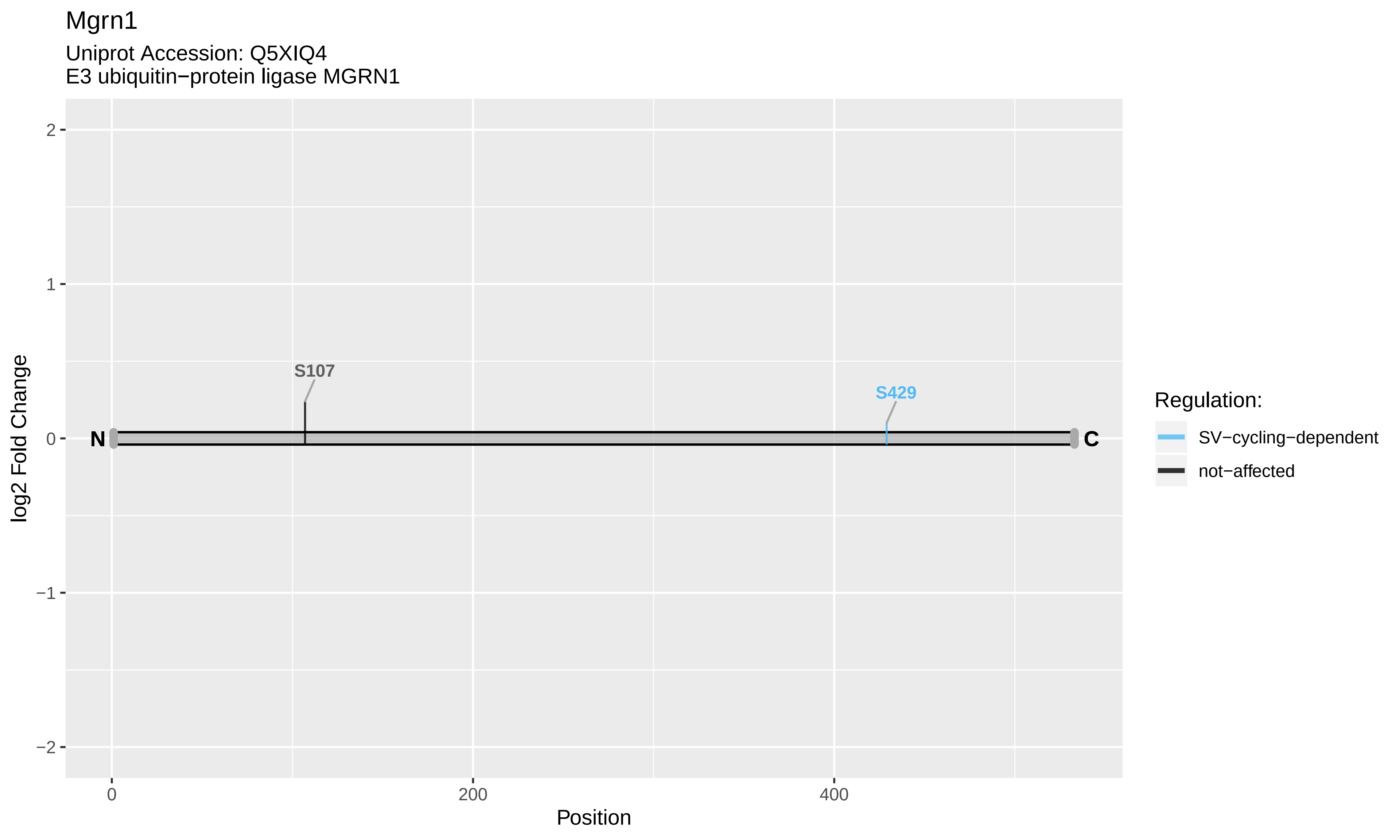
Mgrn1
Uniprot Accession: Q5XIQ4
E3 ubiquitin−protein ligase MGRN1
log2 Fold Change
2
1
0
−1
−2
N
C
S107
S429
0
200
400
Position
Regulation:
SV−cycling−dependent
not−affected

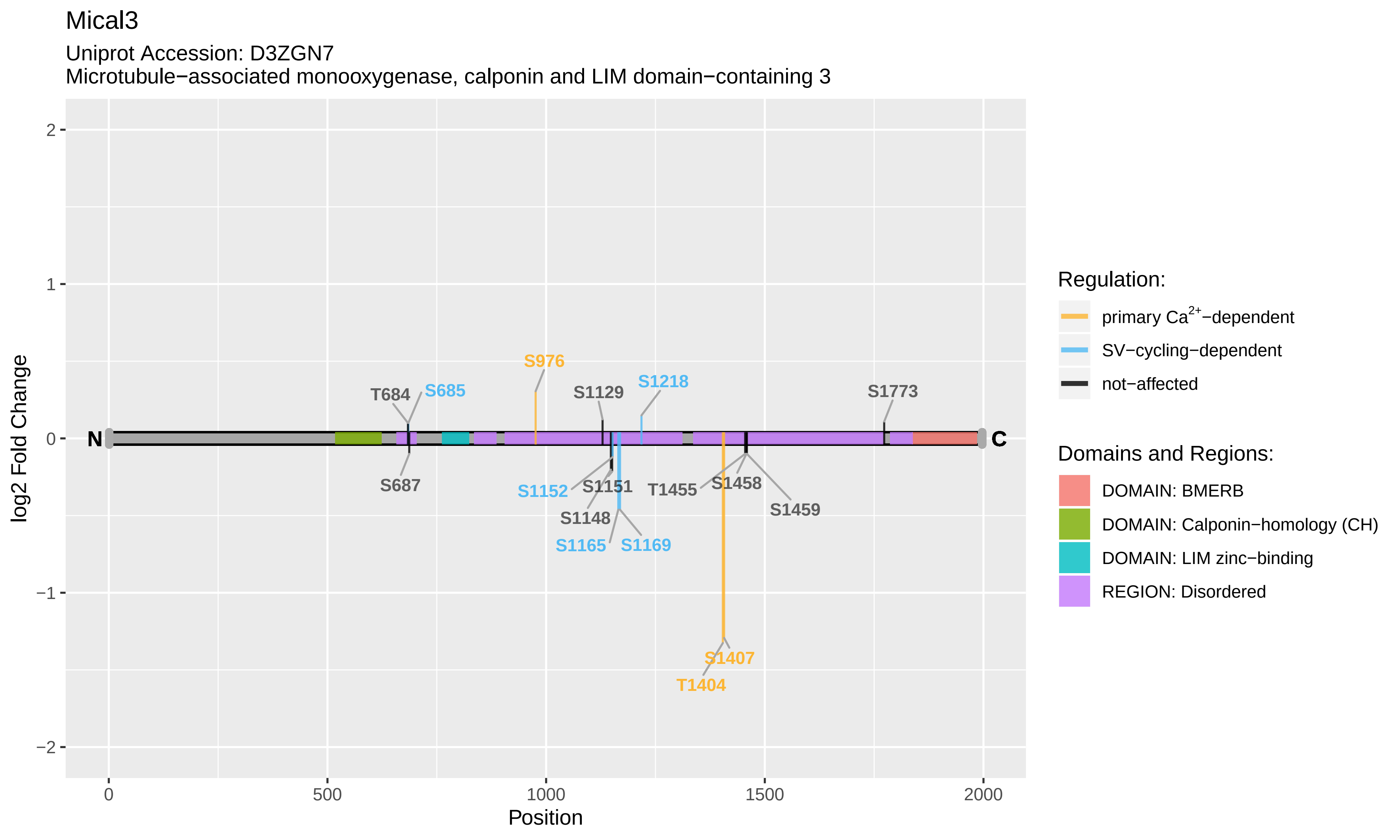
Mical3
Uniprot Accession: D3ZGN7
Microtubule−associated monooxygenase, calponin and LIM domain−containing 3
log2 Fold Change
Position
N
C
S976
S1218
T684
S685
S1129
S1773
S687
S1152
S1151
T1455
S1458
S1459
S1148
S1165
S1169
S1407
T1404
Regulation:
primary Ca2+−dependent
SV−cycling−dependent
not−affected
Domains and Regions:
DOMAIN: BMERB
DOMAIN: Calponin−homology (CH)
DOMAIN: LIM zinc−binding
REGION: Disordered

Mief1

Uniprot Accession: Q5XIS8
Mitochondrial dynamics protein MID51

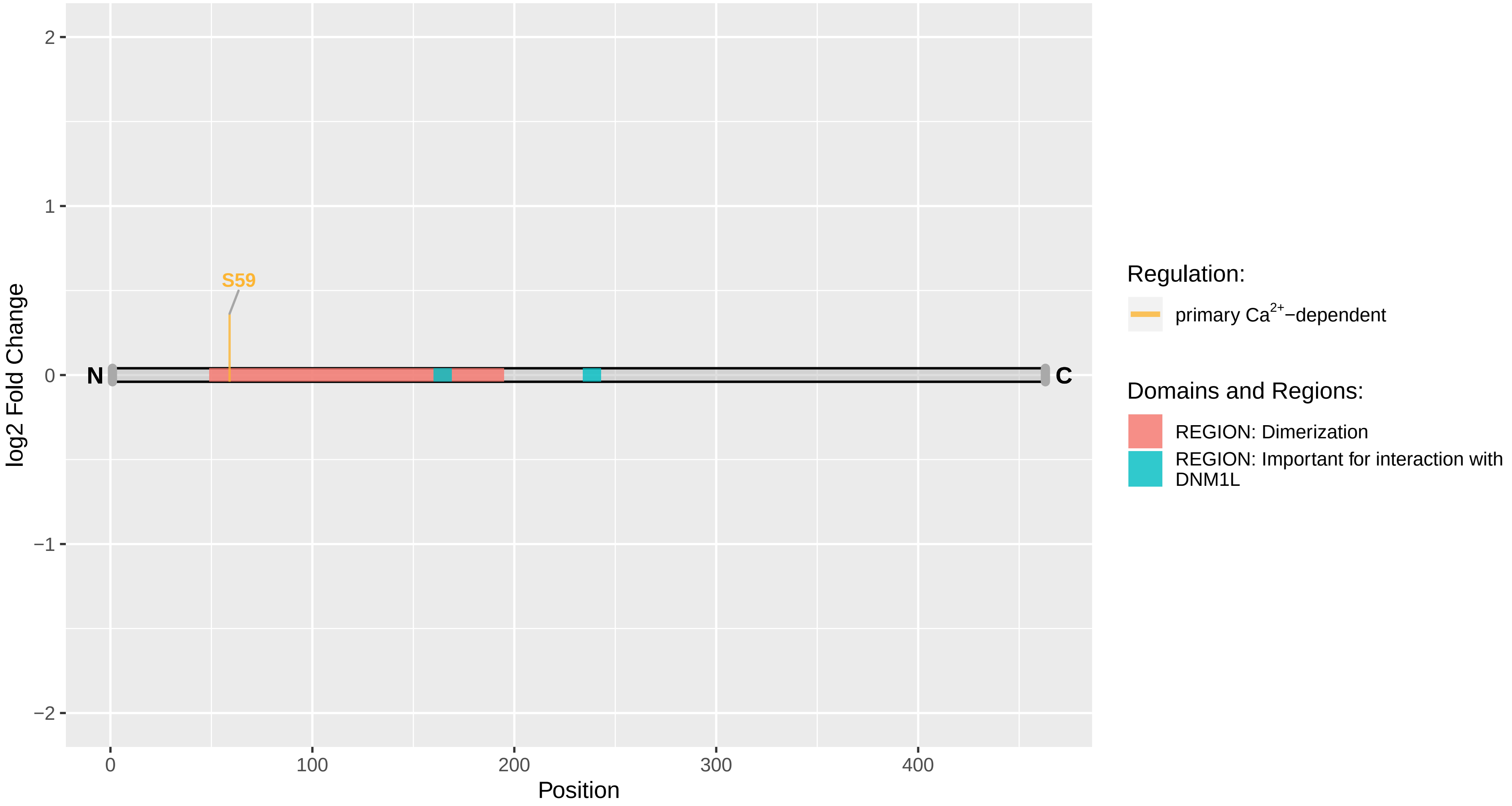

Miga2

Uniprot Accession: A0A0G2KAE6
Mitoguardin 2

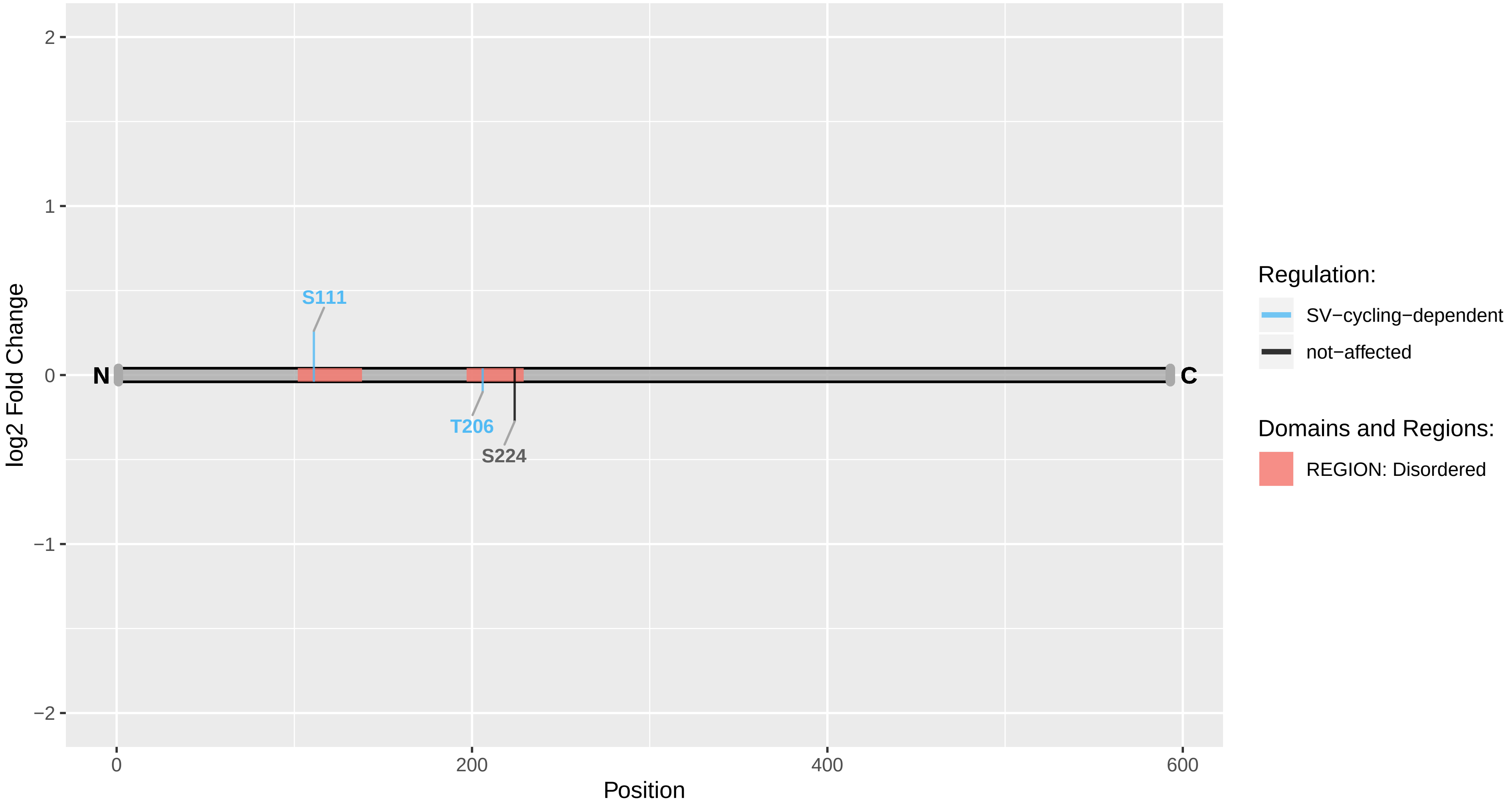

Mindy1

Uniprot Accession: Q5BJQ2
Ubiquitin carboxyl−terminal hydrolase MINDY−1

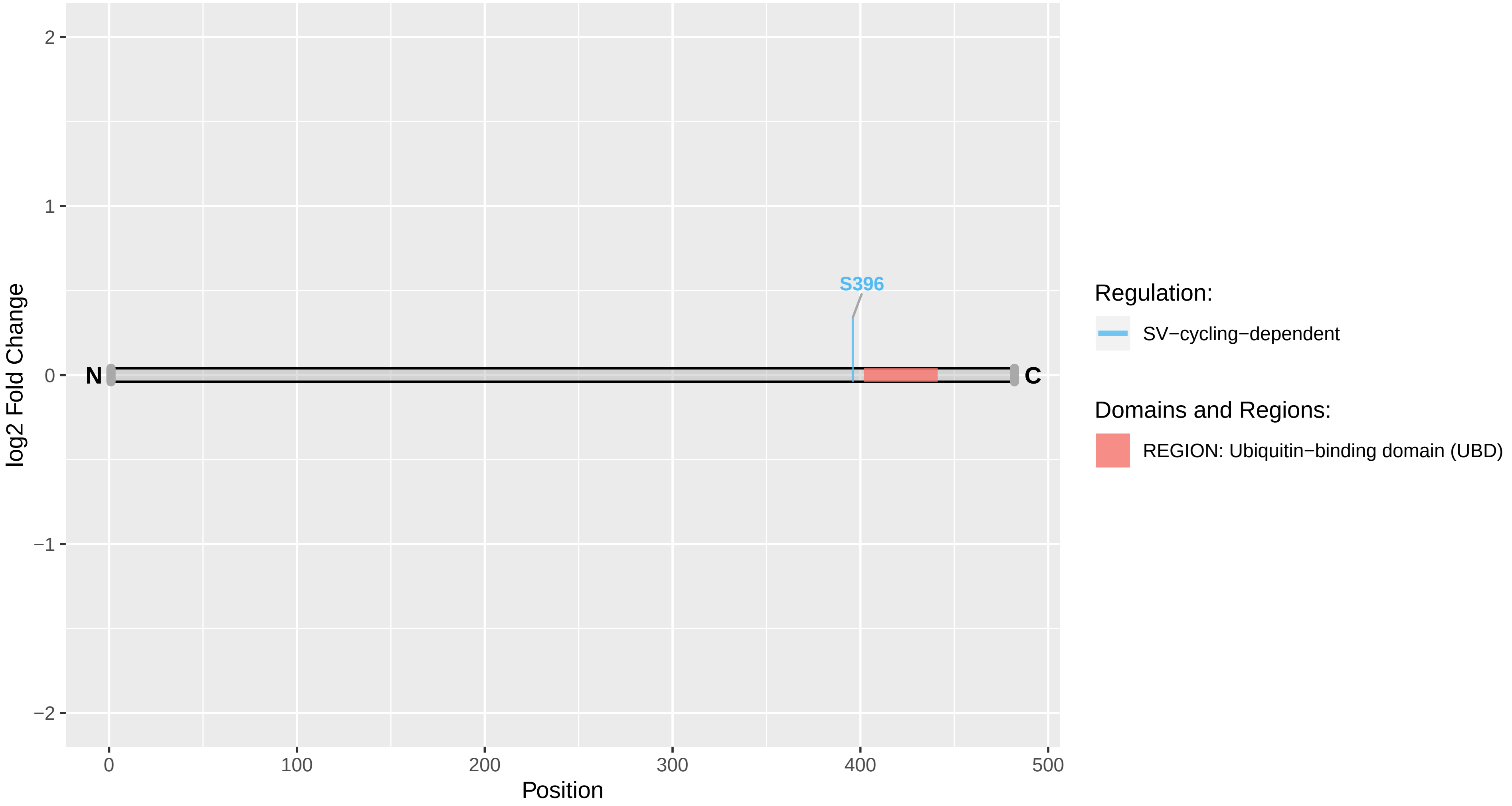

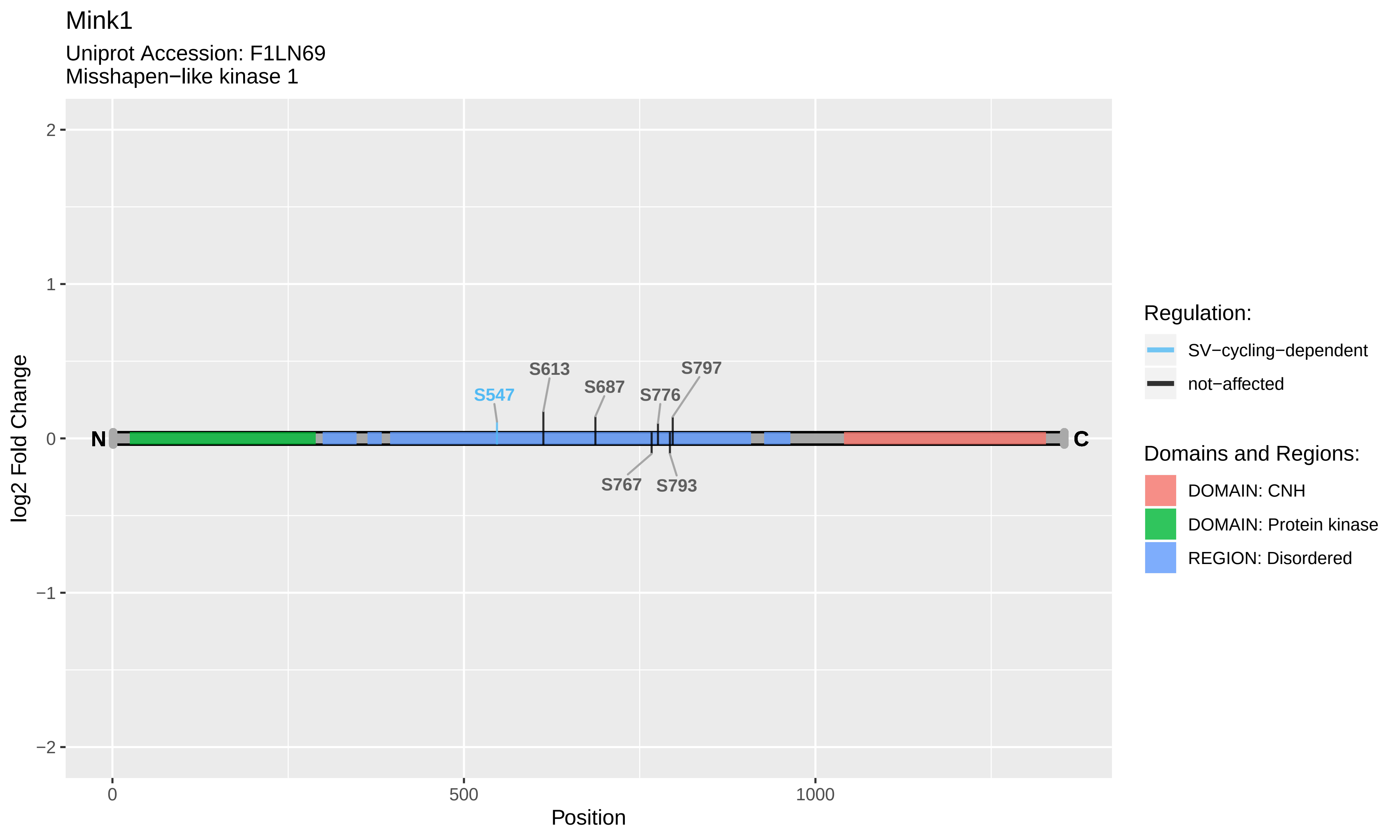

Mink1
Uniprot Accession: F1LN69
Misshapen−like kinase 1
log2 Fold Change
2
1
0
−1
−2
Position
0
500
1000
N
C
S547
S613
S687
S776
S797
S767
S793
Regulation:
SV−cycling−dependent
not−affected
Domains and Regions:
DOMAIN: CNH
DOMAIN: Protein kinase
REGION: Disordered

Mlc1

Uniprot Accession: D4ABB2
Megalencephalic leukoencephalopathy with subcortical cysts 1

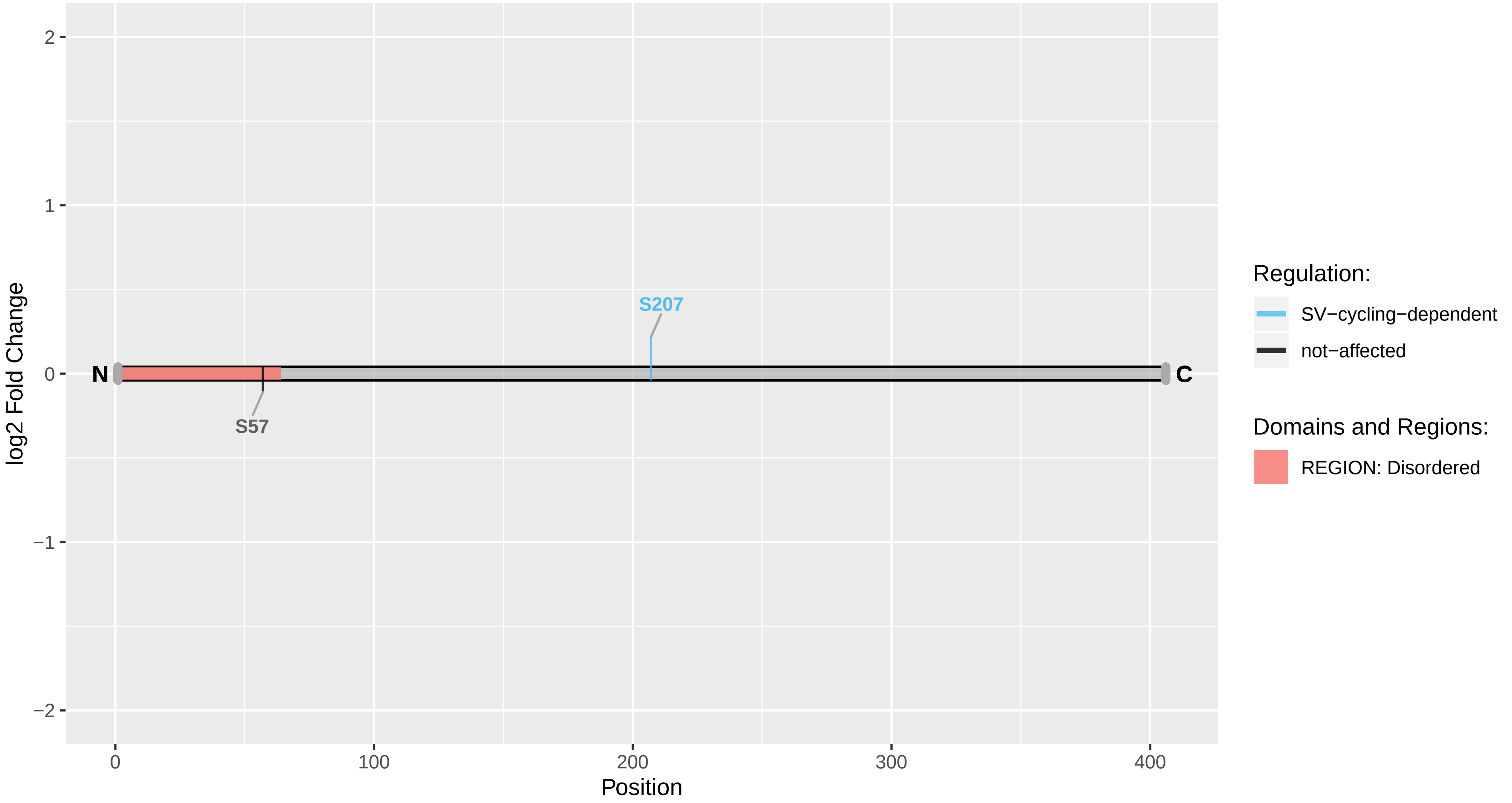

Mobp

Uniprot Accession: Q63327
Myelin−associated oligodendrocyte basic protein

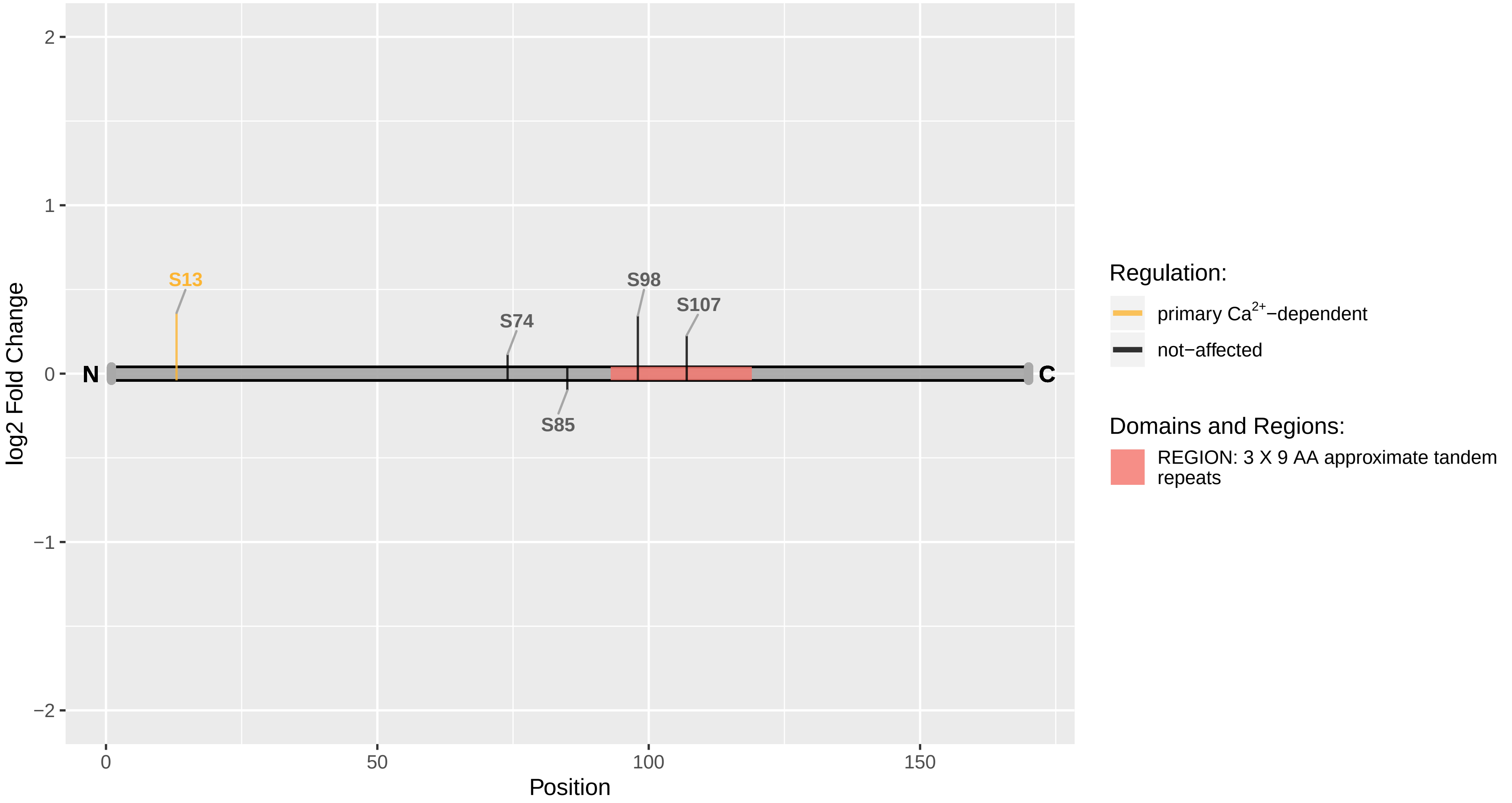

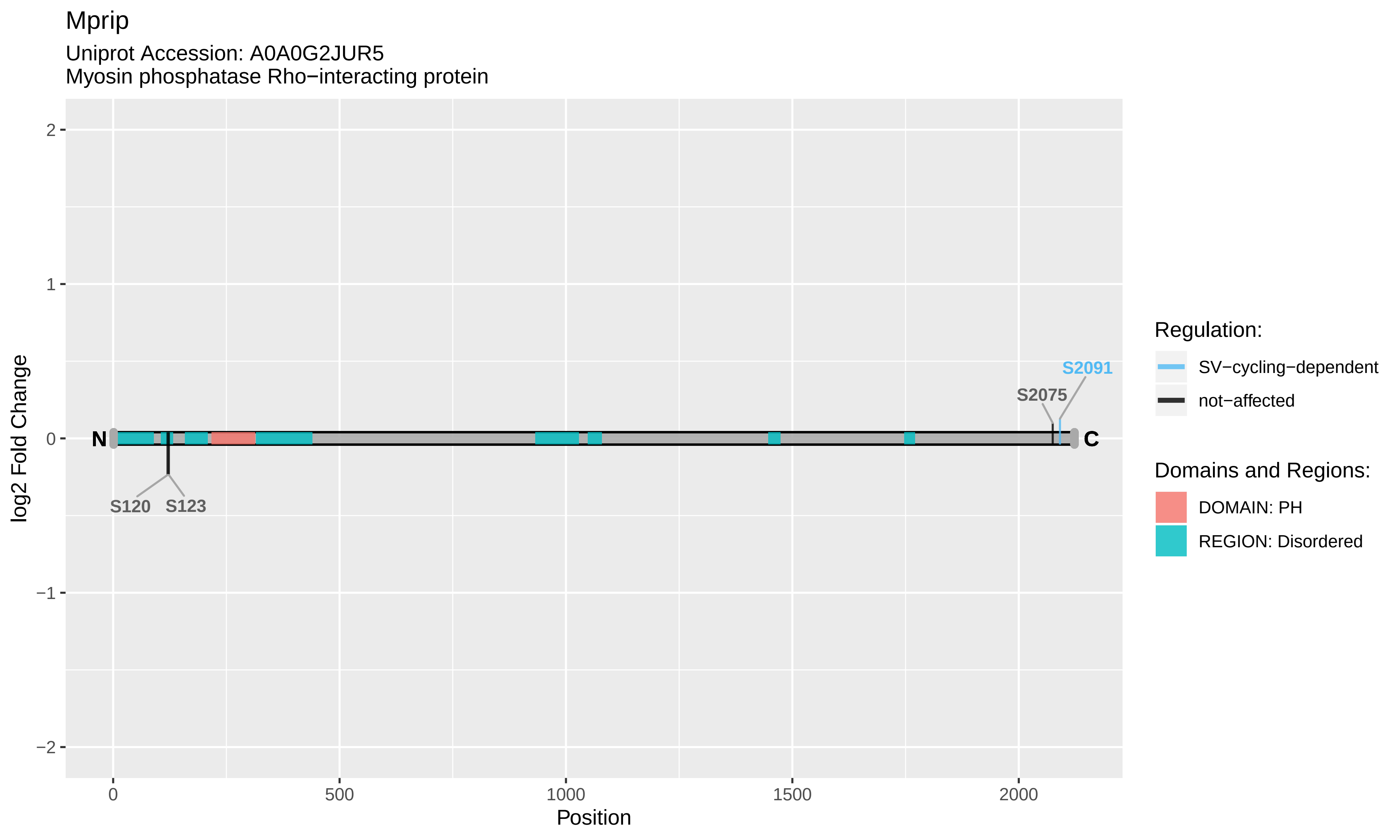

Mprip
Uniprot Accession: A0A0G2JUR5
Myosin phosphatase Rho−interacting protein
log2 Fold Change
Position
N
C
S120
S123
S2075
S2091
Regulation:
SV−cycling−dependent
not−affected
Domains and Regions:
DOMAIN: PH
REGION: Disordered
2
1
0
−1
−2
0
500
1000
1500
2000

Mrtfa

Uniprot Accession: A0A0G2JXC5
Megakaryoblastic leukemia (translocation) 1

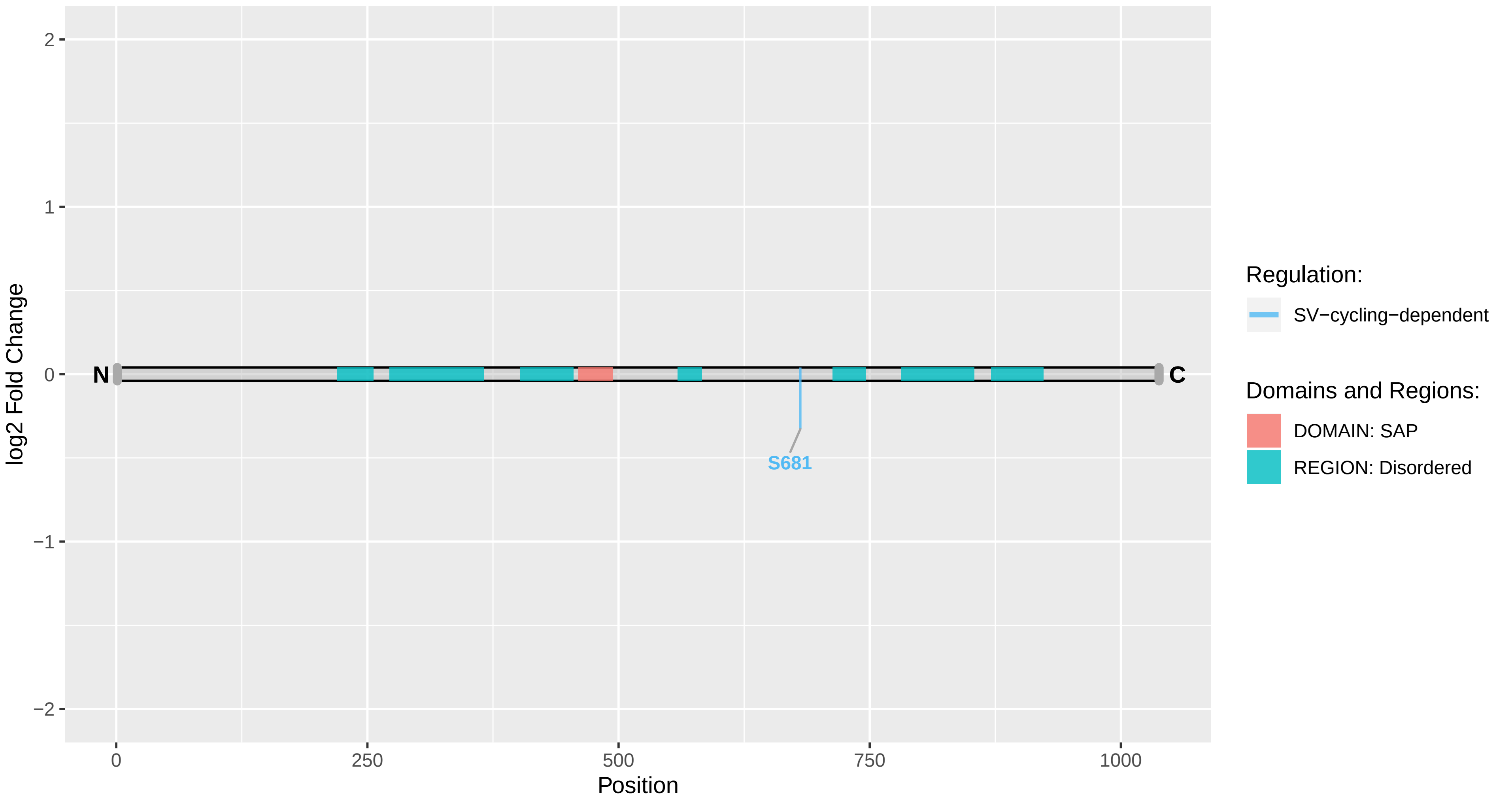

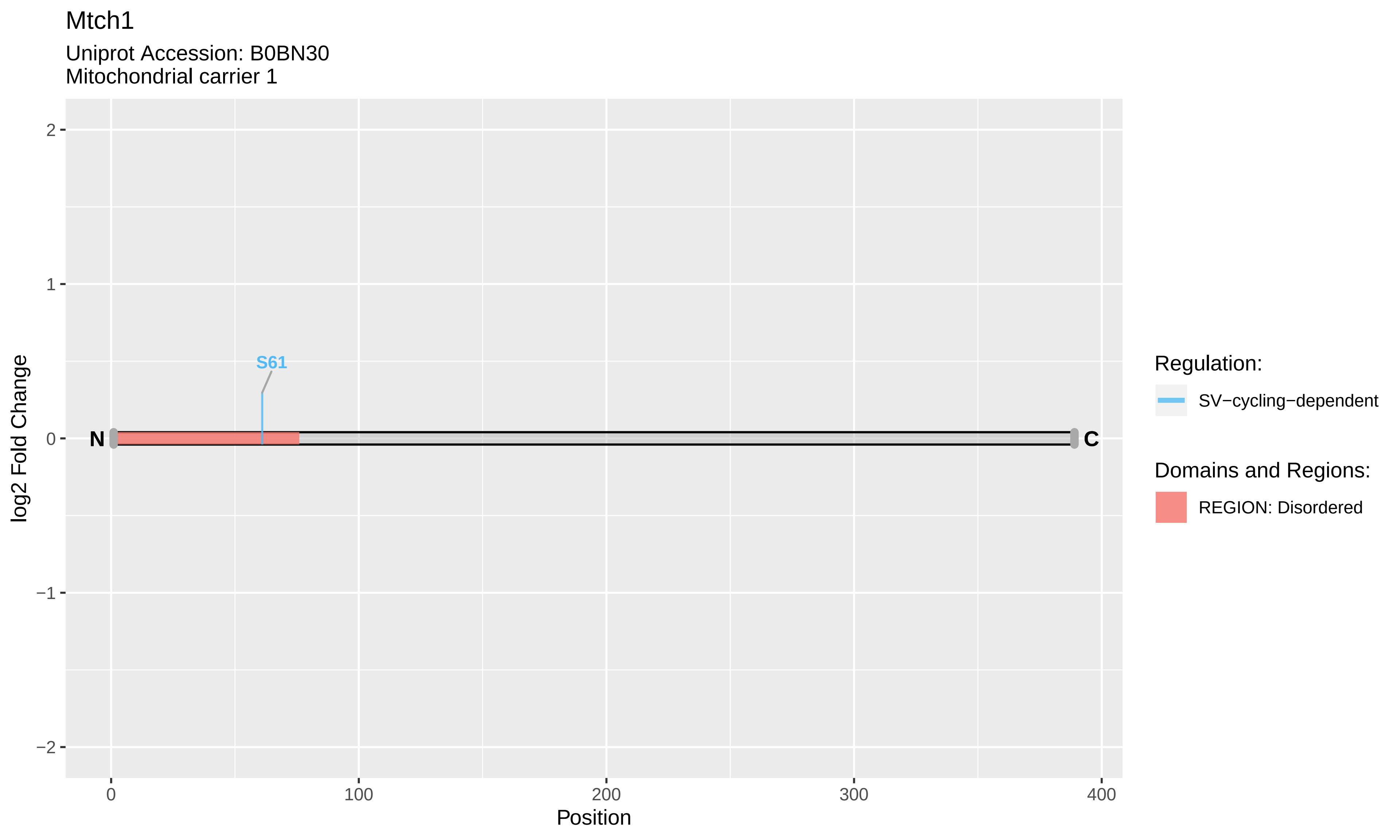

Mtch1
Uniprot Accession: B0BN30
Mitochondrial carrier 1
S61
N
C
2
1
0
−1
−2
0
100
200
300
400
Position
log2 Fold Change
Regulation:
SV−cycling−dependent
Domains and Regions:
REGION: Disordered

Mtcl1

Uniprot Accession: D4A5D4
Microtubule crosslinking factor 1

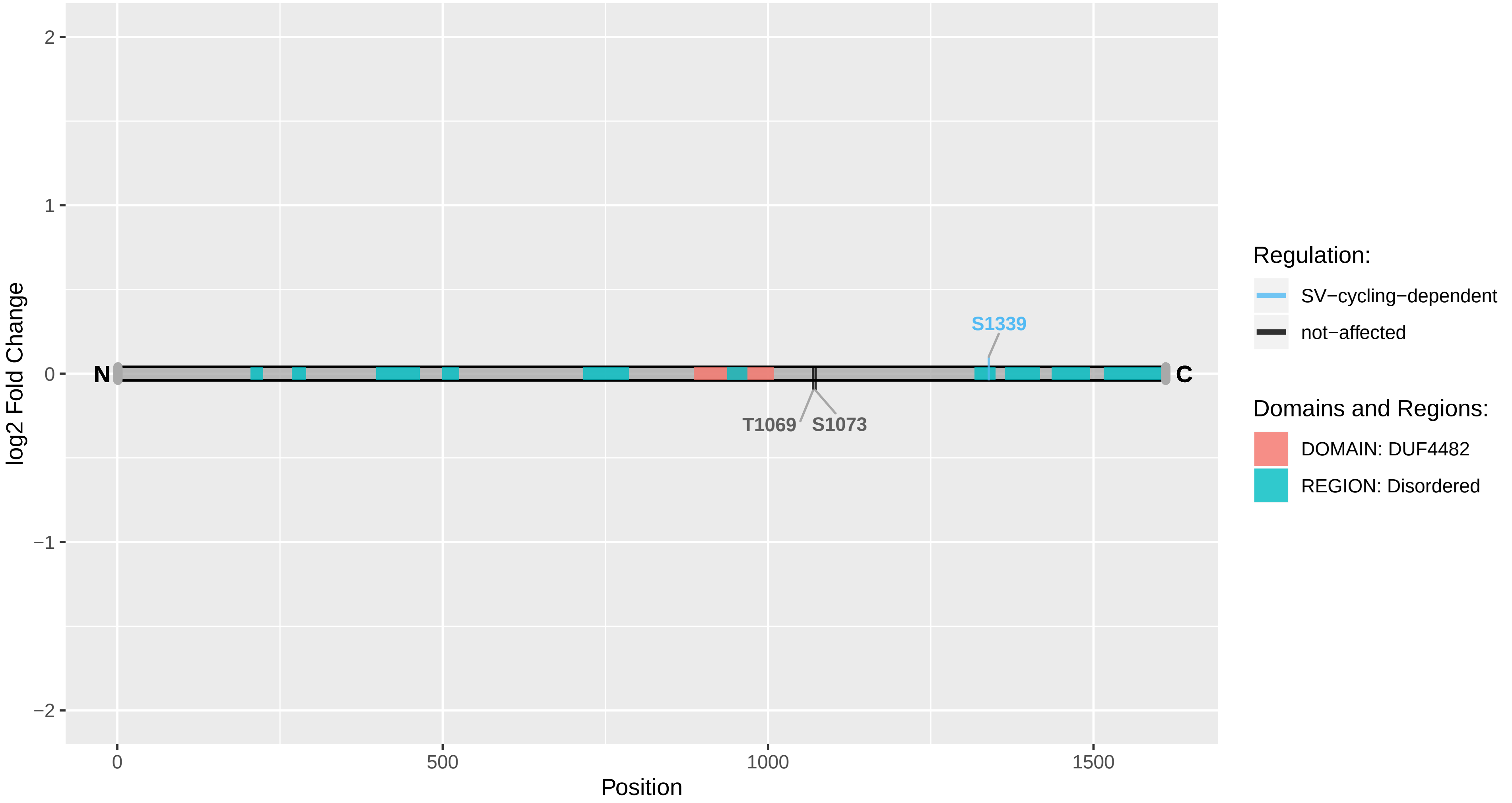

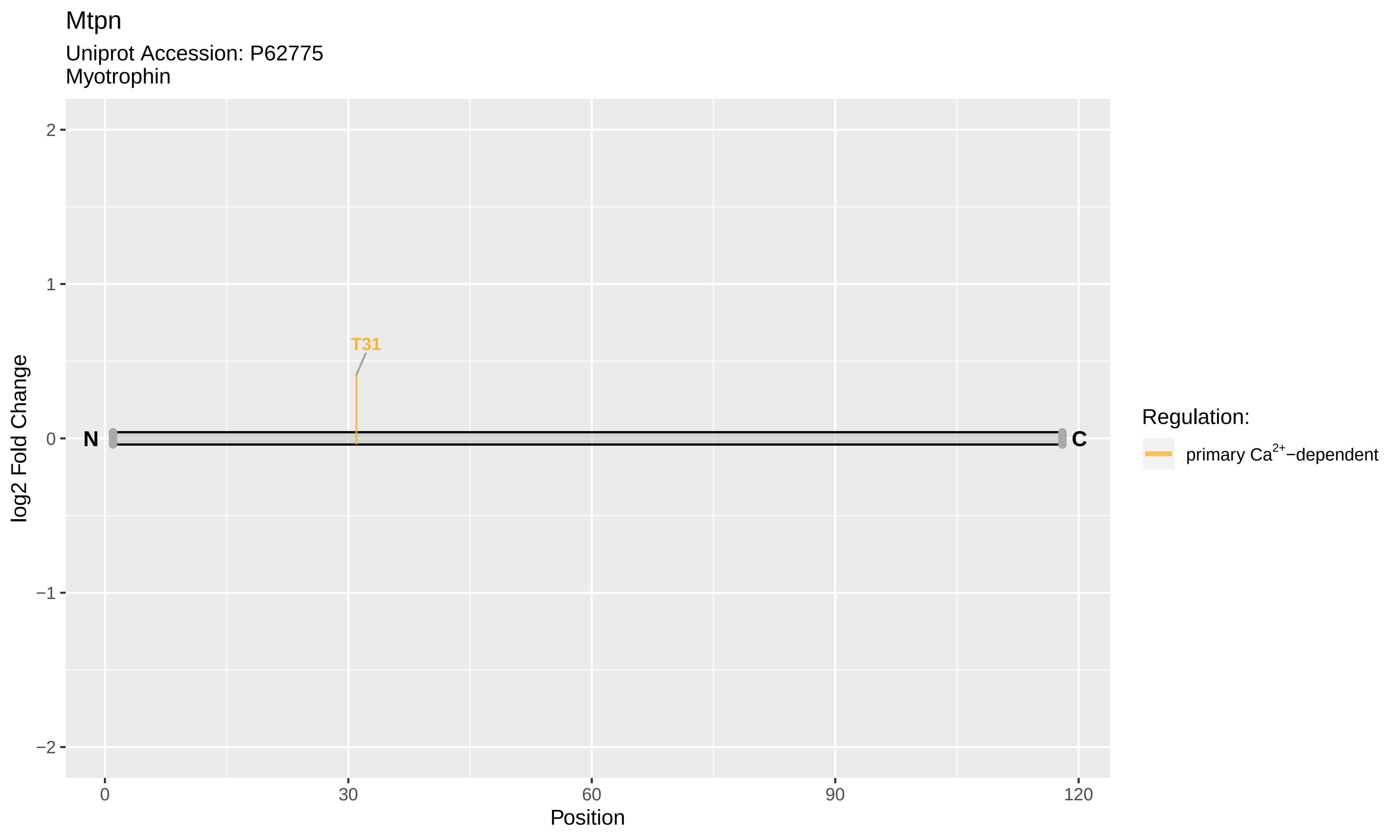

Mtpn
Uniprot Accession: P62775
Myotrophin
T31
N
C
2
1
0
−1
−2
0
30
60
90
120
log2 Fold Change
Position
Regulation:
primary Ca2+−dependent

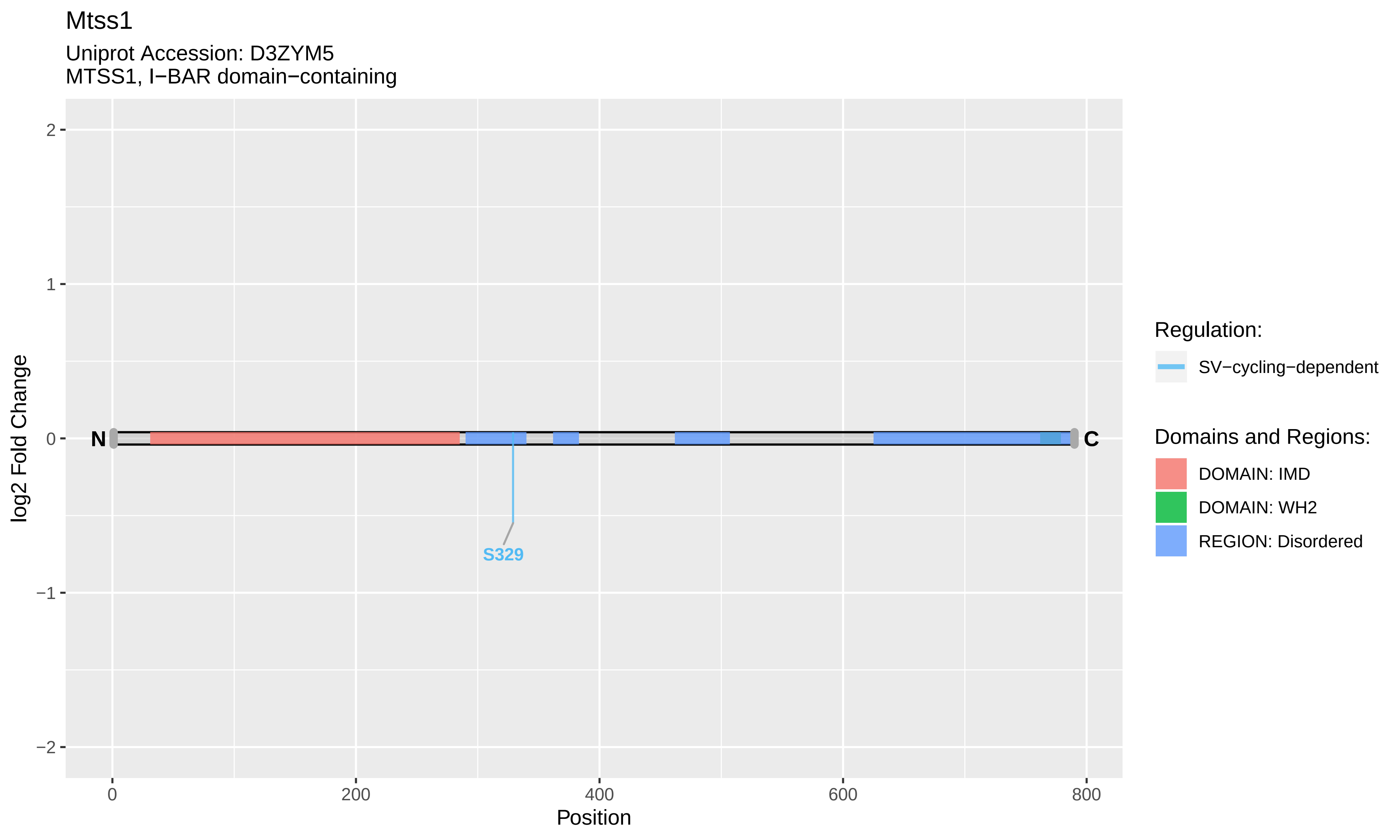

Mtss1
Uniprot Accession: D3ZYM5
MTSS1, I−BAR domain−containing
log2 Fold Change
2
1
0
−1
−2
N
C
S329
0
200
400
600
800
Position
Regulation:
SV−cycling−dependent
Domains and Regions:
DOMAIN: IMD
DOMAIN: WH2
REGION: Disordered

Mtss1l

Uniprot Accession: D4A3S6
MTSS1L, I−BAR domain−containing

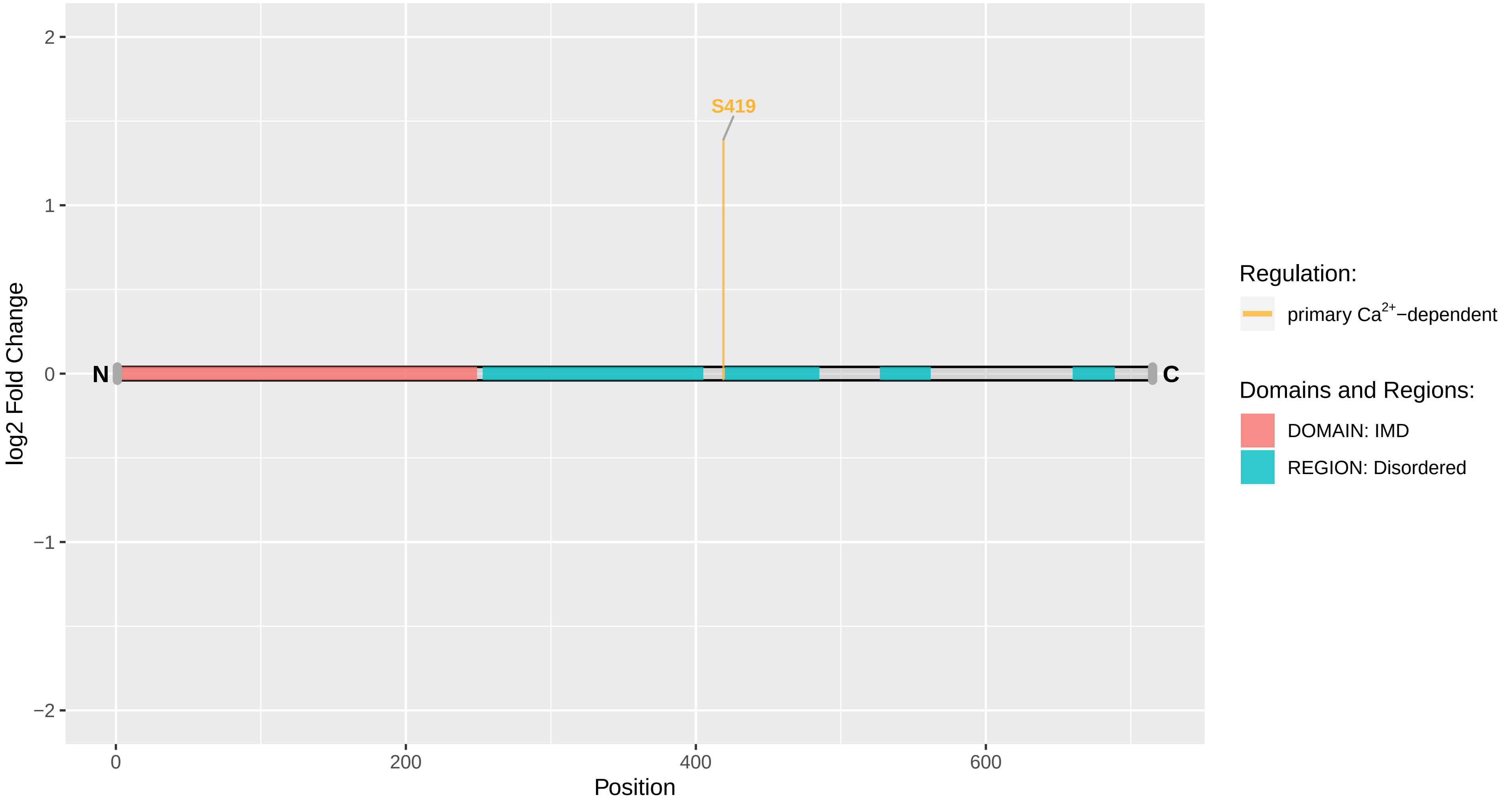

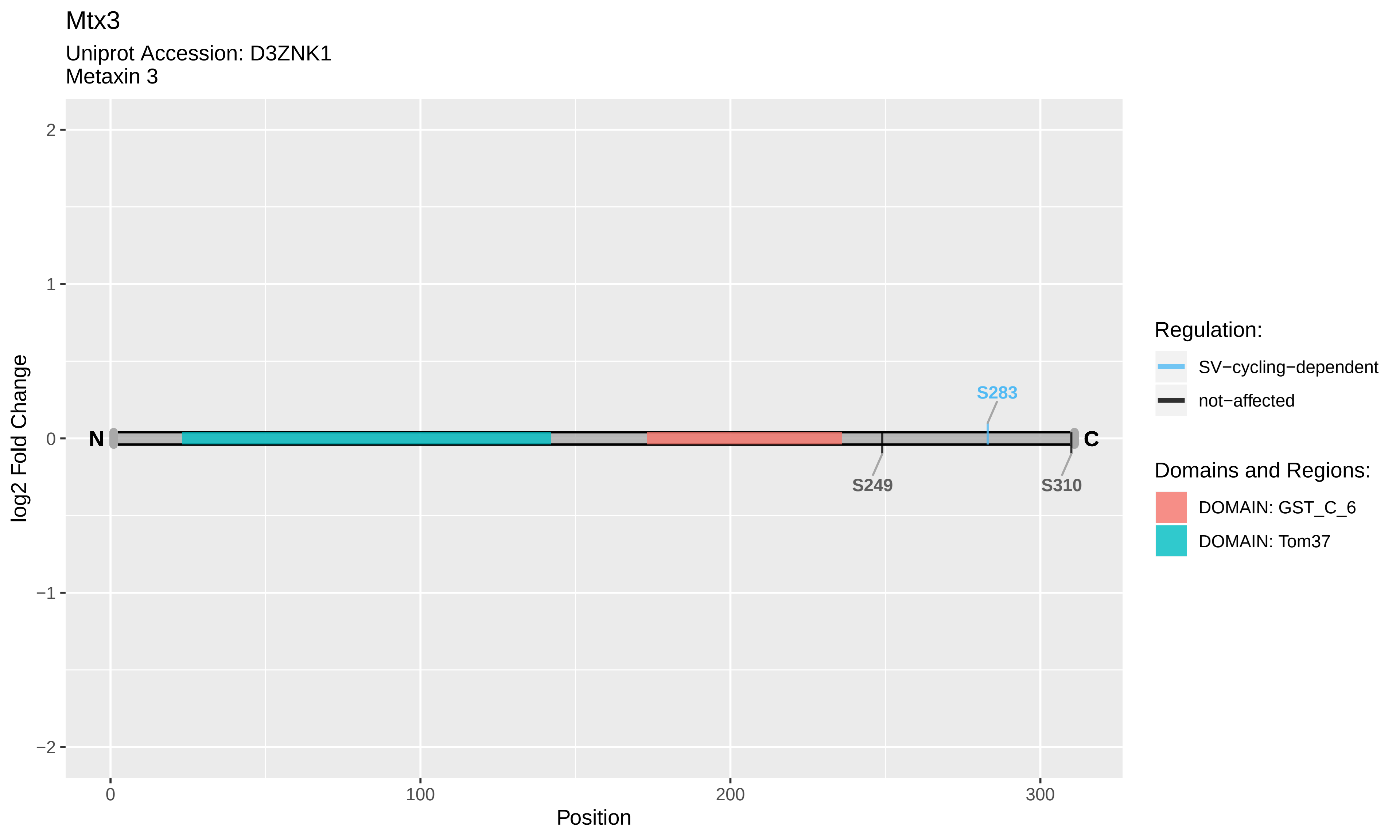
Mtx3
Uniprot Accession: D3ZNK1
Metaxin 3
log2 Fold Change
Position
N
C
S283
S249
S310
2
1
0
−1
−2
0
100
200
300
Regulation:
SV−cycling−dependent
not−affected
Domains and Regions:
DOMAIN: GST_C_6
DOMAIN: Tom37

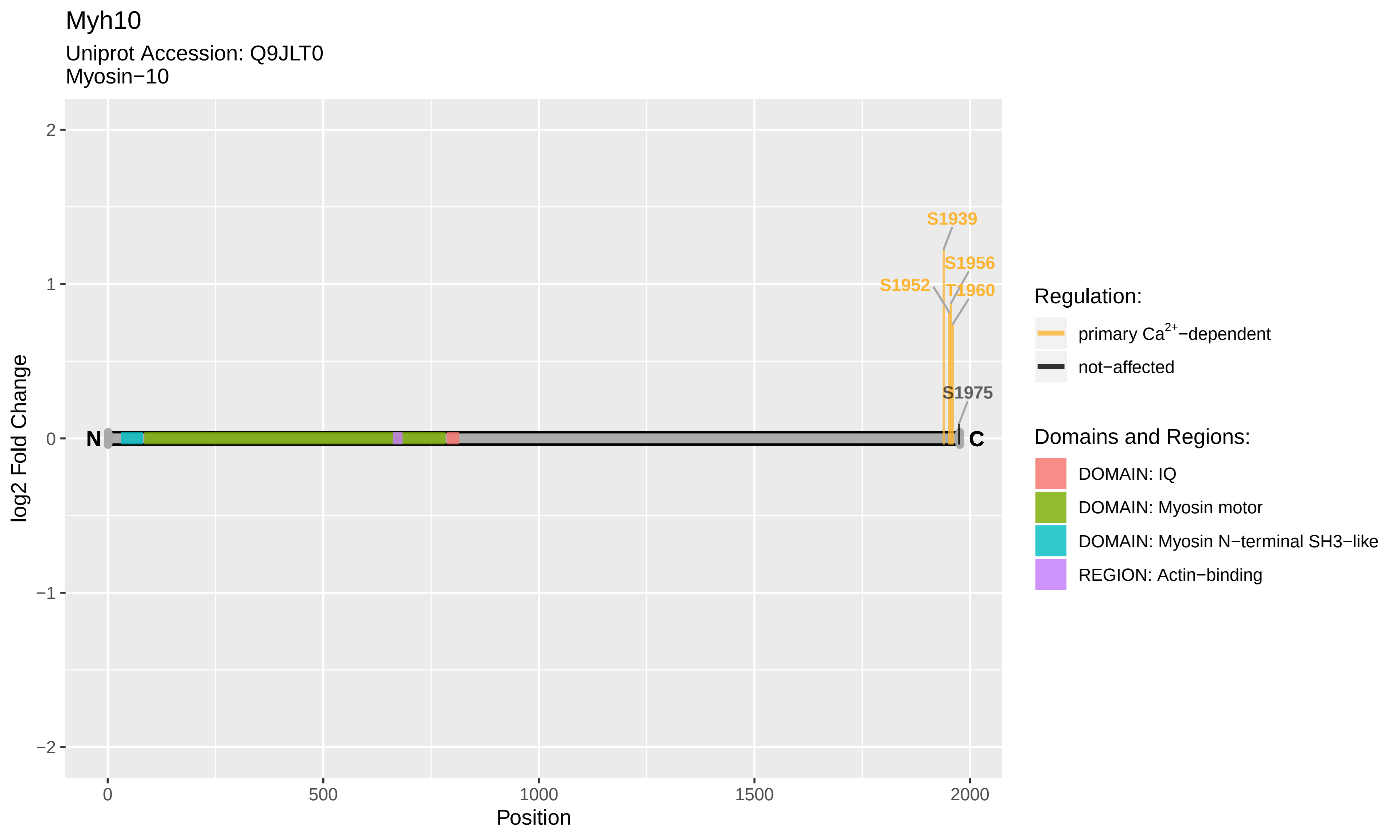

Myh10
Uniprot Accession: Q9JLT0
Myosin−10
S1939
S1956
S1952
T1960
S1975
N
C
log2 Fold Change
Position
2
1
0
−1
−2
0
500
1000
1500
2000
Regulation:
primary Ca2+−dependent
not−affected
Domains and Regions:
DOMAIN: IQ
DOMAIN: Myosin motor
DOMAIN: Myosin N−terminal SH3−like
REGION: Actin−binding

Myo5a

Uniprot Accession: Q9QYF3
Unconventional myosin−Va

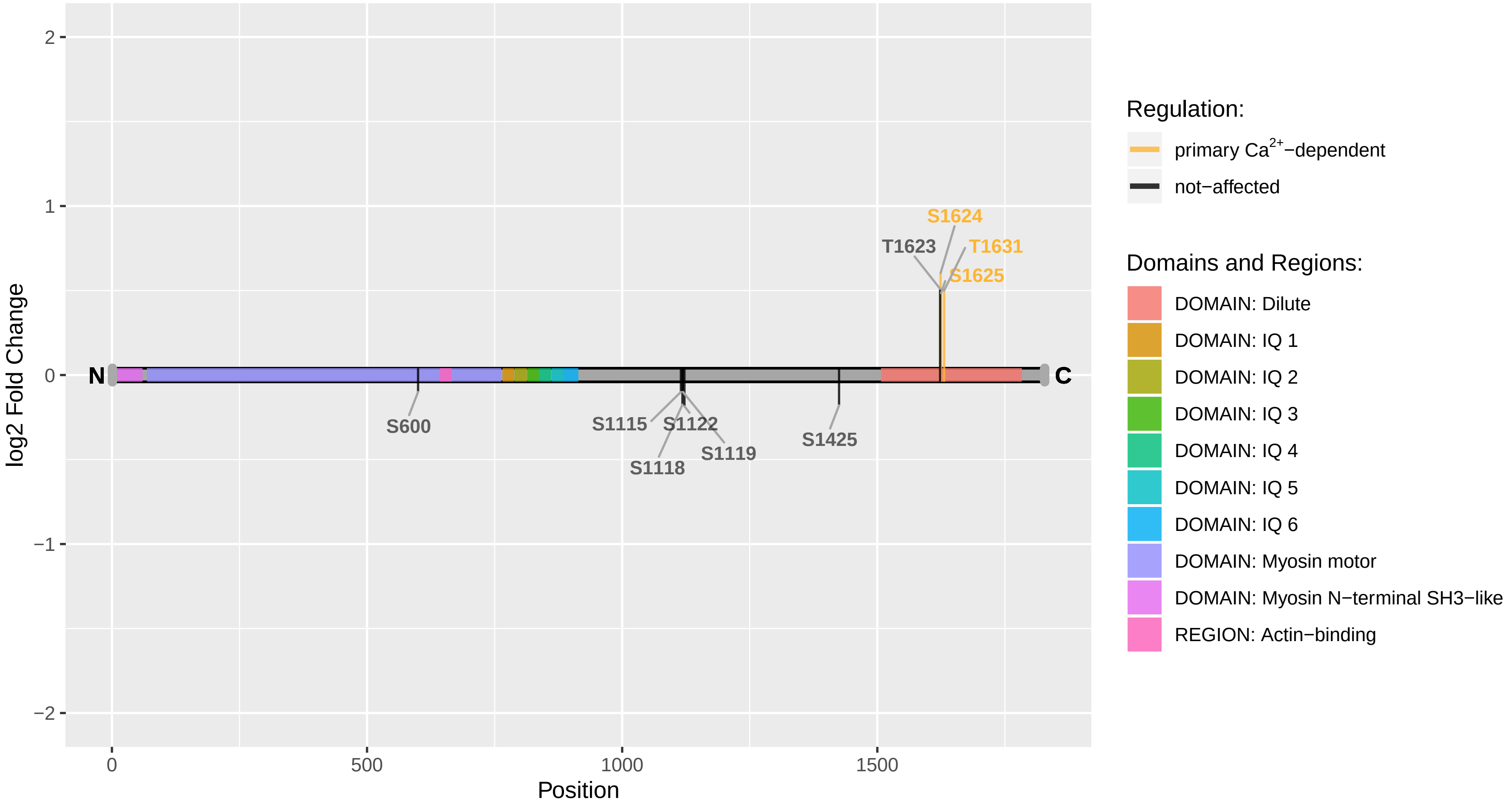

Napepld

Uniprot Accession: Q769K2
N−acyl−phosphatidylethanolamine−hydrolyzing phospholipase D

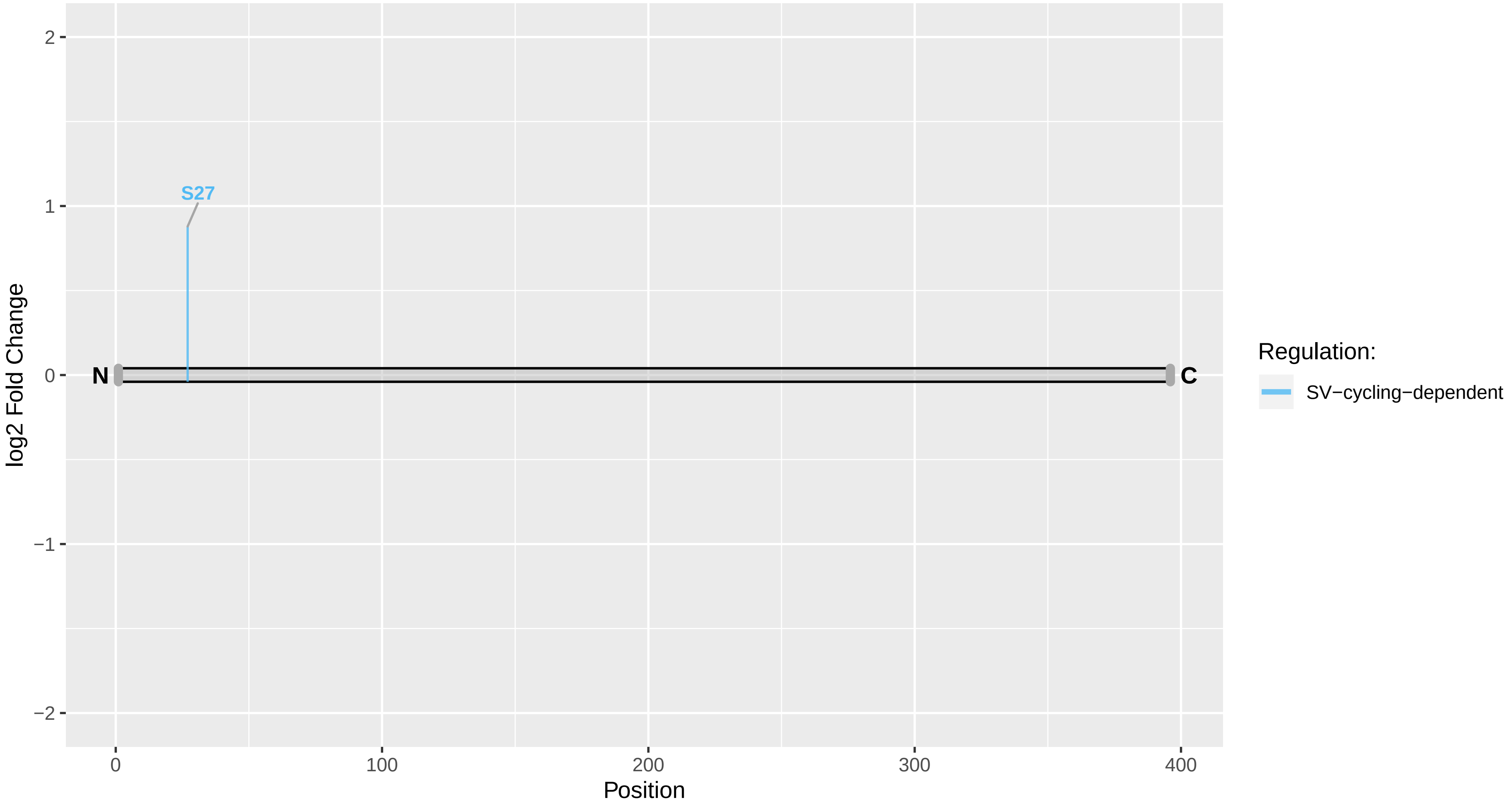

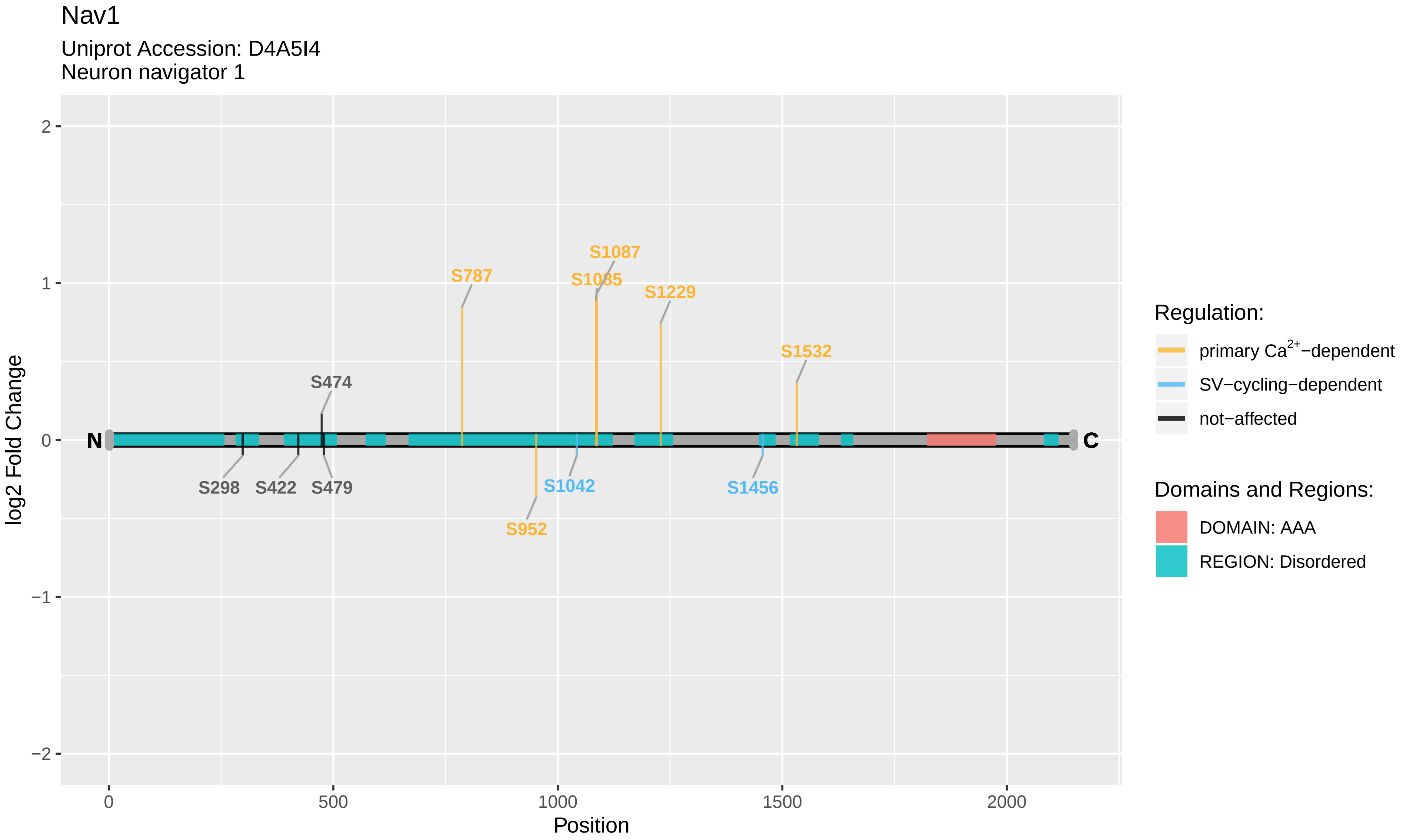

Nav1
Uniprot Accession: D4A5I4
Neuron navigator 1
log2 Fold Change
Position
2
1
0
−1
−2
0
500
1000
1500
2000
N
C
S474
S298
S422
S479
S787
S952
S1042
S1085
S1087
S1229
S1456
S1532
Regulation:
primary Ca^{2+}−dependent
SV−cycling−dependent
not−affected
Domains and Regions:
DOMAIN: AAA
REGION: Disordered

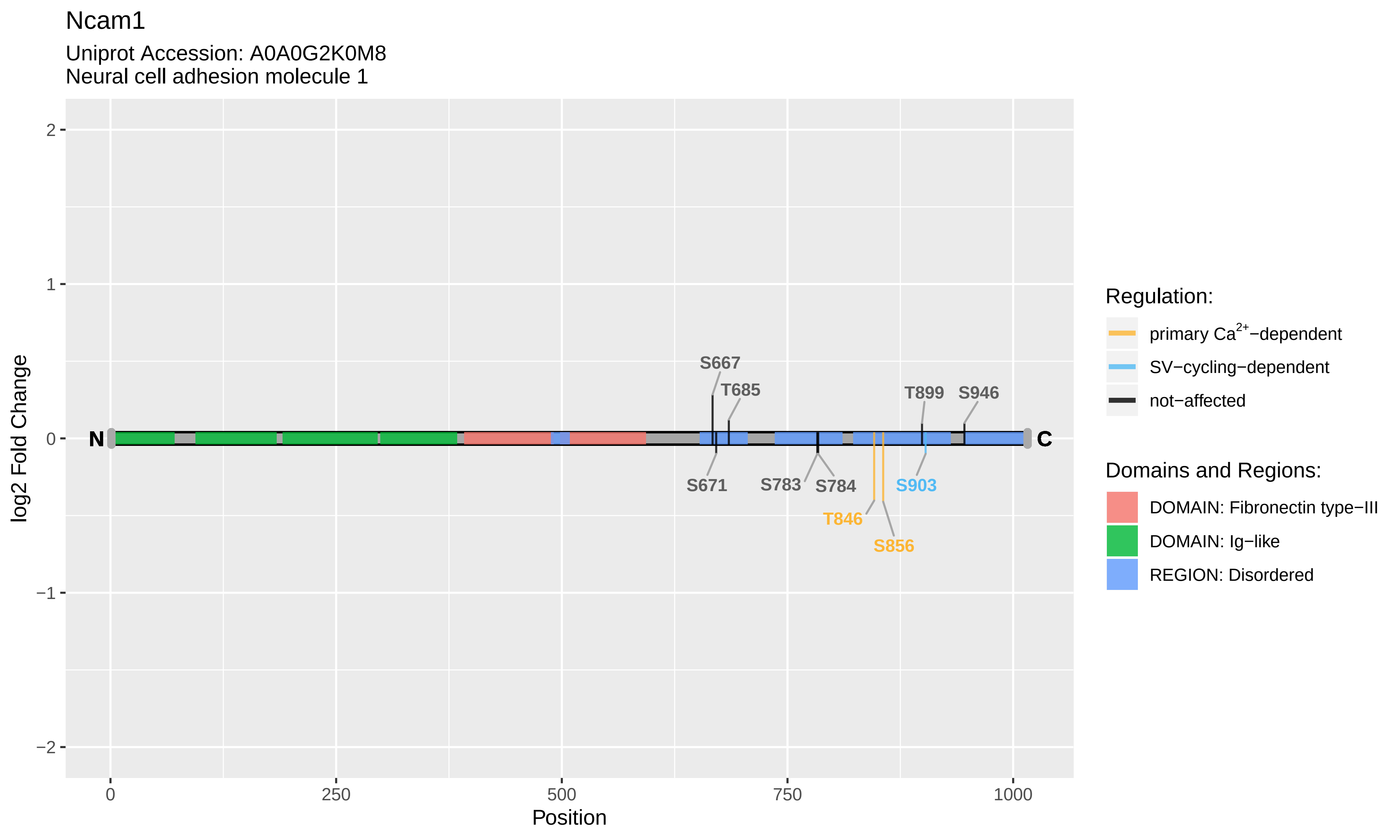

Ncam1
Uniprot Accession: A0A0G2K0M8
Neural cell adhesion molecule 1
log2 Fold Change
Position
N
C
S667
T685
T899
S946
S671
S783
S784
S903
T846
S856
Regulation:
primary Ca2+−dependent
SV−cycling−dependent
not−affected
Domains and Regions:
DOMAIN: Fibronectin type−III
DOMAIN: Ig−like
REGION: Disordered

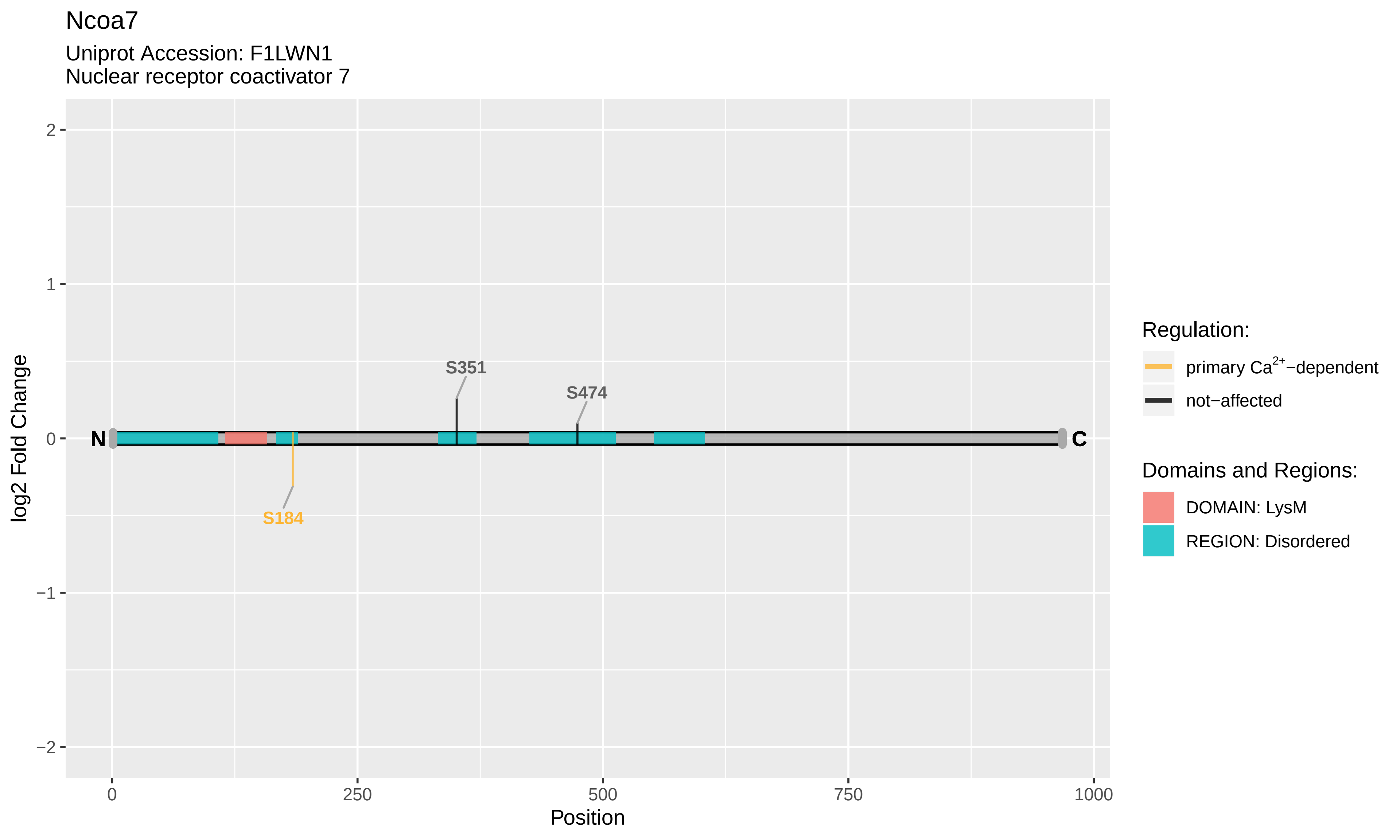

Ncoa7
Uniprot Accession: F1LWN1
Nuclear receptor coactivator 7
log2 Fold Change
2
1
0
−1
−2
N
C
S351
S474
S184
0
250
500
750
1000
Position
Regulation:
primary Ca2+−dependent
not−affected
Domains and Regions:
DOMAIN: LysM
REGION: Disordered

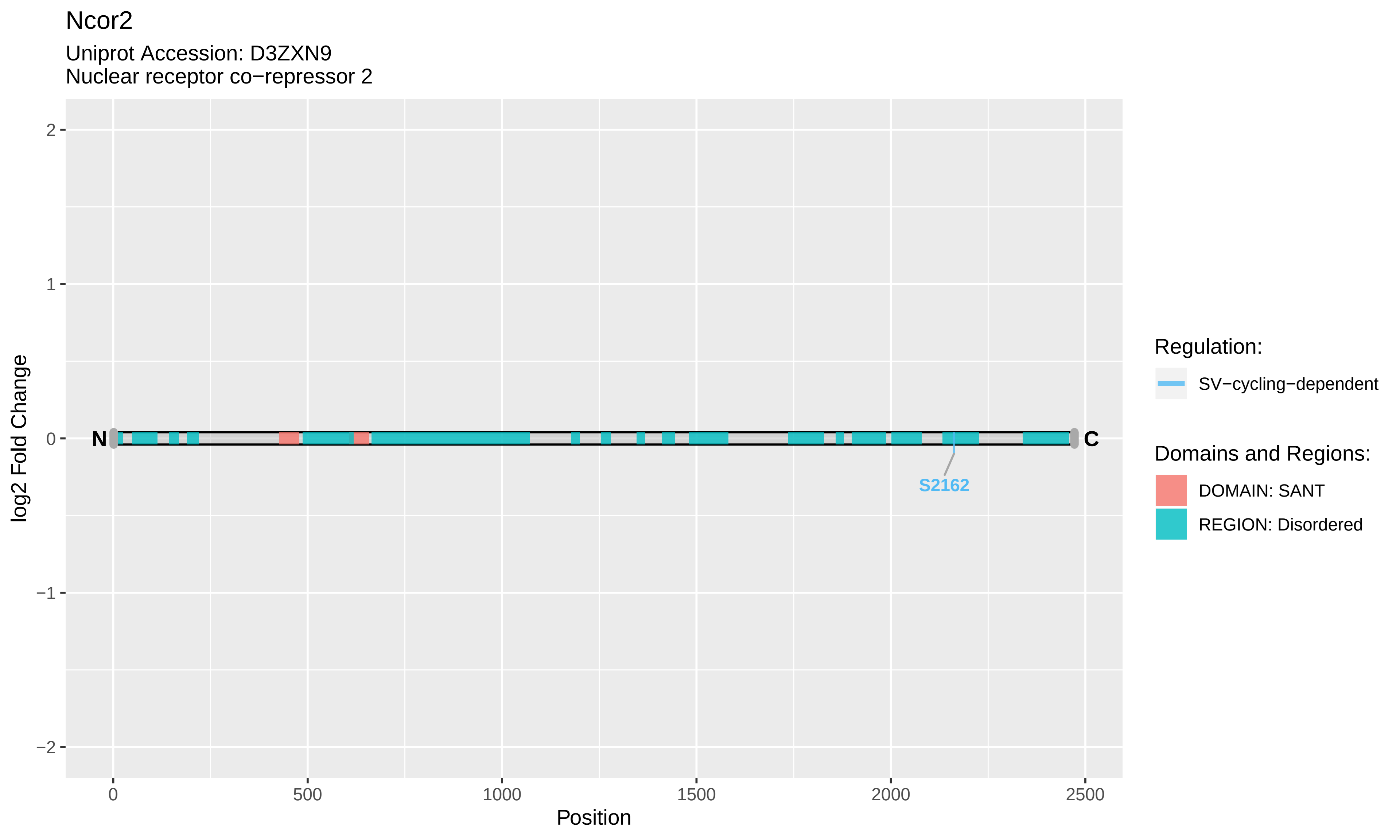

Ncor2
Uniprot Accession: D3ZXN9
Nuclear receptor co−repressor 2
log2 Fold Change
Position
N
C
S2162
Regulation:
SV−cycling−dependent
Domains and Regions:
DOMAIN: SANT
REGION: Disordered

Ndrg2

Uniprot Accession: Q8VBU2
Protein NDRG2

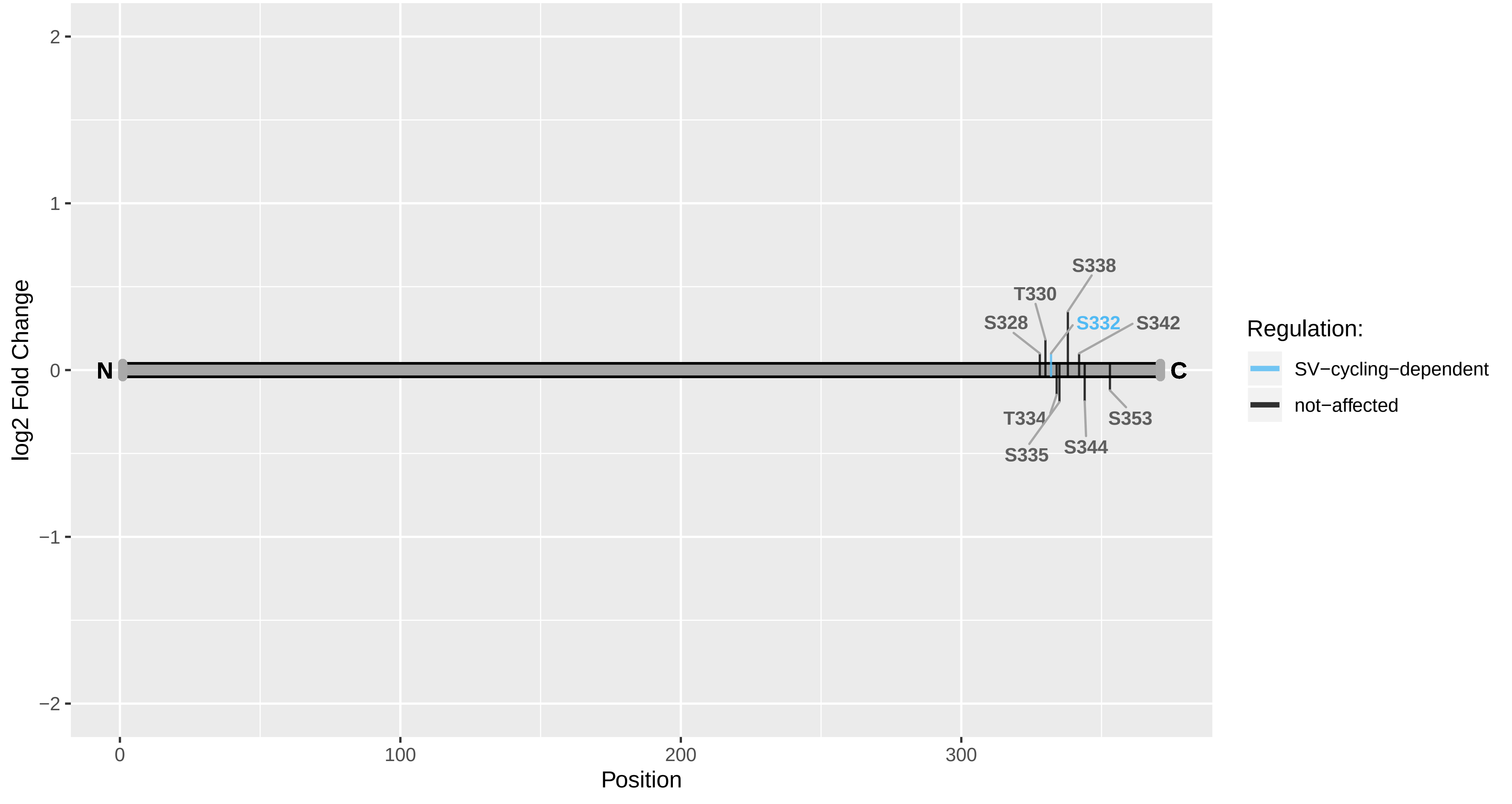

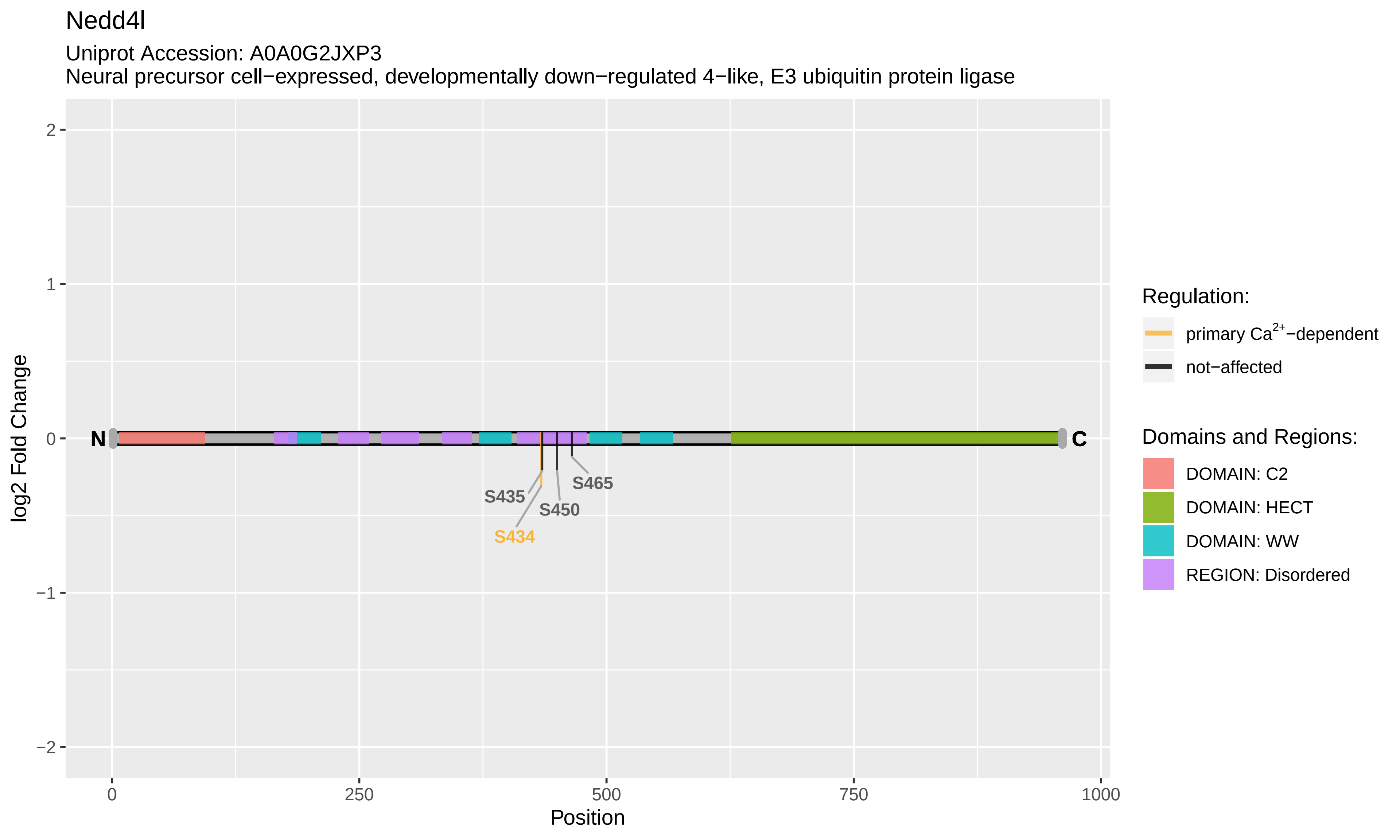

Nedd4l
Uniprot Accession: A0A0G2JXP3
Neural precursor cell−expressed, developmentally down−regulated 4−like, E3 ubiquitin protein ligase
log2 Fold Change
2
1
0
−1
−2
N
C
S465
S435
S450
S434
0
250
500
750
1000
Position
Regulation:
primary Ca2+−dependent
not−affected
Domains and Regions:
DOMAIN: C2
DOMAIN: HECT
DOMAIN: WW
REGION: Disordered

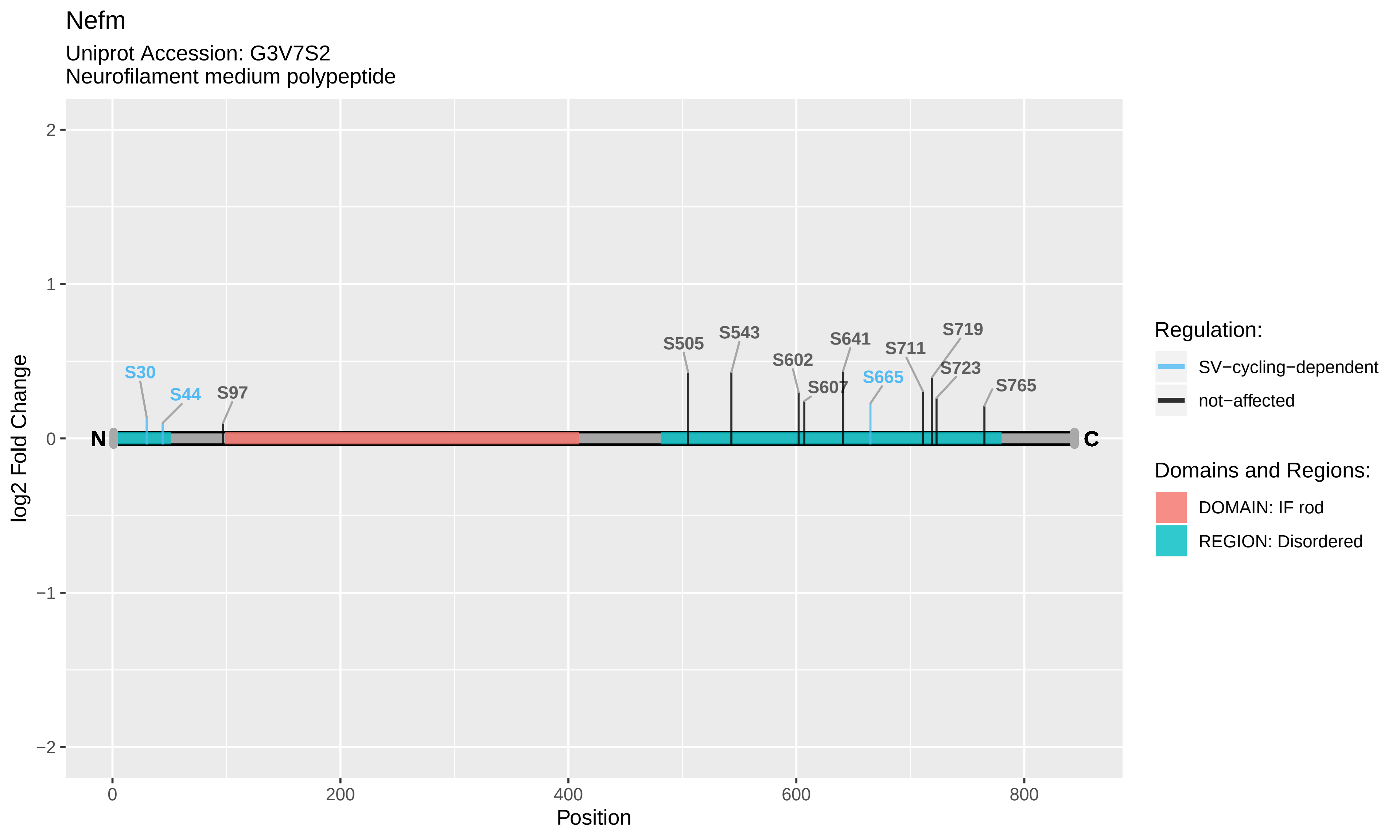
Nefm
Uniprot Accession: G3V7S2
Neurofilament medium polypeptide
log2 Fold Change
2
1
0
−1
−2
N
C
S30
S44
S97
S505
S543
S602
S607
S641
S665
S711
S719
S723
S765
0
200
400
600
800
Position
Regulation:
SV−cycling−dependent
not−affected
Domains and Regions:
DOMAIN: IF rod
REGION: Disordered

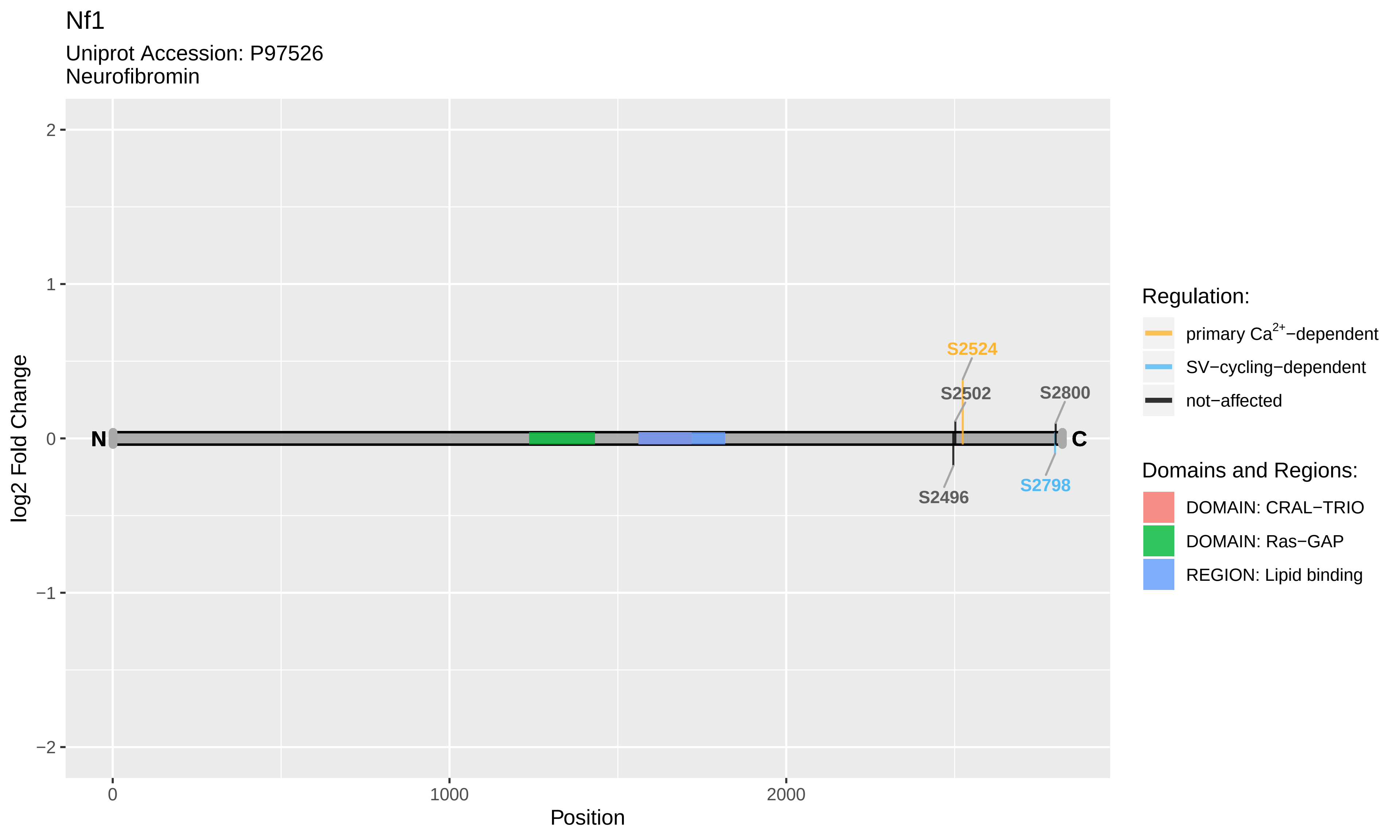

Nf1
Uniprot Accession: P97526
Neurofibromin
log2 Fold Change
2
1
0
−1
−2
0
1000
2000
Position
N
C
S2524
S2502
S2800
S2496
S2798
Regulation:
primary Ca2+−dependent
SV−cycling−dependent
not−affected
Domains and Regions:
DOMAIN: CRAL−TRIO
DOMAIN: Ras−GAP
REGION: Lipid binding

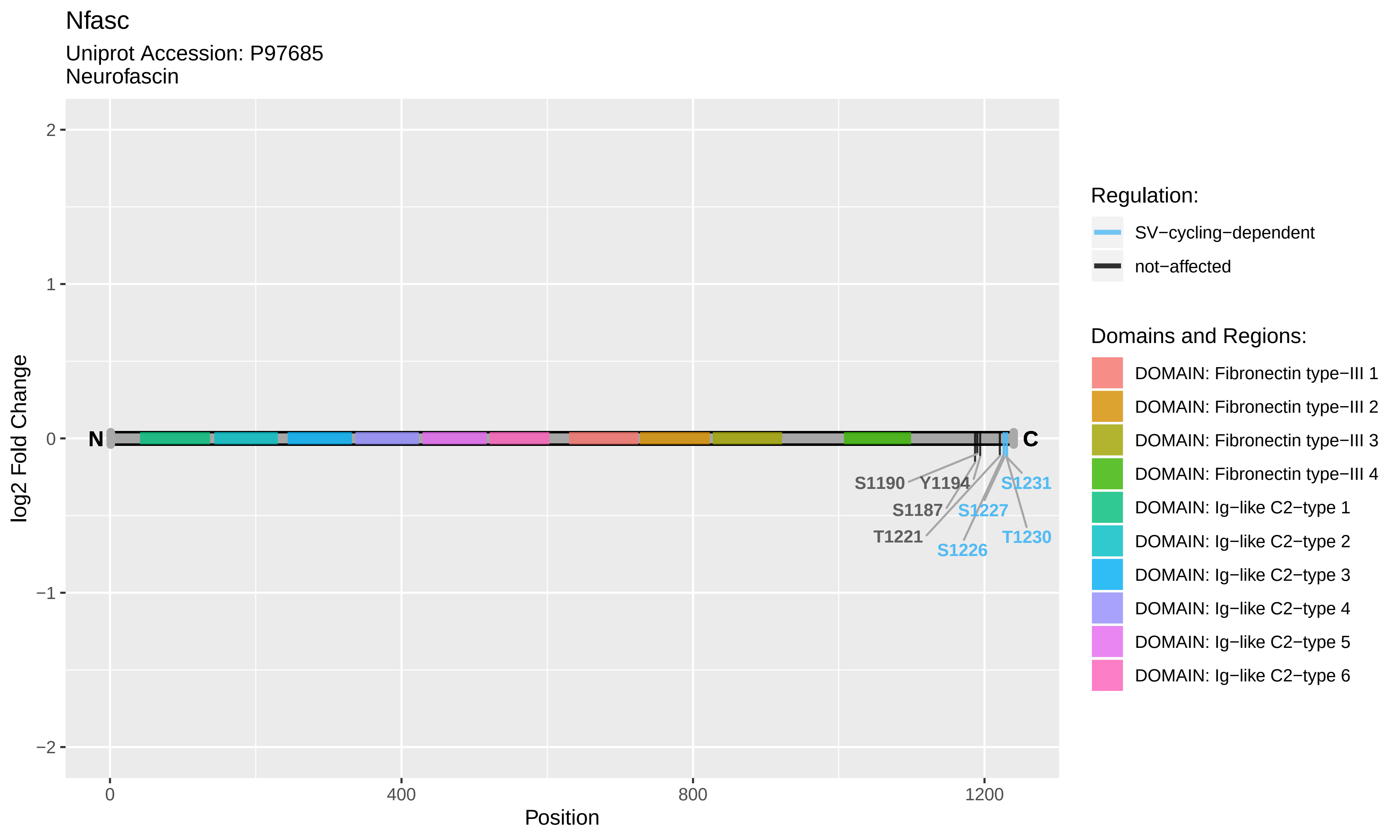

Nfasc
Uniprot Accession: P97685
Neurofascin
log2 Fold Change
2
1
0
−1
−2
N
C
S1190
Y1194
S1231
S1187
S1227
T1221
T1230
S1226
0
400
800
1200
Position
Regulation:
SV−cycling−dependent
not−affected
Domains and Regions:
DOMAIN: Fibronectin type−III 1
DOMAIN: Fibronectin type−III 2
DOMAIN: Fibronectin type−III 3
DOMAIN: Fibronectin type−III 4
DOMAIN: Ig−like C2−type 1
DOMAIN: Ig−like C2−type 2
DOMAIN: Ig−like C2−type 3
DOMAIN: Ig−like C2−type 4
DOMAIN: Ig−like C2−type 5
DOMAIN: Ig−like C2−type 6

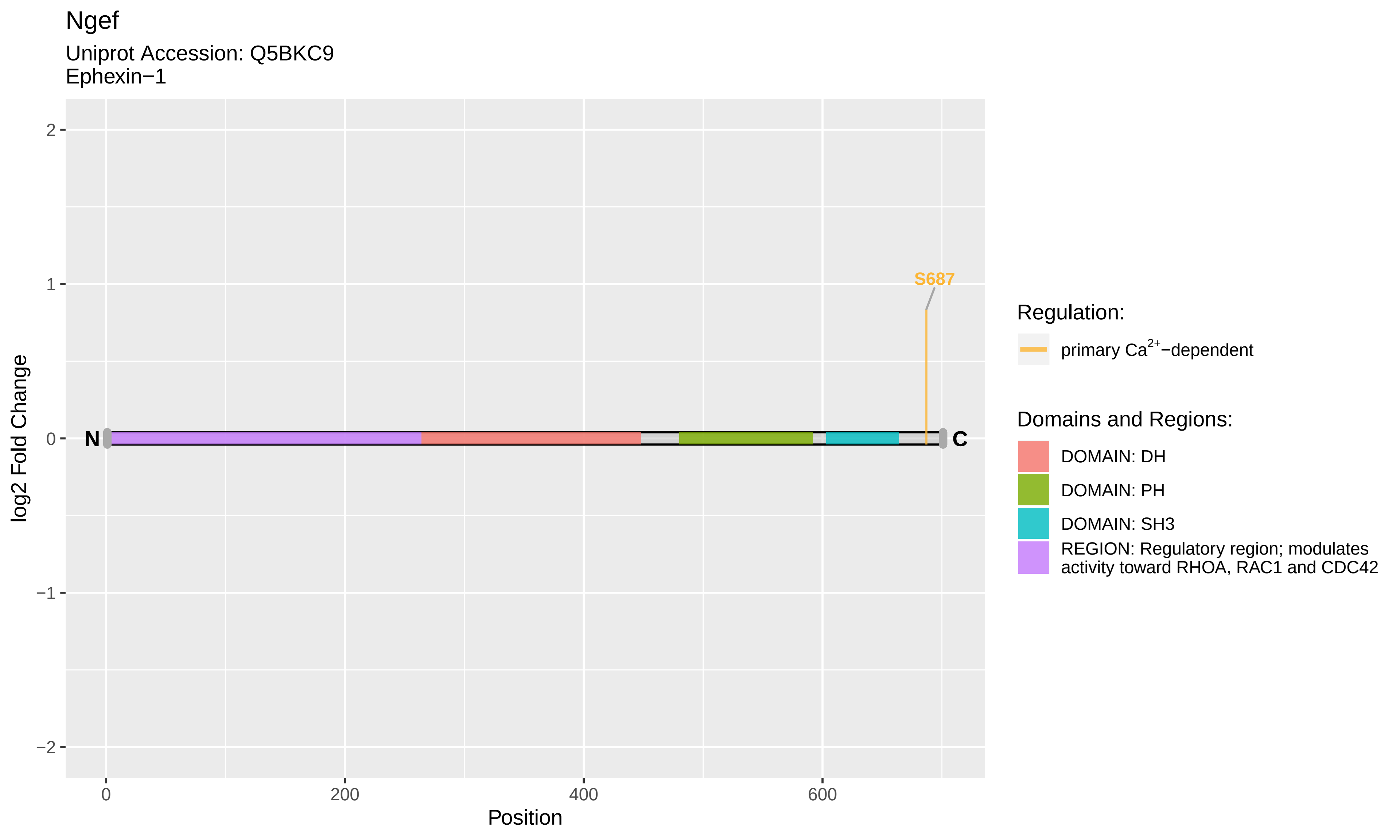

Ngef
Uniprot Accession: Q5BKC9
Ephexin−1
S687
N
C
log2 Fold Change
Position
2
1
0
−1
−2
0
200
400
600
Regulation:
primary Ca2+−dependent
Domains and Regions:
DOMAIN: DH
DOMAIN: PH
DOMAIN: SH3
REGION: Regulatory region; modulates activity toward RHOA, RAC1 and CDC42

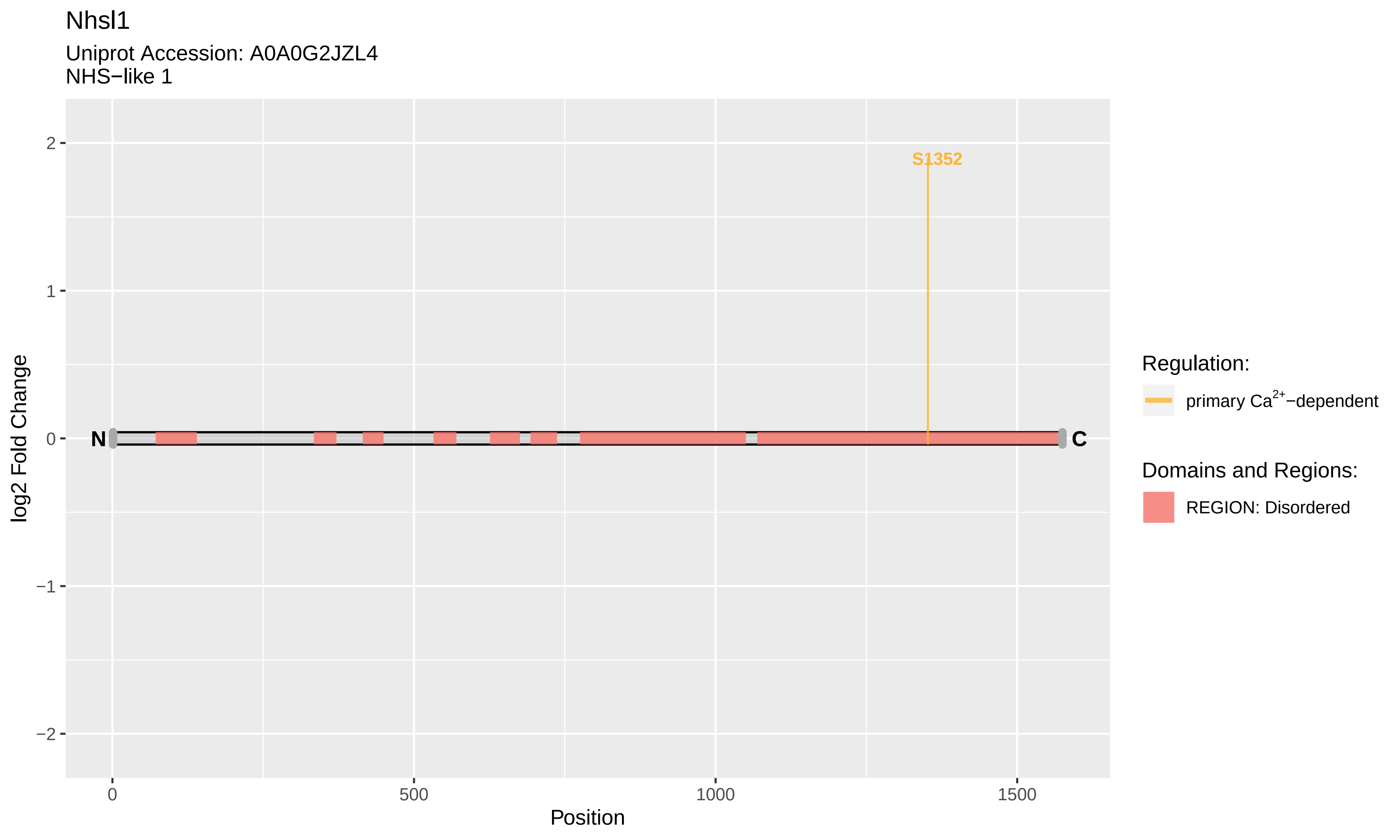

Nhsl1
Uniprot Accession: A0A0G2JZL4
NHS−like 1
S1352
N
C
2
1
0
−1
−2
log2 Fold Change
0
500
1000
1500
Position
Regulation:
primary Ca2+−dependent
Domains and Regions:
REGION: Disordered

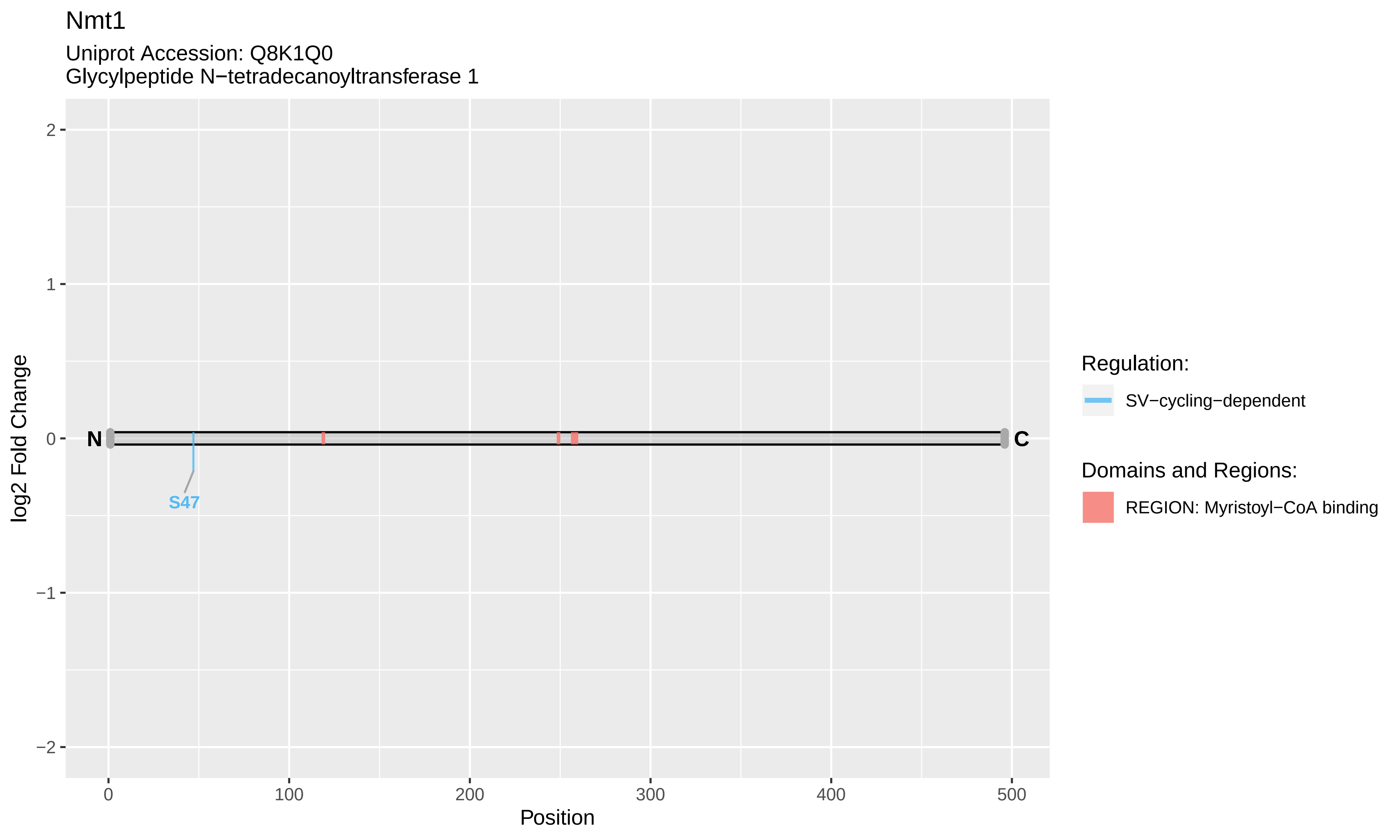
Nmt1
Uniprot Accession: Q8K1Q0
Glycylpeptide N−tetradecanoyltransferase 1
log2 Fold Change
2
1
0
−1
−2
N
C
S47
0
100
200
300
400
500
Position
Regulation:
SV−cycling−dependent
Domains and Regions:
REGION: Myristoyl−CoA binding

Npdc1

Uniprot Accession: Q6AY81
Neural proliferation, differentiation and control, 1

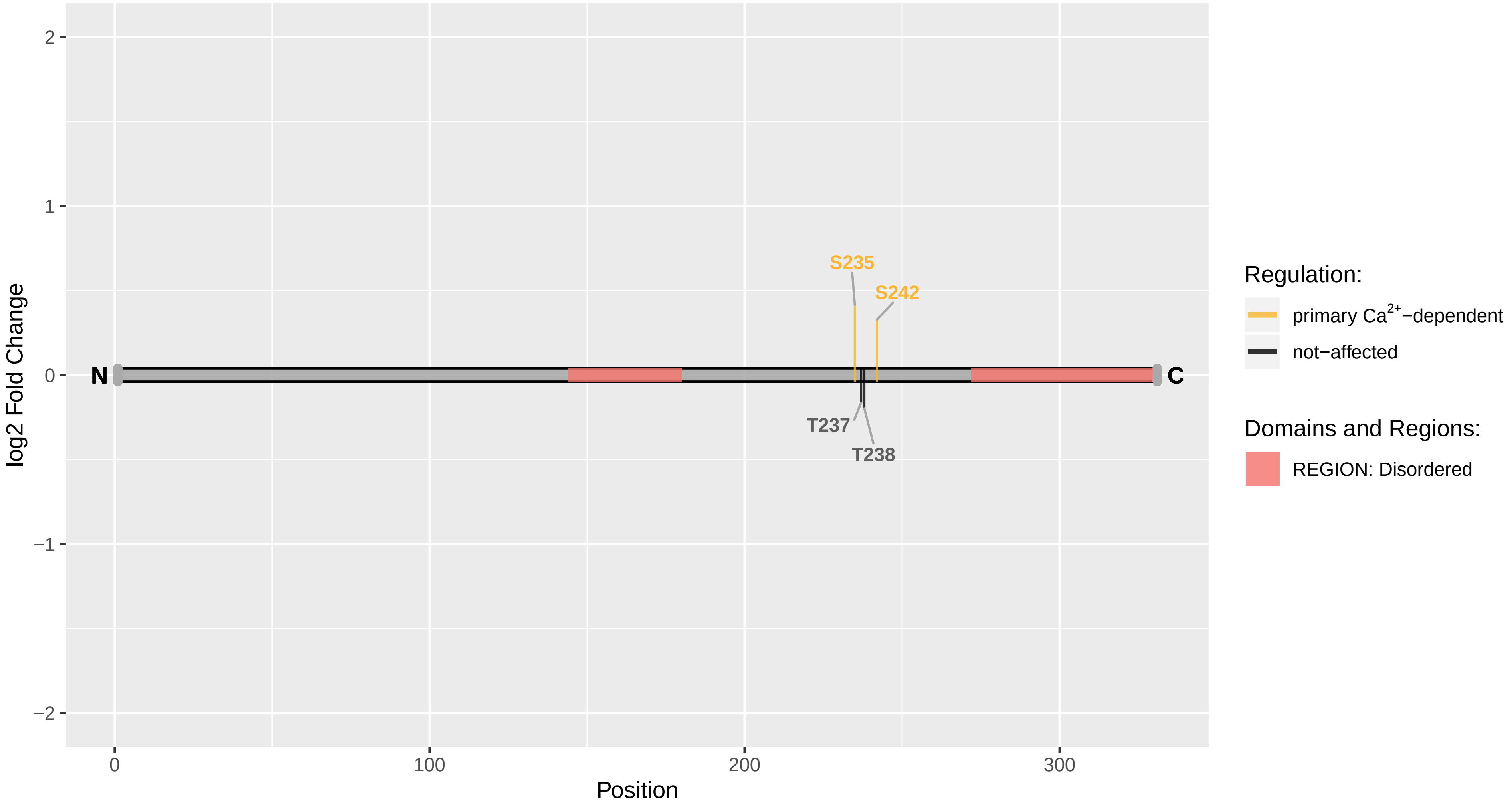

Nptxr

Uniprot Accession: O35764
Neuronal pentraxin receptor

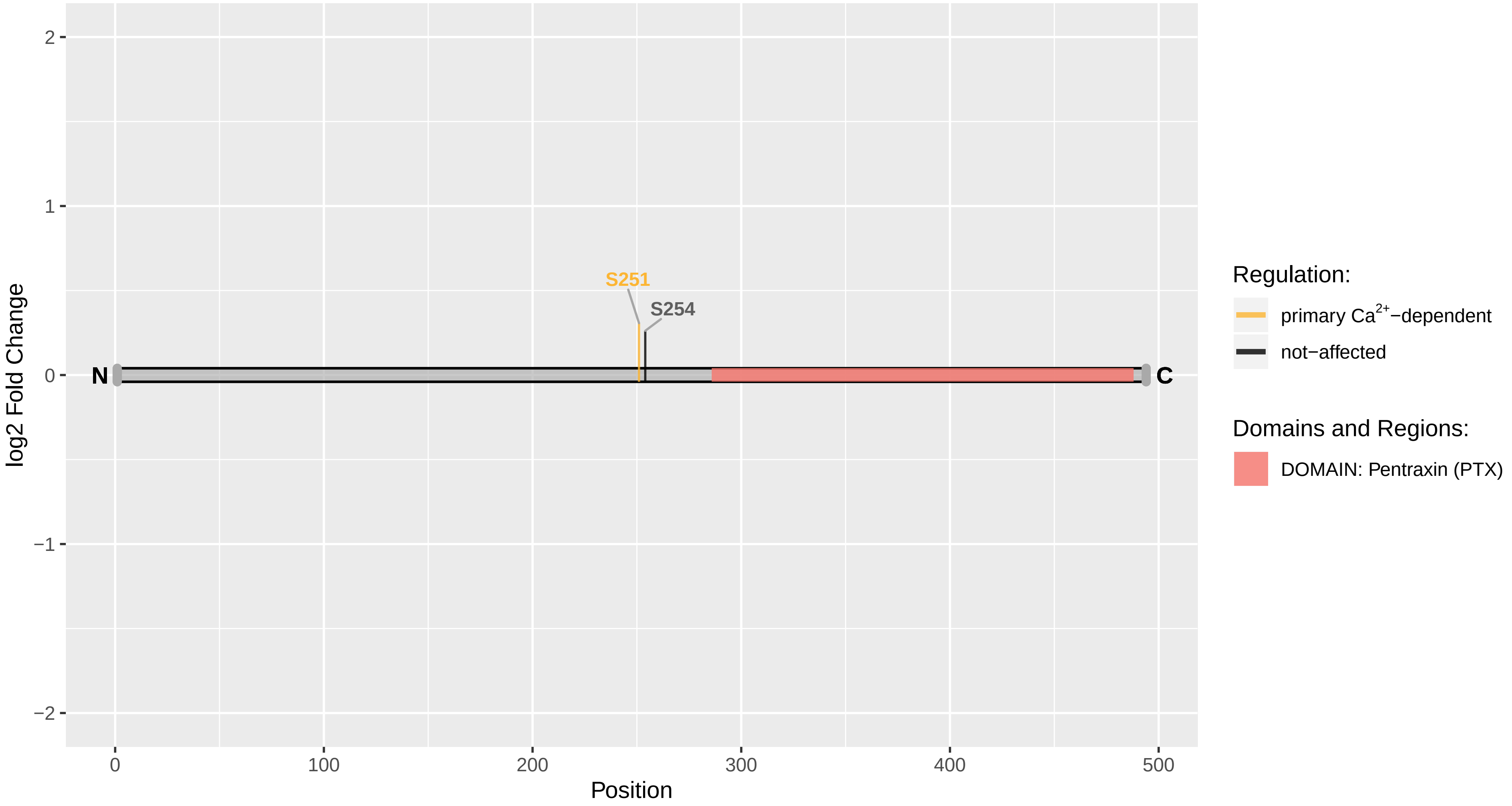

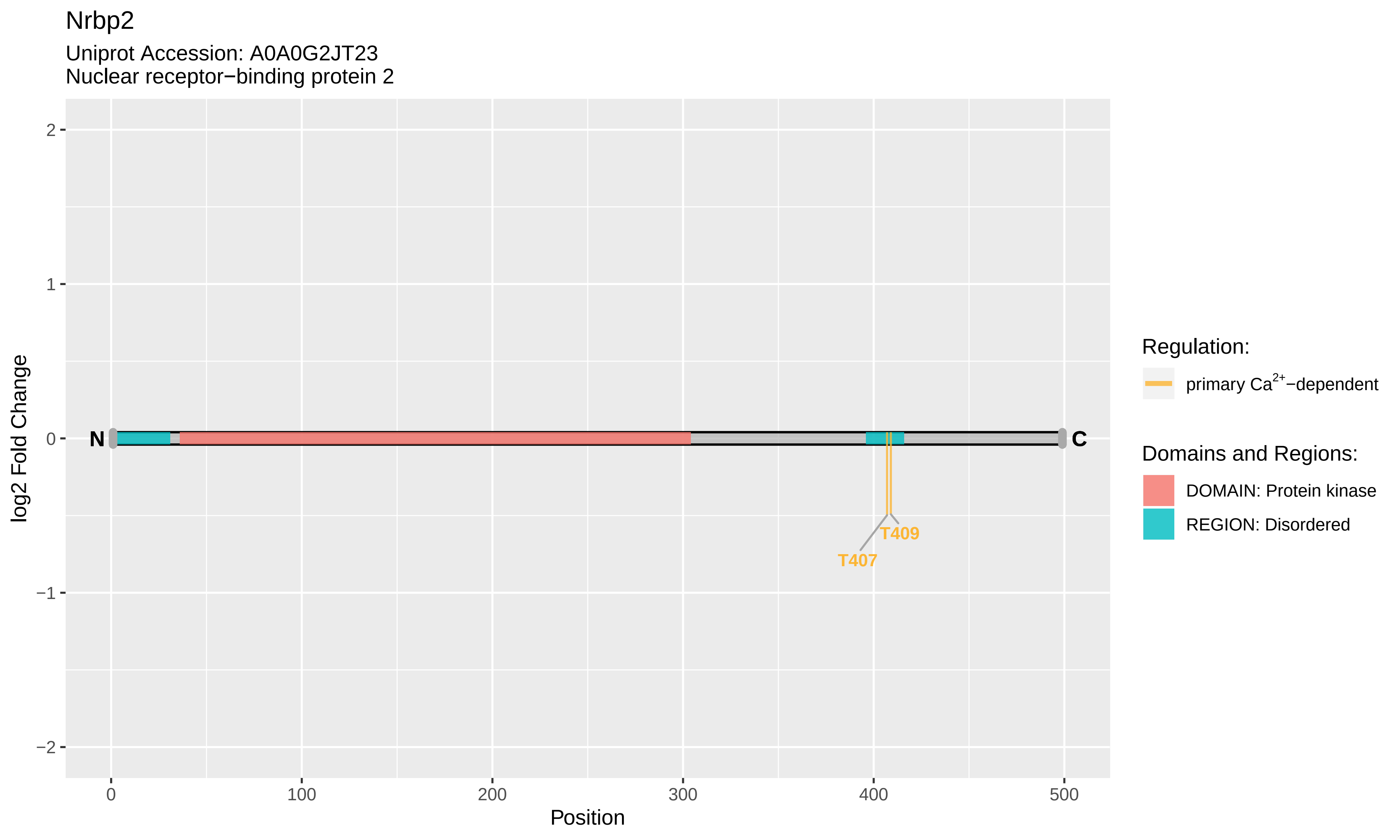
Nrbp2
Uniprot Accession: A0A0G2JT23
Nuclear receptor−binding protein 2
log2 Fold Change
2
1
0
−1
−2
N
C
T409
T407
0
100
200
300
400
500
Position
Regulation:
primary Ca2+−dependent
Domains and Regions:
DOMAIN: Protein kinase
REGION: Disordered

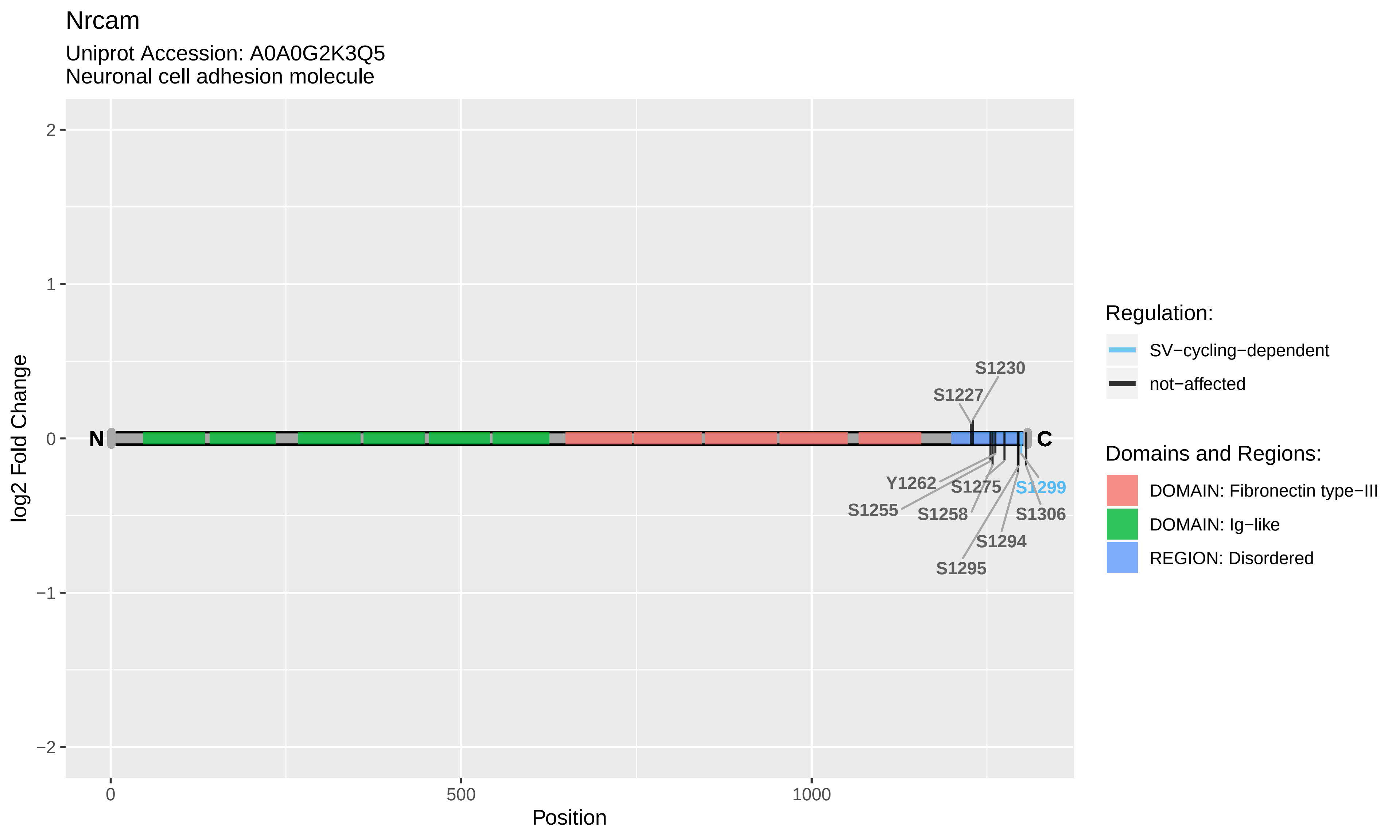

Nrcam
Uniprot Accession: A0A0G2K3Q5
Neuronal cell adhesion molecule
log2 Fold Change
2
1
0
−1
−2
N
C
S1230
S1227
Y1262
S1275
S1299
S1255
S1258
S1306
S1294
S1295
0
500
1000
Position
Regulation:
SV−cycling−dependent
not−affected
Domains and Regions:
DOMAIN: Fibronectin type−III
DOMAIN: Ig−like
REGION: Disordered

Nrdc

Uniprot Accession: P47245
Nardilysin

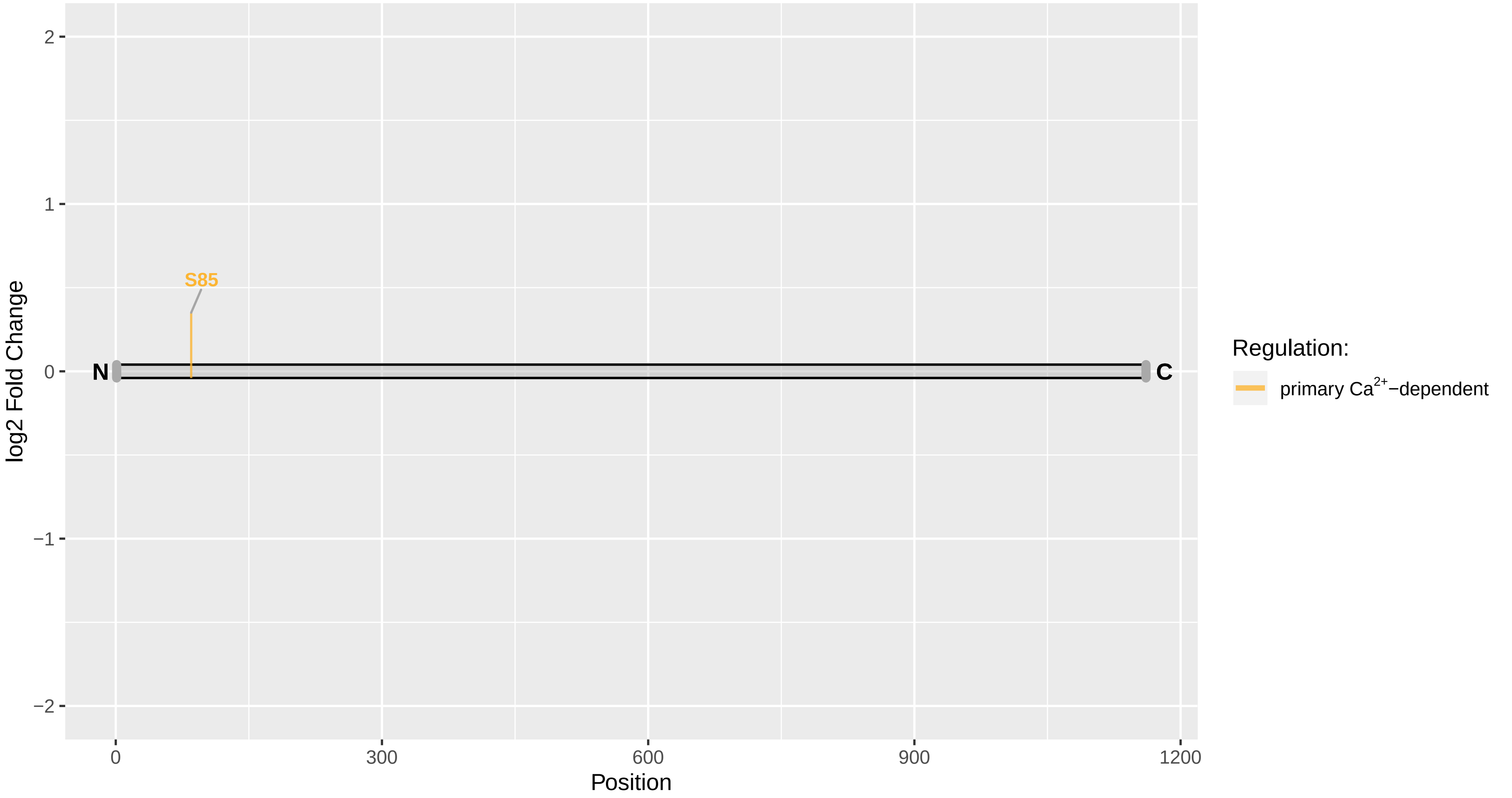

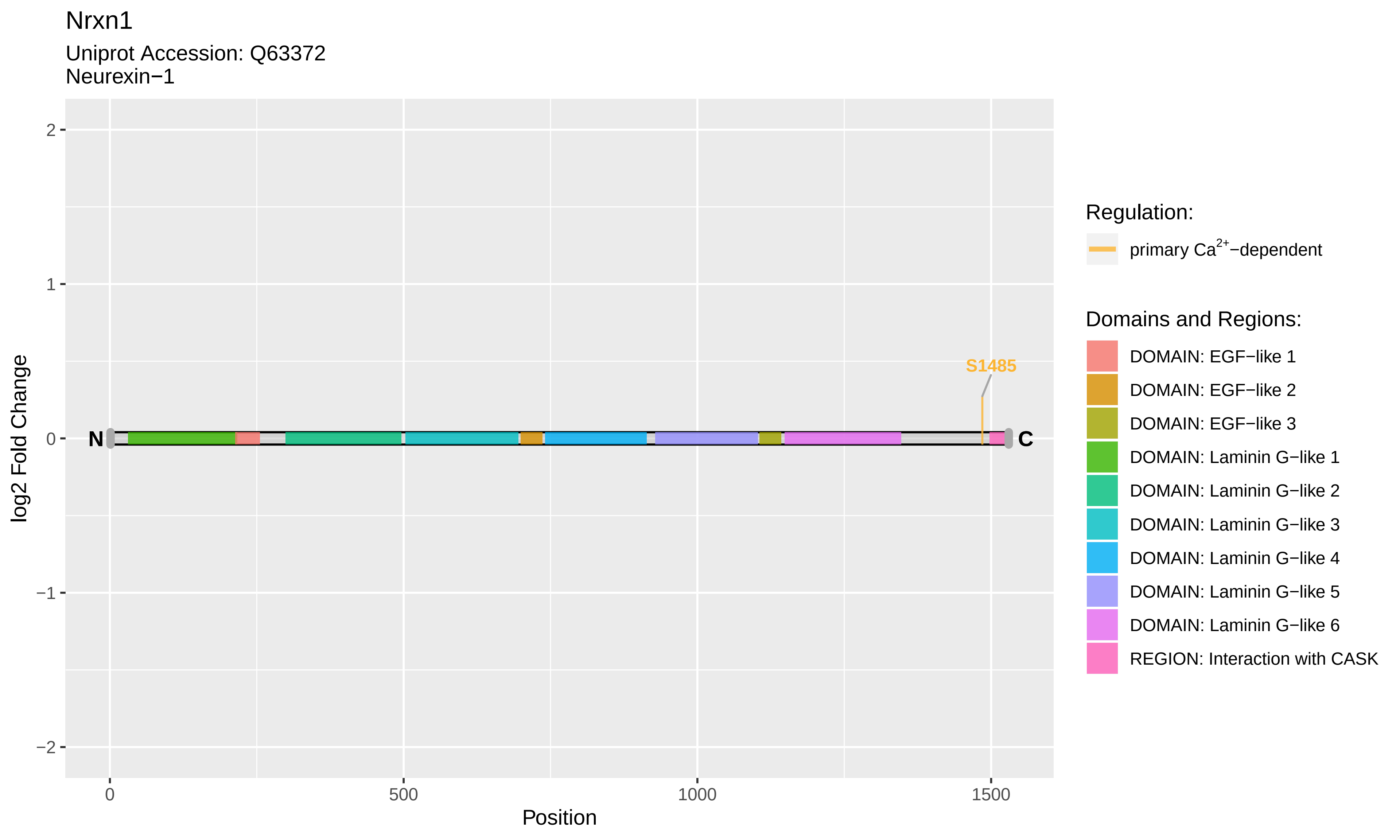

Nrxn1
Uniprot Accession: Q63372
Neurexin−1
S1485
N
C
log2 Fold Change
2
1
0
−1
−2
0
500
1000
1500
Position
Regulation:
primary Ca2+−dependent
Domains and Regions:
DOMAIN: EGF−like 1
DOMAIN: EGF−like 2
DOMAIN: EGF−like 3
DOMAIN: Laminin G−like 1
DOMAIN: Laminin G−like 2
DOMAIN: Laminin G−like 3
DOMAIN: Laminin G−like 4
DOMAIN: Laminin G−like 5
DOMAIN: Laminin G−like 6
REGION: Interaction with CASK

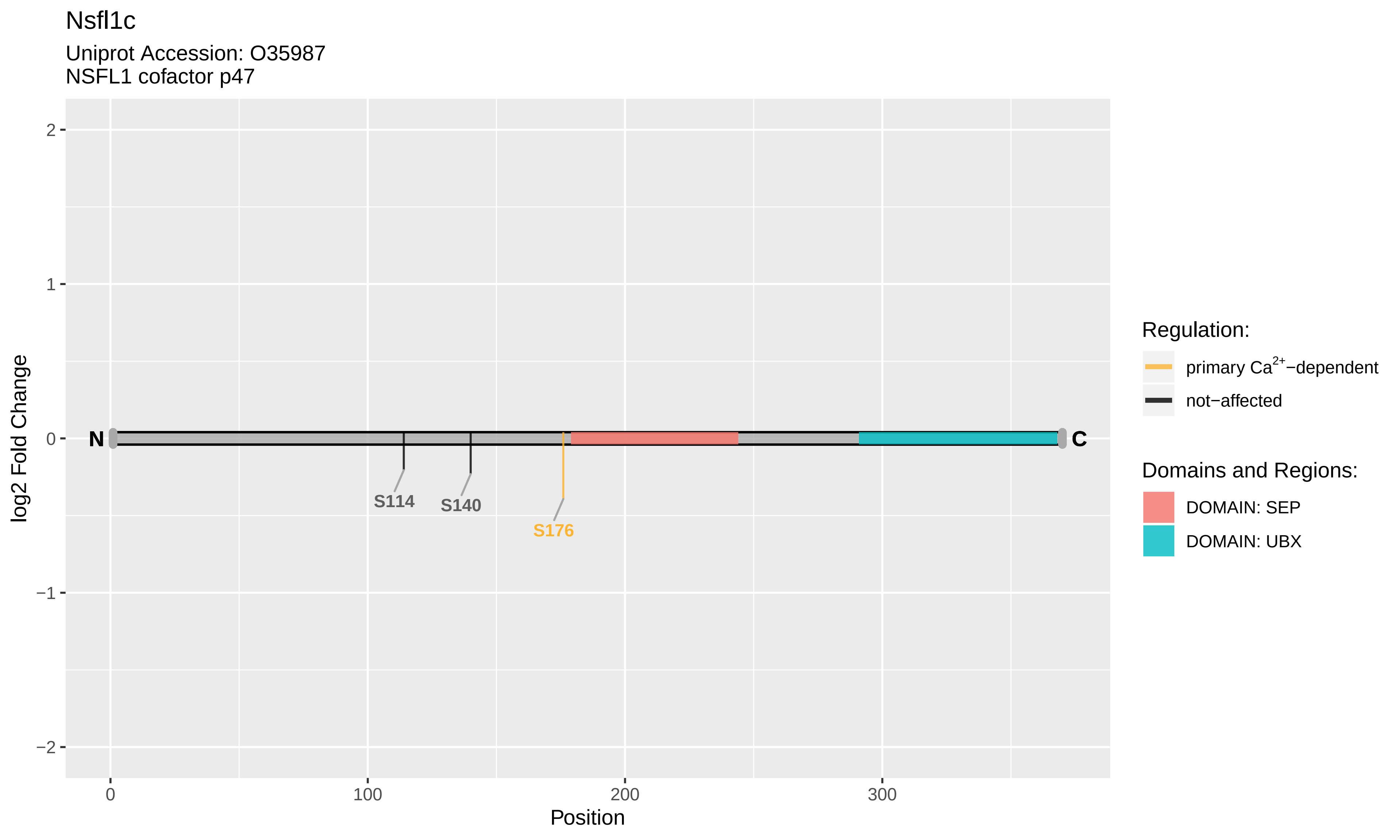

Nsfl1c
Uniprot Accession: O35987
NSFL1 cofactor p47
log2 Fold Change
2
1
0
−1
−2
N
C
S114
S140
S176
0
100
200
300
Position
Regulation:
primary Ca2+−dependent
not−affected
Domains and Regions:
DOMAIN: SEP
DOMAIN: UBX

Nufip2

Uniprot Accession: D3ZC82
Nuclear FMR1−interacting protein 2

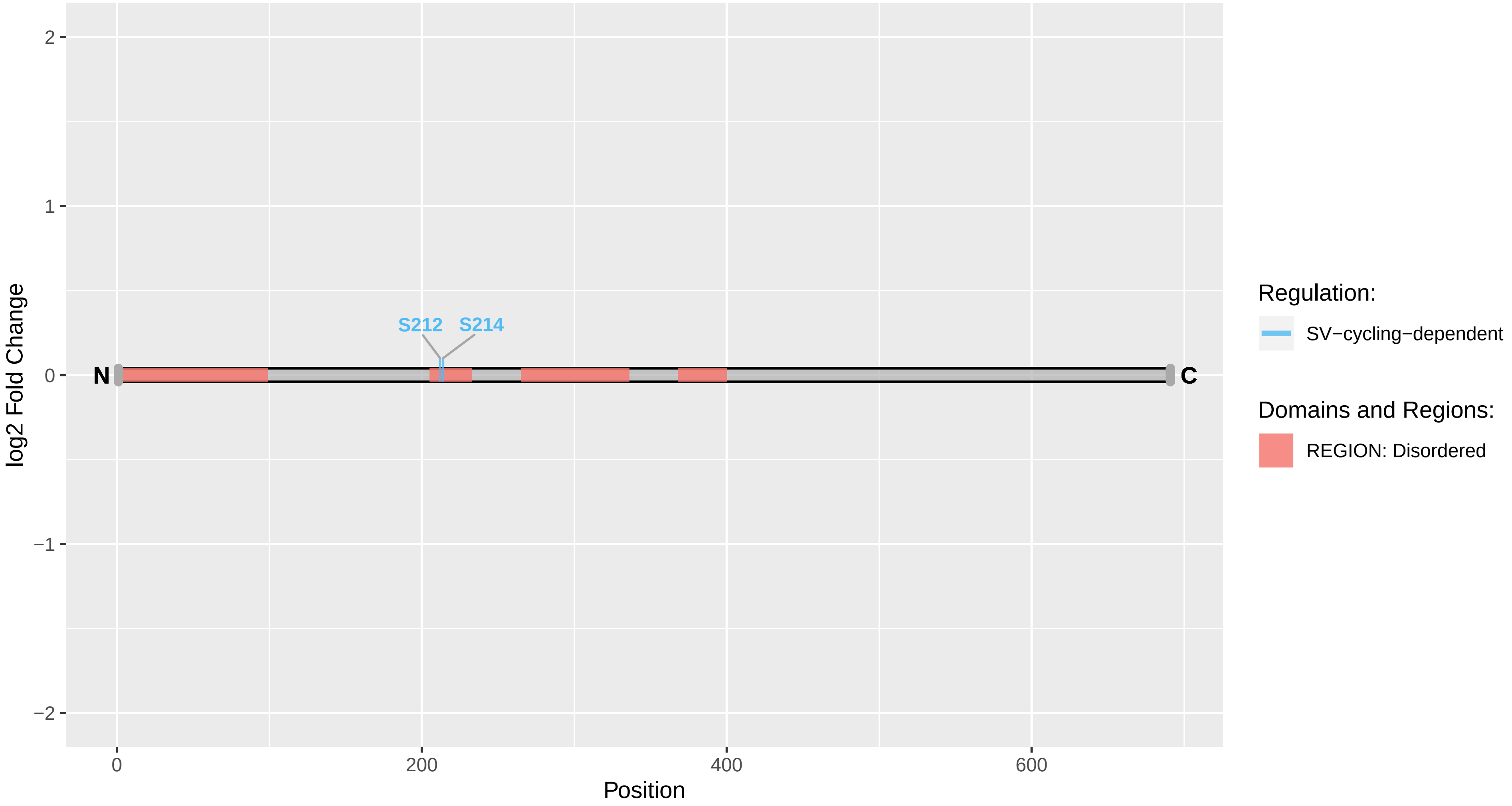

Numb

Uniprot Accession: Q2LC84
Protein numb homolog

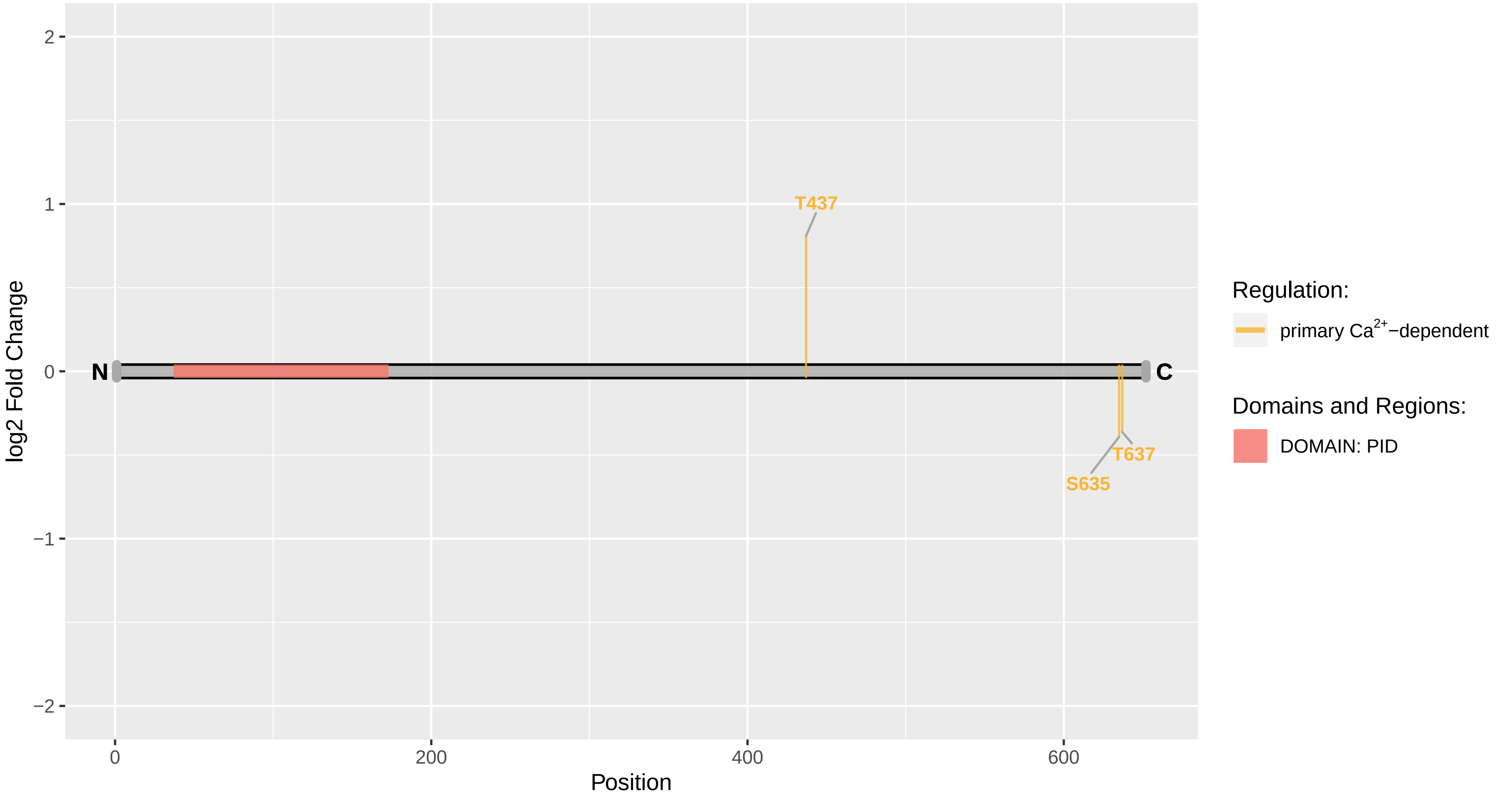

Numbl

Uniprot Accession: A1L1I3
Numb−like protein

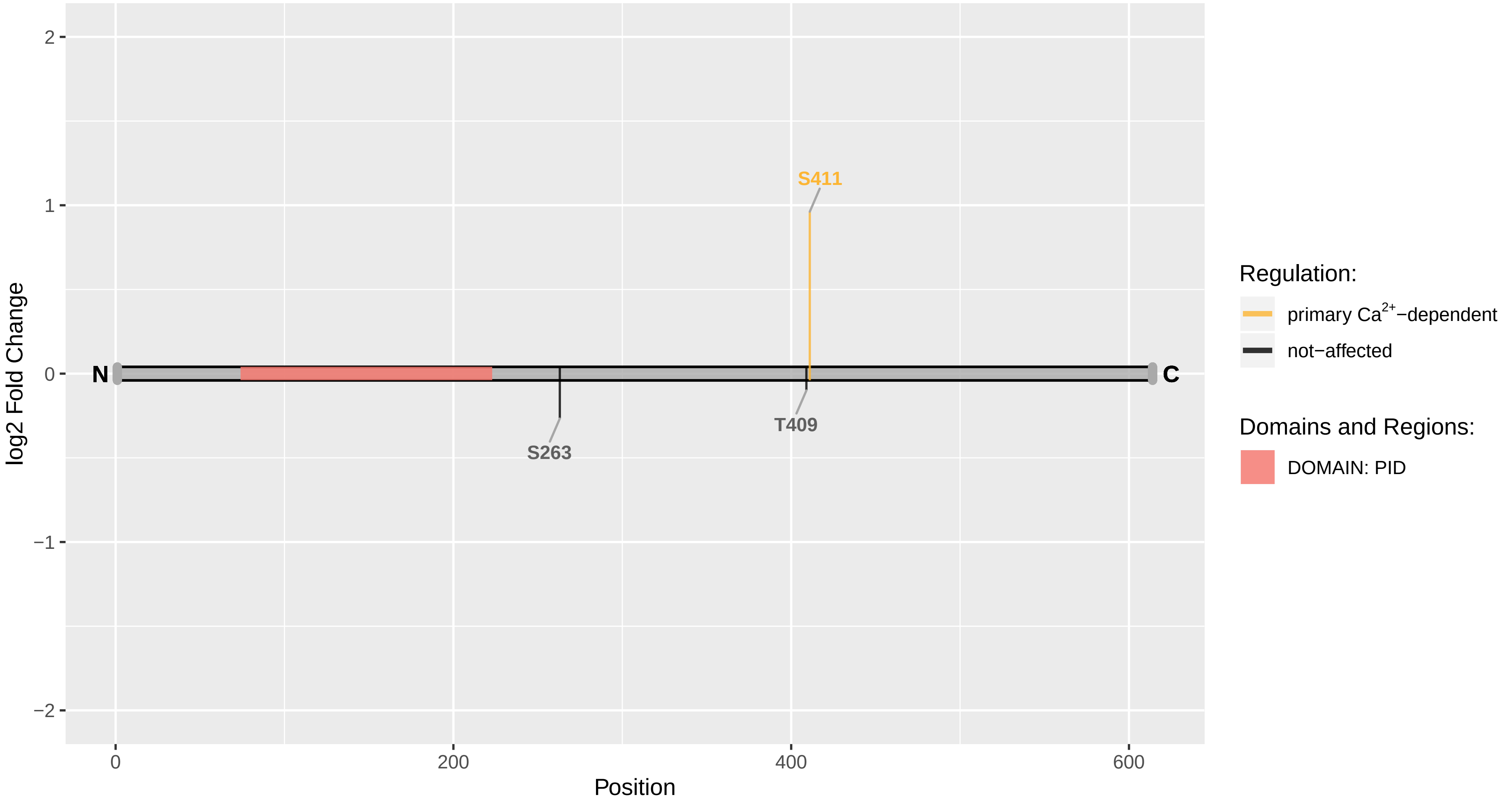

Ociad1

Uniprot Accession: Q5XIG4
OCIA domain−containing protein 1

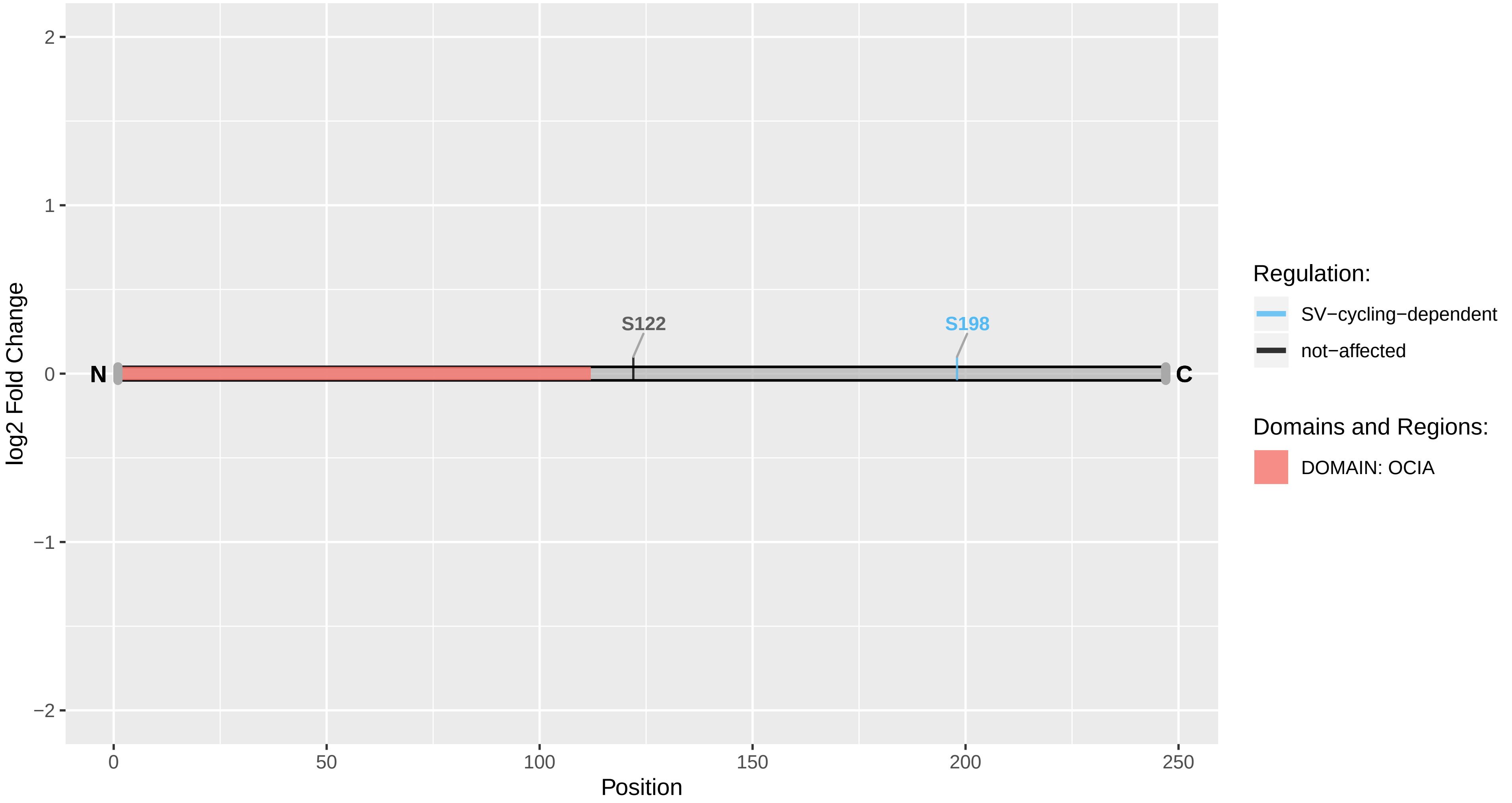

Oprm1

Uniprot Accession: P33535
Mu−type opioid receptor

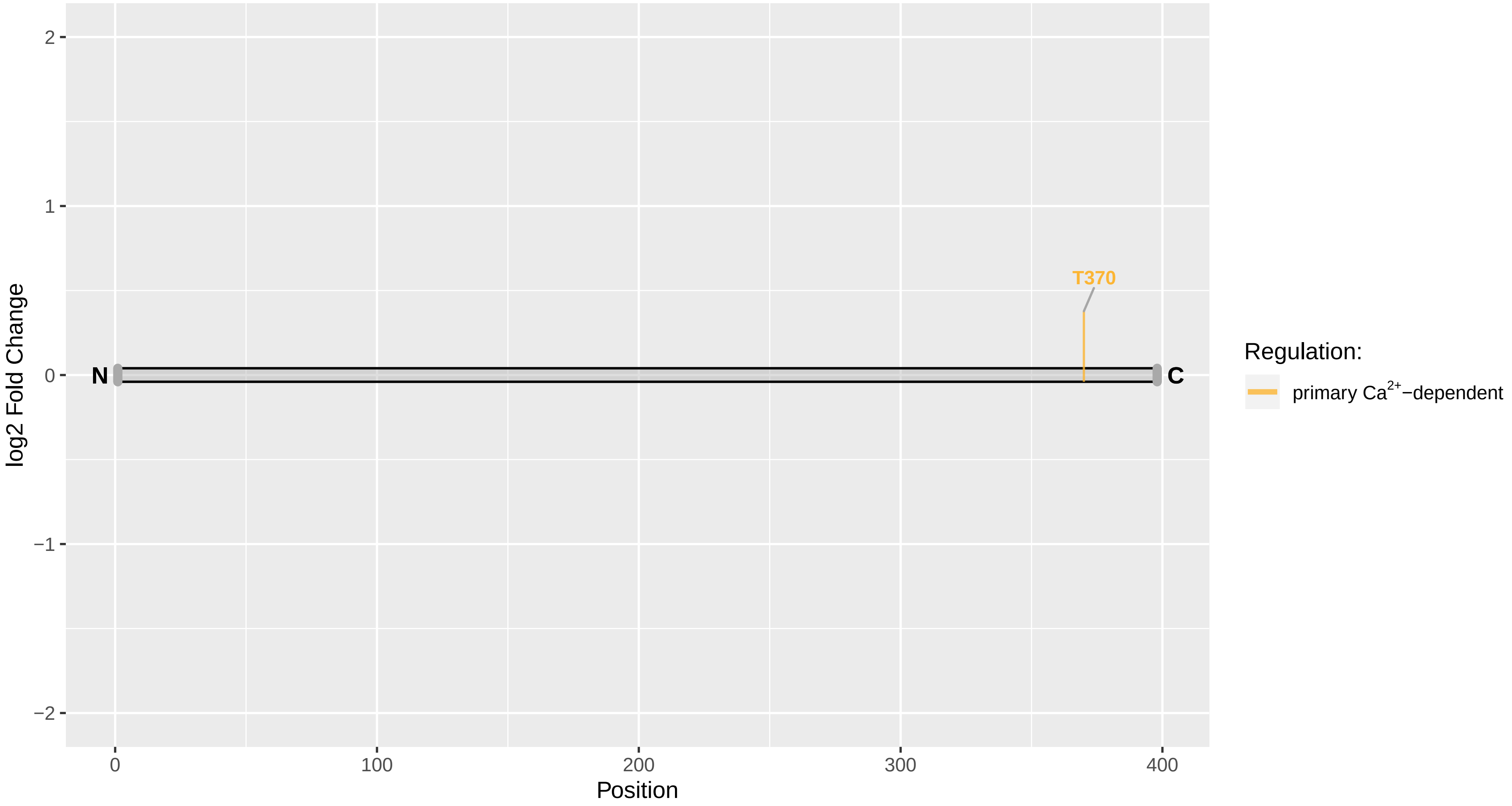

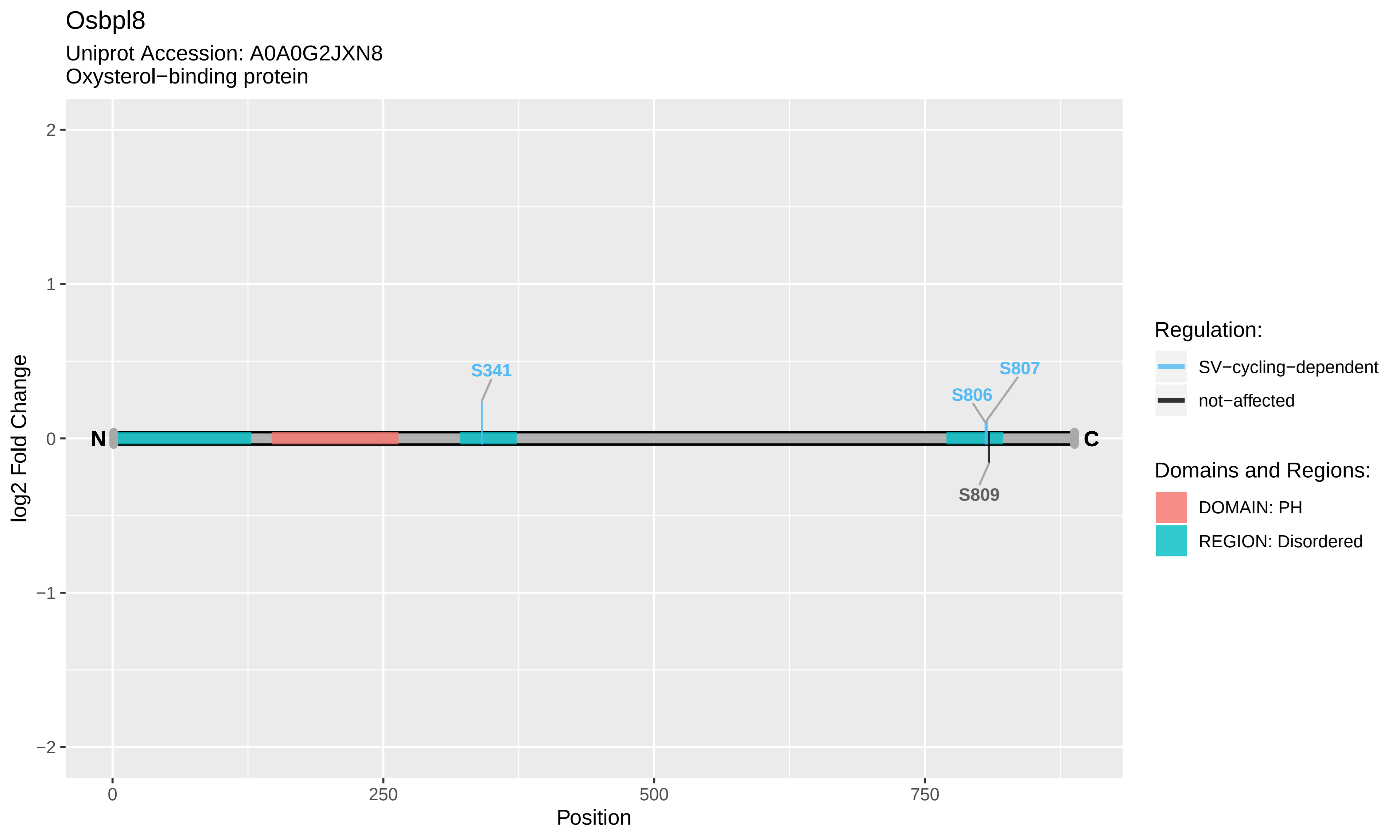
Osbpl8
Uniprot Accession: A0A0G2JXN8
Oxysterol−binding protein
log2 Fold Change
2
1
0
−1
−2
0
250
500
750
Position
N
C
S341
S806
S807
S809
Regulation:
SV−cycling−dependent
not−affected
Domains and Regions:
DOMAIN: PH
REGION: Disordered

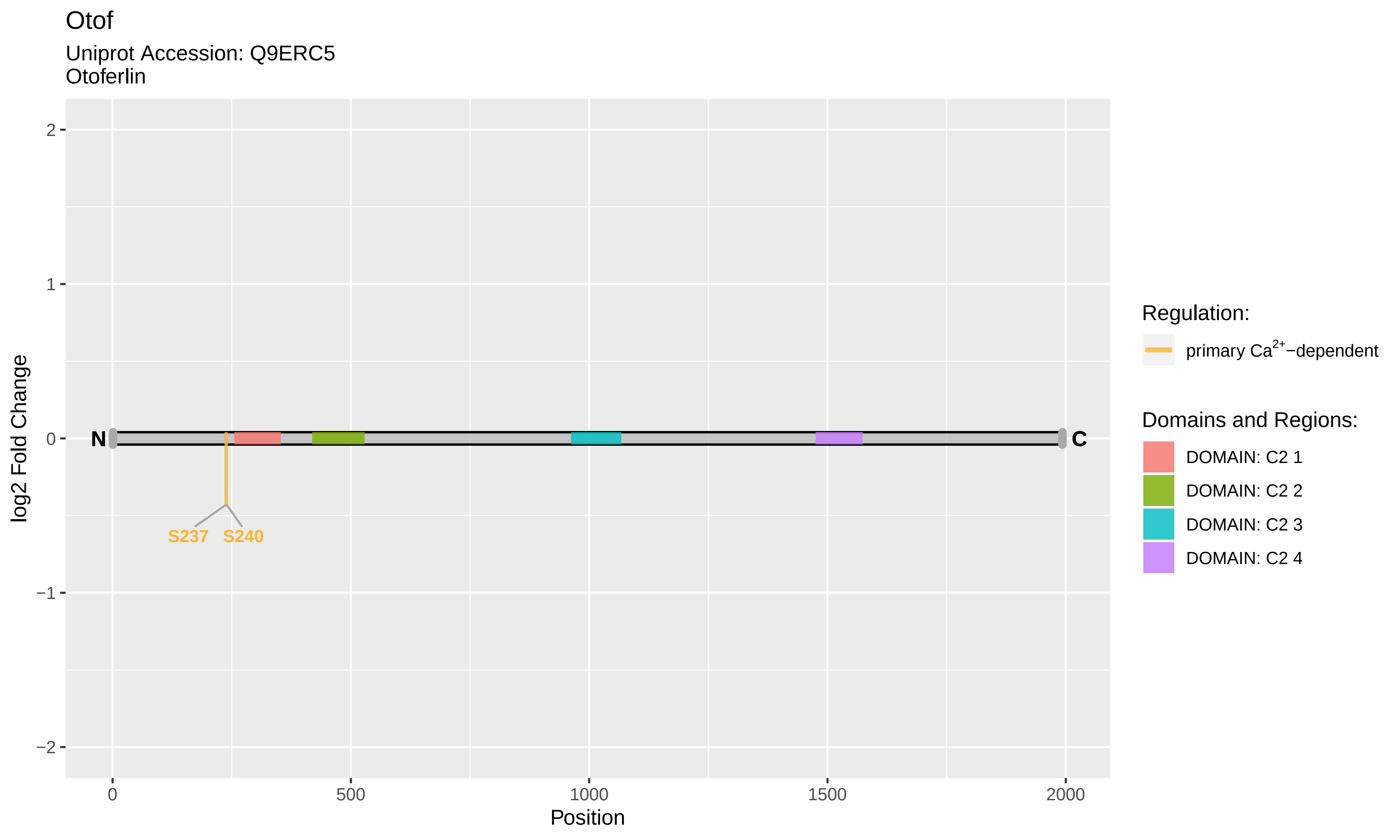

Otof
Uniprot Accession: Q9ERC5
Otoferlin
N
C
S237
S240
log2 Fold Change
Position
2
1
0
−1
−2
0
500
1000
1500
2000
Regulation:
primary Ca2+−dependent
Domains and Regions:
DOMAIN: C2 1
DOMAIN: C2 2
DOMAIN: C2 3
DOMAIN: C2 4

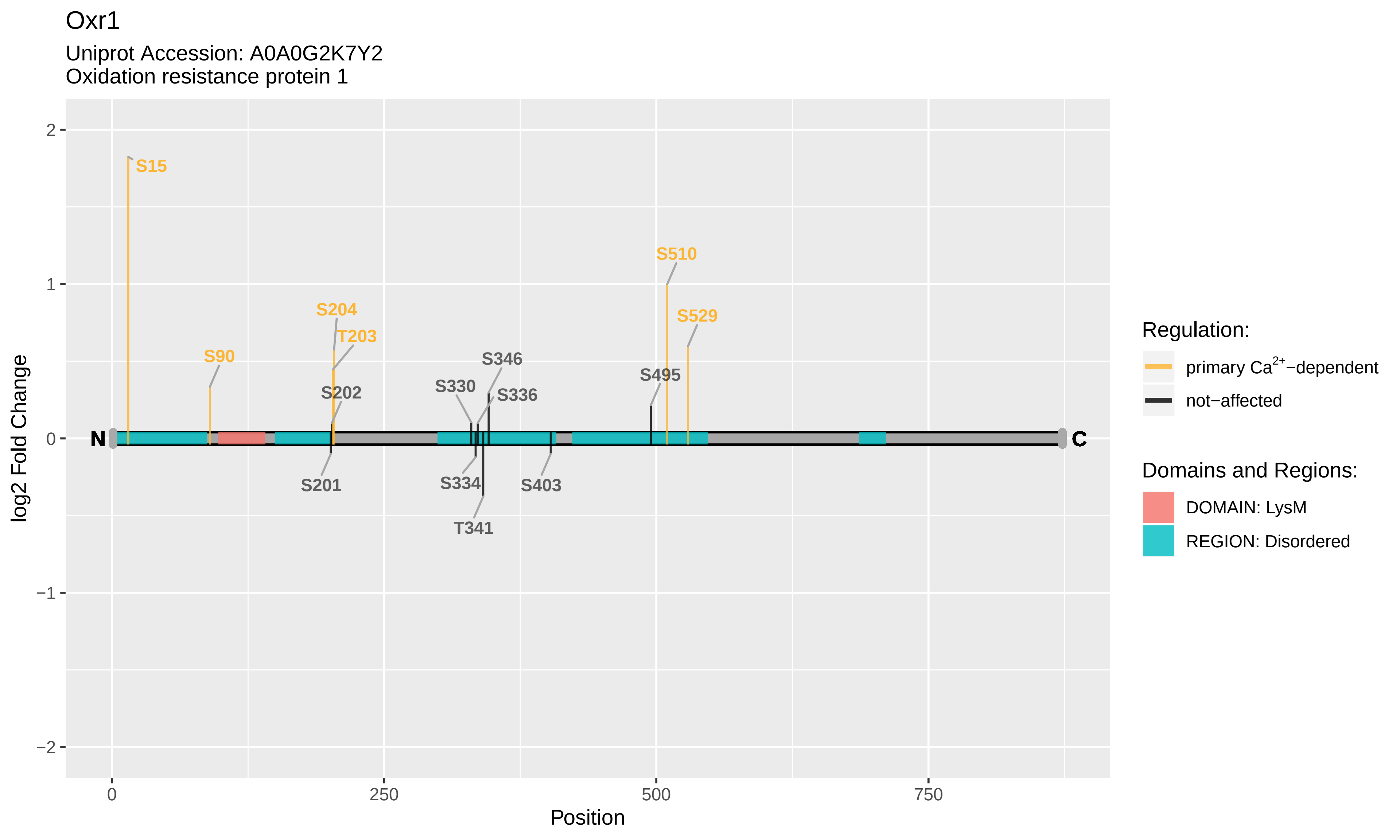

Oxr1
Uniprot Accession: A0A0G2K7Y2
Oxidation resistance protein 1
log2 Fold Change
Position
2
1
0
−1
−2
0
250
500
750
N
C
S15
S90
S204
T203
S202
S201
S330
S346
S336
S334
T341
S403
S495
S510
S529
Regulation:
primary Ca2+−dependent
not−affected
Domains and Regions:
DOMAIN: LysM
REGION: Disordered

Oxsr1

Uniprot Accession: D3ZUC9
Oxidative stress−responsive kinase 1

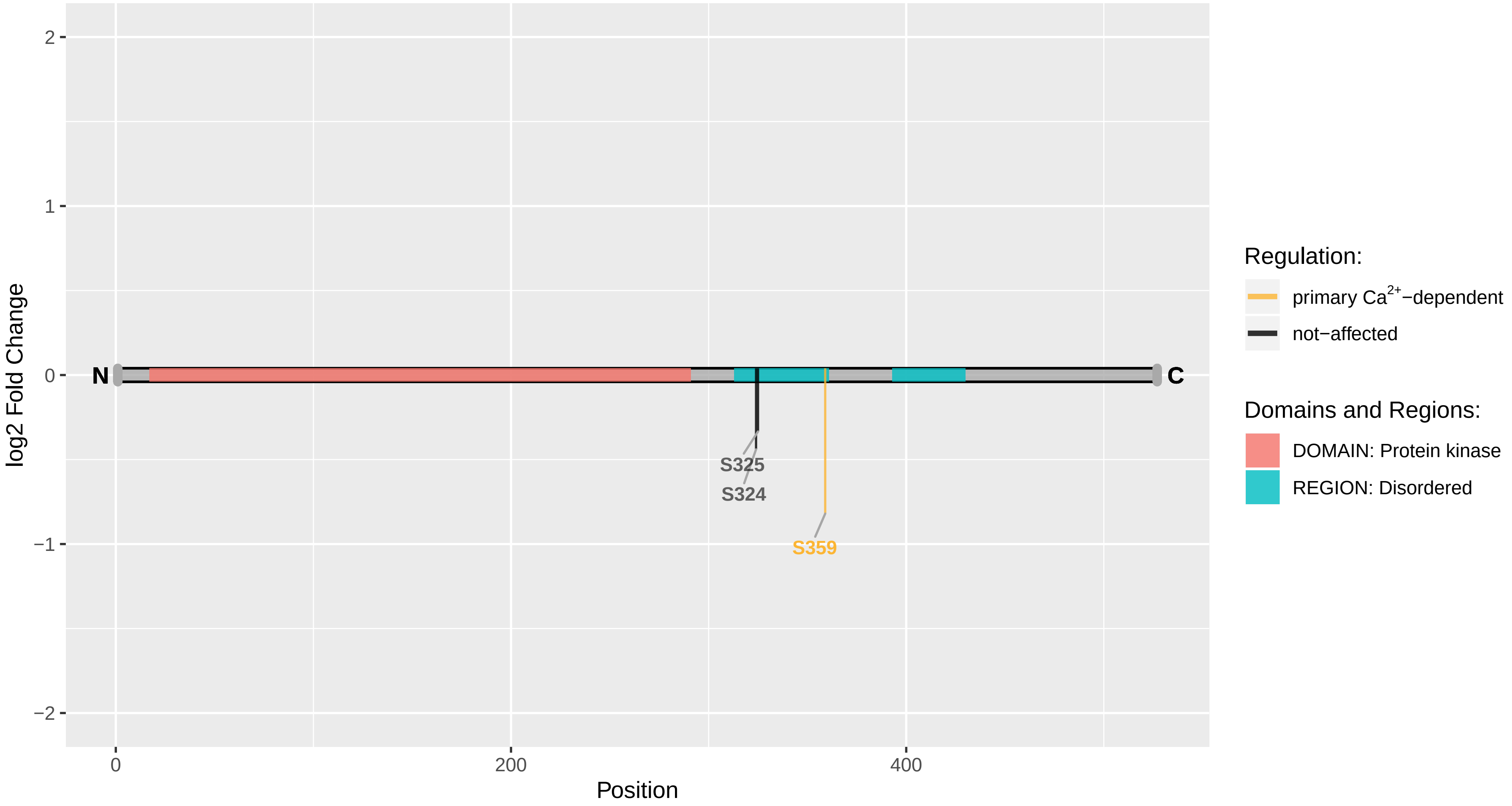

P33monox

Uniprot Accession: Q5U2R6
Putative monooxygenase p33MONOX

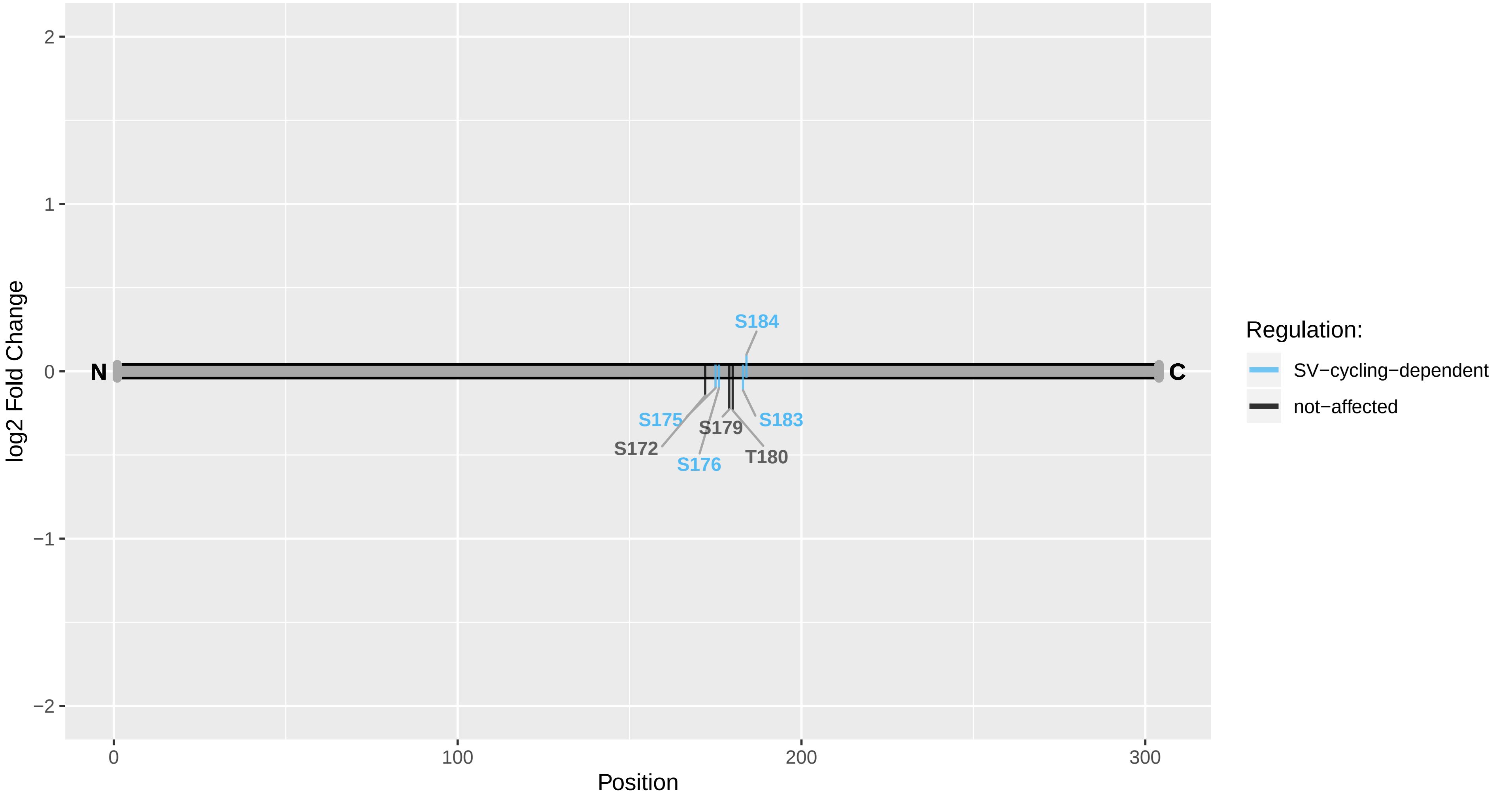

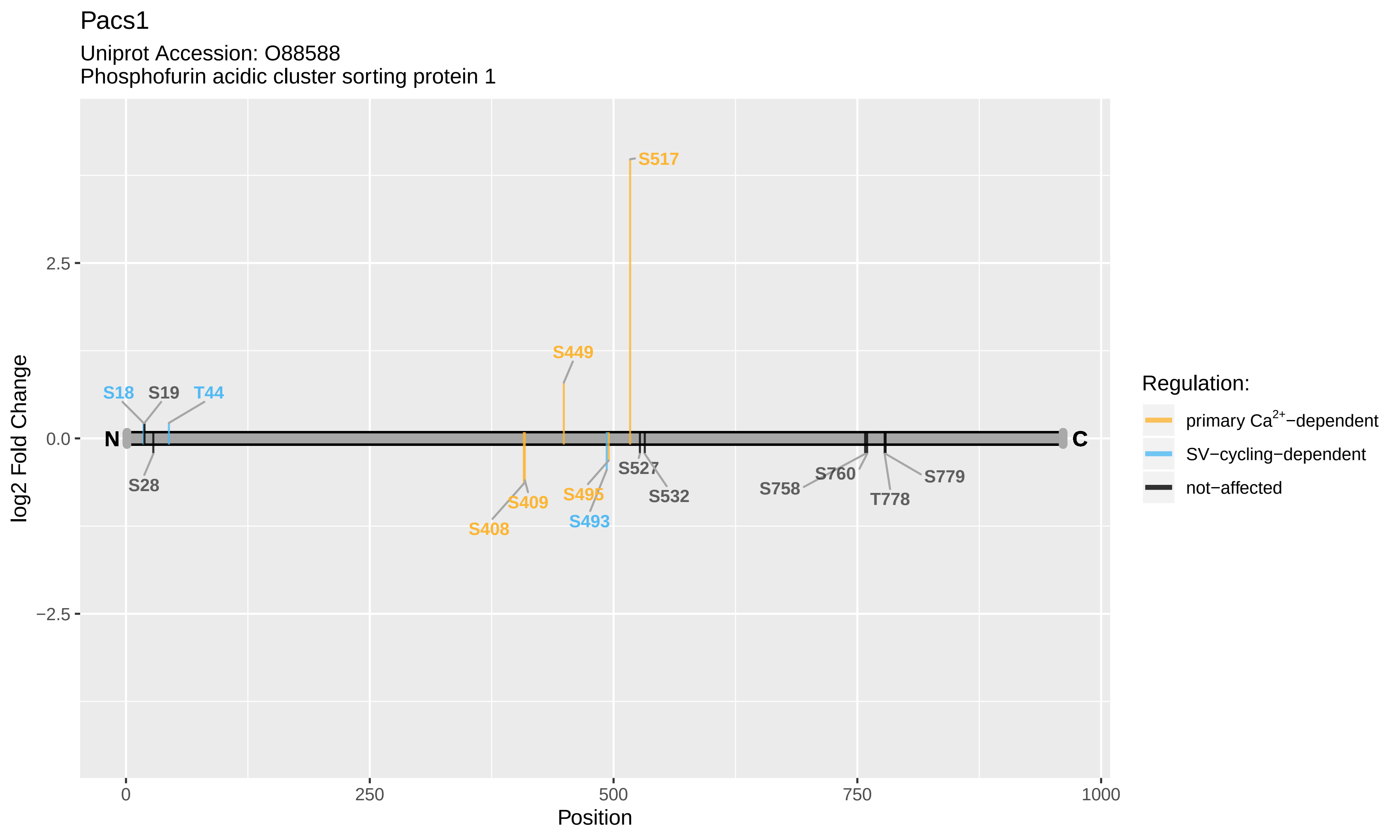

Pacs1
Uniprot Accession: O88588
Phosphofurin acidic cluster sorting protein 1
S517
S449
S18
S19
T44
N
C
S28
S408
S409
S495
S493
S527
S532
S758
S760
T778
S779
2.5
0.0
−2.5
0
250
500
750
1000
log2 Fold Change
Position
Regulation:
primary Ca^{2+}−dependent
SV−cycling−dependent
not−affected

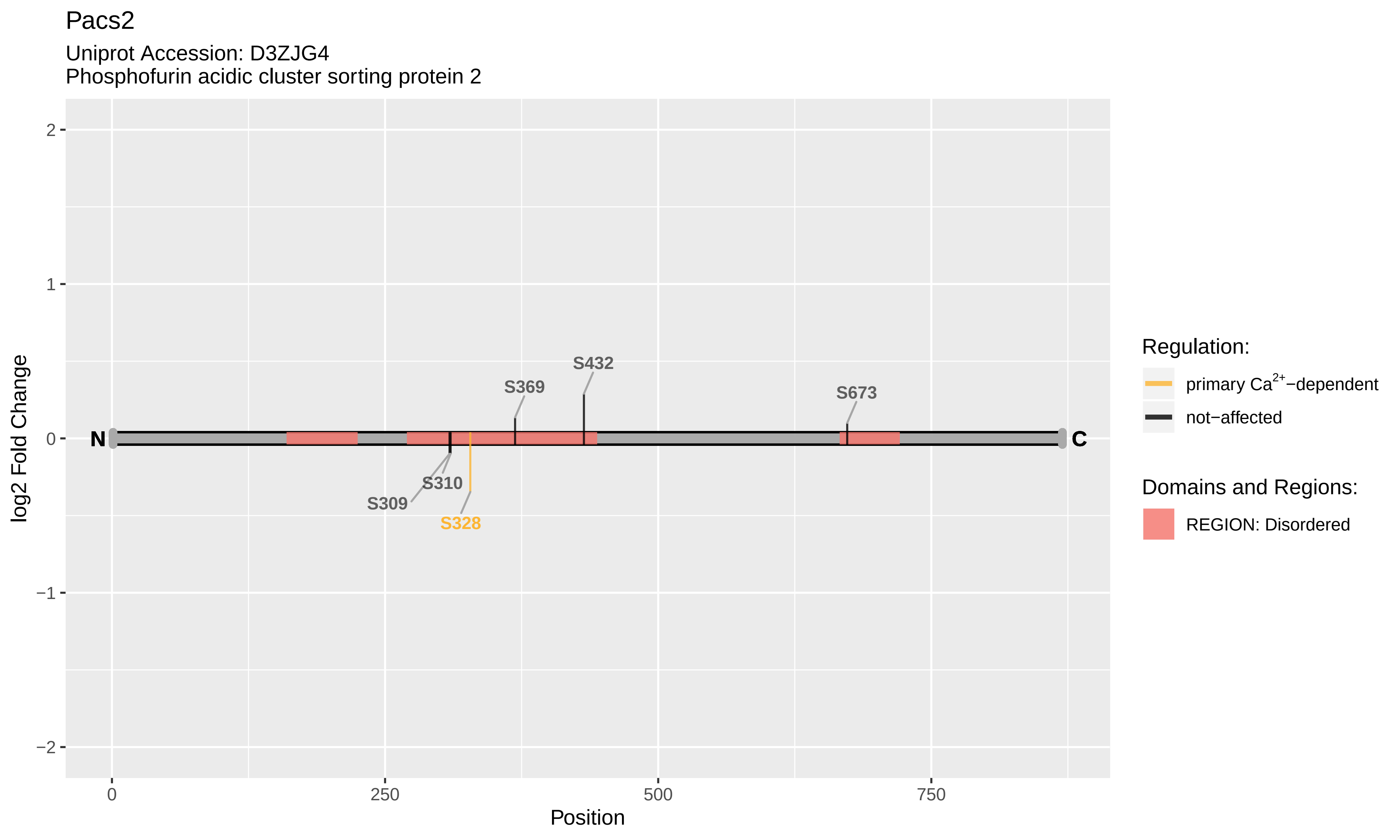
Pacs2
Uniprot Accession: D3ZJG4
Phosphofurin acidic cluster sorting protein 2
log2 Fold Change
2
1
0
−1
−2
N
C
S432
S369
S673
S310
S309
S328
0
250
500
750
Position
Regulation:
primary Ca2+−dependent
not−affected
Domains and Regions:
REGION: Disordered

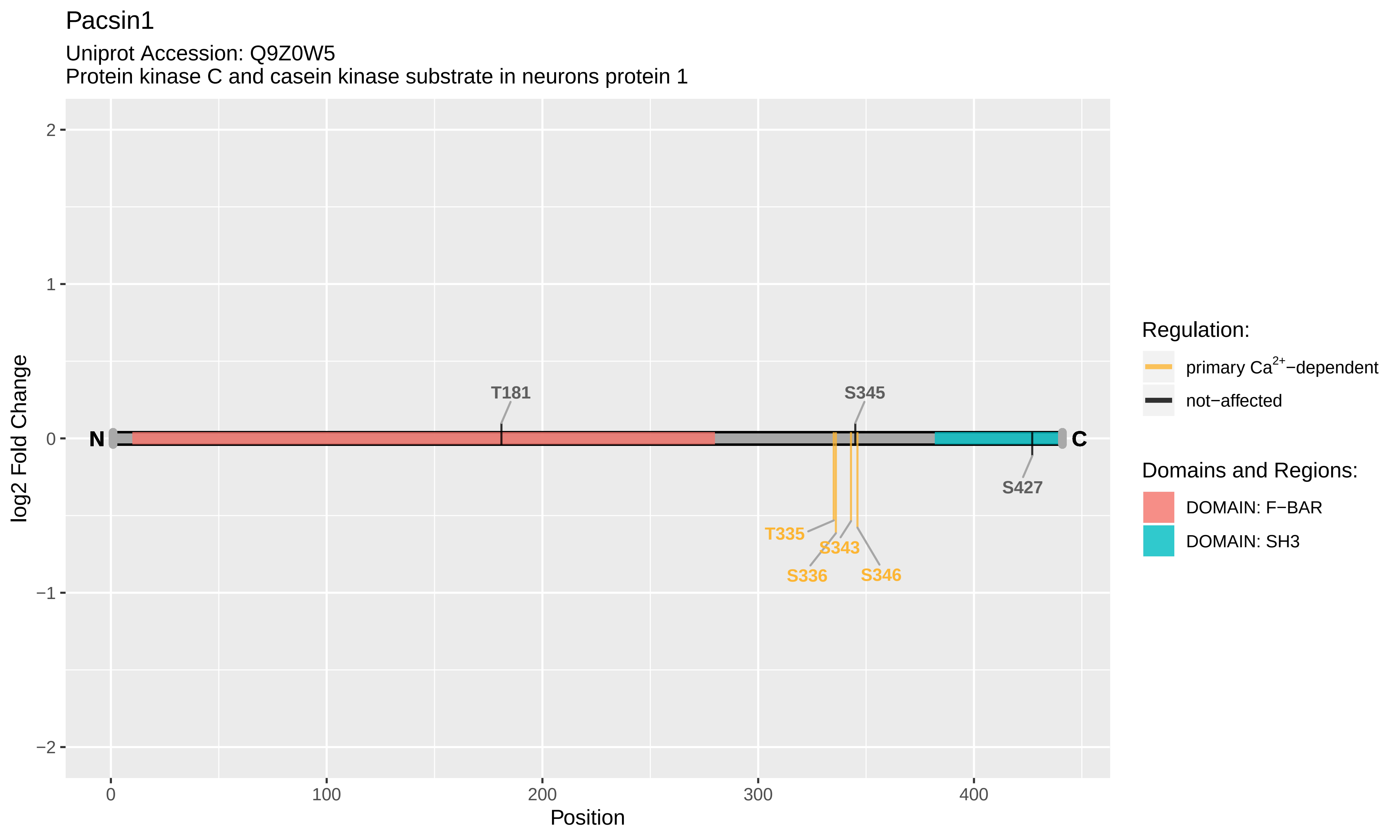

Pacsin1
Uniprot Accession: Q9Z0W5
Protein kinase C and casein kinase substrate in neurons protein 1
log2 Fold Change
2
1
0
−1
−2
N
C
T181
S345
S427
T335
S343
S336
S346
0
100
200
300
400
Position
Regulation:
primary Ca2+−dependent
not−affected
Domains and Regions:
DOMAIN: F−BAR
DOMAIN: SH3

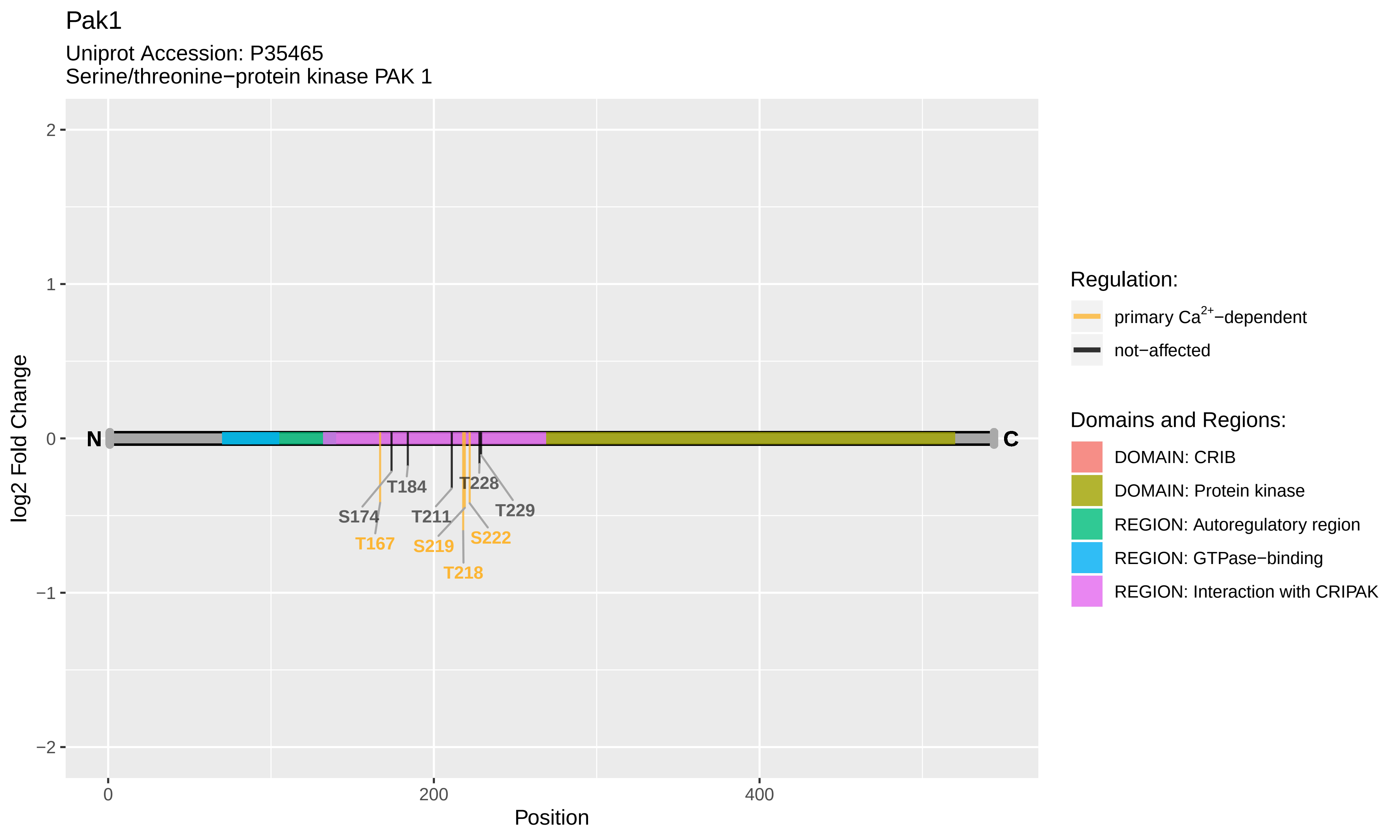

Pak1
Uniprot Accession: P35465
Serine/threonine−protein kinase PAK 1
log2 Fold Change
2
1
0
−1
−2
N
C
T184
T228
S174
T211
T229
T167
S219
S222
T218
0
200
400
Position
Regulation:
primary Ca2+−dependent
not−affected
Domains and Regions:
DOMAIN: CRIB
DOMAIN: Protein kinase
REGION: Autoregulatory region
REGION: GTPase−binding
REGION: Interaction with CRIPAK

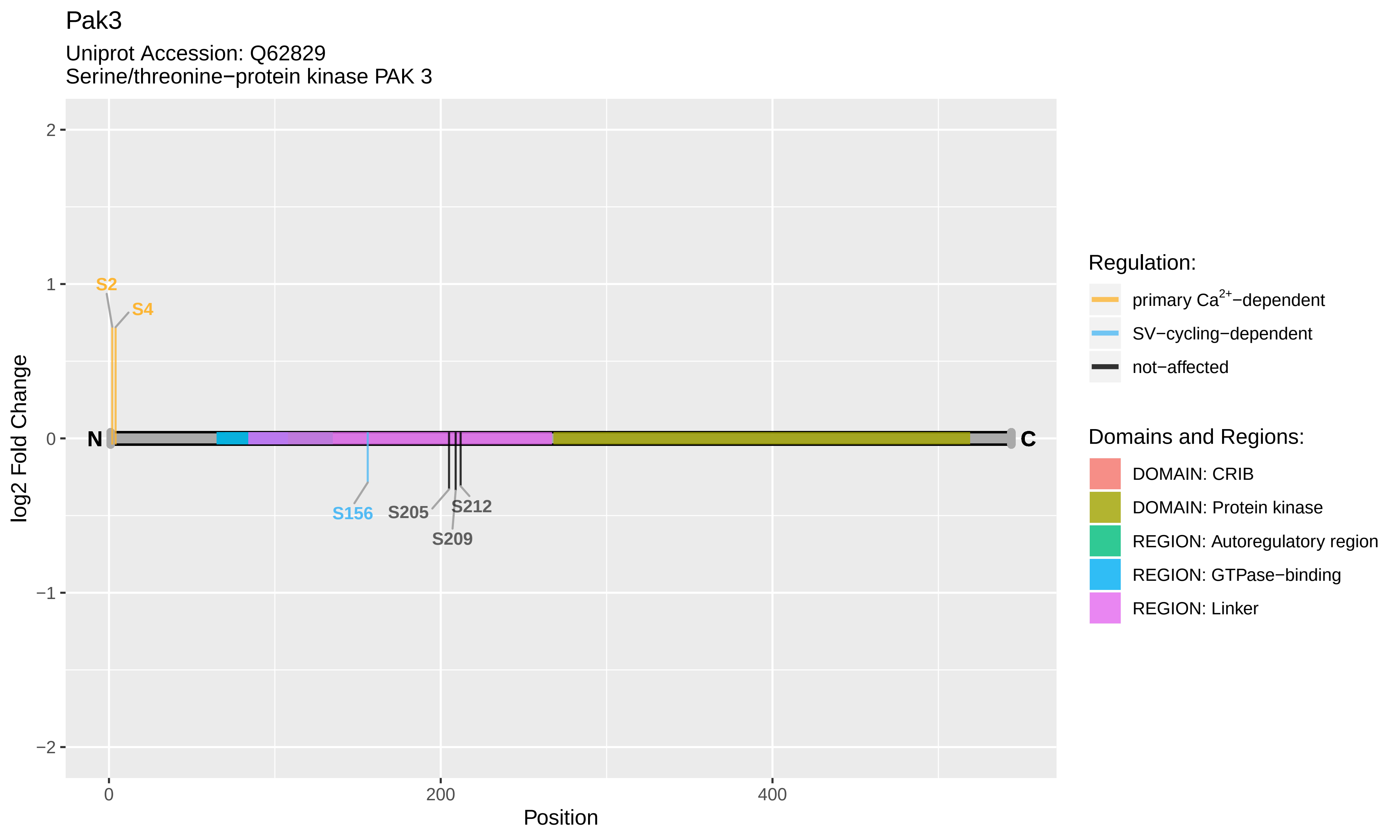

Pak3
Uniprot Accession: Q62829
Serine/threonine−protein kinase PAK 3
S2
S4
N
C
S156
S205
S212
S209
log2 Fold Change
2
1
0
−1
−2
0
200
400
Position
Regulation:
primary Ca2+−dependent
SV−cycling−dependent
not−affected
Domains and Regions:
DOMAIN: CRIB
DOMAIN: Protein kinase
REGION: Autoregulatory region
REGION: GTPase−binding
REGION: Linker

Palm

Uniprot Accession: Q920Q0
Paralemmin−1

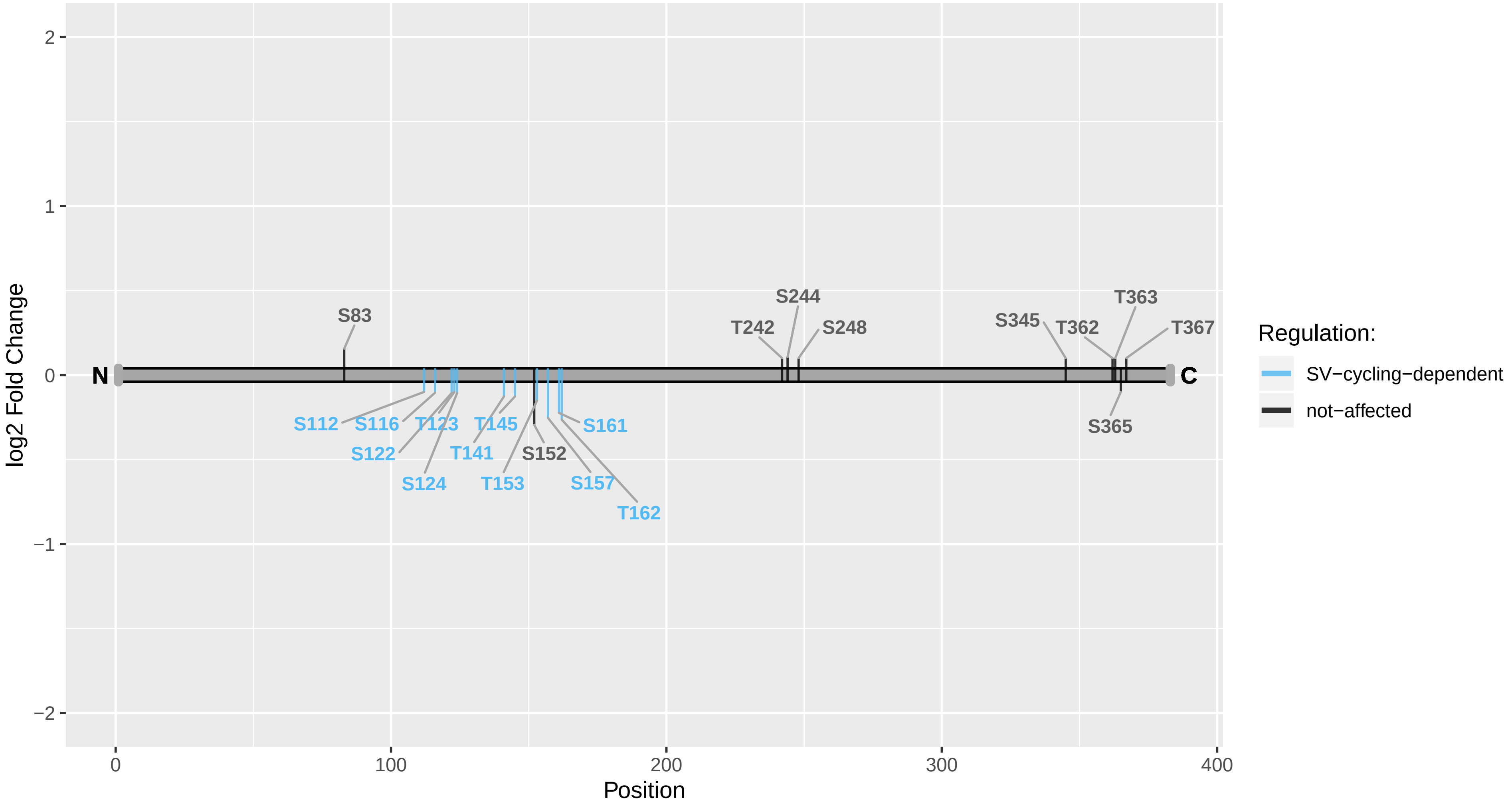

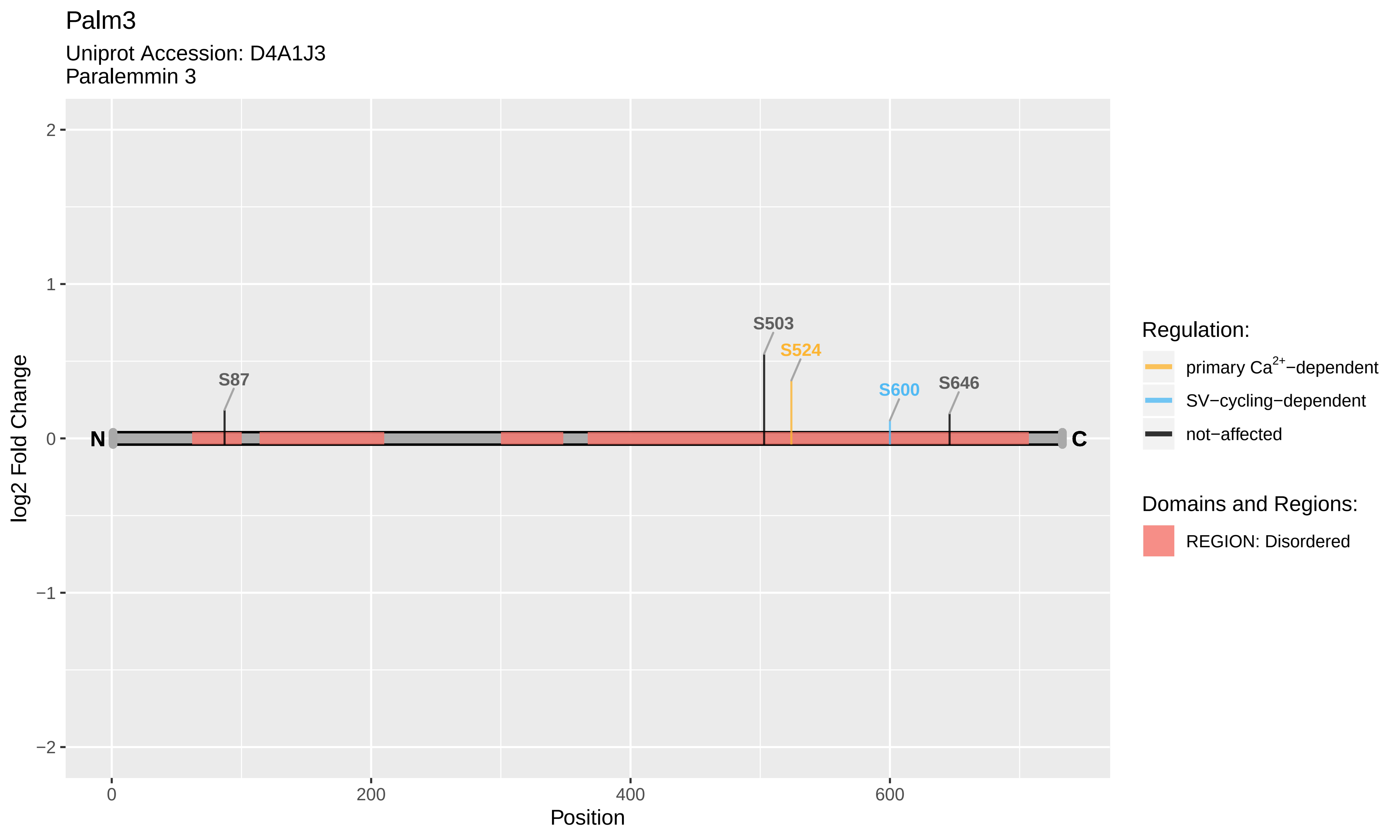

Palm3
Uniprot Accession: D4A1J3
Paralemmin 3
log2 Fold Change
Position
N
C
S87
S503
S524
S600
S646
Regulation:
primary Ca2+−dependent
SV−cycling−dependent
not−affected
Domains and Regions:
REGION: Disordered

Palmd

Uniprot Accession: Q4KM62
Palmdelphin

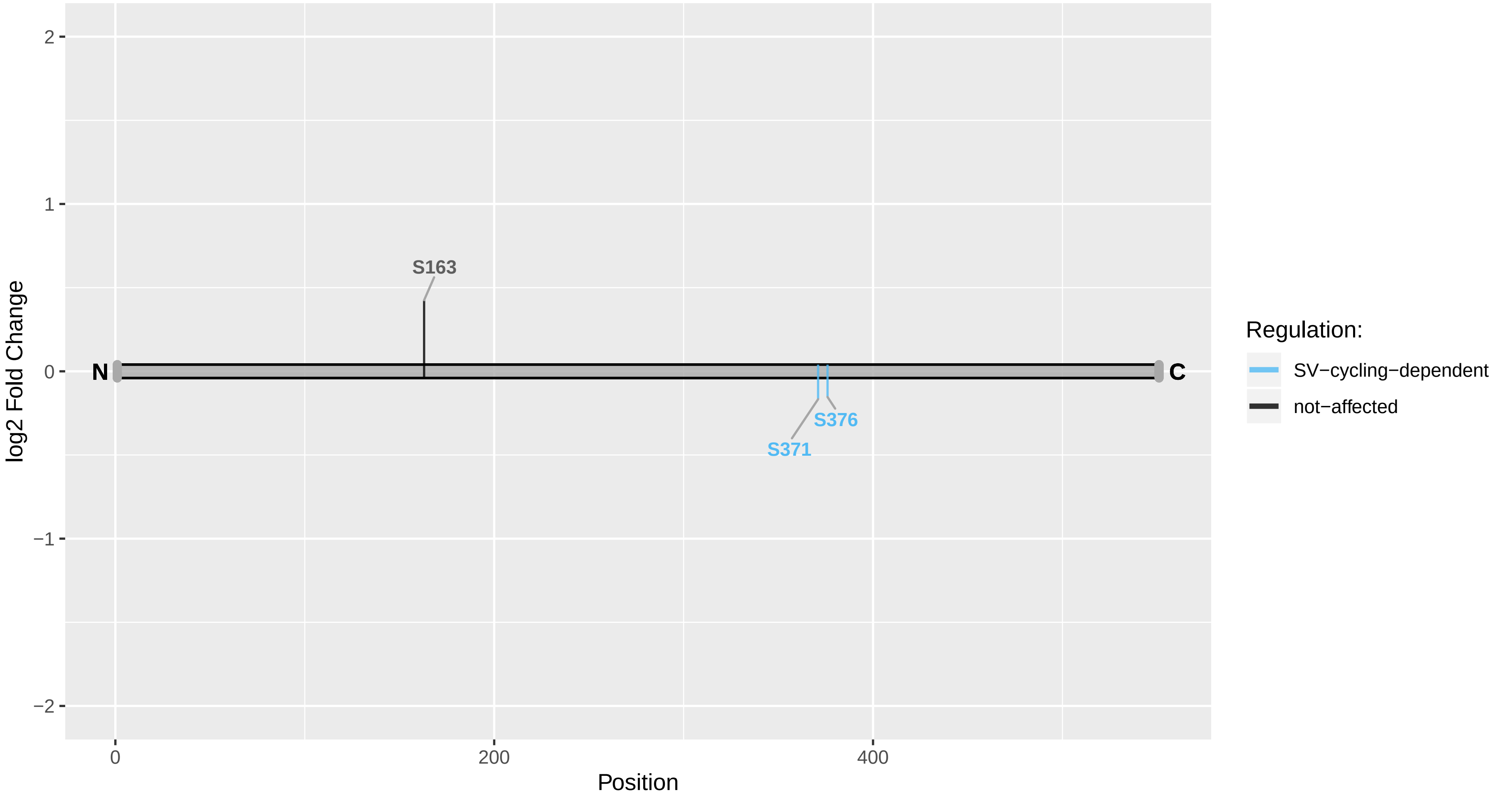

Pank4

Uniprot Accession: G3V7T3
Pantothenate kinase 4

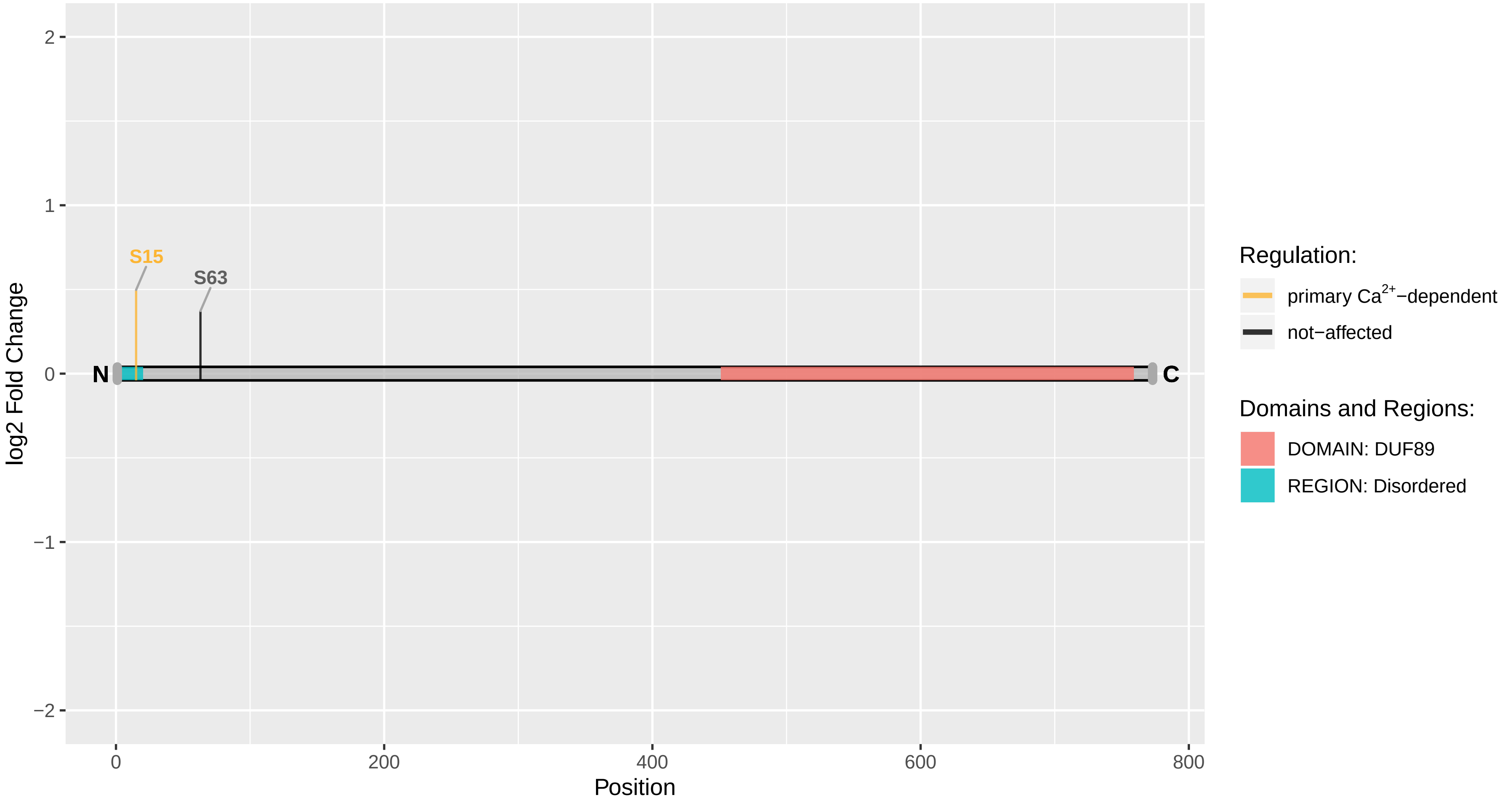

Parp9

Uniprot Accession: A1A5Q1
Poly [ADP−ribose] polymerase

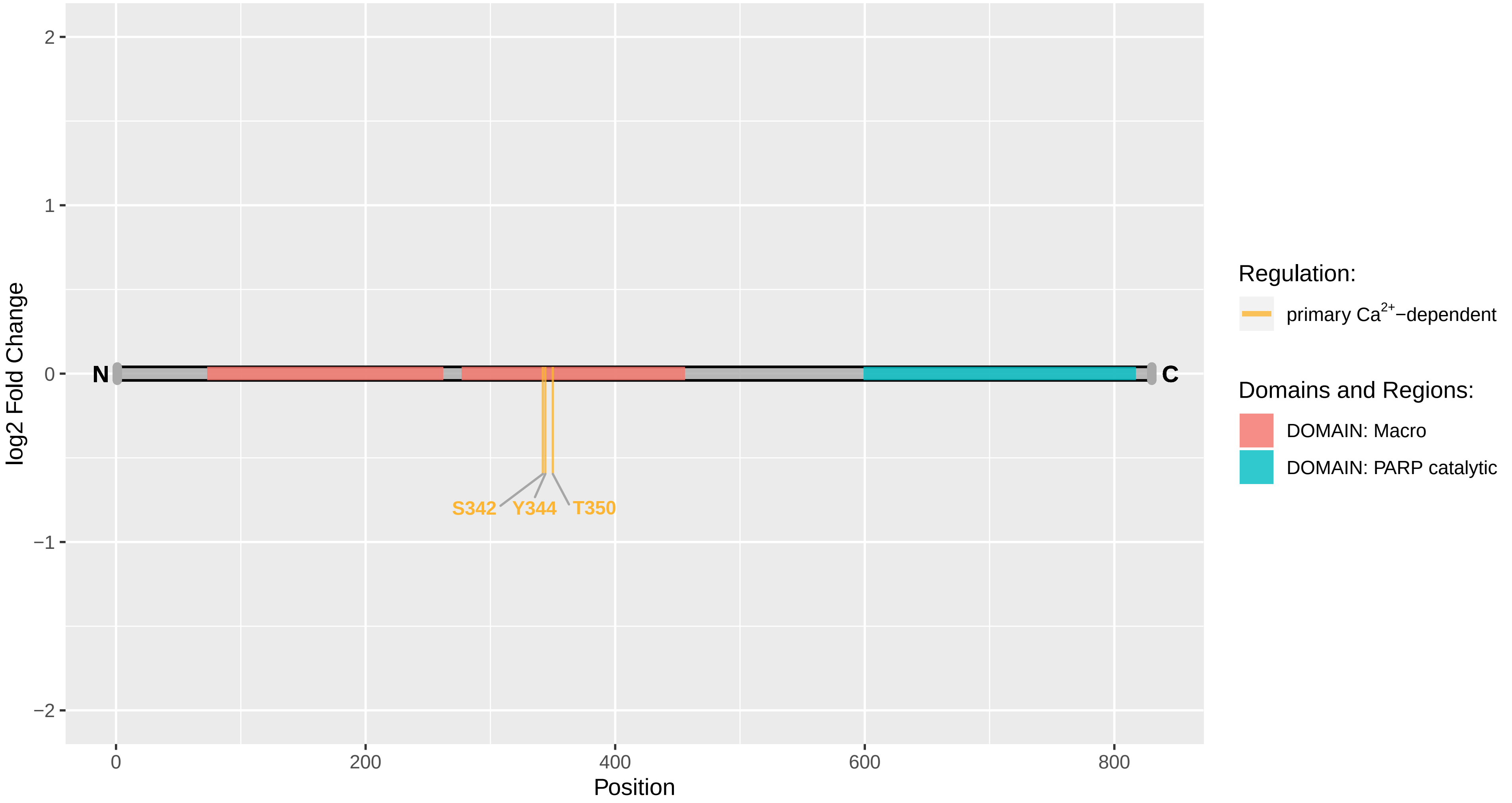

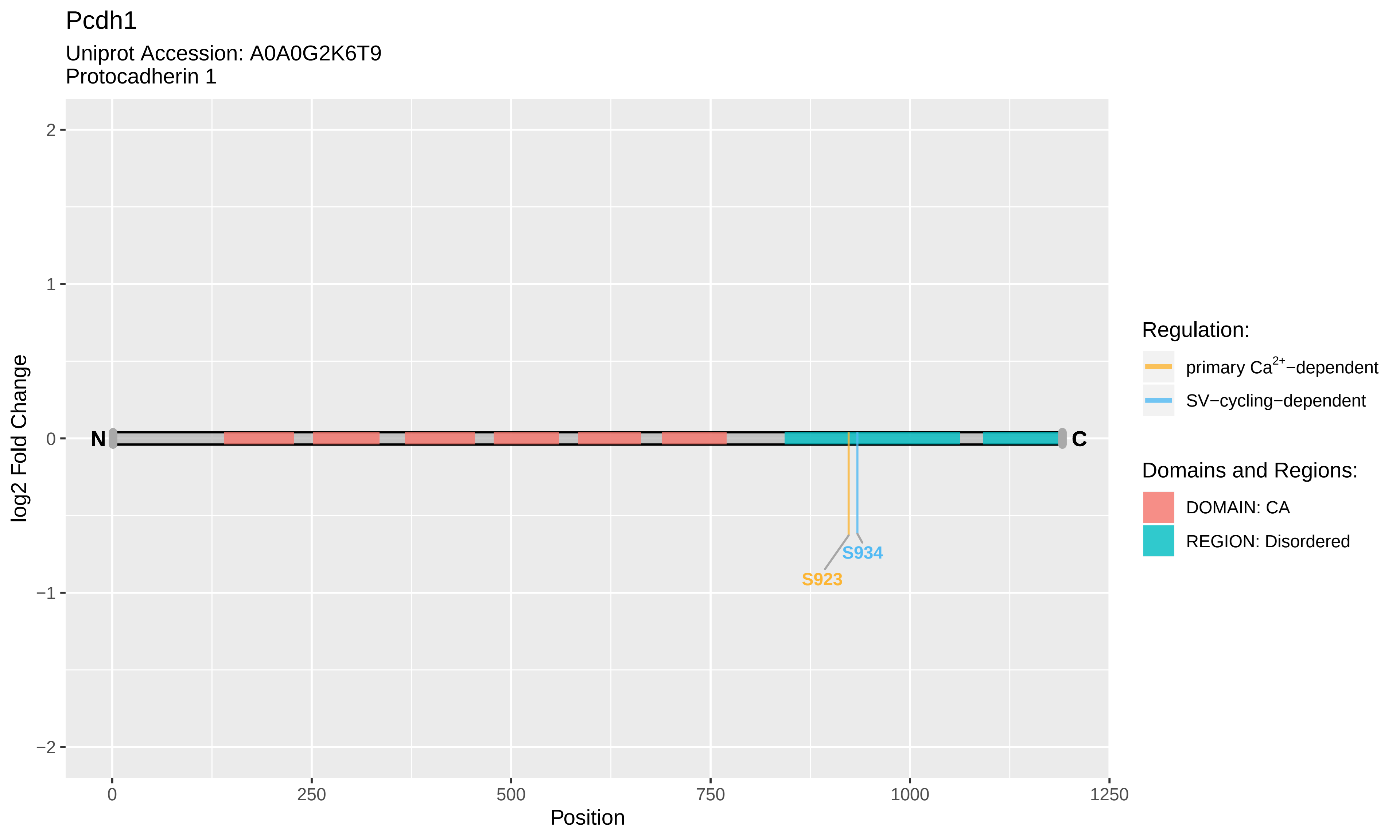

Pcdh1
Uniprot Accession: A0A0G2K6T9
Protocadherin 1
2
1
0
−1
−2
log2 Fold Change
N
C
S934
S923
0
250
500
750
1000
1250
Position
Regulation:
primary Ca2+−dependent
SV−cycling−dependent
Domains and Regions:
DOMAIN: CA
REGION: Disordered

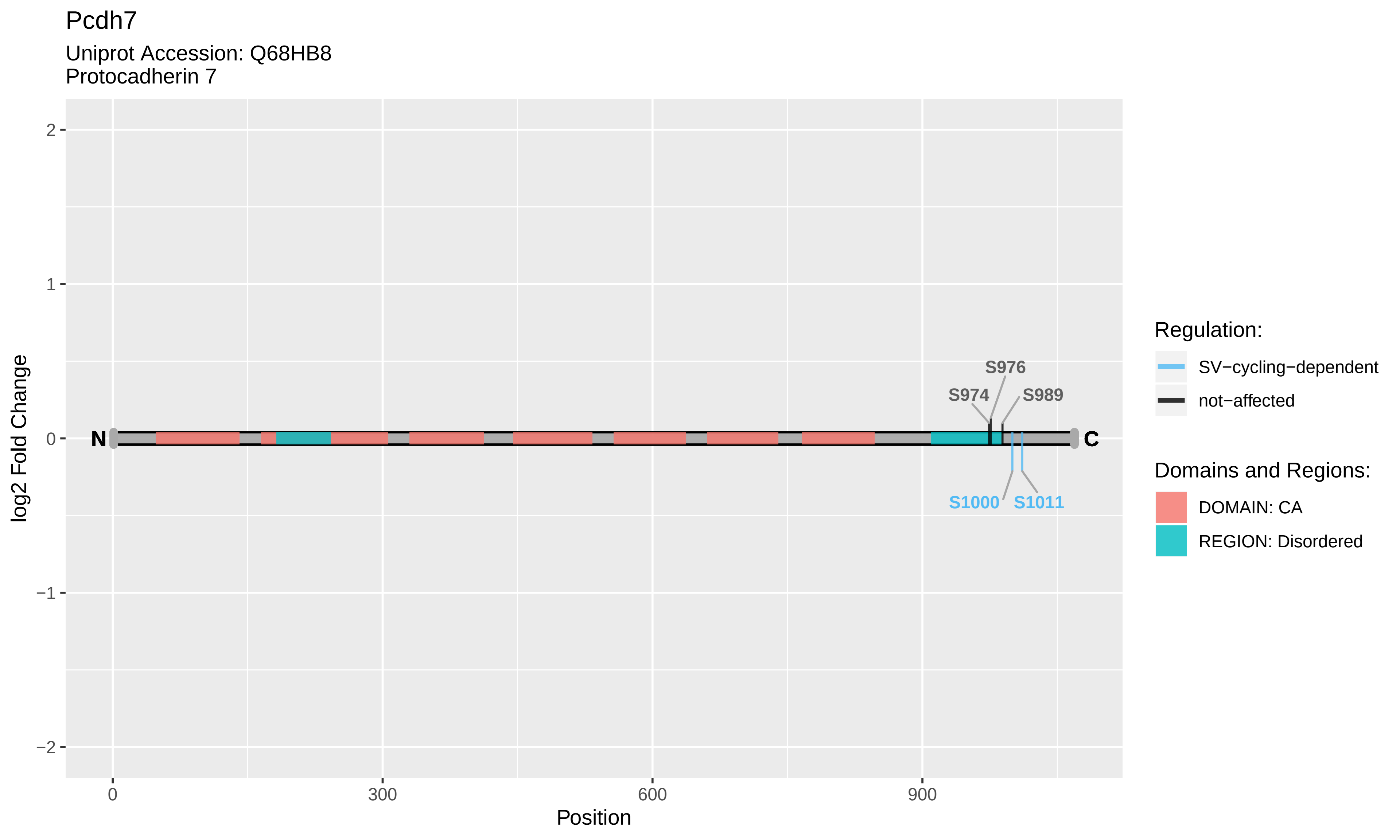

Pcdh7
Uniprot Accession: Q68HB8
Protocadherin 7
log2 Fold Change
2
1
0
−1
−2
N
C
S976
S974
S989
S1000
S1011
0
300
600
900
Position
Regulation:
SV−cycling−dependent
not−affected
Domains and Regions:
DOMAIN: CA
REGION: Disordered

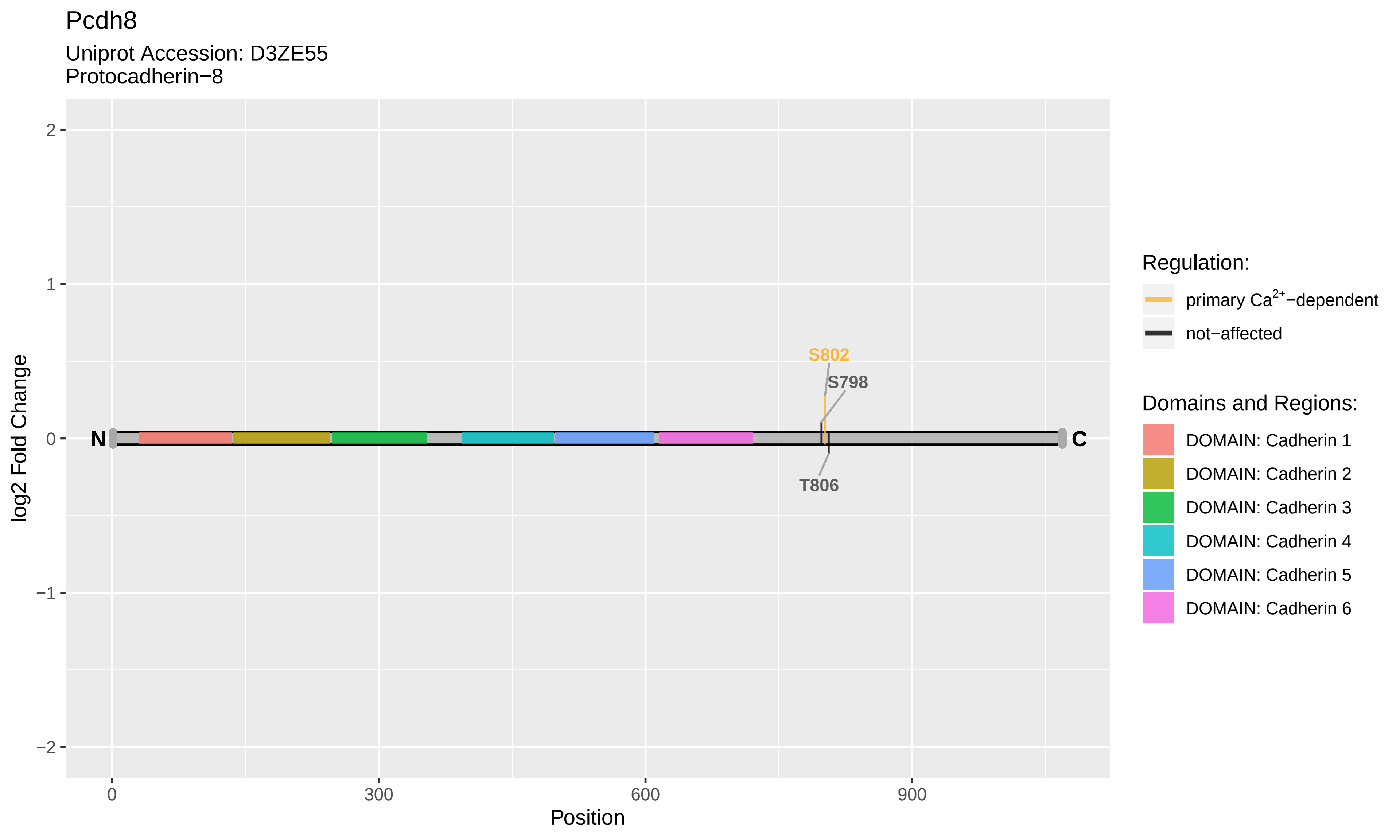
Pcdh8
Uniprot Accession: D3ZE55
Protocadherin−8
log2 Fold Change
Position
2
1
0
−1
−2
0
300
600
900
N
C
S802
S798
T806
Regulation:
primary Ca2+−dependent
not−affected
Domains and Regions:
DOMAIN: Cadherin 1
DOMAIN: Cadherin 2
DOMAIN: Cadherin 3
DOMAIN: Cadherin 4
DOMAIN: Cadherin 5
DOMAIN: Cadherin 6

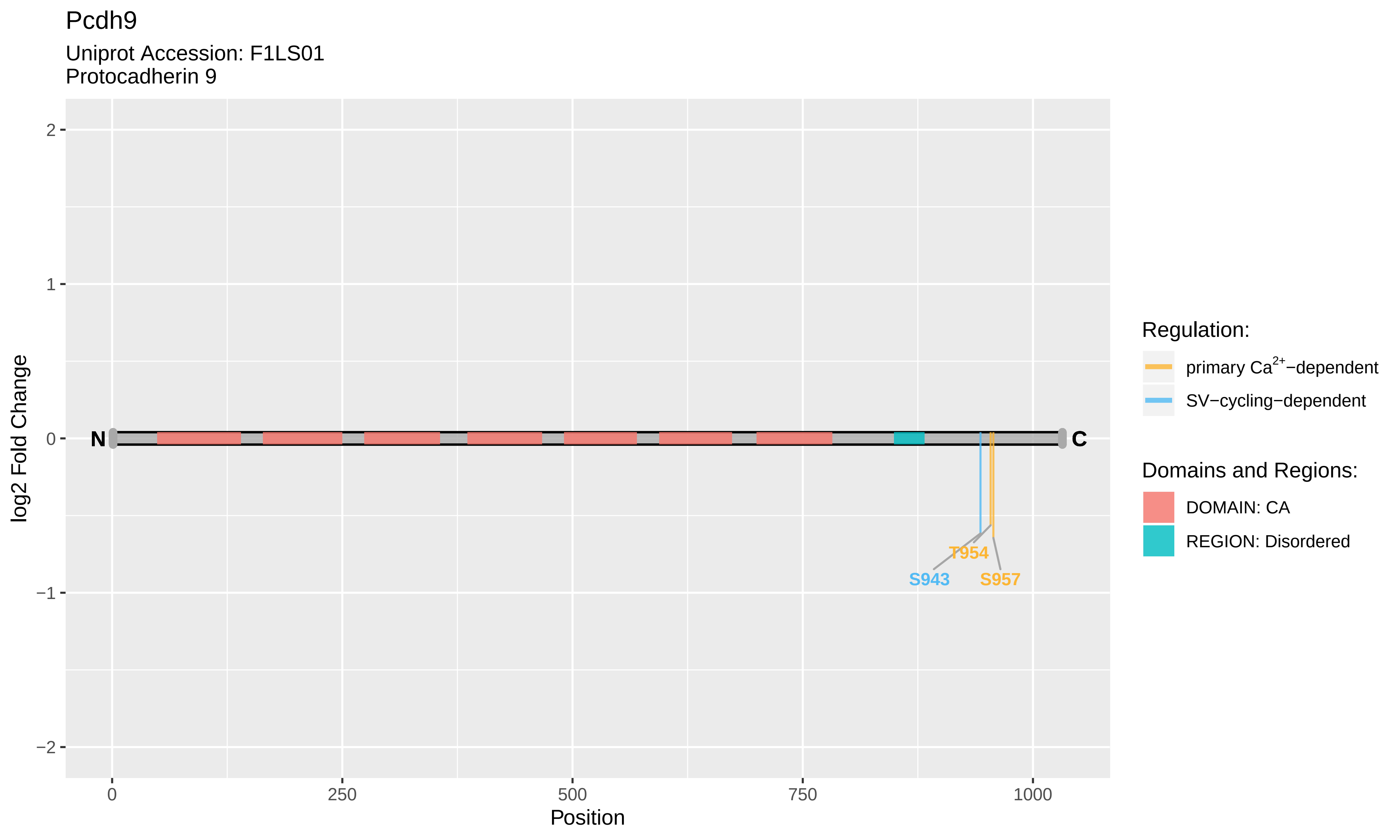

Pcdh9
Uniprot Accession: F1LS01
Protocadherin 9
log2 Fold Change
2
1
0
−1
−2
N
C
T954
S943
S957
0
250
500
750
1000
Position
Regulation:
primary Ca2+−dependent
SV−cycling−dependent
Domains and Regions:
DOMAIN: CA
REGION: Disordered

Pclo

Uniprot Accession: F1M7V4
Protein piccolo

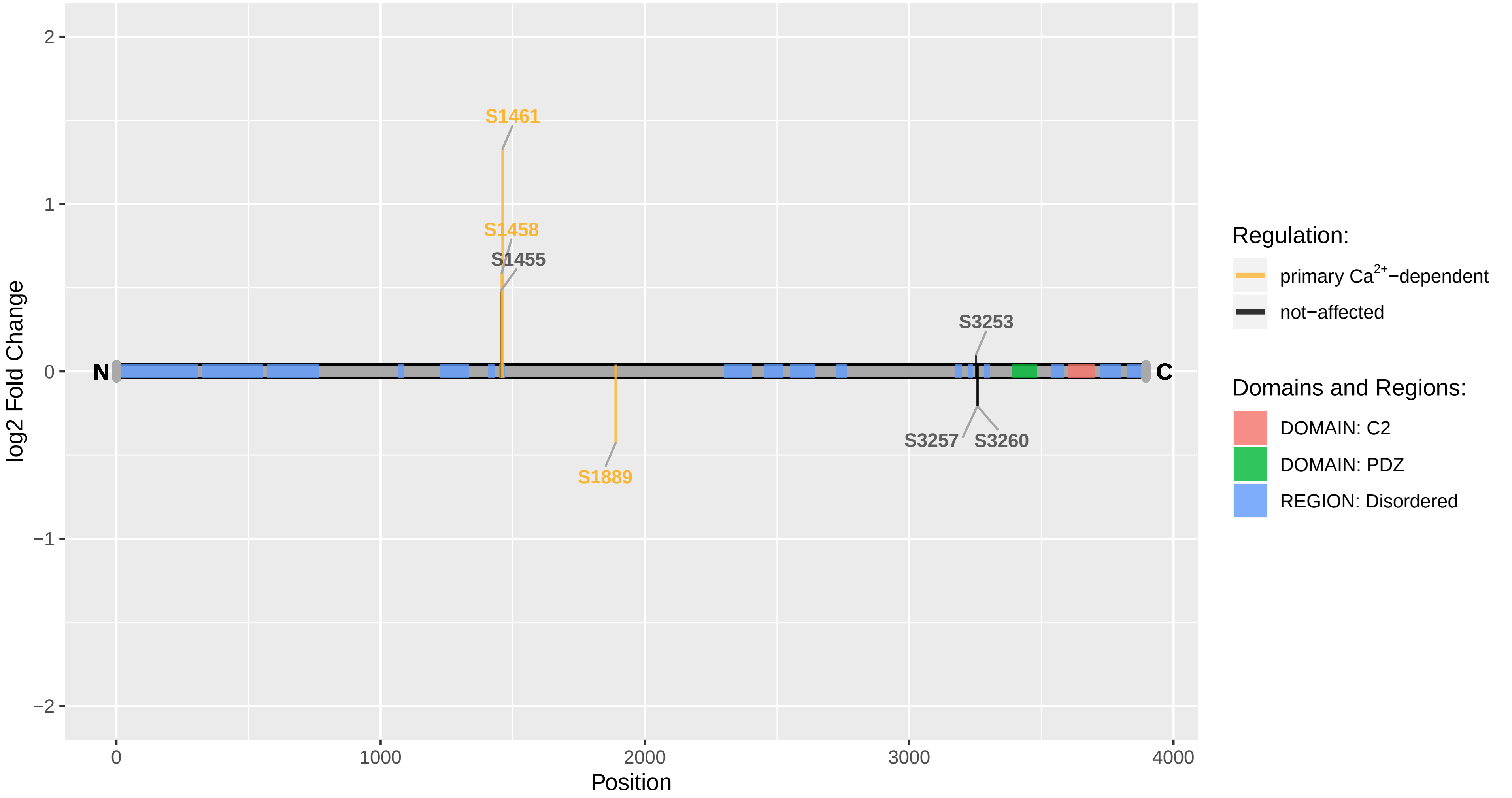

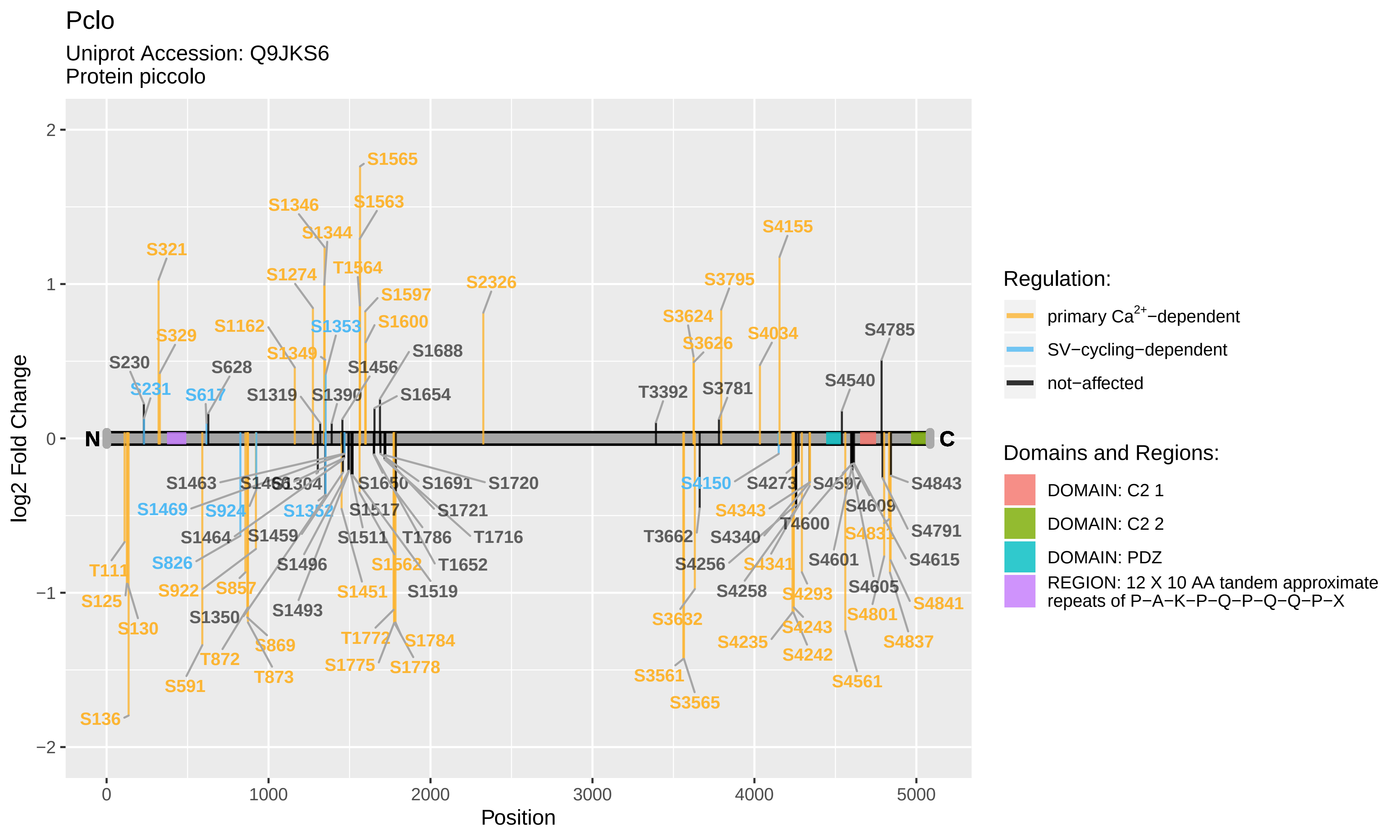

Pclo
Uniprot Accession: Q9JKS6
Protein piccolo
log2 Fold Change
Position
N
C
S1565
S1563
S1346
S1344
S321
T1564
S1274
S1597
S2326
S4155
S3795
S3624
S3626
S4034
S4785
S1162
S1353
S1600
S329
S1349
S1688
S230
S628
S1456
S4540
S231
S617
S1319
S1390
S1654
T3392
S3781
S1463
S1466
S1304
S1650
S1691
S1720
S4150
S4273
S4597
S4843
S1469
S924
S1352
S1517
S1721
S4343
S4609
S1464
S1459
S1511
T1786
T1716
T3662
S4340
T4600
S4831
S4791
S826
S1496
S1562
T1652
S4256
S4341
S4601
S4615
T111
S922
S857
S1451
S1519
S4258
S4293
S4605
S125
S1350
S1493
S3632
S4801
S4841
S130
T1772
S1784
S4235
S4243
S4837
S869
T872
S1775
S1778
S4242
T873
S591
S3561
S4561
S136
S3565
Regulation:
primary Ca2+−dependent
SV−cycling−dependent
not−affected
Domains and Regions:
DOMAIN: C2 1
DOMAIN: C2 2
DOMAIN: PDZ
REGION: 12 X 10 AA tandem approximate repeats of P−A−K−P−Q−P−Q−Q−P−X
2
1
0
−1
−2
0
1000
2000
3000
4000
5000

Pcm1

Uniprot Accession: A0A0G2K0X1
Pericentriolar material 1

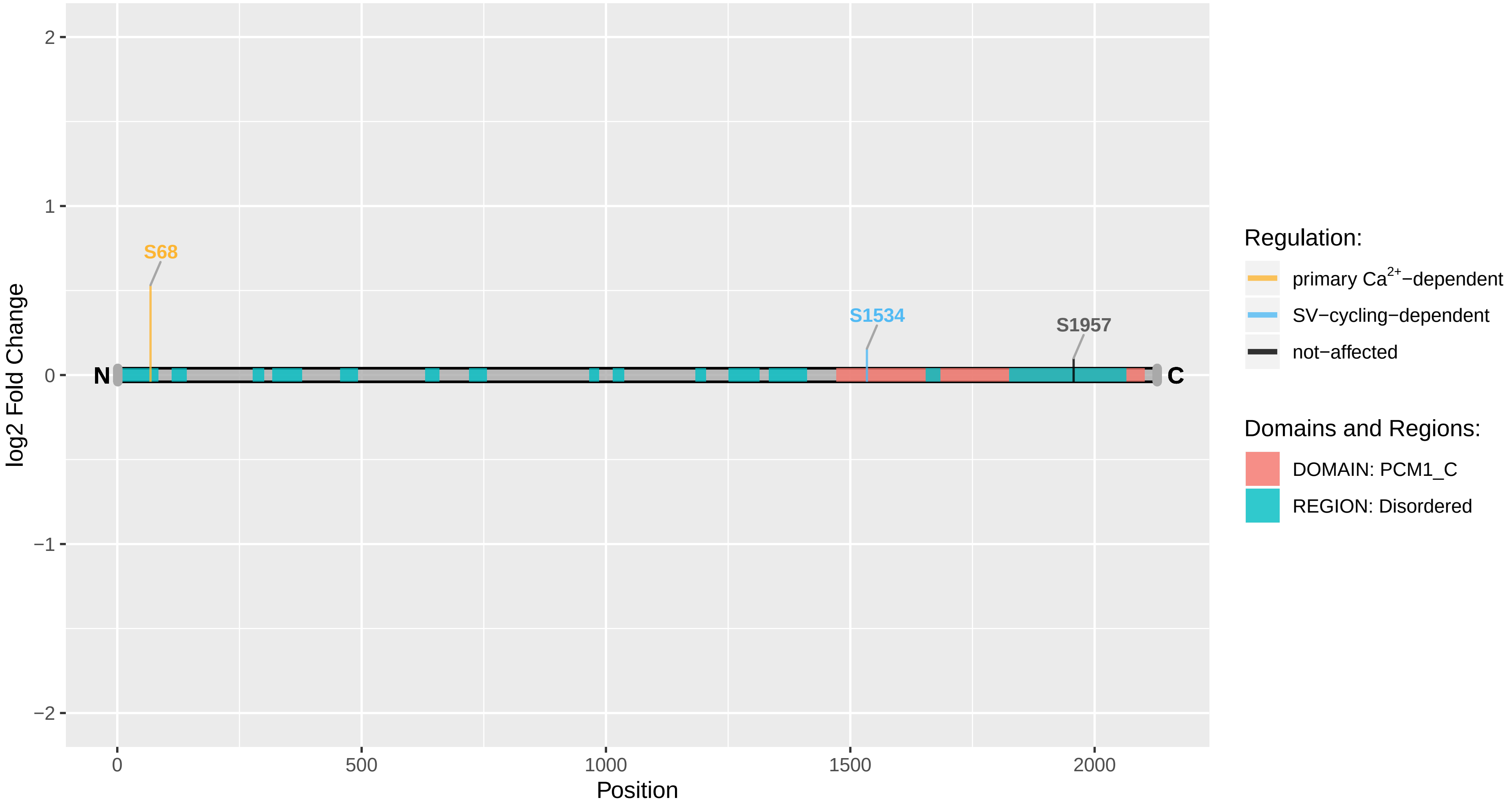

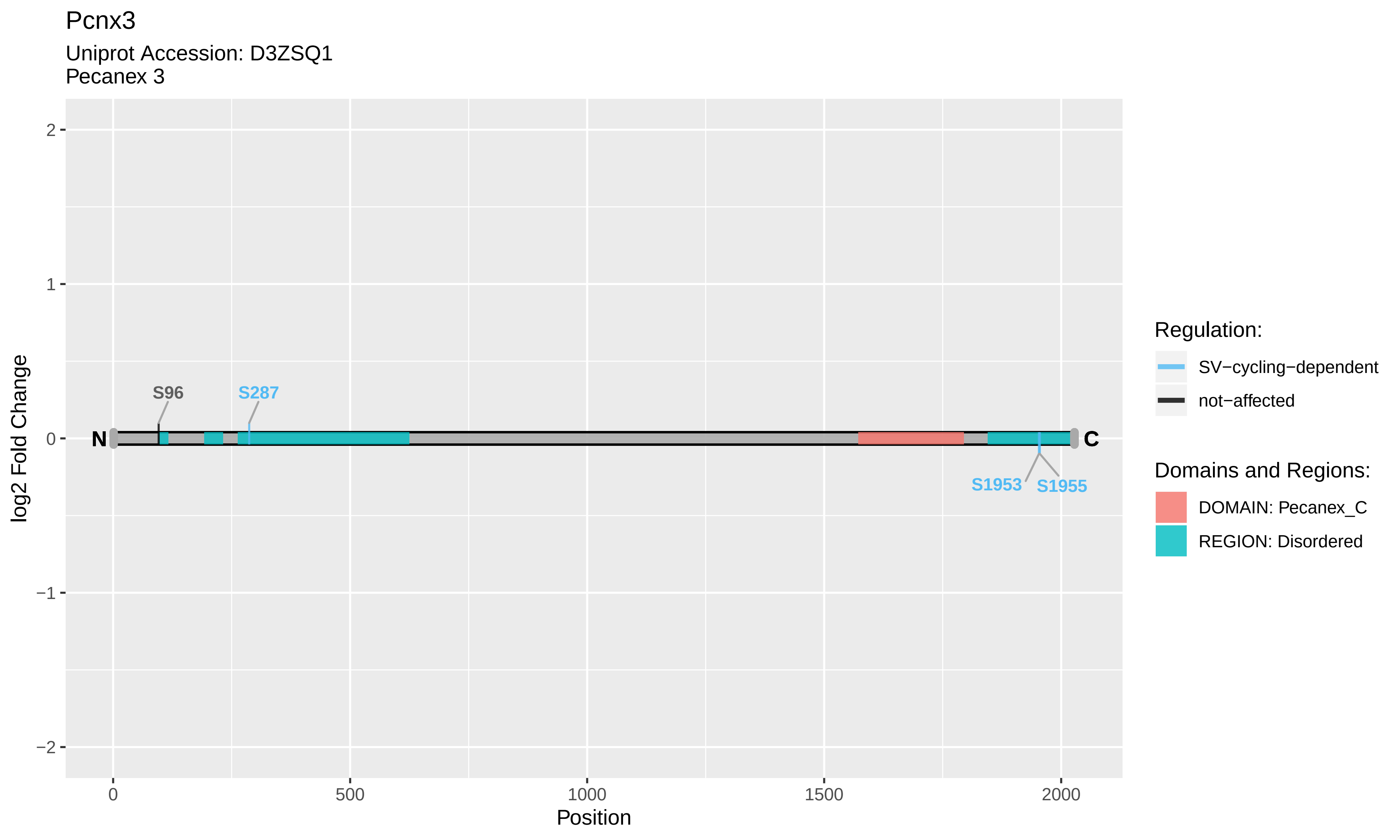

Pcnx3
Uniprot Accession: D3ZSQ1
Pecanex 3
log2 Fold Change
Position
N
C
S96
S287
S1953
S1955
2
1
0
−1
−2
0
500
1000
1500
2000
Regulation:
SV−cycling−dependent
not−affected
Domains and Regions:
DOMAIN: Pecanex_C
REGION: Disordered

Pcp2

Uniprot Accession: D3ZXP8
Purkinje cell protein 2

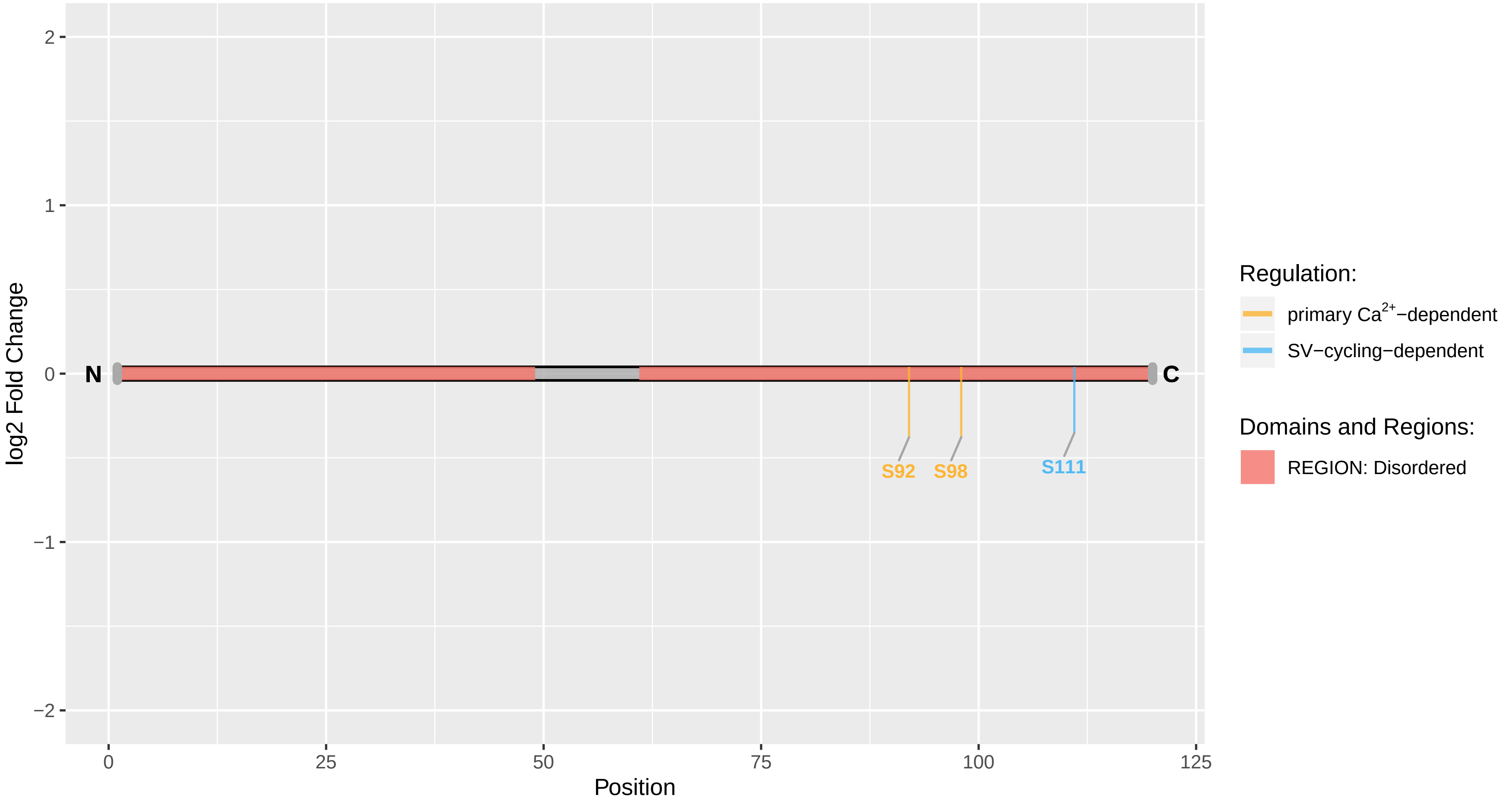

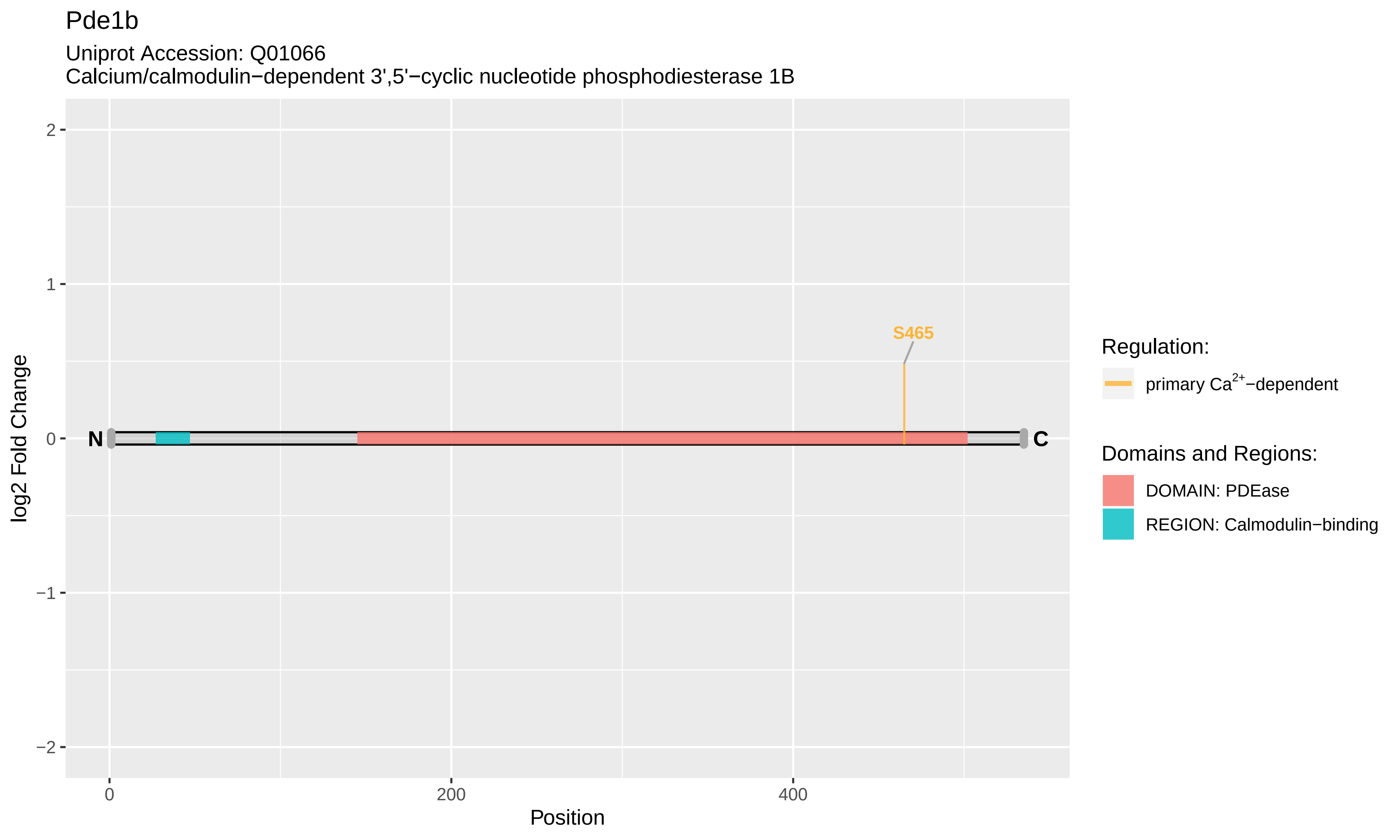

Pde1b
Uniprot Accession: Q01066
Calcium/calmodulin−dependent 3',5'−cyclic nucleotide phosphodiesterase 1B
2
1
0
−1
−2
log2 Fold Change
N
C
S465
0
200
400
Position
Regulation:
primary Ca2+−dependent
Domains and Regions:
DOMAIN: PDEase
REGION: Calmodulin−binding

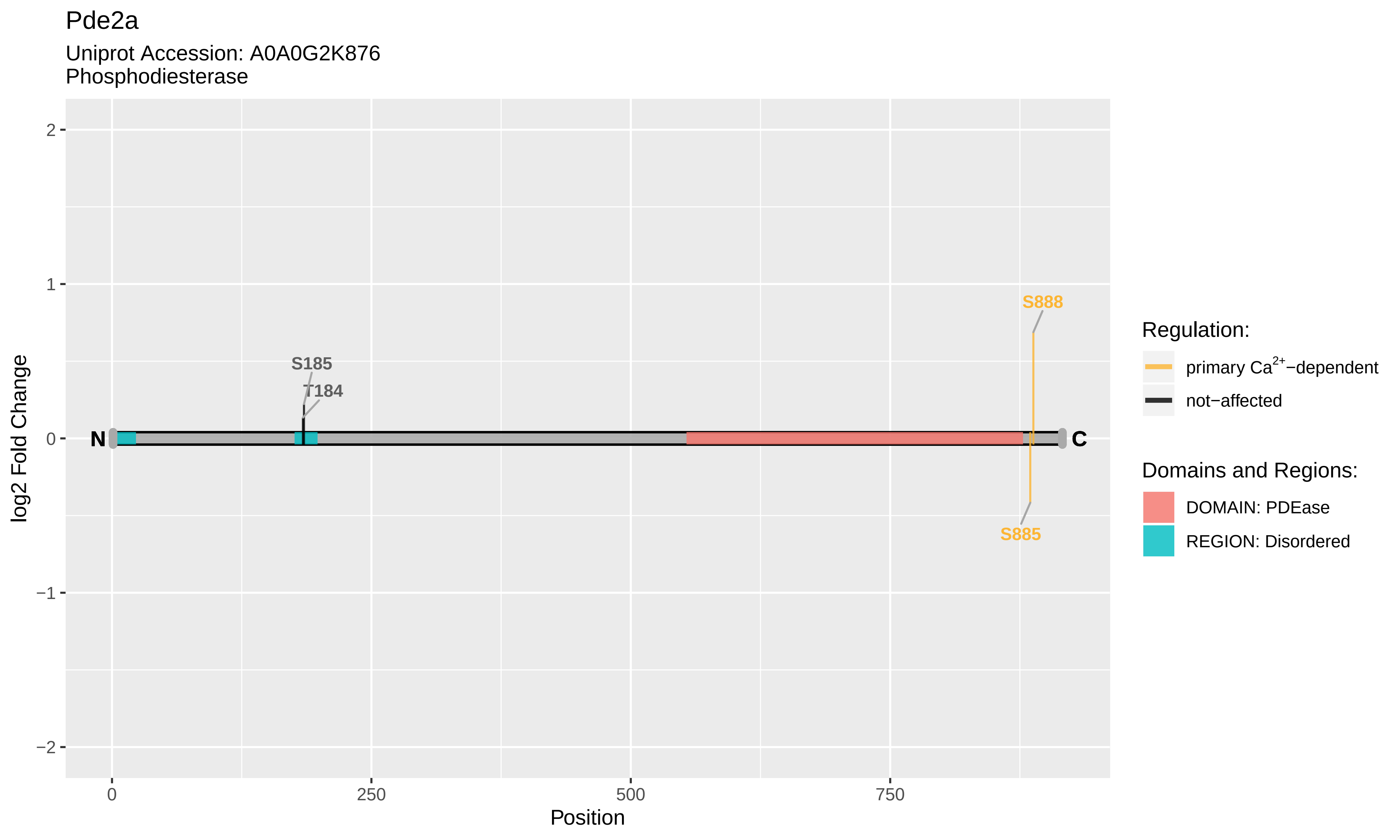
Pde2a
Uniprot Accession: A0A0G2K876
Phosphodiesterase
log2 Fold Change
2
1
0
−1
−2
Position
0
250
500
750
N
C
S185
T184
S888
S885
Regulation:
primary Ca2+−dependent
not−affected
Domains and Regions:
DOMAIN: PDEase
REGION: Disordered

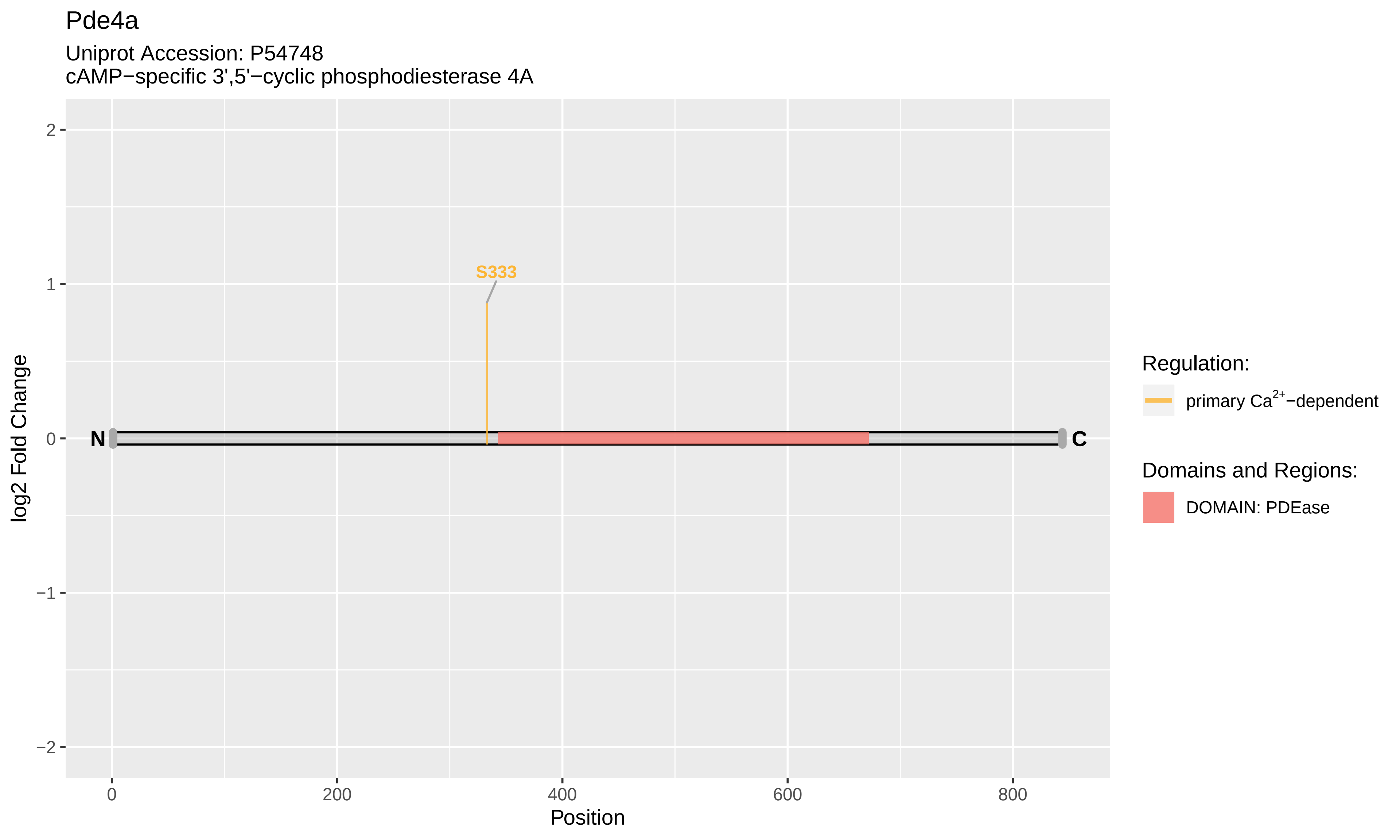

Pde4a
Uniprot Accession: P54748
cAMP−specific 3',5'−cyclic phosphodiesterase 4A
S333
N
C
2
1
0
−1
−2
log2 Fold Change
0
200
400
600
800
Position
Regulation:
primary Ca2+−dependent
Domains and Regions:
DOMAIN: PDEase

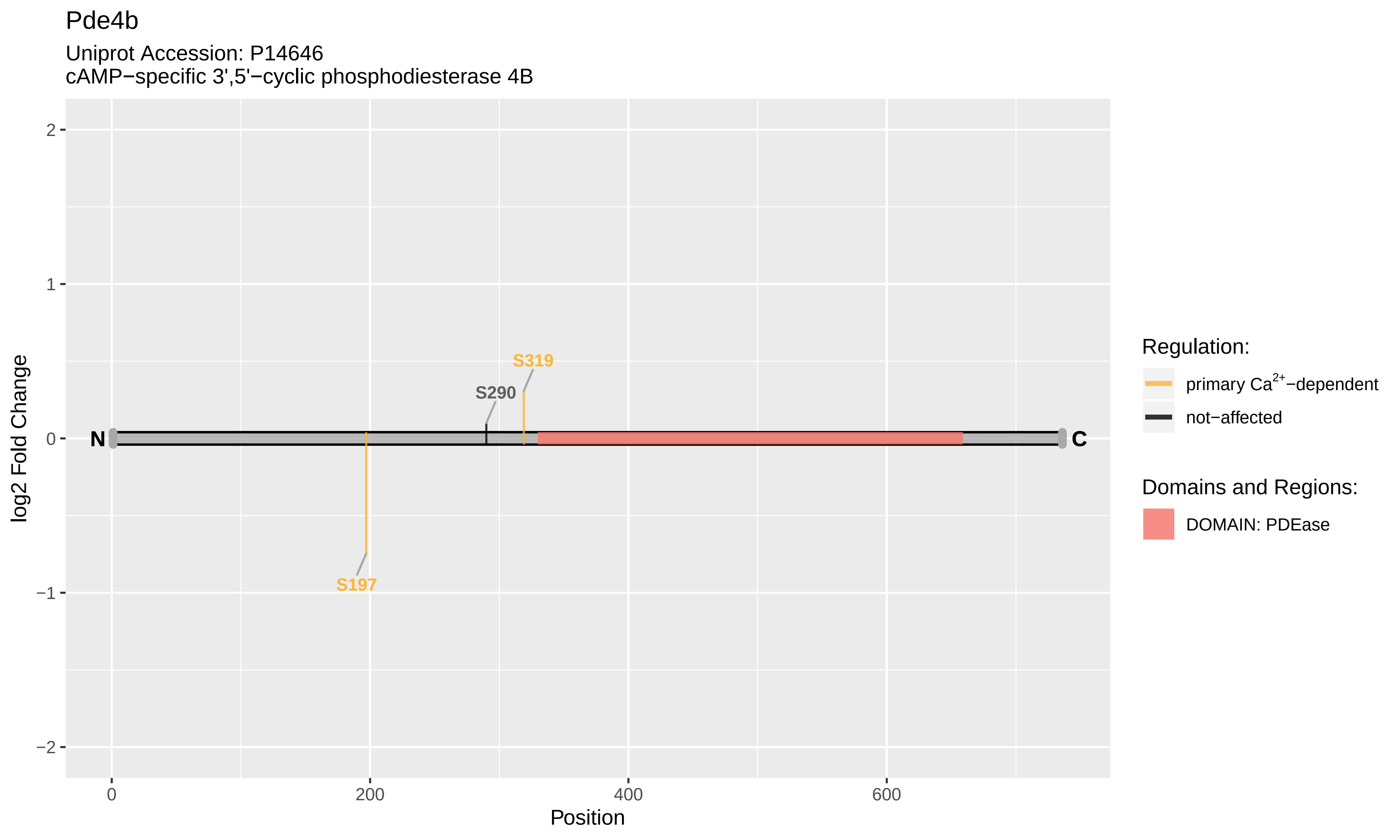
Pde4b
Uniprot Accession: P14646
cAMP−specific 3',5'−cyclic phosphodiesterase 4B
S319
S290
S197
N
C
log2 Fold Change
2
1
0
−1
−2
0
200
400
600
Position
Regulation:
primary Ca2+−dependent
not−affected
Domains and Regions:
DOMAIN: PDEase

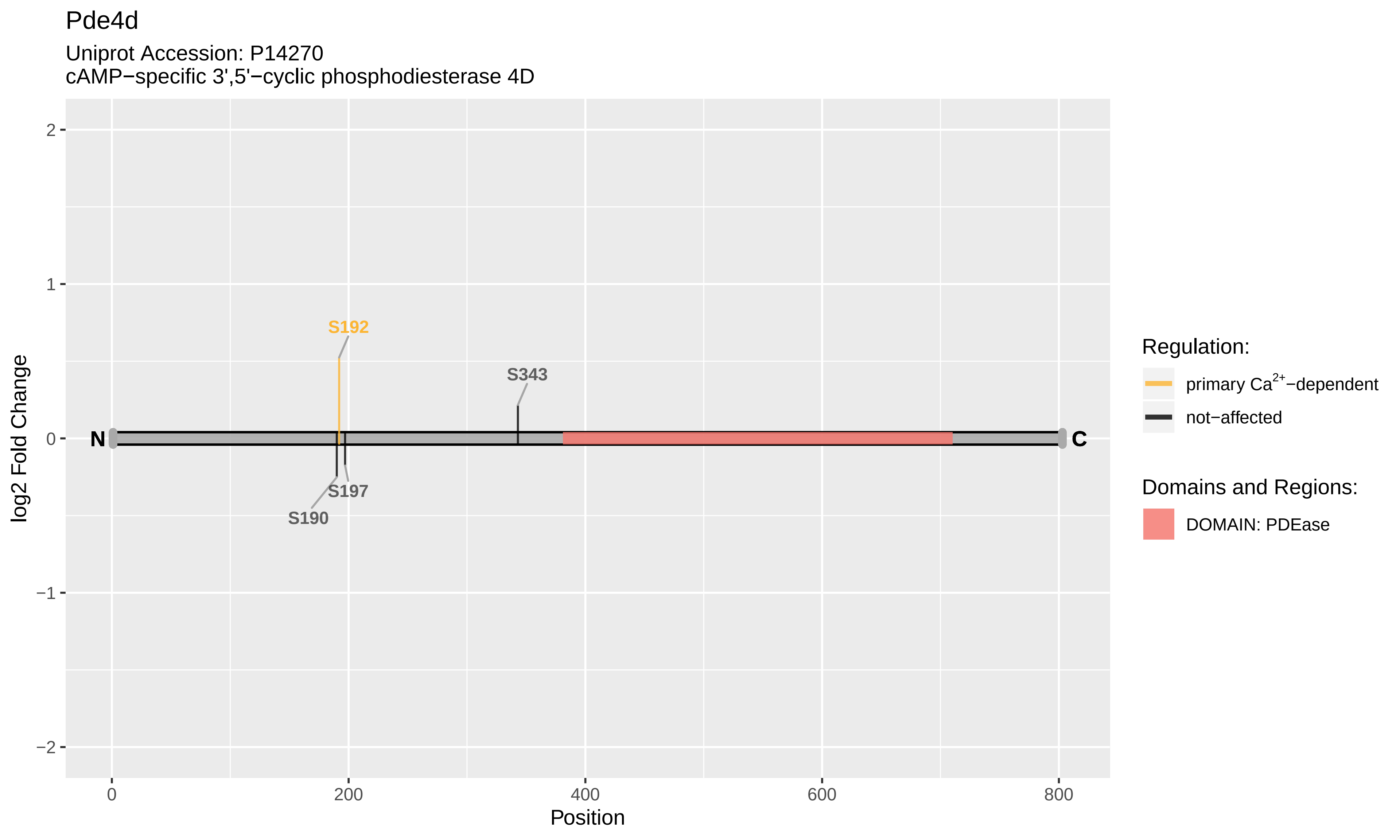

Pde4d
Uniprot Accession: P14270
cAMP−specific 3',5'−cyclic phosphodiesterase 4D
S192
S343
S197
S190
N
C
log2 Fold Change
Position
2
1
0
−1
−2
0
200
400
600
800
Regulation:
primary Ca2+−dependent
not−affected
Domains and Regions:
DOMAIN: PDEase

Pdha1

Uniprot Accession: P26284
Pyruvate dehydrogenase E1 component subunit alpha, somatic form, mitochondrial

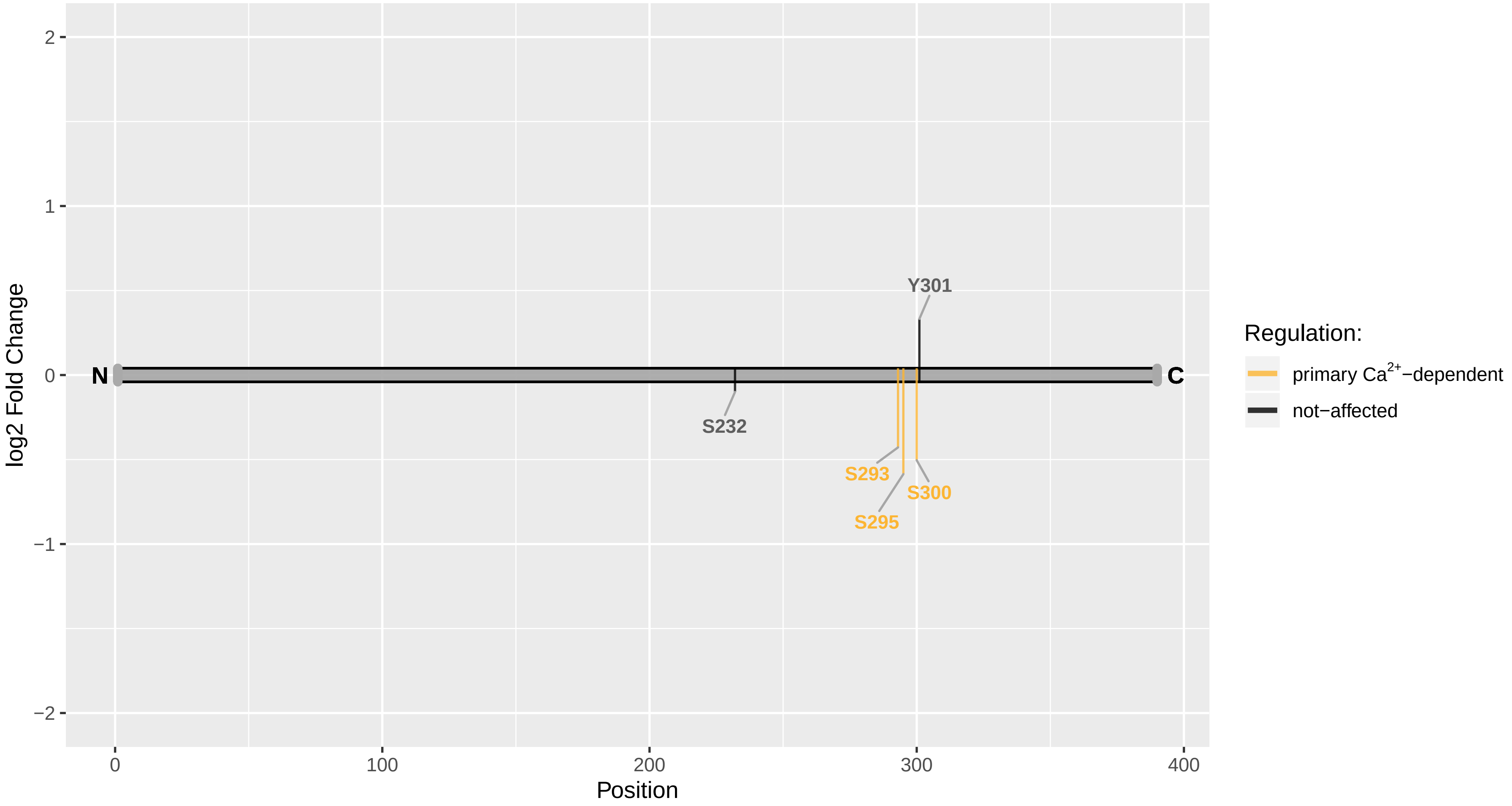

Pdlim5

Uniprot Accession: Q62920
PDZ and LIM domain protein 5

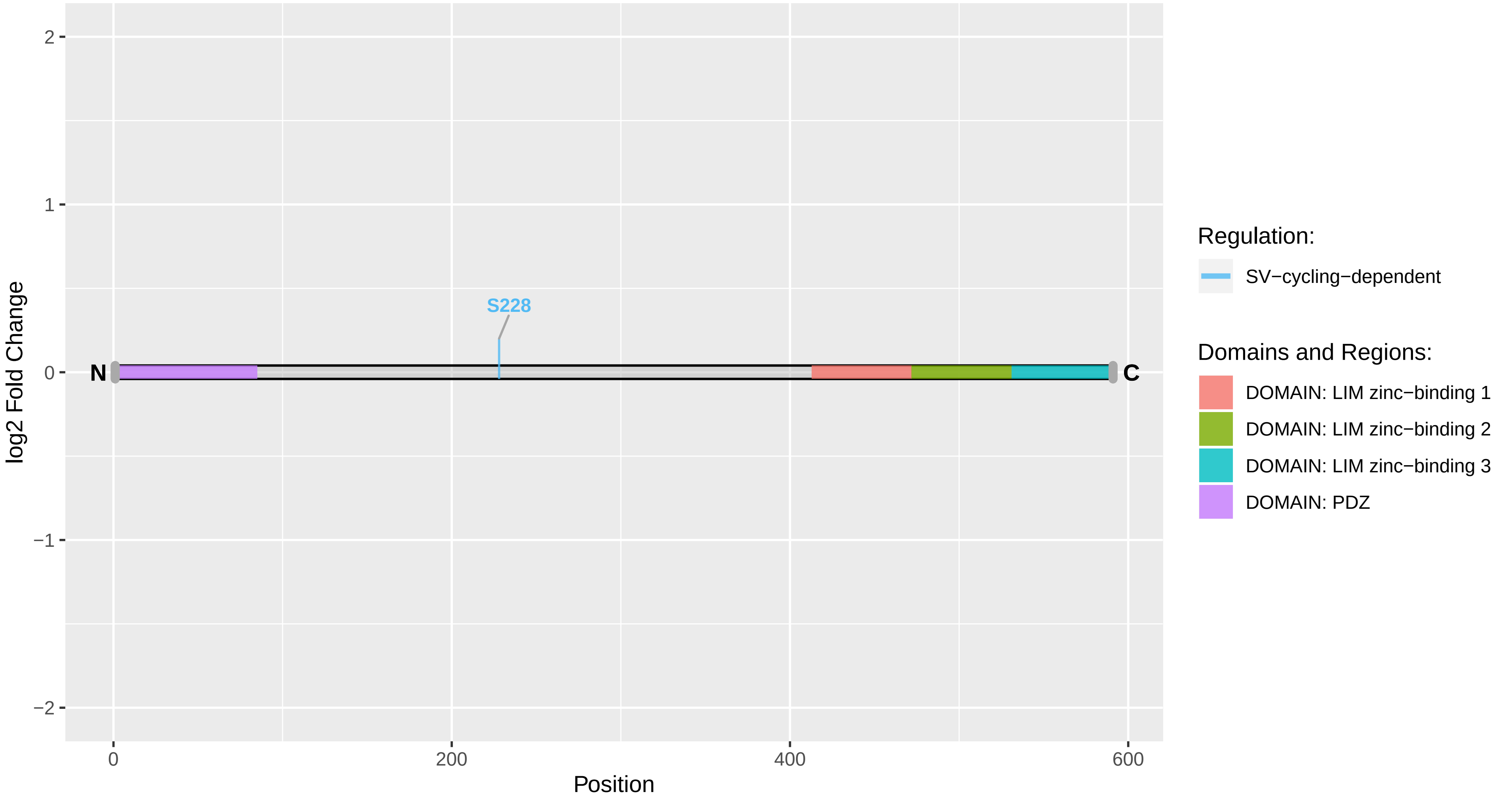

Pdpk1

Uniprot Accession: O55173
3−phosphoinositide−dependent protein kinase 1

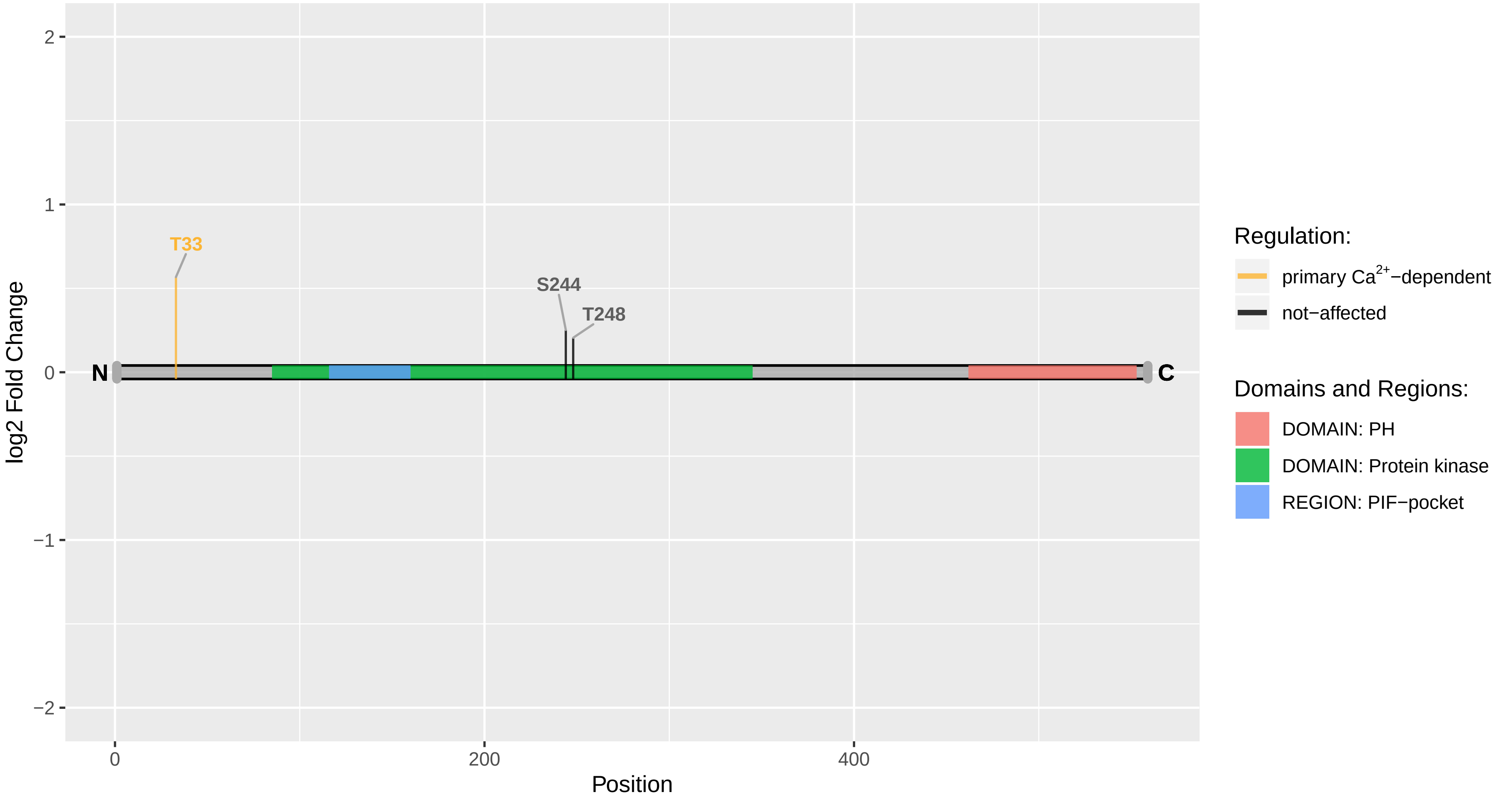

Pdzd8

Uniprot Accession: D3ZXY2
PDZ domain containing 8 (Predicted)

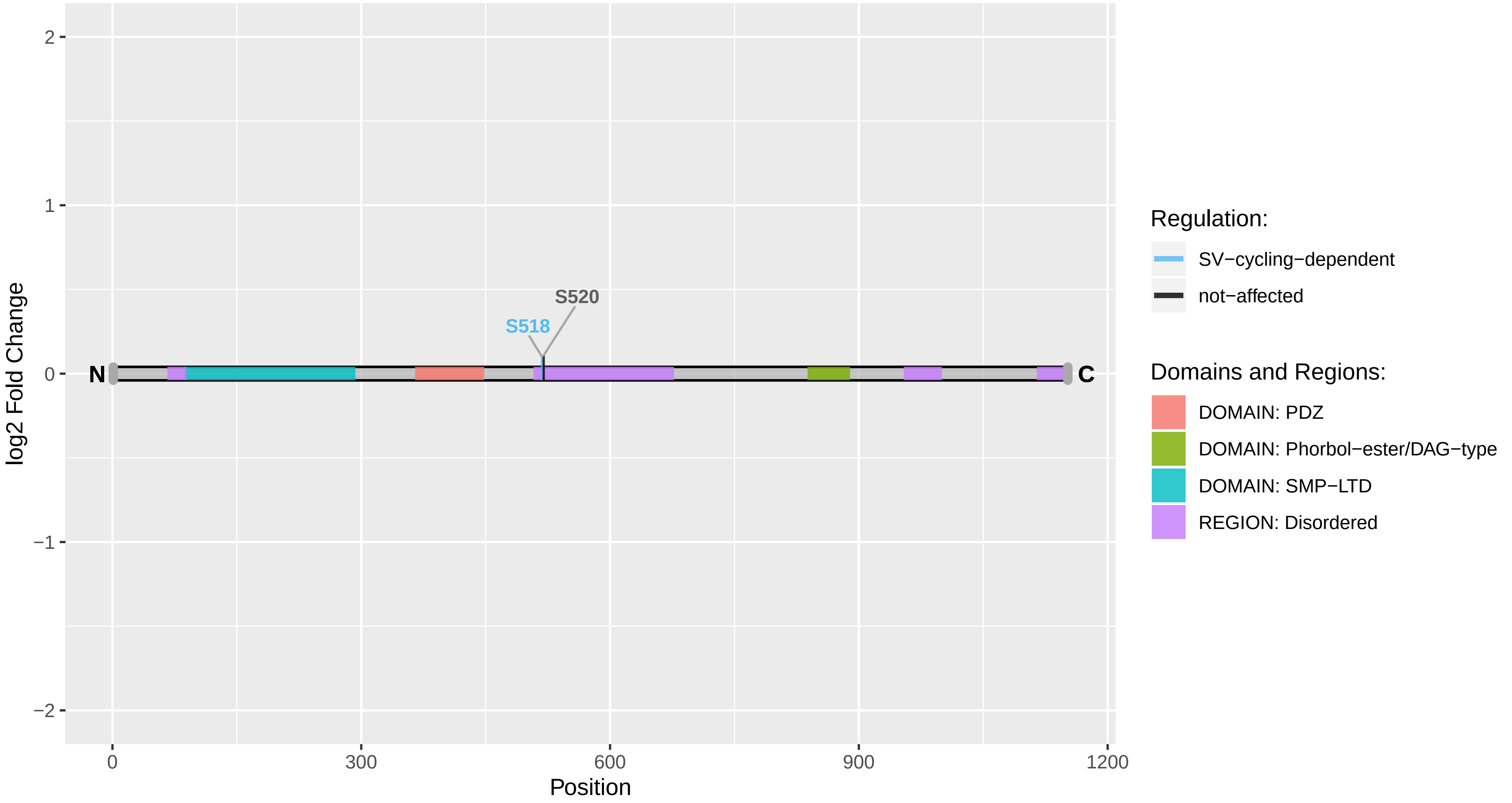

Pex10

Uniprot Accession: D3ZC81
Peroxisomal biogenesis factor 10

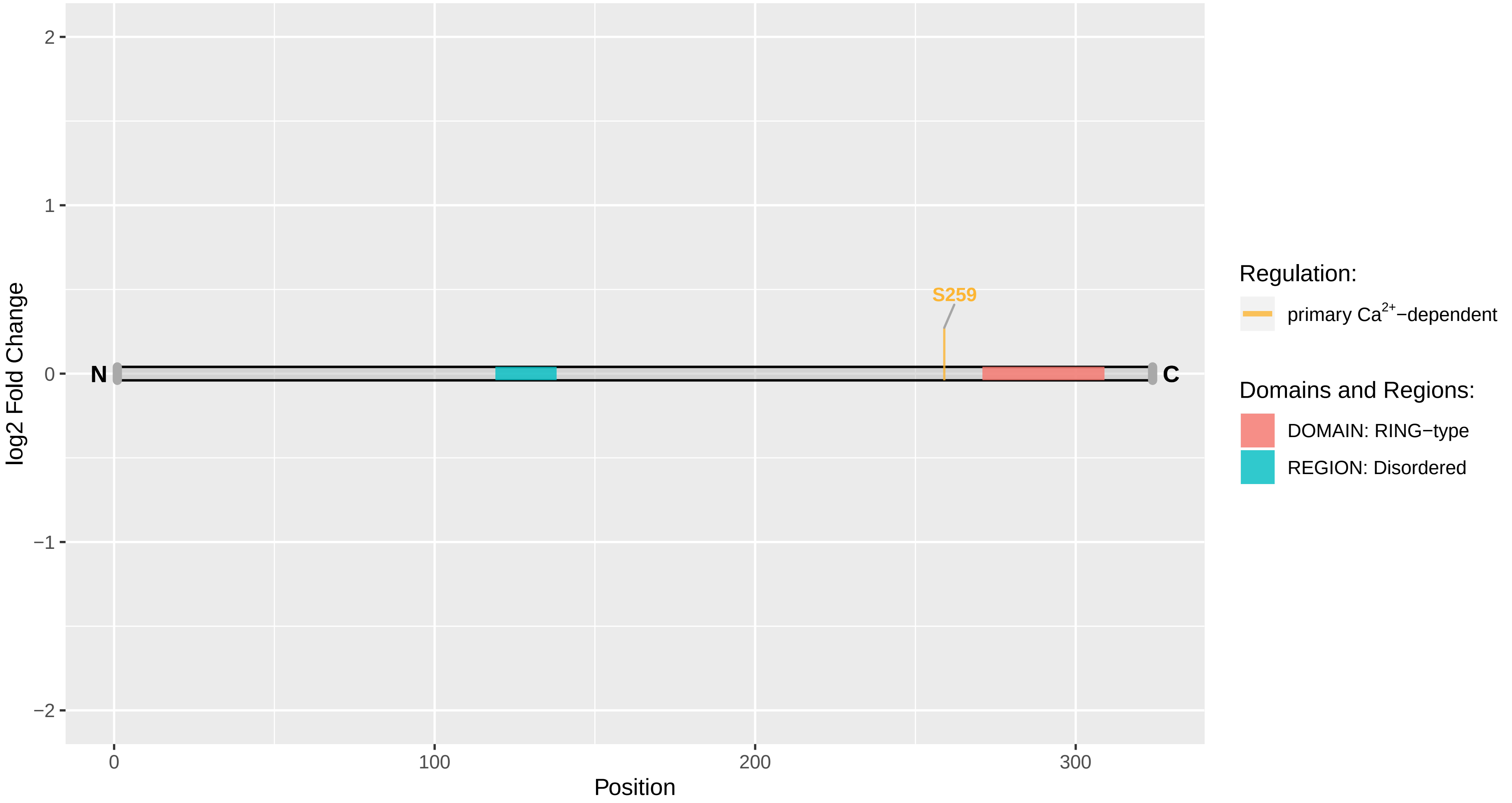

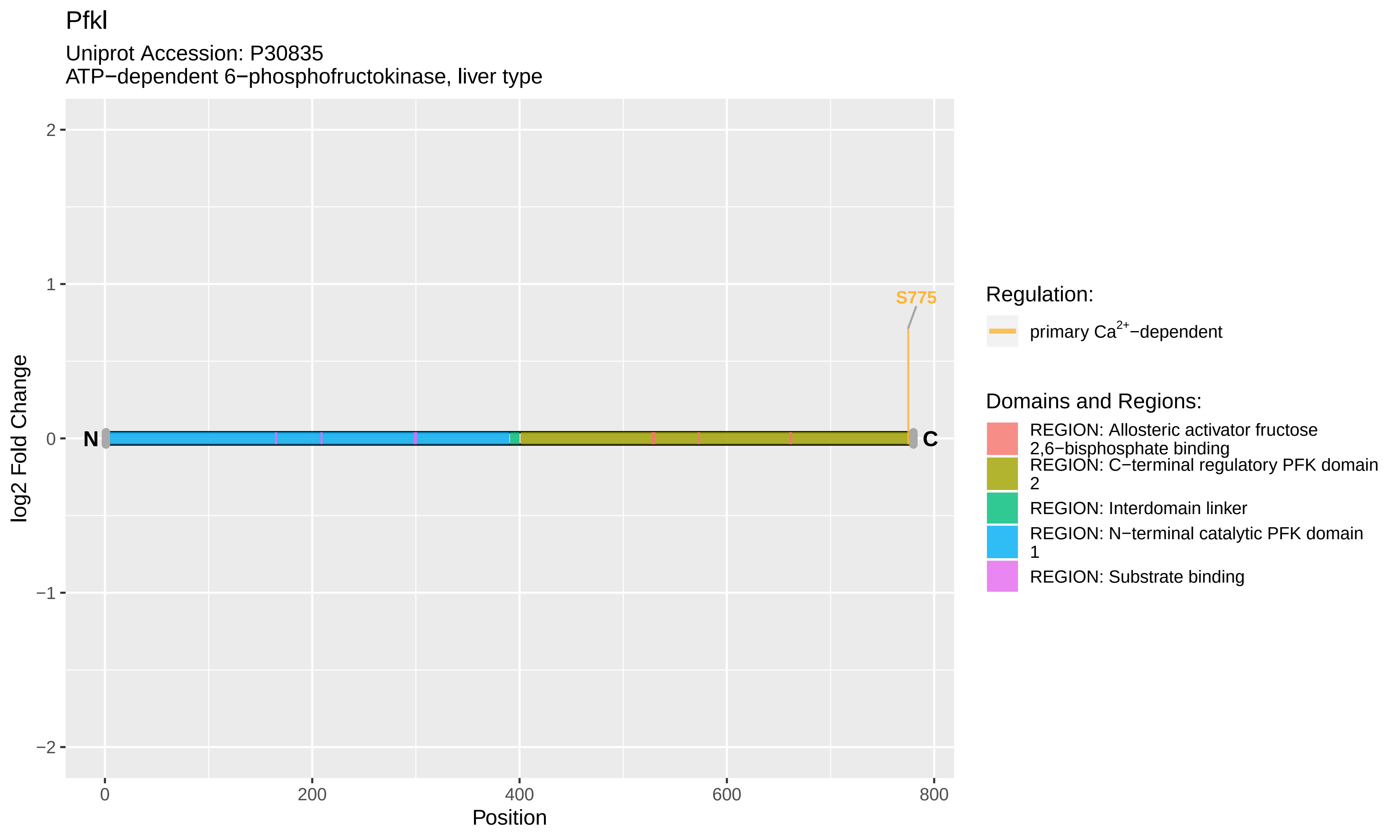
Pfkl
Uniprot Accession: P30835
ATP−dependent 6−phosphofructokinase, liver type
log2 Fold Change
2
1
0
−1
−2
0
200
400
600
800
Position
N
C
S775
Regulation:
primary Ca2+−dependent
Domains and Regions:
REGION: Allosteric activator fructose 2,6−bisphosphate binding
REGION: C−terminal regulatory PFK domain 2
REGION: Interdomain linker
REGION: N−terminal catalytic PFK domain 1
REGION: Substrate binding

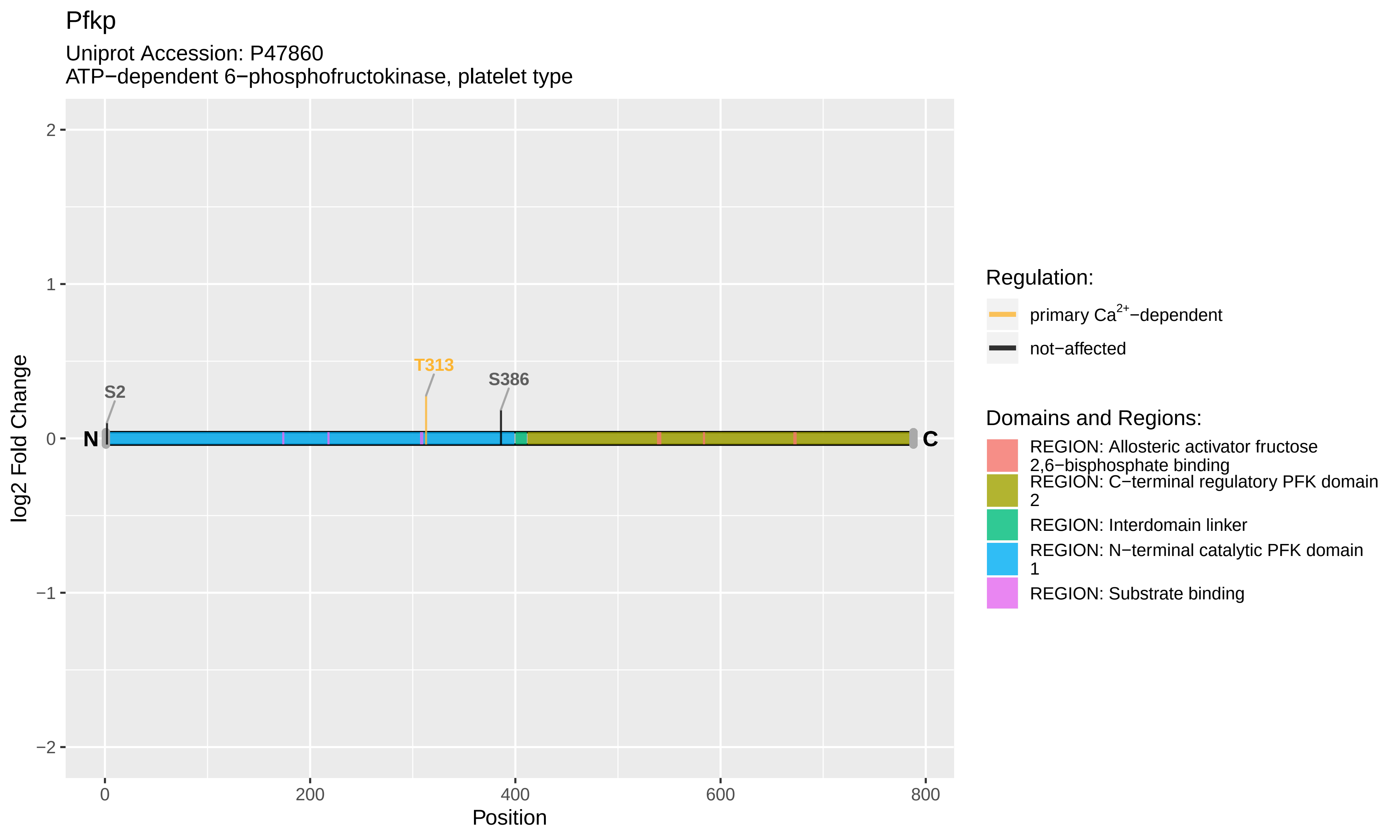

Pfkp
Uniprot Accession: P47860
ATP−dependent 6−phosphofructokinase, platelet type
log2 Fold Change
Position
S2
T313
S386
N
C
Regulation:
primary Ca2+−dependent
not−affected
Domains and Regions:
REGION: Allosteric activator fructose 2,6−bisphosphate binding
REGION: C−terminal regulatory PFK domain 2
REGION: Interdomain linker
REGION: N−terminal catalytic PFK domain 1
REGION: Substrate binding

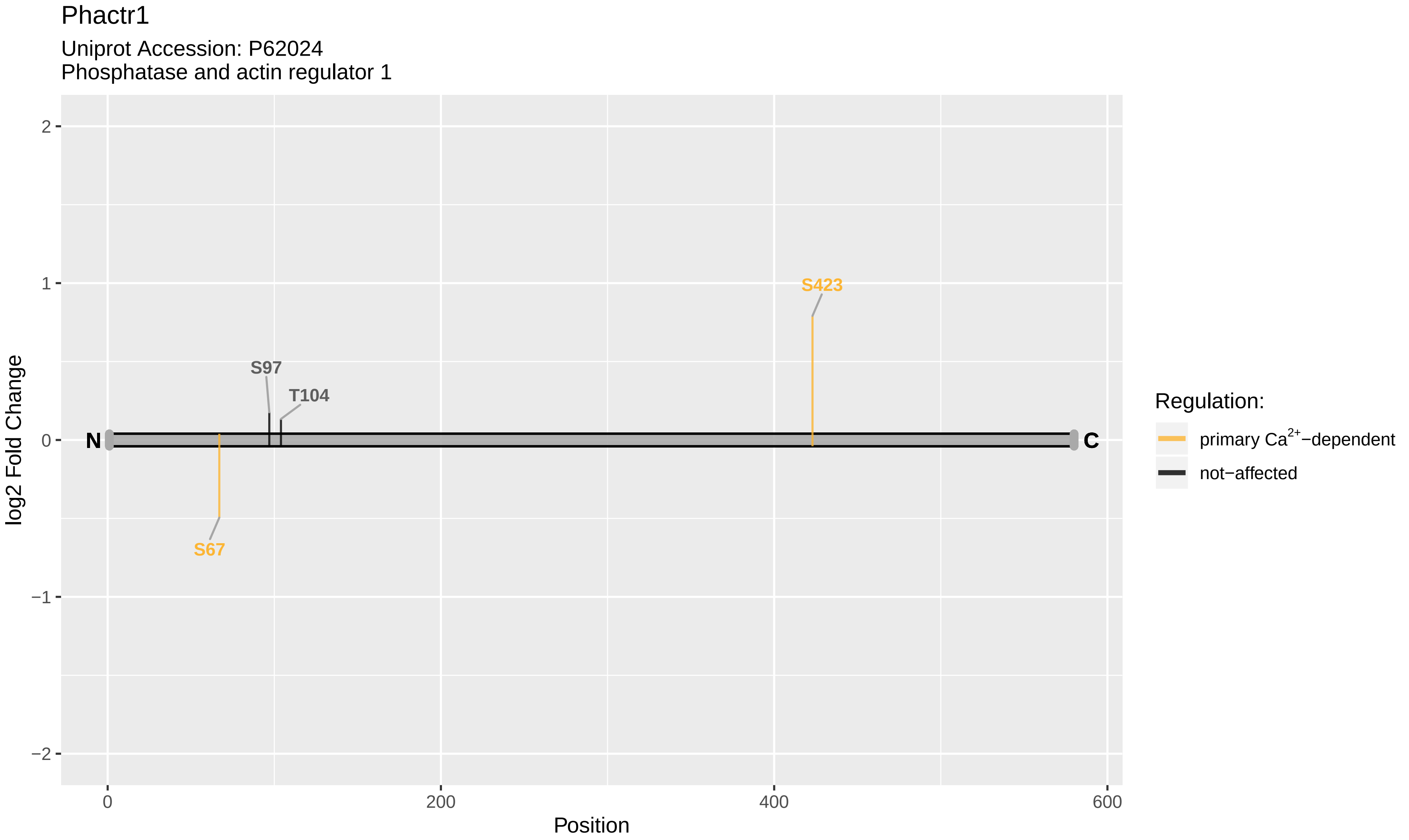

Phactr1
Uniprot Accession: P62024
Phosphatase and actin regulator 1
S423
S97
T104
N
C
S67
2
1
0
−1
−2
log2 Fold Change
0
200
400
600
Position
Regulation:
primary Ca2+−dependent
not−affected

Phactr2

Uniprot Accession: A0A0G2K8Q4
Phosphatase and actin regulator

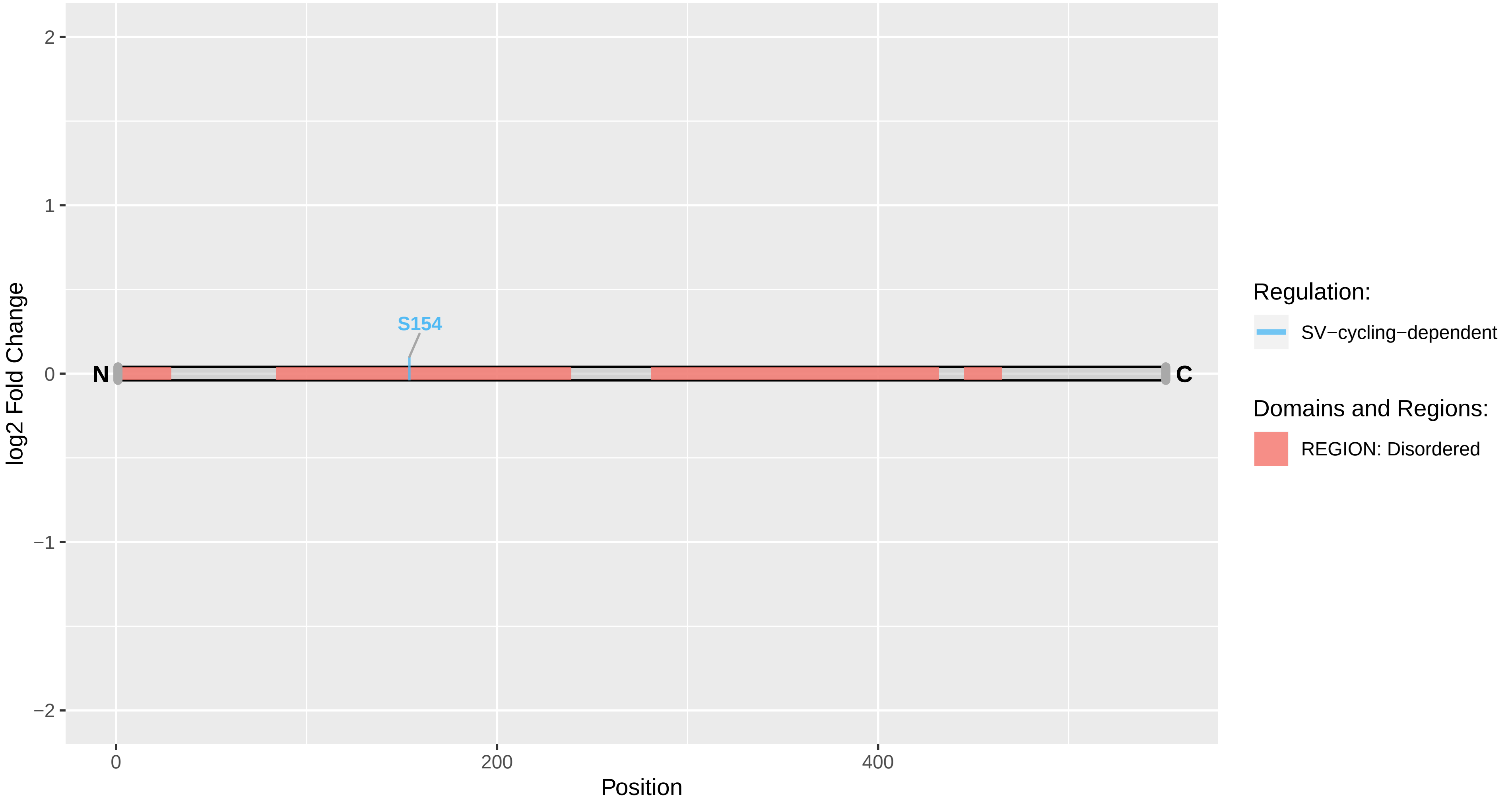

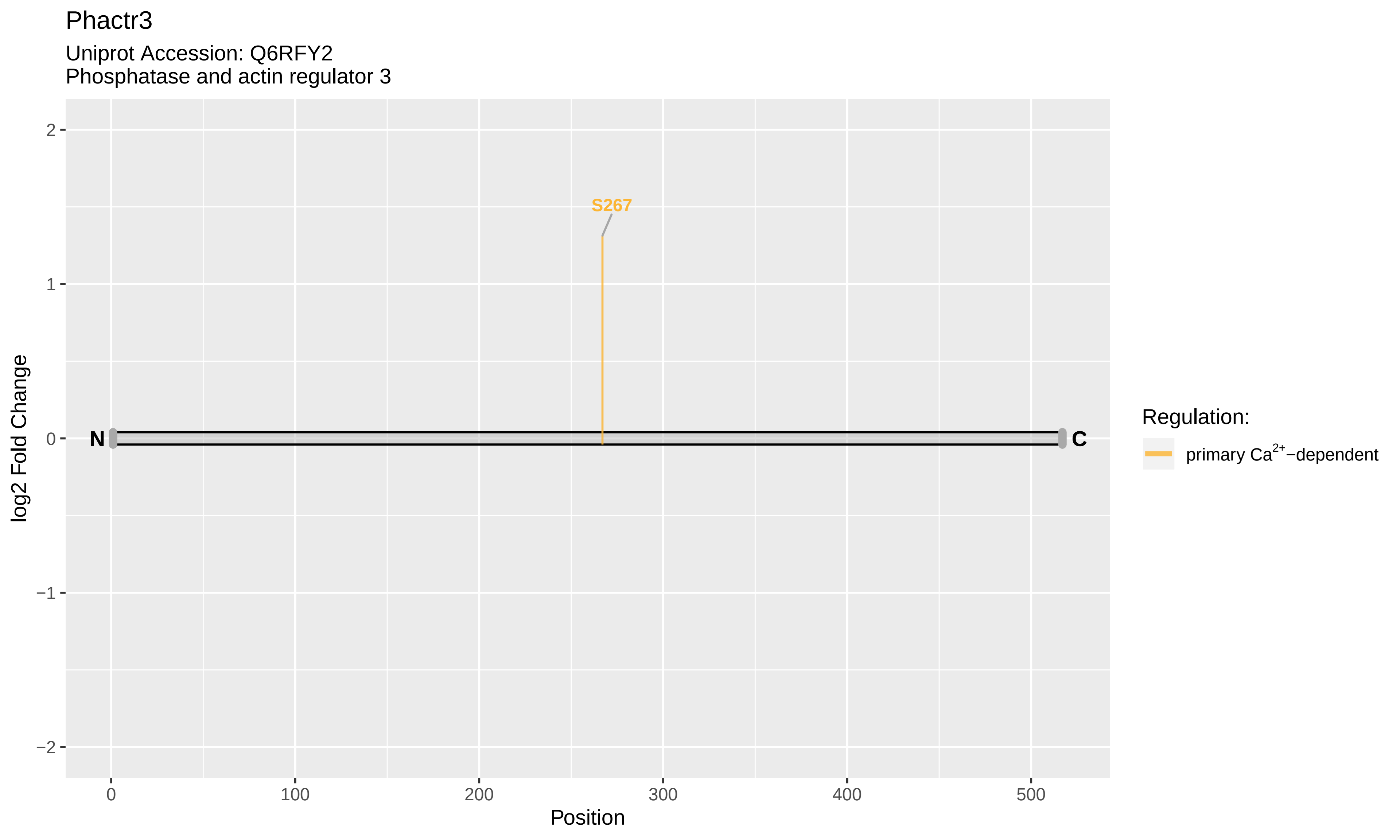
Phactr3
Uniprot Accession: Q6RFY2
Phosphatase and actin regulator 3
S267
N
C
2
1
0
−1
−2
0
100
200
300
400
500
log2 Fold Change
Position
Regulation:
primary Ca2+−dependent

Phb2

Uniprot Accession: Q5XIH7
Prohibitin−2

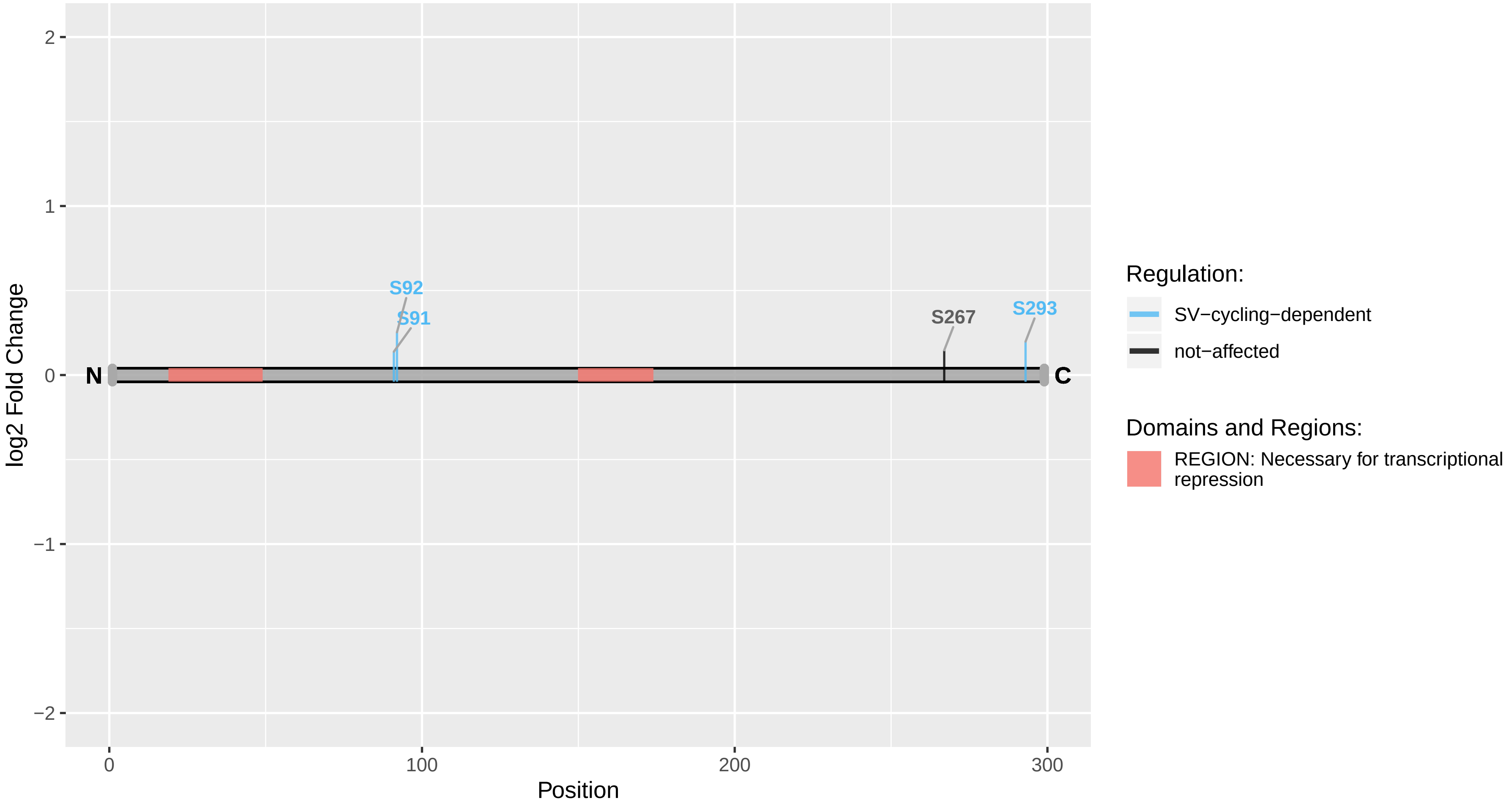

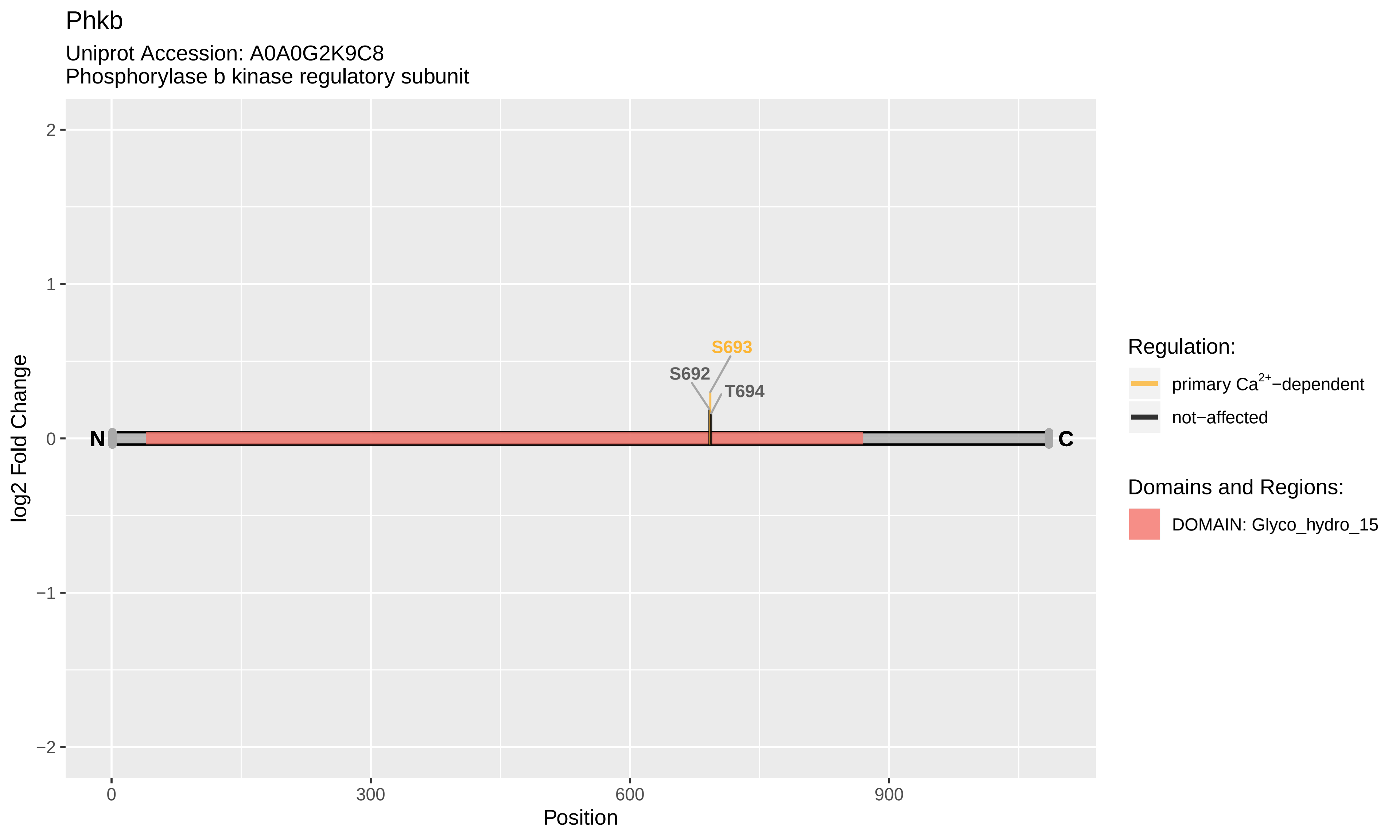
Phkb
Uniprot Accession: A0A0G2K9C8
Phosphorylase b kinase regulatory subunit
S693
S692
T694
N
C
2
1
0
−1
−2
log2 Fold Change
0
300
600
900
Position
Regulation:
primary Ca2+−dependent
not−affected
Domains and Regions:
DOMAIN: Glyco_hydro_15

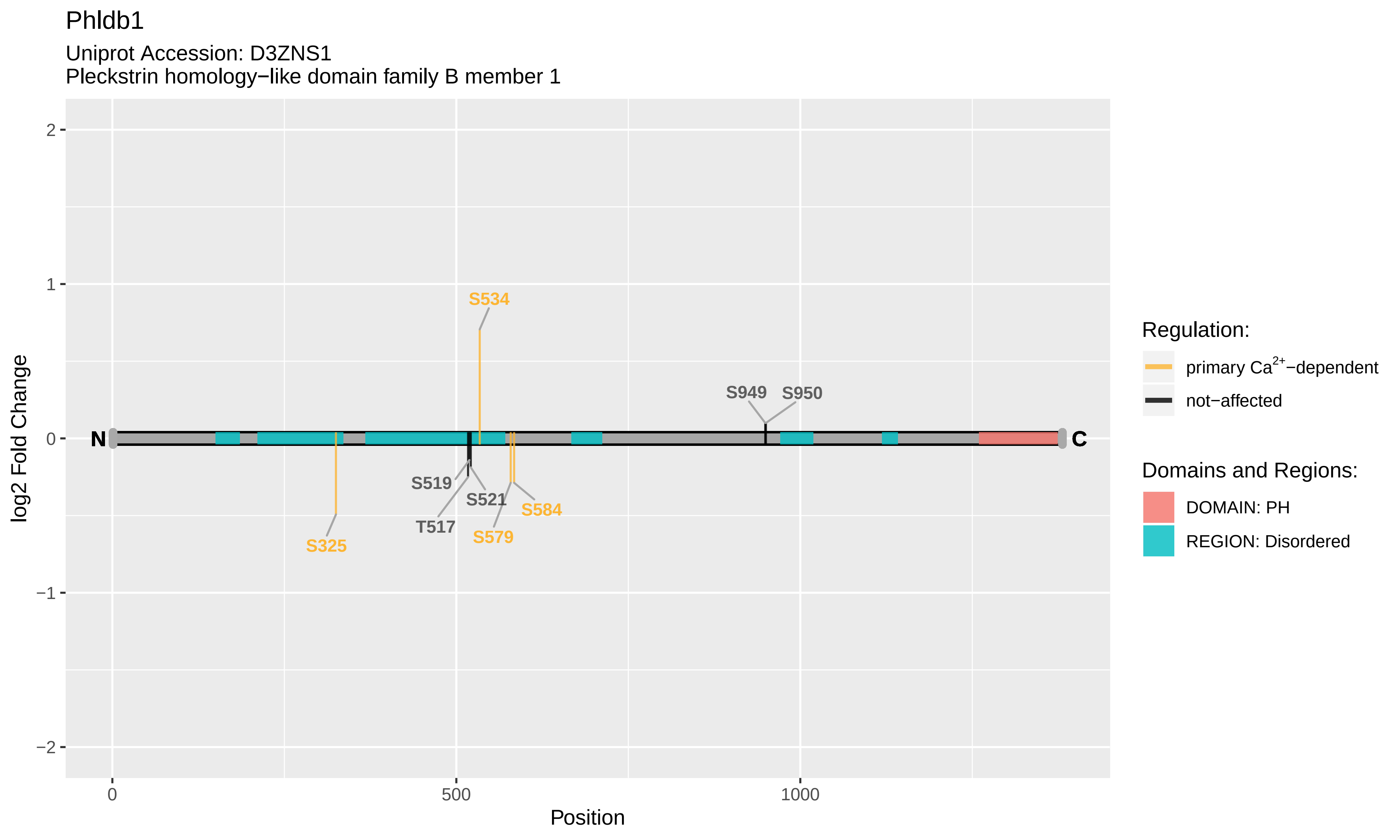

Phldb1
Uniprot Accession: D3ZNS1
Pleckstrin homology−like domain family B member 1
log2 Fold Change
Position
2
1
0
−1
−2
0
500
1000
N
C
S534
S949
S950
S519
S521
T517
S584
S579
S325
Regulation:
primary Ca2+−dependent
not−affected
Domains and Regions:
DOMAIN: PH
REGION: Disordered

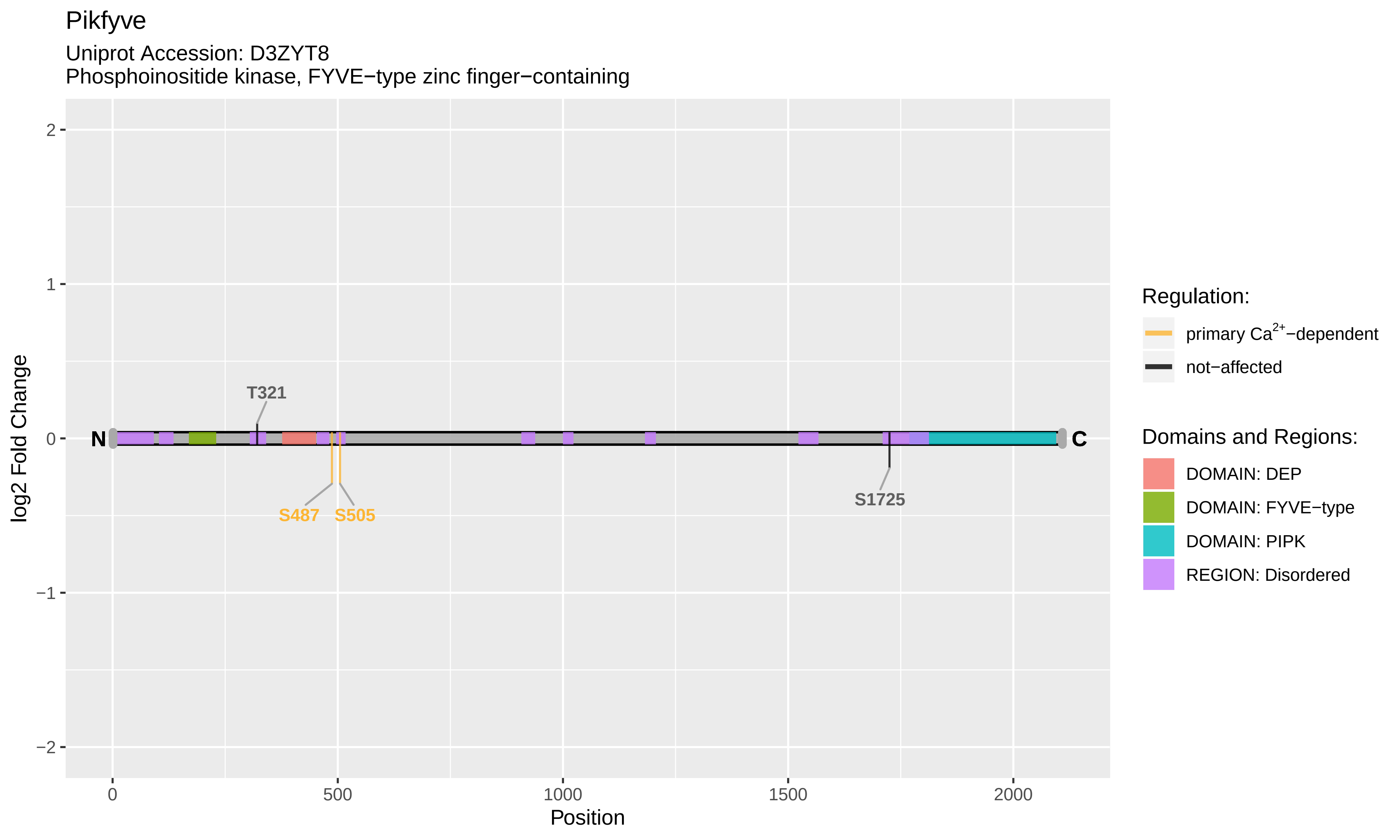
Pikfyve
Uniprot Accession: D3ZYT8
Phosphoinositide kinase, FYVE−type zinc finger−containing
log2 Fold Change
Position
N
C
T321
S487
S505
S1725
Regulation:
primary Ca^{2+}−dependent
not−affected
Domains and Regions:
DOMAIN: DEP
DOMAIN: FYVE−type
DOMAIN: PIPK
REGION: Disordered

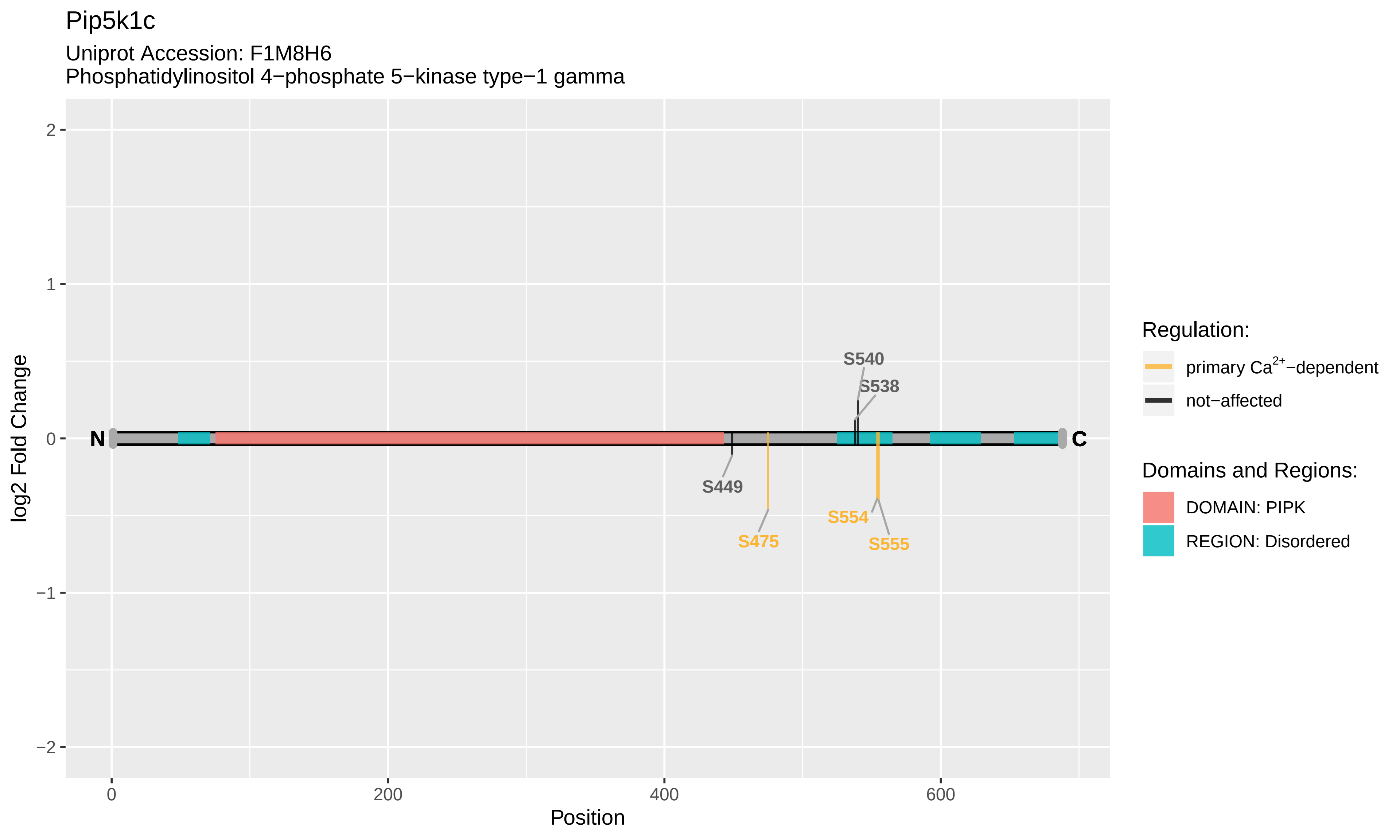
Pip5k1c
Uniprot Accession: F1M8H6
Phosphatidylinositol 4−phosphate 5−kinase type−1 gamma
log2 Fold Change
2
1
0
−1
−2
N
C
S540
S538
S449
S475
S554
S555
0
200
400
600
Position
Regulation:
primary Ca2+−dependent
not−affected
Domains and Regions:
DOMAIN: PIPK
REGION: Disordered

Pitpnm2

Uniprot Accession: F1LXD6
Phosphatidylinositol transfer protein, membrane−associated 2

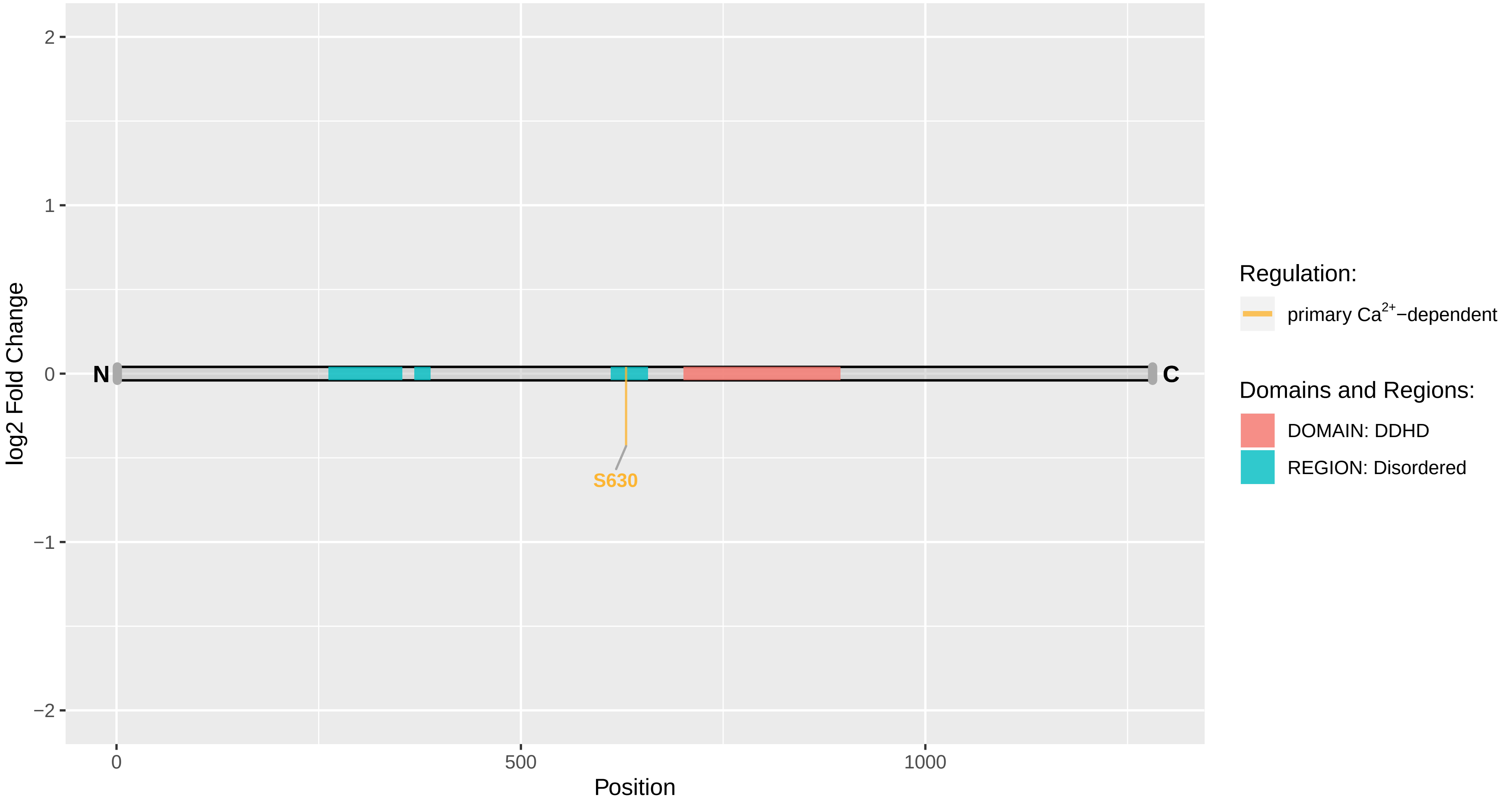

Pja1

Uniprot Accession: A0A0U1RRV0
Praja ring finger ubiquitin ligase 1 (Fragment)

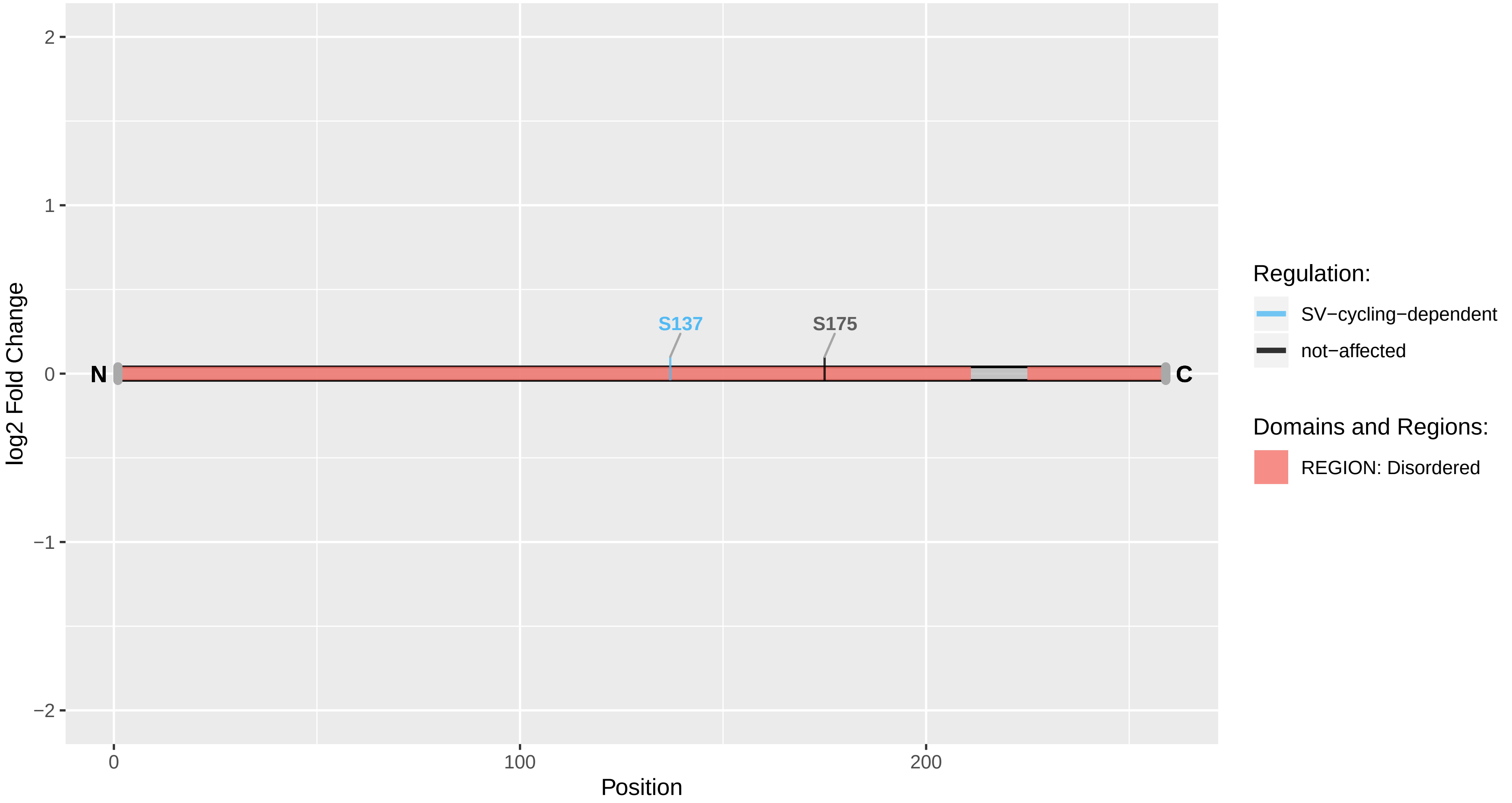

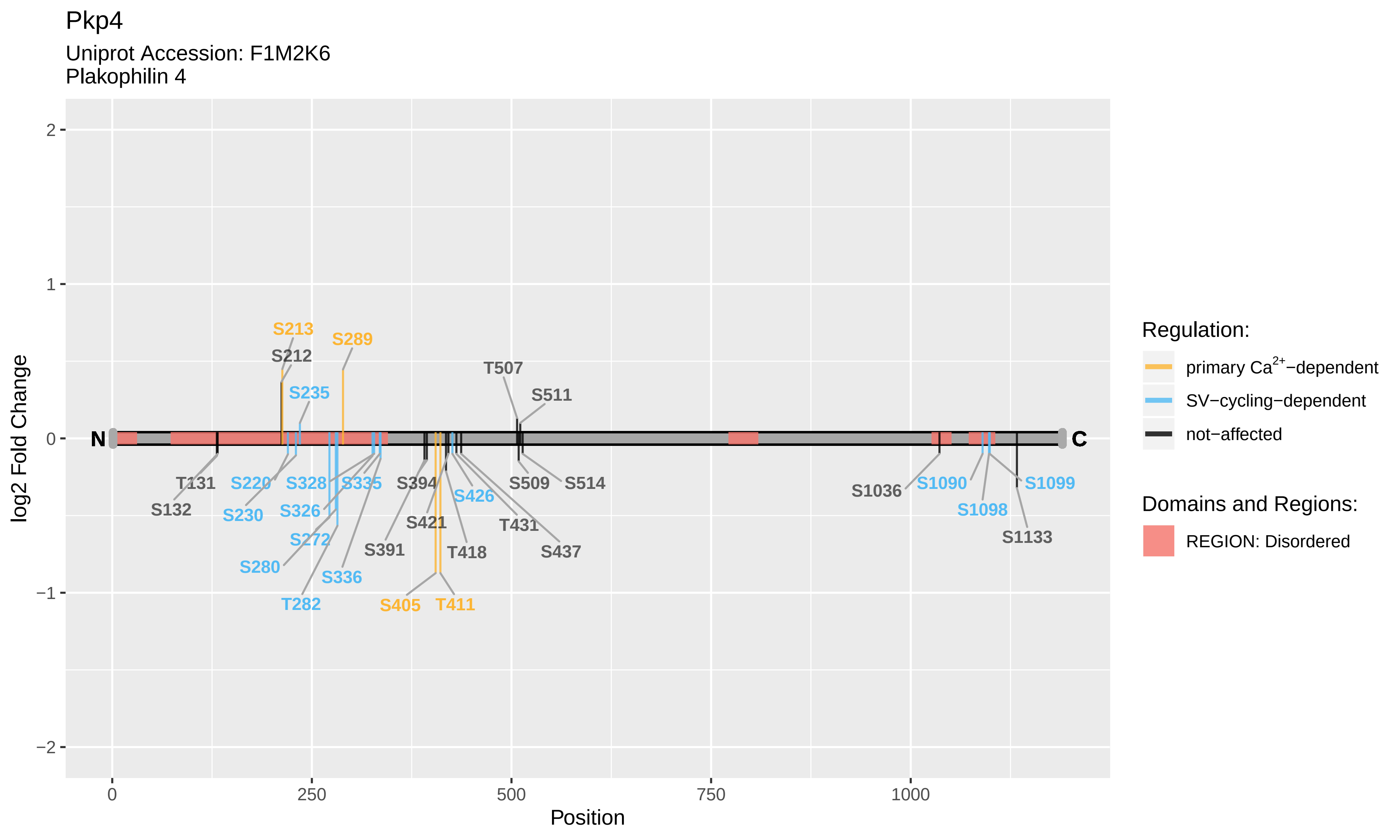
Pkp4
Uniprot Accession: F1M2K6
Plakophilin 4
log2 Fold Change
Position
N
C
S213
S212
S289
S235
T507
S511
T131
S132
S220
S230
S328
S326
S335
S272
S280
T282
S336
S394
S391
S421
S426
T418
S405
T411
T431
S437
S509
S514
S1036
S1090
S1098
S1099
S1133
Regulation:
primary Ca^{2+}−dependent
SV−cycling−dependent
not−affected
Domains and Regions:
REGION: Disordered

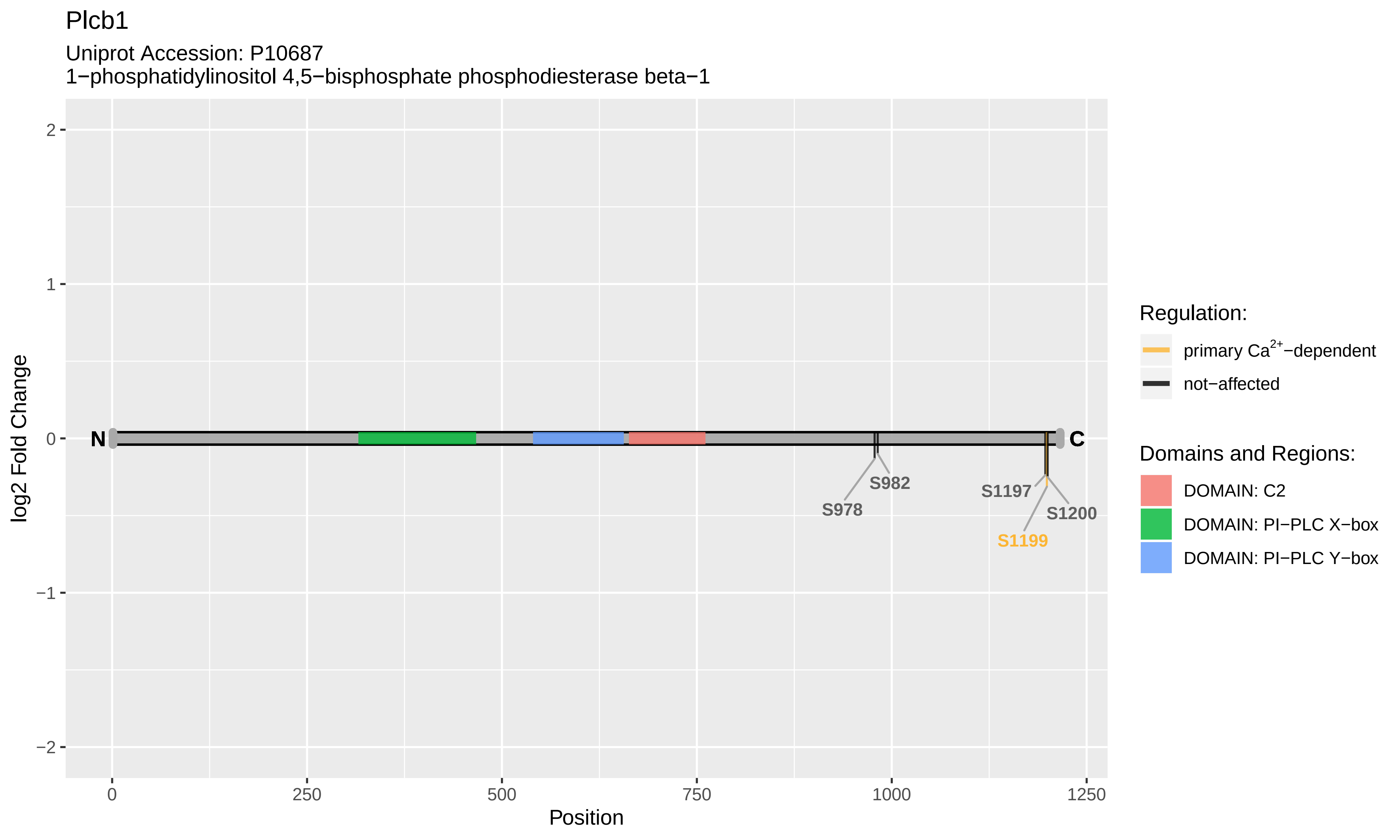
Plcb1
Uniprot Accession: P10687
1−phosphatidylinositol 4,5−bisphosphate phosphodiesterase beta−1
log2 Fold Change
Position
N
C
S978
S982
S1197
S1199
S1200
2
1
0
−1
−2
0
250
500
750
1000
1250
Regulation:
primary Ca2+−dependent
not−affected
Domains and Regions:
DOMAIN: C2
DOMAIN: PI−PLC X−box
DOMAIN: PI−PLC Y−box

Plcg1

Uniprot Accession: P10686
1−phosphatidylinositol 4,5−bisphosphate phosphodiesterase gamma−1

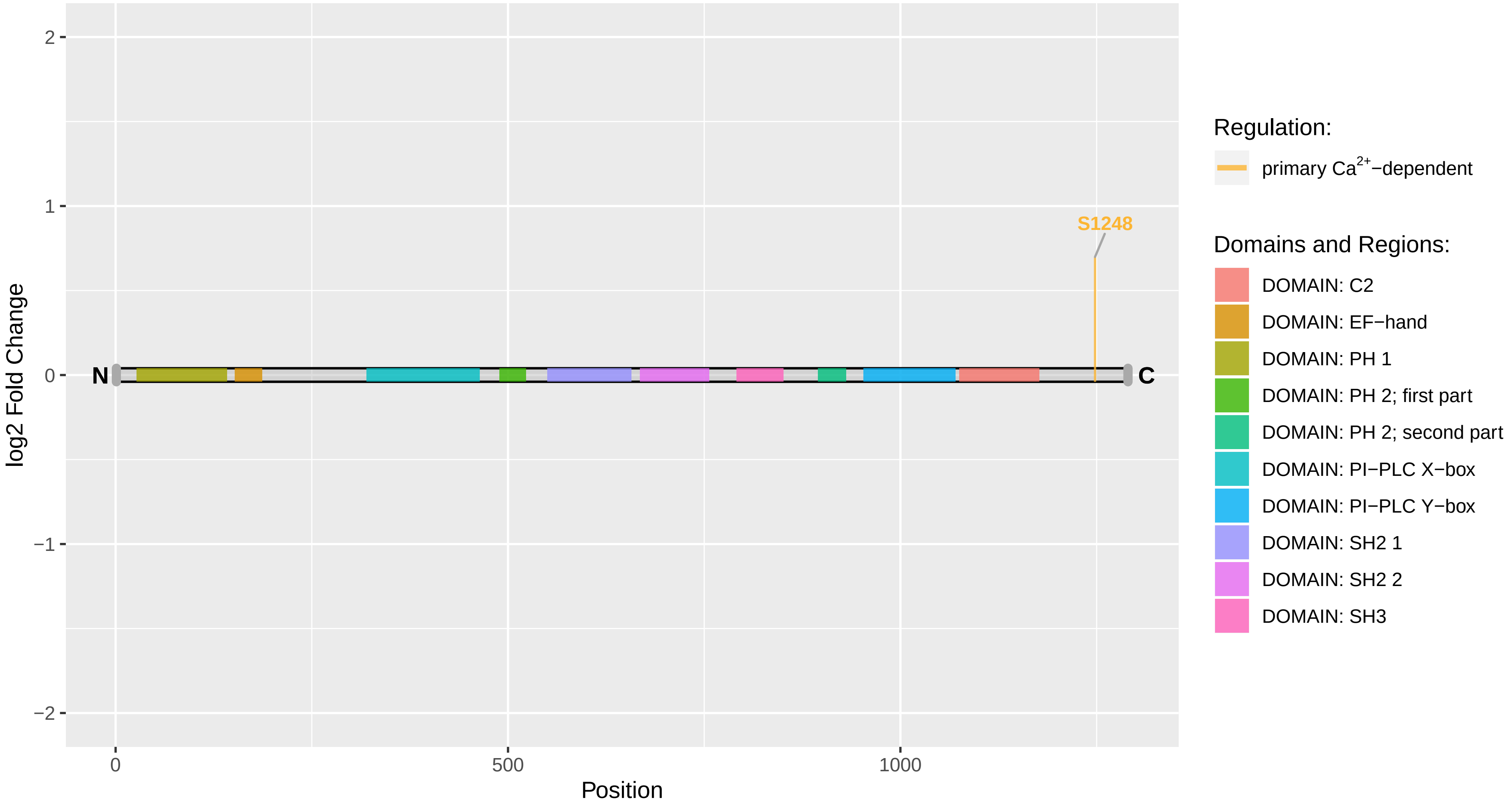

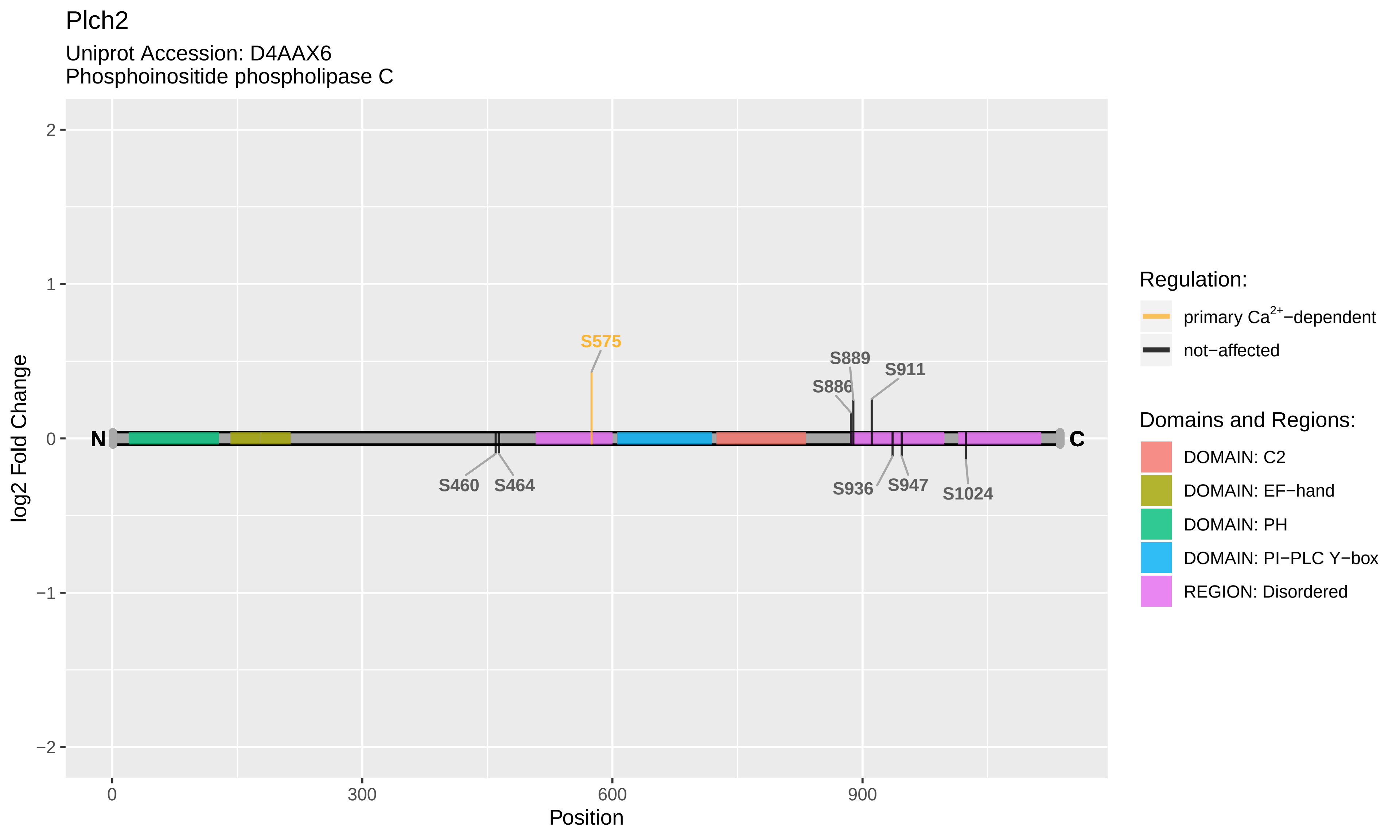

Plch2
Uniprot Accession: D4AAX6
Phosphoinositide phospholipase C
log2 Fold Change
Position
2
1
0
−1
−2
0
300
600
900
N
C
S575
S889
S911
S886
S460
S464
S936
S947
S1024
Regulation:
primary Ca2+−dependent
not−affected
Domains and Regions:
DOMAIN: C2
DOMAIN: EF−hand
DOMAIN: PH
DOMAIN: PI−PLC Y−box
REGION: Disordered

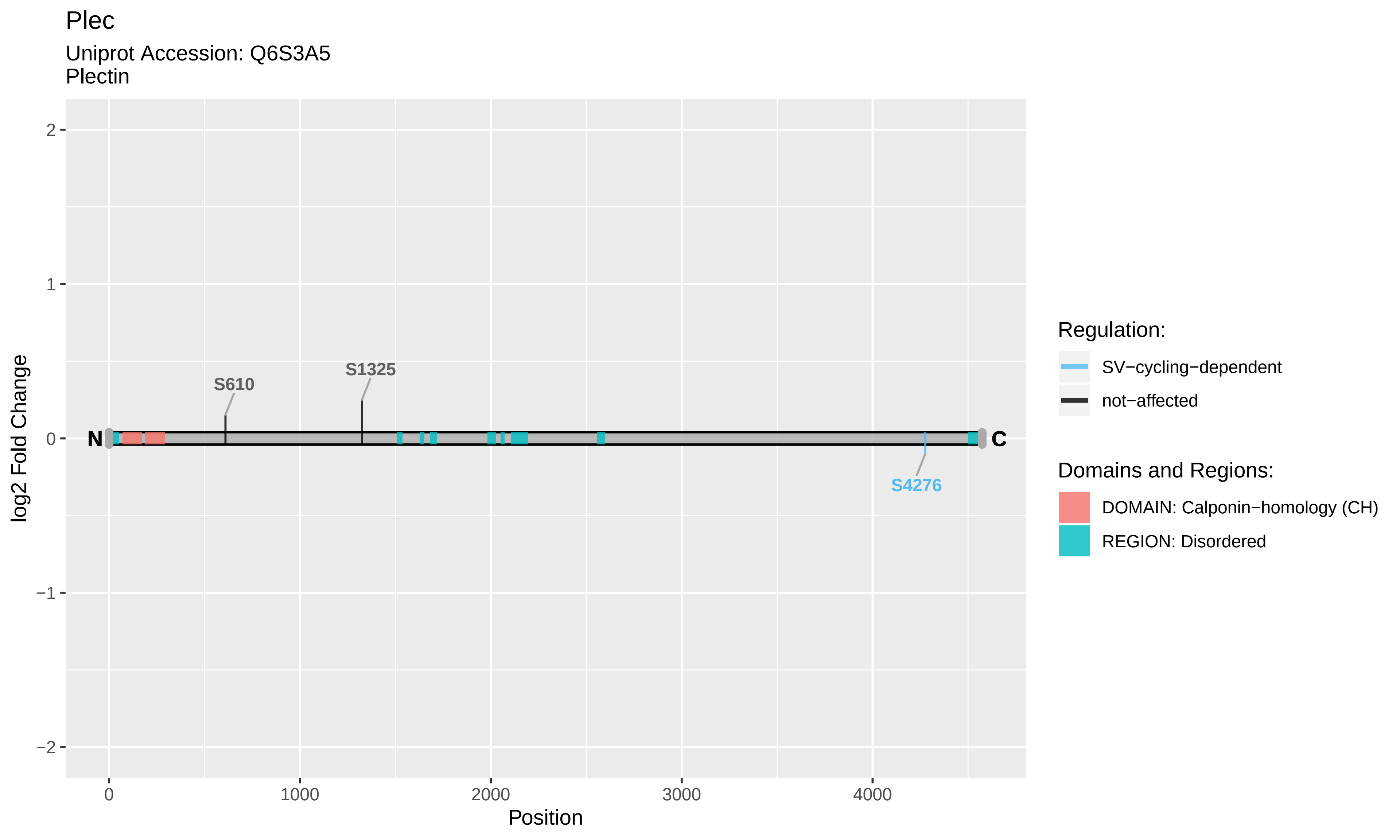
Plec
Uniprot Accession: Q6S3A5
Plectin
log2 Fold Change
2
1
0
−1
−2
N
C
S610
S1325
S4276
0
1000
2000
3000
4000
Position
Regulation:
SV−cycling−dependent
not−affected
Domains and Regions:
DOMAIN: Calponin−homology (CH)
REGION: Disordered

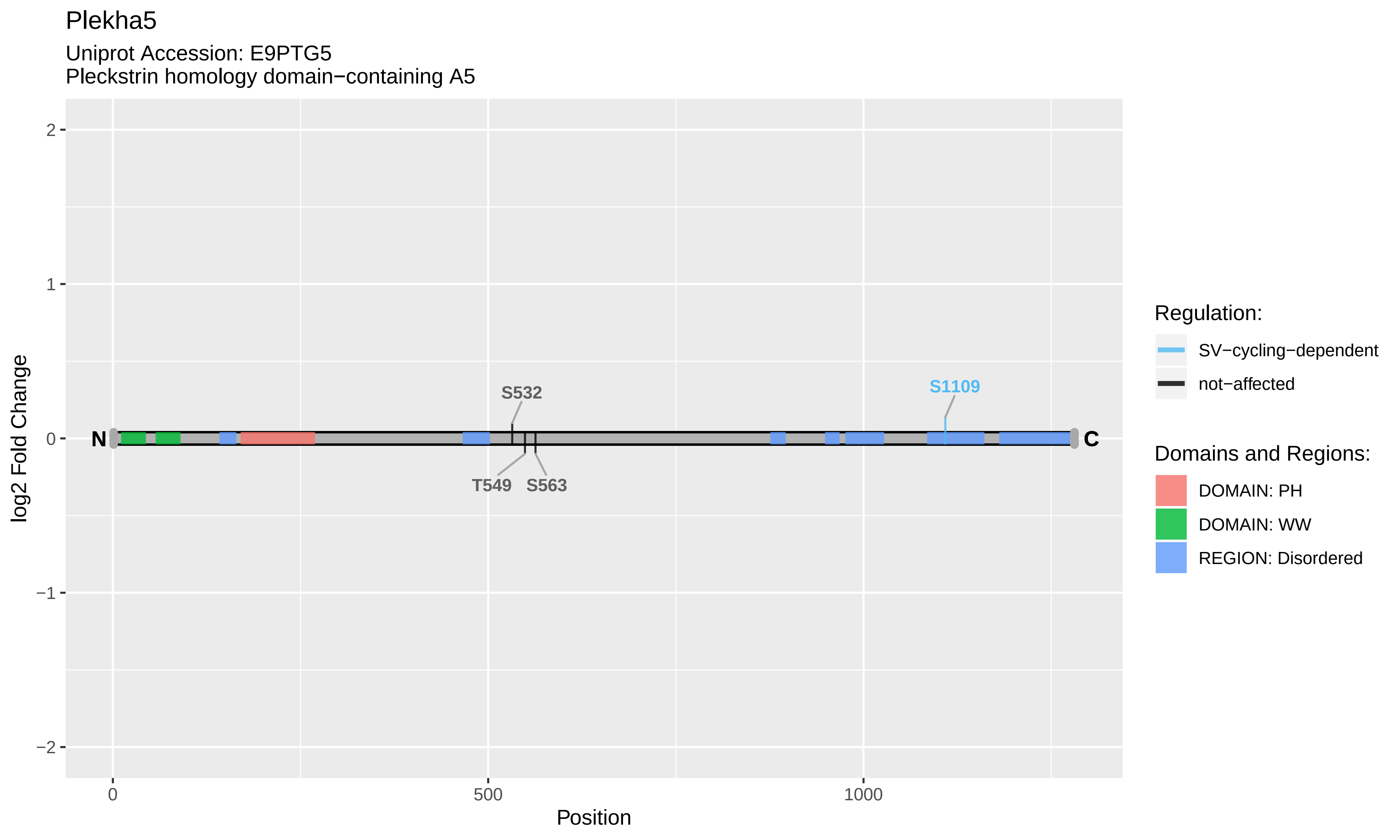

Plekha5
Uniprot Accession: E9PTG5
Pleckstrin homology domain−containing A5
log2 Fold Change
2
1
0
−1
−2
N
C
S532
T549
S563
S1109
0
500
1000
Position
Regulation:
SV−cycling−dependent
not−affected
Domains and Regions:
DOMAIN: PH
DOMAIN: WW
REGION: Disordered

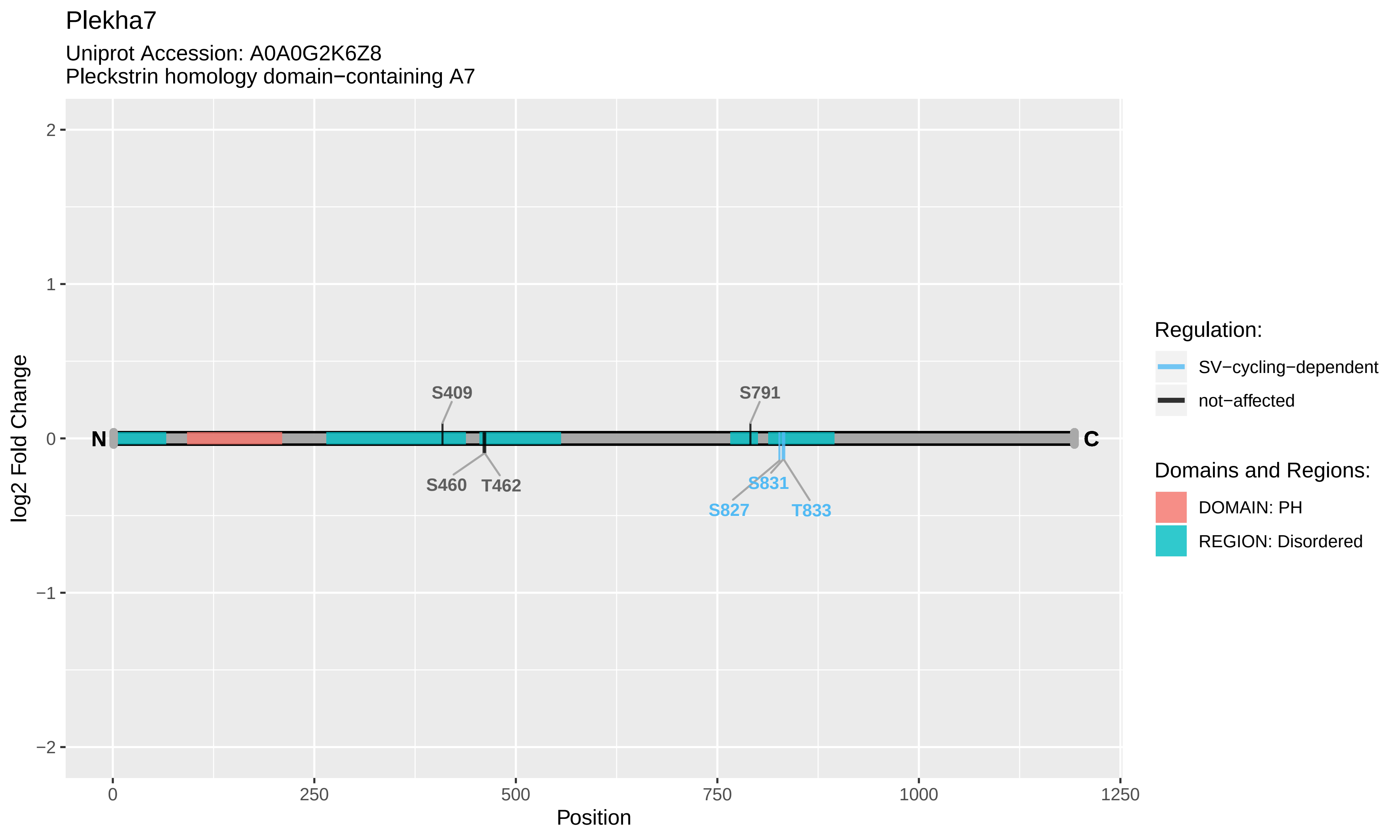

Plekha7
Uniprot Accession: A0A0G2K6Z8
Pleckstrin homology domain−containing A7
log2 Fold Change
2
1
0
−1
−2
N
C
S409
S791
S460
T462
S831
S827
T833
0
250
500
750
1000
1250
Position
Regulation:
SV−cycling−dependent
not−affected
Domains and Regions:
DOMAIN: PH
REGION: Disordered

Plp1

Uniprot Accession: P60203
Myelin proteolipid protein

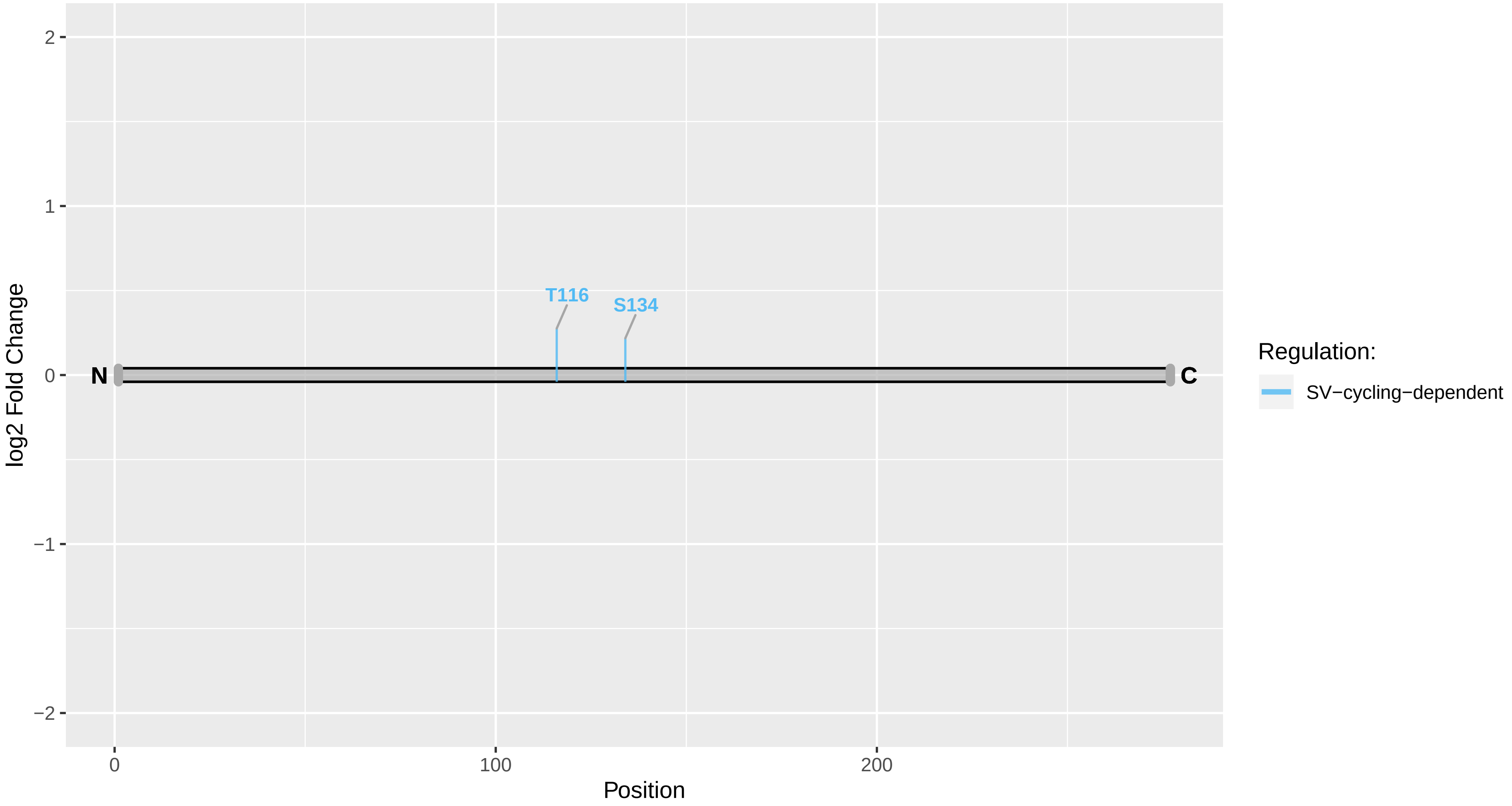

Plpp6

Uniprot Accession: Q66H88
Phospholipid phosphatase 6

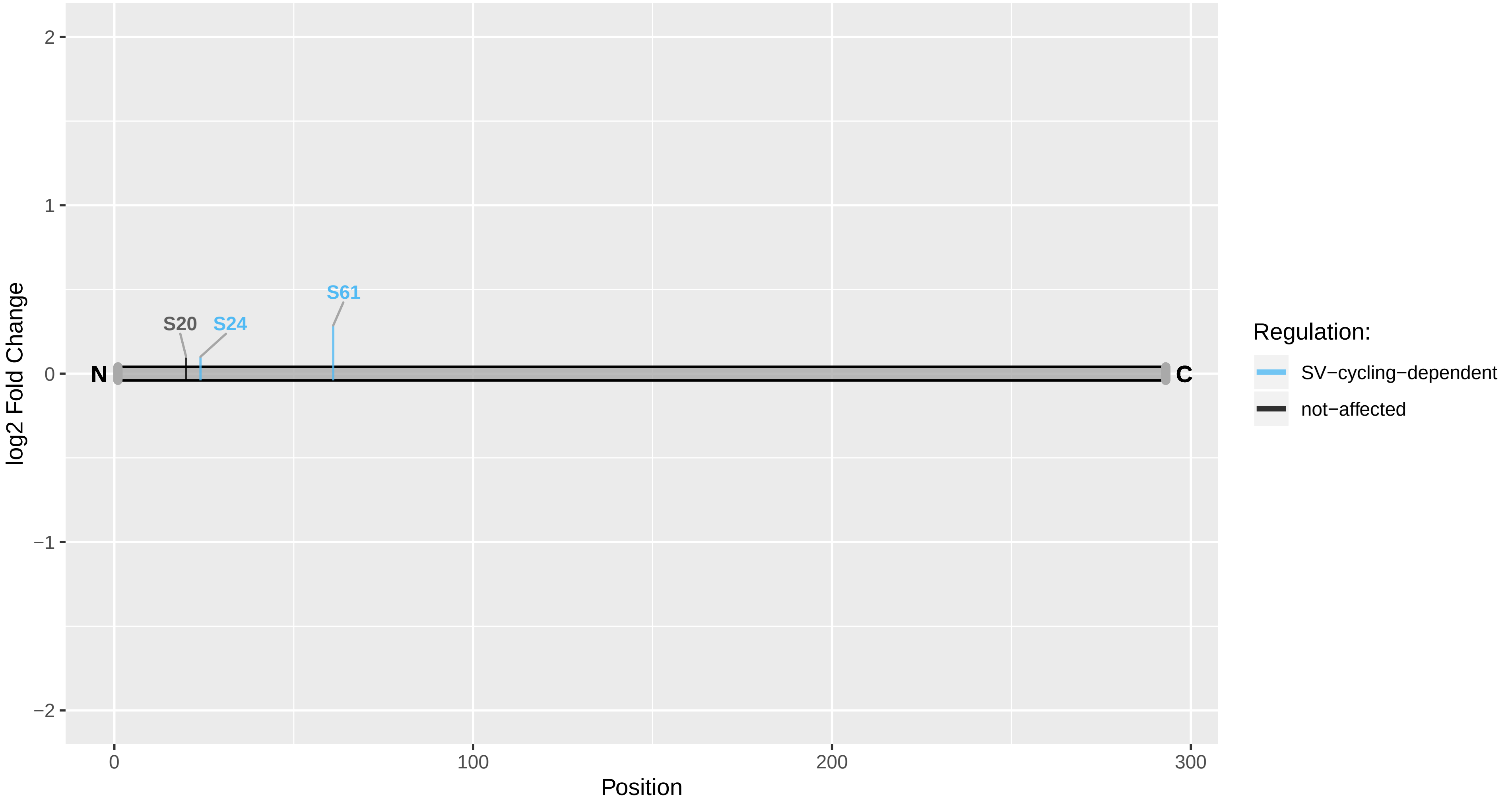

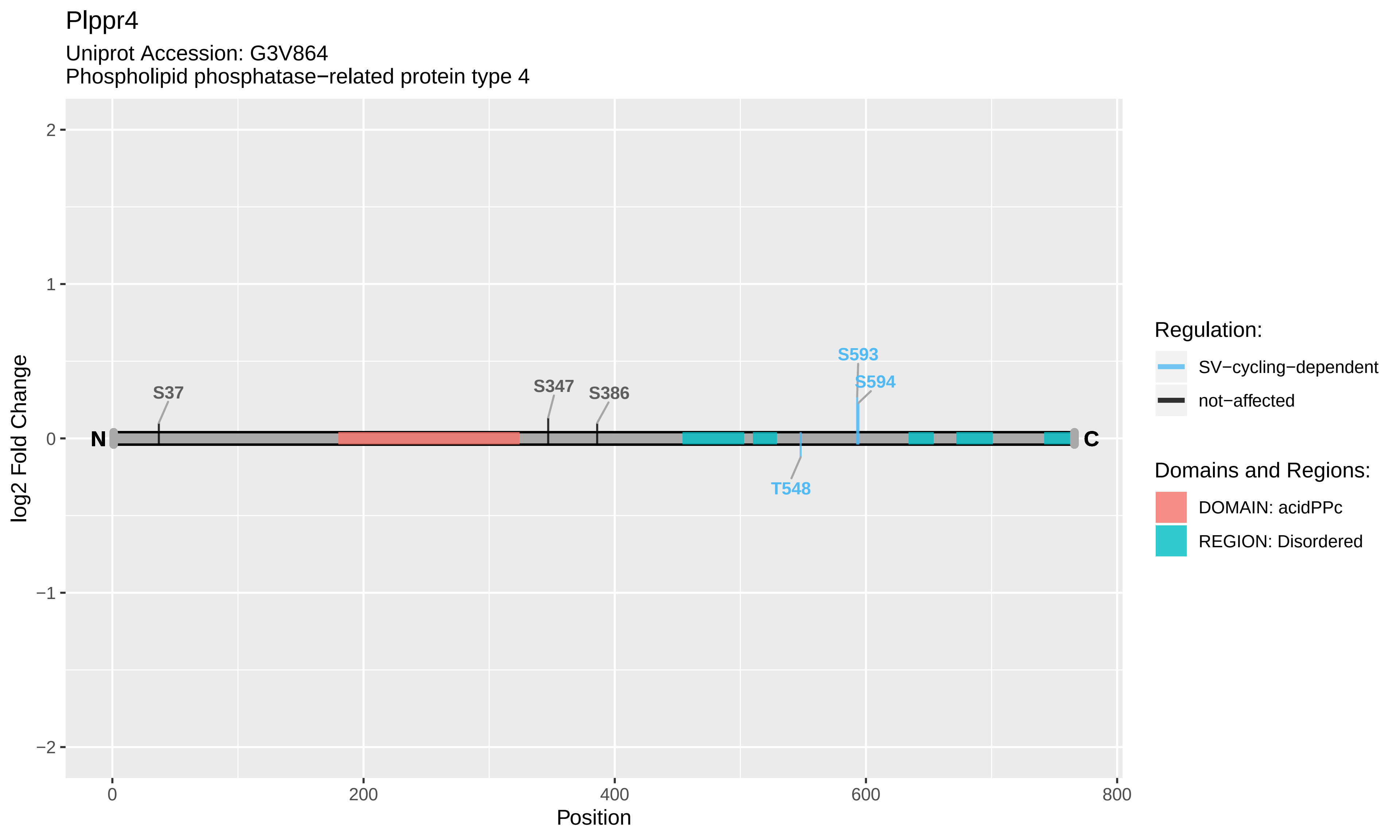

Plppr4
Uniprot Accession: G3V864
Phospholipid phosphatase−related protein type 4
log2 Fold Change
Position
N
C
S37
S347
S386
S593
S594
T548
2
1
0
−1
−2
0
200
400
600
800
Regulation:
SV−cycling−dependent
not−affected
Domains and Regions:
DOMAIN: acidPPc
REGION: Disordered

Pnma8b

Uniprot Accession: D3ZQN3
PNMA family member 8B

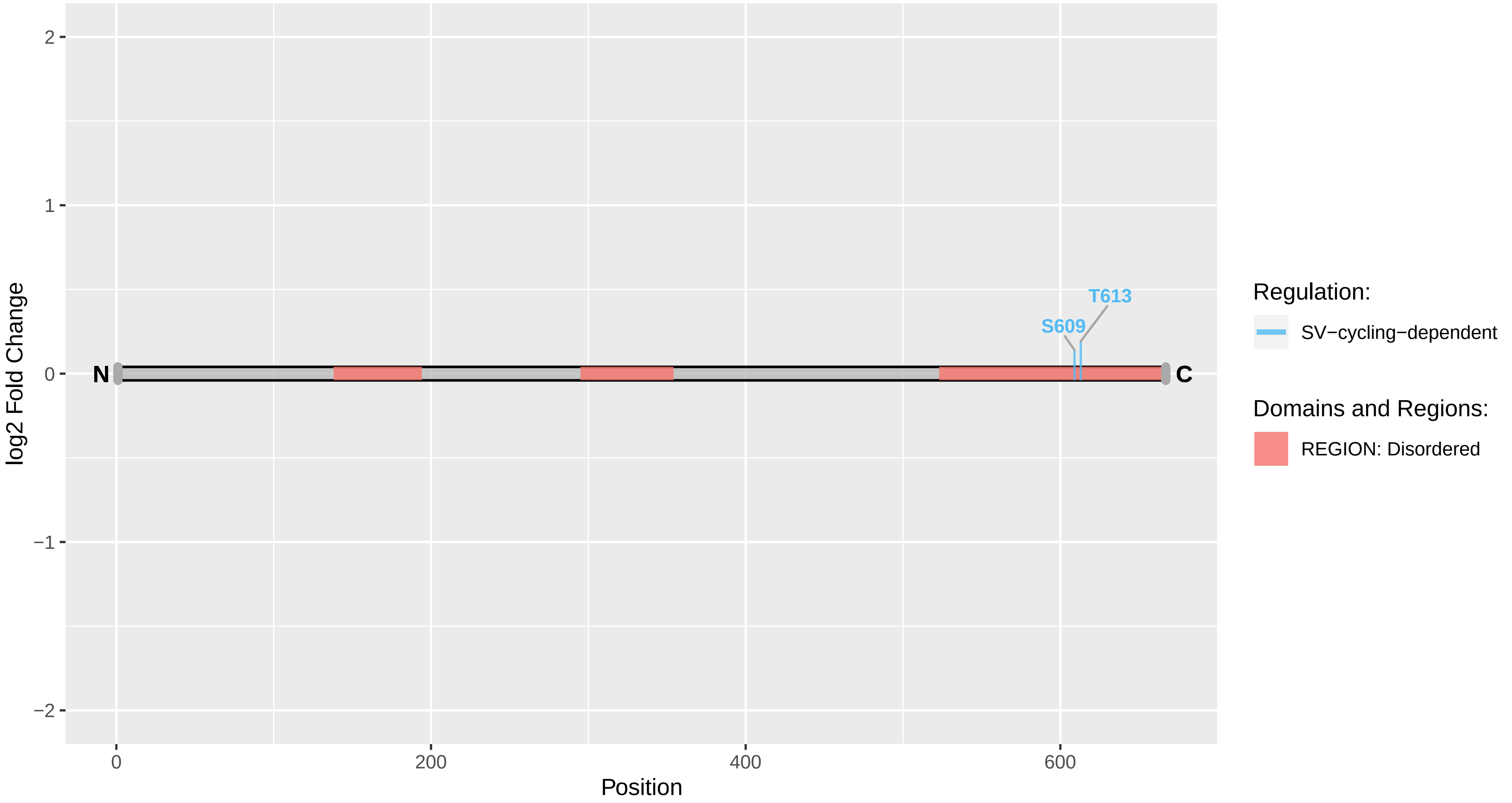

Pom121l2

Uniprot Accession: D3ZGH9
POM121 transmembrane nucleoporin−like 2

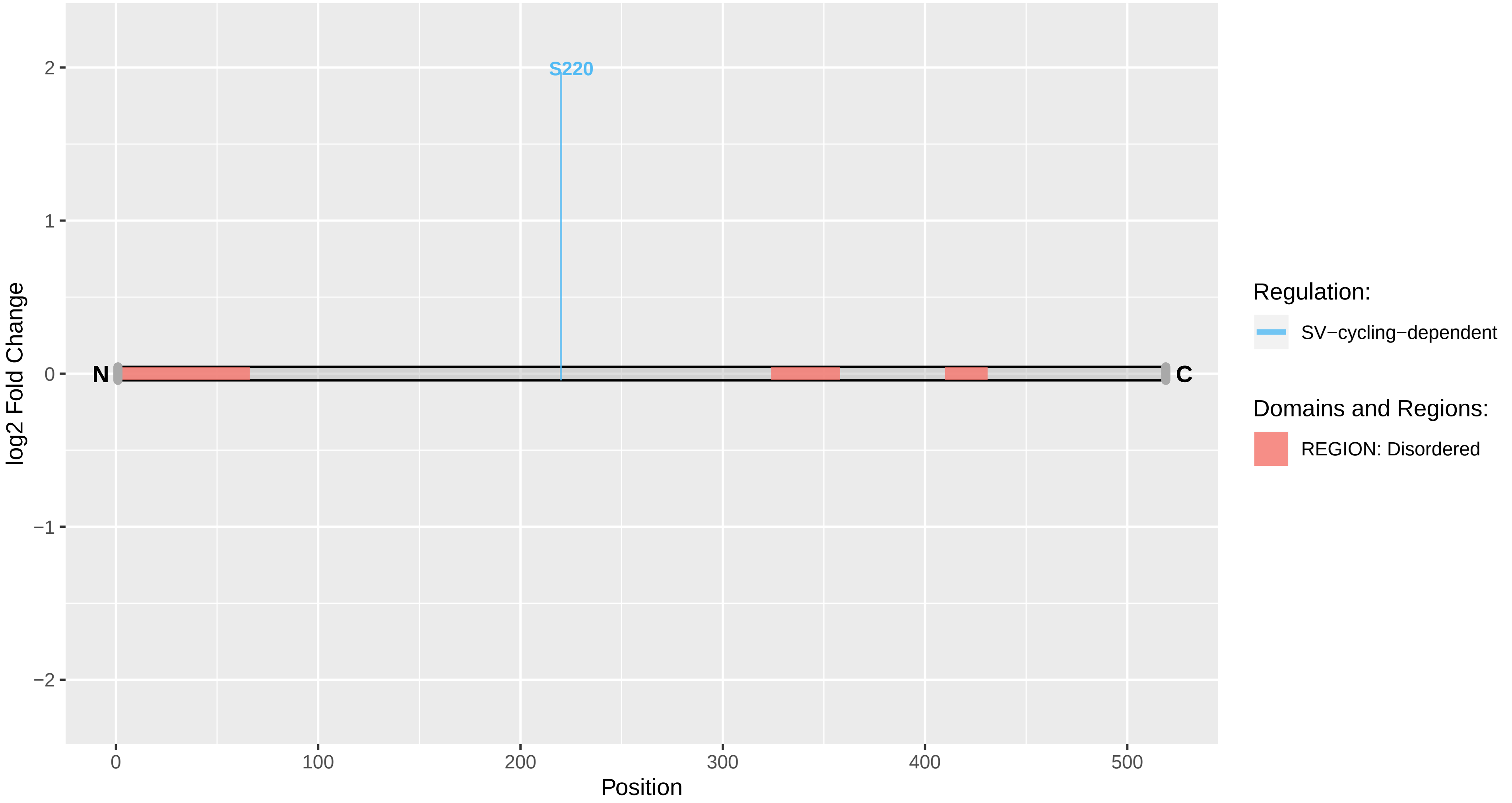

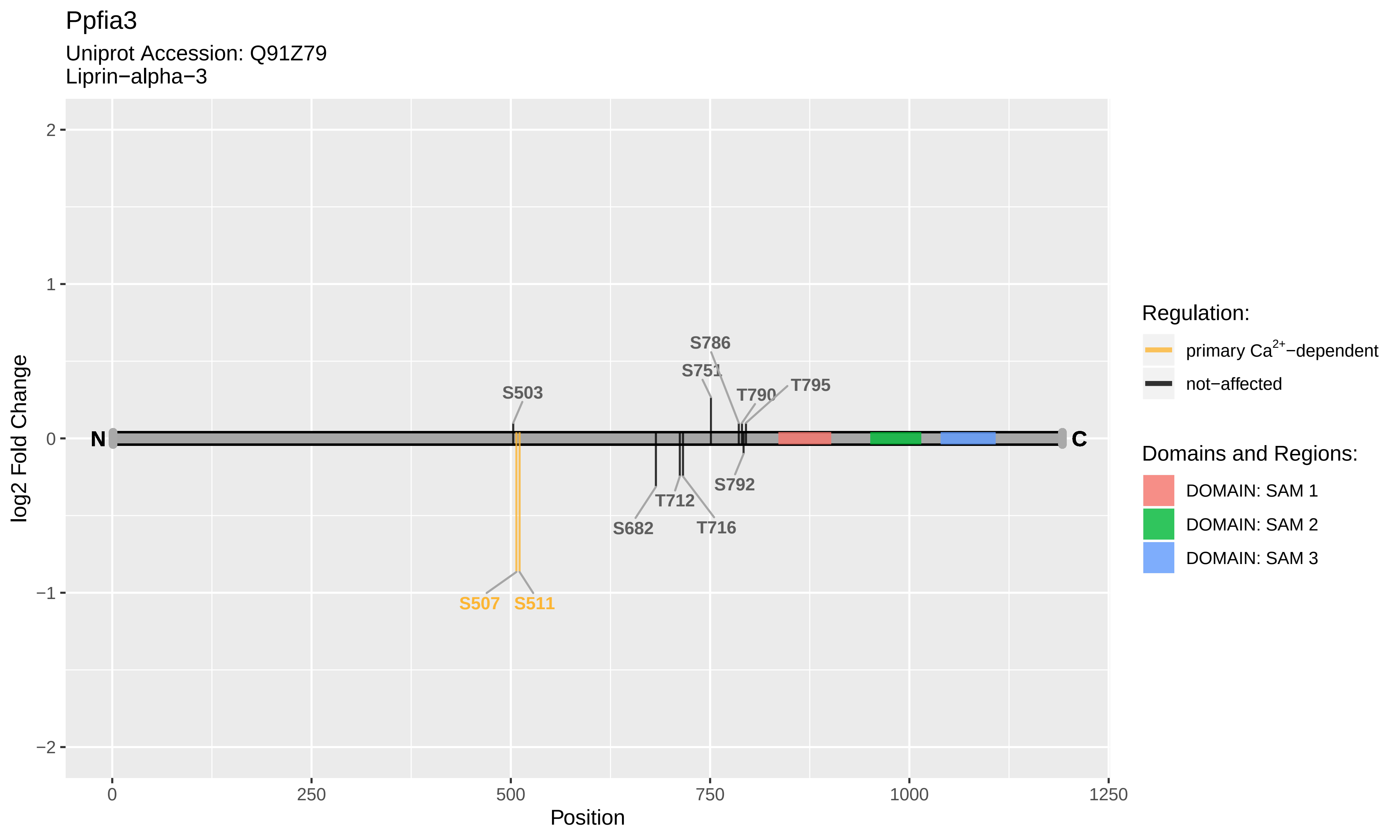

Ppfia3
Uniprot Accession: Q91Z79
Liprin−alpha−3
log2 Fold Change
Position
N
C
S786
S751
S503
T790
T795
S792
T712
S682
T716
S507
S511
2
1
0
−1
−2
0
250
500
750
1000
1250
Regulation:
primary Ca2+−dependent
not−affected
Domains and Regions:
DOMAIN: SAM 1
DOMAIN: SAM 2
DOMAIN: SAM 3

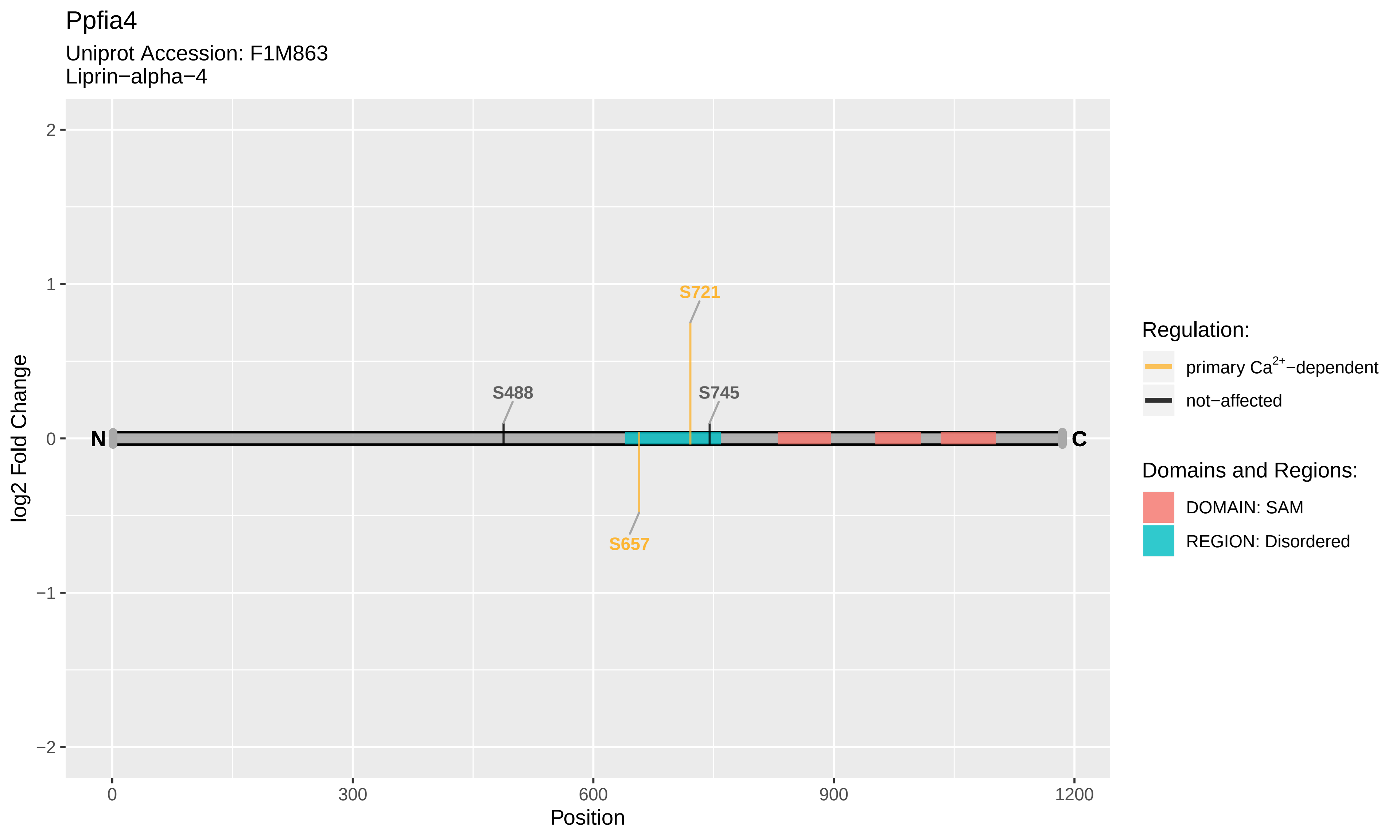

Ppfia4
Uniprot Accession: F1M863
Liprin−alpha−4
log2 Fold Change
2
1
0
−1
−2
N
C
S488
S721
S745
S657
0
300
600
900
1200
Position
Regulation:
primary Ca2+−dependent
not−affected
Domains and Regions:
DOMAIN: SAM
REGION: Disordered

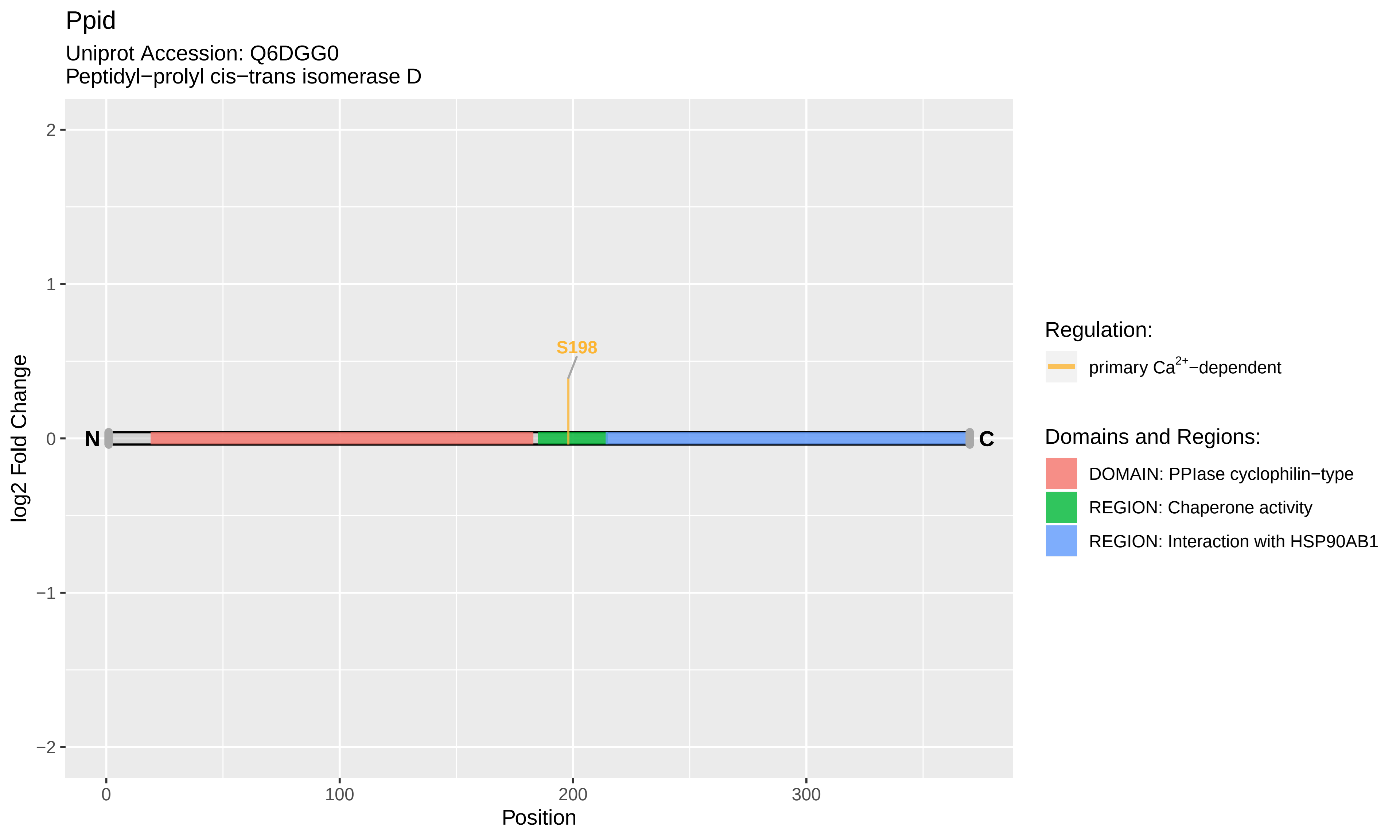

Ppid
Uniprot Accession: Q6DGG0
Peptidyl−prolyl cis−trans isomerase D
log2 Fold Change
2
1
0
−1
−2
N
C
S198
0
100
200
300
Position
Regulation:
primary Ca2+−dependent
Domains and Regions:
DOMAIN: PPIase cyclophilin−type
REGION: Chaperone activity
REGION: Interaction with HSP90AB1

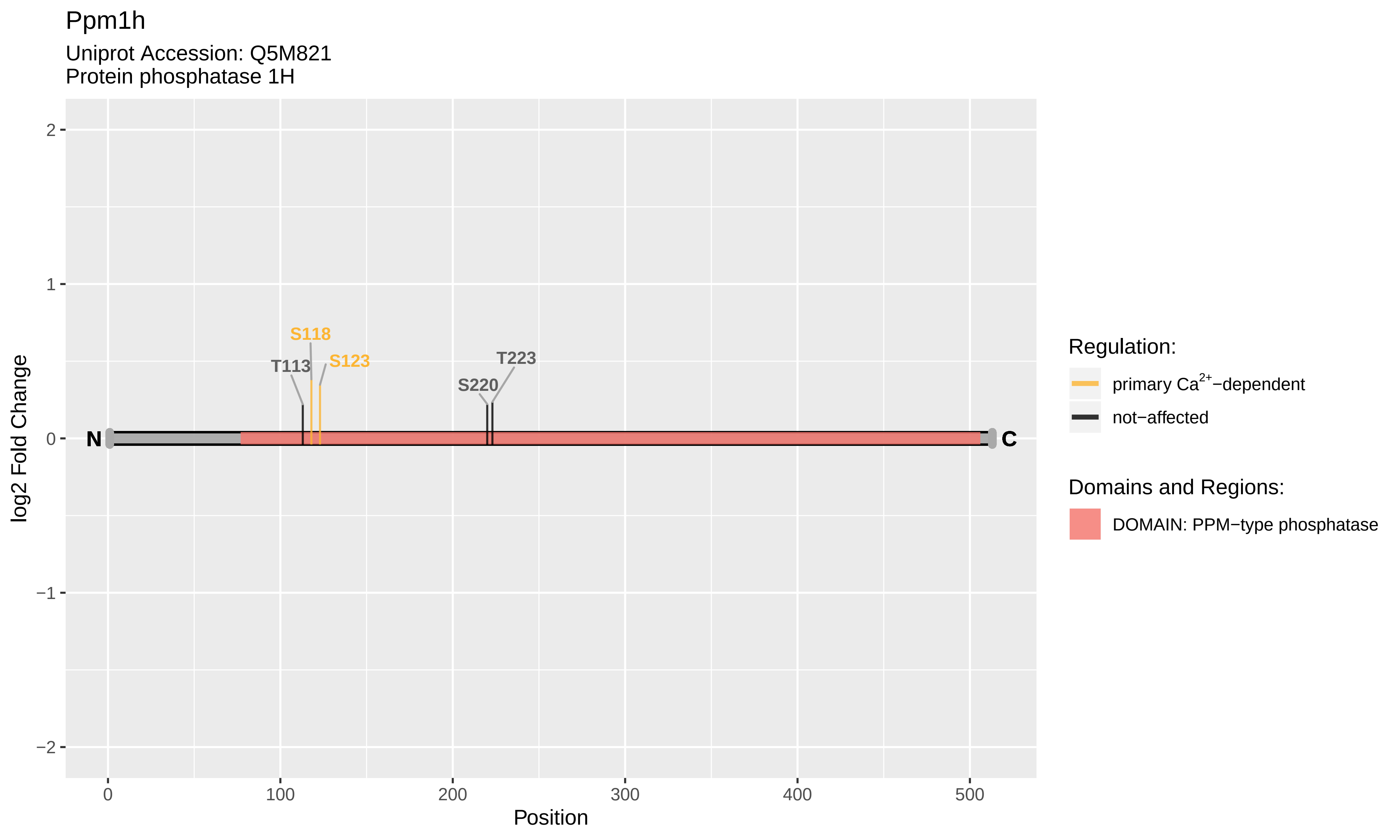

Ppm1h
Uniprot Accession: Q5M821
Protein phosphatase 1H
S118
S123
T113
T223
S220
N
C
2
1
0
−1
−2
log2 Fold Change
0
100
200
300
400
500
Position
Regulation:
primary Ca2+−dependent
not−affected
Domains and Regions:
DOMAIN: PPM−type phosphatase

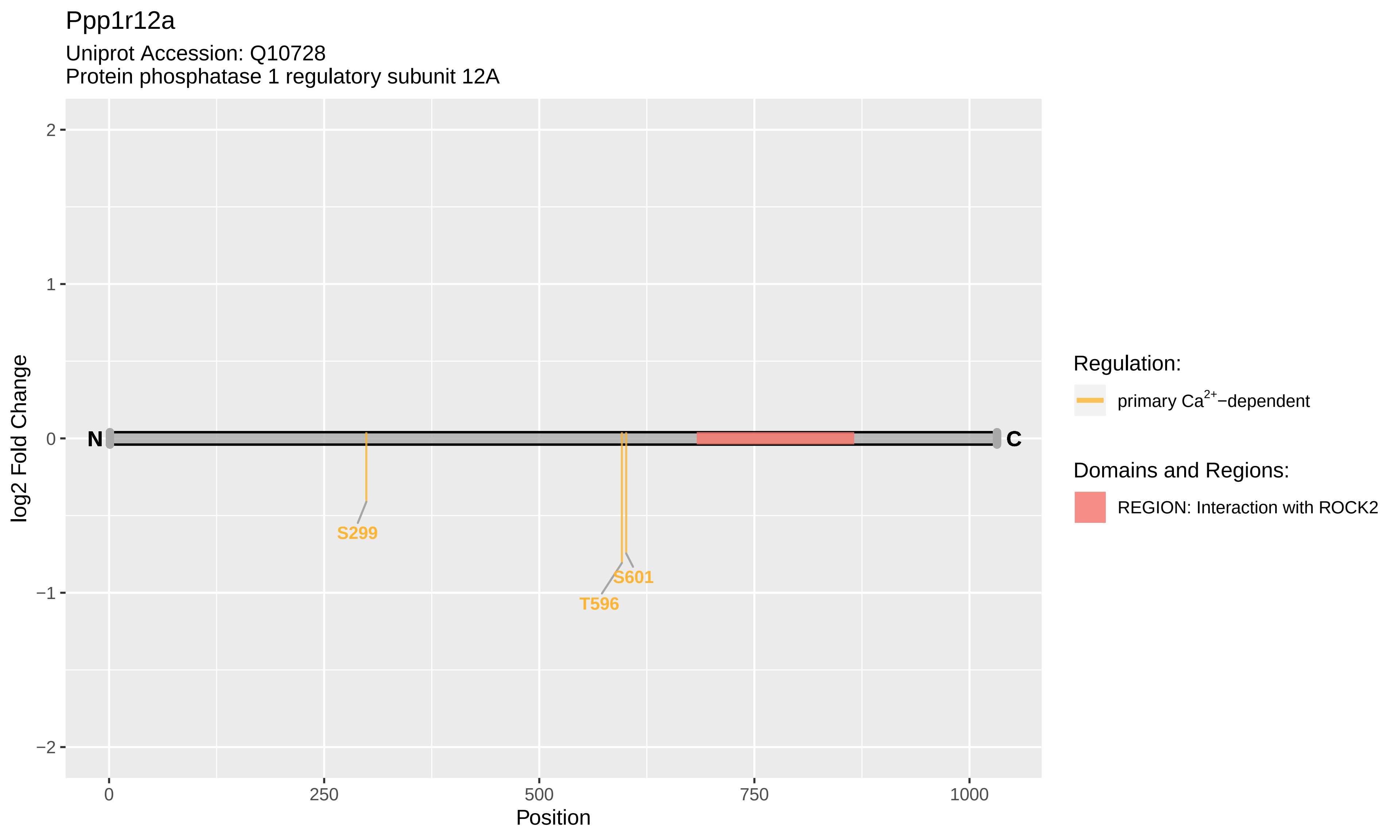

Ppp1r12a
Uniprot Accession: Q10728
Protein phosphatase 1 regulatory subunit 12A
log2 Fold Change
2
1
0
−1
−2
N
C
S299
T596
S601
0
250
500
750
1000
Position
Regulation:
primary Ca2+−dependent
Domains and Regions:
REGION: Interaction with ROCK2

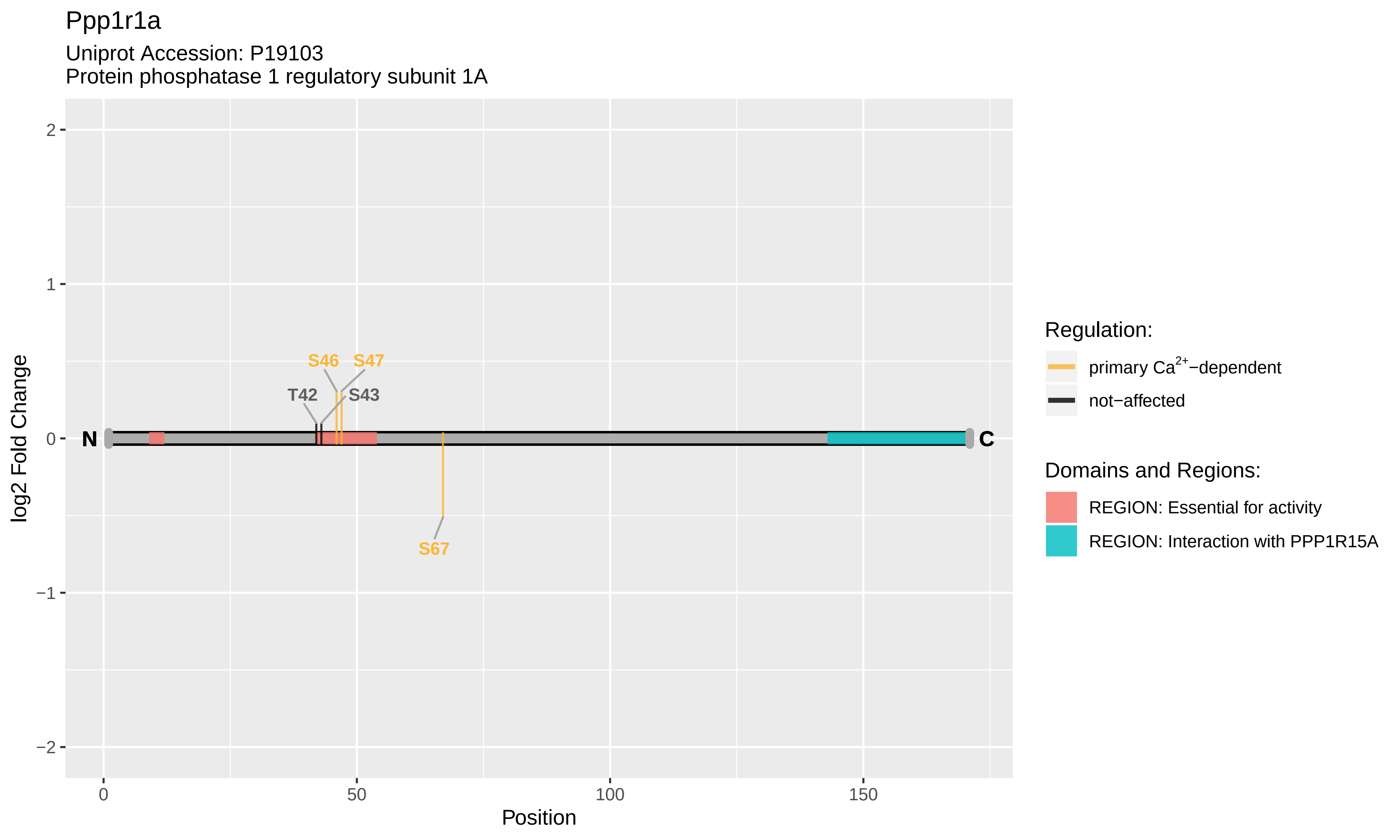

Ppp1r1a
Uniprot Accession: P19103
Protein phosphatase 1 regulatory subunit 1A
log2 Fold Change
Position
S46
S47
T42
S43
S67
N
C
2
1
0
−1
−2
0
50
100
150
Regulation:
primary Ca2+−dependent
not−affected
Domains and Regions:
REGION: Essential for activity
REGION: Interaction with PPP1R15A

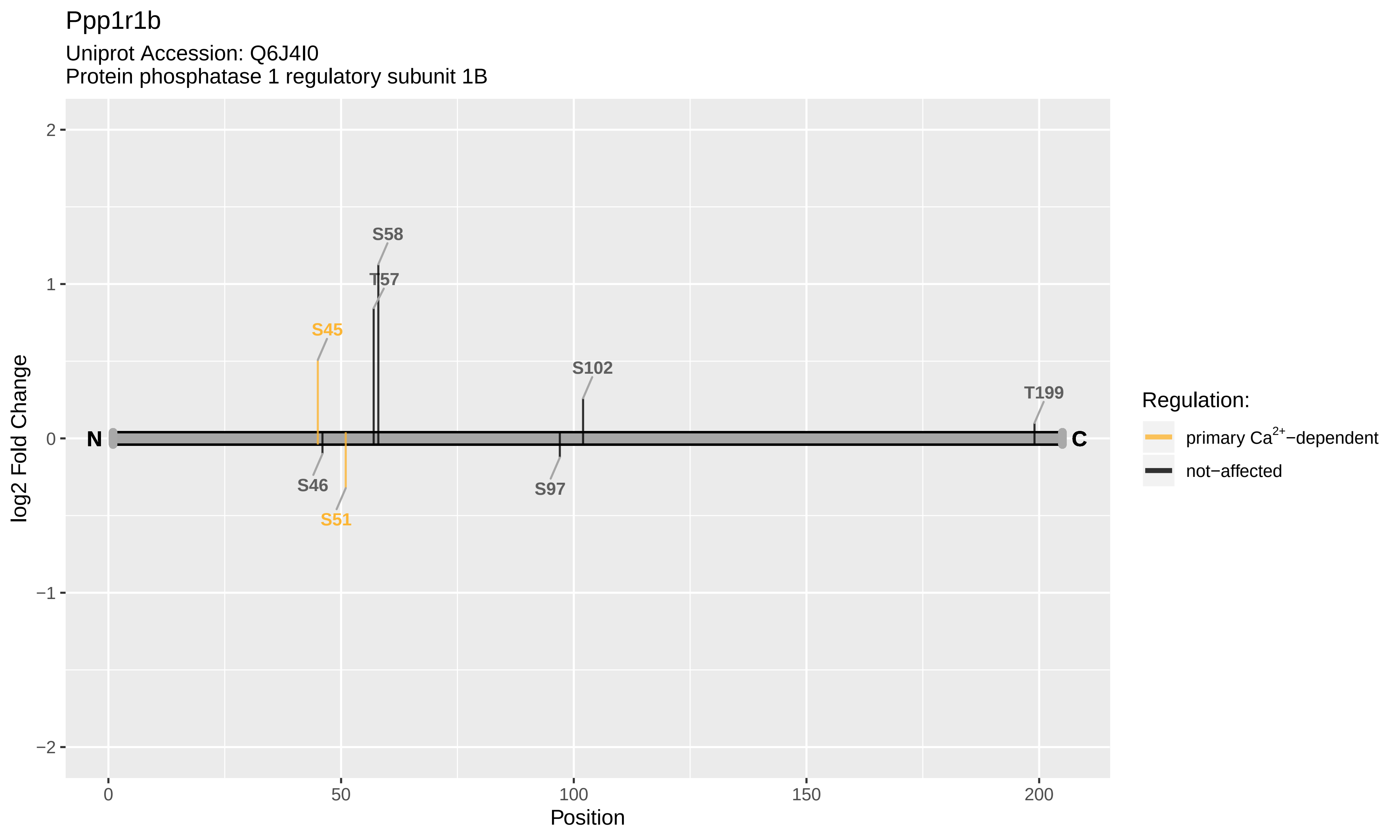
Ppp1r1b
Uniprot Accession: Q6J4I0
Protein phosphatase 1 regulatory subunit 1B
log2 Fold Change
2
1
0
−1
−2
N
C
S58
T57
S45
S102
T199
S46
S51
S97
0
50
100
150
200
Position
Regulation:
primary Ca2+−dependent
not−affected

Ppp1r2

Uniprot Accession: P50411
Protein phosphatase inhibitor 2

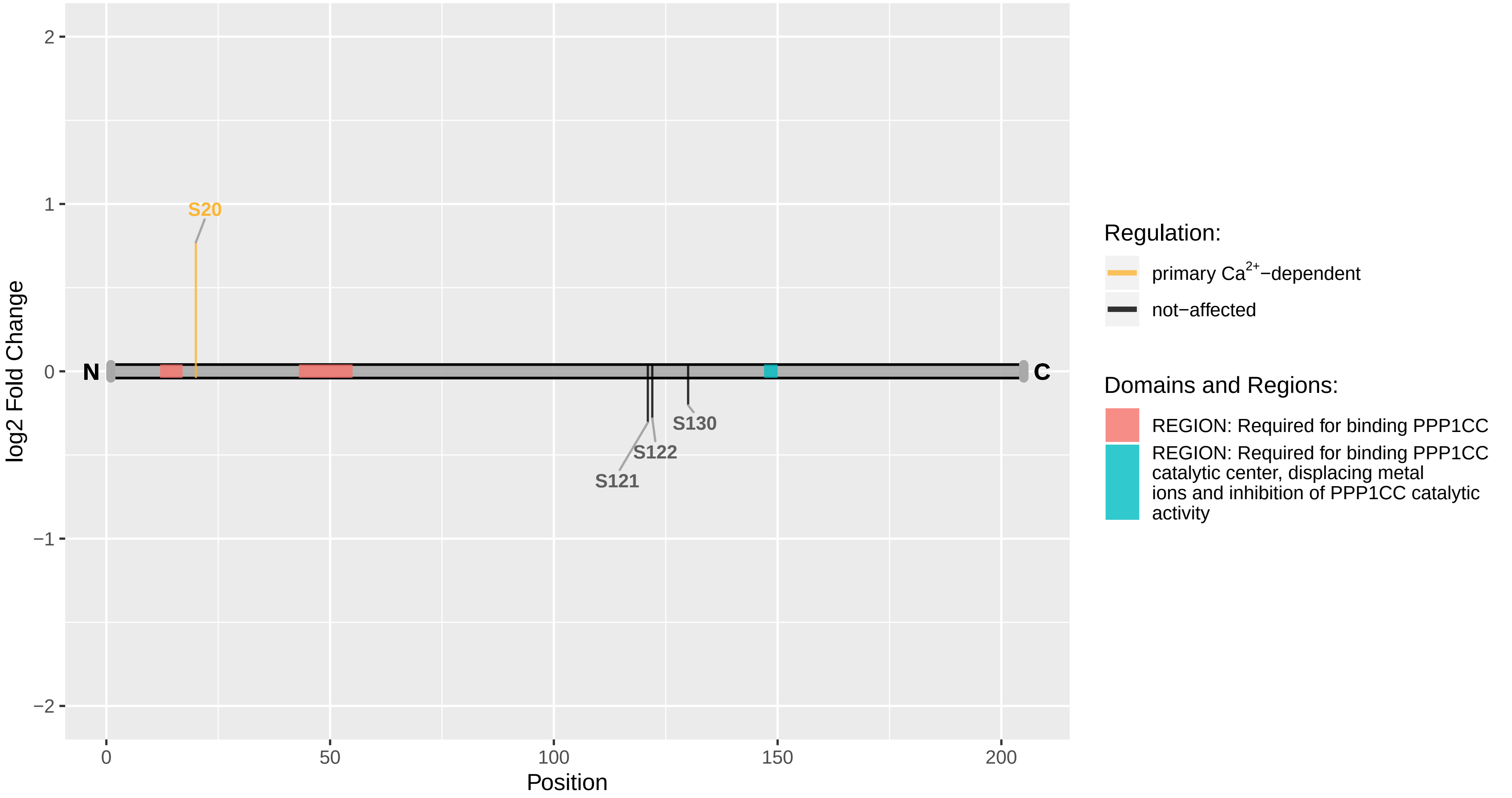

Ppp1r21

Uniprot Accession: F1LYZ8
Protein phosphatase 1, regulatory subunit 21

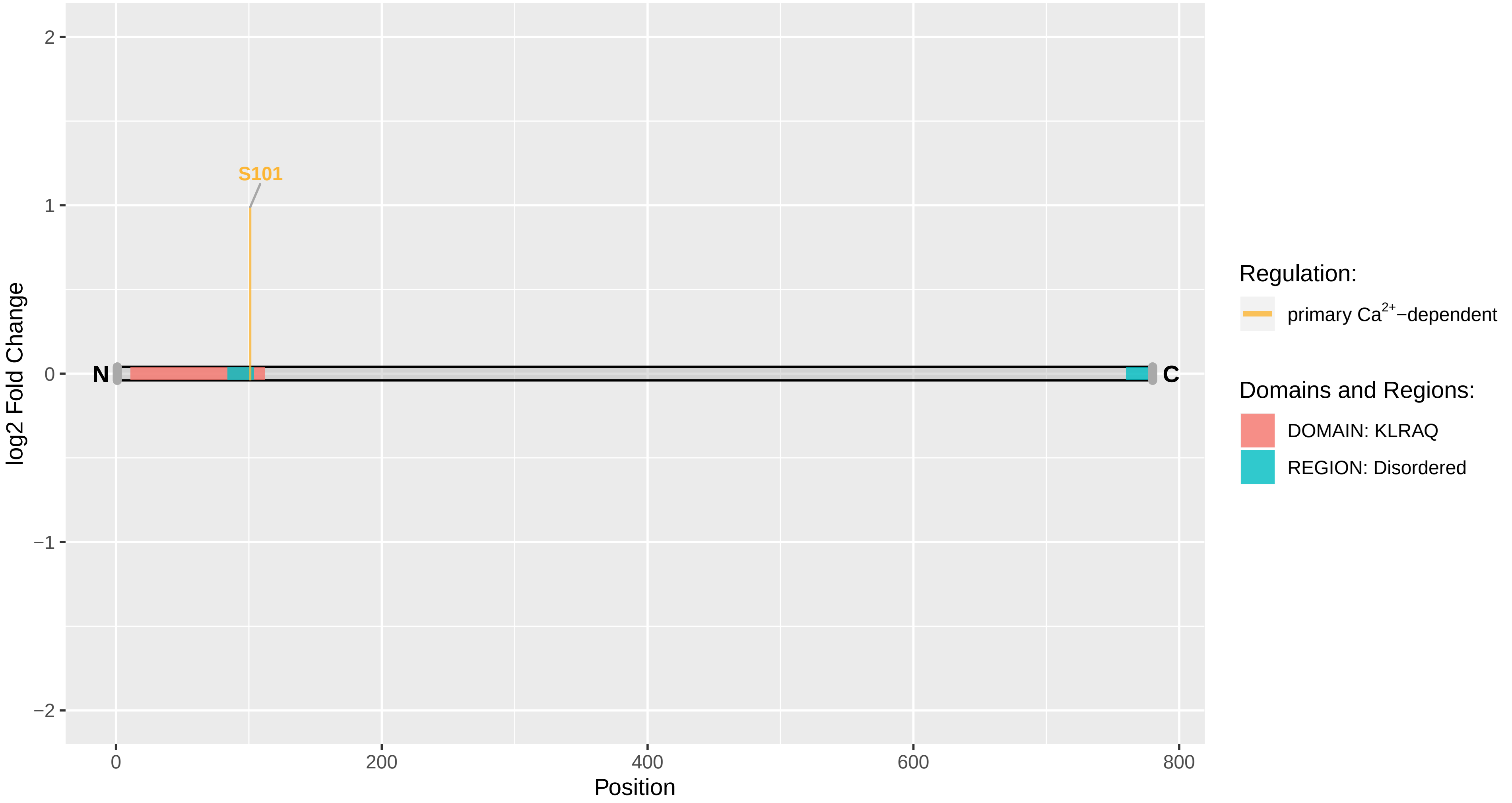

Ppp1r3e

Uniprot Accession: P0C7L8
Protein phosphatase 1 regulatory subunit 3E

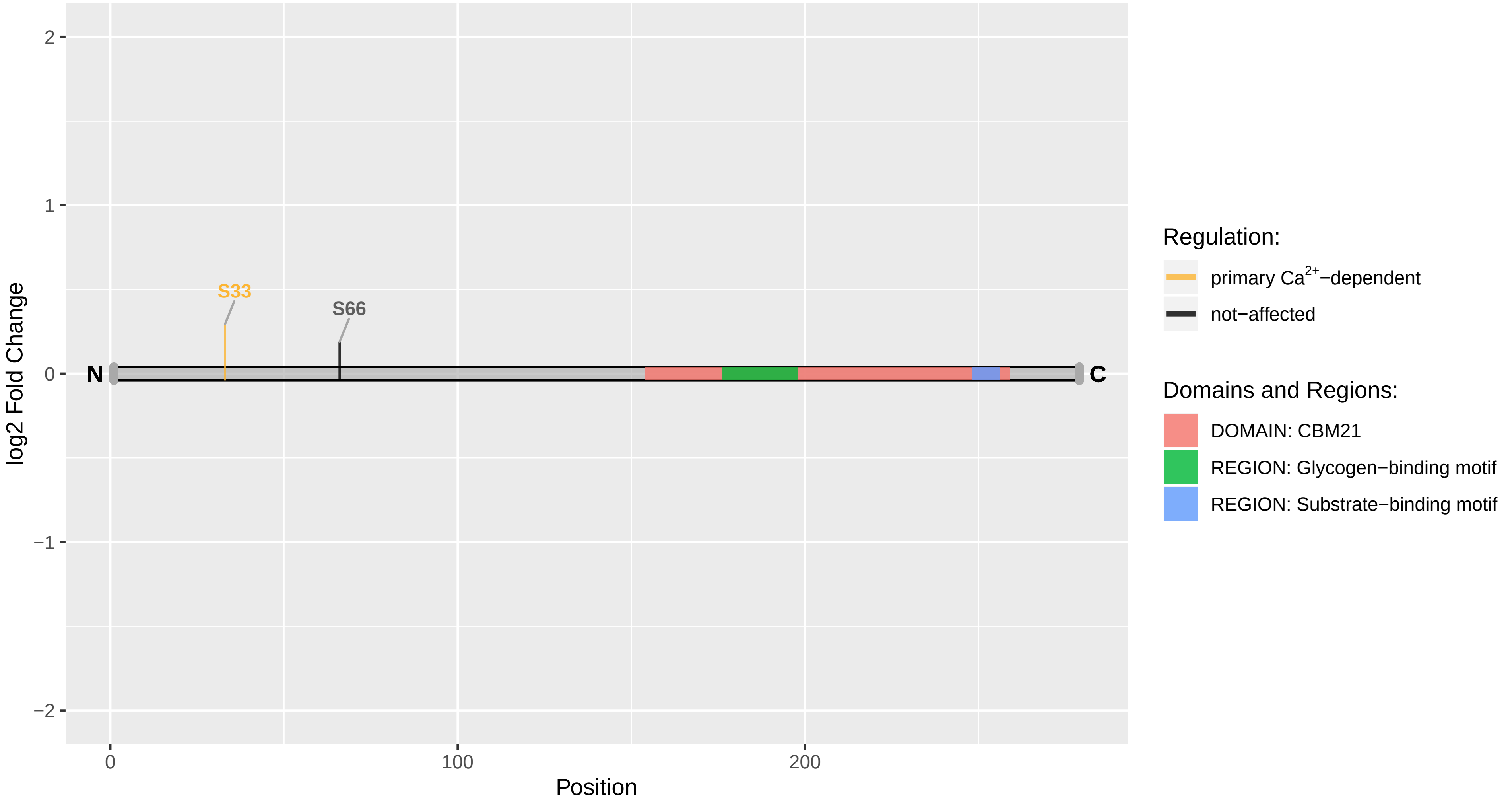

Ppp1r7

Uniprot Accession: Q5HZV9
Protein phosphatase 1 regulatory subunit 7

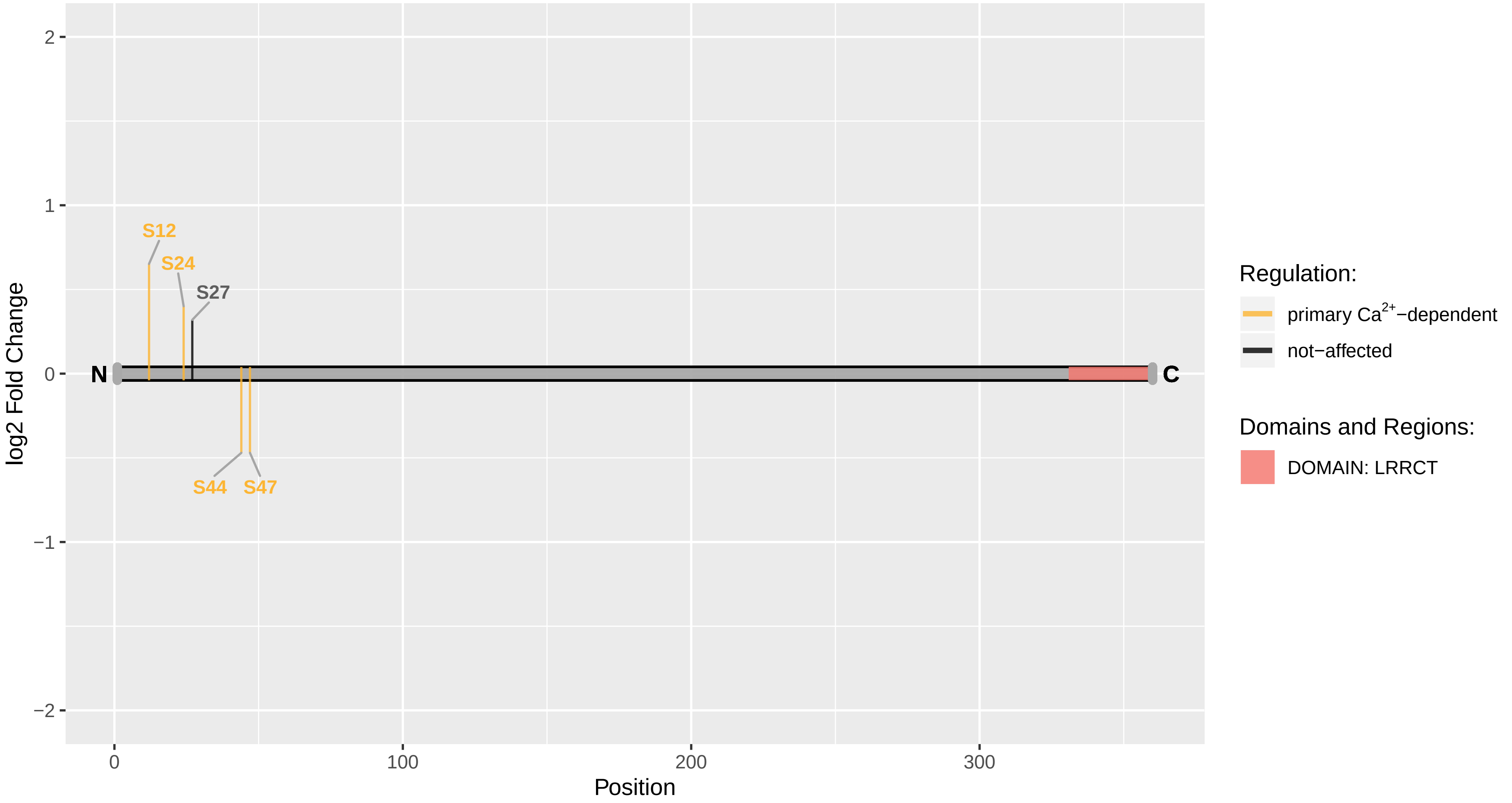

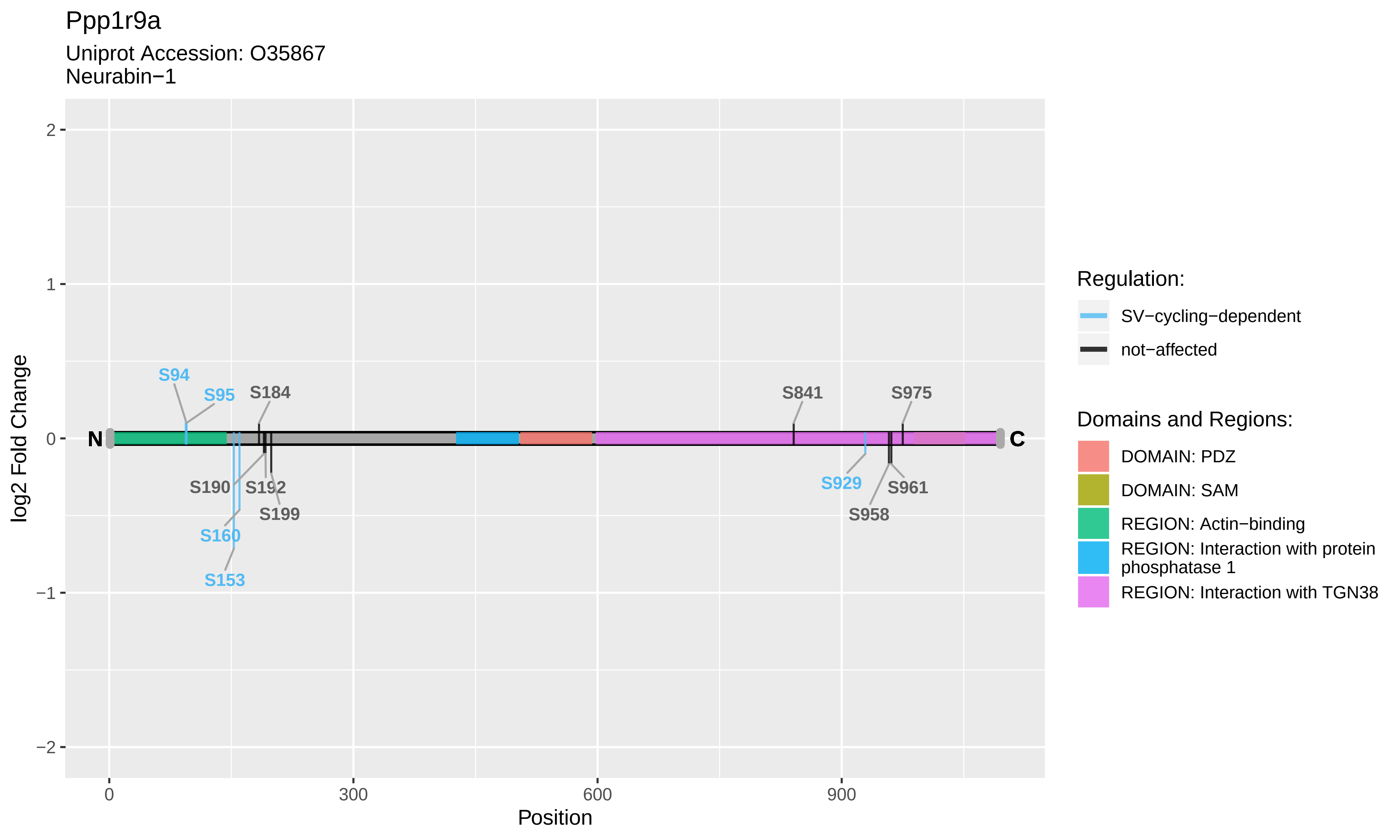
Ppp1r9a
Uniprot Accession: O35867
Neurabin−1
log2 Fold Change
Position
2
1
0
−1
−2
0
300
600
900
N
C
S94
S95
S184
S190
S192
S199
S160
S153
S841
S975
S929
S961
S958
Regulation:
SV−cycling−dependent
not−affected
Domains and Regions:
DOMAIN: PDZ
DOMAIN: SAM
REGION: Actin−binding
REGION: Interaction with protein phosphatase 1
REGION: Interaction with TGN38

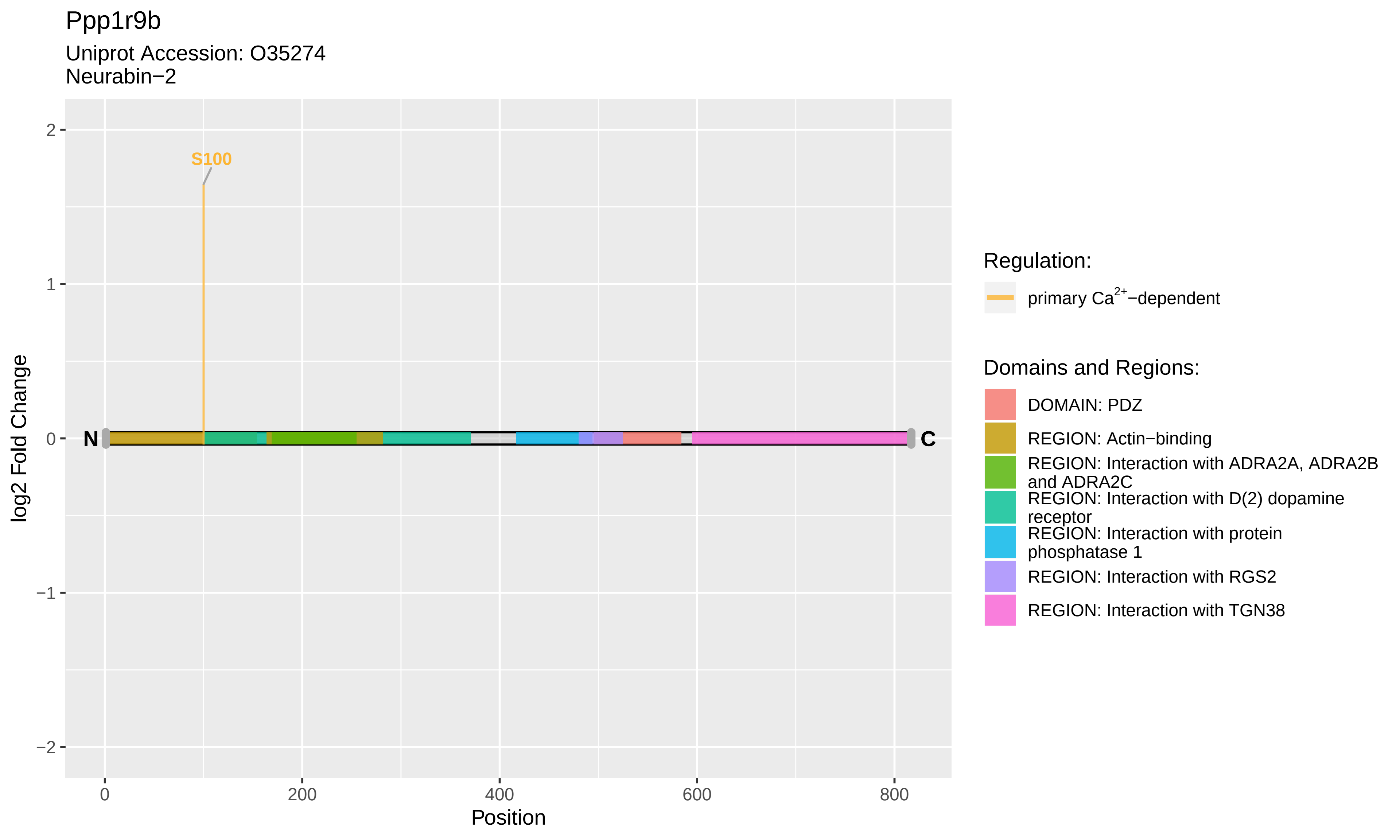

Ppp1r9b
Uniprot Accession: O35274
Neurabin−2
S100
N
C
log2 Fold Change
Position
2
1
0
−1
−2
0
200
400
600
800
Regulation:
primary Ca2+−dependent
Domains and Regions:
DOMAIN: PDZ
REGION: Actin−binding
REGION: Interaction with ADRA2A, ADRA2B and ADRA2C
REGION: Interaction with D(2) dopamine receptor
REGION: Interaction with protein phosphatase 1
REGION: Interaction with RGS2
REGION: Interaction with TGN38

Ppp2r5c

Uniprot Accession: D4A1A5
Serine/threonine−protein phosphatase 2A 56 kDa regulatory subunit

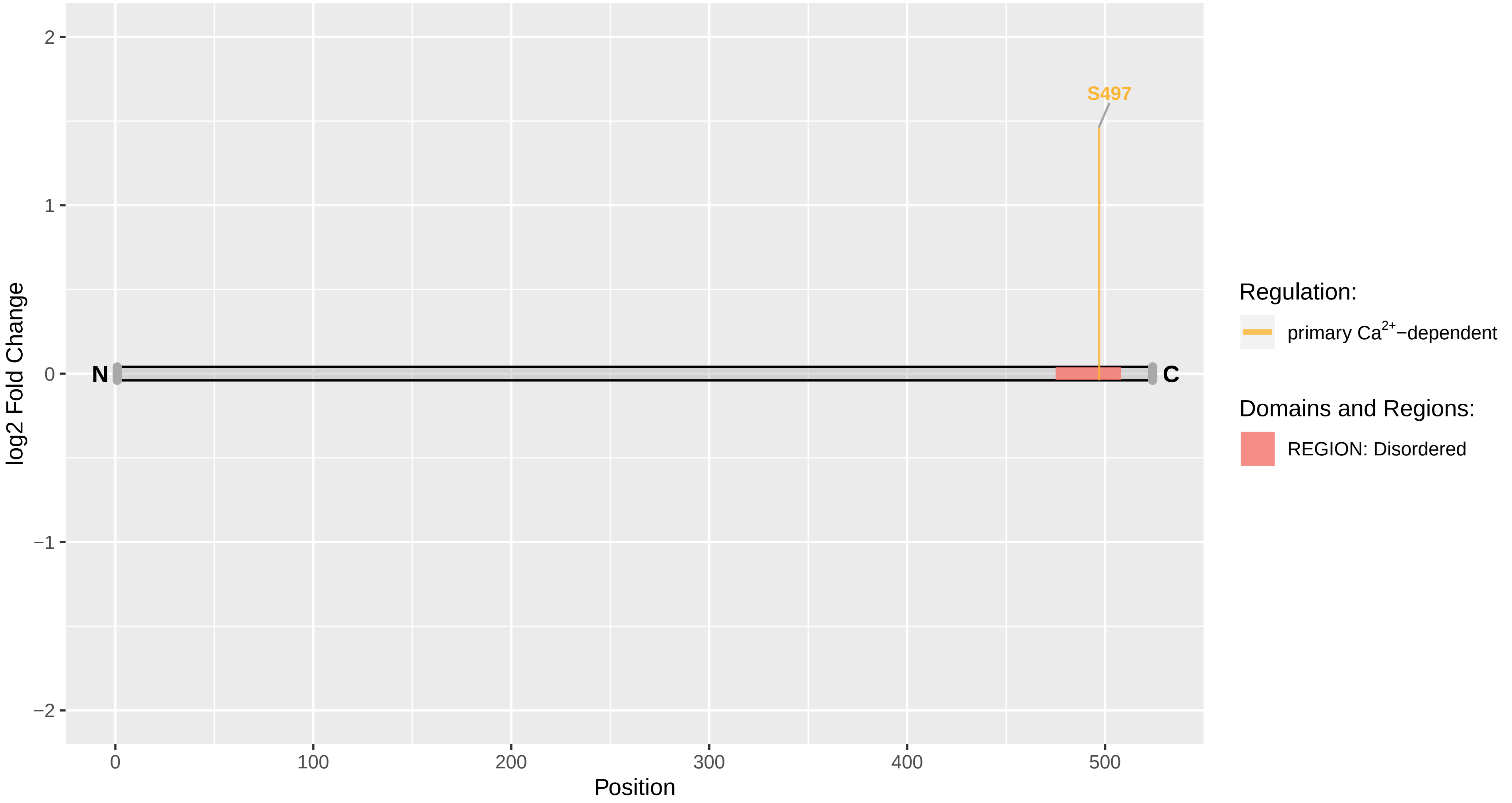

Ppp3ca

Uniprot Accession: P63329
Serine/threonine−protein phosphatase 2B catalytic subunit alpha isoform

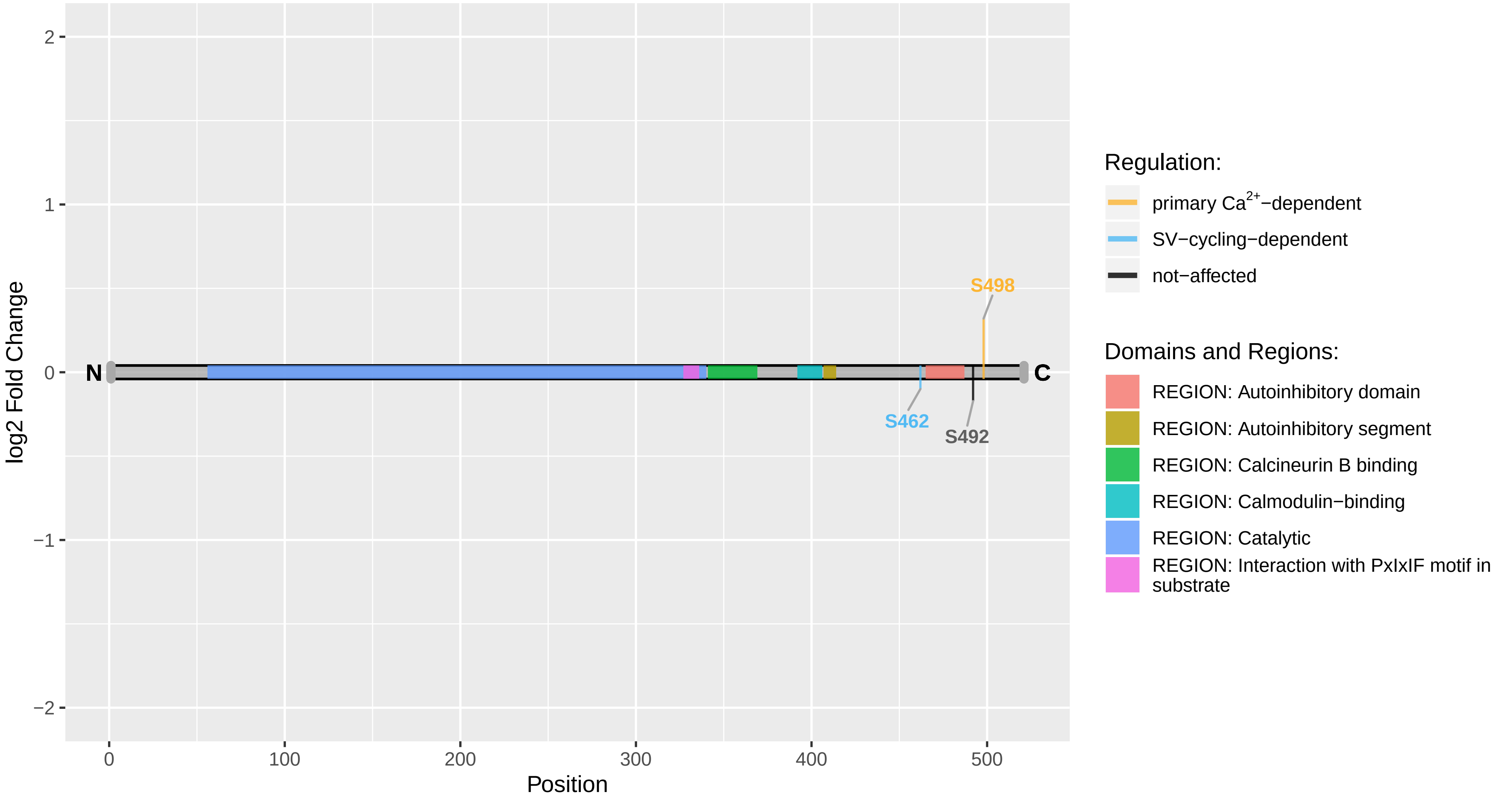

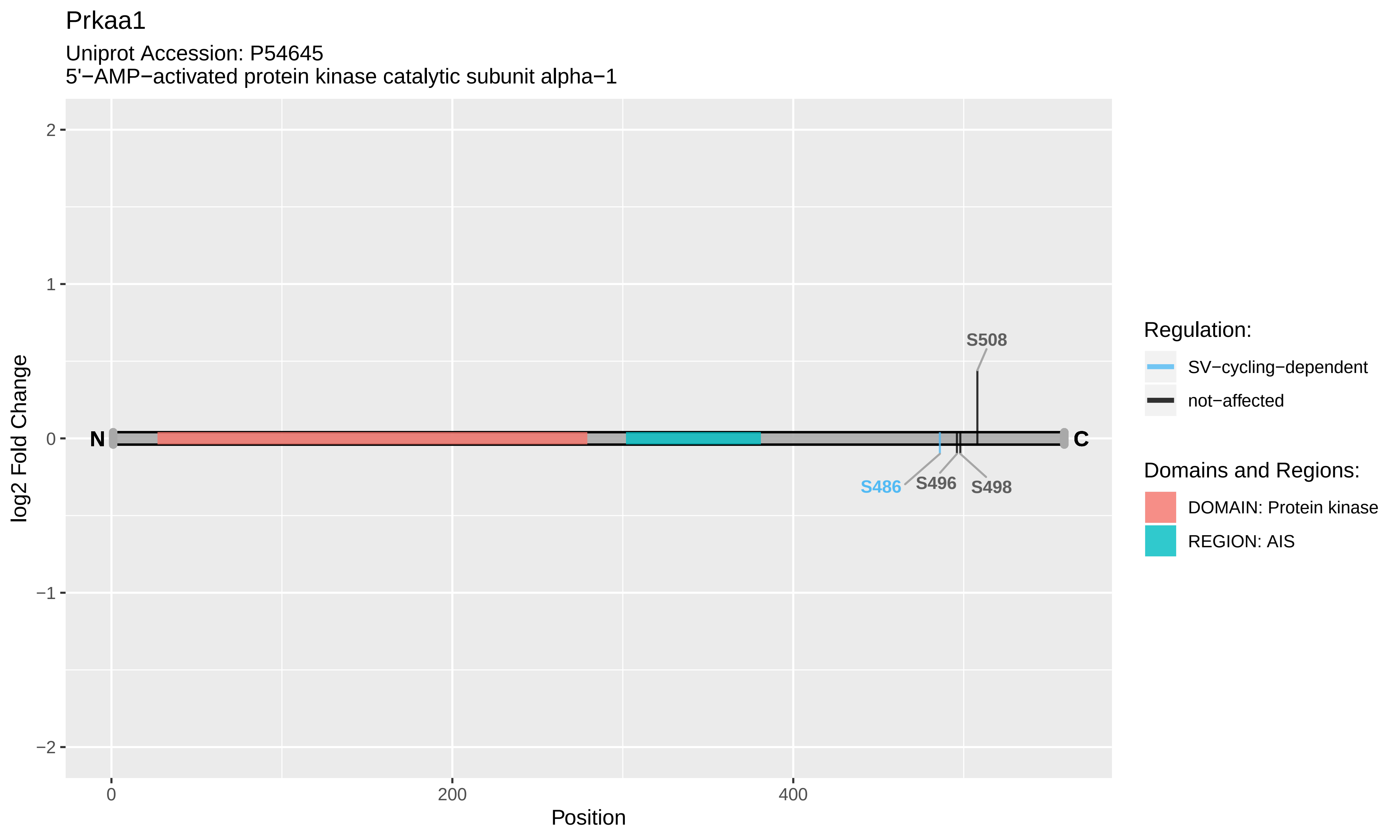

Prrkaa1
Uniprot Accession: P54645
5'−AMP−activated protein kinase catalytic subunit alpha−1
log2 Fold Change
Position
2
1
0
−1
−2
0
200
400
N
C
S508
S486
S496
S498
Regulation:
SV−cycling−dependent
not−affected
Domains and Regions:
DOMAIN: Protein kinase
REGION: AIS

Prkab1

Uniprot Accession: P80386
5'−AMP−activated protein kinase subunit beta−1

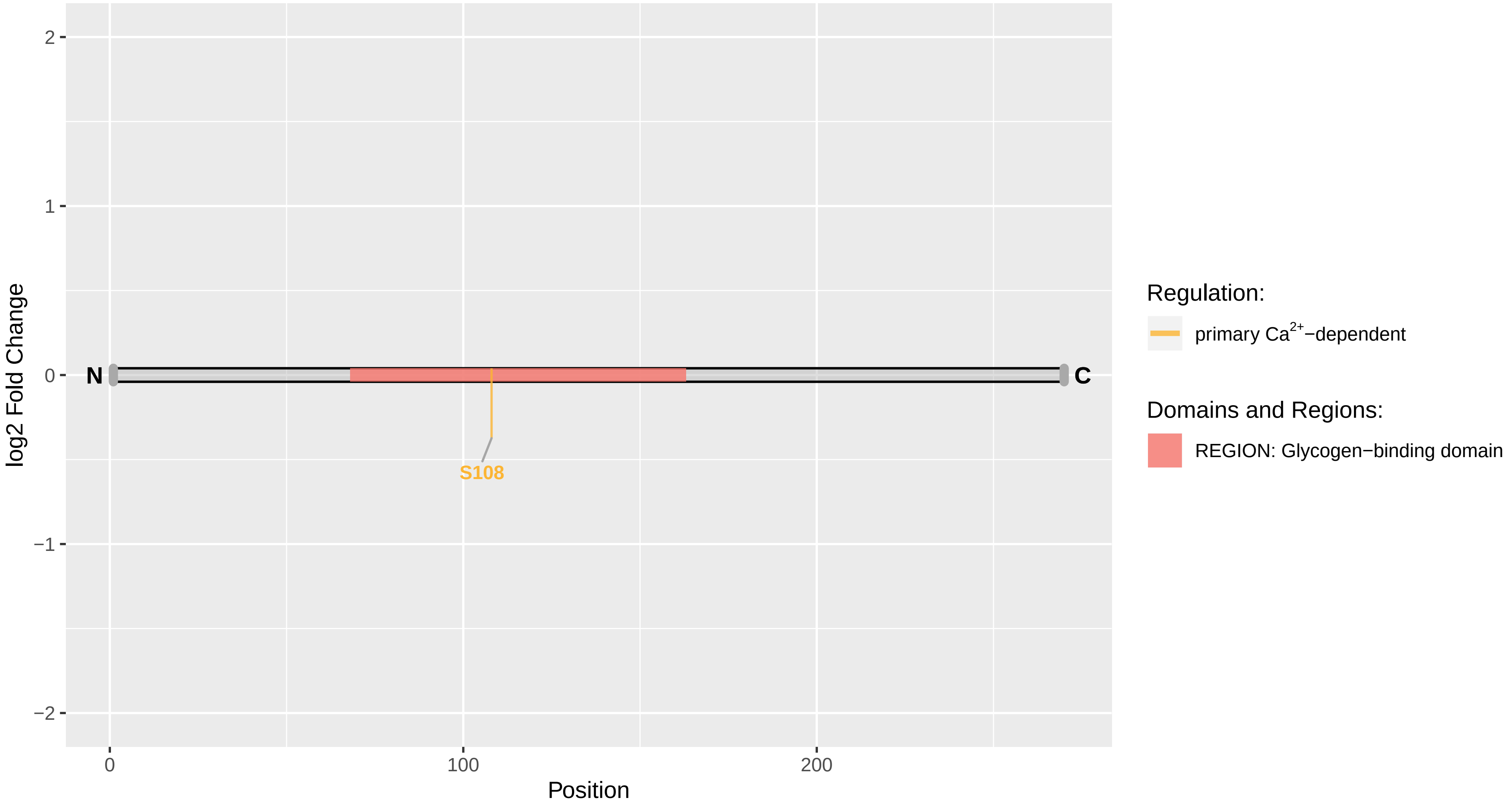

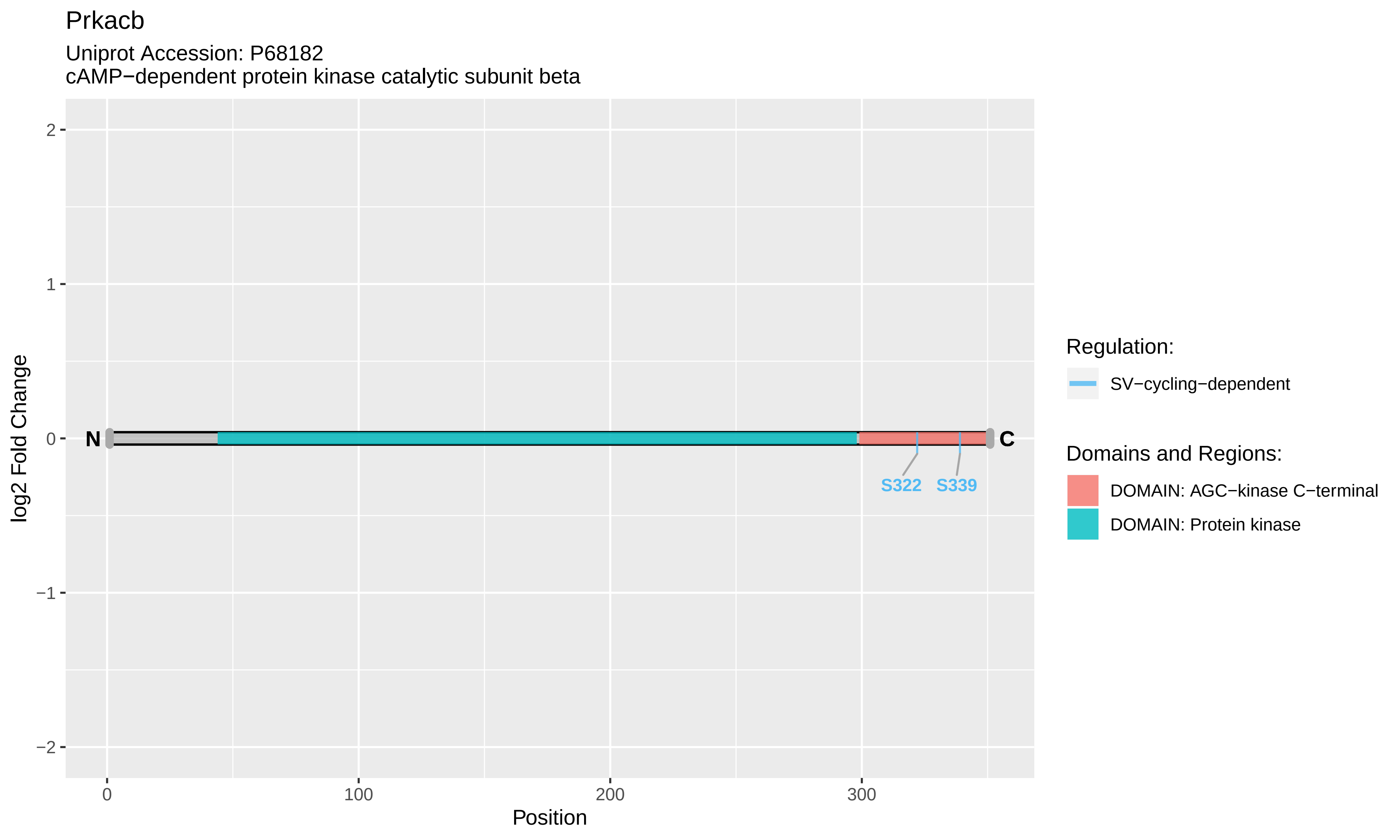
Prkacb
Uniprot Accession: P68182
cAMP−dependent protein kinase catalytic subunit beta
log2 Fold Change
2
1
0
−1
−2
N
C
S322
S339
0
100
200
300
Position
Regulation:
SV−cycling−dependent
Domains and Regions:
DOMAIN: AGC−kinase C−terminal
DOMAIN: Protein kinase

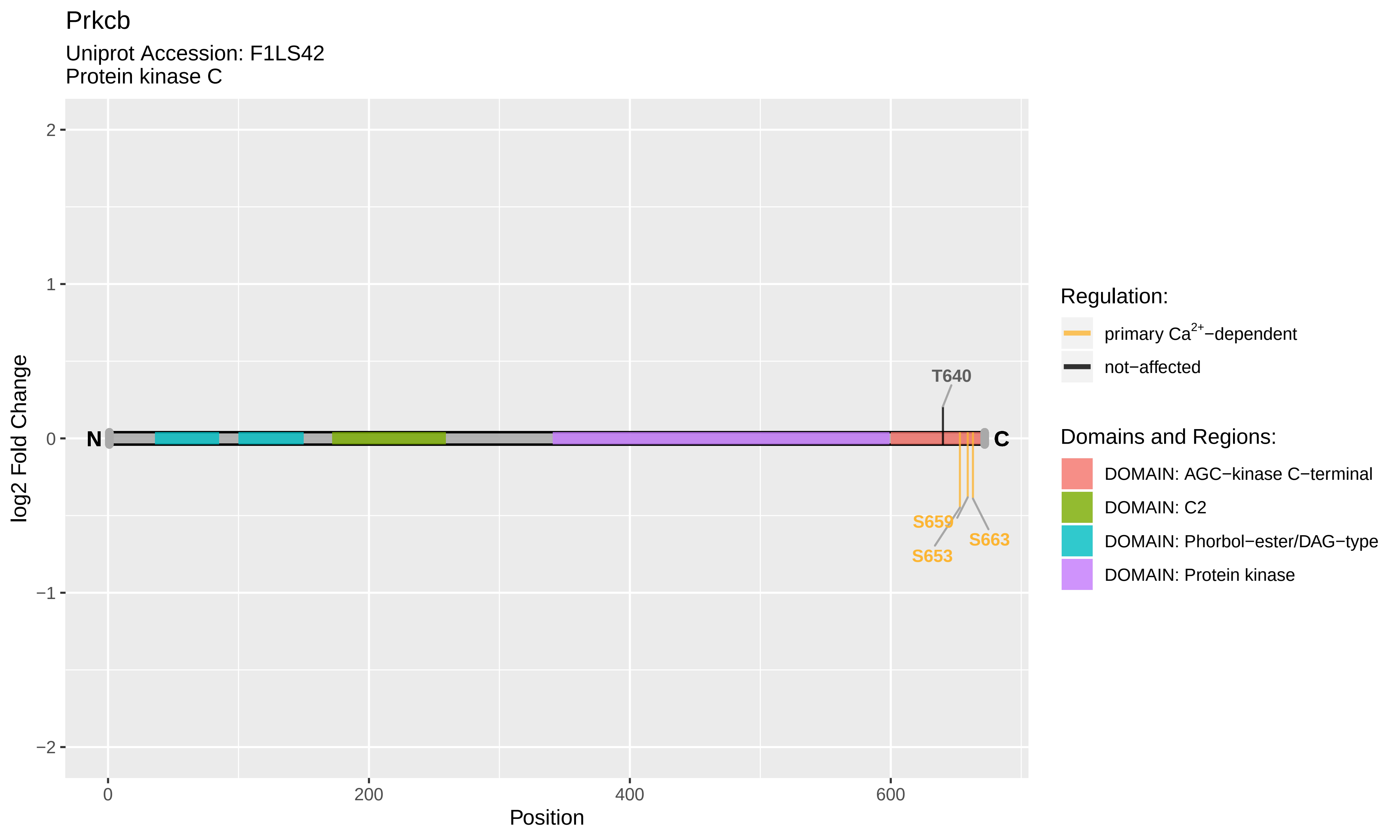

Prkcb
Uniprot Accession: F1LS42
Protein kinase C
log2 Fold Change
Position
N
C
T640
S659
S653
S663
Regulation:
primary Ca2+−dependent
not−affected
Domains and Regions:
DOMAIN: AGC−kinase C−terminal
DOMAIN: C2
DOMAIN: Phorbol−ester/DAG−type
DOMAIN: Protein kinase

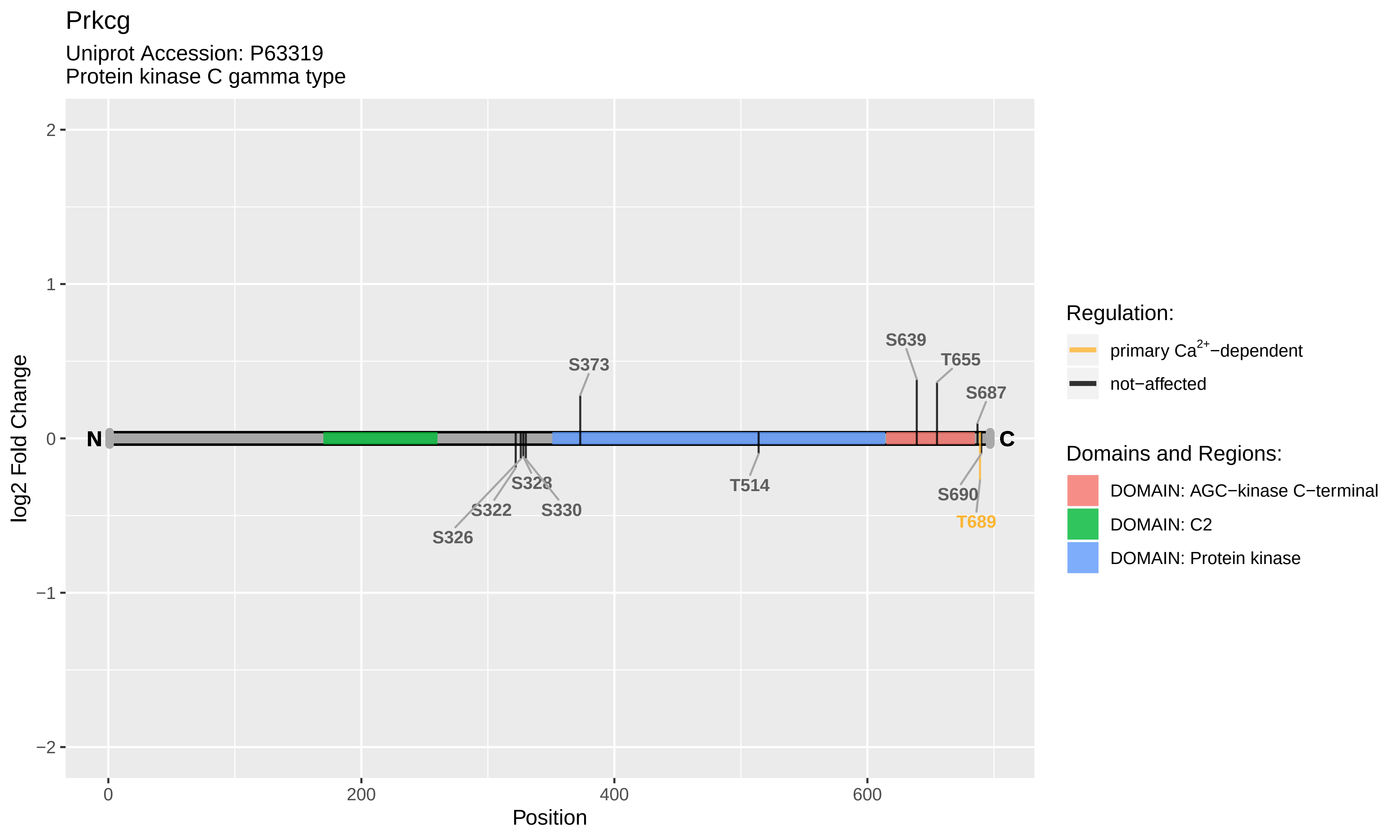

Prkcg
Uniprot Accession: P63319
Protein kinase C gamma type
log2 Fold Change
Position
N
C
S373
S639
T655
S687
S328
S322
S330
S326
T514
S690
T689
Regulation:
primary Ca2+−dependent
not−affected
Domains and Regions:
DOMAIN: AGC−kinase C−terminal
DOMAIN: C2
DOMAIN: Protein kinase

Prpf38b

Uniprot Accession: Q6AXY7
Pre−mRNA−splicing factor 38B

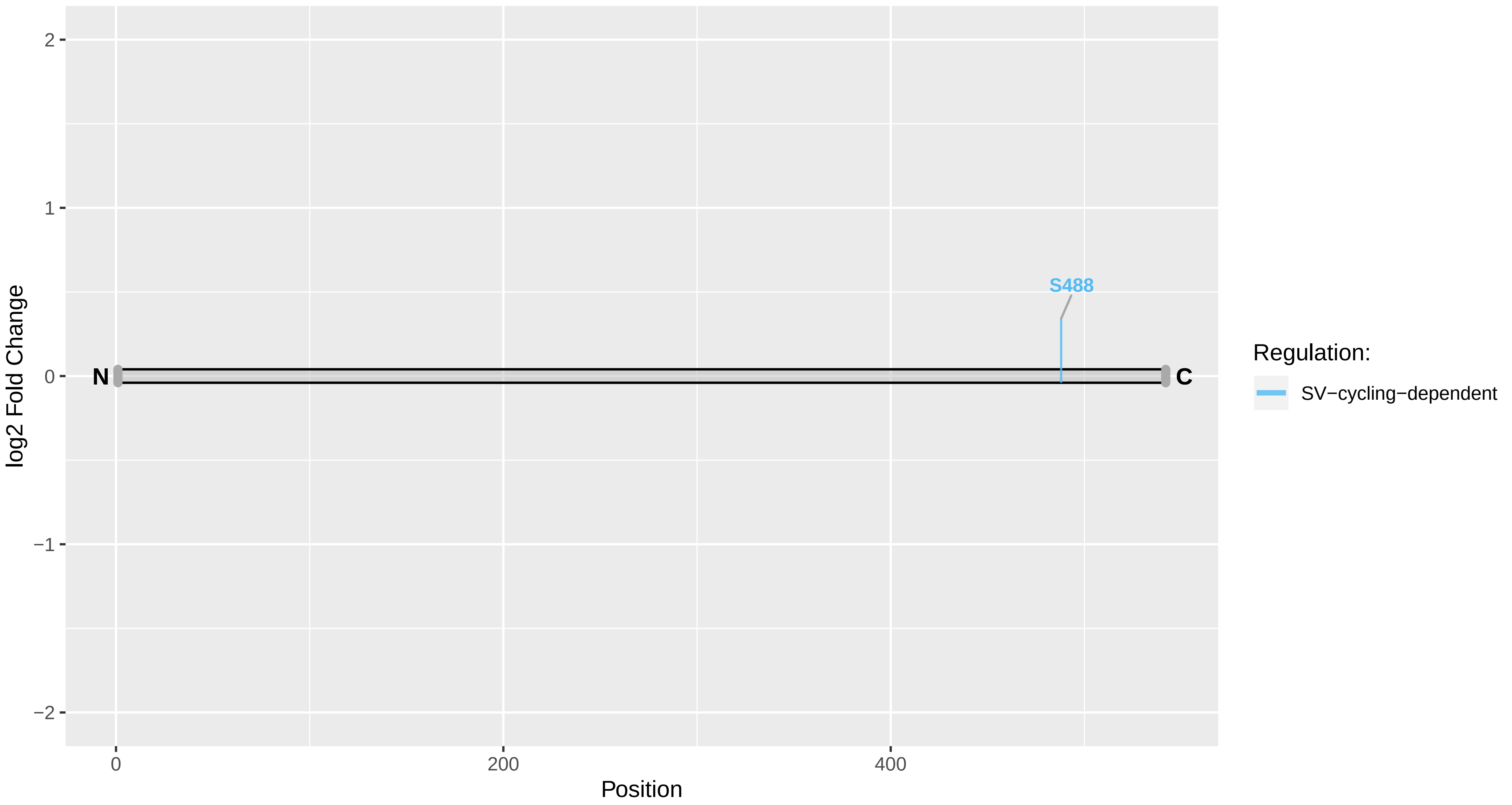

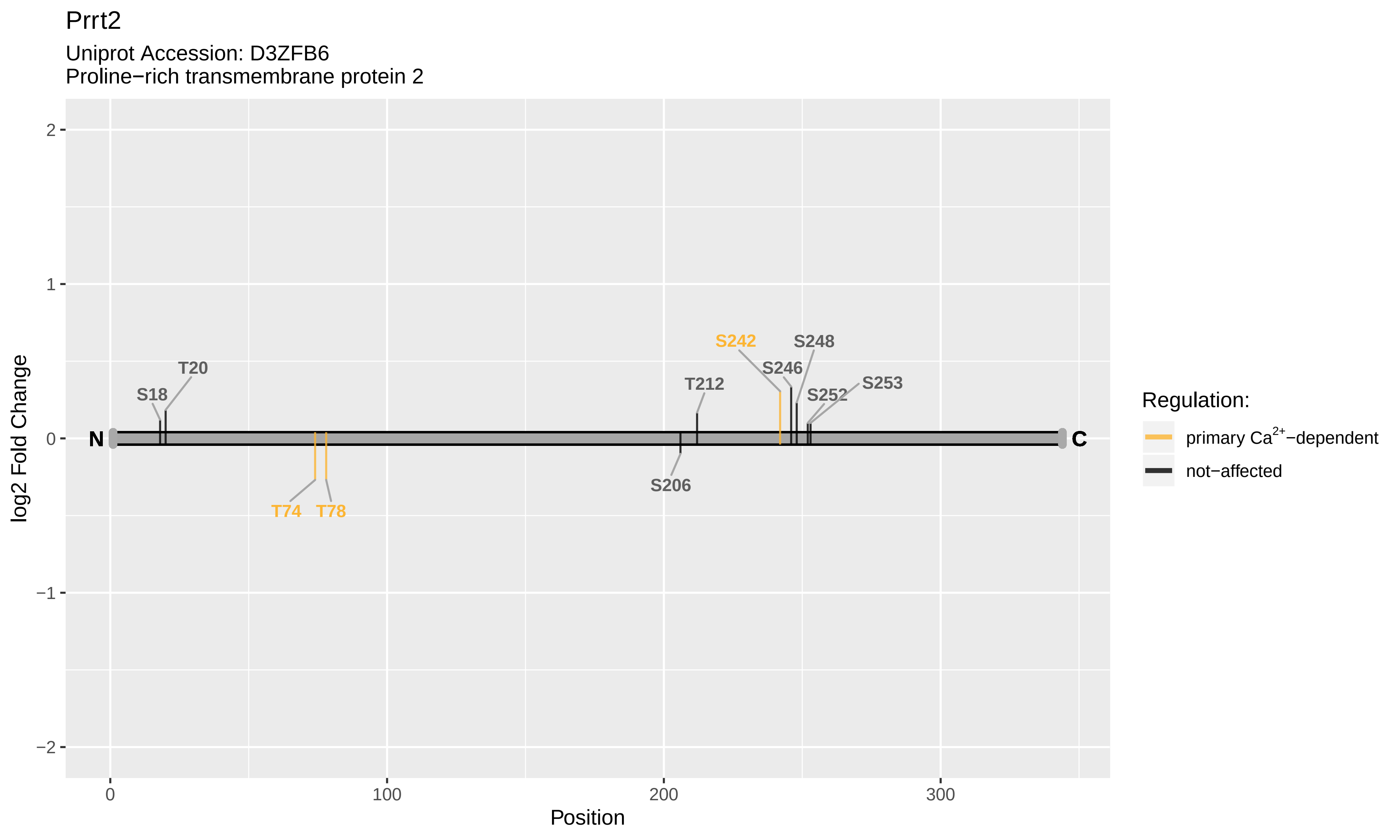
Prrt2
Uniprot Accession: D3ZFB6
Proline−rich transmembrane protein 2
log2 Fold Change
Position
N
C
S18
T20
T74
T78
S206
T212
S242
S246
S248
S252
S253
Regulation:
primary Ca2+−dependent
not−affected
2
1
0
−1
−2
0
100
200
300

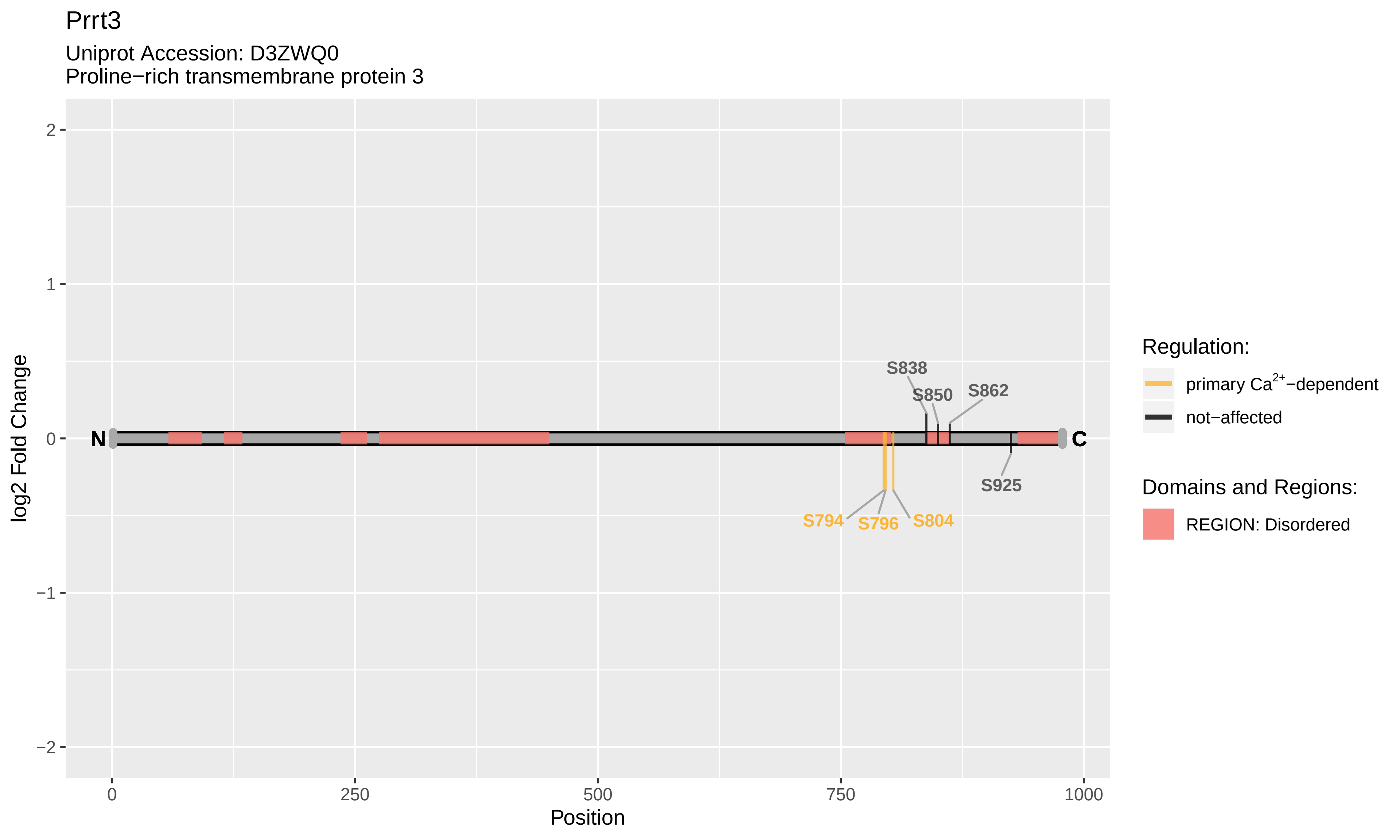

Prrt3
Uniprot Accession: D3ZWQ0
Proline−rich transmembrane protein 3
log2 Fold Change
Position
N
C
S838
S850
S862
S925
S794
S796
S804
Regulation:
primary Ca2+−dependent
not−affected
Domains and Regions:
REGION: Disordered
2
1
0
−1
−2
0
250
500
750
1000

Psd

Uniprot Accession: G3V8J5
PH and SEC7 domain−containing protein 1

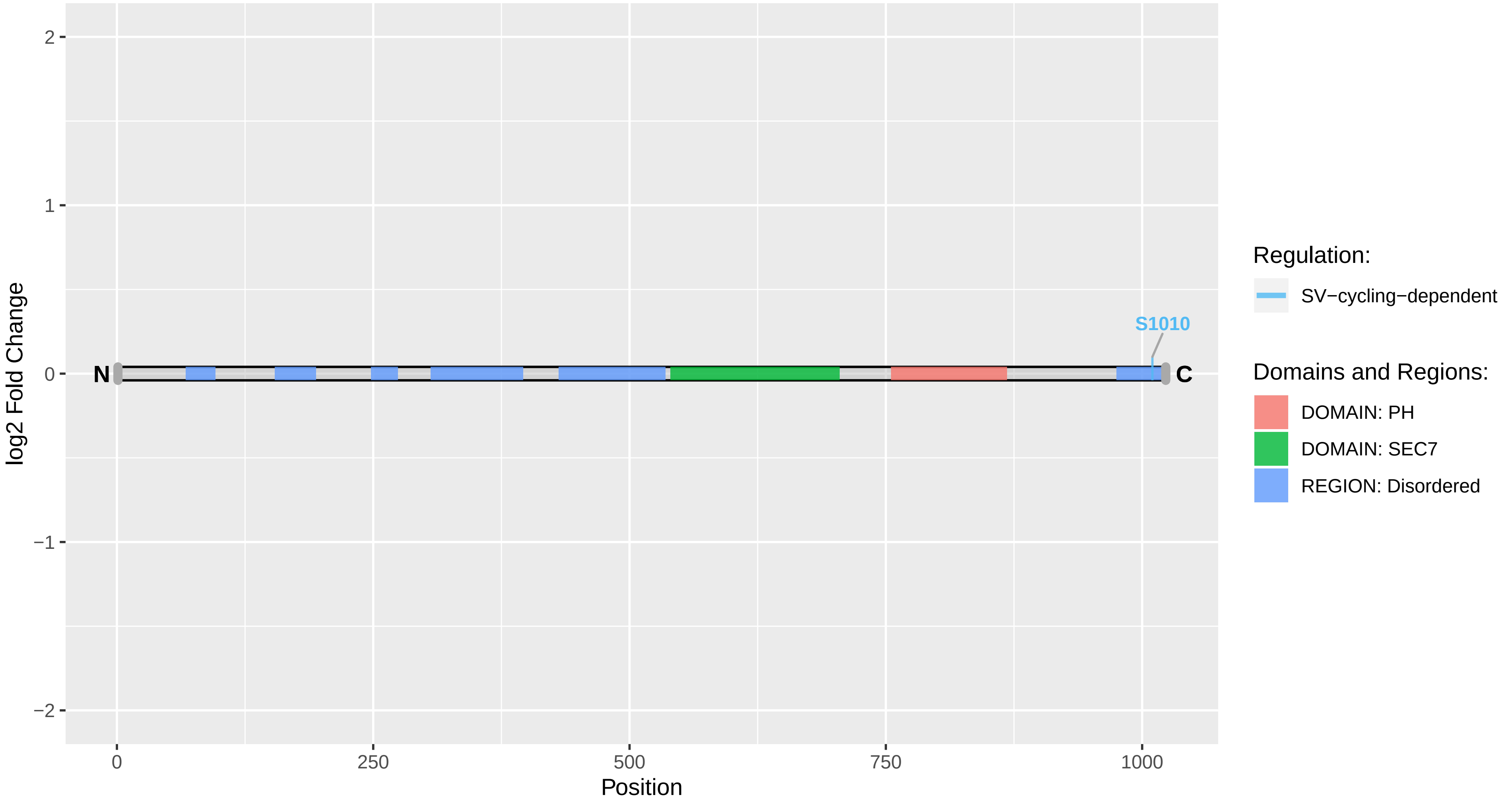

Psmd1

Uniprot Accession: O88761
26S proteasome non−ATPase regulatory subunit 1

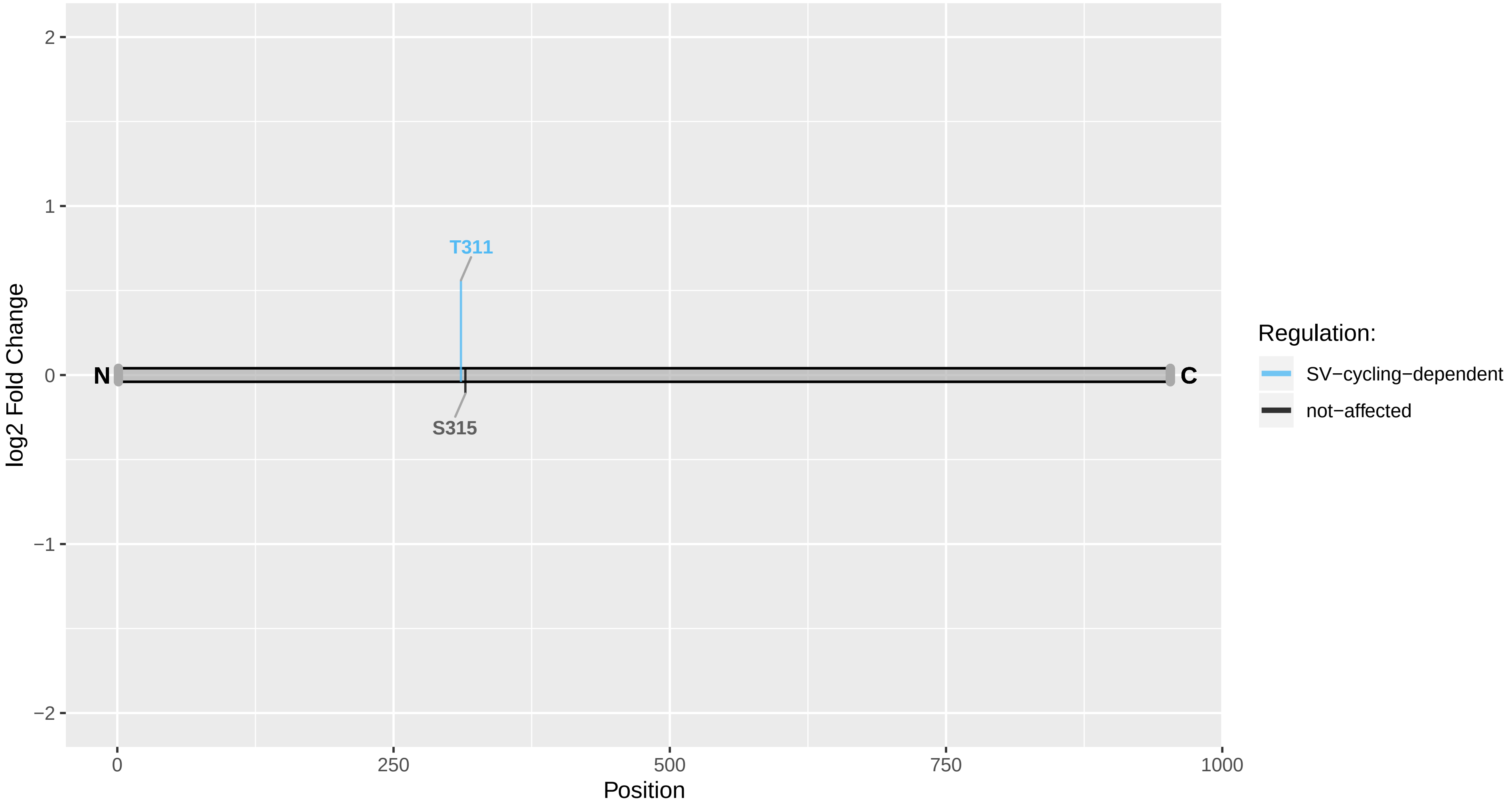

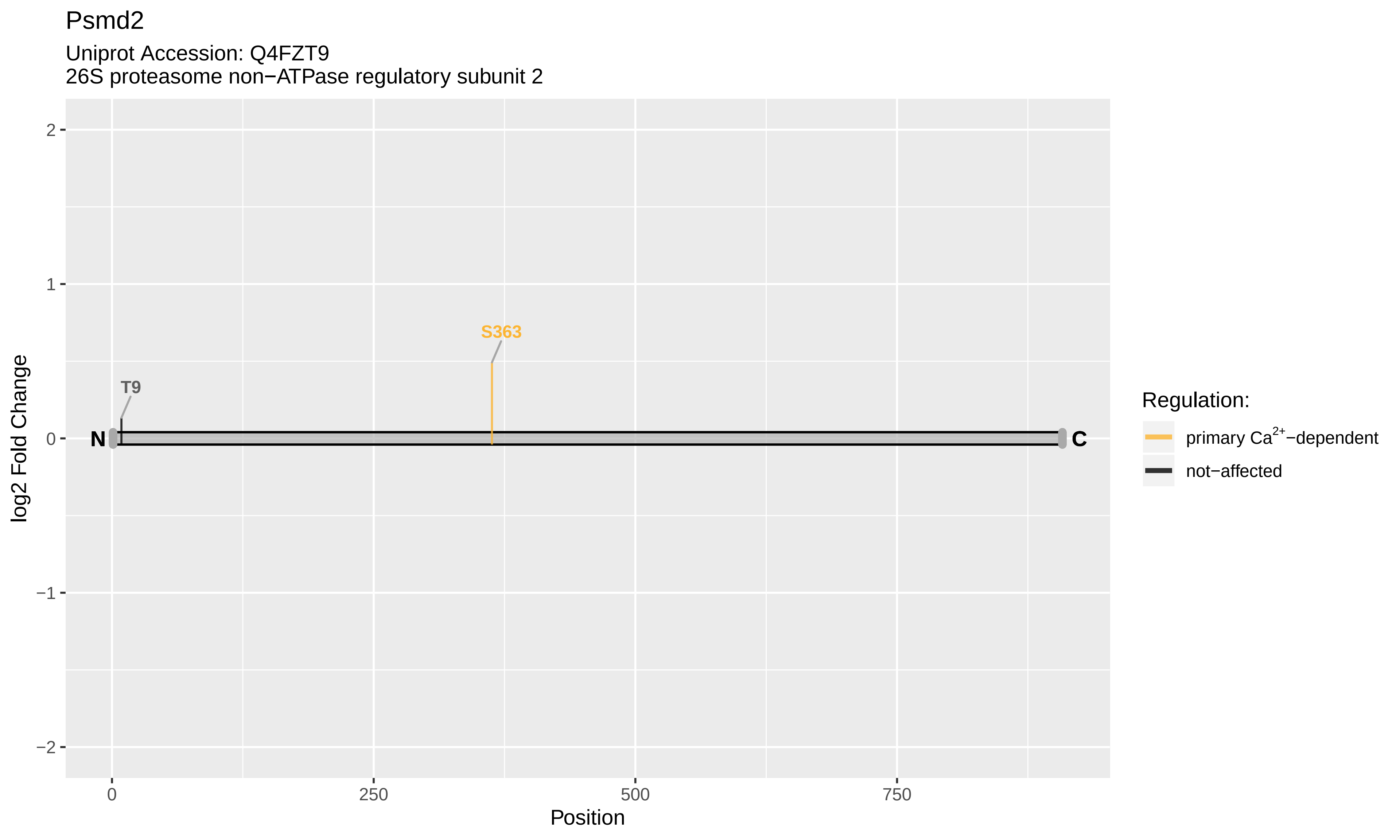

Psmd2
Uniprot Accession: Q4FZT9
26S proteasome non−ATPase regulatory subunit 2
log2 Fold Change
2
1
0
−1
−2
S363
T9
N
C
0
250
500
750
Position
Regulation:
primary Ca2+−dependent
not−affected

Ptprd

Uniprot Accession: M0RB22
Protein tyrosine phosphatase, receptor type, D

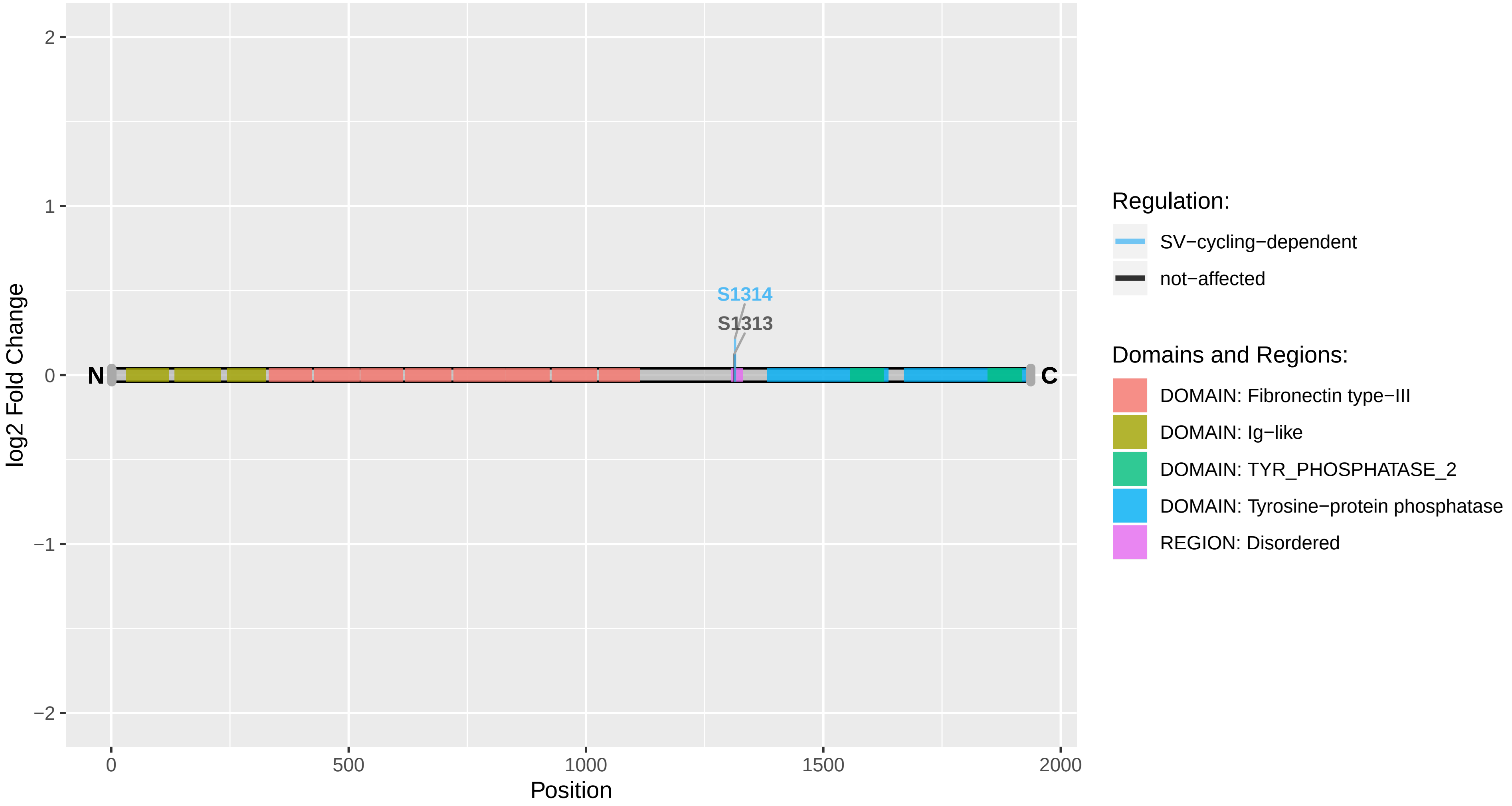

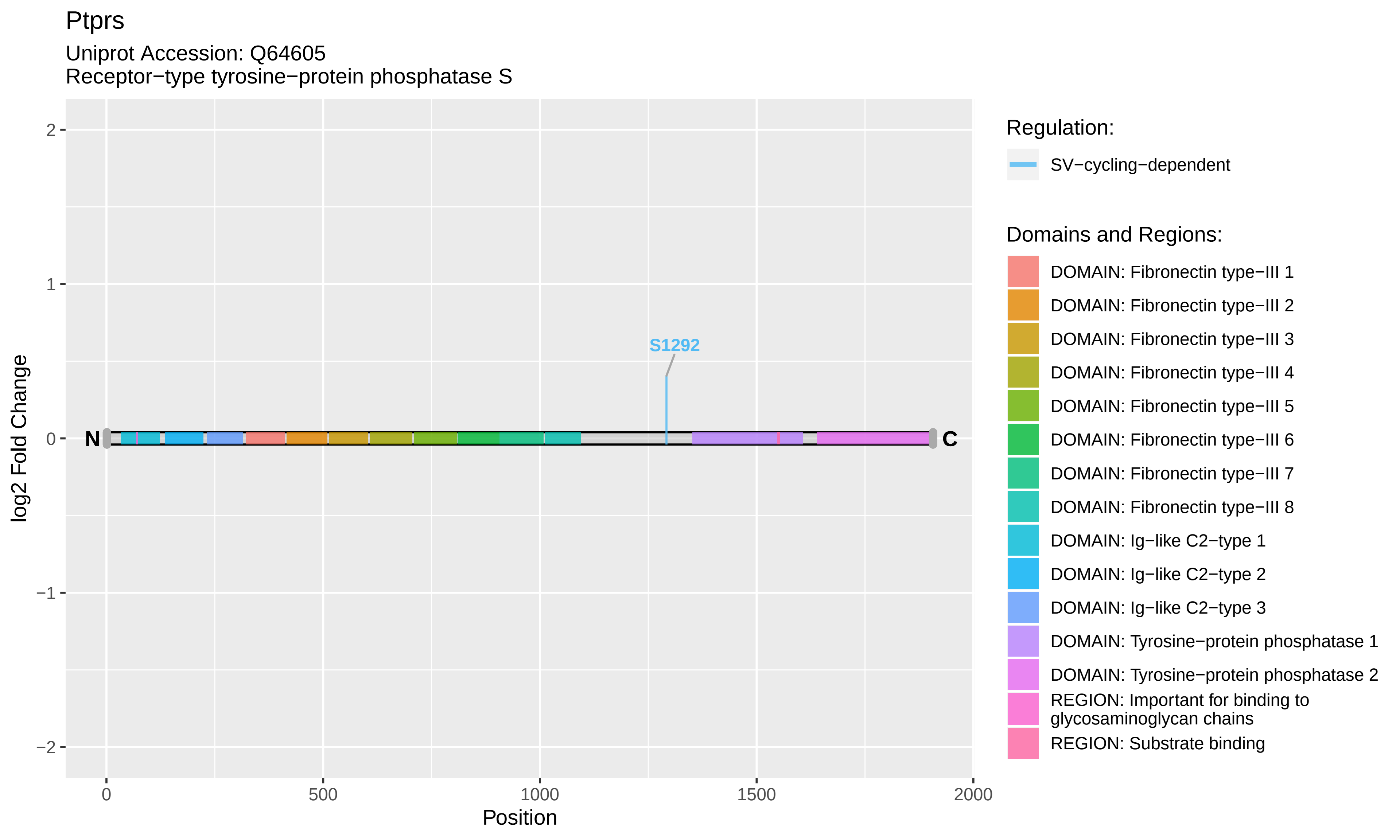

Ptprs
Uniprot Accession: Q64605
Receptor−type tyrosine−protein phosphatase S
log2 Fold Change
2
1
0
−1
−2
N
C
S1292
0
500
1000
1500
2000
Position
Regulation:
SV−cycling−dependent
Domains and Regions:
DOMAIN: Fibronectin type−III 1
DOMAIN: Fibronectin type−III 2
DOMAIN: Fibronectin type−III 3
DOMAIN: Fibronectin type−III 4
DOMAIN: Fibronectin type−III 5
DOMAIN: Fibronectin type−III 6
DOMAIN: Fibronectin type−III 7
DOMAIN: Fibronectin type−III 8
DOMAIN: Ig−like C2−type 1
DOMAIN: Ig−like C2−type 2
DOMAIN: Ig−like C2−type 3
DOMAIN: Tyrosine−protein phosphatase 1
DOMAIN: Tyrosine−protein phosphatase 2
REGION: Important for binding to glycosaminoglycan chains
REGION: Substrate binding

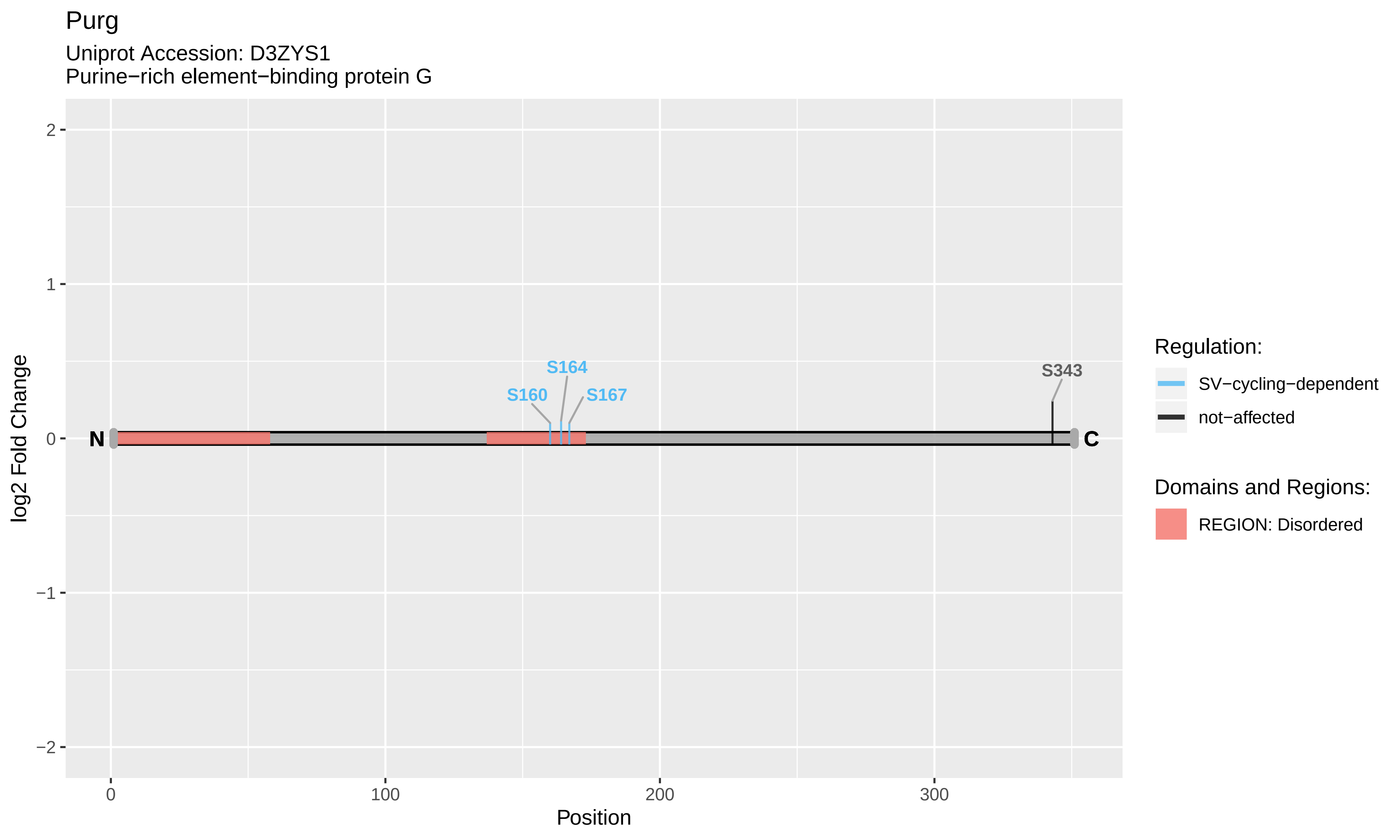

Purg
Uniprot Accession: D3ZYS1
Purine−rich element−binding protein G
log2 Fold Change
Position
S160
S164
S167
S343
N
C
2
1
0
−1
−2
0
100
200
300
Regulation:
SV−cycling−dependent
not−affected
Domains and Regions:
REGION: Disordered

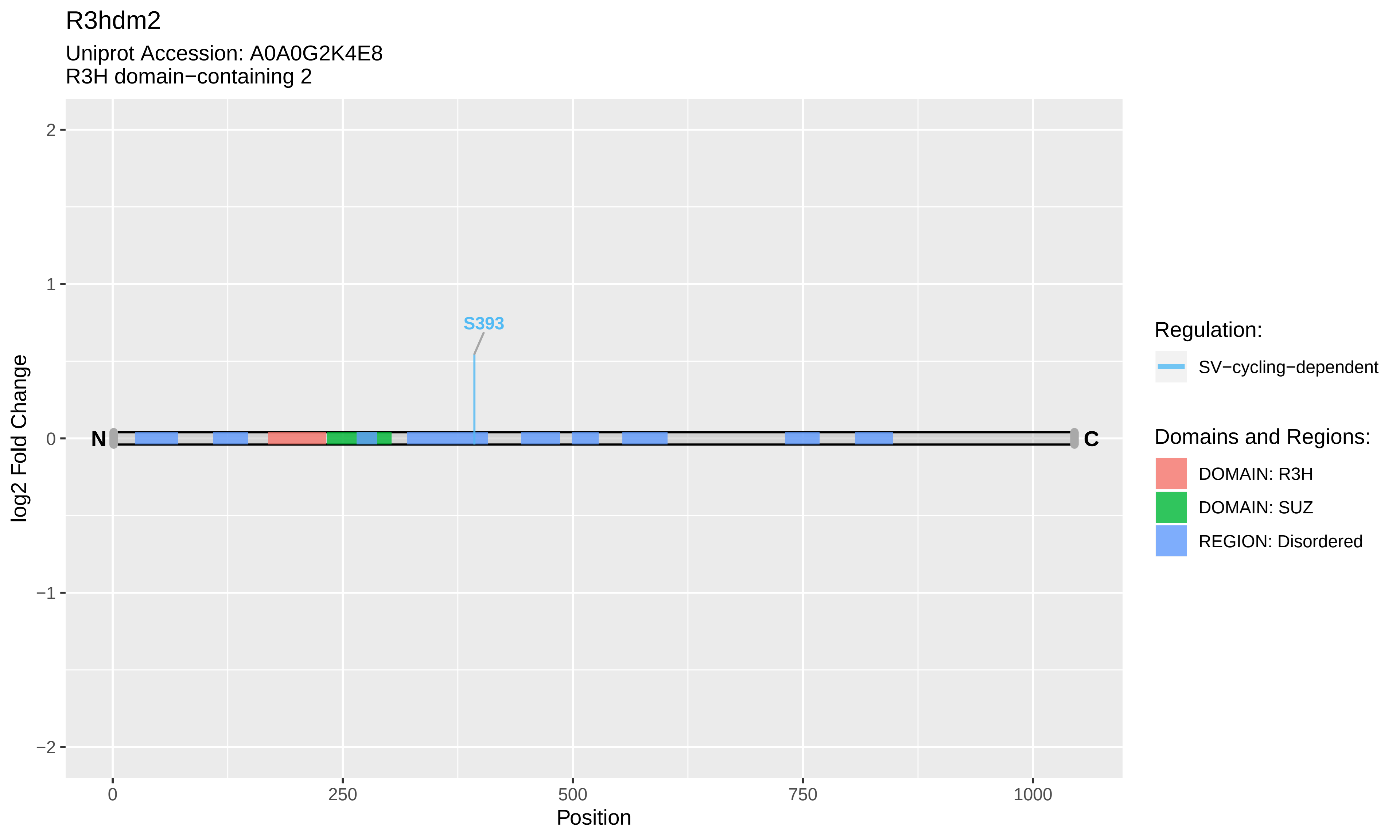

R3hdm2
Uniprot Accession: A0A0G2K4E8
R3H domain−containing 2
S393
N
C
2
1
0
−1
−2
log2 Fold Change
0
250
500
750
1000
Position
Regulation:
SV−cycling−dependent
Domains and Regions:
DOMAIN: R3H
DOMAIN: SUZ
REGION: Disordered

RGD1305178

Uniprot Accession: A0A096MJX5
Similar to Hypothetical protein MGC11690

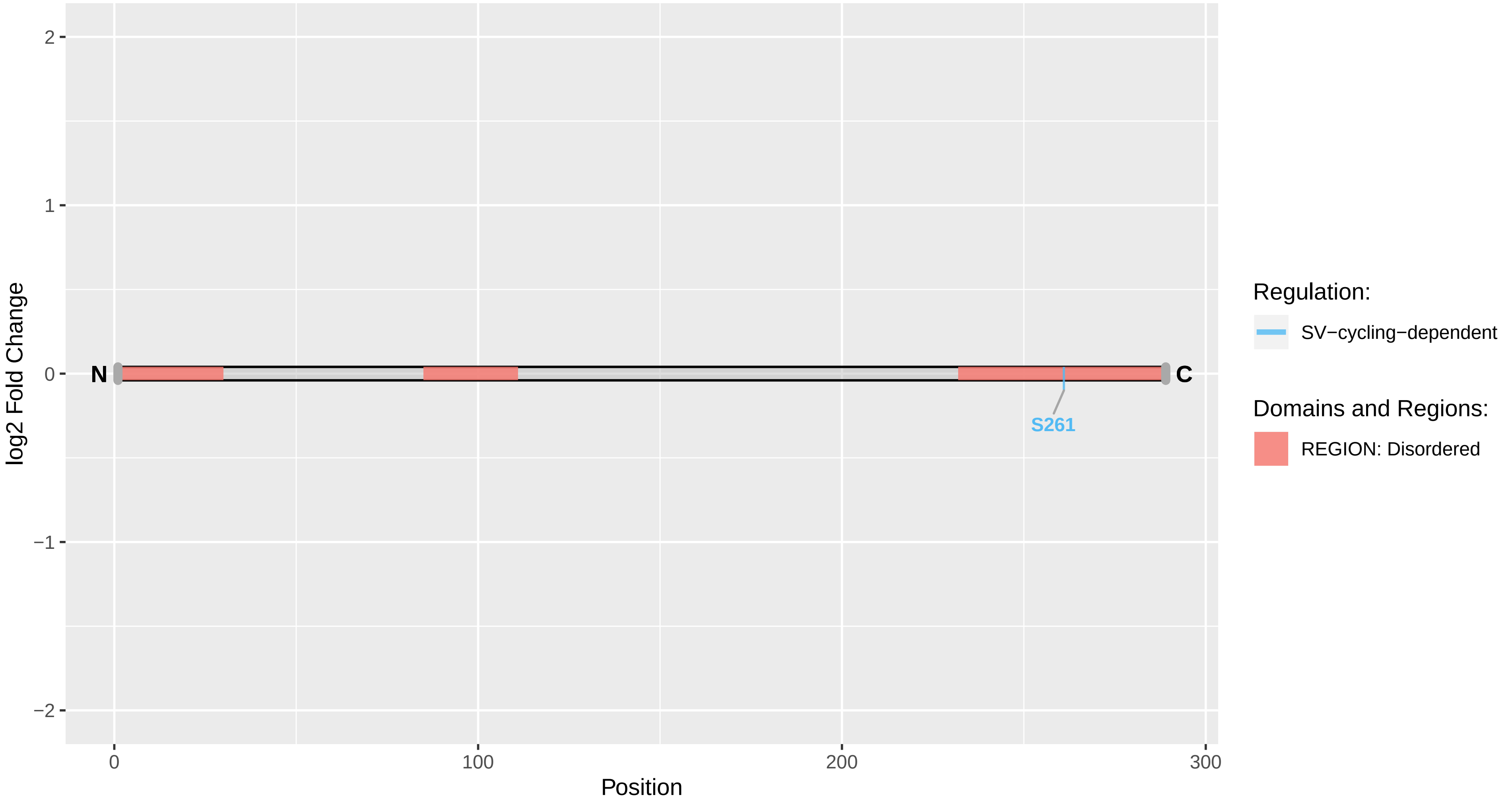

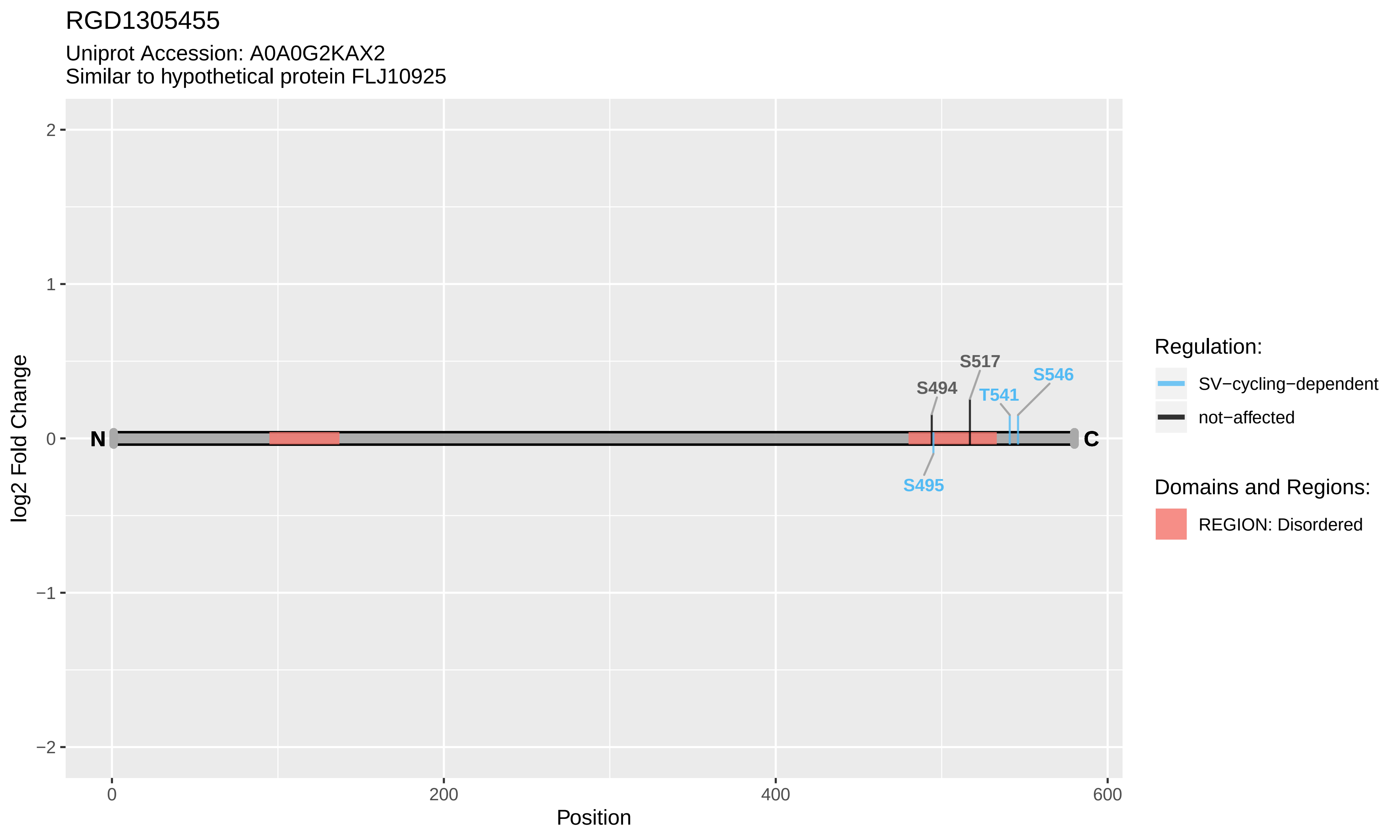

RGD1305455
Uniprot Accession: A0A0G2KAX2
Similar to hypothetical protein FLJ10925
log2 Fold Change
2
1
0
−1
−2
N
C
S494
S517
T541
S546
S495
0
200
400
600
Position
Regulation:
SV−cycling−dependent
not−affected
Domains and Regions:
REGION: Disordered

RGD1306271

Uniprot Accession: D3Z9D0
Similar to KIAA1549 protein

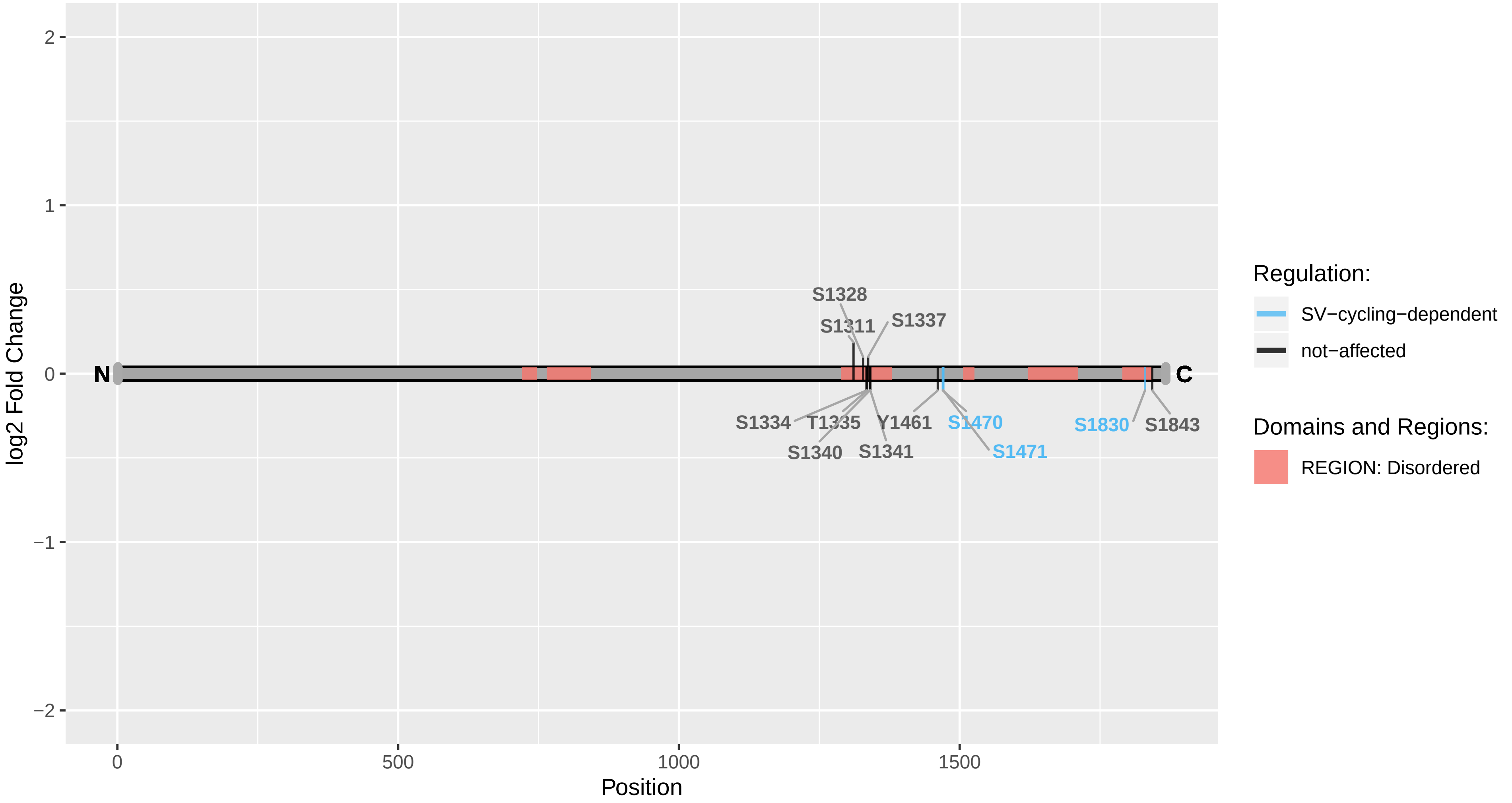

RGD1306556

Uniprot Accession: A0A0G2JZ88
Similar to hypothetical protein A530094D01

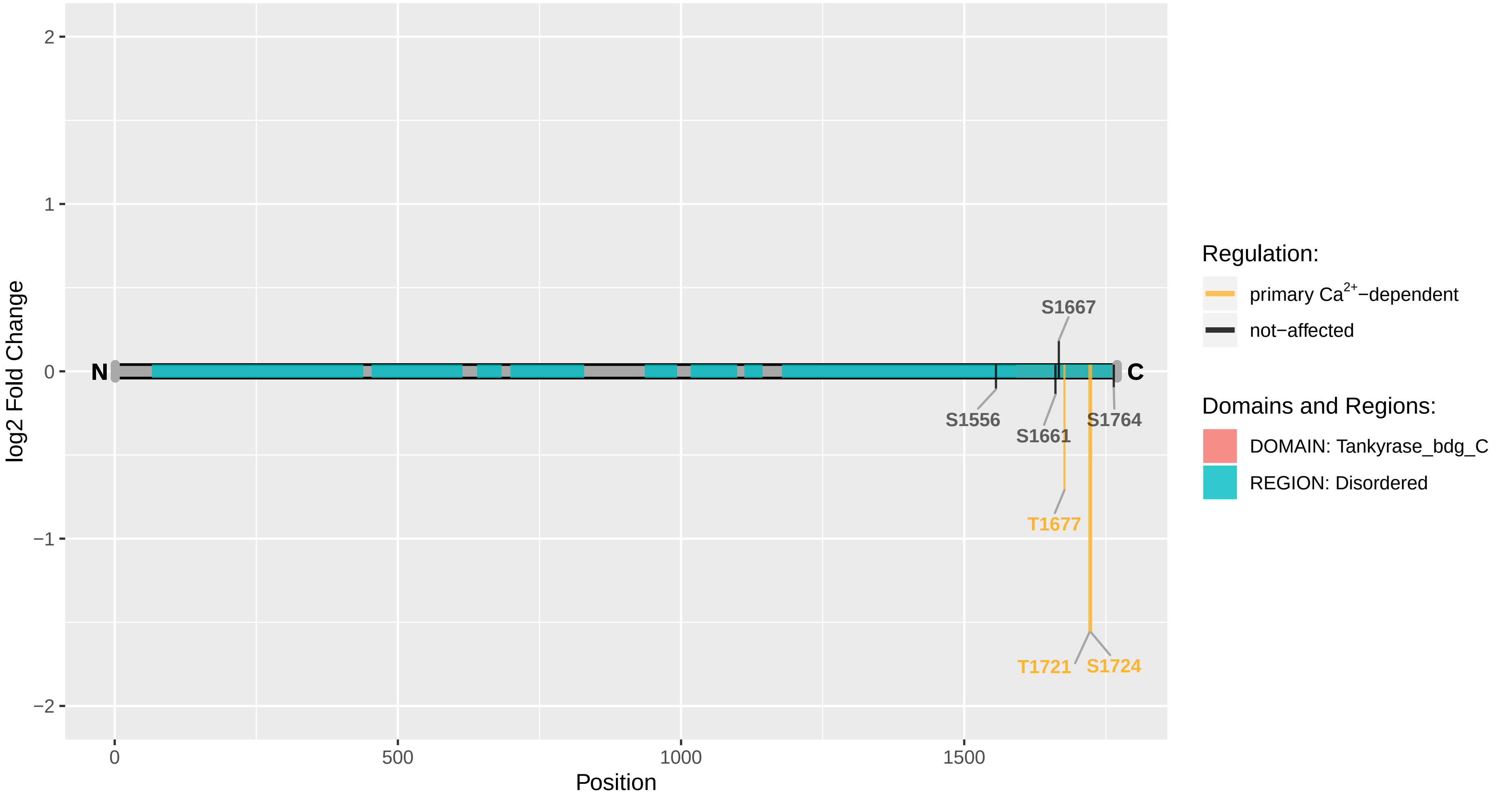

RGD1307100

Uniprot Accession: A0A096MJT6
Similar to RIKEN cDNA D630029K19

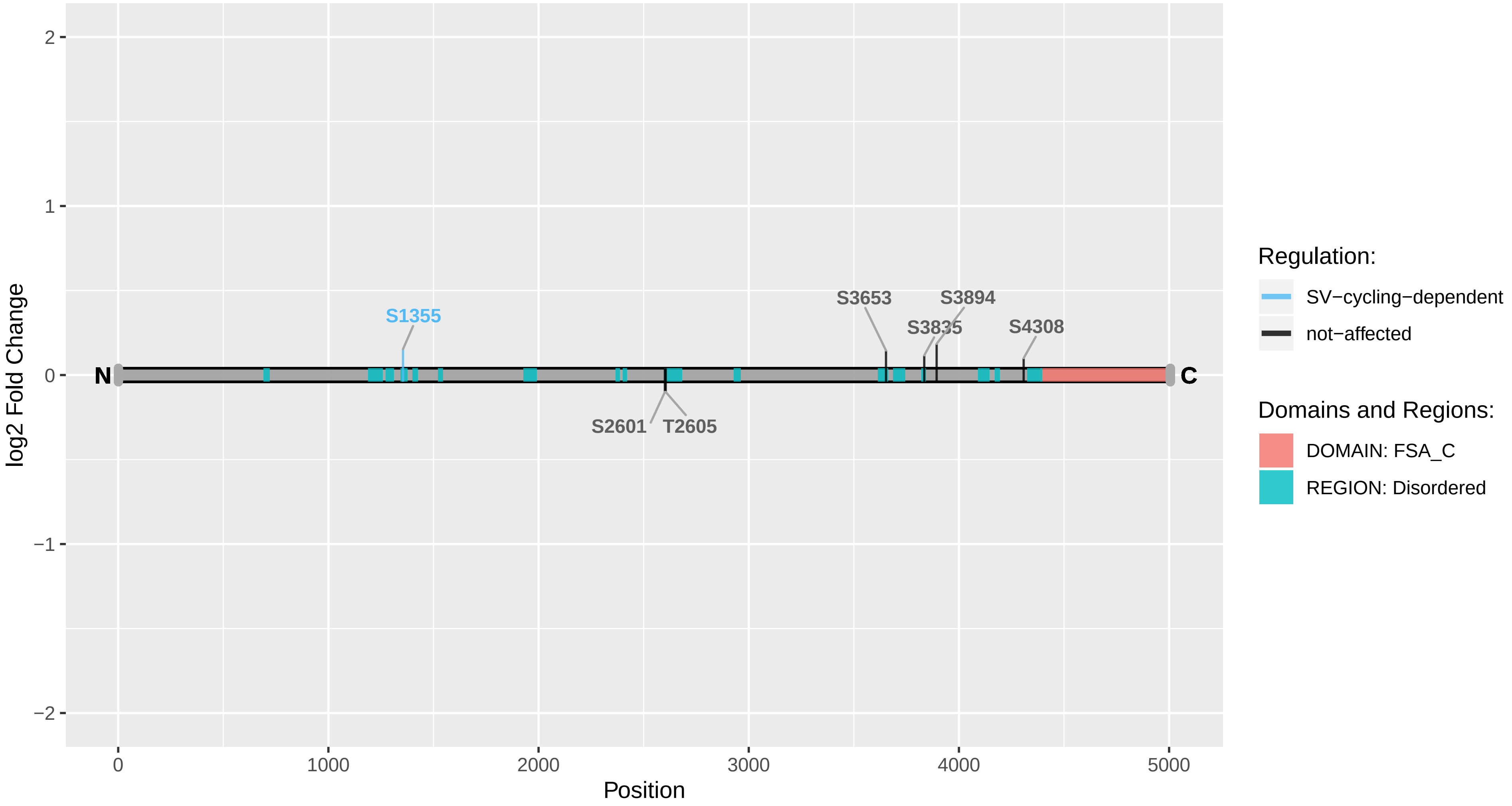

RGD1308601

Uniprot Accession: D4A3X1
Similar to hypothetical protein

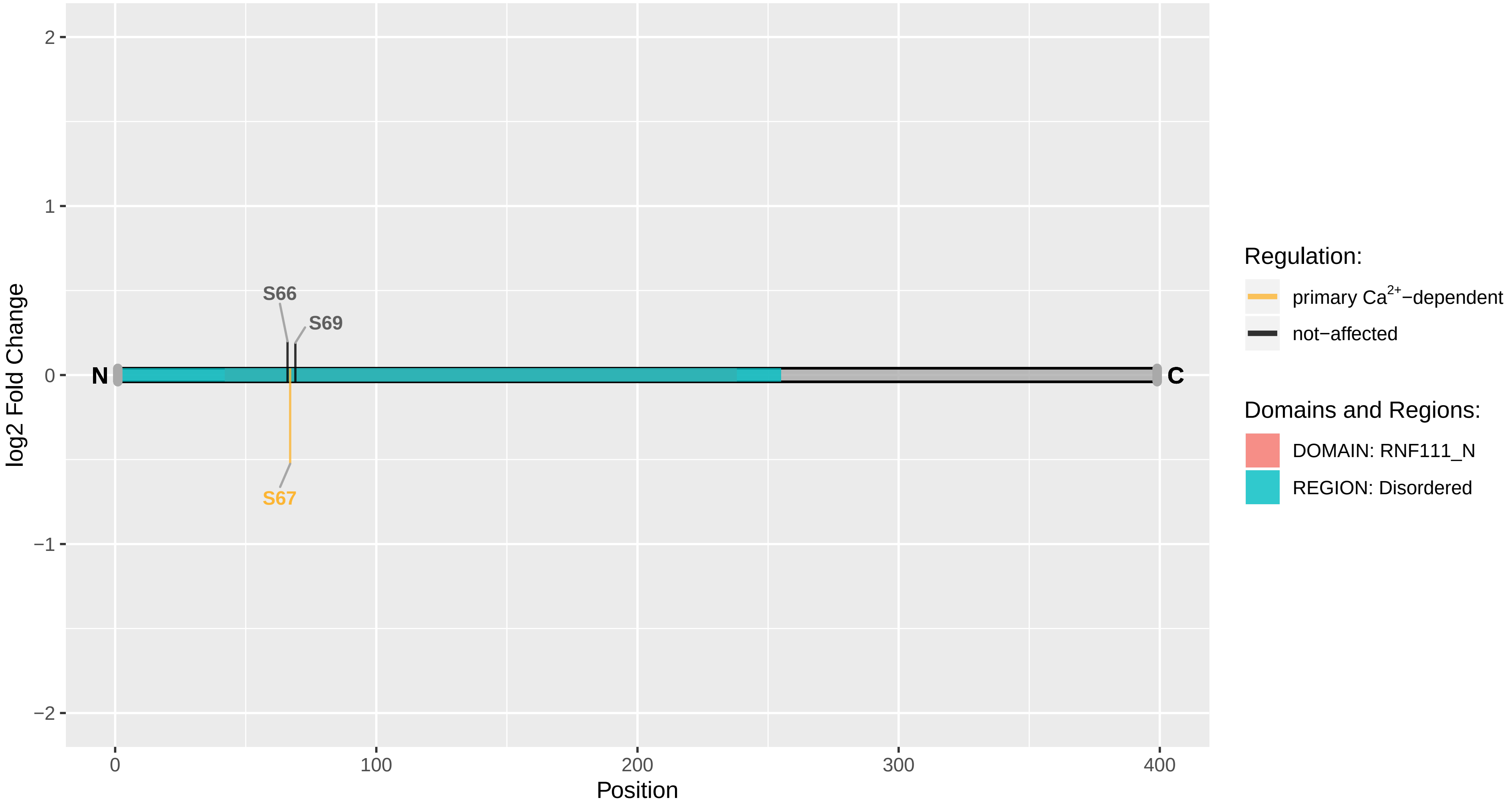

RGD1310819

Uniprot Accession: D3ZBU7
Similar to putative protein (5S487)

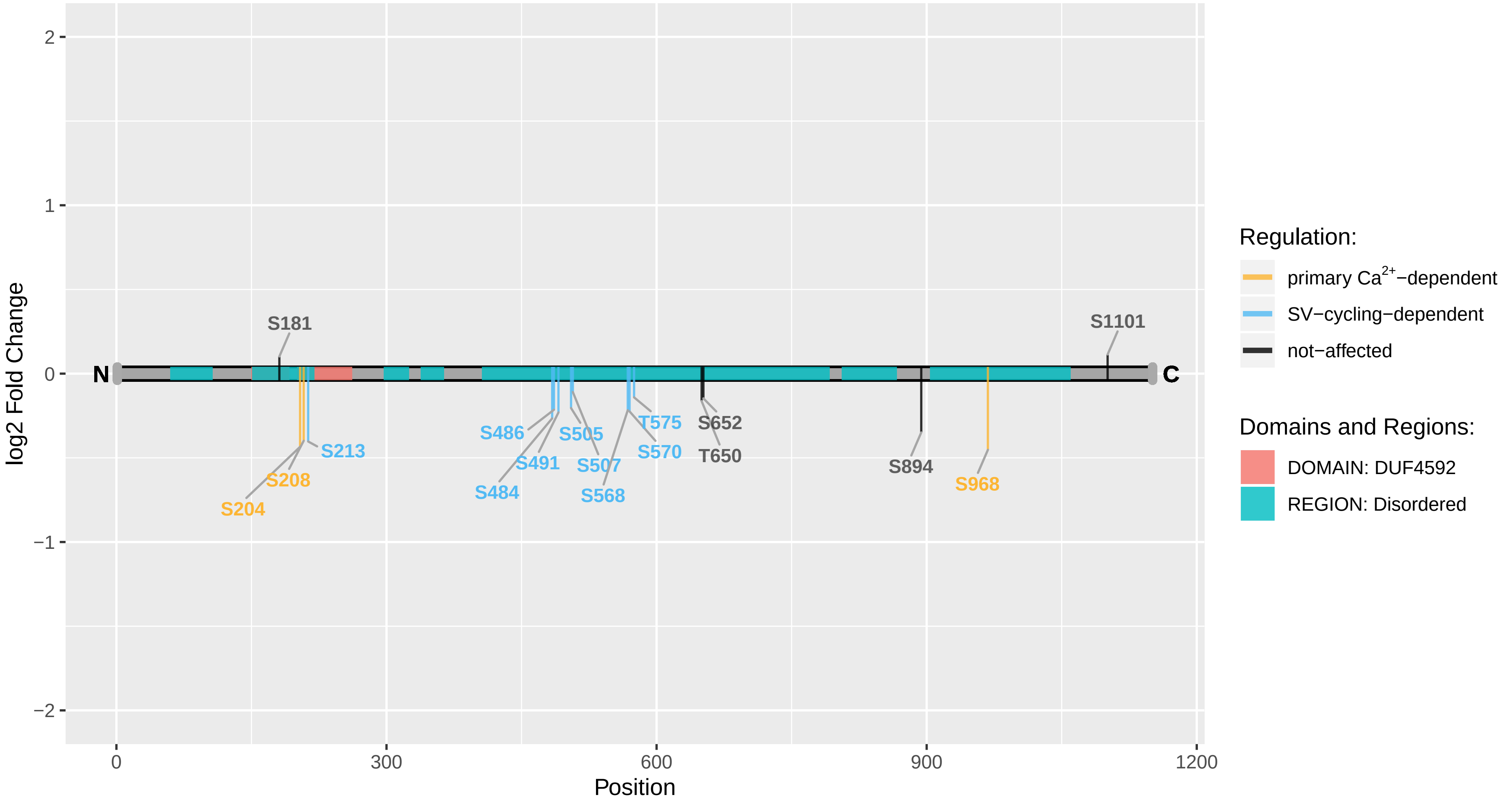

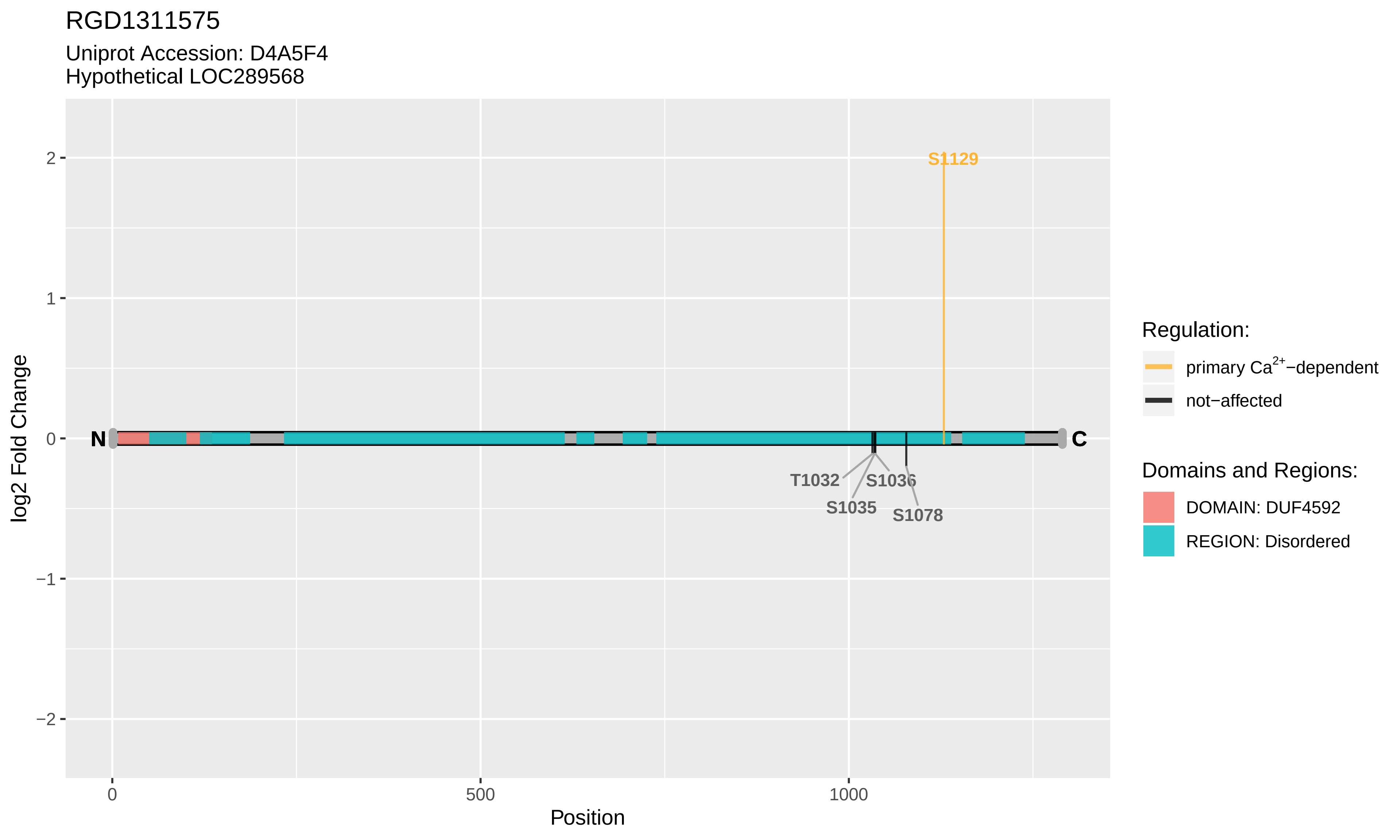

RGD1311575
Uniprot Accession: D4A5F4
Hypothetical LOC289568
S1129
N
C
T1032
S1036
S1035
S1078
log2 Fold Change
2
1
0
−1
−2
0
500
1000
Position
Regulation:
primary Ca2+−dependent
not−affected
Domains and Regions:
DOMAIN: DUF4592
REGION: Disordered

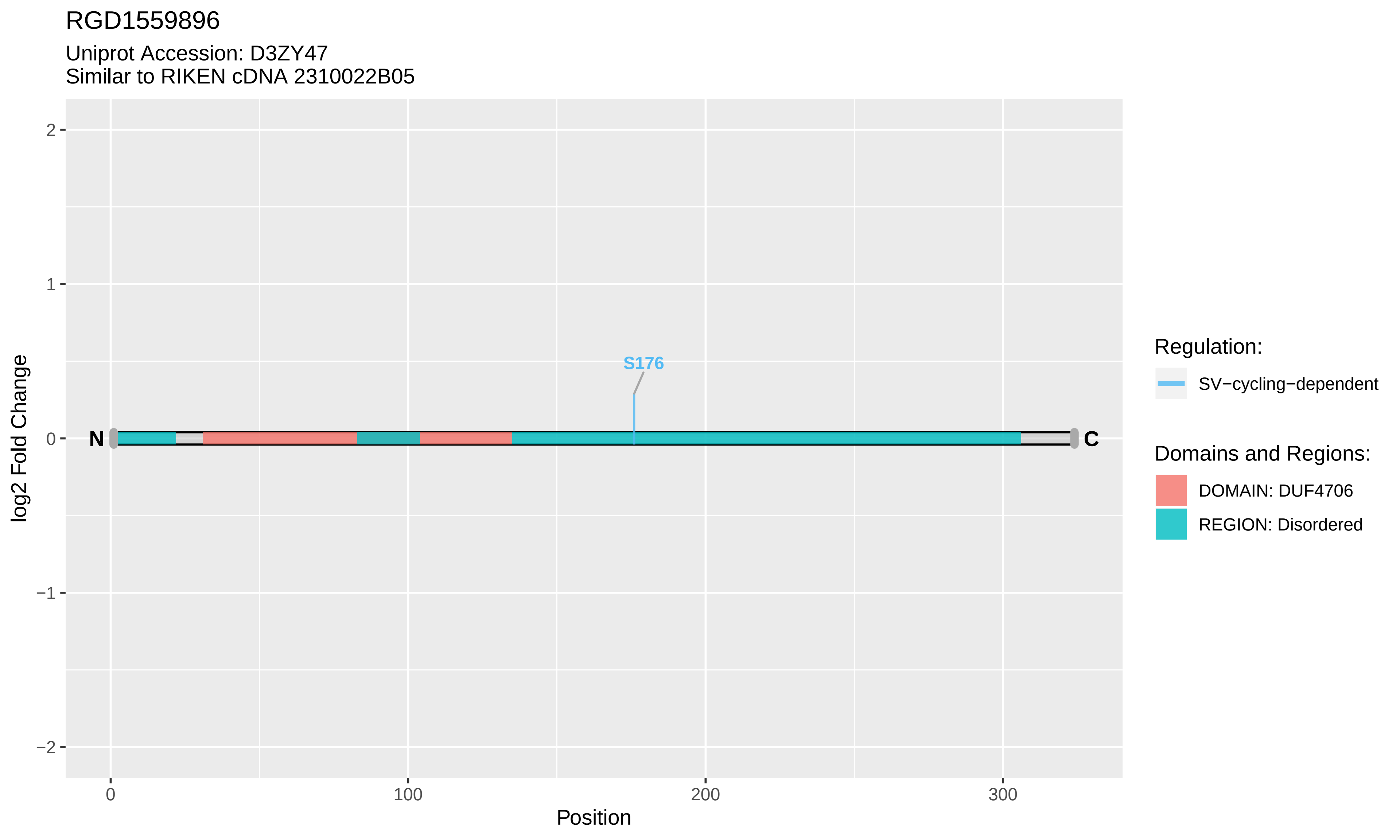
RGD1559896
Uniprot Accession: D3ZY47
Similar to RIKEN cDNA 2310022B05
log2 Fold Change
Position
S176
N
C
2
1
0
−1
−2
0
100
200
300
Regulation:
SV−cycling−dependent
Domains and Regions:
DOMAIN: DUF4706
REGION: Disordered

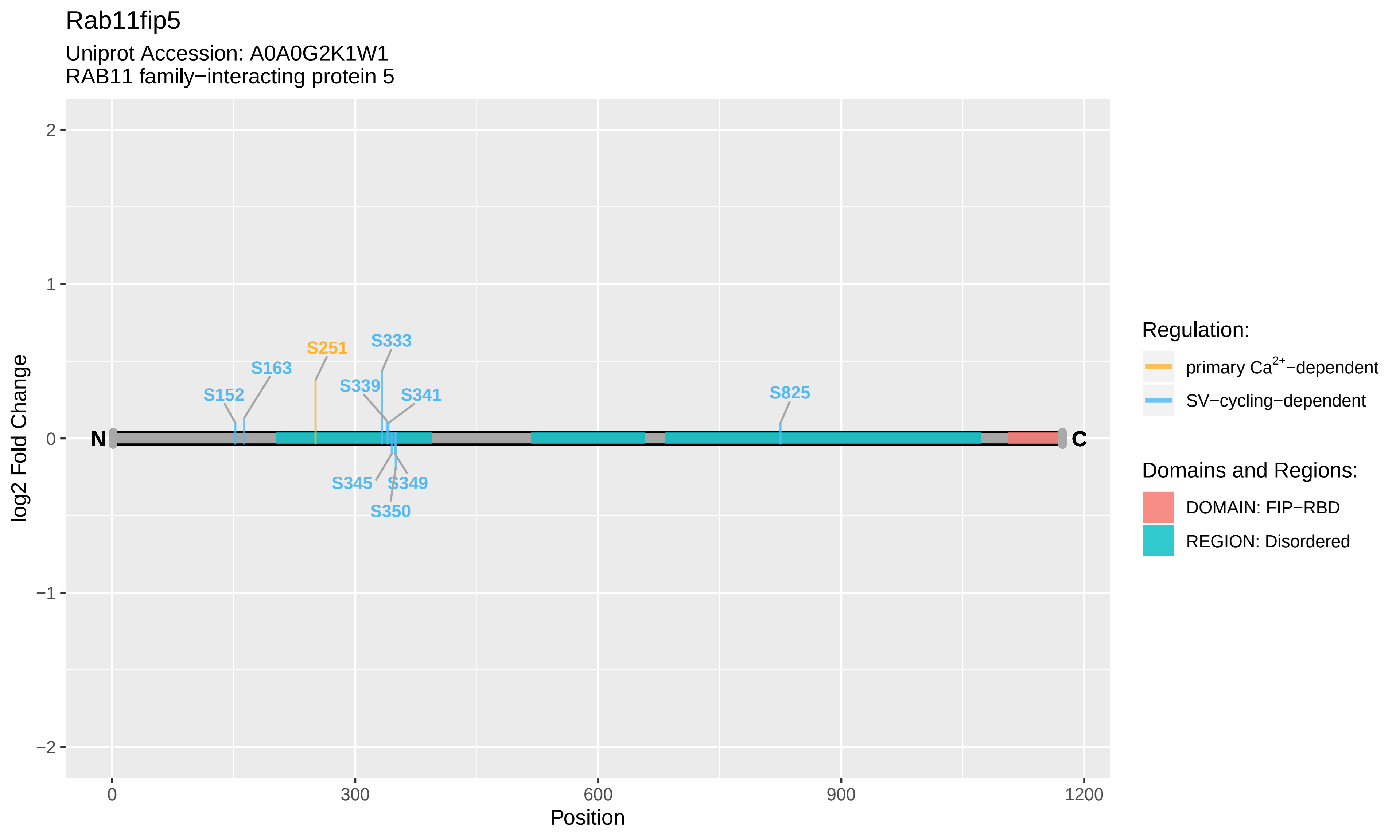

Rab11fip5
Uniprot Accession: A0A0G2K1W1
RAB11 family−interacting protein 5
log2 Fold Change
Position
N
C
S152
S163
S251
S333
S339
S341
S345
S349
S350
S825
Regulation:
primary Ca2+−dependent
SV−cycling−dependent
Domains and Regions:
DOMAIN: FIP−RBD
REGION: Disordered

Rab1A

Uniprot Accession: Q6NYB7
Ras−related protein Rab−1A

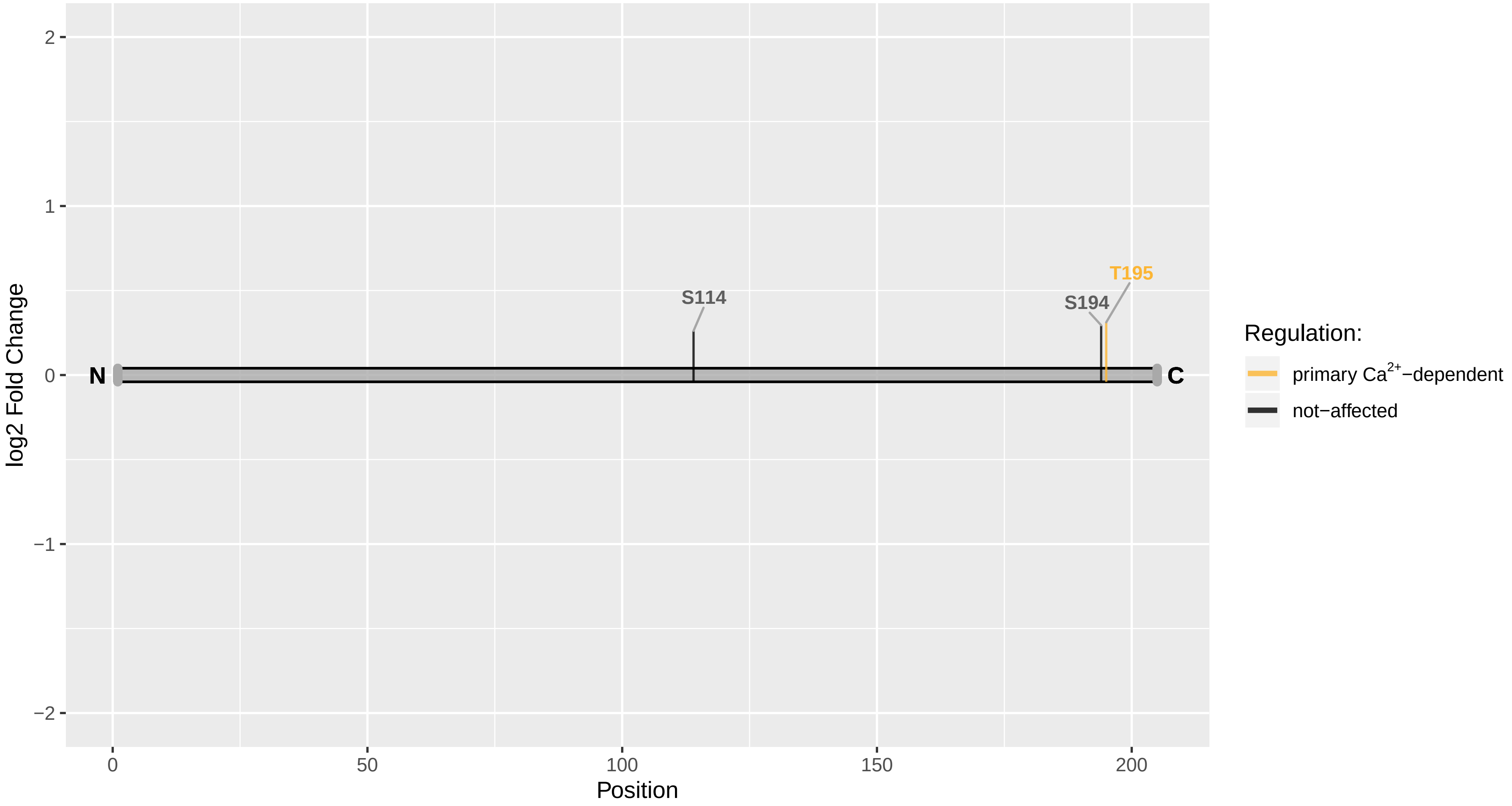

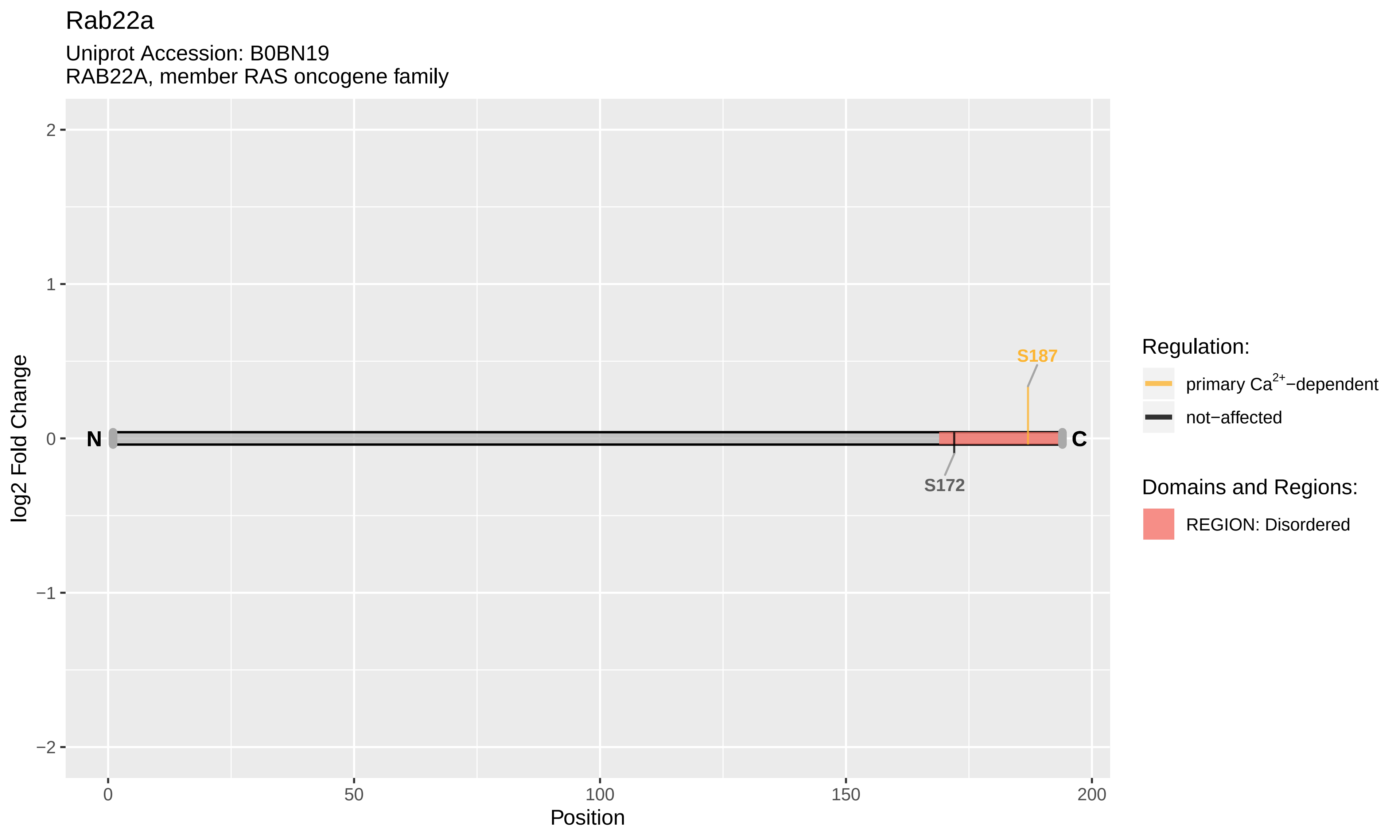

Rab22a
Uniprot Accession: B0BN19
RAB22A, member RAS oncogene family
log2 Fold Change
2
1
0
−1
−2
N
C
S187
S172
0
50
100
150
200
Position
Regulation:
primary Ca2+−dependent
not−affected
Domains and Regions:
REGION: Disordered

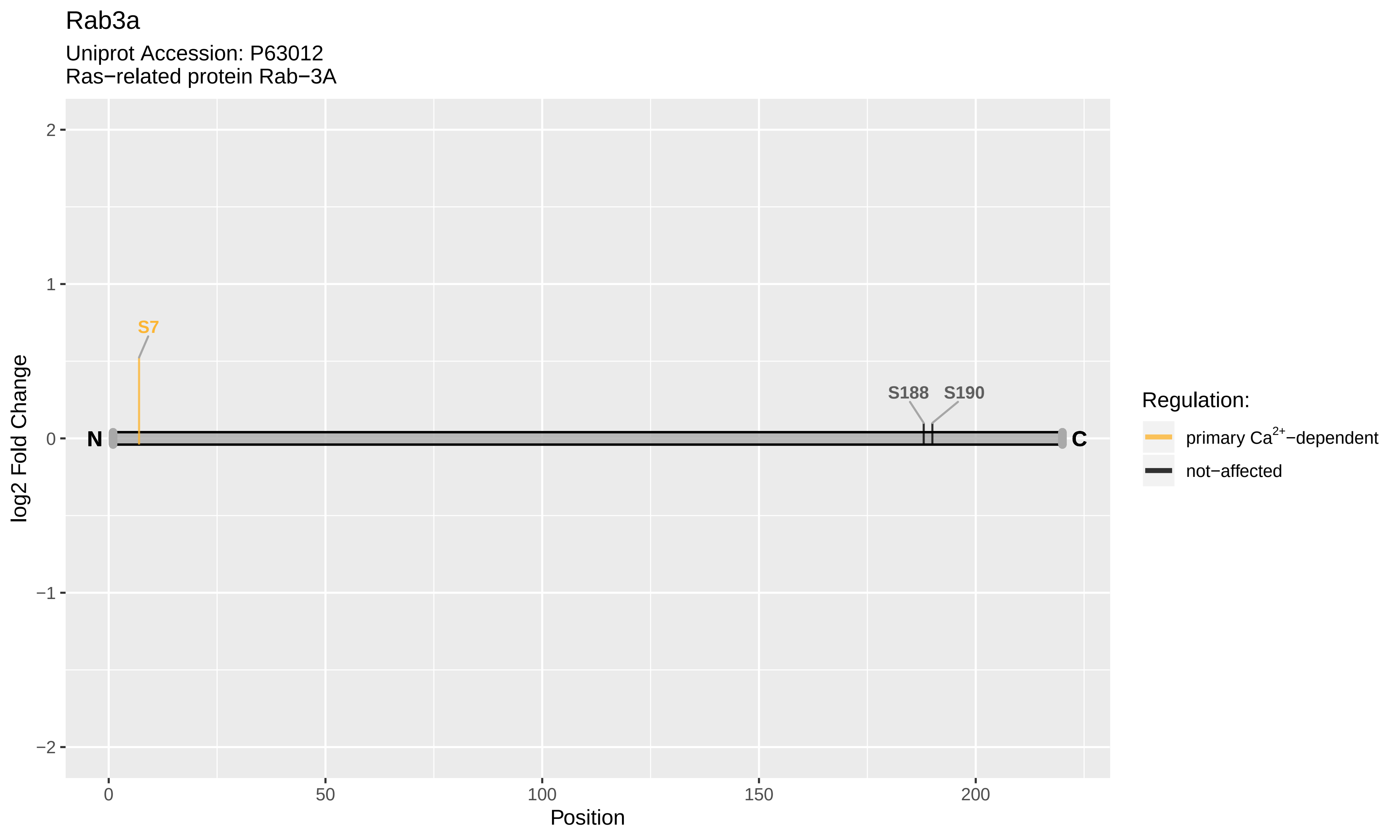
Rab3a
Uniprot Accession: P63012
Ras−related protein Rab−3A
S7
S188
S190
N
C
log2 Fold Change
Position
Regulation:
primary Ca^{2+}−dependent
not−affected

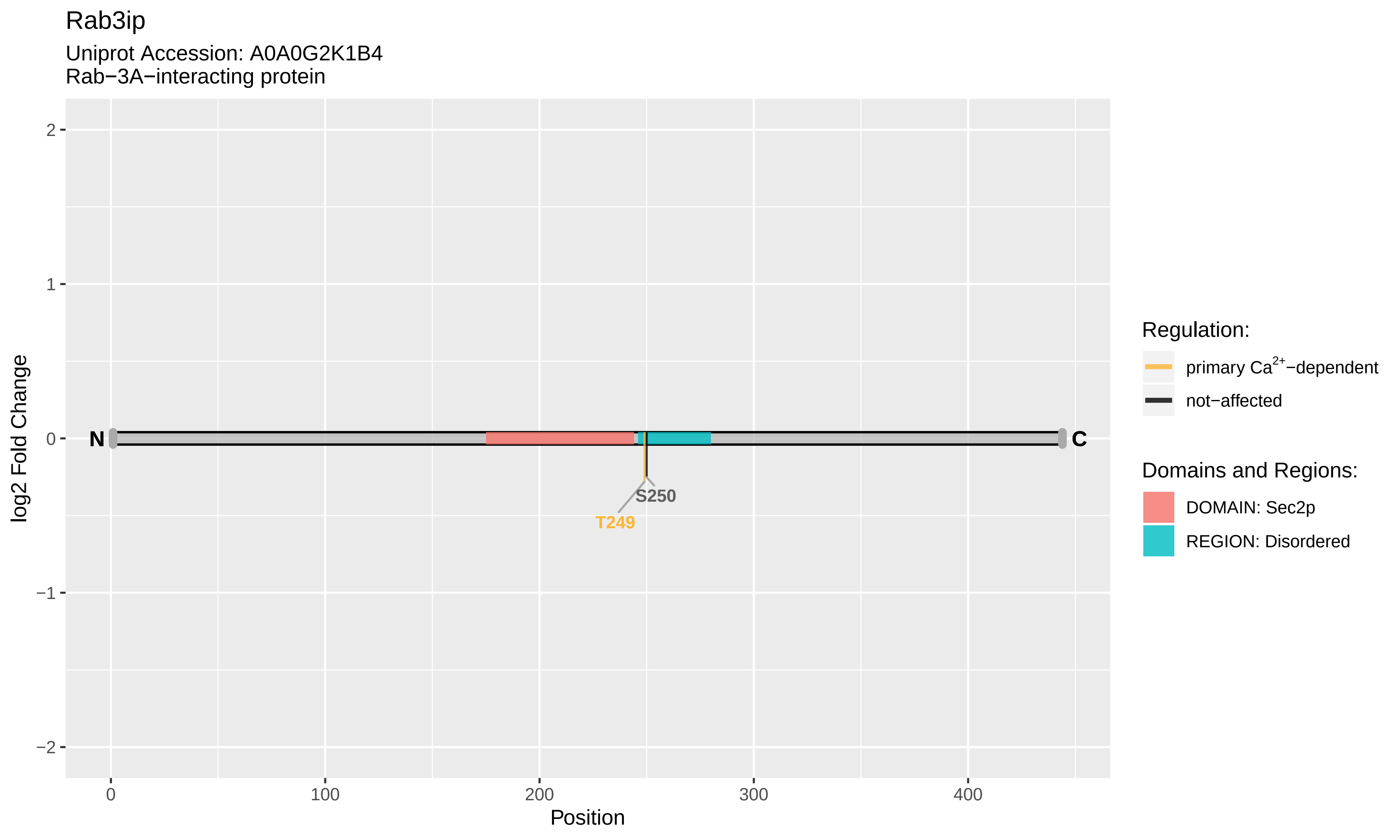
Rab3ip
Uniprot Accession: A0A0G2K1B4
Rab−3A−interacting protein
log2 Fold Change
2
1
0
−1
−2
N
C
S250
T249
0
100
200
300
400
Position
Regulation:
primary Ca2+−dependent
not−affected
Domains and Regions:
DOMAIN: Sec2p
REGION: Disordered

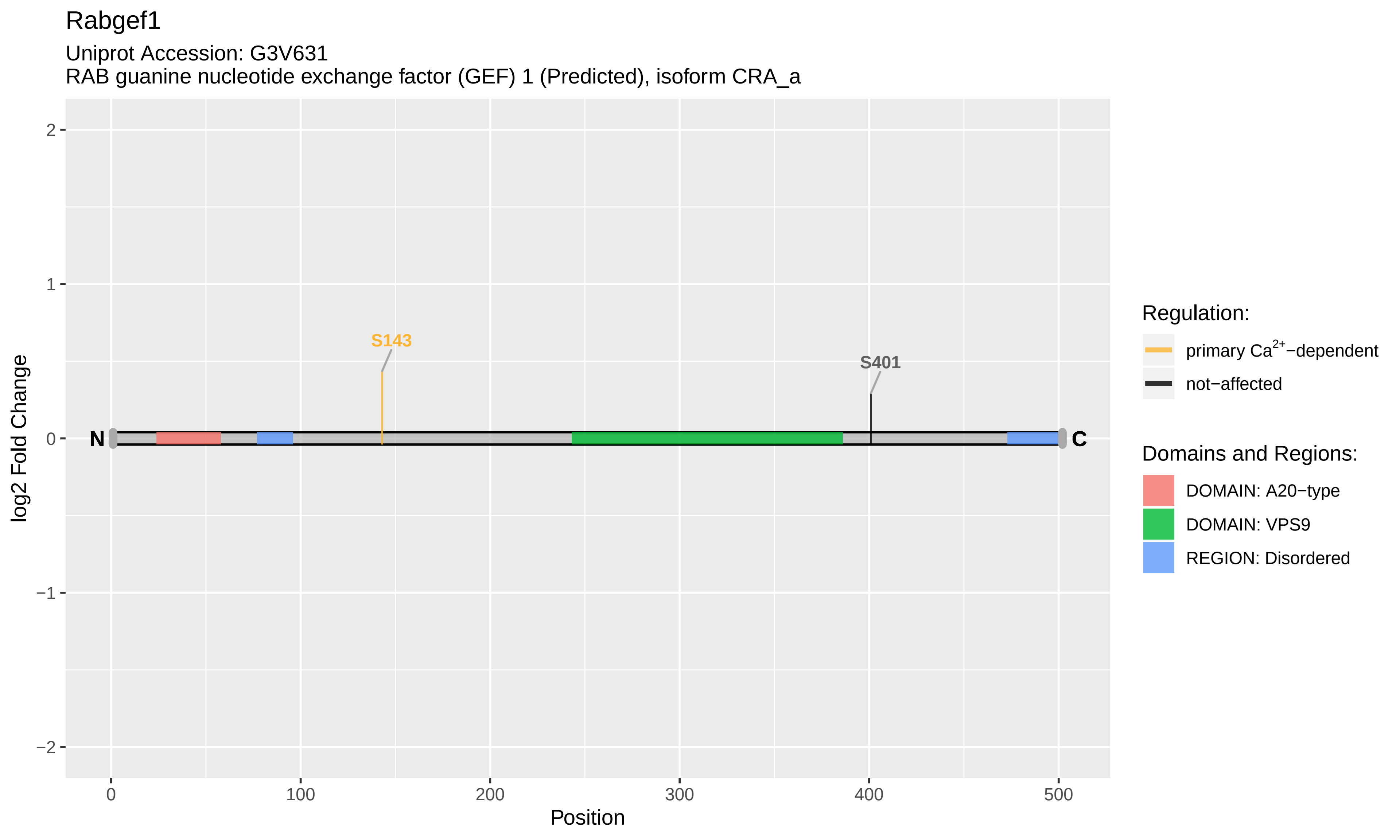
Rabgef1
Uniprot Accession: G3V631
RAB guanine nucleotide exchange factor (GEF) 1 (Predicted), isoform CRA_a
S143
S401
N
C
2
1
0
−1
−2
log2 Fold Change
0
100
200
300
400
500
Position
Regulation:
primary Ca2+−dependent
not−affected
Domains and Regions:
DOMAIN: A20−type
DOMAIN: VPS9
REGION: Disordered

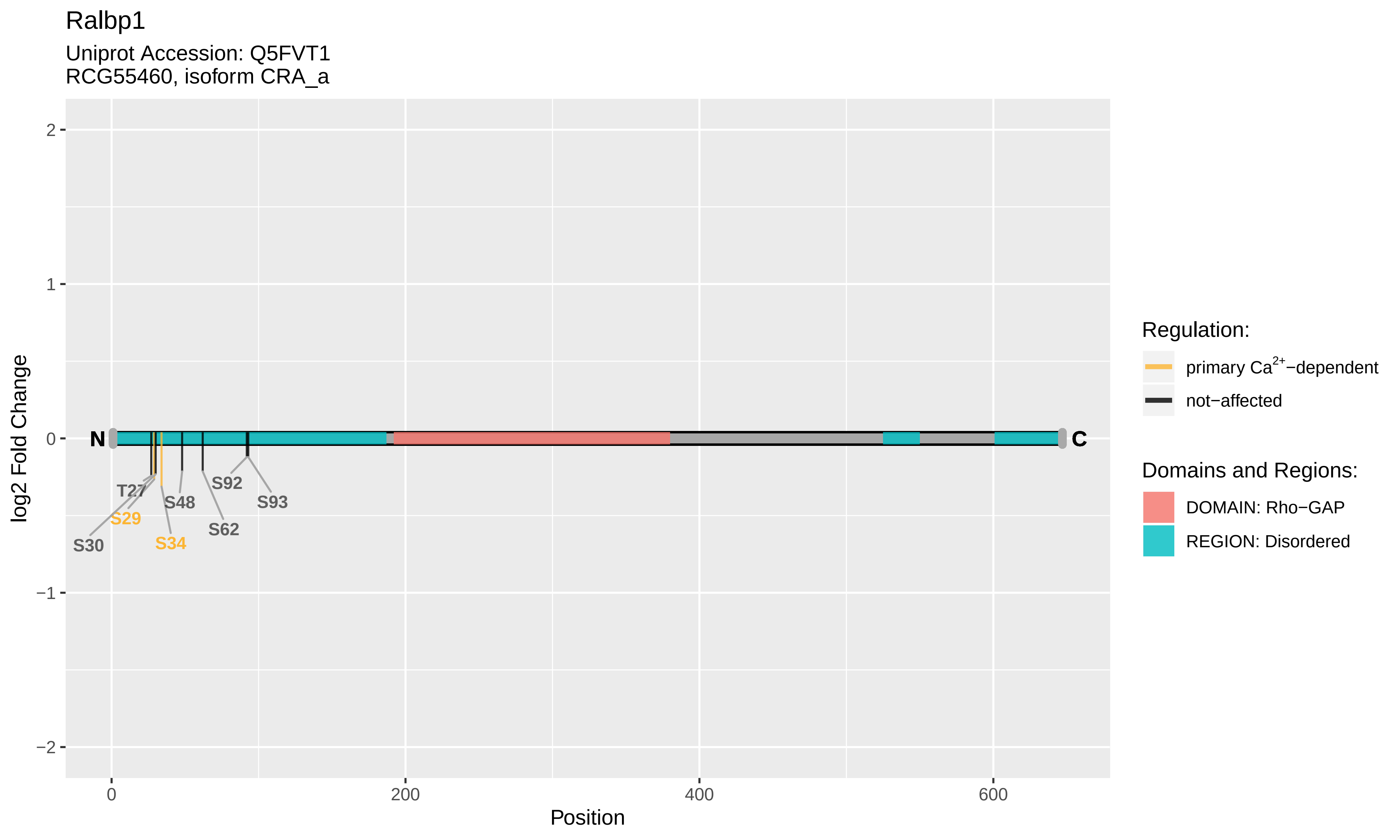

Ralbp1
Uniprot Accession: Q5FVT1
RCG55460, isoform CRA_a
log2 Fold Change
2
1
0
−1
−2
N
C
T27
S29
S30
S34
S48
S62
S92
S93
0
200
400
600
Position
Regulation:
primary Ca2+−dependent
not−affected
Domains and Regions:
DOMAIN: Rho−GAP
REGION: Disordered

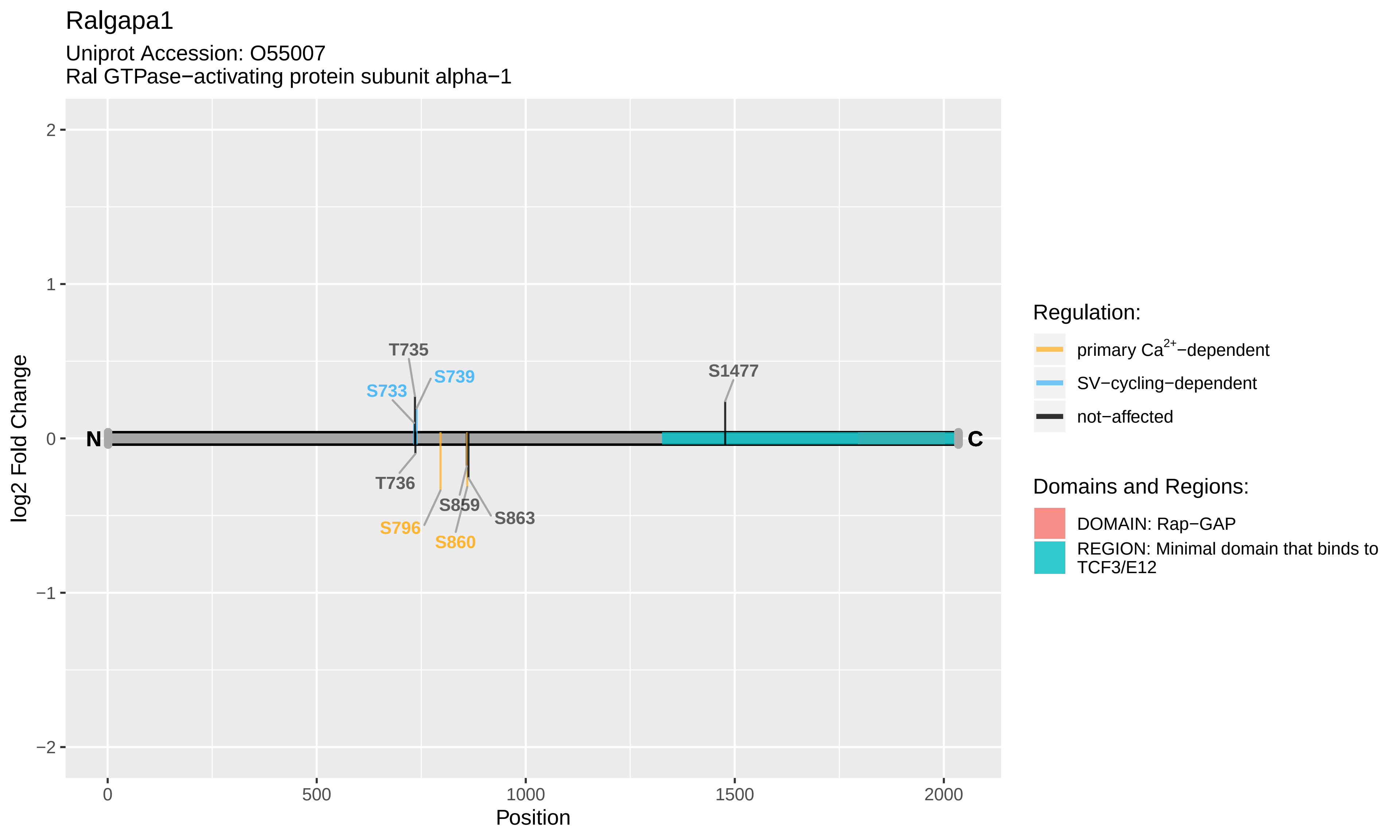
Ralgapa1
Uniprot Accession: O55007
Ral GTPase−activating protein subunit alpha−1
log2 Fold Change
2
1
0
−1
−2
N
C
T735
S739
S733
S1477
T736
S859
S863
S796
S860
0
500
1000
1500
2000
Position
Regulation:
primary Ca2+−dependent
SV−cycling−dependent
not−affected
Domains and Regions:
DOMAIN: Rap−GAP
REGION: Minimal domain that binds to TCF3/E12

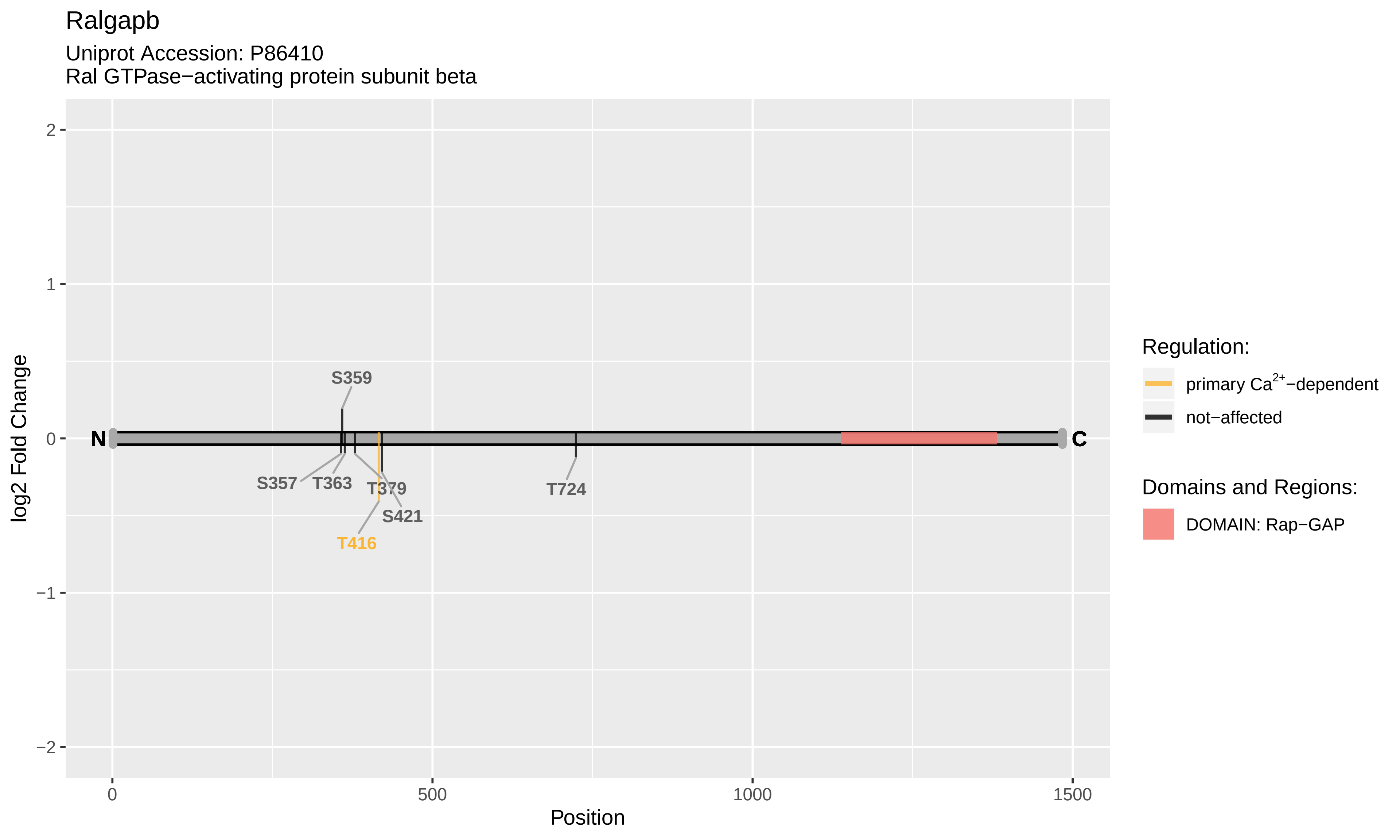

Ralgapb
Uniprot Accession: P86410
Ral GTPase−activating protein subunit beta
log2 Fold Change
2
1
0
−1
−2
N
C
S359
S357
T363
T379
S421
T416
T724
0
500
1000
1500
Position
Regulation:
primary Ca2+−dependent
not−affected
Domains and Regions:
DOMAIN: Rap−GAP

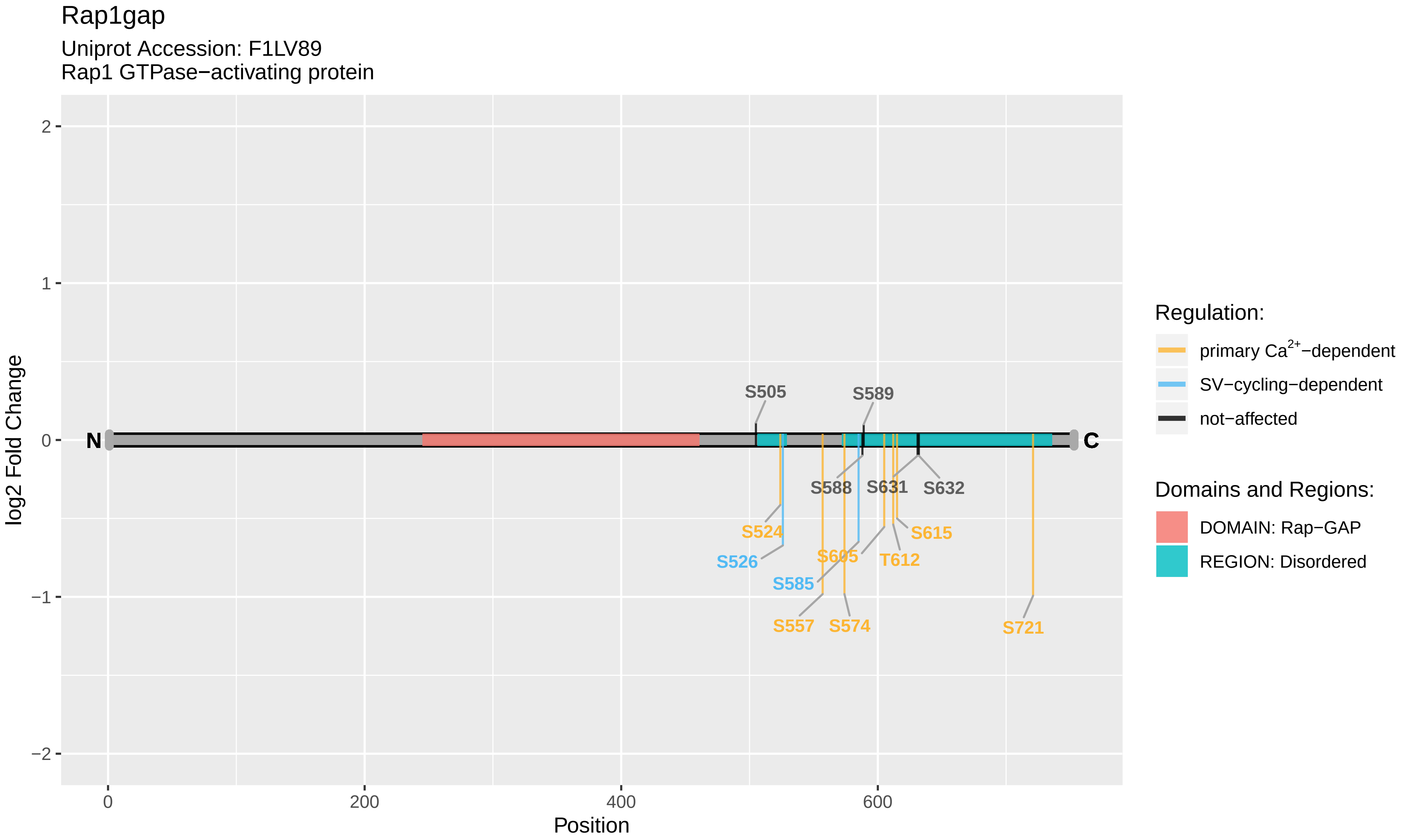

Rap1gap
Uniprot Accession: F1LV89
Rap1 GTPase−activating protein
log2 Fold Change
2
1
0
−1
−2
N
C
S505
S589
S588
S631
S632
S524
S526
S605
T612
S615
S585
S557
S574
S721
0
200
400
600
Position
Regulation:
primary Ca2+−dependent
SV−cycling−dependent
not−affected
Domains and Regions:
DOMAIN: Rap−GAP
REGION: Disordered

Rap1gap2

Uniprot Accession: A0A0G2K0S7
RAP1 GTPase−activating protein 2

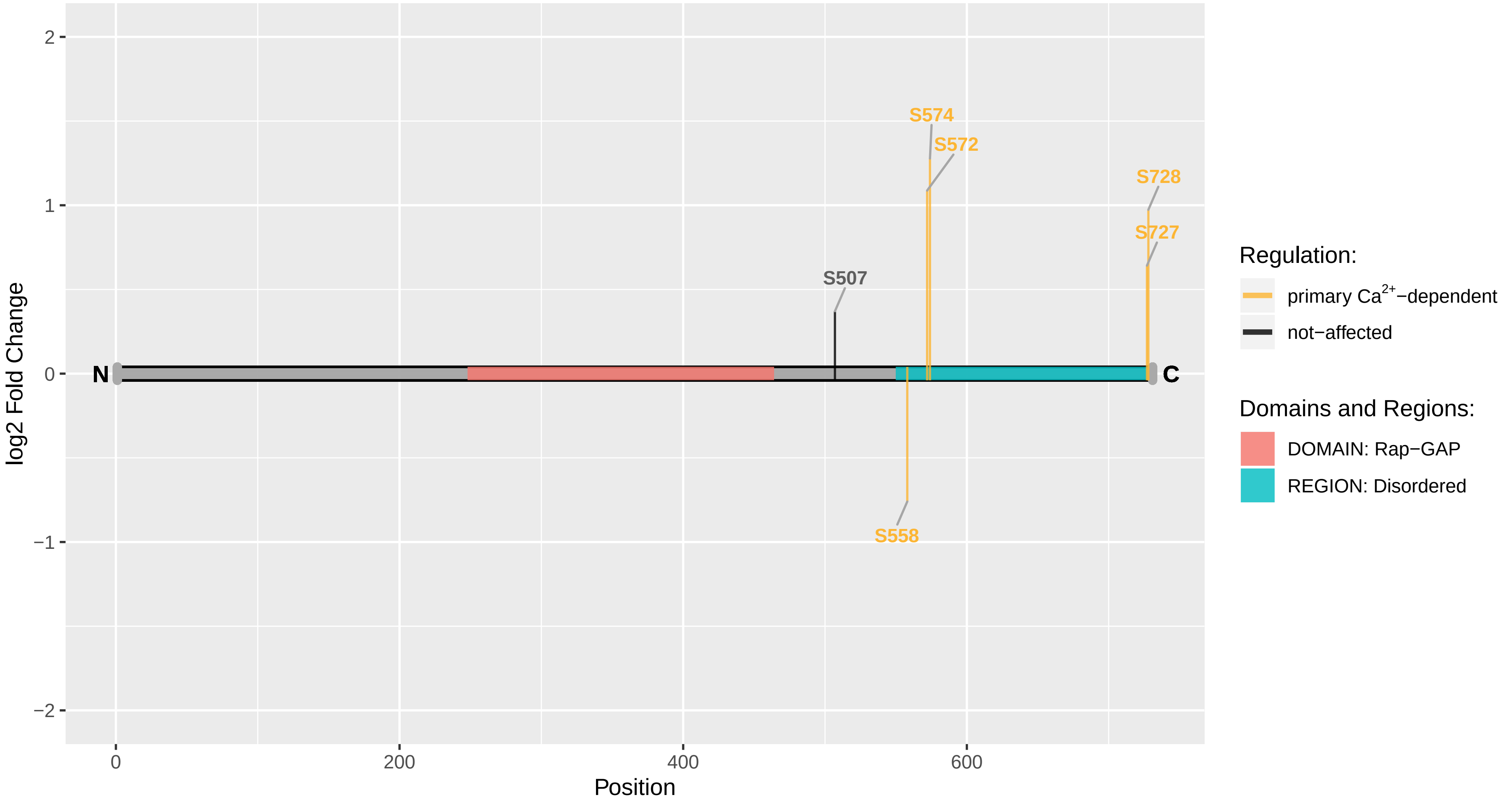

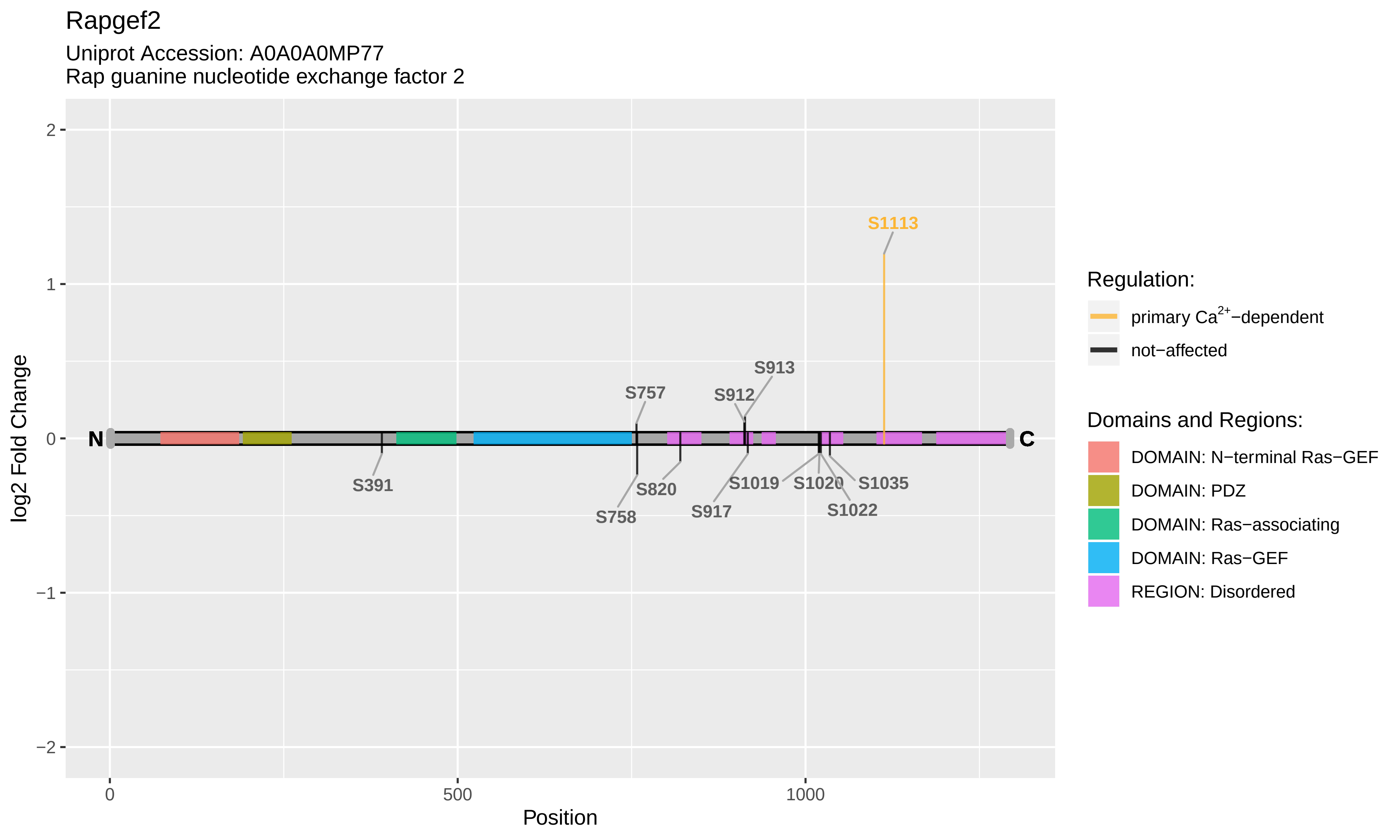

Rapgef2
Uniprot Accession: A0A0A0MP77
Rap guanine nucleotide exchange factor 2
log2 Fold Change
Position
N
C
S1113
S913
S912
S757
S391
S820
S758
S917
S1019
S1020
S1035
S1022
Regulation:
primary Ca2+−dependent
not−affected
Domains and Regions:
DOMAIN: N−terminal Ras−GEF
DOMAIN: PDZ
DOMAIN: Ras−associating
DOMAIN: Ras−GEF
REGION: Disordered

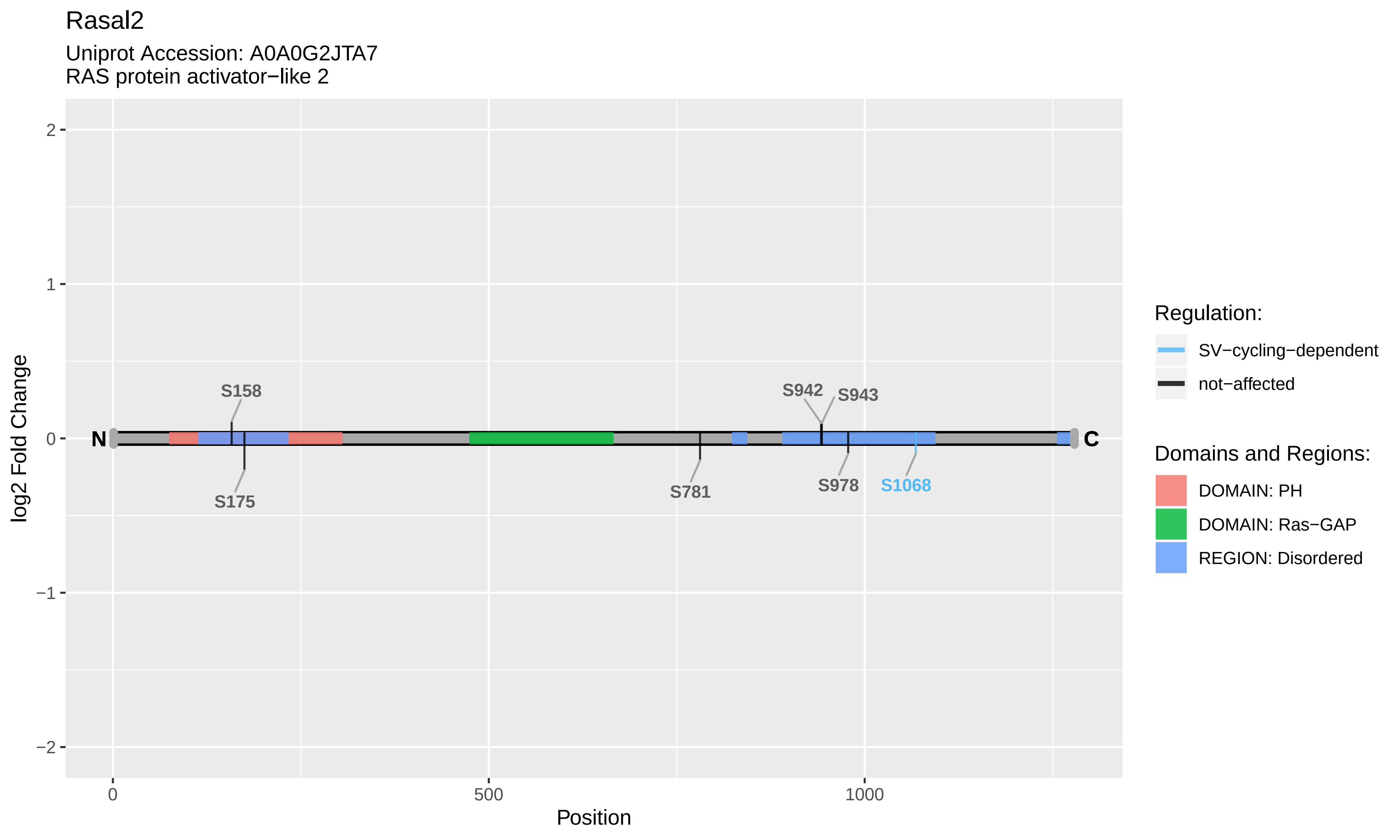

Rasal2
Uniprot Accession: A0A0G2JTA7
RAS protein activator−like 2
log2 Fold Change
2
1
0
−1
−2
N
C
S158
S175
S781
S942
S943
S978
S1068
0
500
1000
Position
Regulation:
SV−cycling−dependent
not−affected
Domains and Regions:
DOMAIN: PH
DOMAIN: Ras−GAP
REGION: Disordered

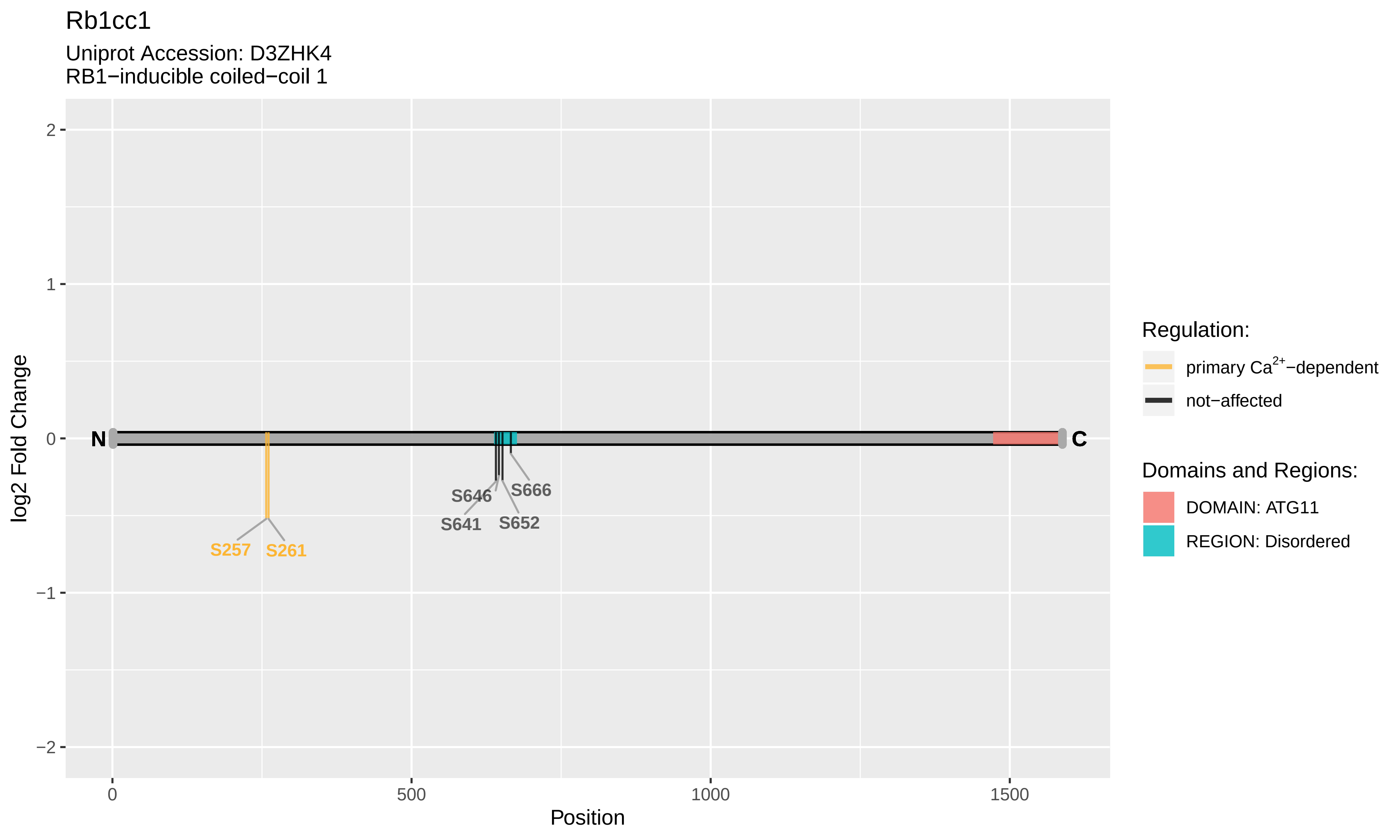

Rb1cc1
Uniprot Accession: D3ZHK4
RB1−inducible coiled−coil 1
log2 Fold Change
2
1
0
−1
−2
N
C
S257
S261
S646
S641
S666
S652
0
500
1000
1500
Position
Regulation:
primary Ca2+−dependent
not−affected
Domains and Regions:
DOMAIN: ATG11
REGION: Disordered

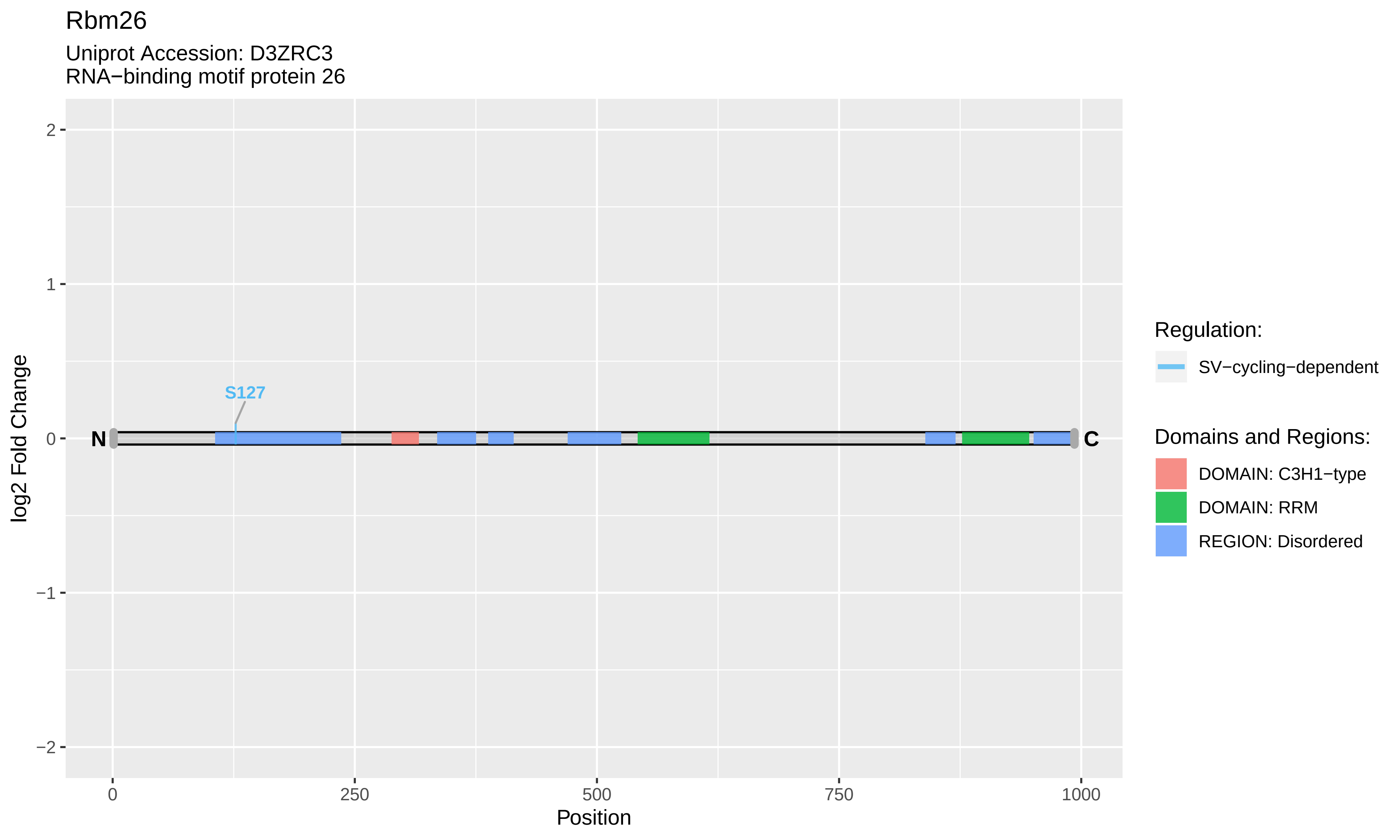

Rbm26
Uniprot Accession: D3ZRC3
RNA−binding motif protein 26
S127
N
C
2
1
0
−1
−2
log2 Fold Change
0
250
500
750
1000
Position
Regulation:
SV−cycling−dependent
Domains and Regions:
DOMAIN: C3H1−type
DOMAIN: RRM
REGION: Disordered

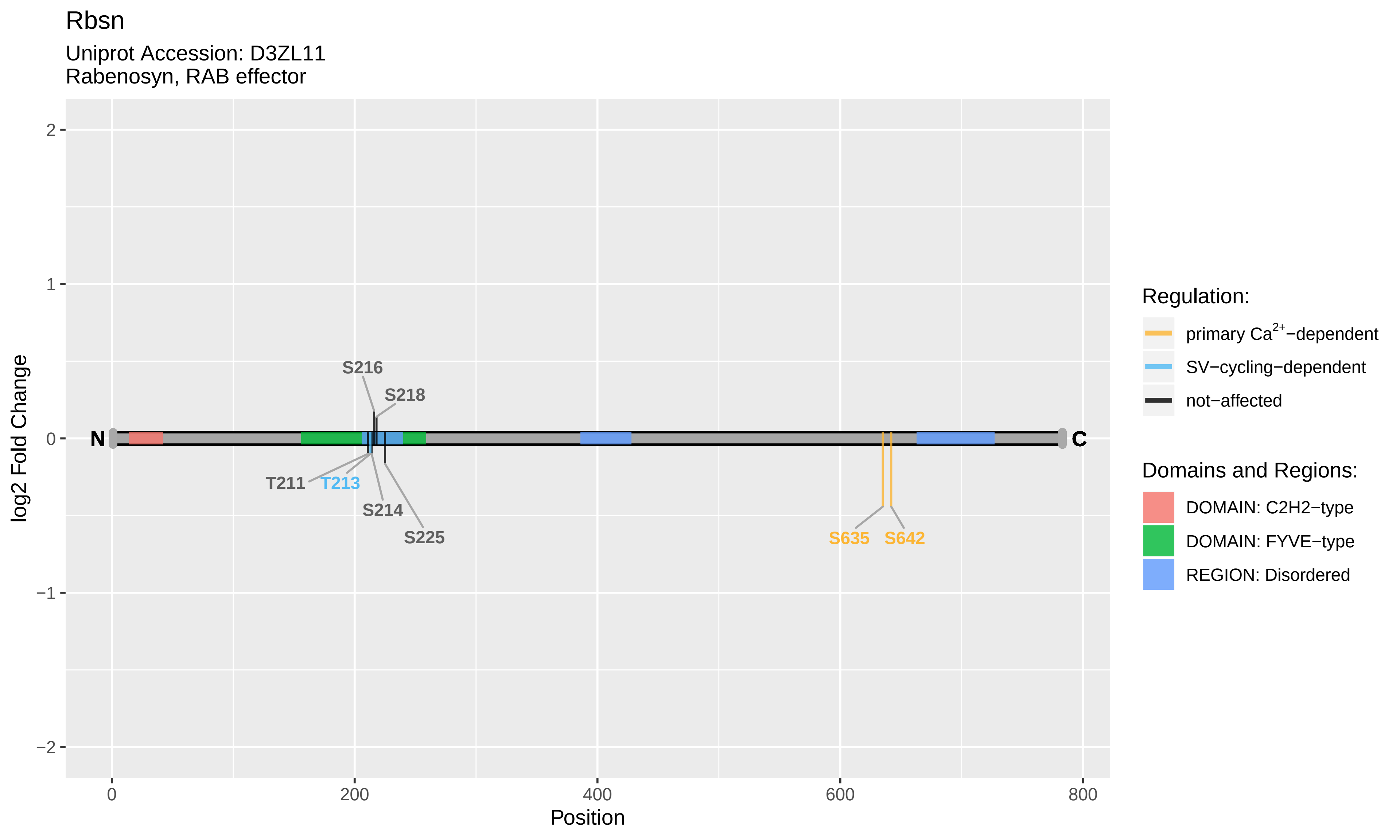
Rbsn
Uniprot Accession: D3ZL11
Rabenosyn, RAB effector
log2 Fold Change
2
1
0
−1
−2
N
C
S216
S218
T211
T213
S214
S225
S635
S642
0
200
400
600
800
Position
Regulation:
primary Ca2+−dependent
SV−cycling−dependent
not−affected
Domains and Regions:
DOMAIN: C2H2−type
DOMAIN: FYVE−type
REGION: Disordered

Relch

Uniprot Accession: D3ZJ01
RAB11−binding and LisH domain, coiled−coil and HEAT repeat−containing

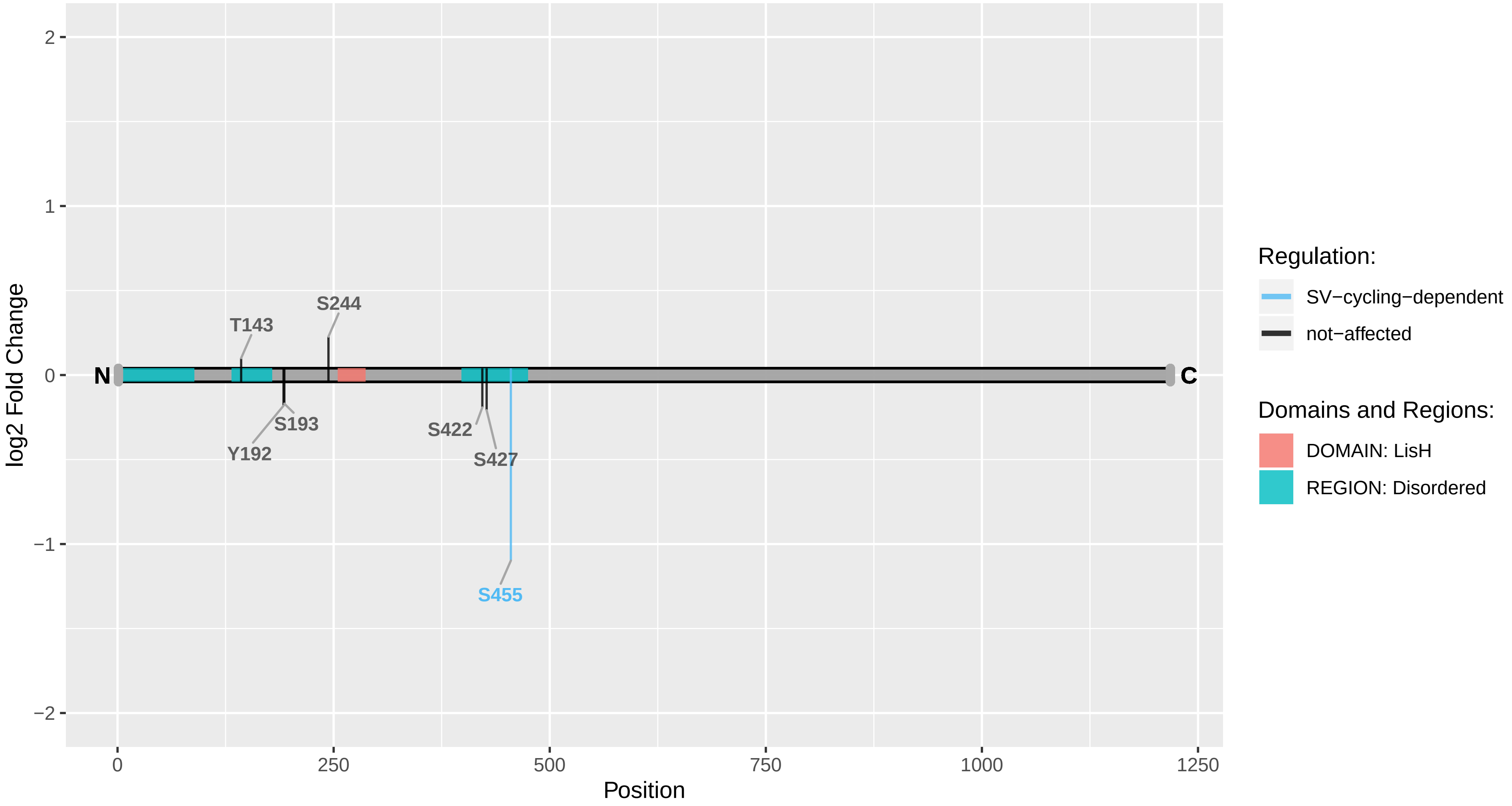

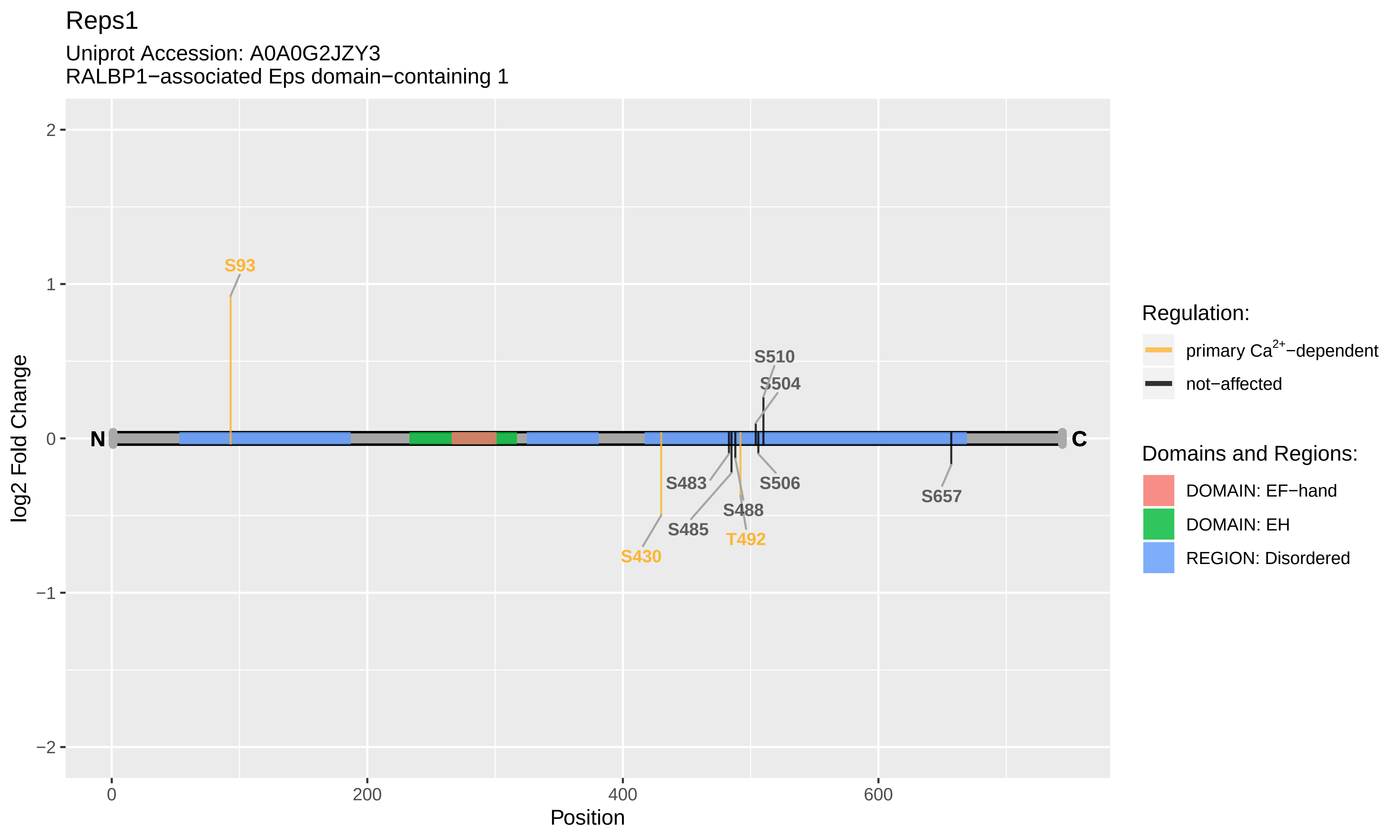

Reps1
Uniprot Accession: A0A0G2JZY3
RALBP1−associated Eps domain−containing 1
S93
S510
S504
S483
S506
S488
S485
T492
S430
S657
N
C
2
1
0
−1
−2
0
200
400
600
log2 Fold Change
Position
Regulation:
primary Ca2+−dependent
not−affected
Domains and Regions:
DOMAIN: EF−hand
DOMAIN: EH
REGION: Disordered

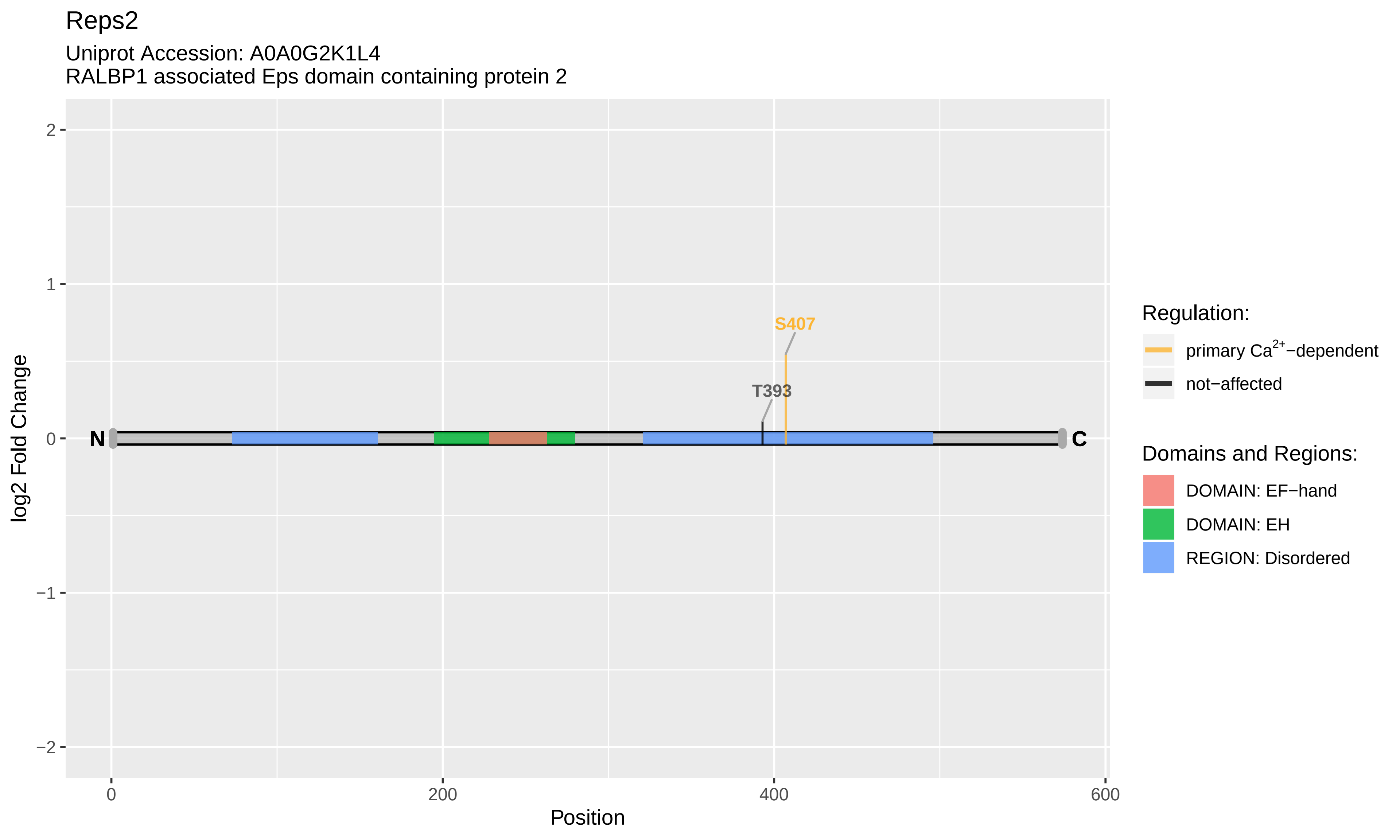
Reps2
Uniprot Accession: A0A0G2K1L4
RALBP1 associated Eps domain containing protein 2
S407
T393
N
C
log2 Fold Change
Position
Regulation:
primary Ca2+−dependent
not−affected
Domains and Regions:
DOMAIN: EF−hand
DOMAIN: EH
REGION: Disordered

Reps2

Uniprot Accession: A0A0G2K285
RALBP1 associated Eps domain containing protein 2

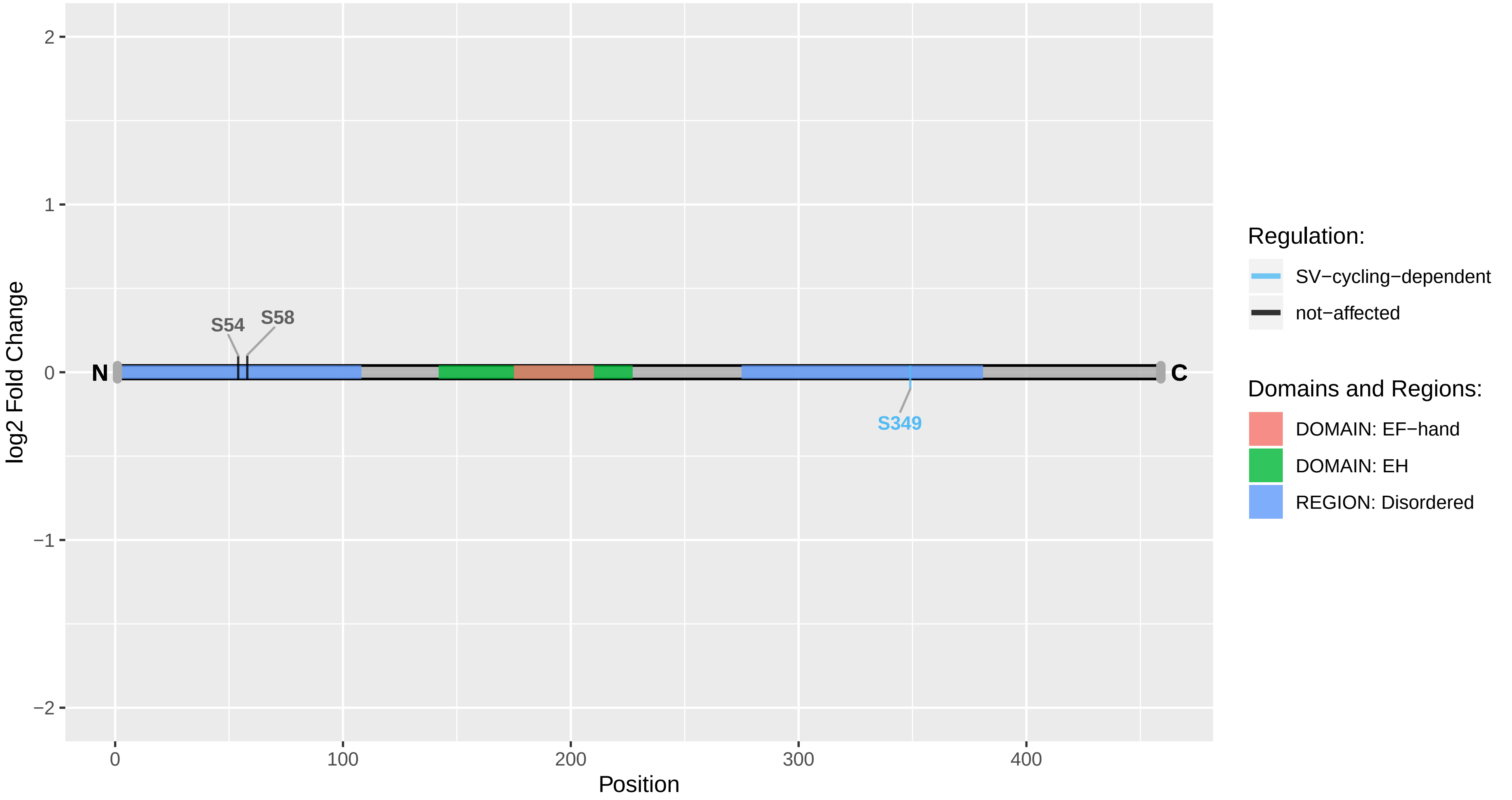

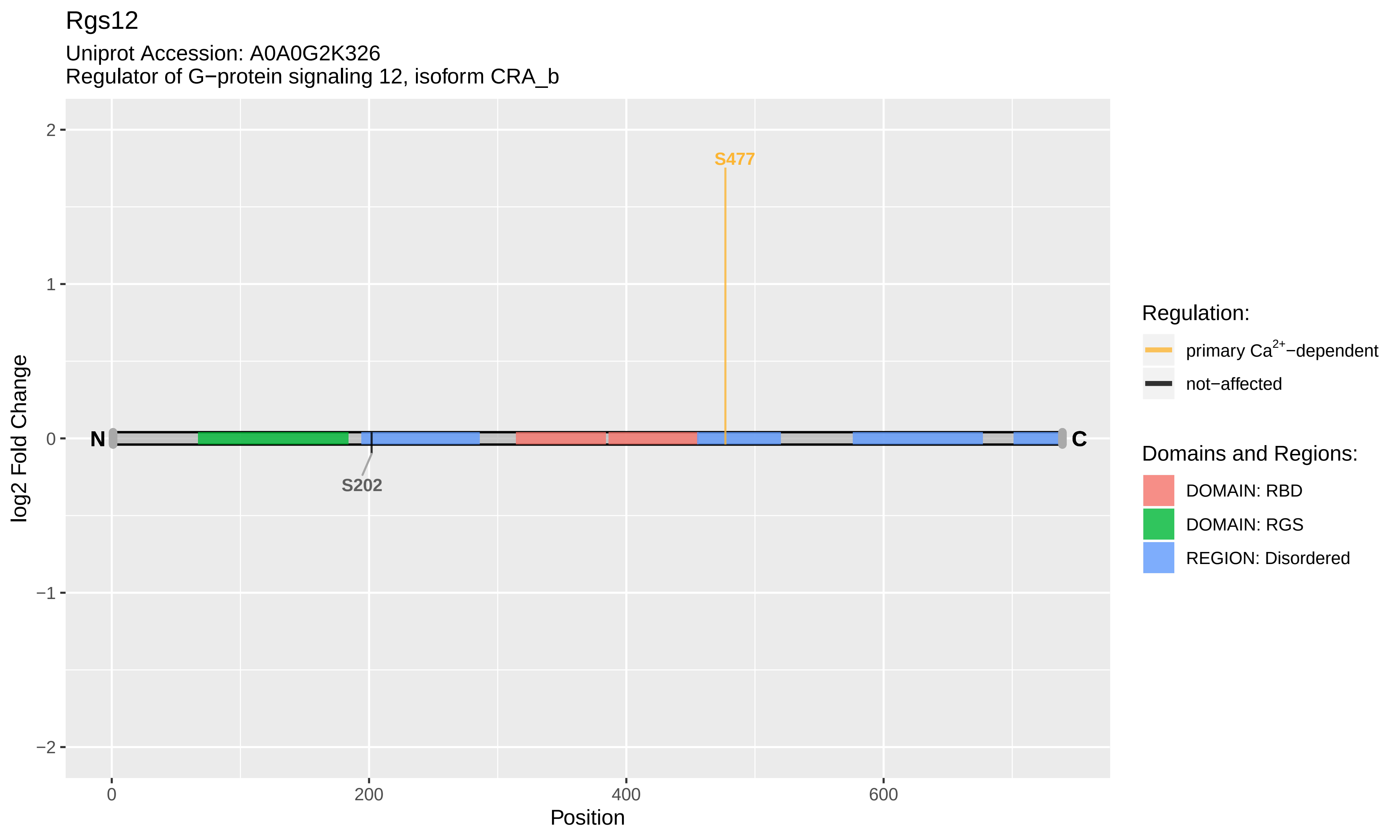
Rgs12
Uniprot Accession: A0A0G2K326
Regulator of G−protein signaling 12, isoform CRA_b
S477
S202
N
C
log2 Fold Change
Position
Regulation:
primary Ca2+−dependent
not−affected
Domains and Regions:
DOMAIN: RBD
DOMAIN: RGS
REGION: Disordered

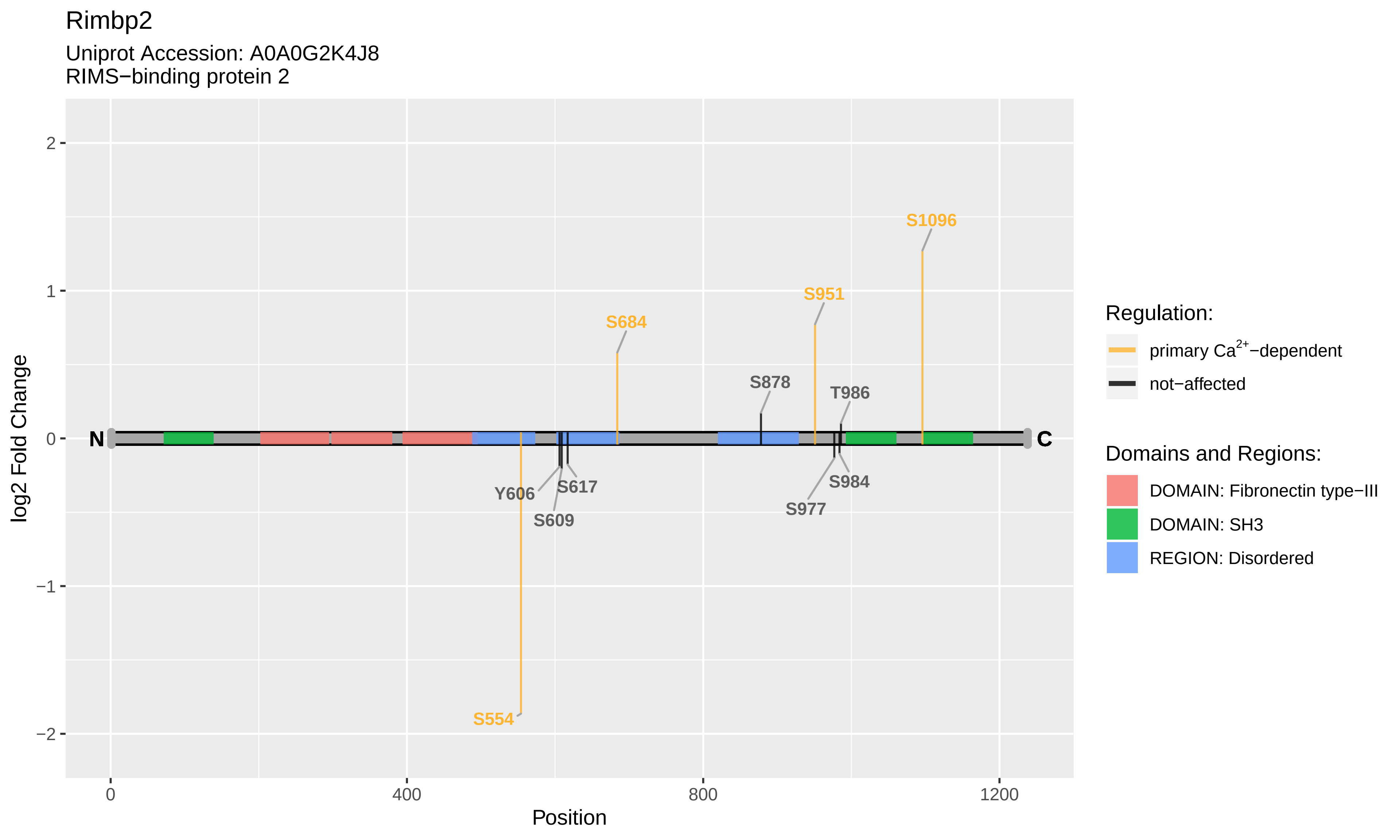

Rimbp2
Uniprot Accession: A0A0G2K4J8
RIMS−binding protein 2
log2 Fold Change
Position
2
1
0
−1
−2
0
400
800
1200
N
C
S1096
S951
S684
S878
T986
Y606
S617
S609
S984
S977
S554
Regulation:
primary Ca2+−dependent
not−affected
Domains and Regions:
DOMAIN: Fibronectin type−III
DOMAIN: SH3
REGION: Disordered

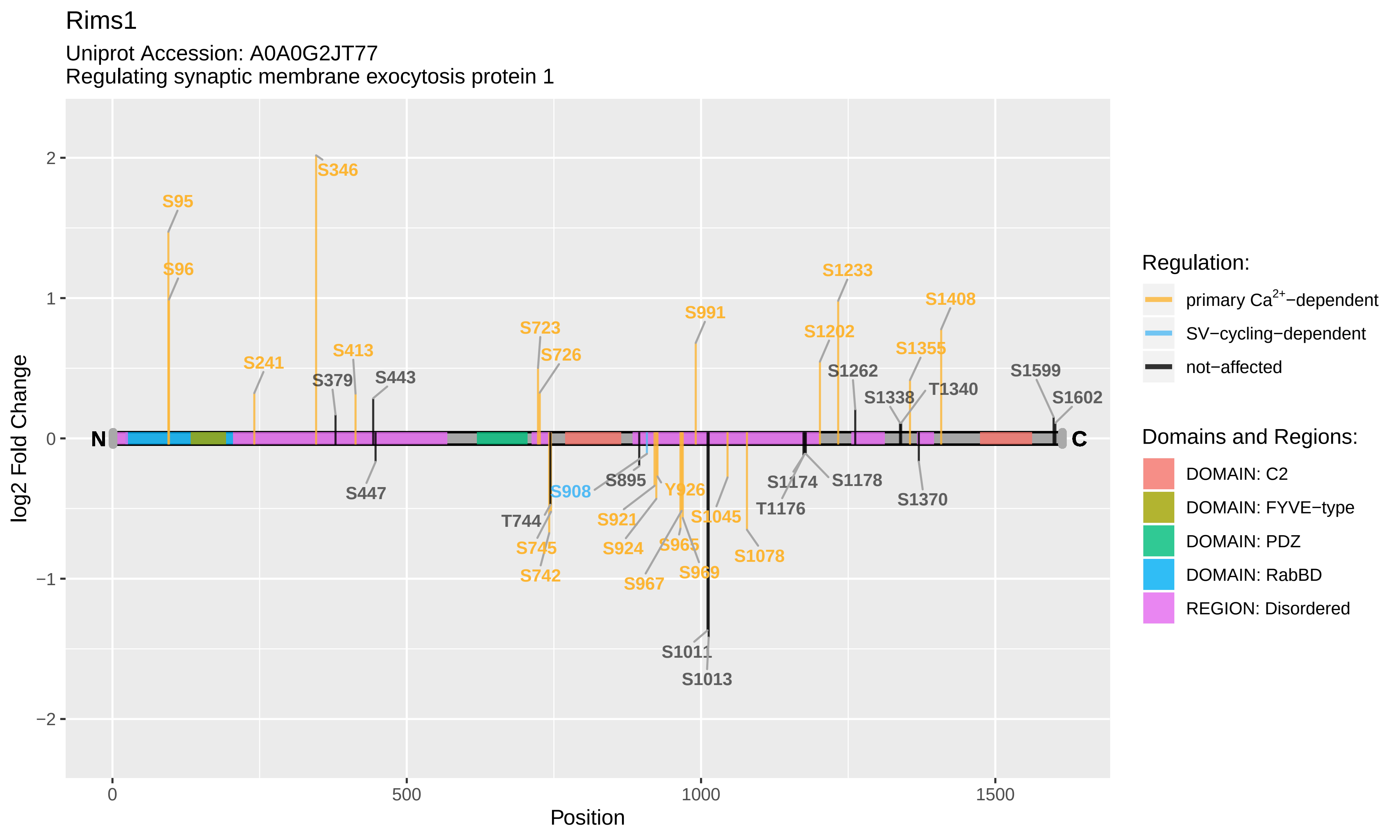

Rims1
Uniprot Accession: A0A0G2JT77
Regulating synaptic membrane exocytosis protein 1
log2 Fold Change
Position
2
1
0
−1
−2
0
500
1000
1500
N
C
S95
S96
S346
S241
S413
S379
S443
S447
S723
S726
T744
S745
S742
S908
S895
S921
S924
Y926
S965
S967
S969
S991
S1011
S1013
S1045
S1078
S1174
T1176
S1178
S1202
S1233
S1262
S1338
T1340
S1355
S1370
S1408
S1599
S1602
Regulation:
primary Ca^{2+}−dependent
SV−cycling−dependent
not−affected
Domains and Regions:
DOMAIN: C2
DOMAIN: FYVE−type
DOMAIN: PDZ
DOMAIN: RabBD
REGION: Disordered

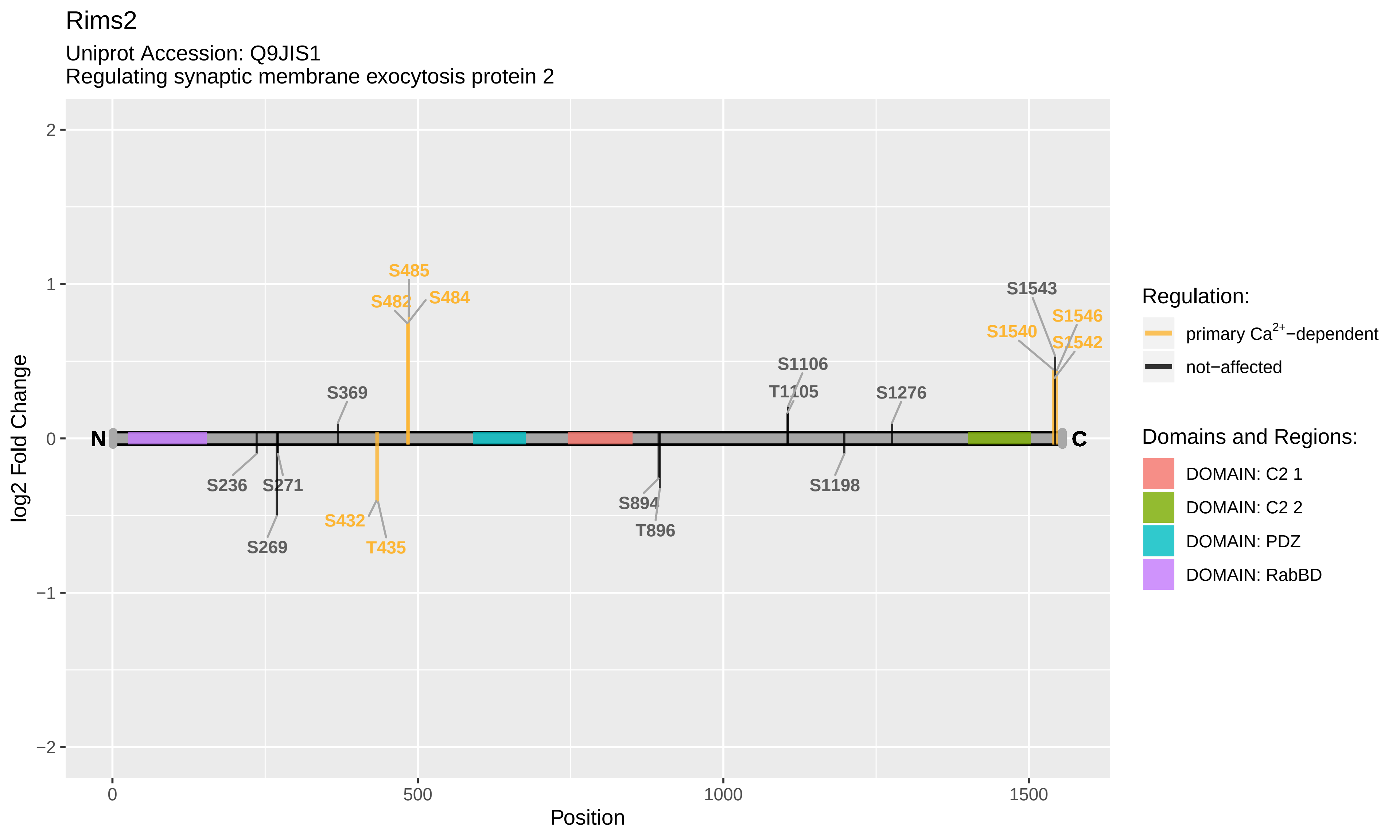

Rims2
Uniprot Accession: Q9JIS1
Regulating synaptic membrane exocytosis protein 2
log2 Fold Change
Position
N
C
S485
S482
S484
S369
S236
S271
S269
S432
T435
S894
T896
S1106
T1105
S1198
S1276
S1543
S1546
S1540
S1542
Regulation:
primary Ca2+−dependent
not−affected
Domains and Regions:
DOMAIN: C2 1
DOMAIN: C2 2
DOMAIN: PDZ
DOMAIN: RabBD

Rmdn2

Uniprot Accession: Q498D5
Regulator of microtubule dynamics protein 2

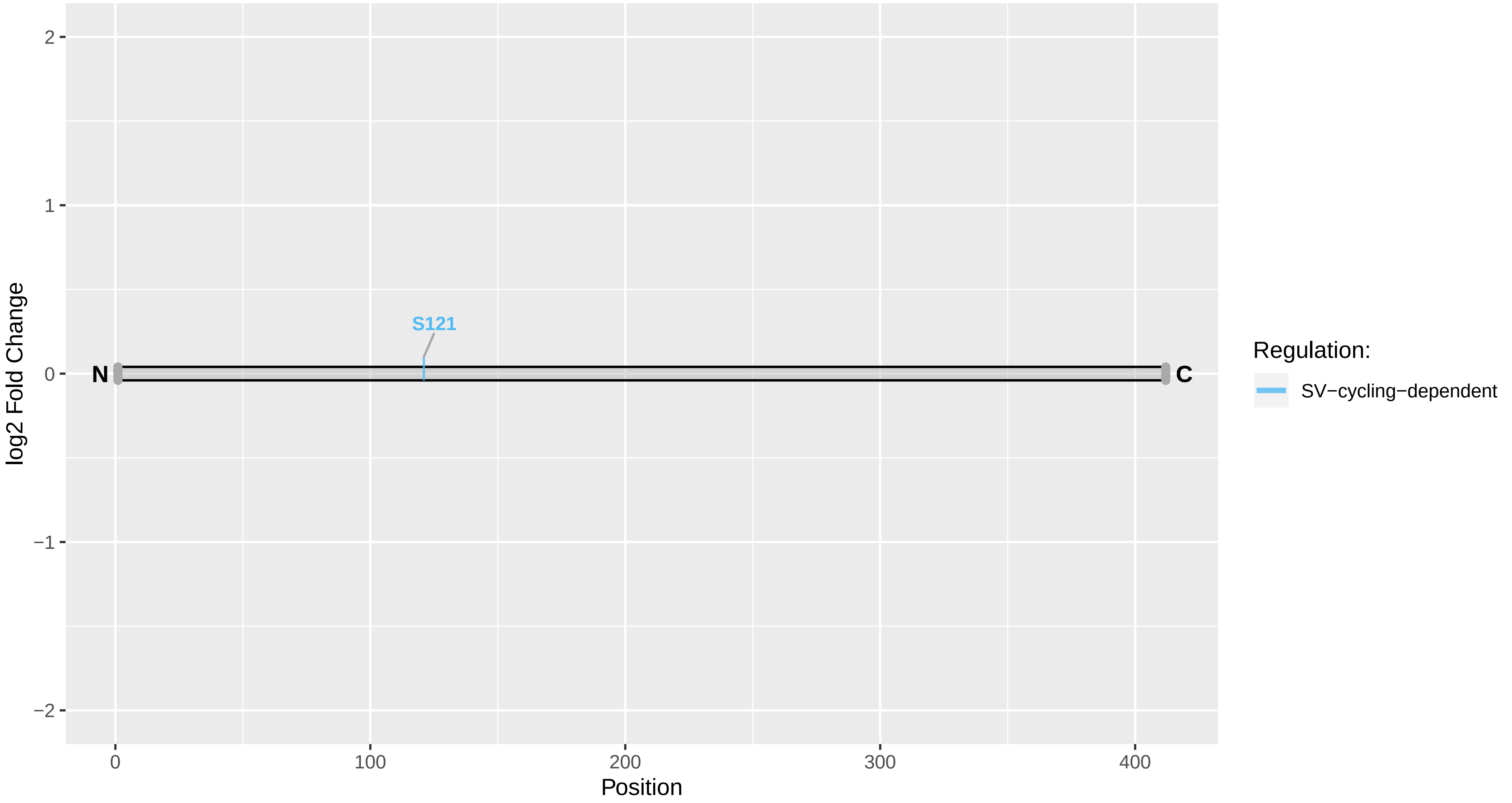

Rmdn3

Uniprot Accession: Q66H15
Regulator of microtubule dynamics protein 3

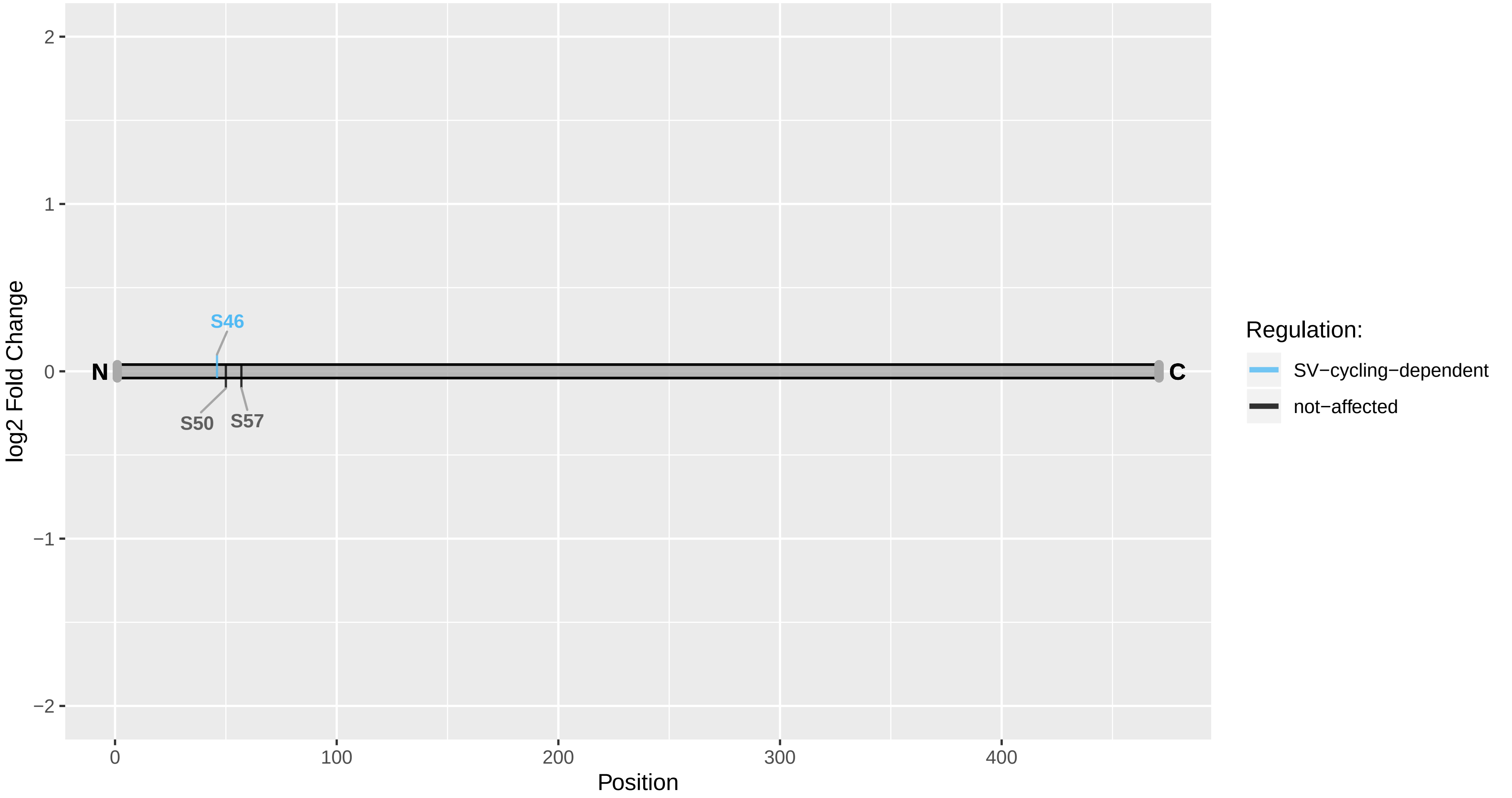

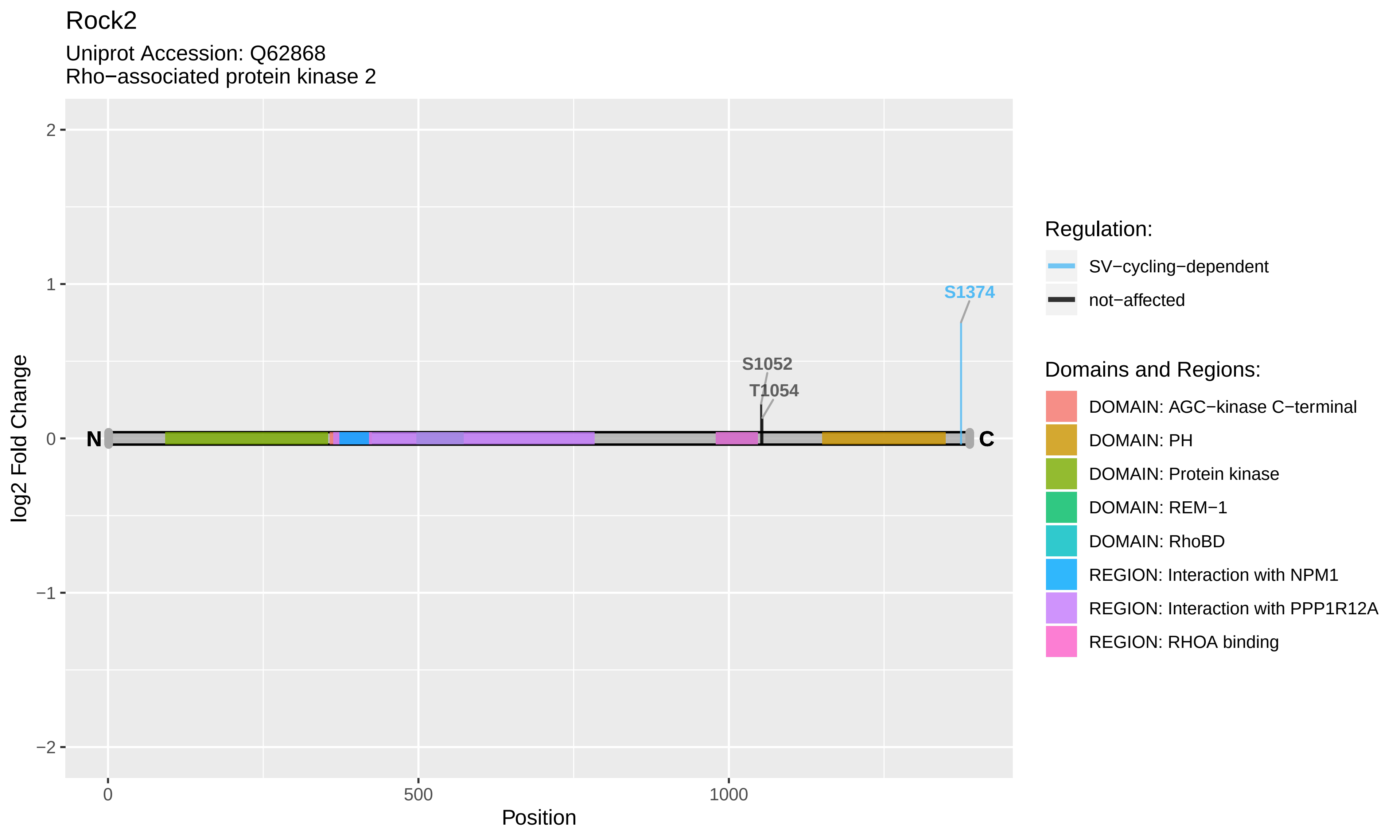
Rock2
Uniprot Accession: Q62868
Rho−associated protein kinase 2
log2 Fold Change
2
1
0
−1
−2
N
C
S1052
T1054
S1374
0
500
1000
Position
Regulation:
SV−cycling−dependent
not−affected
Domains and Regions:
DOMAIN: AGC−kinase C−terminal
DOMAIN: PH
DOMAIN: Protein kinase
DOMAIN: REM−1
DOMAIN: RhoBD
REGION: Interaction with NPM1
REGION: Interaction with PPP1R12A
REGION: RHOA binding

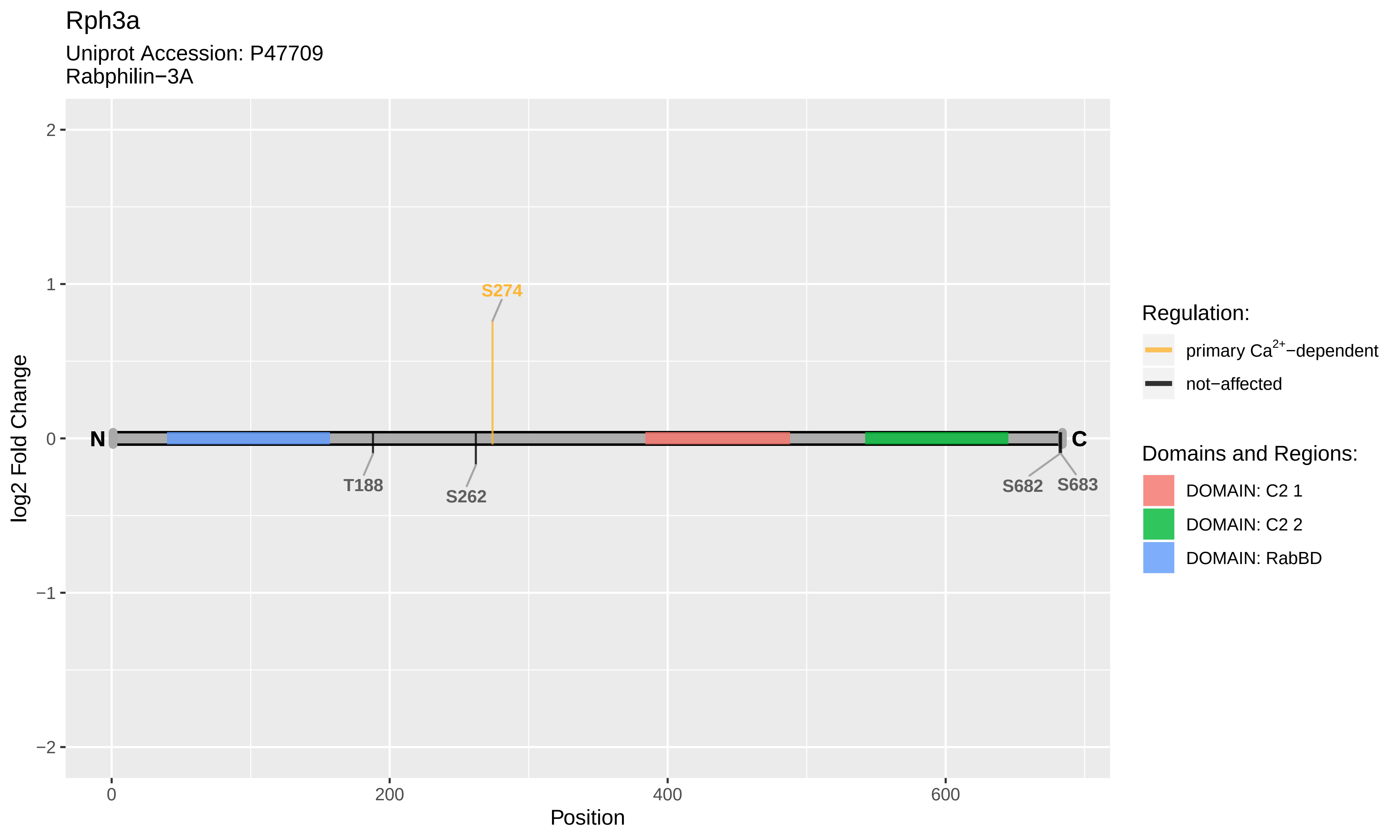

Rph3a
Uniprot Accession: P47709
Rabphilin−3A
S274
T188
S262
S682
S683
N
C
2
1
0
−1
−2
0
200
400
600
log2 Fold Change
Position
Regulation:
primary Ca2+−dependent
not−affected
Domains and Regions:
DOMAIN: C2 1
DOMAIN: C2 2
DOMAIN: RabBD

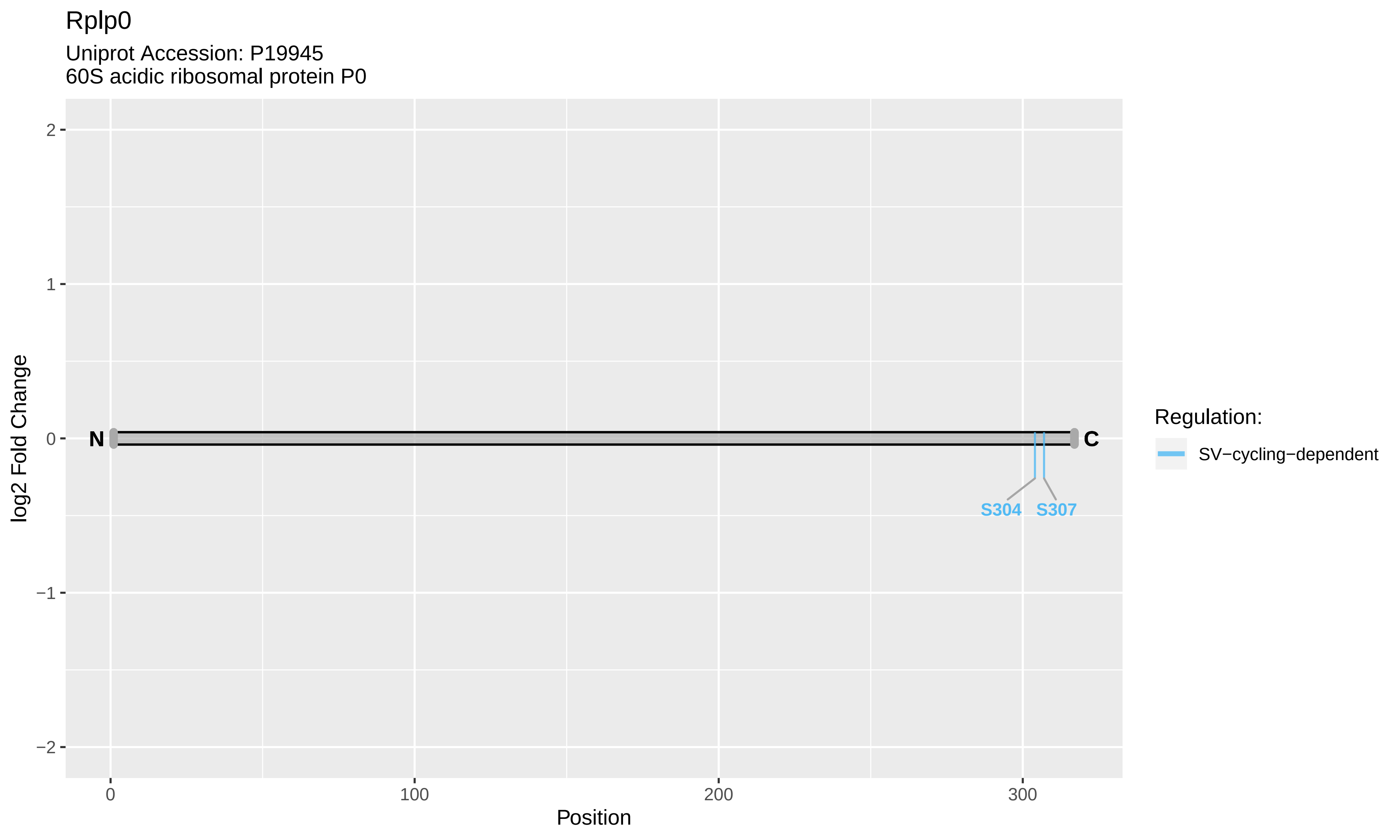

Rplp0
Uniprot Accession: P19945
60S acidic ribosomal protein P0
log2 Fold Change
2
1
0
−1
−2
N
C
S304
S307
0
100
200
300
Position
Regulation:
SV−cycling−dependent

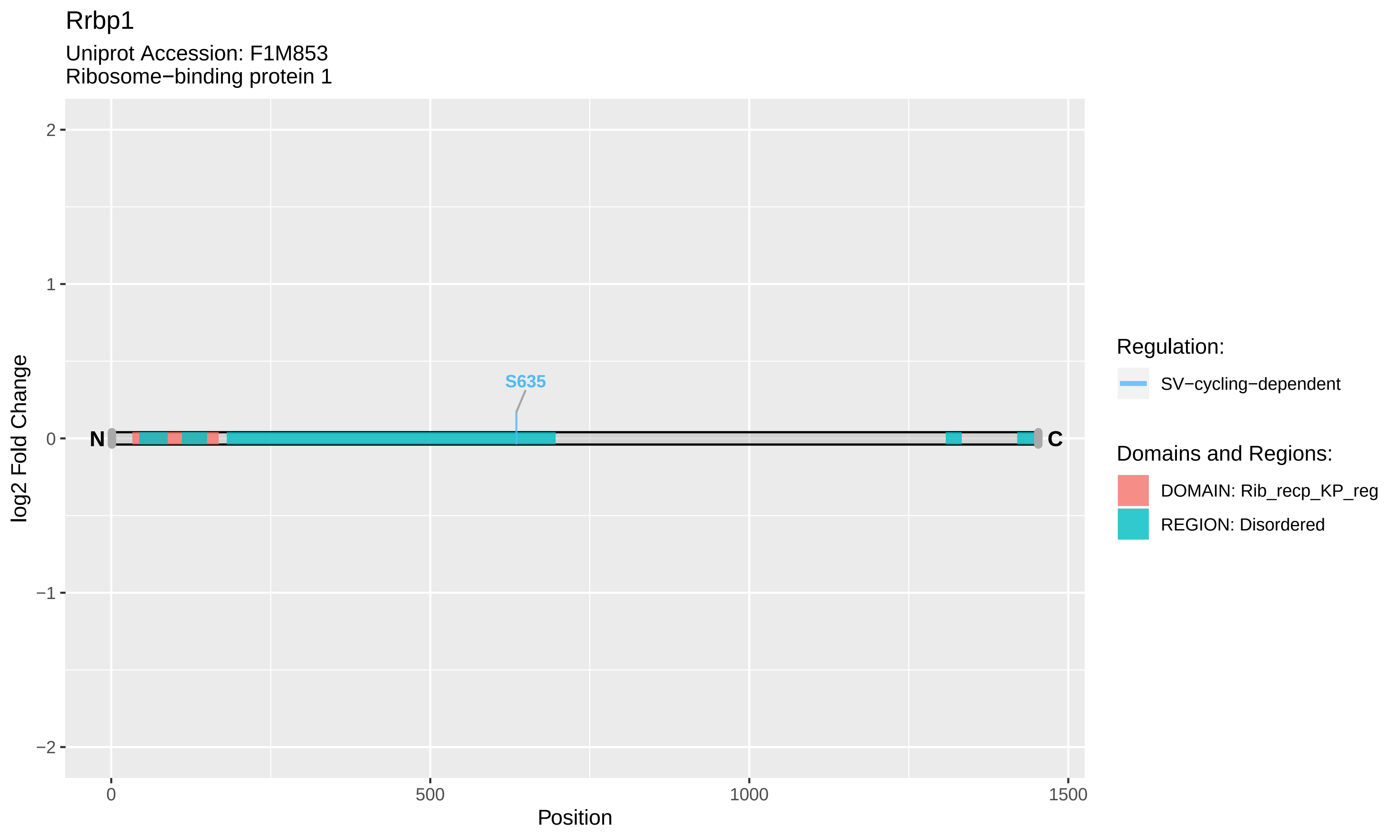
Rrbp1
Uniprot Accession: F1M853
Ribosome−binding protein 1
S635
N
C
log2 Fold Change
2
1
0
−1
−2
0
500
1000
1500
Position
Regulation:
SV−cycling−dependent
Domains and Regions:
DOMAIN: Rib_recp_KP_reg
REGION: Disordered

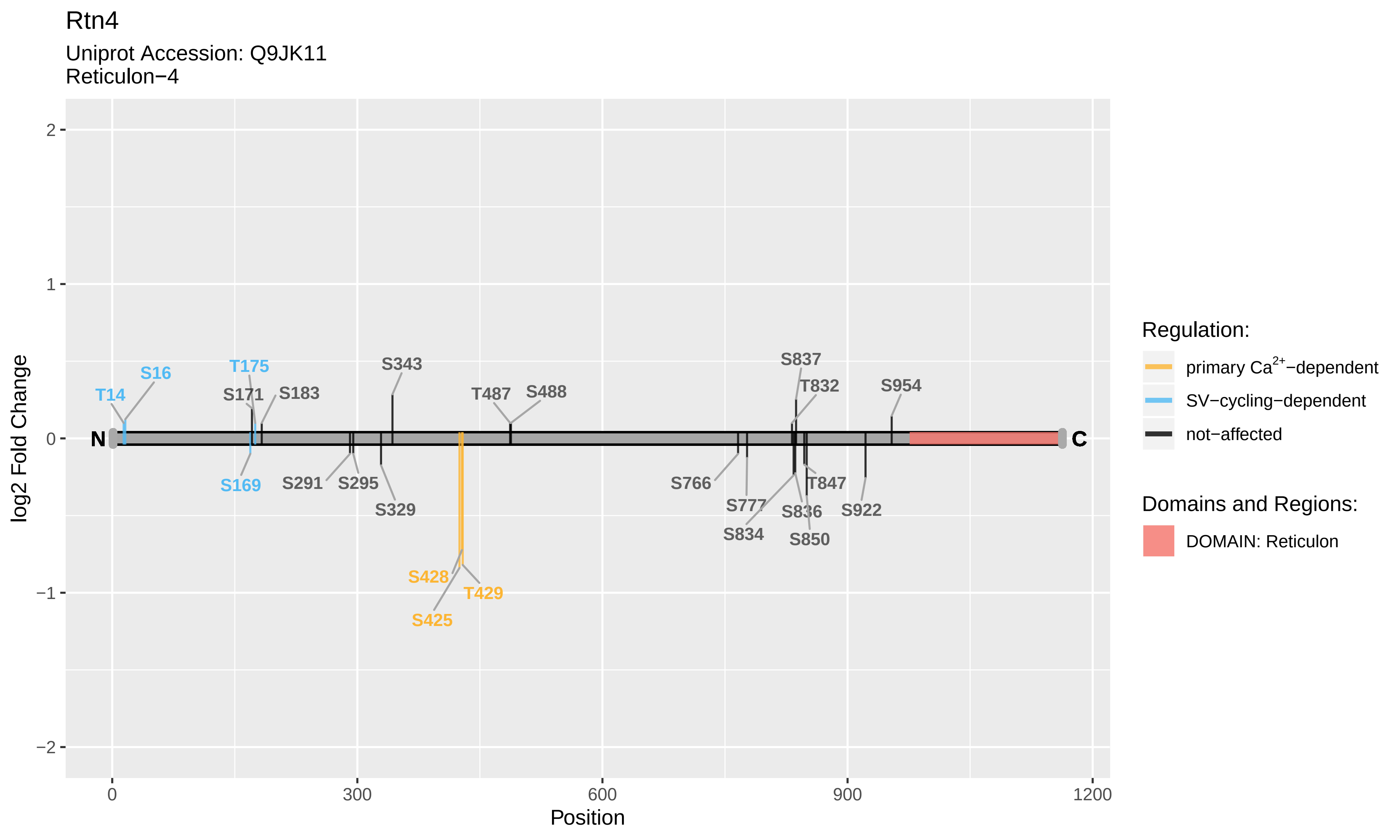

Rtn4
Uniprot Accession: Q9JK11
Reticulon−4
log2 Fold Change
Position
N
C
T14
S16
T175
S171
S183
S169
S291
S295
S329
S343
T487
S488
S428
T429
S425
S766
S777
S834
S836
S837
T832
T847
S850
S922
S954
Regulation:
primary Ca2+−dependent
SV−cycling−dependent
not−affected
Domains and Regions:
DOMAIN: Reticulon

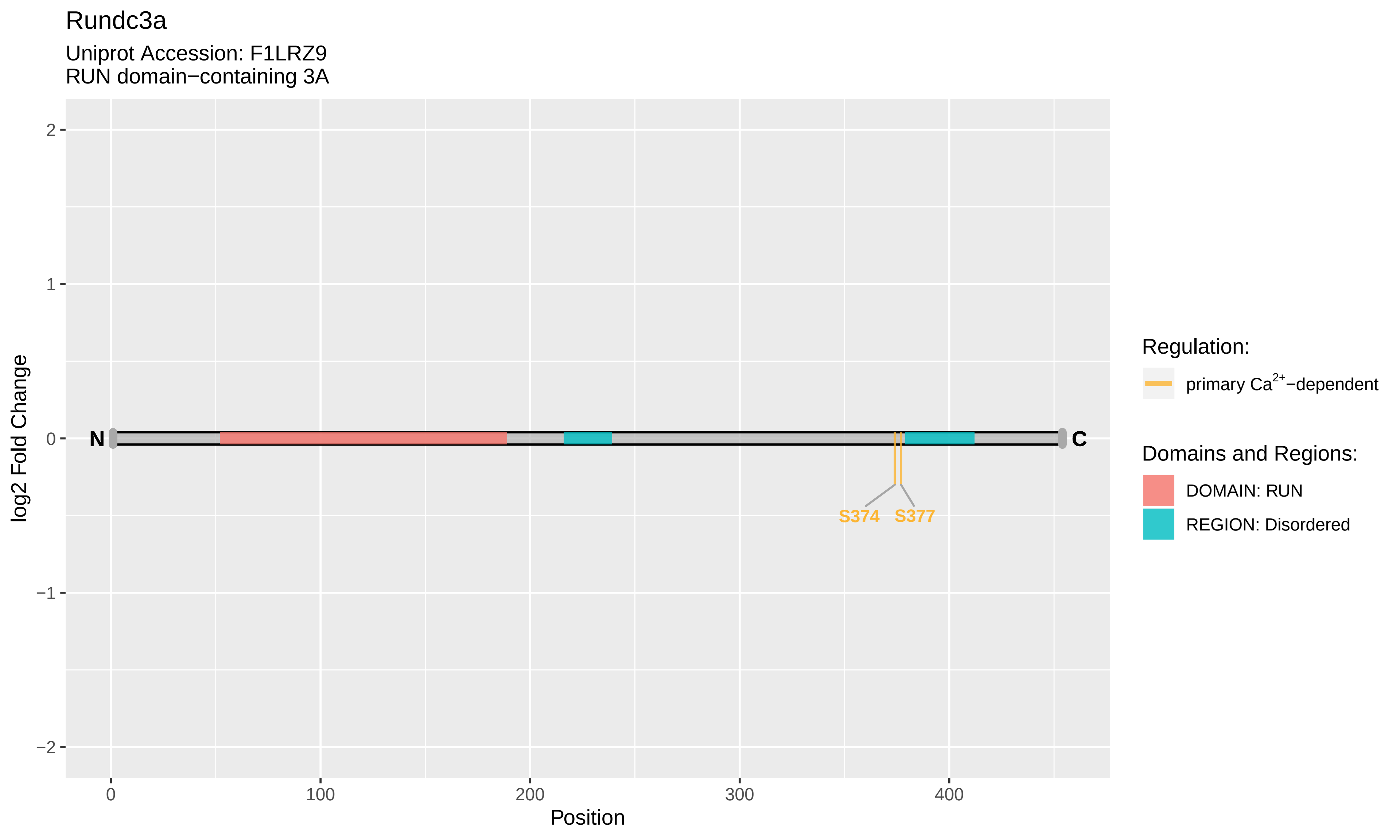

Rundc3a
Uniprot Accession: F1LRZ9
RUN domain−containing 3A
log2 Fold Change
2
1
0
−1
−2
N
C
S374
S377
0
100
200
300
400
Position
Regulation:
primary Ca2+−dependent
Domains and Regions:
DOMAIN: RUN
REGION: Disordered

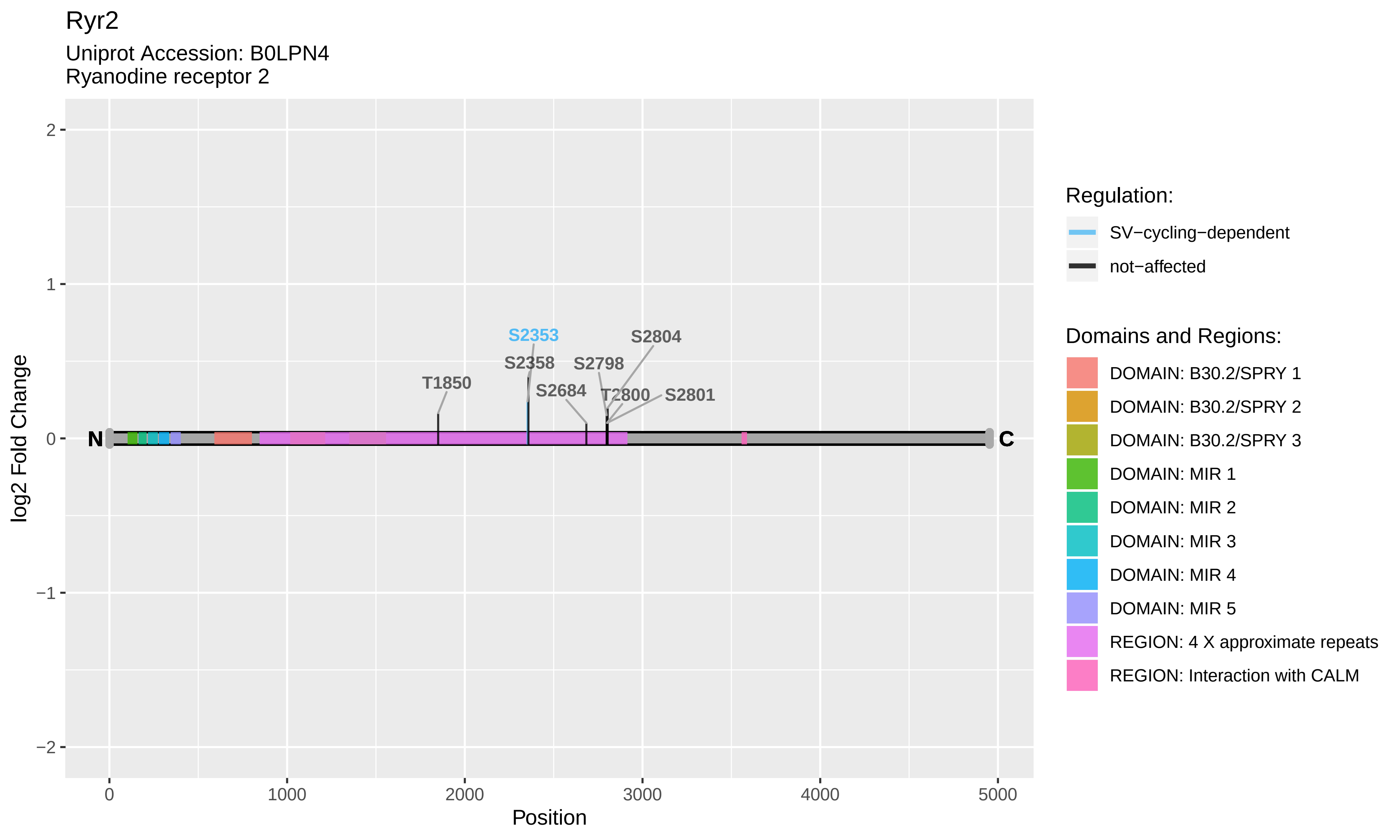

Ryr2
Uniprot Accession: B0LPN4
Ryanodine receptor 2
S2353
S2804
S2358
S2798
T1850
S2684
T2800
S2801
N
C
2
1
0
−1
−2
log2 Fold Change
0
1000
2000
3000
4000
5000
Position
Regulation:
SV−cycling−dependent
not−affected
Domains and Regions:
DOMAIN: B30.2/SPRY 1
DOMAIN: B30.2/SPRY 2
DOMAIN: B30.2/SPRY 3
DOMAIN: MIR 1
DOMAIN: MIR 2
DOMAIN: MIR 3
DOMAIN: MIR 4
DOMAIN: MIR 5
REGION: 4 X approximate repeats
REGION: Interaction with CALM

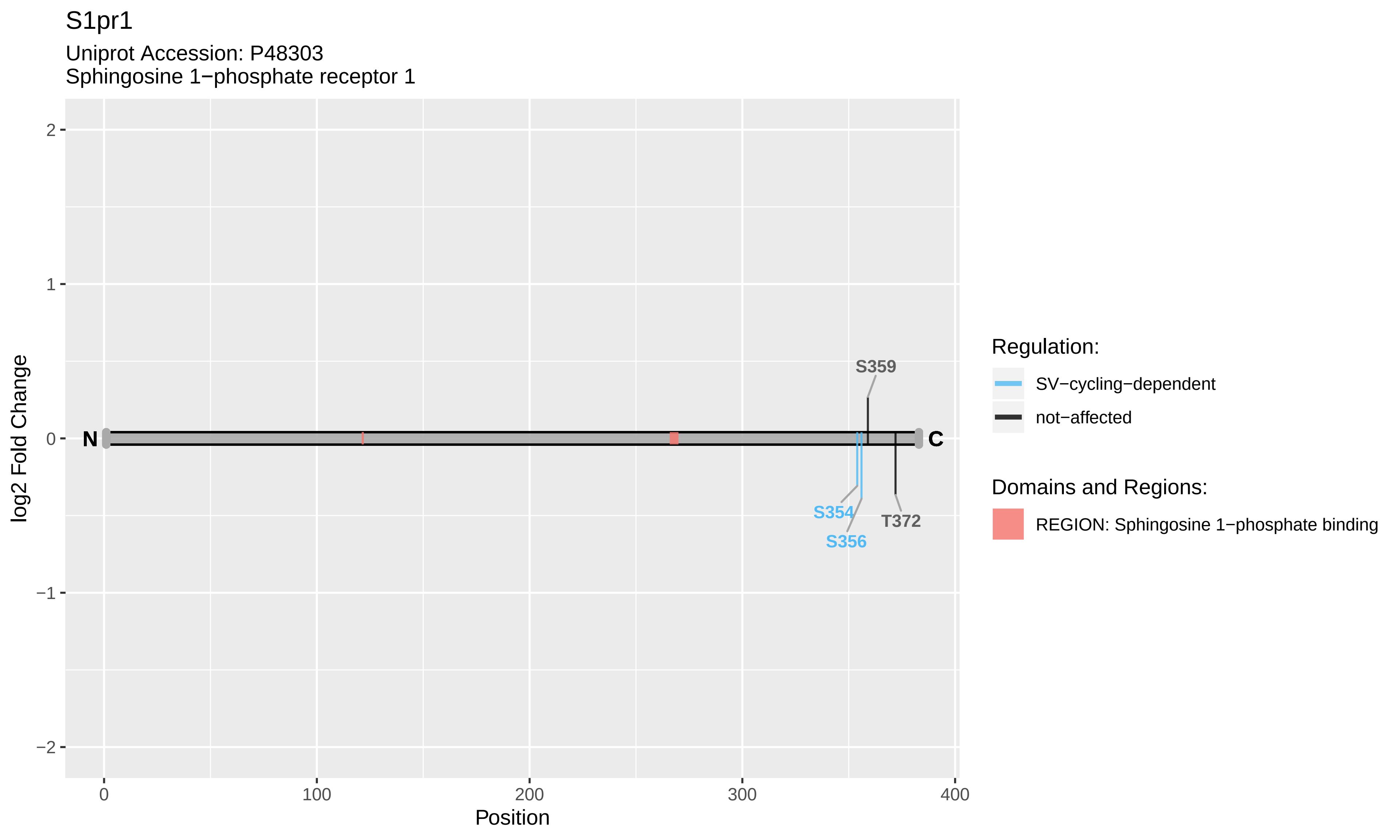

S1pr1
Uniprot Accession: P48303
Sphingosine 1−phosphate receptor 1
log2 Fold Change
Position
N
C
S359
S354
S356
T372
2
1
0
−1
−2
0
100
200
300
400
Regulation:
SV−cycling−dependent
not−affected
Domains and Regions:
REGION: Sphingosine 1−phosphate binding

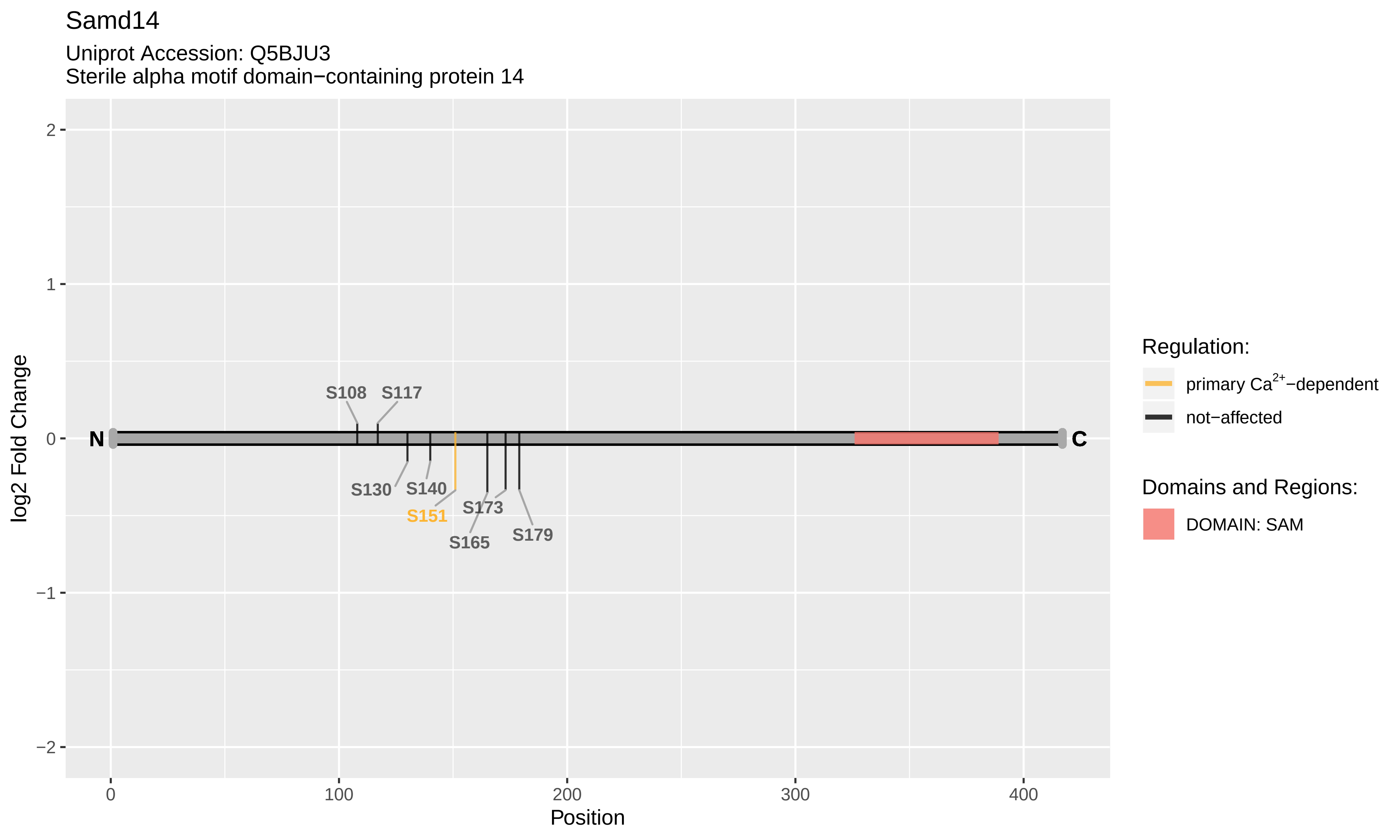
Samd14
Uniprot Accession: Q5BJU3
Sterile alpha motif domain−containing protein 14
log2 Fold Change
Position
N
C
S108
S117
S130
S140
S151
S165
S173
S179
Regulation:
primary Ca2+−dependent
not−affected
Domains and Regions:
DOMAIN: SAM

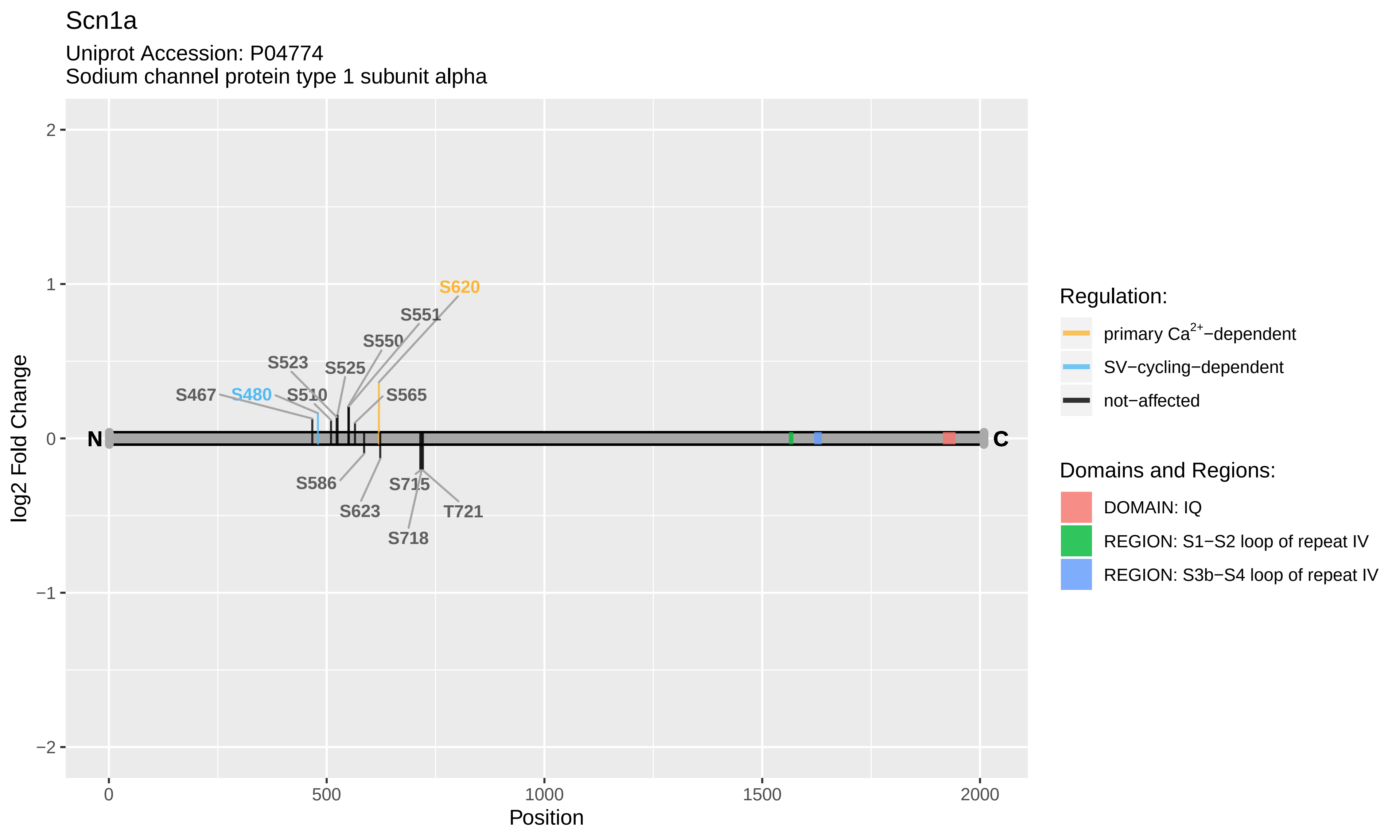

Scn1a
Uniprot Accession: P04774
Sodium channel protein type 1 subunit alpha
log2 Fold Change
Position
N
C
S467
S480
S510
S523
S525
S550
S551
S620
S565
S586
S623
S715
S718
T721
Regulation:
primary Ca2+−dependent
SV−cycling−dependent
not−affected
Domains and Regions:
DOMAIN: IQ
REGION: S1−S2 loop of repeat IV
REGION: S3b−S4 loop of repeat IV

Scn2a

Uniprot Accession: F1M9G9
Sodium channel protein

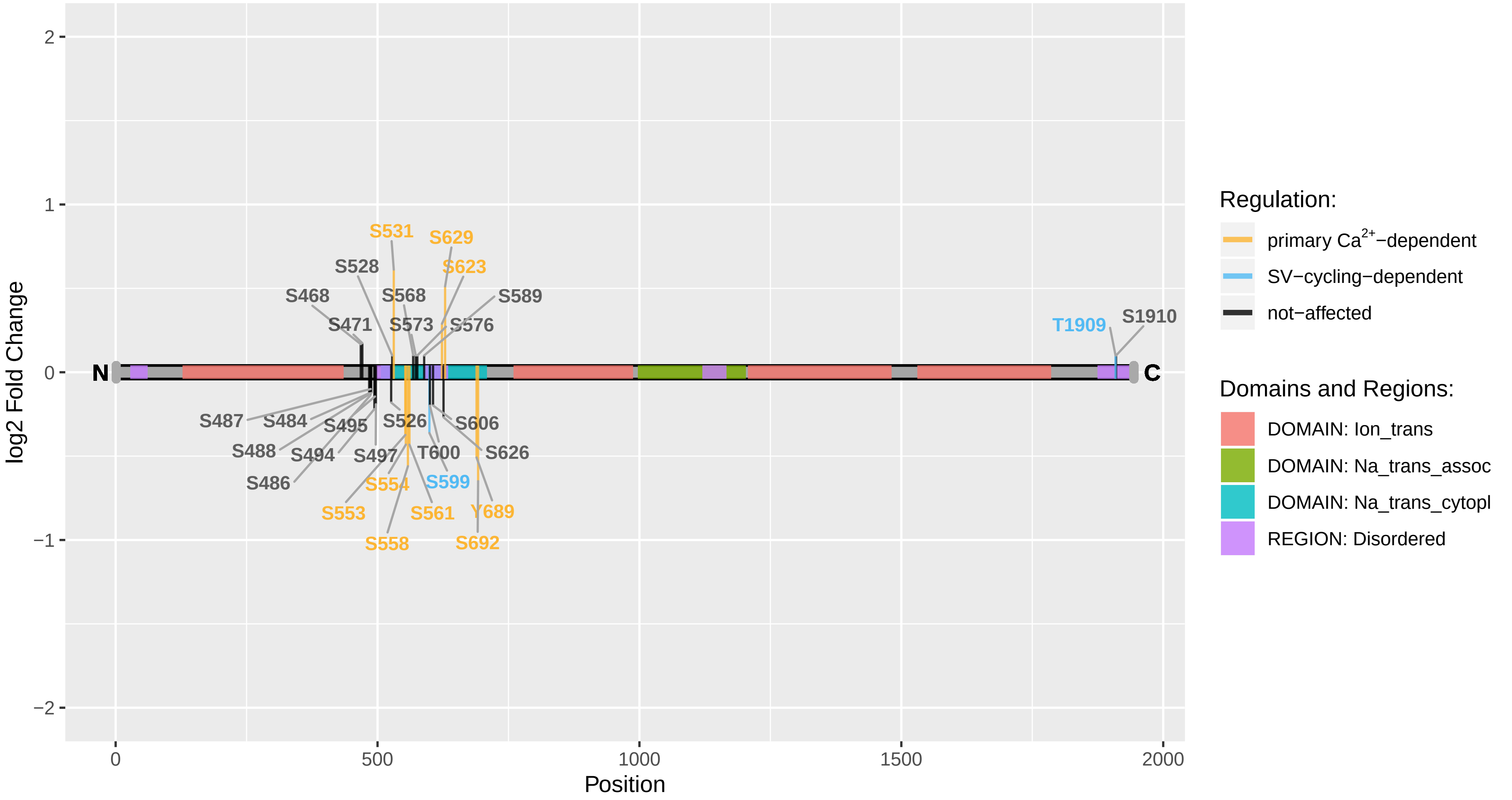

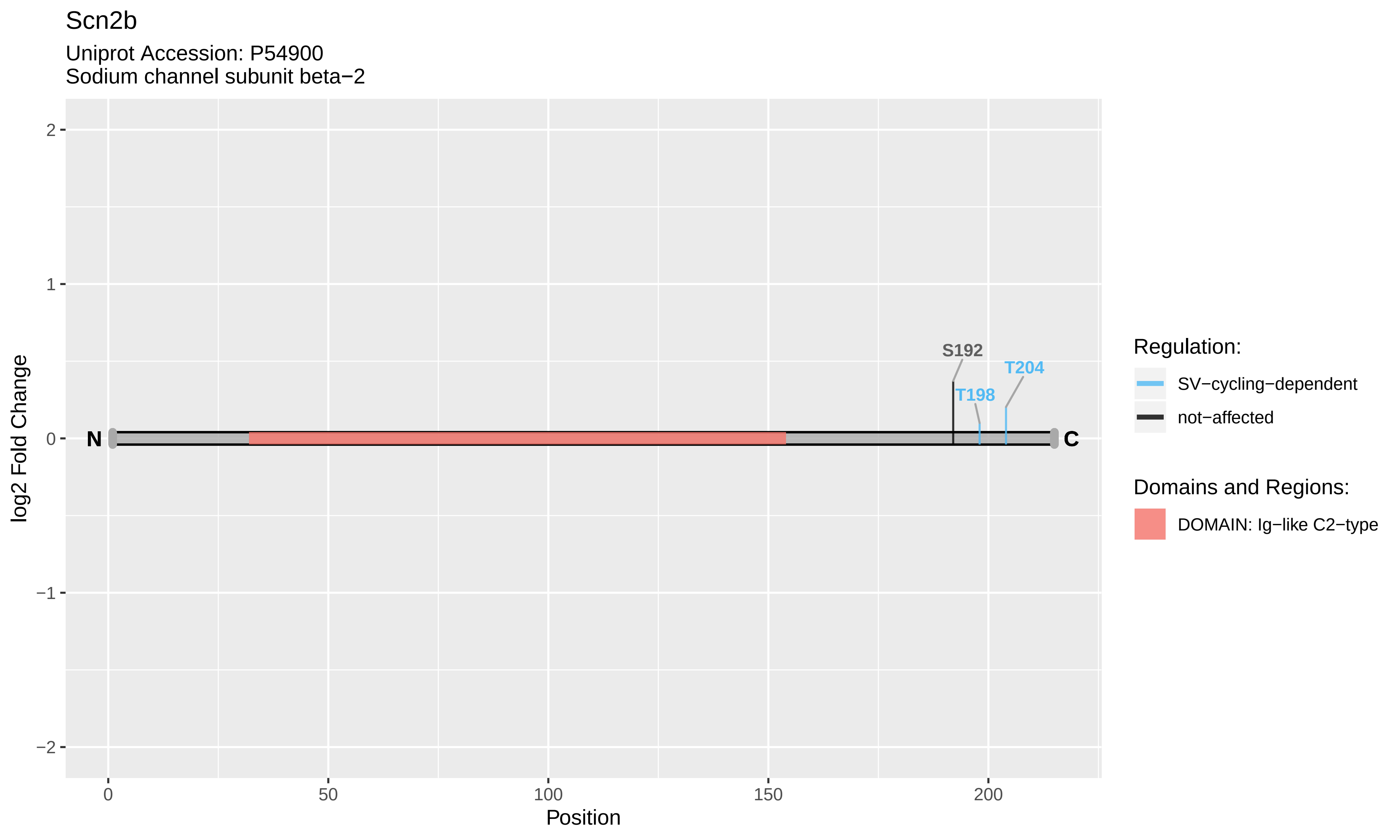

Scn2b
Uniprot Accession: P54900
Sodium channel subunit beta−2
S192
T204
T198
N
C
2
1
0
−1
−2
log2 Fold Change
0
50
100
150
200
Position
Regulation:
SV−cycling−dependent
not−affected
Domains and Regions:
DOMAIN: Ig−like C2−type

Scyl2

Uniprot Accession: A0A0G2JVC2
SCY1−like pseudokinase 2

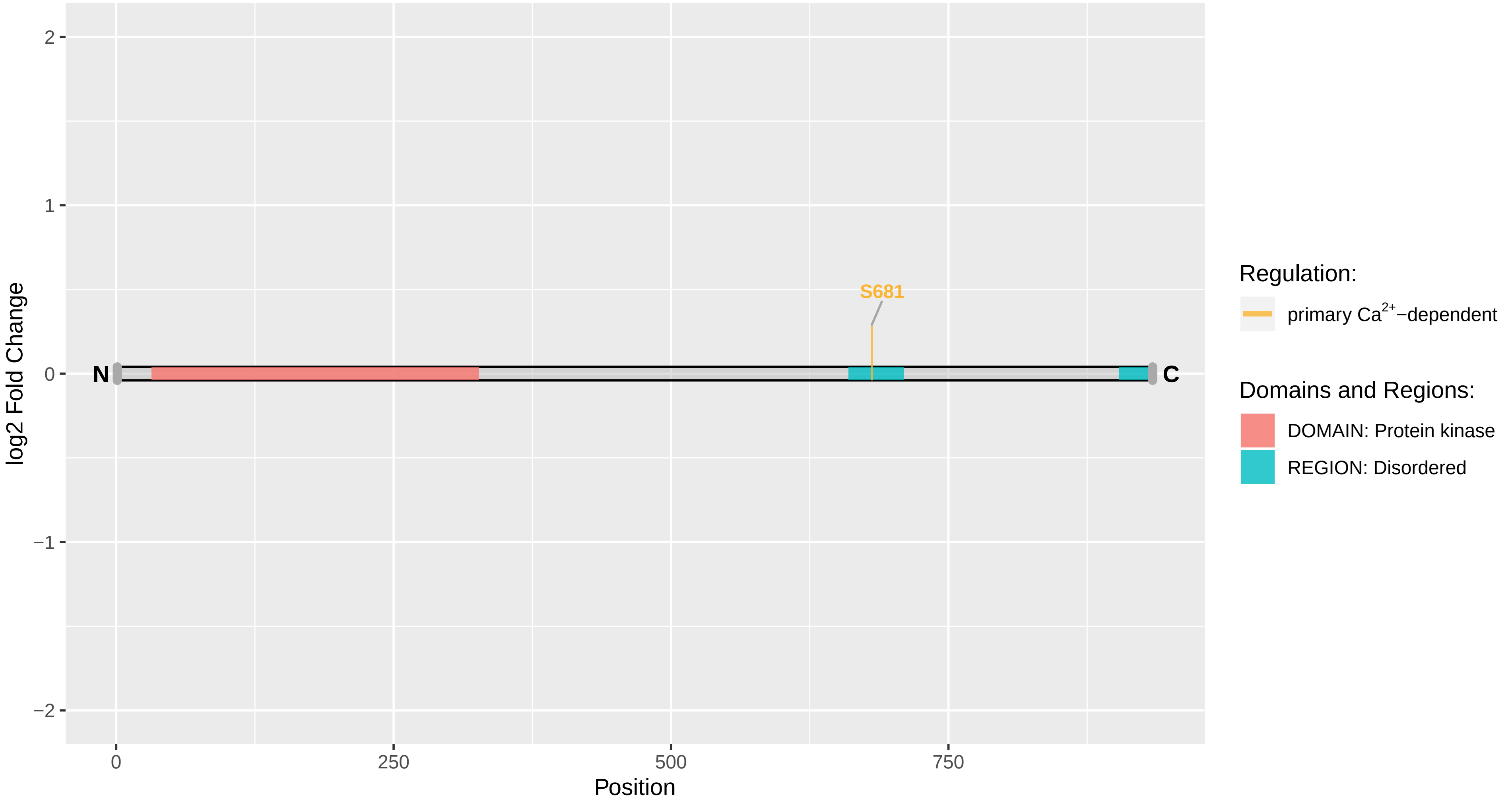

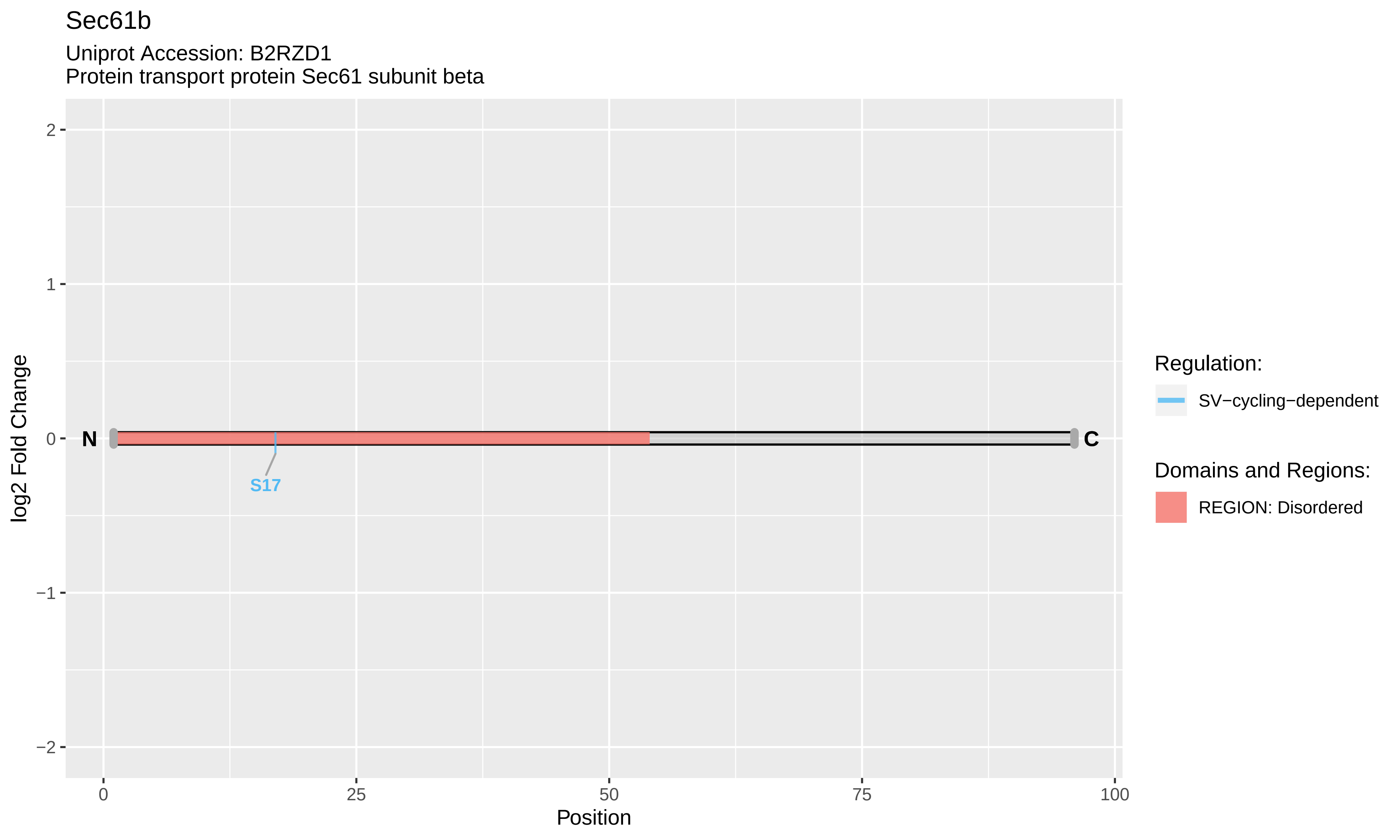
Sec61b
Uniprot Accession: B2RZD1
Protein transport protein Sec61 subunit beta
log2 Fold Change
2
1
0
−1
−2
N
C
S17
0
25
50
75
100
Position
Regulation:
SV−cycling−dependent
Domains and Regions:
REGION: Disordered

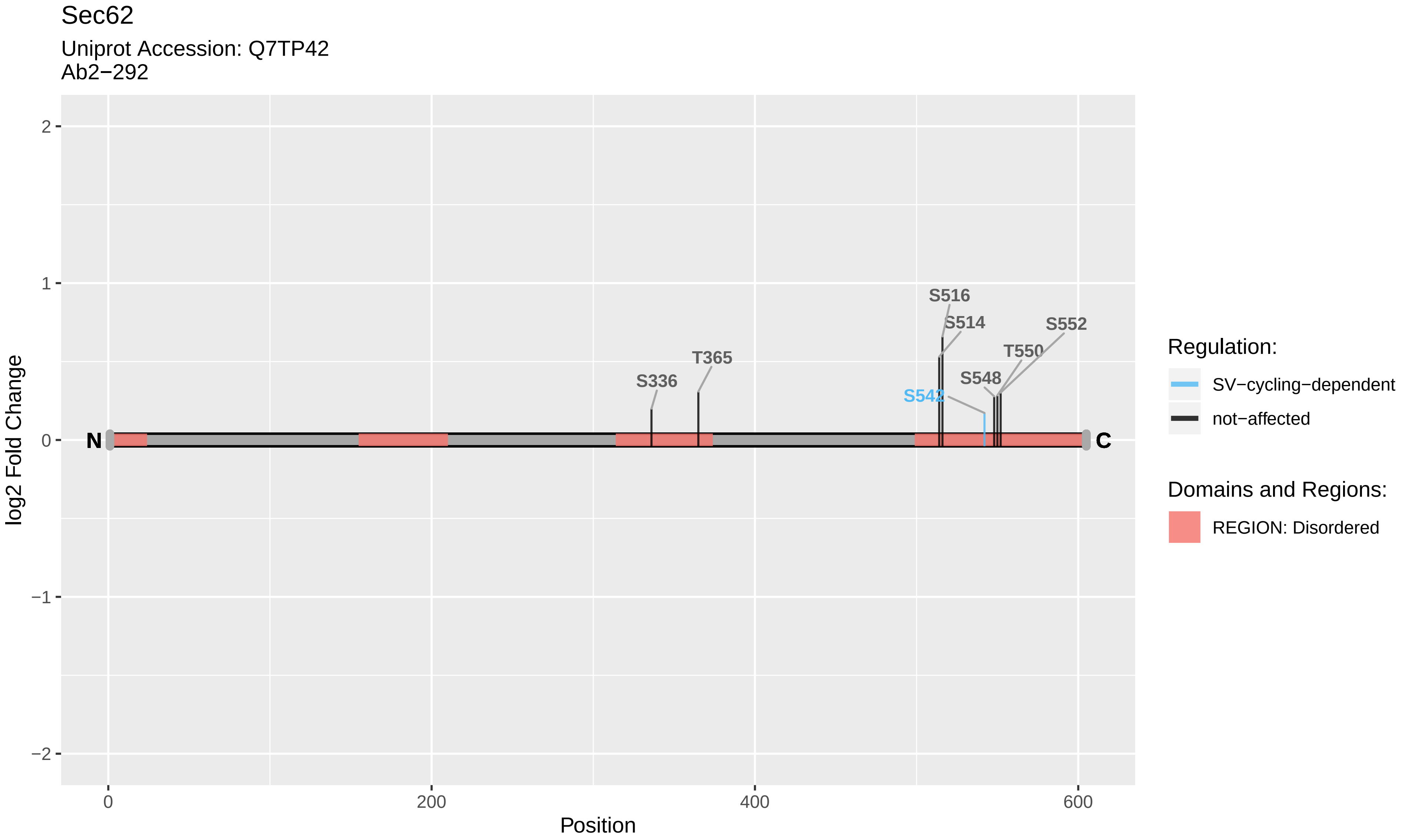
Sec62
Uniprot Accession: Q7TP42
Ab2−292
log2 Fold Change
Position
N
C
S336
T365
S516
S514
S552
T550
S548
S542
Regulation:
SV−cycling−dependent
not−affected
Domains and Regions:
REGION: Disordered

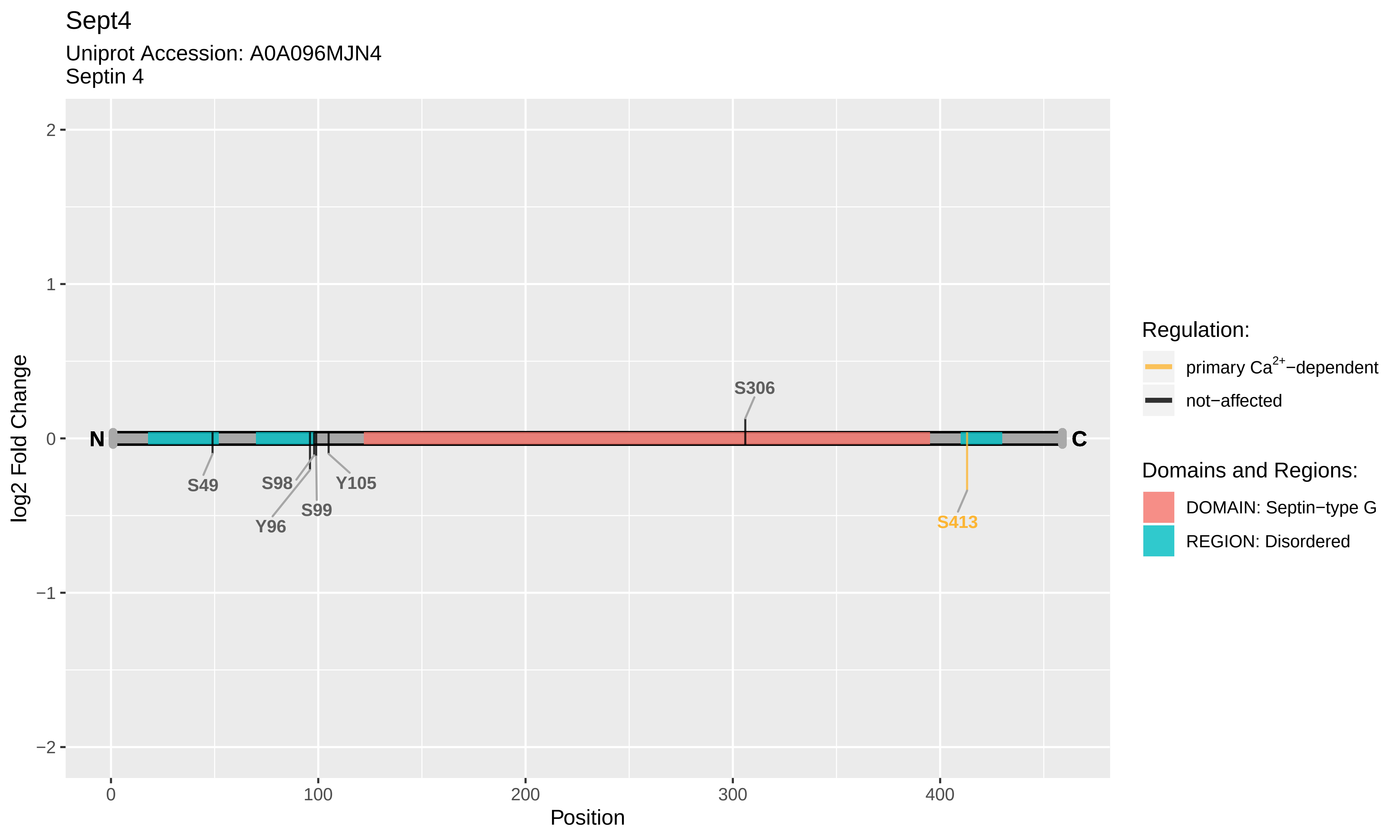
Sept4
Uniprot Accession: A0A096MJN4
Septin 4
log2 Fold Change
Position
N
C
S306
S49
S98
Y105
S99
Y96
S413
Regulation:
primary Ca2+−dependent
not−affected
Domains and Regions:
DOMAIN: Septin−type G
REGION: Disordered
2
1
0
−1
−2
0
100
200
300
400

Sept5

Uniprot Accession: D3ZDH8
Septin 5, isoform CRA_d

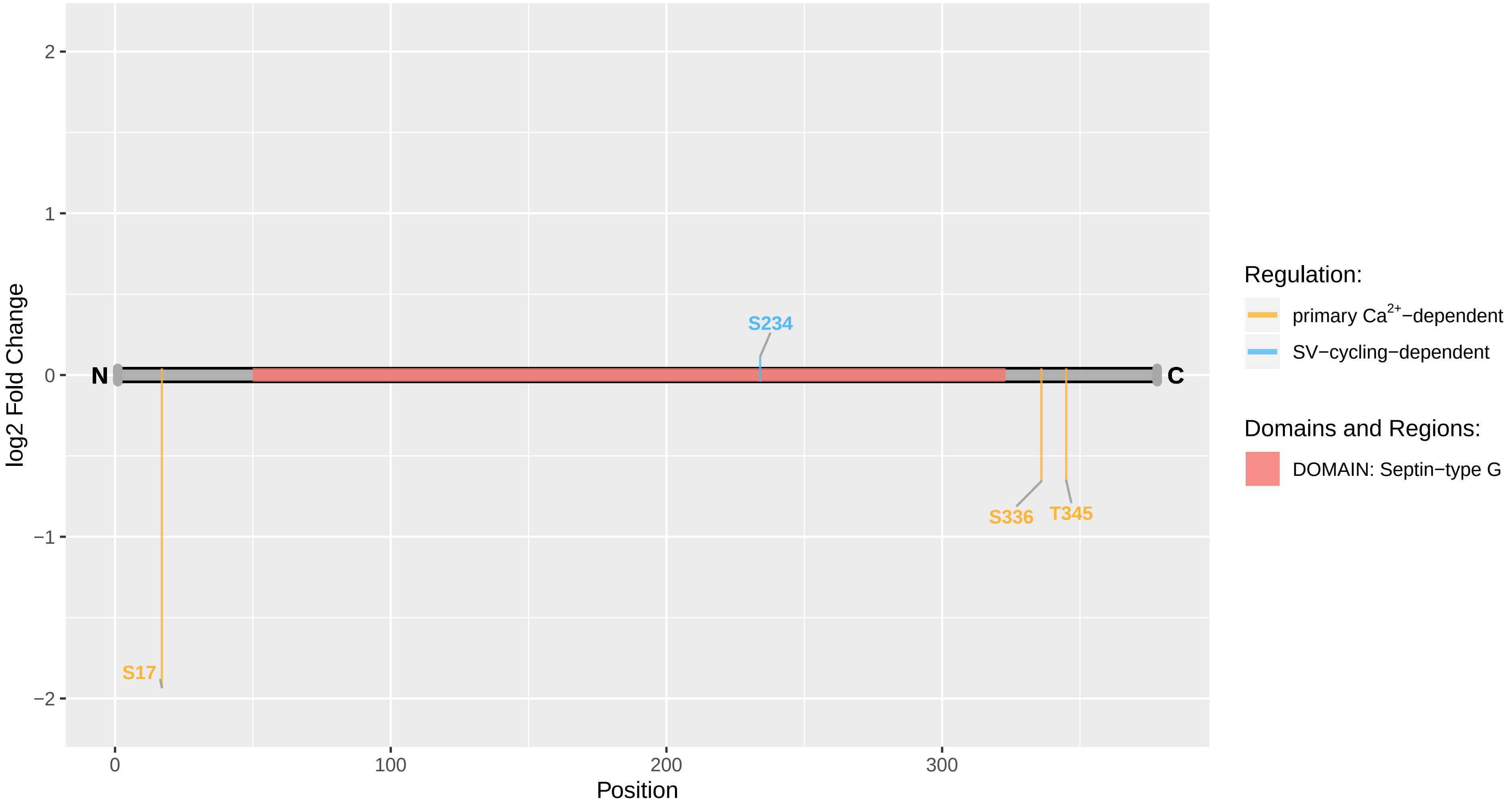

Sept6

Uniprot Accession: A0A0U1RRT8
Septin 6

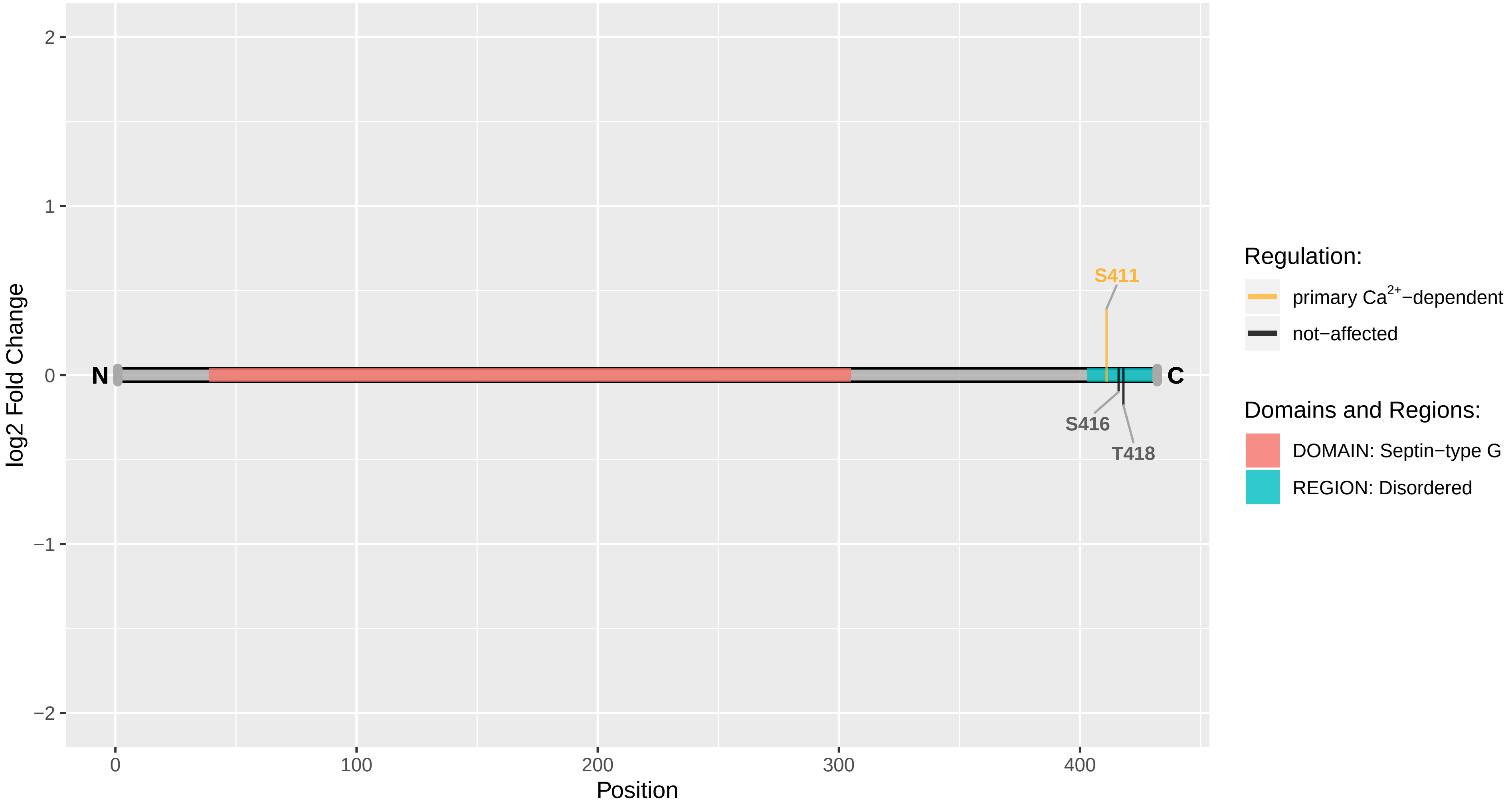

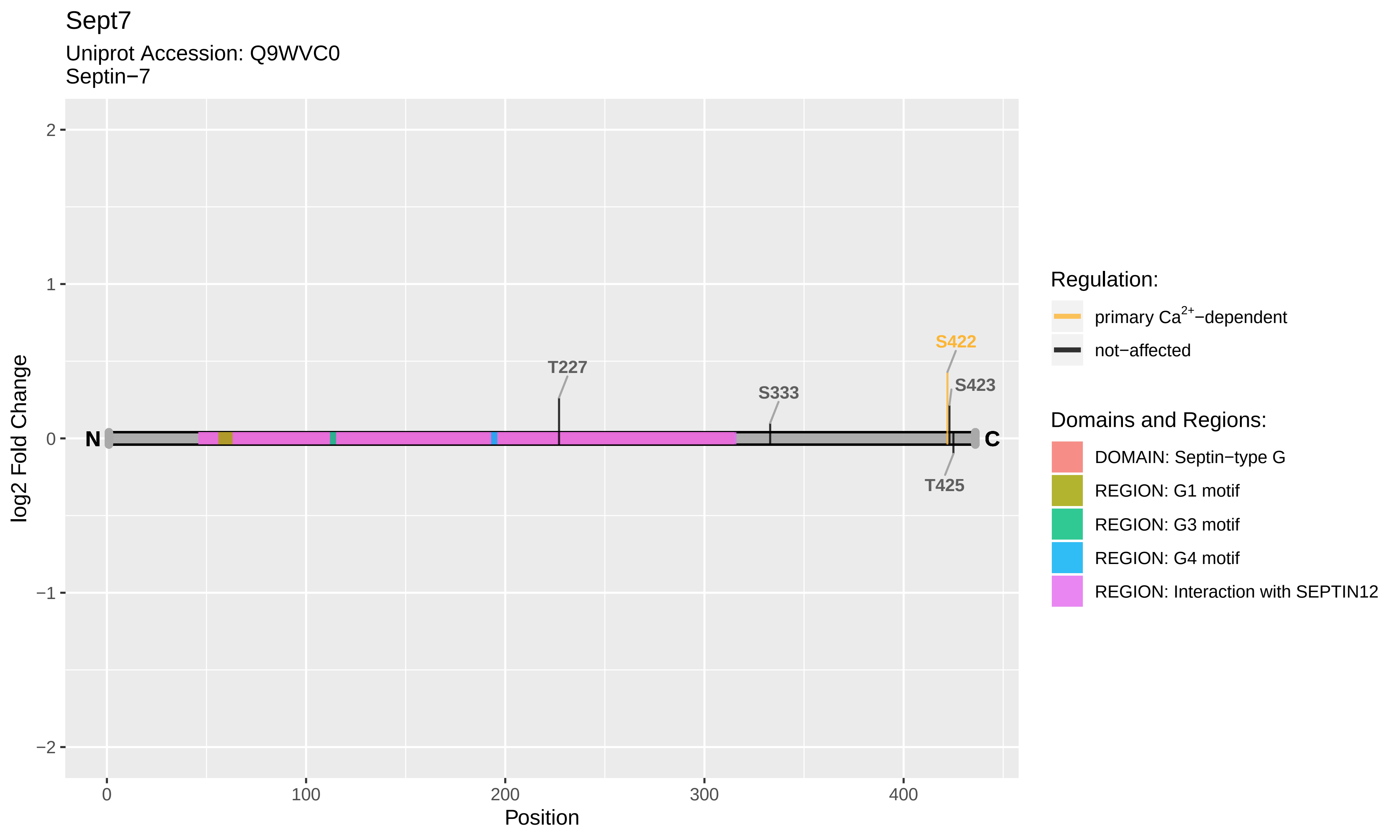
Sept7
Uniprot Accession: Q9WVC0
Septin−7
log2 Fold Change
Position
N
C
T227
S333
S422
S423
T425
Regulation:
primary Ca2+−dependent
not−affected
Domains and Regions:
DOMAIN: Septin−type G
REGION: G1 motif
REGION: G3 motif
REGION: G4 motif
REGION: Interaction with SEPTIN12
2
1
0
−1
−2
0
100
200
300
400

Serbp1

Uniprot Accession: Q6AXS5
Plasminogen activator inhibitor 1 RNA−binding protein

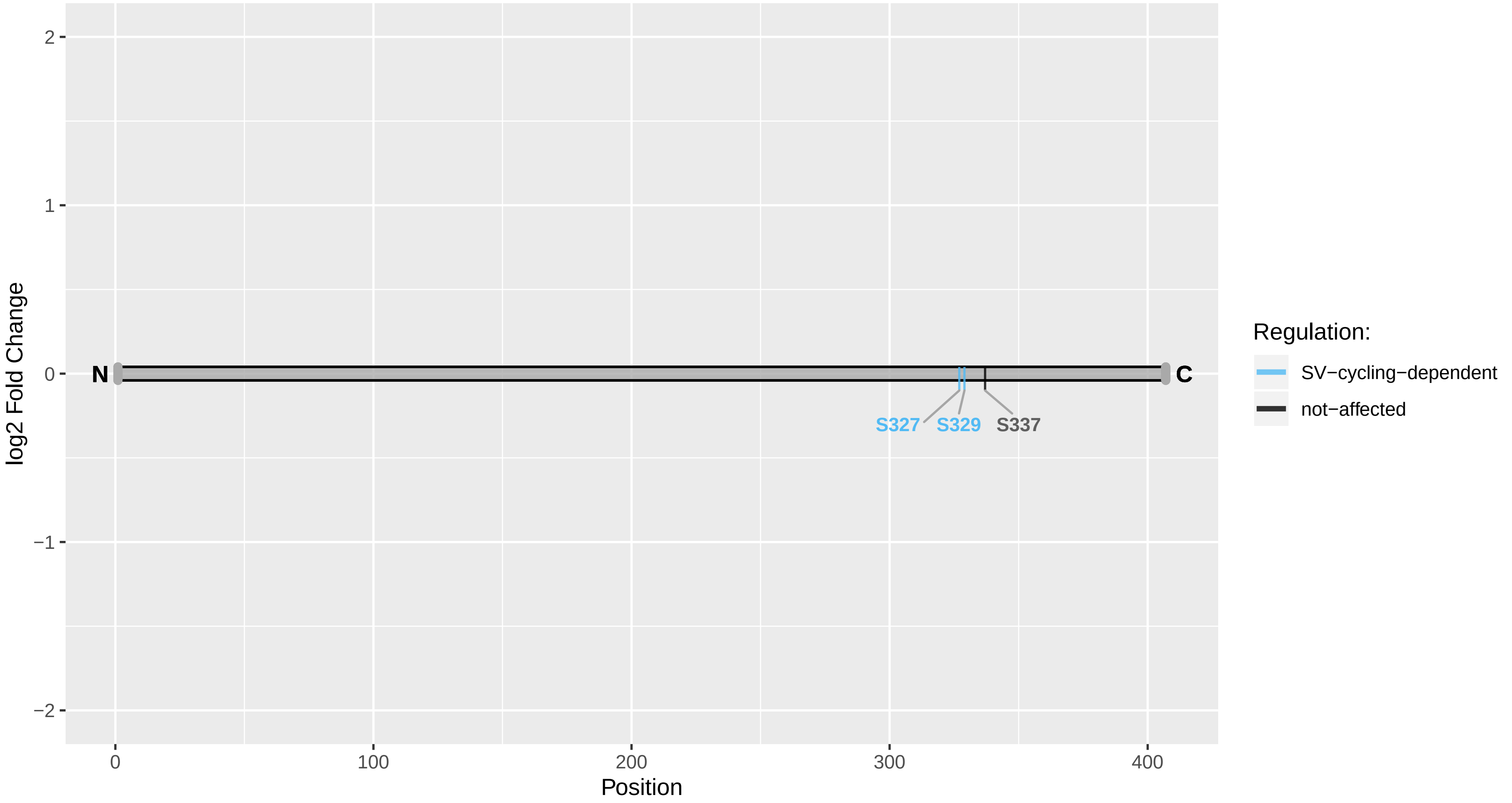

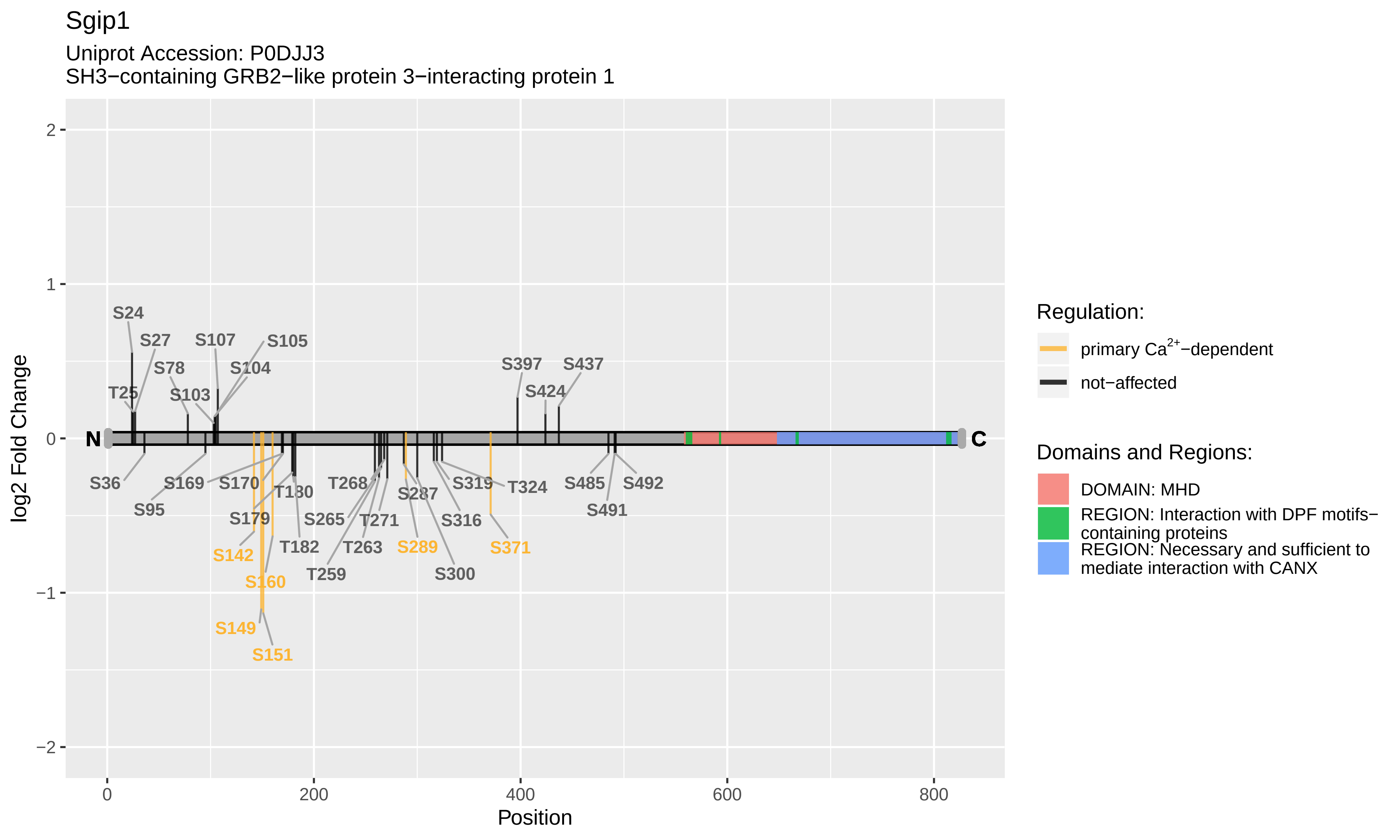
Sgip1
Uniprot Accession: P0DJJ3
SH3−containing GRB2−like protein 3−interacting protein 1
log2 Fold Change
Position
N
C
S24
S27
S78
T25
S103
S107
S104
S105
S397
S437
S424
S36
S95
S169
S170
T180
S179
S142
S160
S149
S151
T182
S265
T268
T271
T263
T259
S287
S289
S300
S319
S316
T324
S371
S485
S492
S491
Regulation:
primary Ca2+−dependent
not−affected
Domains and Regions:
DOMAIN: MHD
REGION: Interaction with DPF motifs−containing proteins
REGION: Necessary and sufficient to mediate interaction with CANX

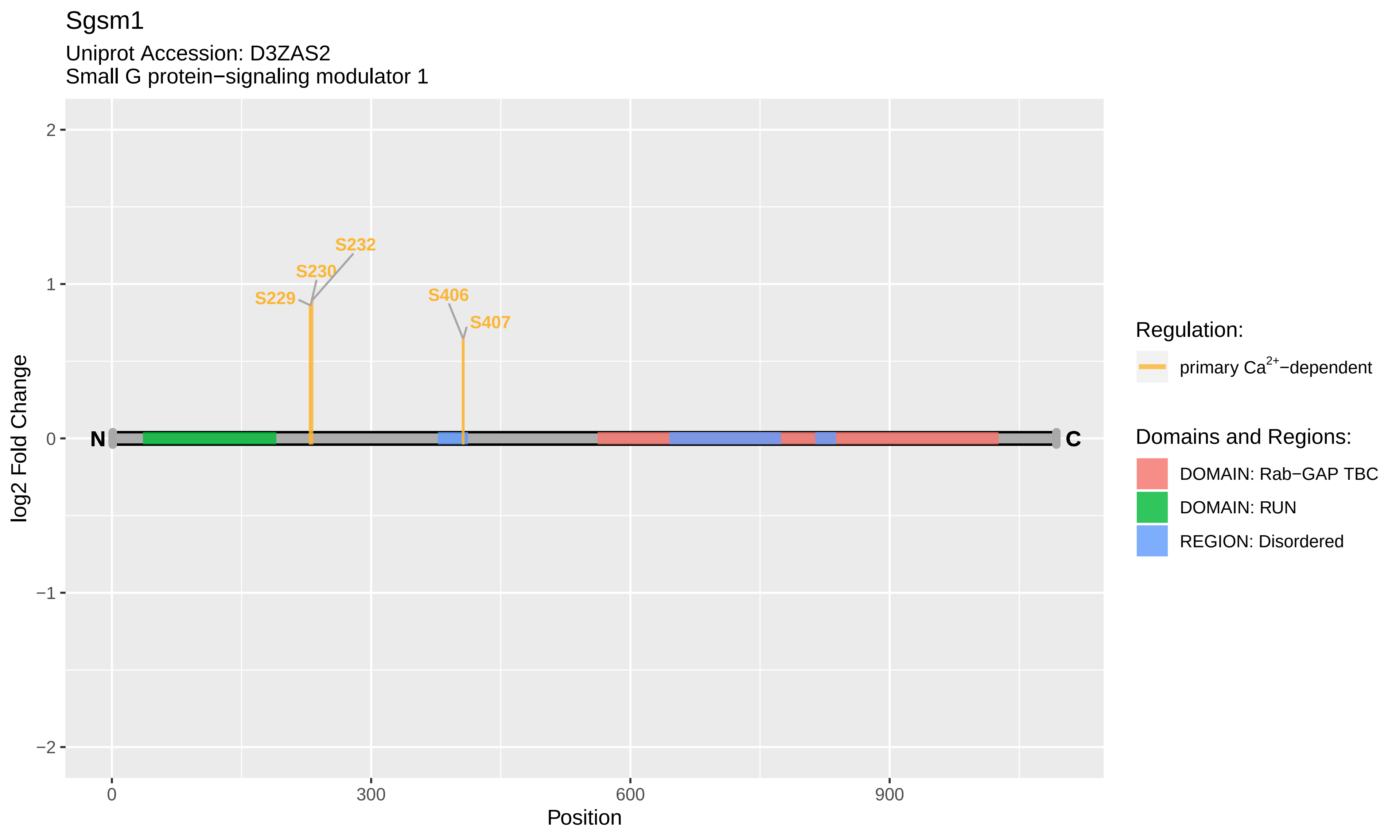

Sgsm1
Uniprot Accession: D3ZAS2
Small G protein−signaling modulator 1
S232
S230
S229
S406
S407
N
C
2
1
0
−1
−2
0
300
600
900
Position
log2 Fold Change
Regulation:
primary Ca2+−dependent
Domains and Regions:
DOMAIN: Rab−GAP TBC
DOMAIN: RUN
REGION: Disordered

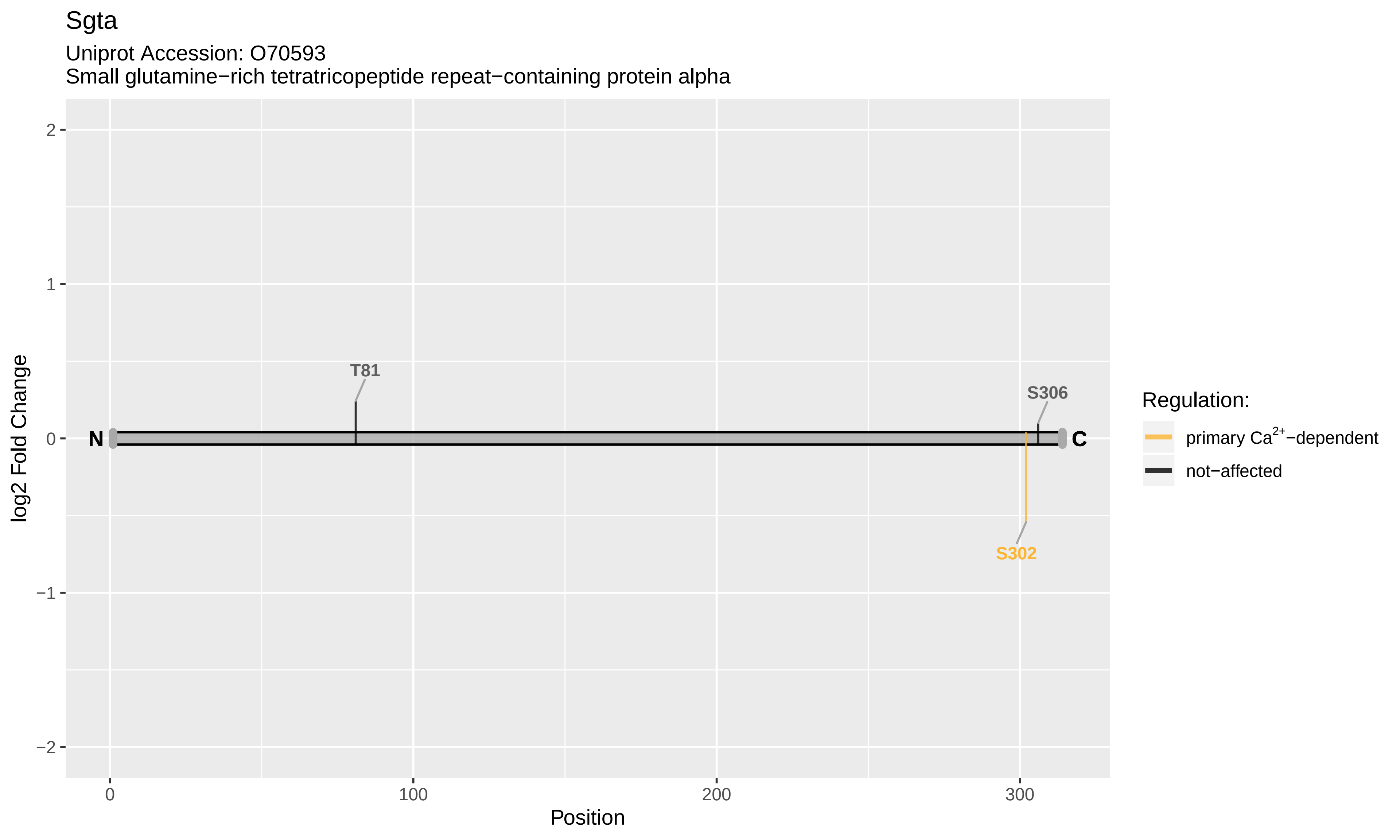

Sgta
Uniprot Accession: O70593
Small glutamine−rich tetratricopeptide repeat−containing protein alpha
T81
S306
S302
N
C
2
1
0
−1
−2
log2 Fold Change
0
100
200
300
Position
Regulation:
primary Ca2+−dependent
not−affected

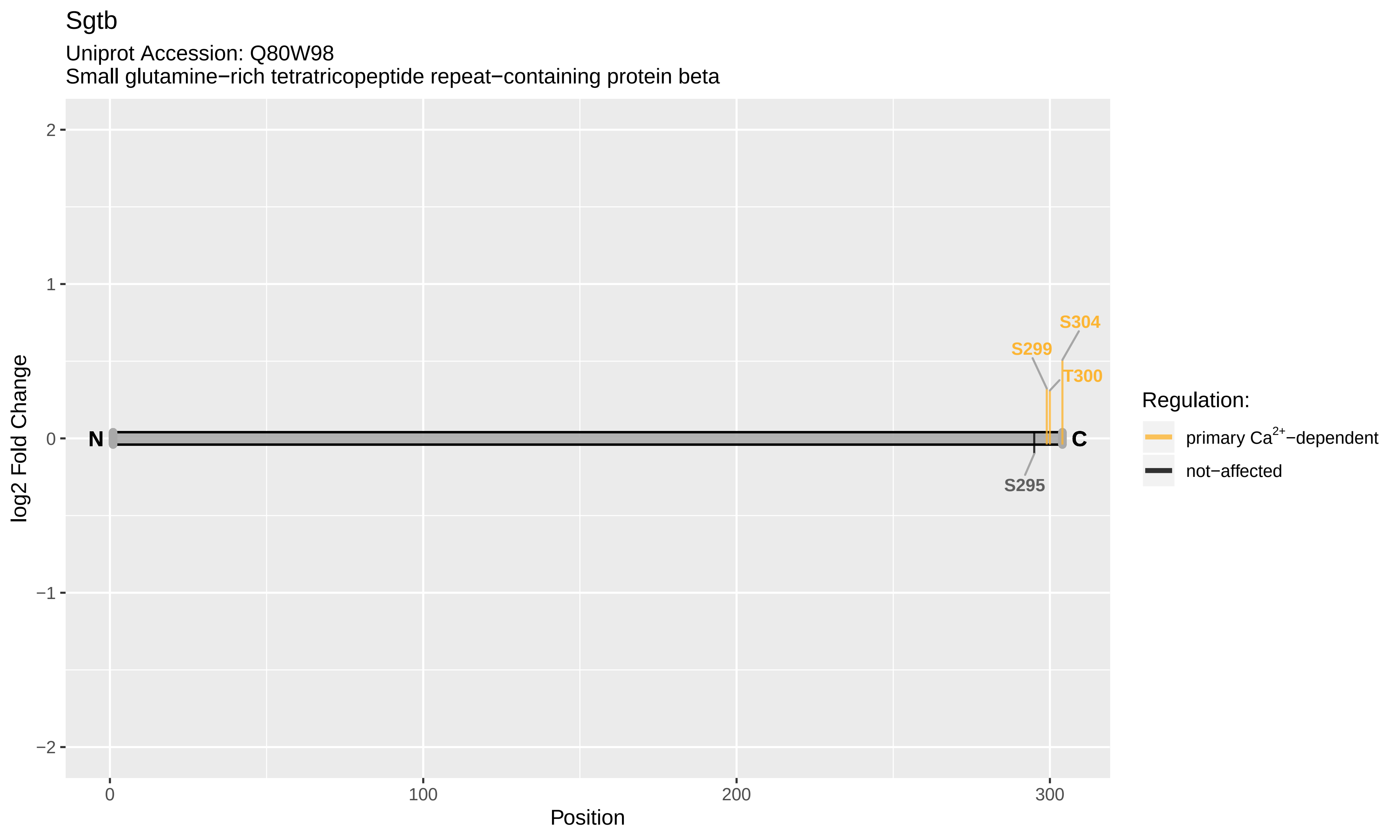
Sgtb
Uniprot Accession: Q80W98
Small glutamine−rich tetratricopeptide repeat−containing protein beta
log2 Fold Change
2
1
0
−1
−2
N
C
S304
S299
T300
S295
0
100
200
300
Position
Regulation:
primary Ca2+−dependent
not−affected

Sh3gl1

Uniprot Accession: O35964
Endophilin−A2

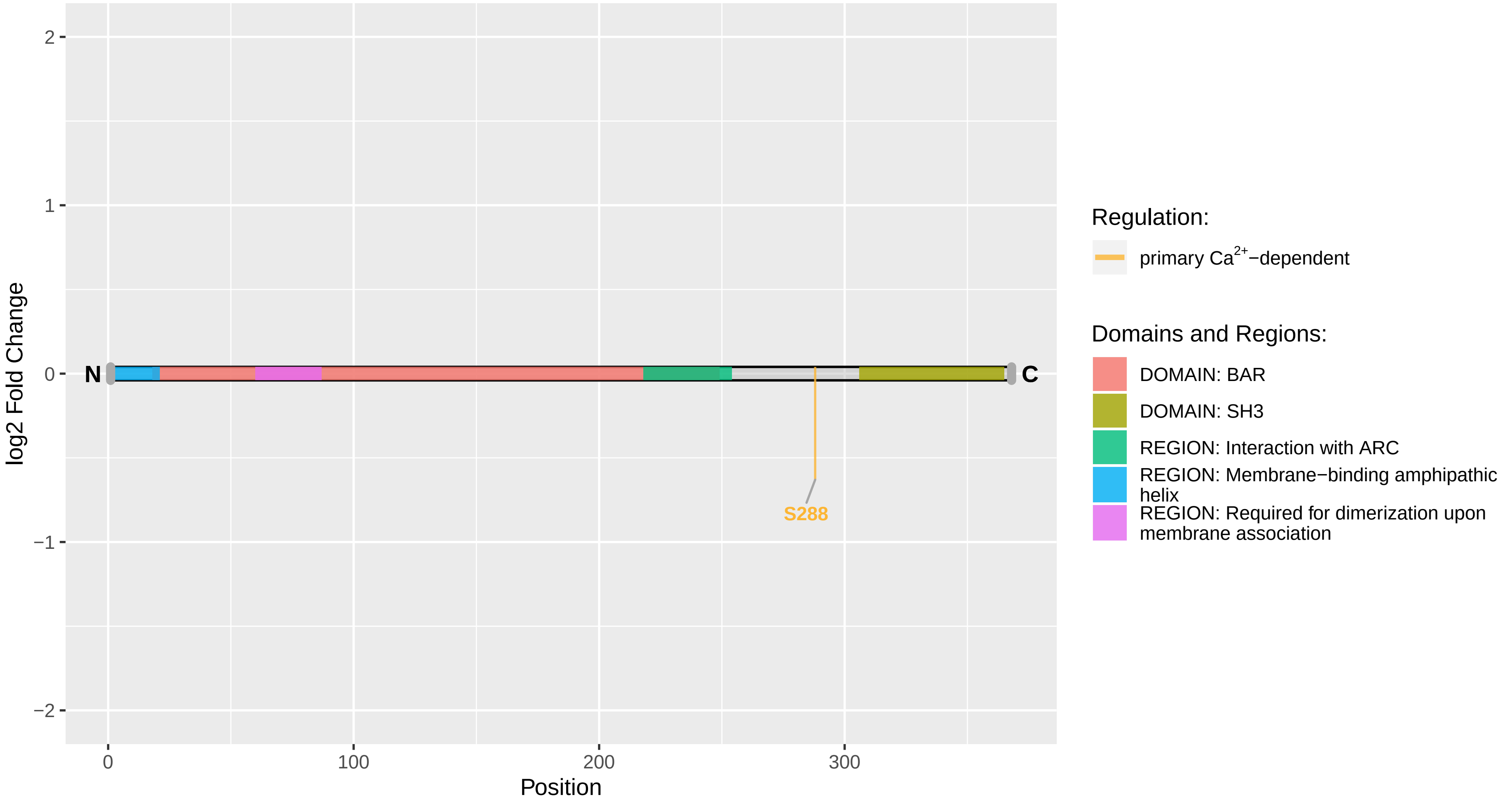

Sh3kbp1

Uniprot Accession: Q925Q9
SH3 domain−containing kinase−binding protein 1

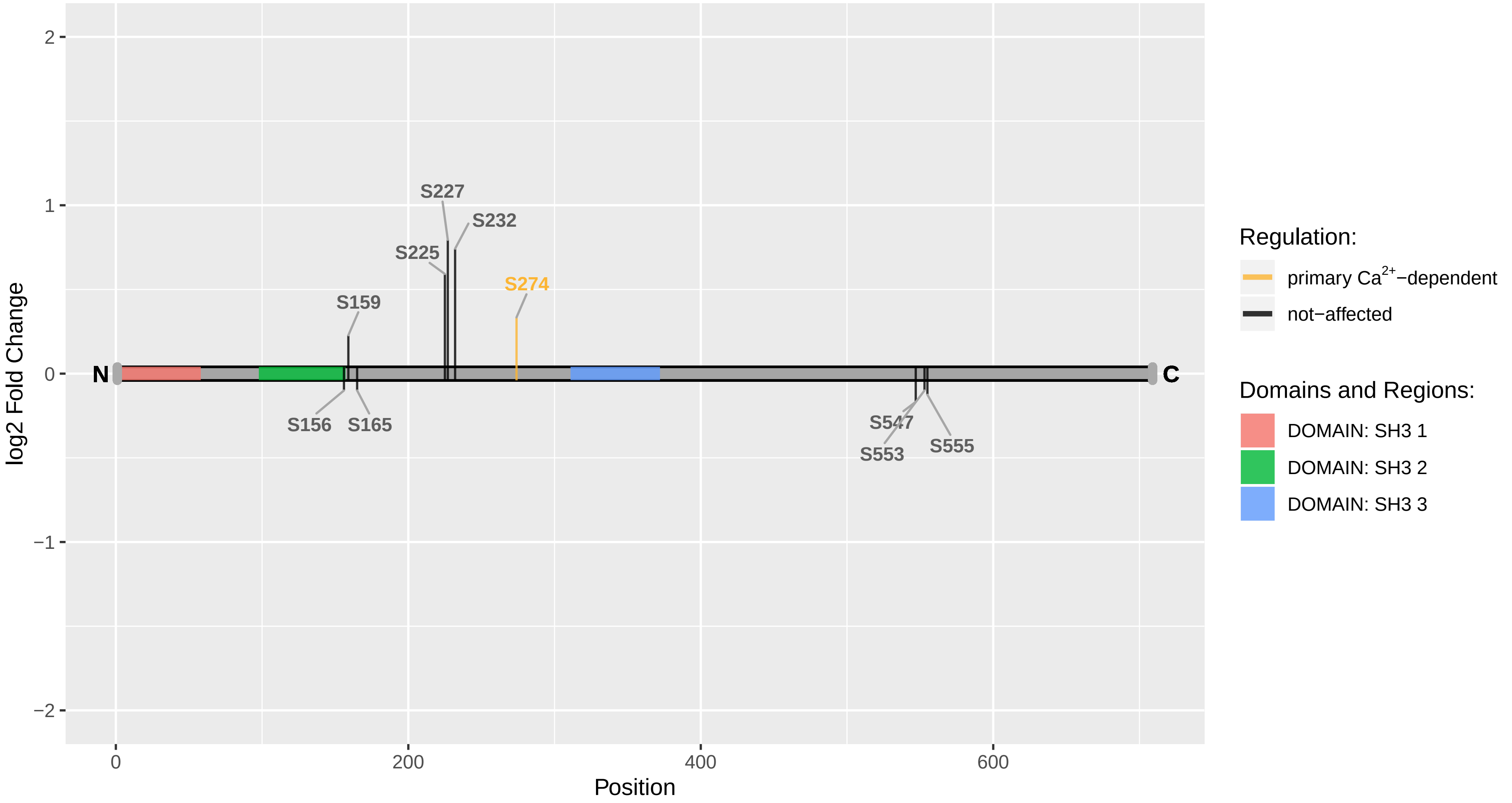

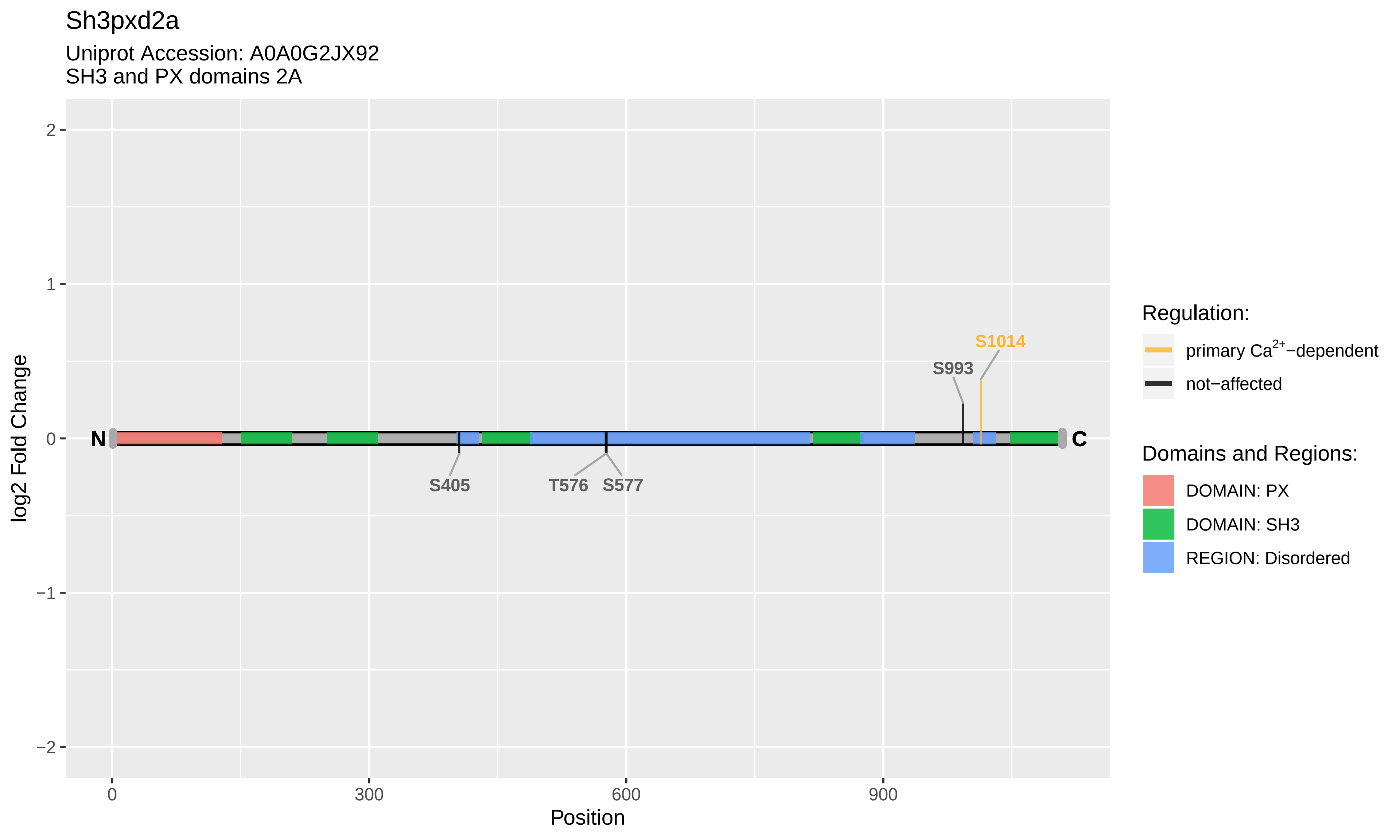

Sh3pxd2a
Uniprot Accession: A0A0G2JX92
SH3 and PX domains 2A
2
1
0
−1
−2
log2 Fold Change
N
C
S1014
S993
S405
T576
S577
0
300
600
900
Position
Regulation:
primary Ca2+−dependent
not−affected
Domains and Regions:
DOMAIN: PX
DOMAIN: SH3
REGION: Disordered

Shank1

Uniprot Accession: Q9WV48
SH3 and multiple ankyrin repeat domains protein 1

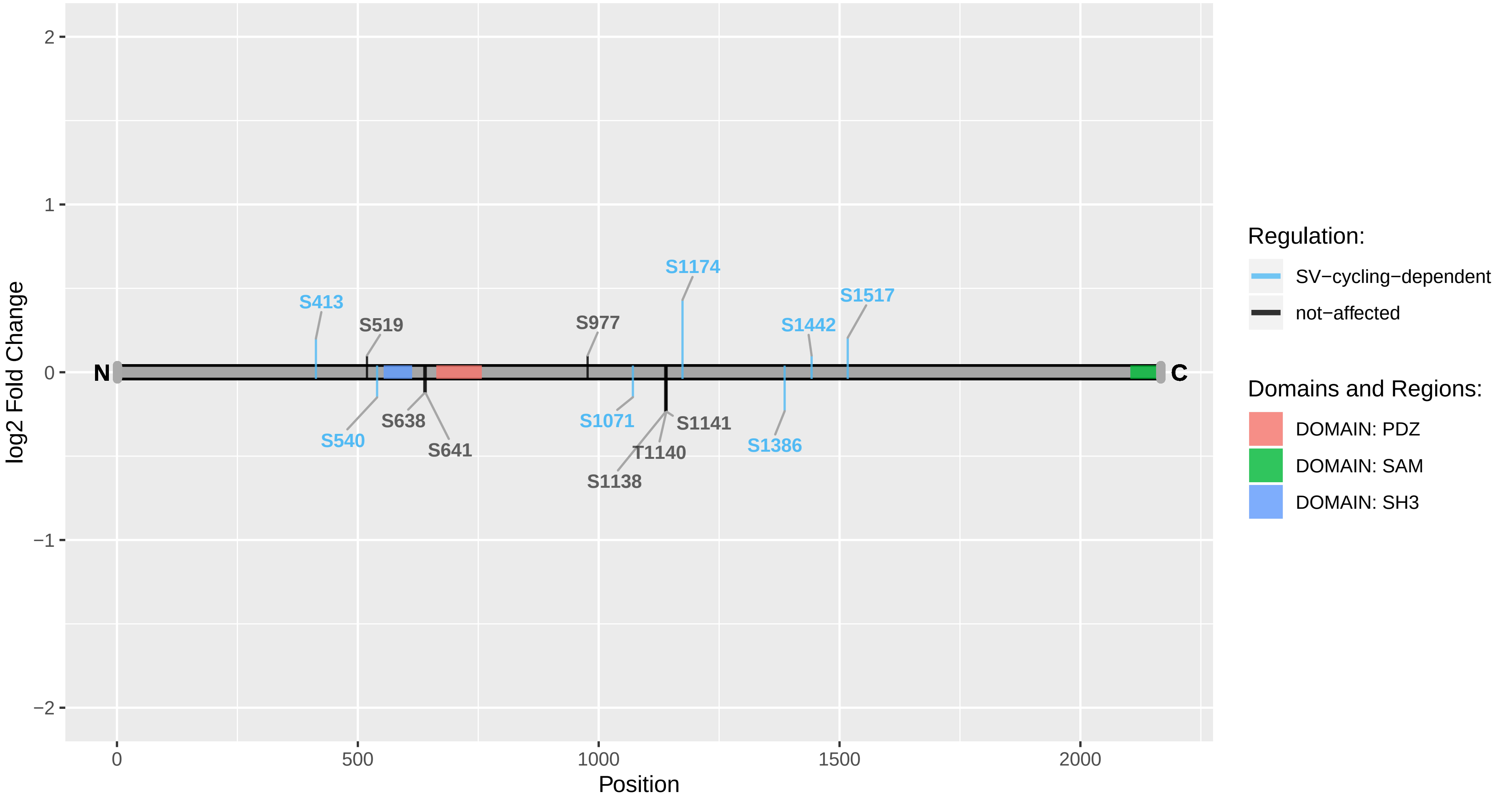

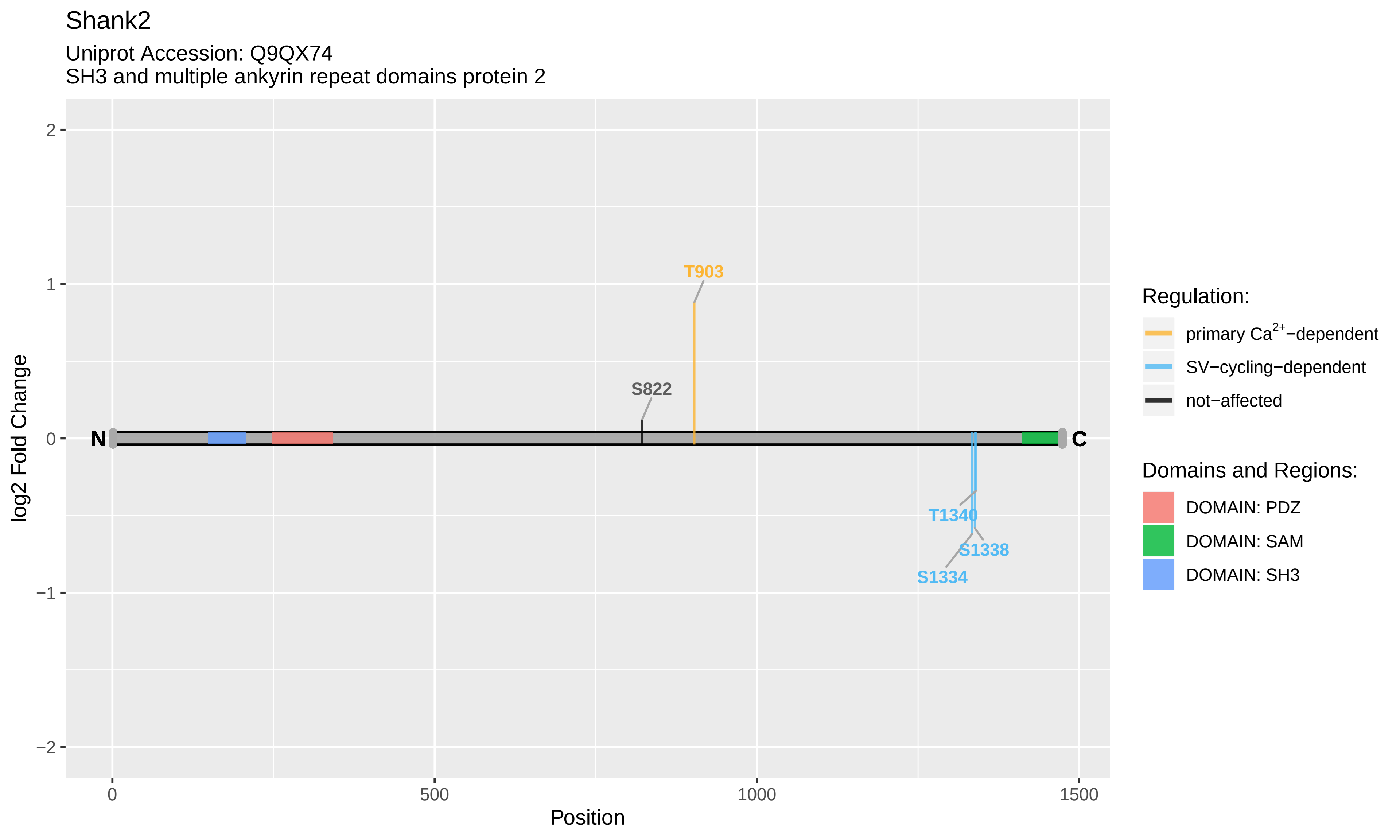

Shank2
Uniprot Accession: Q9QX74
SH3 and multiple ankyrin repeat domains protein 2
T903
S822
N
C
T1340
S1338
S1334
2
1
0
−1
−2
log2 Fold Change
0
500
1000
1500
Position
Regulation:
primary Ca2+−dependent
SV−cycling−dependent
not−affected
Domains and Regions:
DOMAIN: PDZ
DOMAIN: SAM
DOMAIN: SH3

Shank3

Uniprot Accession: Q9JLU4
SH3 and multiple ankyrin repeat domains protein 3

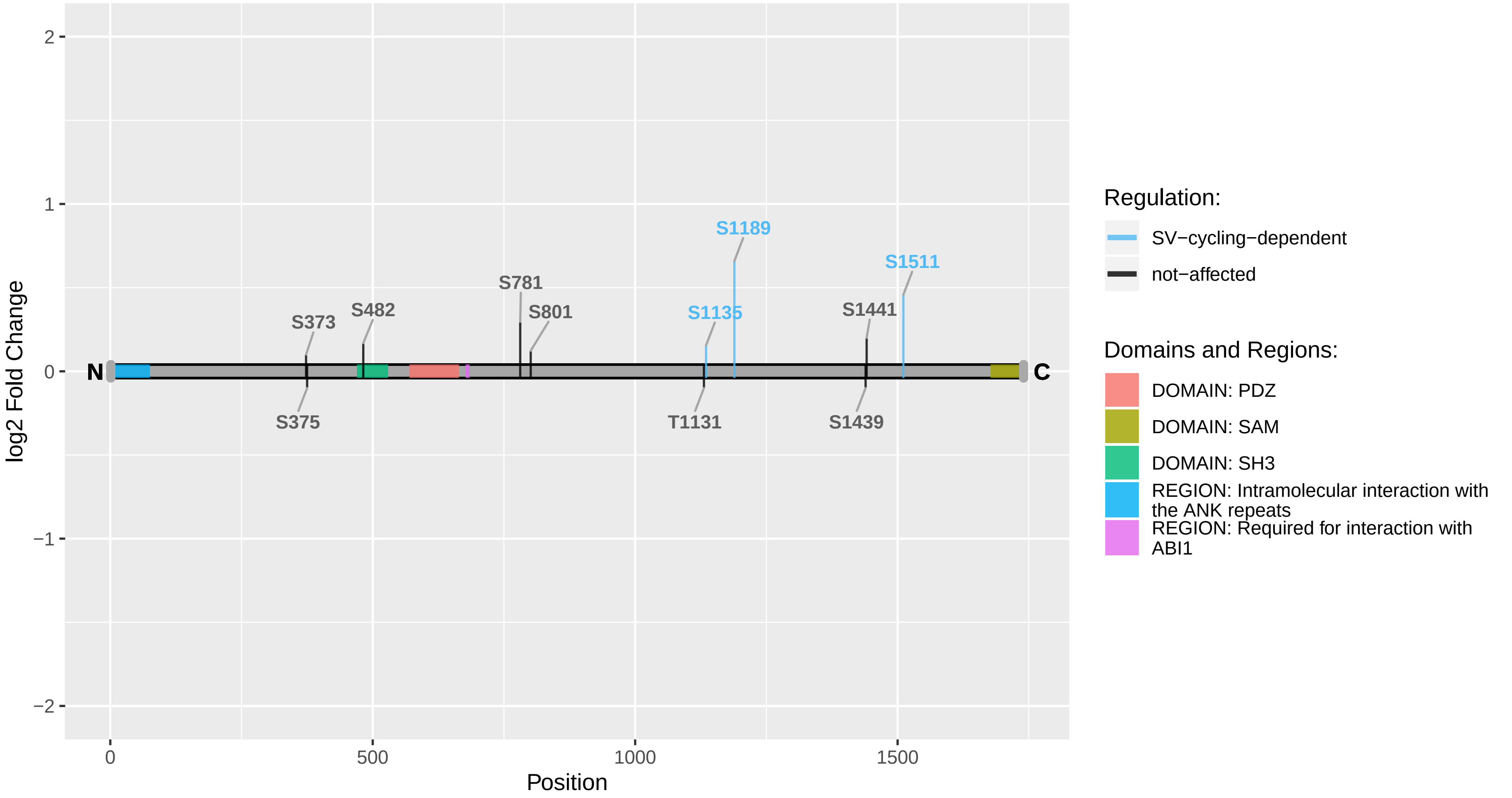

Shroom2

Uniprot Accession: Q7TP36
Protein Shroom2

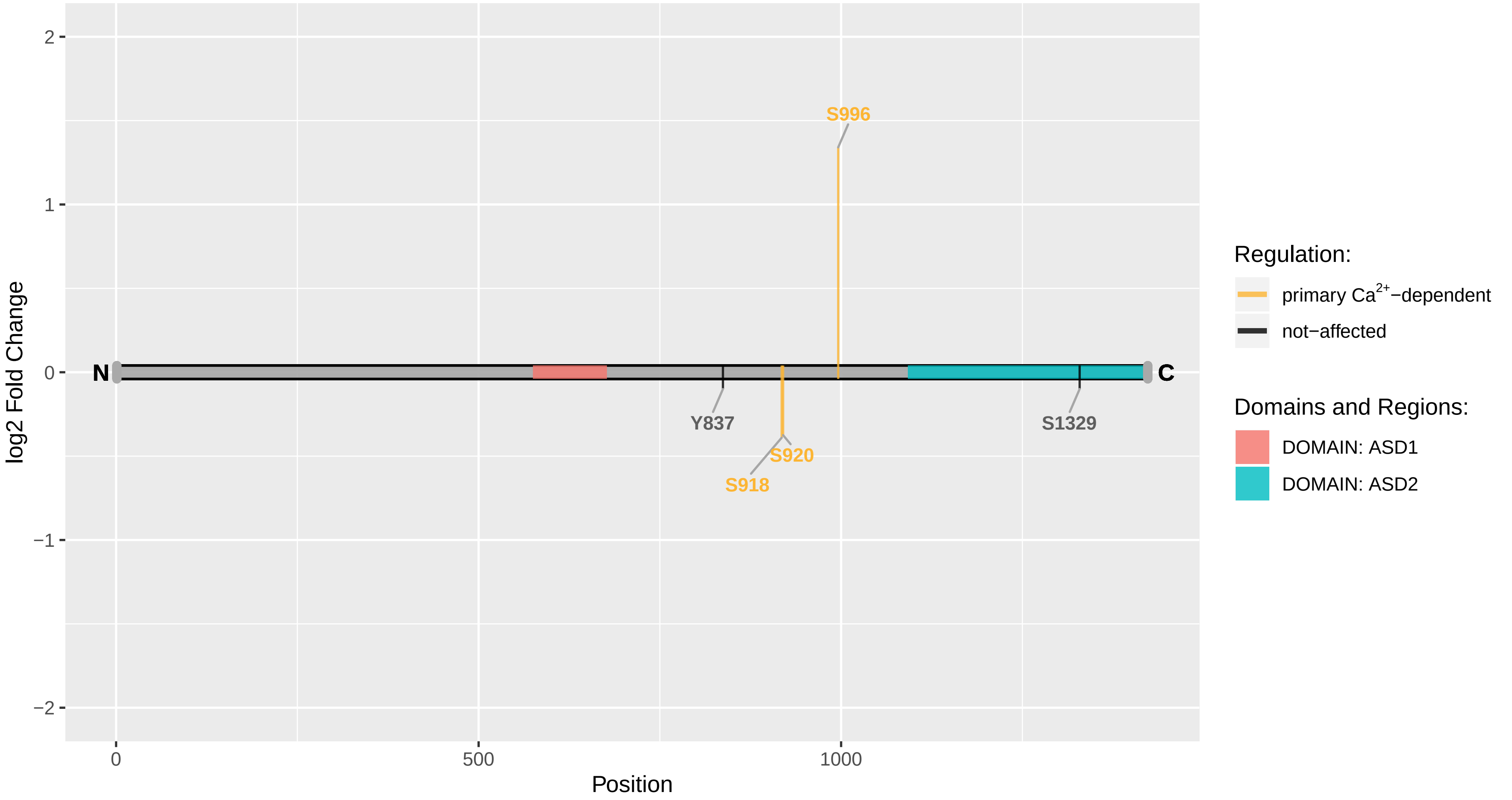

Shtn1

Uniprot Accession: A0MZ67
Shootin−1

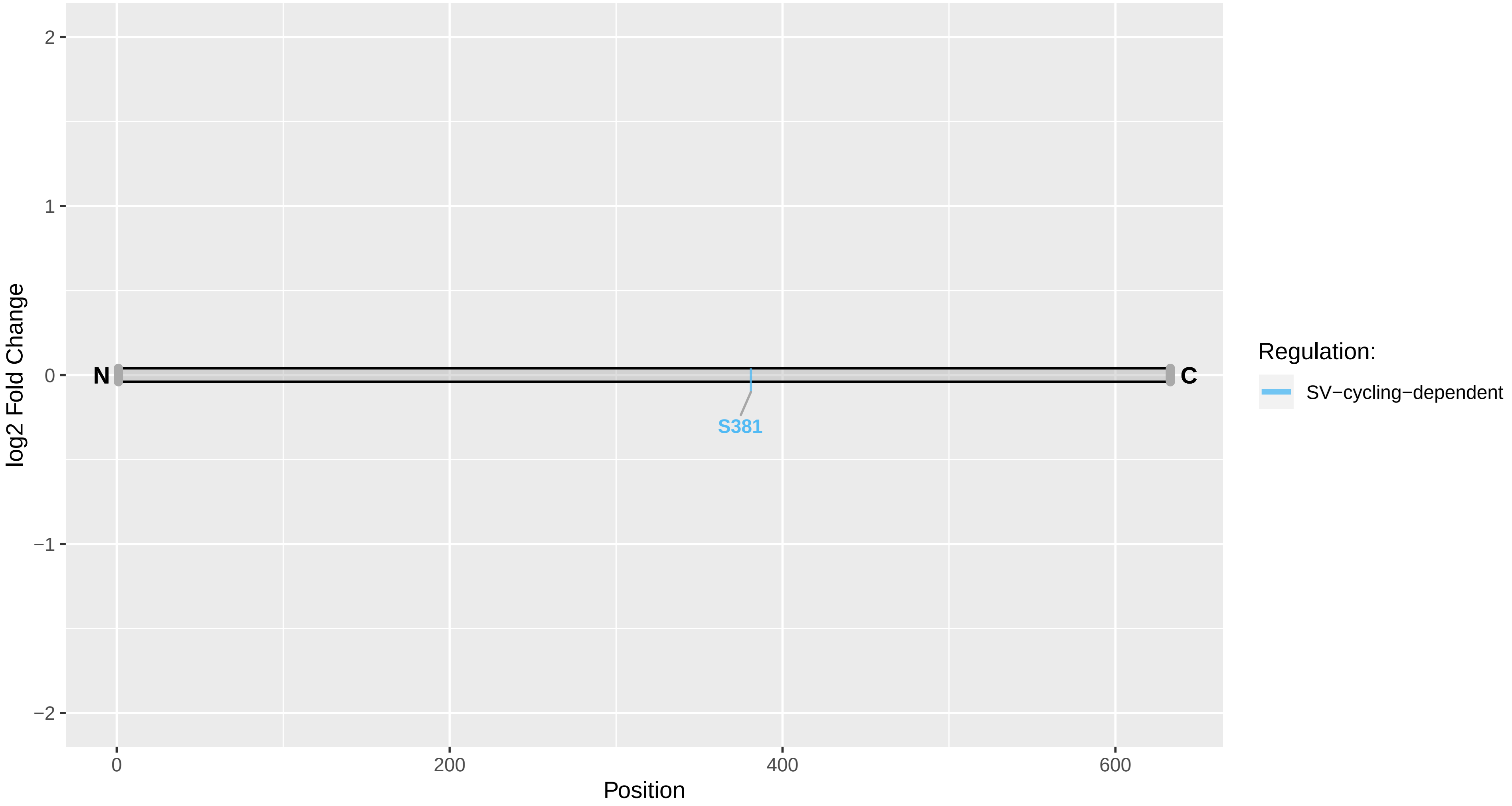

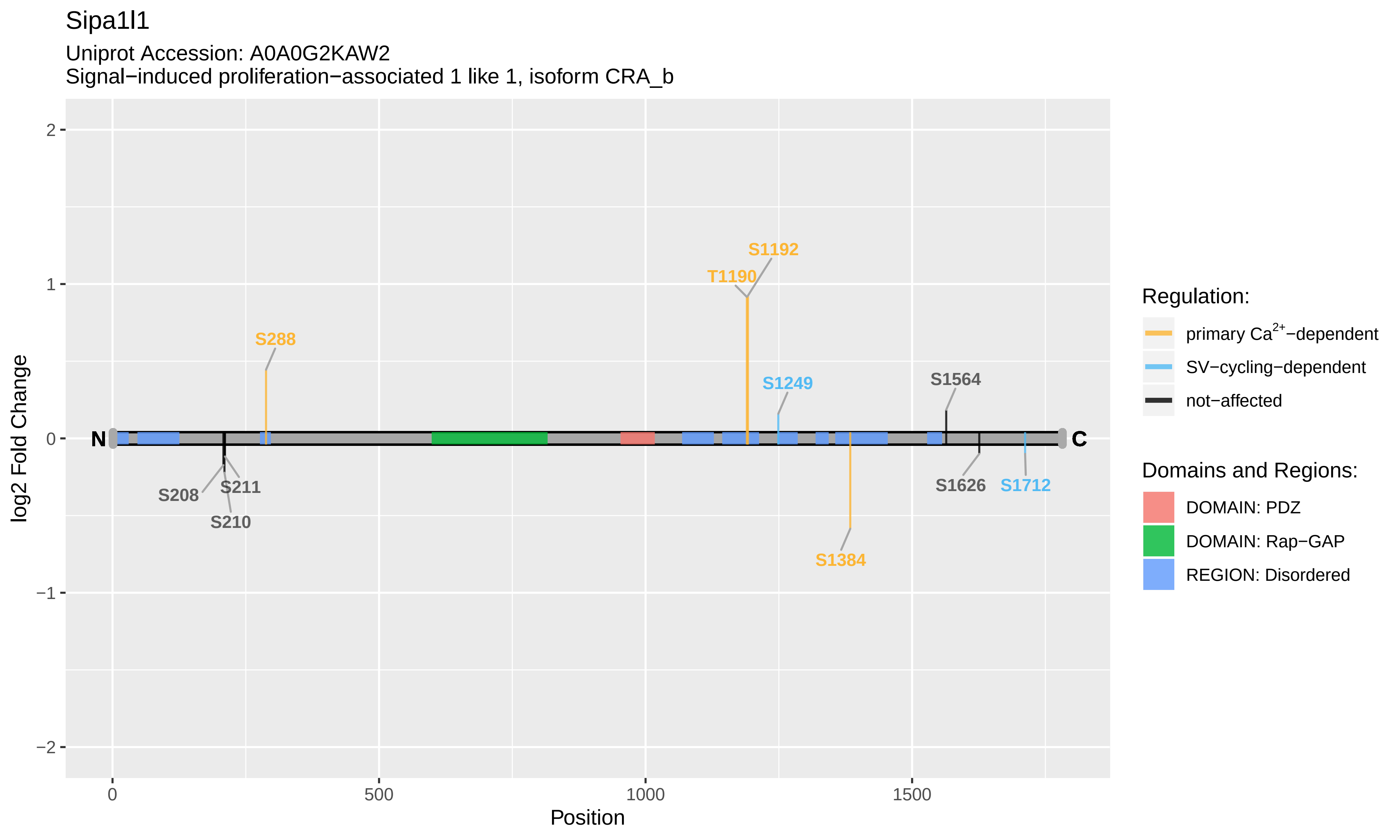
Sipa1l1
Uniprot Accession: A0A0G2KAW2
Signal−induced proliferation−associated 1 like 1, isoform CRA_b
log2 Fold Change
Position
N
C
S1192
T1190
S288
S1249
S1564
S208
S211
S210
S1384
S1626
S1712
Regulation:
primary Ca2+−dependent
SV−cycling−dependent
not−affected
Domains and Regions:
DOMAIN: PDZ
DOMAIN: Rap−GAP
REGION: Disordered

Sipa1l1

Uniprot Accession: O35412
Signal−induced proliferation−associated 1−like protein 1

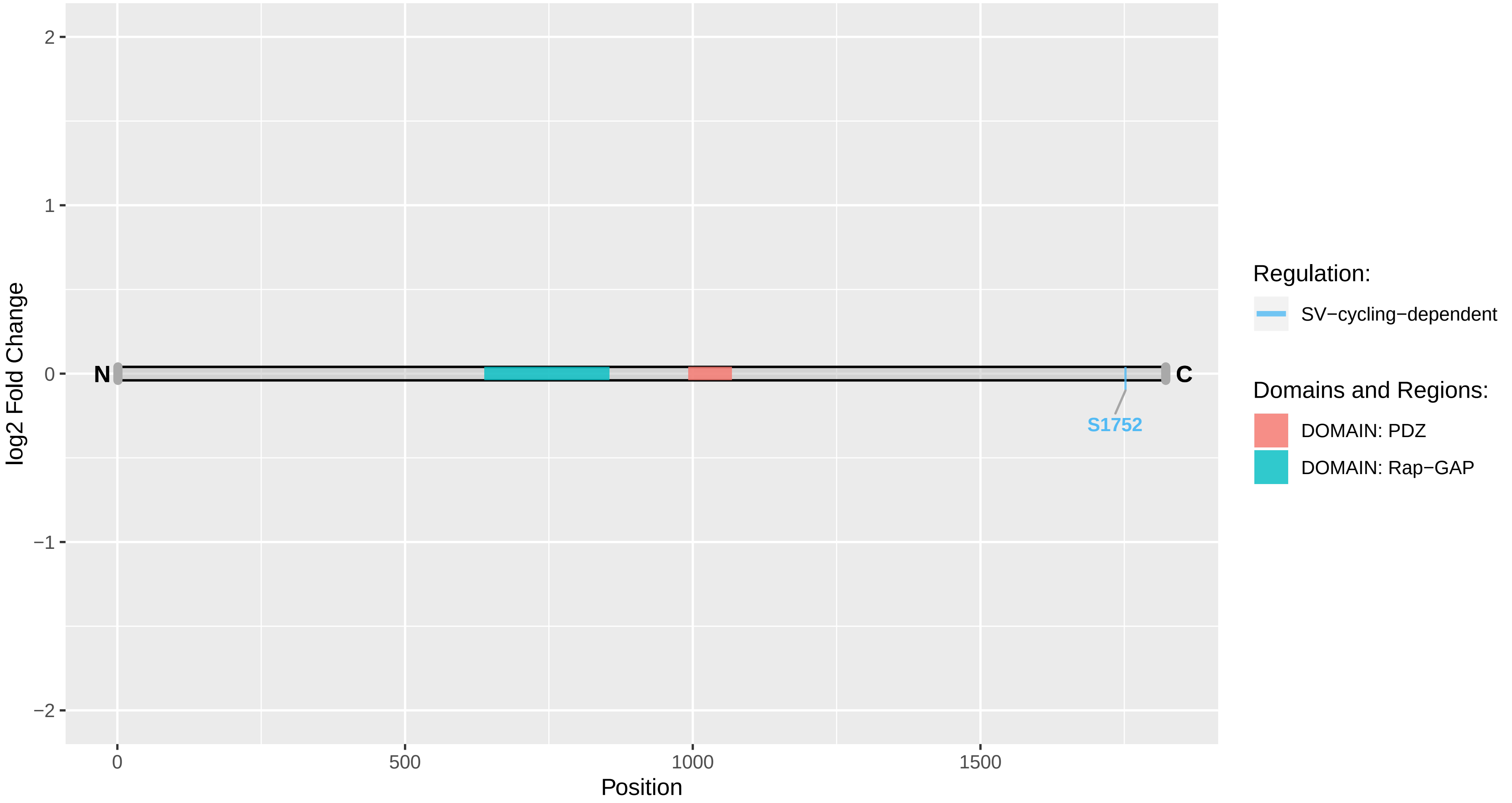

Sipa1l3

Uniprot Accession: F1LYG2
RCG54282

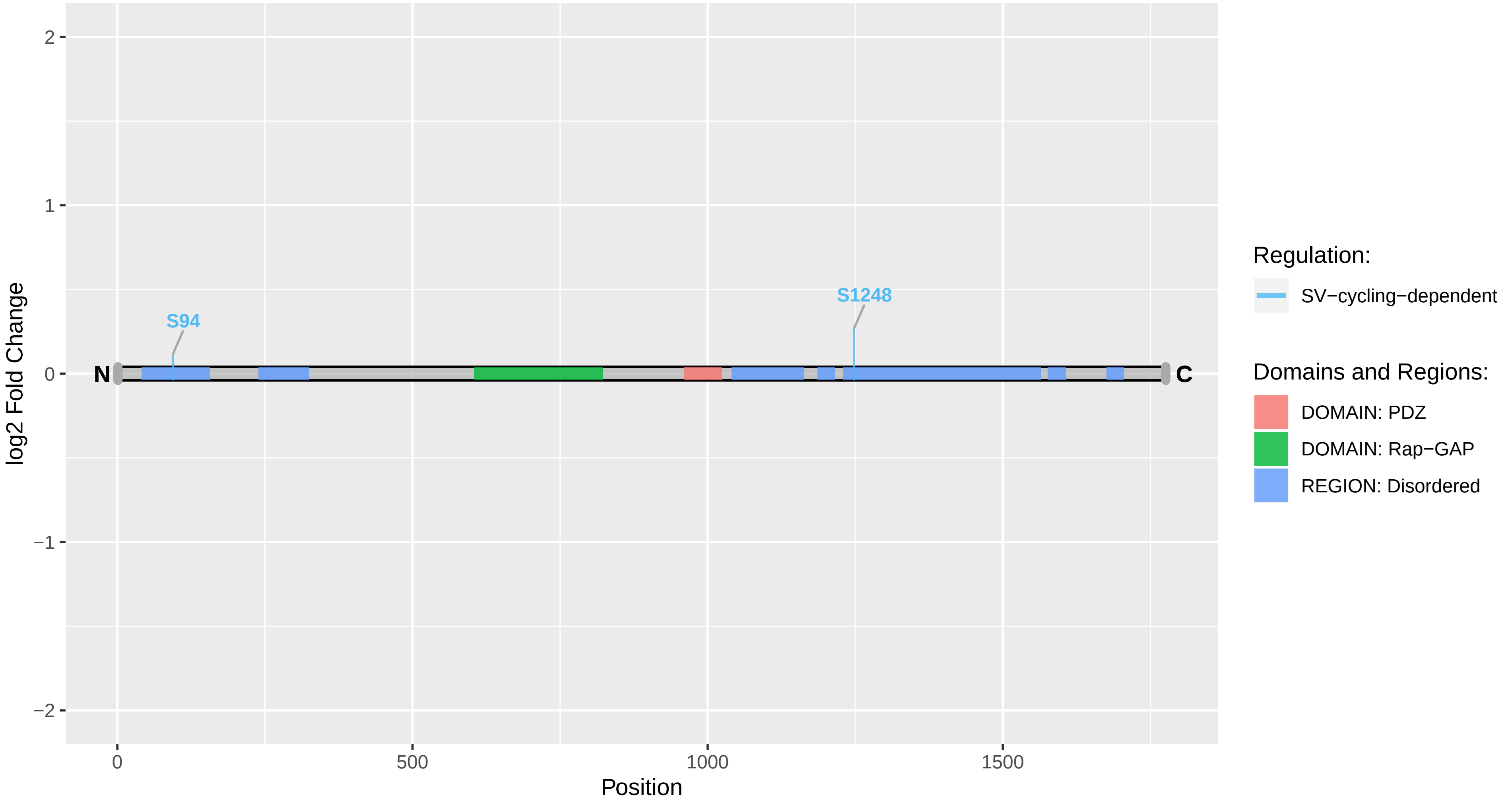

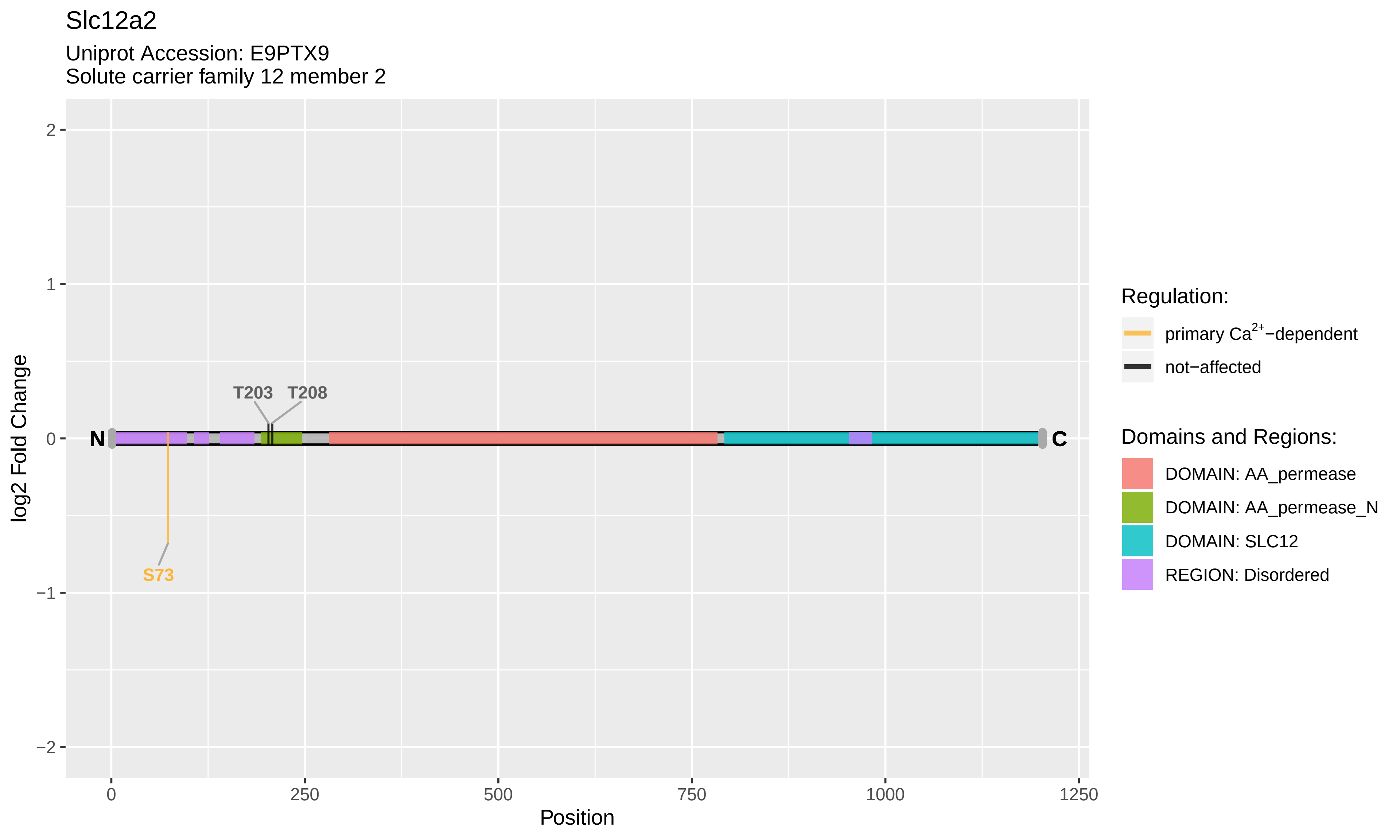
Slc12a2
Uniprot Accession: E9PTX9
Solute carrier family 12 member 2
log2 Fold Change
Position
N
C
T203
T208
S73
Regulation:
primary Ca2+−dependent
not−affected
Domains and Regions:
DOMAIN: AA_permease
DOMAIN: AA_permease_N
DOMAIN: SLC12
REGION: Disordered

Slc12a5

Uniprot Accession: Q63633
Solute carrier family 12 member 5

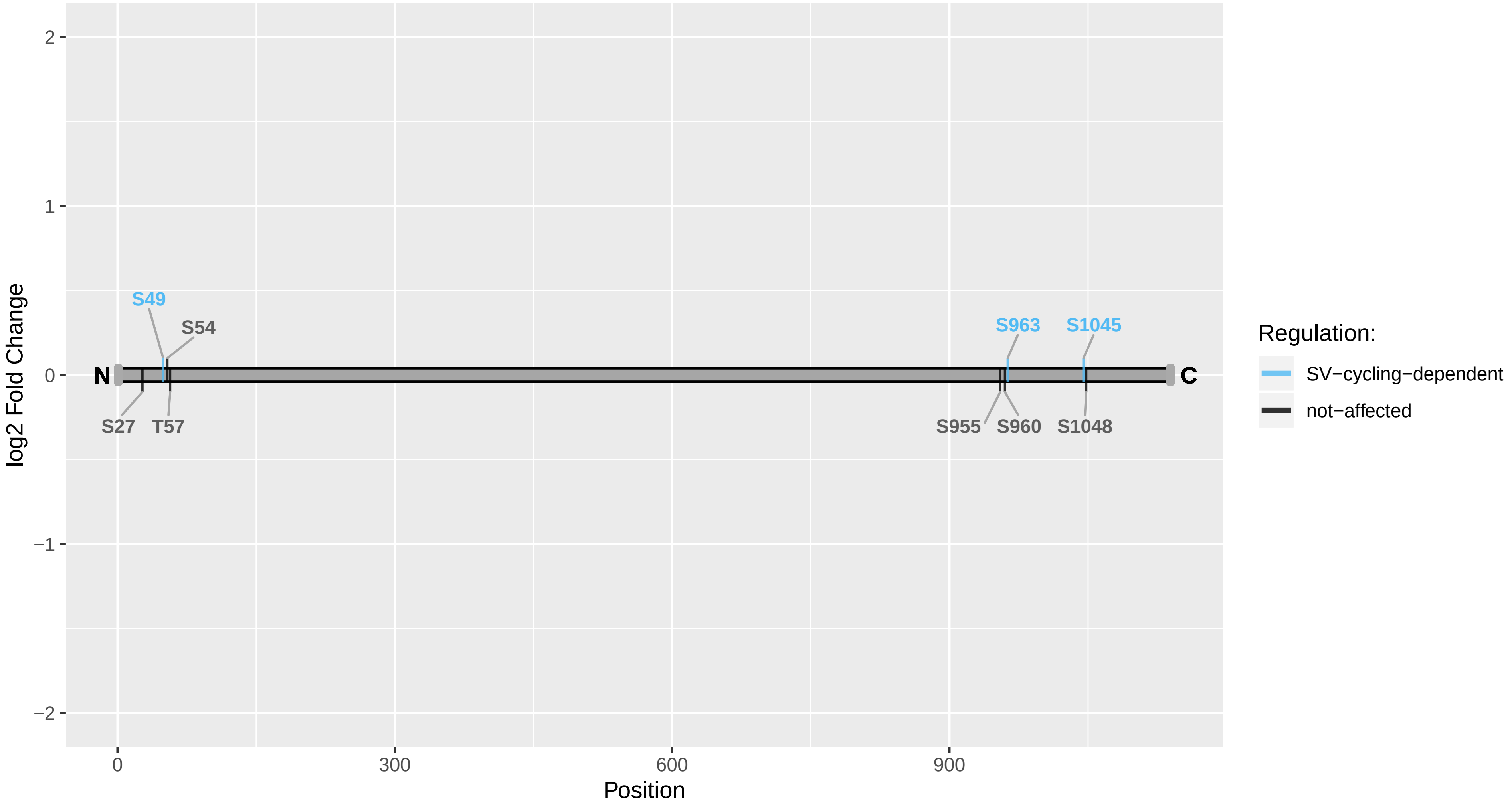

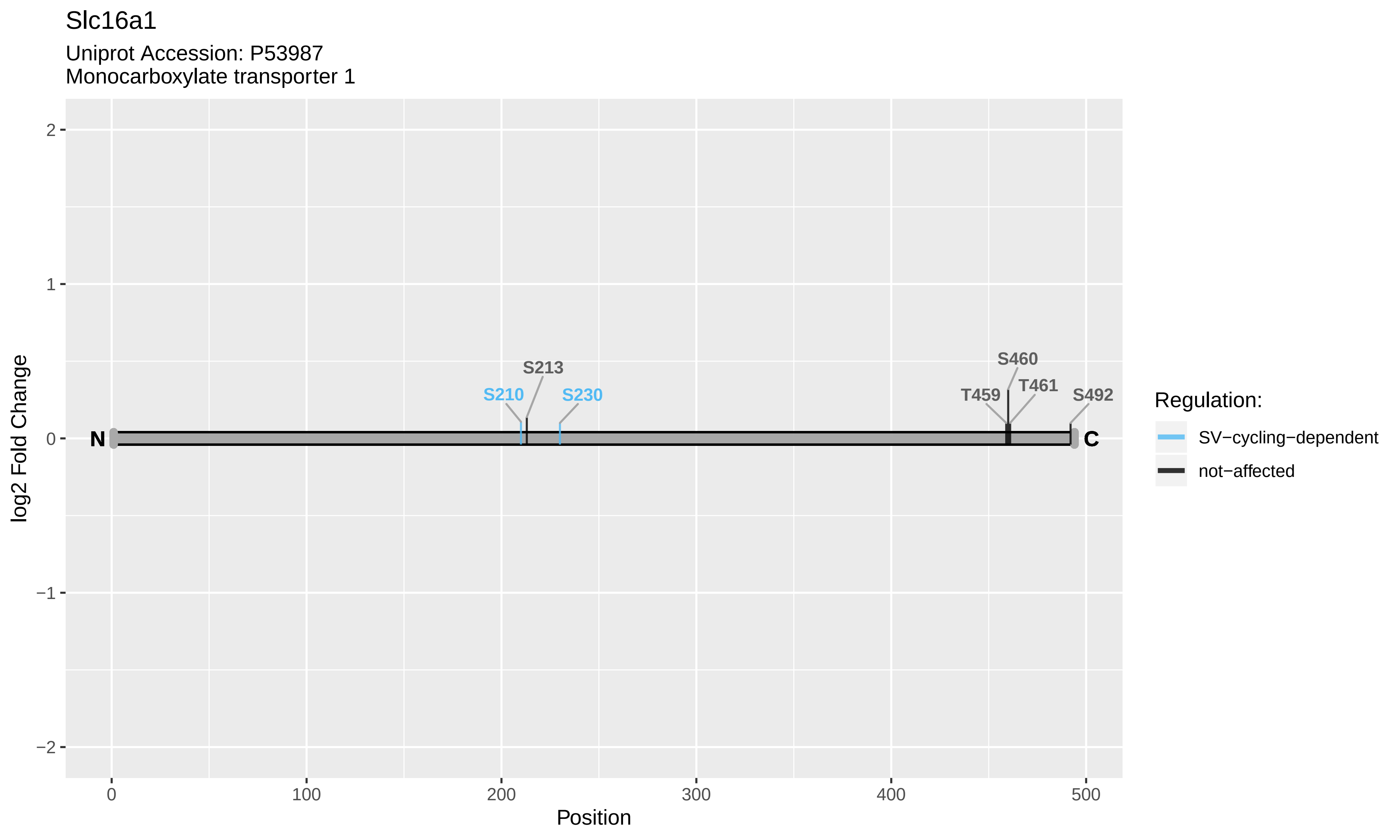
Slc16a1
Uniprot Accession: P53987
Monocarboxylate transporter 1
log2 Fold Change
Position
N
C
S210
S213
S230
T459
S460
T461
S492
2
1
0
−1
−2
0
100
200
300
400
500
Regulation:
SV−cycling−dependent
not−affected

Slc20a2

Uniprot Accession: Q63488
Sodium−dependent phosphate transporter 2

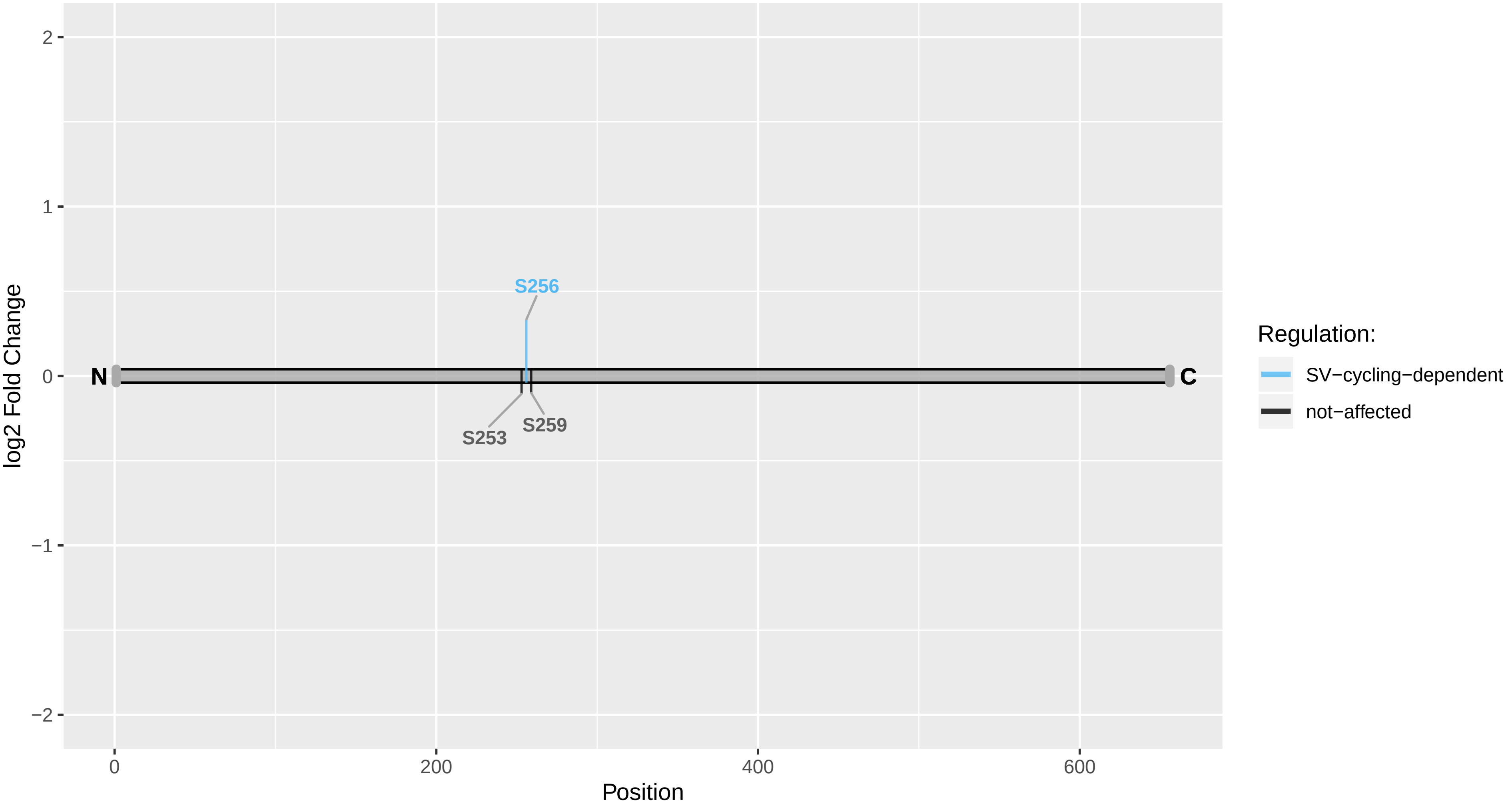

Slc24a2

Uniprot Accession: O54701
Sodium/potassium/calcium exchanger 2

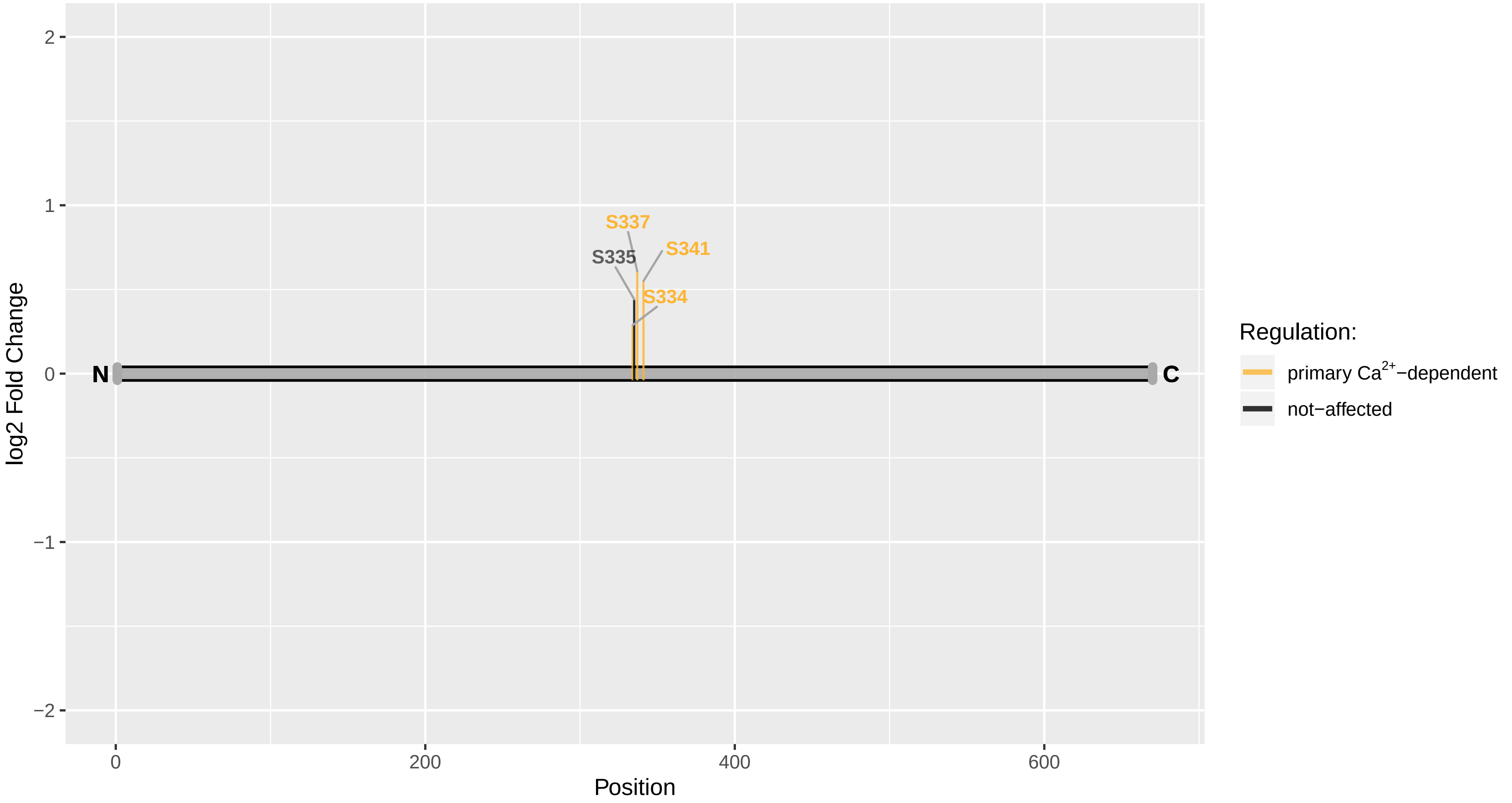

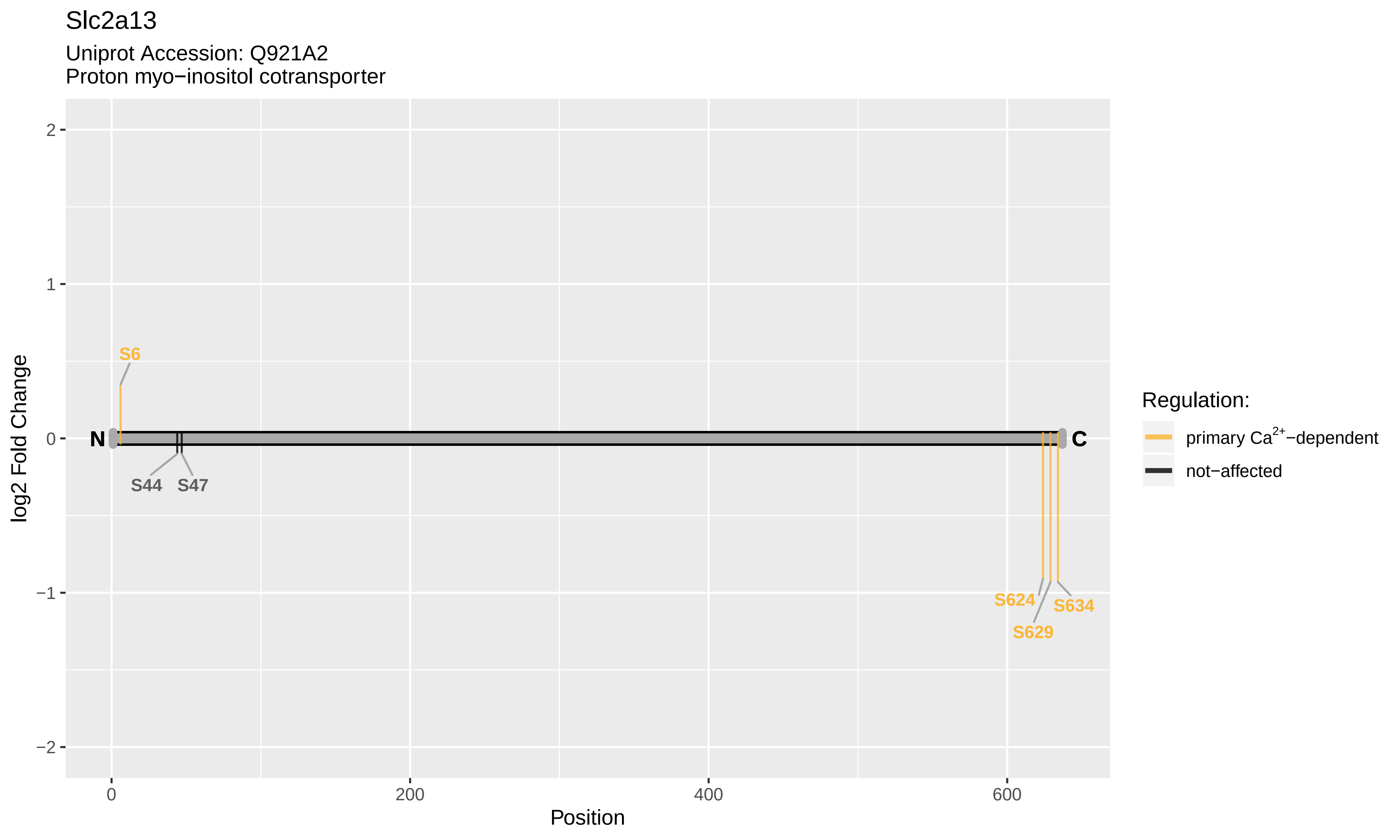

Slc2a13
Uniprot Accession: Q921A2
Proton myo−inositol cotransporter
log2 Fold Change
Position
2
1
0
−1
−2
0
200
400
600
N
C
S6
S44
S47
S624
S629
S634
Regulation:
primary Ca2+−dependent
not−affected

Slc30a3

Uniprot Accession: Q6QIX3
Zinc transporter 3

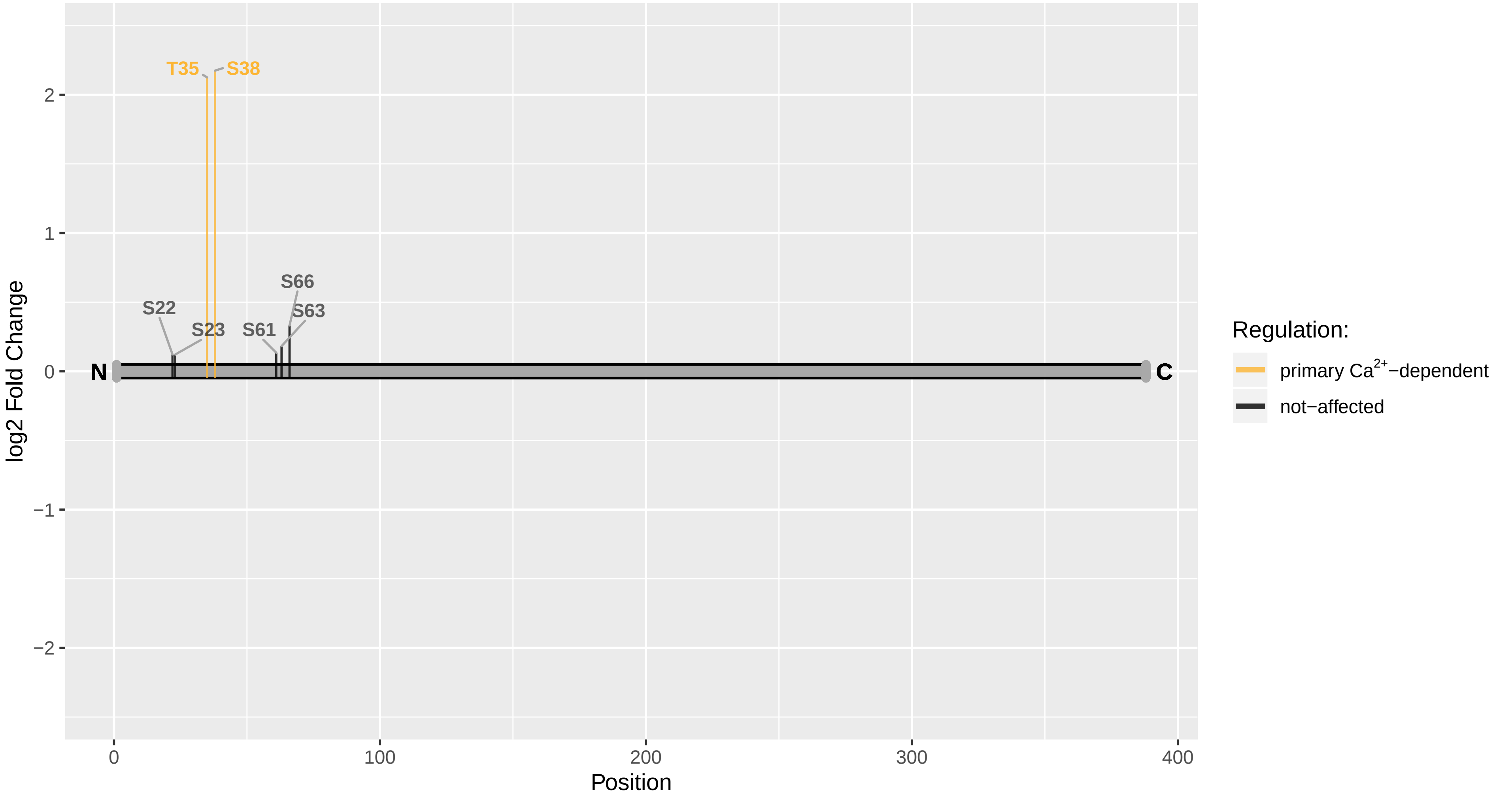

Slc39a10

Uniprot Accession: D4A517
Solute carrier family 39 (Zinc transporter), member 10 (Predicted), isoform CRA_a

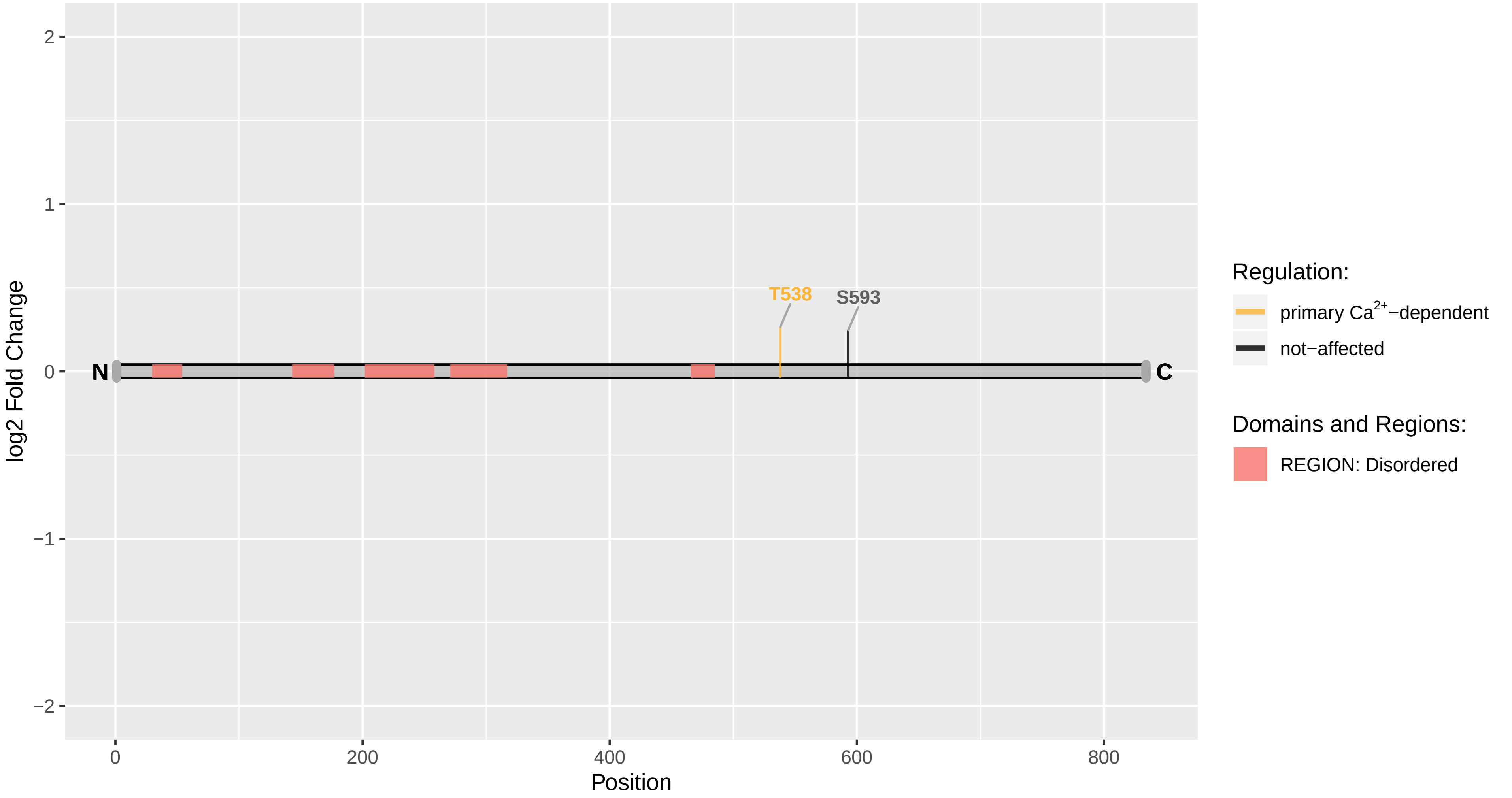

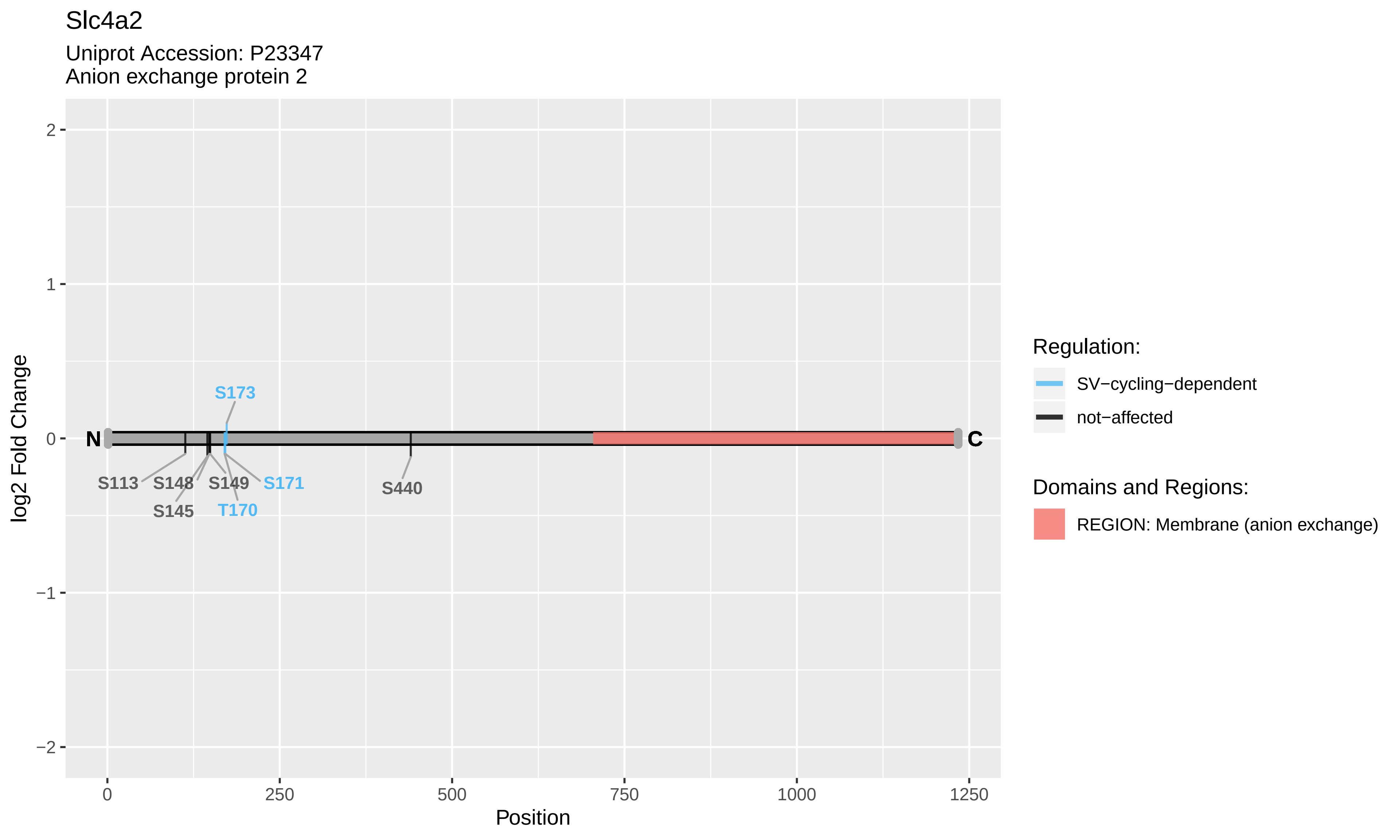

Slc4a2
Uniprot Accession: P23347
Anion exchange protein 2
log2 Fold Change
2
1
0
−1
−2
N
C
S173
S113
S148
S149
S171
S145
T170
S440
0
250
500
750
1000
1250
Position
Regulation:
SV−cycling−dependent
not−affected
Domains and Regions:
REGION: Membrane (anion exchange)

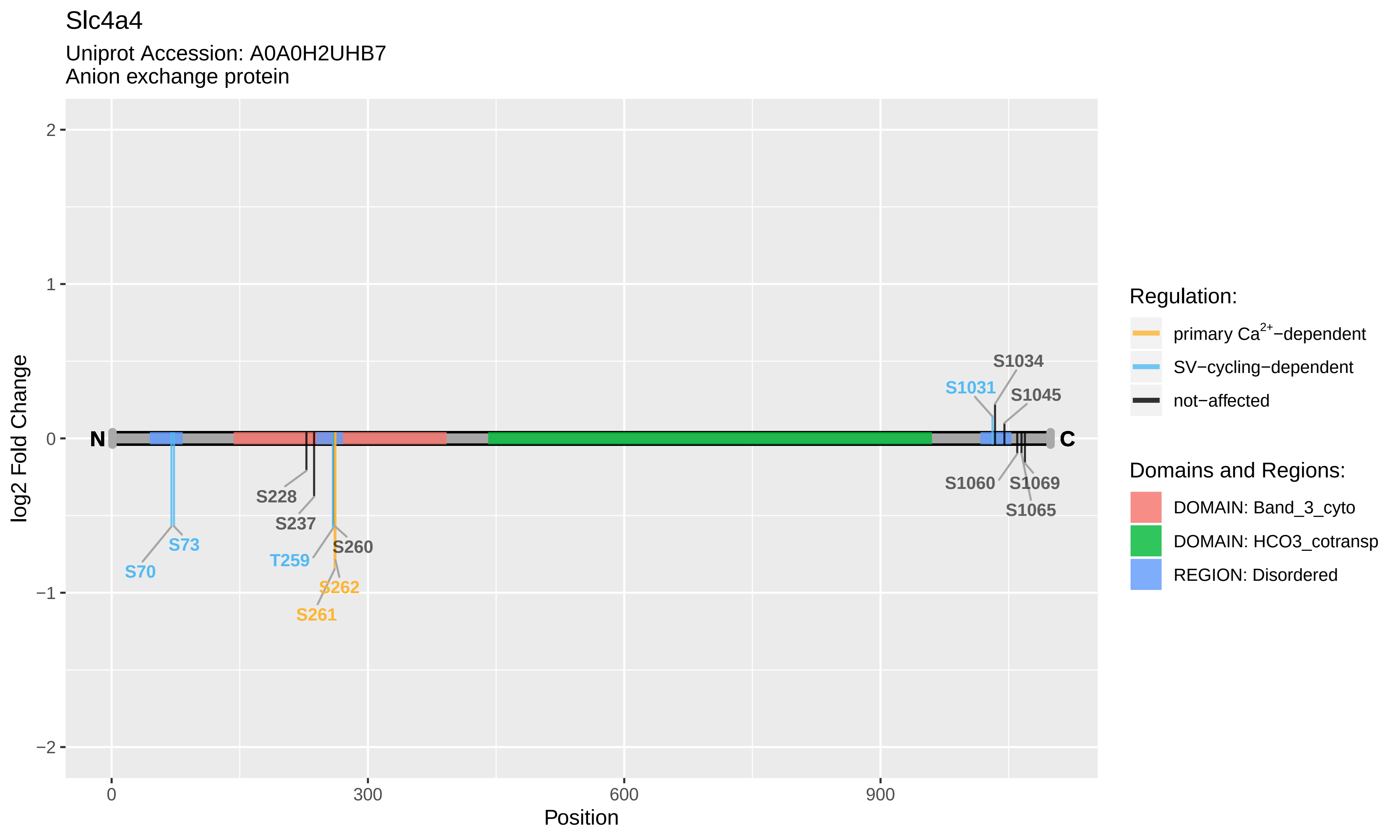

Slc4a4
Uniprot Accession: A0A0H2UHB7
Anion exchange protein
log2 Fold Change
2
1
0
−1
−2
N
C
S1034
S1031
S1045
S1060
S1069
S1065
S228
S237
S73
S70
T259
S260
S262
S261
0
300
600
900
Position
Regulation:
primary Ca2+−dependent
SV−cycling−dependent
not−affected
Domains and Regions:
DOMAIN: Band_3_cyto
DOMAIN: HCO3_cotransp
REGION: Disordered

Slc4a7

Uniprot Accession: Q9R1N3
Sodium bicarbonate cotransporter 3

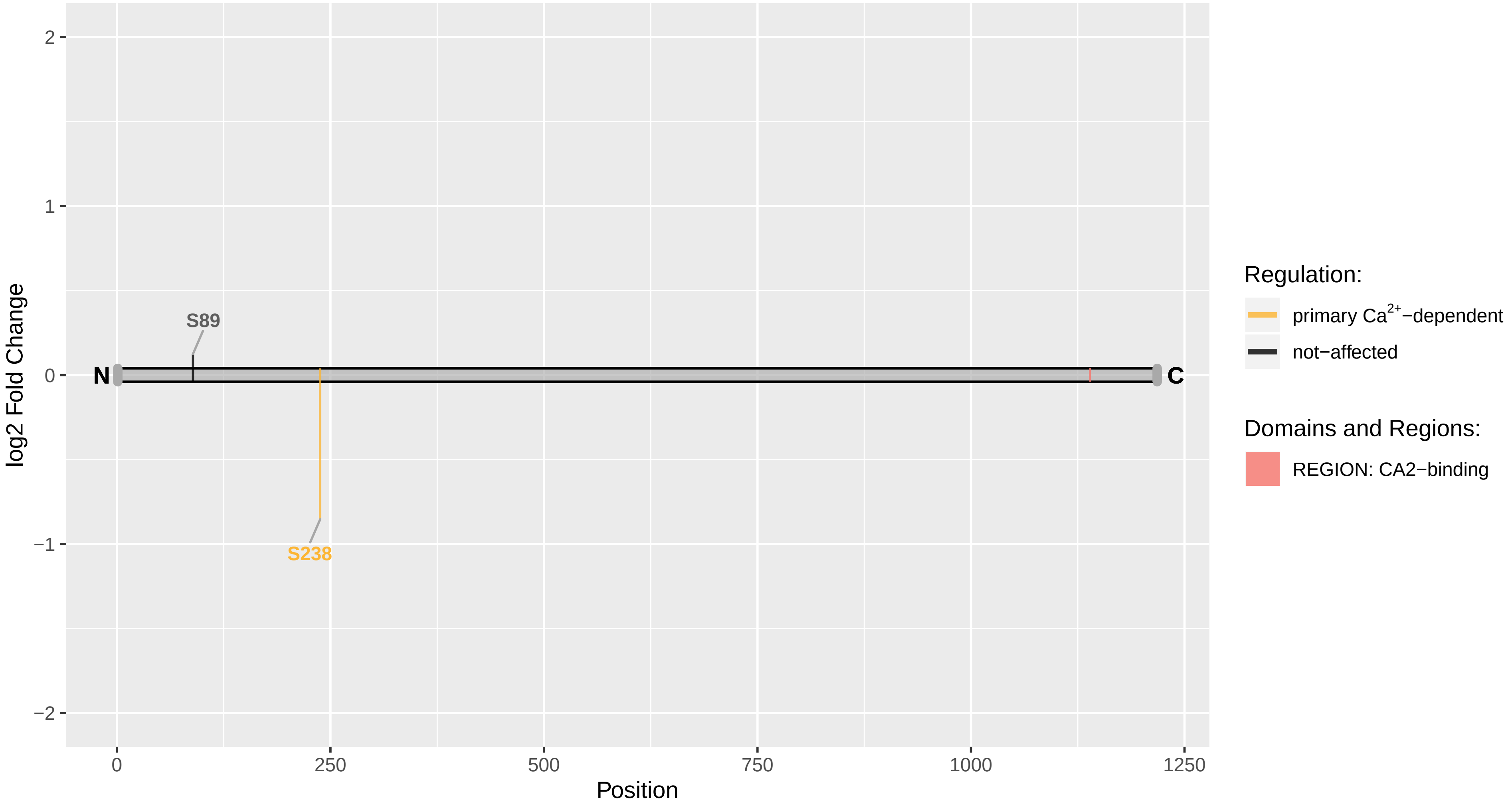

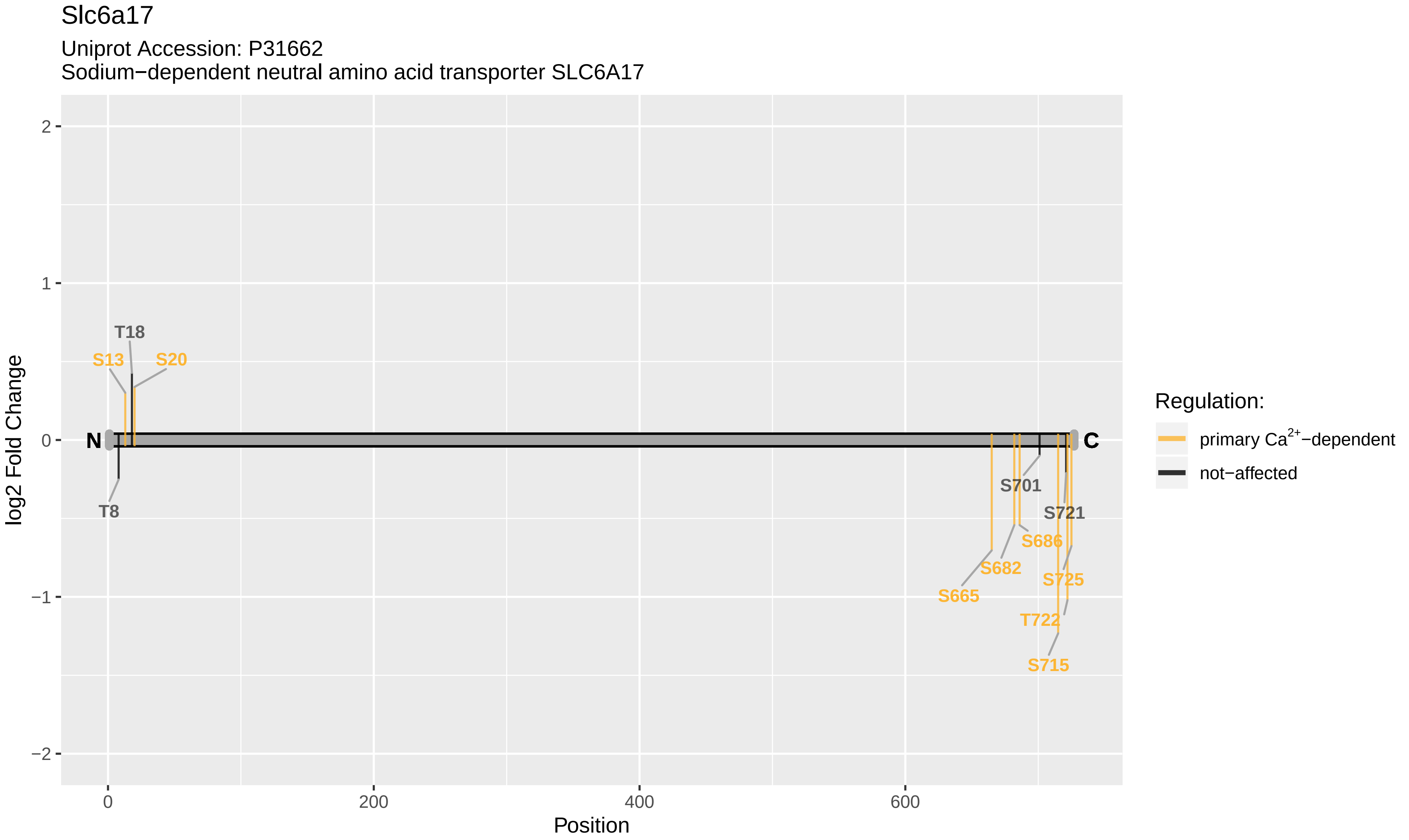

Slc6a17
Uniprot Accession: P31662
Sodium−dependent neutral amino acid transporter SLC6A17
log2 Fold Change
Position
N
C
T18
S13
S20
T8
S701
S721
S686
S682
S725
S665
T722
S715
Regulation:
primary Ca2+−dependent
not−affected

Slc7a8

Uniprot Accession: Q9WVR6
Large neutral amino acids transporter small subunit 2

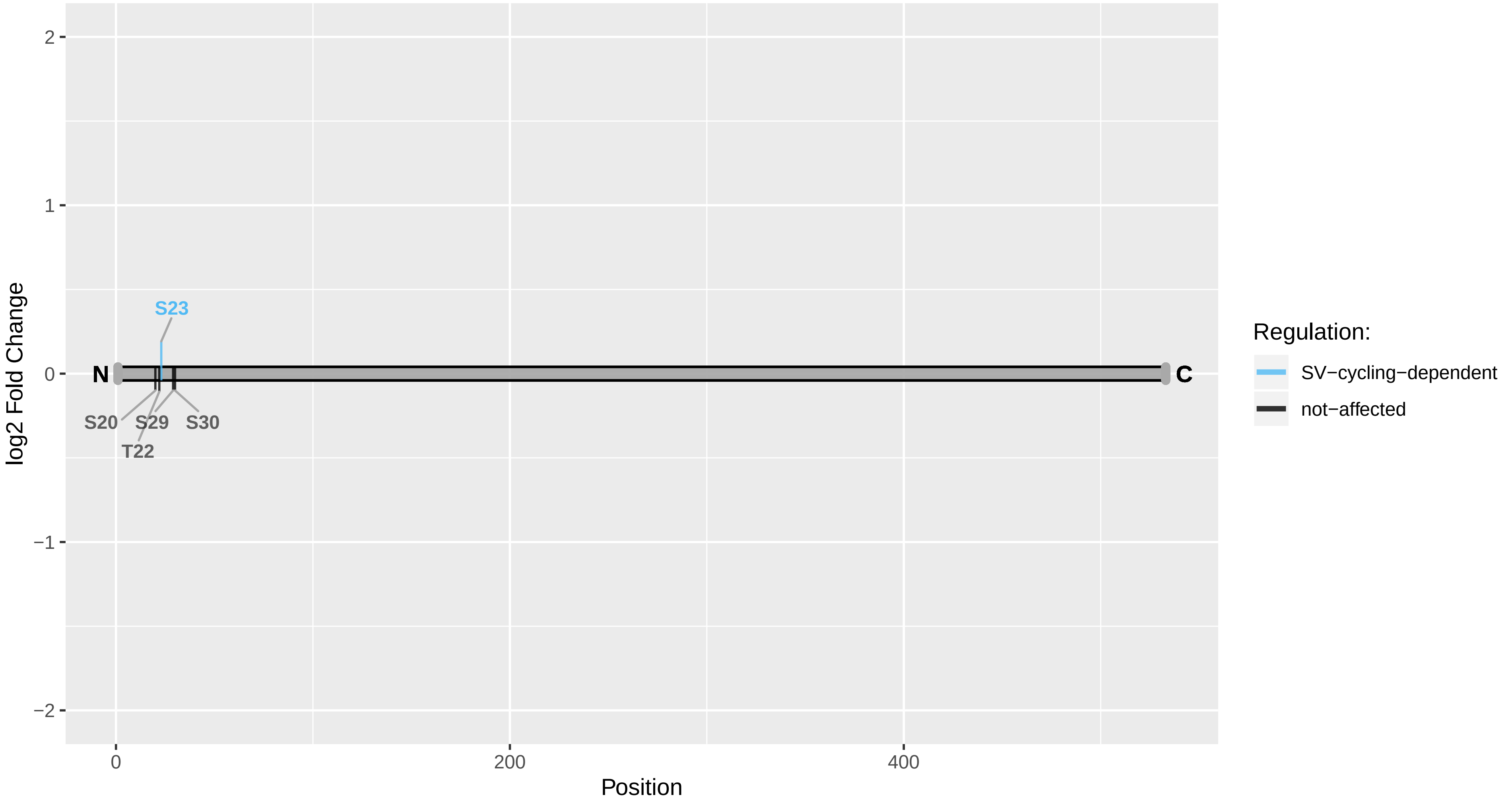

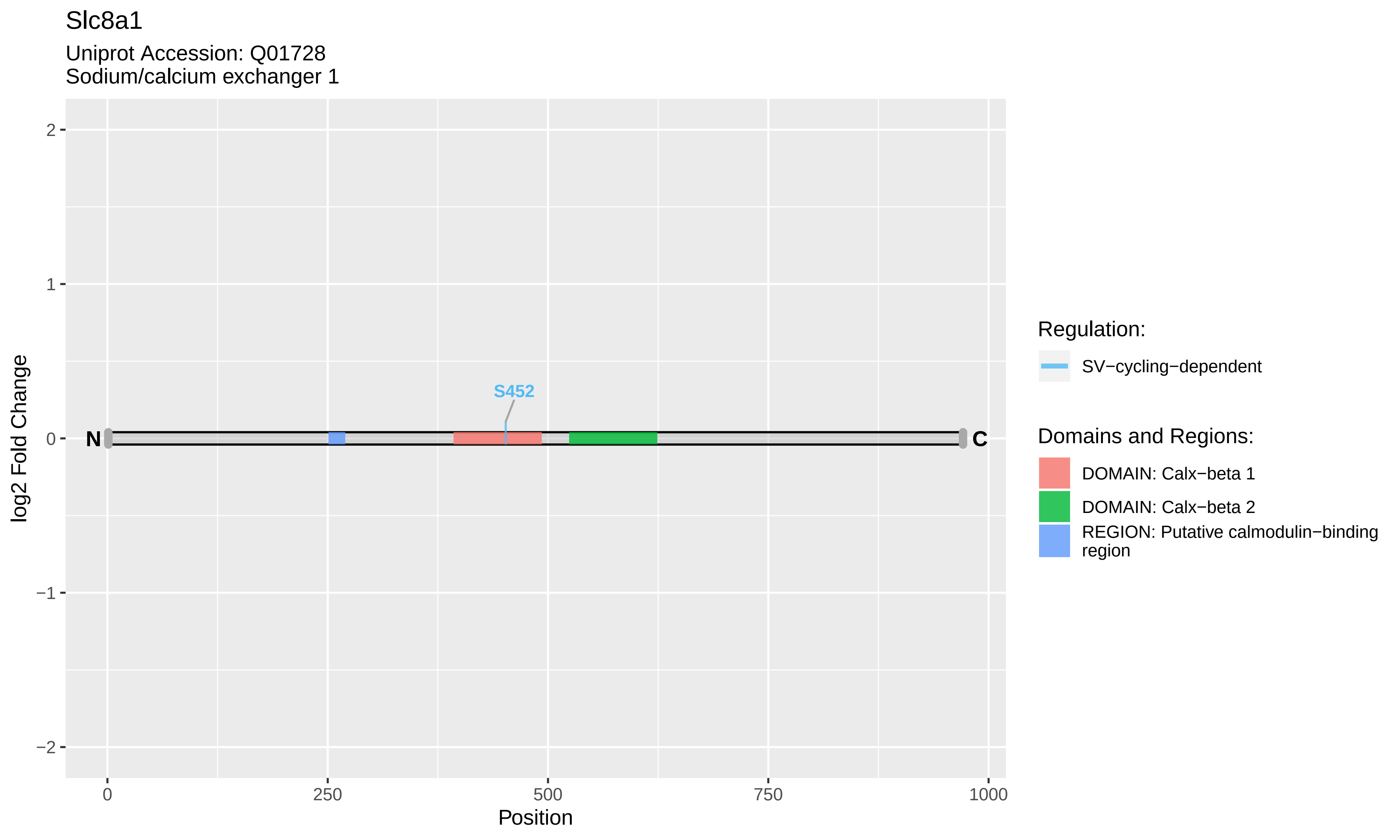

Slc8a1
Uniprot Accession: Q01728
Sodium/calcium exchanger 1
S452
N
C
2
1
0
−1
−2
log2 Fold Change
0
250
500
750
1000
Position
Regulation:
SV−cycling−dependent
Domains and Regions:
DOMAIN: Calx−beta 1
DOMAIN: Calx−beta 2
REGION: Putative calmodulin−binding region

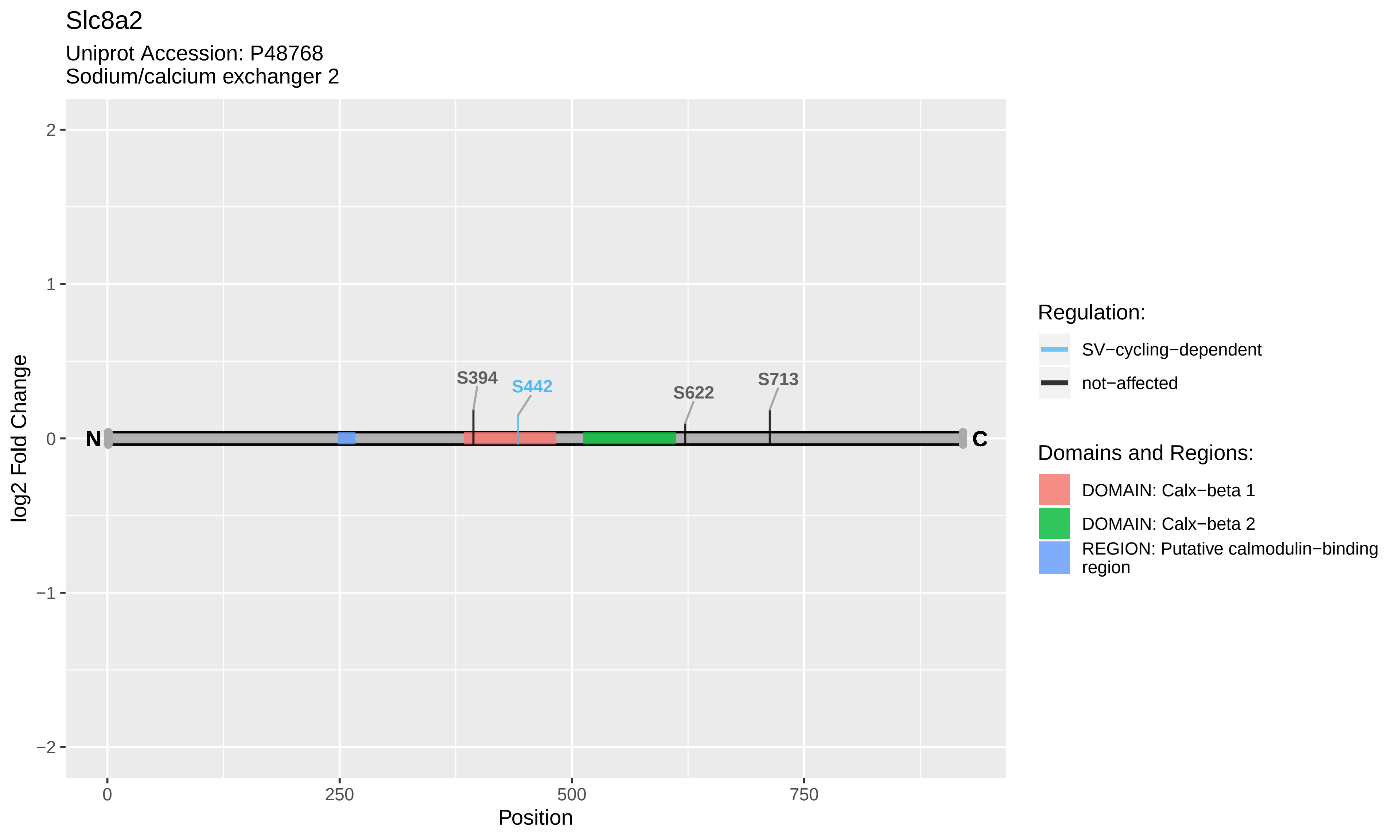

Slc8a2
Uniprot Accession: P48768
Sodium/calcium exchanger 2
log2 Fold Change
2
1
0
−1
−2
N
C
S394
S442
S622
S713
0
250
500
750
Position
Regulation:
SV−cycling−dependent
not−affected
Domains and Regions:
DOMAIN: Calx−beta 1
DOMAIN: Calx−beta 2
REGION: Putative calmodulin−binding region

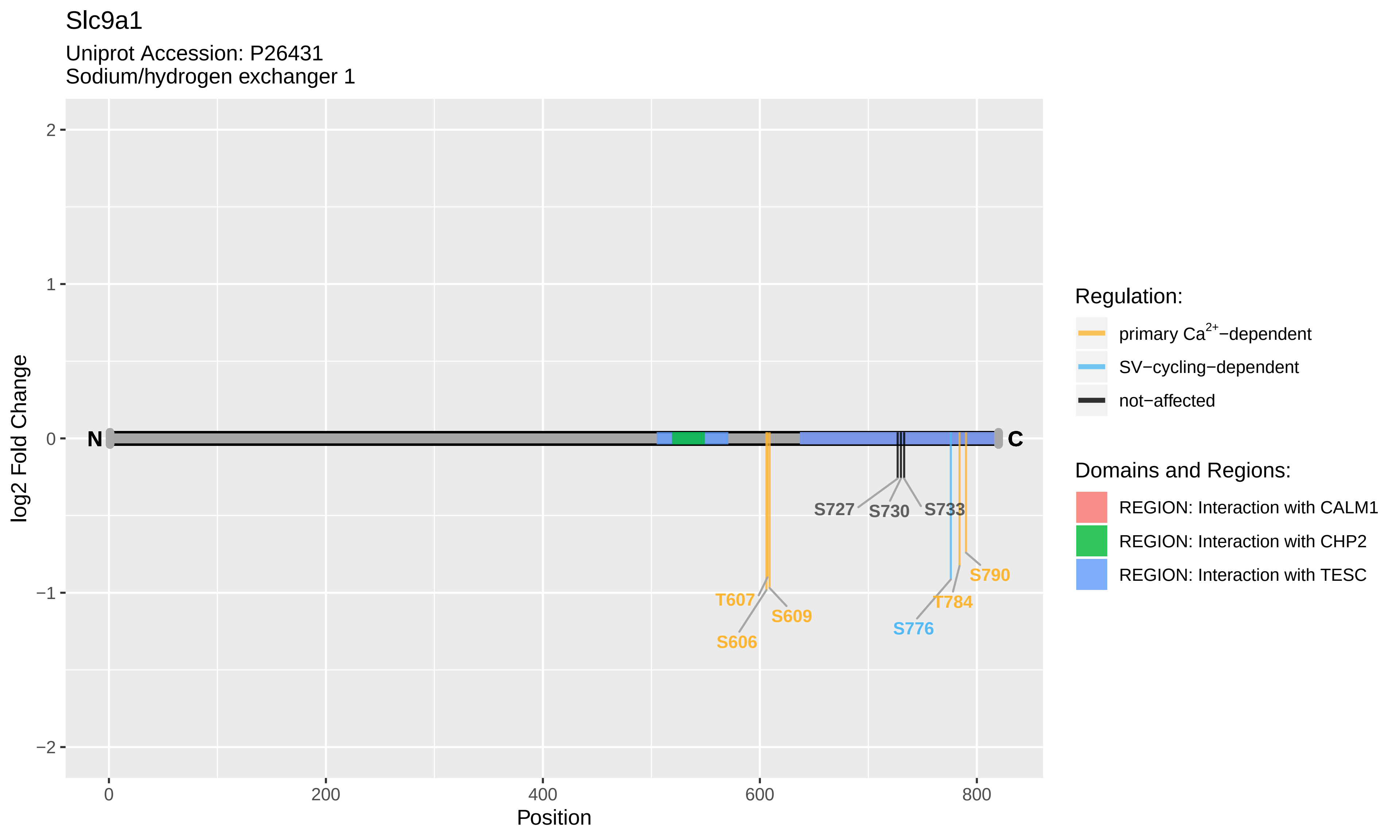

Slc9a1
Uniprot Accession: P26431
Sodium/hydrogen exchanger 1
log2 Fold Change
2
1
0
−1
−2
N
C
S727
S730
S733
S790
T607
S609
T784
S776
S606
0
200
400
600
800
Position
Regulation:
primary Ca2+−dependent
SV−cycling−dependent
not−affected
Domains and Regions:
REGION: Interaction with CALM1
REGION: Interaction with CHP2
REGION: Interaction with TESC

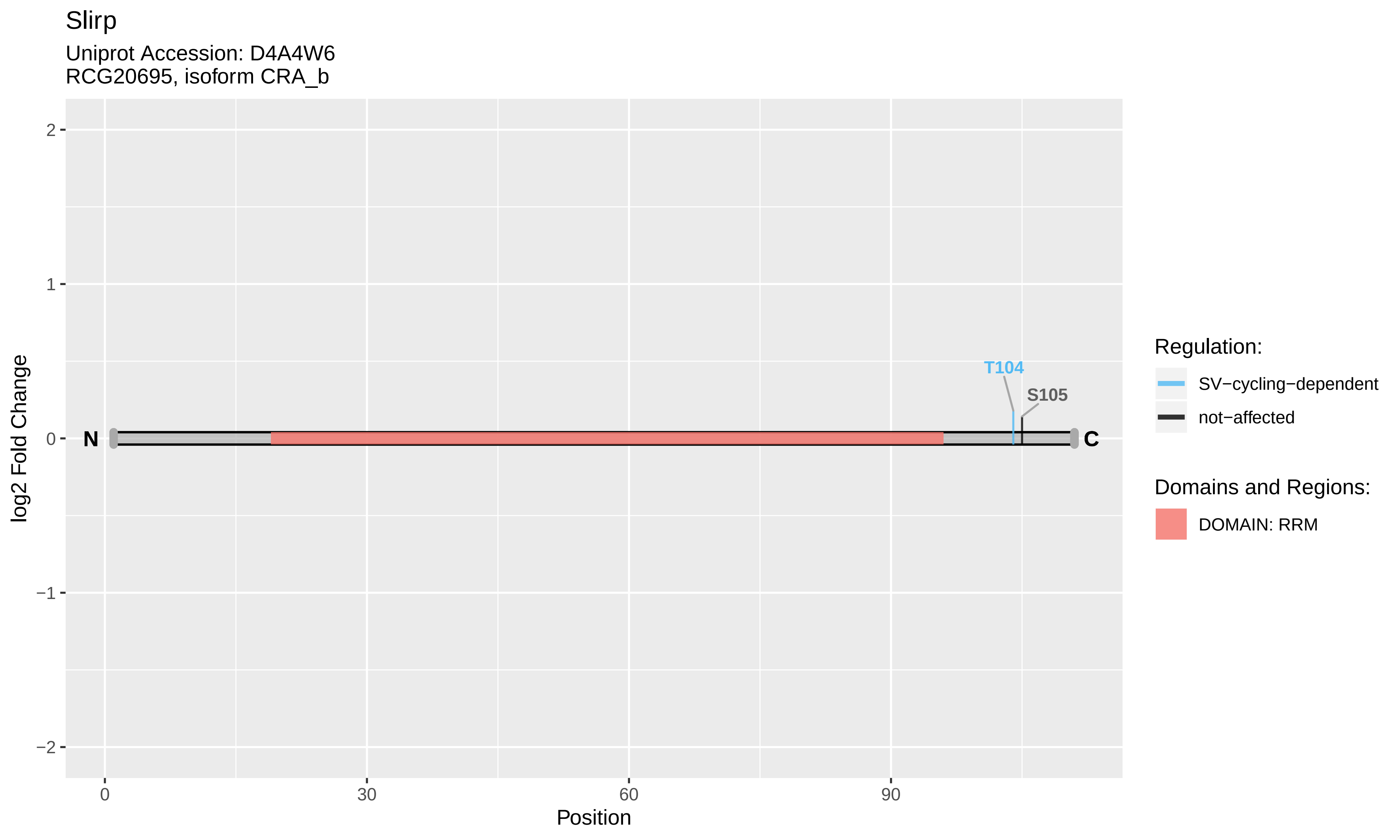

Slirp
Uniprot Accession: D4A4W6
RCG20695, isoform CRA_b
log2 Fold Change
Position
N
C
T104
S105
Regulation:
SV−cycling−dependent
not−affected
Domains and Regions:
DOMAIN: RRM

Slk

Uniprot Accession: G3V7I8
RCG57812, isoform CRA_b

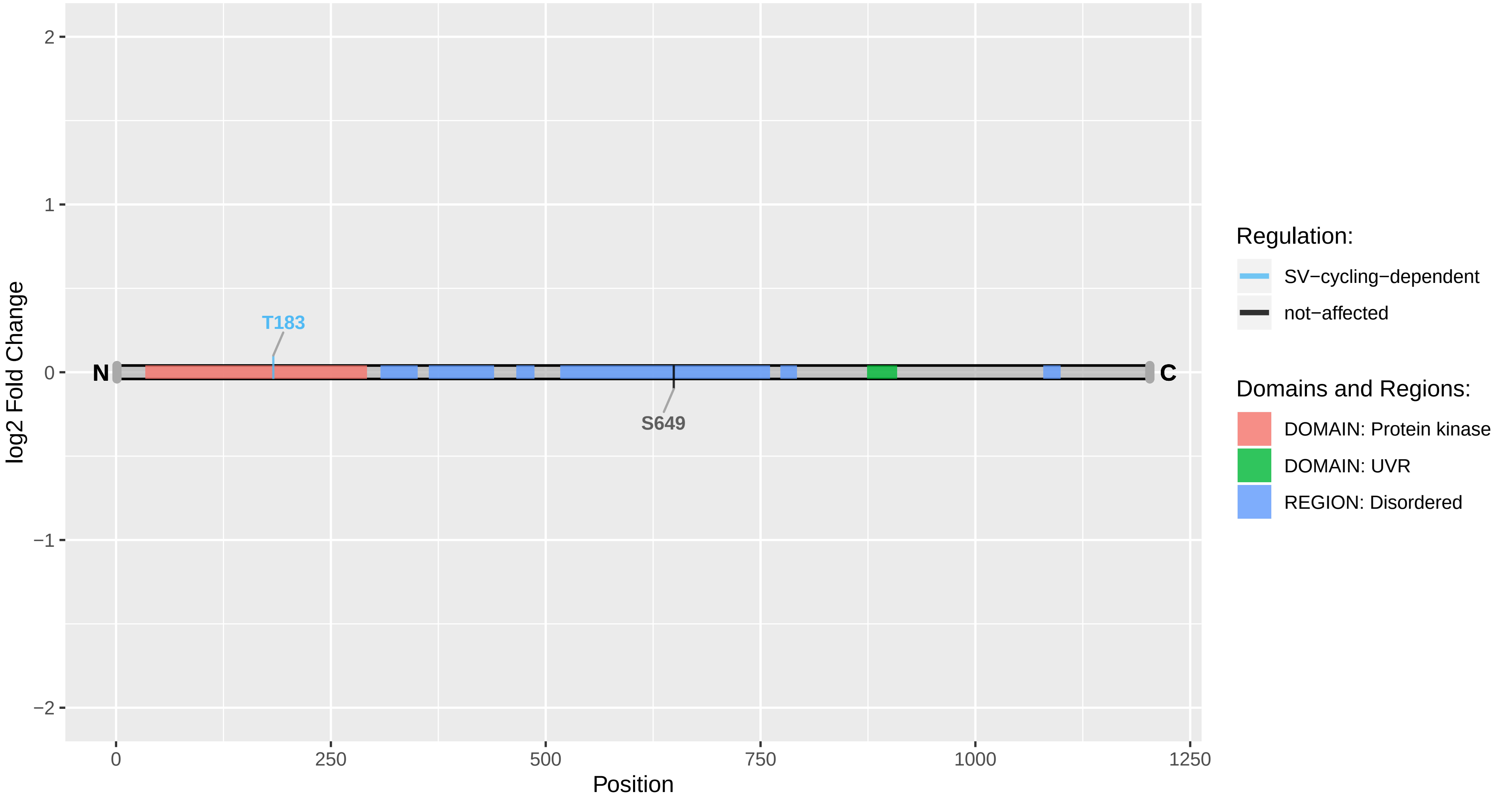

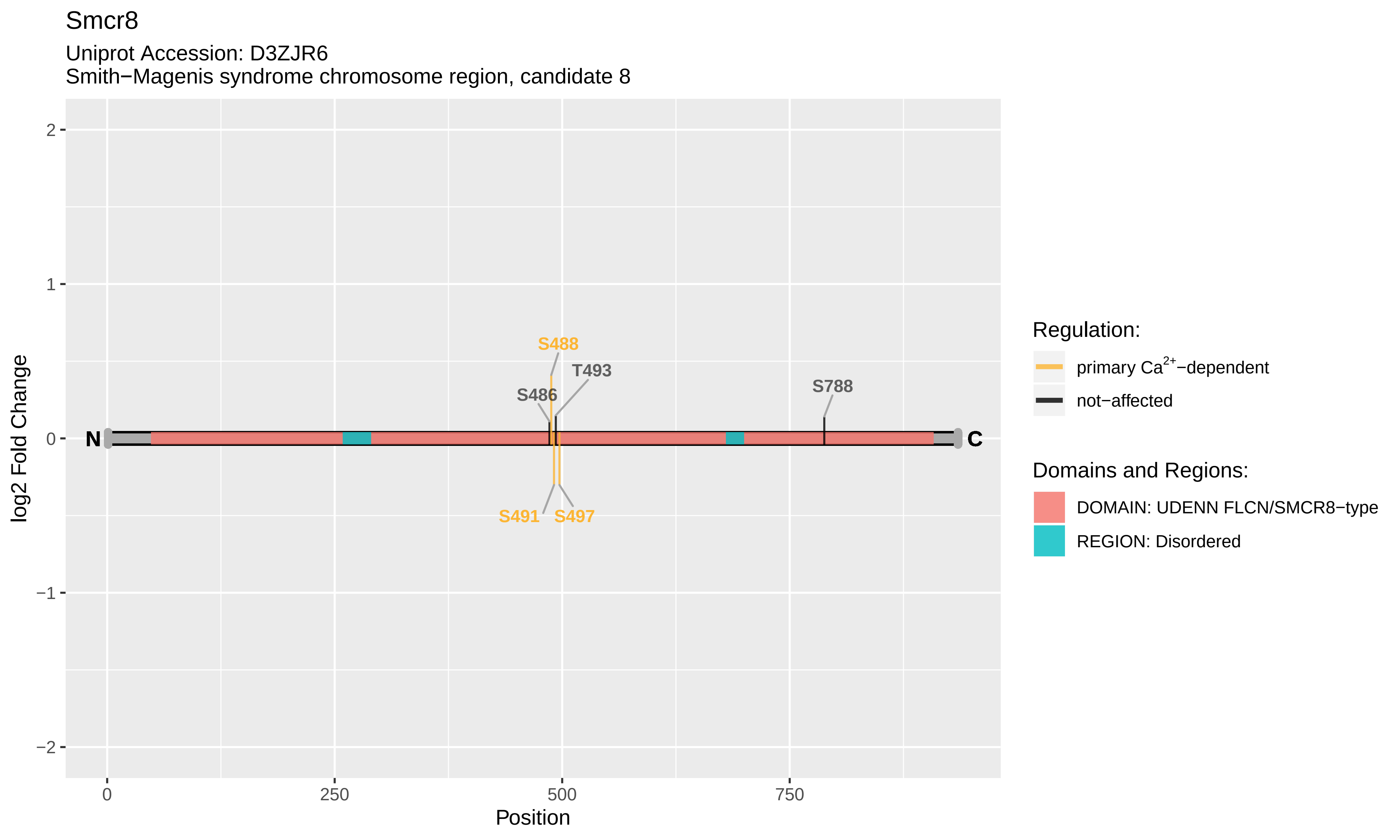

Smcr8
Uniprot Accession: D3ZJR6
Smith−Magenis syndrome chromosome region, candidate 8
S488
T493
S486
S788
N
C
S491
S497
log2 Fold Change
Position
Regulation:
primary Ca2+−dependent
not−affected
Domains and Regions:
DOMAIN: UDENN FLCN/SMCR8−type
REGION: Disordered

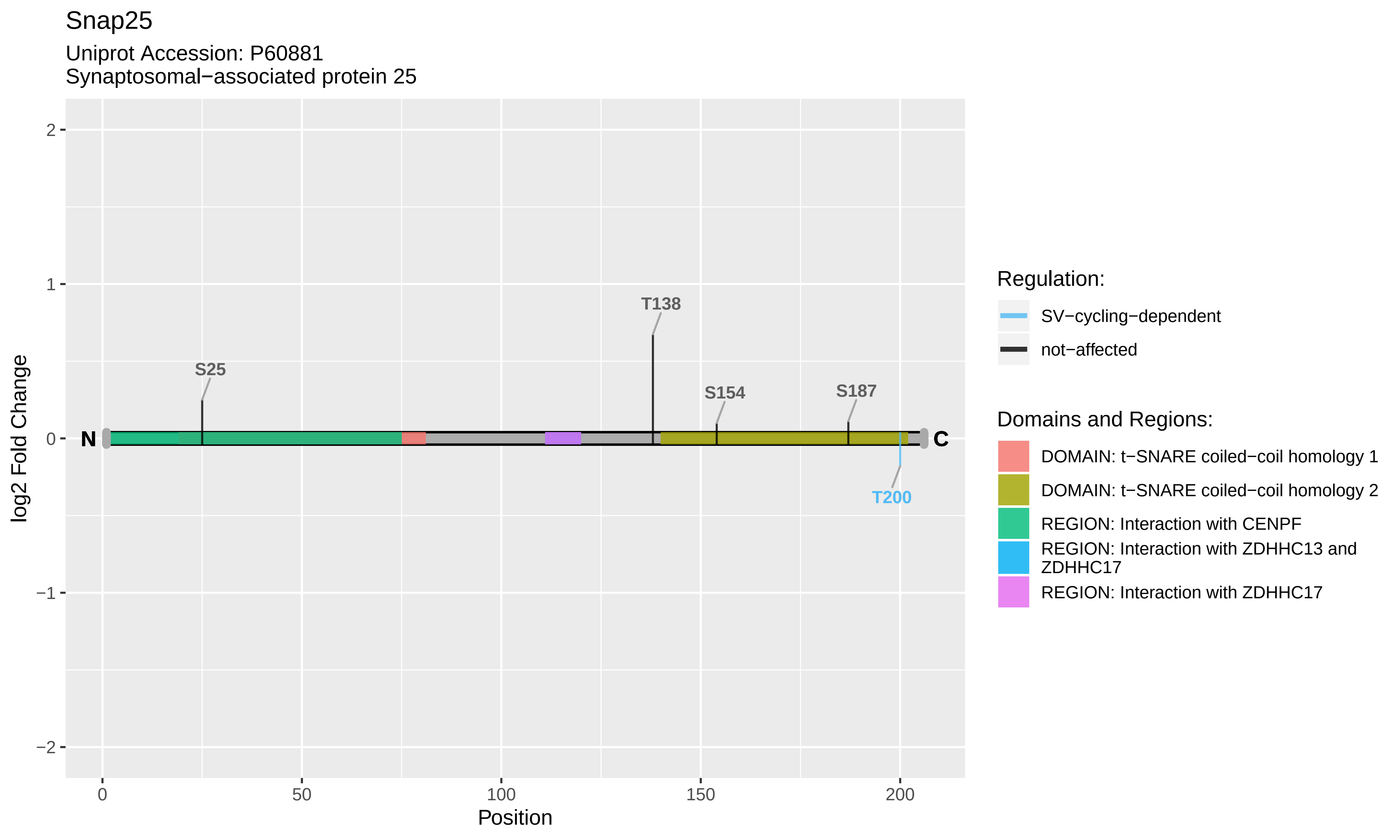
Snap25
Uniprot Accession: P60881
Synaptosomal−associated protein 25
2
1
0
−1
−2
log2 Fold Change
N
C
S25
T138
S154
S187
T200
0
50
100
150
200
Position
Regulation:
SV−cycling−dependent
not−affected
Domains and Regions:
DOMAIN: t−SNARE coiled−coil homology 1
DOMAIN: t−SNARE coiled−coil homology 2
REGION: Interaction with CENPF
REGION: Interaction with ZDHHC13 and ZDHHC17
REGION: Interaction with ZDHHC17

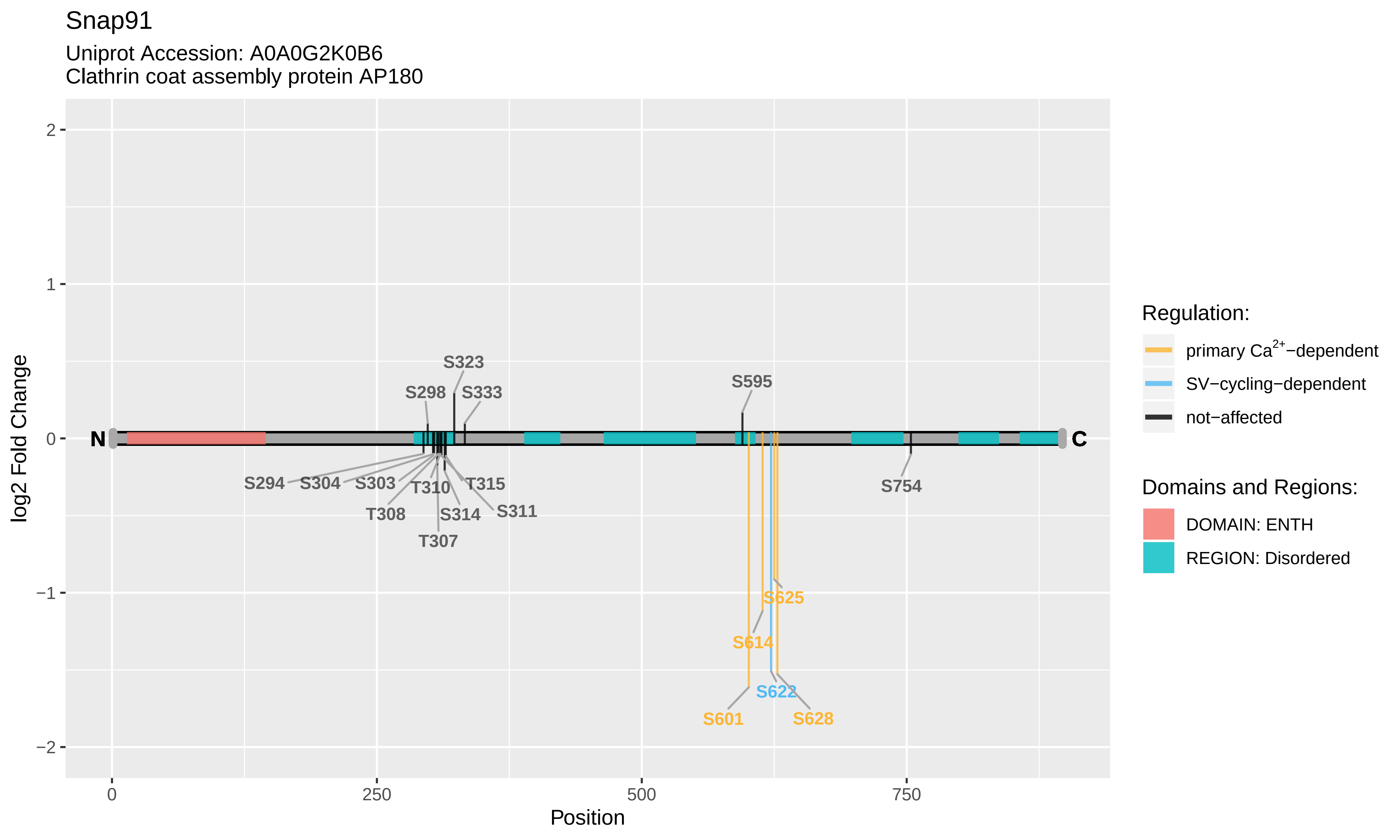

Snap91
Uniprot Accession: A0A0G2K0B6
Clathrin coat assembly protein AP180
2
1
0
−1
−2
log2 Fold Change
N
C
S323
S298
S333
S595
S294
S304
S303
T310
T315
T308
S314
S311
T307
S754
S625
S614
S622
S601
S628
0
250
500
750
Position
Regulation:
primary Ca2+−dependent
SV−cycling−dependent
not−affected
Domains and Regions:
DOMAIN: ENTH
REGION: Disordered

Snph

Uniprot Accession: B5DF41
Syntaphilin

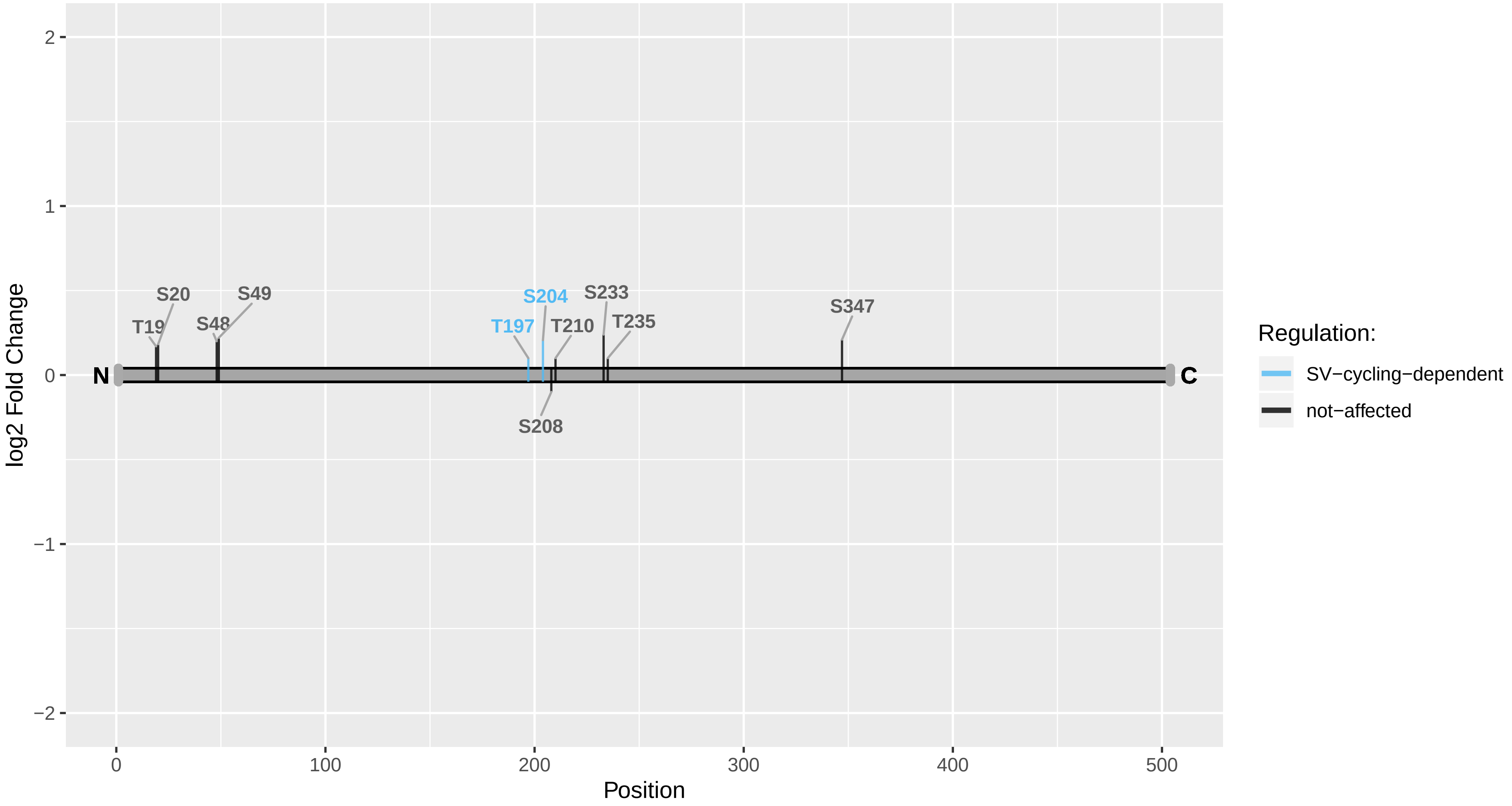

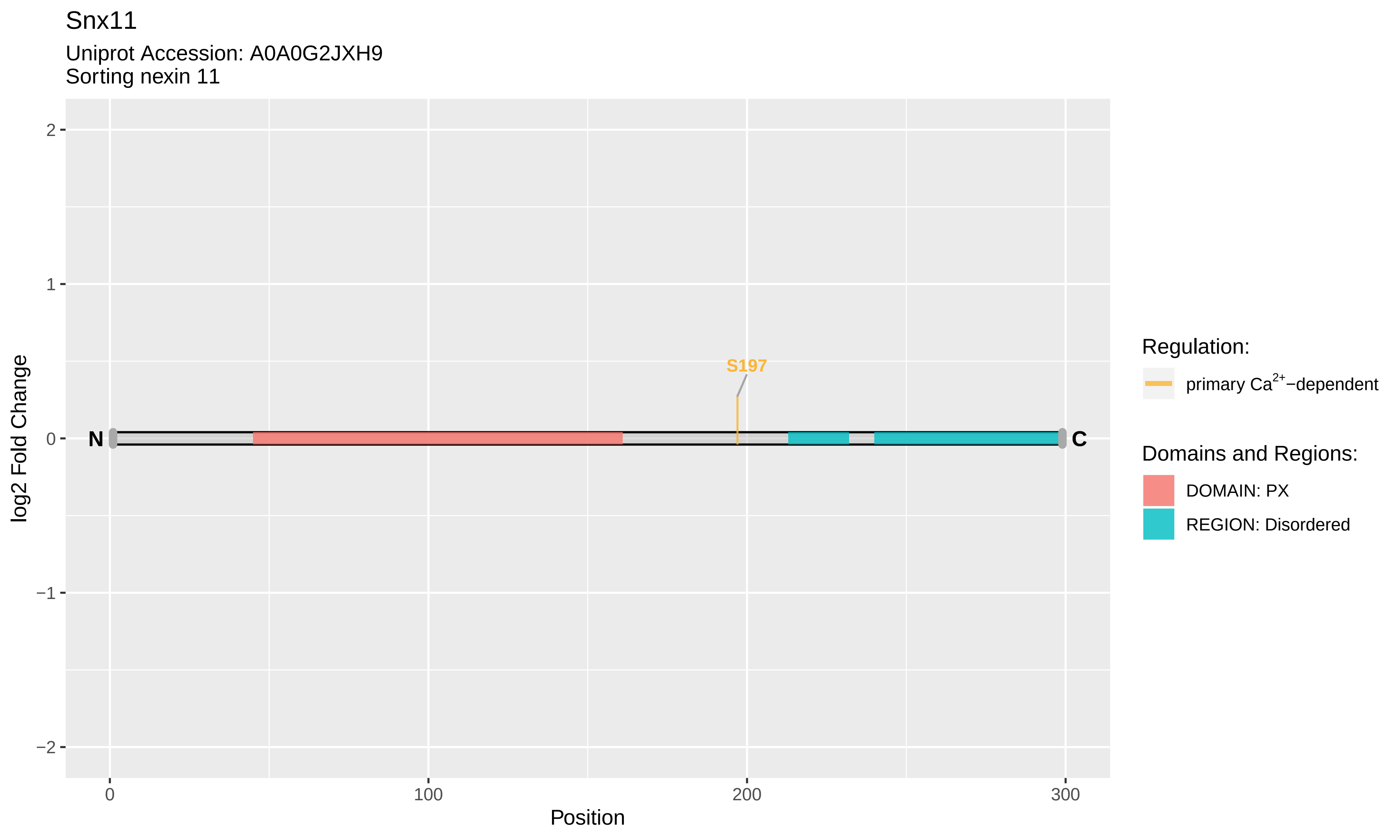

Snx11
Uniprot Accession: A0A0G2JXH9
Sorting nexin 11
log2 Fold Change
Position
S197
N
C
Regulation:
primary Ca2+−dependent
Domains and Regions:
DOMAIN: PX
REGION: Disordered

Snx16

Uniprot Accession: P57769
Sorting nexin−16

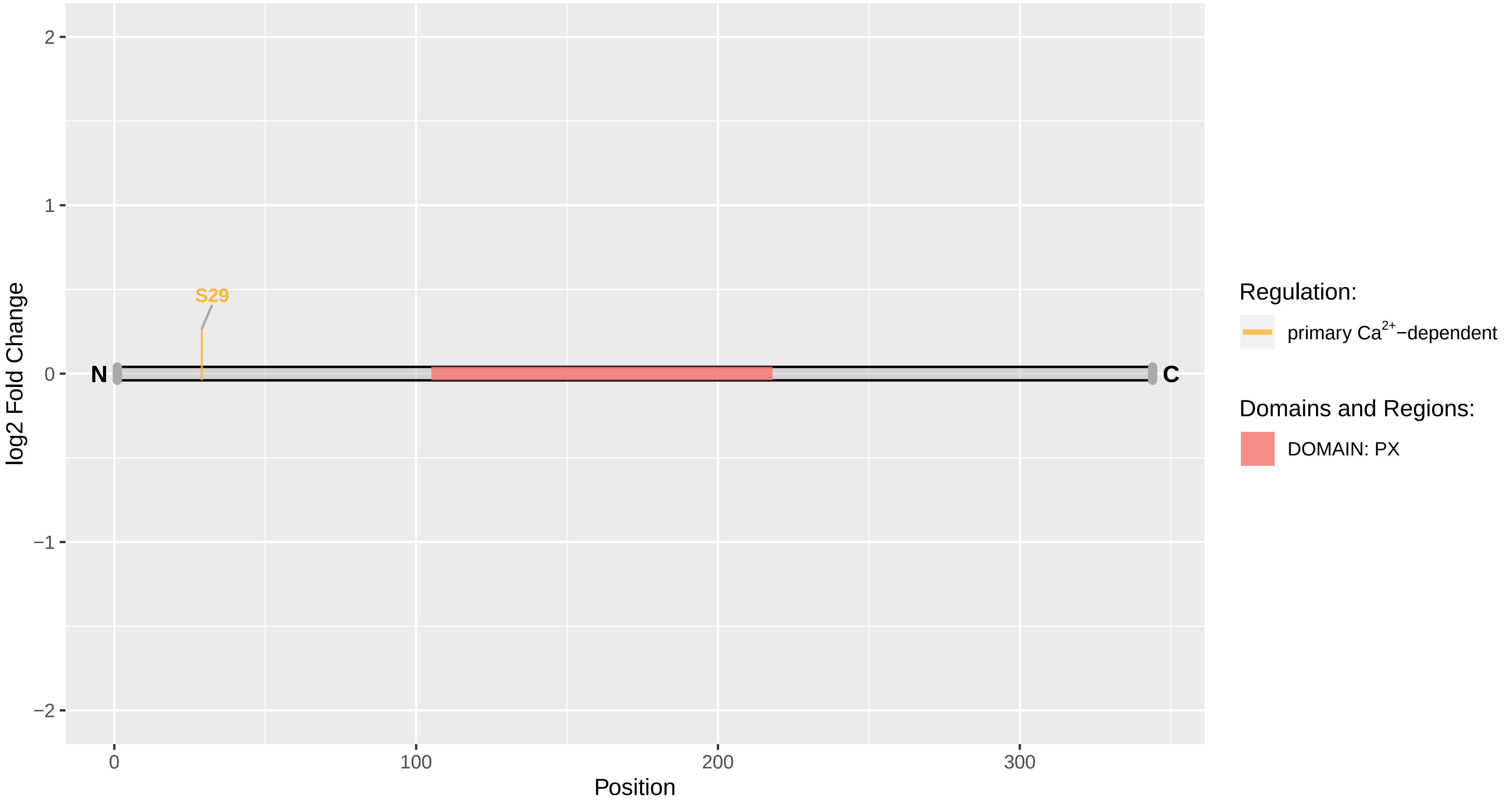

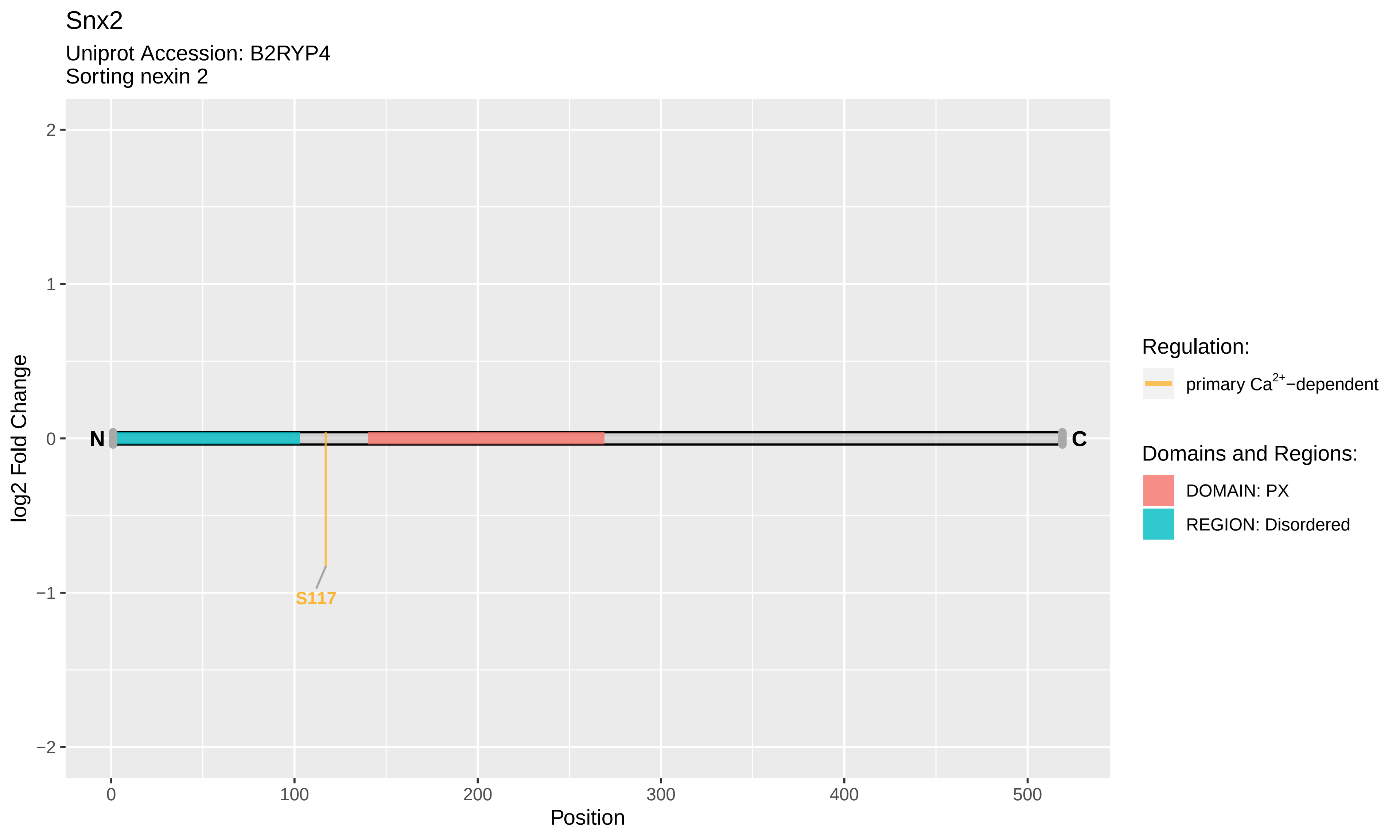

Snx2
Uniprot Accession: B2RYP4
Sorting nexin 2
log2 Fold Change
2
1
0
−1
−2
N
C
S117
0
100
200
300
400
500
Position
Regulation:
primary Ca2+−dependent
Domains and Regions:
DOMAIN: PX
REGION: Disordered

Soga3

Uniprot Accession: D4A0A1
SOGA family member 3

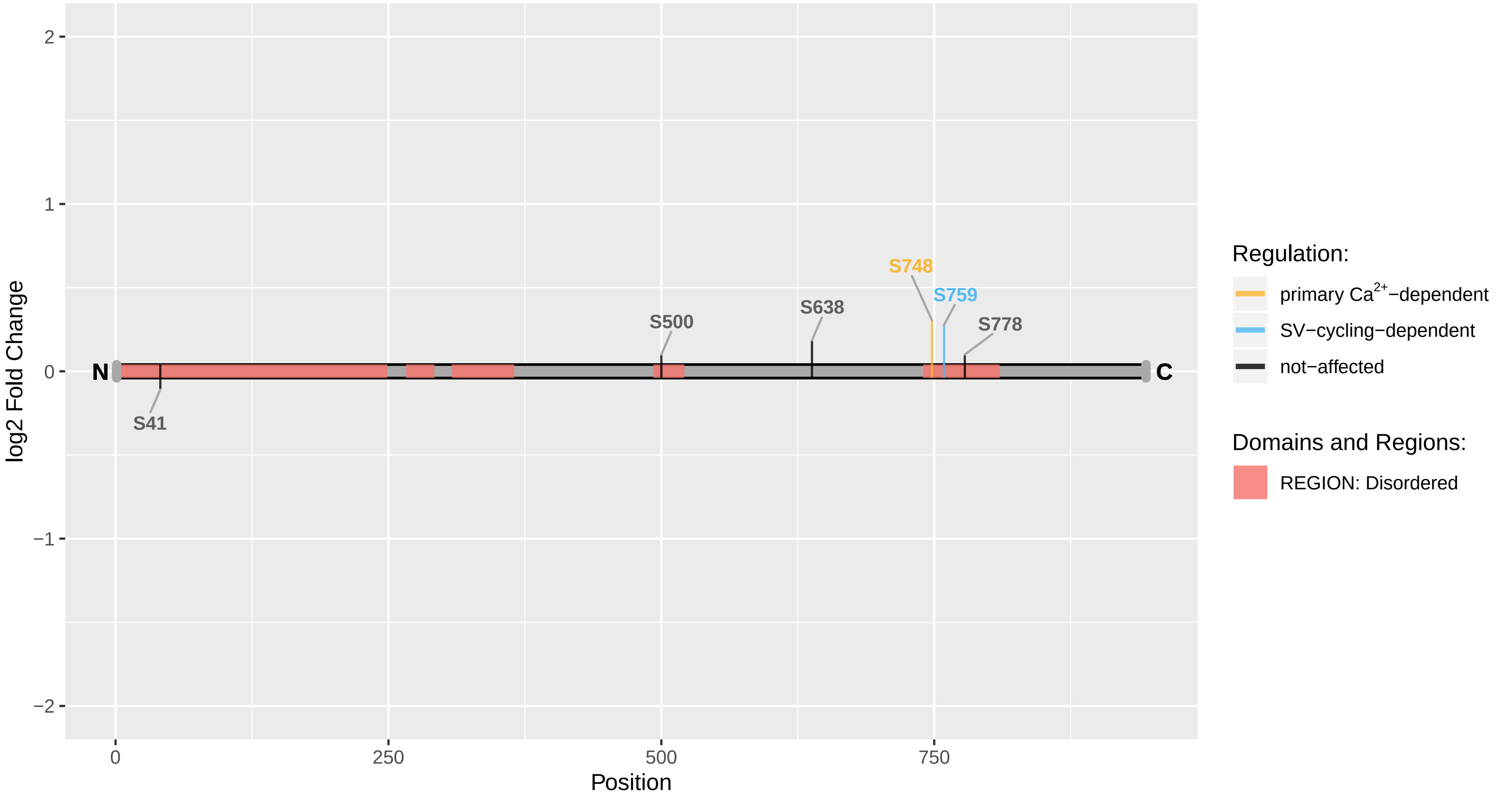

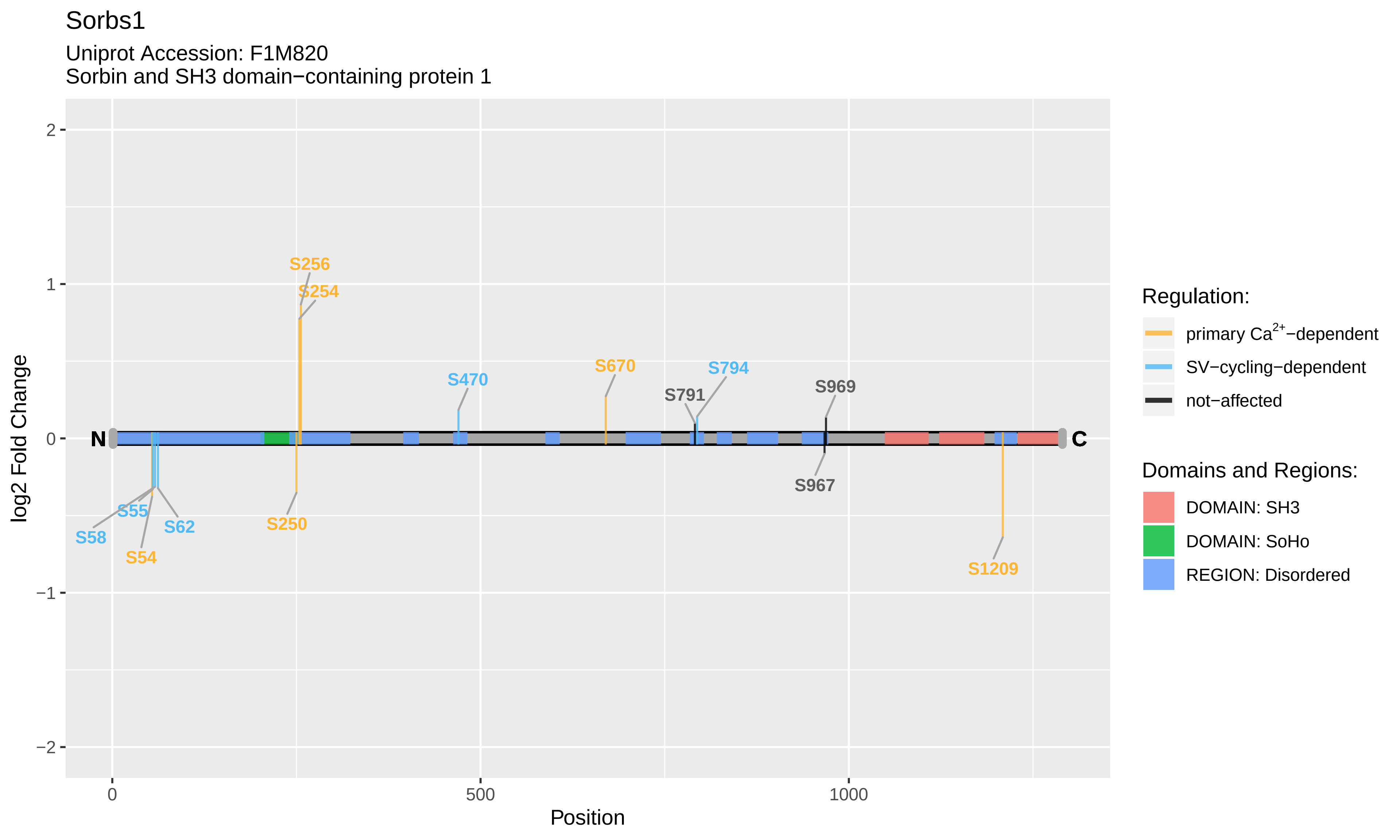
Sorbs1
Uniprot Accession: F1M820
Sorbin and SH3 domain−containing protein 1
log2 Fold Change
Position
2
1
0
−1
−2
0
500
1000
N
C
S256
S254
S470
S670
S794
S791
S969
S967
S55
S58
S62
S54
S250
S1209
Regulation:
primary Ca2+−dependent
SV−cycling−dependent
not−affected
Domains and Regions:
DOMAIN: SH3
DOMAIN: SoHo
REGION: Disordered

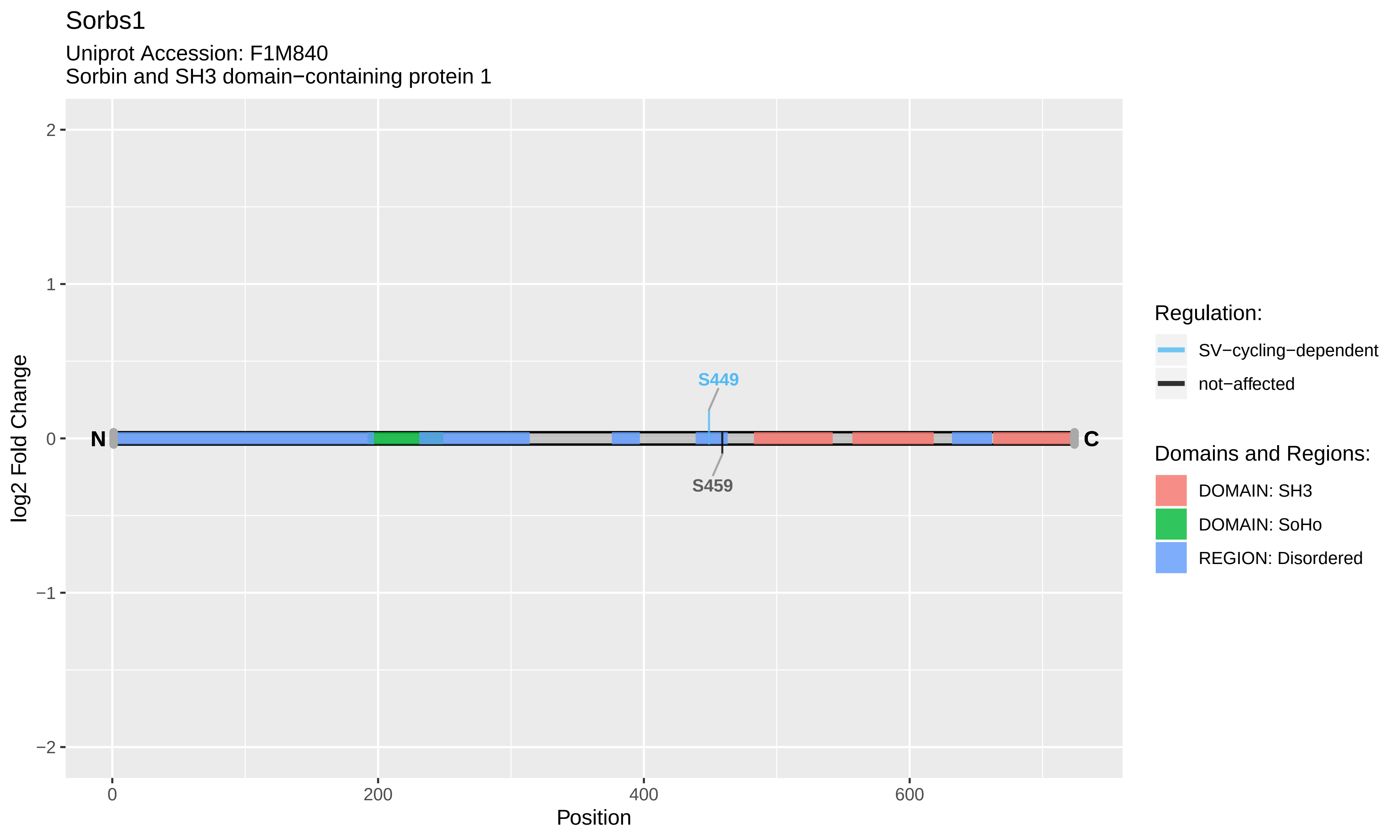

Sorbs1
Uniprot Accession: F1M840
Sorbin and SH3 domain−containing protein 1
S449
S459
N
C
log2 Fold Change
2
1
0
−1
−2
0
200
400
600
Position
Regulation:
SV−cycling−dependent
not−affected
Domains and Regions:
DOMAIN: SH3
DOMAIN: SoHo
REGION: Disordered

Sorbs2

Uniprot Accession: F1LPM3
Sorbin and SH3 domain−containing protein 2

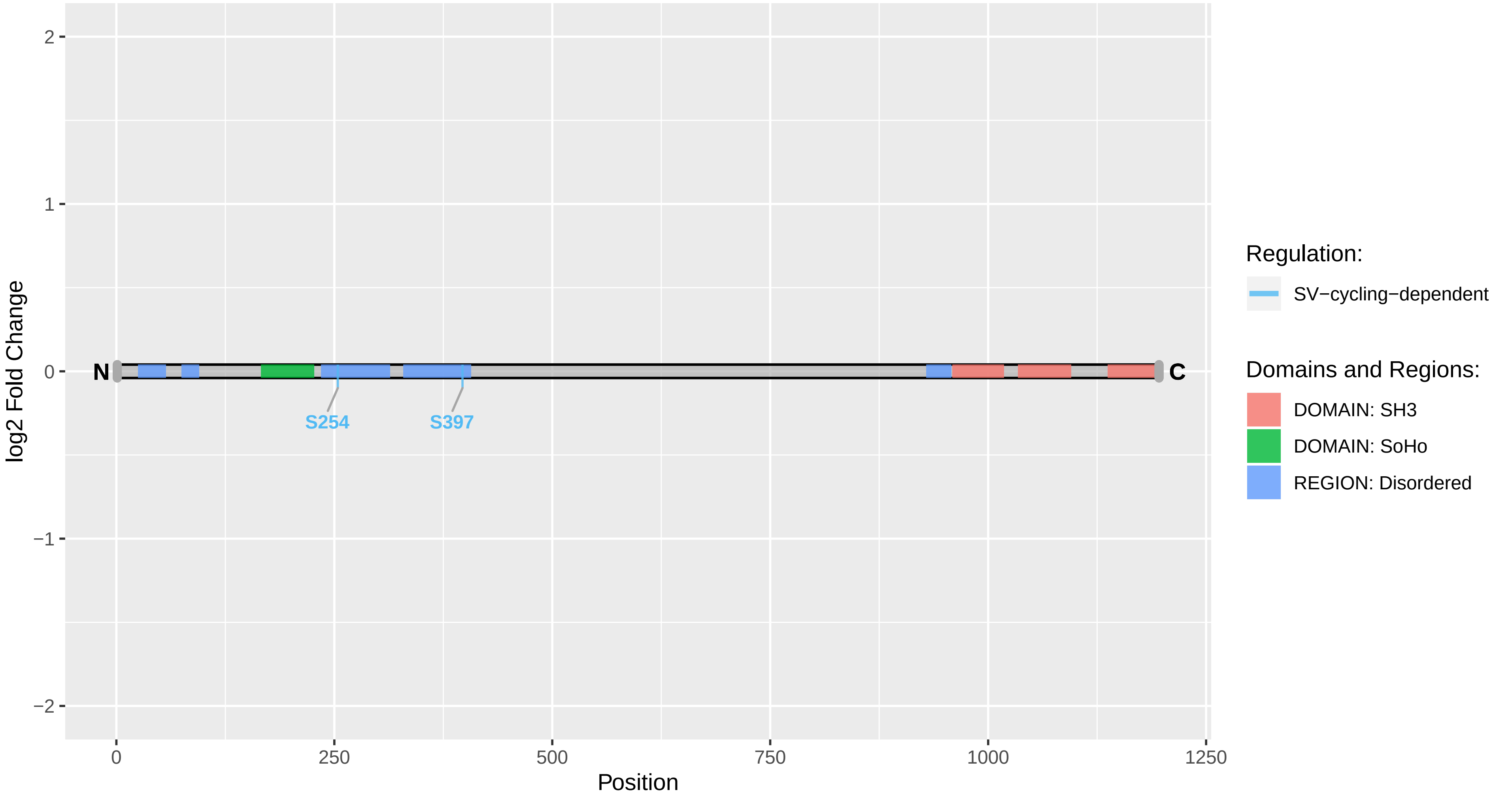

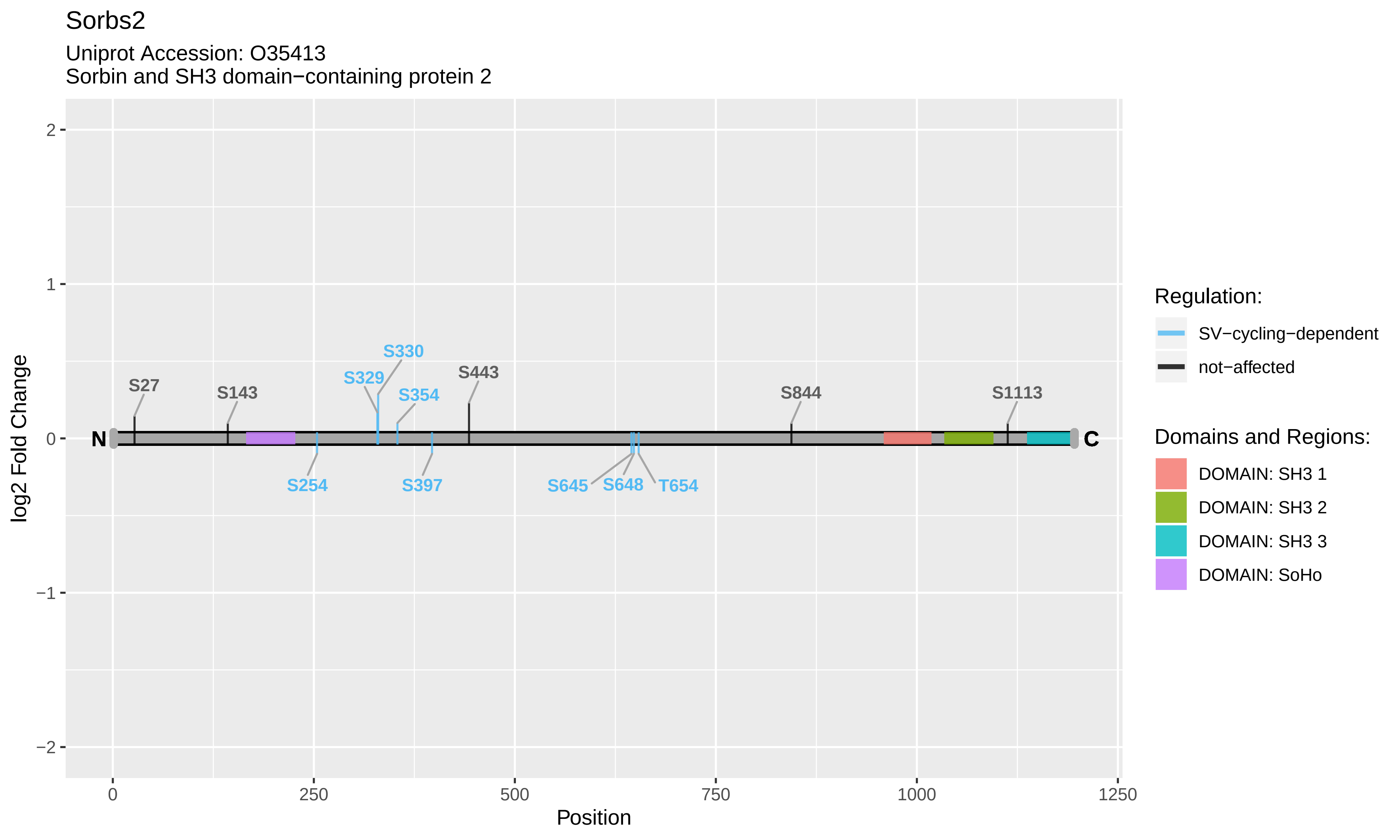
Sorbs2
Uniprot Accession: O35413
Sorbin and SH3 domain−containing protein 2
log2 Fold Change
2
1
0
−1
−2
N
C
S27
S143
S329
S330
S354
S443
S844
S1113
S254
S397
S645
S648
T654
0
250
500
750
1000
1250
Position
Regulation:
SV−cycling−dependent
not−affected
Domains and Regions:
DOMAIN: SH3 1
DOMAIN: SH3 2
DOMAIN: SH3 3
DOMAIN: SoHo

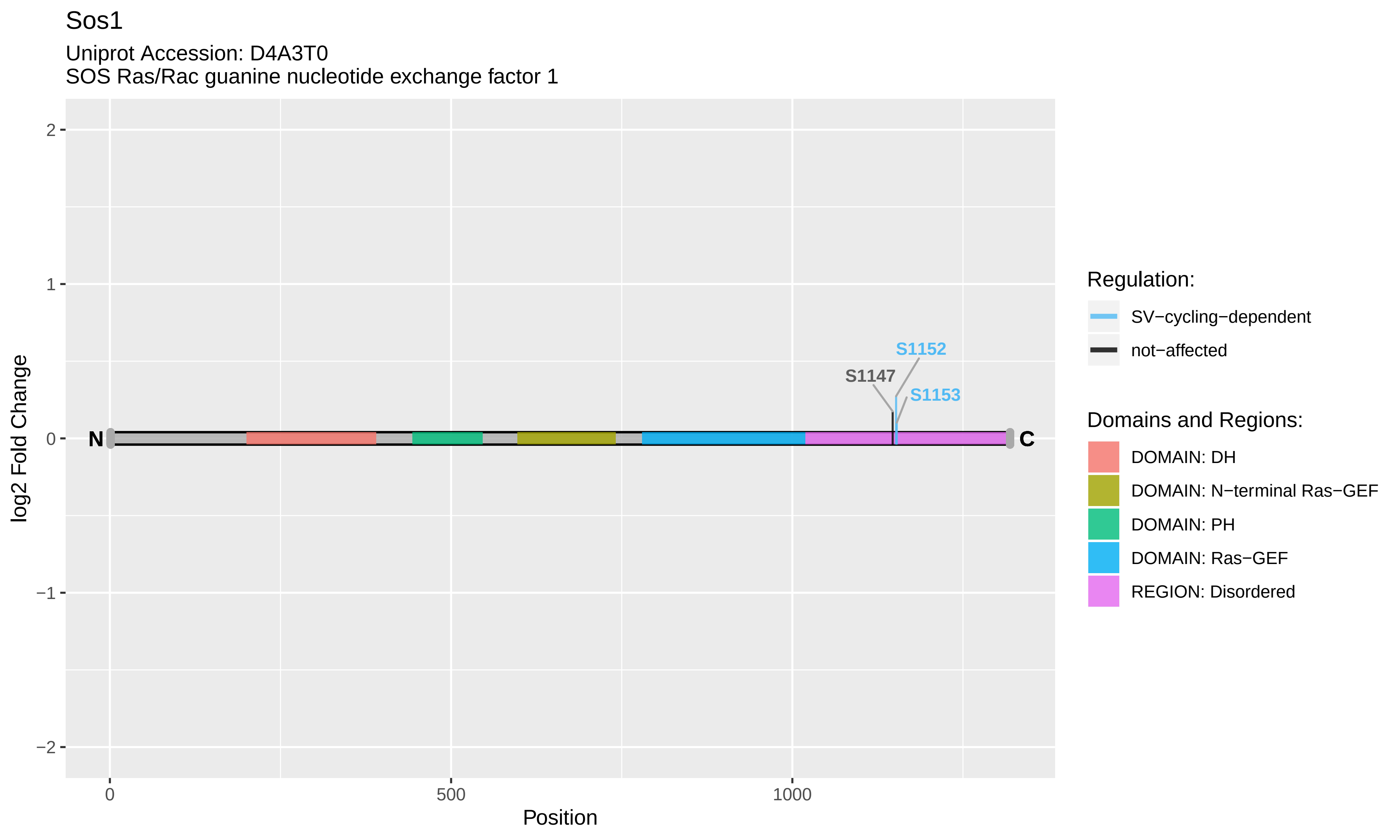
Sos1
Uniprot Accession: D4A3T0
SOS Ras/Rac guanine nucleotide exchange factor 1
log2 Fold Change
2
1
0
−1
−2
0
500
1000
Position
N
C
S1152
S1147
S1153
Regulation:
SV−cycling−dependent
not−affected
Domains and Regions:
DOMAIN: DH
DOMAIN: N−terminal Ras−GEF
DOMAIN: PH
DOMAIN: Ras−GEF
REGION: Disordered

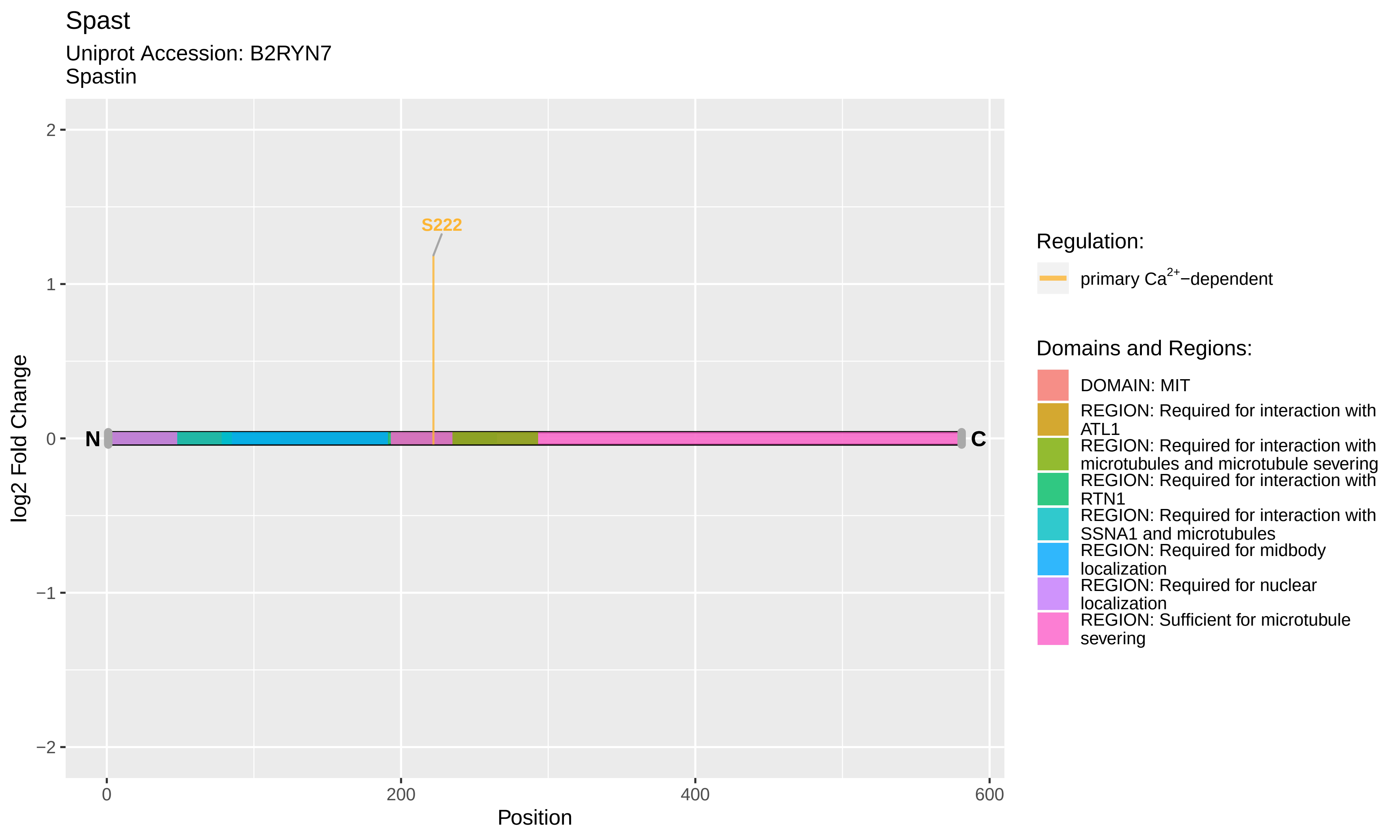

Spast
Uniprot Accession: B2RYN7
Spastin
S222
N
C
log2 Fold Change
2
1
0
−1
−2
0
200
400
600
Position
Regulation:
primary Ca^{2+}−dependent
Domains and Regions:
DOMAIN: MIT
REGION: Required for interaction with ATL1
REGION: Required for interaction with microtubules and microtubule severing
REGION: Required for interaction with RTN1
REGION: Required for interaction with SSNA1 and microtubules
REGION: Required for midbody localization
REGION: Required for nuclear localization
REGION: Sufficient for microtubule severing

Specc1

Uniprot Accession: A0A0G2K5D7
Sperm antigen with calponin homology and coiled−coil domains 1

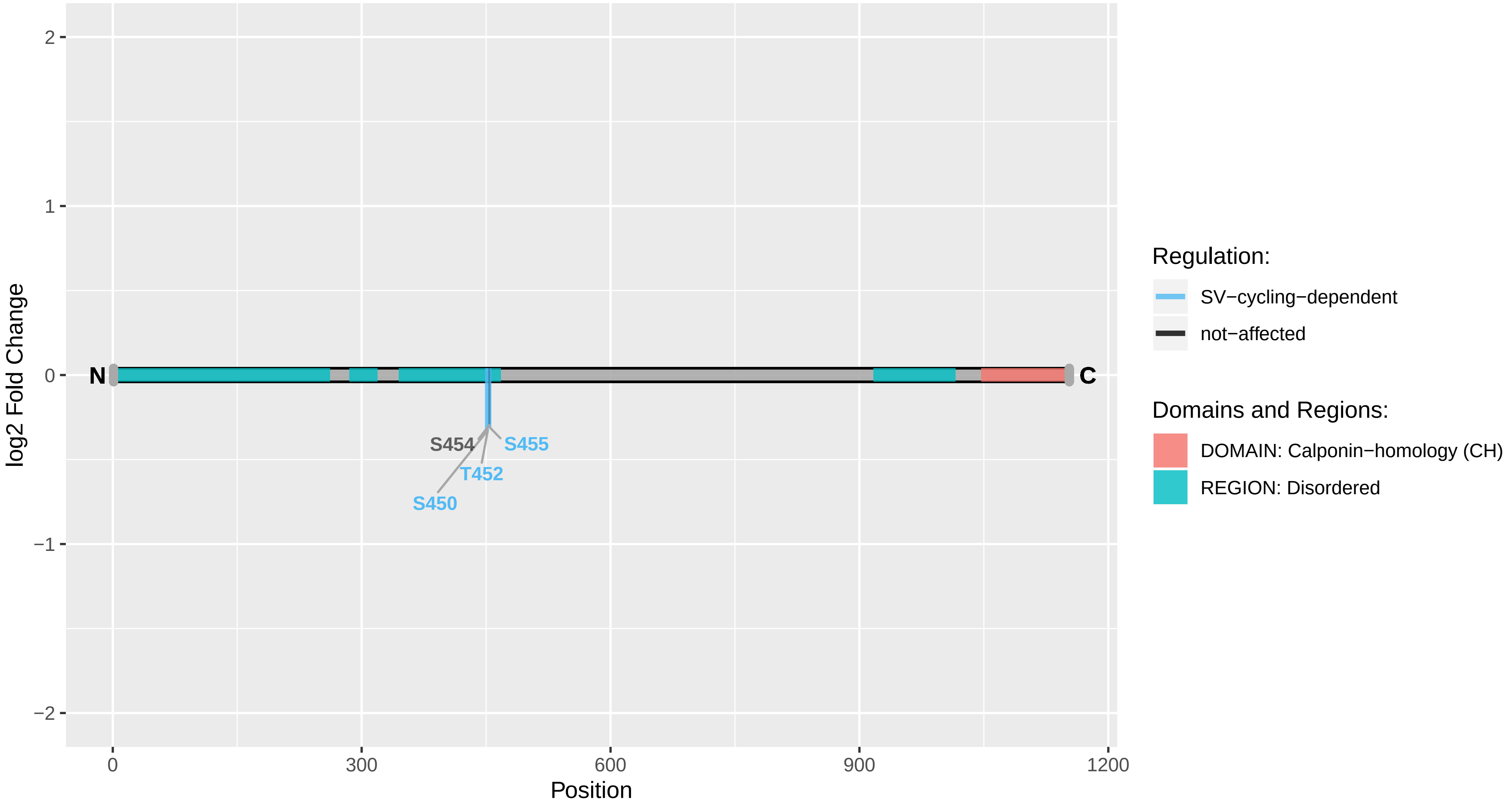

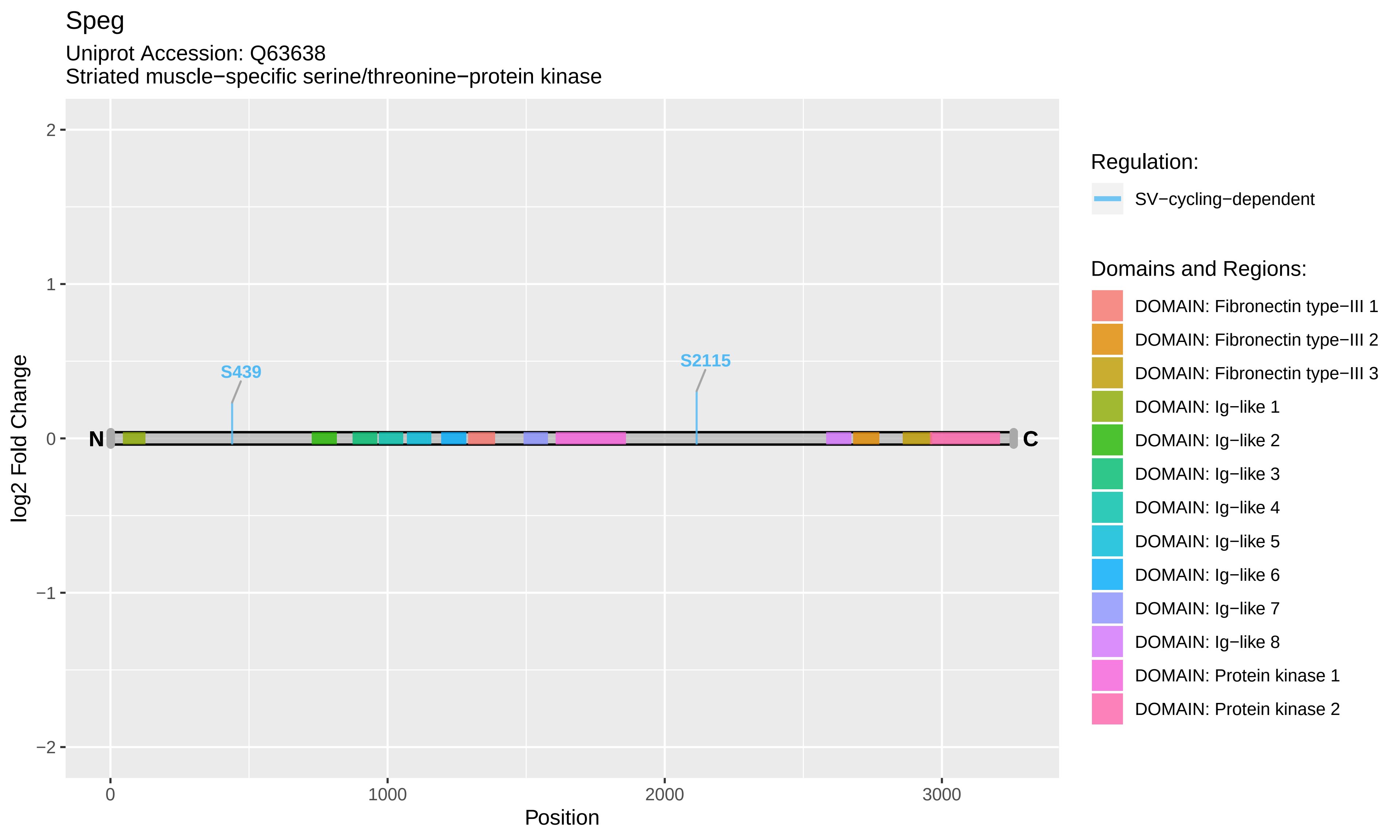
Speg
Uniprot Accession: Q63638
Striated muscle−specific serine/threonine−protein kinase
S439
S2115
N
C
log2 Fold Change
2
1
0
−1
−2
0
1000
2000
3000
Position
Regulation:
SV−cycling−dependent
Domains and Regions:
DOMAIN: Fibronectin type−III 1
DOMAIN: Fibronectin type−III 2
DOMAIN: Fibronectin type−III 3
DOMAIN: Ig−like 1
DOMAIN: Ig−like 2
DOMAIN: Ig−like 3
DOMAIN: Ig−like 4
DOMAIN: Ig−like 5
DOMAIN: Ig−like 6
DOMAIN: Ig−like 7
DOMAIN: Ig−like 8
DOMAIN: Protein kinase 1
DOMAIN: Protein kinase 2

Spire1

Uniprot Accession: D3ZEX7
Spire homolog 1 (Drosophila) (Predicted), isoform CRA_b

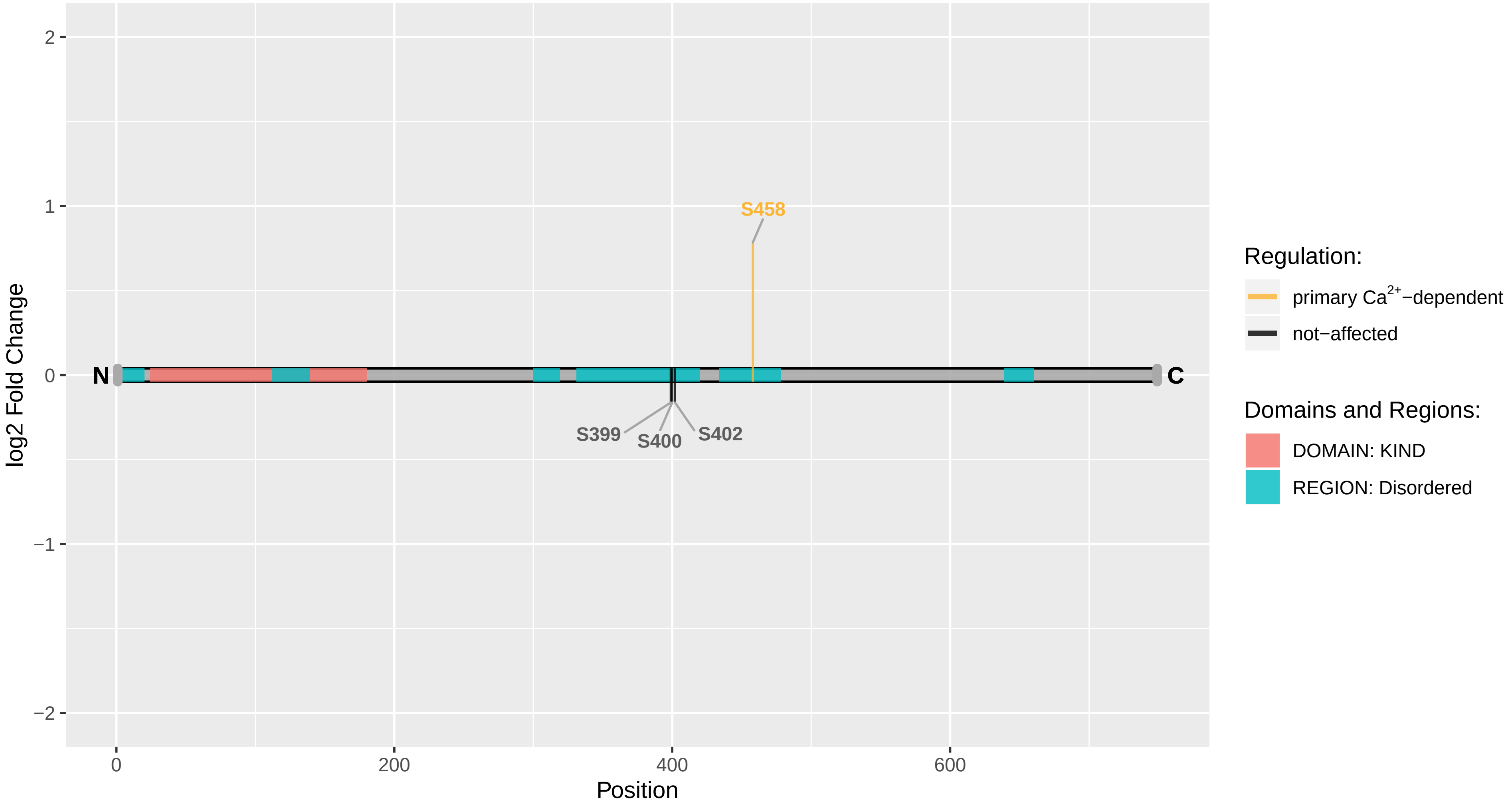

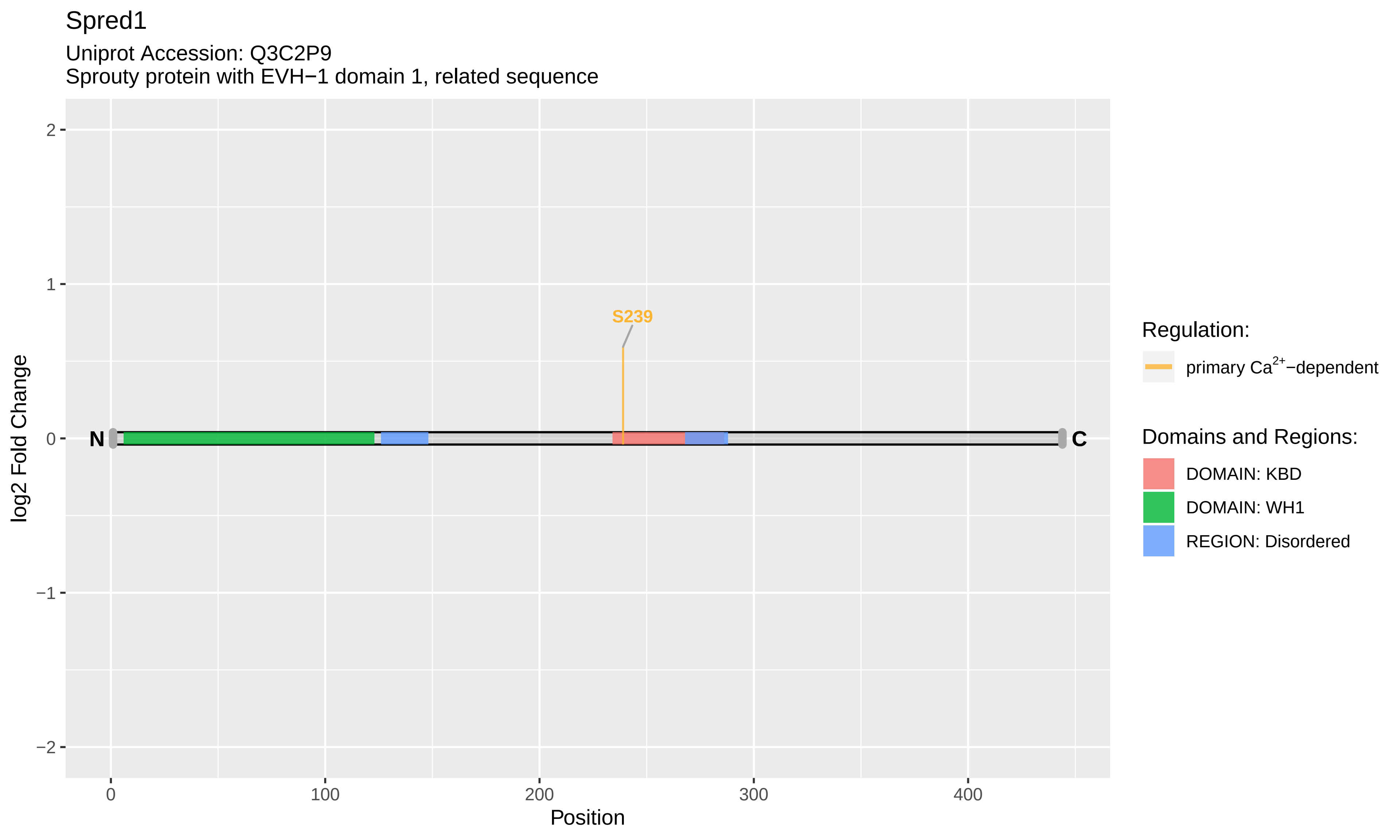

Spred1
Uniprot Accession: Q3C2P9
Sprouty protein with EVH−1 domain 1, related sequence
S239
N
C
2
1
0
−1
−2
log2 Fold Change
0
100
200
300
400
Position
Regulation:
primary Ca2+−dependent
Domains and Regions:
DOMAIN: KBD
DOMAIN: WH1
REGION: Disordered

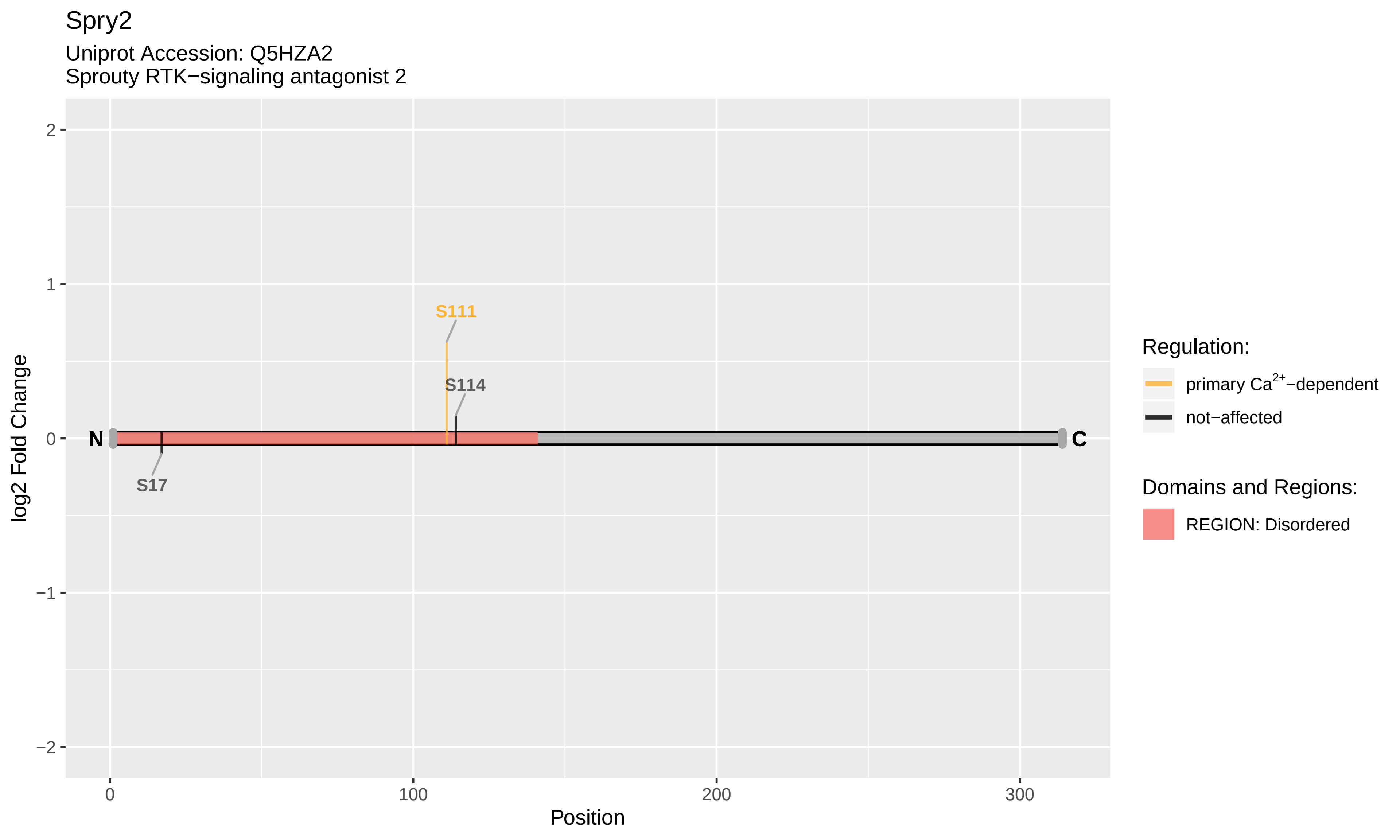

Spry2
Uniprot Accession: Q5HZA2
Sprouty RTK−signaling antagonist 2
log2 Fold Change
2
1
0
−1
−2
S111
S114
S17
N
C
0
100
200
300
Position
Regulation:
primary Ca2+−dependent
not−affected
Domains and Regions:
REGION: Disordered

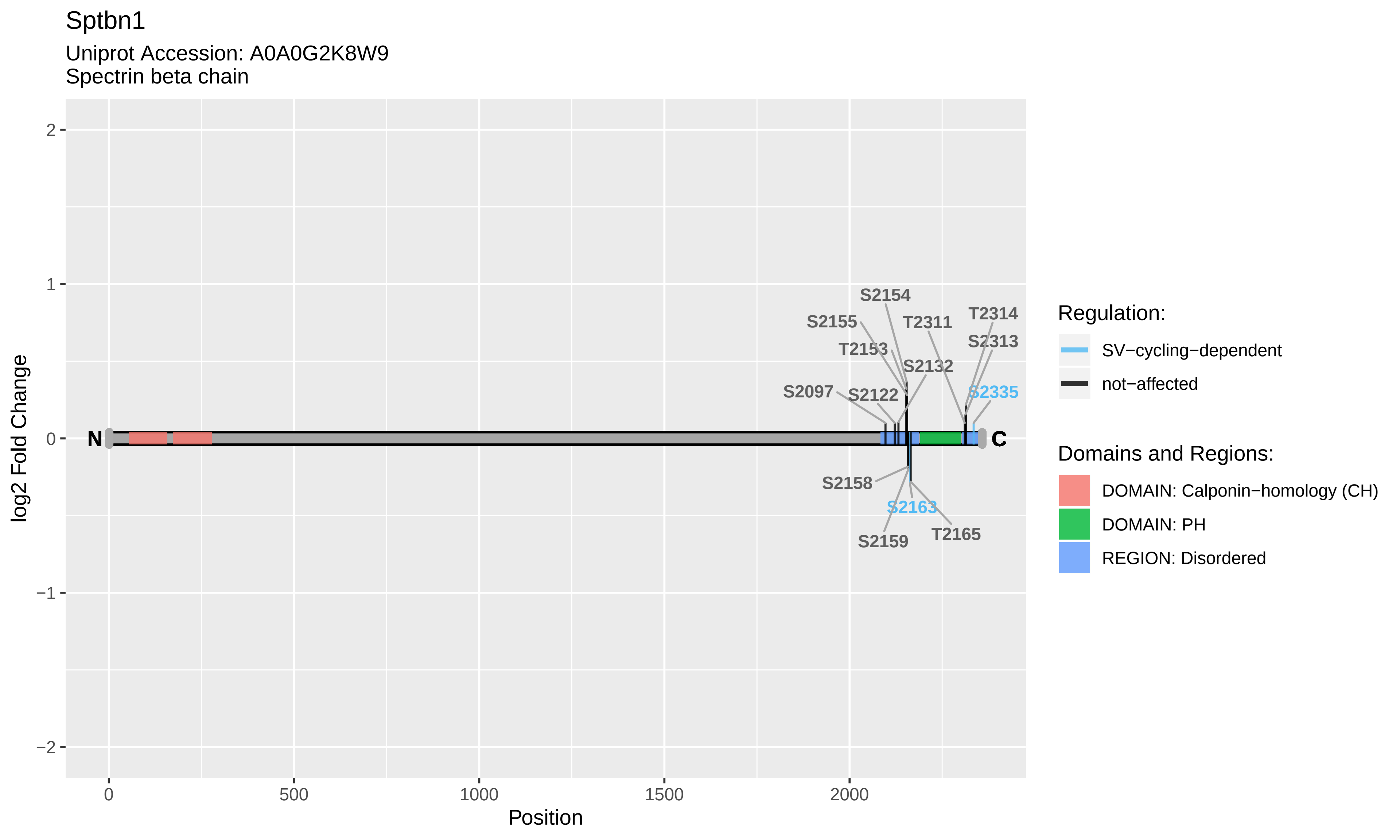
Sptbn1
Uniprot Accession: A0A0G2K8W9
Spectrin beta chain
log2 Fold Change
Position
N
C
S2154
S2155
T2153
S2132
S2097
S2122
T2311
T2314
S2313
S2335
S2158
S2163
S2159
T2165
2
1
0
−1
−2
0
500
1000
1500
2000
Regulation:
SV−cycling−dependent
not−affected
Domains and Regions:
DOMAIN: Calponin−homology (CH)
DOMAIN: PH
REGION: Disordered

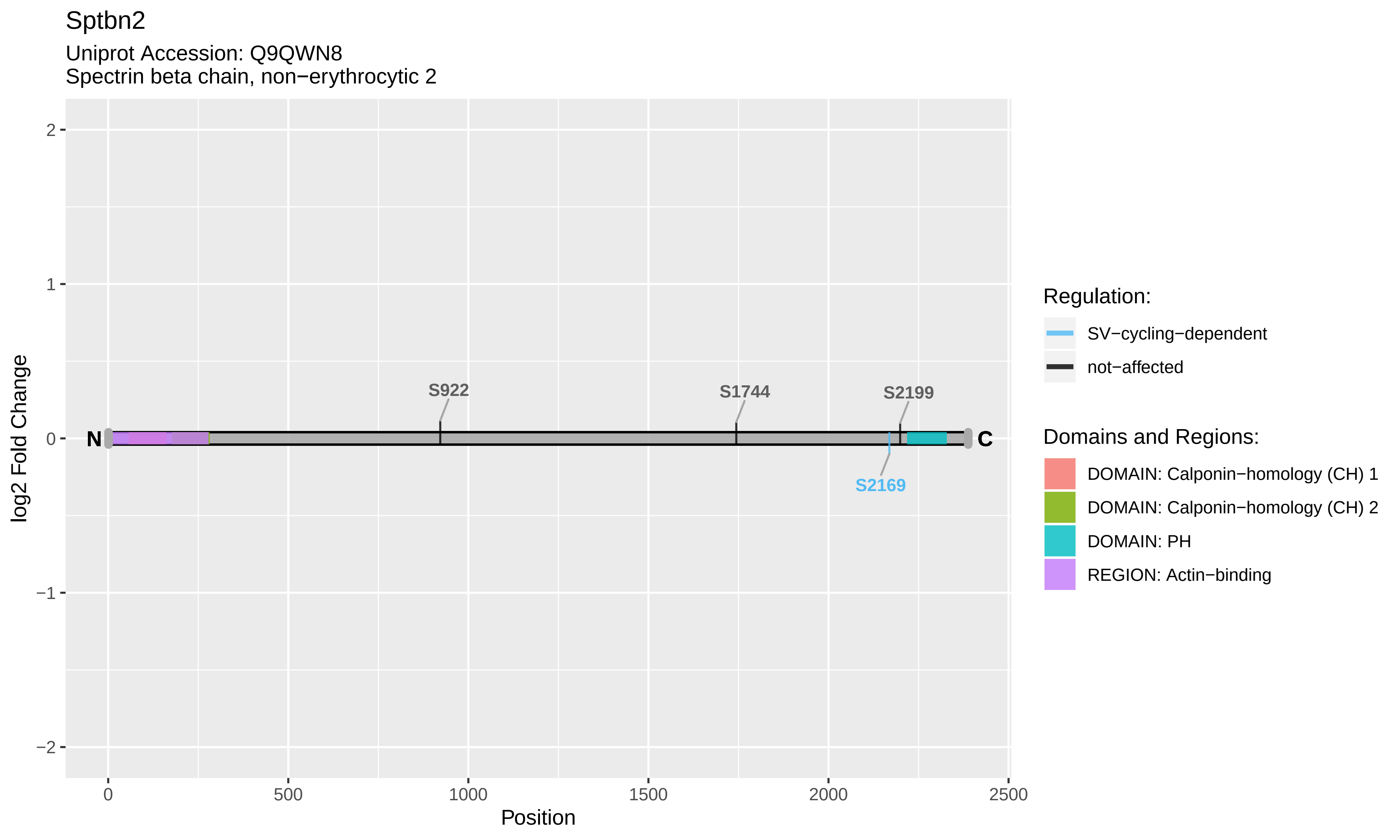

Sptbn2
Uniprot Accession: Q9QWN8
Spectrin beta chain, non−erythrocytic 2
log2 Fold Change
2
1
0
−1
−2
0
500
1000
1500
2000
2500
Position
N
C
S922
S1744
S2199
S2169
Regulation:
SV−cycling−dependent
not−affected
Domains and Regions:
DOMAIN: Calponin−homology (CH) 1
DOMAIN: Calponin−homology (CH) 2
DOMAIN: PH
REGION: Actin−binding

Sptbn4

Uniprot Accession: A0A0G2K677
Spectrin beta chain

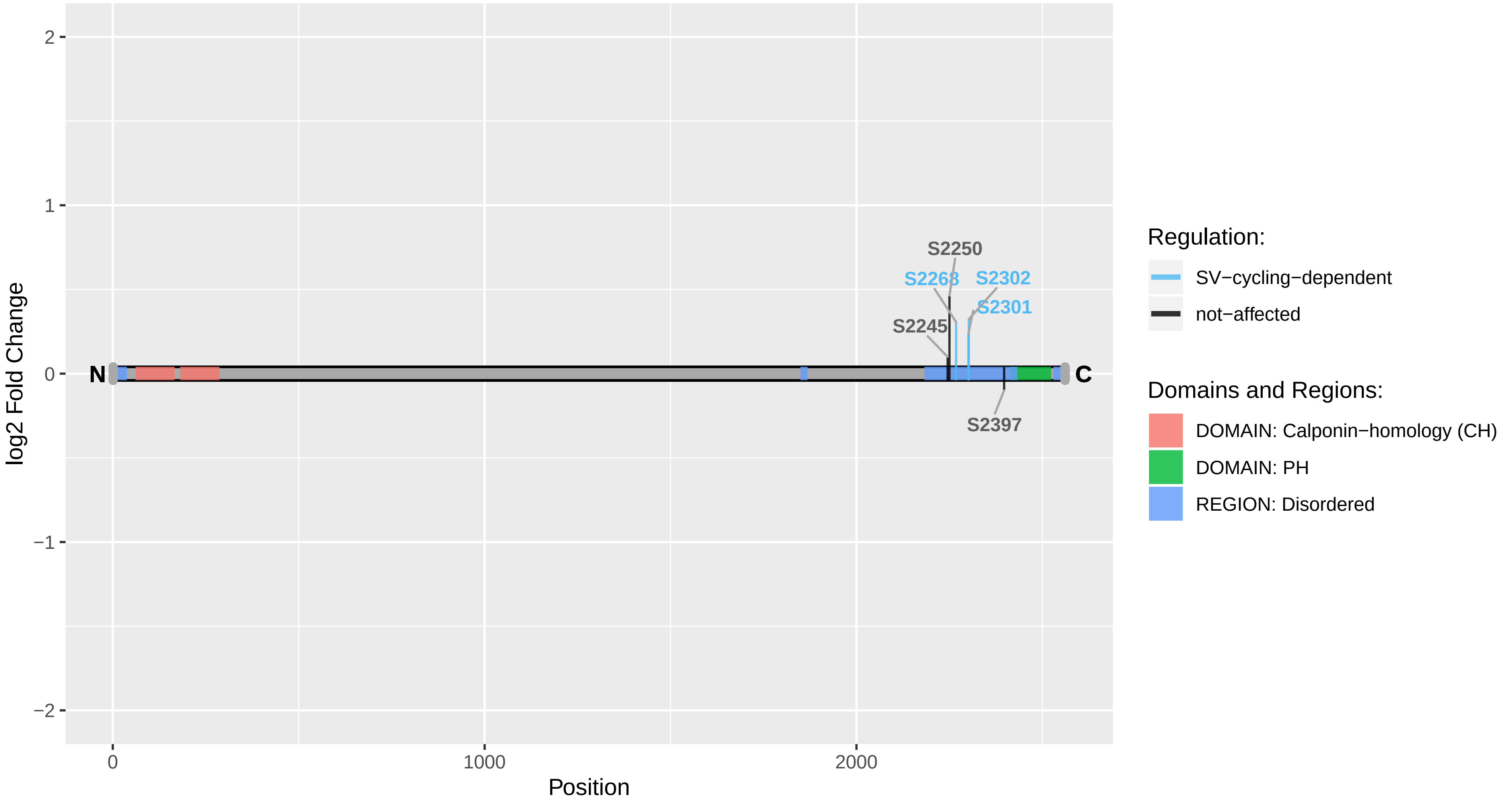

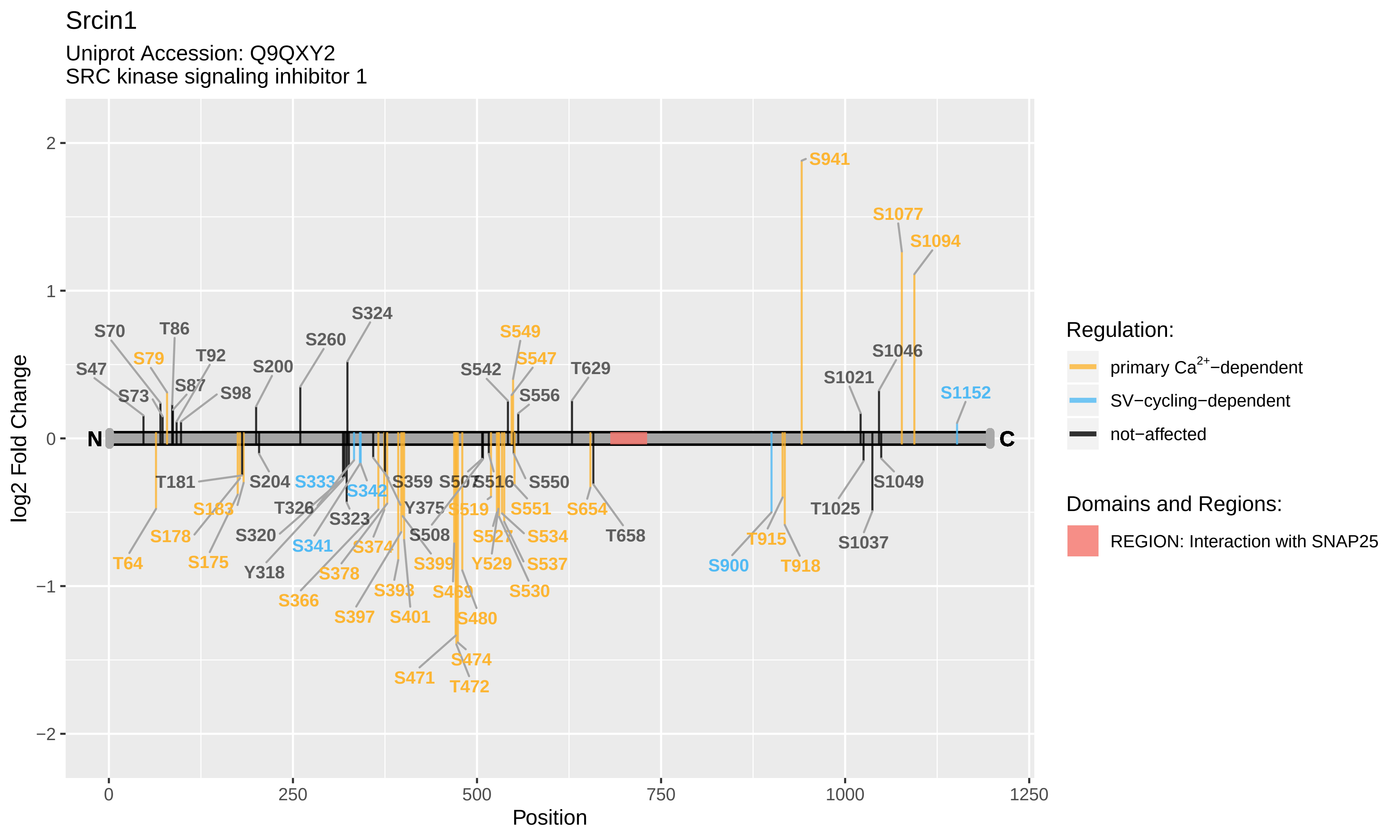

Srcin1
Uniprot Accession: Q9QXY2
SRC kinase signaling inhibitor 1
log2 Fold Change
Position
N
C
S941
S1077
S1094
S324
S70
T86
S260
S549
S547
S79
T92
S200
S47
S87
S542
T629
S73
S98
S556
S1046
S1021
S1152
T181
S204
S333
S342
S359
S507
S516
S550
S1049
S183
T326
Y375
S519
S551
S654
T1025
S323
S178
S320
S508
S527
S534
T658
S1037
T915
T64
S175
S341
S374
S399
Y529
S537
S900
T918
Y318
S378
S393
S469
S530
S366
S397
S401
S480
S474
S471
T472
Regulation:
primary Ca2+−dependent
SV−cycling−dependent
not−affected
Domains and Regions:
REGION: Interaction with SNAP25

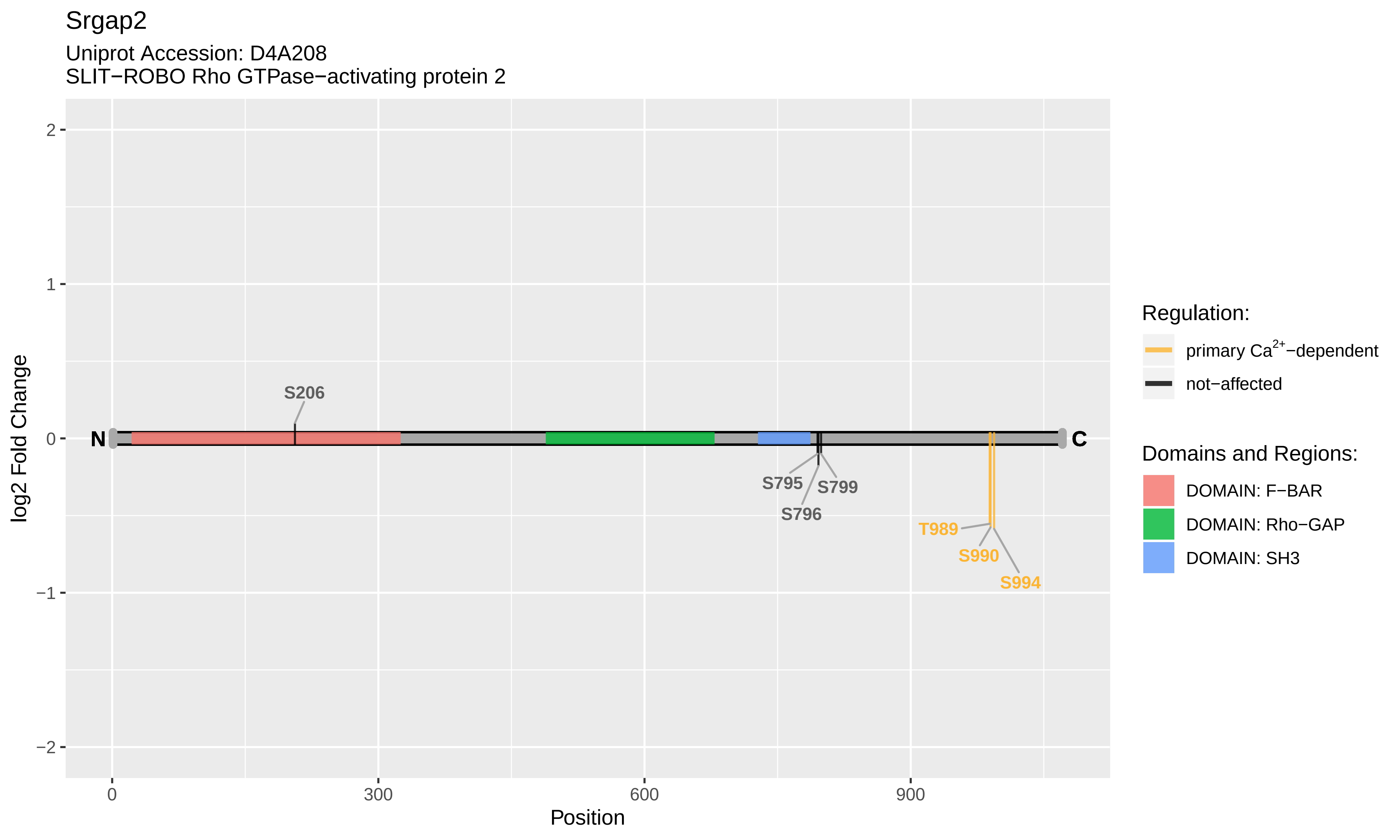

Srgap2
Uniprot Accession: D4A208
SLIT−ROBO Rho GTPase−activating protein 2
log2 Fold Change
Position
2
1
0
−1
−2
0
300
600
900
N
C
S206
S795
S796
S799
T989
S990
S994
Regulation:
primary Ca2+−dependent
not−affected
Domains and Regions:
DOMAIN: F−BAR
DOMAIN: Rho−GAP
DOMAIN: SH3

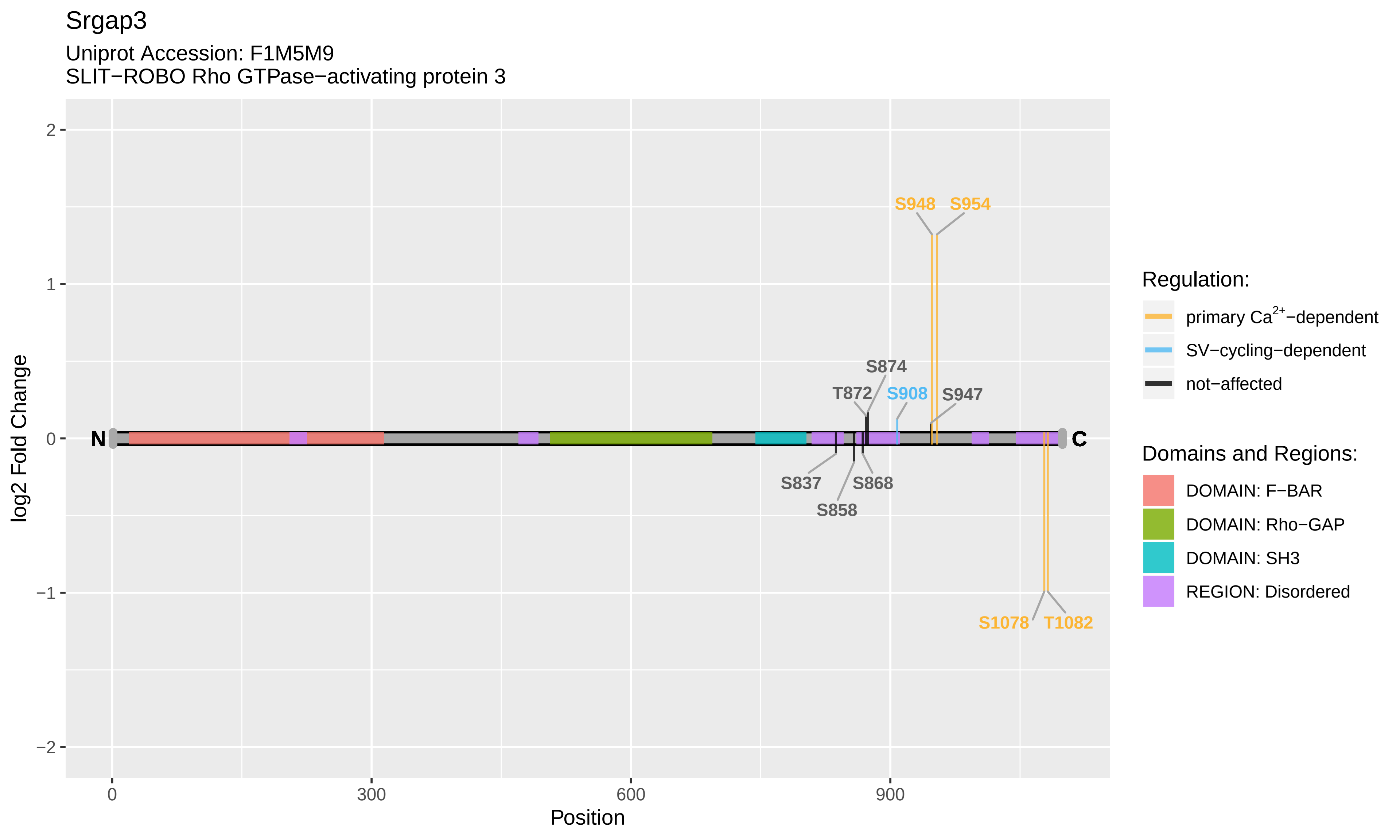

Srgap3
Uniprot Accession: F1M5M9
SLIT−ROBO Rho GTPase−activating protein 3
log2 Fold Change
2
1
0
−1
−2
0
300
600
900
Position
N
C
S948
S954
S874
T872
S908
S947
S837
S868
S858
S1078
T1082
Regulation:
primary Ca2+−dependent
SV−cycling−dependent
not−affected
Domains and Regions:
DOMAIN: F−BAR
DOMAIN: Rho−GAP
DOMAIN: SH3
REGION: Disordered

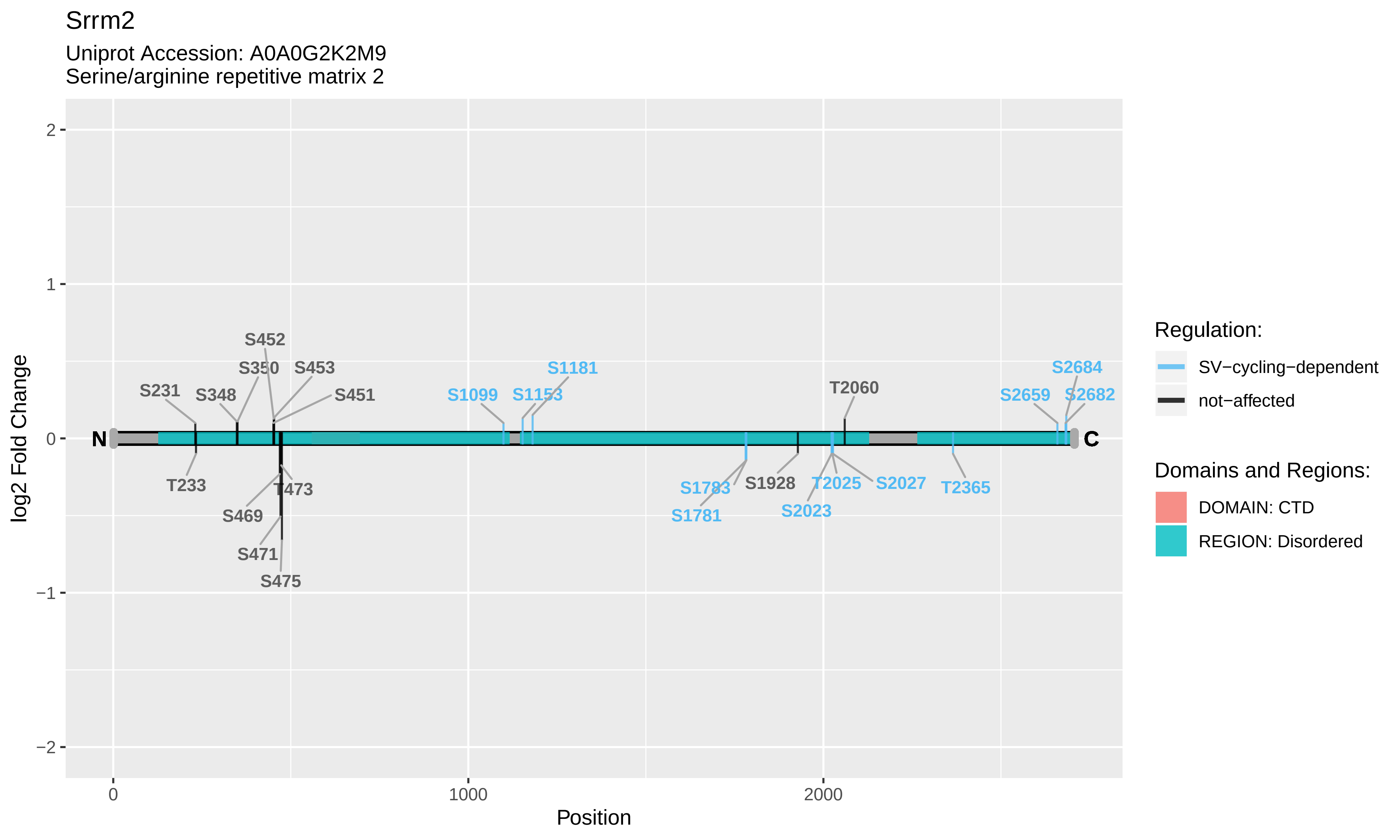

Srrm2
Uniprot Accession: A0A0G2K2M9
Serine/arginine repetitive matrix 2
log2 Fold Change
2
1
0
−1
−2
N
C
S452
S350
S453
S231
S348
S451
T233
S469
T473
S471
S475
S1099
S1153
S1181
S1783
S1781
S1928
T2060
T2025
S2023
S2027
T2365
S2659
S2684
S2682
0
1000
2000
Position
Regulation:
SV−cycling−dependent
not−affected
Domains and Regions:
DOMAIN: CTD
REGION: Disordered

Ssh3

Uniprot Accession: Q5XIS1
Protein phosphatase Slingshot homolog 3

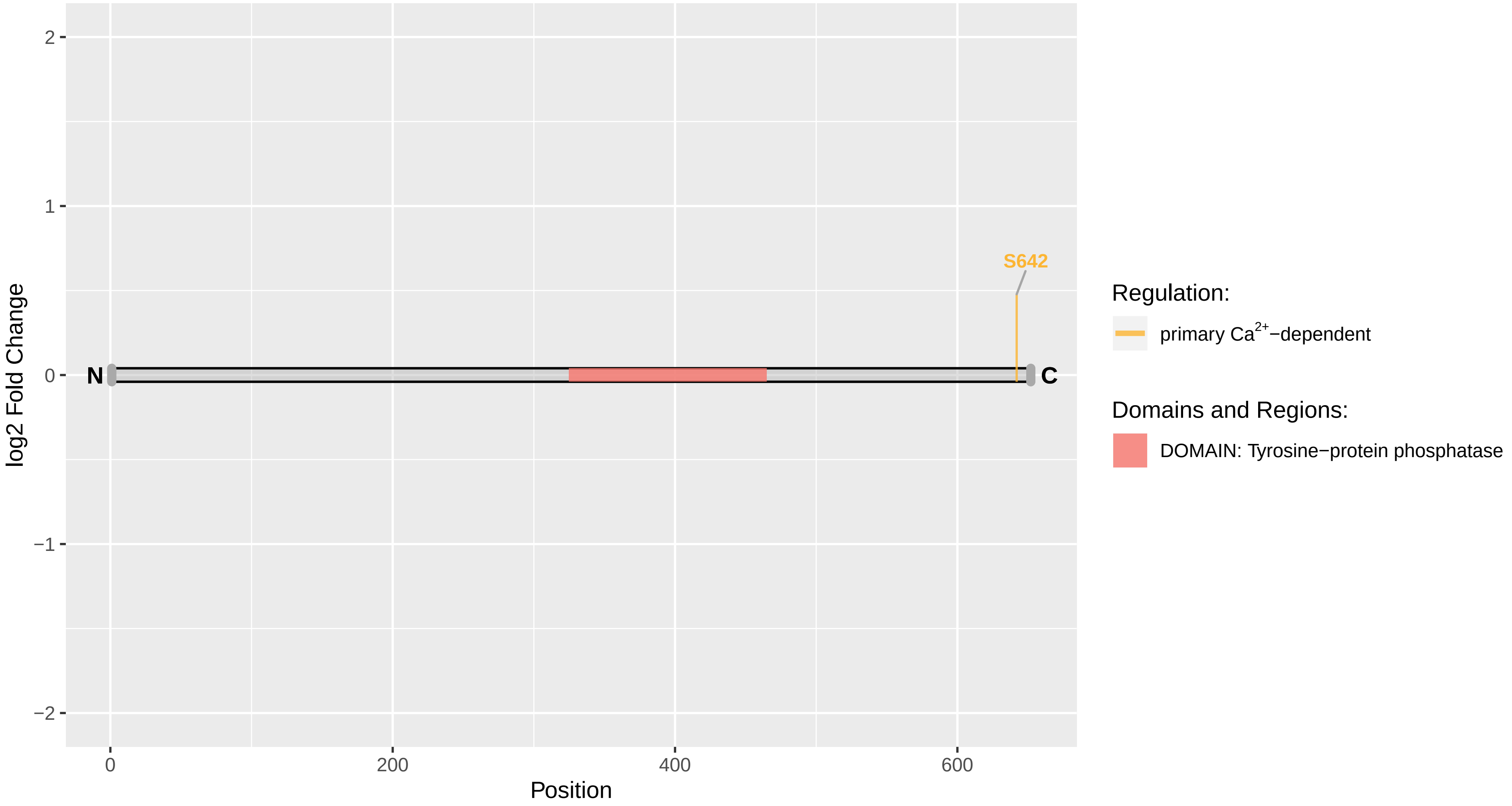

Ssr3

Uniprot Accession: Q08013
Translocon−associated protein subunit gamma

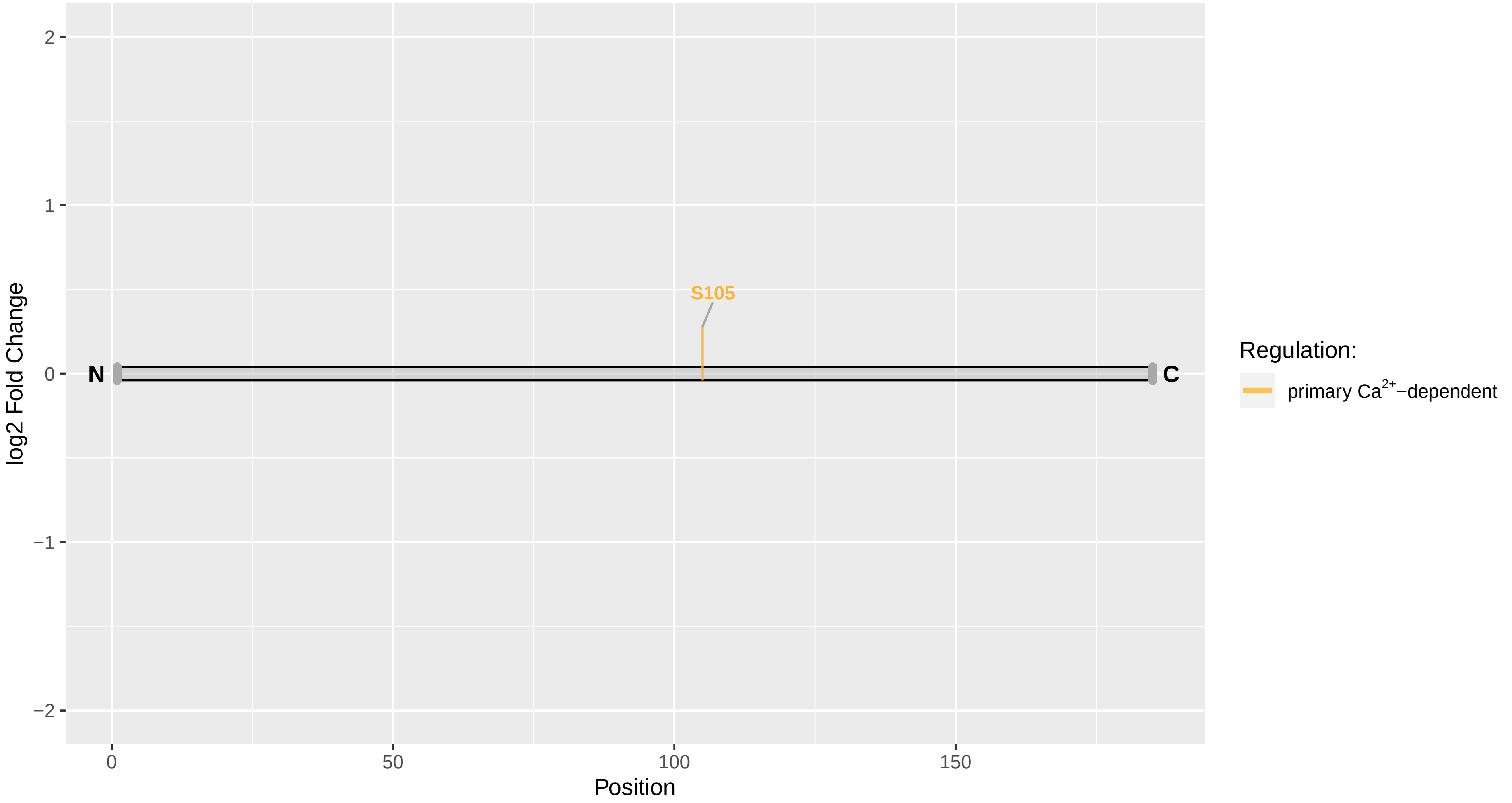

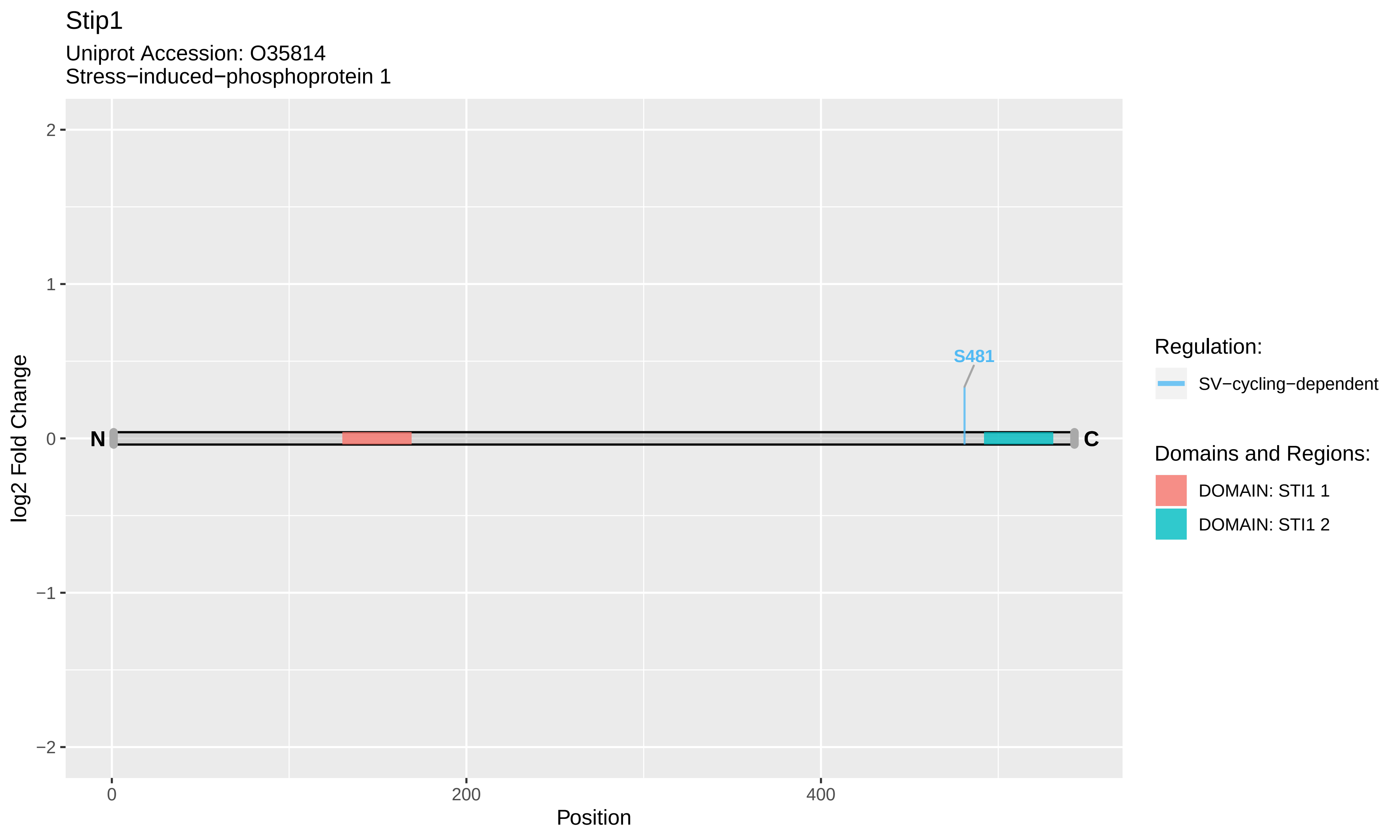

Stip1
Uniprot Accession: O35814
Stress−induced−phosphoprotein 1
log2 Fold Change
2
1
0
−1
−2
N
C
S481
0
200
400
Position
Regulation:
SV−cycling−dependent
Domains and Regions:
DOMAIN: STI1 1
DOMAIN: STI1 2

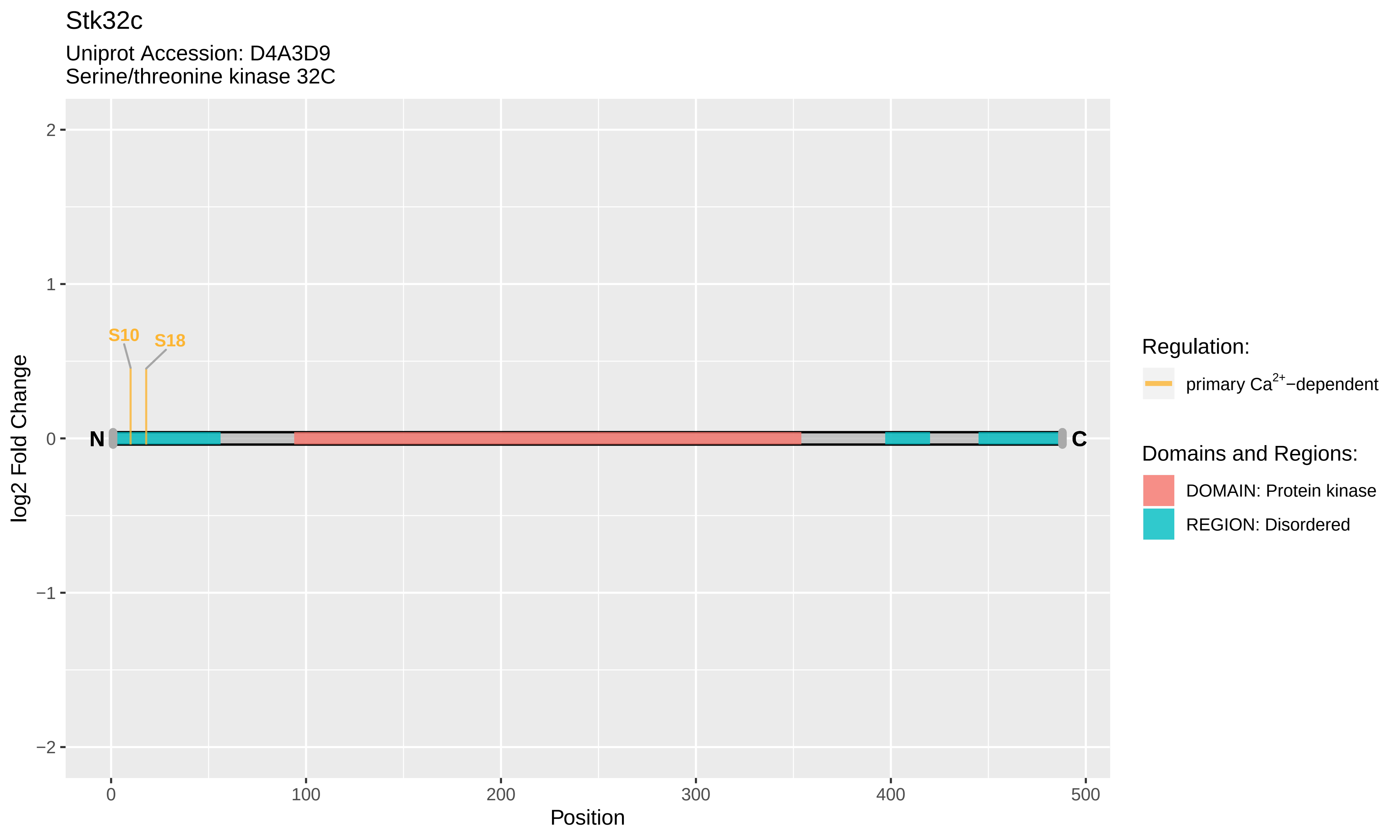

Stk32c
Uniprot Accession: D4A3D9
Serine/threonine kinase 32C
S10
S18
N
C
2
1
0
−1
−2
log2 Fold Change
0
100
200
300
400
500
Position
Regulation:
primary Ca2+−dependent
Domains and Regions:
DOMAIN: Protein kinase
REGION: Disordered

Stmn1

Uniprot Accession: P13668
Stathmin

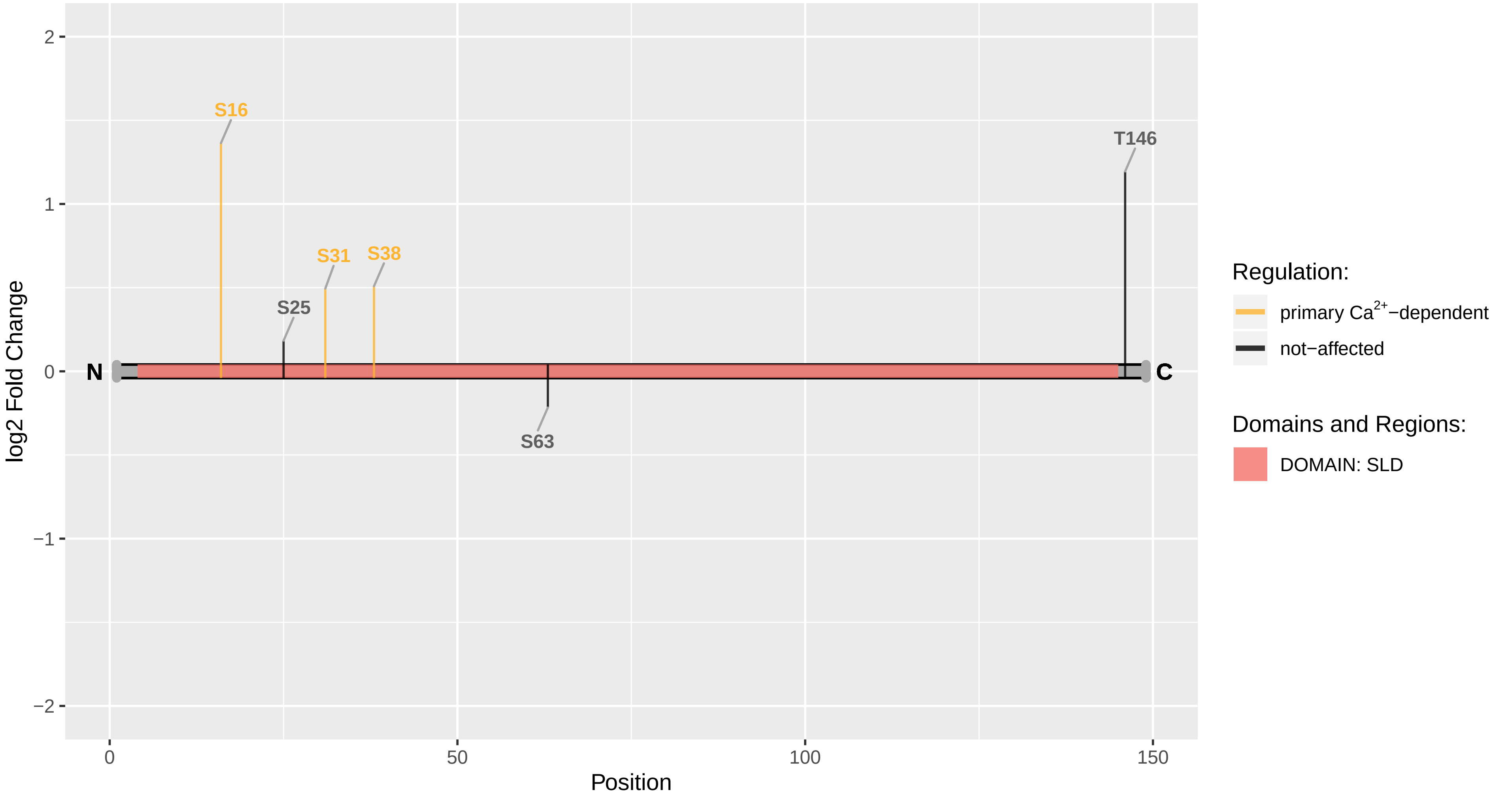

Strip2

Uniprot Accession: A0A0G2JT26
Striatin interacting protein 2

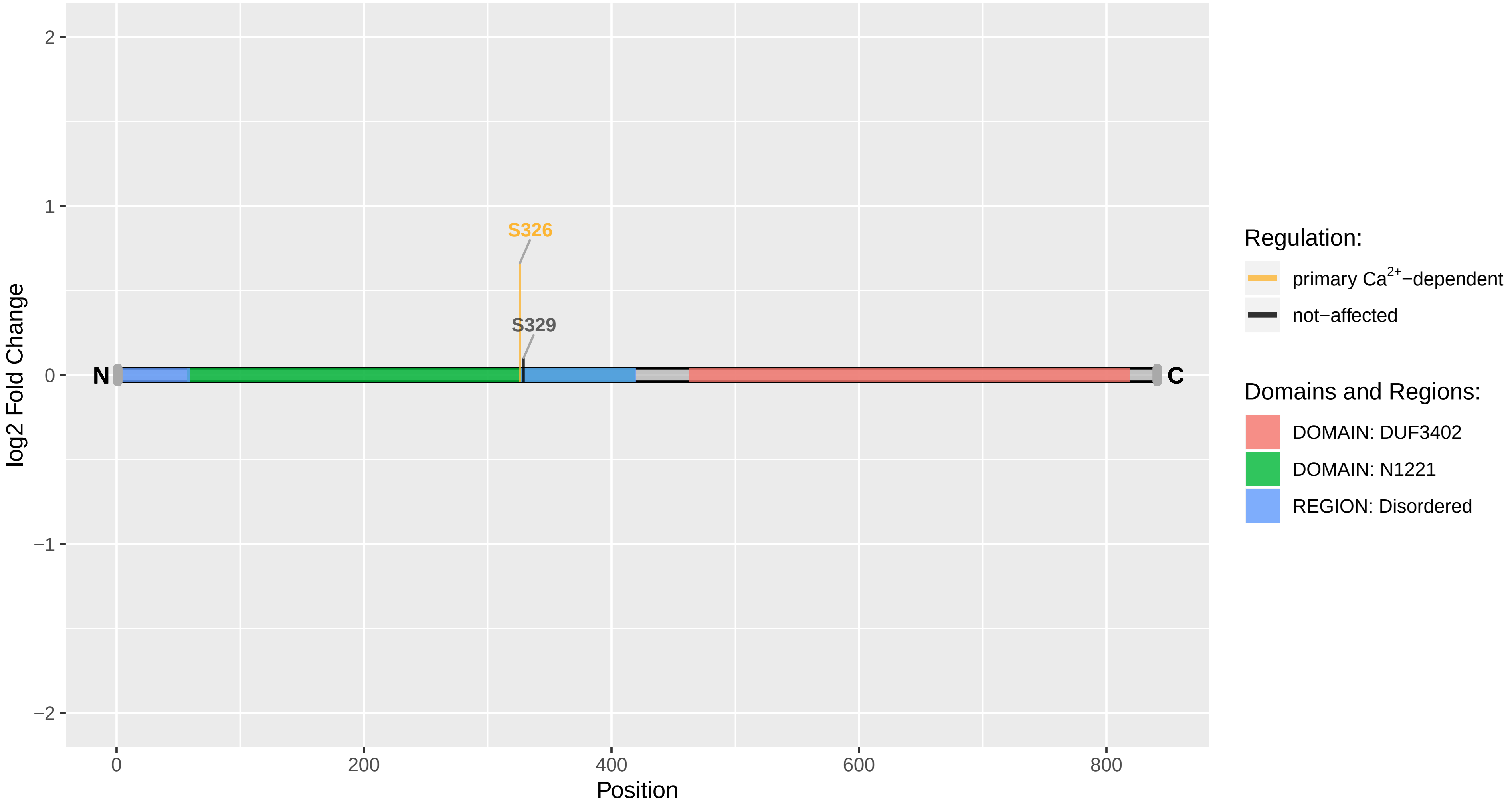

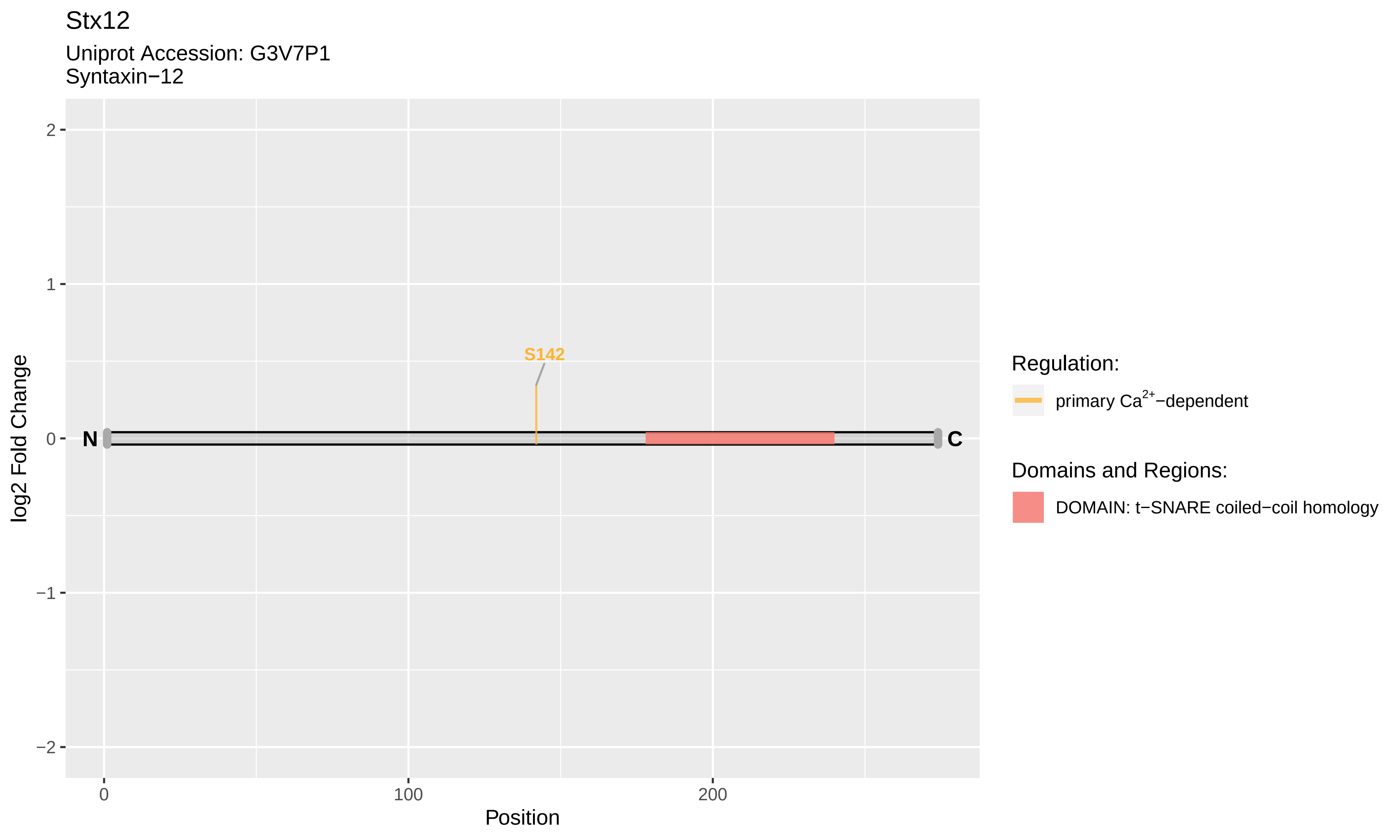
Stx12
Uniprot Accession: G3V7P1
Syntaxin−12
S142
N
C
2
1
0
−1
−2
log2 Fold Change
0
100
200
Position
Regulation:
primary Ca2+−dependent
Domains and Regions:
DOMAIN: t−SNARE coiled−coil homology

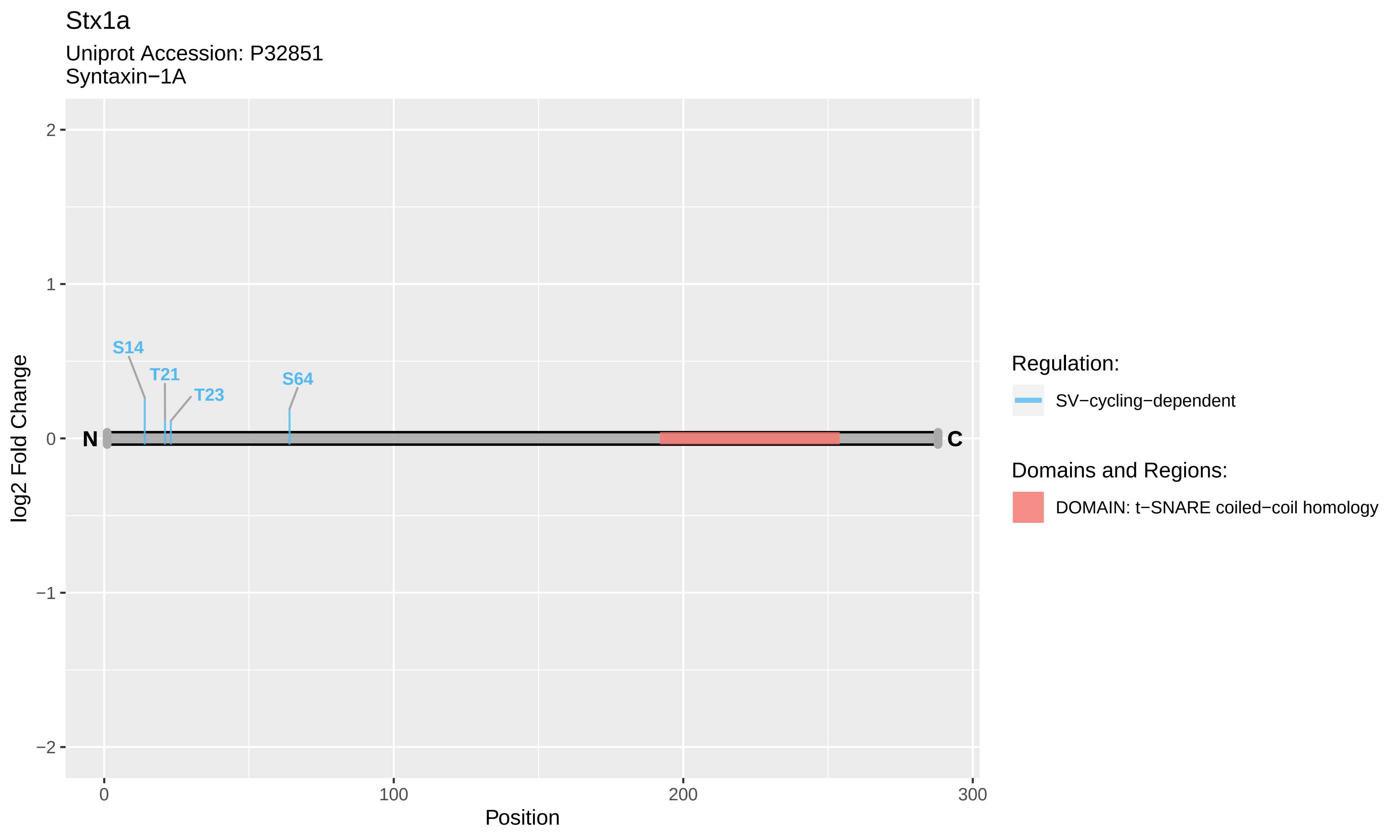
Stx1a
Uniprot Accession: P32851
Syntaxin−1A
2
1
0
−1
−2
log2 Fold Change
S14
T21
T23
S64
N
C
0
100
200
300
Position
Regulation:
SV−cycling−dependent
Domains and Regions:
DOMAIN: t−SNARE coiled−coil homology

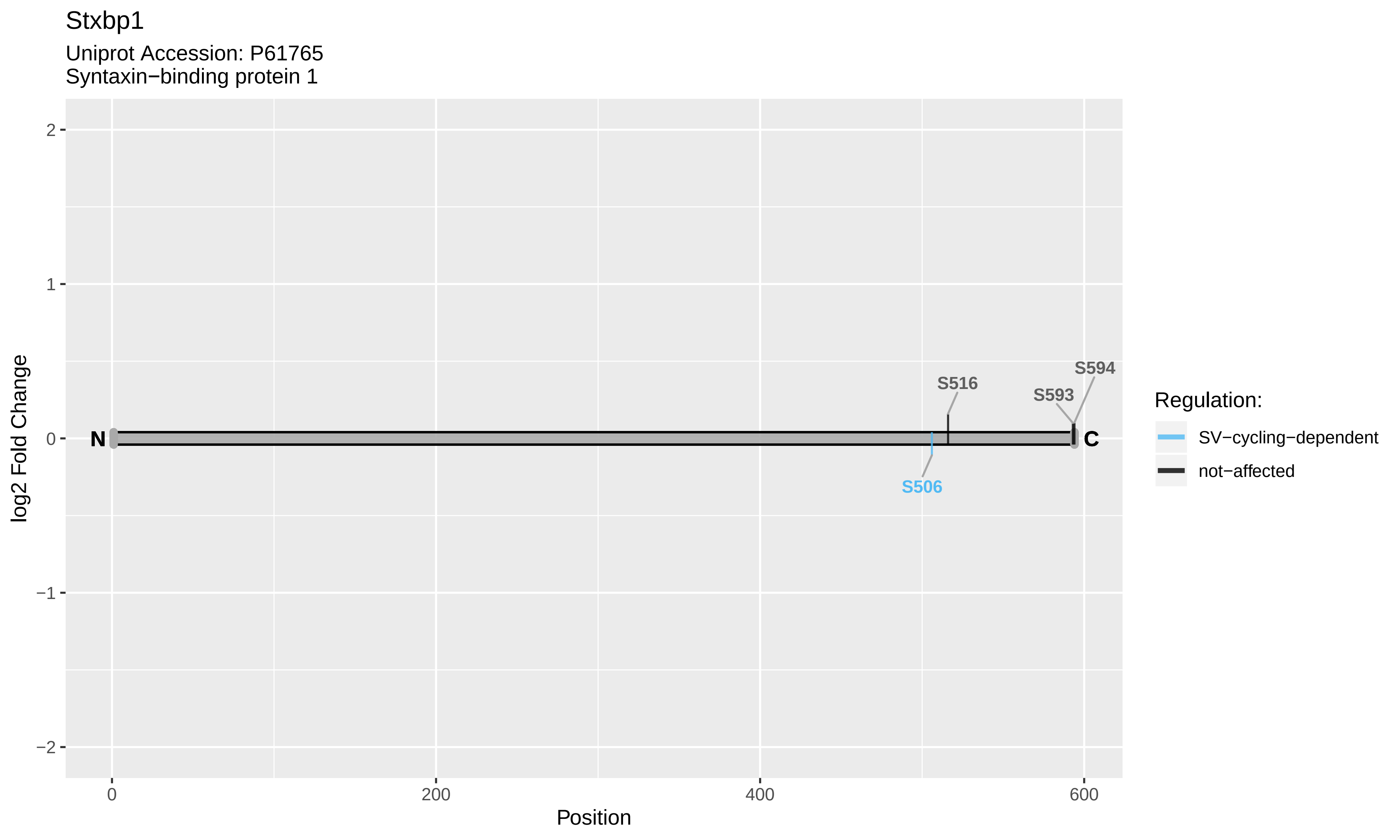

Stxbp1
Uniprot Accession: P61765
Syntaxin−binding protein 1
log2 Fold Change
2
1
0
−1
−2
N
C
S516
S593
S594
S506
0
200
400
600
Position
Regulation:
SV−cycling−dependent
not−affected

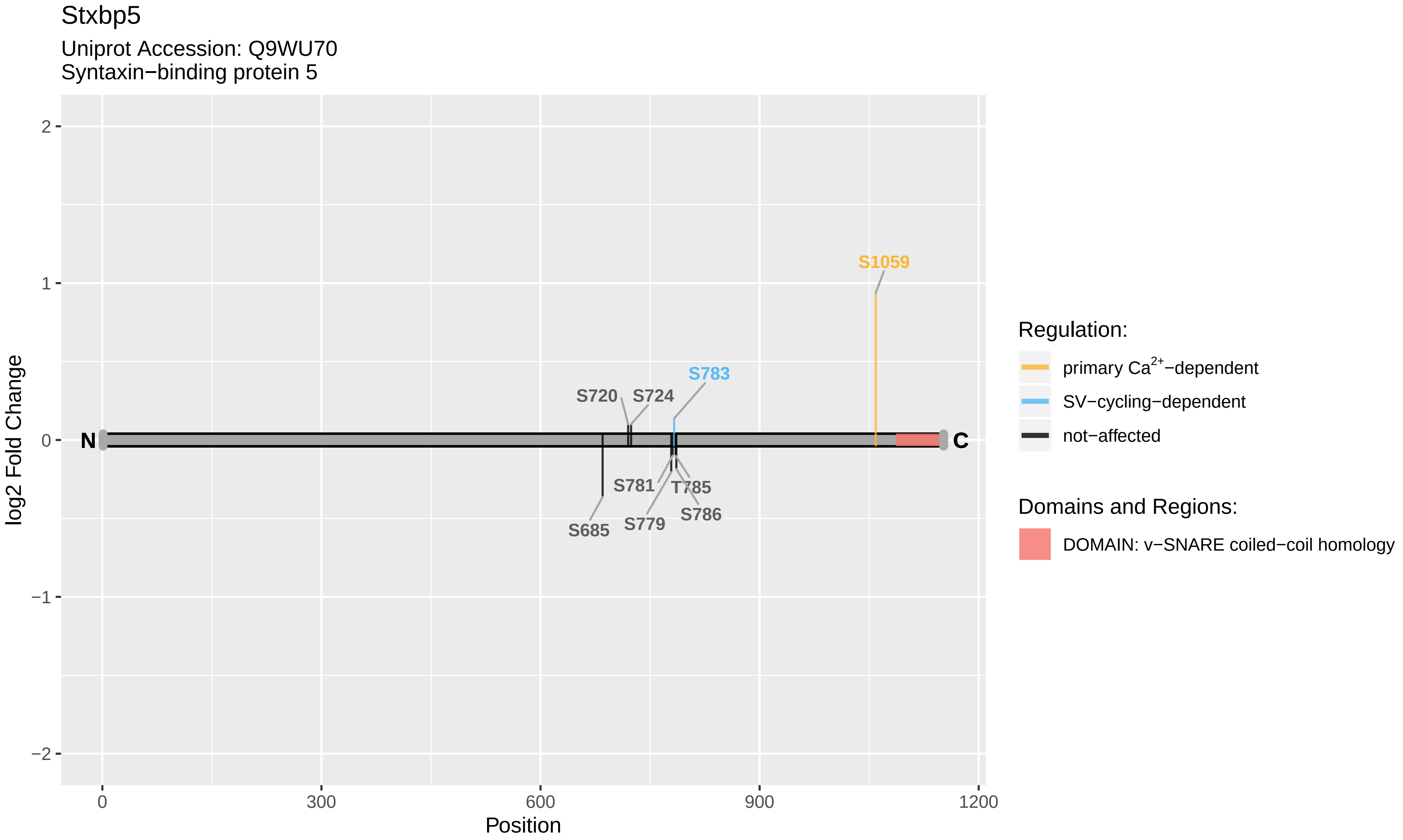
Stxbp5
Uniprot Accession: Q9WU70
Syntaxin−binding protein 5
log2 Fold Change
Position
N
C
S1059
S783
S720
S724
S781
T785
S786
S685
S779
2
1
0
−1
−2
0
300
600
900
1200
Regulation:
primary Ca2+−dependent
SV−cycling−dependent
not−affected
Domains and Regions:
DOMAIN: v−SNARE coiled−coil homology

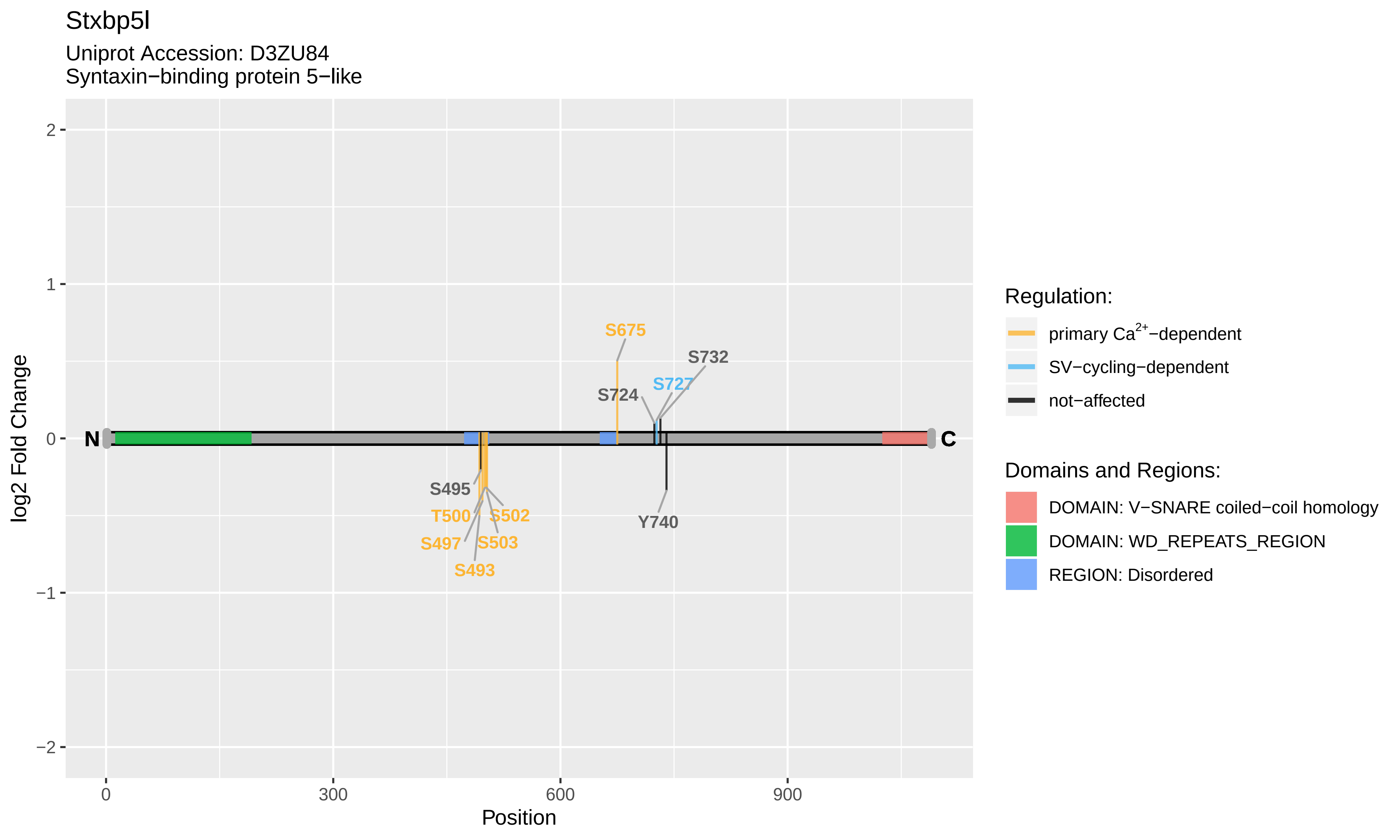

Stxbp5l
Uniprot Accession: D3ZU84
Syntaxin−binding protein 5−like
log2 Fold Change
Position
N
C
S675
S732
S727
S724
S495
T500
S502
S497
S503
S493
Y740
Regulation:
primary Ca2+−dependent
SV−cycling−dependent
not−affected
Domains and Regions:
DOMAIN: V−SNARE coiled−coil homology
DOMAIN: WD_REPEATS_REGION
REGION: Disordered

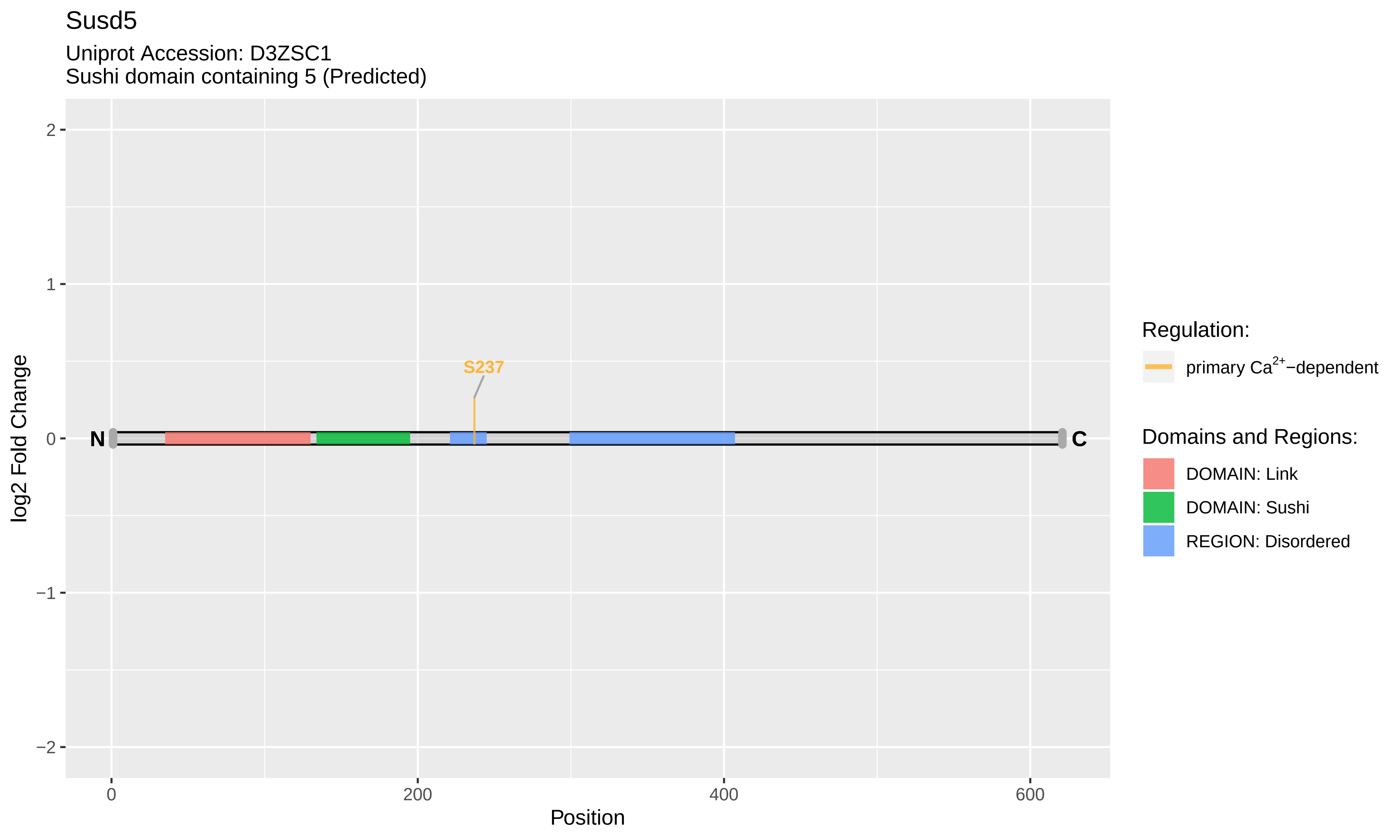

Susd5
Uniprot Accession: D3ZSC1
Sushi domain containing 5 (Predicted)
S237
N
C
log2 Fold Change
2
1
0
−1
−2
0
200
400
600
Position
Regulation:
primary Ca2+−dependent
Domains and Regions:
DOMAIN: Link
DOMAIN: Sushi
REGION: Disordered

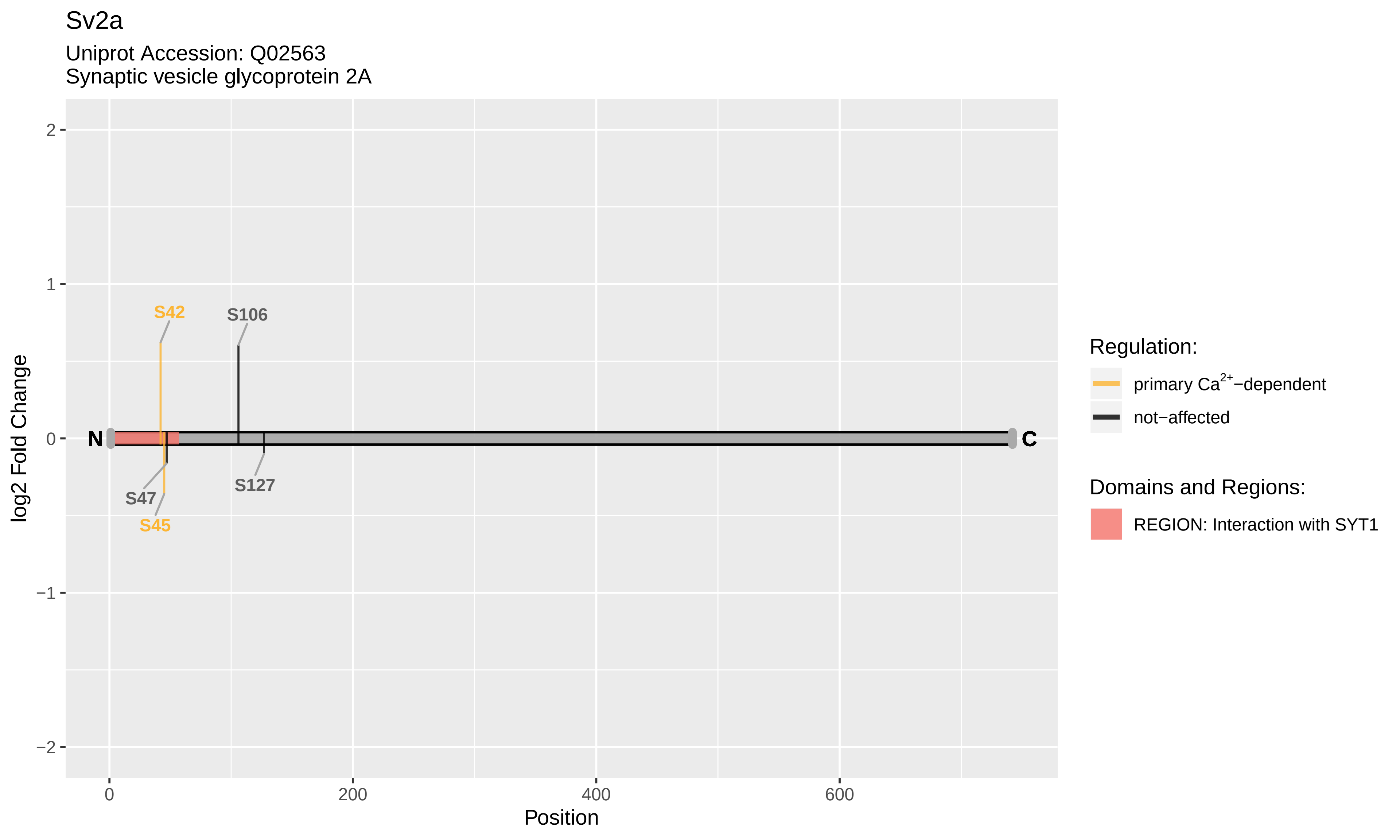

Sv2a
Uniprot Accession: Q02563
Synaptic vesicle glycoprotein 2A
log2 Fold Change
Position
2
1
0
−1
−2
0
200
400
600
N
C
S42
S106
S47
S45
S127
Regulation:
primary Ca2+−dependent
not−affected
Domains and Regions:
REGION: Interaction with SYT1

Svop

Uniprot Accession: Q9Z2I7
Synaptic vesicle 2−related protein

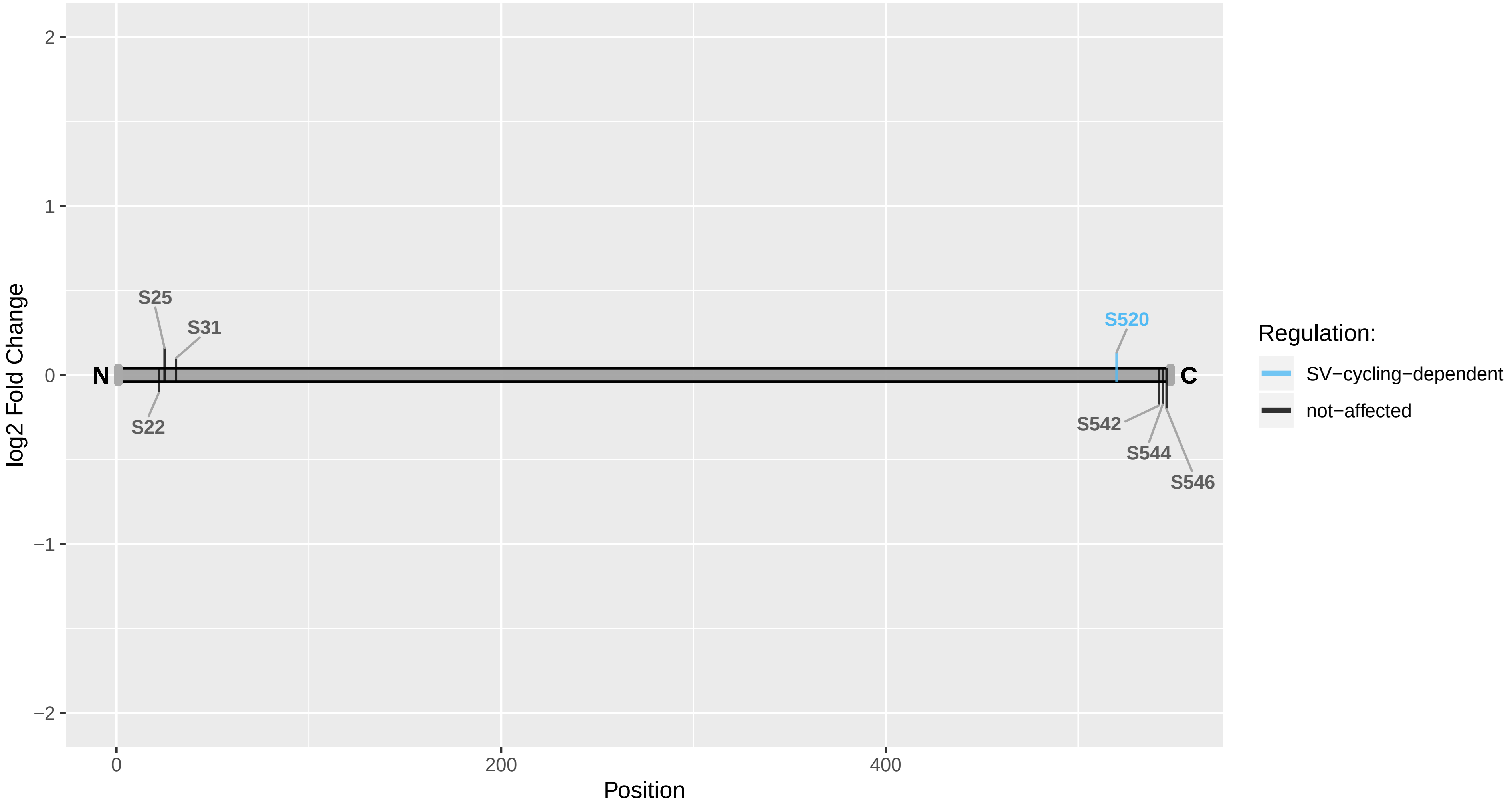

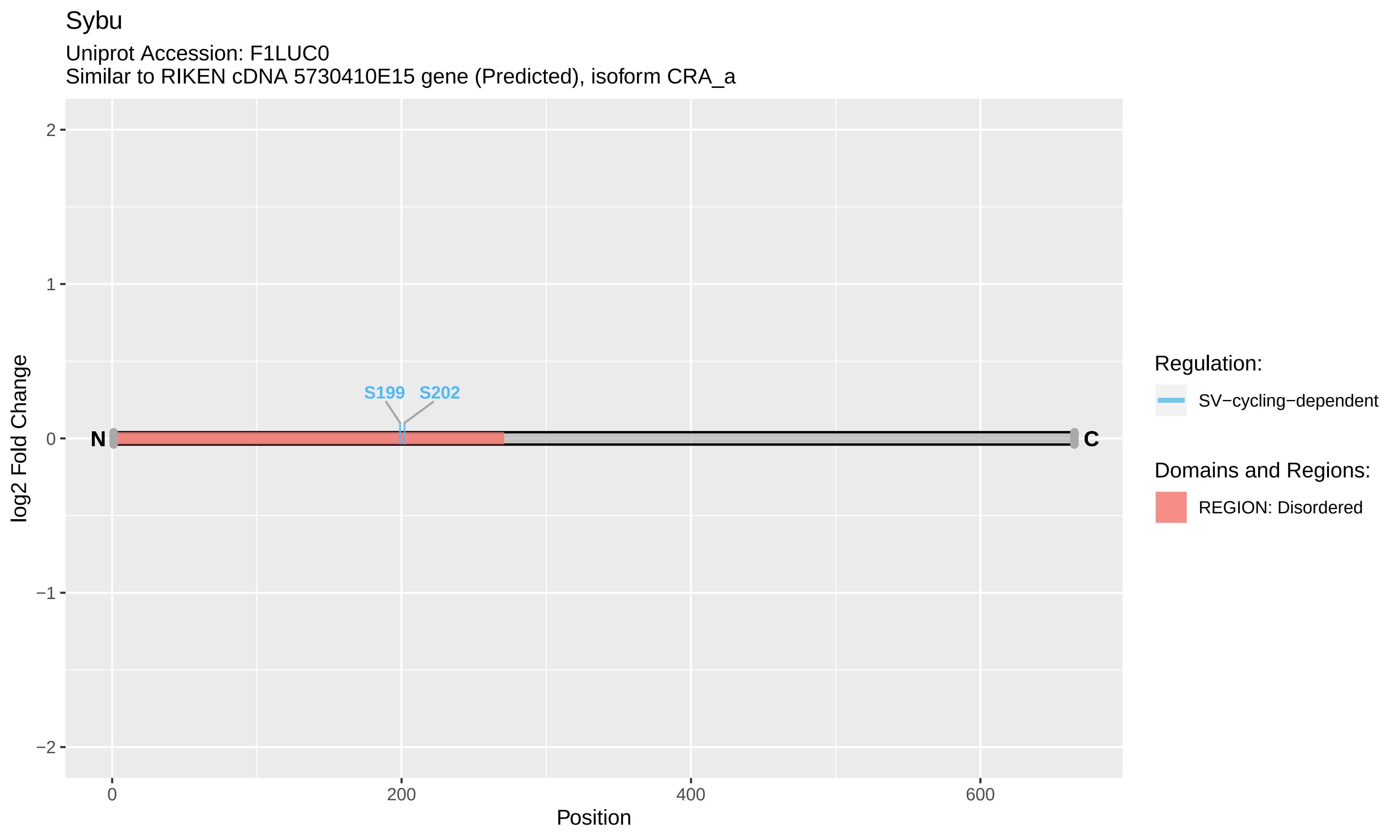
Sybu
Uniprot Accession: F1LUC0
Similar to RIKEN cDNA 5730410E15 gene (Predicted), isoform CRA_a
S199
S202
N
C
2
1
0
−1
−2
log2 Fold Change
0
200
400
600
Position
Regulation:
SV−cycling−dependent
Domains and Regions:
REGION: Disordered

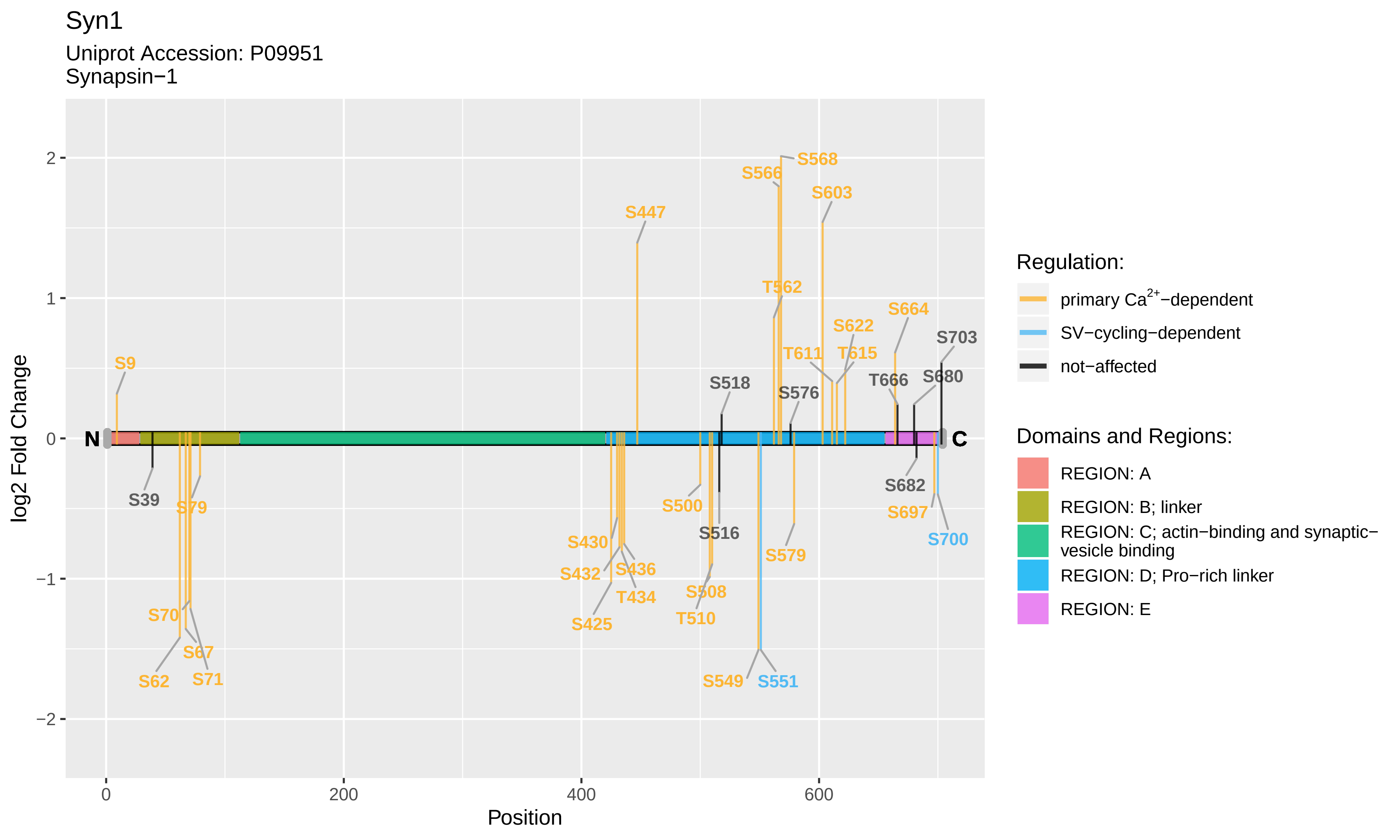

Syn1
Uniprot Accession: P09951
Synapsin−1
log2 Fold Change
Position
N
C
S9
S39
S79
S70
S67
S62
S71
S447
S430
S432
S436
T434
S425
S500
S516
S508
T510
S518
S566
S568
S603
T562
S549
S551
S576
S579
T611
T615
S622
S664
T666
S680
S703
S682
S697
S700
Regulation:
primary Ca2+−dependent
SV−cycling−dependent
not−affected
Domains and Regions:
REGION: A
REGION: B; linker
REGION: C; actin−binding and synaptic−vesicle binding
REGION: D; Pro−rich linker
REGION: E

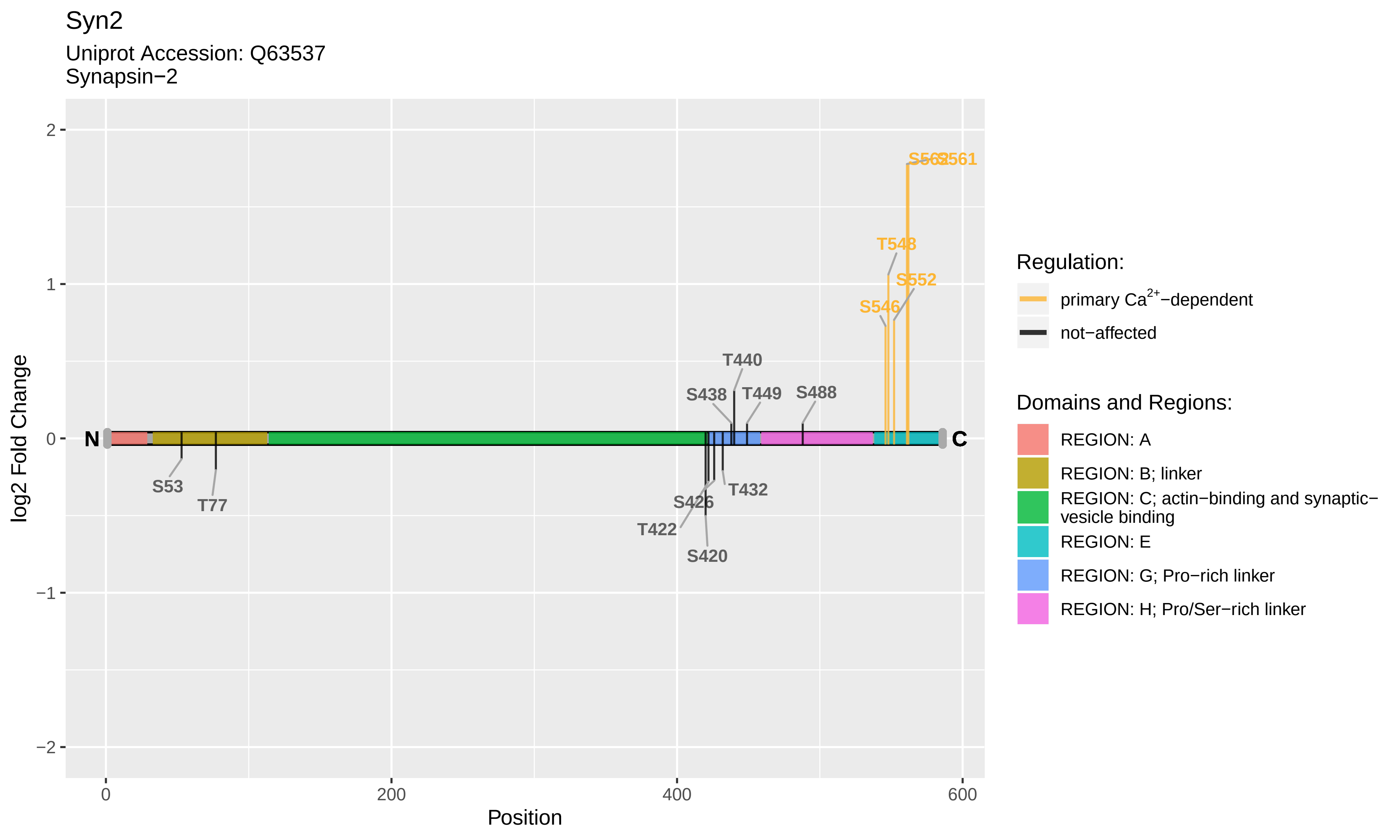

Syn2
Uniprot Accession: Q63537
Synapsin−2
log2 Fold Change
Position
N
C
S53
T77
S420
T422
S426
T432
S438
T440
T449
S488
S546
T548
S552
S561
S563
Regulation:
primary Ca2+−dependent
not−affected
Domains and Regions:
REGION: A
REGION: B; linker
REGION: C; actin−binding and synaptic−vesicle binding
REGION: E
REGION: G; Pro−rich linker
REGION: H; Pro/Ser−rich linker

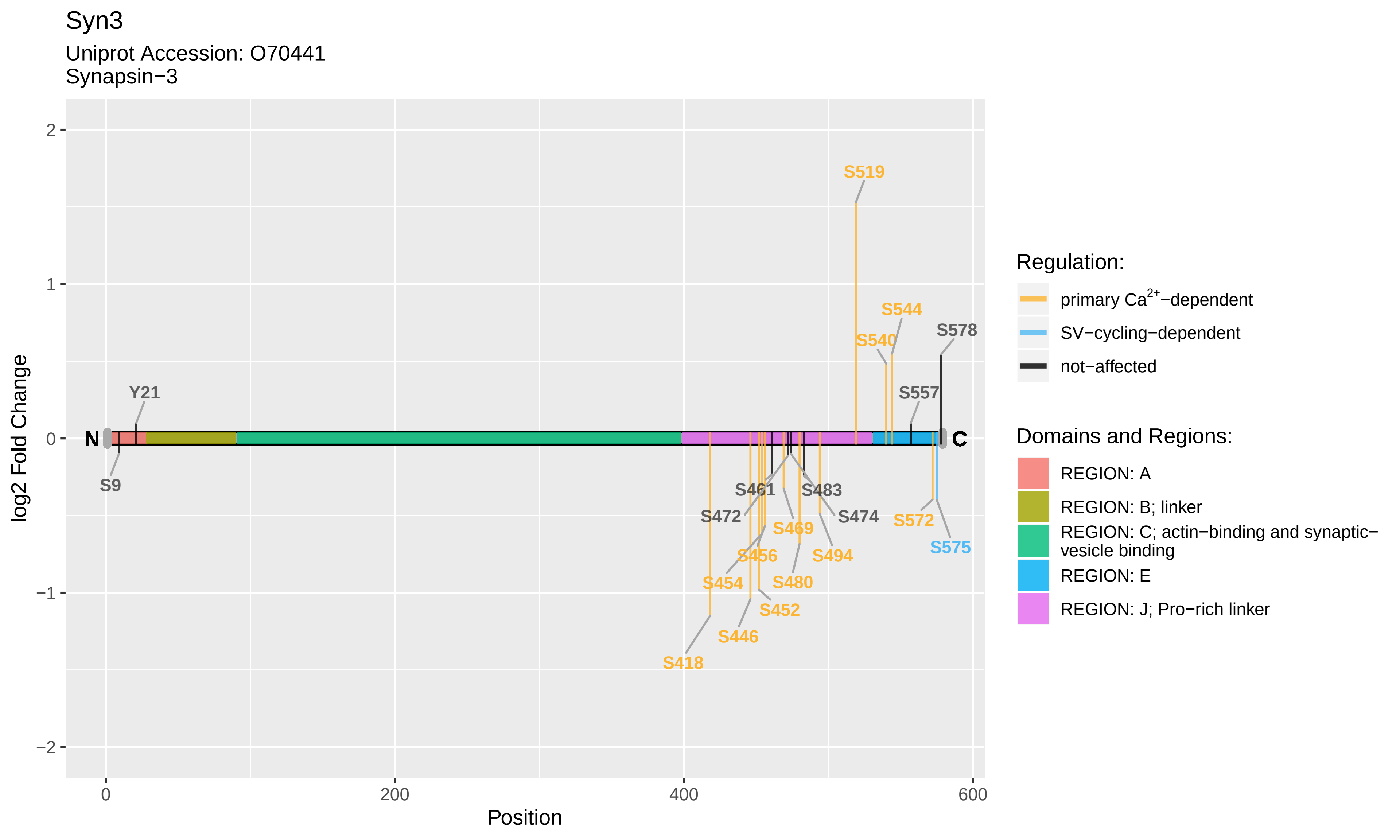

Syn3
Uniprot Accession: O70441
Synapsin−3
log2 Fold Change
Position
N
C
Y21
S9
S519
S544
S540
S578
S557
S461
S483
S472
S474
S469
S572
S575
S456
S494
S454
S480
S452
S446
S418
Regulation:
primary Ca^{2+}−dependent
SV−cycling−dependent
not−affected
Domains and Regions:
REGION: A
REGION: B; linker
REGION: C; actin−binding and synaptic−vesicle binding
REGION: E
REGION: J; Pro−rich linker

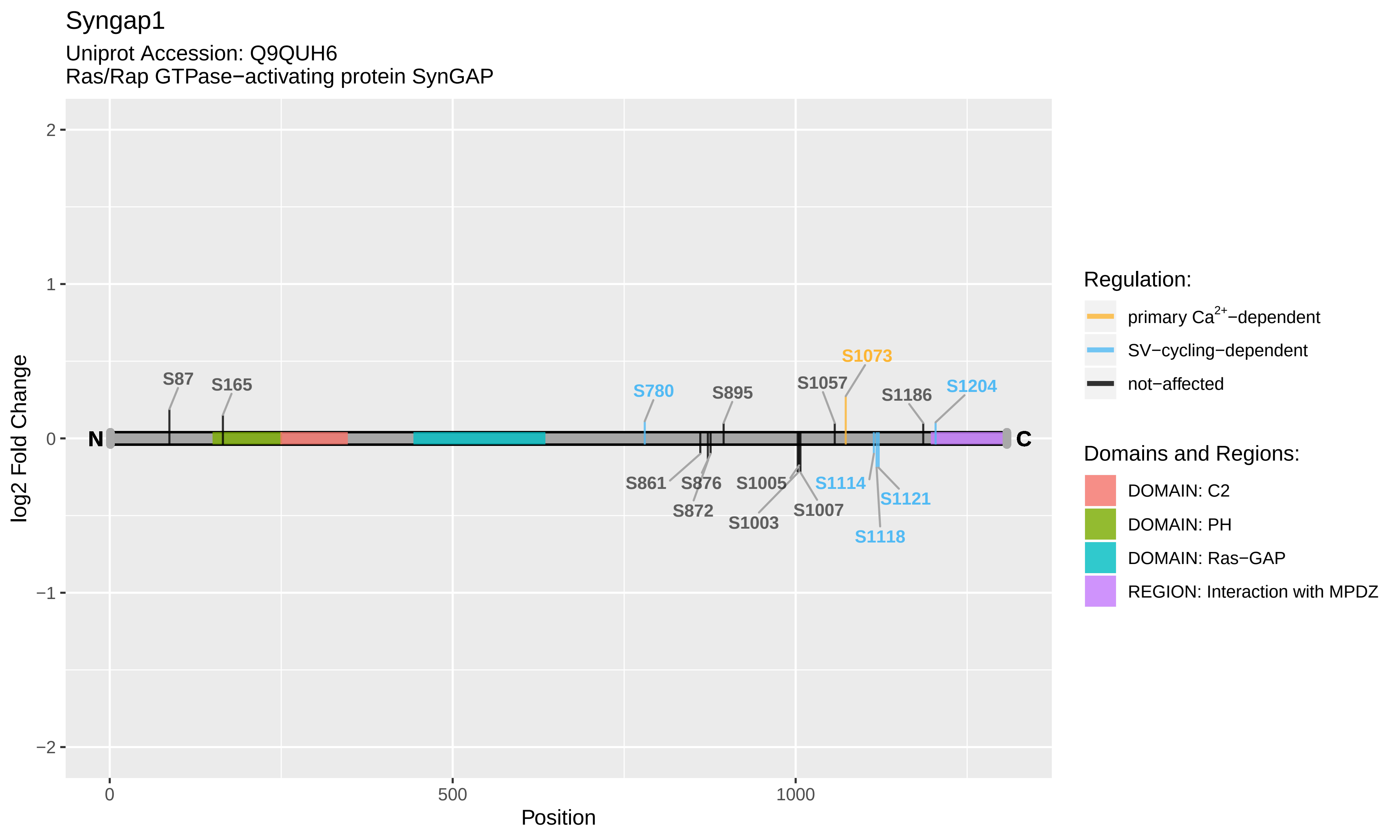

Syngap1
Uniprot Accession: Q9QUH6
Ras/Rap GTPase−activating protein SynGAP
S87
S165
S780
S895
S1057
S1073
S1186
S1204
N
C
S861
S876
S872
S1005
S1003
S1007
S1114
S1121
S1118
2
1
0
−1
−2
0
500
1000
log2 Fold Change
Position
Regulation:
primary Ca2+−dependent
SV−cycling−dependent
not−affected
Domains and Regions:
DOMAIN: C2
DOMAIN: PH
DOMAIN: Ras−GAP
REGION: Interaction with MPDZ

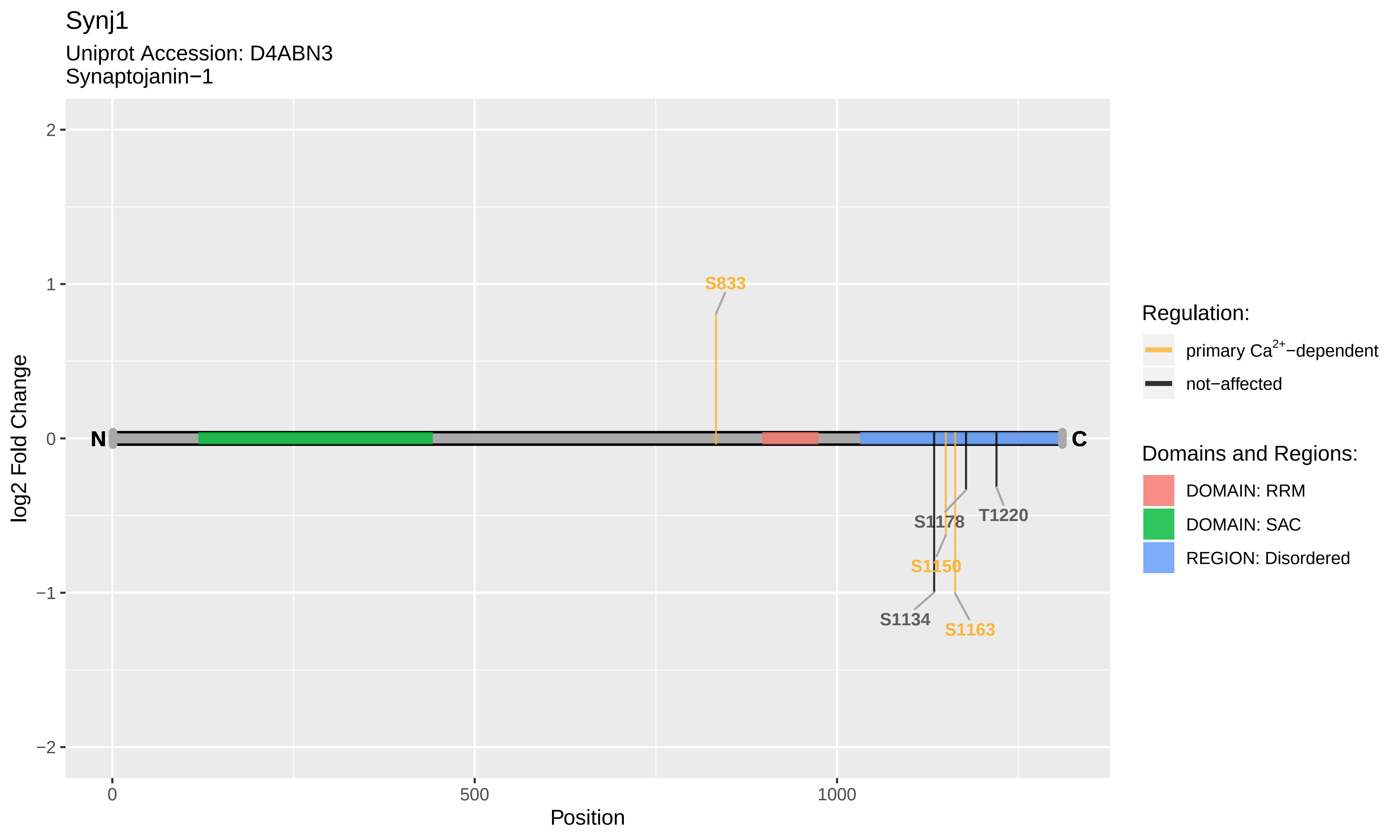

Synj1
Uniprot Accession: D4ABN3
Synaptojanin−1
log2 Fold Change
Position
2
1
0
−1
−2
0
500
1000
N
C
S833
S1178
T1220
S1150
S1134
S1163
Regulation:
primary Ca2+−dependent
not−affected
Domains and Regions:
DOMAIN: RRM
DOMAIN: SAC
REGION: Disordered

Synpo

Uniprot Accession: Q9Z327
Synaptopodin

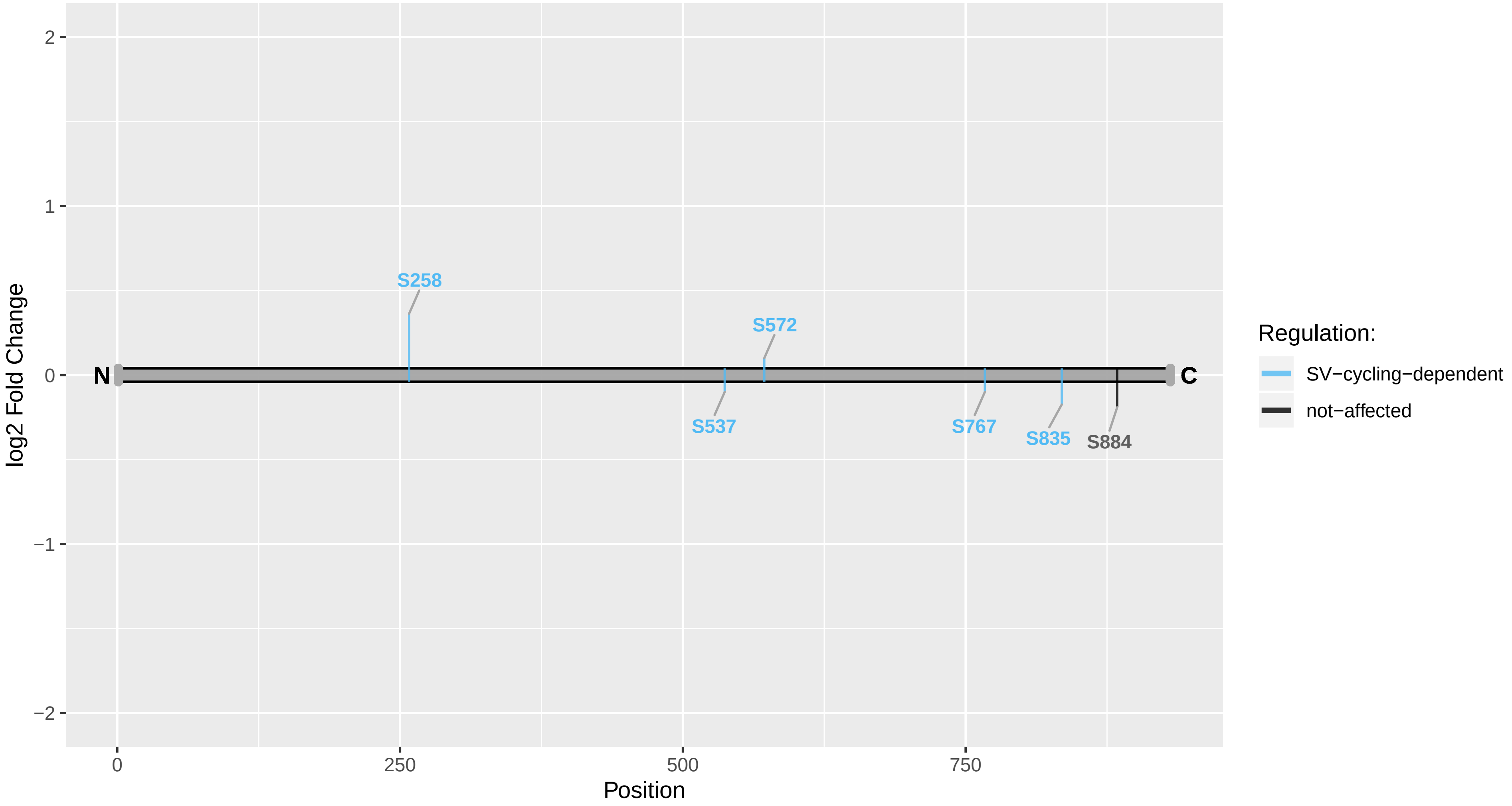

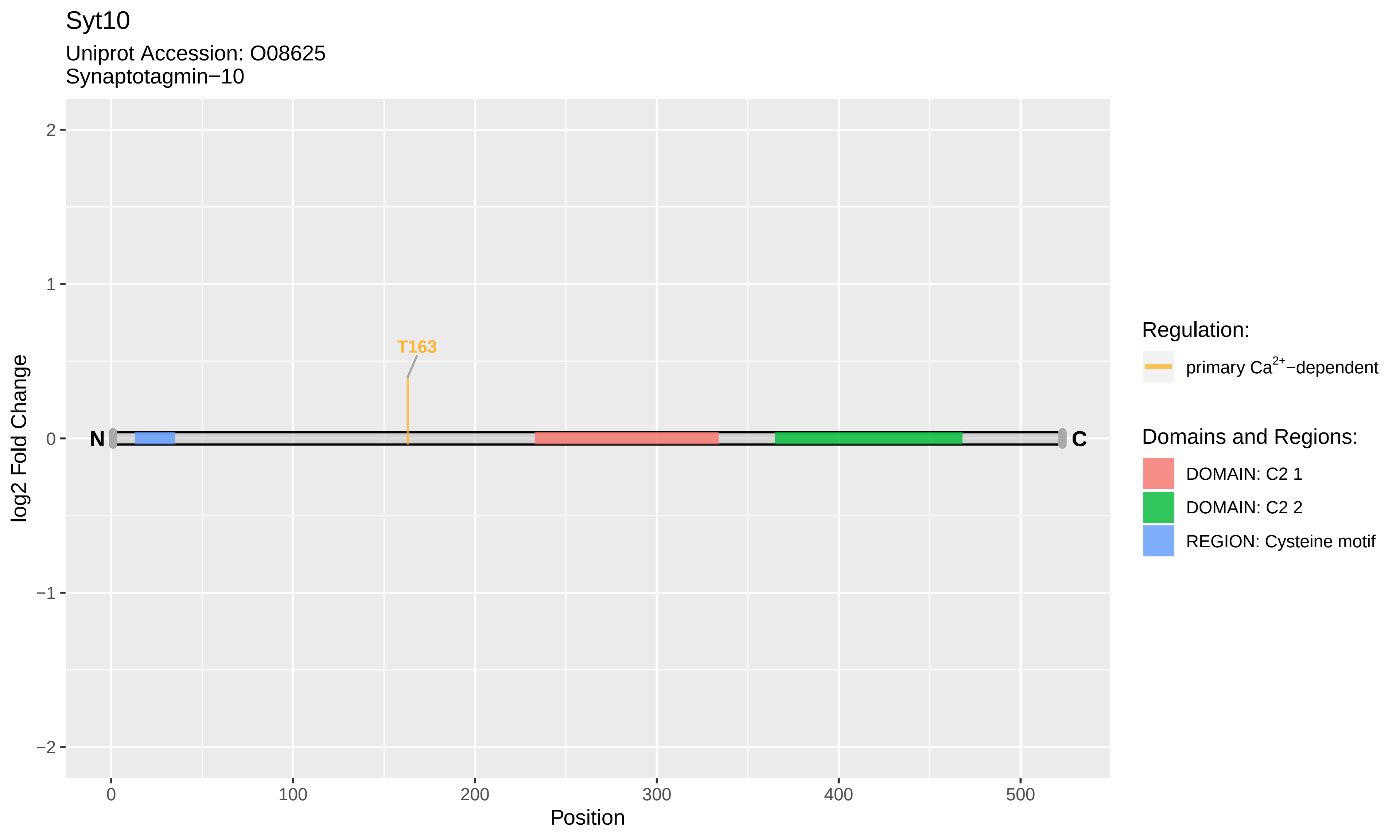

Syt10
Uniprot Accession: O08625
Synaptotagmin−10
T163
N
C
log2 Fold Change
Position
Regulation:
primary Ca2+−dependent
Domains and Regions:
DOMAIN: C2 1
DOMAIN: C2 2
REGION: Cysteine motif

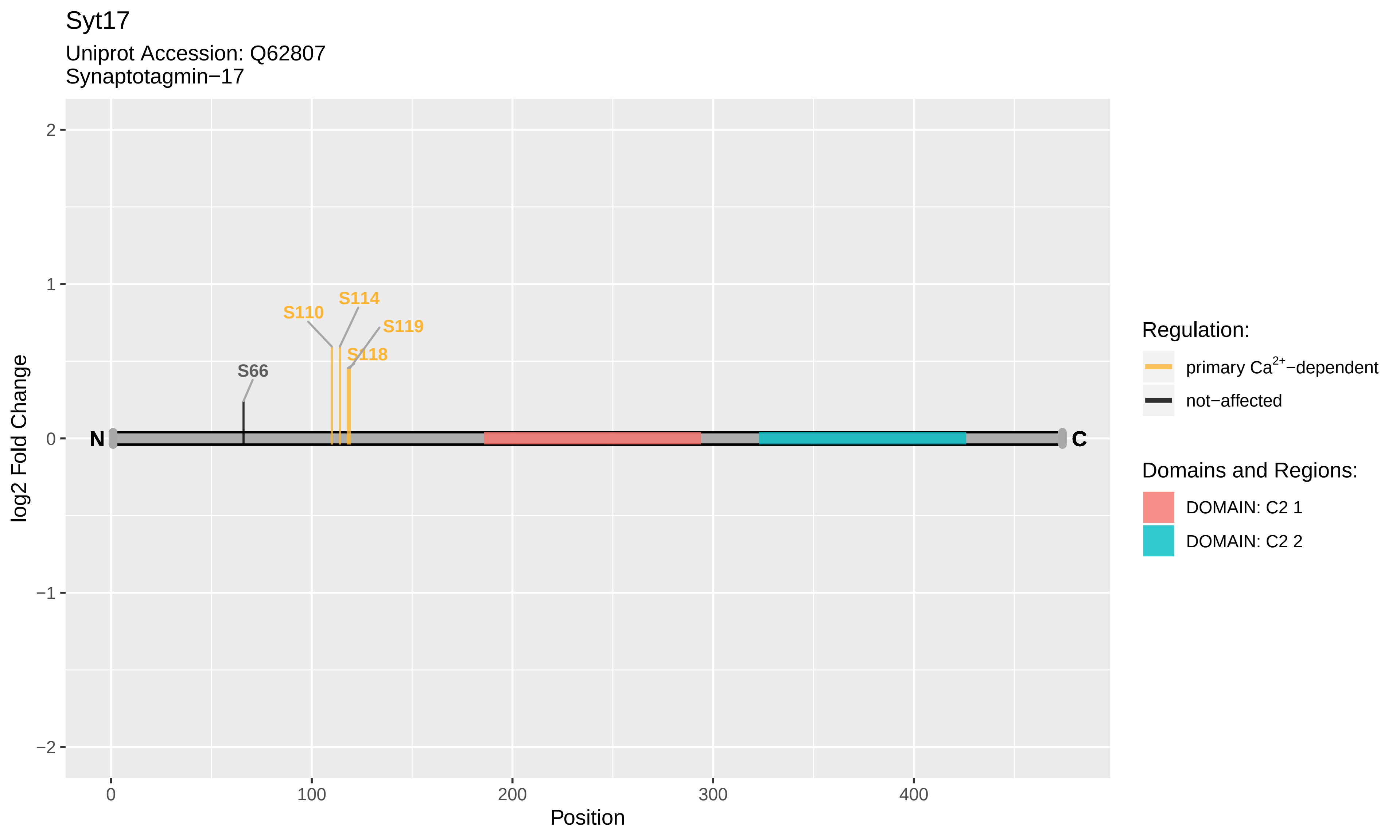
Syt17
Uniprot Accession: Q62807
Synaptotagmin−17
S66
S110
S114
S118
S119
N
C
log2 Fold Change
Position
2
1
0
−1
−2
0
100
200
300
400
Regulation:
primary Ca2+−dependent
not−affected
Domains and Regions:
DOMAIN: C2 1
DOMAIN: C2 2

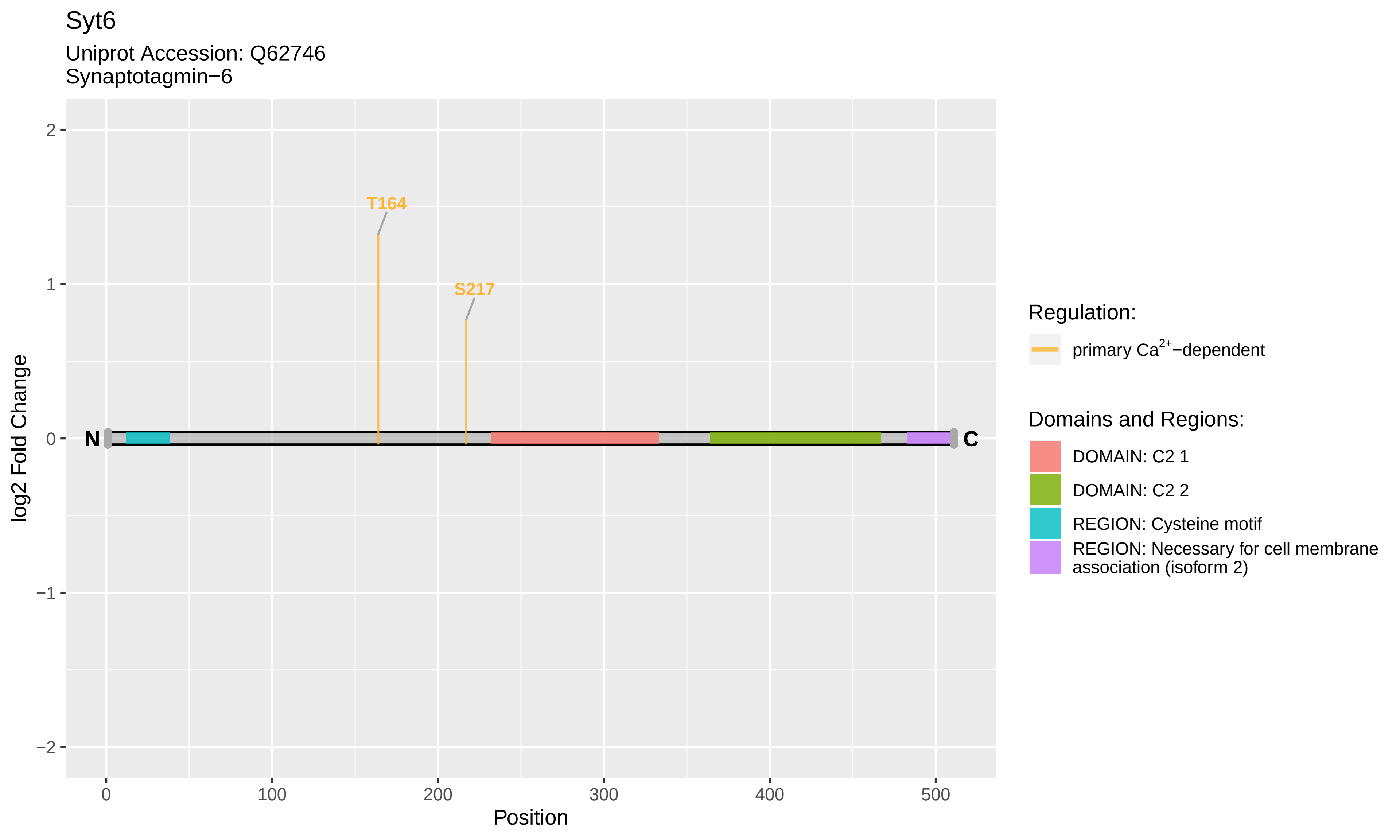

Syt6
Uniprot Accession: Q62746
Synaptotagmin−6
T164
S217
N
C
log2 Fold Change
Position
2
1
0
−1
−2
0
100
200
300
400
500
Regulation:
primary Ca2+−dependent
Domains and Regions:
DOMAIN: C2 1
DOMAIN: C2 2
REGION: Cysteine motif
REGION: Necessary for cell membrane association (isoform 2)

Syt9

Uniprot Accession: Q925C0
Synaptotagmin−9

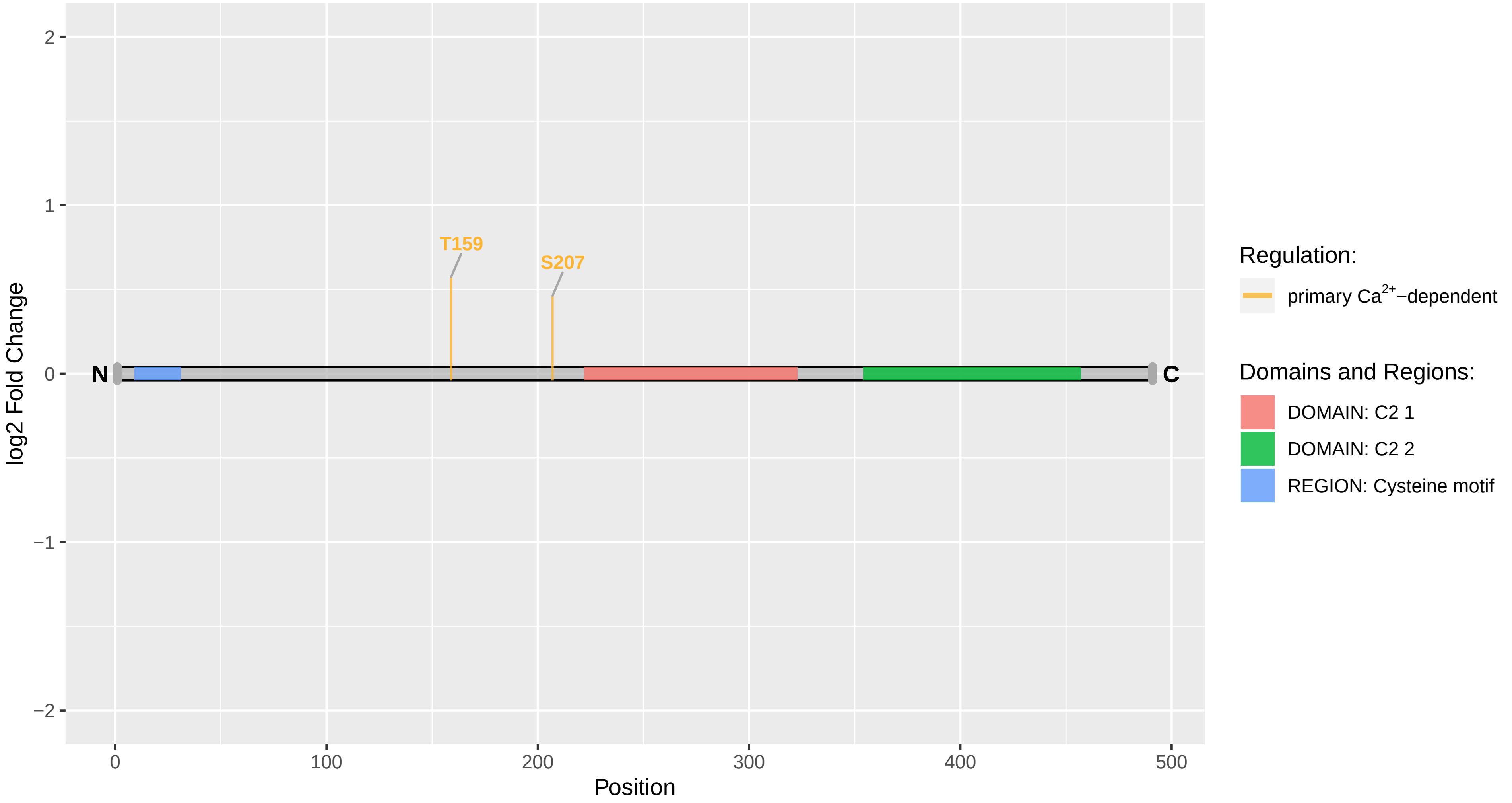

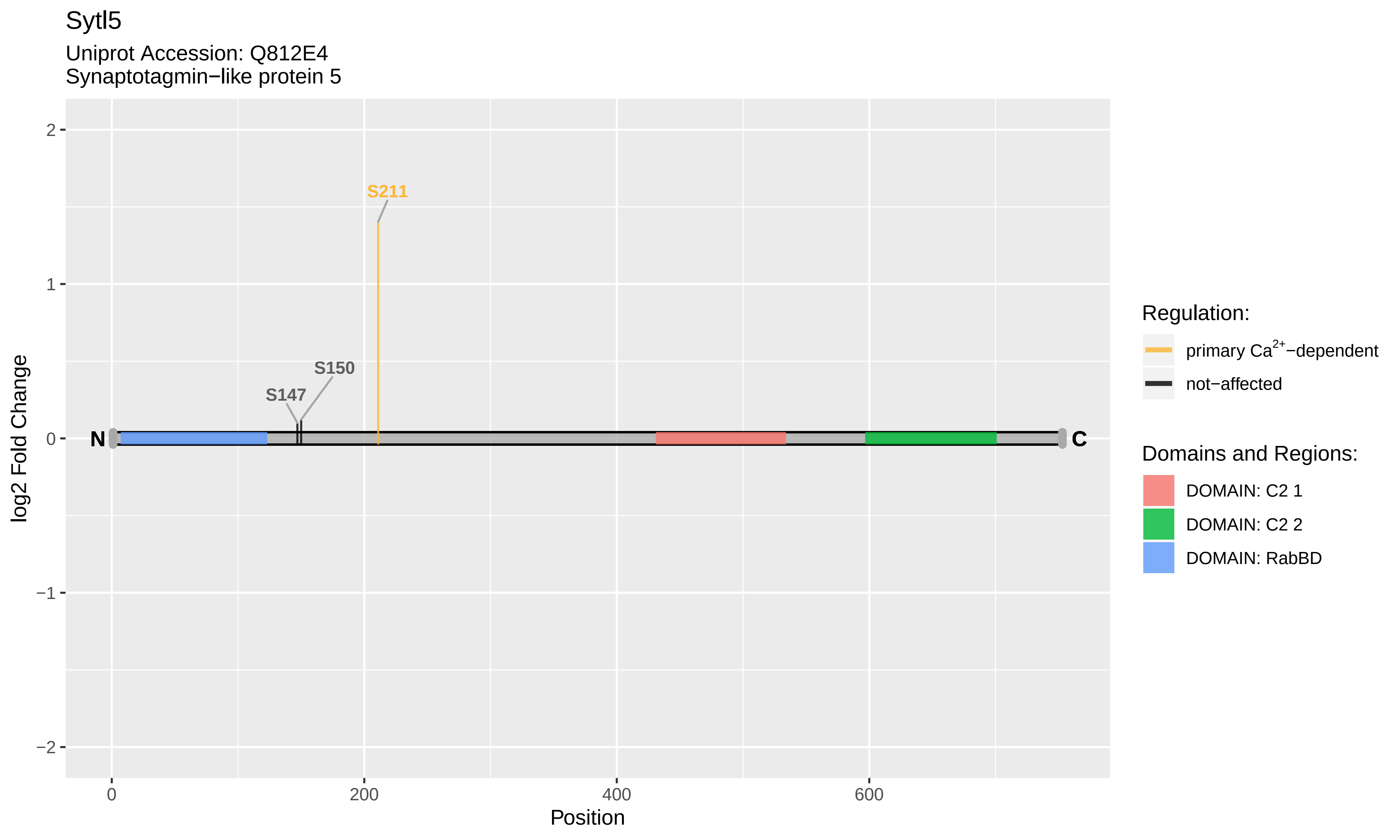

Sytl5
Uniprot Accession: Q812E4
Synaptotagmin−like protein 5
S211
S150
S147
N
C
log2 Fold Change
2
1
0
−1
−2
0
200
400
600
Position
Regulation:
primary Ca2+−dependent
not−affected
Domains and Regions:
DOMAIN: C2 1
DOMAIN: C2 2
DOMAIN: RabBD

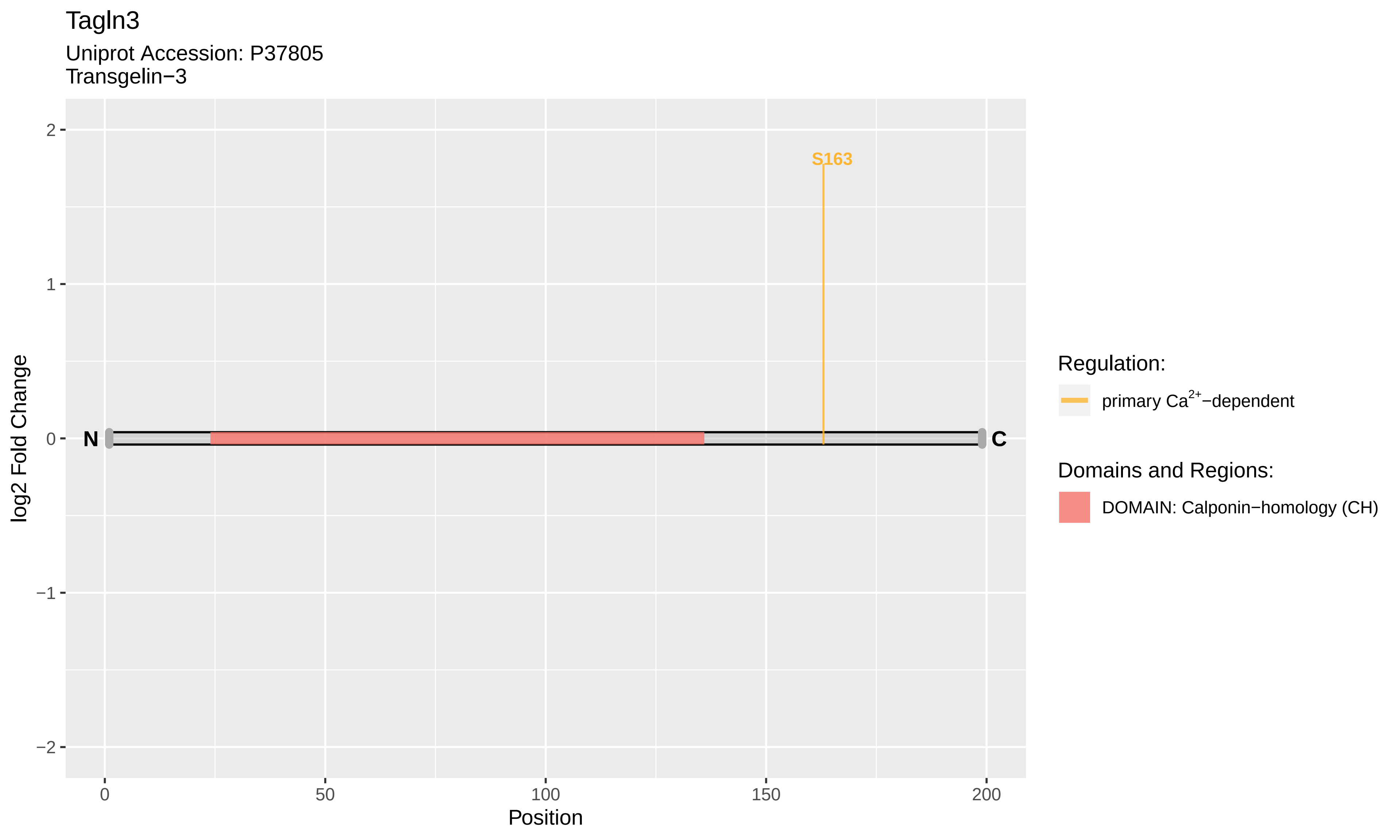

Tagln3
Uniprot Accession: P37805
Transgelin−3
S163
N
C
log2 Fold Change
2
1
0
−1
−2
0
50
100
150
200
Position
Regulation:
primary Ca2+−dependent
Domains and Regions:
DOMAIN: Calponin−homology (CH)

Tanc1

Uniprot Accession: G3V917
Protein TANC1

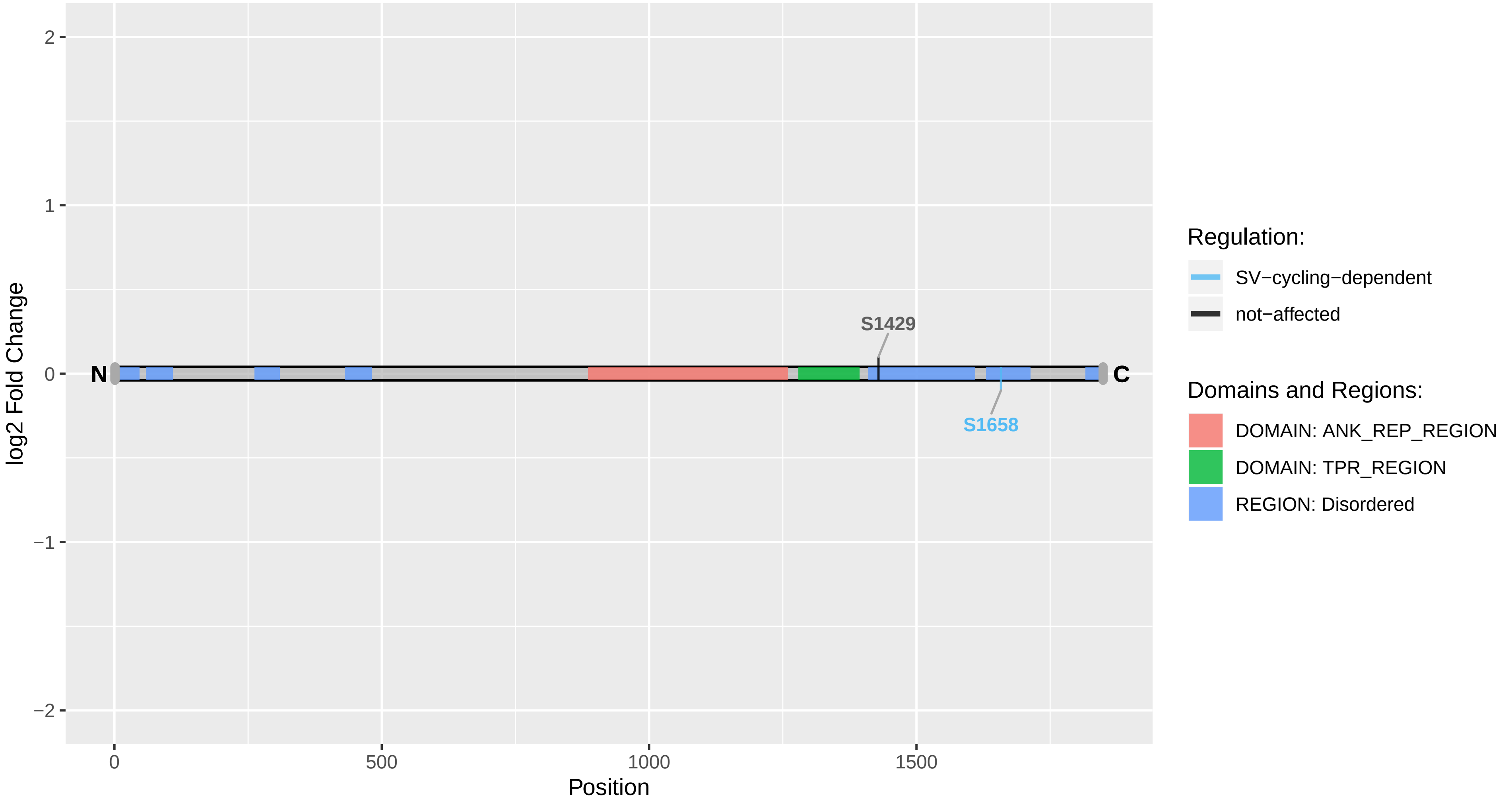

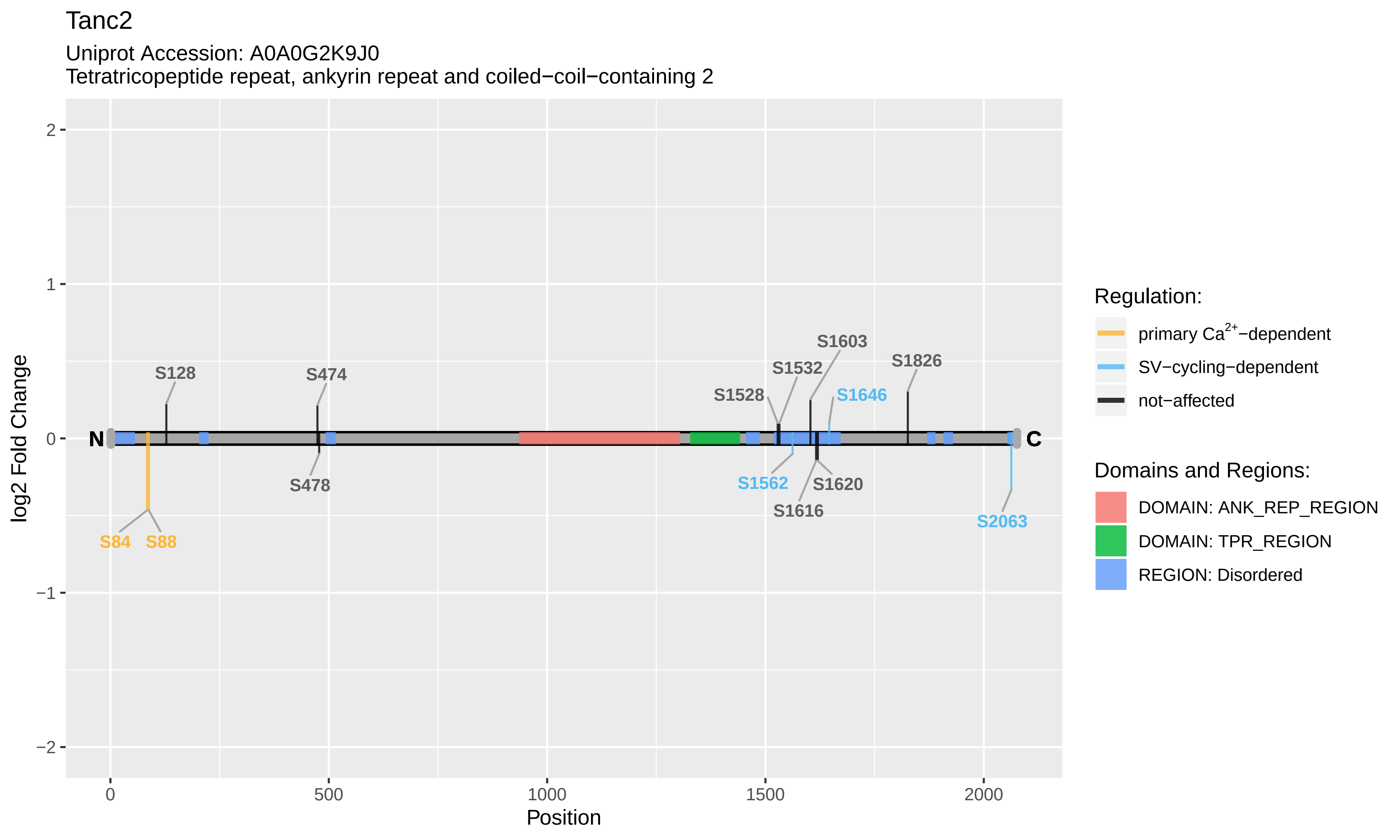

Tanc2
Uniprot Accession: A0A0G2K9J0
Tetratricopeptide repeat, ankyrin repeat and coiled−coil−containing 2
log2 Fold Change
Position
N
C
S128
S474
S478
S84
S88
S1528
S1532
S1603
S1646
S1826
S1562
S1616
S1620
S2063
Regulation:
primary Ca2+−dependent
SV−cycling−dependent
not−affected
Domains and Regions:
DOMAIN: ANK_REP_REGION
DOMAIN: TPR_REGION
REGION: Disordered

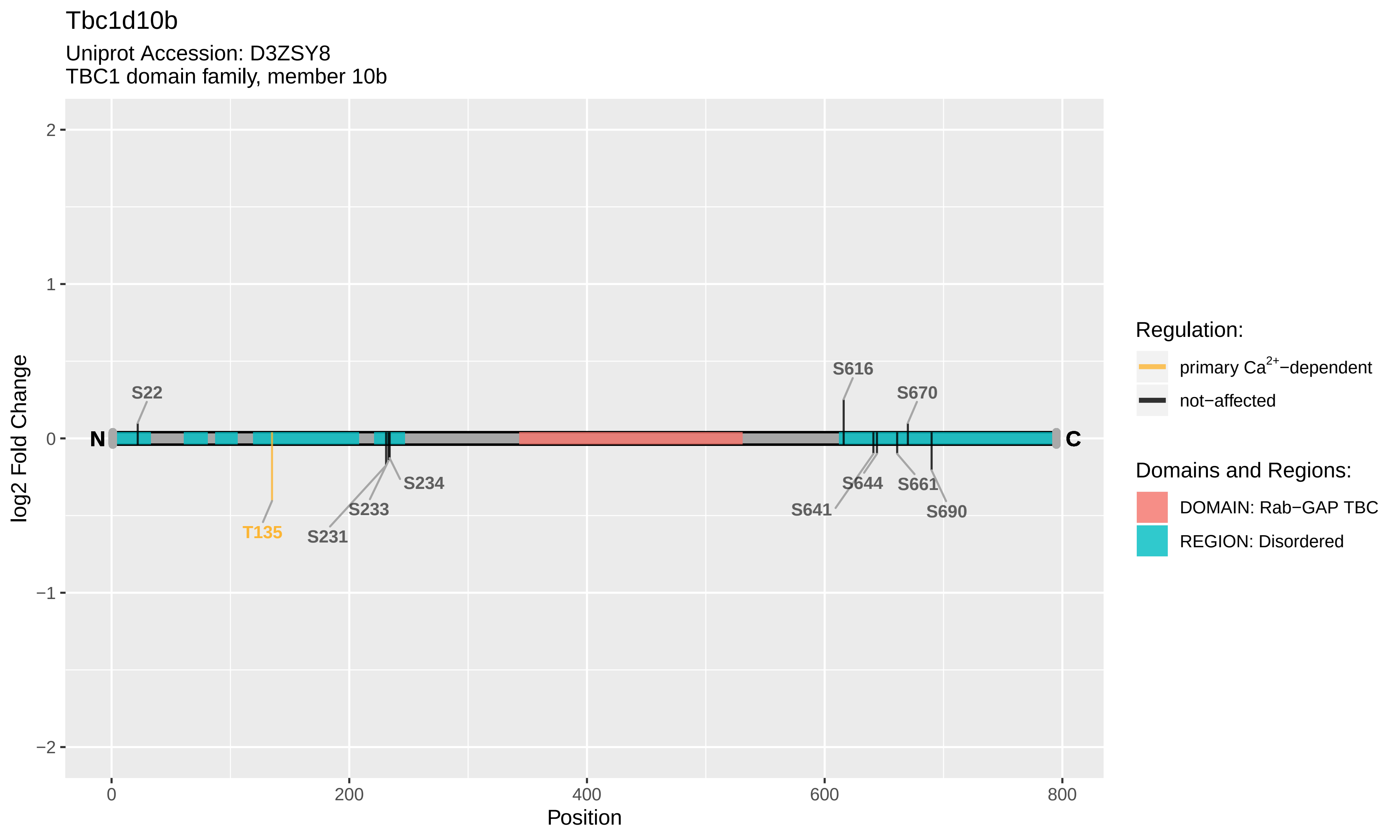

Tbc1d10b
Uniprot Accession: D3ZSY8
TBC1 domain family, member 10b
log2 Fold Change
Position
N
C
S22
T135
S231
S233
S234
S616
S641
S644
S661
S670
S690
Regulation:
primary Ca2+−dependent
not−affected
Domains and Regions:
DOMAIN: Rab−GAP TBC
REGION: Disordered

Tbc1d22b

Uniprot Accession: A0A0G2JWM9
TBC1 domain family, member 22B

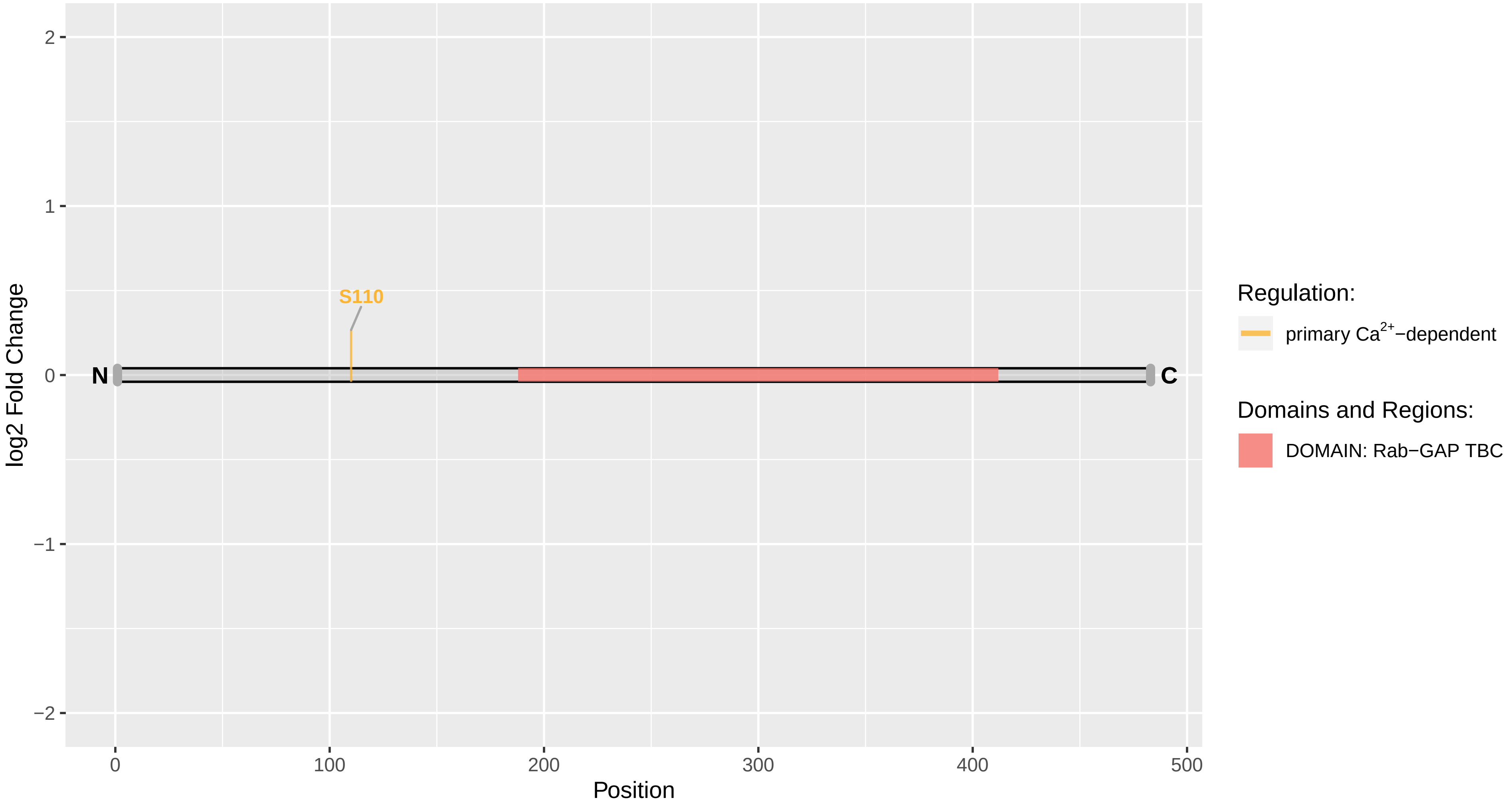

Tbc1d24

Uniprot Accession: A0A0G2K5B0
TBC1 domain family, member 24

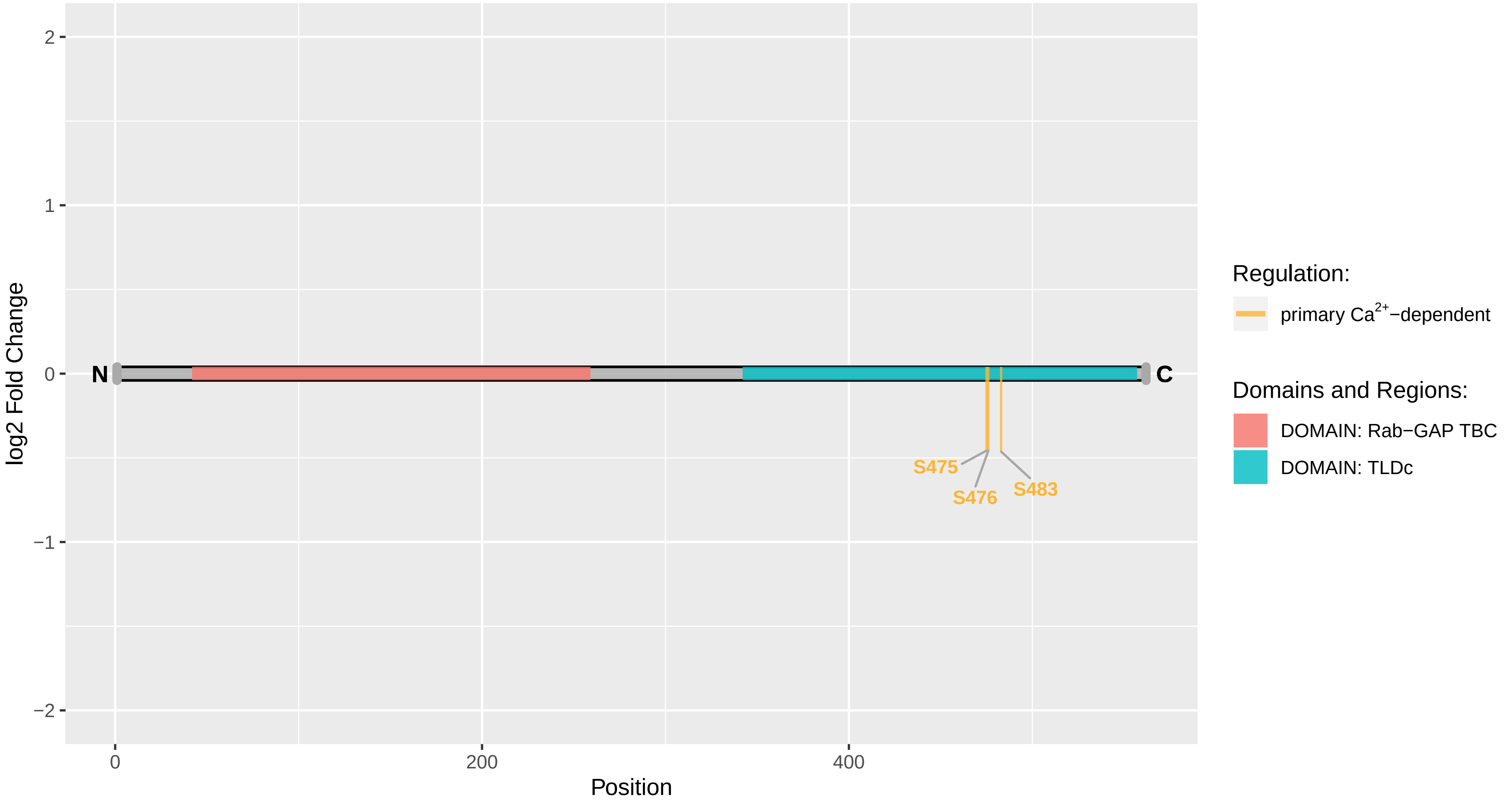

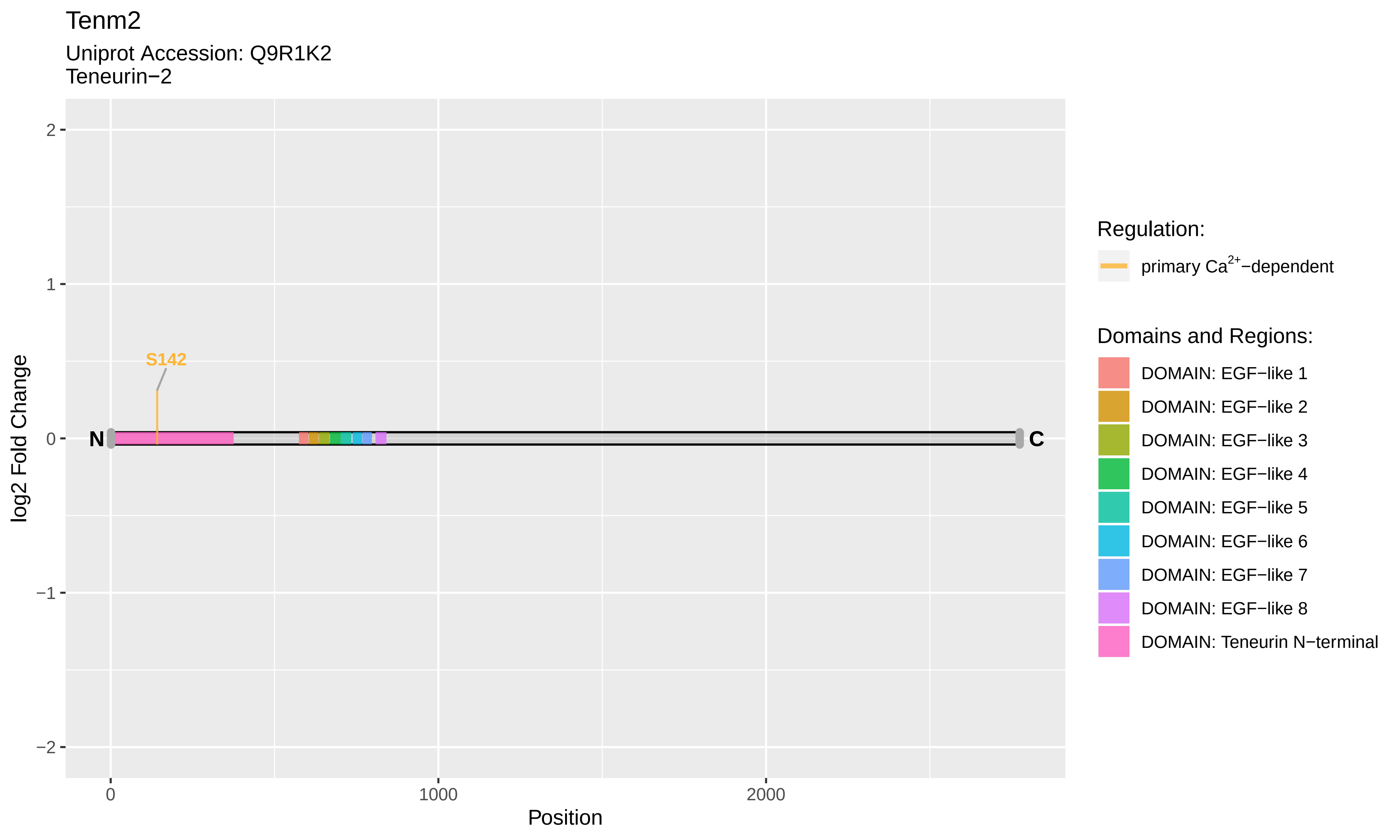
Tenm2
Uniprot Accession: Q9R1K2
Teneurin−2
log2 Fold Change
2
1
0
−1
−2
S142
N
C
0
1000
2000
Position
Regulation:
primary Ca2+−dependent
Domains and Regions:
DOMAIN: EGF−like 1
DOMAIN: EGF−like 2
DOMAIN: EGF−like 3
DOMAIN: EGF−like 4
DOMAIN: EGF−like 5
DOMAIN: EGF−like 6
DOMAIN: EGF−like 7
DOMAIN: EGF−like 8
DOMAIN: Teneurin N−terminal

Tex2

Uniprot Accession: G3V9L1
Testis expressed gene 2

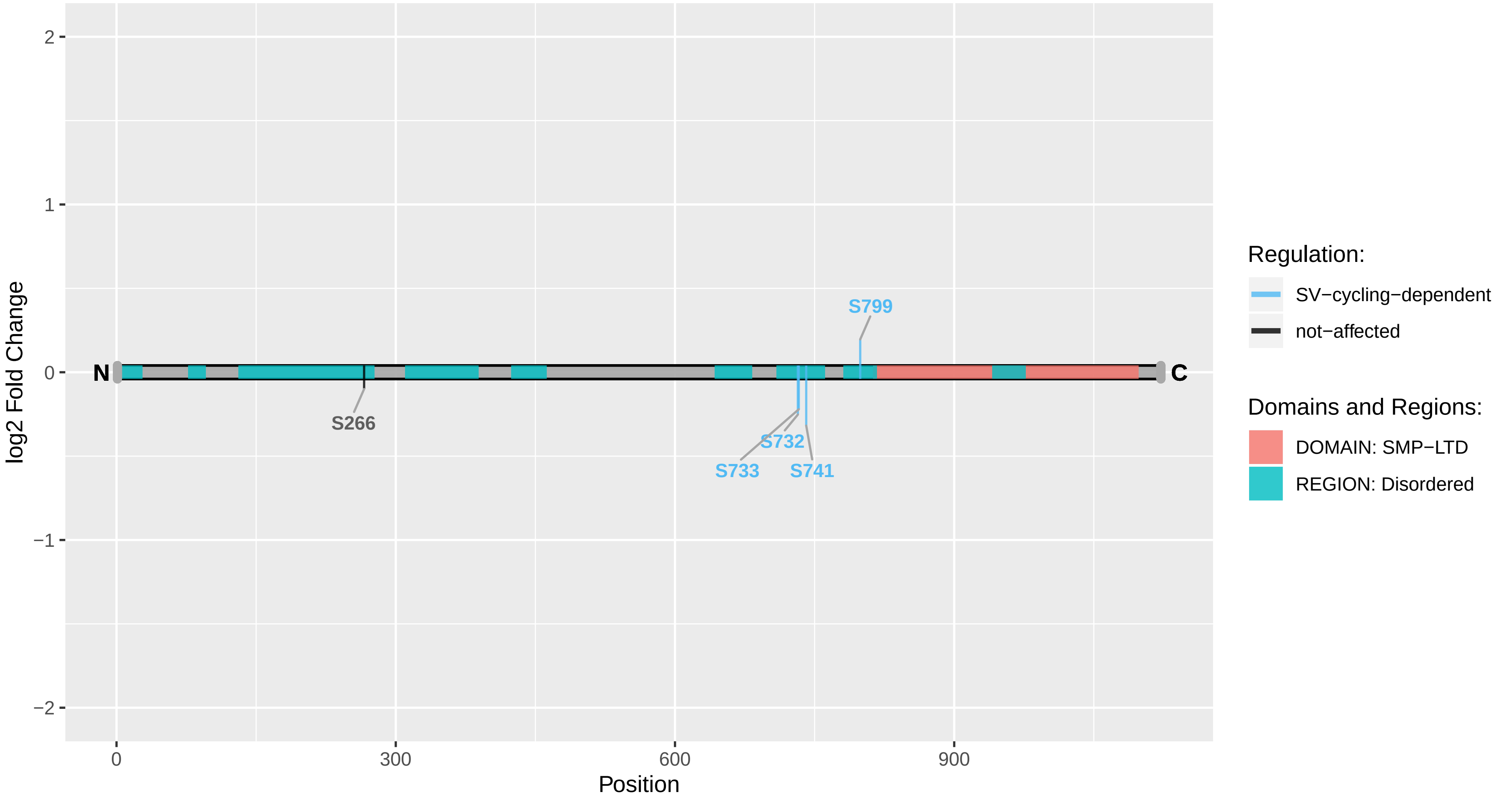

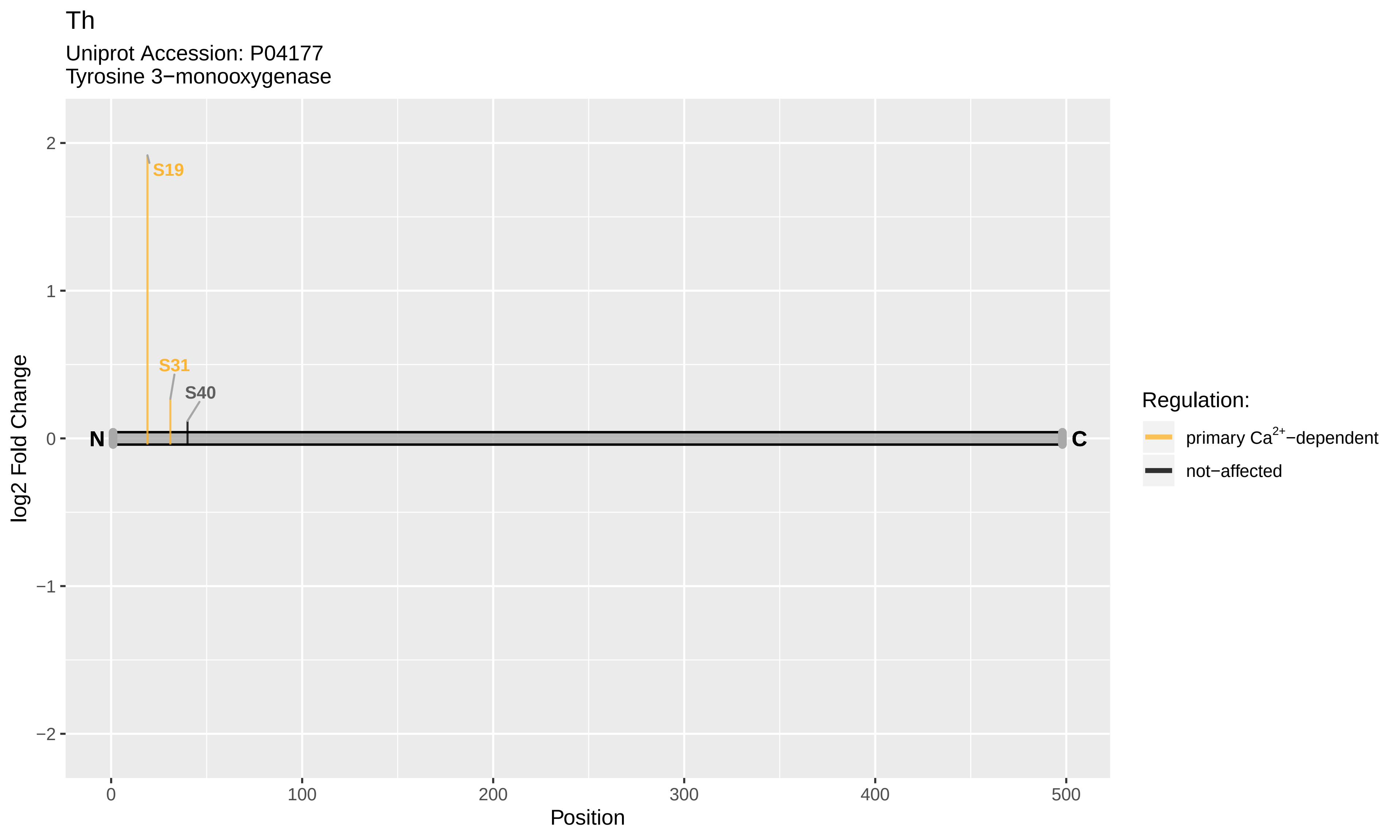

Th
Uniprot Accession: P04177
Tyrosine 3−monooxygenase
S19
S31
S40
N
C
2
1
0
−1
−2
log2 Fold Change
0
100
200
300
400
500
Position
Regulation:
primary Ca2+−dependent
not−affected

Tjp1

Uniprot Accession: A0A0G2K2P5
Tight junction protein ZO−1

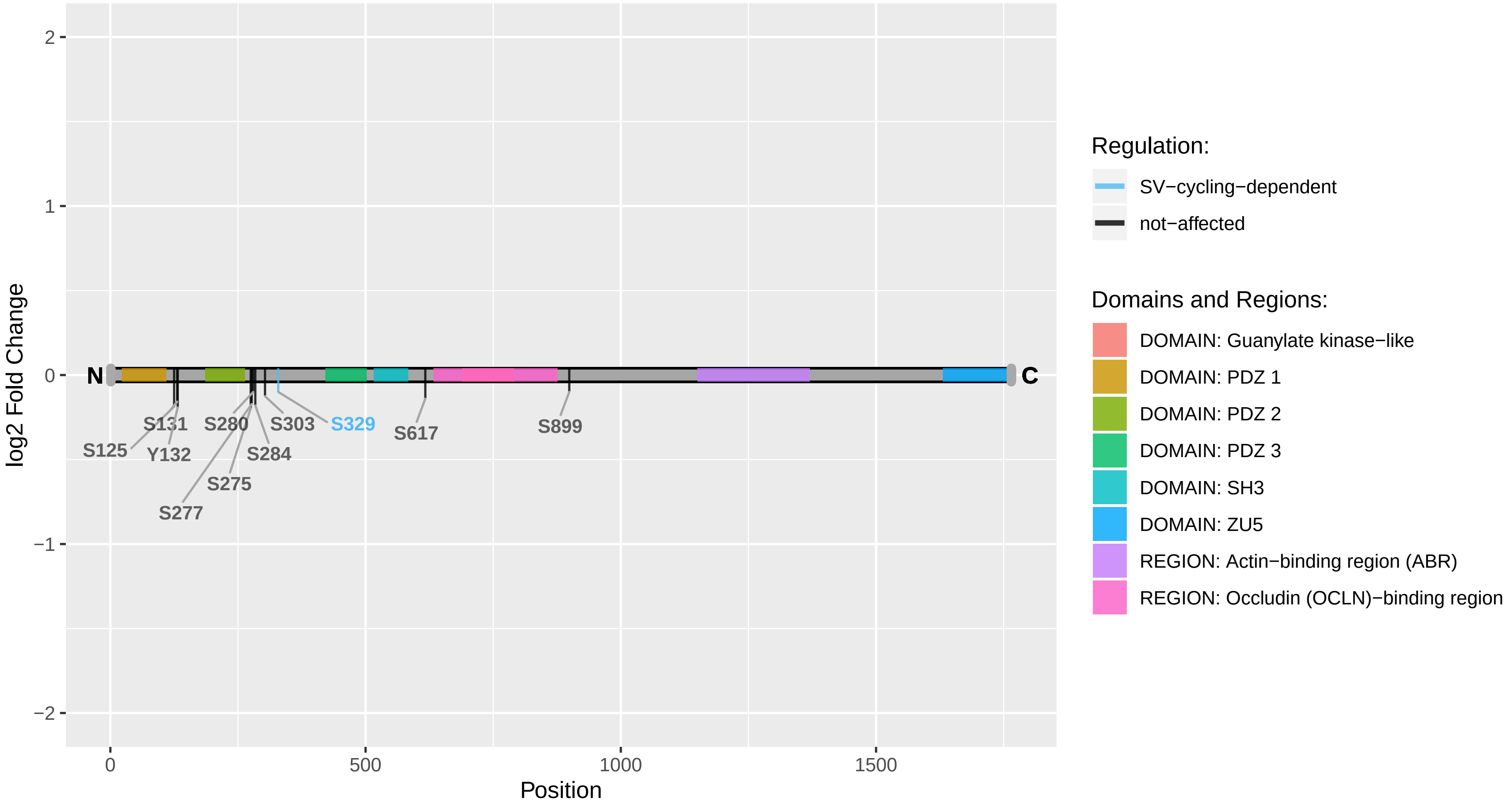

Tjp2

Uniprot Accession: Q3ZB99
Tight junction protein 2

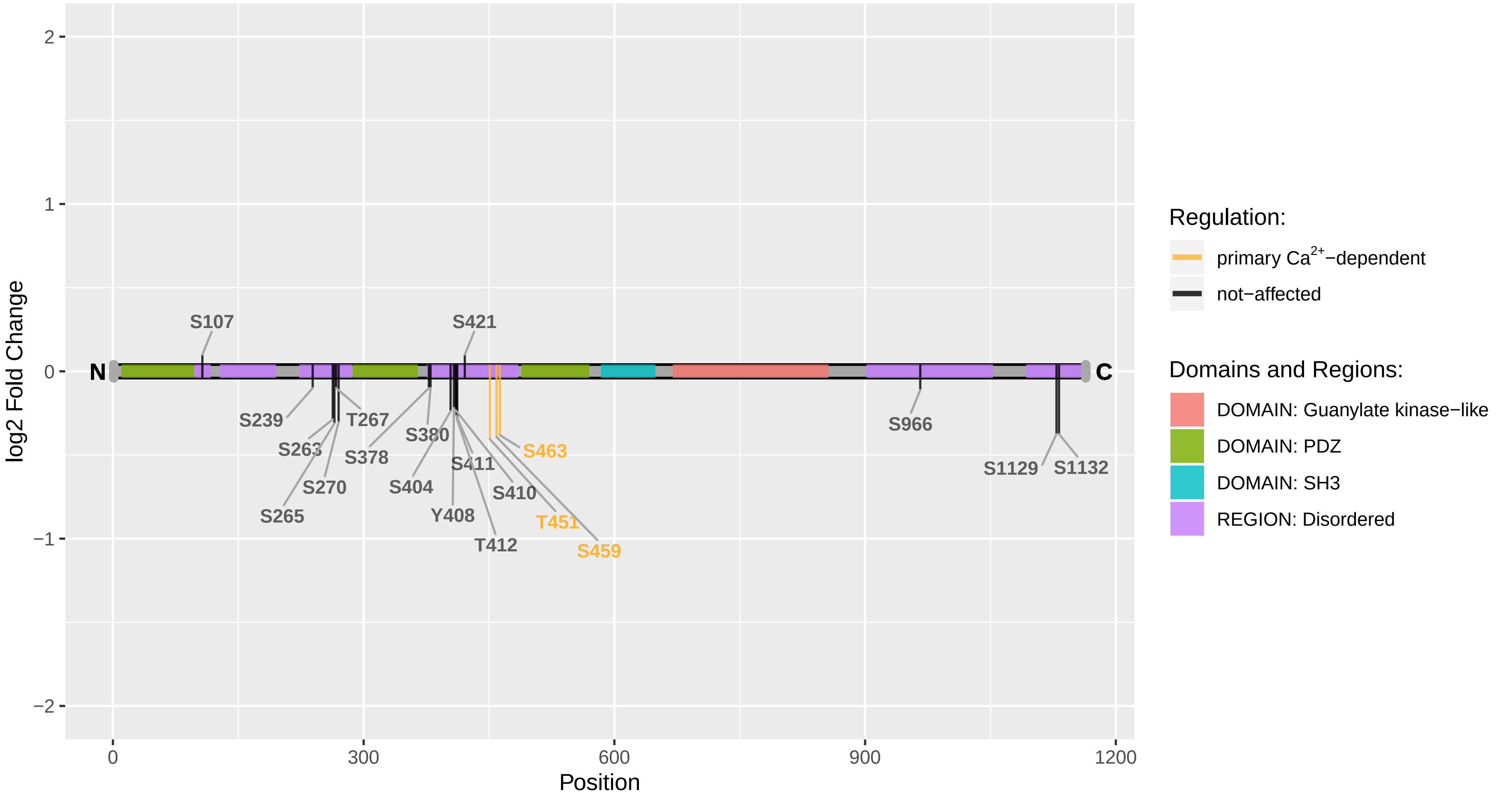

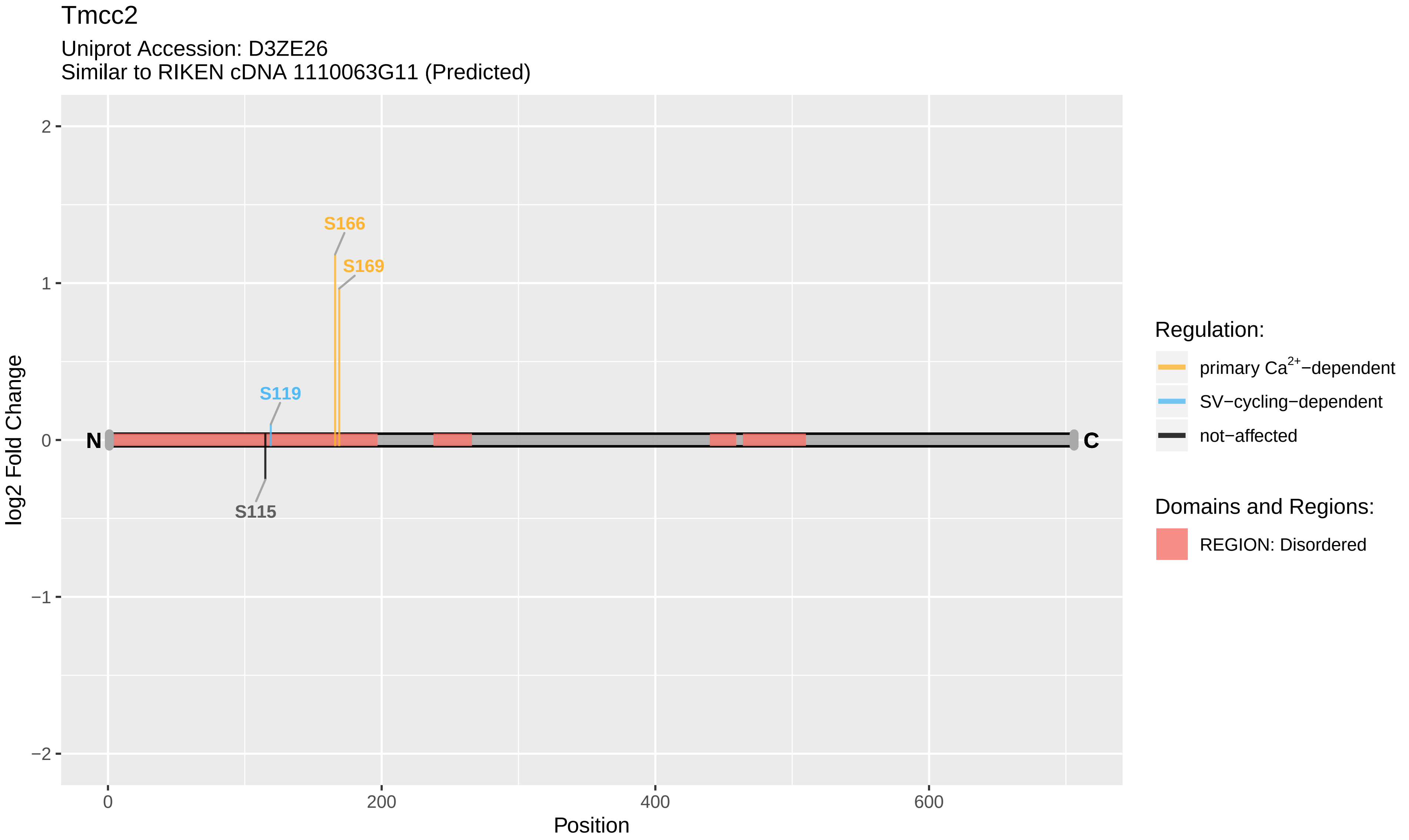

Tmcc2
Uniprot Accession: D3ZE26
Similar to RIKEN cDNA 1110063G11 (Predicted)
S166
S169
S119
S115
N
C
2
1
0
−1
−2
log2 Fold Change
0
200
400
600
Position
Regulation:
primary Ca2+−dependent
SV−cycling−dependent
not−affected
Domains and Regions:
REGION: Disordered

Tmem163

Uniprot Accession: A9CMA6
Transmembrane protein 163

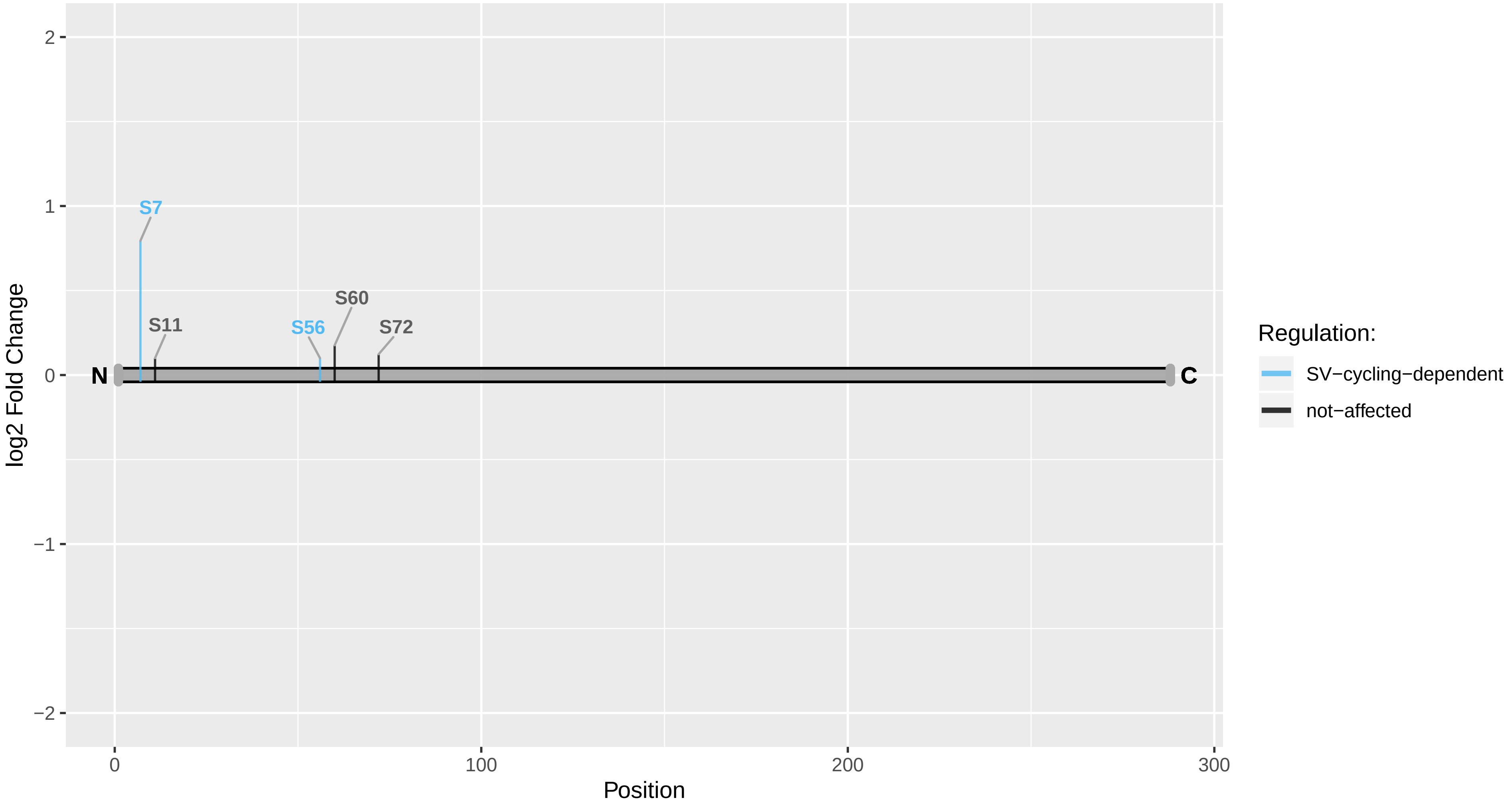

Tmem266

Uniprot Accession: D3ZYW8
Similar to expressed sequence AI118078 (Predicted)

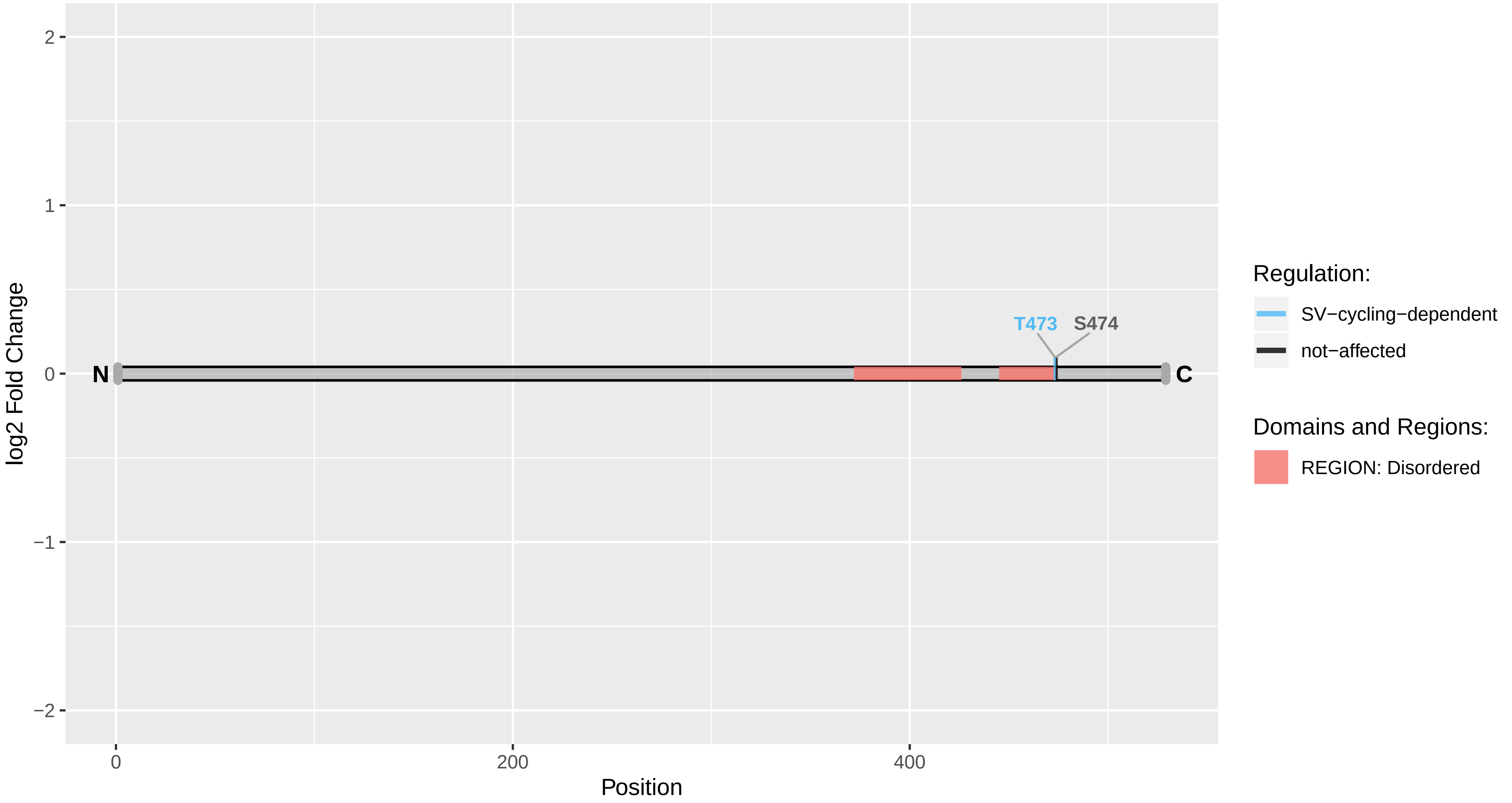

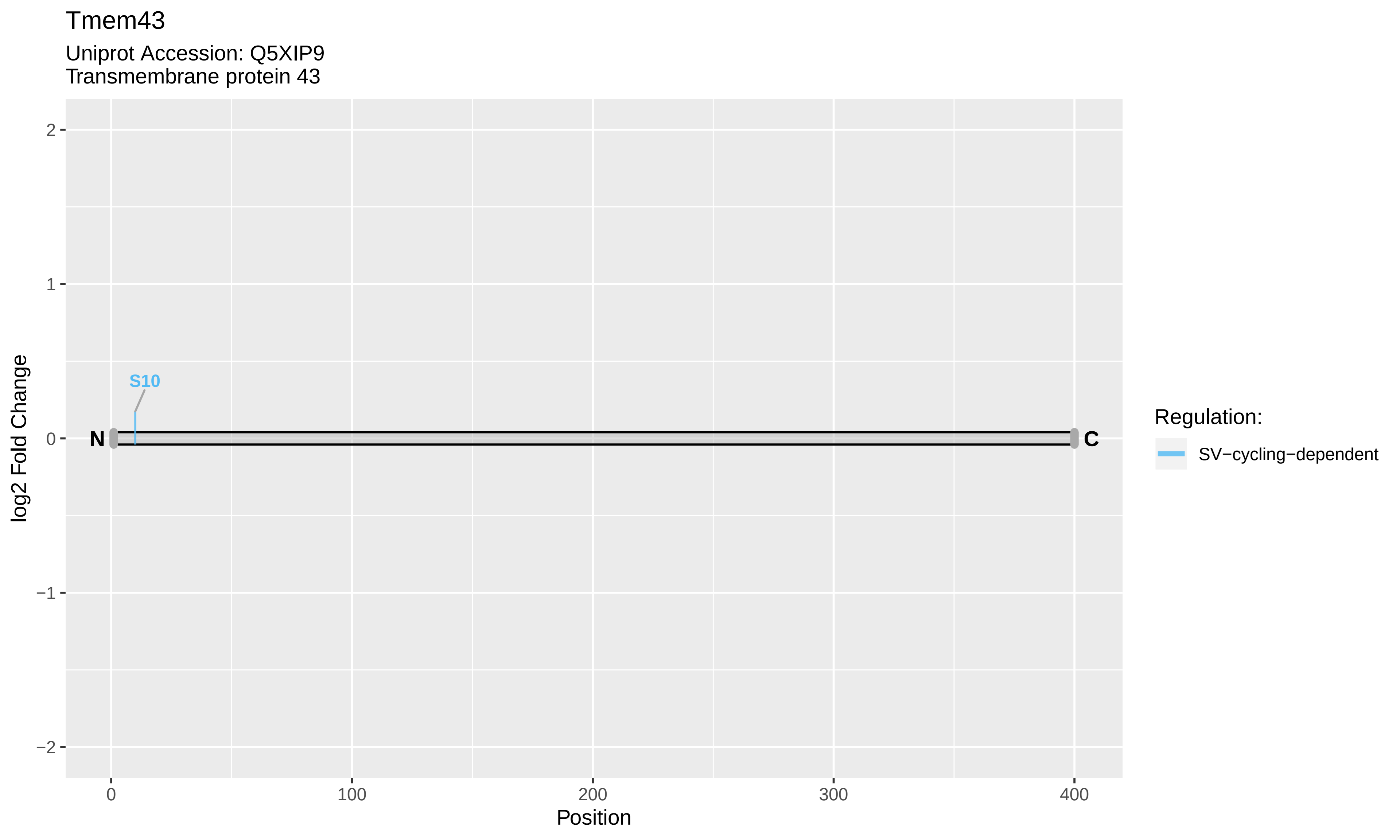

Tmem43
Uniprot Accession: Q5XIP9
Transmembrane protein 43
S10
N
C
2
1
0
−1
−2
0
100
200
300
400
Position
log2 Fold Change
Regulation:
SV−cycling−dependent

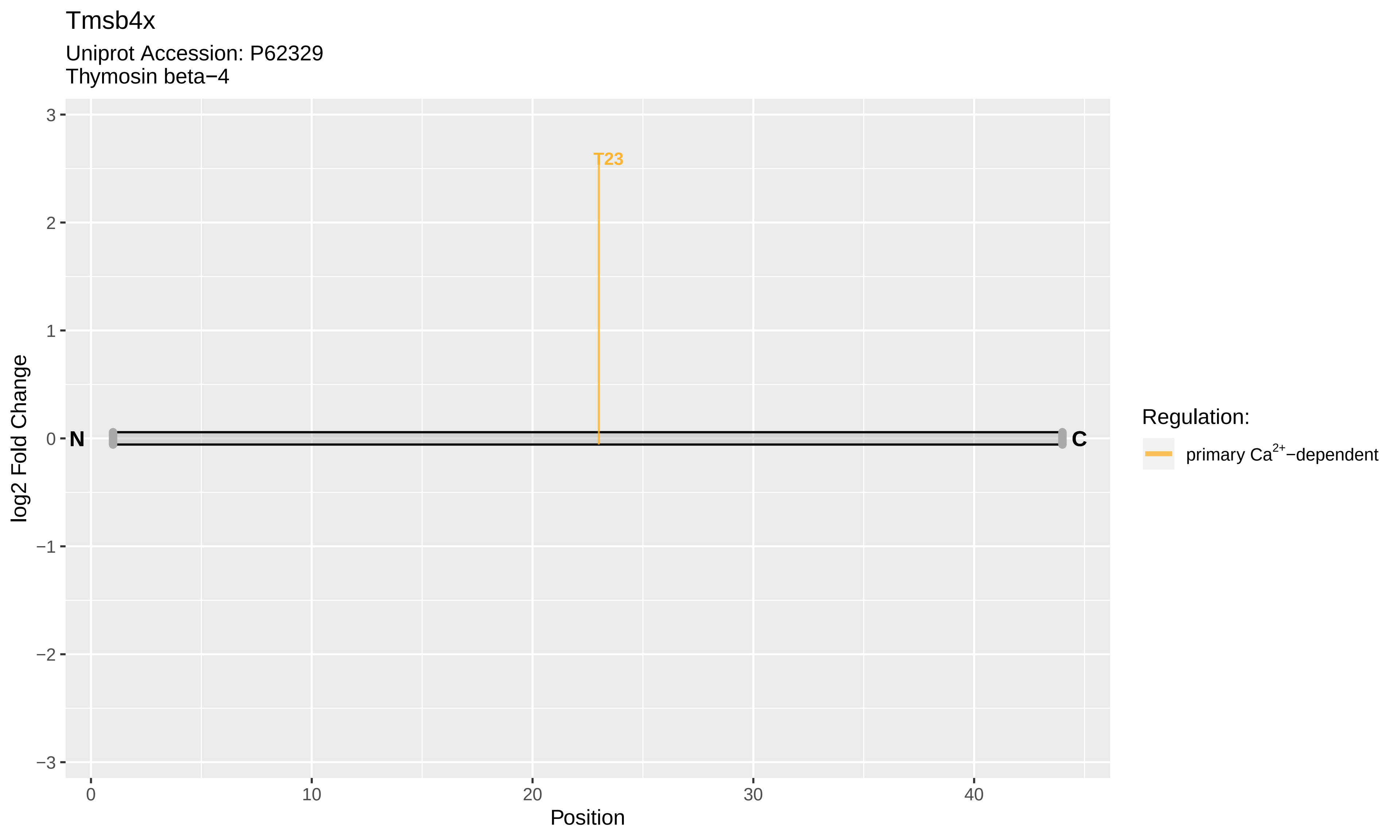

Tmsb4x
Uniprot Accession: P62329
Thymosin beta−4
T23
N
C
log2 Fold Change
Position
Regulation:
primary Ca2+−dependent

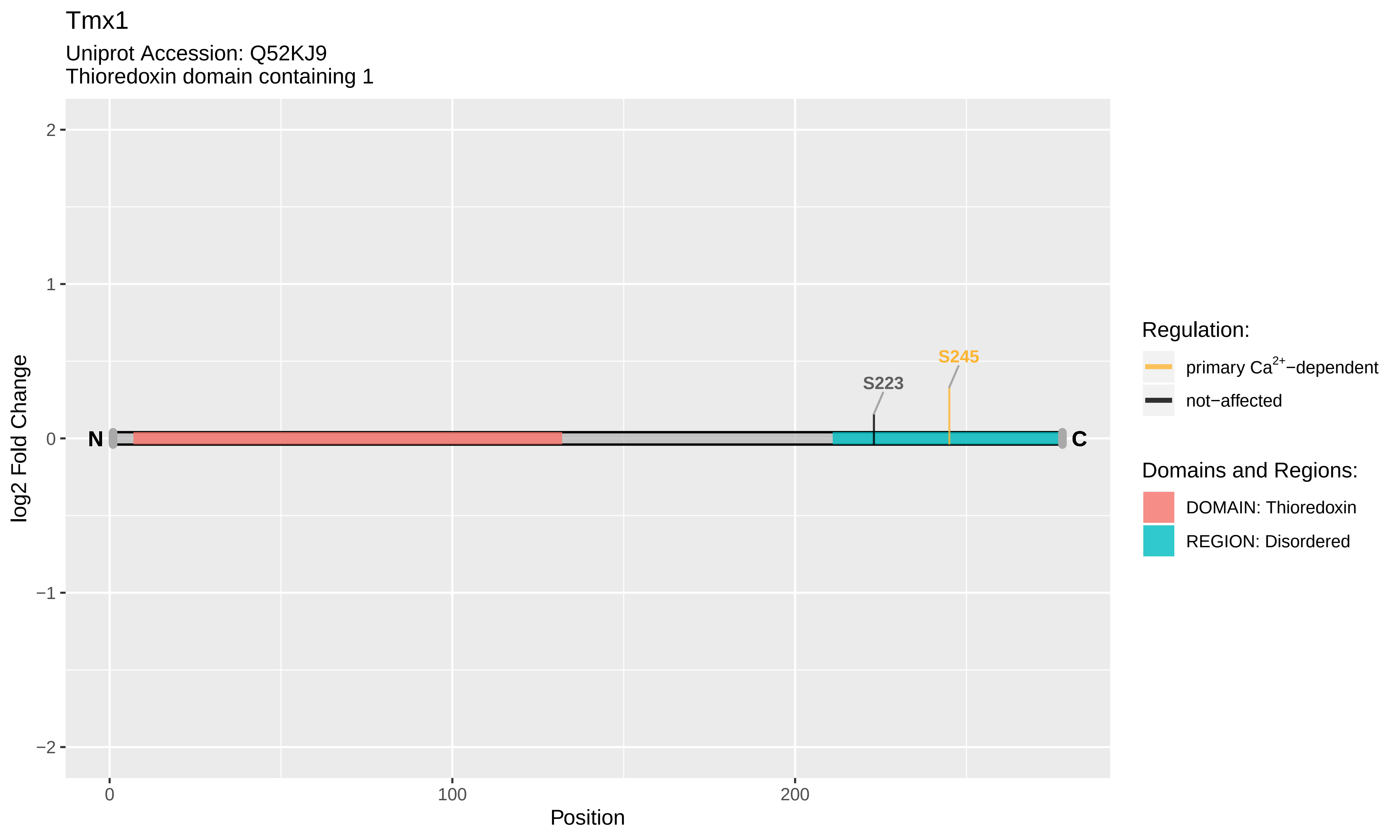

Tmx1
Uniprot Accession: Q52KJ9
Thioredoxin domain containing 1
log2 Fold Change
2
1
0
−1
−2
N
C
S223
S245
0
100
200
Position
Regulation:
primary Ca2+−dependent
not−affected
Domains and Regions:
DOMAIN: Thioredoxin
REGION: Disordered

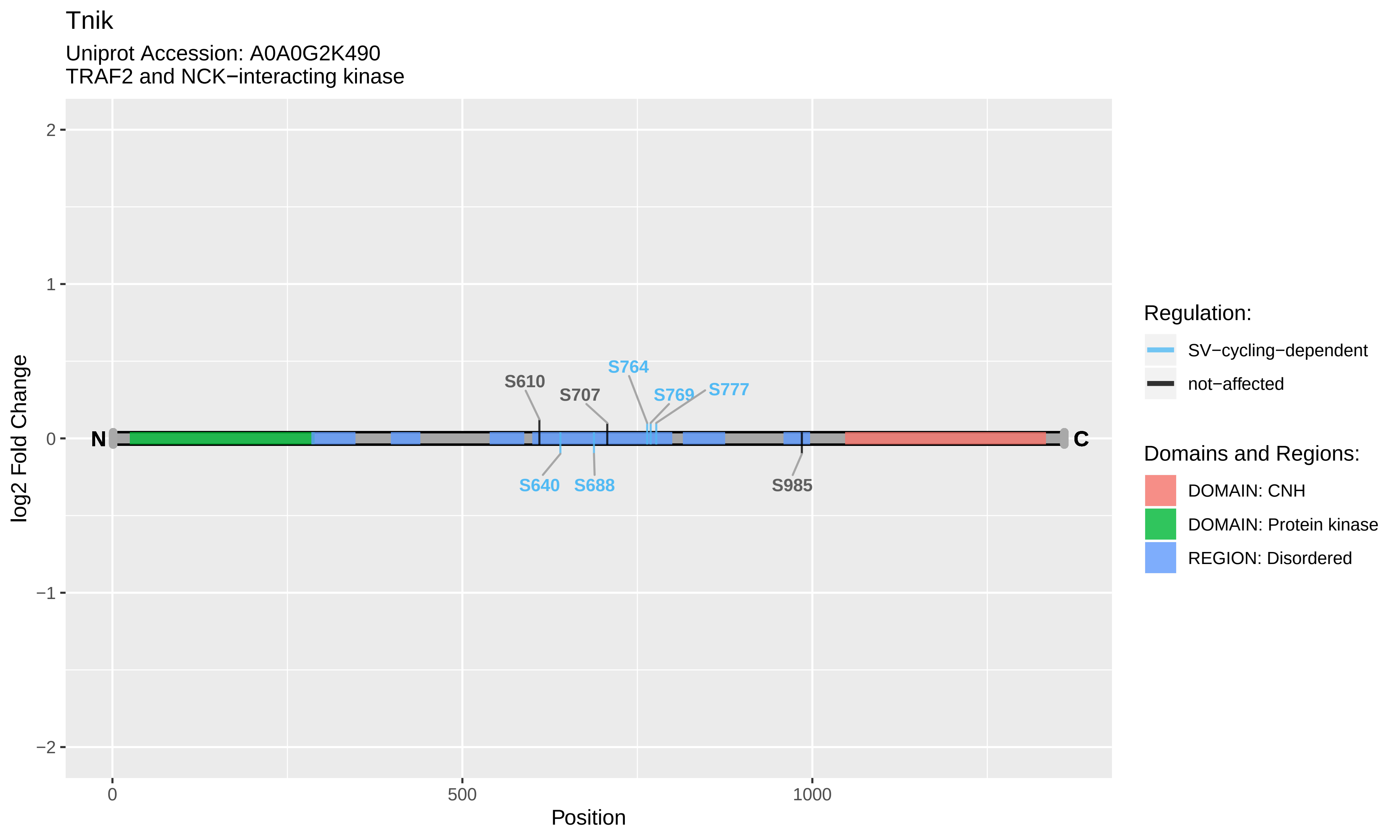

Tnik
Uniprot Accession: A0A0G2K490
TRAF2 and NCK−interacting kinase
log2 Fold Change
2
1
0
−1
−2
N
C
S610
S707
S764
S769
S777
S640
S688
S985
0
500
1000
Position
Regulation:
SV−cycling−dependent
not−affected
Domains and Regions:
DOMAIN: CNH
DOMAIN: Protein kinase
REGION: Disordered

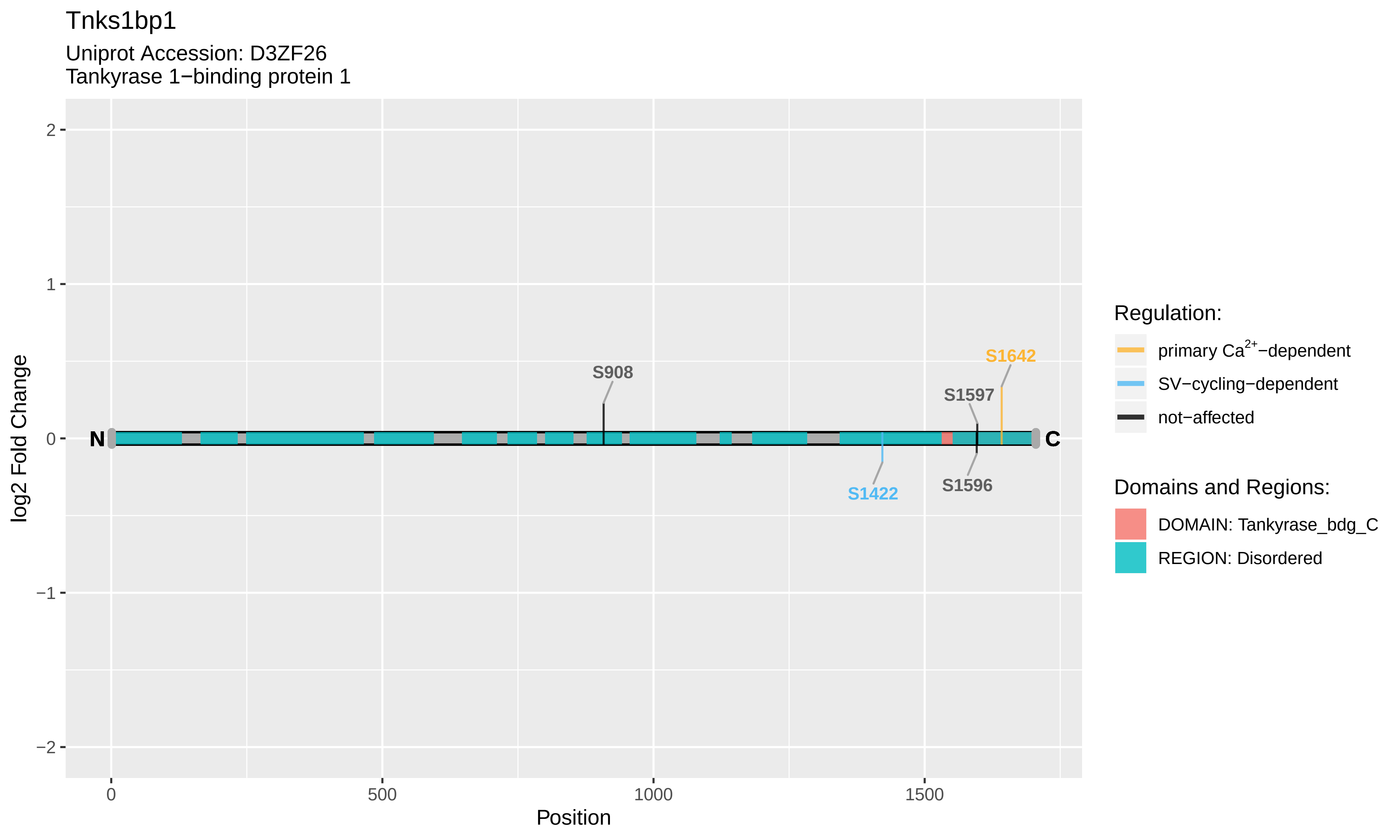

Tnks1bp1
Uniprot Accession: D3ZF26
Tankyrase 1−binding protein 1
S908
S1642
S1597
S1596
S1422
N
C
2
1
0
−1
−2
0
500
1000
1500
Position
log2 Fold Change
Regulation:
primary Ca2+−dependent
SV−cycling−dependent
not−affected
Domains and Regions:
DOMAIN: Tankyrase_bdg_C
REGION: Disordered

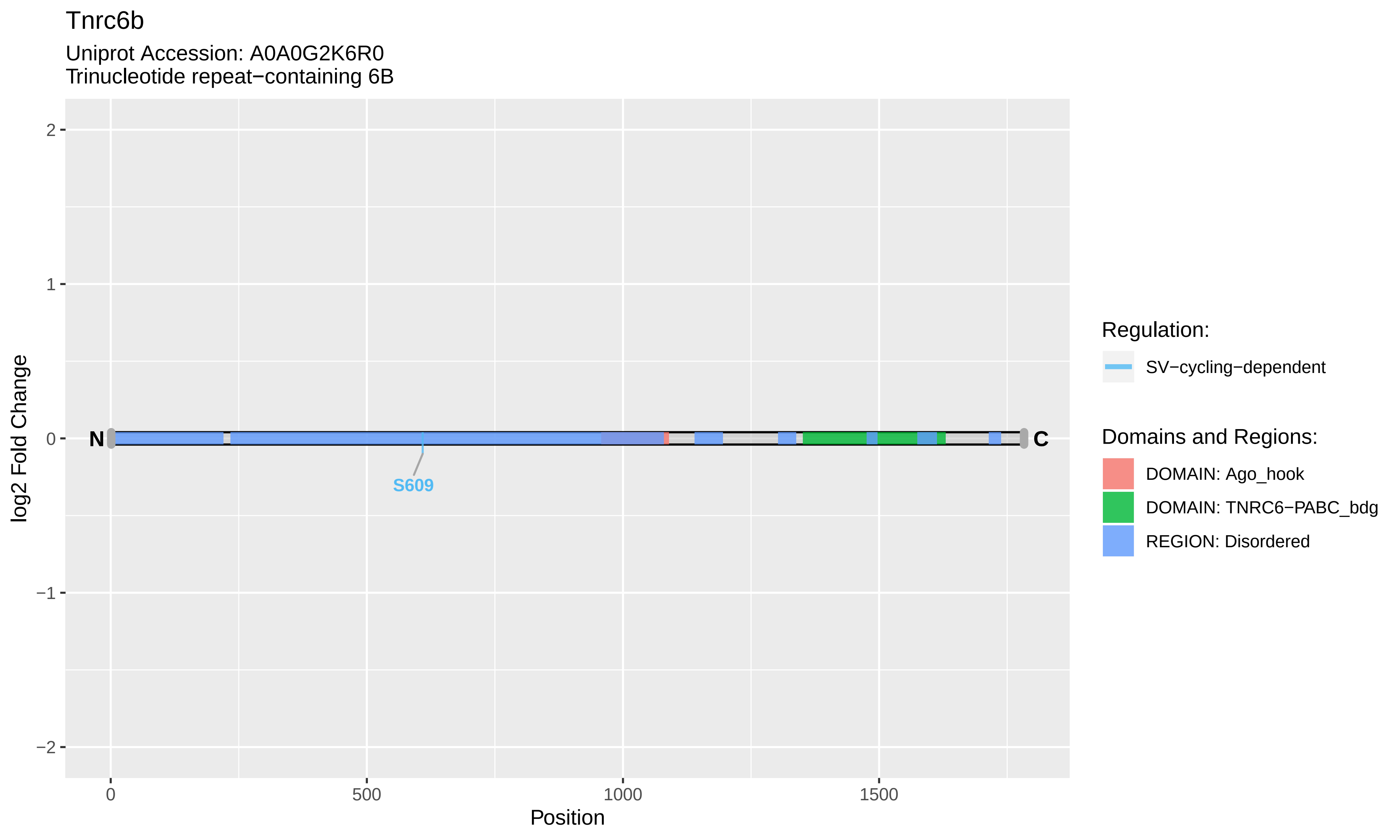

Tnrc6b
Uniprot Accession: A0A0G2K6R0
Trinucleotide repeat−containing 6B
log2 Fold Change
2
1
0
−1
−2
N
C
S609
0
500
1000
1500
Position
Regulation:
SV−cycling−dependent
Domains and Regions:
DOMAIN: Ago_hook
DOMAIN: TNRC6−PABC_bdg
REGION: Disordered

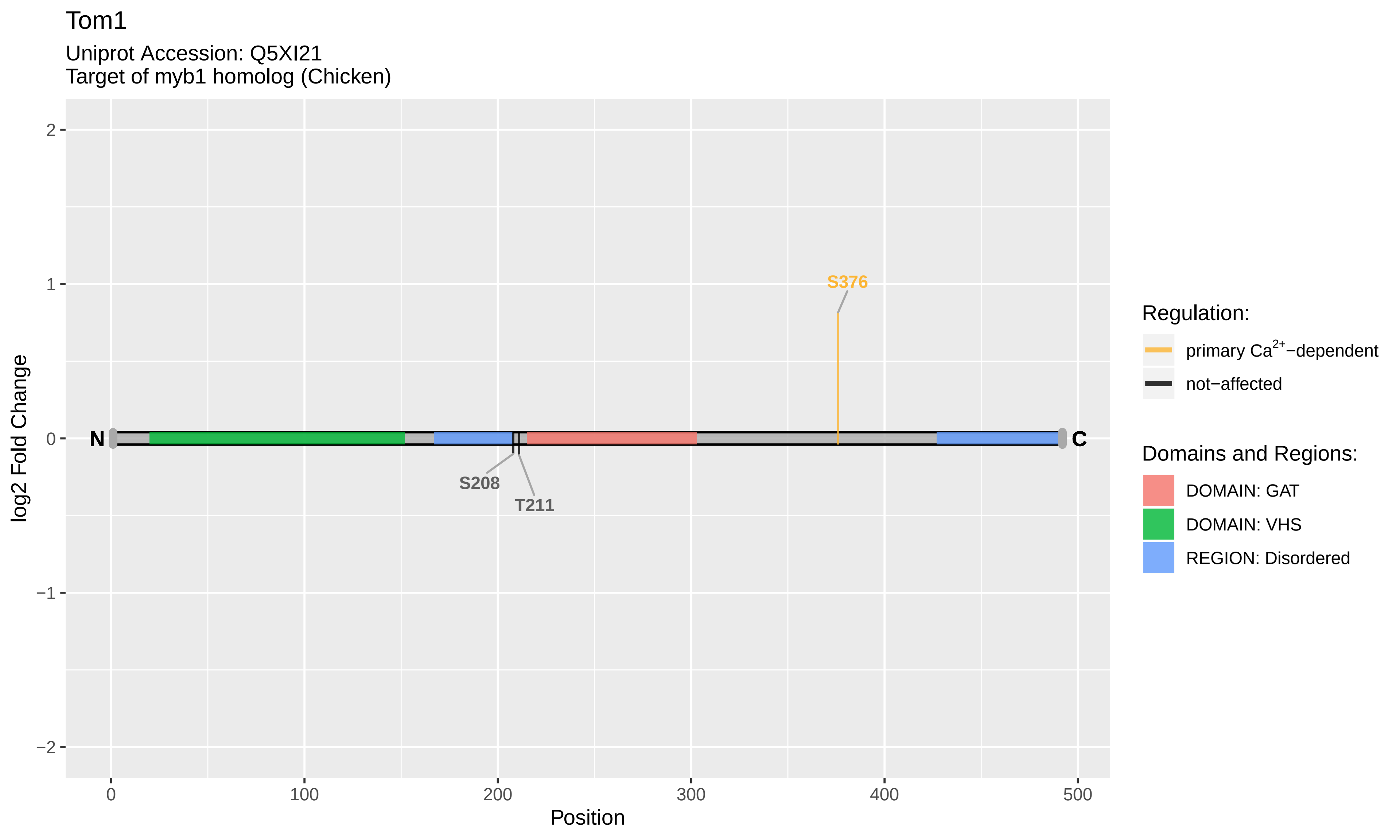

Tom1
Uniprot Accession: Q5XI21
Target of myb1 homolog (Chicken)
log2 Fold Change
Position
N
C
S376
S208
T211
Regulation:
primary Ca2+−dependent
not−affected
Domains and Regions:
DOMAIN: GAT
DOMAIN: VHS
REGION: Disordered

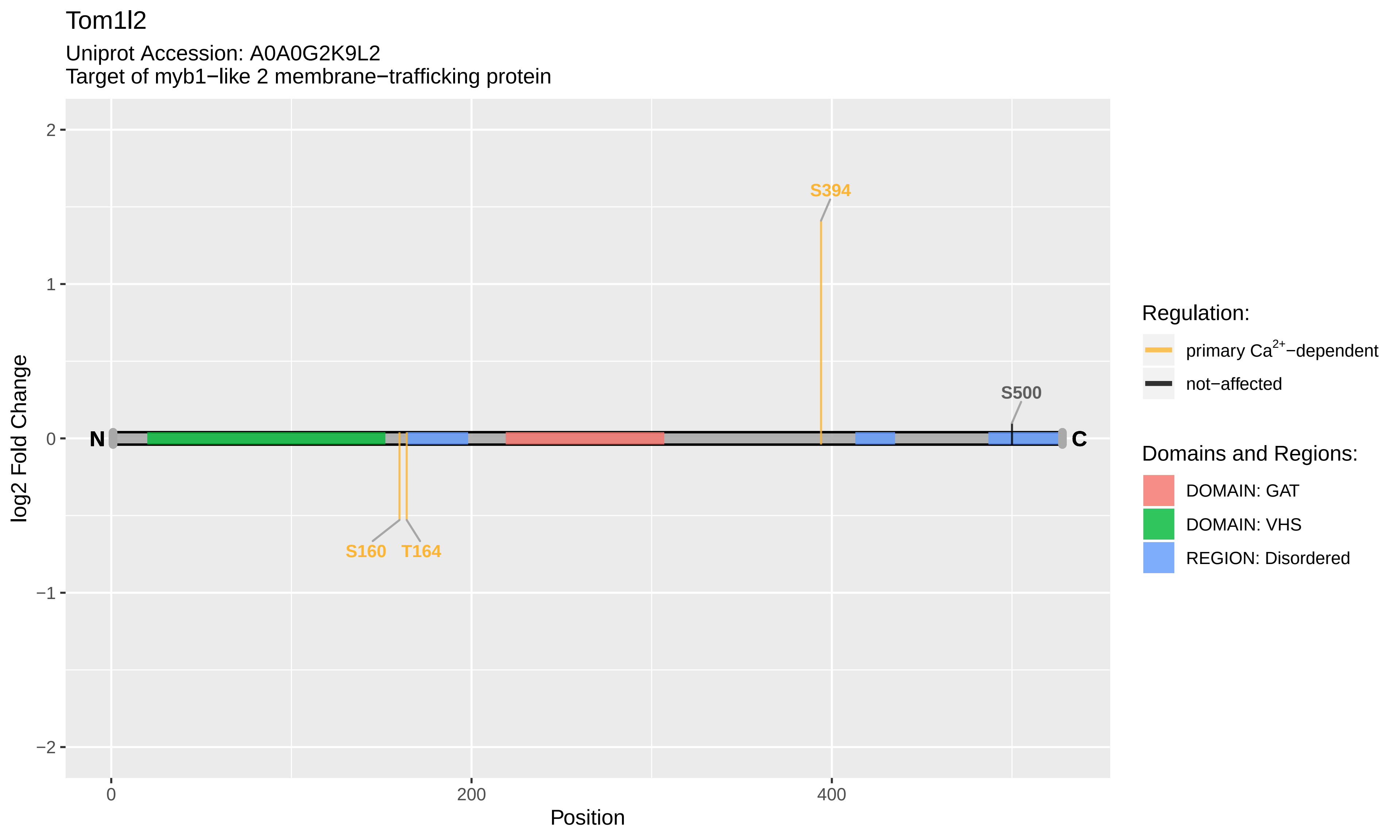
Tom1l2
Uniprot Accession: A0A0G2K9L2
Target of myb1−like 2 membrane−trafficking protein
S394
S500
S160
T164
N
C
log2 Fold Change
Position
2
1
0
−1
−2
0
200
400
Regulation:
primary Ca2+−dependent
not−affected
Domains and Regions:
DOMAIN: GAT
DOMAIN: VHS
REGION: Disordered

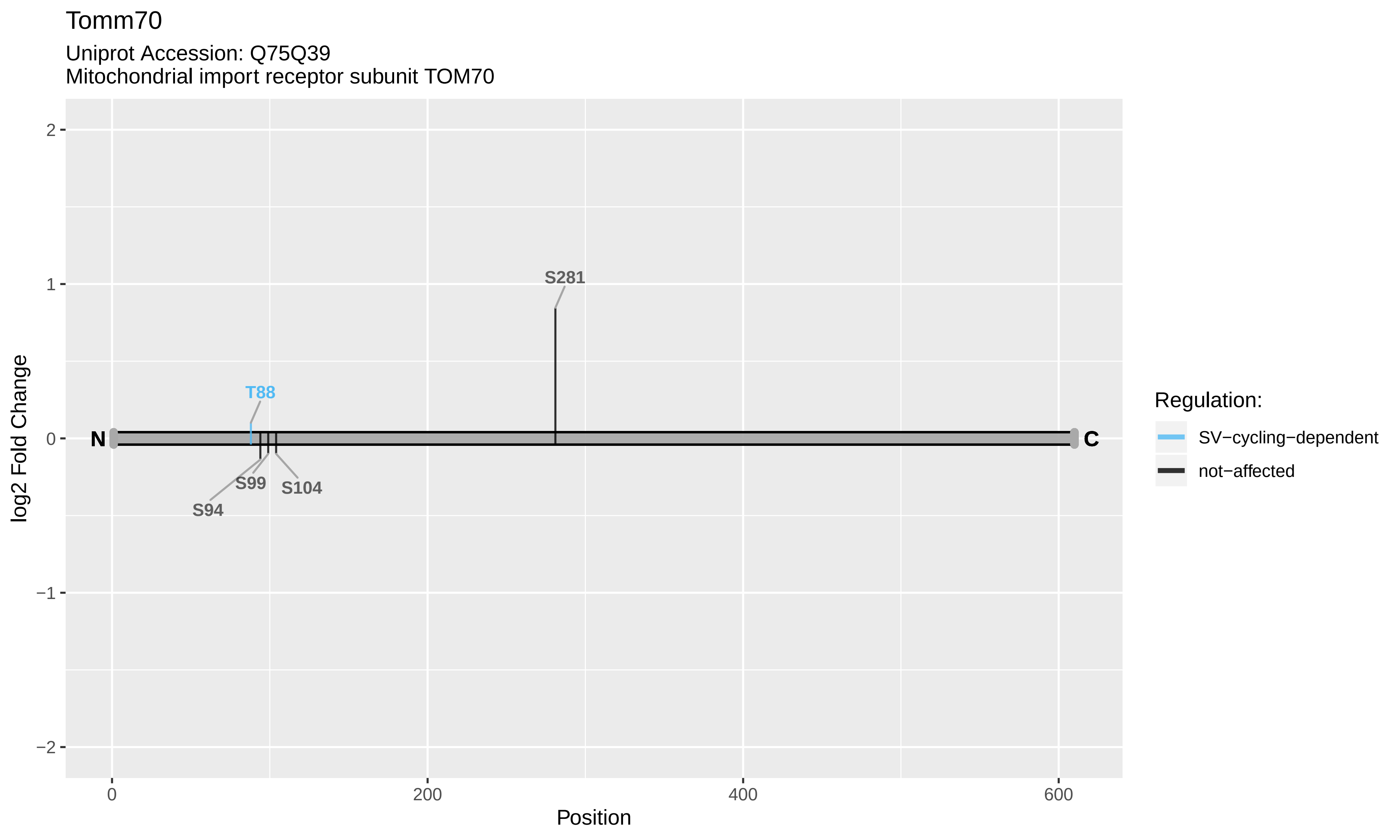

Tomm70
Uniprot Accession: Q75Q39
Mitochondrial import receptor subunit TOM70
log2 Fold Change
Position
2
1
0
−1
−2
0
200
400
600
N
C
T88
S281
S94
S99
S104
Regulation:
SV−cycling−dependent
not−affected

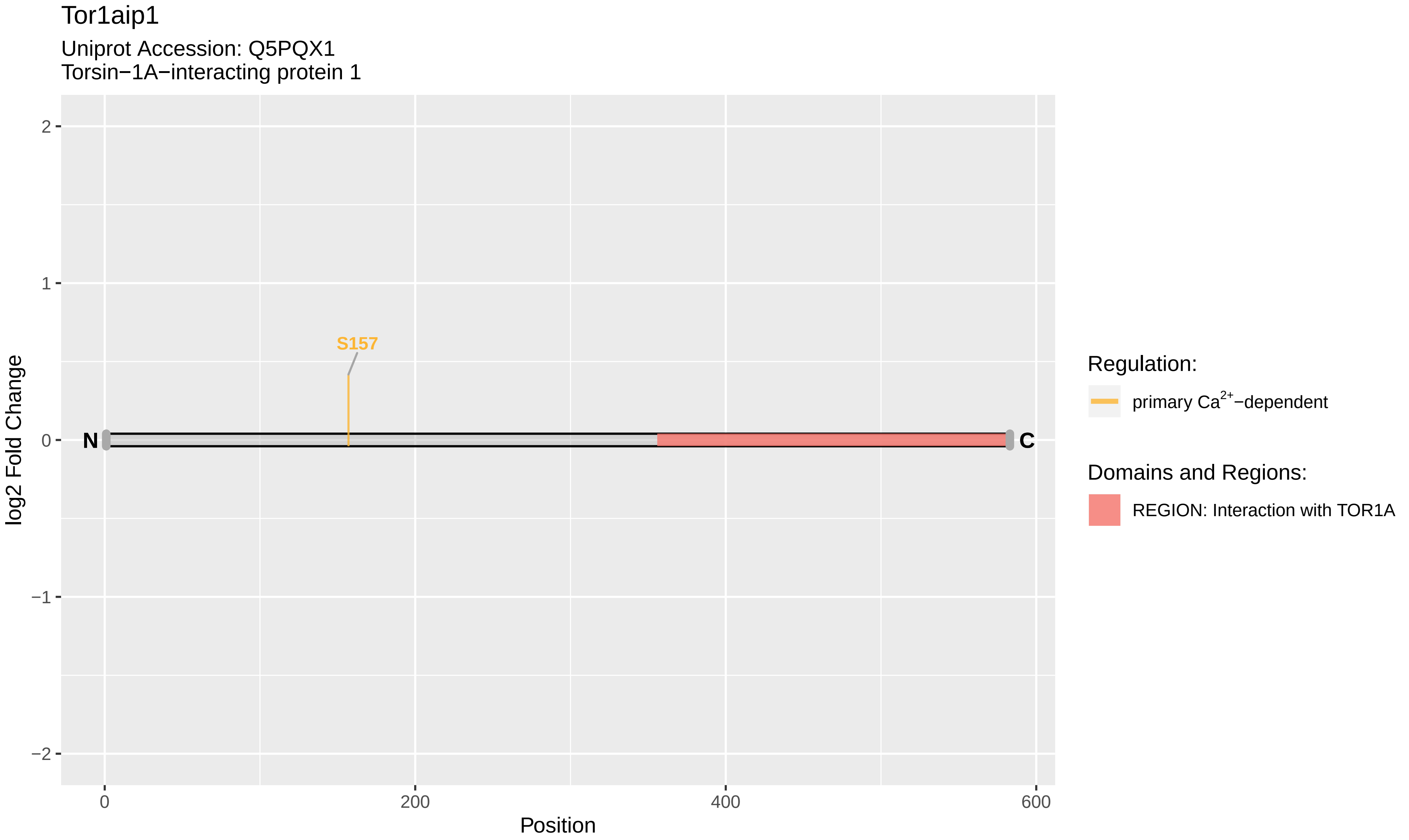

Tor1aip1
Uniprot Accession: Q5PQX1
Torsin−1A−interacting protein 1
S157
N
C
2
1
0
−1
−2
log2 Fold Change
0
200
400
600
Position
Regulation:
primary Ca2+−dependent
Domains and Regions:
REGION: Interaction with TOR1A

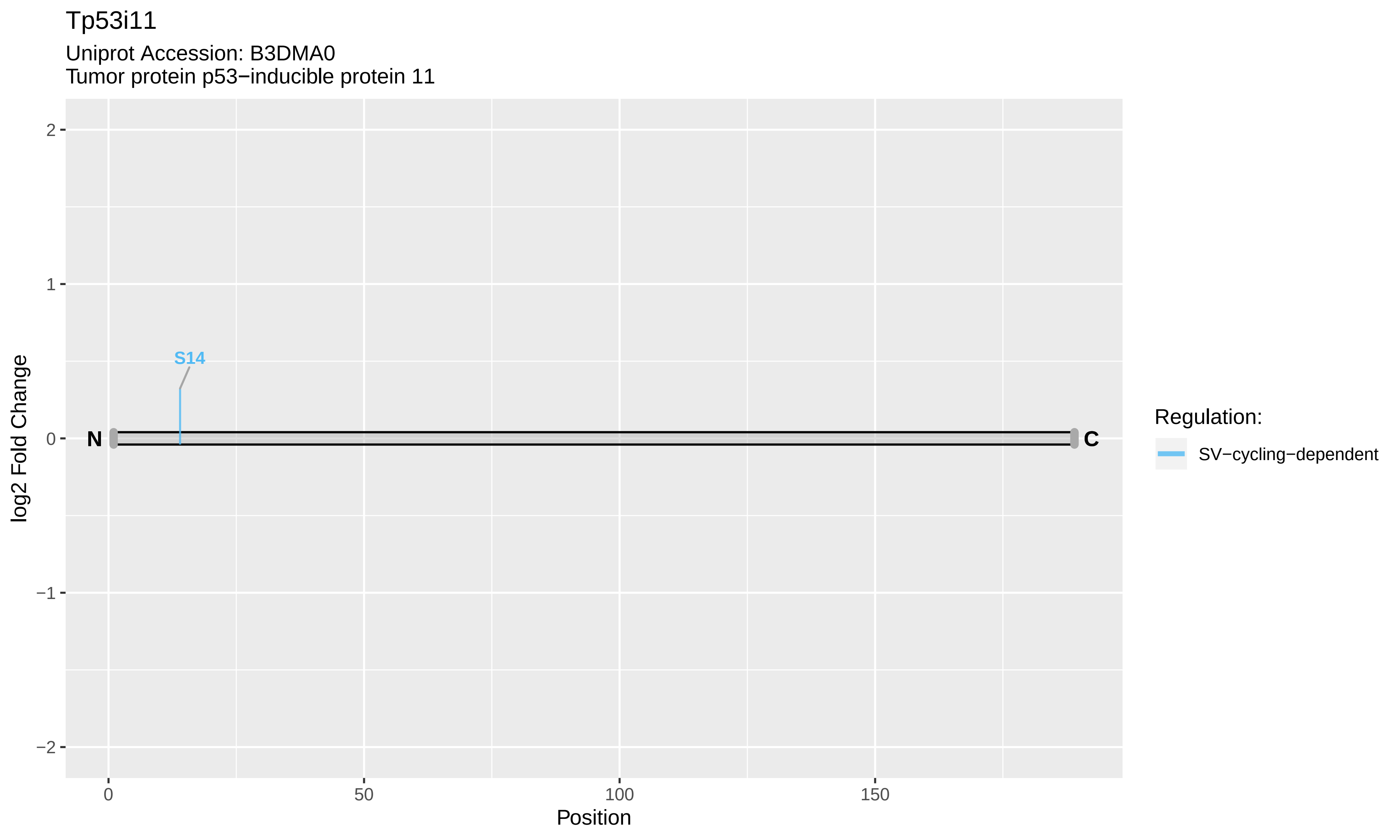
Tp53i11
Uniprot Accession: B3DMA0
Tumor protein p53−inducible protein 11
S14
N
C
log2 Fold Change
2
1
0
−1
−2
0
50
100
150
Position
Regulation:
SV−cycling−dependent

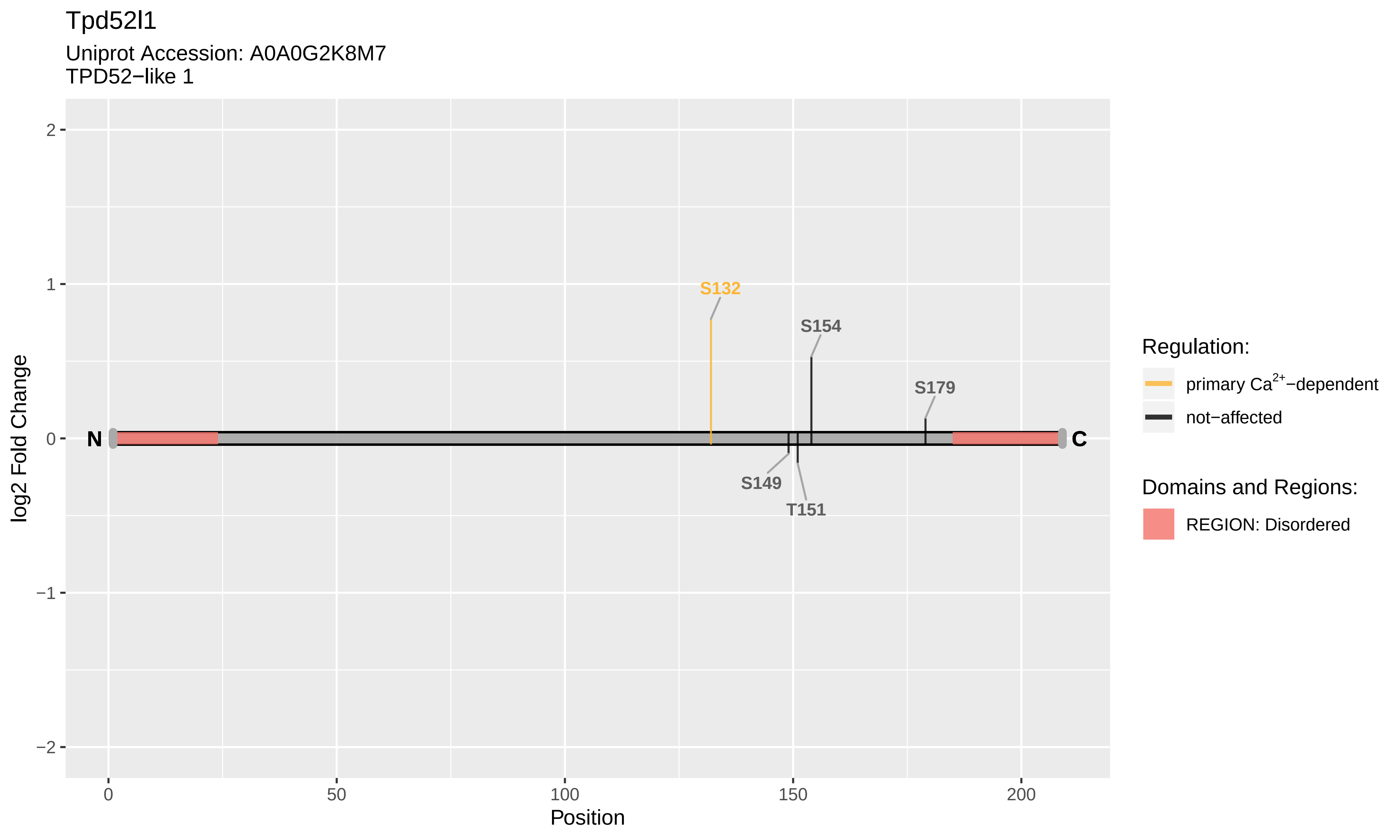

Tpd52l1
Uniprot Accession: A0A0G2K8M7
TPD52−like 1
log2 Fold Change
Position
N
C
S132
S154
S179
S149
T151
Regulation:
primary Ca2+−dependent
not−affected
Domains and Regions:
REGION: Disordered
2
1
0
−1
−2
0
50
100
150
200

Tpd52l2

Uniprot Accession: Q6PCT3
Tumor protein D54

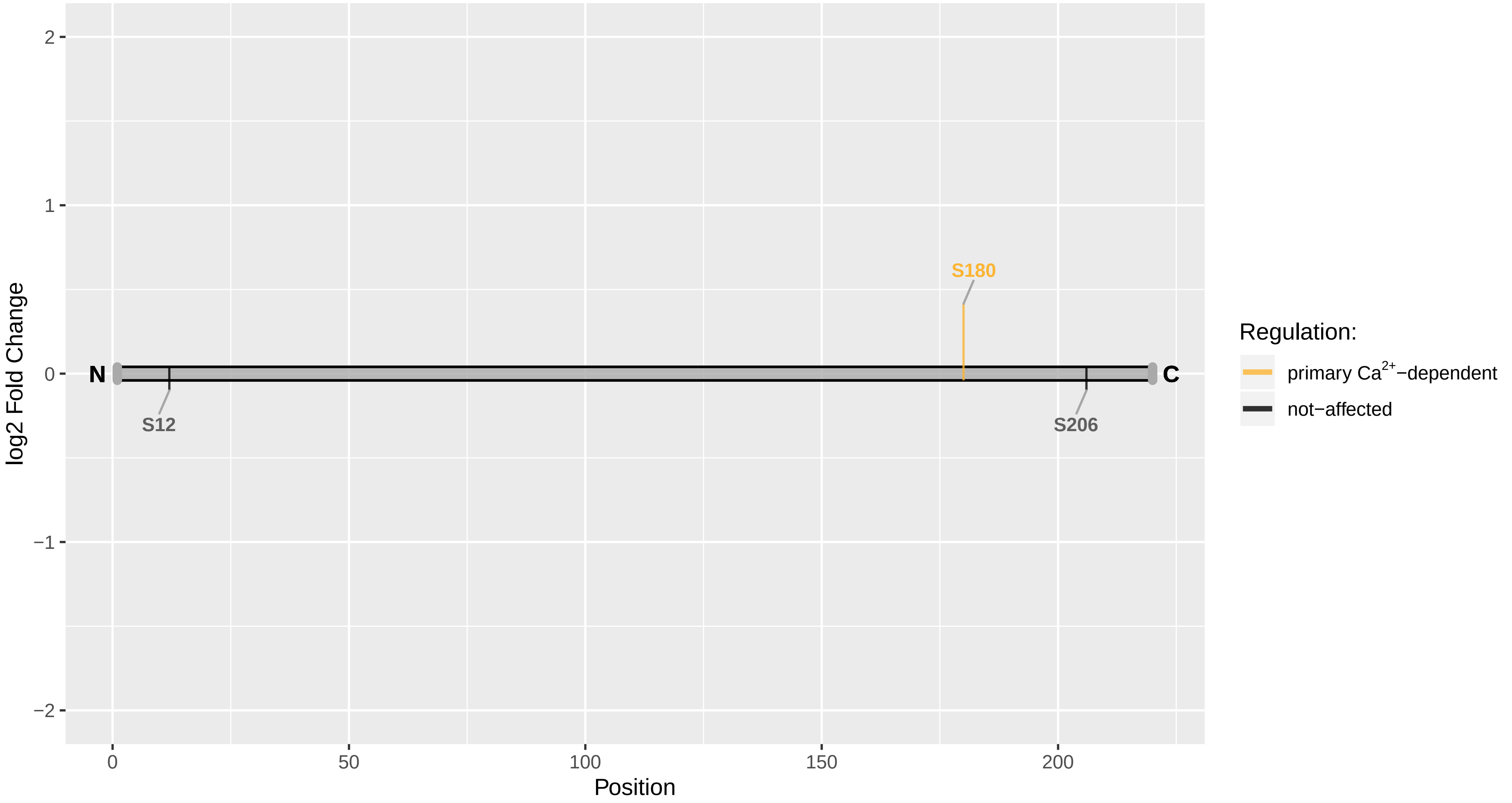

Tppp

Uniprot Accession: A0A0G2K2D6
Tubulin polymerization−promoting protein

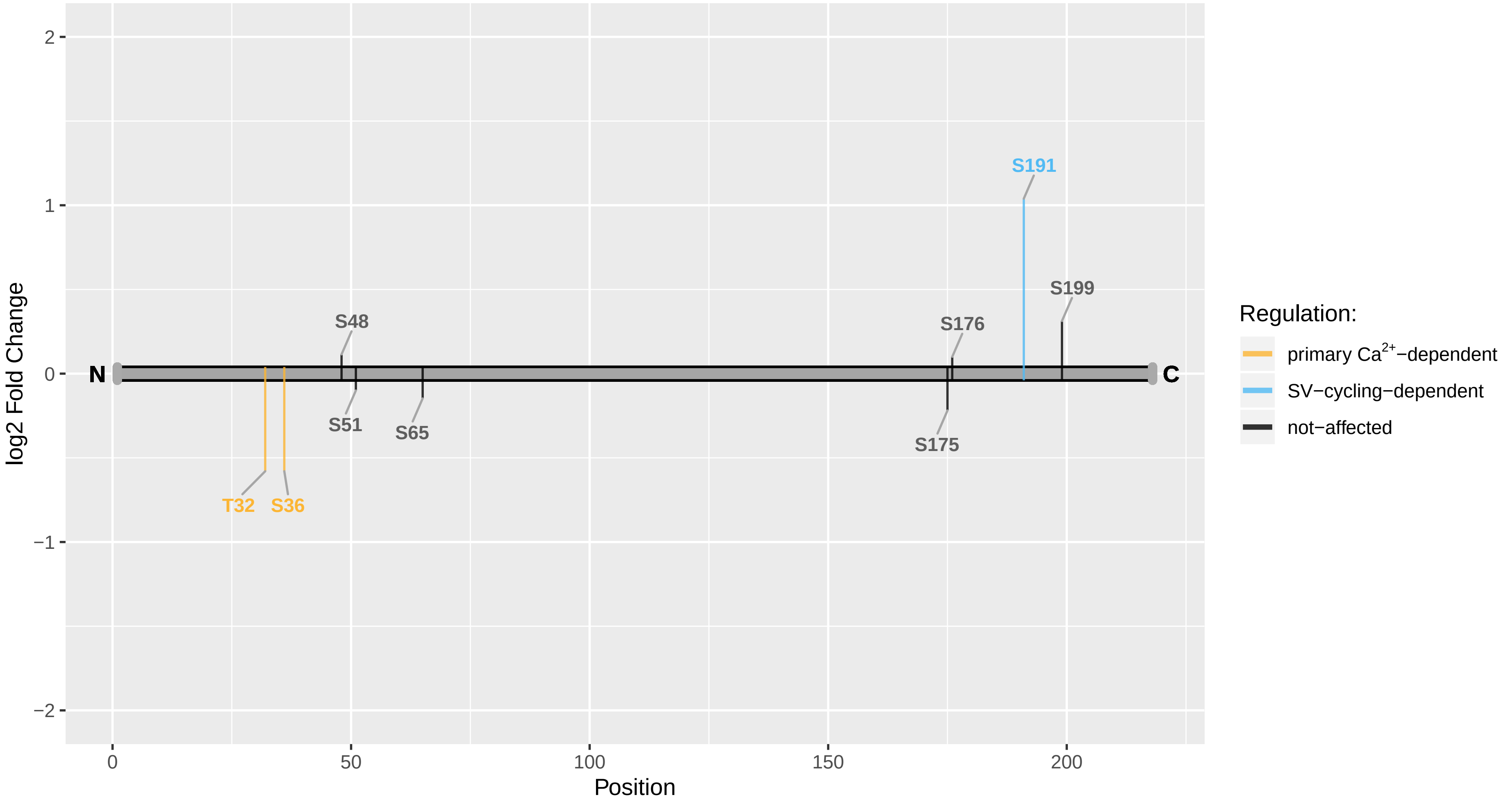

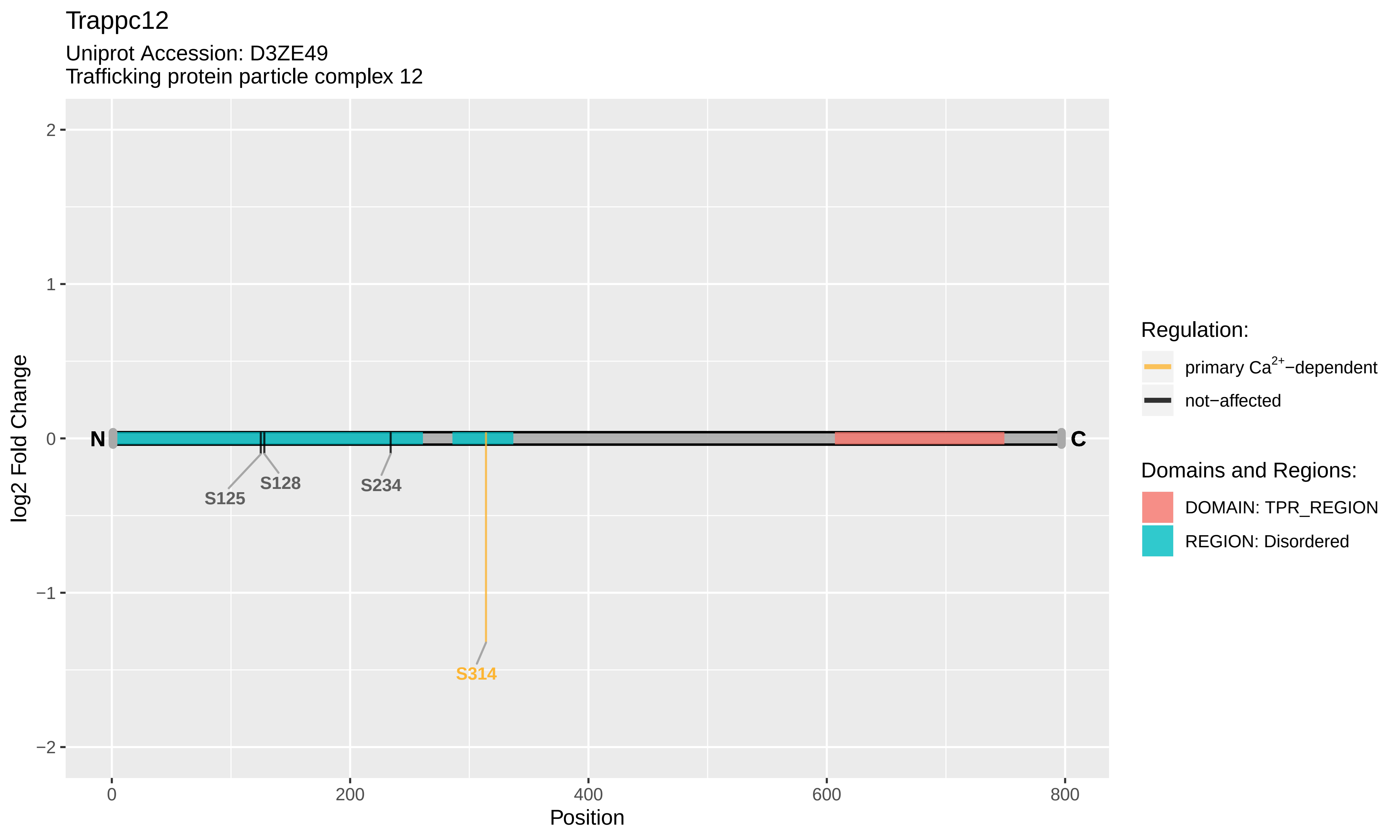

Trappc12
Uniprot Accession: D3ZE49
Trafficking protein particle complex 12
2
1
0
−1
−2
log2 Fold Change
N
C
S125
S128
S234
S314
0
200
400
600
800
Position
Regulation:
primary Ca2+−dependent
not−affected
Domains and Regions:
DOMAIN: TPR_REGION
REGION: Disordered

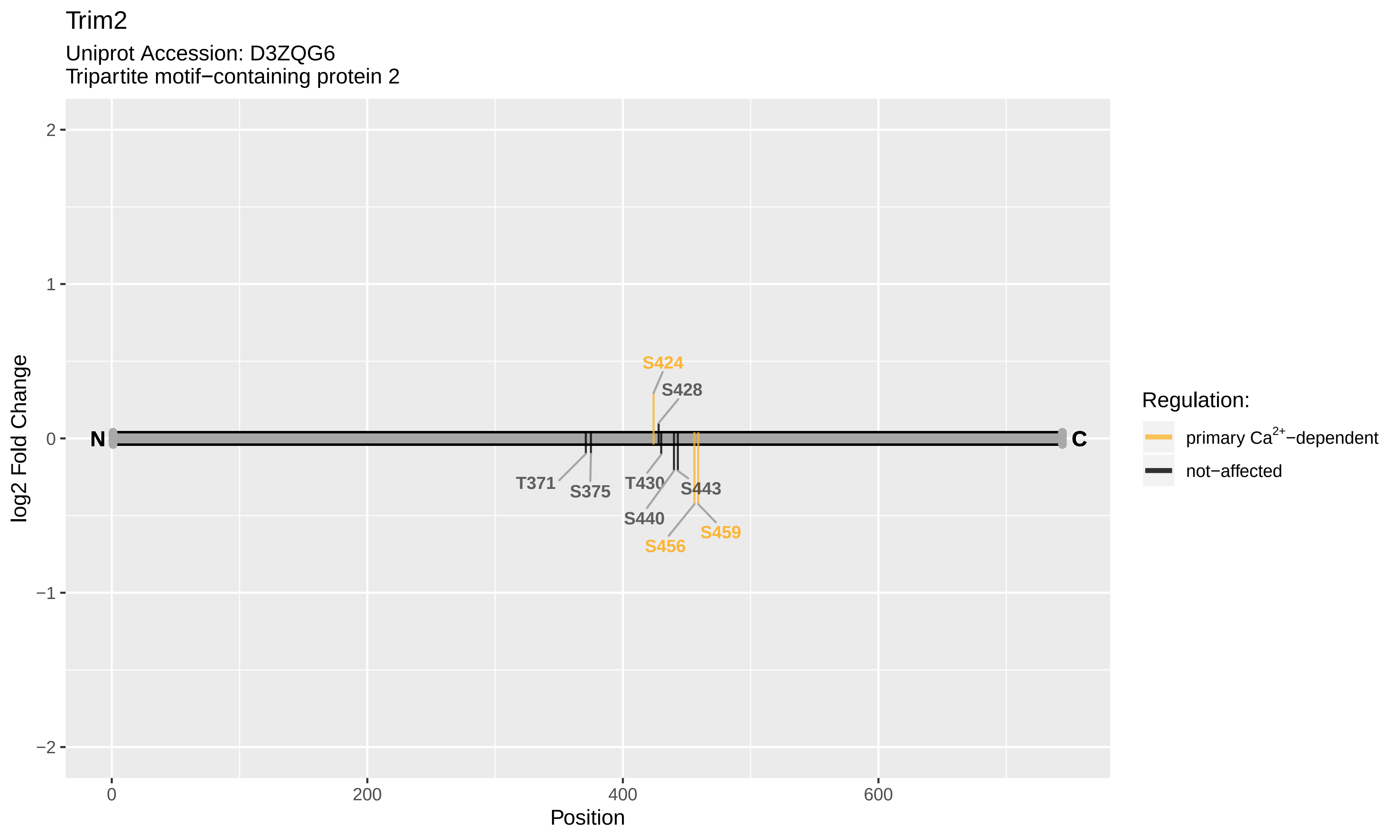

Trim2
Uniprot Accession: D3ZQG6
Tripartite motif−containing protein 2
log2 Fold Change
2
1
0
−1
−2
N
C
S424
S428
T371
S375
T430
S443
S440
S459
S456
0
200
400
600
Position
Regulation:
primary Ca2+−dependent
not−affected

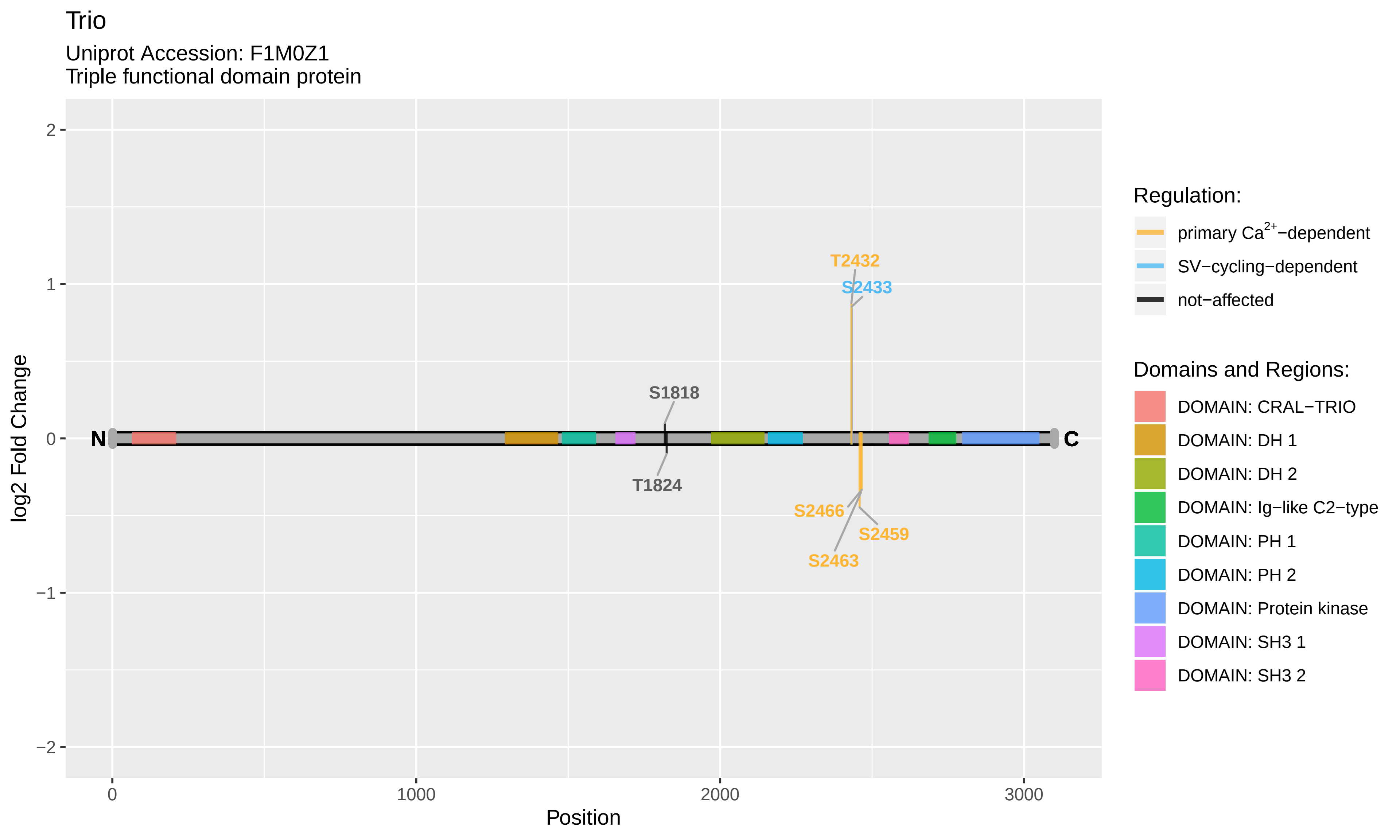

Trio
Uniprot Accession: F1M0Z1
Triple functional domain protein
T2432
S2433
S1818
T1824
S2466
S2459
S2463
N
C
log2 Fold Change
Position
2
1
0
−1
−2
0
1000
2000
3000
Regulation:
primary Ca2+−dependent
SV−cycling−dependent
not−affected
Domains and Regions:
DOMAIN: CRAL−TRIO
DOMAIN: DH 1
DOMAIN: DH 2
DOMAIN: Ig−like C2−type
DOMAIN: PH 1
DOMAIN: PH 2
DOMAIN: Protein kinase
DOMAIN: SH3 1
DOMAIN: SH3 2

Trip12

Uniprot Accession: F1LP64
E3 ubiquitin−protein ligase TRIP12

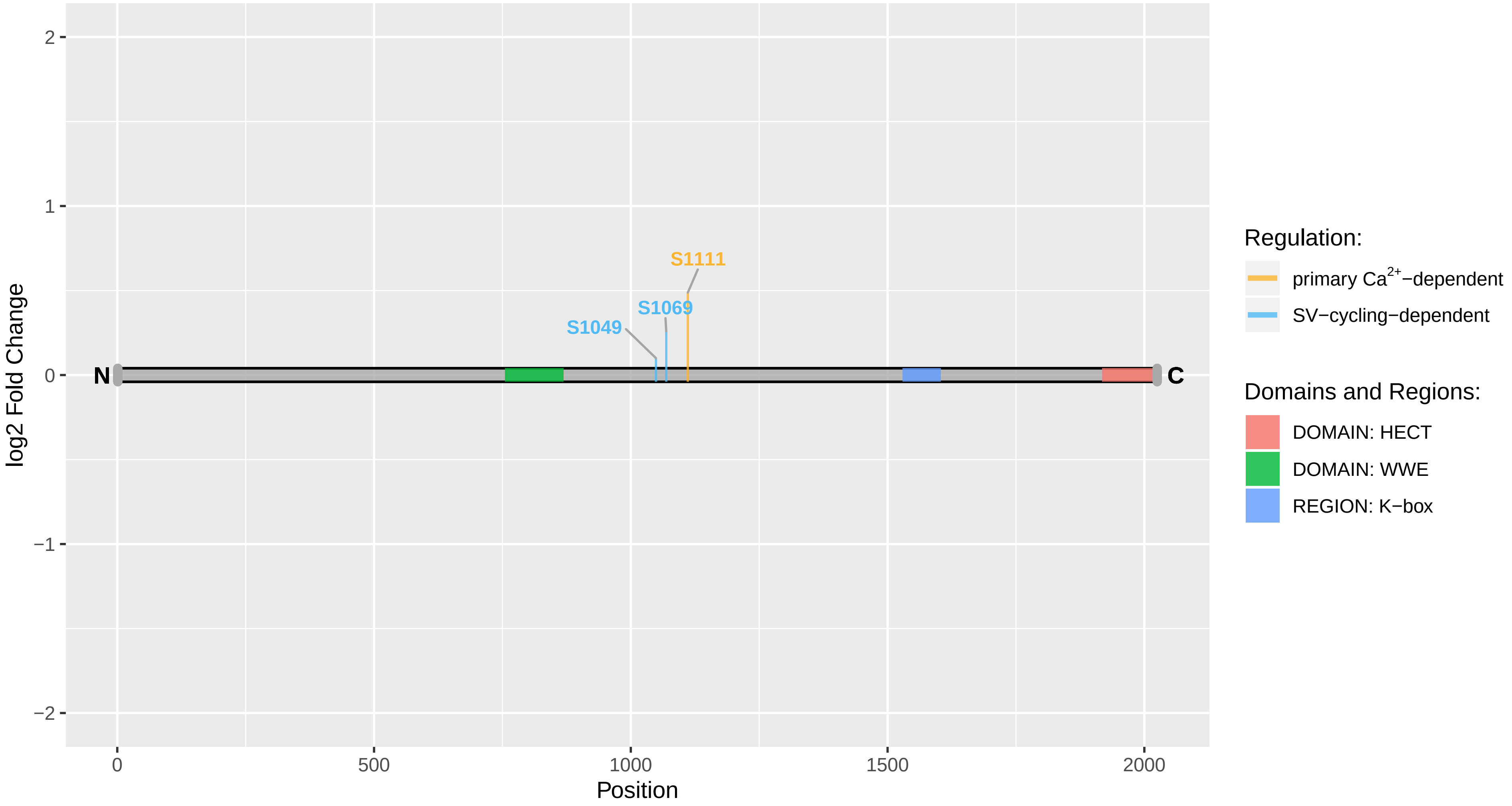

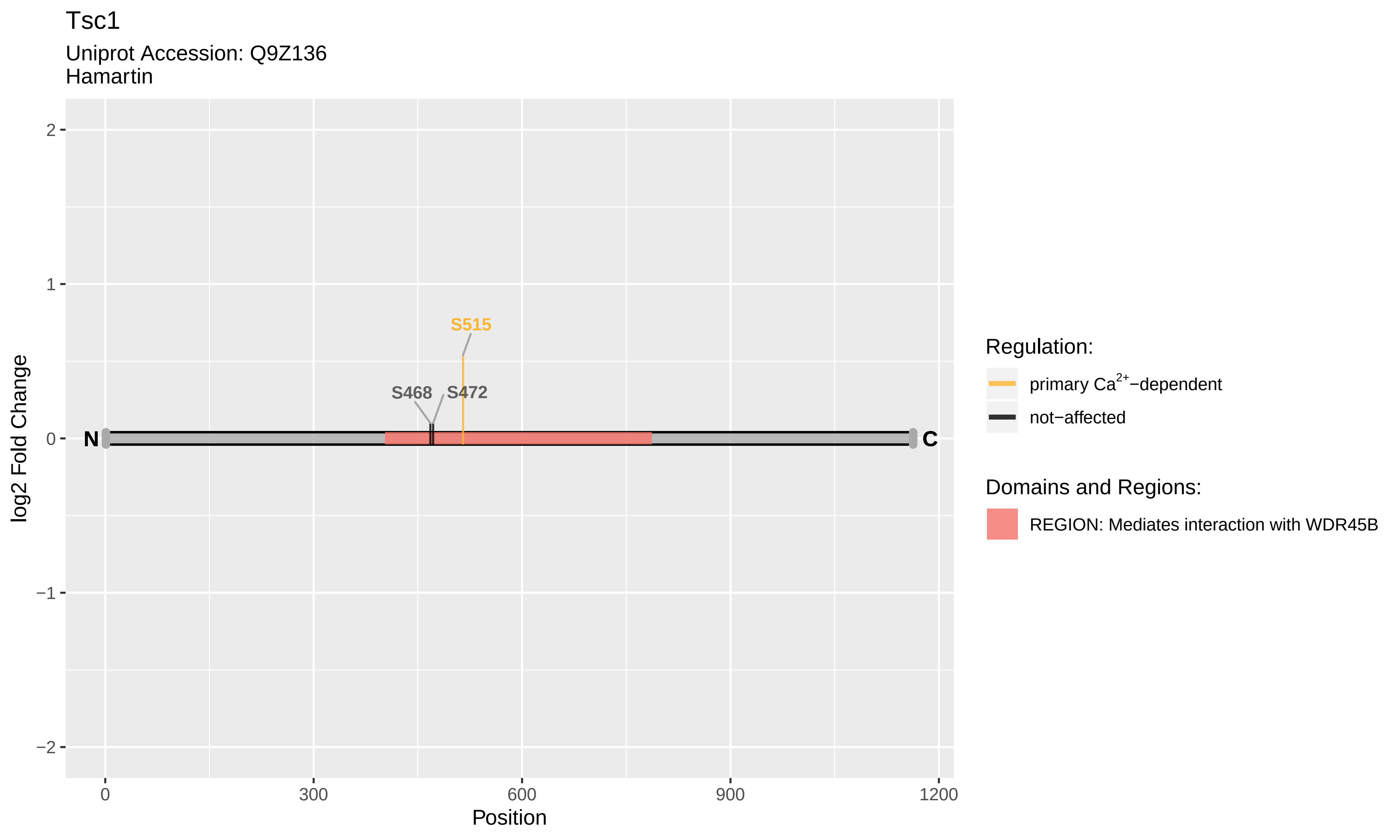

Tsc1
Uniprot Accession: Q9Z136
Hamartin
S515
S468
S472
N
C
log2 Fold Change
2
1
0
−1
−2
0
300
600
900
1200
Position
Regulation:
primary Ca2+−dependent
not−affected
Domains and Regions:
REGION: Mediates interaction with WDR45B

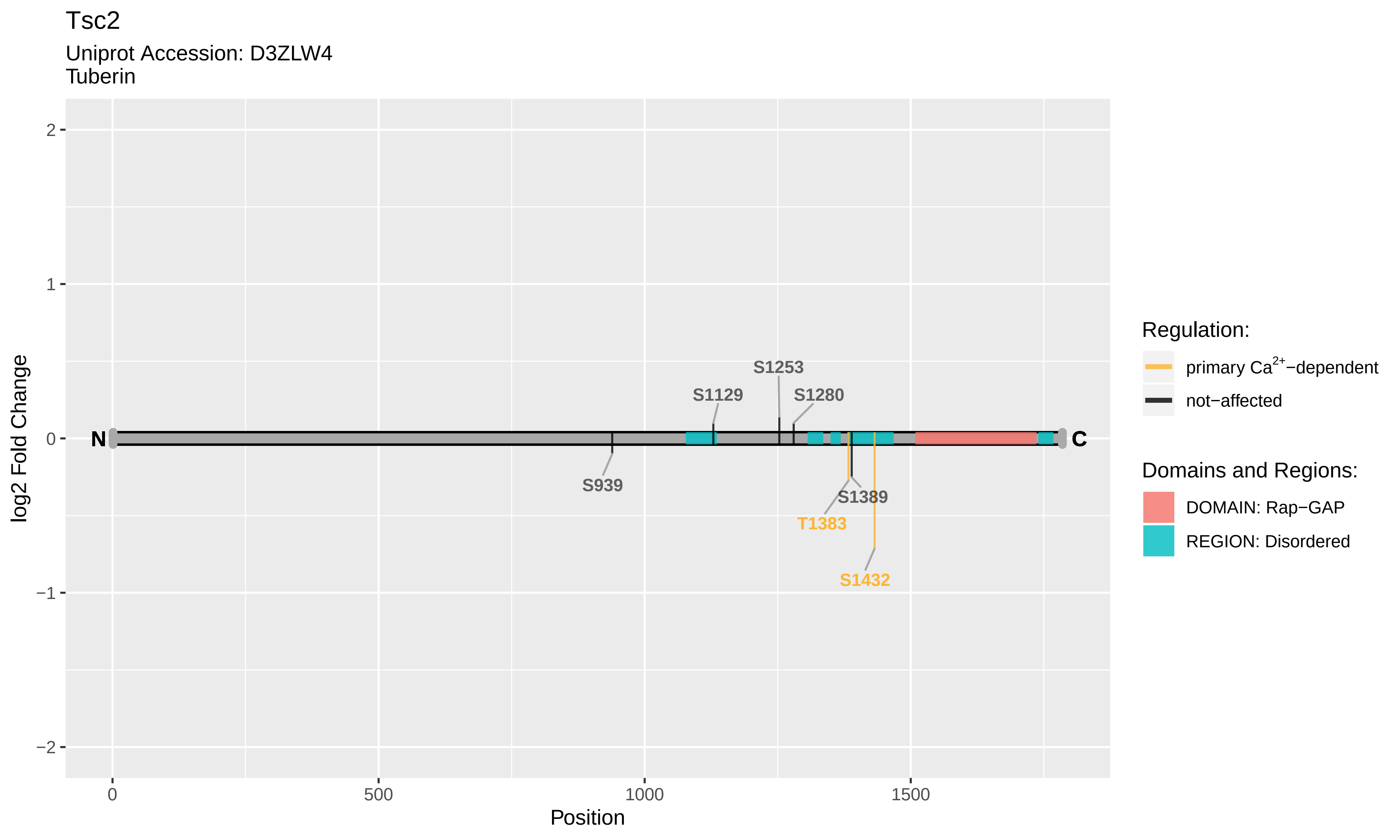

Tsc2
Uniprot Accession: D3ZLW4
Tuberin
log2 Fold Change
Position
N
C
S939
S1129
S1253
S1280
S1389
T1383
S1432
2
1
0
−1
−2
0
500
1000
1500
Regulation:
primary Ca2+−dependent
not−affected
Domains and Regions:
DOMAIN: Rap−GAP
REGION: Disordered

Ttbk1

Uniprot Accession: D3ZAU7
Tau tubulin kinase 1

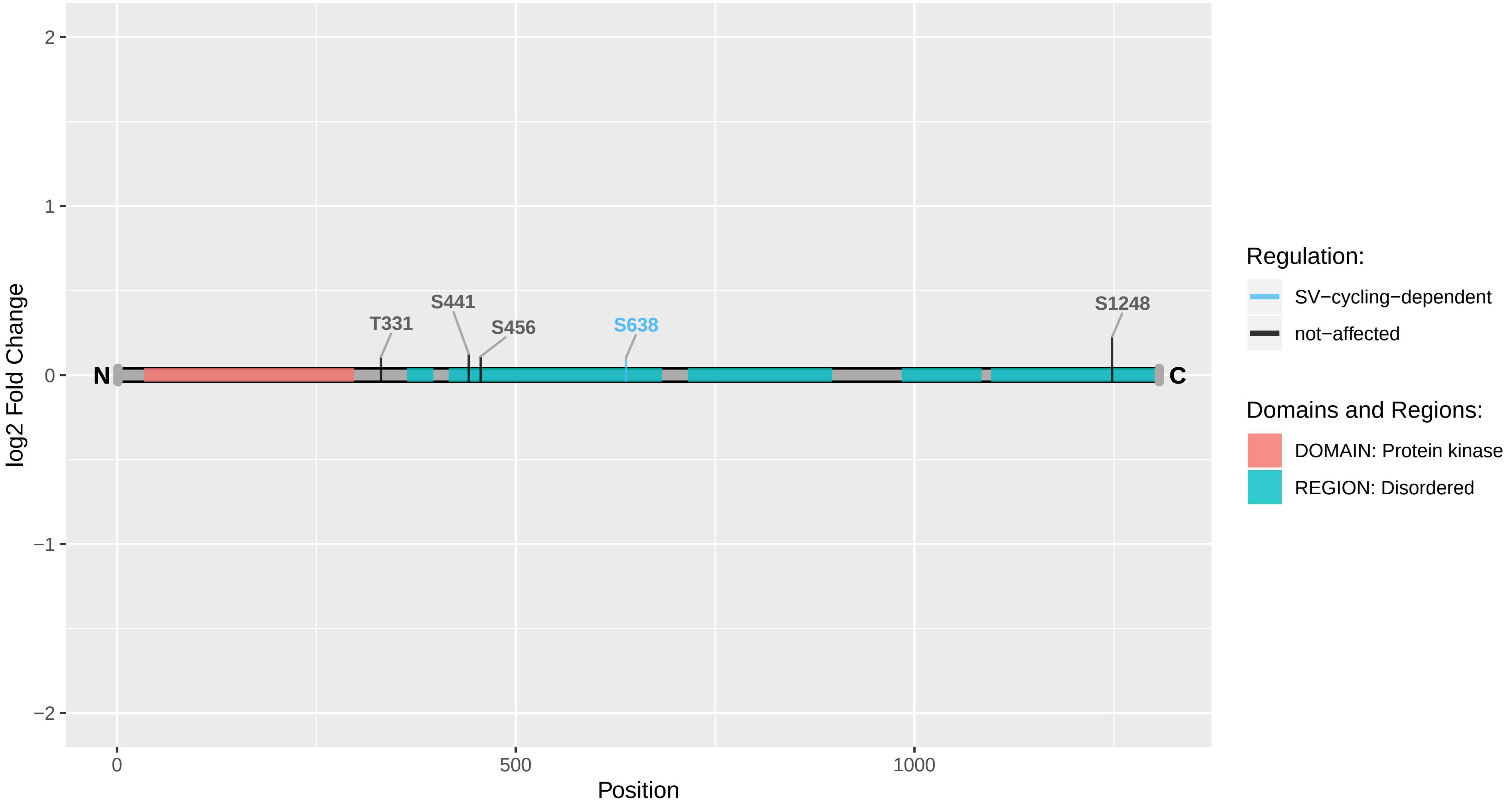

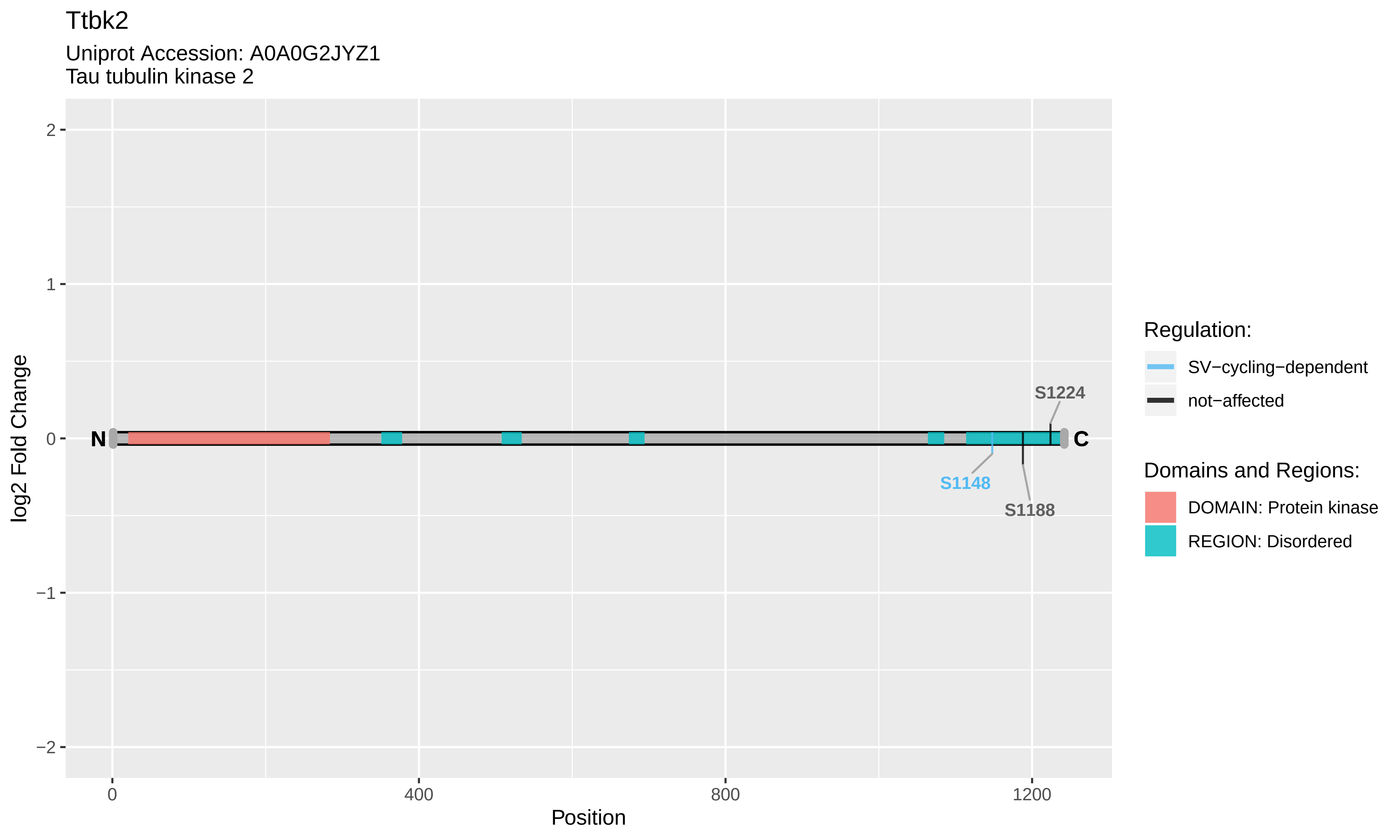

Ttbk2
Uniprot Accession: A0A0G2JYZ1
Tau tubulin kinase 2
log2 Fold Change
2
1
0
−1
−2
N
C
S1224
S1148
S1188
0
400
800
1200
Position
Regulation:
SV−cycling−dependent
not−affected
Domains and Regions:
DOMAIN: Protein kinase
REGION: Disordered

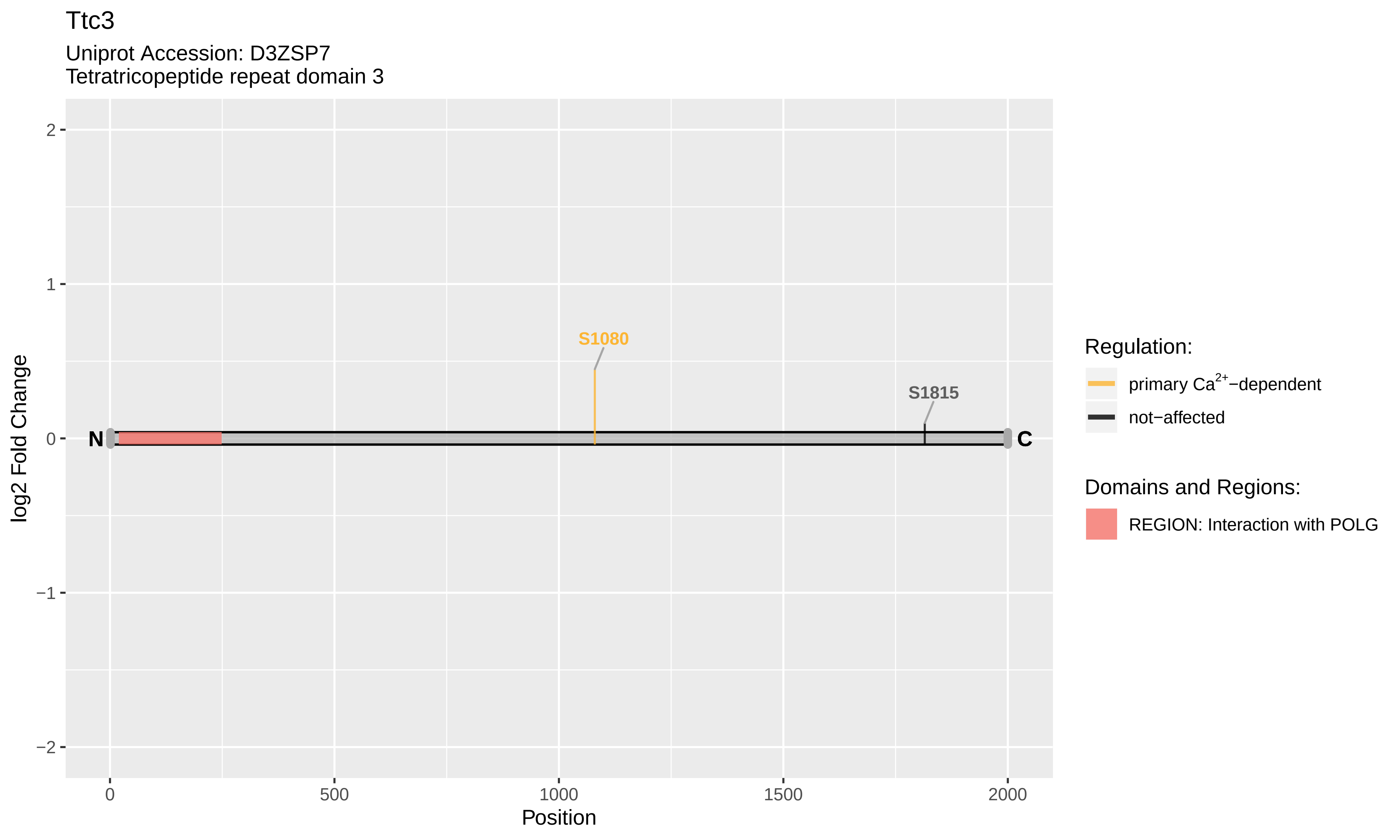

Ttc3
Uniprot Accession: D3ZSP7
Tetratricopeptide repeat domain 3
S1080
S1815
N
C
2
1
0
−1
−2
log2 Fold Change
0
500
1000
1500
2000
Position
Regulation:
primary Ca2+−dependent
not−affected
Domains and Regions:
REGION: Interaction with POLG

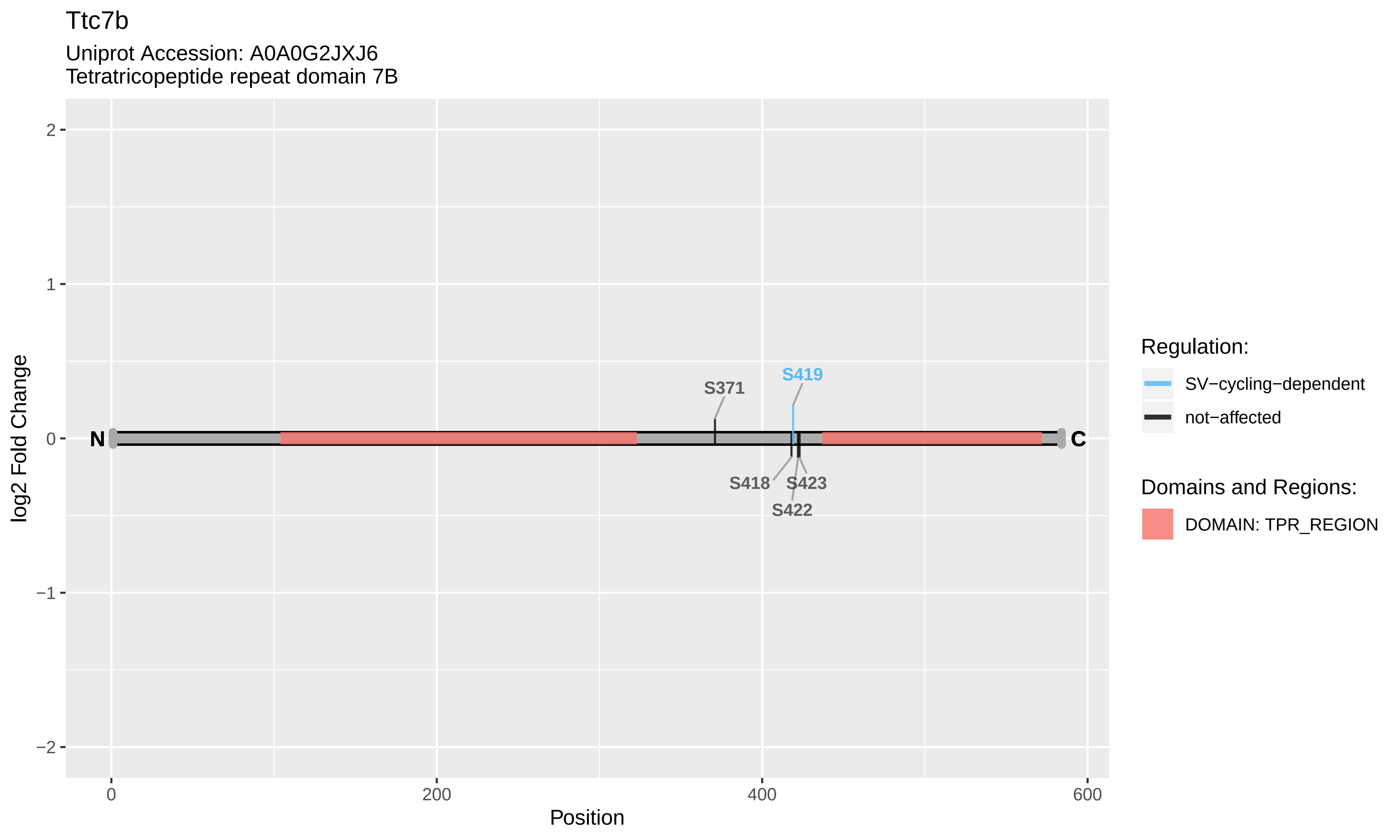

Ttc7b
Uniprot Accession: A0A0G2JXJ6
Tetratricopeptide repeat domain 7B
log2 Fold Change
Position
N
C
S371
S419
S418
S423
S422
Regulation:
SV−cycling−dependent
not−affected
Domains and Regions:
DOMAIN: TPR_REGION

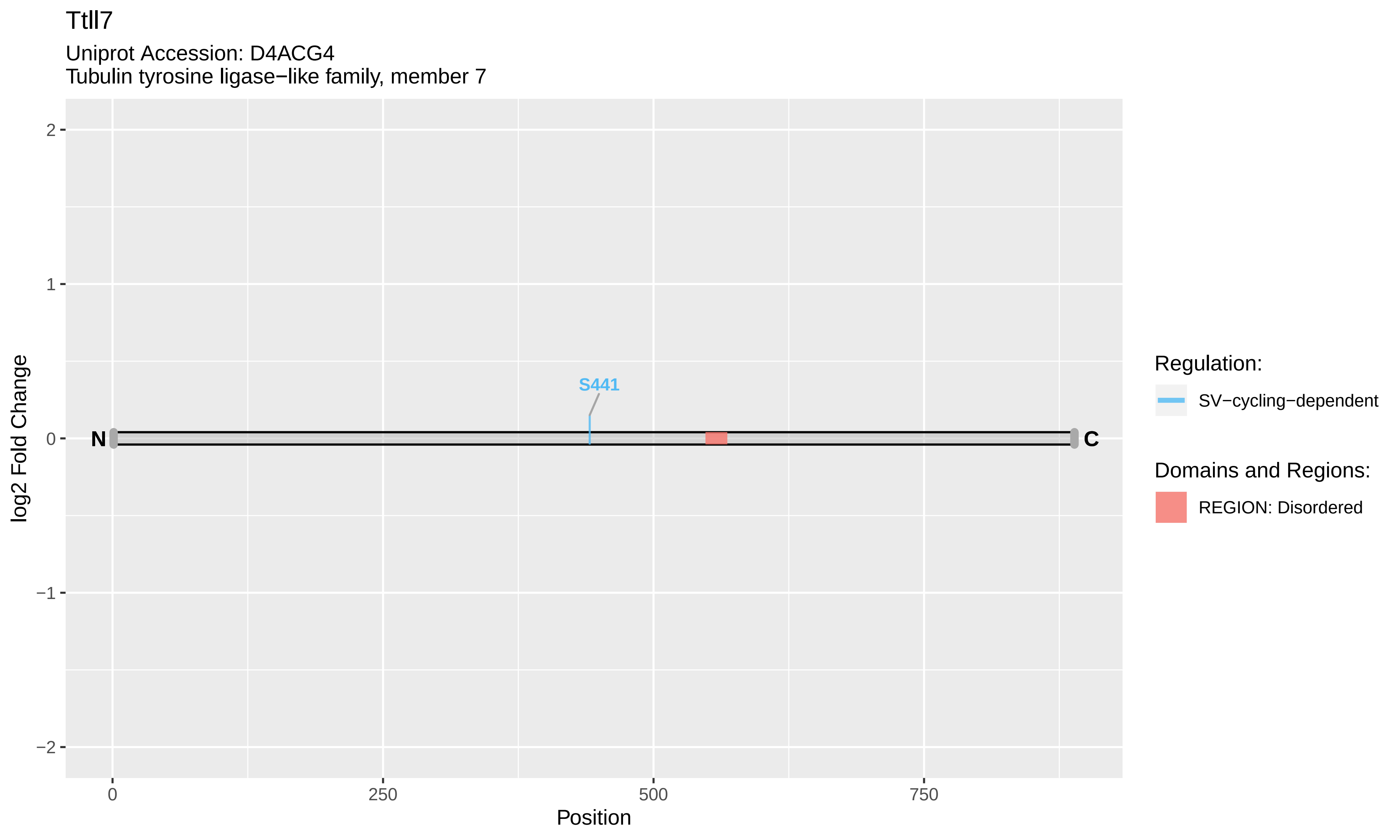
Ttll7
Uniprot Accession: D4ACG4
Tubulin tyrosine ligase−like family, member 7
S441
N
C
2
1
0
−1
−2
log2 Fold Change
0
250
500
750
Position
Regulation:
SV−cycling−dependent
Domains and Regions:
REGION: Disordered

Tubb5

Uniprot Accession: P69897
Tubulin beta−5 chain

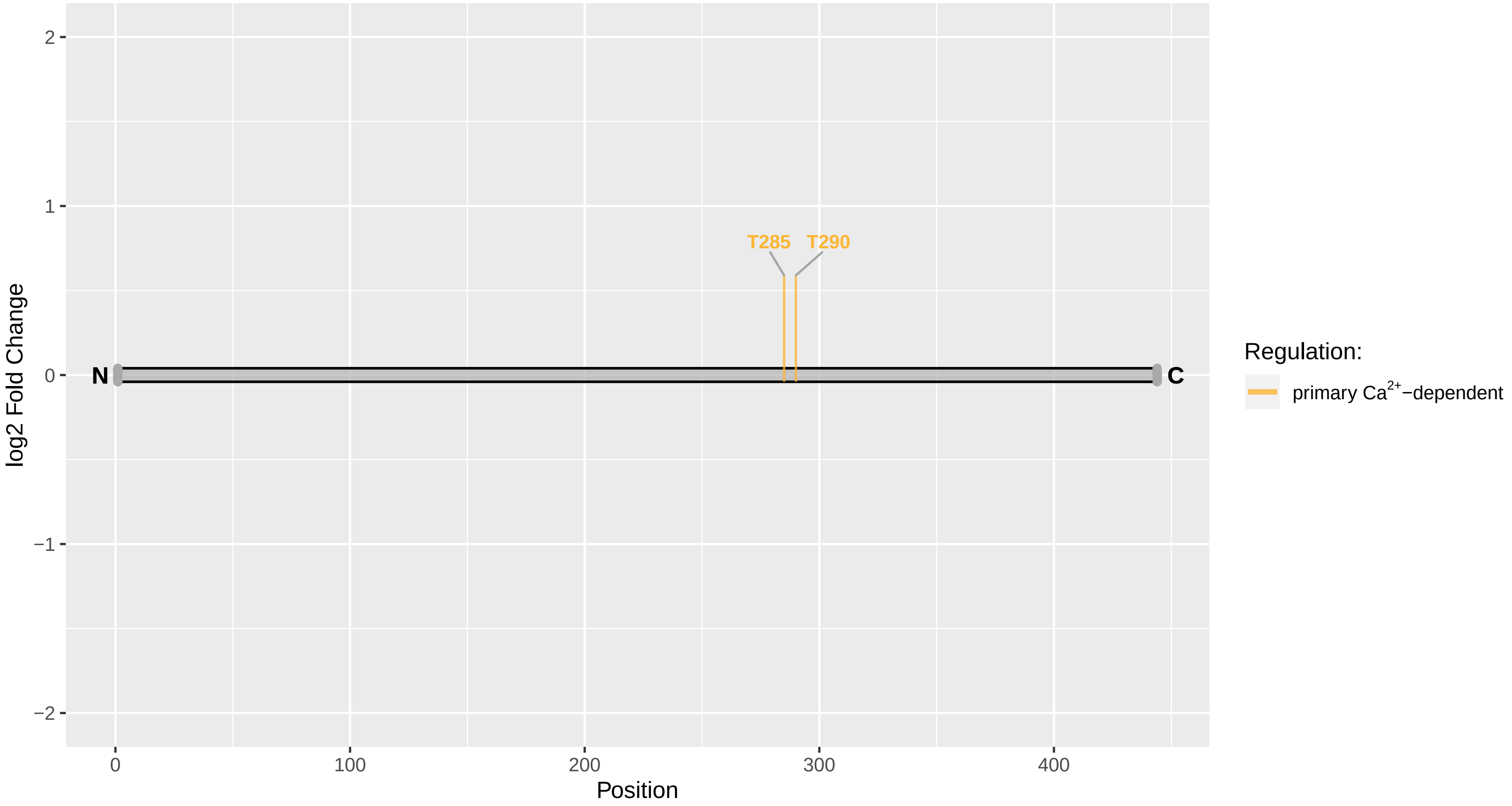

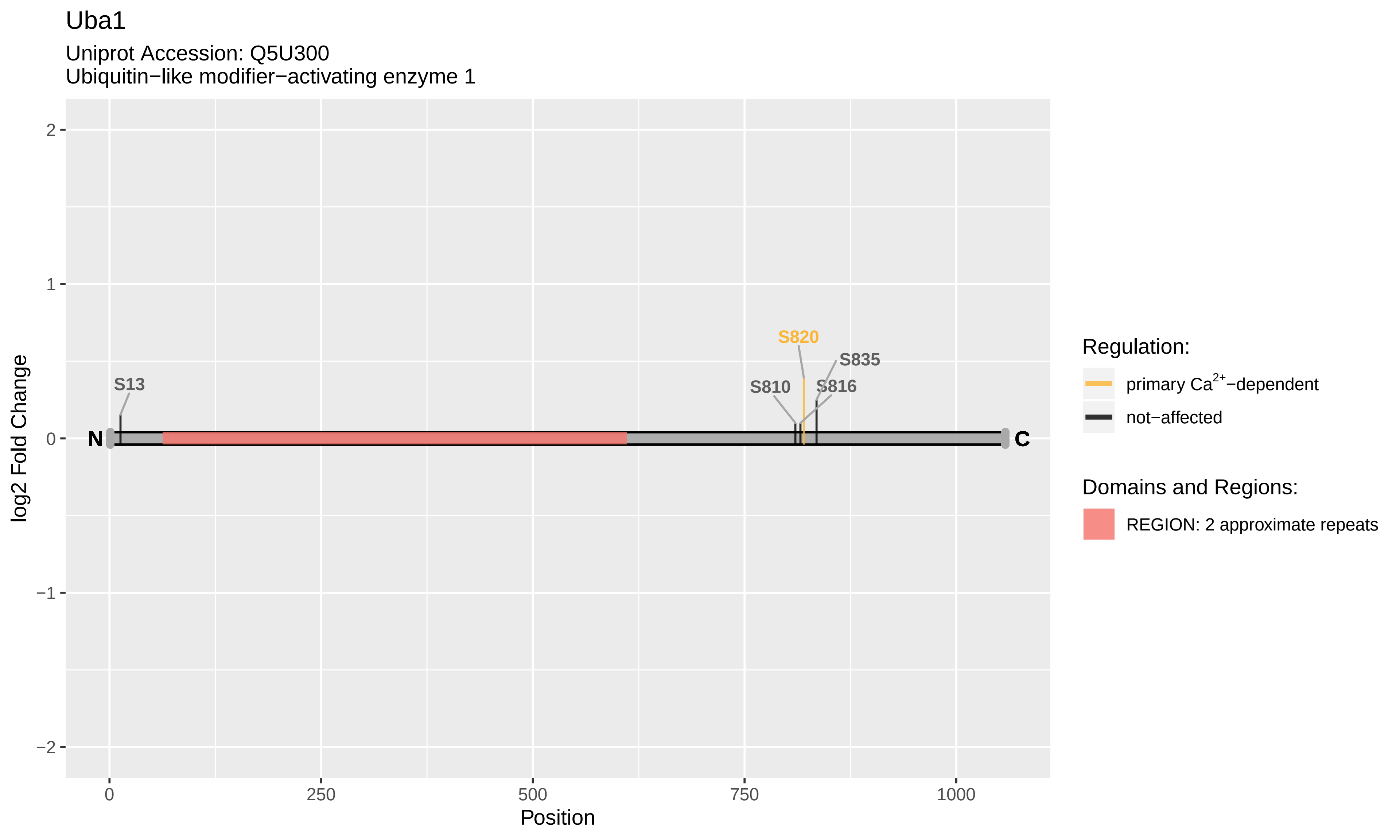

Uba1
Uniprot Accession: Q5U300
Ubiquitin−like modifier−activating enzyme 1
log2 Fold Change
2
1
0
−1
−2
N
C
S13
S810
S820
S816
S835
0
250
500
750
1000
Position
Regulation:
primary Ca2+−dependent
not−affected
Domains and Regions:
REGION: 2 approximate repeats

Uba5

Uniprot Accession: Q5M7A4
Ubiquitin−like modifier−activating enzyme 5

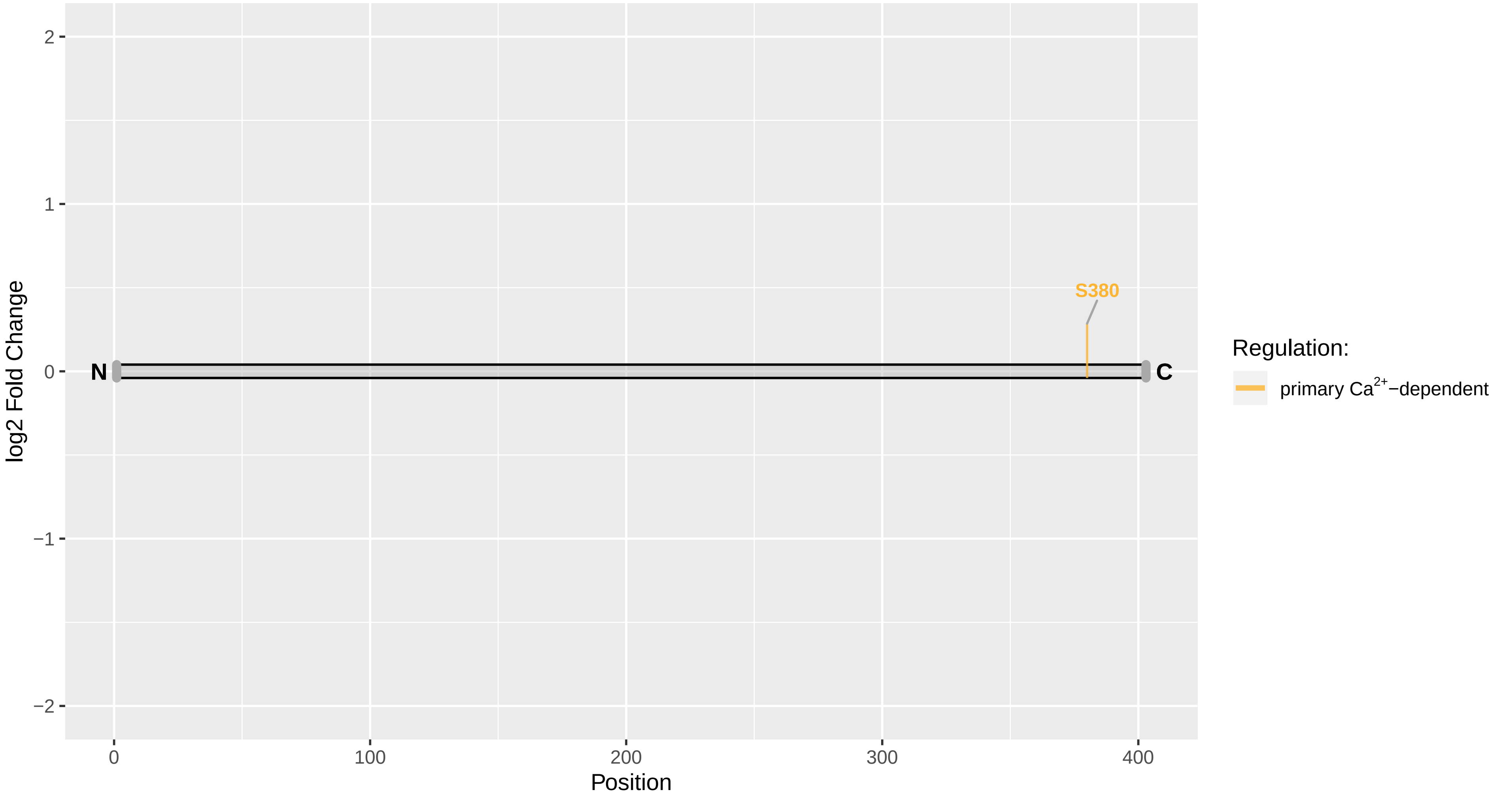

Ubap2l

Uniprot Accession: A0A0G2JYC6
RCG62582, isoform CRA_c

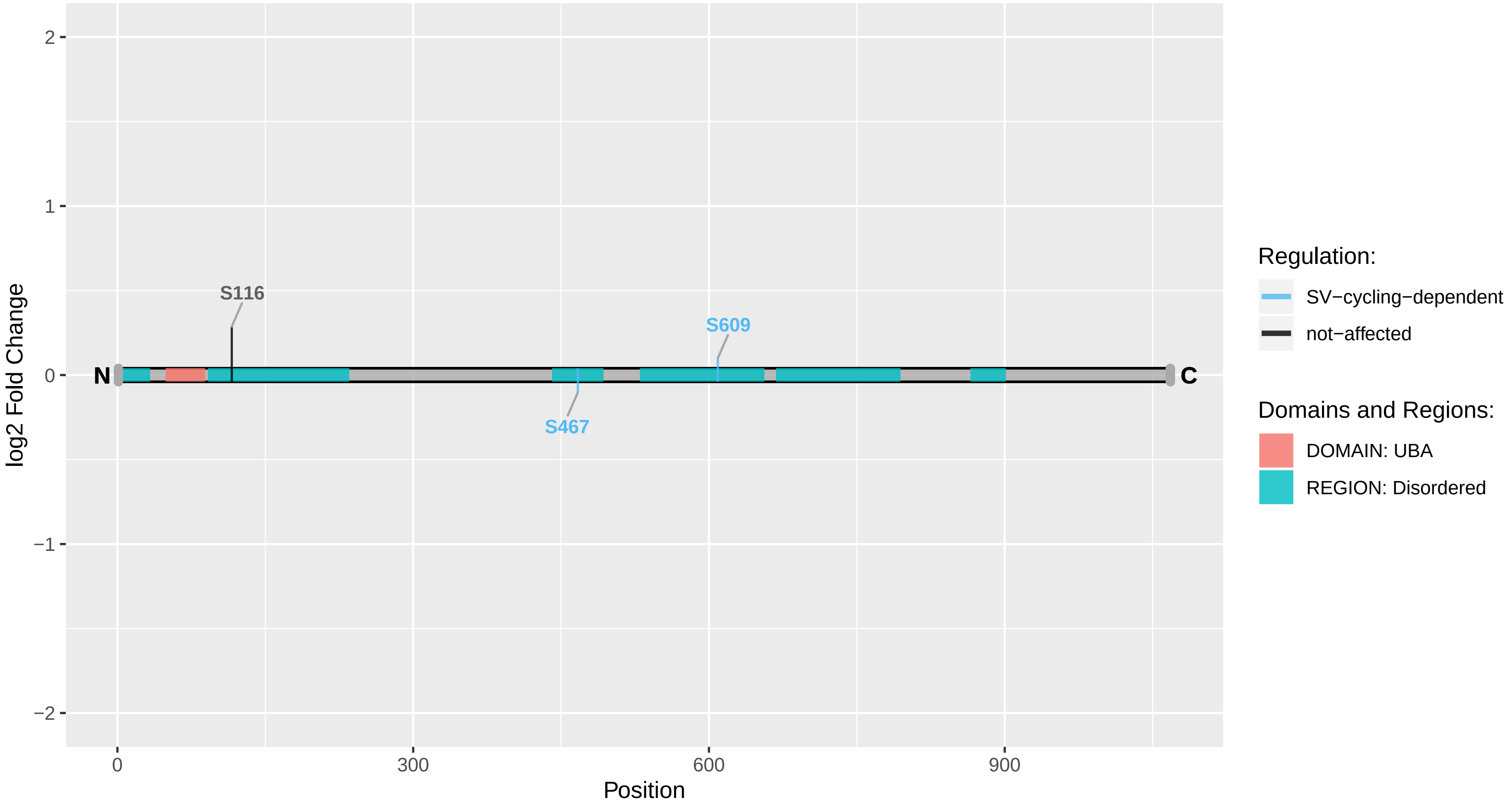

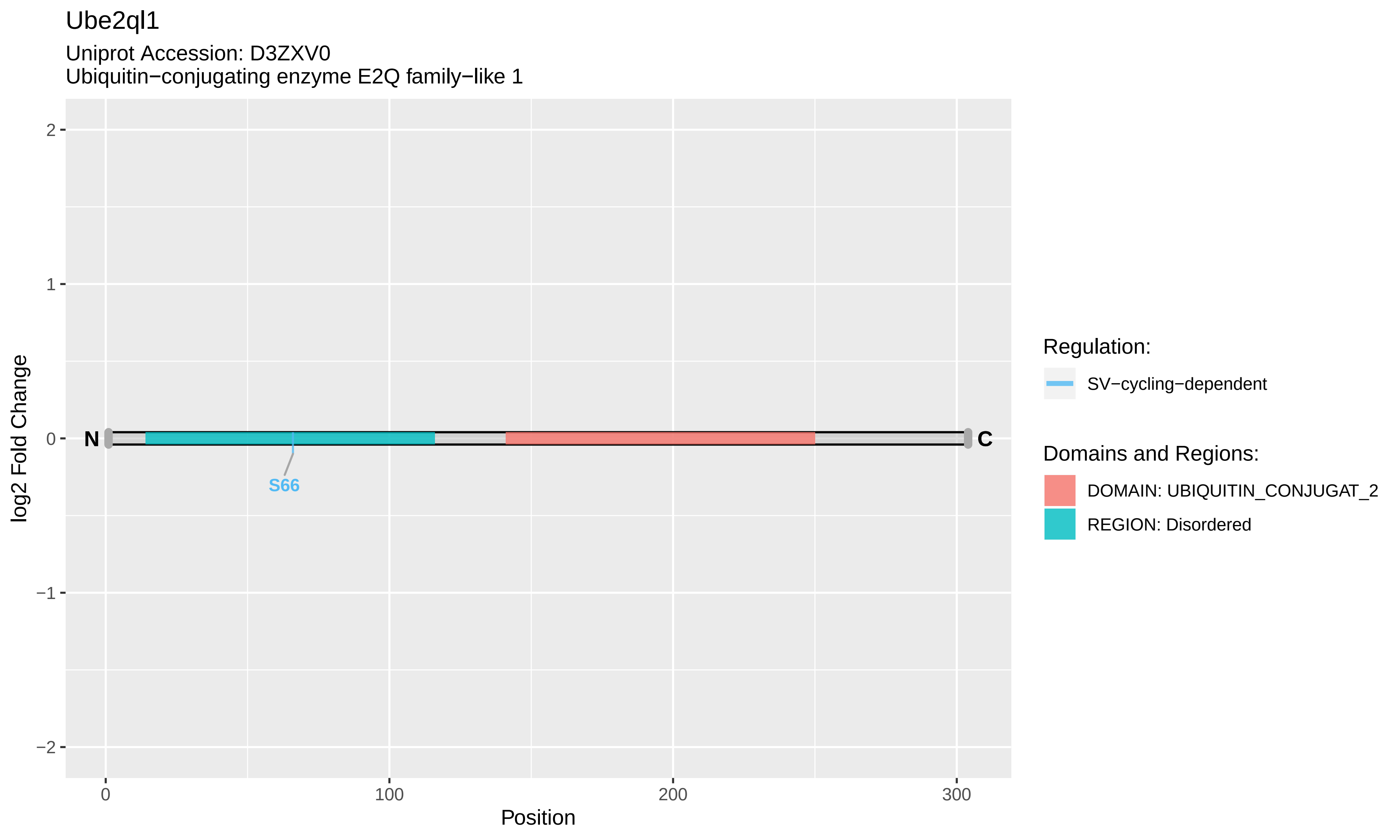

Ube2ql1
Uniprot Accession: D3ZXV0
Ubiquitin−conjugating enzyme E2Q family−like 1
log2 Fold Change
2
1
0
−1
−2
N
C
S66
0
100
200
300
Position
Regulation:
SV−cycling−dependent
Domains and Regions:
DOMAIN: UBIQUITIN_CONJUGAT_2
REGION: Disordered

Ubxn2b

Uniprot Accession: P0C627
UBX domain−containing protein 2B

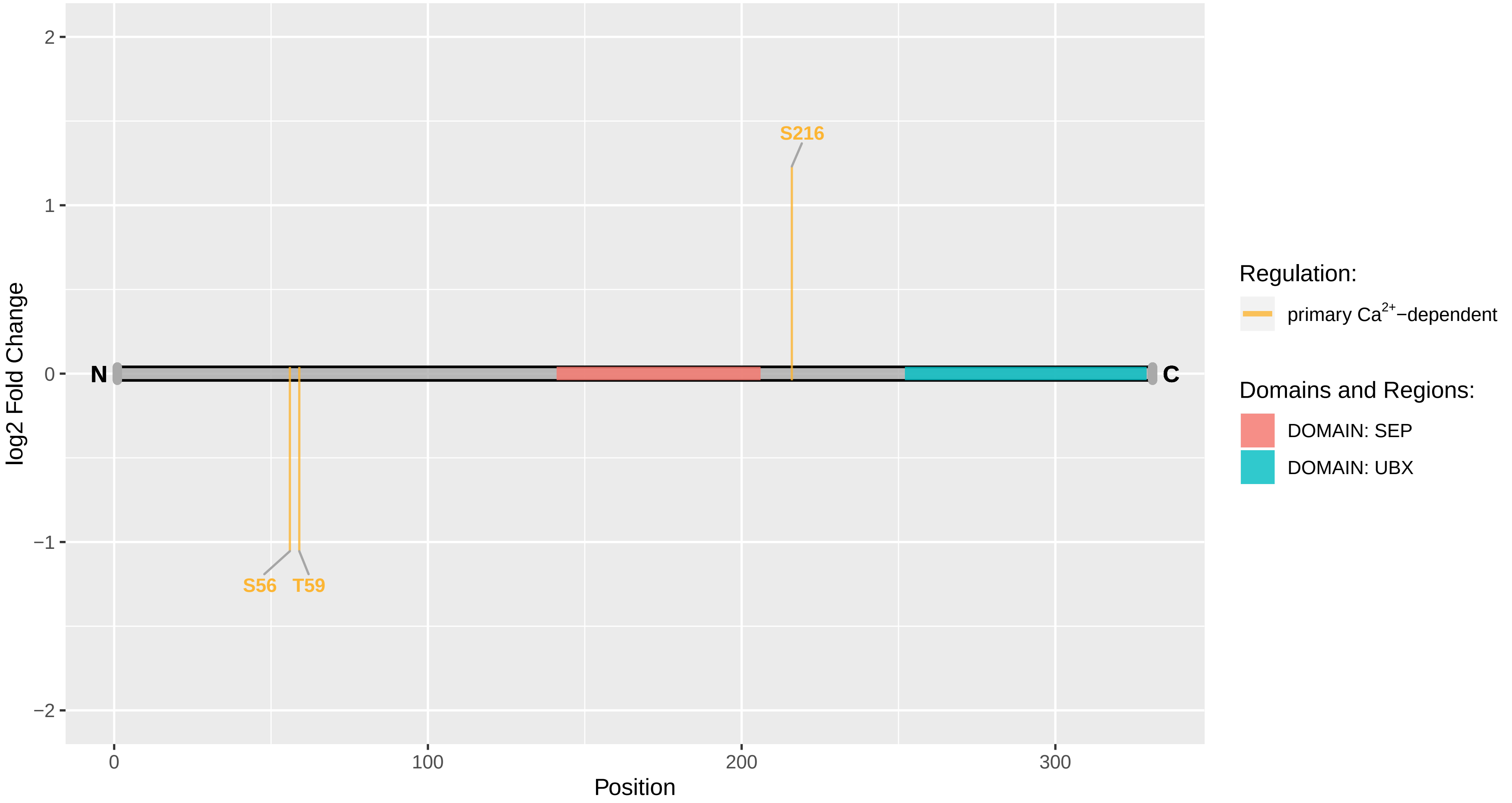

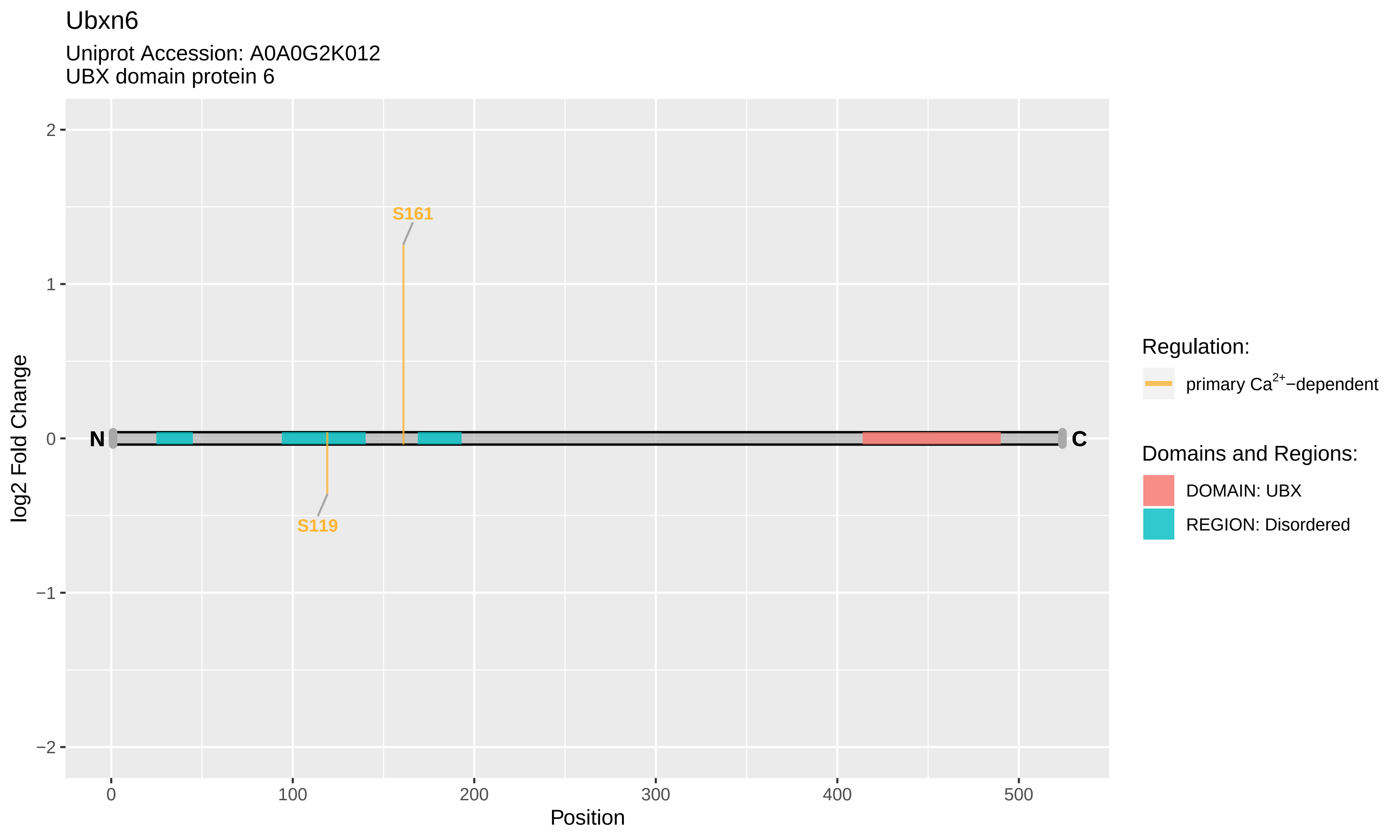

Ubxn6
Uniprot Accession: A0A0G2K012
UBX domain protein 6
S161
S119
N
C
log2 Fold Change
Position
2
1
0
−1
−2
0
100
200
300
400
500
Regulation:
primary Ca2+−dependent
Domains and Regions:
DOMAIN: UBX
REGION: Disordered

Uhrf1bp1l

Uniprot Accession: M0R8V0
UHRF1−binding protein 1−like

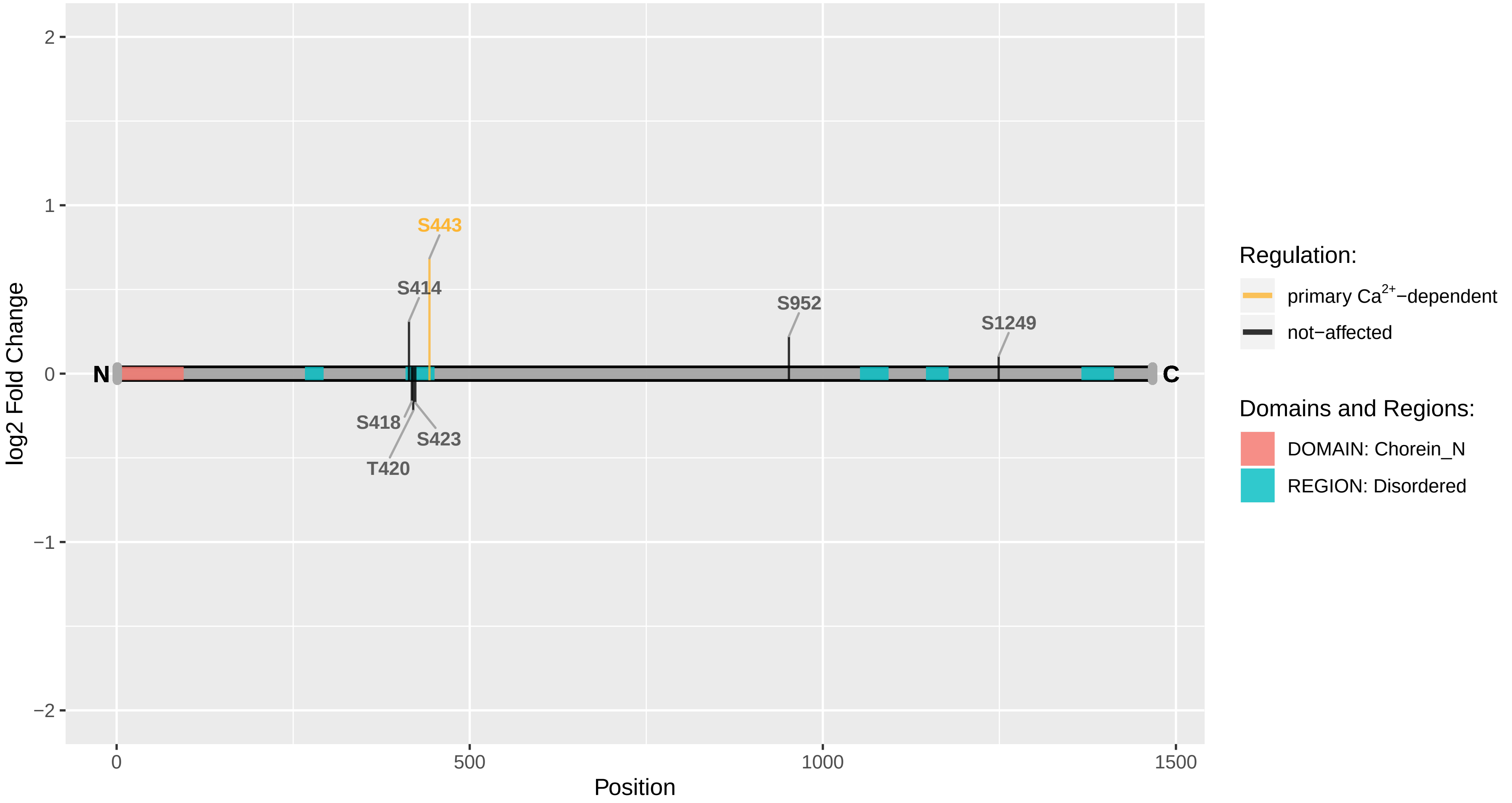

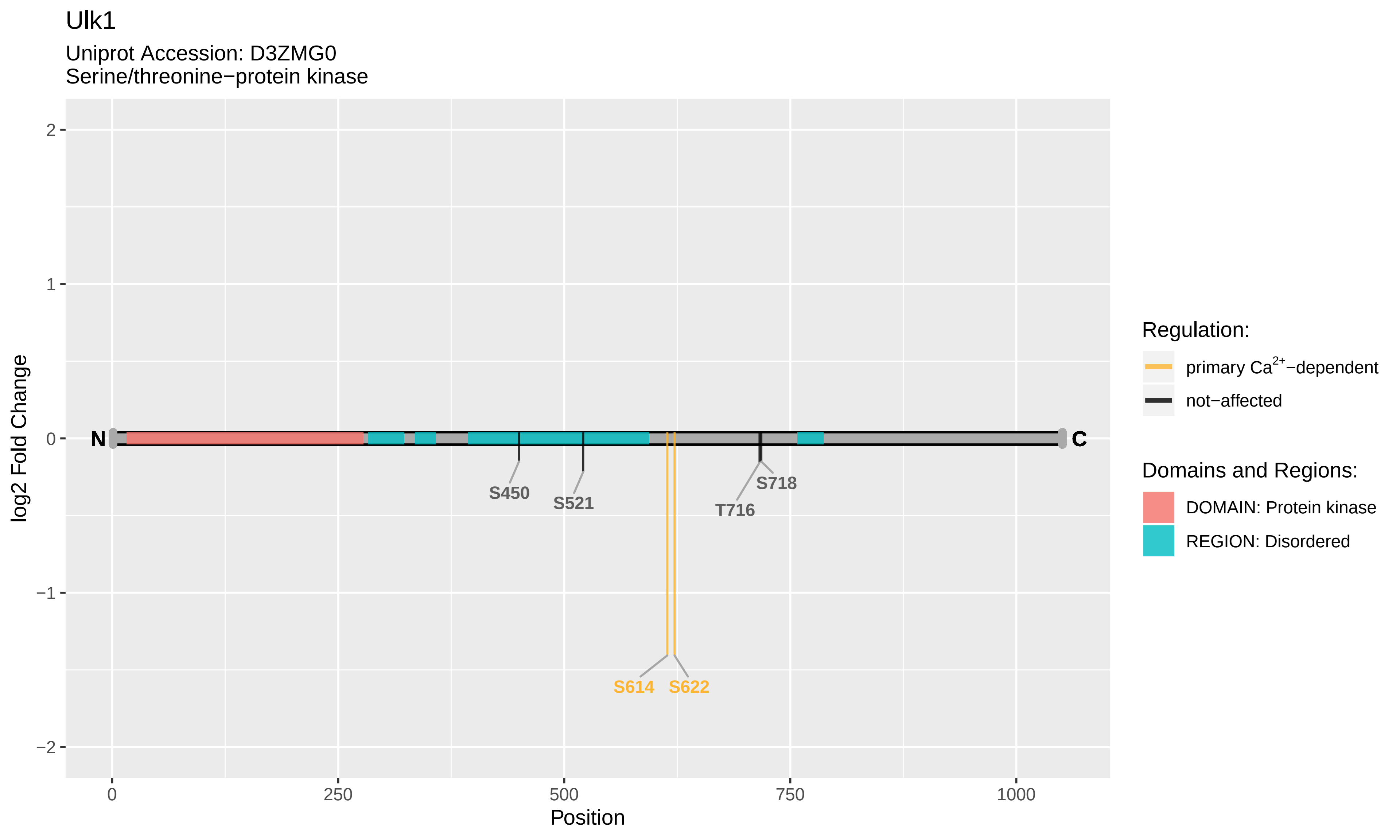
Ulk1
Uniprot Accession: D3ZMG0
Serine/threonine−protein kinase
log2 Fold Change
2
1
0
−1
−2
N
C
S450
S521
S614
S622
T716
S718
0
250
500
750
1000
Position
Regulation:
primary Ca2+−dependent
not−affected
Domains and Regions:
DOMAIN: Protein kinase
REGION: Disordered

Uprt

Uniprot Accession: D4ABC7
Uracil phosphoribosyltransferase homolog

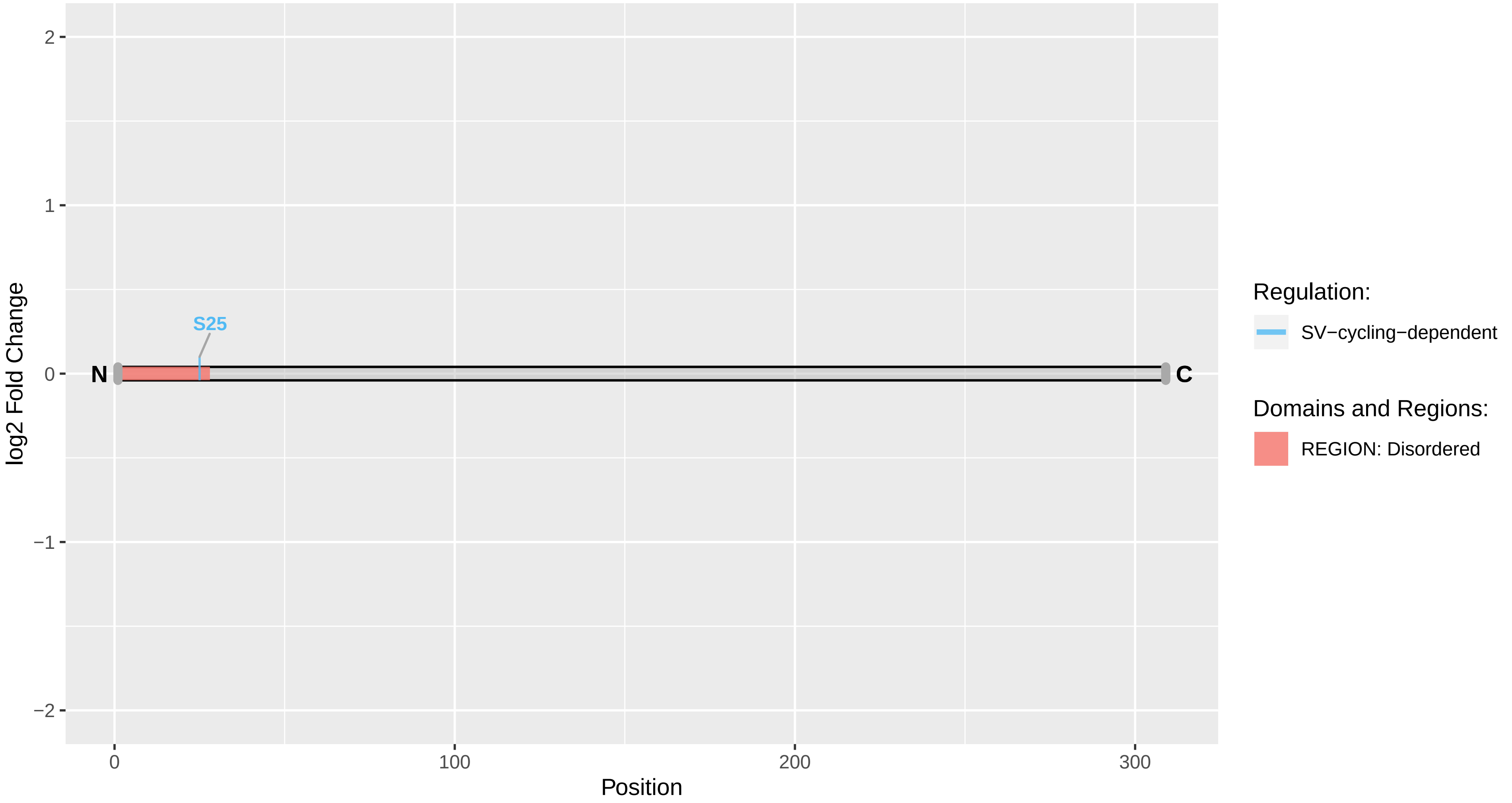

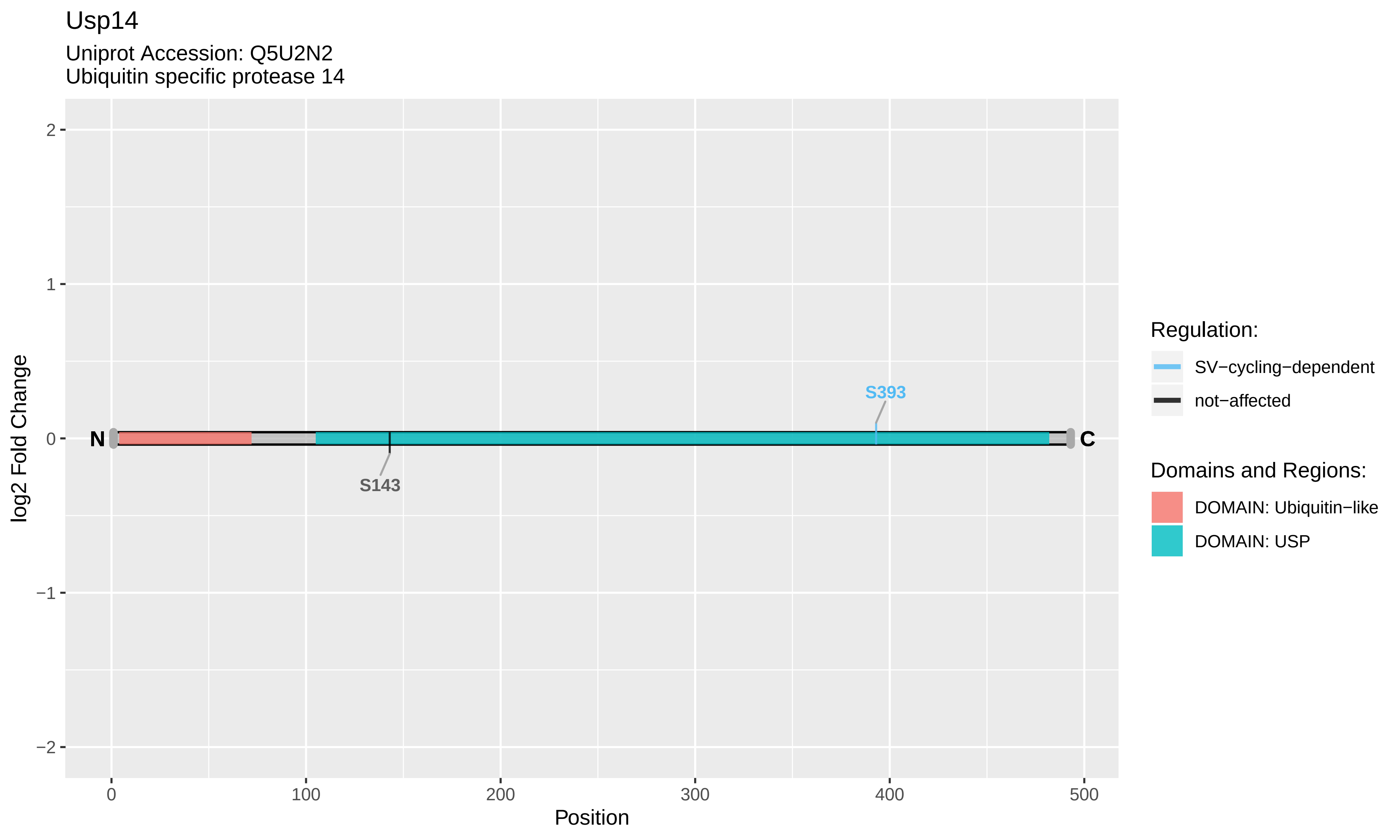
Usp14
Uniprot Accession: Q5U2N2
Ubiquitin specific protease 14
log2 Fold Change
2
1
0
−1
−2
N
C
S393
S143
0
100
200
300
400
500
Position
Regulation:
SV−cycling−dependent
not−affected
Domains and Regions:
DOMAIN: Ubiquitin−like
DOMAIN: USP

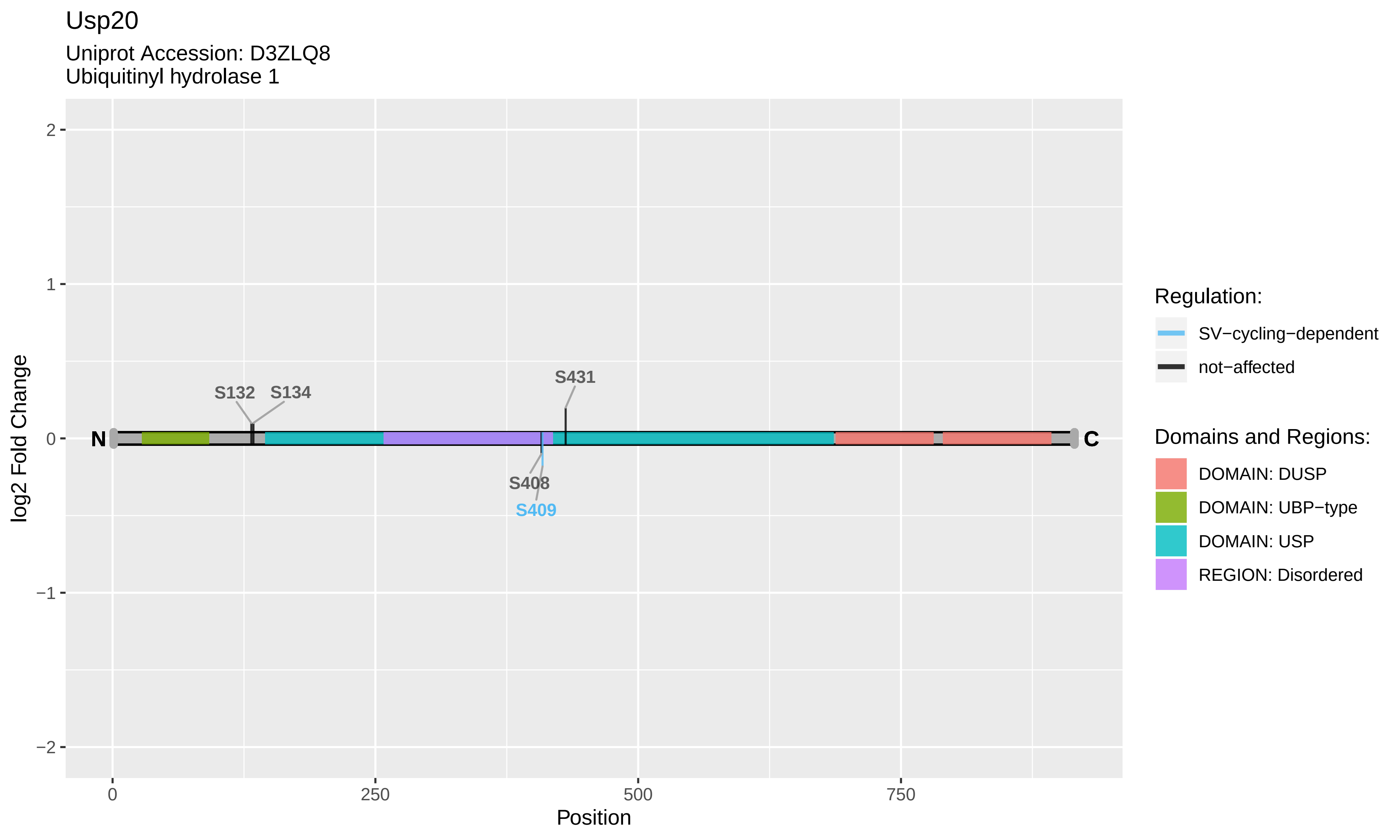

Usp20
Uniprot Accession: D3ZLQ8
Ubiquitinyl hydrolase 1
log2 Fold Change
Position
N
C
S132
S134
S431
S408
S409
2
1
0
−1
−2
0
250
500
750
Regulation:
SV−cycling−dependent
not−affected
Domains and Regions:
DOMAIN: DUSP
DOMAIN: UBP−type
DOMAIN: USP
REGION: Disordered

Usp32

Uniprot Accession: A0A0G2K952
Ubiquitin−specific peptidase 32

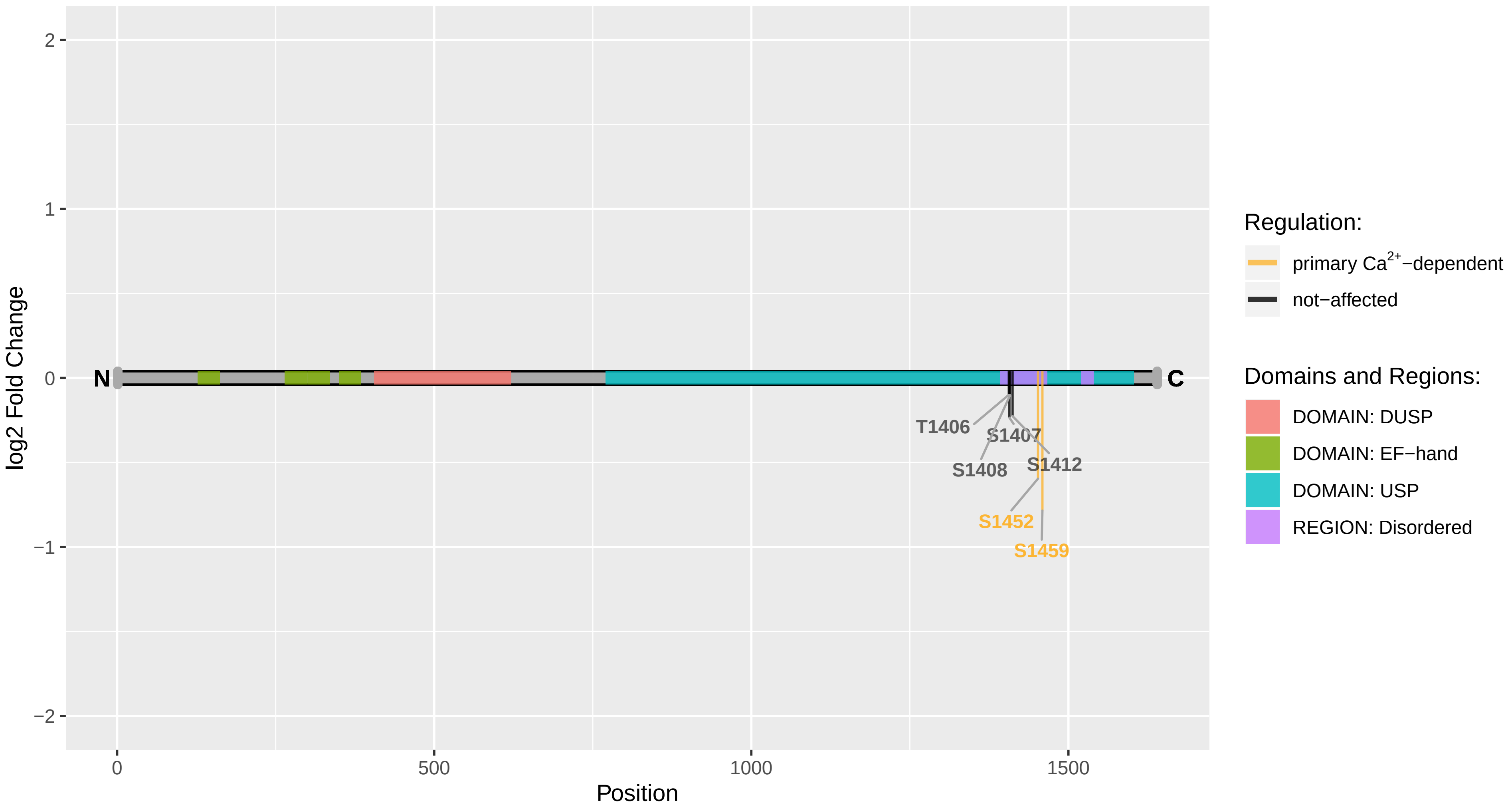

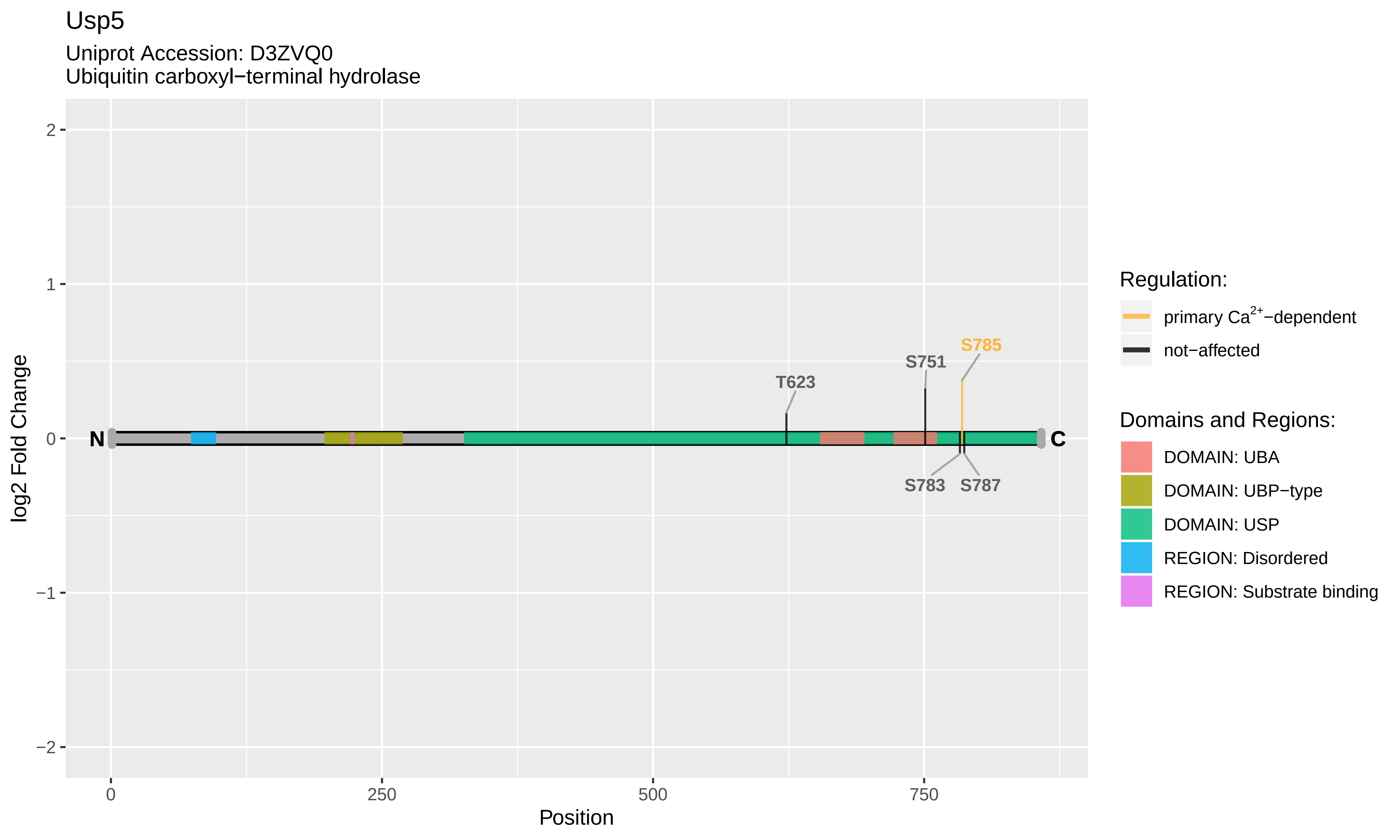

Usp5
Uniprot Accession: D3ZVQ0
Ubiquitin carboxyl−terminal hydrolase
log2 Fold Change
Position
2
1
0
−1
−2
0
250
500
750
N
C
T623
S751
S785
S783
S787
Regulation:
primary Ca2+−dependent
not−affected
Domains and Regions:
DOMAIN: UBA
DOMAIN: UBP−type
DOMAIN: USP
REGION: Disordered
REGION: Substrate binding

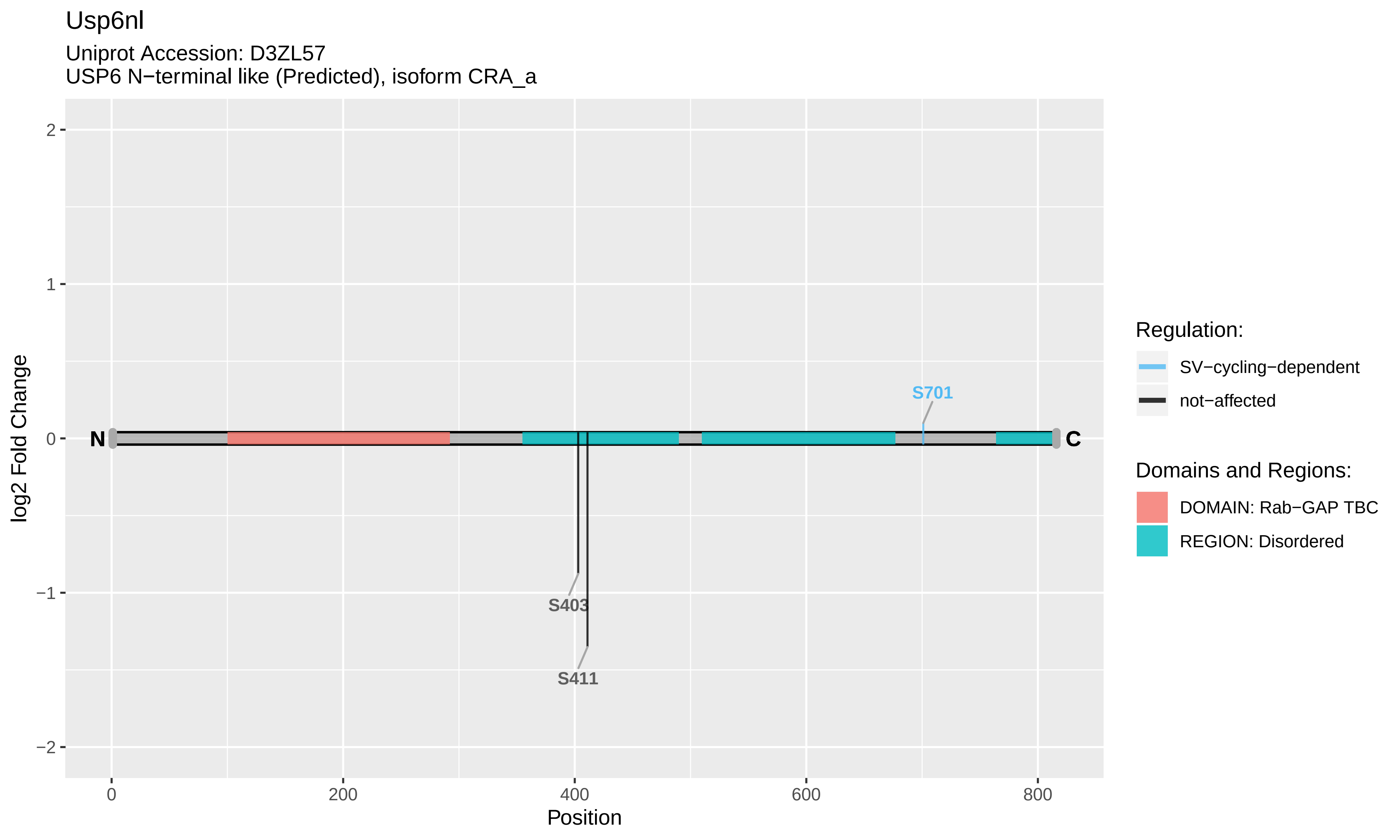

Usp6nl
Uniprot Accession: D3ZL57
USP6 N−terminal like (Predicted), isoform CRA_a
log2 Fold Change
Position
N
C
S701
S403
S411
Regulation:
SV−cycling−dependent
not−affected
Domains and Regions:
DOMAIN: Rab−GAP TBC
REGION: Disordered

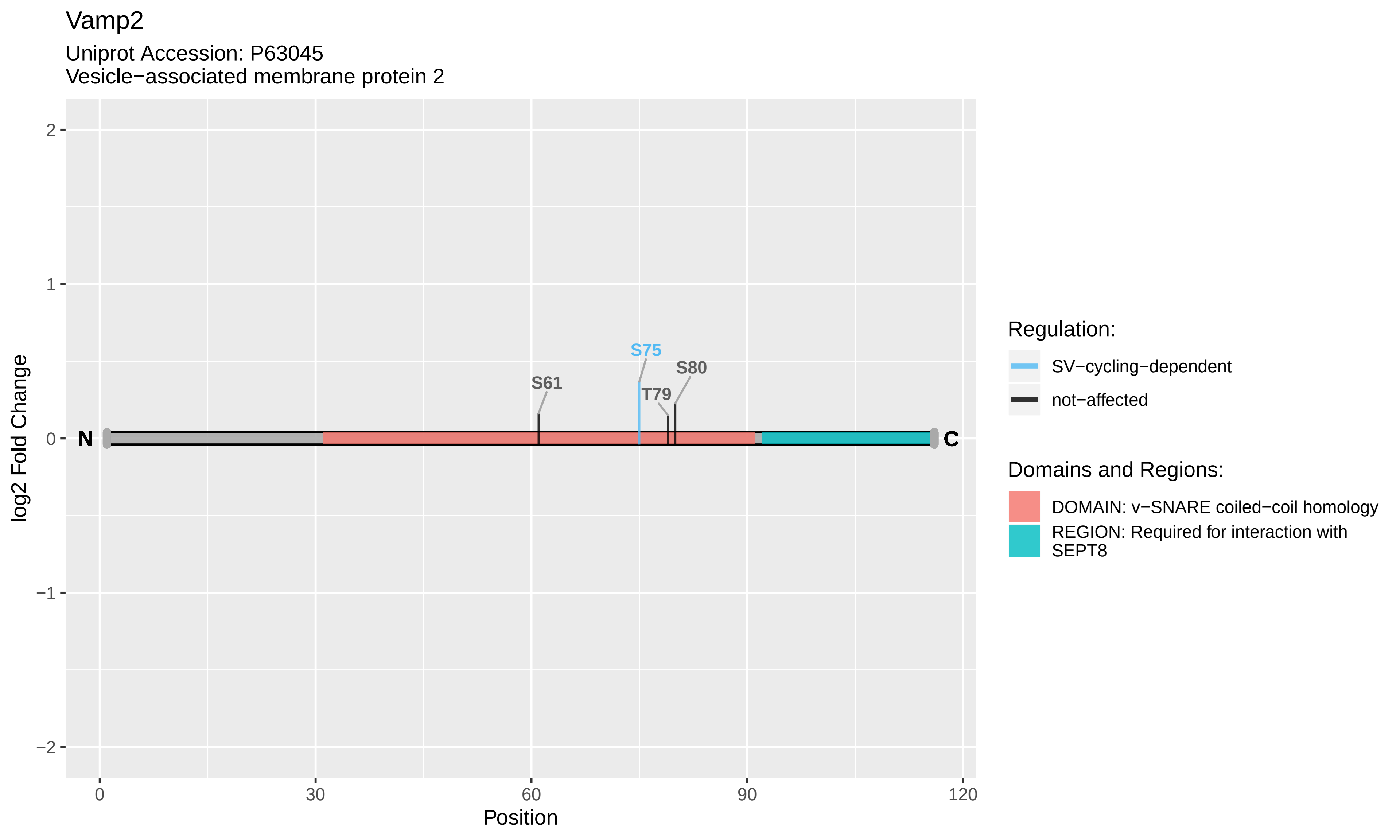

Vamp2
Uniprot Accession: P63045
Vesicle−associated membrane protein 2
log2 Fold Change
2
1
0
−1
−2
0
30
60
90
120
Position
N
C
S61
S75
T79
S80
Regulation:
SV−cycling−dependent
not−affected
Domains and Regions:
DOMAIN: v−SNARE coiled−coil homology
REGION: Required for interaction with SEPT8

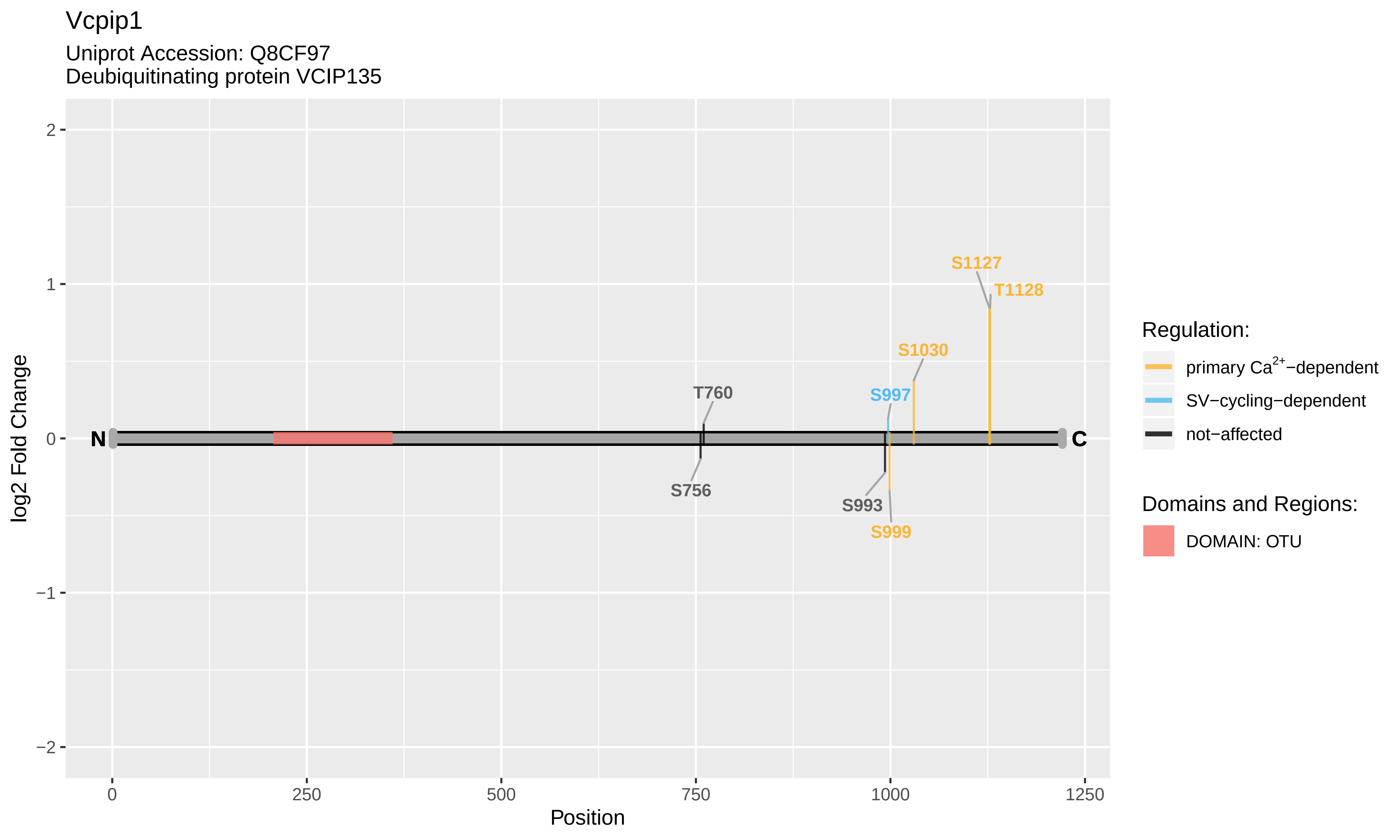

Vcpip1
Uniprot Accession: Q8CF97
Deubiquitinating protein VCIP135
log2 Fold Change
Position
N
C
T760
S756
S997
S993
S999
S1030
S1127
T1128
Regulation:
primary Ca2+−dependent
SV−cycling−dependent
not−affected
Domains and Regions:
DOMAIN: OTU

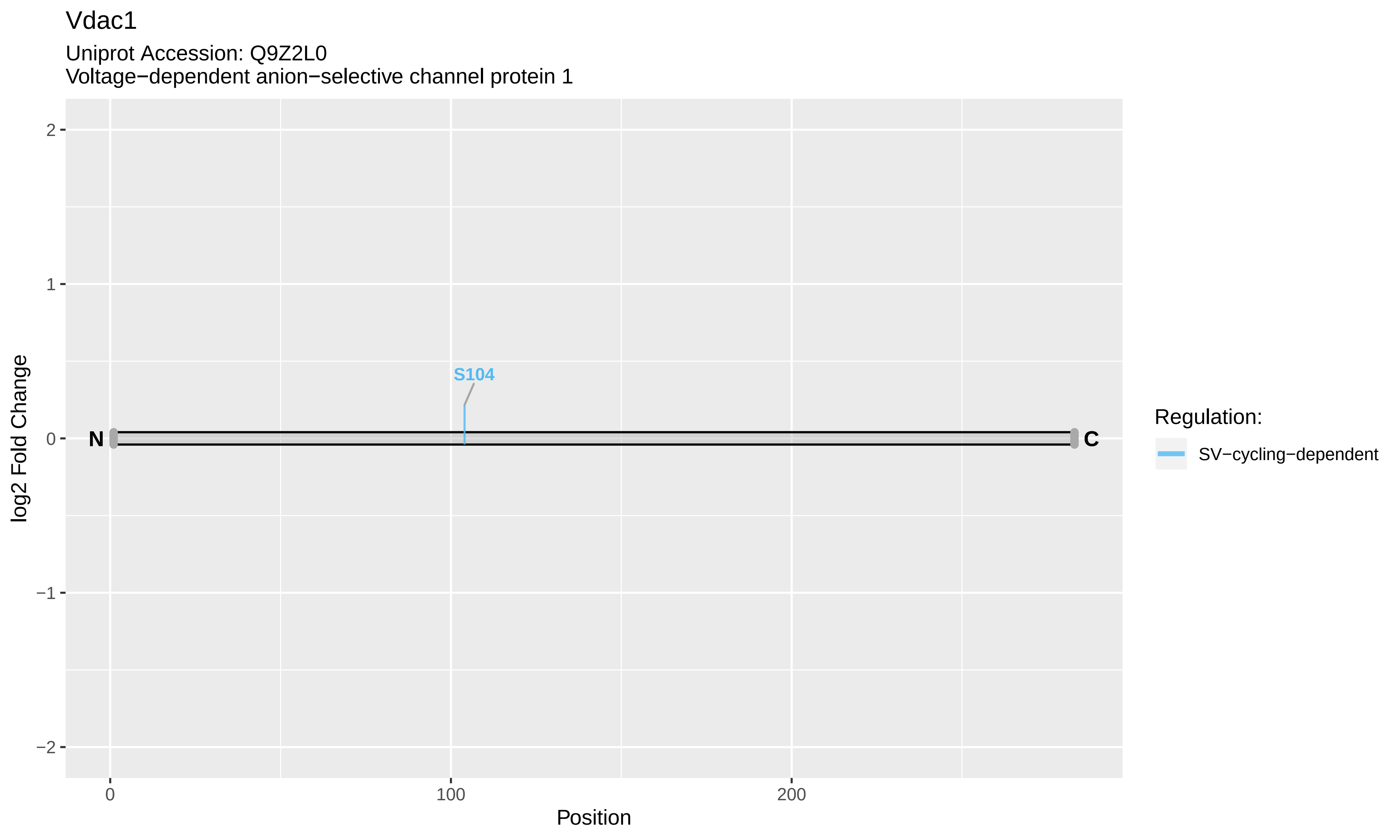

Vdac1
Uniprot Accession: Q9Z2L0
Voltage−dependent anion−selective channel protein 1
S104
N
C
2
1
0
−1
−2
log2 Fold Change
0
100
200
Position
Regulation:
SV−cycling−dependent

Vps26b

Uniprot Accession: B1WBS4
Similar to Vacuolar protein sorting 26 homolog (VPS26 protein homolog), isoform CRA_a

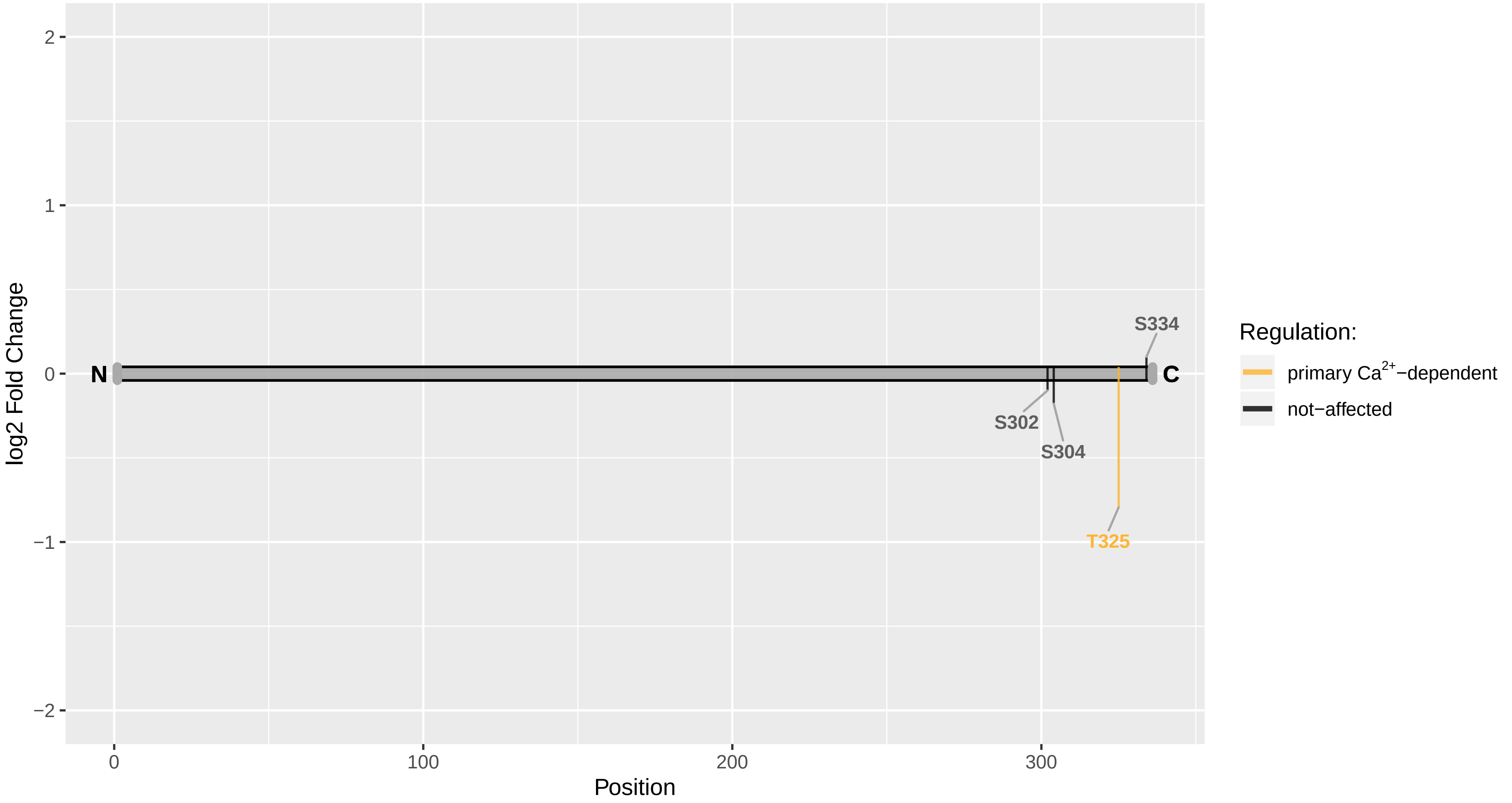

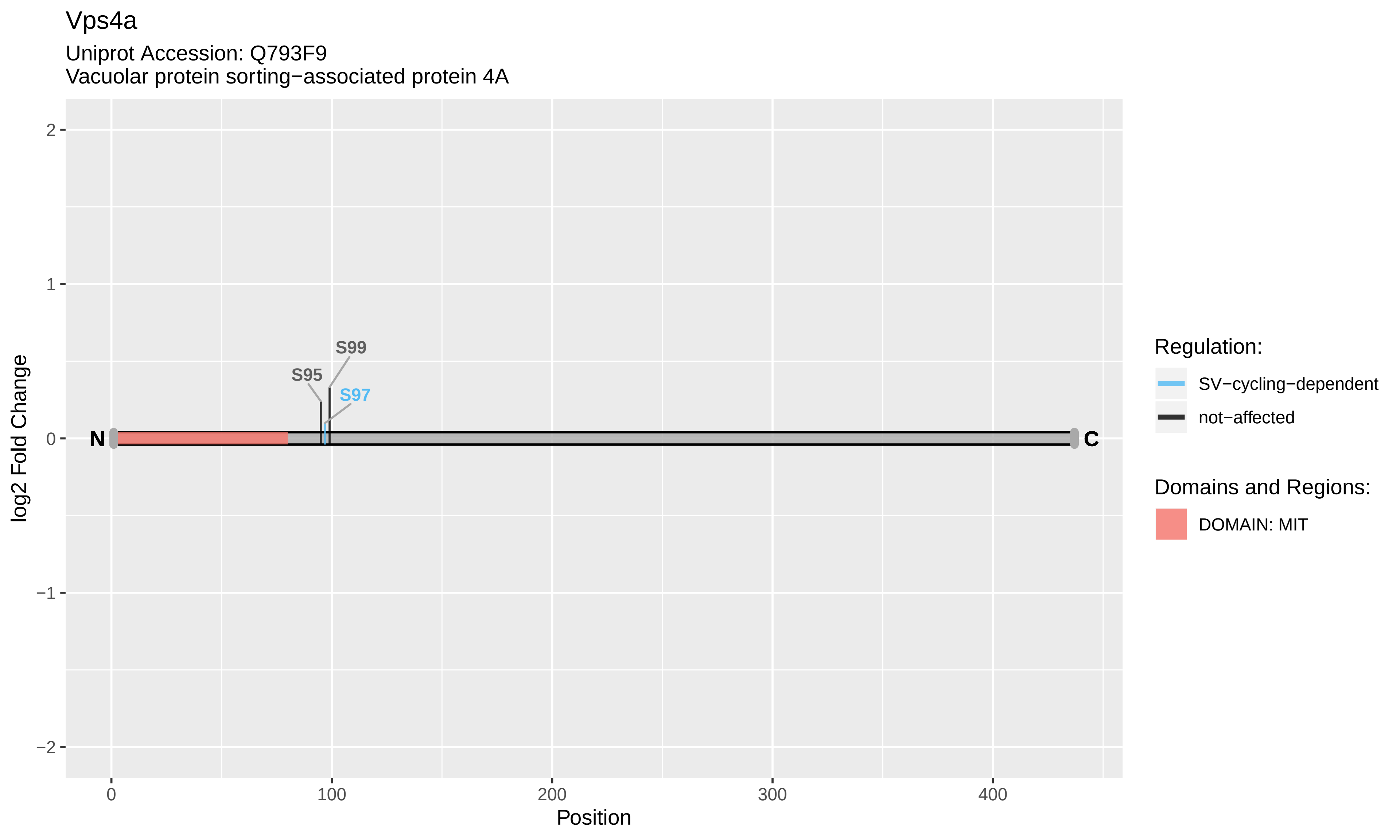
Vps4a
Uniprot Accession: Q793F9
Vacuolar protein sorting−associated protein 4A
log2 Fold Change
2
1
0
−1
−2
N
C
S95
S99
S97
0
100
200
300
400
Position
Regulation:
SV−cycling−dependent
not−affected
Domains and Regions:
DOMAIN: MIT

Vps50

Uniprot Accession: F1LSG8
Syndetin

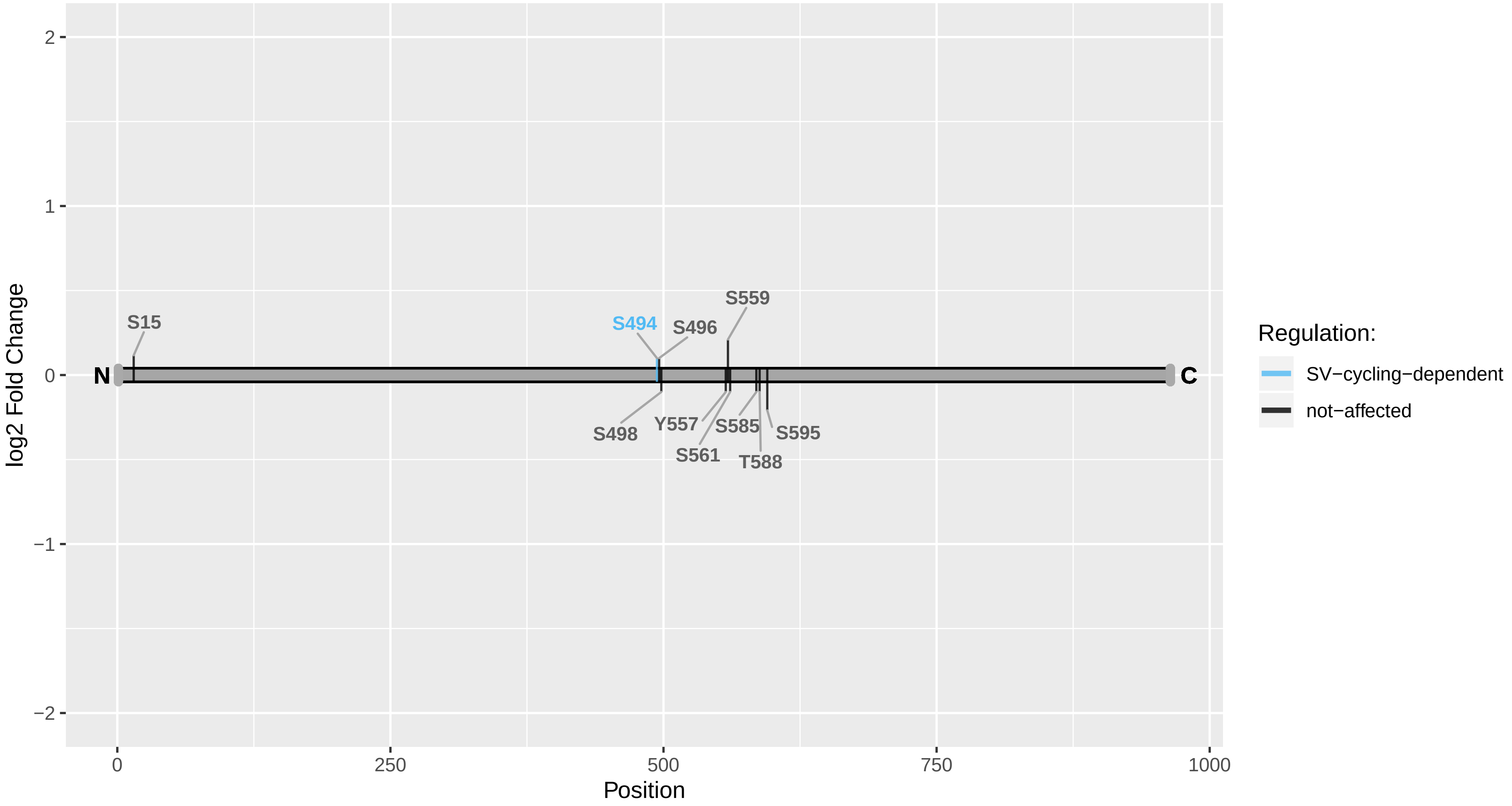

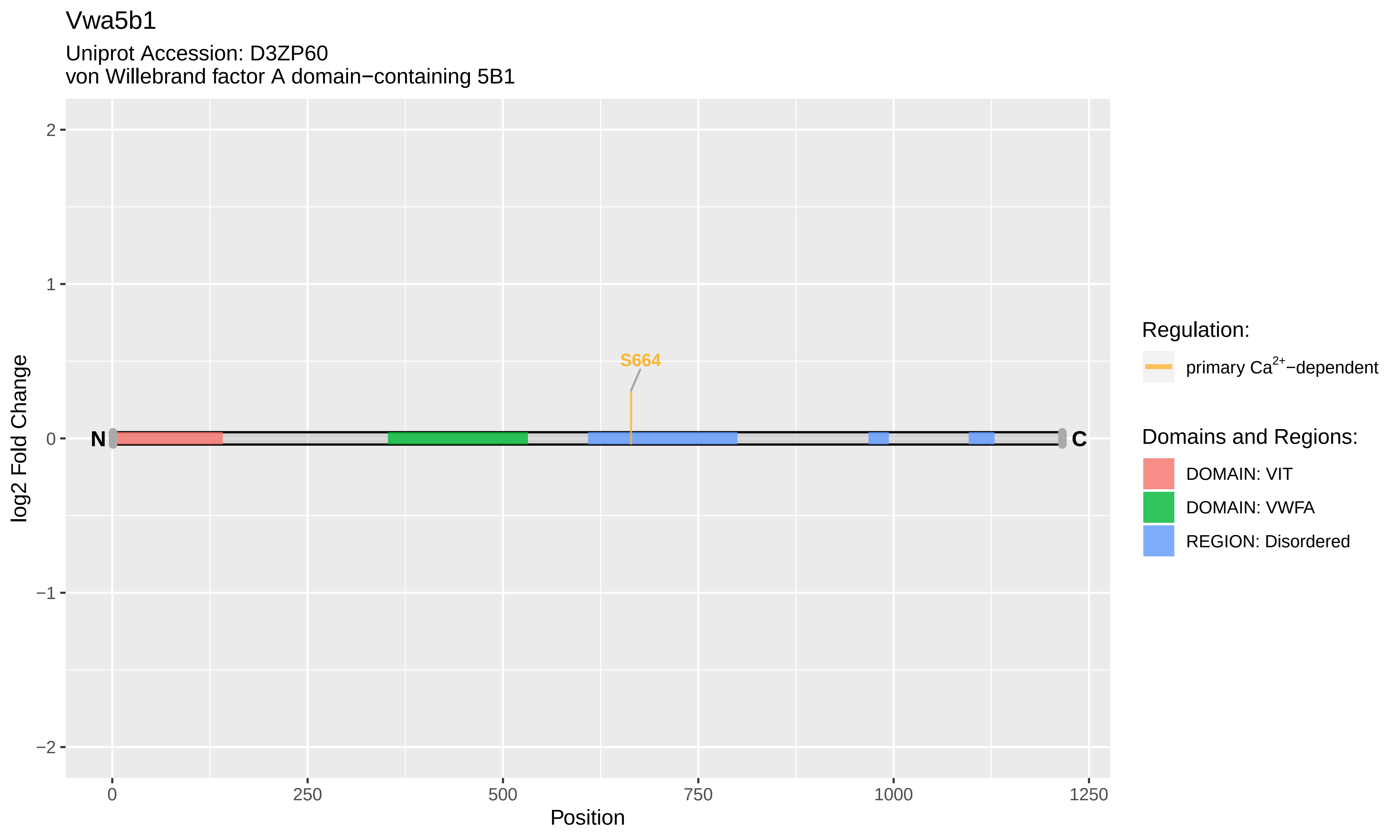

Vwa5b1
Uniprot Accession: D3ZP60
von Willebrand factor A domain−containing 5B1
S664
N
C
2
1
0
−1
−2
log2 Fold Change
0
250
500
750
1000
1250
Position
Regulation:
primary Ca2+−dependent
Domains and Regions:
DOMAIN: VIT
DOMAIN: VWFA
REGION: Disordered

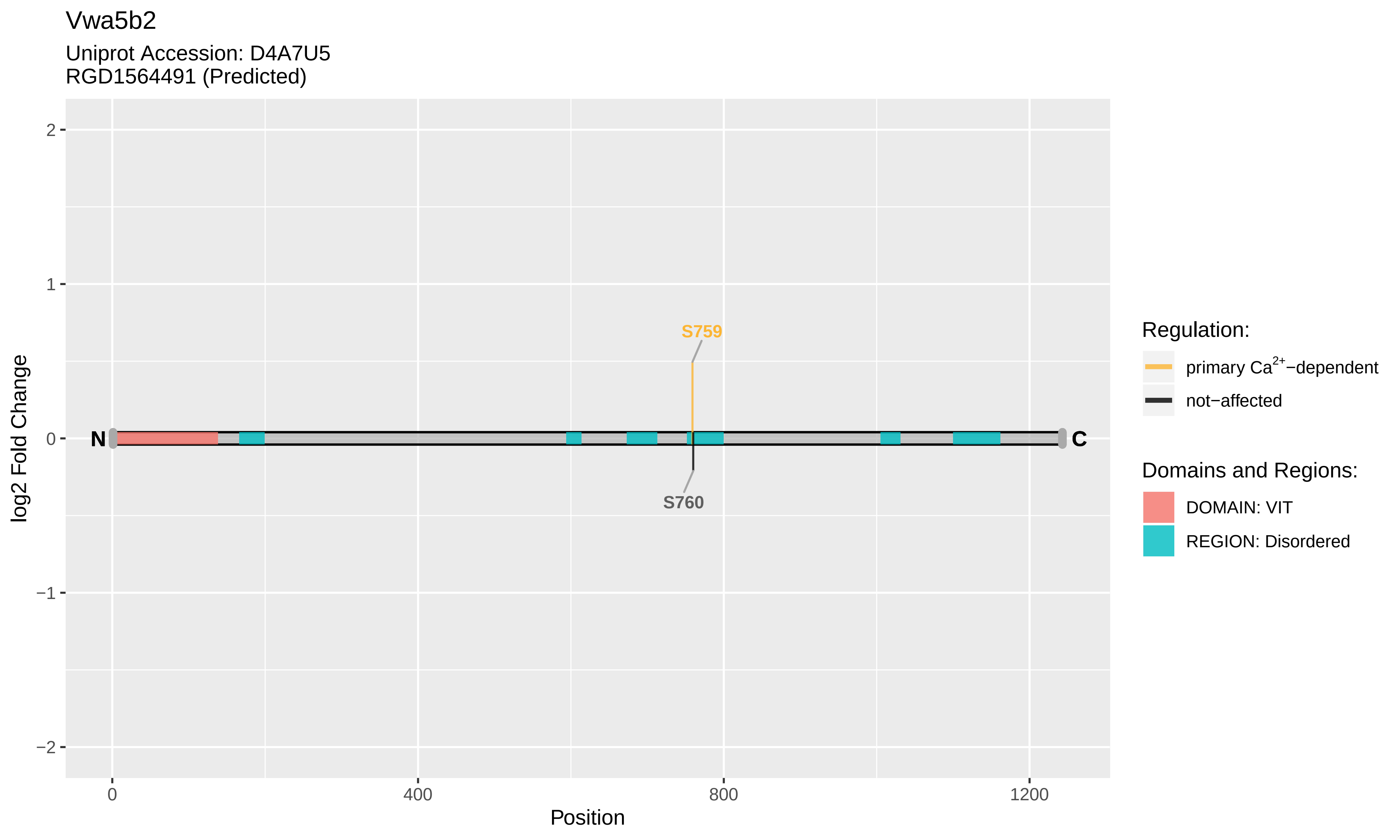

Vwa5b2
Uniprot Accession: D4A7U5
RGD1564491 (Predicted)
2
1
0
−1
−2
log2 Fold Change
N
C
S759
S760
0
400
800
1200
Position
Regulation:
primary Ca2+−dependent
not−affected
Domains and Regions:
DOMAIN: VIT
REGION: Disordered

Wasf3

Uniprot Accession: M0R7F3
WAS protein family, member 3

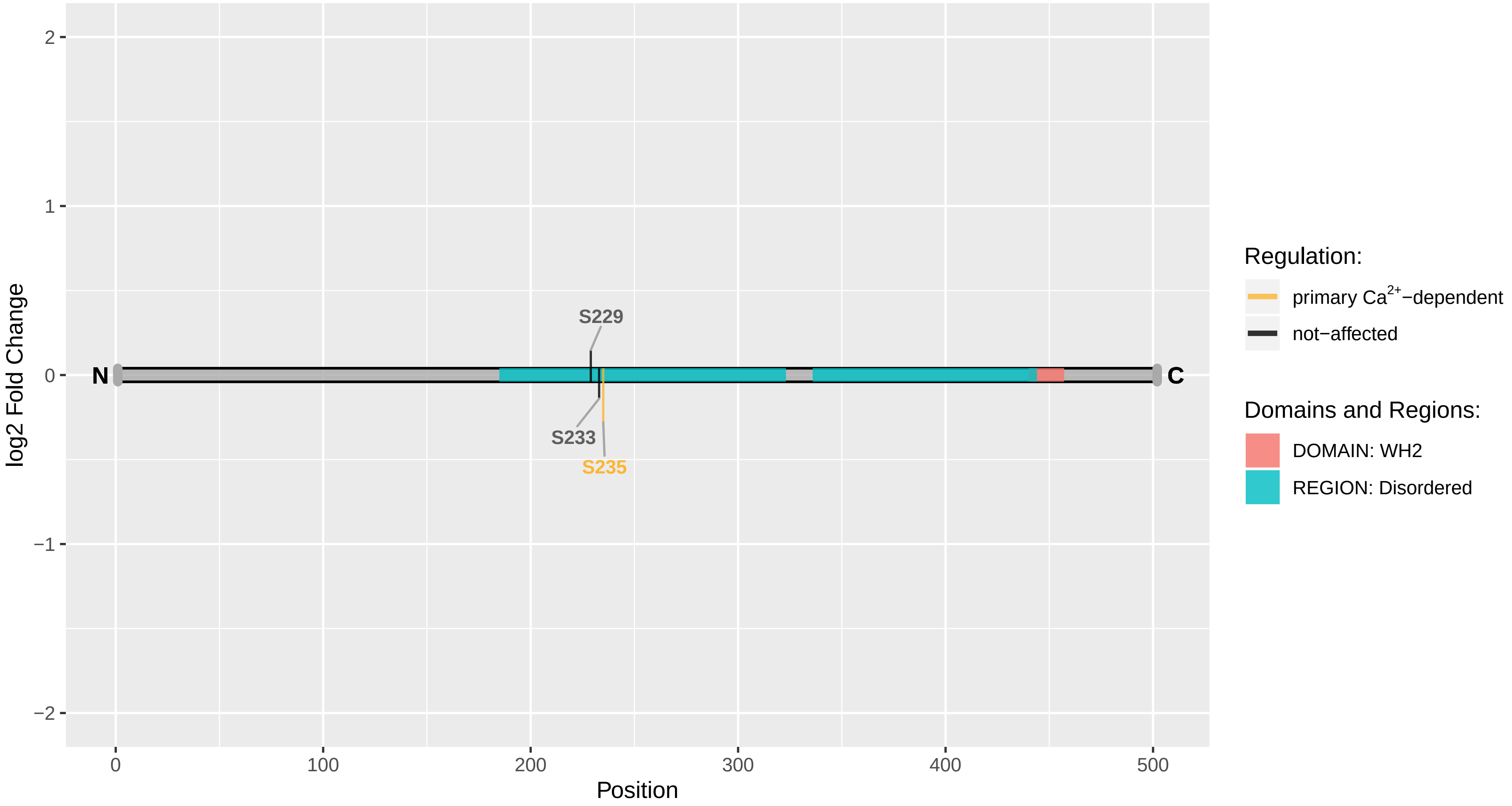

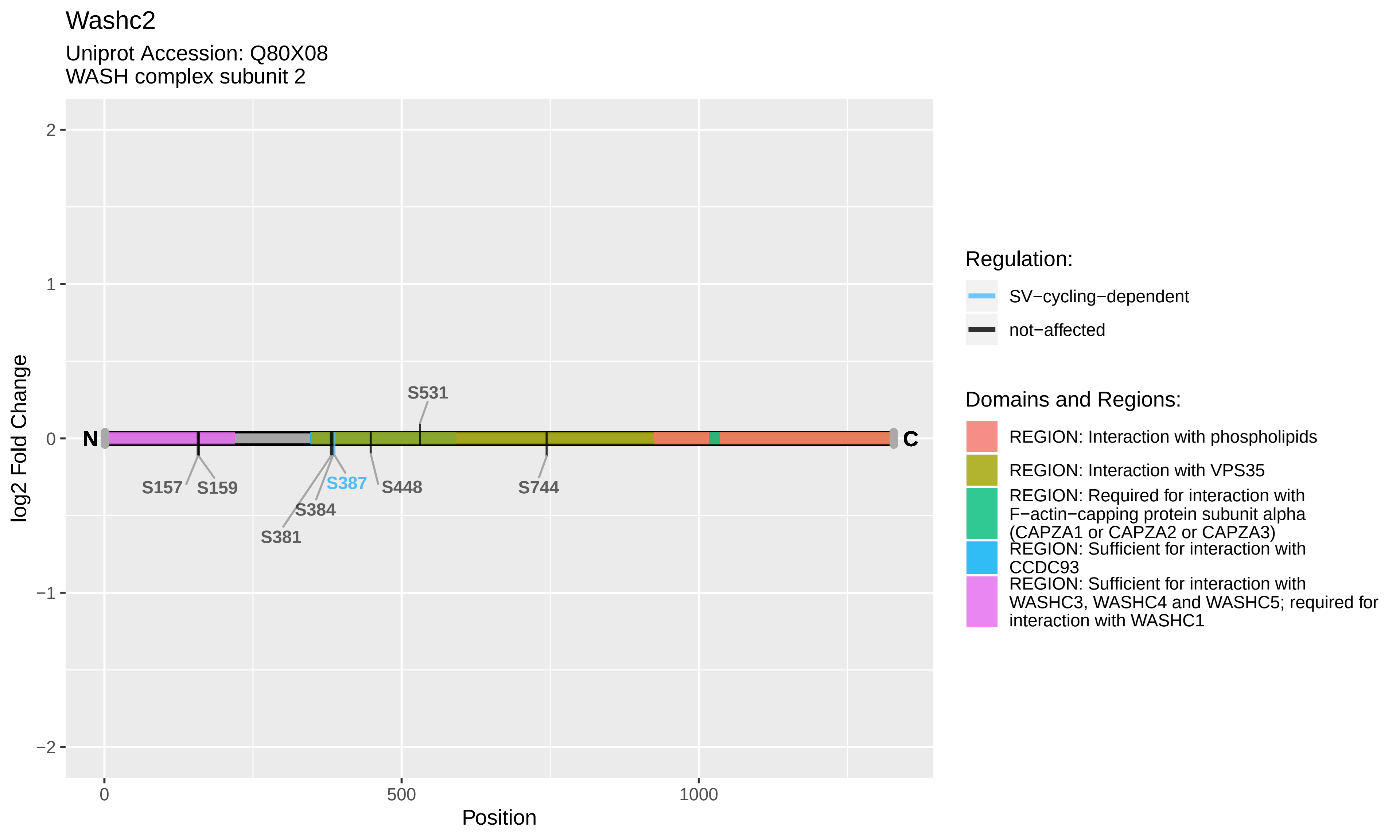

Washc2
Uniprot Accession: Q80X08
WASH complex subunit 2
log2 Fold Change
2
1
0
−1
−2
N
C
S531
S157
S159
S387
S448
S744
S384
S381
0
500
1000
Position
Regulation:
SV−cycling−dependent
not−affected
Domains and Regions:
REGION: Interaction with phospholipids
REGION: Interaction with VPS35
REGION: Required for interaction with F−actin−capping protein subunit alpha (CAPZA1 or CAPZA2 or CAPZA3)
REGION: Sufficient for interaction with CCDC93
REGION: Sufficient for interaction with WASHC3, WASHC4 and WASHC5; required for interaction with WASHC1

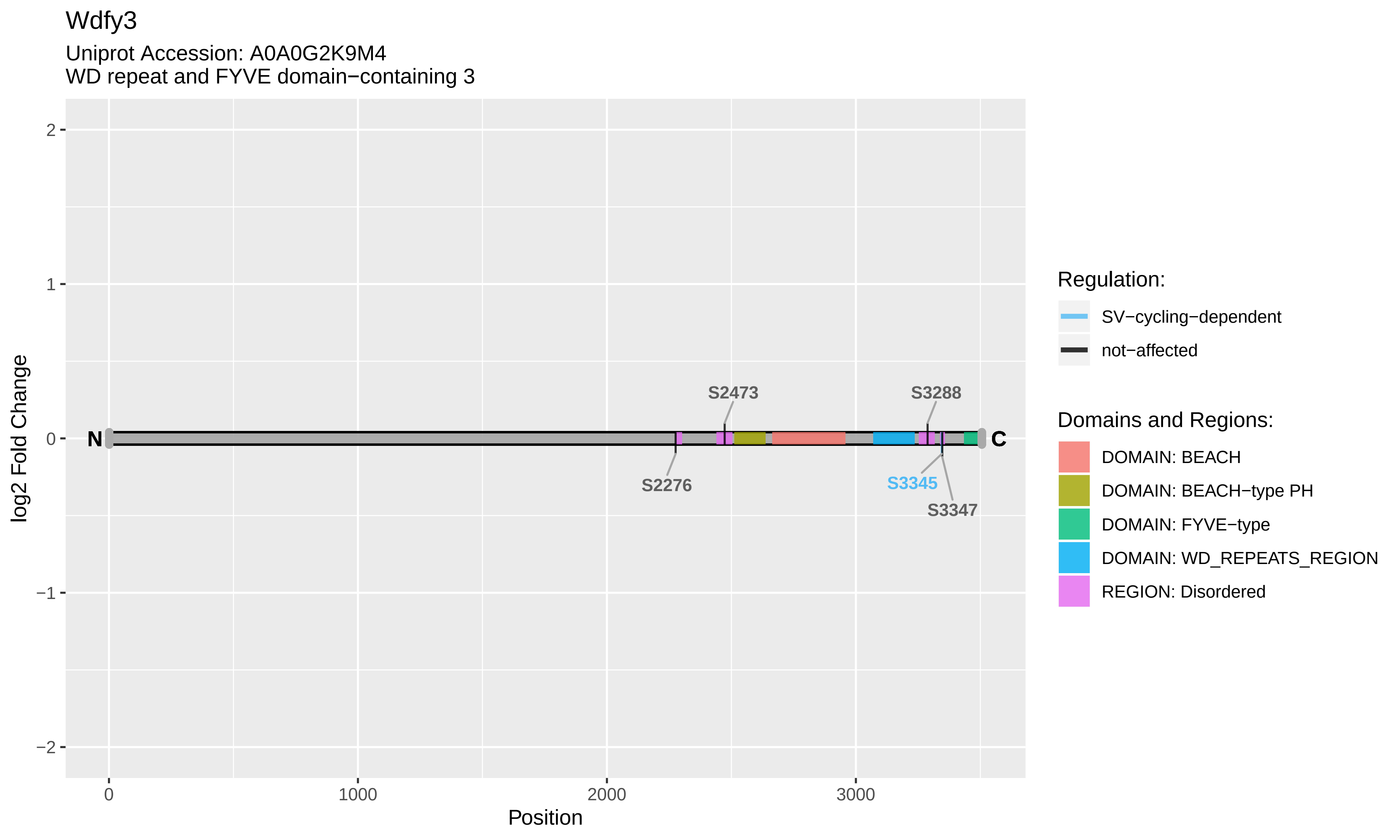

Wdfy3
Uniprot Accession: A0A0G2K9M4
WD repeat and FYVE domain−containing 3
2
1
0
−1
−2
log2 Fold Change
0
1000
2000
3000
Position
N
C
S2473
S3288
S2276
S3345
S3347
Regulation:
SV−cycling−dependent
not−affected
Domains and Regions:
DOMAIN: BEACH
DOMAIN: BEACH−type PH
DOMAIN: FYVE−type
DOMAIN: WD_REPEATS_REGION
REGION: Disordered

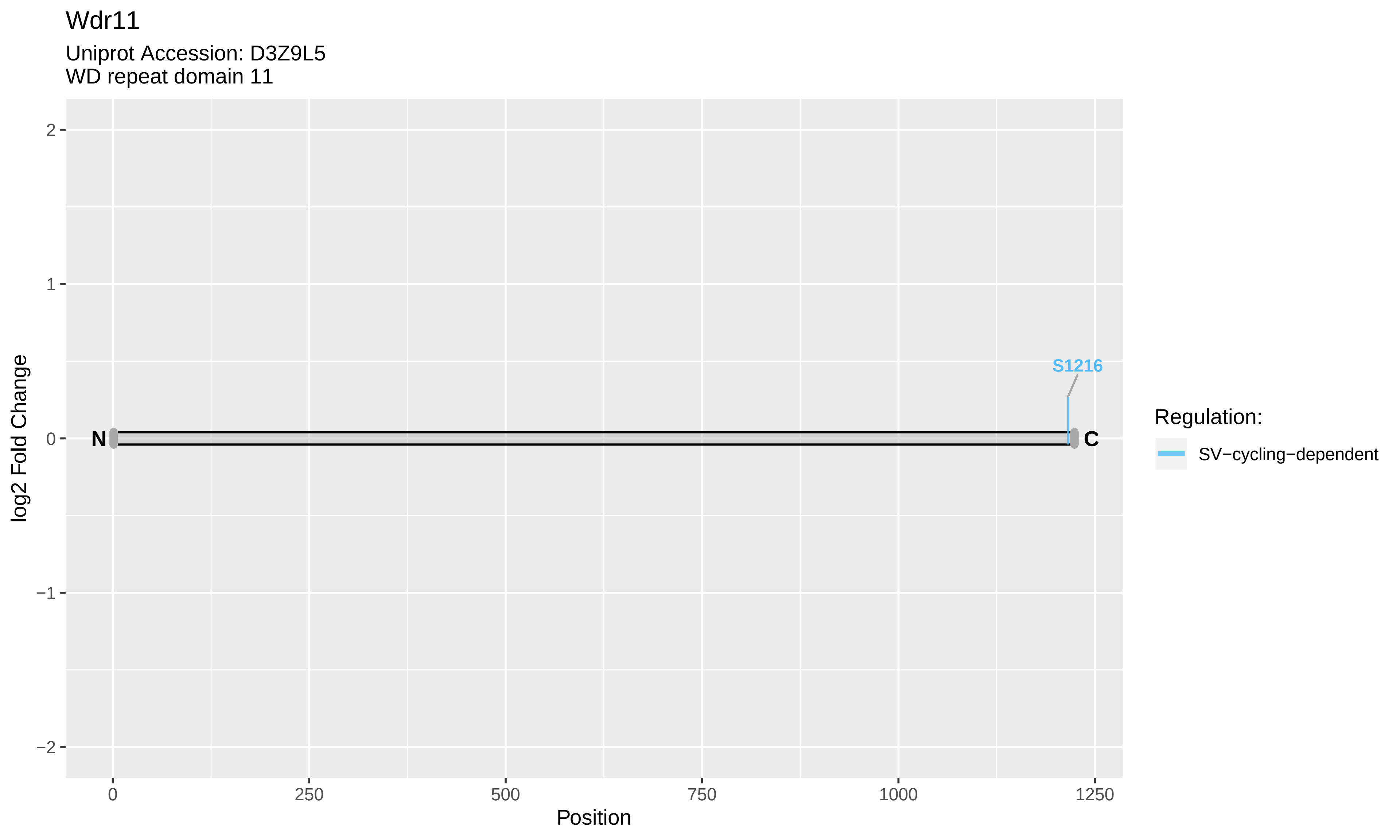

Wdr11
Uniprot Accession: D3Z9L5
WD repeat domain 11
S1216
N
C
2
1
0
−1
−2
0
250
500
750
1000
1250
Position
log2 Fold Change
Regulation:
SV−cycling−dependent

Wdr37

Uniprot Accession: D3ZQ02
WD repeat domain 37

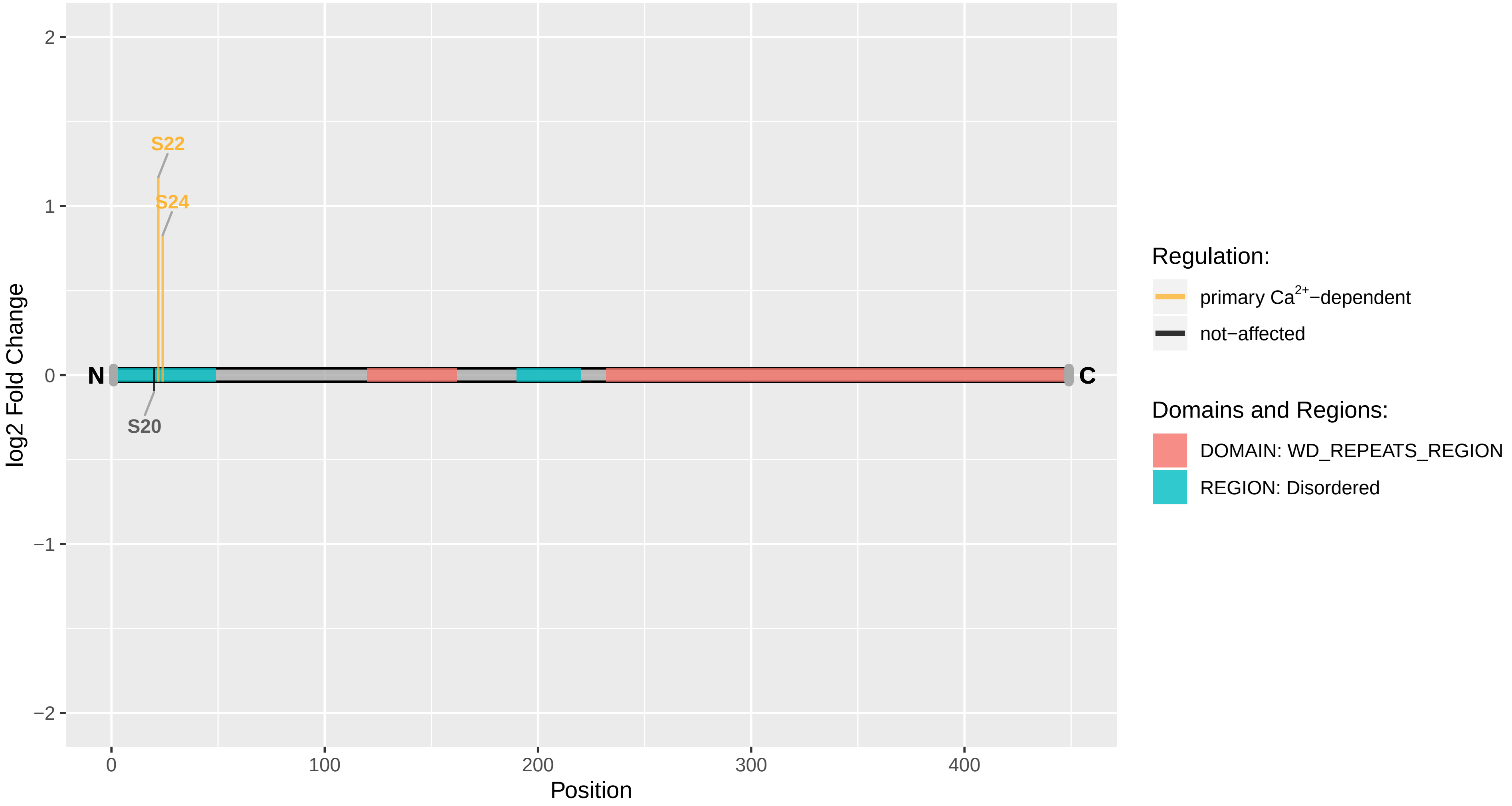

Wdr47

Uniprot Accession: G3V9M3
RCG28460

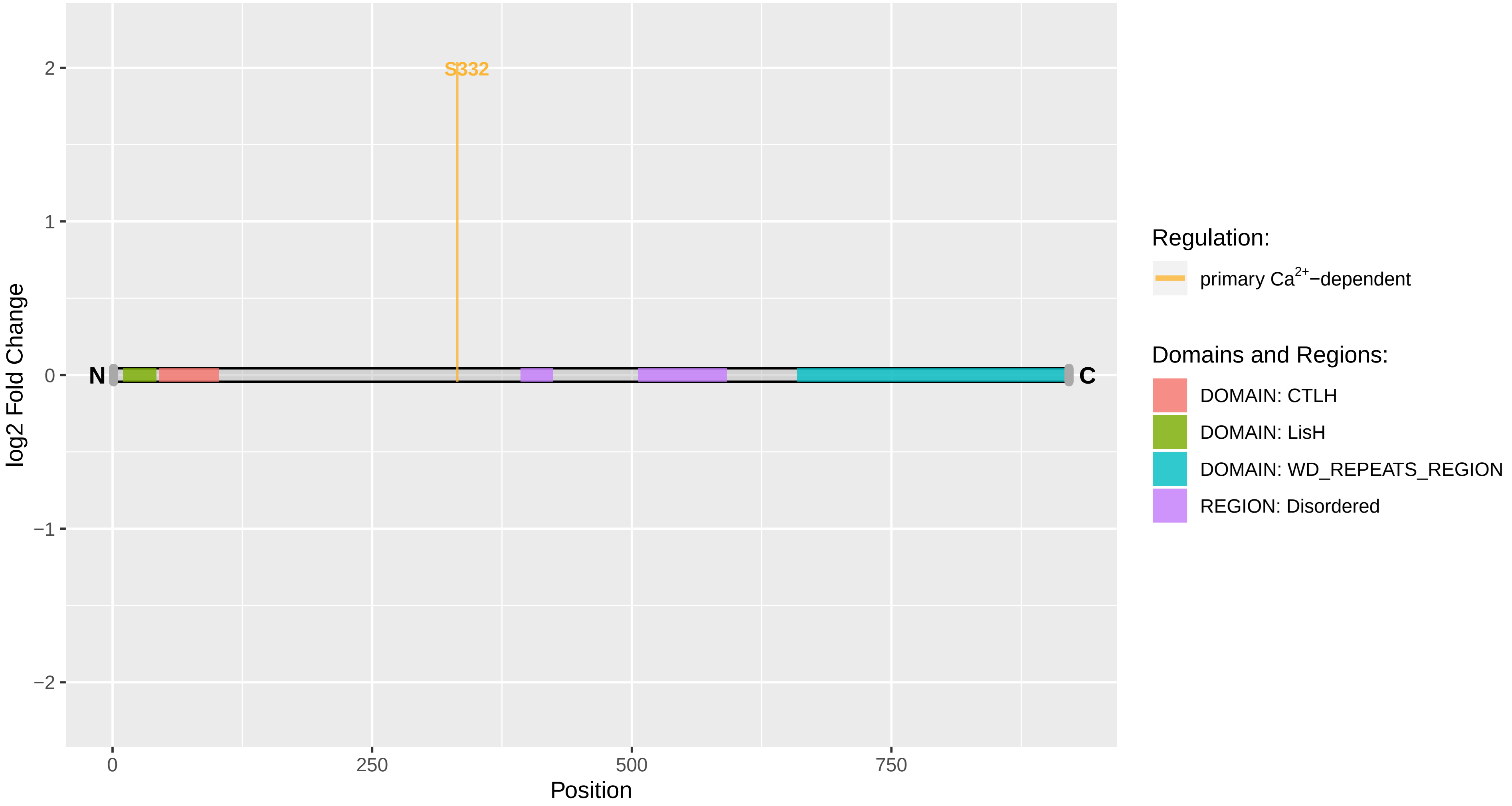

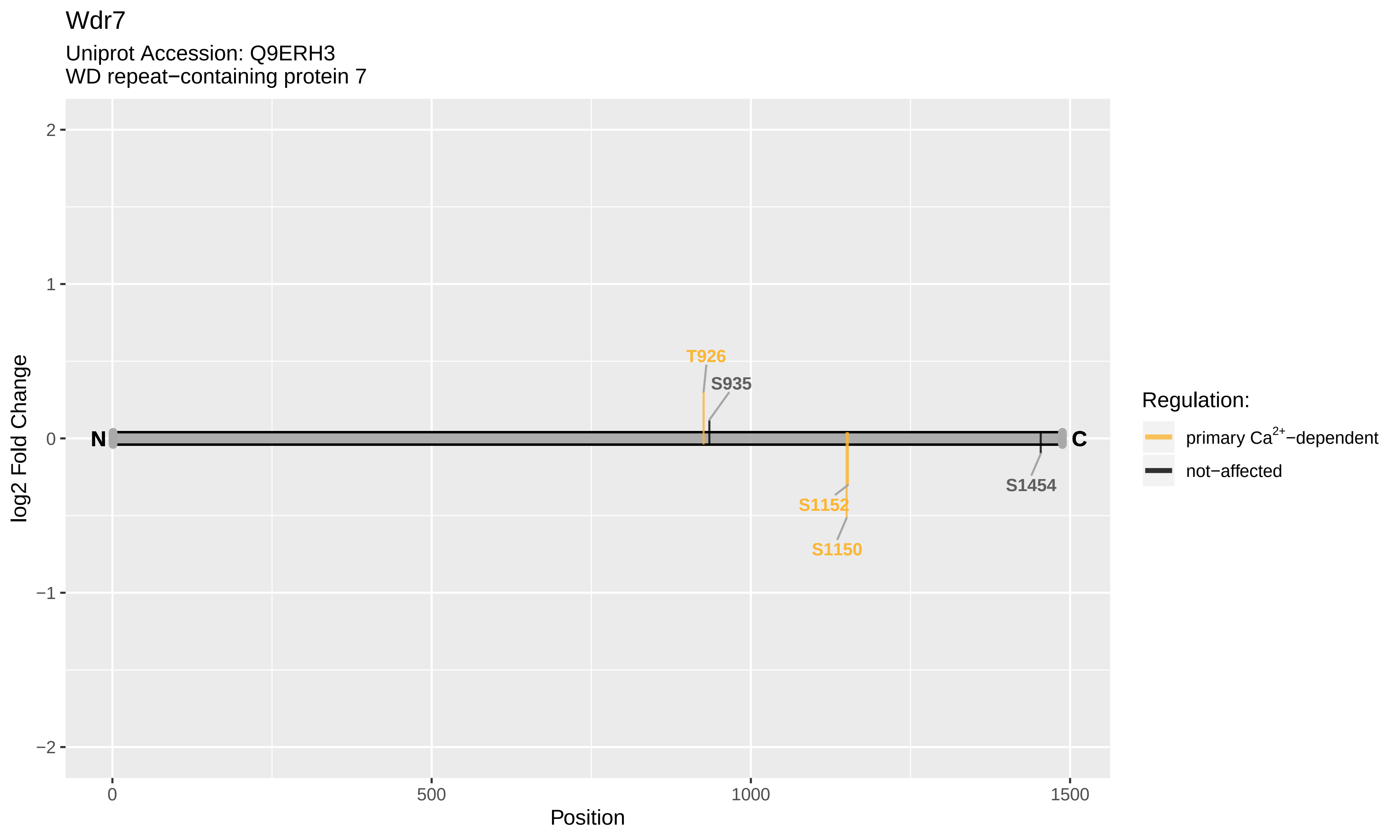

Wdr7
Uniprot Accession: Q9ERH3
WD repeat−containing protein 7
log2 Fold Change
2
1
0
−1
−2
N
C
T926
S935
S1152
S1150
S1454
0
500
1000
1500
Position
Regulation:
primary Ca2+−dependent
not−affected

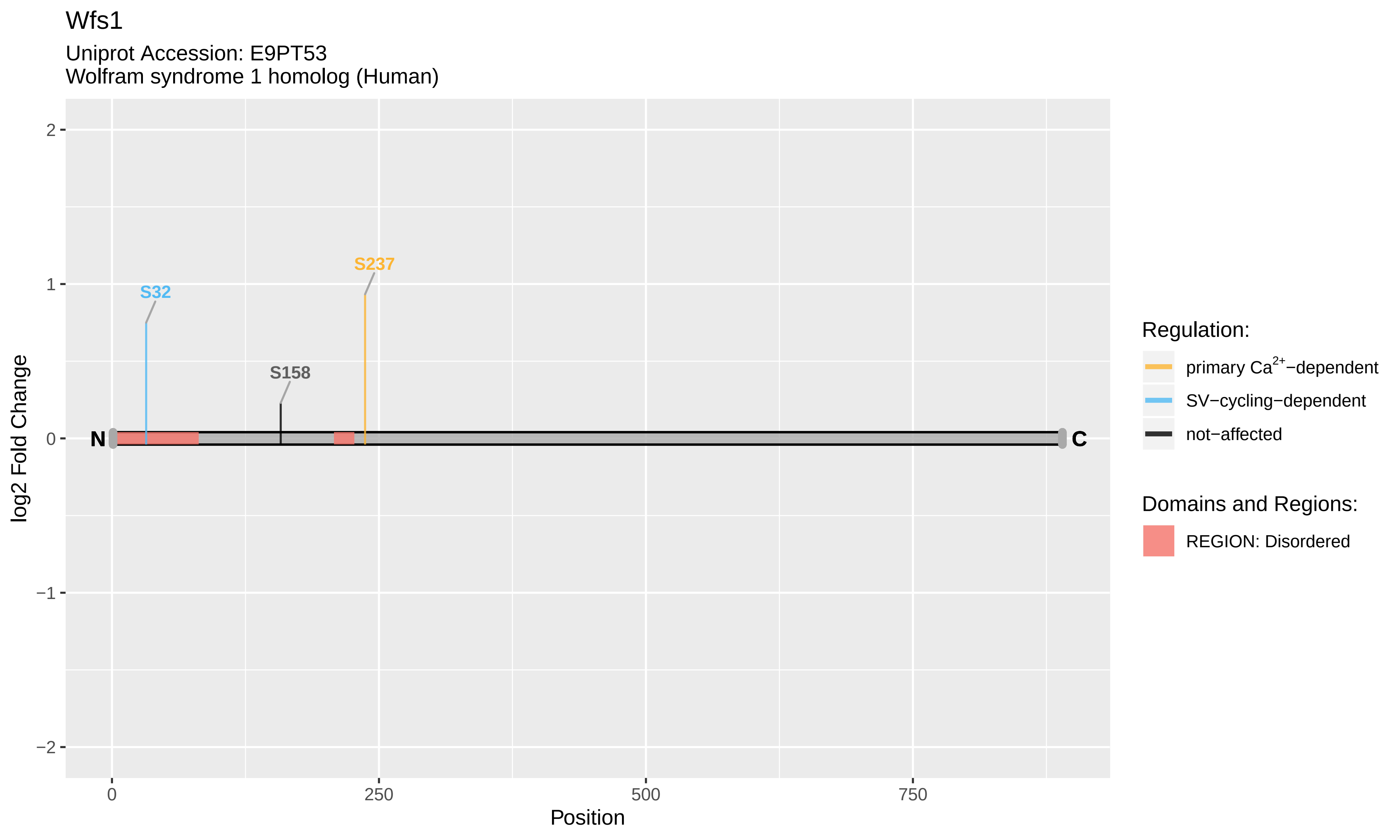

Wfs1
Uniprot Accession: E9PT53
Wolfram syndrome 1 homolog (Human)
log2 Fold Change
2
1
0
−1
−2
S32
S237
S158
N
C
0
250
500
750
Position
Regulation:
primary Ca2+−dependent
SV−cycling−dependent
not−affected
Domains and Regions:
REGION: Disordered

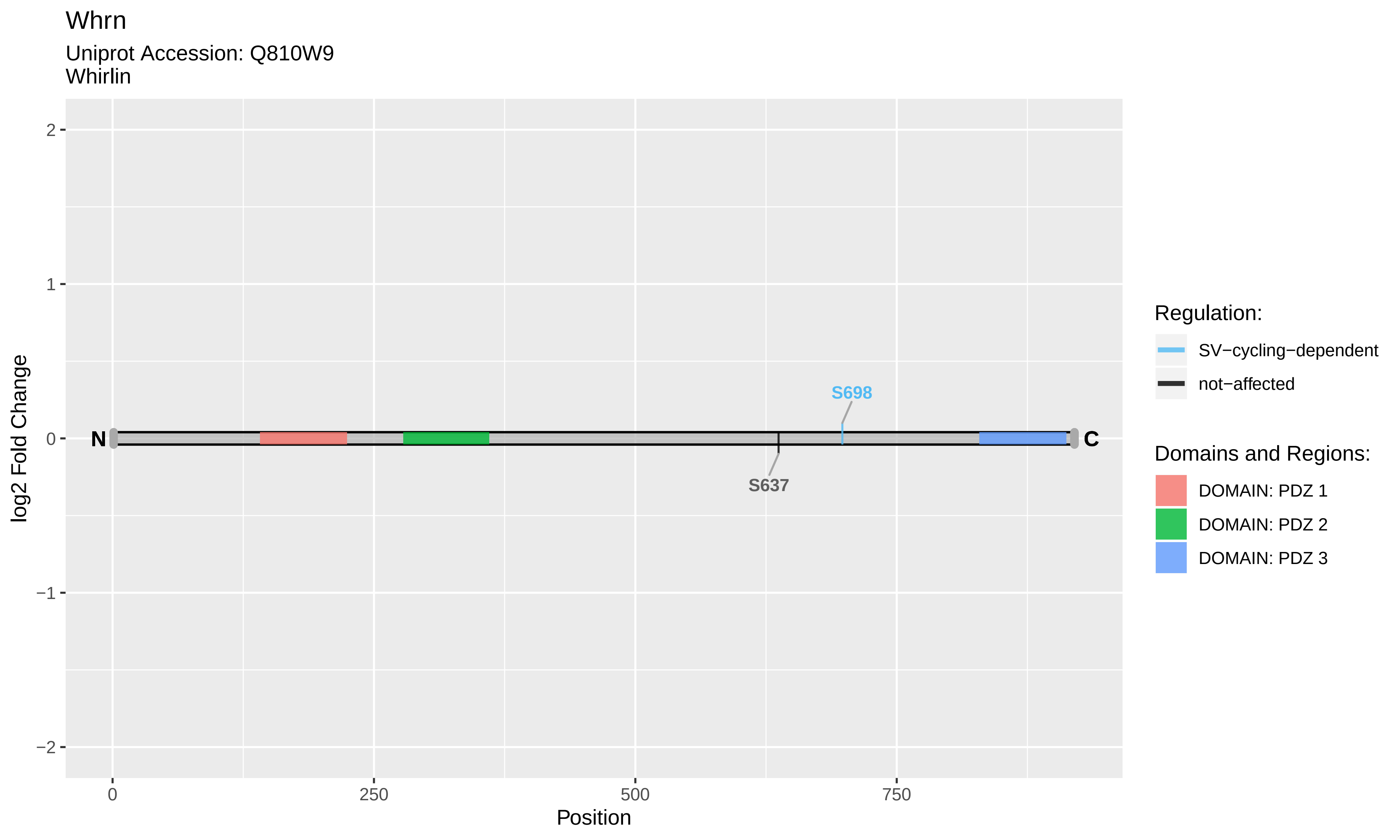
Whrn
Uniprot Accession: Q810W9
Whirlin
log2 Fold Change
2
1
0
−1
−2
N
C
S698
S637
0
250
500
750
Position
Regulation:
SV−cycling−dependent
not−affected
Domains and Regions:
DOMAIN: PDZ 1
DOMAIN: PDZ 2
DOMAIN: PDZ 3

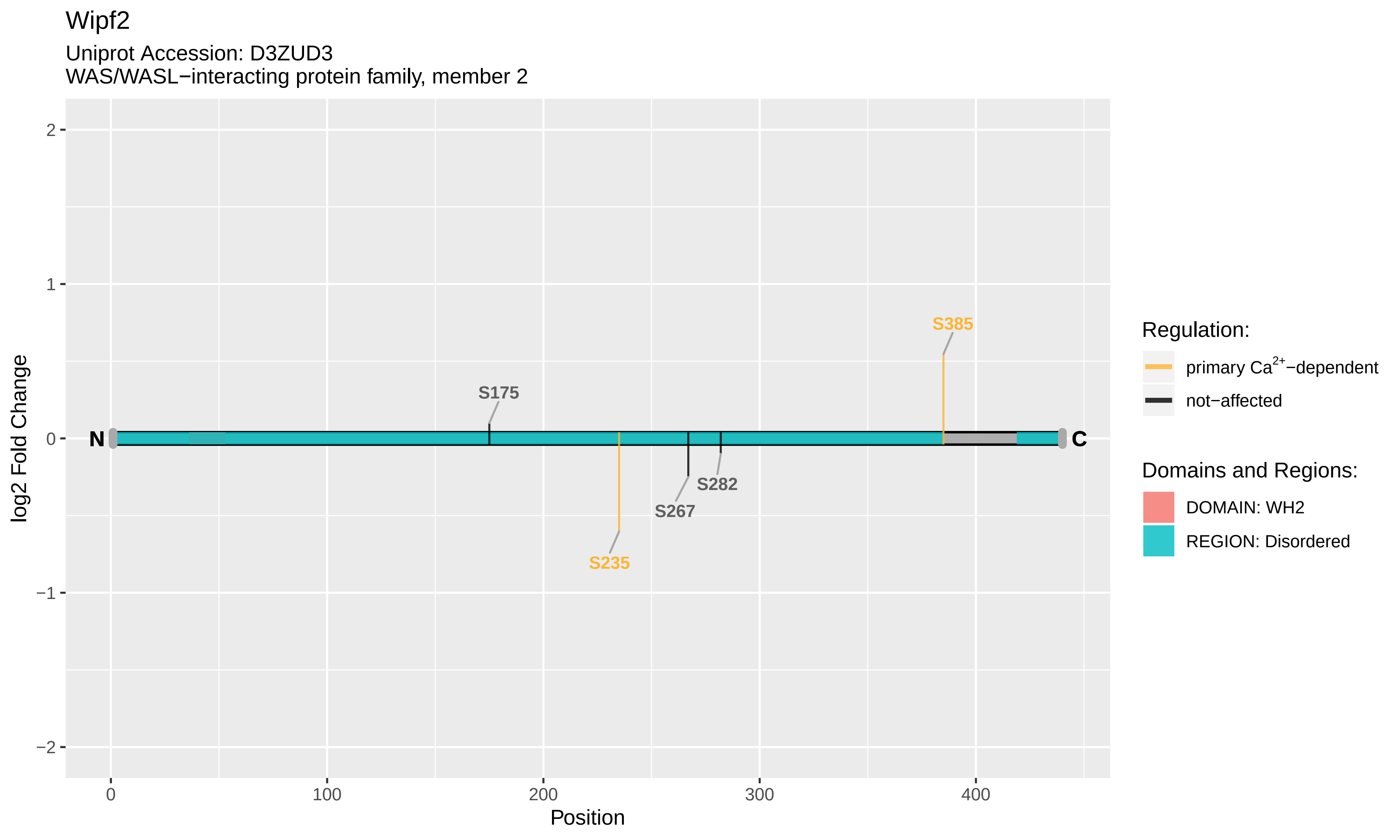

Wipf2
Uniprot Accession: D3ZUD3
WAS/WASL−interacting protein family, member 2
log2 Fold Change
2
1
0
−1
−2
N
C
S175
S235
S267
S282
S385
0
100
200
300
400
Position
Regulation:
primary Ca2+−dependent
not−affected
Domains and Regions:
DOMAIN: WH2
REGION: Disordered

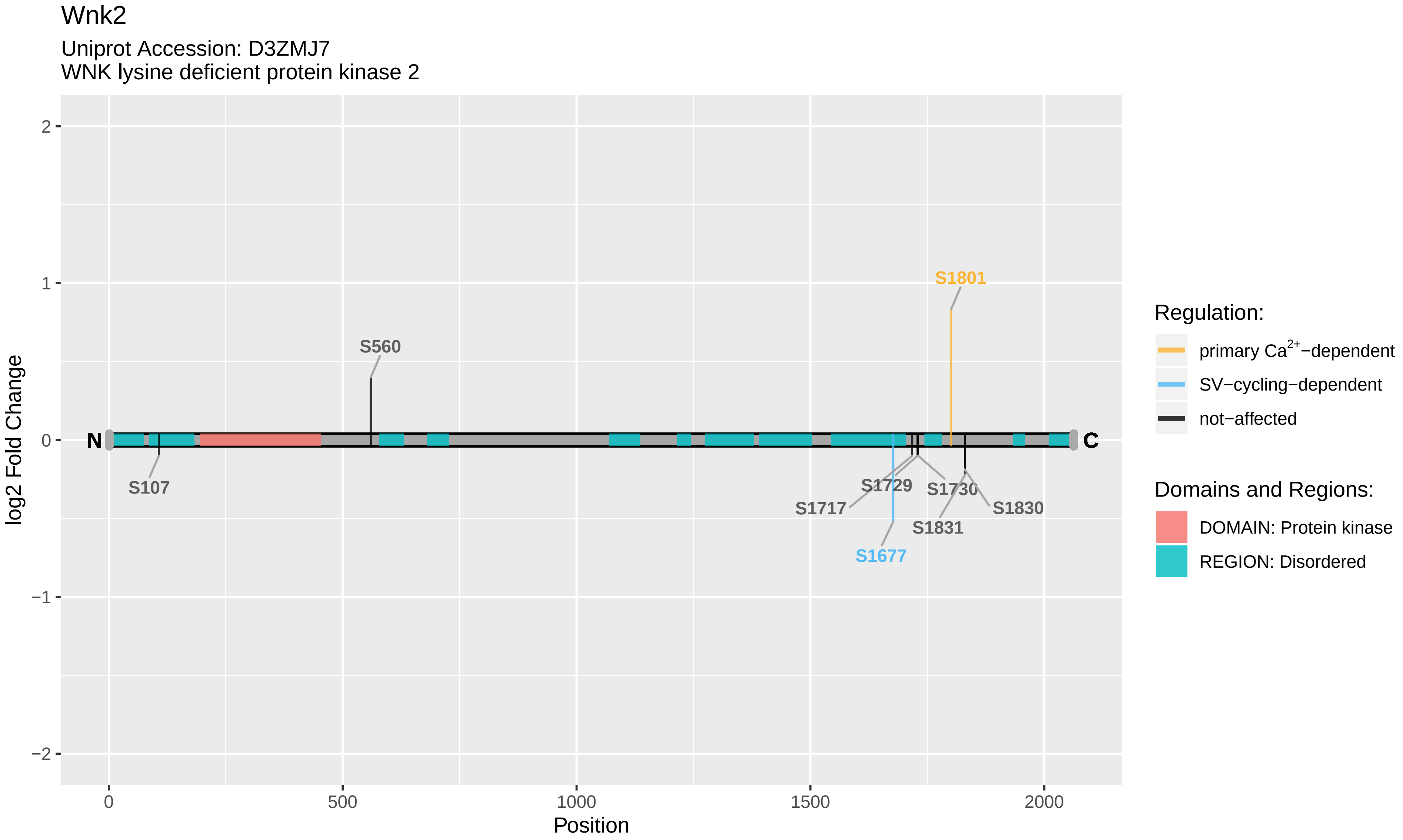

Wnk2
Uniprot Accession: D3ZMJ7
WNK lysine deficient protein kinase 2
S1801
S560
N
C
S107
S1729
S1730
S1717
S1830
S1831
S1677
log2 Fold Change
2
1
0
−1
−2
0
500
1000
1500
2000
Position
Regulation:
primary Ca2+−dependent
SV−cycling−dependent
not−affected
Domains and Regions:
DOMAIN: Protein kinase
REGION: Disordered

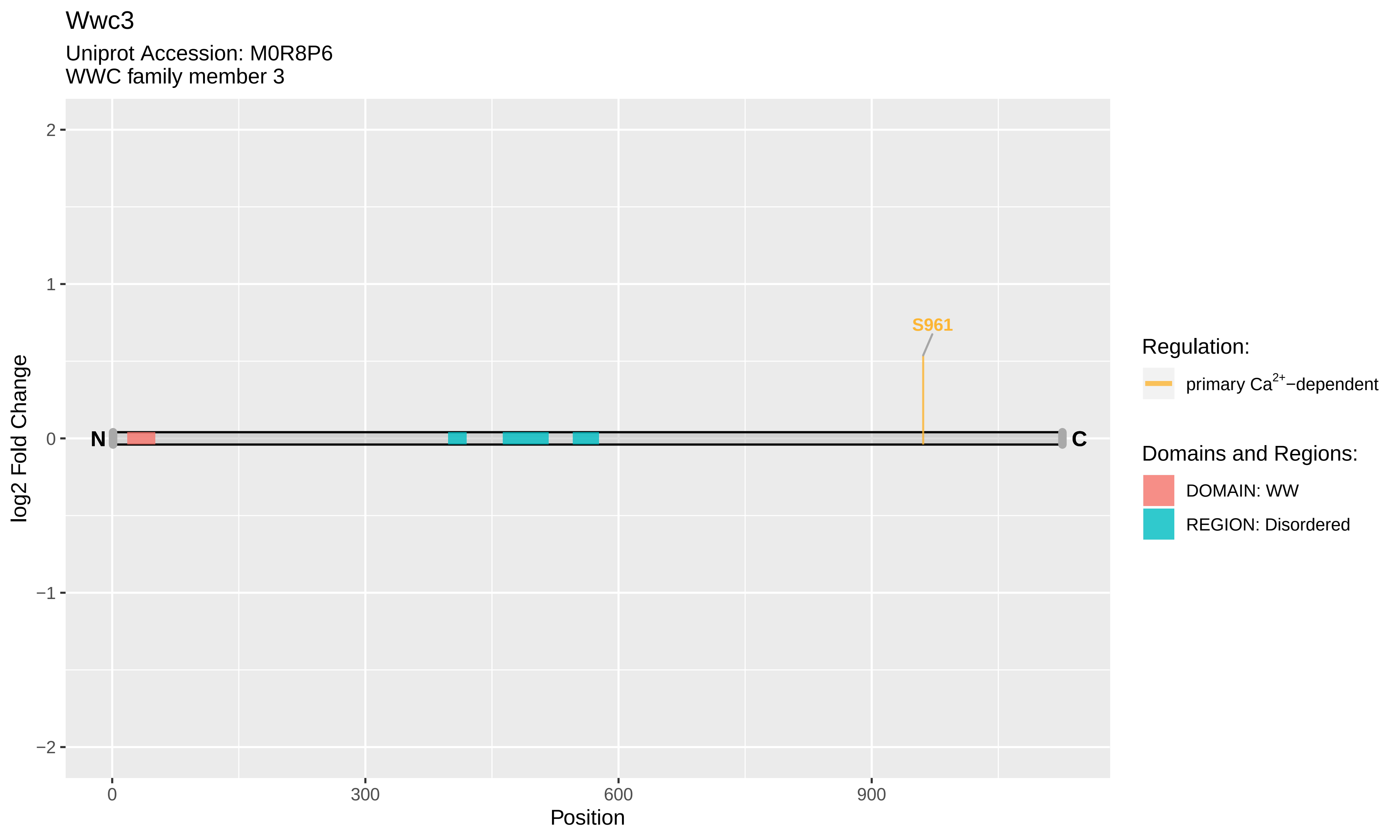

Wwc3
Uniprot Accession: M0R8P6
WWC family member 3
S961
N
C
log2 Fold Change
Position
2
1
0
−1
−2
0
300
600
900
Regulation:
primary Ca2+−dependent
Domains and Regions:
DOMAIN: WW
REGION: Disordered

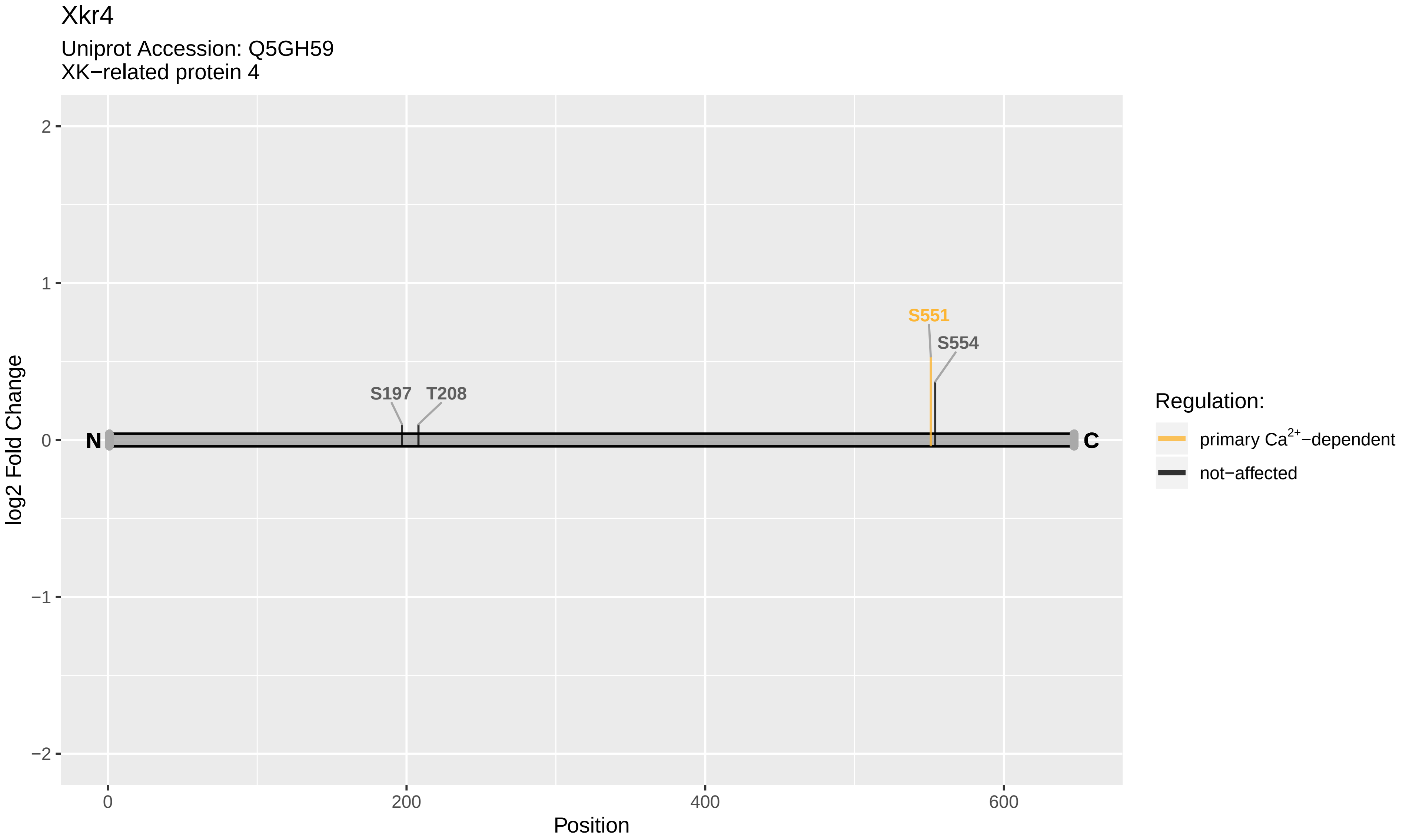

Xkr4
Uniprot Accession: Q5GH59
XK−related protein 4
log2 Fold Change
2
1
0
−1
−2
N
C
S197
T208
S551
S554
0
200
400
600
Position
Regulation:
primary Ca2+−dependent
not−affected

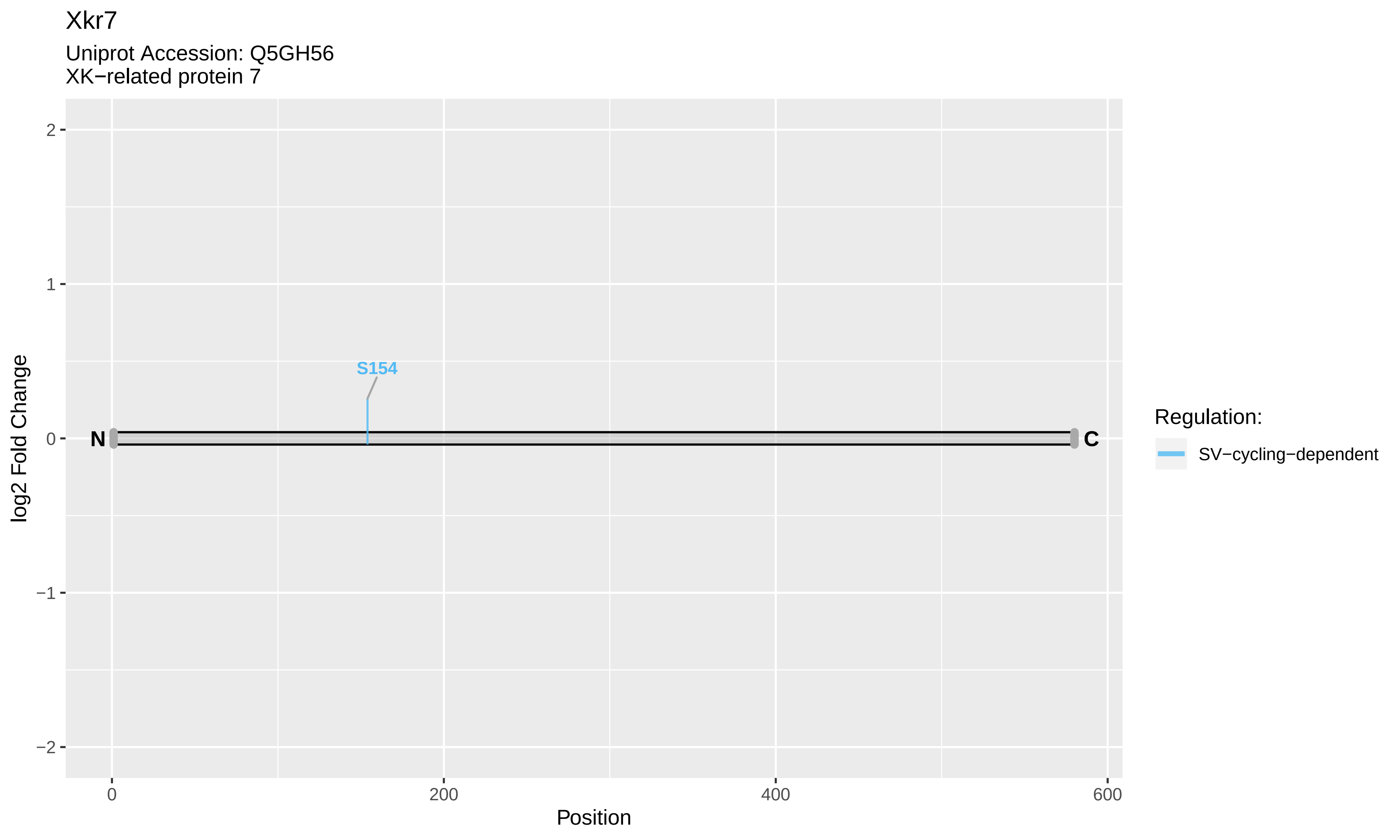
Xkr7
Uniprot Accession: Q5GH56
XK−related protein 7
log2 Fold Change
2
1
0
−1
−2
N
C
S154
0
200
400
600
Position
Regulation:
SV−cycling−dependent

Ywhag

Uniprot Accession: P61983
14−3−3 protein gamma

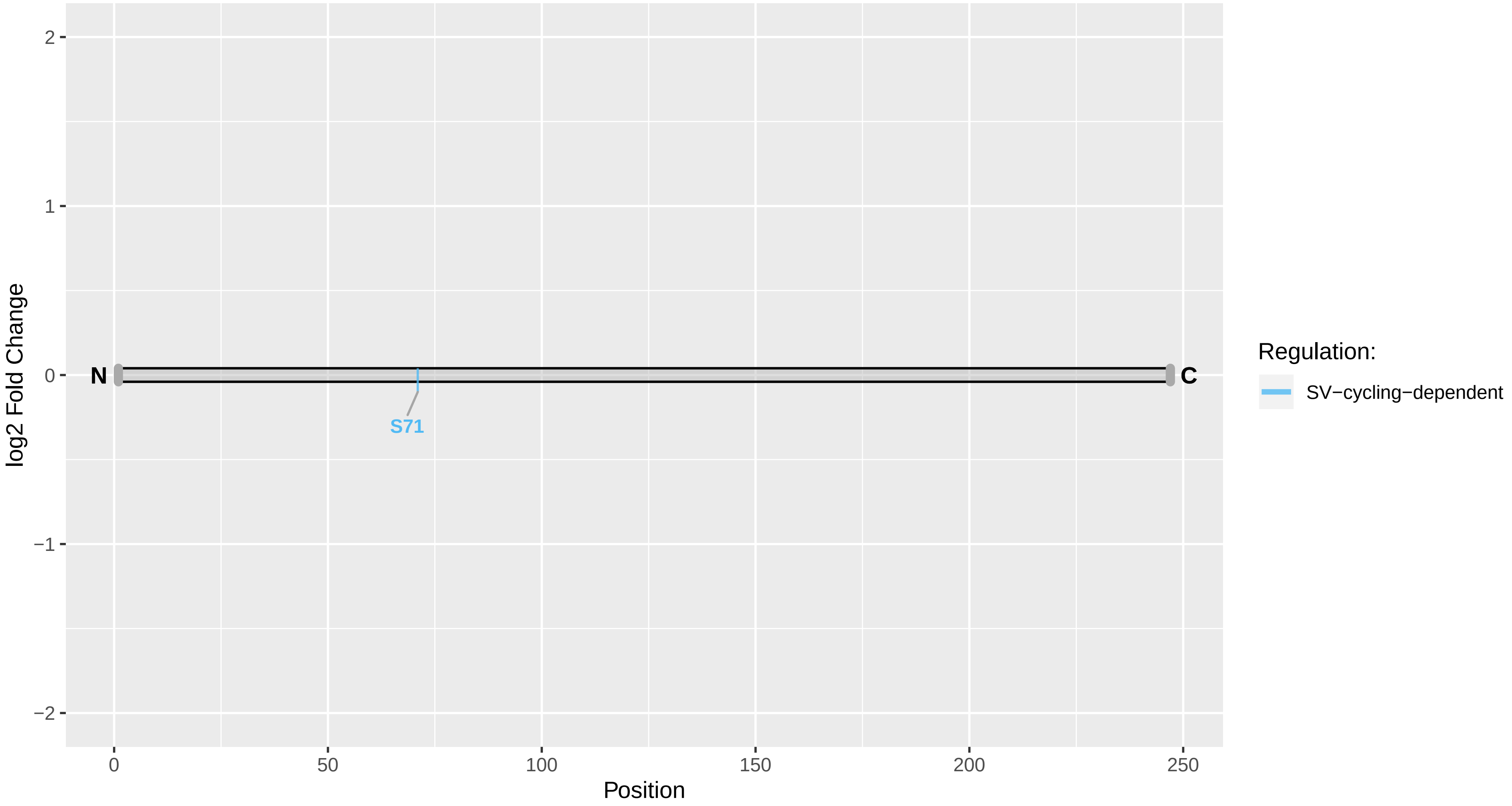

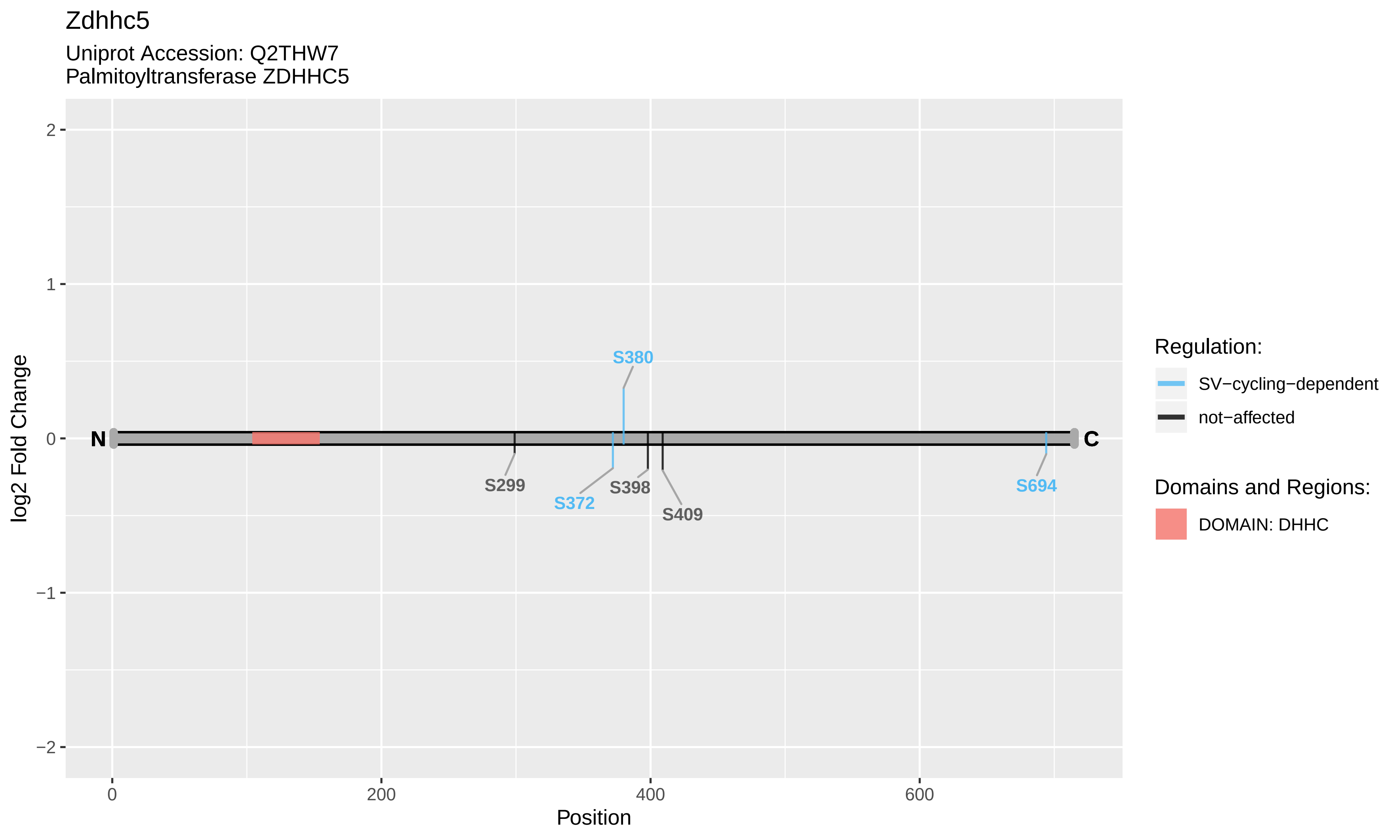
Zdhhc5
Uniprot Accession: Q2THW7
Palmitoyltransferase ZDHHC5
log2 Fold Change
2
1
0
−1
−2
N
C
S380
S299
S372
S398
S409
S694
0
200
400
600
Position
Regulation:
SV−cycling−dependent
not−affected
Domains and Regions:
DOMAIN: DHHC

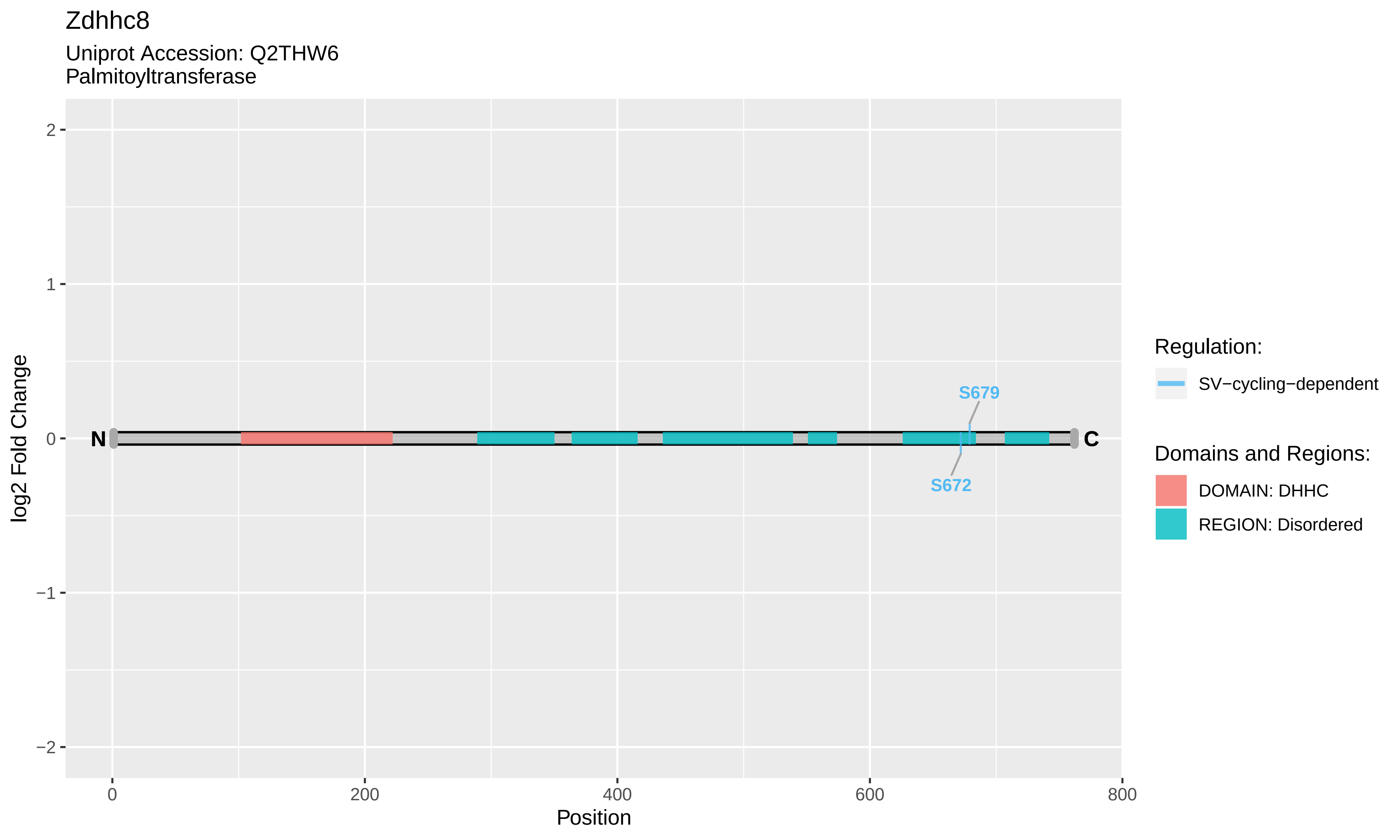

Zdhhc8
Uniprot Accession: Q2THW6
Palmitoyltransferase
log2 Fold Change
2
1
0
−1
−2
N
C
S679
S672
0
200
400
600
800
Position
Regulation:
SV−cycling−dependent
Domains and Regions:
DOMAIN: DHHC
REGION: Disordered

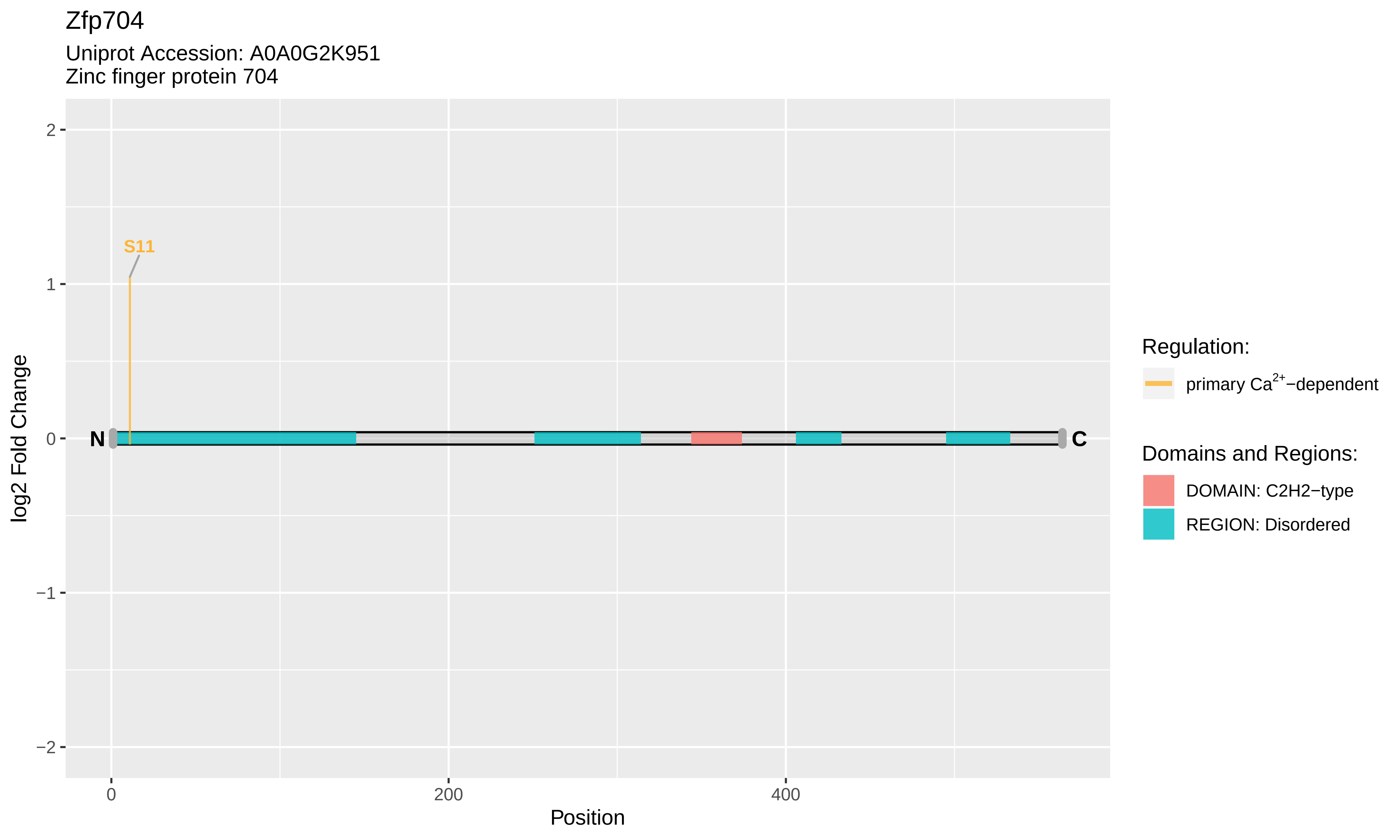

Zfp704
Uniprot Accession: A0A0G2K951
Zinc finger protein 704
S11
N
C
log2 Fold Change
2
1
0
−1
−2
0
200
400
Position
Regulation:
primary Ca2+−dependent
Domains and Regions:
DOMAIN: C2H2−type
REGION: Disordered

Znf365

Uniprot Accession: Q5PQS2
Protein ZNF365

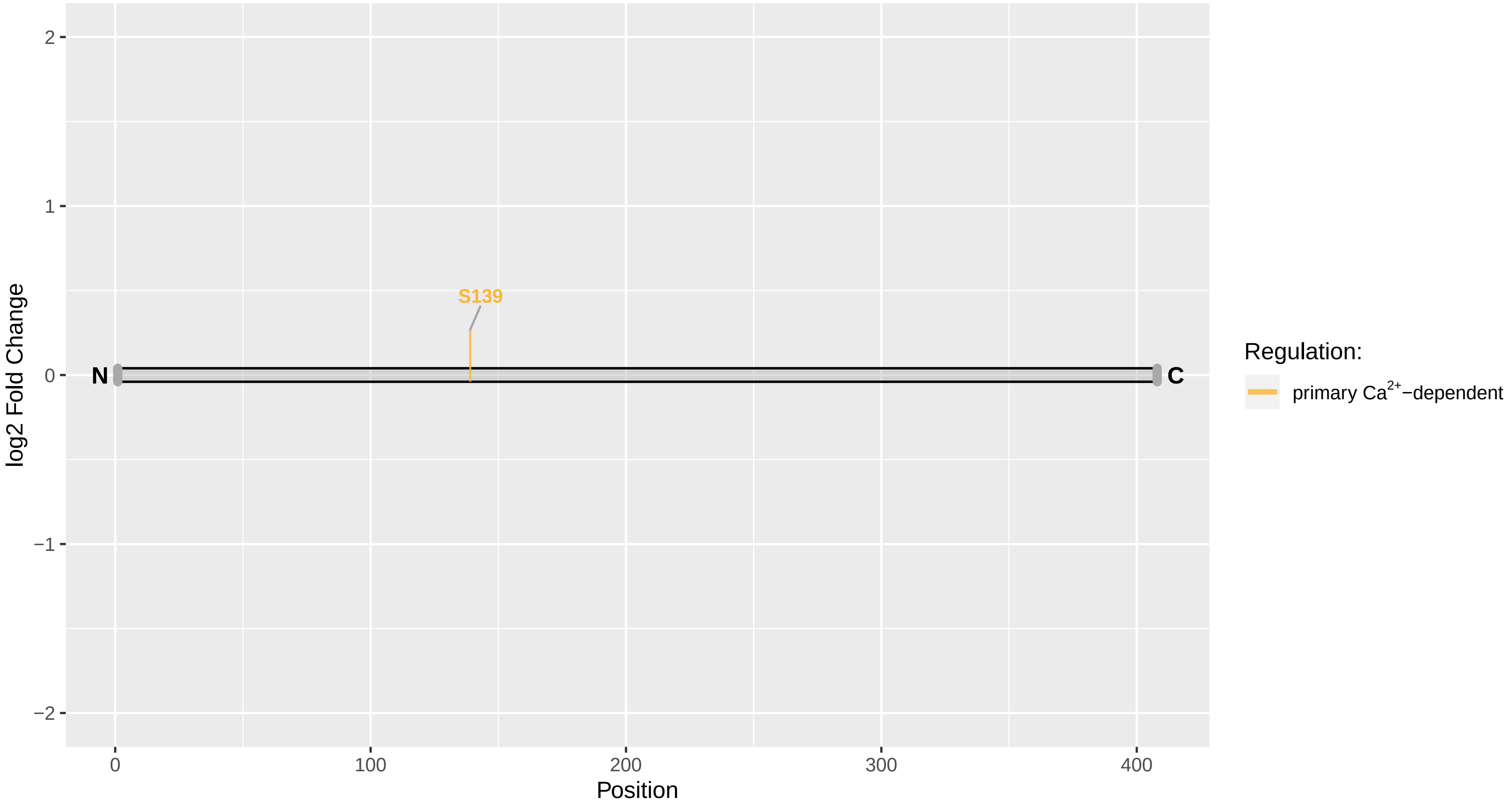

Znf622

Uniprot Accession: Q7TM96
Zinc finger protein 622

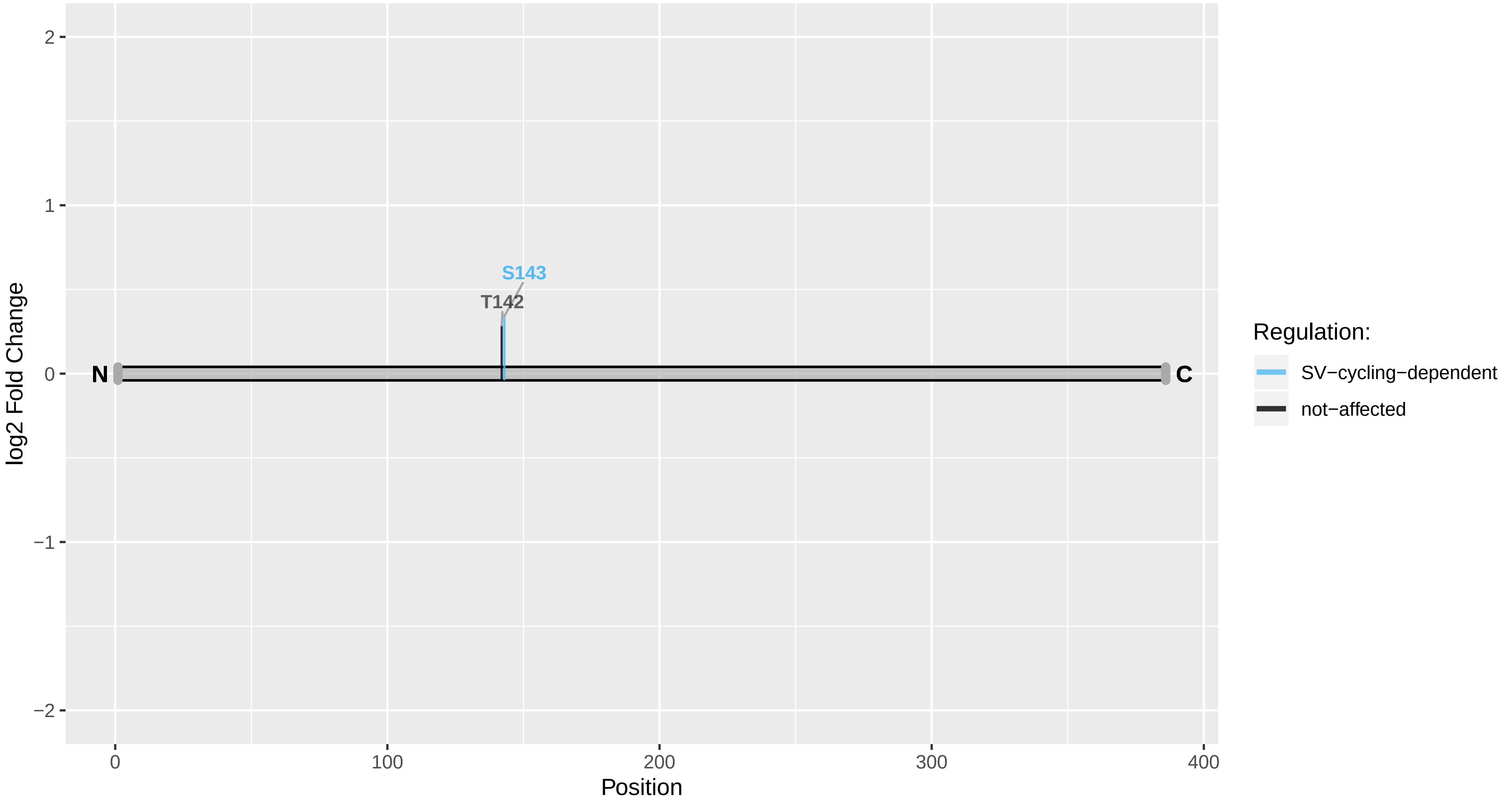

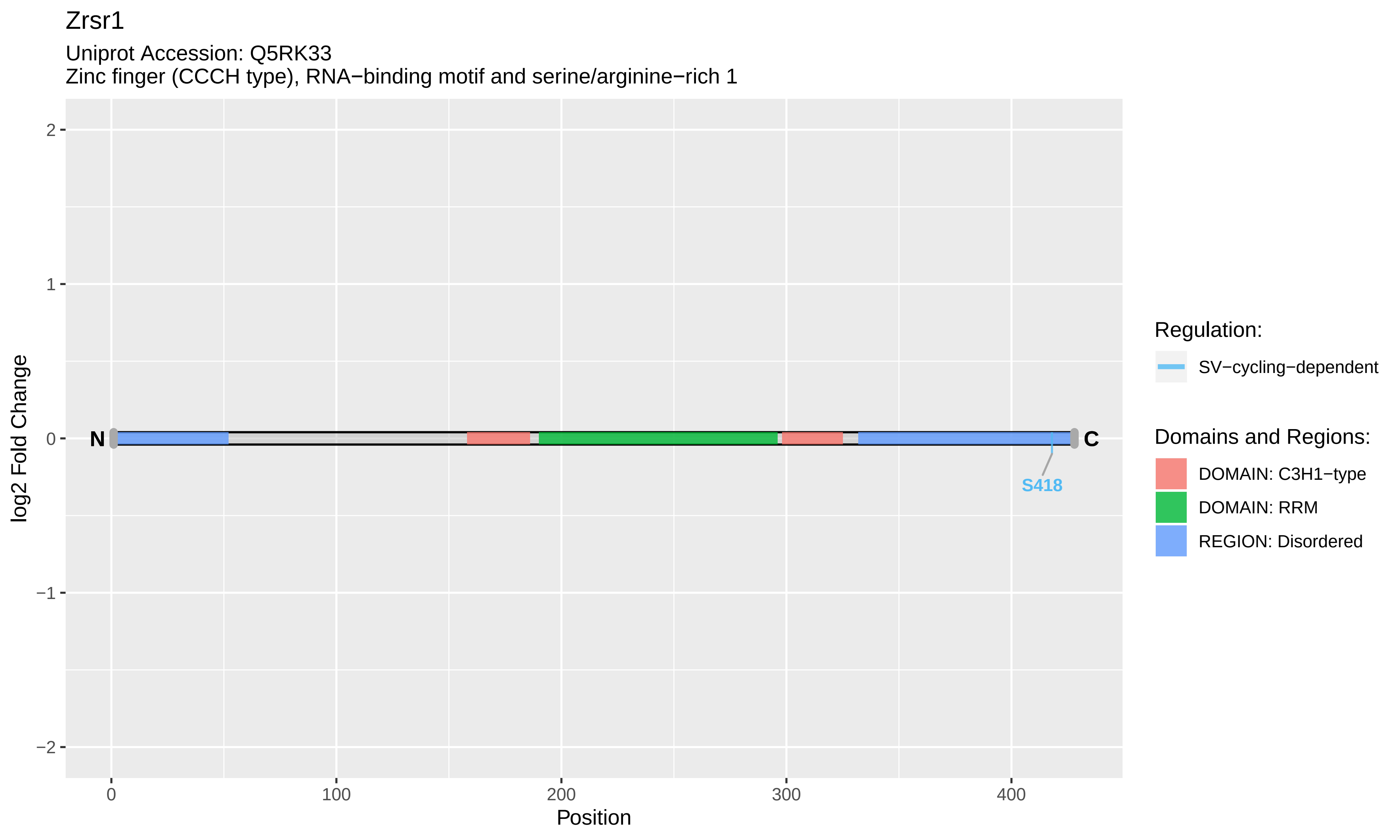
Zrsr1
Uniprot Accession: Q5RK33
Zinc finger (CCCH type), RNA−binding motif and serine/arginine−rich 1
log2 Fold Change
2
1
0
−1
−2
N
C
S418
0
100
200
300
400
Position
Regulation:
SV−cycling−dependent
Domains and Regions:
DOMAIN: C3H1−type
DOMAIN: RRM
REGION: Disordered
