# Supplementary material for: Protein Phosphorylation in Depolarized Synaptosomes: Dissecting Primary Effects of Calcium from Synaptic Vesicle Cycling
Source: Mol Cell Proteomics. 2021 Feb 12;20:100061. doi: 10.1016/j.mcpro.2021.100061 (PMC7995663; doi:10.1016/j.mcpro.2021.100061)
Supplement: Supplemental Material 8 [file mmc8.pdf]

Table of contents:

Supplementary Notes:

Supplementary Note 1: *C. botulinum* cell culture supernatant activity assessment

Supplementary Figures：

Figure S1: Glutamate release assay and Ca^{2+}-levels in cytoplasm.

Figure S2: Kinase groups that might be responsible for the observed phosphorylation changes.

Figure S3: Dependence of phosphorylation sites on calcium influx and SV-cycling.

Figure S4: Site occupancy assessment.

Figure S5: Changes in phosphorylation site intensities for Calcium/calmodulin-dependent kinase 2 alpha, beta, gamma, and delta.

Figure S6: Changes in phosphorylation site intensities for Mitogen-activated protein kinase 1, 3, and Protein kinase C beta.

Figure S7: Phosphatases and their putative substrates

Figure S8: Manual annotation of proteins carrying regulated phosphorylation sites.

Figure S9: Changes in phosphorylation site intensities for Regulating synaptic membrane exocytosis (RIM) protein 1, 2, and RIM-binding protein 2

Figure S10: Changes in phosphorylation site intensities for Dynamin 1, Clathrin coat assembly protein AP180, and Amphiphysin.

Figure S11: Changes in phosphorylation site intensities for Synapsin 1, 2, and 3.

Figure S12: Changes in phosphorylation site intensities for Spectrin beta chain.

Figure S13: Changes in phosphorylation site intensities for Adducin alpha, beta, and gamma.

Figure S14: Changes in phosphorylation site intensities for Potassium voltage-gated channel subfamily A member 2, and subfamily B member 1 and 2.

Figure S15: Changes in phosphorylation site intensities for Potassium voltage-gated channel subfamily D member 2, subfamily KQT member 2, Potassium/sodium hyperpolarization-activated cyclic nucleotide-gated channel 1 and 2.

Figure S16: Regulated phosphorylation sites on selected proteins

Figure S17: Changes in phosphorylation site intensities for Calcium-activated potassium channel subunit alpha 1, Potassium voltage-gated channel subfamily H member 1, and Potassium voltage-gated channel subfamily Q member 5.

Figure S18: Changes in phosphorylation site intensities for Syntaxin 1A, Vesicle-associated membrane protein 2, Syntaxin-binding protein 1, and Cannabinoid receptor 1.

Figure S19: Proteomic profiling of *C. botulinum* cell culture supernatants.

Figure S20: Phosphoproteome analysis of BoNT-treated HeLa nuclear extract.

Figure S21: Proteomics analysis of Mock- or BoNT-treated synaptosomes.

Supplementary Tables:

Table S1: Number of regulated phosphorylation events per predicted kinase group in Ca *vs.* EGTA experiment.

Table S2: Enriched gene ontology biological function terms based on synapse-specific SynGO database

Table S3 Number of regulated phosphorylation events per predicted kinase group in Mock *vs*. BoNT experiment.

Table S4: Number of regulated phosphorylation events per predicted kinase group in Ca *vs.* EGTA and Mock *vs.* BoNT experiments.

Supplementary Data:

"SupplData01_Phosphosite_Intensities.xlsx"
Phosphorylation site intensities used for the assessment of differential protein phosphorylation in Ca *vs.* EGTA and Mock *vs.* BoNT experiments

"SupplData02_Proteingroups_Intensities_MockBoNT_AD.xlsx"
Protein group intensities used for the assessment of the differential protein content in depolarized synaptosomes following Mock (A+D) or BoNT (A+D) treatment.

"SupplData03_Proteingroups_Intensities_MockBoNT_CB.xlsx"
Protein group intensities used for the assessment of the differential protein content in depolarized synaptosomes following Mock (C1+B) or BoNT (C1+B) treatment.

"SupplData04_Protein_classification.xlsx"
Classification of proteins based on the classification of the regulated phosphorylation sites they carry.

"SupplData05_Phosphoproteins_regulated_CaEGTA.pdf"
Representation of log_2 fold changes of the phosphorylation site intensities per protein sequence in depolarized synaptosomes following incubation with Ca^{2+} or EGTA. Only proteins with significantly regulated phosphorylation sites are shown.

"SupplData06_Phosphoproteins_regulated_MockBoNT.pdf"
Representation of log_2 fold changes of the phosphorylation site intensities per protein sequence in depolarized synaptosomes following incubation with Mock or BoNT. Only proteins with significantly regulated phosphorylation sites are shown.

Supplementary Note

C. botulinum cell culture supernatant activity assessment

BoNT-treatment of synaptosomes negatively affected glutamate release and did not compromise the increase in cytoplasmatic Ca^{2+}-concentration following KCl-stimulation (**Figure S1B, C**). Proteome profiling of the *C. botulinum* cell culture supernatants used in the study (**Figure S19**) identified BoNTs among the most abundant proteins. However, we could detect presence of a putative protease and two putative phosphatases in the cell culture supernatants as well. To exclude unspecific phosphatase activity, we treated nuclear extract of HeLa cells with a combination of BoNT/A-D and subsequently performed a total quantitative phosphoproteome analysis. The analysis did not reveal significant phosphatase activity that could be attributed to the treatment with *C. botulinum* cell culture supernatants (**Figure S20**). To exclude unspecific protease activity, we analyzed peptides in the unbound fraction following TiO_2-enrichment of phosphorylated peptides. The analysis did not show strong changes in protein intensities in synaptosomes following BoNT-treatment (**Figure S21**). Although the intensities of some proteins seem to be slightly affected by BoNT/C1+ BoNT/B treatment, the observed mild effect is not at all comparable with the extreme effect that BoNT-treatment has on the phosphorylation of synaptic proteins. Neither have we seen a significant reduction in the level of SNARE proteins (**Figure S4E, F**). We, therefore, hypothesized that only a minor pool of SNARE proteins is cleaved following BoNT-treatment. SNARE proteins that reside inside non-functional synaptosomes would be protected from cleavage and mask the reduction in SNARE-proteins. We also cannot exclude some non-proteolytic effects of BoNT since such effects were previously discussed [1]. Important for this study, BoNT-treatment dampened glutamate release without compromising calcium influx, which allowed us to investigate calcium and SV-cycling effects on protein phosphorylation in synaptosomes.

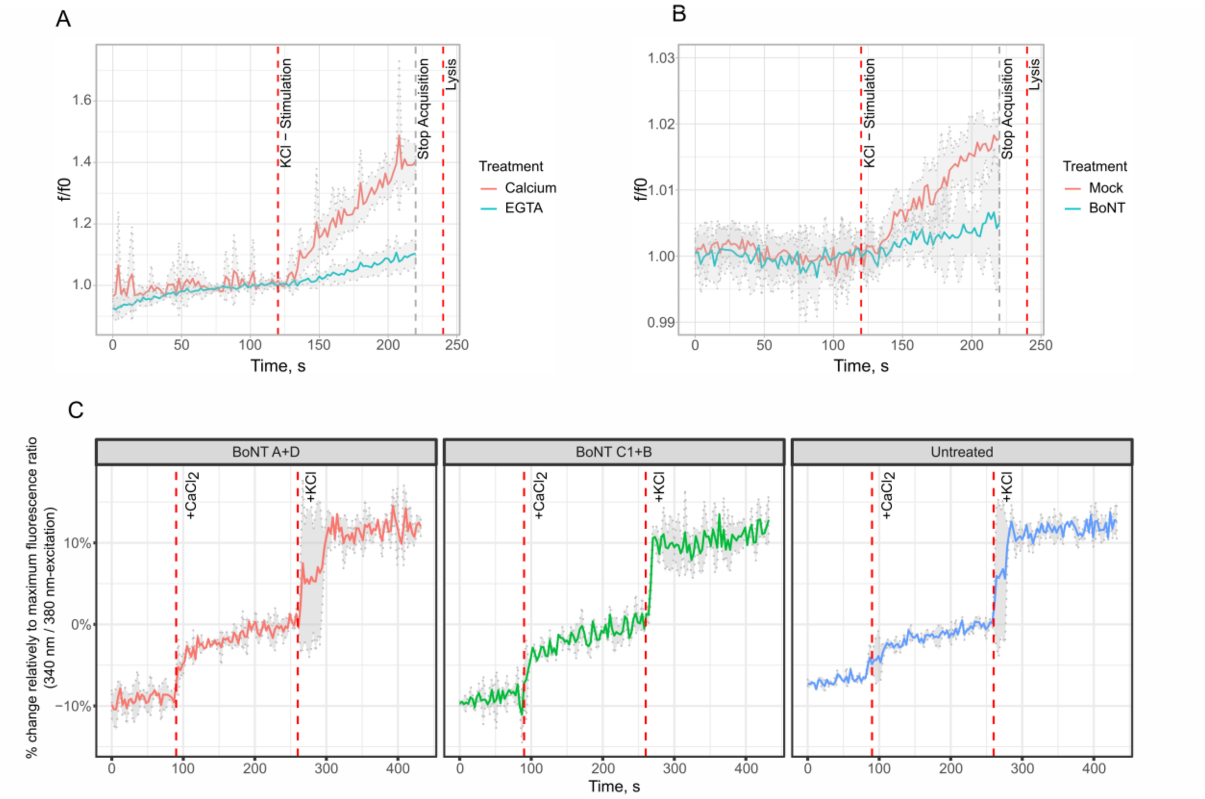

Figure S1 Glutamate release assay and Ca²⁺-levels in cytoplasm. *(**A**) Glutamate release following depolarization with potassium chloride in the presence of* Ca^{2+} *ions or EGTA. X-axis: time in seconds, Y-axis: normalized fluorescence intensity (f0 = fluorescence intensity before KCl-stimulation). Red line: synaptosomes stimulated in the presence of* Ca^{2+} *(average of 6 replicates). Green line: synaptosomes stimulated in the presence of EGTA (average of 6 replicates). Shaded grey area corresponds to the standard deviation at each time point. (**B**) Glutamate release of Mock- or BoNT- treated synaptosomes. X-axis: time in seconds, Y-axis: fluorescent intensity (f) normalized by fluorescence intensity before KCl-stimulation (f0). Red line: mock-treated synaptosomes (average of 6 replicates). Green line: BoNT-treated synaptosomes (average of 6 replicates). Shaded grey area corresponds to the standard deviation at each time point. Due to the presence of an interfering agent in the cell culture supernatants of C. botulinum, the magnitude of the fluorescence is reduced and experiences increased fluctuations. (**C**) Cytoplasmatic calcium levels in BoNT or untreated synaptosomes. Synaptosomes were pre-incubated with BoNT/A and BoNT/D, BoNT/C1 and BoNT/B or an equal volume of sodium-containing buffer (untreated). Changes in cytoplasmic calcium levels were monitored with Fura2-AM dye using an approach previously introduced elsewhere [2]. Fluorescence ratios were calculated by dividing fluorescence signal at 340 nm excitation wavelength by the signal at 380 nm excitation. Fluorescence ratio was normalized by the maximum fluorescence ratio after synaptosomal lysis. The normalized fluorescence ratio before KCl stimulation was subtracted to reveal proportional changes in cytoplasmatic* Ca^{2+}*-levels.*

A

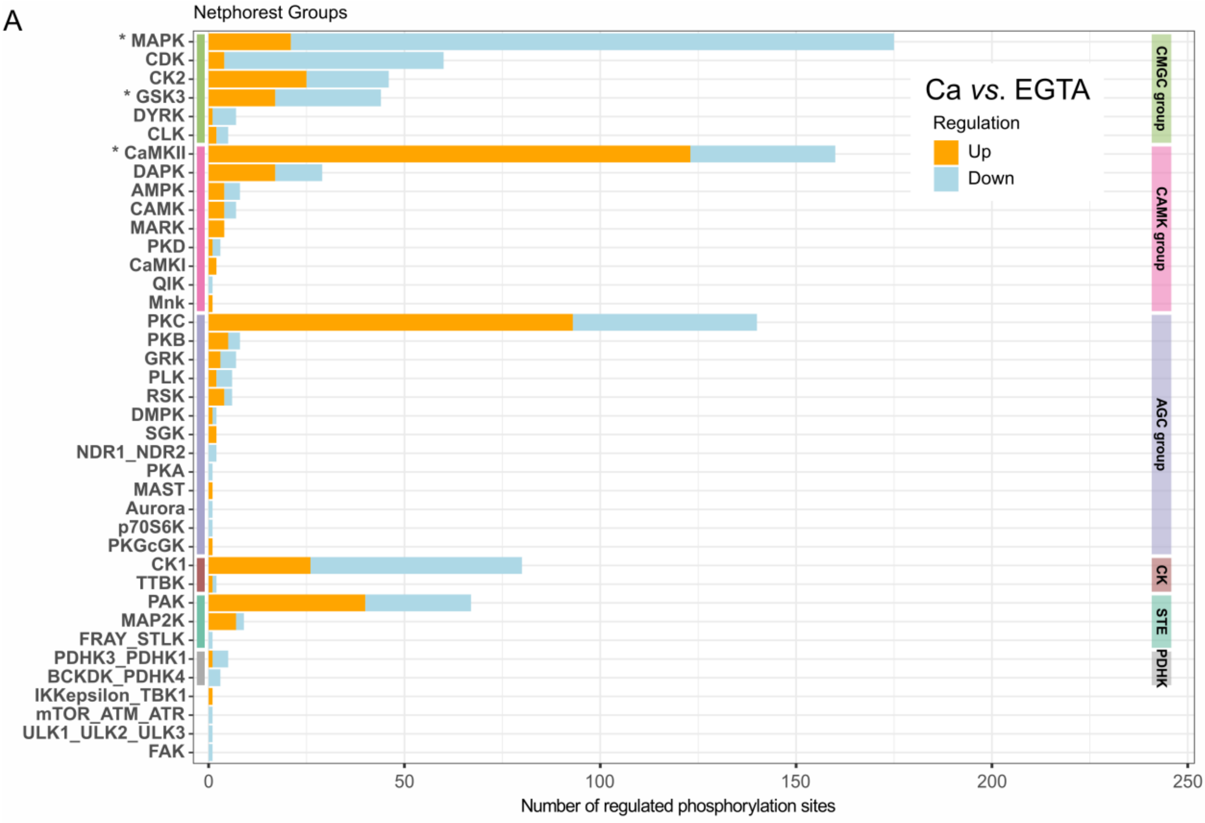

Netphorest Groups
* MAPK
CDK
CK2
* GSK3
DYRK
CLK
* CaMKII
DAPK
AMPK
CAMK
MARK
PKD
CaMKI
QIK
Mnk
PKC
PKB
GRK
PLK
RSK
DMPK
SGK
NDR1_NDR2
PKA
MAST
Aurora
p70S6K
PKGcGK
CK1
TTBK
PAK
MAP2K
FRAY_STLK
PDHK3_PDHK1
BCKDK_PDHK4
IKKepsilon_TBK1
mTOR_ATM_ATR
ULK1_ULK2_ULK3
FAK
0
50
100
150
200
250
Number of regulated phosphorylation sites
Ca vs. EGTA
Regulation
Up
Down
CMGC group
CAMK group
AGC group
CK
STE
PDHK

B

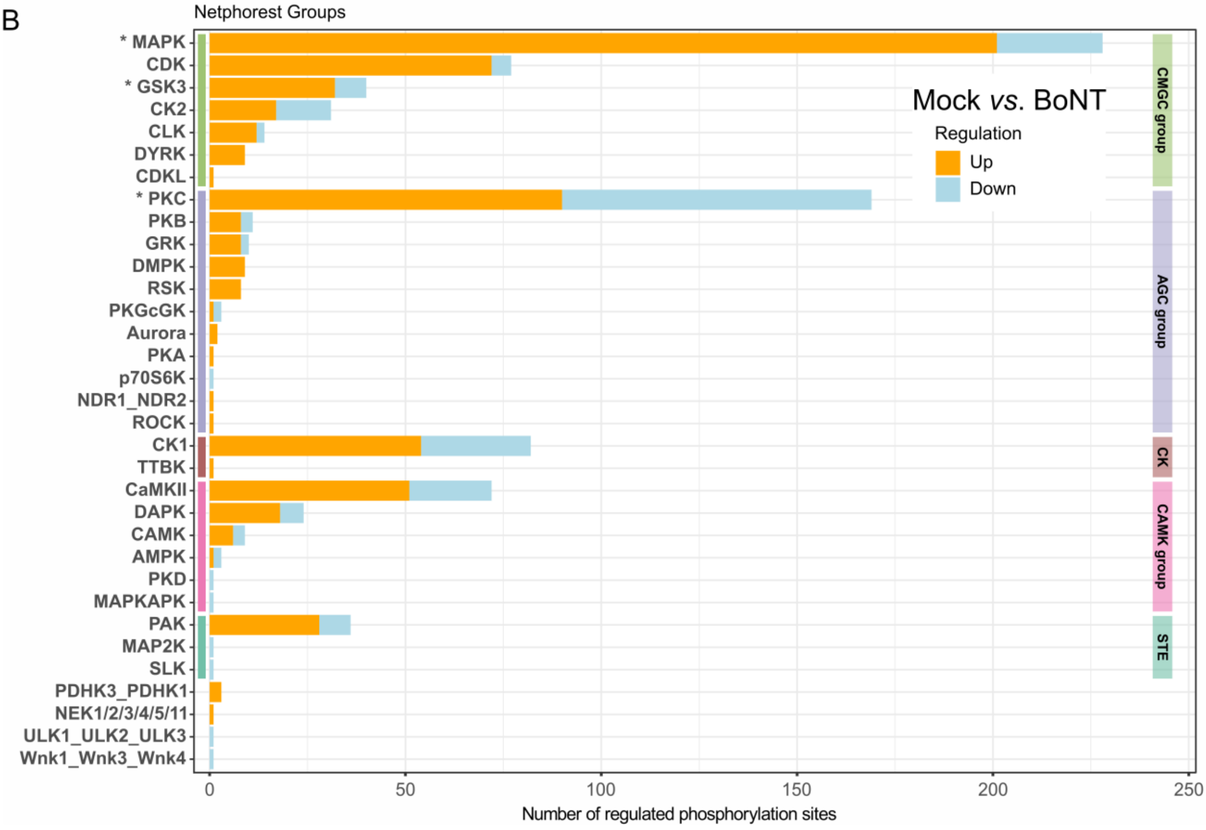

Netphorest Groups
* MAPK
CDK
* GSK3
CK2
CLK
DYRK
CDKL
* PKC
PKB
GRK
DMPK
RSK
PKGcGK
Aurora
PKA
p70S6K
NDR1_NDR2
ROCK
CK1
TTBK
CaMKII
DAPK
CAMK
AMPK
PKD
MAPKAPK
PAK
MAP2K
SLK
PDHK3_PDHK1
NEK1/2/3/4/5/11
ULK1_ULK2_ULK3
Wnk1_Wnk3_Wnk4
0
50
100
150
200
250
Number of regulated phosphorylation sites
Mock vs. BoNT
Regulation
Up
Down
CMGC group
AGC group
CK
CAMK group
STE

***Figure S2: Kinase groups that might be responsible for the observed phosphorylation changes**. (**A**, **B**) Extended versions of Figure 3E and F, respectively. The stacked bar graphs show the total number of significantly regulated phosphorylation events in Ca vs. EGTA (a) or Mock vs. BoNT (b) experiments that can be a result of known (PhosphositePlus database [3]) or predicted (NetworKIN [4, 5]) kinase-substrate interactions. Orange bars depict the number of phosphorylation events that show significant intensity increase in Ca^{2+} or Mock-treated synaptosomes versus respective control (EGTA or BoNT). Blue bars correspond to down-regulated sites, respectively. The kinase classification (Netphorest Groups) follows one introduced by Netphorest [4] and uses its second-level group annotation. Colored horizontal bars delineate the highest-level group in the kinase classification. Asterisks mark kinase groups which are predicted to control significantly more phosphorylation sites than expected as based on predicted kinase-substrate interactions in human proteome (Fisher's exact test Benjamini-Hochberg adjusted p-value < 0.01).). Abbreviations: AMPK, AMP-activated protein kinase; BCKDK, branched chain ketoacid dehydrogenase kinase; CaMKII calcium-calmodulin kinase 2; CDK, cyclin-dependent kinase; CDKL, cyclin-dependent-like kinase; CK1, casein kinase 1; CK2, casein kinase 2; CLK, SRPK1 and Clk/Sty protein kinase; DAPK, death associated protein kinase; DMPK, DYRK, dual-specificity tyrosine (Y) phosphorylation-regulated kinase; FAK, focal adhesion kinase GRK, G protein coupled receptor kinase; GSK3, glycogen synthase kinase 3; MAPK, mitogen activated protein kinase; MAP2K, mitogen activated protein kinase kinase; MAPKAPK, MAP kinase activated protein kinase; MARK, microtubule associated kinases; Mnk, MAPK-interacting protein kinases; NDR, nuclear dbf2-related kinase; NEK, NIMA (never in mitosis A)-related kinase; PAK, p21 activated kinase; MAST, microtubule-associated serine/threonine-protein kinase; PKA, protein kinase A; PKB, protein kinase B; PKC, protein kinase C; PKD, protein kinase D; PDHK, pyruvate dehydrogenase kinase; PKGcGK, cGMP-dependent protein kinase; QIK, Qin-induced kinase; ROCK, Rho-associated protein kinase; RSK, ribosomal S6 kinase; SGK, serum and glucocorticoid regulated kinase; STLK, STE20-like serine/threonine-protein kinase; TBK, TANK-binding kinase; TTBK, tau-tubulin kinase; ULK, Unc-51 like autophagy activating kinase; Wnk, with-no-lysine (K) kinase.*

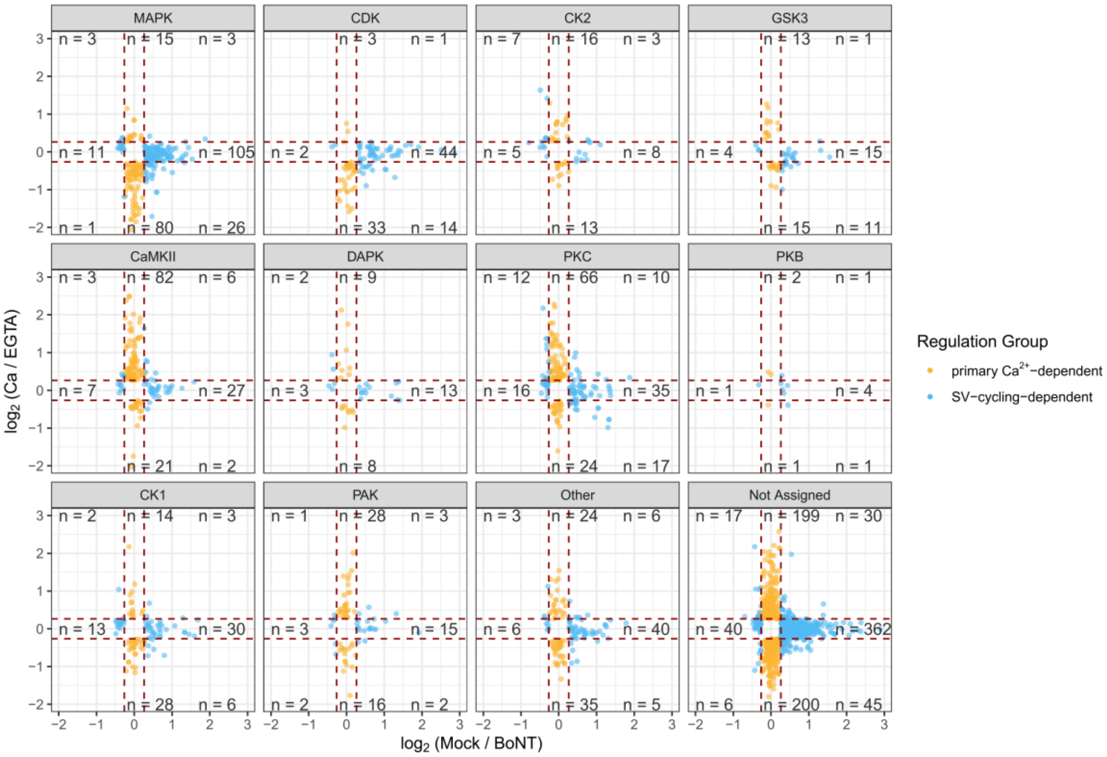

Figure S3: Dependence of phosphorylation sites on calcium influx and SV-cycling. *An extended version of Figure 4A: categorization of phosphorylation events based on the magnitude of the log_2 fold changes in Mock vs. BoNT experiment (x-axis) and log_2 fold changes in Ca vs. EGTA experiment (y-axis). Phosphorylation events were further segregated based on the known / predicted kinase class. The kinase classification (Netphorest Groups) follows one introduced by Netphorest [4] and uses its second-level group annotation. "Other" category contains phosphorylation evens attributed to smaller kinase classes. Only events quantified in both experiments are shown, and their q-values satisfy a q-value threshold of < 0.01. The events are classified into two regulation groups: (i) "primary Ca^{2+}-dependent" (sites with no significant or not-quantified SV-cycling effect, i.e. absolute log_2 (Mock/BoNT) < 0.263 and absolute log_2 (Ca/EGTA) > 0.263), shown as orange dots; and (ii) "SV-cycling-dependent" (absolute log_2 (Mock/BoNT) > 0.263).*

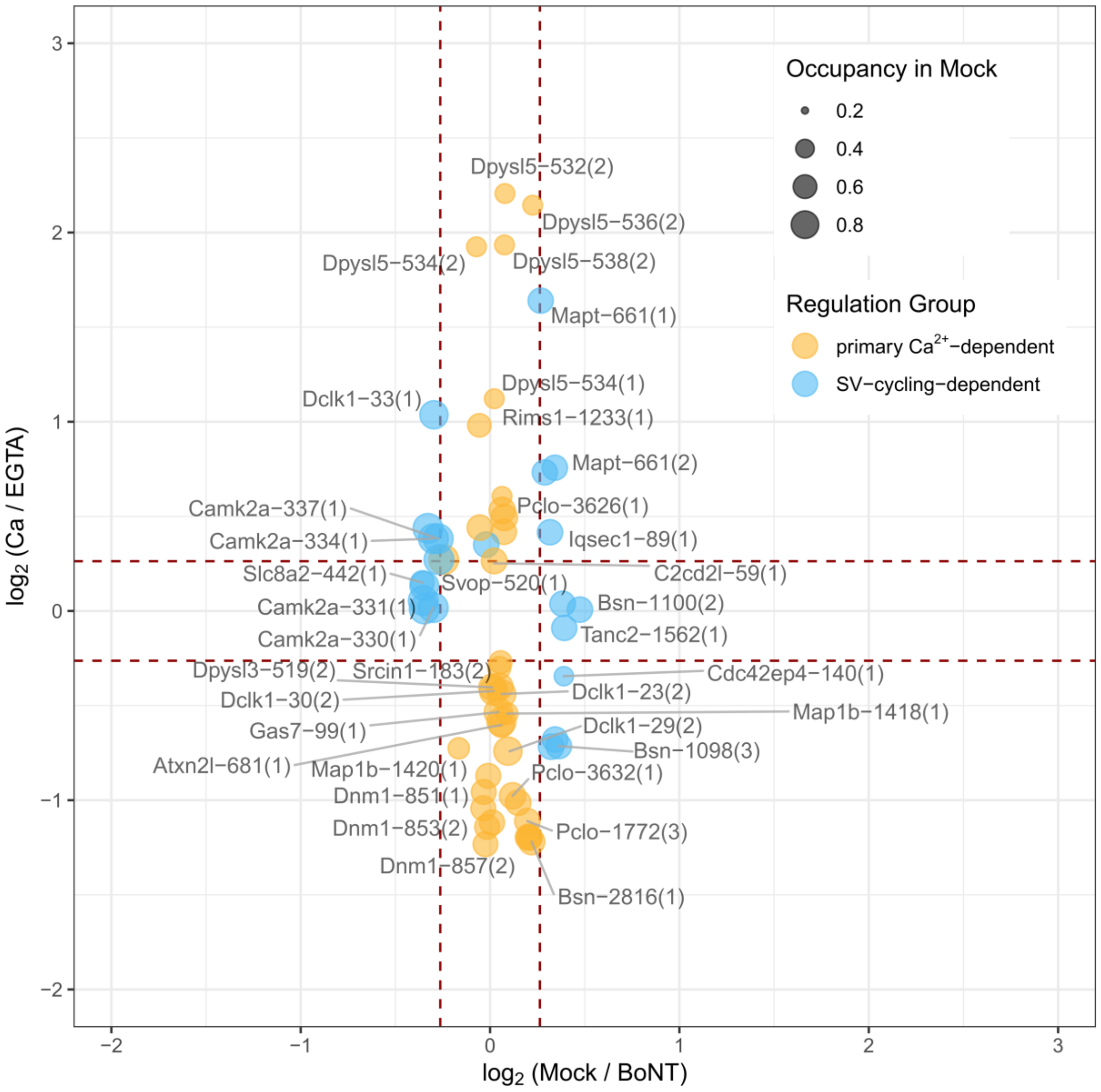

Figure S4: Site occupancy assessment. *Site occupancies were calculated using the 3D linear model as suggested by Hoegrebe et al [6] and are based on the observed intensity changes in phosphorylated peptide/non-phosphorylated peptide pairs and total protein intensity in Mock/BoNT experiments. Only occupancy estimations with a p value below 0.1 were considered.*

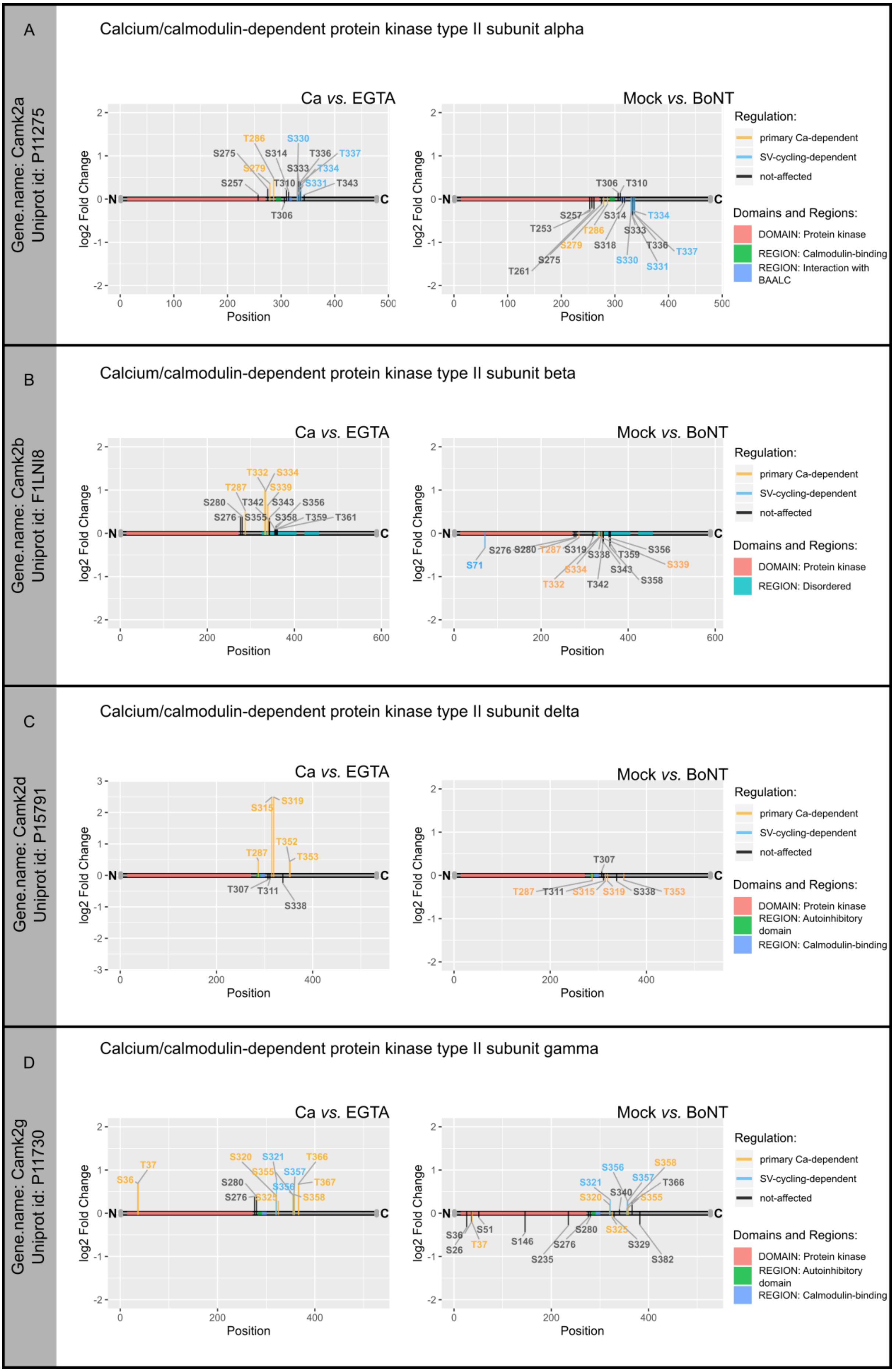
A
Gene.name: Camk2a
Uniprot id: P11275
Calcium/calmodulin-dependent protein kinase type II subunit alpha
Ca vs. EGTA
Mock vs. BoNT
log2 Fold Change
Position
T286 S330
S275 S314 T336 T337
S279 S333 T334
S257 T310 S331 T343
T306
T306 T310
S257 S314 T334
T253 T286 S333
S279 S318 T336 T337
S275 S330
T261 S331
Regulation:
primary Ca-dependent
SV-cycling-dependent
not-affected
Domains and Regions:
DOMAIN: Protein kinase
REGION: Calmodulin-binding
REGION: Interaction with BAALC
B
Gene.name: Camk2b
Uniprot id: F1LNI8
Calcium/calmodulin-dependent protein kinase type II subunit beta
Ca vs. EGTA
Mock vs. BoNT
log2 Fold Change
Position
T332 S334
T287 S339
S280 T342 S343 S356
S276 S355 S358 T359 T361
S276 S280 T287 S319 S356
S71 S338 T359 S339
S334 S343
T332 T342 S358
Regulation:
primary Ca-dependent
SV-cycling-dependent
not-affected
Domains and Regions:
DOMAIN: Protein kinase
REGION: Disordered
C
Gene.name: Camk2d
Uniprot id: P15791
Calcium/calmodulin-dependent protein kinase type II subunit delta
Ca vs. EGTA
Mock vs. BoNT
log2 Fold Change
Position
S315 S319
T352
T287 T353
T307 T311
S338
T307
T287 T311 S315 S319 S338 T353
Regulation:
primary Ca-dependent
SV-cycling-dependent
not-affected
Domains and Regions:
DOMAIN: Protein kinase
REGION: Autoinhibitory domain
REGION: Calmodulin-binding
D
Gene.name: Camk2g
Uniprot id: P11730
Calcium/calmodulin-dependent protein kinase type II subunit gamma
Ca vs. EGTA
Mock vs. BoNT
log2 Fold Change
Position
T37 S320 S321 T366
S36 S355 S357
S280 S356 T367
S276 S325 S358
S356 S358
S357
S321 S340 T366
S320 S355
S36 S51 S280 S325
S26 T37 S146 S276 S329
S235 S382
Regulation:
primary Ca-dependent
SV-cycling-dependent
not-affected
Domains and Regions:
DOMAIN: Protein kinase
REGION: Autoinhibitory domain
REGION: Calmodulin-binding

Figure S5: Changes in phosphorylation site intensities for Calcium/calmodulin-dependent kinase 2 alpha, beta, gamma, and delta. *X-axis shows positions of modified amino acids. A grey horizontal bar represents a protein sequence with its N- and C termini denoted as "N" and "C". Colored segments mark positions of domains and regions on protein sequence as annotated in Uniprot [7]. Changes in phosphorylation site intensities are expressed as log_2 fold changes (y-axis). Non-significant changes in phosphorylation site intensities are colored black. Significant changes are shown in orange or blue, depending on the classification of the site as "primarily Ca-dependent", or "SV-cycling-dependent", respectively. If a phosphorylation site was quantified on peptides that were singly, doubly or multiply phosphorylated (also known as "multiplicity"), the multiplicity with the highest magnitude of log_2 fold change is depicted. Proteins are grouped by the gene name and Uniprot identifier.*

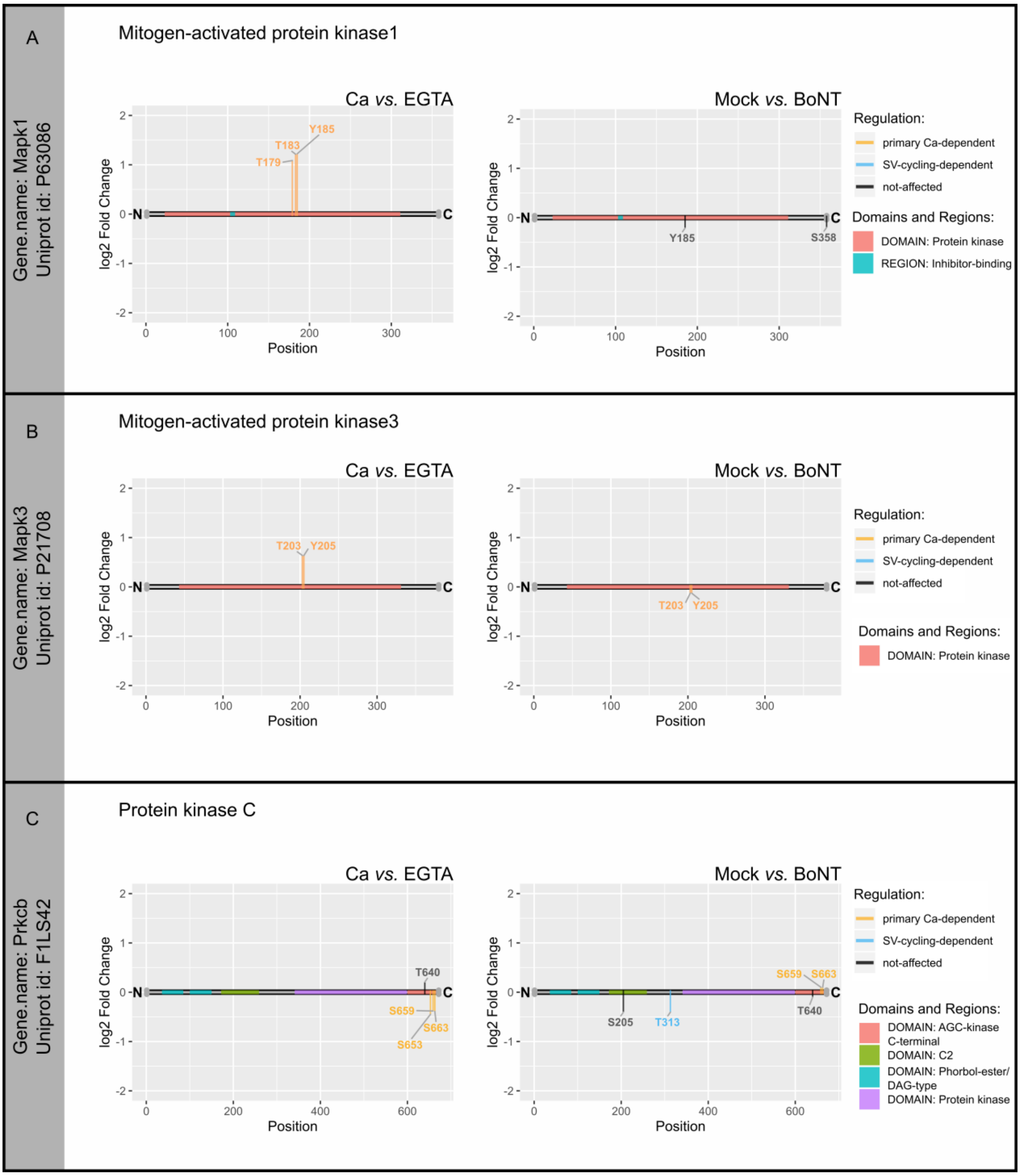

***Figure S6: Changes in phosphorylation site intensities for Mitogen-activated protein kinase 1, 3, and Protein kinase C beta**. X-axis shows positions of modified amino acids. A grey horizontal bar represents a protein sequence with its N- and C termini denoted as “N” and “C”. Colored segments mark positions of domains and regions on protein sequence as annotated in Uniprot [7]. Changes in phosphorylation site intensities are expressed as $\log_2$ fold changes (y-axis). Non-significant changes in phosphorylation site intensities are colored black. Significant changes are shown in orange or blue, depending on the classification of the site as “primarily Ca-dependent”, or “SV-cycling-dependent”, respectively. If a phosphorylation site was quantified on peptides that were singly, doubly or multiply phosphorylated (also known as “multiplicity”), the multiplicity with the highest magnitude of $\log_2$ fold change is depicted. Proteins are grouped by the gene name and Uniprot identifier.*

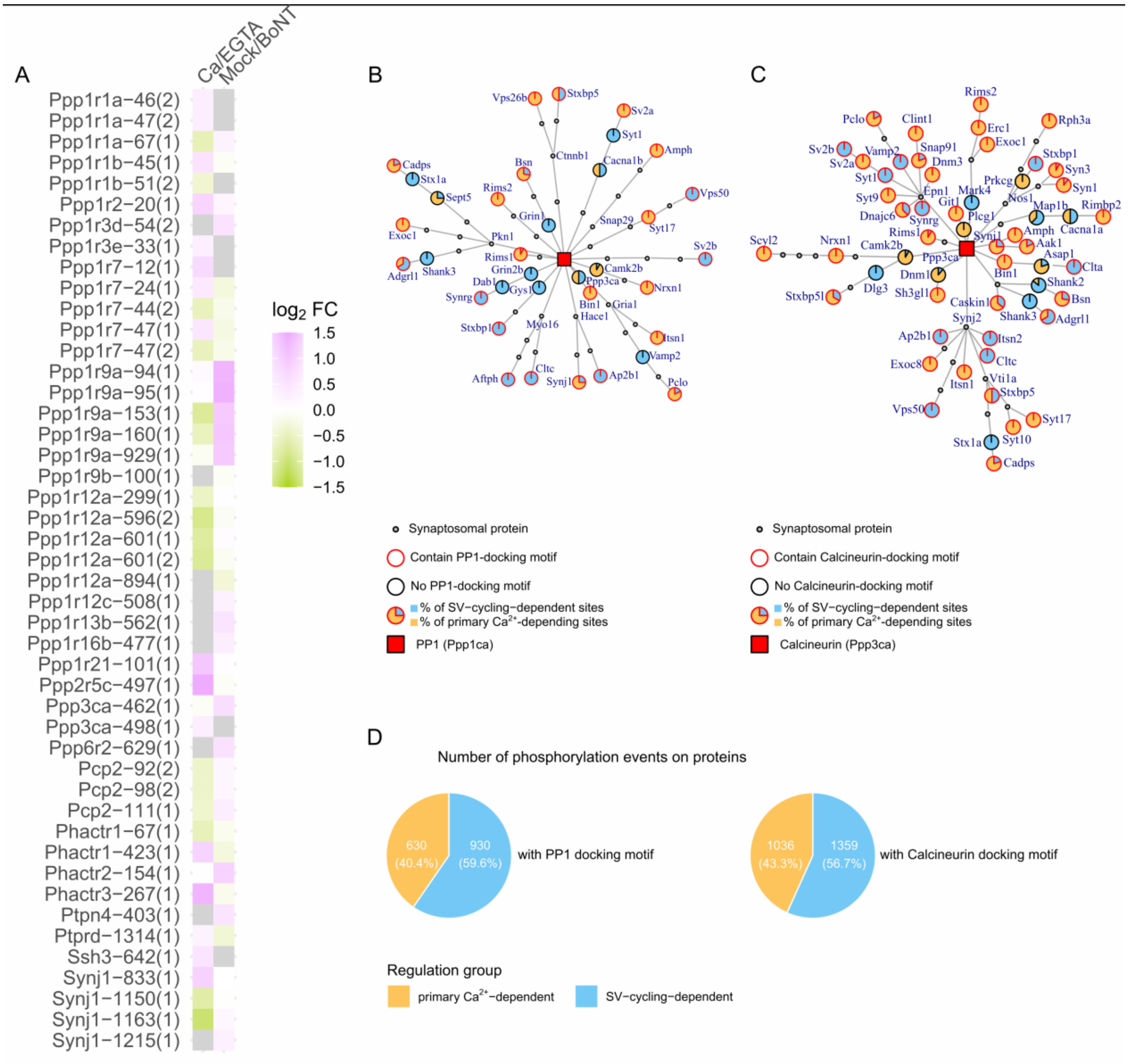

Figure S7: Phosphatases and their putative substrates. A. *Significantly regulated phosphorylation events on phosphatases and phosphatase-related proteins in Ca/EGTA or Mock/BoNT experiment. Phosphorylation events are designated as gene name followed by the amino acid position and phosphorylation multiplicity (in brackets).* ***B & C.*** *Protein-Protein Interaction map between phosphatase (PP1 in B and Calcineurin in C) and selected phosphoproteins involved in endo/exocytosis and containing PP1 (B) or Calcineurin (C) docking motif (shown as blue/orange colored circles with red edges). Docking motifs described in [8] were used for the analysis. Phosphoproteins that did not contain docking motifs are shown as blue/orange colored circles with black edges. The blue/orange area within a circle correspond to a percentage of sites classified as "primary Ca^{2+}-dependent" (orange) or "SV-cycling-dependent" (blue). Synaptosomal proteins not containing significantly regulated phosphorylation sites are shown as small grey circles. Phosphatases (PP1 or Calcineurin) are represented by red squares. Protein-protein interactions were extracted from String database [9] and included interactions between synaptosomal proteins which are experimentally supported or annotated in manually curated databases.* ***D.*** *Number of of "primary Ca^{2+}-dependent" (orange) and "SV-cycling-dependent" (blue) phosphorylation events on proteins containing a PP1 (E) or Calcineurin (F) docking motif [8].*

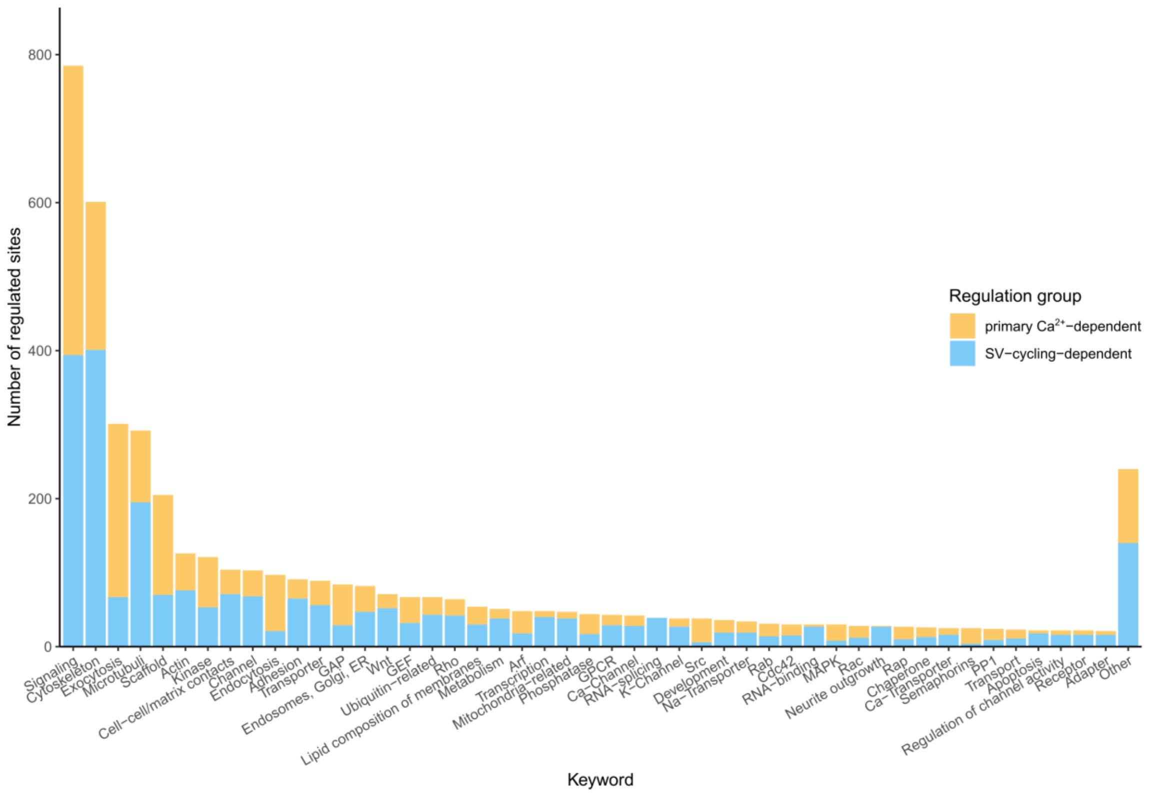

Figure S8: Manual annotation of proteins carrying regulated phosphorylation sites. *Key-words were assigned to proteins carrying regulated phosphorylation sites that specify a function / localization the protein might be related to. One protein can have one or more terms assigned. Number of significantly regulated phosphorylation sites for each term is shown as stacked bars with colors representing regulation group of the phosphorylation sites. Arf stays for ADP-ribosylation factor small GTPase; ER ~ endoplasmatic reticulum; GAP ~ GTPase activating protein; GEF ~ GTP exchanging factor; GPCR ~ G-protein coupled receptor; PP1 ~ protein phosphatase-1*

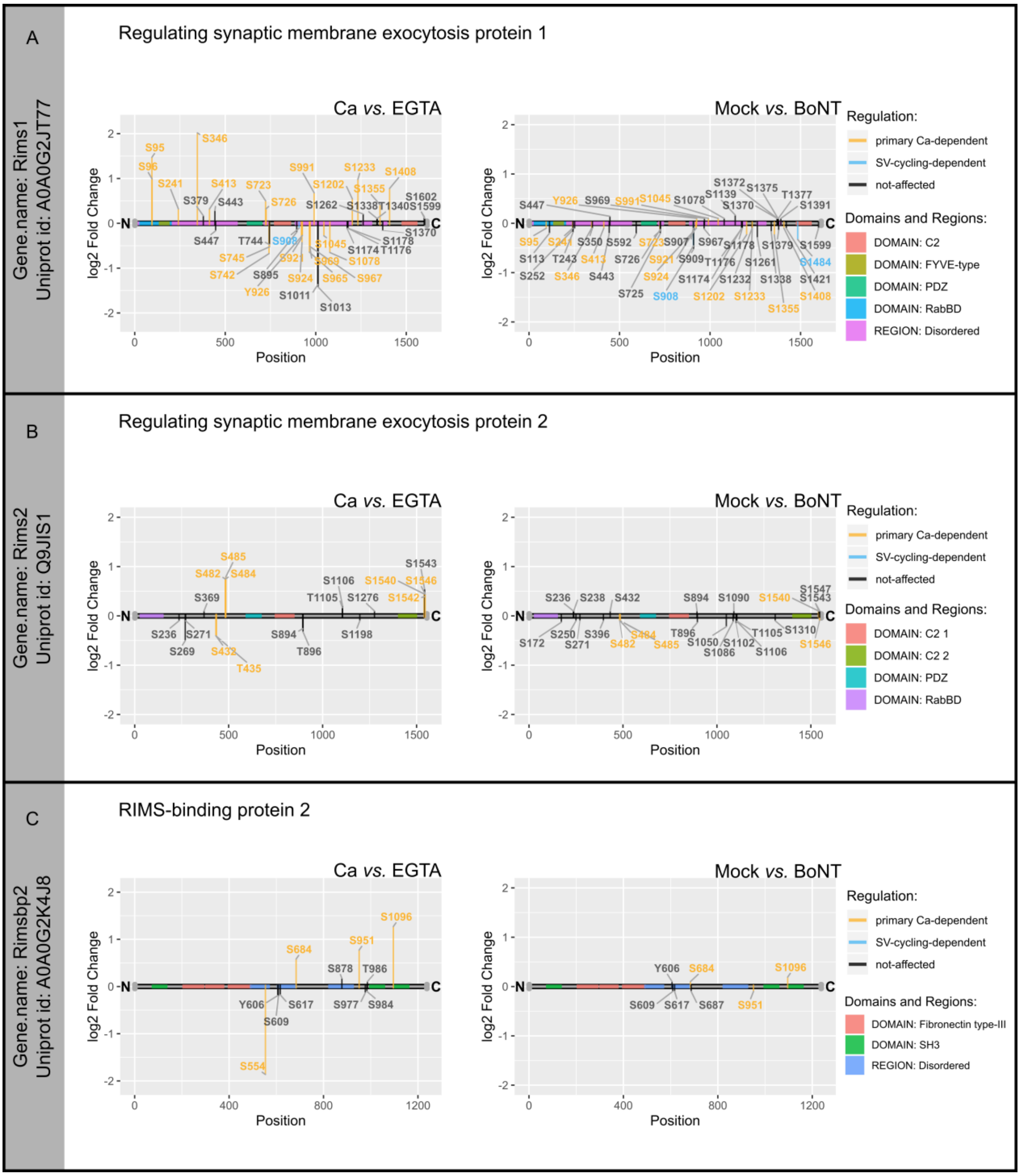

Figure S9: Changes in phosphorylation site intensities for Regulating synaptic membrane exocytosis (RIM) protein 1, 2, and RIM-binding protein 2*. X-axis shows positions of modified amino acids. A grey horizontal bar represents a protein sequence with its N- and C termini denoted as "N" and "C". Colored segments mark positions of domains and regions on protein sequence as annotated in Uniprot [7]. Changes in phosphorylation site intensities are expressed as log$_2$ fold changes (y-axis). Non-significant changes in phosphorylation site intensities are colored black. Significant changes are shown in orange or blue, depending on the classification of the site as "primarily Ca-dependent", or "SV-cycling-dependent", respectively. If a phosphorylation site was quantified on peptides that were singly, doubly or multiply phosphorylated (also known as "multiplicity"), the multiplicity with the highest magnitude of log$_2$ fold change is depicted. Proteins are grouped by the gene name and Uniprot identifier.*

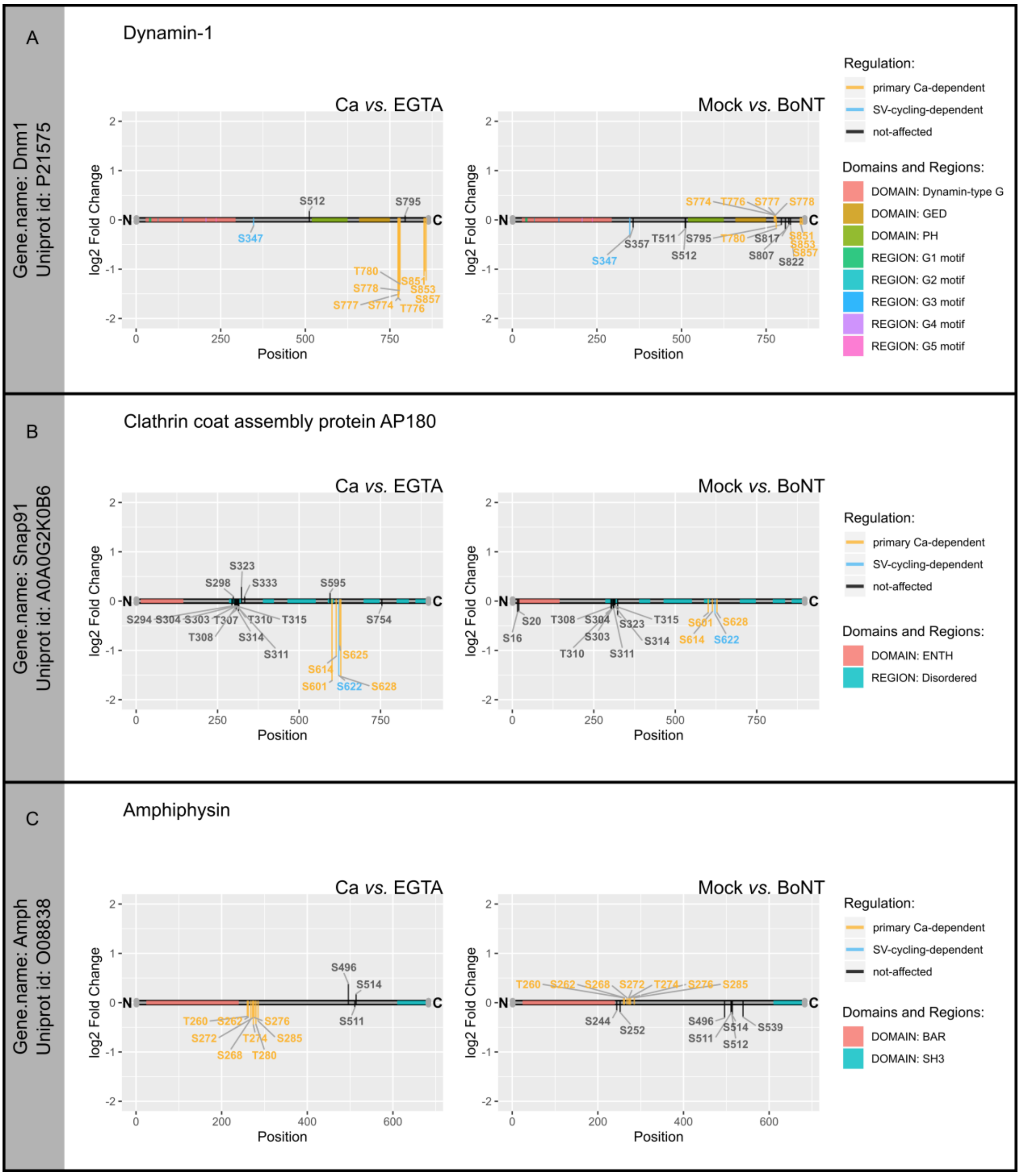

Figure S10: Changes in phosphorylation site intensities for Dynamin-1, Clathrin coat assembly protein AP180, and Amphiphysin. *X-axis shows positions of modified amino acids. A grey horizontal bar represents a protein sequence with its N- and C termini denoted as "N" and "C". Colored segments mark positions of domains and regions on protein sequence as annotated in Uniprot [7]. Changes in phosphorylation site intensities are expressed as log_2 fold changes (y-axis). Non-significant changes in phosphorylation site intensities are colored black. Significant changes are shown in orange or blue, depending on the classification of the site as "primarily Ca-dependent", or "SV-cycling-dependent", respectively. If a phosphorylation site was quantified on peptides that were singly, doubly or multiply phosphorylated (also known as "multiplicity"), the multiplicity with the highest magnitude of log_2 fold change is depicted. Proteins are grouped by the gene name and Uniprot identifier.*

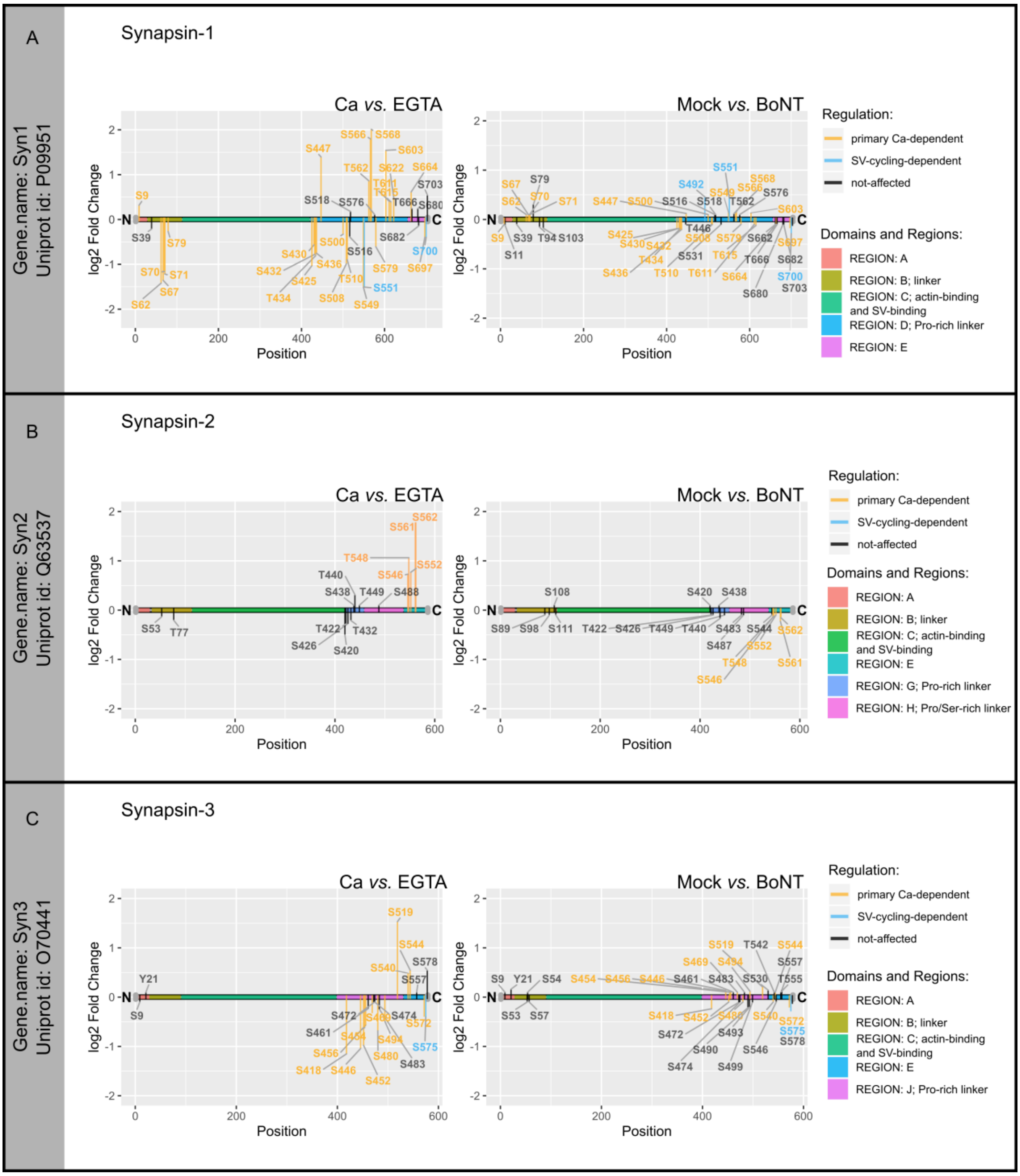

Figure S11: Changes in phosphorylation site intensities for Synapsin-1, 2, and 3*. X-axis shows positions of modified amino acids. A grey horizontal bar represents a protein sequence with its N- and C termini denoted as "N" and "C". Colored segments mark positions of domains and regions on protein sequence as annotated in Uniprot [7]. Changes in phosphorylation site intensities are expressed as log_2 fold changes (y-axis). Non-significant changes in phosphorylation site intensities are colored black. Significant changes are shown in orange or blue, depending on the classification of the site as "primarily Ca-dependent", or "SV-cycling-dependent", respectively. If a phosphorylation site was quantified on peptides that were singly, doubly or multiply phosphorylated (also known as "multiplicity"), the multiplicity with the highest magnitude of log_2 fold change is depicted. Proteins are grouped by the gene name and Uniprot identifier.*

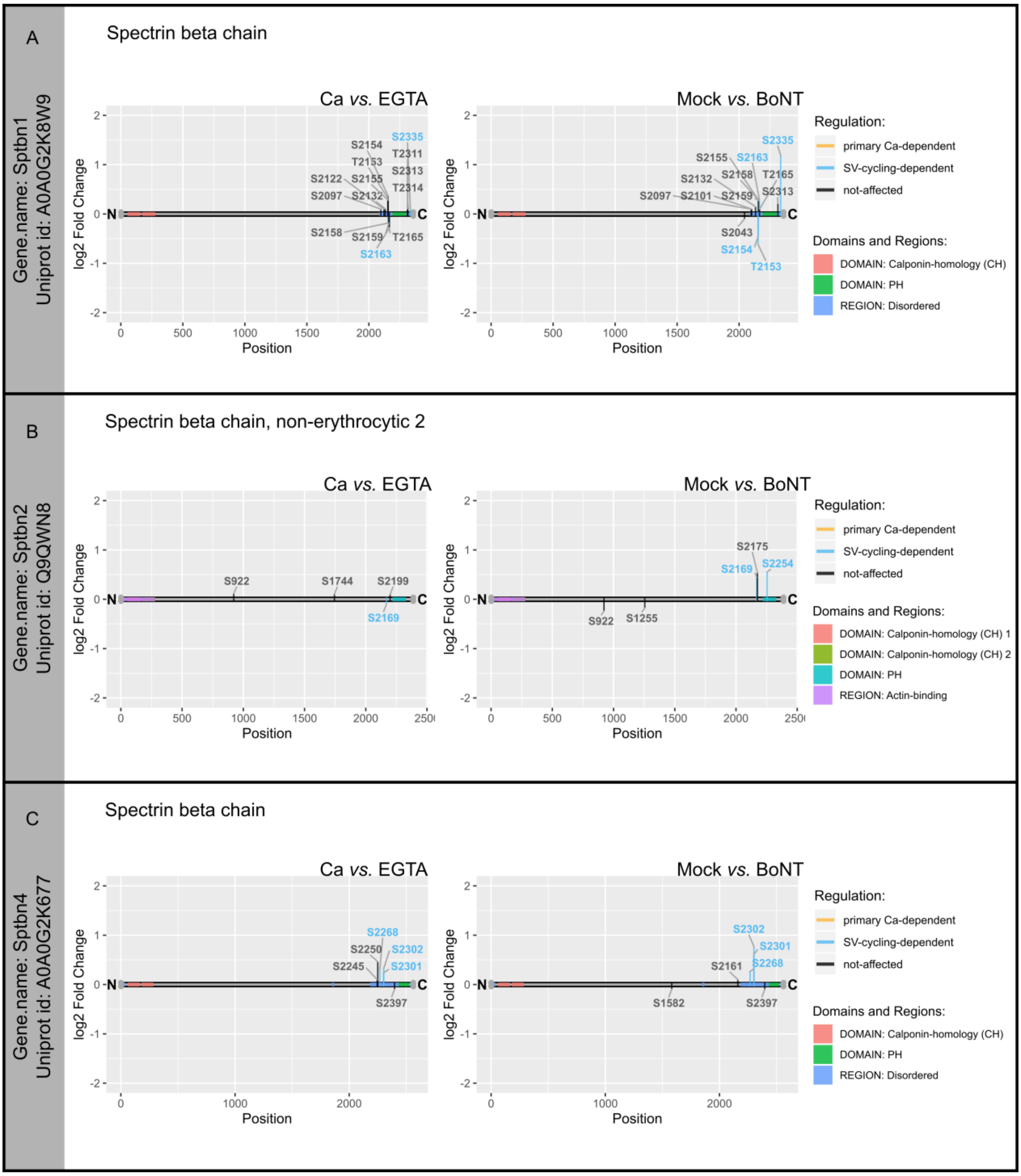

***Figure S12: Changes in phosphorylation site intensities for Spectrin beta chain**. X-axis shows positions of modified amino acids. A grey horizontal bar represents a protein sequence with its N- and C termini denoted as "N" and "C". Colored segments mark positions of domains and regions on protein sequence as annotated in Uniprot [7]. Changes in phosphorylation site intensities are expressed as log_2 fold changes (y-axis). Non-significant changes in phosphorylation site intensities are colored black. Significant changes are shown in orange or blue, depending on the classification of the site as "primarily Ca-dependent", or "SV-cycling-dependent", respectively. If a phosphorylation site was quantified on peptides that were singly, doubly or multiply phosphorylated (also known as "multiplicity"), the multiplicity with the highest magnitude of log_2 fold change is depicted. Proteins are grouped by the gene name and Uniprot identifier.*

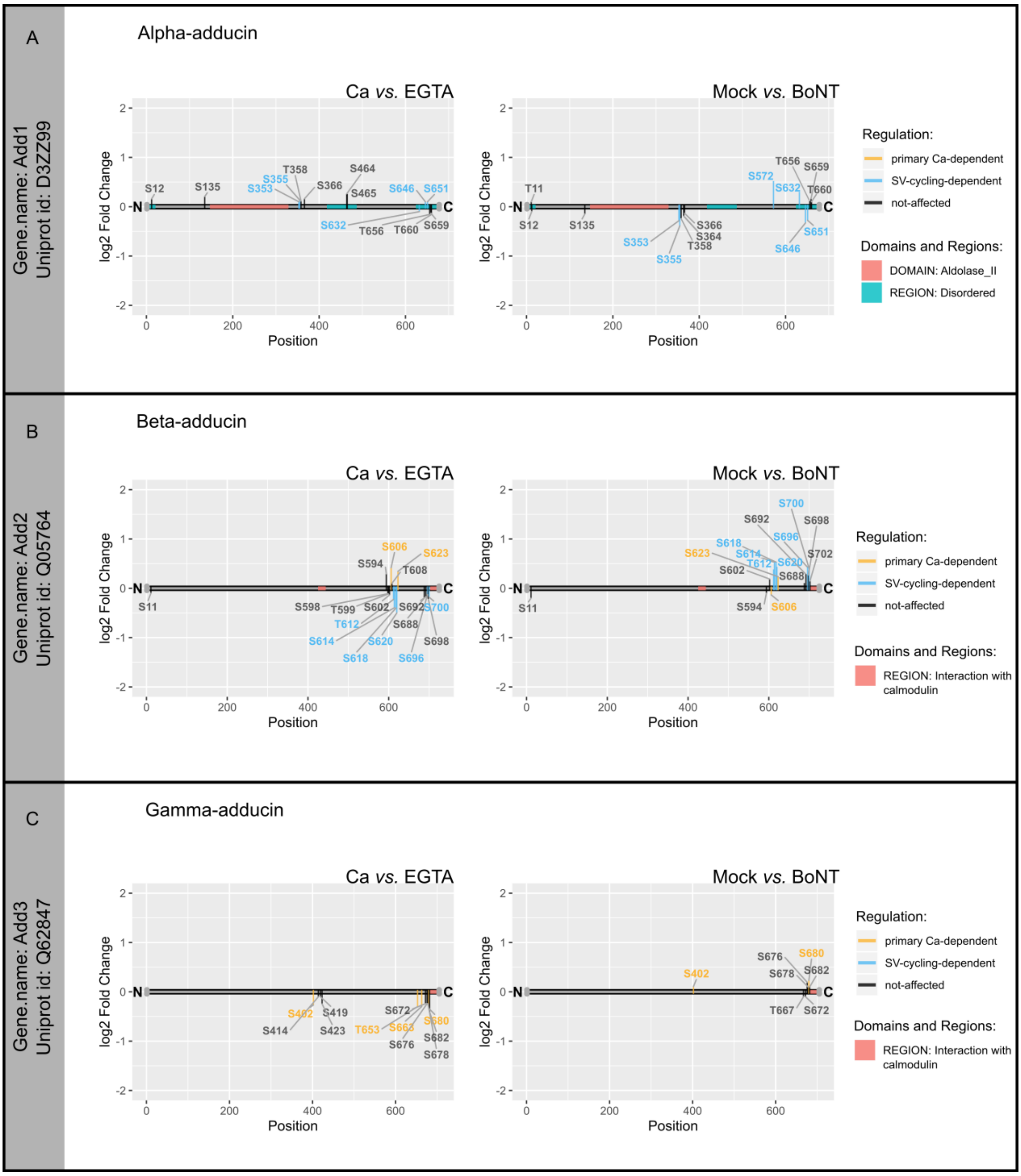

***Figure S13: Changes in phosphorylation site intensities for Adducin alpha, beta, and gamma**. X-axis shows positions of modified amino acids. A grey horizontal bar represents a protein sequence with its N- and C termini denoted as "N" and "C". Colored segments mark positions of domains and regions on protein sequence as annotated in Uniprot [7]. Changes in phosphorylation site intensities are expressed as log$_2$ fold changes (y-axis). Non-significant changes in phosphorylation site intensities are colored black. Significant changes are shown in orange or blue, depending on the classification of the site as "primarily Ca-dependent", or "SV-cycling-dependent", respectively. If a phosphorylation site was quantified on peptides that were singly, doubly or multiply phosphorylated (also known as "multiplicity"), the multiplicity with the highest magnitude of log$_2$ fold change is depicted. Proteins are grouped by the gene name and Uniprot identifier.*

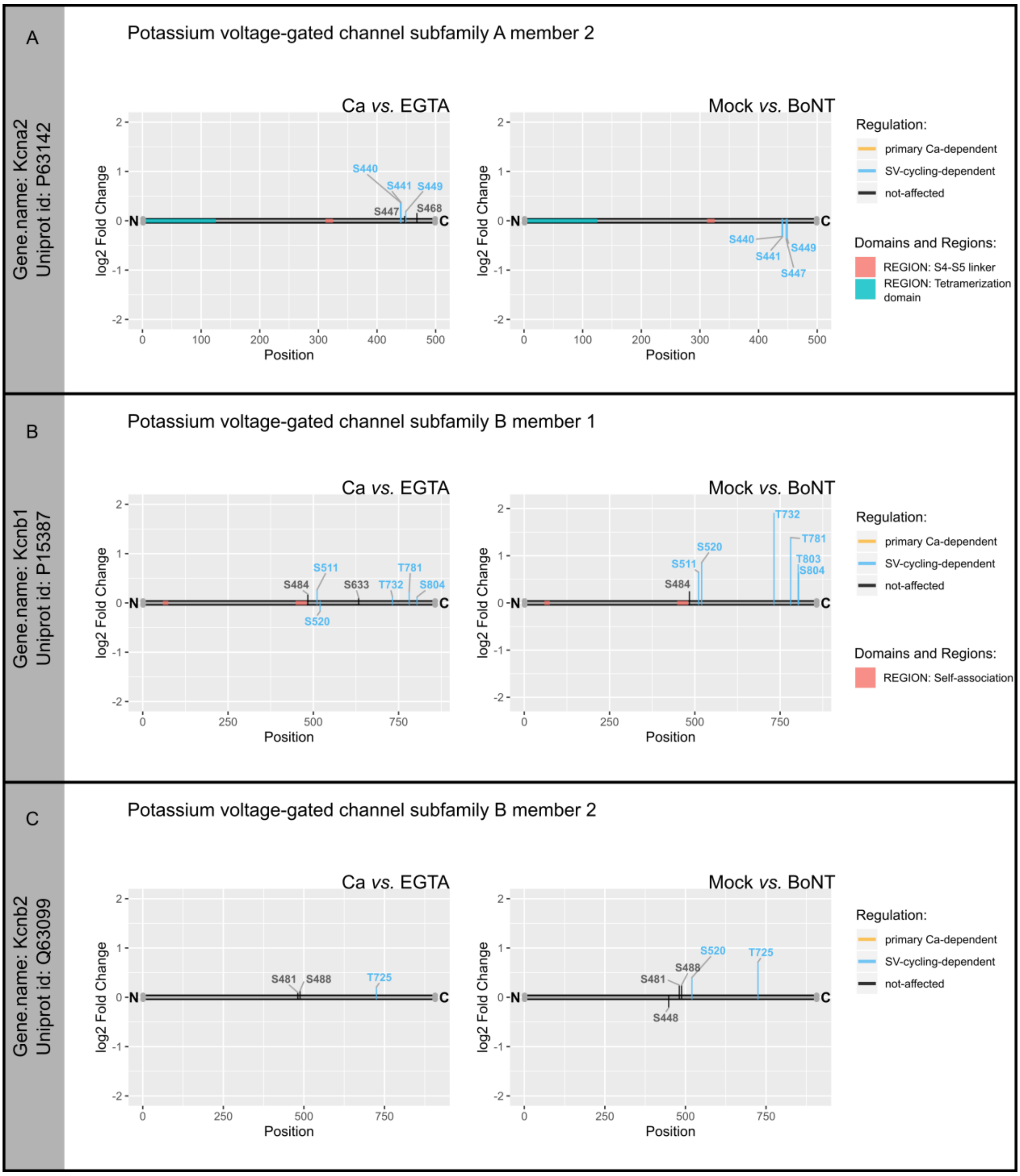

Figure S14: Changes in phosphorylation site intensities for Potassium voltage-gated channel subfamily A member 2, and subfamily B member 1 and 2*. X-axis shows positions of modified amino acids. A grey horizontal bar represents a protein sequence with its N- and C termini denoted as "N" and "C". Colored segments mark positions of domains and regions on protein sequence as annotated in Uniprot [7]. Changes in phosphorylation site intensities are expressed as $\log_2$ fold changes (y-axis). Non-significant changes in phosphorylation site intensities are colored black. Significant changes are shown in orange or blue, depending on the classification of the site as "primarily Ca-dependent", or "SV-cycling-dependent", respectively. If a phosphorylation site was quantified on peptides that were singly, doubly or multiply phosphorylated (also known as "multiplicity"), the multiplicity with the highest magnitude of $\log_2$ fold change is depicted. Proteins are grouped by the gene name and Uniprot identifier.*

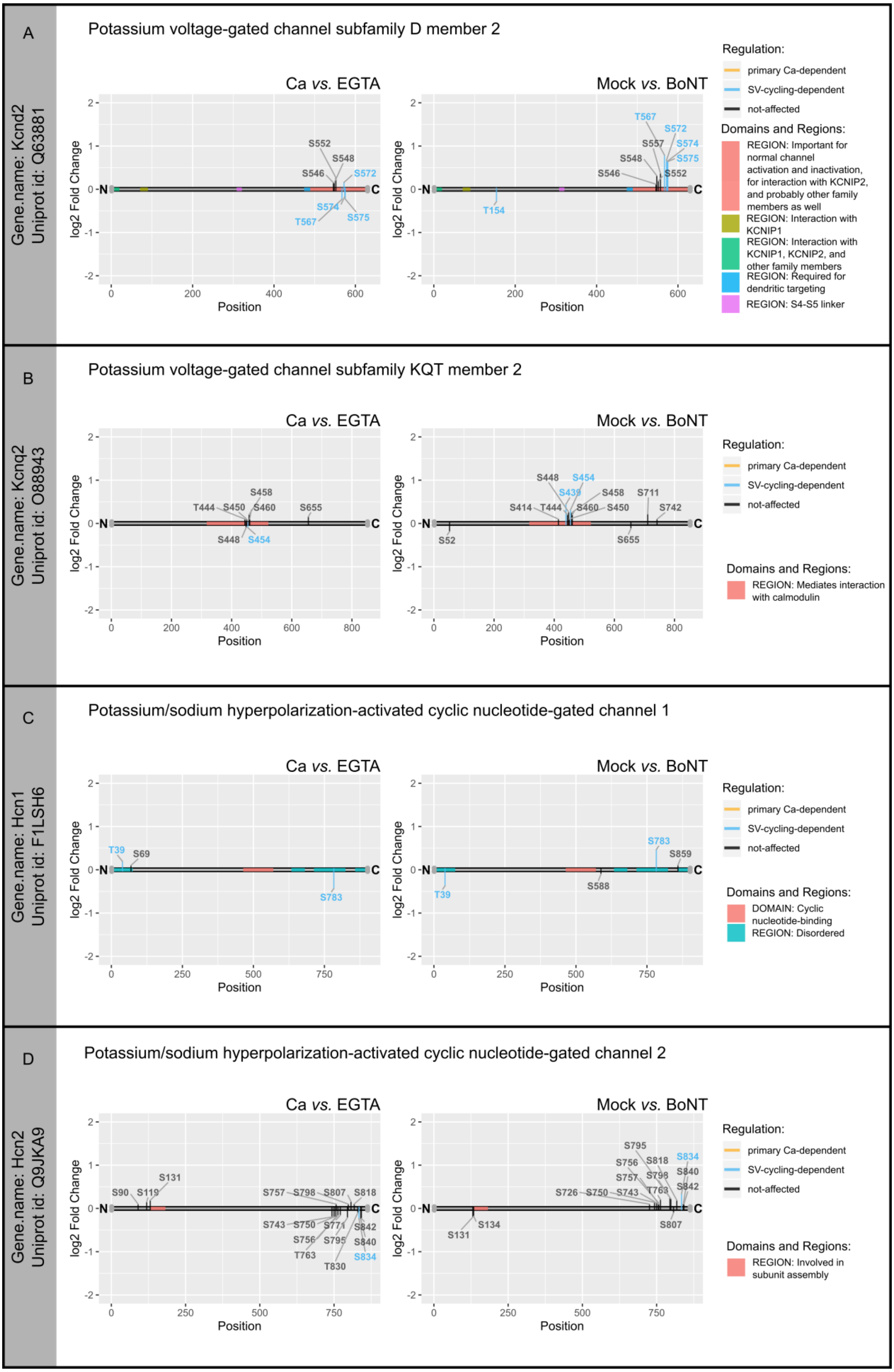

A
Gene.name: Kcnd2
Uniprot id: Q63881
Potassium voltage-gated channel subfamily D member 2
Ca vs. EGTA
Mock vs. BoNT
log2 Fold Change
Position
S552 S548 S546 S572 S574 T567 S575
T567 S572 S557 S574 S548 S575 S546 S552 T154
Regulation:
primary Ca-dependent
SV-cycling-dependent
not-affected
Domains and Regions:
REGION: Important for normal channel activation and inactivation, for interaction with KCNIP2, and probably other family members as well
REGION: Interaction with KCNIP1
REGION: Interaction with KCNIP1, KCNIP2, and other family members
REGION: Required for dendritic targeting
REGION: S4-S5 linker
B
Gene.name: Kcnq2
Uniprot id: O88943
Potassium voltage-gated channel subfamily KQT member 2
Ca vs. EGTA
Mock vs. BoNT
S458 T444 S450 S460 S655 S448 S454
S448 S454 S439 S458 S711 S414 T444 S460 S450 S742 S52 S655
Regulation:
primary Ca-dependent
SV-cycling-dependent
not-affected
Domains and Regions:
REGION: Mediates interaction with calmodulin
C
Gene.name: Hcn1
Uniprot id: F1LSH6
Potassium/sodium hyperpolarization-activated cyclic nucleotide-gated channel 1
Ca vs. EGTA
Mock vs. BoNT
T39 S69 S783
S783 S859 T39 S588
Regulation:
primary Ca-dependent
SV-cycling-dependent
not-affected
Domains and Regions:
DOMAIN: Cyclic nucleotide-binding
REGION: Disordered
D
Gene.name: Hcn2
Uniprot id: Q9JKA9
Potassium/sodium hyperpolarization-activated cyclic nucleotide-gated channel 2
Ca vs. EGTA
Mock vs. BoNT
S131 S90 S119 S757 S798 S807 S818 S743 S750 S771 S842 S756 S795 S840 T763 S834 T830
S795 S834 S756 S818 S840 S757 S798 S842 S726 S750 S743 T763 S807 S134 S131
Regulation:
primary Ca-dependent
SV-cycling-dependent
not-affected
Domains and Regions:
REGION: Involved in subunit assembly

Figure S15: Changes in phosphorylation site intensities for Potassium voltage-gated channel subfamily D member 2, subfamily KQT member 2, Potassium/sodium hyperpolarization-activated cyclic nucleotide-gated channel 1 and 2. *X-axis shows positions of modified amino acids. A grey horizontal bar represents a protein sequence with its N- and C termini denoted as "N" and "C". Colored segments mark positions of domains and regions on protein sequence as annotated in Uniprot [7]. Changes in phosphorylation site intensities are expressed as* log_2 *fold changes (y-axis). Non-significant changes in phosphorylation site intensities are colored black. Significant changes are shown in orange or blue, depending on the classification of the site as "primarily Ca-dependent", or "SV-cycling-dependent", respectively. If a phosphorylation site was quantified on peptides that were singly, doubly or multiply phosphorylated (also known as "multiplicity"), the multiplicity with the highest magnitude of* log_2 *fold change is depicted. Proteins are grouped by the gene name and Uniprot identifier.*

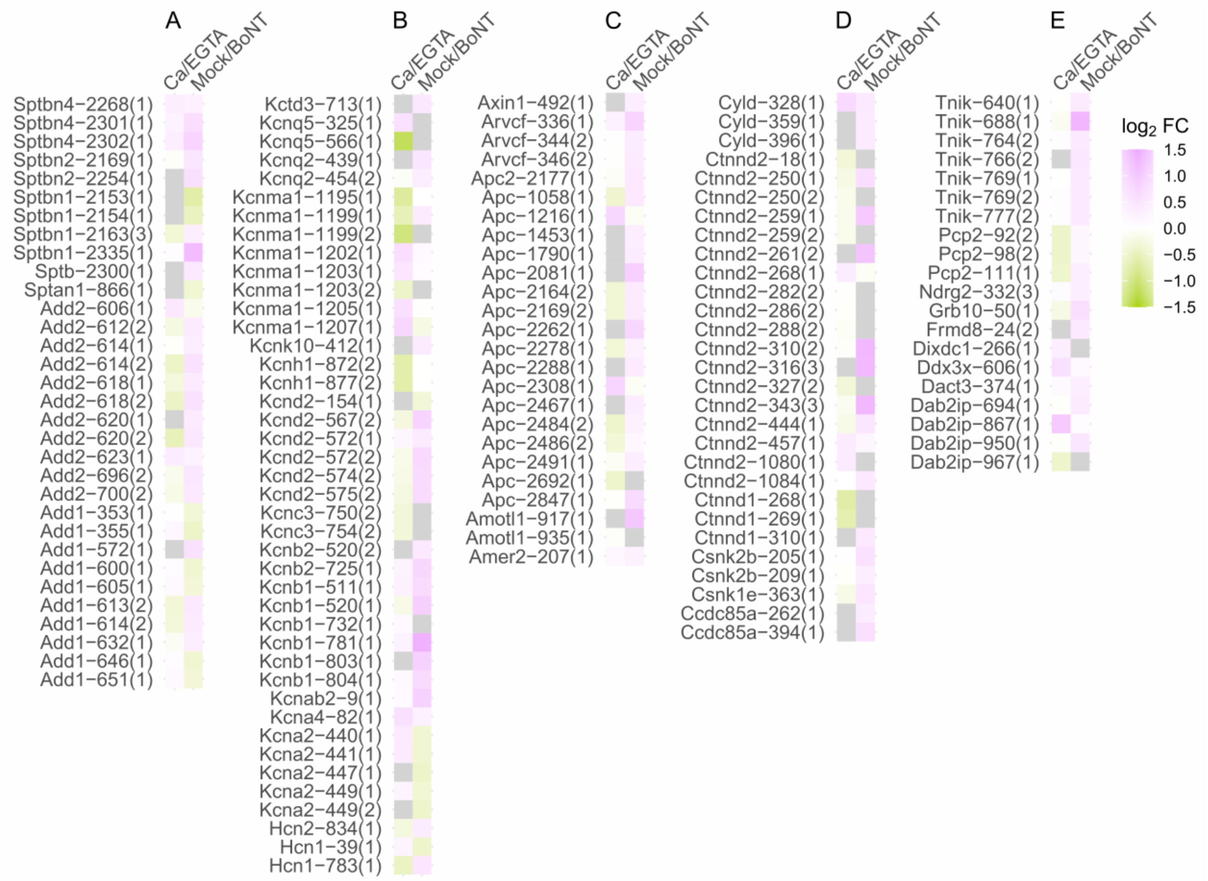

Figure S16: Regulated phosphorylation sites on selected proteins. A. *Regulated sites on cytoskeleton-related proteins spectrin and adducing.* ***B.*** *Regulated sites on potassium-channel proteins.* ***C-D.*** *Regulated sites on Wnt-pathway-related proteins. Phosphorylation events are designated as gene name followed by the amino acid position and phosphorylation multiplicity (in brackets).*

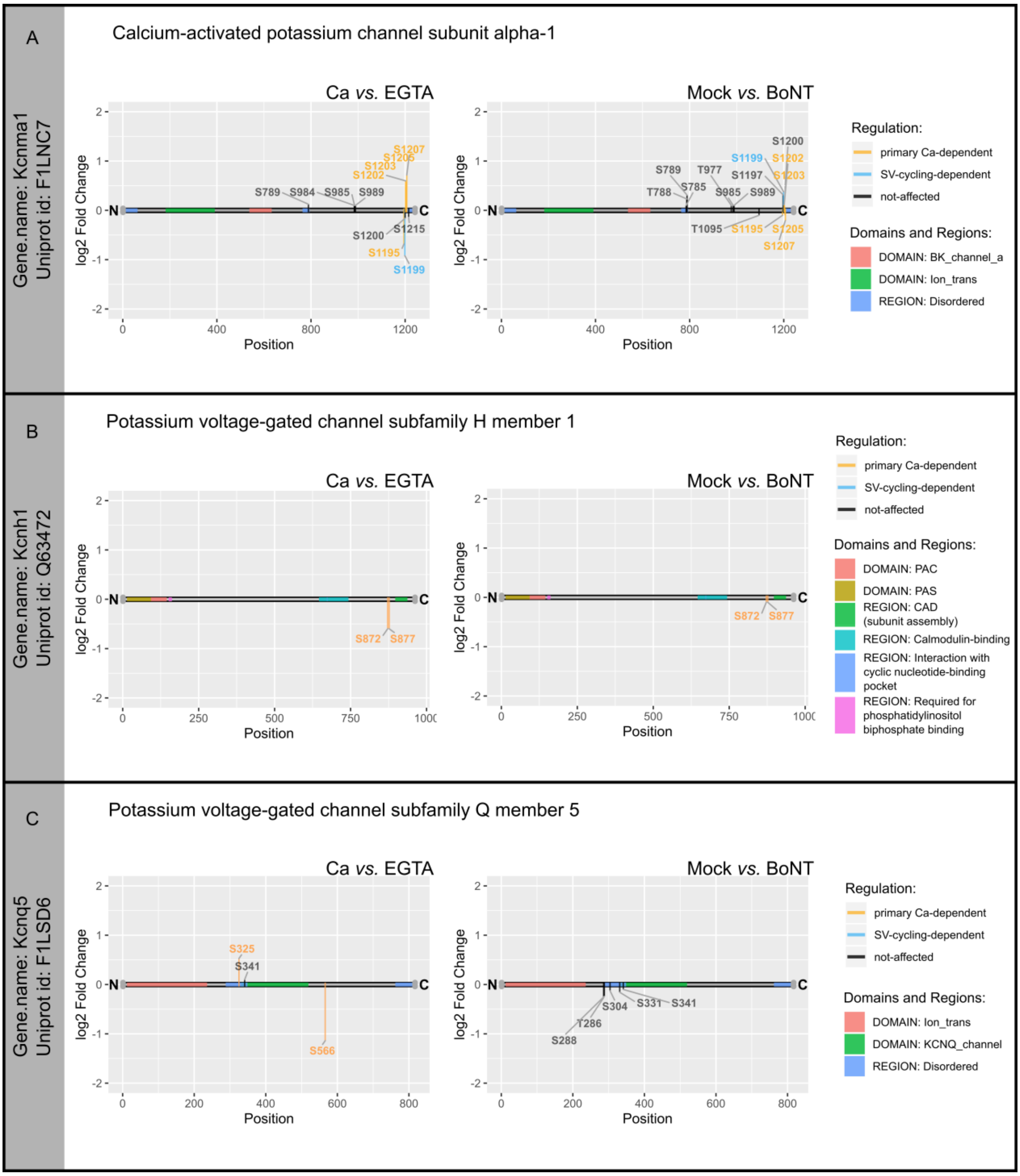

Figure S17: Changes in phosphorylation site intensities for Calcium-activated potassium channel subunit alpha-1, Potassium voltage-gated channel subfamily H member 1, and Potassium voltage-gated channel subfamily Q member 5*. X-axis shows positions of modified amino acids. A grey horizontal bar represents a protein sequence with its N- and C termini denoted as "N" and "C". Colored segments mark positions of domains and regions on protein sequence as annotated in Uniprot [7]. Changes in phosphorylation site intensities are expressed as log_2 fold changes (y-axis). Non-significant changes in phosphorylation site intensities are colored black. Significant changes are shown in orange or blue, depending on the classification of the site as "primarily Ca-dependent", or "SV-cycling-dependent", respectively. If a phosphorylation site was quantified on peptides that were singly, doubly or multiply phosphorylated (also known as "multiplicity"), the multiplicity with the highest magnitude of log_2 fold change is depicted. Proteins are grouped by the gene name and Uniprot identifier.*

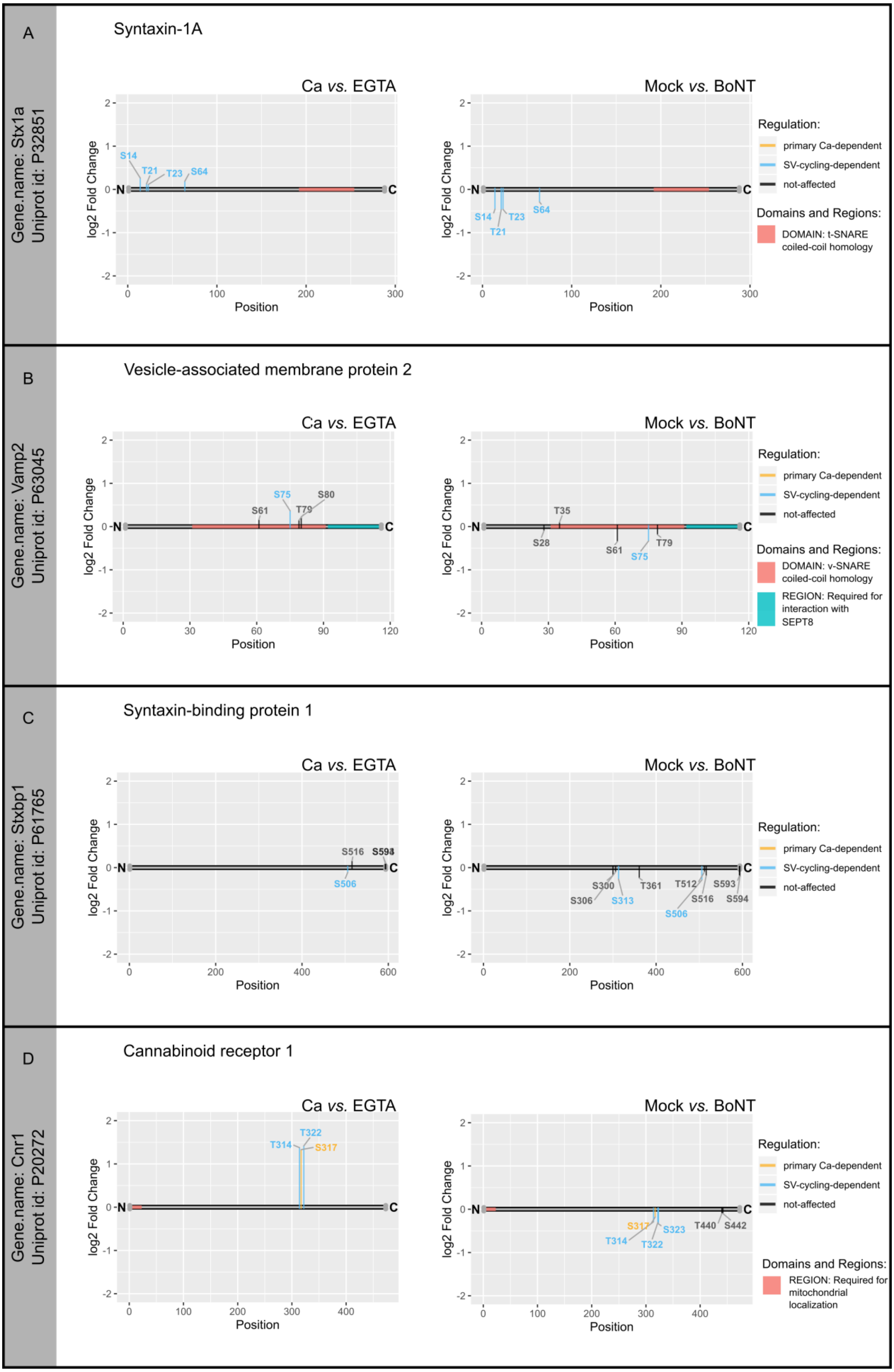
A
Gene.name: Stx1a
Uniprot id: P32851
Syntaxin-1A
Ca vs. EGTA
Mock vs. BoNT
log2 Fold Change
Position
S14
T21
T23
S64
Regulation:
primary Ca-dependent
SV-cycling-dependent
not-affected
Domains and Regions:
DOMAIN: t-SNARE coiled-coil homology
B
Gene.name: Vamp2
Uniprot id: P63045
Vesicle-associated membrane protein 2
S75
S80
S61
T79
T35
S28
DOMAIN: v-SNARE coiled-coil homology
REGION: Required for interaction with SEPT8
C
Gene.name: Stxbp1
Uniprot id: P61765
Syntaxin-binding protein 1
S516
S506
S300
S306
S313
T361
T512
S593
S594
D
Gene.name: Cnr1
Uniprot id: P20272
Cannabinoid receptor 1
T322
T314
S317
S323
T440
S442
REGION: Required for mitochondrial localization

Figure S18: Changes in phosphorylation site intensities for Syntaxin-1A, Vesicle-associated membrane protein 2, Syntaxin-binding protein 1, and Cannabinoid receptor 1*. X-axis shows positions of modified amino acids. A grey horizontal bar represents a protein sequence with its N- and C termini denoted as "N" and "C". Colored segments mark positions of domains and regions on protein sequence as annotated in Uniprot [7]. Changes in phosphorylation site intensities are expressed as log_2 fold changes (y-axis). Non-significant changes in phosphorylation site intensities are colored black. Significant changes are shown in orange or blue, depending on the classification of the site as "primarily Ca-dependent", or "SV-cycling-dependent", respectively. If a phosphorylation site was quantified on peptides that were singly, doubly or multiply phosphorylated (also known as "multiplicity"), the multiplicity with the highest magnitude of log_2 fold change is depicted. Proteins are grouped by the gene name and Uniprot identifier.*

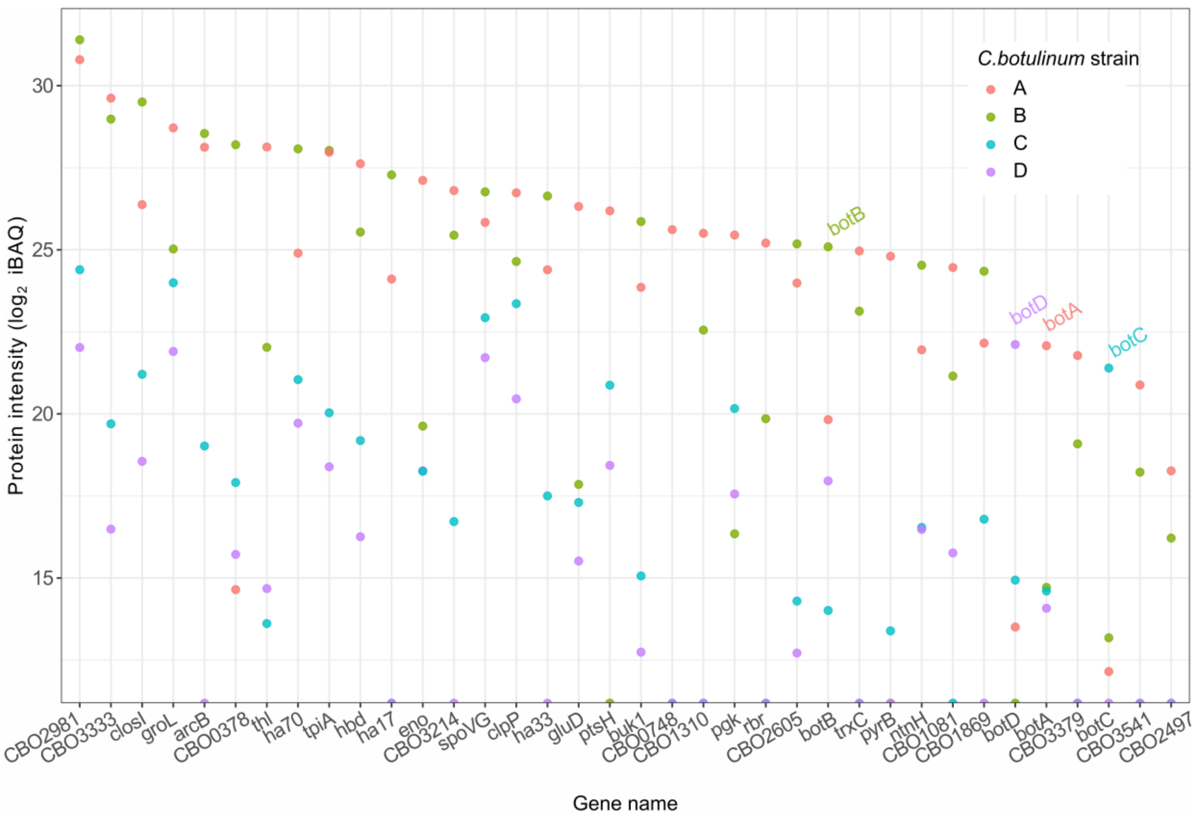

Figure S19: Proteomic profiling of C. botulinum cell culture supernatants. *Protein content in C. botulinum cell culture supernatants was assessed using iBAQ values [10]. Top 30 most intense proteins, botulinum toxins (botA, botB, botC, botD), as well as putative protease (CBO3541) and putative phosphatases (CBO3379, CBO2497) that were identified in the cell culture supernatants are ranked based on iBAQ intensity. Colors encode C.botulinum strains: A, red; B, green; C, blue; D, violet.*

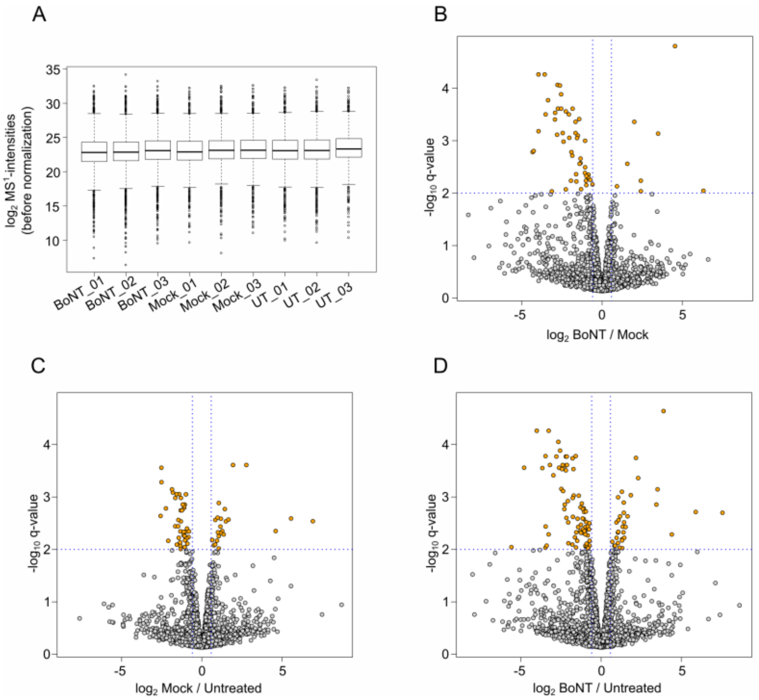

Figure S20: Phosphoproteome analysis of BoNT-treated HeLa nuclear extract. *Unspecific activity of C. botulinum cell culture supernatants was tested in nuclear extract of HeLa cells. Nuclear extract was treated for 1.5 h with a combination of the four C. botulinum cell culture supernatants, BoNT A-D (BoNT); heat-inactivated BoNT A-D (Mock), or buffer (Untreated, UT). Afterwards, proteins were digested with trypsin and phosphorylated peptides were enriched using TiO_2-beads. Precursor intensities in MS^1 scans were used to assess intensities of phosphorylated sites. (**A**) Box-whisker plot of $\log_2$ phosphorylation site intensities before normalization. Boxes indicate 25% and 75% quantiles (interquartile range, IQR), vertical lines indicate medians, and whiskers indicate data within 1.5 × IQR. (**B-D**) Differences in phosphorylation site intensities were assessed using limma package [11]. Volcano plots of $-\log_{10}$ q-values vs. $\log_2$ fold change: BoNT vs. Mock (b), Mock vs. Untreated (c), BoNT vs. Untreated (d). Orange dots represent phosphorylation events with a q-value of < 0.01 and an absolute $\log_2$ FC of at least 0.586.*

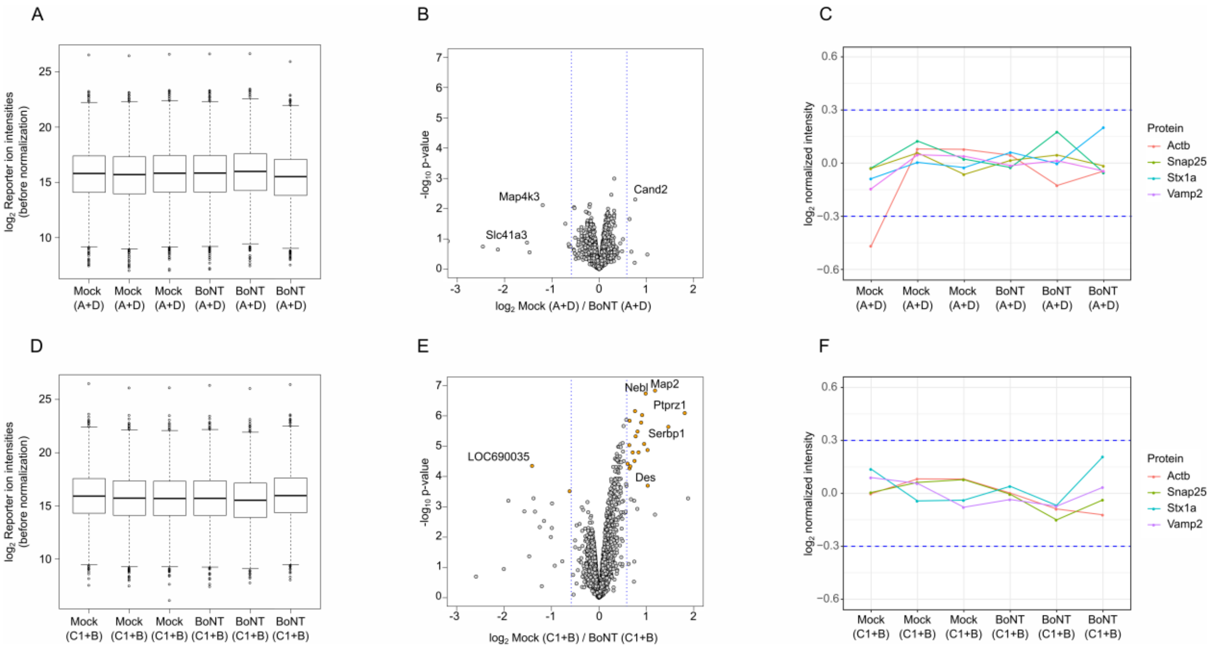

Figure S21: Proteomics analysis of Mock- or BoNT-treated synaptosomes. *Protein content in Mock- or BoNT-treated synaptosomes was assessed using TMT6-labeled peptides in the unbound fraction (not-phosphorylated peptides) after phosphopeptide enrichment. Following C. botulinum cell culture supernatants were used: (**A-C**) C. botulinum A+D, (**D-E**) C. botulinum C1+B. (**A**, **D**) Box-whisker plot of* $\log_2$ *reporter ion intensities before normalization. Boxes indicate 25% and 75% quantiles (interquartile range, IQR), vertical lines indicate medians, and whiskers indicate data within 1.5* $\times$ *IQR. (**B**, **E**) Differential protein content was assessed using limma package [11]. Volcano plots of* $-\log_{10}$ *p-values vs.* $\log_2$ *Mock/BoNT. Orange dots: proteins with a Benjamini-Hochberg adjusted p-value of < 0.01 and an absolute* $\log_2FC$ *of at least 0.586. (**C**, **E**)* $\log_2$ *normalized reporter ion intensities of selected proteins, actin-beta (Actb), Snap25, syntaxin-1a (Stx1a), Vamp2.*

Table S1: Number of regulated phosphorylation events per predicted kinase group in Ca vs. EGTA experiment. *"Sites in" show number of phosphorylation events that can be regulated by a respective kinase group as determined in the Ca vs. EGTA experiment. "Sites out" contains the number of phosphorylation events possibly regulated by other kinases. Similarly, "Sites in bcgr" and "Sites out bcgr" summarize the number of predicted substrate-kinase relations in the human proteome (used as background). Fisher's exact test was applied to test enrichment of substrate-kinase relationships as compared to predicted substrate-kinase relations in the human proteome. "p.val" and "p.adj" are raw and Benjamini-Hochberg adjusted p-values, respectively.*

Kinase group	Sites in	Sites out	Sites in bcgr	Sites out bcgr	p.val	p.adj
MAPK	175	728	6329	34101	0.003	0.009
CaMKII	160	743	2764	37666	0.000	0.000
PKC	141	762	5783	34647	0.269	0.377
CK1	80	823	4292	36138	0.100	0.201
PAK	67	836	3317	37113	0.425	0.518
CDK	60	843	3278	37152	0.122	0.221
CK2	46	857	2457	37973	0.258	0.371
GSK3	44	859	970	39460	0.000	0.000
DAPK	29	874	2043	38387	0.011	0.026
MAP2K	9	894	1967	38463	0.000	0.000
AMPK	8	895	196	40234	0.091	0.196
PKB	8	895	601	39829	0.162	0.283
CAMK	7	896	261	40169	0.531	0.627
GRK	7	896	798	39632	0.007	0.018
RSK	6	897	276	40154	1.000	1.000
PLK	6	897	929	39501	0.000	0.001
PDHK3_PDHK1	5	898	250	40180	1.000	1.000
CLK	5	898	771	39659	0.001	0.004
MARK	4	899	279	40151	0.537	0.627
SGK	3	900	69	40361	0.208	0.315
BCKDK_PDHK4	3	900	74	40356	0.237	0.349
PKD	3	900	193	40237	0.804	0.868
CaMKI	2	901	36	40394	0.201	0.314
DMPK	2	901	198	40232	0.335	0.446
p70S6K	1	902	49	40381	1.000	1.000
PKGcGK	1	902	414	40016	0.002	0.006
PKA	1	902	425	40005	0.002	0.006
mTOR_ATM_ATR	1	902	507	39923	0.000	0.001
Aurora	1	902	904	39526	0.000	0.000

Table S2: Enriched gene ontology biological function terms based on synapse-specific SynGO database [12]. *Proteins carrying regulated phosphorylation sites as determined in Ca vs. EGTA experiments were annotated using the synapse specific SynGO database. Significantly enriched (FDR < 0.001) GO biological function terms are presented together with the hierarchical structure. Note that only a part of proteins carrying regulated phosphorylation sites could be annotated using SynGO database (161 out of 463 unique genes).*

GO term ID	GO term name - hierarchical structure	GSEA FDR corrected p-value
SYNGO:synprocess	process in the synapse	5.58E-54
SYNGO:presynprocess	├─ process in the presynapse	9.91E-32
GO:0099509	│ ├─ regulation of presynaptic cytosolic calcium levels	8.86E-09
GO:0099626	│ │ └─ voltage-gated calcium channel activity involved in regulation of presynaptic cytosolic calcium levels	3.02E-05
GO:0099508	│ │ ├─ voltage-gated ion channel activity involved in regulation of presynaptic membrane potential	2.38E-04
GO:0099504	│ ├─ synaptic vesicle cycle	2.42E-27
GO:0097091	│ │ ├─ synaptic vesicle clustering	3.02E-05
GO:0016079	│ │ ├─ synaptic vesicle exocytosis	4.57E-17
GO:0099502	│ │ │ ├─ calcium-dependent activation of synaptic vesicle fusion	8.08E-08
GO:0150037	│ │ │ │ └─ regulation of calcium-dependent activation of synaptic vesicle fusion	7.22E-08
GO:2000300	│ │ │ ├─ regulation of synaptic vesicle exocytosis	1.13E-05
GO:0016081	│ │ │ ├─ synaptic vesicle docking	1.41E-06
GO:0016082	│ │ │ └─ synaptic vesicle priming	6.78E-09
GO:0048488	│ │ └─ synaptic vesicle endocytosis	8.40E-08
GO:0099525	│ ├─ presynaptic dense core vesicle exocytosis	7.59E-04
SYNGO:postsynprocess	├─ process in the postsynapse	7.05E-11
GO:0099072	│ ├─ regulation of postsynaptic membrane neurotransmitter receptor levels	2.16E-11
GO:0099645	│ │ ├─ neurotransmitter receptor localization to postsynaptic specialization membrane	1.38E-06
GO:0098884	│ │ └─ postsynaptic neurotransmitter receptor endocytosis	6.86E-04
GO:0099537	│ ├─ trans-synaptic signaling	3.84E-10
GO:0099542	│ │ │ └─ trans-synaptic signaling by endocannabinoid	1.27E-04
GO:0007268	│ │ └─ chemical synaptic transmission	7.22E-08
GO:0050804	│ │ ├─ modulation of chemical synaptic transmission	1.41E-06
GO:0050808	├─ synapse organization	1.53E-24
GO:0099173	│ ├─ postsynapse organization	7.98E-04
GO:0099175	│ │ ├─ regulation of postsynapse organization	7.69E-04
GO:0099010	│ │ └─ modification of postsynaptic structure	1.10E-06
GO:0098885	│ │ └─ modification of postsynaptic actin cytoskeleton	4.06E-06
GO:1905274	│ │ └─ regulation of modification of postsynaptic actin cytoskeleton	2.61E-04
GO:0098918	│ ├─ structural constituent of synapse	2.45E-13
GO:0098882	│ │ │ ├─ structural constituent of active zone	1.01E-07
GO:0099186	│ │ └─ structural constituent of postsynapse	1.80E-07
GO:0098919	│ │ └─ structural constituent of postsynaptic density	6.09E-05
GO:0007416	│ ├─ synapse assembly	2.38E-04
GO:0099188	│ └─ postsynaptic cytoskeleton organization	1.38E-06
GO:0098974	│ ├─ postsynaptic actin cytoskeleton organization	4.06E-06

Table S3 Number of regulated phosphorylation events per predicted kinase group in Mock vs BoNT experiment. *"Sites in" show number of phosphorylation events that can be regulated by a respective kinase group as determined in the Mock vs. BoNT experiment. "Sites out" contains the number of phosphorylation events possibly regulated by other kinases. Similarly, "Sites in bcgr" and "Sites out bcgr" summarize the number of predicted substrate-kinase relations in human proteome (used as background). Fisher's exact test was applied to test enrichment of substrate-kinase relationships as compared to predicted substrate-kinase relations in the human proteome. "p.val" and "p.adj" are raw and Benjamini-Hochberg adjusted p-values, respectively.*

**Kinase group**	**Sites in**	**Sites out**	**Sites in bcgr**	**Sites out bcgr**	**p.val**	**p.adj**
MAPK	229	628	6320	34052	0.000	0.000
PKC	169	688	5933	34439	0.000	0.000
CK1	82	775	4486	35886	0.169	0.287
CDK	78	779	3324	37048	0.347	0.452
CaMKII	72	785	2812	37560	0.104	0.201
GSK3	40	817	984	39388	0.000	0.001
PAK	36	821	3407	36965	0.000	0.000
CK2	32	825	2565	37807	0.001	0.004
DAPK	24	833	2076	38296	0.001	0.004
CLK	14	843	773	39599	0.704	0.804
PKB	11	846	603	39769	0.775	0.868
GRK	10	847	794	39578	0.104	0.201
DMPK	9	848	171	40201	0.013	0.031
RSK	9	848	245	40127	0.116	0.216
CAMK	9	848	273	40099	0.202	0.314
AMPK	3	854	208	40164	0.806	0.868
PDHK3_PDHK1	3	854	266	40106	0.387	0.493
PKGcGK	3	854	404	39968	0.054	0.120
Aurora	2	855	1078	39294	0.000	0.000
MAPKAPK	1	856	18	40354	0.329	0.446
SLK	1	856	23	40349	0.396	0.493
p70S6K	1	856	50	40322	1.000	1.000
PKD	1	856	191	40181	0.196	0.314
ROCK	1	856	355	40017	0.008	0.021
PKA	1	856	430	39942	0.002	0.006
NEK1_NEK5_NEK3_NEK4_NEK11_NEK2	1	856	601	39771	0.000	0.000
MAP2K	1	856	1982	38390	0.000	0.000

Table S4: Number of regulated phosphorylation events per predicted kinase group in Ca vs. EGTA and Mock vs BoNT experiments. *"CaEGTA" and "MockBoNT" show the number of the regulated phosphorylation events per kinase group in Ca^{2+} vs. EGTA and Mock vs. BoNT experiments, respectively. "Total CaEGTA" and "Total MockBoNT" show the total number of the regulated phosphorylation events with a predicted substrate-kinase relationship in respective experiment. Fisher's exact test was used to test the difference in the occurrence of predicted substrate-kinase relationships in Ca vs. EGTA and Mock vs. BoNT experiments. "p.val" is a p-value of the respective test. "p.adj.BH" is the p-value after Benjamini-Hochberg correction.*

Kinase group	CaEGTA	Total CaEGTA	MockBoTN	Total MockBoTN	p.val	p.adj
CaMKII	160	903	72	857	0.000	0.000
MAPK	175	903	229	857	0.004	0.039
PAK	67	903	36	857	0.008	0.051
MAP2K	9	903	1	857	0.022	0.101
PLK	6	903	0	857	0.031	0.101
DMPK	2	903	9	857	0.034	0.101
CLK	5	903	14	857	0.037	0.101
PKC	141	903	169	857	0.065	0.153
CDK	60	903	78	857	0.078	0.164
CK2	46	903	32	857	0.204	0.387
AMPK	8	903	3	857	0.227	0.393
RSK	6	903	9	857	0.443	0.669
GRK	7	903	10	857	0.470	0.669
PKB	8	903	11	857	0.493	0.669
DAPK	29	903	24	857	0.677	0.719
CAMK	7	903	9	857	0.620	0.719
DYRK	7	903	9	857	0.620	0.719
CK1	80	903	82	857	0.682	0.719
GSK3	44	903	40	857	0.911	0.911

References

1. Popoff, M.R. and Poulain, B. (2010) Bacterial toxins and the nervous system: neurotoxins and multipotential toxins interacting with neuronal cells. *Toxins* 2, 683-737.
2. Nicholls, D.G., Sihra, T.S. and Sanchez-Prieto, J. (1987) Calcium-dependent and-independent release of glutamate from synaptosomes monitored by continuous fluorometry. *Journal of neurochemistry* 49, 50-57.
3. Hornbeck, P.V., Zhang, B., Murray, B., Kornhauser, J.M., Latham, V. and Skrzypek, E. (2015) PhosphoSitePlus, 2014: mutations, PTMs and recalibrations. *Nucleic acids research* 43, D512-D520.
4. Miller, M.L., Jensen, L.J., Diella, F., Jørgensen, C., Tinti, M., Li, L., Hsiung, M., Parker, S.A., Bordeaux, J., Sicheritz-Ponten, T. and Olhovsky, M., 2008. Linear motif atlas for phosphorylation-dependent signaling. *Science signaling*, *1*(35), ra2-ra2.
5. Horn, H., Schoof, E.M., Kim, J., Robin, X., Miller, M.L., Diella, F., Palma, A., Cesareni, G., Jensen, L.J. and Linding, R. (2014) KinomeXplorer: an integrated platform for kinome biology studies. *Nature methods* 11, 603.
6. Hogrebe, A., von Stechow, L., Bekker-Jensen, D.B., Weinert, B.T., Kelstrup, C.D. and Olsen, J.V. (2018) Benchmarking common quantification strategies for large-scale phosphoproteomics. *Nature communications*, *9*(1), 1-13
7. UniProt Consortium (2019) UniProt: a worldwide hub of protein knowledge. *Nucleic acids research* 47, D506-D515
8. Roy, J. and Cyert, M.S. (2009) Cracking the phosphatase code: docking interactions determine substrate specificity. *Science signaling*, *2*(100), re9-re9.
9. Szklarczyk, D., Gable, A. L., Lyon, D., Junge, A., Wyder, S., Huerta-Cepas, J., Simonovic, M., Doncheva, N. T., Morris, J. H., and Bork, P. (2019) STRING v11: protein–protein association networks with increased coverage, supporting functional discovery in genome-wide experimental datasets. *Nucleic acids research* 47, D607-D613
10. Schwanhäusser, B., Busse, D., Li, N., Dittmar, G., Schuchhardt, J., Wolf, J., Chen, W. and Selbach, M. (2011) Global quantification of mammalian gene expression control. *Nature* 473, 337-342.
11. Smyth, G.K. (2005) Limma: linear models for microarray data. In *Bioinformatics and computational biology solutions using R and Bioconductor*, 397-420, Springer, New York, NY.
12. Koopmans, F., van Nierop, P., Andres-Alonso, M., Byrnes, A., Cijsouw, T., Coba, M.P., Cornelisse, L.N., Farrell, R.J., Goldschmidt, H.L., Howrigan, D.P. and Hussain, N.K. (2019) SynGO: an evidence-based, expert-curated knowledge base for the synapse. *Neuron* 103(2), 217-234.
